# SwitchFinder – a novel method and query facility for discovering dynamic gene expression patterns

**DOI:** 10.1186/s12859-016-1391-0

**Published:** 2016-12-15

**Authors:** Svetlana Bulashevska, Colin Priest, Daniel Speicher, Jörg Zimmermann, Frank Westermann, Armin B. Cremers

**Affiliations:** 1 B-IT Bonn-Aachen International Center for Information Technology, University of Bonn, Dahlmannstr. 2, Bonn, 53113 Germany; 2 Sigma Plus Consulting Pty Ltd, Crows Nest, 2065 NSW Australia; 3 Institute of Computer Science, University of Bonn, Roemerstr. 164, Bonn, 53117 Germany; 4 Neuroblastoma Genomics Group, German Cancer Research Center (DKFZ), Im Neuenheimer Feld 280, Heidelberg, 69120 Germany

**Keywords:** Time-series analysis, Dynamic patterns of gene expression, Change-point problem, Change-point modeling, Bayesian modeling, MCMC, Gibbs sampling, Neuroblastoma, ATRA-induced differentiation

## Abstract

**Background:**

Biological systems and processes are highly dynamic. To gain insights into their functioning time-resolved measurements are necessary. Time-resolved gene expression data captures temporal behaviour of the genes genome-wide under various biological conditions: in response to stimuli, during cell cycle, differentiation or developmental programs. Dissecting dynamic gene expression patterns from this data may shed light on the functioning of the gene regulatory system. The present approach facilitates this discovery. The fundamental idea behind it is the following: there are change-points (switches) in the gene behaviour separating intervals of increasing and decreasing activity, whereas the intervals may have different durations. Elucidating the switch-points is important for the identification of biologically meanigfull features and patterns of the gene dynamics.

**Results:**

We developed a statistical method, called SwitchFinder, for the analysis of time-series data, in particular gene expression data, based on a change-point model. Fitting the model to the gene expression time-courses indicates switch-points between increasing and decreasing activities of each gene. Two types of the model - based on linear and on generalized logistic function - were used to capture the data between the switch-points. Model inference was facilitated with the Bayesian methodology using Markov chain Monte Carlo (MCMC) technique Gibbs sampling. Further on, we introduced features of the switch-points: *growth*, *decay*, *spike* and *cleft*, which reflect important dynamic aspects. With this, the gene expression profiles are represented in a qualitative manner - as sets of the dynamic features at their onset-times. We developed a Web application of the approach, enabling to put queries to the gene expression time-courses and to deduce groups of genes with common dynamic patterns.

SwitchFinder was applied to our original data - the gene expression time-series measured in neuroblastoma cell line upon treatment with all-*trans* retinoic acid (ATRA). The analysis revealed eight patterns of the gene expression responses to ATRA, indicating the induction of the BMP, WNT, Notch, FGF and NTRK-receptor signaling pathways involved in cell differentiation, as well as the repression of the cell-cycle related genes.

**Conclusions:**

SwitchFinder is a novel approach to the analysis of biological time-series data, supporting inference and interactive exploration of its inherent dynamic patterns, hence facilitating biological discovery process. SwitchFinder is freely available at https://newbioinformatics.eu/switchfinder.

**Electronic supplementary material:**

The online version of this article (doi:10.1186/s12859-016-1391-0) contains supplementary material, which is available to authorized users.

## Background

Time-resolved measurements are performed to study the dynamics of biological processes e.g. the dynamics of gene expression in response to treatments, upon induction of a transcription factor, during cell cycle or embryonic development. The temporal response patterns may shed light on coordination and regulation of the genes, aiding the inference of gene regulatory networks. Several methods for the analysis of the time-course gene expression data were developed, reviewed in [1], however, major challenges remain. The time courses are mainly short, hindering the inference of complex models with many parameters. The Markov model-based methods [2, 3] rely on the assumption that the underlying process is a) Markovian and b) stationary: a) the state of a gene at each time-point depends only on the state of the system at the previous time-point and b) the probability of a transition from one time-point to the next is constant for all time-points. The biological relevance of these assumptions is questionable. The gene regulatory circuits permanently rewire – the genes switch between different regimes of activity, whereas the durations of the regimes may have different length. In fact, these are the turning points of gene behaviour that have biological relevance and are important to elucidate. The gene expression data is likewise sampled at the irregularly spaced time-points with a hope to capture real biological events. The sparse irregular sampling generates spiky, saw-toothed data, presenting a difficulty for smooth interpolations. To overcome this, in [4] the use of piecewise constant functions was advocated.

The most common purpose of the time-resolved gene expression data analysis was to derive groups of genes with similar dynamical responses. Model-based clustering [5] executes simultaneously two tasks: fitting a model to gene expression profiles and grouping the genes based on the parameters of the fitted models. However, relations between genes across time may have only a fragmentary character like e.g. immediate-early responses to stimulation. Modeling the expression profile of an individual gene might be more appropriate. Even at the risk of over-fitting, this has an advantage of capturing unique features of the gene temporal behaviour. In [6], a mathematical model of response dynamics - the *impulse model* - was proposed for fitting the individual gene profile. The model contains seven biologically relevant parameters, emphasizing important aspects of the gene dynamics e.g. point of induction. In [7], the model was used in an integrative clustering-modeling approach.

In the present approach, called *SwitchFinder*, a time-series model is proposed that explicitly assumes the existence of the switch-points (*switches*) between intervals of increasing and decreasing activities, which are interpolated with linear or generalized logistic function. Fitting the model to the time-resolved gene expression data implies the prediction of the switch-points of individual genes.

Our approach has origin in the change-point modelling, that has been widely applied in engineering, ecology, economics and finance [8–14]. The fundamental idea is: the model is characterized by a number of discrete regimes, within which different model parameters apply. The model switches from one regime to another and the characteristics of the observations change according to the particular regime. Assessing the locations of the change-points (called in the literature switch-points, breakpoints, structural breaks or thresholds) may give valuable insights into the modelled process. Various approaches to the change-point problem for models with different assumptions were proposed. To mention are diagnostic methods based on testing with e.g. Schwartz’s Bayesian Information Criterion (BIC) [15], iterative fitting procedures for segmented regressions [16, 17], non-parametric smoothers [11] and dynamic programming algorithms [18–20]. Bayesian approach to multiple change-points problem dates back to [21] and was further elaborated by e.g. [10, 22, 23]. In [24], the product partition model was used in the Bayesian framework, see also [25]. In [26], the multiple change-points model was formulated in terms of a latent discrete-state variable indicating the regimes and evolving as a discrete time, discrete-state Markov process governed by a transition probability matrix. The model was estimated with Markov chain Monte Carlo (MCMC) sampling. Bayesian methodology is valuable for the inference of the change-point models, since it treats the change-points locations as parameters to be estimated in the same framework as the other model parameters. A MCMC technique Gibbs sampling proved to be especially attractive for the Bayesian inference [27].

The central interest of the present work was the inference of the switch-points indicating changes between the regimes of the gene activity. Our model represents a series of switch-points (peaks and troughs), joined by lines or logistic curves. We developed a Gibbs sampling procedure for the Bayesian inference of the model.

The switch-points elucidated by the analysis may indicate an onset of features like Growth or Decay, introduced here to capture substantial dynamic properties of the gene behaviour. Knowing onset-times of the dynamic features enables to represent the gene profiles in a qualitative manner. This is utilized in our approach to perform partitioning of the genes into groups with common dynamic patterns. The present approach inspires to put queries to the gene set like for example: which genes have peaks/troughs of their activity at certain time points? Which genes exhibit growth or decay at the particular onset-times? The Web application of the approach provides the query interface and grants to a human expert a possibility to query the time-resolved data, facilitating the biological discovery process.

The present approach decouples the two tasks – statistically fitting individual genetic profiles and grouping of them. We do not regard the gene data set as multivariate time-series data, as was done in [28]. The rationale behind this is that an individual gene dynamics is not only governed by the system dynamics, but by some external factors remaining “behind the scene” (e.g. chromatin modifications, post-transcriptional modifications, protein degradation). In [28], the authors segmented multivariate biological time-series with the help of the fused LASSO regression, based on the assumption that the data at each time-point (response) is explained with the preceding time-points (regressors), except at the breakpoints, for which the preceding time-points have negligible explanatory power. The authors then clustered the gene profiles in each segment. To the contrary, we first seek to detect the change-points in each gene behaviour, then the change-points can be used to get insights into the dependency structure of the system.

In the next section, we introduce the model and its Bayesian formulation.

## Methods

### The model

**Fig. 1:**
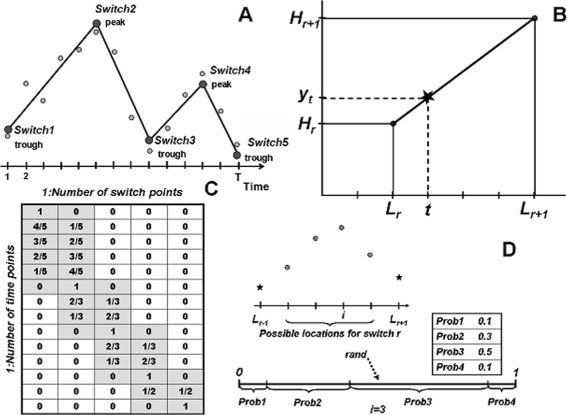
Illustration of the modeling. **a** Example of a model with four regimes and five switches. **b** When the switch locations are known, the data within each regime is fitted with a line - linear interpolation. **c** Design matrix for the model in A, calculated using linear factors, specifies the linear regression needed to determine the switch heights. **d** Roulette method for sampling of a switch location between the neighbouring switches, based on the calculated probabilities of four possible locations

Figure 1, A illustrates the model for an exemplary gene expression profile with *T*=14 measurements. The model contains *N*=5 switches at time-points 1, 6, 9, 12, 14 (*switch locations*) of the following types: *trough*, *peak*, *trough*, *peak* and *trough*. The switches separate intervals of increasing and decreasing activities of the gene, called *regimes*. The model assumes that the data within the regimes is interpolated with linear functions. The goal of the present method is to infer the most probable time-points of switches between the regimes while fitting the model to the time-series data.

Let *r* be the regime index: *r*=1,…,*N*−1. We denote the locations of the switches with *L*_*r*_ (*N*-dimensional vector) and the *y*-values at these locations (*switch heights*) with *H*_*r*_. The model assumes that the data values at time-points between the switches are determined by the linear interpolation. Figure 1,b displays one time interval with time-points *t*∈{*L*_*r*_,…,*L*_*r*+1_}. The interpolated value at the time-point *t* is denoted by *y*_*t*_. Due to the linearity property, the following proportion is valid: $\frac {t-L_{r} }{L_{r+1} -L_{r}} =\frac {y_{t}-H_{r}}{H_{r+1}-H_{r}}$. Solving this equation for *y*_*t*_, while denoting with $LF:=\frac {L_{r+1} -t}{L_{r+1}-L_{r}}$ (*linear factor*), we get:

1 $$\begin{array}{@{}rcl@{}} y_{t}=H_{r} \cdot LF+H_{r+1} \cdot \left(1-LF\right)  \\ {\text{for}}~t\in \left[L_{r},\ldots,L_{r+1} \right].\quad  \end{array} $$

If *Y*=(*y*_*t*_)_*t*=1,…,*T*_ is the data, the set of Eq. (1) for all intervals *r* specifies a *linear regression model* with the *N*-dimensional vector of parameters H=(*H*_*r*_)_*r*=1,…*N*_. So the model underlying our approach is specified as: *Y*=*X*·*H*+*e*, where *X* is the (*T*×*N*)-dimensional *design matrix*, defined with the help of the linear factors for all *t* and all *r* (see the matrix in Fig. 1,c for the model in Fig. 1,a). Vector *e* is the *error term*, which can be written as: *e*=*σ*·*e*_*t*_, *e*_*t*_∼N(0,1), where *σ* is the standard deviation of the error term. The parameters of the model to be estimated in course of the model inference are: locations of the switches *L*_*r*_, *r*=2,…,*N*−1, the heights of the switches *H*_*r*_,*r*=1,…,*N* and *σ*. (For simplicity of the modelling, the first and the last time-points of the time-series are always labelled as switches).

If switch locations *L*_*r*_ are known, the linear regression model is specified and can be fitted to the data *Y* by the **Ordinary Least Squares** (**OLS**) method. Then, the parameters of the model (i.e. the switch heights) can be determined by: H**=**(*X* ^*T*^ *X*)^−1^ *X* ^*T*^ *Y*. The fitted values under the model are calculated by: *Y*_*fitted*_=*X*·*H*.

In the following, for the sake of simplicity, we use a common notation for the linear regression model: *β* instead of *H*. Let the linear regression model be formulated as follows:

2 $$ Y=X\beta+e,\quad e\sim N\left(0,\sigma^{2} I\right).  $$

The *N*-dimensional vector of regression coefficients *β* and the standard deviation *σ* are parameters to be estimated.

### Model inference

Probabilistic inference of the model (estimation of the switch locations and the parameters *β* and *σ*) was facilitated by the Bayesian methodology. Within a Bayesian framework, inference about parameters of a model, *θ*, is made based on its *posterior* distribution given the data, *p*(*θ*|*Y*), using the proportionality: *p*(*θ*|*Y*)∝*L*(*θ*|*Y*)*p*(*θ*), where *L*(*θ*|*Y*) is the *likelihood* function and *p*(*θ*) is the *prior* distribution of the parameters. Since the direct Bayesian inference of the present model is infeasible, the Markov chain Monte Carlo (MCMC) technique **Gibbs sampling** presents an attractive possibility. Gibbs sampling reduces a problem of sampling from a complex posterior distribution to a series of more tractable subtasks of sampling from simpler, lower-dimensional distributions, simulations from which can be done using standard functions [29, 30]. Namely, Gibbs sampling iteratively generates samples from *full conditional posterior distributions* as outlined below.

Suppose the model has *k* parameters *θ*=(*θ*_1_,…,*θ*_*k*_). Given an arbitrary set of starting values $(\theta _{2}^{(0)},\ldots,\theta _{k}^{(0)})$, consider the following steps:

$${}\begin{array}{l} \text{Step~ 1.~Draw} \theta_{1}^{(1)} \text{from}~ p\left(\theta_{1} \left|\theta_{2}^{(0)} \right.,\ldots,\theta_{k}^{(0)},Y\right) \\ \text{Step~ 2.~ Draw} \theta_{2}^{(1)} \text{from}~ p\left(\theta_{2} \left|\theta_{1}^{(1)} \right.,\theta_{3}^{(0)},\ldots,\theta_{k}^{(0)},Y\right) \\ \text{Step~3.~ Draw} \theta_{3}^{(1)} \text{from}~p\left(\theta_{3} \left|\theta_{1}^{(1)} \right.,\theta_{2}^{(1)},\theta_{4}^{(0)},\ldots,\theta_{k}^{(0)},Y\right) \\ \vdots\\ \text{Step~ k.~Draw} \theta_{k}^{(1)} \text{from}~ p\left(\theta_{k} \left|\theta_{1}^{(1)} \right.,\ldots,\theta_{k-1}^{(1)},Y\right) \end{array} $$

Steps 1 through *k* are repeated *J* times, where *J* is the number of iterations, to obtain the samples $(\theta _{1}^{(j)},\theta _{2}^{(j)},\ldots,\theta _{k}^{(j)}),\; j=1,\ldots,J$. The distribution $p(\theta _{i} \left |\theta _{1}^{(j)} \right.,\ldots,\theta _{i-1}^{(j)},\theta _{i+1}^{(j-1)},\ldots,\theta _{k}^{(j-1)},Y)$ is called the *full conditional posterior distribution*. If *J* is large enough, after some *L*, the Gibbs sampler has converged [29]. Then the joint and marginal distributions of *θ*_1_,…,*θ*_*k*_ can be approximated by the empirical distributions of the simulated values. E.g. the mean of the marginal distribution of *θ*_*i*_ may be calculated by:

$$\frac{\sum_{j=1}^{J-L}\theta_{i}^{L+j} }{J-L}. $$

In the following, we derive the conditional posterior distributions of *β* and *σ* ^2^.

#### Conditional distribution of *β*, given *σ*^2^.

Assume *σ* ^2^ is known. We prescribe a multivariate normal distribution for the parameter *β*. Let the prior distribution of *β* is given by:

*β*|*σ* ^2^∼*N*(*β*_0_,*Σ*_0_), where the vector *β*_0_ and the covariance matrix *Σ*_0_ are known. The prior density can be written as:

$$\begin{array}{@{}rcl@{}} p\left(\beta \left|\sigma^{2} \right. \right)= \\ \left(2\pi \right)^{-\frac{N}{2}} \left|\Sigma_{0} \right|^{-\frac{1}{2}} \exp \left\{-\frac{1}{2} \left(\beta -\beta_{0} \right)\Sigma_{0}^{-1} \left(\beta -\beta_{0} \right)\right\} \\ \propto \exp \left\{-\frac{1}{2} \left(\beta -\beta_{0} \right)\Sigma_{0}^{-1} \left(\beta -\beta_{0} \right)\right\}. \end{array} $$

Because of the assumption of normality in (2), the likelihood function is given by:

3 $$\begin{array}{@{}rcl@{}} L\left(\beta,\sigma^{2} \left|Y\right. \right)= \\ \left(2\pi \sigma^{2} \right)^{-\frac{T}{2}} \exp \left\{-\frac{1}{2\sigma^{2}} \left(Y-X\beta \right)^{T} \left(Y-X\beta \right)\right\} \\ \propto \exp \left\{-\frac{1}{2\sigma^{2}} \left(Y-X\beta \right)^{T} \left(Y-X\beta \right)\right\}.  \end{array} $$

Combining the prior density and the likelihood function, the posterior distribution of *β*, conditional on *σ* ^2^, is specified by the following normal distribution (see [31], Chapter 7; [32]):

$$\begin{array}{l} \beta \left|\sigma^{2} \right.,Y\sim N\left(\beta_{1},\Sigma_{1} \right), \text{where}\\ \beta_{1} =\left(\Sigma_{0}^{-1} +\sigma^{-2} X^{T} X\right)^{-1} \left(\Sigma_{0}^{-1} \beta_{0} +\sigma^{-2} X^{T} Y\right),\\ \Sigma_{1} =\left(\Sigma_{0}^{-1} +\sigma^{-2} X^{T} X\right)^{-1}. \end{array} $$

In case of an uninformative prior i.e. when *β*_0_ is the vector of nulls and *Σ*_0_ contains big values, the Bayesian estimate of the probability distribution of *β* is analogous to the distribution of the *best linear unbiased estimator* of *β* obtained by the OLS method. Namely, the unbiased estimator of *β* is a normally distributed random variable [31]:

$$\beta_{unbiased} \sim \mathrm{N} \left(\left(X^{T} X\right)^{-1} X^{T} Y,\sigma^{2} \left(X^{T} X\right)^{-1} \right). $$

So, we can use *β*_*fitted*_=(*X* ^*T*^ *X*)^−1^ *X* ^*T*^ *Y* and *Σ*=*σ* ^2^(*X* ^*T*^ *X*)^−1^ as the mean and the covariance matrix for sampling the values of *β*.

If the mean vector *μ* and the covariance matrix *Σ* of the multivariate normal distribution are known, a commonly used method for generating values from this distribution is the following. Identify matrix *A*, which is the Cholesky decomposition i.e. *AA* ^*T*^=*Σ*, then the sample value is calculated as: *μ*+*AE*, where *E* is an *N*-dimensional vector of standard normal variables sampled from N(0,1).

While sampling *β*, rejection sampling was used to ensure the validity of the new model: only models with alternating troughs and peaks and non-degenerate (i.e. with each data point as switch or with a regime having low amplitude) are admissible.

#### Conditional distribution of *σ*^2^, given *β*

Assume *β* is known. The usual specification for the distribution of *σ* ^2^ is the *inverted Gamma* distribution (because this is the natural conjugate prior for normal likelihood). So, $\frac {1}{\sigma ^{2} }$ should be Gamma-distributed. Let the prior distribution of $\frac {1}{\sigma ^{2} }$ has the form: $\frac {1}{\sigma ^{2}} \left |\beta \right. \sim \Gamma \left (\frac {\nu _{0} }{2},\frac {\delta _{0} }{2} \right)$, where *ν*_0_ and *δ*_0_ are known, so

$$p\left(\frac{1}{\sigma^{2}} \left|\beta \right. \right)\propto \left(\frac{1}{\sigma^{2}} \right)^{\frac{\nu_{0} }{2} -1} \exp \left(-\frac{\delta_{0} }{2\sigma^{2}} \right). $$

The likelihood function is given by (3). Multiplying the prior density and the likelihood gives the following posterior density:

$$p\left(\frac{1}{\sigma^{2}} \left|\beta,Y\right. \right)\sim \left(\frac{1}{\sigma^{2}} \right)^{\frac{\nu_{1} }{2} -1} \exp \left(-\frac{\delta_{1} }{2\sigma^{2}} \right) $$

that is also of a Gamma form, suggesting the following posterior distribution of $\frac {1}{\sigma ^{2} }$:

$$\begin{array}{l} \frac{1}{\sigma^{2}} \left|\beta \right.,Y\sim \Gamma \left(\frac{\nu_{1} }{2},\frac{\delta_{1} }{2} \right), \text{where}\\ \nu_{1} =\nu_{0}+T,\\ \delta_{1} =\delta_{0} +\left(Y-X\beta \right)^{T} \left(Y-X\beta \right). \end{array} $$

It can be shown that in case of uninformative priors (*ν*_0_=0, *δ*_0_=1) this distribution is analogous to the distribution of the unbiased estimator of *σ* ^2^, determined by the OLS method. If $\sigma _{unbiased}^{2} $is the unbiased estimator of *σ* ^2^, then it is distributed as (see [31]):

$$\sigma_{unbiased}^{2} \sim \frac{\sigma_{fitted}^{2} }{T-M} \chi^{2} \left(T-M\right), $$

where *M* is the number of regressors in the model (here, *M=1*), *χ* ^2^ is the chi-squared distribution. So, we can use $\frac {\sigma _{fitted}^{2} }{T-1} \chi ^{2} \left (T-1\right)$ for sampling the values for *σ* ^2^, where *σ*_*fitted*_ is calculated from data.

#### Sampling switch locations, given all the other information

While sampling a location for a switch *r*, we assume that the locations of the previous and the subsequent switches are known, so the possible choices lie in the interval *i*∈{*L*_*r*−1_+1,…,*L*_*r*+1_−1} representing a finite number of possibilities. Figure 1,d illustrates the approach. For each possible value *i*, by Bayes theorem, the posterior probability of the switch taking the particular location is the following: $p\left (L_{r} =i\left |Y\right. \right)=\frac {L\left (Y\left |L_{r} =i\right. \right)p\left (L_{r} =i\right)}{P(Y)} $,where *p*(*L*_*r*_=*i*) is the prior probability, *L*(*Y*|*L*_*r*_=*i*) is the likelihood of data, given the particular location. It can be written: $P(Y)=\sum _{j}L\left (Y\left |L_{r} =j\right. \right)p\left (L_{r} =j\right) $. If we assume the uninformative prior, the probabilities *p*(*L*_*r*_=*j*) are the same for all *j*i.e. *p*(*L*_*r*_=*j*)=*p*(*L*_*r*_=*i*). Thus, the following formula for the calculation of probabilities of the possible switch locations results:

$$p\left(L_{r} =i\left|Y\right. \right)=\frac{L\left(Y\left|L_{r} =i\right. \right)}{\sum_{j}L\left(Y\left|L_{r} =j\right. \right)}. $$

The likelihood of data, given the particular location, can be calculated as the product of the probabilities of making the error $\left (e_{t} \right)_{t\in \left \{L_{r-1} +1,\ldots,L_{r+1} -1\right \}} $, where each error is calculated as: $e_{t} =\frac {Y^{t} -Y_{fitted}^{t} }{\sigma } $. Note that the error is standard normal distributed: *e*_*t*_∼N(0,1), so we can use the R function *pnorm*(*e*_*t*_)to obtain the individual probabilities (the number of probabilities is *L*_*r*+1_−*L*_*r*−1_−1).

Once we have *p*(*L*_*r*_=*i*|*Y*) i.e. the probabilities of each possible location given all the other information (let denote them with *probs*), we can sample an integer value with these probabilities by the Roulette selection method (Fig. 1,d). I.e. a random value *rand* is generated from the uniform distribution and $i := 1+max\left \{m\left |\Sigma _{i=1}^{m}probs_{i} < rand \right.\right \}$ will be taken as the sampled value for the switch location.

**Fig. 2:**
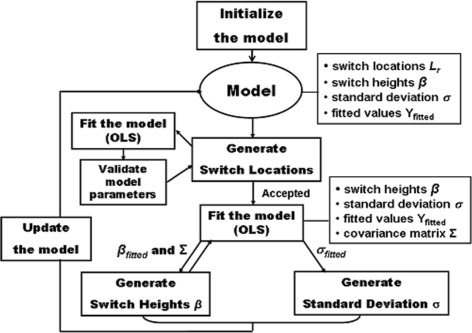
Workflow of the algorithm. The algorithm presents the iterative sampling of the model parameters in the Gibbs procedure. Parameter values (switch locations, heights, standard deviation) and fitted data are stored for each current model. The generated switch locations are accepted only if they produce a valid model. The parameters of the fitted model (switch heights and covariance matrix) are used to generate new sample values for the switch heights. Then, upon model fit, the fitted standard deviation is used to produce a sample value for the standard deviation. The next iteration with the updated model proceeds, new switch locations will be generated

The workflow of the algorithm in Fig. 2 represents the repeated sampling of the model parameters in course of the MCMC iterations. In each run, the algorithm first allocates the switch-points and then fits the model, providing necessary quantities for the sampling of new values for the model parameters. Only switch locations that generate valid models are accepted.

The number of the switch-points, with which the MCMC procedure is initialized, is calculated with the exploratory non-parametric technique LOESS [33], originally LOWESS (LOcally Weighted Scatter-plot smoother). It is a method for fitting a smooth curve between two variables. The procedure performs weighted polynomial regression for only a subset of observations i.e the fitting at point *t* is weighted toward the data nearest to *t*. The distance to *t*, that is considered near to it, is controlled by the parameter *span*. When span is less than 1, it represents the proportion of the total data included within each subset. More details can be found in the description of the R function *loess*, used in this work. The polynomial for the regression equations here was quadratic (degree 2). LOESS fits a non-linear smoothing curve to the data, helping to reveal structural patterns in it. We use the fitting data to calculate local minima and maxima along the curve suggesting the number of the switch-points. Higher values of the span produce smoother curves, hence, the number of the switch-points decreases. Setting for the span is found in an iterative procedure. Starting with the small span 0.1, a curve is fitted to the data while increasing the span by a small amount (0.05) until none of the local minima and maxima are located immediately adjacent. The last number of the minima and maxima (added with 2 for the first and the last time-points) yields the number of the switch-points.

We call the presented model Model_Lin to distinguish it from the Model_Logit described in the next section.

#### Modeling with the generalized logistic function (Model_Logit)

Sometimes the increasing/decreasing activity of a gene exhibits a saturated behaviour, stabilizing with time. To model this, the generalised logistic function was used. We assume that in each time interval {*L*_*trough*_,*L*_*peak*_}between two switch points, which are a trough and a peak, the fitted data lies on a logistic (sigmoid) curve and is calculated as follows:

4 $$ Y_{fitted} (t)=H_{trough} +FL\left(prop_{t} \right)\cdot \left(H_{peak} -H_{trough}\right)   $$

where *H*_*trough*_ and *H*_*peak*_ are switch heights, *prop*_*t*_ is the proportional location of the time-point *t* with respect to the trough and is calculated as $prop_{t} :=\frac {t-L_{trough}}{L_{peak} -L_{trough}}$. *FL* is the *generalized logistic function* defined as (see [34]):

$$y(t)=\frac{K}{\left(1+Qe^{-ab\left(t-t_{0} \right)} \right)^{{1\left/\right. b}} }, $$

which is the solution of the differential (*Richard’s growth* equation: $\frac {\partial y}{\partial t} =ay\left [1-\left (\frac {y}{K} \right)^{b} \right ]$ with initial condition *y*(*t*_0_)=*y*_0_, where $Q=-1+\left (\frac {K}{y_{0}} \right)^{b} $.

The parameter *b* allows the shape of the sigmoid curve to vary flexibly. *K* is the maximum observable value of y, in our case *K=1*. In the present work, we used the following parameterization (*y*_0_=0.001 is a small value):

$$\begin{array}{l} FL\left(prop_{t} \right):= \\ \frac{1}{\left(1+\left(-1+\left(\frac{1}{0.001} \right)^{{1\left/\right. \kappa}} \right)\cdot \exp \left(-1\cdot prop_{t} \cdot B\right)\right)^{\kappa}}  \end{array} $$

*B* plays a role of the growth rate. Note that the linear transformation of a logistic curve in Eq. (4) is also a logistic curve. With this transformation the lower and upper asymptotic heights of the logistic curve *FL* (0 and 1) are moved to be the trough and the peak values, respectively. Equation (4) can be rewritten as:

*Y*_*fitted*_(*t*)=(1−*FL*(*prop*_*t*_))·*H*_*trough*_+*FL*(*prop*_*t*_)·*H*_*peak*_. Then

5 $$ Y\sim \left(1-FL\left(prop_{t} \right)\right)\cdot H_{trough} +FL\left(prop_{t} \right)\cdot H_{peak}  $$

represents a linear regression model (see Additional file 1, supplementary text). Fitting the model to the data *Y* (R function *lm*) facilitates calculation of the switch heights, analogously to the Model_Lin described above. The generalized logistic transformation of the proportional location of each time-point between the neighbouring trough and peak allows for flexible modelling of the gene expression increase/decrease within time intervals of different length. Sampling of the logistic function parameters *B* and *κ* was executed with the help of *bootstrapping* ([35]) as follows. First, when switch locations and heights are known, Eq. (4) is rewritten as: $\frac {Y(t)-H_{{trough}} }{H_{{peak}} -H_{{trough}}} ={FL} \left ({prop}_{t} \right)$. Denoting the left-hand side with *propy*_*t*_ yields:

6 $$ propy_{t} =FL\left(prop_{t} \right).  $$

Thus, the parameters *B* and *κ* are estimated with the nonlinear least squares method (R function *nls*) - by fitting the nonlinear function *FL* to the data *x*=*prop*_*t*_, *y*=*propy*_*t*_. So, for the current model in each MCMC iteration, the design matrix is constructed and the linear regression model (5) is fitted to the data *Y* to calculate the switch heights *H*. Then *Y*_*fitted*_ is calculated by (4). Further on, the residuals $E^{t}=Y^{t}-Y_{fitted}^{t}$ are calculated and the bootstrap samples of the residuals $E_{b}=\left \{{E_{b}^{1}},\ldots,{E_{b}^{T}}\right \}$ are used to calculate the bootstrapped values *Y*_*b*_=*Y*_*fitted*_+*E*_*b*_, which are then fitted by (6) to obtain the samples of the parameters *B* and *κ*. Apart from that, the workflow of the MCMC-based inference of the Model_Logit is analogous to that of the Model_Lin depicted in Fig. 2. One-regime models, presenting just logistic increase or decrease, are termed here Logit_Up and Logit_Down.

### SwitchFinder as Web Application

**Fig. 3:**
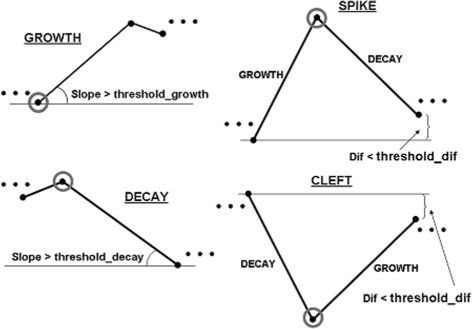
Features of the switch-points defined in SwitchFinder: Growth, Decay, Spike and Cleft. Features are assigned to the switch-points to capture meaningfull properties of the time-series. The feature Growth is assigned to the switches of types trough, if the corresponding Slope is greater than the Threshold with the default value threshold_growth. The user is able to adjust the Threshold for selecting more striking Growth-effects. The feature Decay is defined similarly. The higher-order feature Spike is assigned to the switch, designated with Decay, if its left-hand side neighbour has the feature Growth and the absolute difference Dif in the gene expression levels of the neighbouring switches is smaller than the Threshold with the default threshold_dif. The aim is to select a really spiking behaviour: after a rapid growth, rapid decay to almost the same level occurs. The feature Cleft designates the opposite behaviour: after a decay, growth to almost the same level occurs

**Fig. 4:**
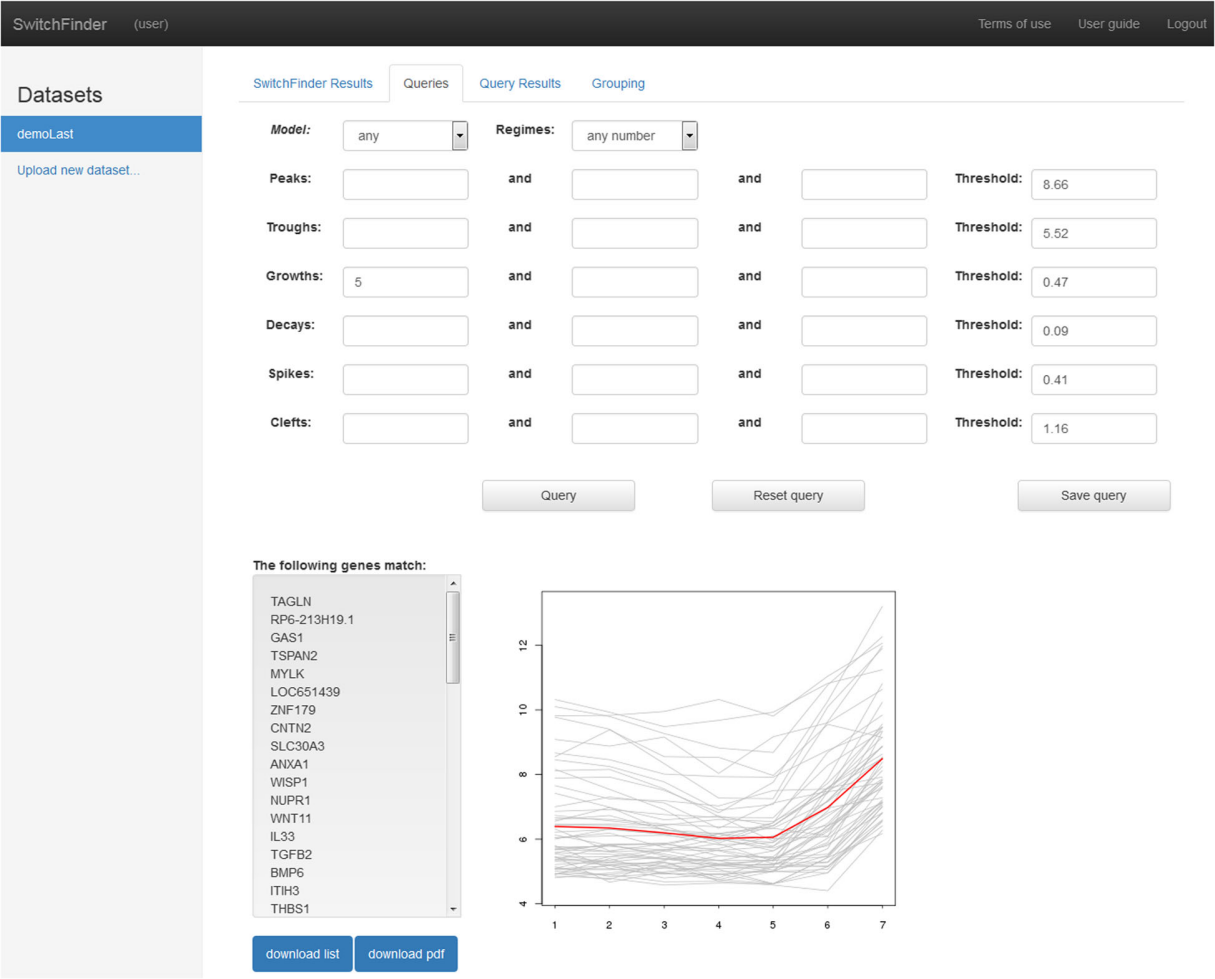
SwitchFinder query interface. Snapshot of the user interface demonstrating the result of the query - gene profiles with the feature Growth at the switch-point at time *t*=5. The Slope of the Growth was greater than the Treshold. It is possible to download the result and to store the query

We developed a Web application of the method SwitchFinder, which provides the user-interface for uploading the time-series data, executing the algorithm and performing queries to the results of the data analysis, thus maintaining a feedback-loop between generation and interpretation of the results. We propose the concept of *features*, assigned to the inferred switch-points, which capture meaningfull properties of the time-series. The basic features *peaks* and *troughs* are the switch-points of the genetic activities deduced by the method directly. The queries are supported: which genes have peaks/troughs at the given time points?. Hence, early, middle and late responses can be elucidated. The user can input thresholds on values of the peaks and troughs to select stronger effects and focus on fewer genes. Further features – *Growth* and *Decay* – designate those troughs and peaks that represent onsets of significant growth or decay of the gene activity as defined with the help of the slope (see Fig. 3). By increasing thresholds for the slopes, stronger effects can be selected. The next-level features - *spikes* and *clefts* - are defined based on the previous-level features using three switch-points (Fig. 3). The query result i.e. the set of genes, which exhibit the given features at the given time-points, is downloadable as the list of genes or the plot (see Fig. 4). Single queries can be logically combined. The default values suggested by the Web application for the thresholds of the slopes are computed as 25%-quantile of the distribution of the slopes over the data. With the default thresholds, the application computes features and represents each gene in form of a *qualitative profile* – as the set of features with their respective onset-times (locations). For a location, its highest-level feature is stored. The profiles that were fitted with a one-regime logistic model additionally obtain features *LogitUp* or *LogitDown*. Grouping of the genes is executed by k-means clustering of the qualitative profiles using the Jaccard similarity [36]. Jaccard measure is especially appropriate for the calculation of similarity between two sets containing different numbers of elements. The concepts of features and qualitative profiles help to reveal groups of genes, organized around remarkable properties of the dynamic behaviour. The suggested grouping is only a platform for further investigations and exploration of the data set. By querying the data set and grouping, meaningful patterns of the dynamic gene expression can be deduced.

## Results and discussion

### Application of SwitchFinder to simulated data

To test robustness of the algorithm SwitchFinder, especially with respect to short time-series data, we generated 10 data sets, each containing 2500 synthetic gene expression profiles of the length T=7. The simulation scheme for a data set was the following. The profiles were generated with standard deviation *sigma=0.2* from the following models: a) Logit_Up (500 samples) and Logit_Down (500 samples) using 10 different combinations of the parameters *κ* and *B*: (0.4,20), (0.5,15), (0.5,20), (1.5,20), (2,8), (8,5), (10,8), (20,5), (20,10), (20,18) including extreme values that challenge the fitting procedure; b) models Model_Lin with one internal switch point of the type *peak* located at *t*=2/4/6 (600 cases) and of the type *trough* located at *t*=2/4/5 (600 cases); c) models Model_Lin with two internal switch points of the types (*peak, trough*) located at time-points *t*=2,5 (200 cases) and of the types (*trough, peak*) located at *t*=2,6 (200 cases); d) model Model_Logit with parameters *κ*=20,*B*=10 and one internal switch at *t*=5 (100 cases). The parameters (heights) of the models were simulated to obtain realistic gene expression values as commonly produced by Agilent technology: sampled from log-normal distribution (*meanlog=2*, *sdlog=0.3*) and truncated to the interval (0,20). The scheme produces biologically realistic data sets with rich dynamic responses. Table 1 demonstrates the results of the application of SwitchFinder to 10 artificial data sets. The goodness-of-fit of a model fitted to a gene expression profile was assessed with the residual standard deviation (RSD). The descriptive statistics of the RSDs for each data set is displayed. The statistics are very stable across the data sets. For the data cases originating from Model_Lin and Model_Logit models, precision and recall were calculated (Prec:=TP/(TP+FP), Recall:=TP/(TP+FN)), to evaluate the accuracy of the prediction of the switch-points. The values were stably good. A small number of functions mismatches occurred (e.g. when data generated from one-regime logistic model was fitted with Model_Lin by the algorithm).

**Table 1:** Results of the application of SwitchFinder to 10 simulated data sets

Mean of	SD of	Precision	Recall	Functions
RSDs	RSDs			mismatches
0.17	0.09	0.93	0.94	0.07
0.17	0.09	0.92	0.94	0.06
0.17	0.10	0.92	0.94	0.06
0.17	0.09	0.93	0.94	0.06
0.17	0.11	0.92	0.94	0.06
0.17	0.10	0.92	0.94	0.06
0.17	0.09	0.91	0.94	0.07
0.17	0.10	0.91	0.93	0.06
0.17	0.09	0.92	0.93	0.06
0.17	0.10	0.92	0.93	0.06

### Application of SwitchFinder to human cell cycle data (long time-series)

**Fig. 5:**
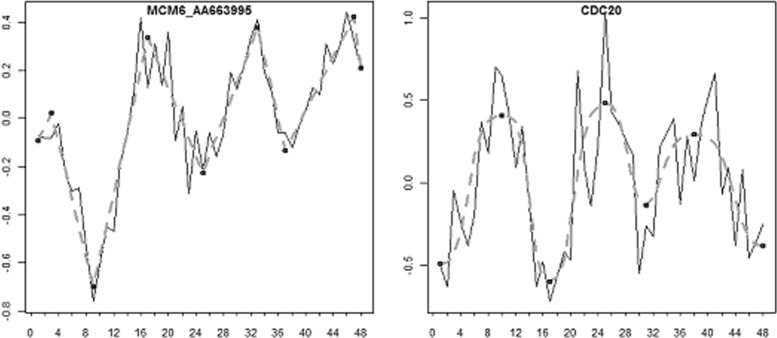
Examples of the fit. SwitchFinder was applied to the time-series of the cell cycle regulated genes from Whitfield et al. The fitted data is presented with dashed lines/curves, the switch-points are depicted in black. The time-course of the gene MCM6 is fitted with Model_Lin, the gene CDC20 showed better fit with Model_Logit

To verify that the algorithm is suitable for long time-series, we applied it to the gene expression data from [37] measured at 48 time-points during cell division cycle in human cancer cell line HeLa. We used the profiles of 66 known cell cycle regulated genes measured upon release of double thymidine block till 46 hours. The fitting results can be observed in Additional file 2. The mean of the residual standard deviations was 0.14 (sd=0.06). Figure 5 demonstrates examples of the fitted profiles.

We sorted the genes by the time-points of their first peaks over the time, peaks with expression around 0 were neglected. The ordering revealed a clear picture of the cyclic activity of the genes and a good separation of G1/S and G2/M cell cycle phases (see supplementary Figure 5 in Additional file 1). Note that the present analysis did not use the assumption of periodicity of the gene expression, which was explicitely introduced into the analysis by [37]. Thus, the use of SwitchFinder allows for explicit temporal ordering of biological events like gene activity peaks. The reconstructed temporal order of the gene activities during the cell cycle demonstrated e.g. that the genes *SLBP*, *MCM6*, *MSH2*, *NUCKS* for their activation need earlier signals.

### Application of SwitchFinder to data from neuroblastoma cell line treated with ATRA (short time-series)

Neuroblastoma is an embryonal tumor arising from the neural crest precursors of the peripheral nervous system. It is supposed that a mechanism underlying this malignancy is the block of cell differentiation, which promotes maintenance of cell stemness and cell proliferation [38]. Differentiation therapies attempt to rescue the suppressed function i.e. to induce differentiation of neuroblastoma cells [39]. The aim of the present application of SwitchFinder was to identify genes involved in neuroblastoma differentiation and to study their expression patterns over time.

**Fig. 6:**
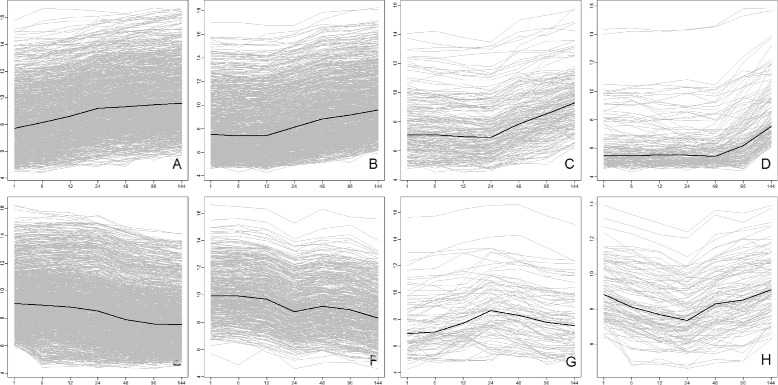
Dynamic patterns of the gene expression response in neuroblastoma cell line to treatment with ATRA. **a** INDUCED_IMMEDIATELY Genes in this group were induced immediately upon treatment with ATRA. **b** INDUCED_12 The activation of these genes by ATRA started at 12 hrs. **c** INDUCED_24 Genes in this group were induced in response to ATRA after 24 hrs. **d** INDUCED_LATE Genes in this group showed late induction: after 48 or 96 hrs. **e** REPRESSED These genes responded to ATRA immediately with the decrease of expression. **f** REPRESSED_CYCLIC These genes, involved in the cell-cycle, were repressed by ATRA. **g** SPIKED Genes in this group responded to ATRA with increase and then decrease of their activity, revealing a peak between 12 and 48 hrs. **h** CLEFTED This group summarizes the genes with a transient response to ATRA i.e their expression declined and then increased. The average gene expression profile for each group is depicted in *black*

We applied the approach to our original data: the gene expression time-series measured at 1, 6, 12, 24, 48, 96, 144 hours (T=7) in neuroblastoma cell line BE(2)-C after treatment with the differentiation agent all-*trans* retinoic acid (ATRA). BE(2)-C (ECACC 95011817, ATCC CRL-2268, [40]) is a clone of the SK-N-BE(2) neuroblastoma cell line established in 1972 (ECACC 95011815, ATCC CRL-2271). The Agilent whole genome 4x44K microarray raw data was background-corrected and quantile-normalized (R package *limma*, [41]). A probe was selected for further analysis if the standard deviation of its expression profile was greater than 0.5 (to exclude probes with insufficient dynamics) and the gene expression in the respective non-treated control was stable. In total, 4422 probes (genes) were selected and fitted by SwitchFinder, from them 3787 probes were assigned to 8 groups representing meaningful dynamic patterns (Fig. 6, Additional files 3: Additional files A-H).

Eight groups of genes, delineated by the analysis, reflect the time-resolved transcriptional response of neuroblastoma genes to the treatment with ATRA. Four groups comprise the activated genes, which were induced: immediately (Fig. 6 a, 883 probes), after 12 hours (B, 869 probes), after 24 hours (C, 184 probes) and after 48 hours (D, 149 probes). The group G (82 probes) summarizes genes with spiked behaviour, mostly at 12 or 48 hrs. Three groups comprise genes repressed by ATRA: the group E (1080 probes) with declining gene expression pattern, the group F (437 probes) with a cyclic decrease and the group H (107 probes) with genes having clefts at different time-points, mostly at 24 hrs. Each group/pattern is characterized by one or more features e.g. onset of *Growth* at the first time-point for the group A. However, the patterns were delineated not solely by the features-based clustering, but also by some additional considerations. Many genes from the group F were fitted with the model Logit_Down as the genes from E, however, their declining cyclic pattern was further discerned by the additional condition: if the expression value at 24 hrs. was lower than at the neighbouring time-points. Further on, the Logit_Up model was a good fit for many activated genes. However, to elucidate the time of induction more precisely, we sorted the profiles by decreasing *k* and decreasing B (parameters of the logistic model), thus obtaining the temporal ordering of the genes starting from steep, early responses via S-formed (bended at 12, 24 hrs.) to convex, late responses.

The functional annotation of the gene groups was executed with the program DAVID [42–44]. Table 2 displays important genes from the group A of immediately induced genes, together with their gene ontology annotations. In suppl. Tables S1-S8 (Additional file 4), the genes and their functional annotations are presented for each group. Our results indicate that the transcriptional response of neuroblastoma cells to the treatment with ATRA is the time-resolved realization of the BMP, Wnt, Notch and FGF signalling, as well as of the G-protein coupled and neurotrophin TRK (NTRK) receptor signalling. This coincides with the gene regulatory programs during differentiation of the neural crest (NC) cells in course of the development of the sympathetic nervous system [45].

**Table 2:** A. INDUCED_IMMEDIATELY

BACH2, BATF2, CREM, CSRNP3, DACH1, EBF1, EGR1/2/3, FOS, FOXC1, GATA6, HES1, HEY1, HIC1, HIF1A, HOXD1/3/8/9/10/13, KDM5B(JARID1B), KLF12, LEF1, MAFB, NCOA3/7, NKX3-1, NR0B1, PBX1, PPARG/D, RARA, SMAD3, SOX4/8/9, TBX2/3, TEAD2, TLE3, TLX2, TULP4, ZFP2, ZNF71/135/436/606/641	GO:000 3700 sequence-specific DNA binding transcription factor activity; GO:0006355 regulation of transcription, DNA-templated; GO:0030154 cell differentiation
AKR1C1/3, BCDO2, CRABP2, CYP26A1/B1, DHRS3, RARA, RBP1, RDH10, SDC4, SP100, STRA6, PPARD/G	GO:0001523 retinoid metabolic process; GO:0042573 retinoic acid metabolic process; GO:0001972 retinoic acid binding; GO:0032526 response to retinoic acid
BMP4, EGR1, GREM2, LEF1	GO:0030509 BMP signaling pathway
DACT3, LEF1, PSEN1, SOX4	GO:0016055 Wnt signaling pathway
FOXC1, HES1, HEY1, HIF1A, MDK, NCOR2, PSEN1, TLE3	GO:0007219 Notch signaling pathway; GO:0005112 Notch binding
ERBB2, IRS2, KITLG, PDGFRA/B, SPRY2/4	GO:0007173 epidermal growth factor receptor signaling
PDGFRA/B, PLAT	GO:0048008 platelet-derived growth factor receptor signaling pathway
NGFR, NTRK1, PCSK5, PLEKHG2, RALB, RIT1	GO:0048011 neurotrophin TRK receptor signaling pathway; GO:0038180 nerve growth factor signaling pathway
DISP1	GO:0007224 smoothened signaling pathway; GO:0008158 hedgehog receptor activity; GO:0009880 embryonic pattern specification
APC2, EML4, KIFAP3, LYST, NEIL2, SPTAN1	GO:0015630 microtubule cytoskeleton
AHNAK, ARPC1B, AVIL, CORO2A, CTTNBP2NL, FAM129B, FGD4/6, FHL2, FLNB, KALRN, LCP1, MYRIP, PDLIM5/7, PPP1R12B, SYNPO/2, TRIOBP, VCL	GO:0015629 actin cytoskeleton
ARHGDIB, CLASP2, CNN2, LIMK1, NUAK2, PAK1, PALM, PFN2, PLK2, RND3, SDCBP, SOX9	GO:0007010 cytoskeleton organization
CEACAM1, GAB2, ITGA1, ITGB8; ADD3, LIMK1, MYADM, MRCL3(MYL12A), TRIO	GO:0007229 integrin-mediated signaling pathway; GO:0005911 cell-cell junction; GO:0040011 locomotion; GO:0016477 cell migration
ANTXR1, ATP1B1, BVES, CALCA, CDH23, CEACAM1, CLSTN3, COL12A1, COMP, FBLIM1, KITLG, NCAM2, NEO1, PCDHB2/4/6/9-11/13/14, PPFIBP1, PSEN1, PVRL2, RET, RND3, SPP1, TGFB1I1, TPBG, TRO,VTN	GO:0007155 cell adhesion; GO:0007411 axon guidance
HIF1A, HTR2B, KITLG, LEF1, RET, SOX8	GO:0001755 neural crest cell migration
EGR2/EGR3, ERBB2, SOX8	GO:0007422 peripheral nervous system development
JARID1B, JARID2	GO:0016568 chromatin modification; GO:0048863 stem cell differentiation
SLIT2, SLITRK6, FLOT1	GO:0035385 Roundabout signaling pathway; GO:0050772 positive regulation of axonogenesis
EPHA2, EPHB3; SEMA6C, SEMA6D	GO:0048013 ephrin receptor signaling pathway; GO:0030215 semaphorin receptor binding; GO:0007411 axon guidance
DCX, DPYSL3, ERBB2, KCNQ2, PSEN1, PTPRO, RRAS, SPTAN1, ST8SIA4; STMN2, TEAD2	GO:0007411 axon guidance; GO:0030426 growth cone; GO:0048666 neuron development
LAMB2, LAMC1	GO:0005605 basal lamina; GO:0031175 neuron projection development
DLG2, GLS, GNG2/8, HCN1, KCNQ2, PANX, RRAS, SDCBP, SST, SYNJ2, SYT2; STX7, STXBP5/6	GO:0007268 synaptic transmission; GO:0019905 syntaxin binding; GO:0045202 synapse
HTR2B, FOS,KALRN, NAB2, NAV2, DCX, RGS9, RTN4, VCL	GO:0007399 nervous system development; neurite branching; GO:0030334 regulation of cell migration
CDKL5	GO:0001764 neuron migration; GO:0050773 regulation of dendrite development; GO:0051726 regulation of cell cycle
BCL2, BOK, CASP4/9, CTSB, NLRP1, SKIL; ANGPT1, CPEB4, CRLF1, F2R, HIF1A, MDK, NTRK1, PSEN1	GO:0006915 apoptotic process; GO:0043524 negative regulation of neuron apoptotic process
ADAM12, ADAMTS9, MMP2/11	GO:0008237 metallopeptidase activity
F2R, GALR1, GPR161, HTR2B, IGF2R, P2RY2, PTGER2, PTGIR	GO:0004930 G-protein coupled receptor activity; GO:0004966 galanin receptor activity; GO:0007218 neuropeptide signaling pathway; GO:0007189 adenylate cyclase-activating G-protein coupled receptor signaling pathway
CRLF1	GO:0005127 ciliary neurotrophic factor receptor binding

The table displays exemplary the genes from the group A and their functional annotations. The group A contains genes that demonstrated immediate increase of expression in response to ATRA

The groups of immediate and early (12 hrs.) responses are very rich on transcription factors involved in determination of cell fates and regulation of embryonic development: *HOXD*-genes, *SOX4/8/9*, *FOXC1*, *FOXO1A*, *BMP4*, *TLE3*, *TLX2* etc., see Table 2. As expected, early induced were the genes involved in retinoic acid metabolism and signalling: *RARA*, *RBP1*, *RDH10*, *SP100*, *CRABP2*, *CYP26A1* and *RDH12*.

The gene *SNAI2*, playing a role in the epithelial-to-mesenchymal transition (EMT), is over-expressed (group A) accompanied with the down-regulation of adherence junction genes like cadherins *CDH4/7/22*, claudin *CLDN11*, cingulin *CGN*, catenins *CTNNA1/2*, as well as of tight and gap junction genes *TJP1* and *GJA5* [46]. Interestingly, the gene *SNAI1*, initially over-expressed as compared to the control, decays under the influence of ATRA. The mesenchymal markers were induced immediately or at 12 hours: fibronectin FN1, fibronectin receptors *ITGB1/3/8*, *FNDC4/5*, vitronectin *VTN* and vimentin *VIM*. Cell polarity regulator *PPARD* and a member of crumbs complex, *CRB1*, belong to the group A. The metalloproteinases *MMP2/11/15* and *ADAM19/22/23*, which facilitate degradation of the extracellular matrix, were active immediately or at 12 hours. Thus, the results indicate a contribution of ATRA to the migratory phenotype of neuroblastoma cells.

Induced immediately were the receptors *NTRK1* and *NGFR* - regulators of the nerve growth factor signalling known to be responsible for the maturation of the peripheral nervous systems through regulation of proliferation, differentiation and survival of neurons [47]. Activated early were the genes responsible for the axon guidance, axonogenesis, neuron projection, neurite outgrowth etc., which participate in the ephrin, semaphorin, plexin and Roundabout signalling: *EFNB2*, *EFNA2/4*, *EPHA2*, *EPHB3*, *SEMA4C/6C*, *PLXNA2/4A*, *SLIT2*, *SLITRK6*. Interestingly, the semaphorin SEMA6A, known to control cell migration, was repressed, although its receptor *PLXNA2* was activated after 12 hrs. Previously, *SEMA6A* was found upregulated in undifferentiated embryonic stem (ES) cells [48]. Further observation: the neuropilin signalling (*NRP1* and *NRP2*, group E) was repressed, together with the ephrin ligand *EFNA1*. In general, a complex spatio-temporal expression of guidance molecules and genes involved in neuron migration was observed. Vast transcriptional changes were induced by ATRA at genes involved in cytoskeleton organization, cell polarization and immune processes. E.g. the chemokine receptor *CXCR4* was induced at 24 hrs. It represents a positive cue for the migration of the NC cells (its ligand *CXCL12* was active after 48 hours). We suppose that canonical Wnt signalling is repressed or delayed upon treatment with ATRA, with non-canonical Wnt signalling taking place: *PPARD/G* and *TLE3* were induced, *TCF7* and *TCF19* were repressed, *DACT3* (antagonist of beta-catenin) and further genes annotated with *negative regulation of canonical Wnt signaling pathway* were induced: *ANKRD6*, *DKK1/2*, *SFRP1*. The gene *WNT11* was activated lately (group D).

A clearly observable effect of ATRA-treatment on NB cells is the repression of genes involved in cell cycle regulation, particularly in G1/S and G2/M transitions of mitotic cell cycle, in cell proliferation, DNA metabolic process, DNA damage response, DNA repair signalling: *MYCN*, *AURKA/B*, *BIRC5*, *CDC2/6*, *CENPF*, *PCNA*, *PLK1/4* etc. (Group F). Furthermore, genes responsible for negative regulation of cell proliferation e.g. *CDKN1A* were active at 12 or 24 hrs. Notably, the gene *ALK*, an important unfavourable prognostic marker in neuroblastoma, was repressed.

To summarize, our study documented a powerful transcriptional effect of ATRA on NB cells. A complex gene regulatory machinery controls the two properties of neural crest cells: ability to extensively migrate and differentiate into numerous derivatives and to maintain multipotency [45]. The role of retinoic acid hereto even in normal organism is still not well understood. In neuroblastoma, the normal properties interfere with the abnormal EMT and migratory characteristics, acquired by tumor cells due to the genomic lesions in several developmental and guidance molecules genes [49]. We suppose that the dynamics of gene expression in neuroblastoma neurogenesis is influenced by the genetic aberrations inherent to this malignancy.

## Conclusions

Identifying dynamic patterns under various biological conditions is crucial for the understanding of a biological system. The patterns reflect the coordination, co-regulation and control of the system components. Identifying temporal changes and patterns of gene expression is important for the inference of gene regulatory networks. We developed a method SwitchFinder for the analysis of time-resolved data, applicable to the gene expression data. The change-point model at the core of the method represents a series of the switch-points between regimes of increasing and decreasing activities, captured by linear or generalized logistic functions. SwitchFinder fits the model to the gene-expression profiles, inferring the switch-points inherent to the gene dynamics. The method exploits Bayesian model inference with the MCMC technique Gibbs sampling. To note, the method is suitable for long, as well as for short time-series.

The advantage of the present approach is the inference of biologically justified and interpretable features of the genetic activity, as well as the possibility of their subjective exploration by researchers with different goals and background knowledge, in different biological scenarios. The Web application of the approach provides the user interface for querying the gene time-series. A flexibility is given to the user to adjust the selection criteria for restricting the results to substantial dynamic phenomena. Actively guiding the data analysis is valuable for biologists, as opposite to an automatic, unsupervised application of a statistical/bioinformatics method. Some *features* of the data might be designated as important by an expert subjectively - beyond those obtained by statistical learning based on statistical characteristics. The features, in a next level of abstraction, can constitute further features or *patterns*. Such a qualitative approach should overcome over-fitting and lead towards biologically meaningful results.

The features-based clustering is preferrable than clustering methods based on distance measures like Euclidian distance or correlation. The latter ignore the dynamic nature of the temporal data and overlook single data points, which represent important changes in the gene behaviour associated with the events of the gene regulatory control.

To mention, the present method is independent of the quantitative expression levels of different genes. It would not miss a relation between the genes with different abundance, but with the same qualitative pattern.

Previously, a platform PESTS was created, making the analysis of some statistical features of the gene profiles accessible via the user interface [50]. Qualitative representation of the gene expression profiles was performed in [51] by the Trend Temporal Abstraction, which transforms the time-series into series of intervals with increasing, decreasing or steady trends. However, the *dominant points*, defining the intervals, were determined by the approximation of the data curves based on thresholds chosen by the user. This makes the algorithm sensitive to noise. The method concentrated on shapes of the gene profiles, rather than on proper timing of the dynamic events. To emphasize, the statistical inference of the prominent time-points in the temporal profiles is of advantage. The Temporal Abstraction clustering was implemented in the software TimeClust [52], together with other clustering methods. Also, Hvidsten et al. [53] performed qualitative representations of the gene expression profiles in terms of templates (increasing, decreasing and constant) over sub-intervals. The authors used such descriptions as attributes in the rough-sets based classification system to relate genes to biological processes. Parameter values for the identification of the templates were chosen experimentally with a purpose to maximize the performance of the whole system. Unlike previous approaches, our qualitative descriptions of the gene-expression profiles are based on the labelling of the switch-points – not of the intervals. With this, gene profiles with activity intervals starting at the same time-point, but having different durations would still have a chance to be assigned to the same group. This might be important for the elucidation of those gene regulatory events like e.g. when the gene group is controlled by a transcription factor. Furthermore, we are able to define higher-order qualitative features of the switch-points like spikes and clefts.

The present method combines quantitative and qualitative characteristics: statistically inferred timing of dynamic events and the qualitative dynamic features.

The approach offers a great flexibility in the induction of biological knowledge from time-series data: the user may explore the gene set by clustering (unsupervised) or interactively (supervised) by putting queries and experimenting with the qualitative features of particular time-points.

The results of the method provide a platform for studying temporal relations like e.g. time delays with the goal to deduce dependencies between the genes. Modelling cellular dynamic responses on the level of pathways and networks can be considered as possible extensions of the approach. Our next goal is to adapt the SwitchFinder to the analysis of RNA-seq time-series.

## Additional files

Additional file 1 This file contains supplementary text and figures with additional explanations to the Model_Logit, as well as the supplementary Figure 5 with the heatmap, displaying the profiles of the cell cycle regulated genes ordered based on the results of the SwitchFinder. (PDF 64 kb)

Additional file 2 Results of the application of SwitchFinder to the cell cycle regulated genes. The gene expression profiles are plotted in black, the fitted lines - in blue, the fitted logistic curves - in red. The switch-points are depicted as dots. (PDF 106 kb)

Additional file 3 Additional file A-H. These files contain the fitting results for the genes from the groups A-H, deduced by SwitchFinder, which represent eight dynamic patterns of the gene expression response to ATRA in neuroblastoma cell line. (ZIP 2457 kb)

Additional file 4 Tables S1-S8. Demonstrating genes from the groups A-H and their functional annotations. (PDF 63 kb)

## Abbreviations

ATRA: all-*trans* retinoic acid
BIC: Bayesian Information Criterion
EMT: epithelial-to-mesenchymal transition
ES: embryonic stem
LASSO: least absolute shrinkage and selection operator
LOESS: LOWESS, locally weighted scatterplot smoothing
MCMC: Markov chain Monte Carlo
NC: neural crest
OLS: ordinary least squares
RNA-seq: RNA sequencing
