# Supplementary material for: SwitchFinder – a novel method and query facility for discovering dynamic gene expression patterns
Source: BMC Bioinformatics. 2016 Dec 15;17:532. doi: 10.1186/s12859-016-1391-0 (PMC5160026; doi:10.1186/s12859-016-1391-0)
Supplement: Additional file 1 — This file contains supplementary text and figures with additional explanations to the Model_Logit, as well as the supplementary Figure 5 with the heatmap, displaying the profiles of the cell cycle regulated genes ordered based on the results of the SwitchFinder. (PDF 64 kb) [file 12859_2016_1391_MOESM1_ESM.pdf]

Additional File 1 for the manuscript by Bulashevska et al.

Modeling with the logistic function (Model_Logit).

Sometimes the increasing/decreasing activity of a gene exhibits a saturated behaviour, stabilizing with time. To model this, the generalized logistic function was used. Supplementary Figure 1 displays a cartoon example of such gene expression profile with *N=4* switches at time-points 1, 7, 11, 16.

Equation $Y \sim \left(1 - FL\left(prop_t\right)\right) \cdot H_{trough} + FL\left(prop_t\right) \cdot H_{peak}$ (see equation 5 in the manuscript) represents a linear regression model with the design matrix as shown exemplary in Suppl. Fig. 3 for the model in Suppl. Fig. 2. Fitting the model to the data *Y* (R function *lm*) facilitates calculation of the switch heights.

Using the generalized logistic transformation of the proportional location of each time-point between the neighbouring trough and peak allows for flexible modelling of the gene expression increase/decrease within time intervals of different length. Suppl. Fig. 4 displays the forms of the curves, defined by the function *FL* with parameters $B=8$ and $\kappa=10$ for the time intervals of lengths 2, 3, …, 15.

Supplementary Figures

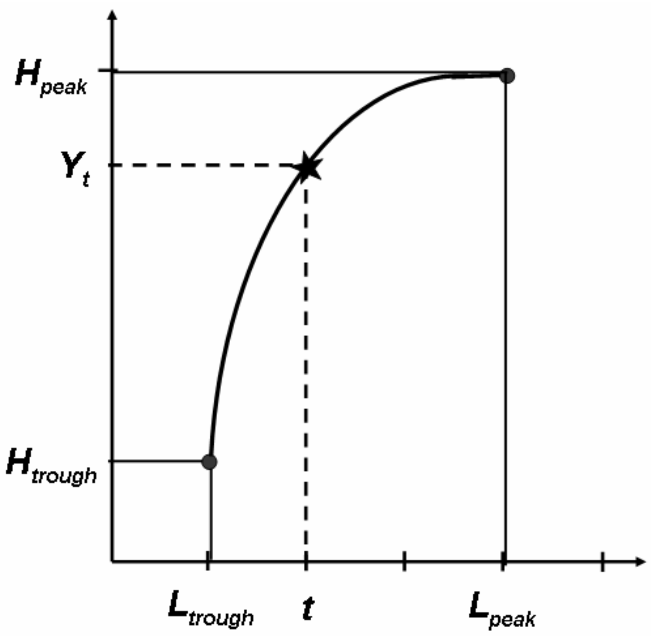

Suppl. Figure 1. Fitting with generalised logistic function between two switch points (trough and peak).

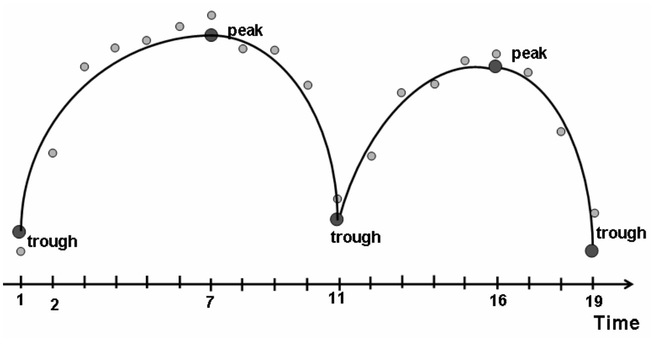

Suppl. Figure 2. Cartoon of a gene expression profile fitted with Model_Logit with 5 switch points.

1:Number of switch points

1:Number of time points

1	0	0	0	0
1-FL(1/6)	FL(1/6)	0	0	0
1-FL(2/6)	FL(2/6)	0	0	0
1-FL(3/6)	FL(3/6)	0	0	0
1-FL(4/6)	FL(4/6)	0	0	0
1-FL(5/6)	FL(5/6)	0	0	0
0	1	0	0	0
0	FL(3/4)	1-FL(3/4)	0	0
0	FL(2/4)	1-FL(2/4)	0	0
0	FL(1/4)	1-FL(1/4)	0	0
0	0	1	0	0
0	0	1-FL(1/5)	FL(1/5)	0
0	0	1-FL(2/5)	FL(2/5)	0
0	0	1-FL(3/5)	FL(3/5)	0
0	0	1-FL(4/5)	FL(4/5)	0
0	0	0	1	0
0	0	0	FL(1/3)	1-FL(1/3)
0	0	0	FL(2/3)	1-FL(2/3)
0	0	0	0	1

Suppl. Figure 3. Design matrix of the linear regression for finding switch heights in the Model_Logit.

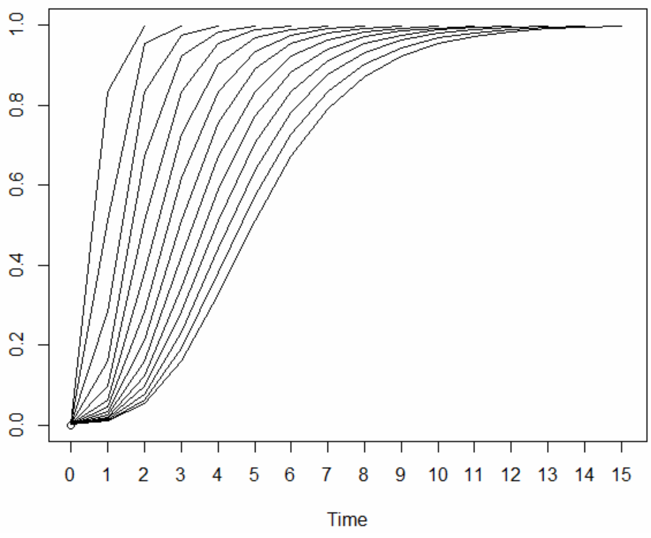

Suppl. Figure 4. Generalized logistic curves in the time intervals of length 2, 3,…,15 calculated as the function FL (B=8, *k*=10) of the proportional locations of time-points within the intervals.

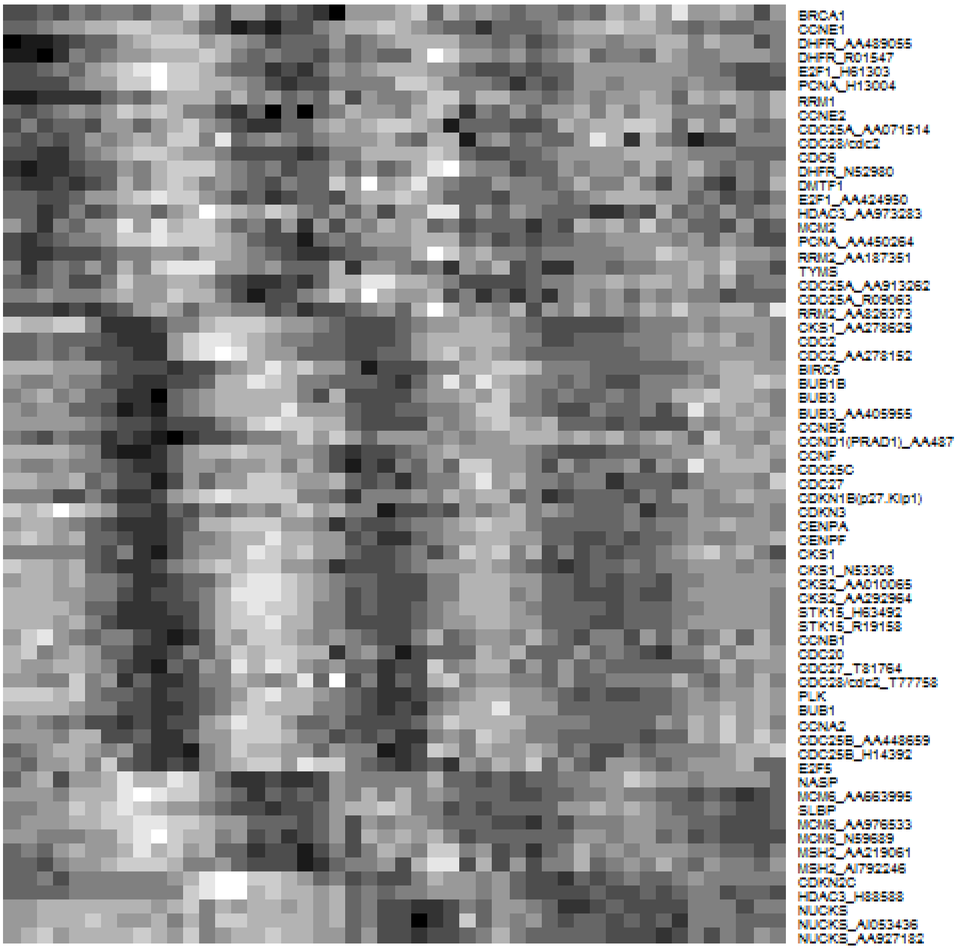

Suppl. Figure 5. Heatmap of the 66 cell cycle regulated genes from Whitfield et al. The genes were ordered by their first peaks over time (the peaks were the switch-points elucidated by SwitchFinder).
