# Supplementary material for: SwitchFinder – a novel method and query facility for discovering dynamic gene expression patterns
Source: BMC Bioinformatics. 2016 Dec 15;17:532. doi: 10.1186/s12859-016-1391-0 (PMC5160026; doi:10.1186/s12859-016-1391-0)
Supplement: Additional file 2 — Results of the application of SwitchFinder to the cell cycle regulated genes. The gene expression profiles are plotted in black, the fitted lines - in blue, the fitted logistic curves - in red. The switch-points are depicted as dots. (PDF 106 kb) [file 12859_2016_1391_MOESM2_ESM.pdf]

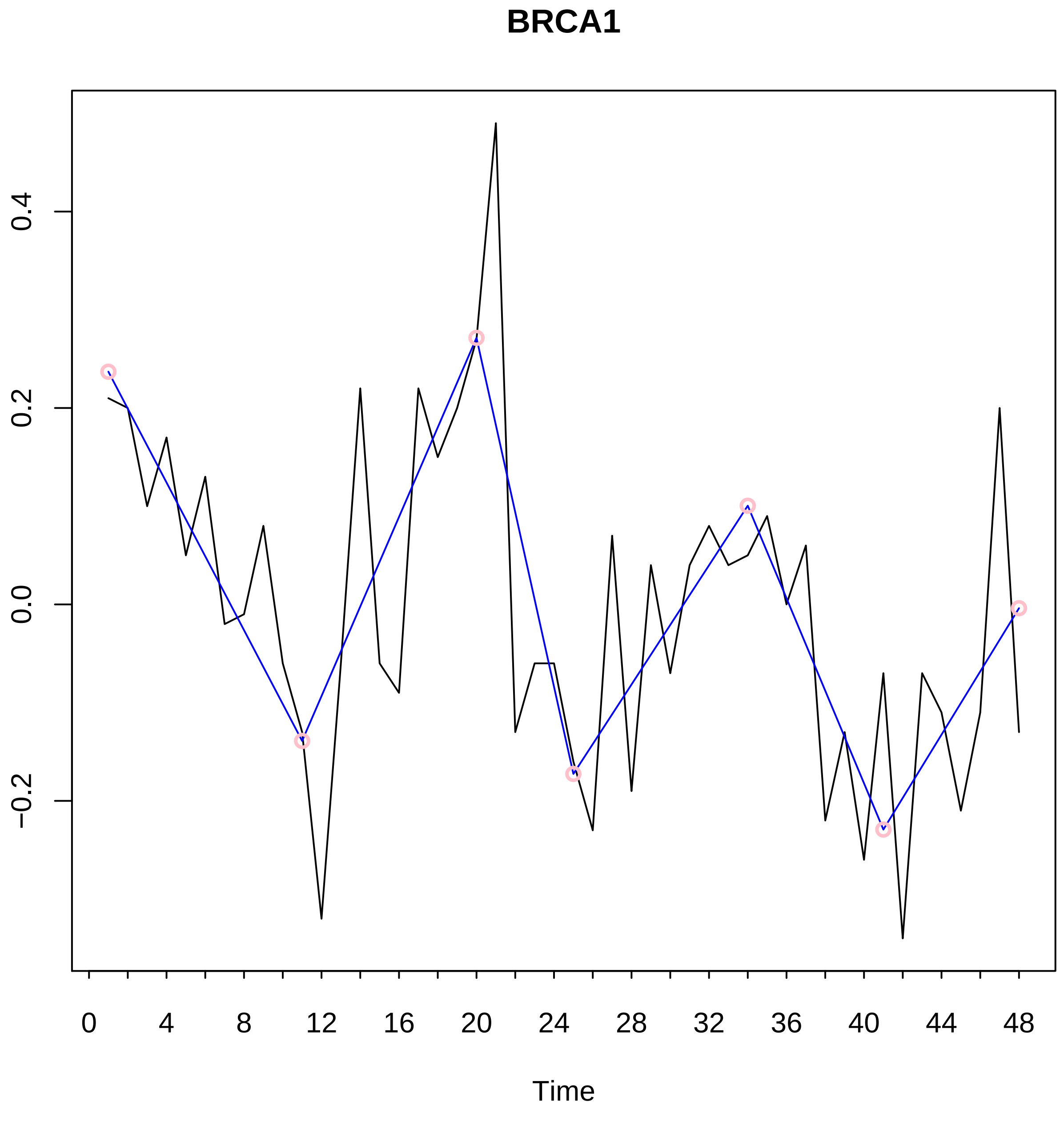
BRCA1
0.4
0.2
0.0
−0.2
0
4
8
12
16
20
24
28
32
36
40
44
48
Time

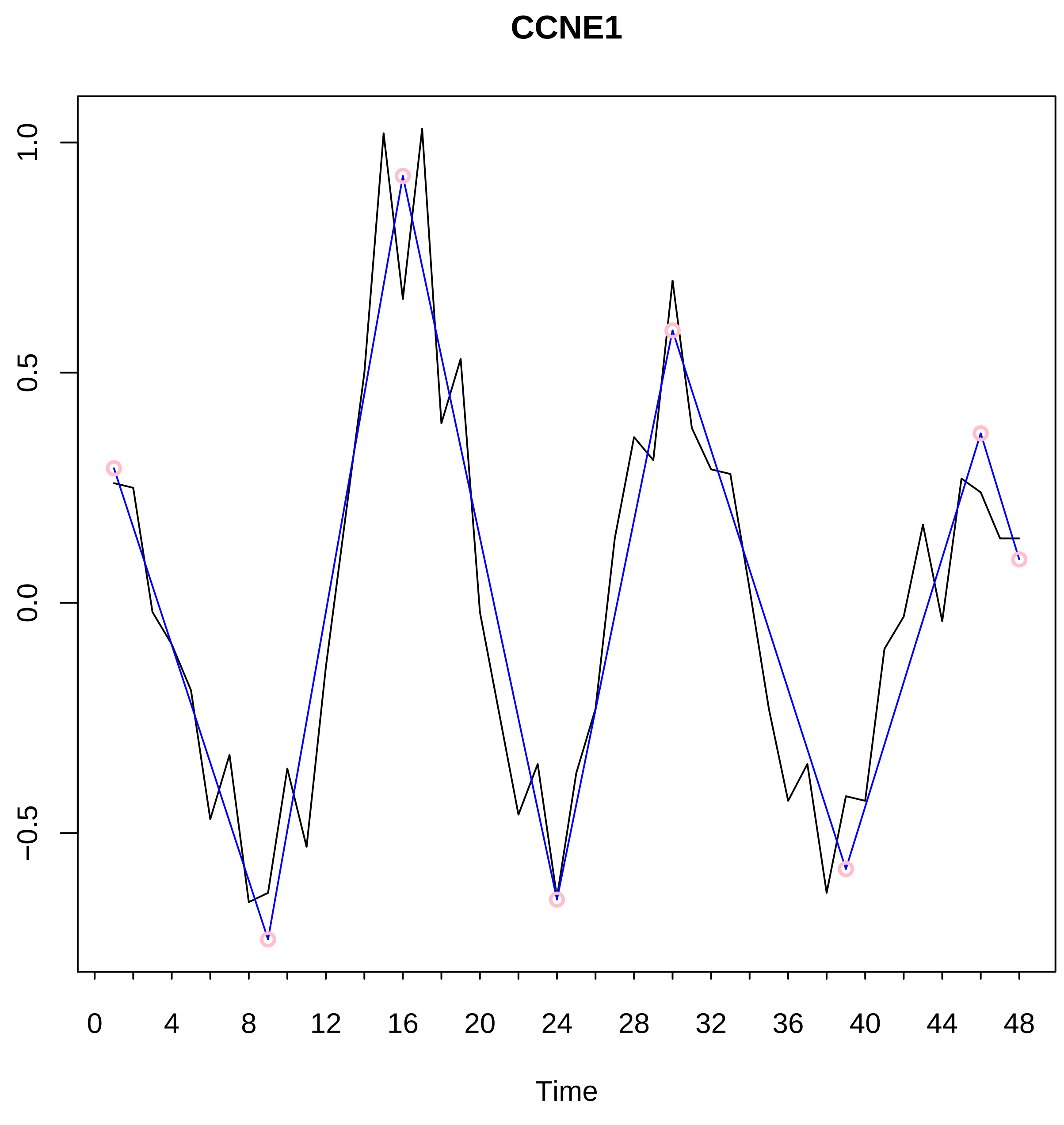
CCNE1
1.0
0.5
0.0
−0.5
0
4
8
12
16
20
24
28
32
36
40
44
48
Time

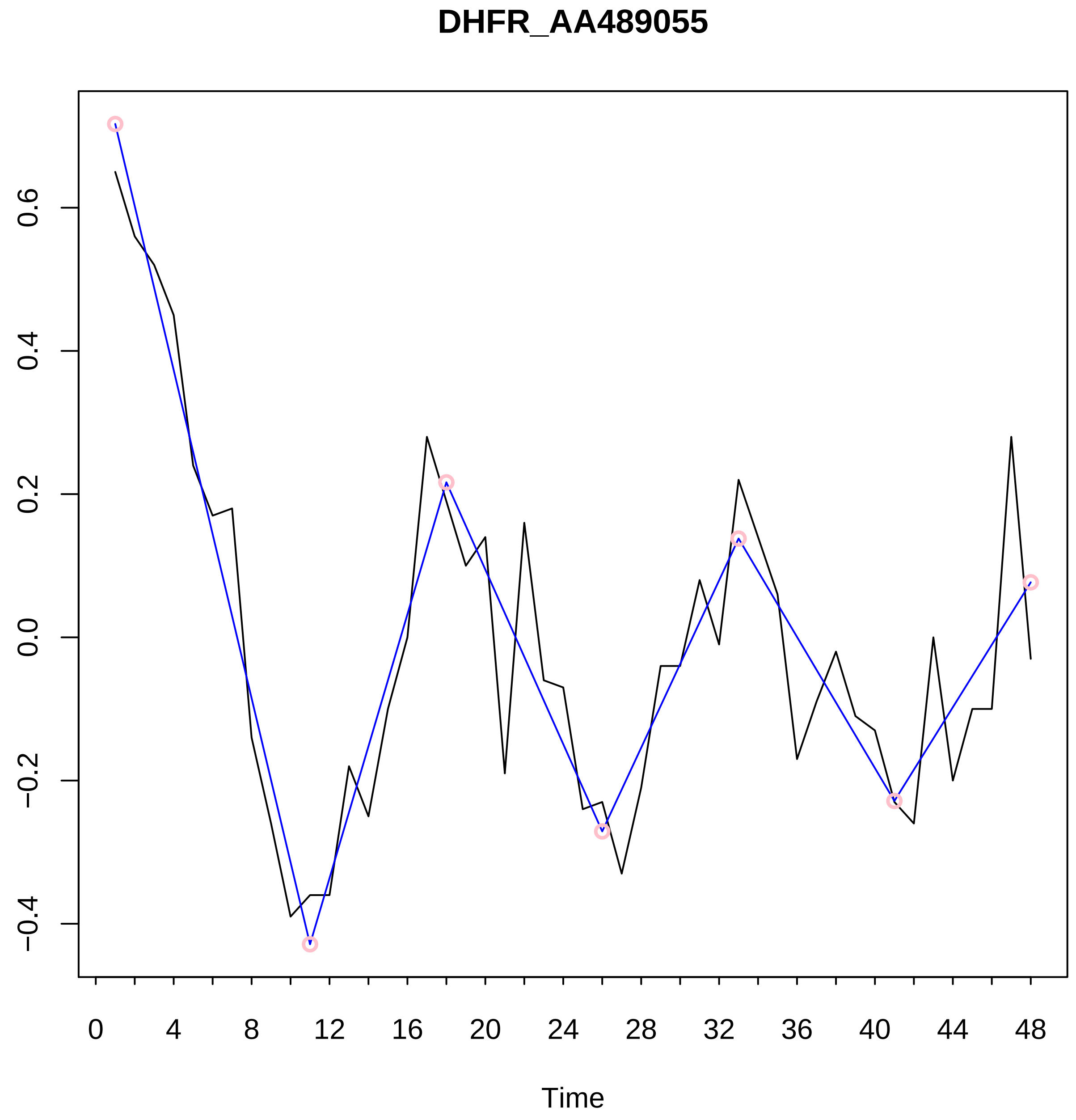

DHFR_AA489055
0.6
0.4
0.2
0.0
−0.2
−0.4
0
4
8
12
16
20
24
28
32
36
40
44
48
Time

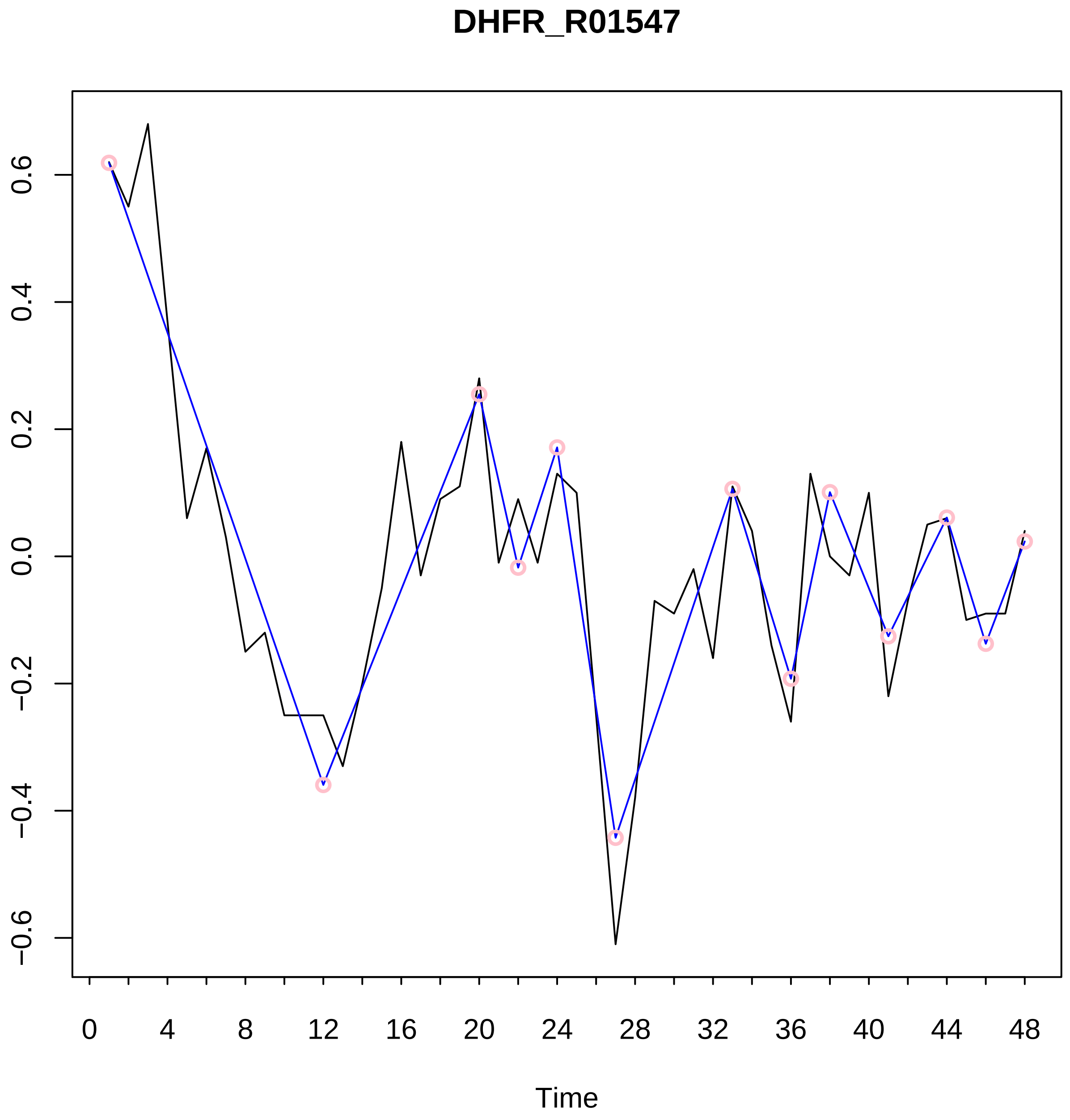

DHFR_R01547
0.6
0.4
0.2
0.0
−0.2
−0.4
−0.6
0
4
8
12
16
20
24
28
32
36
40
44
48
Time

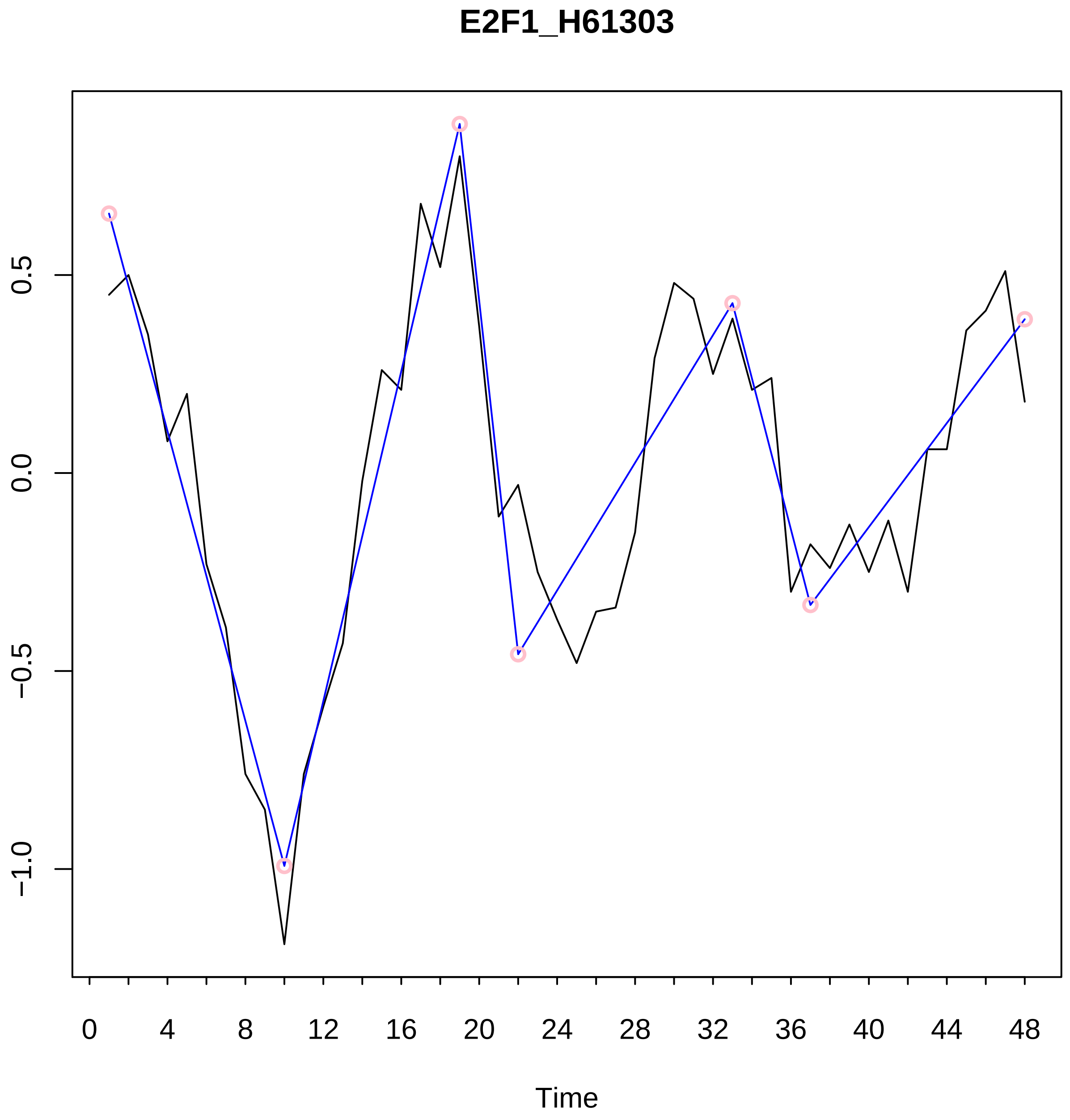
E2F1_H61303
0.5
0.0
−0.5
−1.0
0
4
8
12
16
20
24
28
32
36
40
44
48
Time

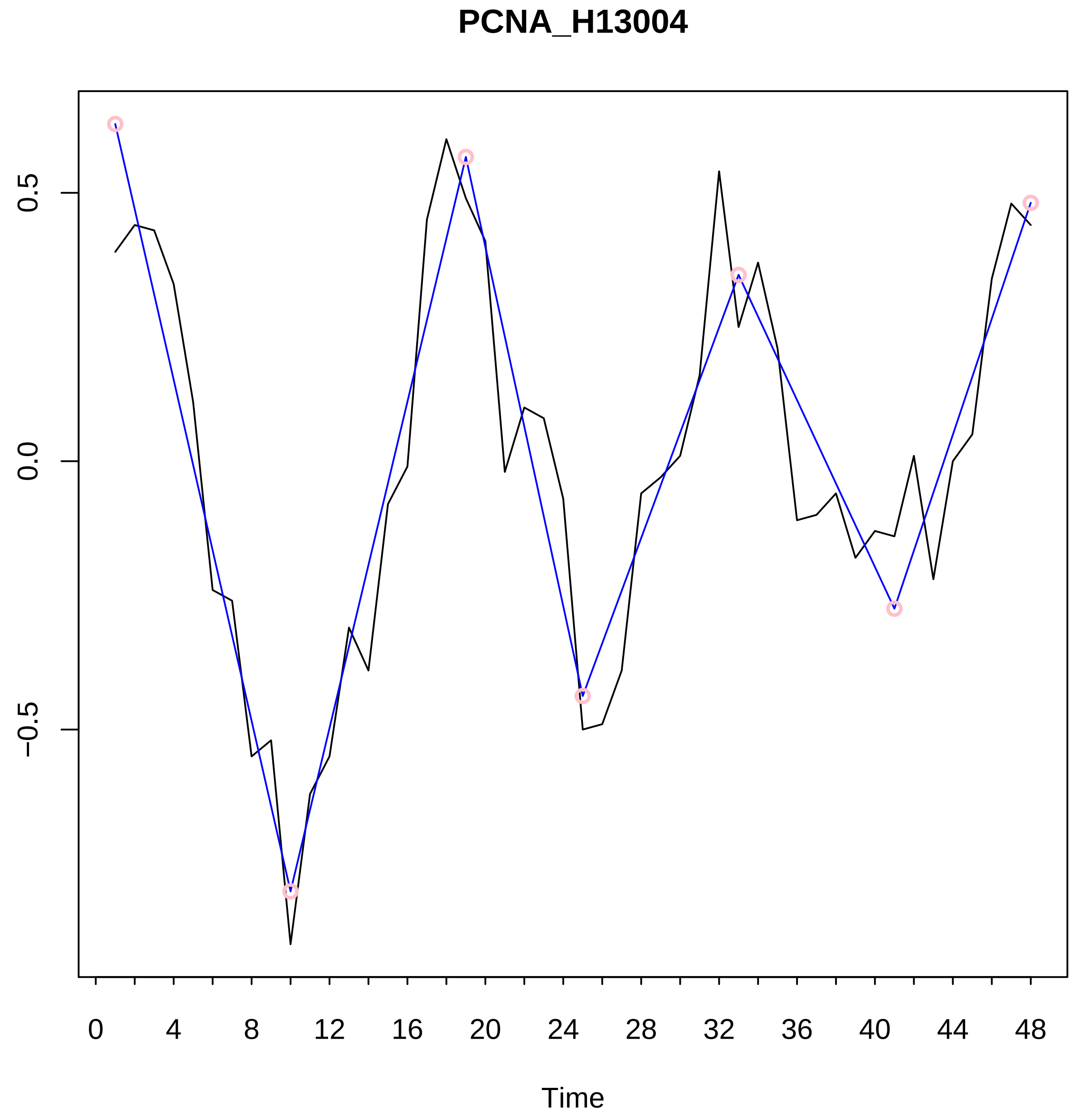

PCNA_H13004
0.5
0.0
−0.5
0
4
8
12
16
20
24
28
32
36
40
44
48
Time

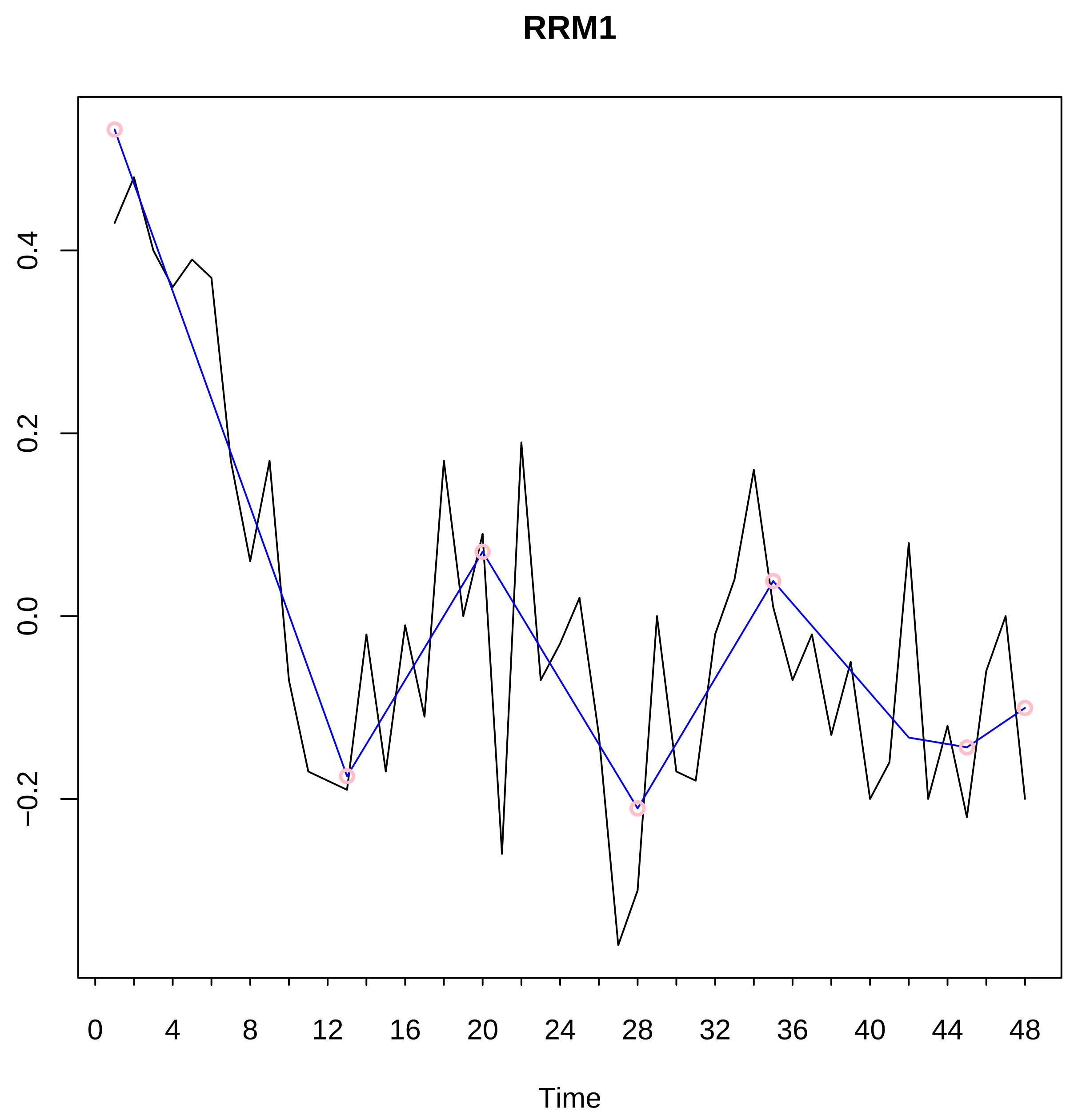
RRM1
0.4
0.2
0.0
−0.2
0
4
8
12
16
20
24
28
32
36
40
44
48
Time

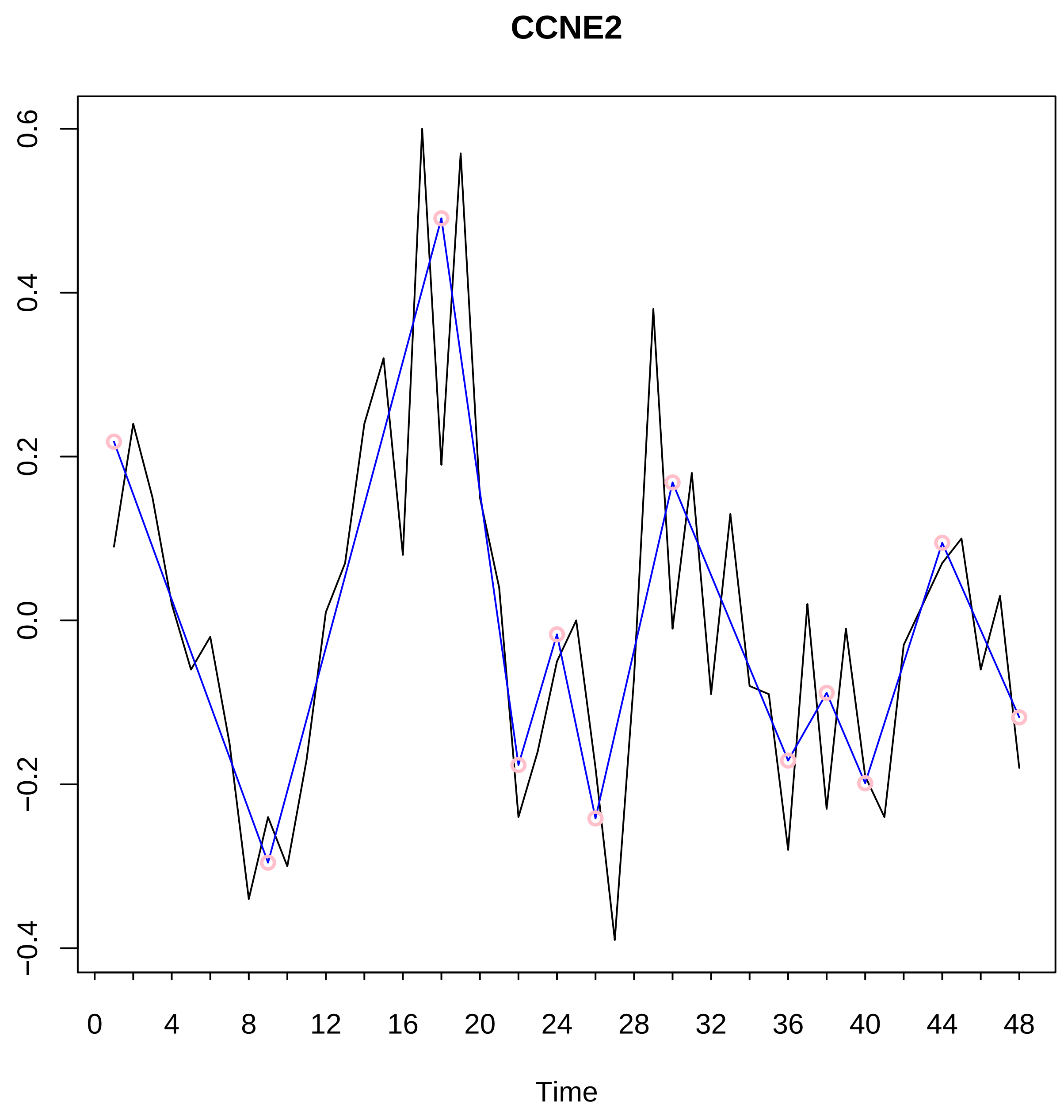
CCNE2
0.6
0.4
0.2
0.0
−0.2
−0.4
0
4
8
12
16
20
24
28
32
36
40
44
48
Time

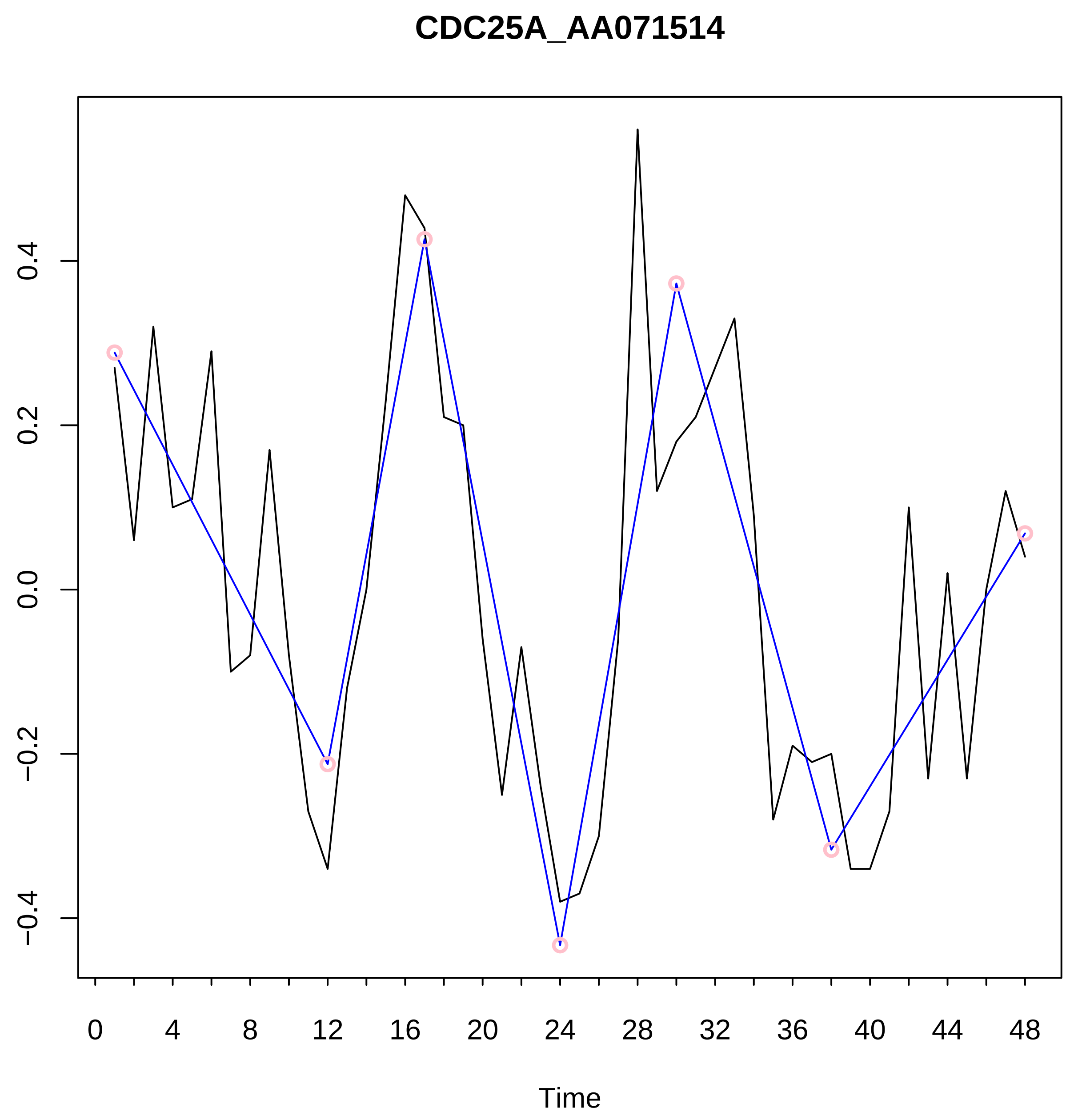

CDC25A_AA071514
0.4
0.2
0.0
−0.2
−0.4
0
4
8
12
16
20
24
28
32
36
40
44
48
Time

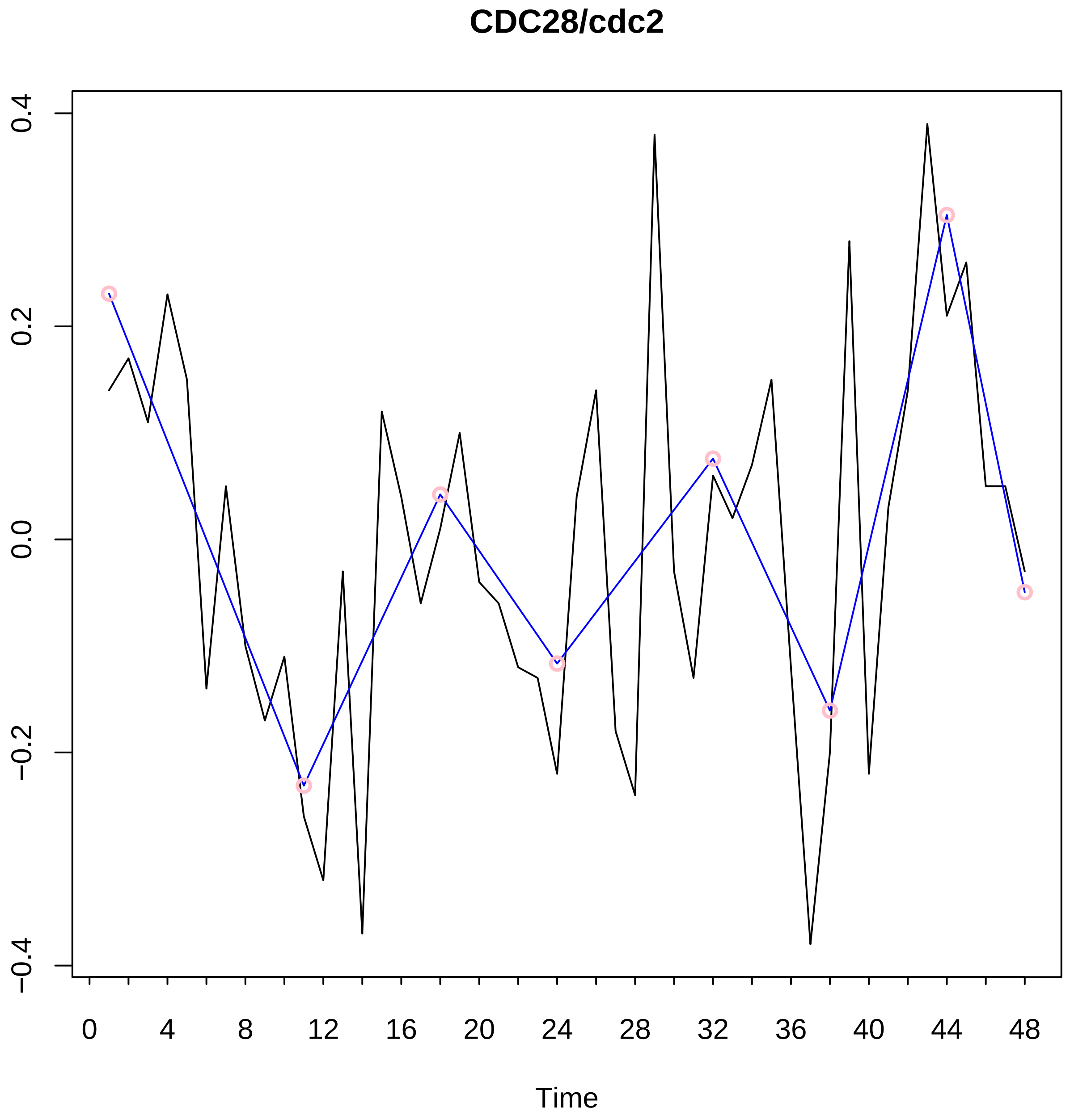
CDC28/cdc2
0.4
0.2
0.0
−0.2
−0.4
0
4
8
12
16
20
24
28
32
36
40
44
48
Time

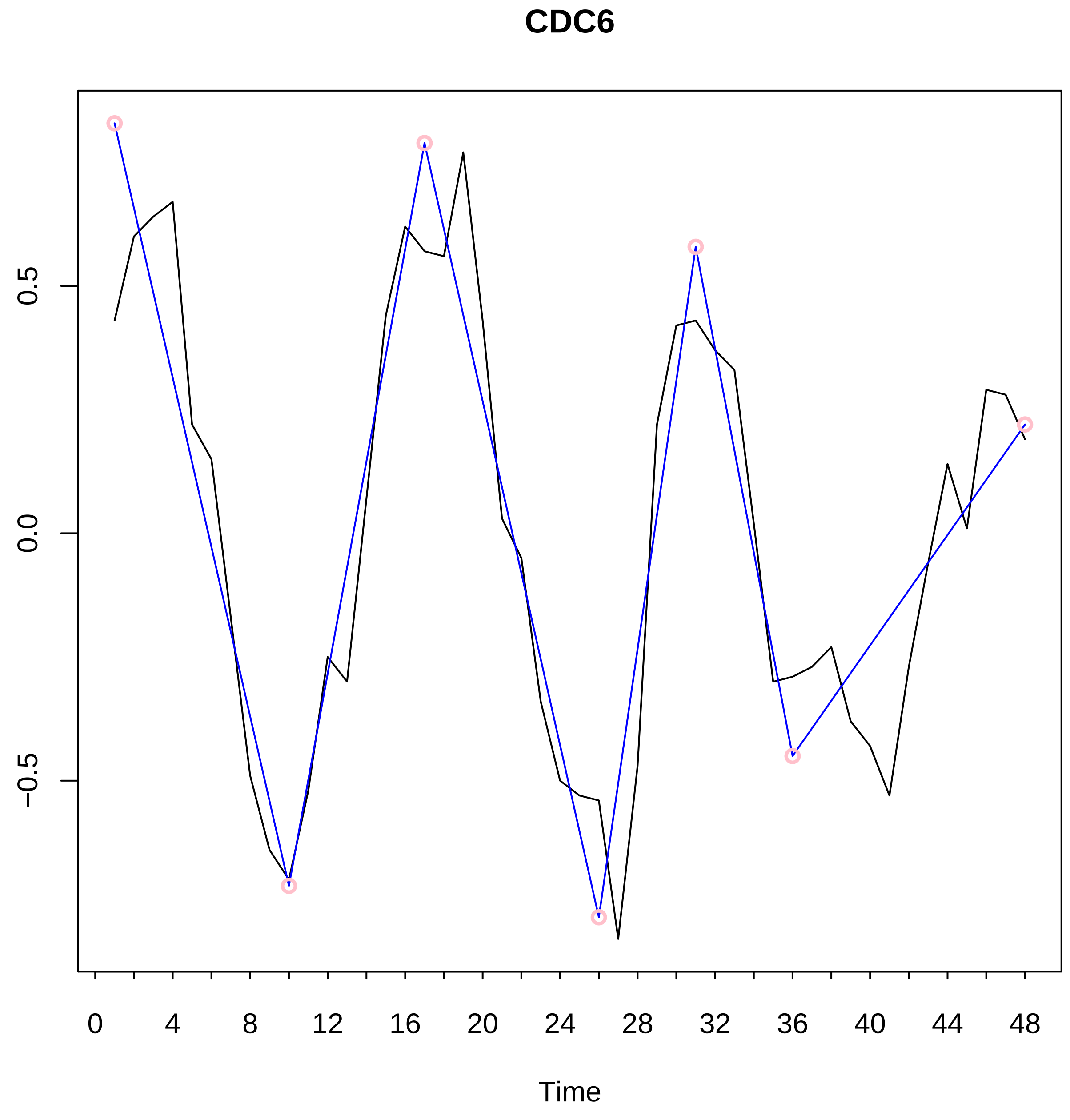

CDC6
0.5
0.0
−0.5
0
4
8
12
16
20
24
28
32
36
40
44
48
Time

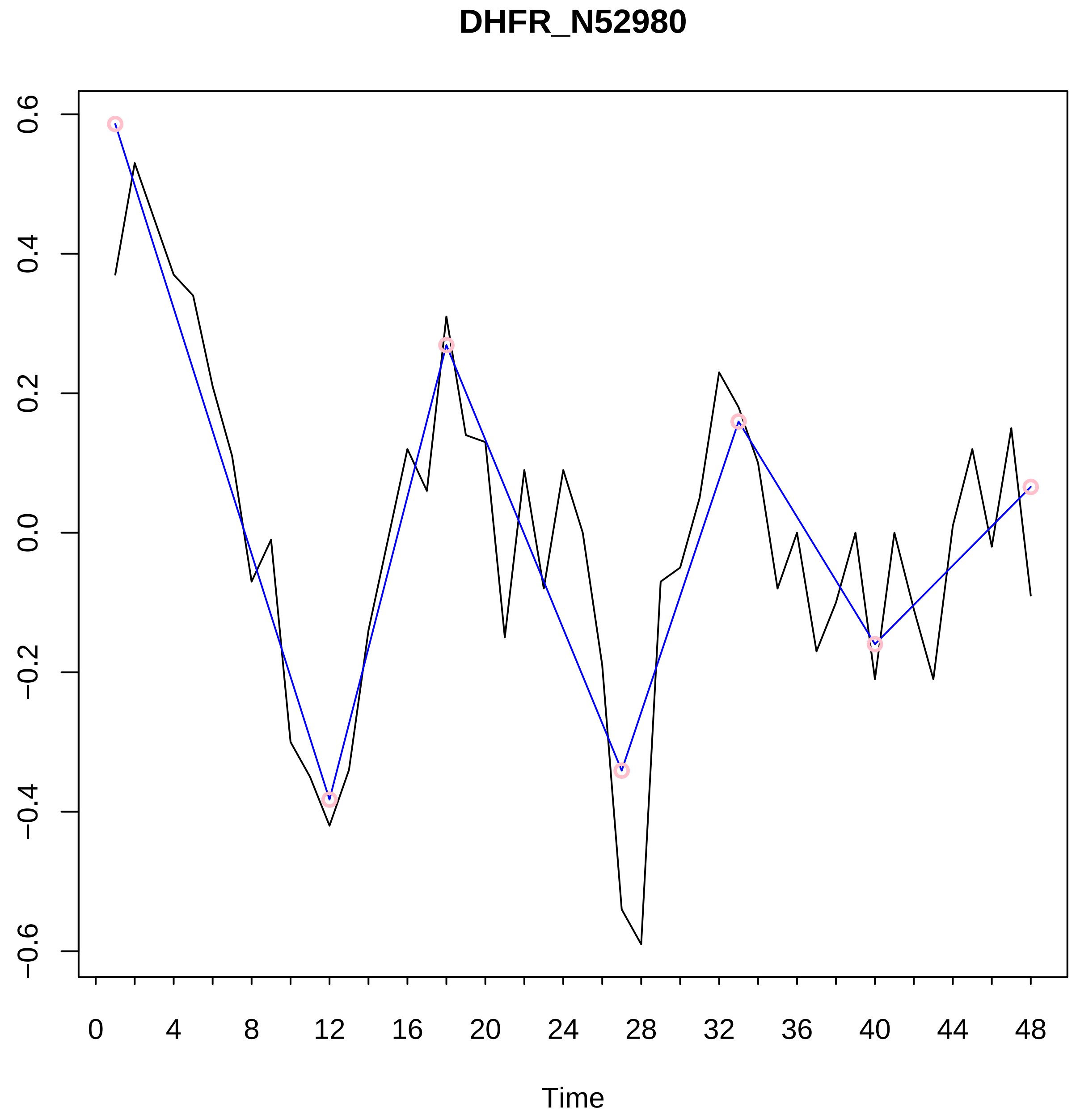
DHFR_N52980
0.6
0.4
0.2
0.0
−0.2
−0.4
−0.6
0
4
8
12
16
20
24
28
32
36
40
44
48
Time

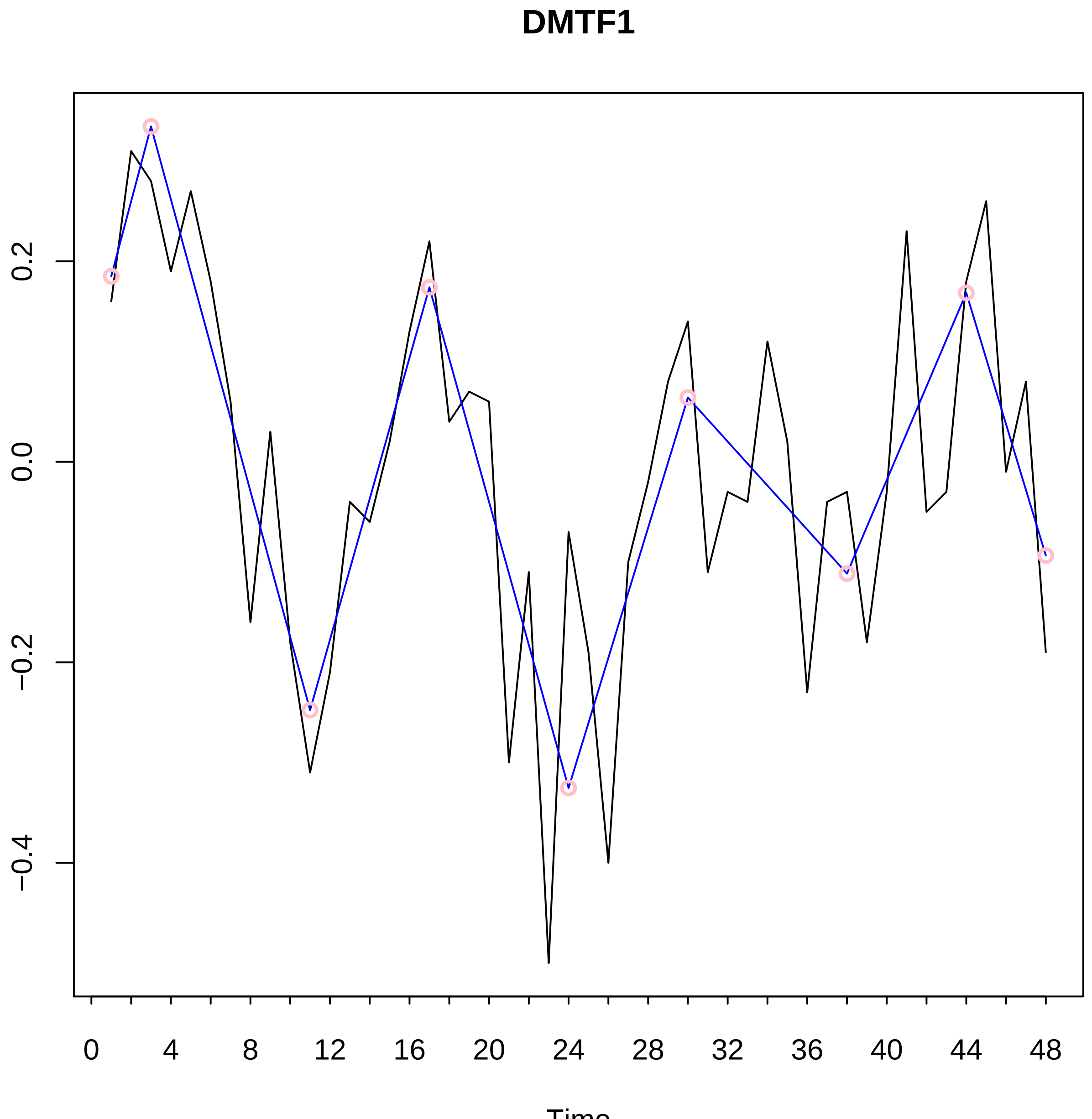

DMTF1
0.2
0.0
−0.2
−0.4
0
4
8
12
16
20
24
28
32
36
40
44
48
Time

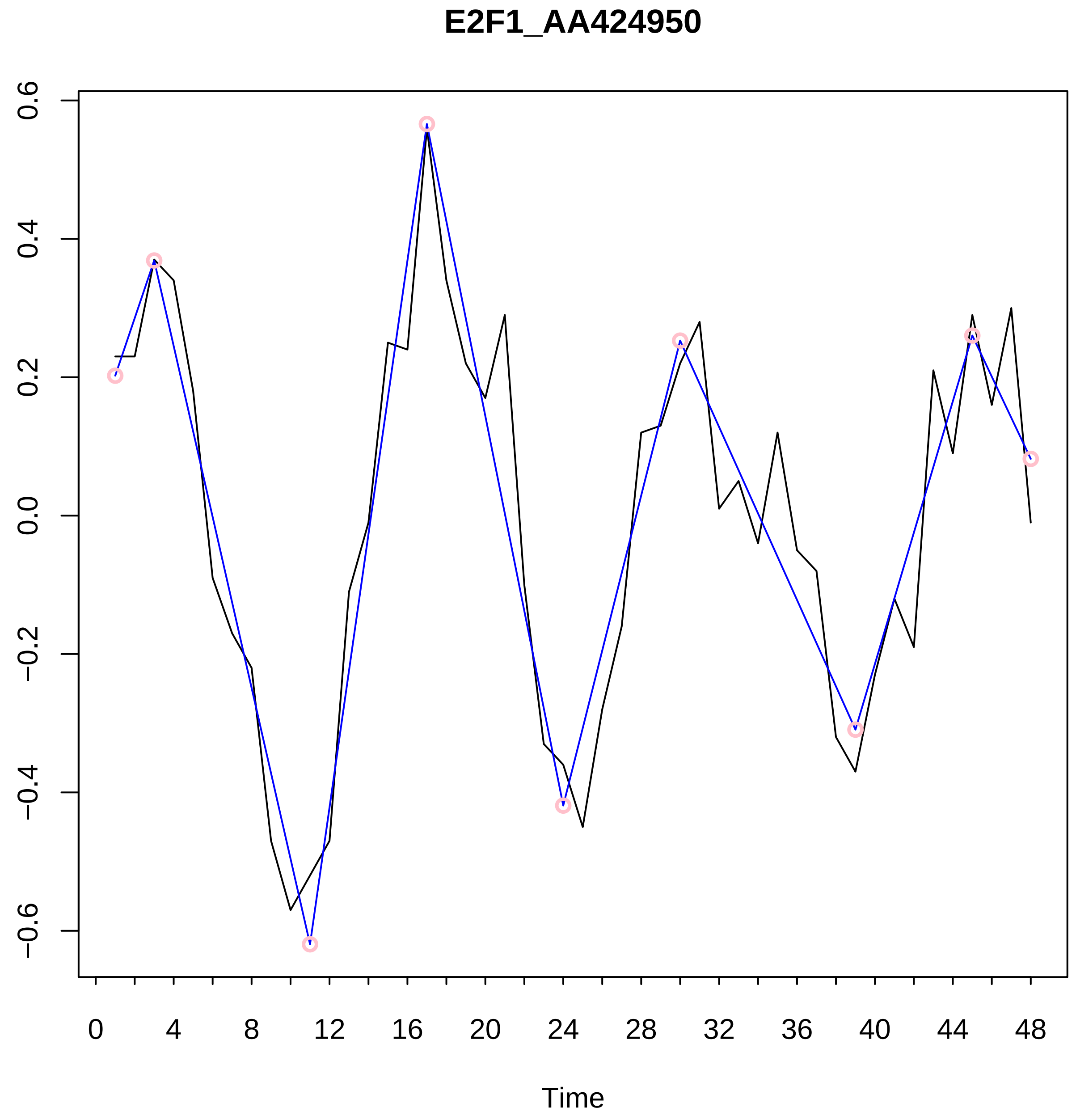
E2F1_AA424950
0.6
0.4
0.2
0.0
−0.2
−0.4
−0.6
0
4
8
12
16
20
24
28
32
36
40
44
48
Time

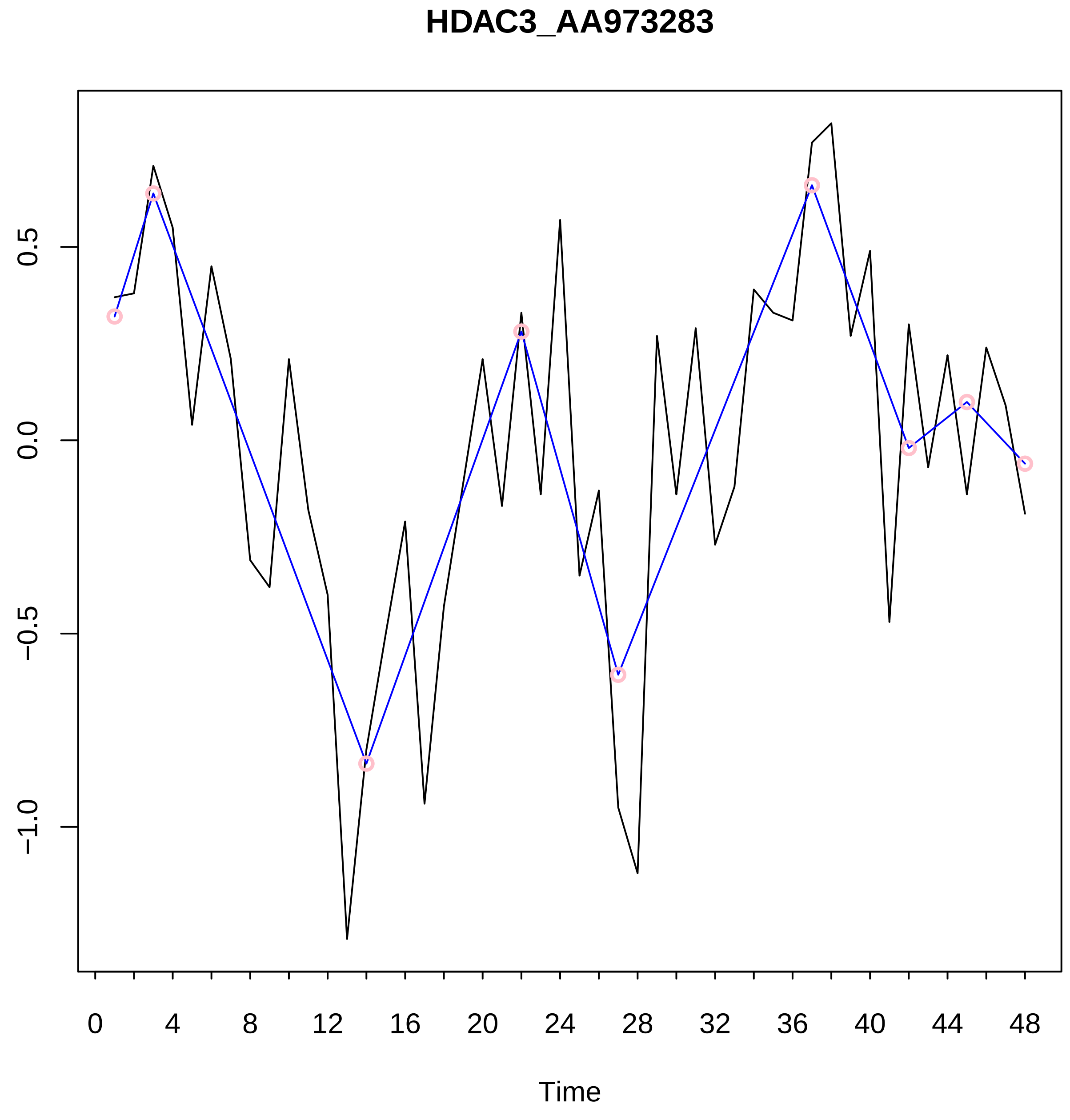
HDAC3_AA973283
0.5
0.0
−0.5
−1.0
0
4
8
12
16
20
24
28
32
36
40
44
48
Time

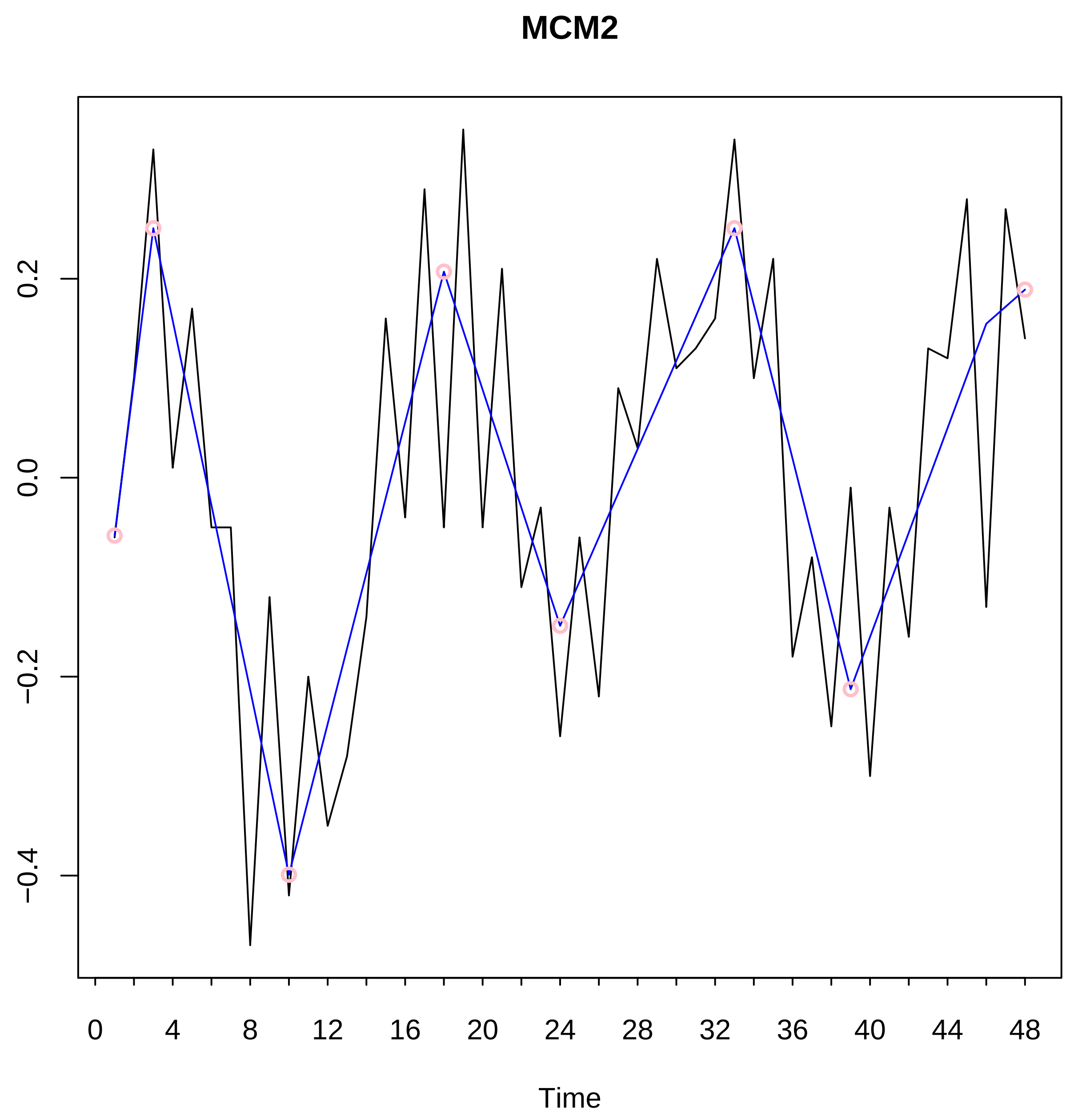
MCM2
0.2
0.0
−0.2
−0.4
0
4
8
12
16
20
24
28
32
36
40
44
48
Time

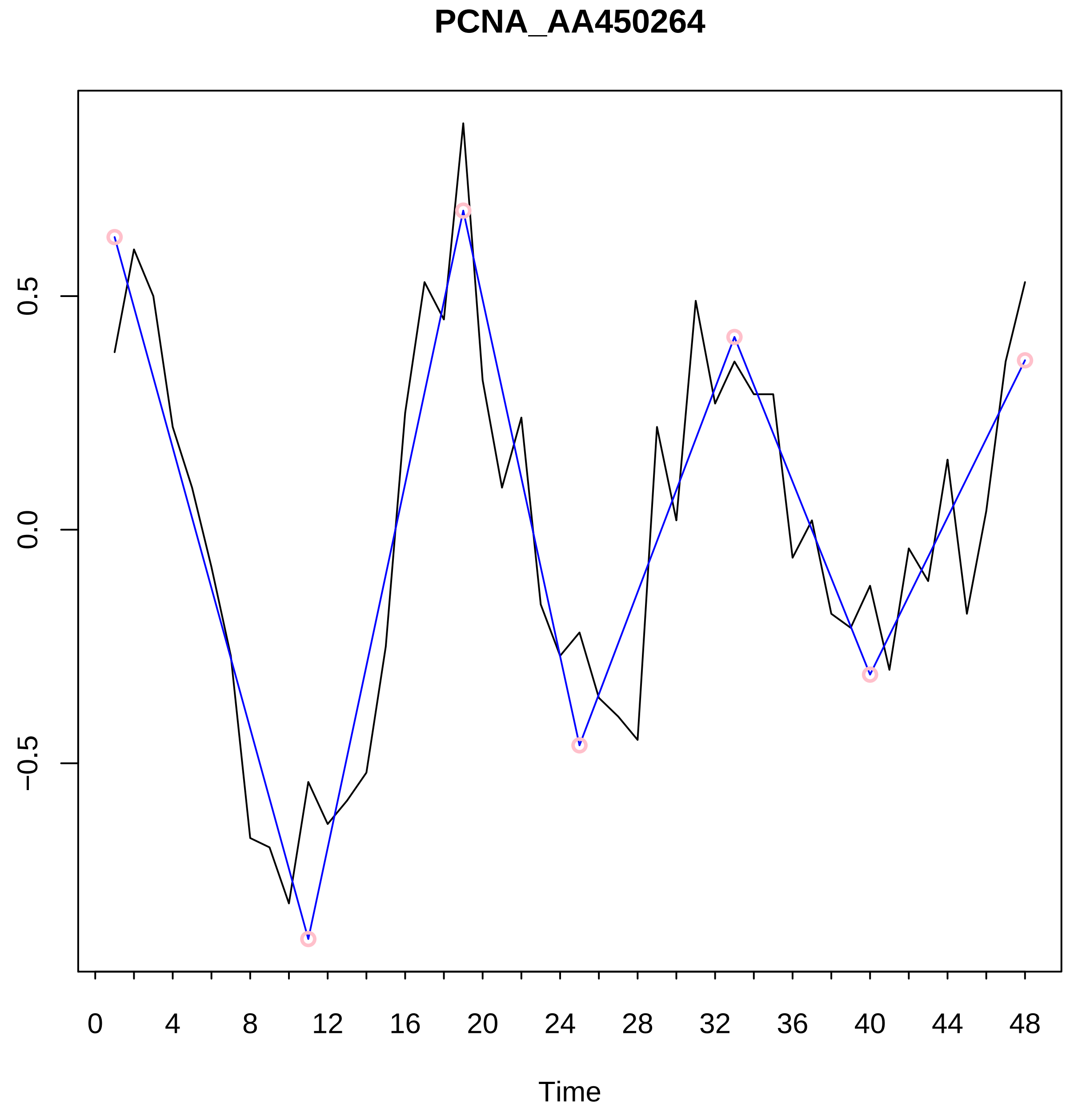

PCNA_AA450264
0.5
0.0
−0.5
0
4
8
12
16
20
24
28
32
36
40
44
48
Time

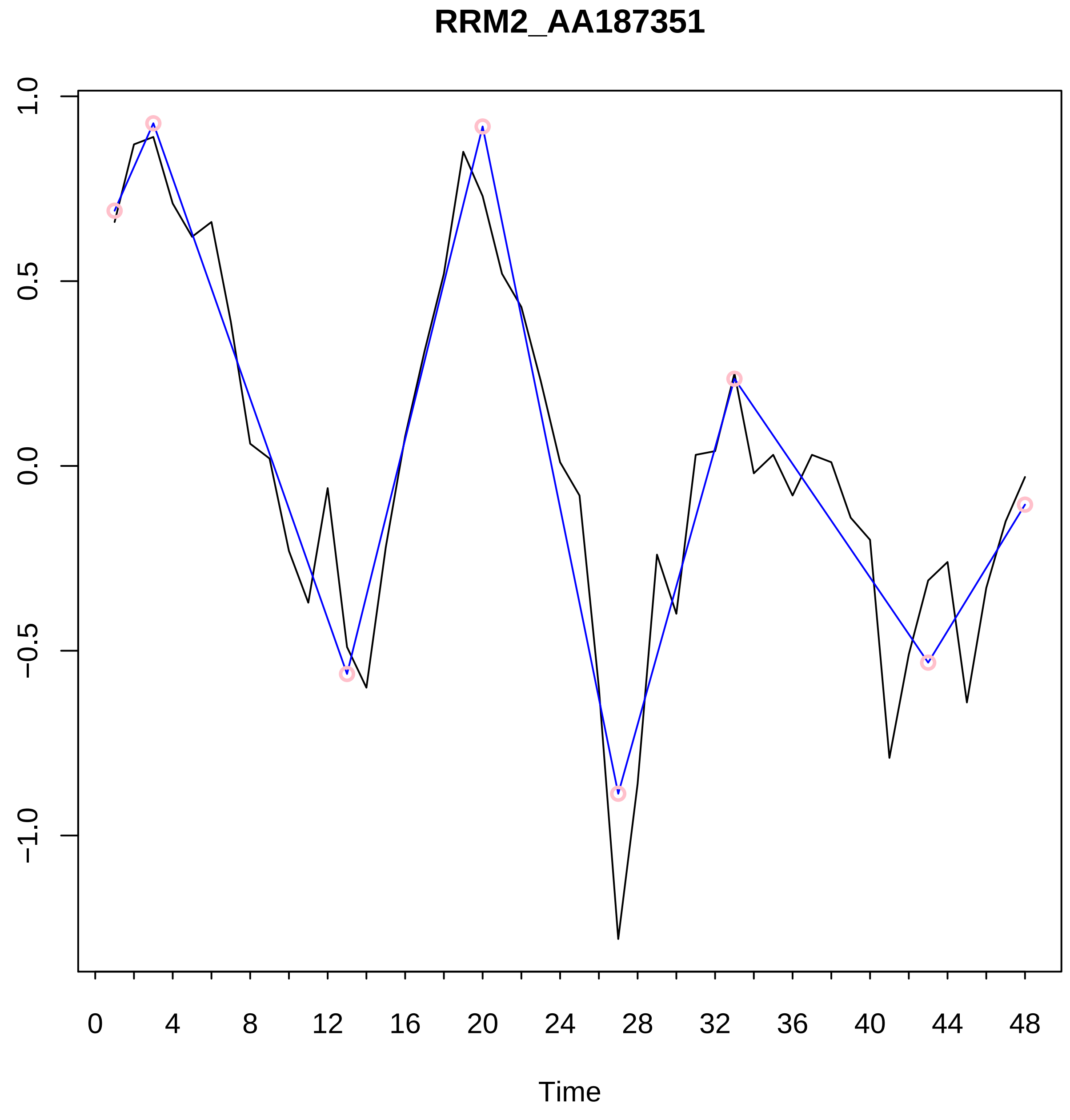
RRM2_AA187351
1.0
0.5
0.0
−0.5
−1.0
0
4
8
12
16
20
24
28
32
36
40
44
48
Time

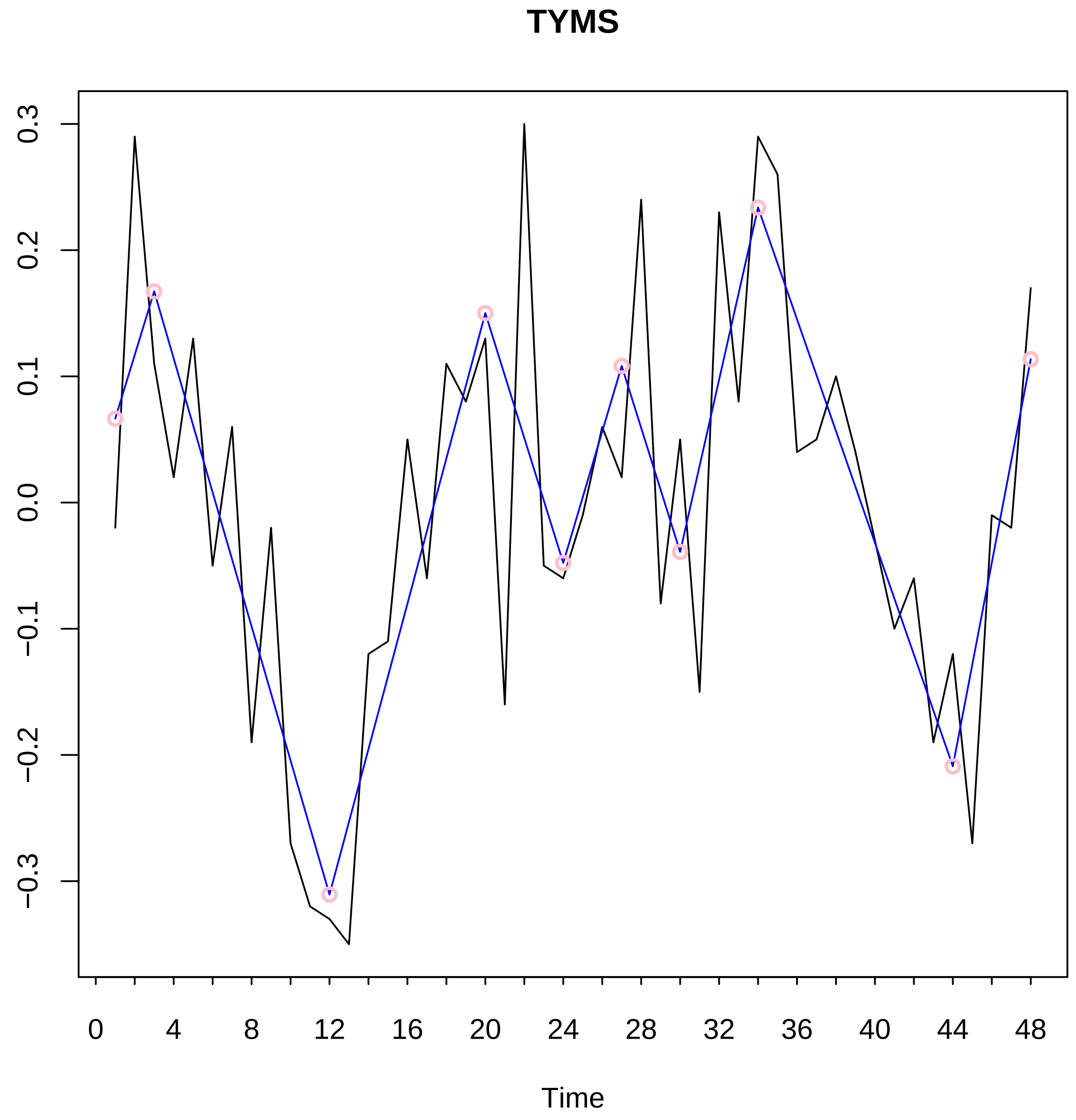
TYMS
0.3
0.2
0.1
0.0
−0.1
−0.2
−0.3
0
4
8
12
16
20
24
28
32
36
40
44
48
Time

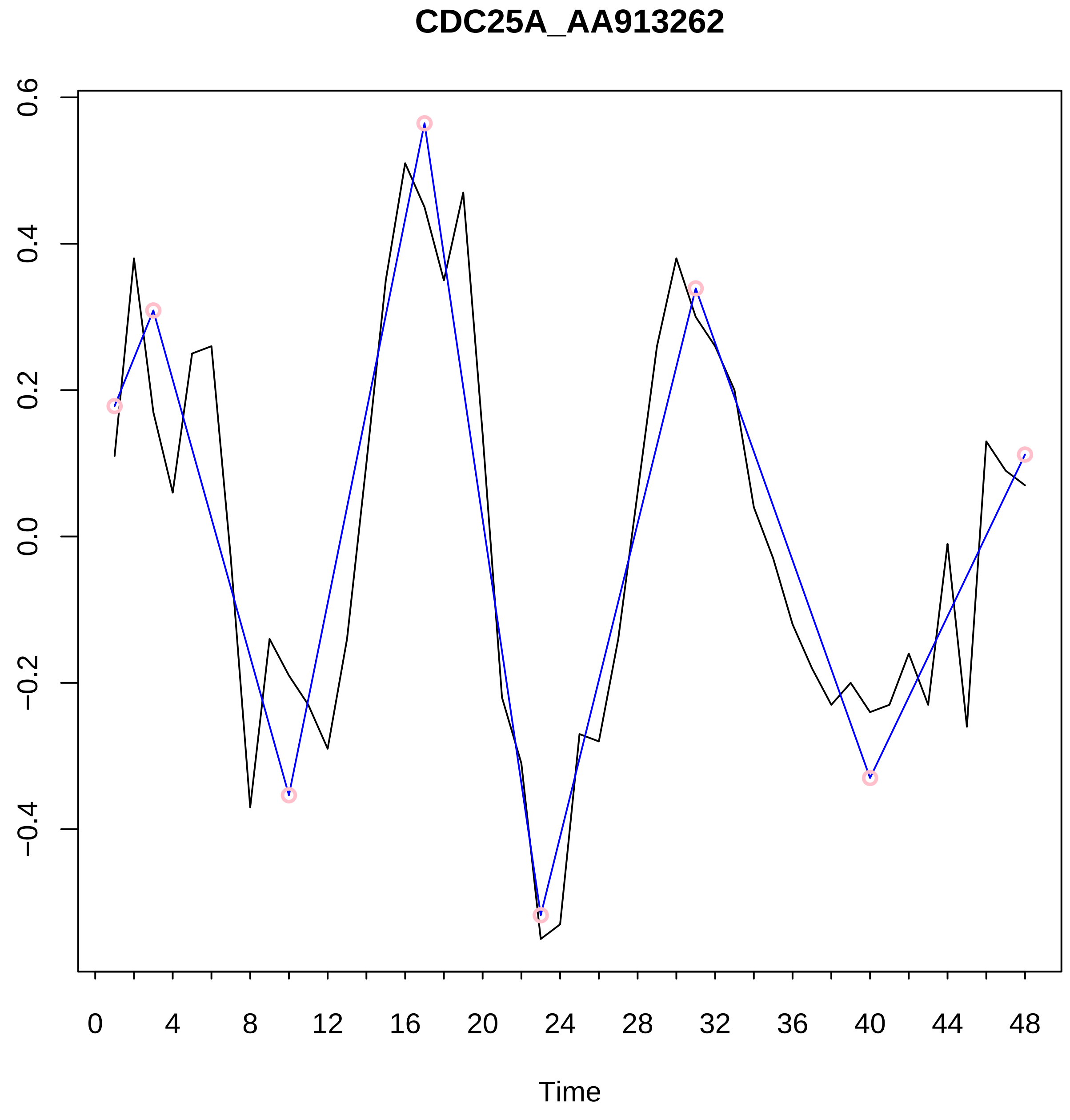
CDC25A_AA913262
0.6
0.4
0.2
0.0
−0.2
−0.4
0
4
8
12
16
20
24
28
32
36
40
44
48
Time

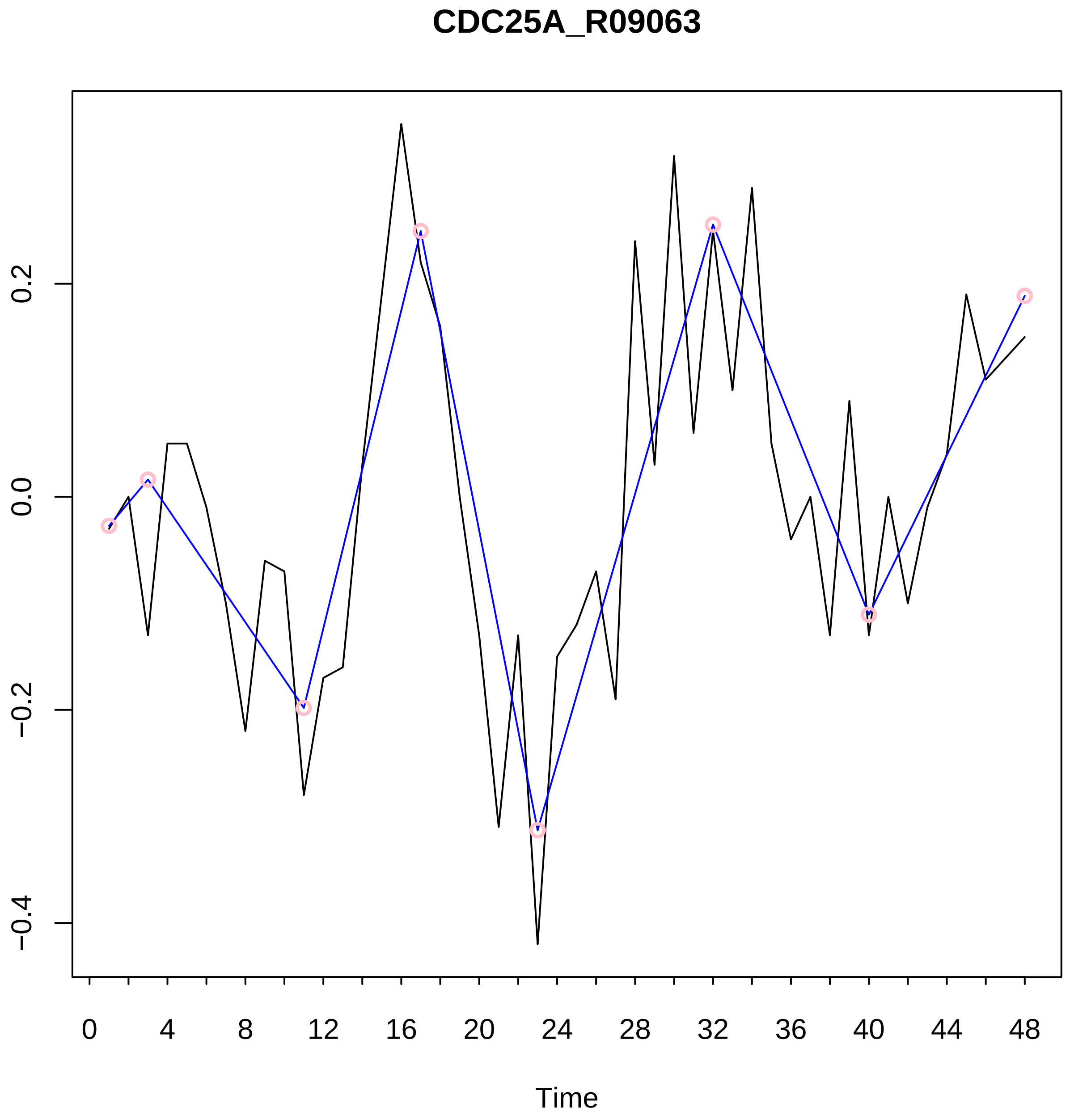
CDC25A_R09063
0.2
0.0
−0.2
−0.4
0
4
8
12
16
20
24
28
32
36
40
44
48
Time

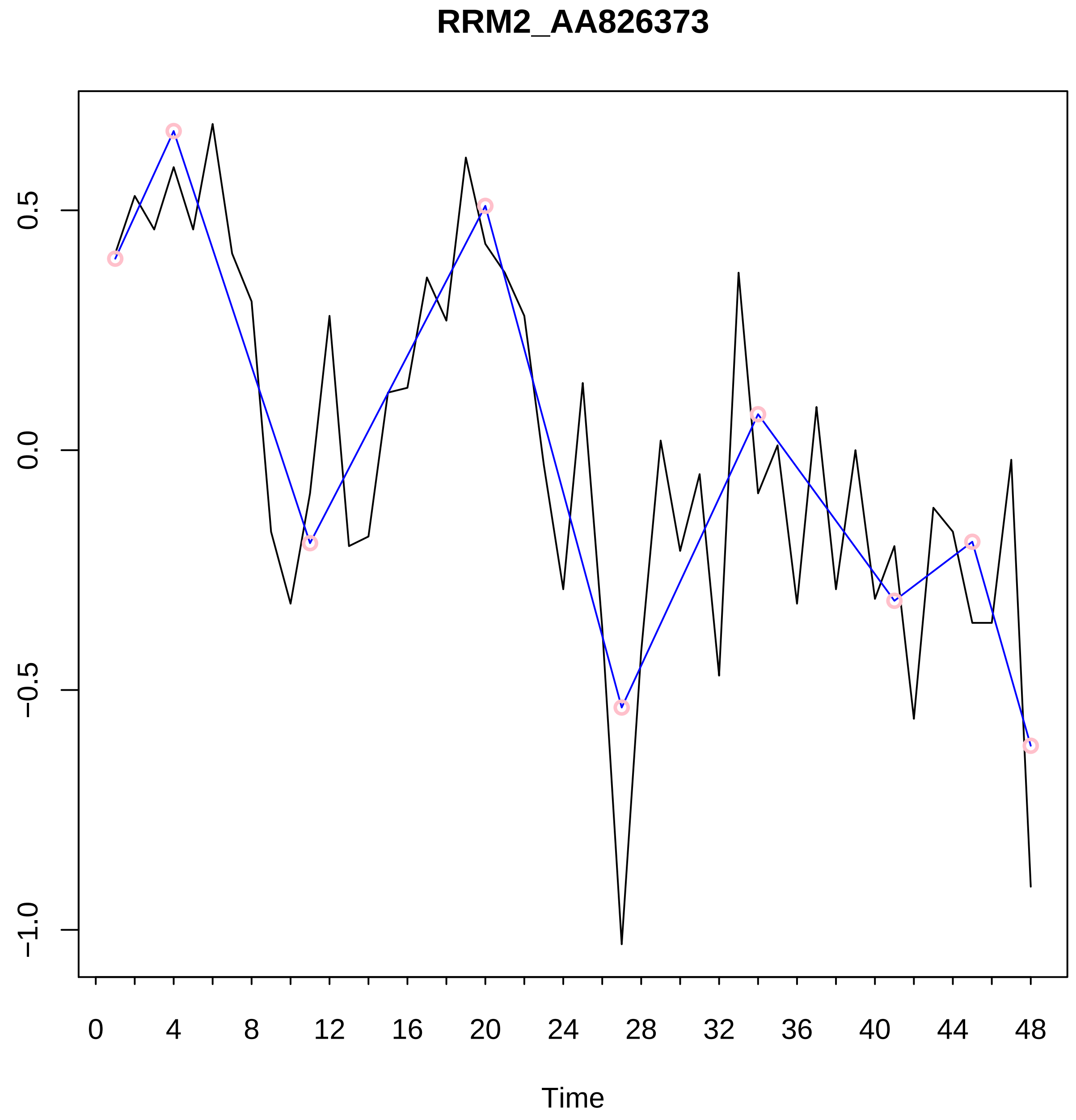
RRM2_AA826373
0.5
0.0
−0.5
−1.0
0
4
8
12
16
20
24
28
32
36
40
44
48
Time

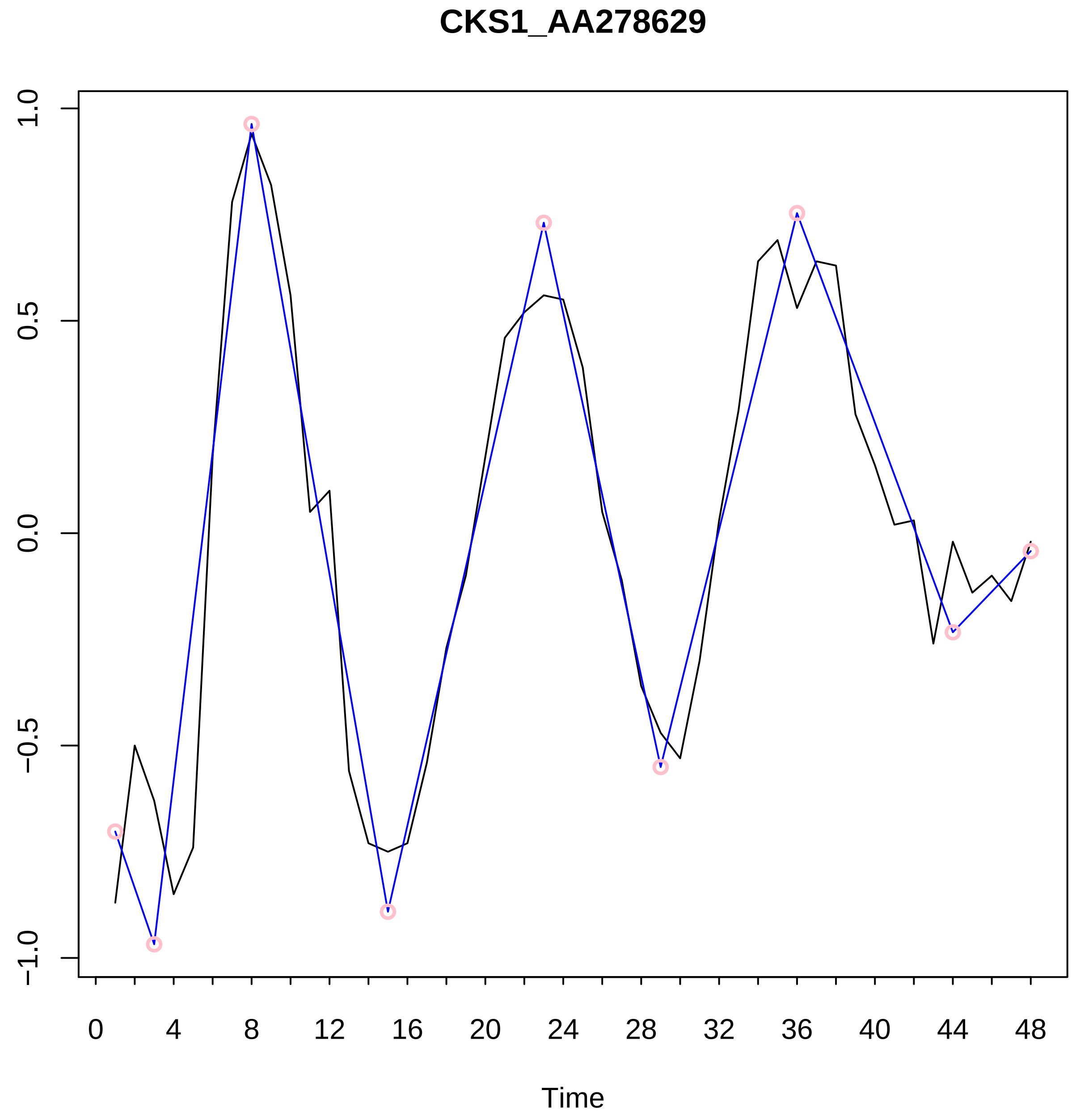

CKS1_AA278629
1.0
0.5
0.0
−0.5
−1.0
0
4
8
12
16
20
24
28
32
36
40
44
48
Time

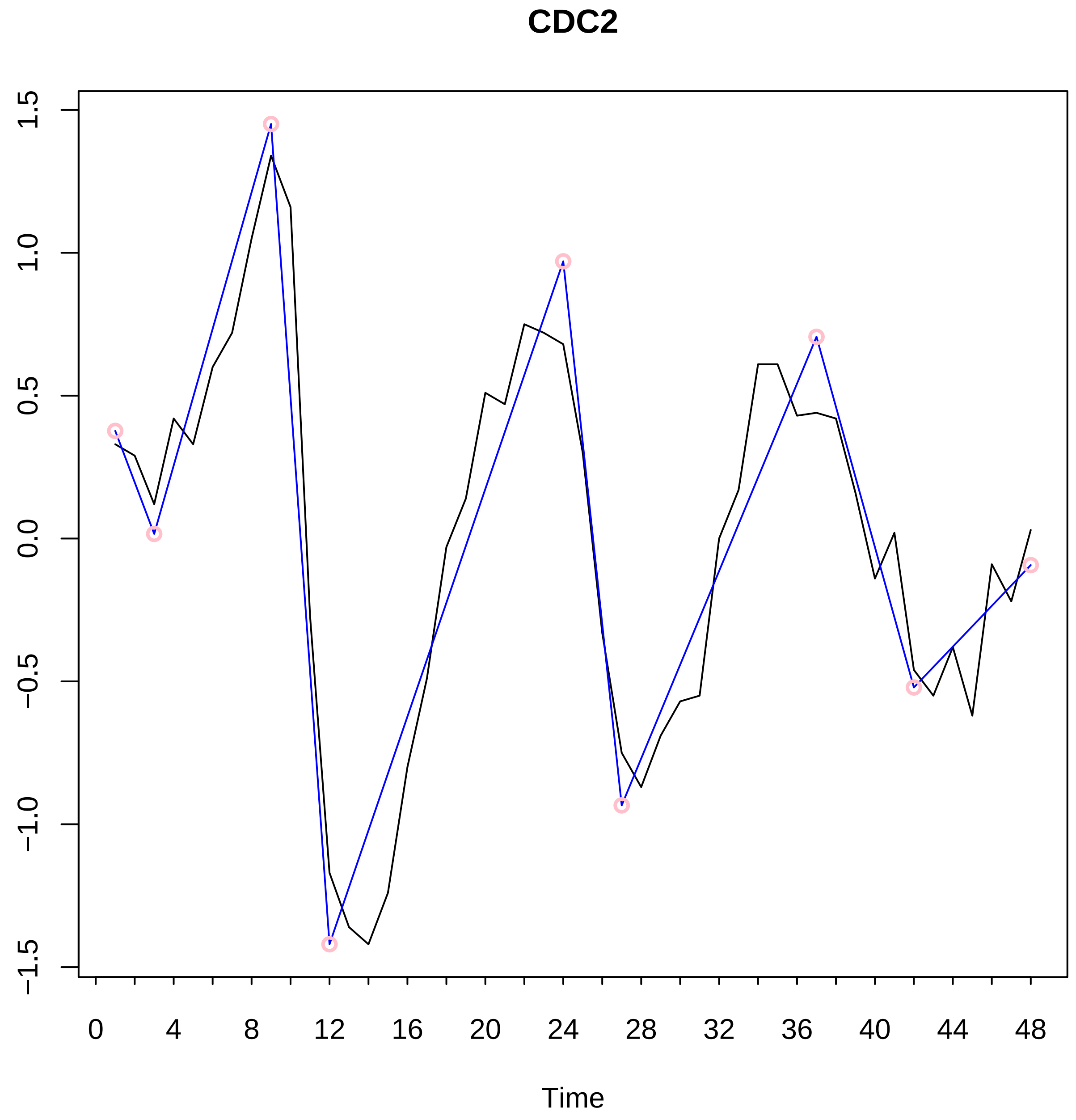
CDC2
1.5
1.0
0.5
0.0
−0.5
−1.0
−1.5
0
4
8
12
16
20
24
28
32
36
40
44
48
Time

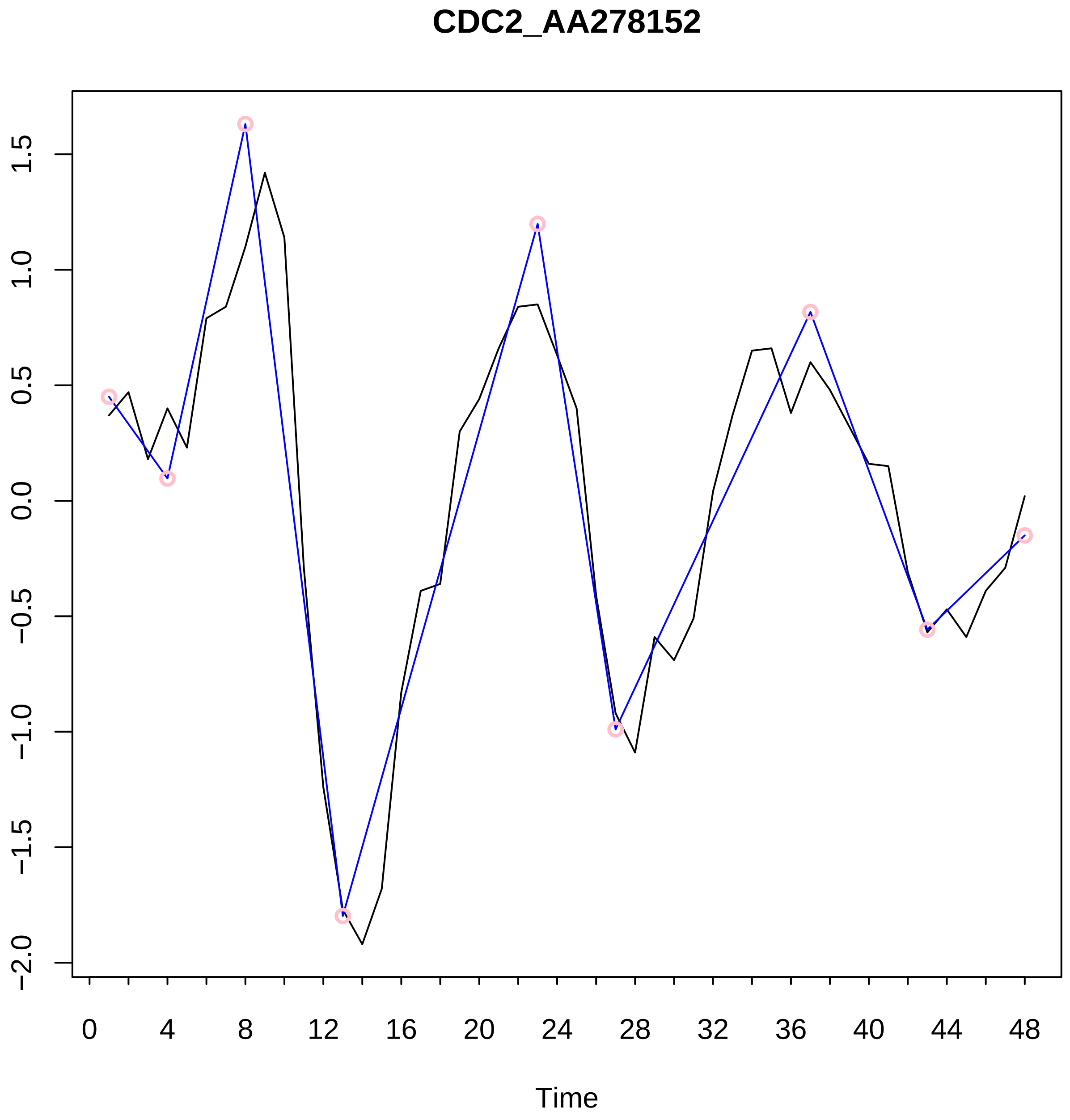

CDC2_AA278152
1.5
1.0
0.5
0.0
−0.5
−1.0
−1.5
−2.0
0
4
8
12
16
20
24
28
32
36
40
44
48
Time

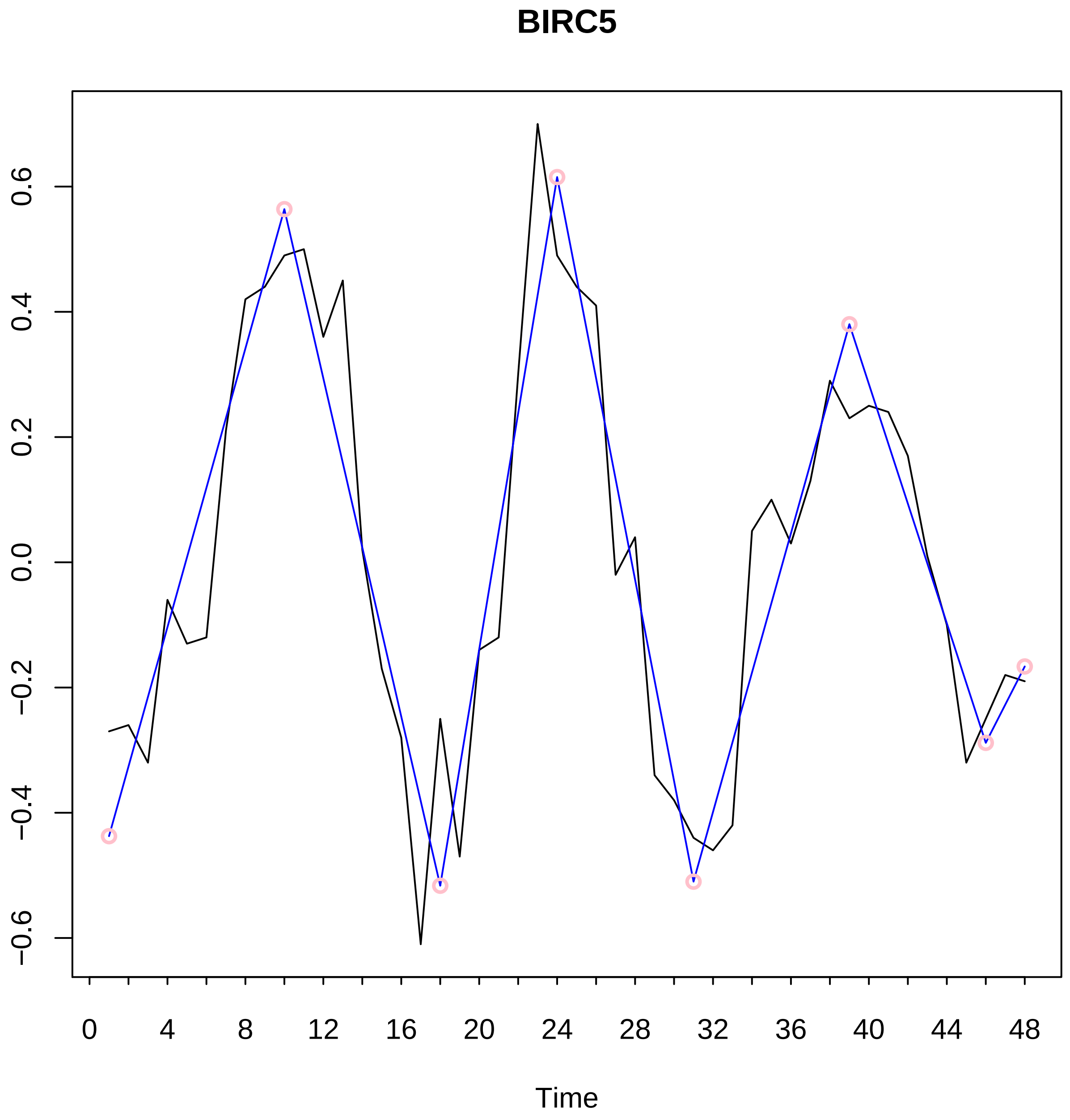

BIRC5
0.6
0.4
0.2
0.0
−0.2
−0.4
−0.6
0
4
8
12
16
20
24
28
32
36
40
44
48
Time

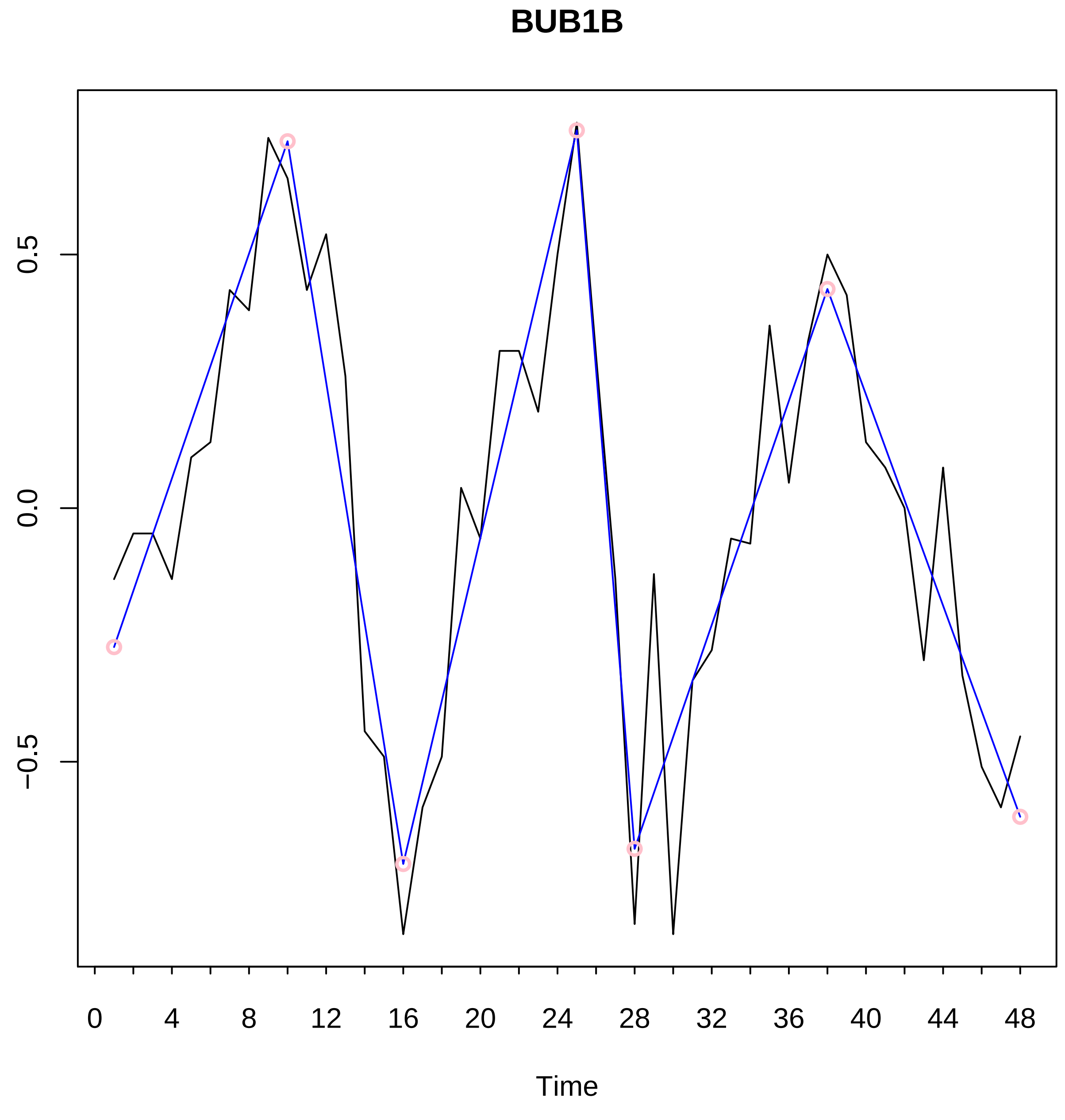
BUB1B
0.5
0.0
−0.5
0
4
8
12
16
20
24
28
32
36
40
44
48
Time

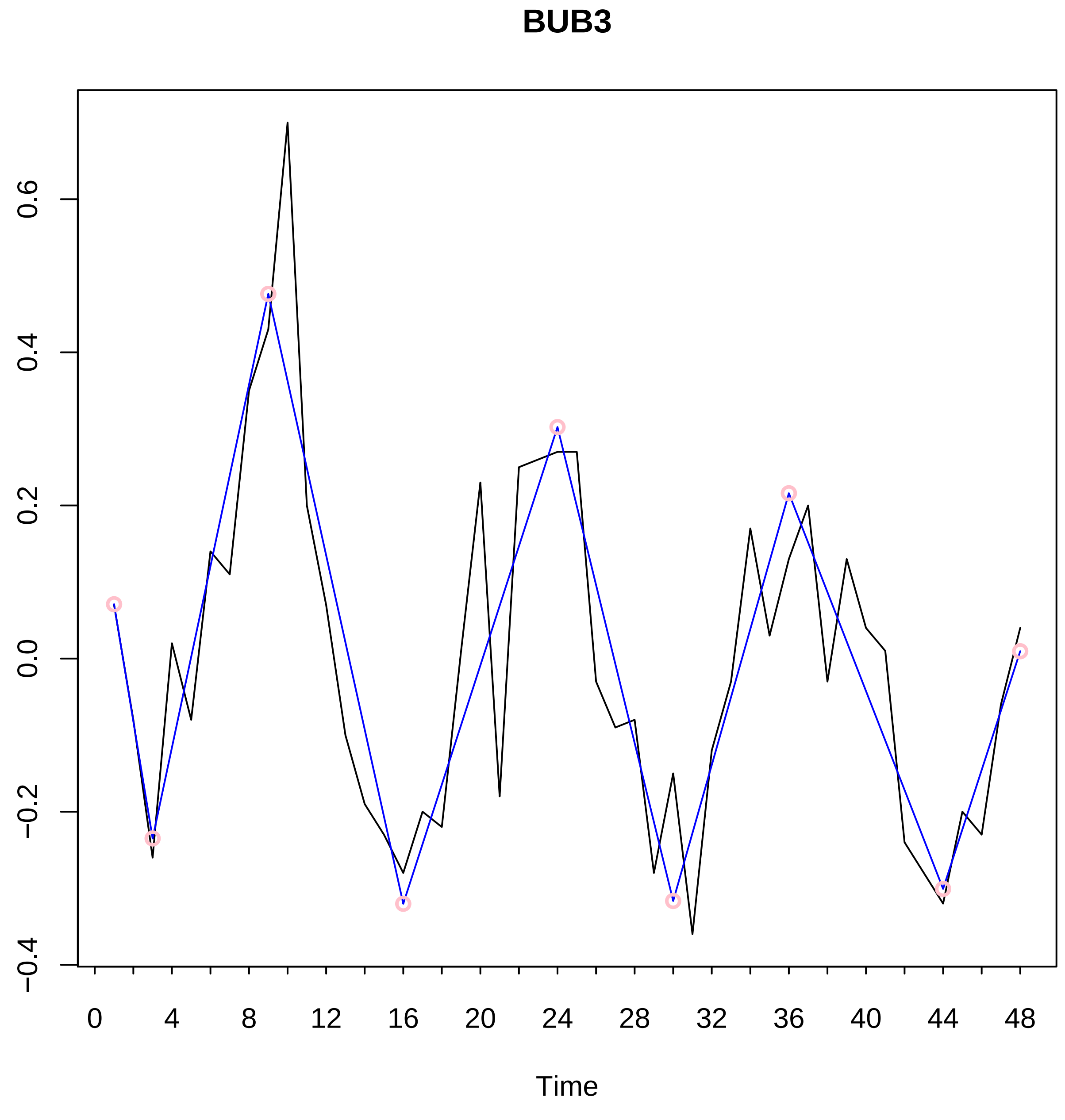
BUB3
0.6
0.4
0.2
0.0
−0.2
−0.4
0
4
8
12
16
20
24
28
32
36
40
44
48
Time

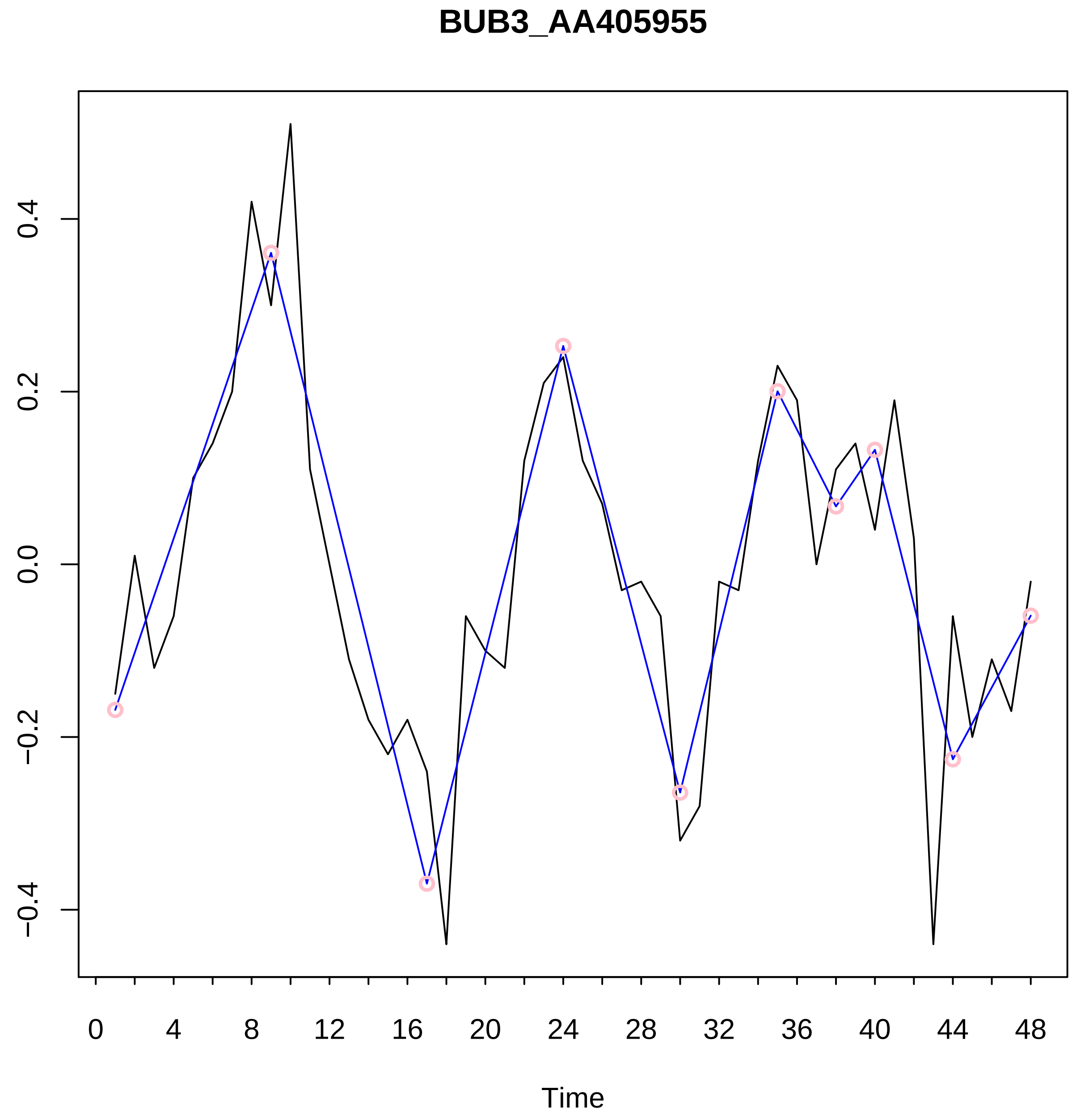
BUB3_AA405955
0.4
0.2
0.0
−0.2
−0.4
0
4
8
12
16
20
24
28
32
36
40
44
48
Time

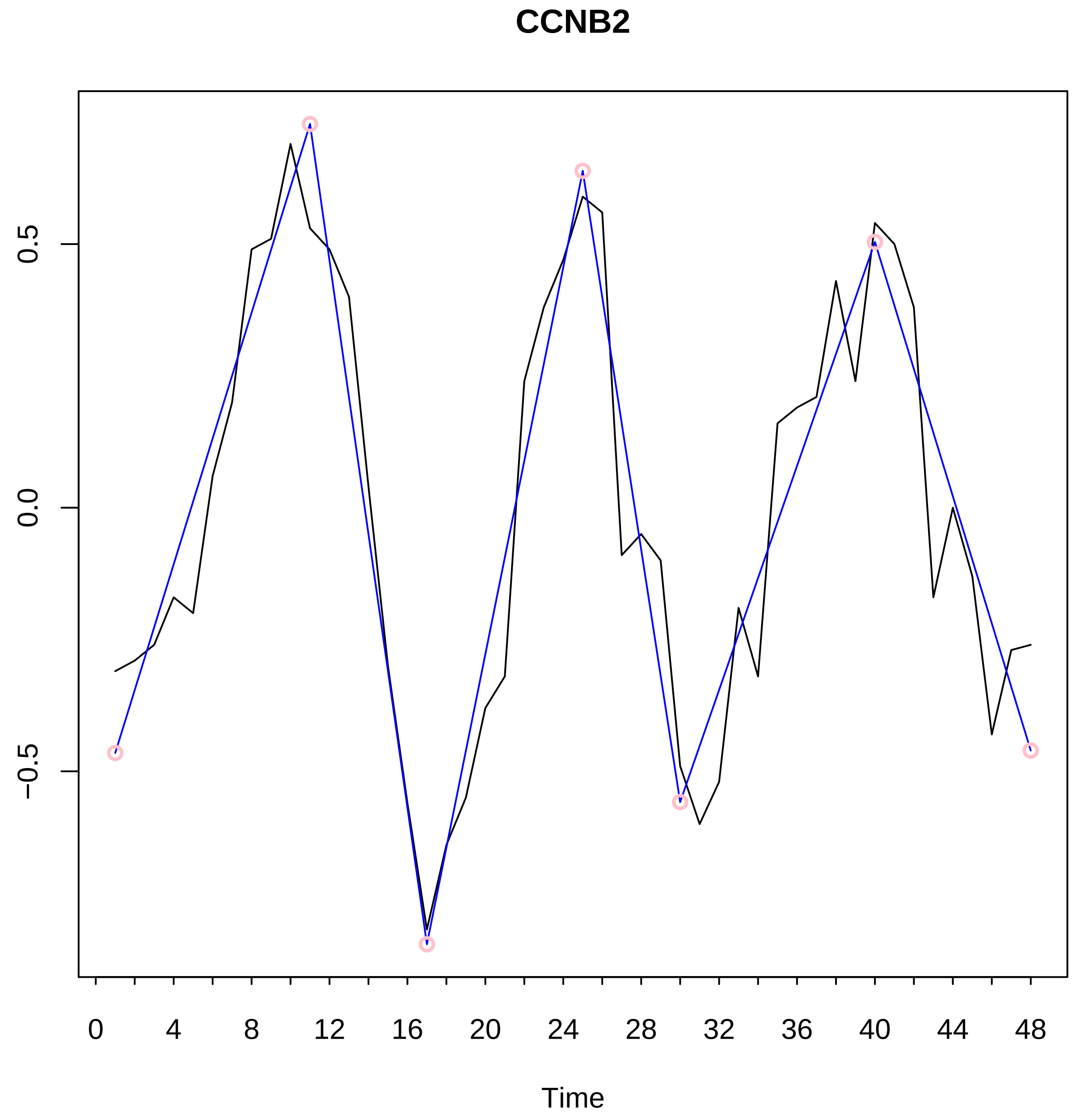

CCNB2
0.5
0.0
−0.5
0
4
8
12
16
20
24
28
32
36
40
44
48
Time

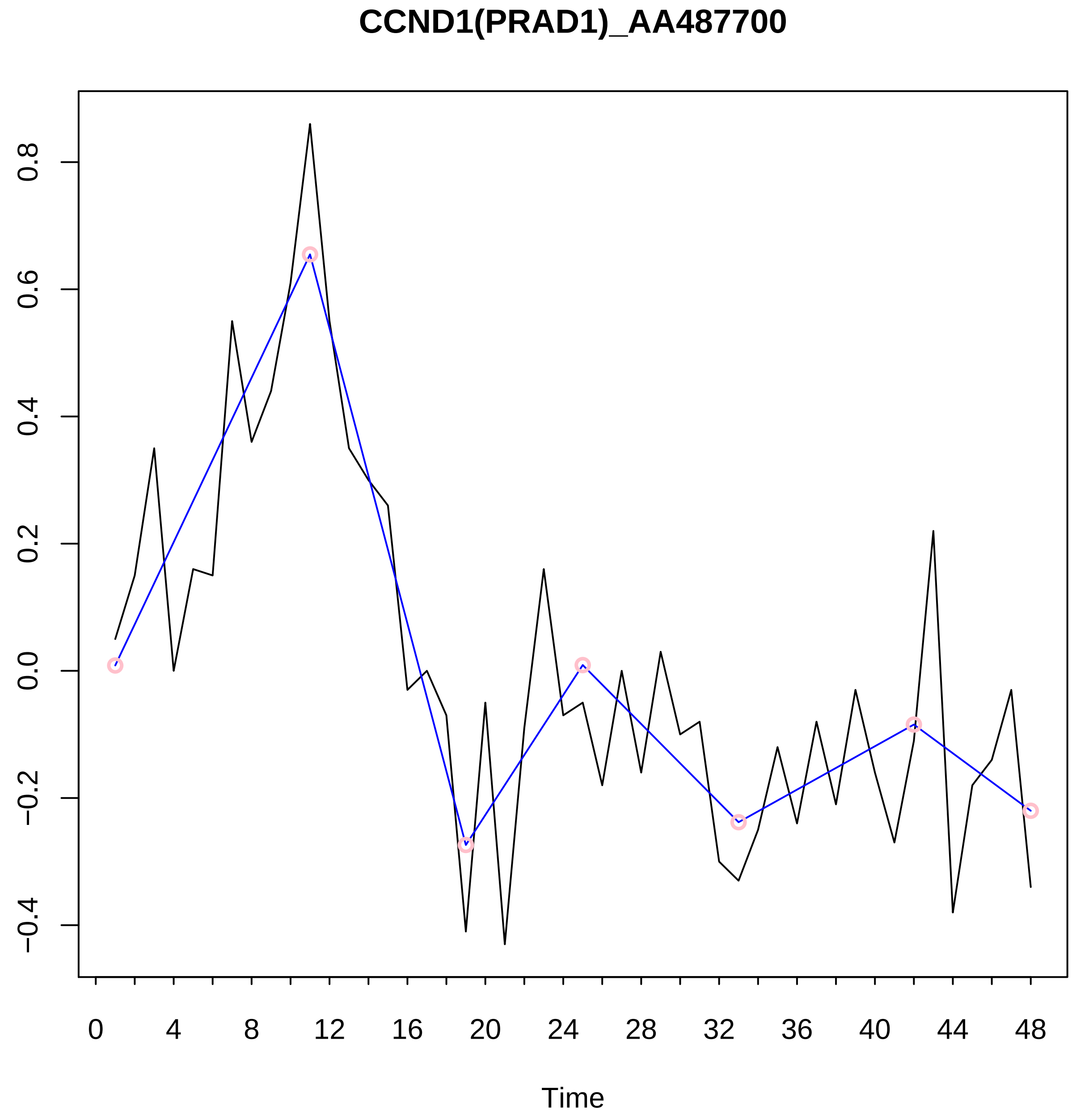

CCND1(PRAD1)_AA487700
0.8
0.6
0.4
0.2
0.0
−0.2
−0.4
0
4
8
12
16
20
24
28
32
36
40
44
48
Time

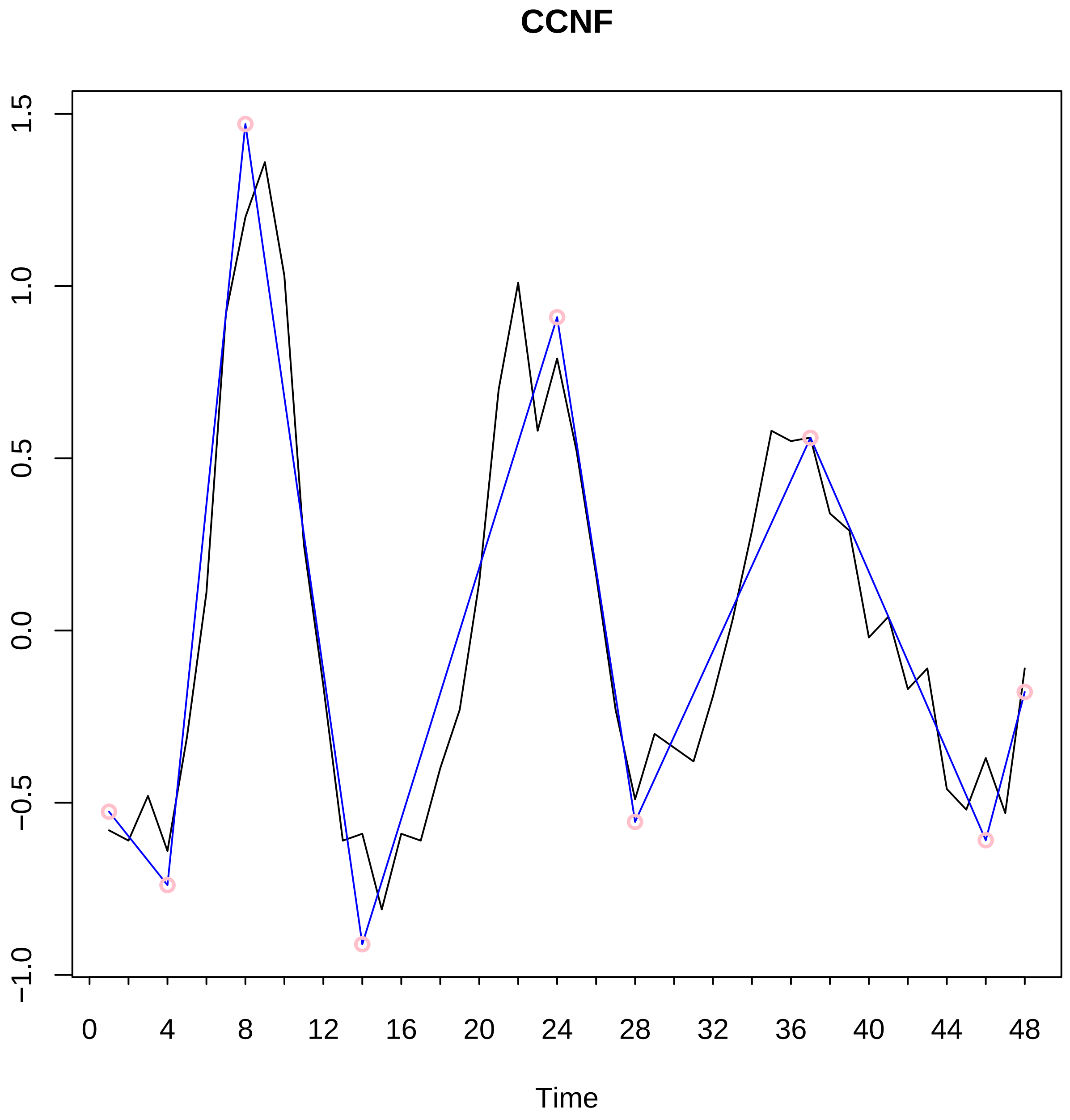

CCNF
1.5
1.0
0.5
0.0
−0.5
−1.0
0
4
8
12
16
20
24
28
32
36
40
44
48
Time

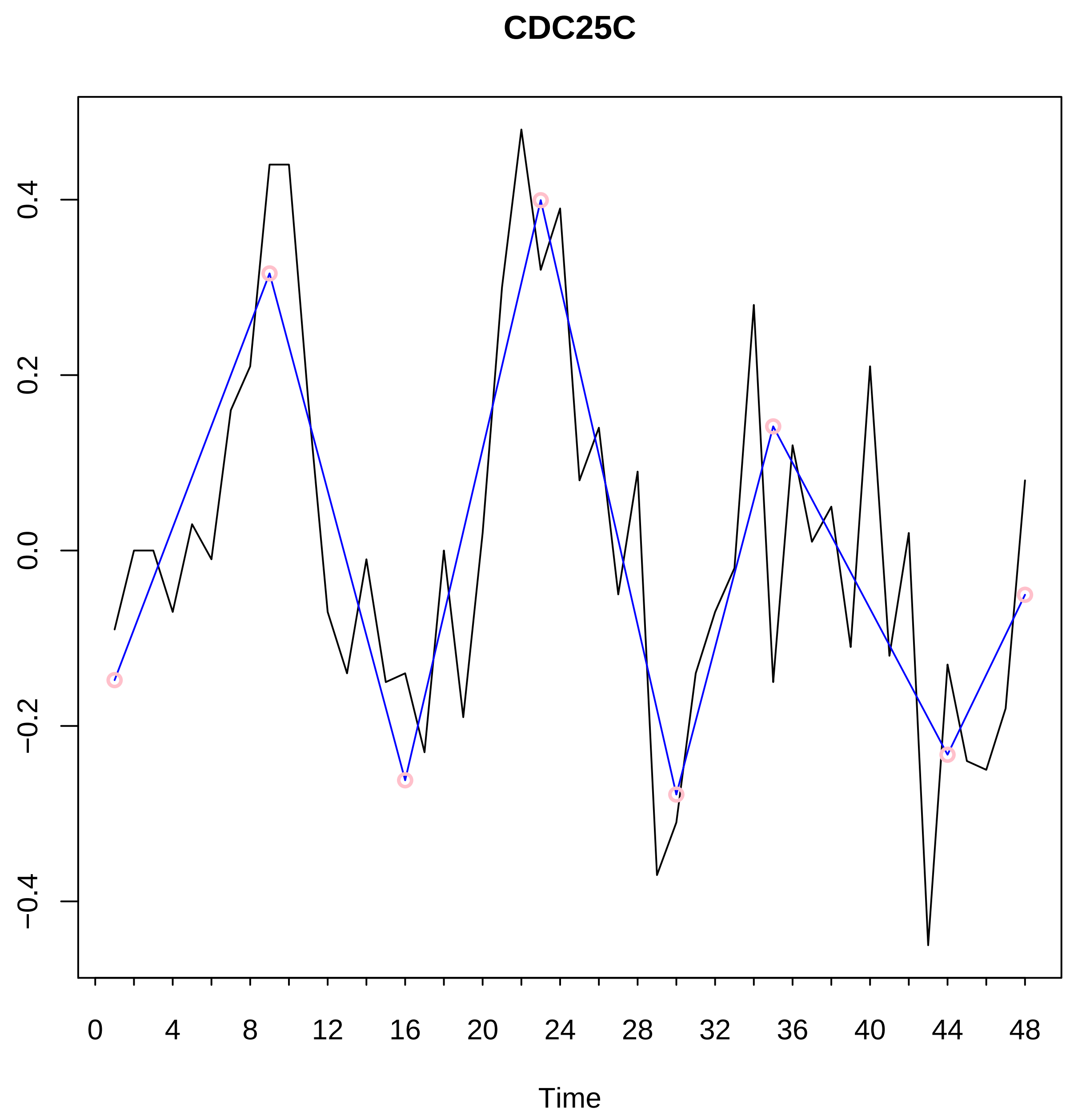
CDC25C
0.4
0.2
0.0
−0.2
−0.4
0
4
8
12
16
20
24
28
32
36
40
44
48
Time

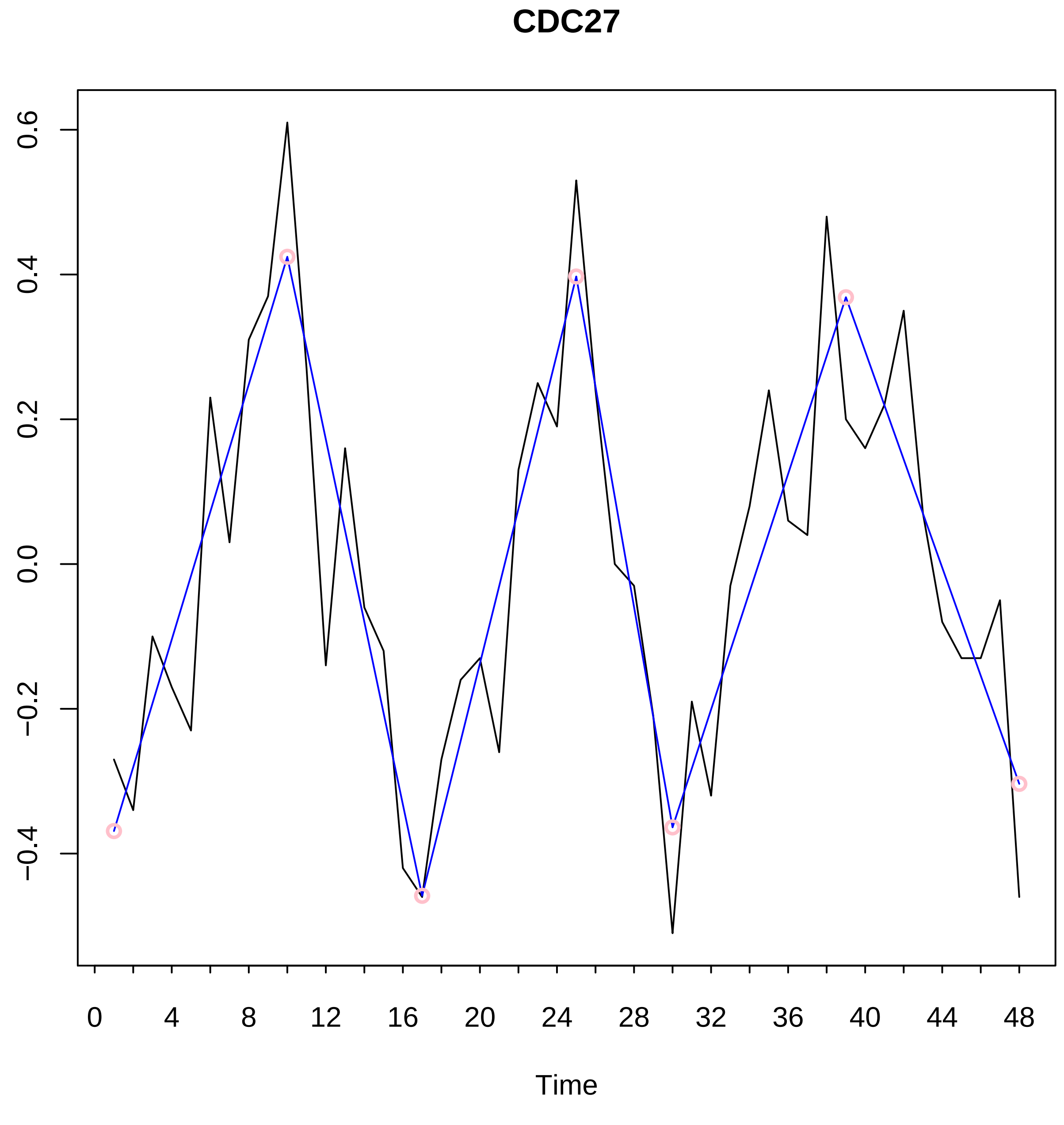
CDC27
0.6
0.4
0.2
0.0
−0.2
−0.4
0
4
8
12
16
20
24
28
32
36
40
44
48
Time

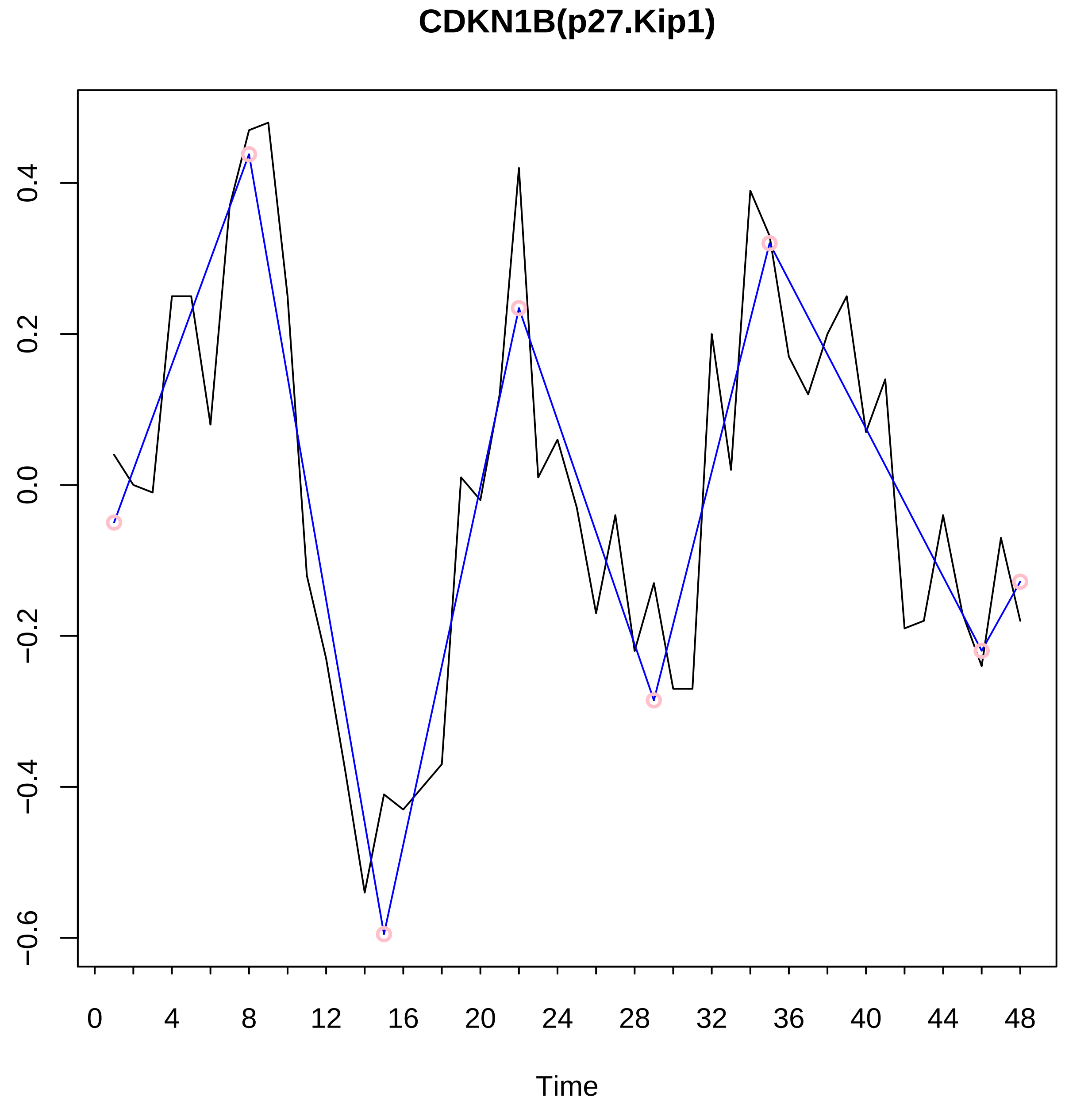
CDKN1B(p27.Kip1)
0.4
0.2
0.0
−0.2
−0.4
−0.6
0
4
8
12
16
20
24
28
32
36
40
44
48
Time

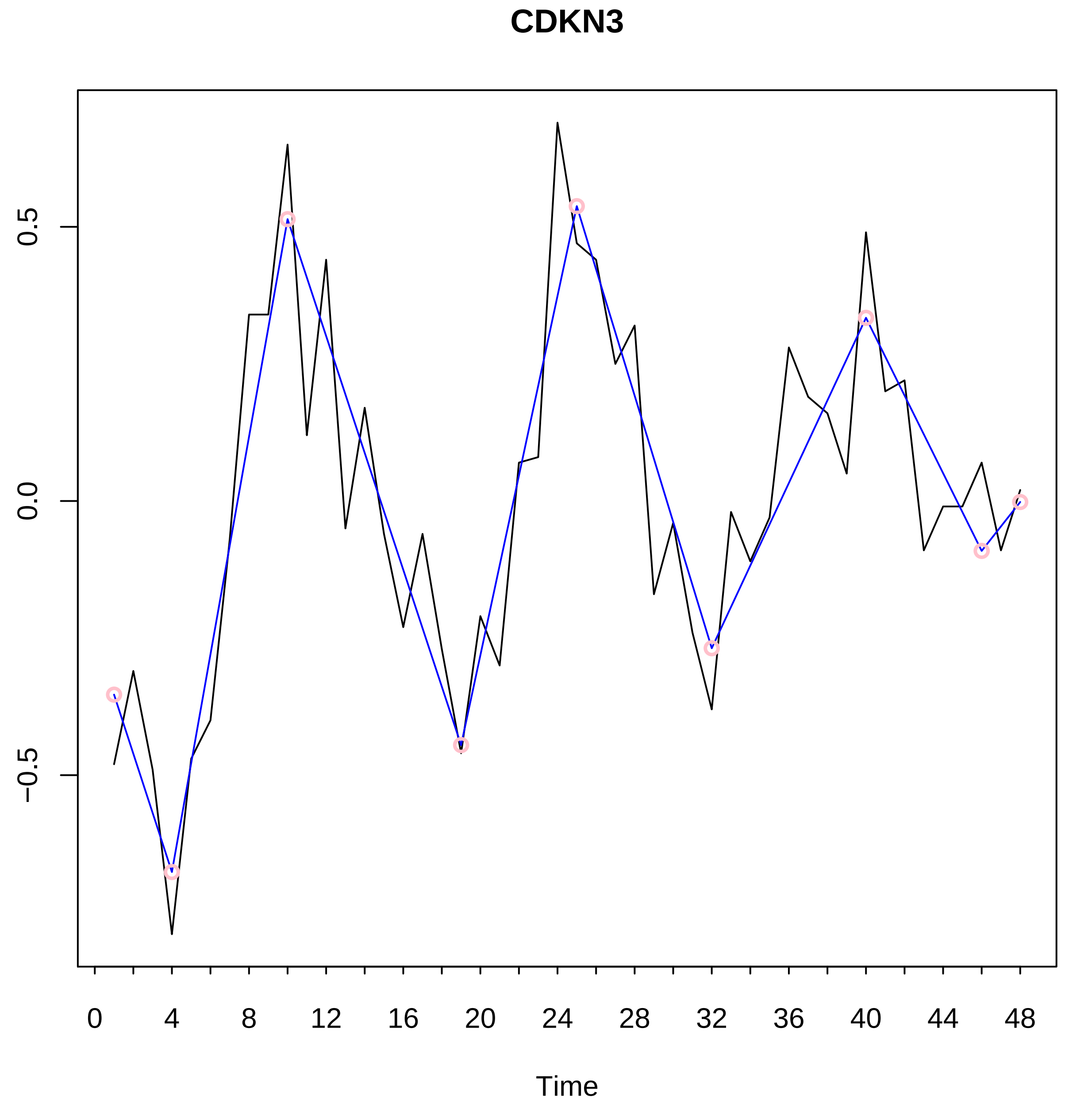
CDKN3
0.5
0.0
−0.5
0
4
8
12
16
20
24
28
32
36
40
44
48
Time

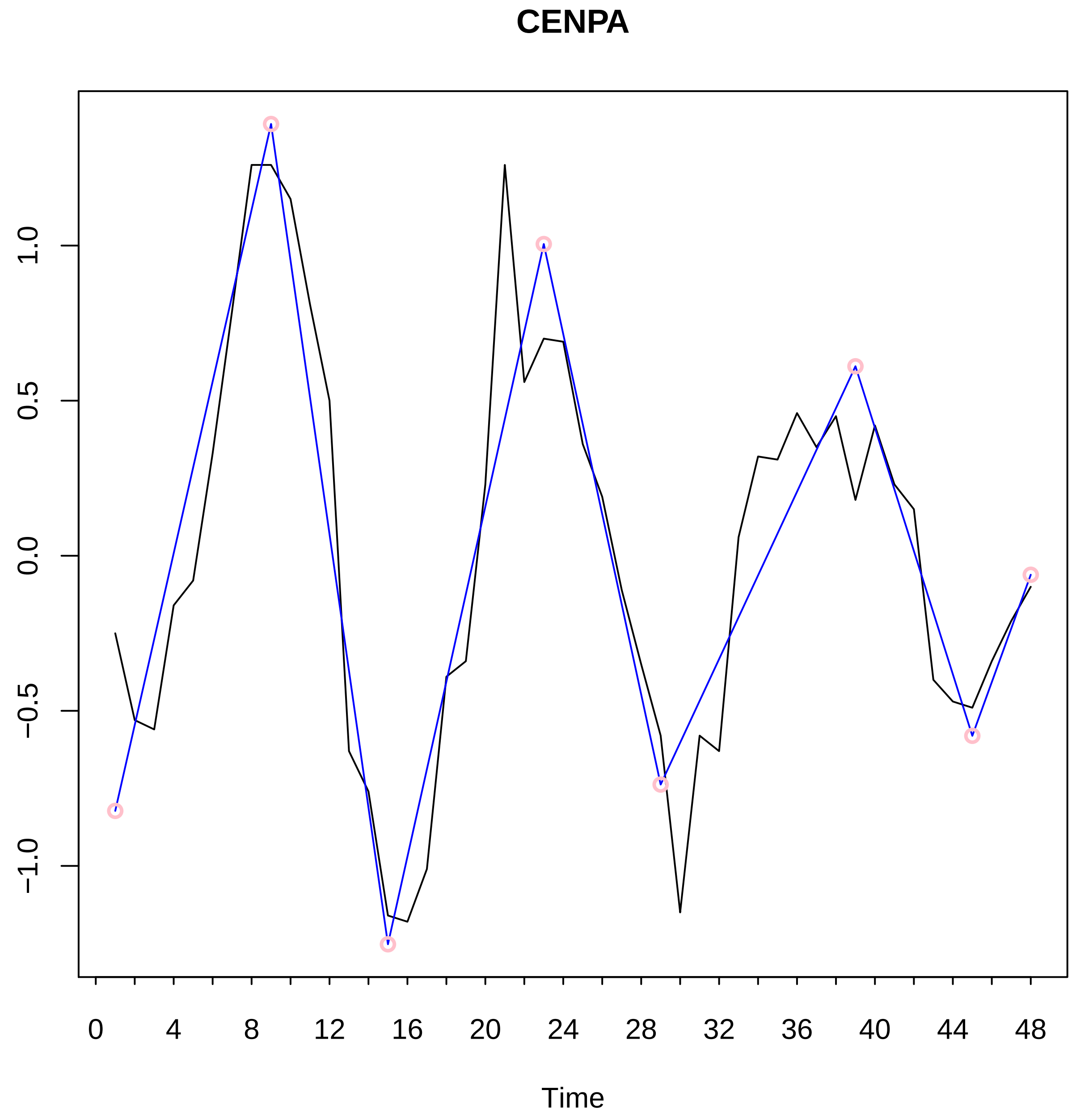
CENPA
1.0
0.5
0.0
−0.5
−1.0
0
4
8
12
16
20
24
28
32
36
40
44
48
Time

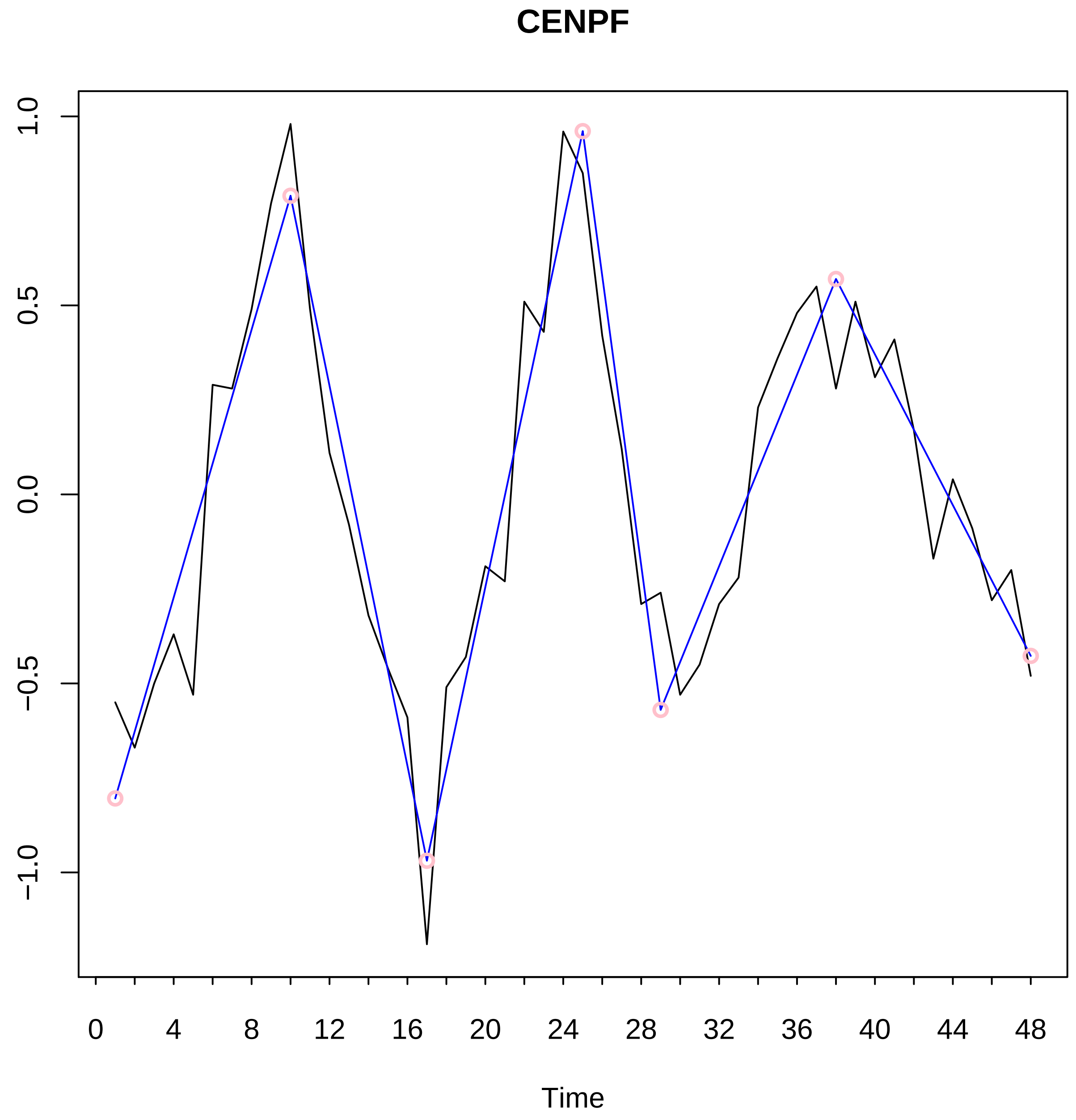
CENPF
1.0
0.5
0.0
−0.5
−1.0
0
4
8
12
16
20
24
28
32
36
40
44
48
Time

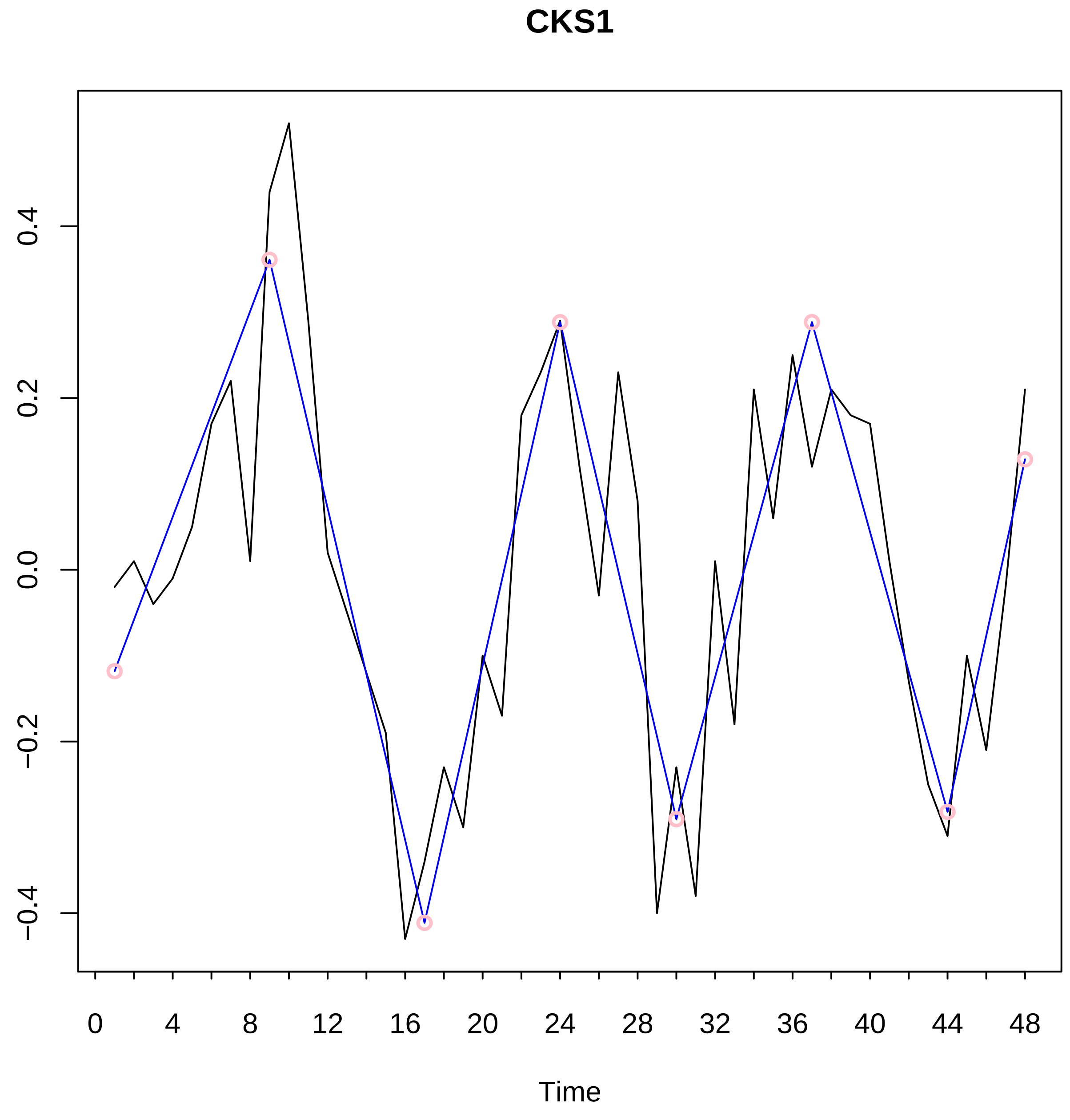
CKS1
0.4
0.2
0.0
−0.2
−0.4
0
4
8
12
16
20
24
28
32
36
40
44
48
Time

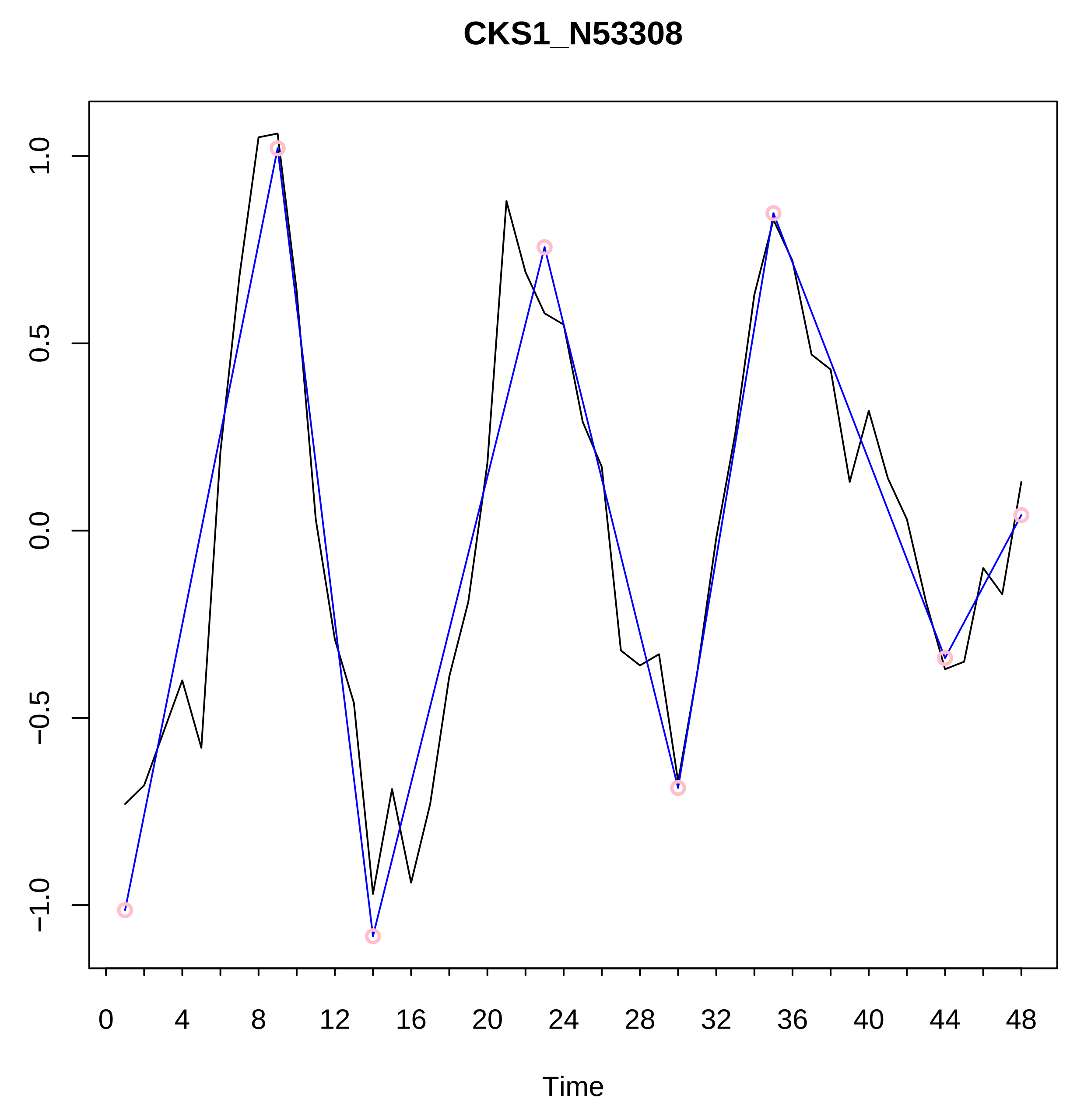
CKS1_N53308
1.0
0.5
0.0
−0.5
−1.0
0
4
8
12
16
20
24
28
32
36
40
44
48
Time

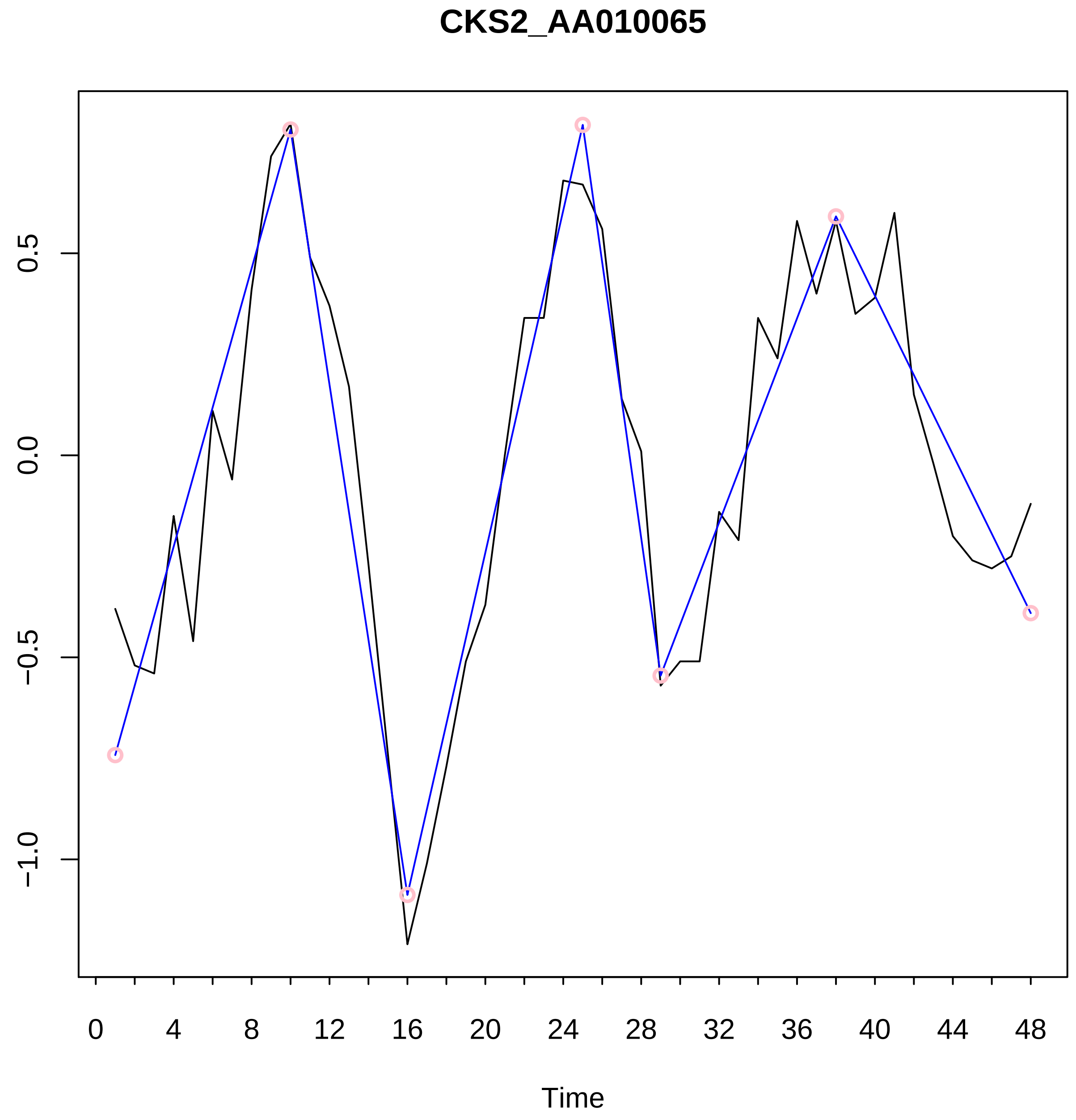

CKS2_AA010065
0.5
0.0
−0.5
−1.0
0
4
8
12
16
20
24
28
32
36
40
44
48
Time

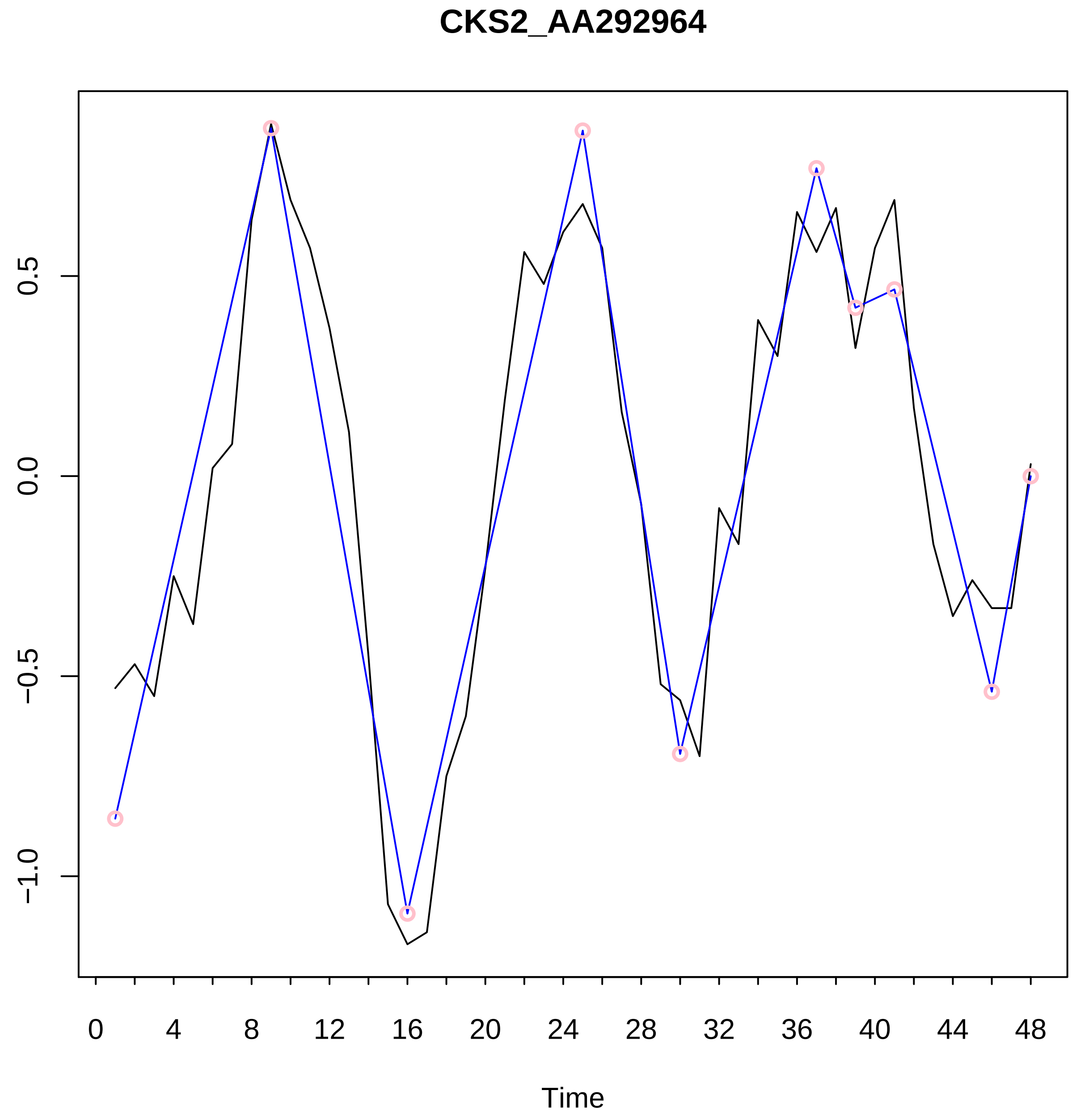

CKS2_AA292964
0.5
0.0
−0.5
−1.0
0
4
8
12
16
20
24
28
32
36
40
44
48
Time

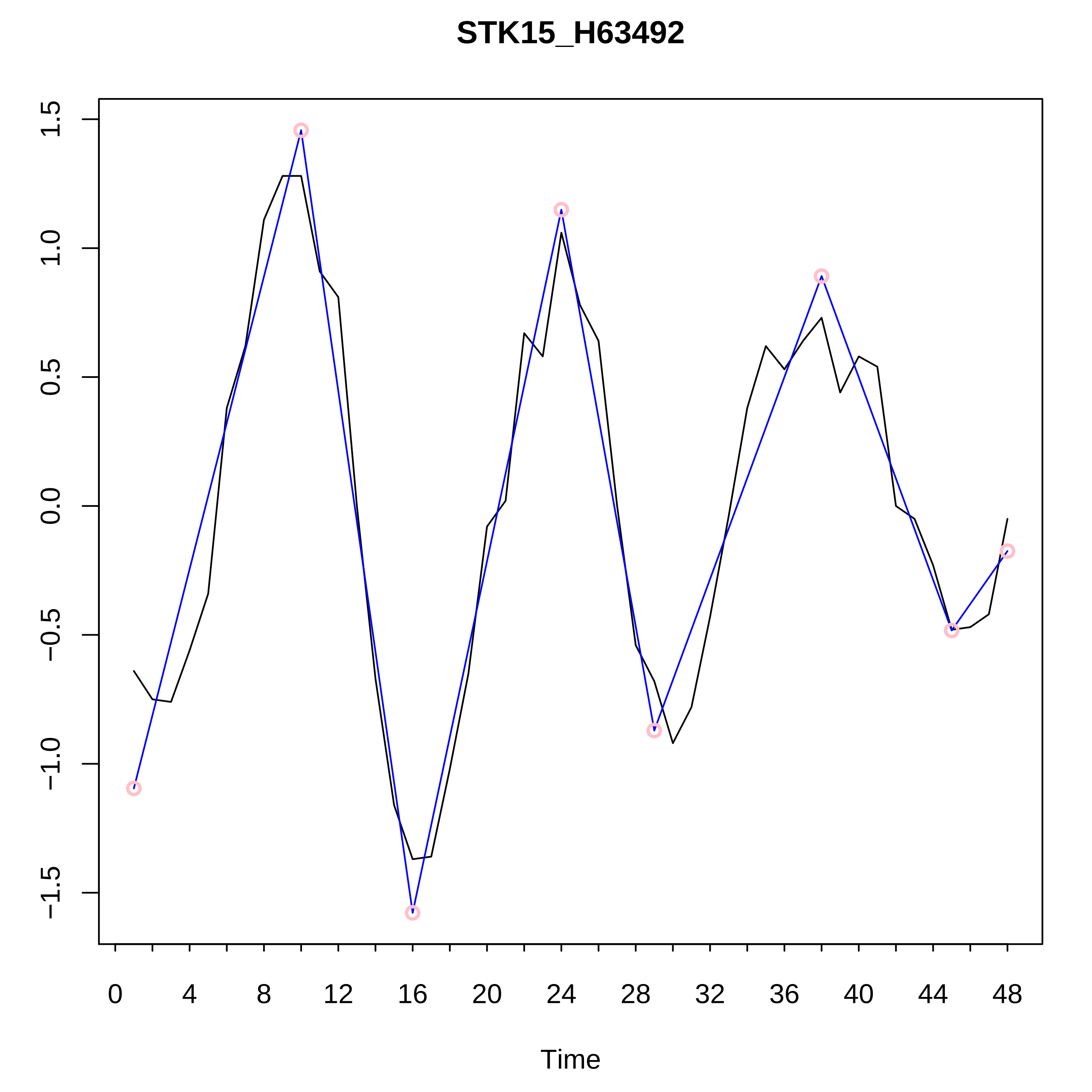
STK15_H63492
1.5
1.0
0.5
0.0
−0.5
−1.0
−1.5
0
4
8
12
16
20
24
28
32
36
40
44
48
Time

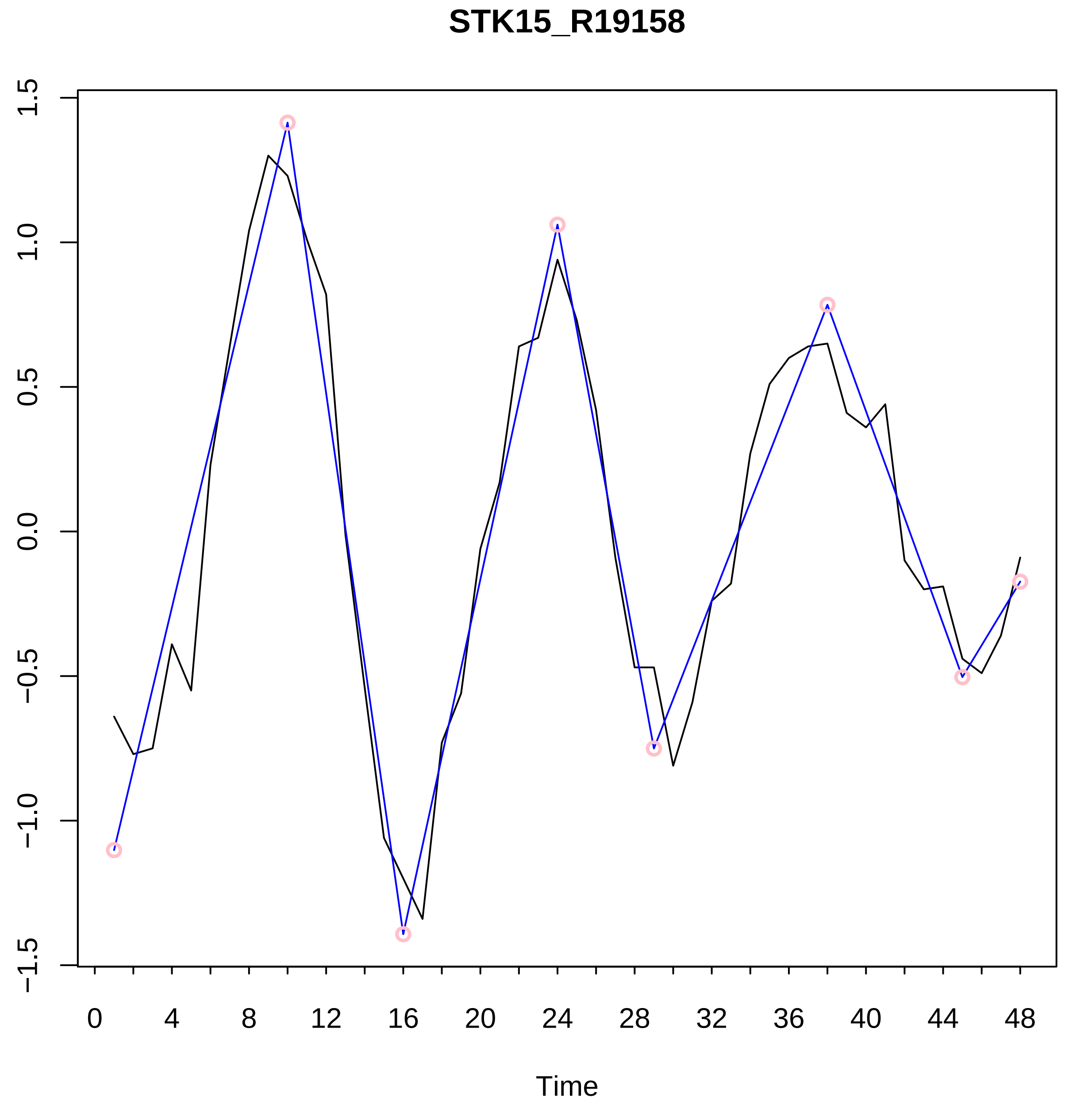
STK15_R19158
−1.5
−1.0
−0.5
0.0
0.5
1.0
1.5
0
4
8
12
16
20
24
28
32
36
40
44
48
Time

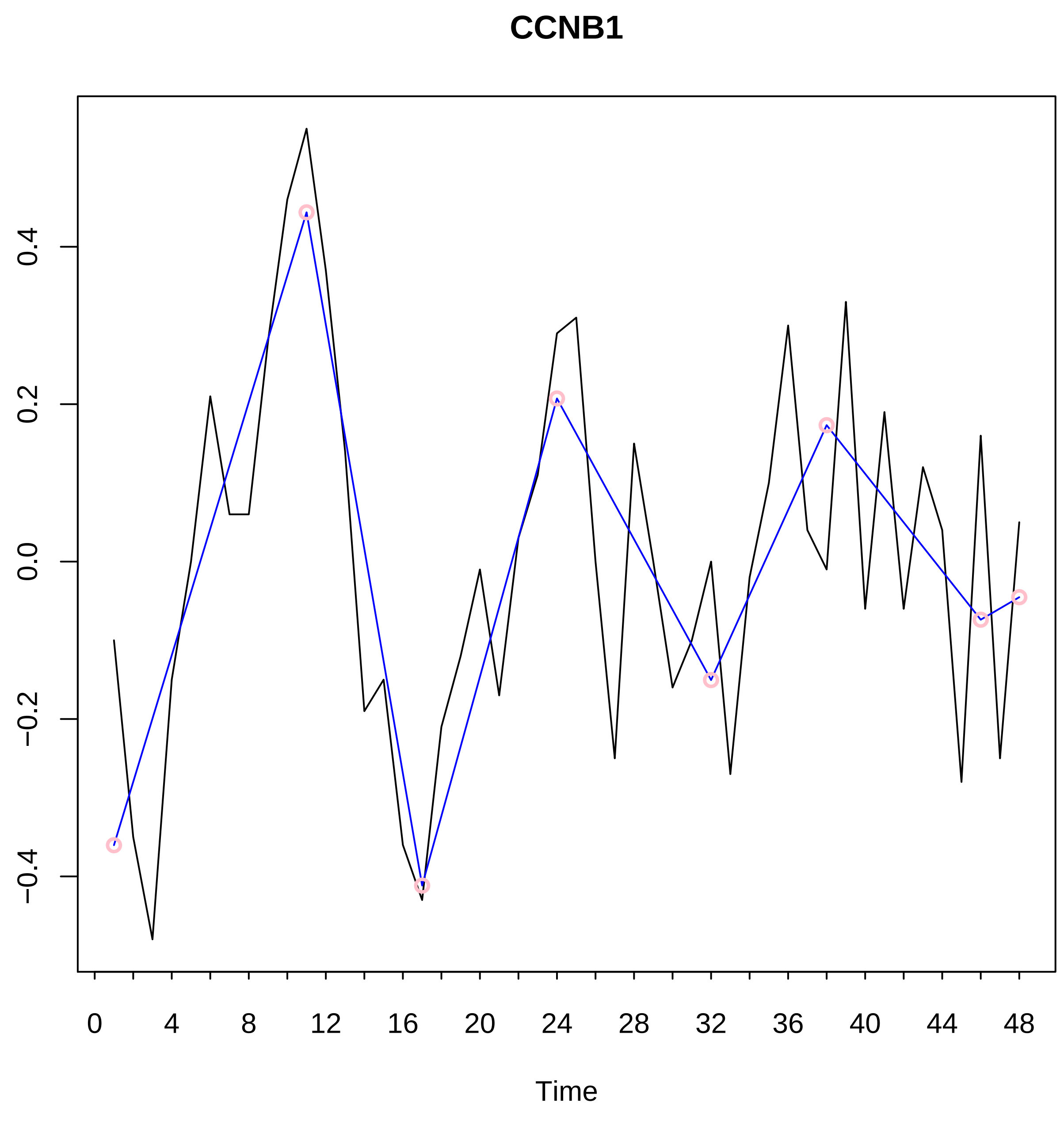
CCNB1
0.4
0.2
0.0
−0.2
−0.4
0
4
8
12
16
20
24
28
32
36
40
44
48
Time

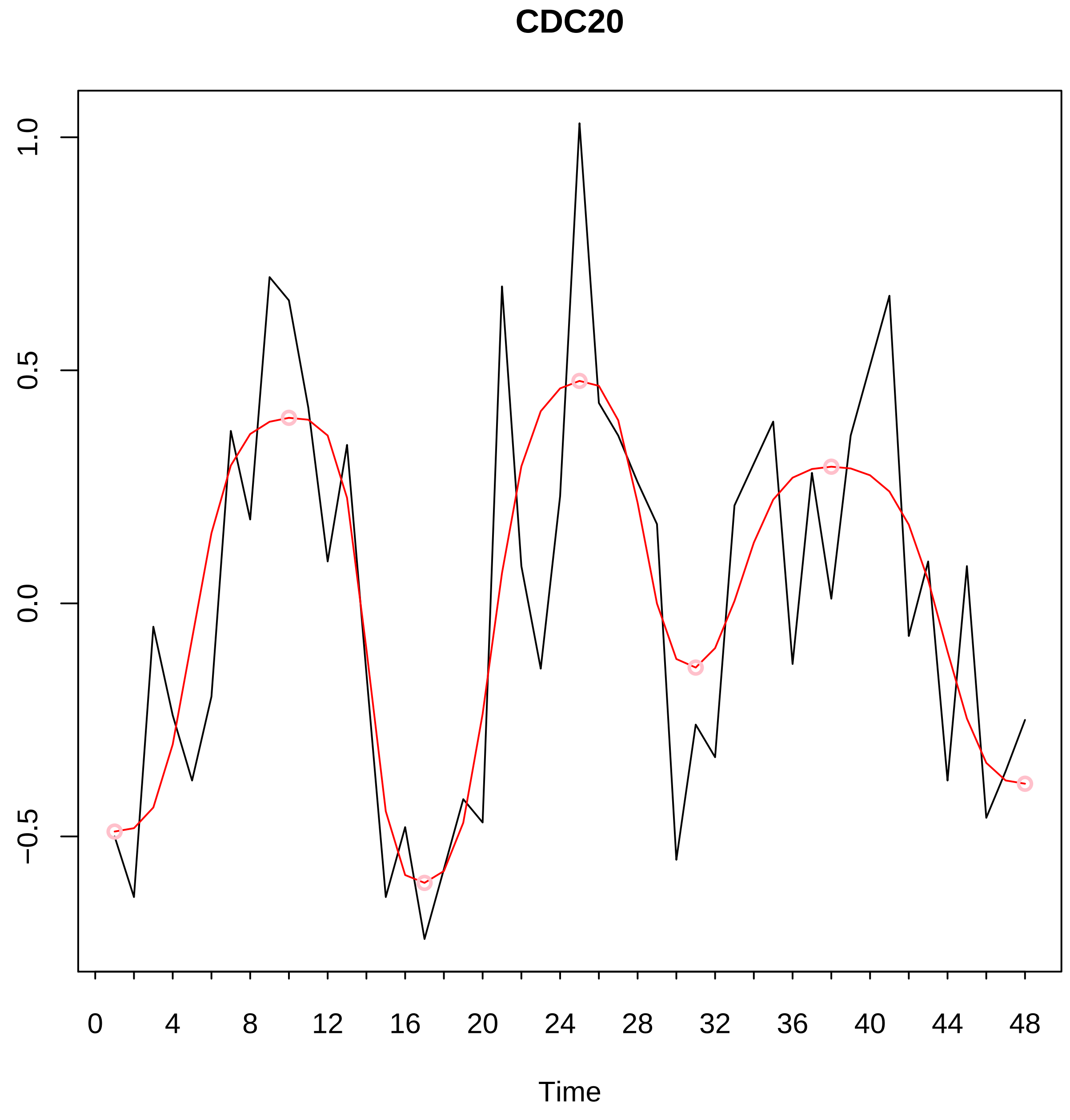
CDC20
1.0
0.5
0.0
−0.5
0
4
8
12
16
20
24
28
32
36
40
44
48
Time

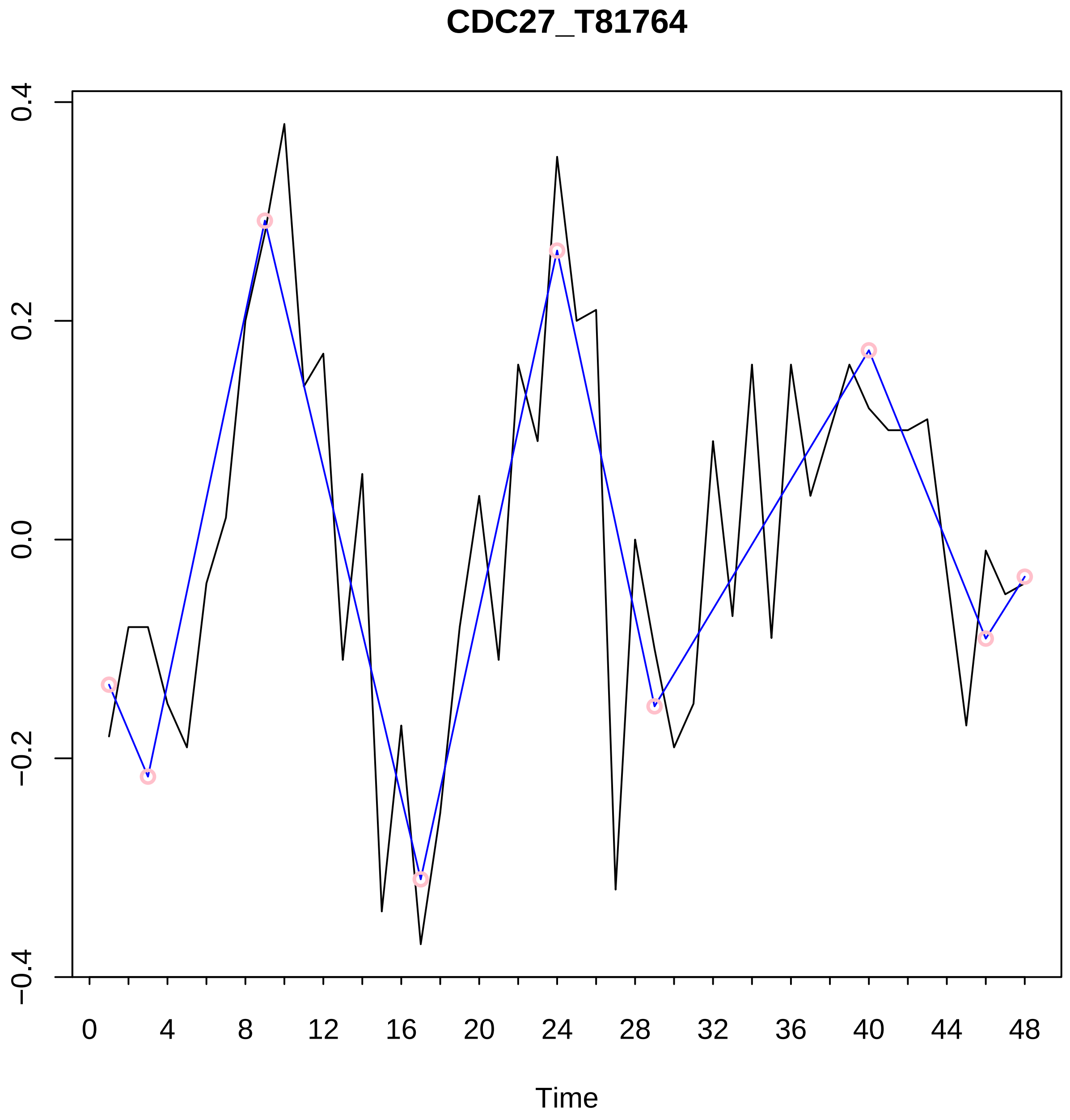
CDC27_T81764
0.4
0.2
0.0
−0.2
−0.4
0
4
8
12
16
20
24
28
32
36
40
44
48
Time

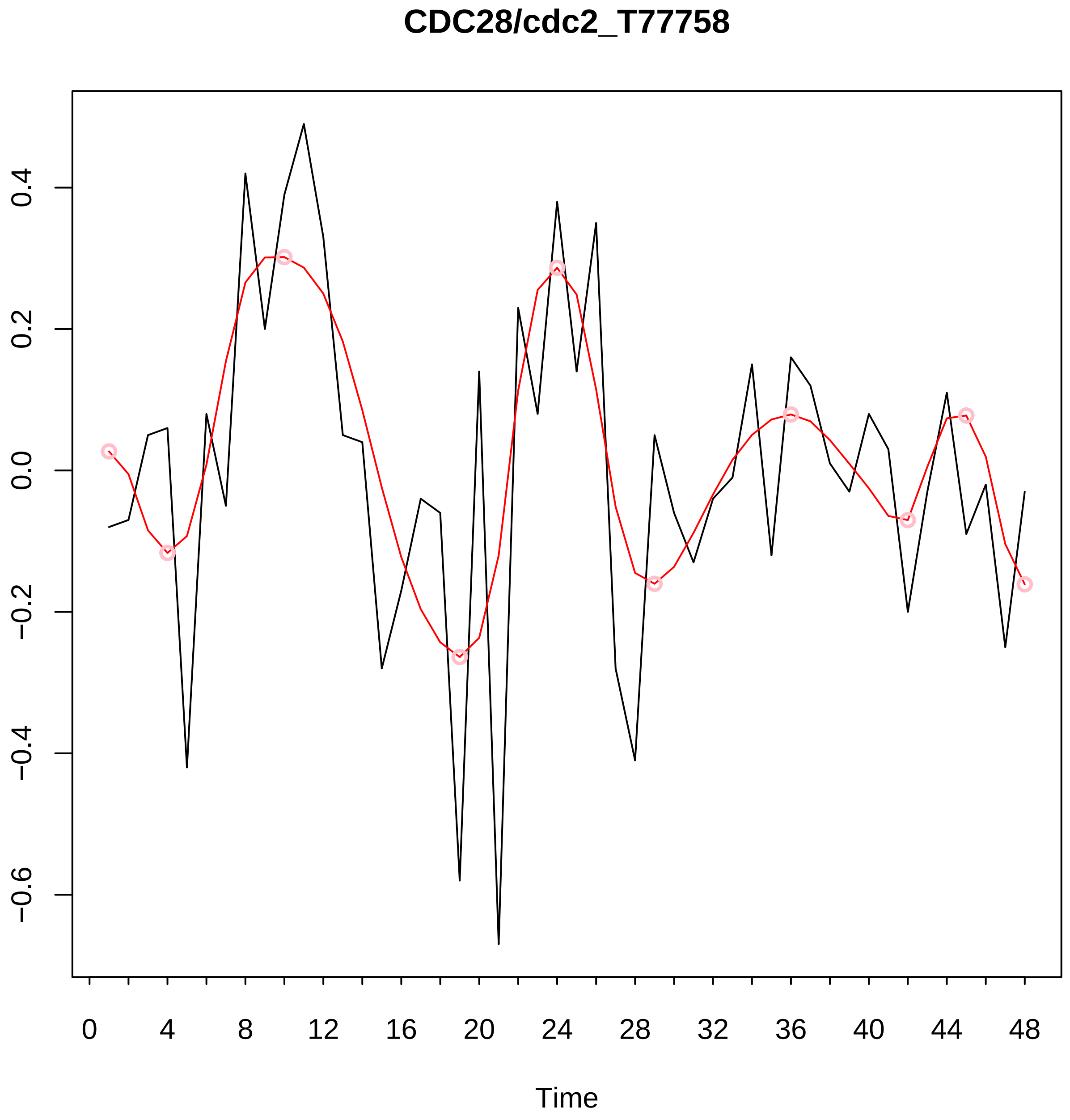
CDC28/cdc2_T77758
0.4
0.2
0.0
−0.2
−0.4
−0.6
0
4
8
12
16
20
24
28
32
36
40
44
48
Time

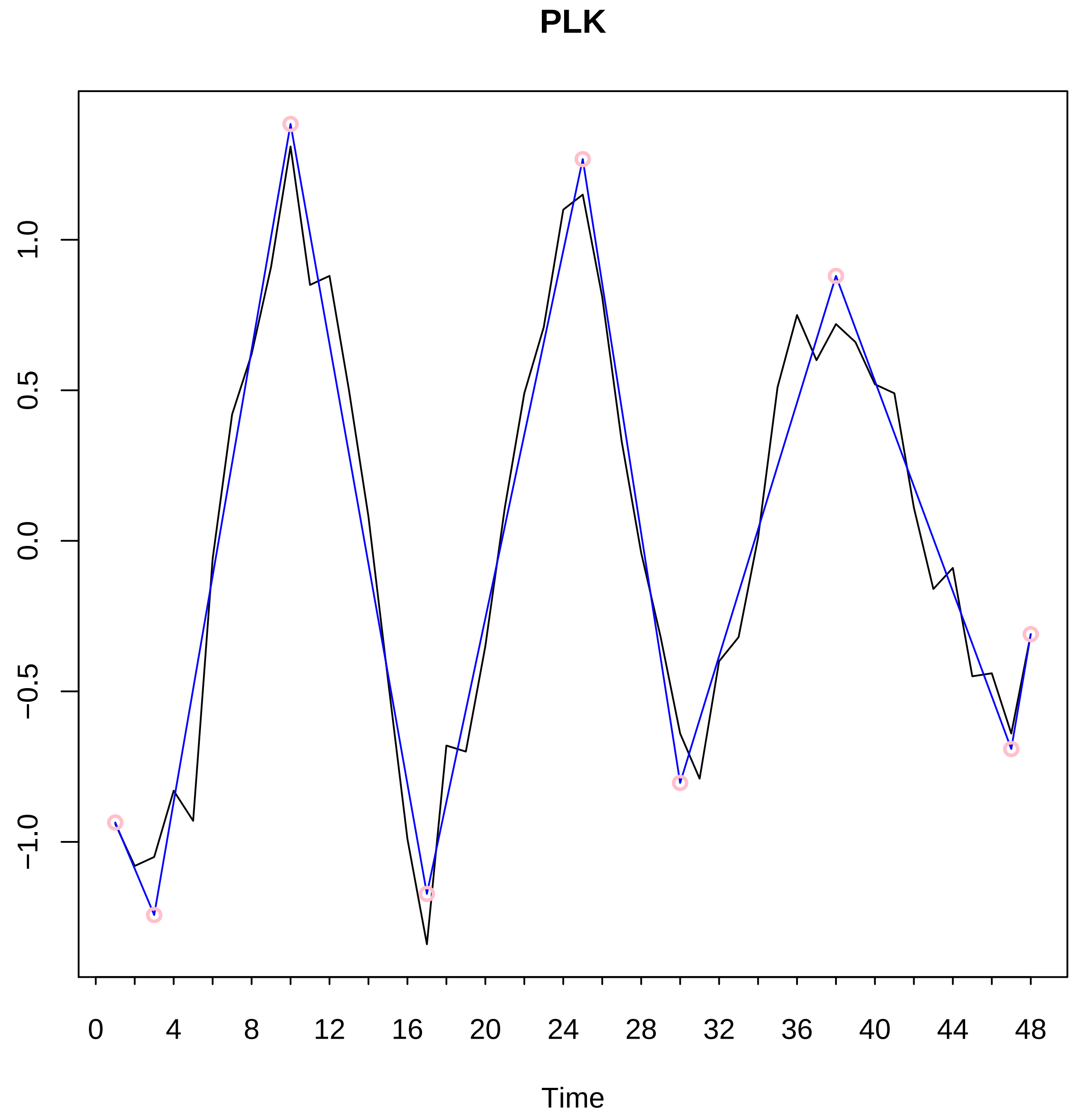
PLK
1.0
0.5
0.0
−0.5
−1.0
0
4
8
12
16
20
24
28
32
36
40
44
48
Time

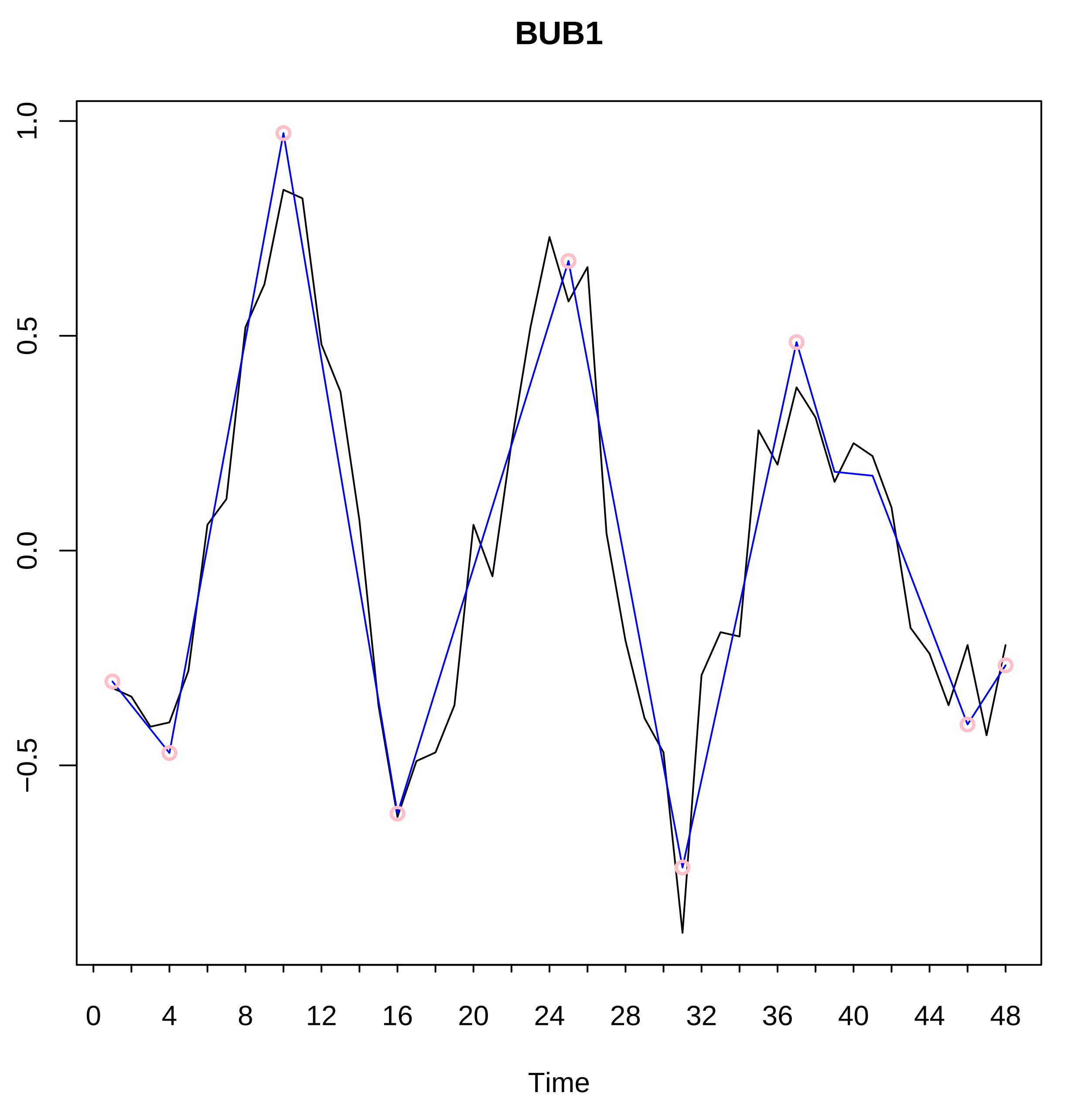
BUB1
1.0
0.5
0.0
−0.5
0
4
8
12
16
20
24
28
32
36
40
44
48
Time

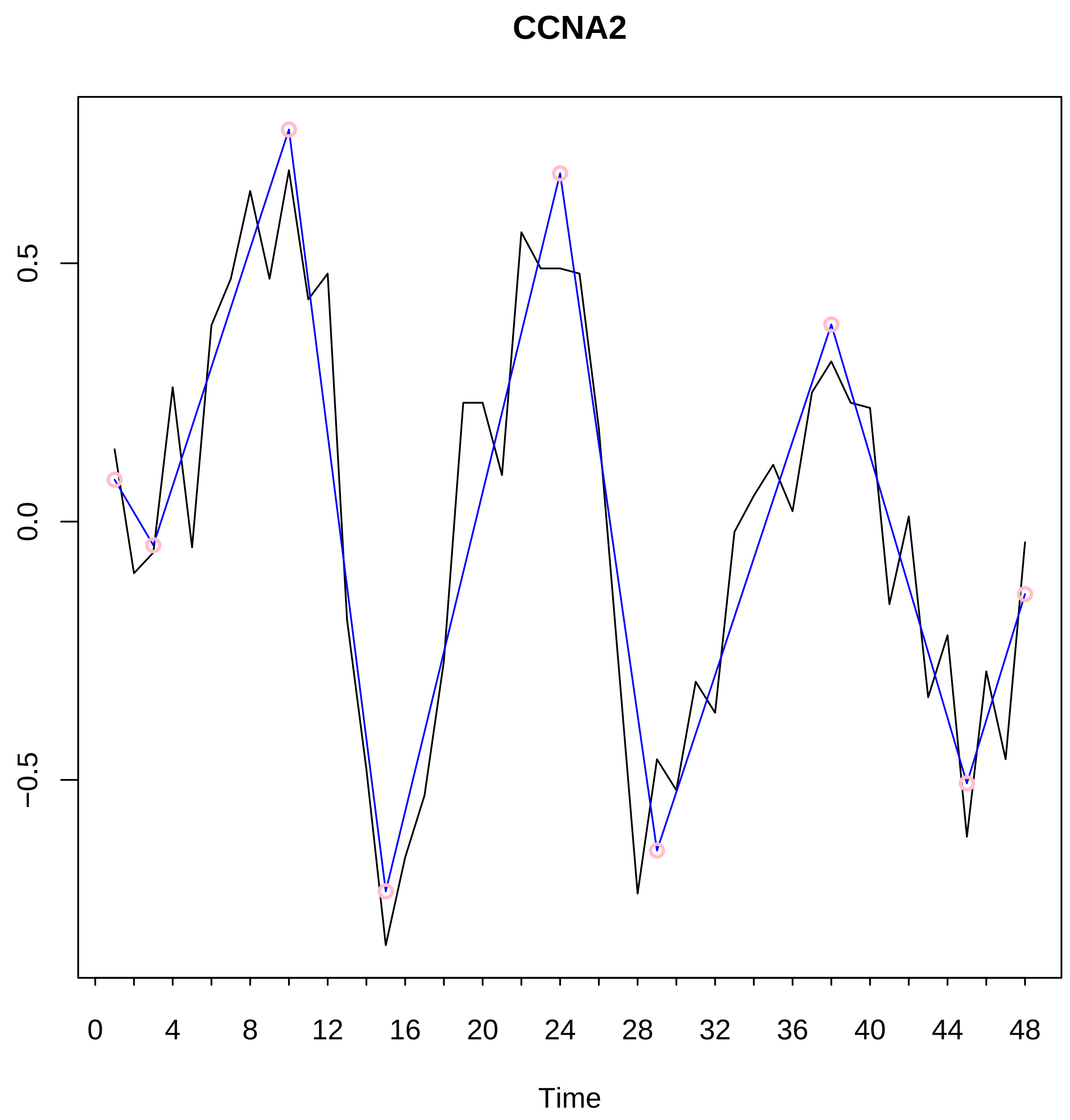

CCNA2
0.5
0.0
−0.5
0
4
8
12
16
20
24
28
32
36
40
44
48
Time

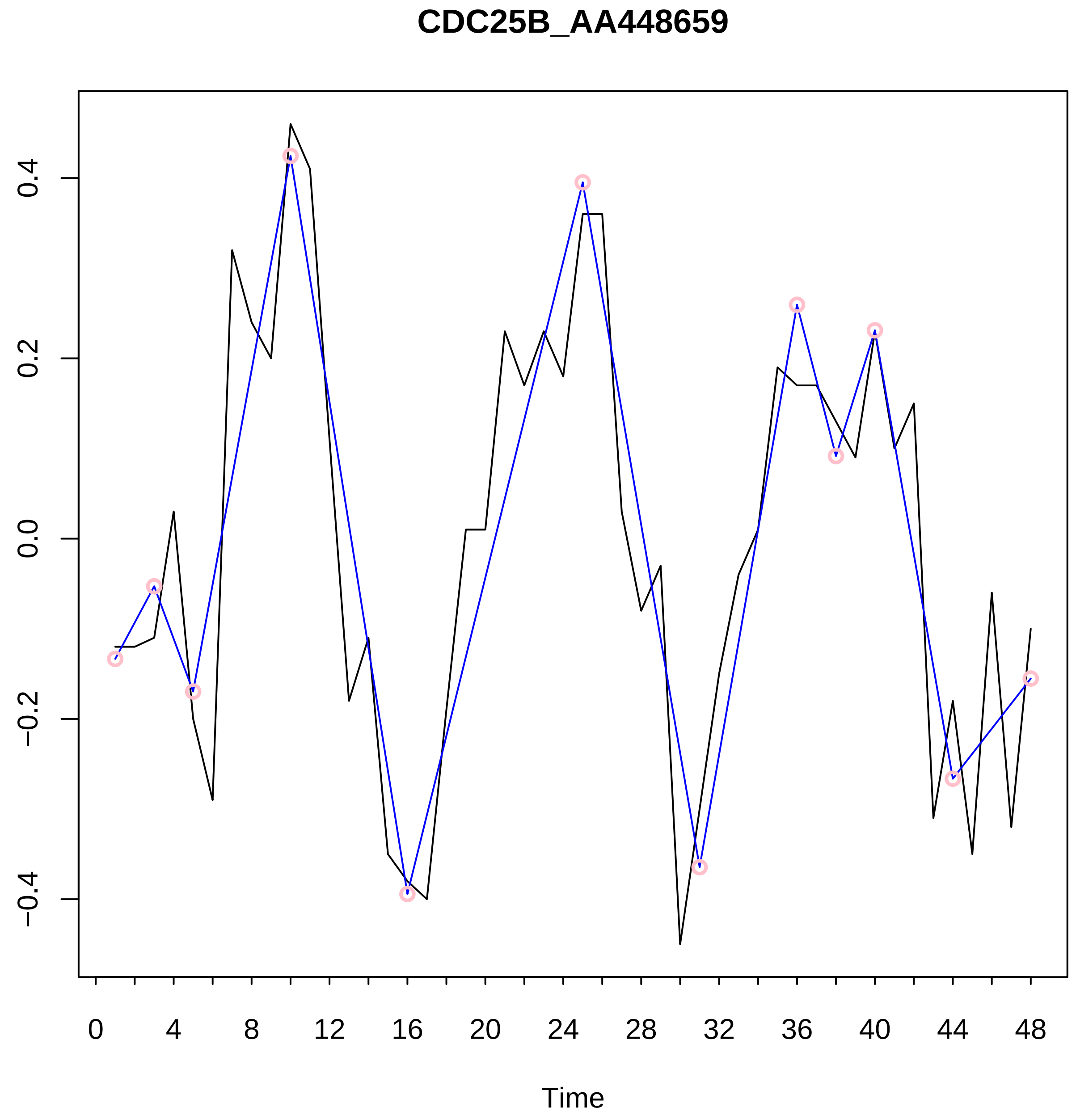

CDC25B_AA448659
0.4
0.2
0.0
−0.2
−0.4
0
4
8
12
16
20
24
28
32
36
40
44
48
Time

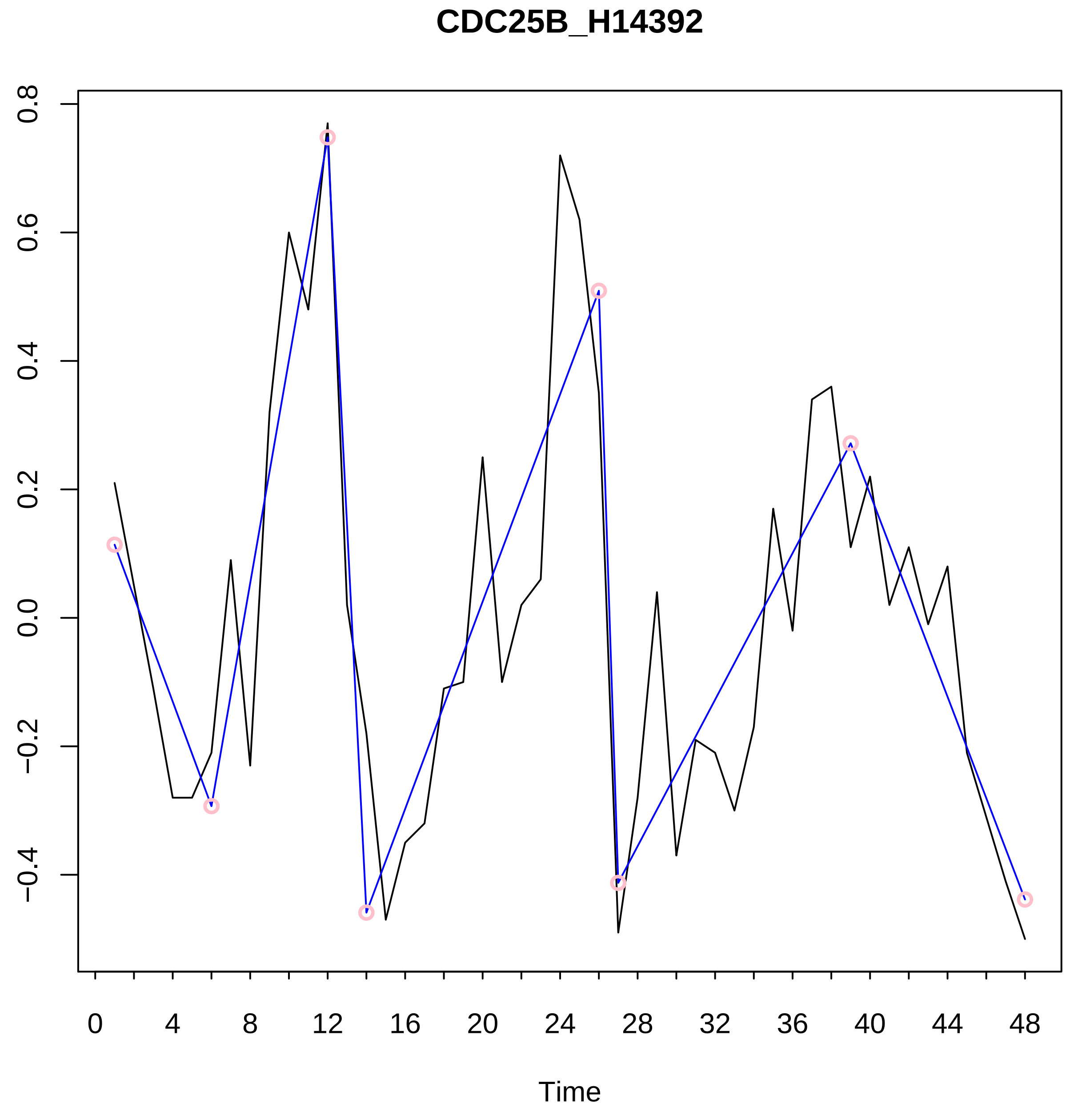

CDC25B_H14392
0.8
0.6
0.4
0.2
0.0
−0.2
−0.4
0
4
8
12
16
20
24
28
32
36
40
44
48
Time

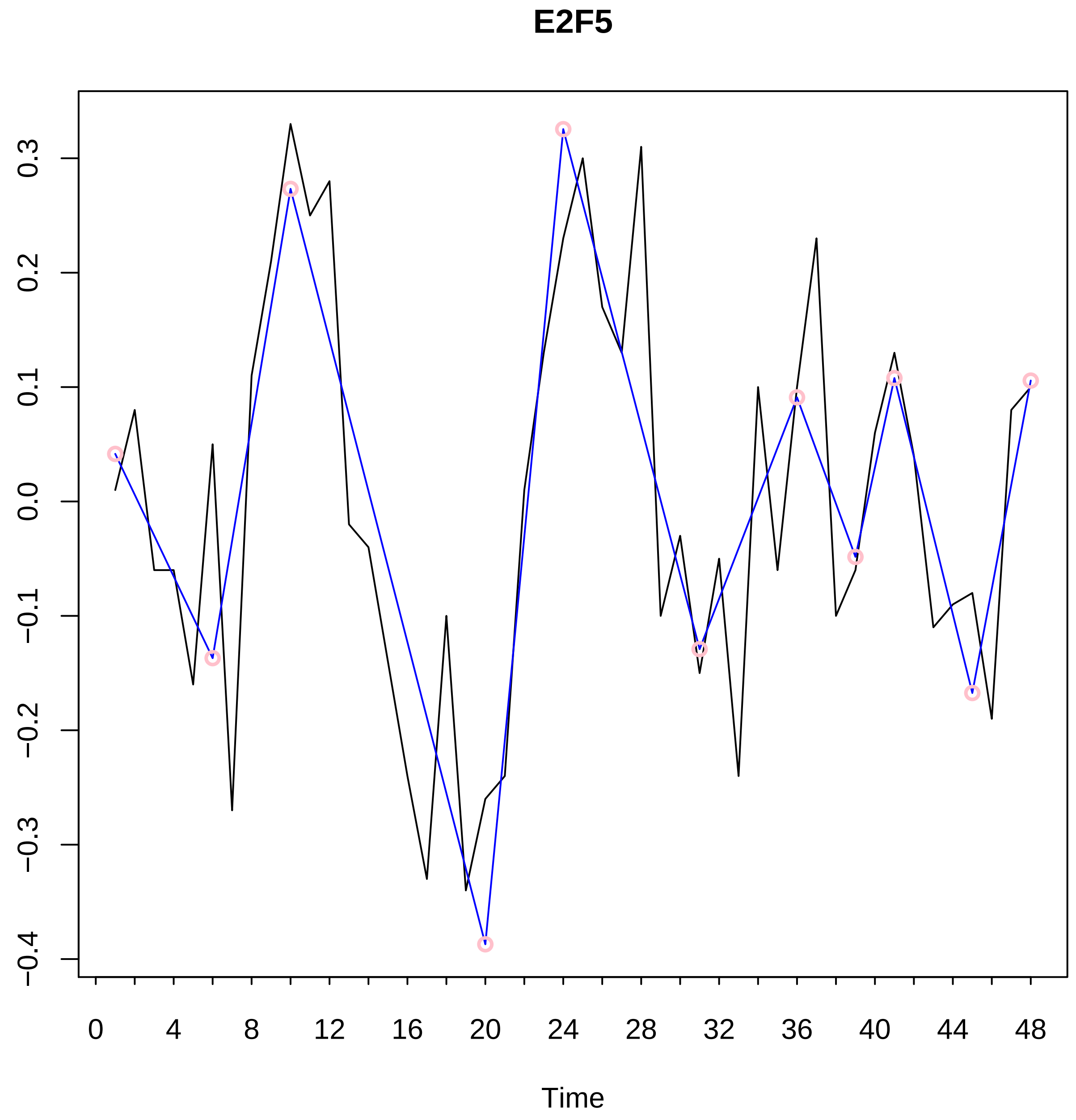

E2F5
0.3
0.2
0.1
0.0
−0.1
−0.2
−0.3
−0.4
0
4
8
12
16
20
24
28
32
36
40
44
48
Time

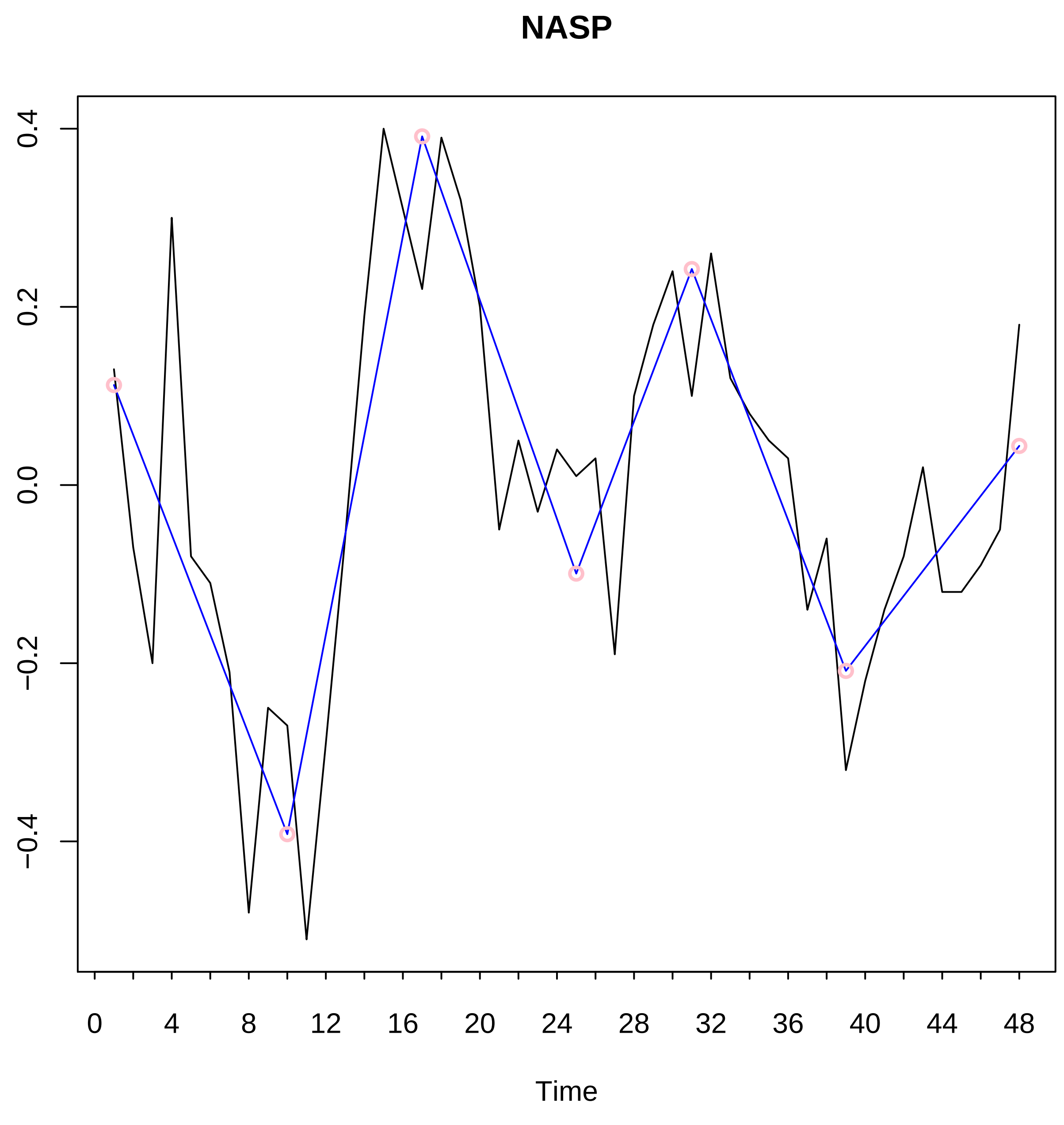
NASP
0.4
0.2
0.0
−0.2
−0.4
0
4
8
12
16
20
24
28
32
36
40
44
48
Time

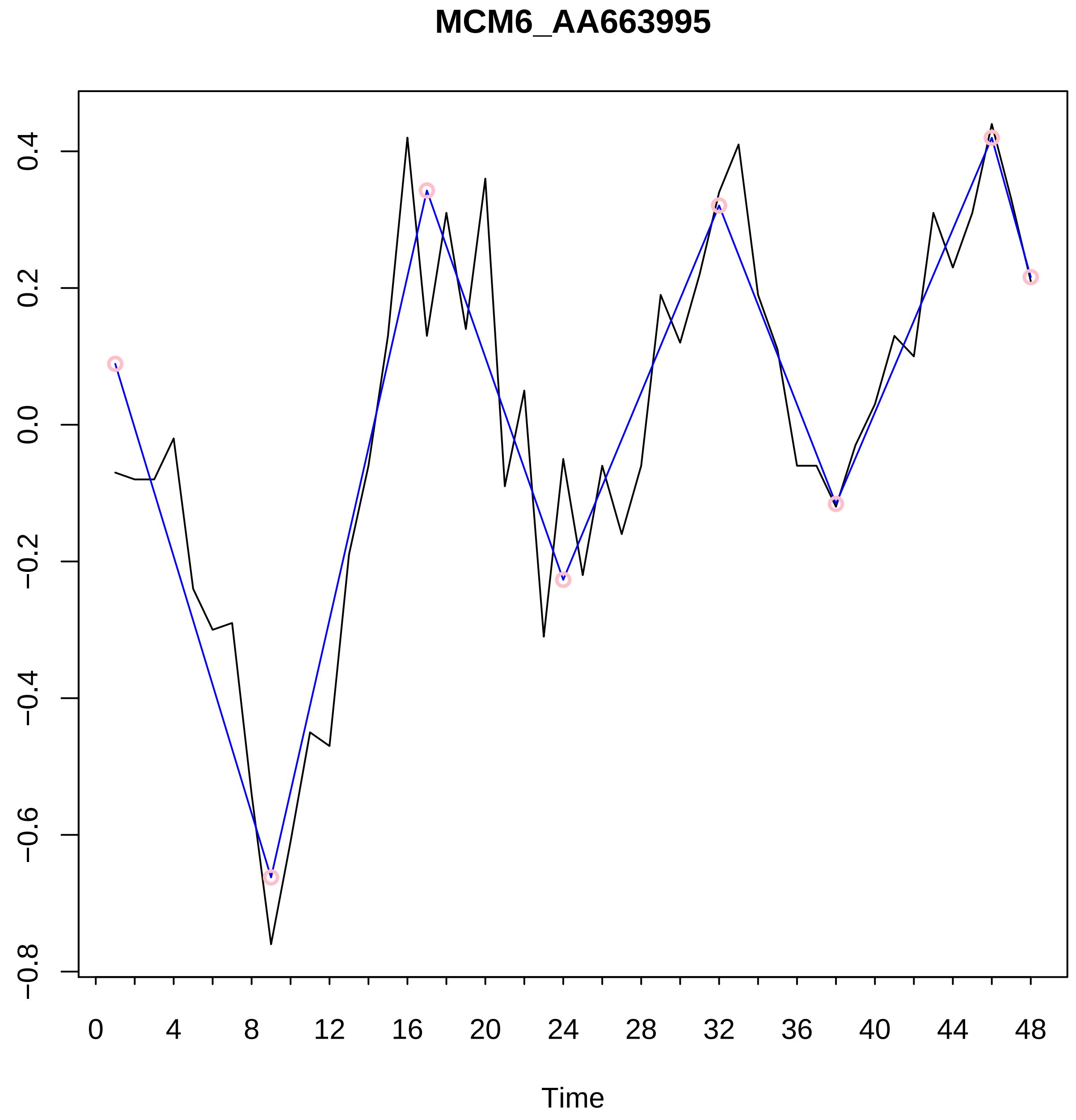
MCM6_AA663995
0.4
0.2
0.0
−0.2
−0.4
−0.6
−0.8
0
4
8
12
16
20
24
28
32
36
40
44
48
Time

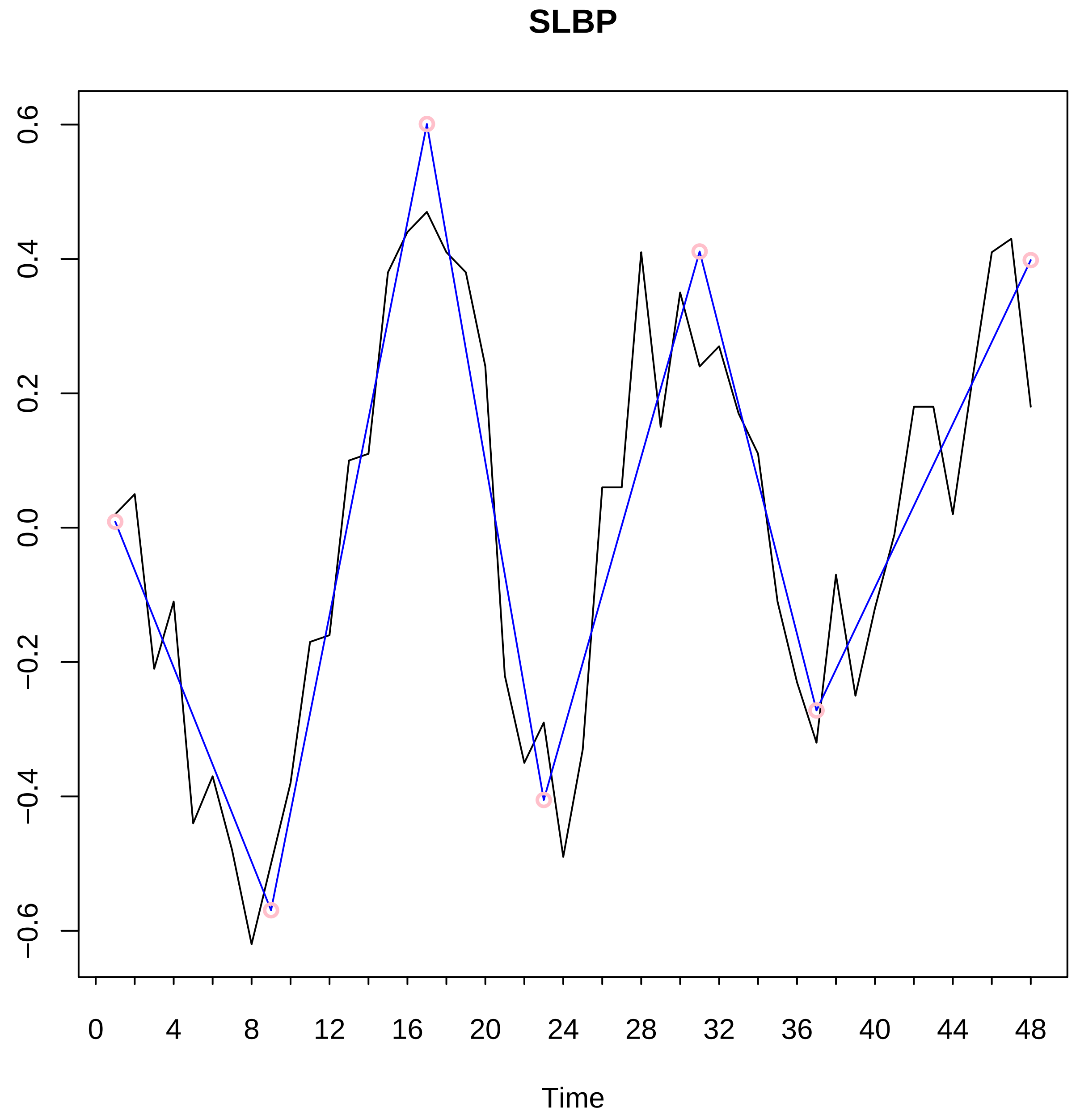
SLBP
0.6
0.4
0.2
0.0
−0.2
−0.4
−0.6
0
4
8
12
16
20
24
28
32
36
40
44
48
Time

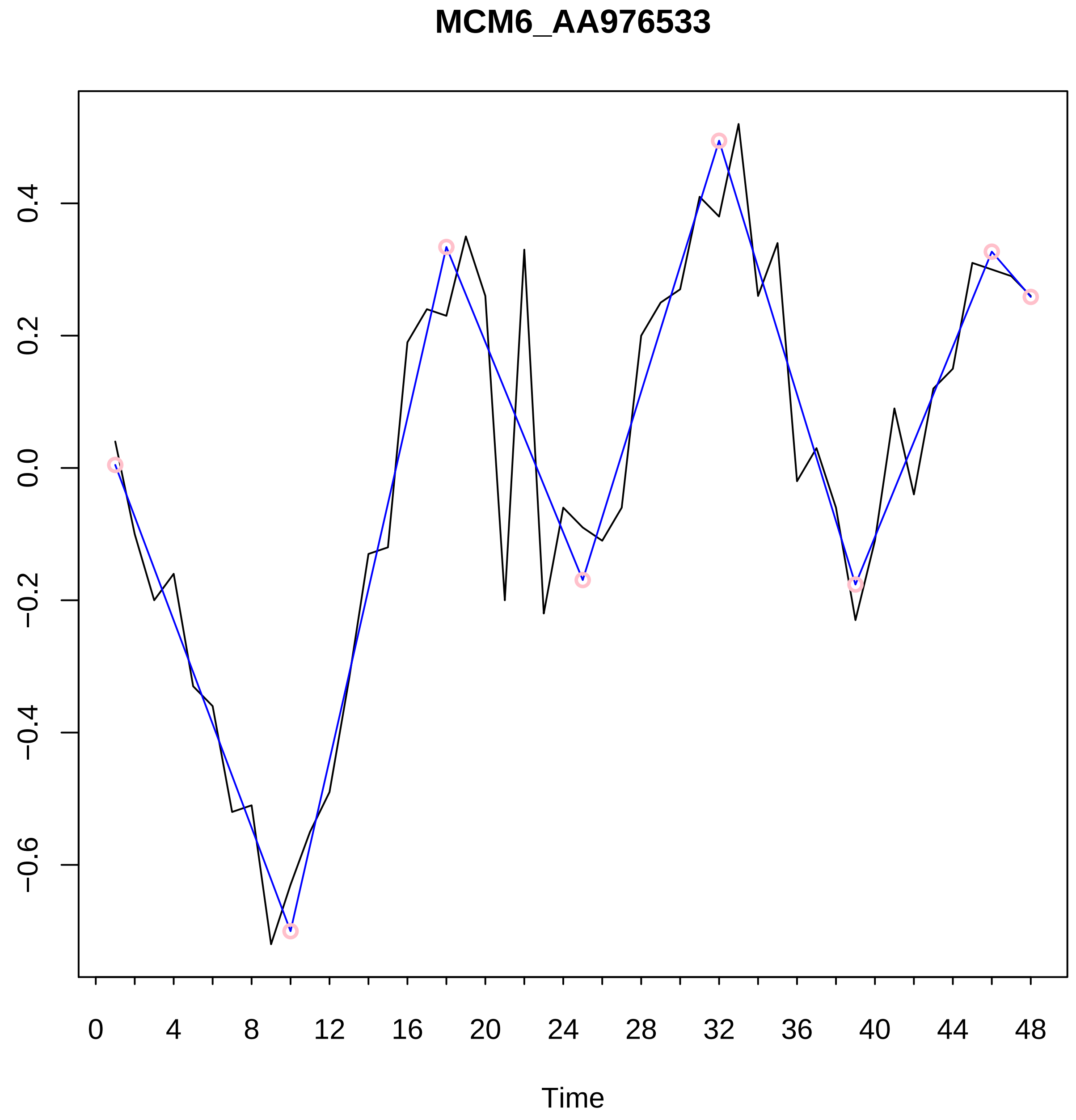
MCM6_AA976533
0.4
0.2
0.0
−0.2
−0.4
−0.6
0
4
8
12
16
20
24
28
32
36
40
44
48
Time

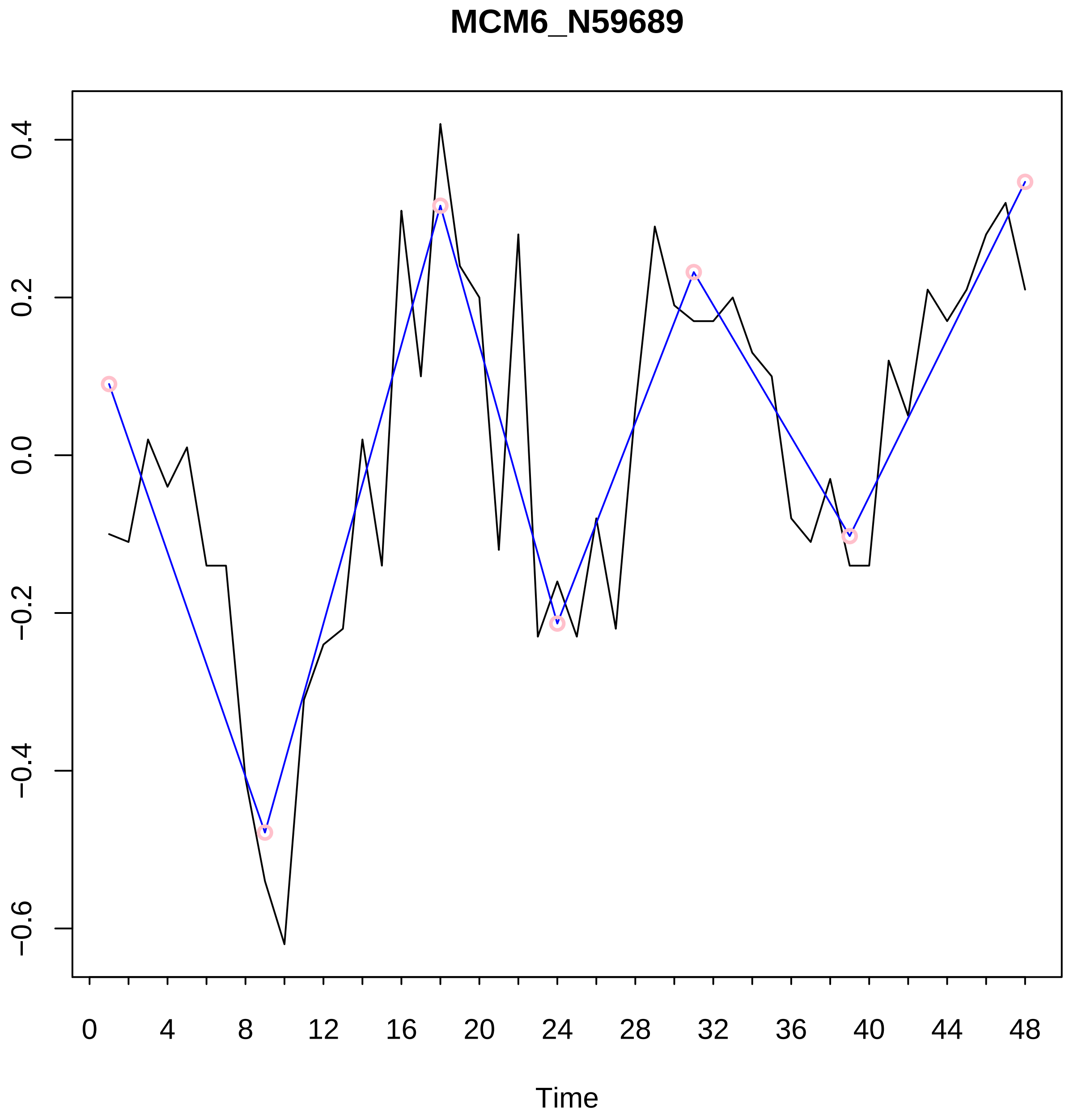

MCM6_N59689
0.4
0.2
0.0
−0.2
−0.4
−0.6
0
4
8
12
16
20
24
28
32
36
40
44
48
Time

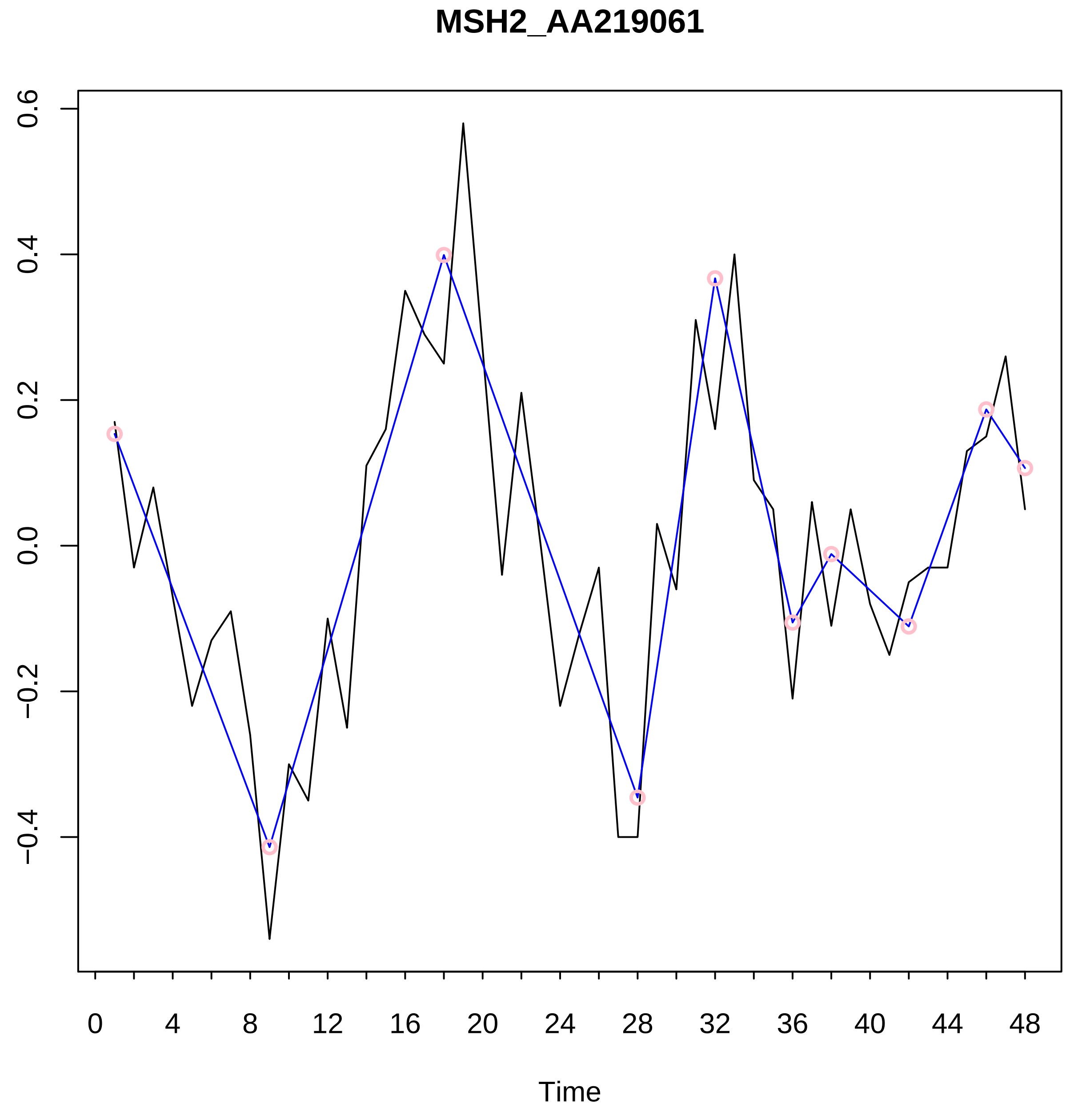
MSH2_AA219061
0.6
0.4
0.2
0.0
−0.2
−0.4
0
4
8
12
16
20
24
28
32
36
40
44
48
Time

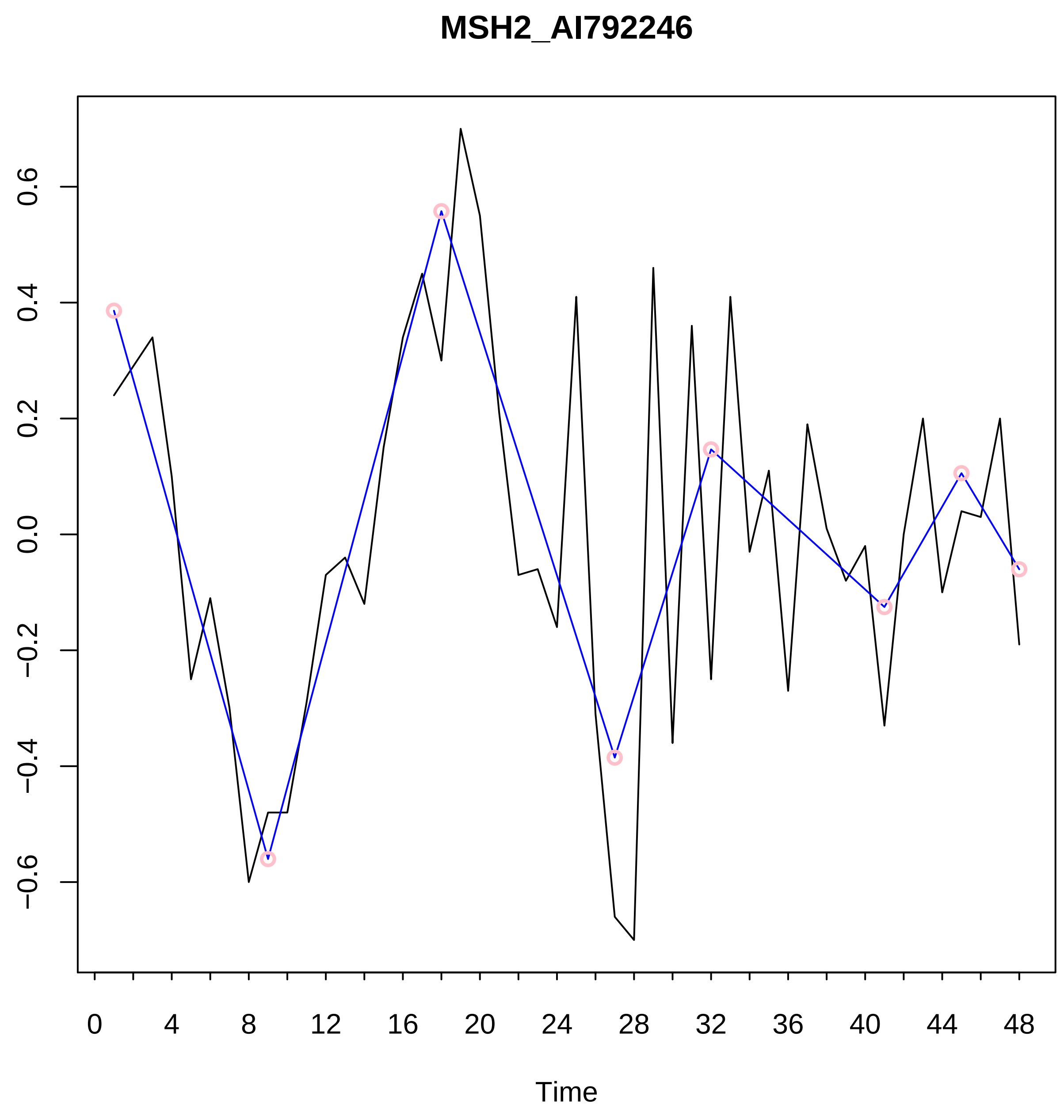
MSH2_AI792246
0.6
0.4
0.2
0.0
−0.2
−0.4
−0.6
0
4
8
12
16
20
24
28
32
36
40
44
48
Time

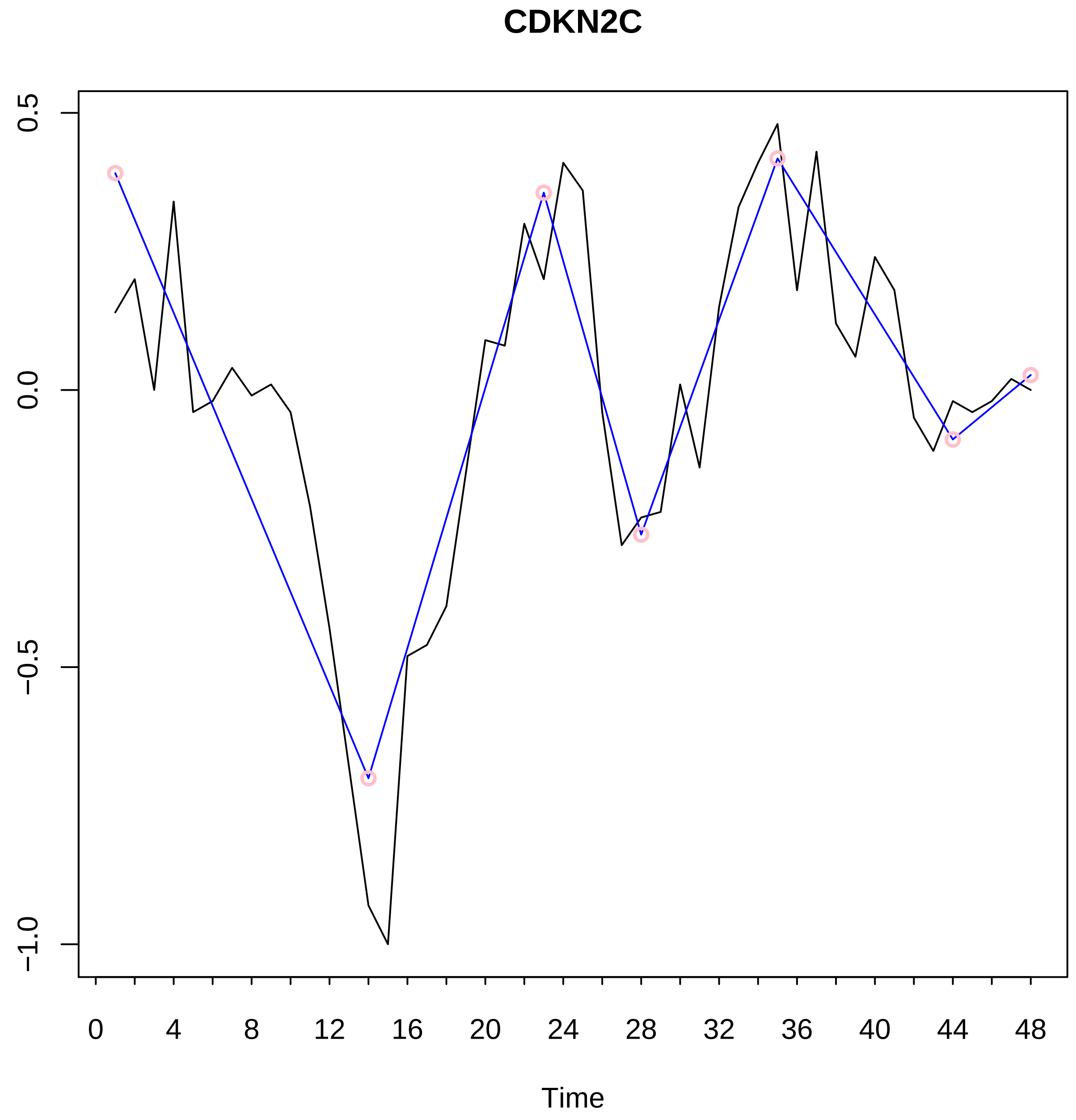
CDKN2C
0.5
0.0
−0.5
−1.0
0
4
8
12
16
20
24
28
32
36
40
44
48
Time

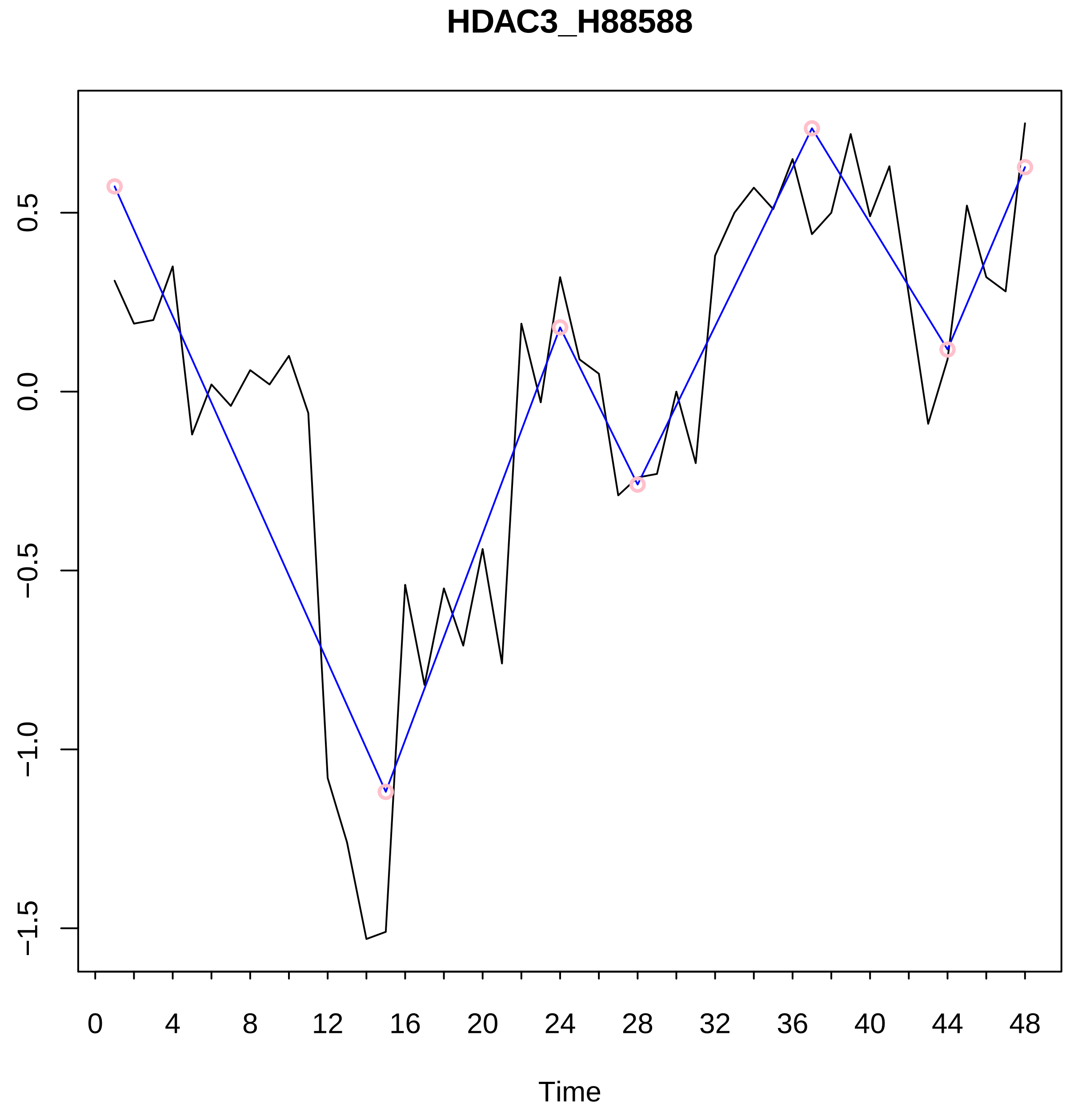
HDAC3_H88588
0.5
0.0
−0.5
−1.0
−1.5
0
4
8
12
16
20
24
28
32
36
40
44
48
Time

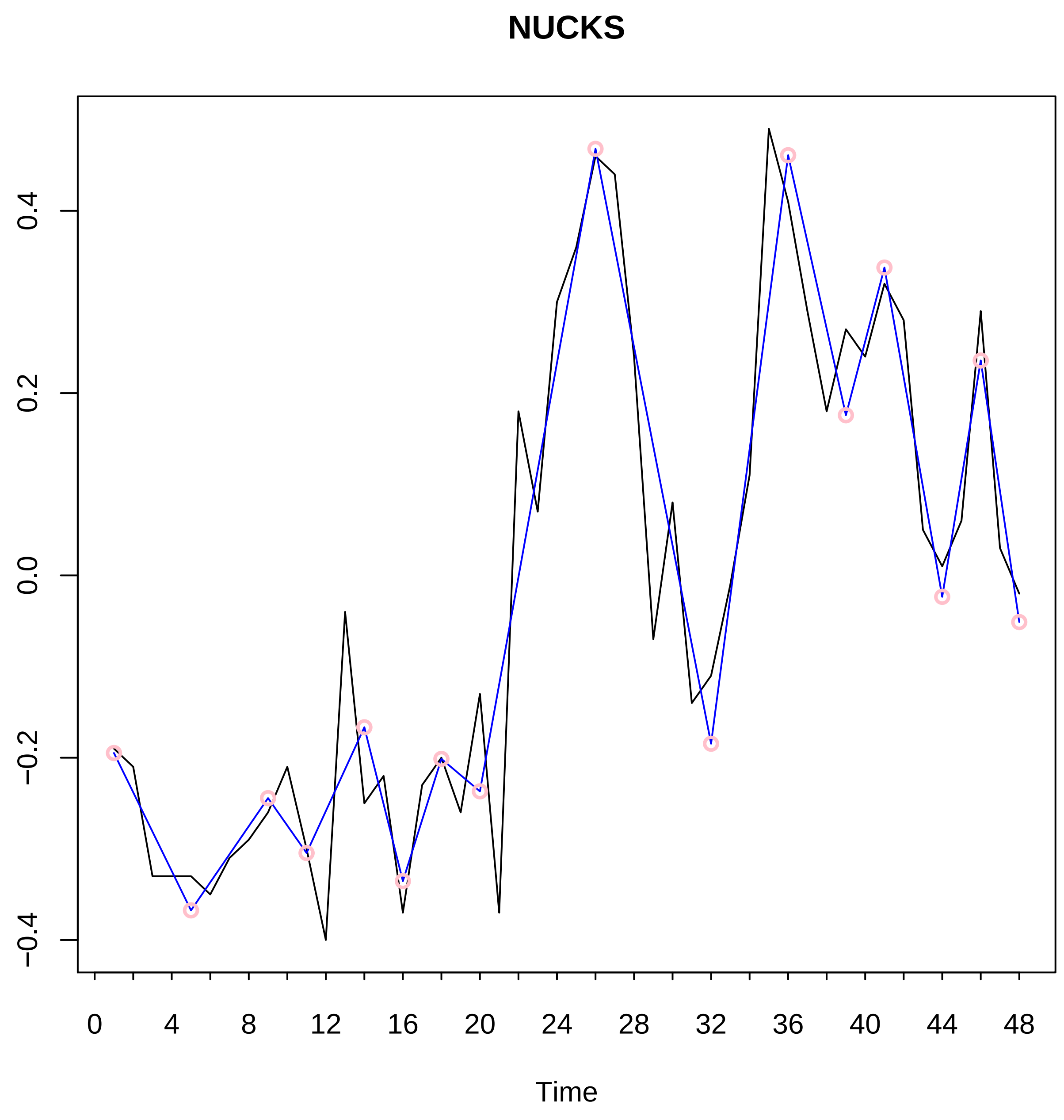
NUCKS
0.4
0.2
0.0
−0.2
−0.4
0
4
8
12
16
20
24
28
32
36
40
44
48
Time

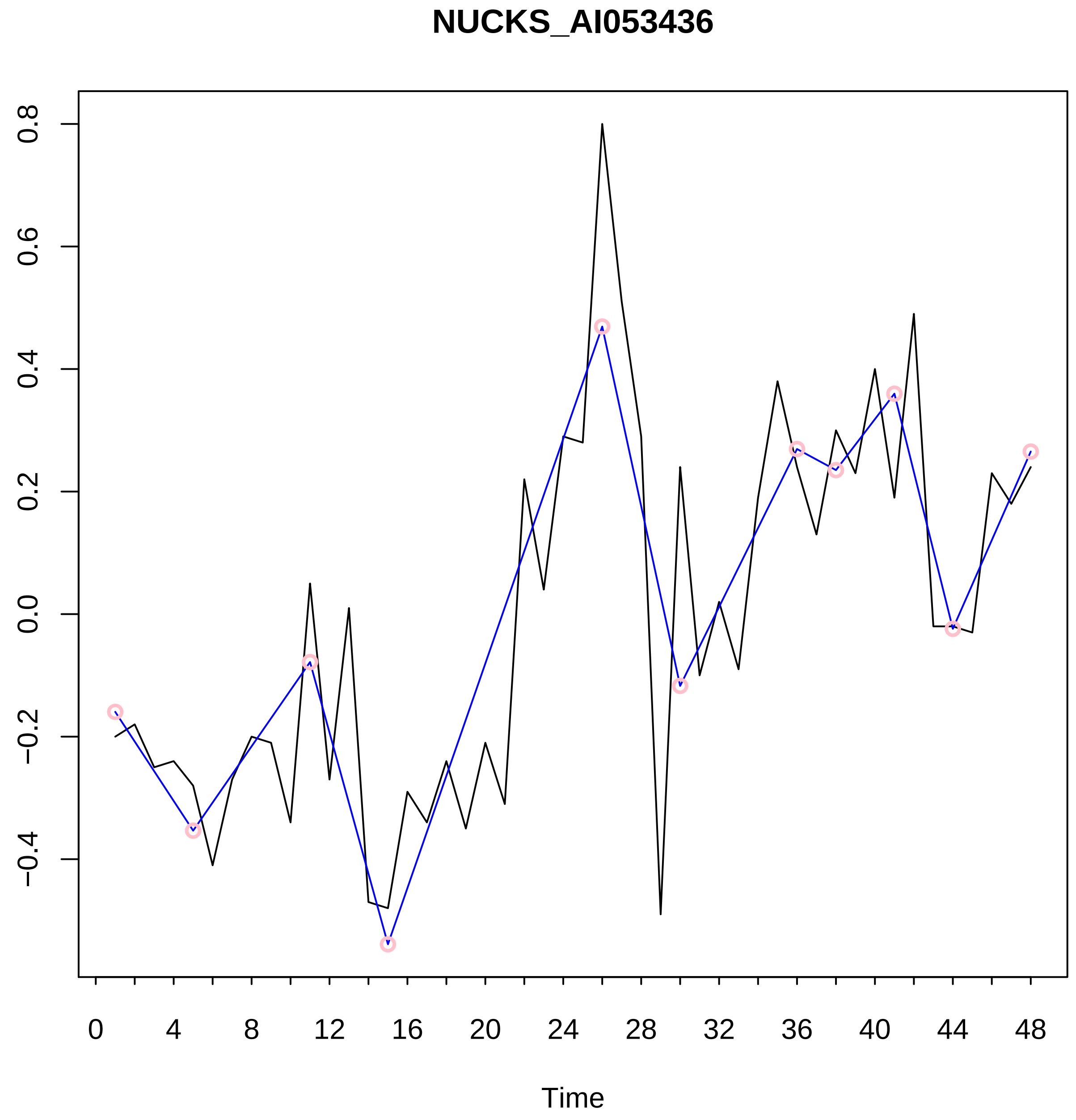
NUCKS_AI053436
0.8
0.6
0.4
0.2
0.0
−0.2
−0.4
0
4
8
12
16
20
24
28
32
36
40
44
48
Time

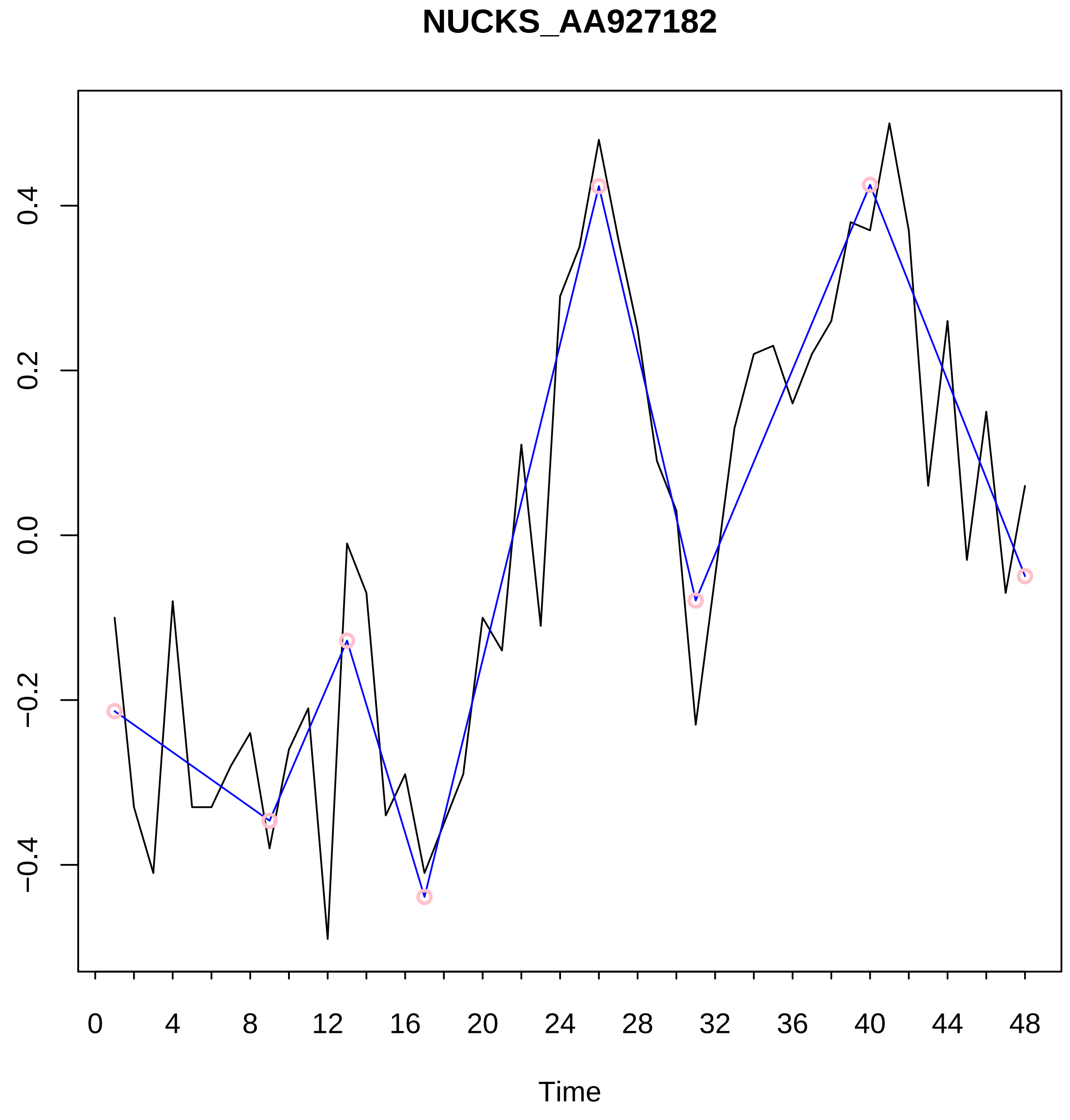
NUCKS_AA927182
0.4
0.2
0.0
−0.2
−0.4
0
4
8
12
16
20
24
28
32
36
40
44
48
Time
