# Supplementary material for: SwitchFinder – a novel method and query facility for discovering dynamic gene expression patterns
Source: BMC Bioinformatics. 2016 Dec 15;17:532. doi: 10.1186/s12859-016-1391-0 (PMC5160026; doi:10.1186/s12859-016-1391-0)

A_23_P206280 GPR56 16q13

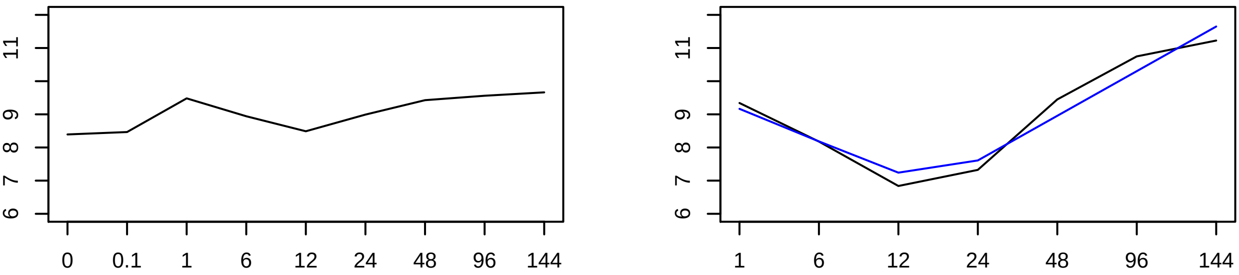

A_23_P211273 TMPRSS3 21q22.3

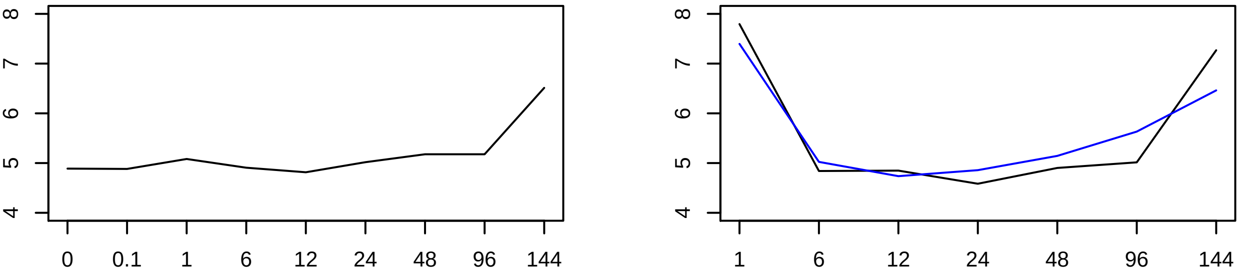

A_23_P24843 MICAL2 11p15.3

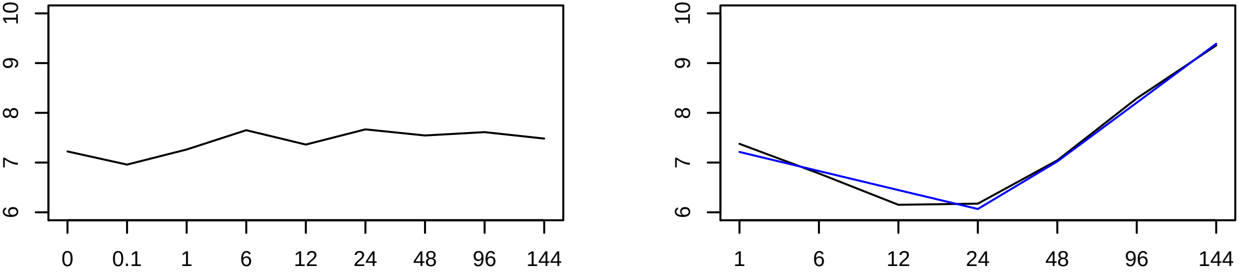

A_23_P206284 GPR56 16q13

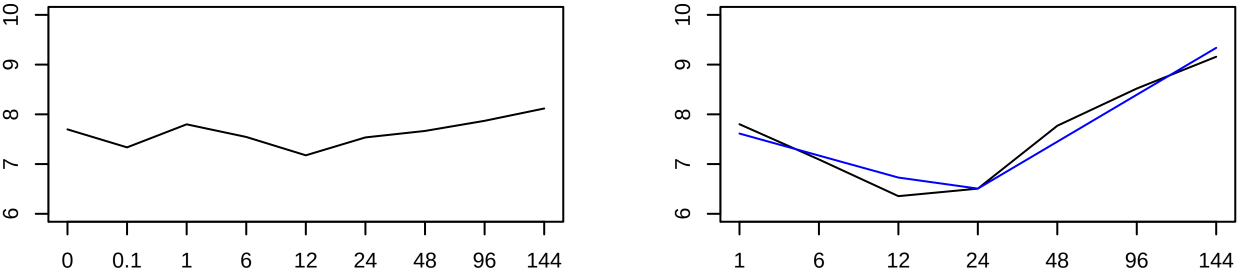

A_23_P65518 DACT1 14q23.1

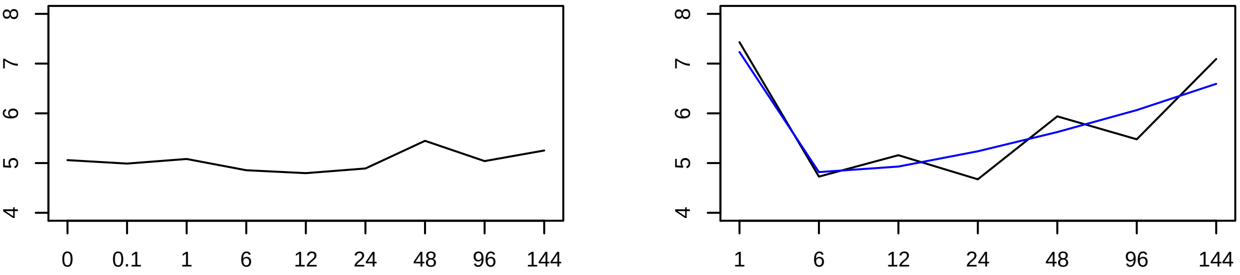

A_23_P70648 HTR1E 6q15

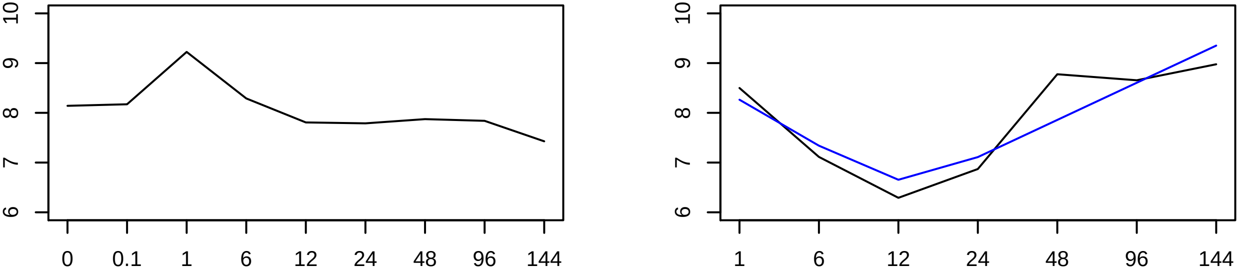

A_23_P5845 KHK 2p23.3

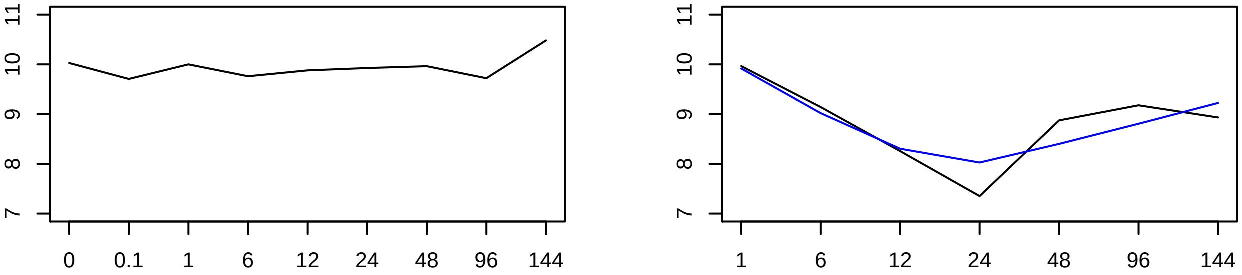

A_23_P255111 THC2545097 NA

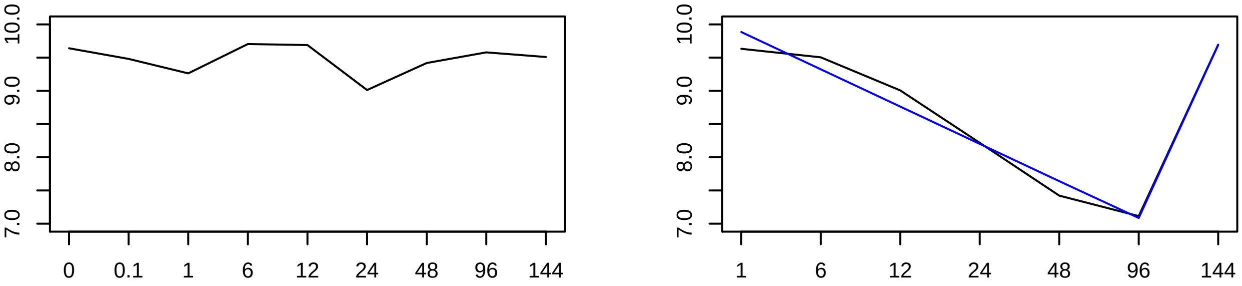

A_23_P52362 SLC18A3 10q11.23

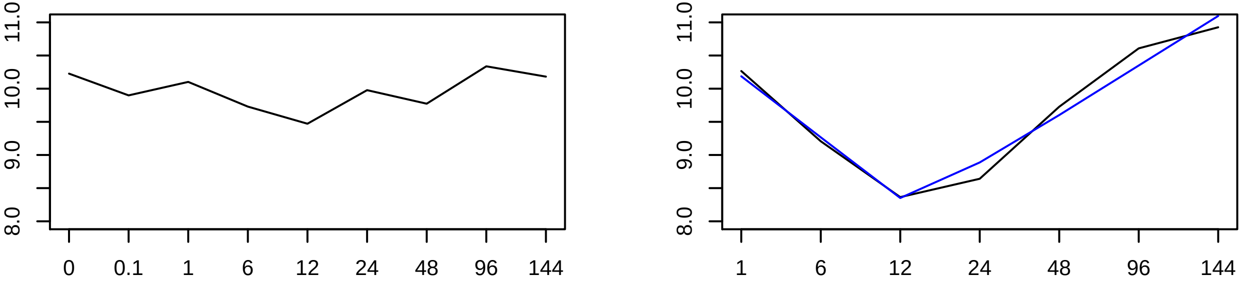

A_32_P170454 HRK 12q24.22

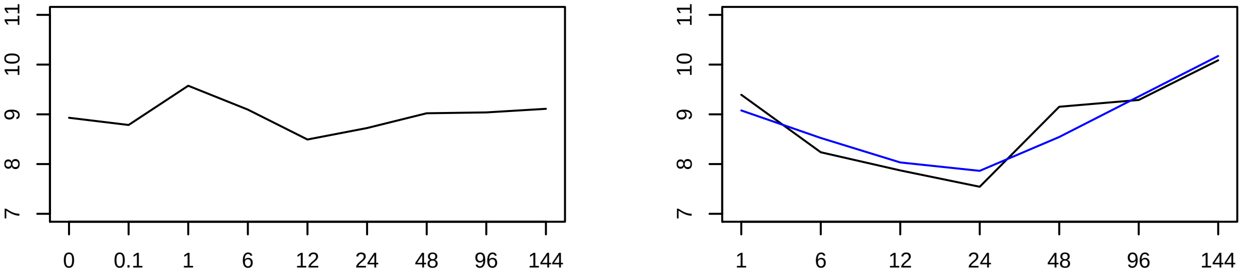

A_32_P117016 ALDH1L2 12q23.3

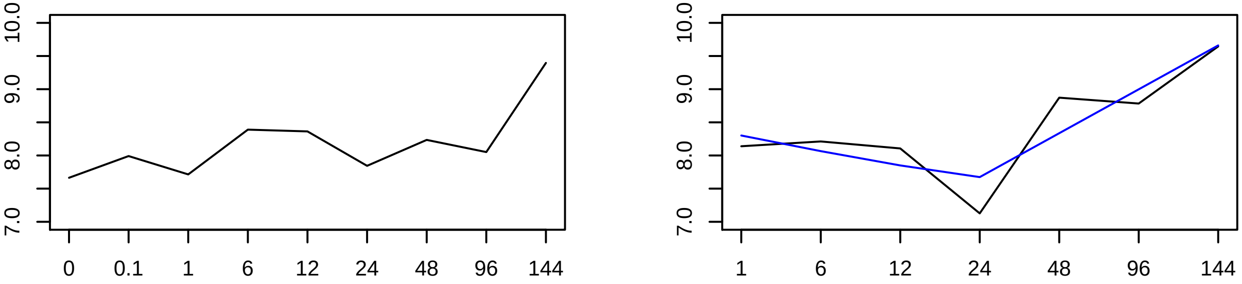

A_32_P29118 SEMA3D 7q21.11

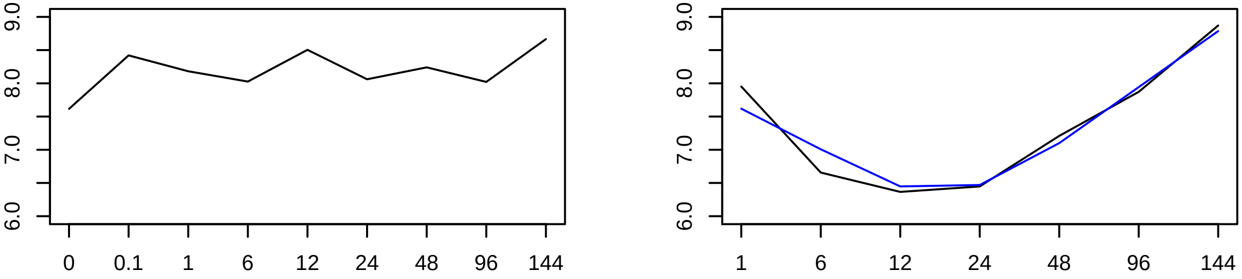

A_23_P140817 PARD6A 16q22.1

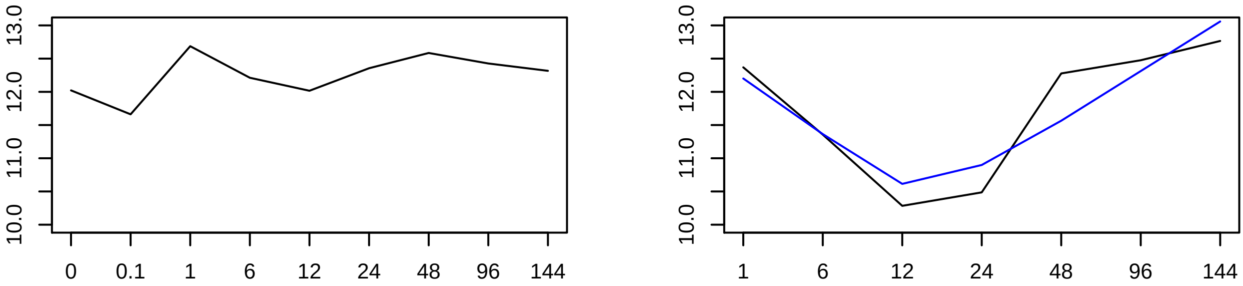

A_23_P126613 AQP10 1q21.3

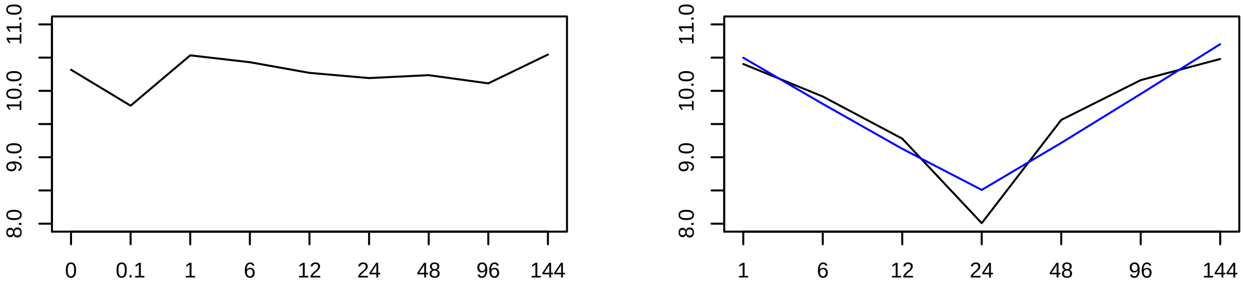

A_23_P215060 PODXL 7q32.3

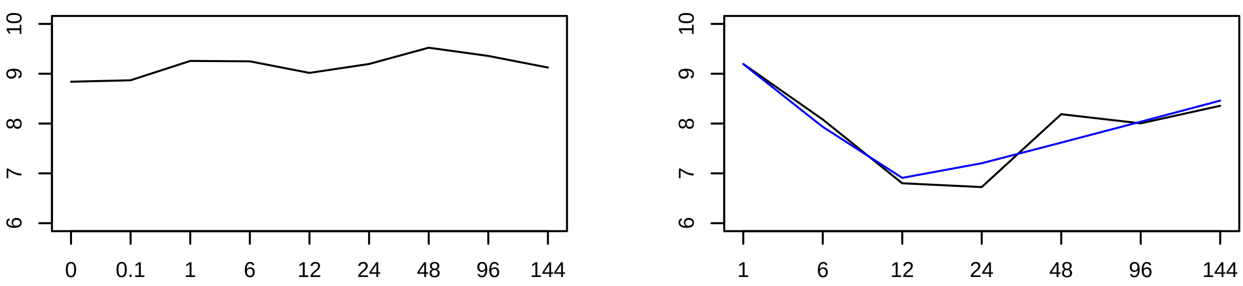

A_23_P32454 TG 8q24.22

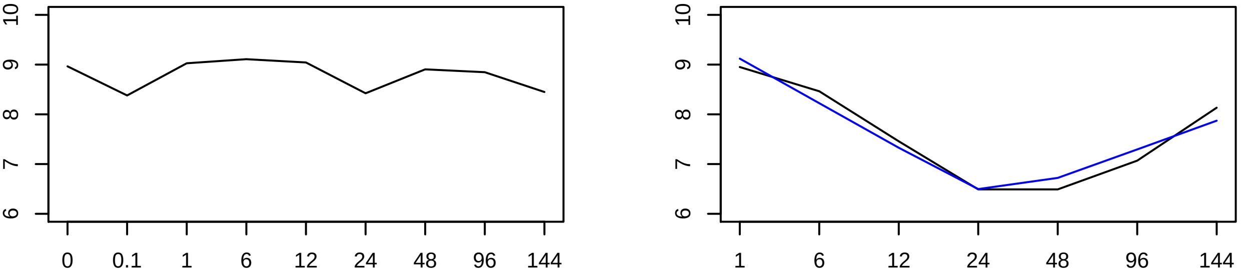

A_23_P65678 FBN1 15q21.1

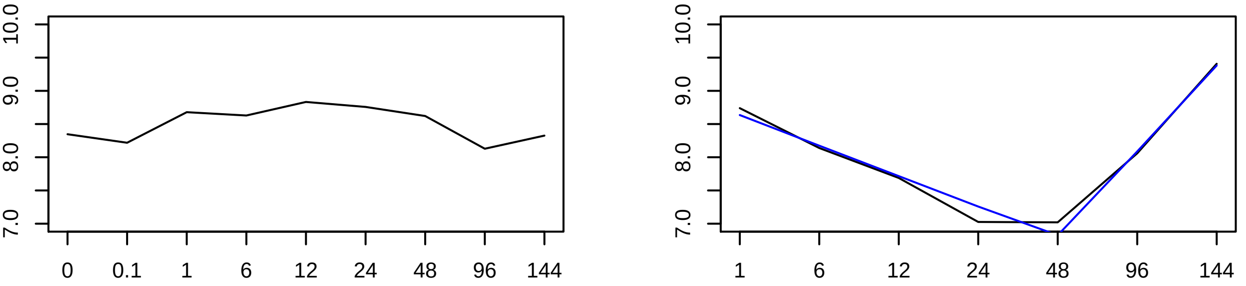

A_23_P138706 ADRA2A 10q25.2

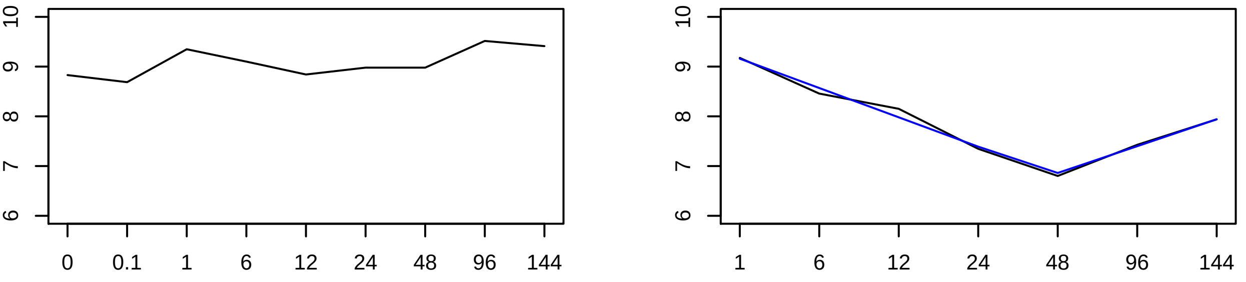

A_23_P128174 RAB3IP 12q15

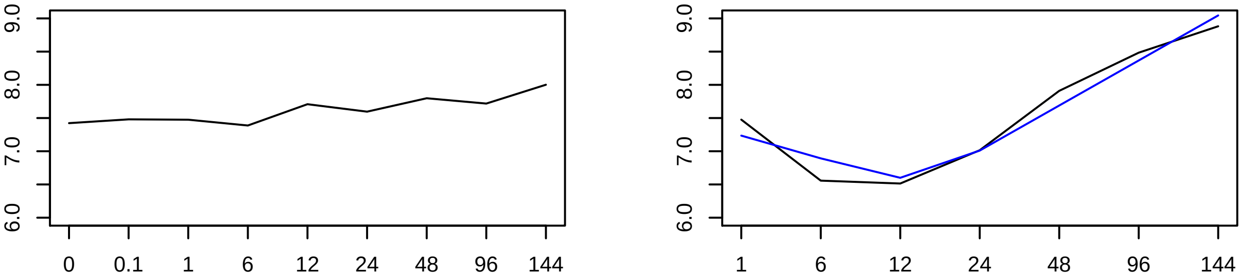

A_32_P24585 SH3PXD2B 5q35.1

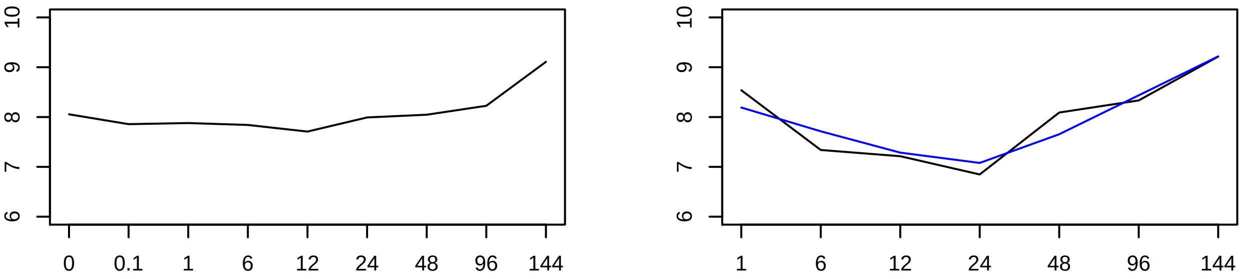

A_23_P211468 TIMP3 22q12.3

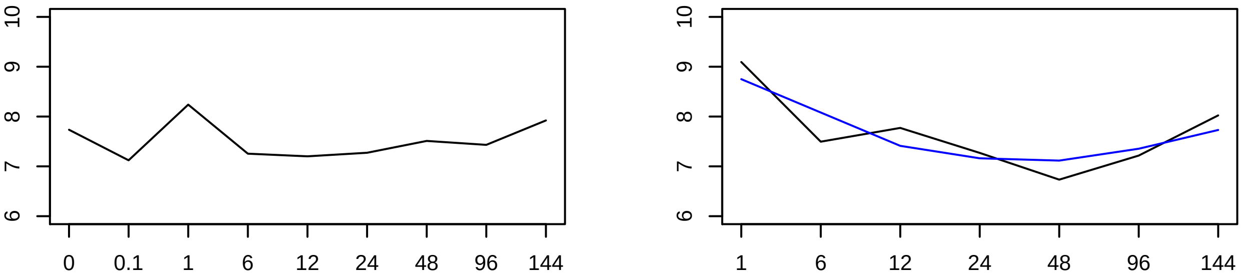

A_32_P47538 BC037919 NA

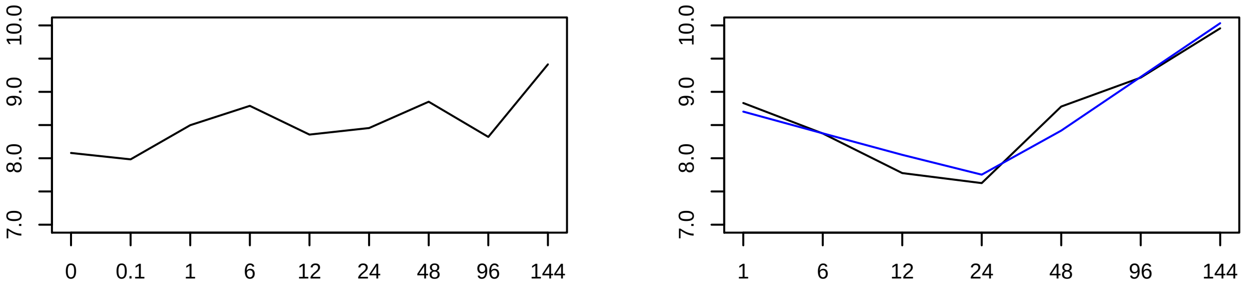

A_32_P168605 PCDH10 4q28.3

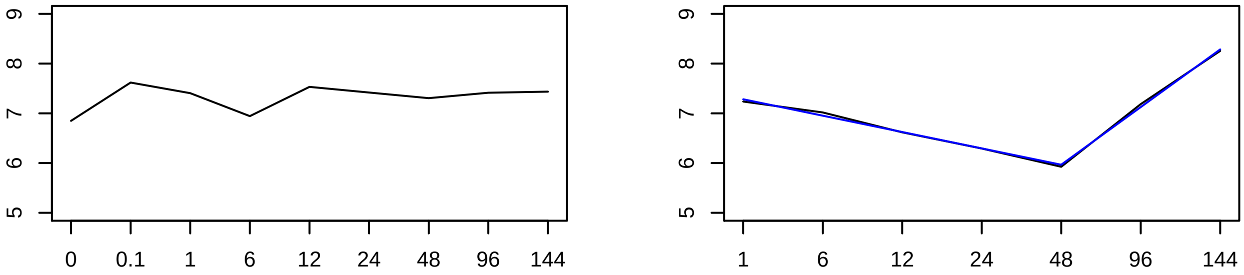

A_23_P131676 CXCR7 2q37.3

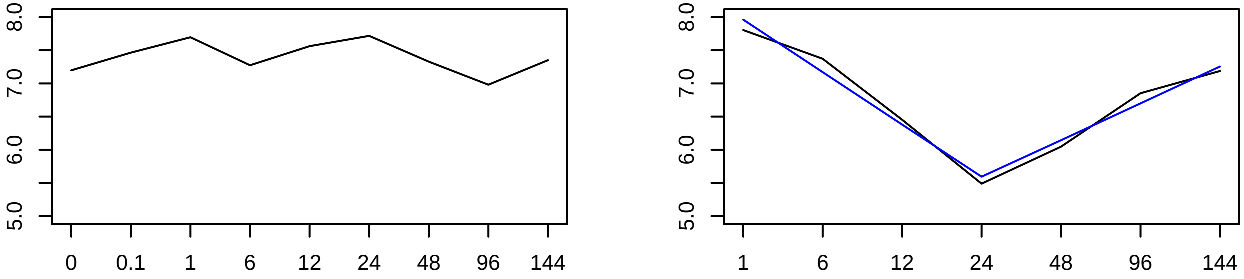

A_23_P119337 ATF5 19q13.33

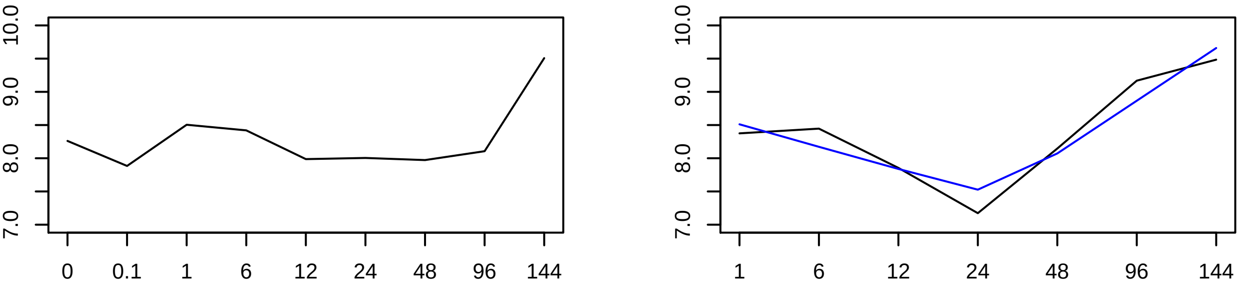

A_23_P349966 TMEM130 7q22.1

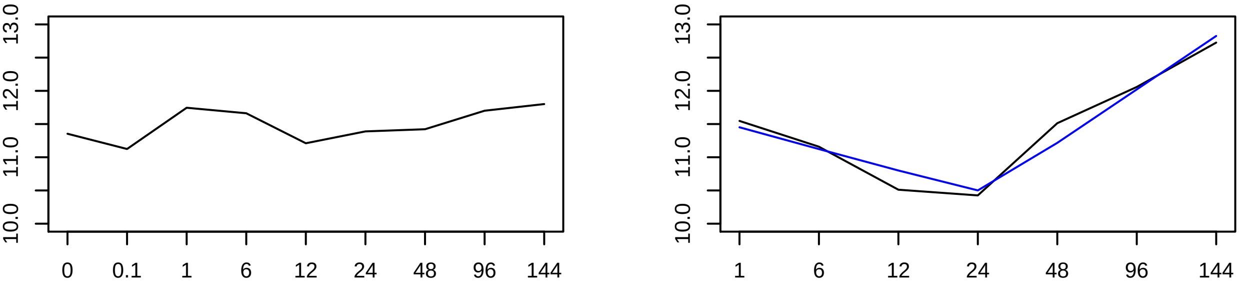

A_23_P376497 IGFBP5 2q35

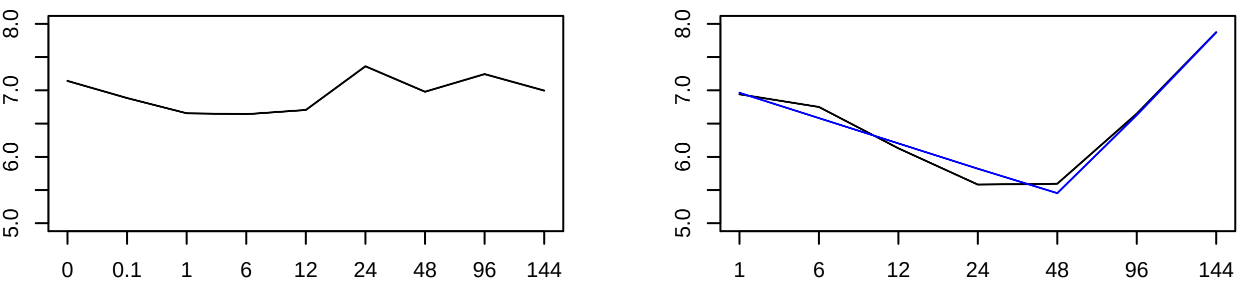

A_23_P382775 BBC3 19q13.32

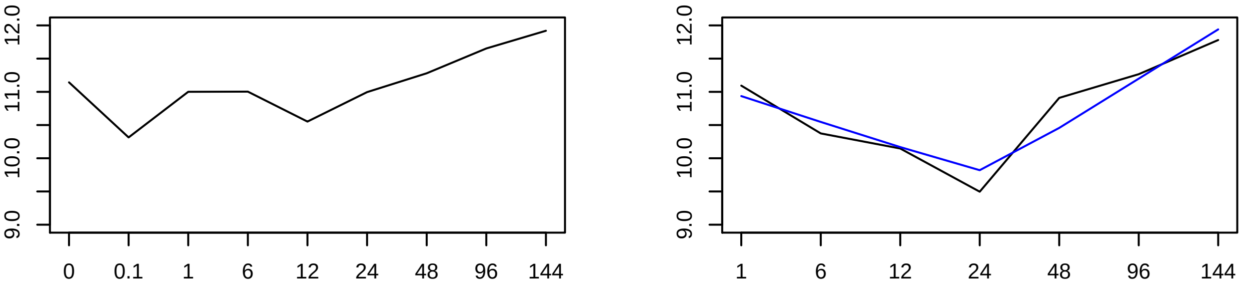

A_23_P819 ISG15 1p36.33

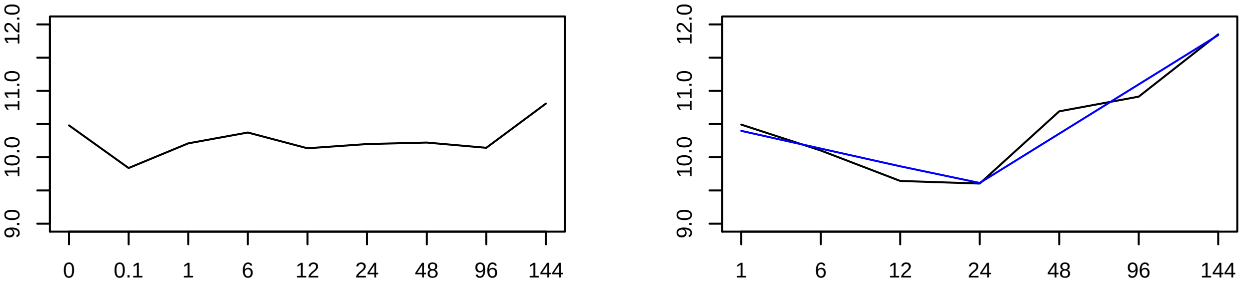

A_23_P128728 ARG2 14q24.1

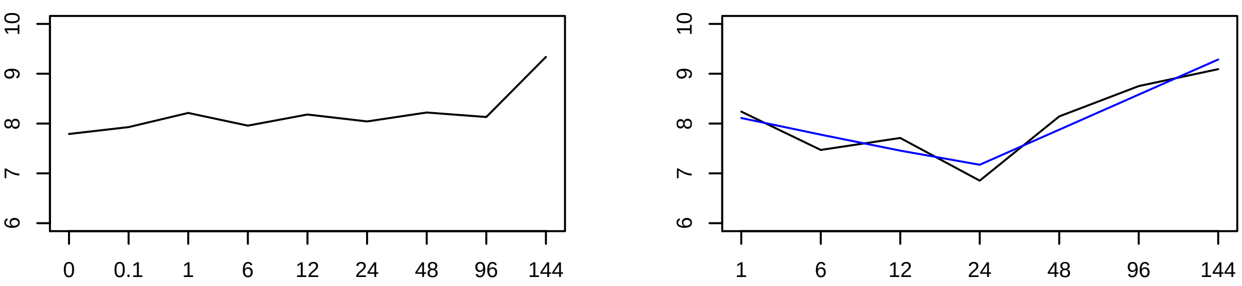

A_23_P32404 ISG20 15q26.1

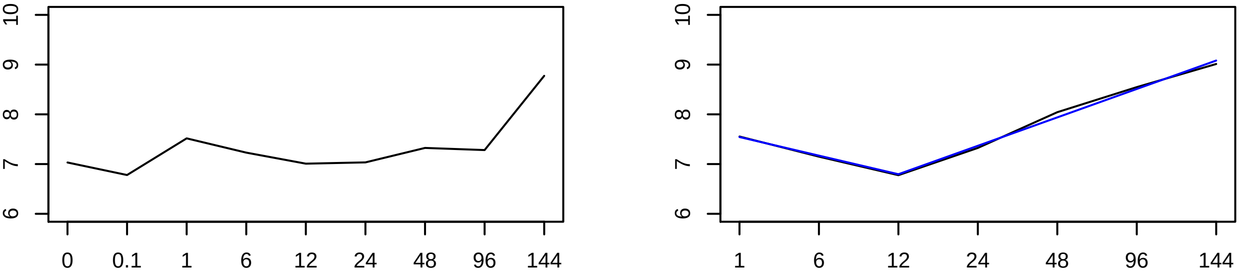

A_23_P46928 PFKP 10p15.2

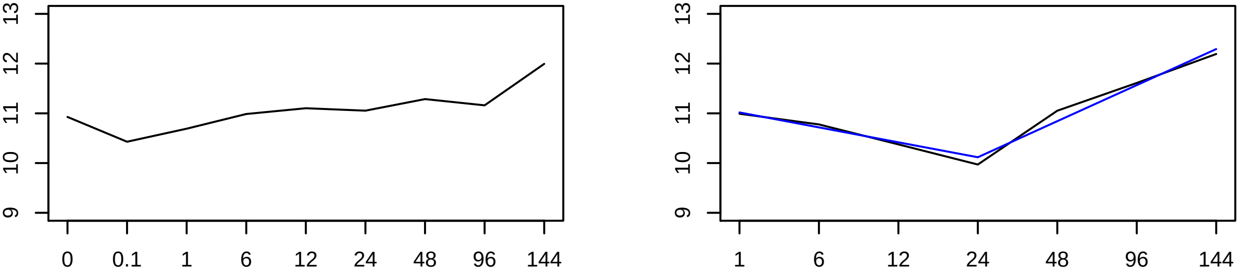

A_23_P141346 MPP3 17q21.31

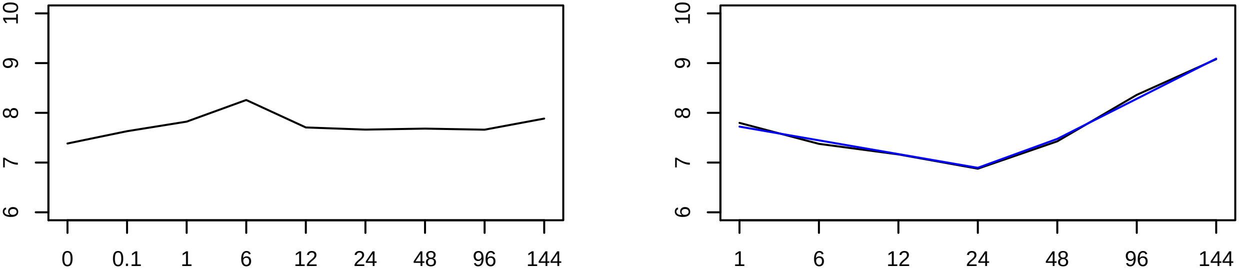

A_23_P257043 GEM 8q22.1

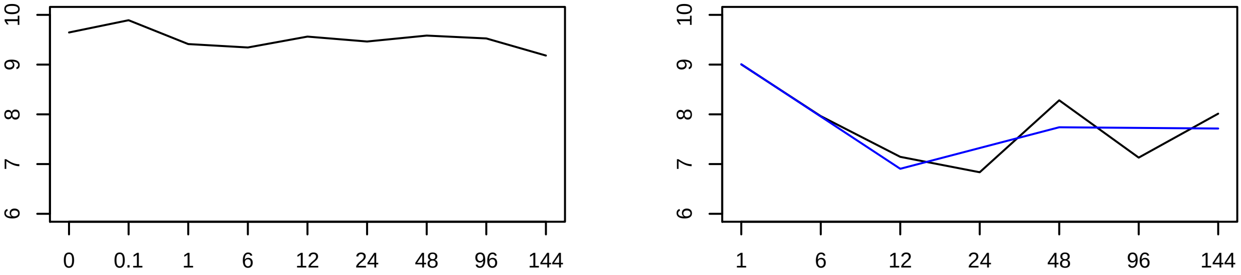

A_23_P345692 IL17D 13q12.11

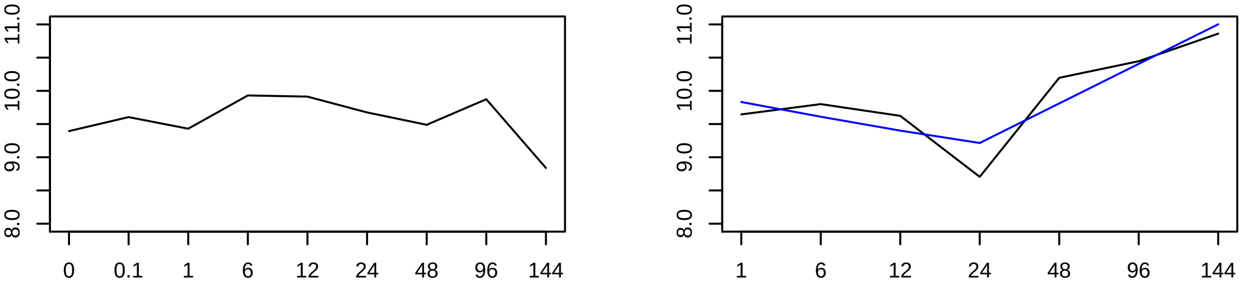

A_32_P119033 PLCXD3 5p13.1

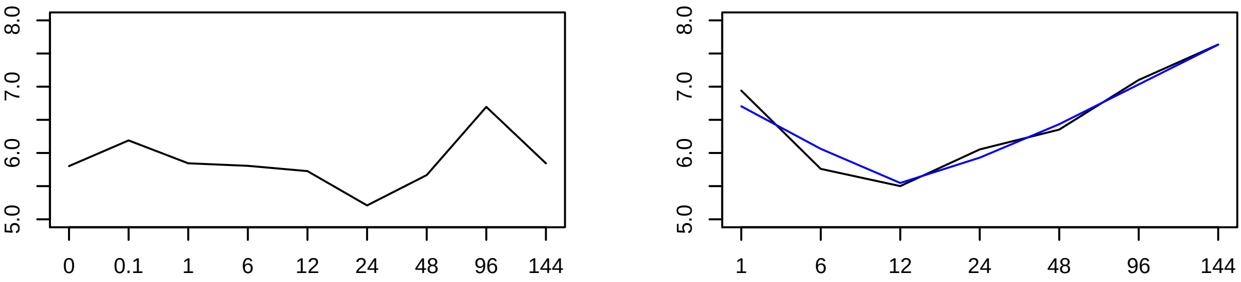

A_23_P76364 CD9 12p13.31

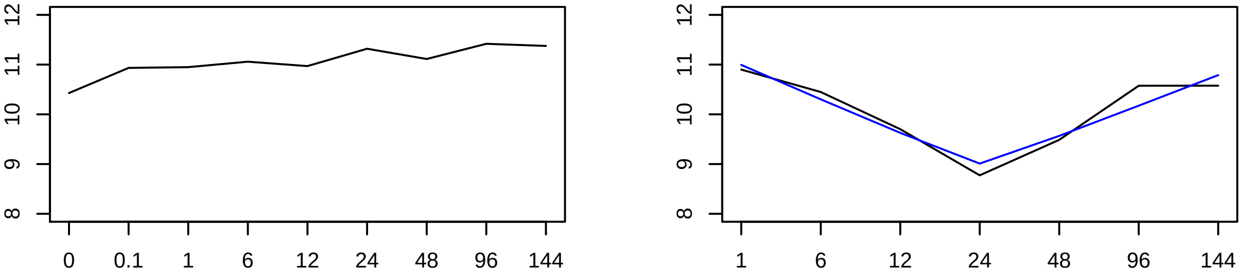

A_23_P110686 STC2 5q35.2

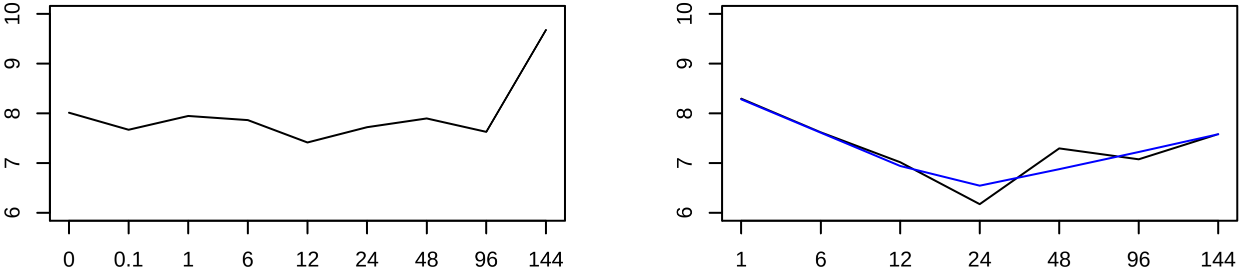

A_24_P24819 AGRIN 1p36.33

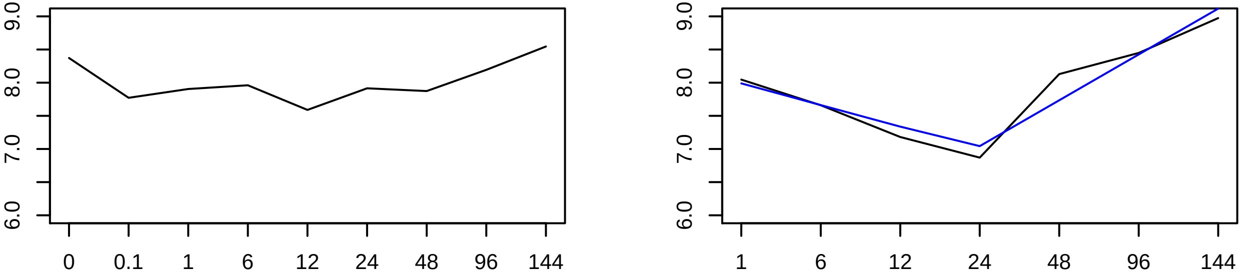

A_32_P40673 A_32_P40673 NA

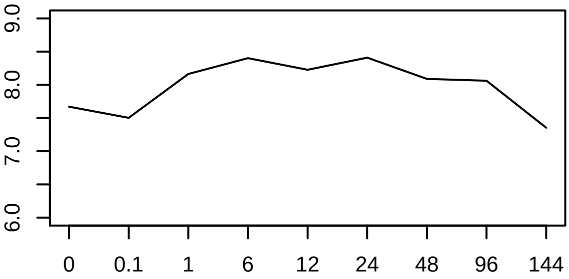

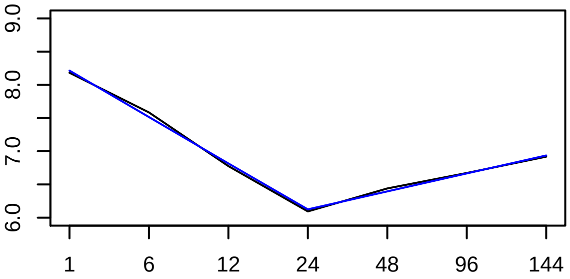

A_23_P117582 JDP2 14q24.3

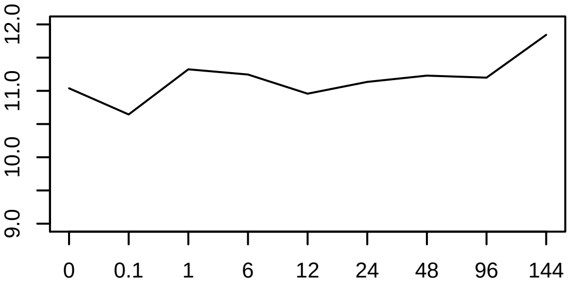

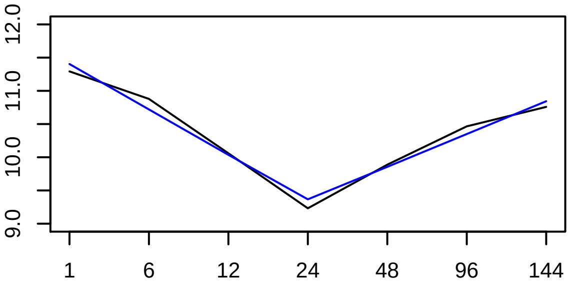

A_23_P140821 PARD6A 16q22.1

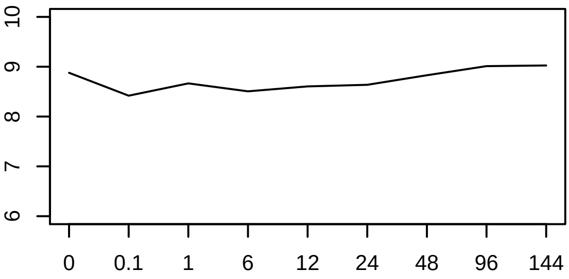

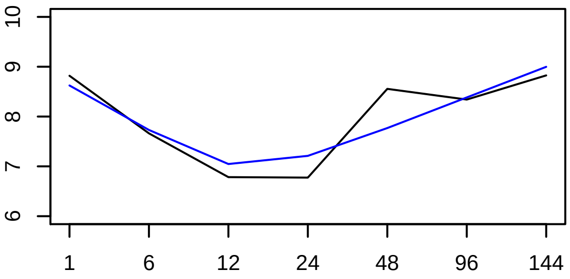

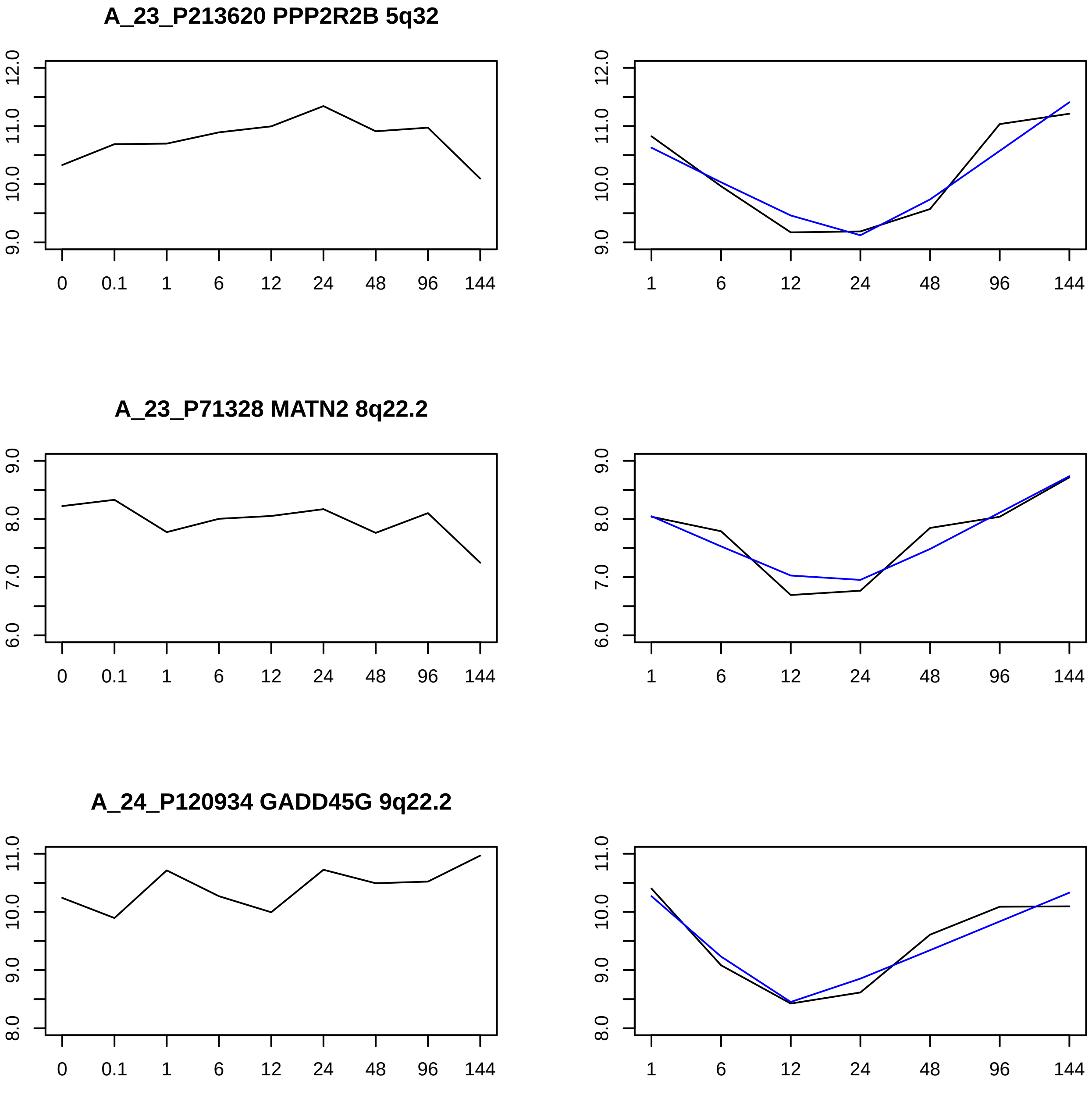

A_23_P213620 PPP2R2B 5q32
9.0
10.0
11.0
12.0
0
0.1
1
6
12
24
48
96
144
A_23_P71328 MATN2 8q22.2
6.0
7.0
8.0
9.0
A_24_P120934 GADD45G 9q22.2
8.0
9.0
10.0
11.0

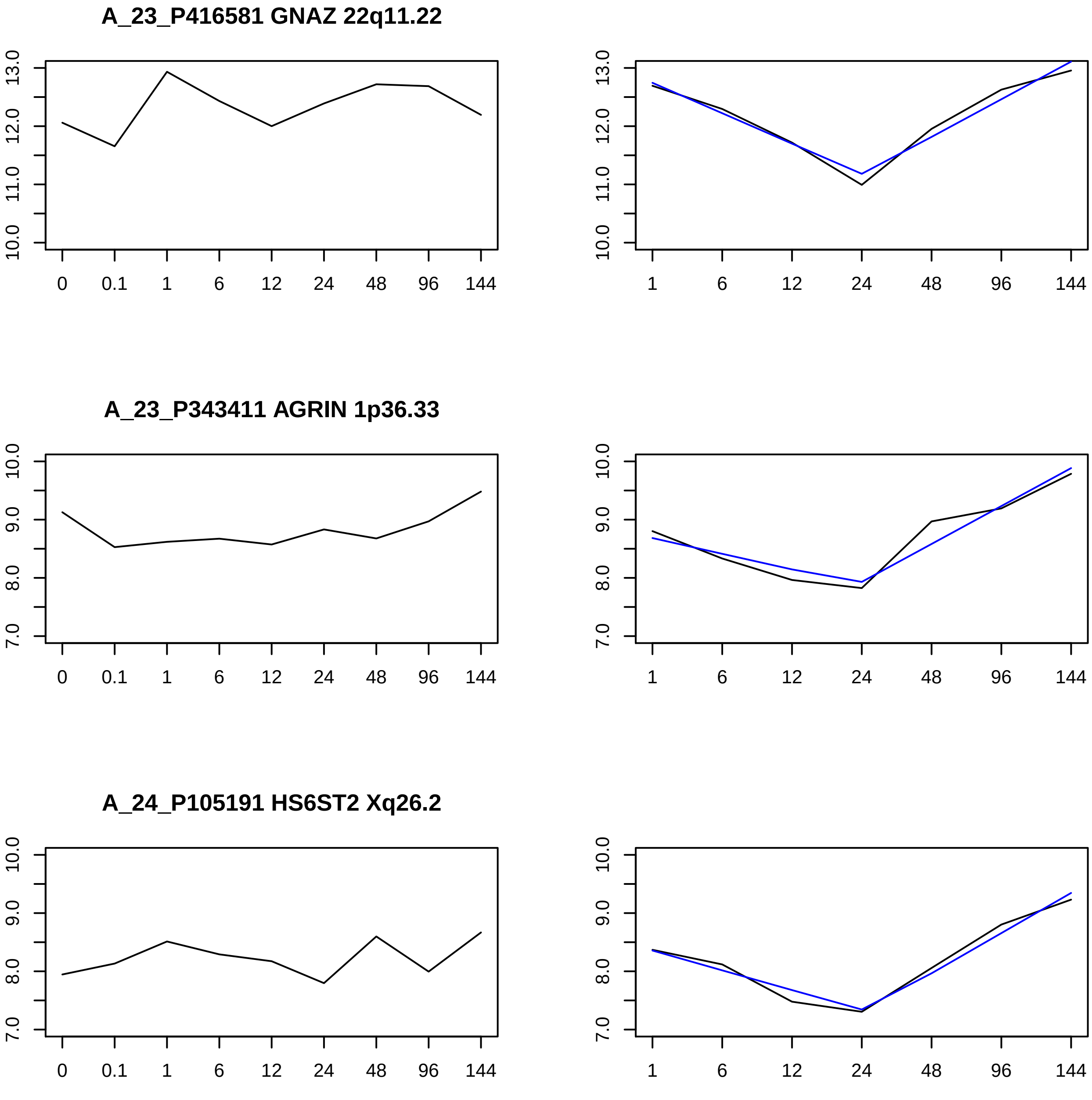
A_23_P416581 GNAZ 22q11.22
10.0
11.0
12.0
13.0
0
0.1
1
6
12
24
48
96
144
1
6
12
24
48
96
144
A_23_P343411 AGRIN 1p36.33
7.0
8.0
9.0
10.0
A_24_P105191 HS6ST2 Xq26.2

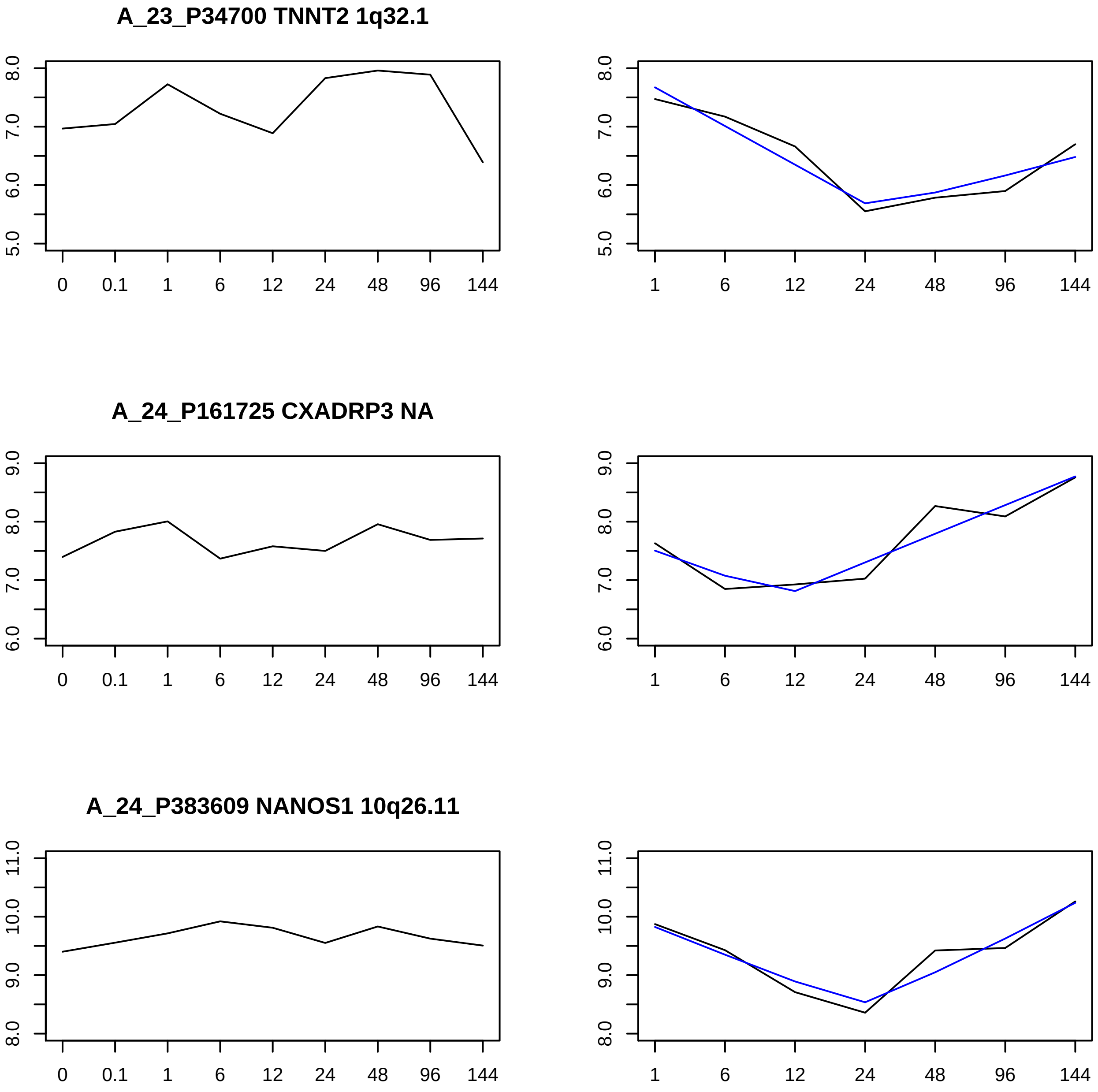
A_23_P34700 TNNT2 1q32.1
5.0 6.0 7.0 8.0
0 0.1 1 6 12 24 48 96 144
5.0 6.0 7.0 8.0
1 6 12 24 48 96 144
A_24_P161725 CXADRP3 NA
6.0 7.0 8.0 9.0
0 0.1 1 6 12 24 48 96 144
6.0 7.0 8.0 9.0
1 6 12 24 48 96 144
A_24_P383609 NANOS1 10q26.11
8.0 9.0 10.0 11.0
0 0.1 1 6 12 24 48 96 144
8.0 9.0 10.0 11.0
1 6 12 24 48 96 144

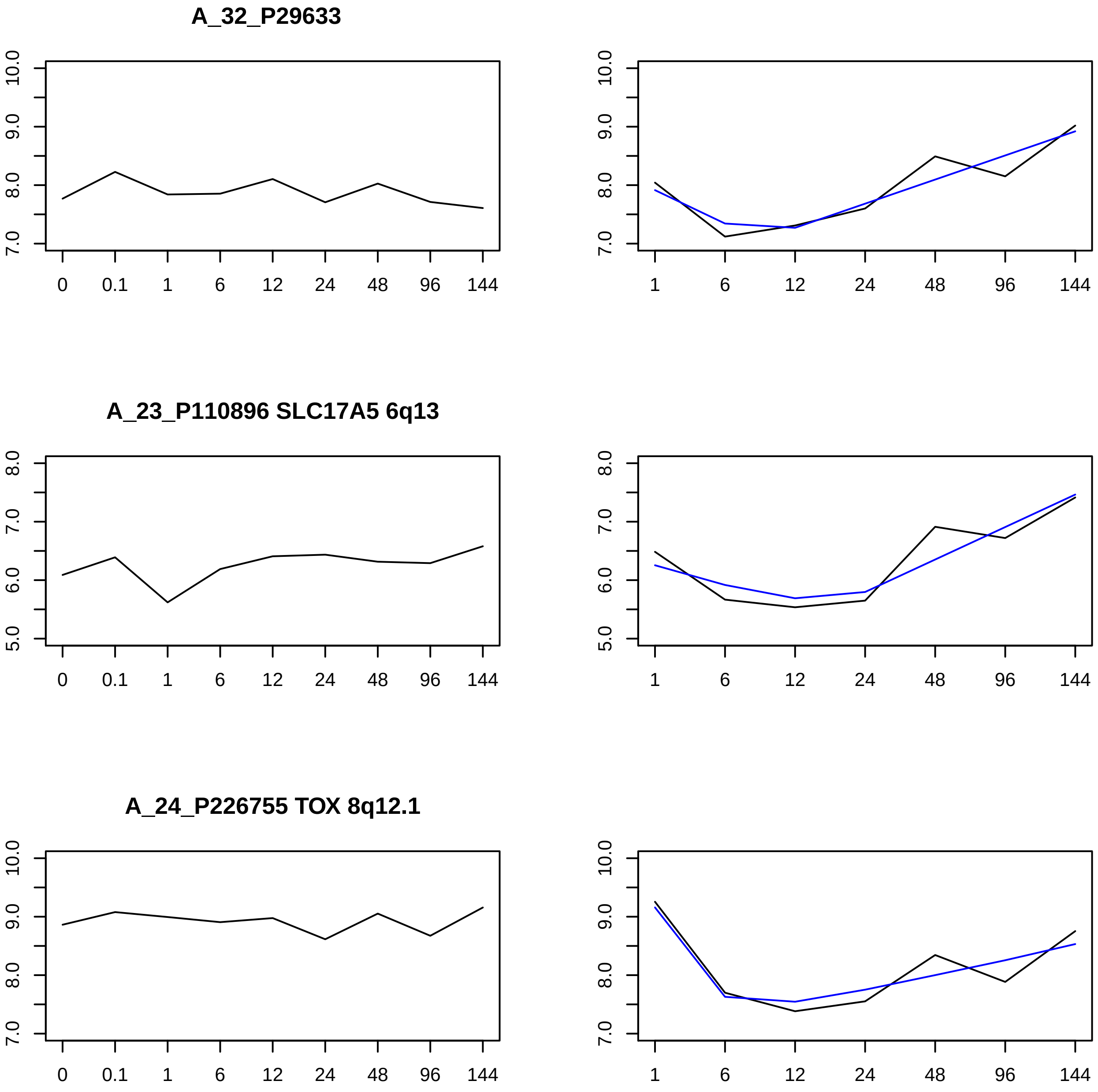

A_32_P29633
A_23_P110896 SLC17A5 6q13
A_24_P226755 TOX 8q12.1
7.0
8.0
9.0
10.0
5.0
6.0
0
0.1
1
6
12
24
48
96
144

A_32_P132317 GPR155 2q31.1

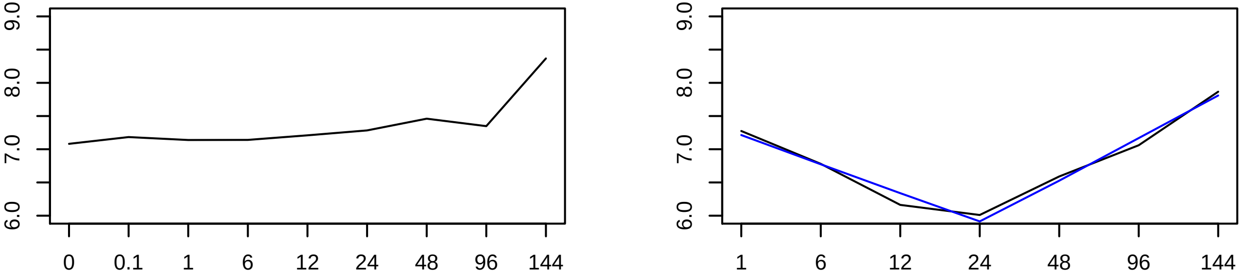

A_23_P98974 LGR5 12q21.1

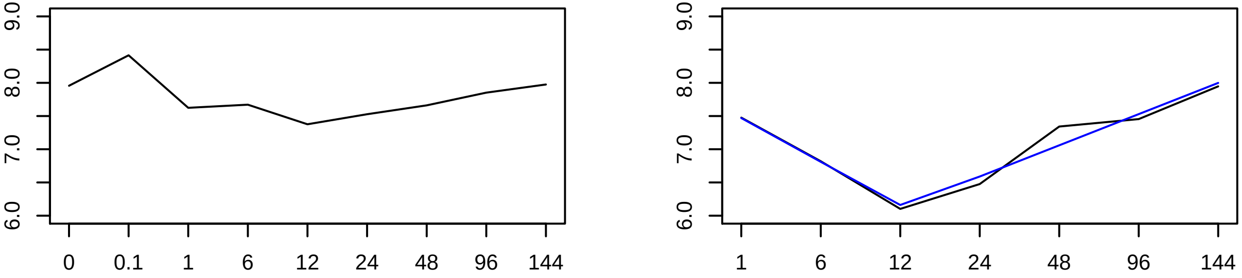

A_23_P74309 NOS1AP 1q23.3

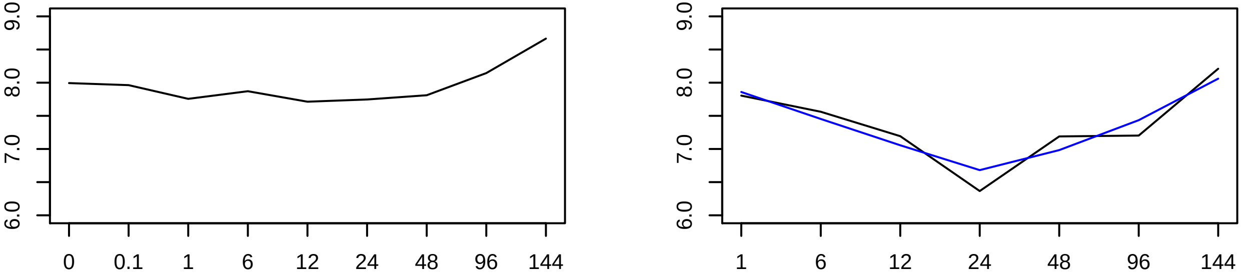

A_23_P146456 CTSL2 9q22.33

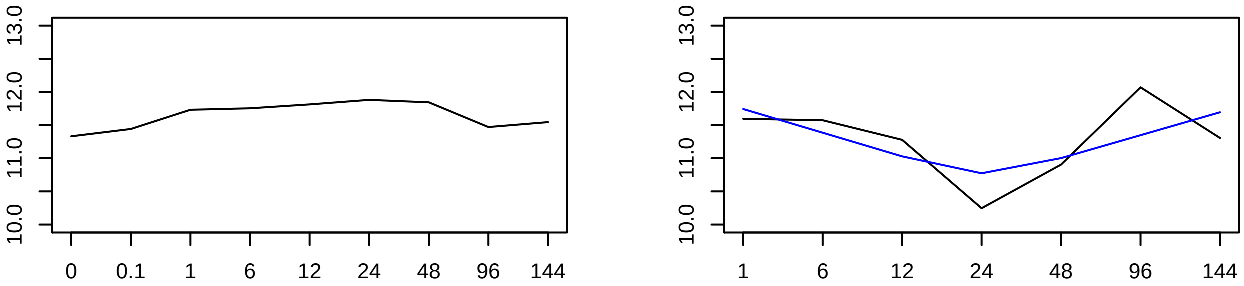

A_24_P918317 DKK3 11p15.3

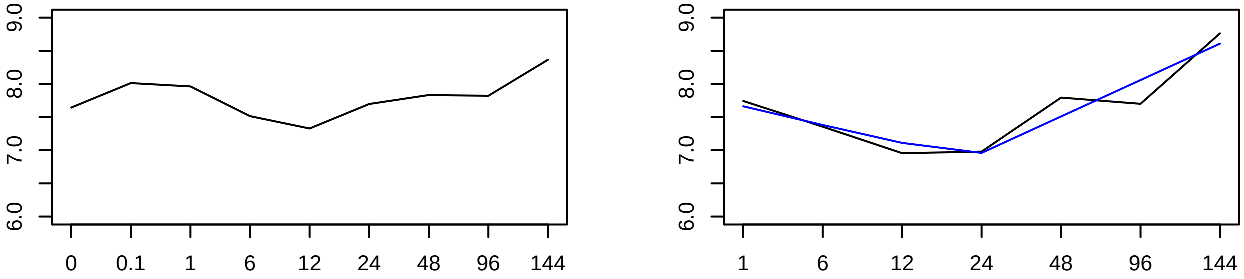

A_32_P156786 HRK 12q24.22

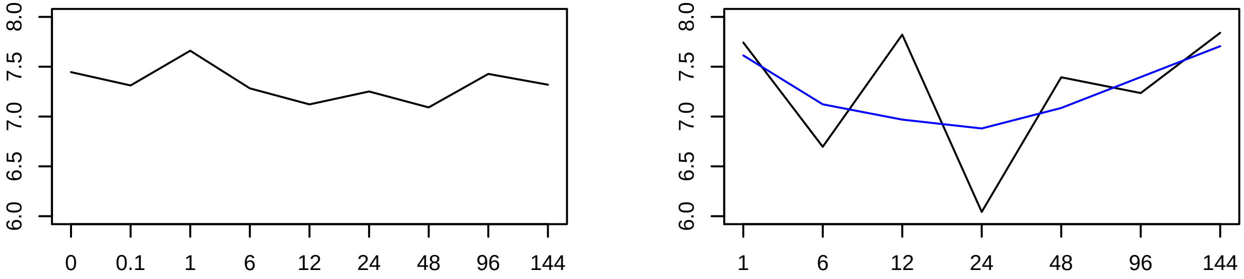

A_23_P155786 SULT1E1 4q13.3

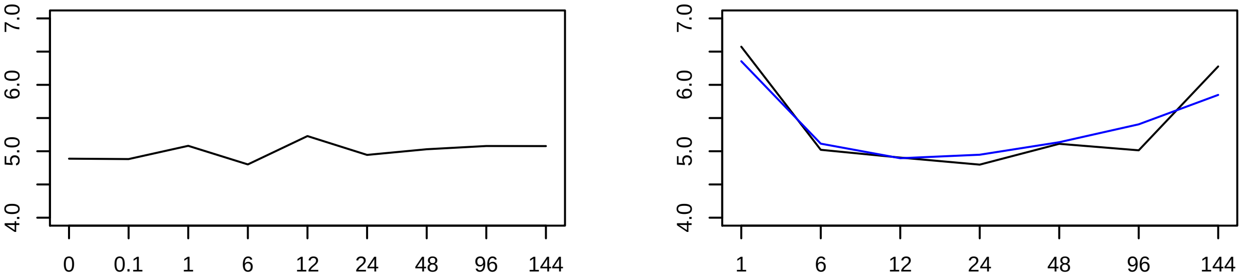

A_23_P132175 RTN4R 22q11.21

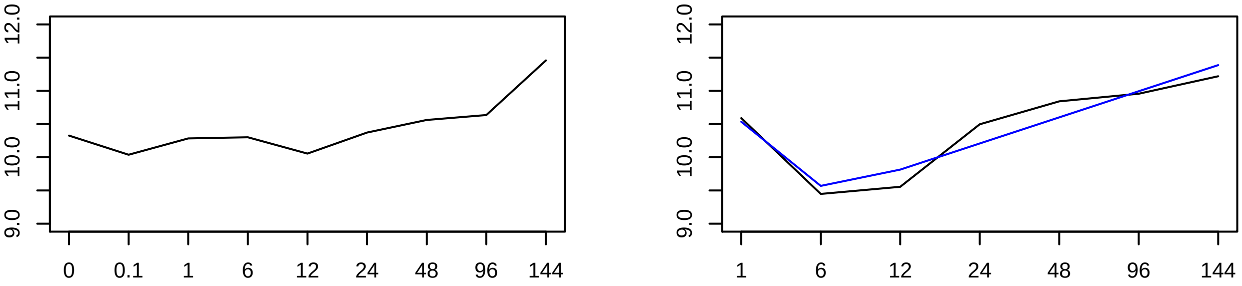

A_32_P209230 CITED4 1p34.2

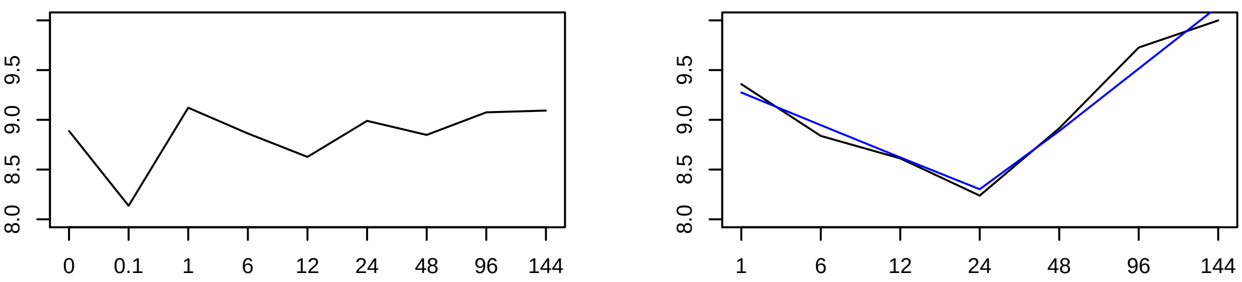

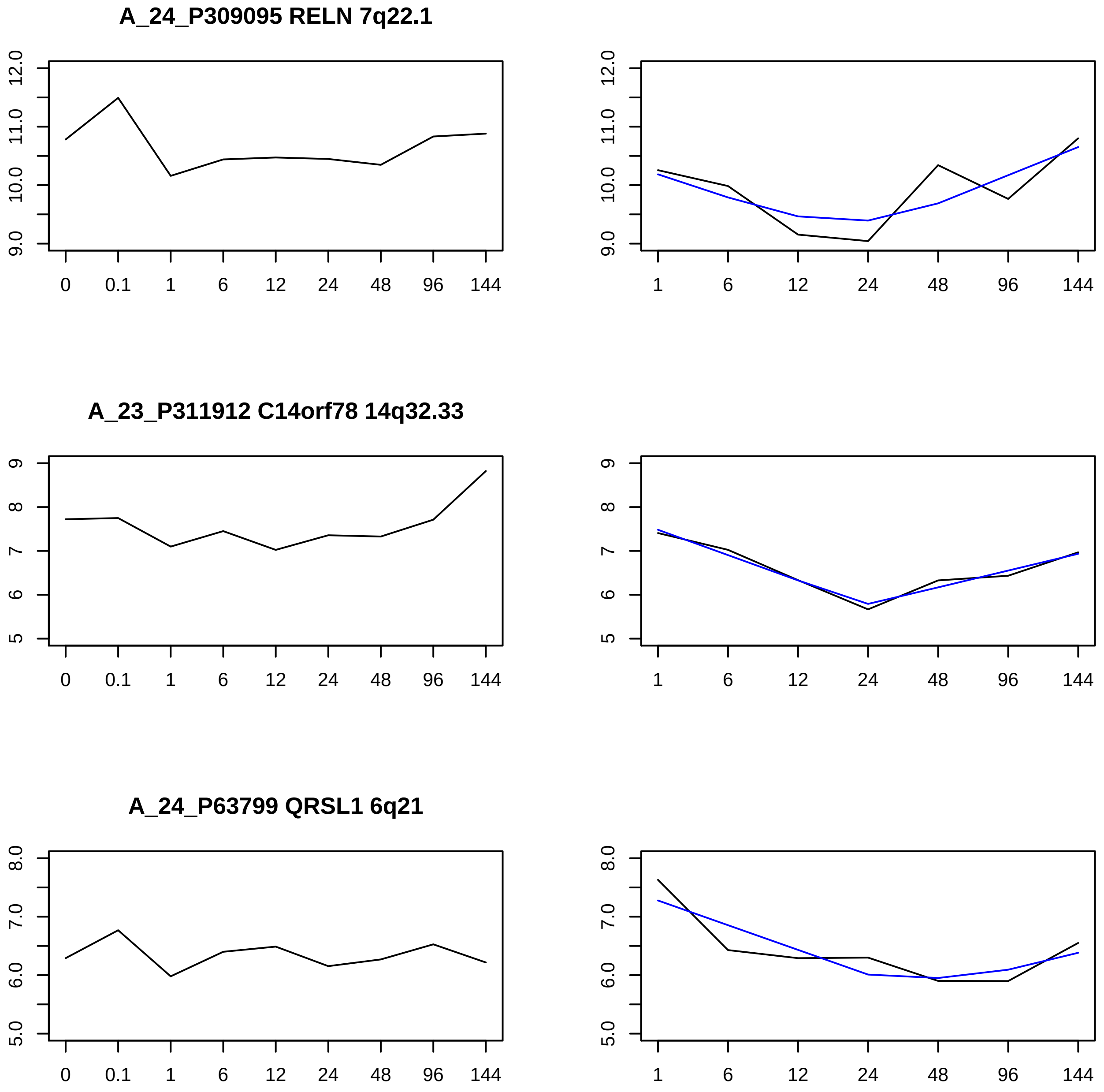

A_24_P309095 RELN 7q22.1
9.0
10.0
11.0
12.0
0
0.1
1
6
12
24
48
96
144
A_23_P311912 C14orf78 14q32.33
5
6
7
8
9
A_24_P63799 QRSL1 6q21
5.0
6.0
7.0
8.0

A_23_P210756 SNAP25 20p12.2

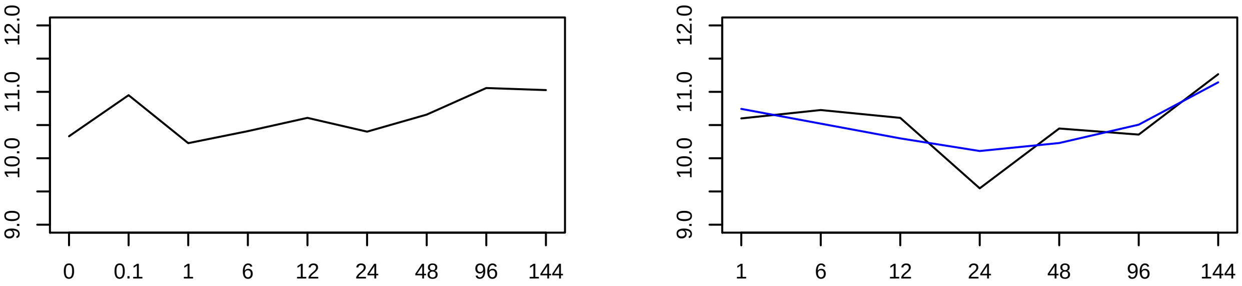

A_23_P407497 NRG1 8p12

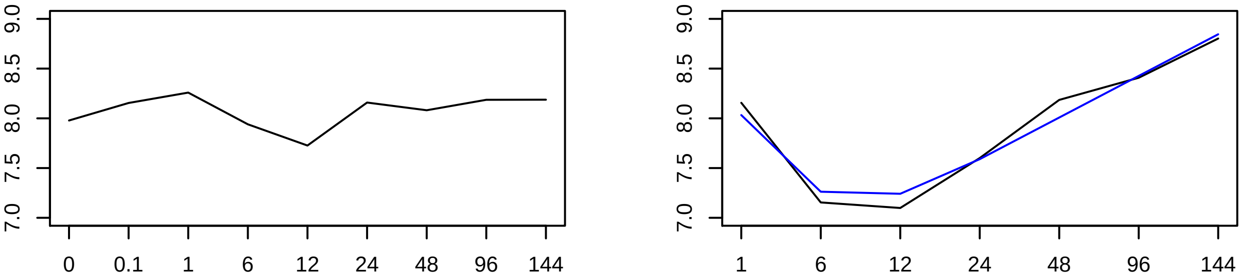

A_23_P127426 TM7SF2 11q13.1

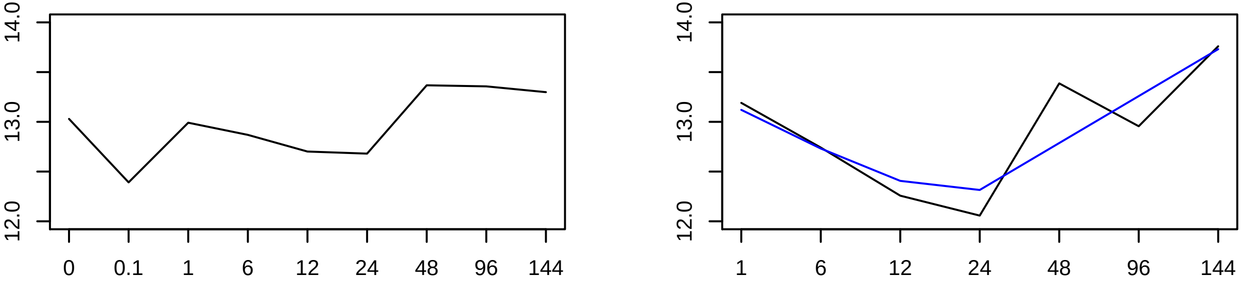

A_23_P25194 HRK 12q24.22

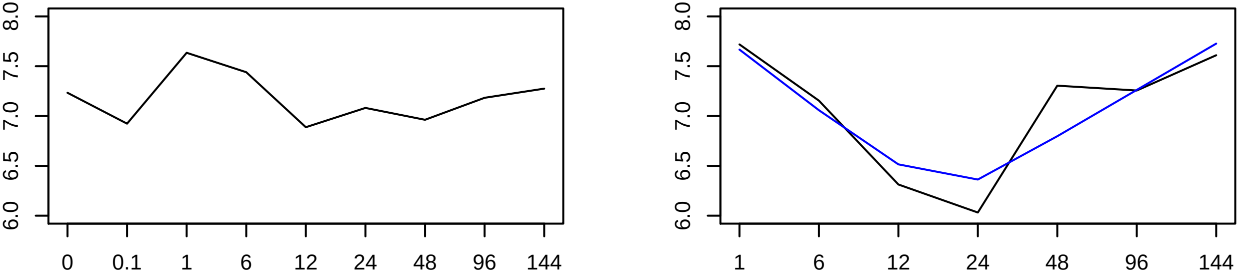

A_23_P115862 COMTD1 10q22.2

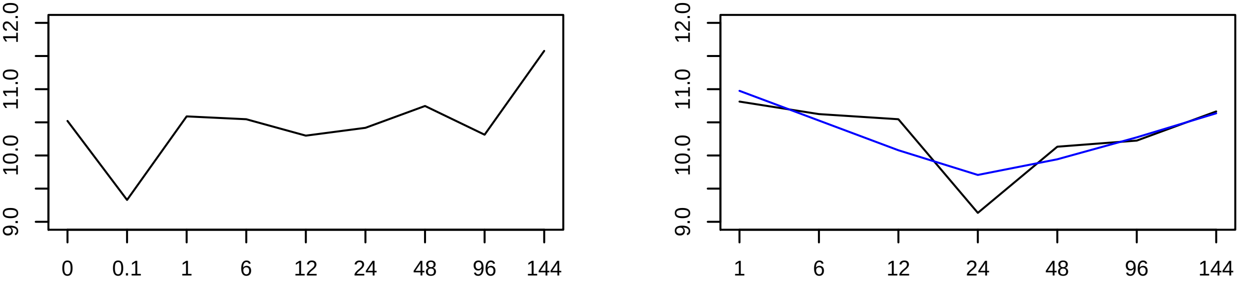

A_23_P33326 ADRA1B 5q33.3

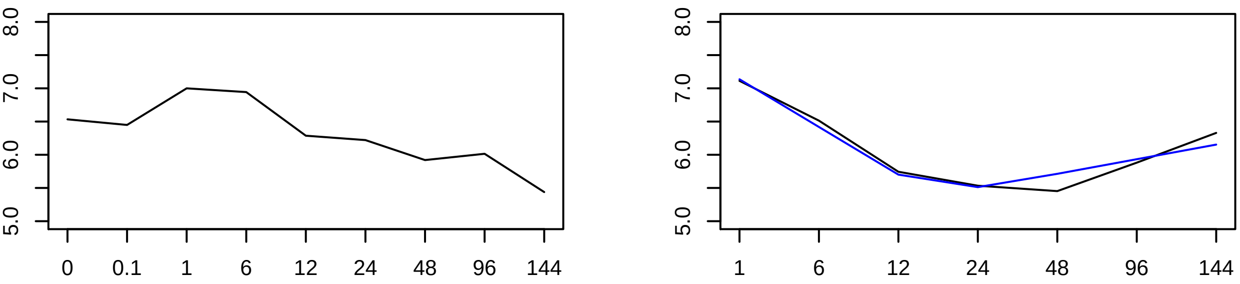

A_23_P9086 ZDHHC2 8p22

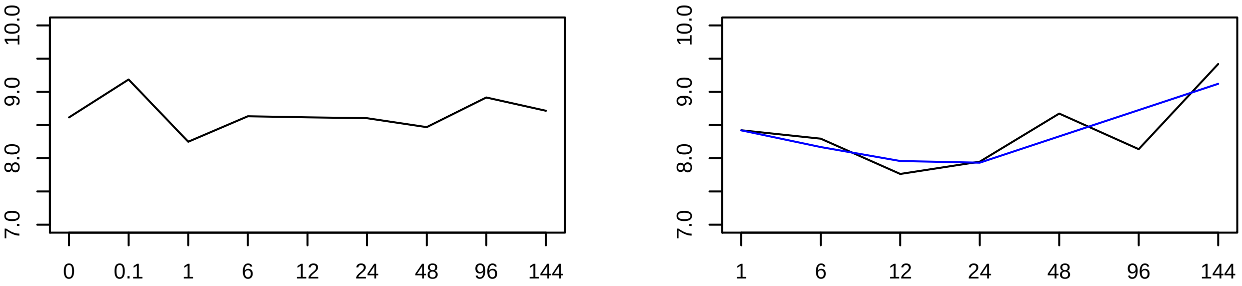

A_23_P158593 COL5A1 9q34.3

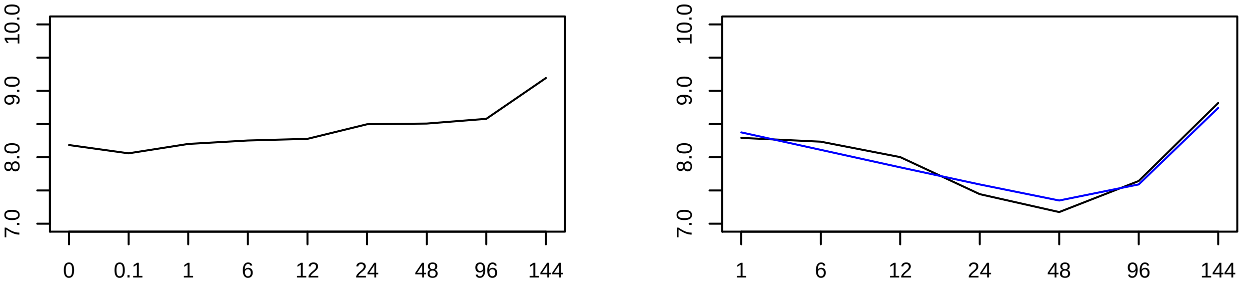

A_23_P3221 SQRDL 15q21.1

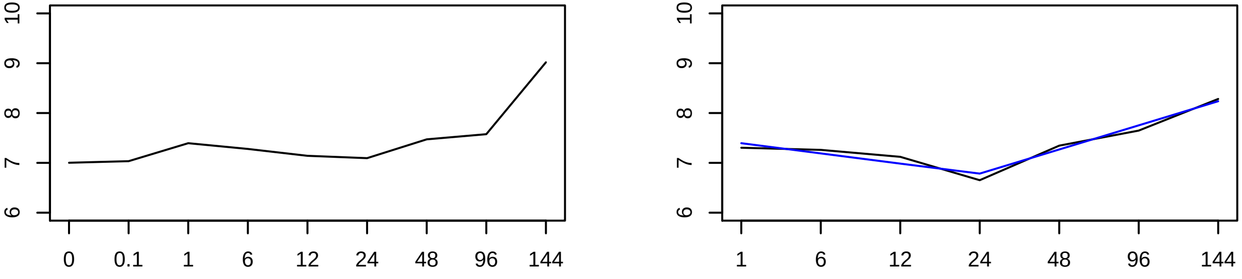

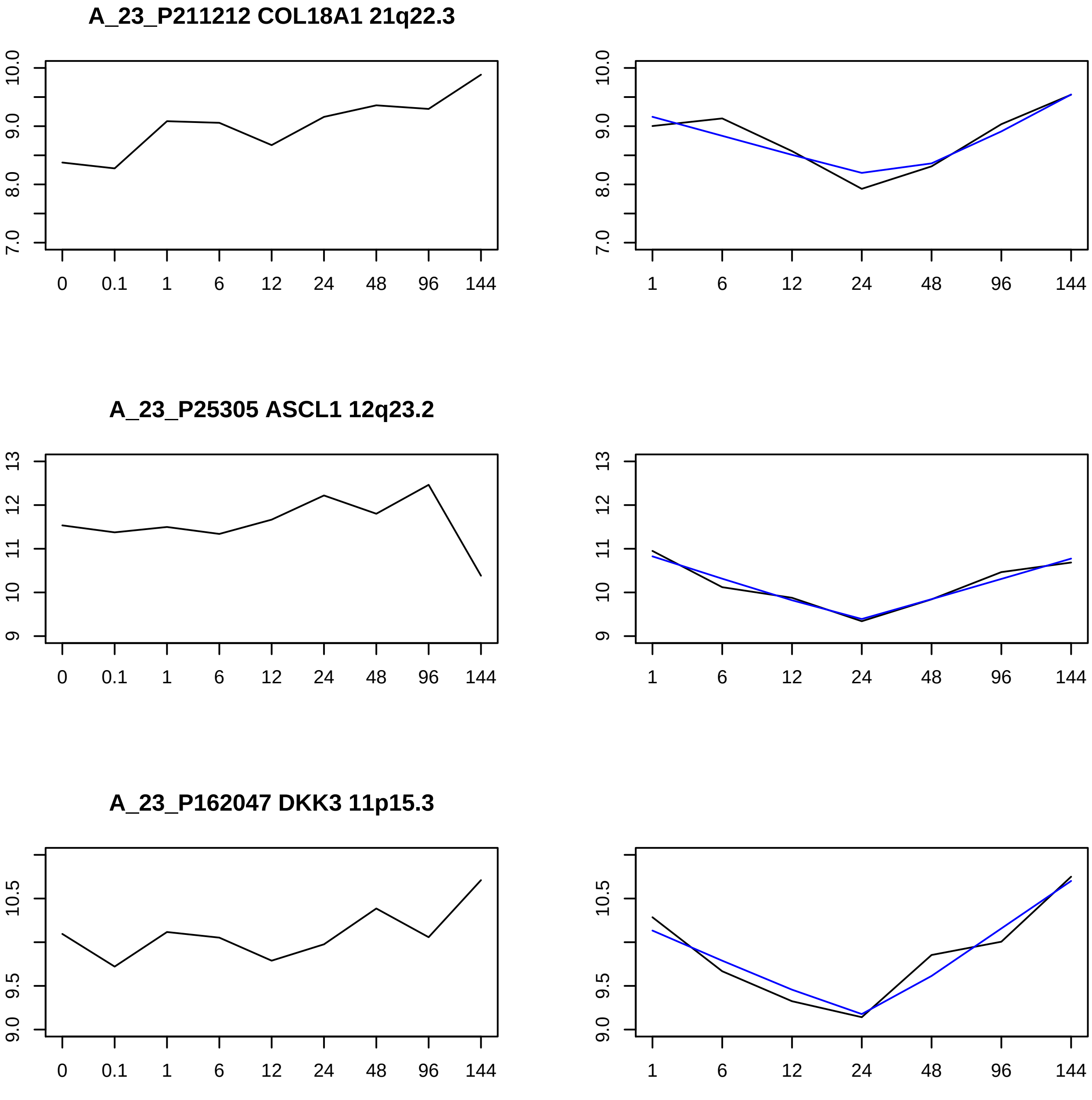
A_23_P211212 COL18A1 21q22.3
7.0
8.0
9.0
10.0
0
0.1
1
6
12
24
48
96
144
A_23_P25305 ASCL1 12q23.2
9
10
11
12
13
A_23_P162047 DKK3 11p15.3
9.0
9.5
10.5

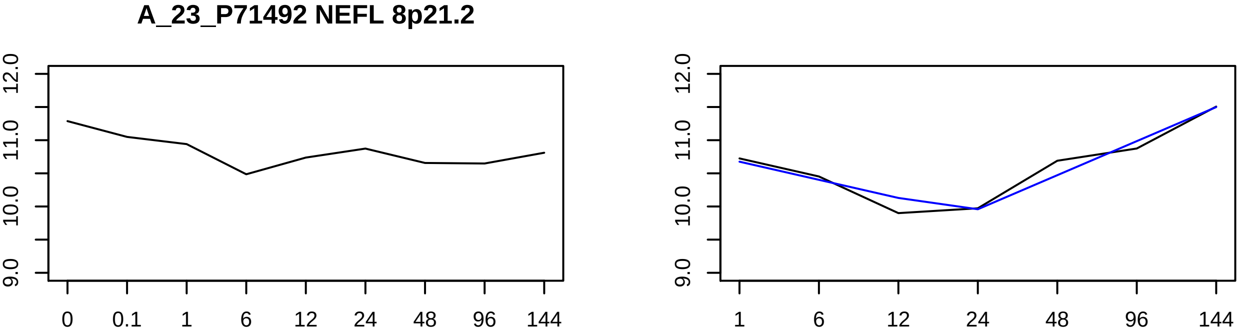
A_23_P71492 NEFL 8p21.2
9.0
10.0
11.0
12.0
0
0.1
1
6
12
24
48
96
144
1
6
12
24
48
96
144

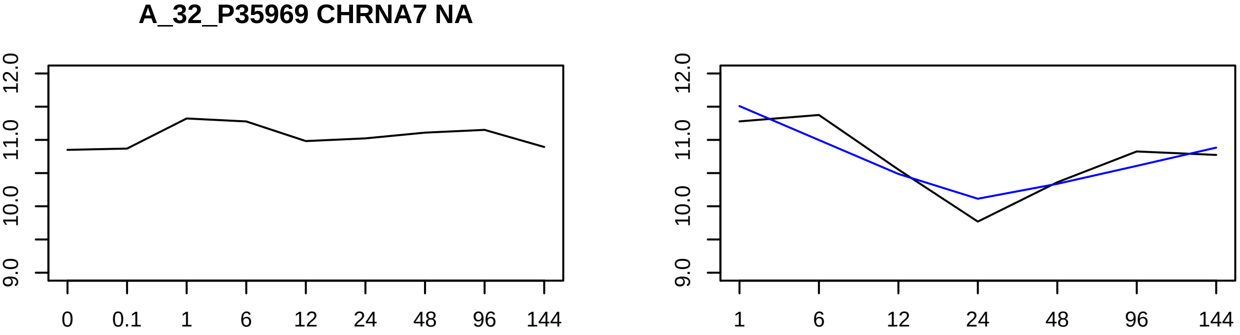
A_32_P35969 CHRNA7 NA
9.0
10.0
11.0
12.0
0
0.1
1
6
12
24
48
96
144
1
6
12
24
48
96
144

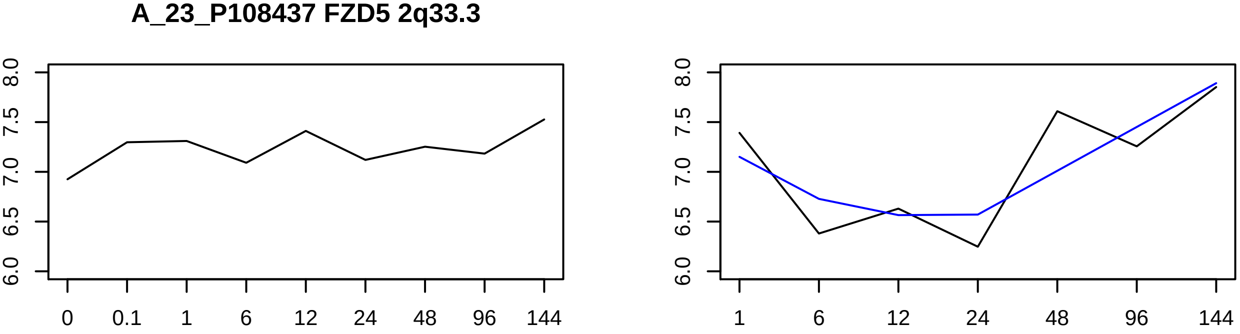
A_23_P108437 FZD5 2q33.3
6.0
6.5
7.0
7.5
8.0
0
0.1
1
6
12
24
48
96
144
1
6
12
24
48
96
144

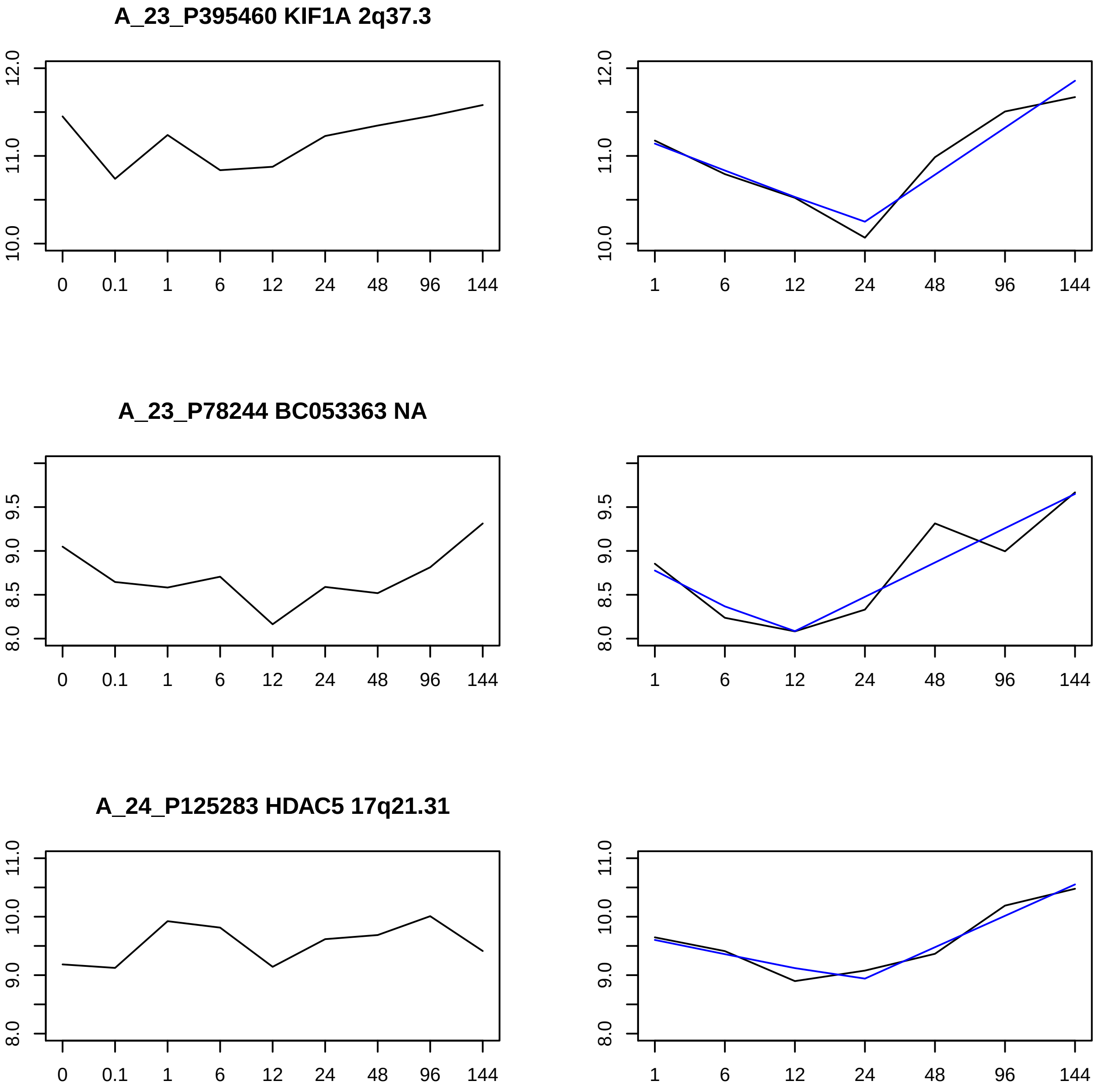
A_23_P395460 KIF1A 2q37.3
10.0
11.0
12.0
0
0.1
1
6
12
24
48
96
144
A_23_P78244 BC053363 NA
8.0
8.5
9.0
9.5
A_24_P125283 HDAC5 17q21.31
8.0
9.0
10.0
11.0

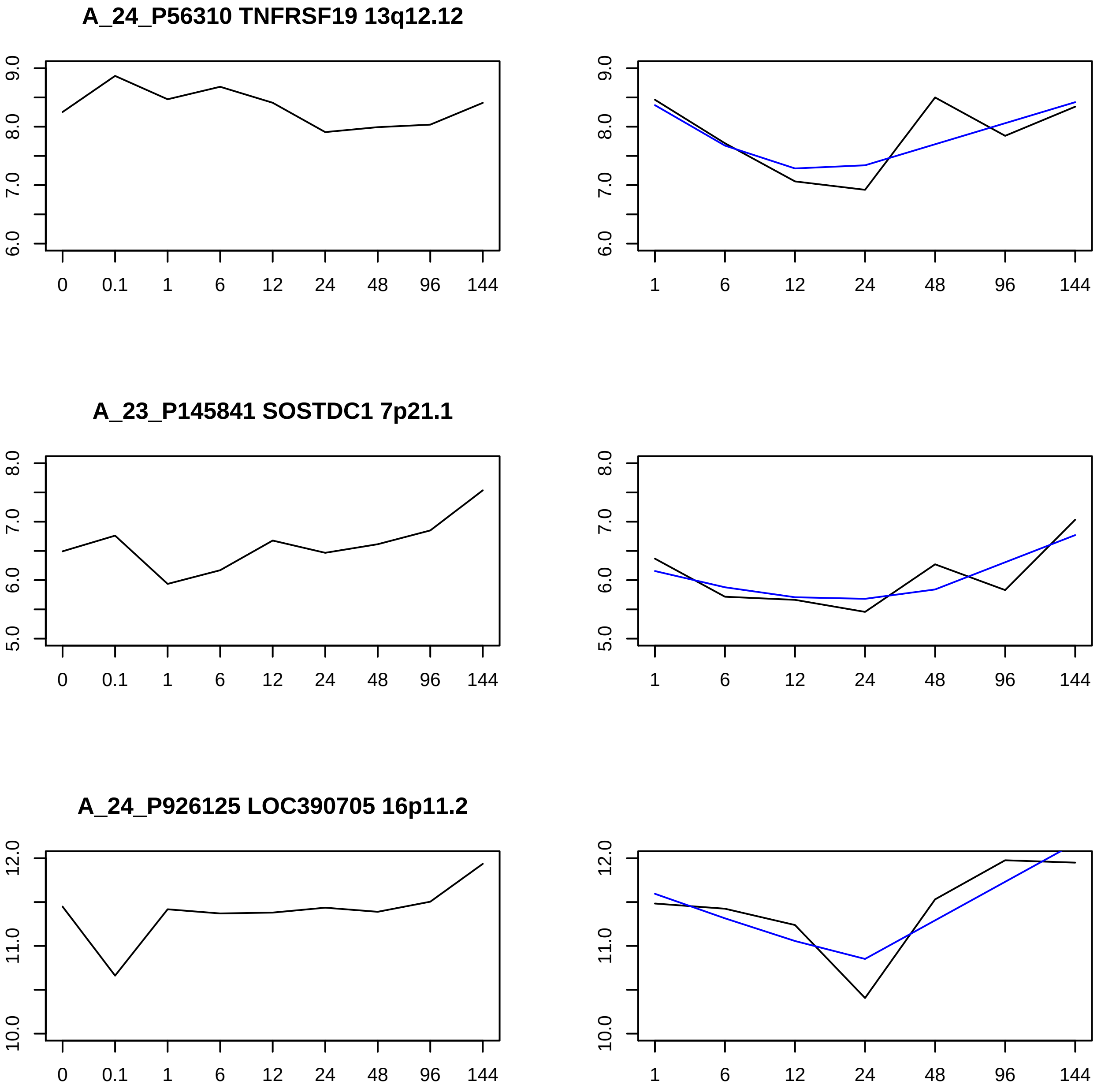
A_24_P56310 TNFRSF19 13q12.12
6.0
7.0
8.0
9.0
0
0.1
1
6
12
24
48
96
144
A_23_P145841 SOSTDC1 7p21.1
5.0
6.0
7.0
8.0
A_24_P926125 LOC390705 16p11.2
10.0
11.0
12.0

A_23_P212617 TFRC 3q29

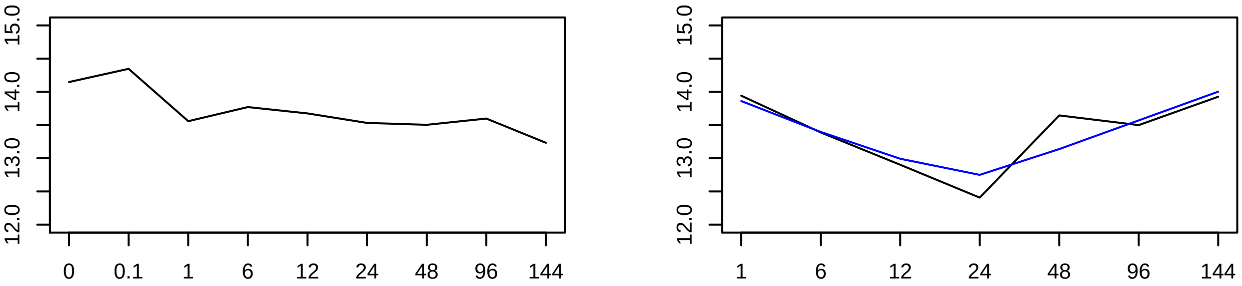

A_24_P201381 FGFBP3 10q23.32

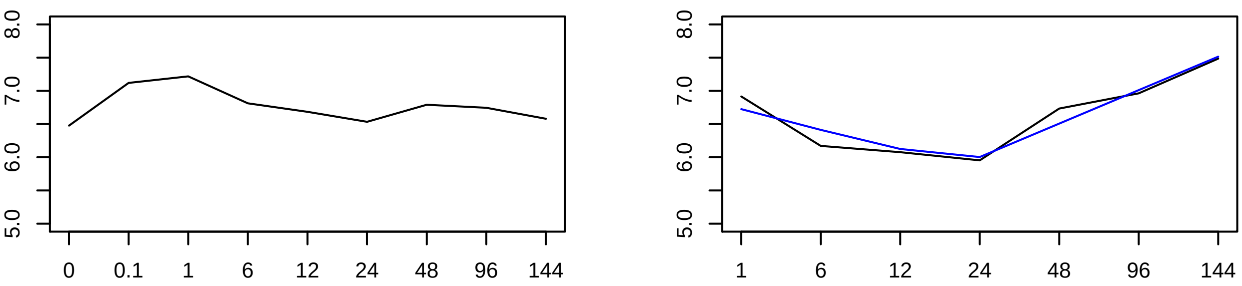

A_23_P3483 CACNA1H 16p13.3

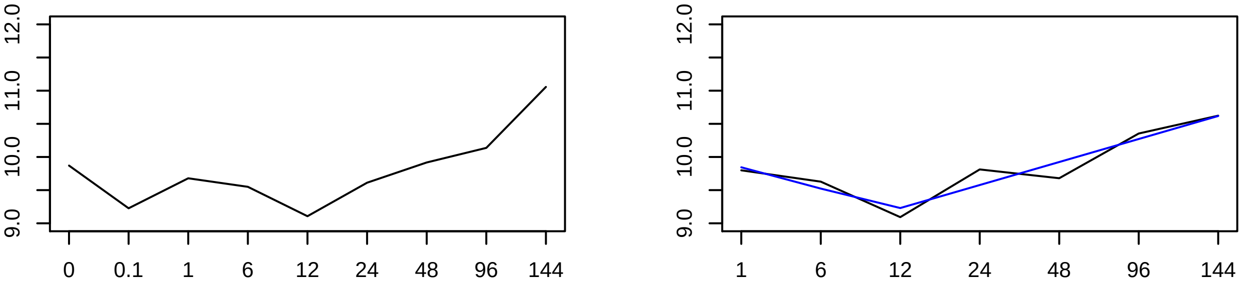

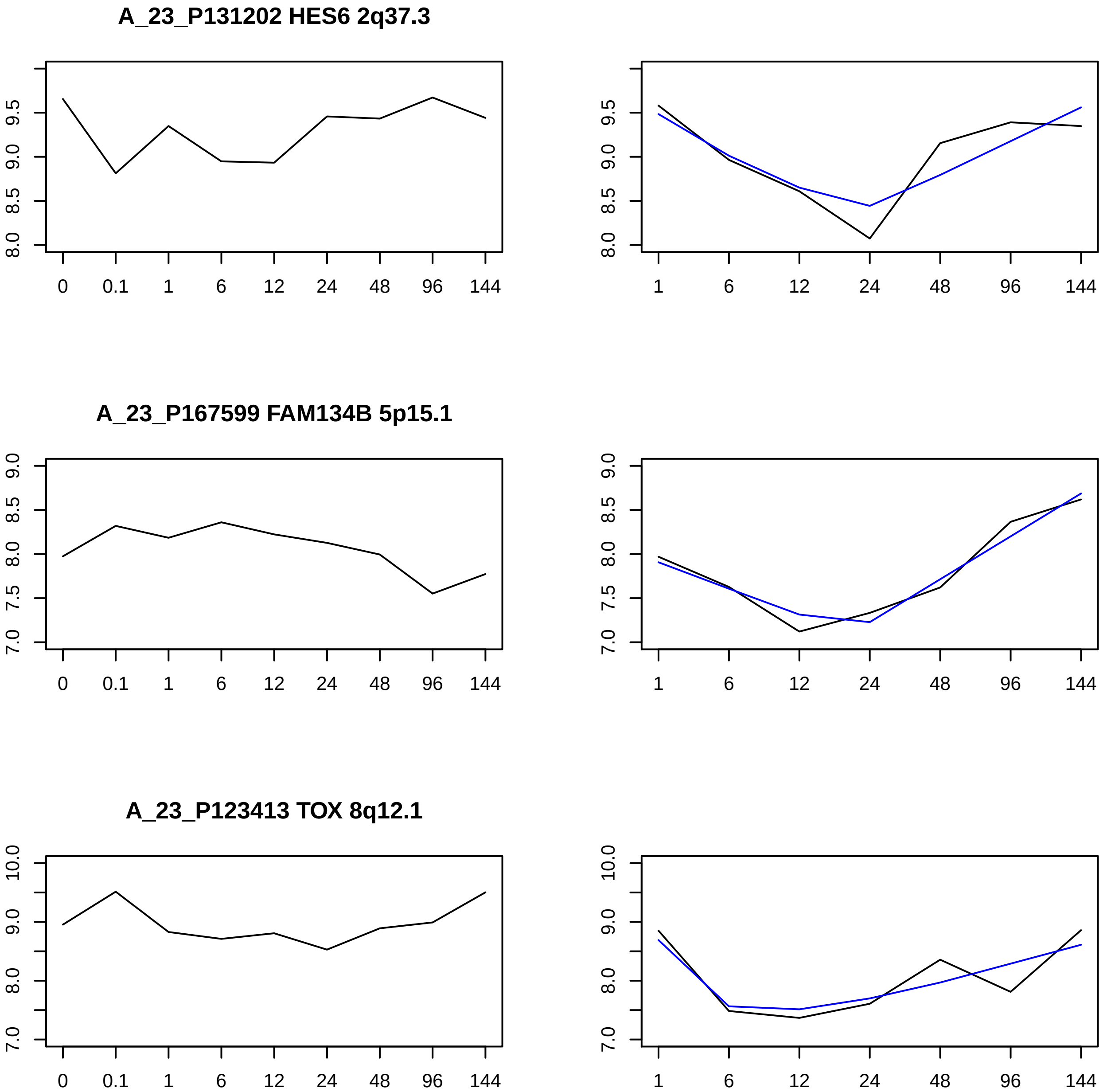

A_23_P131202 HES6 2q37.3
8.0
8.5
9.0
9.5
0
0.1
1
6
12
24
48
96
144
A_23_P167599 FAM134B 5p15.1
7.0
7.5
8.0
8.5
9.0
A_23_P123413 TOX 8q12.1
10.0

A_24_P286687 ZDHHC2 8p22

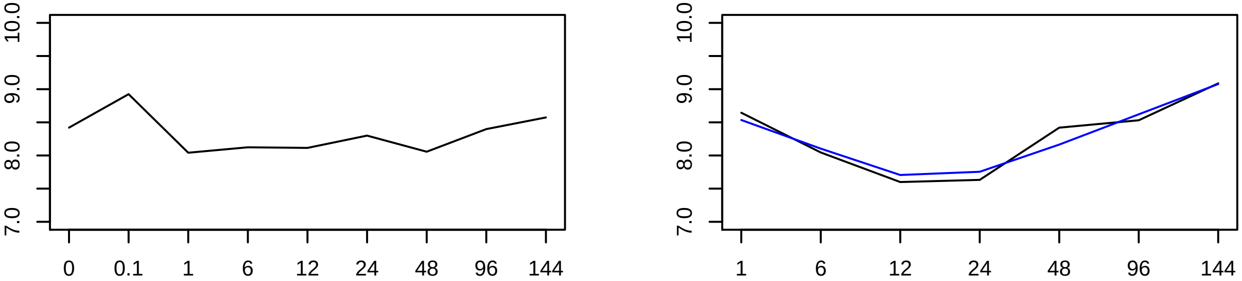

A_23_P55917 SYT3 19q13.33

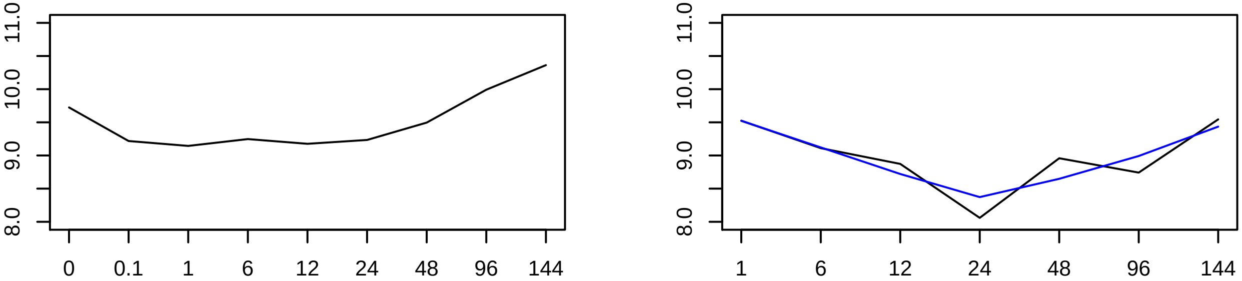

A_23_P42435 DTNBP1 6p22.3

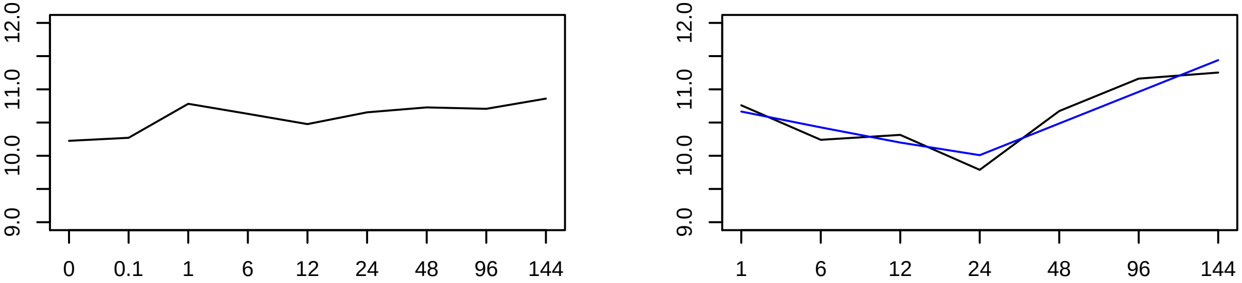

A_23_P103511 ENST00000367932 NA

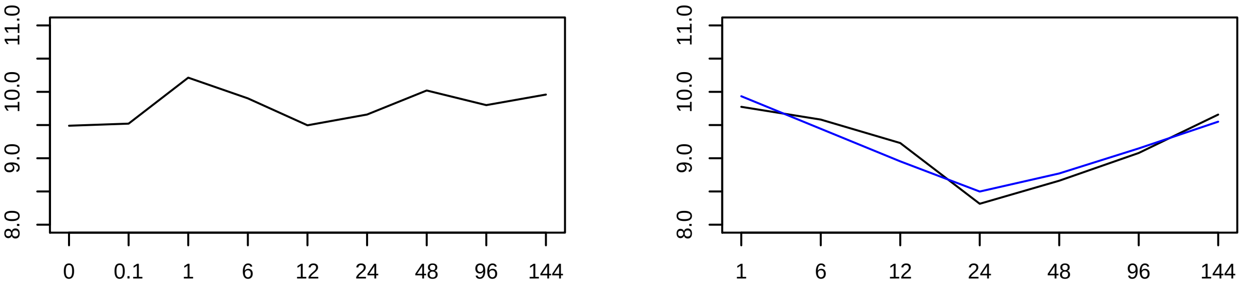

A_32_P122951 A_32_P122951 NA

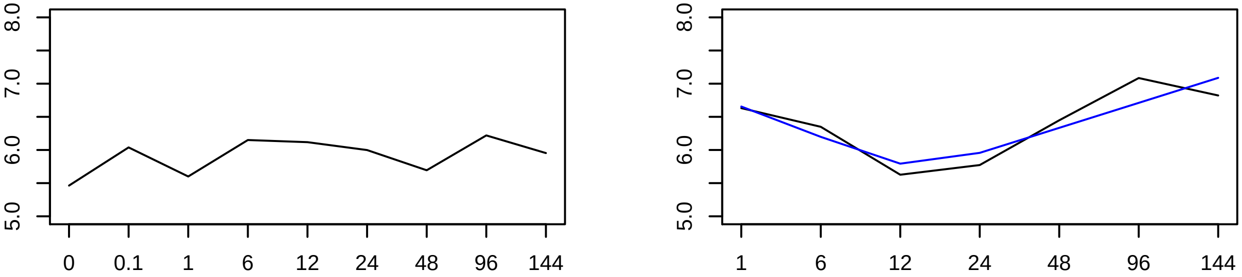

A_23_P380181 LMO4 1p22.3

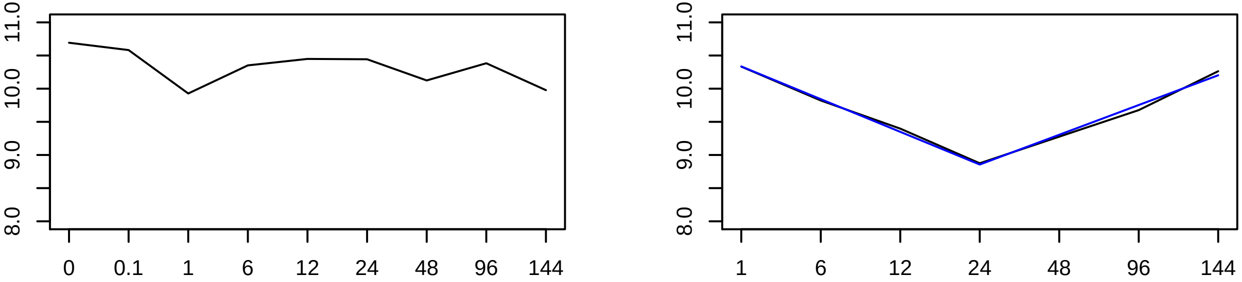

A_23_P90510 REEP6 19p13.3

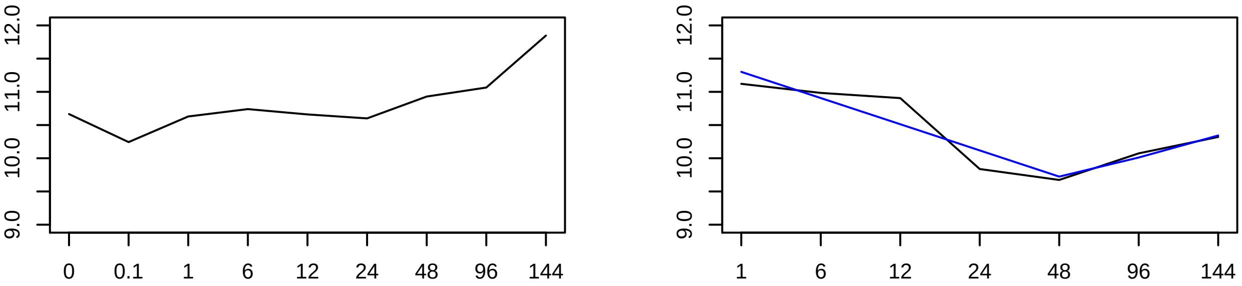

A_23_P257057 THEM6 8q24.3

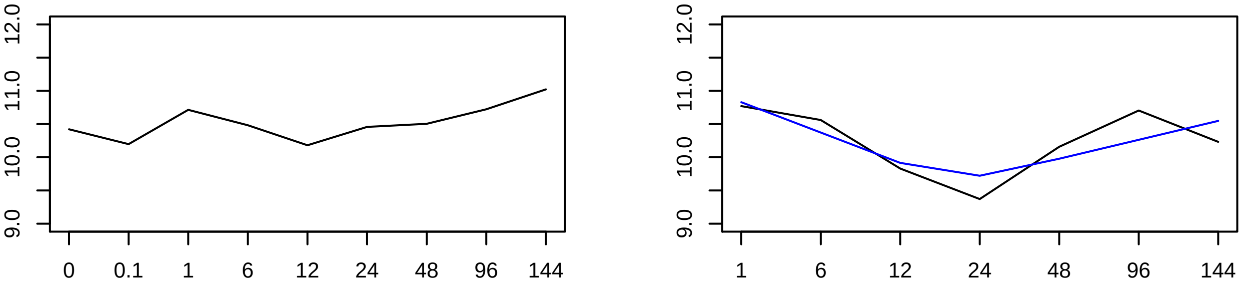

A_23_P14986 HSD11B2 16q22.1

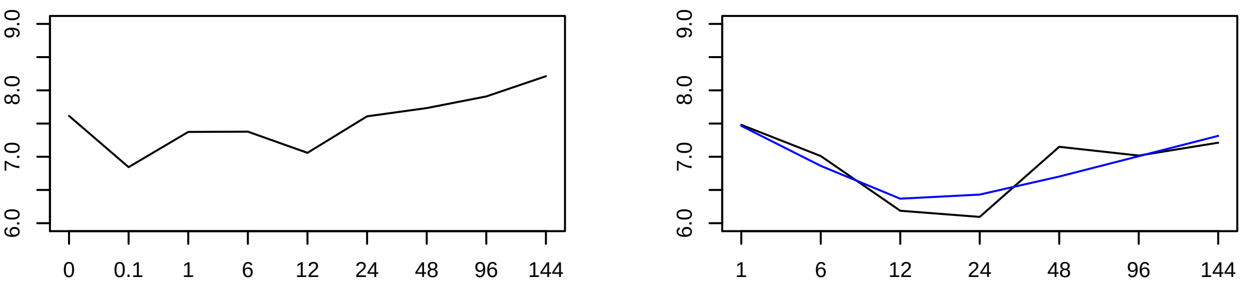

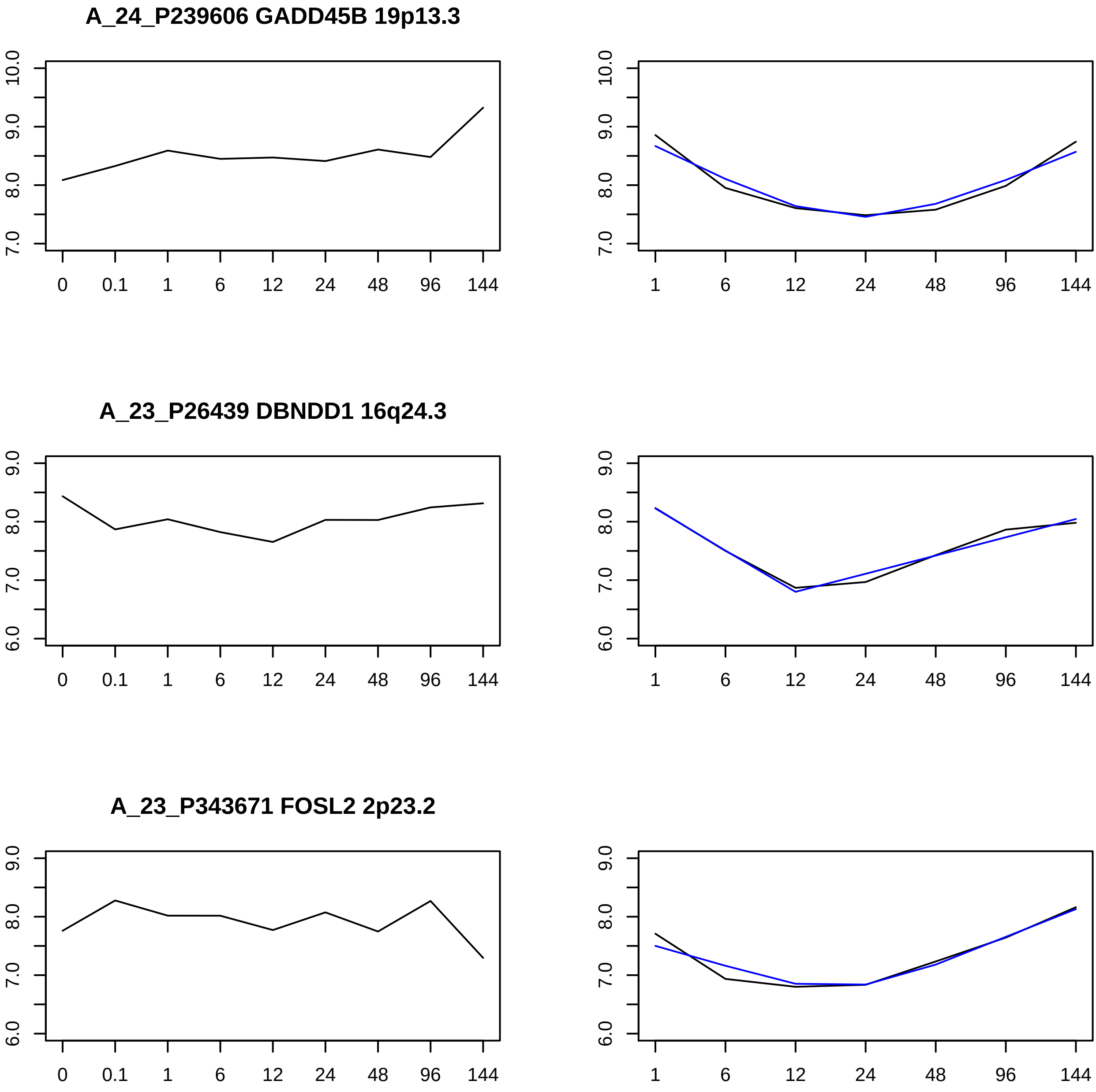
A_24_P239606 GADD45B 19p13.3
10.0
9.0
8.0
7.0
0
0.1
1
6
12
24
48
96
144
A_23_P26439 DBNDD1 16q24.3
A_23_P343671 FOSL2 2p23.2
6.0

A_23_P127565 LAYN 11q23.1

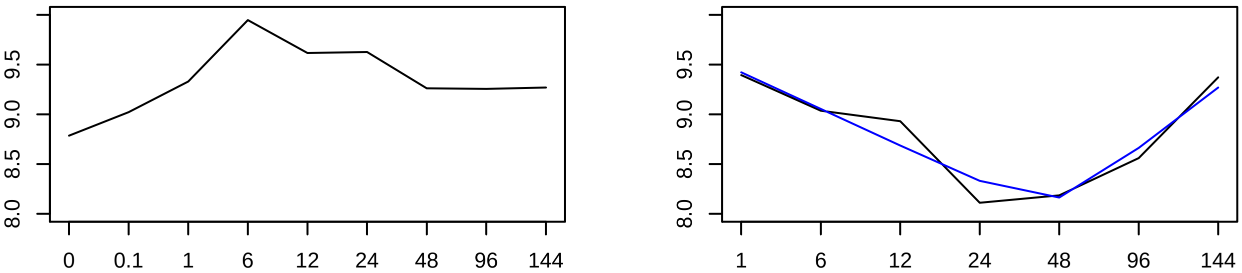

A_23_P375494 CEBPA 19q13.11

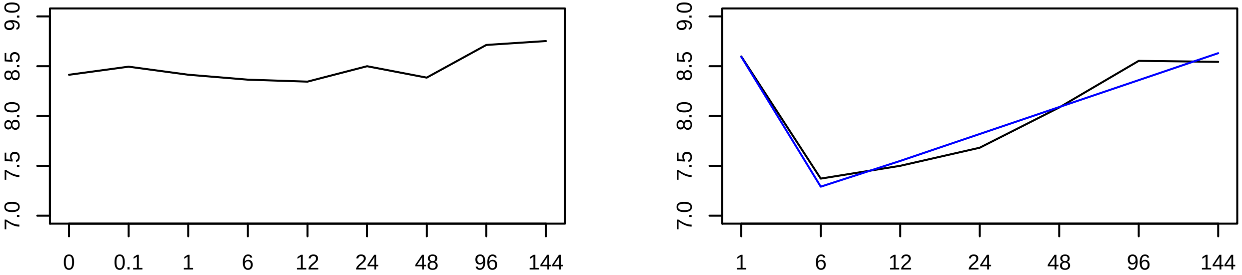

Supplement: Additional file 3 — Additional file A-H. These files contain the fitting results for the genes from the groups A-H, deduced by SwitchFinder, which represent eight dynamic patterns of the gene expression response to ATRA in neuroblastoma cell line. (ZIP 2457 kb) [file 12859_2016_1391_MOESM3_ESM.zip › AdditionalFile_G.pdf]
