# Supplementary material for: SwitchFinder – a novel method and query facility for discovering dynamic gene expression patterns
Source: BMC Bioinformatics. 2016 Dec 15;17:532. doi: 10.1186/s12859-016-1391-0 (PMC5160026; doi:10.1186/s12859-016-1391-0)

A_23_P85800 CD52 1p36.11

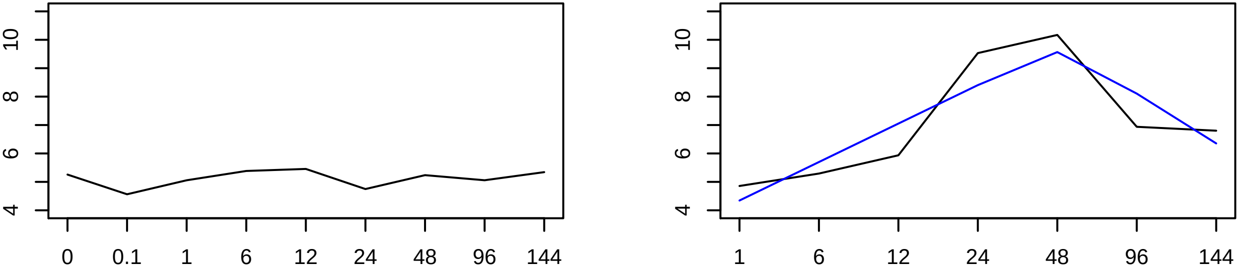

A_24_P766716 CMKLR1 NA

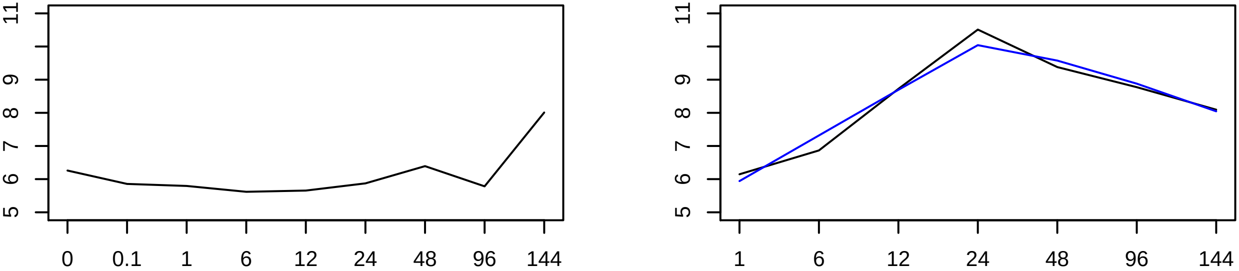

A_24_P95723 KIAA0125 14q32.33

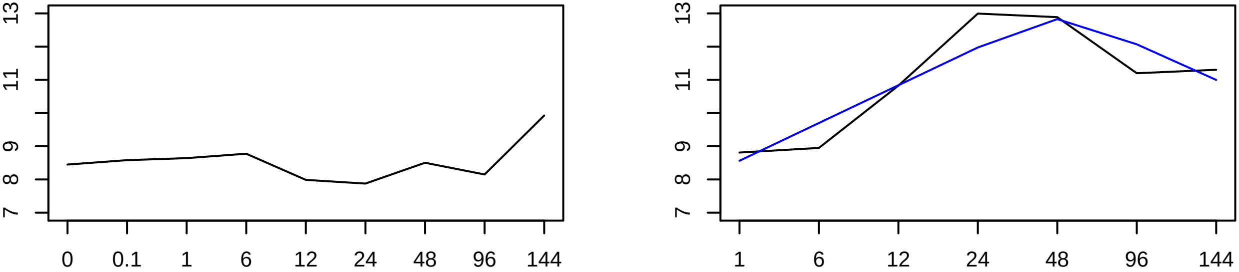

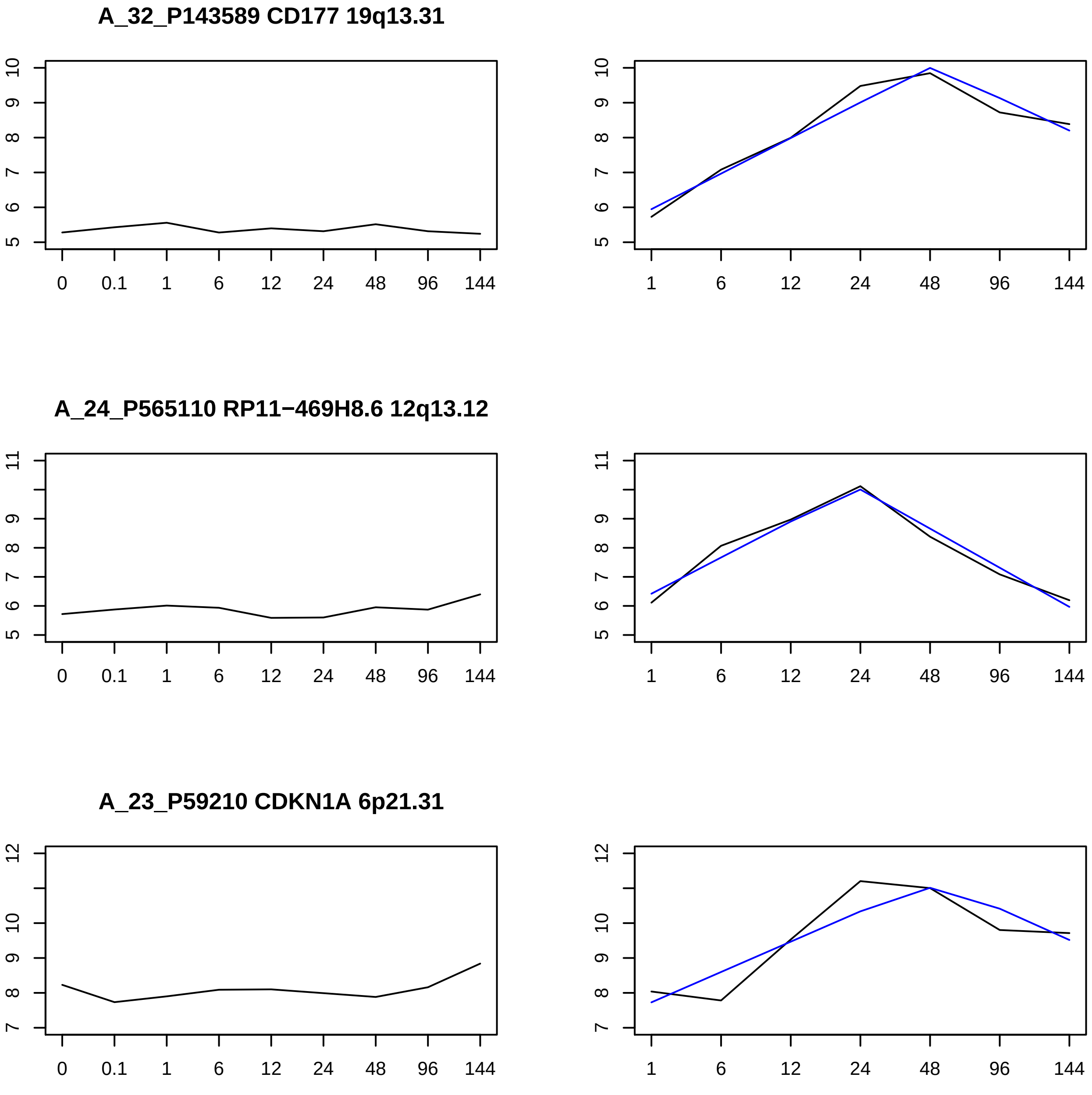
A_32_P143589 CD177 19q13.31
A_24_P565110 RP11−469H8.6 12q13.12
A_23_P59210 CDKN1A 6p21.31
0
0.1
1
6
12
24
48
96
144

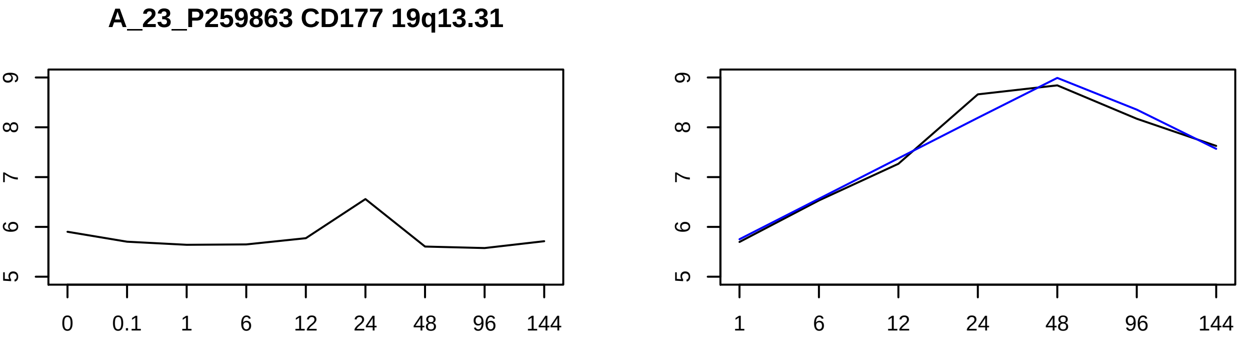
A_23_P259863 CD177 19q13.31
5
6
7
8
9
0
0.1
1
6
12
24
48
96
144
1
6
12
24
48
96
144

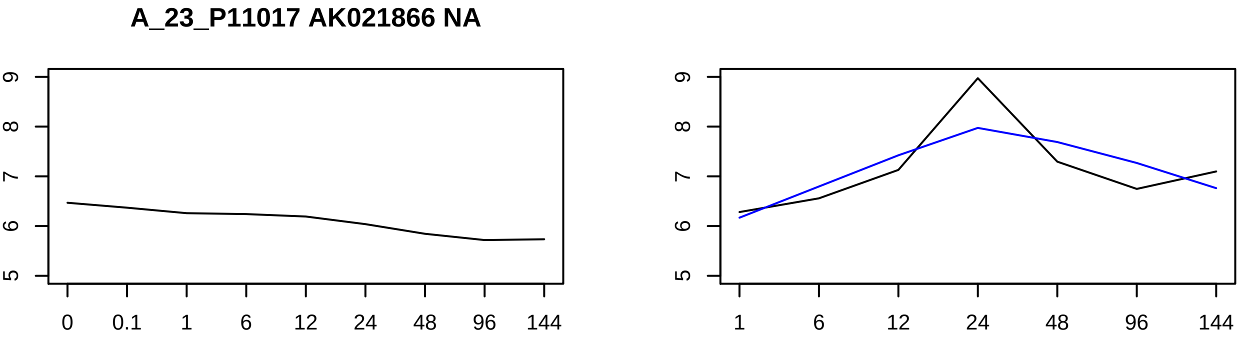
A_23_P11017 AK021866 NA
5
6
7
8
9
0
0.1
1
6
12
24
48
96
144
1
6
12
24
48
96
144

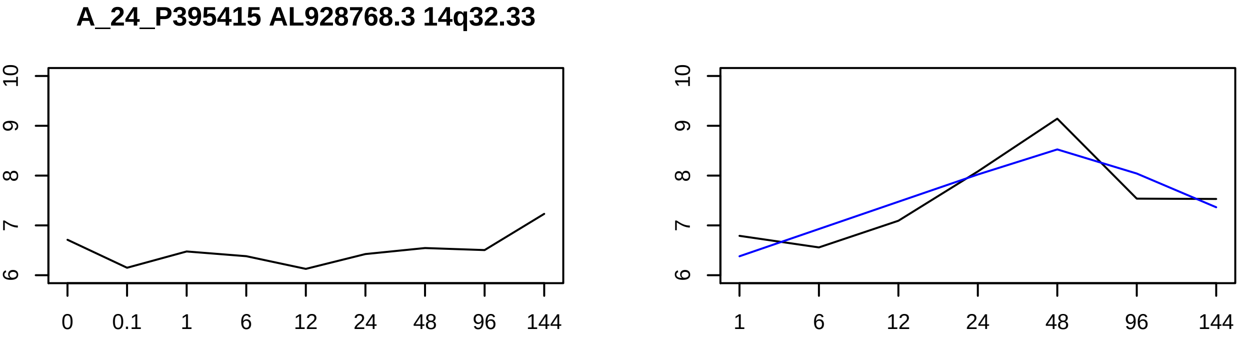
A_24_P395415 AL928768.3 14q32.33
6
7
8
9
10
0
0.1
1
6
12
24
48
96
144
1
6
12
24
48
96
144

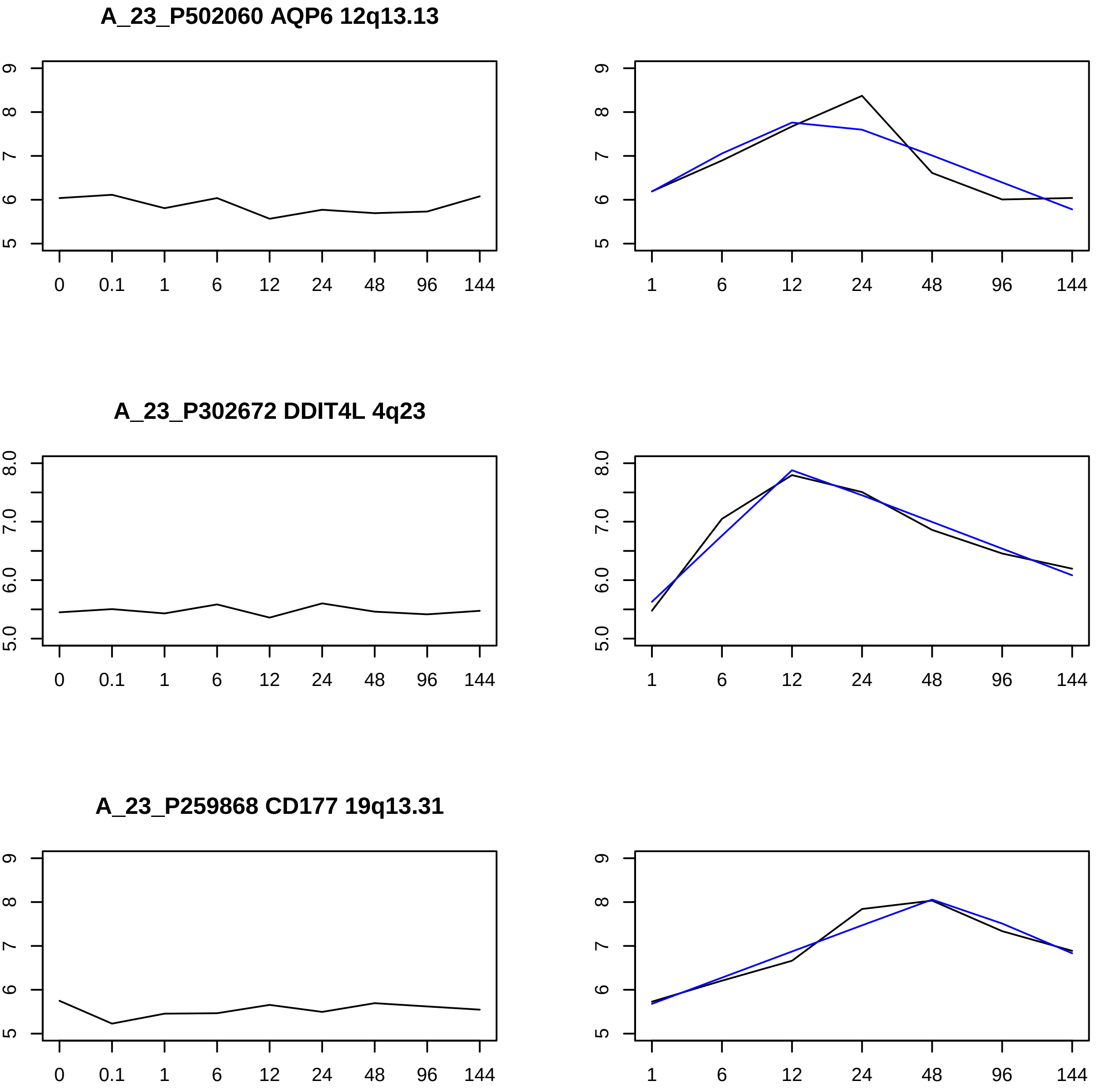
A_23_P502060 AQP6 12q13.13
A_23_P302672 DDIT4L 4q23
A_23_P259868 CD177 19q13.31
0
0.1
1
6
12
24
48
96
144
5
6
7
8
9
5.0
6.0
7.0
8.0

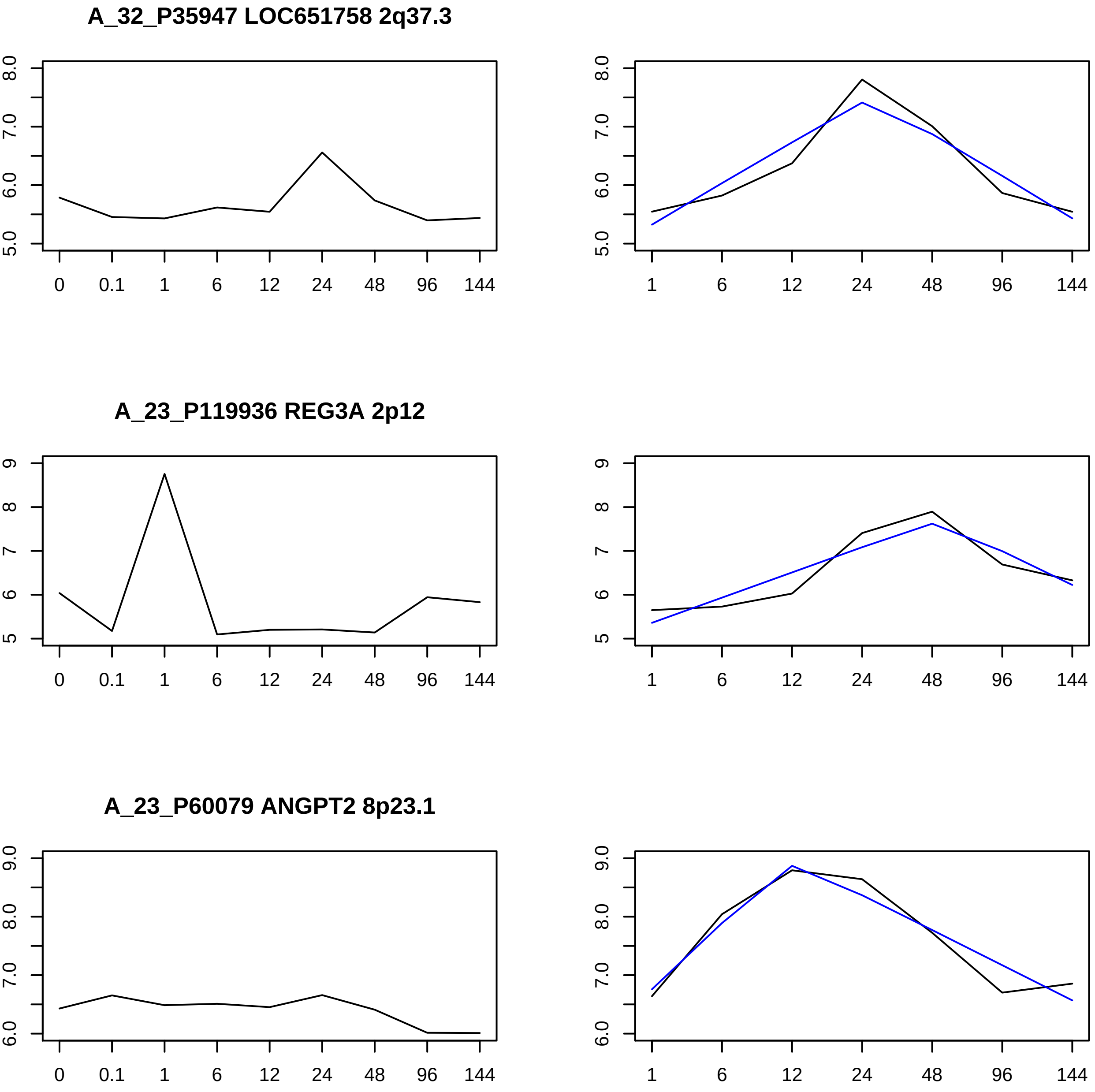
A_32_P35947 LOC651758 2q37.3
5.0
6.0
7.0
8.0
0
0.1
1
6
12
24
48
96
144
1
6
12
24
48
96
144
A_23_P119936 REG3A 2p12
5
6
7
8
9
A_23_P60079 ANGPT2 8p23.1
6.0
7.0
8.0
9.0

A_24_P112941 LRRN6A 15q24.3

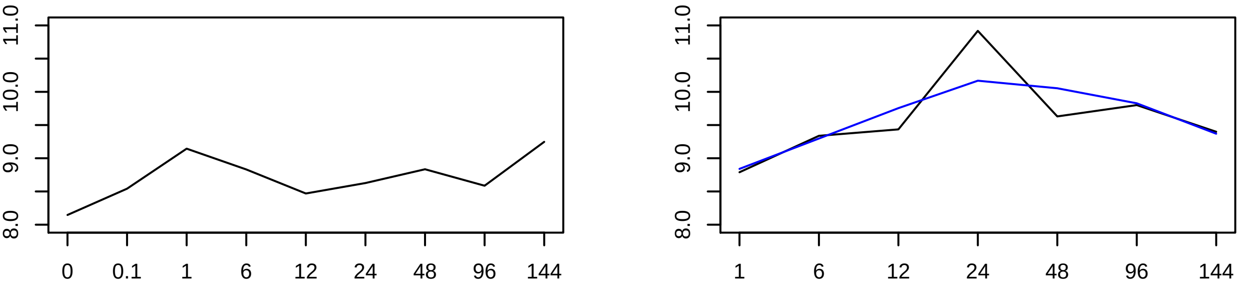

A_23_P130906 TEX101 19q13.31

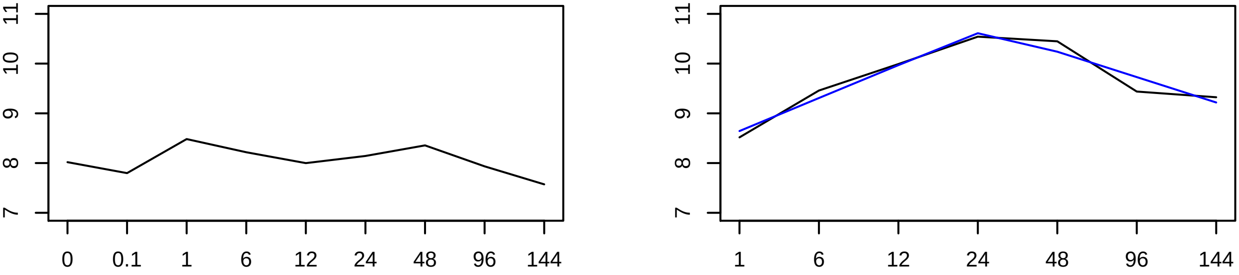

A_24_P203000 IL2RB 22q12.3

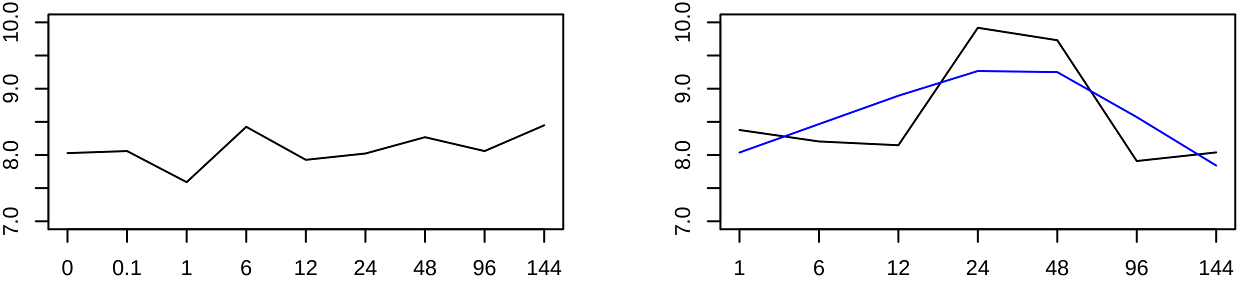

A_23_P218774 RAC2 22q13.1

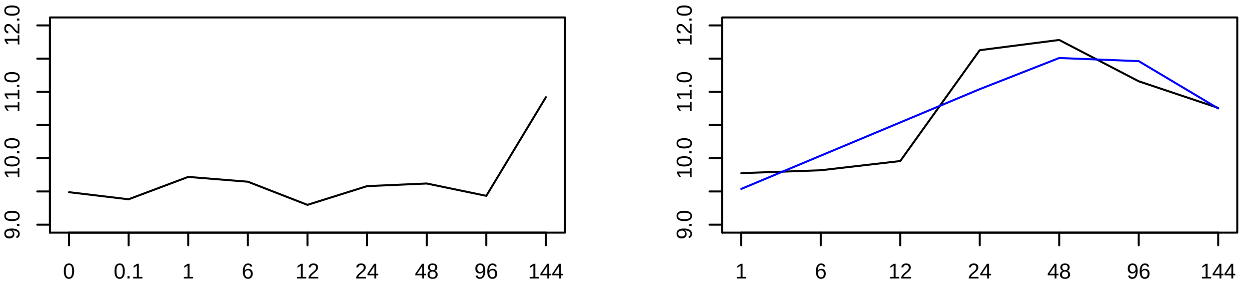

A_23_P408913 TTC30B 2q31.2

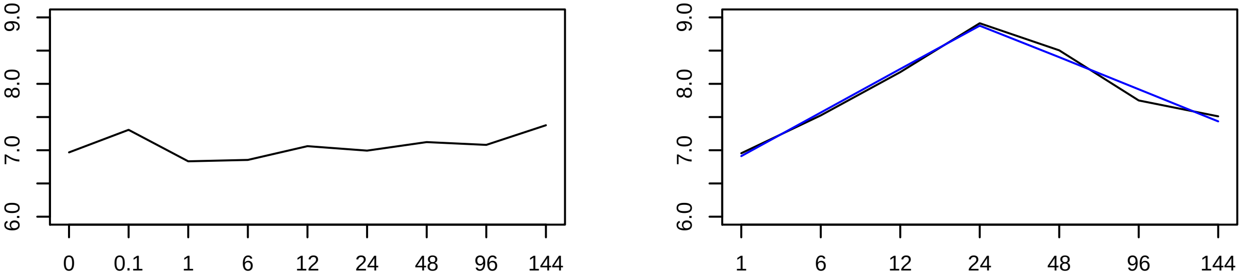

A_24_P406754 LOXL4 10q24.2

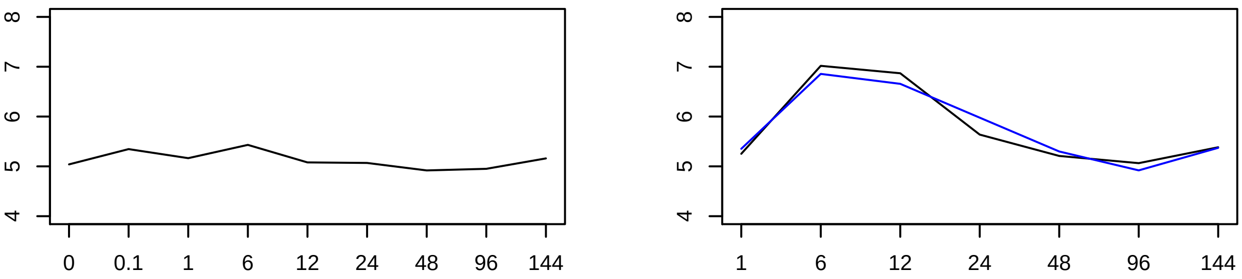

A_24_P29401 PIK3R1 5q13.1

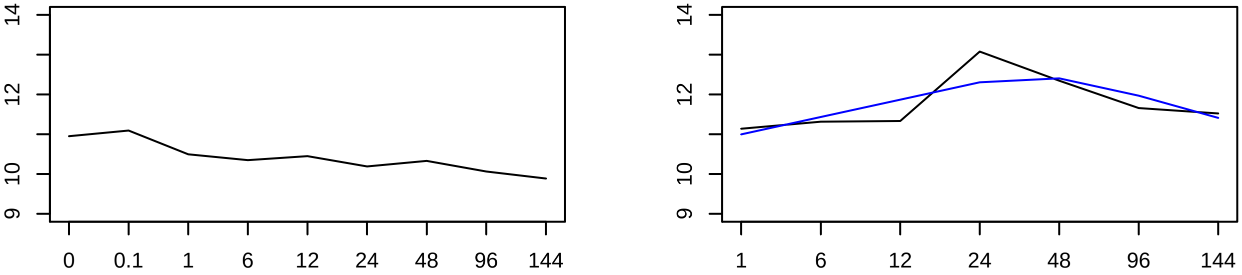

A_23_P217845 RGS16 1q25.3

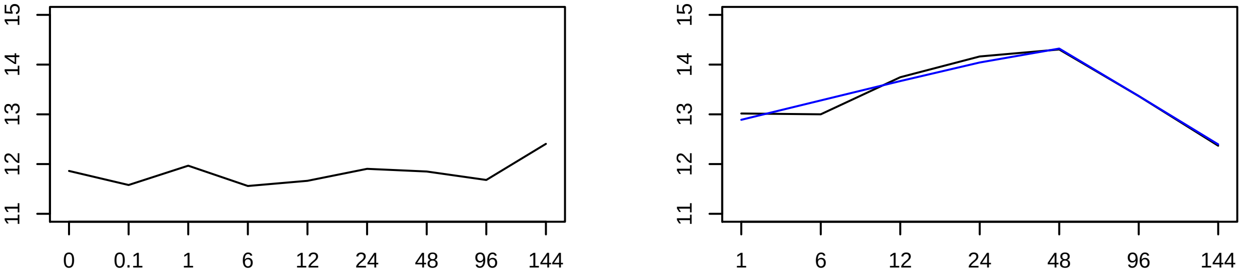

A_24_P239668 ZNF37A 10p11.21

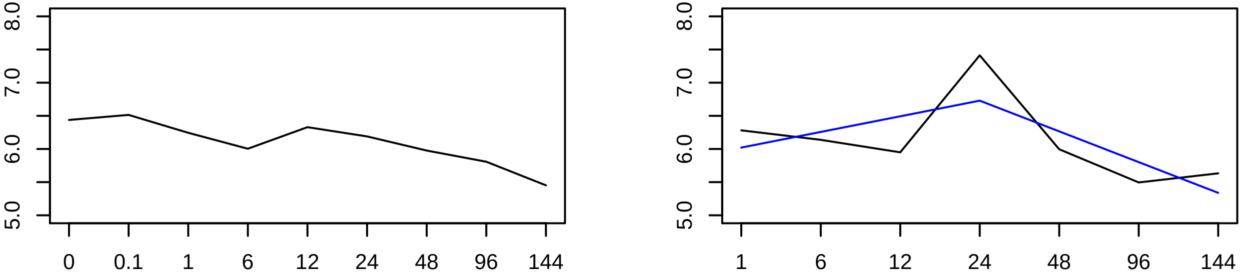

A_23_P421379 IGF2 11p15.5

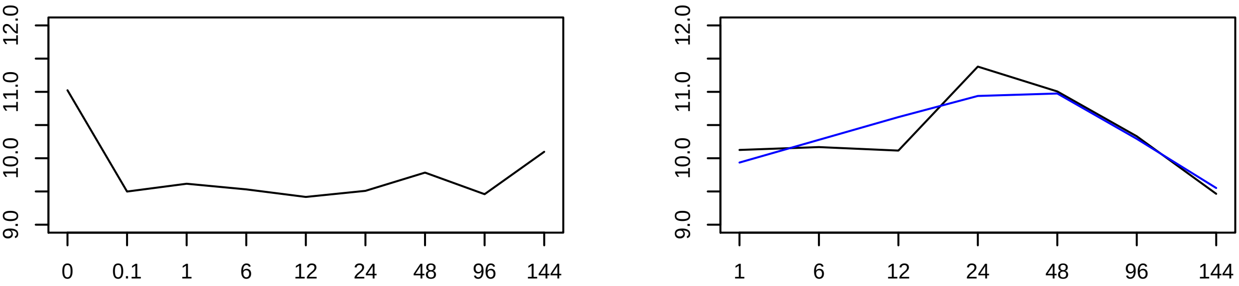

A_24_P12573 CCL26 7q11.23

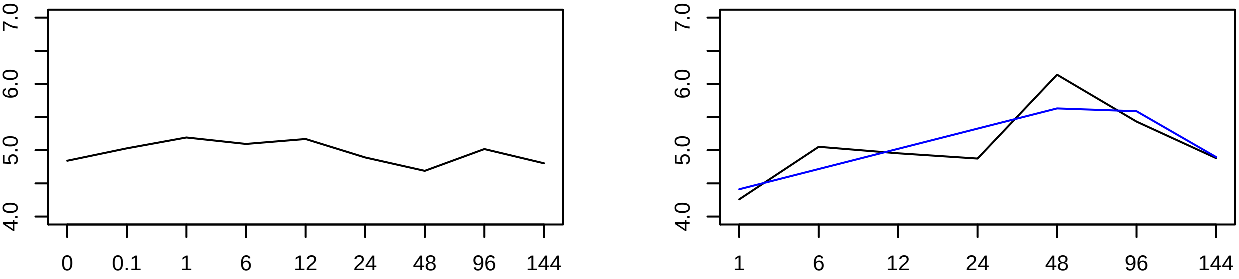

A_24_P934145 MYH6 14q11.2

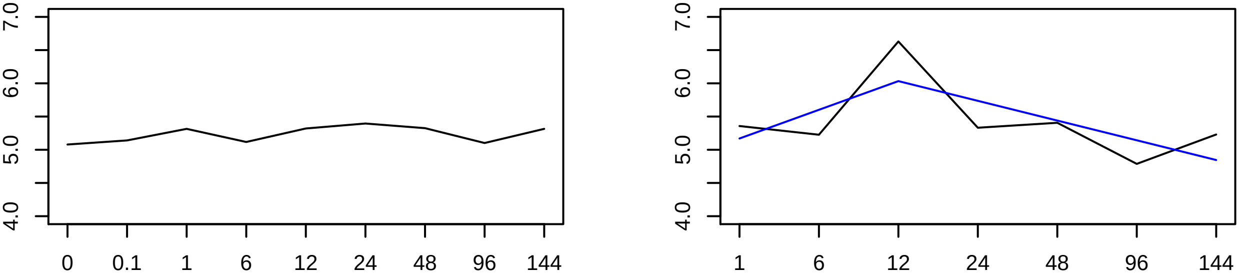

A_23_P15542 HSD17B1 17q21.31

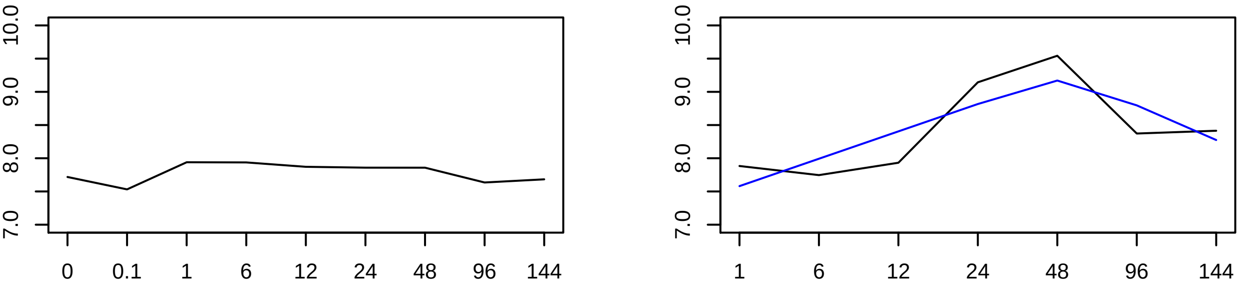

A_32_P145477 BX350256 NA

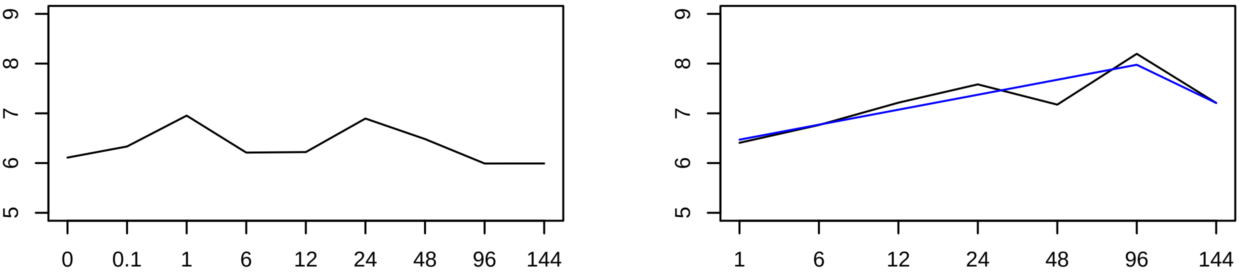

A_23_P218770 RAC2 22q13.1

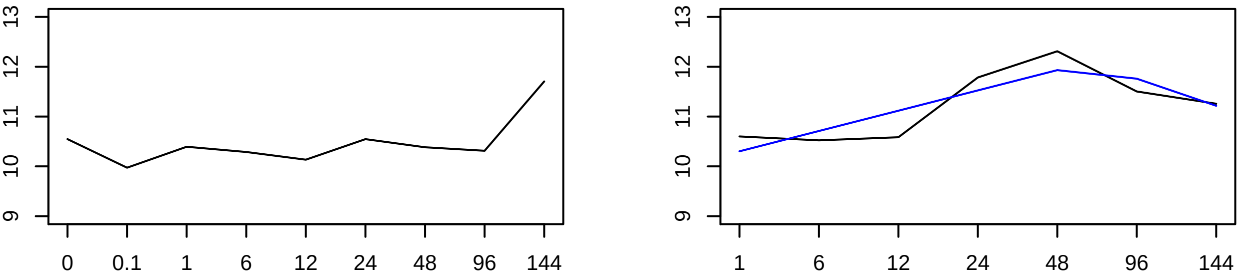

A_23_P156087 GHR 5p12

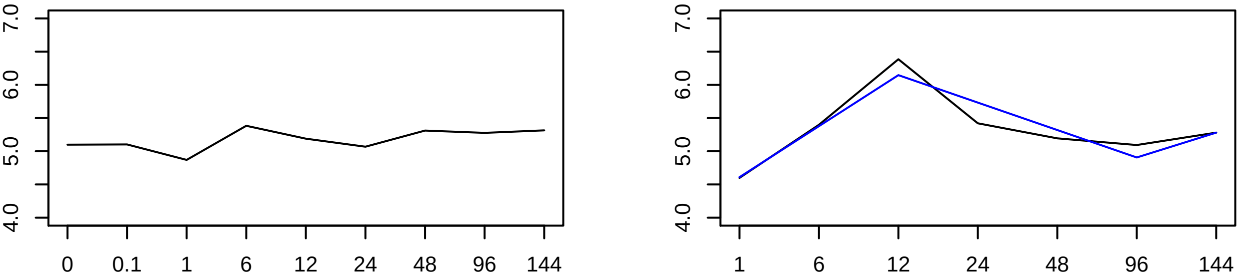

A_24_P85169 BTBD9 6p21.2

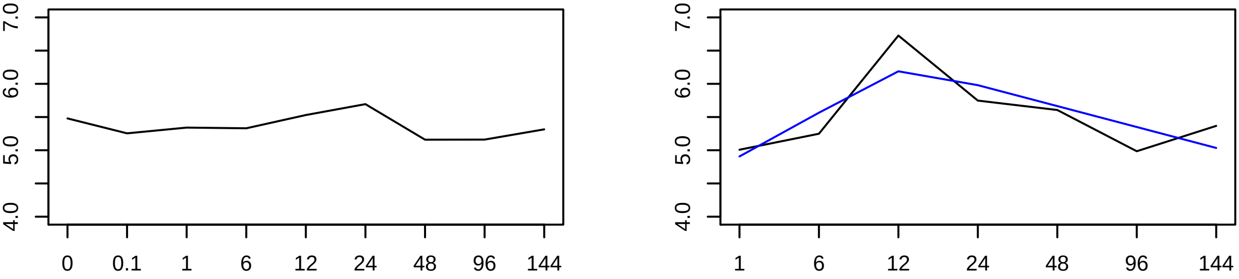

A_23_P62901 BTG2 1q32.1

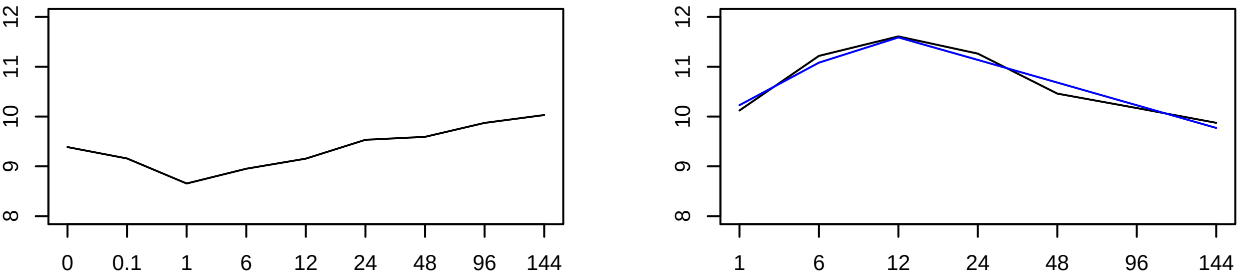

A_24_P251411 TMEM87B 2q13

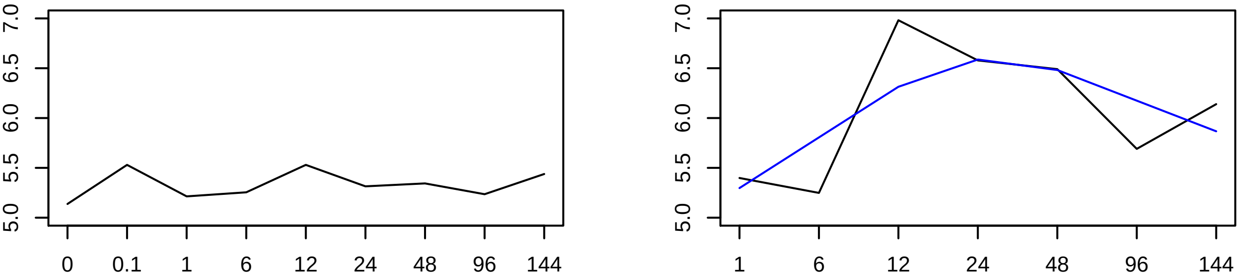

A_23_P168828 KLF10 8q22.3

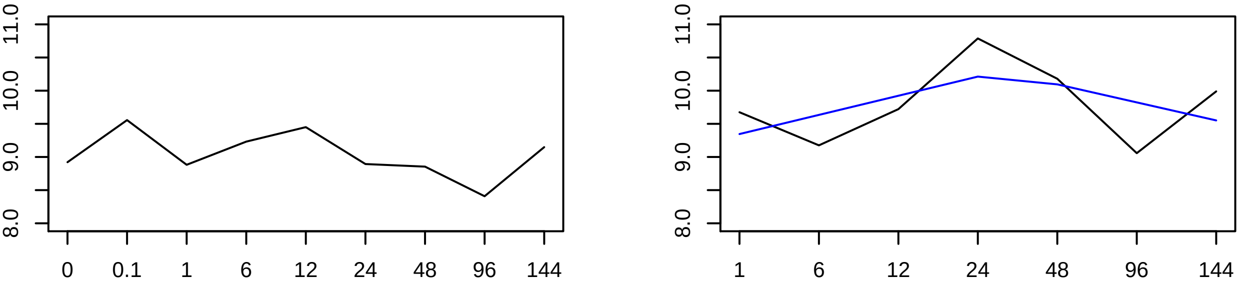

A_23_P25706 CLMN 14q32.13

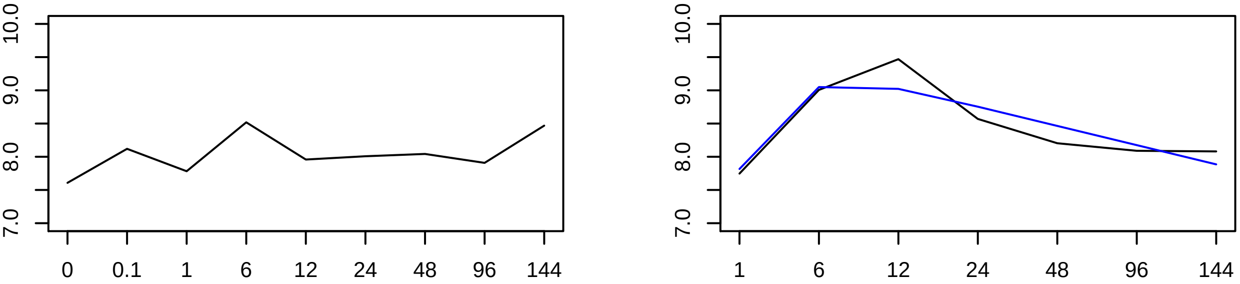

A_24_P179479 PRKD3 2p22.2

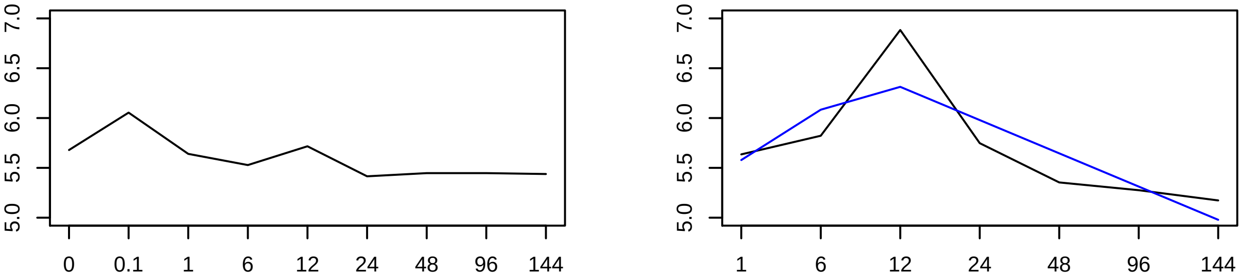

A_24_P924920 ABHD2 15q26.1

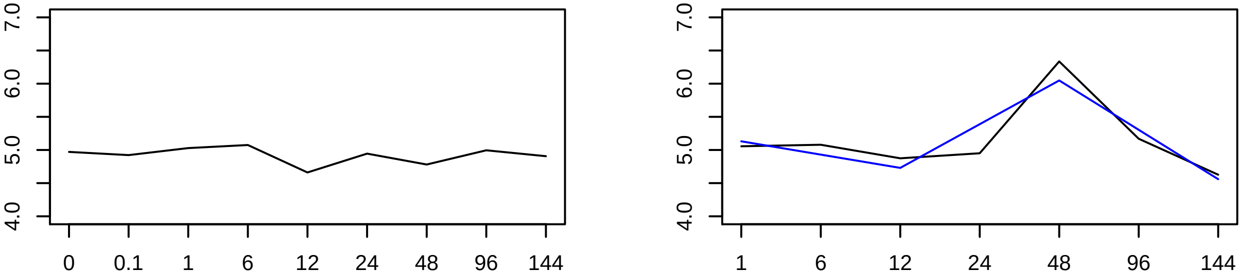

A_24_P568190 GUSBP1 5q13.2

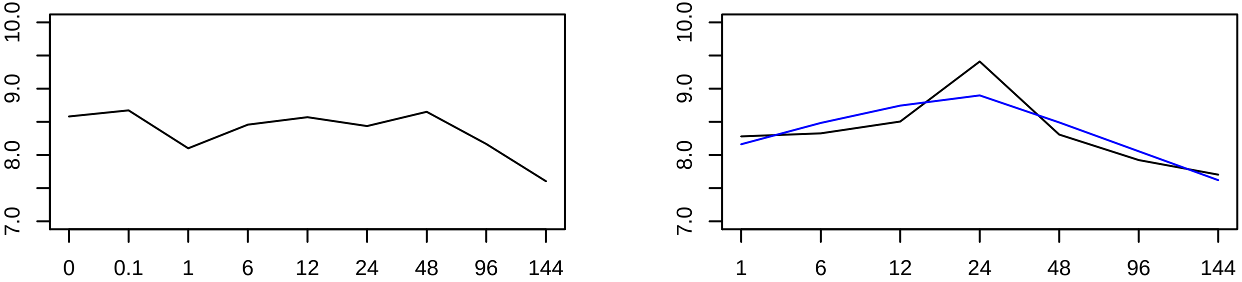

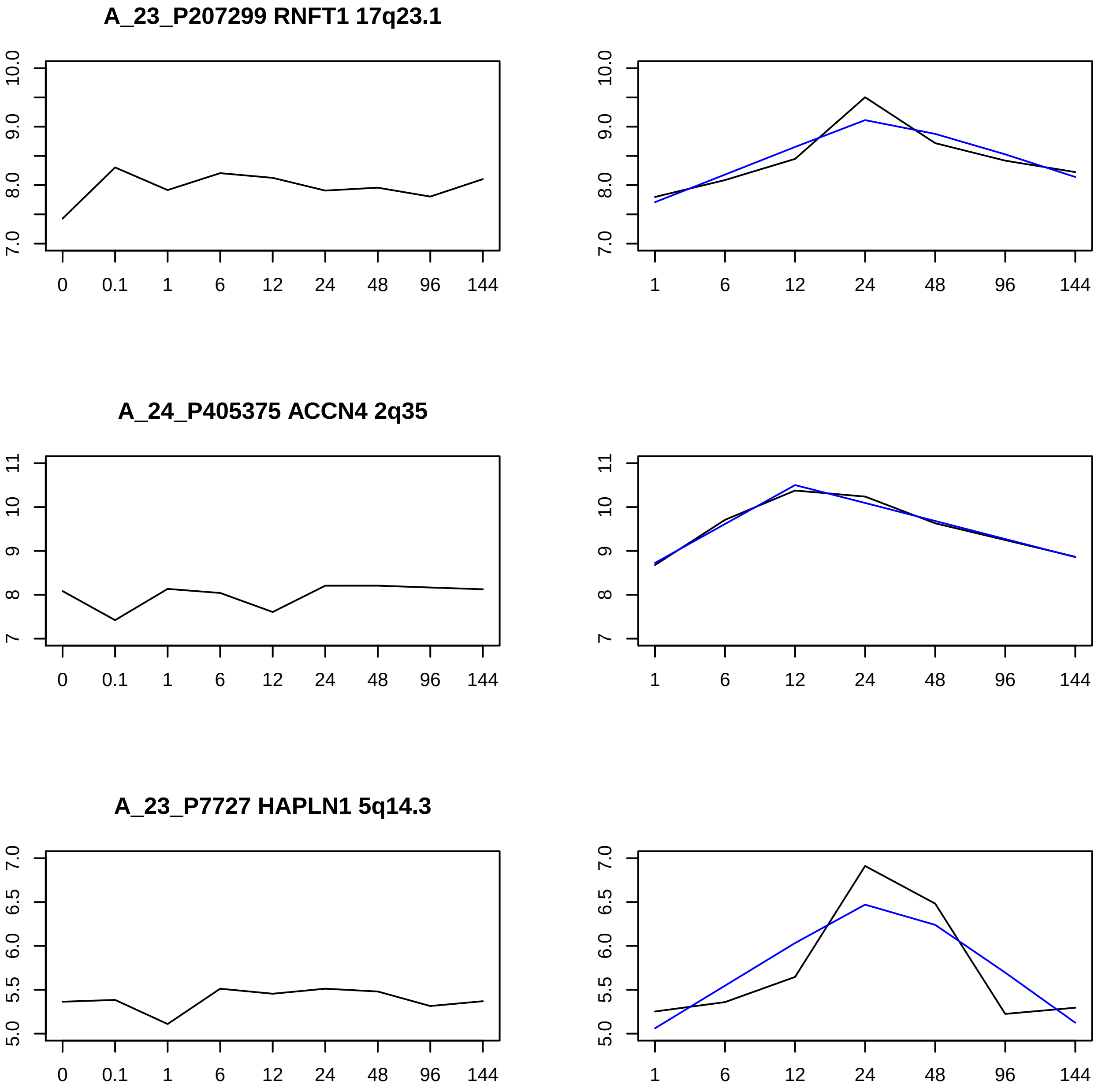
A_23_P207299 RNFT1 17q23.1
7.0
8.0
9.0
10.0
0
0.1
1
6
12
24
48
96
144
1
6
12
24
48
96
144
A_24_P405375 ACCN4 2q35
7
8
9
10
11
A_23_P7727 HAPLN1 5q14.3
5.0
5.5
6.0
6.5
7.0

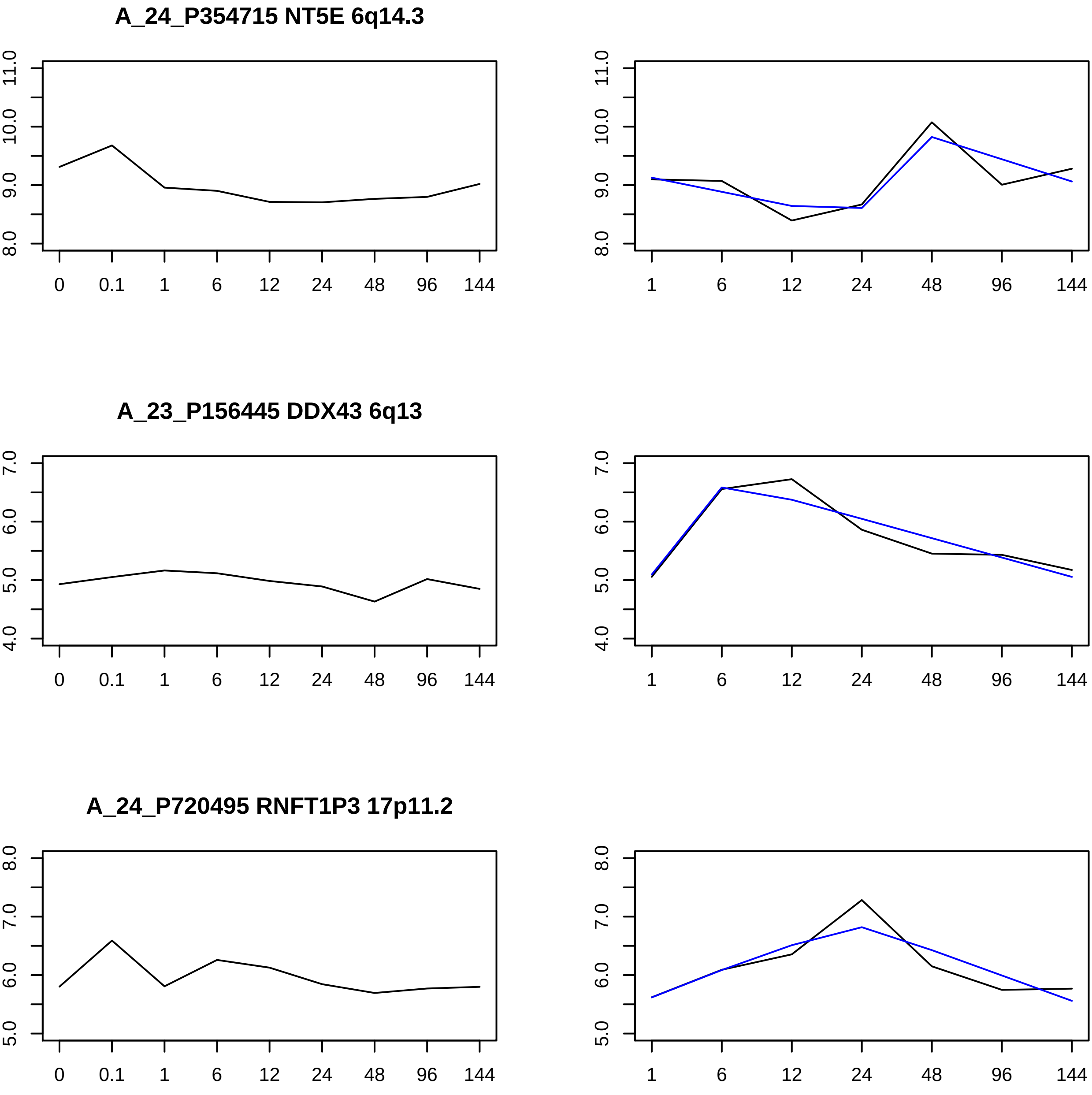
A_24_P354715 NT5E 6q14.3
8.0
9.0
10.0
11.0
0
0.1
1
6
12
24
48
96
144
A_23_P156445 DDX43 6q13
4.0
5.0
6.0
7.0
A_24_P720495 RNFT1P3 17p11.2
5.0
6.0
7.0
8.0

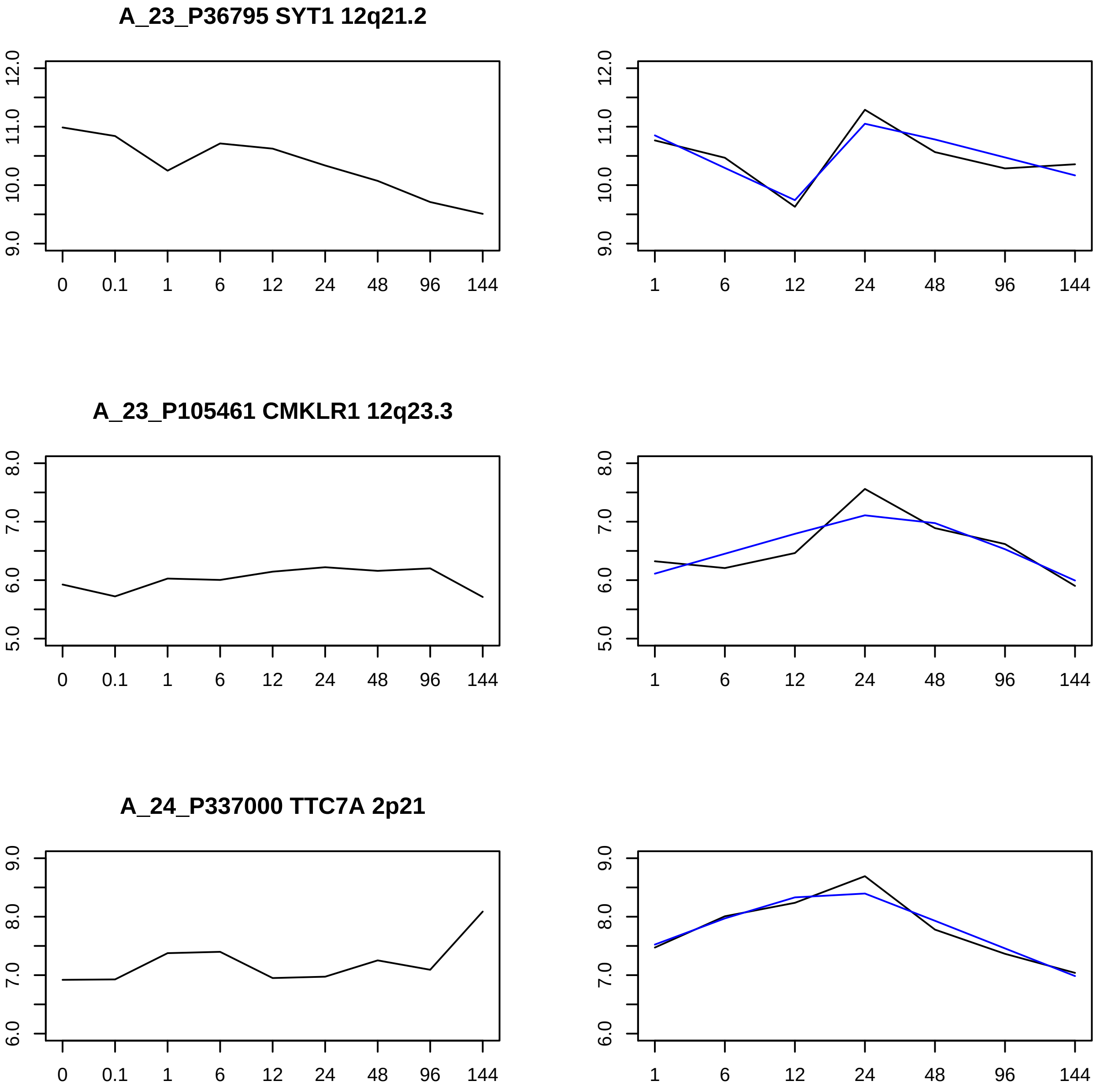
A_23_P36795 SYT1 12q21.2
A_23_P105461 CMKLR1 12q23.3
A_24_P337000 TTC7A 2p21
9.0 10.0 11.0 12.0
5.0 6.0 7.0 8.0
6.0 7.0 8.0 9.0
0 0.1 1 6 12 24 48 96 144
1 6 12 24 48 96 144

A_23_P119916 WNT6 2q35

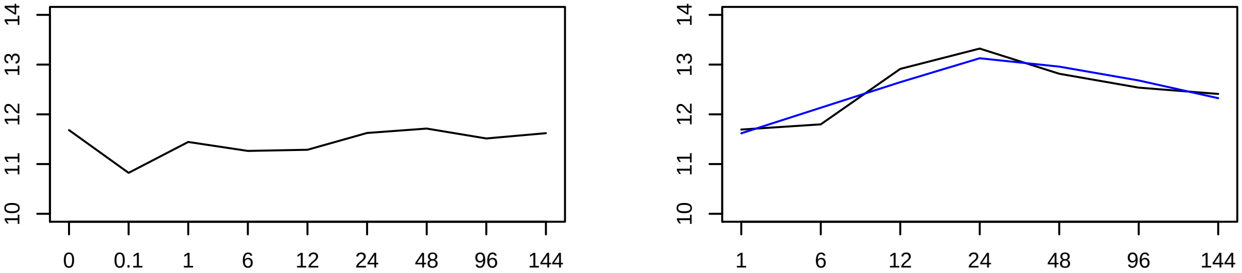

A_24_P918752 CHD7 8q12.2

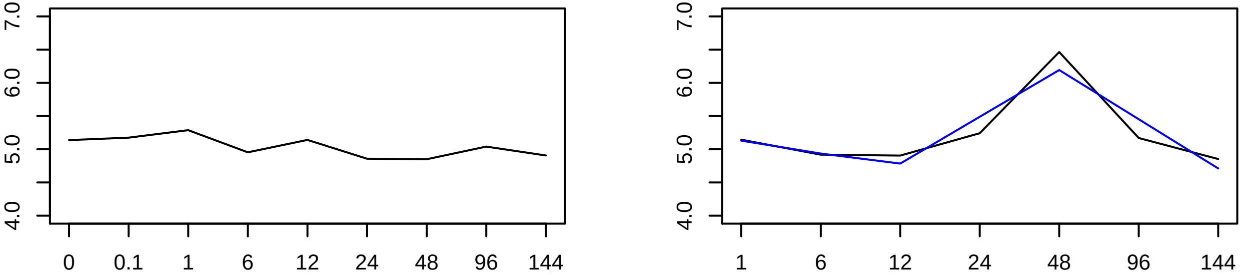

A_23_P129425 TSNAXIP1 16q22.1

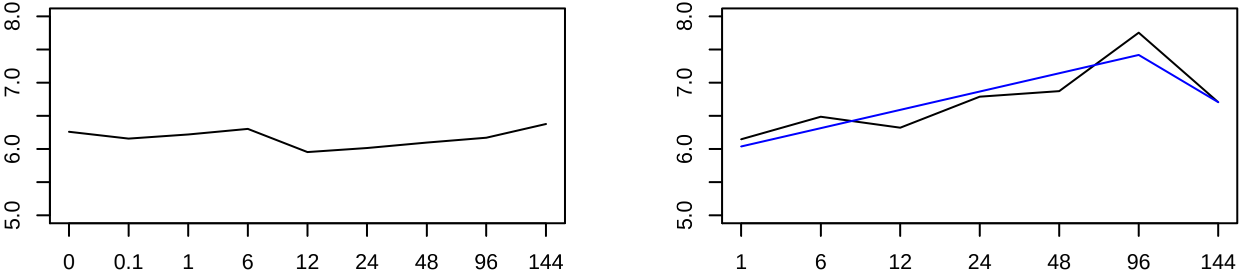

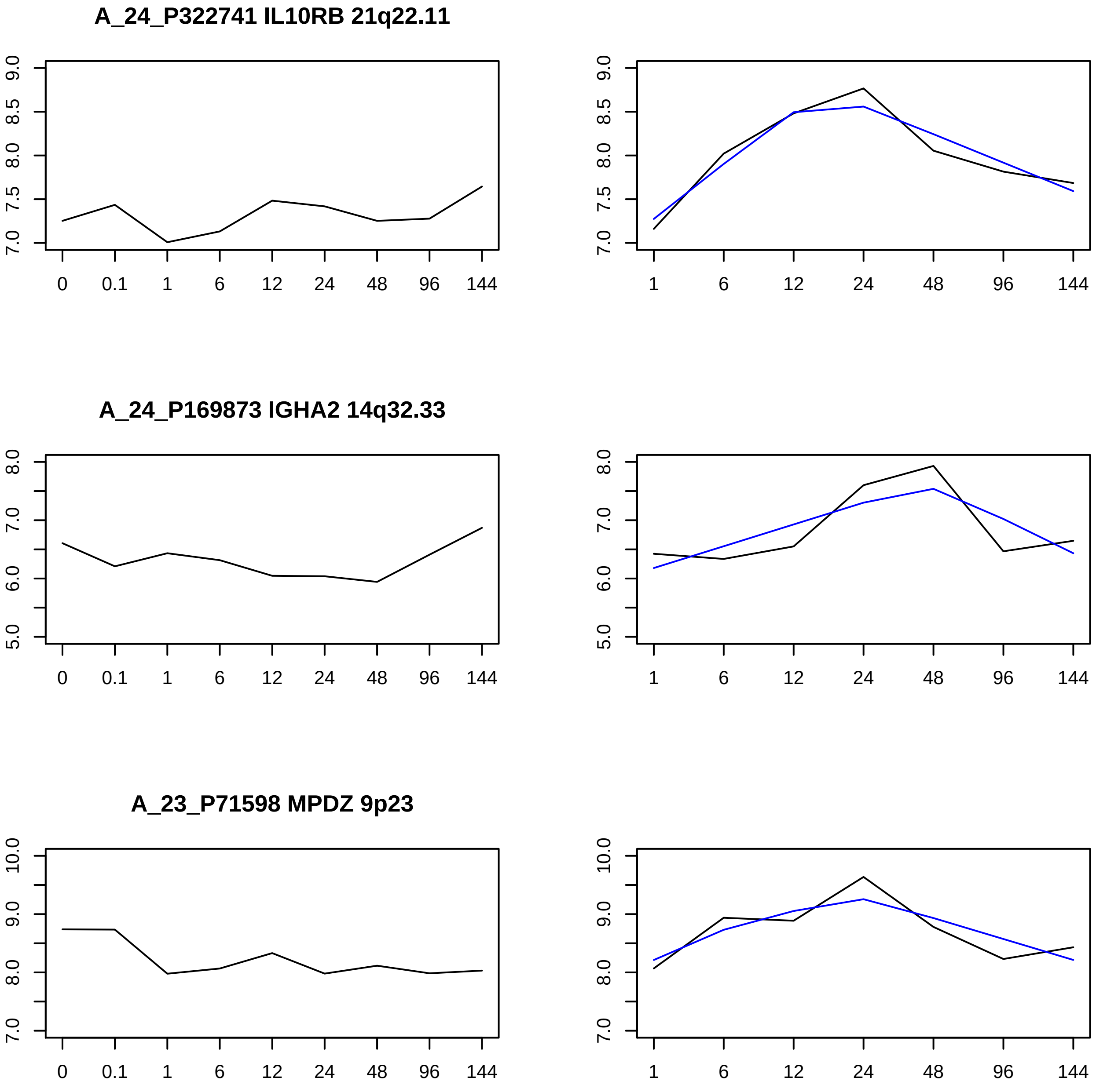
A_24_P322741 IL10RB 21q22.11
7.0
7.5
8.0
8.5
9.0
0
0.1
1
6
12
24
48
96
144
A_24_P169873 IGHA2 14q32.33
5.0
6.0
7.0
8.0
A_23_P71598 MPDZ 9p23
7.0
8.0
9.0
10.0

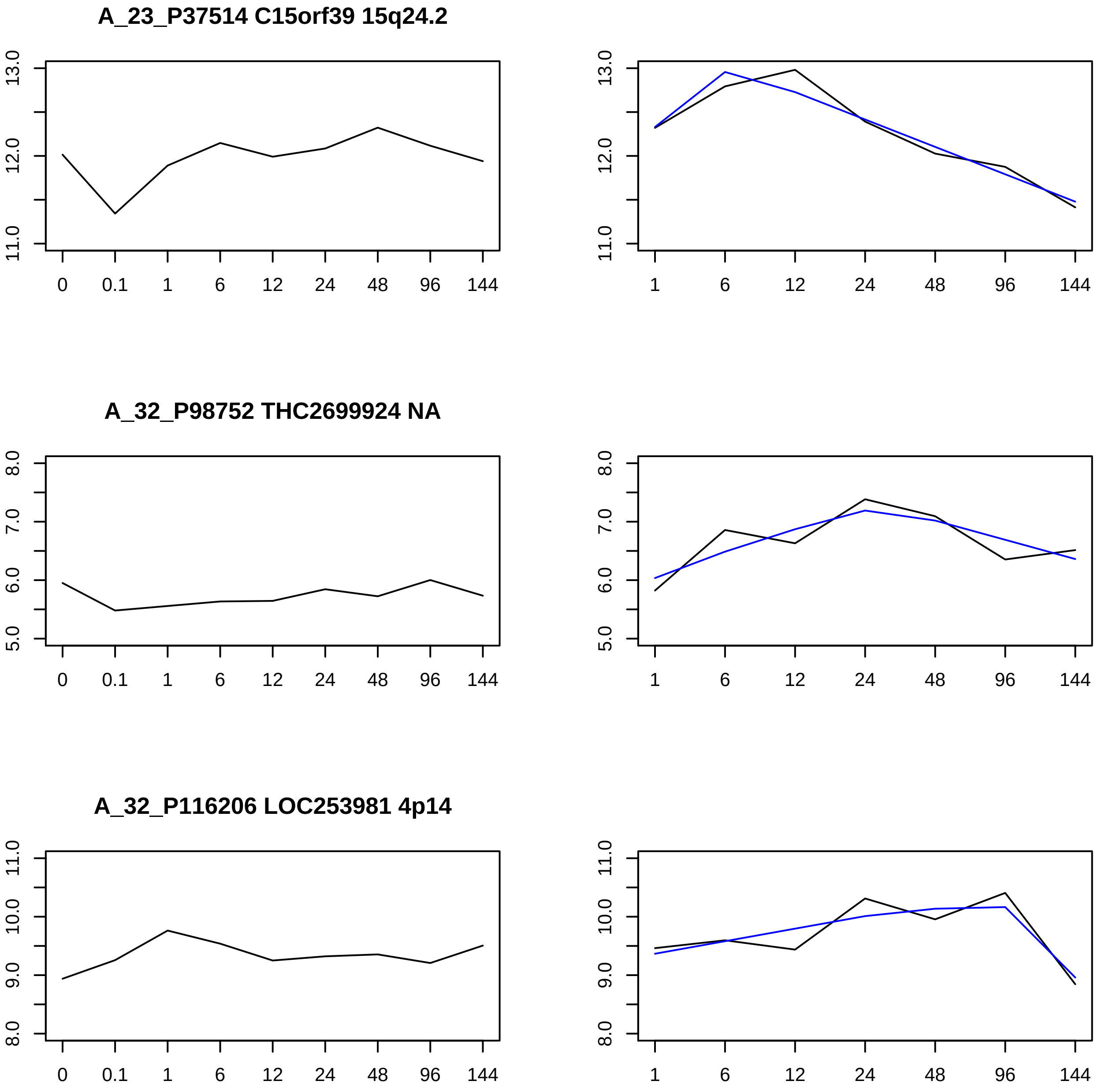
A_23_P37514 C15orf39 15q24.2
11.0
12.0
13.0
0 0.1 1 6 12 24 48 96 144
1 6 12 24 48 96 144
A_32_P98752 THC2699924 NA
5.0
6.0
7.0
8.0
0 0.1 1 6 12 24 48 96 144
1 6 12 24 48 96 144
A_32_P116206 LOC253981 4p14
8.0
9.0
10.0
11.0
0 0.1 1 6 12 24 48 96 144
1 6 12 24 48 96 144

A_24_P170983 ESPNL 2q37.3

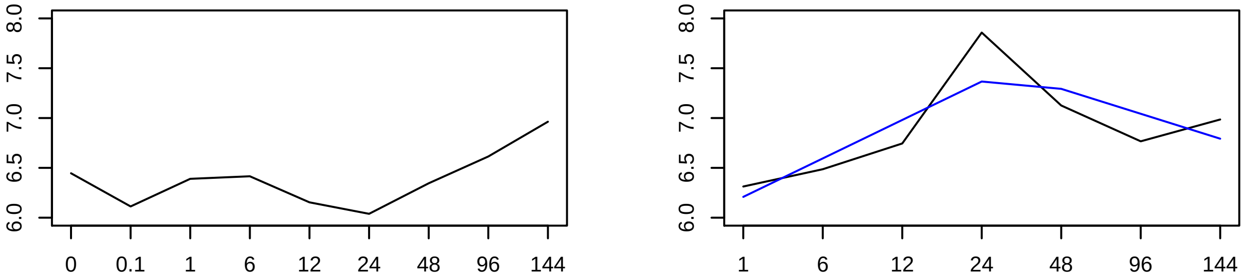

A_24_P450092 LOC254057 3q21.3

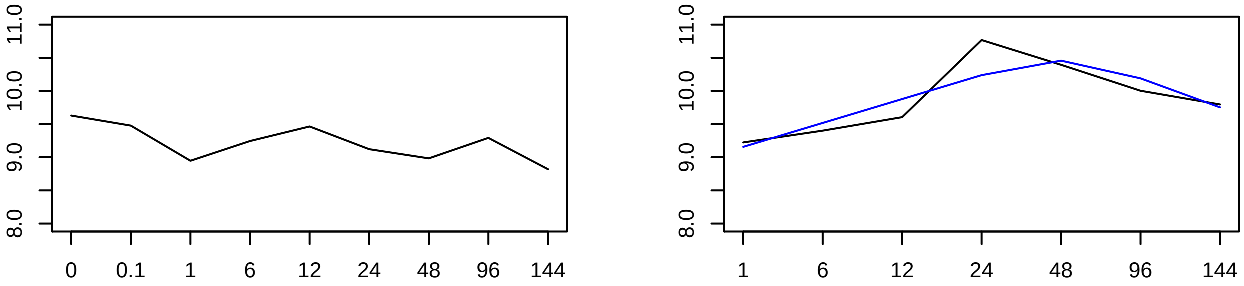

A_24_P673786 PIP5K2A 10p12.2

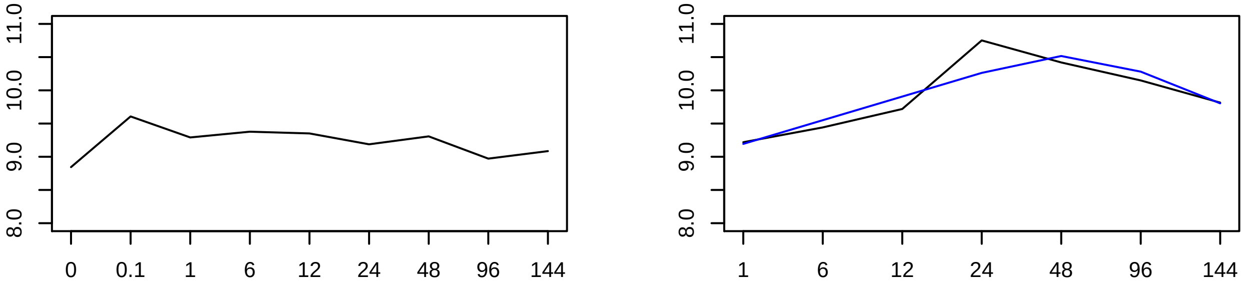

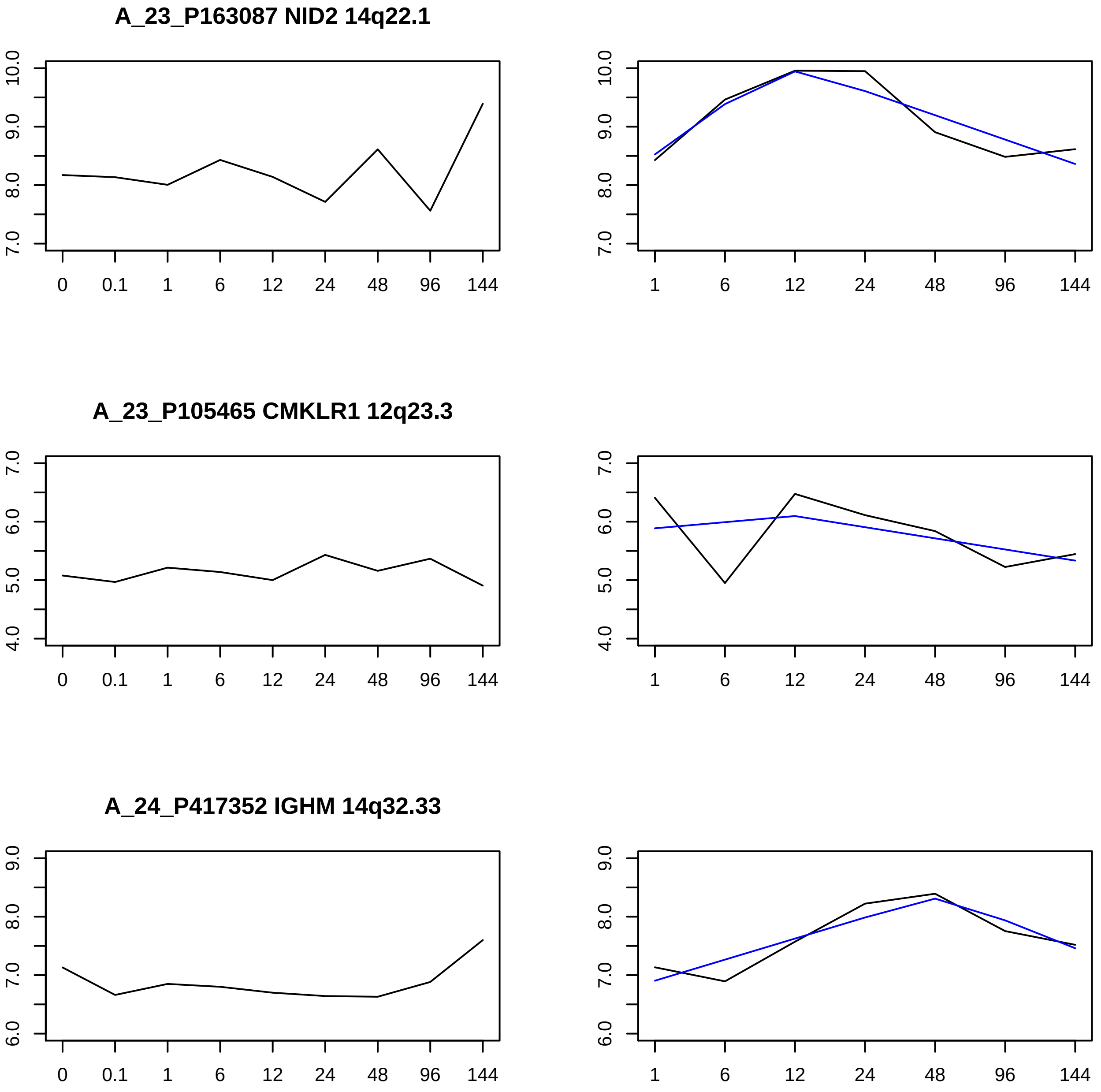
A_23_P163087 NID2 14q22.1
7.0
8.0
9.0
10.0
0
0.1
1
6
12
24
48
96
144
7.0
8.0
9.0
10.0
1
6
12
24
48
96
144
A_23_P105465 CMKLR1 12q23.3
4.0
5.0
6.0
7.0
0
0.1
1
6
12
24
48
96
144
4.0
5.0
6.0
7.0
1
6
12
24
48
96
144
A_24_P417352 IGHM 14q32.33
6.0
7.0
8.0
9.0
0
0.1
1
6
12
24
48
96
144
6.0
7.0
8.0
9.0
1
6
12
24
48
96
144

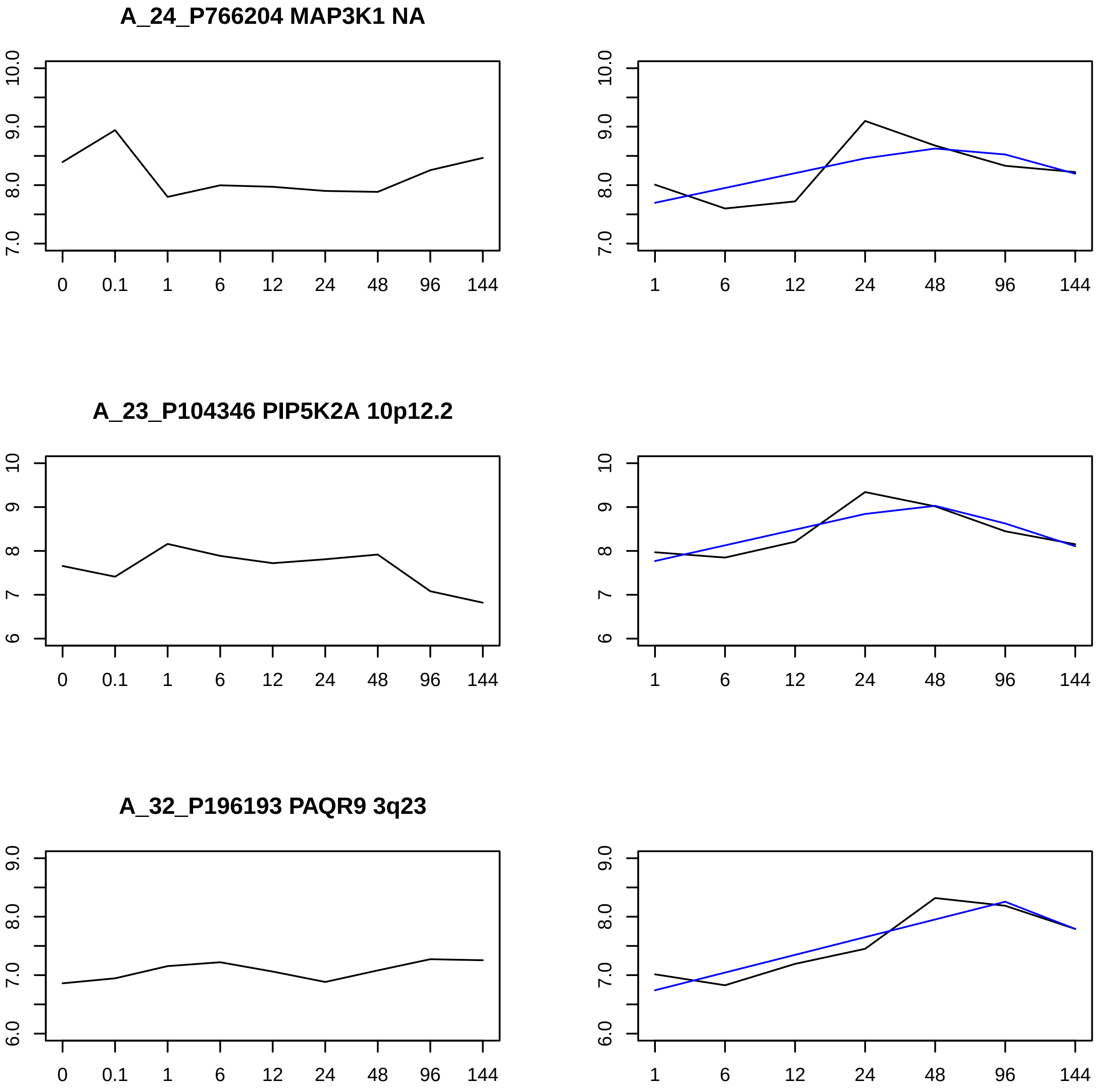
A_24_P766204 MAP3K1 NA
7.0
8.0
9.0
10.0
0
0.1
1
6
12
24
48
96
144
A_23_P104346 PIP5K2A 10p12.2
6
7
8
9
10
A_32_P196193 PAQR9 3q23
6.0
7.0
8.0
9.0

A_23_P320578 RGS16 1q25.3

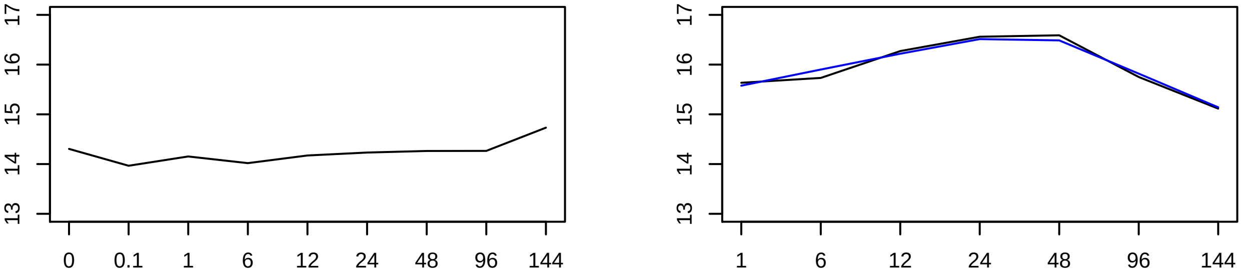

A_24_P260443 THBS4 5q14.1

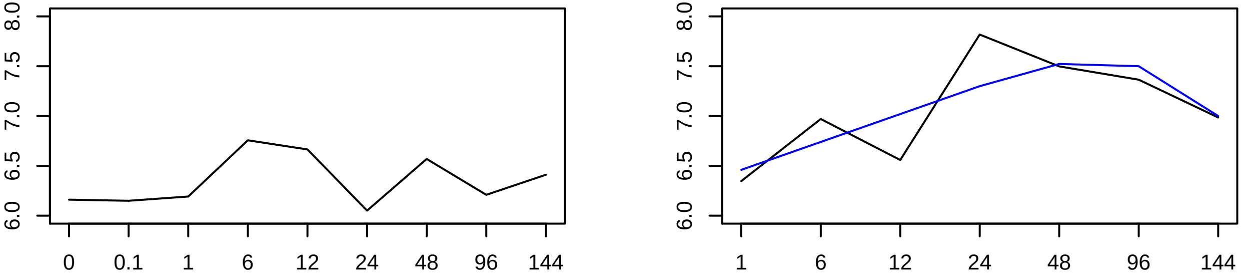

A_24_P548264 THC2510261 NA

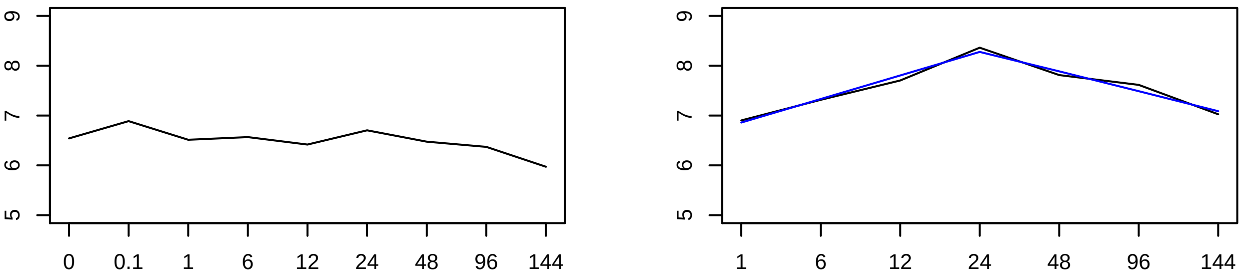

A_24_P6933 TPCN2 NA

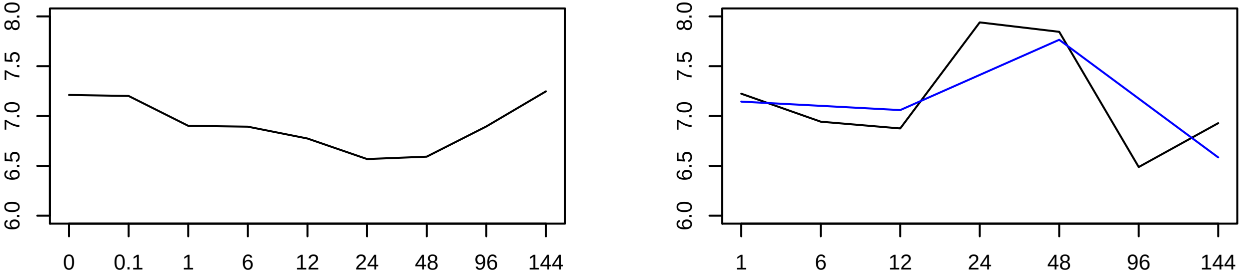

A_23_P206661 NQO1 16q22.1

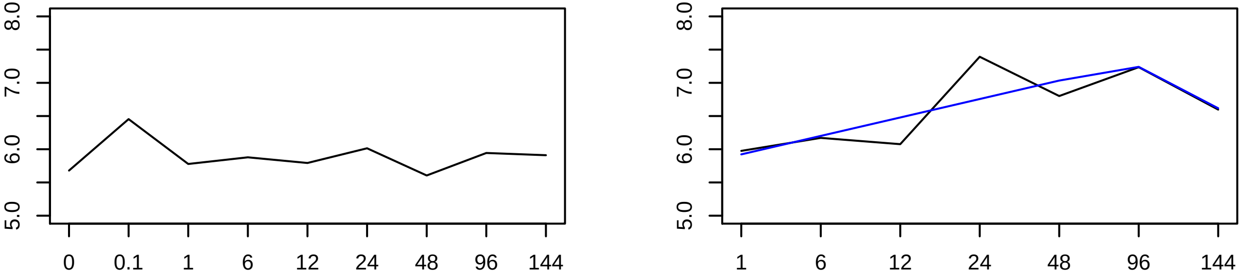

A_23_P436284 OSTbeta 15q22.31

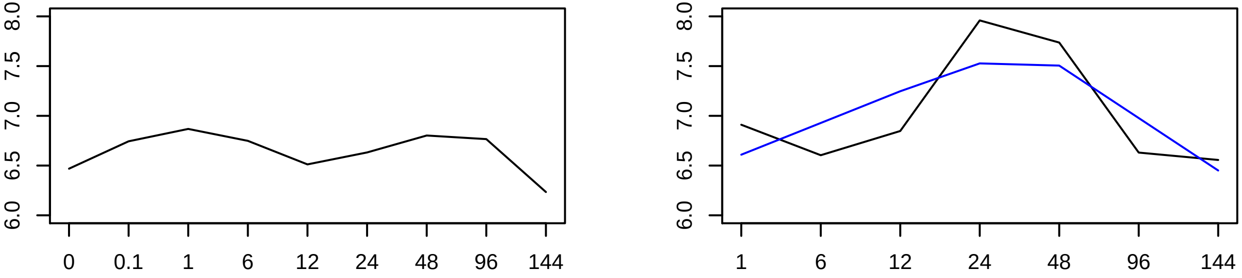

A_23_P144980 PIK3R1 5q13.1

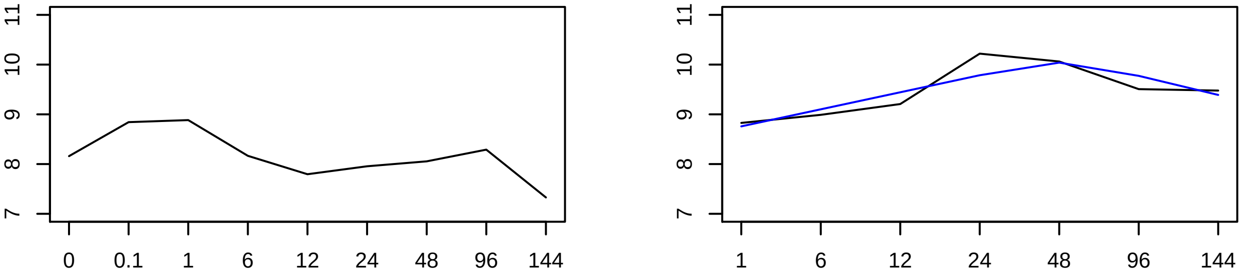

A_32_P184746 THC2715480 NA

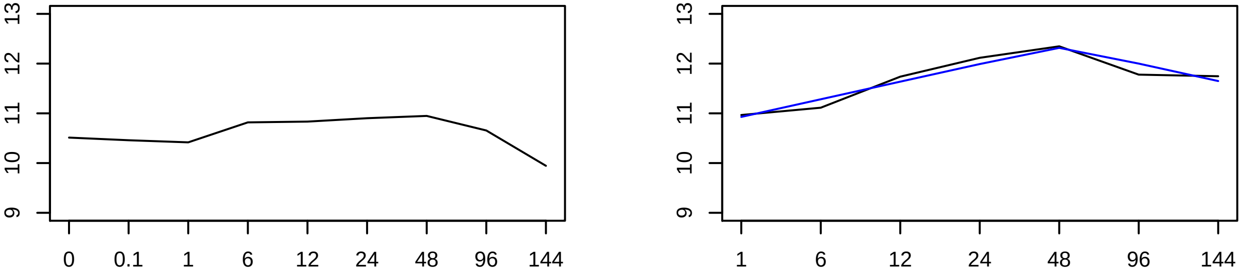

A_23_P155057 PSCD4 22q13.1

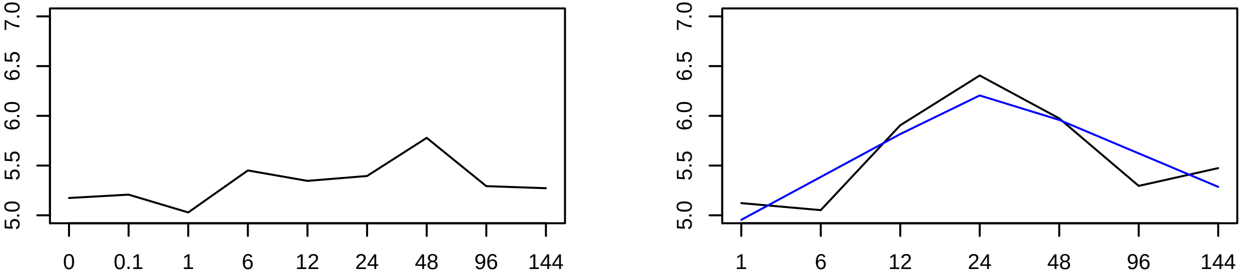

A_23_P64611 P2RY6 11q13.4

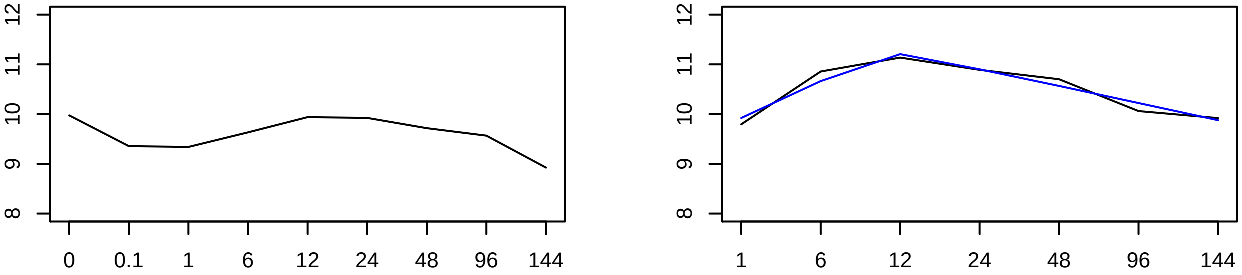

A_32_P112752 LOC440084 12p13.2

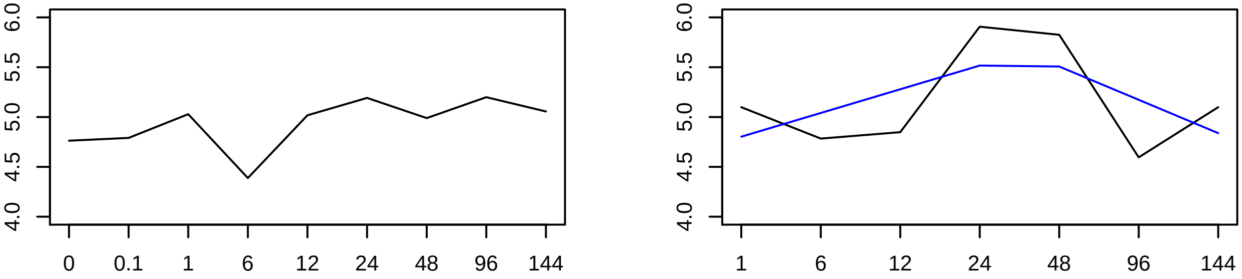

A_23_P131611 TTC30A 2q31.2

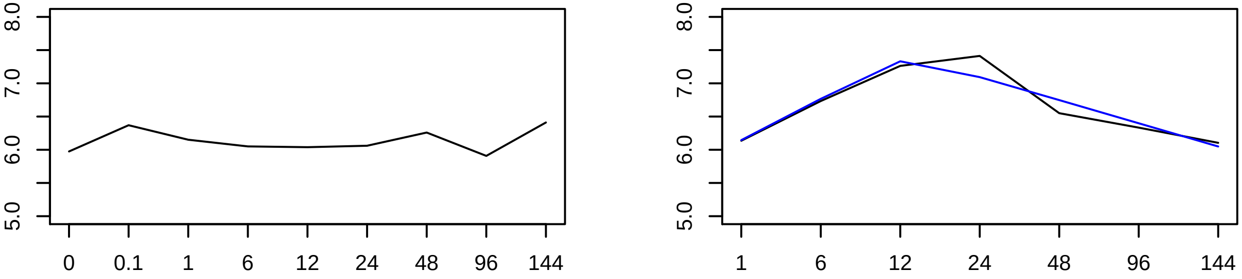

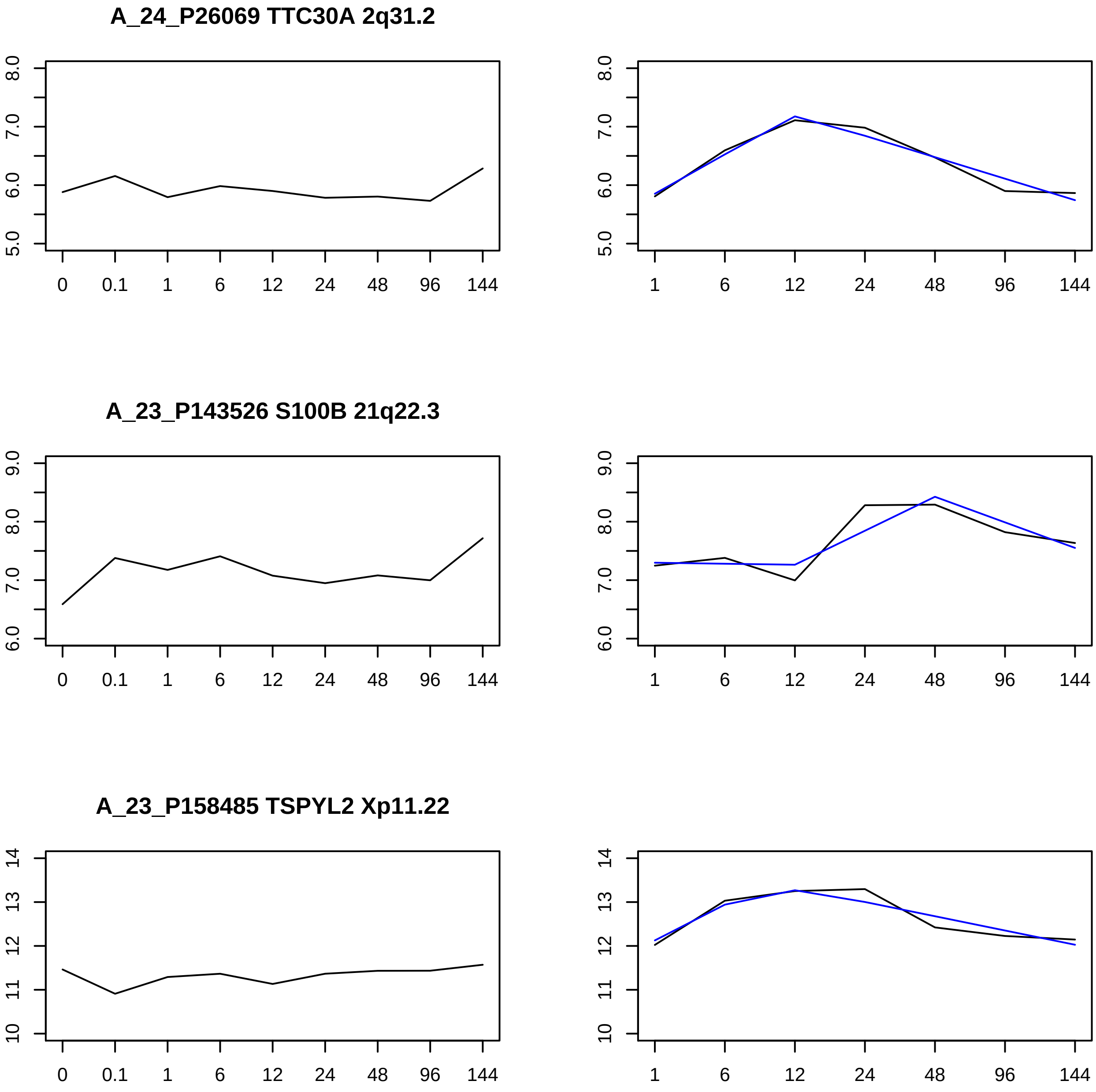
A_24_P26069 TTC30A 2q31.2
5.0
6.0
7.0
8.0
0
0.1
1
6
12
24
48
96
144
5.0
6.0
7.0
8.0
1
6
12
24
48
96
144
A_23_P143526 S100B 21q22.3
6.0
7.0
8.0
9.0
0
0.1
1
6
12
24
48
96
144
6.0
7.0
8.0
9.0
1
6
12
24
48
96
144
A_23_P158485 TSPYL2 Xp11.22
10
11
12
13
14
0
0.1
1
6
12
24
48
96
144
10
11
12
13
14
1
6
12
24
48
96
144

A_23_P170608 TSPYL2 Xp11.22

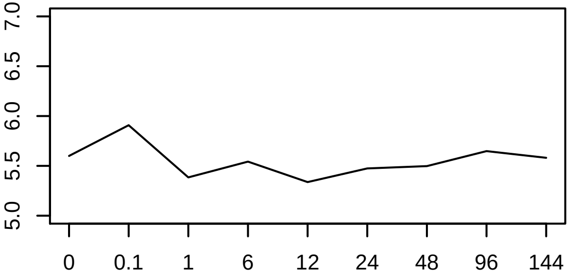

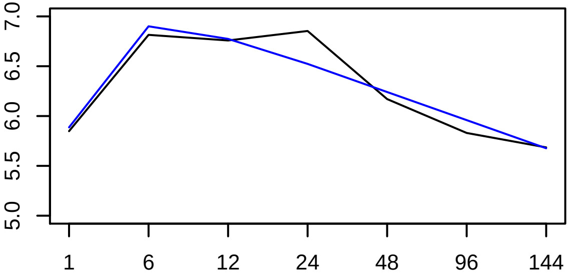

Supplement: Additional file 3 — Additional file A-H. These files contain the fitting results for the genes from the groups A-H, deduced by SwitchFinder, which represent eight dynamic patterns of the gene expression response to ATRA in neuroblastoma cell line. (ZIP 2457 kb) [file 12859_2016_1391_MOESM3_ESM.zip › AdditionalFile_H.pdf]
