# Supplementary material for: SwitchFinder – a novel method and query facility for discovering dynamic gene expression patterns
Source: BMC Bioinformatics. 2016 Dec 15;17:532. doi: 10.1186/s12859-016-1391-0 (PMC5160026; doi:10.1186/s12859-016-1391-0)

**A_23_P84399 CNTNAP2 7q36.1**

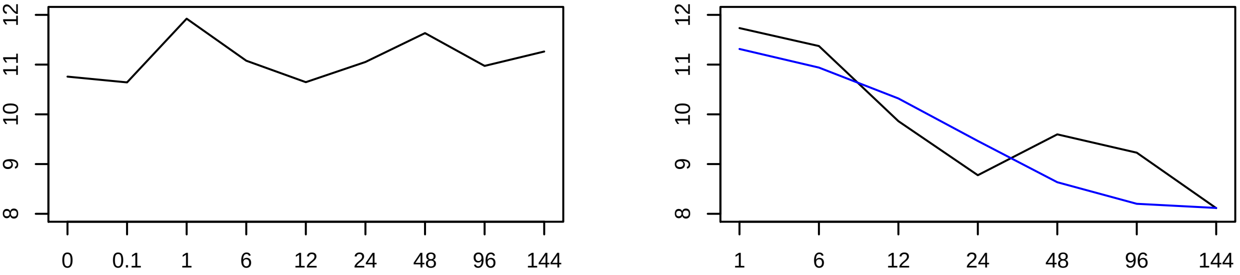


**A_24_P174793 PCSK1 5q15**

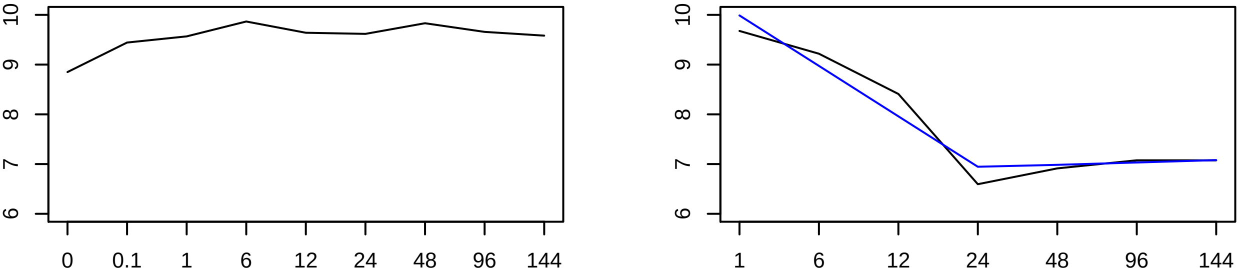


**A_23_P87310 LMO1 11p15.4**

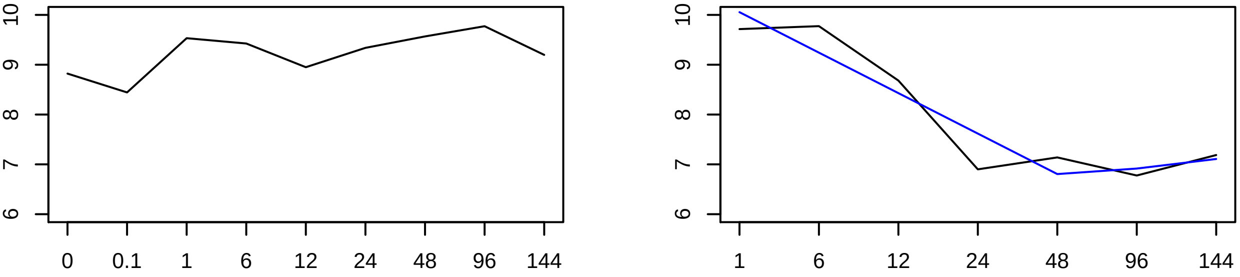

**A_23_P422851 CABLES1 18q11.2**

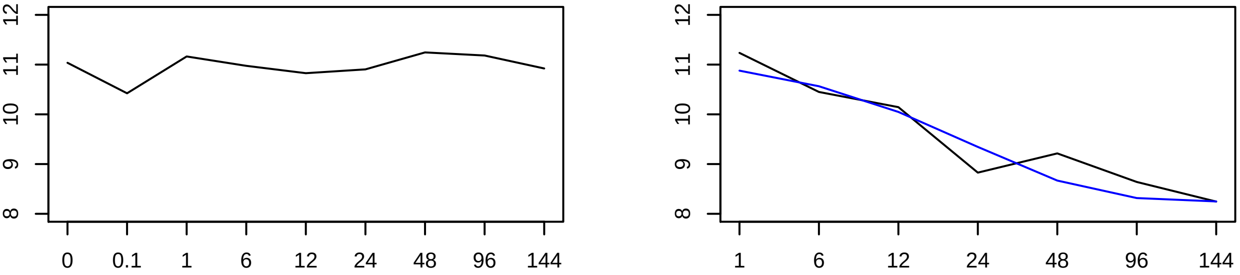


**A_23_P74449 GLOXD1 1p34.1**

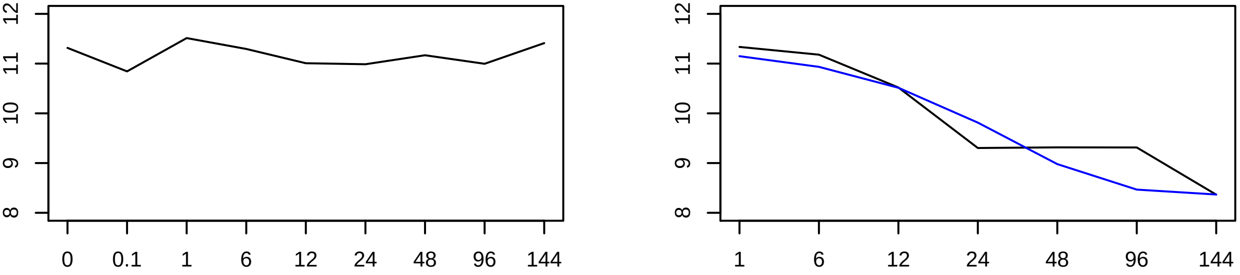


**A_23_P26426 CPNE7 16q24.3**

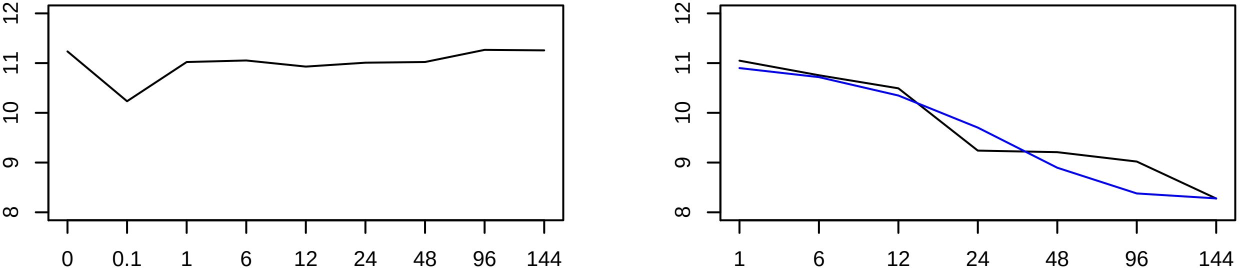

**A_23_P35995 ASAM 11q24.1**

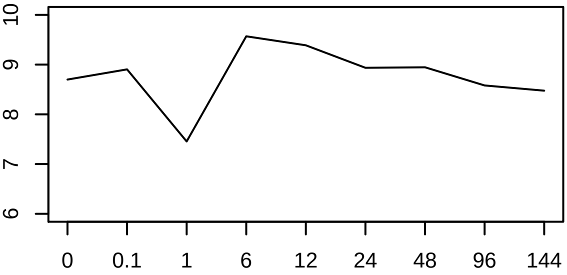


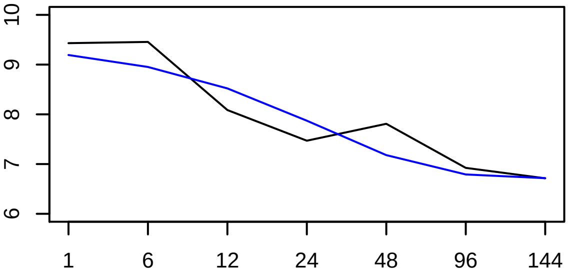


**A_24_P274795 CDCA7L 7p15.3**

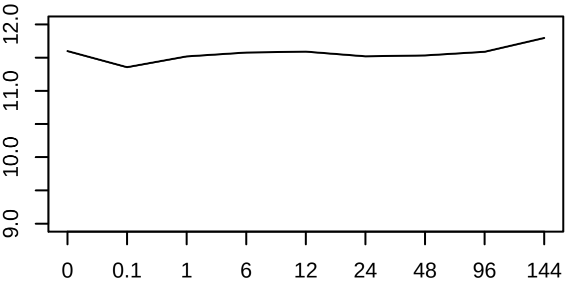


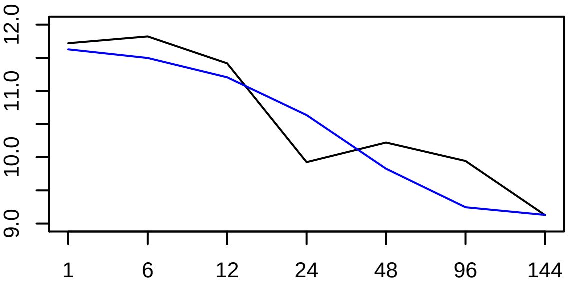


**A_23_P215976 CCNE2 8q22.1**

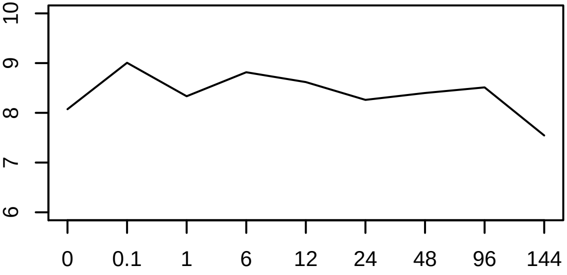


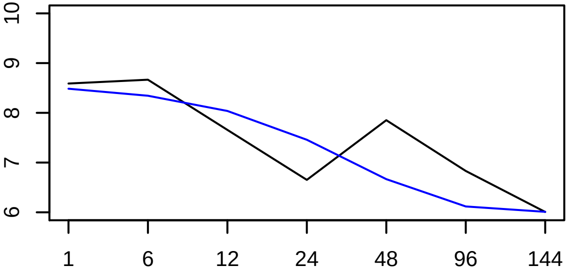

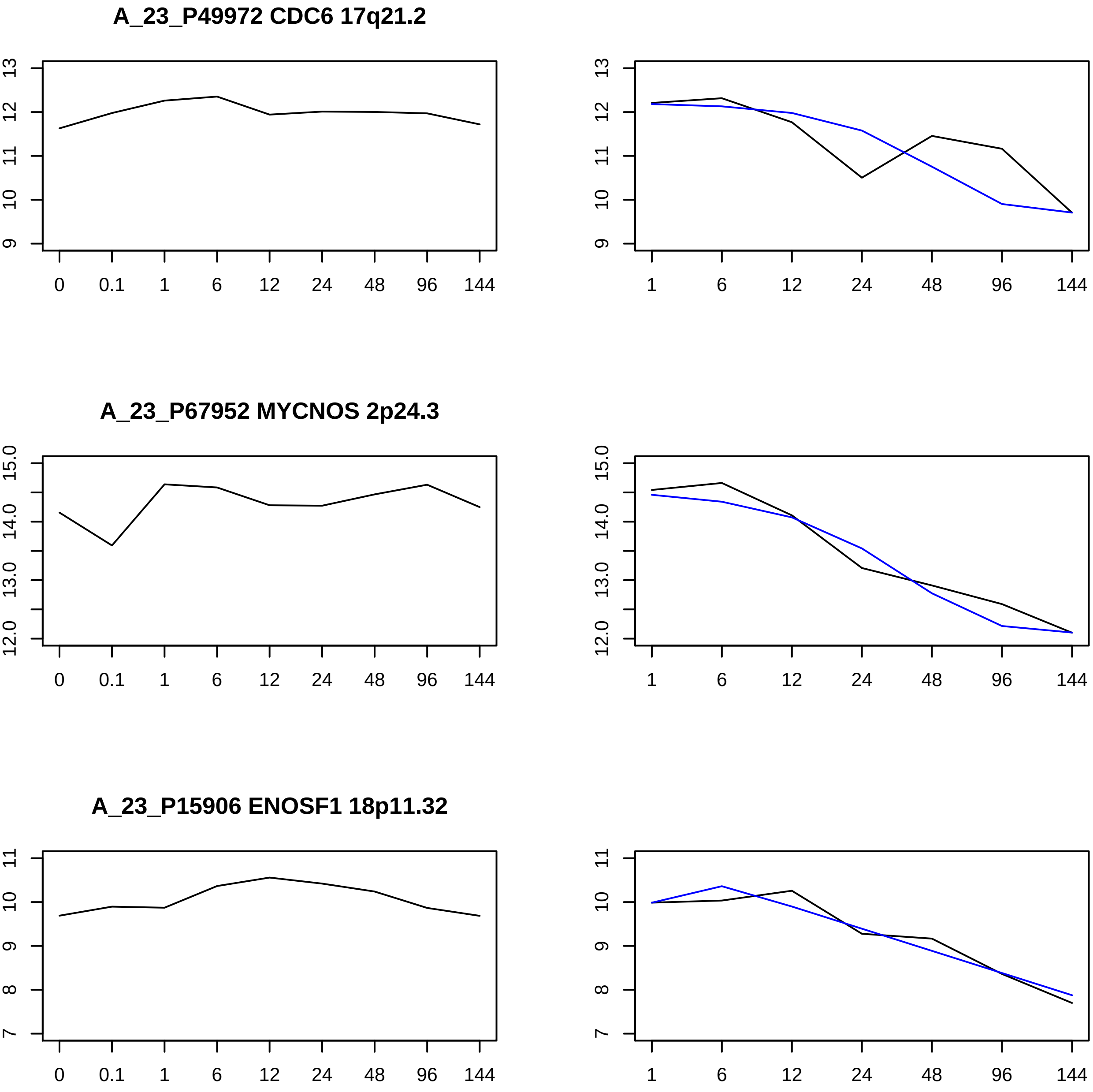
A_23_P49972 CDC6 17q21.2
9
10
11
12
13
0
0.1
1
6
12
24
48
96
144
1
6
12
24
48
96
144
A_23_P67952 MYCNOS 2p24.3
12.0
13.0
14.0
15.0
A_23_P15906 ENOSF1 18p11.32
7
8
9
10
11

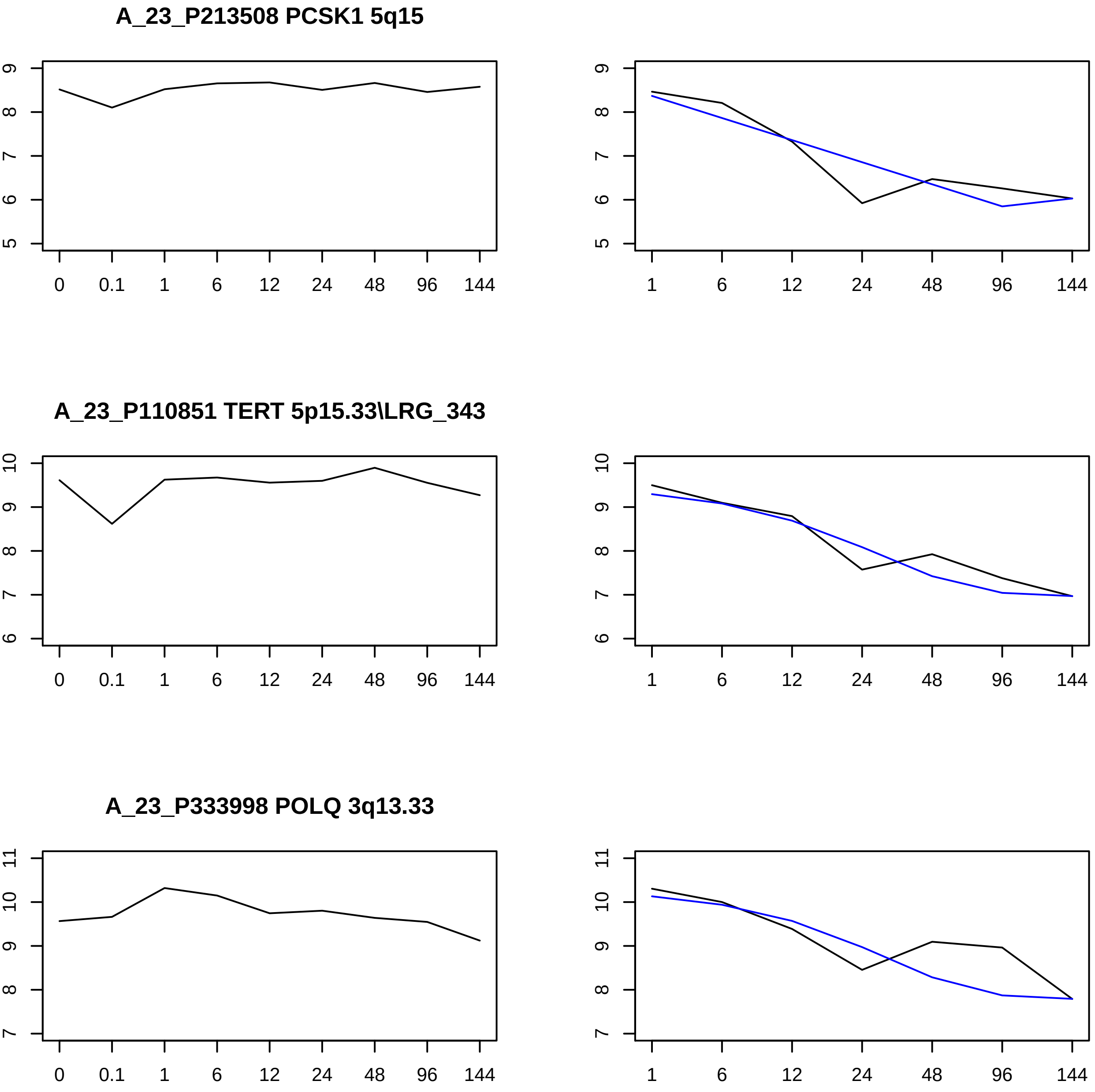
A_23_P213508 PCSK1 5q15
5 6 7 8 9
0 0.1 1 6 12 24 48 96 144
1 6 12 24 48 96 144
A_23_P110851 TERT 5p15.33\LRG_343
6 7 8 9 10
0 0.1 1 6 12 24 48 96 144
1 6 12 24 48 96 144
A_23_P333998 POLQ 3q13.33
7 8 9 10 11
0 0.1 1 6 12 24 48 96 144
1 6 12 24 48 96 144

A_23_P408955 E2F2 1p36.12

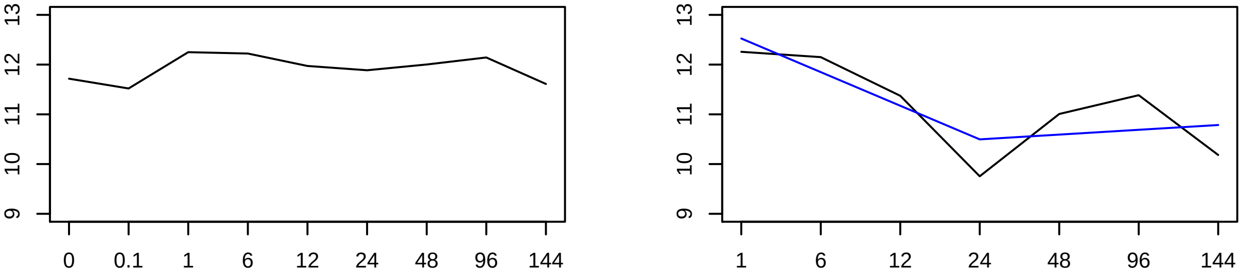


A_23_P163099 POLE2 14q22.1

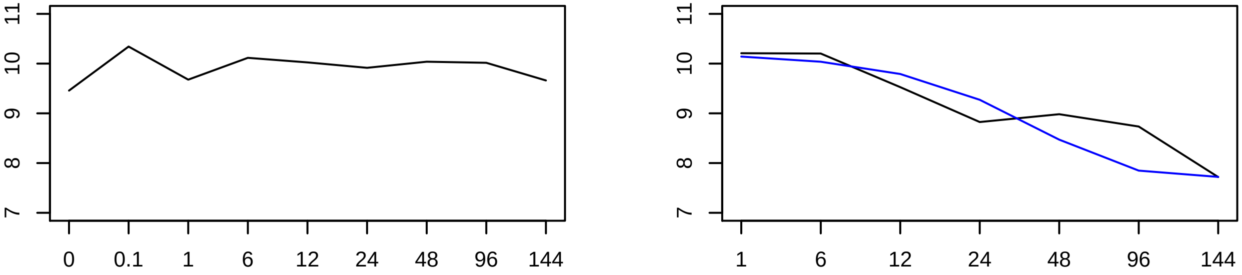


A_23_P360626 LOC201164 17p11.2

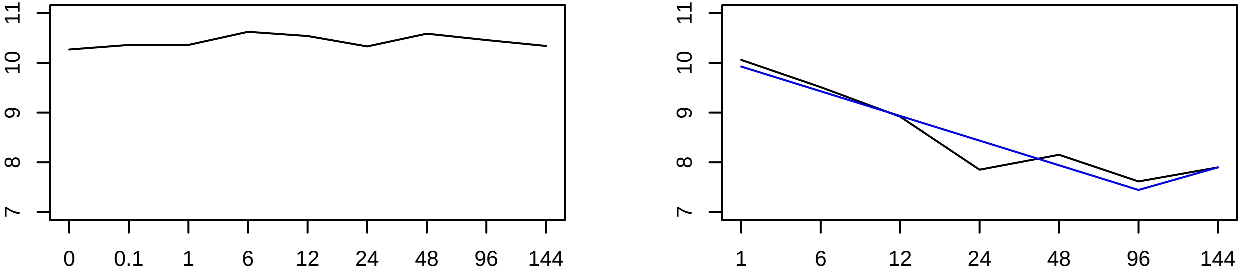

**A_24_P592871 CB250445 NA**

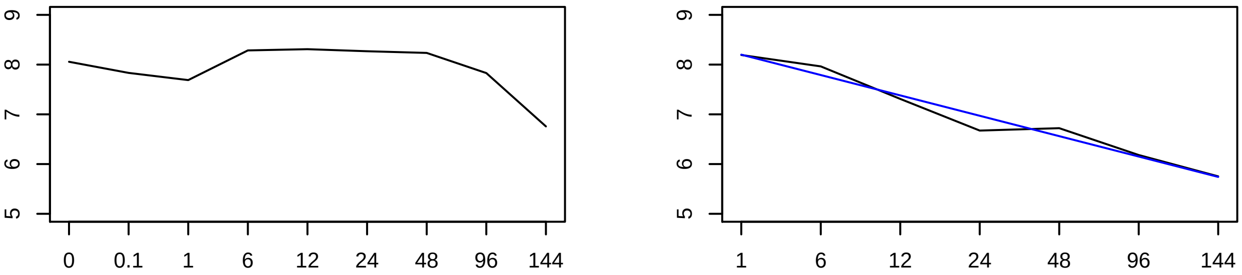


**A_23_P354864 CCDC78 16p13.3**

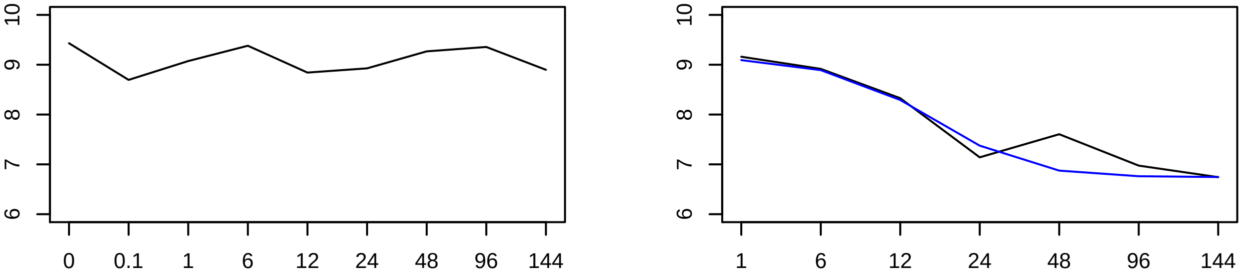


**A_32_P231226 AK095046 NA**

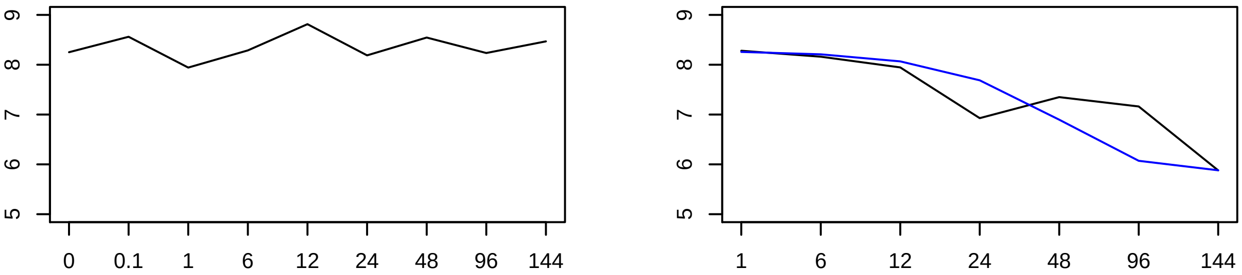

**A_32_P30874 RP3−406A7.7 6q23.3**

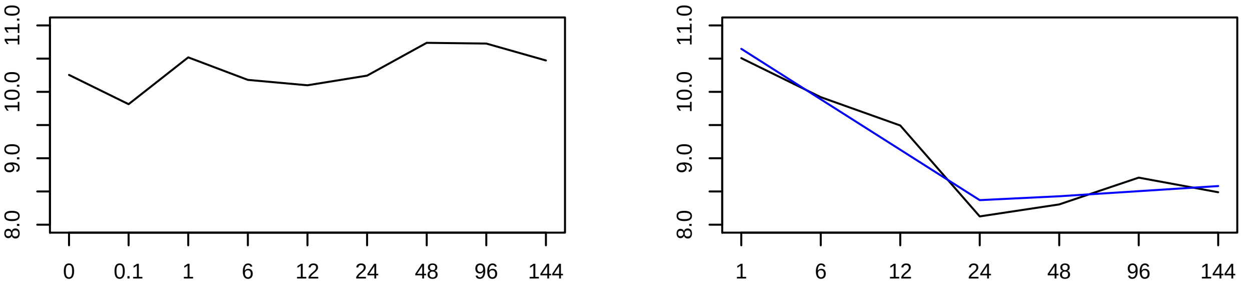


**A_24_P160773 ANKRD18B 9p13.3**

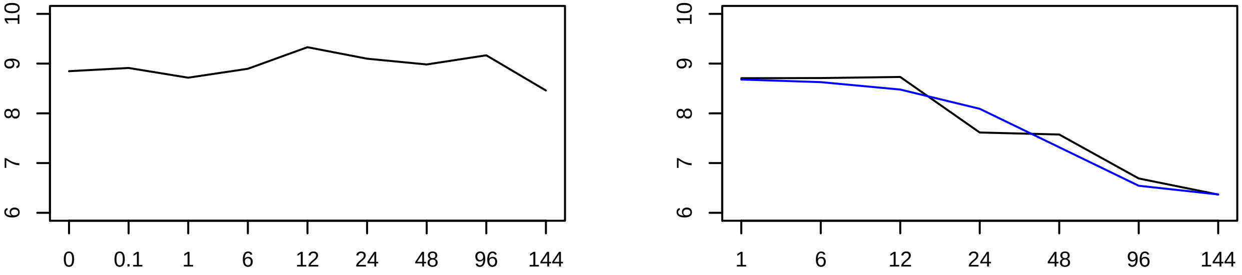


**A_23_P369201 SCARB1 12q24.31**

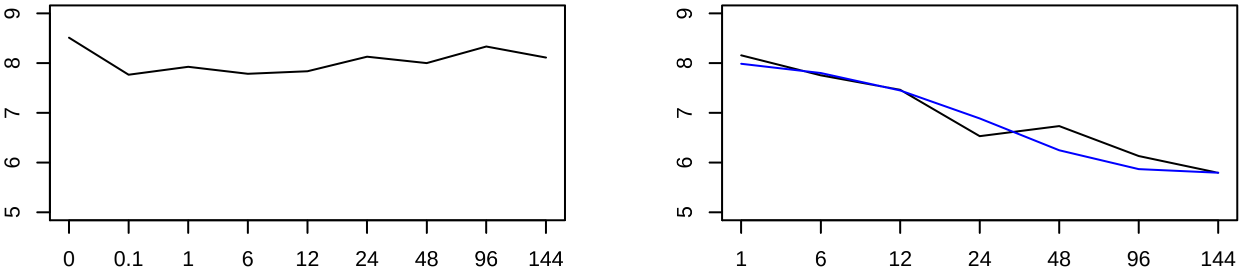

**A_32_P220519 CEP152 15q21.1**

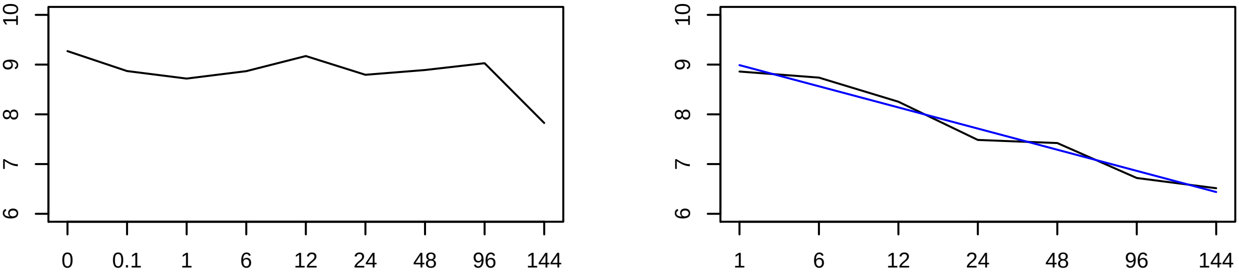


**A_24_P75056 POLD3 11q13.4**

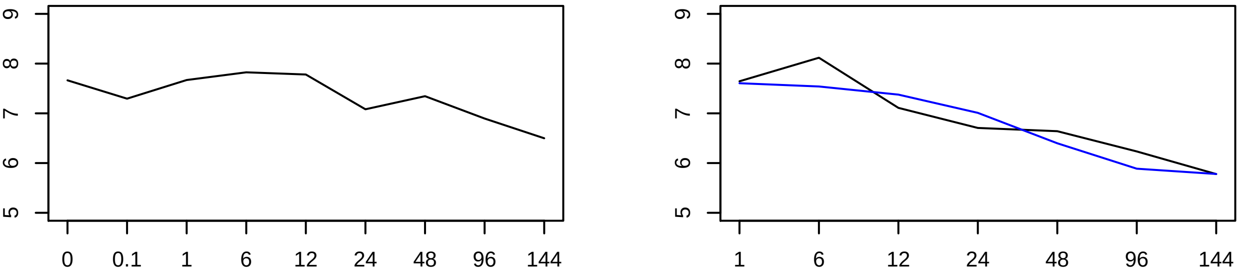


**A_24_P260639 HIST1H1D 6p22.1**

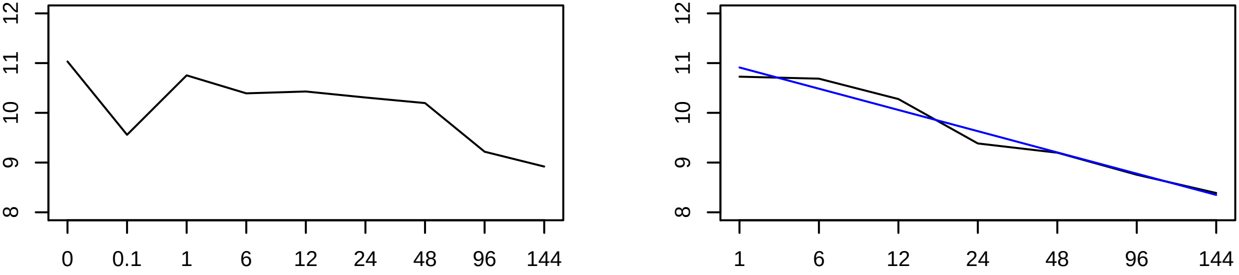

**A_23_P10385 DTL 1q32.3**

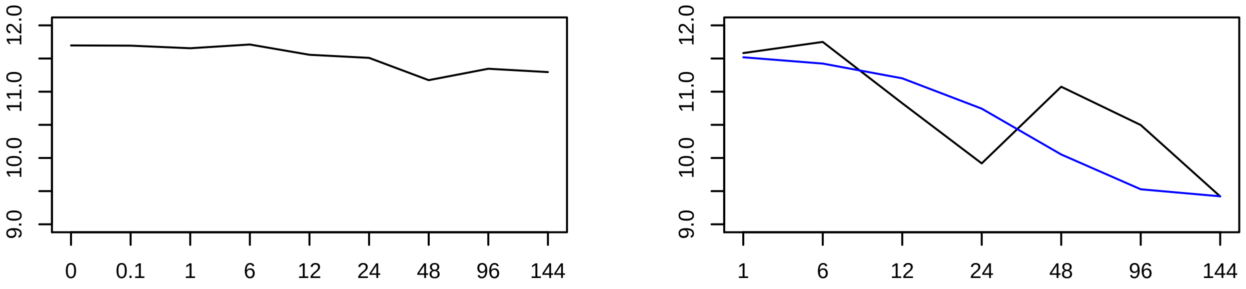


**A_32_P218671 THC2672257 NA**

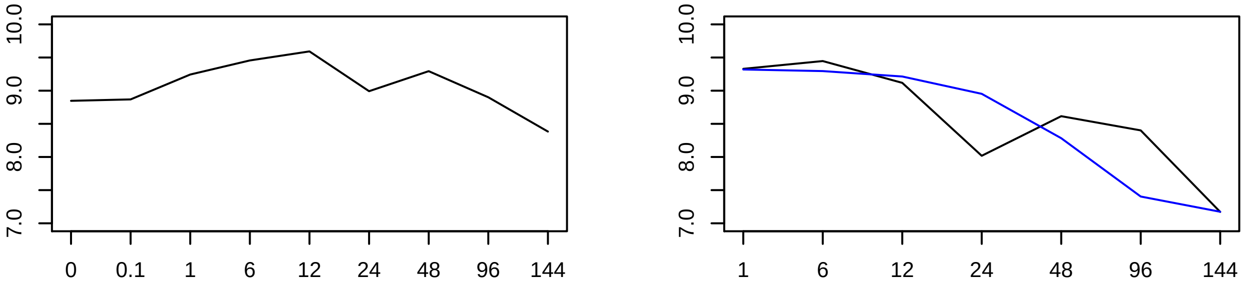


**A_24_P211151 EXOSC5 19q13.2**

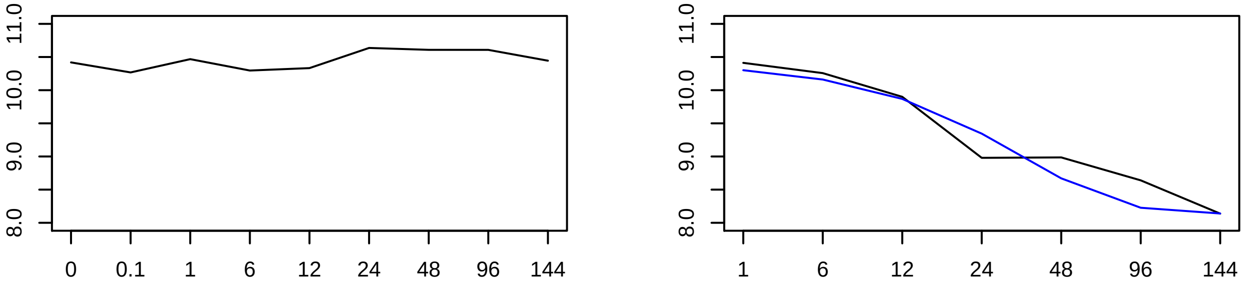

**A_23_P250385 HIST1H1B 6p22.1**

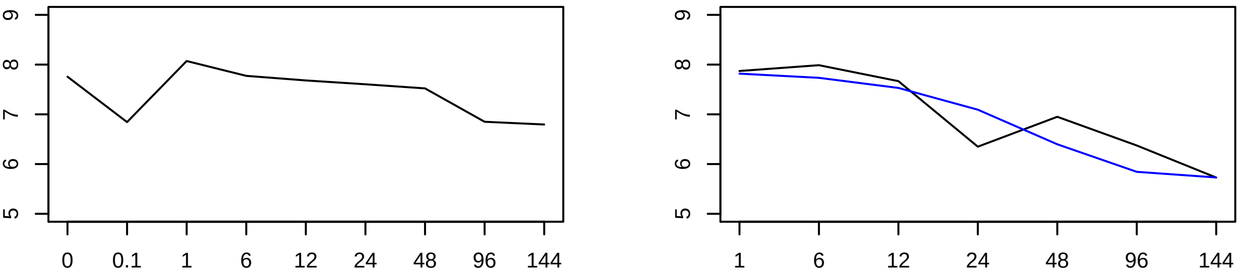


**A_23_P57306 CHAF1B 21q22.13**

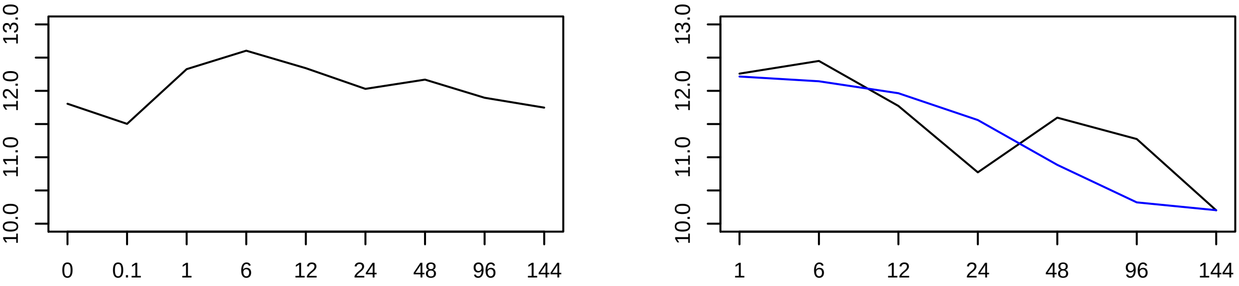


**A_23_P80032 E2F1 20q11.22**

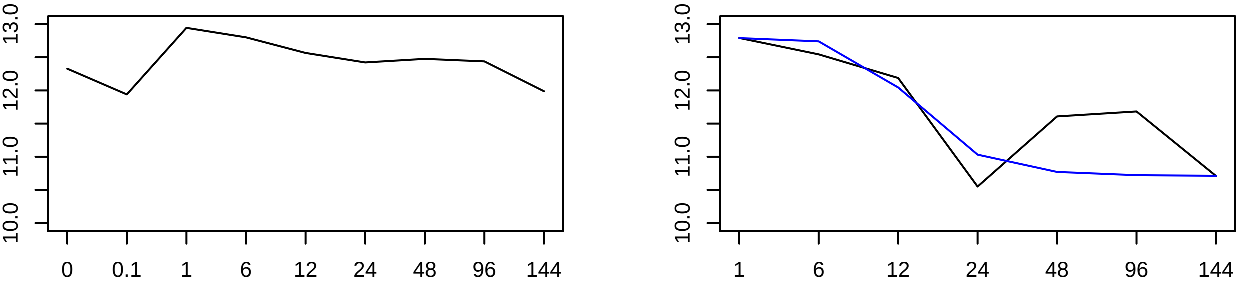

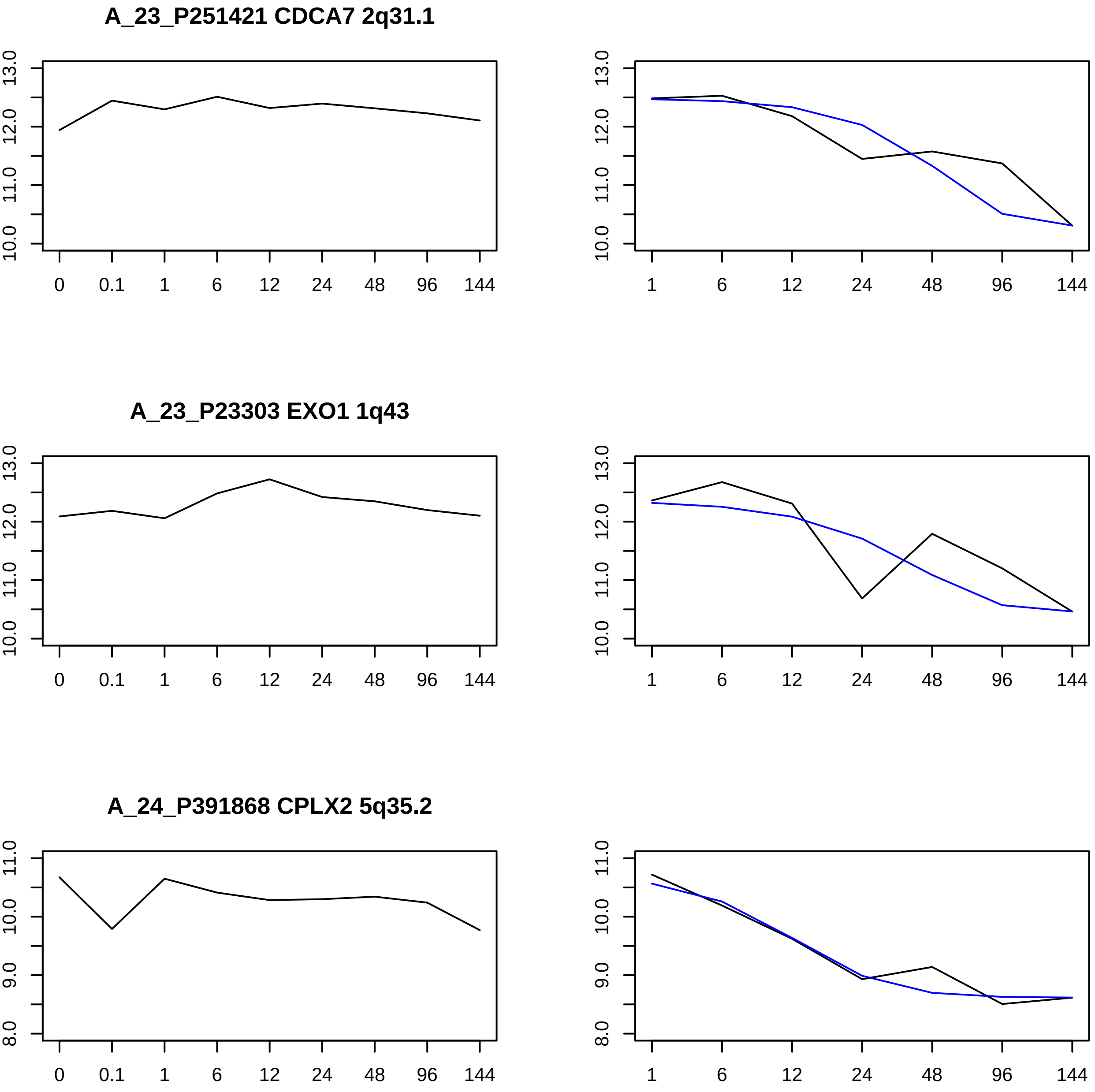
A_23_P251421 CDCA7 2q31.1
A_23_P23303 EXO1 1q43
A_24_P391868 CPLX2 5q35.2
13.0
12.0
11.0
10.0
9.0
8.0
0
0.1
1
6
12
24
48
96
144

**A_23_P207400 BRCA1 17q21.31**

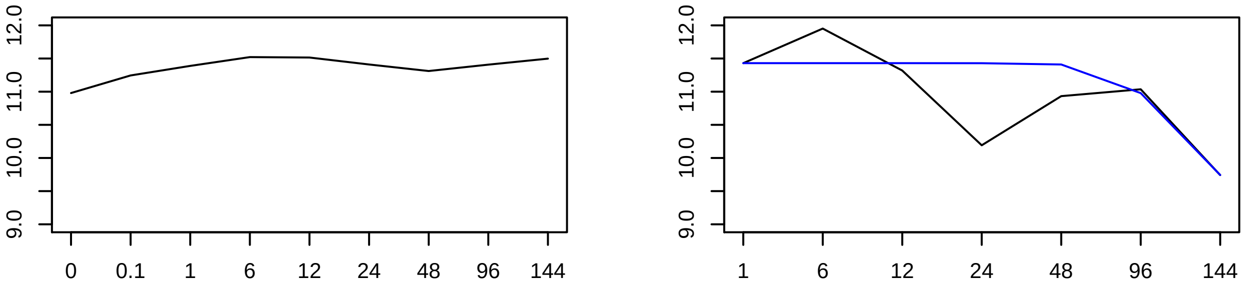


**A_23_P9779 IRX5 16q12.2**

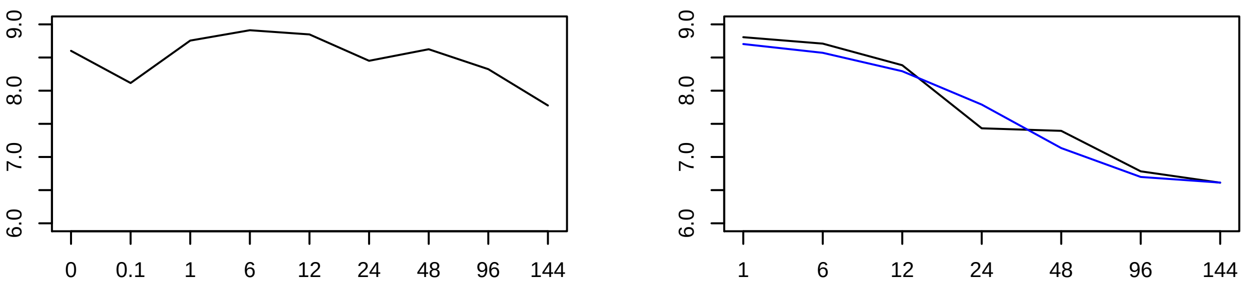


**A_24_P127462 PCNAP3 Xp11.3**

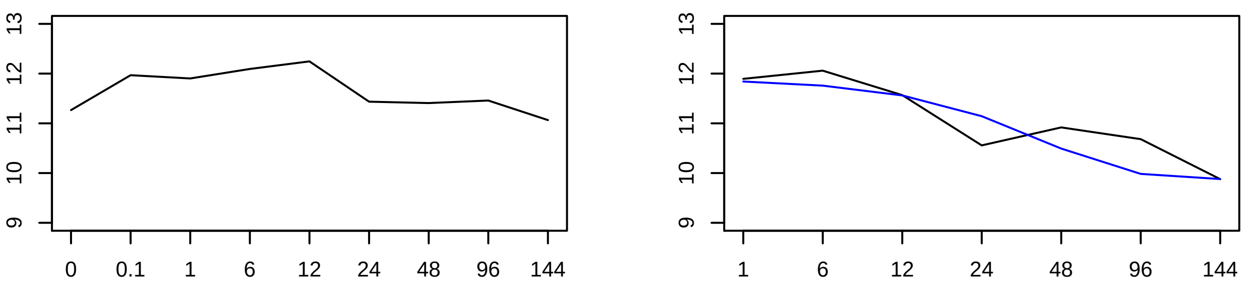

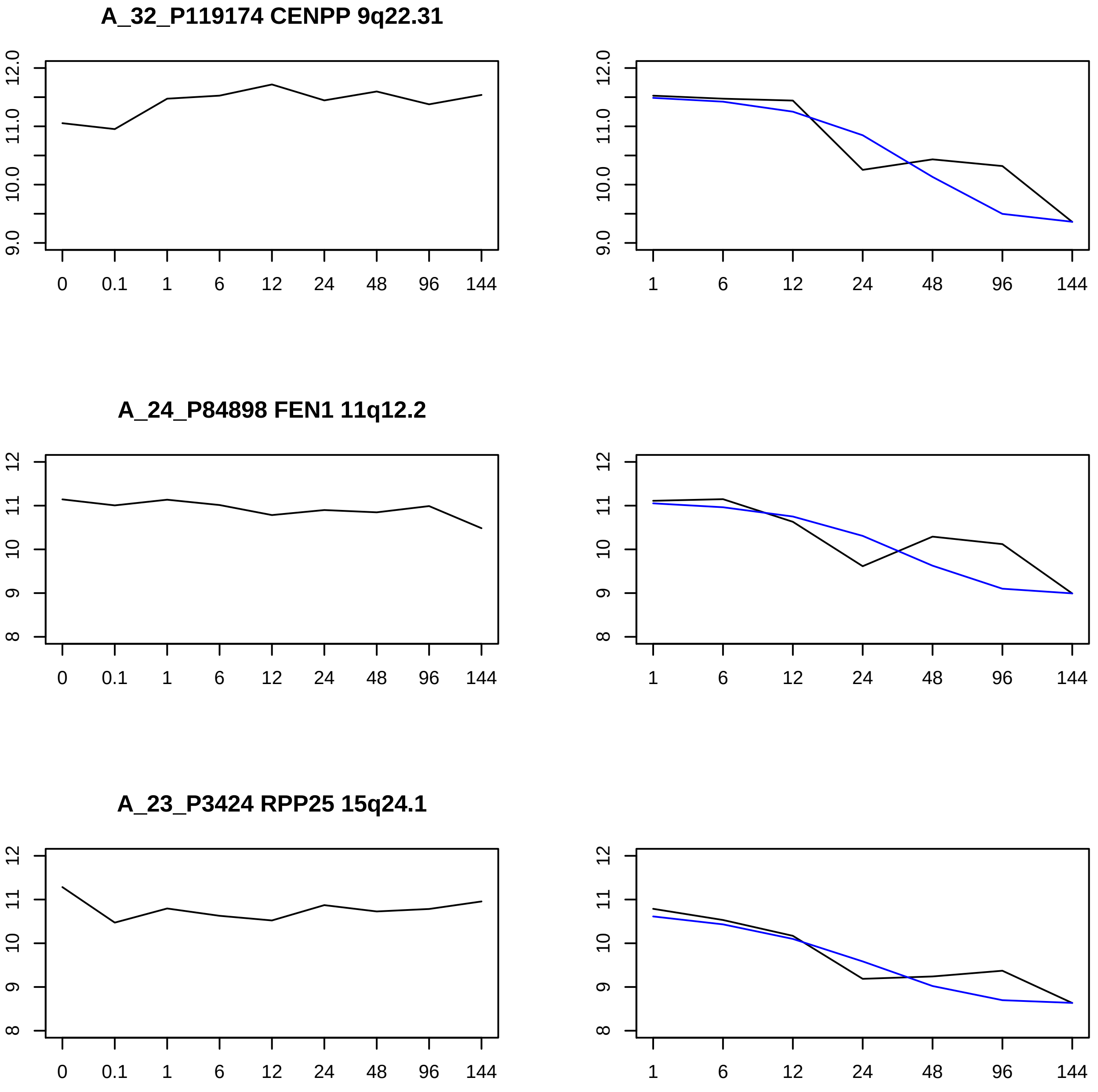

A_32_P119174 CENPP 9q22.31
9.0
10.0
11.0
12.0
0
0.1
1
6
12
24
48
96
144
A_24_P84898 FEN1 11q12.2
8
9
10
11
12
A_23_P3424 RPP25 15q24.1

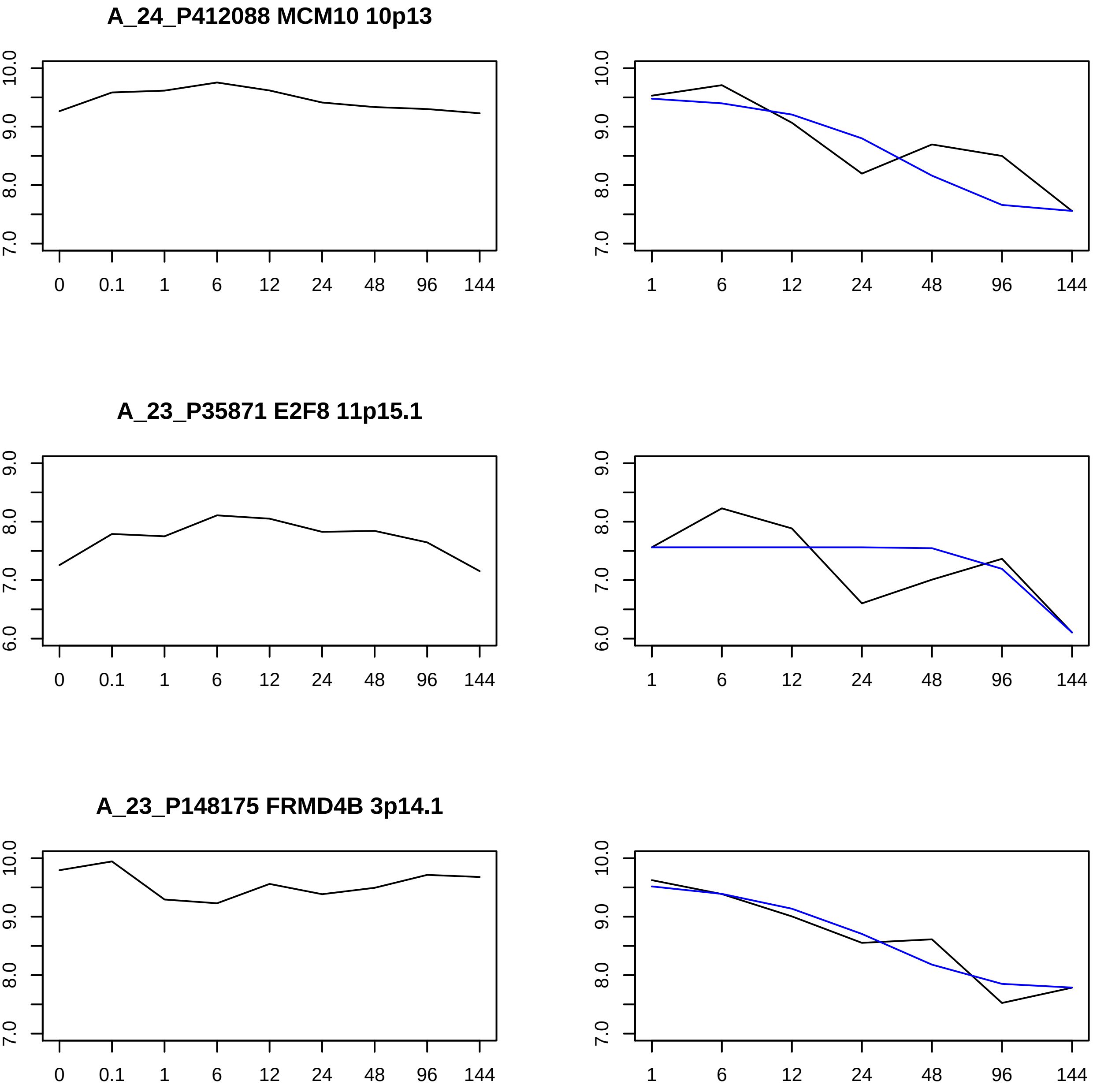

A_24_P412088 MCM10 10p13
A_23_P35871 E2F8 11p15.1
A_23_P148175 FRMD4B 3p14.1
7.0
8.0
9.0
10.0
6.0
0
0.1
1
6
12
24
48
96
144

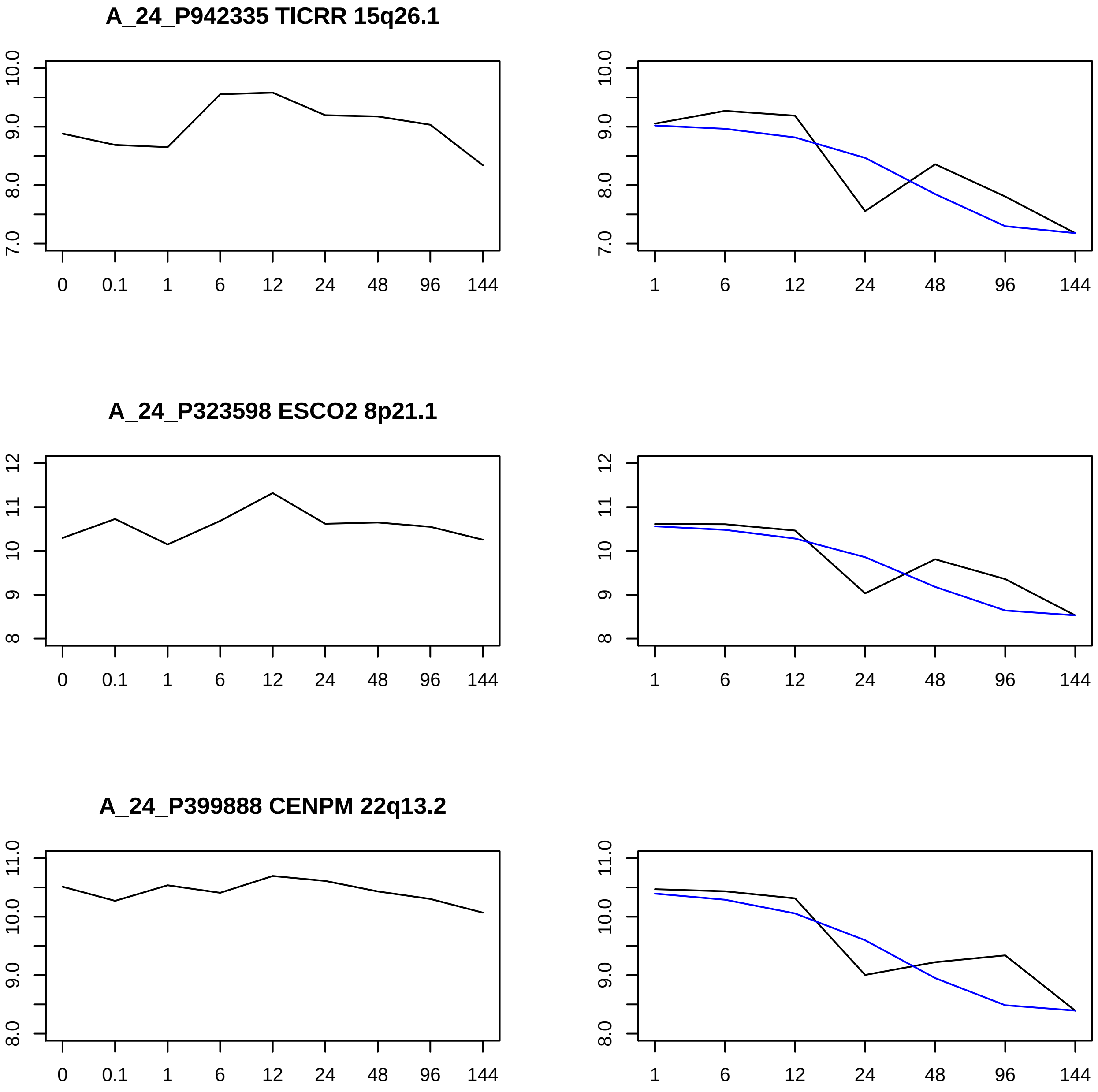
A_24_P942335 TICRR 15q26.1
7.0
8.0
9.0
10.0
0
0.1
1
6
12
24
48
96
144
1
6
12
24
48
96
144
A_24_P323598 ESCO2 8p21.1
8
9
10
11
12
A_24_P399888 CENPM 22q13.2
8.0
9.0
10.0
11.0

**A_23_P165937 DSN1 20q11.23**

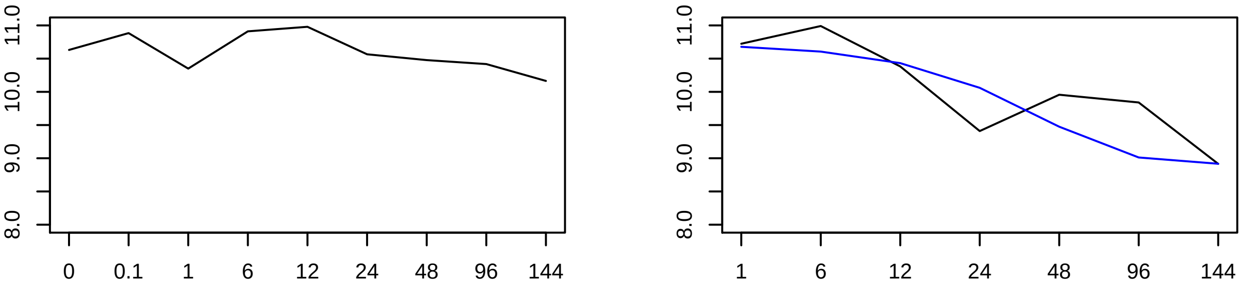


**A_24_P117528 PRPS2 Xp22.2**

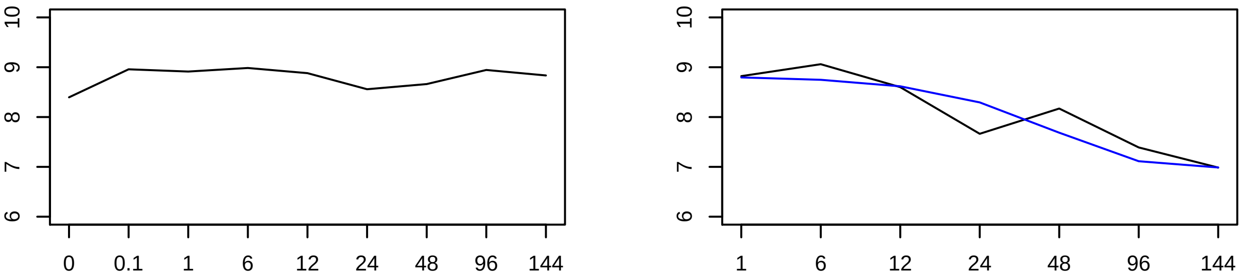


**A_23_P370989 MCM4 8q11.21**

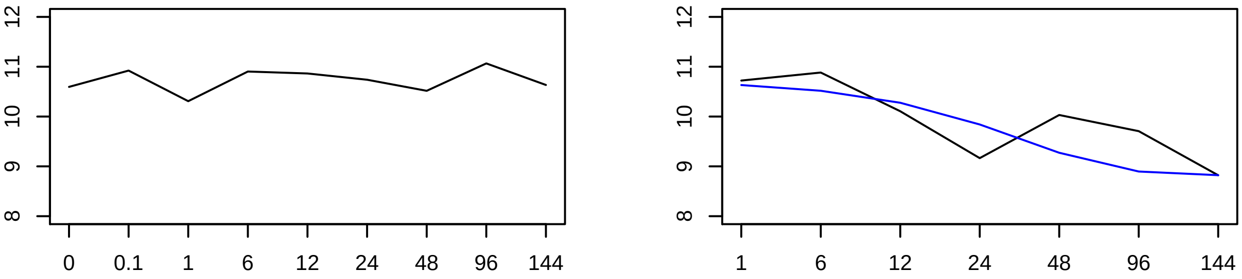

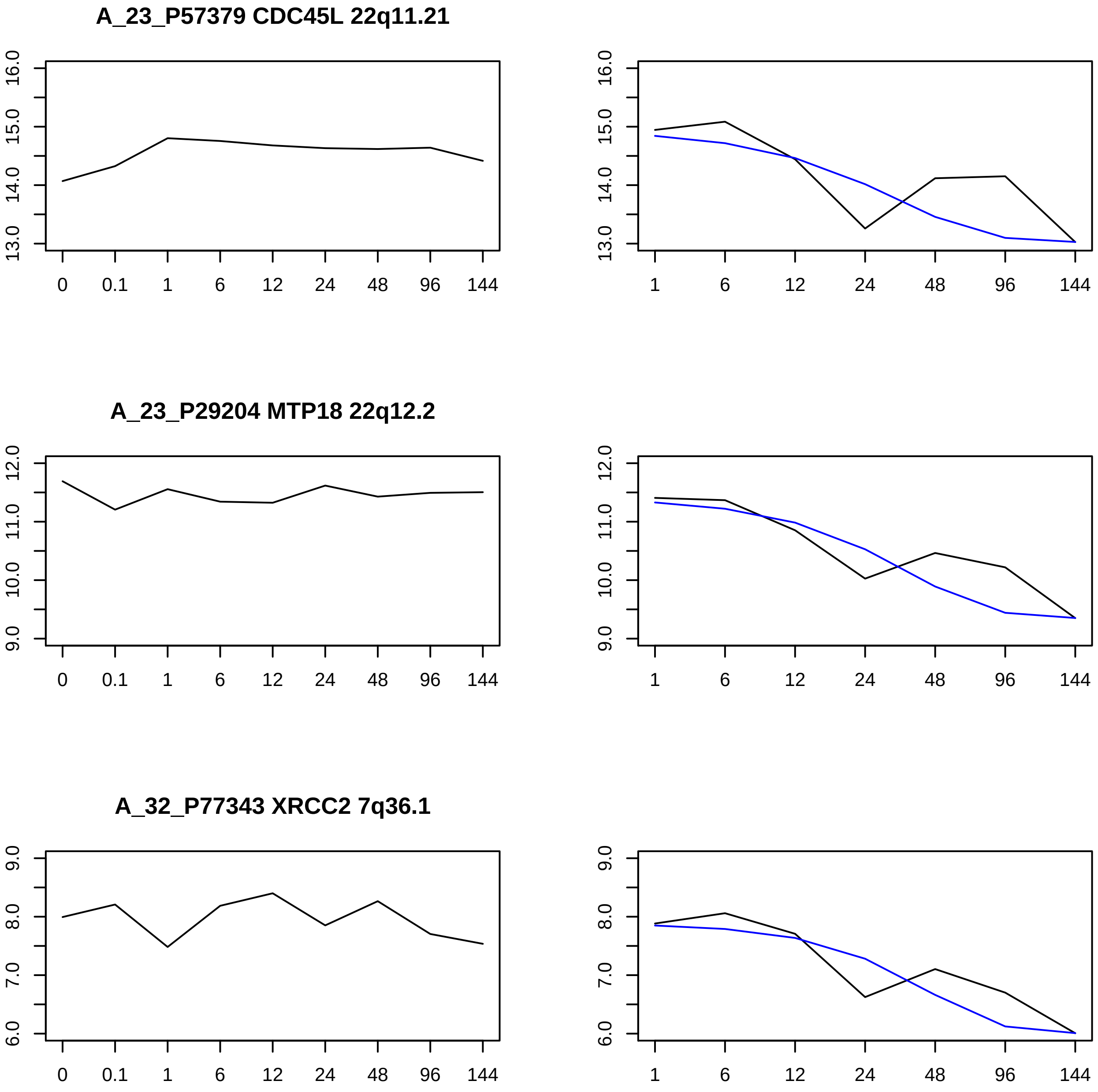
A_23_P57379 CDC45L 22q11.21
13.0
14.0
15.0
16.0
0
0.1
1
6
12
24
48
96
144
1
6
12
24
48
96
144
A_23_P29204 MTP18 22q12.2
9.0
10.0
11.0
12.0
A_32_P77343 XRCC2 7q36.1
6.0
7.0
8.0
9.0

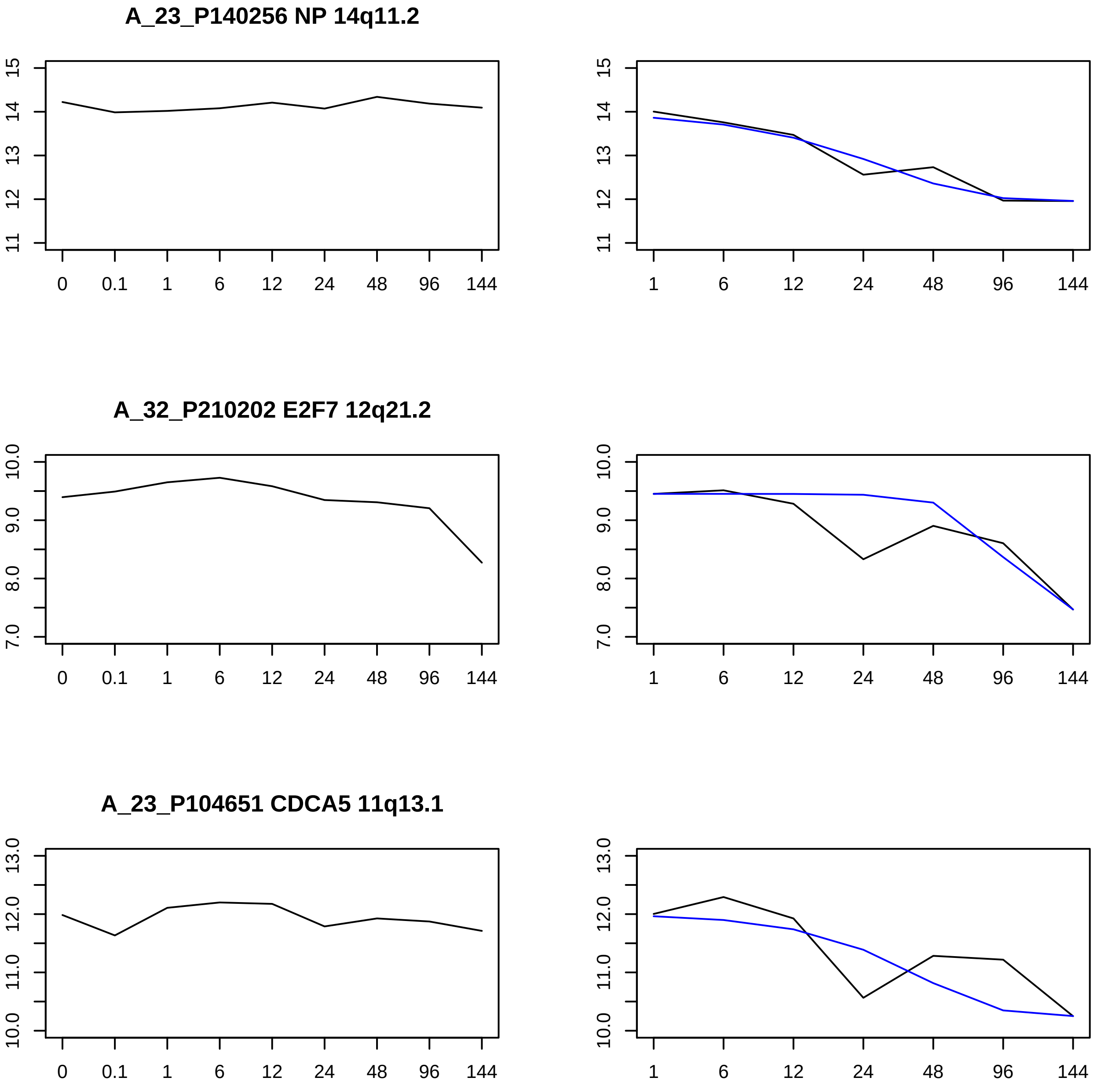
A_23_P140256 NP 14q11.2
A_32_P210202 E2F7 12q21.2
A_23_P104651 CDCA5 11q13.1
0 0.1 1 6 12 24 48 96 144
1 6 12 24 48 96 144
11 12 13 14 15
7.0 8.0 9.0 10.0
10.0 11.0 12.0 13.0

**A_23_P146830 SLC25A10 17q25.3**

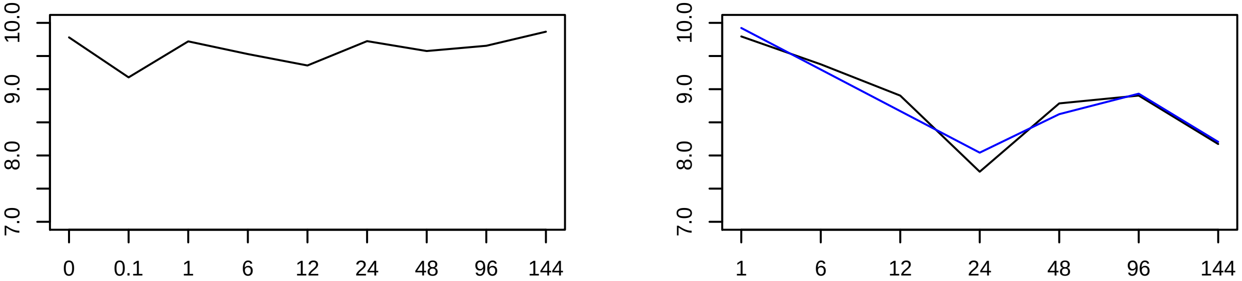


**A_23_P37704 CDT1 16q24.3**

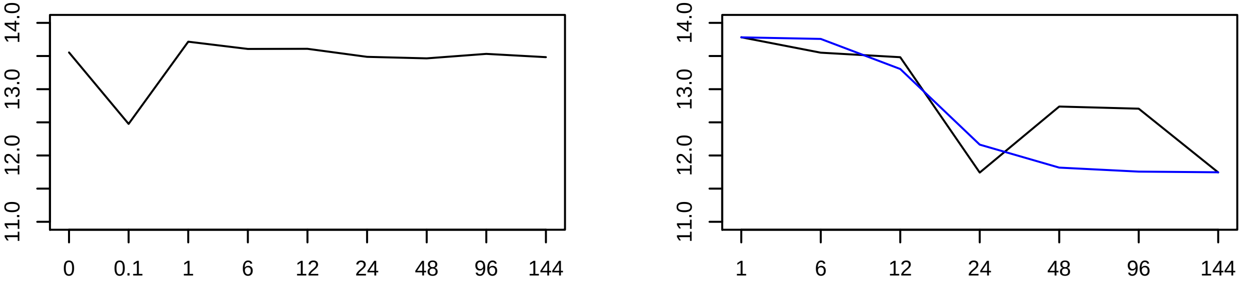


**A_32_P150891 DIAPH3 13q21.2**

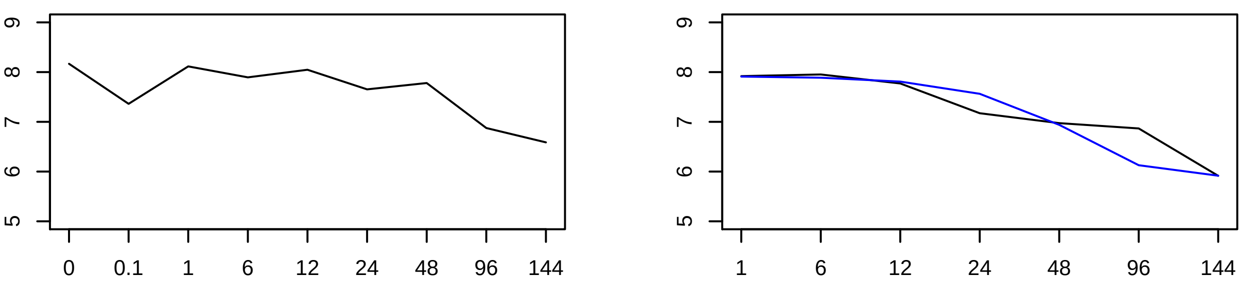

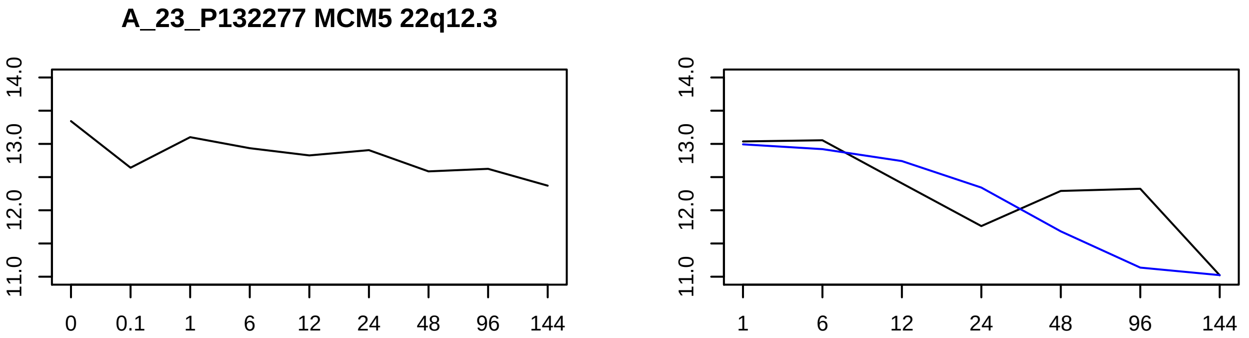
A_23_P132277 MCM5 22q12.3
11.0
12.0
13.0
14.0
0
0.1
1
6
12
24
48
96
144
1
6
12
24
48
96
144

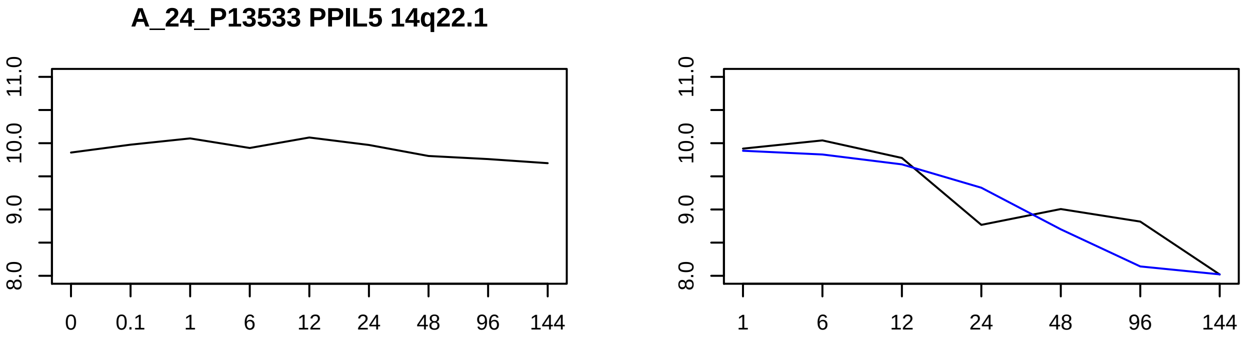
A_24_P13533 PPIL5 14q22.1
8.0
9.0
10.0
11.0
0
0.1
1
6
12
24
48
96
144
1
6
12
24
48
96
144

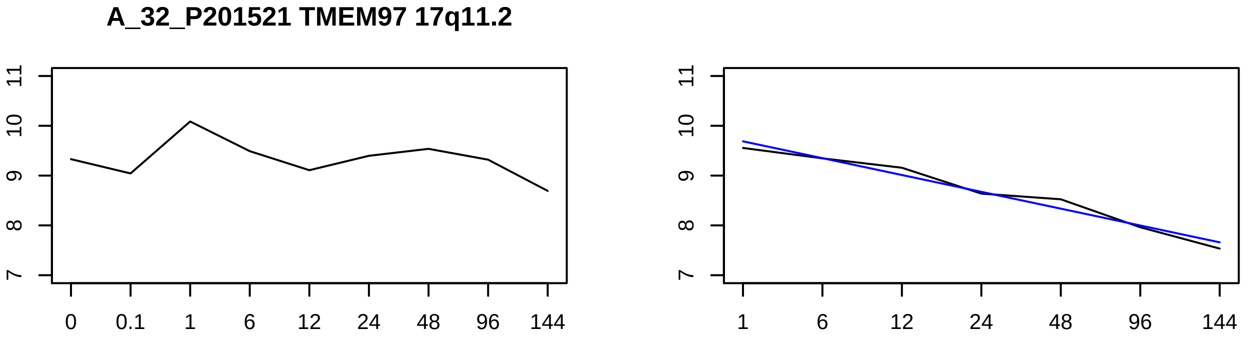
A_32_P201521 TMEM97 17q11.2
7
8
9
10
11
0
0.1
1
6
12
24
48
96
144
1
6
12
24
48
96
144

**A_24_P195400 LOC391247 20q11.23**

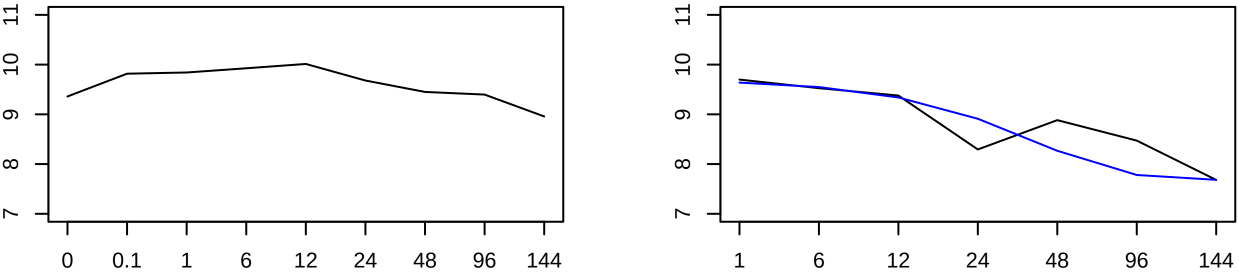


**A_23_P161474 MCM10 10p13**

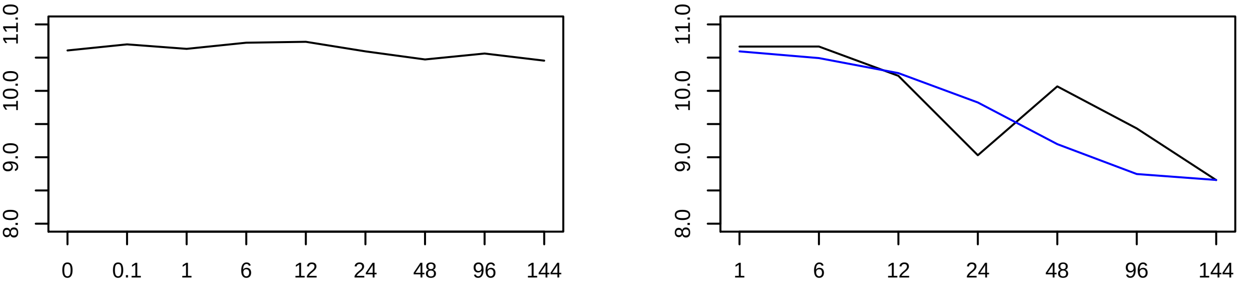


**A_24_P176374 CDT1 16q24.3**

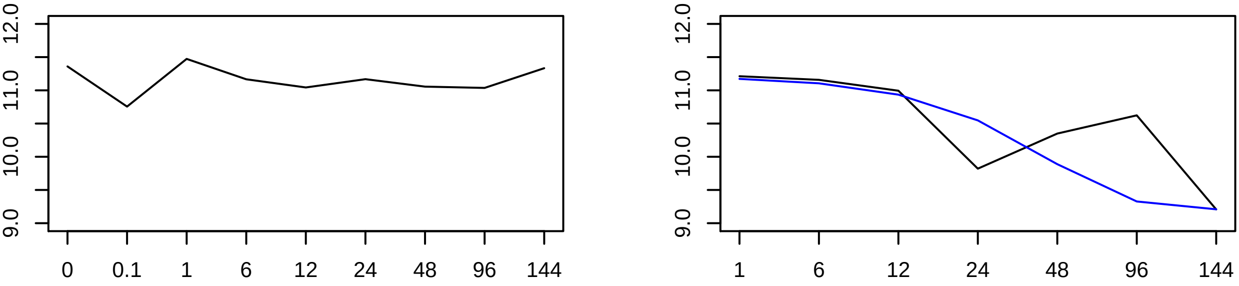

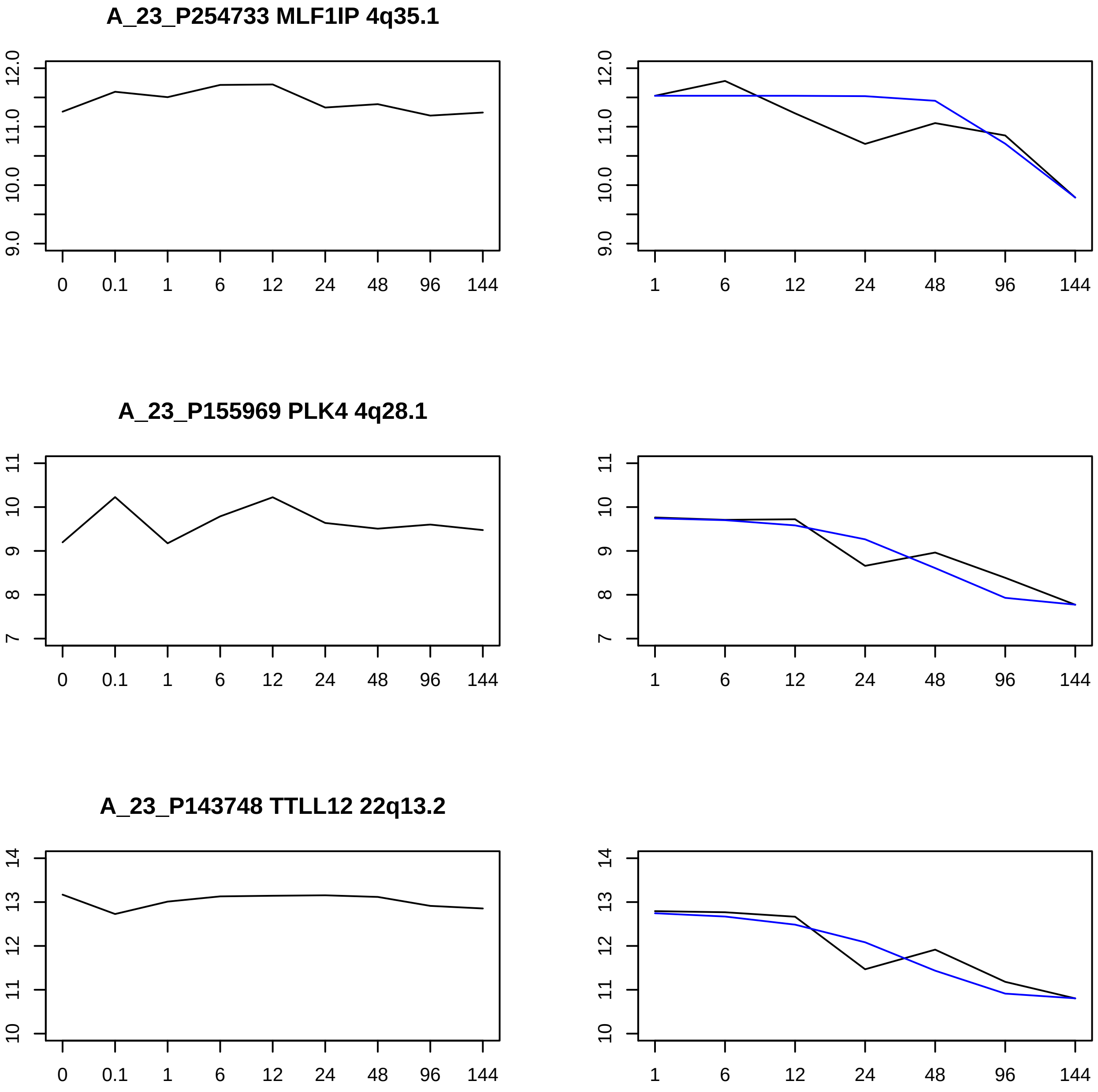

A_23_P254733 MLF1IP 4q35.1
9.0 10.0 11.0 12.0
0 0.1 1 6 12 24 48 96 144
1 6 12 24 48 96 144
A_23_P155969 PLK4 4q28.1
7 8 9 10 11
0 0.1 1 6 12 24 48 96 144
1 6 12 24 48 96 144
A_23_P143748 TTLL12 22q13.2
10 11 12 13 14
0 0.1 1 6 12 24 48 96 144
1 6 12 24 48 96 144

**A_23_P170541 KCNH6 17q23.3**

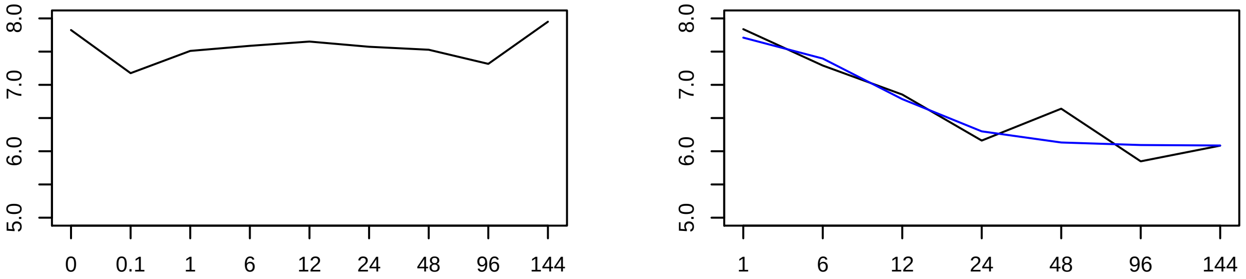


**A_23_P25313 BRI3BP 12q24.31**

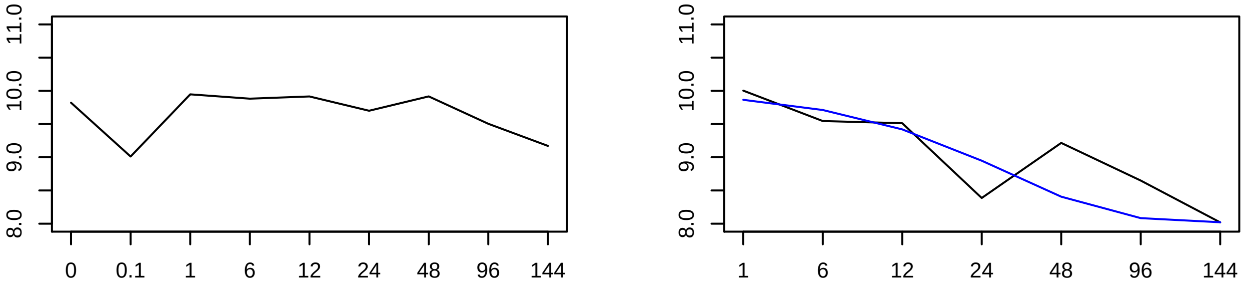


**A_23_P200015 AK5 1p31.1**

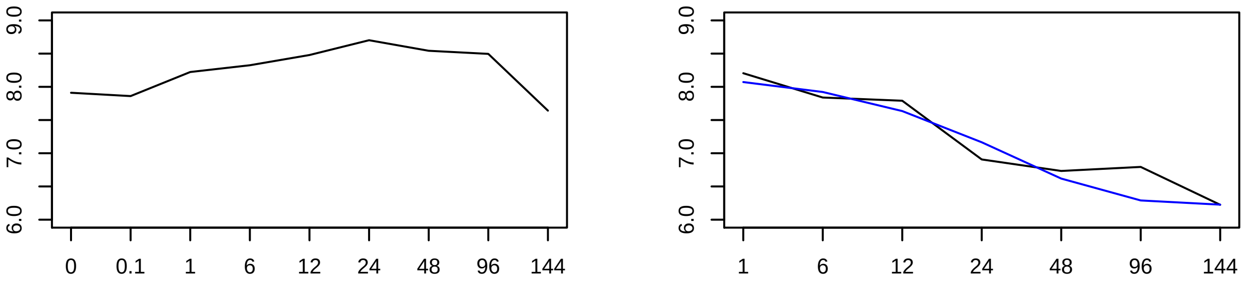

**A_23_P253752 FAM54A 6q23.3**

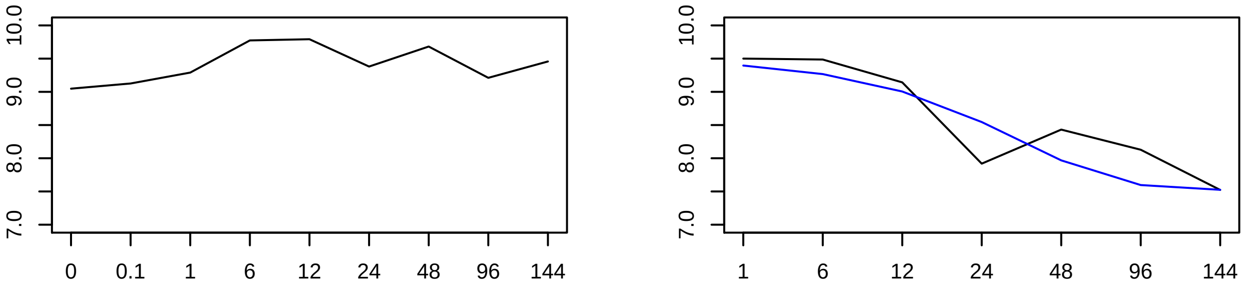


**A_24_P397107 CDC25A 3p21.31**

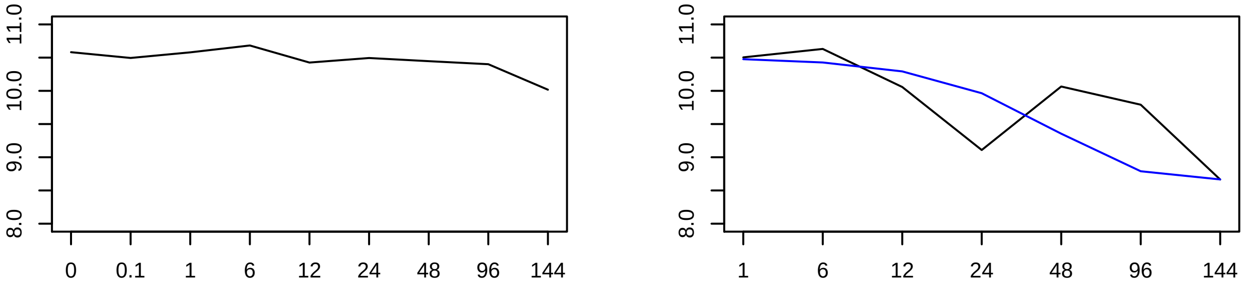


**A_24_P73158 FEN1 11q12.2**

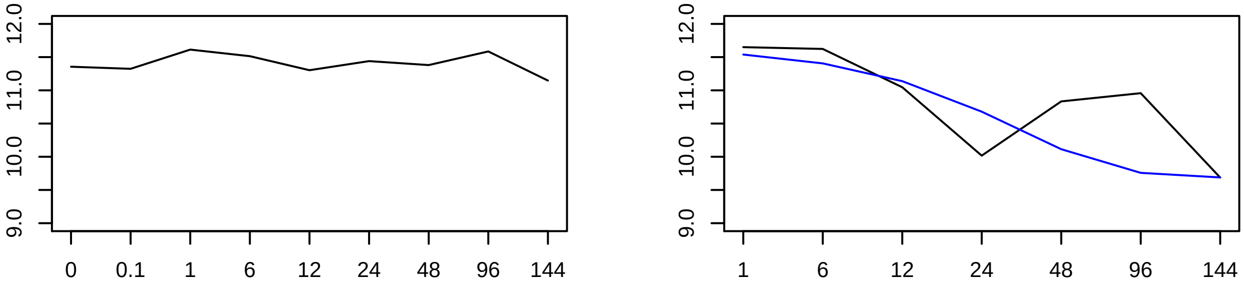

**A_23_P212159 NUP210 3p25.1**

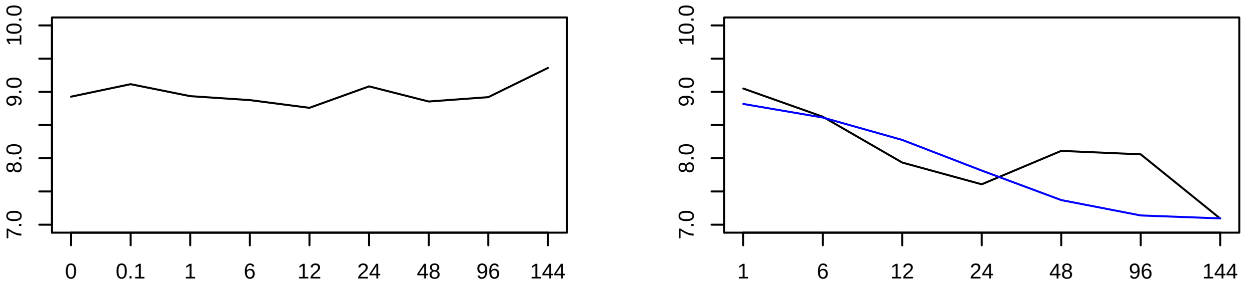


**A_32_P200934 HELLS 10q23.33**

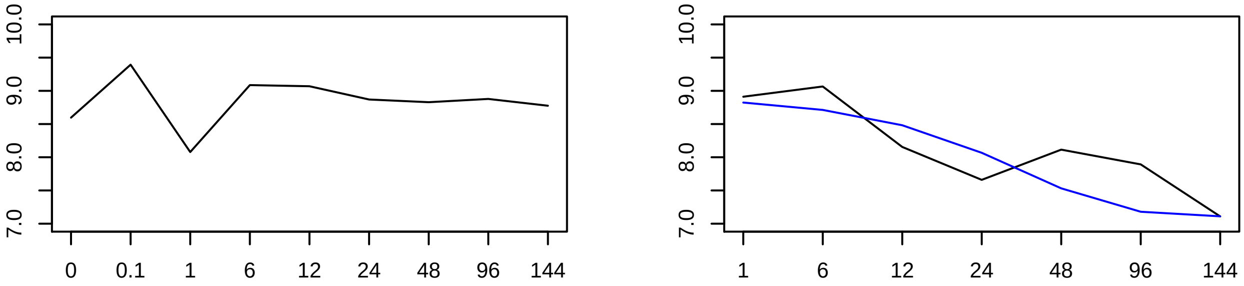


**A_32_P158543 MYCN 2p24.3**

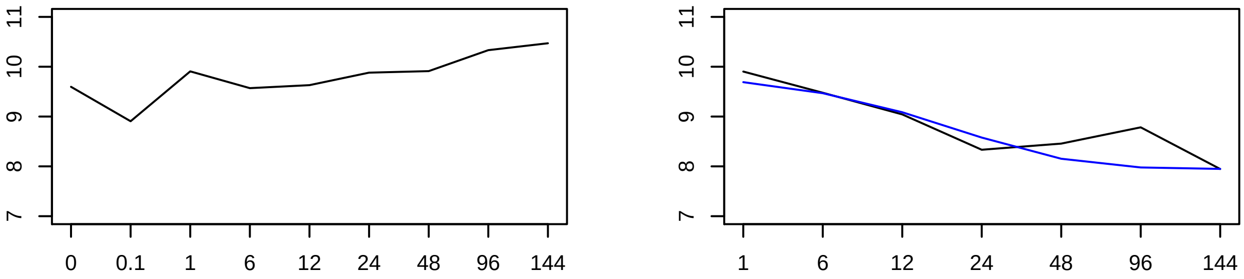

**A_23_P138507 CDC2 10q21.2**

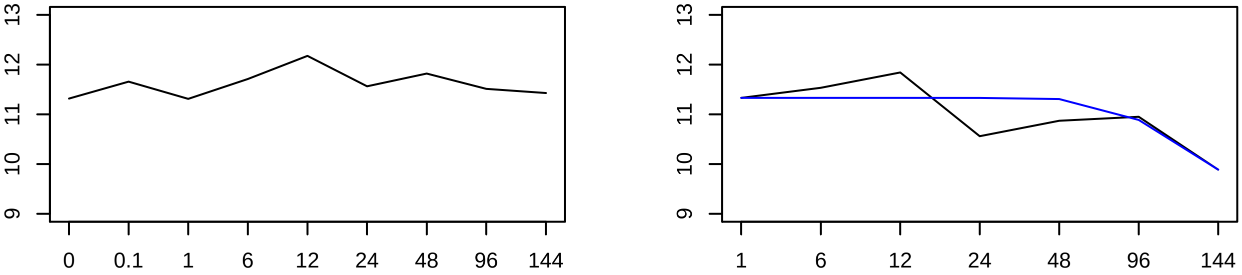


**A_23_P218827 POLQ 3q13.33**

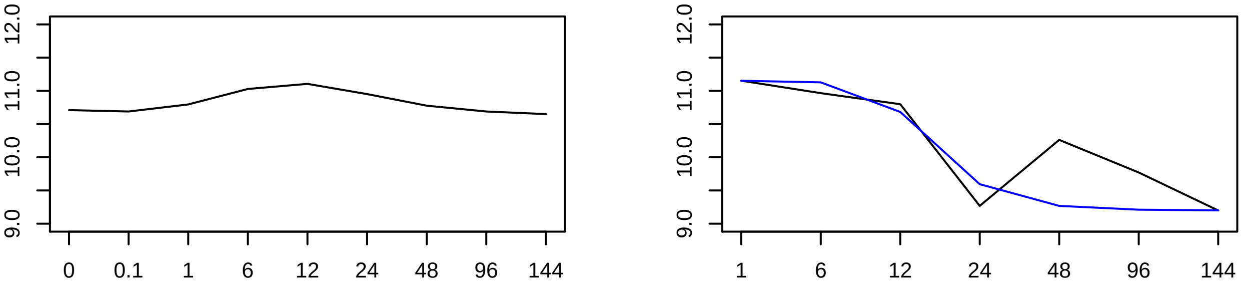


**A_23_P95802 CNTNAP2 7q36.1**

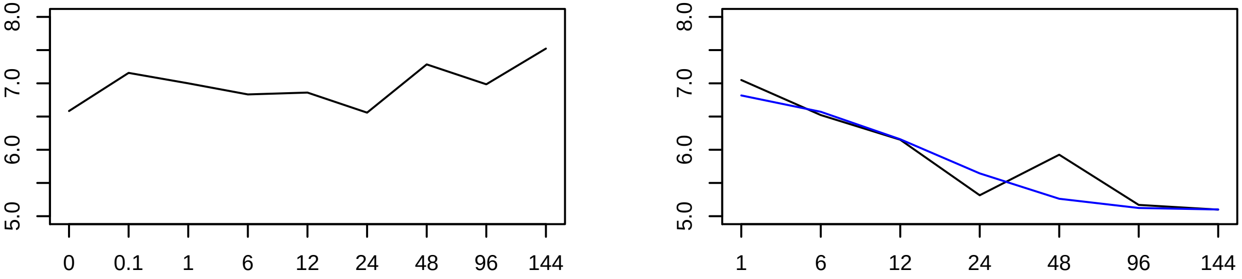

**A_23_P60271 SMC2 9q31.1**

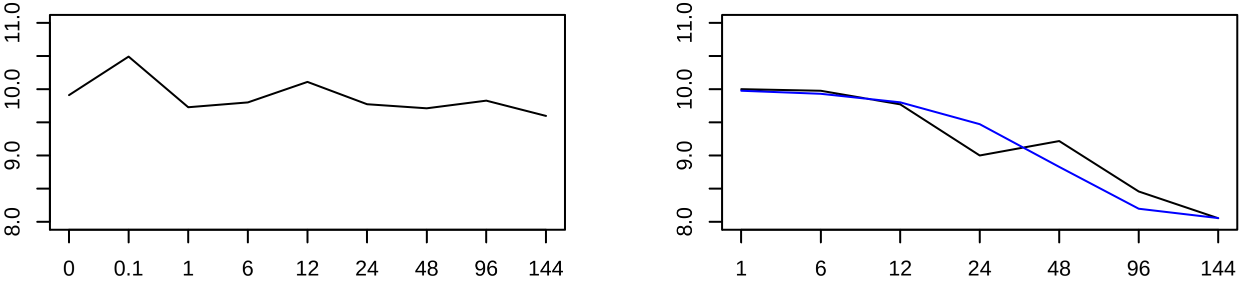


**A_23_P208880 UHRF1 19p13.3**

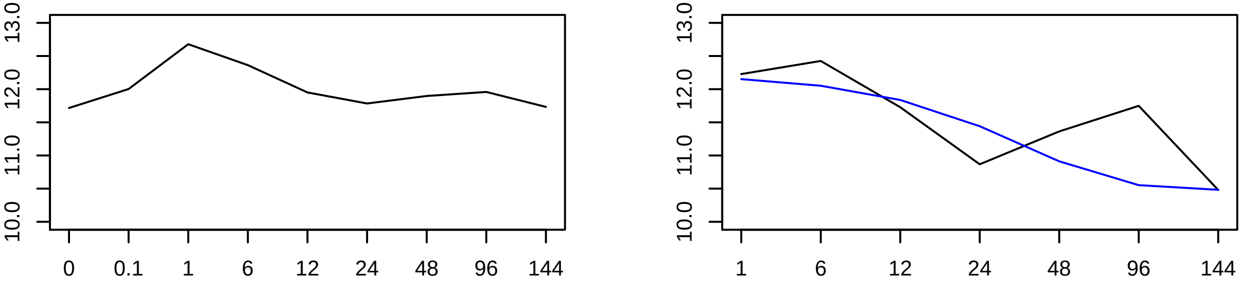


**A_32_P211188 LOC153346 5q33.1**

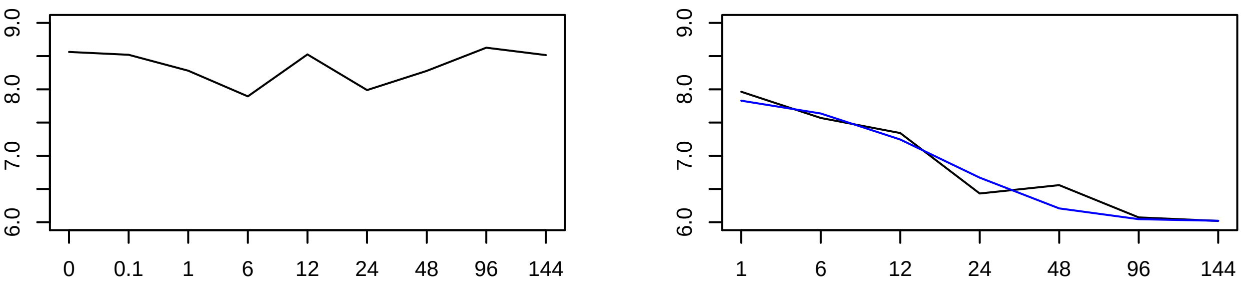

**A_23_P50096 TYMS 18p11.32**

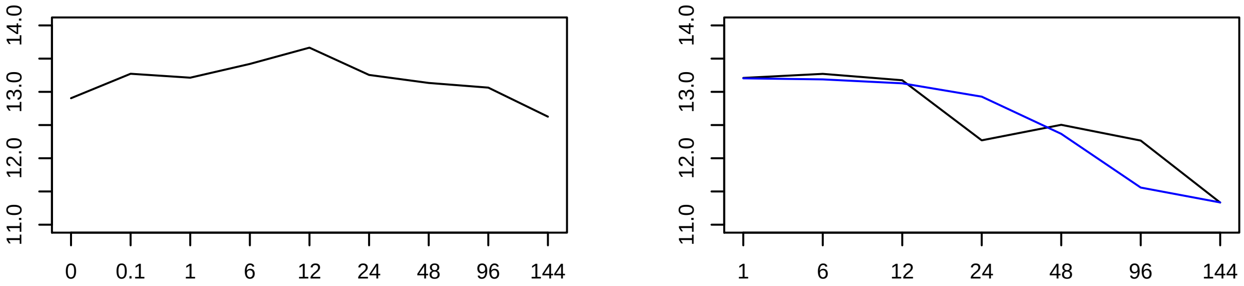


**A_23_P118246 GINS2 16q24.1**

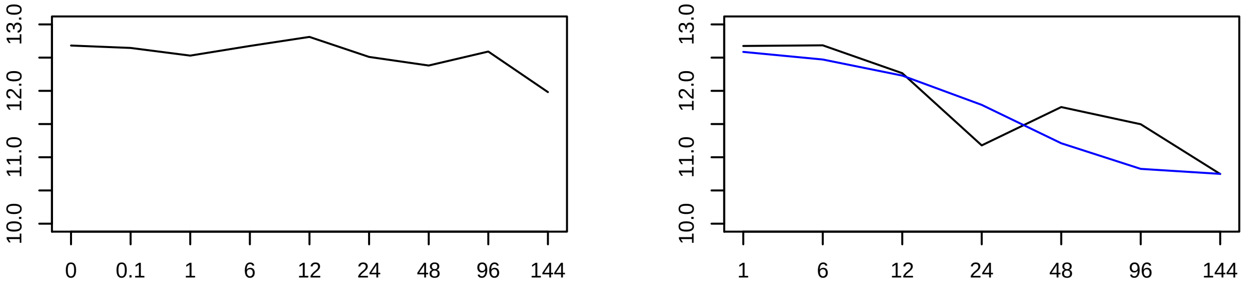


**A_23_P502654 SHMT1 17p11.2**

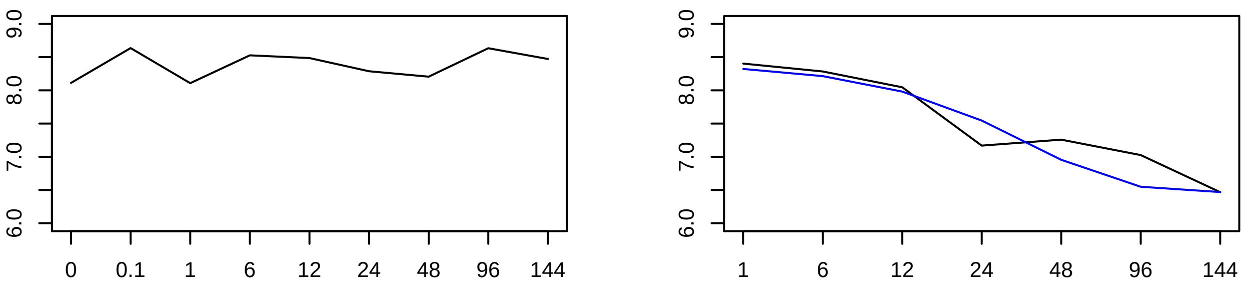

**A_23_P155711 NEIL3 4q34.3**

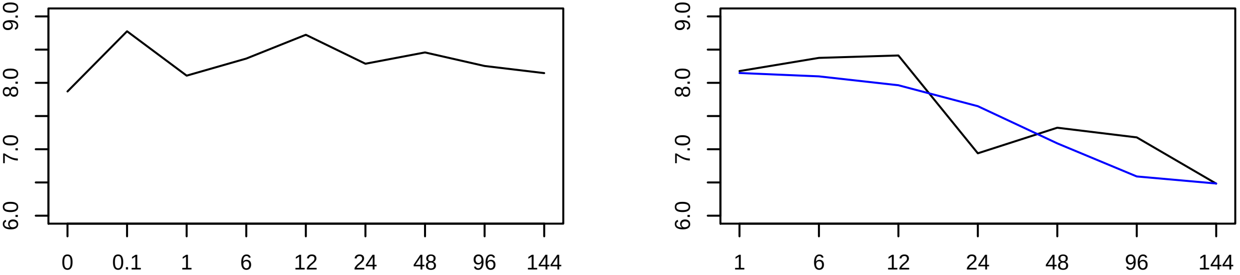


**A_23_P410587 PHF17 4q28.2**

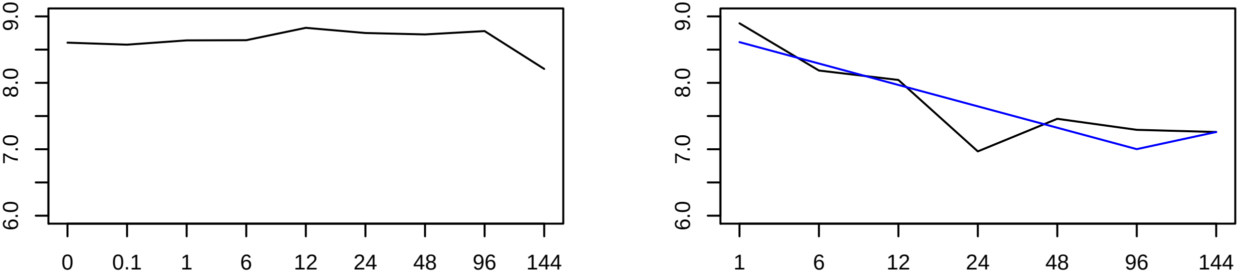


**A_32_P138032 C1orf61 1q22**

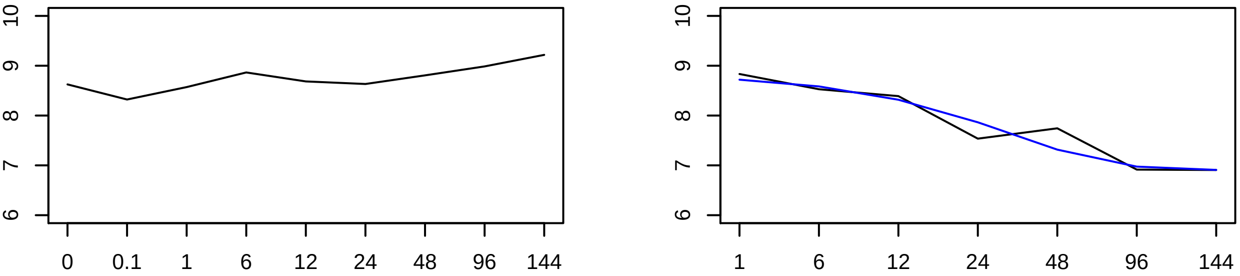

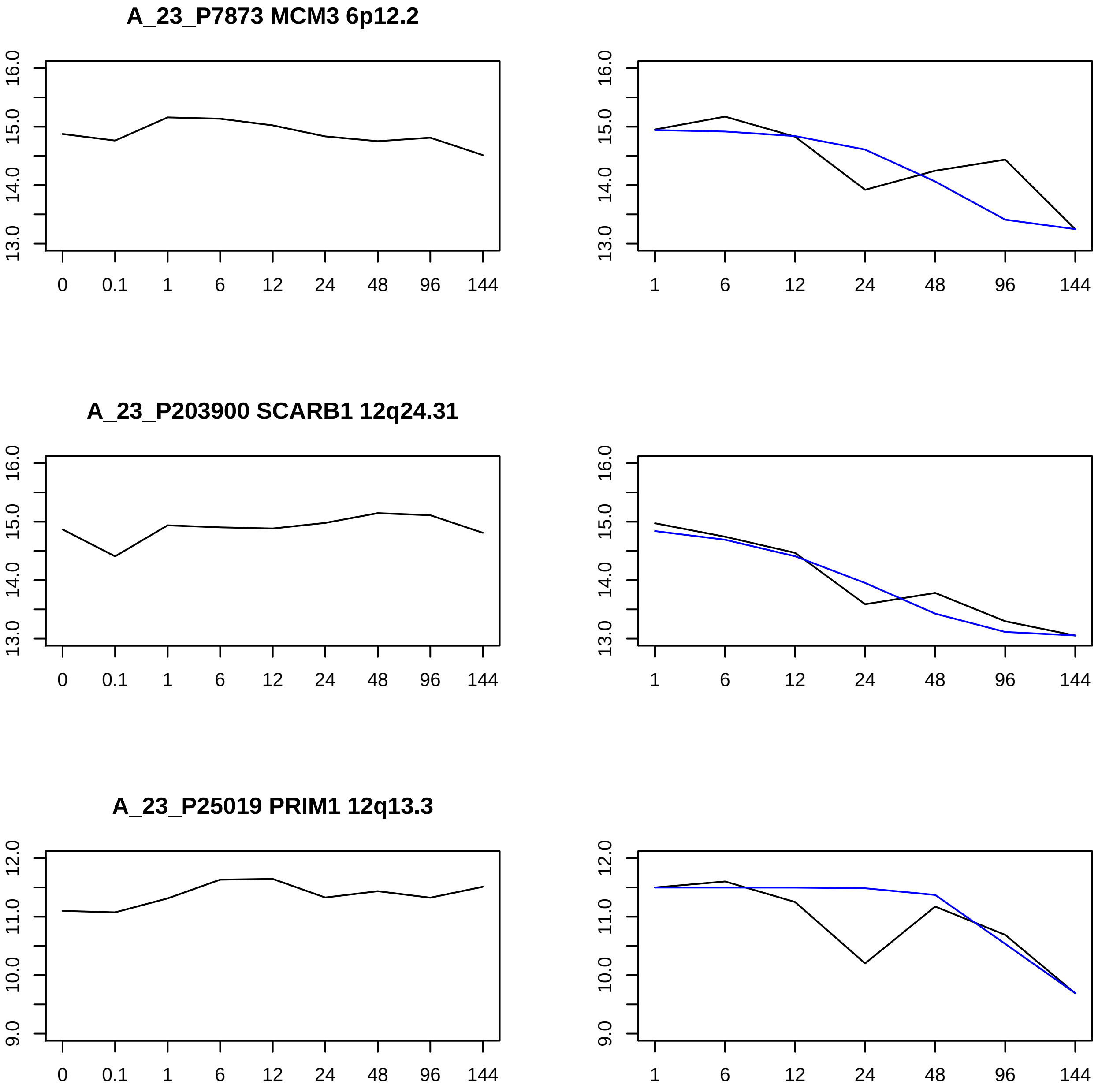
A_23_P7873 MCM3 6p12.2
13.0
14.0
15.0
16.0
0
0.1
1
6
12
24
48
96
144
1
6
12
24
48
96
144
A_23_P203900 SCARB1 12q24.31
13.0
14.0
15.0
16.0
0
0.1
1
6
12
24
48
96
144
1
6
12
24
48
96
144
A_23_P25019 PRIM1 12q13.3
9.0
10.0
11.0
12.0
0
0.1
1
6
12
24
48
96
144
1
6
12
24
48
96
144

**A_23_P117797 CLN6 15q23**

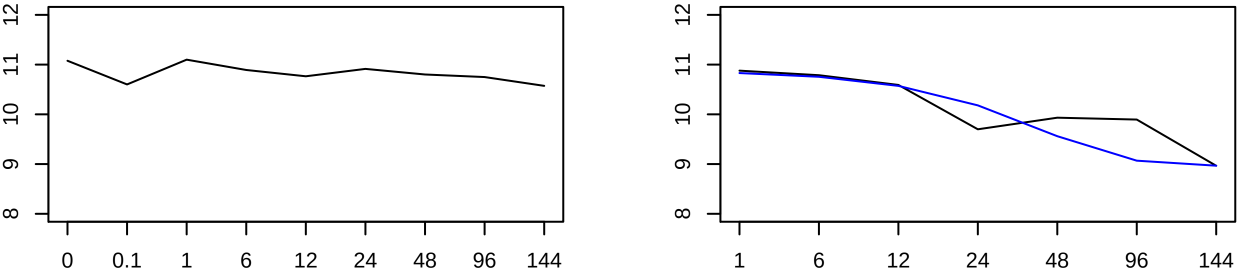


**A_23_P111860 FLJ10324 7p22.1**

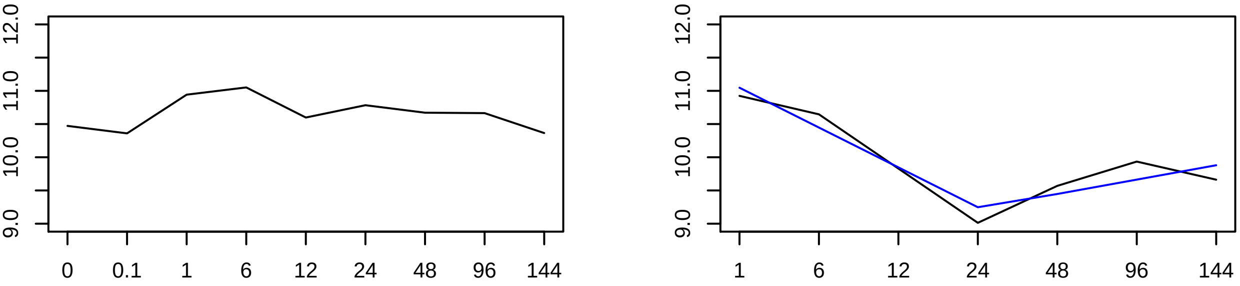


**A_23_P398515 PKMYT1 16p13.3**

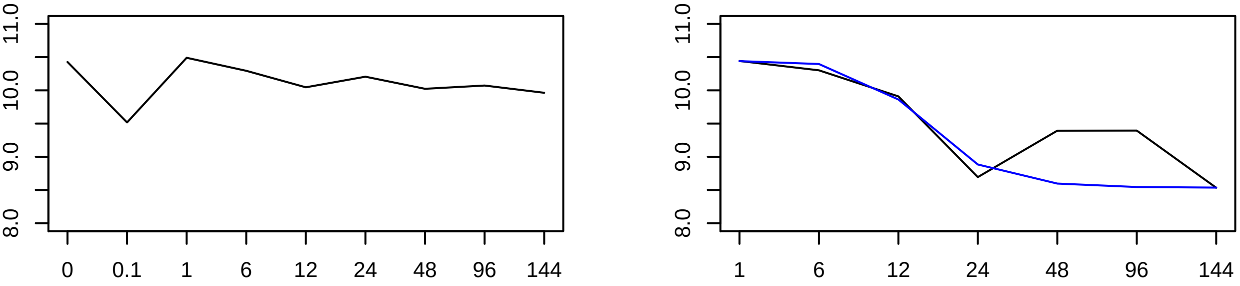

A_24_P48057 IRX5 16q12.2

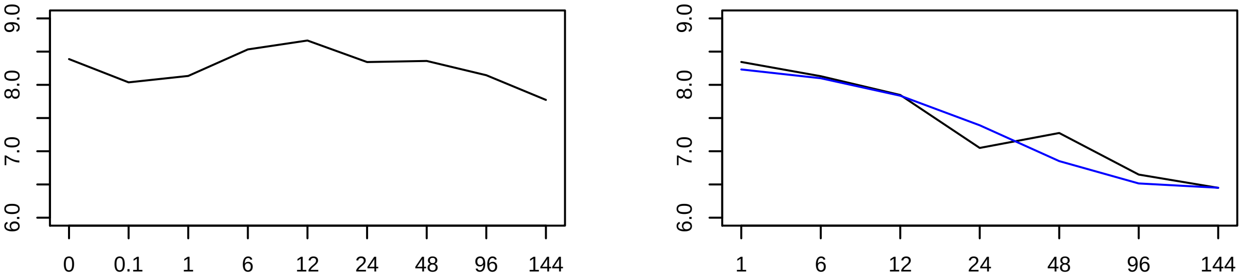


A_23_P202104 PPIF 10q22.3

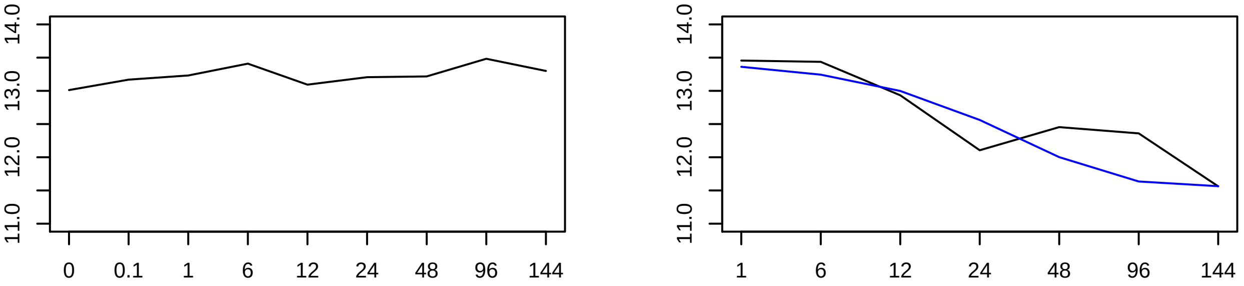


A_24_P105102 PKMYT1 16p13.3

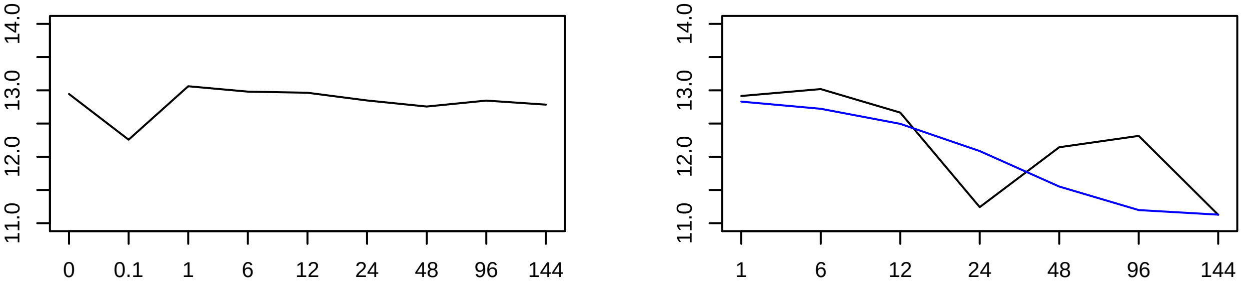

**A_24_P324449 ABCB10 1q42.13**

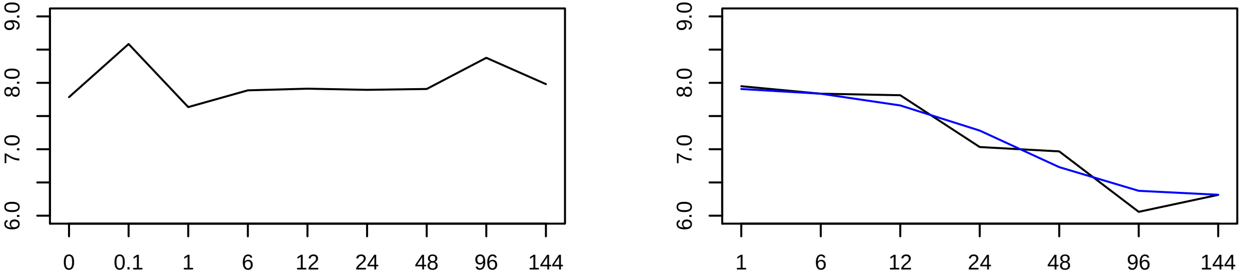


**A_24_P273014 A_24_P273014 NA**

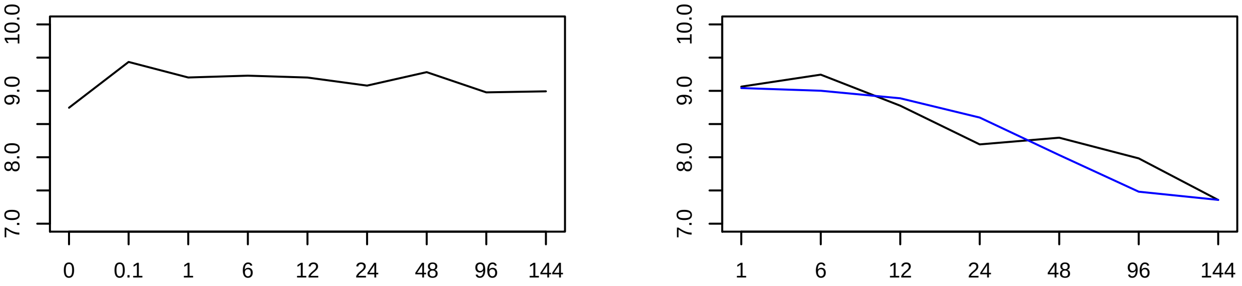


**A_23_P99292 RAD51AP1 12p13.32**

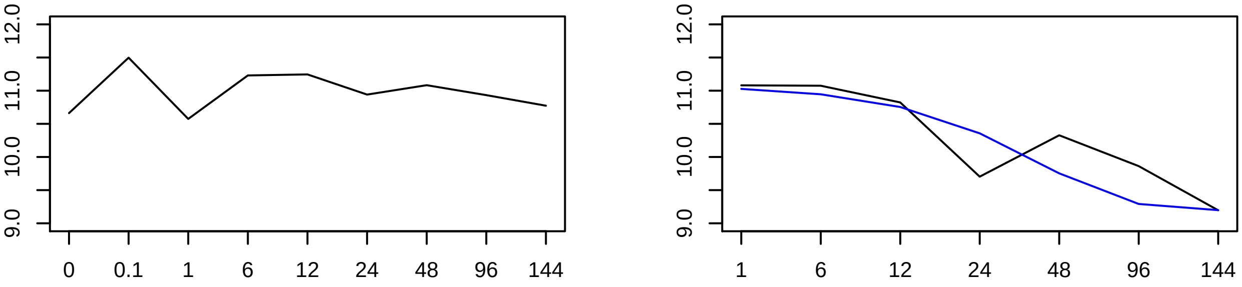

**A_23_P73348 IRX6 16q12.2**

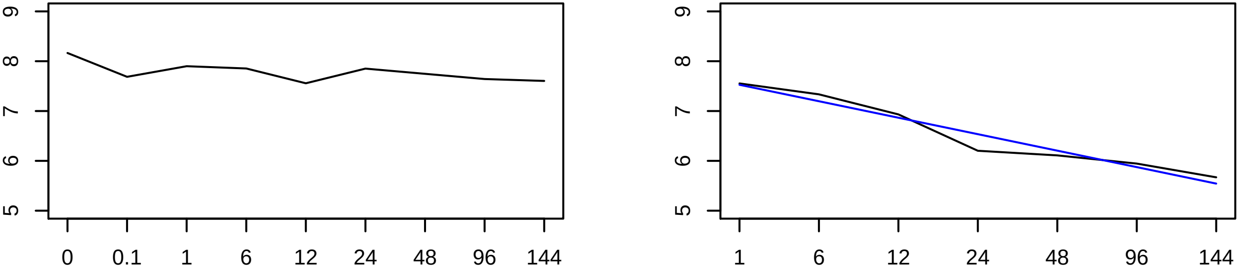


**A_23_P4503 ENOSF1 18p11.32**

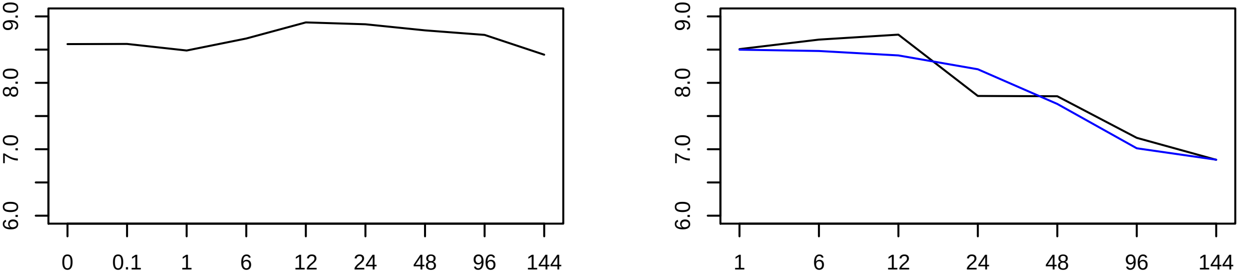


**A_24_P200427 PAICS 4q12**

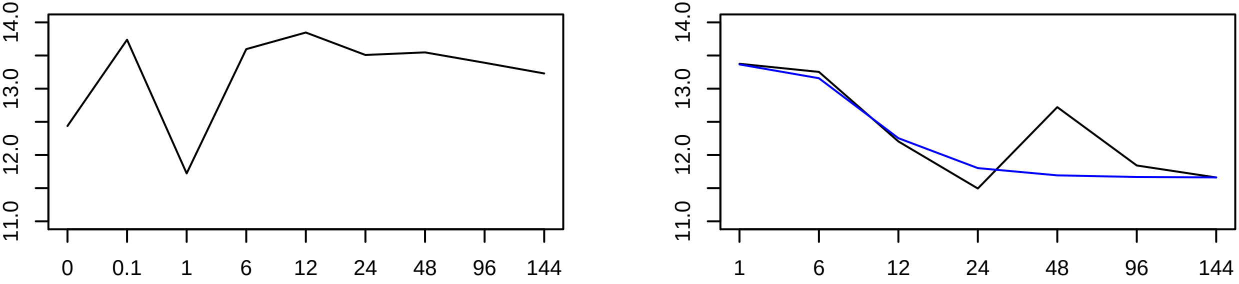

**A_23_P379794 PIGW 17q12**

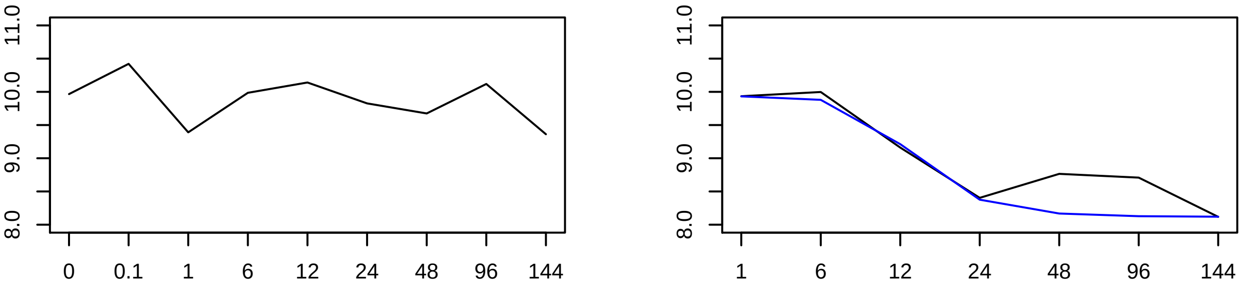


**A_23_P18598 PI4K2B 4p15.2**

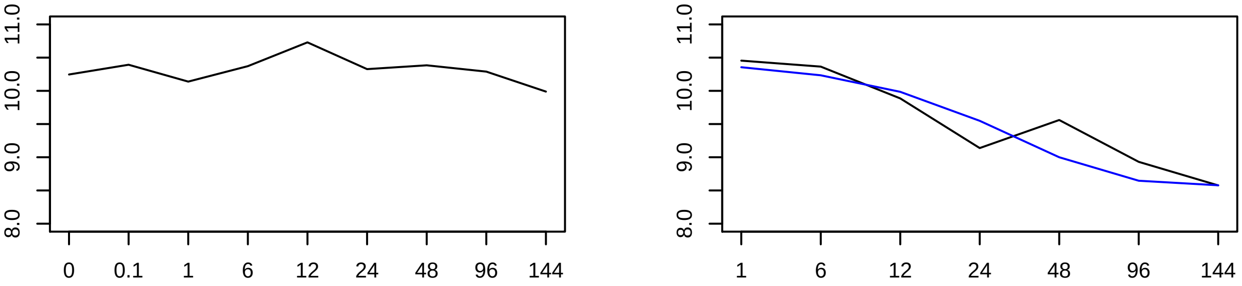


**A_23_P93823 RFC2 7q11.23**

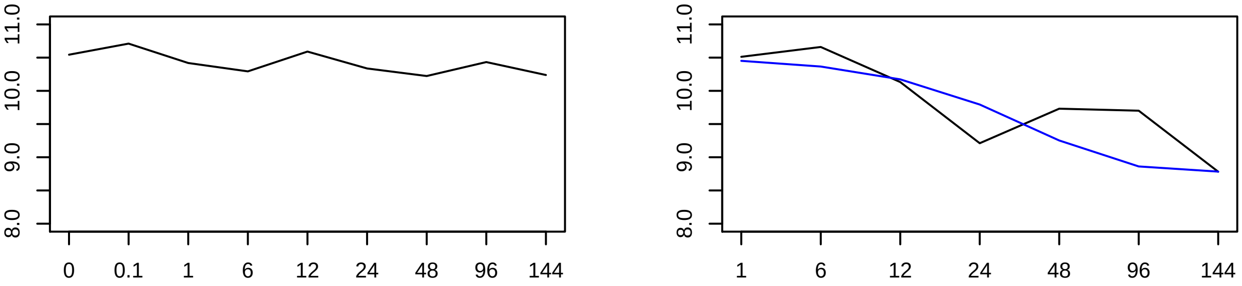

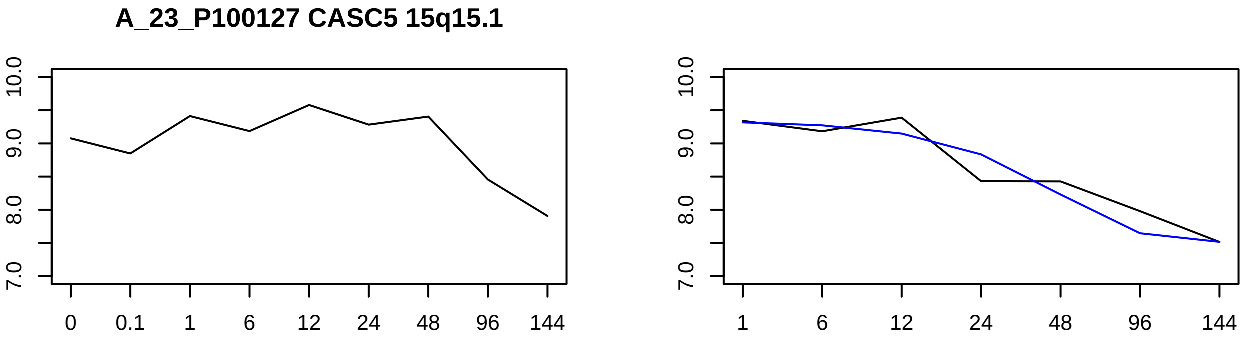

A_23_P100127 CASC5 15q15.1
7.0
8.0
9.0
10.0
0
0.1
1
6
12
24
48
96
144


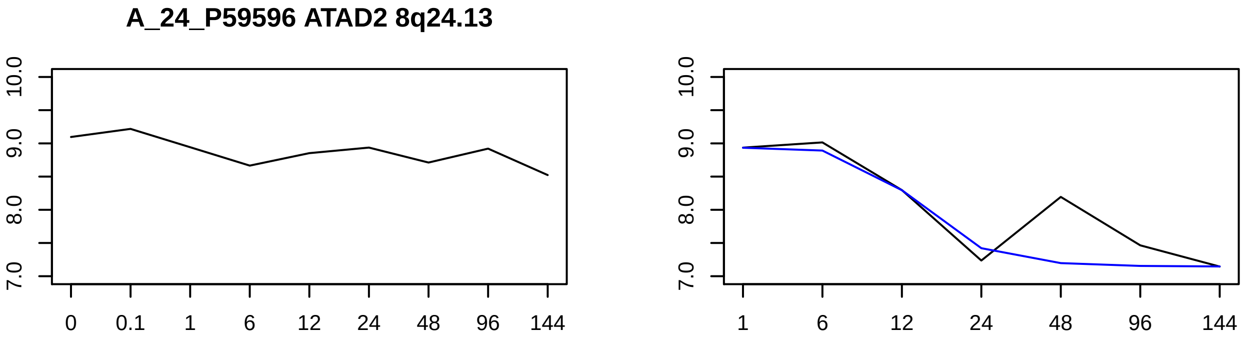

A_24_P59596 ATAD2 8q24.13
7.0
8.0
9.0
10.0
0
0.1
1
6
12
24
48
96
144


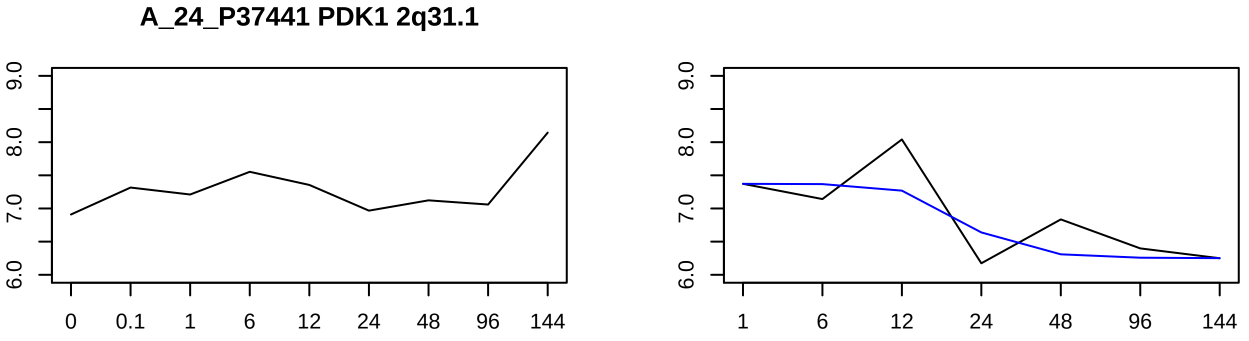

A_24_P37441 PDK1 2q31.1
6.0
7.0
8.0
9.0
0
0.1
1
6
12
24
48
96
144

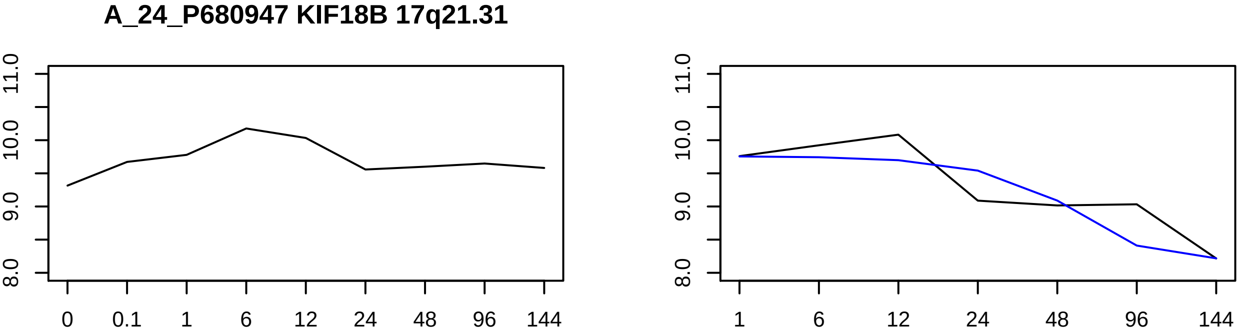
A_24_P680947 KIF18B 17q21.31
8.0
9.0
10.0
11.0
0
0.1
1
6
12
24
48
96
144
1
6
12
24
48
96
144

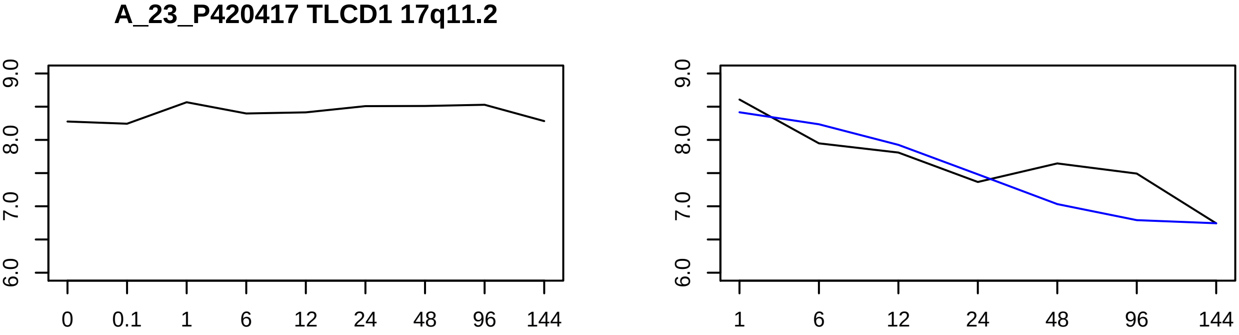
A_23_P420417 TLCD1 17q11.2
6.0
7.0
8.0
9.0
0
0.1
1
6
12
24
48
96
144
1
6
12
24
48
96
144

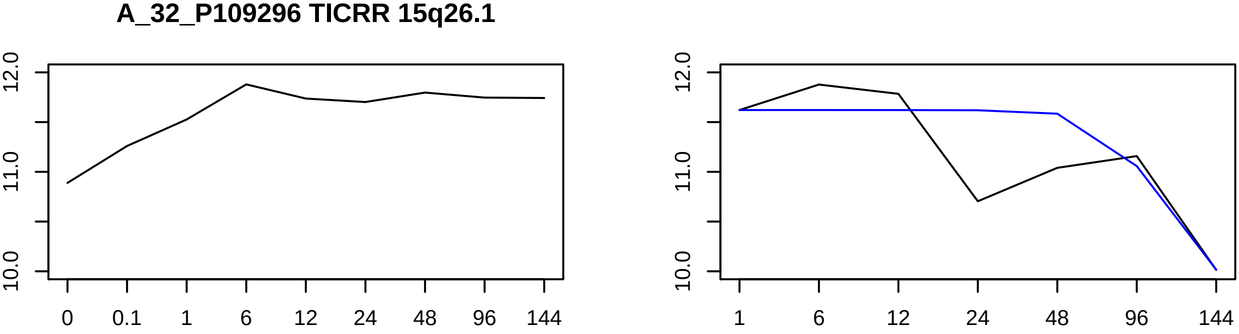
A_32_P109296 TICRR 15q26.1
10.0
11.0
12.0
0
0.1
1
6
12
24
48
96
144
1
6
12
24
48
96
144

**A_23_P161615 POLA2 11q13.1**

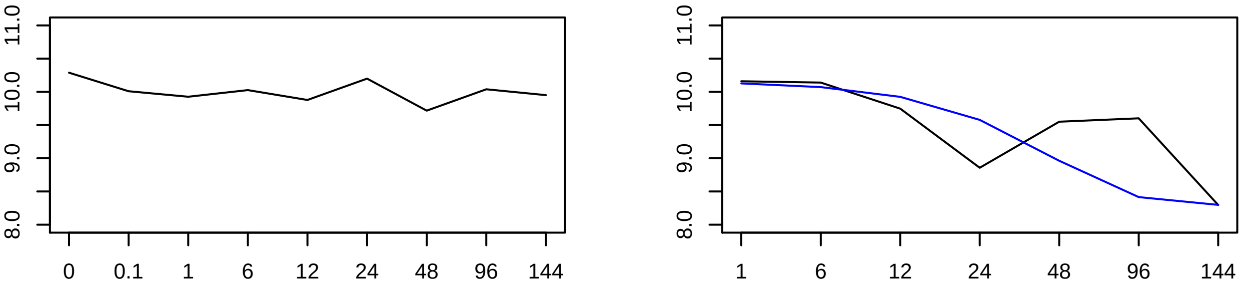


**A_23_P90612 MCM6 2q21.3**

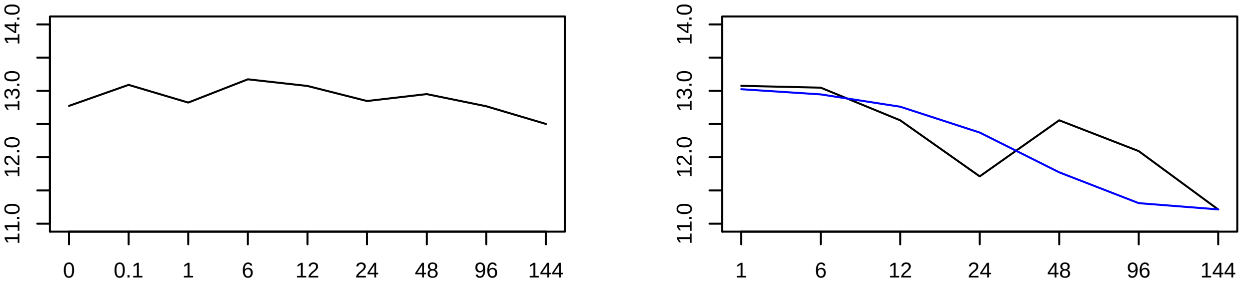


**A_32_P182439 POLD3 11q13.4**

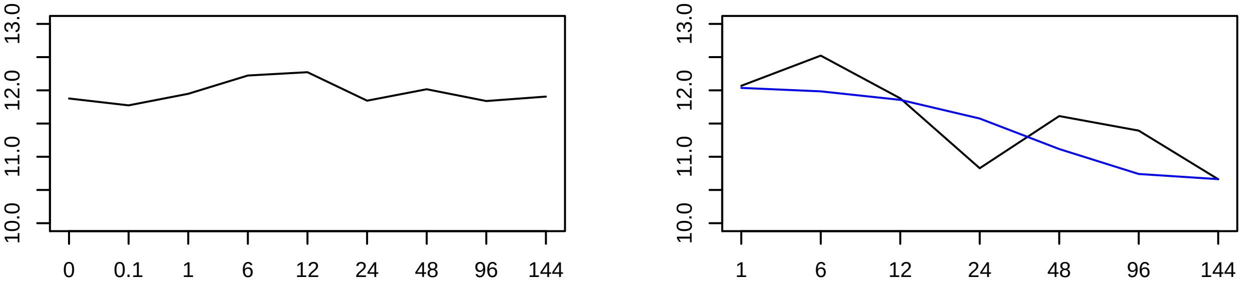

**A_32_P95914 C6orf167 6q16.1**

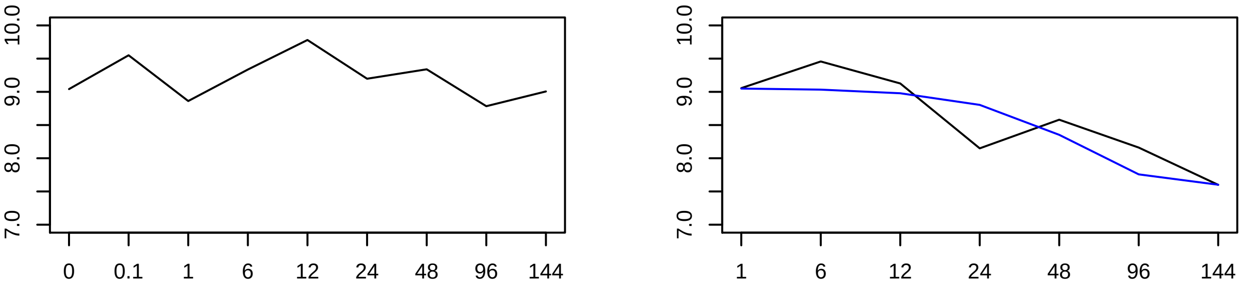


**A_24_P159323 GINS3 16q21**

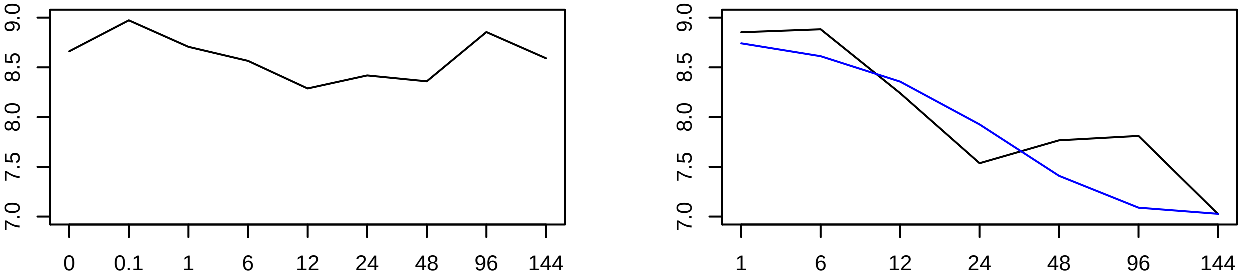


**A_23_P95302 RFC5 12q24.23**

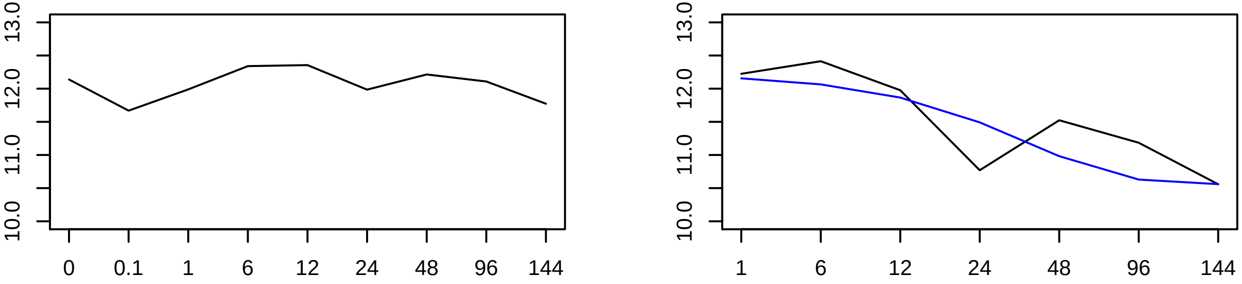

**A_32_P177539 IRX6 16q12.2**

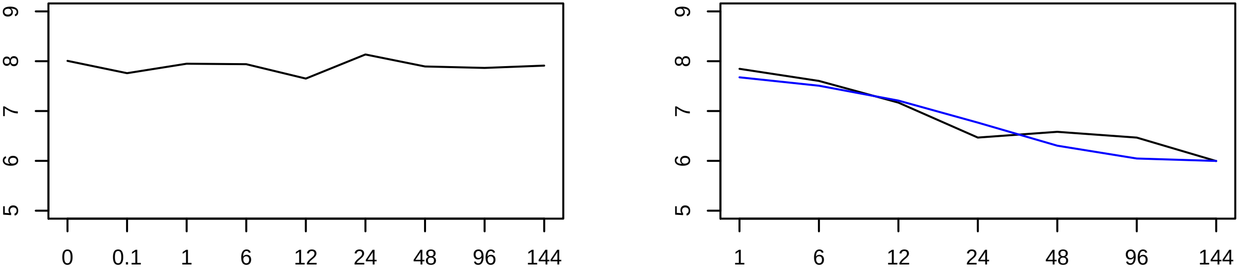


**A_24_P398585 UNG 12q24.11**

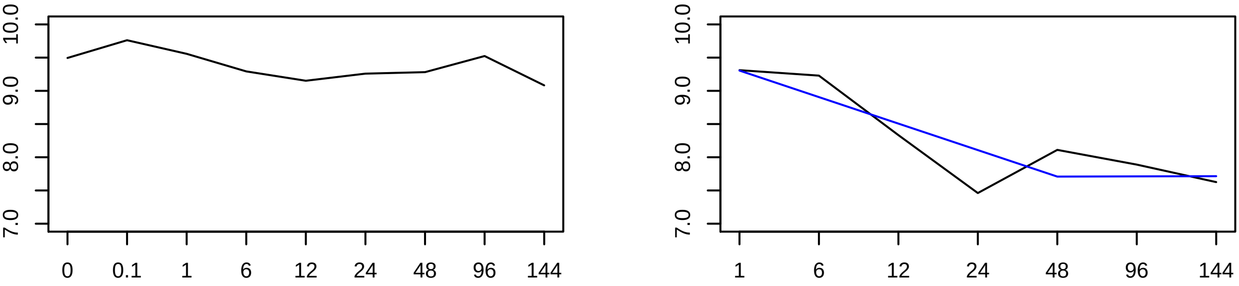


**A_23_P216068 ATAD2 8q24.13**

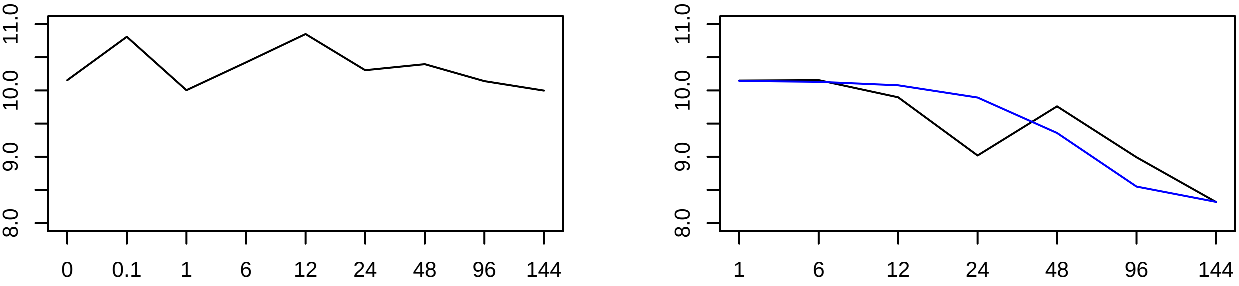

**A_23_P202100 A_23_P202100 NA**

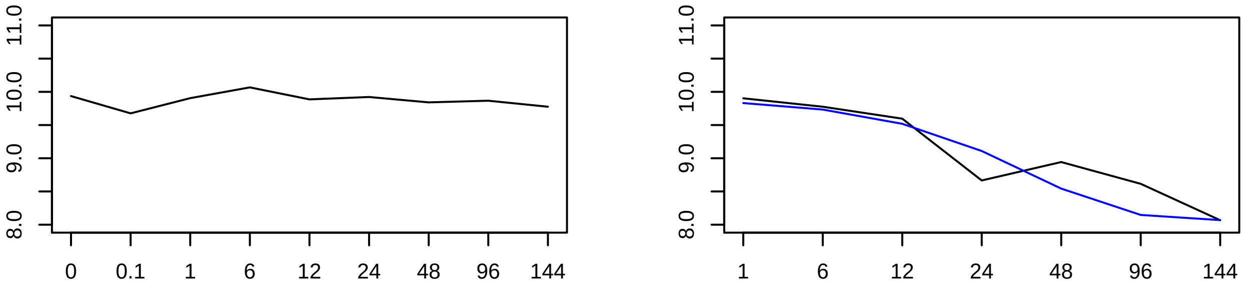


**A_23_P7582 TCF7 5q31.1**

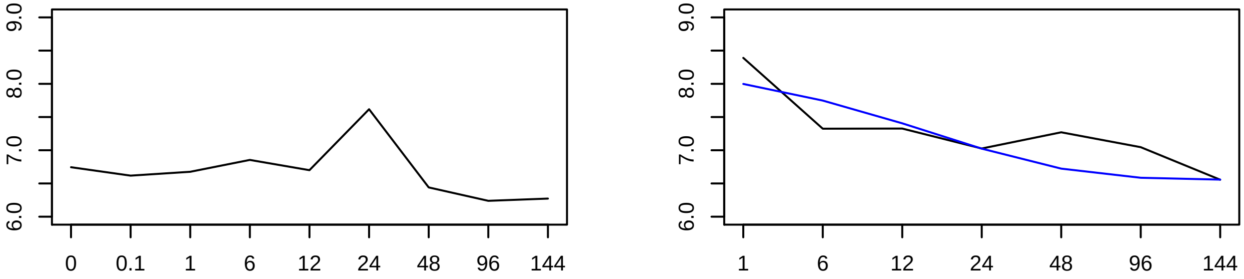


**A_23_P385861 CDCA2 8p21.2**

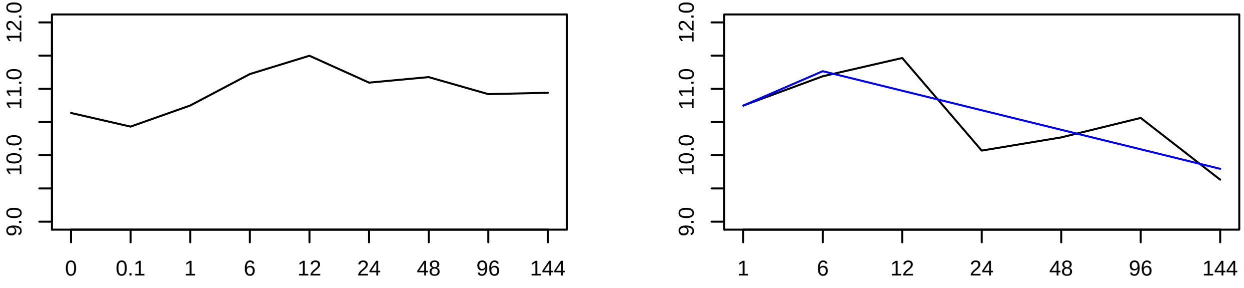

A_23_P57709 PCOLCE2 3q23

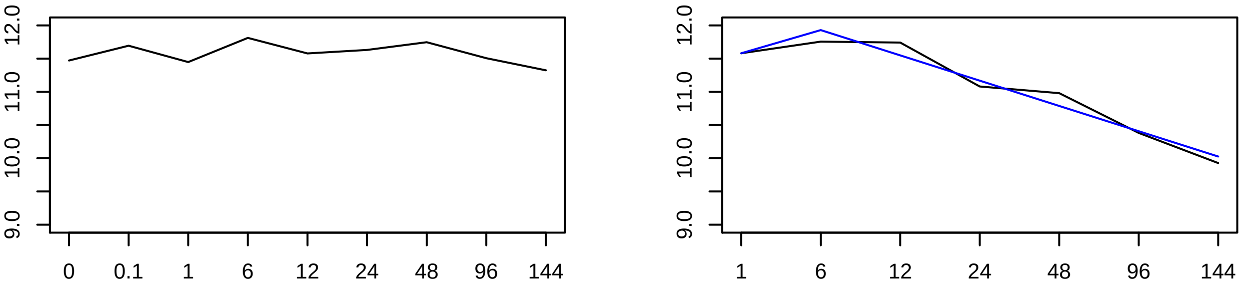


A_23_P411833 WDR90 16p13.3

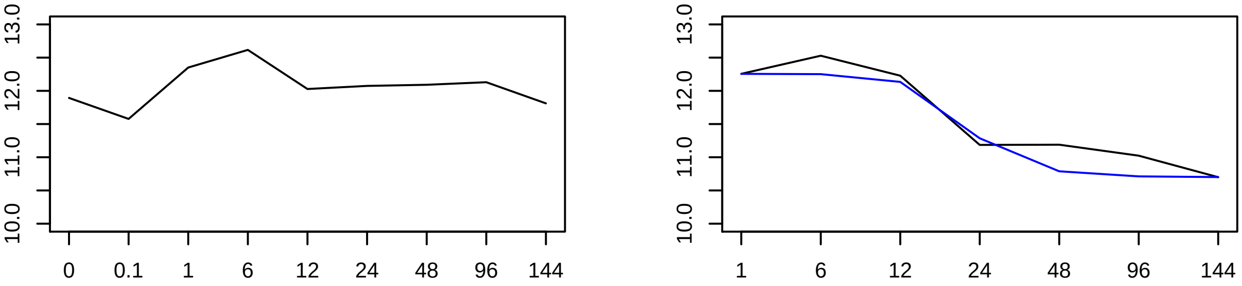


A_24_P171549 CDCA7 2q31.1

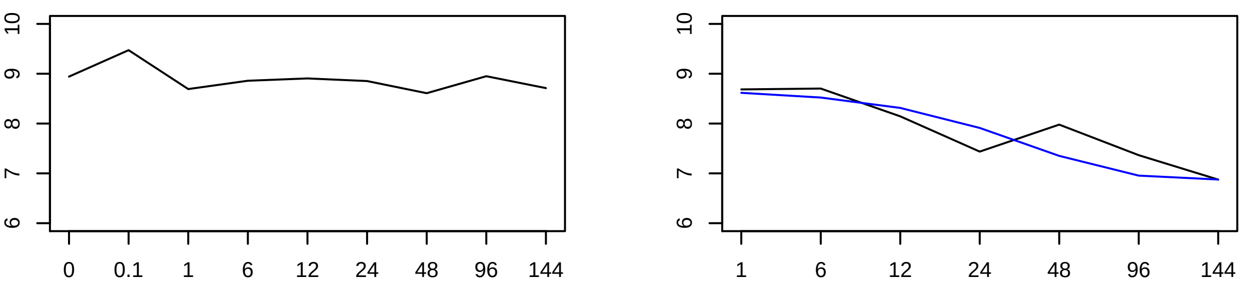

**A_23_P167256 PHF17 4q28.2**

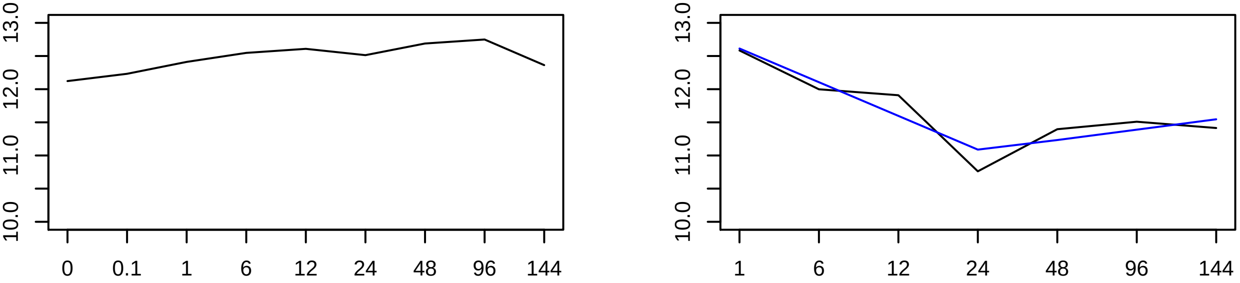


**A_32_P19806 DNAH11 7p15.3**

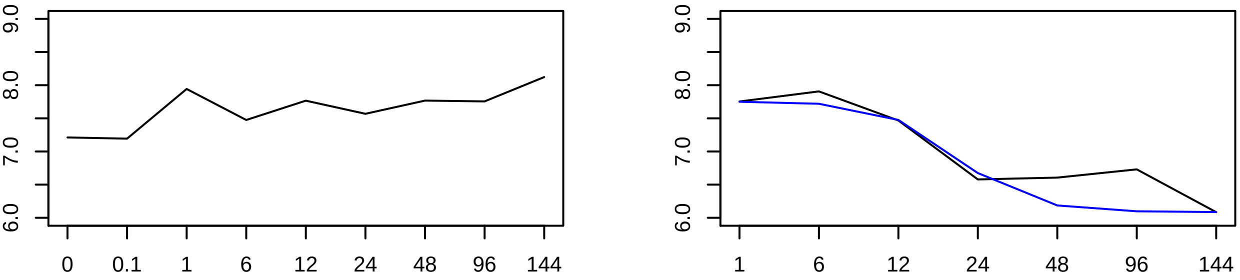


**A_24_P305556 MCM8 20p12.3**

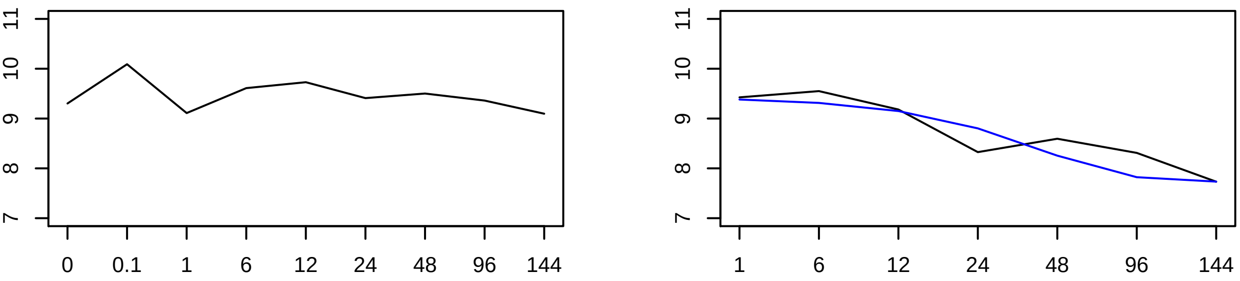

**A_23_P99452 BRCA2 13q13.1**

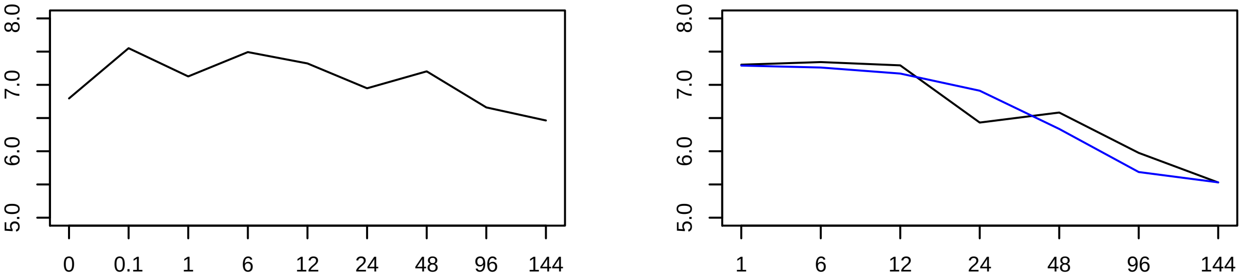


**A_32_P93996 CENPP 9q22.31**

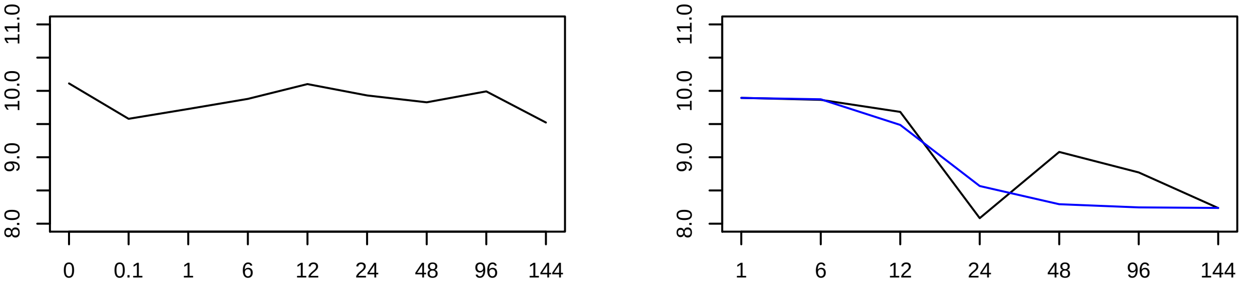


**A_23_P27656 C19orf48 19q13.33**

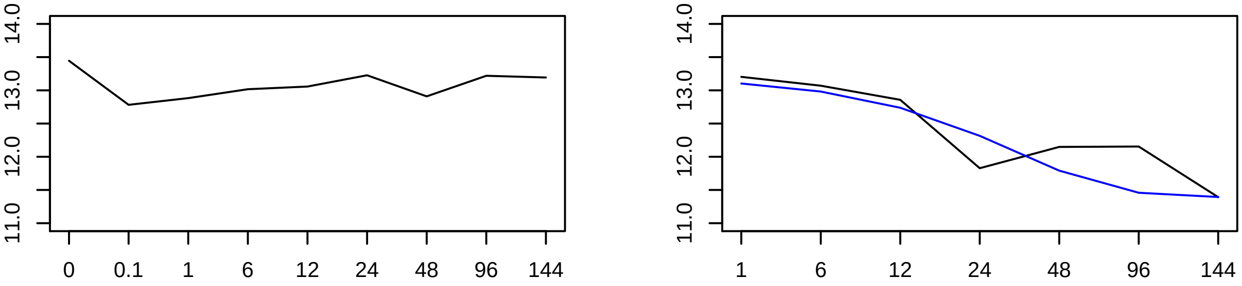

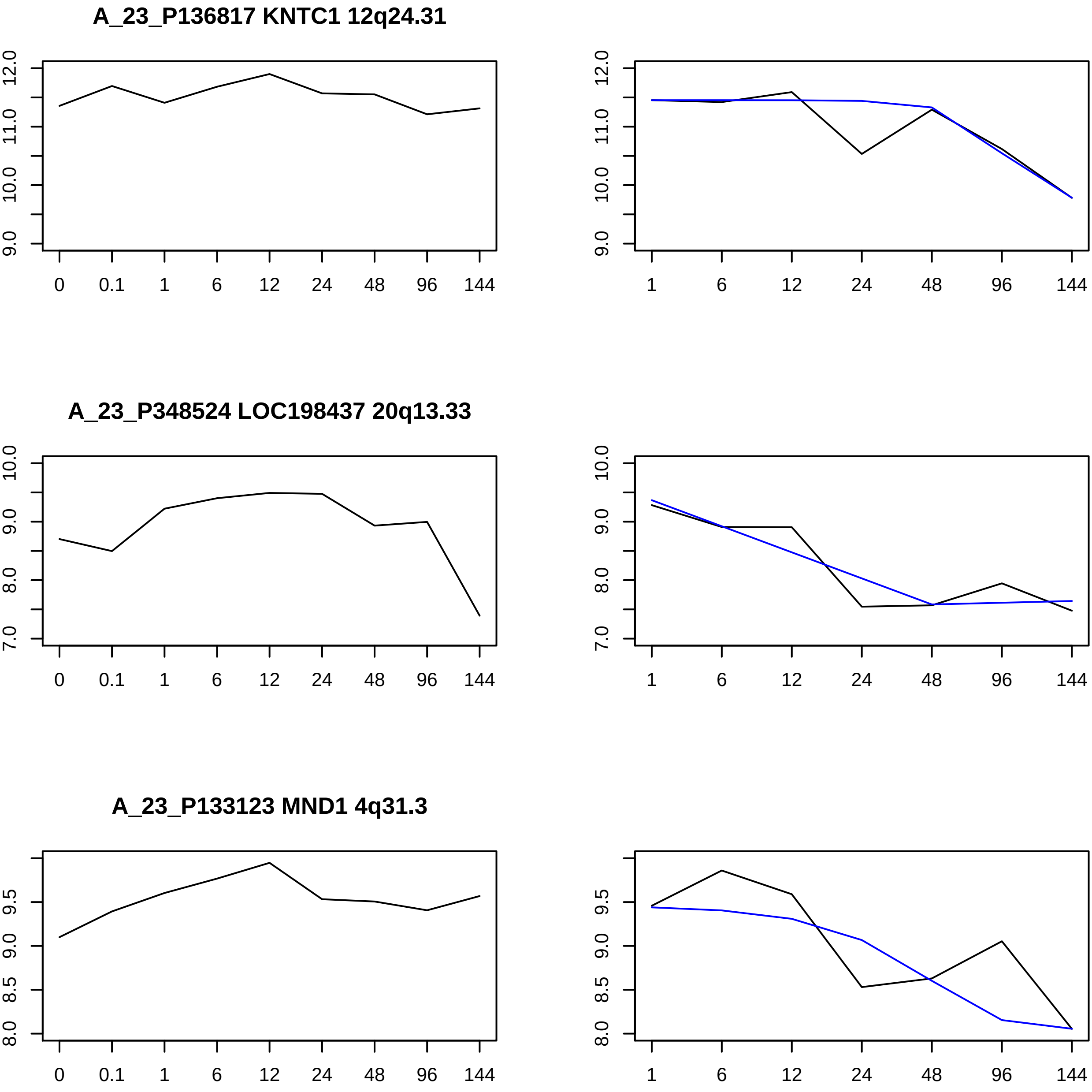
A_23_P136817 KNTC1 12q24.31
9.0
10.0
11.0
12.0
0
0.1
1
6
12
24
48
96
144
1
6
12
24
48
96
144
A_23_P348524 LOC198437 20q13.33
7.0
8.0
9.0
10.0
A_23_P133123 MND1 4q31.3
8.0
8.5
9.0
9.5

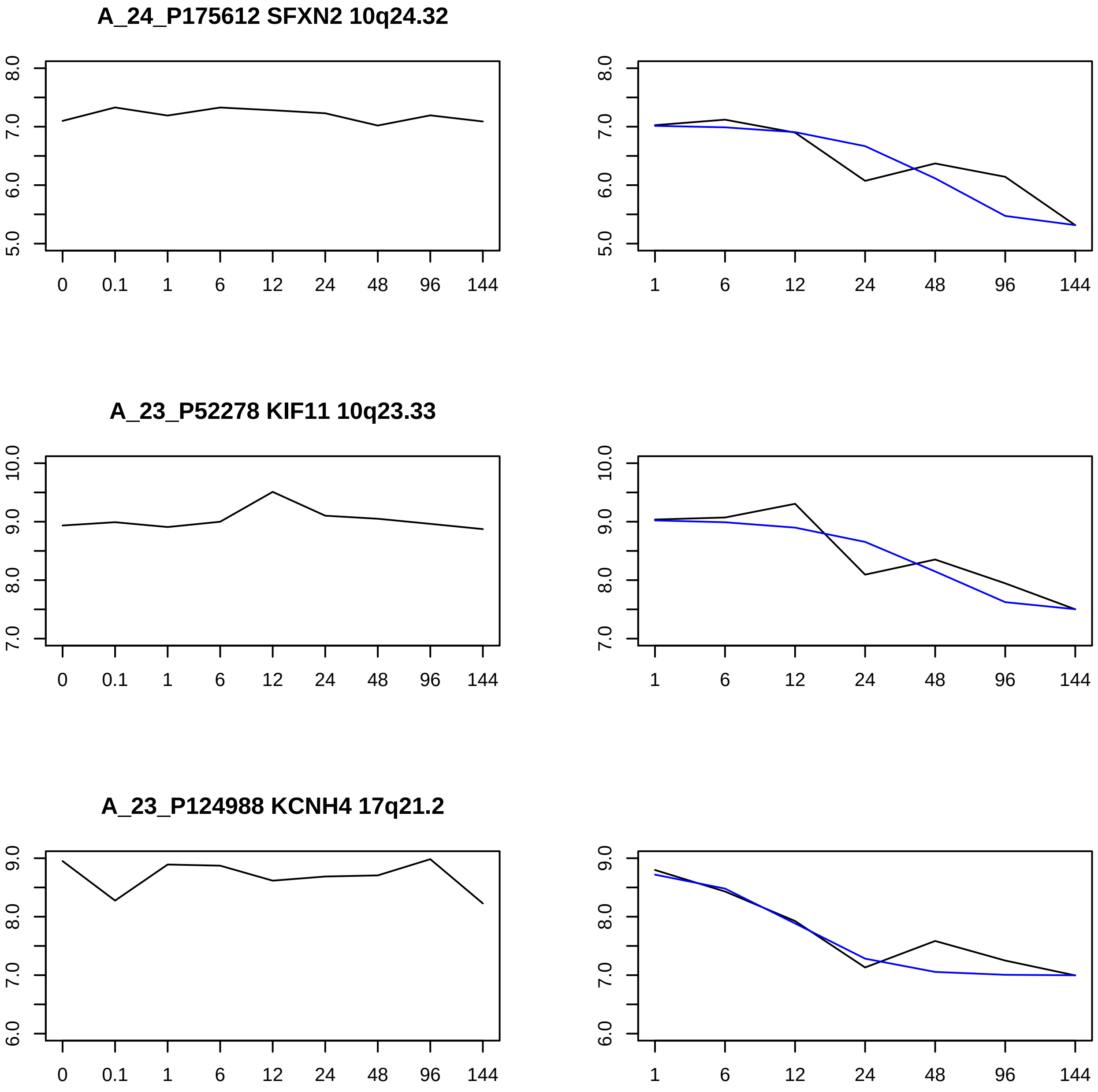

A_24_P175612 SFXN2 10q24.32
5.0 6.0 7.0 8.0
0 0.1 1 6 12 24 48 96 144
5.0 6.0 7.0 8.0
1 6 12 24 48 96 144
A_23_P52278 KIF11 10q23.33
7.0 8.0 9.0 10.0
0 0.1 1 6 12 24 48 96 144
7.0 8.0 9.0 10.0
1 6 12 24 48 96 144
A_23_P124988 KCNH4 17q21.2
6.0 7.0 8.0 9.0
0 0.1 1 6 12 24 48 96 144
6.0 7.0 8.0 9.0
1 6 12 24 48 96 144

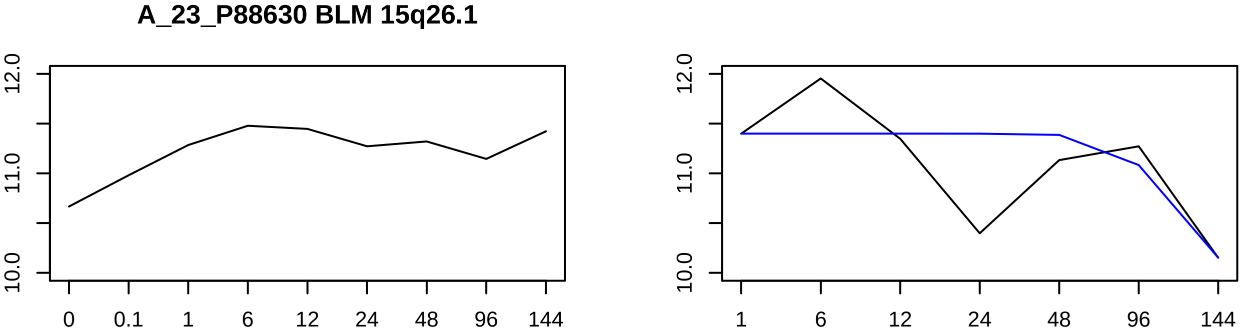
A_23_P88630 BLM 15q26.1
10.0
11.0
12.0
0
0.1
1
6
12
24
48
96
144


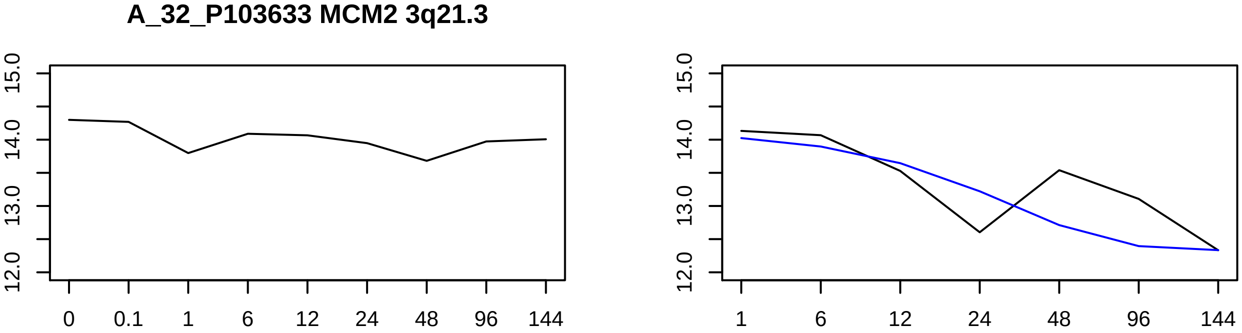
A_32_P103633 MCM2 3q21.3
12.0
13.0
14.0
15.0
0
0.1
1
6
12
24
48
96
144


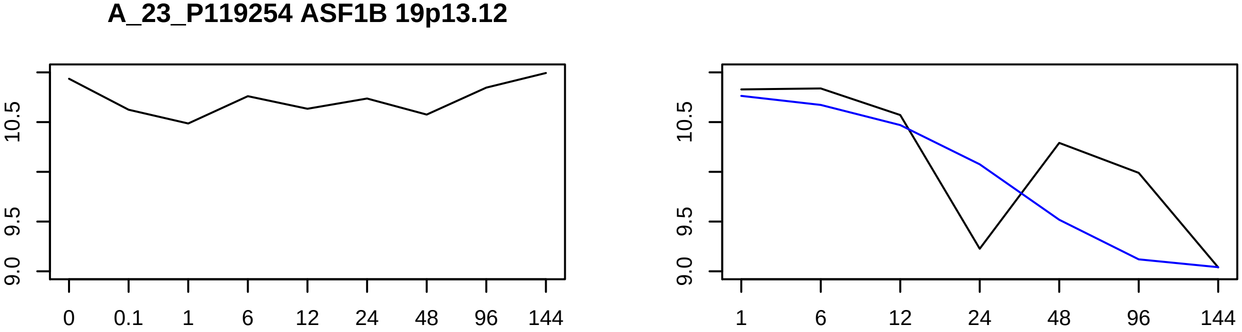
A_23_P119254 ASF1B 19p13.12
9.0
9.5
10.5
0
0.1
1
6
12
24
48
96
144

**A_24_P190168 TMEM97 17q11.2**

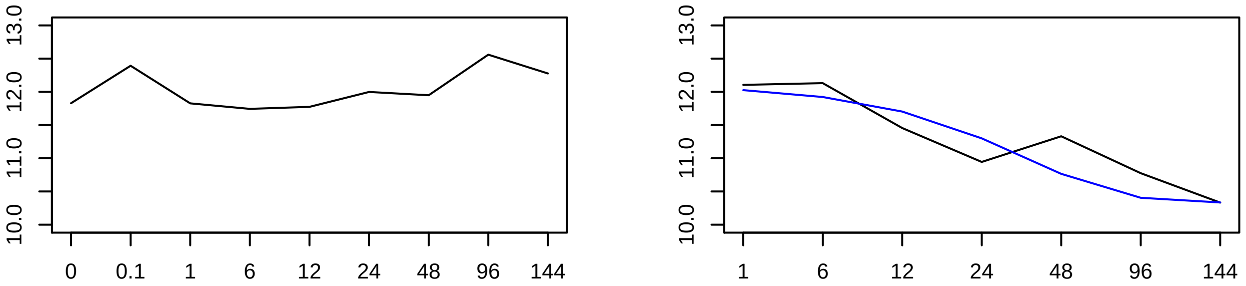


**A_23_P373992 WDR90 16p13.3**

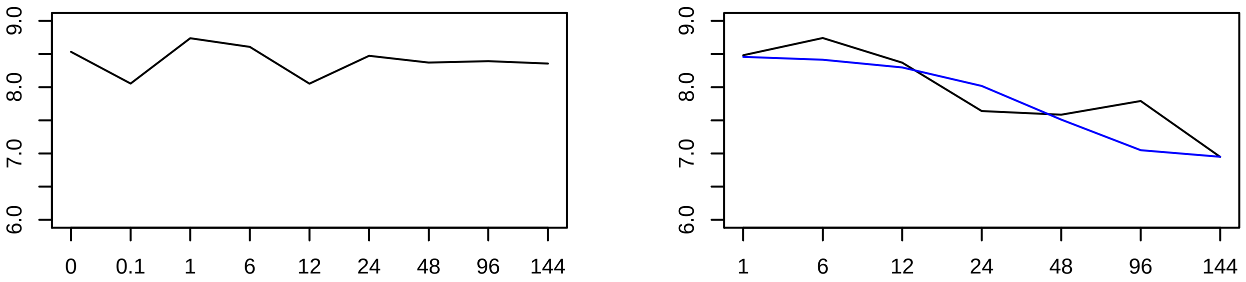


**A_24_P323434 CDCA2 8p21.2**

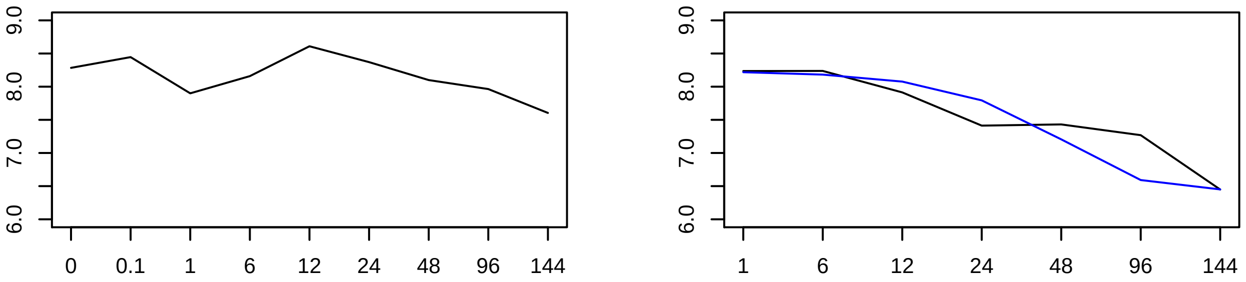

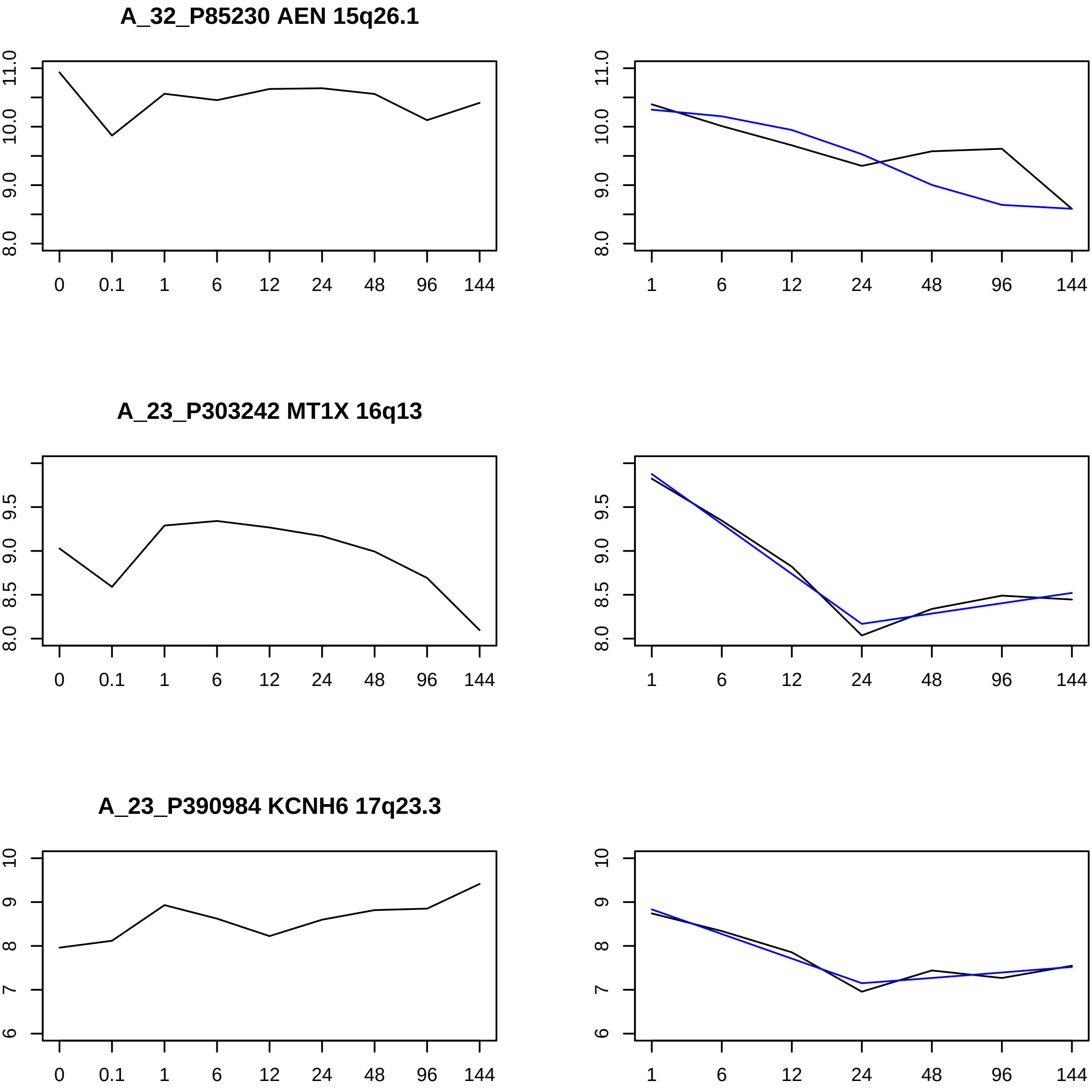

A_32_P85230 AEN 15q26.1
A_23_P303242 MT1X 16q13
A_23_P390984 KCNH6 17q23.3

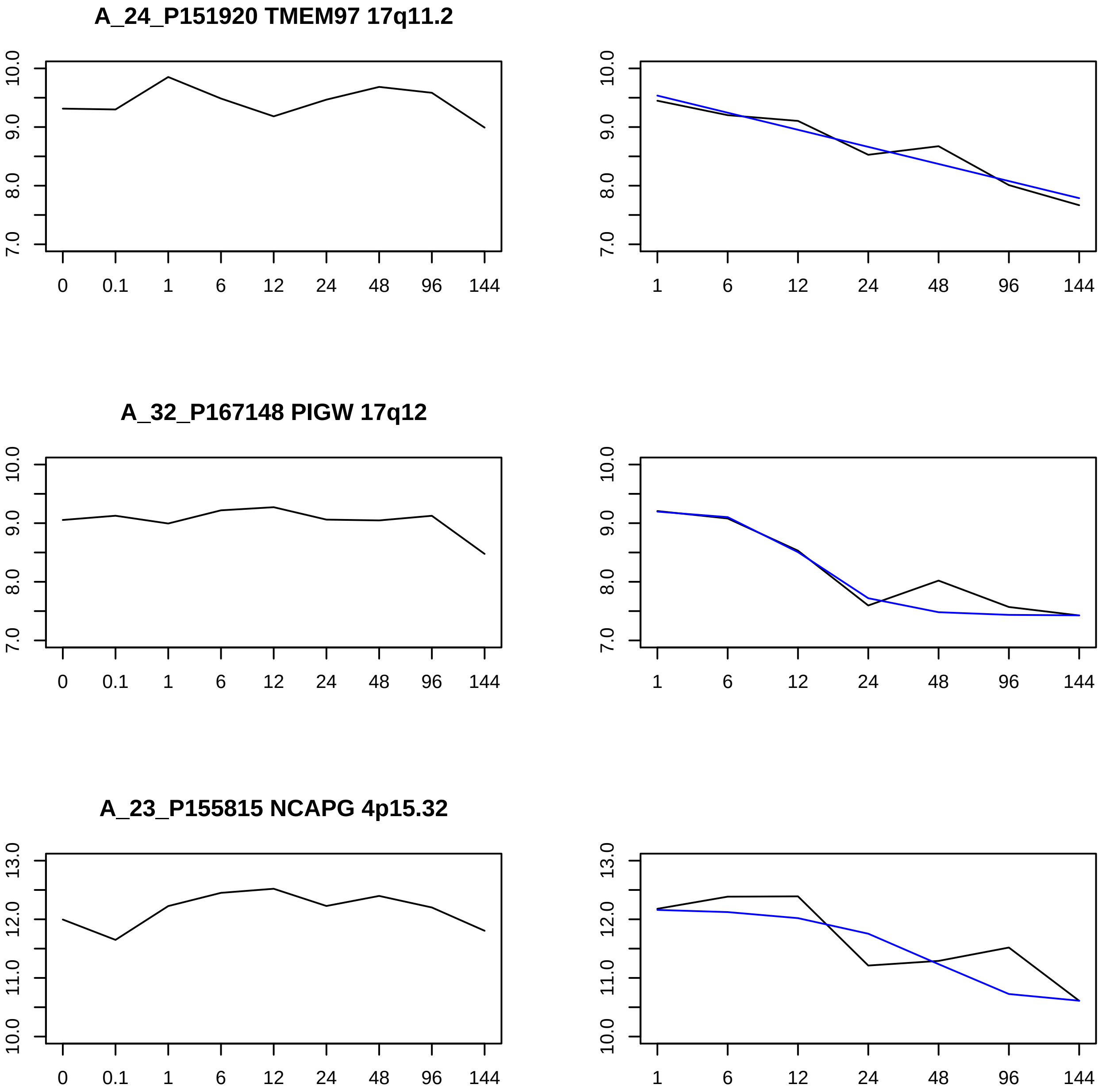

A_24_P151920 TMEM97 17q11.2
A_32_P167148 PIGW 17q12
A_23_P155815 NCAPG 4p15.32
7.0
8.0
9.0
10.0
11.0
12.0
13.0
0
0.1
1
6
12
24
48
96
144

**A_23_P216108 ANK1 8p11.21**

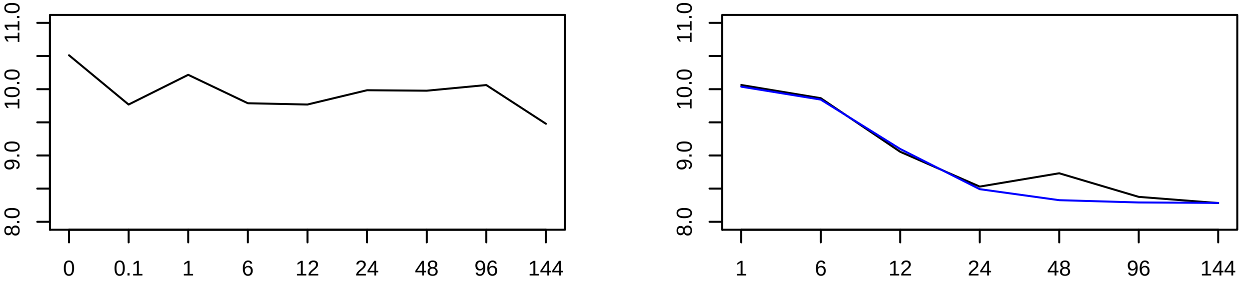


**A_24_P125096 MT1X 16q13**

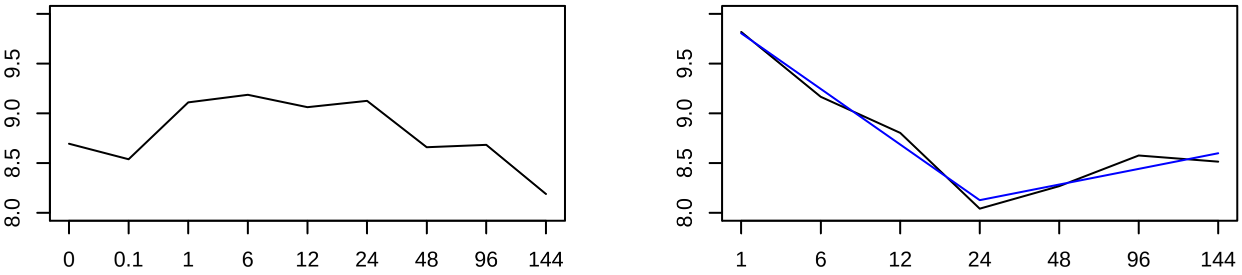


**A_23_P415443 NCAPH 2q11.2**

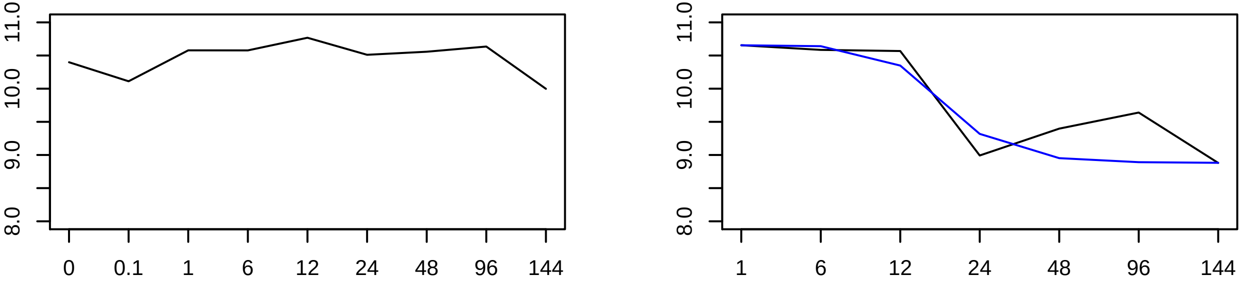

**A_32_P16625 LOC728688 12p12.2**

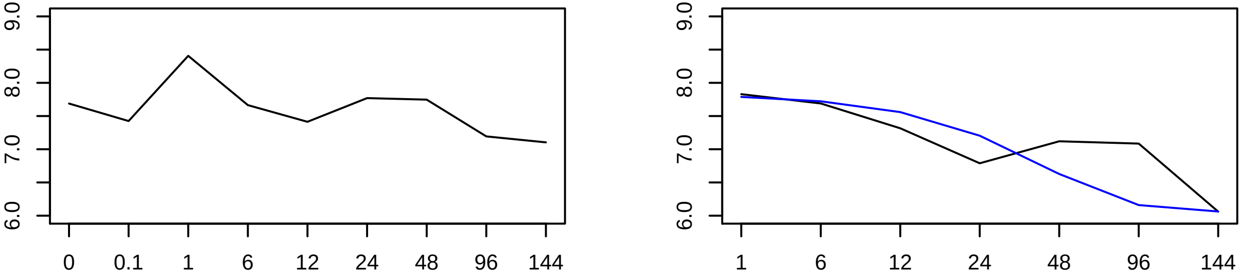


**A_32_P96719 SHCBP1 16q11.2**

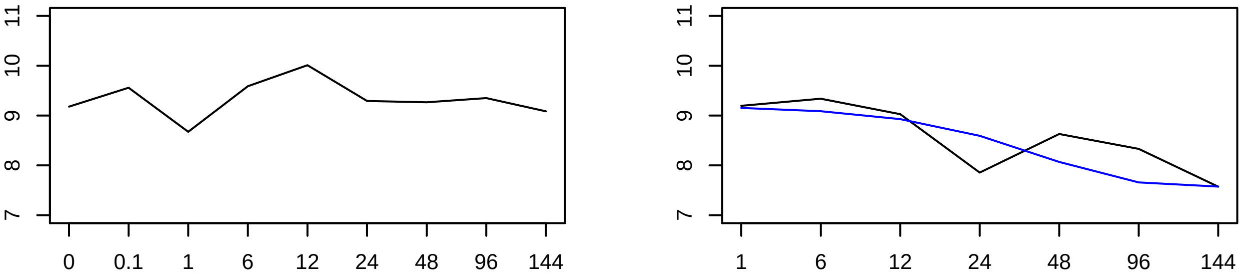


**A_23_P96325 ERCC6L Xq13.1**

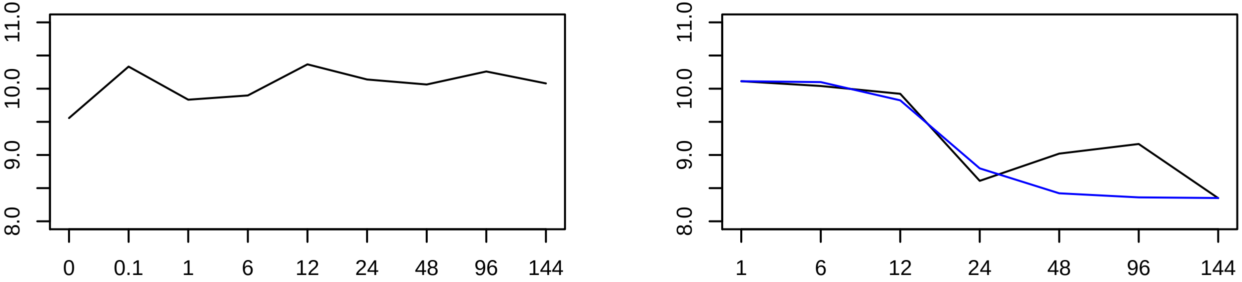

**A_23_P379614 OIP5 15q15.1**

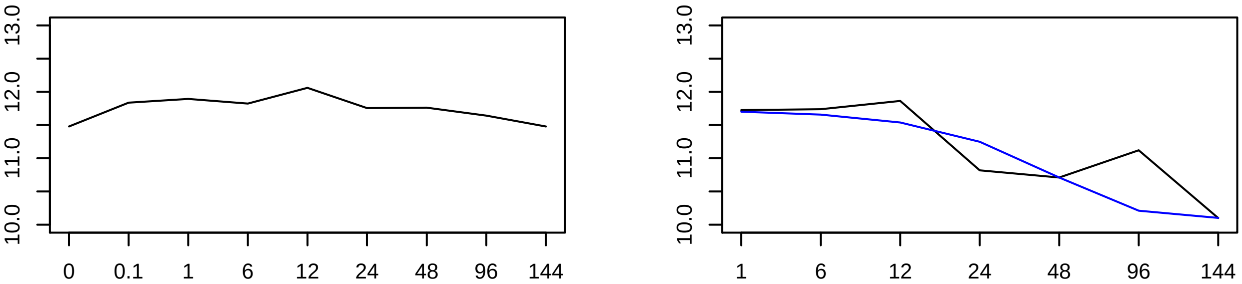


**A_23_P123544 CHRAC1 8q24.3**

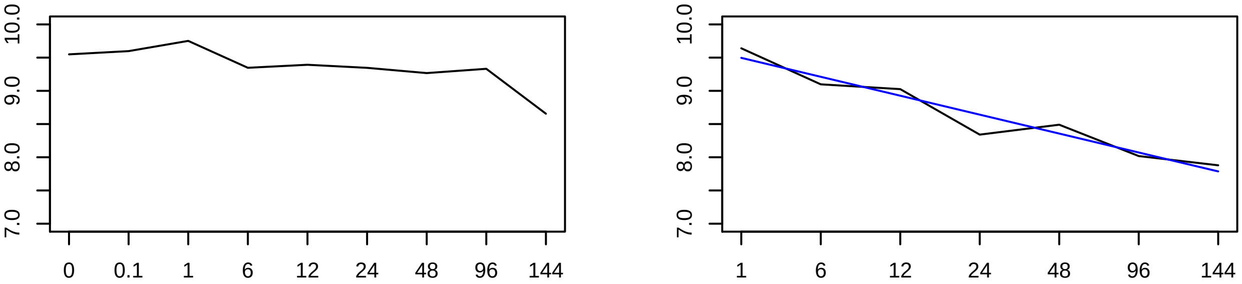


**A_23_P429491 C11orf82 11q14.1**

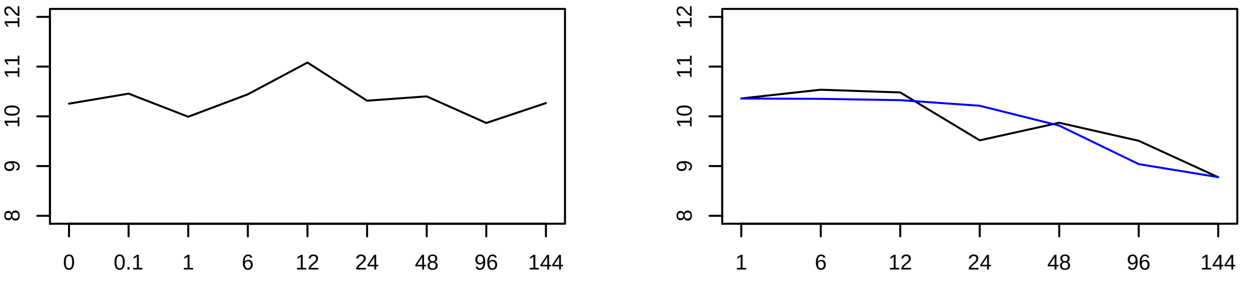

**A_23_P140088 RNASEH2B 13q14.3**

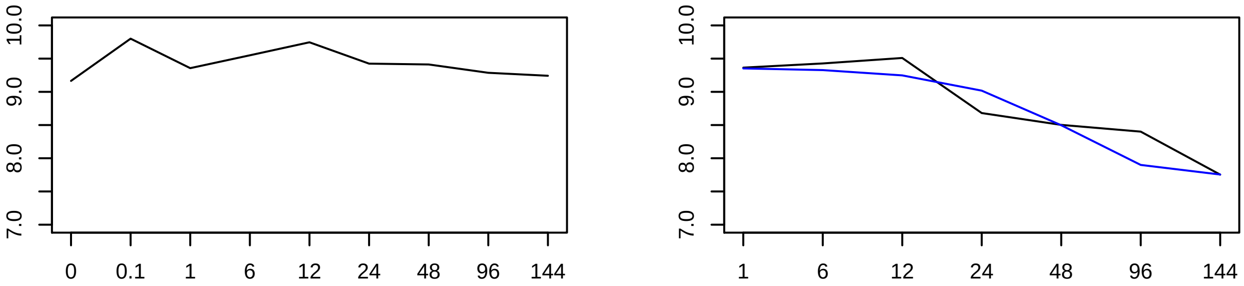


**A_23_P123308 TEX15 8p12**

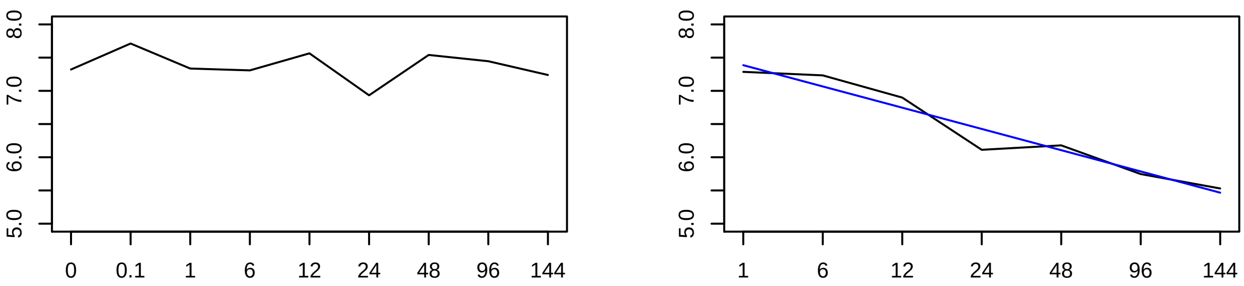


**A_24_P529168 C13orf25 13q31.3**

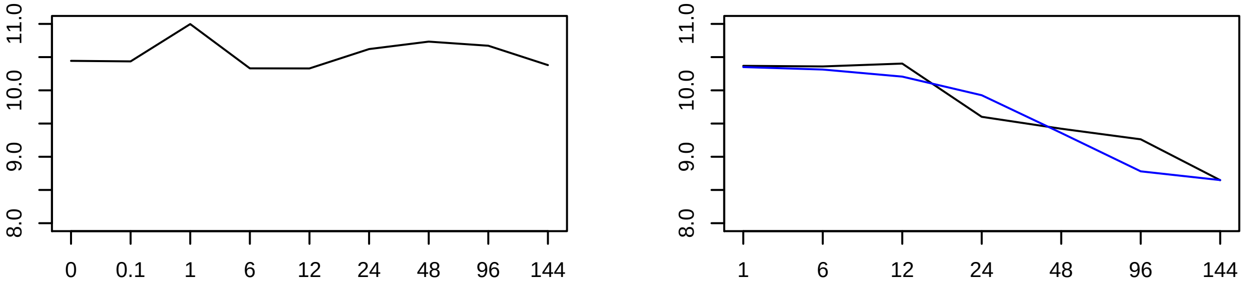

**A_32_P126832 CD511705 NA**

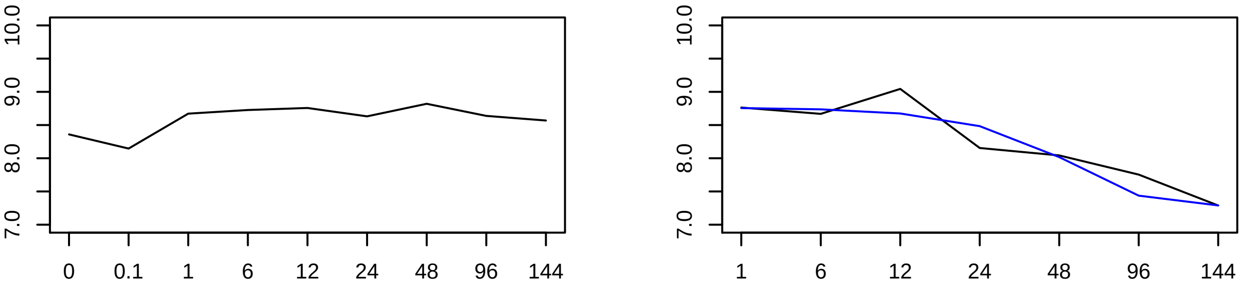


**A_23_P63789 ZWINT 10q21.1**

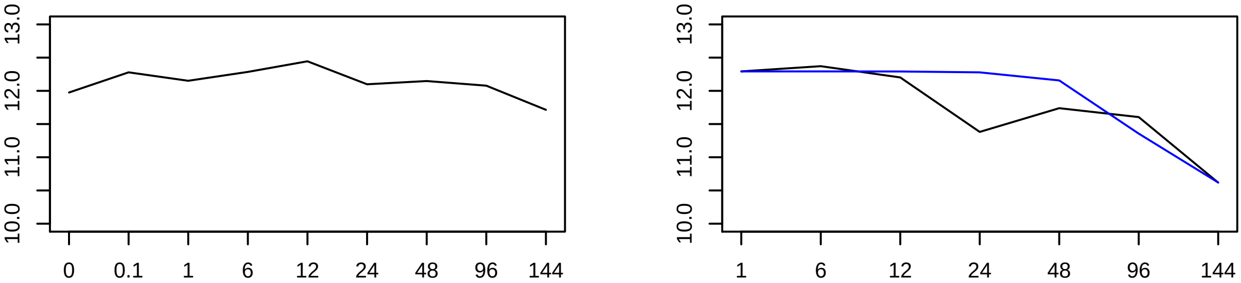


**A_24_P99071 RANBP5 13q32.2**

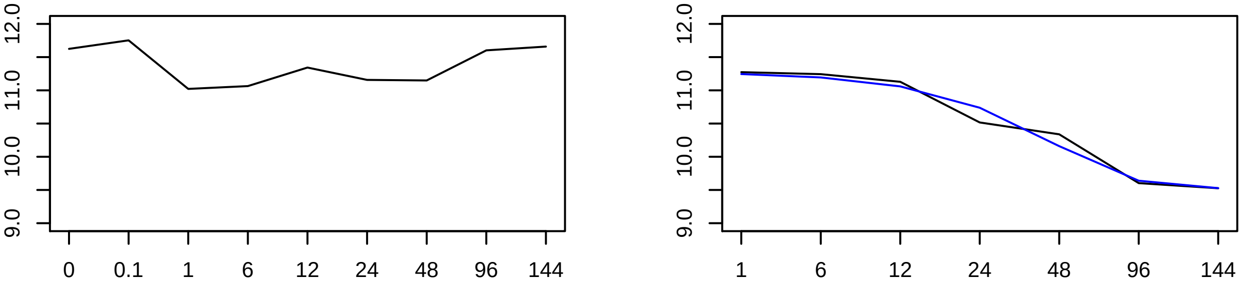

A_23_P46309 SNHG3−RCC1 1p35.3

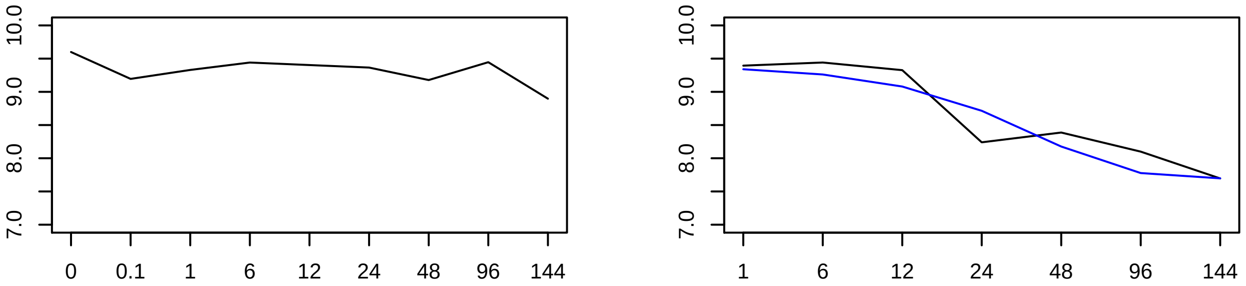


A_23_P152136 GINS3 16q21

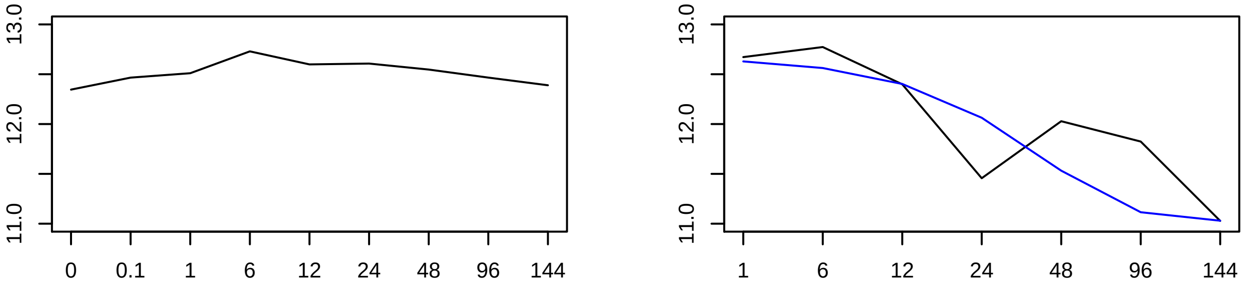


A_23_P136805 ARHGAP11A 15q13.3

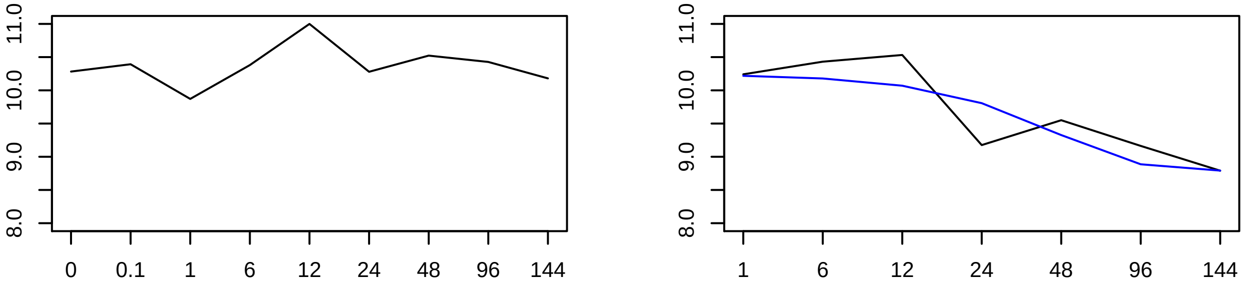

**A_23_P32707 ESPL1 12q13.13**

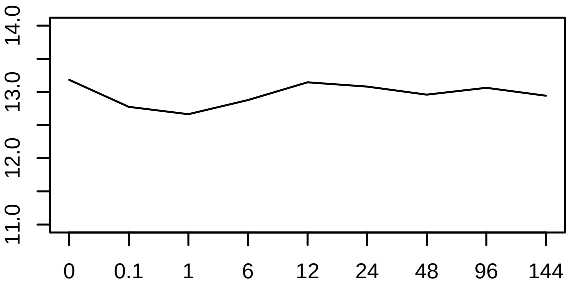


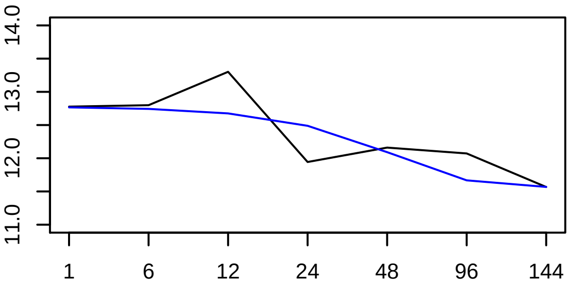


**A_23_P353717 RMI2 16p13.13**

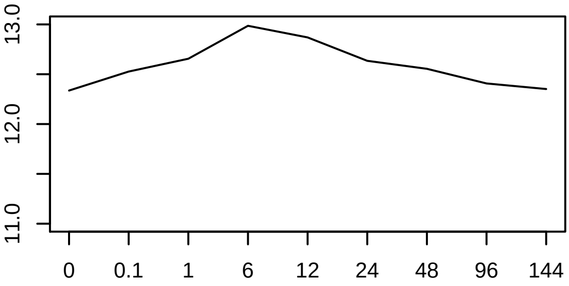


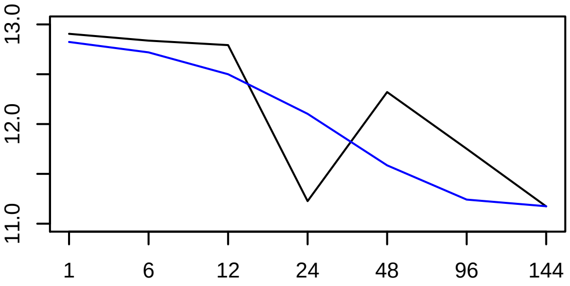


**A_23_P12816 HELLS 10q23.33**

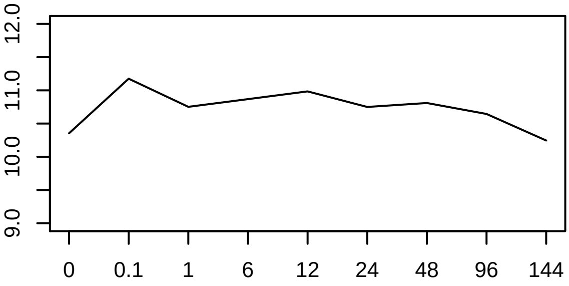


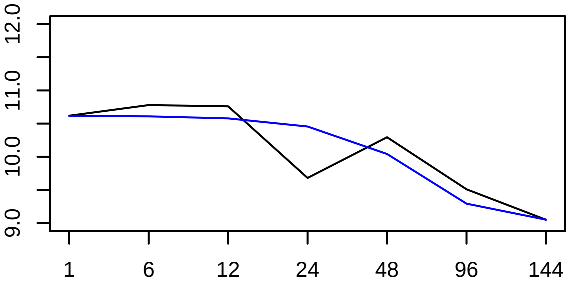

**A_24_P56363 CAB39L 13q14.2**

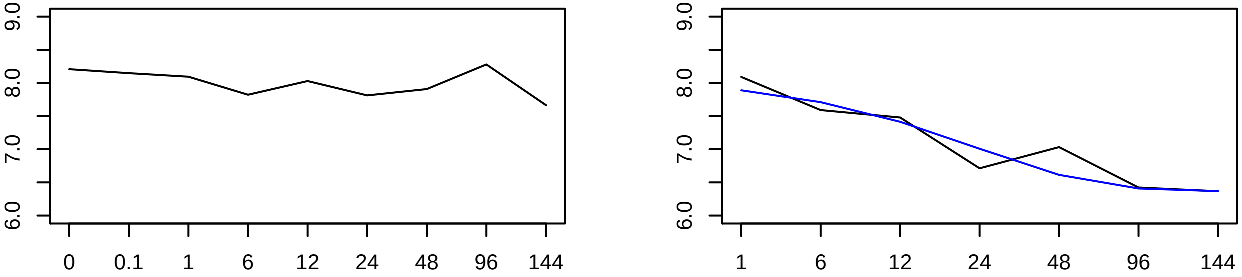


**A_23_P19712 GMNN 6p22.2**

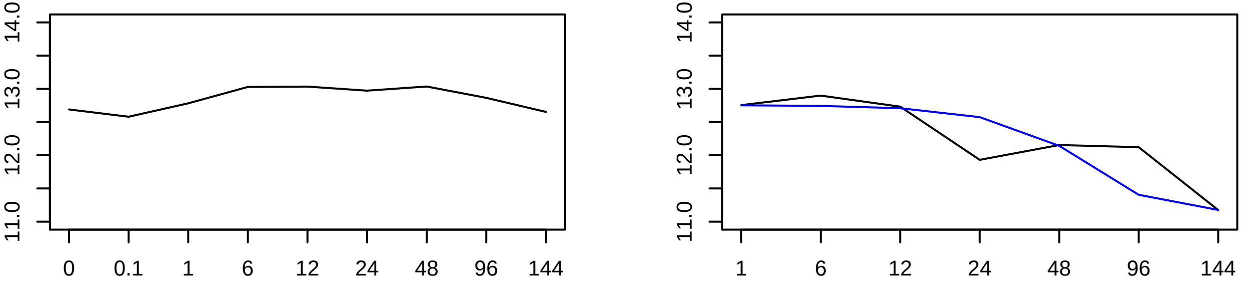


**A_32_P62997 PBK 8p21.1**

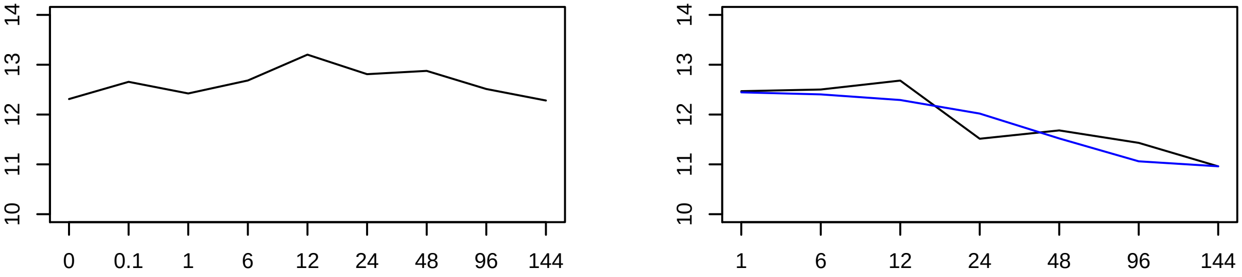

A_24_P314571 SPBC24 19p13.2

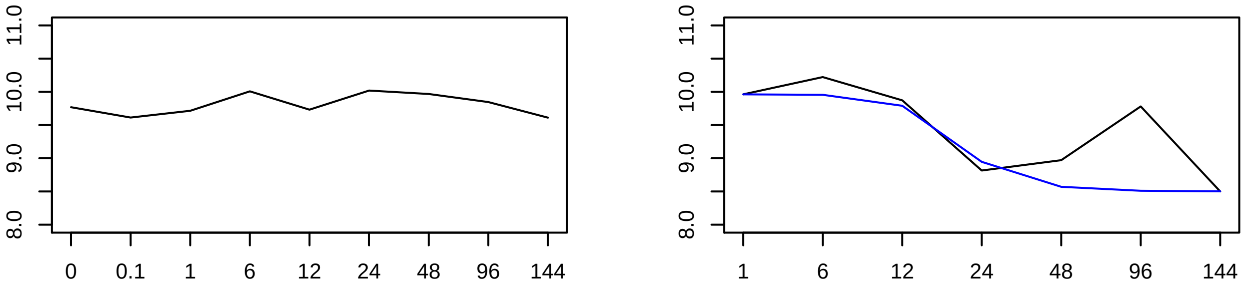


A_23_P203949 DDX11 12p11.21

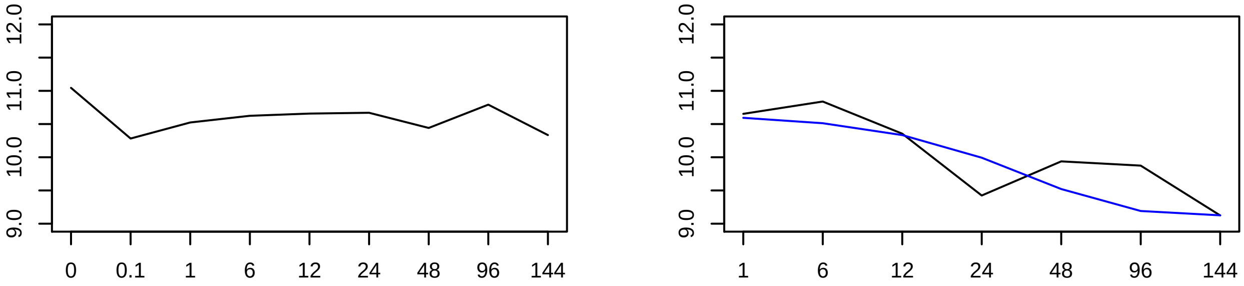


A_23_P216396 EXOSC2 9q34.12

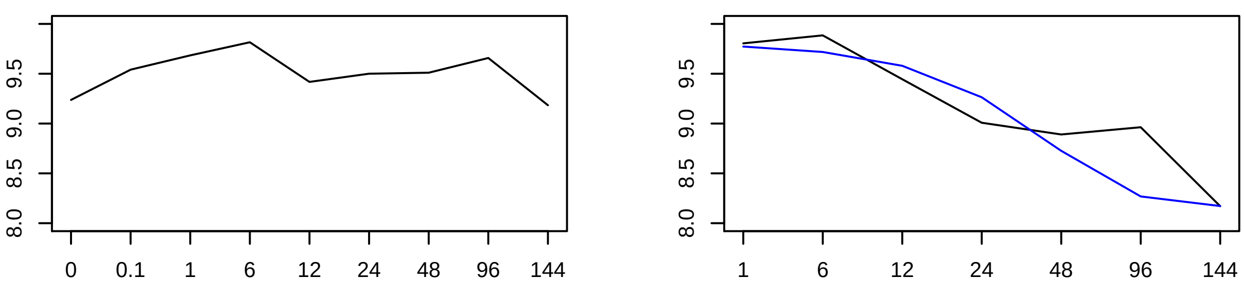

**A_23_P53198 DGAT2 11q13.5**

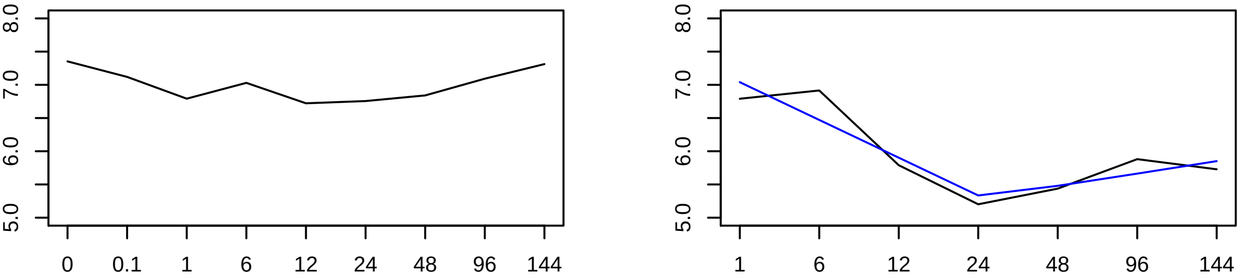


**A_23_P403081 C5orf34 5p12**

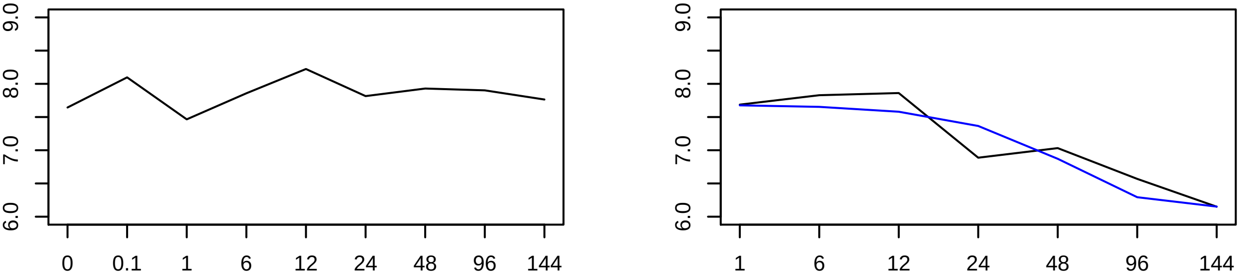


**A_24_P257099 DKFZp762E1312 2q37.1**

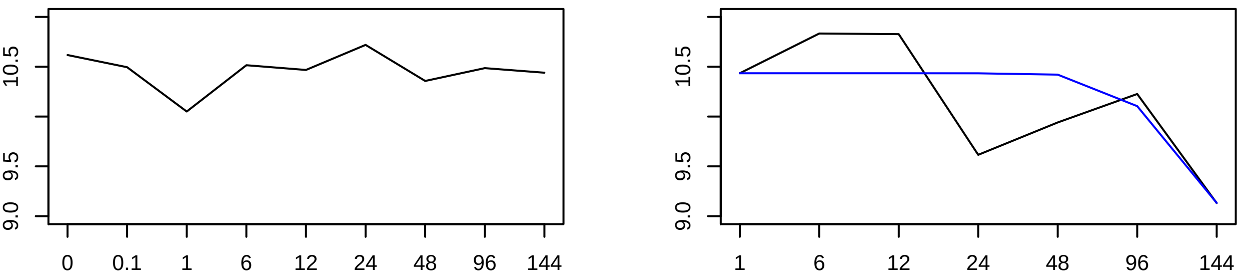

**A_23_P320250 MYOHD1 17q12**

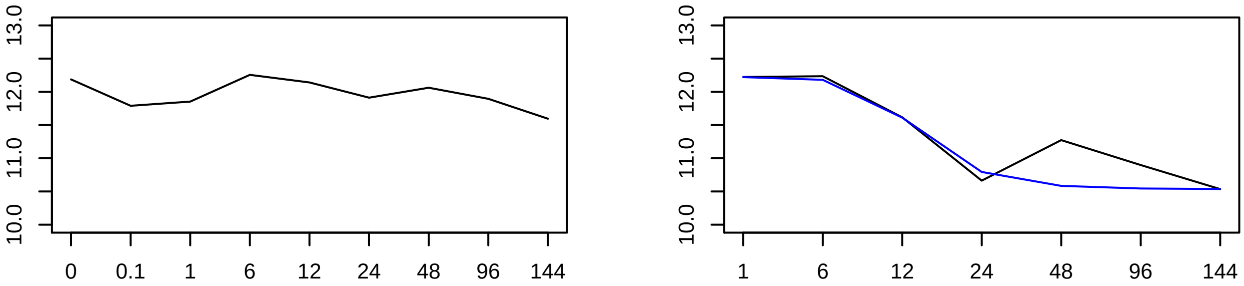


**A_24_P254285 MGME1 20p11.23**

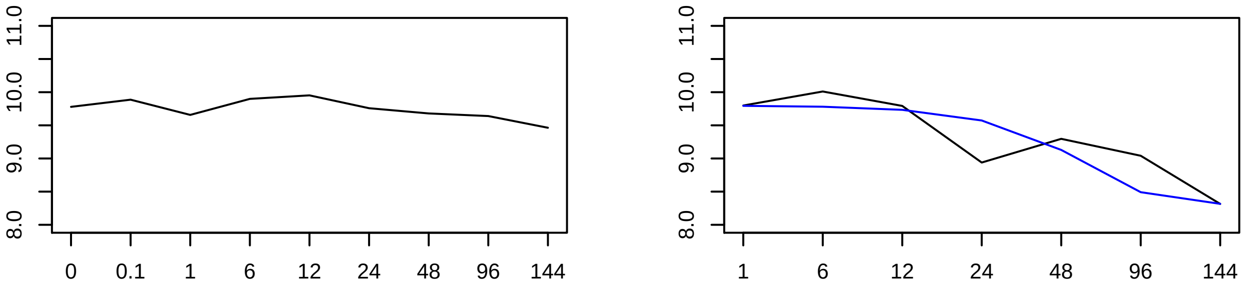


**A_23_P53363 XRCC6BP1 12q14.1**

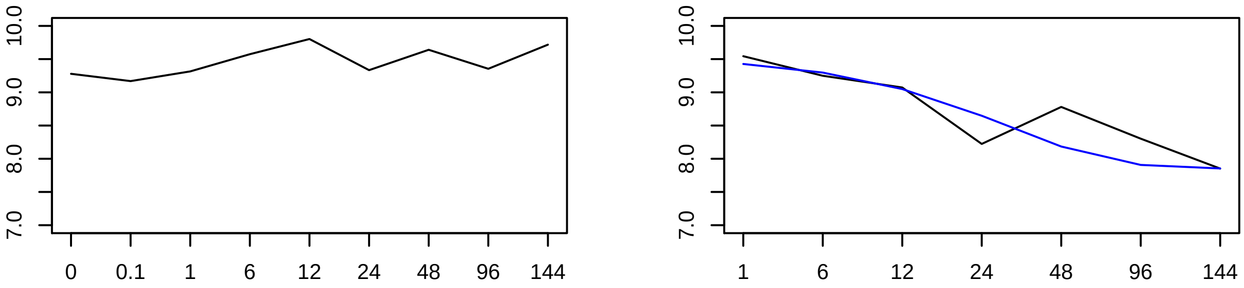

**A_24_P414419 HADH 4q25**

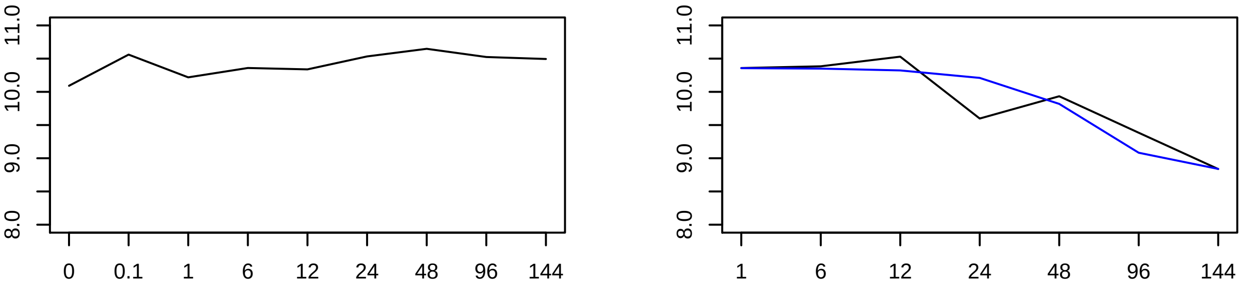


**A_23_P25354 P2RX7 12q24.31**

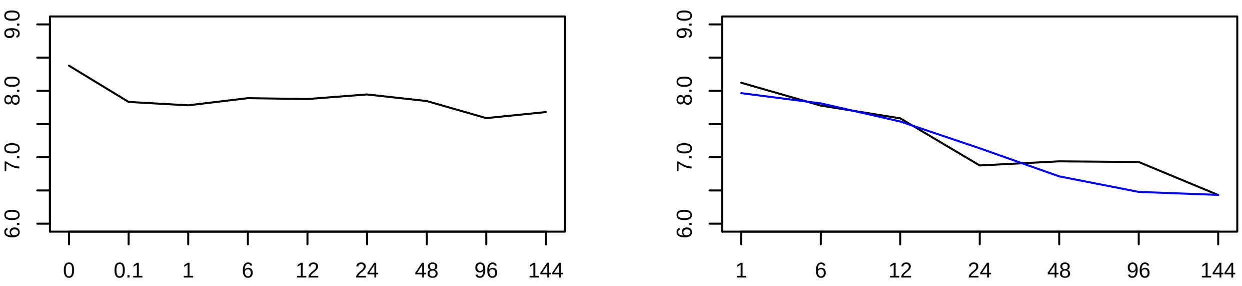


**A_23_P214908 MTHFD1L 6q25.1**

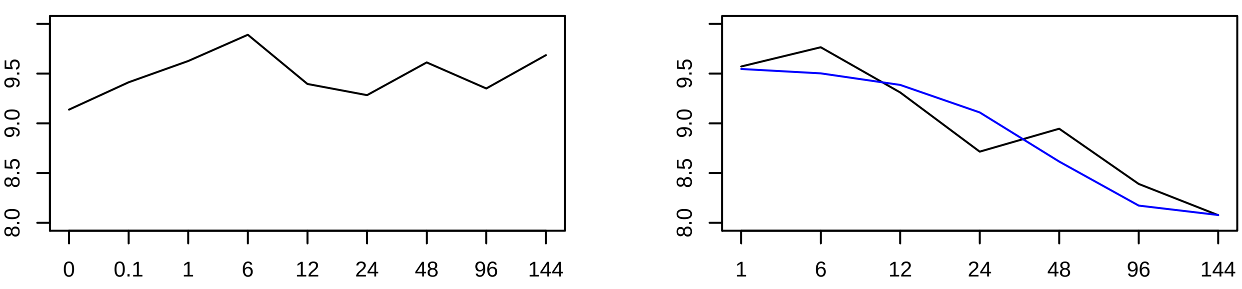

**A_23_P9458 POLR1E 9p13.2**

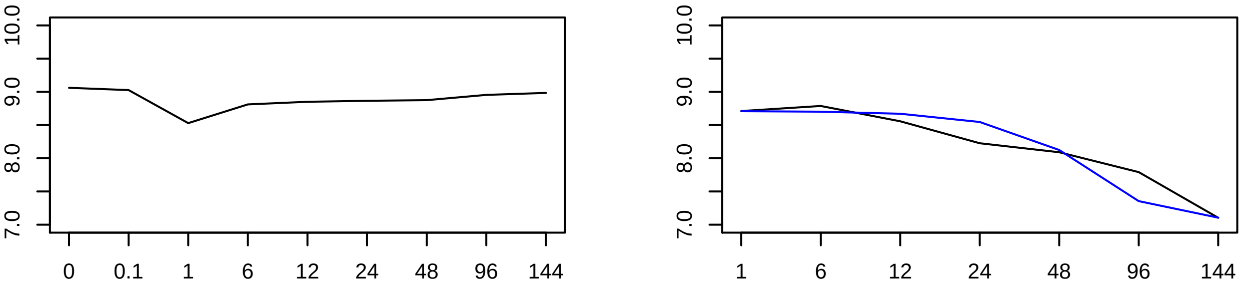


**A_23_P121423 CDC25A 3p21.31**

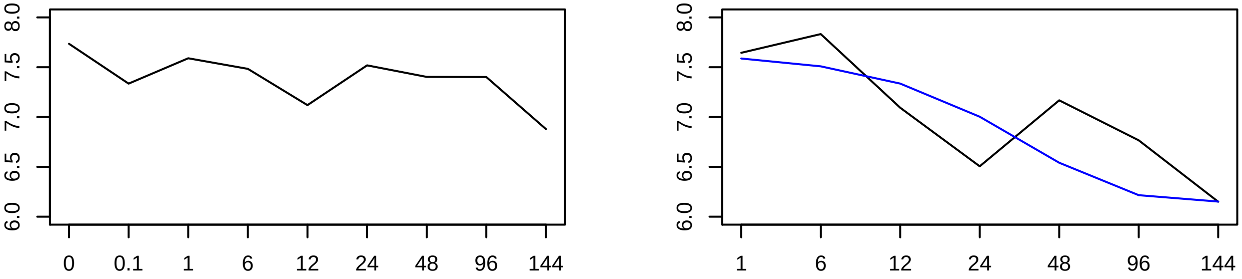


**A_23_P166526 RIBC2 22q13.31**

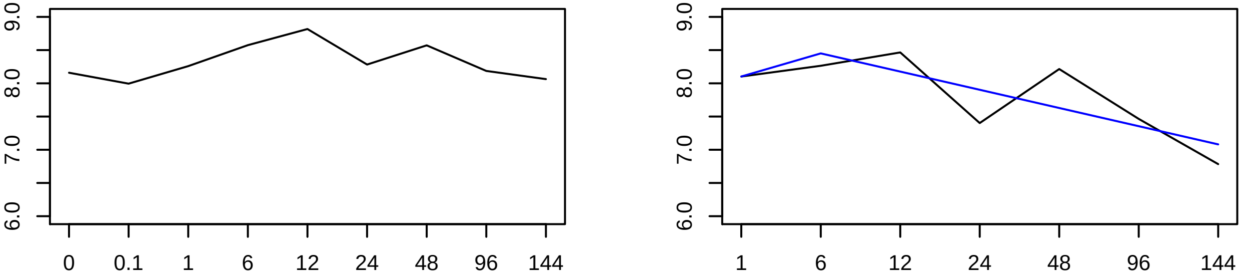

**A_23_P168747 NCAPG2 7q36.3**

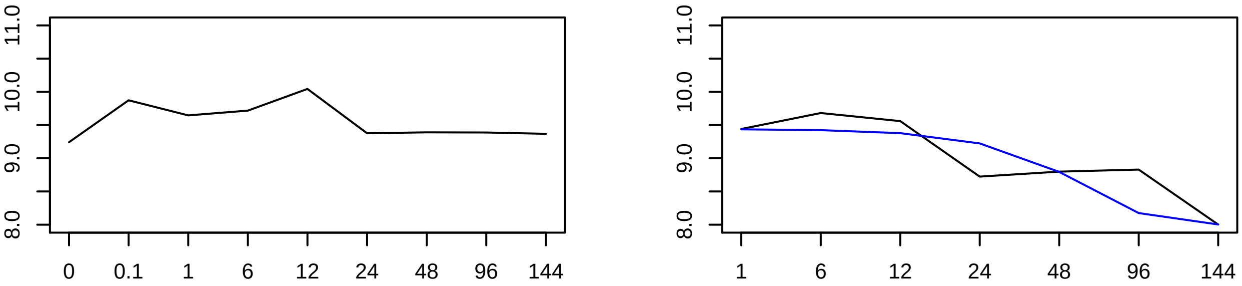


**A_23_P117274 MIPEP 13q12.12**

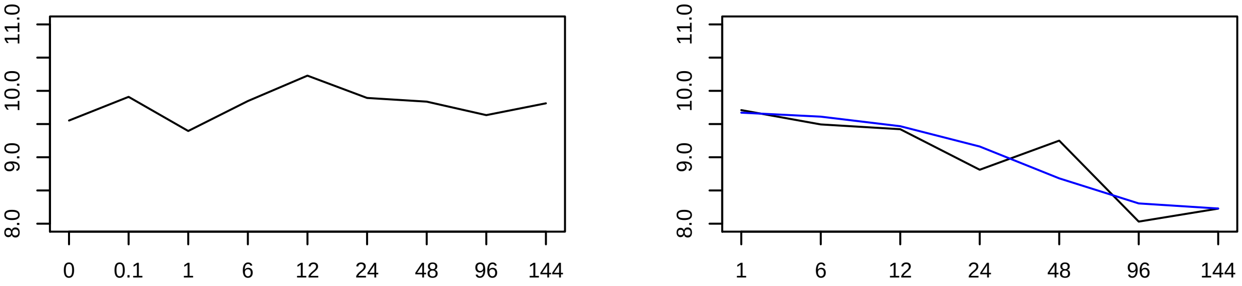


**A_23_P308731 ZNF207 17q11.2**

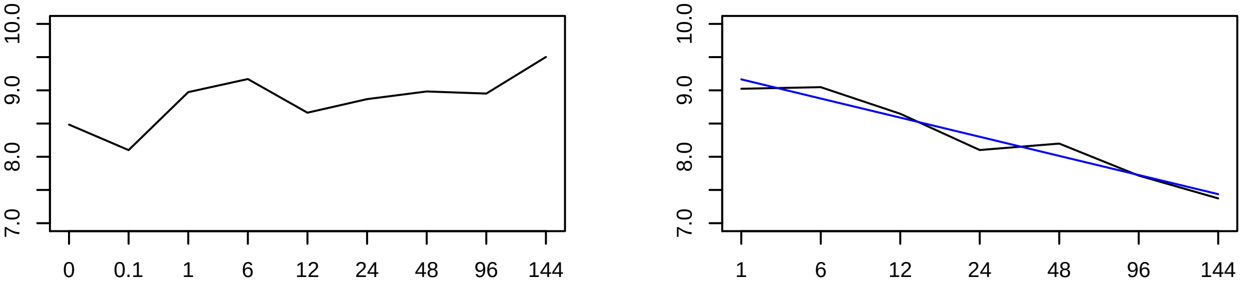

**A_23_P28886 PCNA 20p12.3**

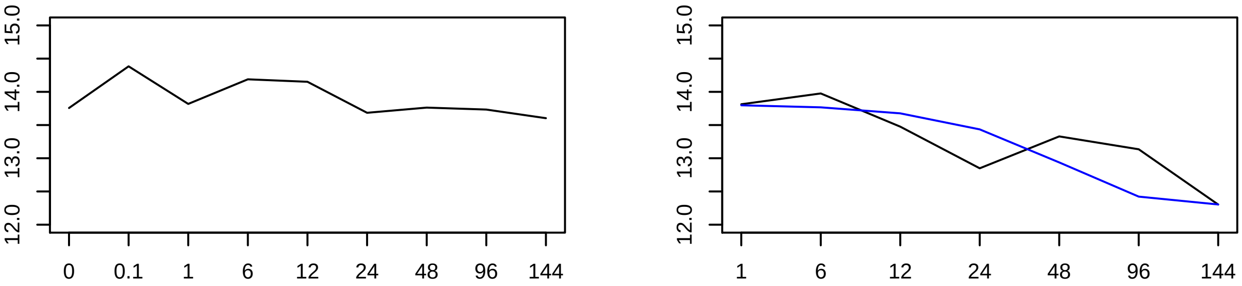


**A_23_P1492 AVPI1 10q24.2**

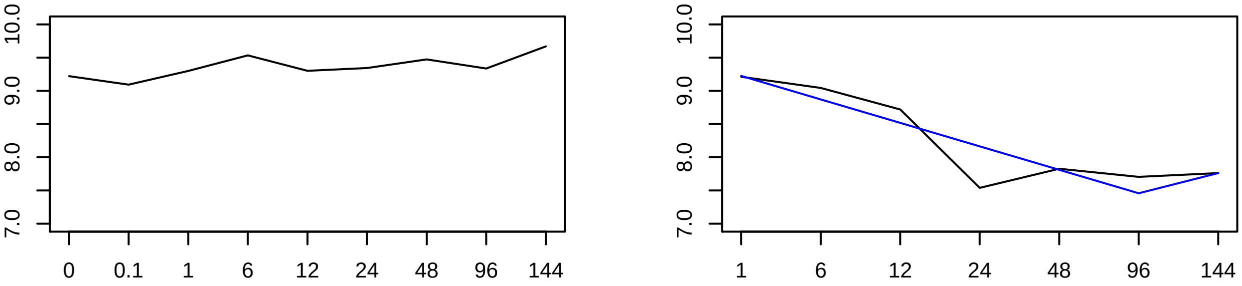


**A_24_P320284 DHFR 5q14.1**

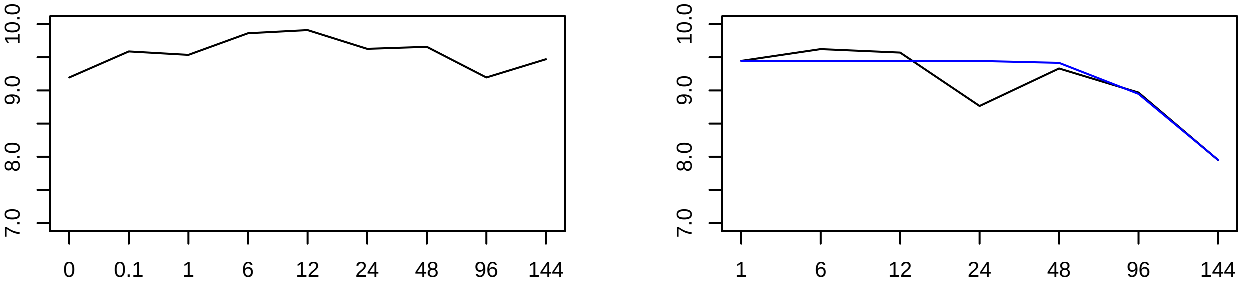

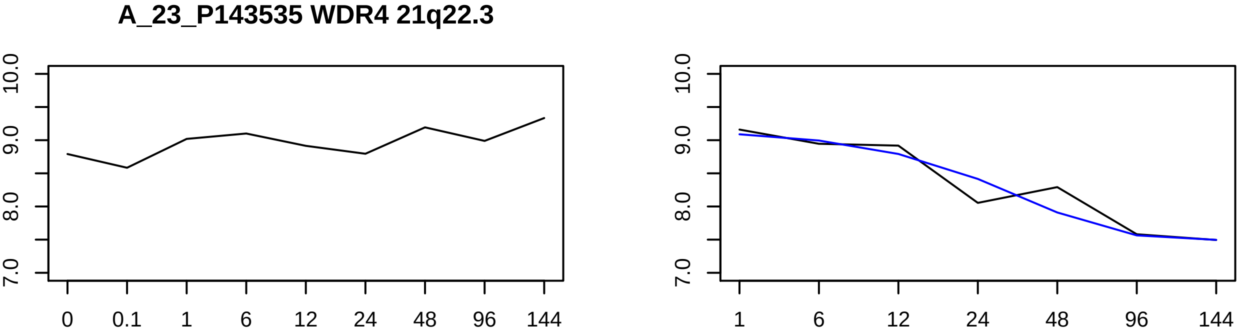
A_23_P143535 WDR4 21q22.3
10.0
9.0
8.0
7.0
0 0.1 1 6 12 24 48 96 144
1 6 12 24 48 96 144

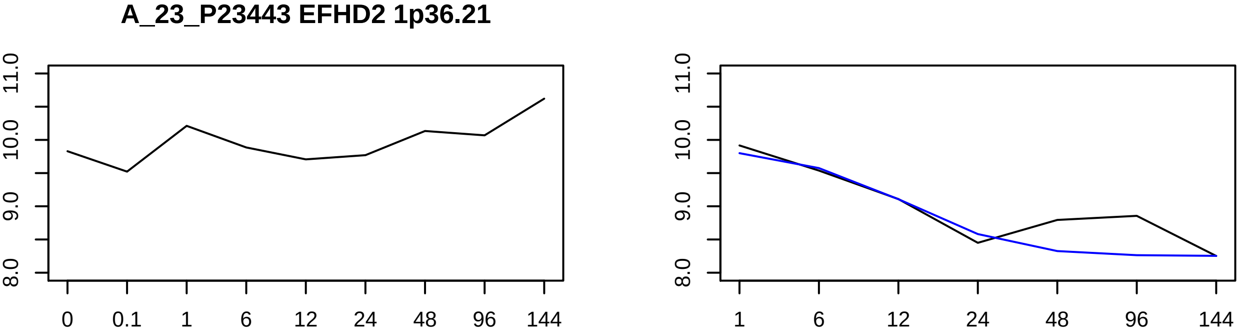
A_23_P23443 EFHD2 1p36.21
11.0
10.0
9.0
8.0
0 0.1 1 6 12 24 48 96 144
1 6 12 24 48 96 144

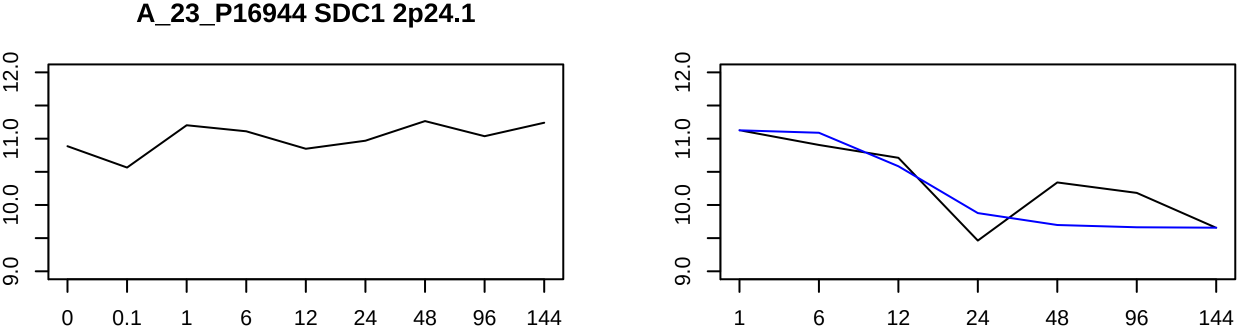
A_23_P16944 SDC1 2p24.1
12.0
11.0
10.0
9.0
0 0.1 1 6 12 24 48 96 144
1 6 12 24 48 96 144

**A_23_P349398 ZAR1 4p12**

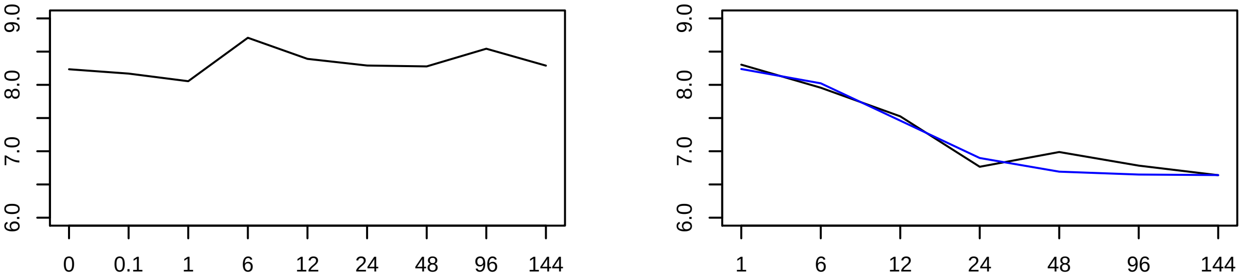


**A_23_P161152 PDSS1 10p12.1**

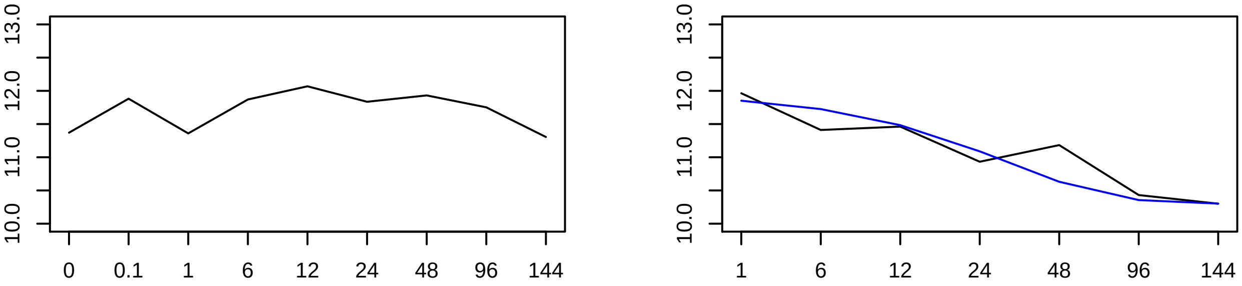


**A_24_P126628 KIAA0286 12q13.3**

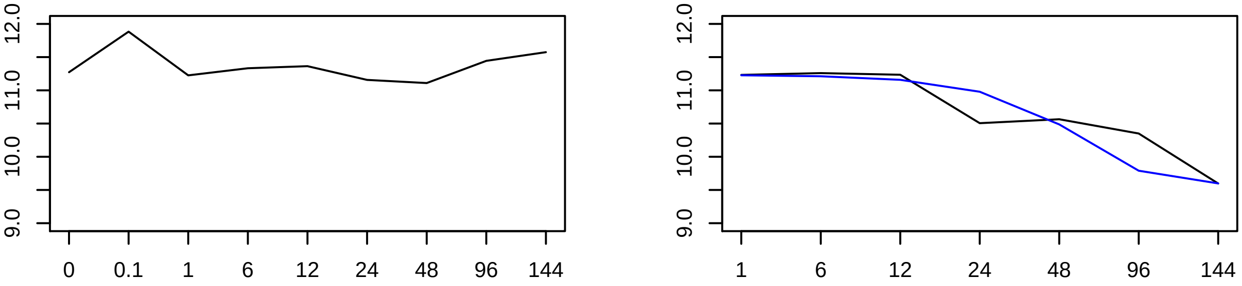

## A_23_P130182 AURKB 17p13.1

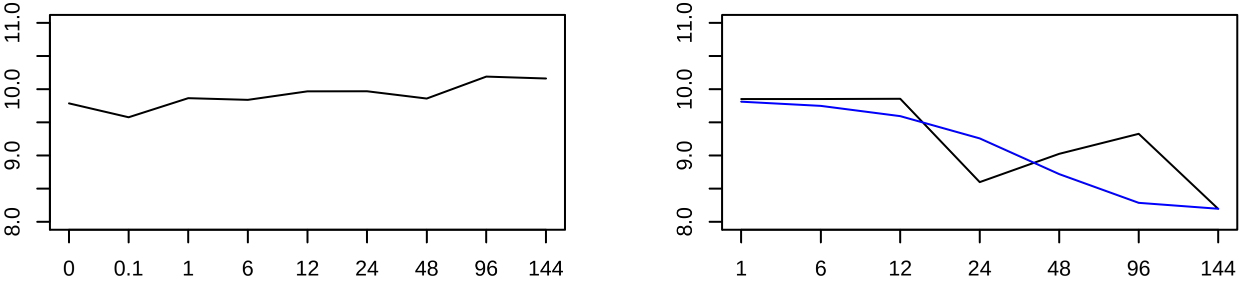


## A_24_P76521 GSG2 17p13.3

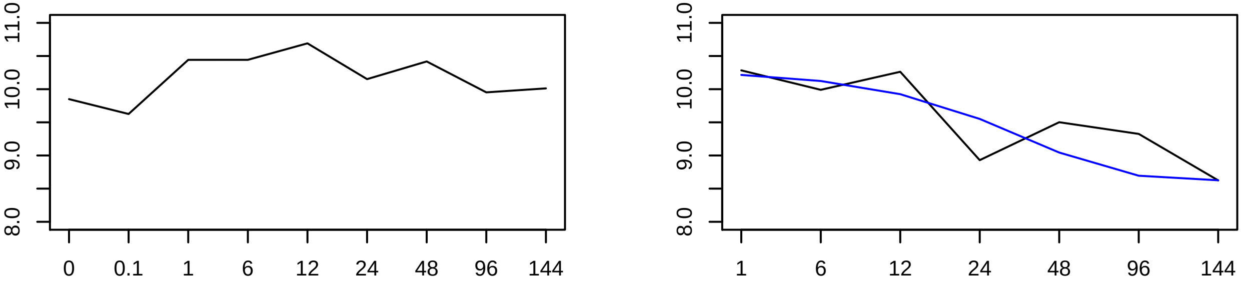


## A_23_P157795 CTNNAL1 9q31.3

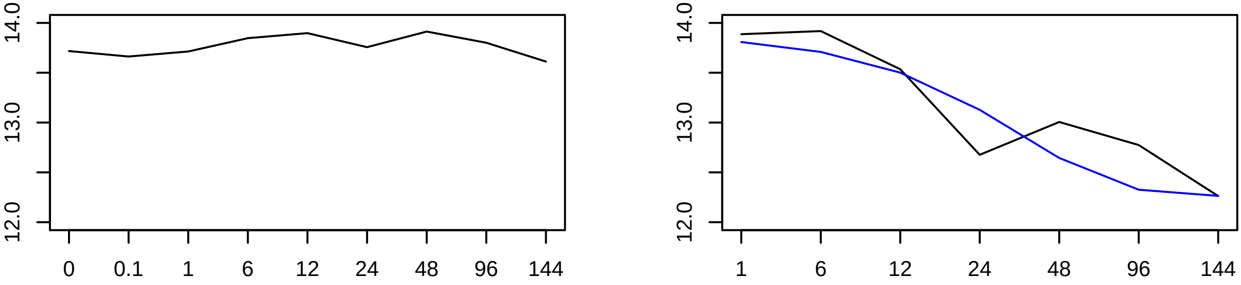

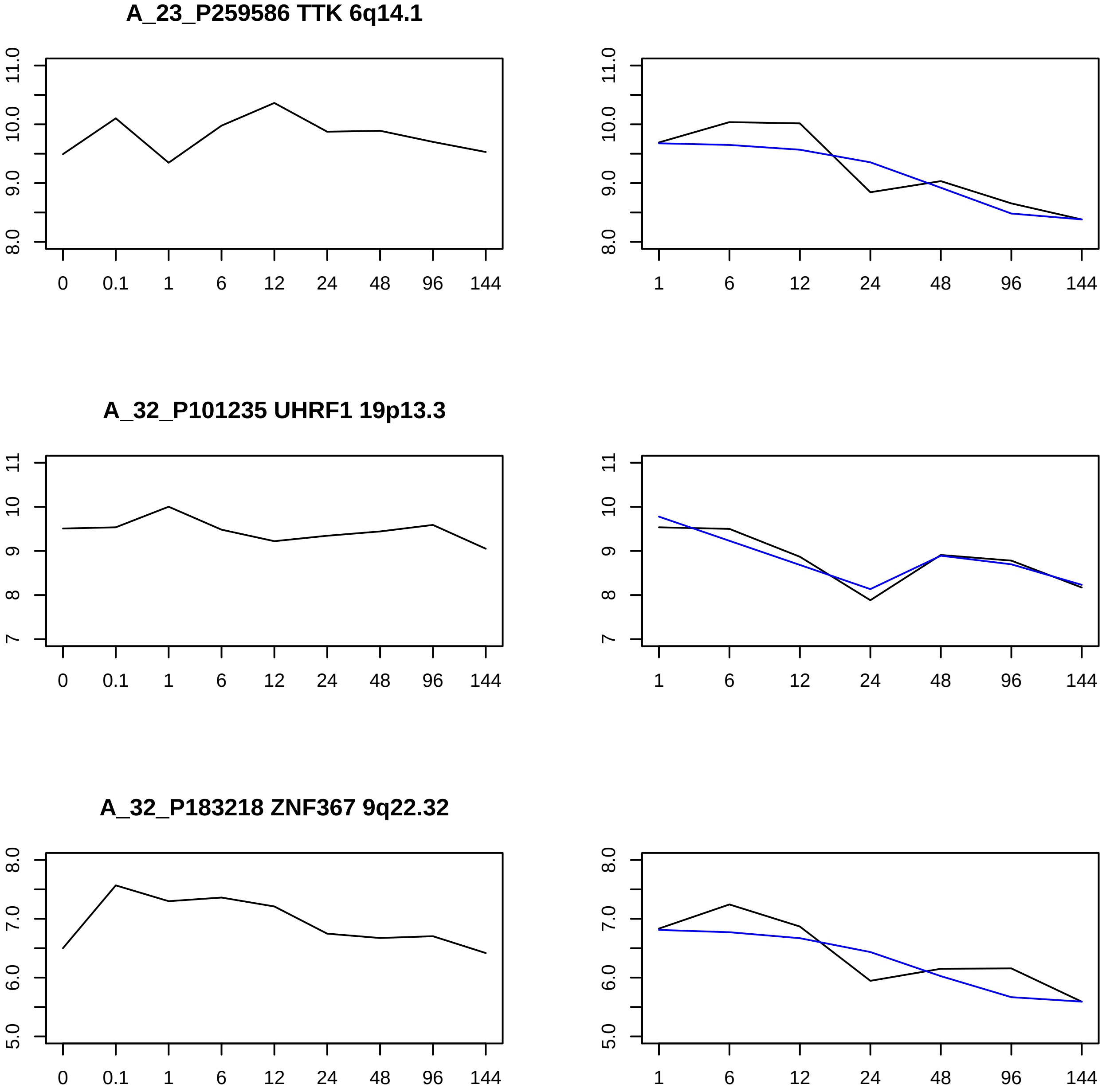
A_23_P259586 TTK 6q14.1
A_32_P101235 UHRF1 19p13.3
A_32_P183218 ZNF367 9q22.32
0
0.1
1
6
12
24
48
96
144
8.0
9.0
10.0
11.0
7
8
9
10
11
5.0
6.0
7.0

**A_23_P26557 C16orf59 16p13.3**

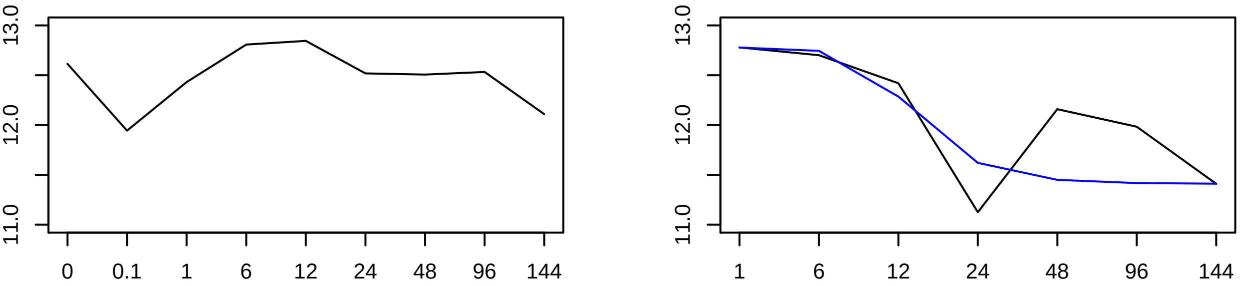


**A_32_P157304 POLD3 11q13.4**

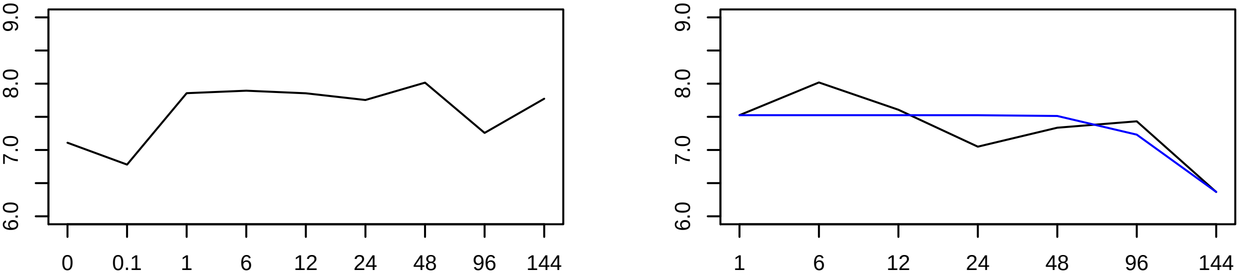


**A_23_P100344 ORC6L 16q11.2**

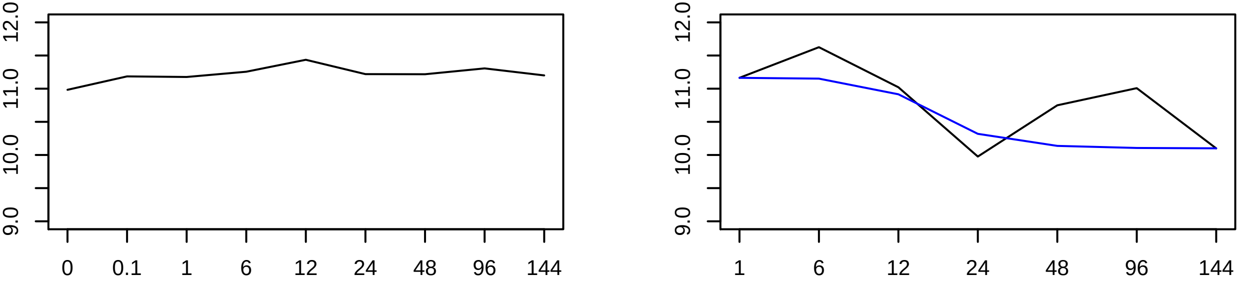

**A_23_P431381 C14orf80 14q32.33**

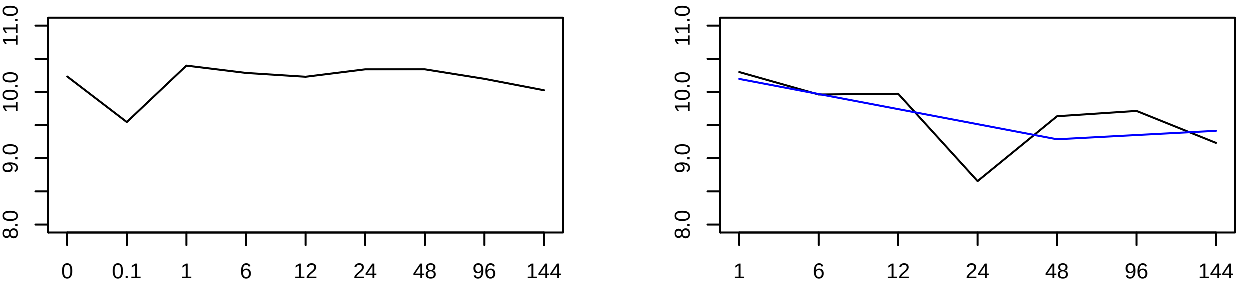


**A_23_P71558 RECQL4 8q24.3**

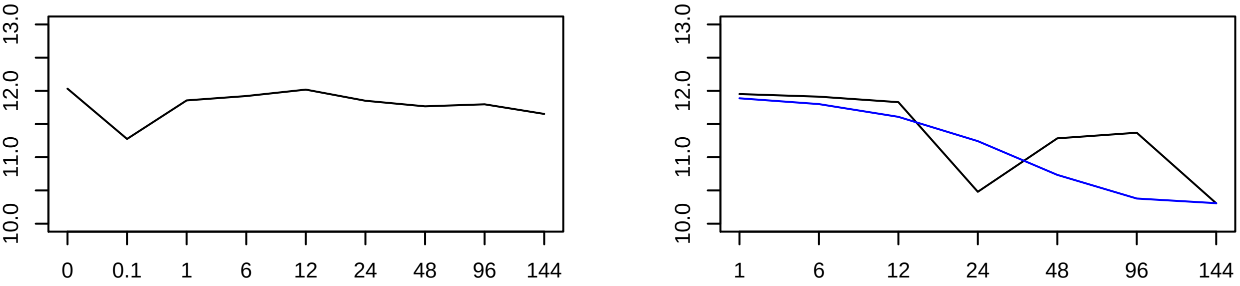


**A_24_P413884 CENPA 2p23.3**

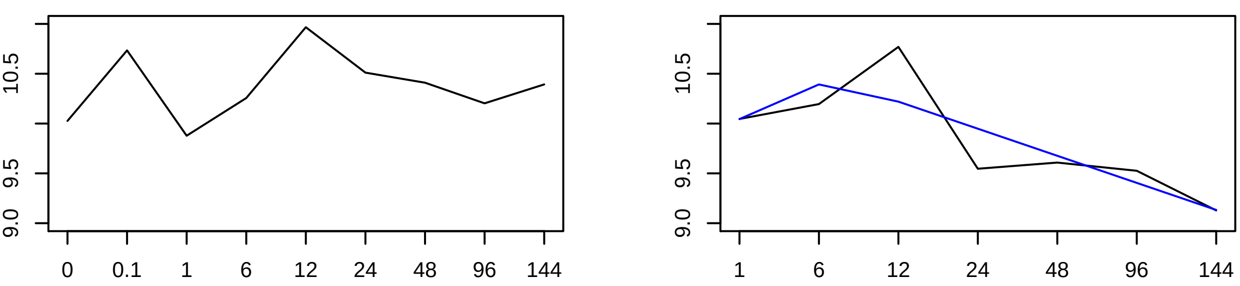

A_23_P252740 DSCC1 8q24.12

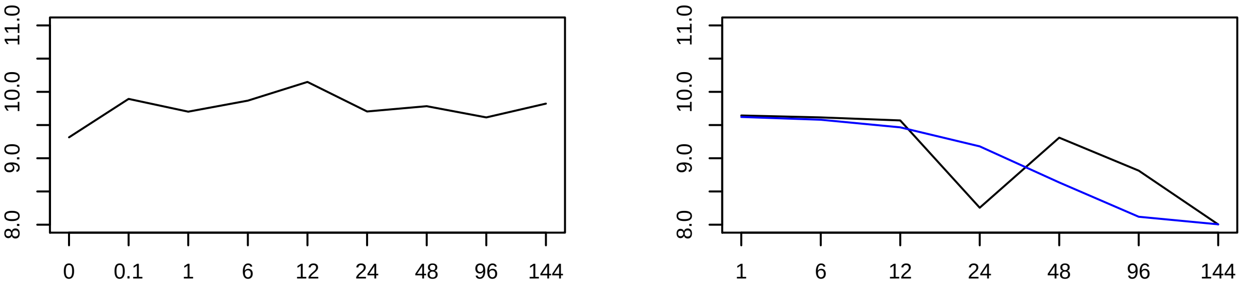


A_23_P134147 ZBTB24 6q21

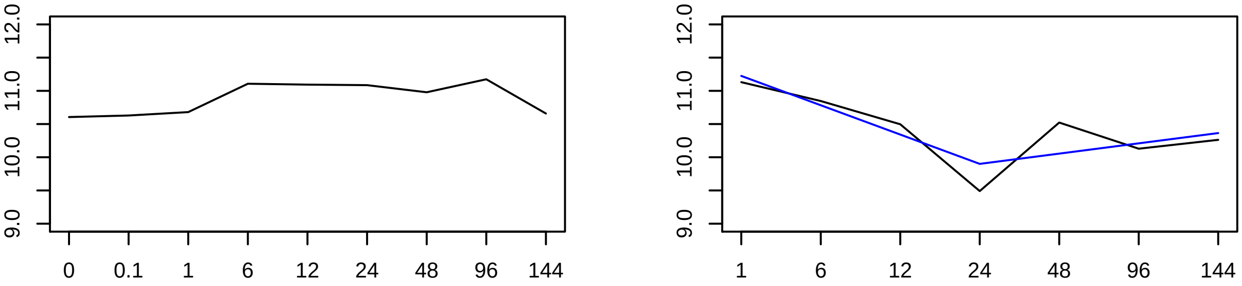


A_23_P213166 C4orf21 4q25

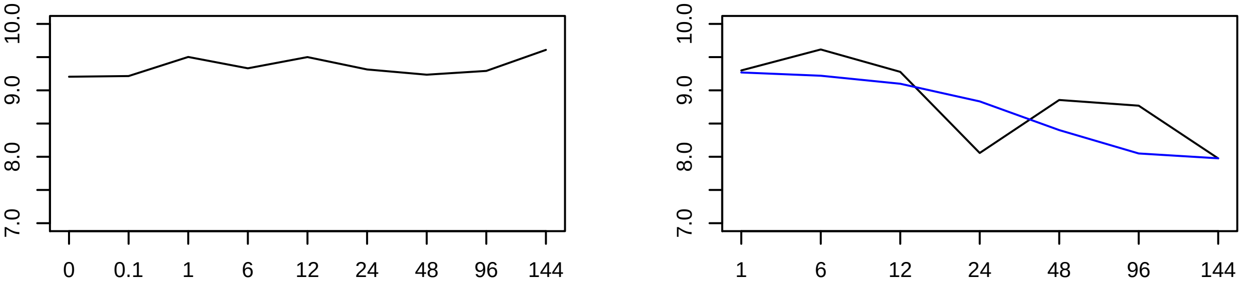

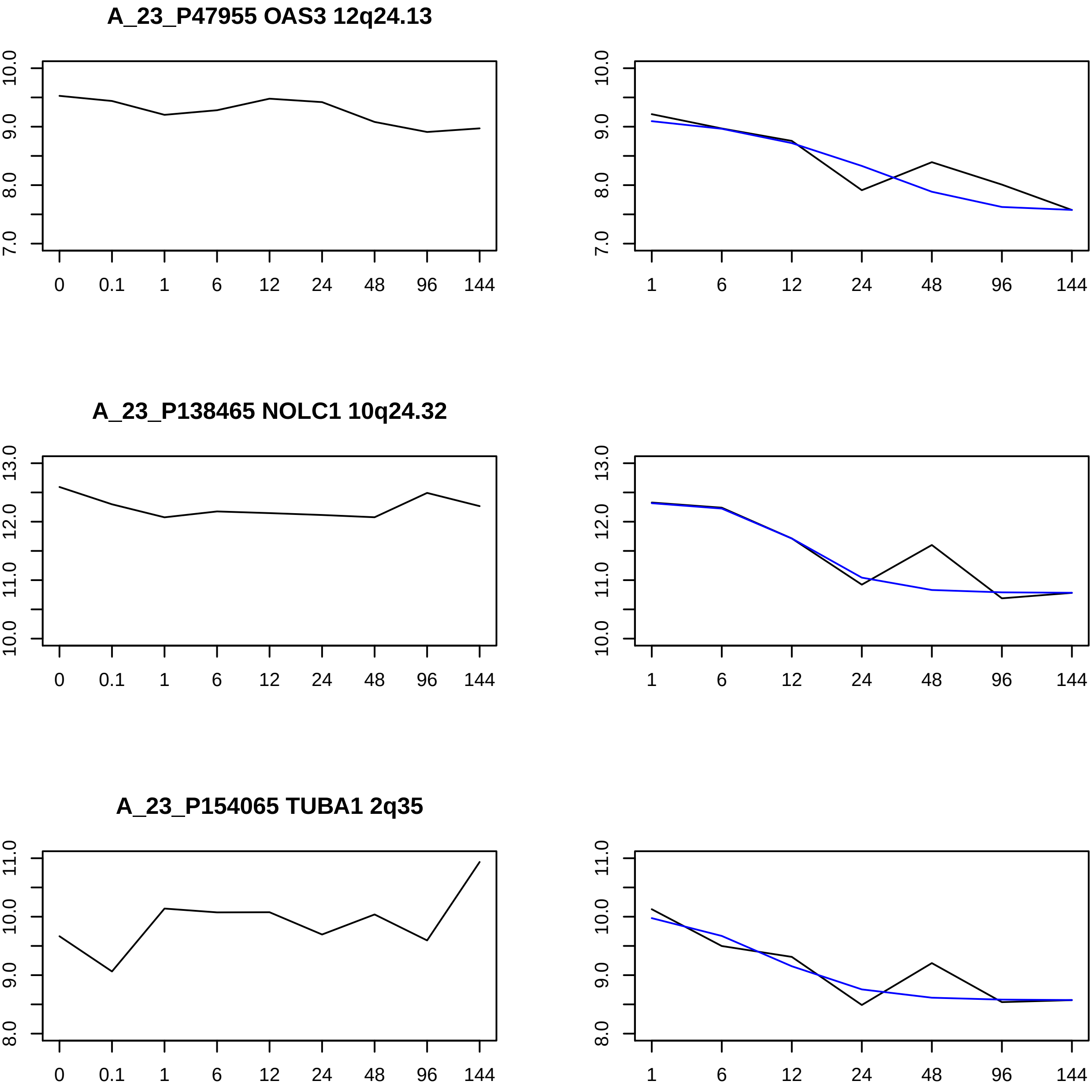
A_23_P47955 OAS3 12q24.13
7.0
8.0
9.0
10.0
0
0.1
1
6
12
24
48
96
144
A_23_P138465 NOLC1 10q24.32
10.0
11.0
12.0
13.0
A_23_P154065 TUBA1 2q35
8.0
9.0
10.0
11.0

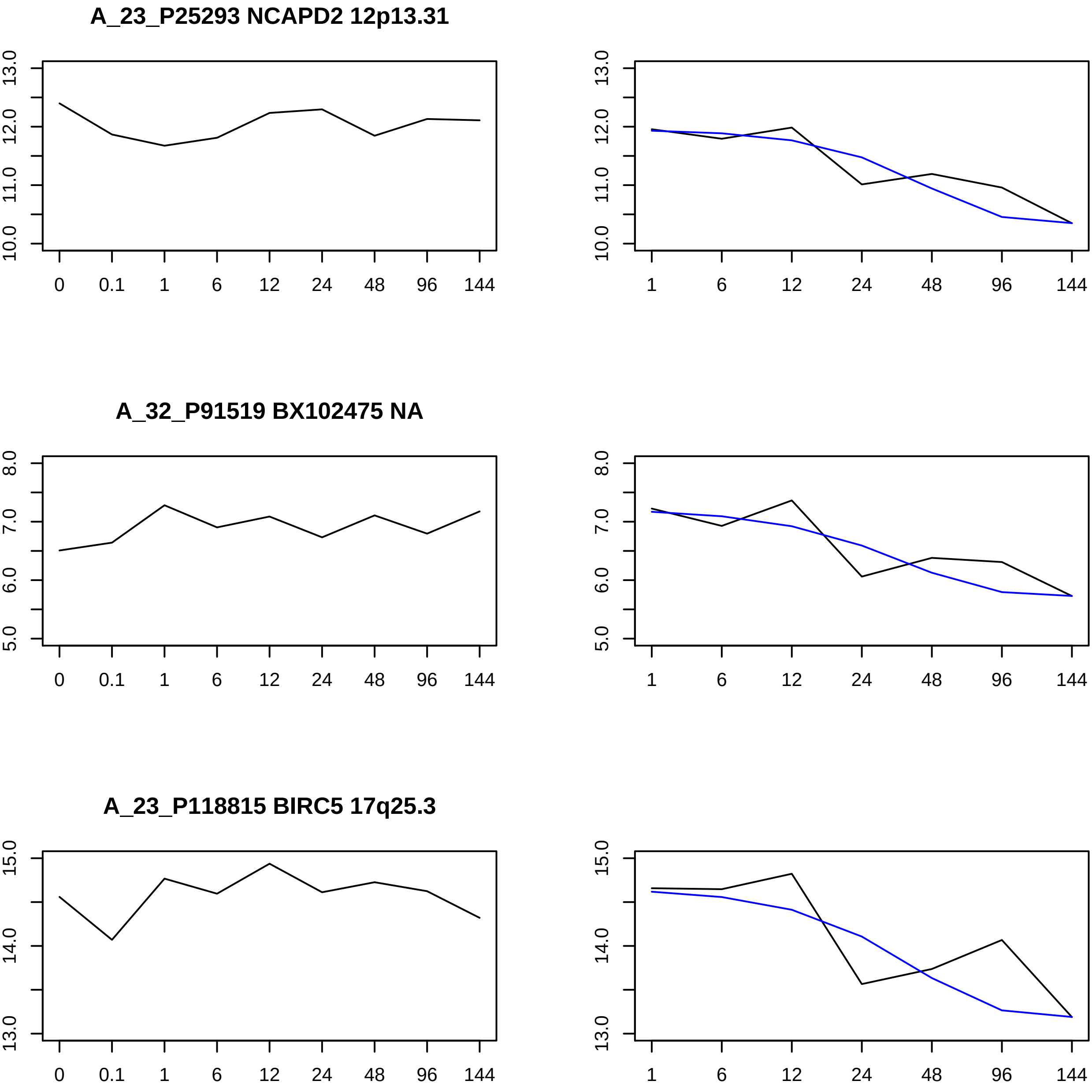

A_23_P25293 NCAPD2 12p13.31
10.0
11.0
12.0
13.0
0
0.1
1
6
12
24
48
96
144
A_32_P91519 BX102475 NA
5.0
6.0
7.0
8.0
A_23_P118815 BIRC5 17q25.3
13.0
14.0
15.0

## A_32_P71447 NCAPD3 11q25

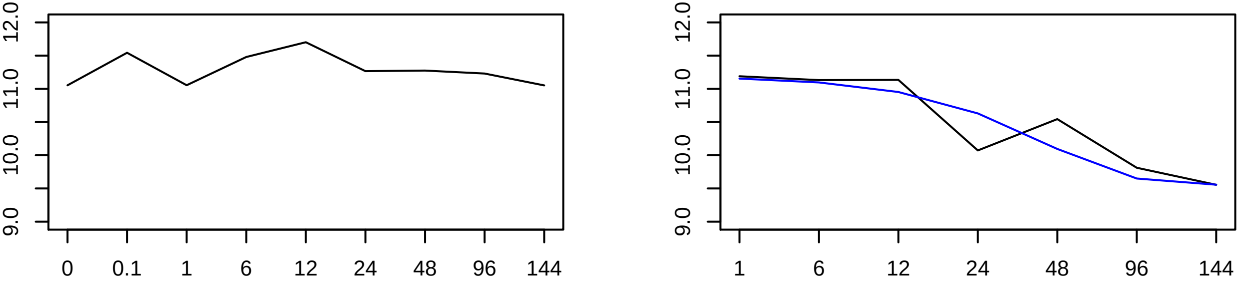


## A_23_P42335 FANCE 6p21.31

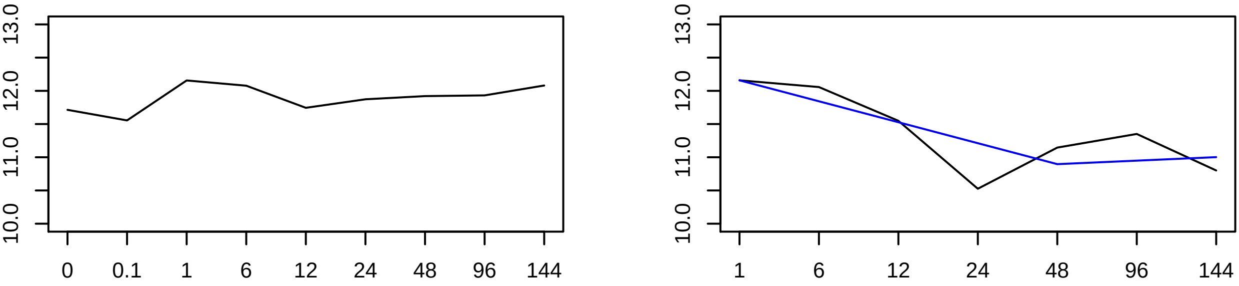


## A_23_P51085 SPBC25 2q24.3

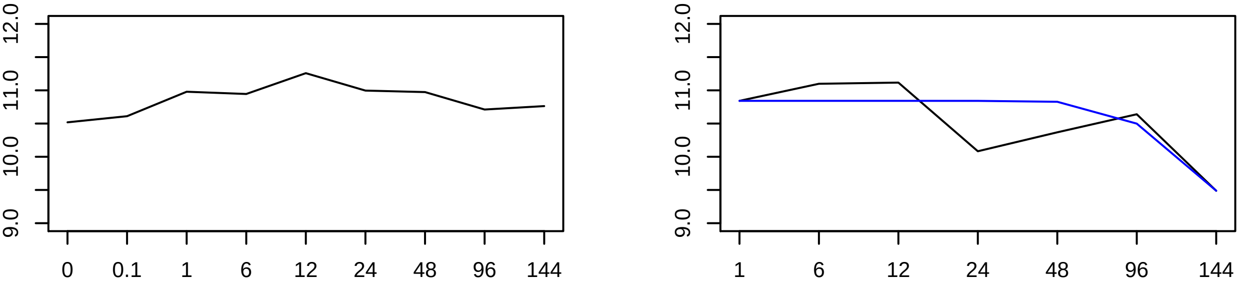

**A_32_P182135 A_32_P182135 NA**

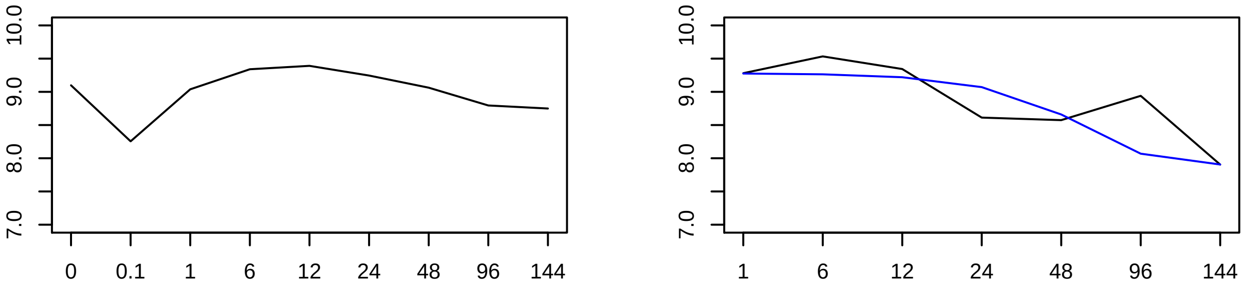


**A_23_P312300 SCGB2A1 11q12.3**

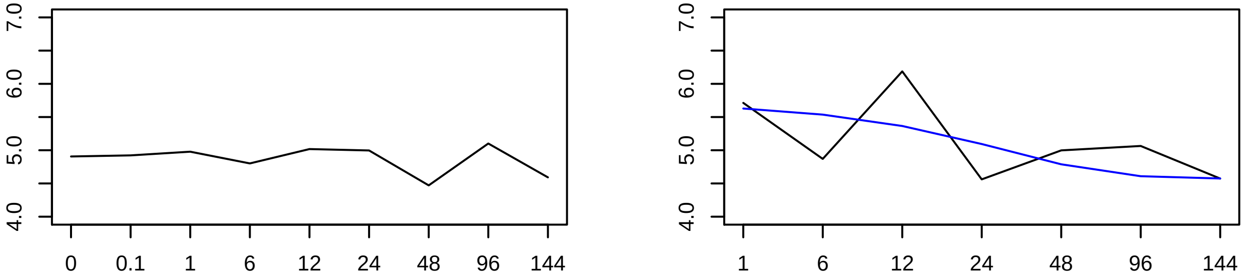


**A_23_P67771 BARD1 2q35**

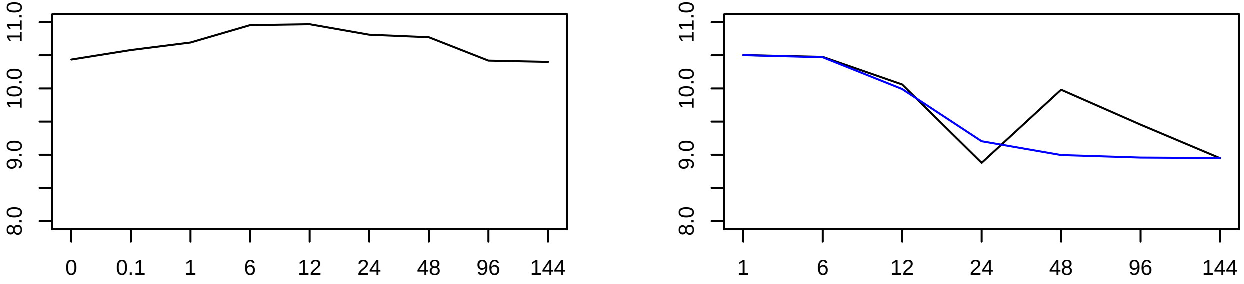

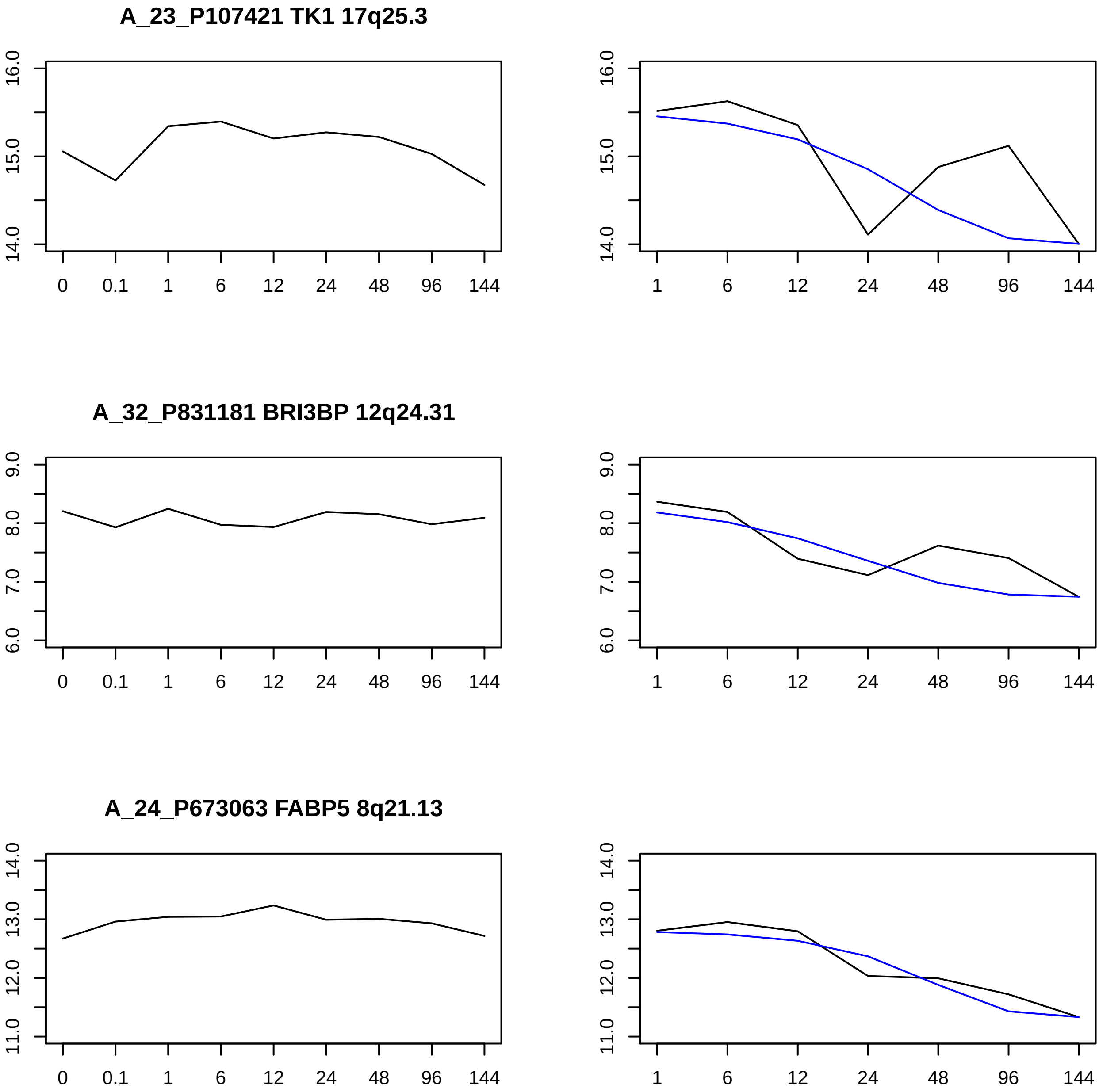
A_23_P107421 TK1 17q25.3
A_32_P831181 BRI3BP 12q24.31
A_24_P673063 FABP5 8q21.13

**A_23_P210853 GINS1 20p11.21**

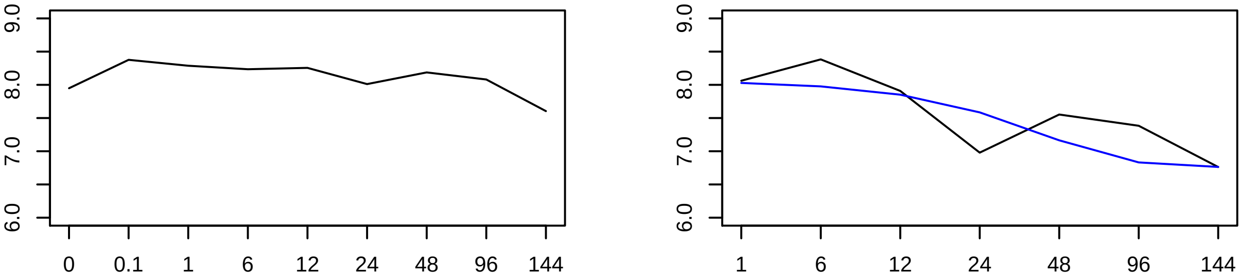


**A_24_P234196 RRM2 2p25.1**

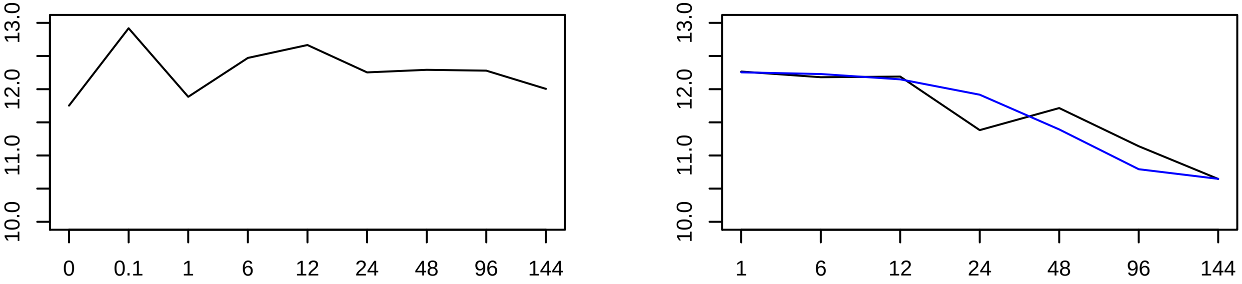


**A_23_P206441 FANCA 16q24.3**

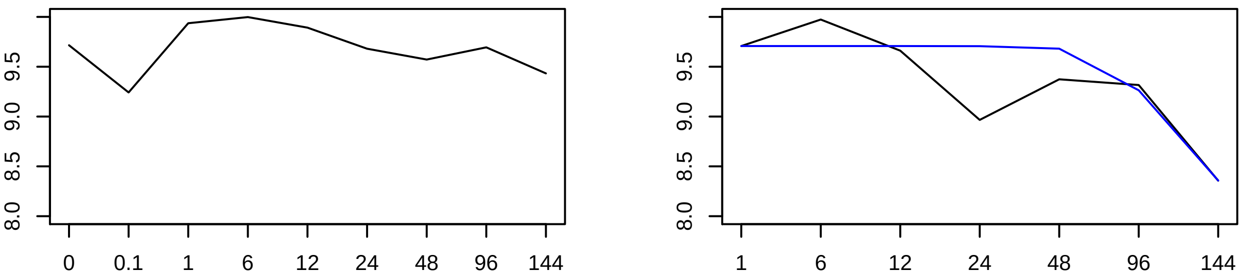

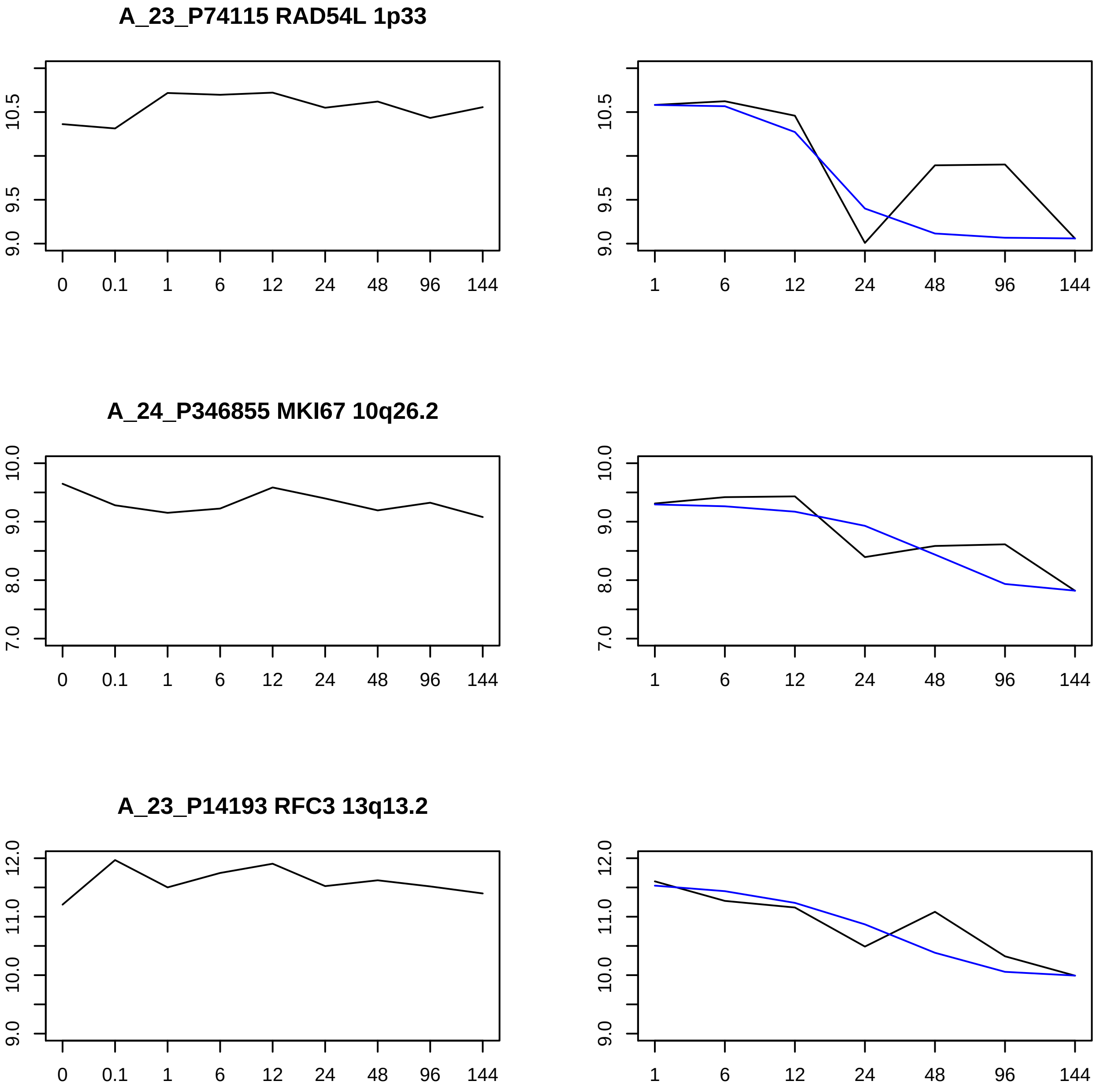

A_23_P74115 RAD54L 1p33
9.0
9.5
10.5
0
0.1
1
6
12
24
48
96
144
1
6
12
24
48
96
144
A_24_P346855 MKI67 10q26.2
7.0
8.0
9.0
10.0
A_23_P14193 RFC3 13q13.2
9.0
10.0
11.0
12.0

**A_23_P34800 NASP 1p34.1**

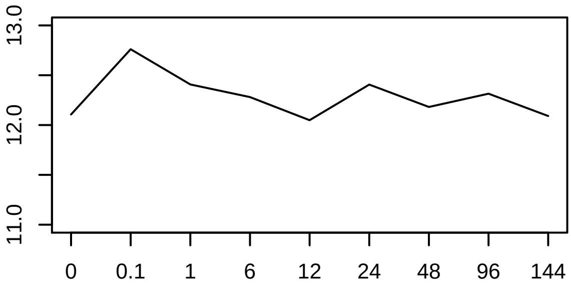


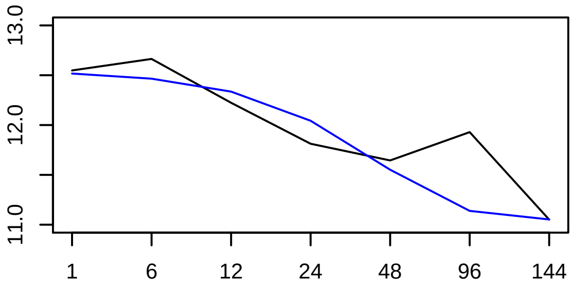


**A_23_P156310 SKP2 5p13.2**

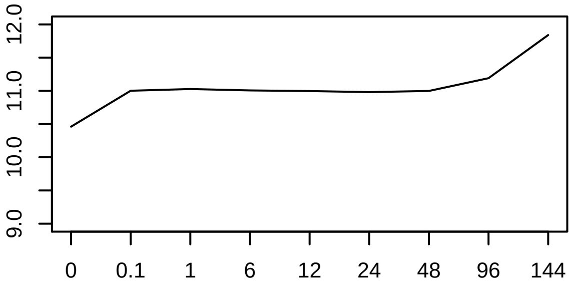


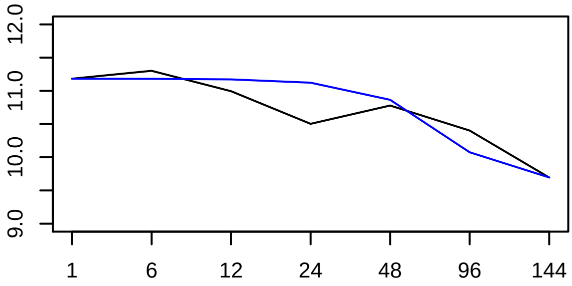


**A_24_P312671 MRE11A 11q21**

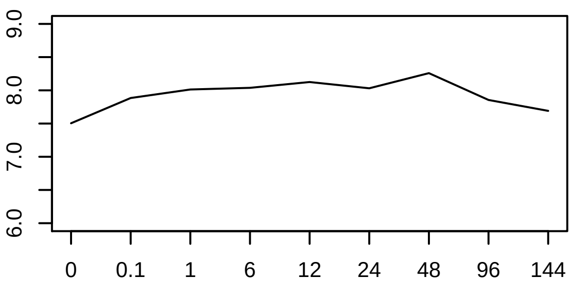


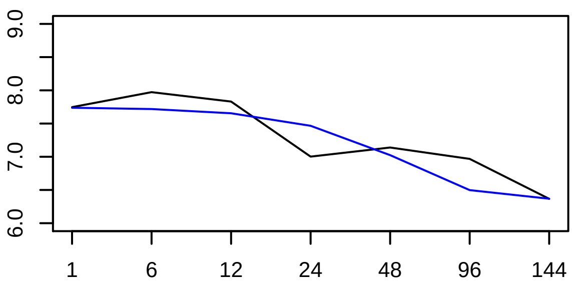

**A_23_P102071 AK027315 NA**

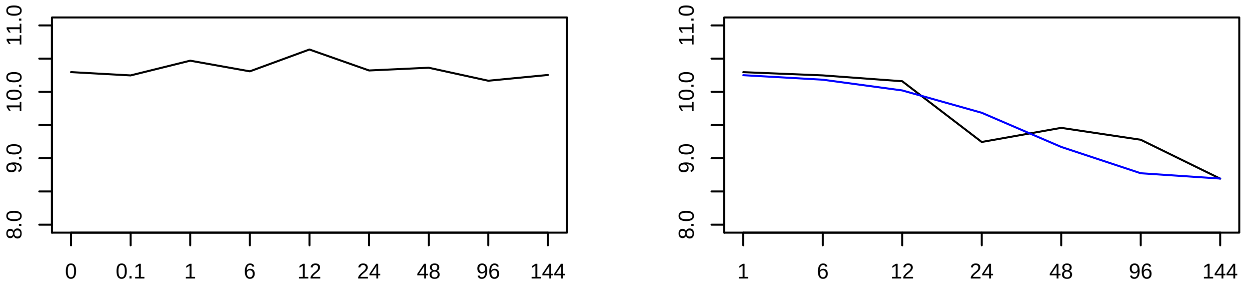


**A_23_P343927 HIST2H2AB 1q21.2**

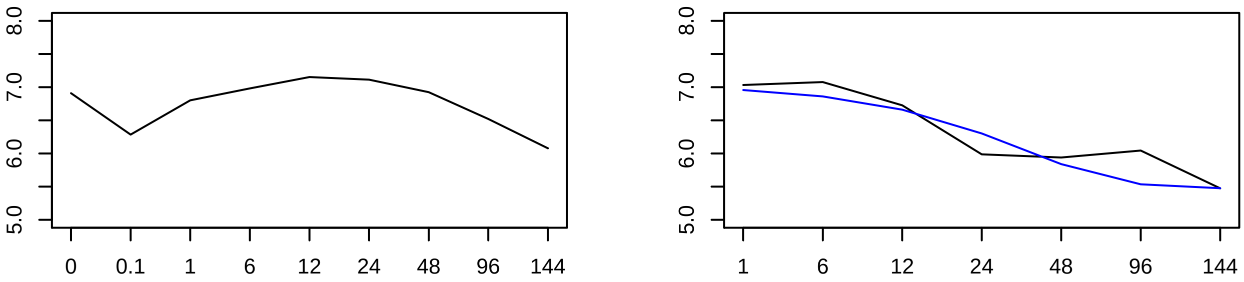


**A_23_P167263 PHF17 4q28.2**

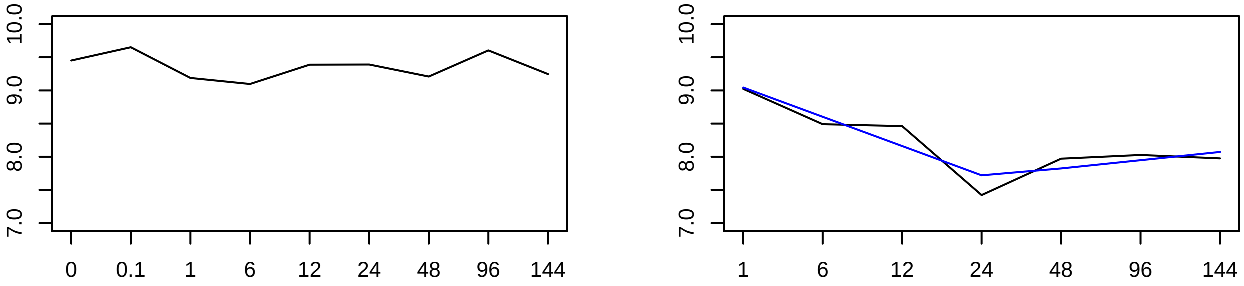

A_23_P162171 MCAM 11q23.3

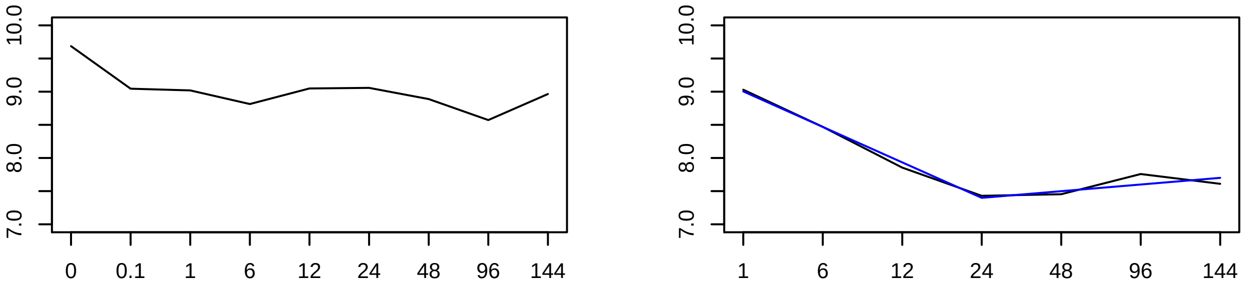


A_24_P274073 ABCB10 1q42.13

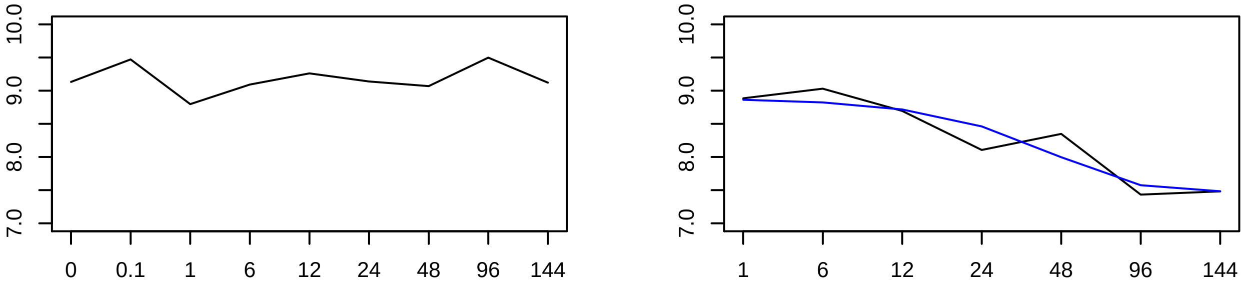


A_23_P384698 LOC442075 3p25.3

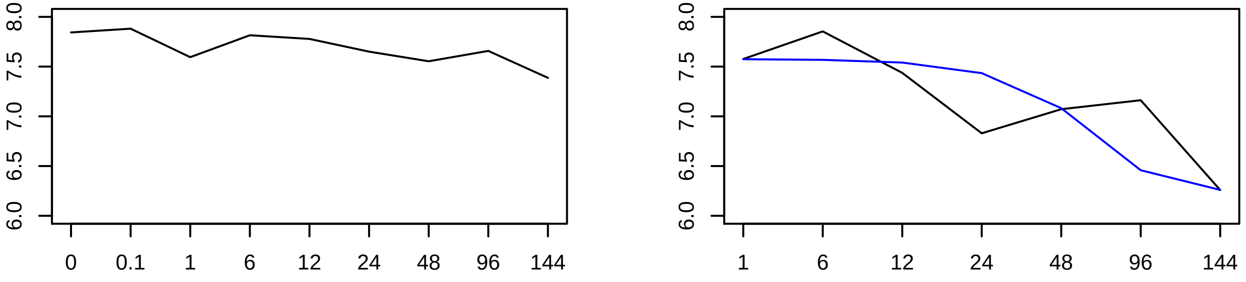

**A_23_P160537 AUNIP 1p36.11**

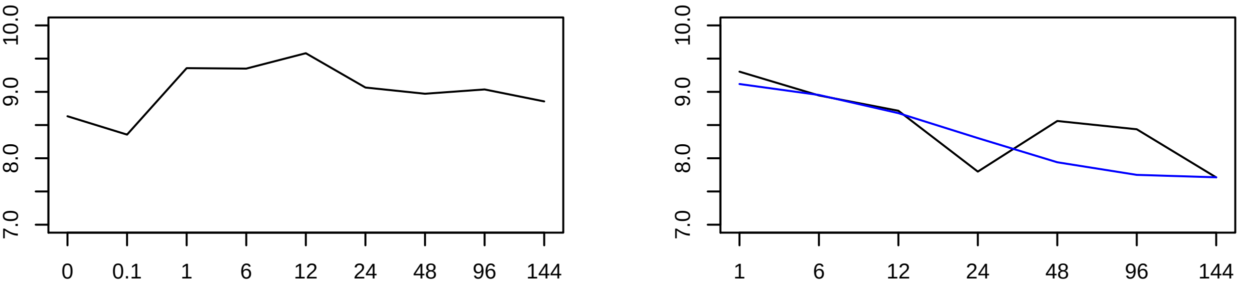


**A_24_P38895 H2AFX 11q23.3**

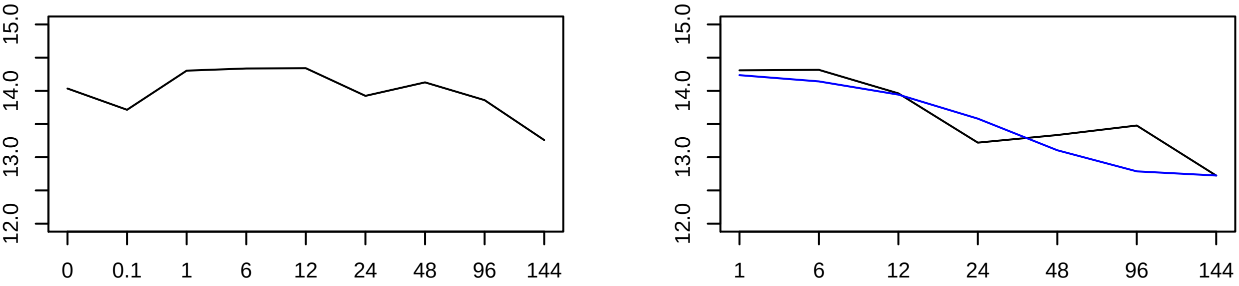


**A_24_P277576 TRIP13 5p15.33**

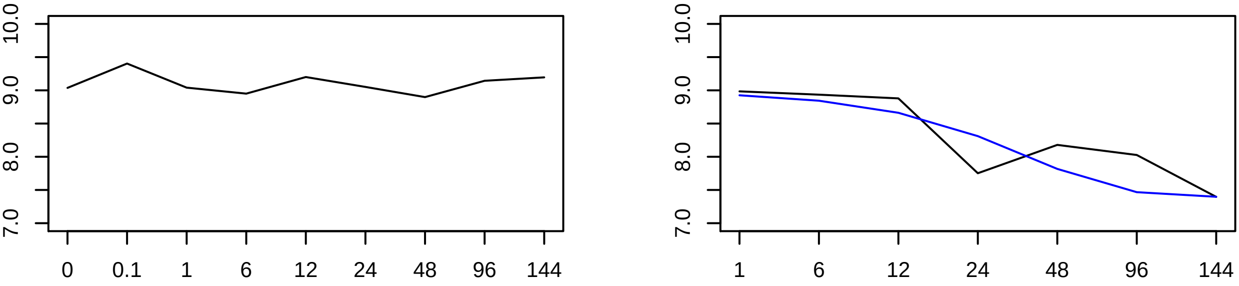

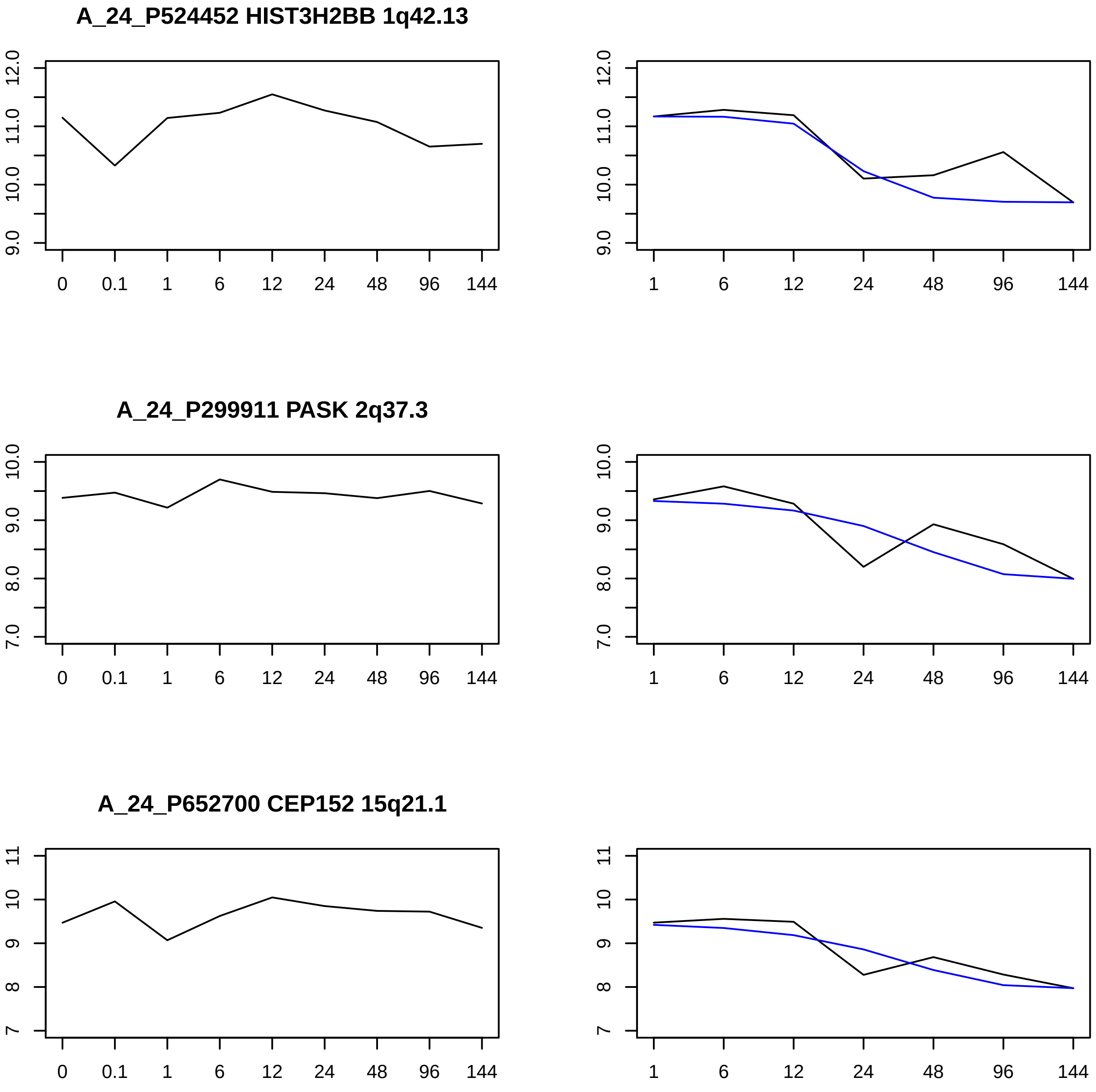
A_24_P524452 HIST3H2BB 1q42.13
9.0
10.0
11.0
12.0
0
0.1
1
6
12
24
48
96
144
1
6
12
24
48
96
144
A_24_P299911 PASK 2q37.3
7.0
8.0
9.0
10.0
A_24_P652700 CEP152 15q21.1
7
8
9
10
11

**A_24_P225616 RRM2 2p25.1**

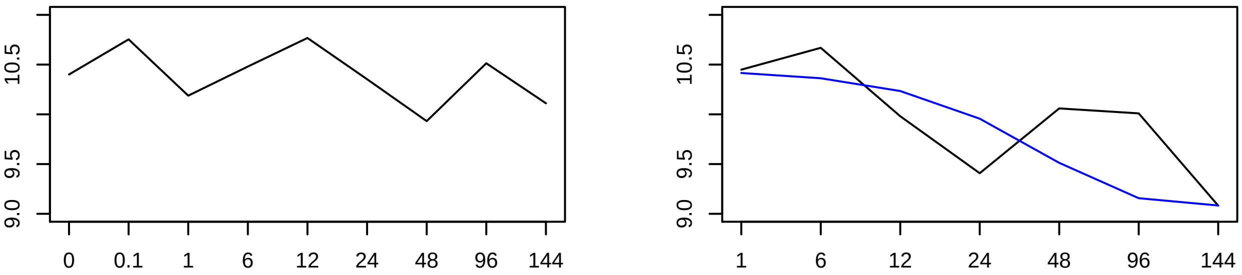


**A_32_P28365 NASP 1p34.1**

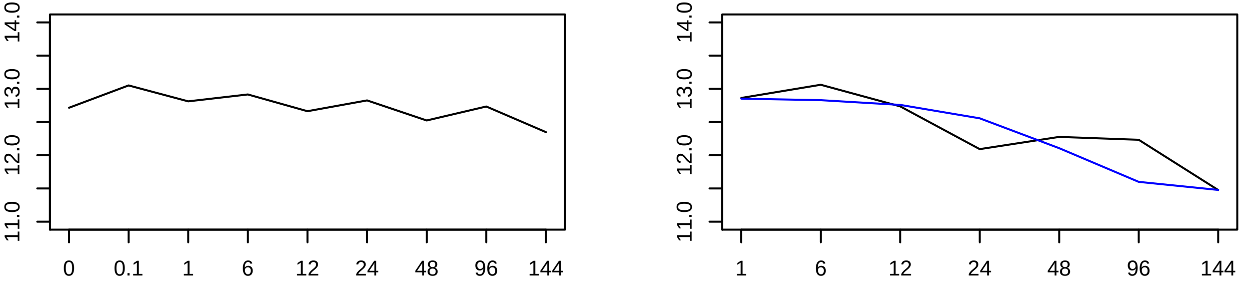


**A_23_P83266 ENDOG 9q34.11**

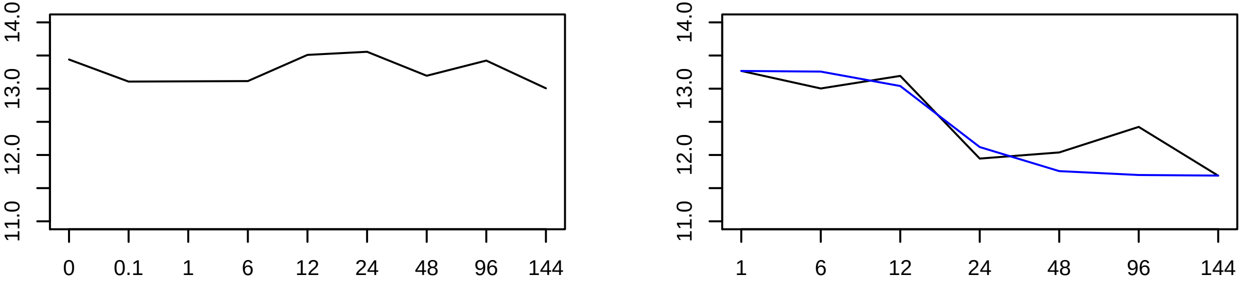

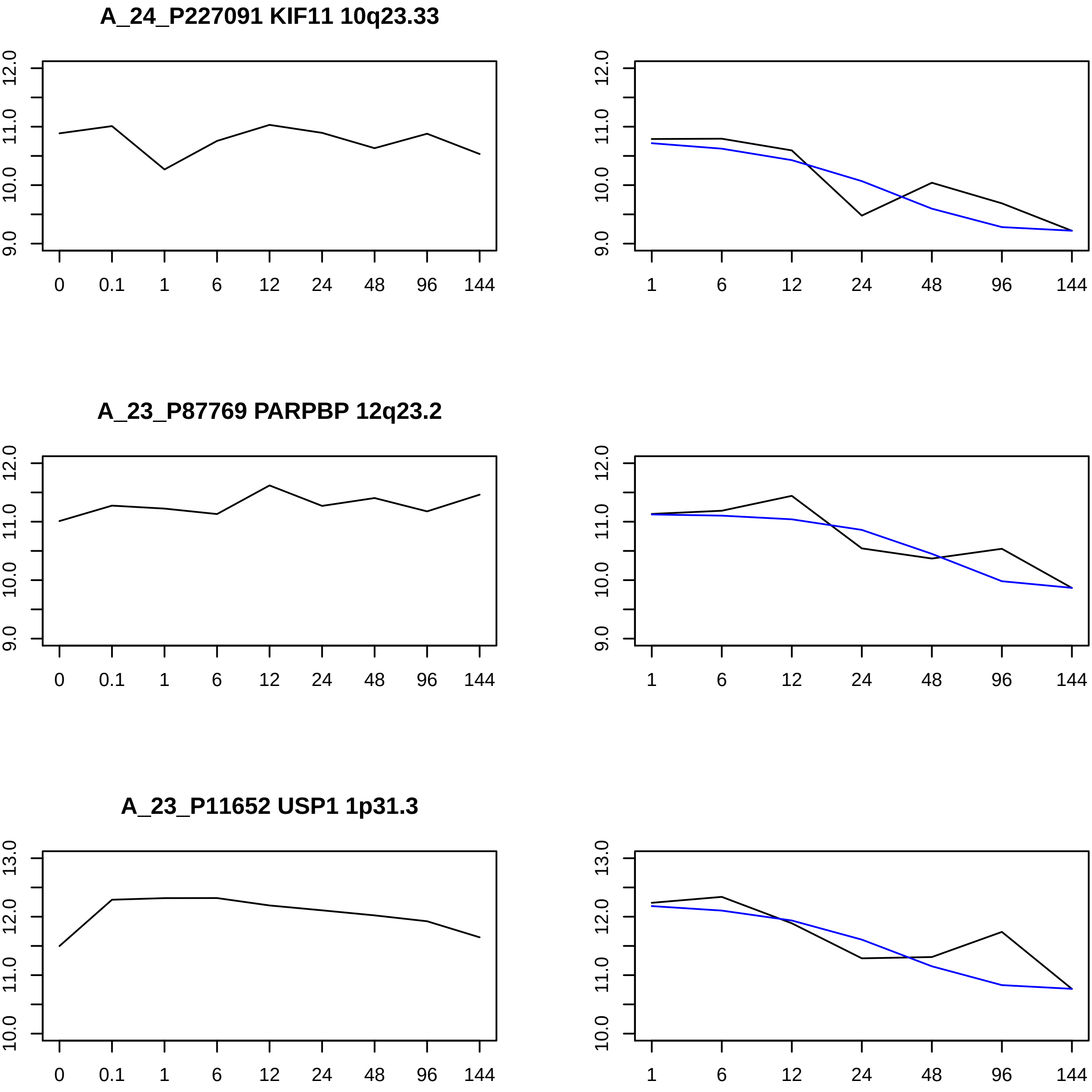
A_24_P227091 KIF11 10q23.33
A_23_P87769 PARPBP 12q23.2
A_23_P11652 USP1 1p31.3
9.0
10.0
11.0
12.0
13.0
0
0.1
1
6
12
24
48
96
144

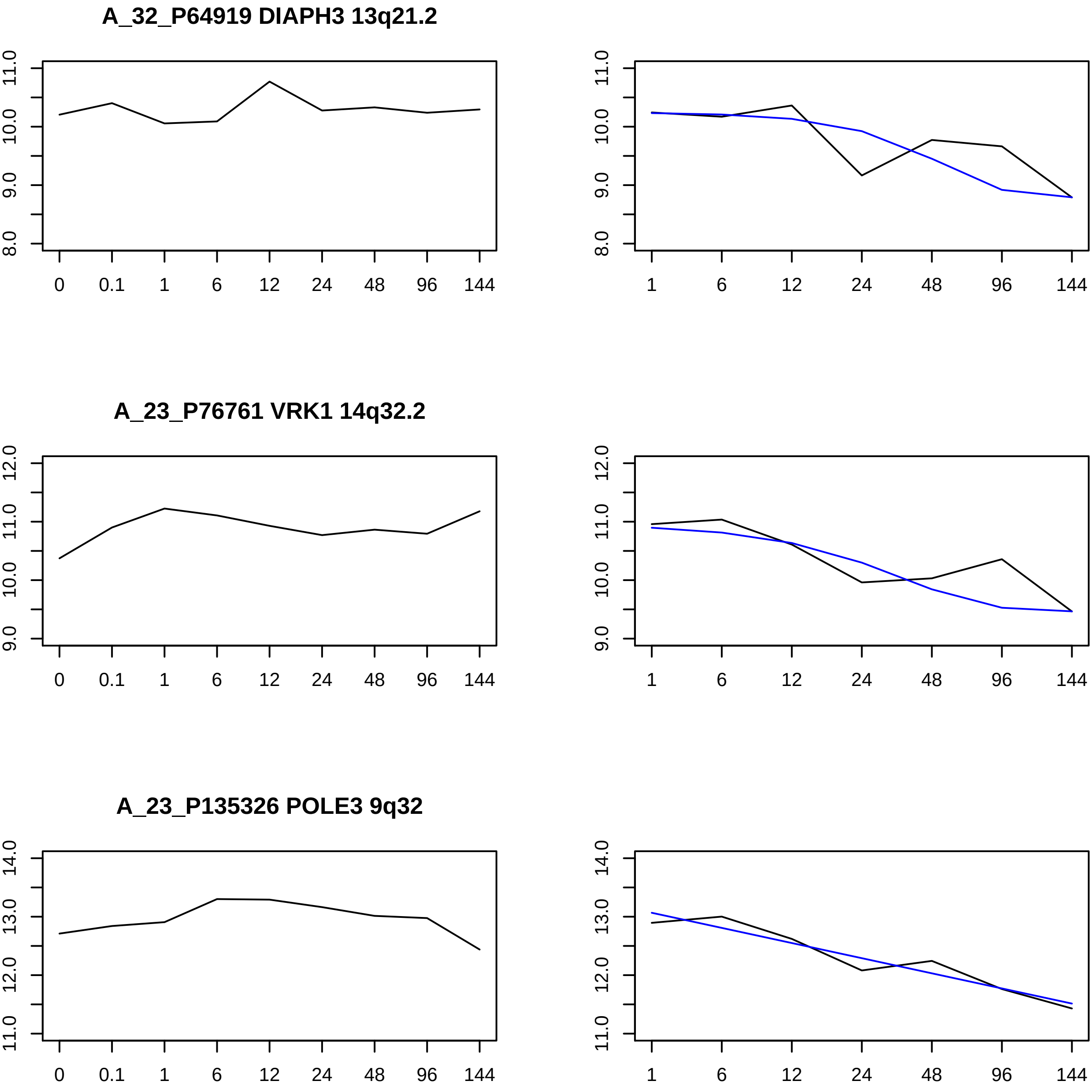
A_32_P64919 DIAPH3 13q21.2
8.0
9.0
10.0
11.0
0
0.1
1
6
12
24
48
96
144
1
6
12
24
48
96
144
A_23_P76761 VRK1 14q32.2
9.0
10.0
11.0
12.0
A_23_P135326 POLE3 9q32
11.0
12.0
13.0
14.0

**A_24_P326660 MCAM 11q23.3**

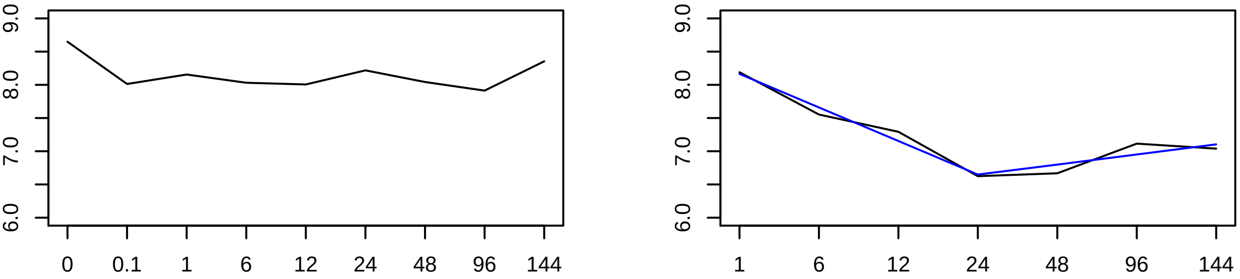


**A_23_P25873 WDHD1 14q22.3**

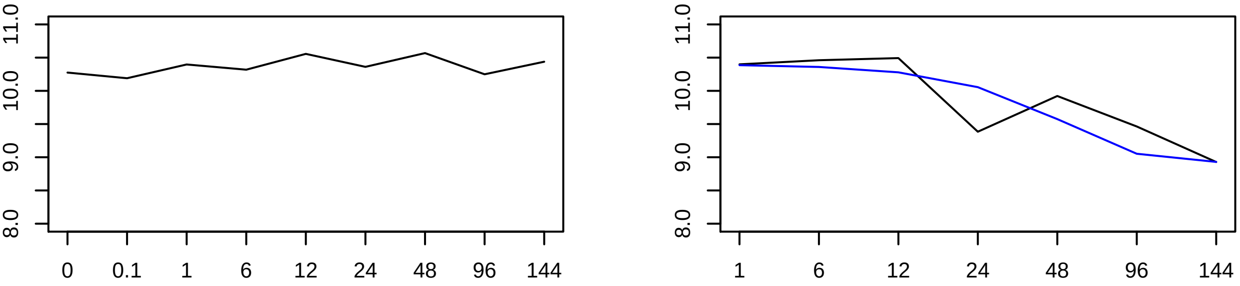


**A_23_P99930 TIPIN 15q22.31**

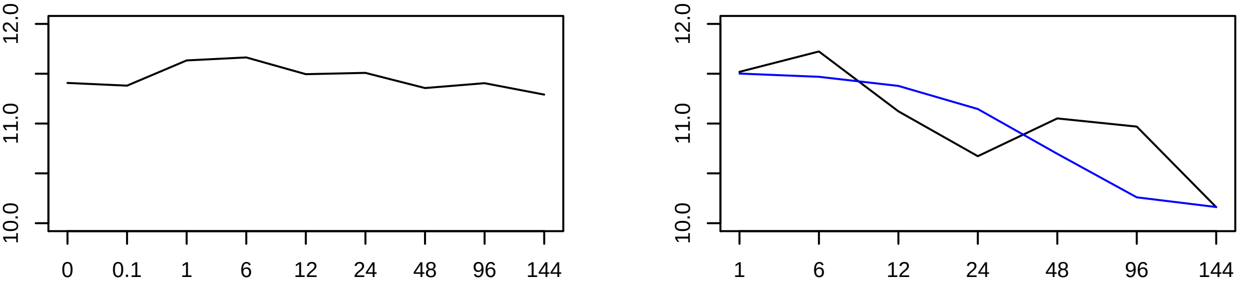

**A_23_P204133 GALNT6 12q13.13**

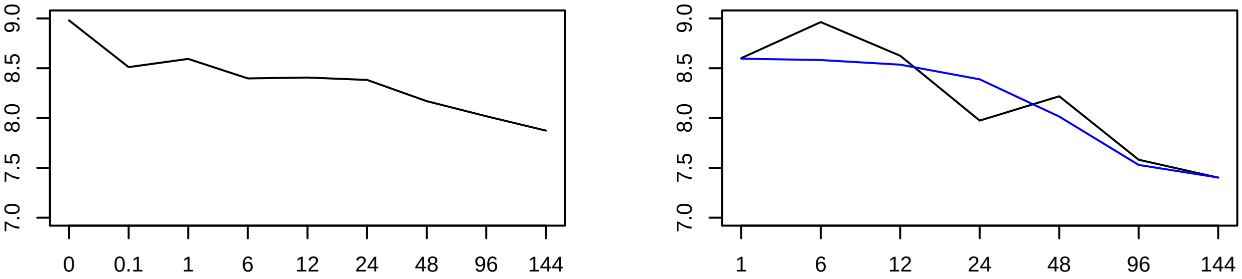


**A_23_P94141 RAD54B 8q22.1**

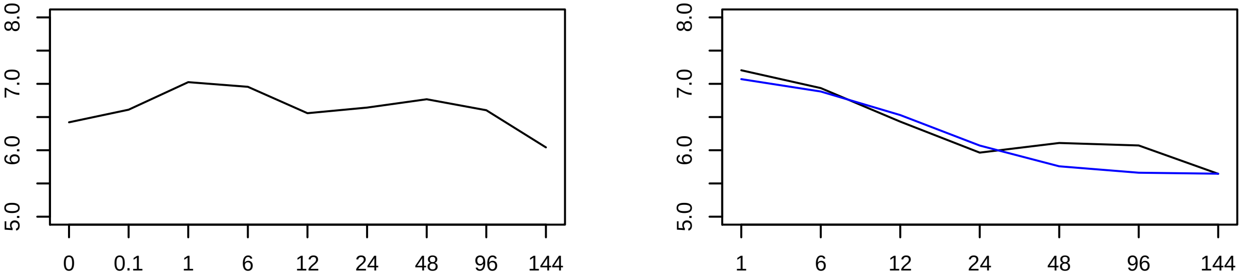


**A_23_P47790 METTL1 12q14.1**

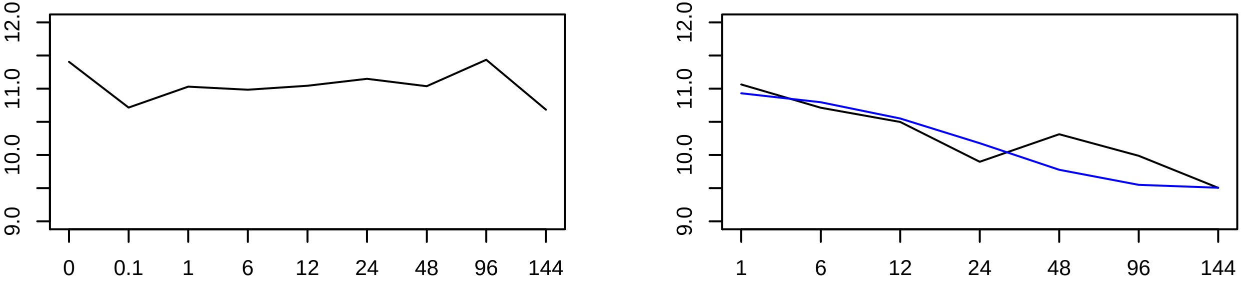

A_24_P319942 SSR3 3q25.31

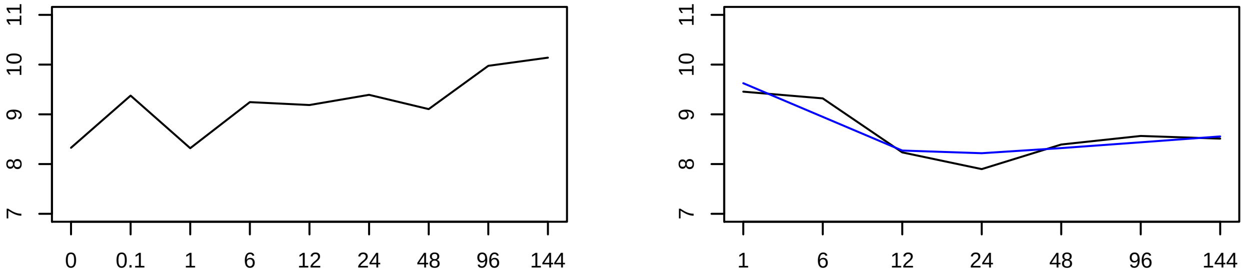


A_23_P57588 GTSE1 22q13.31

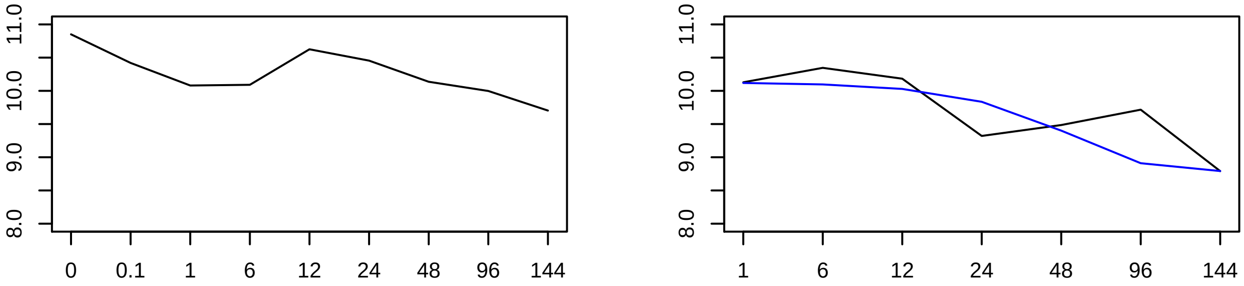


A_23_P435029 ENST00000369158 NA

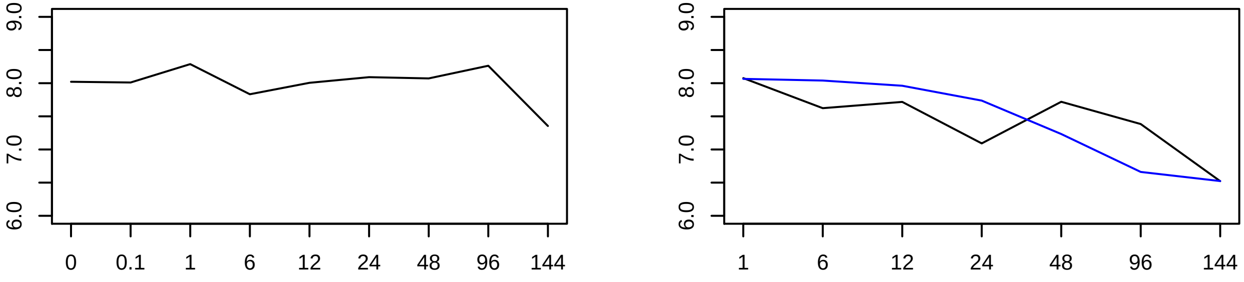

**A_23_P68547 MCM8 20p12.3**

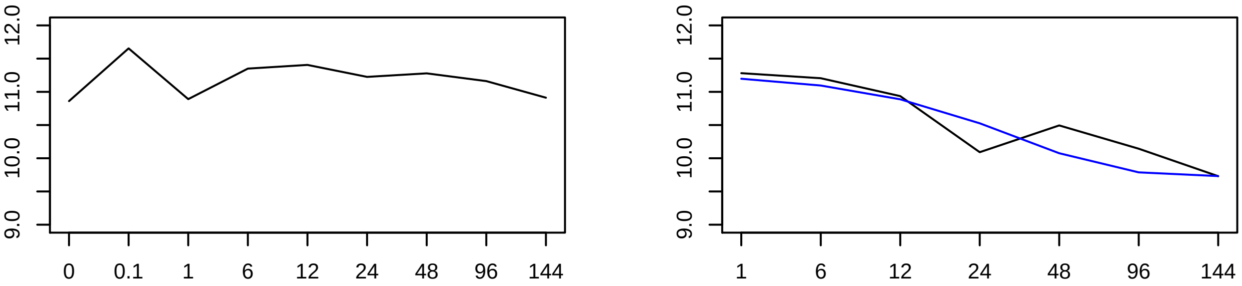


**A_23_P332326 ARHGEF19 1p36.13**

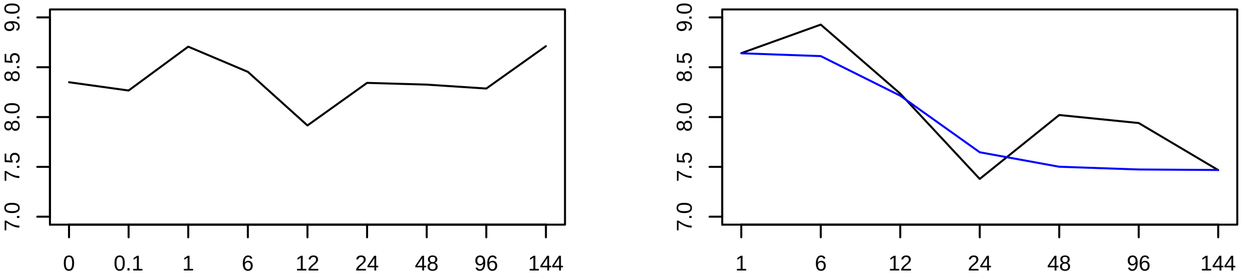


**A_23_P101551 BCAT2 19q13.33**

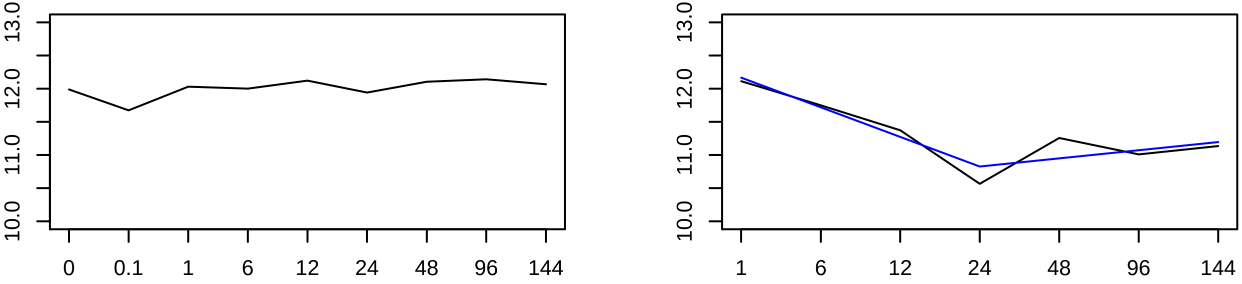

**A_23_P92132 IFRD2 3p21.31**

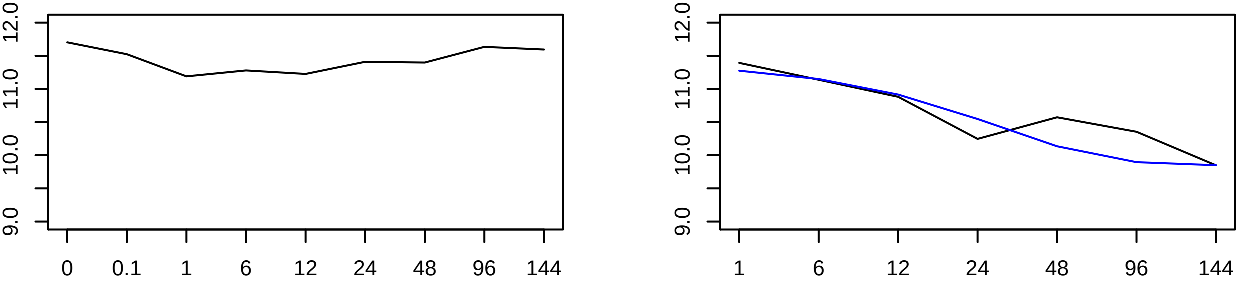


**A_23_P88095 TBC1D4 13q22.2**

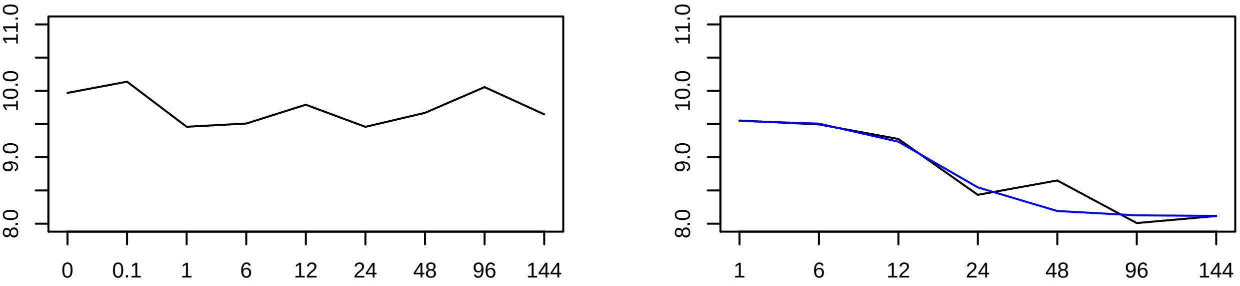


**A_23_P218879 TREX1 3p21.31**

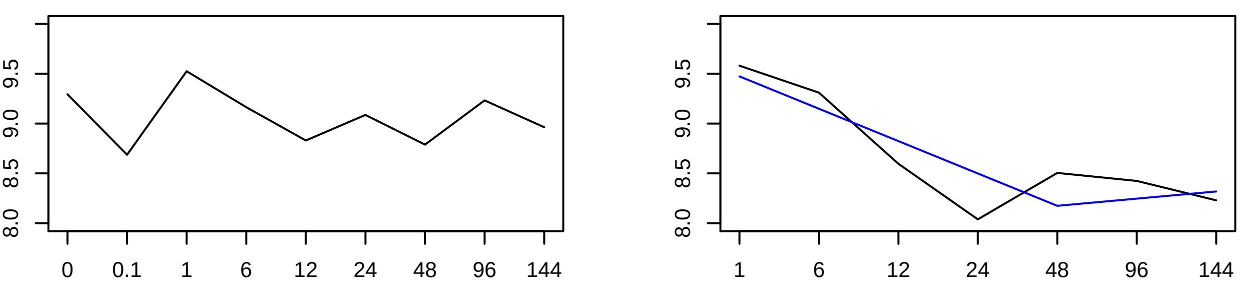

A_23_P41280 PAICS 4q12

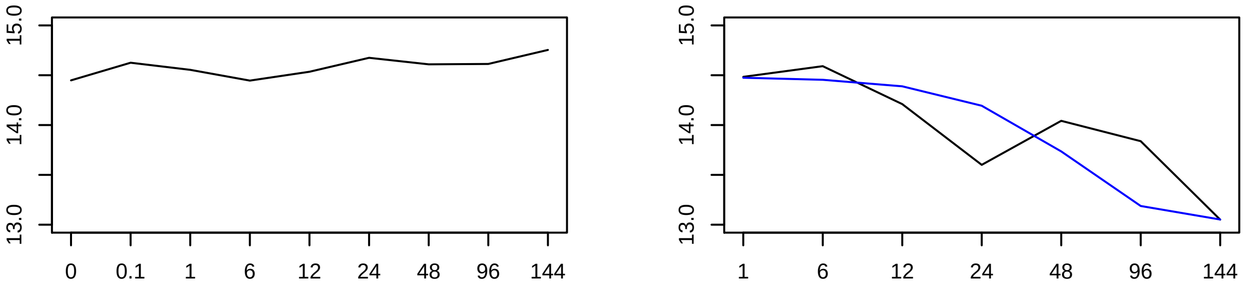


A_23_P52986 VWCE 11q12.2

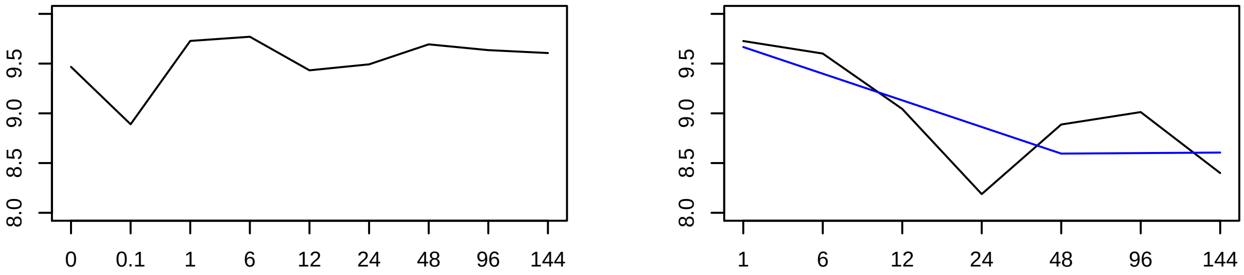


A_32_P100464 THC2691500 NA

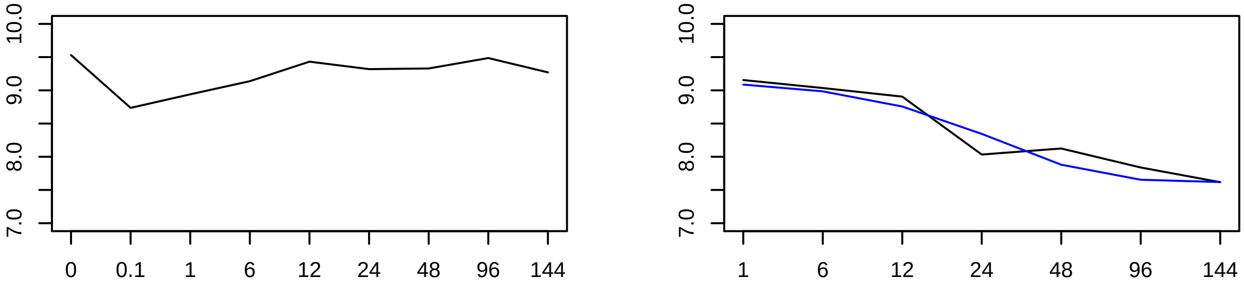

**A_24_P354488 ASAHL 4q21.1**

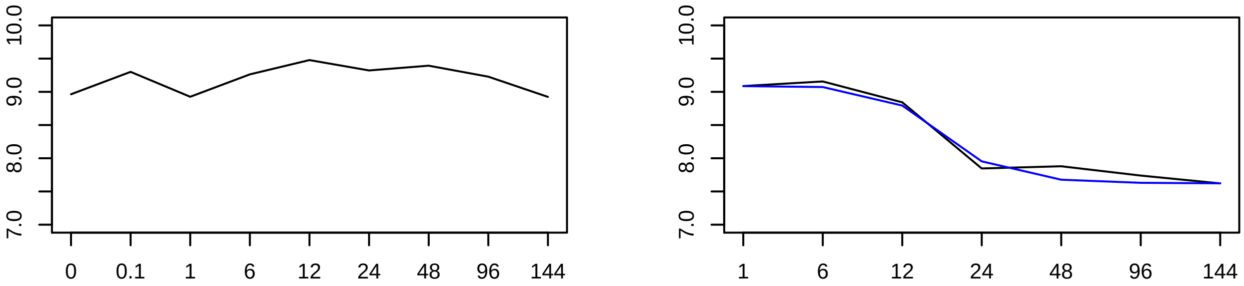


**A_23_P203947 DDX11 12p11.21**

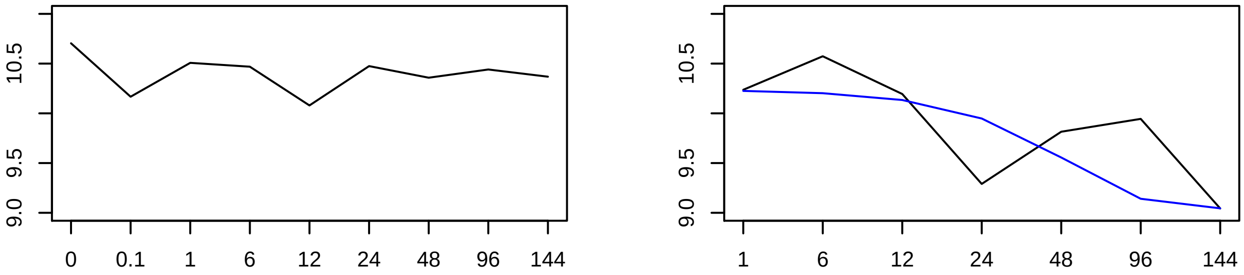


**A_24_P565908 LOC646091 2q33.3**

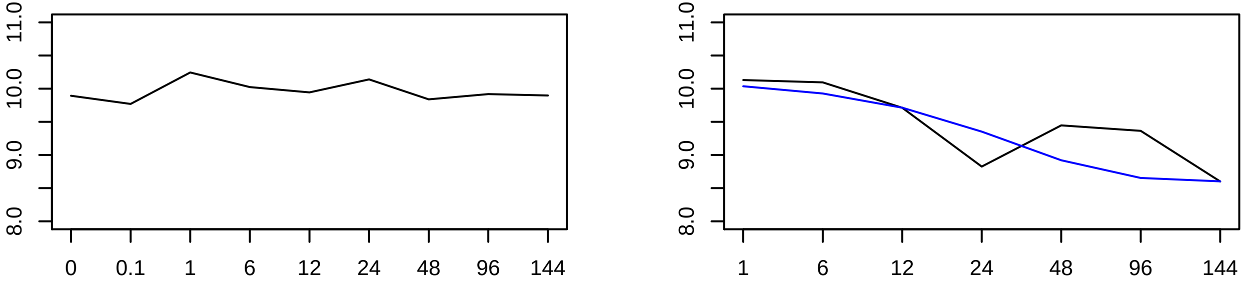

**A_24_P83678 C6orf167 6q16.1**

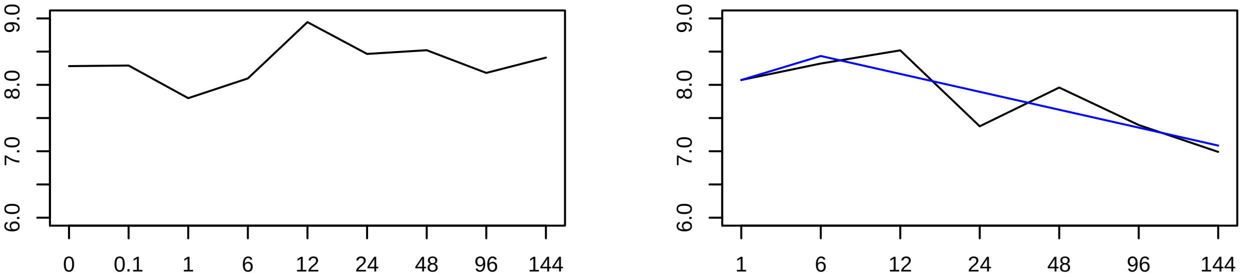


**A_23_P23765 ITGB3BP 1p31.3**

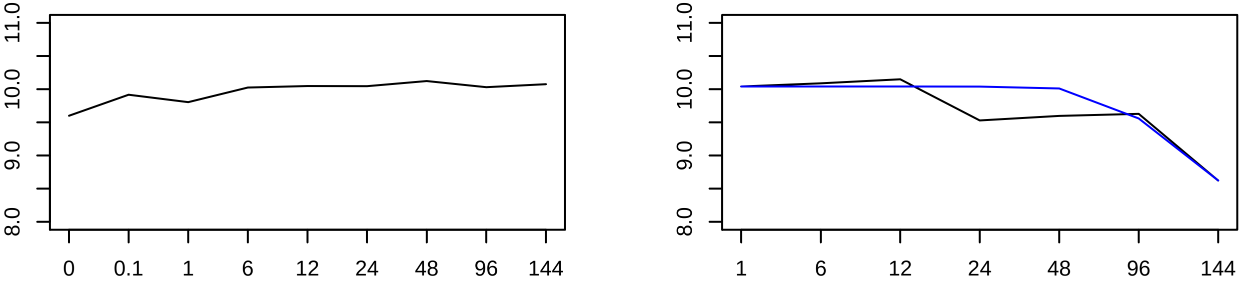


**A_32_P31165 BC000986 NA**

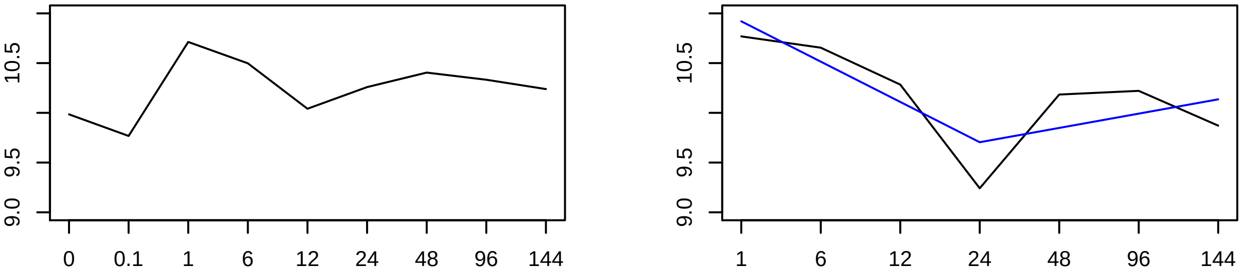

## A_23_P7976 HIST1H1E 6p22.1

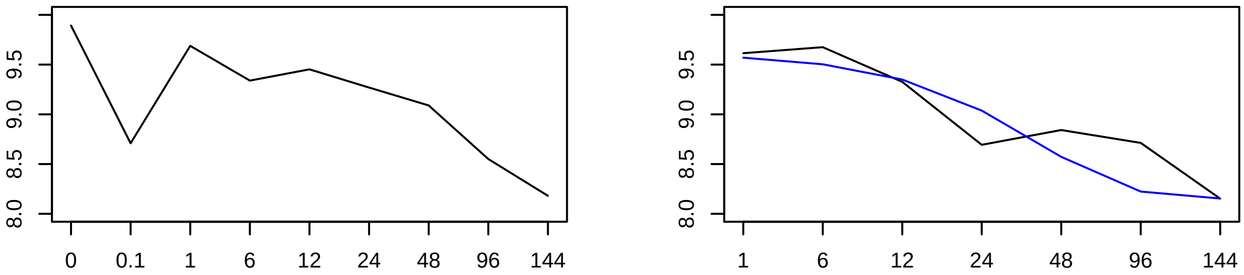


## A_24_P99090 CKAP2 13q14.3

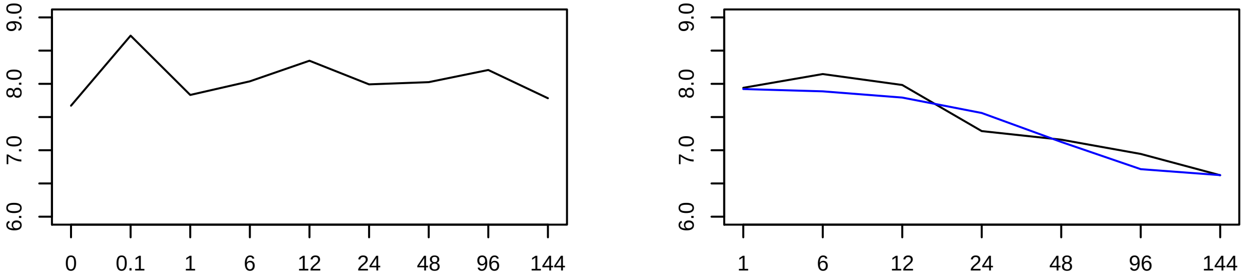


## A_23_P43597 KIF24 9p13.3

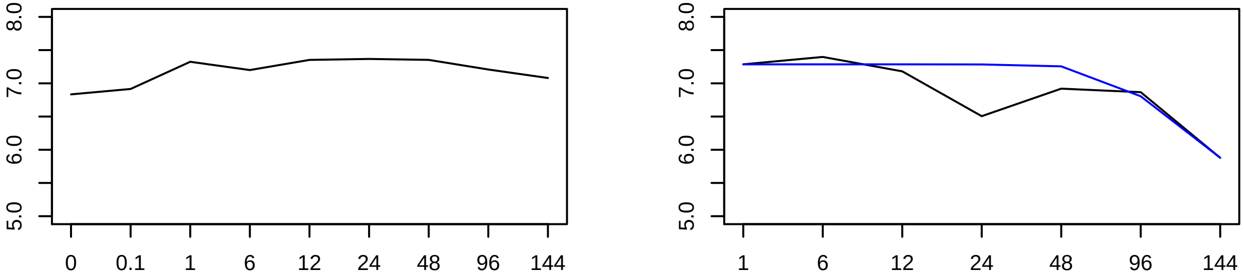

**A_24_P383680 A_24_P383680 NA**

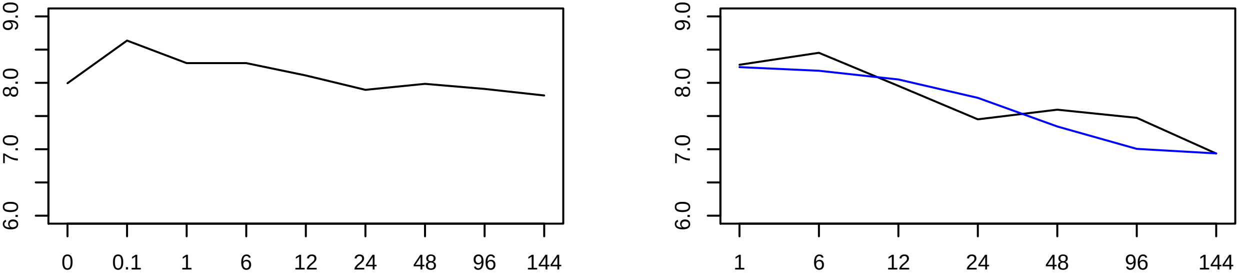


**A_24_P287941 PSMC3IP 17q21.31**

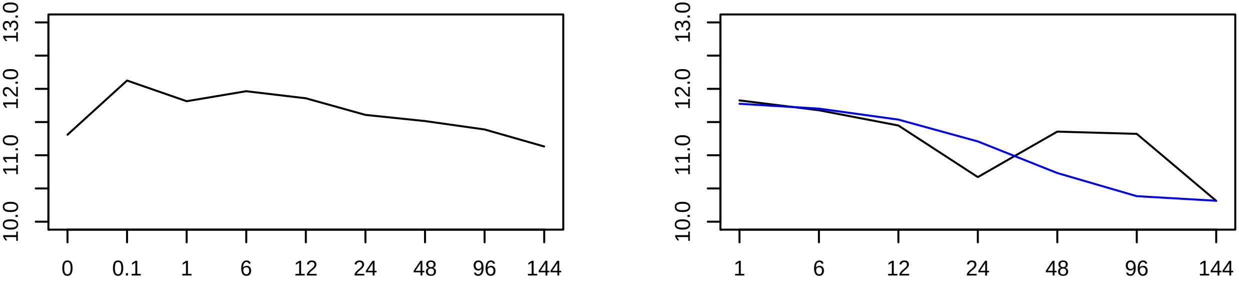


**A_23_P7101 SLBP 4p16.3**

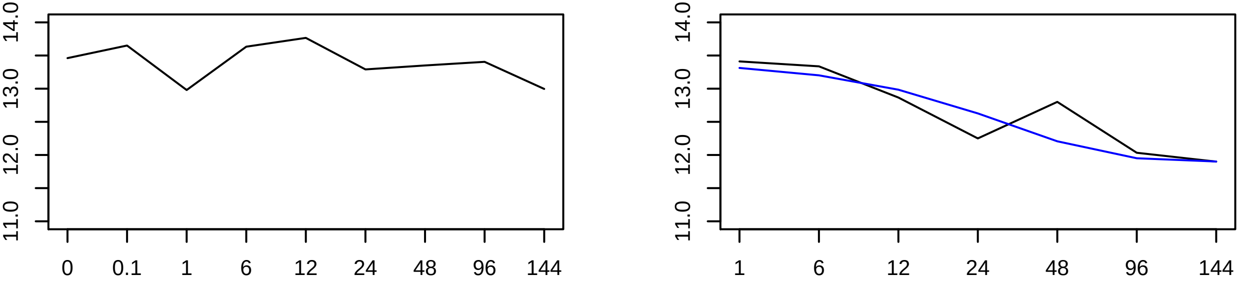

A_23_P66732 GSG2 17p13.3

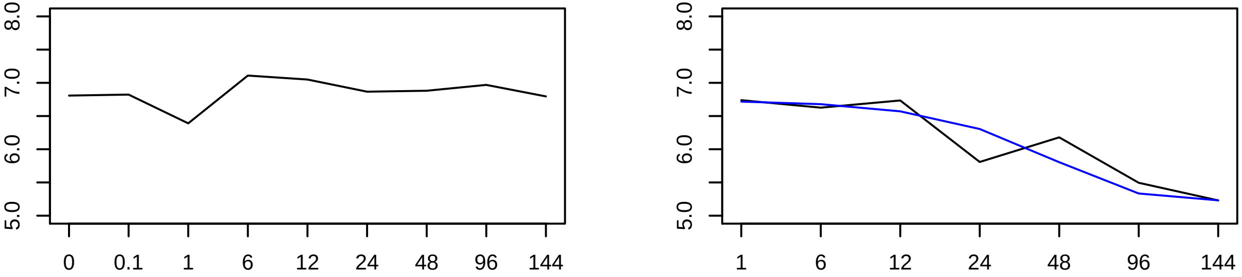


A_24_P266037 DIMT1L 5q12.1

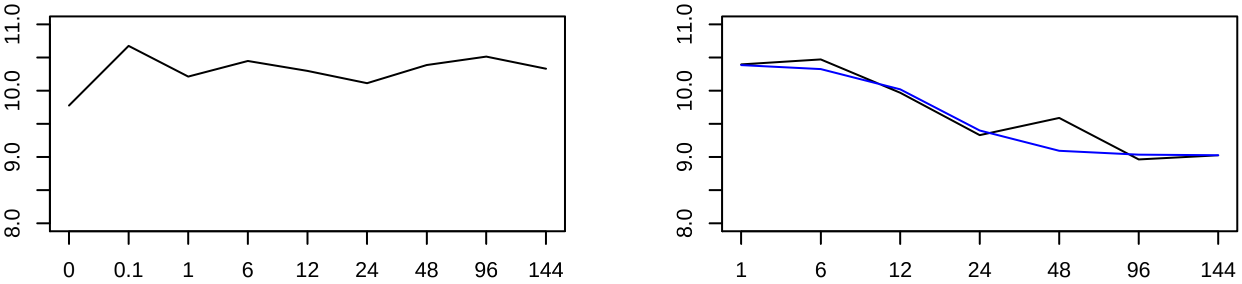


A_24_P53519 CHAF1A 19p13.3

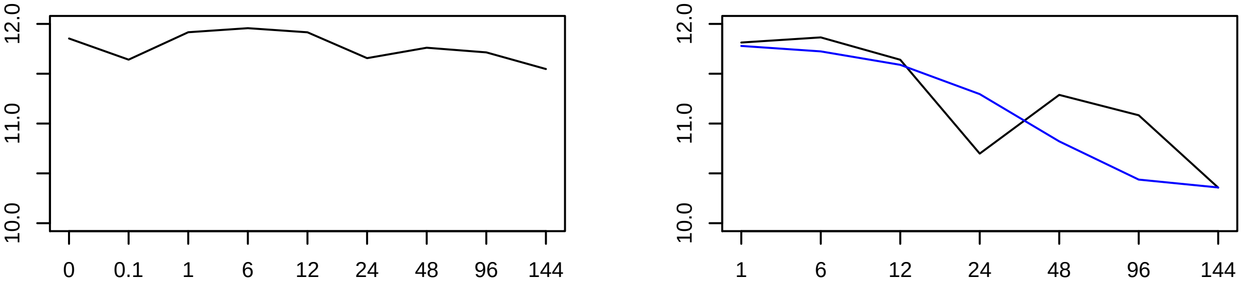

**A_23_P375104 KIAA1794 15q26.1**

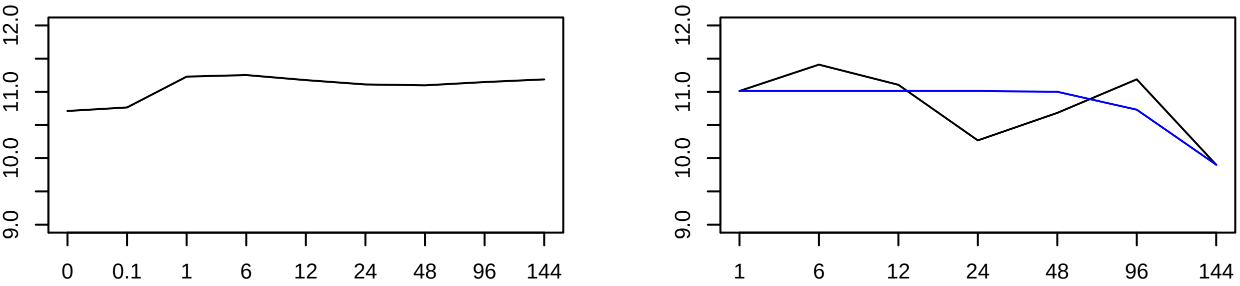


**A_24_P14156 KNTC2 18p11.32**

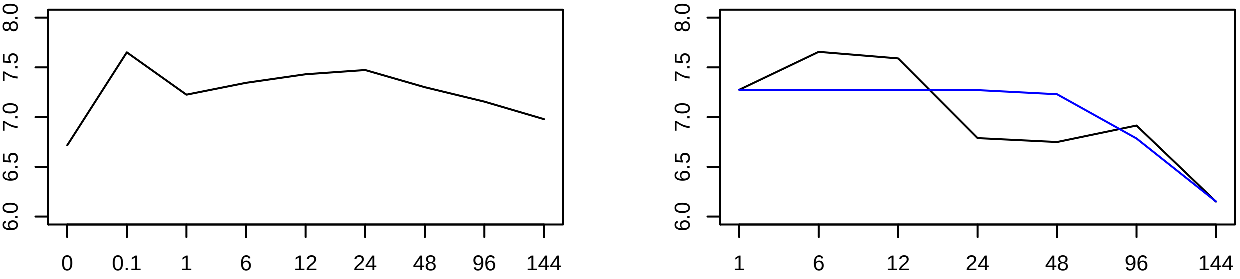


**A_24_P409500 BC000986 NA**

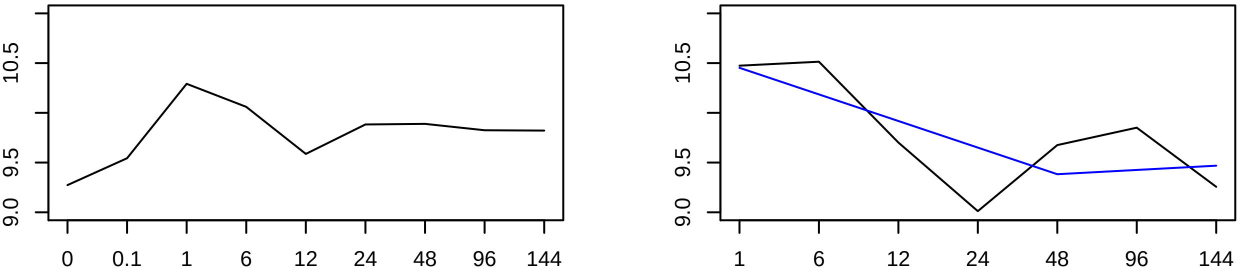

**A_23_P311144 CCDC138 2q13**

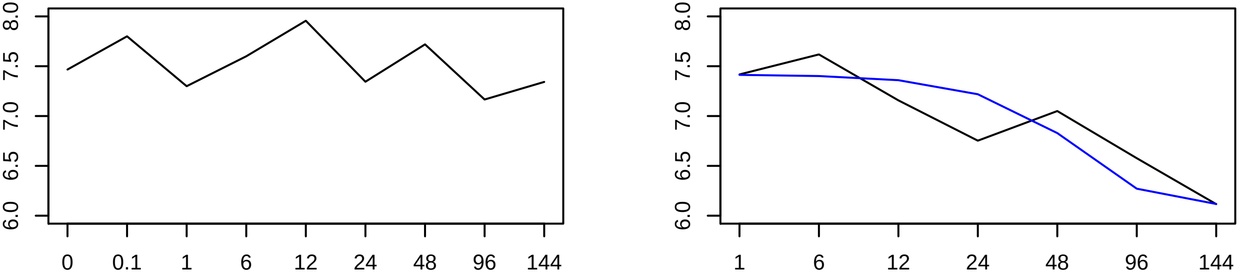


**A_23_P87351 RRM1 11p15.4**

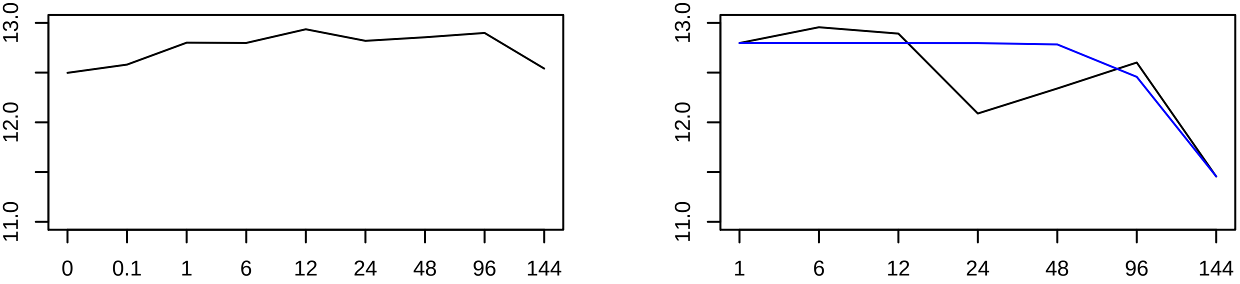


**A_24_P222192 TMEM48 1p32.3**

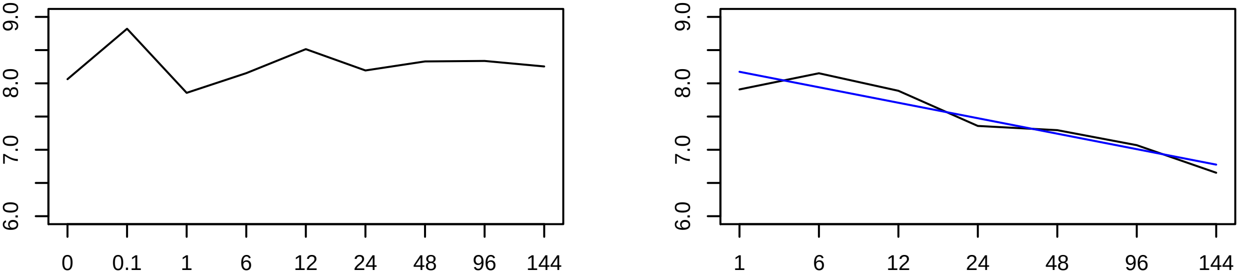

**A_23_P70328 CENPQ 6p12.3**

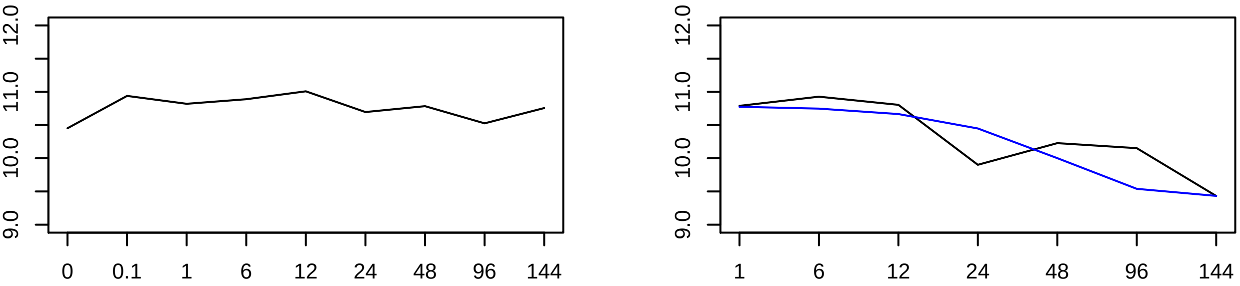


**A_23_P70249 CDC25C 5q31.2**

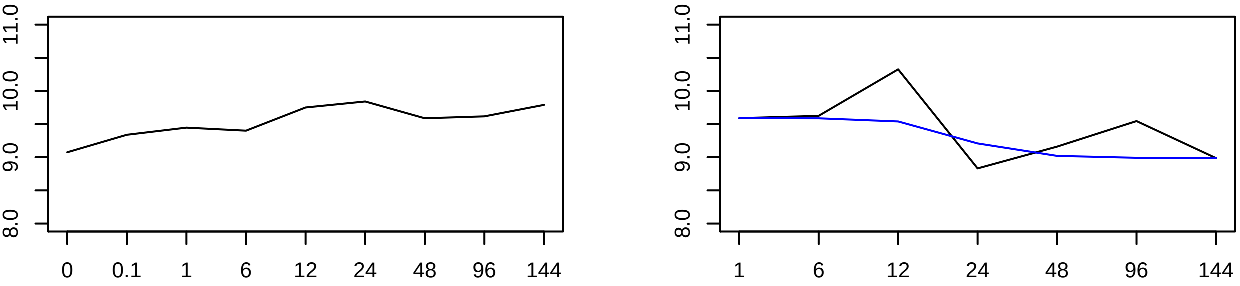


**A_23_P50108 KNTC2 18p11.32**

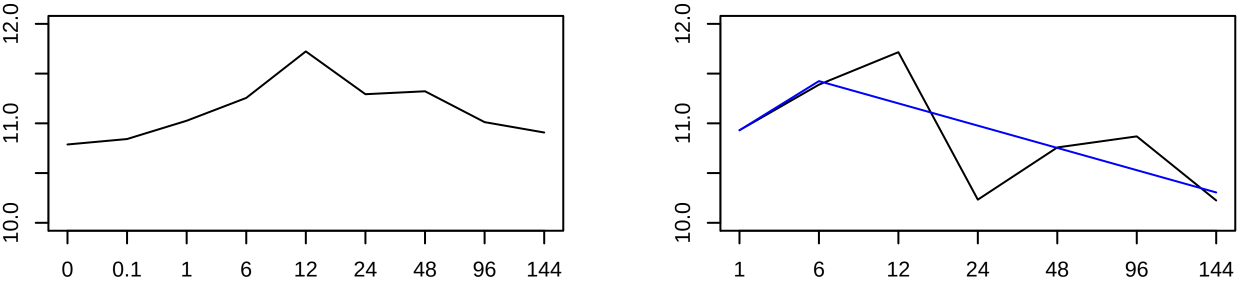

A_24_P296254 ARHGAP11A 15q13.3

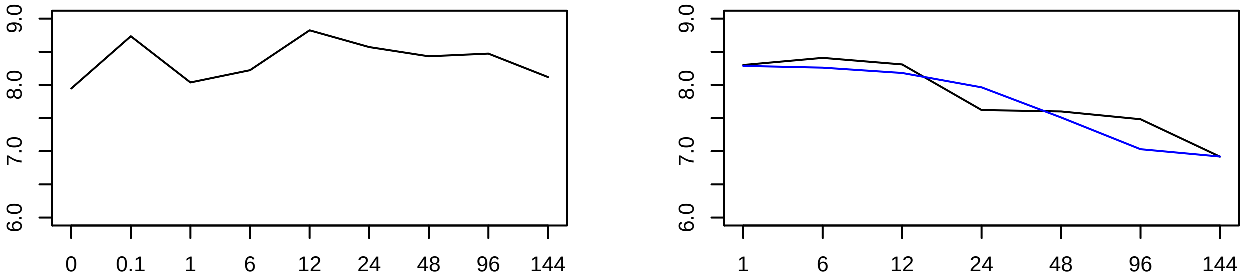


A_32_P208136 MMS22L 6q16.1

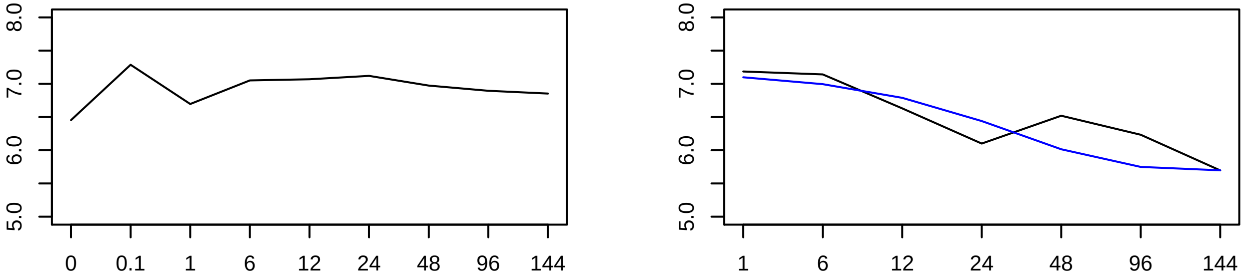


A_24_P316363 GTF3C2 2p23.3

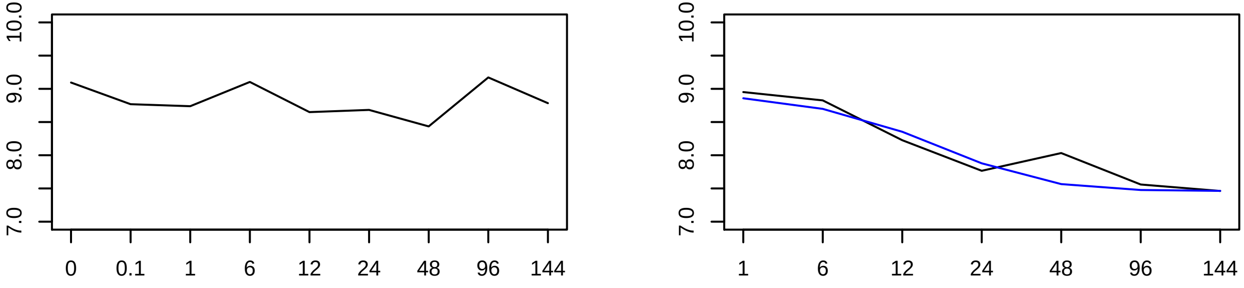

**A_23_P33613 XTP3TPA 16p11.2**

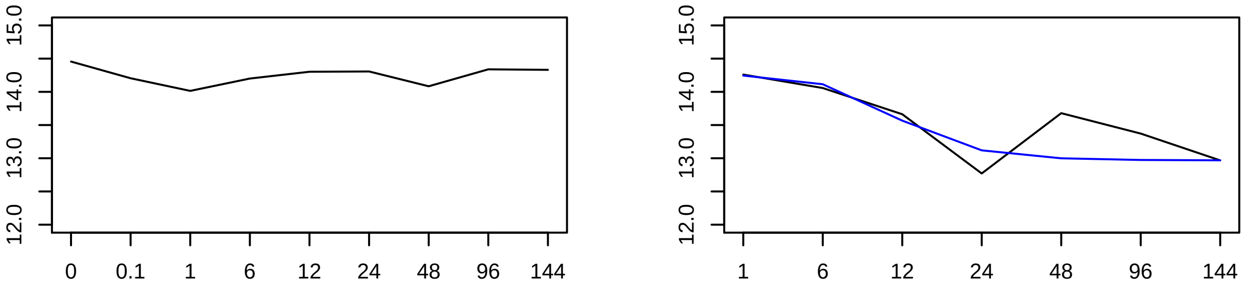


**A_23_P88362 PPIL5 14q22.1**

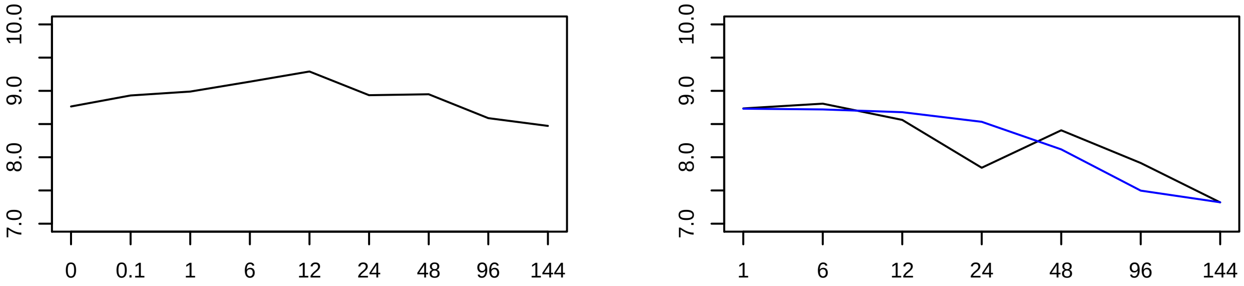


**A_24_P15702 LOC389386 6p21.31**

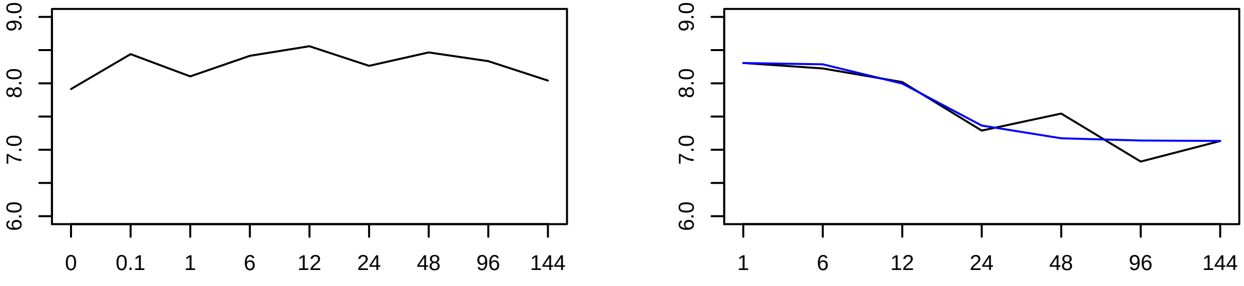

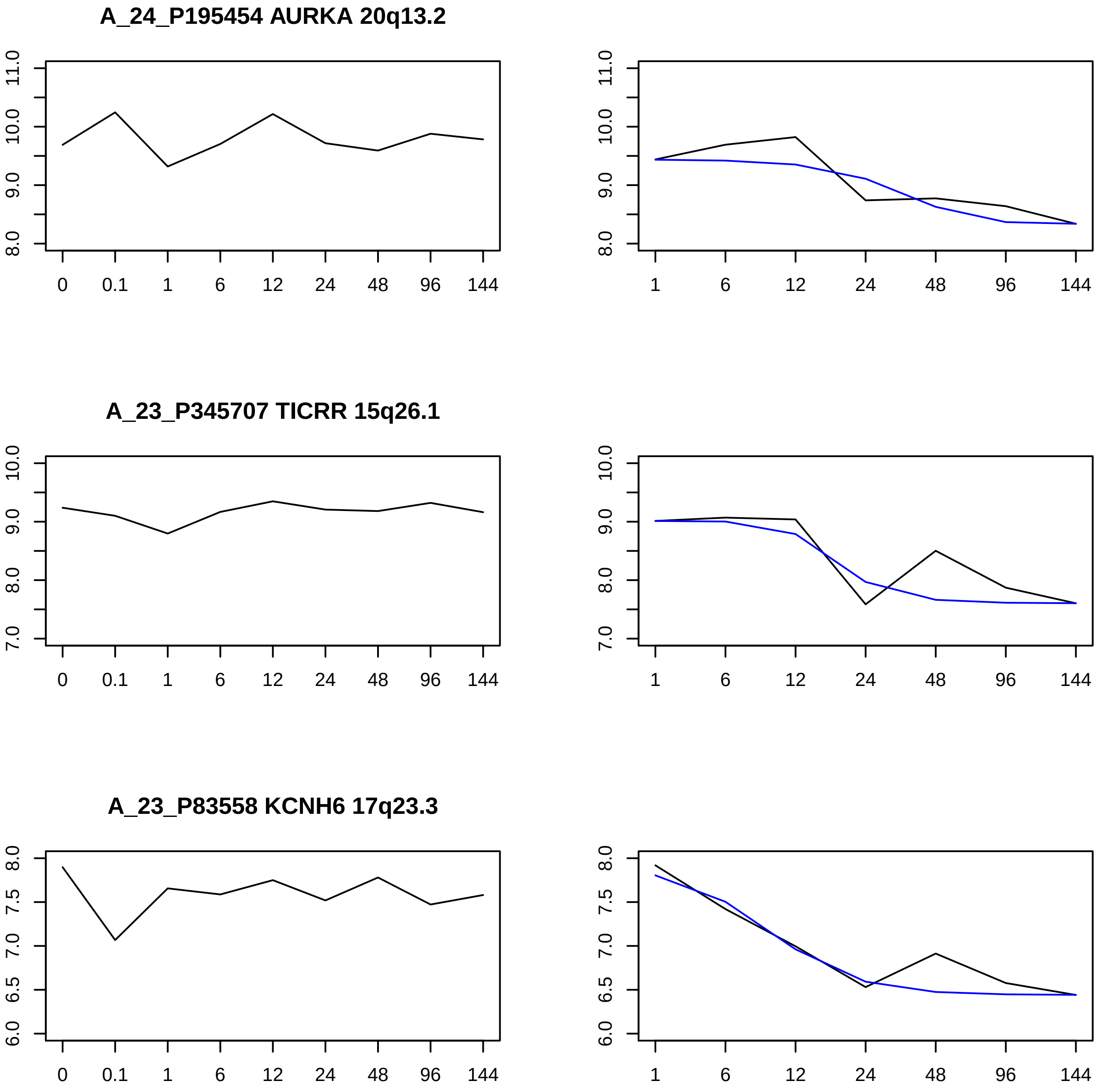
A_24_P195454 AURKA 20q13.2
A_23_P345707 TICRR 15q26.1
A_23_P83558 KCNH6 17q23.3
0 0.1 1 6 12 24 48 96 144
1 6 12 24 48 96 144
8.0 9.0 10.0 11.0
7.0 8.0 9.0 10.0
6.0 6.5 7.0 7.5 8.0

A_23_P45799 ORC1L 1p32.3

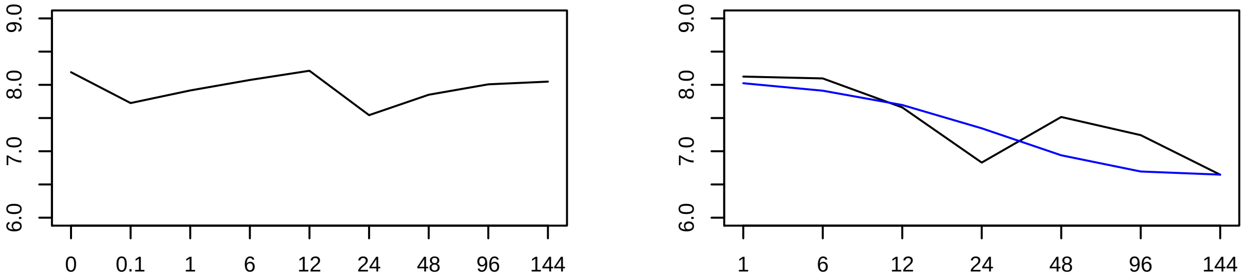


A_23_P118834 TOP2A 17q21.2

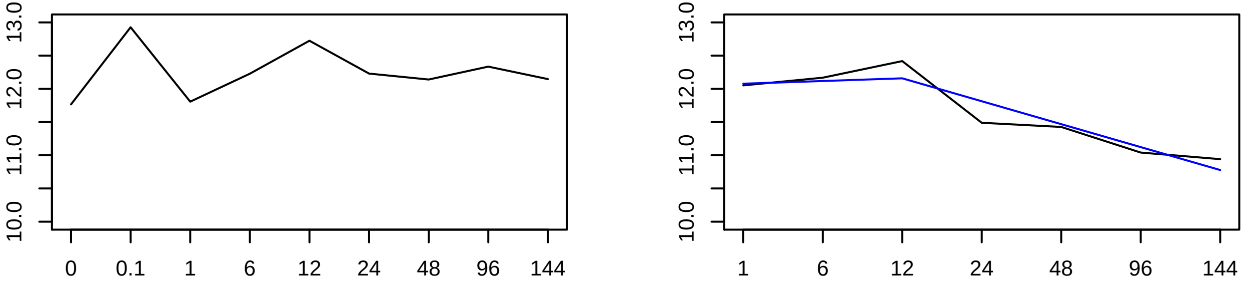


A_24_P235049 MTHFD1L 6q25.1

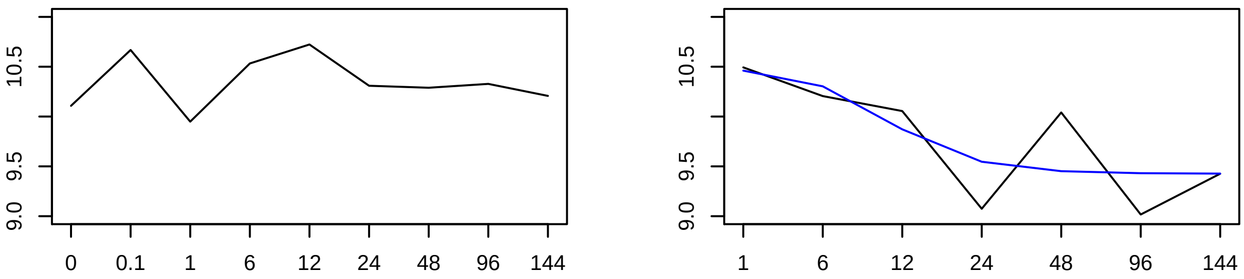

**A_23_P47788 METTL1 12q14.1**

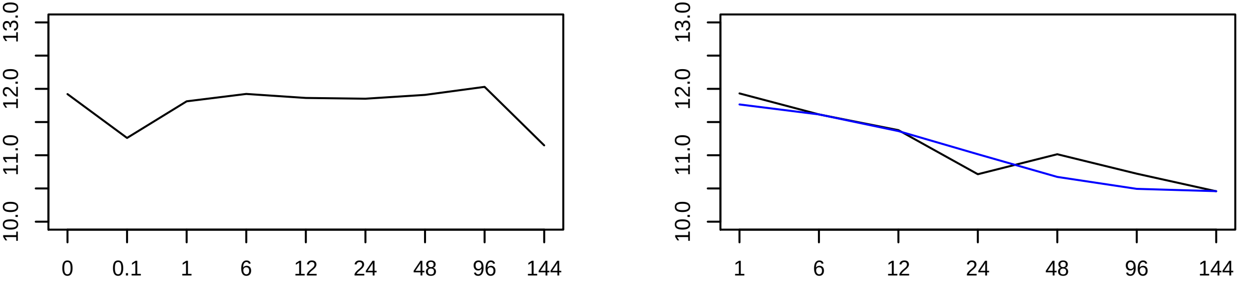


**A_24_P592544 BC107568 NA**

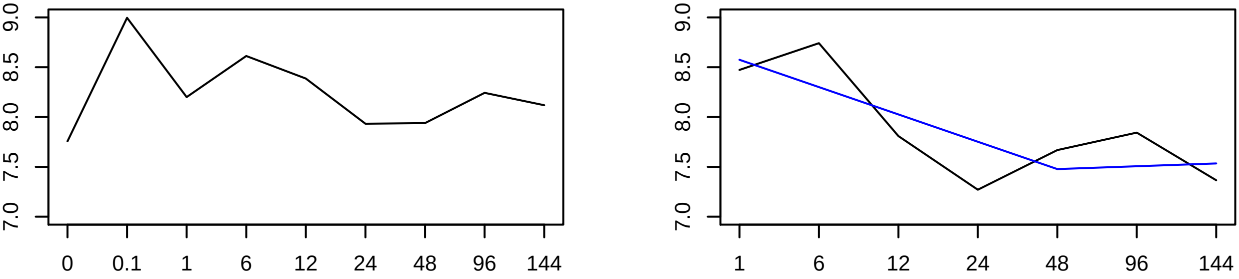


**A_23_P88731 RAD51 15q15.1**

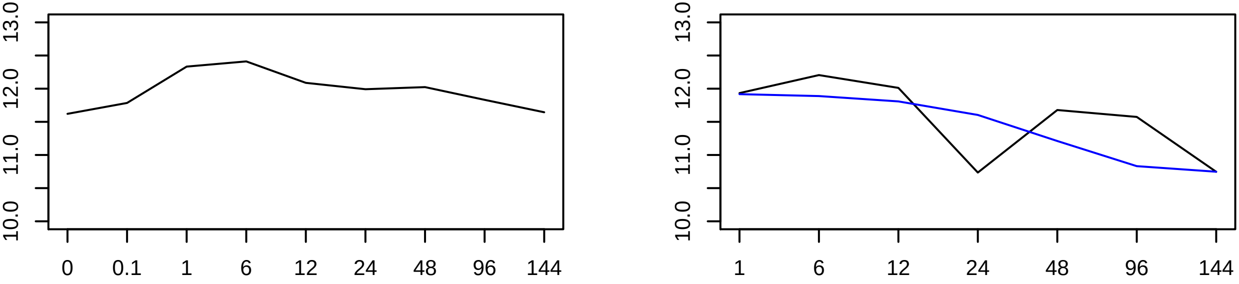

**A_23_P146637 OPRS1 9p13.3**

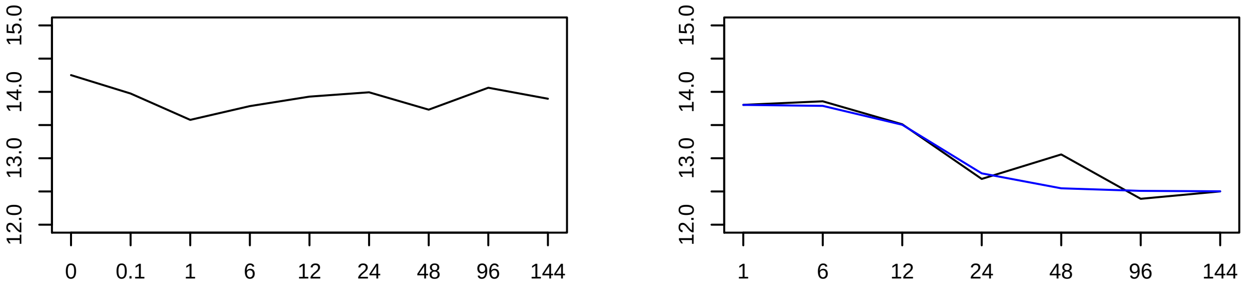


**A_23_P50990 CENPO 2p23.3**

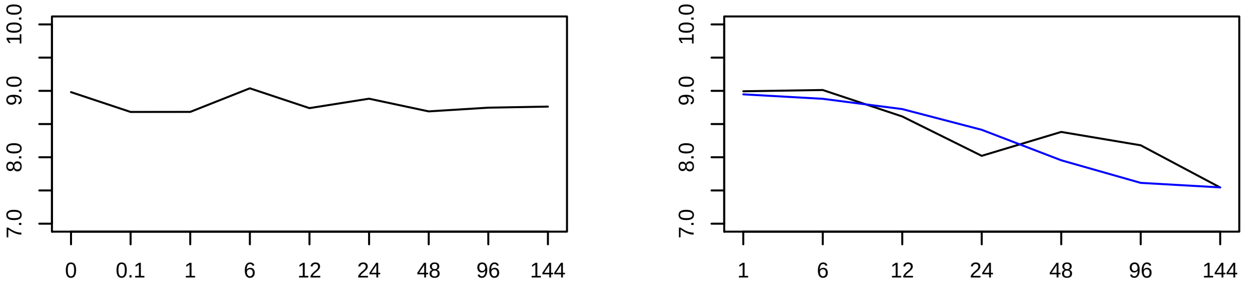


**A_23_P106024 JAG2 14q32.33**

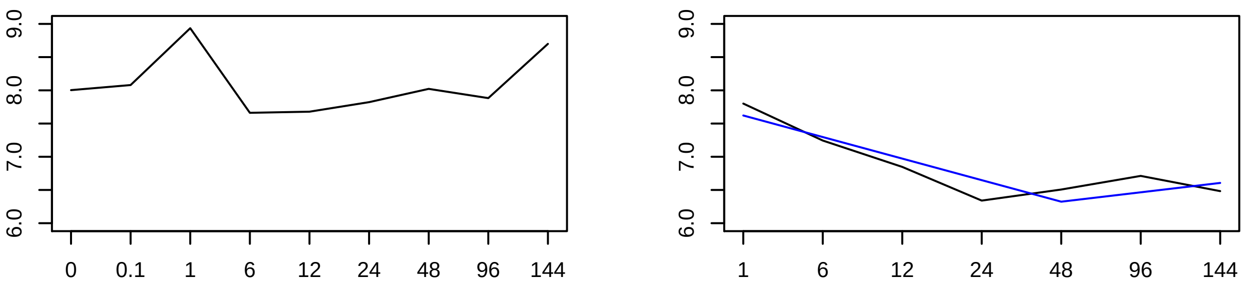

**A_24_P21715 RAD9A 11q13.1**

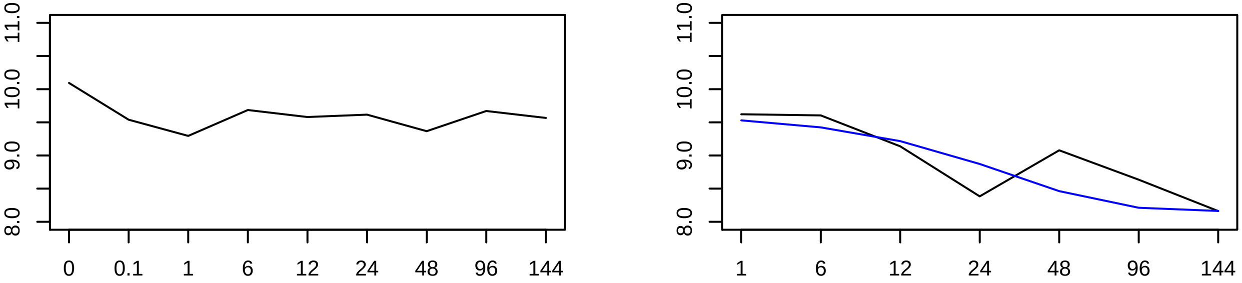


**A_32_P95729 KIAA1794 15q26.1**

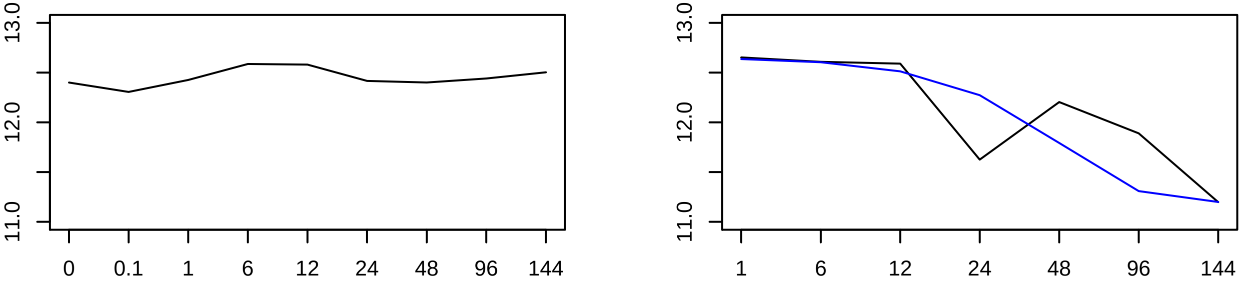


**A_24_P322354 SKA1 18q21.1**

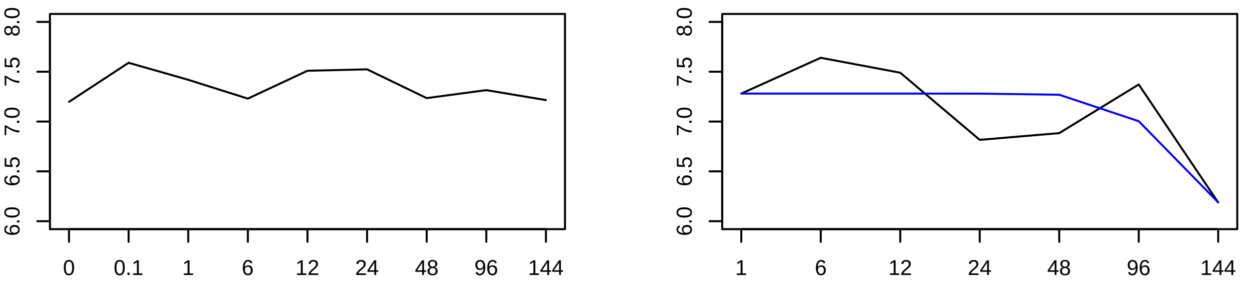

A_23_P117494 MTHFD1 14q23.2

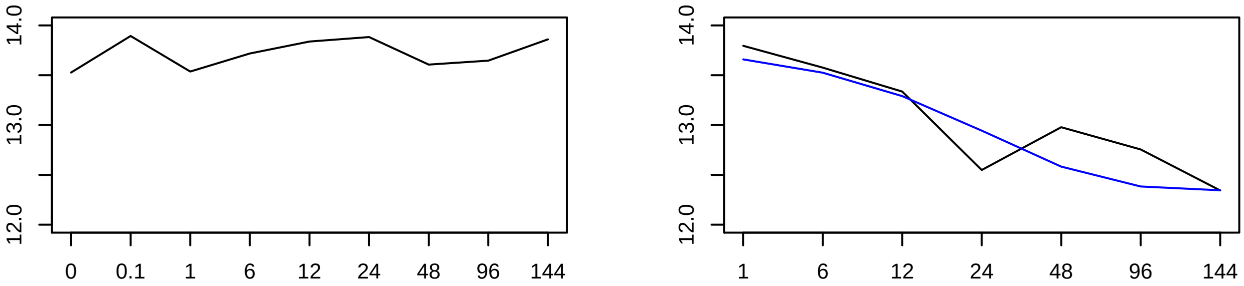


A_23_P339705 WDR62 19q13.12

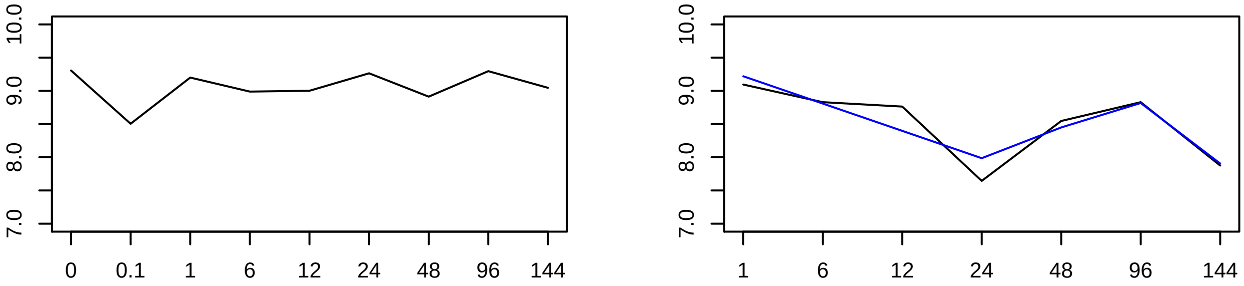


A_32_P51119 STOX1 10q21.3

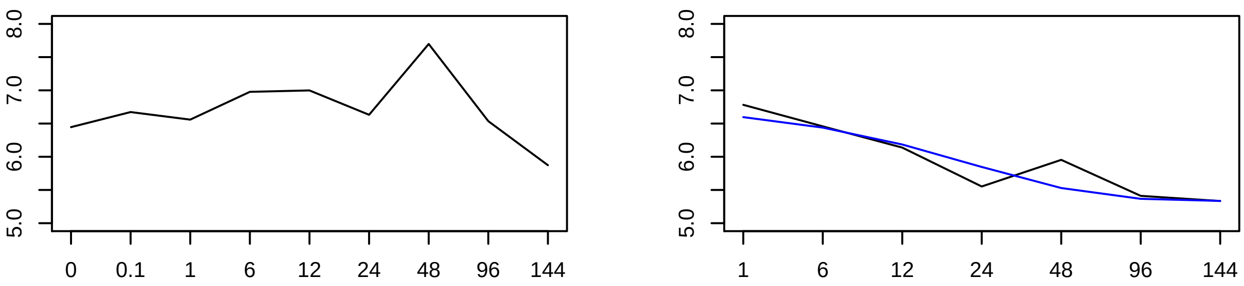

**A_23_P348298 SAC3D1 11q13.1**

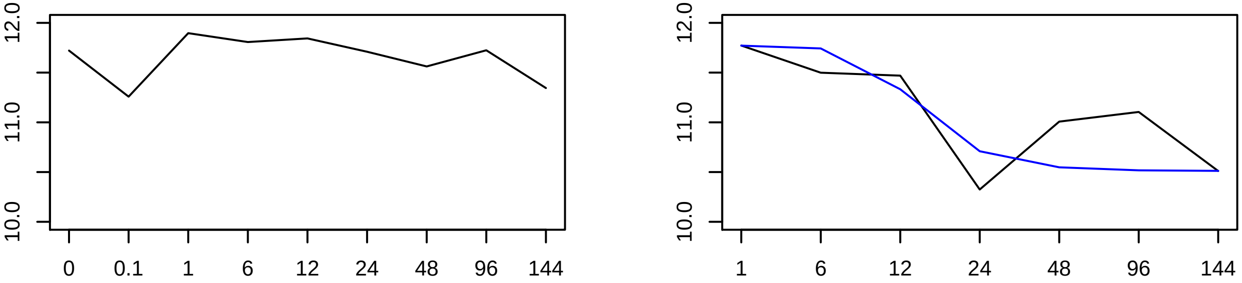


**A_23_P102769 MGME1 20p11.23**

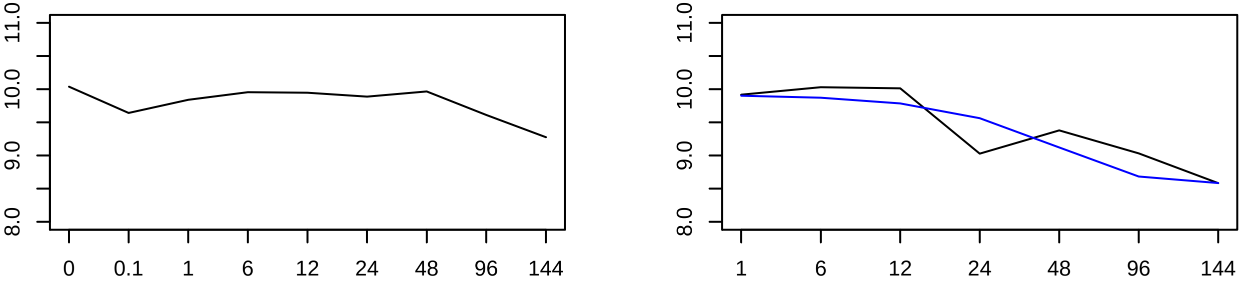


**A_23_P159355 POLE 12q24.33**

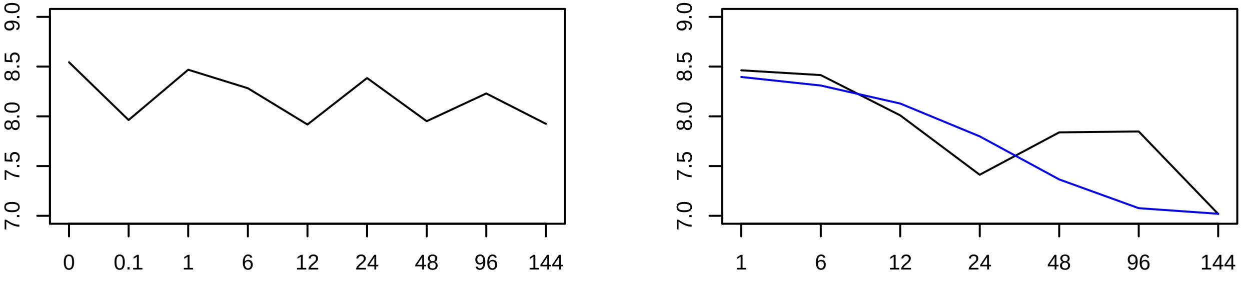

**A_24_P416079 NUSAP1 15q15.1**

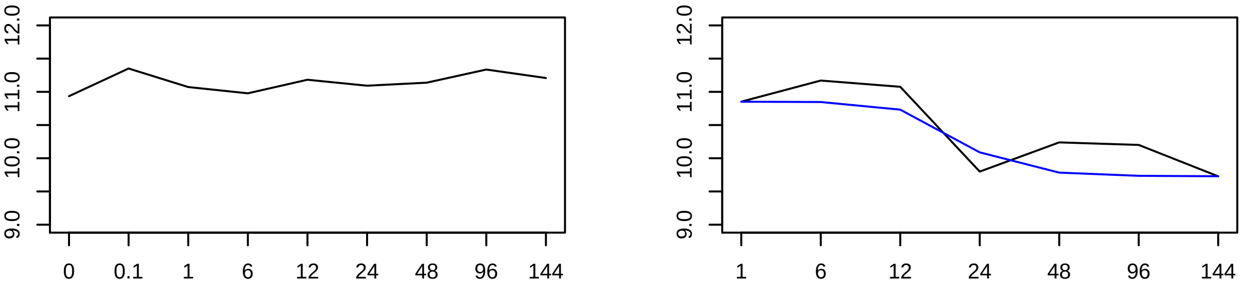


**A_23_P426511 LOC91431 4q25**

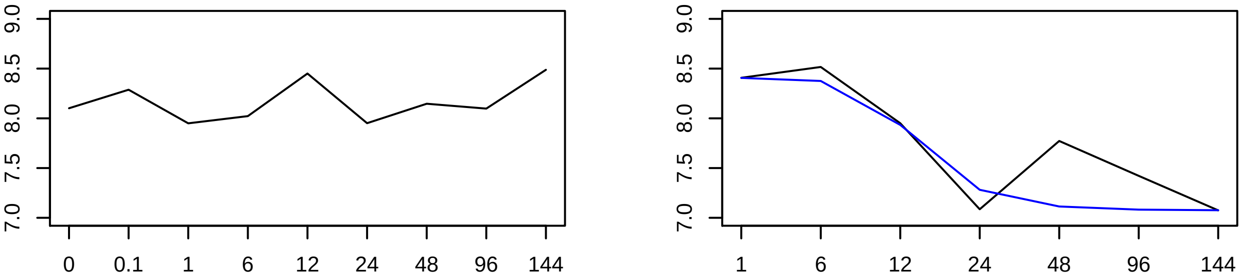


**A_23_P96641 PRPS2 Xp22.2**

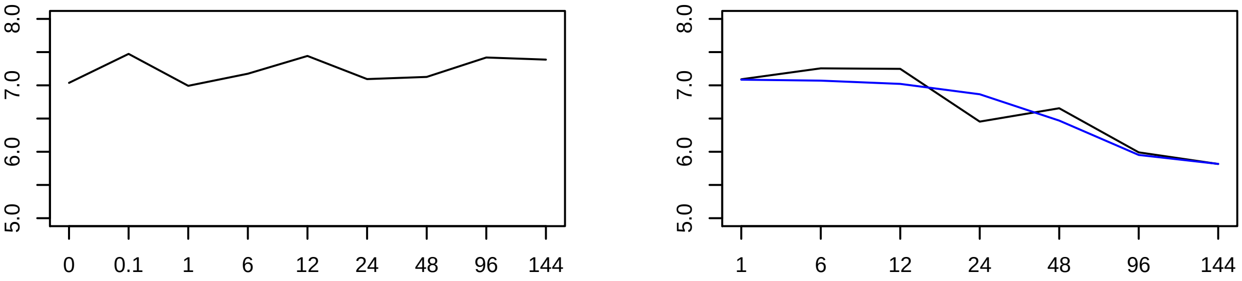

**A_24_P343095 DHFR 5q14.1**

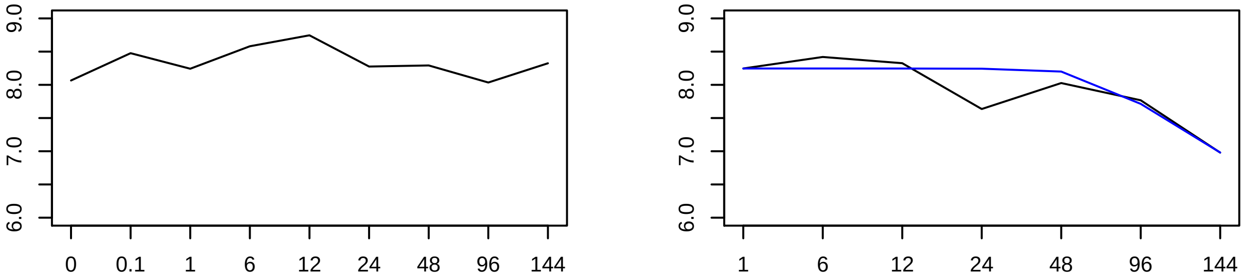


**A_23_P208310 CD3EAP 19q13.32**

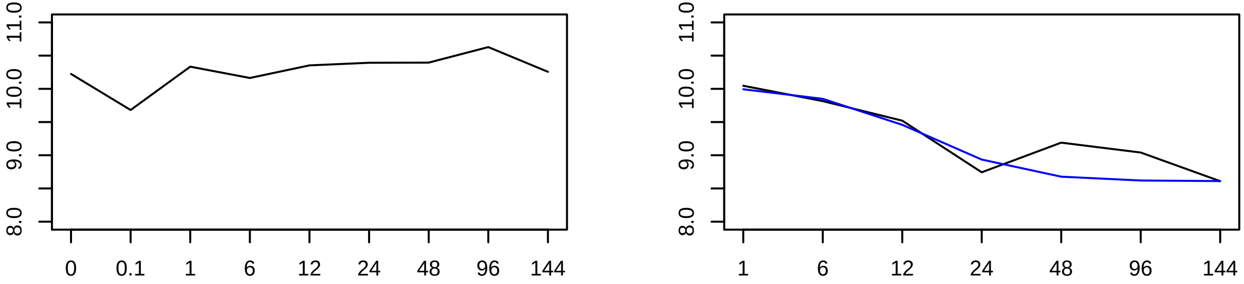


**A_24_P483083 TFDP1 13q34**

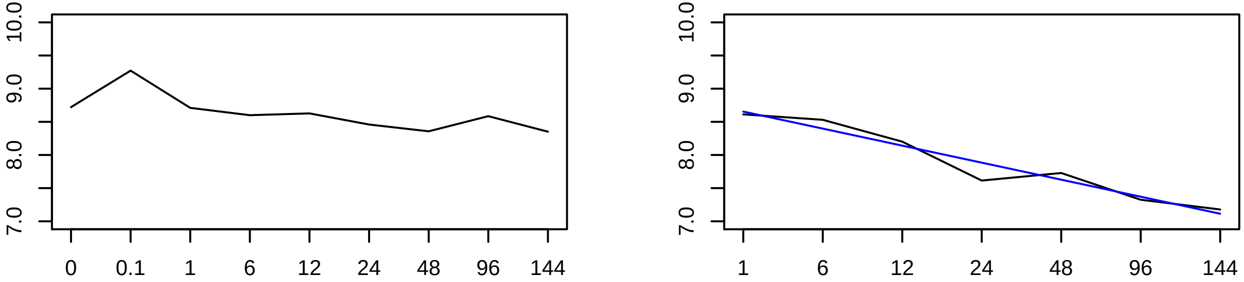

**A_23_P74914 KIAA0133 1q42.13**

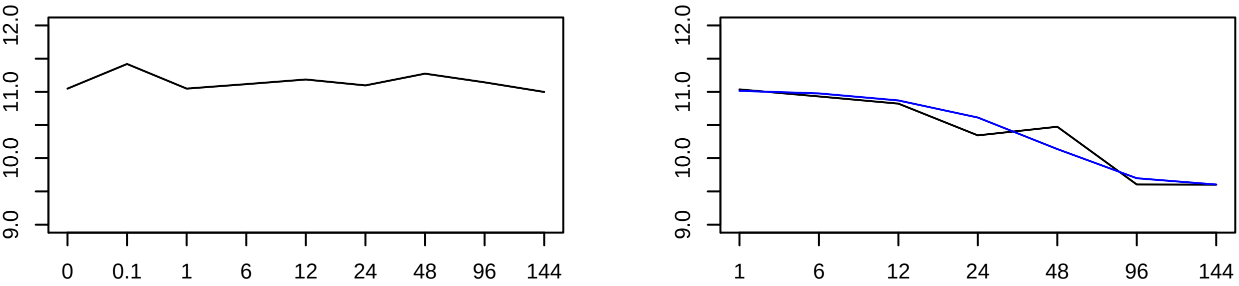


**A_23_P150189 MRE11A 11q21**

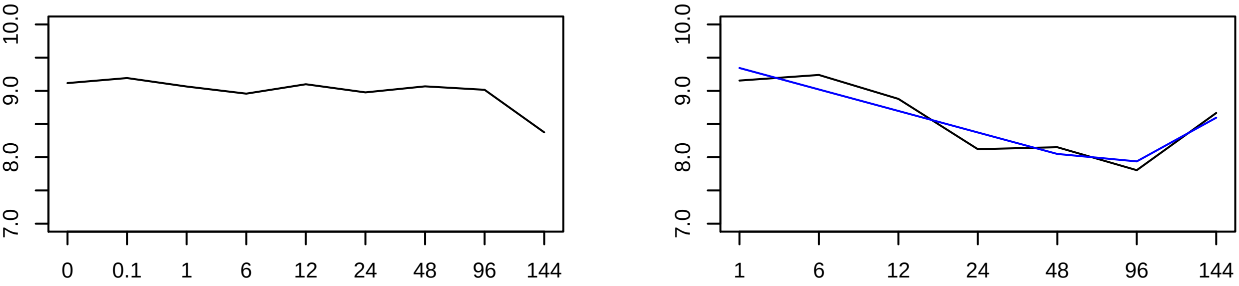


**A_23_P50376 ZNF331 19q13.41**

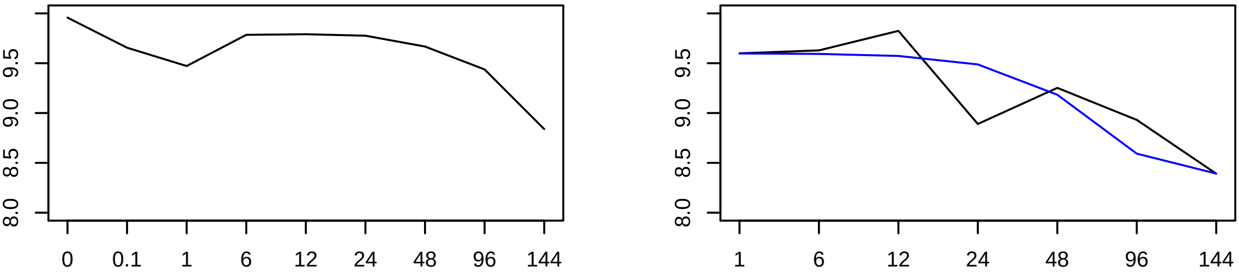

**A_23_P97161 TTF2 1p13.1**

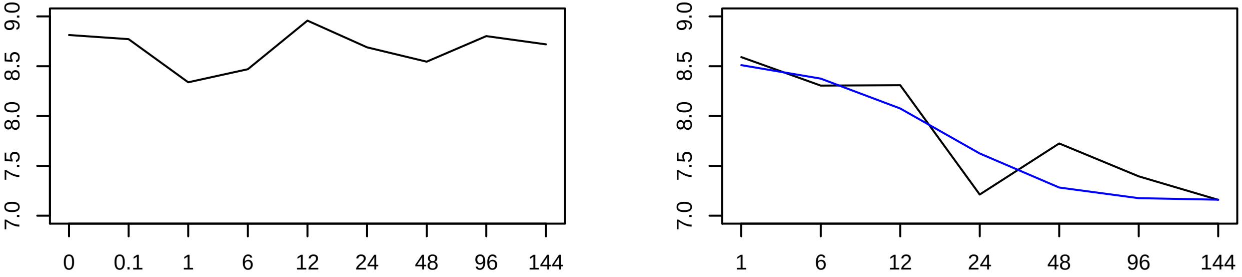


**A_23_P162719 DIAPH3 13q21.2**

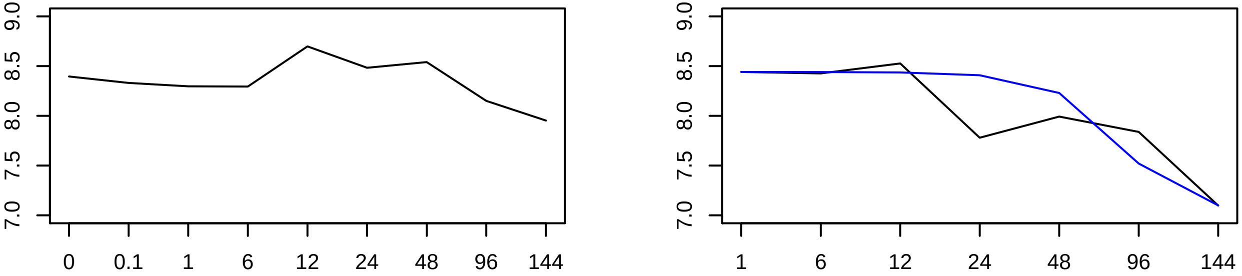


**A_23_P339480 HAT1 2q31.1**

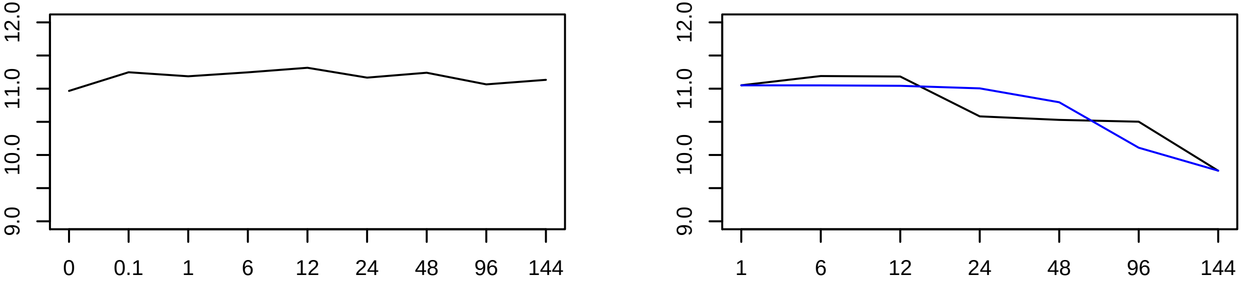

**A_23_P216355 NFKBIL2 8q24.3**

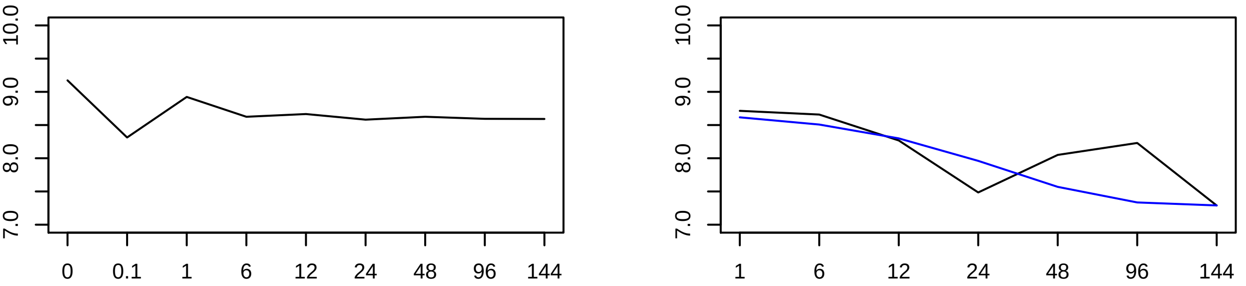


**A_24_P280868 FAM86B1 8p23.1**

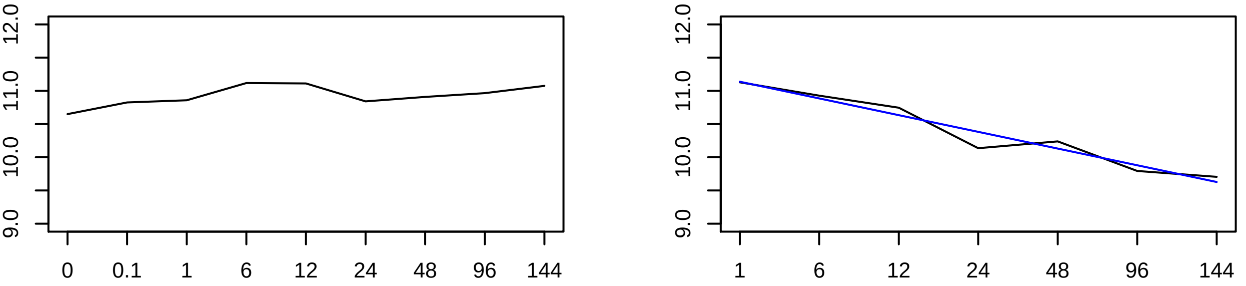


**A_24_P258051 MASTL 10p12.1**

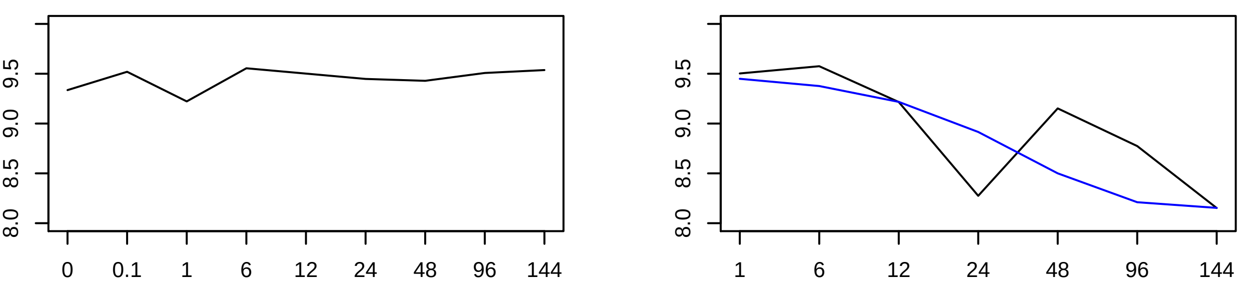

A_32_P147090 ARHGAP11A 15q13.3

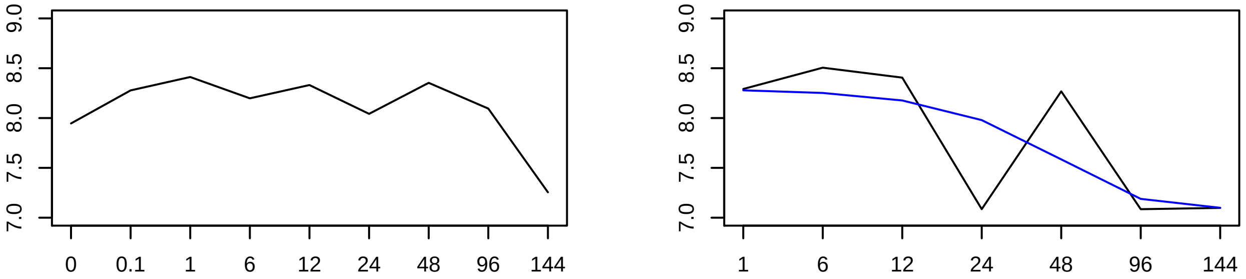


A_24_P158385 ZMYND19 9q34.3

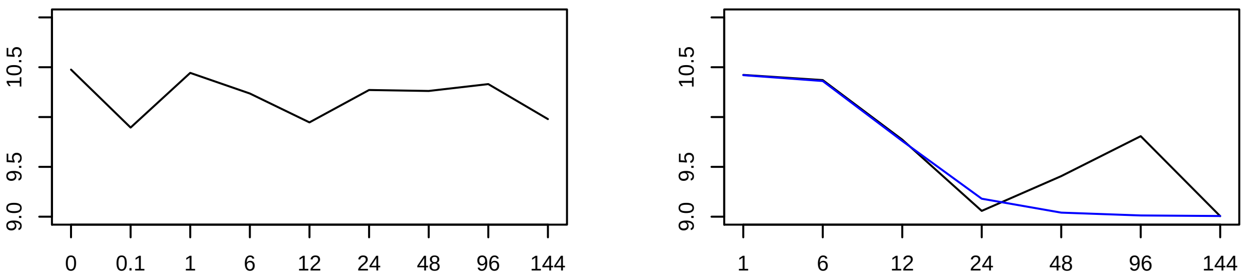


A_24_P218979 CDCA3 12p13.31

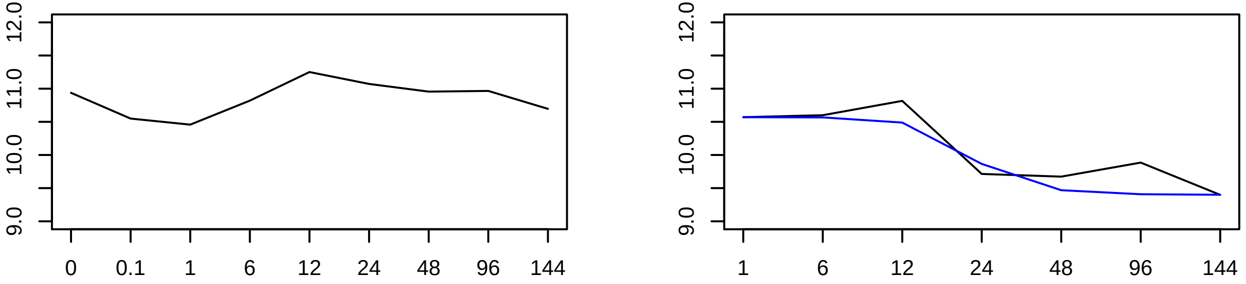

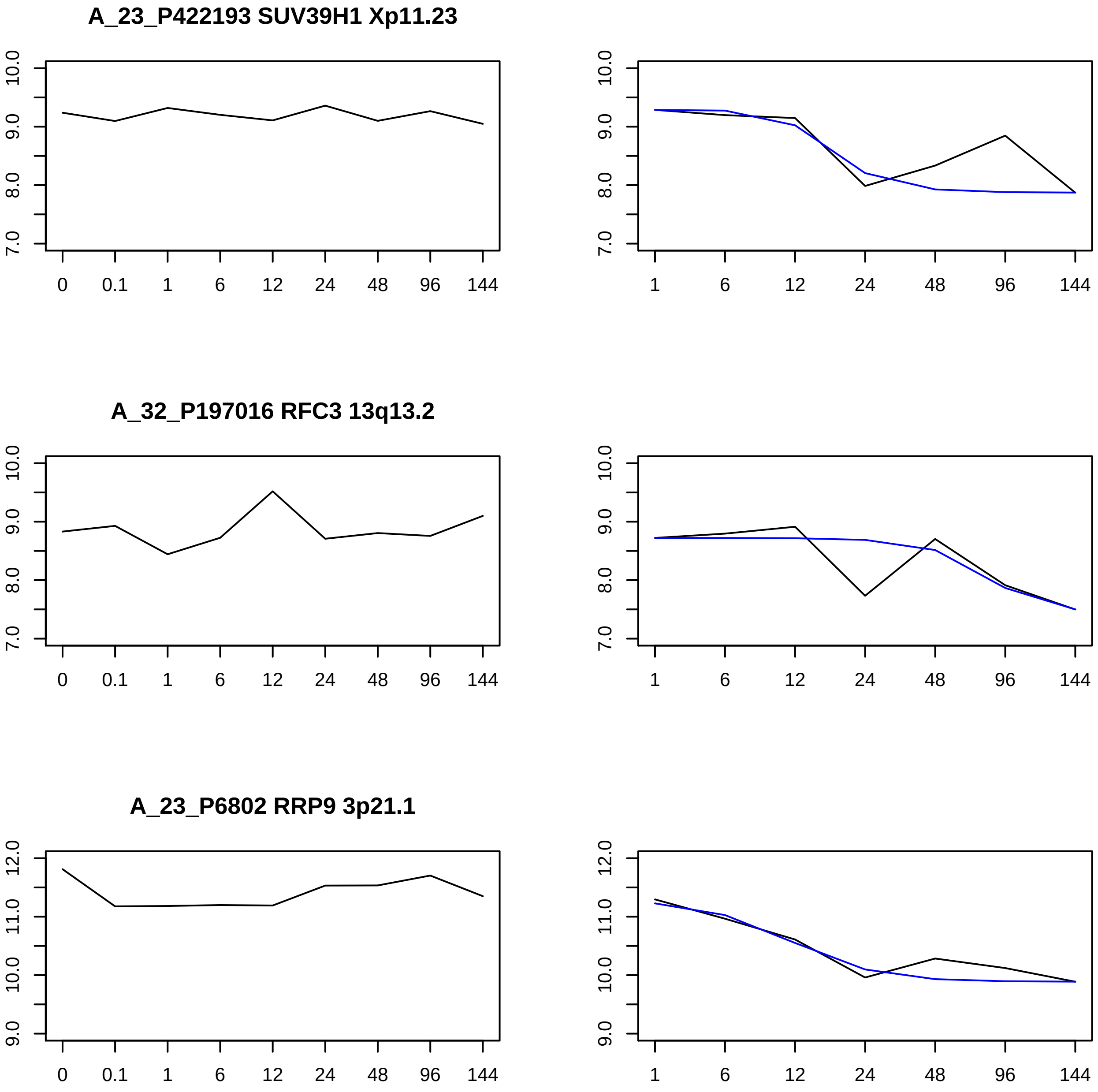
A_23_P422193 SUV39H1 Xp11.23
A_32_P197016 RFC3 13q13.2
A_23_P6802 RRP9 3p21.1

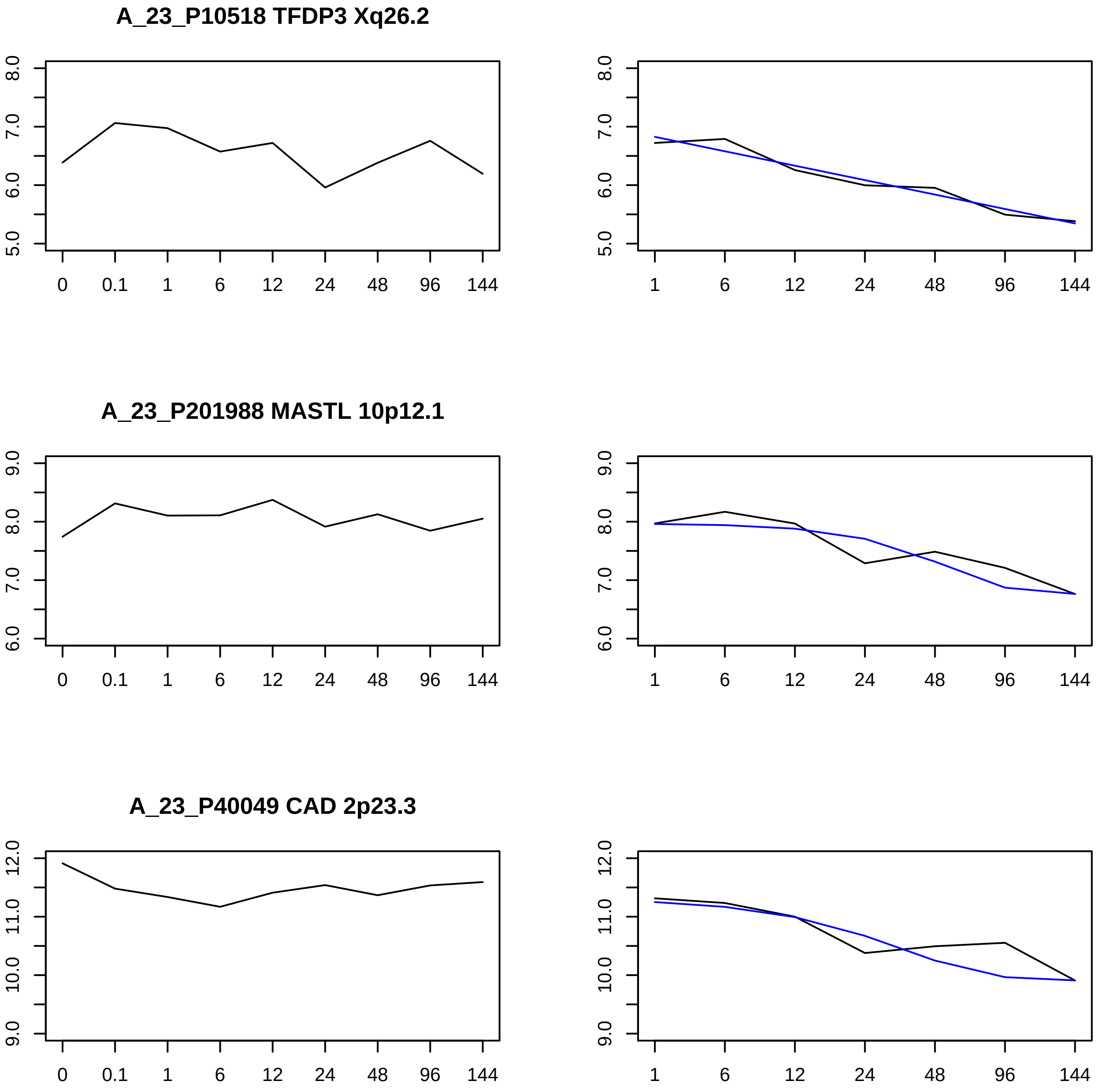
A_23_P10518 TFDP3 Xq26.2
5.0
6.0
7.0
8.0
0
0.1
1
6
12
24
48
96
144
A_23_P201988 MASTL 10p12.1
6.0
7.0
8.0
9.0
A_23_P40049 CAD 2p23.3
9.0
10.0
11.0
12.0

**A_32_P199301 TFDP1 13q34**

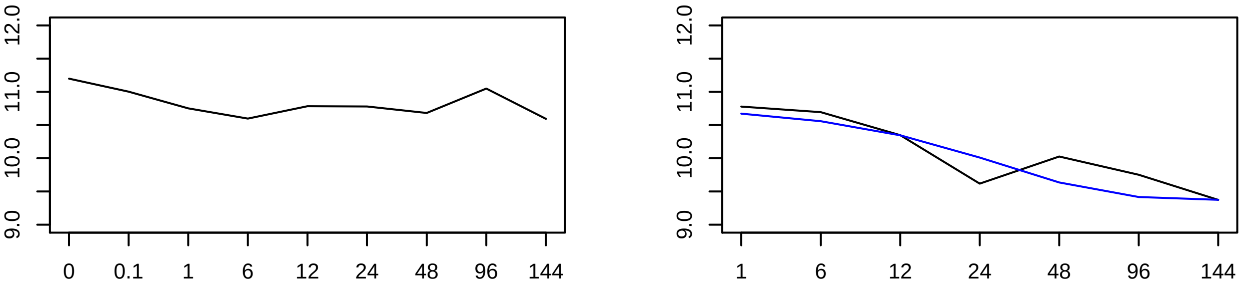


**A_23_P50456 POLD1 19q13.33**

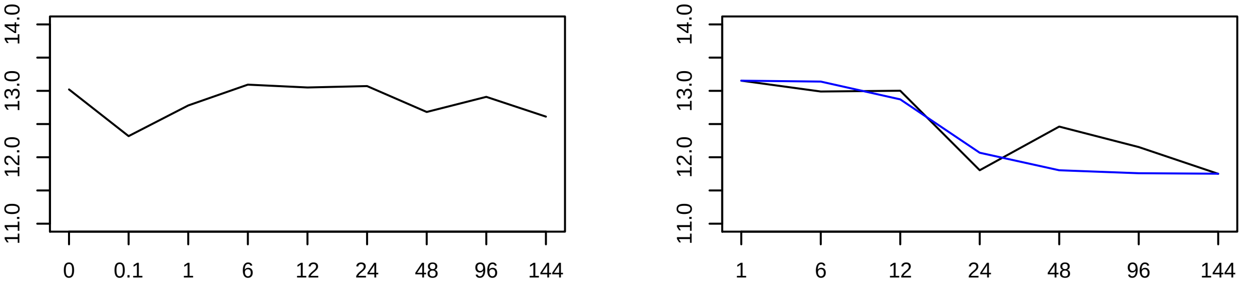


**A_23_P256297 KIAA0179 21q22.3**

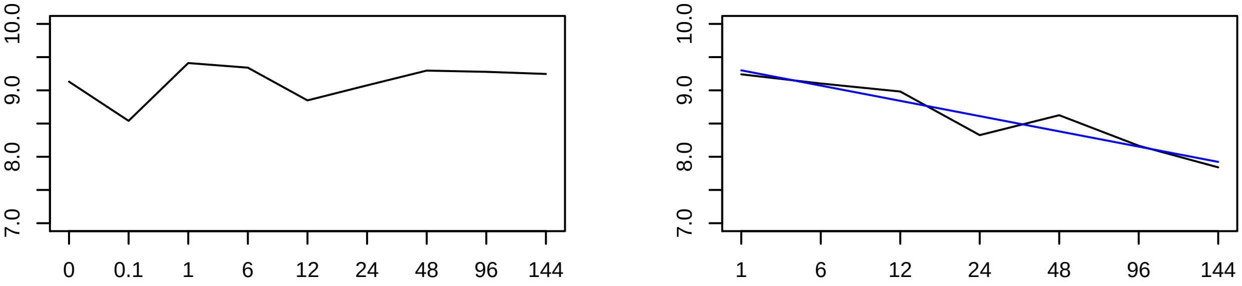

**A_23_P129075 WDR76 15q15.3**

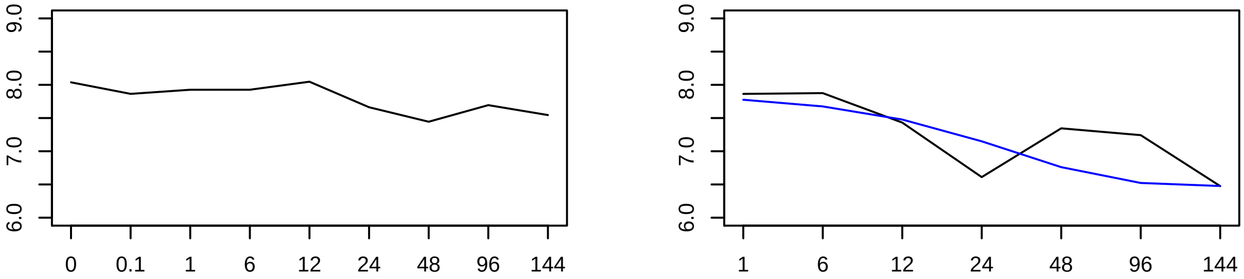


**A_23_P168014 HIST1H2AJ 6p22.1**

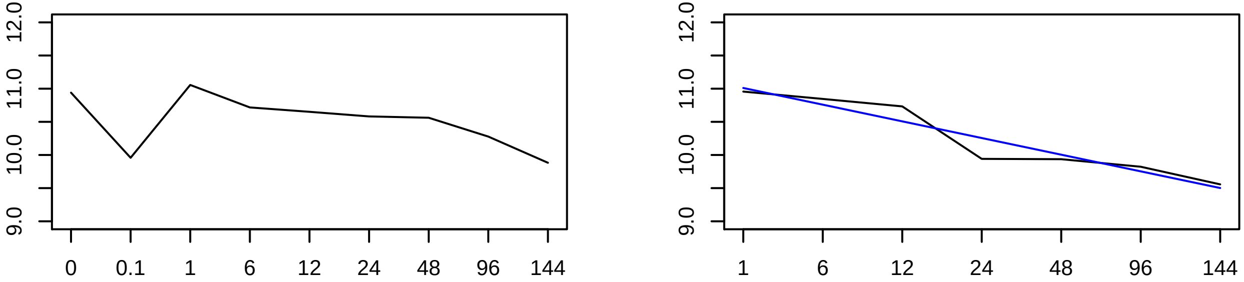


**A_23_P50081 IMPA2 18p11.21**

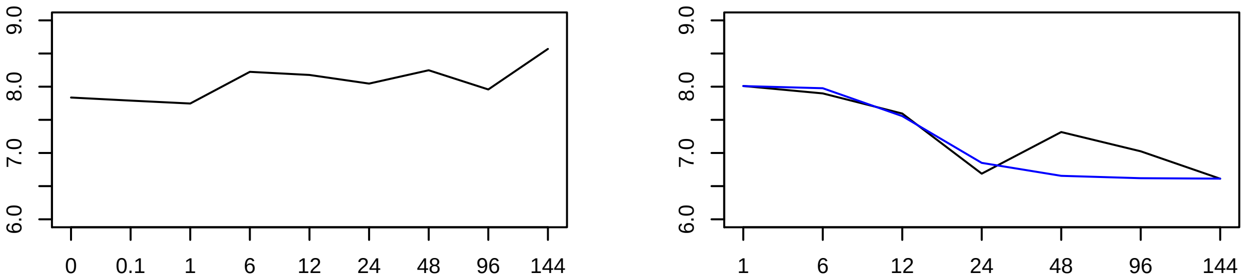

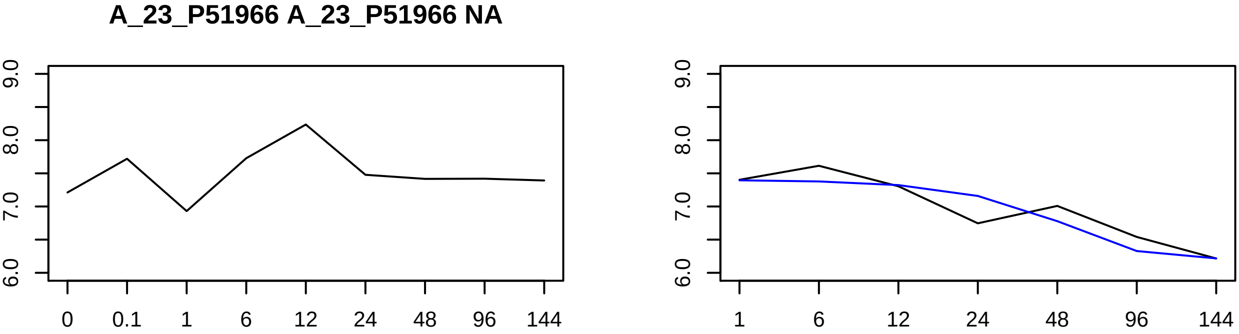
A_23_P51966 A_23_P51966 NA
6.0 7.0 8.0 9.0
0 0.1 1 6 12 24 48 96 144
1 6 12 24 48 96 144

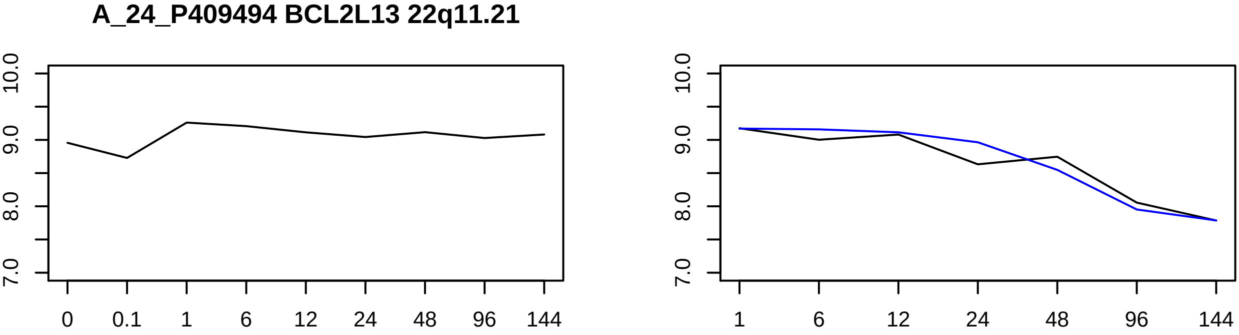
A_24_P409494 BCL2L13 22q11.21
7.0 8.0 9.0 10.0
0 0.1 1 6 12 24 48 96 144
1 6 12 24 48 96 144

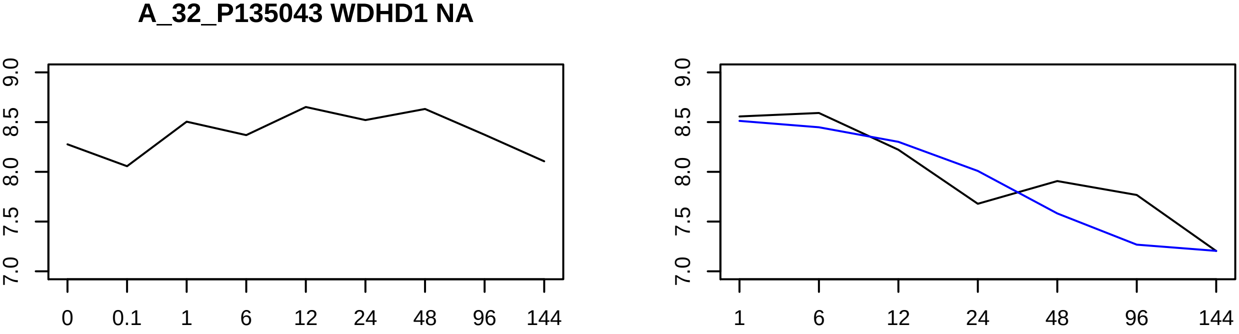
A_32_P135043 WDHD1 NA
7.0 7.5 8.0 8.5 9.0
0 0.1 1 6 12 24 48 96 144
1 6 12 24 48 96 144

**A_23_P162476 CDCA3 12p13.31**

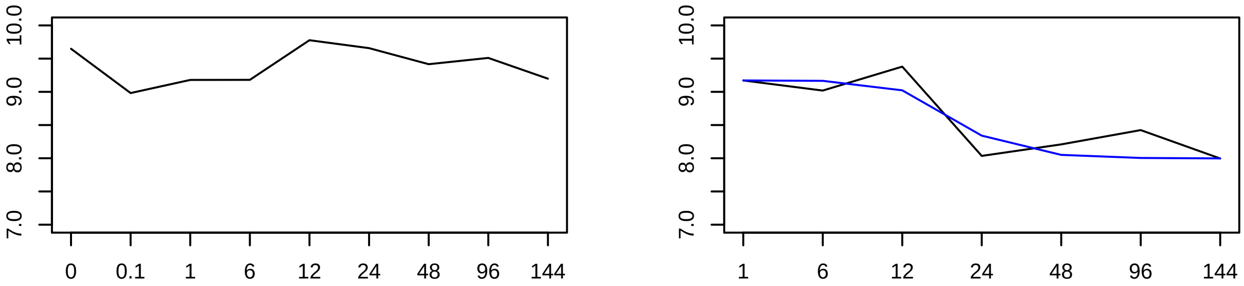


**A_24_P402588 BCL11A 2p16.1**

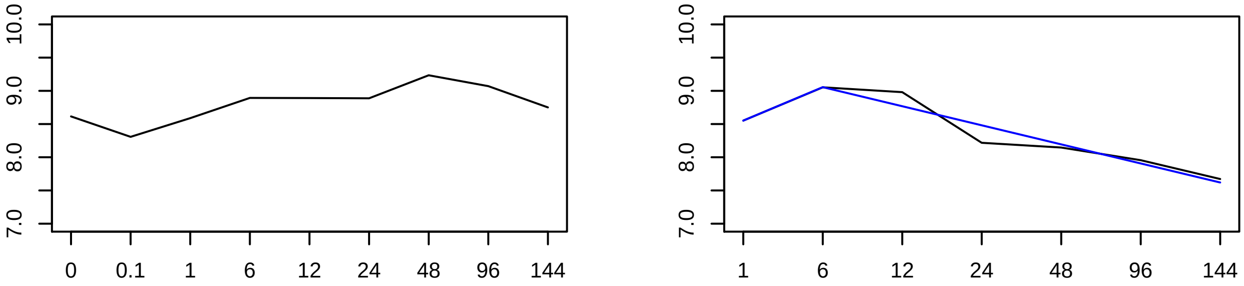


**A_23_P107073 RPA1 17p13.3**

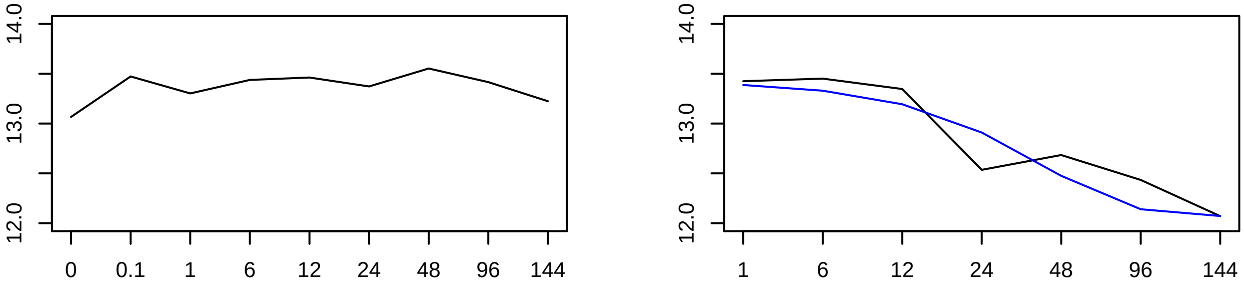

**A_23_P433690 ZNF331 19q13.41**

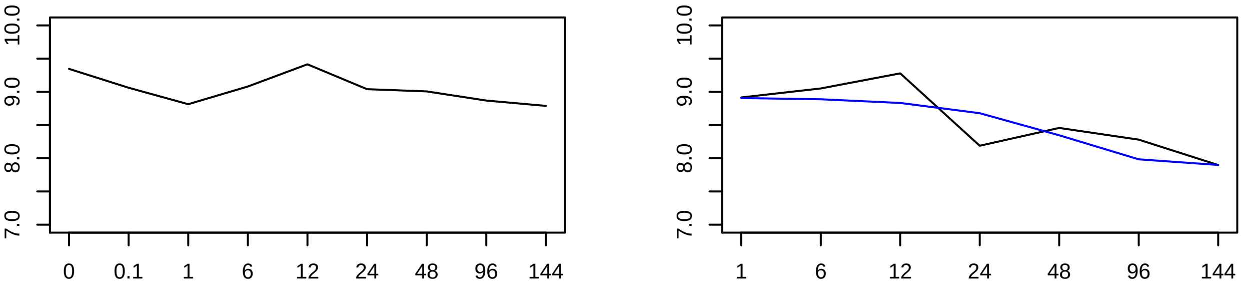


**A_23_P65041 RACGAP1P 12q12**

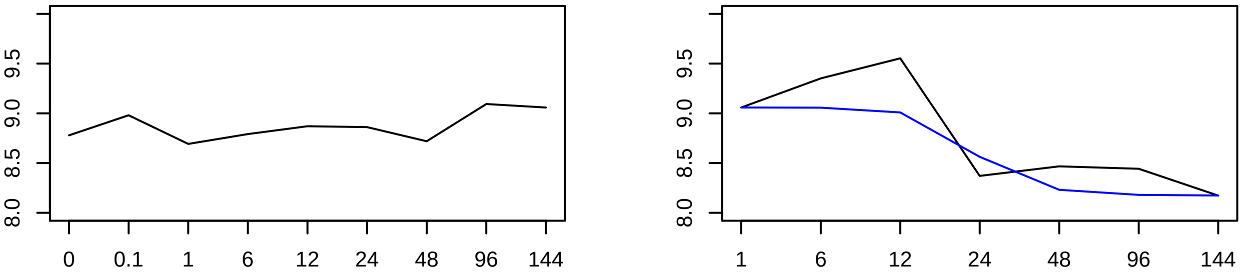


**A_23_P159390 TOPBP1 3q22.1**

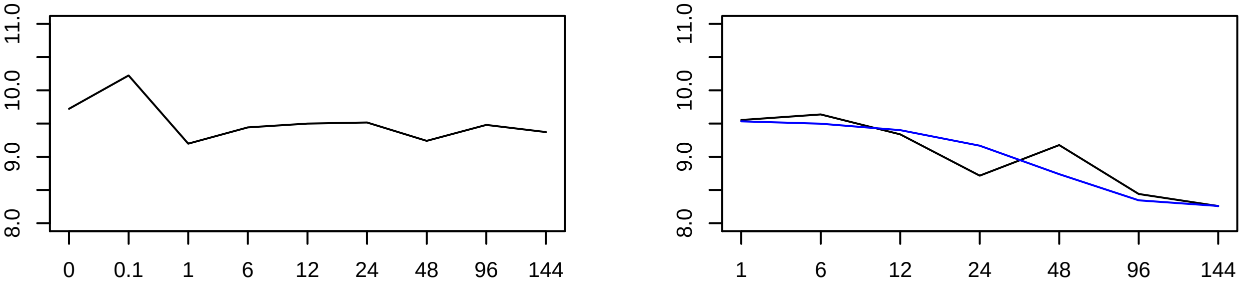

**A_23_P214907 MTHFD1L 6q25.1**

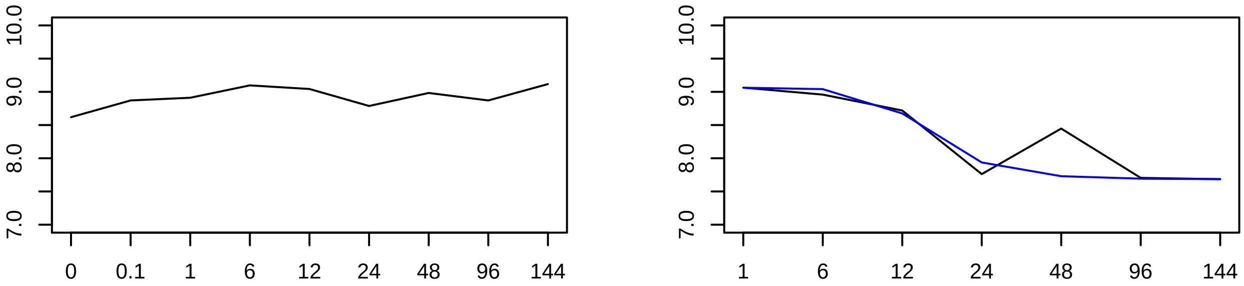


**A_23_P9894 PRMT3 11p15.1**

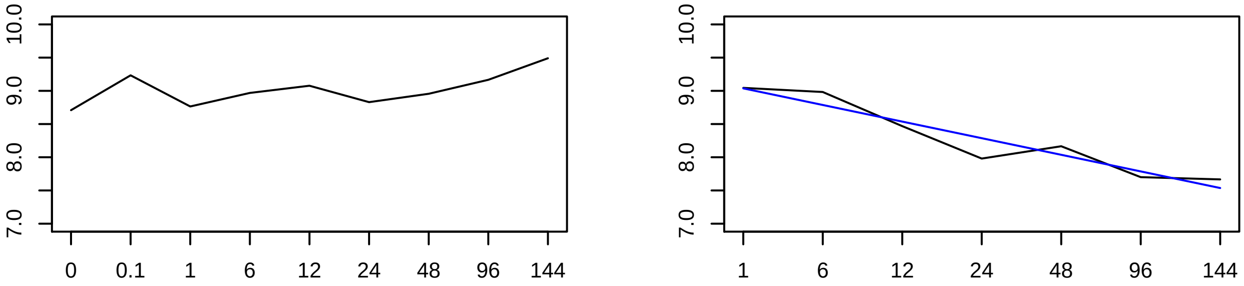


**A_32_P30004 AF086044 NA**

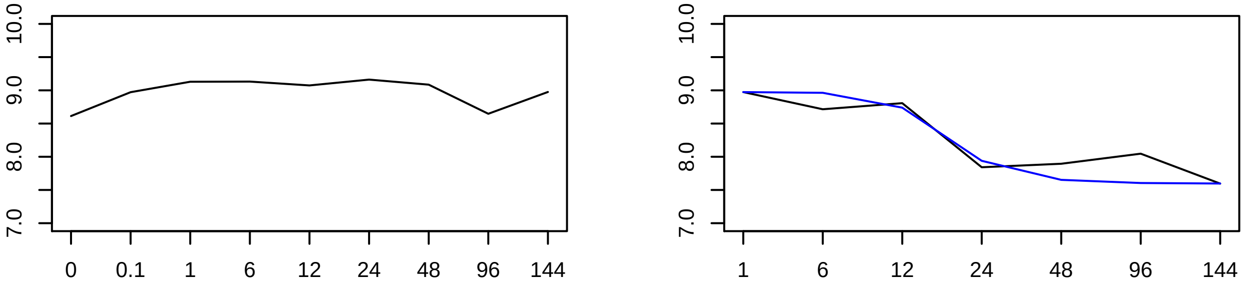

**A_23_P66872 GEMIN4 17p13.3**

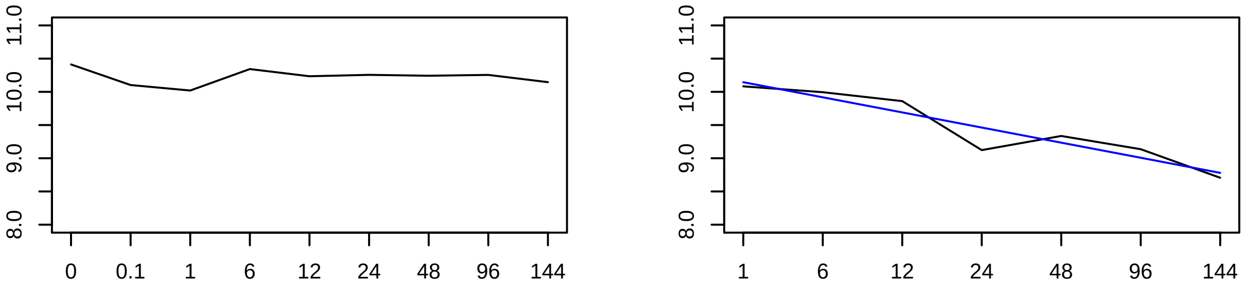


**A_23_P420551 CIT 12q24.23**

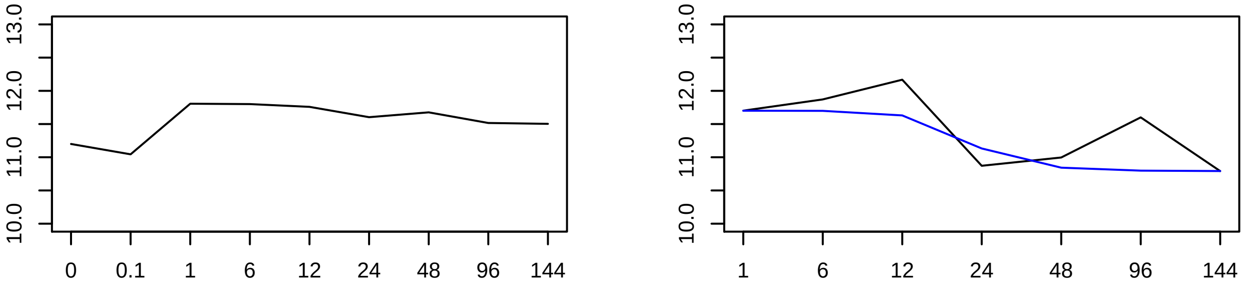


**A_32_P24165 FANCD2 3p25.3**

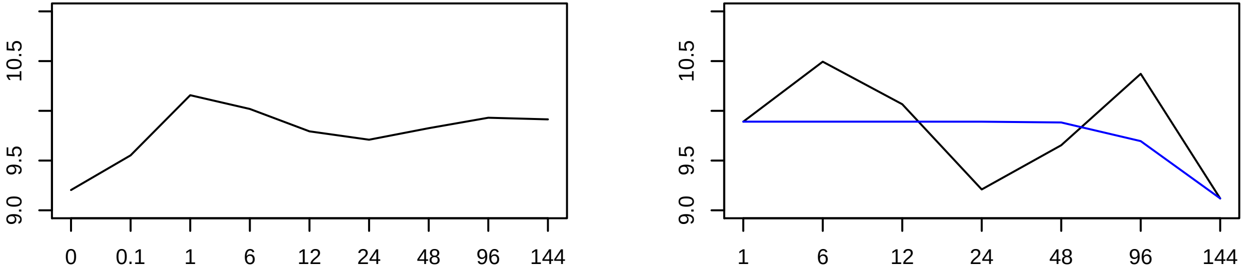

**A_23_P109452 CHEK2 22q12.1**

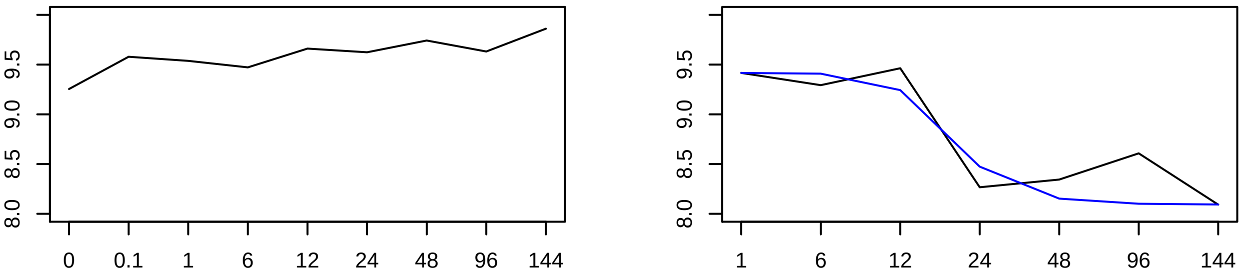


**A_23_P71644 FANCG 9p13.3**

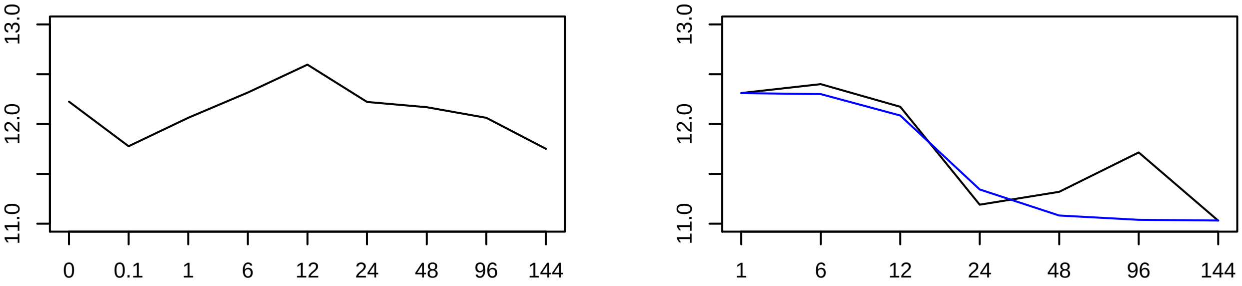


**A_23_P45970 C1orf109 1p34.3**

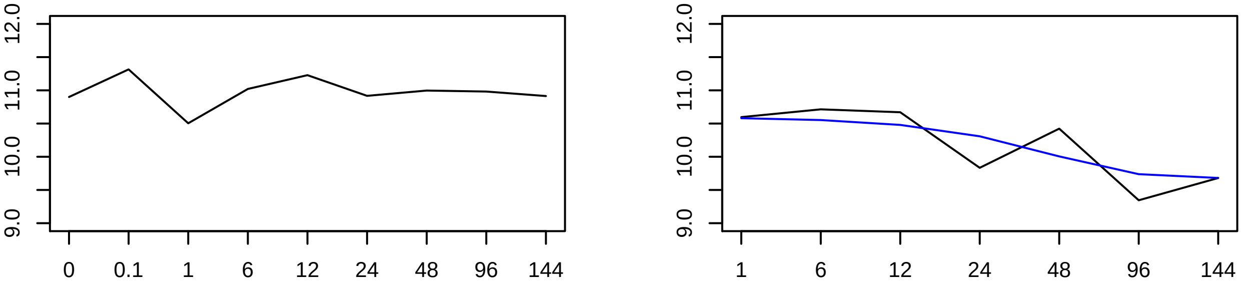

**A_23_P375 CDCA8 1p34.3**

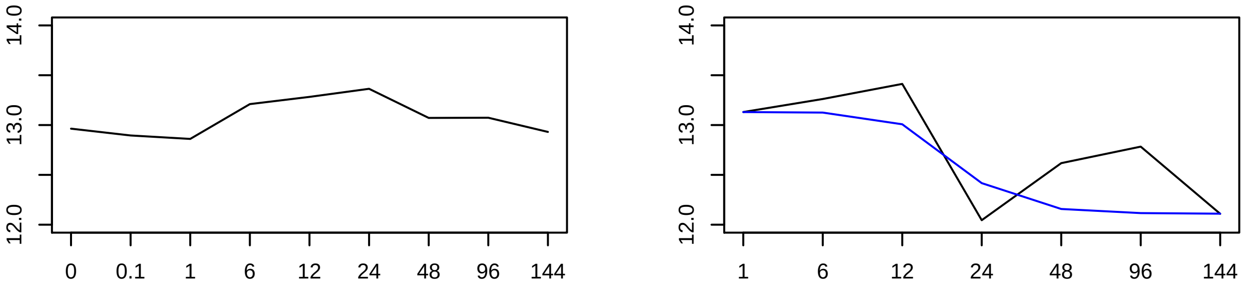


**A_32_P179746 THC2676284 NA**

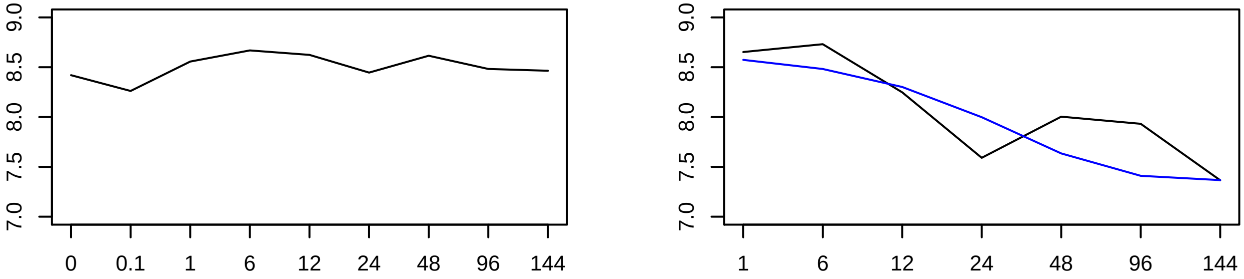


**A_32_P192823 PRPS1L1 7p21.1**

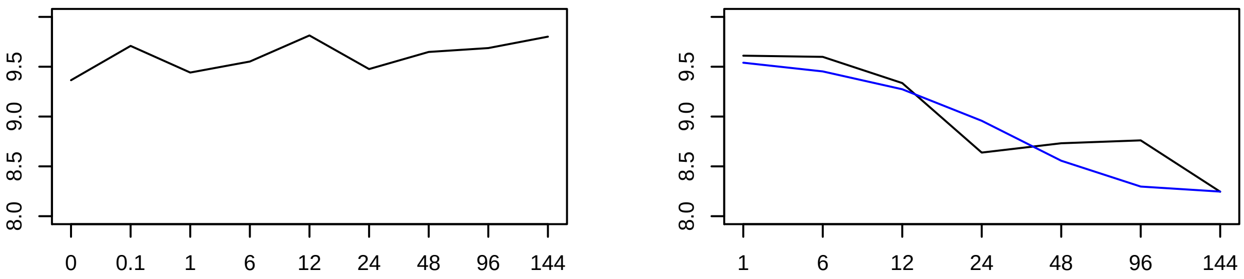

**A_23_P430201 CEP128 14q31.1**

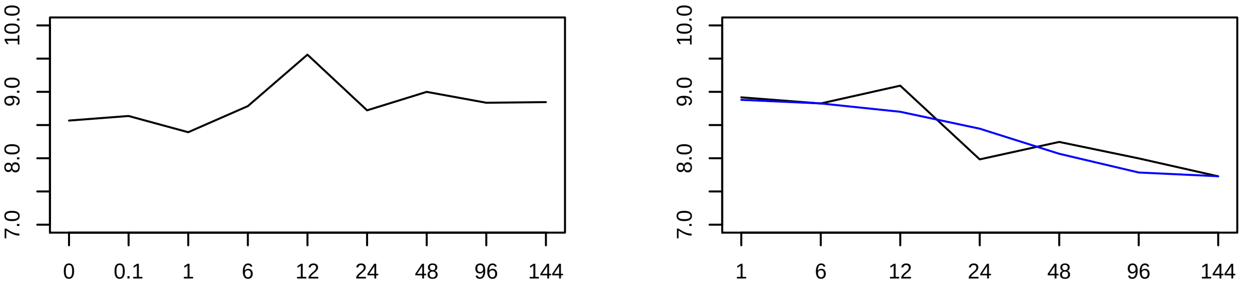


**A_23_P205449 CDCA4 14q32.33**

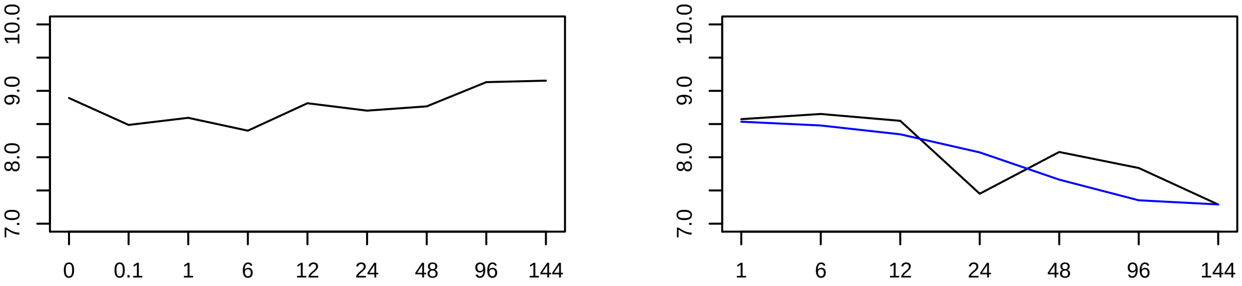


**A_23_P201357 ATAD3A 1p36.33**

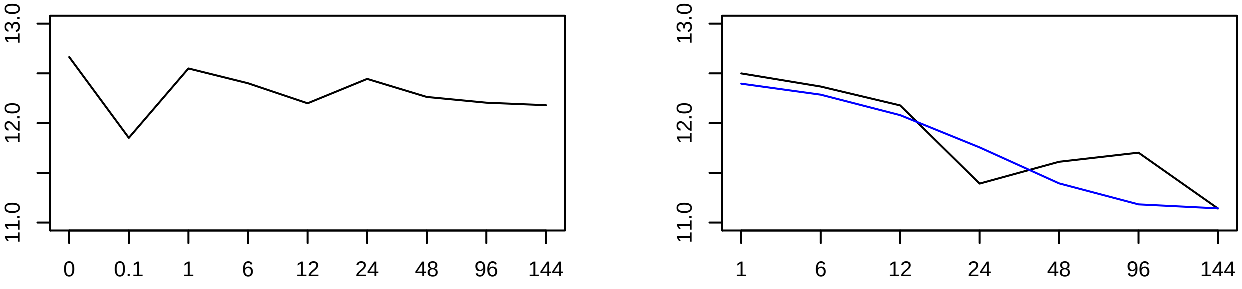

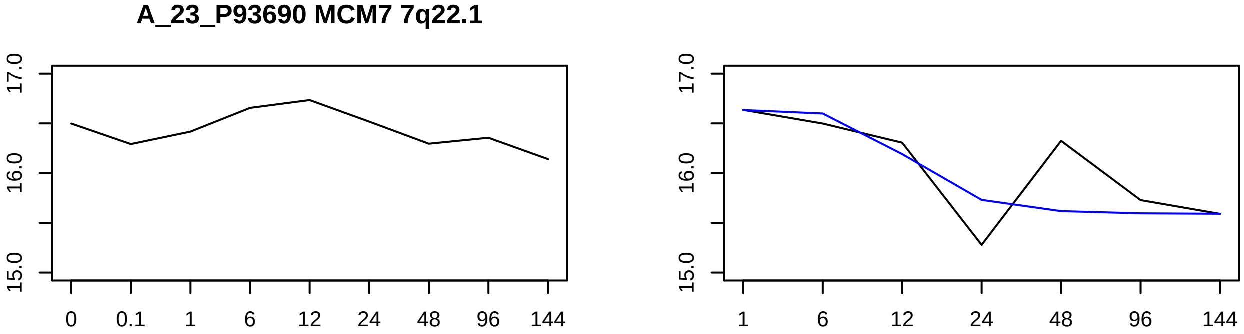
A_23_P93690 MCM7 7q22.1
15.0
16.0
17.0
0
0.1
1
6
12
24
48
96
144
1
6
12
24
48
96
144

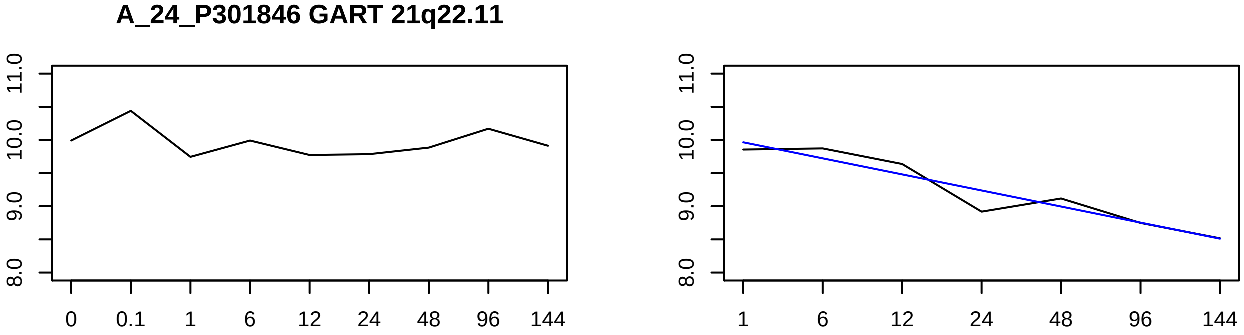
A_24_P301846 GART 21q22.11
8.0
9.0
10.0
11.0
0
0.1
1
6
12
24
48
96
144
1
6
12
24
48
96
144

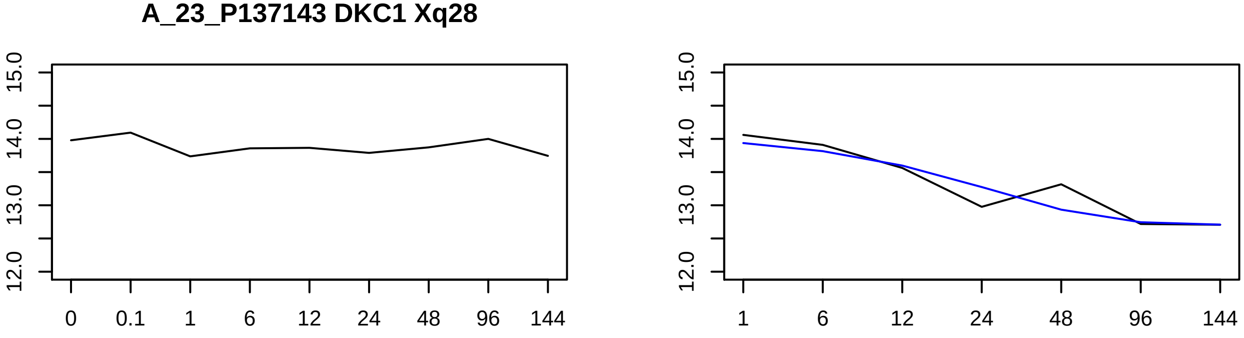
A_23_P137143 DKC1 Xq28
12.0
13.0
14.0
15.0
0
0.1
1
6
12
24
48
96
144
1
6
12
24
48
96
144

**A_24_P123347 PPAT 4q12**

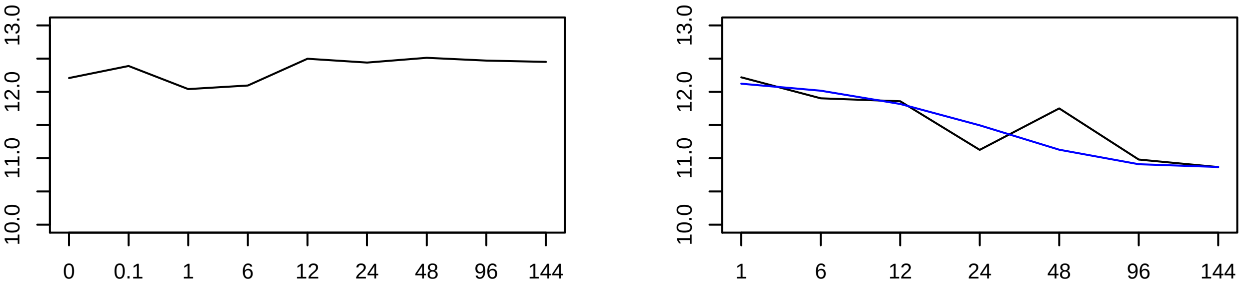


**A_23_P15844 BRIP1 17q23.2**

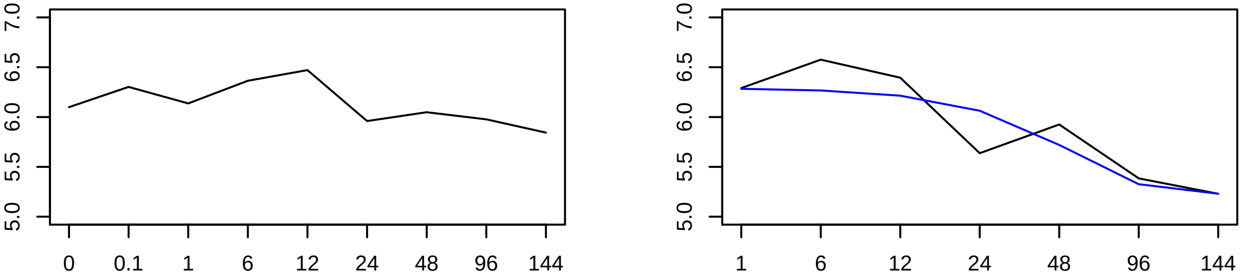


**A_23_P80902 KIF15 3p21.31**

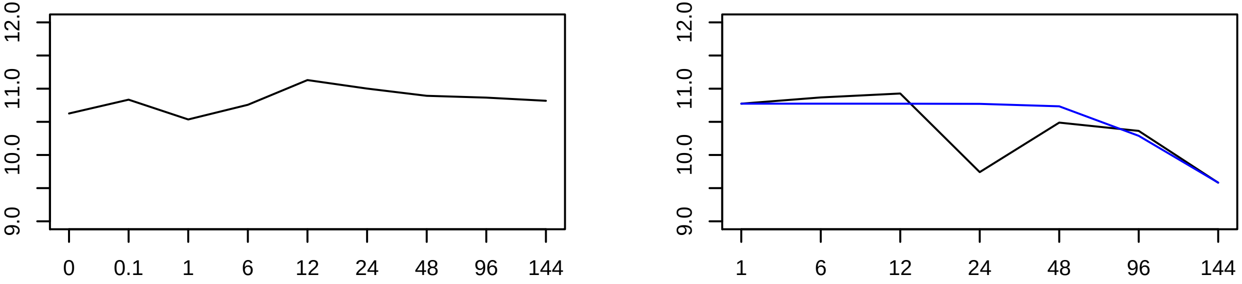

**A_23_P89755 RNF138 18q12.1**

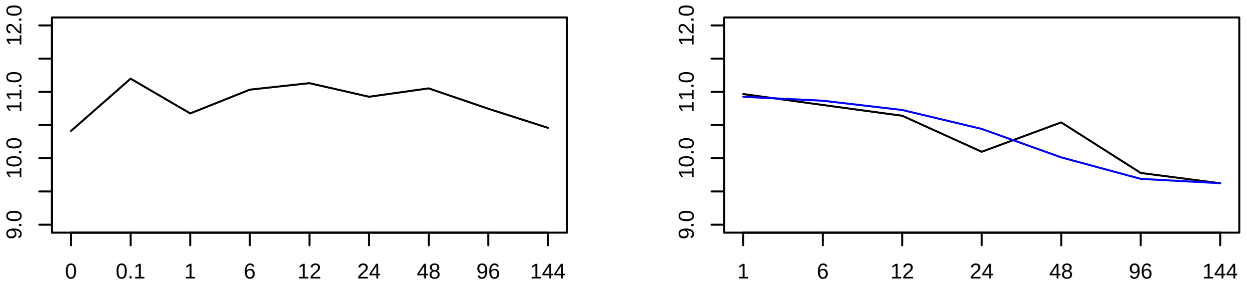


**A_32_P135243 MTHFD1L 6q25.1**

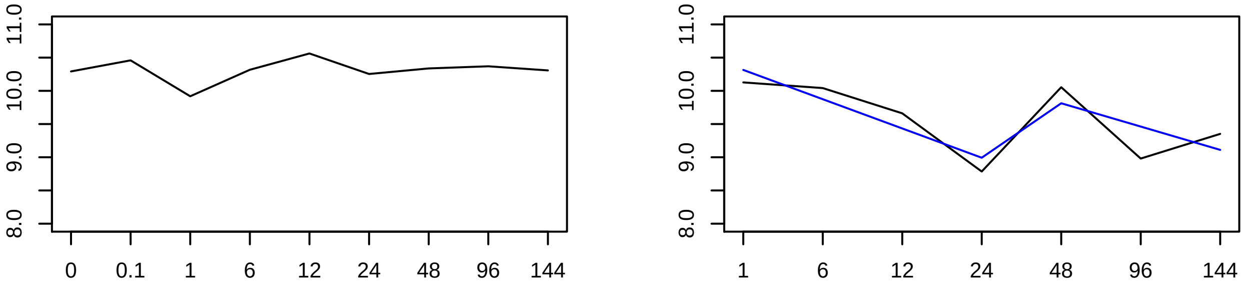


**A_23_P410625 ZNF367 9q22.32**

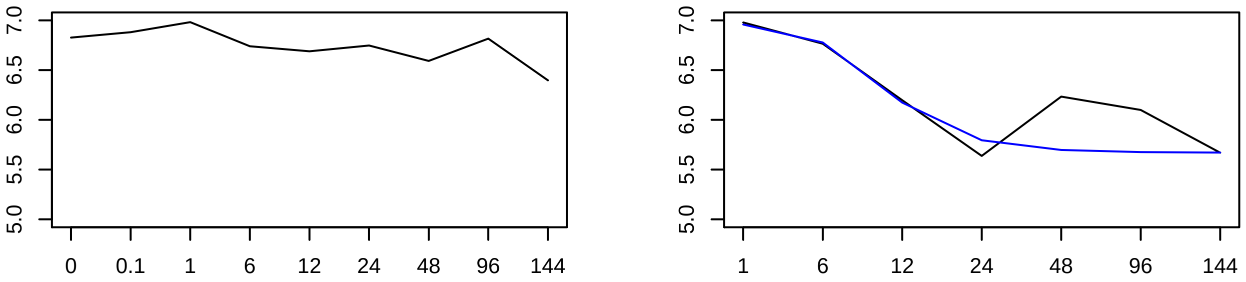

**A_23_P89509 SPAG5 17q11.2**

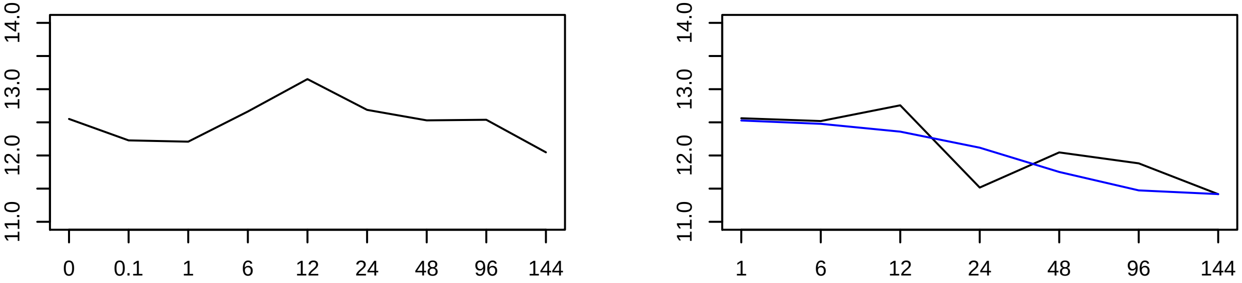


**A_32_P127153 SORD 15q21.1**

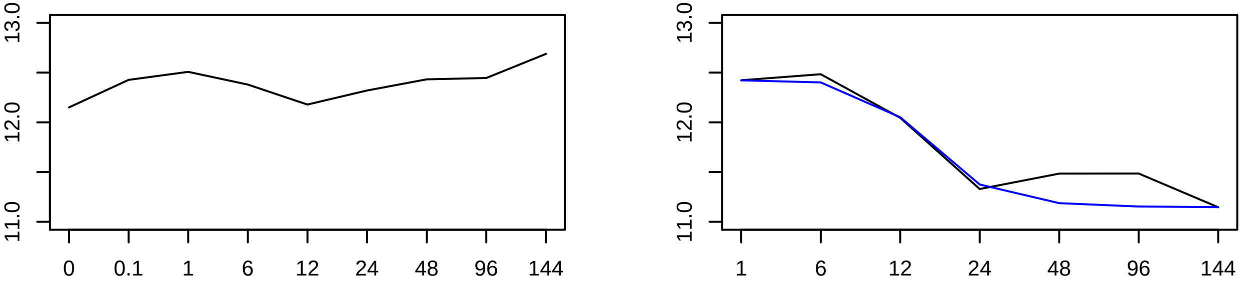


**A_23_P28733 RBL1 20q11.23**

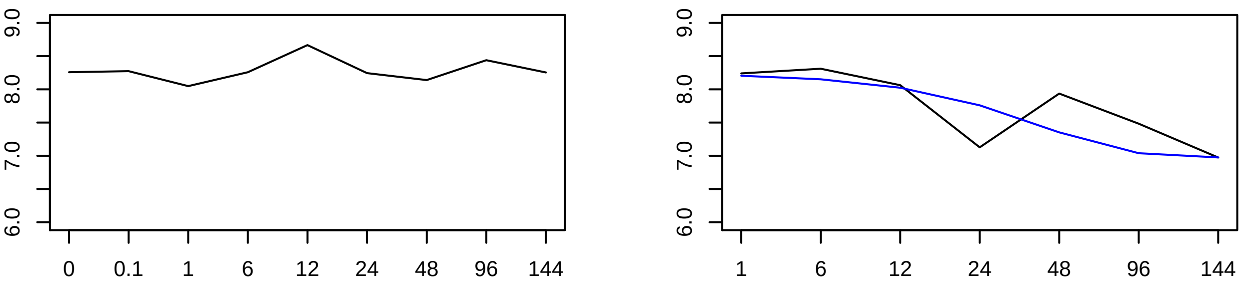

**A_23_P354208 WDR81 17p13.3**

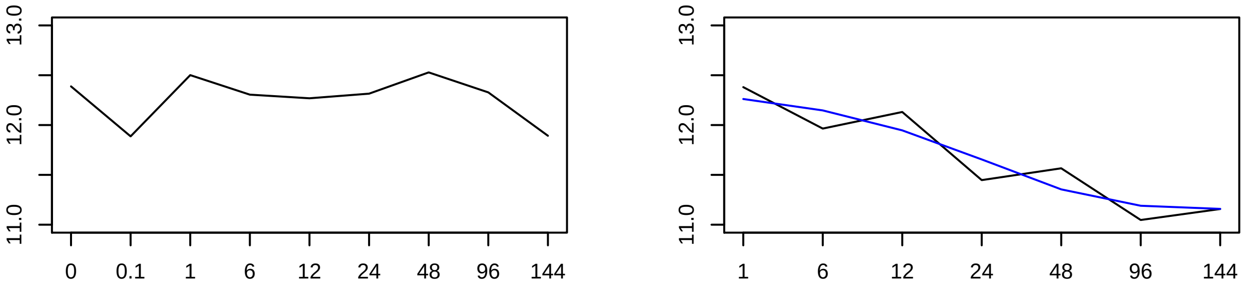


**A_23_P154675 SNRPB 20p13**

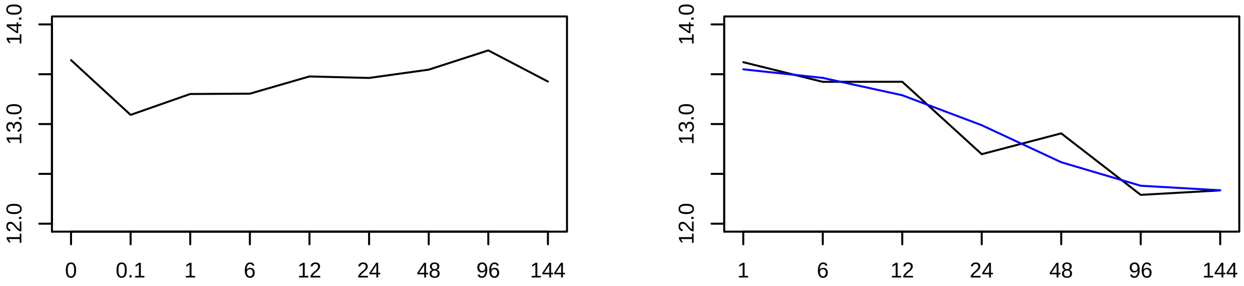


**A_23_P152284 C16orf33 16p13.3**

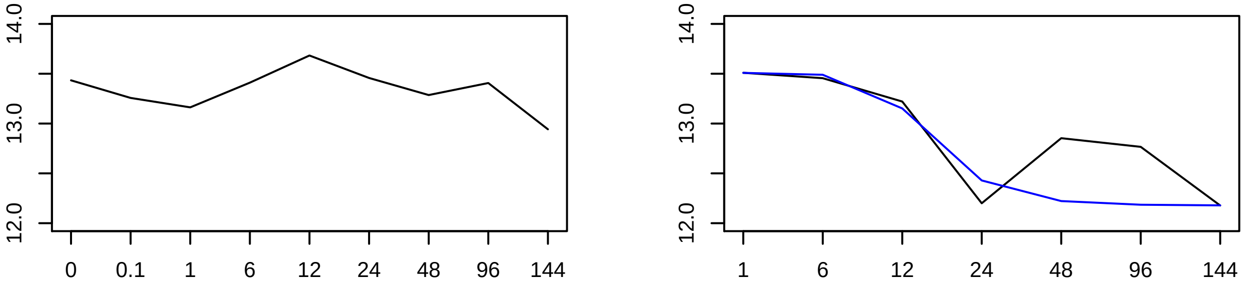

**A_23_P118174 PLK1 16p12.1**

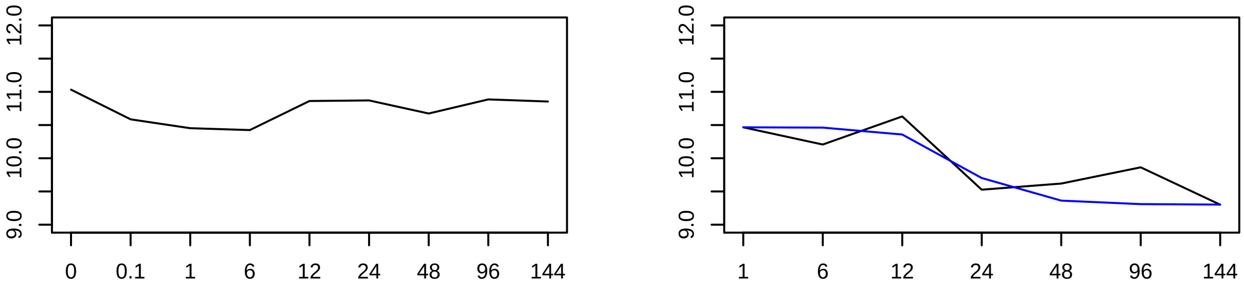


**A_23_P92441 MAD2L1 4q27**

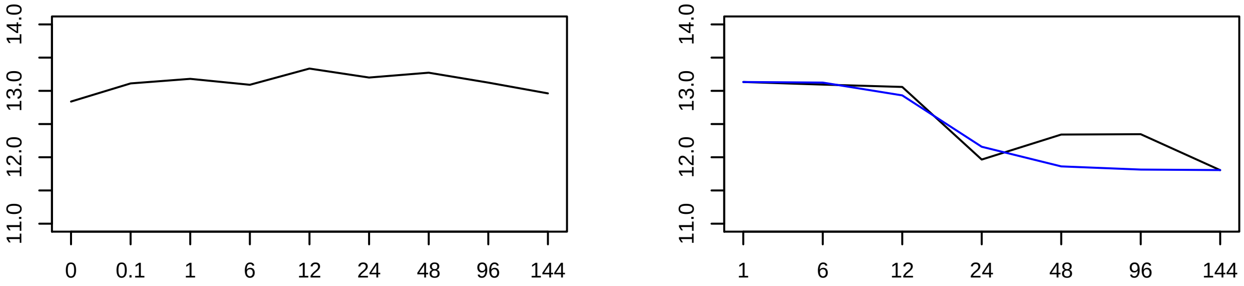


**A_23_P93258 HIST1H3B 6p22.1**

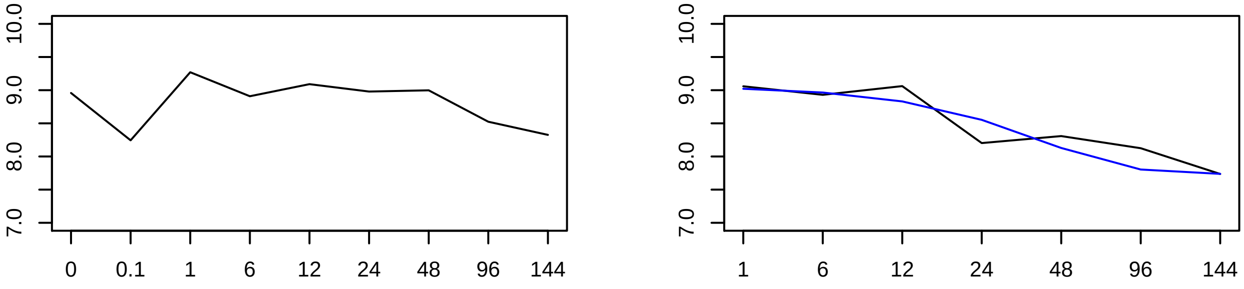

A_23_P254612 DBF4 7q21.12

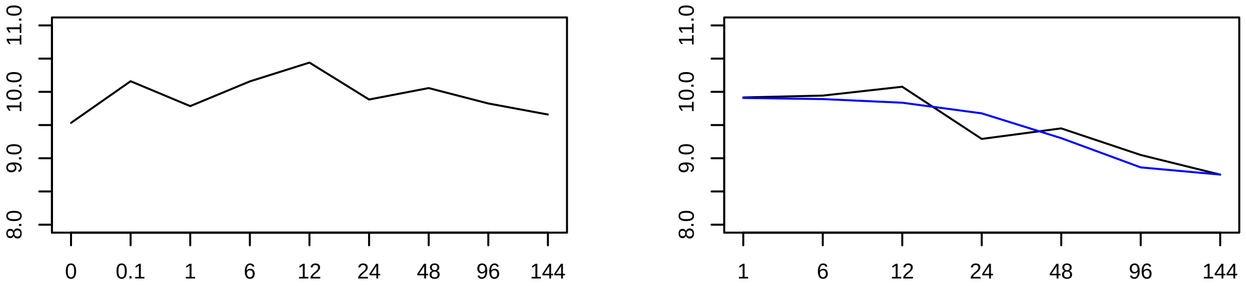


A_24_P174563 GRPEL1 4p16.1

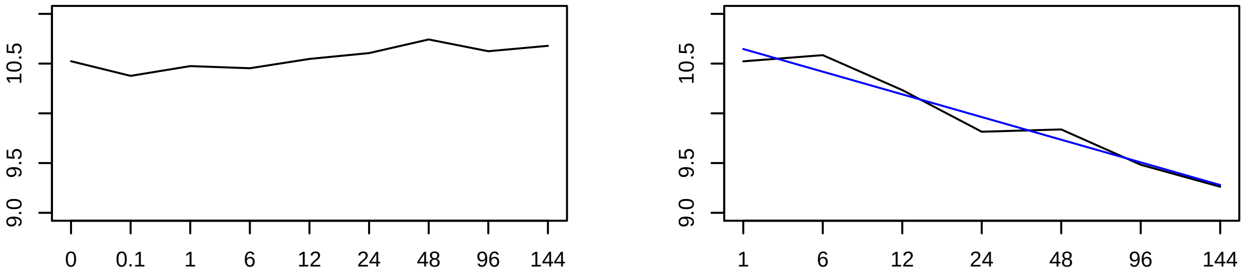


A_32_P69536 AC019129.2 2p21

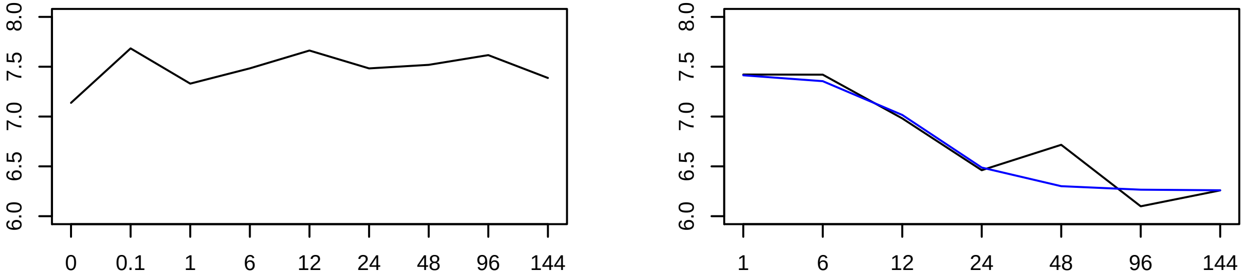

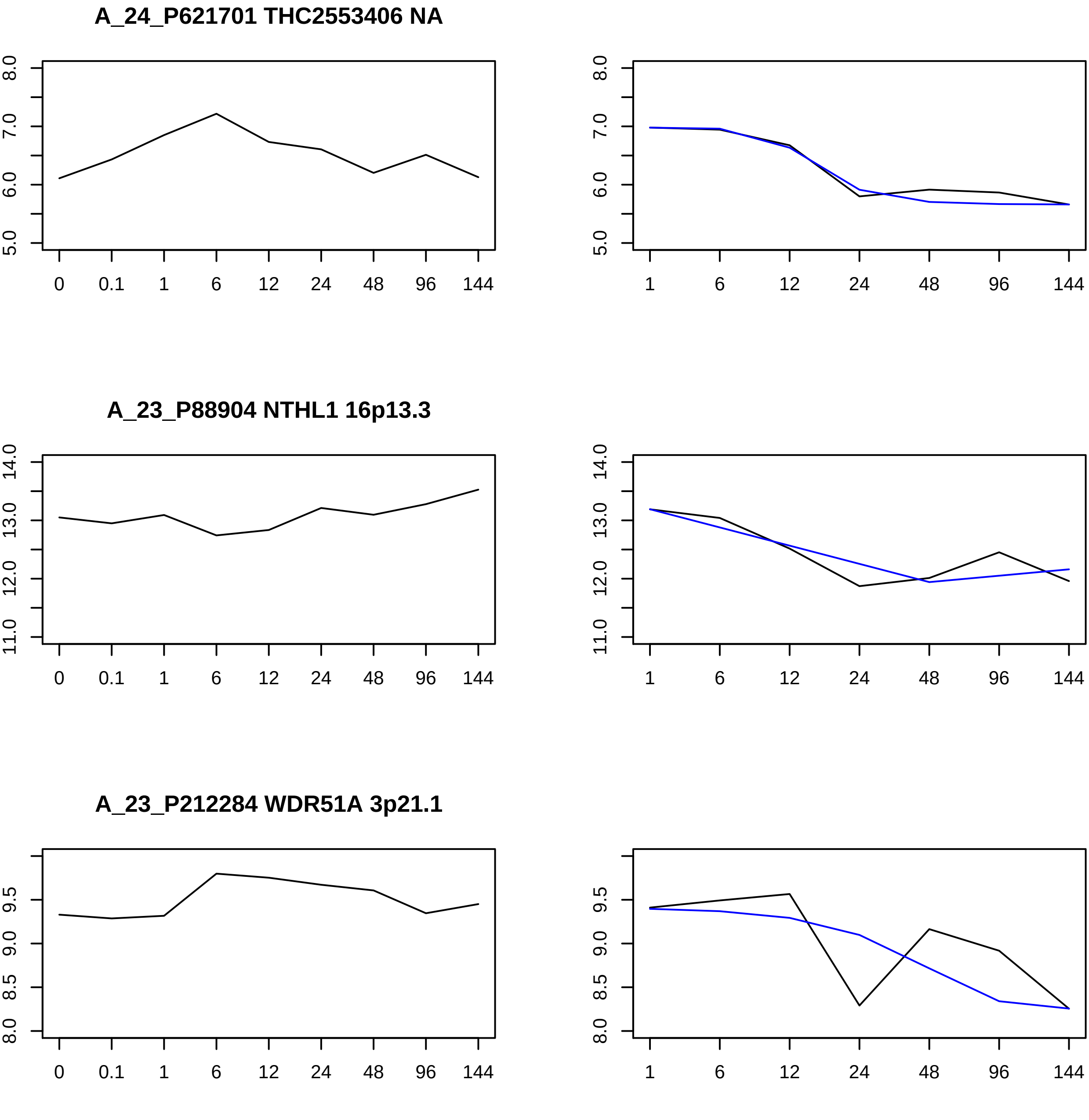
A_24_P621701 THC2553406 NA
5.0
6.0
7.0
8.0
0
0.1
1
6
12
24
48
96
144
A_23_P88904 NTHL1 16p13.3
11.0
12.0
13.0
14.0
A_23_P212284 WDR51A 3p21.1
8.0
8.5
9.0
9.5

**A_24_P123658 RPL7L1 6p21.1**

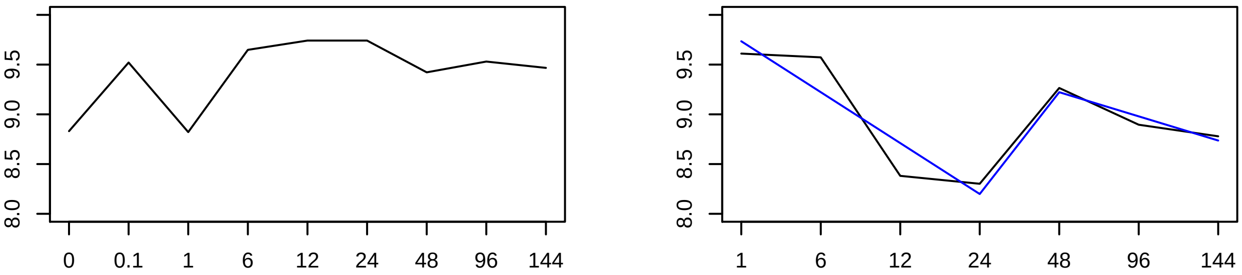


**A_23_P93321 TCF19 6p21.33**

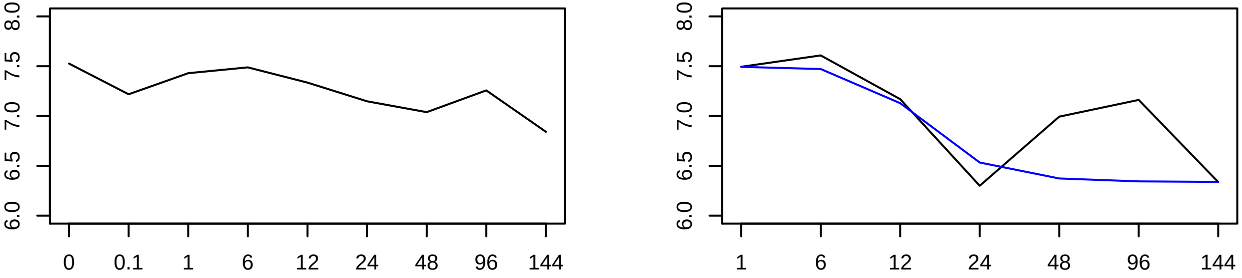


**A_23_P36305 ATG16L2 11q13.4**

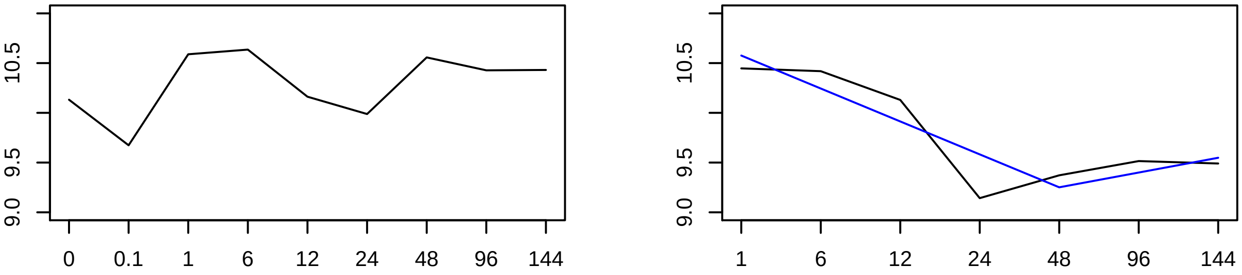

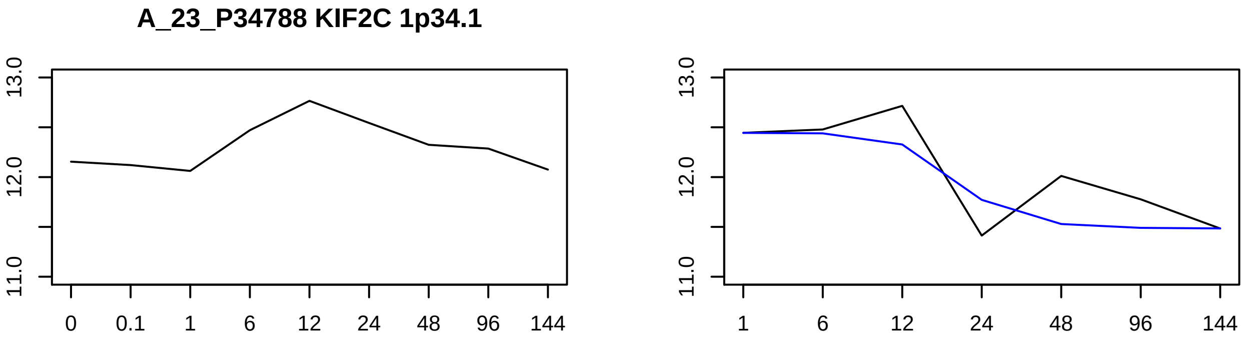
A_23_P34788 KIF2C 1p34.1
11.0
12.0
13.0
0
0.1
1
6
12
24
48
96
144
1
6
12
24
48
96
144

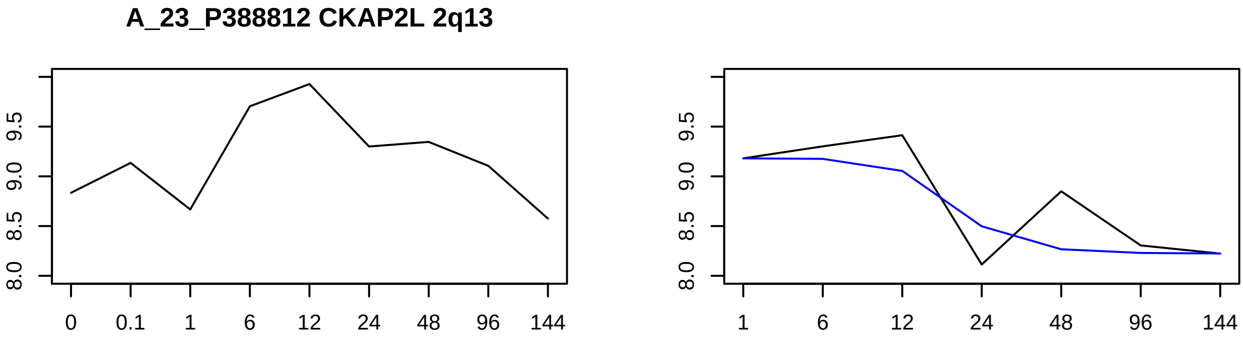
A_23_P388812 CKAP2L 2q13
8.0
8.5
9.0
9.5
0
0.1
1
6
12
24
48
96
144
1
6
12
24
48
96
144

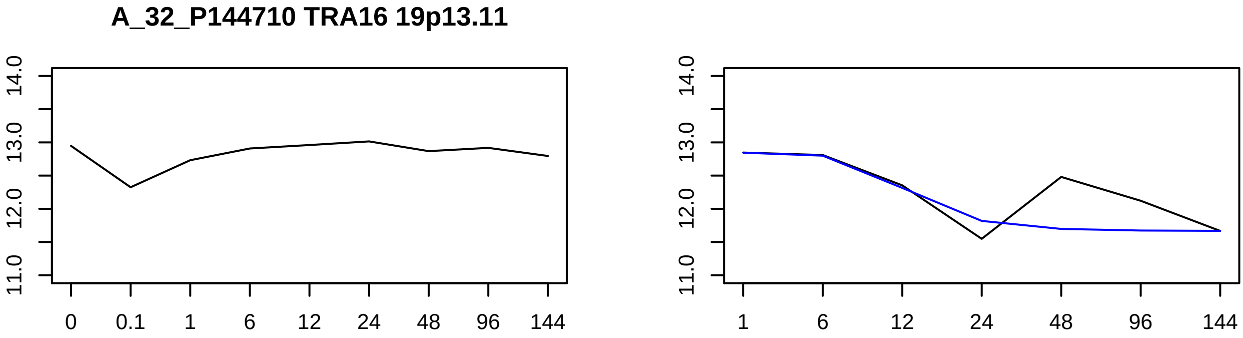
A_32_P144710 TRA16 19p13.11
11.0
12.0
13.0
14.0
0
0.1
1
6
12
24
48
96
144
1
6
12
24
48
96
144

**A_32_P44274 CHTF18 16p13.3**

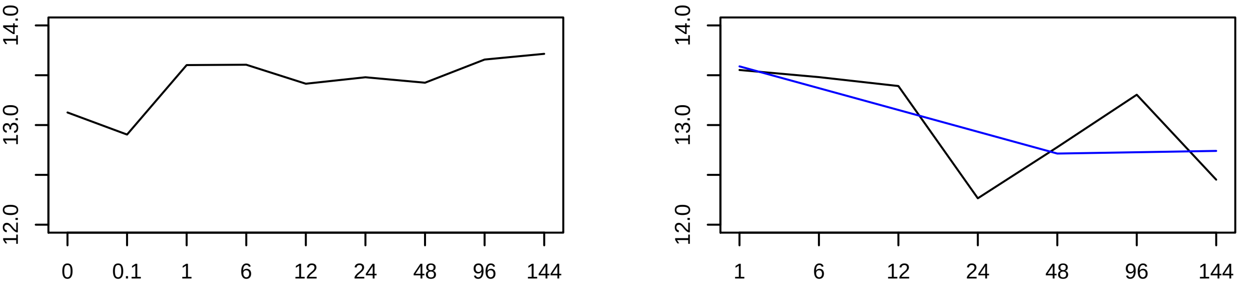


**A_23_P146347 FAM29A 9p22.1**

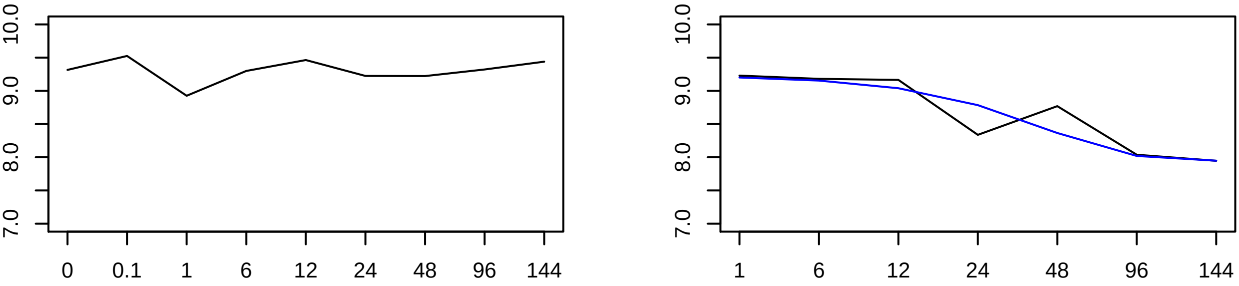


**A_24_P202512 RNF138 18q12.1**

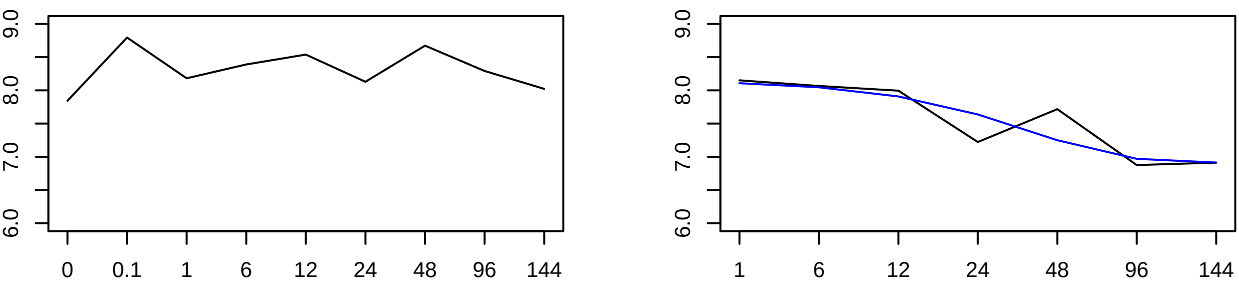

A_23_P205393 C14orf130 14q32.12

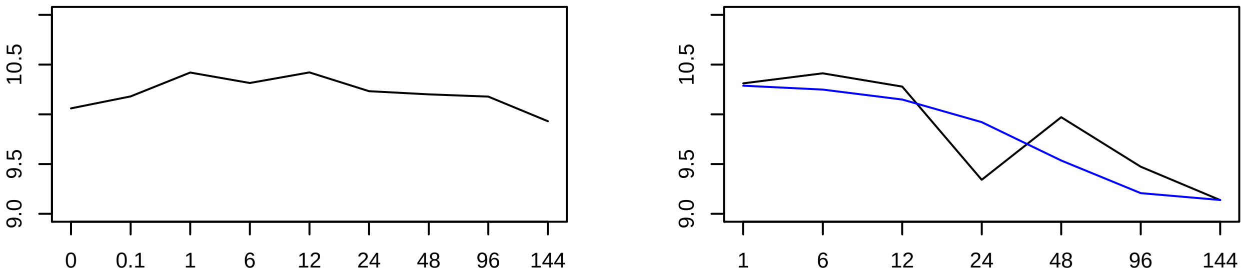


A_23_P74349 NUF2 1q23.3

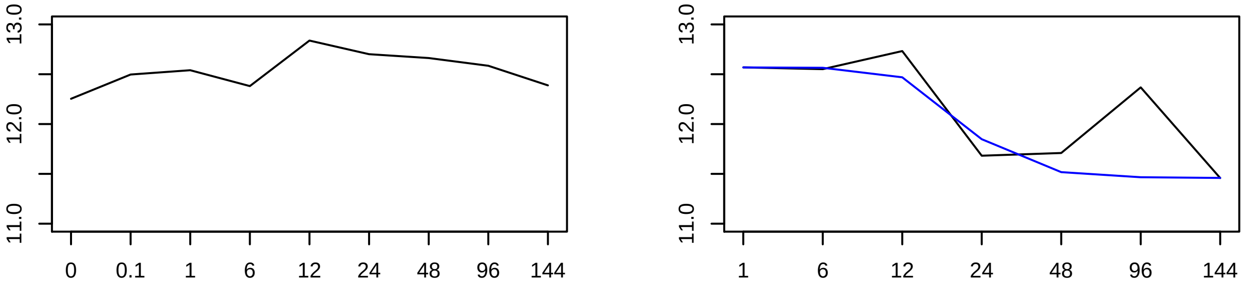


A_24_P162485 ANK1 8p11.21

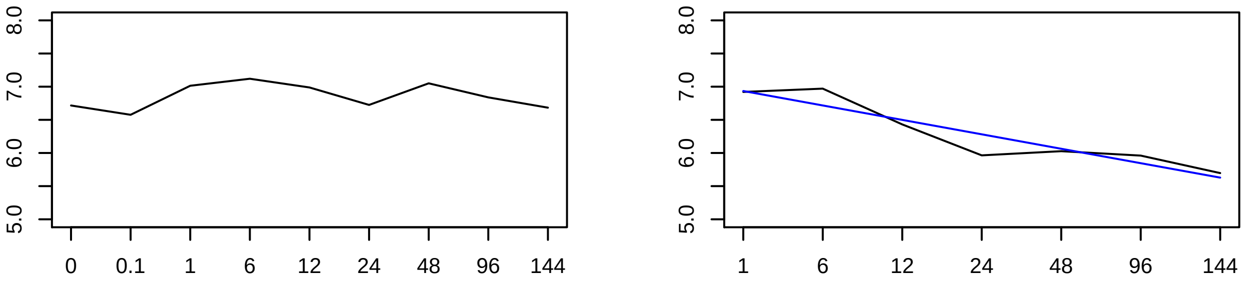

A_23_P143190 MYBL2 20q13.12

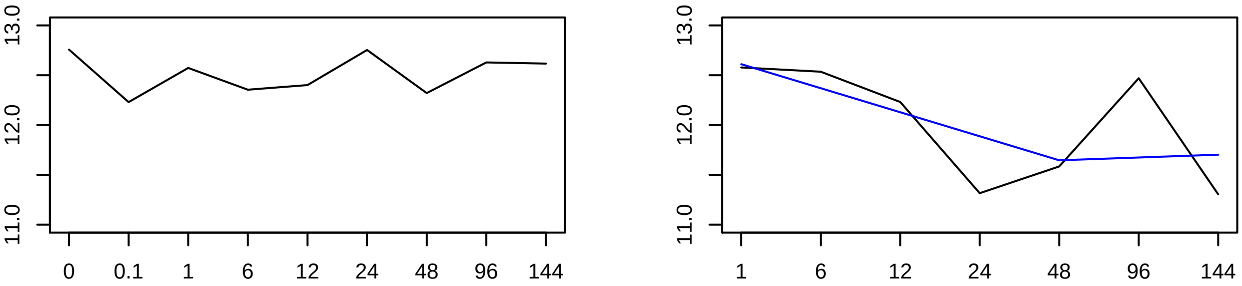


A_23_P58321 CCNA2 4q27

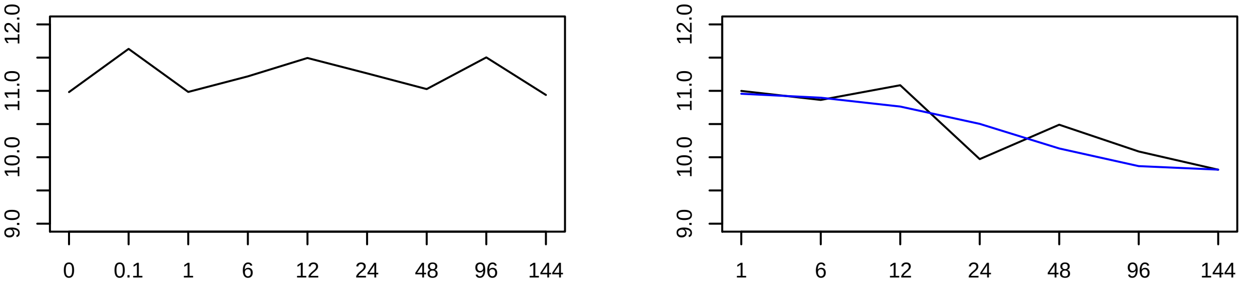


A_24_P133488 CDCA4 14q32.33

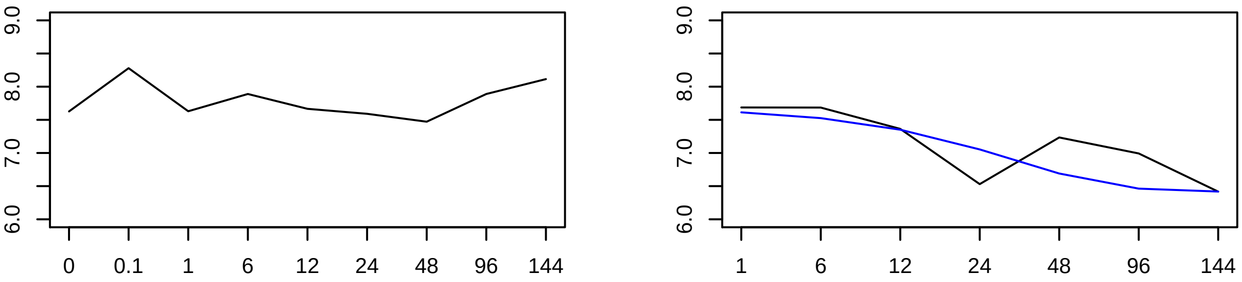

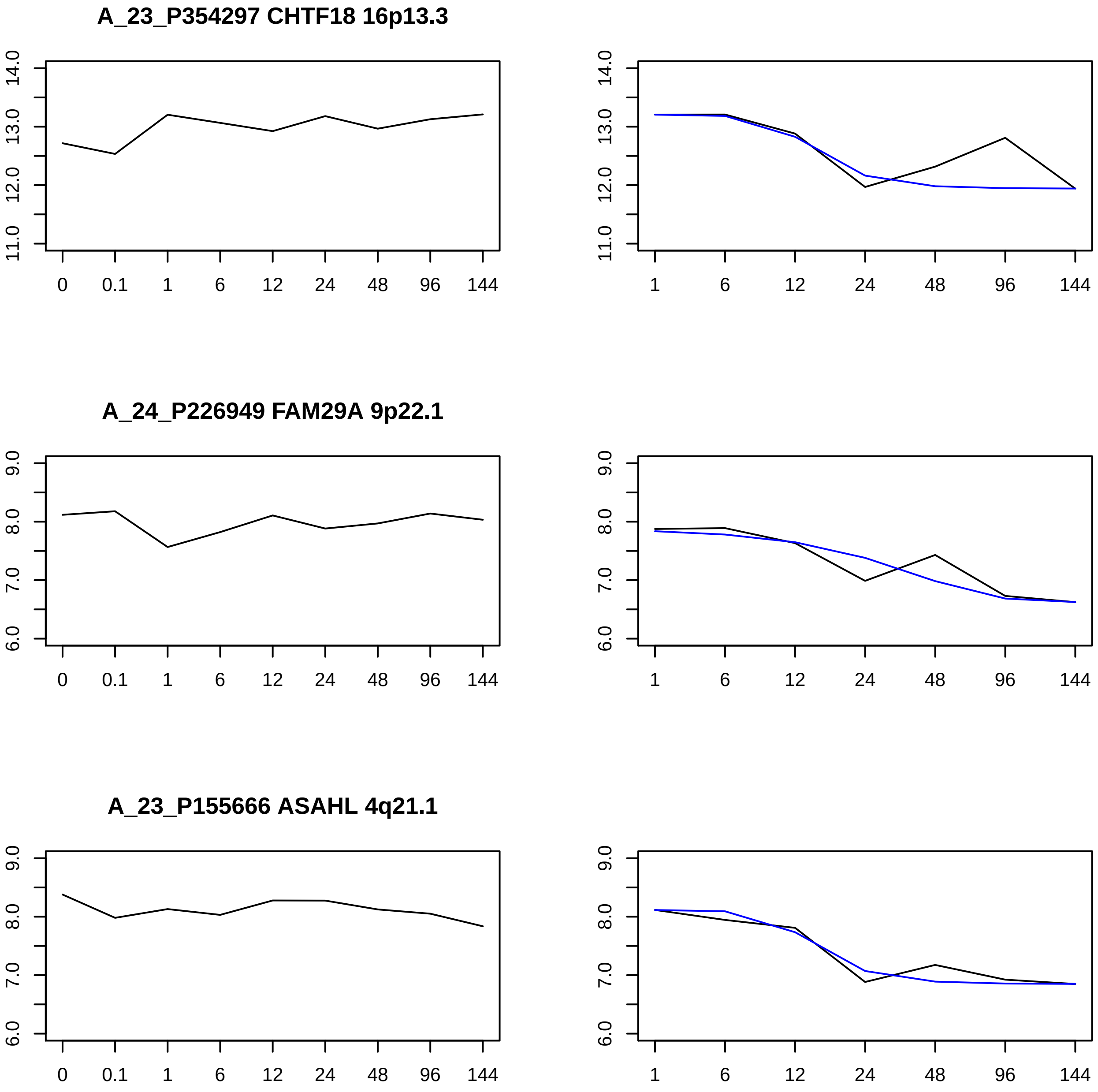
A_23_P354297 CHTF18 16p13.3
11.0
12.0
13.0
14.0
0
0.1
1
6
12
24
48
96
144
A_24_P226949 FAM29A 9p22.1
6.0
7.0
8.0
9.0
A_23_P155666 ASAHL 4q21.1

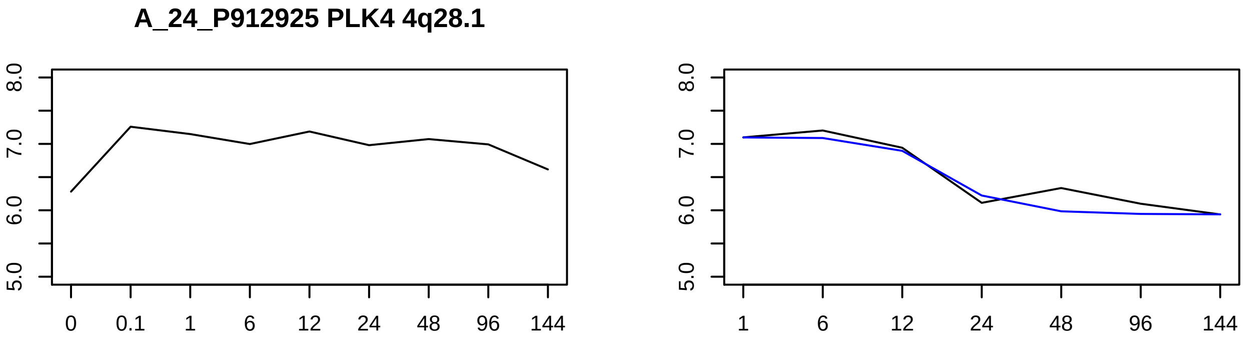
A_24_P912925 PLK4 4q28.1
5.0
6.0
7.0
8.0
0
0.1
1
6
12
24
48
96
144
1
6
12
24
48
96
144

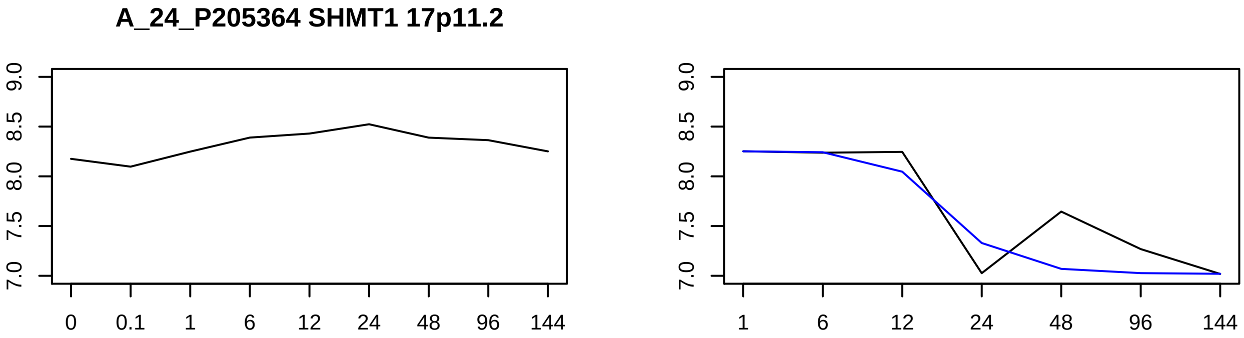
A_24_P205364 SHMT1 17p11.2
7.0
7.5
8.0
8.5
9.0
0
0.1
1
6
12
24
48
96
144
1
6
12
24
48
96
144

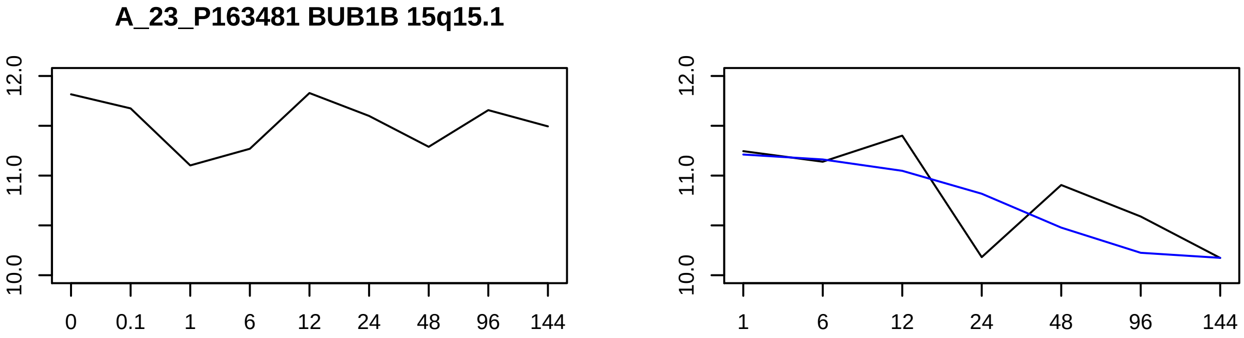
A_23_P163481 BUB1B 15q15.1
10.0
11.0
12.0
0
0.1
1
6
12
24
48
96
144
1
6
12
24
48
96
144

**A_32_P206698 CKS1B 1q21.3**

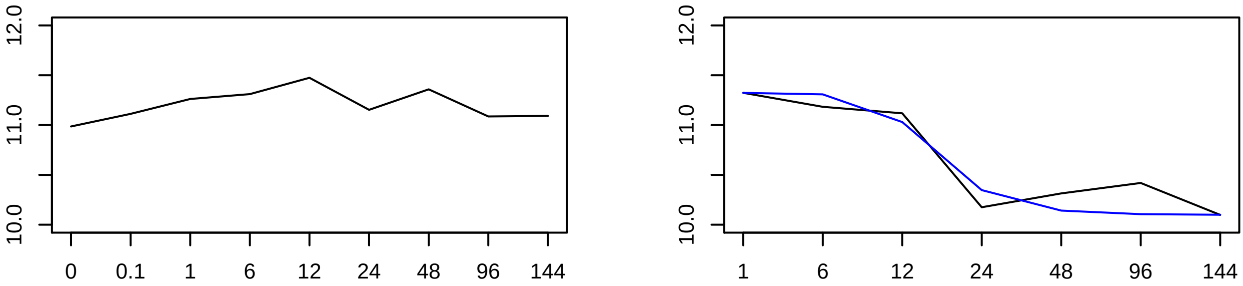


**A_23_P104065 ICMT 1p36.31**

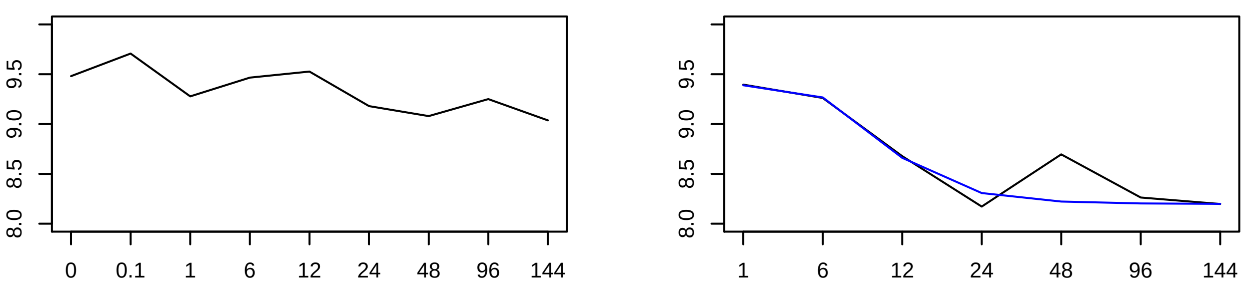


**A_23_P91328 NOL5A 20p13**

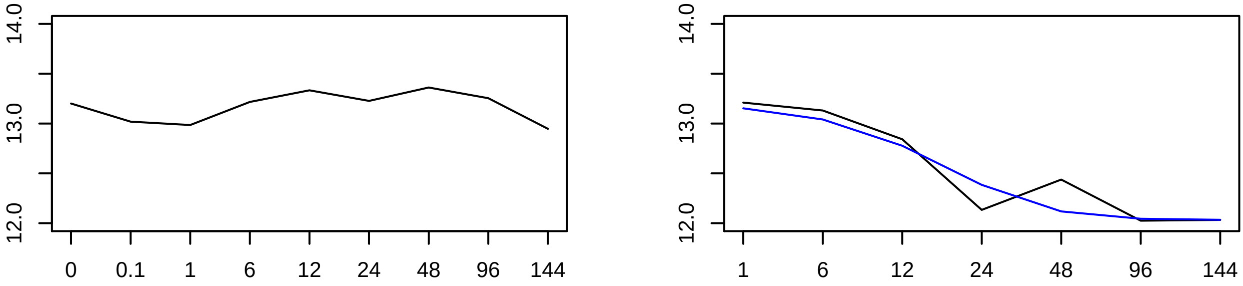

**A_23_P50455 POLD1 19q13.33**

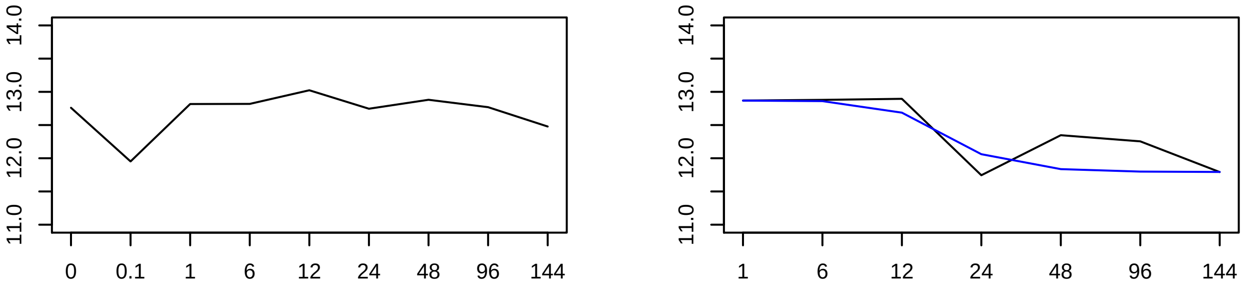


**A_23_P202029 SPFH1 10q24.2**

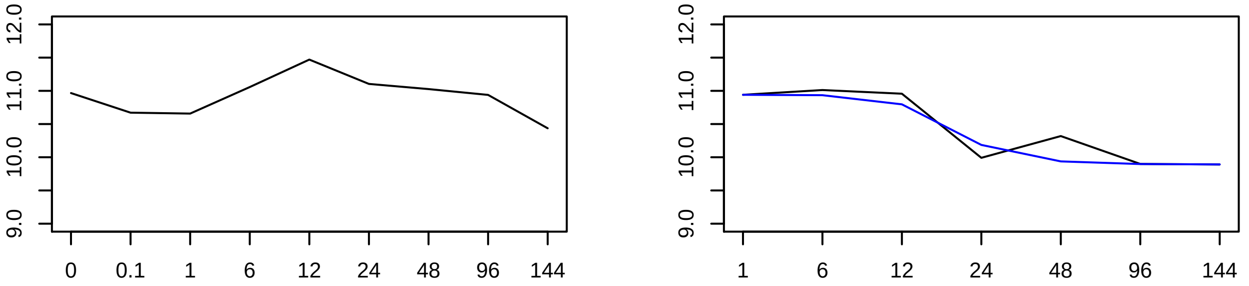

Supplement: Additional file 3 — Additional file A-H. These files contain the fitting results for the genes from the groups A-H, deduced by SwitchFinder, which represent eight dynamic patterns of the gene expression response to ATRA in neuroblastoma cell line. (ZIP 2457 kb) [file 12859_2016_1391_MOESM3_ESM.zip › AdditionalFile_F.pdf]
