# Supplementary material for: SwitchFinder – a novel method and query facility for discovering dynamic gene expression patterns
Source: BMC Bioinformatics. 2016 Dec 15;17:532. doi: 10.1186/s12859-016-1391-0 (PMC5160026; doi:10.1186/s12859-016-1391-0)
Supplement: Additional file 3 — Additional file A-H. These files contain the fitting results for the genes from the groups A-H, deduced by SwitchFinder, which represent eight dynamic patterns of the gene expression response to ATRA in neuroblastoma cell line. (ZIP 2457 kb) [file 12859_2016_1391_MOESM3_ESM.zip › AdditionalFile_E.pdf]

A_24_P236251 DLK1 14q32.2

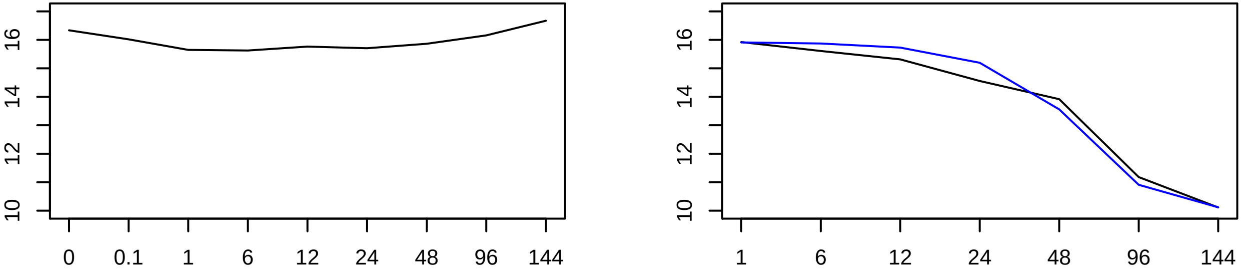

A_32_P111072 THC2781239 NA

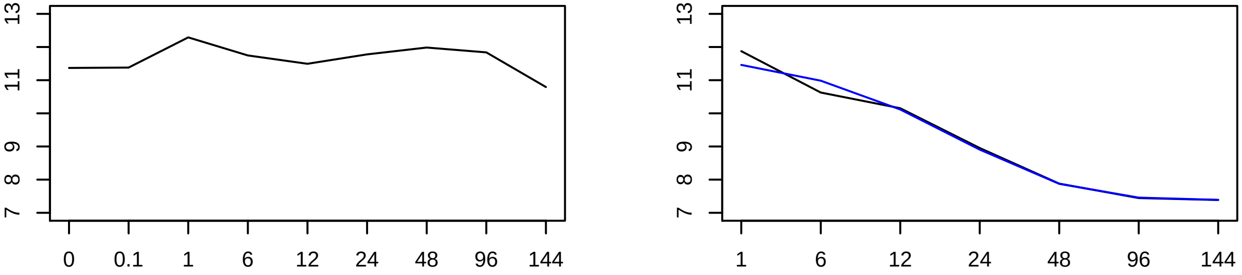

A_23_P168909 ZFPM2 8q23.1

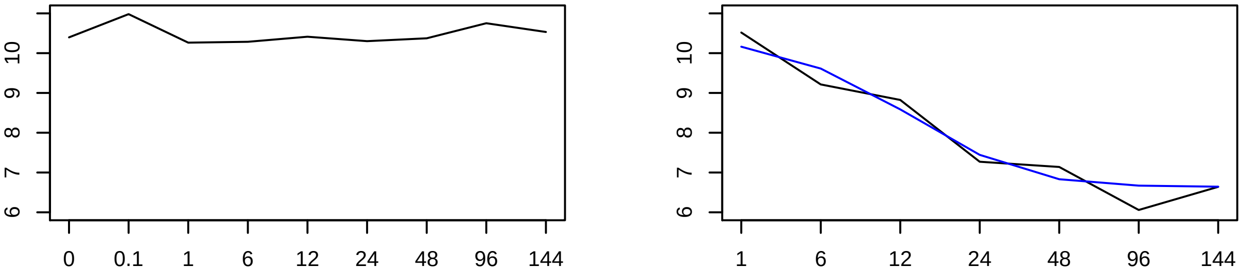

A_32_P222241 A_32_P222241 NA

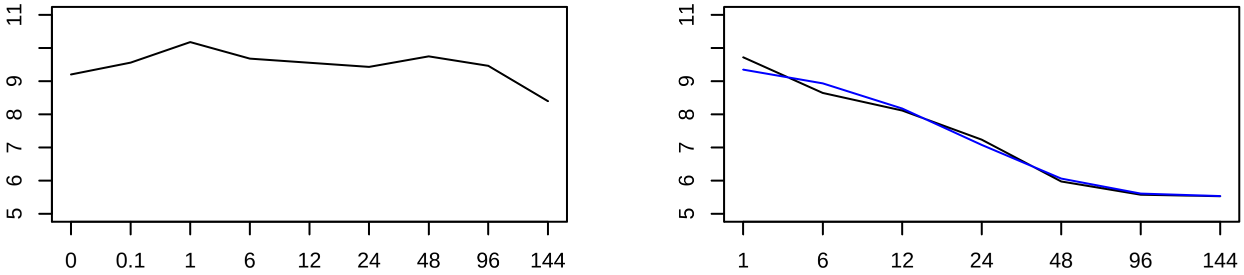

A_24_P850187 A_24_P850187 NA

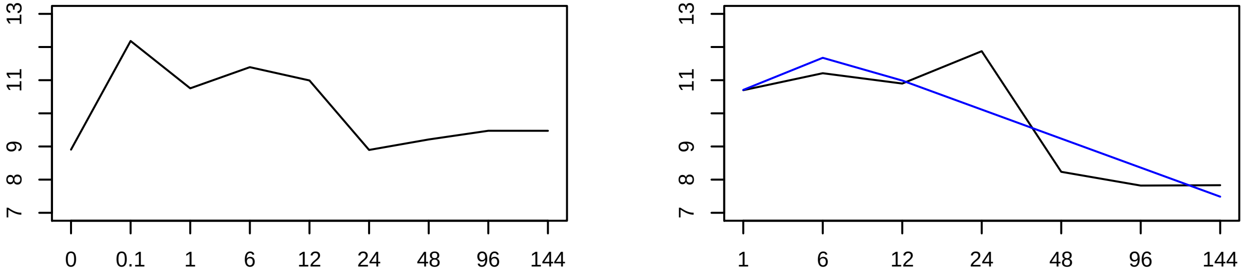

A_23_P251151 NELL1 11p15.1

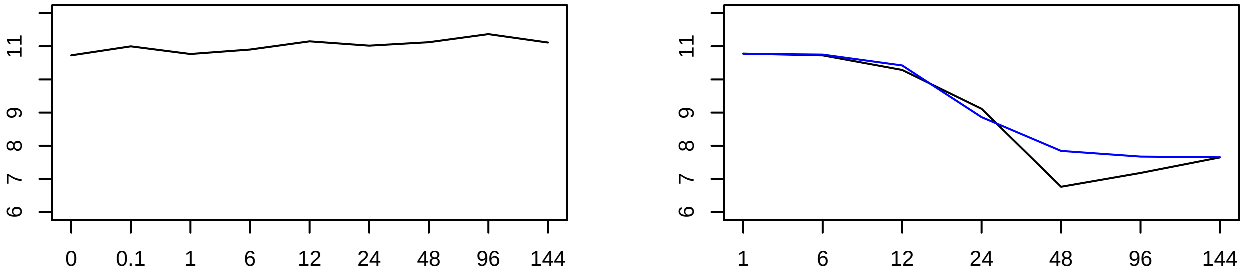

A_32_P51005 AL834342 NA

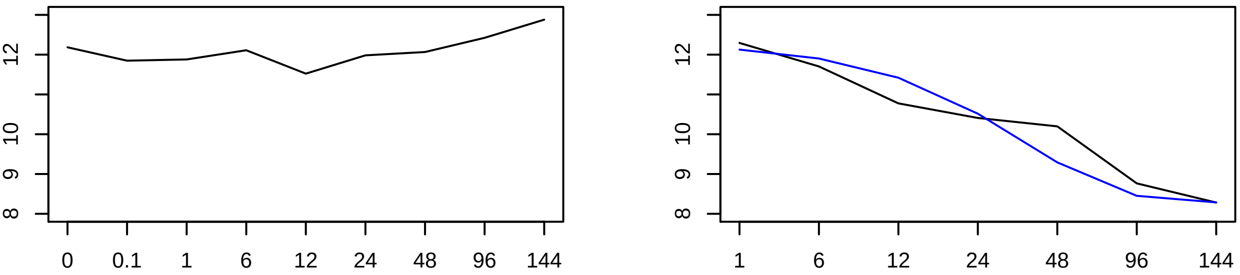

A_23_P98399 HTR3A 11q23.2

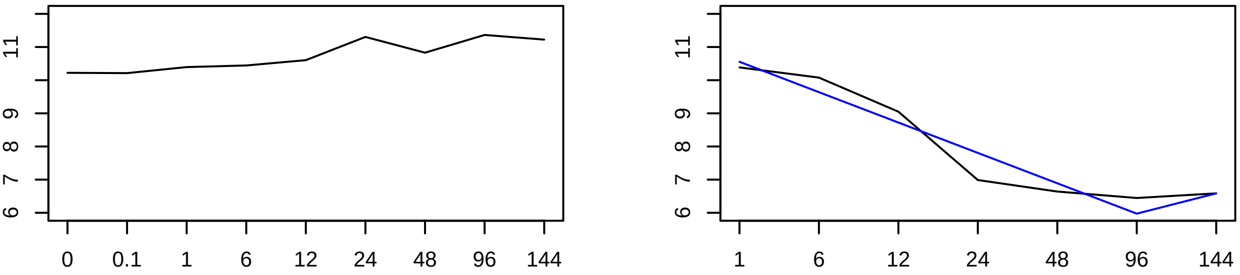

A_23_P154605 SULF2 20q13.12

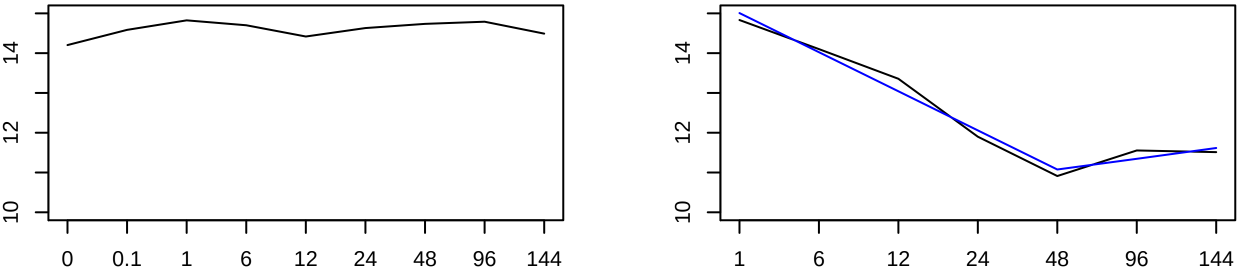

A_32_P94176 THC2713710 NA

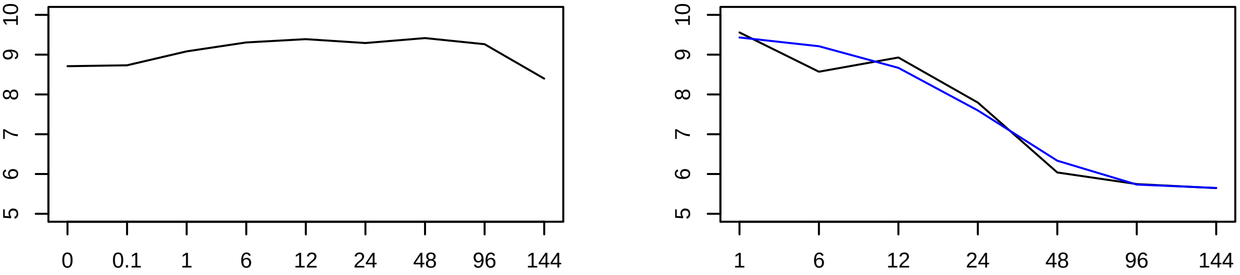

A_23_P414654 RAB37 17q25.1

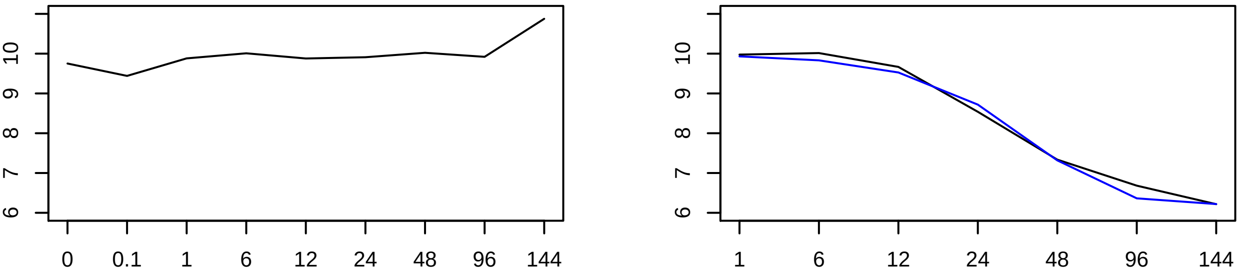

A_23_P56369 ABCA12 2q35

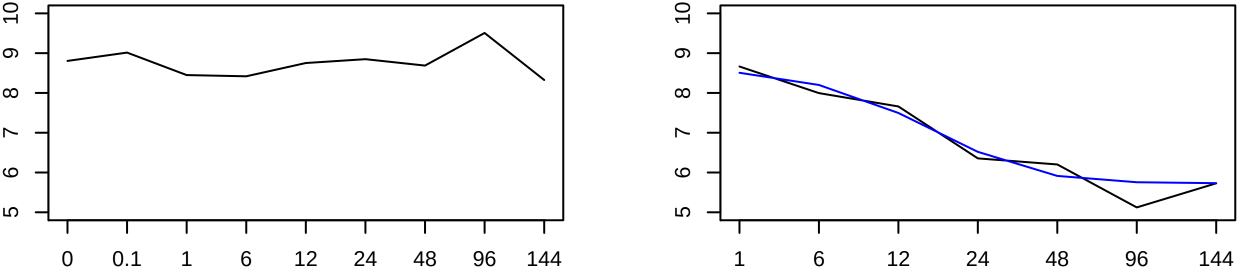

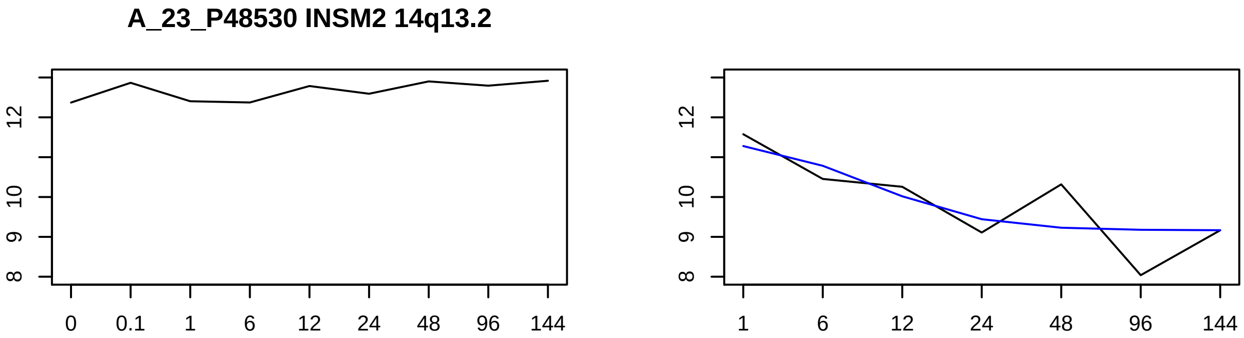
A_23_P48530 INSM2 14q13.2
8
9
10
12
0
0.1
1
6
12
24
48
96
144
8
9
10
12
1
6
12
24
48
96
144

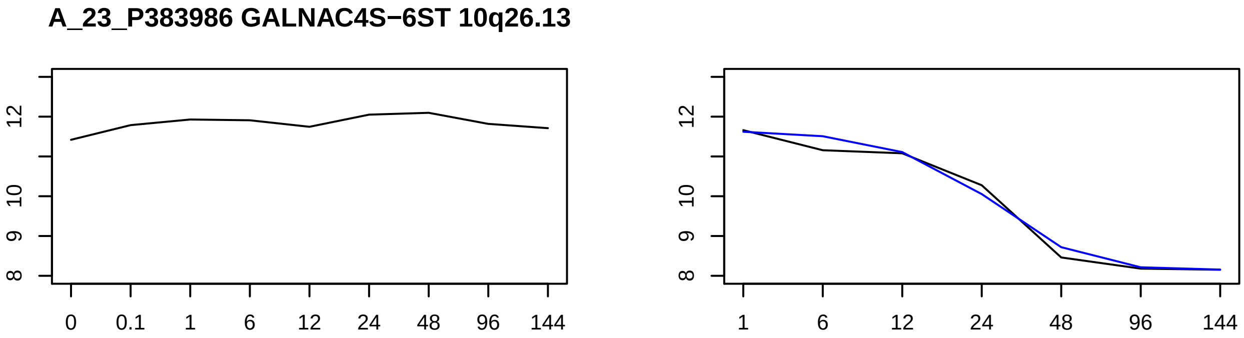
A_23_P383986 GALNAC4S−6ST 10q26.13
8
9
10
12
0
0.1
1
6
12
24
48
96
144
8
9
10
12
1
6
12
24
48
96
144

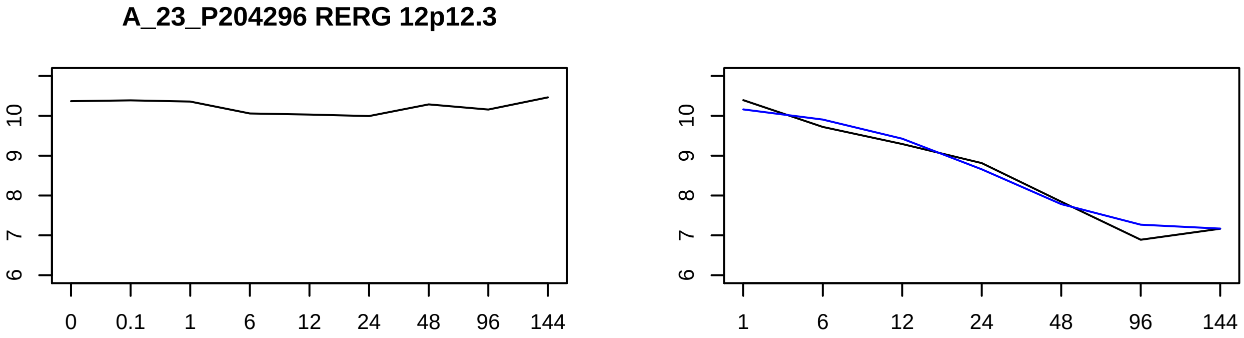
A_23_P204296 RERG 12p12.3
6
7
8
9
10
0
0.1
1
6
12
24
48
96
144
6
7
8
9
10
1
6
12
24
48
96
144

A_24_P321525 RERG 12p12.3

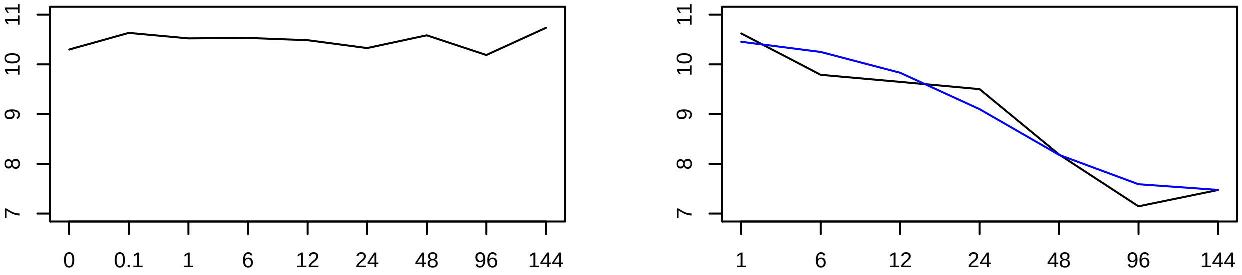

A_23_P32805 GRID1 10q23.1

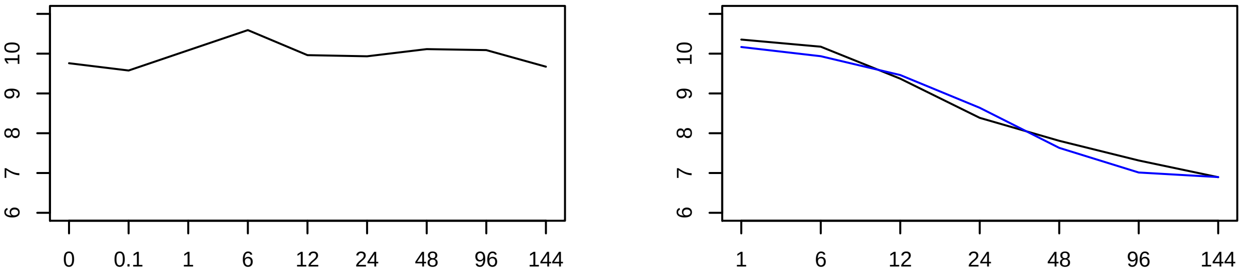

A_23_P210581 KCNG1 20q13.13

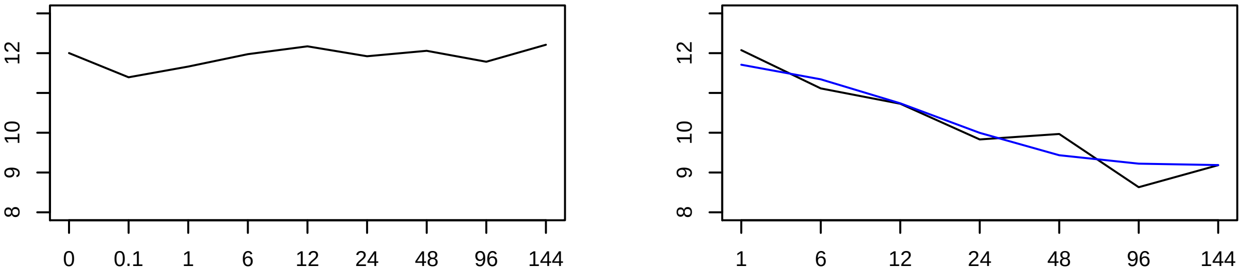

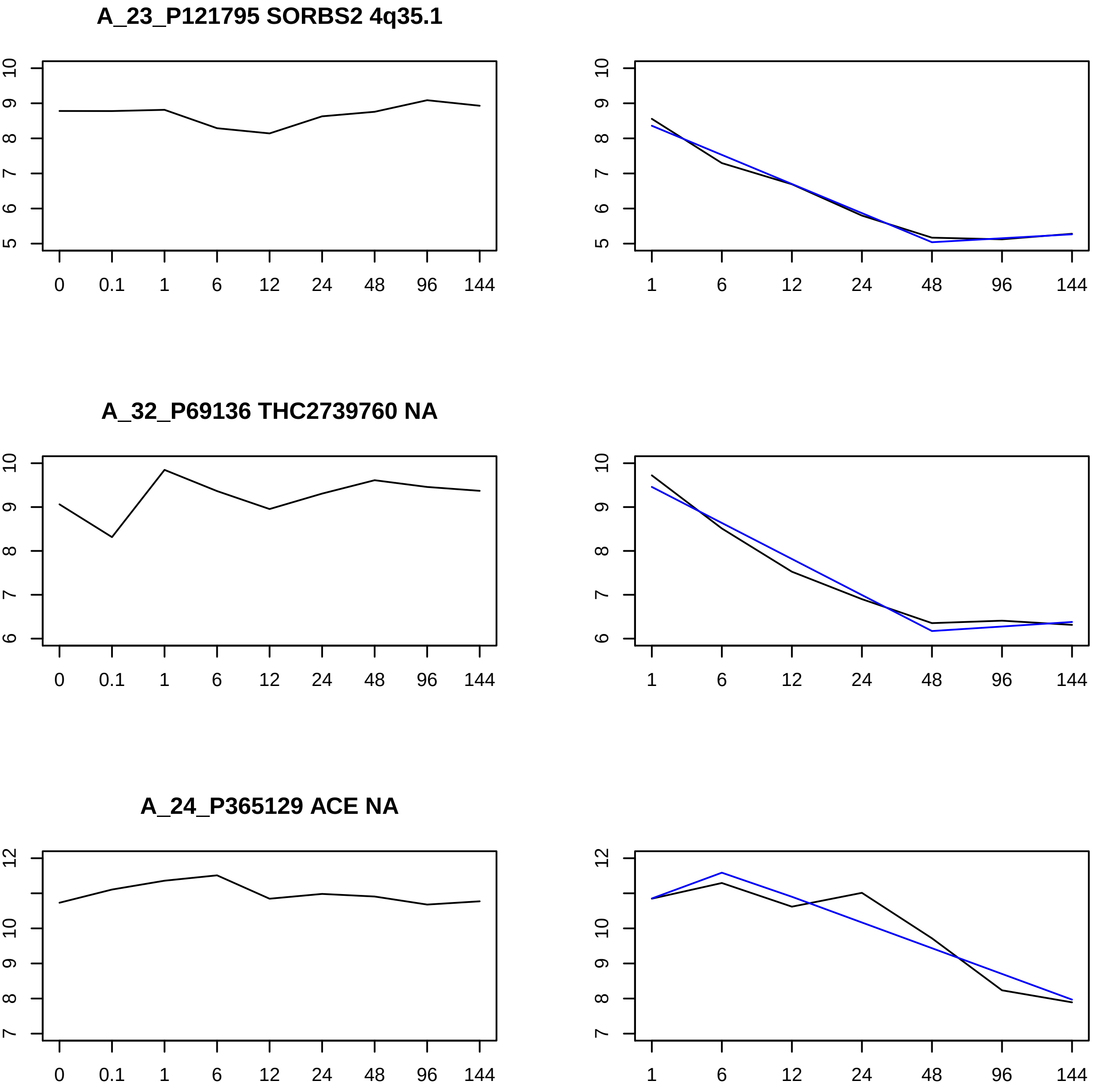
A_23_P121795 SORBS2 4q35.1
A_32_P69136 THC2739760 NA
A_24_P365129 ACE NA
0 0.1 1 6 12 24 48 96 144
1 6 12 24 48 96 144
5 6 7 8 9 10
6 7 8 9 10
7 8 9 10 12

A_23_P135239 TLE1 9q21.31

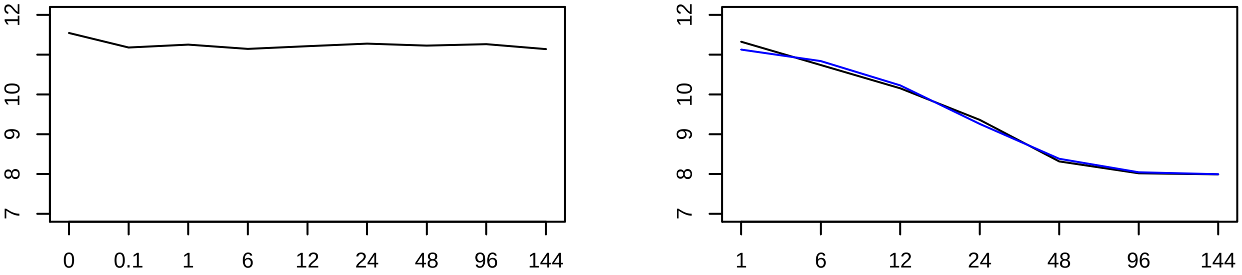

A_32_P82409 NAP1L1 12q21.2

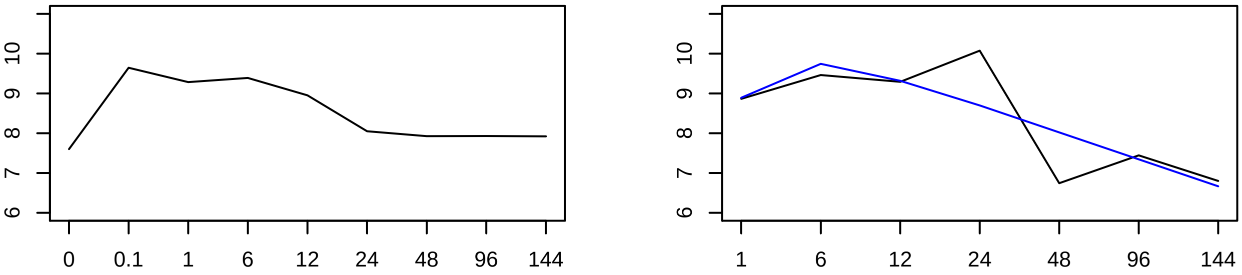

A_32_P148538 LPPR4 1p21.2

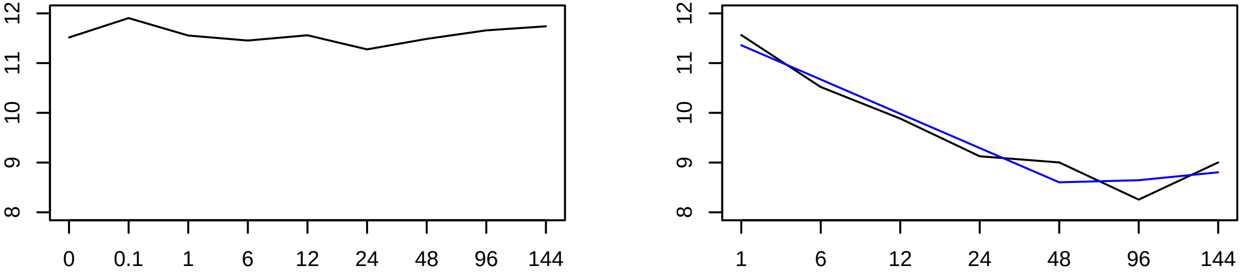

A_23_P58770 HAND1 5q33.2

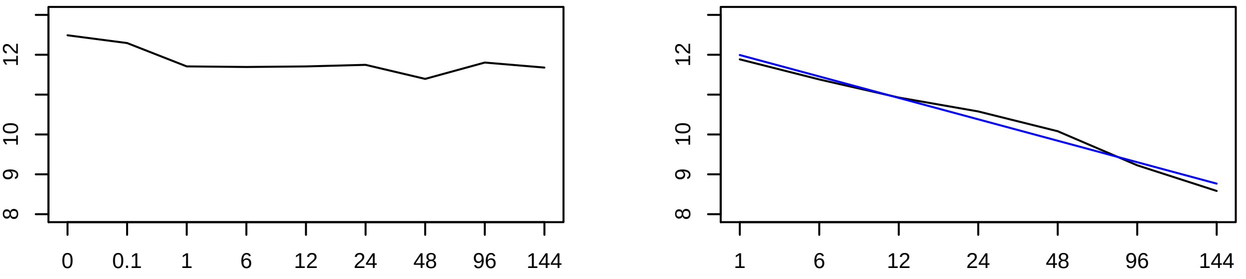

A_24_P300021 SHOX2 3q25.32

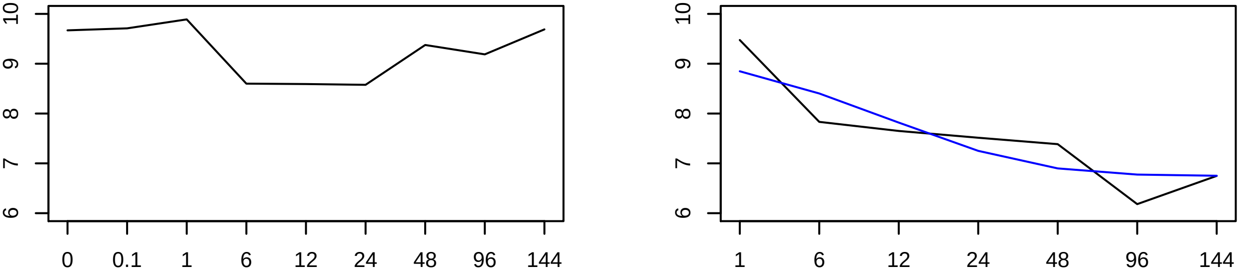

A_24_P286114 SLC1A3 5p13.2

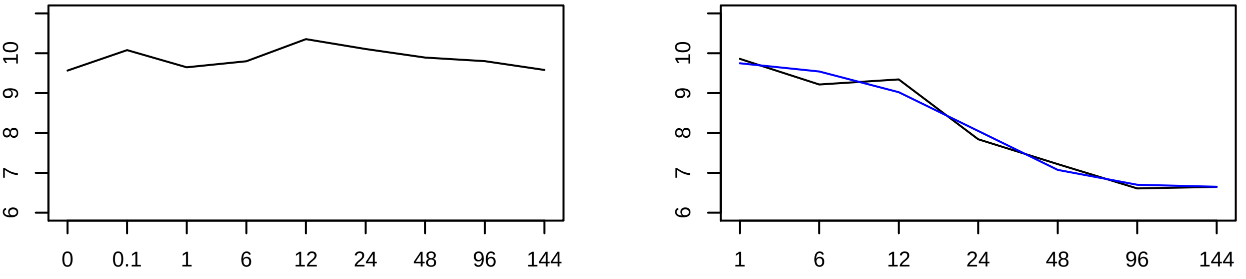

A_23_P131846 SNAI1 20q13.13

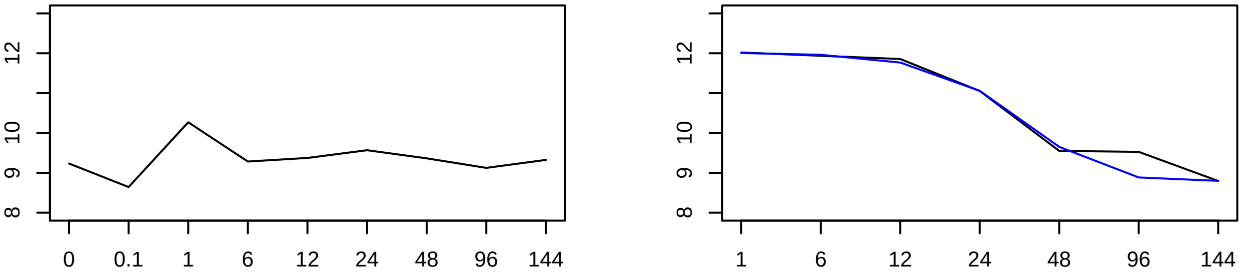

A_23_P153676 TLE2 19p13.3

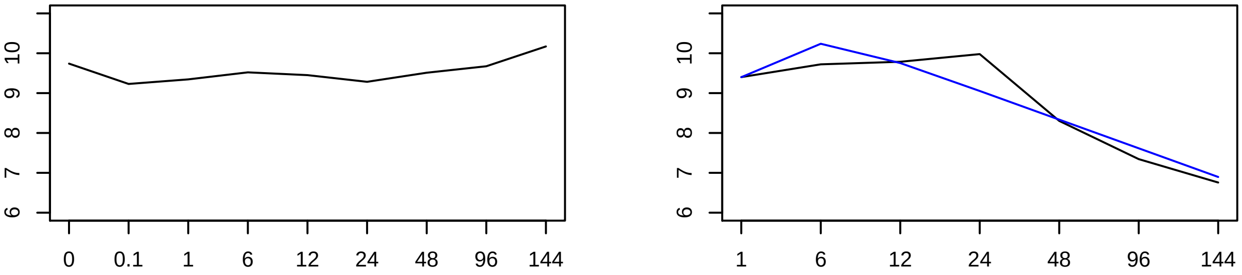

A_24_P870799 GRIK3 1p34.3

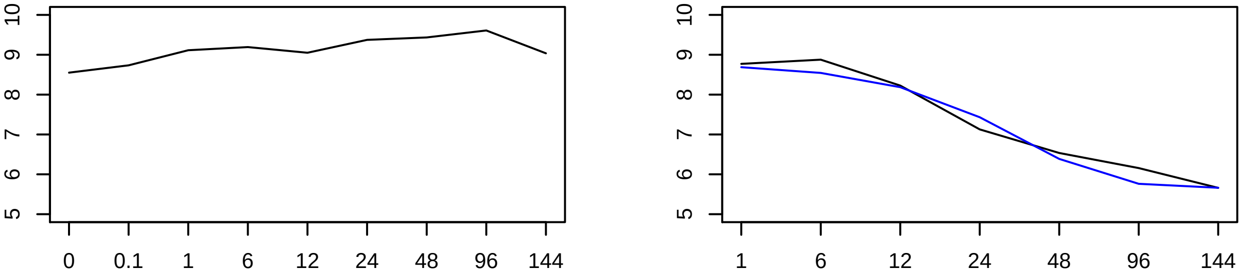

A_32_P122715 THC2656519 NA

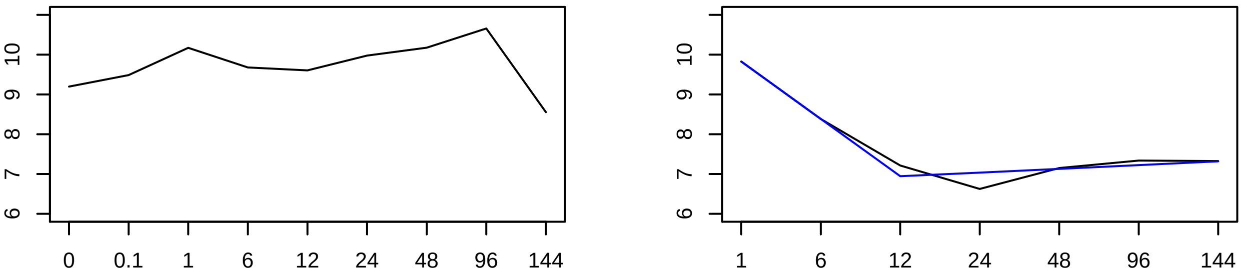

A_23_P124384 SHOX2 3q25.32

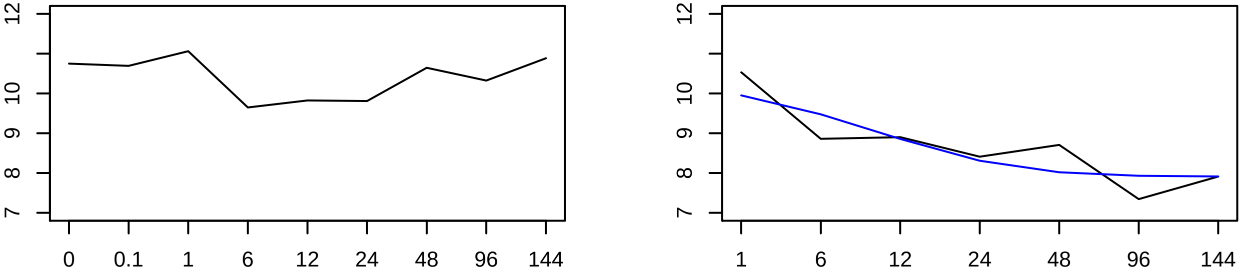

A_32_P37875 THC2665223 NA

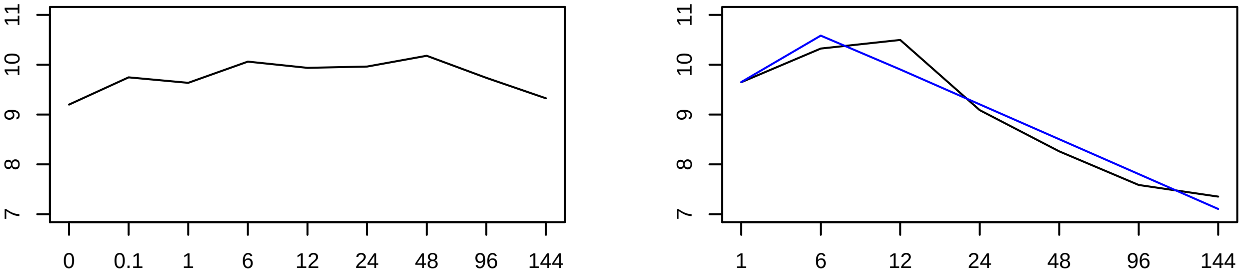

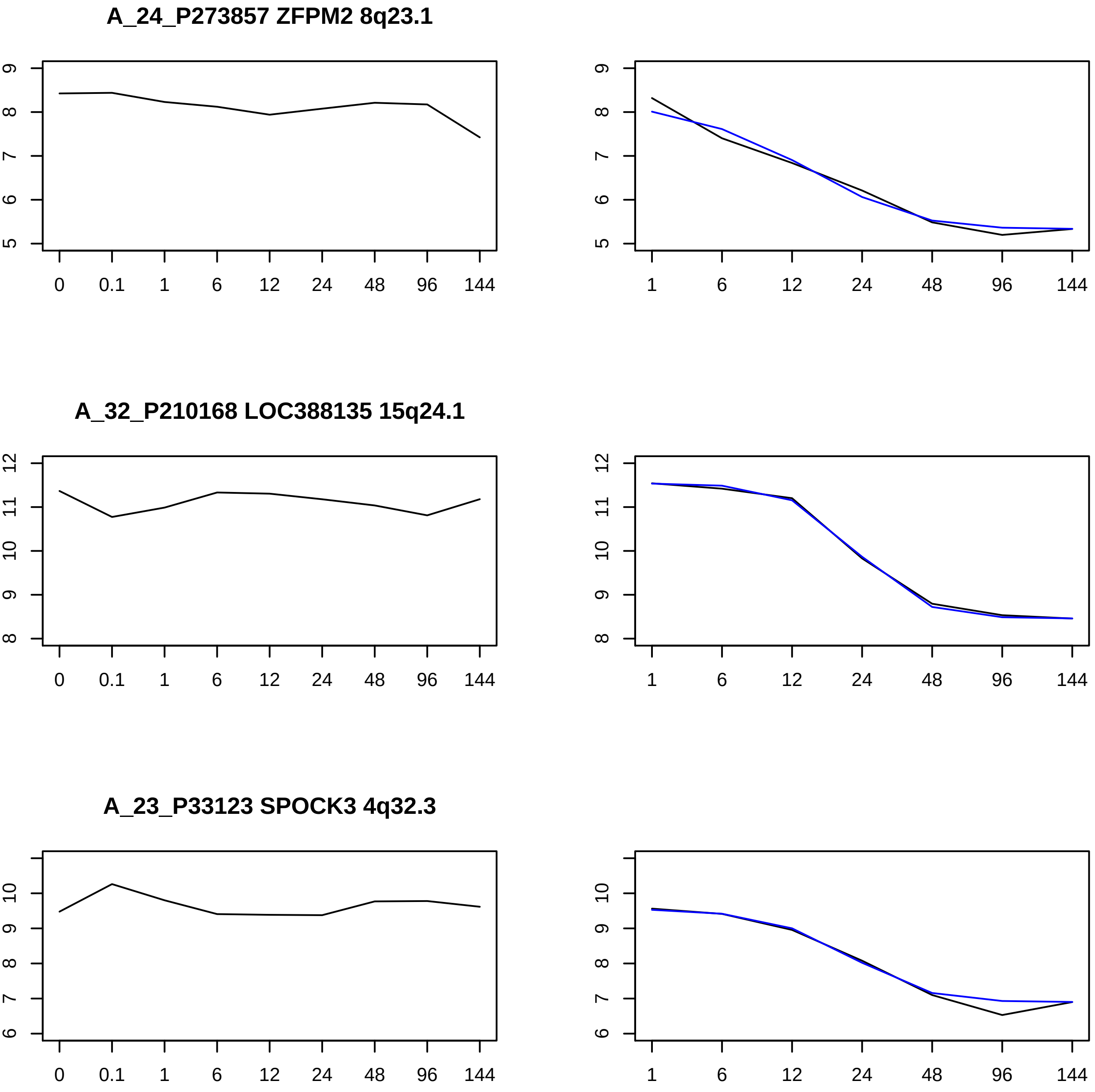
A_24_P273857 ZFPM2 8q23.1
A_32_P210168 LOC388135 15q24.1
A_23_P33123 SPOCK3 4q32.3
0
0.1
1
6
12
24
48
96
144

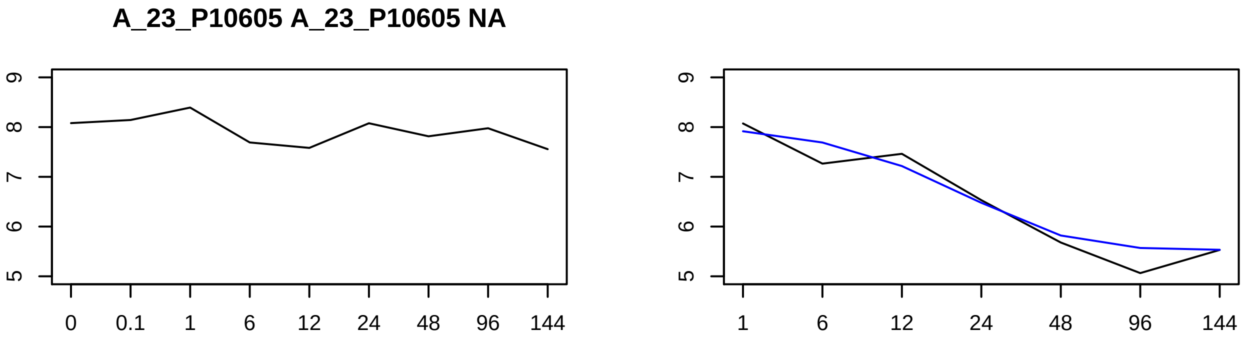
A_23_P10605 A_23_P10605 NA
5
6
7
8
9
0
0.1
1
6
12
24
48
96
144
1
6
12
24
48
96
144

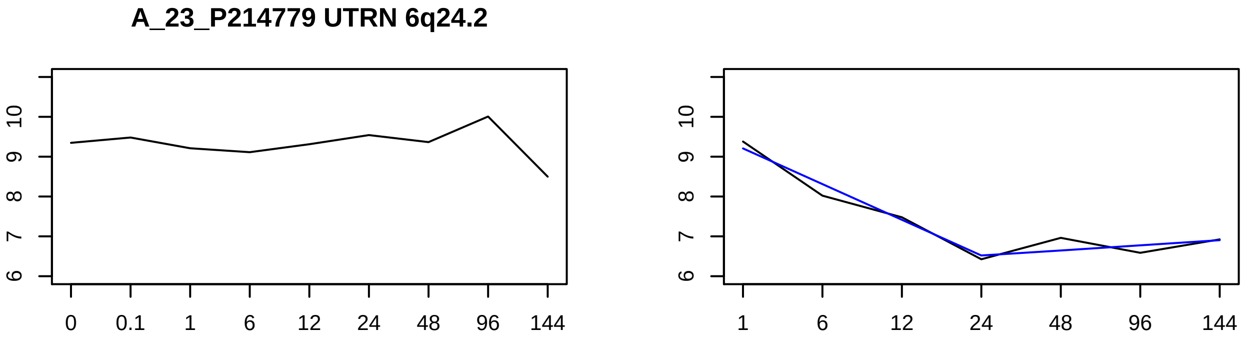
A_23_P214779 UTRN 6q24.2
6
7
8
9
10
0
0.1
1
6
12
24
48
96
144
1
6
12
24
48
96
144

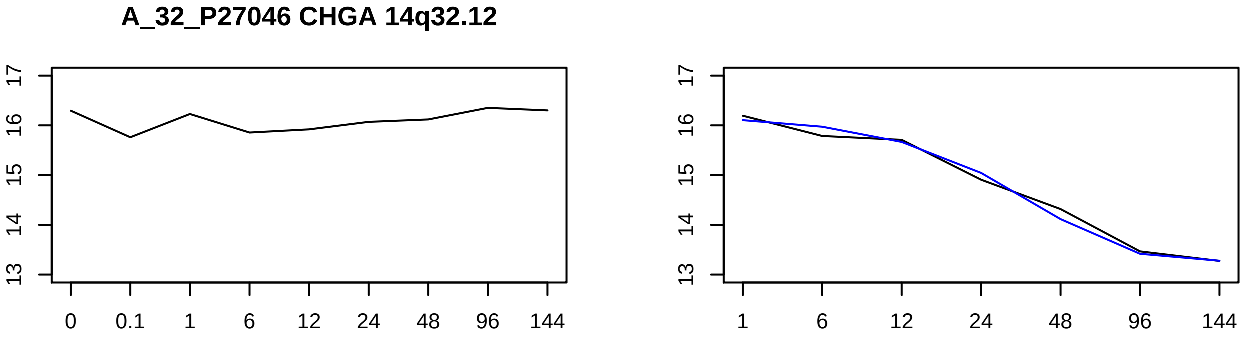
A_32_P27046 CHGA 14q32.12
13
14
15
16
17
0
0.1
1
6
12
24
48
96
144
1
6
12
24
48
96
144

A_24_P280897 LOC388532 19q13.11

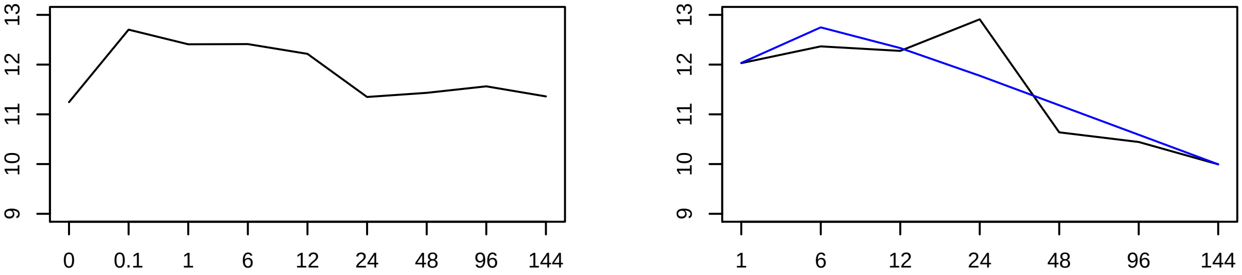

A_24_P298604 LOC731599 4p16.1

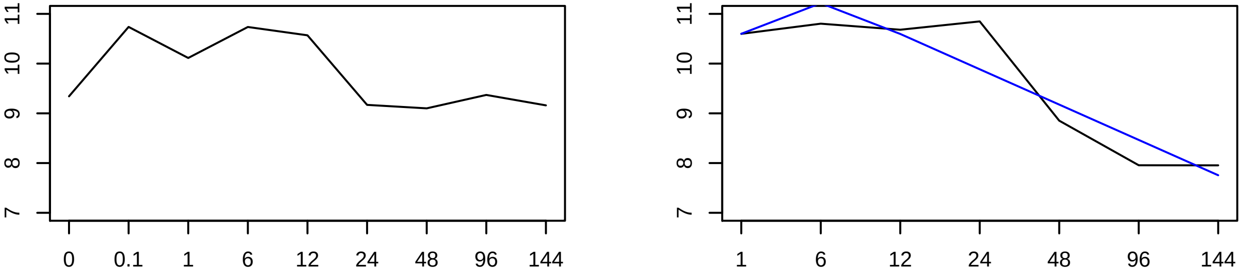

A_24_P912136 AKT3 4q35.1

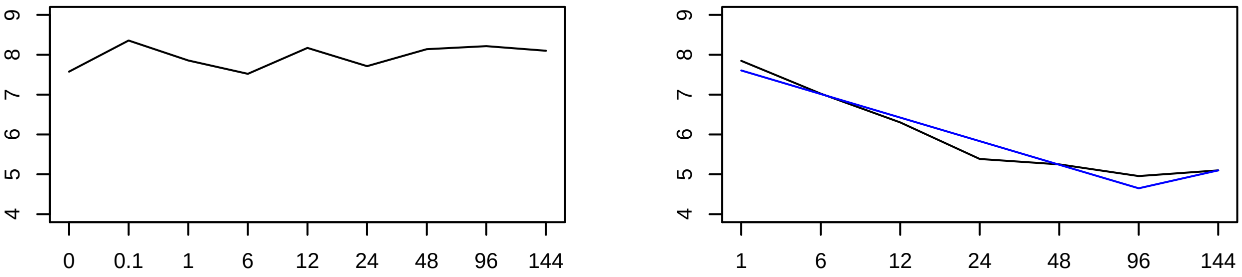

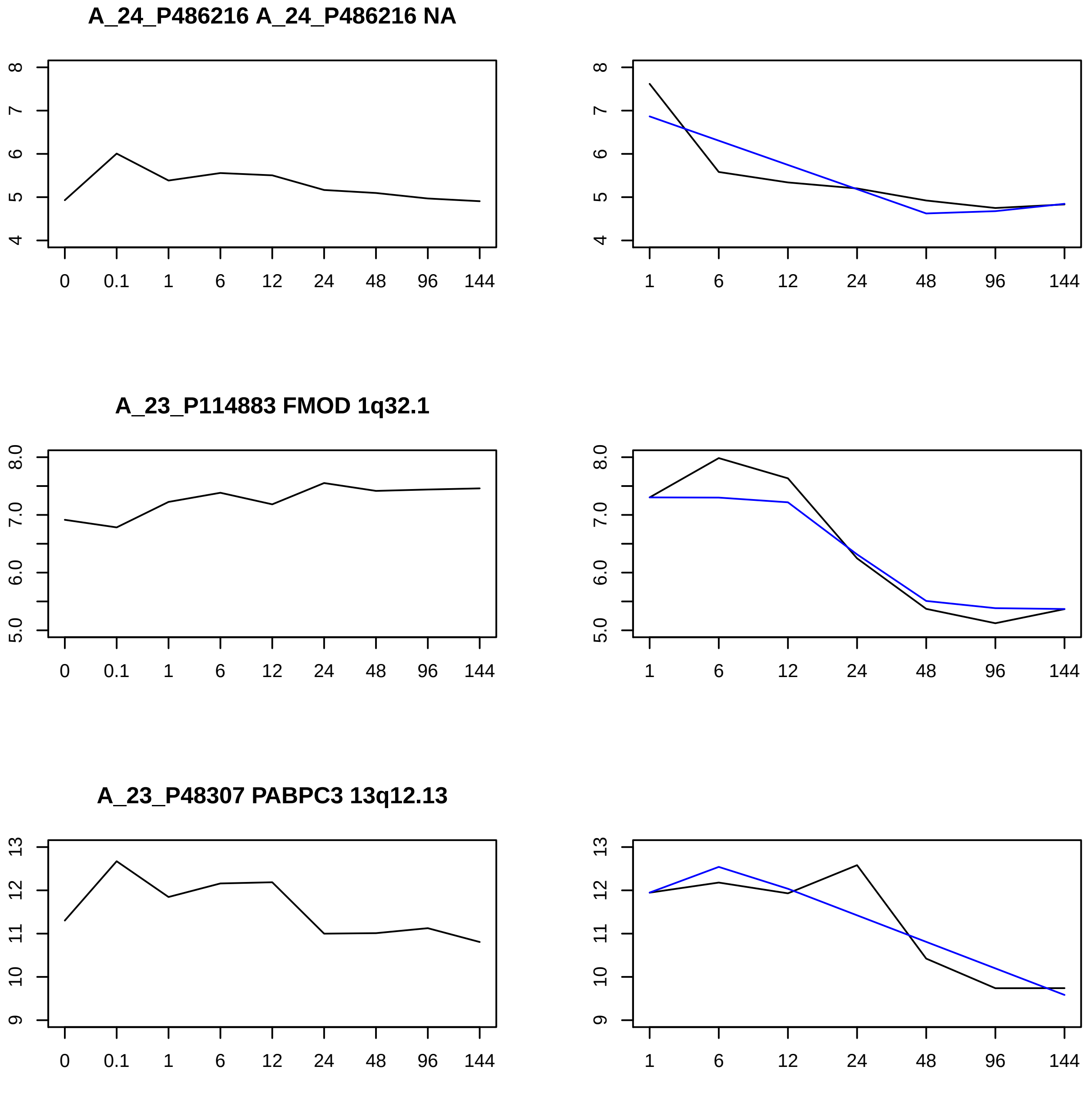
A_24_P486216 A_24_P486216 NA
4
5
6
7
8
0
0.1
1
6
12
24
48
96
144
A_23_P114883 FMOD 1q32.1
5.0
6.0
7.0
8.0
A_23_P48307 PABPC3 13q12.13
9
10
11
12
13

A_24_P153800 SUPT16H 14q11.2

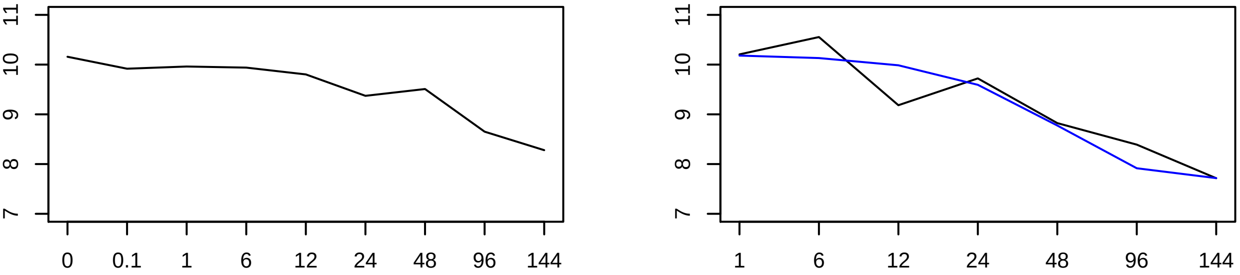

A_23_P27306 COLEC12 18p11.32

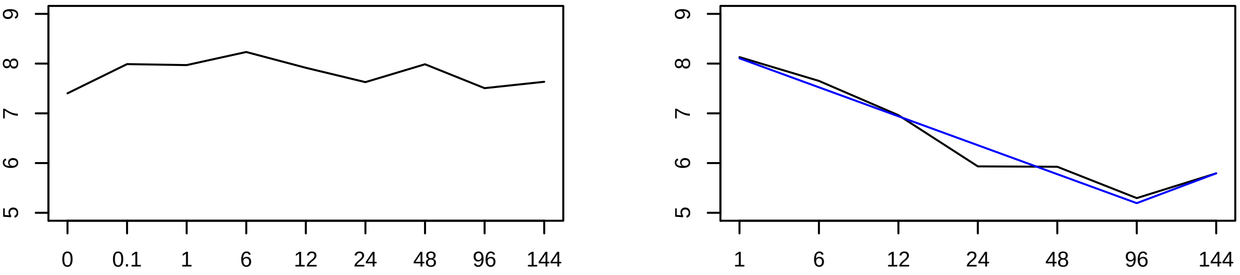

A_23_P109184 INSM1 20p11.23

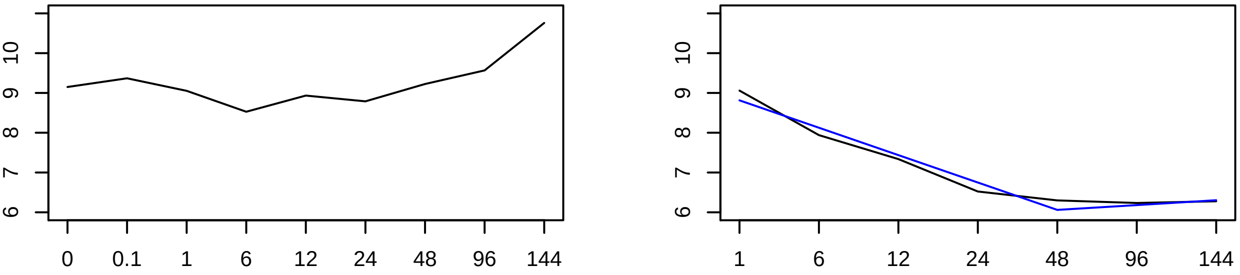

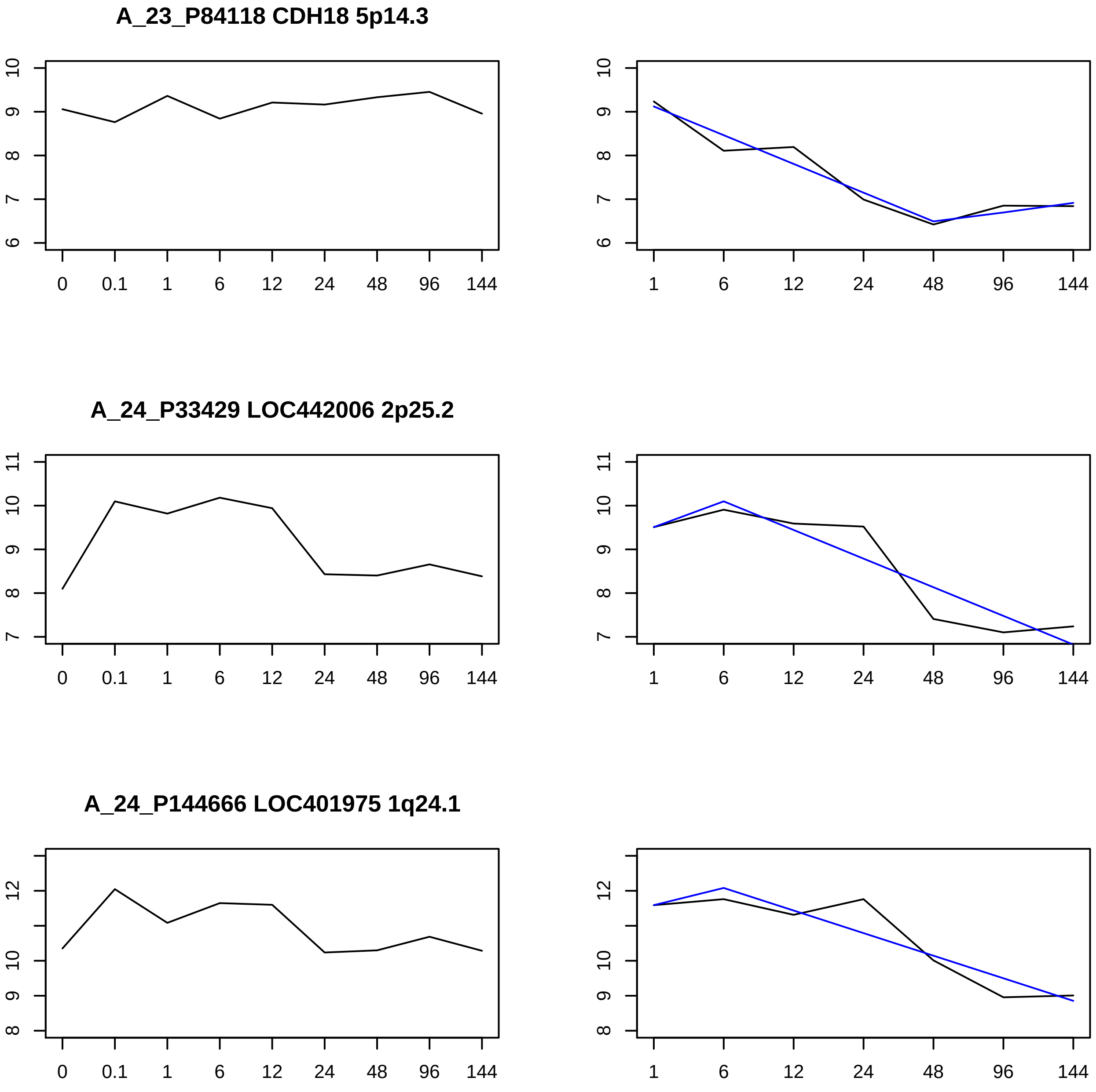
A_23_P84118 CDH18 5p14.3
A_24_P33429 LOC442006 2p25.2
A_24_P144666 LOC401975 1q24.1
0
0.1
1
6
12
24
48
96
144

A_32_P524014 UTRN 6q24.2

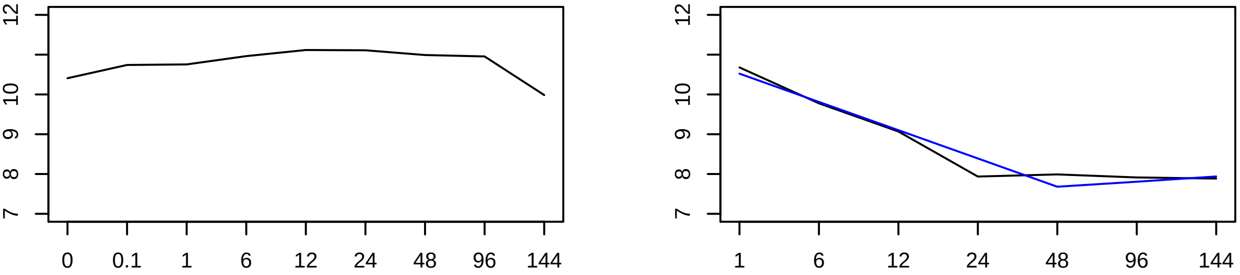

A_24_P93624 RAB3IP 12q15

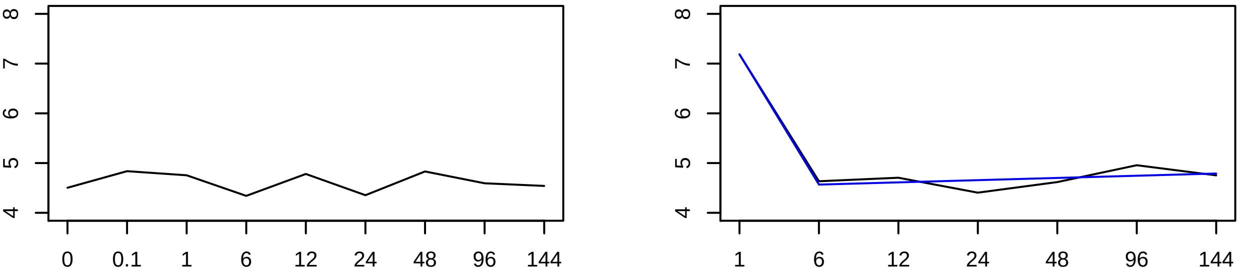

A_24_P707102 THC2713242 NA

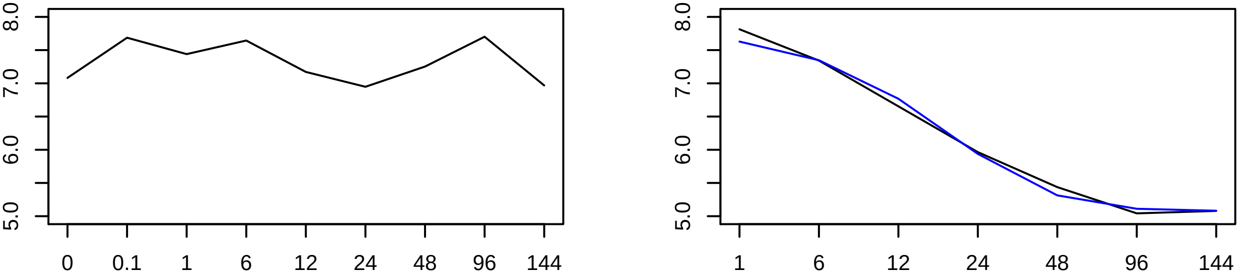

A_24_P928052 NRP1 10p11.22

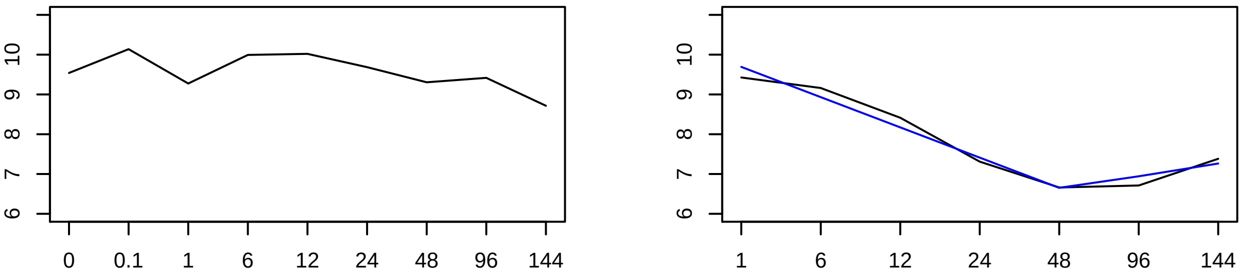

A_24_P652786 THC2533996 NA

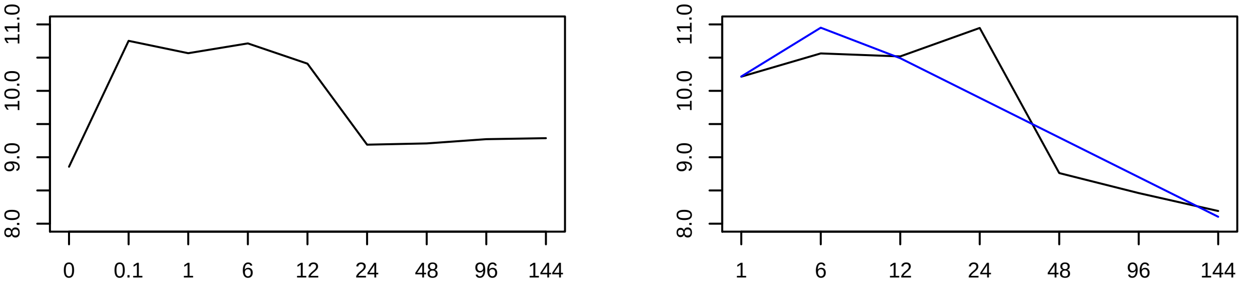

A_23_P67847 GALNT14 2p23.1

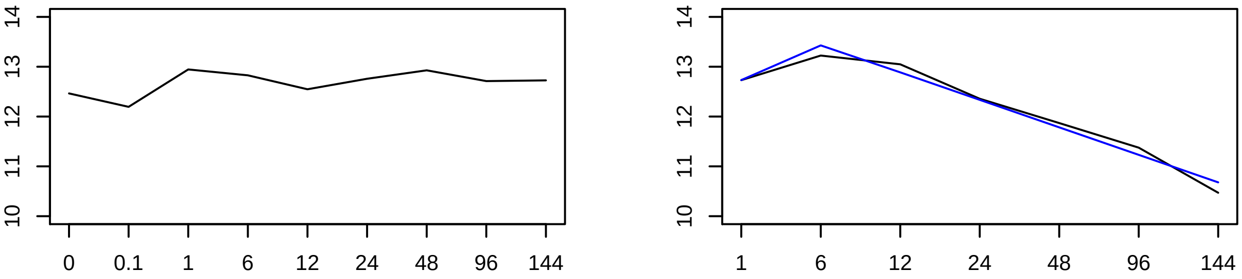

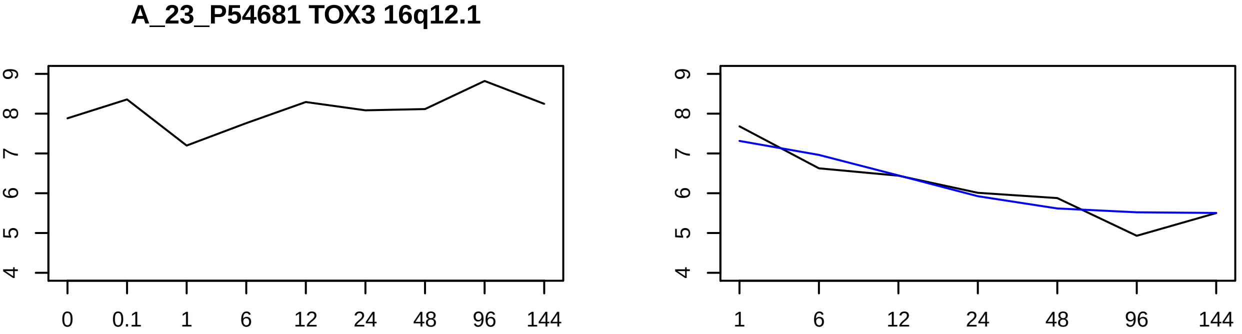
A_23_P54681 TOX3 16q12.1
4 5 6 7 8 9
0 0.1 1 6 12 24 48 96 144
1 6 12 24 48 96 144

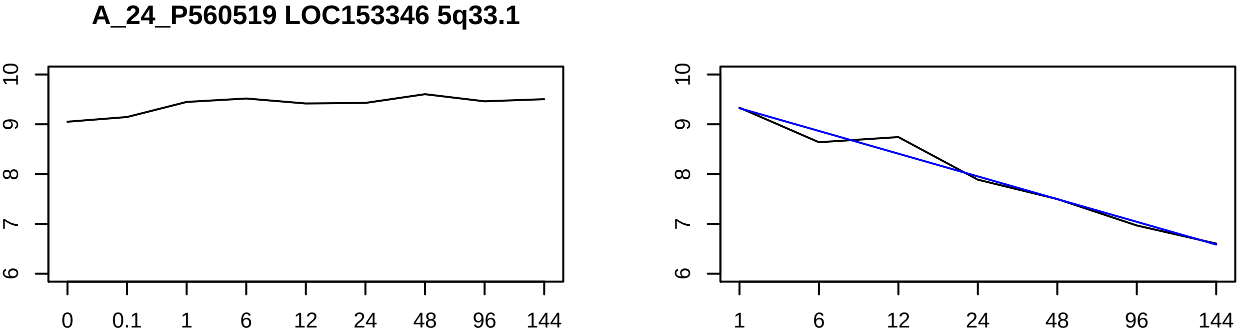
A_24_P560519 LOC153346 5q33.1
6 7 8 9 10
0 0.1 1 6 12 24 48 96 144
1 6 12 24 48 96 144

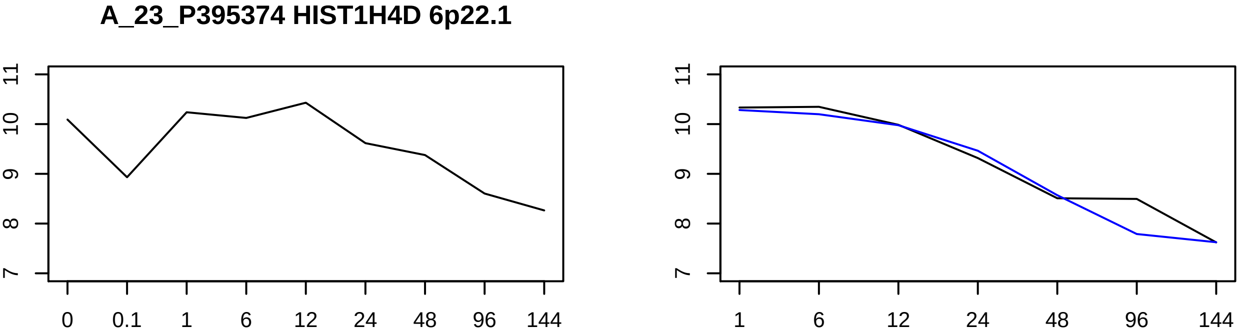
A_23_P395374 HIST1H4D 6p22.1
7 8 9 10 11
0 0.1 1 6 12 24 48 96 144
1 6 12 24 48 96 144

A_23_P353035 IGFBP7 4q12

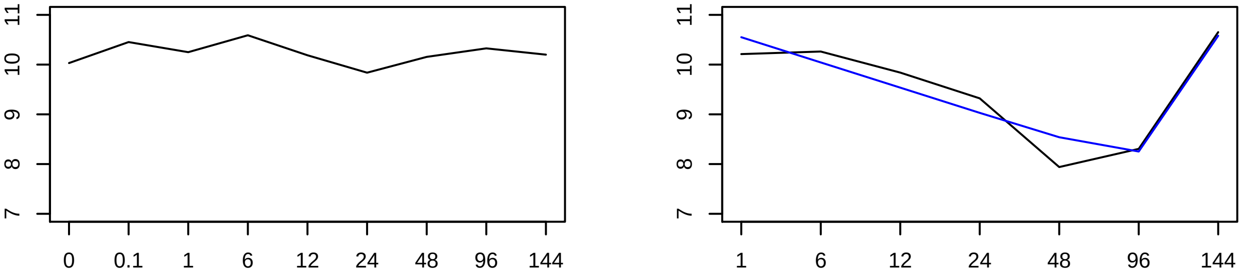

A_24_P20327 KLF15 3q21.2

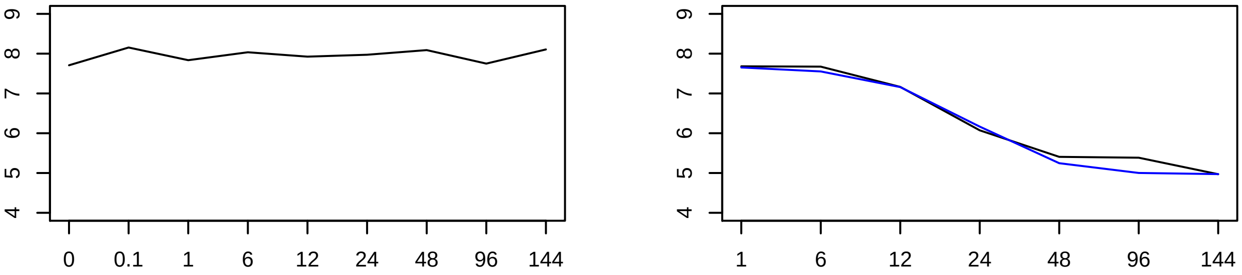

A_24_P914479 SNX5 20p11.23

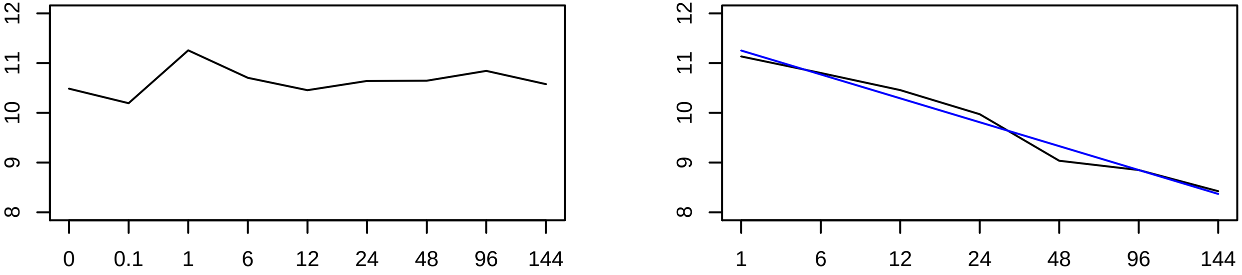

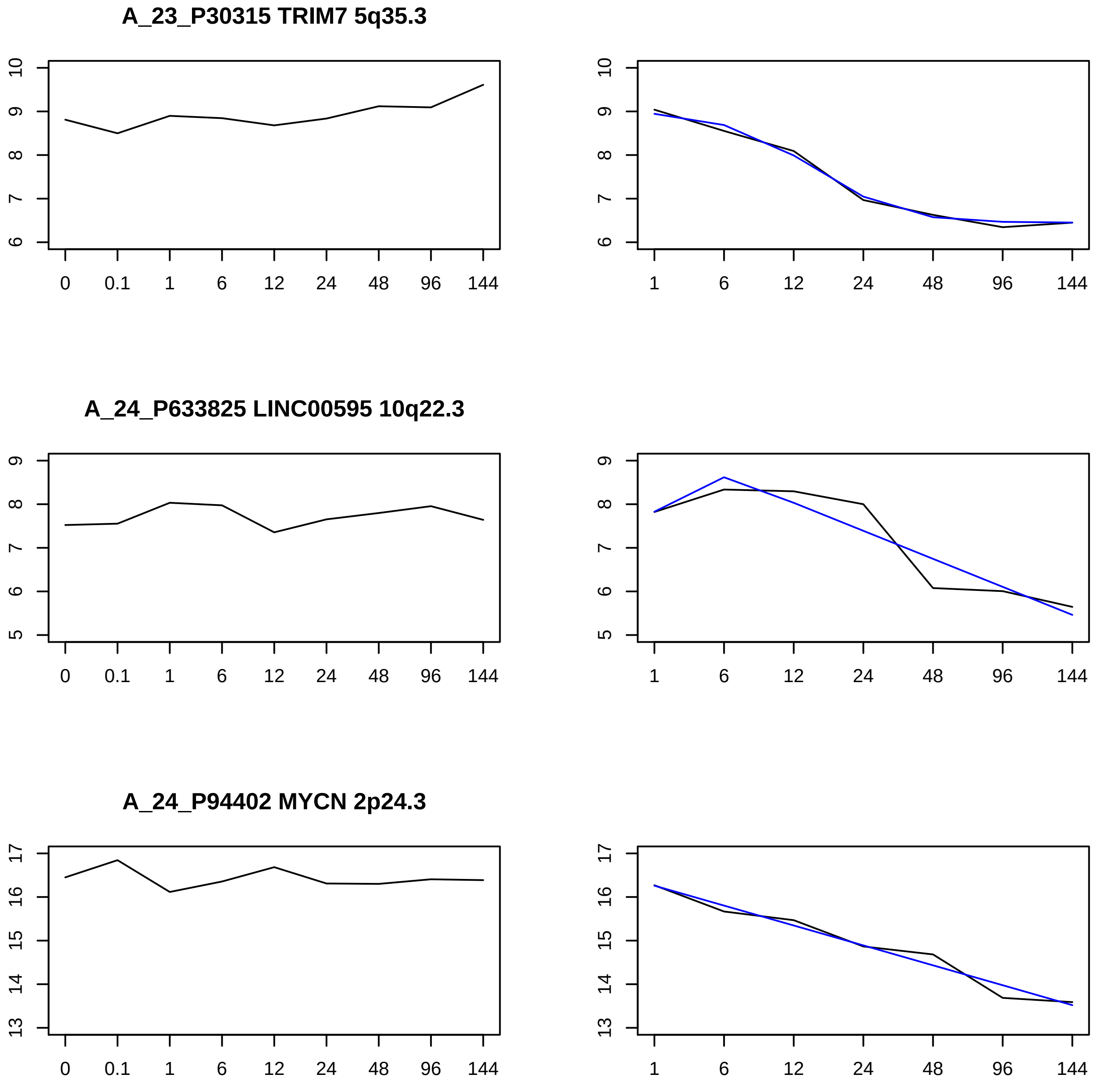
A_23_P30315 TRIM7 5q35.3
6 7 8 9 10
0 0.1 1 6 12 24 48 96 144
1 6 12 24 48 96 144
A_24_P633825 LINC00595 10q22.3
5 6 7 8 9
0 0.1 1 6 12 24 48 96 144
1 6 12 24 48 96 144
A_24_P94402 MYCN 2p24.3
13 14 15 16 17
0 0.1 1 6 12 24 48 96 144
1 6 12 24 48 96 144

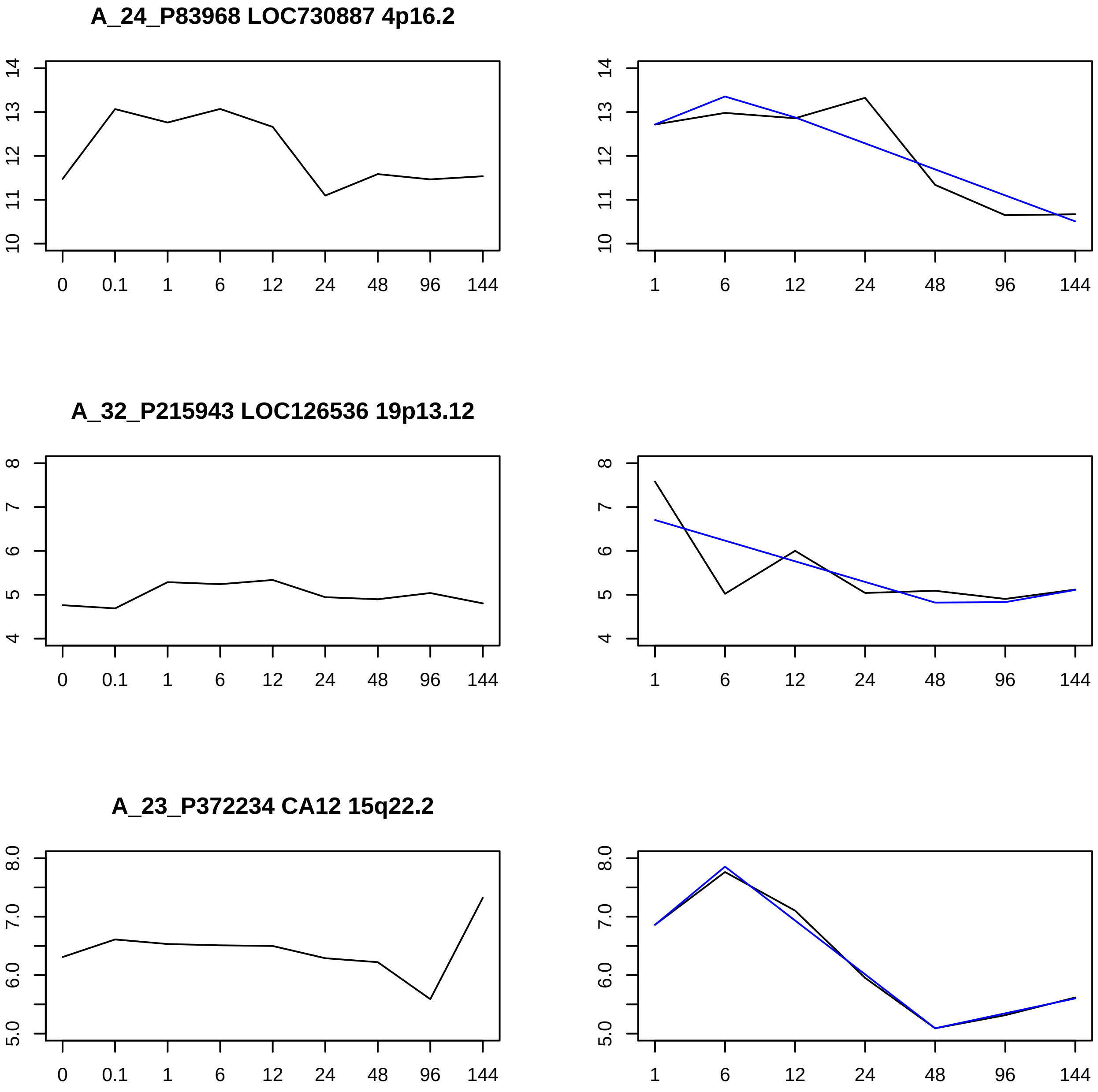
A_24_P83968 LOC730887 4p16.2
10
11
12
13
14
0
0.1
1
6
12
24
48
96
144
1
6
12
24
48
96
144
A_32_P215943 LOC126536 19p13.12
4
5
6
7
8
A_23_P372234 CA12 15q22.2
5.0
6.0
7.0
8.0

A_32_P187009 SERINC5 NA

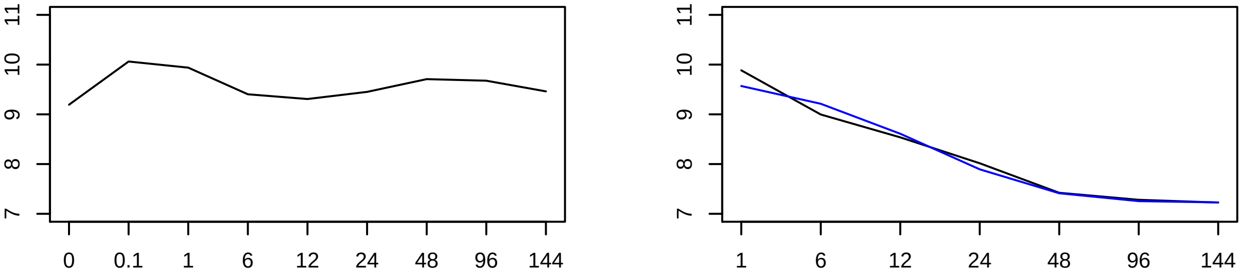

A_24_P324084 A_24_P324084 NA

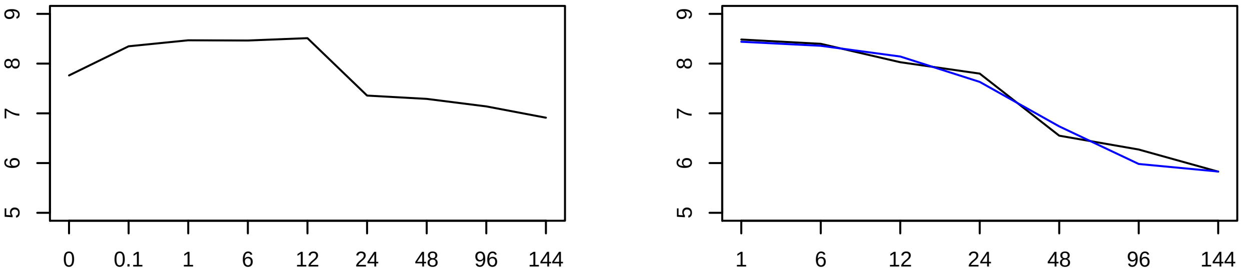

A_24_P418517 LOC390411 13q21.33

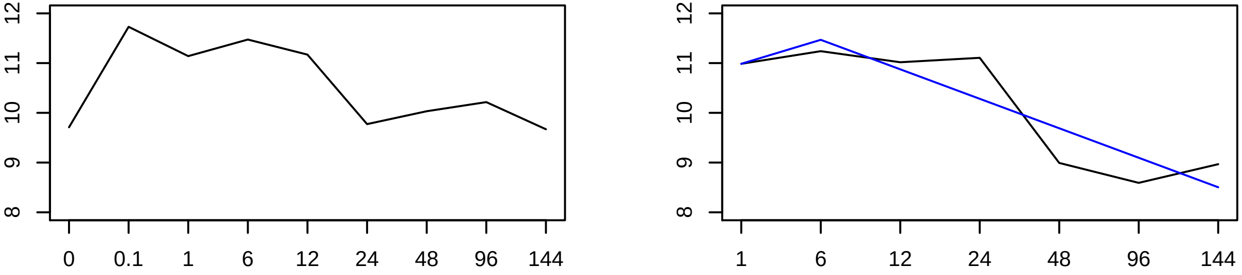

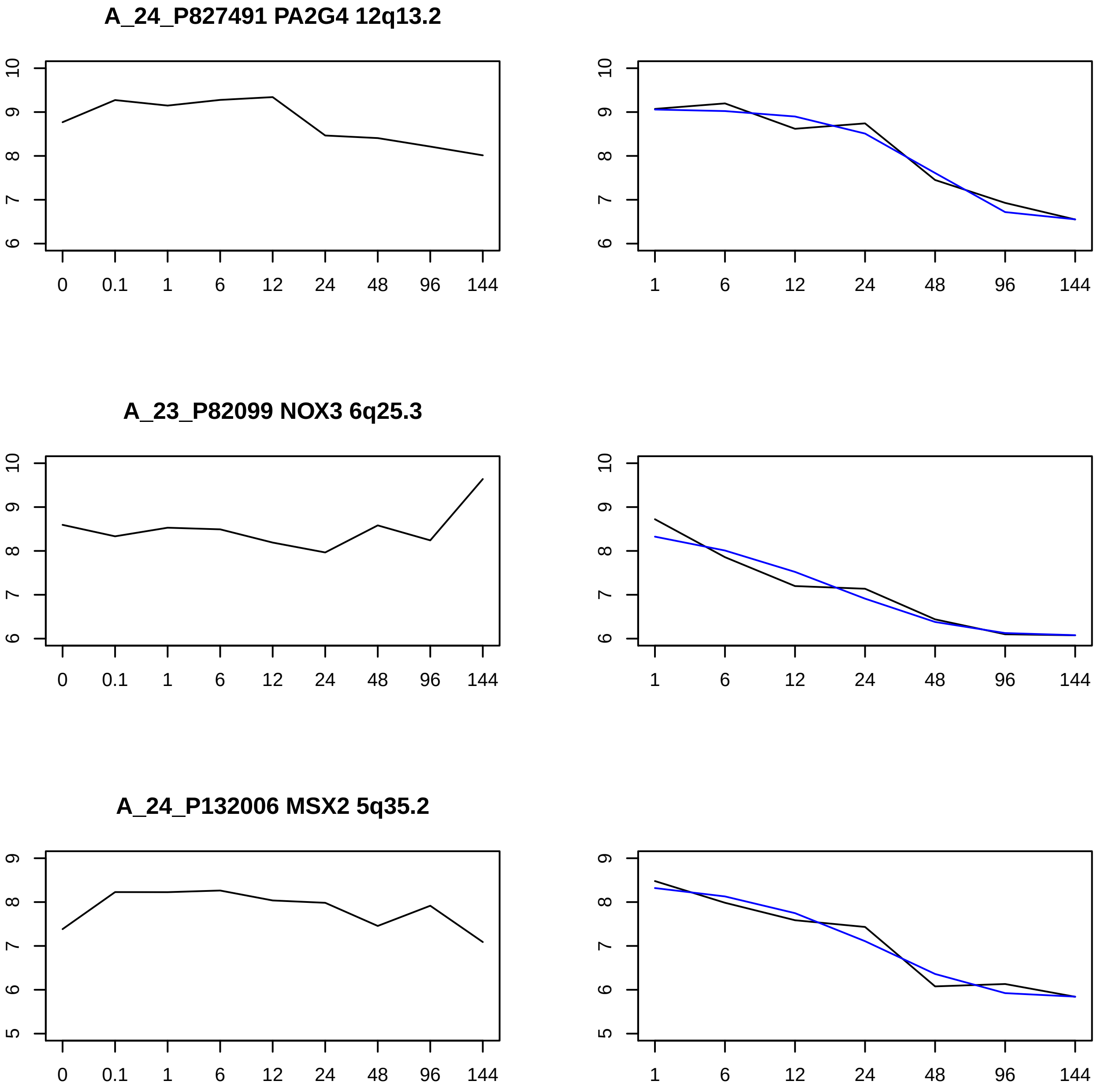
A_24_P827491 PA2G4 12q13.2
A_23_P82099 NOX3 6q25.3
A_24_P132006 MSX2 5q35.2
0
0.1
1
6
12
24
48
96
144
5
6
7
8
9
10

A_32_P160127 EBF3 10q26.3

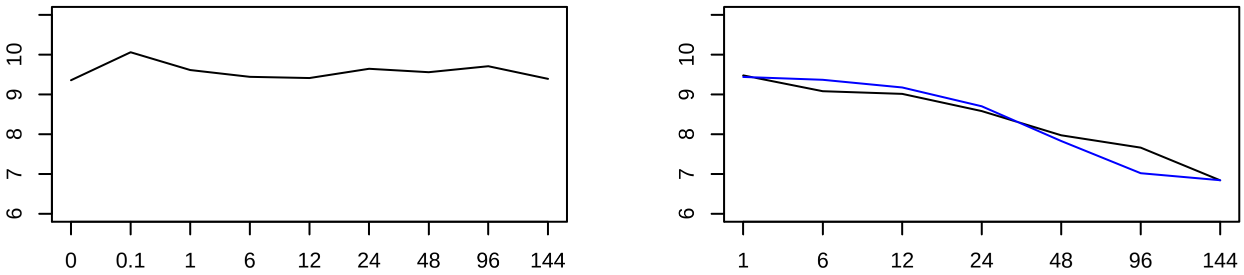

A_24_P24972 RPL22P11 NA

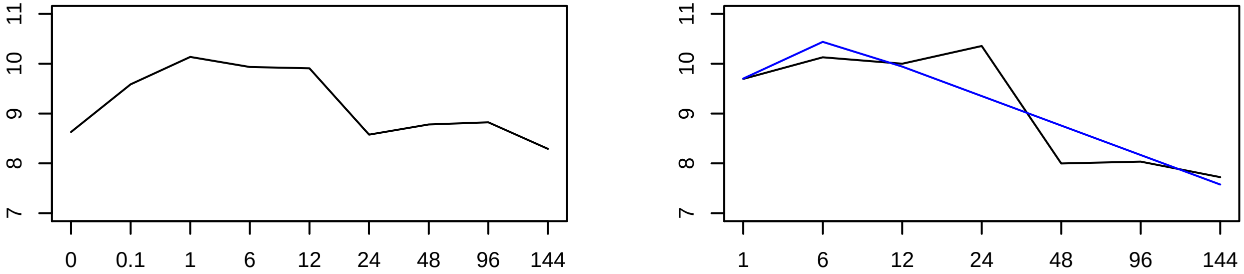

A_23_P213959 PPARGC1B 5q33.1

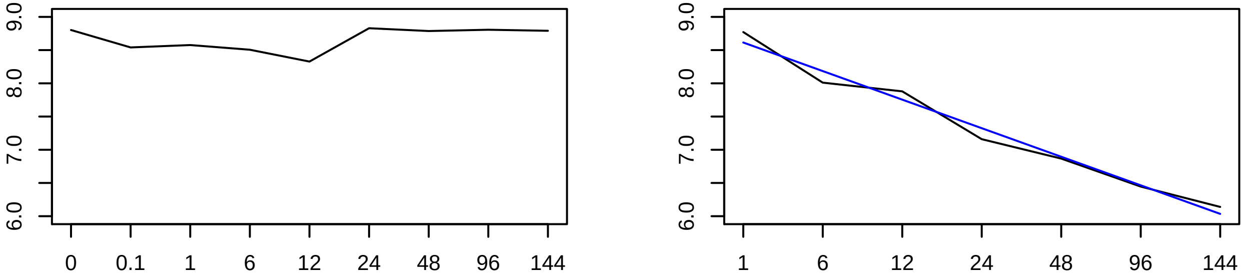

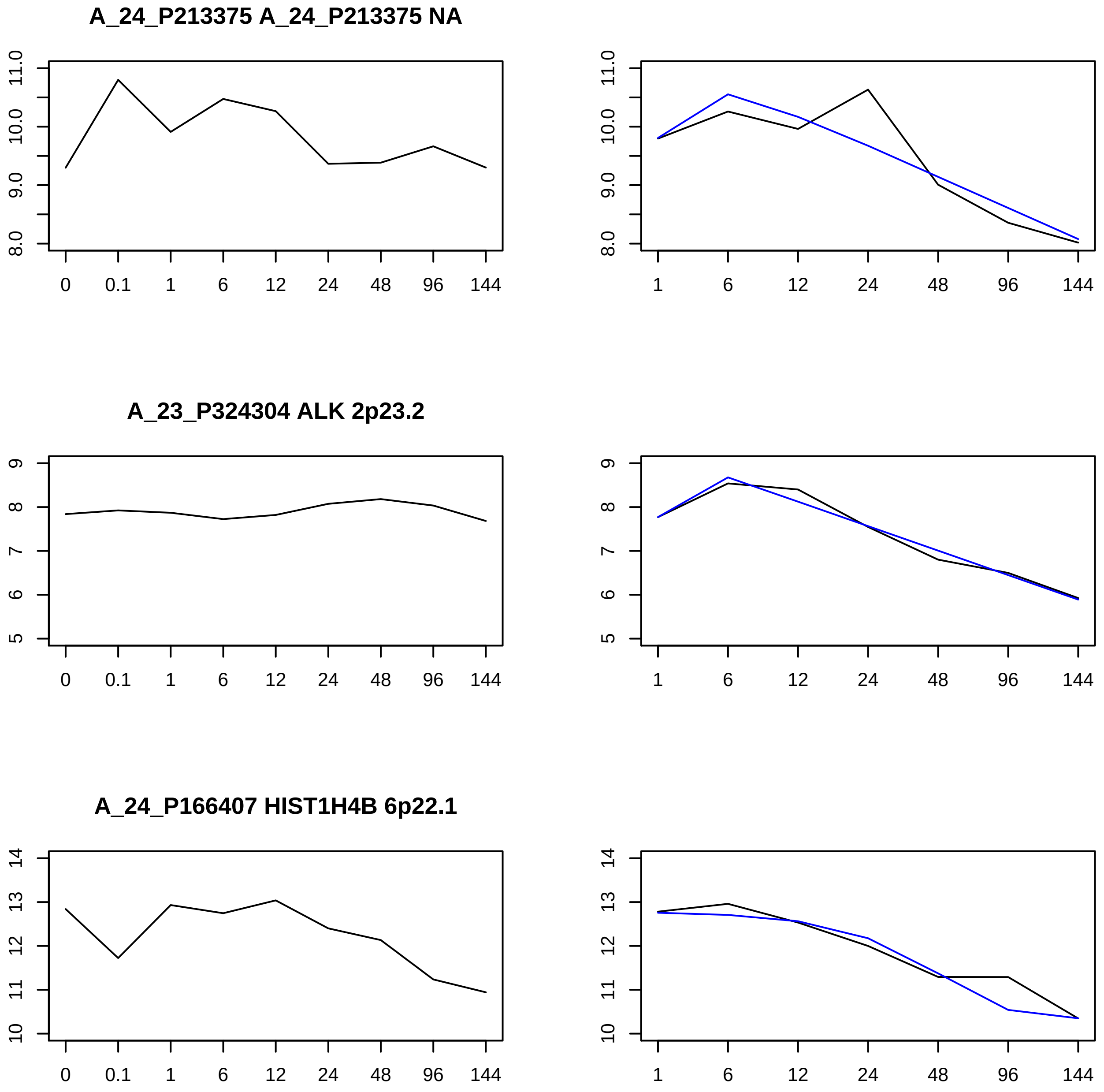
A_24_P213375 A_24_P213375 NA
8.0 9.0 10.0 11.0
0 0.1 1 6 12 24 48 96 144
8.0 9.0 10.0 11.0
1 6 12 24 48 96 144
A_23_P324304 ALK 2p23.2
5 6 7 8 9
0 0.1 1 6 12 24 48 96 144
5 6 7 8 9
1 6 12 24 48 96 144
A_24_P166407 HIST1H4B 6p22.1
10 11 12 13 14
0 0.1 1 6 12 24 48 96 144
10 11 12 13 14
1 6 12 24 48 96 144

A_24_P937435 RBED1 2p11.2

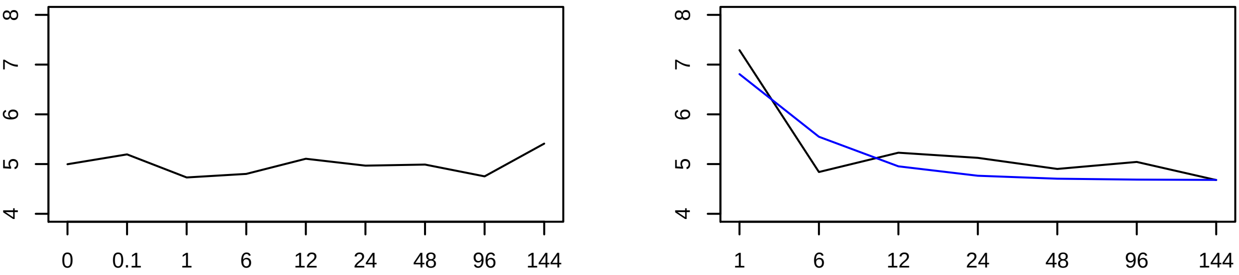

A_23_P431933 CAMKK1 17p13.2

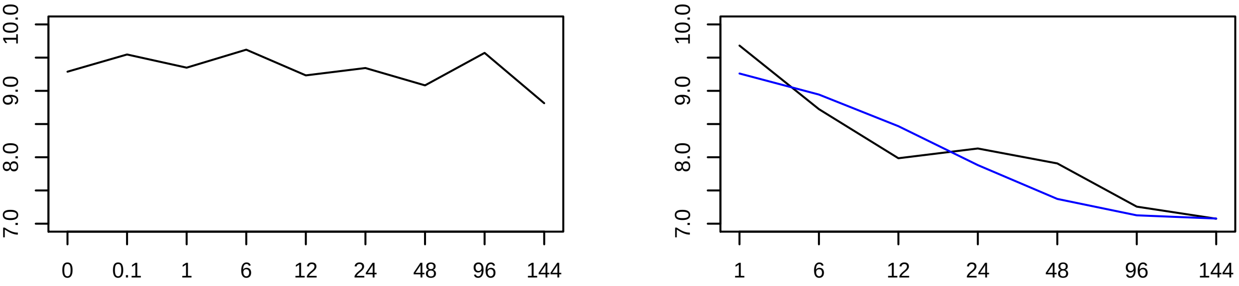

A_24_P349596 LOC646980 1p21.2

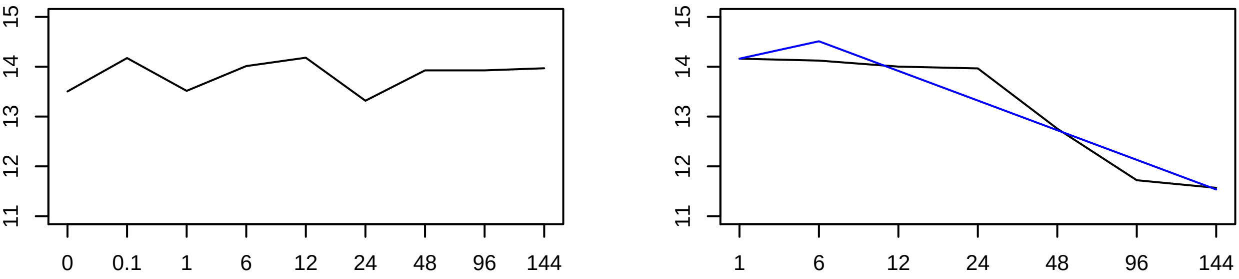

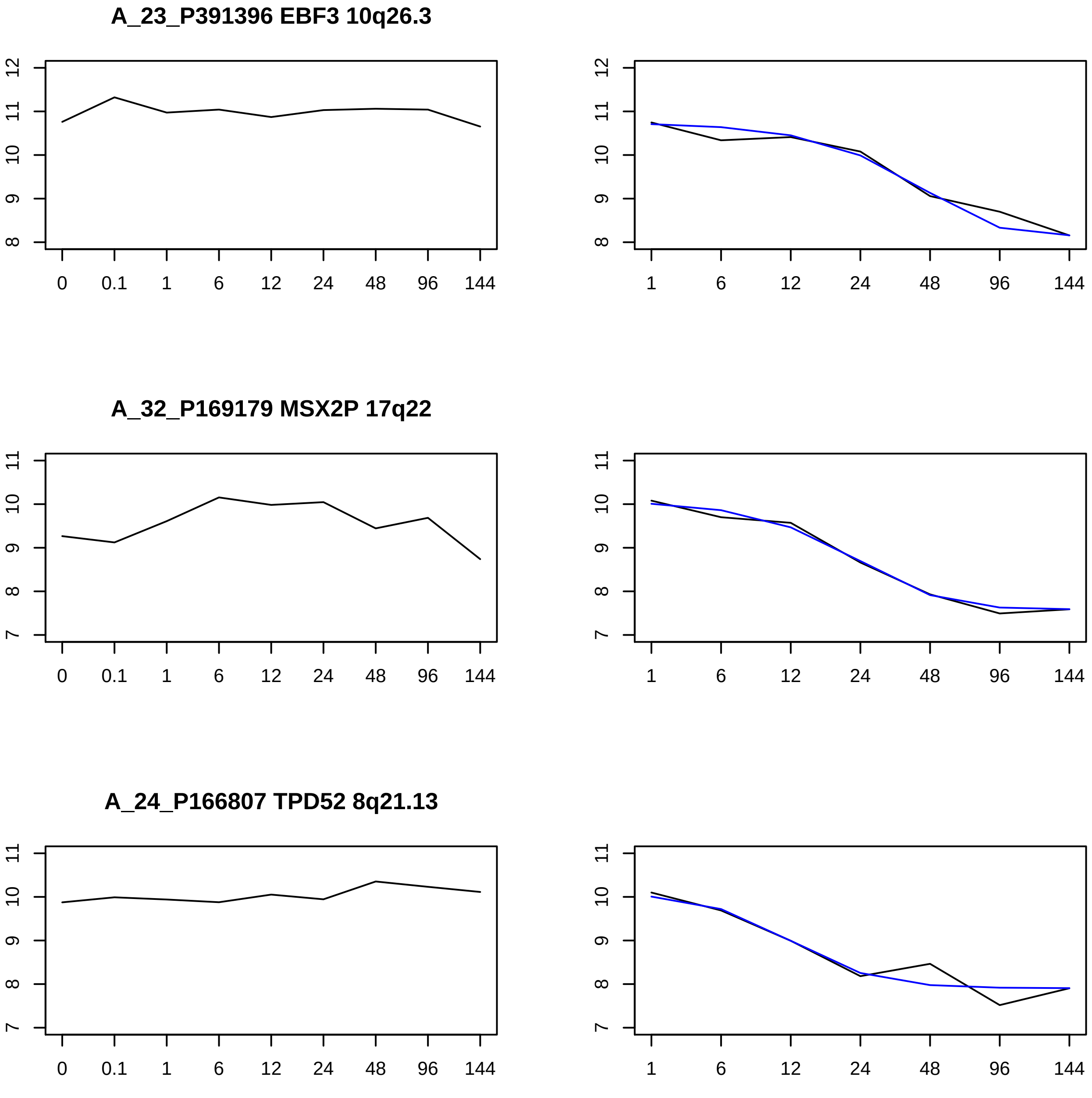
A_23_P391396 EBF3 10q26.3
8 9 10 11 12
0 0.1 1 6 12 24 48 96 144
1 6 12 24 48 96 144
A_32_P169179 MSX2P 17q22
7 8 9 10 11
0 0.1 1 6 12 24 48 96 144
1 6 12 24 48 96 144
A_24_P166807 TPD52 8q21.13
7 8 9 10 11
0 0.1 1 6 12 24 48 96 144
1 6 12 24 48 96 144

A_23_P340218 FLJ35773 17p13.1

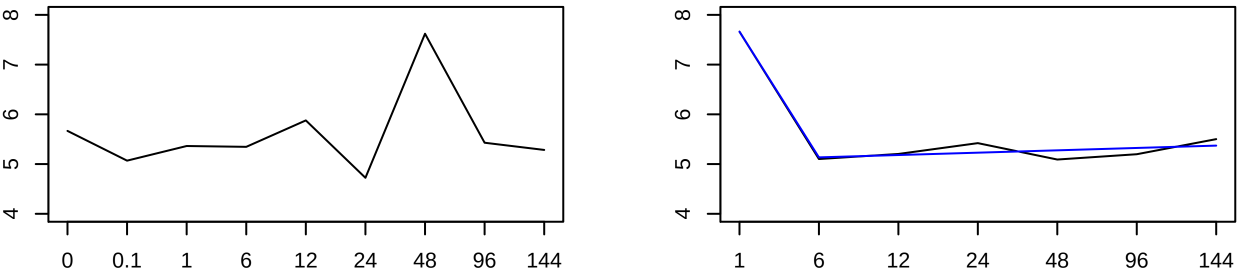

A_24_P384411 A_24_P384411 NA

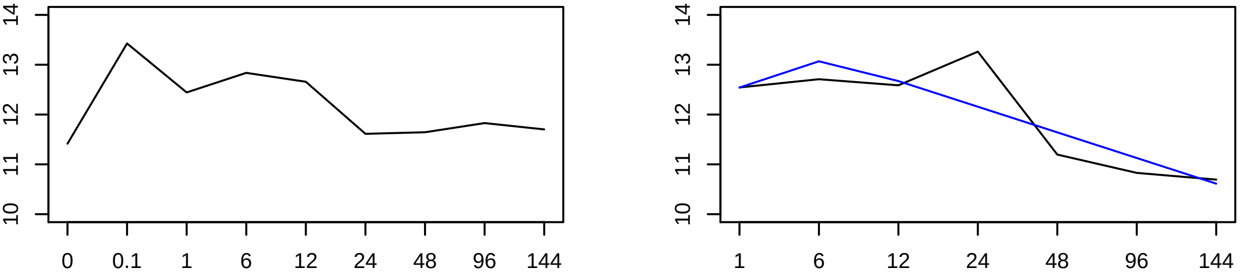

A_32_P50973 THC2643327 NA

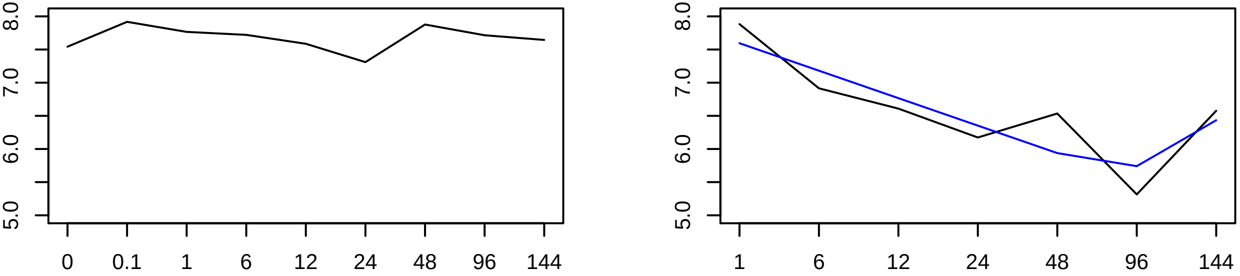

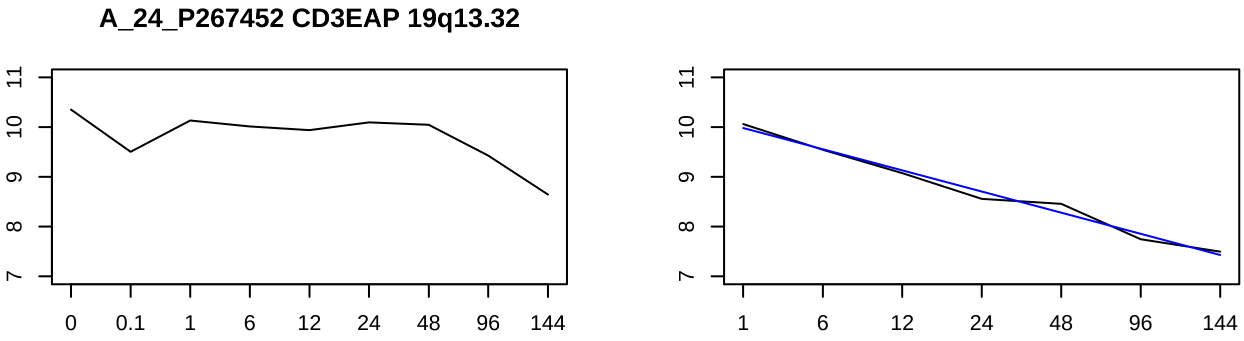
A_24_P267452 CD3EAP 19q13.32
7 8 9 10 11
0 0.1 1 6 12 24 48 96 144
7 8 9 10 11
1 6 12 24 48 96 144

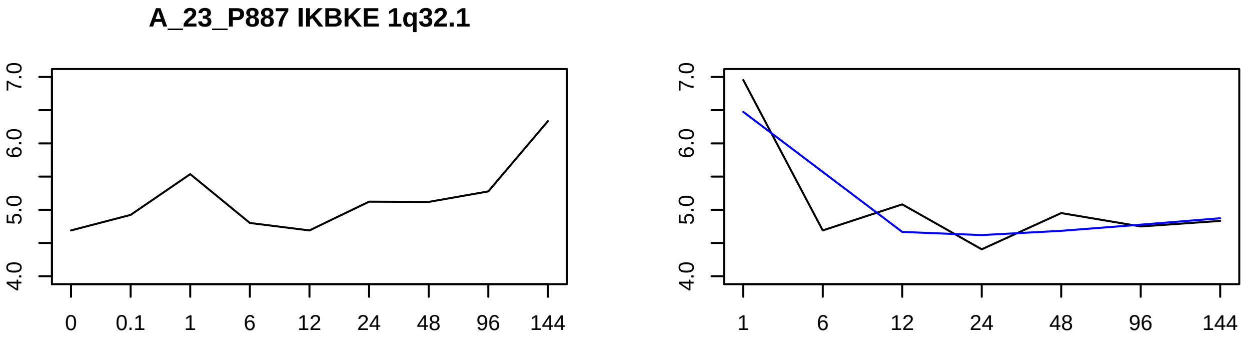
A_23_P887 IKBKE 1q32.1
4.0 5.0 6.0 7.0
0 0.1 1 6 12 24 48 96 144
4.0 5.0 6.0 7.0
1 6 12 24 48 96 144

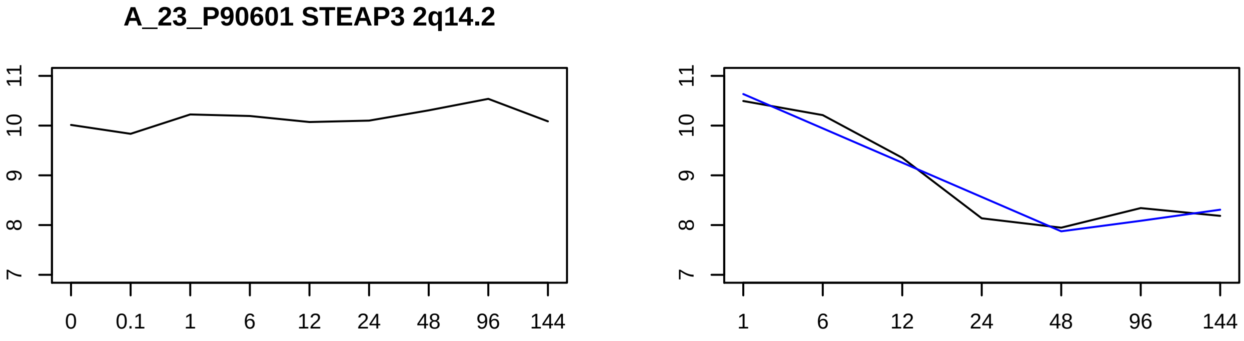
A_23_P90601 STEAP3 2q14.2
7 8 9 10 11
0 0.1 1 6 12 24 48 96 144
7 8 9 10 11
1 6 12 24 48 96 144

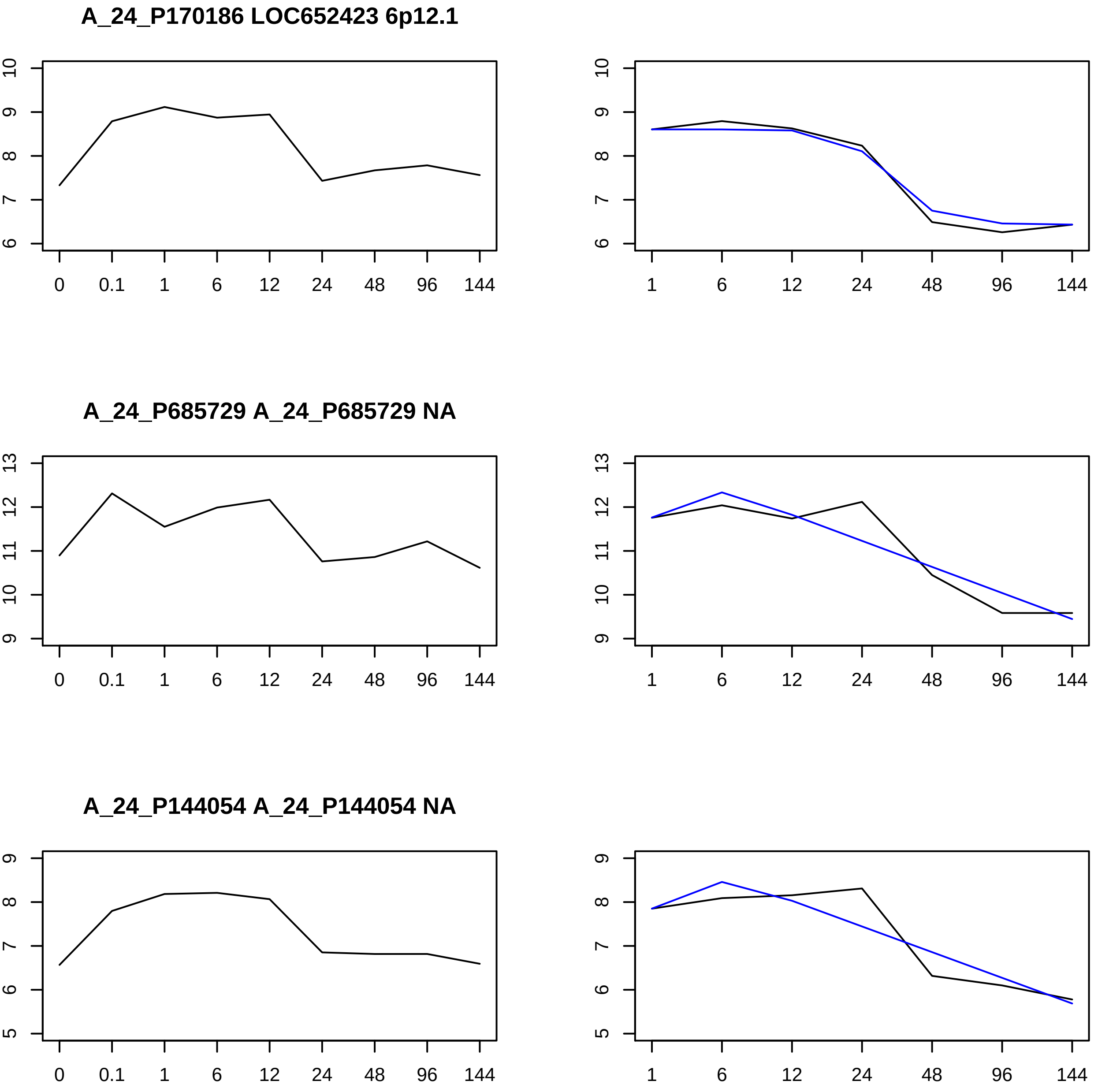
A_24_P170186 LOC652423 6p12.1
6 7 8 9 10
0 0.1 1 6 12 24 48 96 144
1 6 12 24 48 96 144
A_24_P685729 A_24_P685729 NA
9 10 11 12 13
0 0.1 1 6 12 24 48 96 144
1 6 12 24 48 96 144
A_24_P144054 A_24_P144054 NA
5 6 7 8 9
0 0.1 1 6 12 24 48 96 144
1 6 12 24 48 96 144

A_23_P7752 SEMA6A 5q23.1

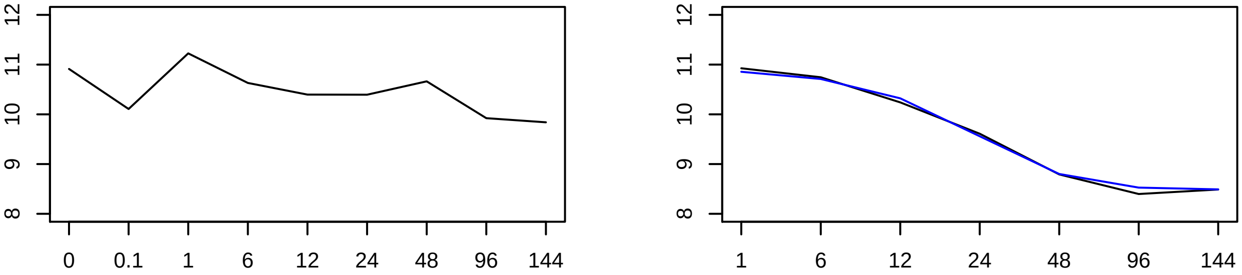

A_24_P306469 LOC257039 3q24

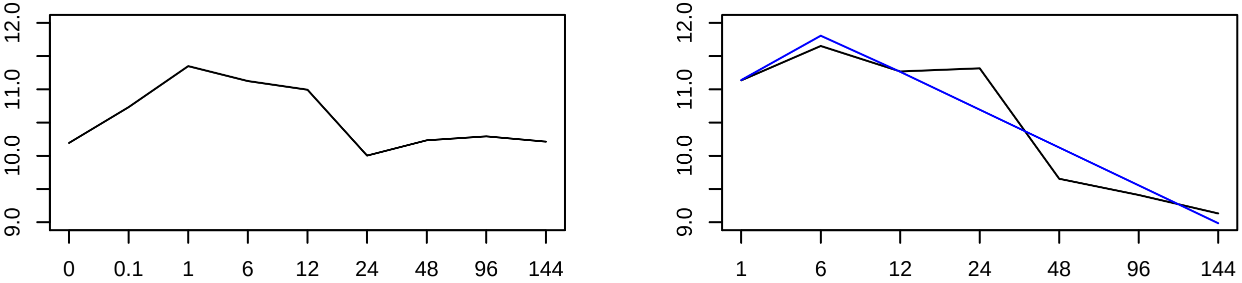

A_23_P5903 SLCO4A1 20q13.33

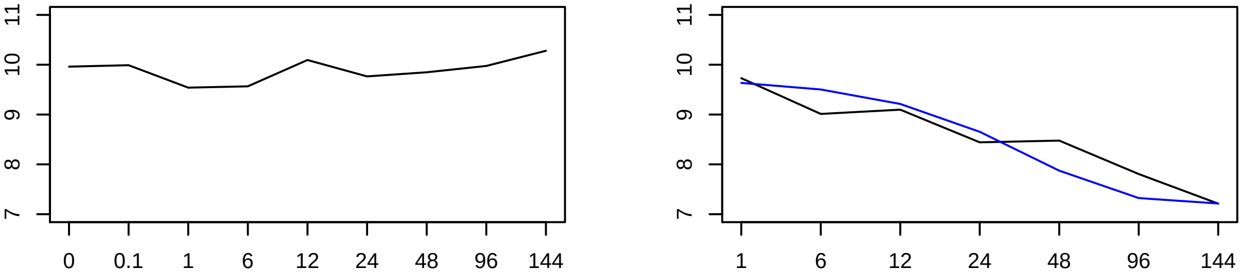

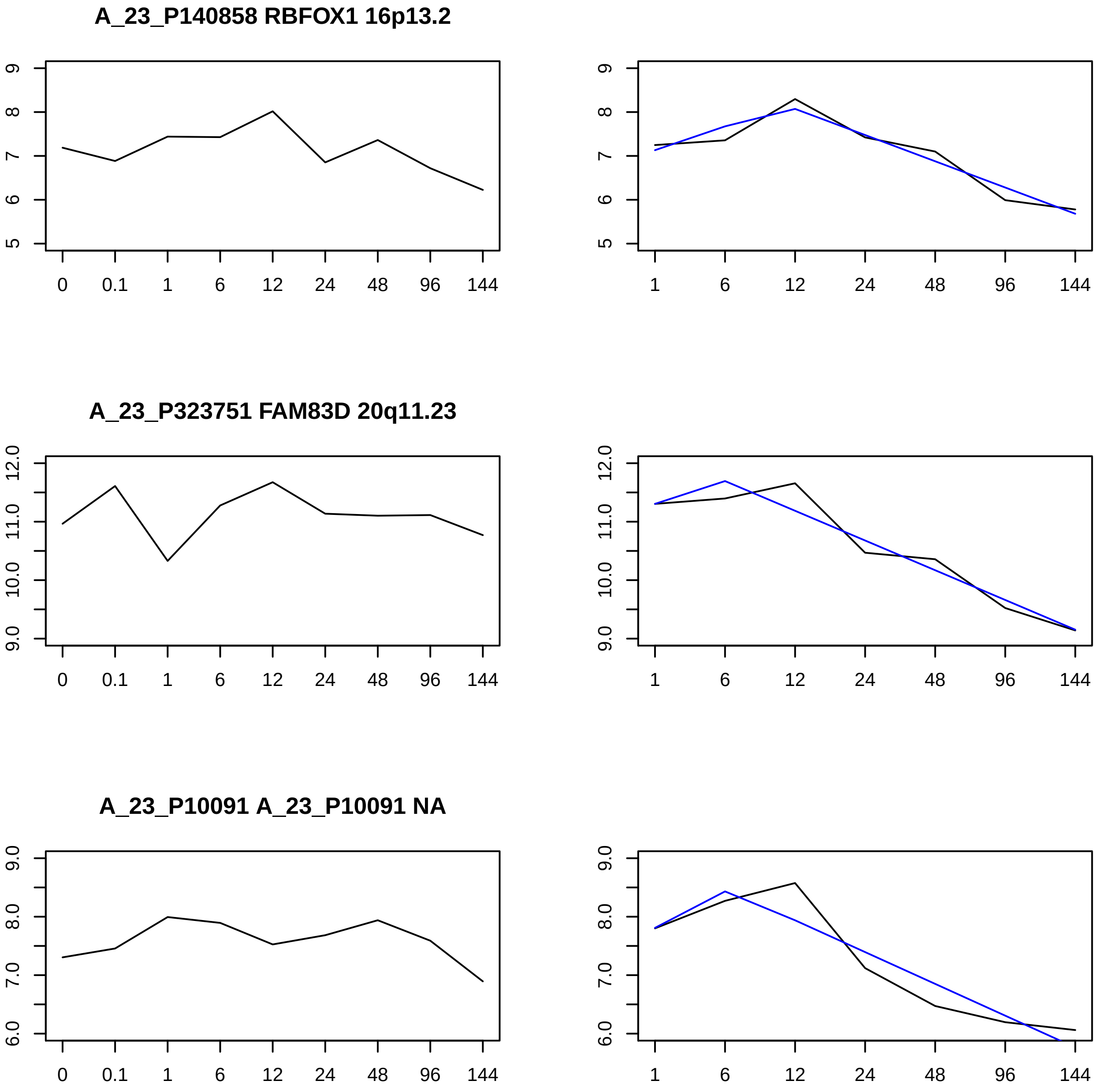
A_23_P140858 RBFOX1 16p13.2
A_23_P323751 FAM83D 20q11.23
A_23_P10091 A_23_P10091 NA
0 0.1 1 6 12 24 48 96 144
1 6 12 24 48 96 144

A_23_P88262 TRAV20 NA

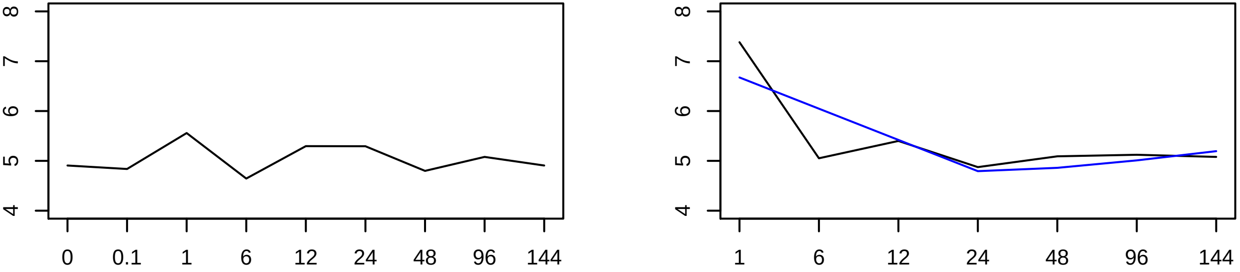

A_23_P346900 CACNA2D2 3p21.31

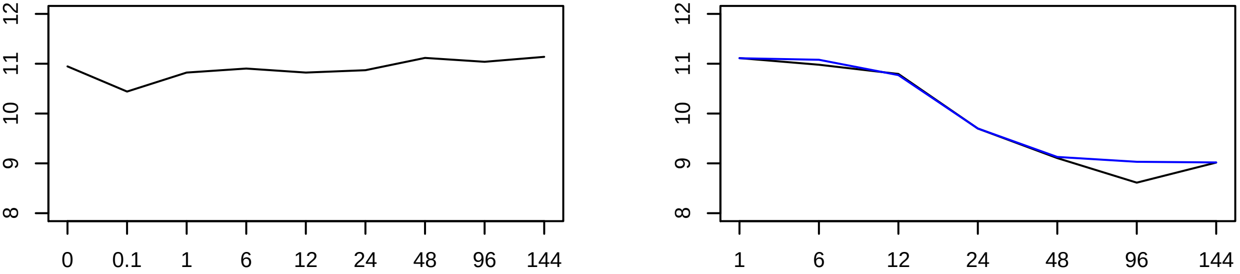

A_32_P186038 THC2736130 NA

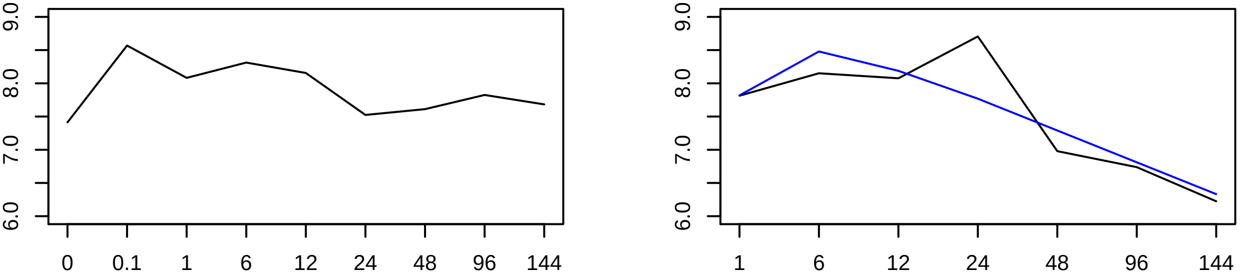

A_24_P418536 LOC343495 1p12

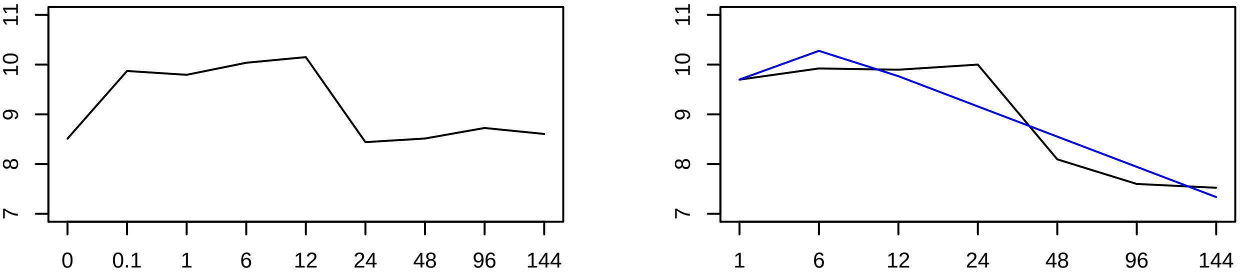

A_24_P298616 A_24_P298616 NA

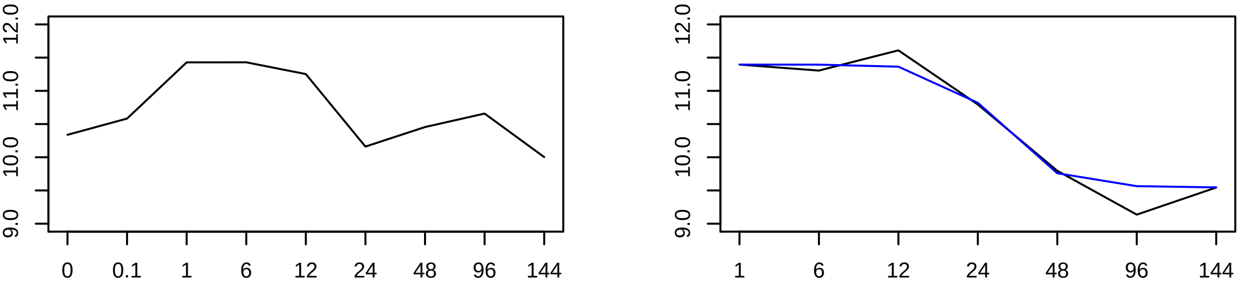

A_24_P315474 LOC646973 2q37.1

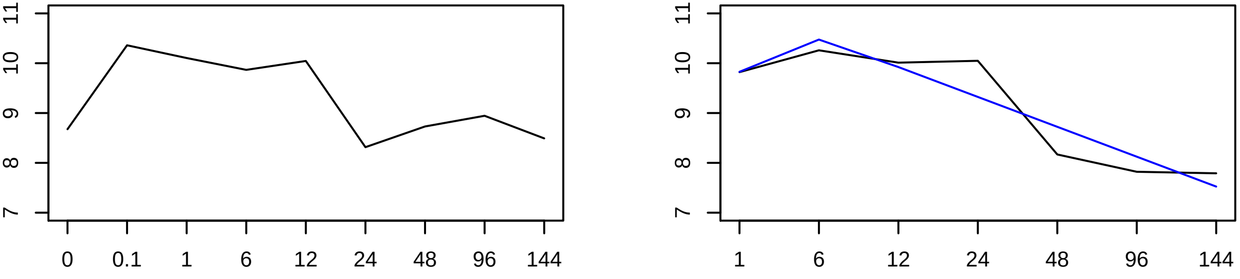

A_24_P25040 A_24_P25040 NA

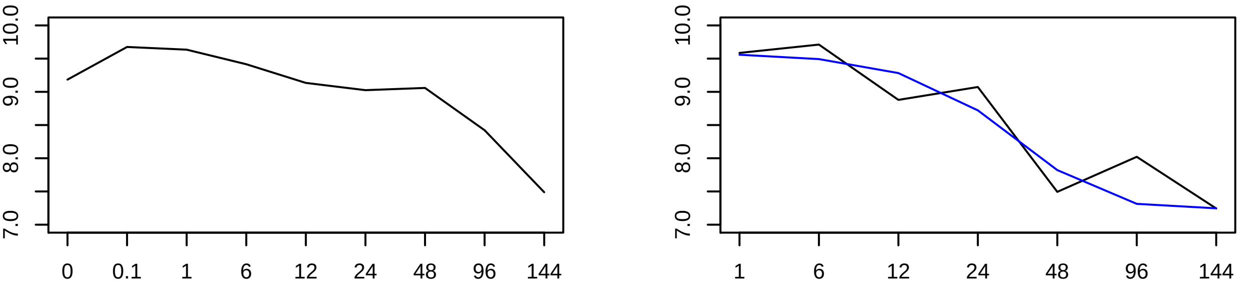

A_24_P358205 A_24_P358205 NA

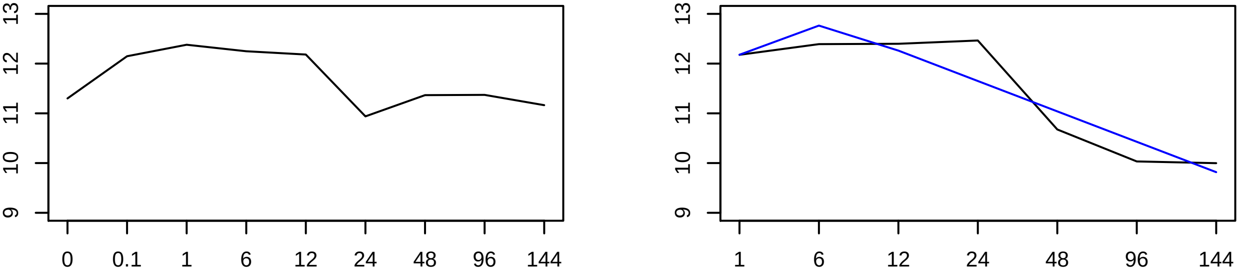

A_24_P118382 A_24_P118382 NA

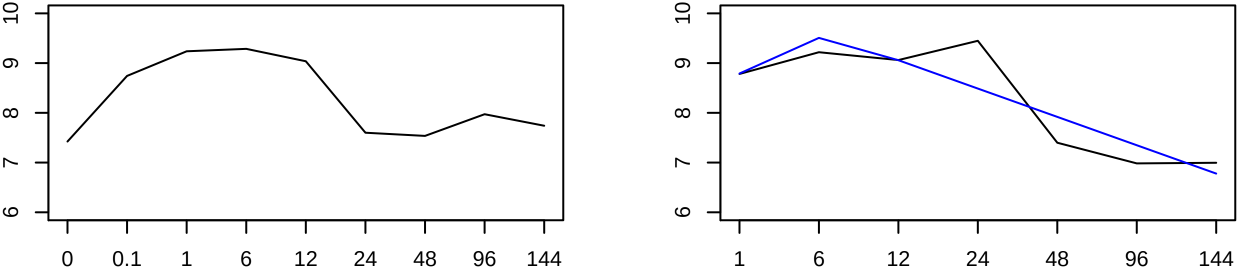

A_24_P409560 LOC649228 5q23.3

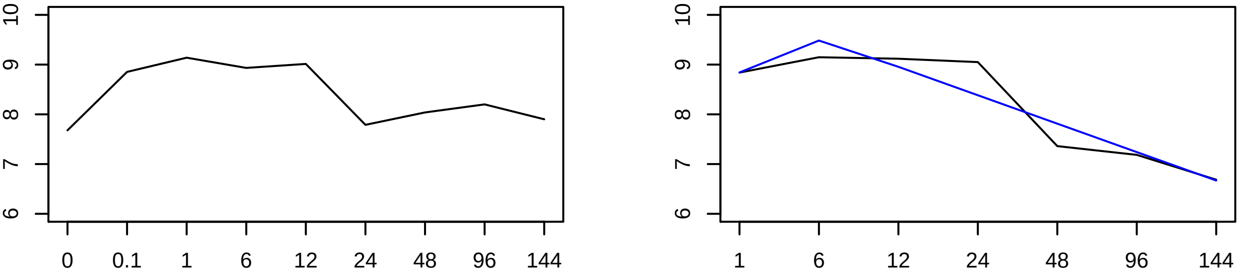

A_23_P59888 NACAP1 8q22.3

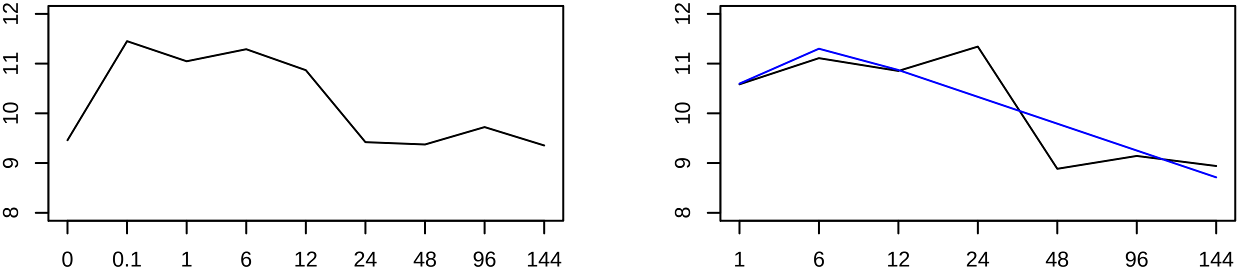

A_24_P6381 SERINC5 5q14.1

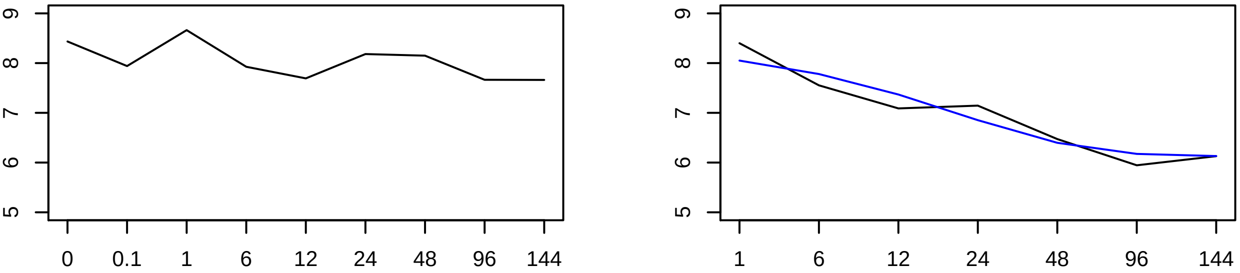

A_23_P74059 NPPA 1p36.22

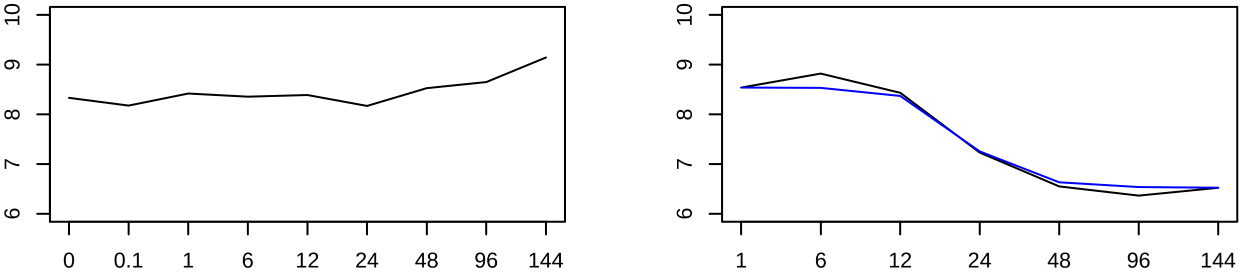

A_23_P304110 SOWAHA 5q31.1

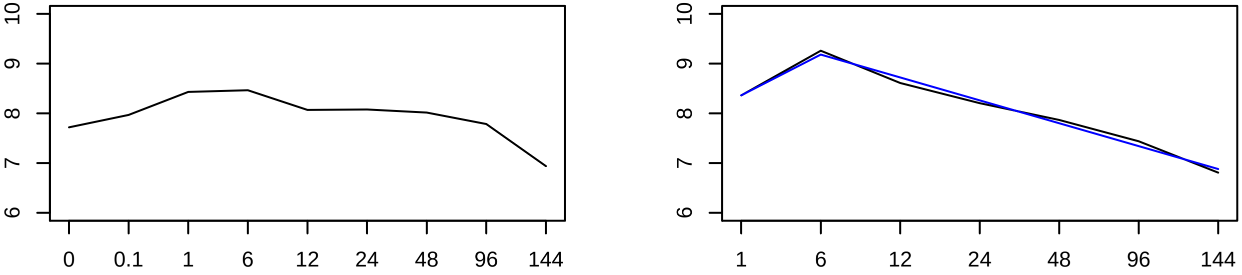

A_24_P323635 LOC137107 8p12

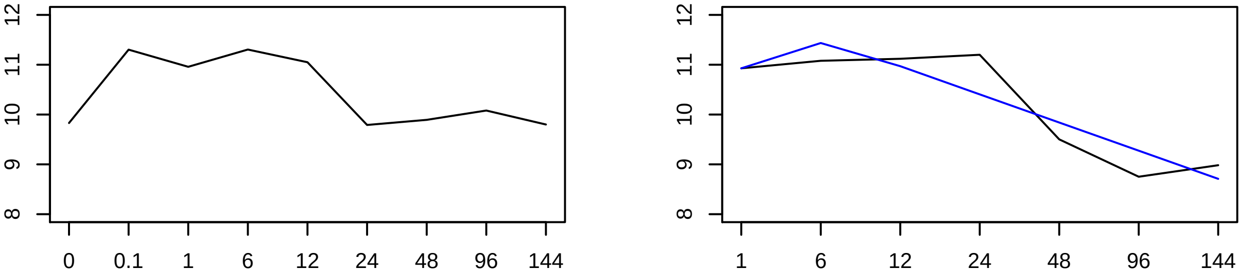

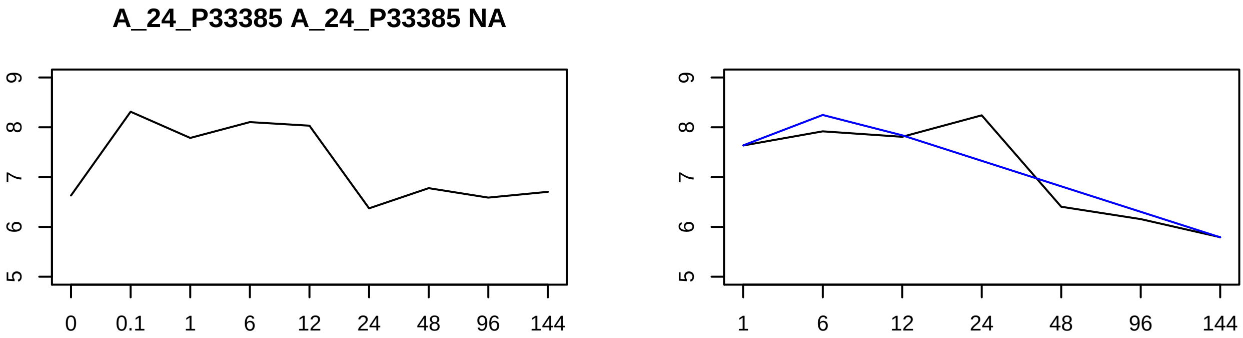
A_24_P33385 A_24_P33385 NA
5 6 7 8 9
0 0.1 1 6 12 24 48 96 144
1 6 12 24 48 96 144

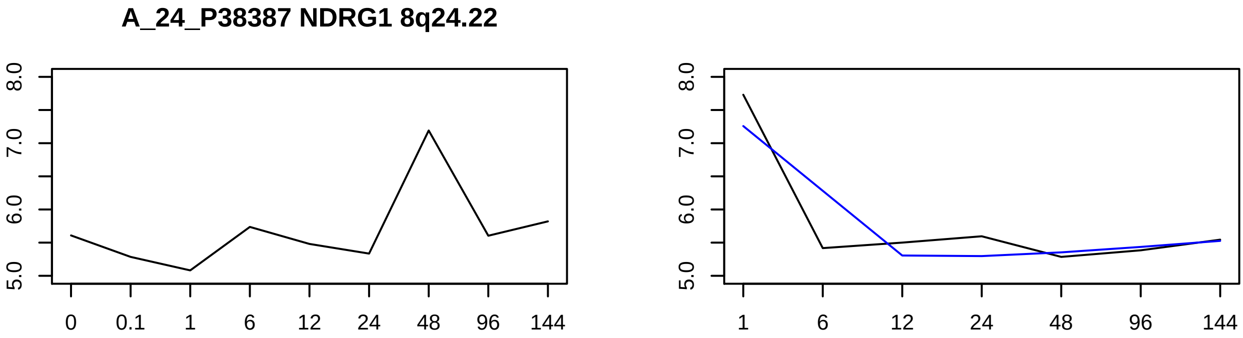
A_24_P38387 NDRG1 8q24.22
5.0 6.0 7.0 8.0
0 0.1 1 6 12 24 48 96 144
1 6 12 24 48 96 144

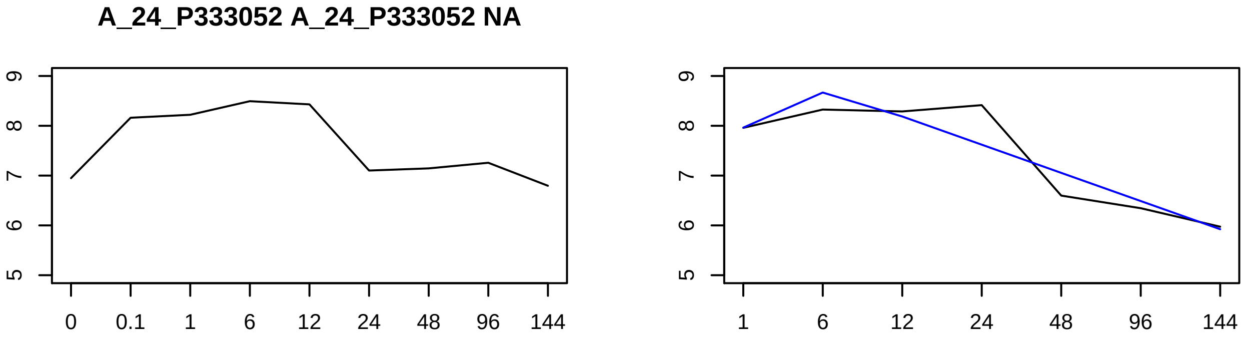
A_24_P333052 A_24_P333052 NA
5 6 7 8 9
0 0.1 1 6 12 24 48 96 144
1 6 12 24 48 96 144

A_24_P916116 A_24_P916116 NA

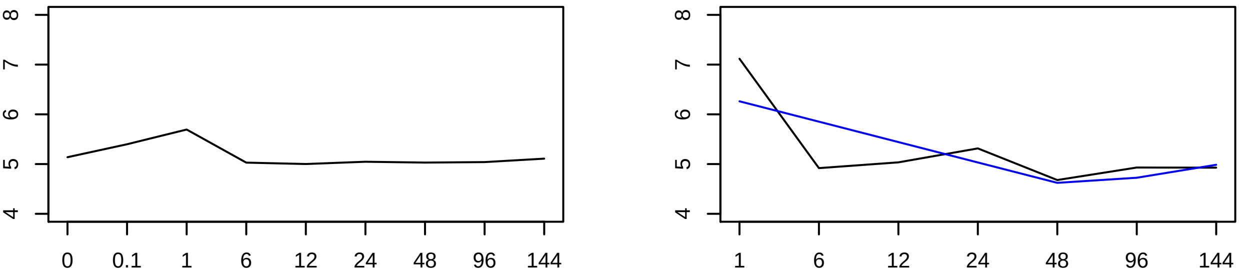

A_24_P337239 FGF5 4q21.21

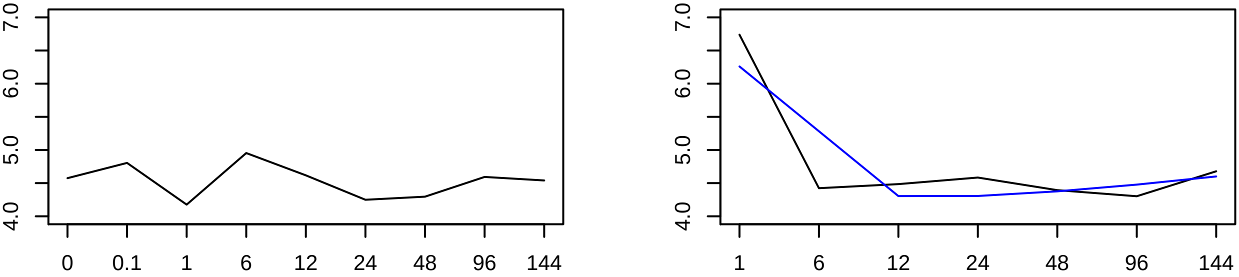

A_24_P938169 RPL22 1p36.31

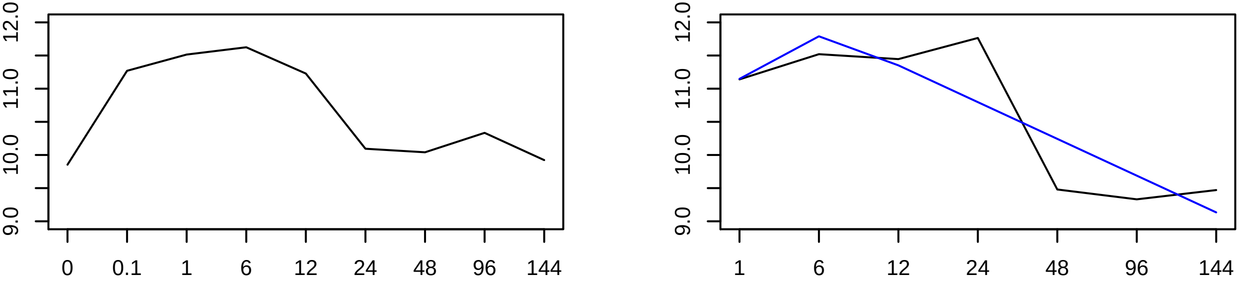

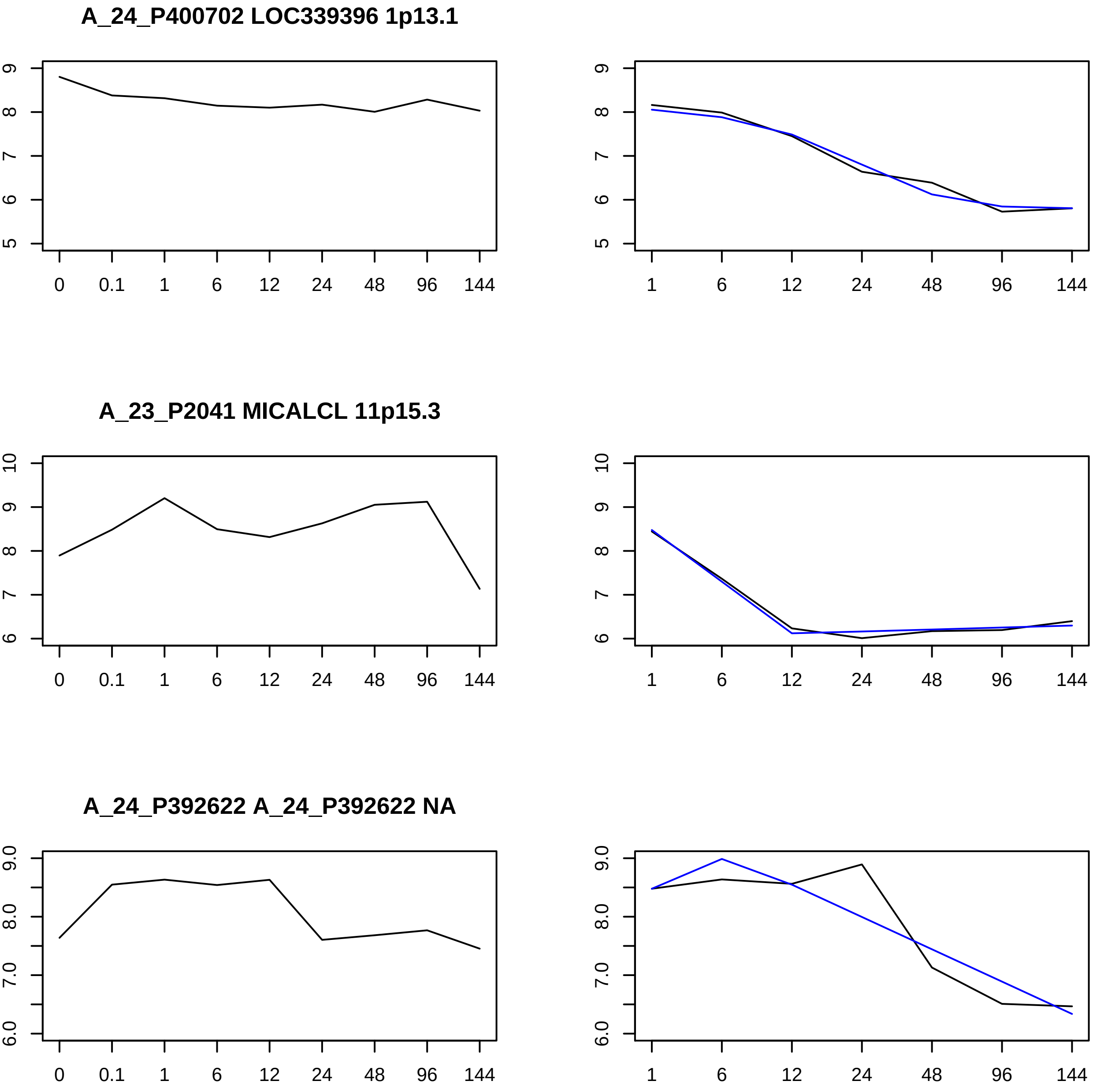

A_24_P400702 LOC339396 1p13.1
5 6 7 8 9
0 0.1 1 6 12 24 48 96 144
1 6 12 24 48 96 144
A_23_P2041 MICALCL 11p15.3
6 7 8 9 10
0 0.1 1 6 12 24 48 96 144
1 6 12 24 48 96 144
A_24_P392622 A_24_P392622 NA
6.0 7.0 8.0 9.0
0 0.1 1 6 12 24 48 96 144
1 6 12 24 48 96 144

A_24_P400751 A_24_P400751 NA

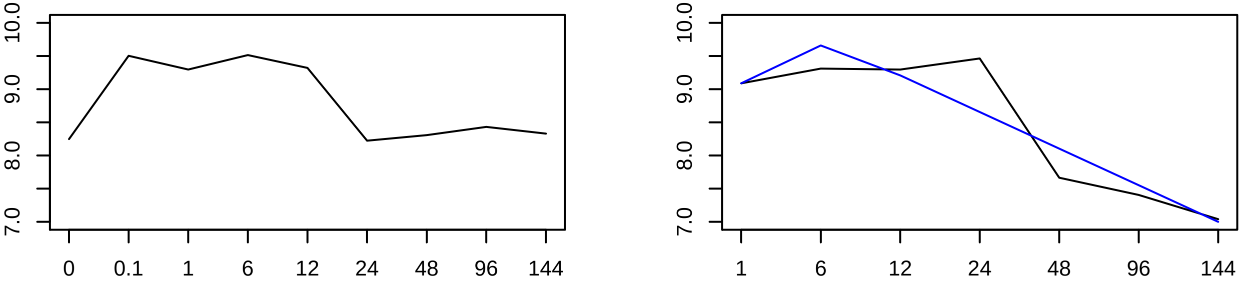

A_24_P306921 LOC124496 16q23.1

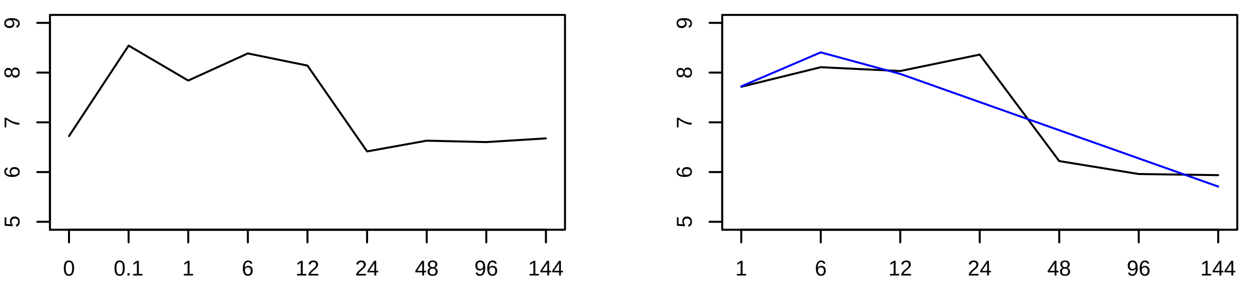

A_24_P59239 A_24_P59239 NA

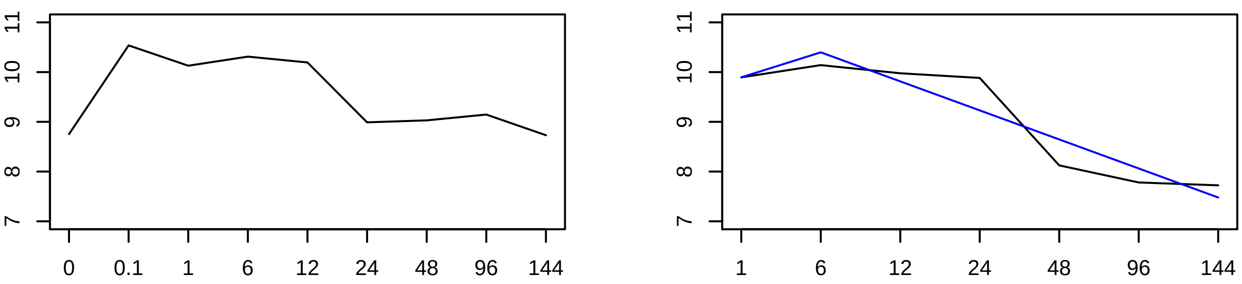

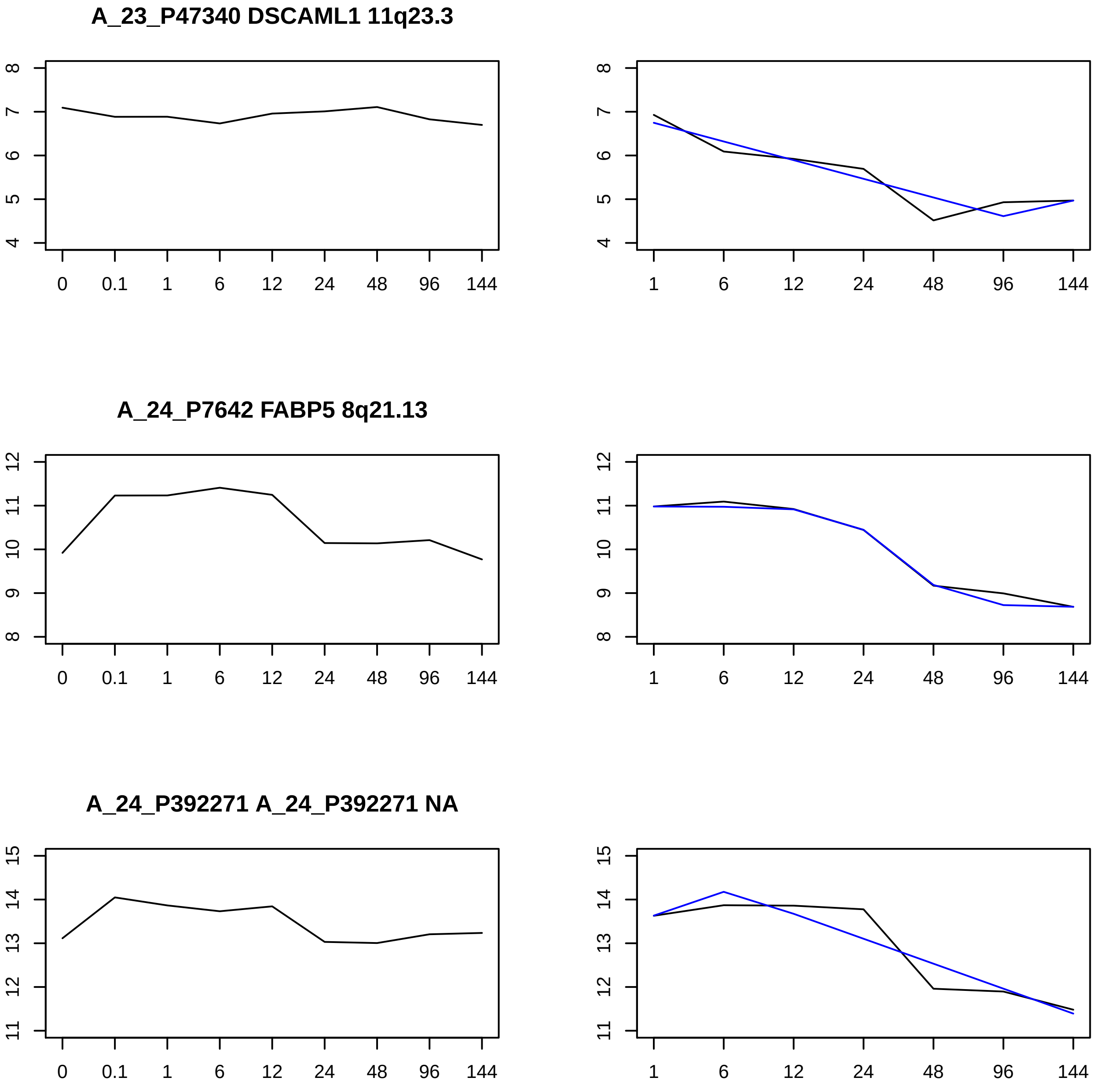

A_23_P47340 DSCAML1 11q23.3
4
5
6
7
8
0
0.1
1
6
12
24
48
96
144
1
6
12
24
48
96
144
A_24_P7642 FABP5 8q21.13
8
9
10
11
12
0
0.1
1
6
12
24
48
96
144
1
6
12
24
48
96
144
A_24_P392271 A_24_P392271 NA
11
12
13
14
15
0
0.1
1
6
12
24
48
96
144
1
6
12
24
48
96
144

A_32_P134968 SPTB 14q23.3

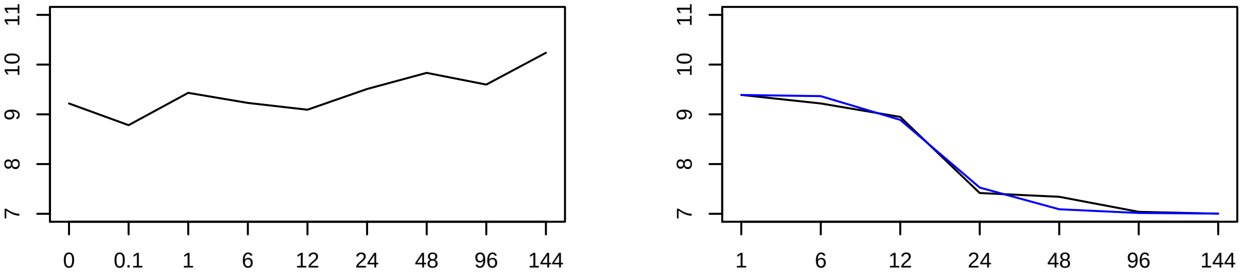

A_24_P382187 IGFBP4 17q21.2

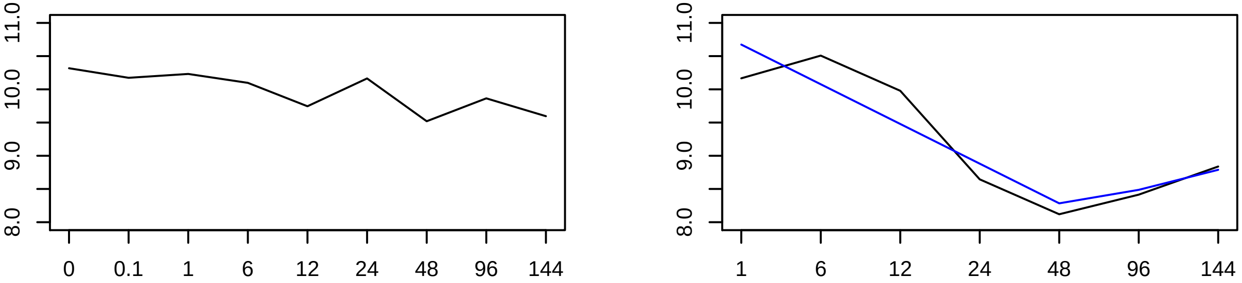

A_32_P34365 AA541413 NA

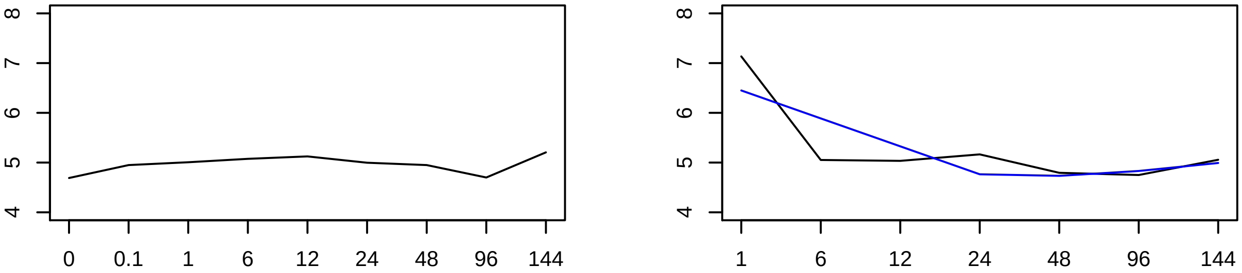

A_24_P24982 A_24_P24982 NA

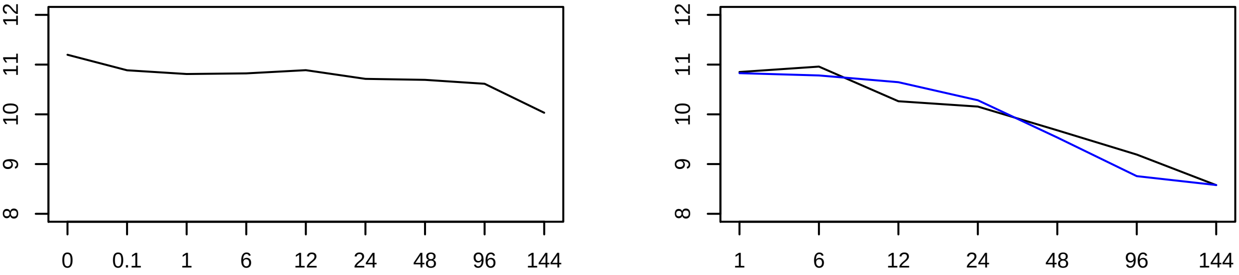

A_24_P675947 RPS3AP5 10q23.1

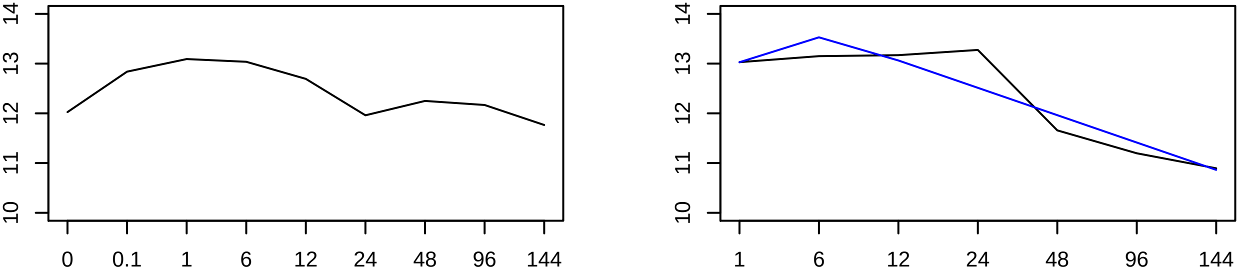

A_24_P41662 A_24_P41662 NA

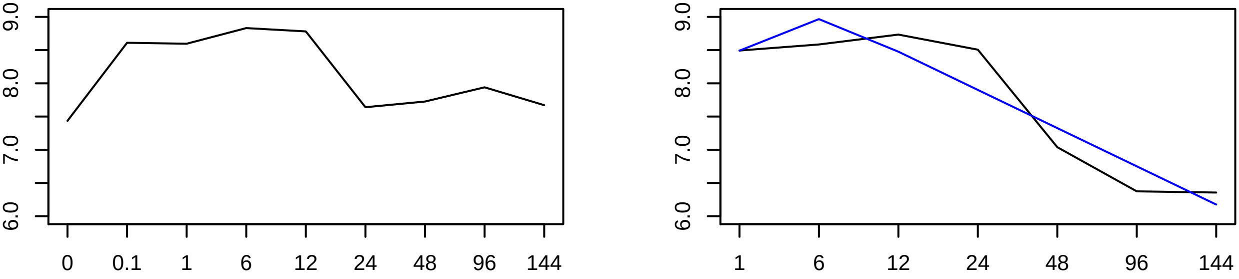

A_23_P210210 EPAS1 2p21

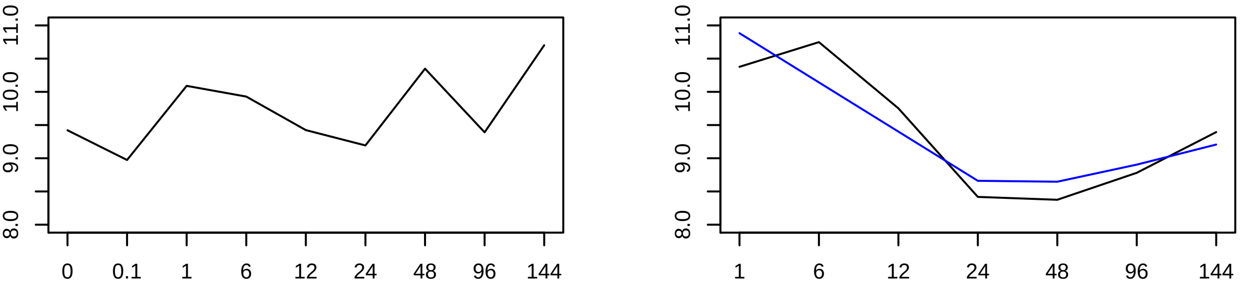

A_23_P127948 ADM 11p15.4

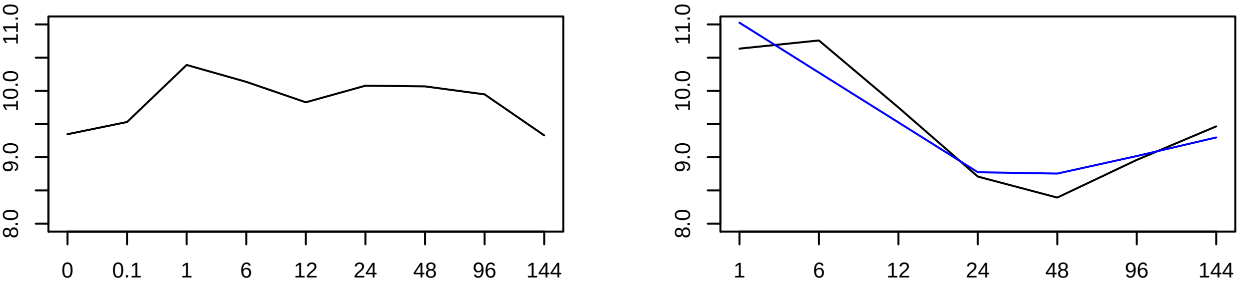

A_24_P58242 ENST00000304016 NA

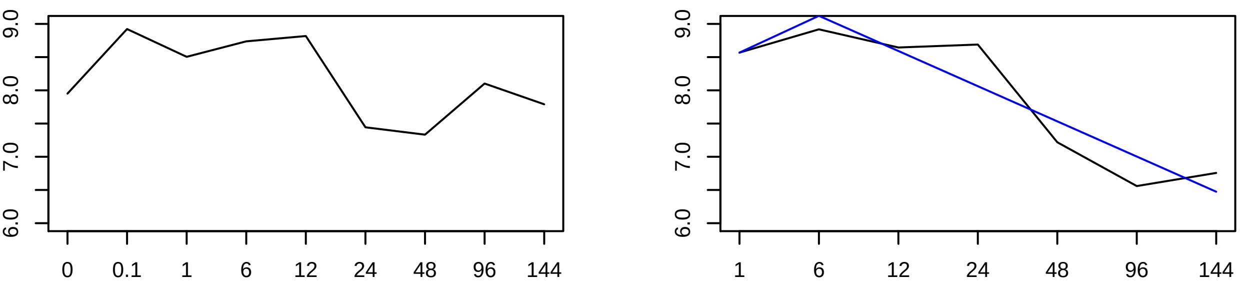

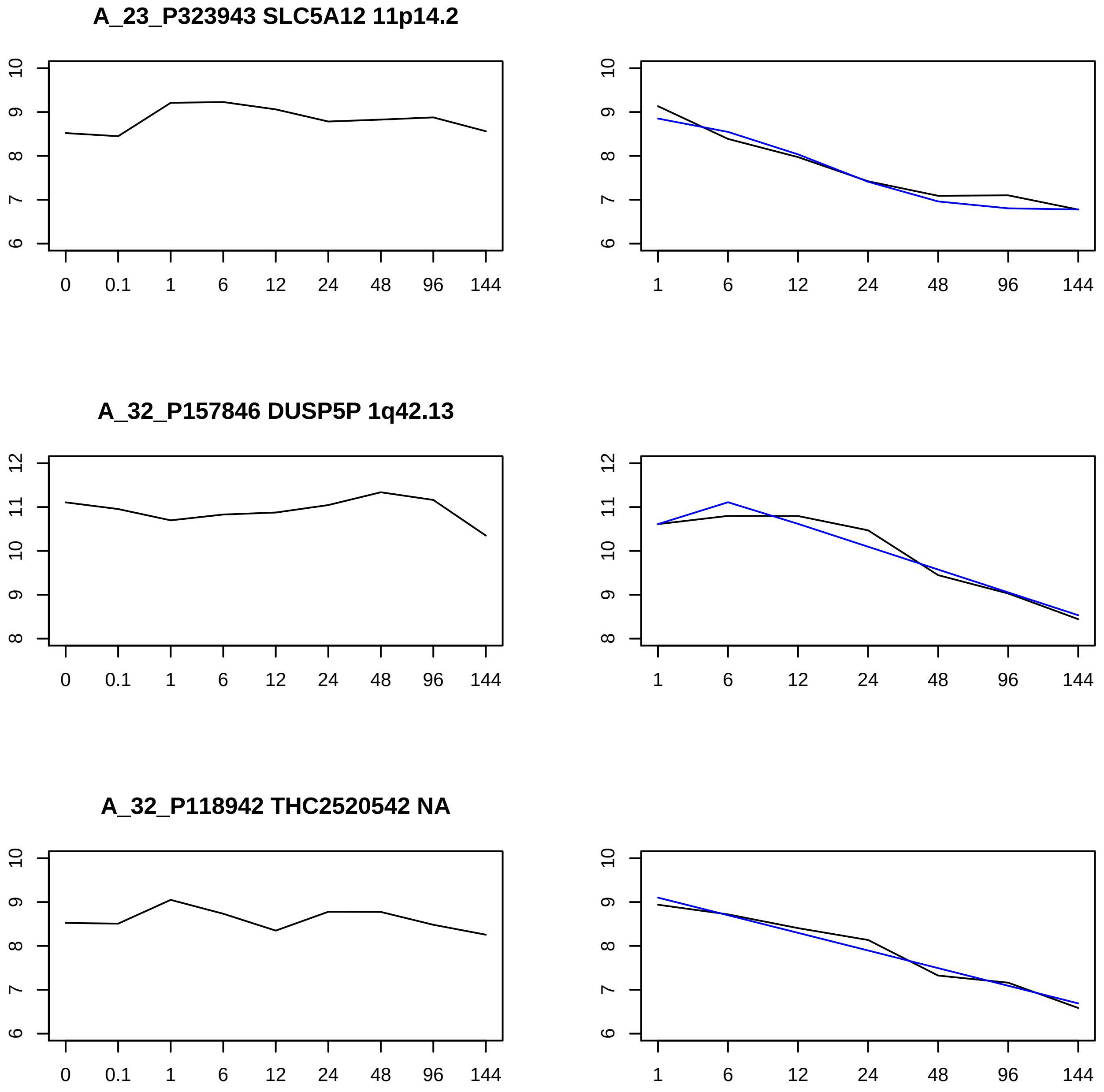
A_23_P323943 SLC5A12 11p14.2
6 7 8 9 10
0 0.1 1 6 12 24 48 96 144
1 6 12 24 48 96 144
A_32_P157846 DUSP5P 1q42.13
8 9 10 11 12
0 0.1 1 6 12 24 48 96 144
1 6 12 24 48 96 144
A_32_P118942 THC2520542 NA
6 7 8 9 10
0 0.1 1 6 12 24 48 96 144
1 6 12 24 48 96 144

A_24_P118281 RPS23P2 4q28.3

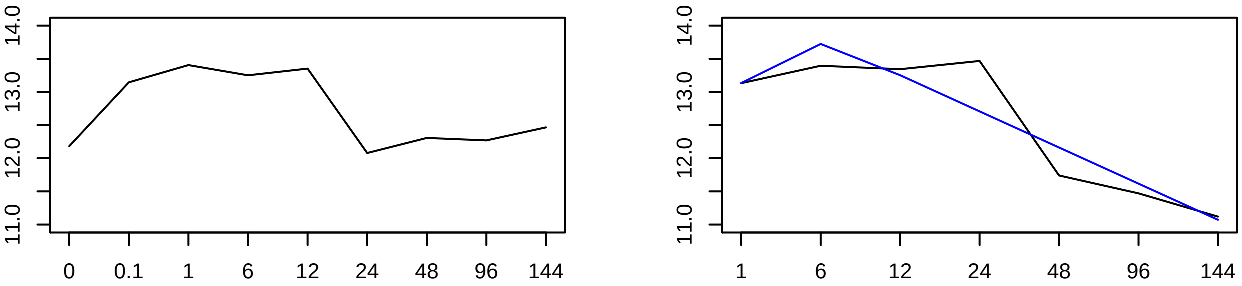

A_24_P93452 A_24_P93452 NA

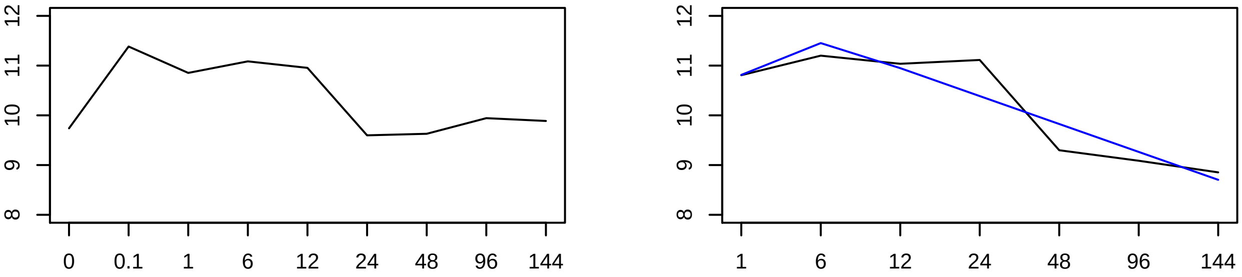

A_24_P41149 ENST00000308118 NA

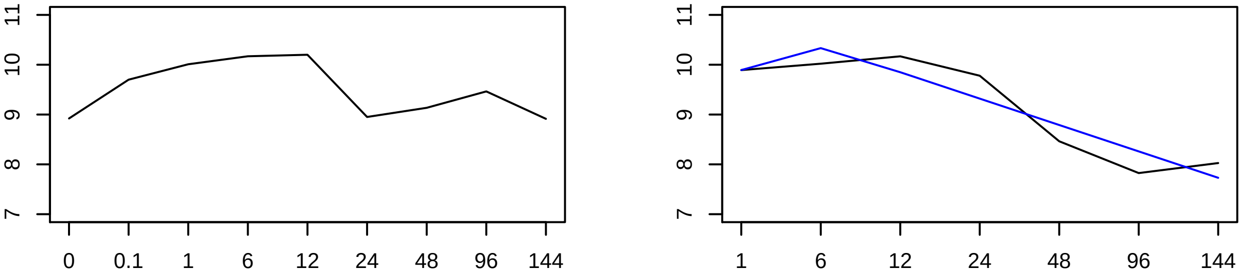

A_24_P127312 RPL17P33 8p21.1

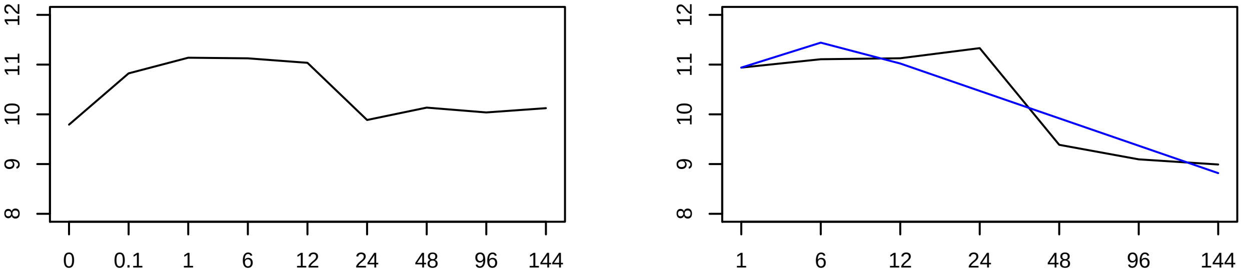

A_24_P101271 A_24_P101271 NA

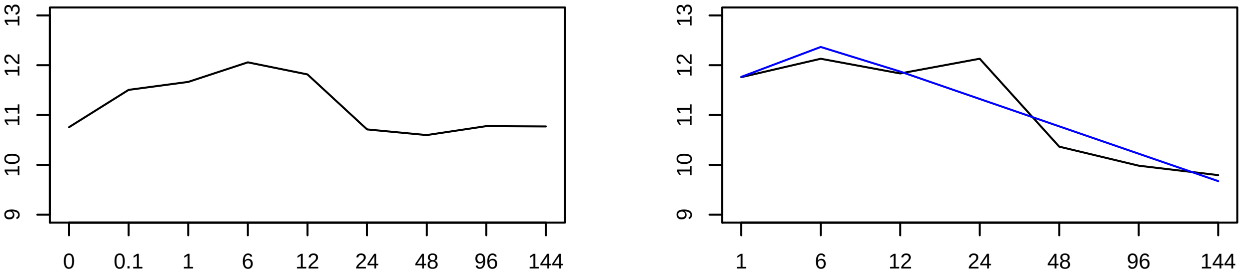

A_32_P65691 THC2686343 NA

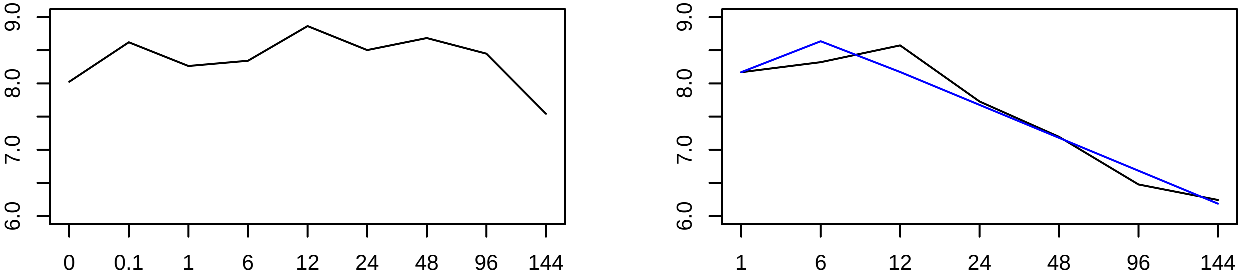

A_24_P58759 LOC402226 5q22.3

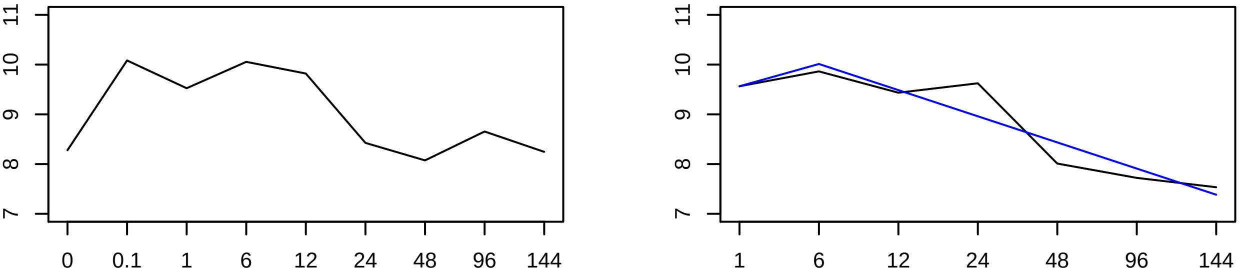

A_32_P216715 AA601031 NA

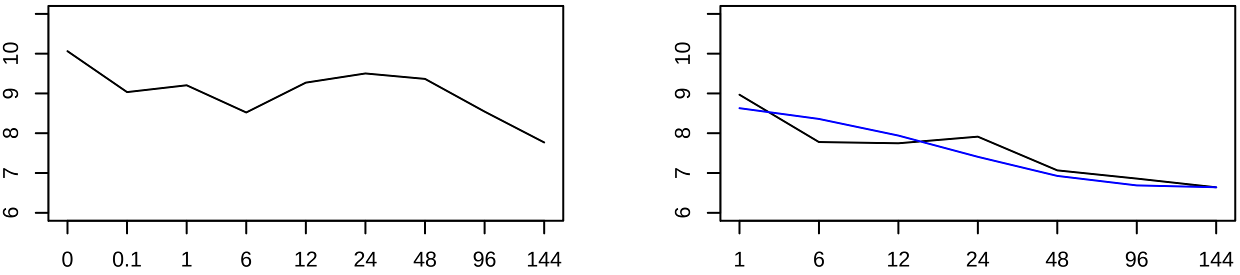

A_24_P75778 LOC392005 7p15.2

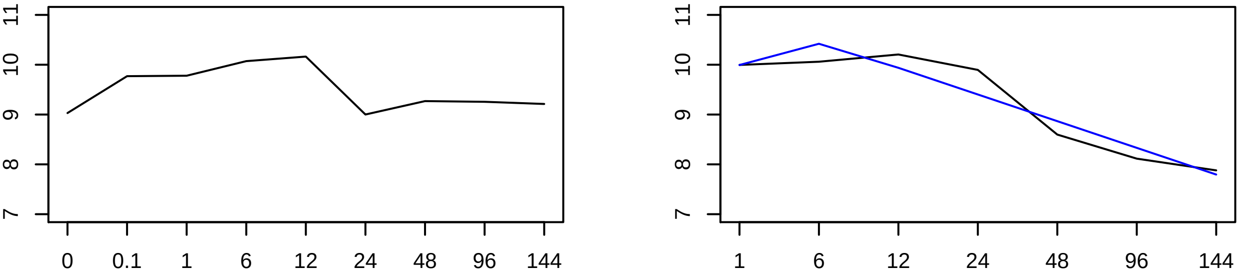

A_23_P334308 KIAA0774 13q12.3

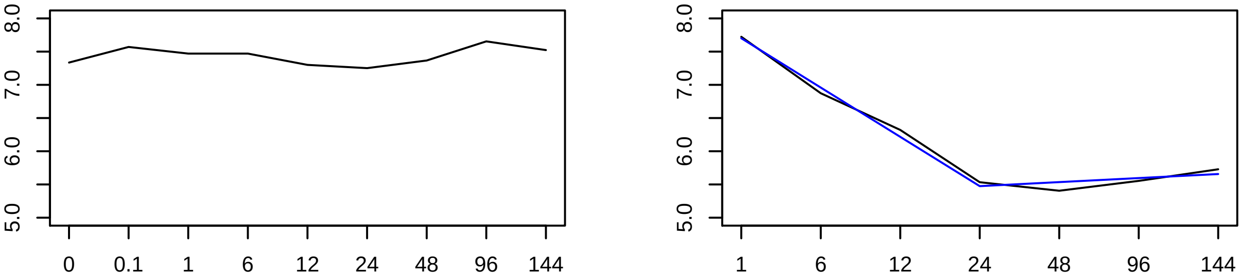

A_24_P289573 A_24_P289573 NA

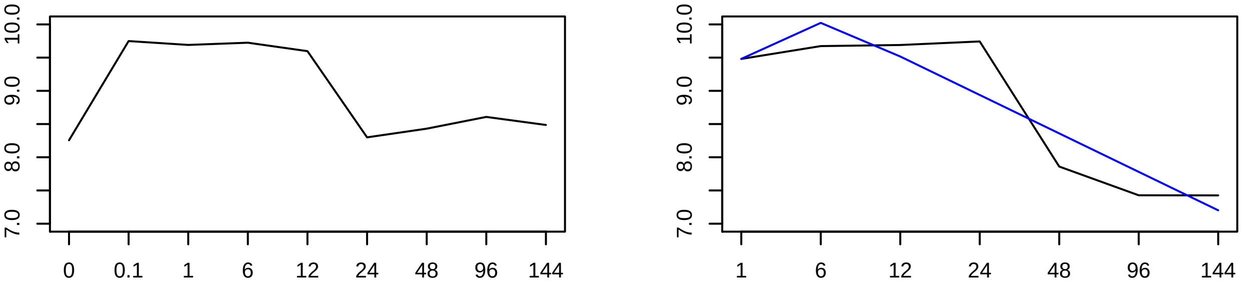

A_24_P937095 SLC30A1 1q32.3

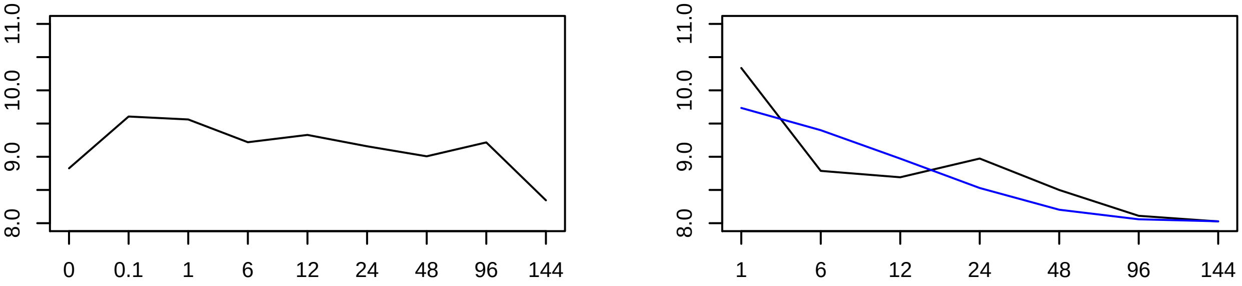

A_23_P381645 EBF3 10q26.3

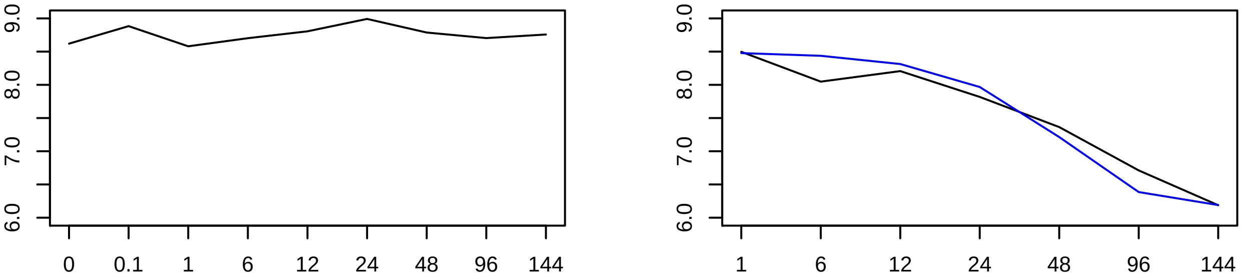

A_24_P856273 A_24_P856273 NA

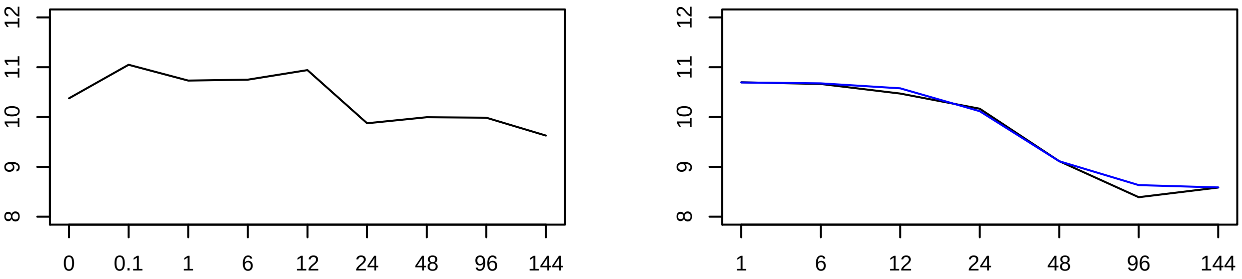

A_24_P400457 CYB5RL NA

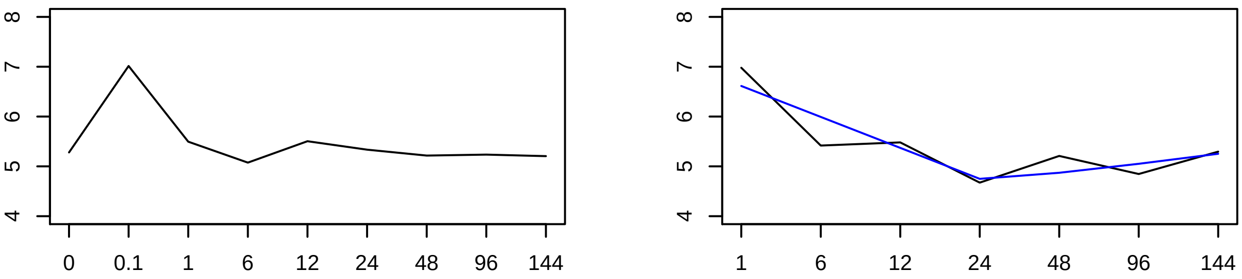

A_23_P306987 SOX7 8p23.1

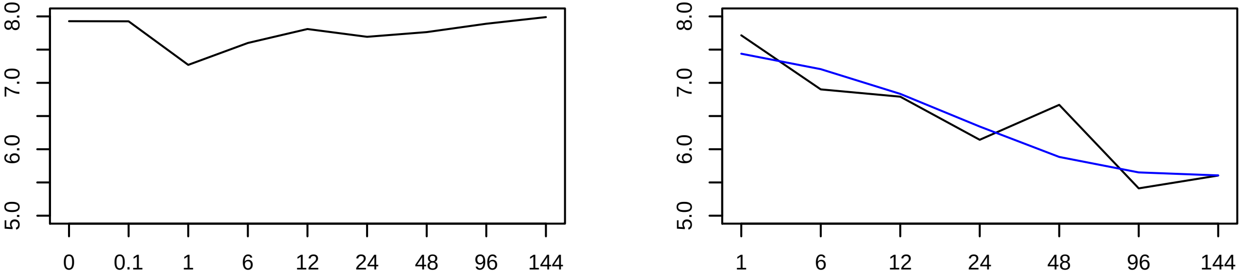

A_24_P94101 PRO2949 8q22.3

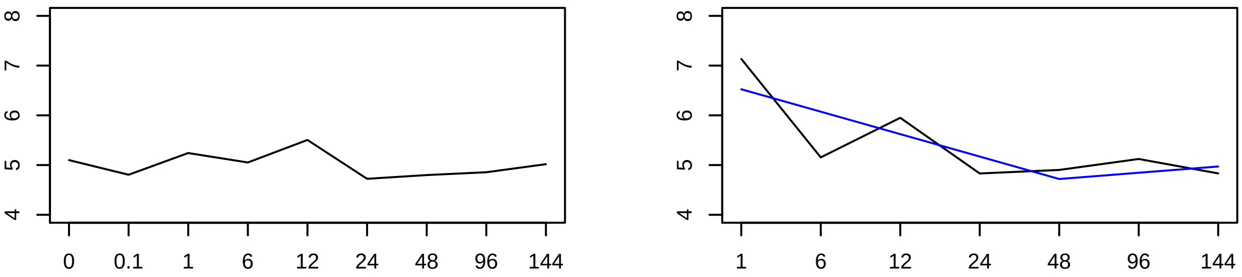

A_24_P255763 A_24_P255763 NA

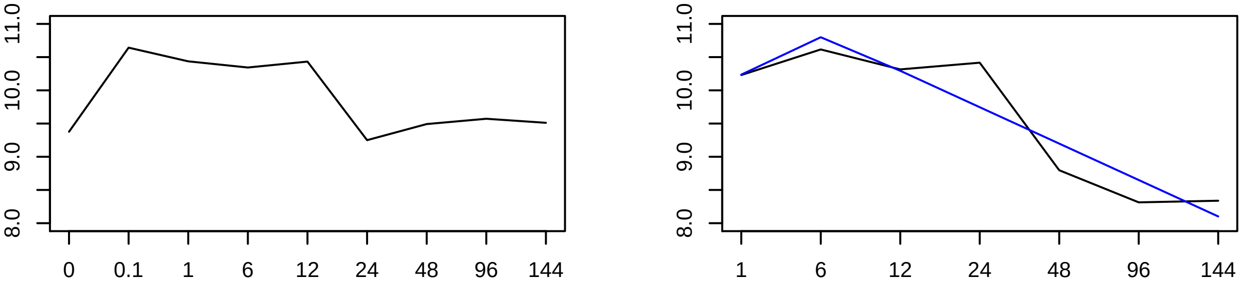

A_24_P204474 A_24_P204474 NA

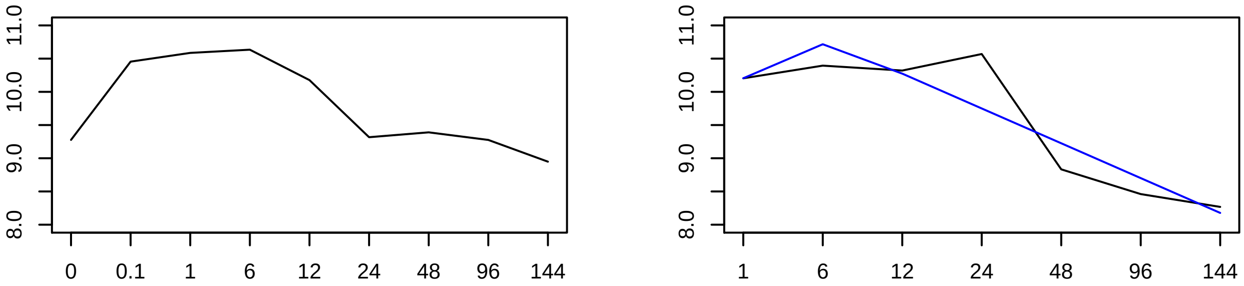

A_24_P289477 LOC729662 1p36.13

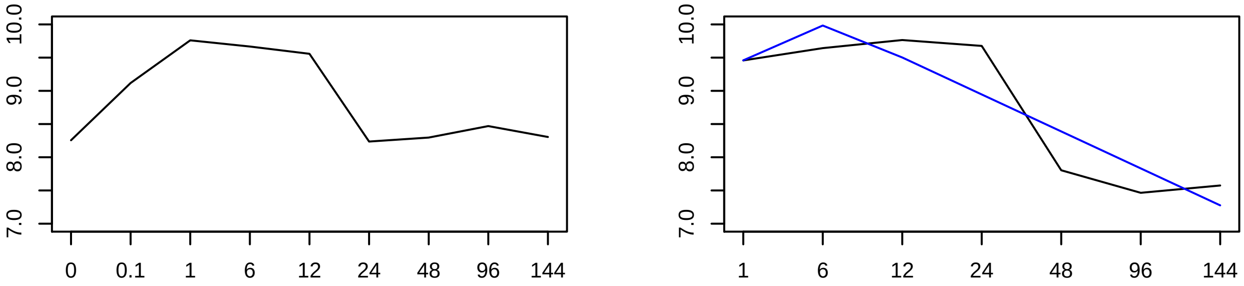

A_24_P367602 DUSP5P 1q42.13

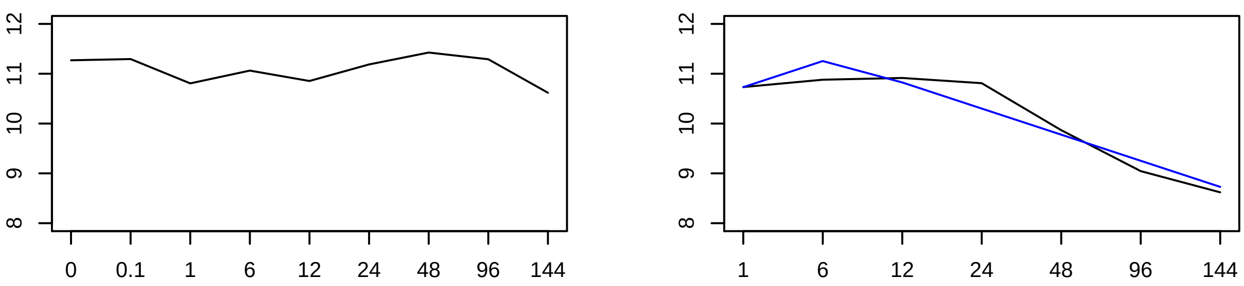

A_23_P23815 SLC30A1 1q32.3

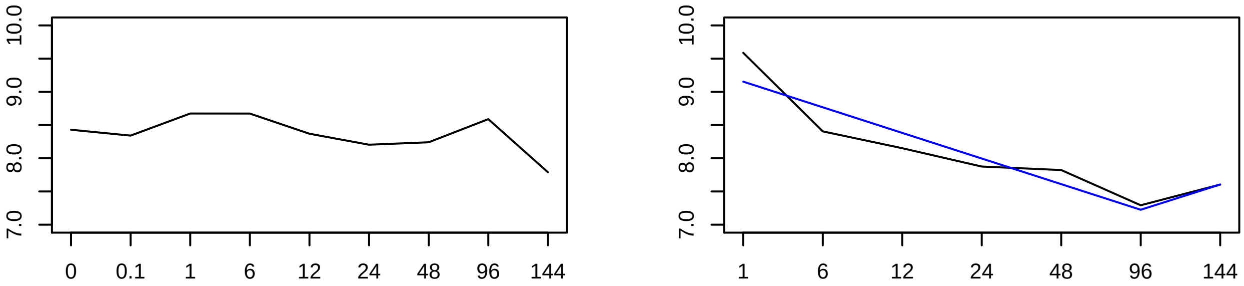

A_23_P166616 AGTR1 3q24

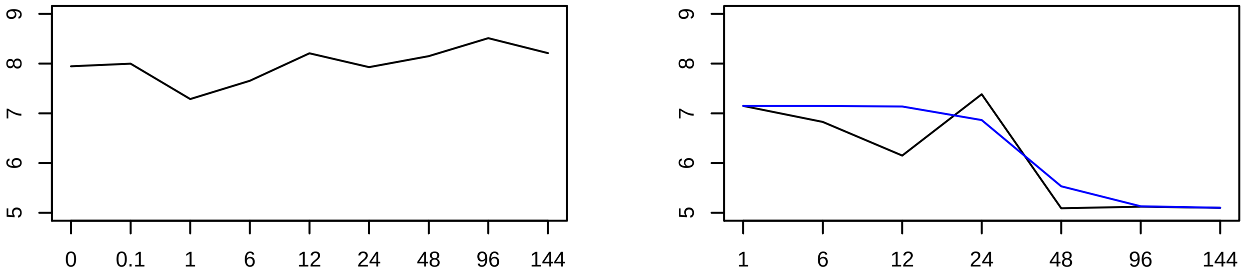

A_24_P780353 A_24_P780353 NA

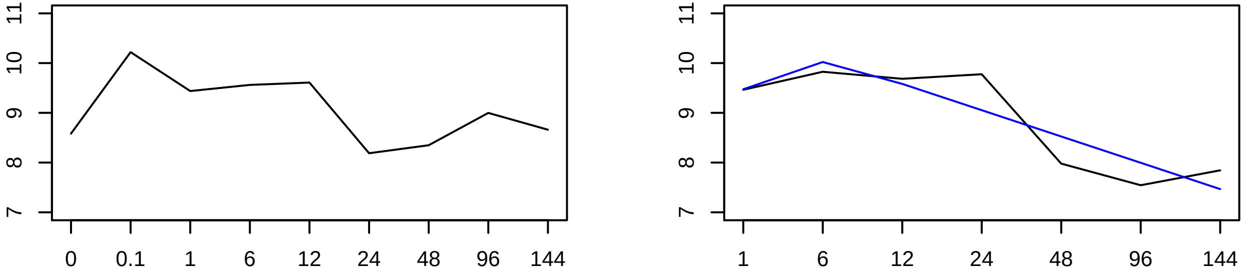

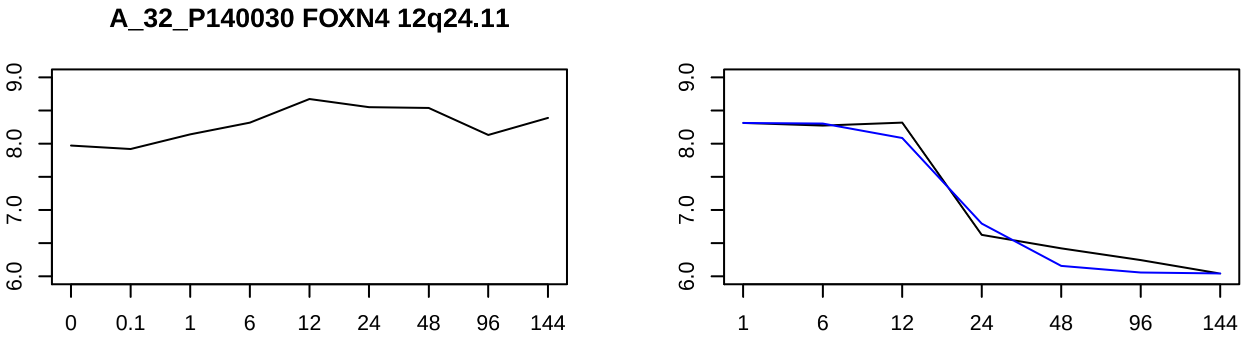
A_32_P140030 FOXN4 12q24.11
6.0 7.0 8.0 9.0
0 0.1 1 6 12 24 48 96 144
1 6 12 24 48 96 144

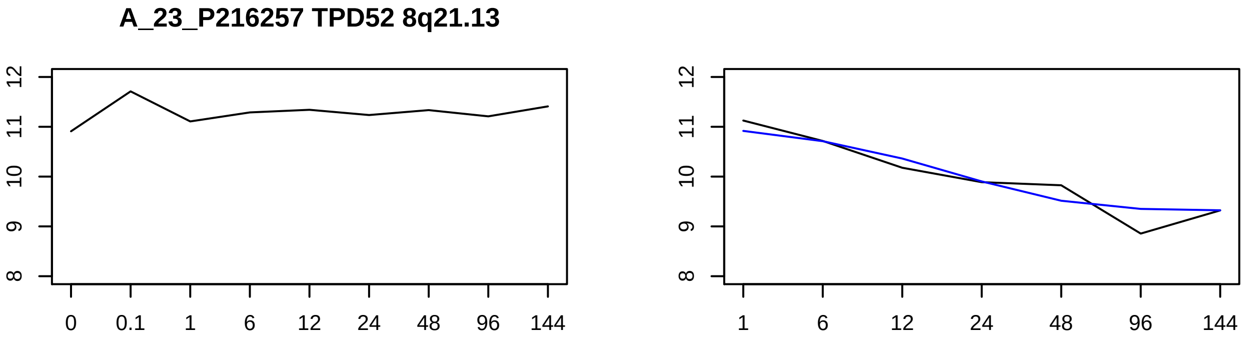
A_23_P216257 TPD52 8q21.13
8 9 10 11 12
0 0.1 1 6 12 24 48 96 144
1 6 12 24 48 96 144

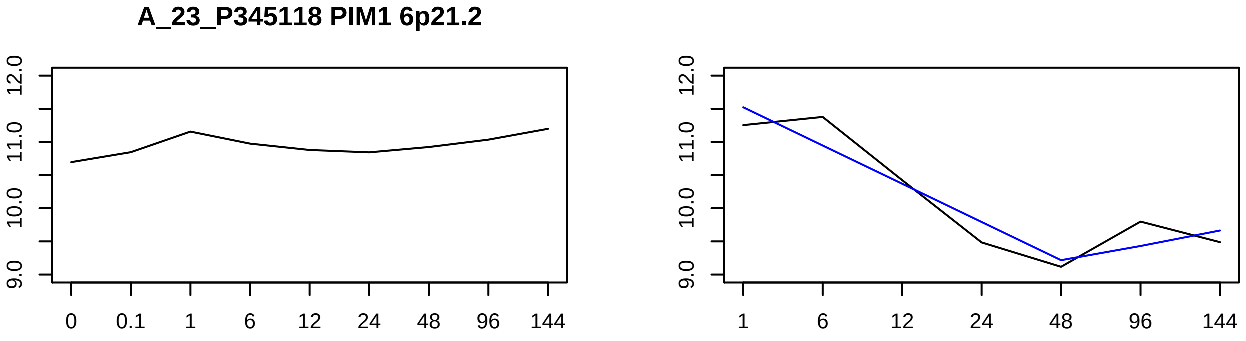
A_23_P345118 PIM1 6p21.2
9.0 10.0 11.0 12.0
0 0.1 1 6 12 24 48 96 144
1 6 12 24 48 96 144

A_23_P85598 CABC1 1q42.13

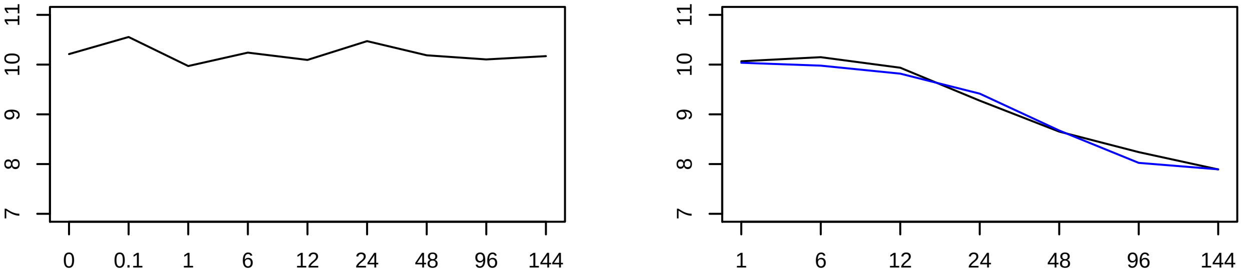

A_23_P111604 NXPH1 7p21.3

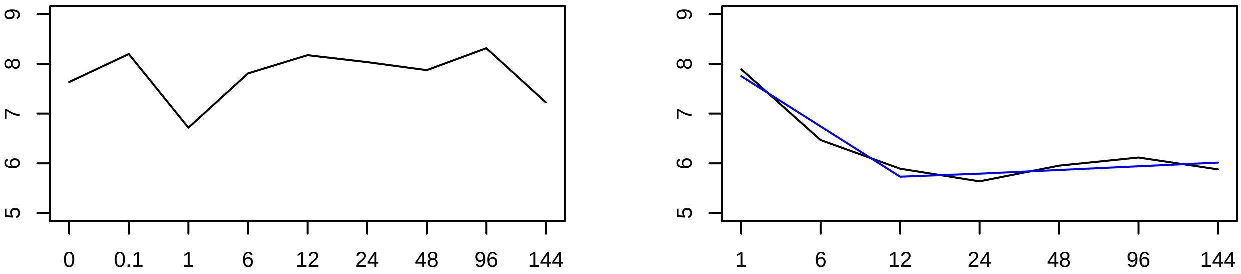

A_24_P358337 A_24_P358337 NA

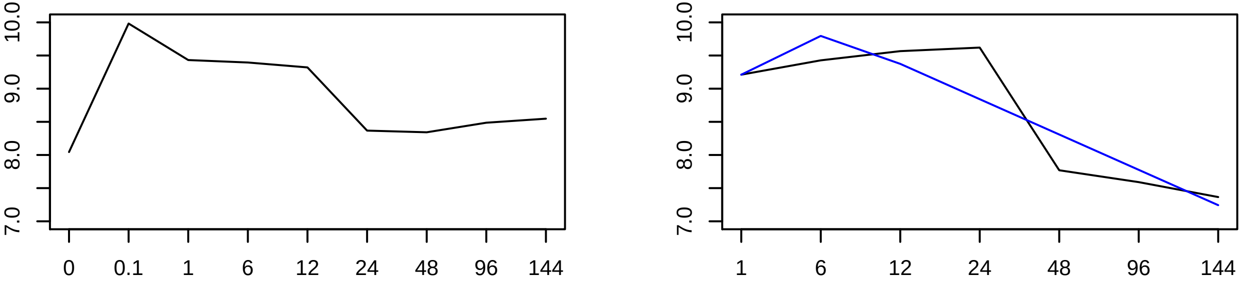

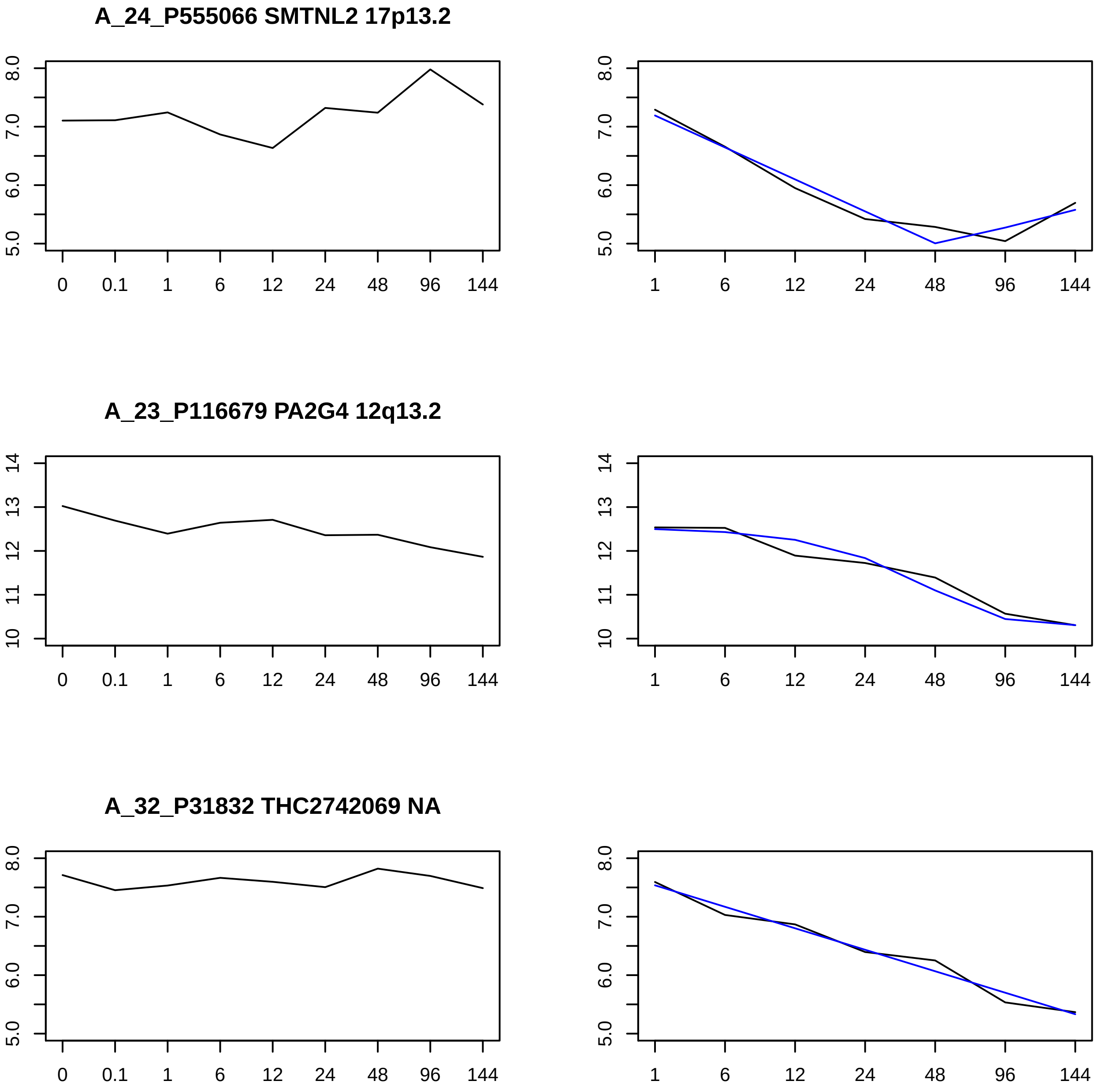
A_24_P555066 SMTNL2 17p13.2
A_23_P116679 PA2G4 12q13.2
A_32_P31832 THC2742069 NA
5.0 6.0 7.0 8.0
10 11 12 13 14
0 0.1 1 6 12 24 48 96 144
1 6 12 24 48 96 144

A_23_P201918 ABCB10 1q42.13

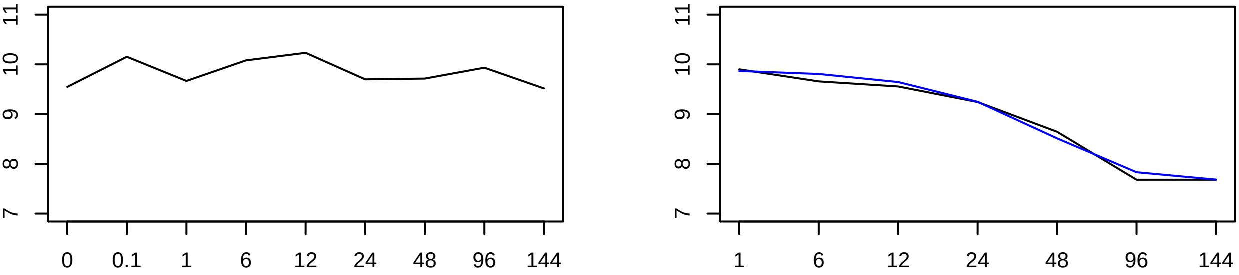

A_24_P264549 A_24_P264549 NA

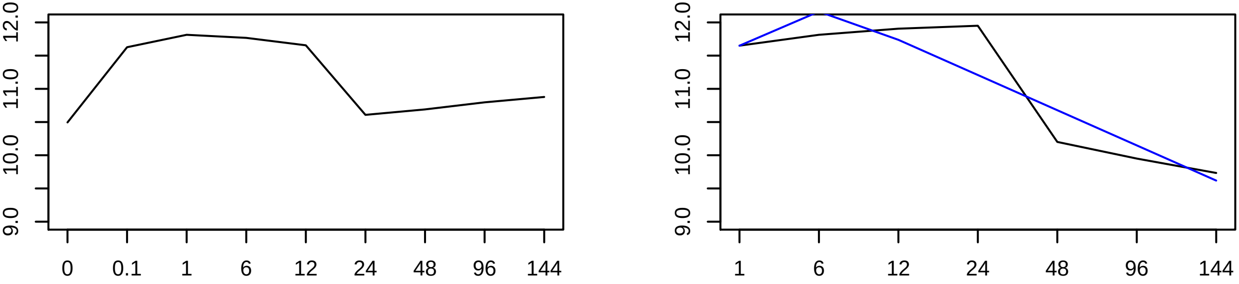

A_32_P211045 DHFR 5q14.1

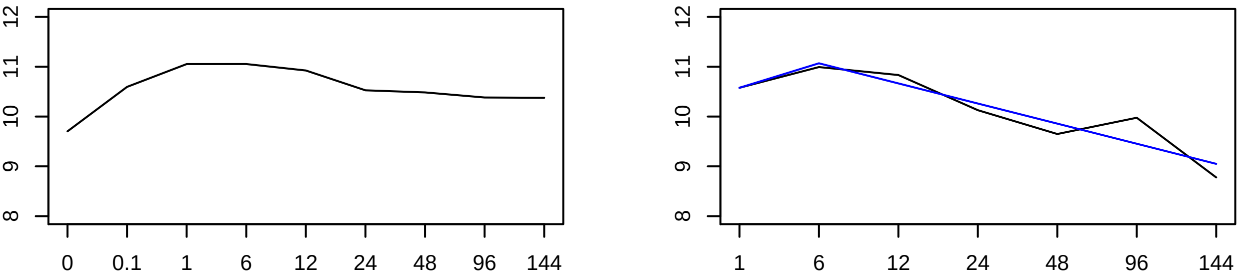

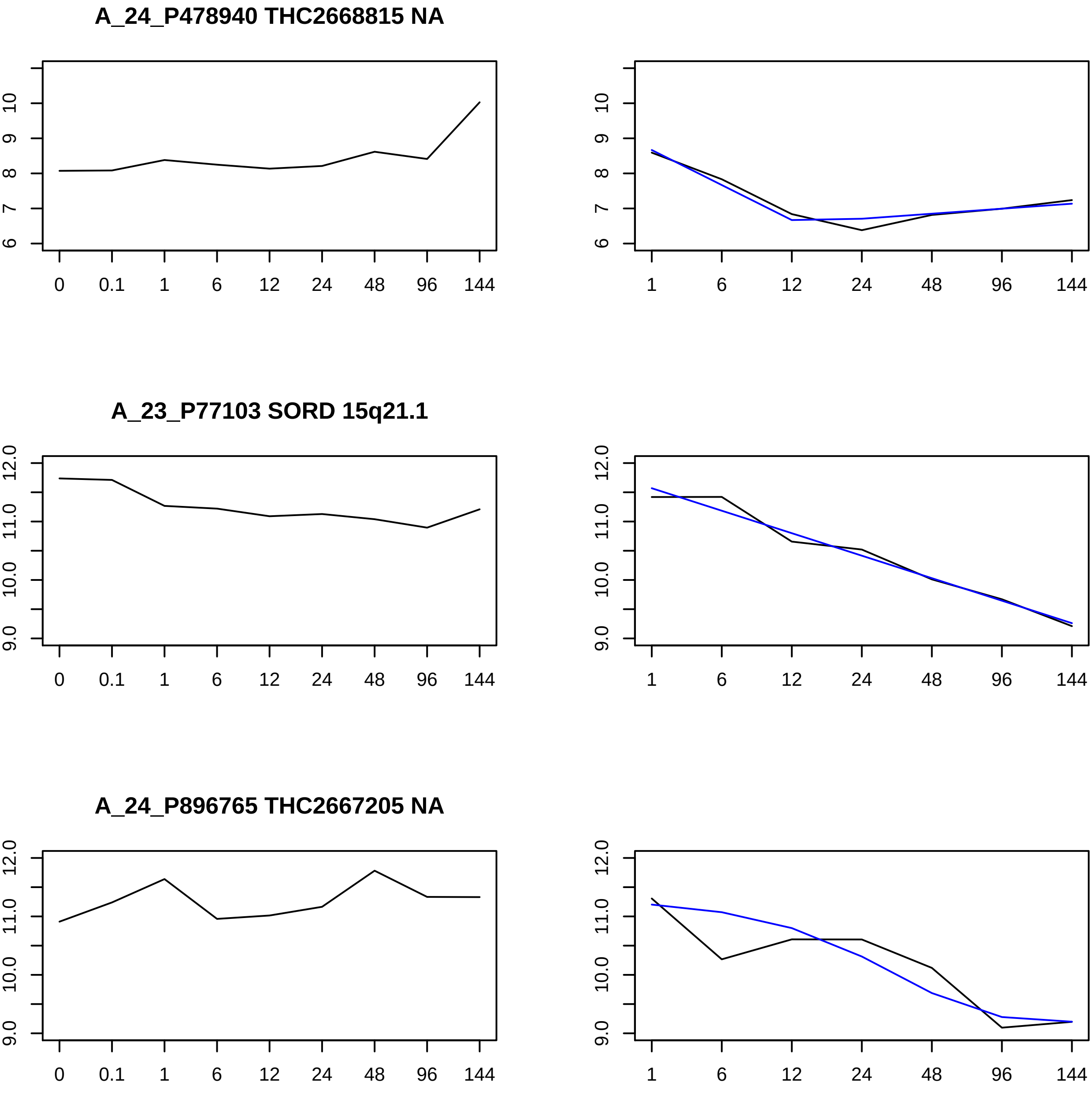
A_24_P478940 THC2668815 NA
6
7
8
9
10
0
0.1
1
6
12
24
48
96
144
A_23_P77103 SORD 15q21.1
9.0
10.0
11.0
12.0
A_24_P896765 THC2667205 NA

A_24_P358390 A_24_P358390 NA

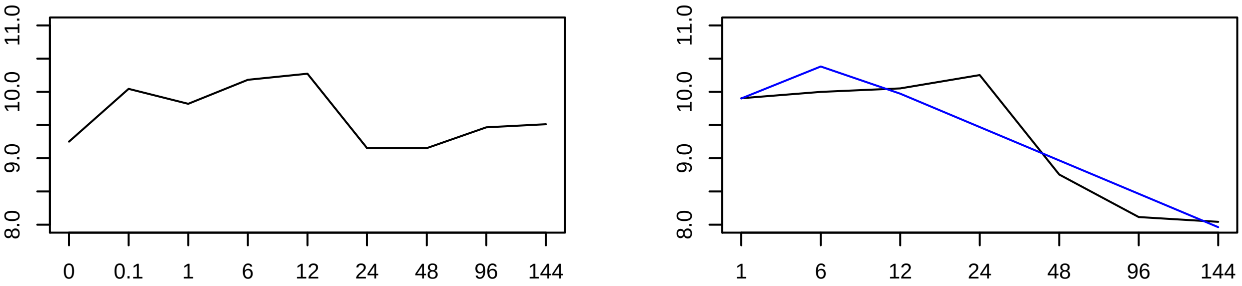

A_32_P23624 ABCB10 1q42.13

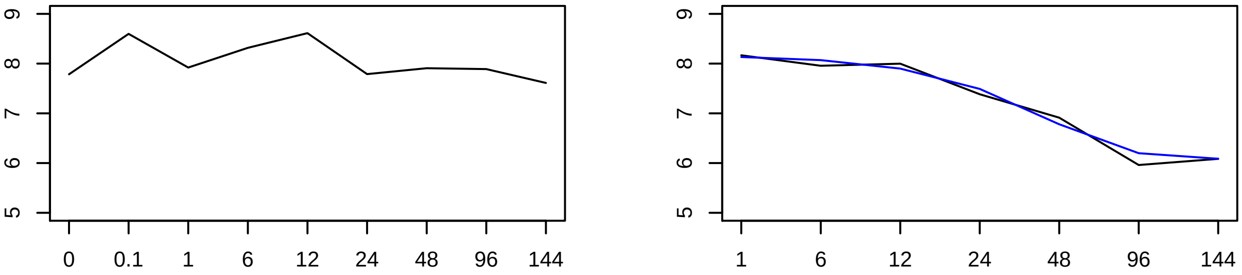

A_23_P422144 FAM43A 3q29

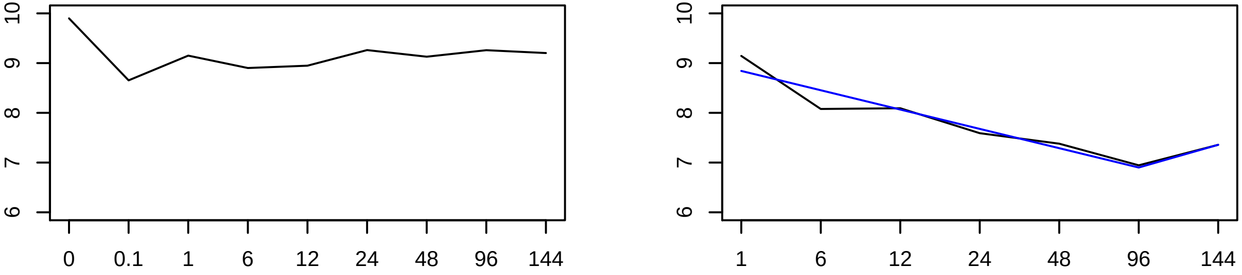

A_24_P926450 THC2537217 NA

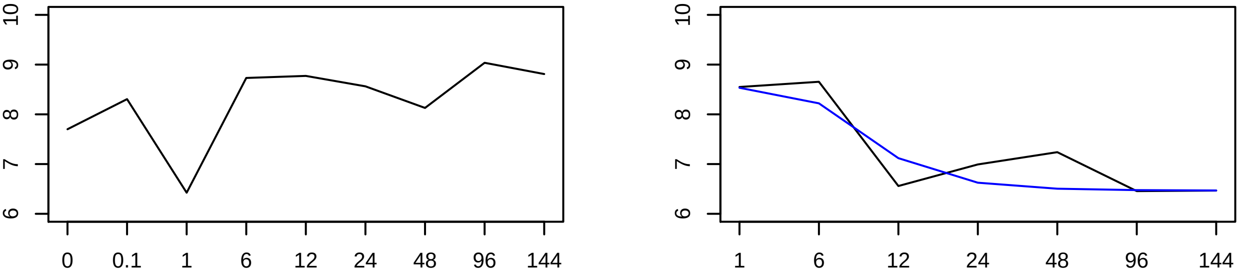

A_24_P213478 SEMA6A 5q23.1

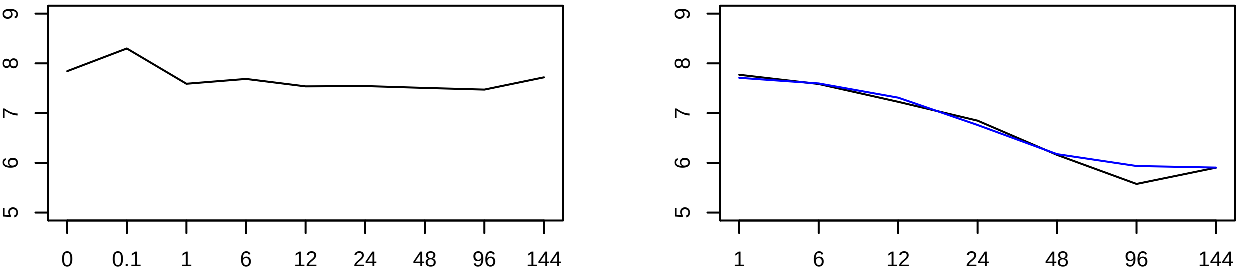

A_24_P383569 LOC391130 1q24.1

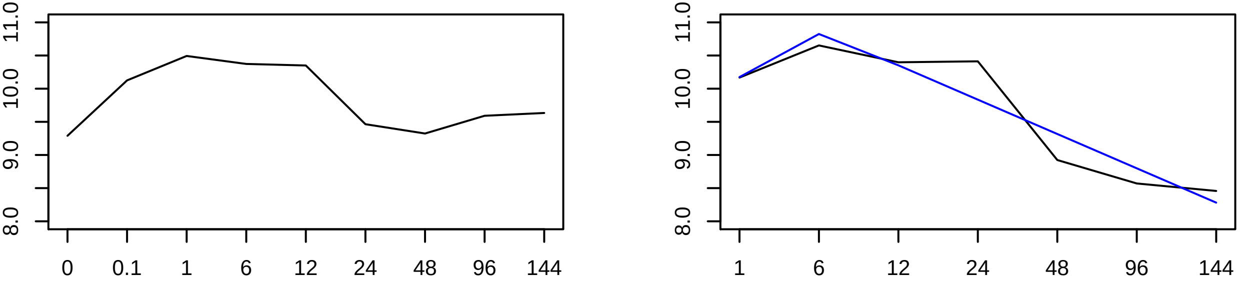

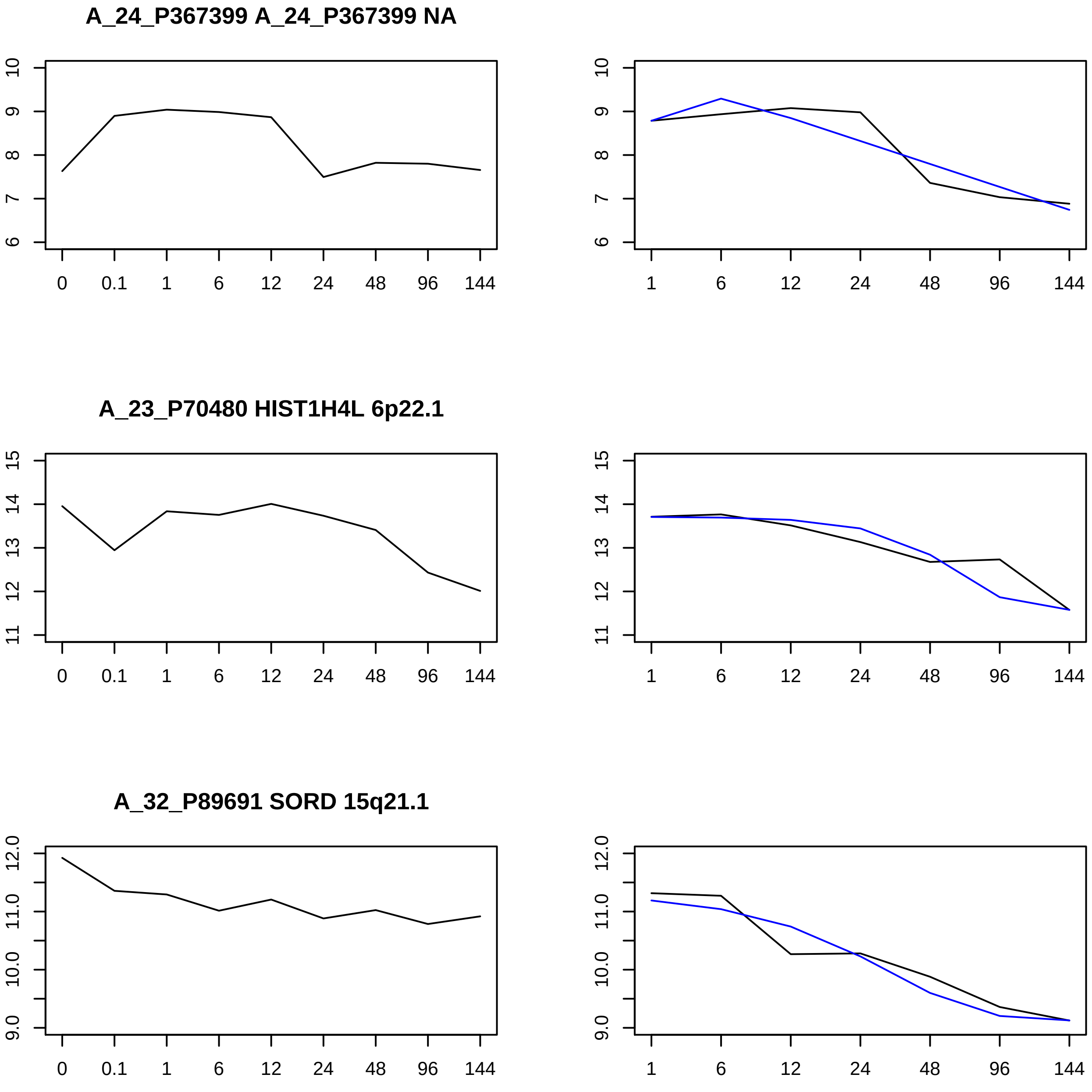
A_24_P367399 A_24_P367399 NA
A_23_P70480 HIST1H4L 6p22.1
A_32_P89691 SORD 15q21.1
0 0.1 1 6 12 24 48 96 144
1 6 12 24 48 96 144
6 7 8 9 10
11 12 13 14 15
9.0 10.0 11.0 12.0

A_24_P204238 tcag7.1239 7q36.1

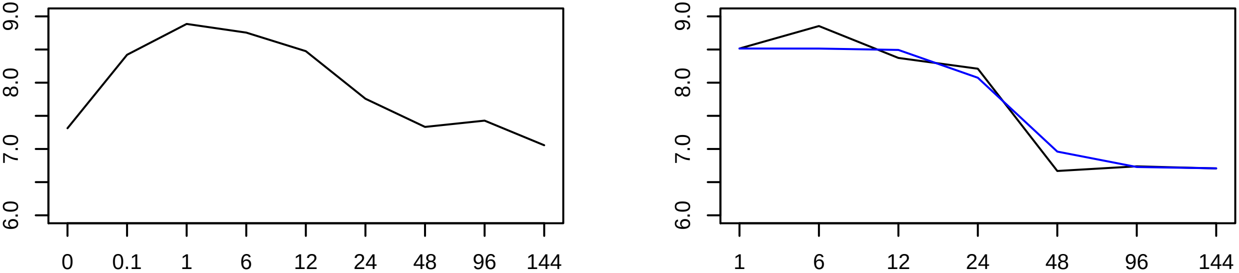

A_24_P641673 A_24_P641673 NA

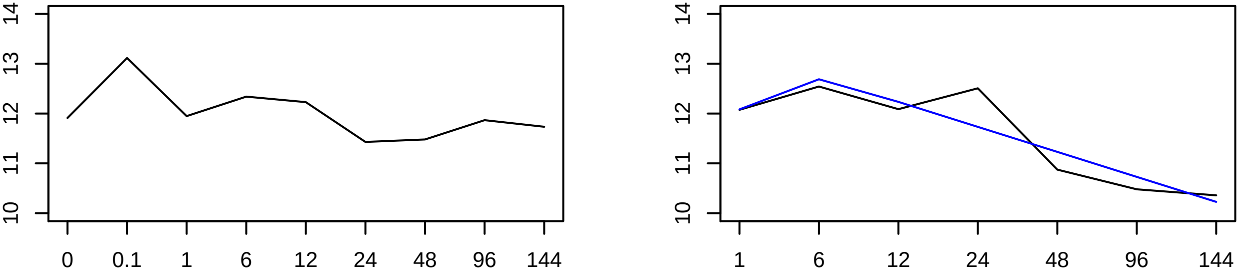

A_23_P17593 CDH4 20q13.33

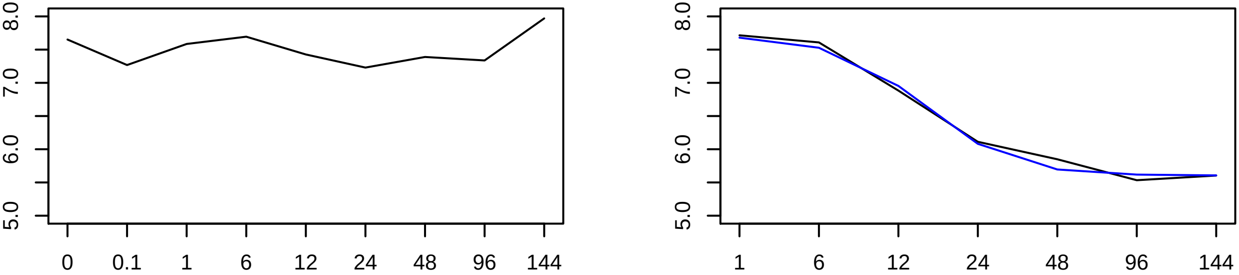

A_24_P75558 LOC646108 Xq13.2

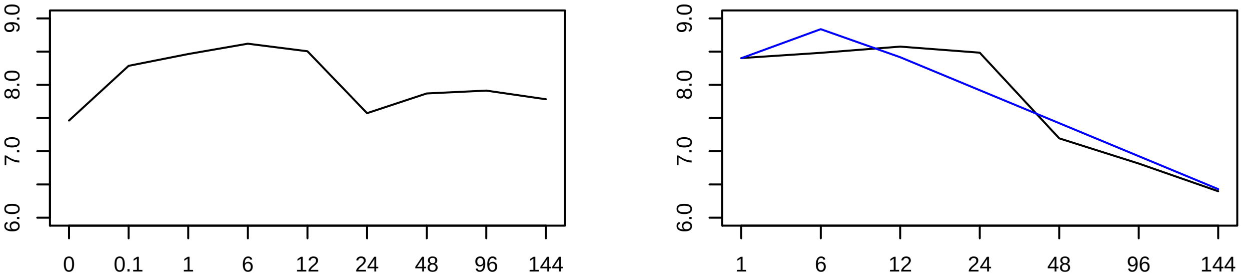

A_32_P200025 A_32_P200025 NA

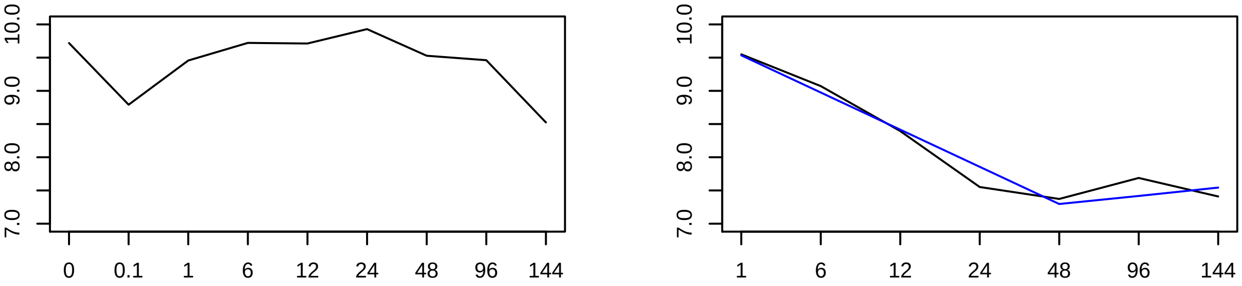

A_23_P250963 SLC1A3 5p13.2

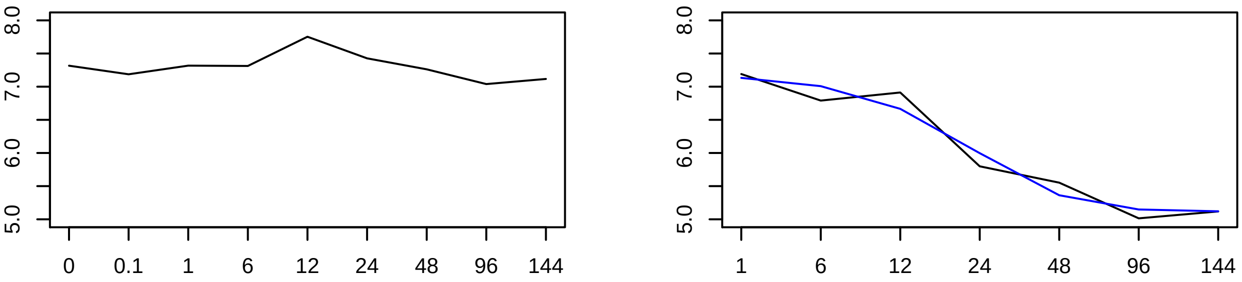

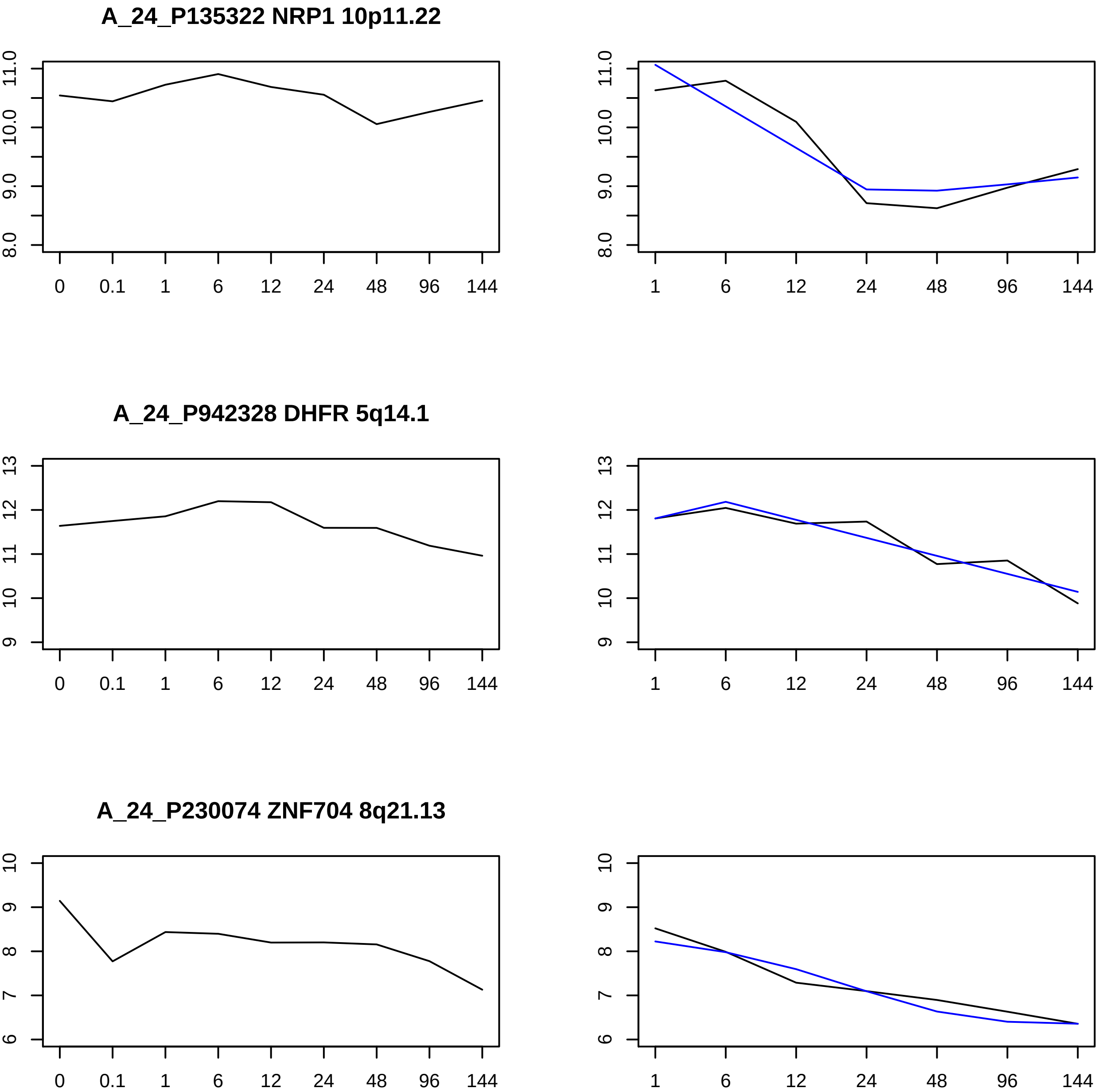
A_24_P135322 NRP1 10p11.22
8.0
9.0
10.0
11.0
0 0.1 1 6 12 24 48 96 144
1 6 12 24 48 96 144
A_24_P942328 DHFR 5q14.1
9
10
11
12
13
0 0.1 1 6 12 24 48 96 144
1 6 12 24 48 96 144
A_24_P230074 ZNF704 8q21.13
6
7
8
9
10
0 0.1 1 6 12 24 48 96 144
1 6 12 24 48 96 144

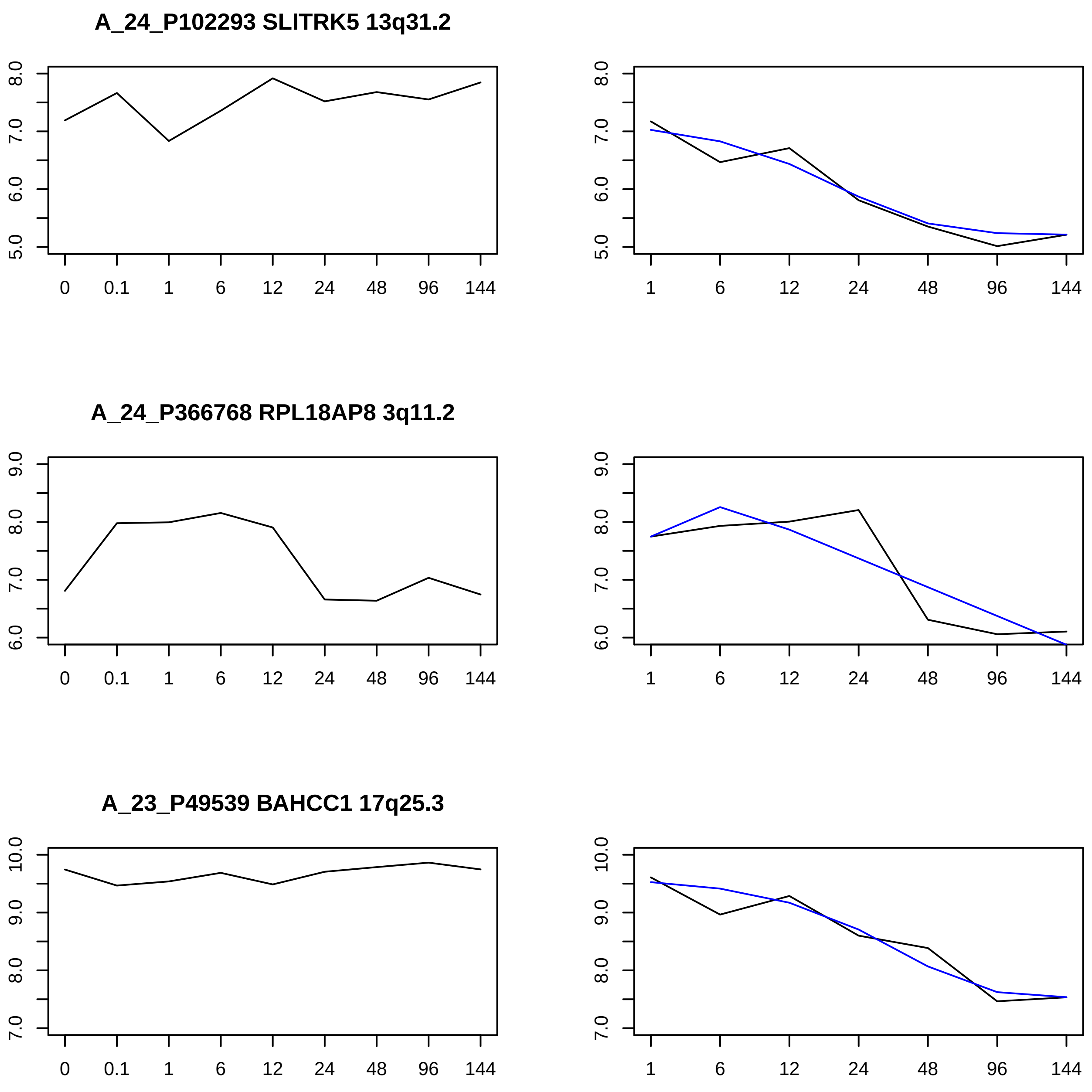
A_24_P102293 SLITRK5 13q31.2
5.0
6.0
7.0
8.0
0
0.1
1
6
12
24
48
96
144
1
6
12
24
48
96
144
A_24_P366768 RPL18AP8 3q11.2
6.0
7.0
8.0
9.0
A_23_P49539 BAHCC1 17q25.3
7.0
8.0
9.0
10.0

A_24_P361896 MT2A 16q13

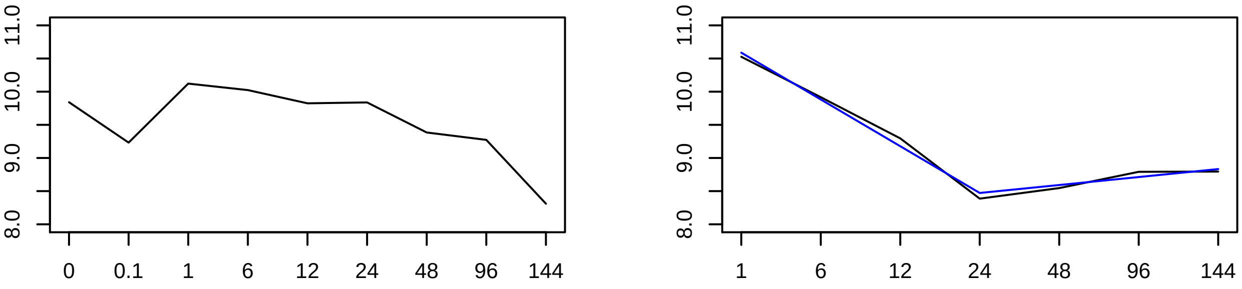

A_32_P128952 A_32_P128952 NA

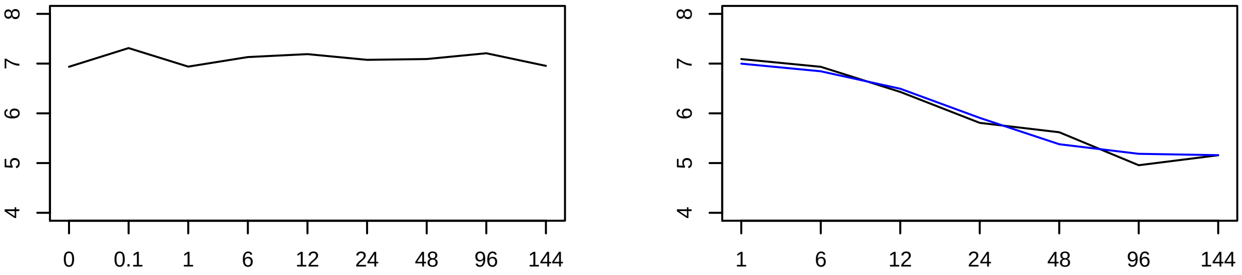

A_24_P50801 NRP2 2q33.3

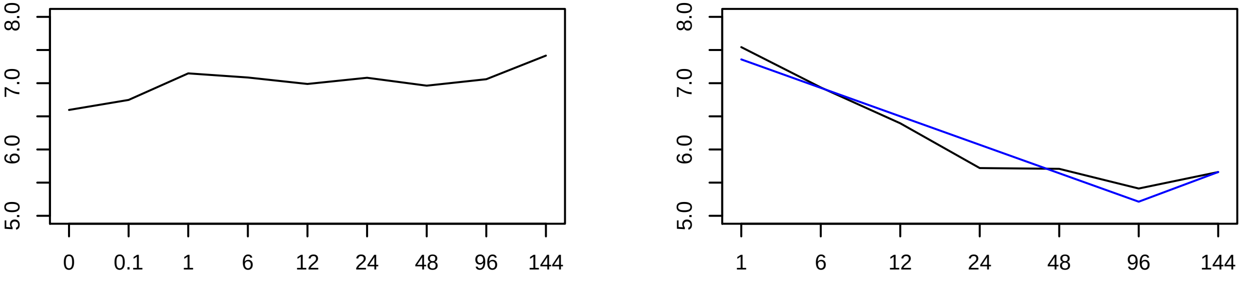

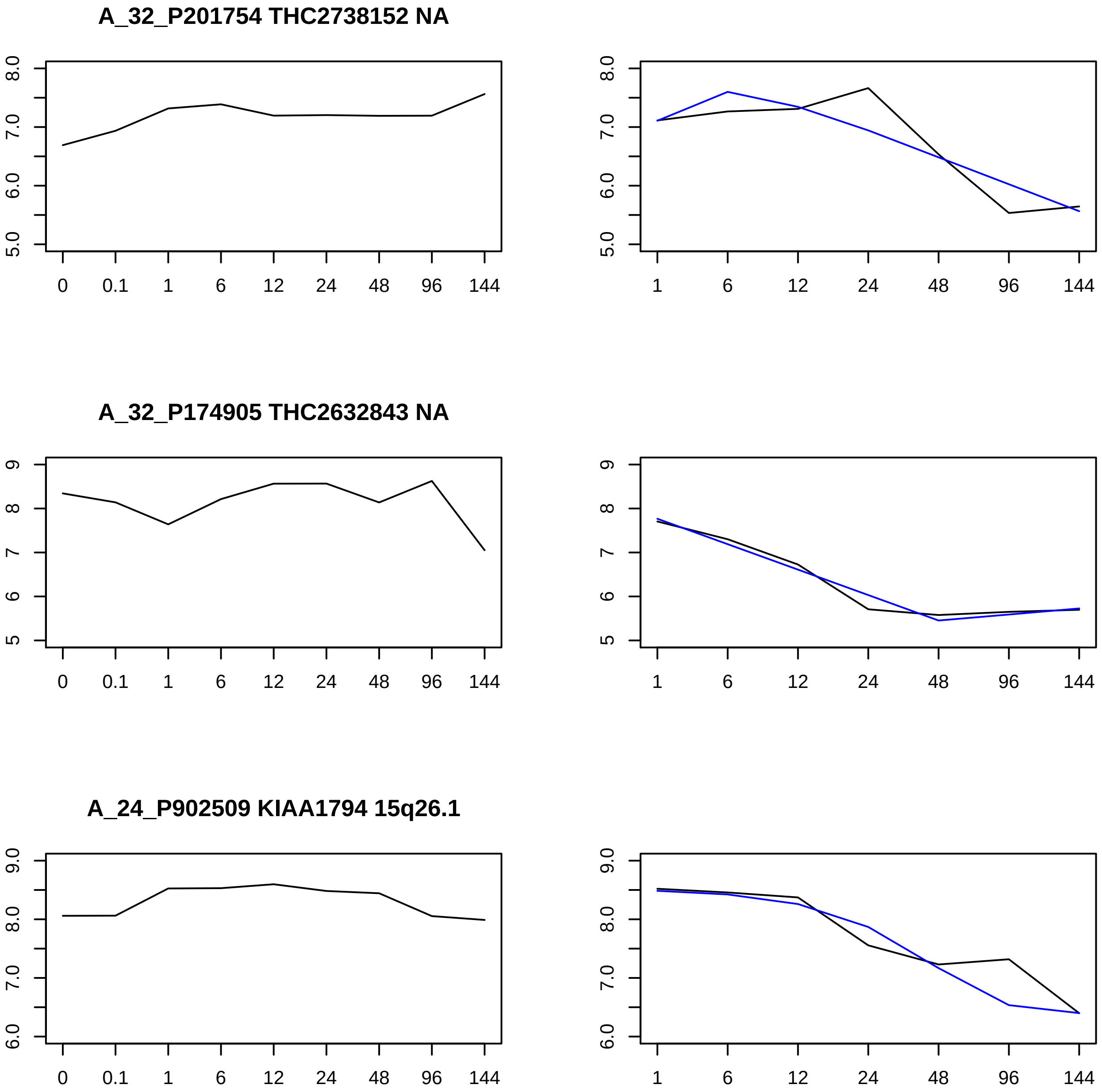
A_32_P201754 THC2738152 NA
5.0
6.0
7.0
8.0
0
0.1
1
6
12
24
48
96
144
A_32_P174905 THC2632843 NA
5
6
7
8
9
A_24_P902509 KIAA1794 15q26.1
6.0
7.0
8.0
9.0

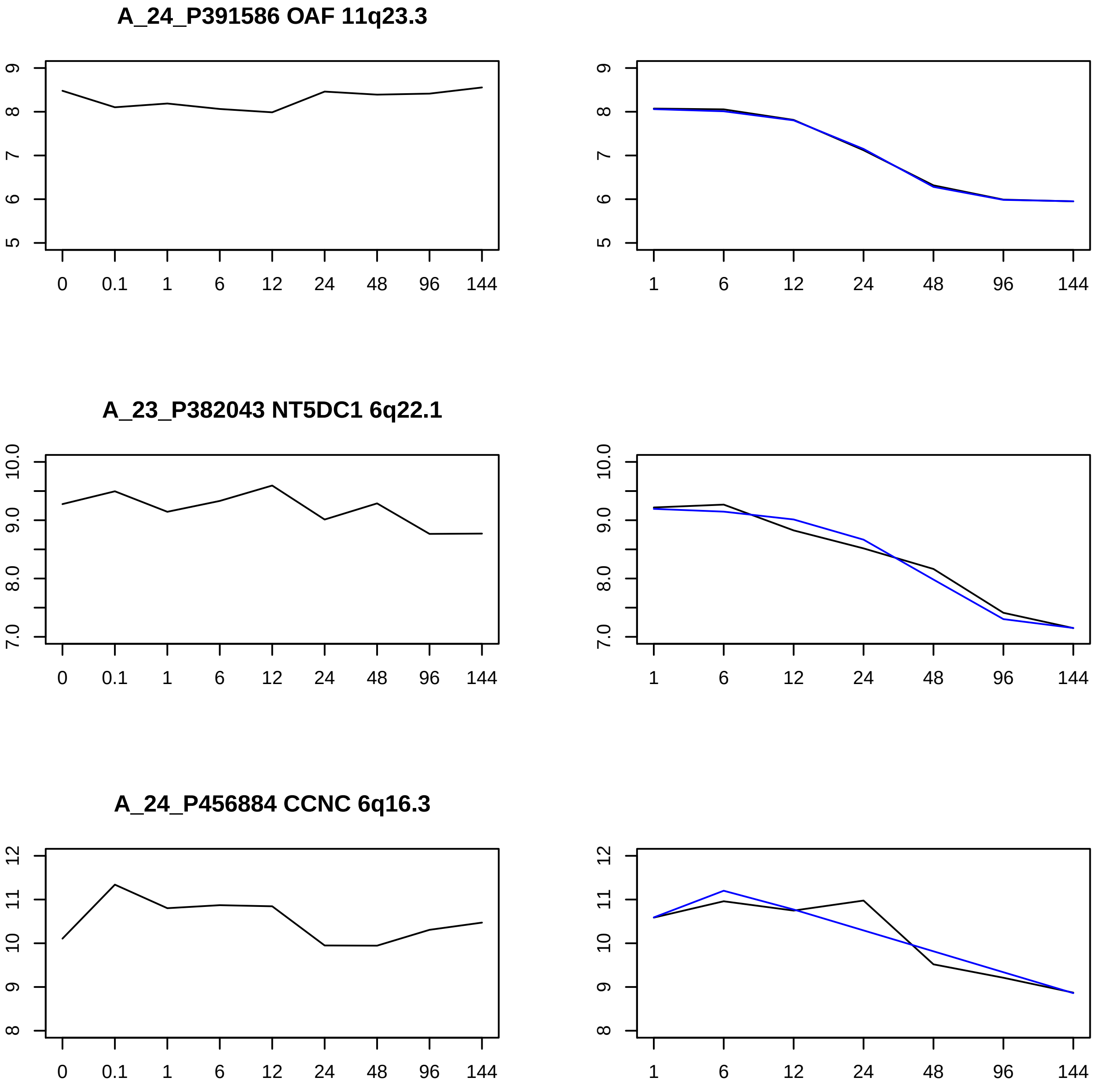

A_24_P391586 OAF 11q23.3
A_23_P382043 NT5DC1 6q22.1
A_24_P456884 CCNC 6q16.3
0 0.1 1 6 12 24 48 96 144
1 6 12 24 48 96 144
5 6 7 8 9
7.0 8.0 9.0 10.0
8 9 10 11 12

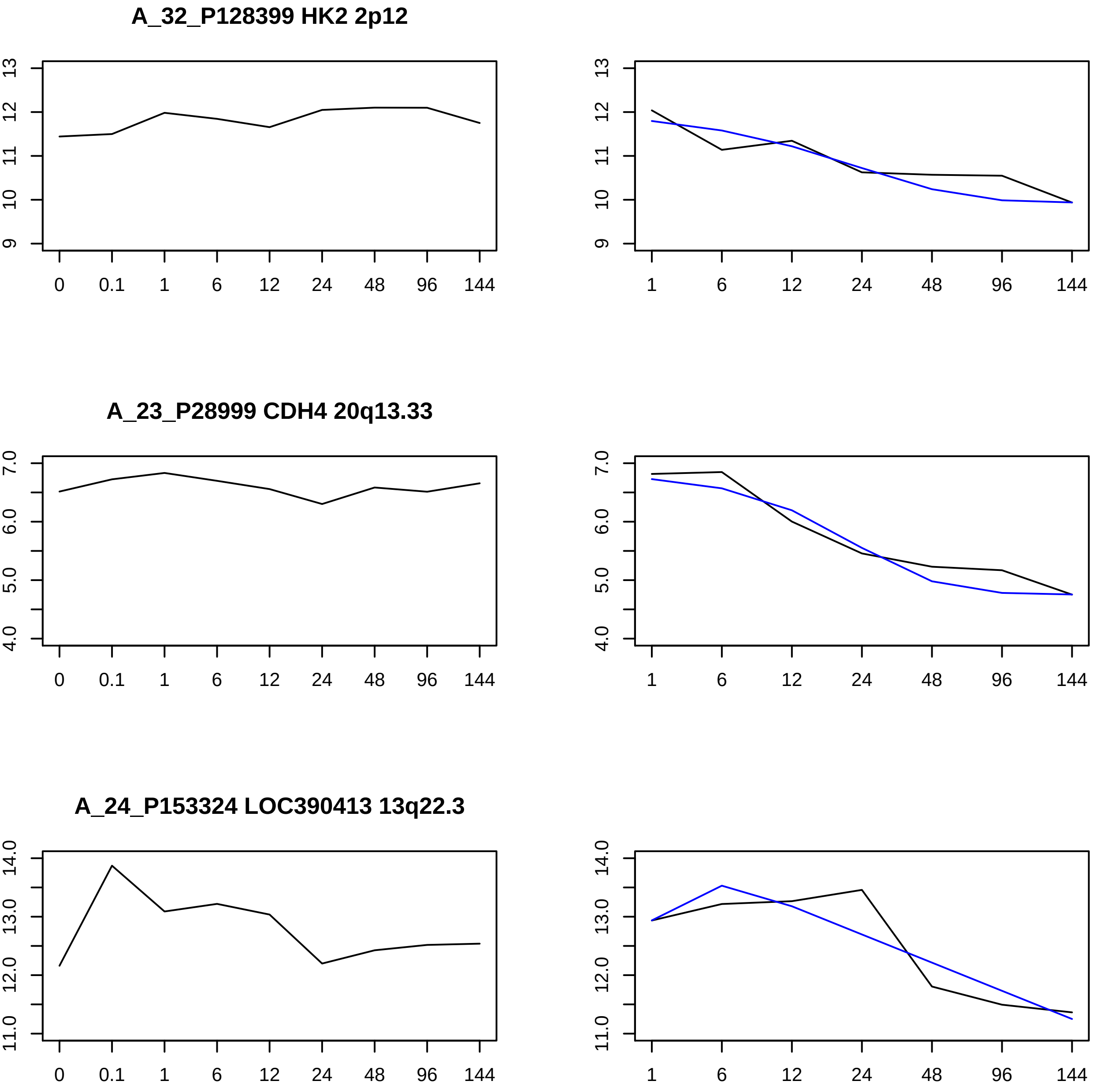

A_32_P128399 HK2 2p12
9
10
11
12
13
0
0.1
1
6
12
24
48
96
144
1
6
12
24
48
96
144
A_23_P28999 CDH4 20q13.33
4.0
5.0
6.0
7.0
0
0.1
1
6
12
24
48
96
144
1
6
12
24
48
96
144
A_24_P153324 LOC390413 13q22.3
11.0
12.0
13.0
14.0
0
0.1
1
6
12
24
48
96
144
1
6
12
24
48
96
144

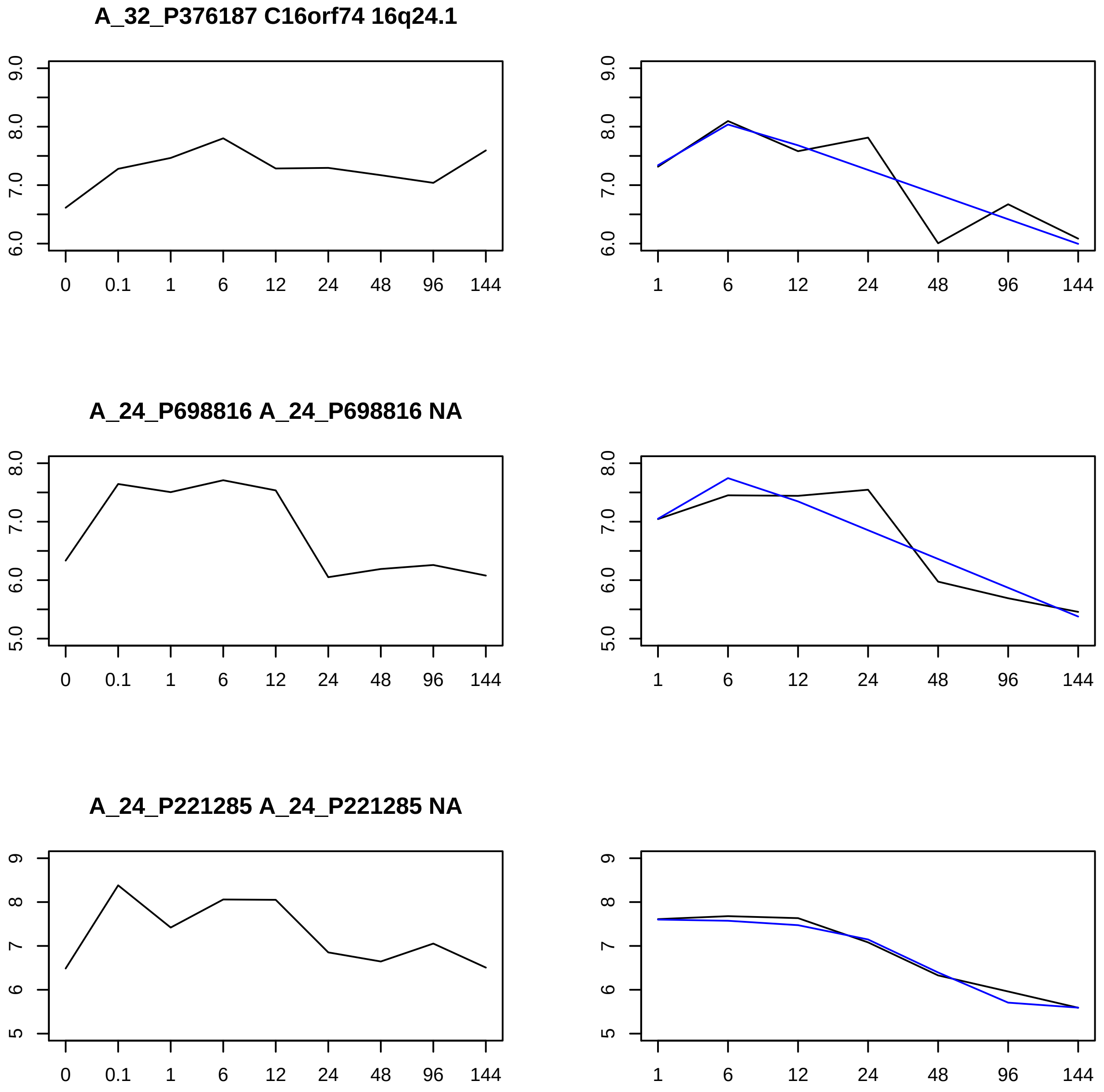
A_32_P376187 C16orf74 16q24.1
6.0
7.0
8.0
9.0
0
0.1
1
6
12
24
48
96
144
1
6
12
24
48
96
144
A_24_P698816 A_24_P698816 NA
5.0
6.0
7.0
8.0
A_24_P221285 A_24_P221285 NA
5
6
7
8
9

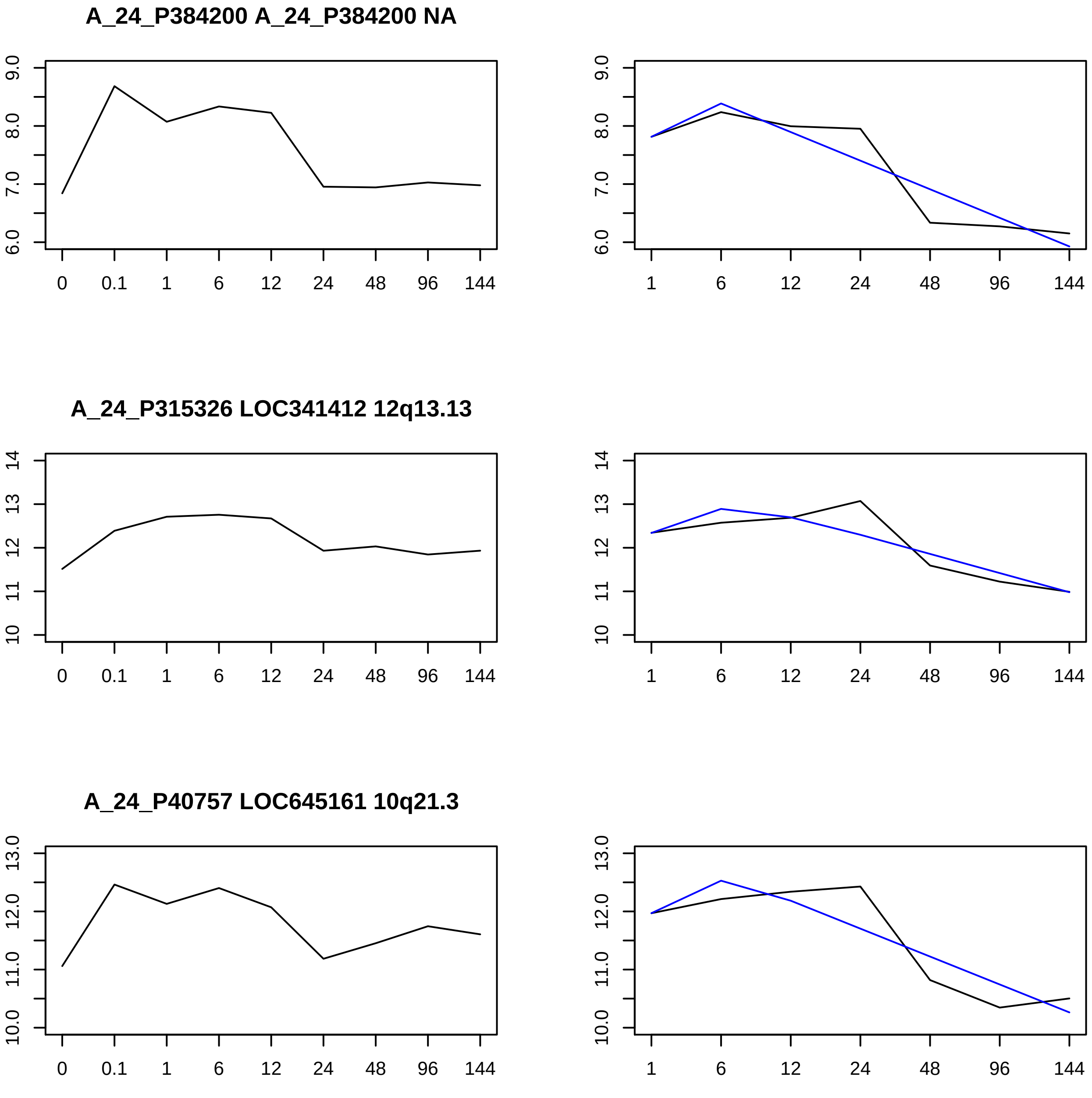
A_24_P384200 A_24_P384200 NA
6.0 7.0 8.0 9.0
0 0.1 1 6 12 24 48 96 144
1 6 12 24 48 96 144
A_24_P315326 LOC341412 12q13.13
10 11 12 13 14
0 0.1 1 6 12 24 48 96 144
1 6 12 24 48 96 144
A_24_P40757 LOC645161 10q21.3
10.0 11.0 12.0 13.0
0 0.1 1 6 12 24 48 96 144
1 6 12 24 48 96 144

A_23_P94133 POP1 8q22.2

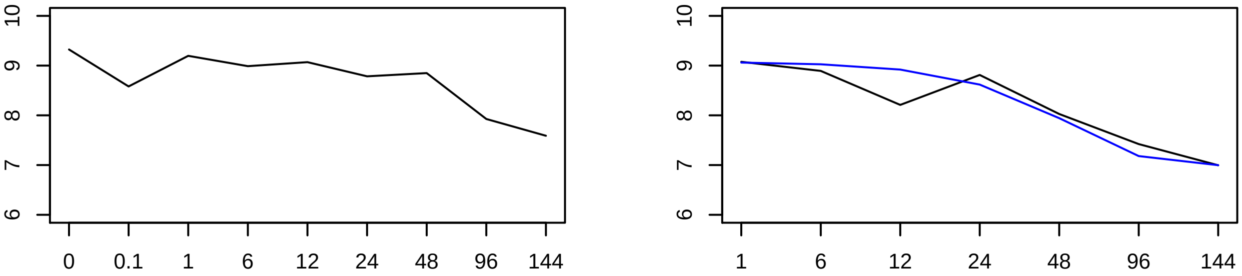

A_23_P205738 BCL11B 14q32.2

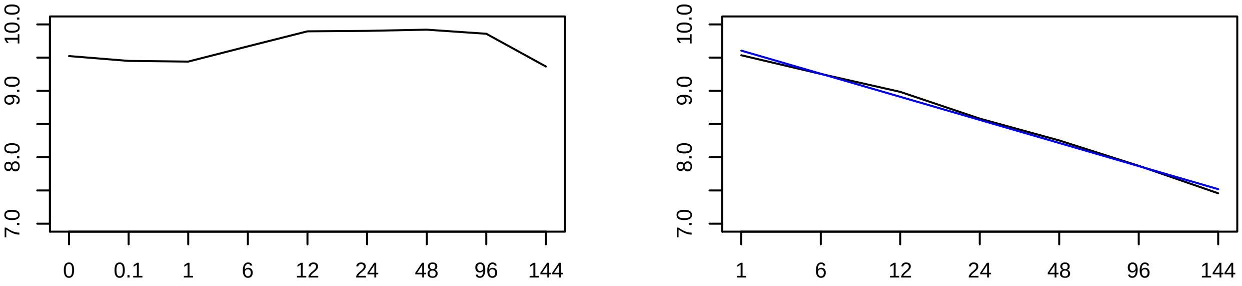

A_24_P922569 PKD1L1 7p12.3

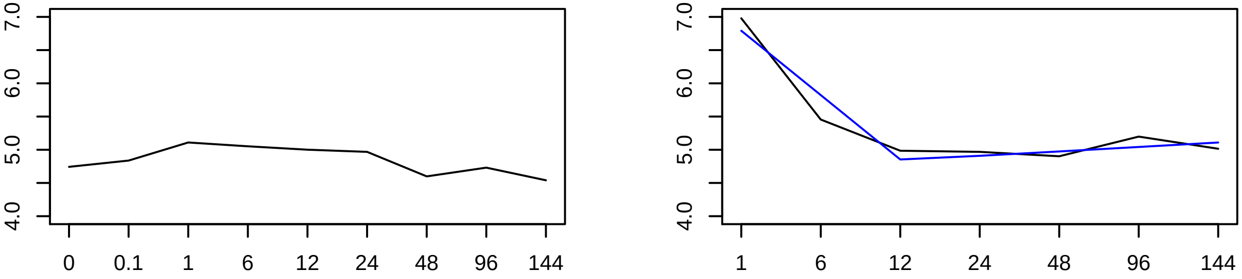

A_24_P505790 ZNF704 8q21.13

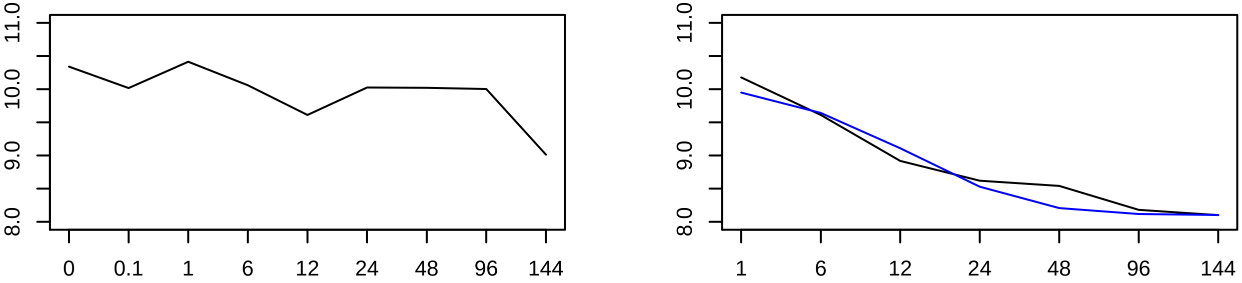

A_23_P2366 NUDT4 12q22

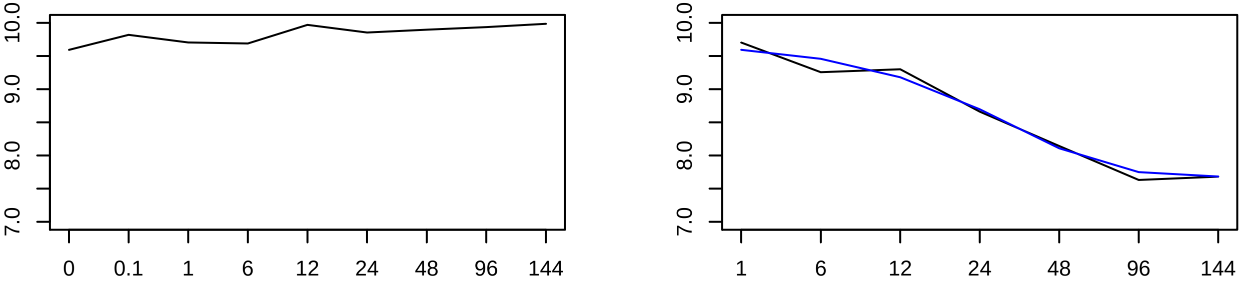

A_24_P913701 THC2558878 NA

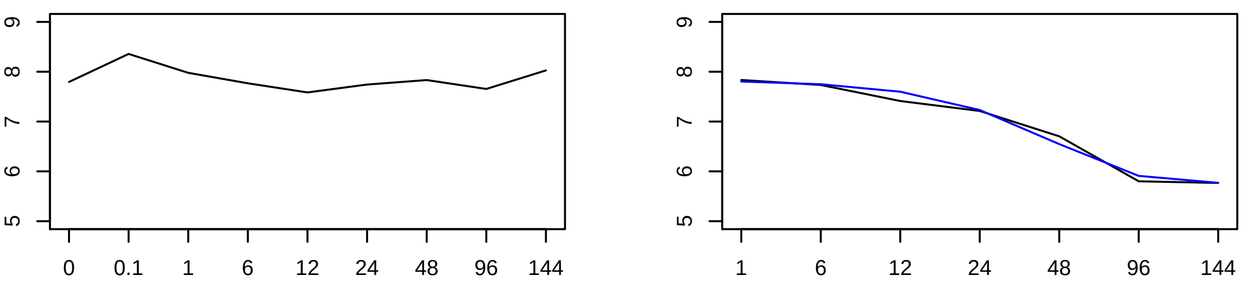

A_23_P106844 MT2A 16q13

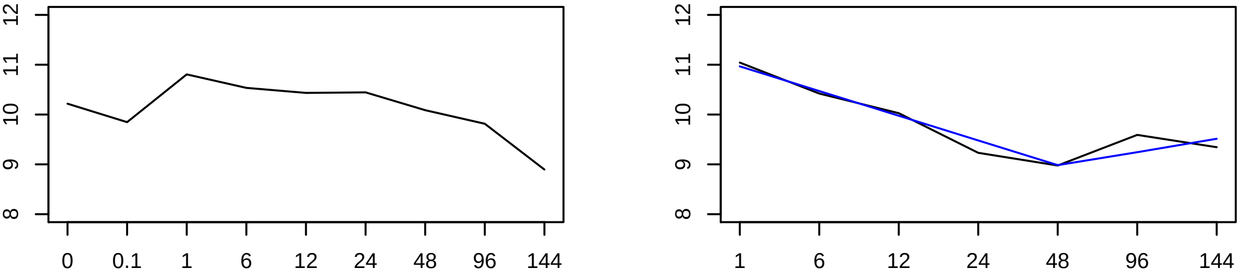

A_24_P384059 LOC440396 17p13.3

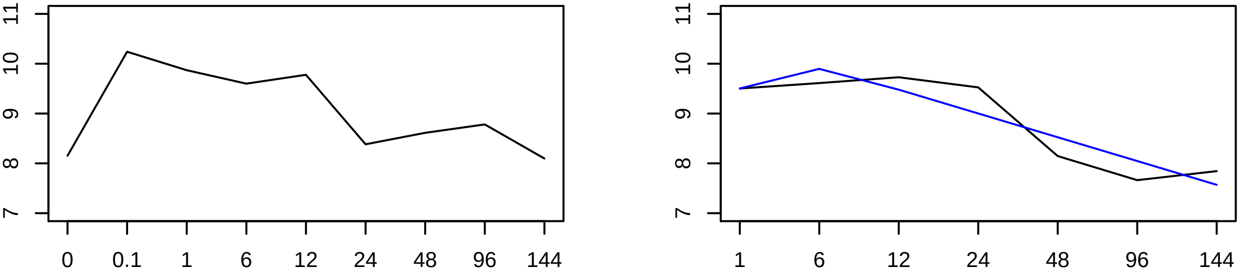

A_24_P807445 A_24_P807445 NA

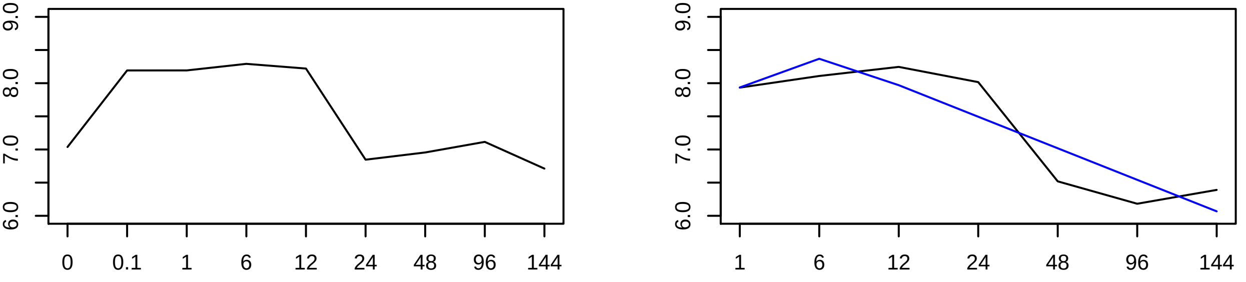

A_32_P145856 ENST00000247761 NA

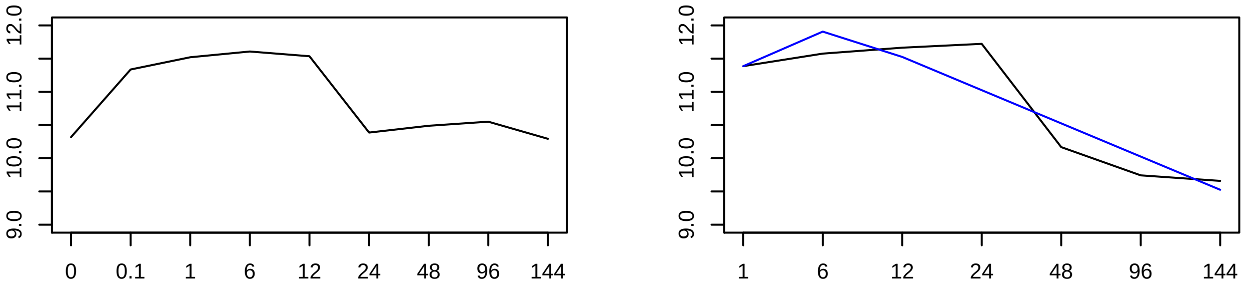

A_23_P145024 ADRB2 5q33.1

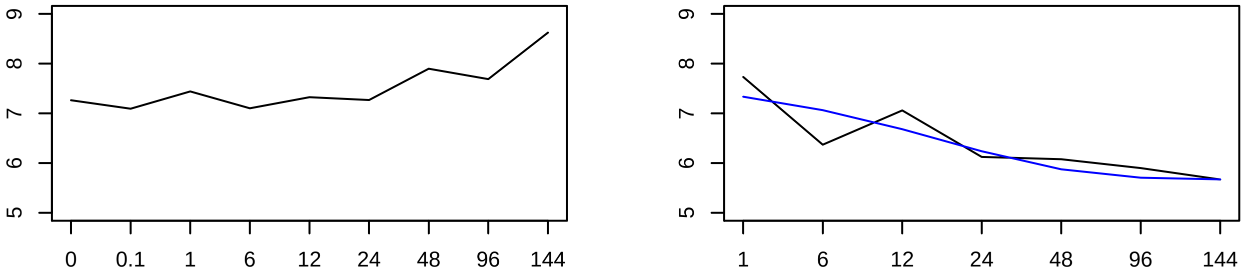

A_23_P206598 THC2726281 NA

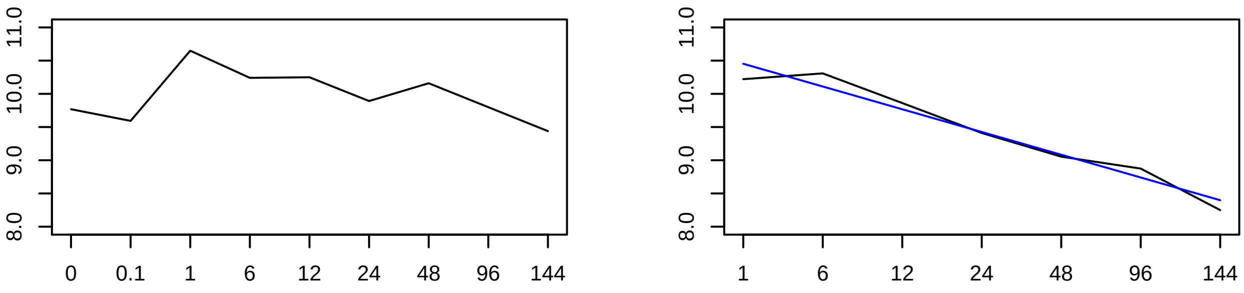

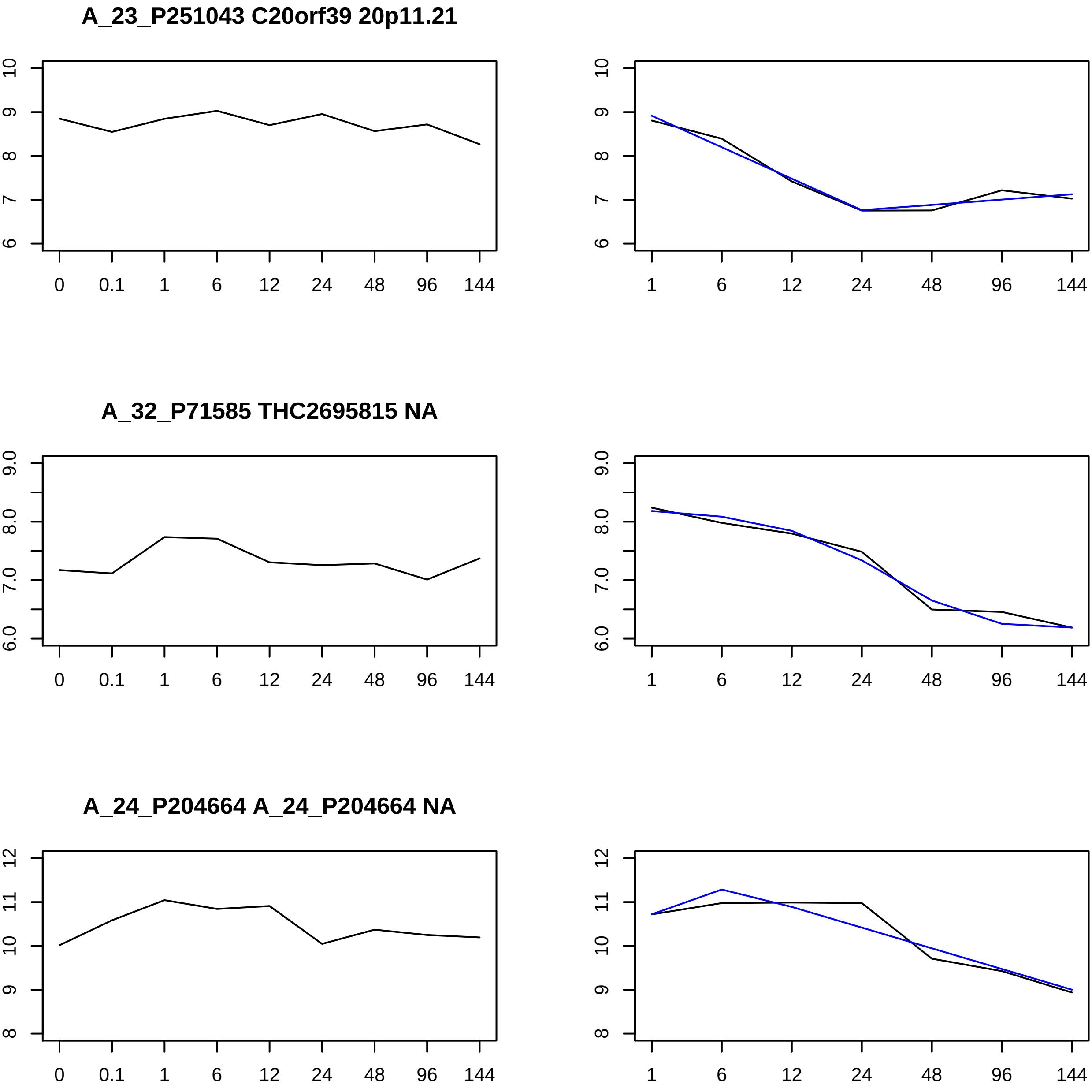
A_23_P251043 C20orf39 20p11.21
6
7
8
9
10
0
0.1
1
6
12
24
48
96
144
1
6
12
24
48
96
144
A_32_P71585 THC2695815 NA
6.0
7.0
8.0
9.0
A_24_P204664 A_24_P204664 NA
8
9
10
11
12

A_24_P375573 A_24_P375573 NA

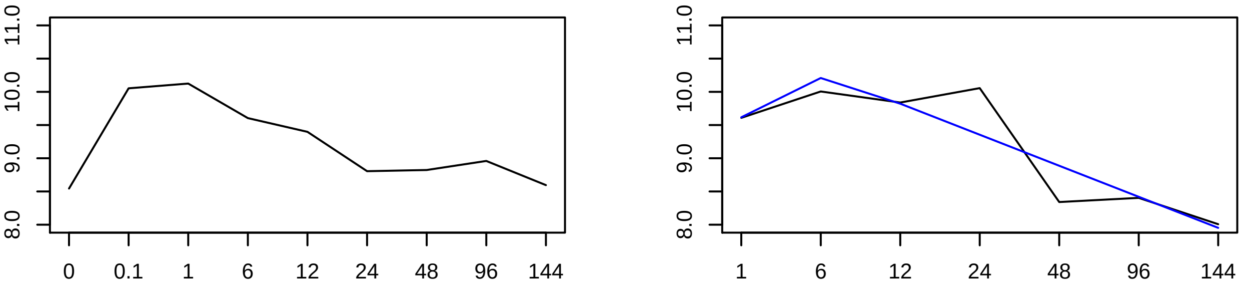

A_24_P477353 A_24_P477353 NA

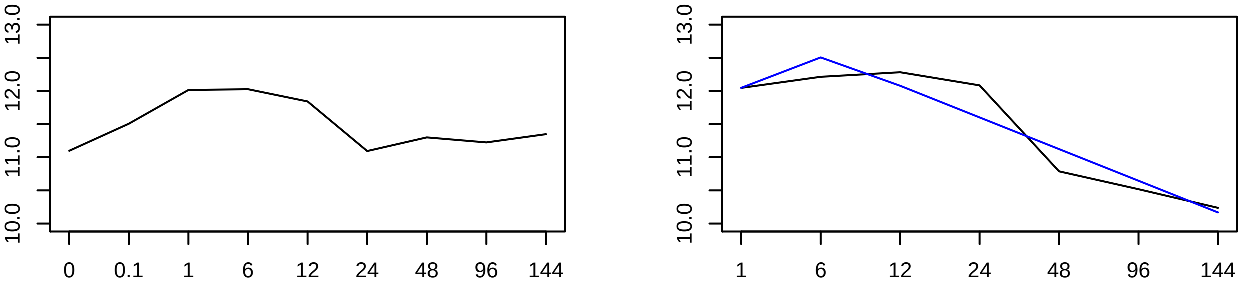

A_24_P169855 LOC645412 19q13.32

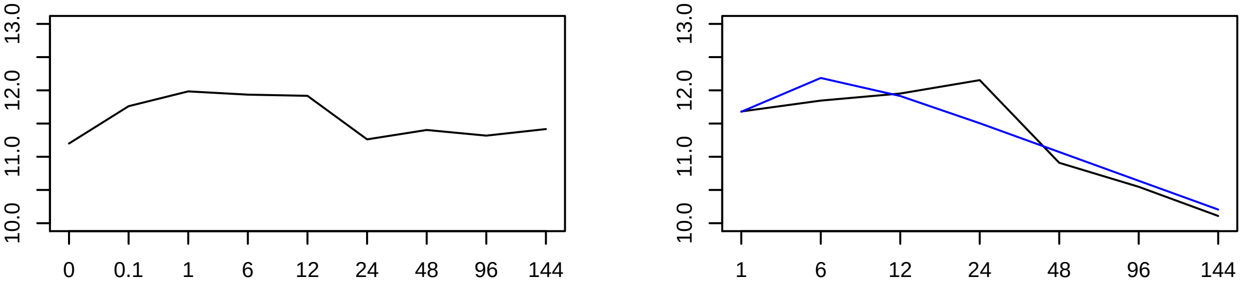

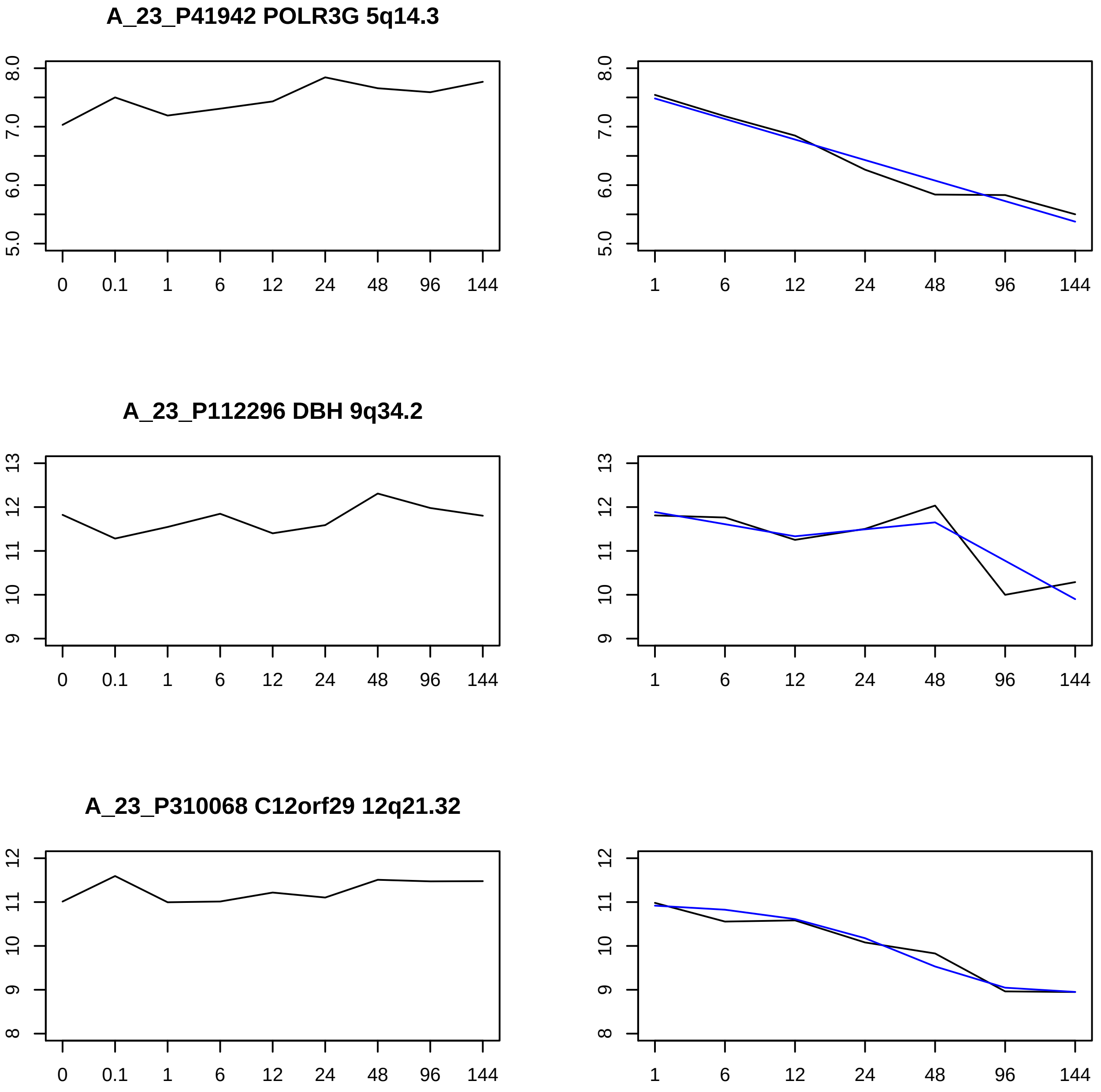

A_23_P41942 POLR3G 5q14.3
5.0 6.0 7.0 8.0
0 0.1 1 6 12 24 48 96 144
1 6 12 24 48 96 144
A_23_P112296 DBH 9q34.2
9 10 11 12 13
0 0.1 1 6 12 24 48 96 144
1 6 12 24 48 96 144
A_23_P310068 C12orf29 12q21.32
8 9 10 11 12
0 0.1 1 6 12 24 48 96 144
1 6 12 24 48 96 144

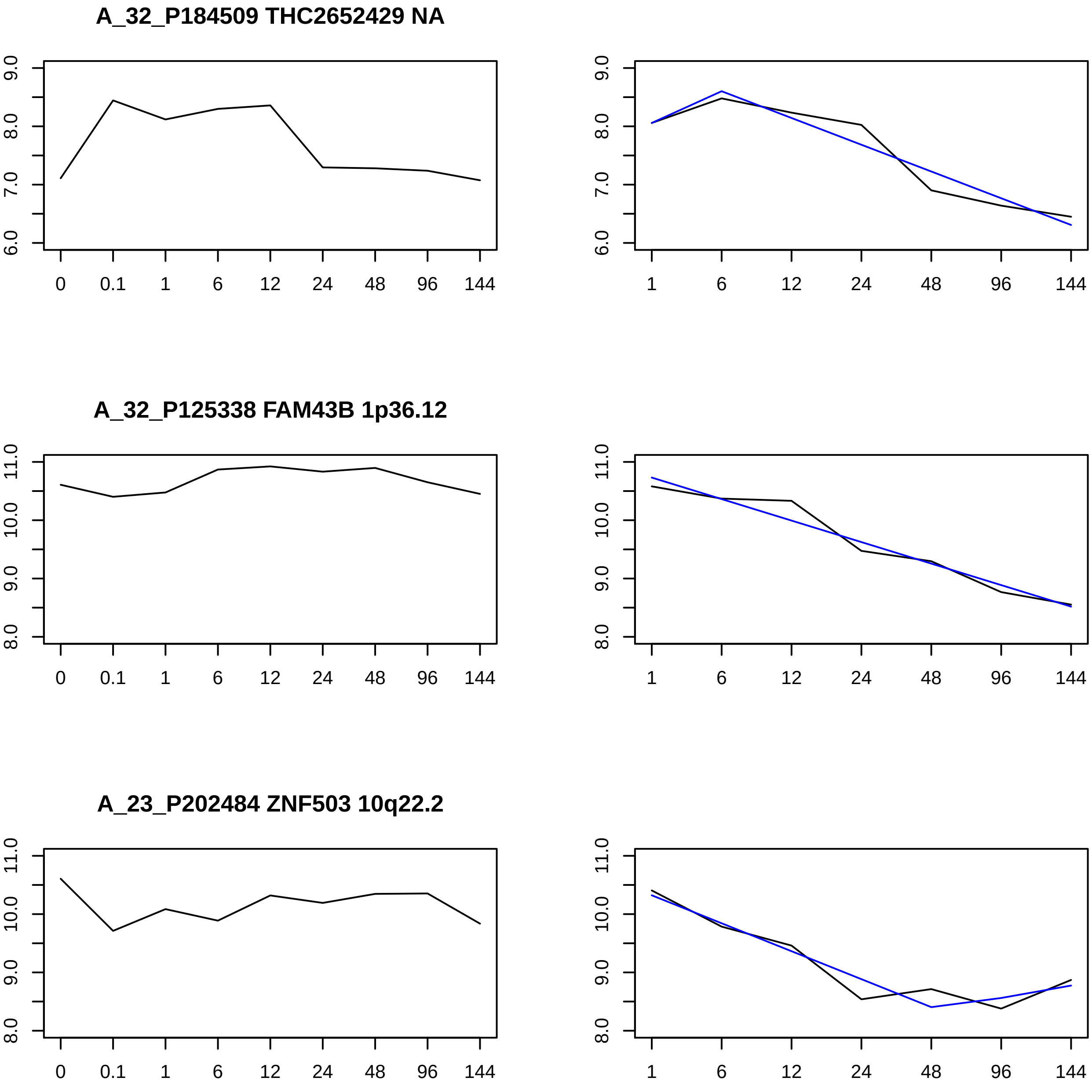
A_32_P184509 THC2652429 NA
A_32_P125338 FAM43B 1p36.12
A_23_P202484 ZNF503 10q22.2
6.0 7.0 8.0 9.0
8.0 9.0 10.0 11.0
0 0.1 1 6 12 24 48 96 144
1 6 12 24 48 96 144

A_23_P415401 KLF9 9q21.11

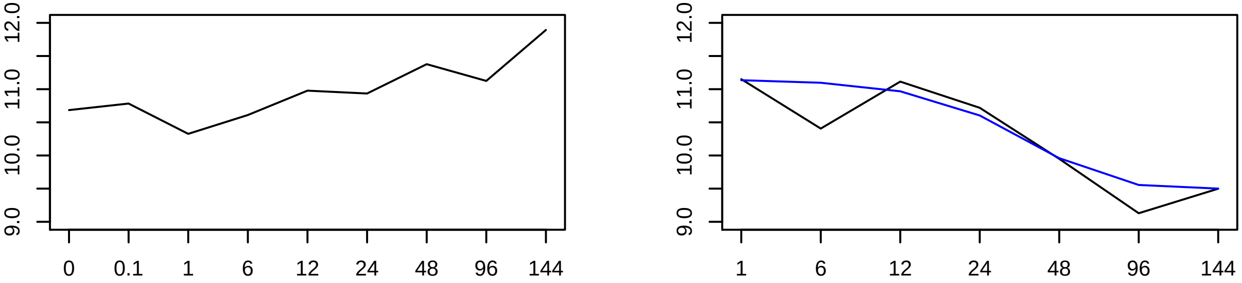

A_24_P171110 HNRPU 1q44

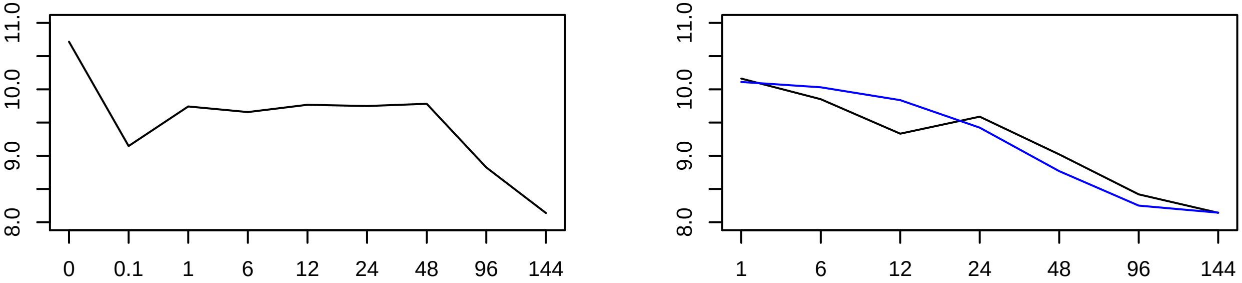

A_32_P36404 PTPRT 20q12

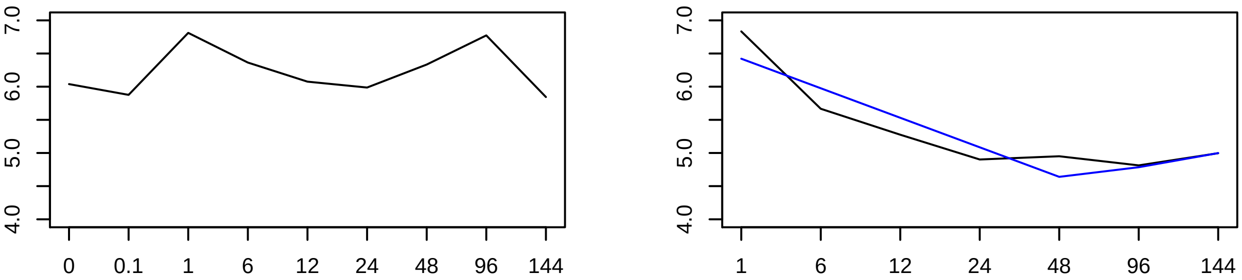

A_24_P270044 ASAM 11q24.1

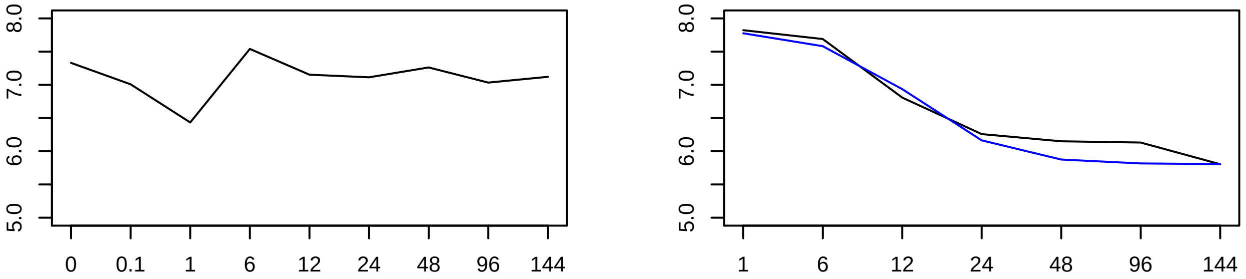

A_23_P40184 CDH22 20q13.12

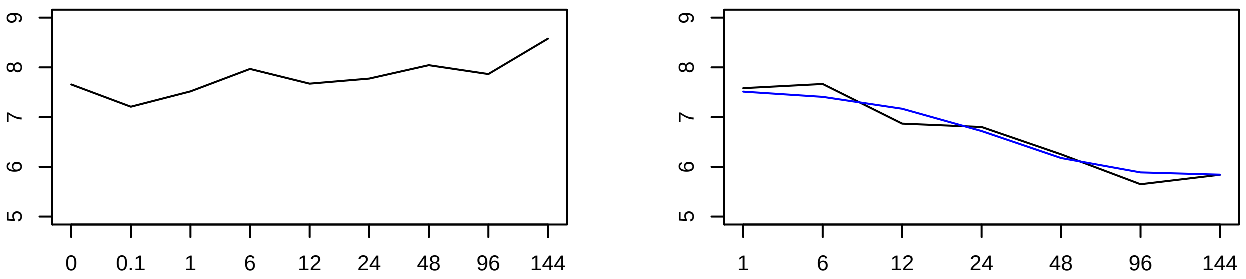

A_24_P383802 LOC391387 2p13.2

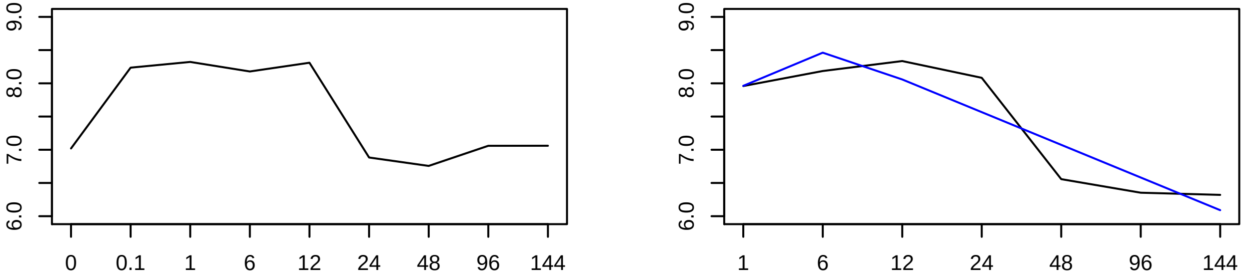

A_24_P392842 A_24_P392842 NA

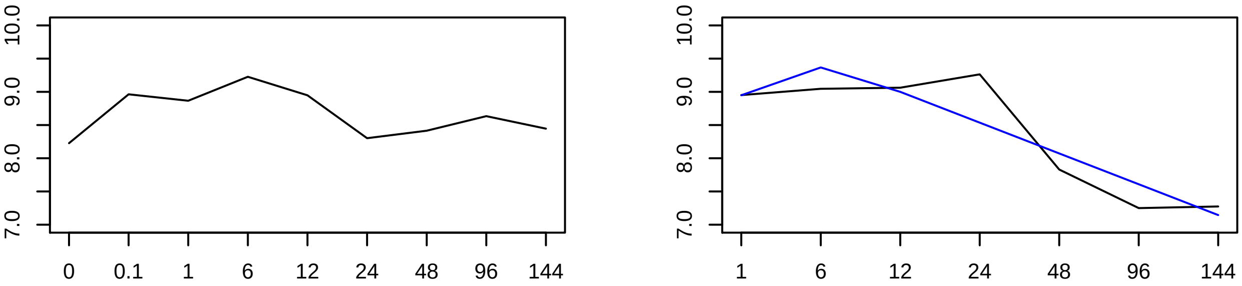

A_32_P32091 YAE1D1 NA

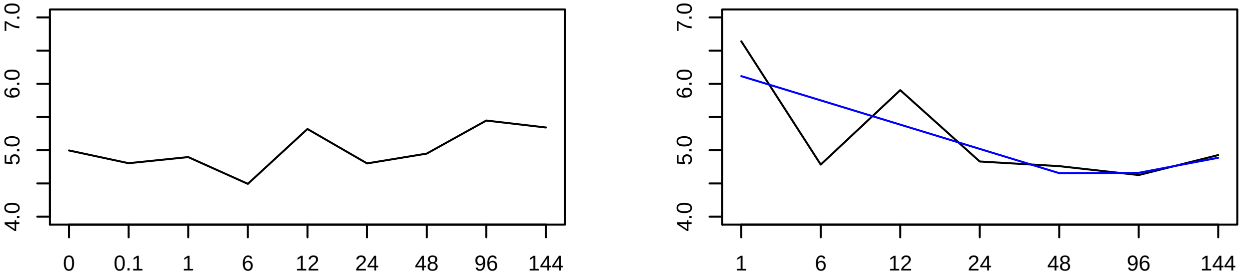

A_23_P418785 STXBP5L 3q13.33

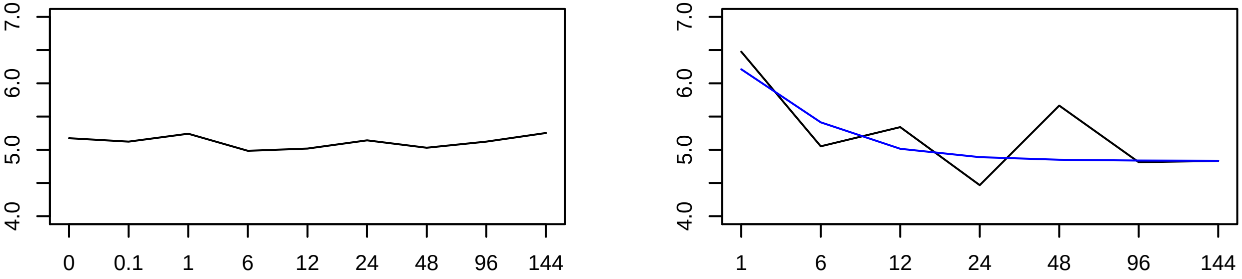

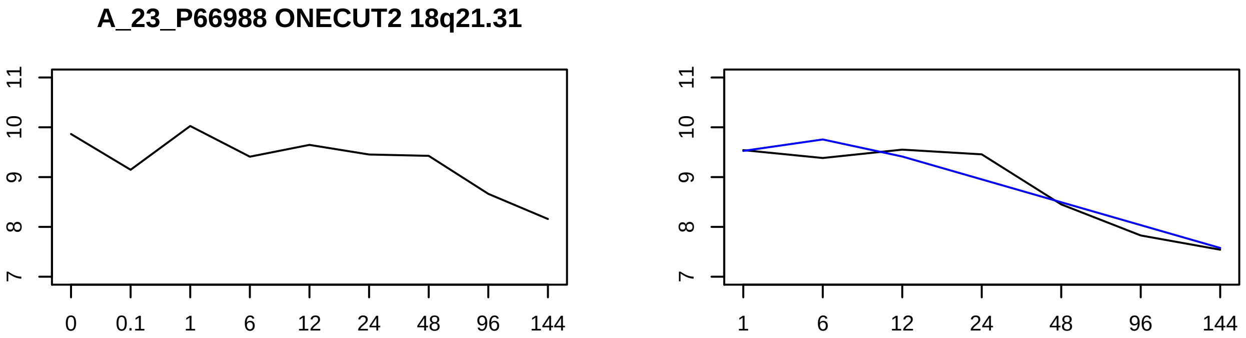
A_23_P66988 ONECUT2 18q21.31
7
8
9
10
11
0
0.1
1
6
12
24
48
96
144
1
6
12
24
48
96
144

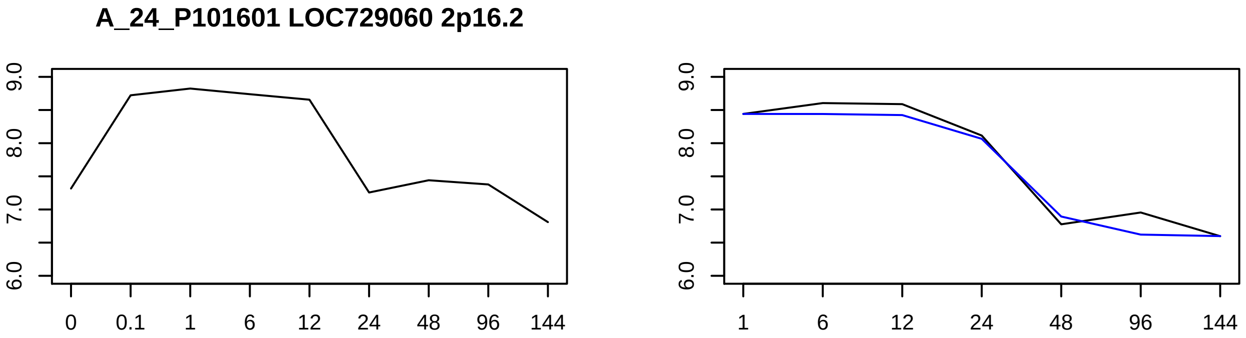
A_24_P101601 LOC729060 2p16.2
6.0
7.0
8.0
9.0
0
0.1
1
6
12
24
48
96
144
1
6
12
24
48
96
144

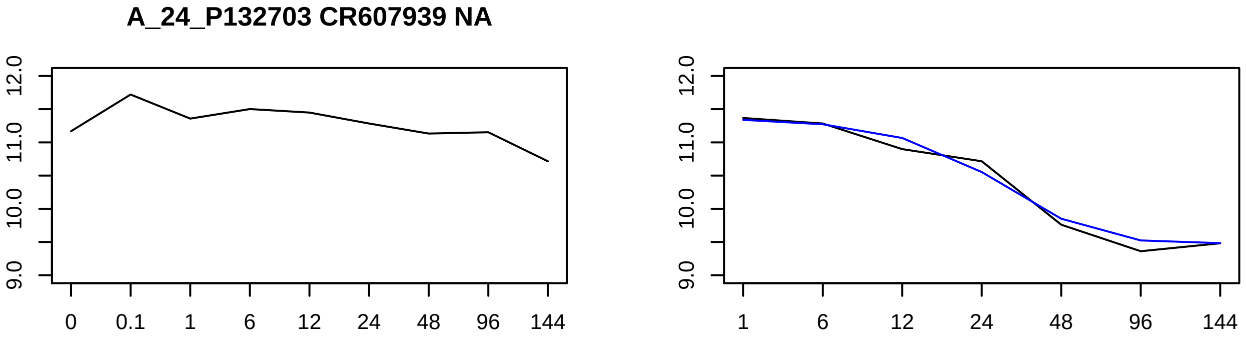
A_24_P132703 CR607939 NA
9.0
10.0
11.0
12.0
0
0.1
1
6
12
24
48
96
144
1
6
12
24
48
96
144

A_32_P113742 RPL21 10q26.12

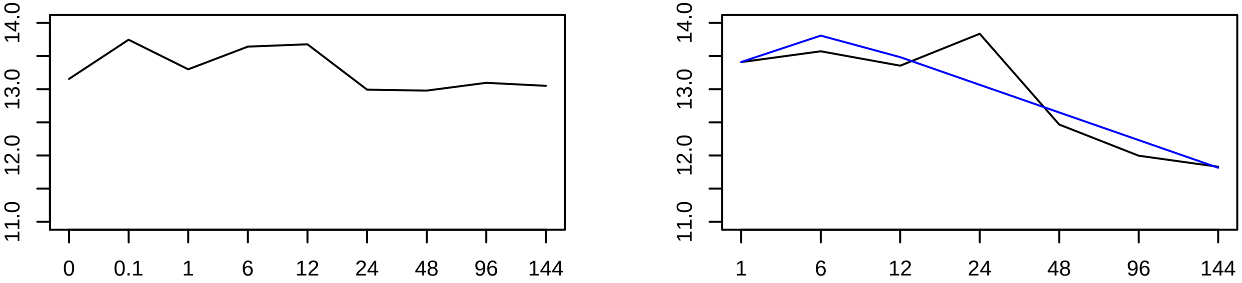

A_24_P43959 FRMD4A 10p13

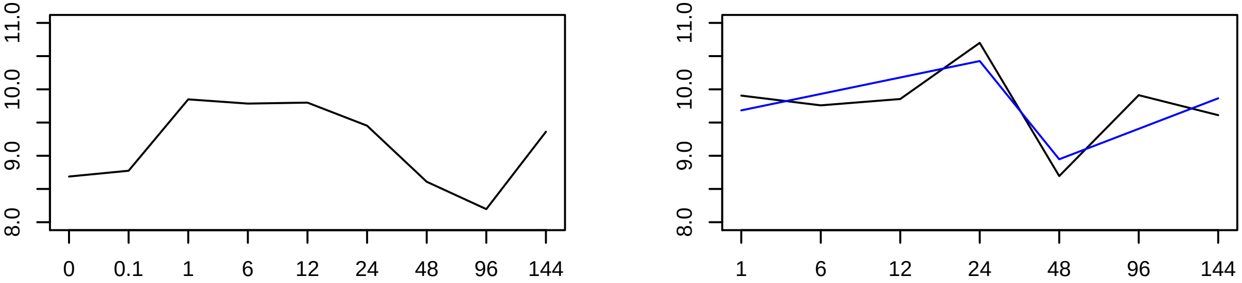

A_23_P113811 A_23_P113811 NA

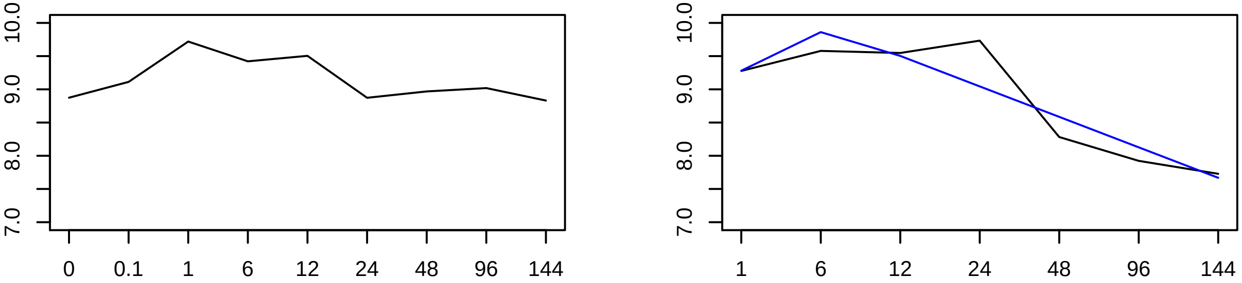

A_24_P76142 HSPD1P10 6q15

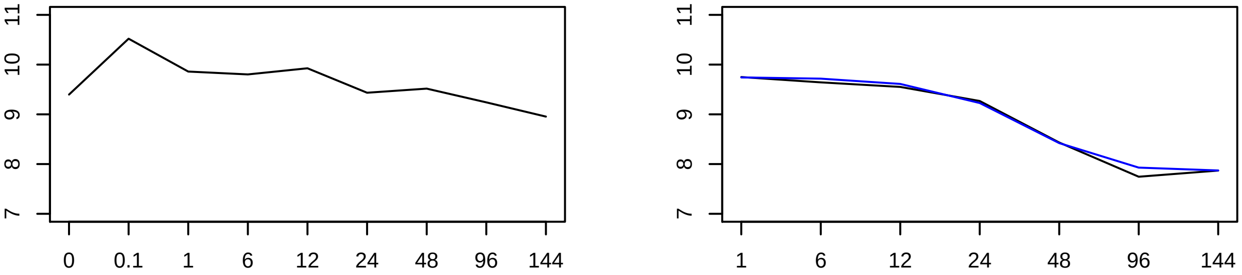

A_32_P110820 THC2662255 NA

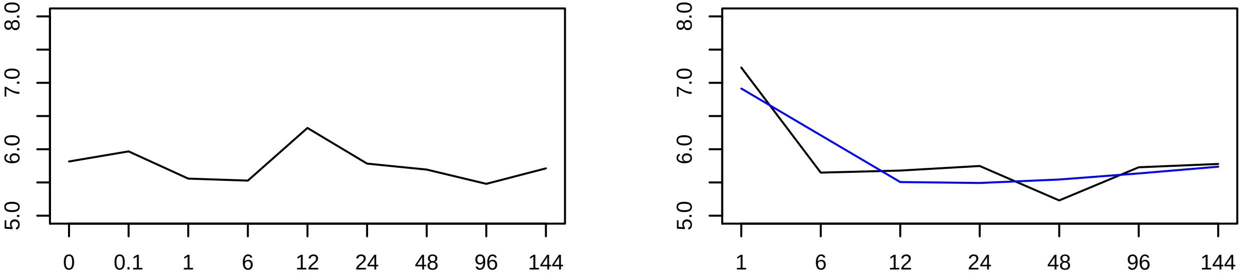

A_24_P298099 A_24_P298099 NA

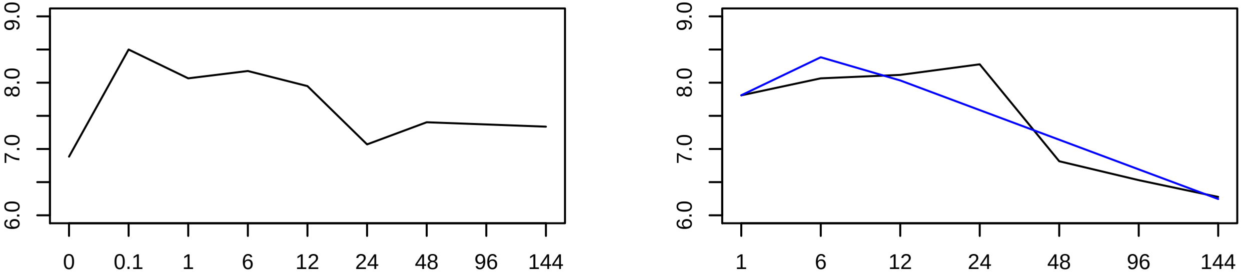

A_23_P571 SLC2A1 1p34.2

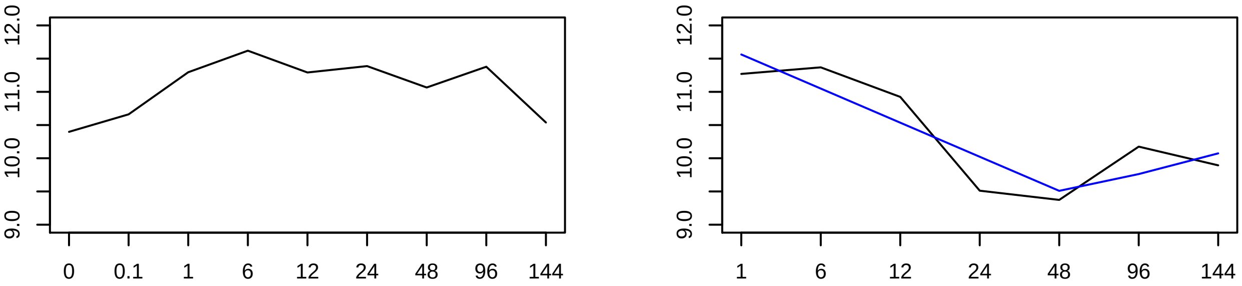

A_24_P170726 LOC645427 17p11.2

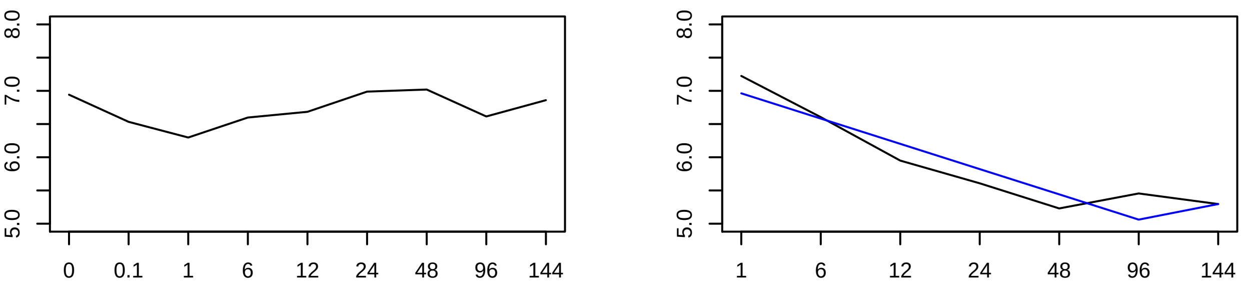

A_23_P205057 PCDH17 13q21.1

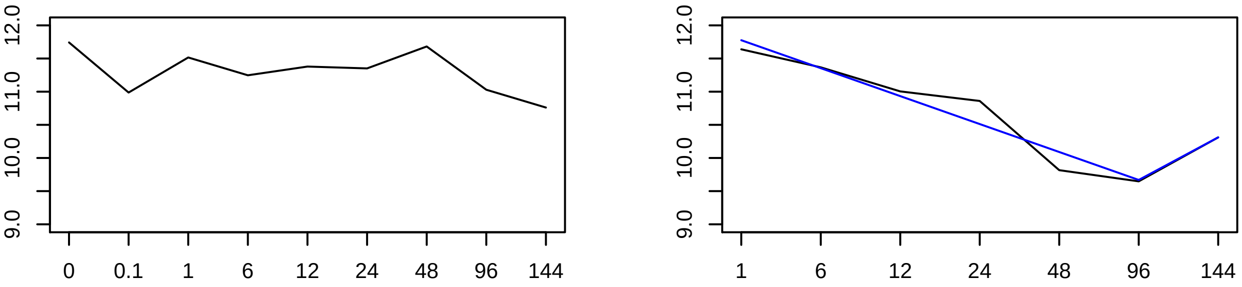

A_23_P422305 SFXN4 10q26.11

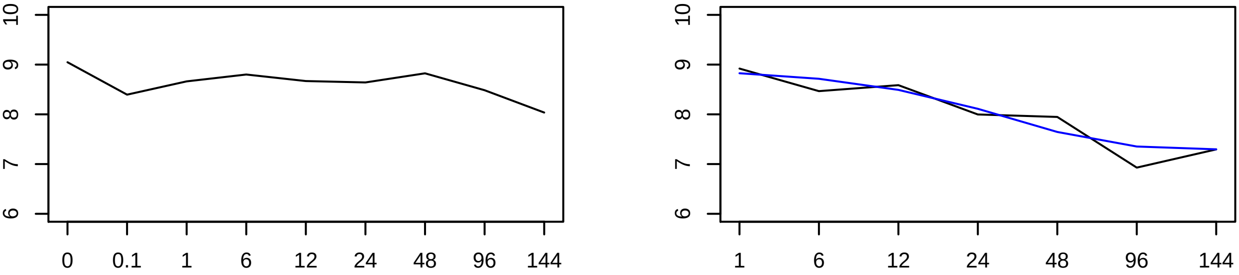

A_24_P698759 A_24_P698759 NA

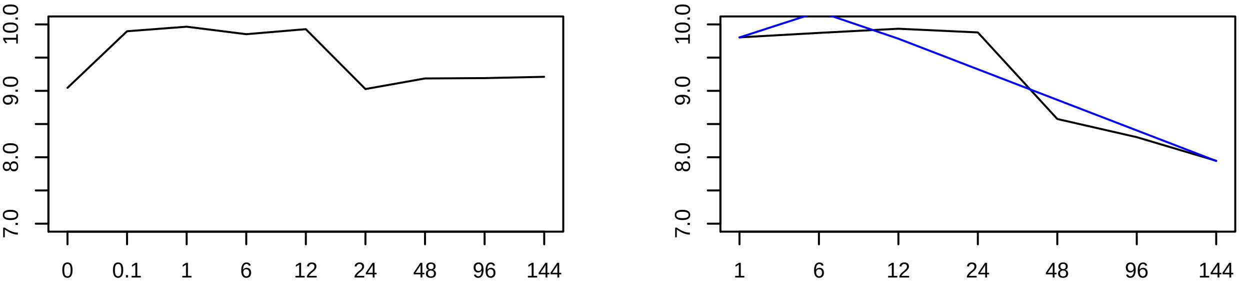

A_24_P392900 A_24_P392900 NA

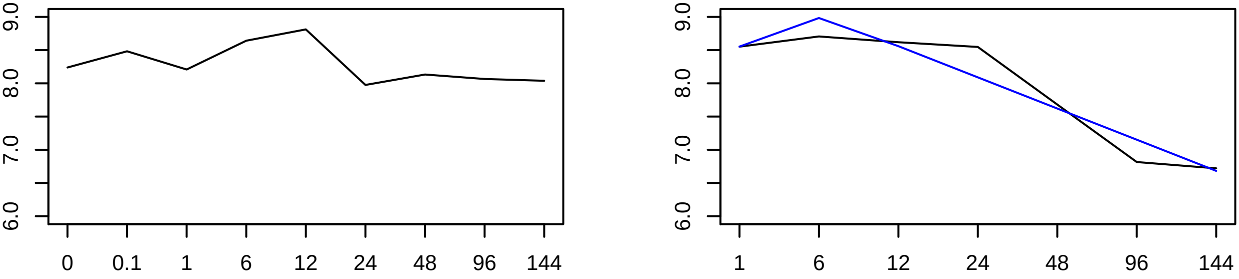

A_24_P126931 A_24_P126931 NA

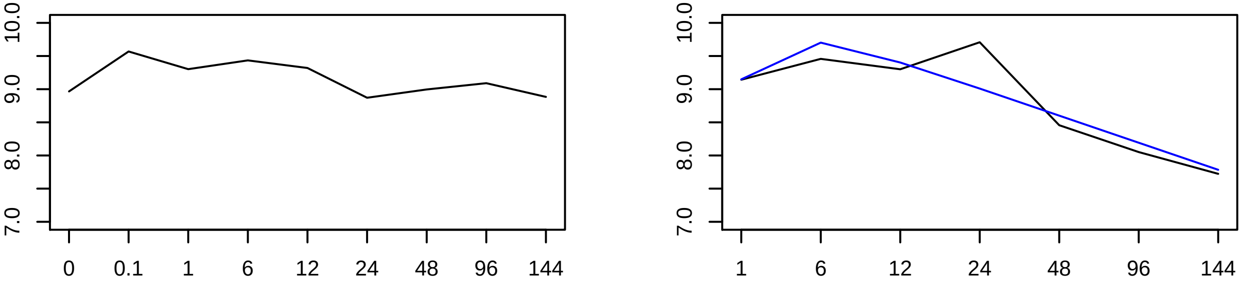

A_24_P911179 ASPM 1q31.3

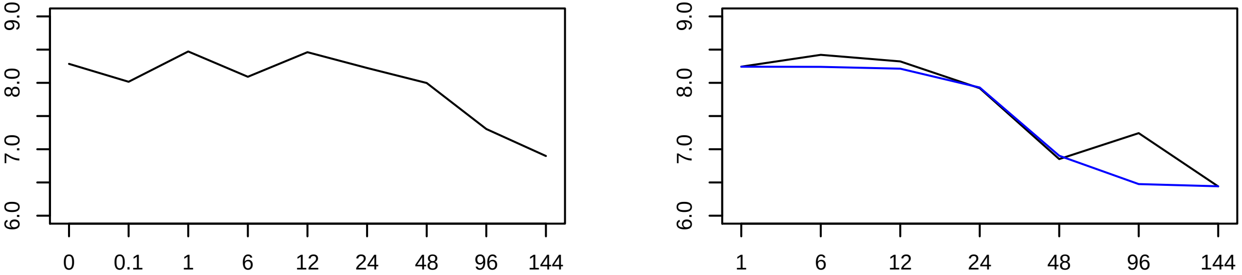

A_23_P315789 RFXAP 13q13.3

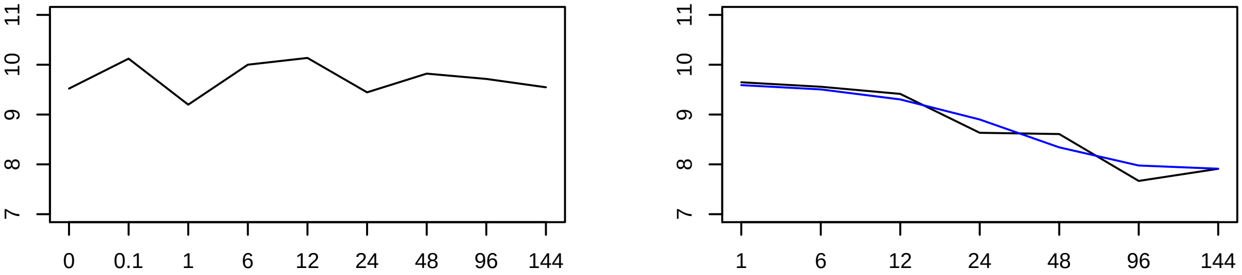

A_32_P100974 RPL24 3q12.3

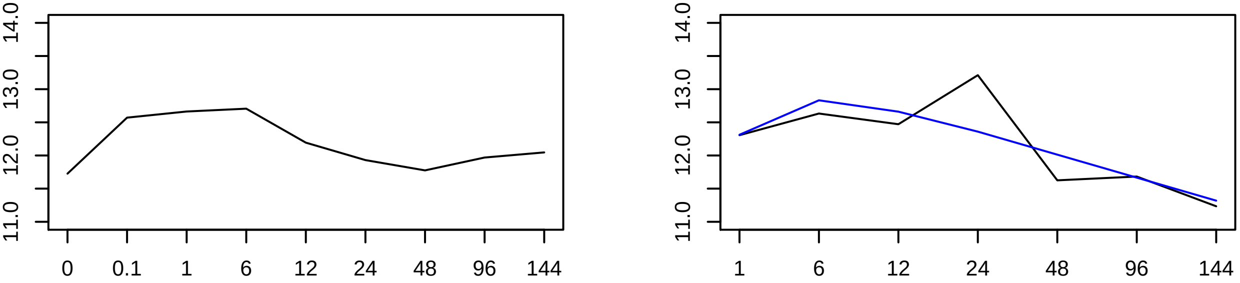

A_24_P460309 THC2657647 NA

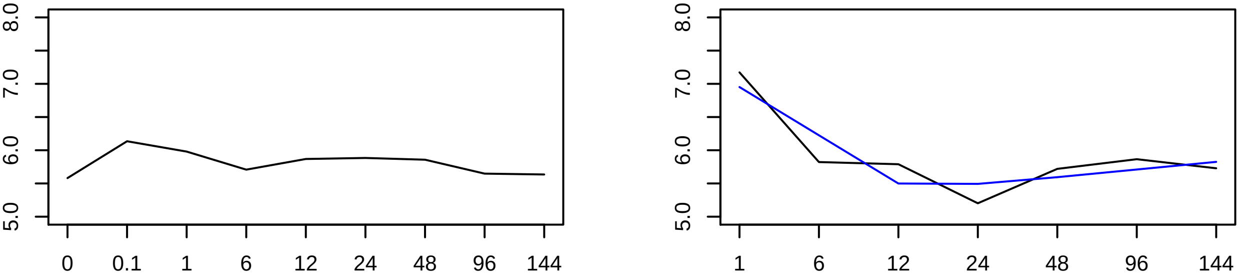

A_32_P153071 VIPR2 7q36.3

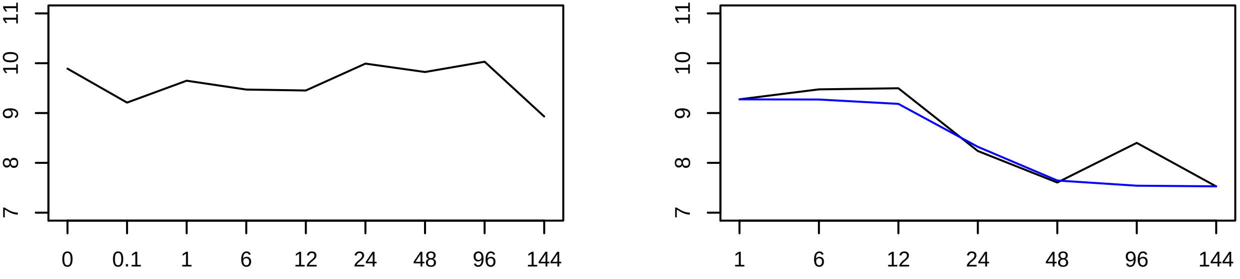

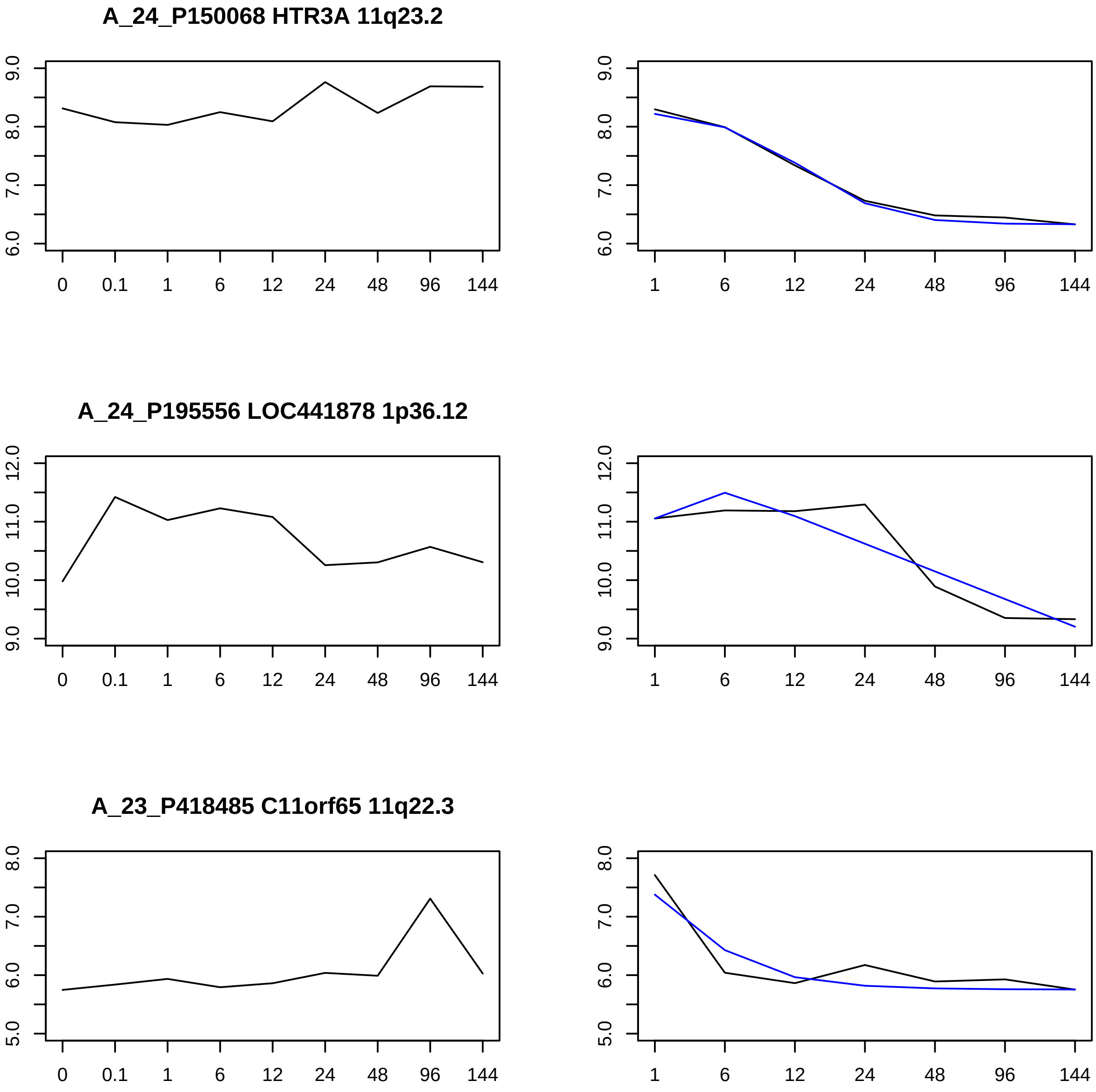
A_24_P150068 HTR3A 11q23.2
6.0
7.0
8.0
9.0
0
0.1
1
6
12
24
48
96
144
1
6
12
24
48
96
144
A_24_P195556 LOC441878 1p36.12
9.0
10.0
11.0
12.0
A_23_P418485 C11orf65 11q22.3
5.0
6.0
7.0
8.0

A_24_P191847 PTCD2 5q13.2

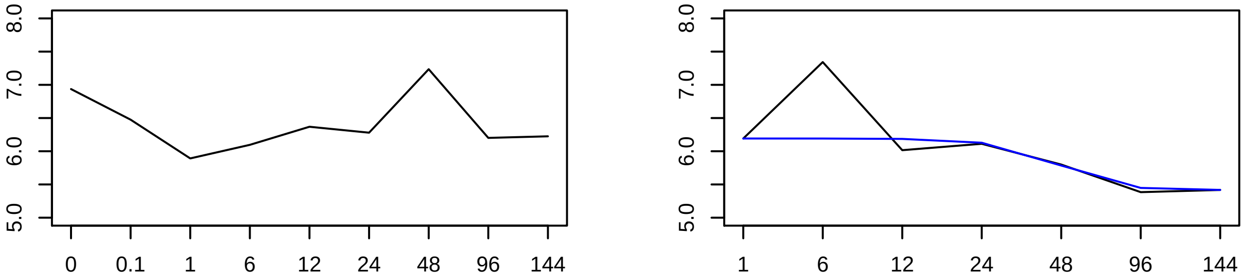

A_23_P126426 DPH2 1p34.1

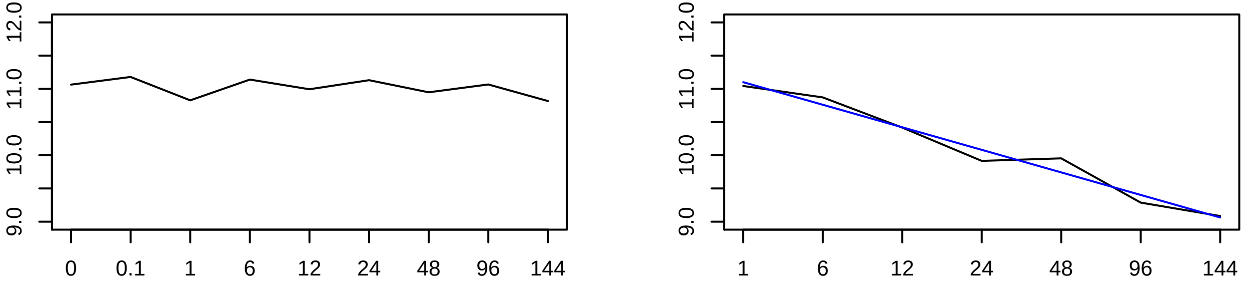

A_24_P135551 LOC130865 2p16.1

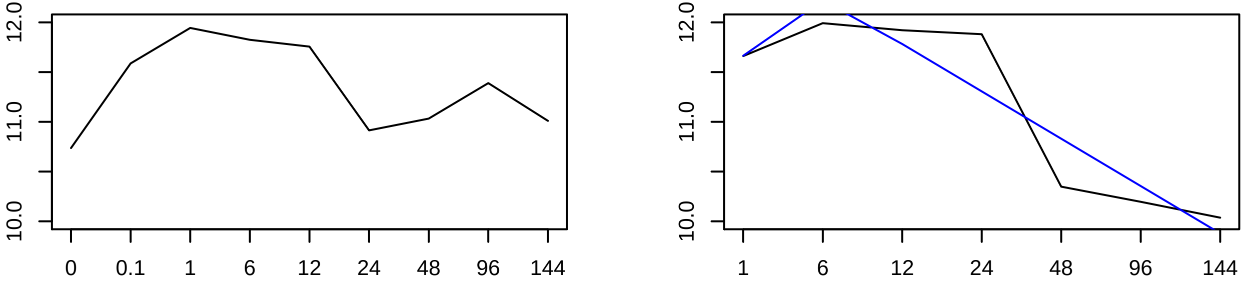

A_24_P126425 FLJ22374 7p15.1

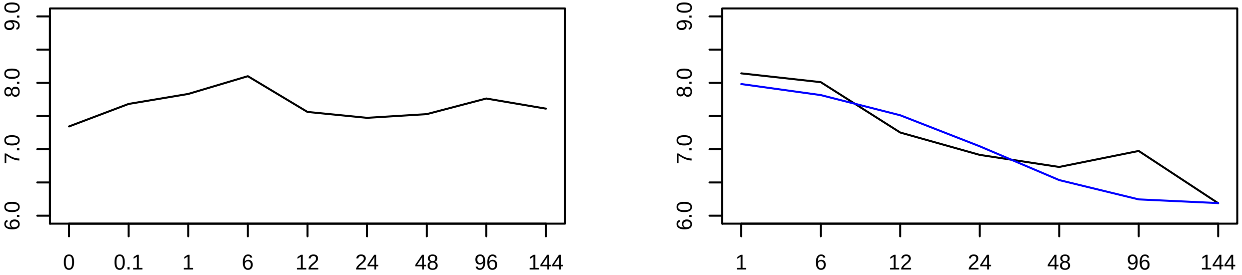

A_23_P101240 VSIG10L NA

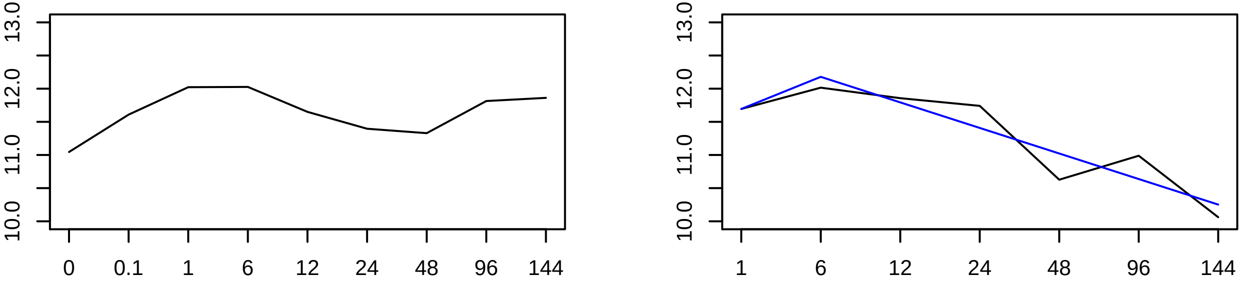

A_24_P410070 A_24_P410070 NA

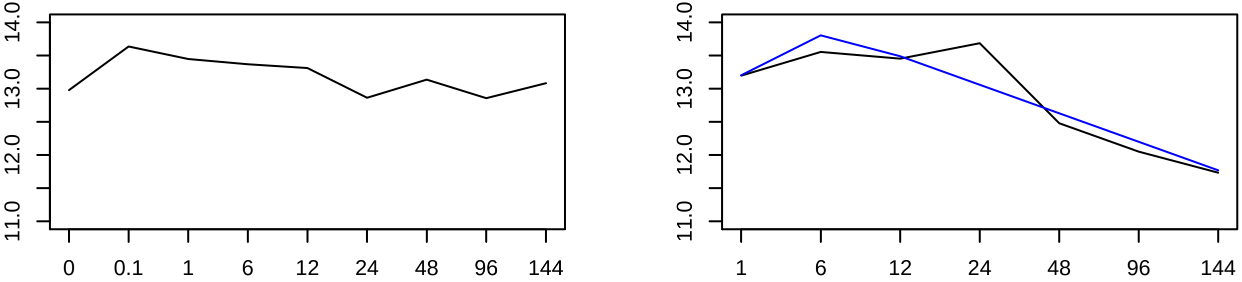

A_23_P59022 TRERF1 6p21.1

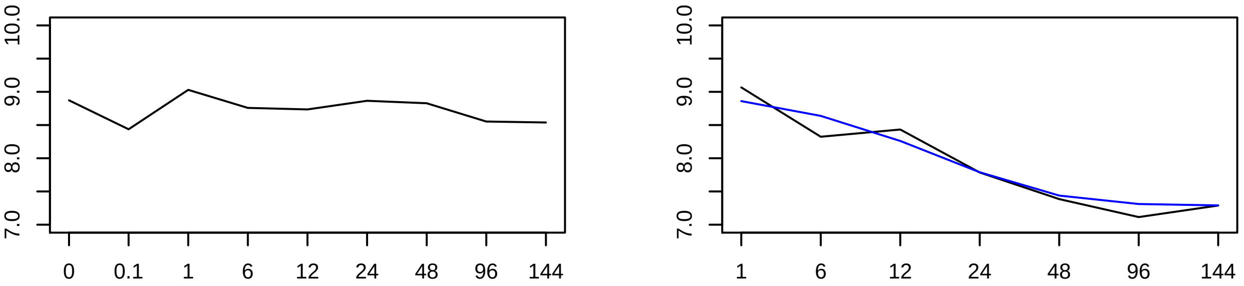

A_23_P12755 LOXL4 10q24.2

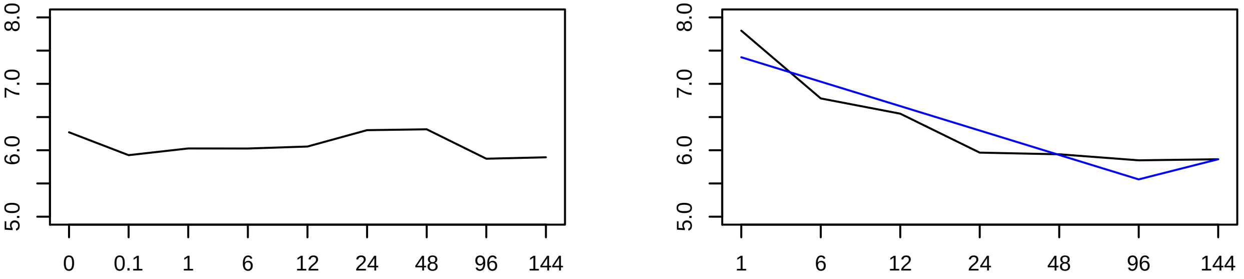

A_24_P634602 BE542385 NA

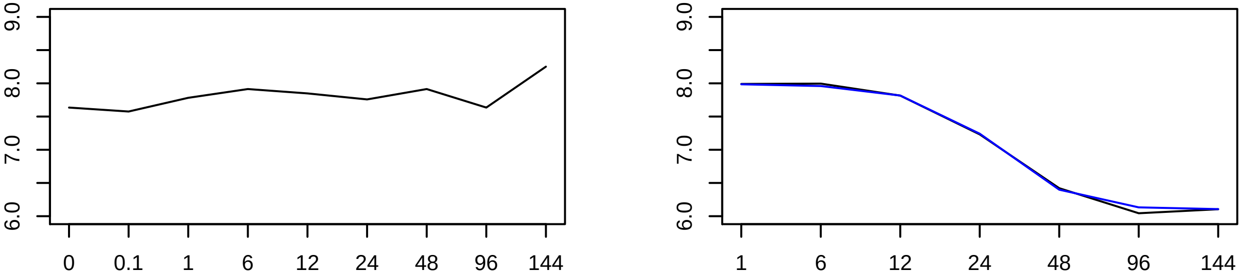

A_24_P281304 A_24_P281304 NA

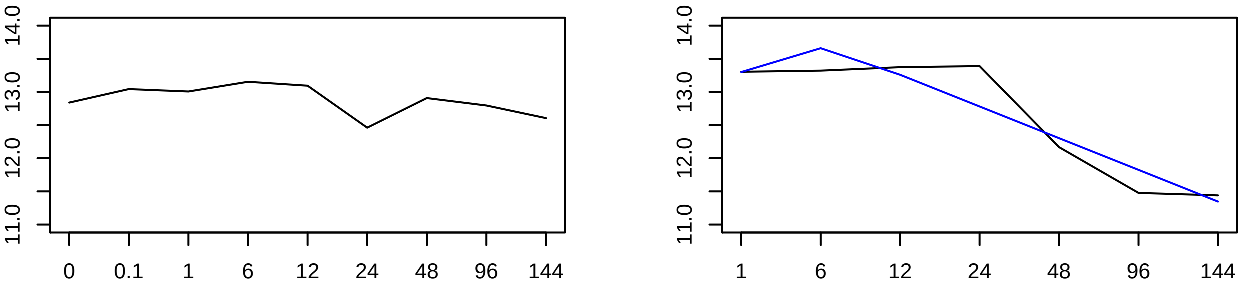

A_24_P325992 LIFR 5p13.1

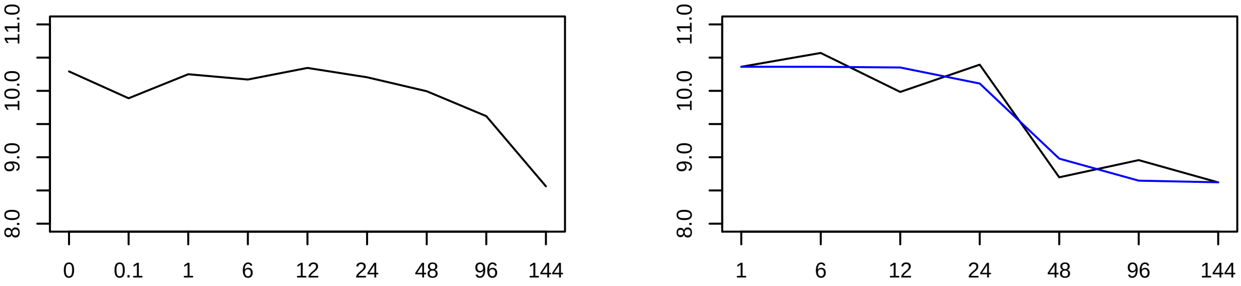

A_23_P31681 NDUFAF6 NA

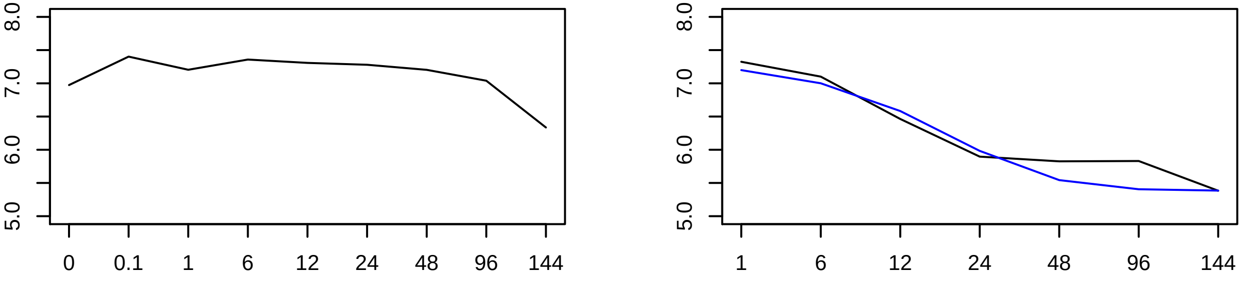

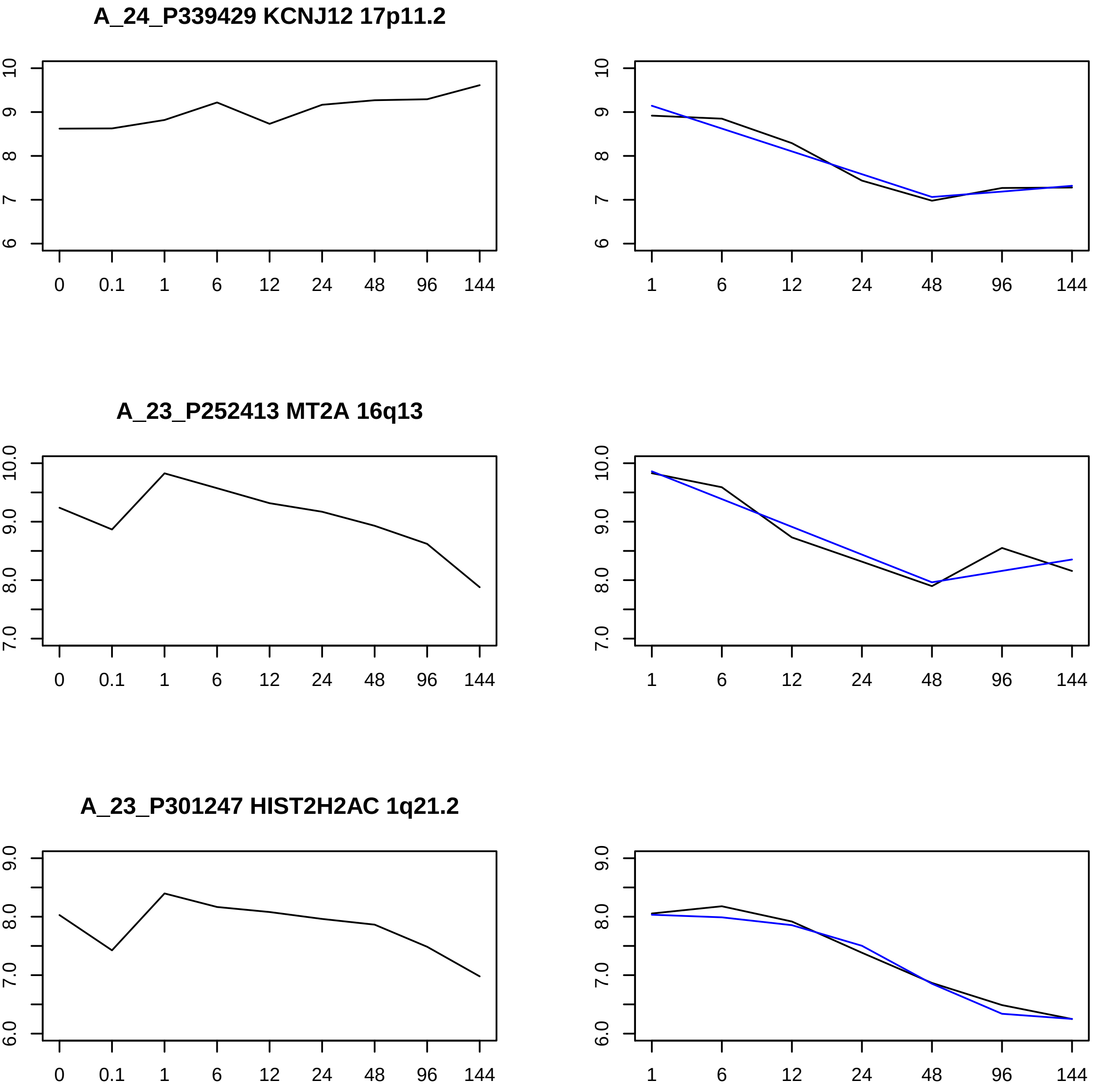
A_24_P339429 KCNJ12 17p11.2
A_23_P252413 MT2A 16q13
A_23_P301247 HIST2H2AC 1q21.2
0 0.1 1 6 12 24 48 96 144
1 6 12 24 48 96 144
6 7 8 9 10
7.0 8.0 9.0 10.0
6.0 7.0 8.0 9.0

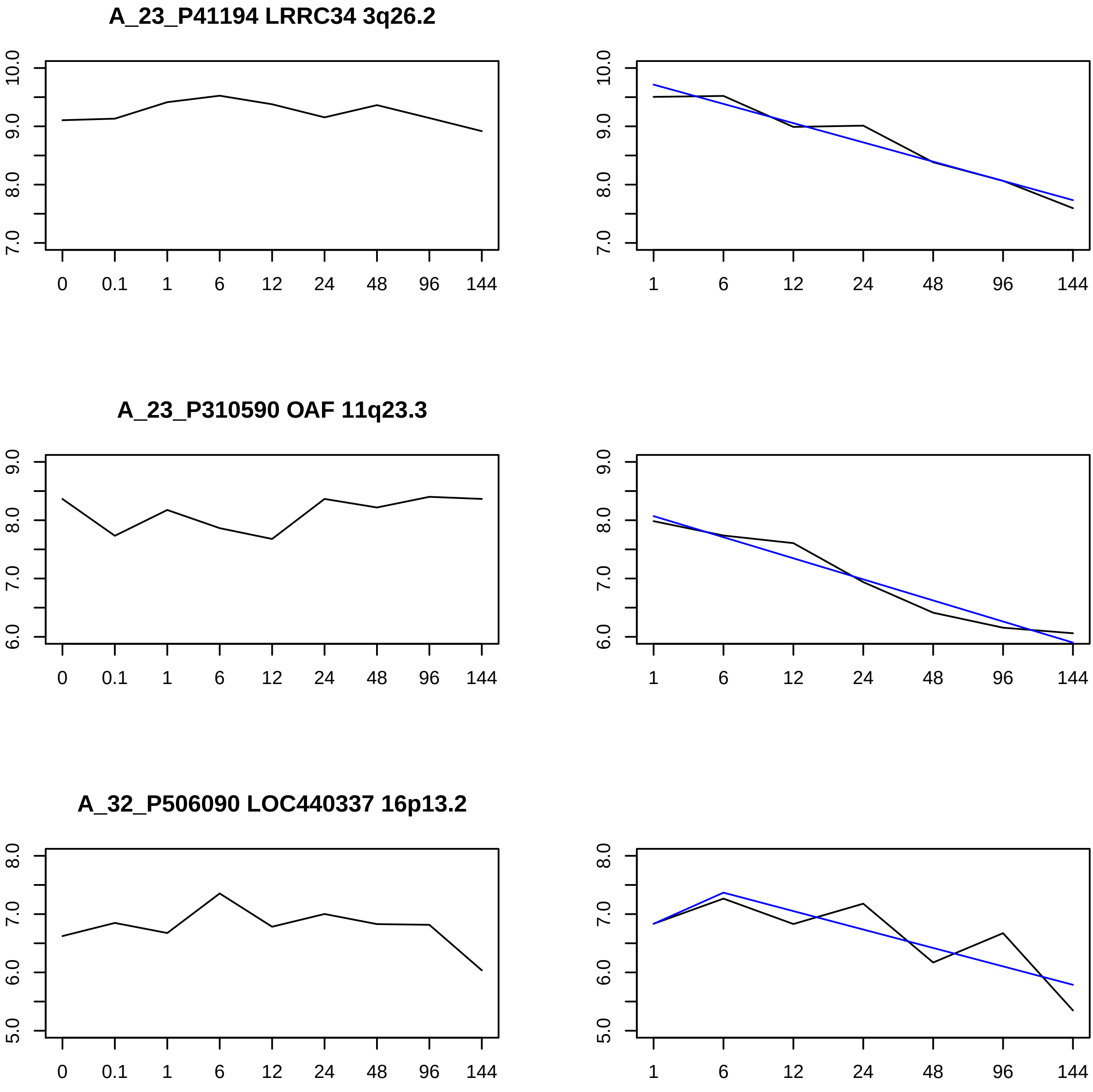
A_23_P41194 LRRC34 3q26.2
10.0
9.0
8.0
7.0
0
0.1
1
6
12
24
48
96
144
A_23_P310590 OAF 11q23.3
9.0
8.0
7.0
6.0
A_32_P506090 LOC440337 16p13.2
8.0
7.0
6.0
5.0

A_24_P730256 RP11−389O22.4 1p13.2

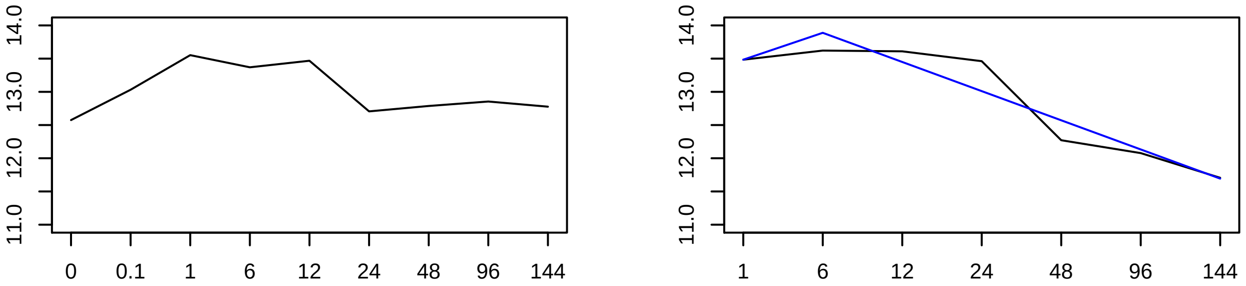

A_24_P936252 DANCR\MIR4449\SNORA26 4q12

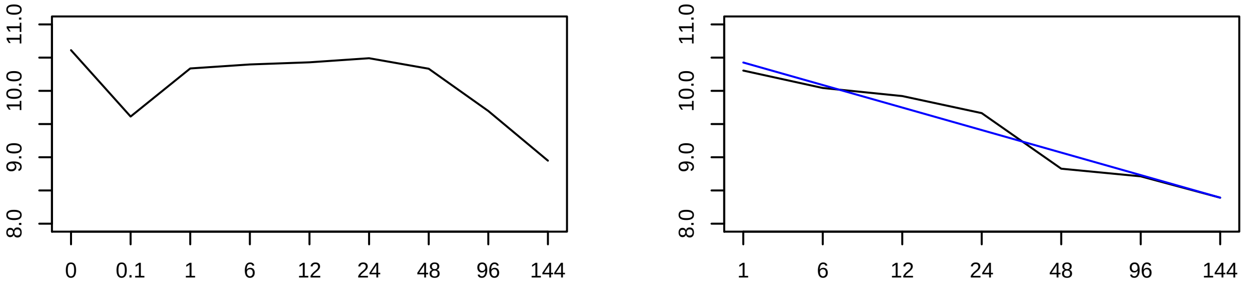

A_23_P75310 ARHGAP22 10q11.22

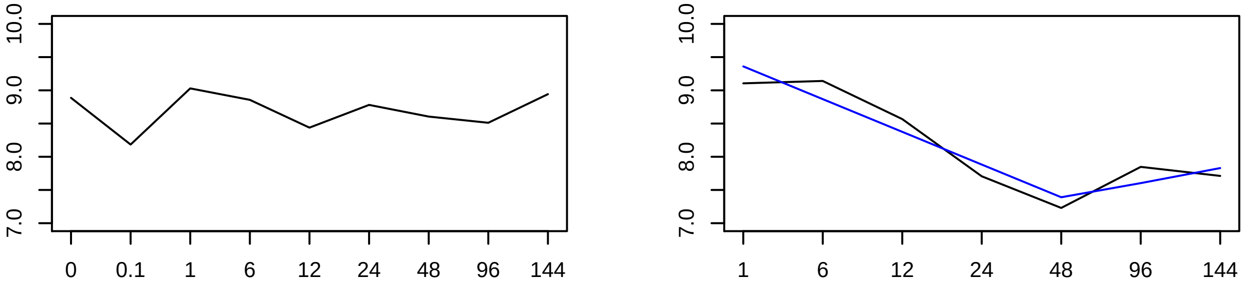

A_24_P477048 A_24_P477048 NA

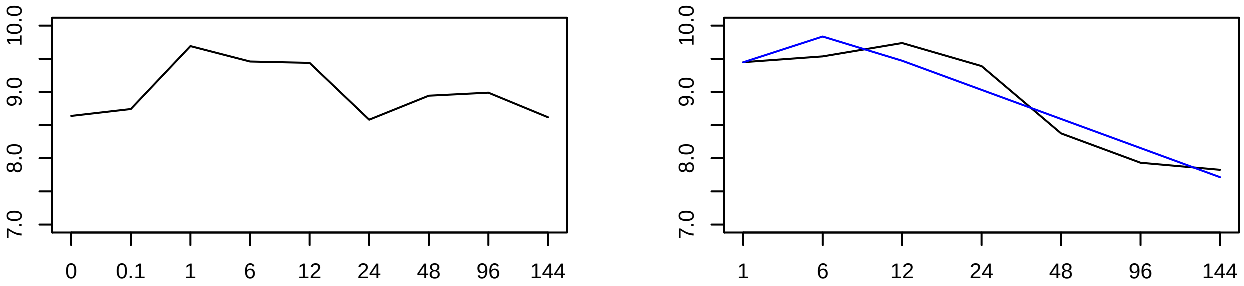

A_23_P18123 NLGN1 3q26.31

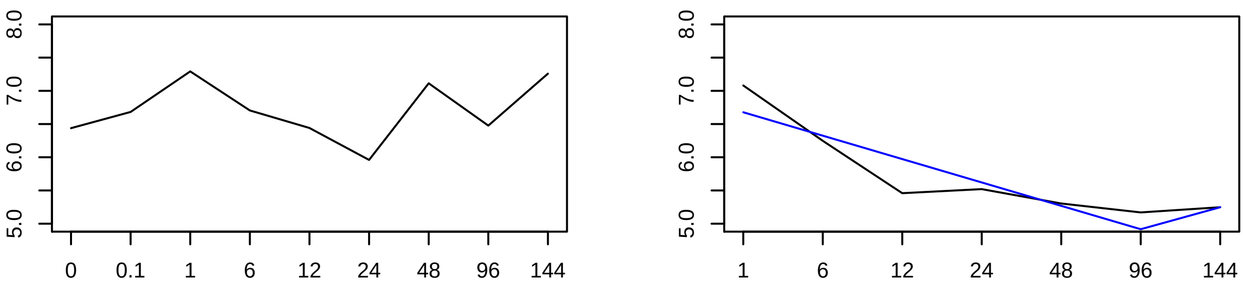

A_23_P212482 SLC9A9 3q24

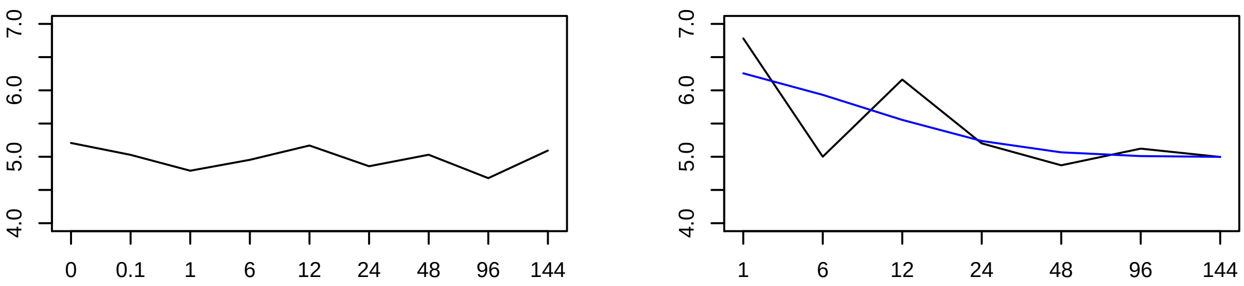

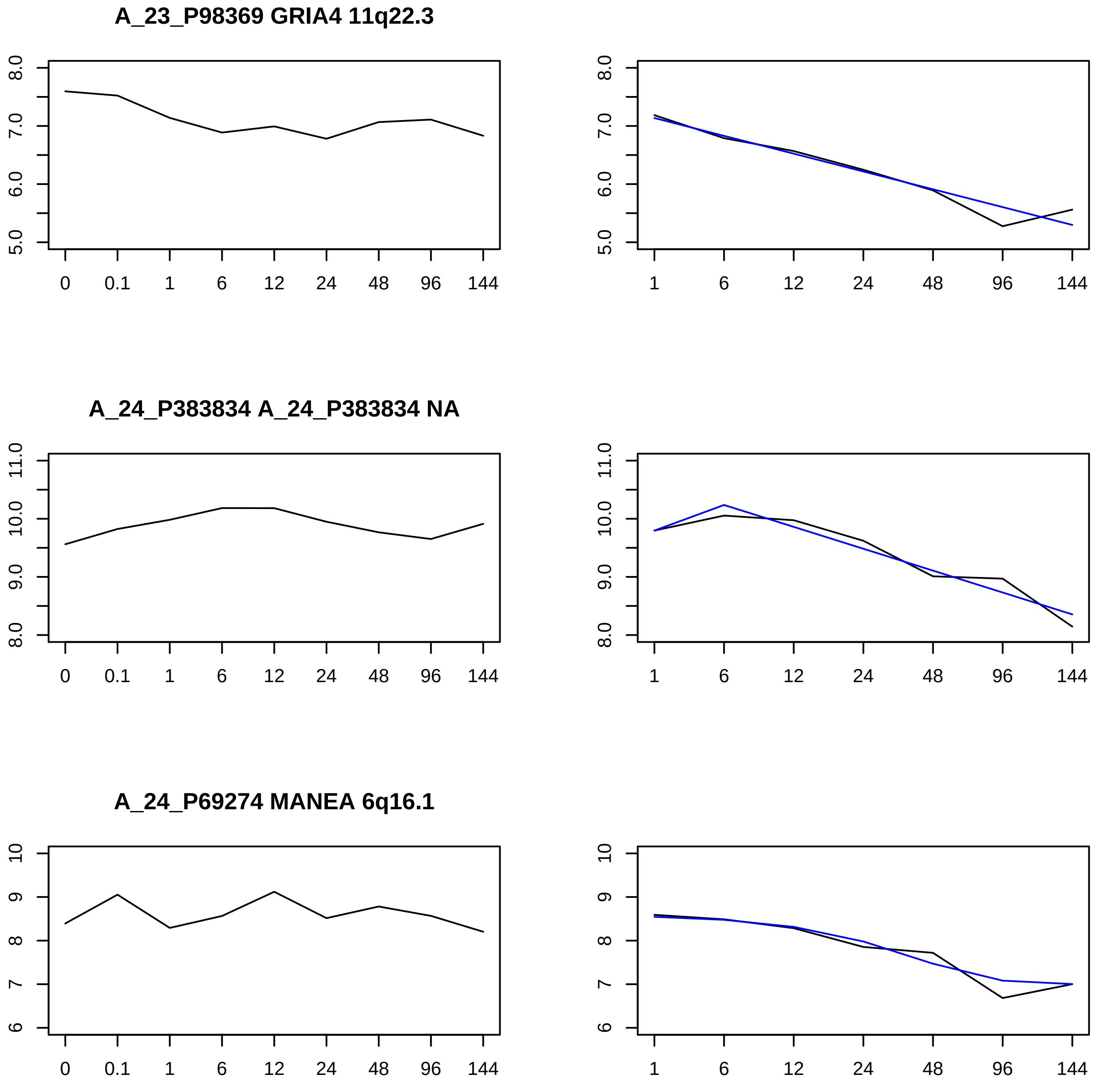

A_23_P98369 GRIA4 11q22.3
5.0
6.0
7.0
8.0
0
0.1
1
6
12
24
48
96
144
1
6
12
24
48
96
144
A_24_P383834 A_24_P383834 NA
8.0
9.0
10.0
11.0
A_24_P69274 MANEA 6q16.1
6
7
8
9
10

A_24_P638453 NCAPD2P1 7p14.3

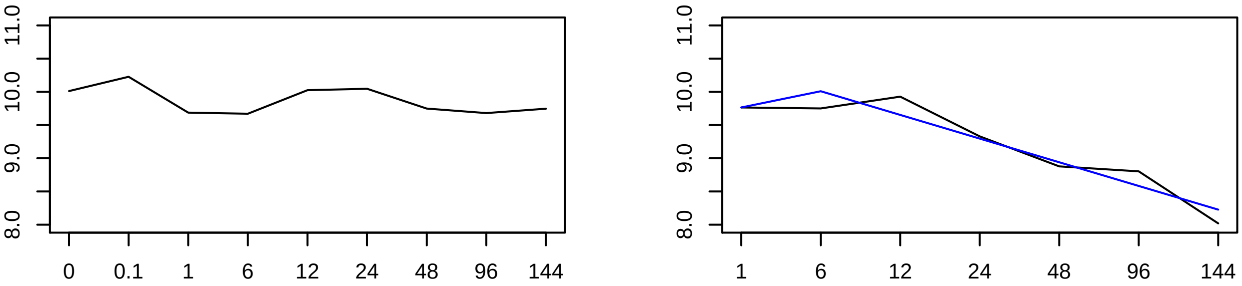

A_24_P217904 TRERF1 6p21.1

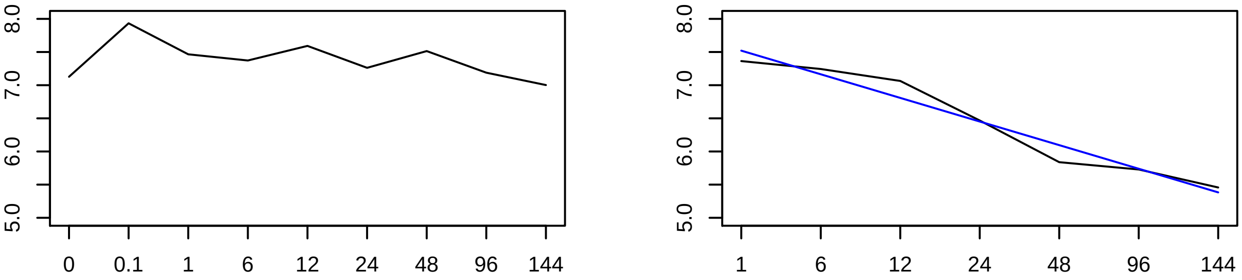

A_24_P505981 A_24_P505981 NA

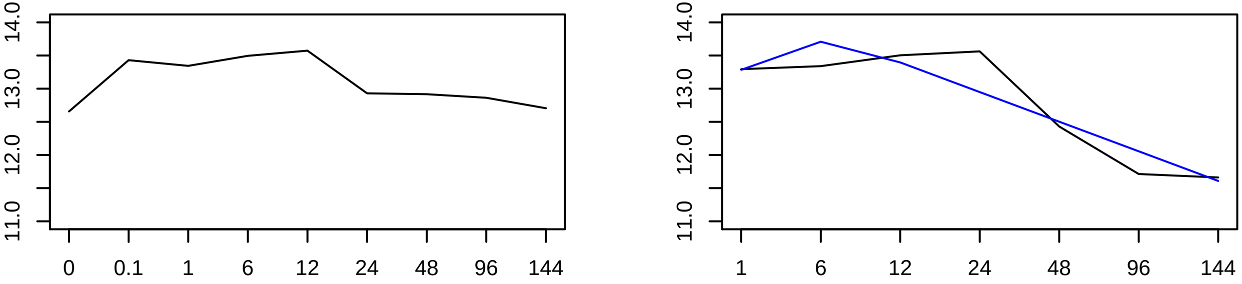

A_32_P68076 EIF3M NA

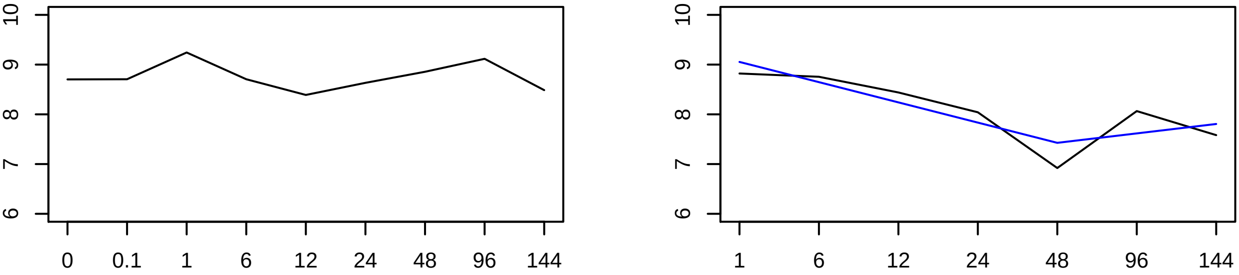

A_32_P15544 PRIMA1 14q32.13

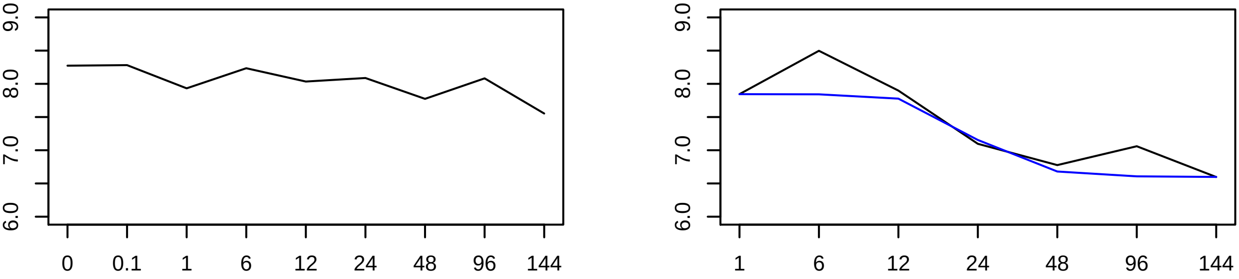

A_32_P542308 RP11−658F2.3

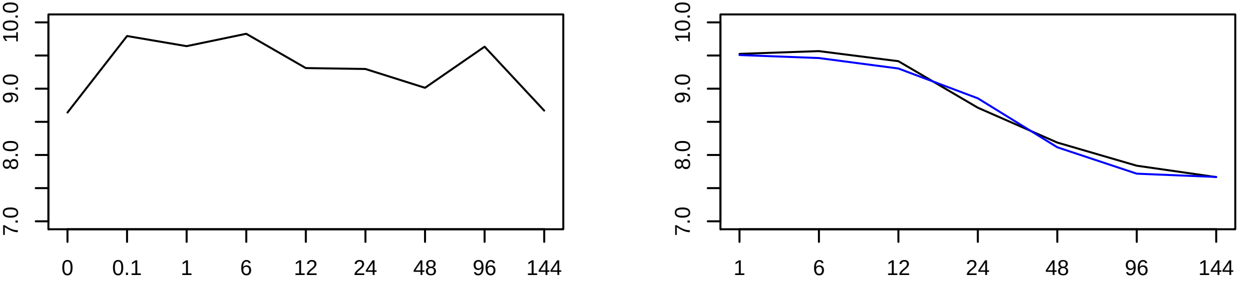

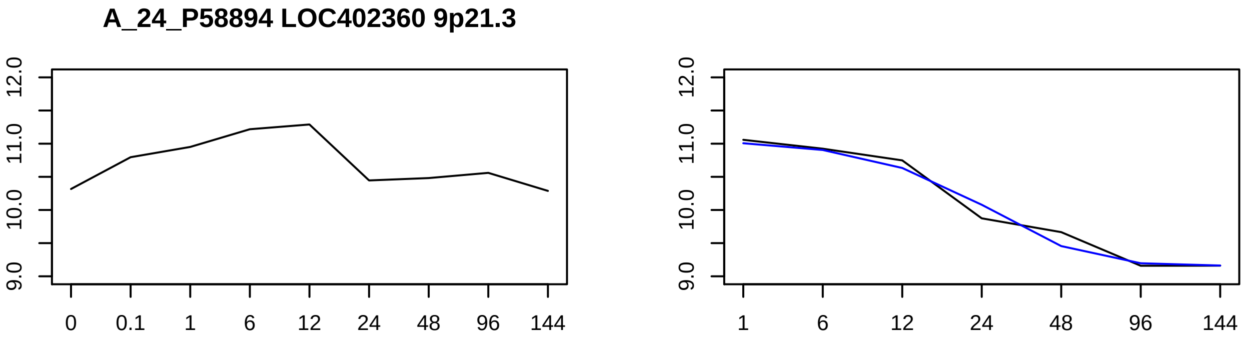
A_24_P58894 LOC402360 9p21.3
9.0
10.0
11.0
12.0
0
0.1
1
6
12
24
48
96
144
1
6
12
24
48
96
144

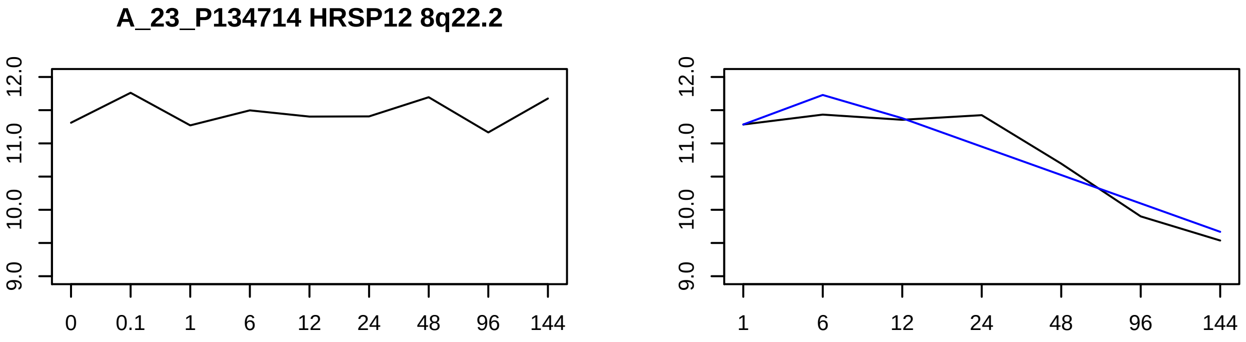
A_23_P134714 HRSP12 8q22.2
9.0
10.0
11.0
12.0
0
0.1
1
6
12
24
48
96
144
1
6
12
24
48
96
144

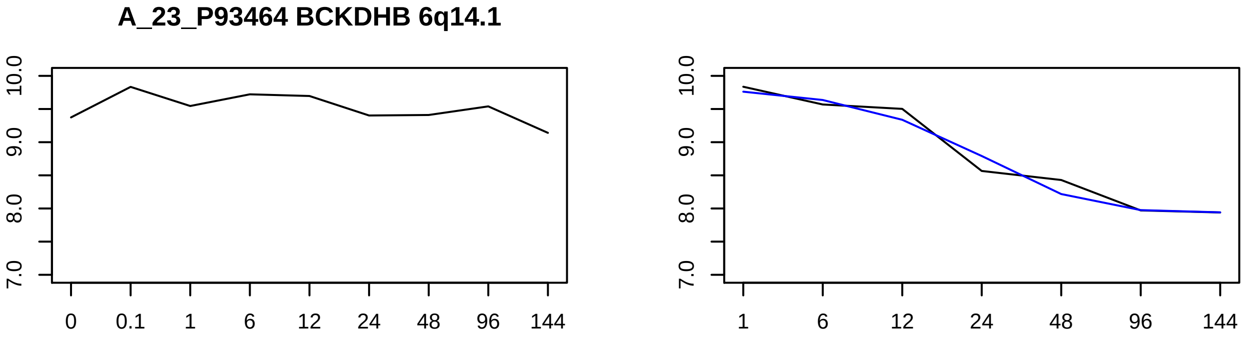
A_23_P93464 BCKDHB 6q14.1
7.0
8.0
9.0
10.0
0
0.1
1
6
12
24
48
96
144
1
6
12
24
48
96
144

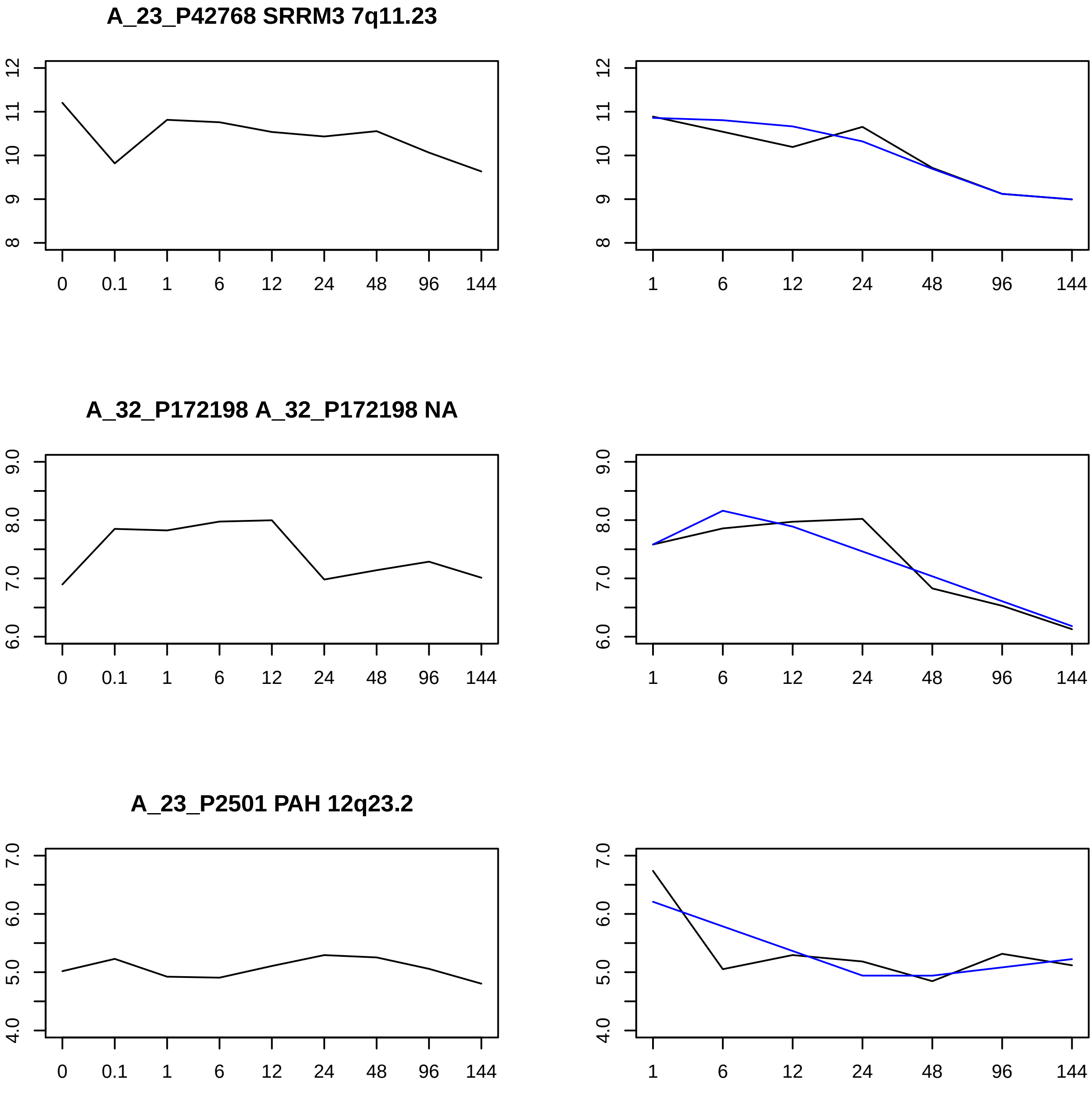
A_23_P42768 SRRM3 7q11.23
8
9
10
11
12
0
0.1
1
6
12
24
48
96
144
A_32_P172198 A_32_P172198 NA
6.0
7.0
8.0
9.0
A_23_P2501 PAH 12q23.2
4.0
5.0
6.0
7.0

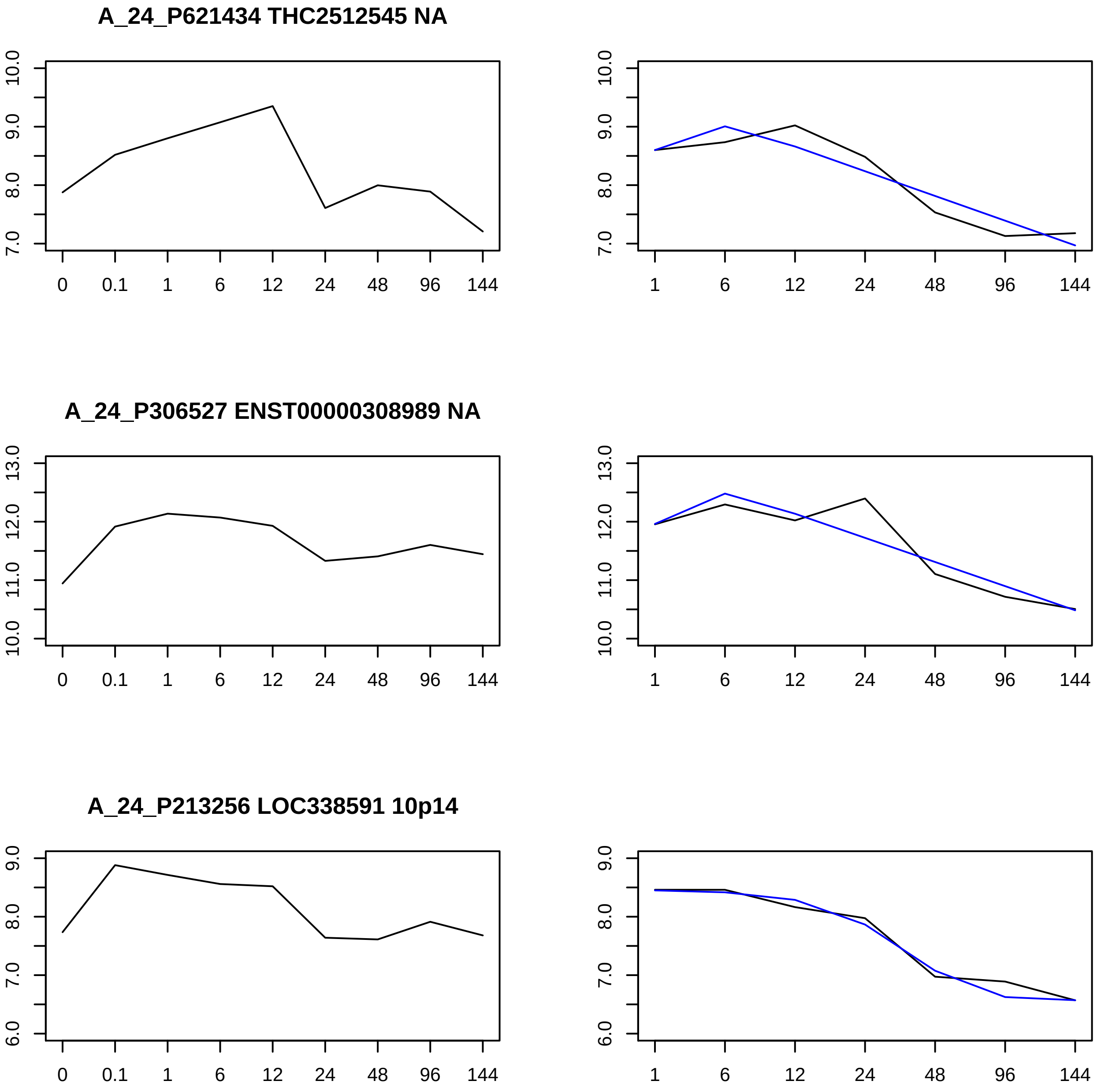
A_24_P621434 THC2512545 NA
A_24_P306527 ENST00000308989 NA
A_24_P213256 LOC338591 10p14
0
0.1
1
6
12
24
48
96
144

A_32_P224566 LIN28B 6q21

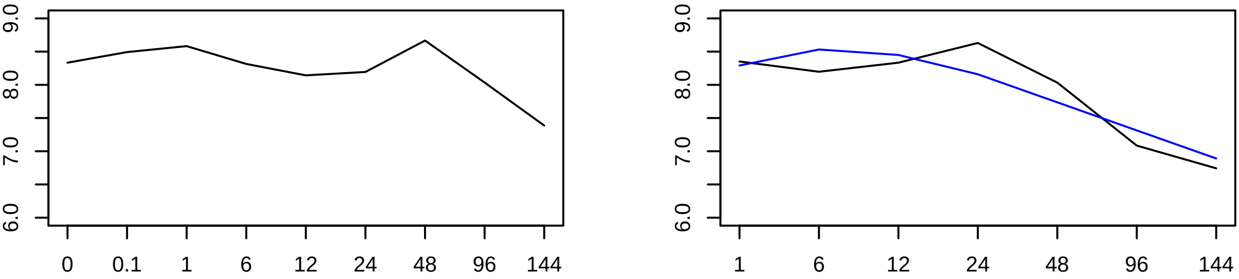

A_24_P332504 RPS17P13 7p14.2

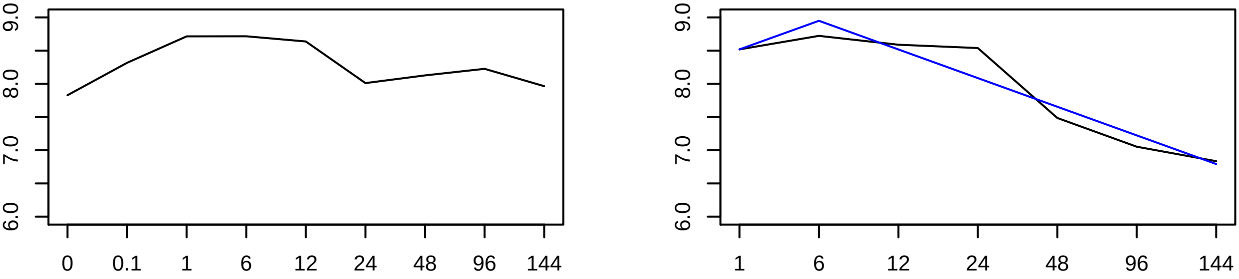

A_24_P910952 LHX9 1q31.3

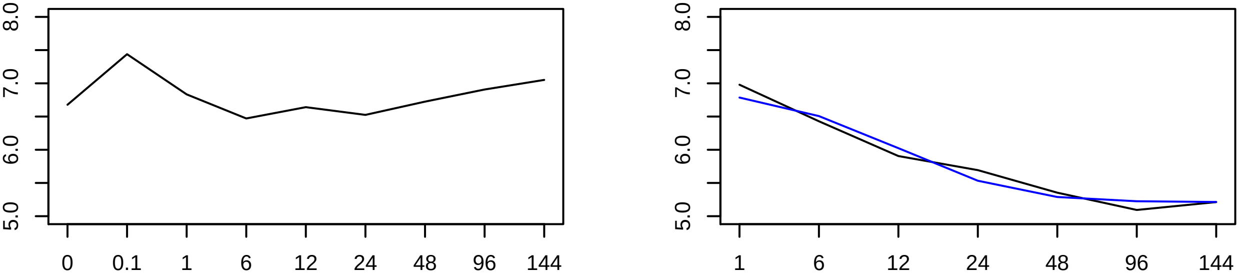

A_23_P71480 DEFB1 8p23.1

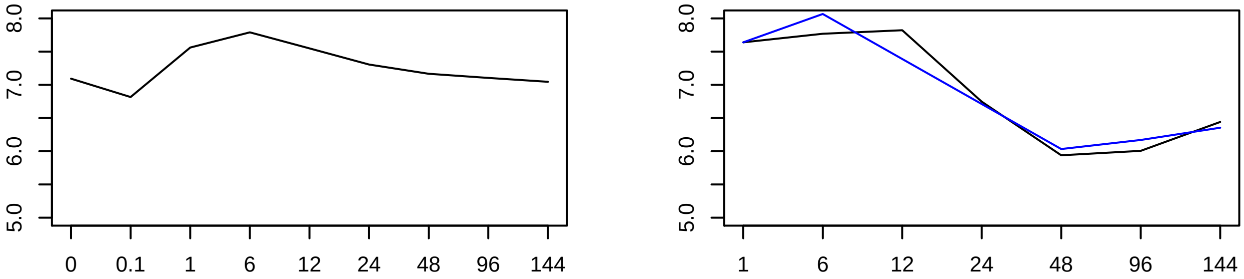

A_23_P94800 S100A4 1q21.3

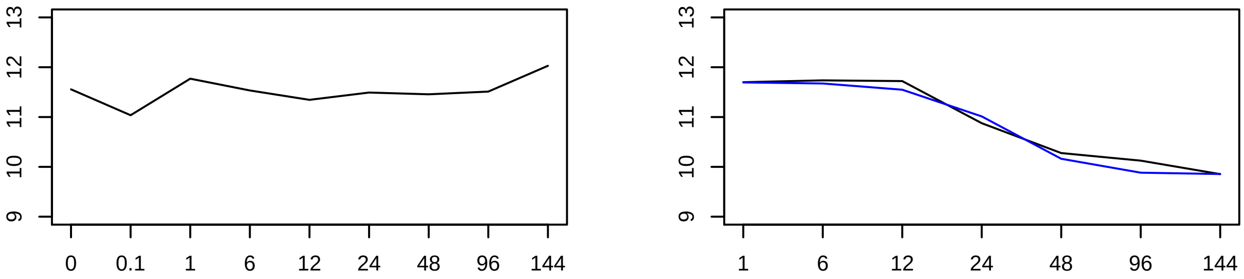

A_32_P137604 ANKRD33B NA

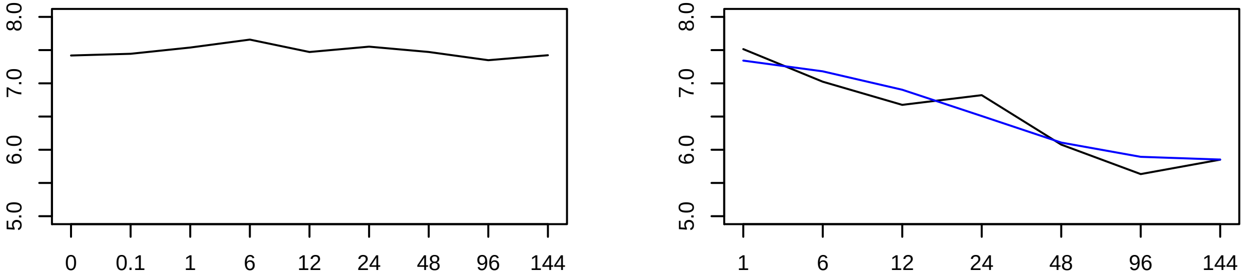

A_24_P226008 MGLL 3q21.3

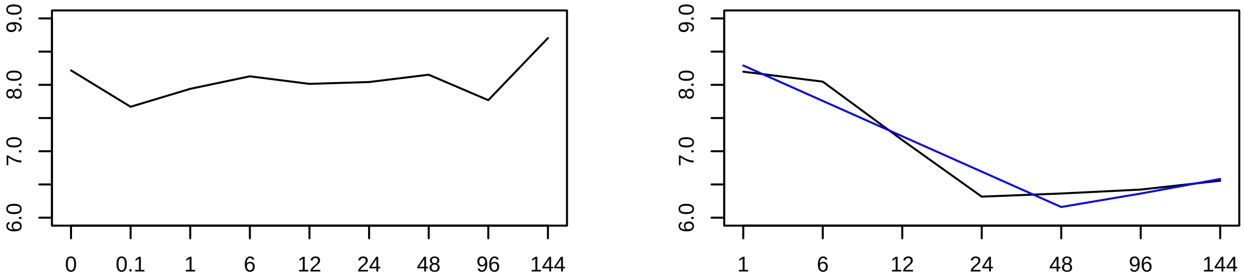

A_24_P195510 LOC400064 12q23.1

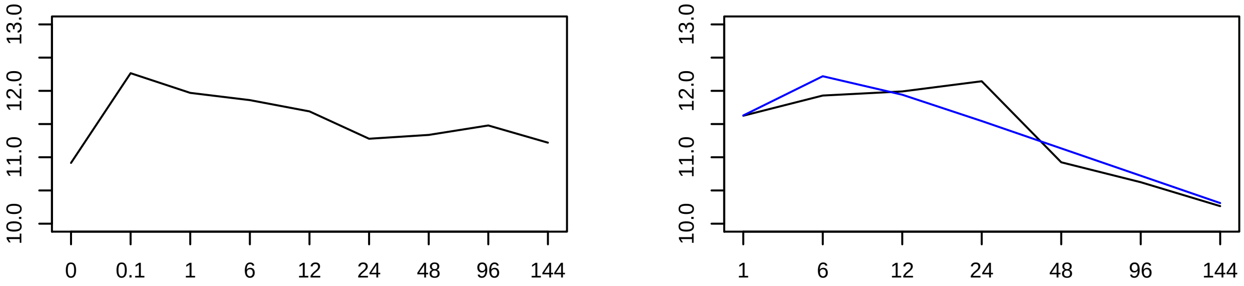

A_24_P178723 A_24_P178723 NA

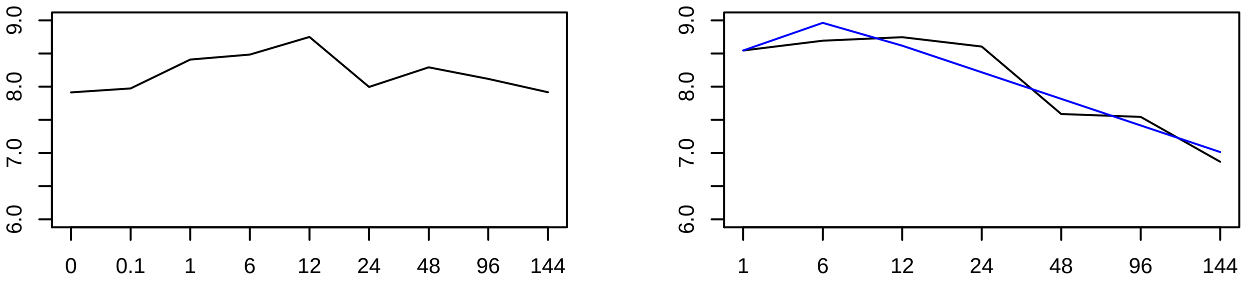

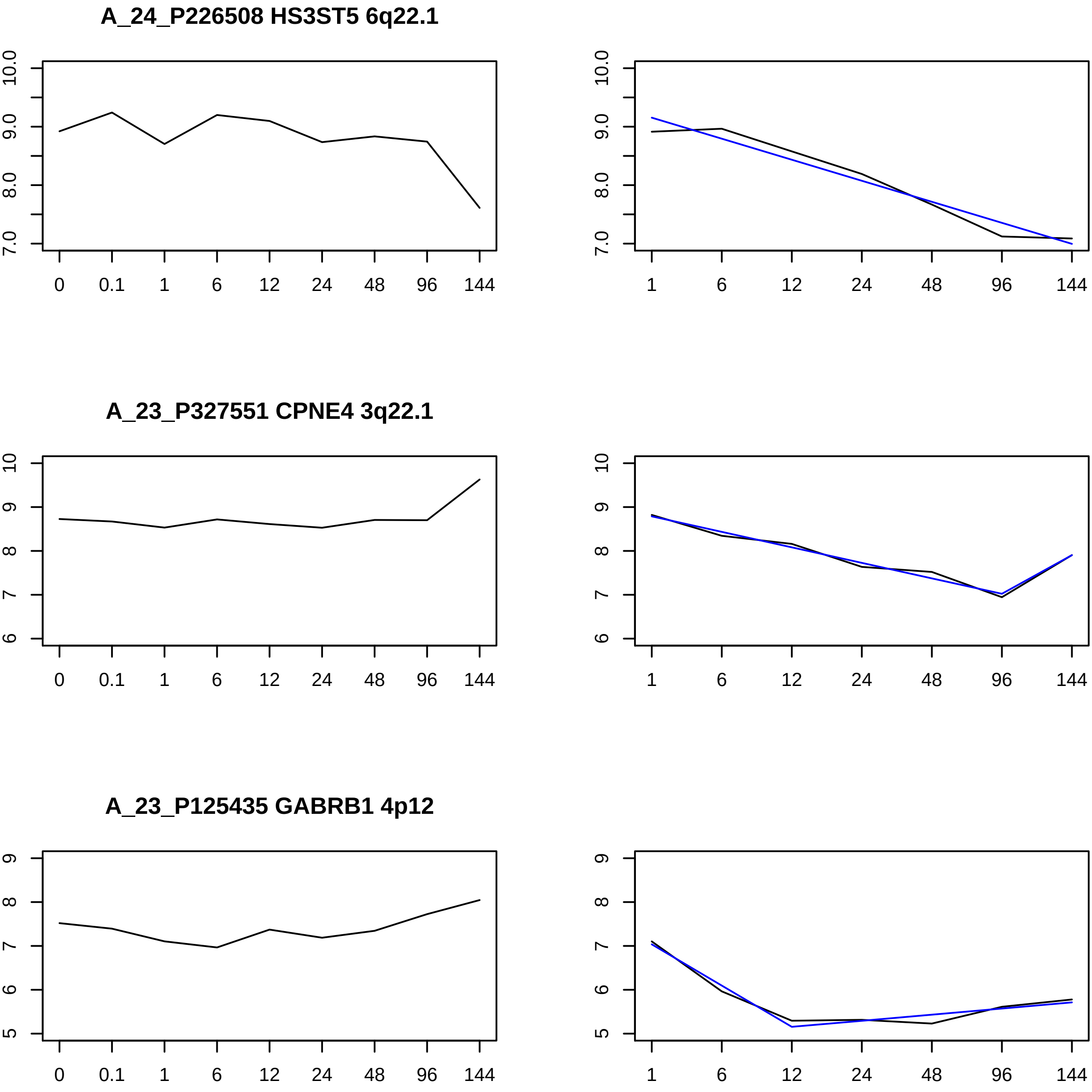

A_24_P226508 HS3ST5 6q22.1
7.0
8.0
9.0
10.0
0
0.1
1
6
12
24
48
96
144
A_23_P327551 CPNE4 3q22.1
6
7
8
9
10
A_23_P125435 GABRB1 4p12
5
6
7
8
9

A_24_P367369 A_24_P367369 NA

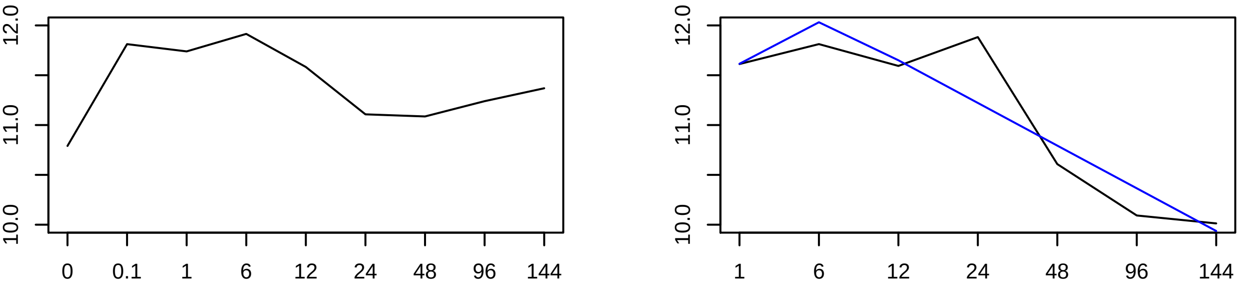

A_24_P350008 A_24_P350008 NA

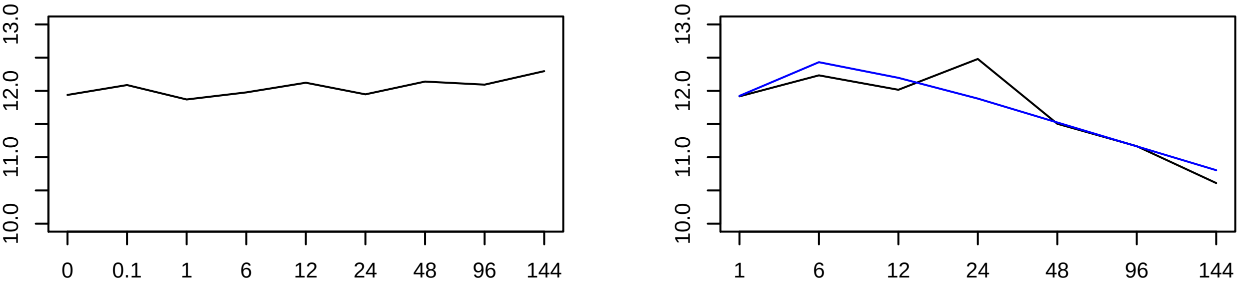

A_23_P375165 TEX19 17q25.3

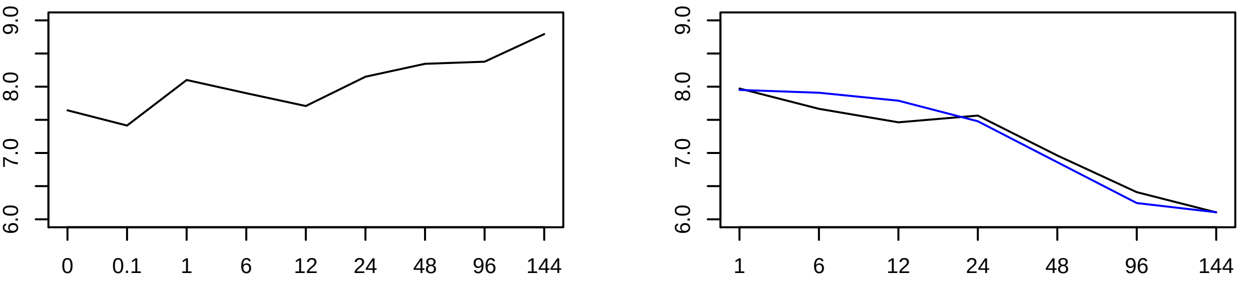

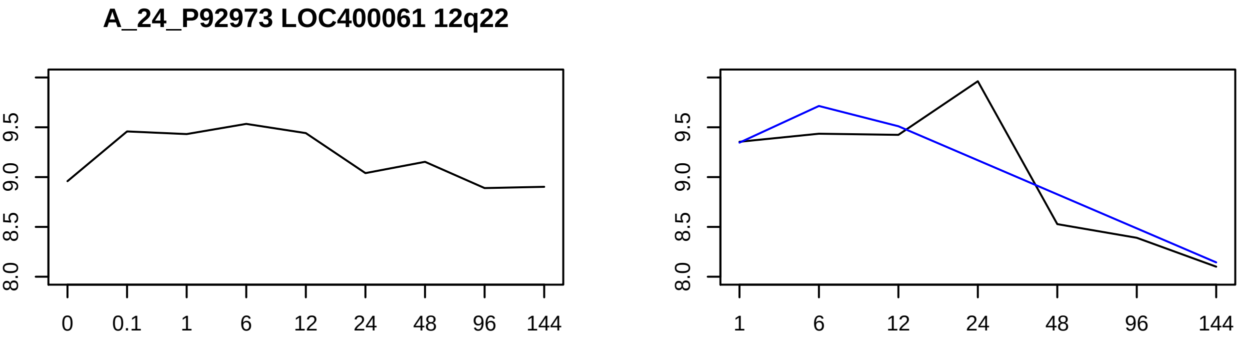
A_24_P92973 LOC400061 12q22
8.0
8.5
9.0
9.5
0
0.1
1
6
12
24
48
96
144

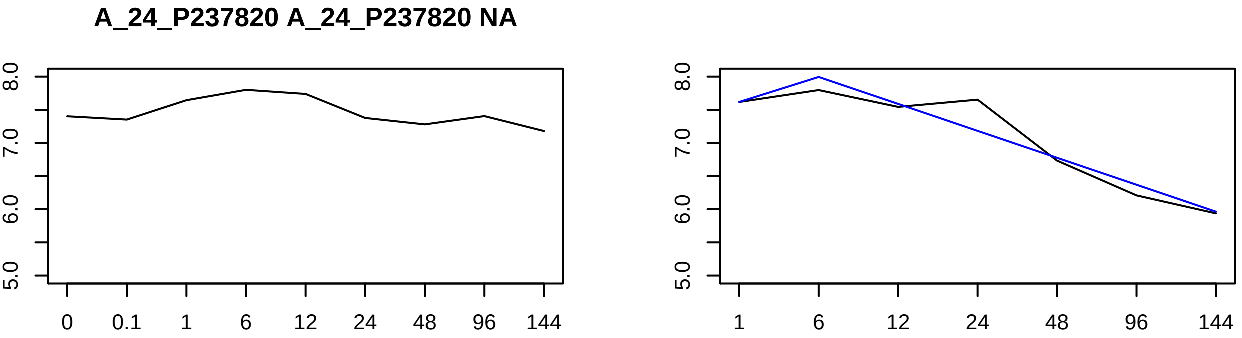
A_24_P237820 A_24_P237820 NA
5.0
6.0
7.0
8.0
0
0.1
1
6
12
24
48
96
144

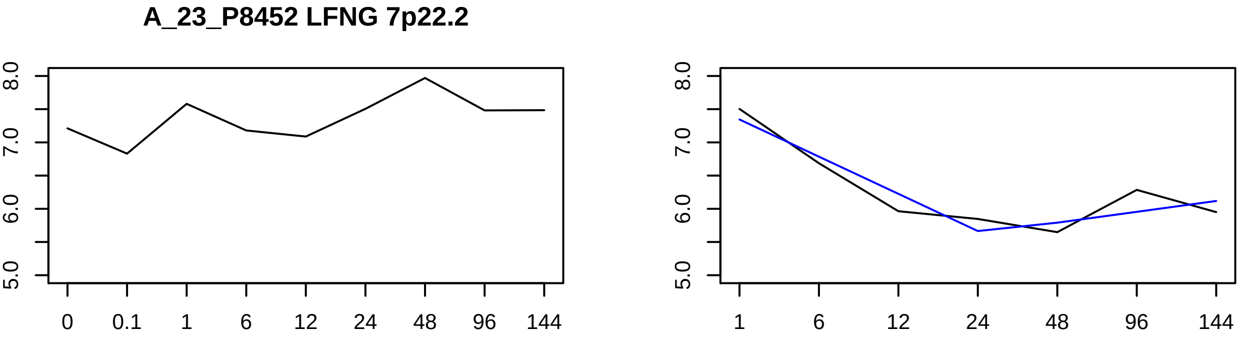
A_23_P8452 LFNG 7p22.2
5.0
6.0
7.0
8.0
0
0.1
1
6
12
24
48
96
144

A_24_P686965 SH2D5 1p36.12

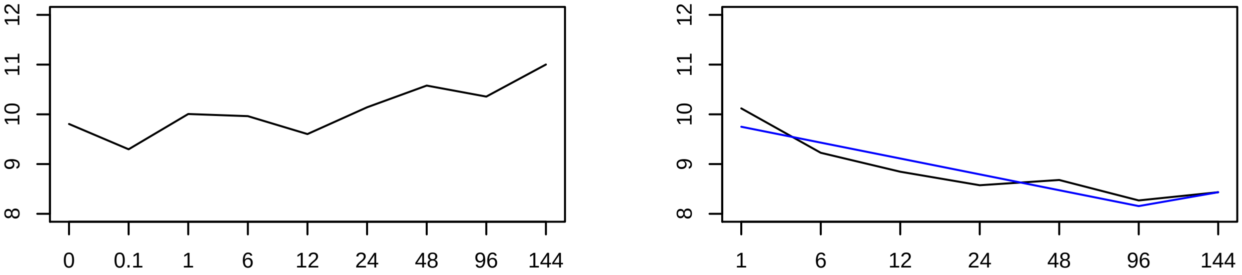

A_24_P50437 BC065737 NA

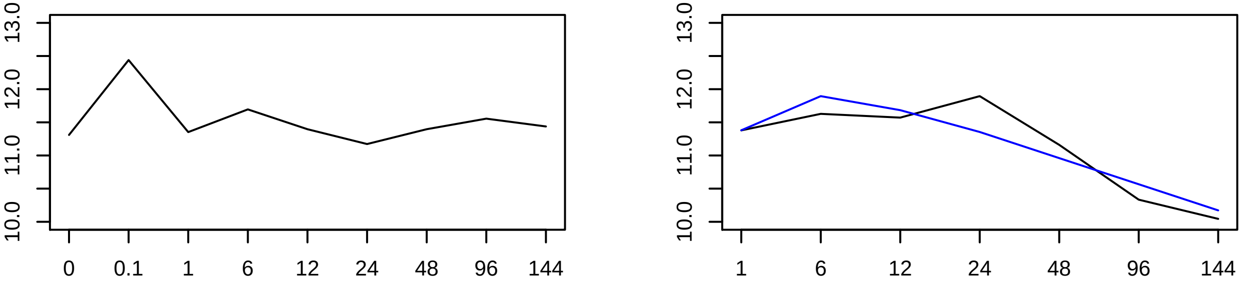

A_24_P409402 LOC643220 5q12.1

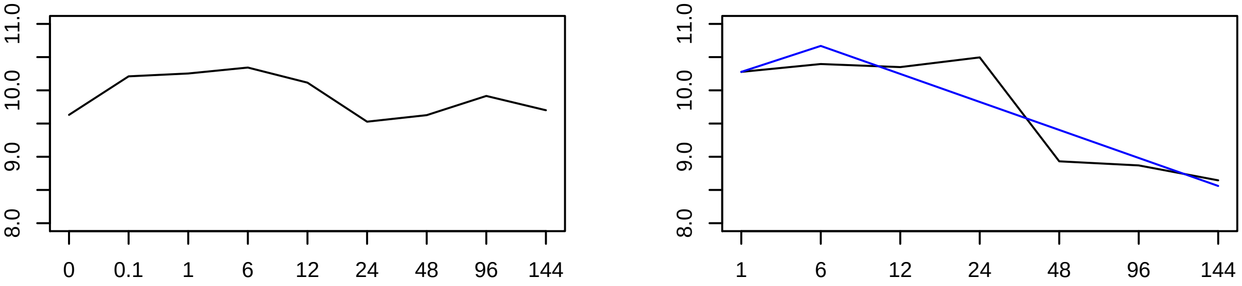

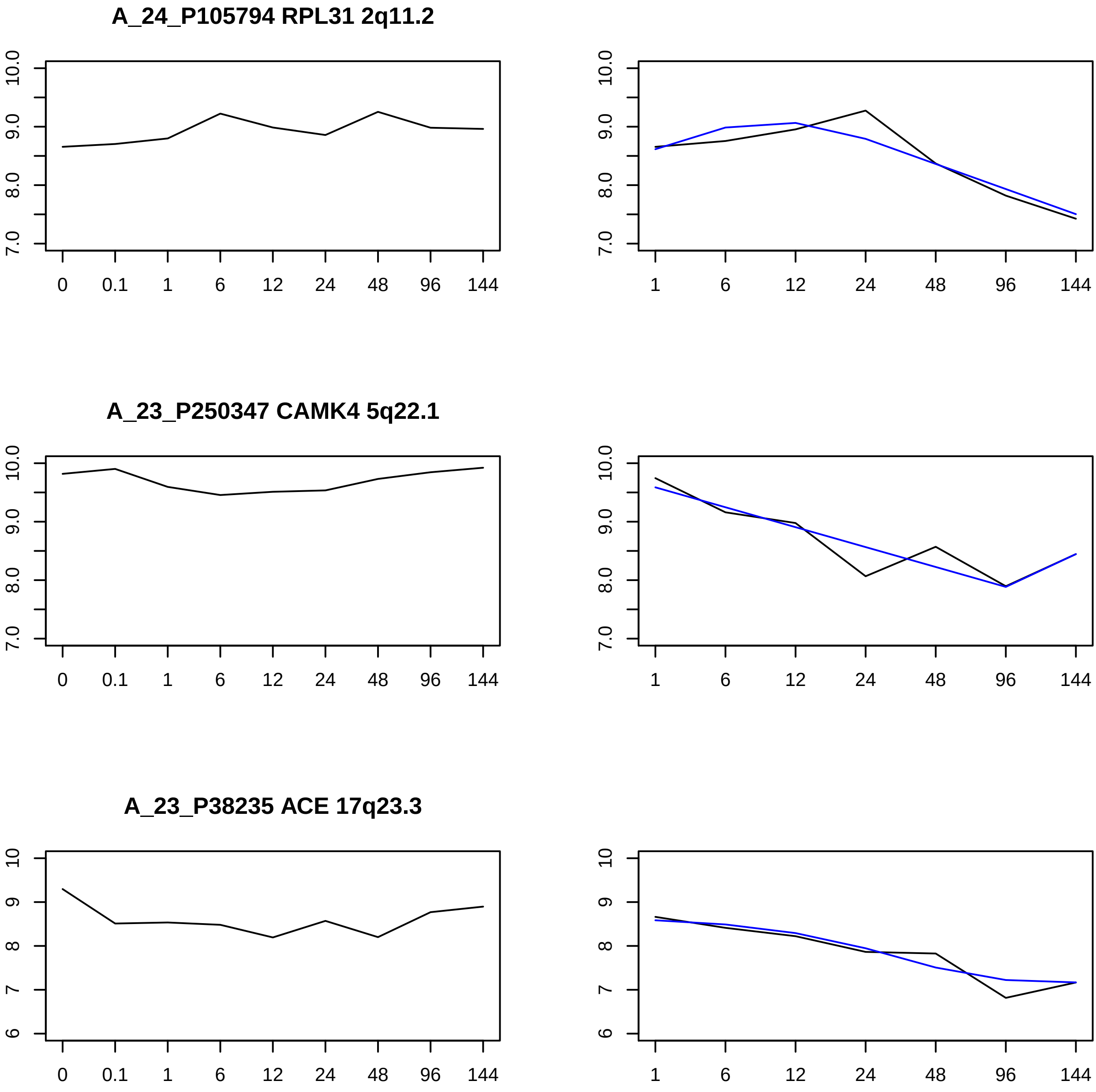
A_24_P105794 RPL31 2q11.2
A_23_P250347 CAMK4 5q22.1
A_23_P38235 ACE 17q23.3
7.0 8.0 9.0 10.0
6 7 8 9 10
0 0.1 1 6 12 24 48 96 144
1 6 12 24 48 96 144

A_32_P42213 A_32_P42213 NA

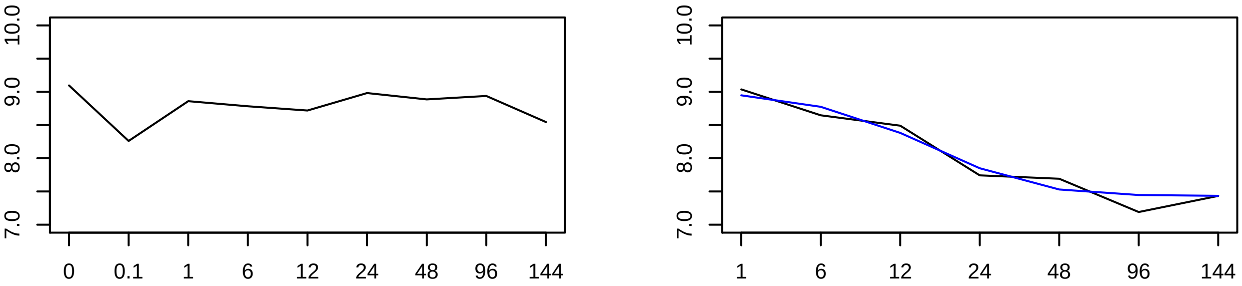

A_32_P29814 THC2785331 NA

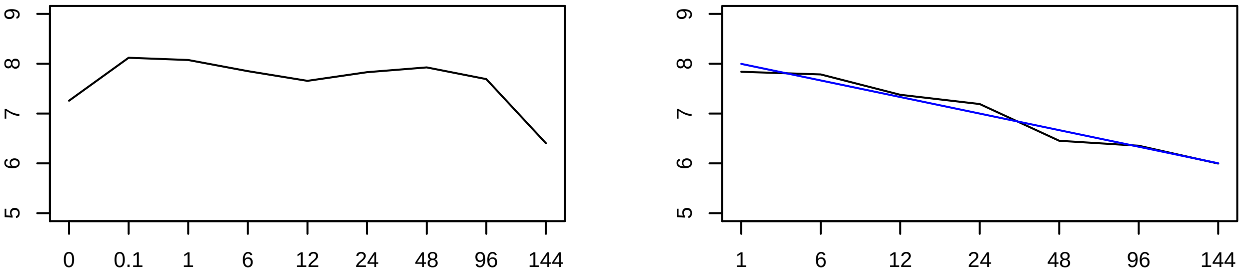

A_32_P10936 CDH12 5p14.3

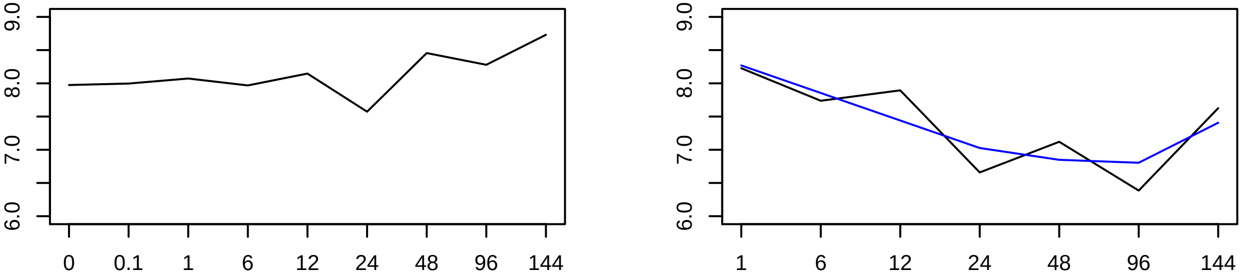

A_24_P140475 SORBS2 4q35.1

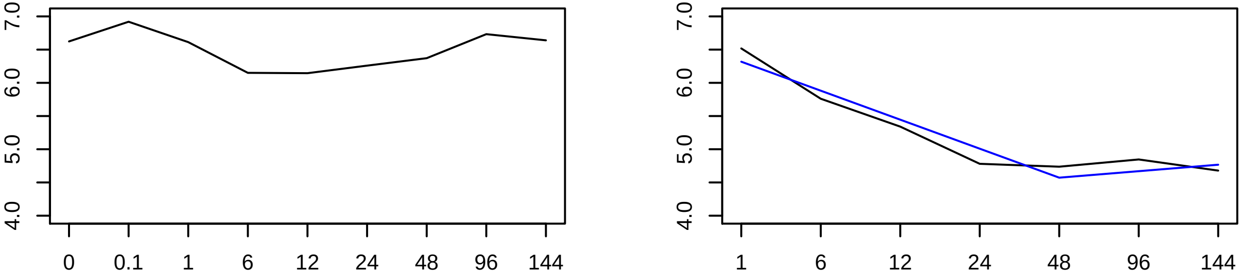

A_24_P41309 CR596550 NA

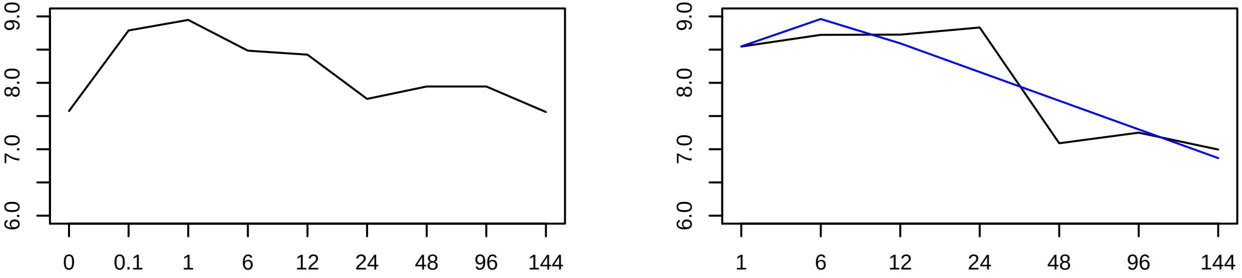

A_24_P109191 A_24_P109191 NA

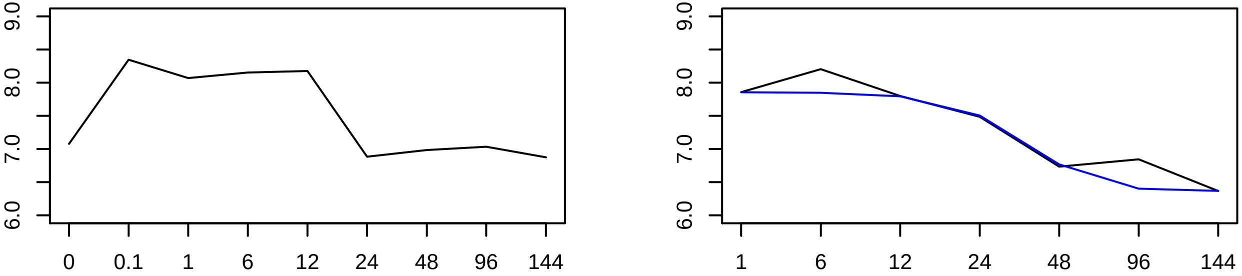

A_24_P24786 RP11−399E6.2 1p34.2

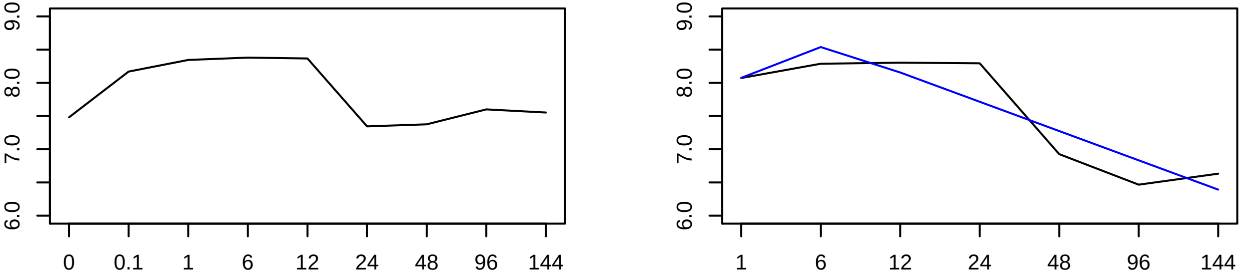

A_24_P323084 C17orf55 17q25.3

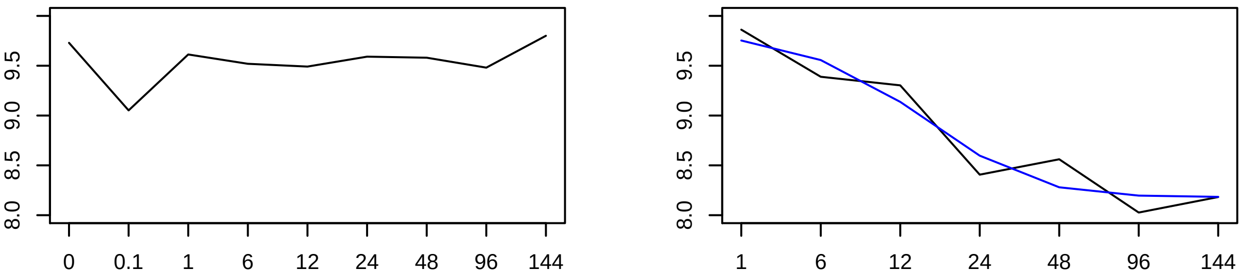

A_24_P132008 MSX2 5q35.2

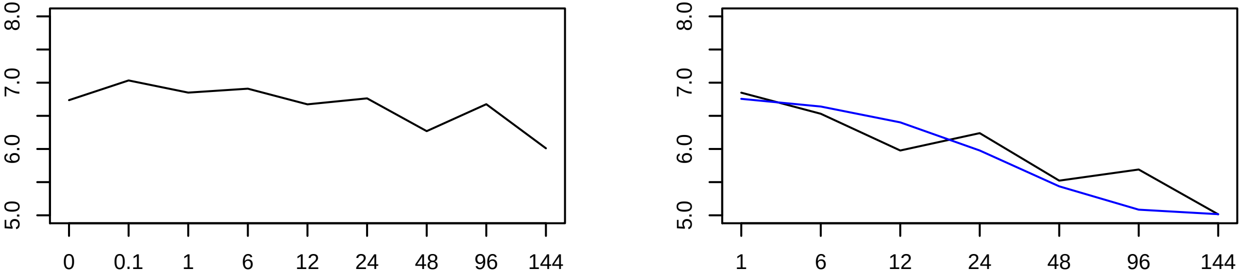

A_24_P238427 RPL13P6 14q32.32

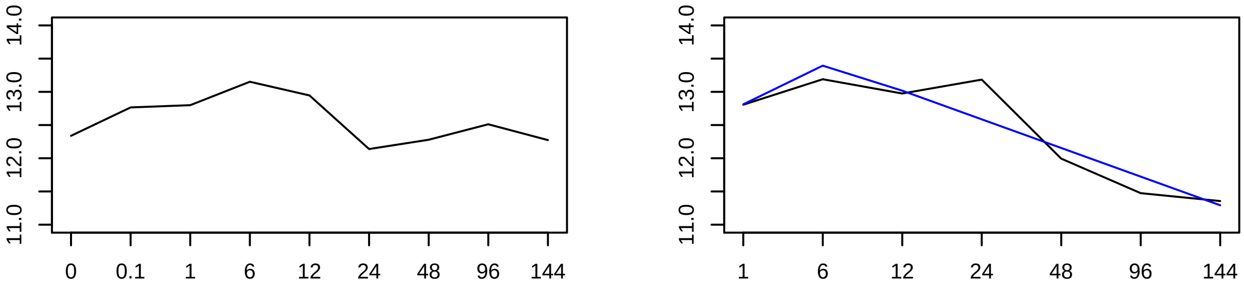

A_24_P15754 TOMM40 19q13.32

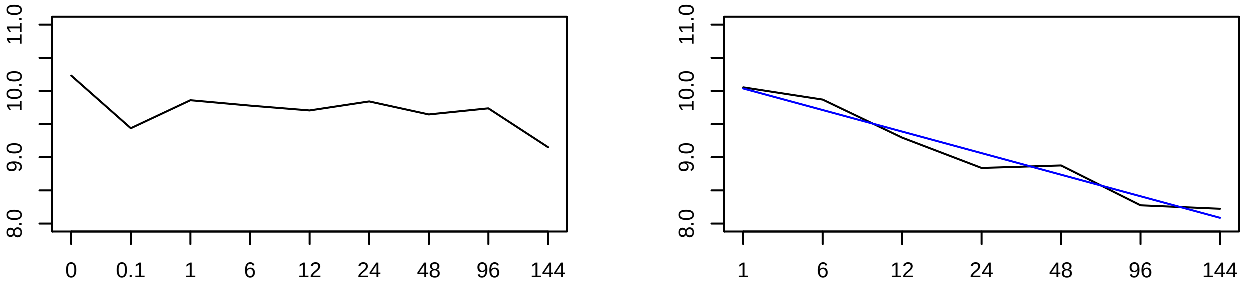

A_32_P215621 AK094991 NA

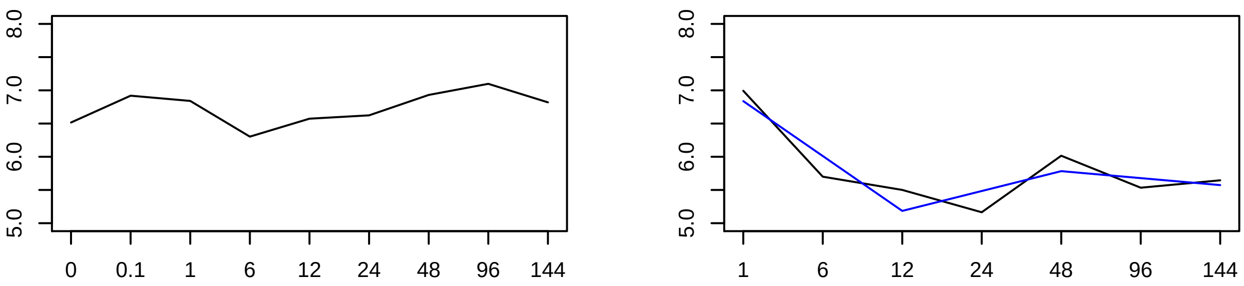

A_23_P423695 MXD4 4p16.3

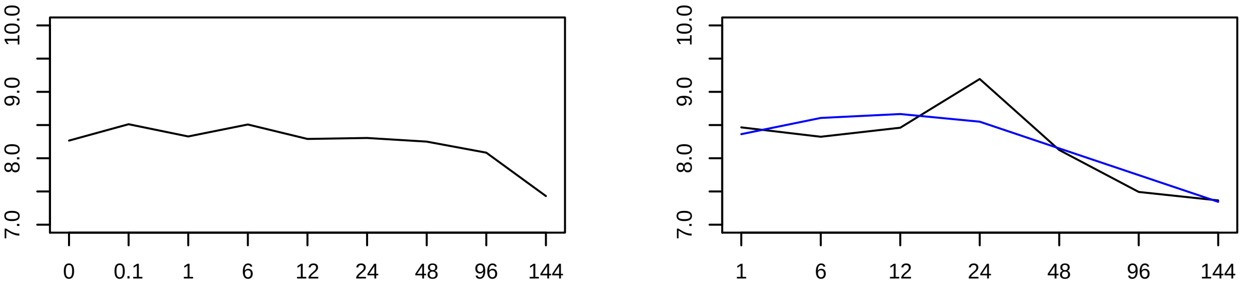

A_24_P41629 LOC402176 4q12

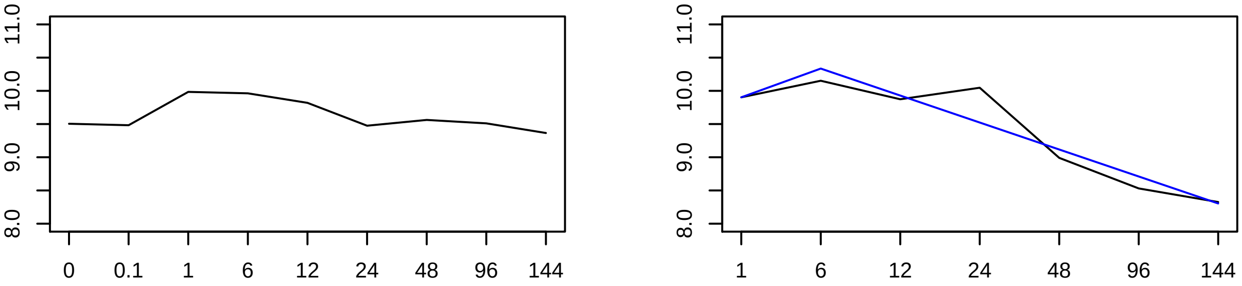

A_23_P147431 LYN 8q12.1

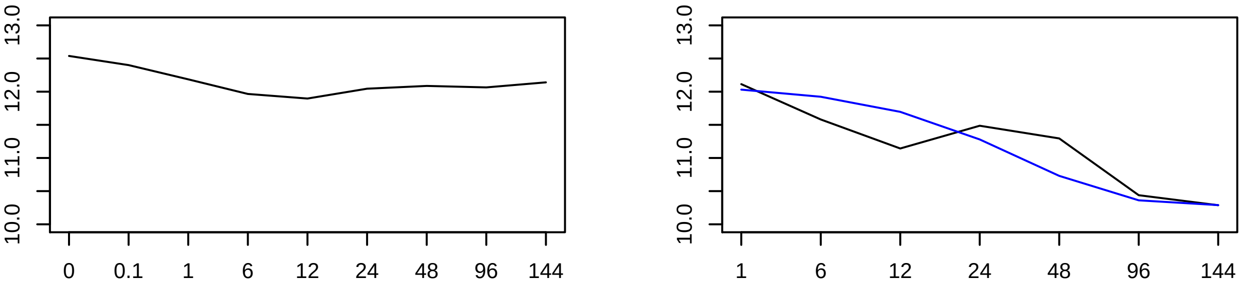

A_23_P255827 FKSG2 8p12

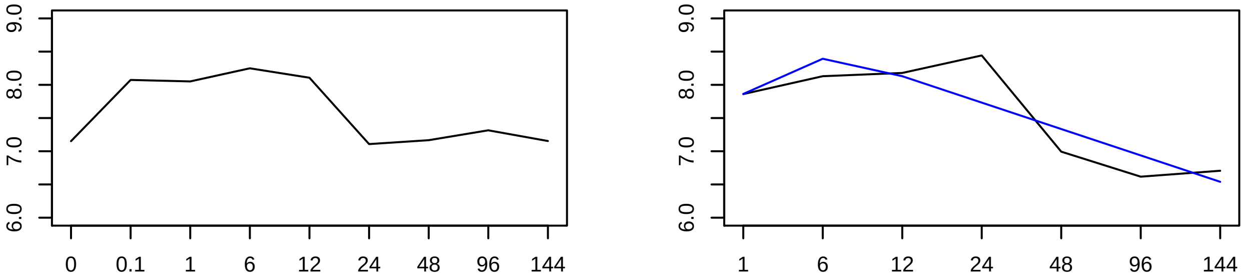

A_23_P338981 CYGB 17q25.1

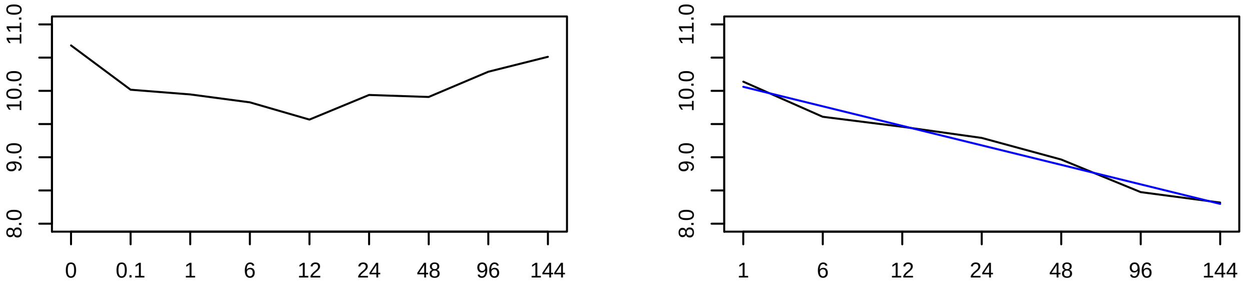

A_23_P258504 TRA@ 14q11.2

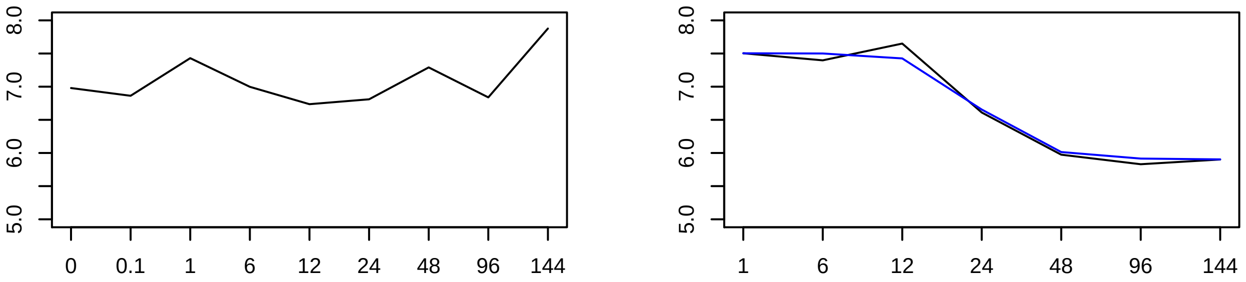

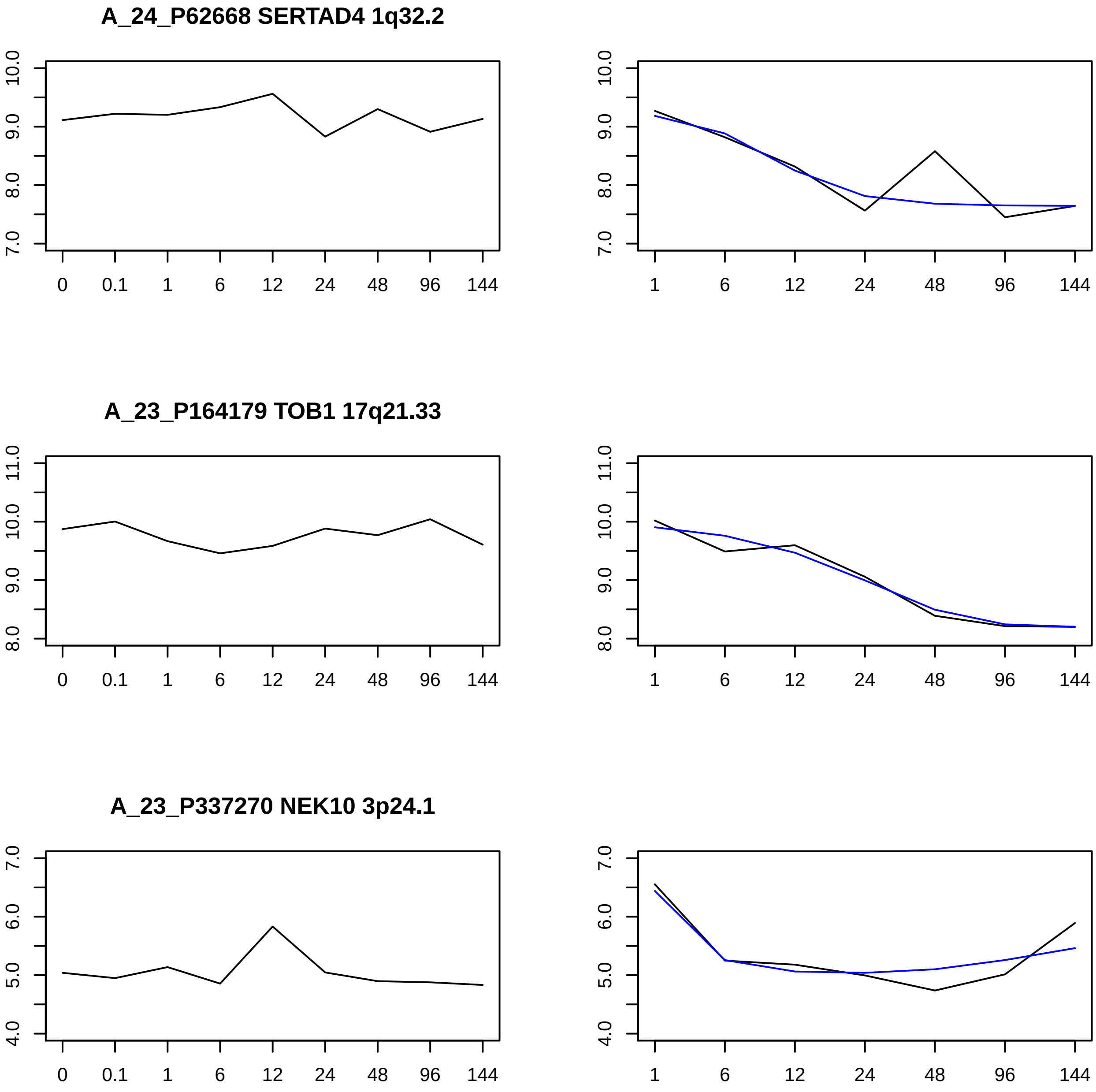
A_24_P62668 SERTAD4 1q32.2
A_23_P164179 TOB1 17q21.33
A_23_P337270 NEK10 3p24.1
7.0 8.0 9.0 10.0
8.0 9.0 10.0 11.0
4.0 5.0 6.0 7.0
0 0.1 1 6 12 24 48 96 144
1 6 12 24 48 96 144

A_24_P85881 GPR113 2p23.3

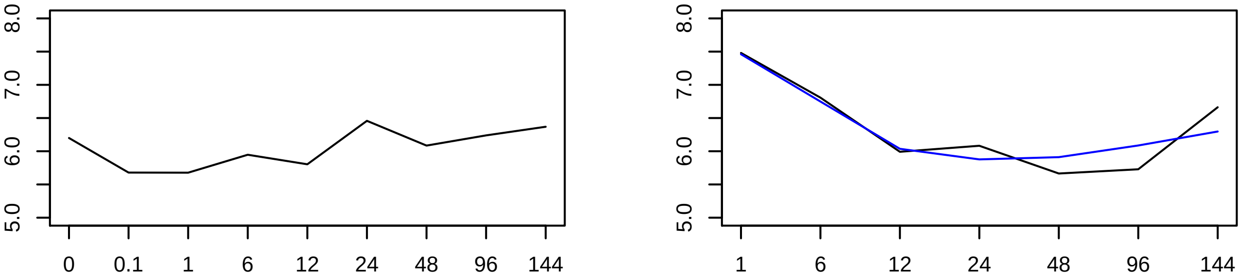

A_32_P514599 HSPD1P6 3p22.2

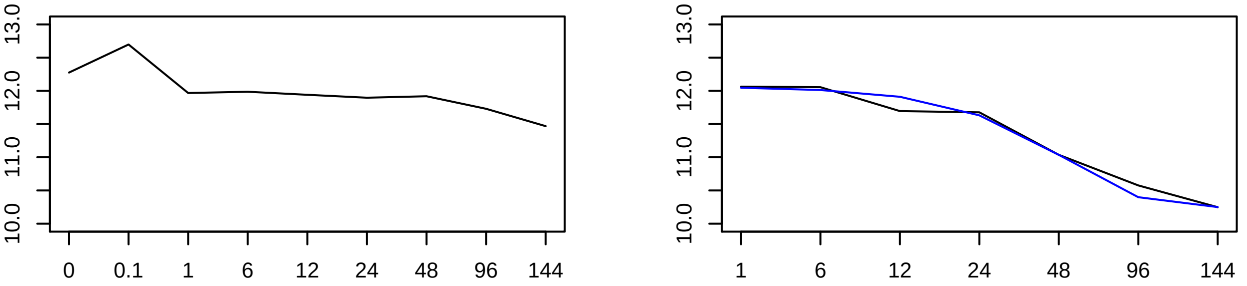

A_23_P82503 PEG10 7q21.3

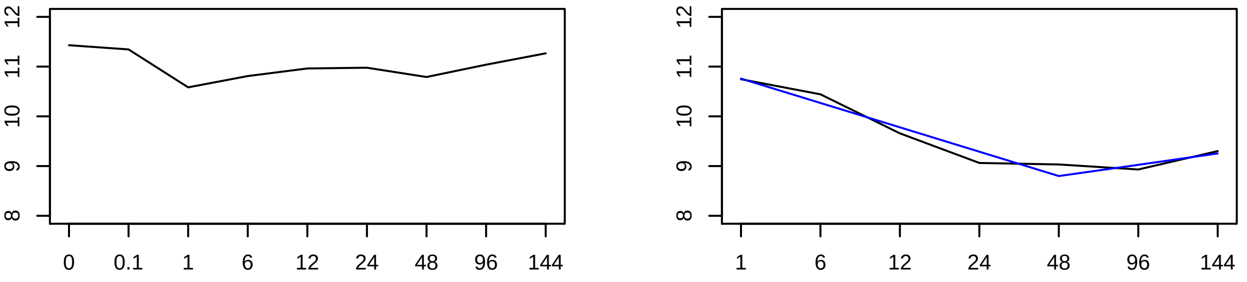

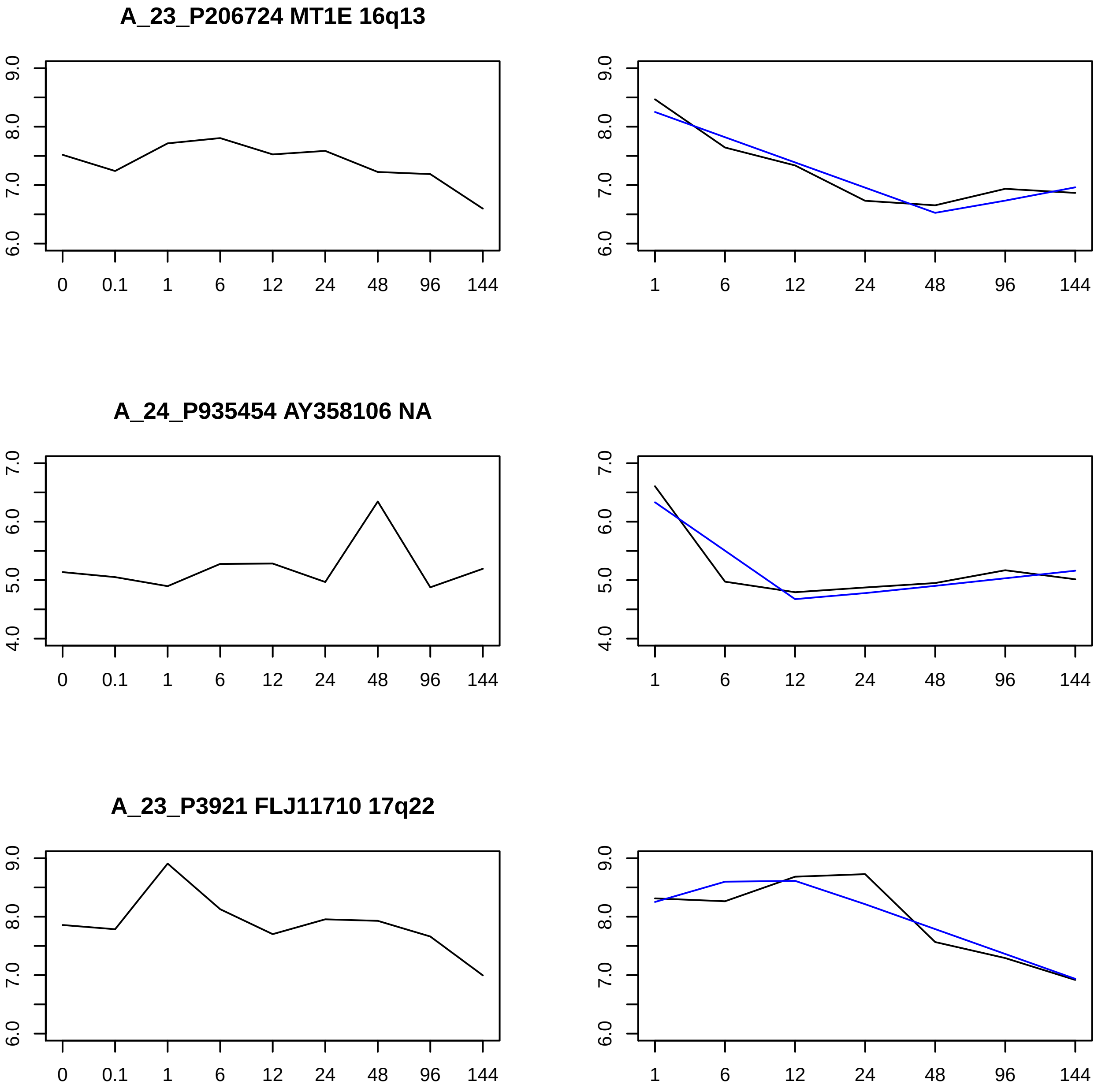
A_23_P206724 MT1E 16q13
A_24_P935454 AY358106 NA
A_23_P3921 FLJ11710 17q22
6.0
7.0
8.0
9.0
4.0
5.0
6.0
7.0
0
0.1
1
6
12
24
48
96
144

A_24_P15114 USP37 2q35

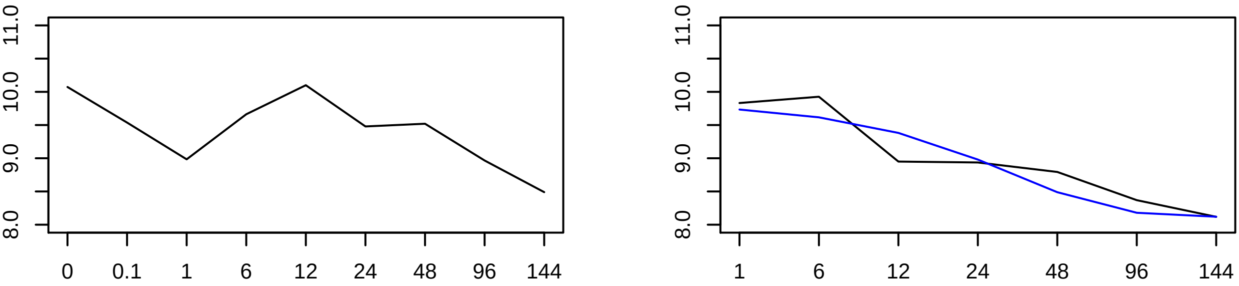

A_32_P139196 C13orf25 13q31.3

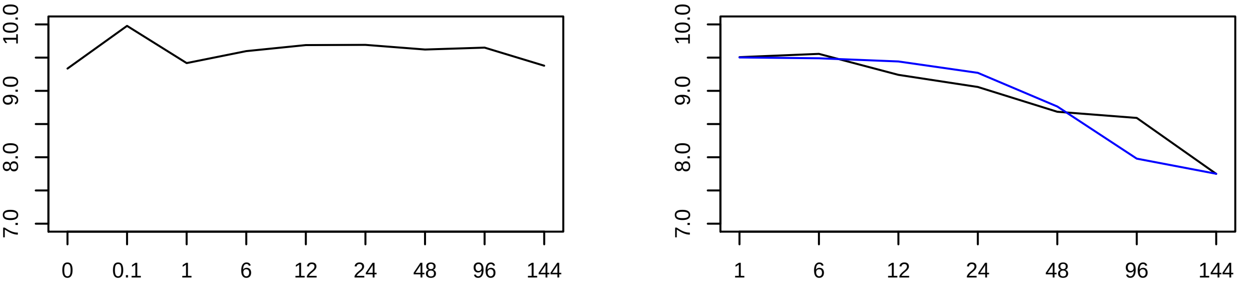

A_24_P178154 A_24_P178154 NA

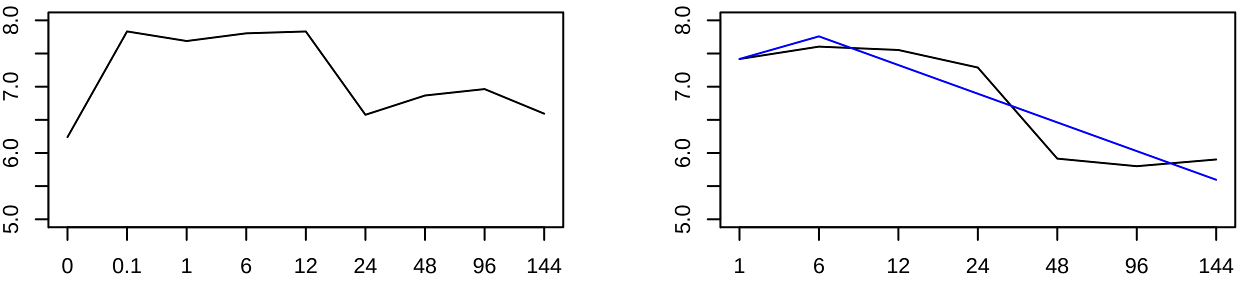

A_24_P524562 THC2563460 NA

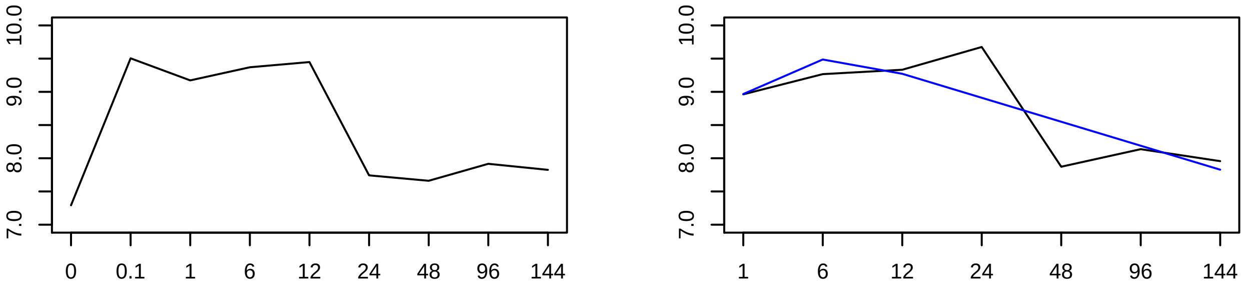

A_24_P323974 A_24_P323974 NA

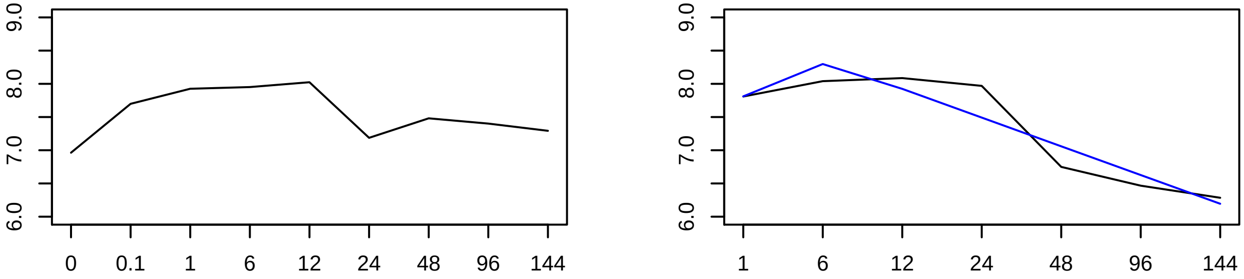

A_24_P230009 A_24_P230009 NA

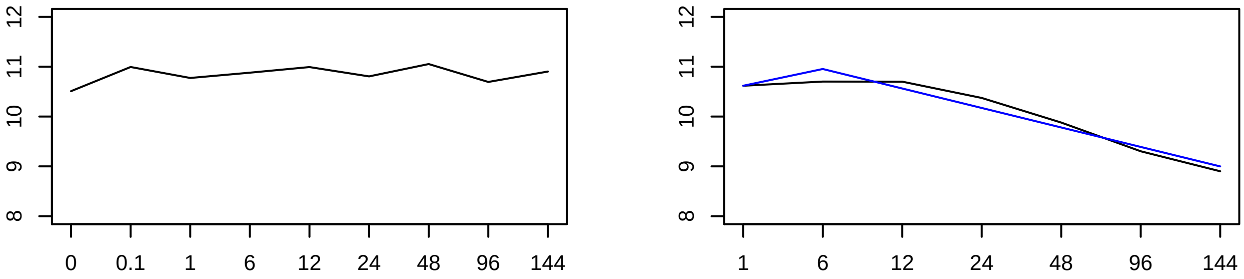

A_23_P213527 GPR150 NA

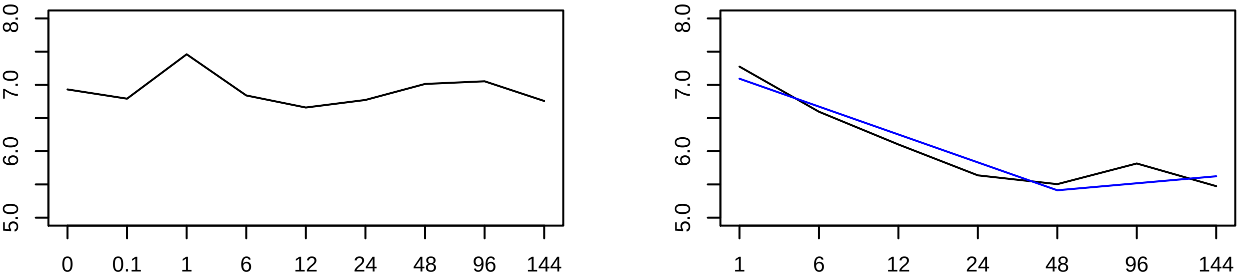

A_23_P20443 LZTS1 8p21.3

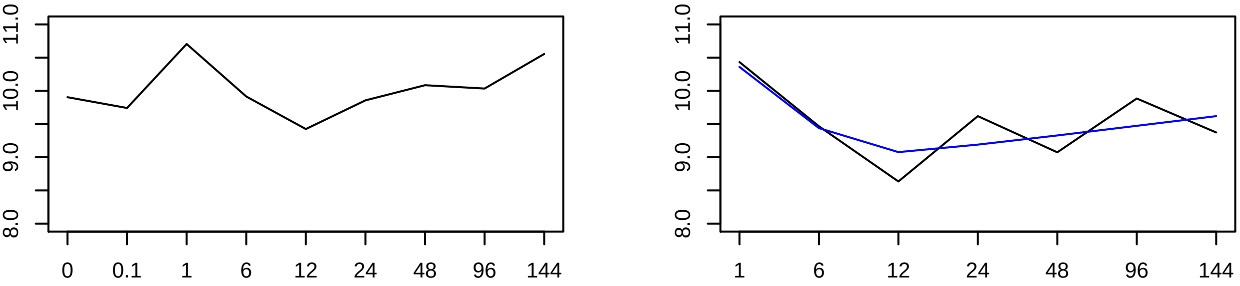

A_24_P357518 RPL21 13q12.2

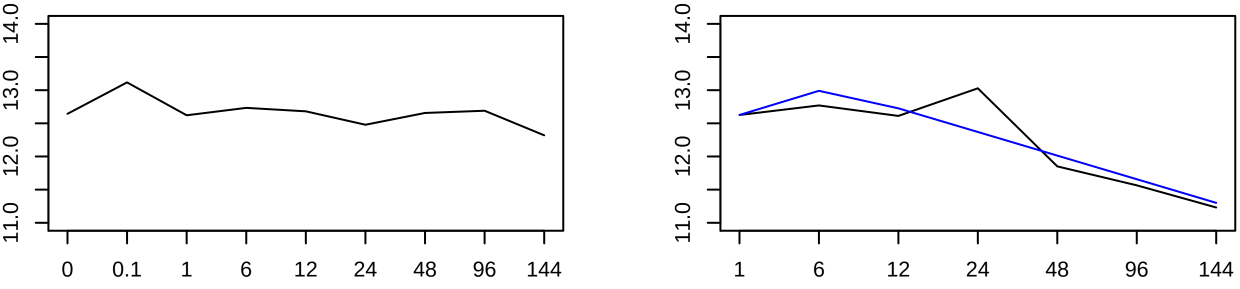

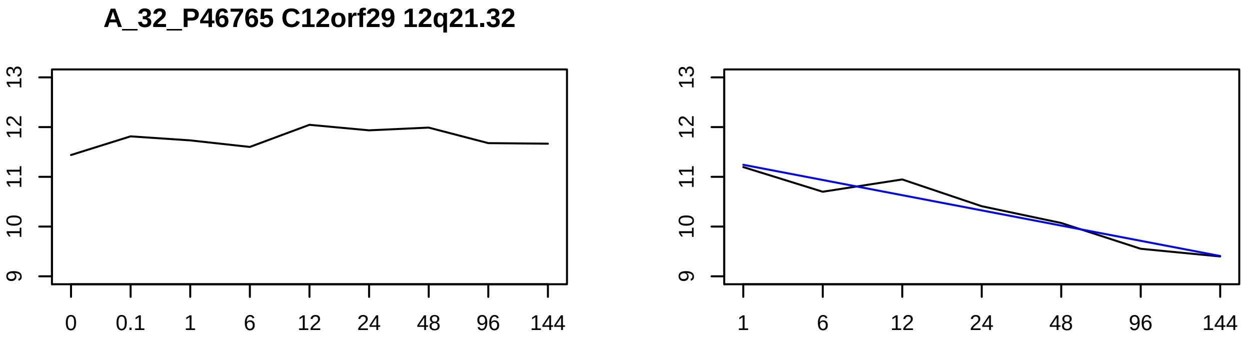
A_32_P46765 C12orf29 12q21.32
9
10
11
12
13
0
0.1
1
6
12
24
48
96
144
1
6
12
24
48
96
144

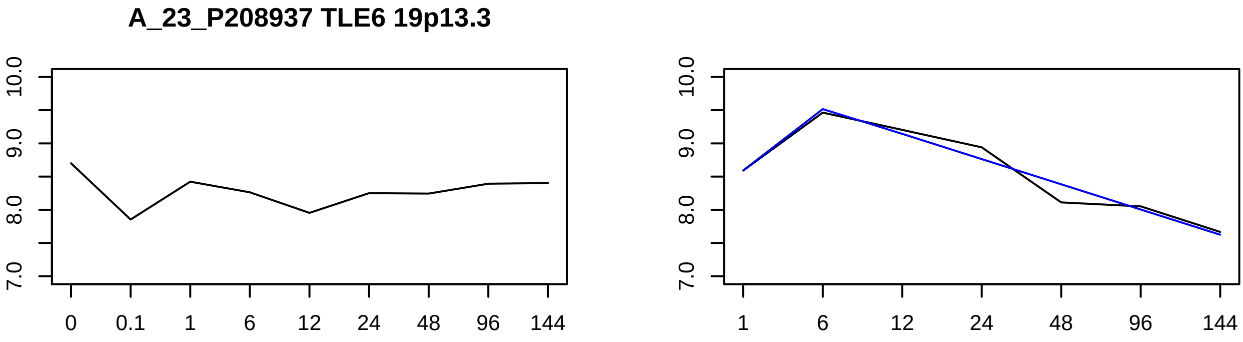
A_23_P208937 TLE6 19p13.3
7.0
8.0
9.0
10.0
0
0.1
1
6
12
24
48
96
144
1
6
12
24
48
96
144

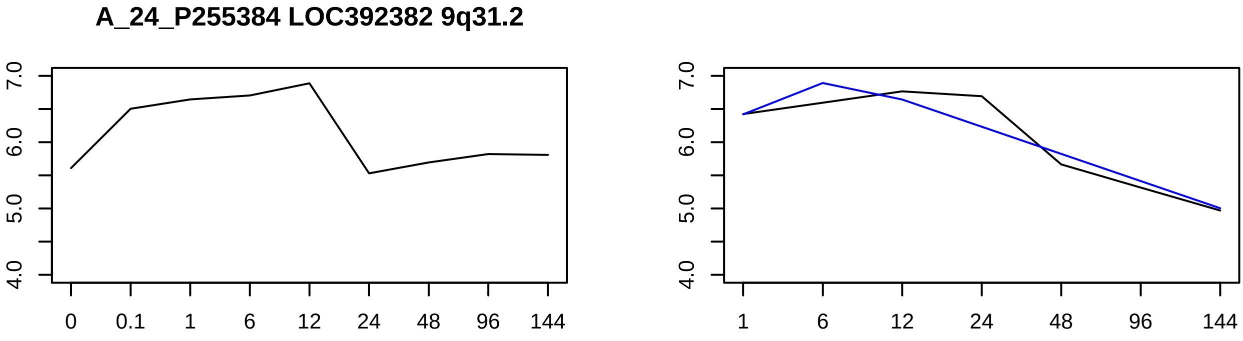
A_24_P255384 LOC392382 9q31.2
4.0
5.0
6.0
7.0
0
0.1
1
6
12
24
48
96
144
1
6
12
24
48
96
144

A_24_P737660 MEG3 14q32.2

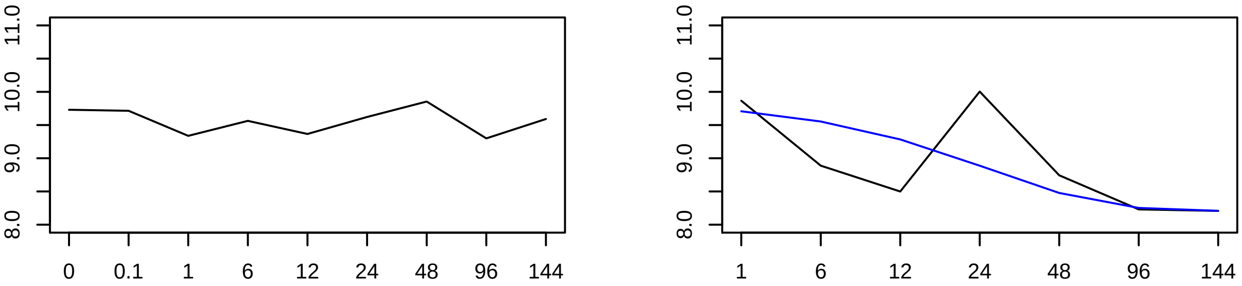

A_32_P136351 SLITRK1 13q31.1

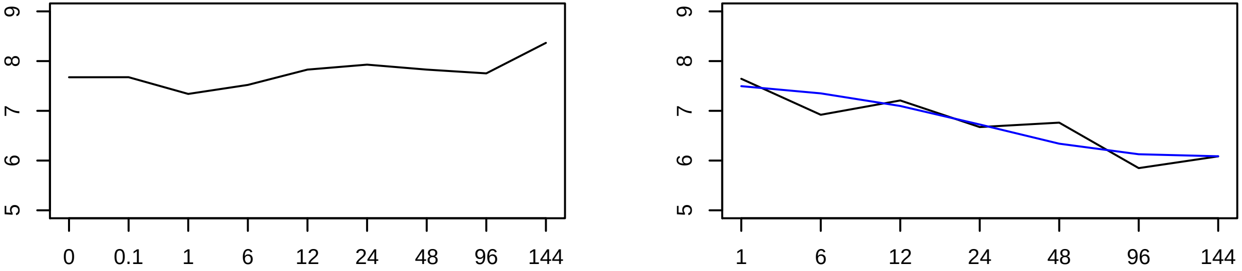

A_23_P117302 AF354444 NA

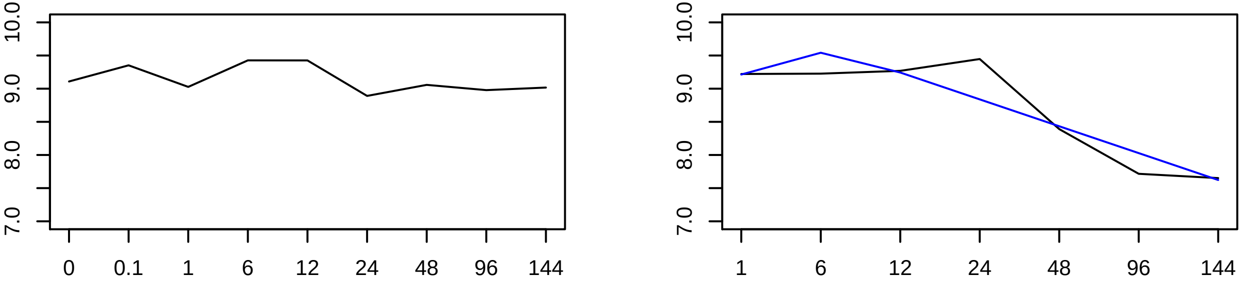

A_24_P204165 A_24_P204165 NA

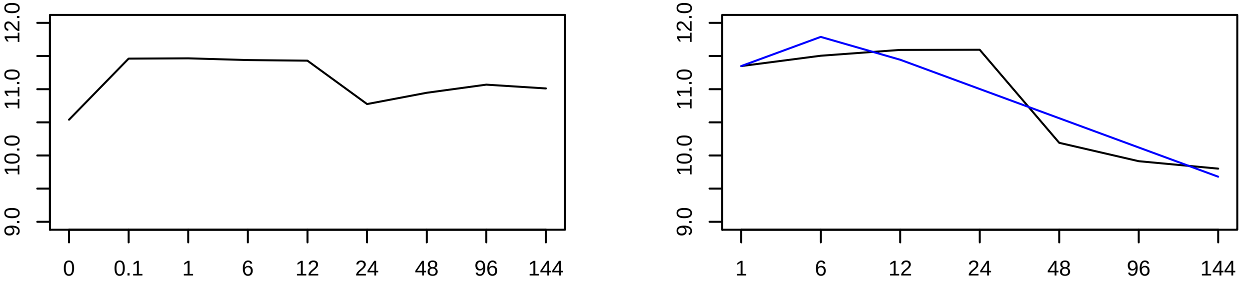

A_24_P910381 A_24_P910381 NA

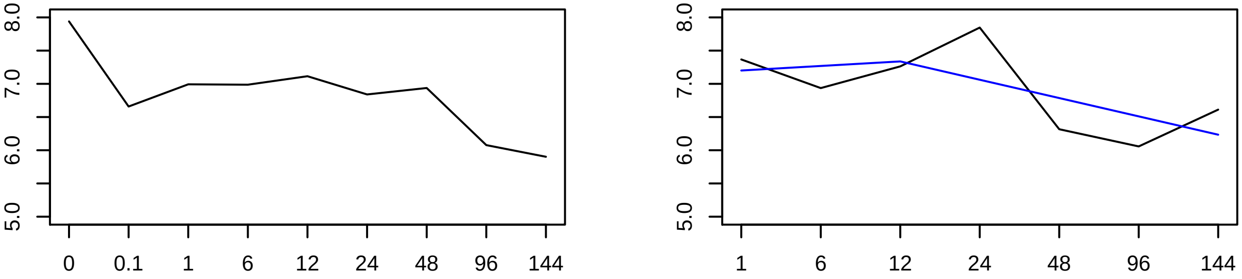

A_24_P545200 GAS5 1q25.1

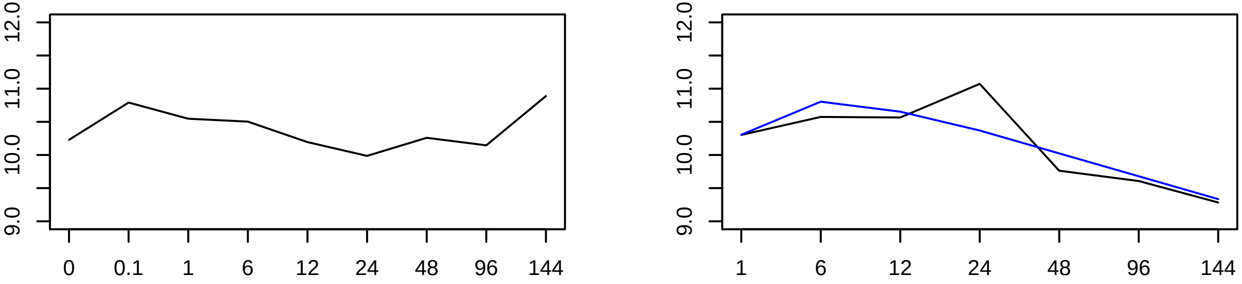

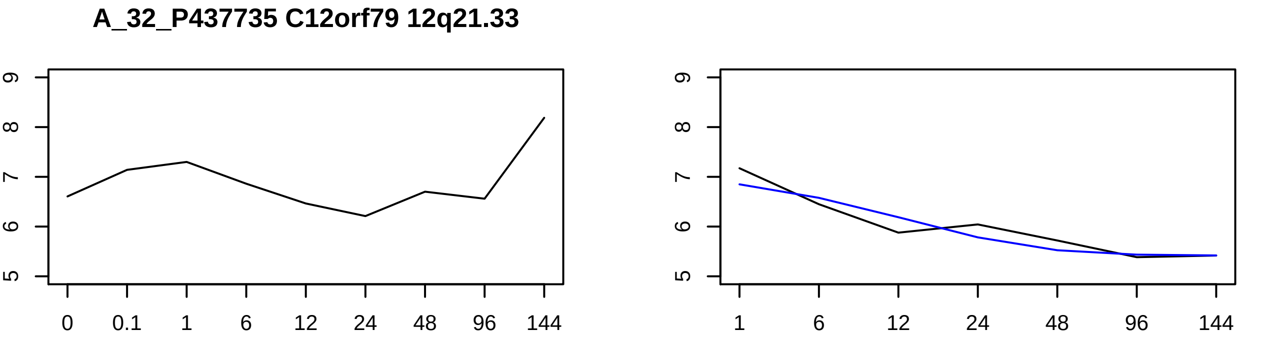
A_32_P437735 C12orf79 12q21.33
5
6
7
8
9
0
0.1
1
6
12
24
48
96
144

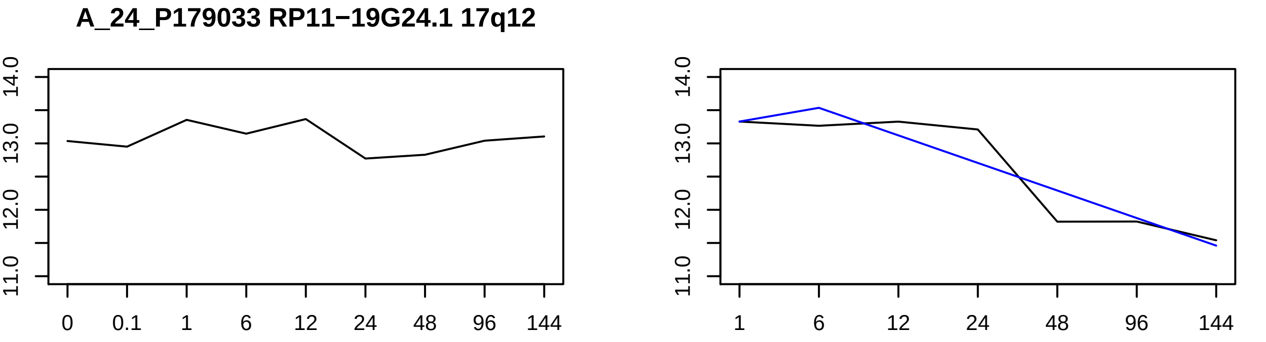
A_24_P179033 RP11−19G24.1 17q12
11.0
12.0
13.0
14.0
0
0.1
1
6
12
24
48
96
144

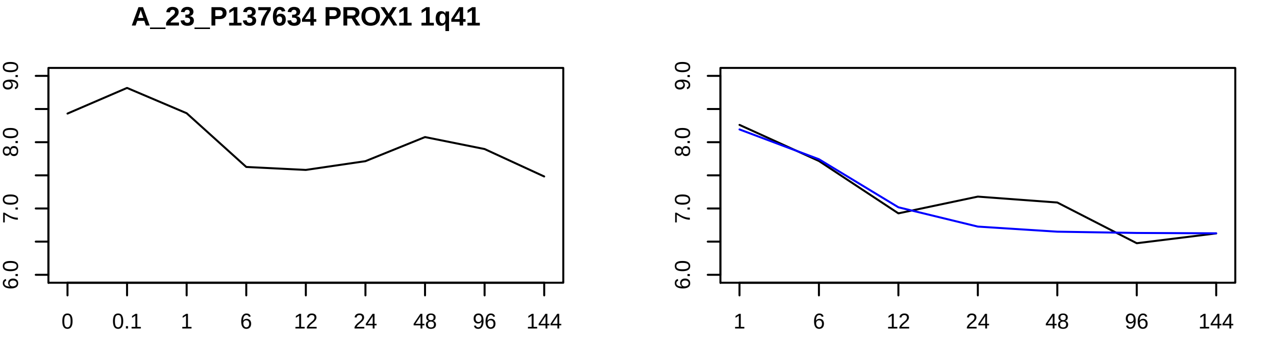
A_23_P137634 PROX1 1q41
6.0
7.0
8.0
9.0
0
0.1
1
6
12
24
48
96
144

A_24_P170874 BC013295 NA

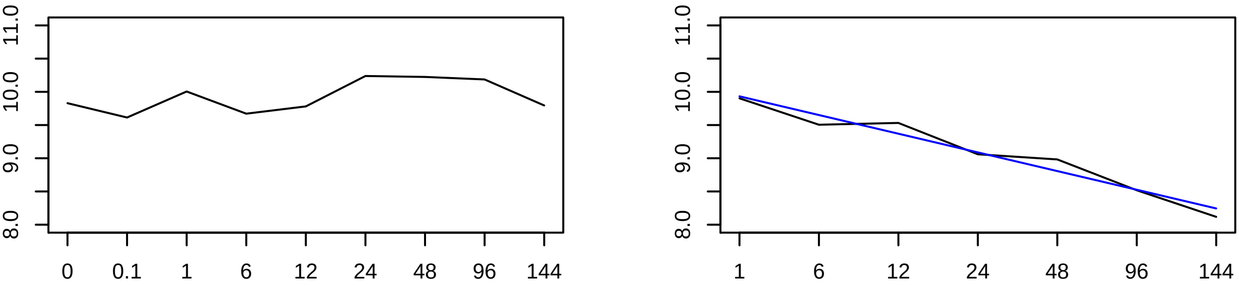

A_24_P75376 ZC3H13 13q14.12

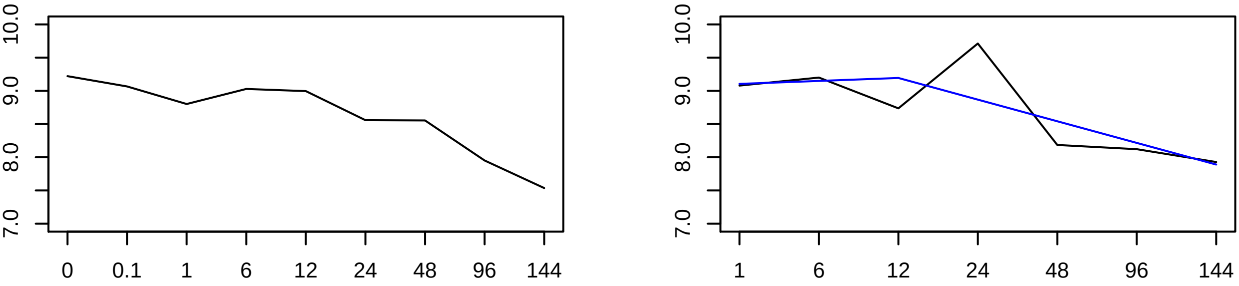

A_24_P126902 A_24_P126902 NA

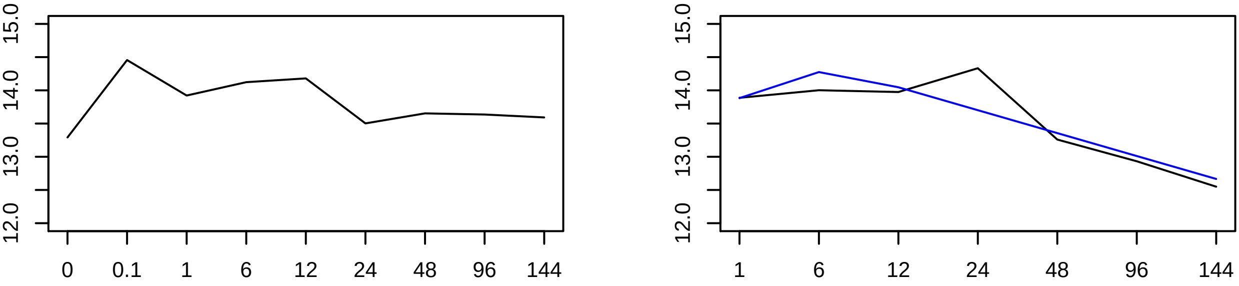

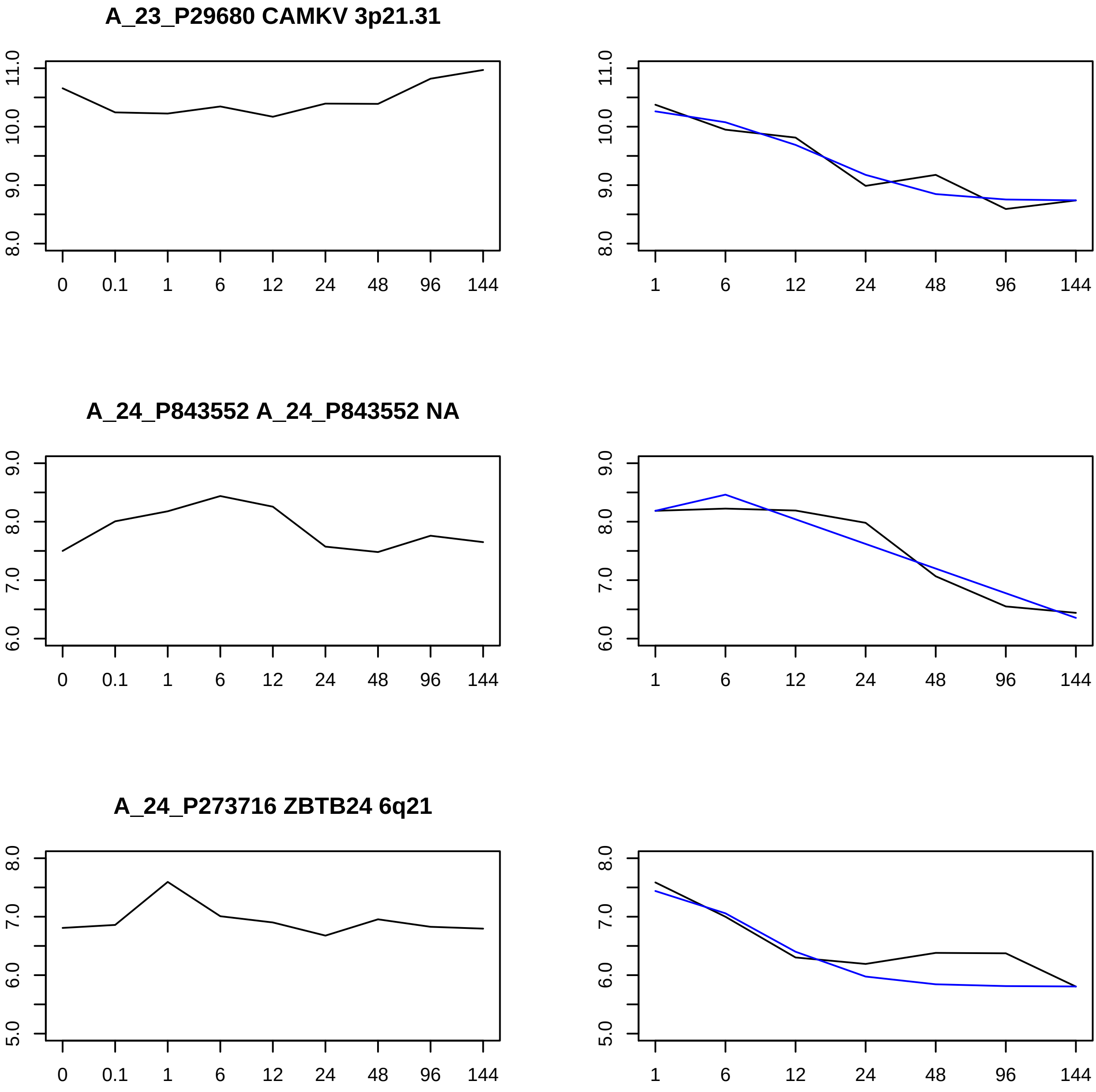
A_23_P29680 CAMKV 3p21.31
8.0
9.0
10.0
11.0
0
0.1
1
6
12
24
48
96
144
1
6
12
24
48
96
144
A_24_P843552 A_24_P843552 NA
6.0
7.0
8.0
9.0
A_24_P273716 ZBTB24 6q21
5.0
6.0
7.0
8.0

A_24_P66932 LOC442293 7p14.2

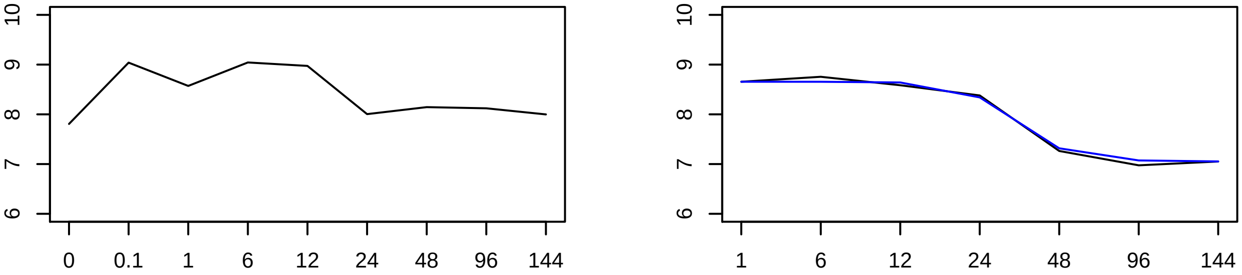

A_32_P224525 LOC131873 3q22.1

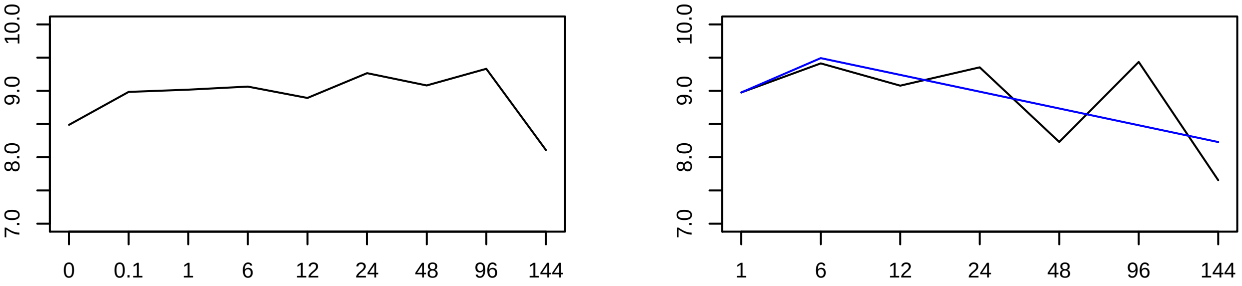

A_32_P38093 ATOH8 NA

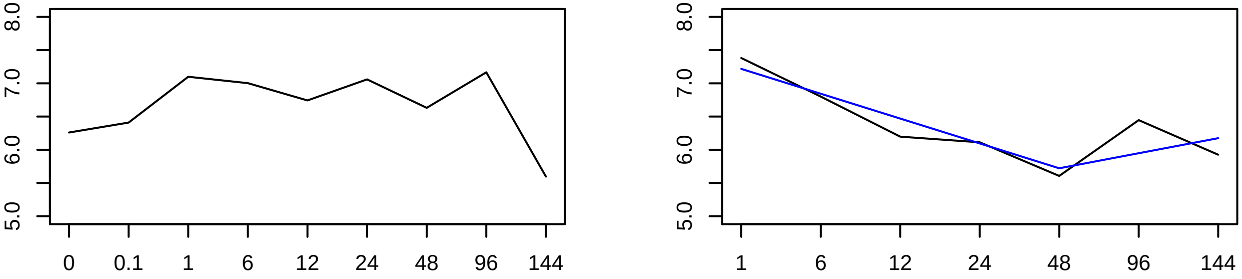

A_24_P61753 KIAA0664 17p13.3

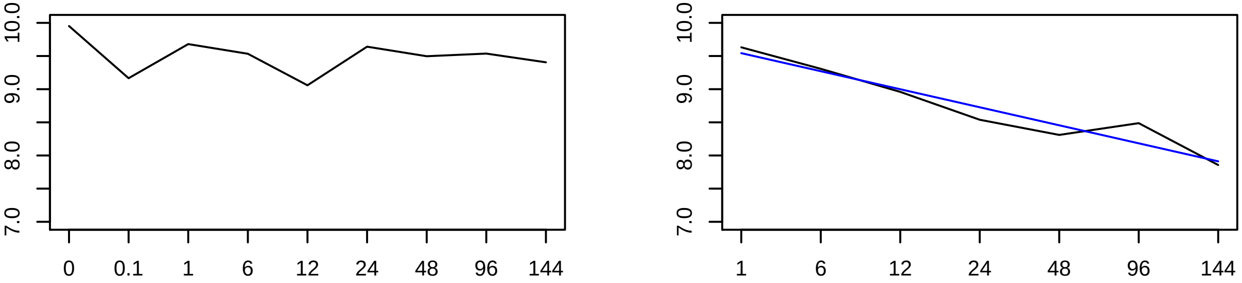

A_23_P200955 A_23_P200955 NA

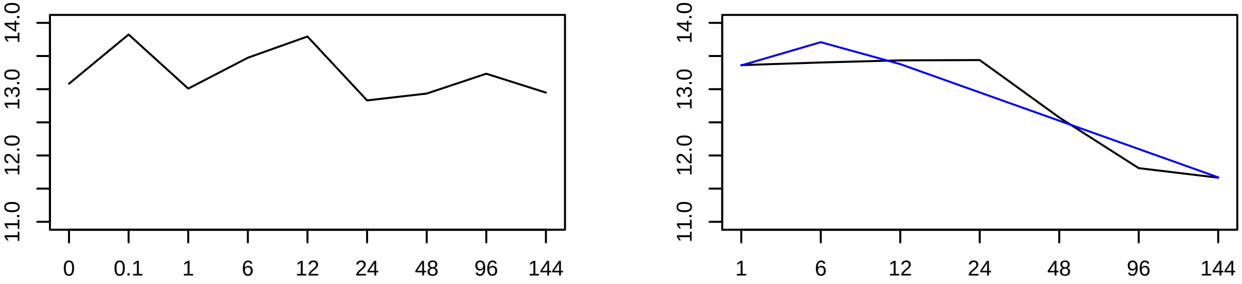

A_24_P357791 RPL36AP6 9q31.2

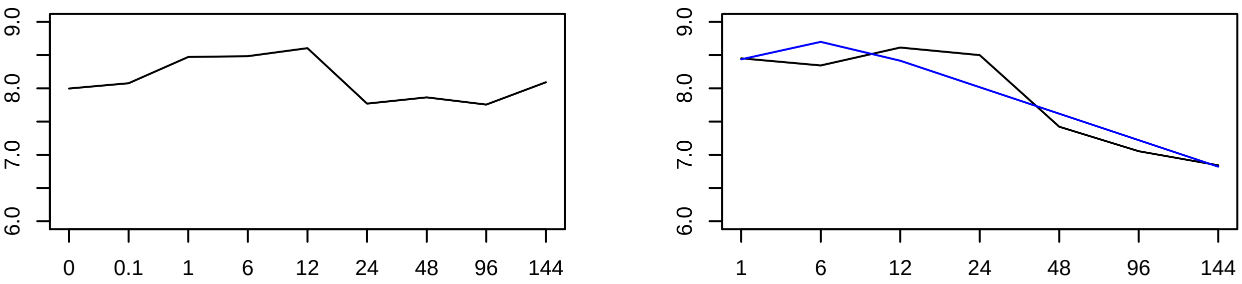

A_23_P96483 ZBTB33 Xq24

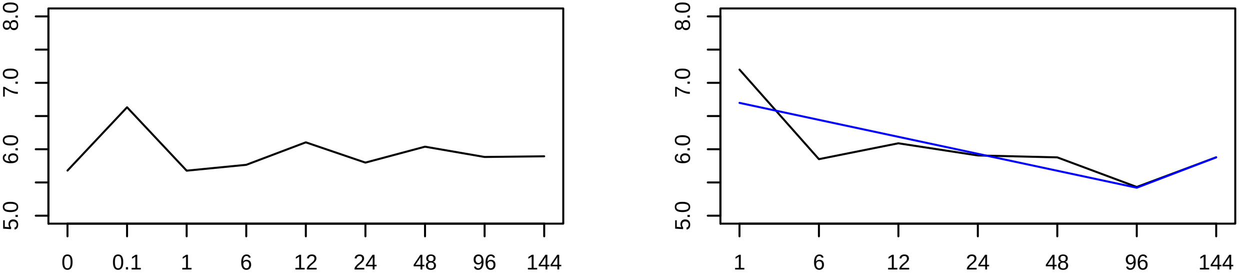

A_24_P272403 BE816155 NA

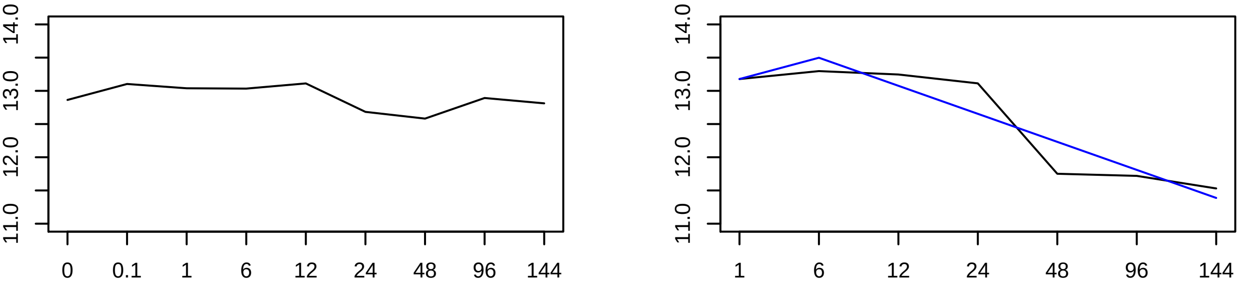

A_24_P857669 SEMA6A NA

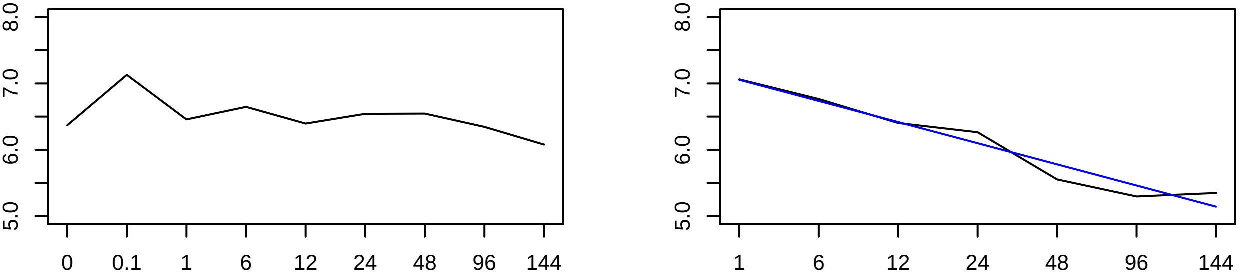

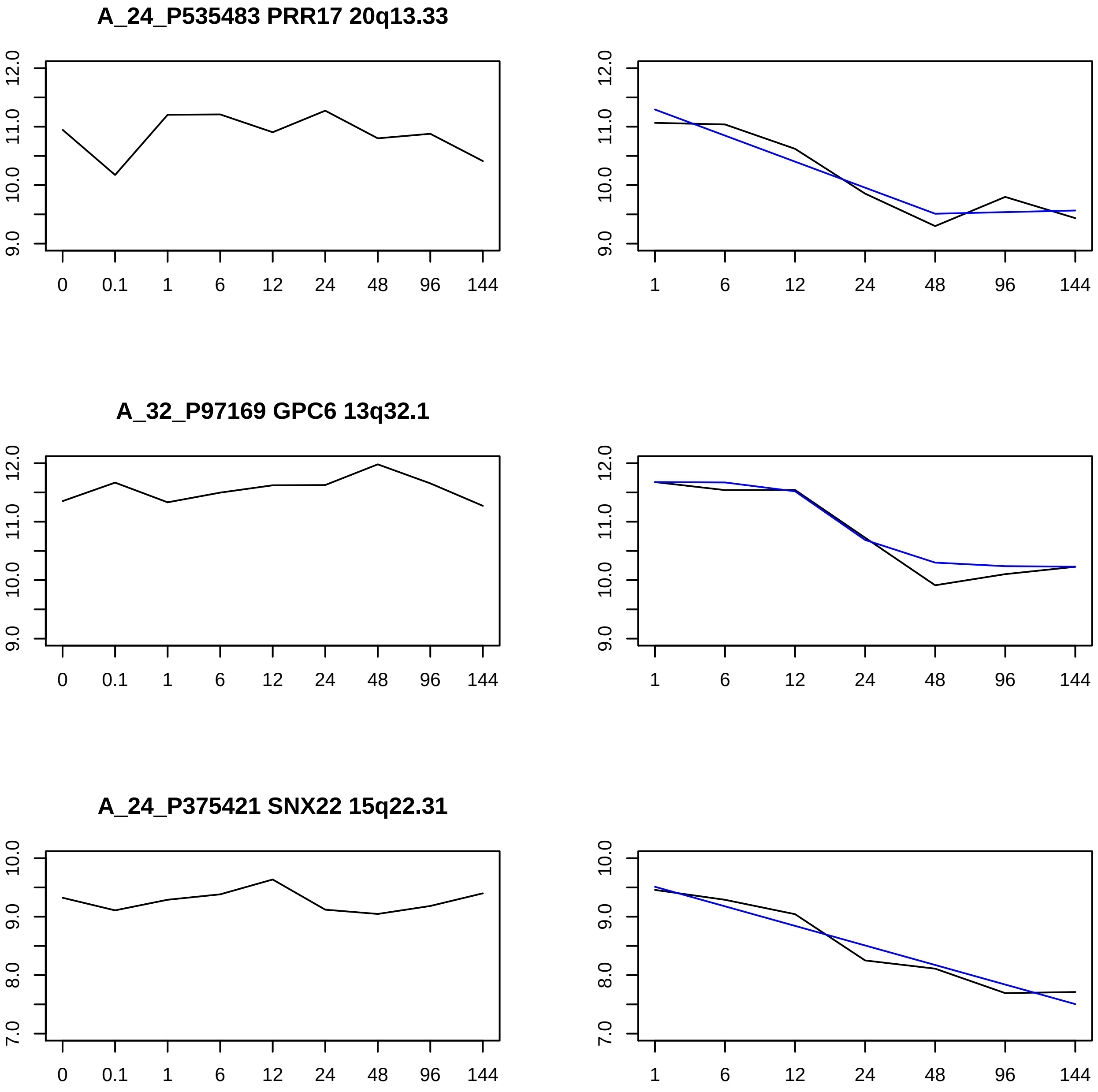
A_24_P535483 PRR17 20q13.33
A_32_P97169 GPC6 13q32.1
A_24_P375421 SNX22 15q22.31

A_23_P259892 ENO1B NA

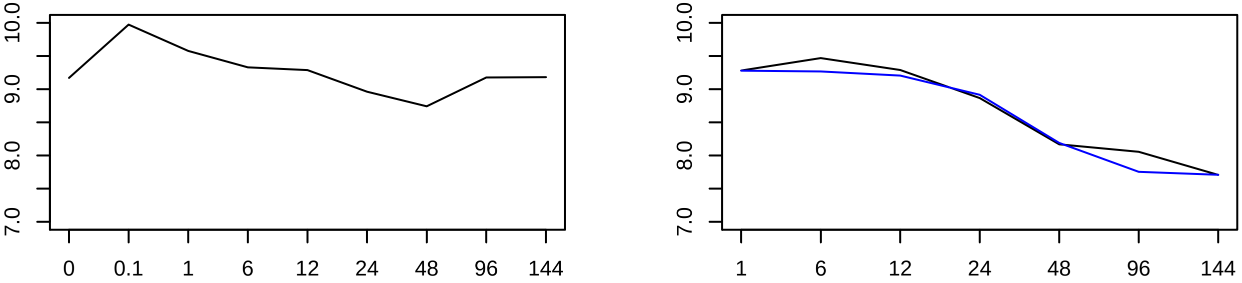

A_32_P53713 CLN3 16p11.2

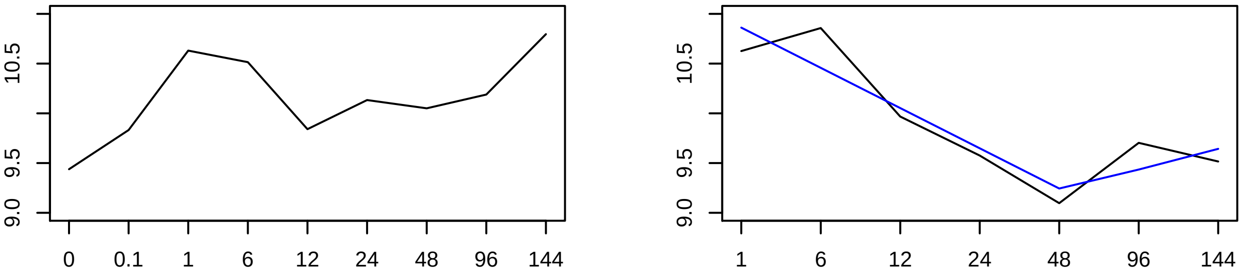

A_32_P138432 LAMB4\NRCAM 7q31.1

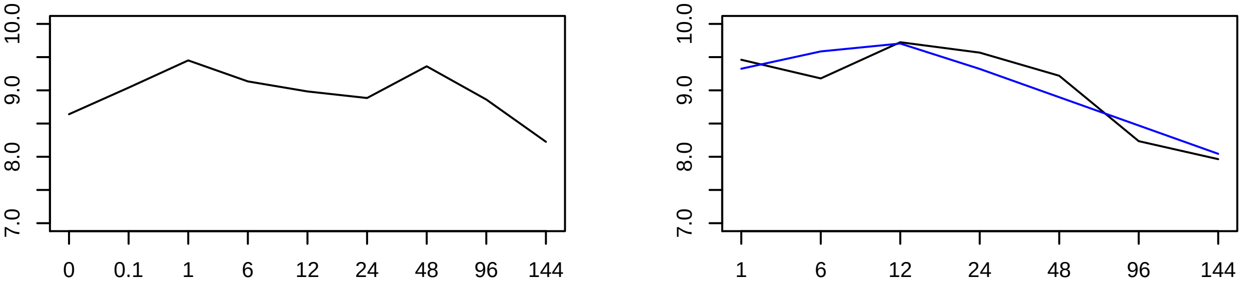

A_24_P350017 ENST00000341824 NA

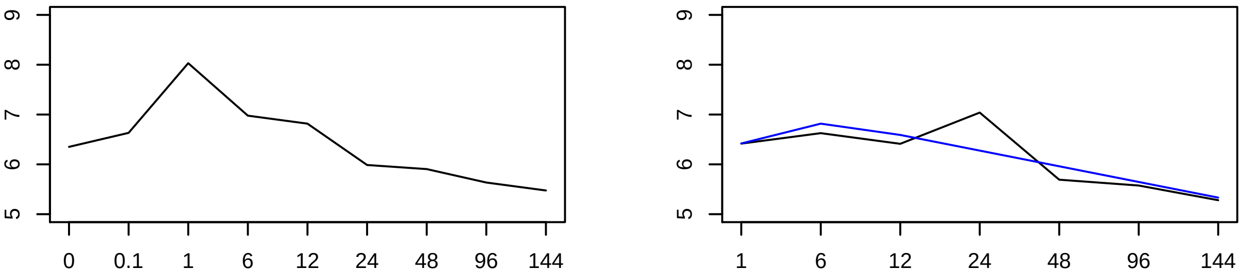

A_24_P76169 AC011933.2 17q25.1

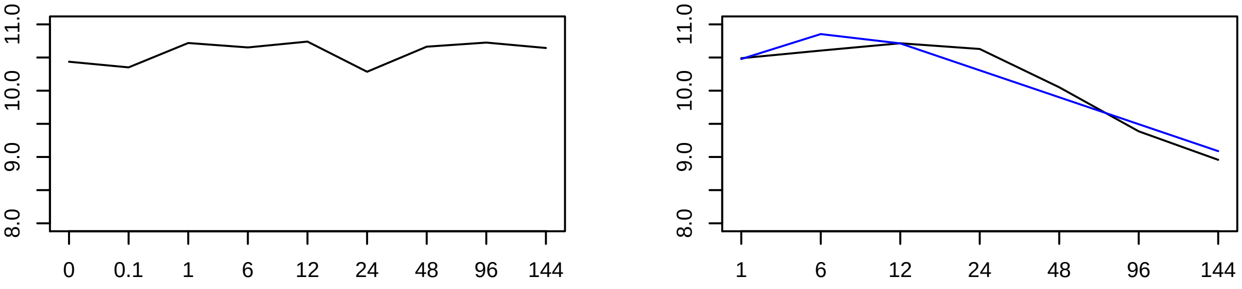

A_24_P409521 A_24_P409521 NA

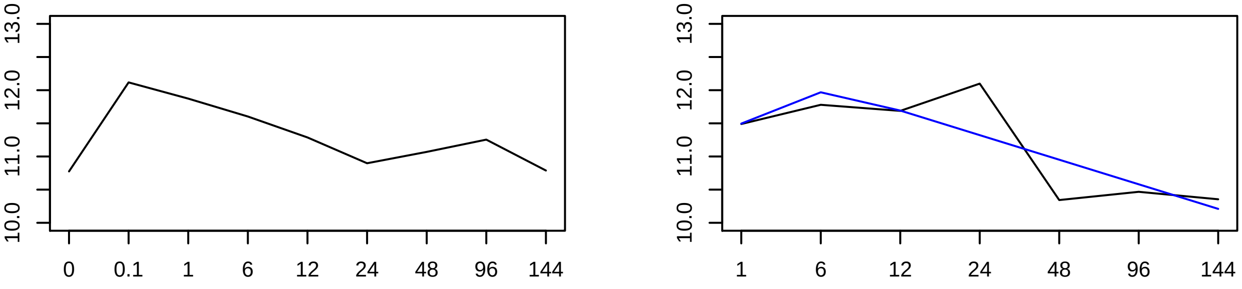

A_23_P59338 PDE7B 6q23.3

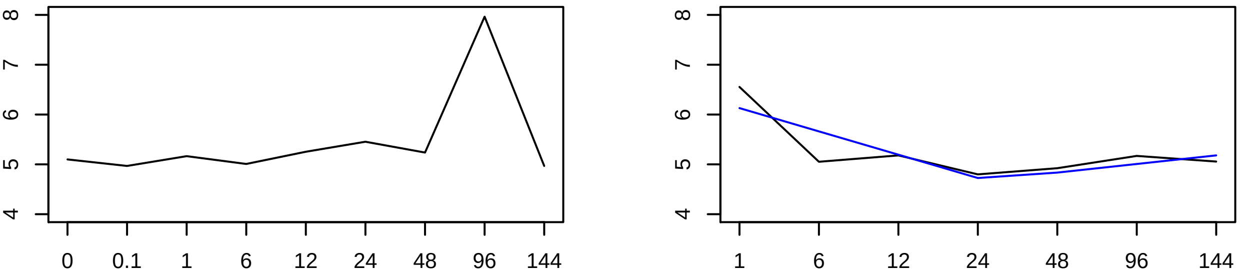

A_24_P280953 RP11−6K23.1 12p12.3

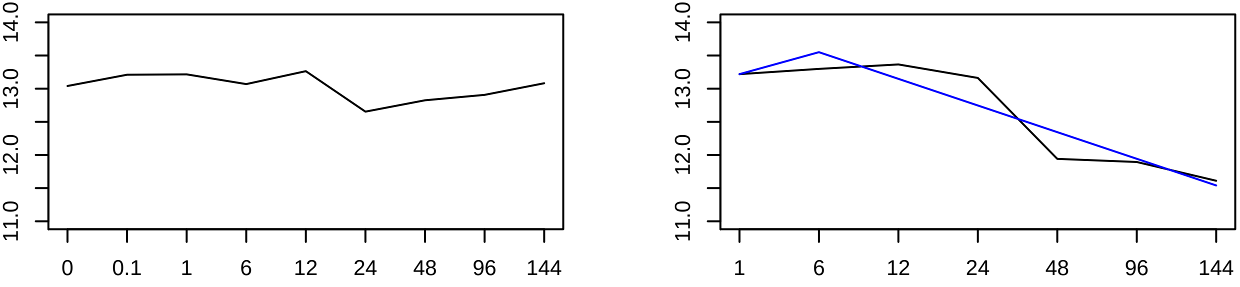

A_24_P111009 SAFB2 19p13.3

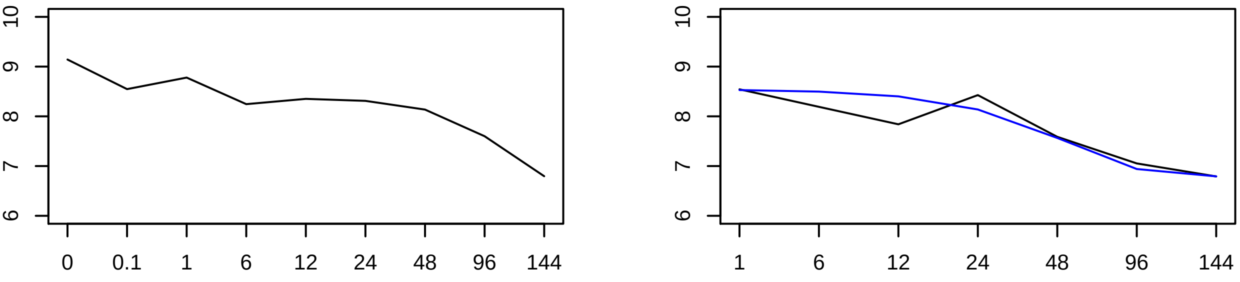

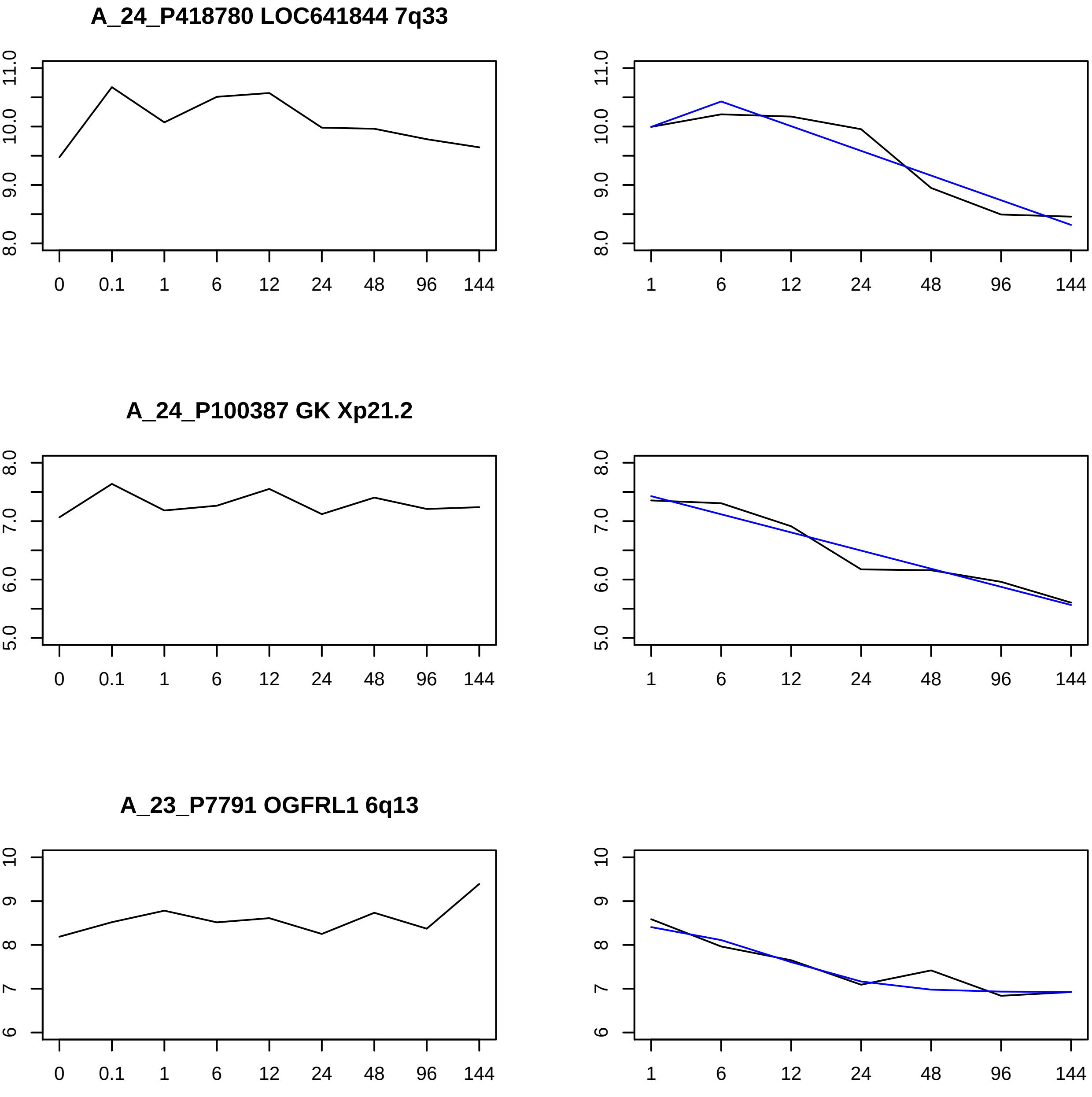
A_24_P418780 LOC641844 7q33
8.0
9.0
10.0
11.0
0
0.1
1
6
12
24
48
96
144
1
6
12
24
48
96
144
A_24_P100387 GK Xp21.2
5.0
6.0
7.0
8.0
A_23_P7791 OGFRL1 6q13
6
7
8
9
10

A_24_P272548 FAM185A NA

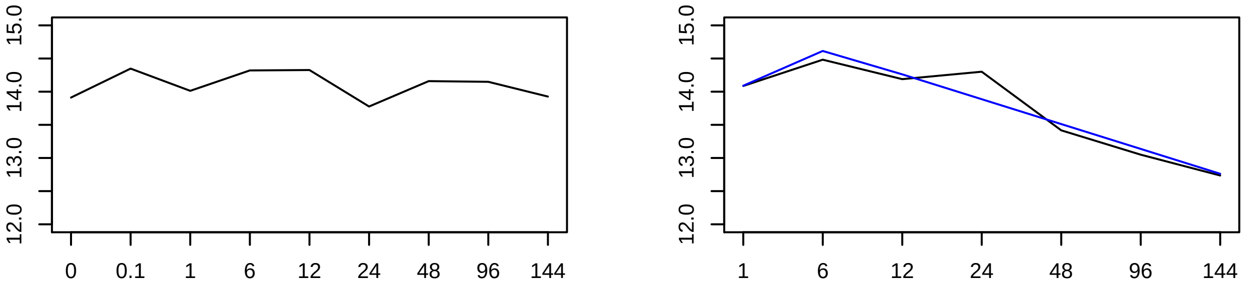

A_24_P186274 IGFBPL1 9p13.1

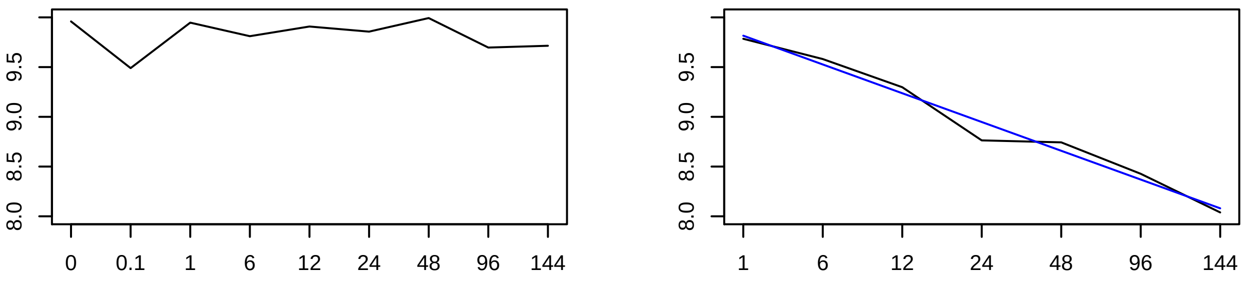

A_23_P203957 TMTC1 12p11.22

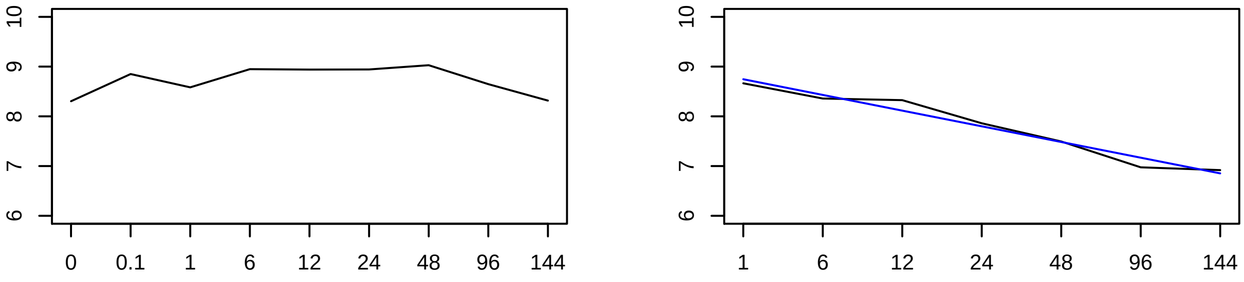

A_24_P229807 LOC652863 Xp22.2

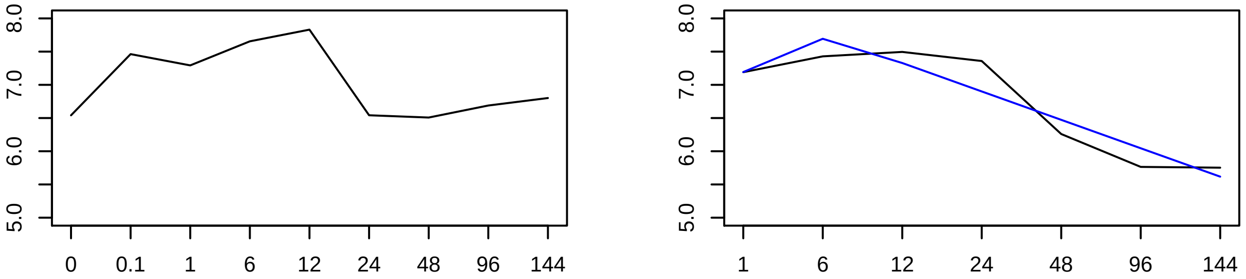

A_24_P32708 LOC401959 1p12

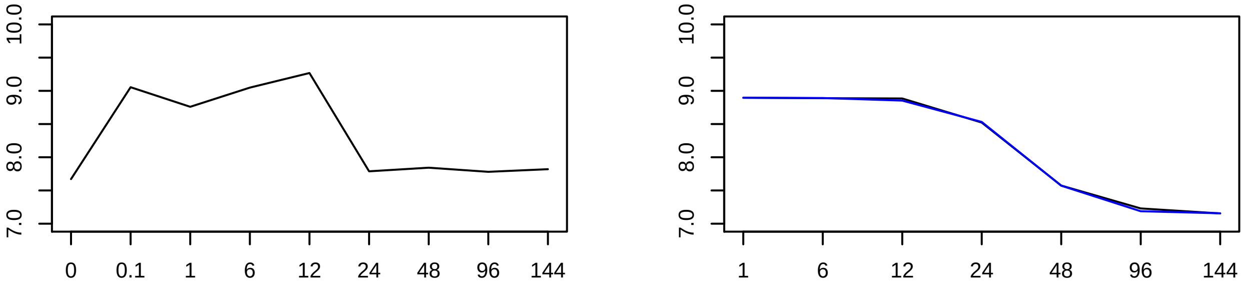

A_32_P34 LOC100131581 NA

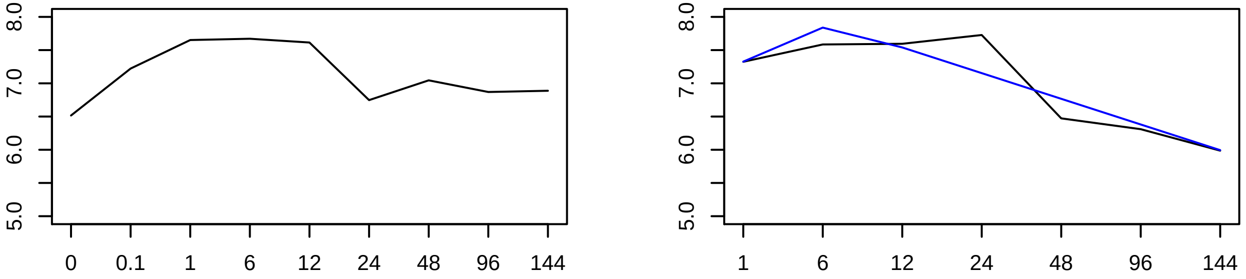

A_32_P147797 A_32_P147797 NA

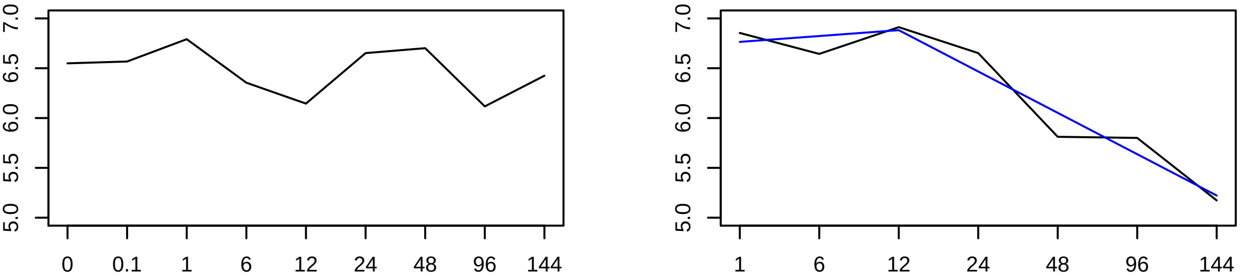

A_23_P146997 CXorf15 Xp22.2

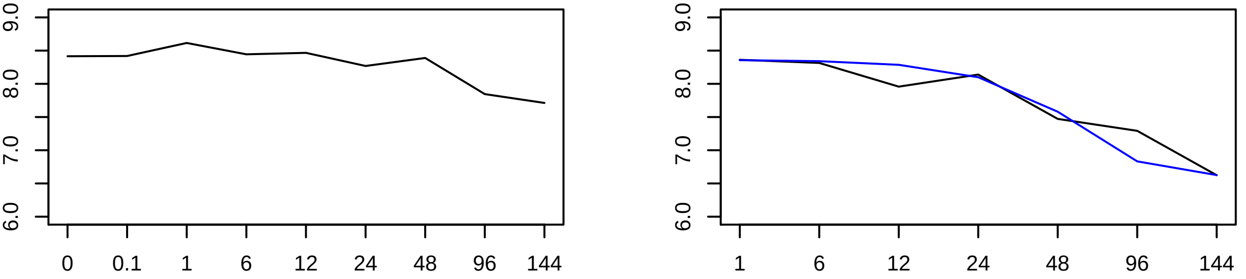

A_23_P202594 C10orf119 10q26.11

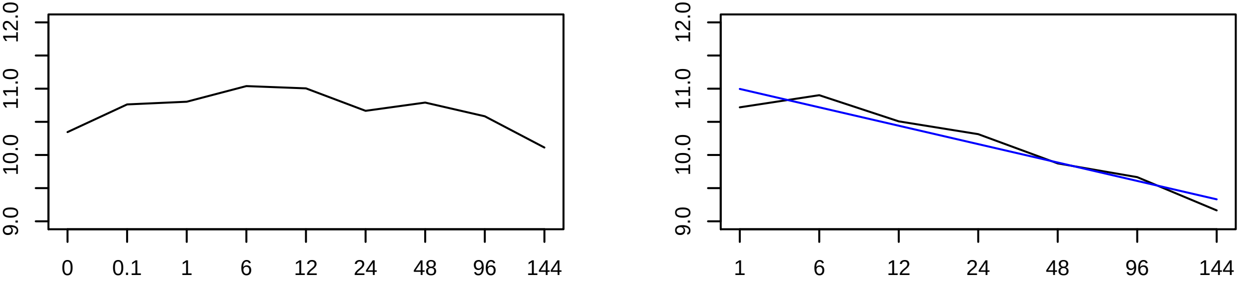

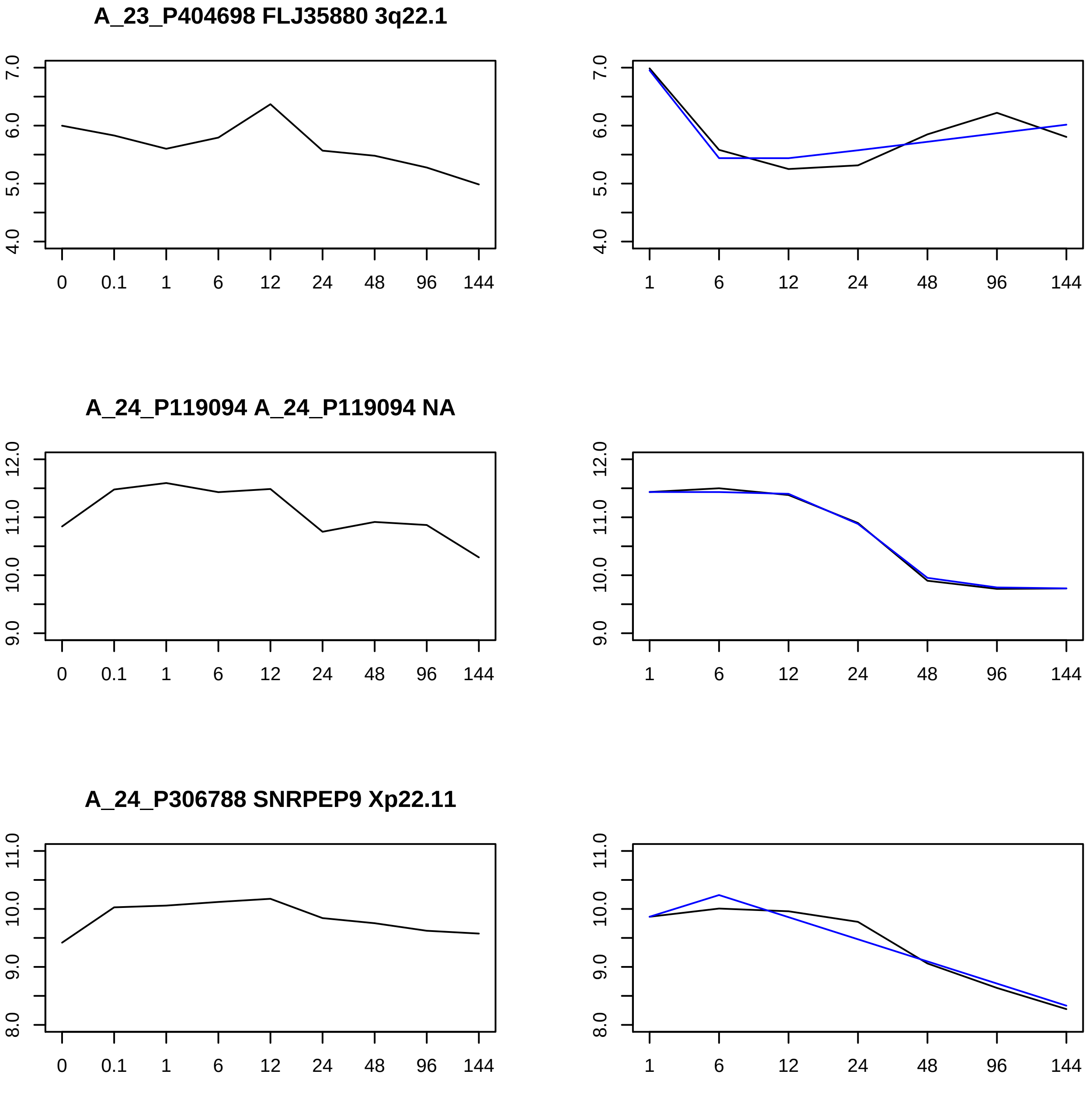

A_23_P404698 FLJ35880 3q22.1
A_24_P119094 A_24_P119094 NA
A_24_P306788 SNRPEP9 Xp22.11
4.0 5.0 6.0 7.0
9.0 10.0 11.0 12.0
8.0 9.0 10.0 11.0
0 0.1 1 6 12 24 48 96 144
1 6 12 24 48 96 144

A_24_P76120 THC2474831 NA

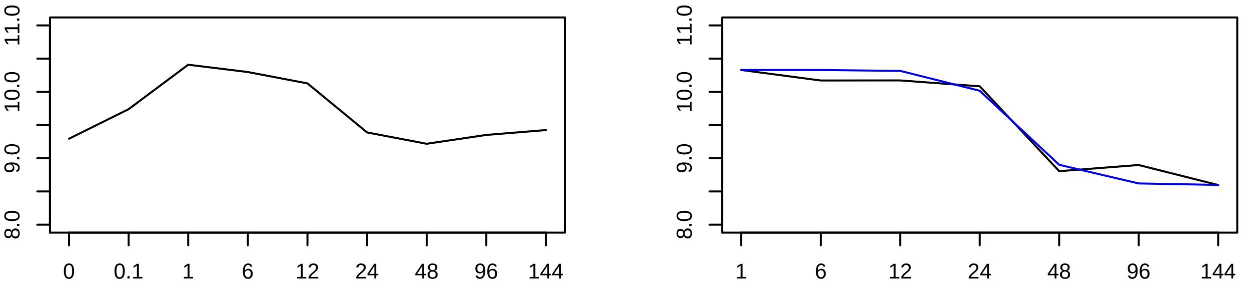

A_23_P41327 LYAR 4p16.2

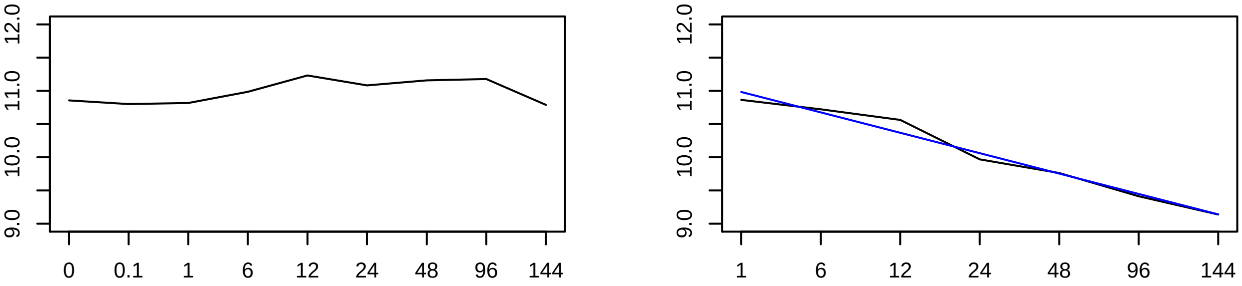

A_23_P344444 ASB17 1p31.1

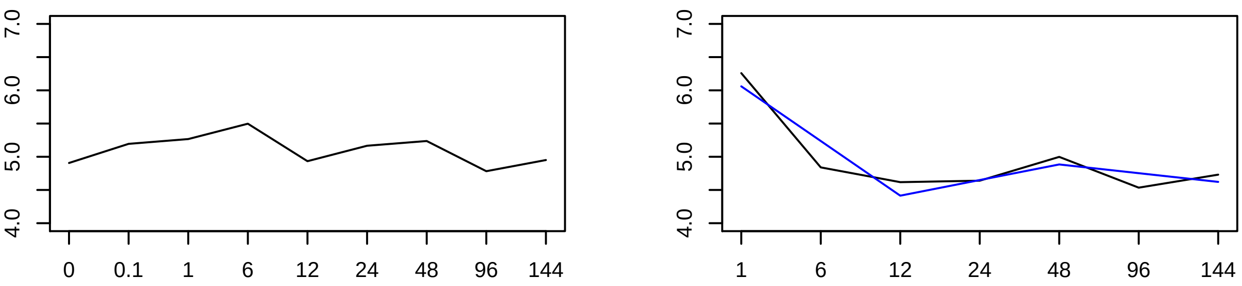

A_32_P135818 RPS3A 4q31.3

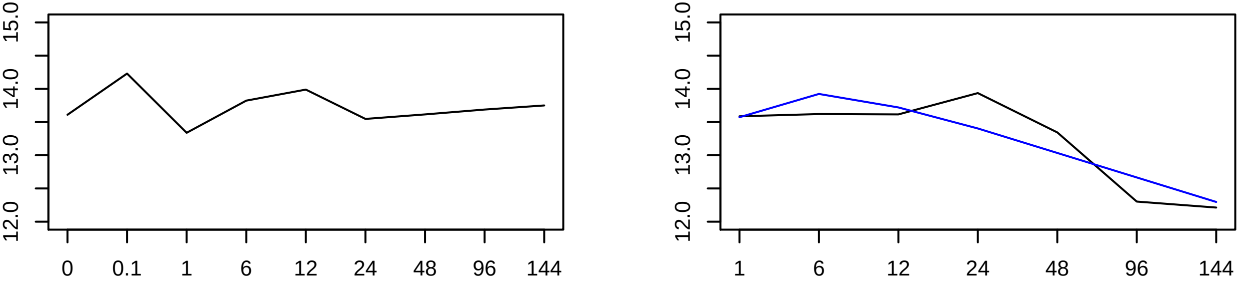

A_24_P92744 RPS2P52 19p13.3

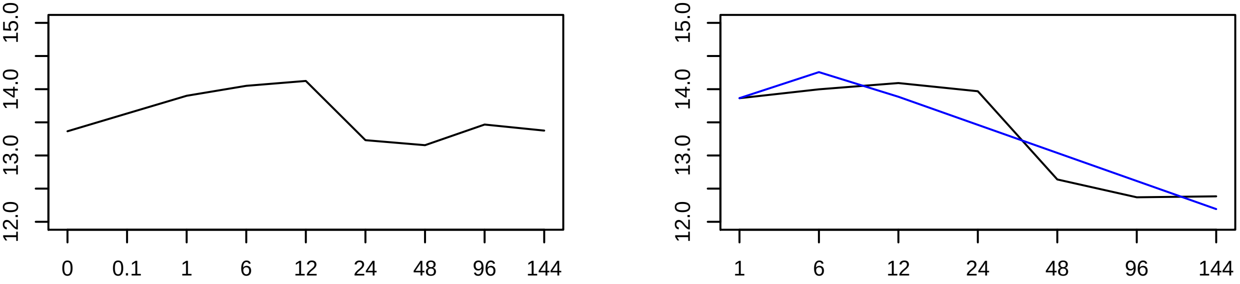

A_23_P318904 SERTAD4 1q32.2

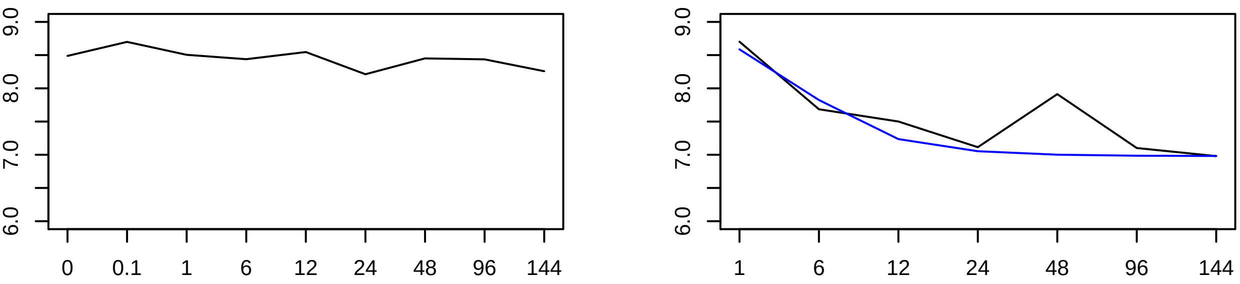

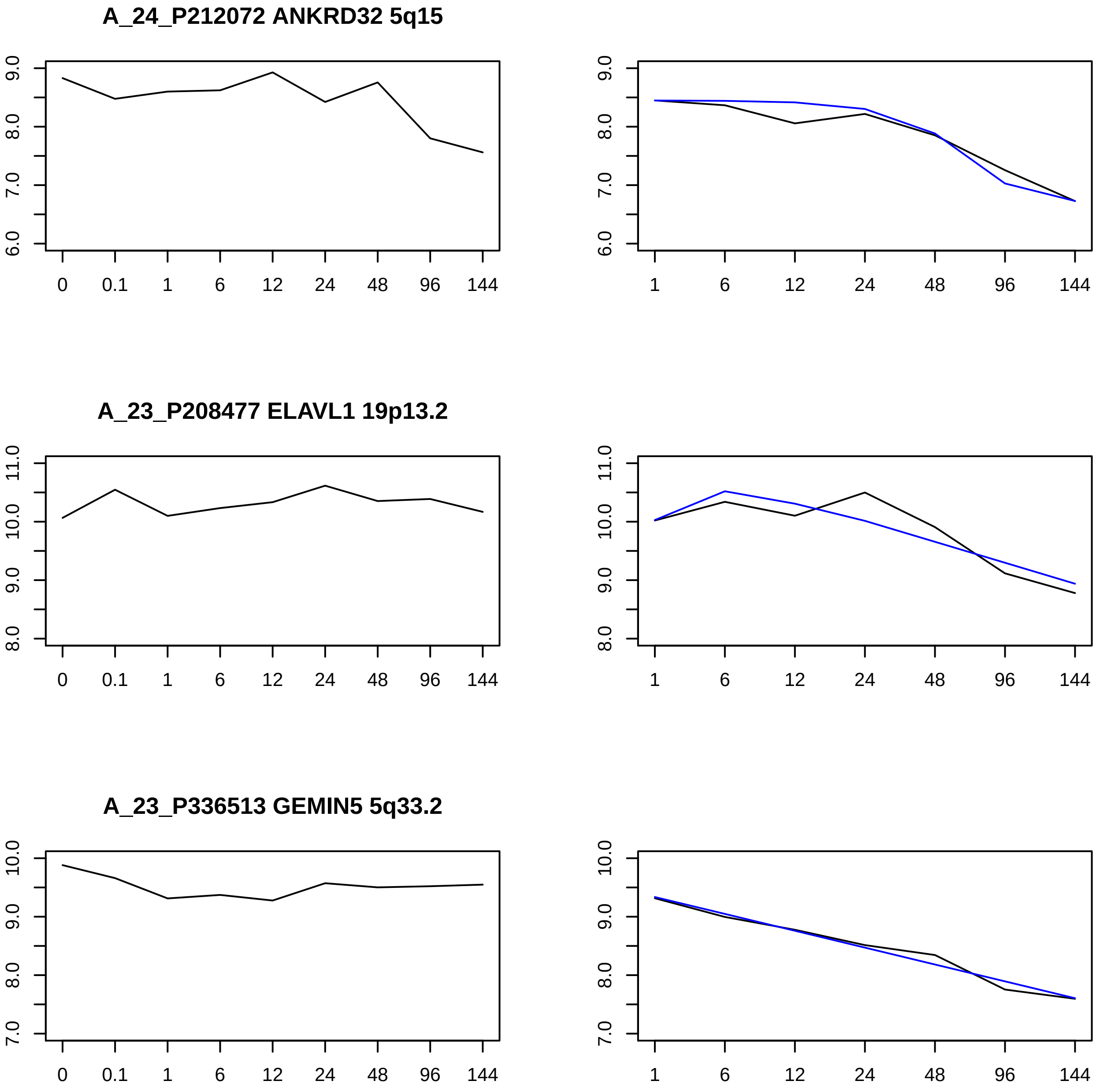
A_24_P212072 ANKRD32 5q15
6.0 7.0 8.0 9.0
0 0.1 1 6 12 24 48 96 144
1 6 12 24 48 96 144
A_23_P208477 ELAVL1 19p13.2
8.0 9.0 10.0 11.0
0 0.1 1 6 12 24 48 96 144
1 6 12 24 48 96 144
A_23_P336513 GEMIN5 5q33.2
7.0 8.0 9.0 10.0
0 0.1 1 6 12 24 48 96 144
1 6 12 24 48 96 144

A_24_P923594 ZNF605 NA

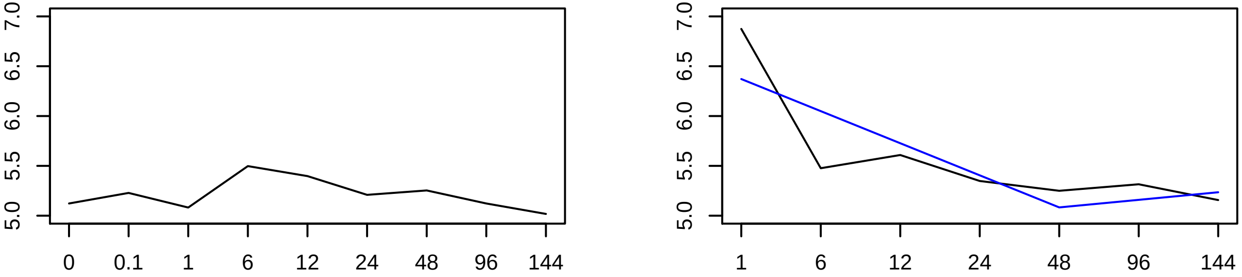

A_24_P878388 RP13−926M18.1 Xp22.31

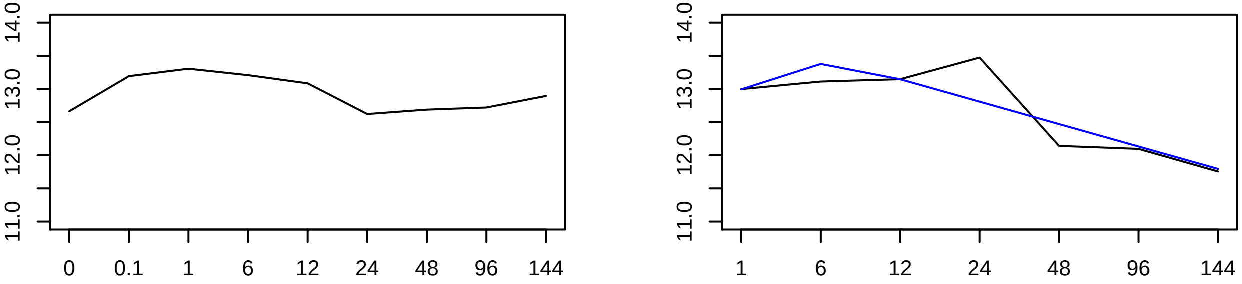

A_23_P202334 FGFR2 10q26.13

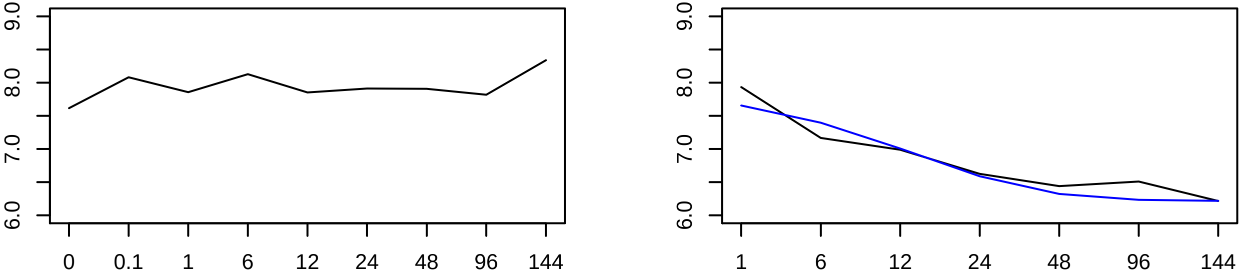

A_23_P162589 VDR 12q13.11

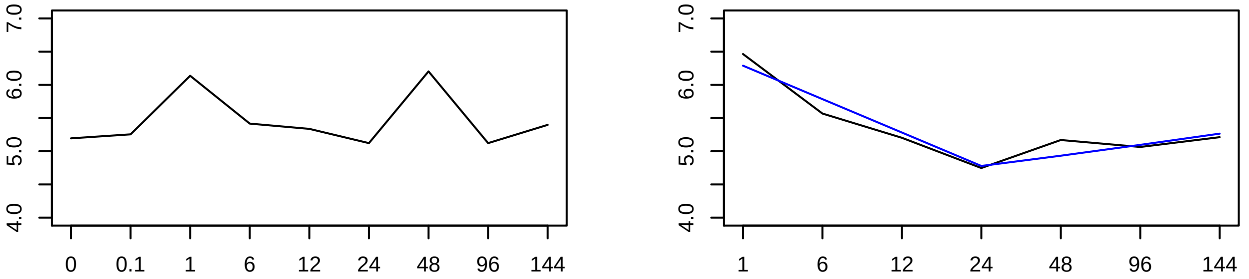

A_24_P452175 A_24_P452175 NA

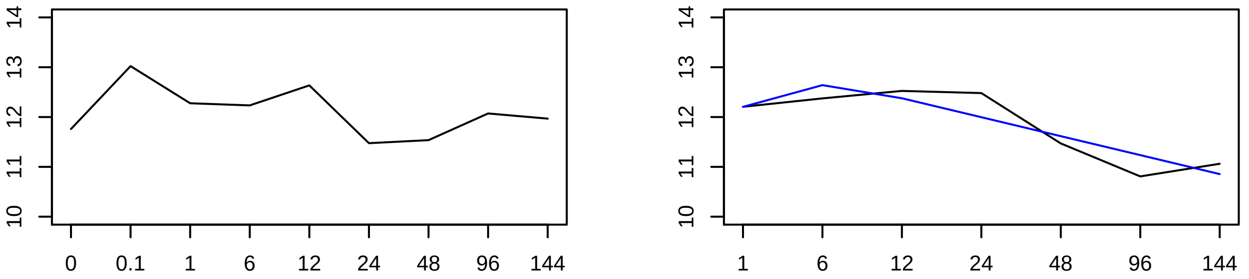

A_23_P353744 LARP2 4q28.2

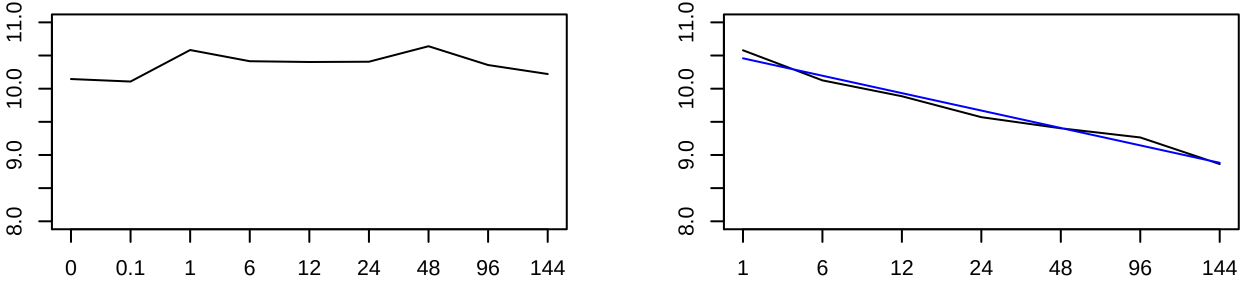

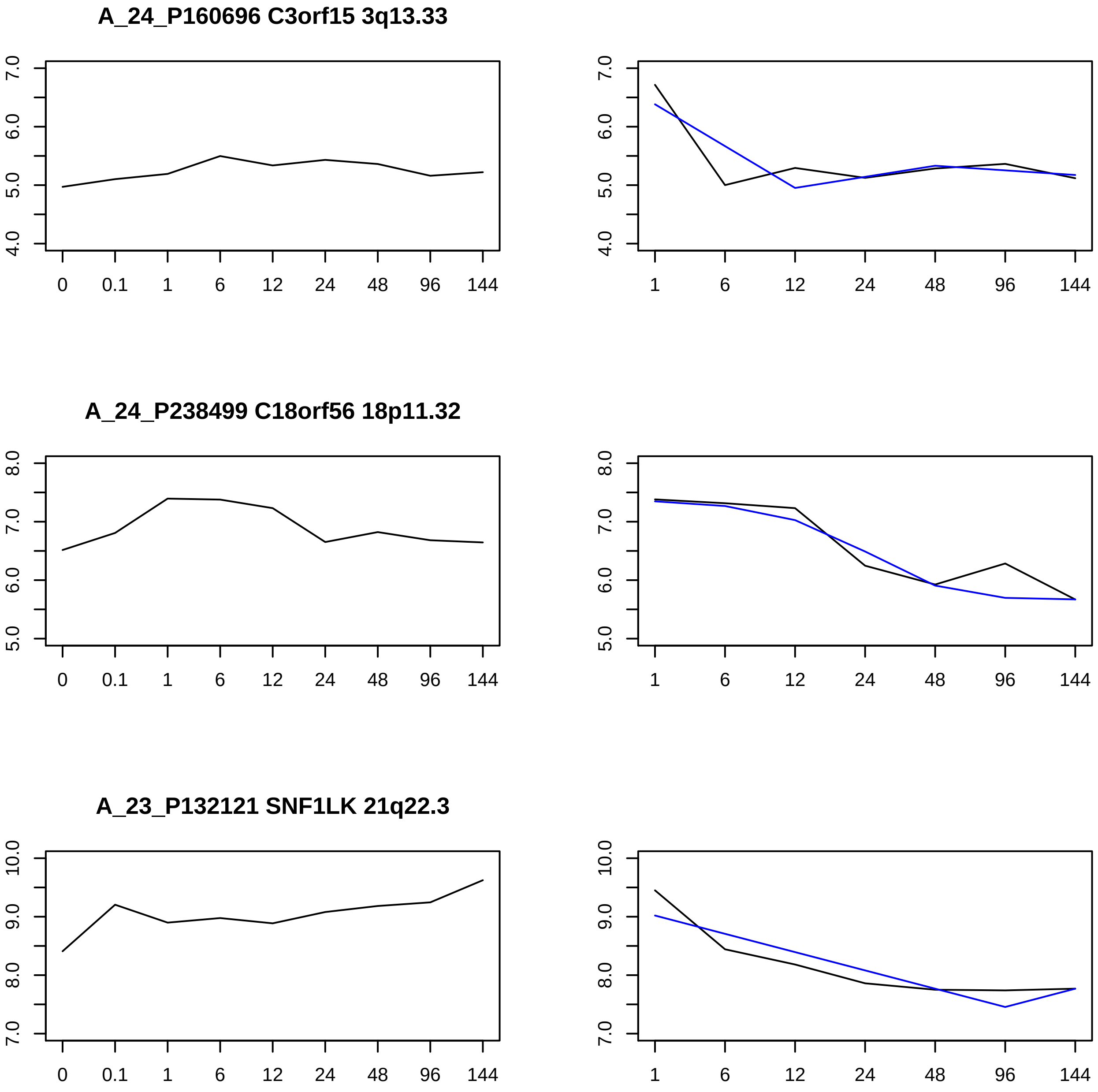
A_24_P160696 C3orf15 3q13.33
A_24_P238499 C18orf56 18p11.32
A_23_P132121 SNF1LK 21q22.3
0 0.1 1 6 12 24 48 96 144
1 6 12 24 48 96 144
4.0 5.0 6.0 7.0
5.0 6.0 7.0 8.0
7.0 8.0 9.0 10.0

A_32_P178696 NUP153 NA

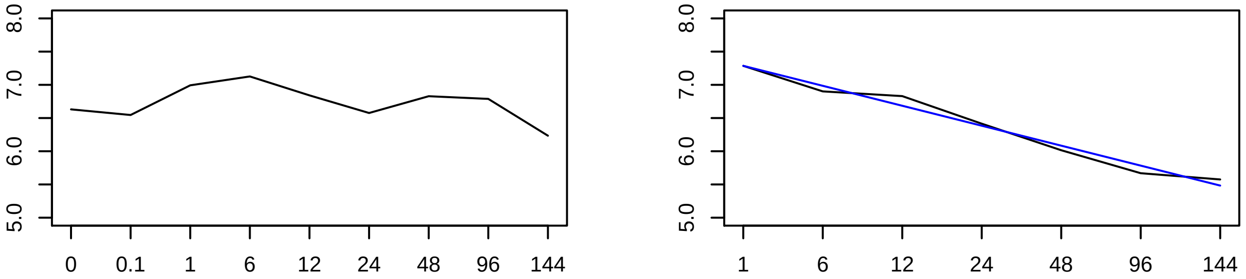

A_24_P823096 AL109708 NA

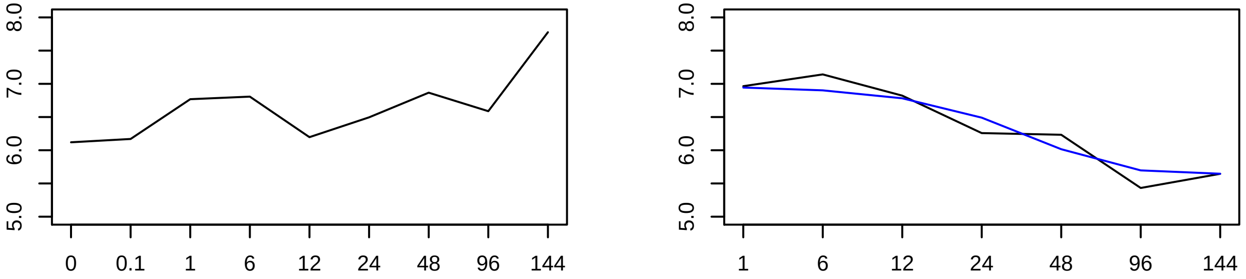

A_24_P832737 A_24_P832737 NA

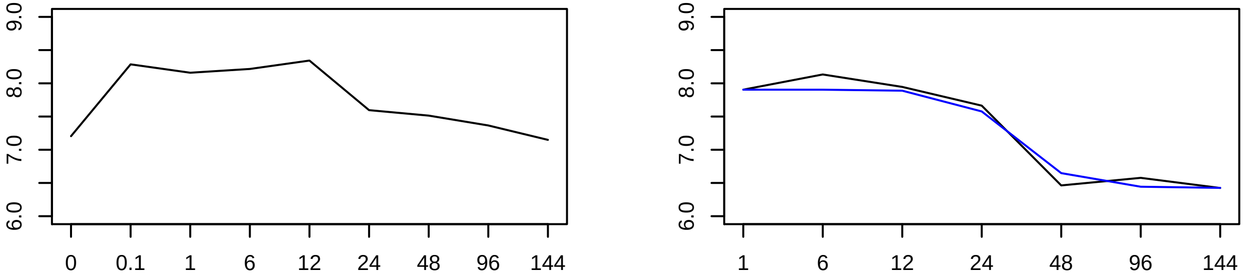

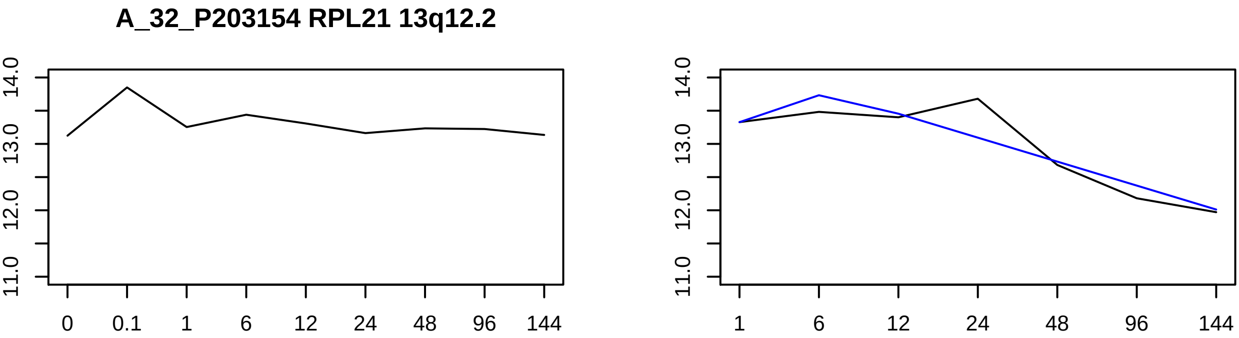
A_32_P203154 RPL21 13q12.2
11.0
12.0
13.0
14.0
0
0.1
1
6
12
24
48
96
144

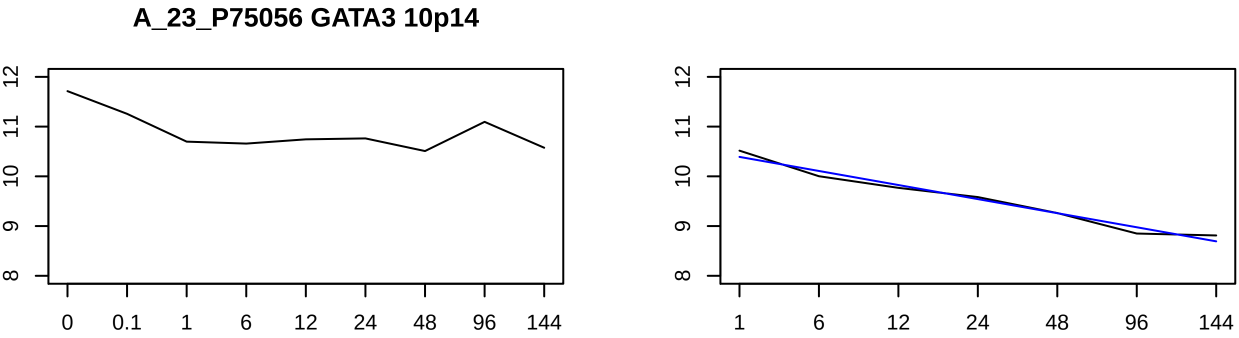
A_23_P75056 GATA3 10p14
8
9
10
11
12
0
0.1
1
6
12
24
48
96
144

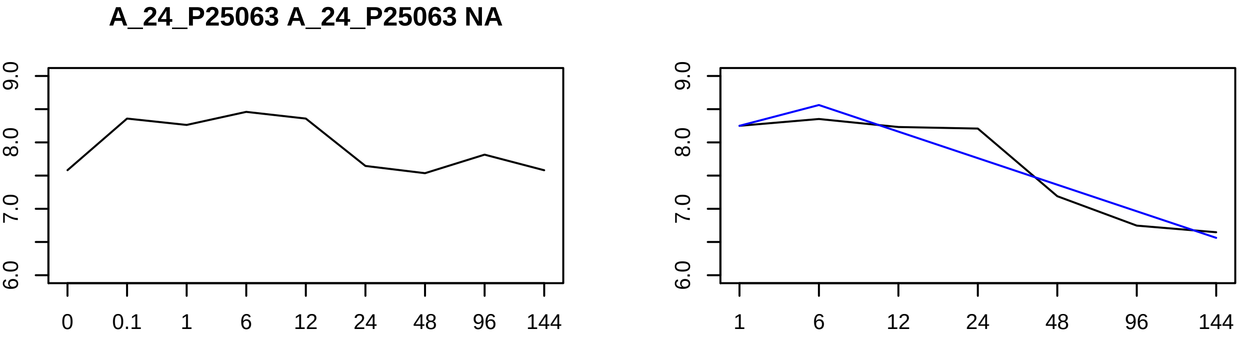
A_24_P25063 A_24_P25063 NA
6.0
7.0
8.0
9.0
0
0.1
1
6
12
24
48
96
144

A_24_P920693 CROP 17q21.33

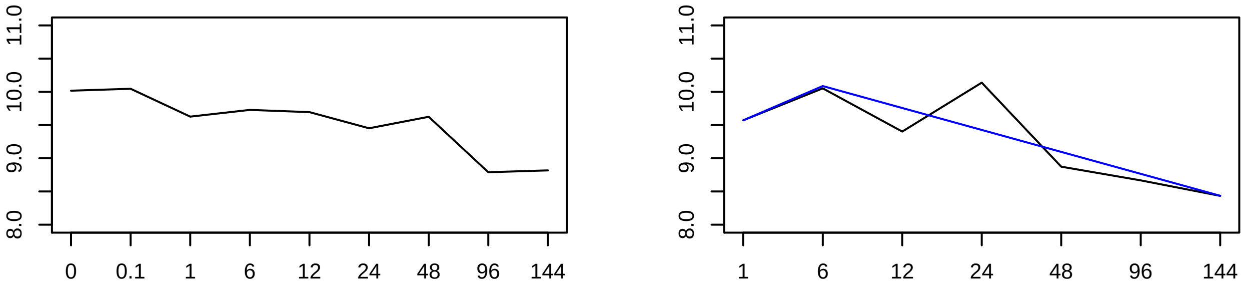

A_24_P306585 ANKRD18CP\ 9q22.33

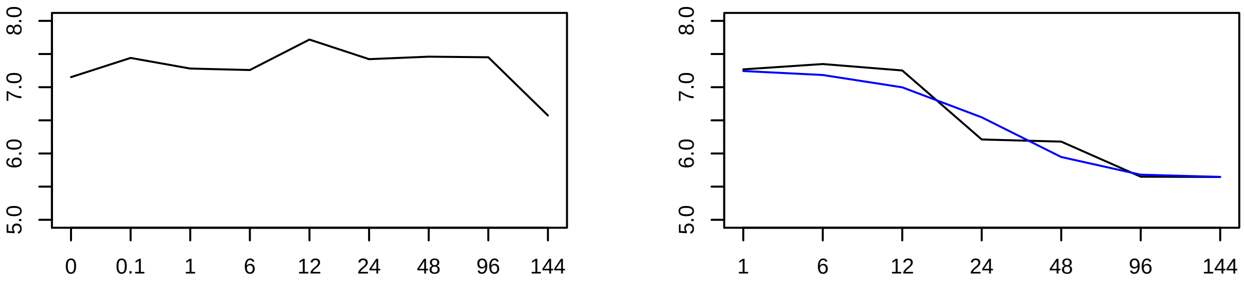

A_24_P92661 LOC401863 16q24.1

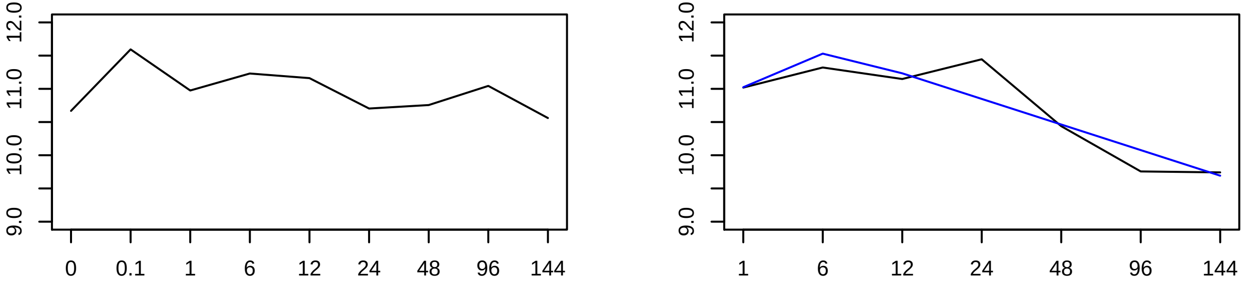

A_32_P76091 HSPD1 2q33.1

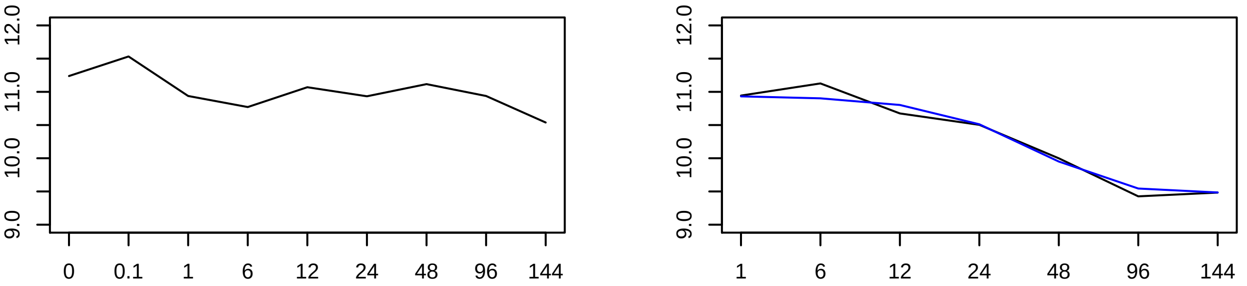

A_24_P75308 LOC650808 14q11.2

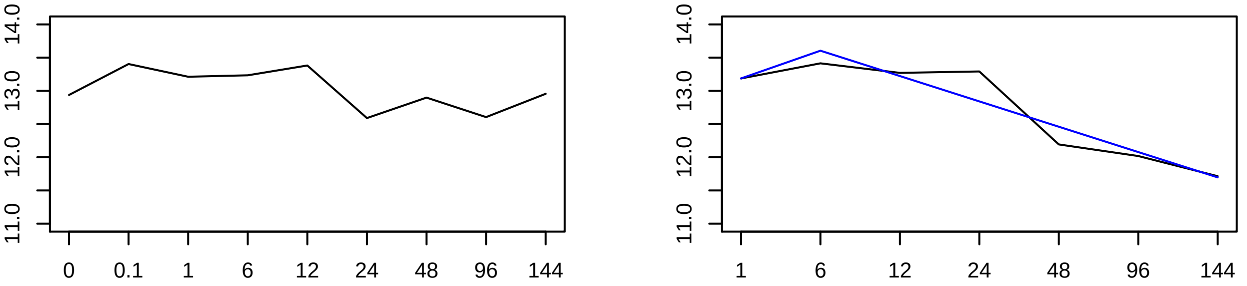

A_32_P70483 THC2712447 NA

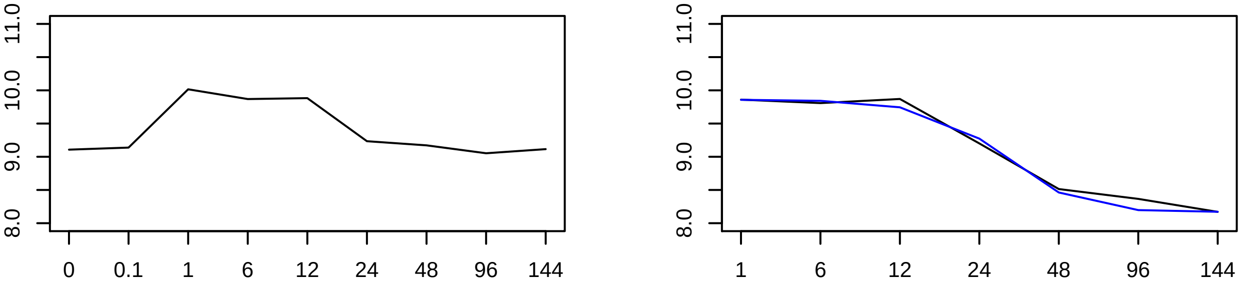

A_23_P254654 CLIC3 9q34.3

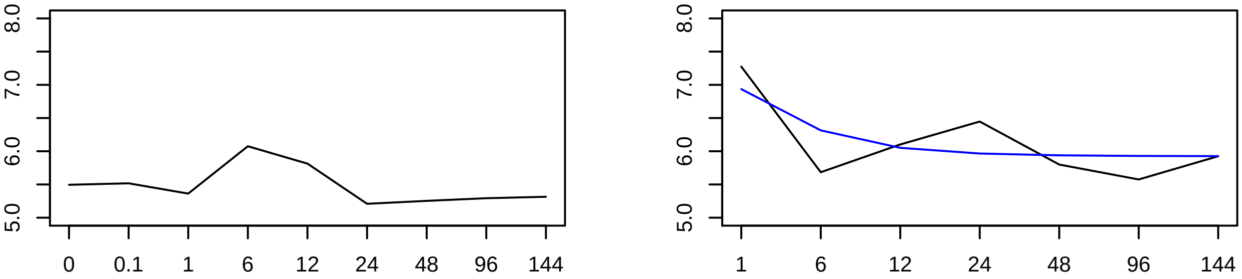

A_24_P902313 BF129169 NA

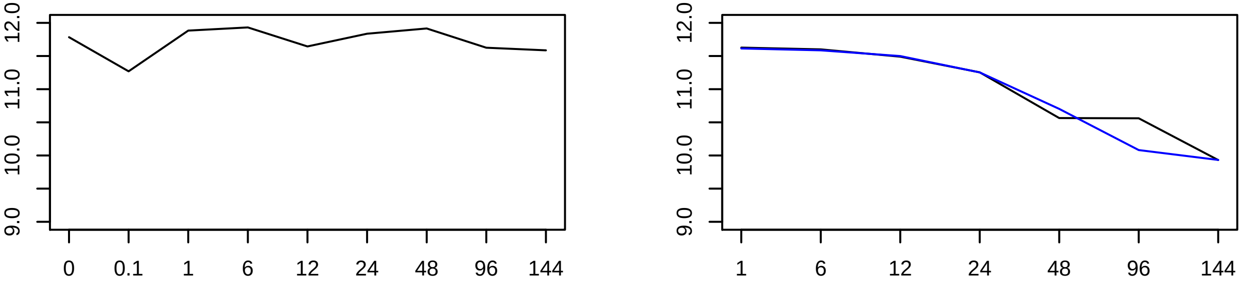

A_23_P94875 INPP5E 9q34.3

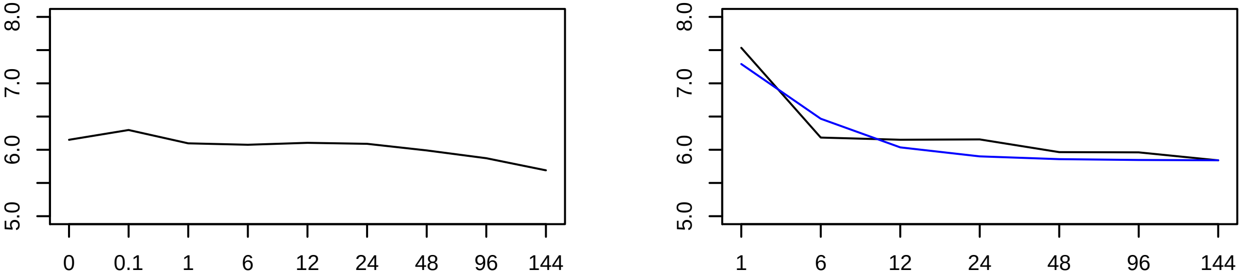

A_23_P167553 DHFR 5q14.1

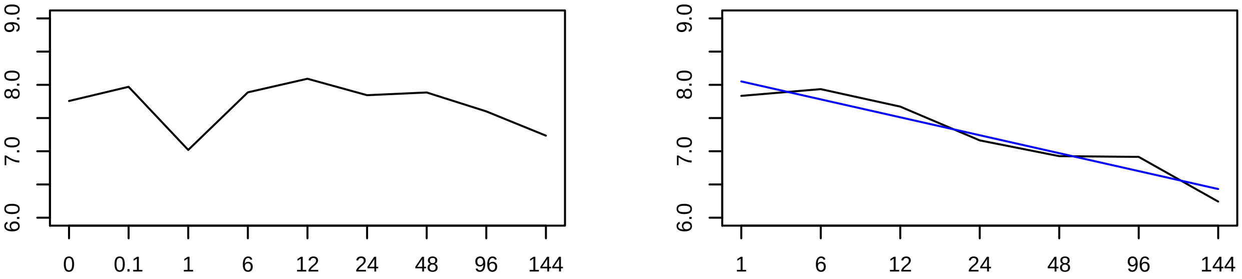

A_23_P147786 RIMS2 8q22.3

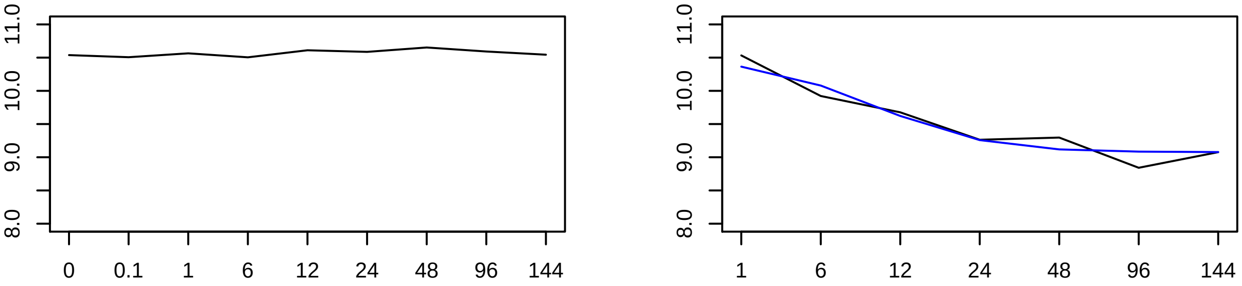

A_24_P375405 A_24_P375405 NA

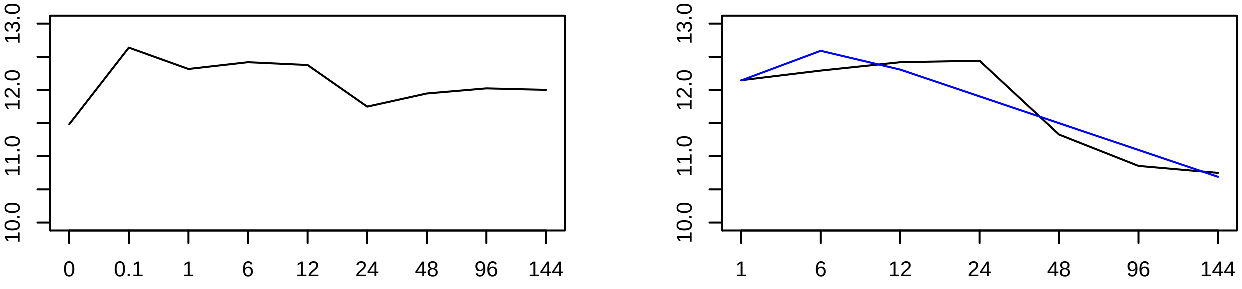

A_24_P554040 BF509345 NA

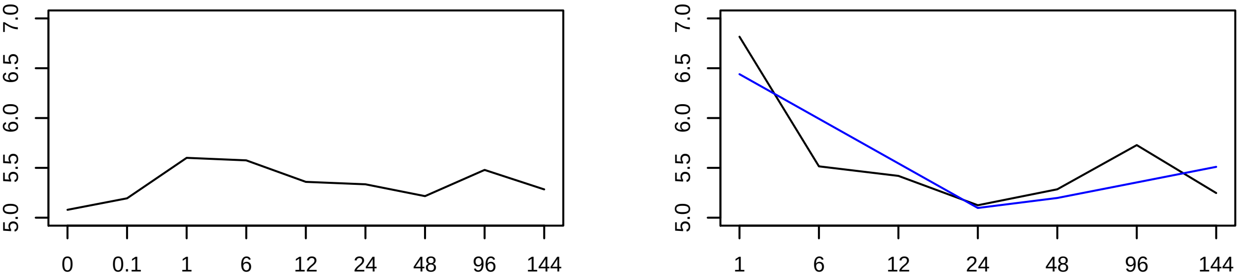

A_23_P4416 ZNF287 17p11.2

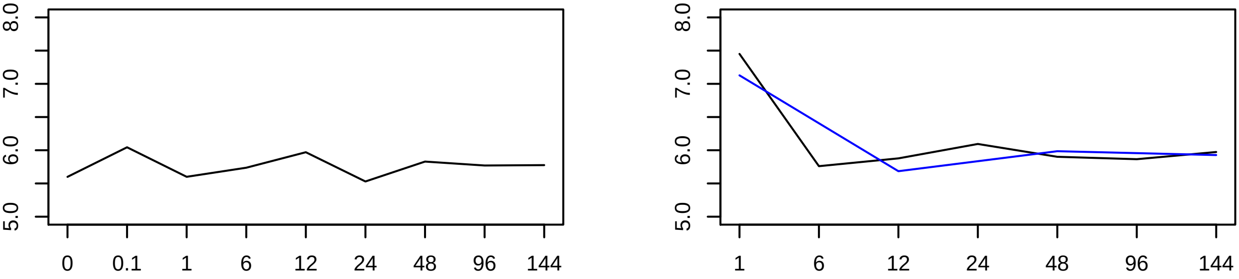

A_24_P289178 C16orf74 16q24.1

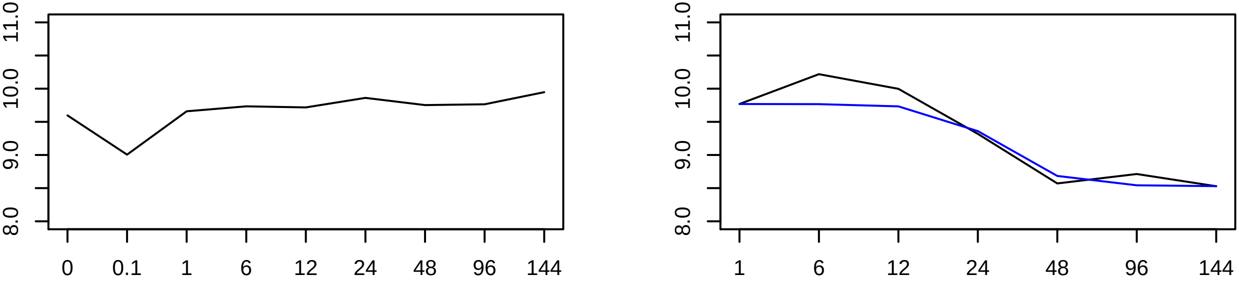

A_23_P22086 LOC649828 NA

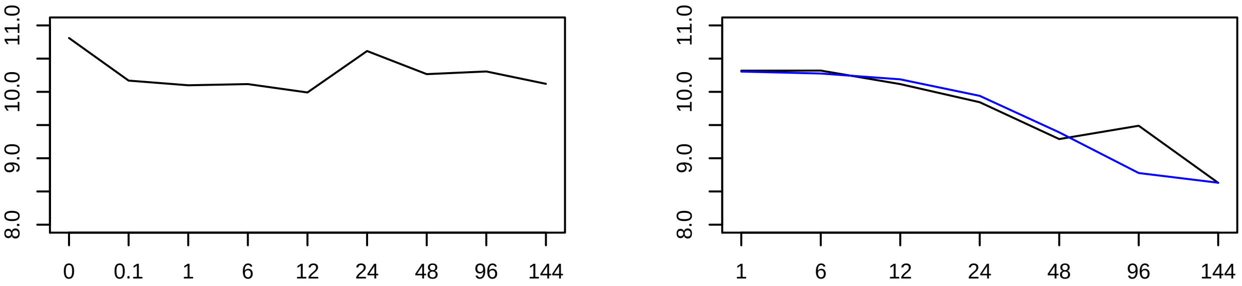

A_24_P393844 DPH2 1p34.1

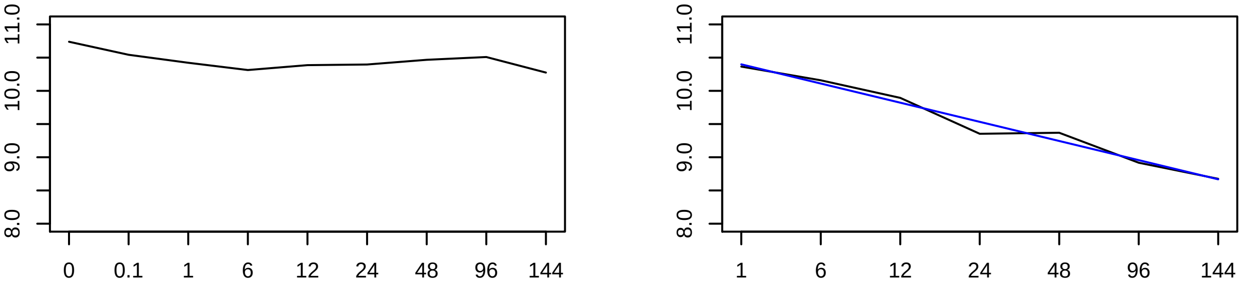

A_24_P171131 DERL1 NA

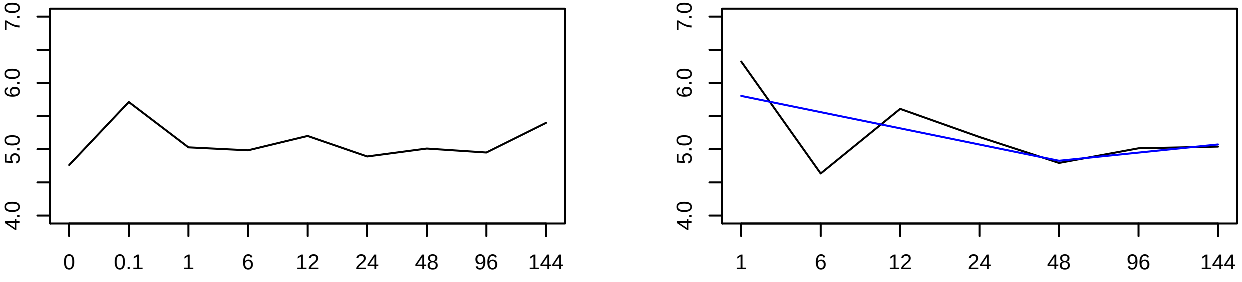

A_24_P917866 SET 9q34.11

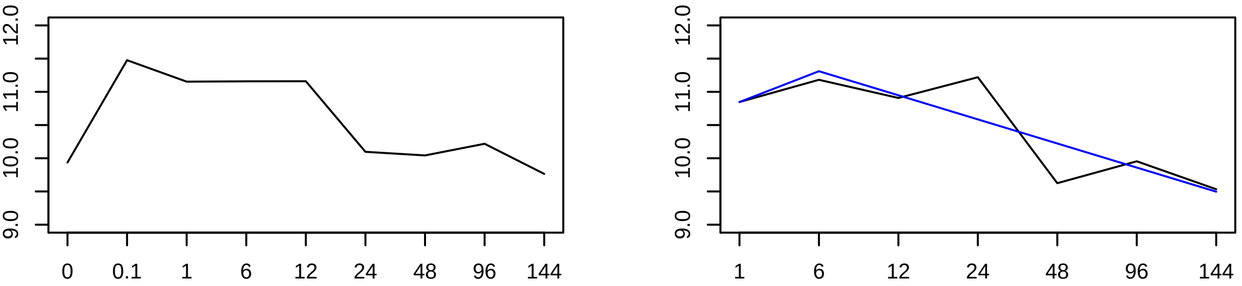

A_23_P361419 DEPDC1B 5q12.1

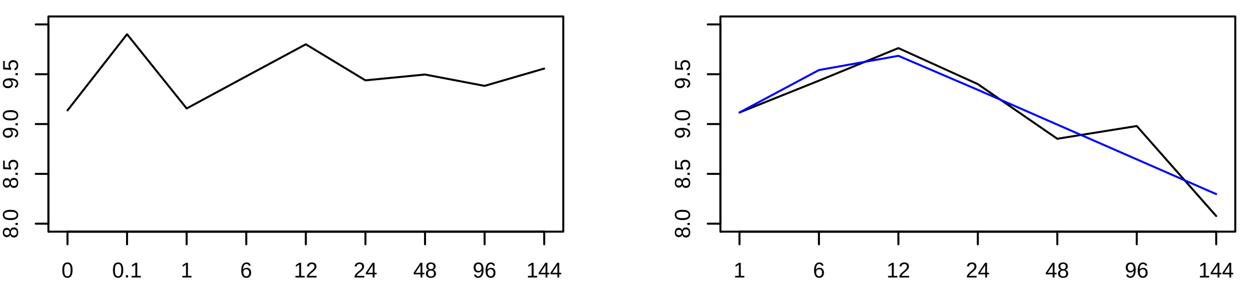

A_32_P81149 RPL14 3p22.1

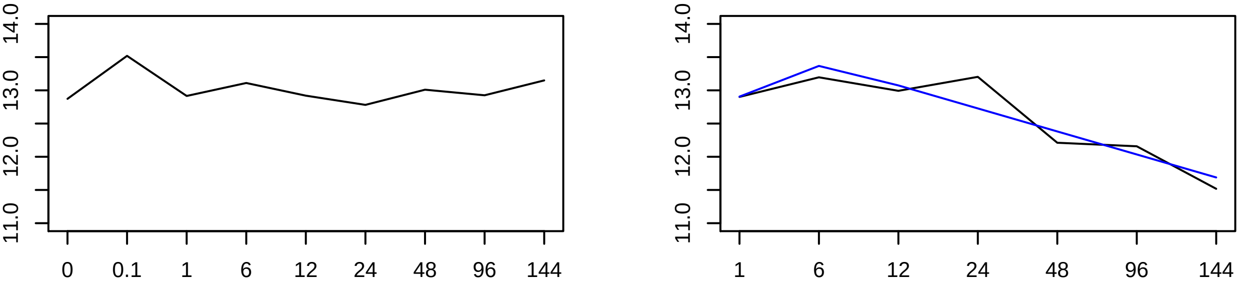

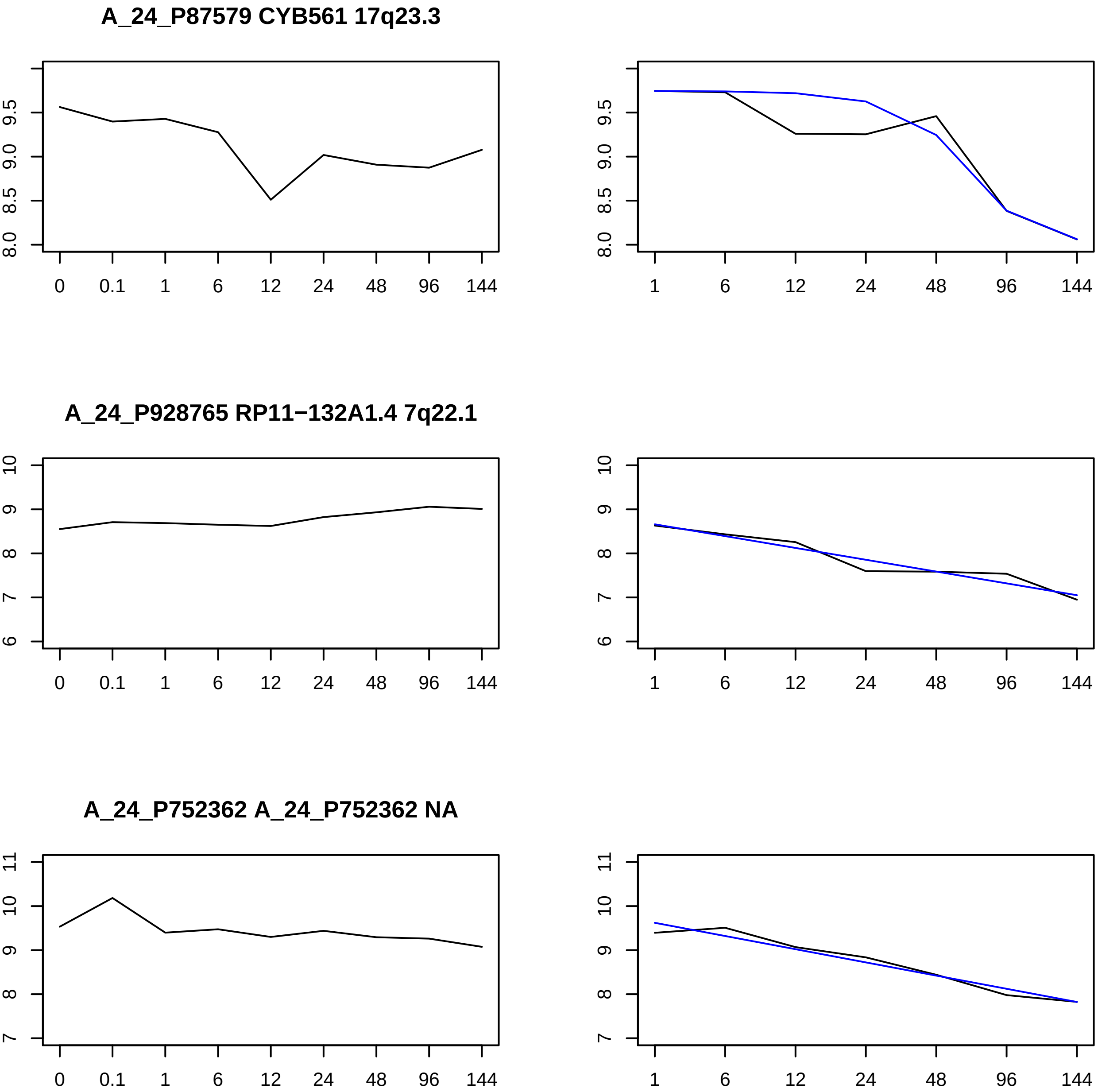
A_24_P87579 CYB561 17q23.3
8.0
8.5
9.0
9.5
0
0.1
1
6
12
24
48
96
144
8.0
8.5
9.0
9.5
1
6
12
24
48
96
144
A_24_P928765 RP11−132A1.4 7q22.1
6
7
8
9
10
0
0.1
1
6
12
24
48
96
144
6
7
8
9
10
1
6
12
24
48
96
144
A_24_P752362 A_24_P752362 NA
7
8
9
10
11
0
0.1
1
6
12
24
48
96
144
7
8
9
10
11
1
6
12
24
48
96
144

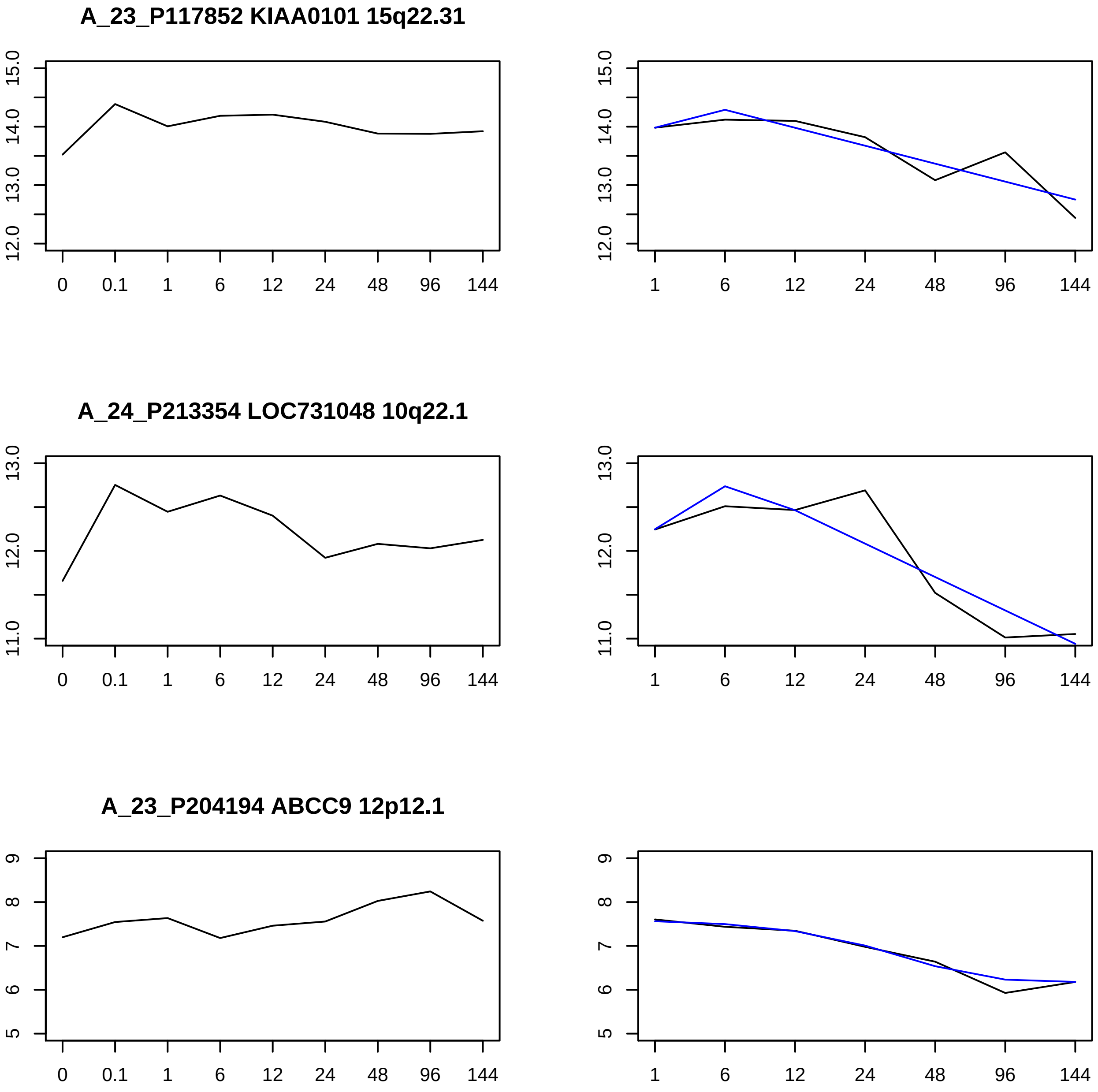
A_23_P117852 KIAA0101 15q22.31
12.0 13.0 14.0 15.0
0 0.1 1 6 12 24 48 96 144
1 6 12 24 48 96 144
A_24_P213354 LOC731048 10q22.1
11.0 12.0 13.0
0 0.1 1 6 12 24 48 96 144
1 6 12 24 48 96 144
A_23_P204194 ABCC9 12p12.1
5 6 7 8 9
0 0.1 1 6 12 24 48 96 144
1 6 12 24 48 96 144

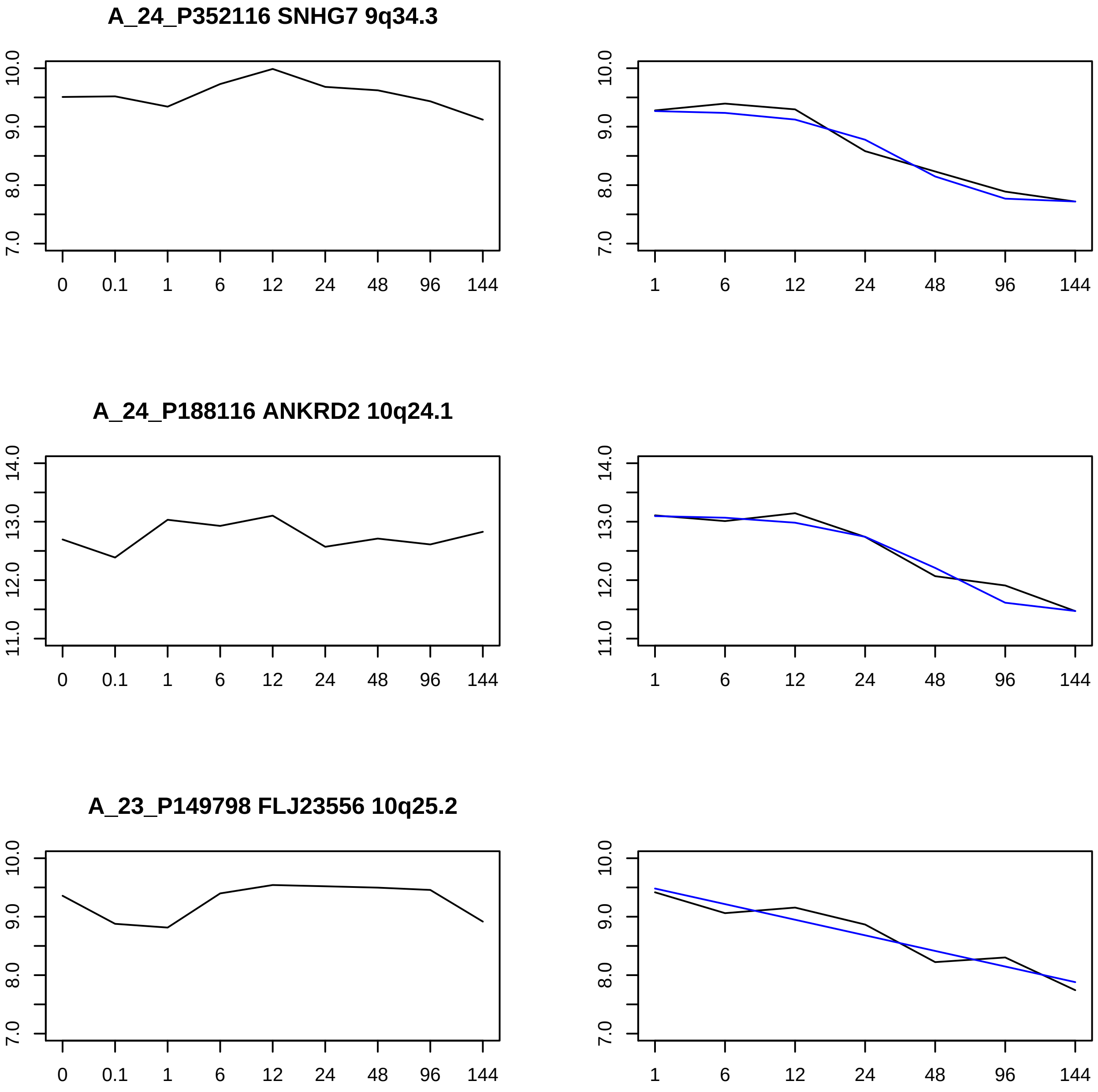
A_24_P352116 SNHG7 9q34.3
A_24_P188116 ANKRD2 10q24.1
A_23_P149798 FLJ23556 10q25.2
7.0 8.0 9.0 10.0
11.0 12.0 13.0 14.0
0 0.1 1 6 12 24 48 96 144
1 6 12 24 48 96 144

A_23_P93940 PGAM2 7p13

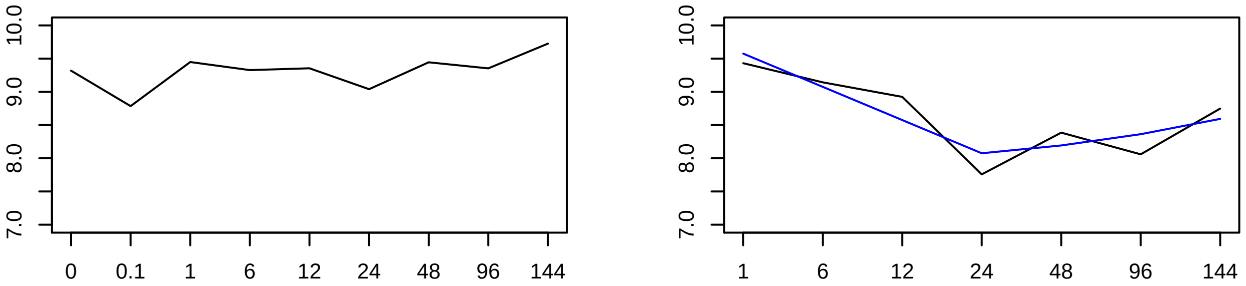

A_24_P383459 RNF157 17q25.1

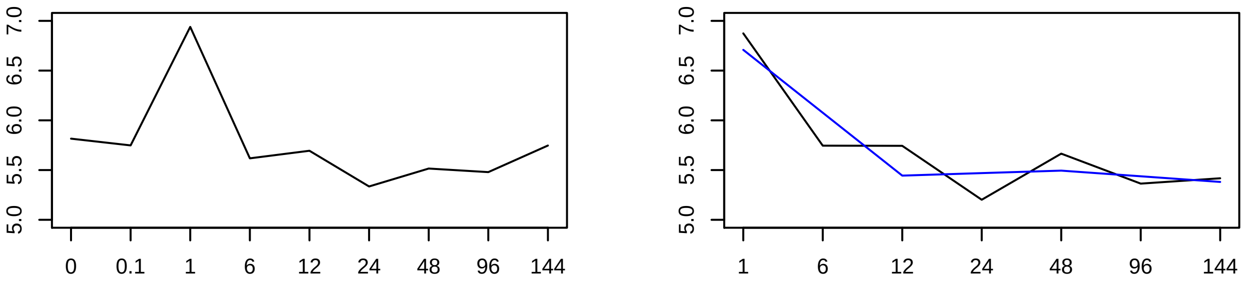

A_23_P149064 PTPRU 1p35.3

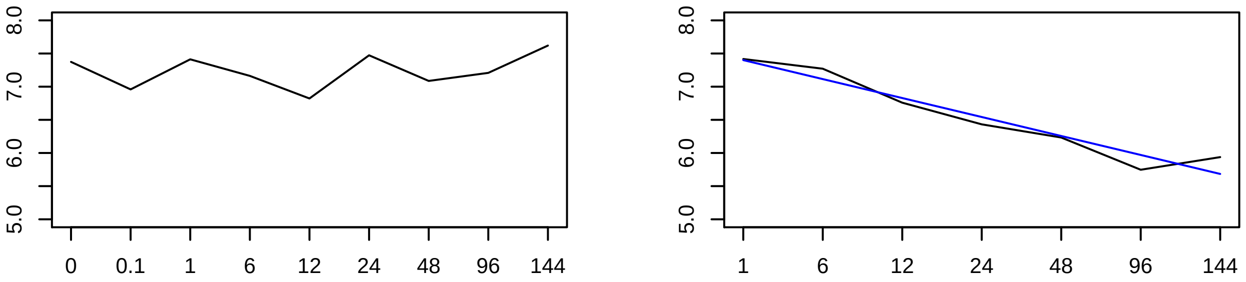

A_23_P66481 RTN4RL1 17p13.3

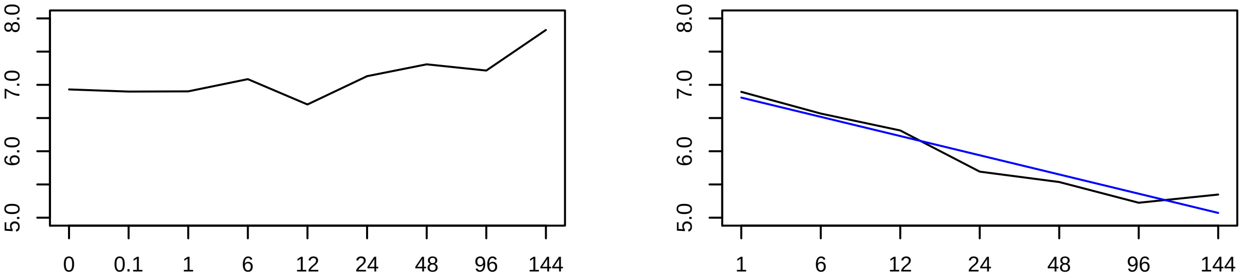

A_23_P400378 GPBAR1 2q35

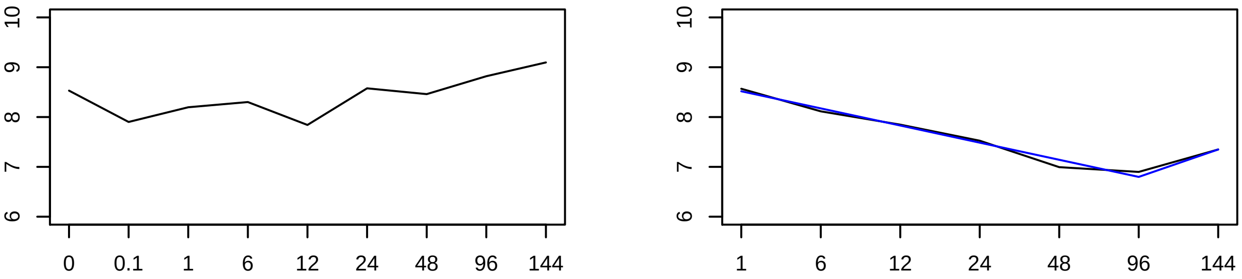

A_32_P498287 SLC7A14 3q26.2

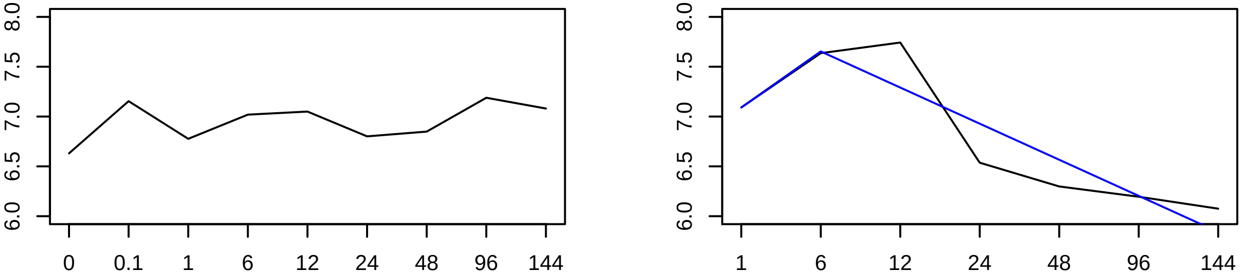

A_23_P40657 GCAT 22q13.1

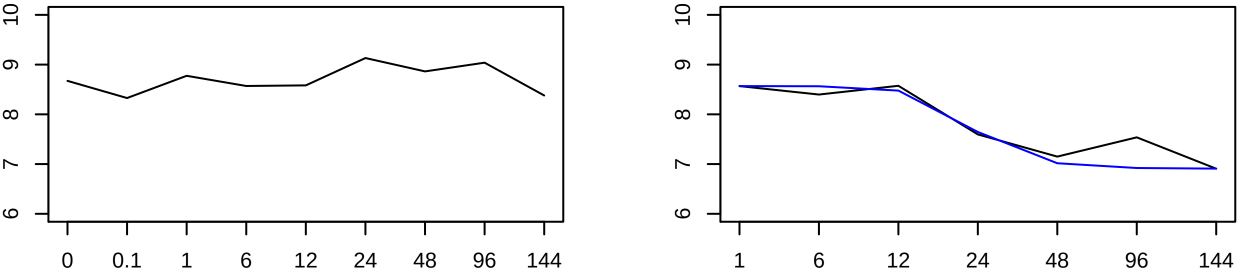

A_23_P16252 KLK1 19q13.33

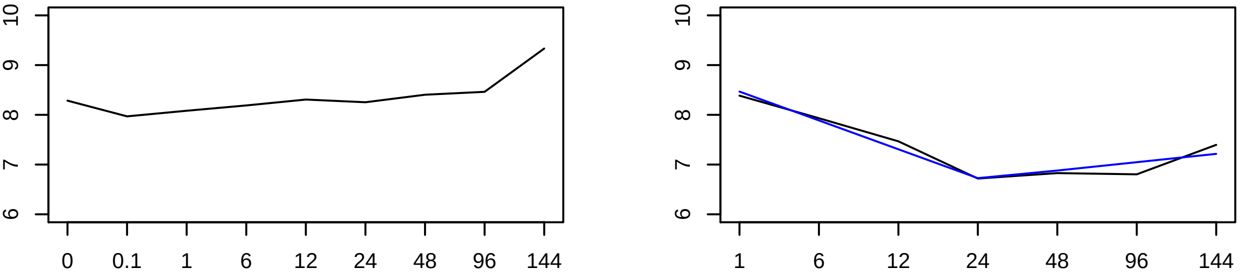

A_23_P413641 PREX1 20q13.13

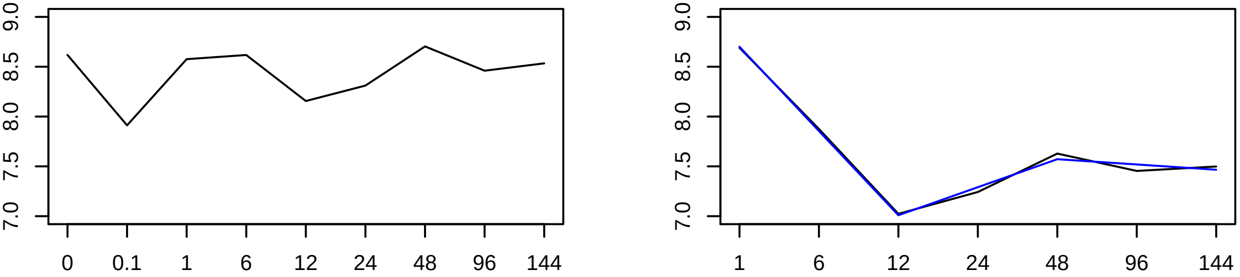

A_23_P317591 SEMA3A 7q21.11

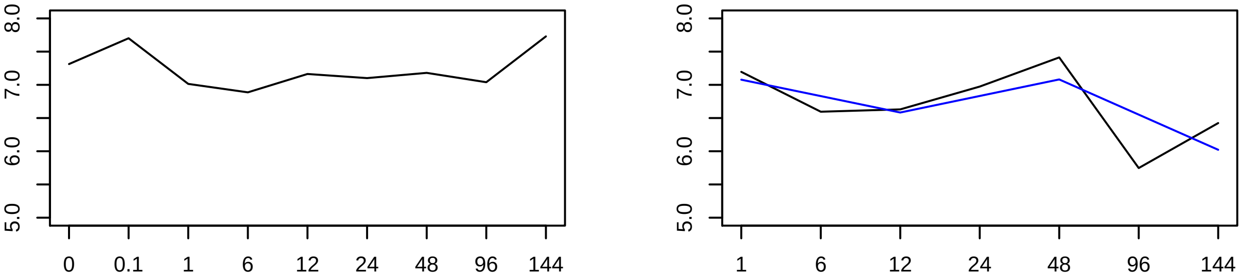

A_23_P77612 KREMEN2 16p13.3

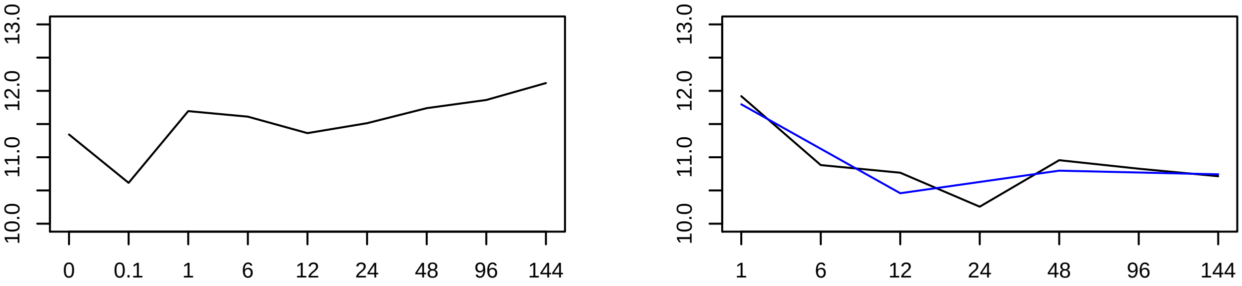

A_24_P34611 SIX3 2p21

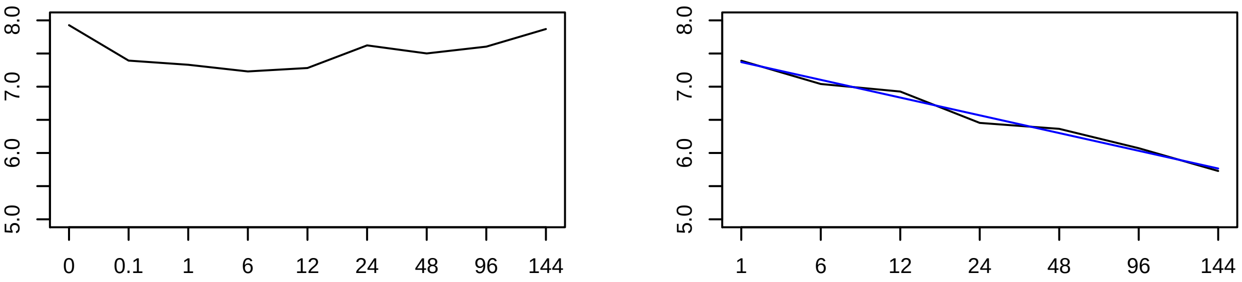

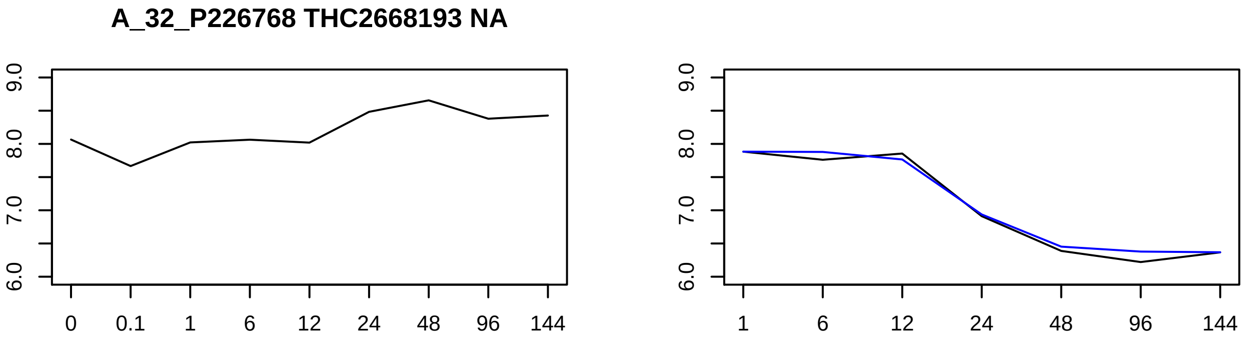
A_32_P226768 THC2668193 NA
6.0 7.0 8.0 9.0
0 0.1 1 6 12 24 48 96 144
1 6 12 24 48 96 144

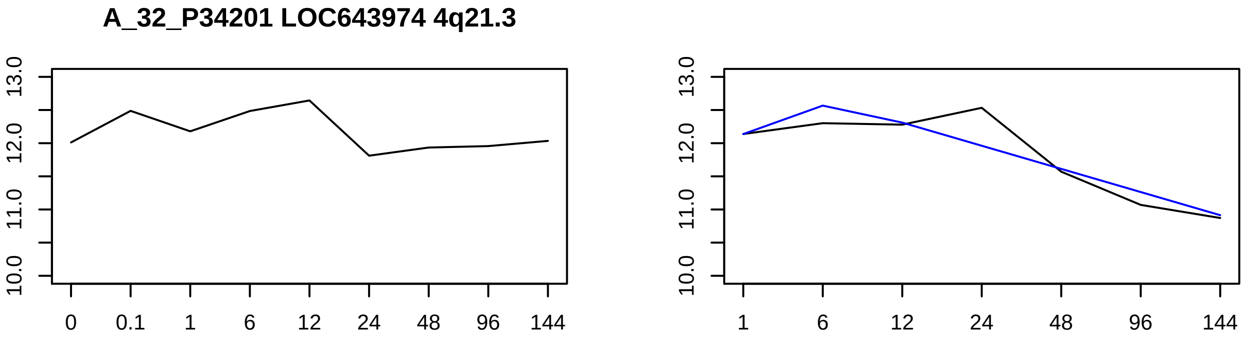
A_32_P34201 LOC643974 4q21.3
10.0 11.0 12.0 13.0
0 0.1 1 6 12 24 48 96 144
1 6 12 24 48 96 144

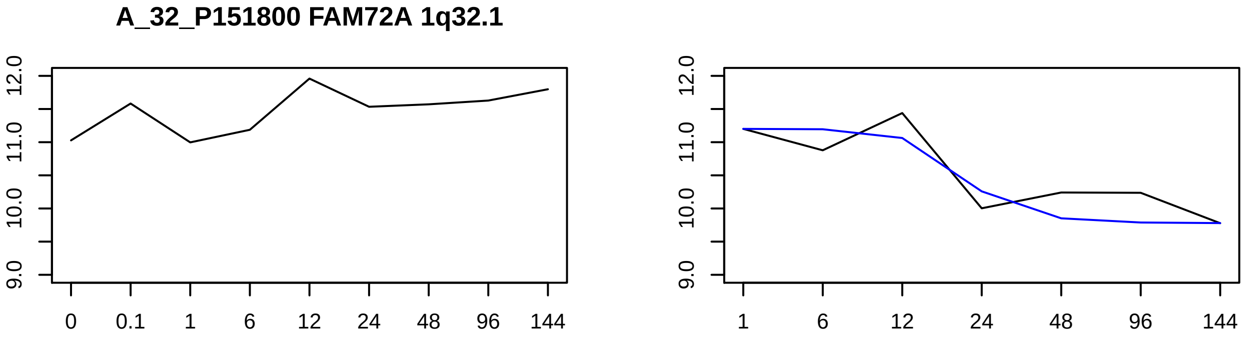
A_32_P151800 FAM72A 1q32.1
9.0 10.0 11.0 12.0
0 0.1 1 6 12 24 48 96 144
1 6 12 24 48 96 144

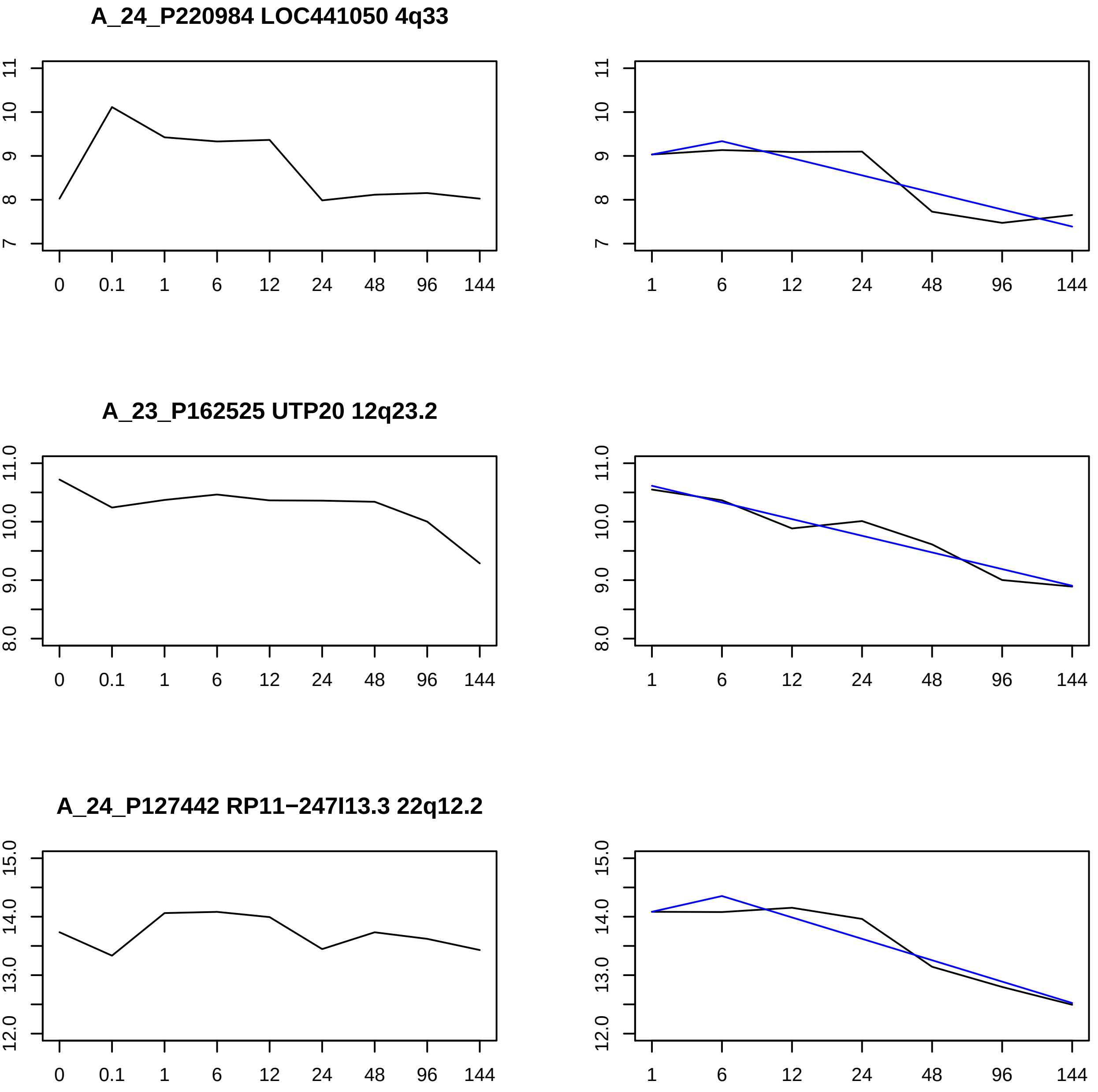
A_24_P220984 LOC441050 4q33
A_23_P162525 UTP20 12q23.2
A_24_P127442 RP11−247I13.3 22q12.2
0 0.1 1 6 12 24 48 96 144
1 6 12 24 48 96 144
7 8 9 10 11
8.0 9.0 10.0 11.0
12.0 13.0 14.0 15.0

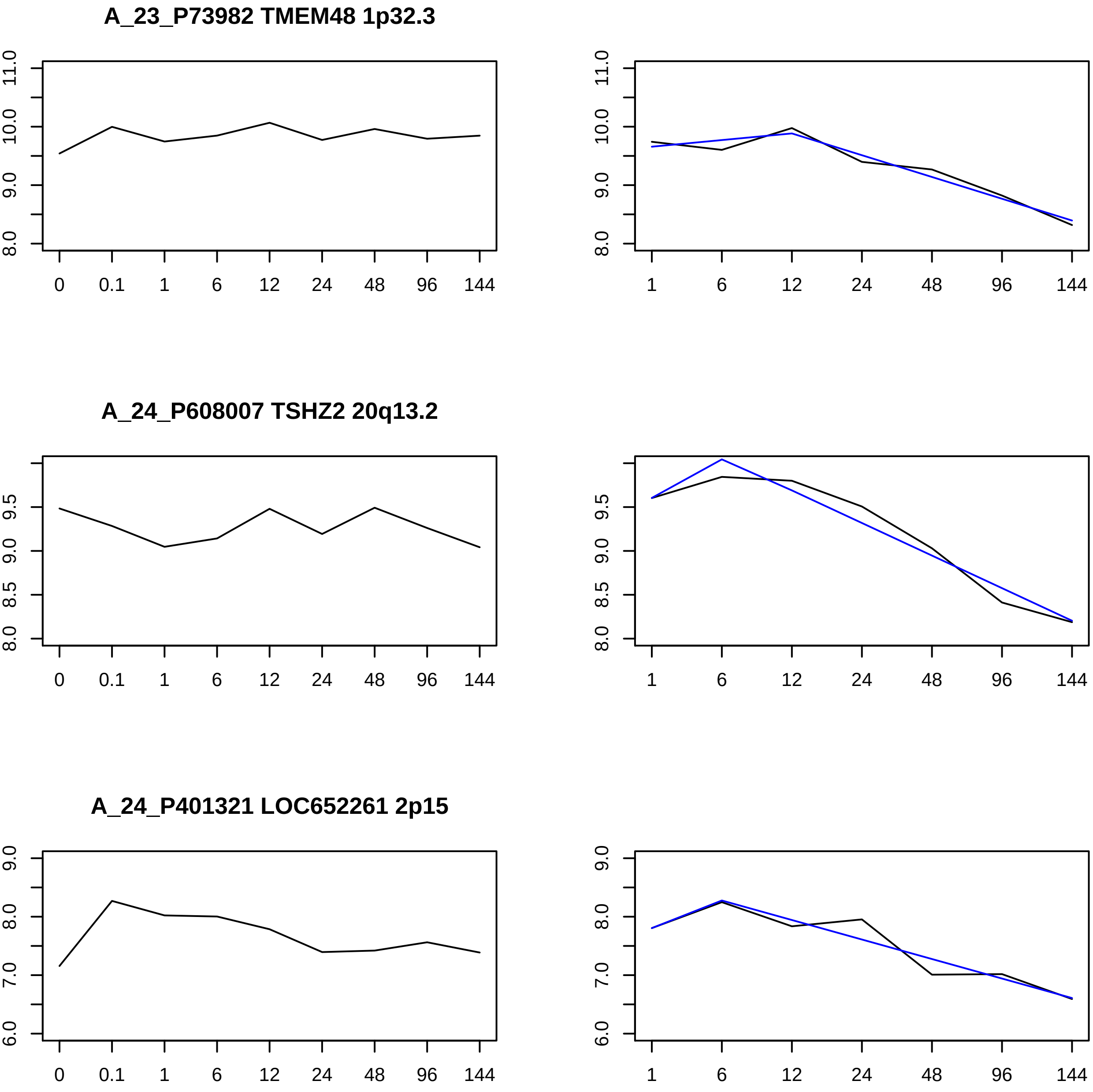
A_23_P73982 TMEM48 1p32.3
A_24_P608007 TSHZ2 20q13.2
A_24_P401321 LOC652261 2p15
0 0.1 1 6 12 24 48 96 144
1 6 12 24 48 96 144
8.0 9.0 10.0 11.0
8.0 8.5 9.0 9.5
6.0 7.0 8.0 9.0

A_24_P392947 A_24_P392947 NA

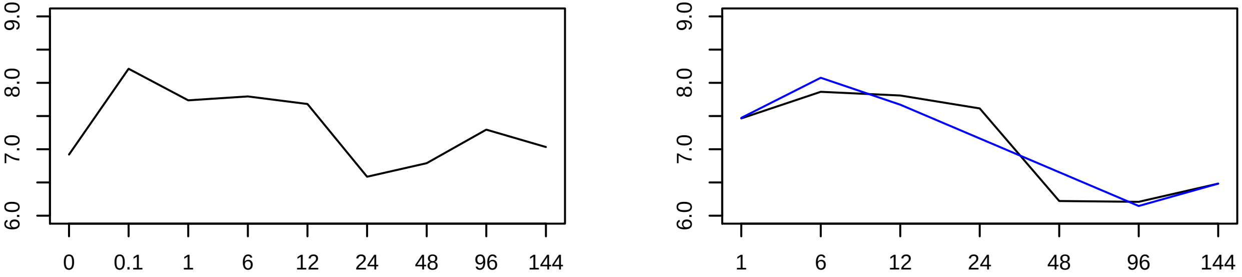

A_24_P246863 LOC729701 15q22.2

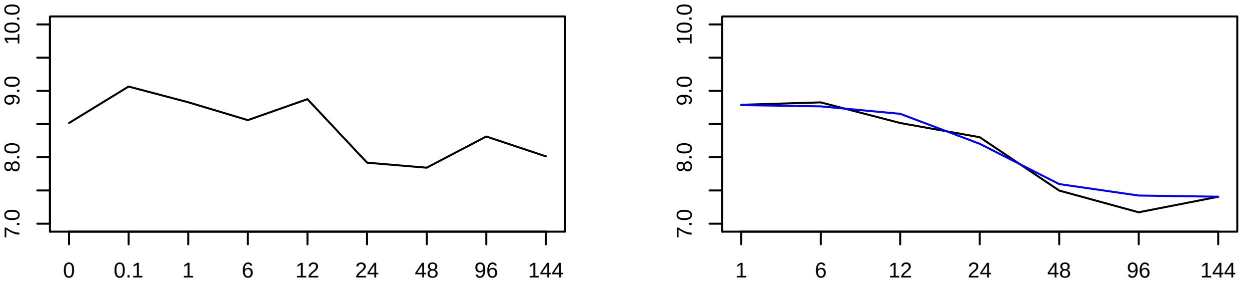

A_23_P94579 SNHG7 9q34.3

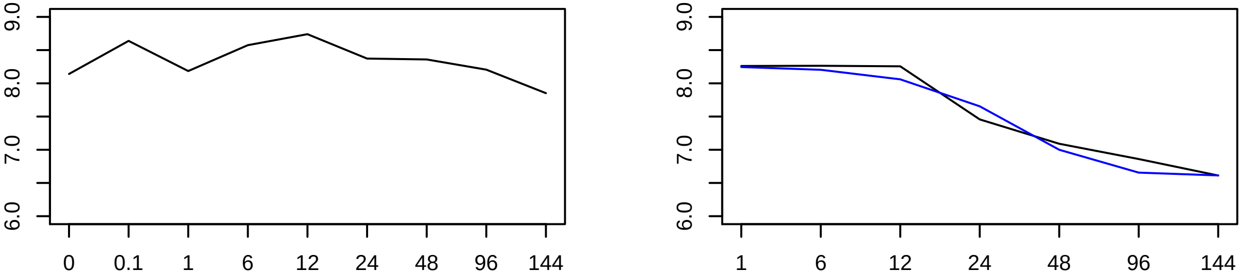

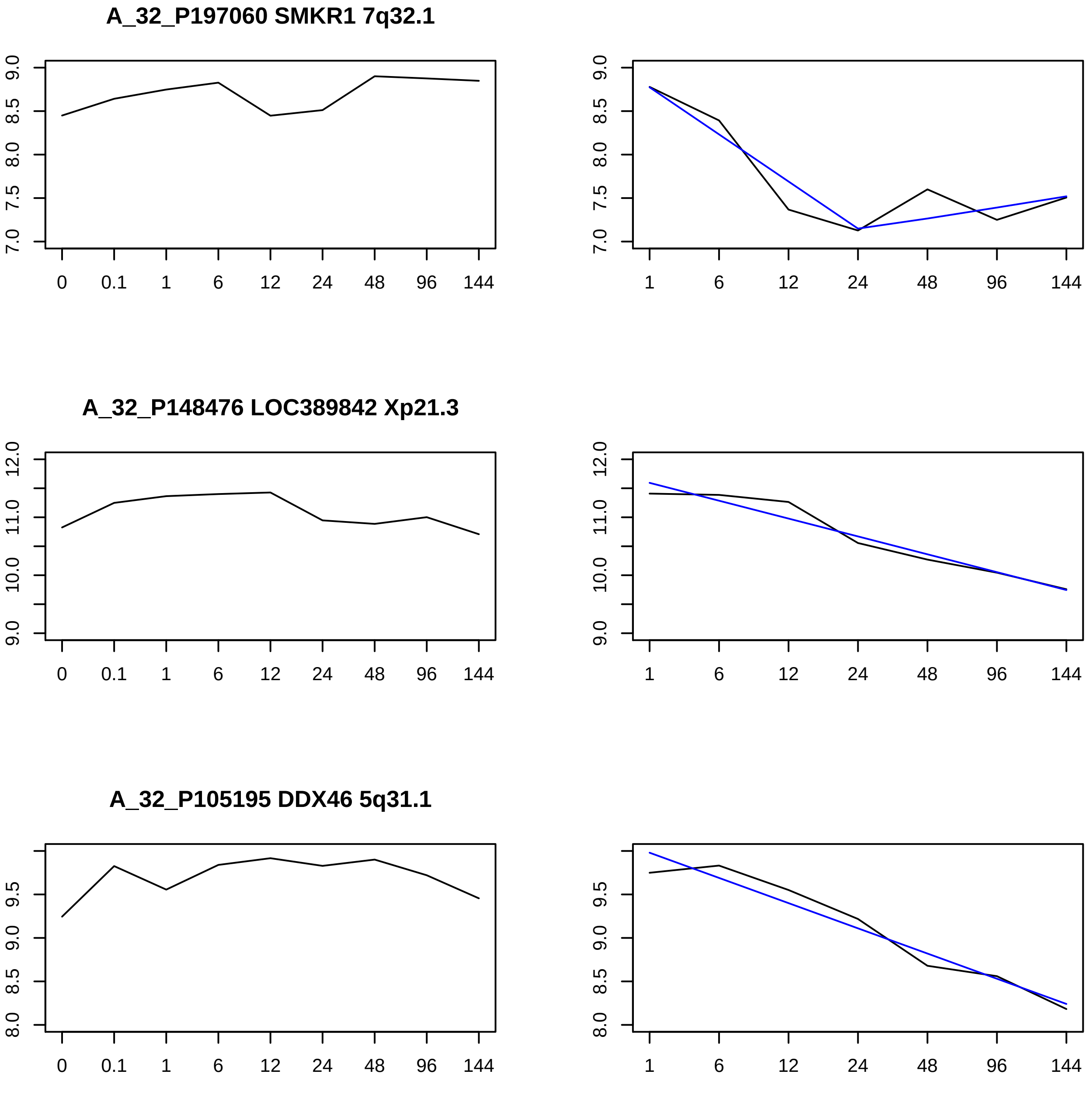
A_32_P197060 SMKR1 7q32.1
7.0
7.5
8.0
8.5
9.0
0
0.1
1
6
12
24
48
96
144
1
6
12
24
48
96
144
A_32_P148476 LOC389842 Xp21.3
9.0
10.0
11.0
12.0
A_32_P105195 DDX46 5q31.1
8.0
8.5
9.0
9.5

A_24_P76358 LOC643981 10q23.33

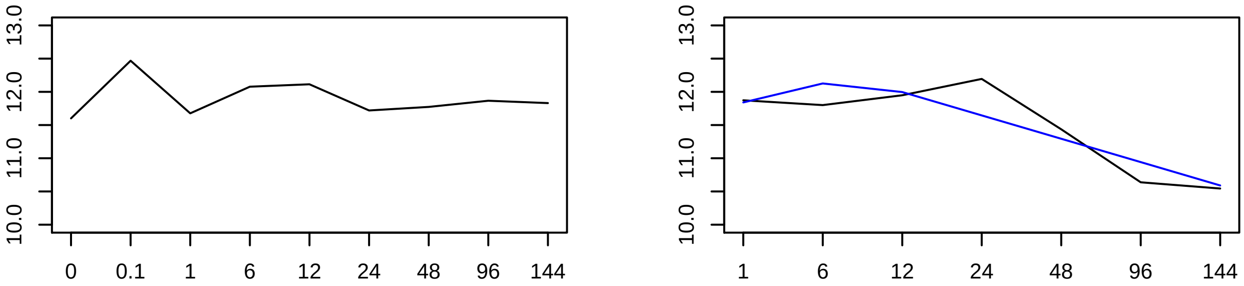

A_23_P137797 RYR2 1q43

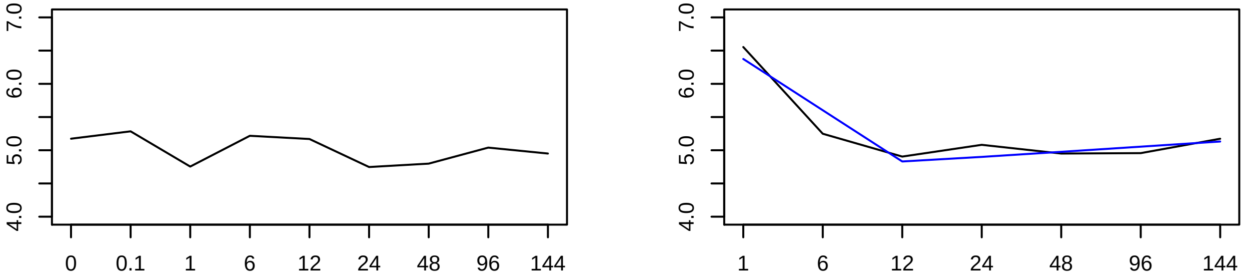

A_24_P464798 A_24_P464798 NA

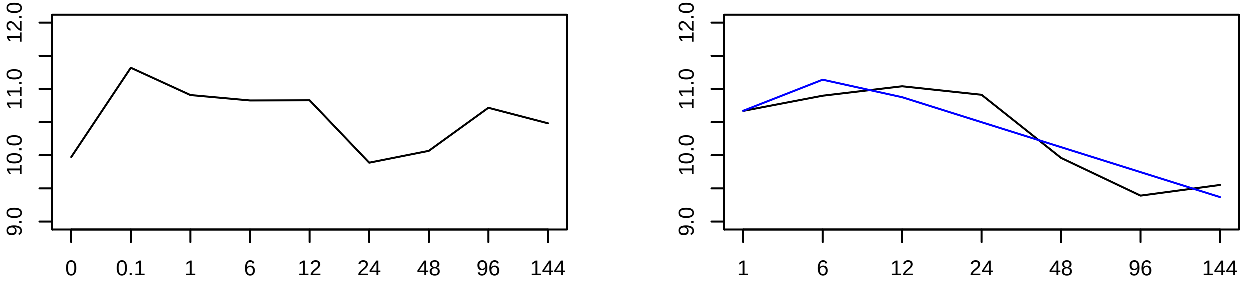

A_23_P257562 SORBS2 4q35.1

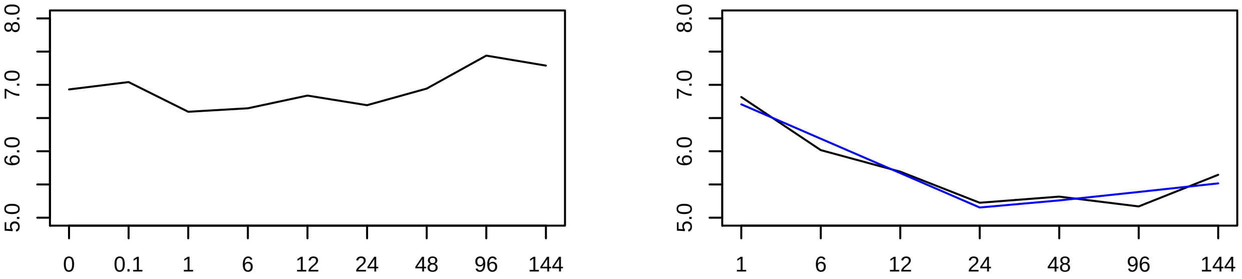

A_24_P392082 ENST00000361800 NA

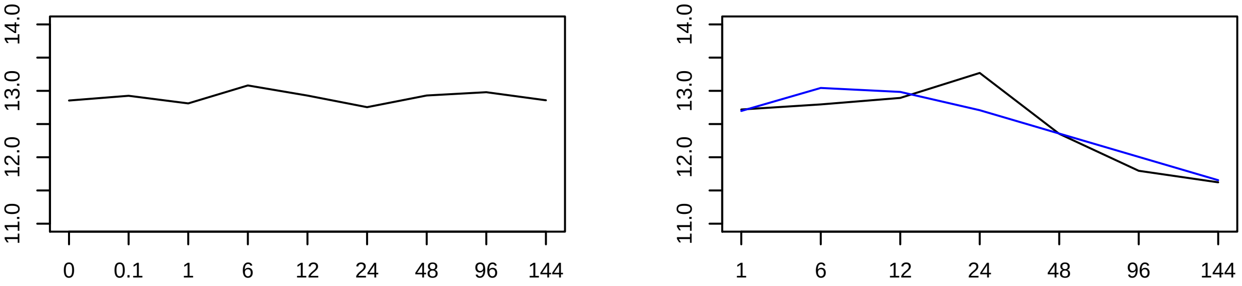

A_23_P150667 KIF18A 11p14.1

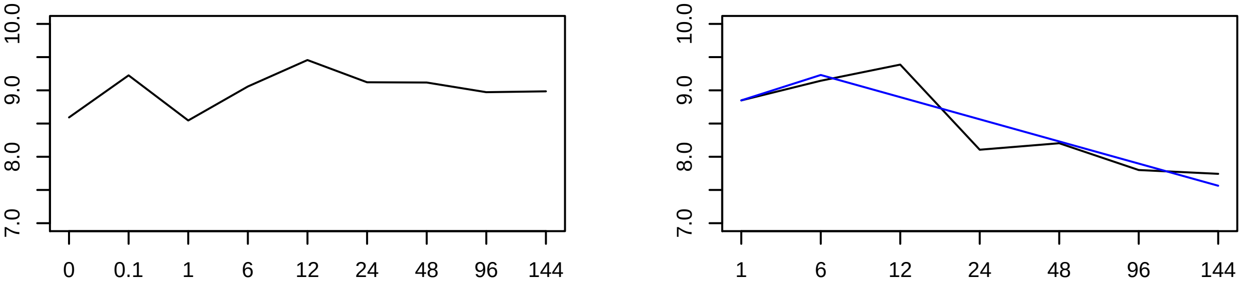

A_24_P45728 CGN 1q21.3

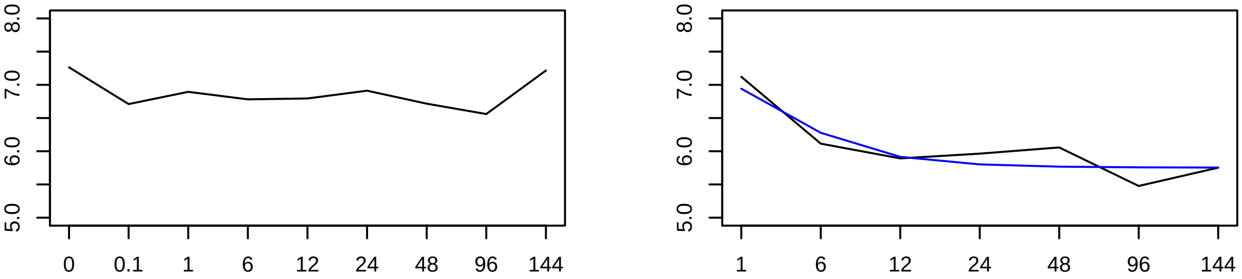

A_24_P333306 AK023737 NA

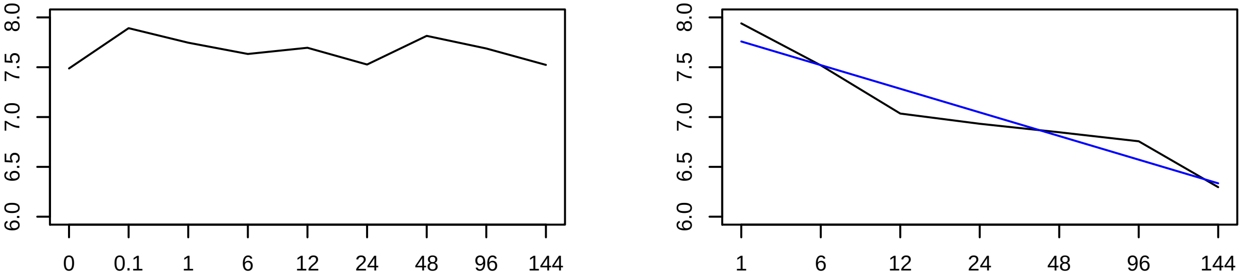

A_32_P48506 NAALADL2−AS3 3q26.31

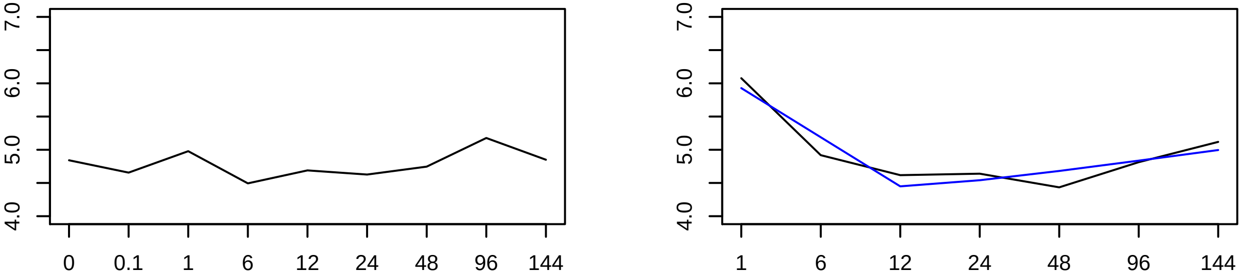

A_32_P113736 THC2693923 NA

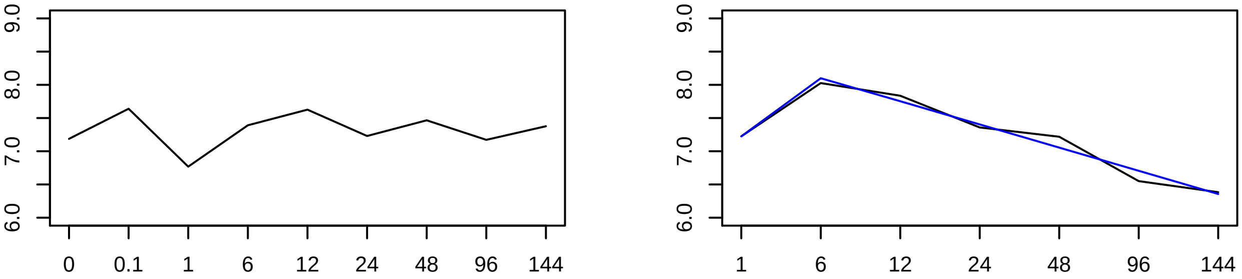

A_24_P366465 A_24_P366465 NA

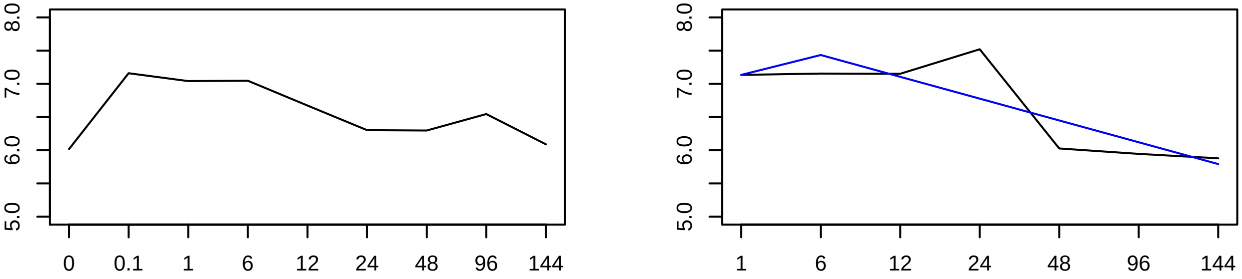

A_24_P38944 CCDC86 11q12.2

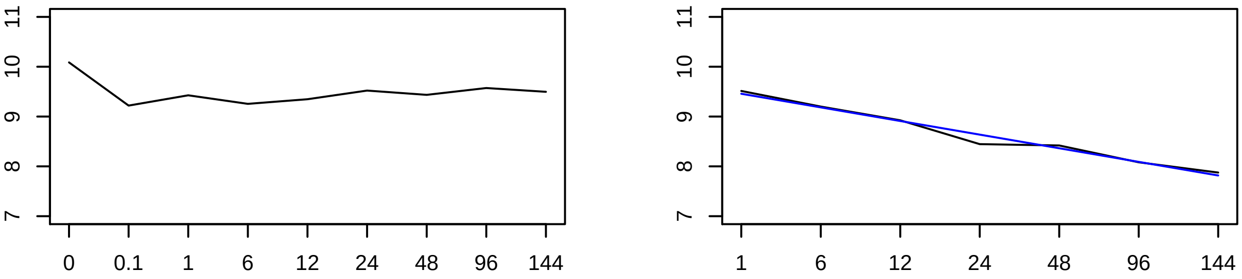

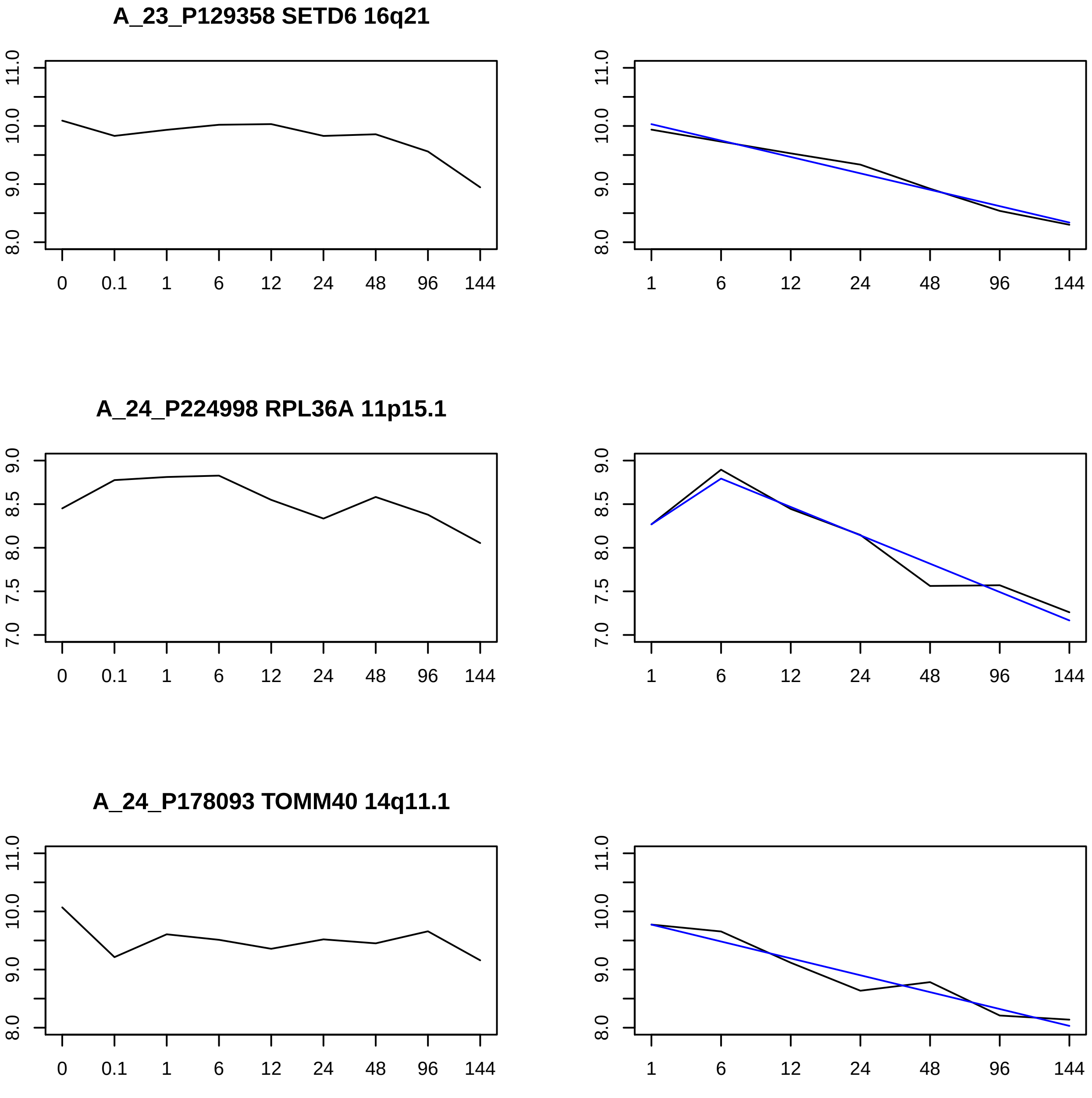
A_23_P129358 SETD6 16q21
A_24_P224998 RPL36A 11p15.1
A_24_P178093 TOMM40 14q11.1
8.0
9.0
10.0
11.0
7.0
7.5
8.0
8.5
9.0
0
0.1
1
6
12
24
48
96
144

A_23_P170337 ALDH4A1 1p36.13

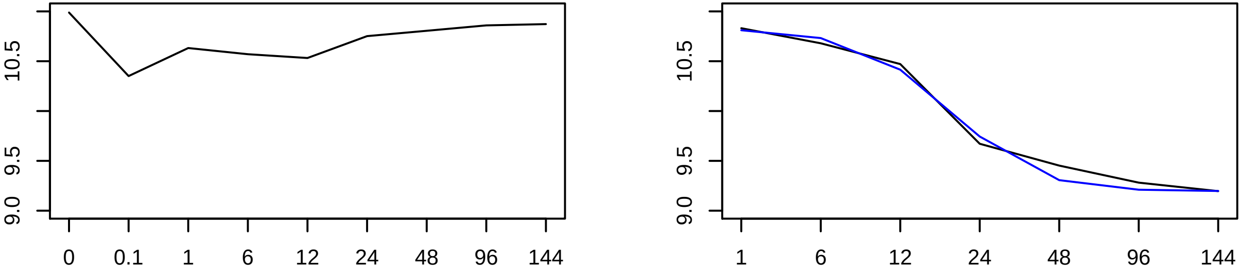

A_24_P856794 AW576858 NA

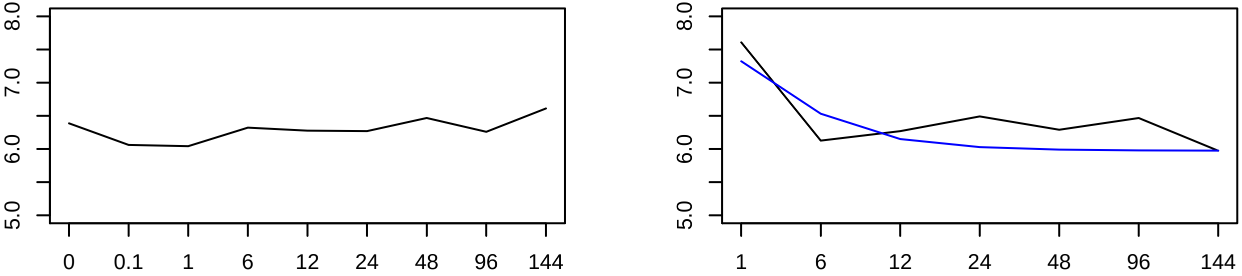

A_23_P203439 KCNC1 11p15.1

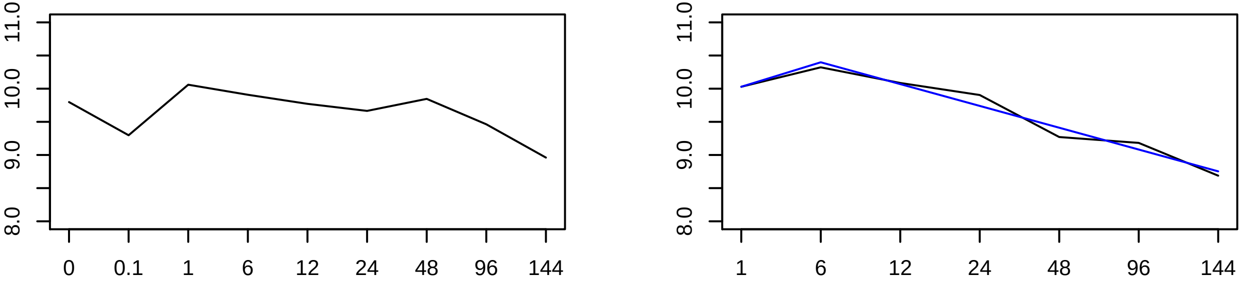

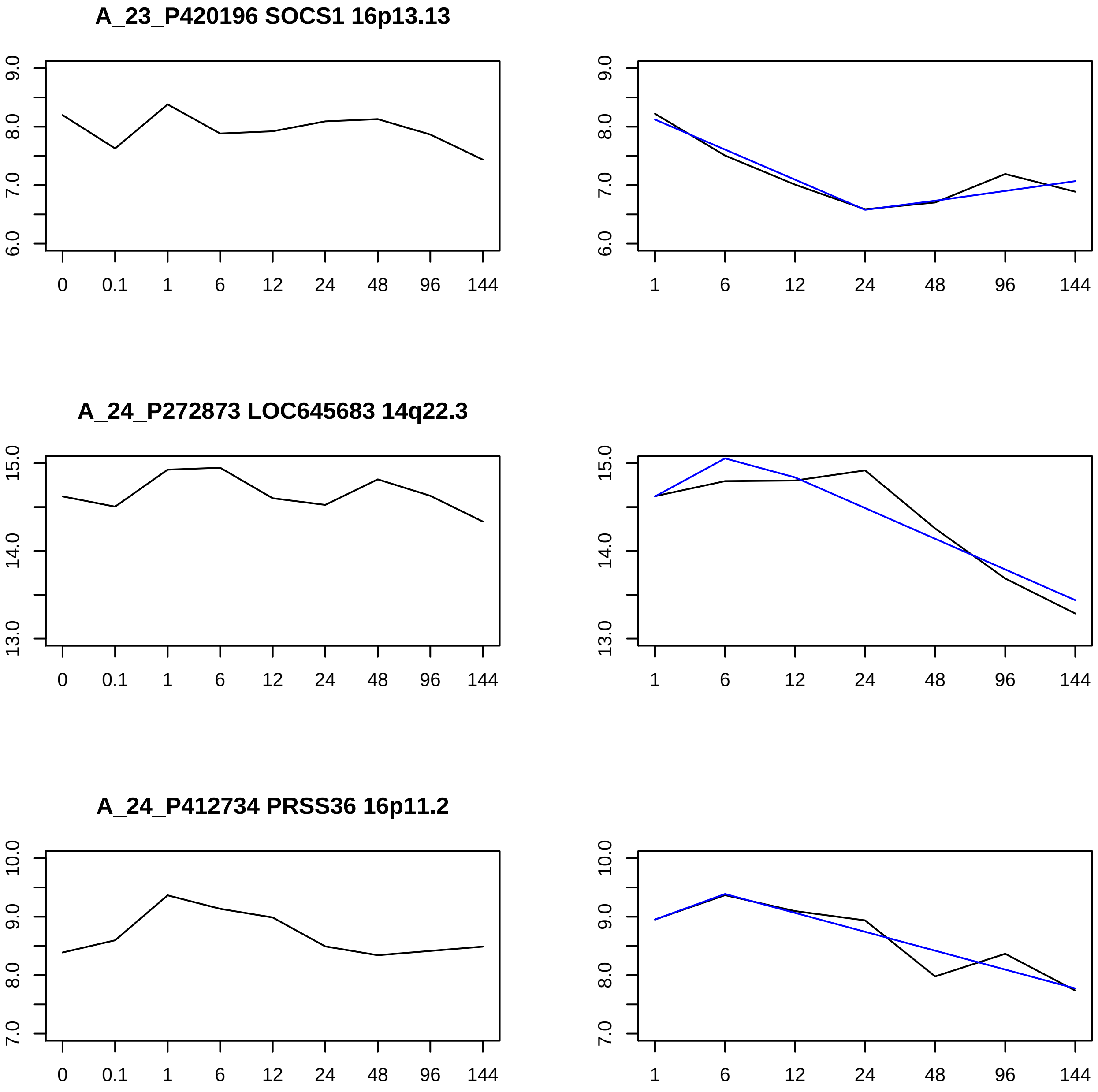
A_23_P420196 SOCS1 16p13.13
6.0
7.0
8.0
9.0
0
0.1
1
6
12
24
48
96
144
1
6
12
24
48
96
144
A_24_P272873 LOC645683 14q22.3
13.0
14.0
15.0
A_24_P412734 PRSS36 16p11.2
7.0
8.0
9.0
10.0

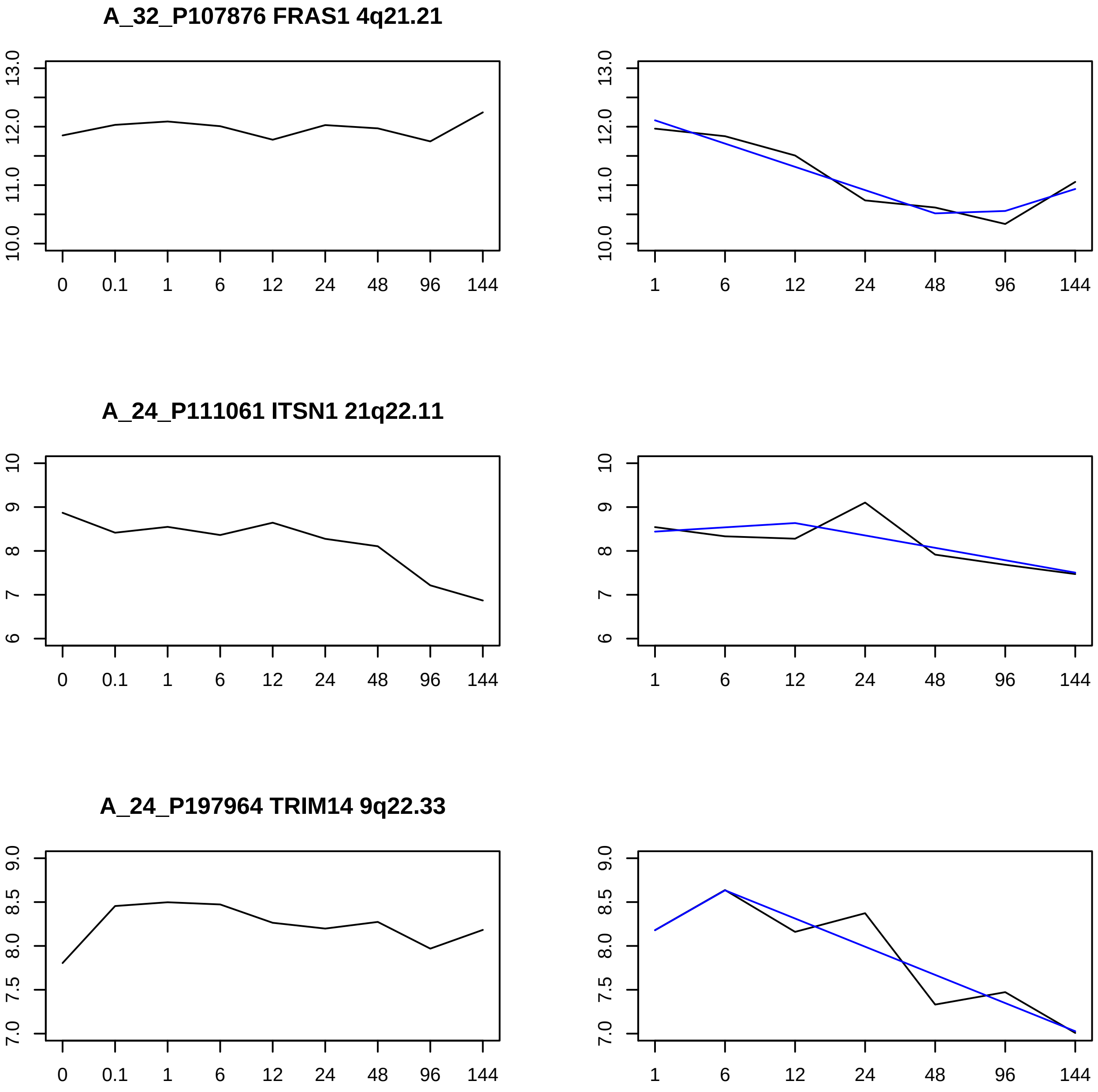
A_32_P107876 FRAS1 4q21.21
10.0
11.0
12.0
13.0
0
0.1
1
6
12
24
48
96
144
A_24_P111061 ITSN1 21q22.11
6
7
8
9
10
A_24_P197964 TRIM14 9q22.33
7.0
7.5
8.0
8.5
9.0

A_24_P135771 RPL35AP30 12q24.23

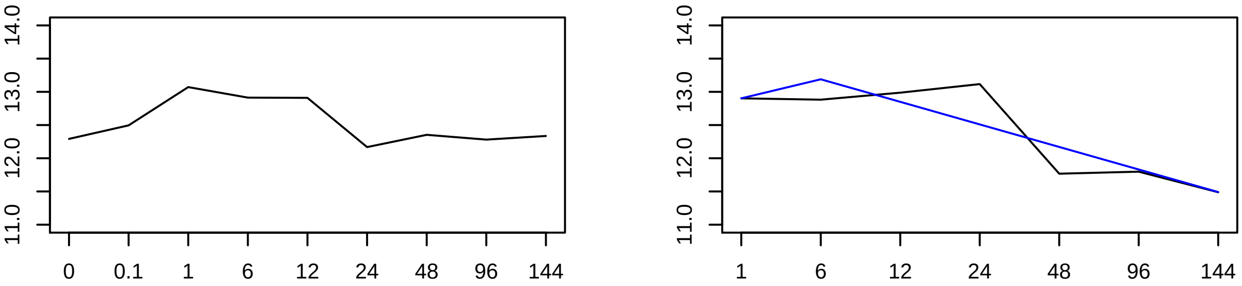

A_24_P264192 SRRM1 1p36.11

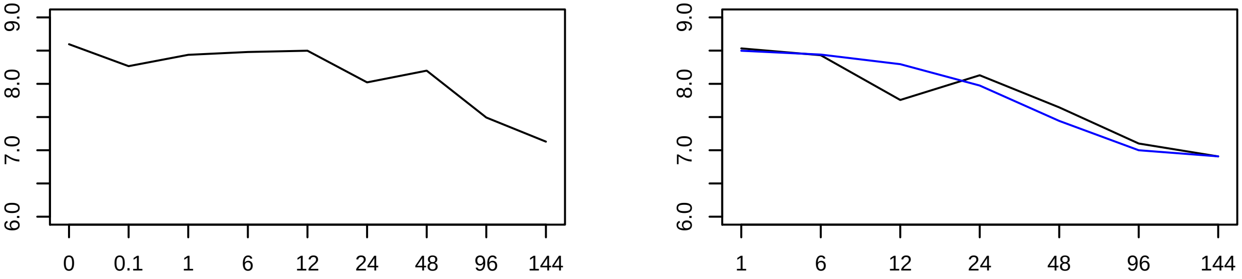

A_24_P943129 CDC20B 5q11.2

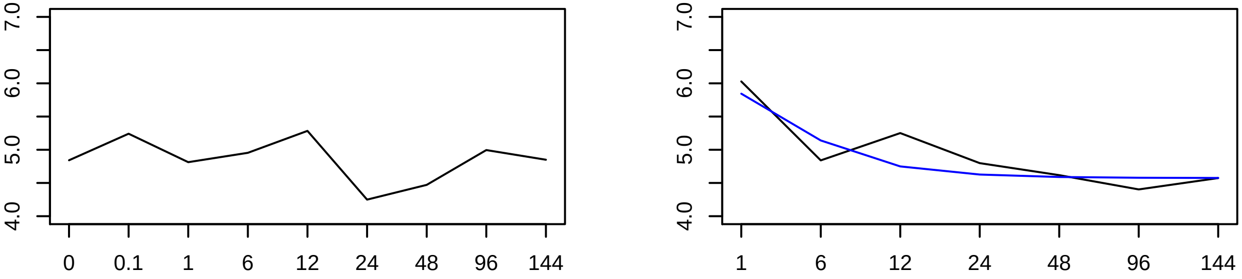

A_23_P92569 WWC2 4q35.1

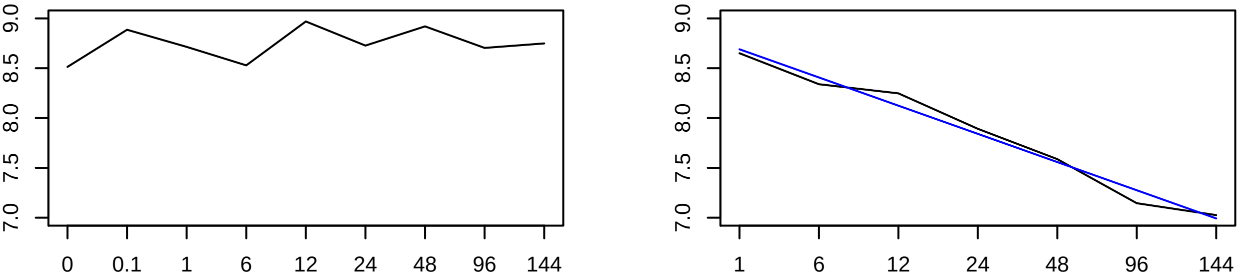

A_23_P317056 ND6 NA

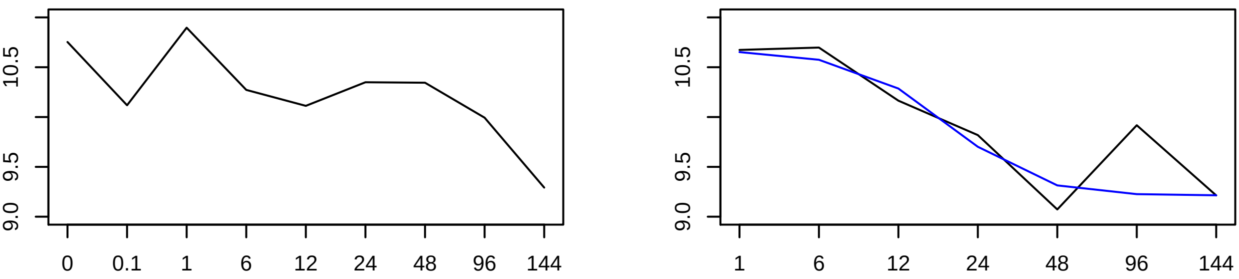

A_32_P168561 THC2634862 NA

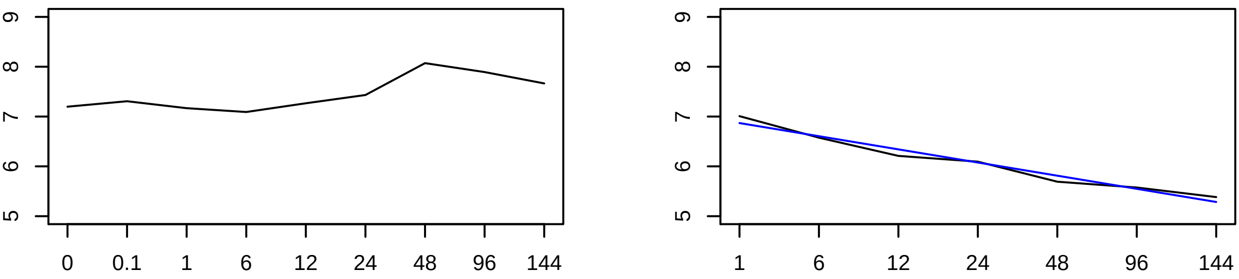

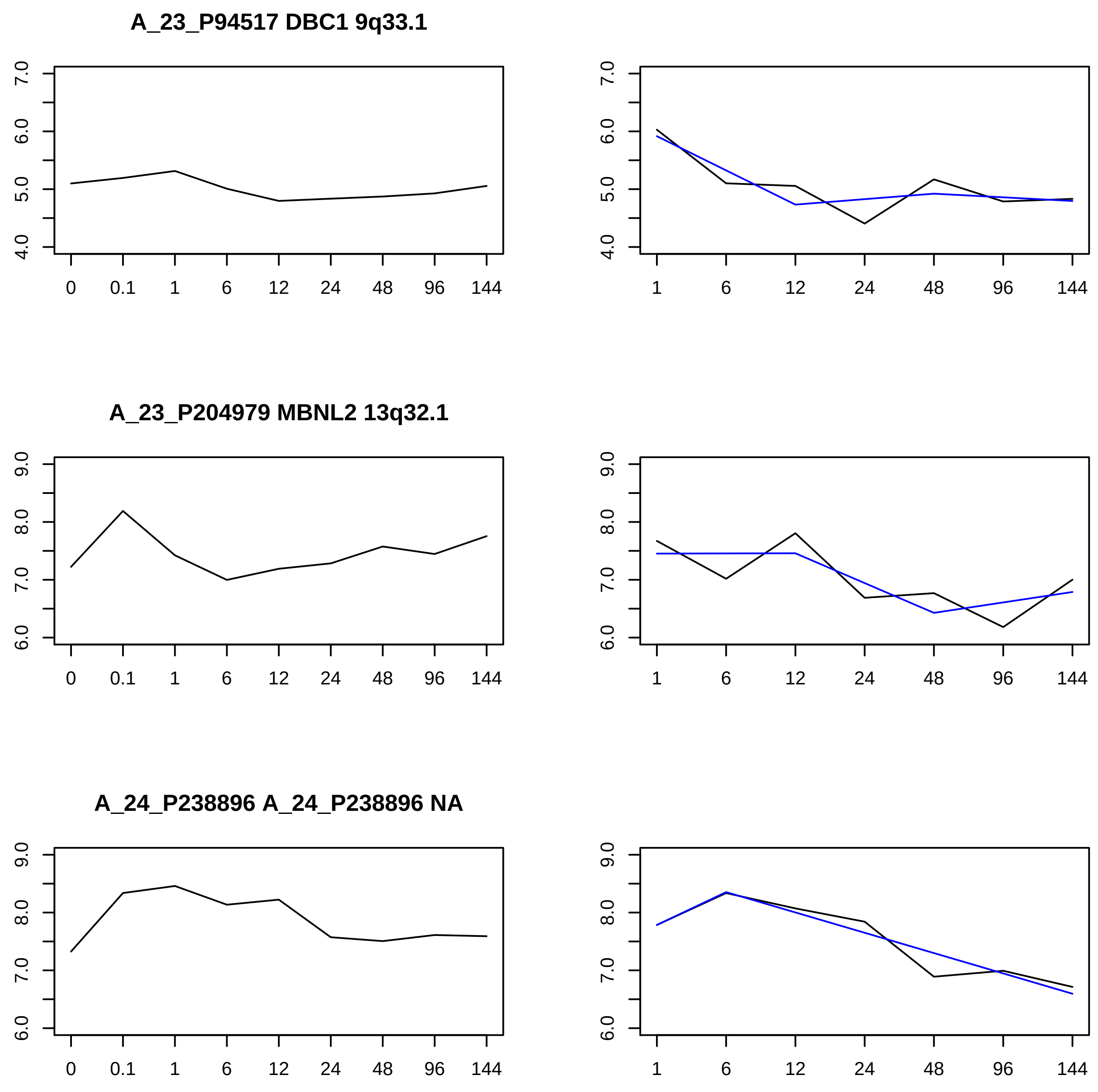

A_23_P94517 DBC1 9q33.1
A_23_P204979 MBNL2 13q32.1
A_24_P238896 A_24_P238896 NA
4.0 5.0 6.0 7.0
6.0 7.0 8.0 9.0
0 0.1 1 6 12 24 48 96 144
1 6 12 24 48 96 144

A_23_P107507 CBX1 17q21.32

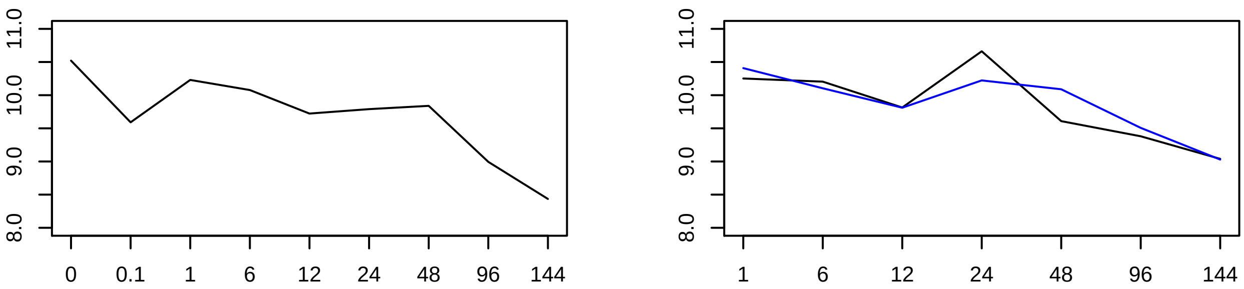

A_23_P41987 GFRA3 5q31.2

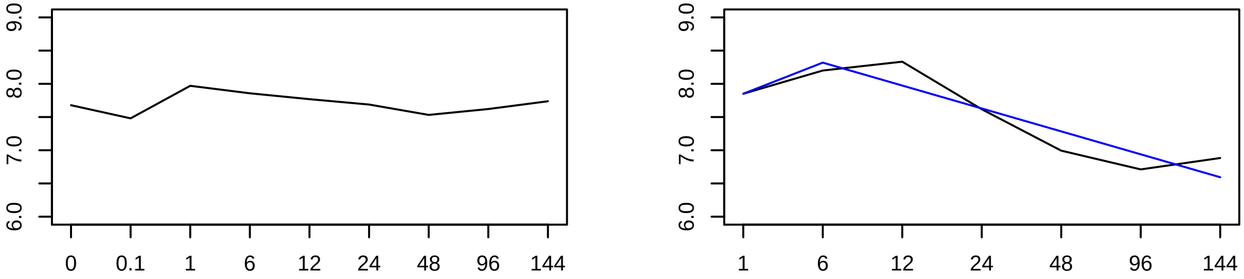

A_24_P265135 UBE3C 7q36.3

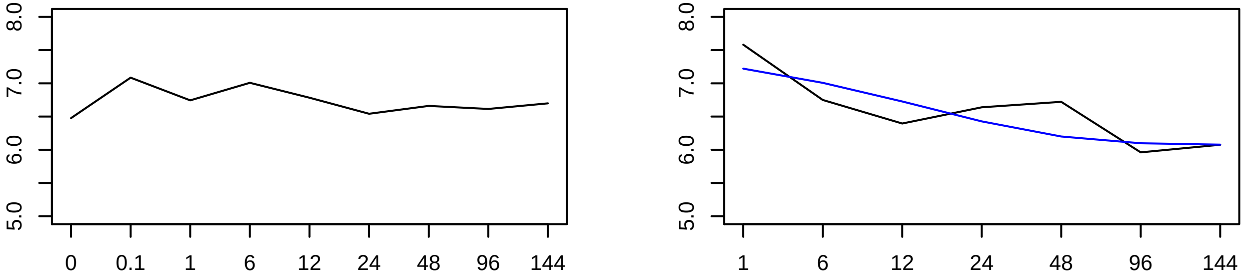

A_24_P418786 RANP8 13q12.11

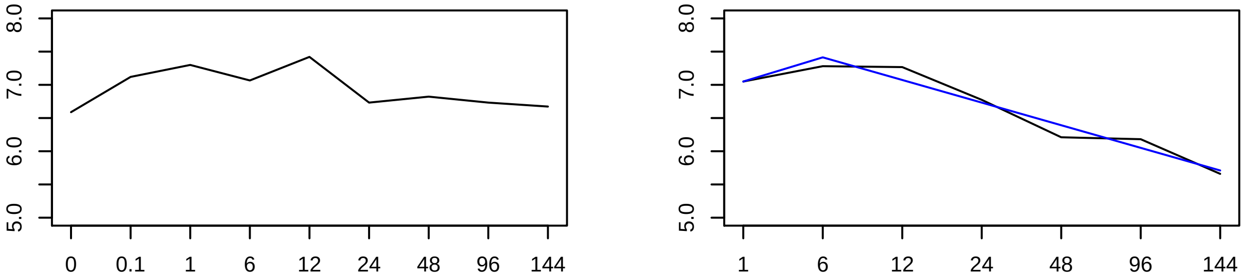

A_32_P157228 AHCTF1 1q44

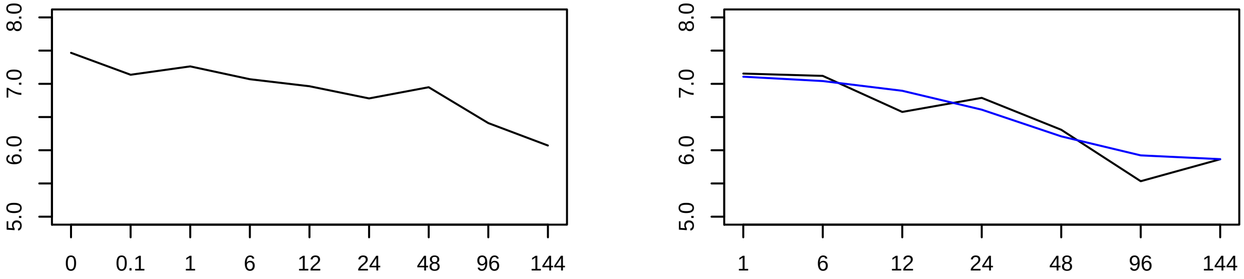

A_32_P61936 ENST00000374860 NA

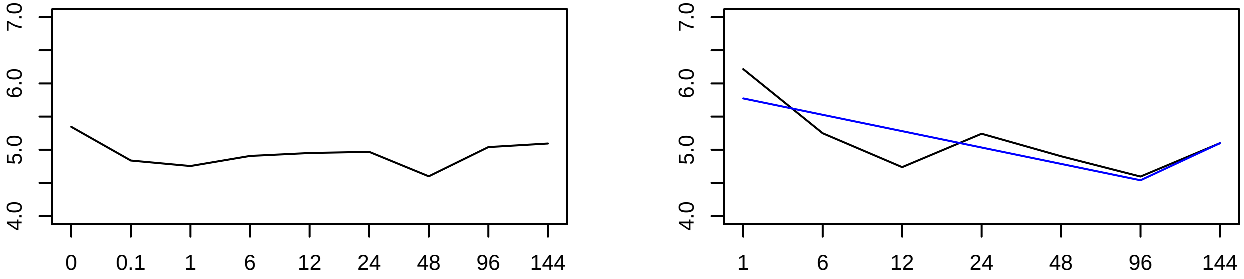

A_24_P84698 LOC124865 17q21.33

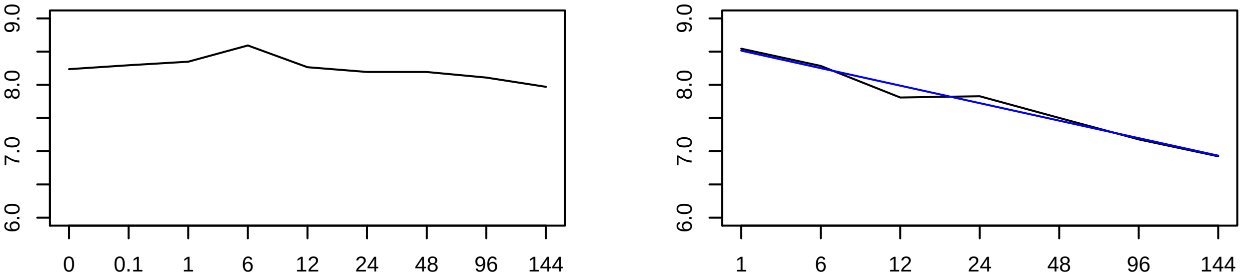

A_24_P333106 ENST00000332696 NA

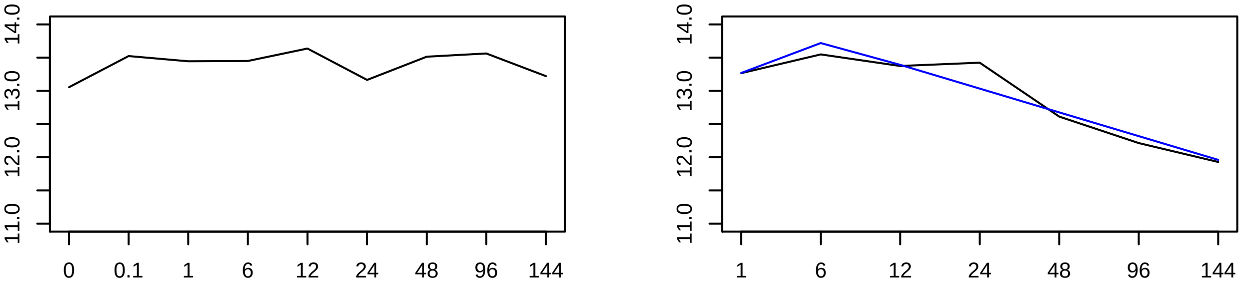

A_24_P127661 A_24_P127661 NA

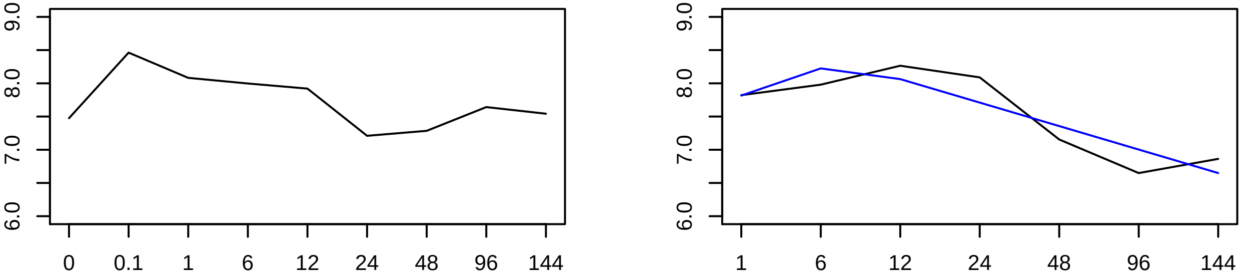

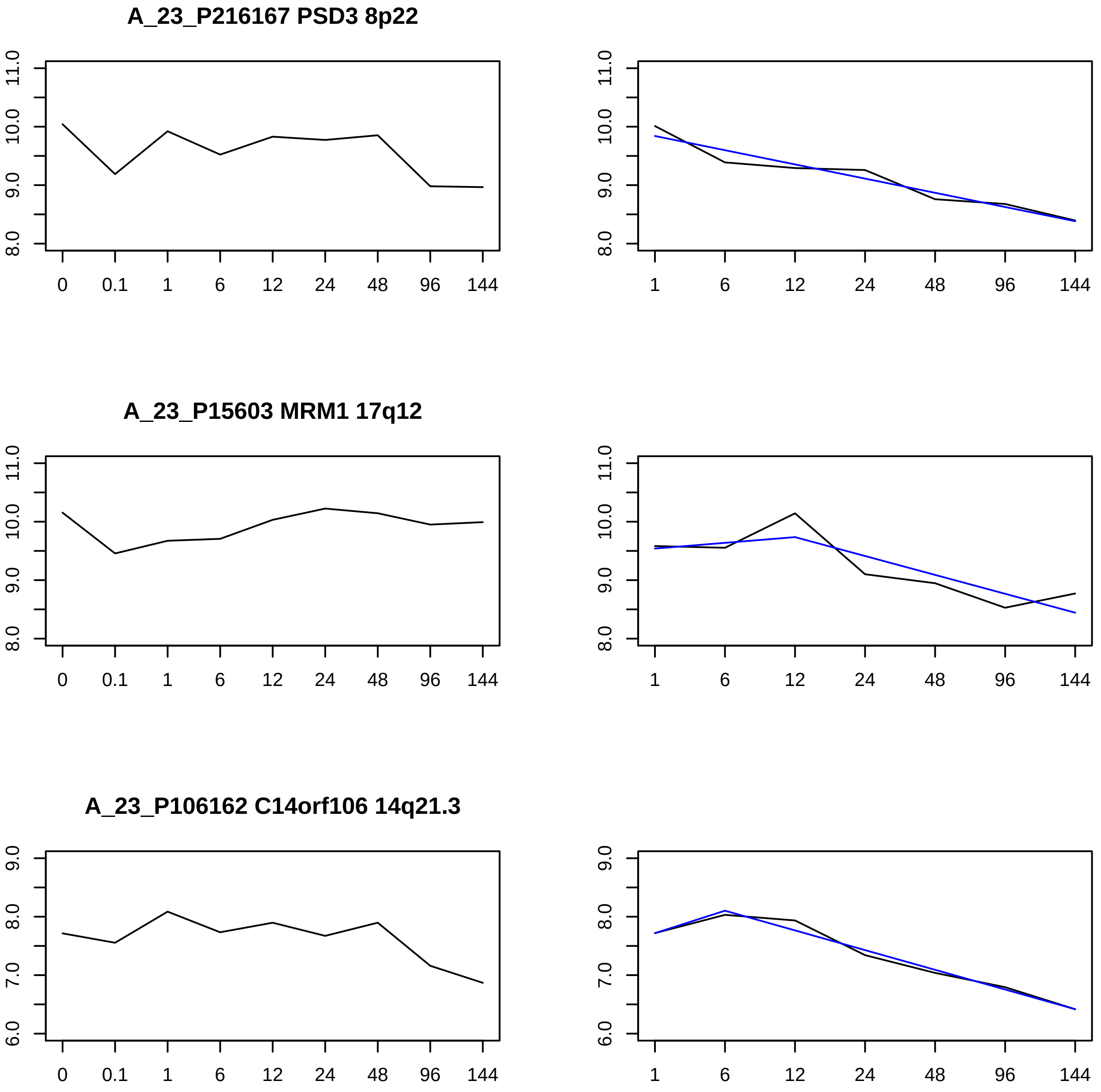
A_23_P216167 PSD3 8p22
A_23_P15603 MRM1 17q12
A_23_P106162 C14orf106 14q21.3
8.0 9.0 10.0 11.0
6.0 7.0 8.0 9.0
0 0.1 1 6 12 24 48 96 144
1 6 12 24 48 96 144

A_24_P307184 LOC400013 12p12.2

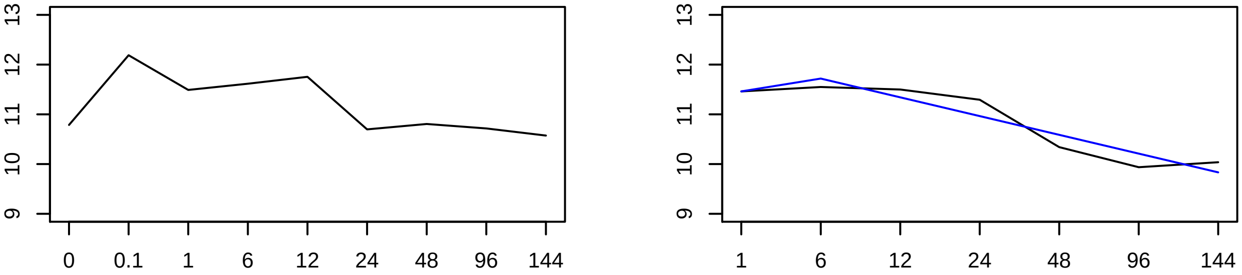

A_23_P58169 NPY2R 4q32.1

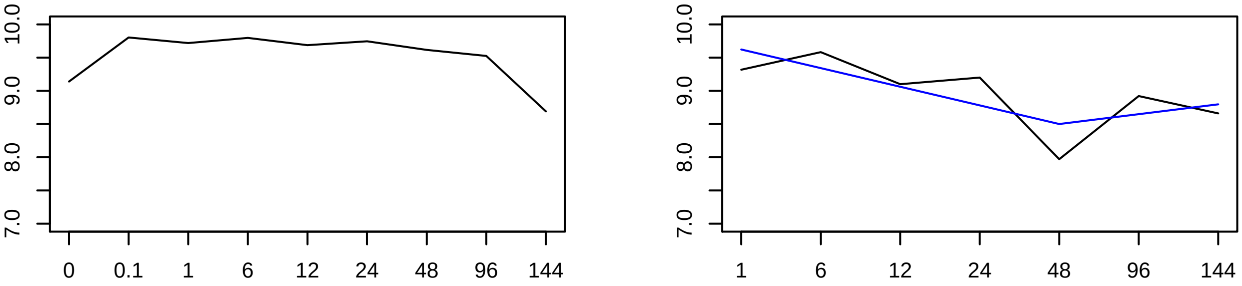

A_24_P374973 A_24_P374973 NA

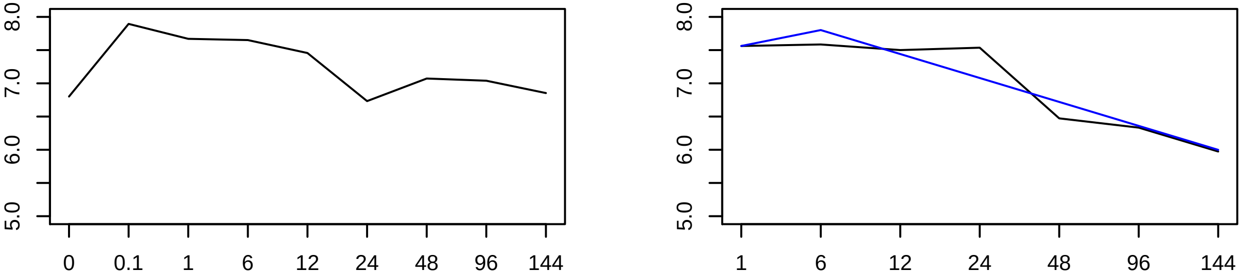

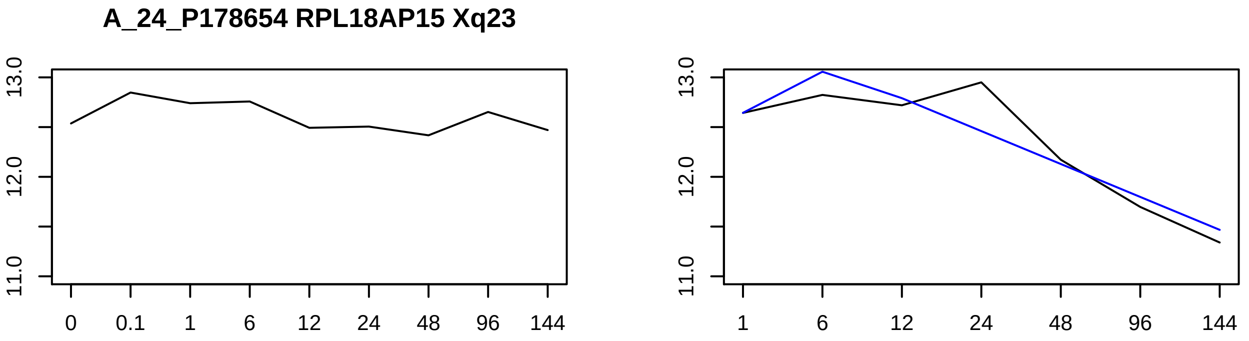
A_24_P178654 RPL18AP15 Xq23
13.0
12.0
11.0
0 0.1 1 6 12 24 48 96 144
1 6 12 24 48 96 144

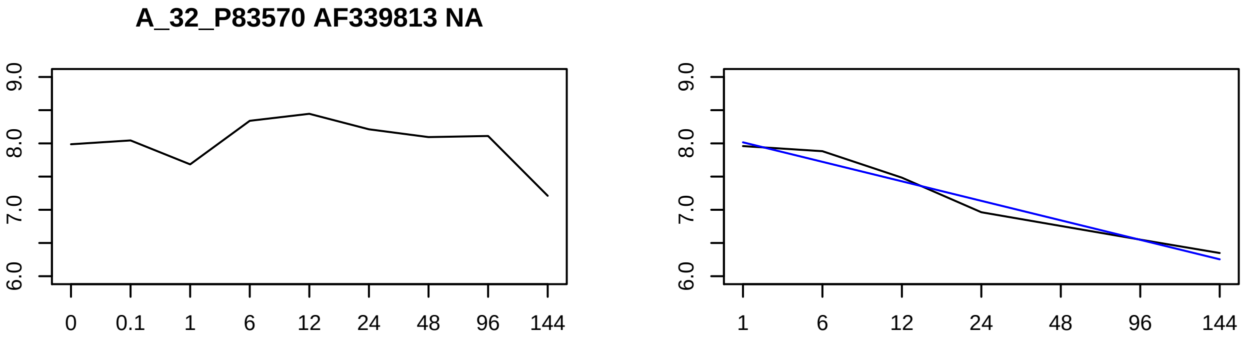
A_32_P83570 AF339813 NA
9.0
8.0
7.0
6.0
0 0.1 1 6 12 24 48 96 144
1 6 12 24 48 96 144

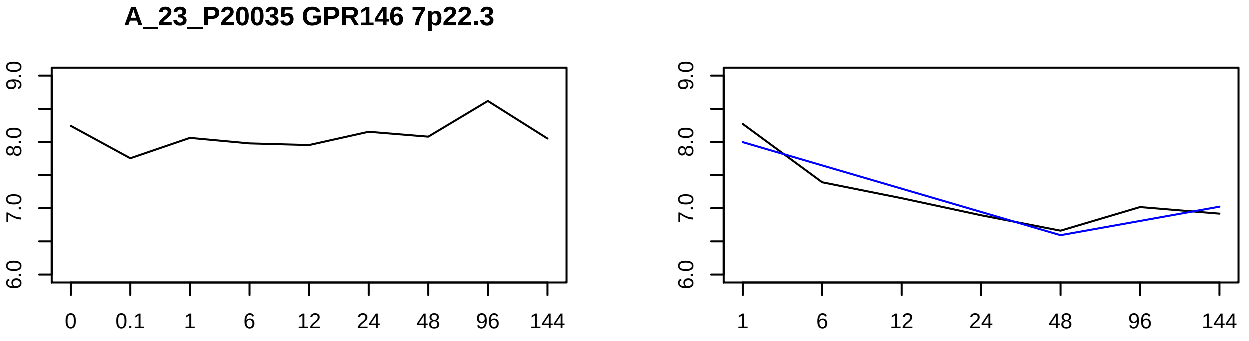
A_23_P20035 GPR146 7p22.3
9.0
8.0
7.0
6.0
0 0.1 1 6 12 24 48 96 144
1 6 12 24 48 96 144

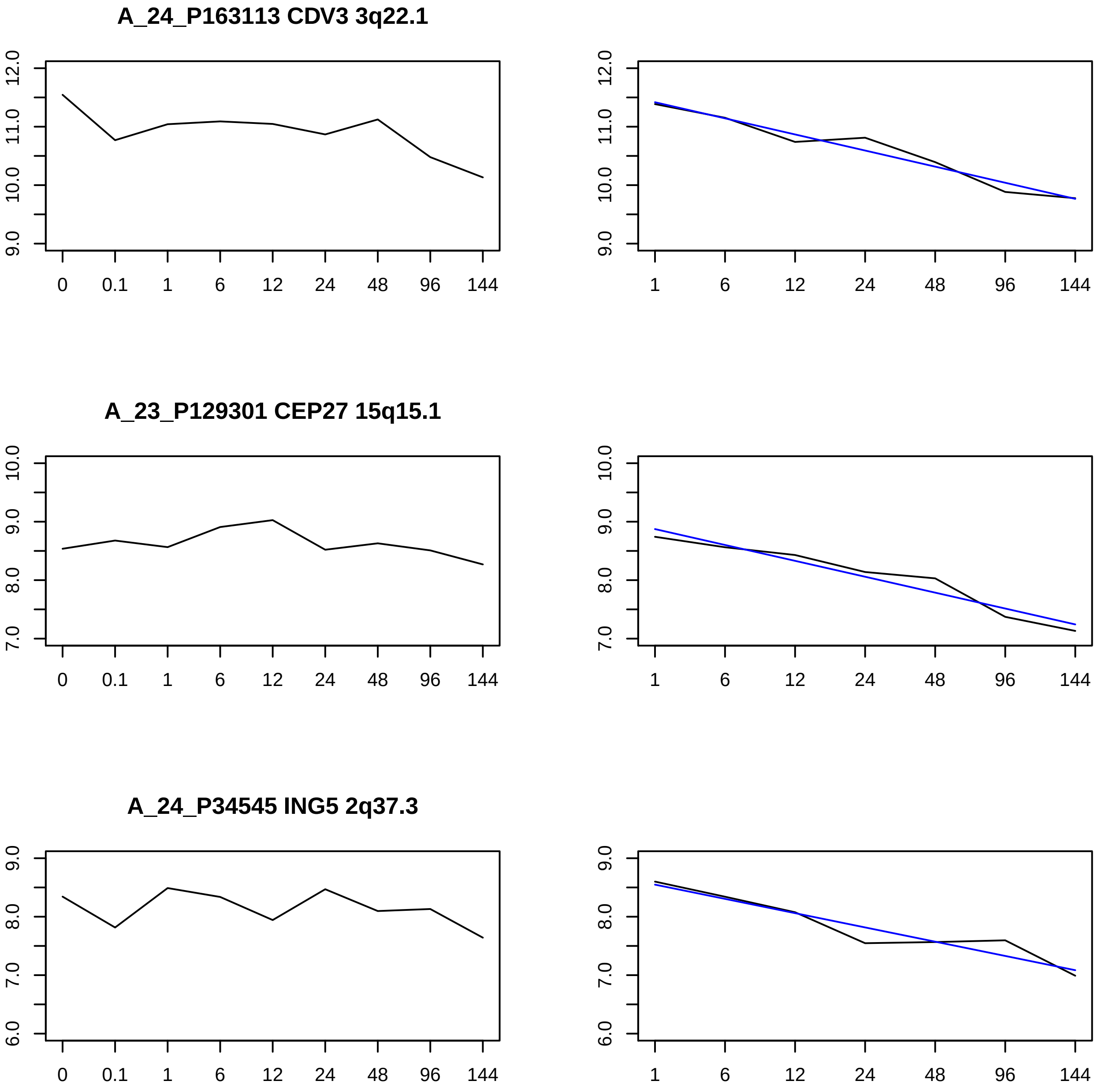

A_24_P163113 CDV3 3q22.1
9.0
10.0
11.0
12.0
0
0.1
1
6
12
24
48
96
144
1
6
12
24
48
96
144
A_23_P129301 CEP27 15q15.1
7.0
8.0
9.0
10.0
A_24_P34545 ING5 2q37.3
6.0
7.0
8.0
9.0

A_24_P360601 NOL8 9q22.31

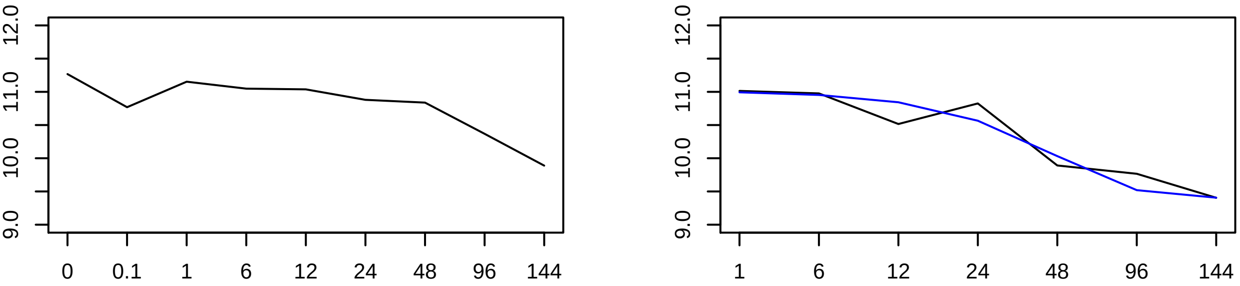

A_23_P80940 PPAT 4q12

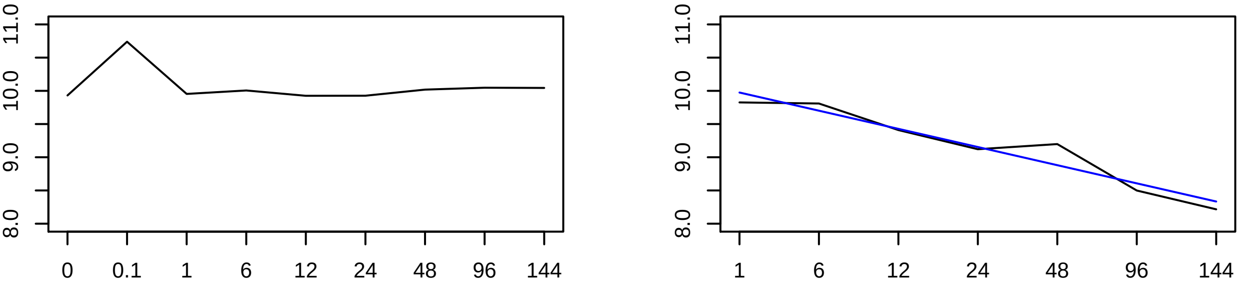

A_23_P305759 ABHD3 18q11.2

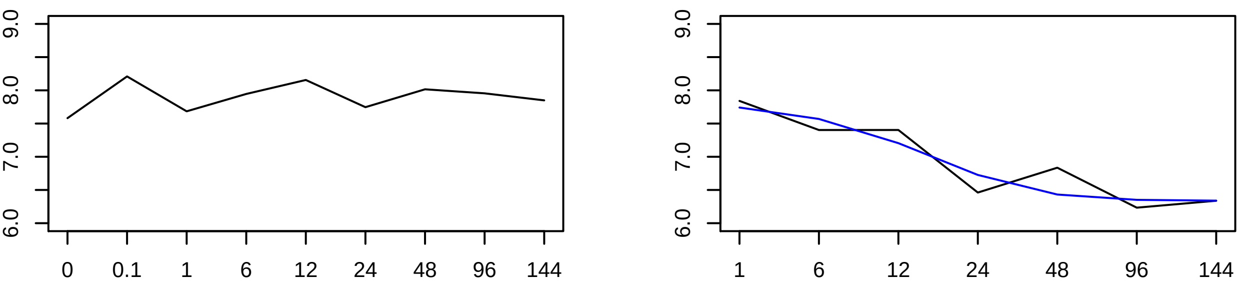

A_32_P6015 HLXB9 7q36.3

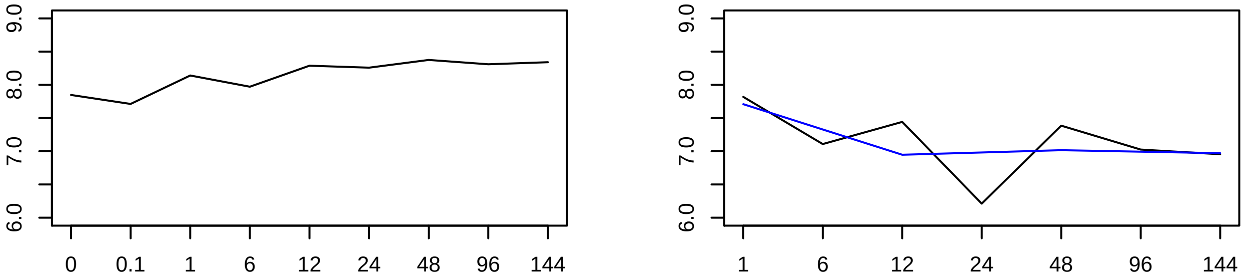

A_24_P21447 SURF6 9q34.2

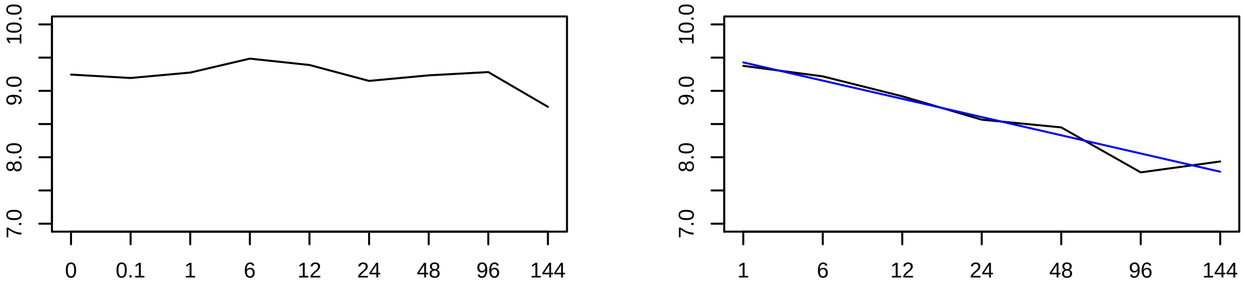

A_24_P230416 A_24_P230416 NA

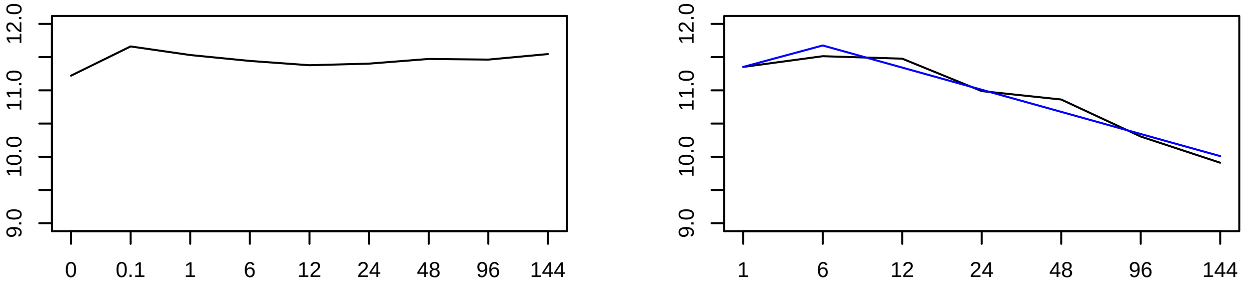

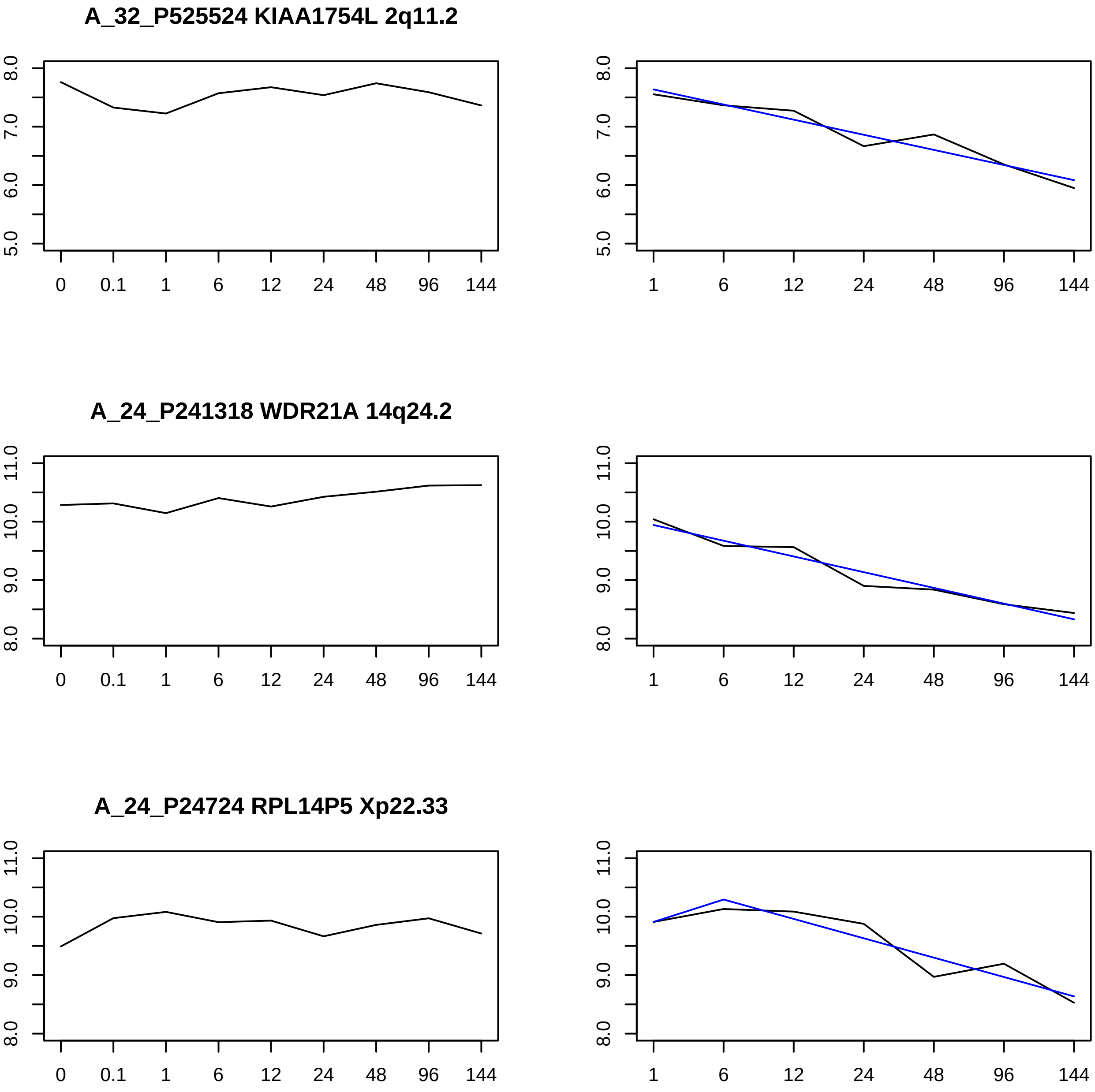
A_32_P525524 KIAA1754L 2q11.2
A_24_P241318 WDR21A 14q24.2
A_24_P24724 RPL14P5 Xp22.33
5.0
6.0
7.0
8.0
8.0
9.0
10.0
11.0
0
0.1
1
6
12
24
48
96
144

A_24_P221601 A_24_P221601 NA

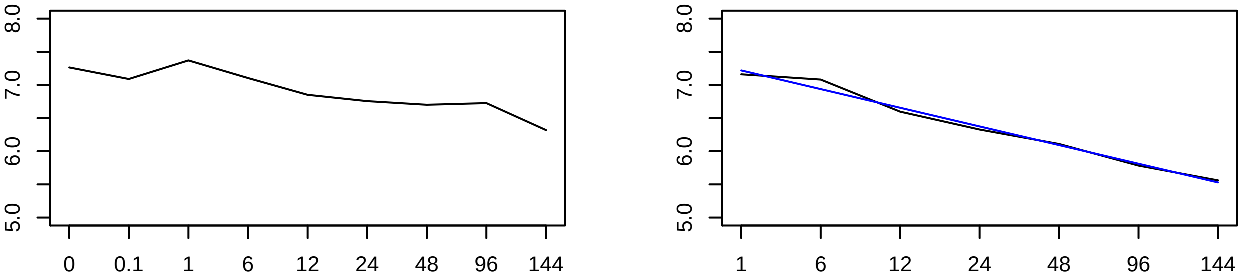

A_24_P273074 A_24_P273074 NA

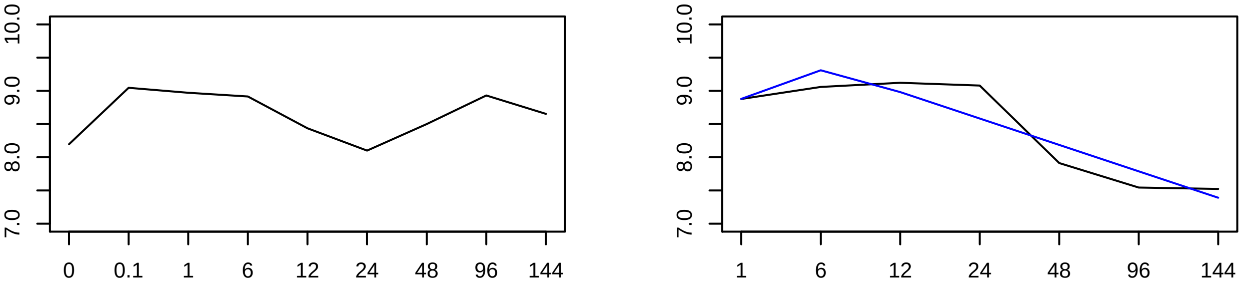

A_23_P402604 PFAS 17p13.1

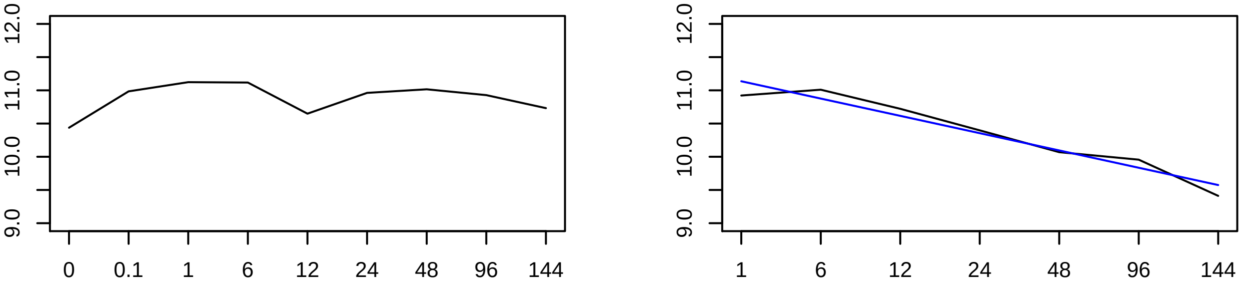

A_23_P39251 LSDP5 19p13.3

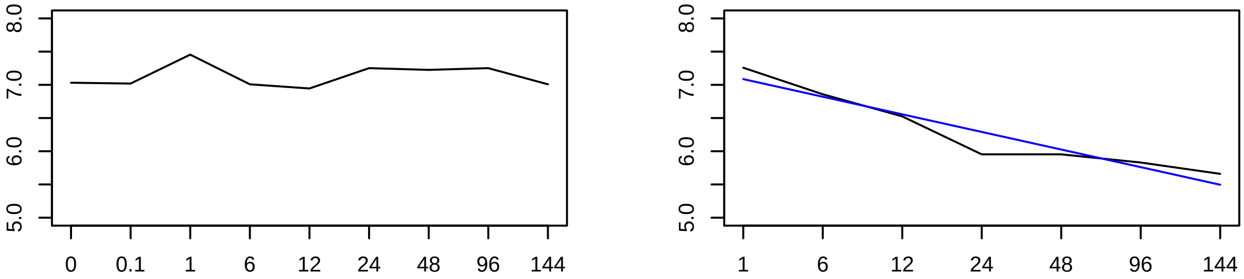

A_24_P24150 A_24_P24150 NA

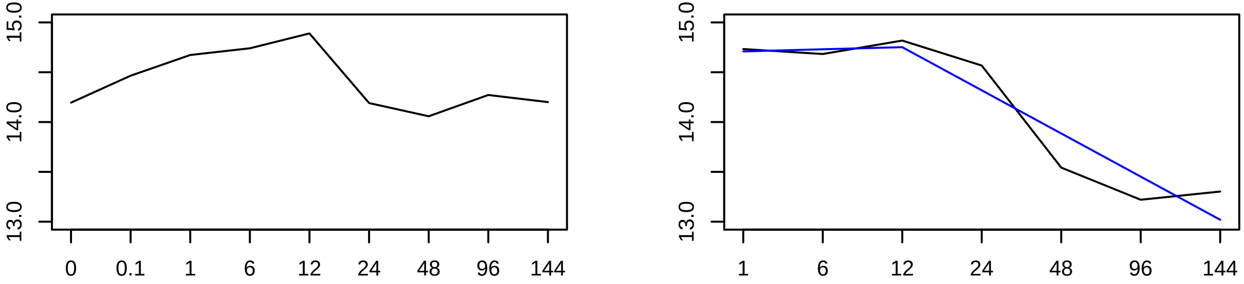

A_24_P188377 CD55 1q32.2

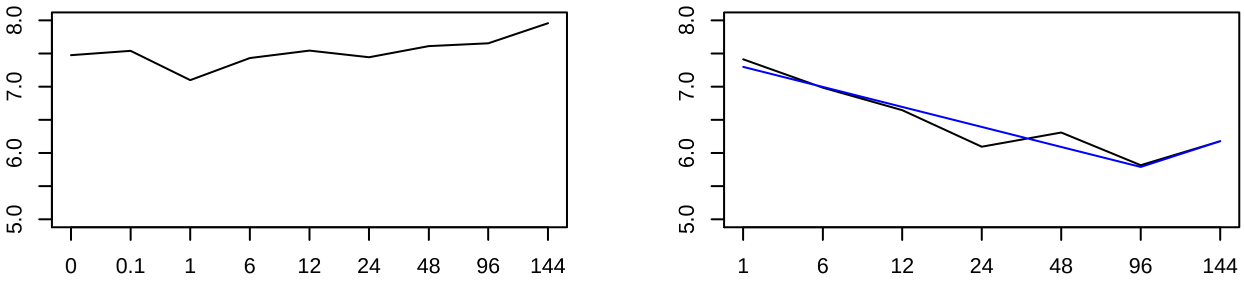

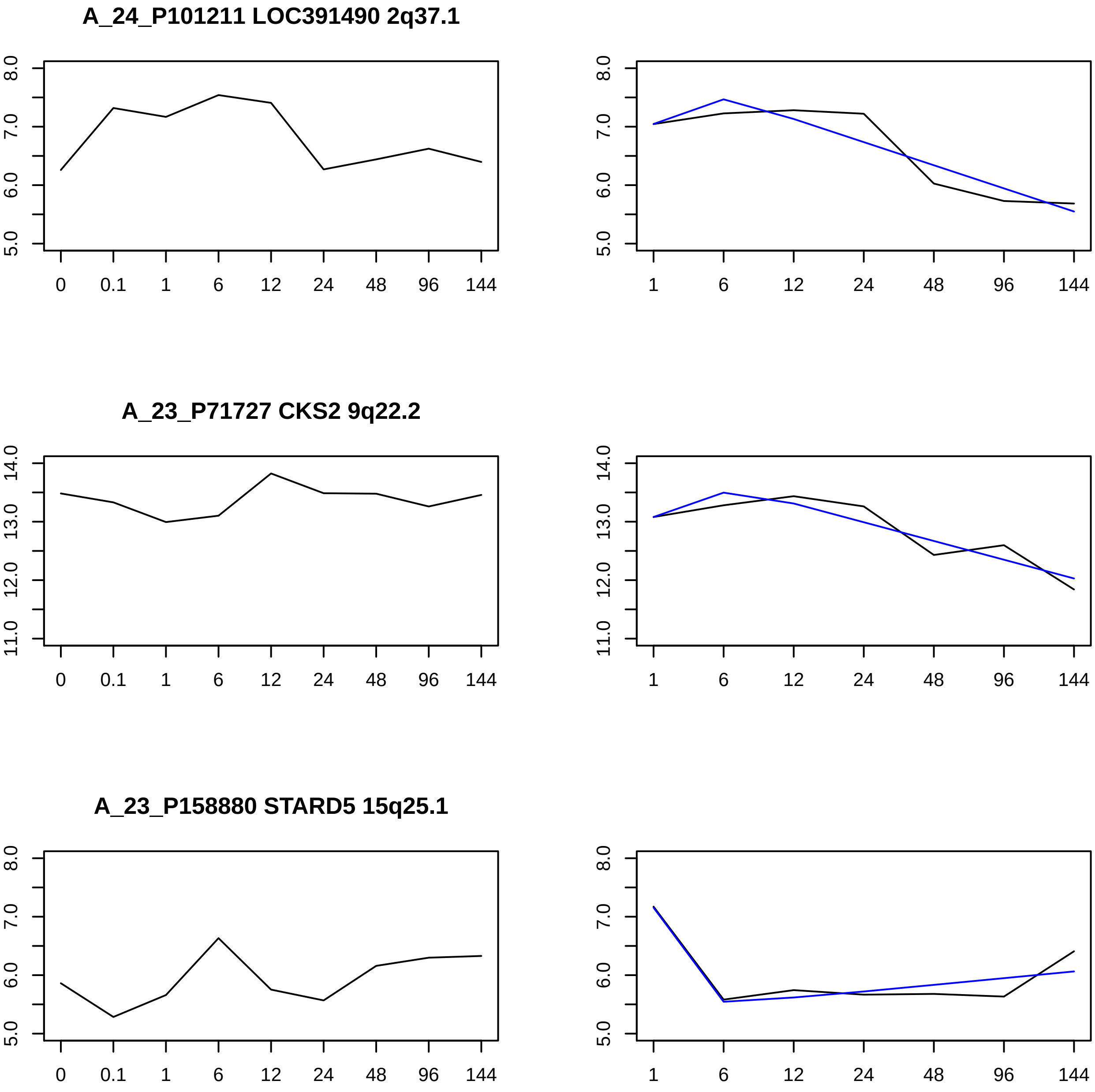
A_24_P101211 LOC391490 2q37.1
A_23_P71727 CKS2 9q22.2
A_23_P158880 STARD5 15q25.1
5.0
6.0
7.0
8.0
11.0
12.0
13.0
14.0
0
0.1
1
6
12
24
48
96
144

A_24_P925559 A_24_P925559 NA

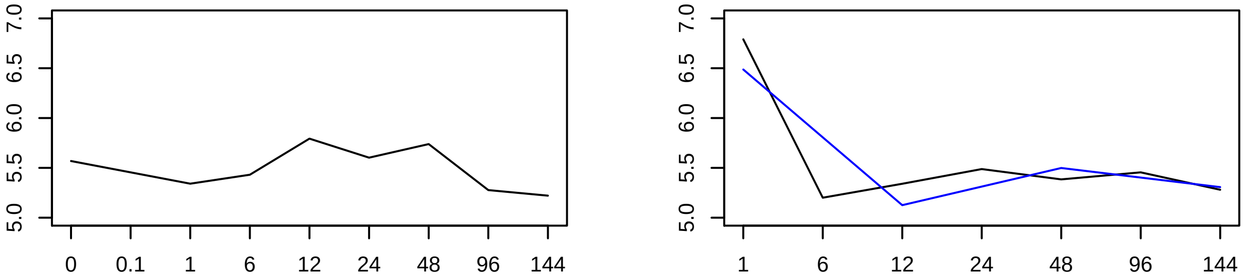

A_24_P462899 CENPW 6q22.32

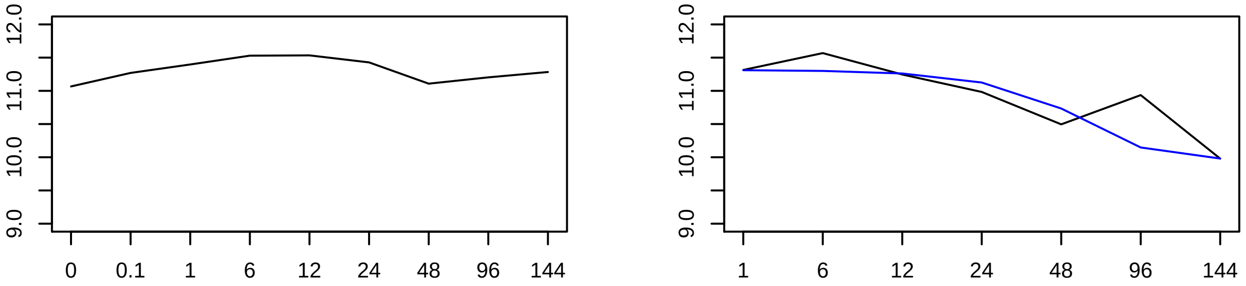

A_24_P392774 HSD17B12 11p11.2

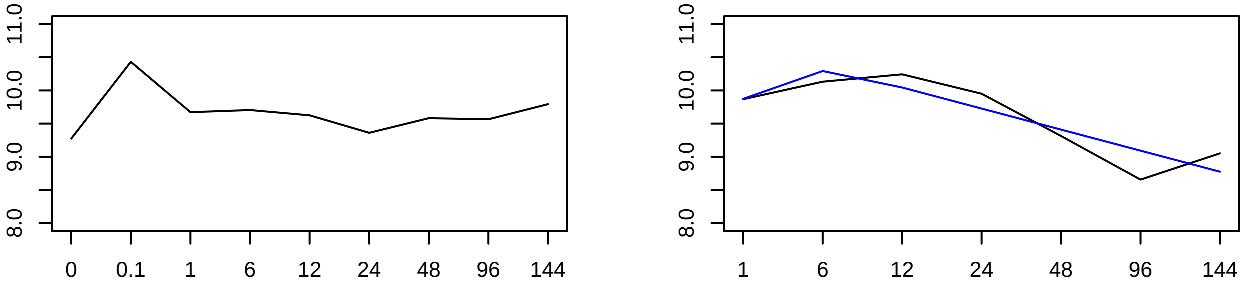

A_23_P125990 E2F2 1p36.12

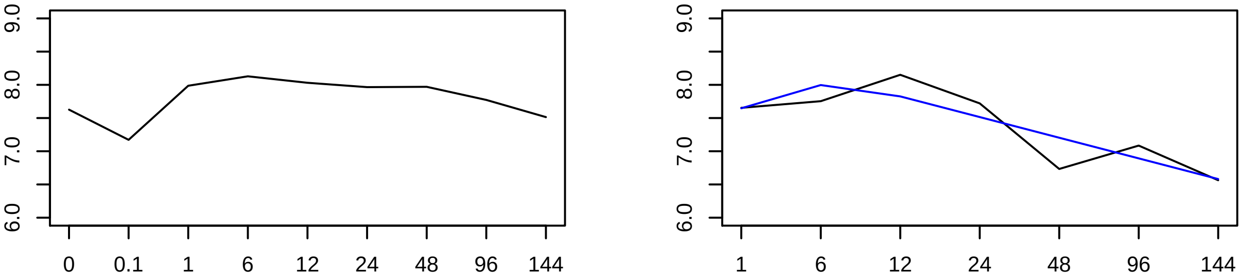

A_24_P681563 A_24_P681563 NA

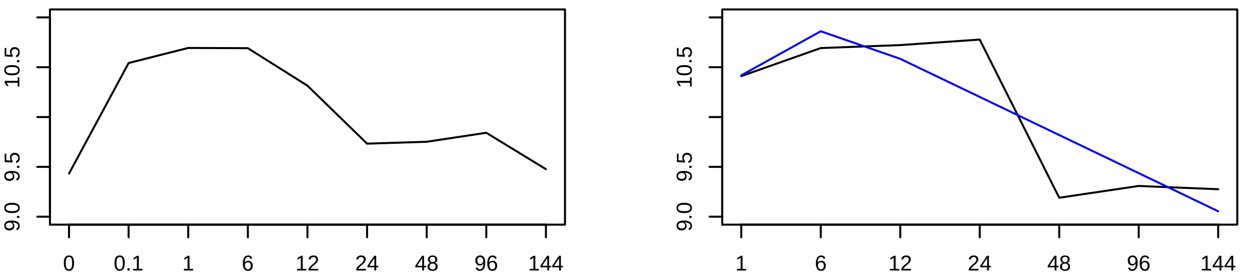

A_24_P392351 THC2541331 NA

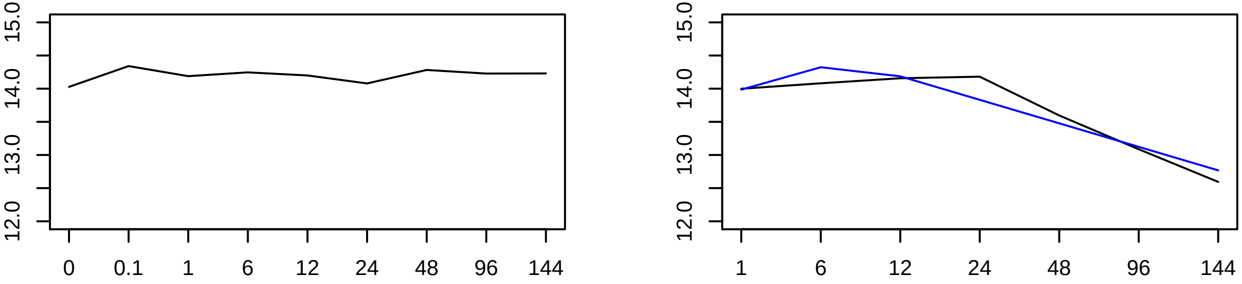

A_24_P152775 LOC442195 6p22.1

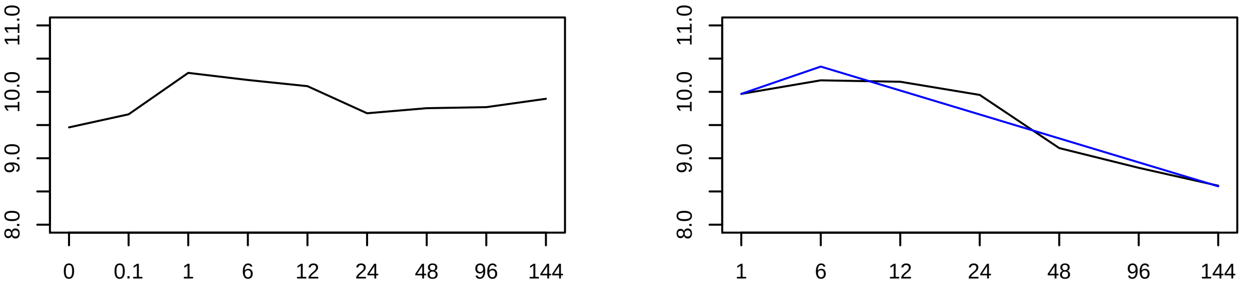

A_24_P941336 TSR1 17p13.3

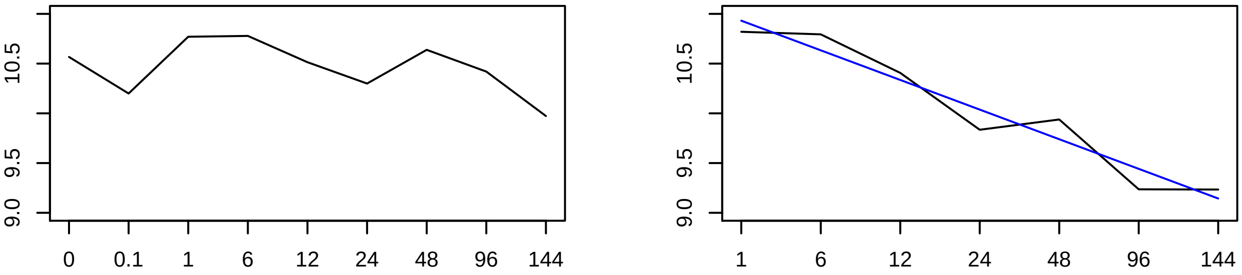

A_23_P403745 LOC92154 16q22.1

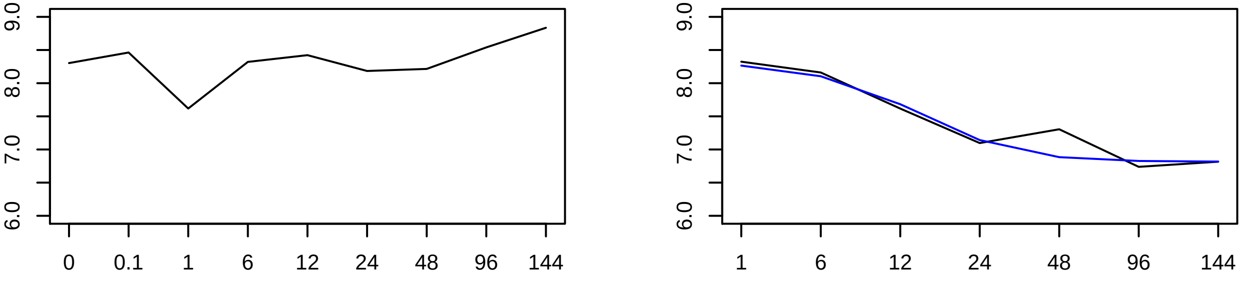

A_32_P219620 EYA4 6q23.2

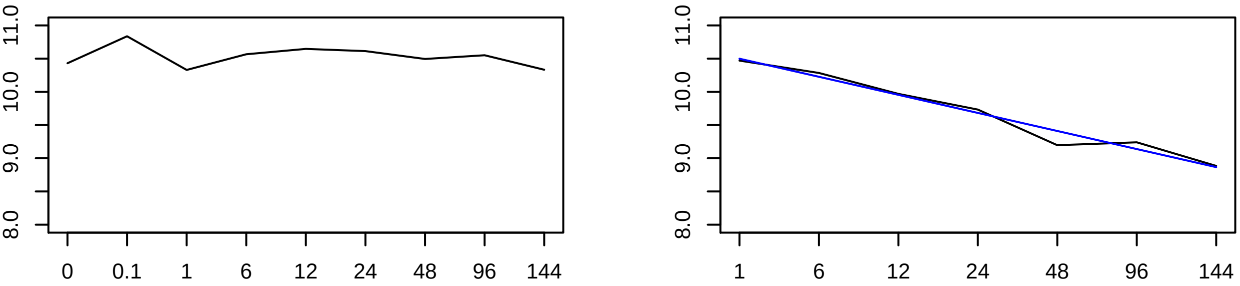

A_32_P150263 SNORA76\SNORD104 17q23.3

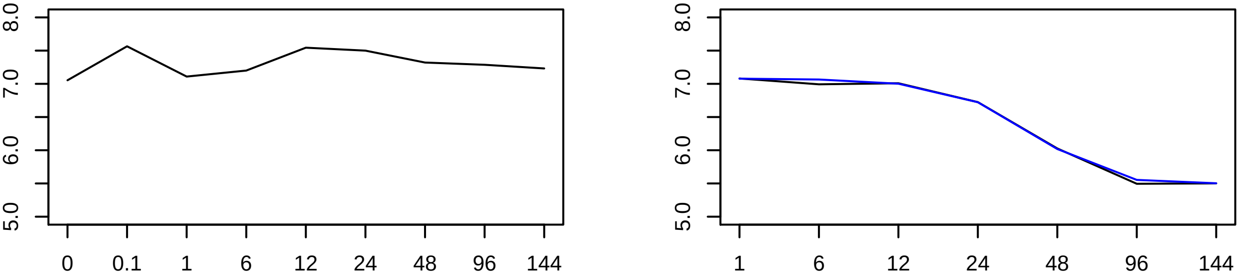

A_24_P804667 LOC751071 11q12.3

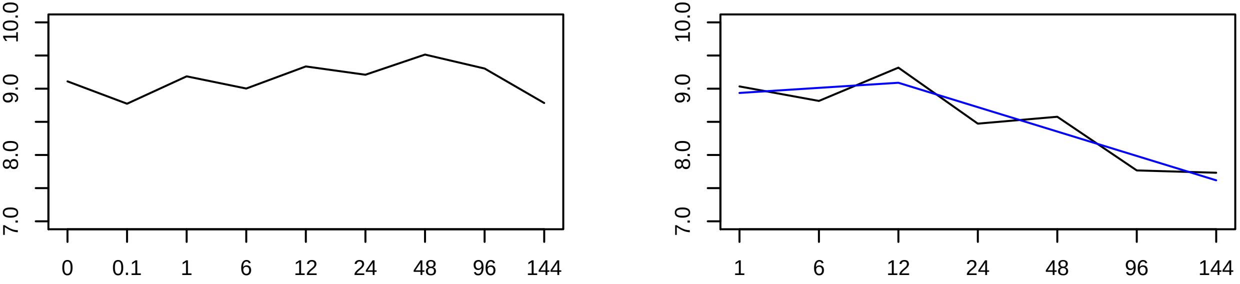

A_24_P118271 A_24_P118271 NA

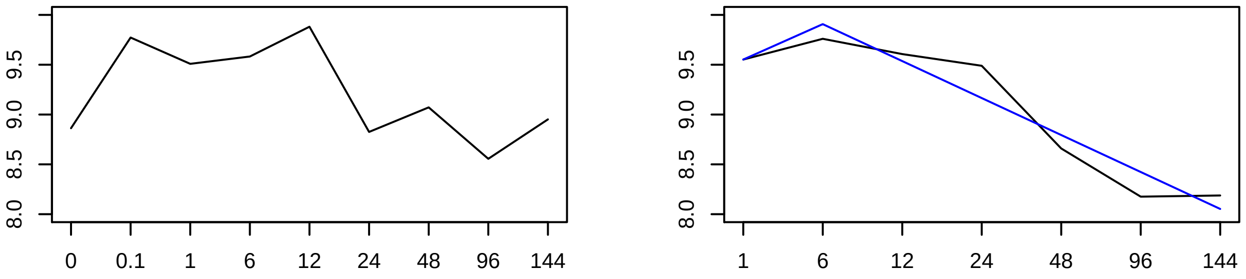

A_32_P83776 C1orf96 1q42.13

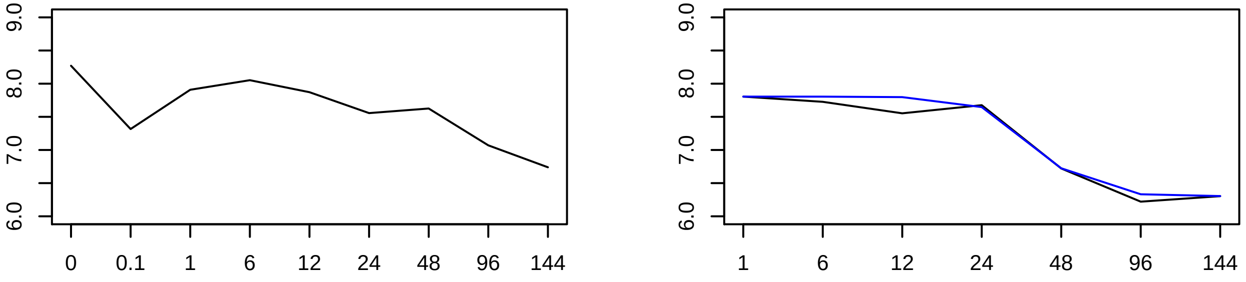

A_23_P54605 RSL1D1 16p13.13

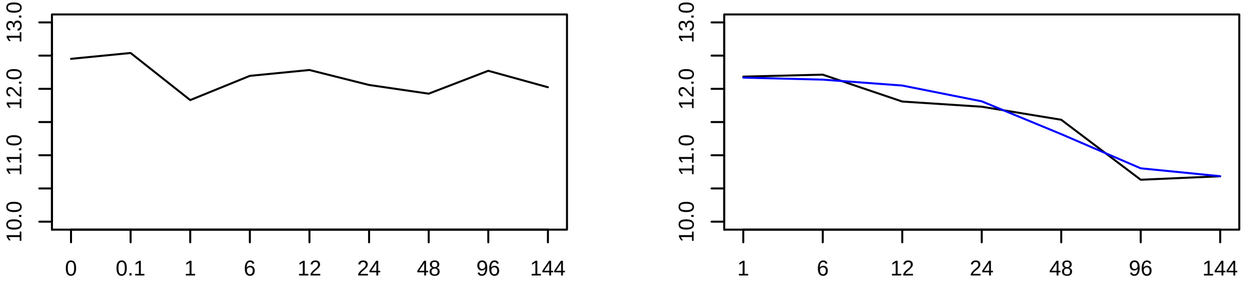

A_24_P126890 RPL9 4p14

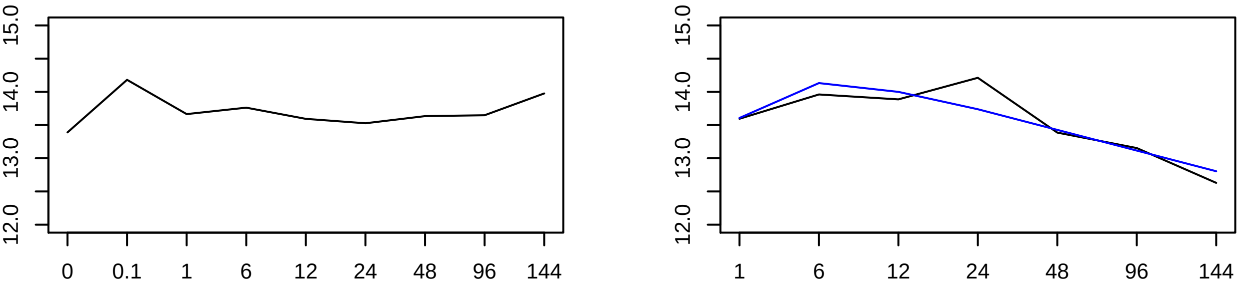

A_24_P71700 ZBTB47 3p22.1

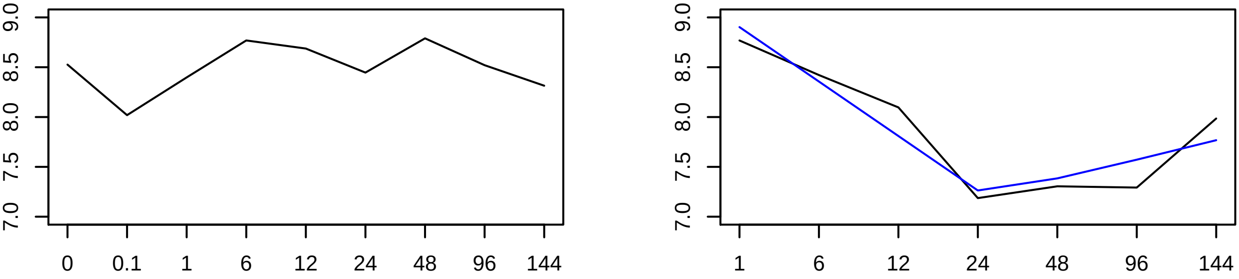

A_23_P255663 MANEA 6q16.1

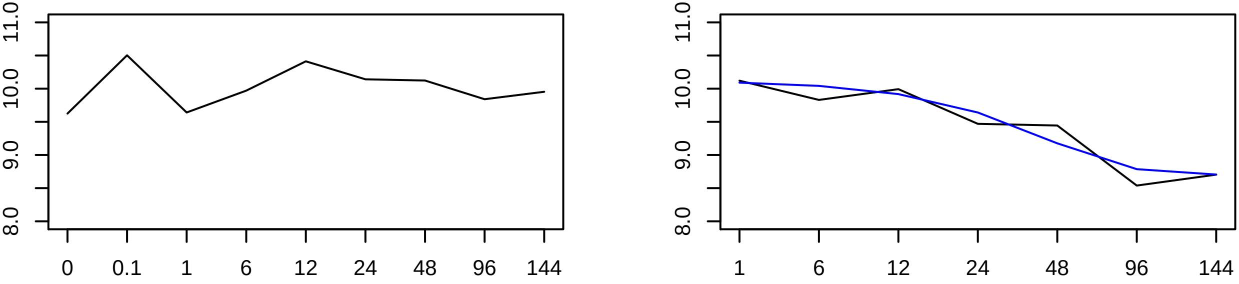

A_32_P130577 BC066989 NA

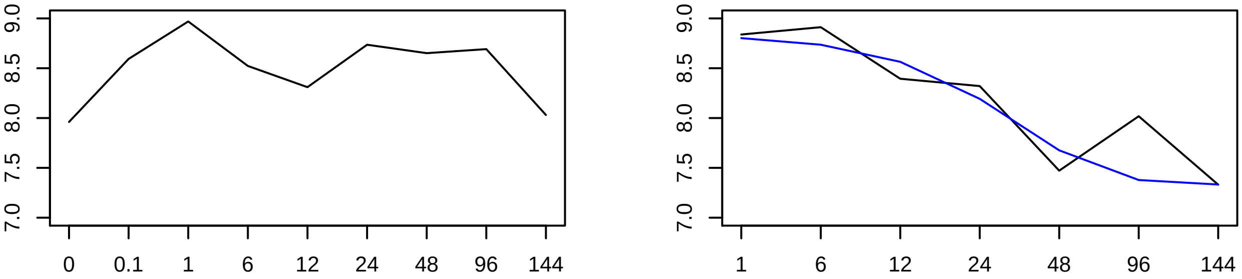

A_24_P324405 ANKRD11 16q24.3

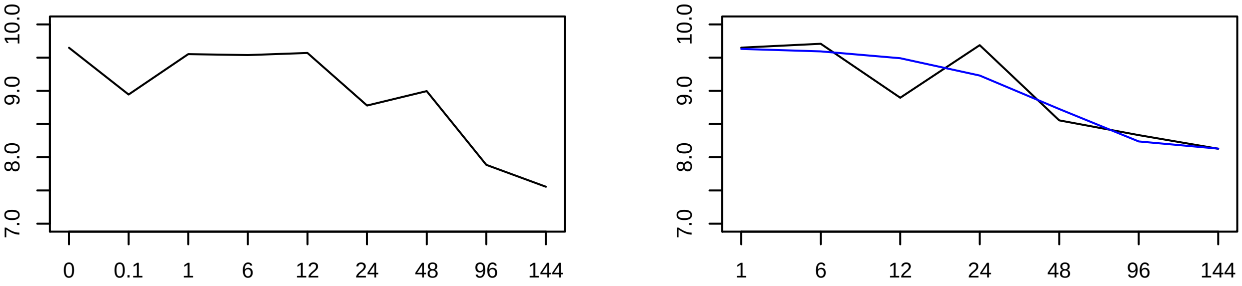

A_24_P160874 DUT 15q21.1

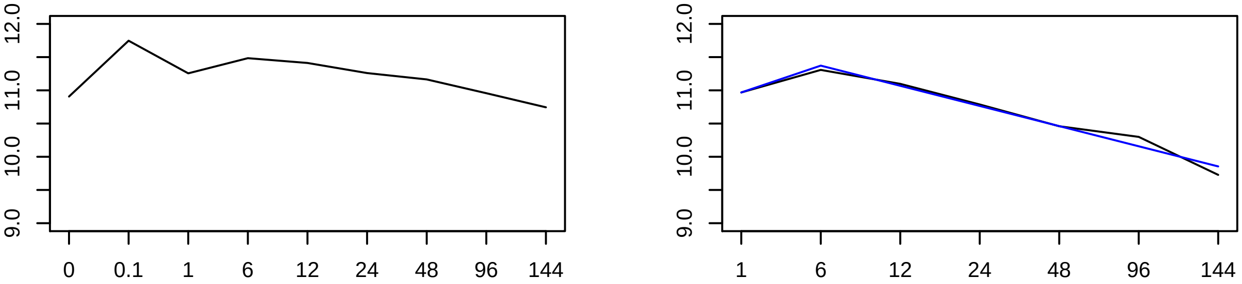

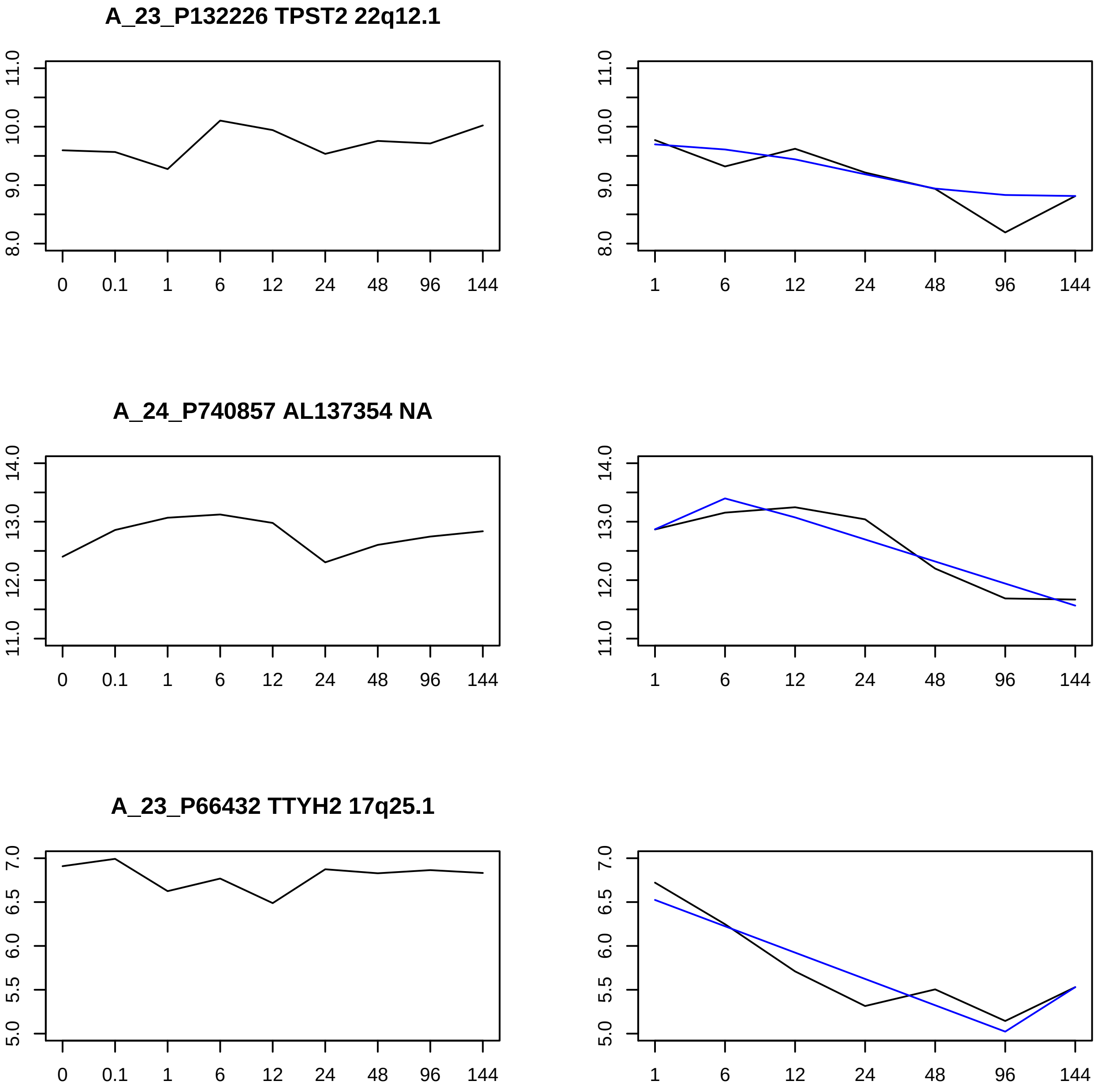

A_23_P132226 TPST2 22q12.1
A_24_P740857 AL137354 NA
A_23_P66432 TTYH2 17q25.1
0
0.1
1
6
12
24
48
96
144
8.0
9.0
10.0
11.0
12.0
13.0
14.0
5.0
5.5
6.0
6.5
7.0

A_24_P49747 HMGB3P24 9p13.3

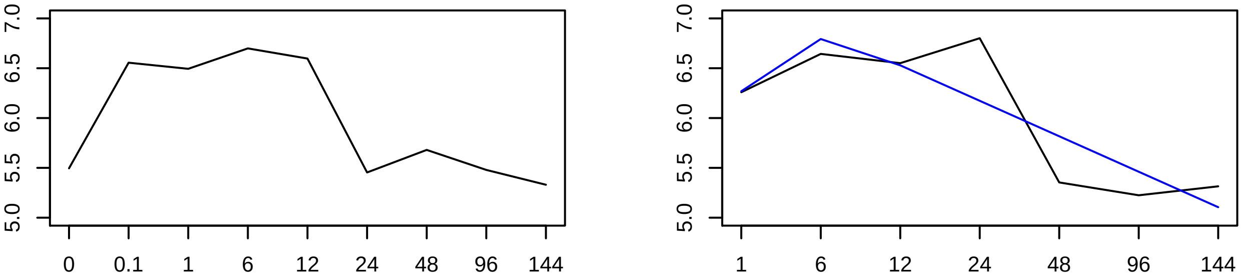

A_23_P30707 RNASET2 6q27

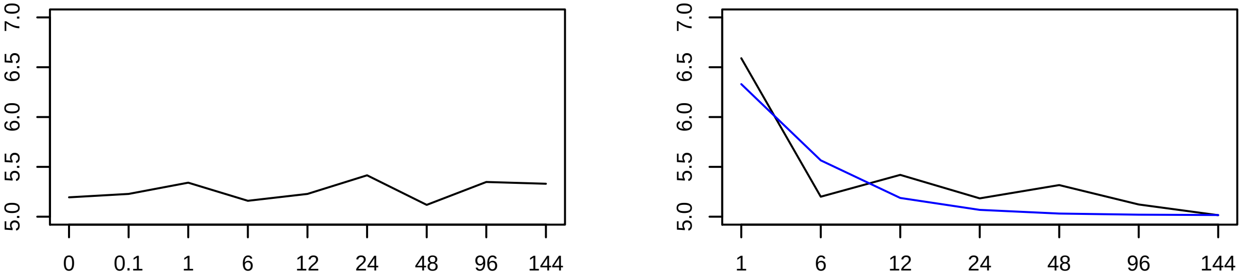

A_24_P392436 A_24_P392436 NA

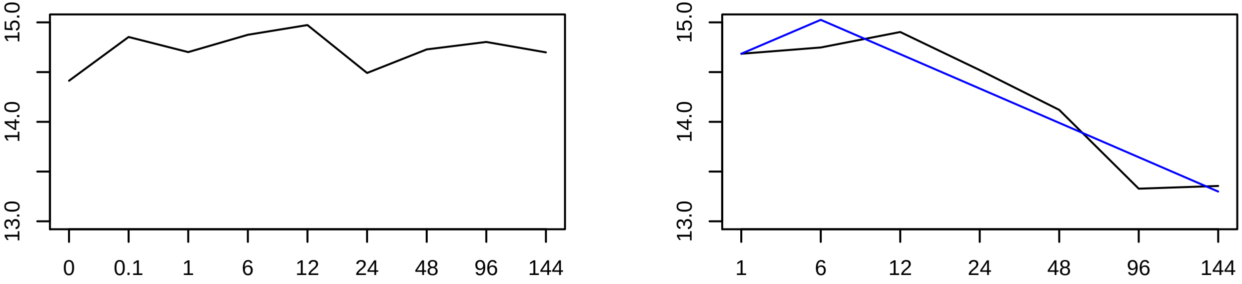

A_32_P205053 UBXD3 1p36.12

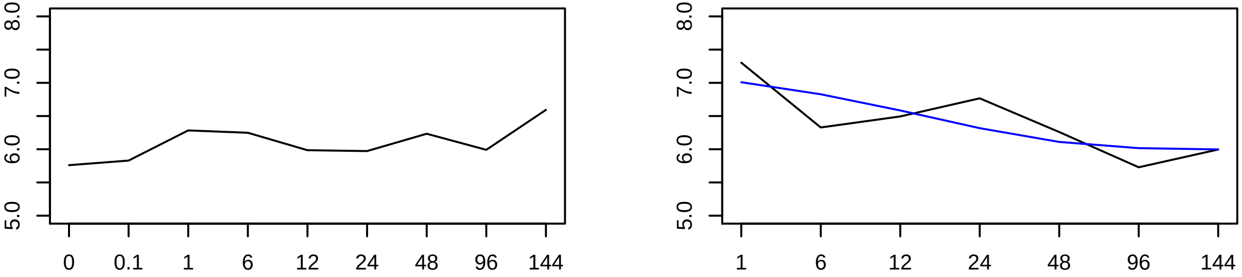

A_24_P417757 A_24_P417757 NA

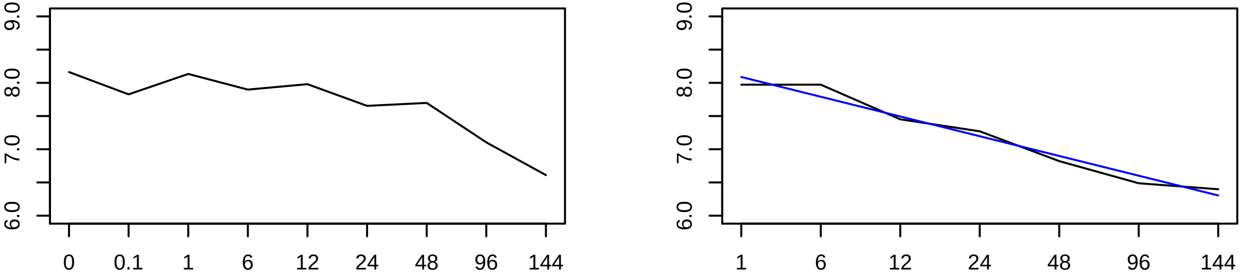

A_23_P416036 UCHL5IP Xq28

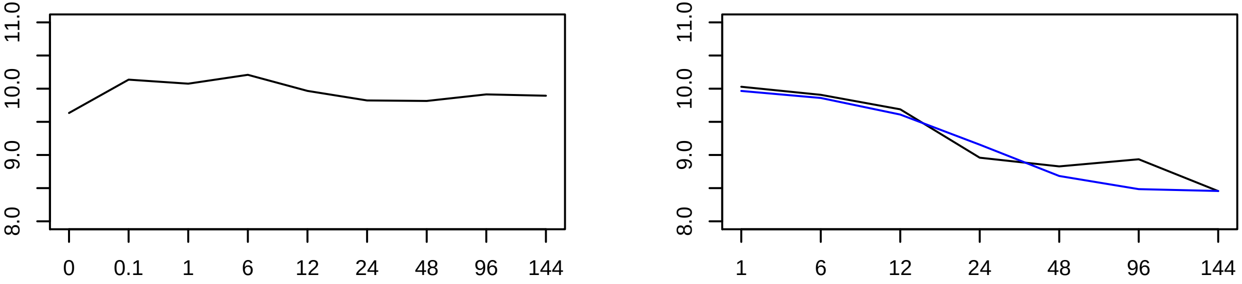

A_32_P172141 CDON 11q24.2

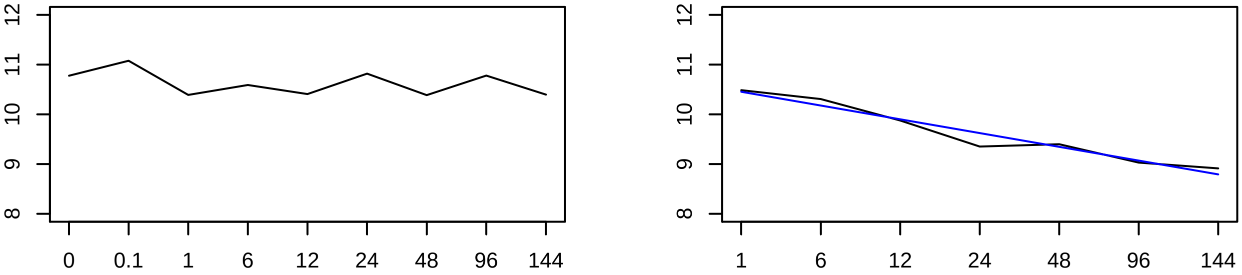

A_24_P761490 A_24_P761490 NA

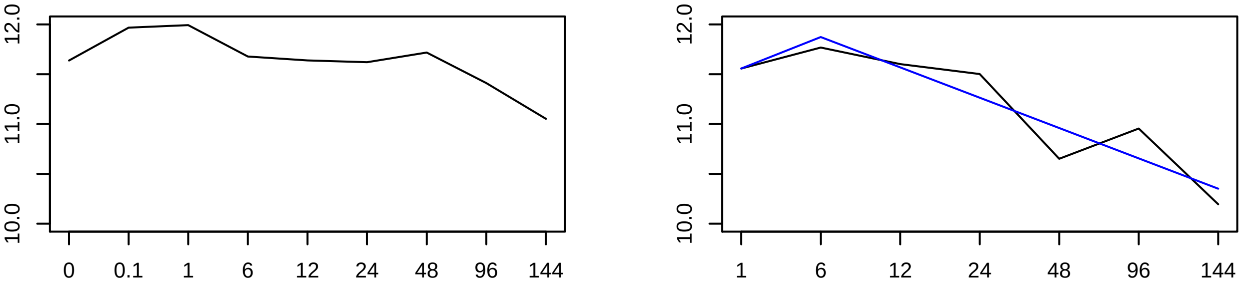

A_24_P309645 TPCN1 12q24.13

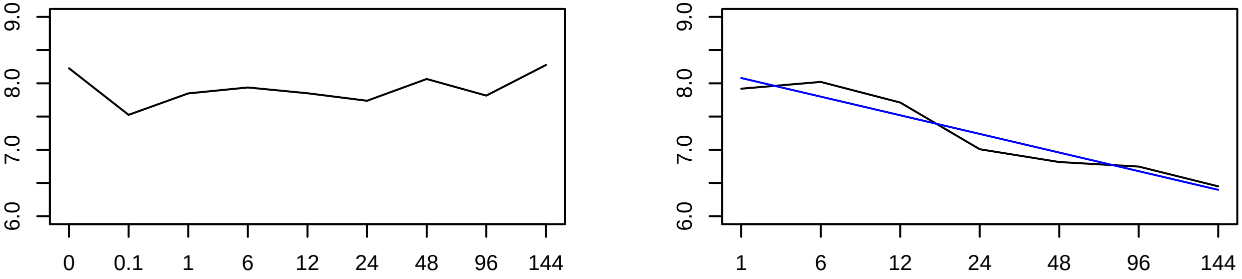

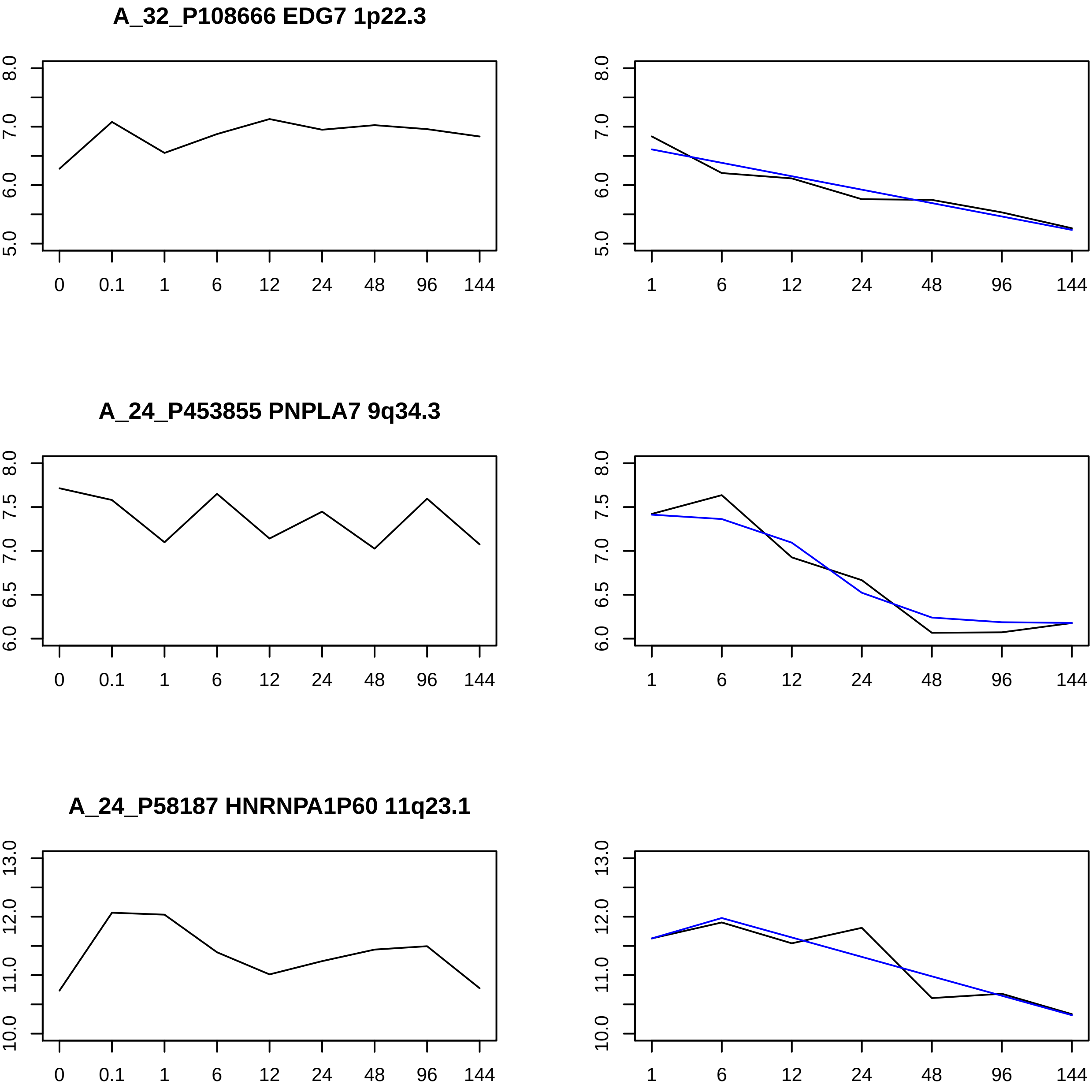
A_32_P108666 EDG7 1p22.3
A_24_P453855 PNPLA7 9q34.3
A_24_P58187 HNRNPA1P60 11q23.1
0 0.1 1 6 12 24 48 96 144
1 6 12 24 48 96 144
5.0 6.0 7.0 8.0
6.0 6.5 7.0 7.5 8.0
10.0 11.0 12.0 13.0

A_24_P891265 A_24_P891265 NA

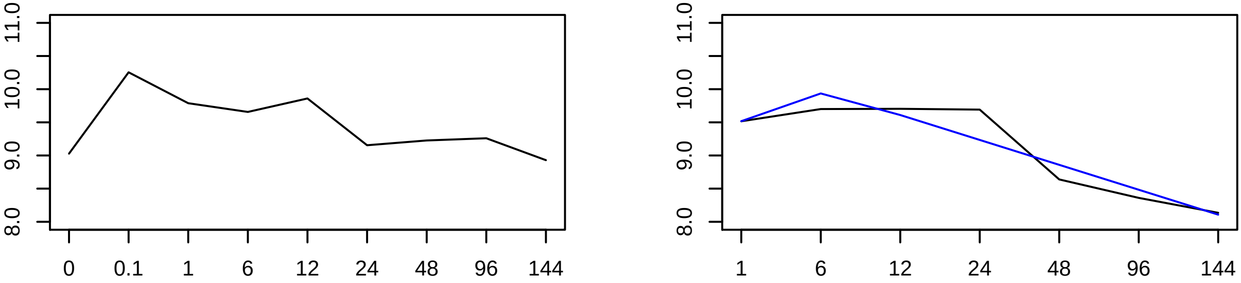

A_24_P289753 A_24_P289753 NA

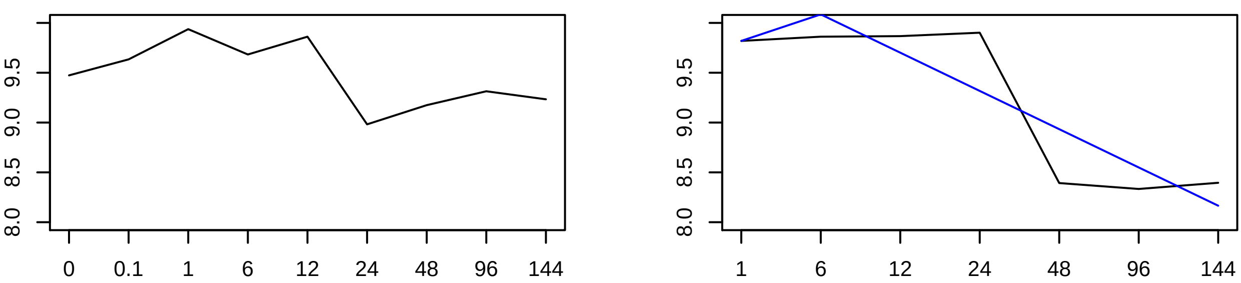

A_32_P25204 PRKDC 8q11.21

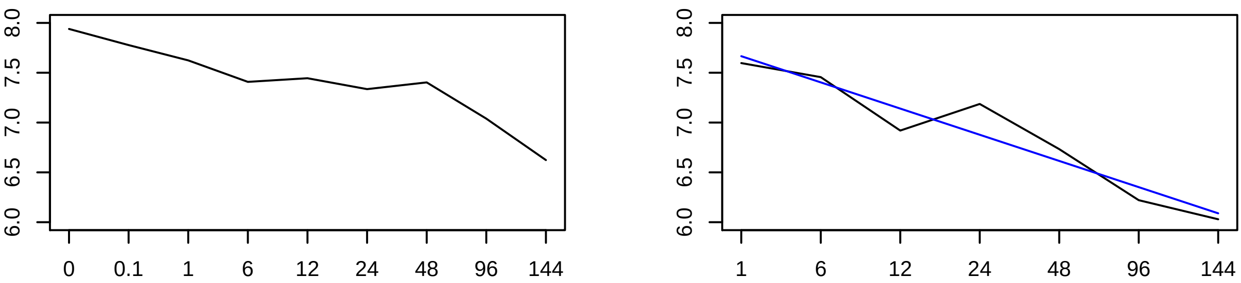

A_24_P576445 TBC1D8 2q11.2

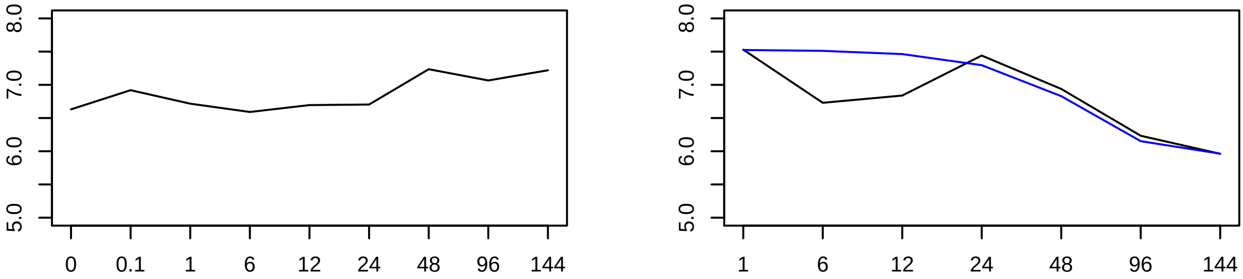

A_32_P197053 BC035417 NA

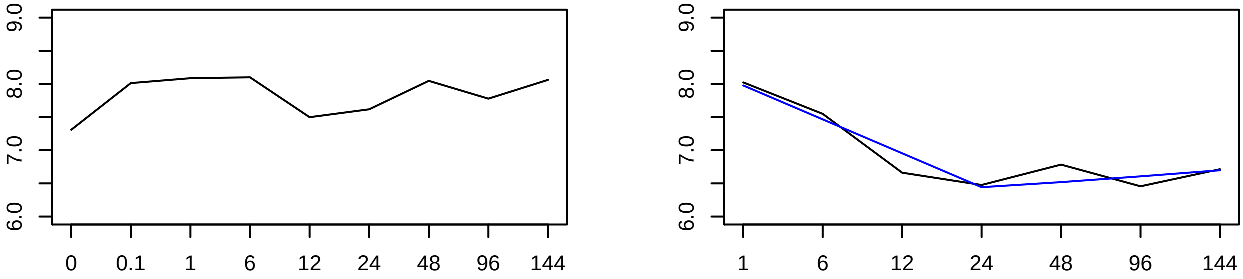

A_32_P34970 THC2533630 NA

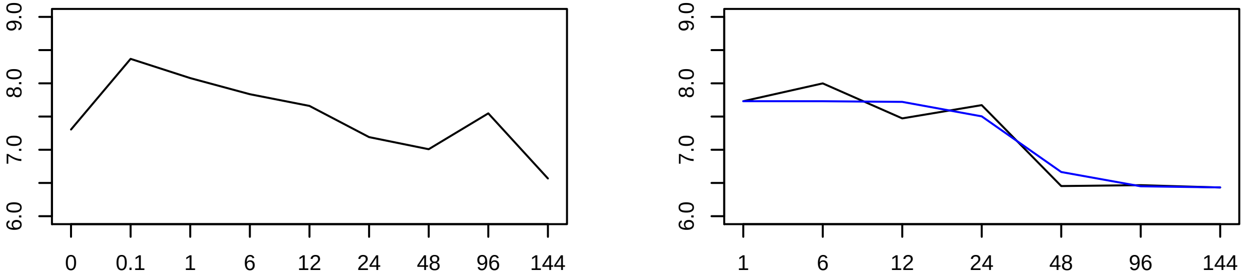

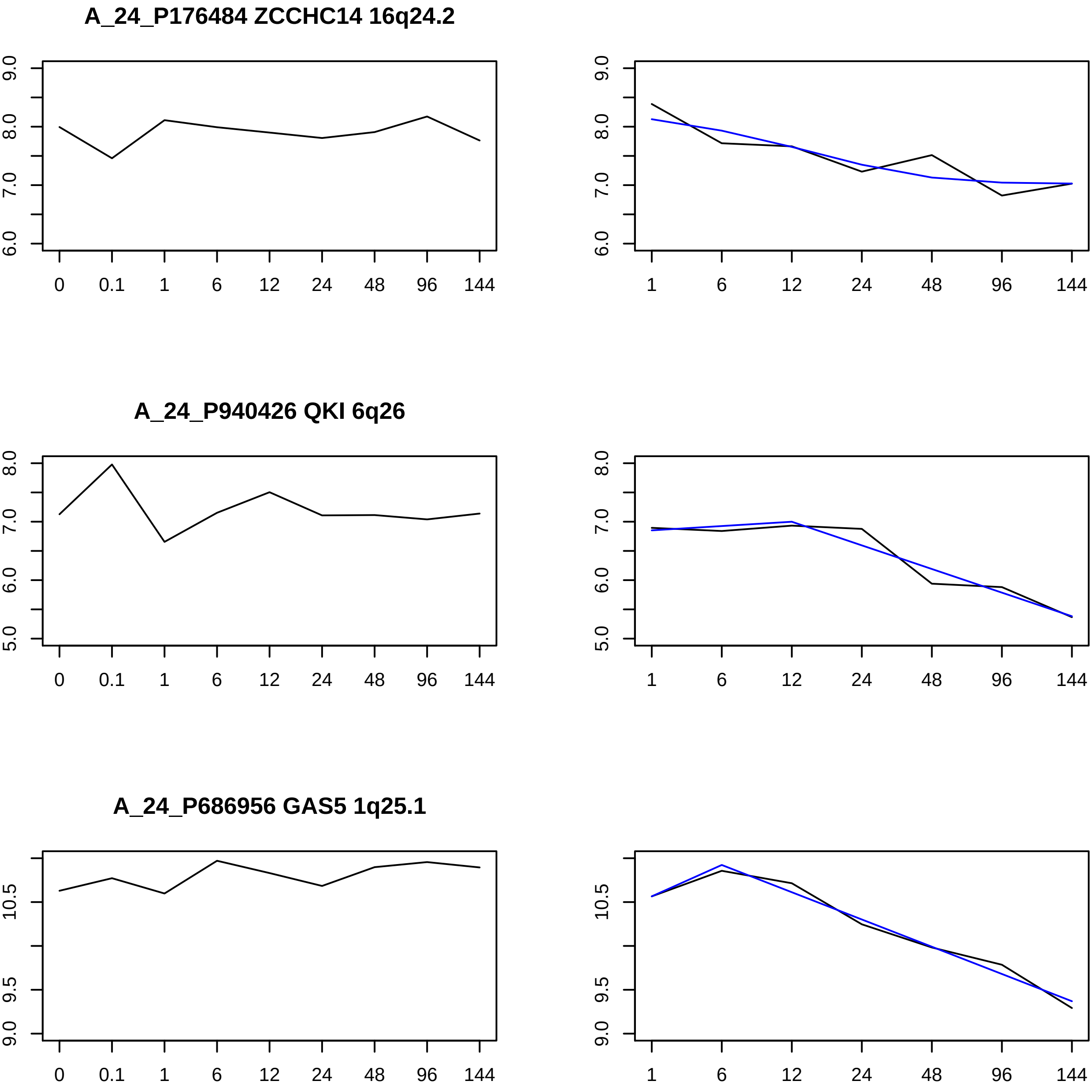
A_24_P176484 ZCCHC14 16q24.2
6.0
7.0
8.0
9.0
0
0.1
1
6
12
24
48
96
144
1
6
12
24
48
96
144
A_24_P940426 QKI 6q26
5.0
6.0
7.0
8.0
A_24_P686956 GAS5 1q25.1
9.0
9.5
10.5

A_23_P312840 SEMA6A 5q23.1

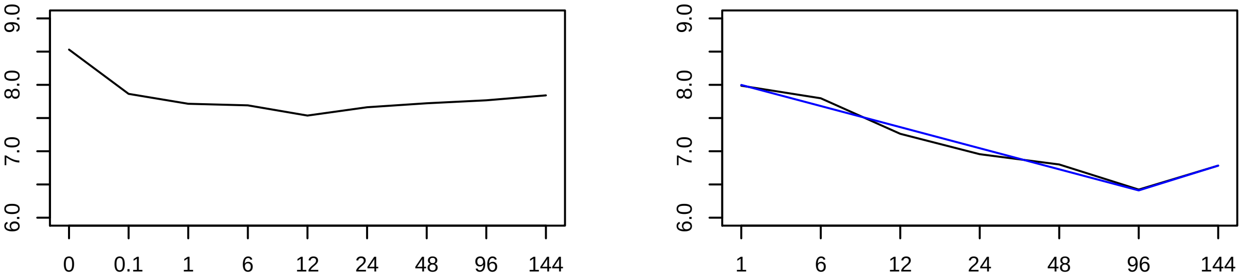

A_23_P78053 FAM117A 17q21.33

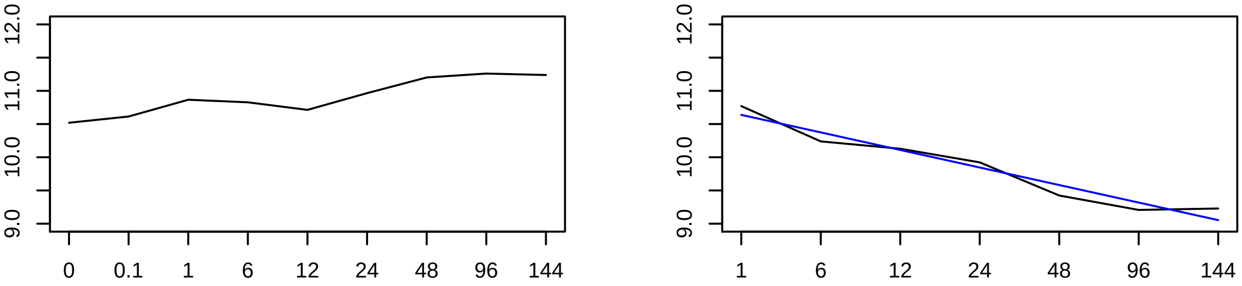

A_32_P31945 ACADSB 10q26.13

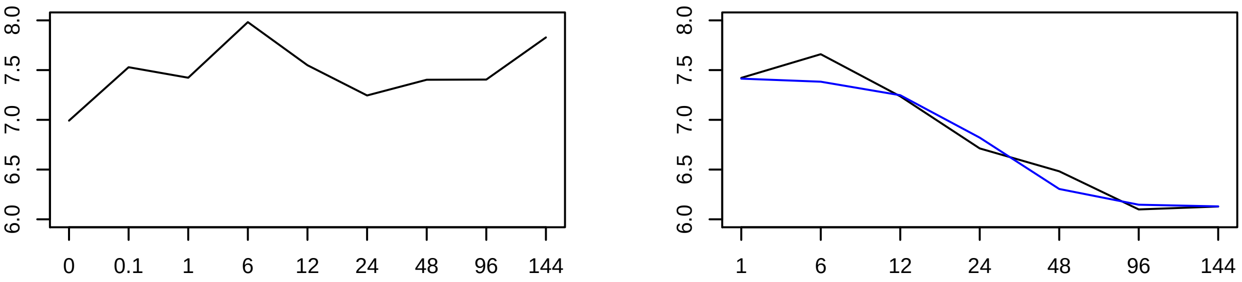

A_23_P257111 FBP1 9q22.32

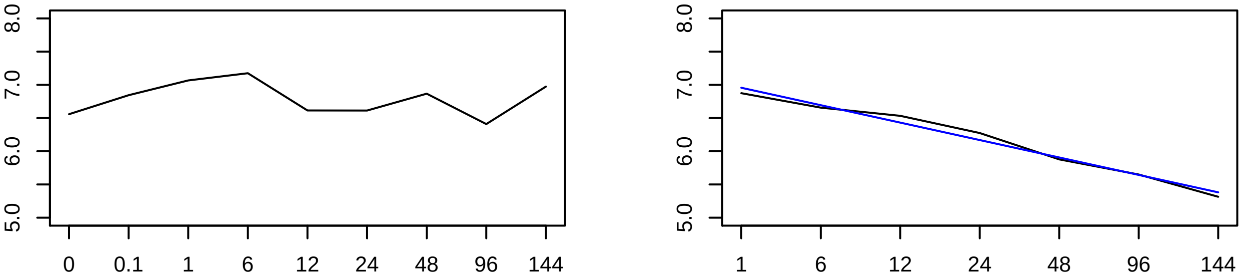

A_24_P616082 A_24_P616082 NA

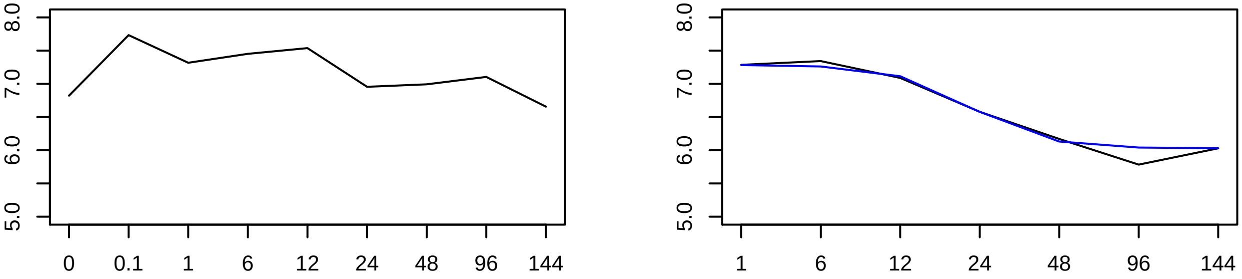

A_23_P416178 NAG6 7q32.1

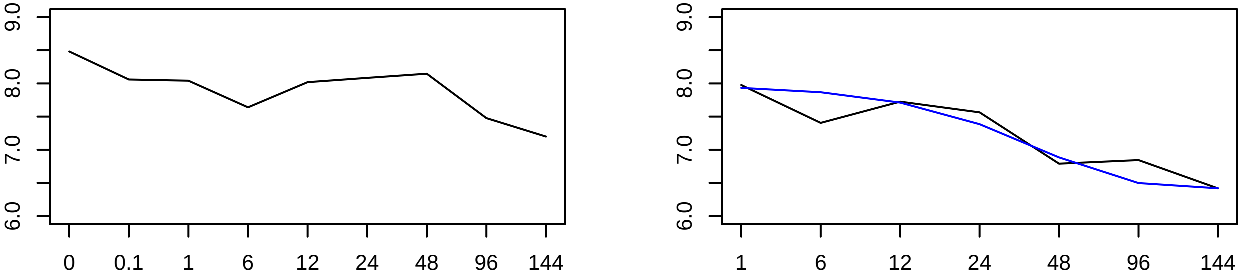

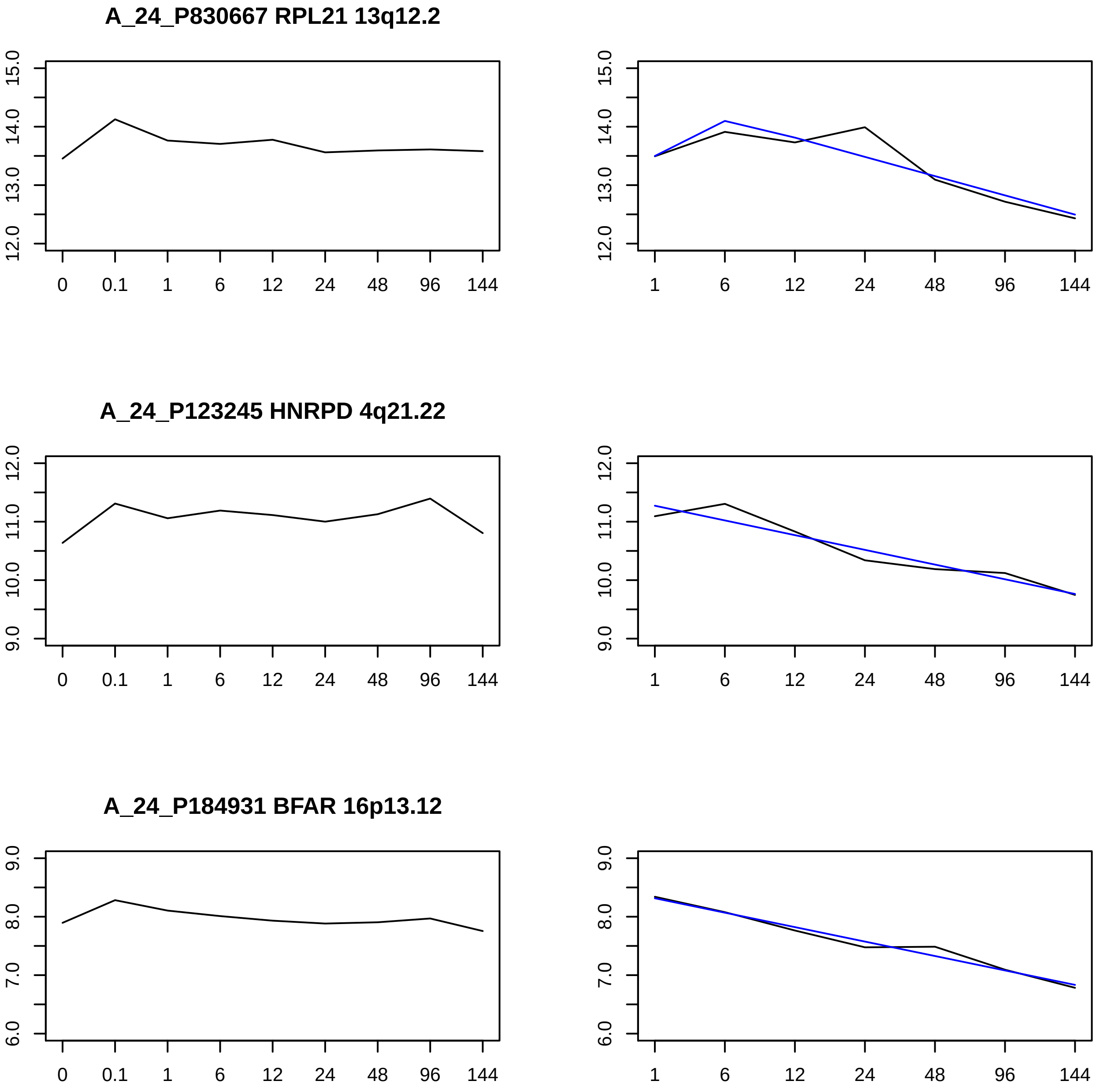
A_24_P830667 RPL21 13q12.2
12.0
13.0
14.0
15.0
0
0.1
1
6
12
24
48
96
144
1
6
12
24
48
96
144
A_24_P123245 HNRPD 4q21.22
9.0
10.0
11.0
12.0
A_24_P184931 BFAR 16p13.12
6.0
7.0
8.0
9.0

A_24_P891276 LOC442292 7p15.2

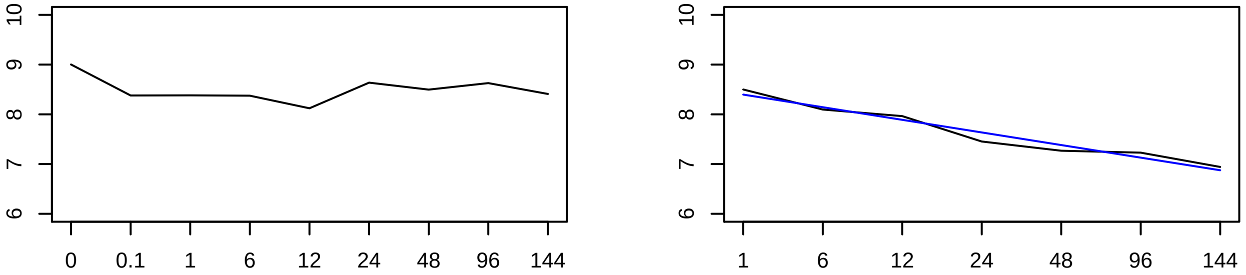

A_32_P211752 THC2645710 NA

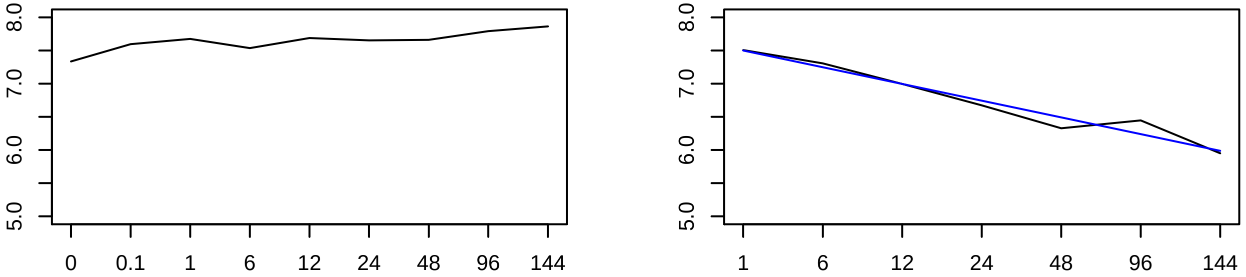

A_23_P138461 PEO1 10q24.31

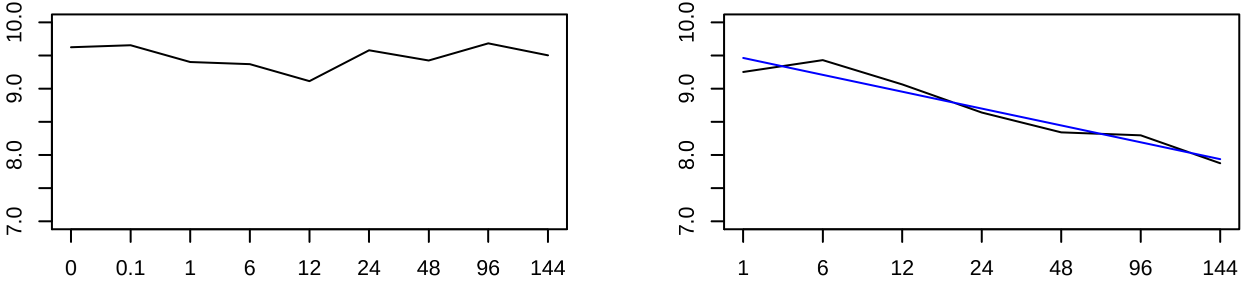

A_24_P366457 A_24_P366457 NA

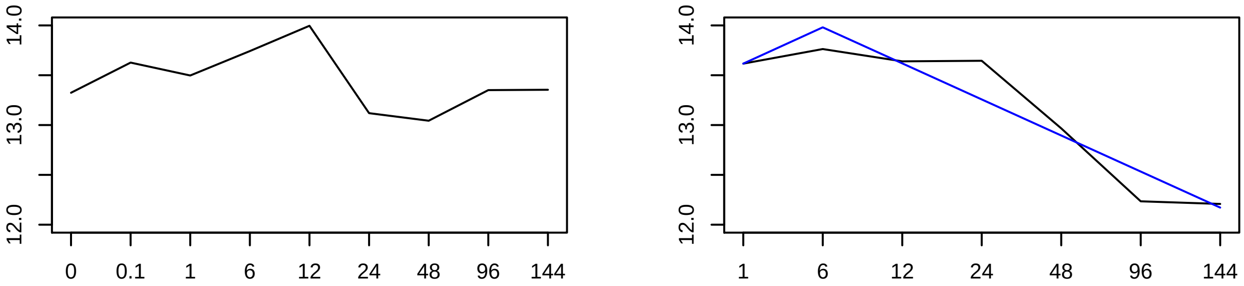

A_23_P371495 TMTC1 12p11.22

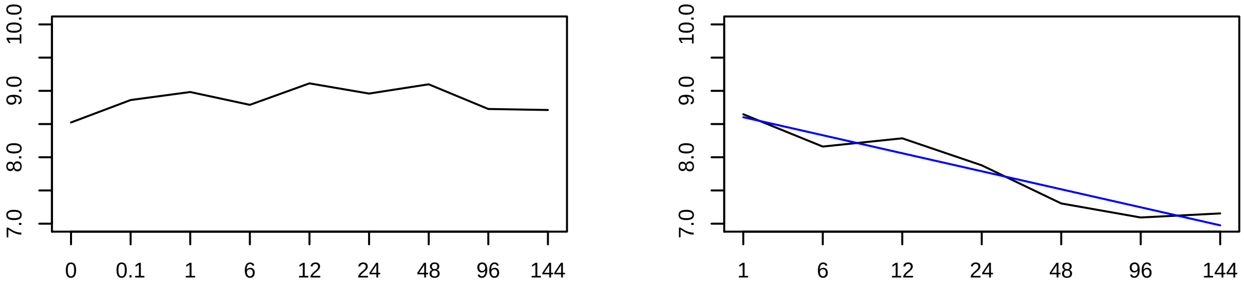

A_23_P9223 C9orf21 9q22.33

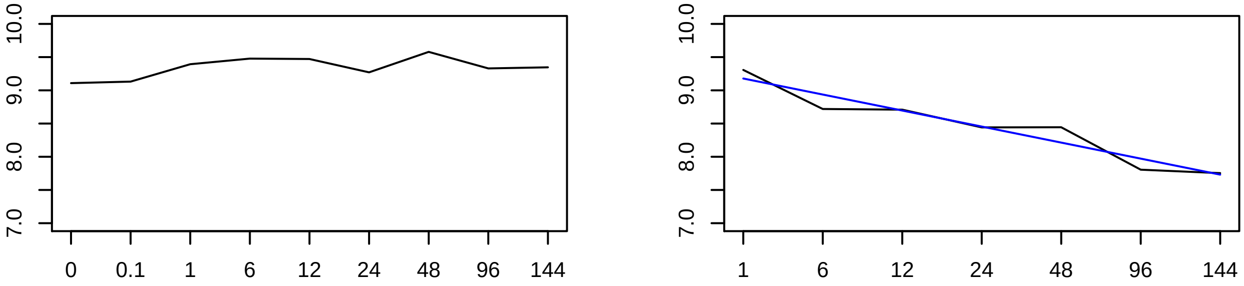

A_23_P22072 DLEU2 13q14.3

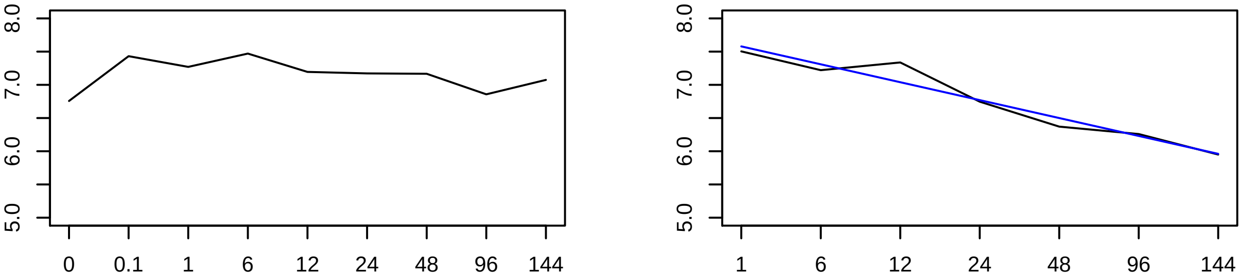

A_24_P101480 A_24_P101480 NA

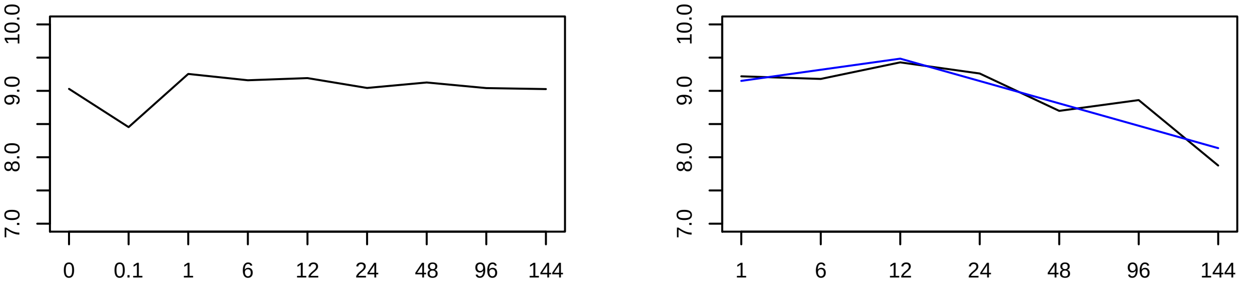

A_23_P87773 PARPBP 12q23.2

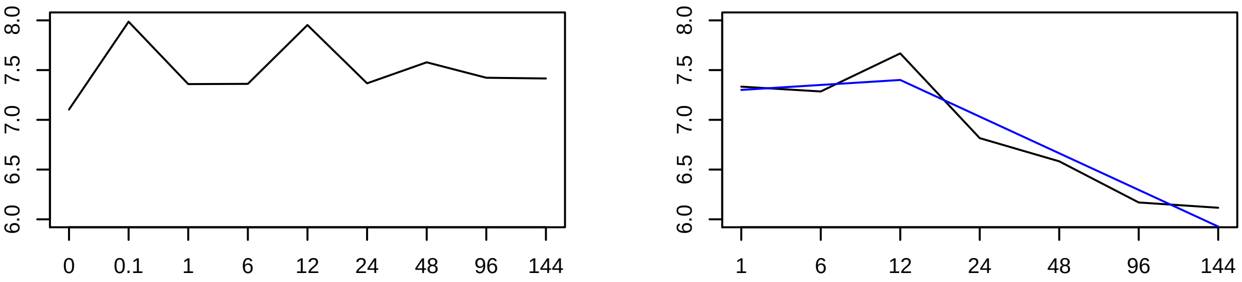

A_23_P46337 DIEXF 1q32.2

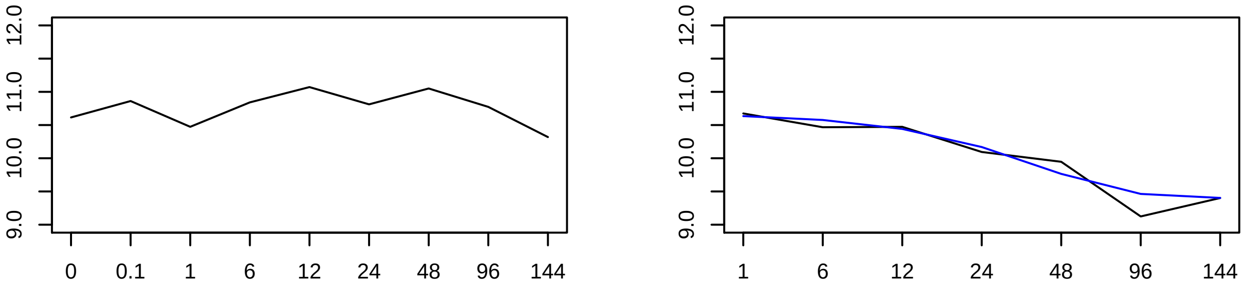

A_24_P280933 LOC647722 14q23.1

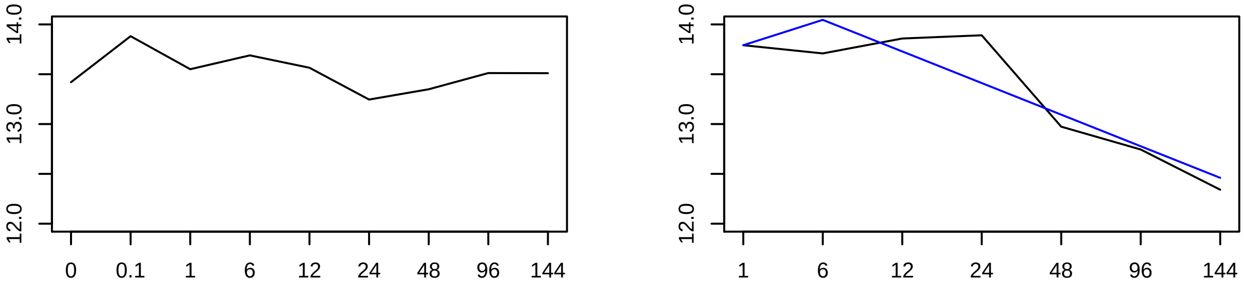

A_23_P350574 FCRLB 1q23.3

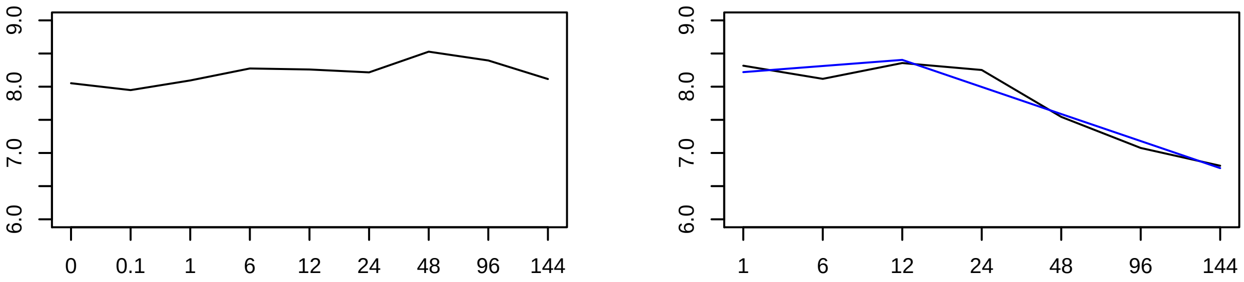

A_24_P88266 PROX1 1q41

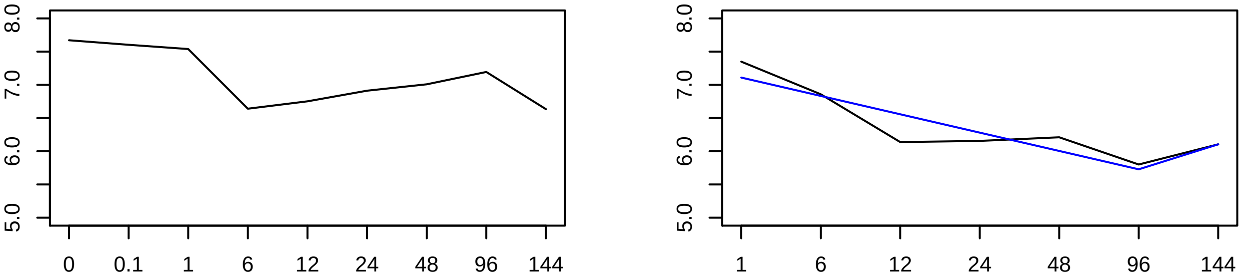

A_24_P32836 A_24_P32836 NA

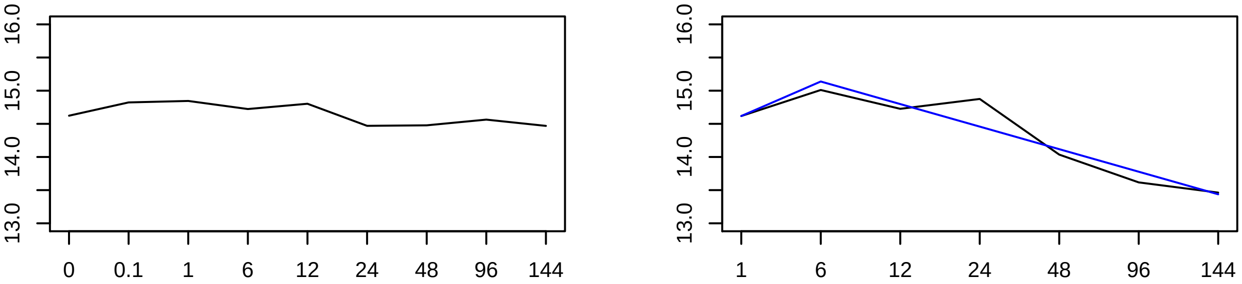

A_23_P370035 WDR21A 14q24.2

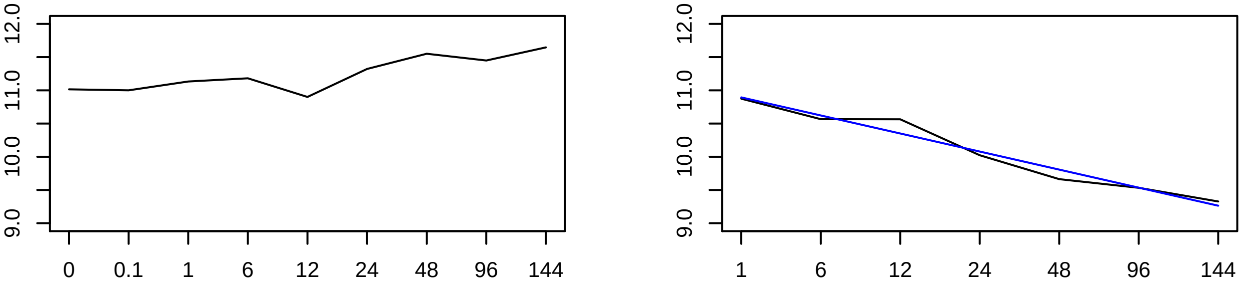

A_24_P50707 LOC645573 18p11.22

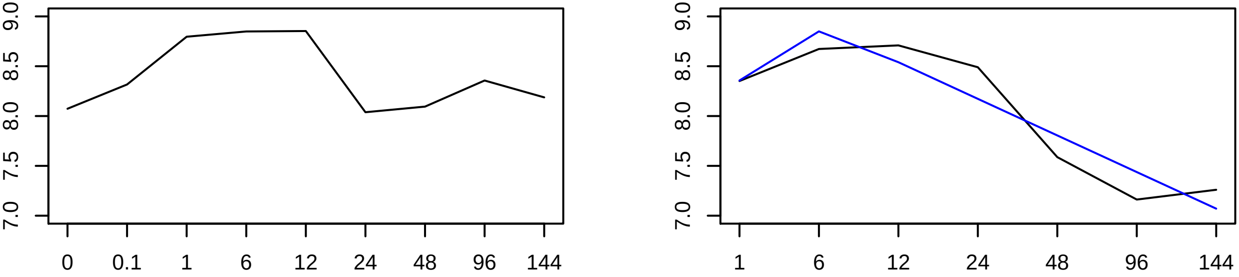

A_23_P96556 GK Xp21.2

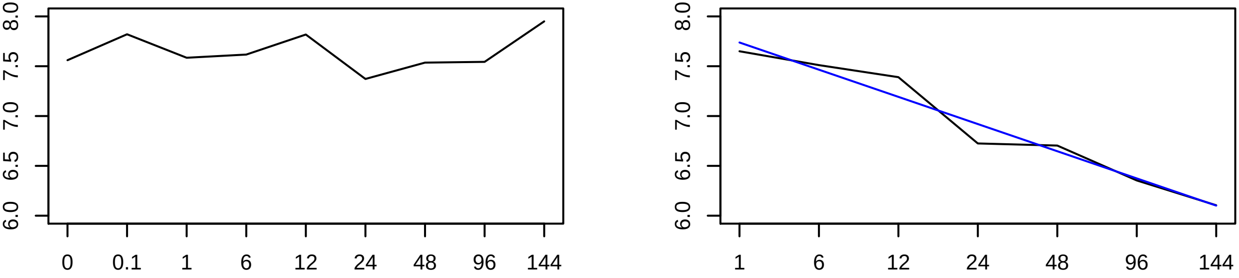

A_32_P94087 LOC402176 4q12

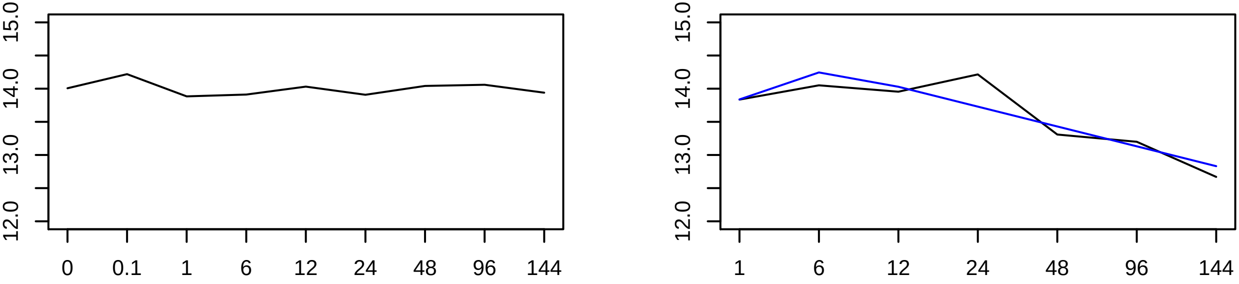

A_24_P289984 A_24_P289984 NA

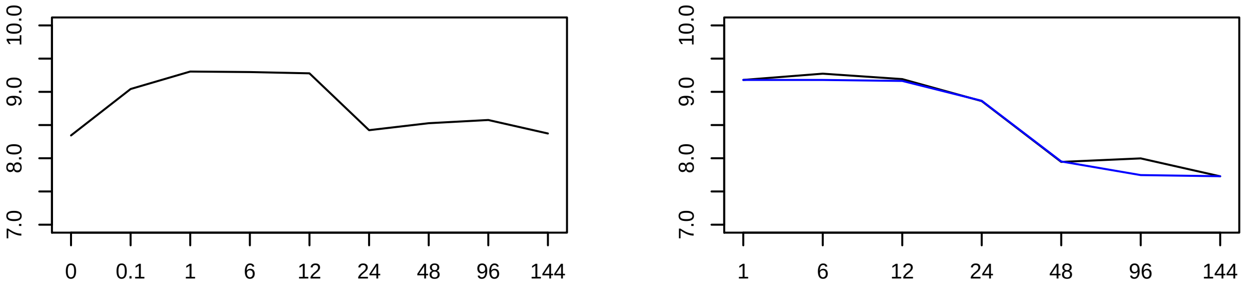

A_23_P76234 RPH3A 12q24.13

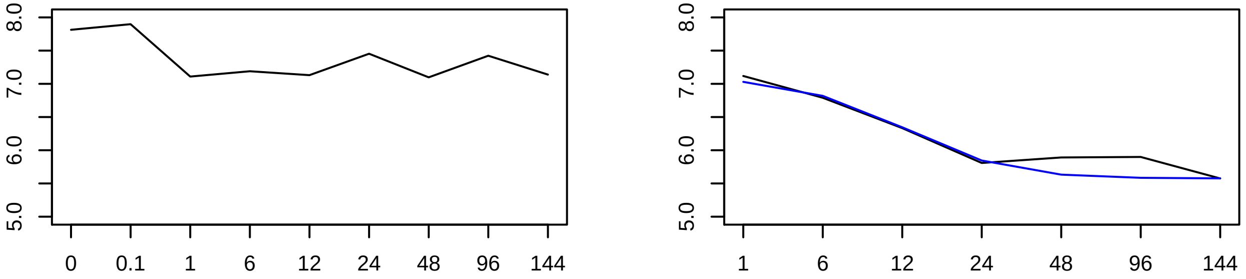

A_23_P86855 LRP16 11q13.1

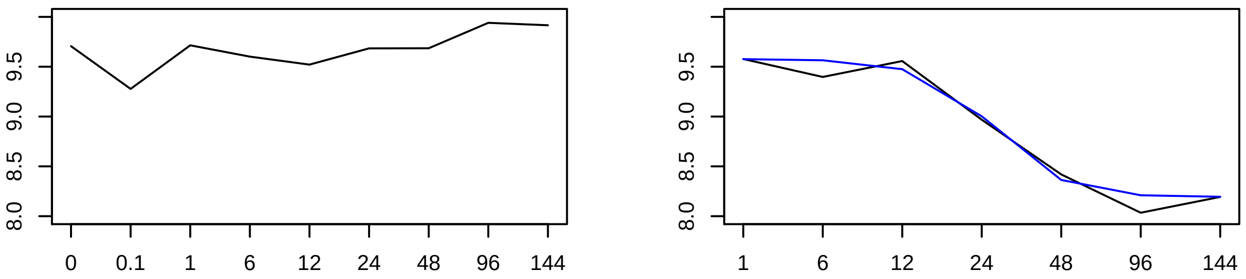

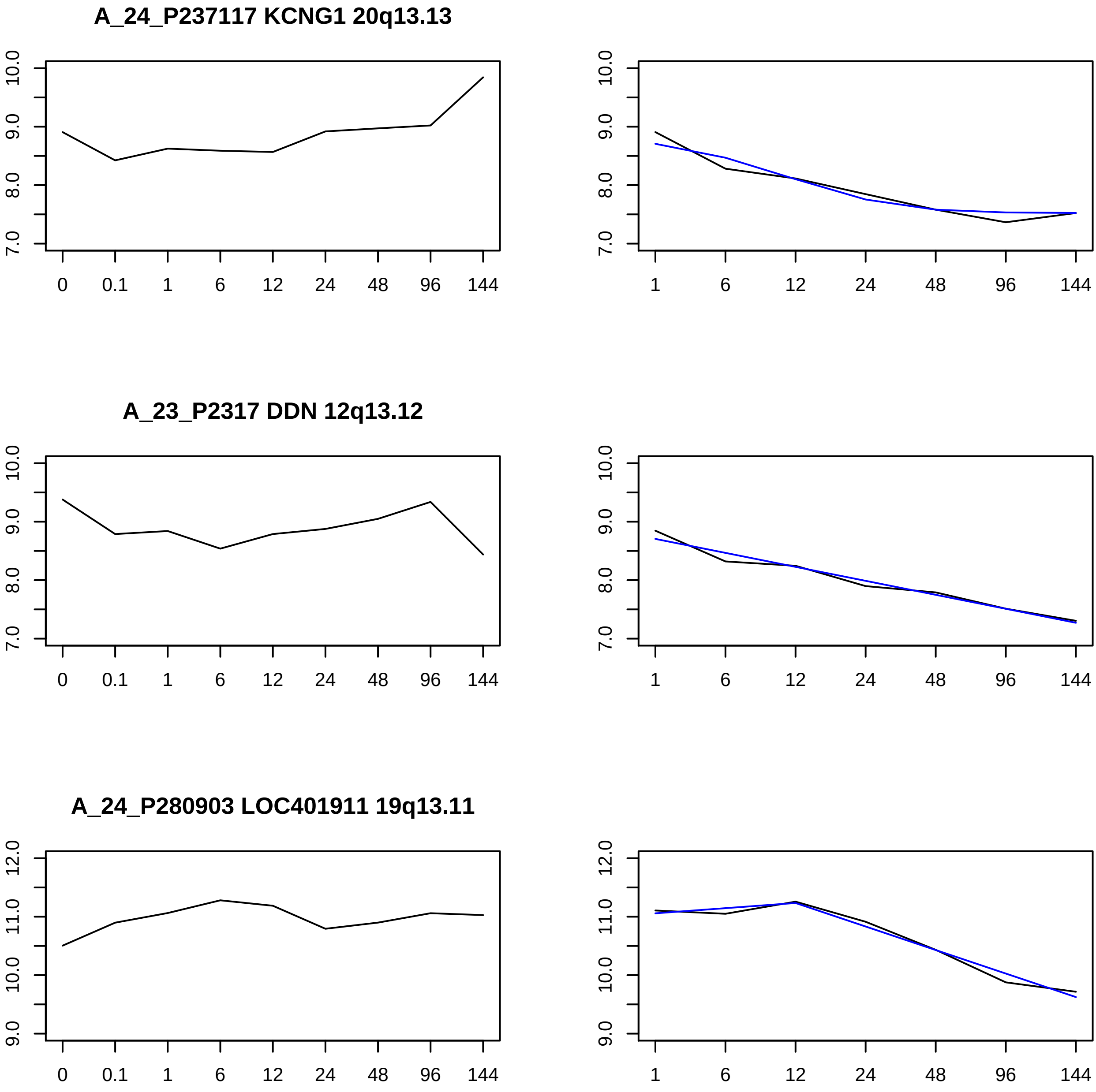
A_24_P237117 KCNG1 20q13.13
7.0
8.0
9.0
10.0
0
0.1
1
6
12
24
48
96
144
1
6
12
24
48
96
144
A_23_P2317 DDN 12q13.12
A_24_P280903 LOC401911 19q13.11
9.0
10.0
11.0
12.0

A_24_P212864 LOC646161 7p21.2

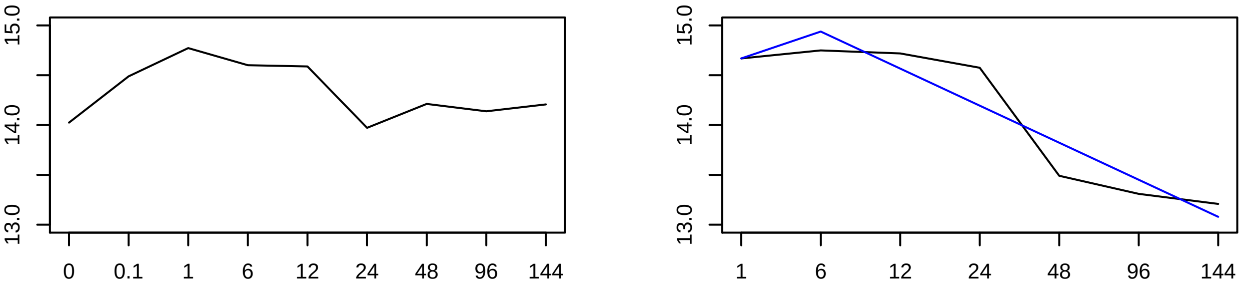

A_24_P392022 FAM86A 16p13.3

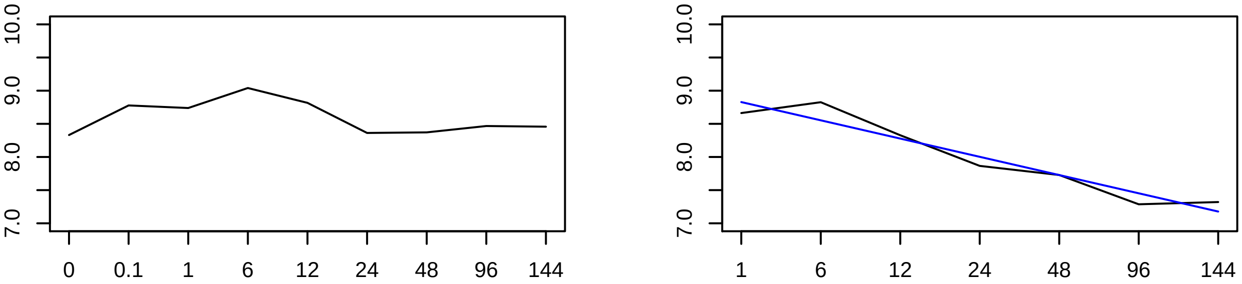

A_32_P154091 LOC389641 8p21.3

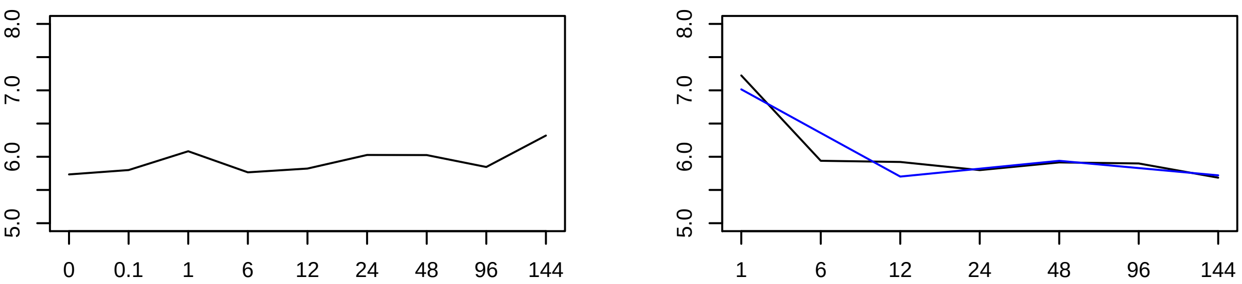

A_23_P427703 MT1L 16q13

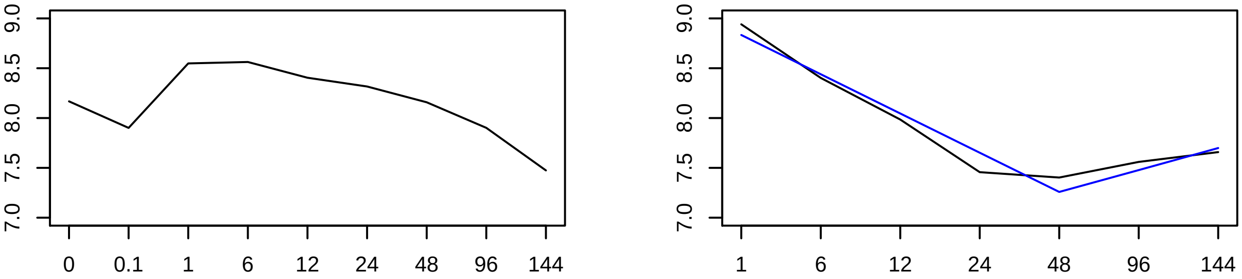

A_24_P459522 AK000144 NA

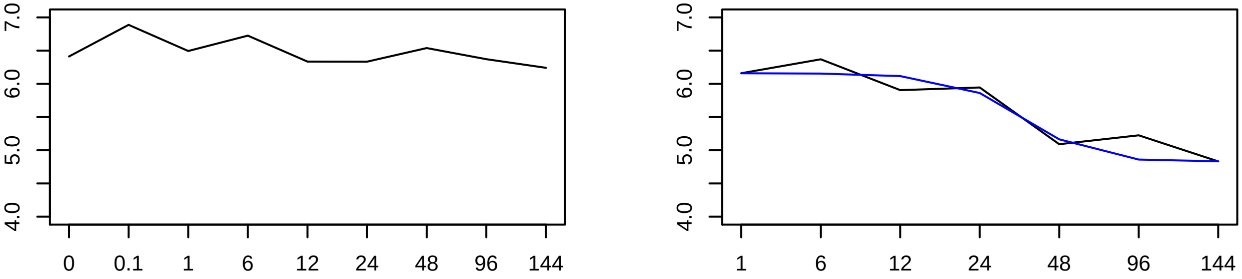

A_23_P160934 ANP32E 1q21.2

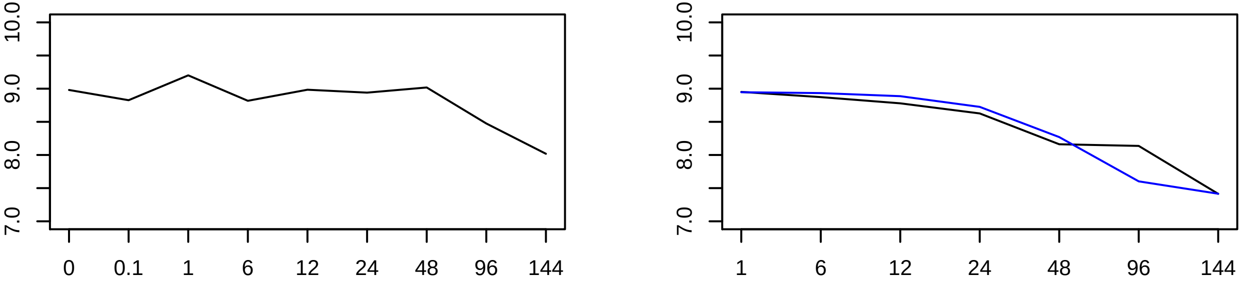

A_32_P20717 HADHAP1 NA

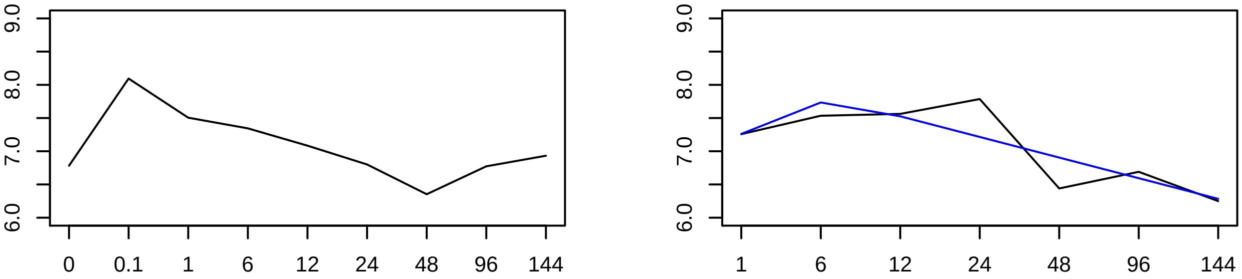

A_24_P134727 TFAM 10q21.1

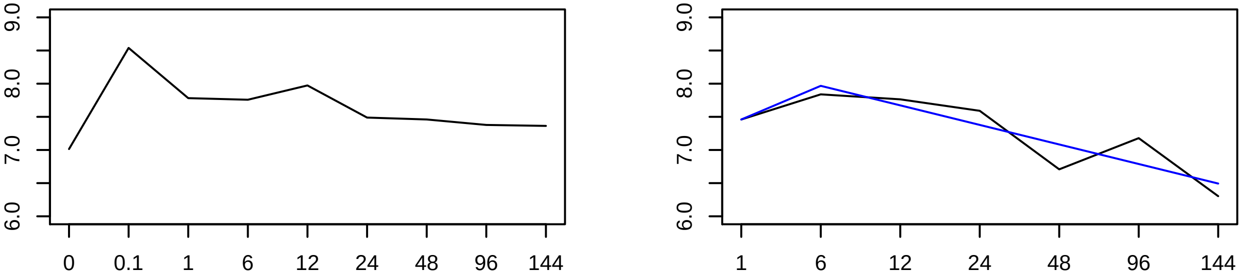

A_24_P160920 RP11−256G5.1 6p21.1

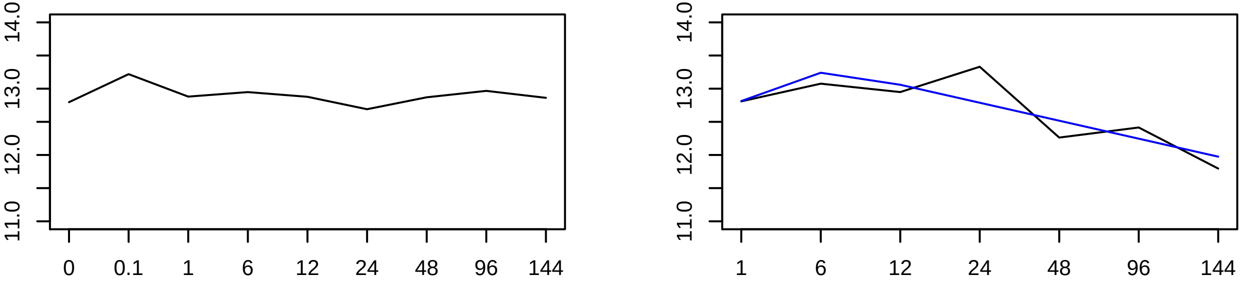

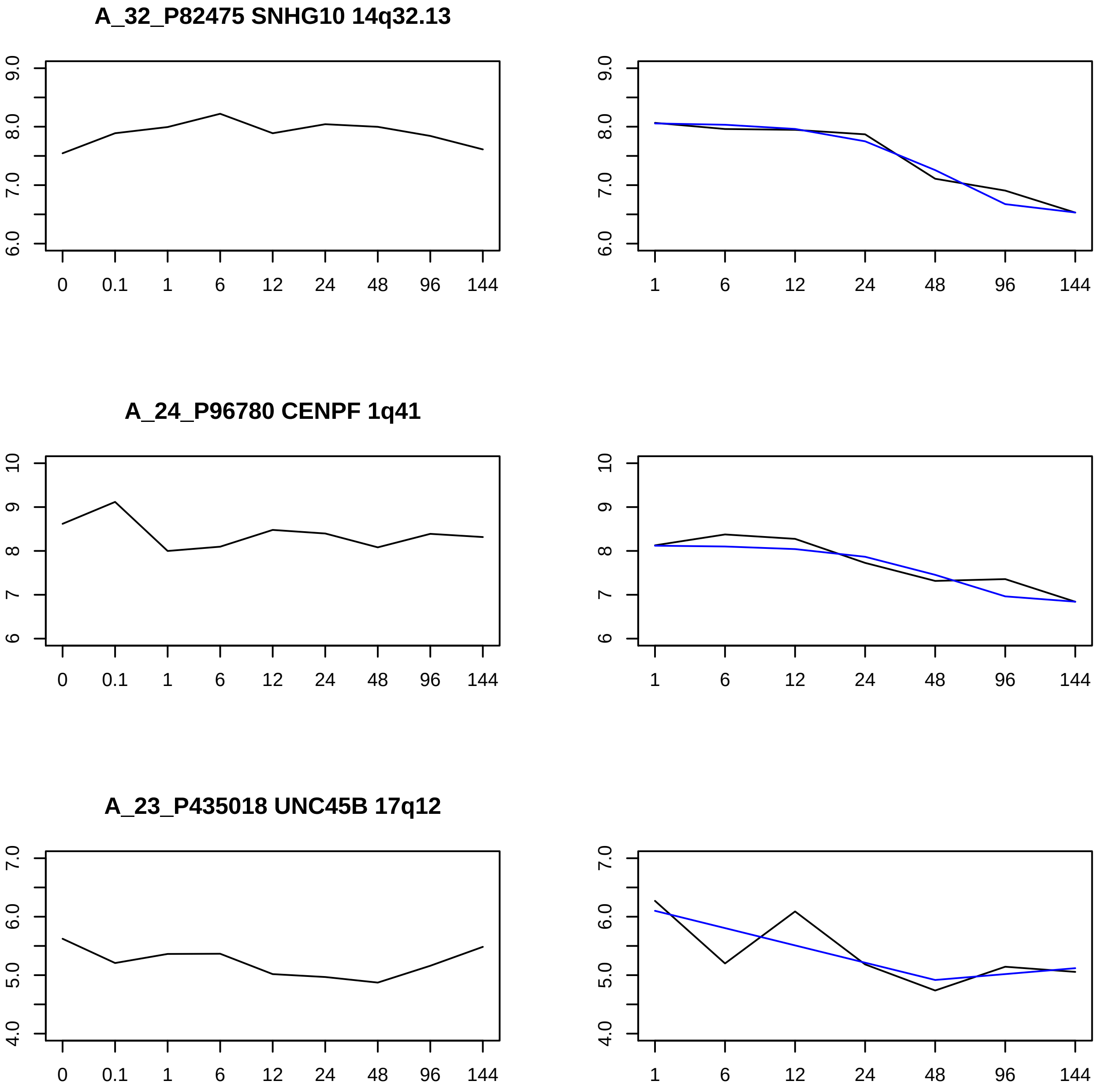

A_32_P82475 SNHG10 14q32.13
6.0
7.0
8.0
9.0
0
0.1
1
6
12
24
48
96
144
A_24_P96780 CENPF 1q41
6
7
8
9
10
A_23_P435018 UNC45B 17q12
4.0
5.0
6.0
7.0

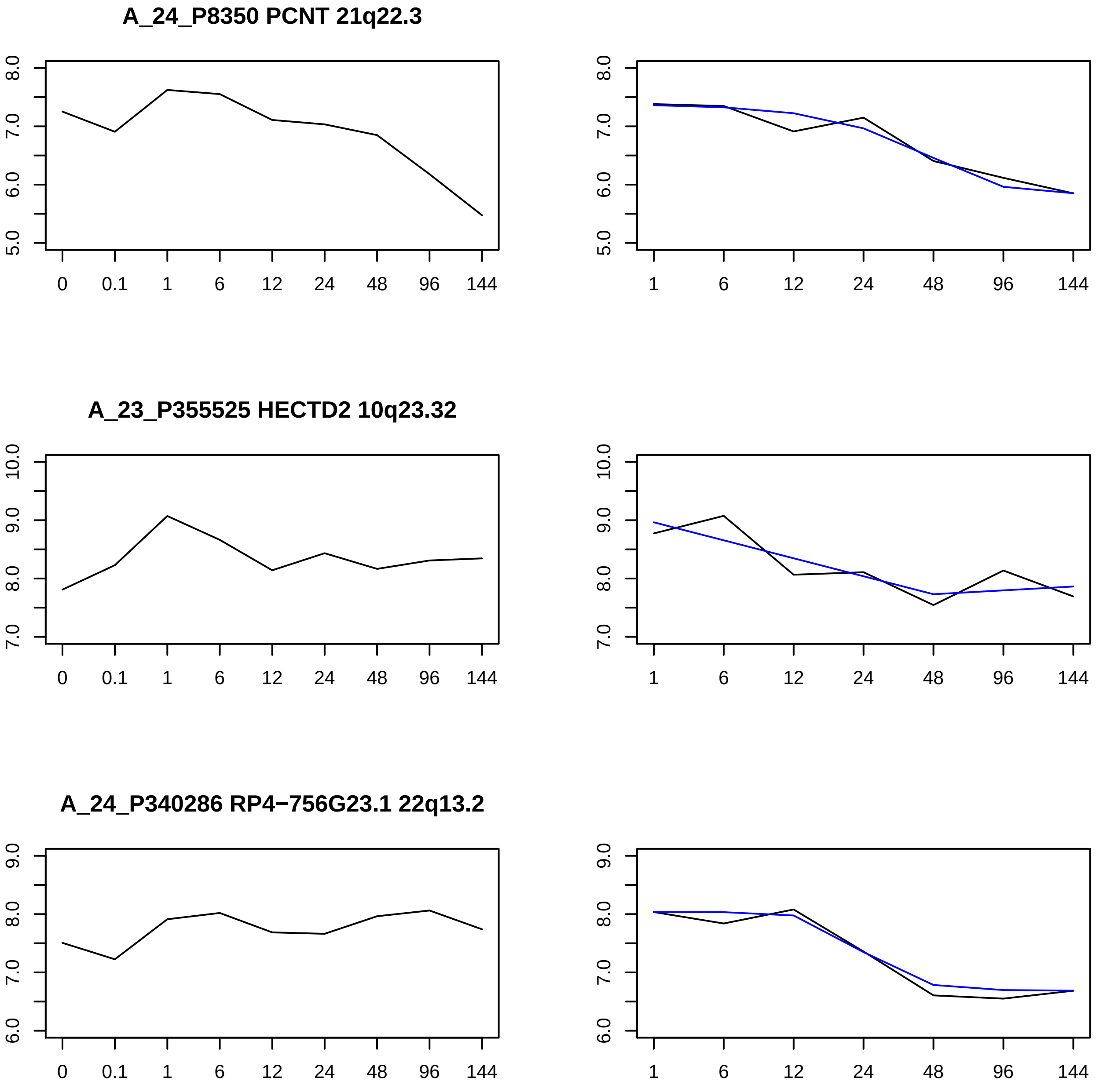

A_24_P8350 PCNT 21q22.3
5.0
6.0
7.0
8.0
0
0.1
1
6
12
24
48
96
144
A_23_P355525 HECTD2 10q23.32
7.0
8.0
9.0
10.0
A_24_P340286 RP4−756G23.1 22q13.2
6.0
7.0
8.0
9.0

A_24_P187921 MANEA 6q16.1

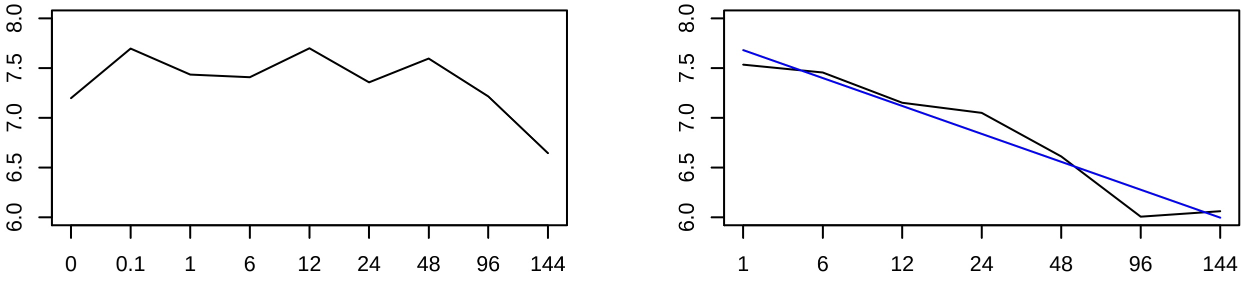

A_32_P361884 MGC34824 2p22.2

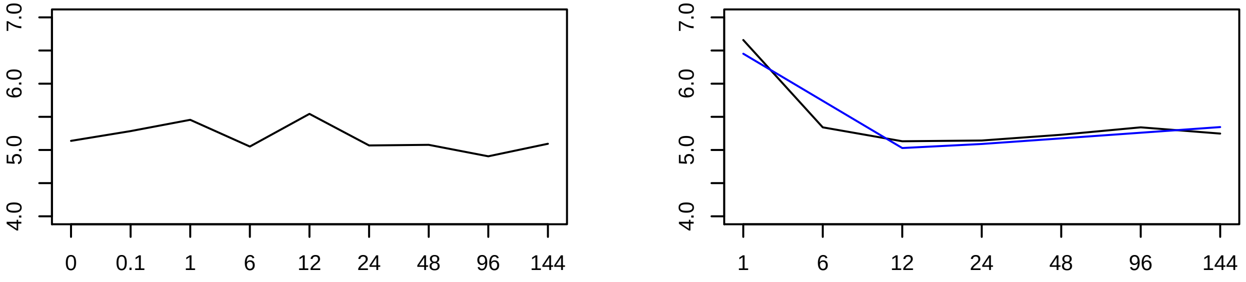

A_23_P129821 KCNJ12 17p11.2

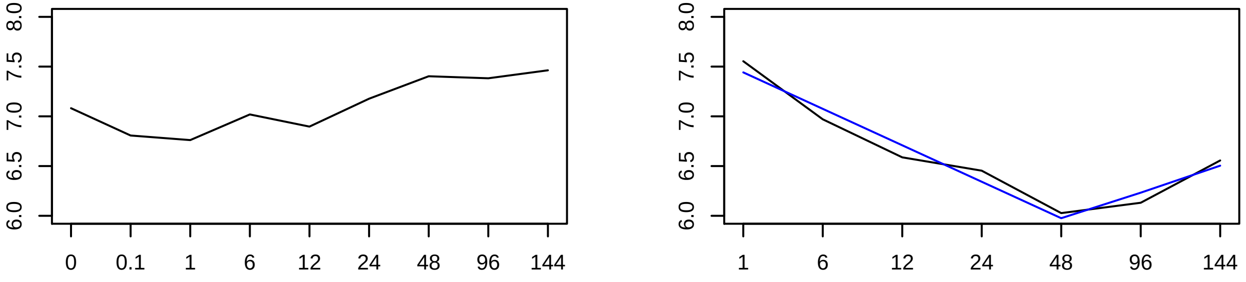

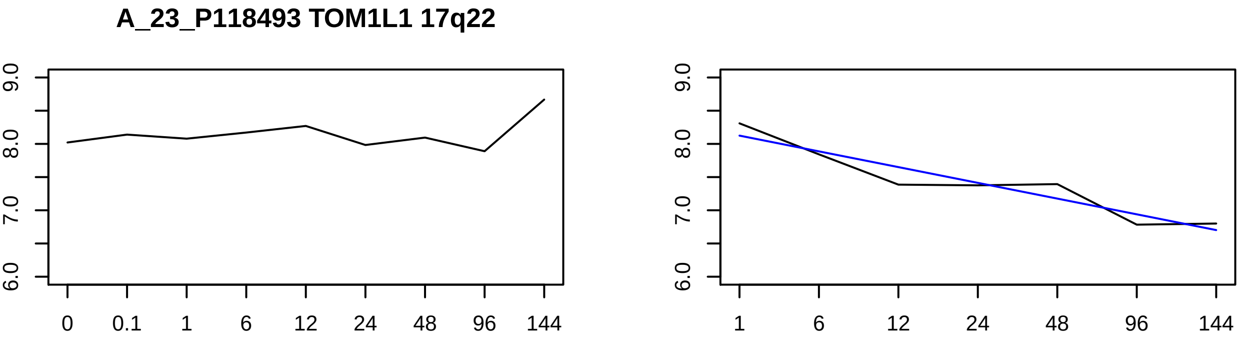
A_23_P118493 TOM1L1 17q22
6.0
7.0
8.0
9.0
0
0.1
1
6
12
24
48
96
144
1
6
12
24
48
96
144

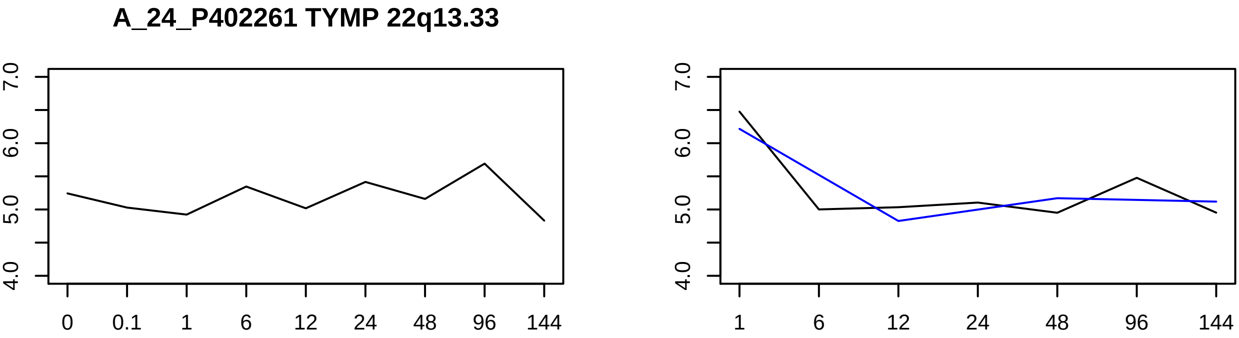
A_24_P402261 TYMP 22q13.33
4.0
5.0
6.0
7.0
0
0.1
1
6
12
24
48
96
144
1
6
12
24
48
96
144

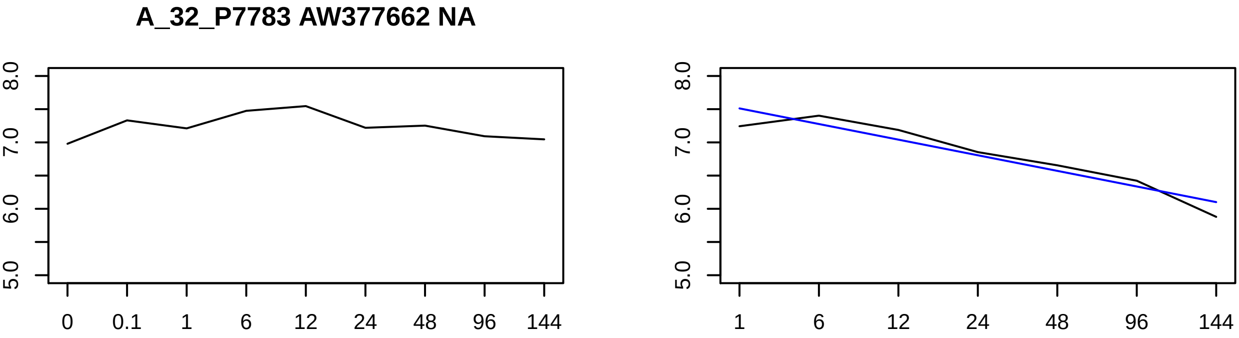
A_32_P7783 AW377662 NA
5.0
6.0
7.0
8.0
0
0.1
1
6
12
24
48
96
144
1
6
12
24
48
96
144

A_32_P22622 C4orf9 4p16.3

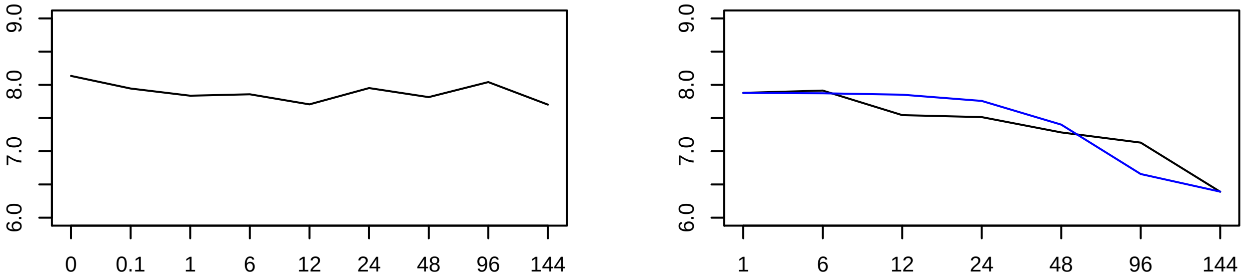

A_23_P212383 SACM1L 3p21.31

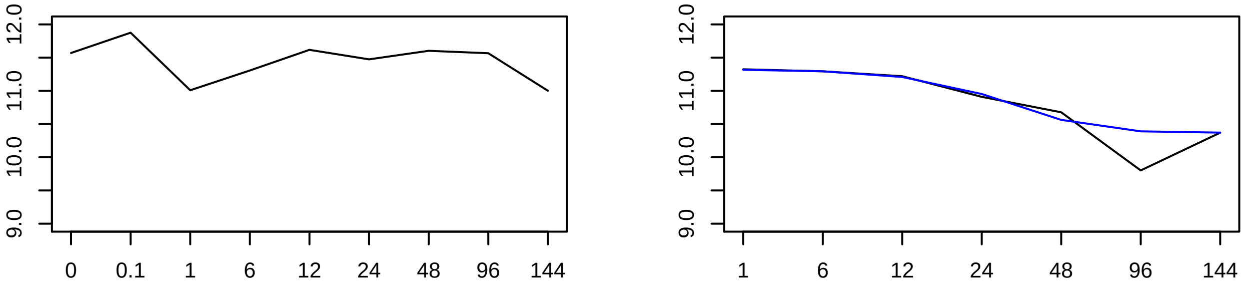

A_23_P213350 A_23_P213350 NA

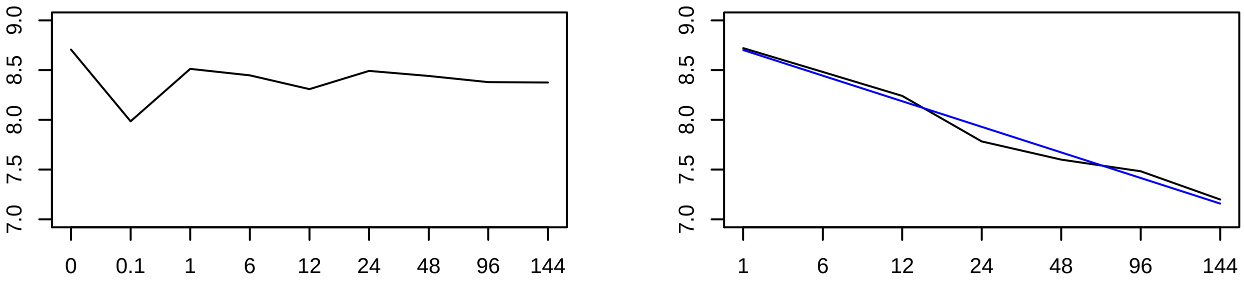

A_23_P256455 RPA3 7p21.3

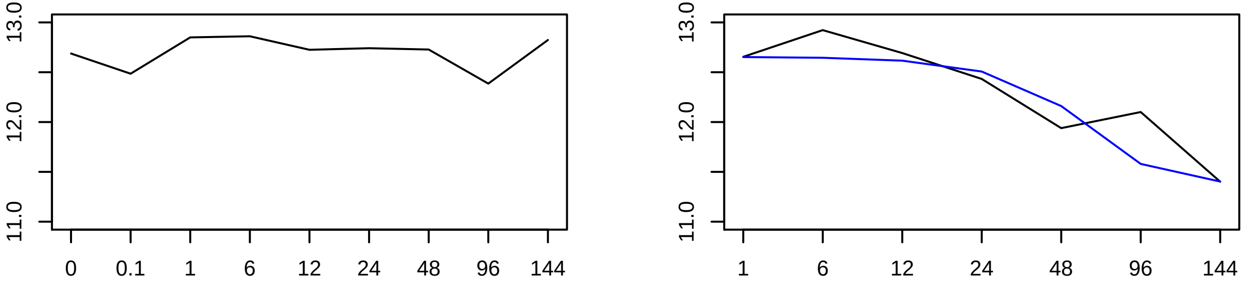

A_23_P153146 CDH7 18q22.1

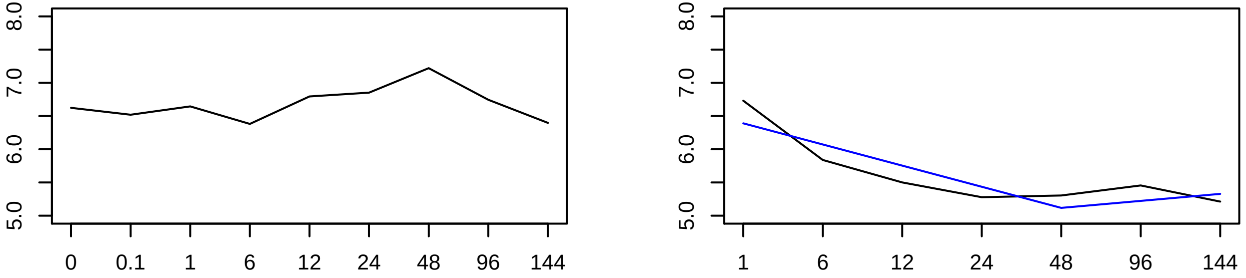

A_32_P5628 A_32_P5628 NA

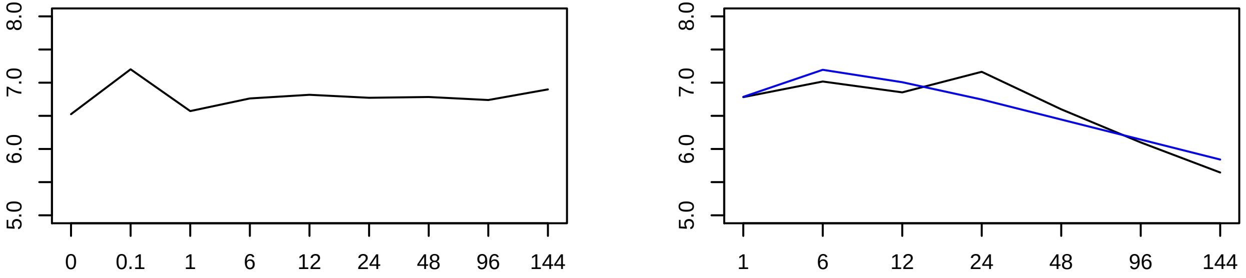

A_23_P379159 BRWD3 Xq21.1

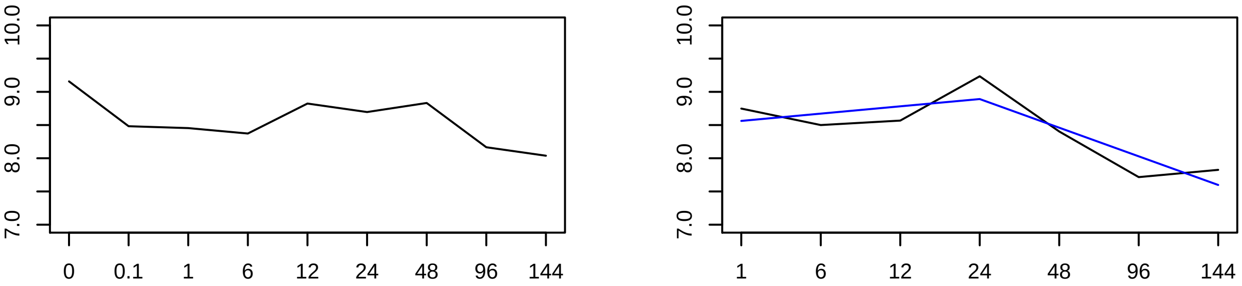

A_23_P165162 HAMP 19q13.12

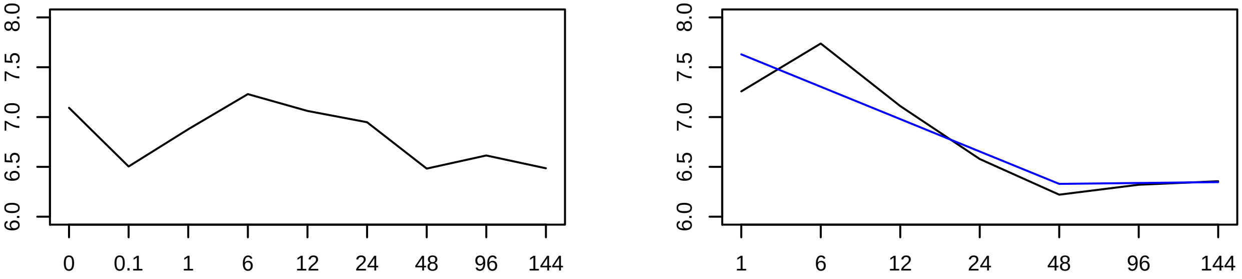

A_24_P50753 NUDT4 12q22

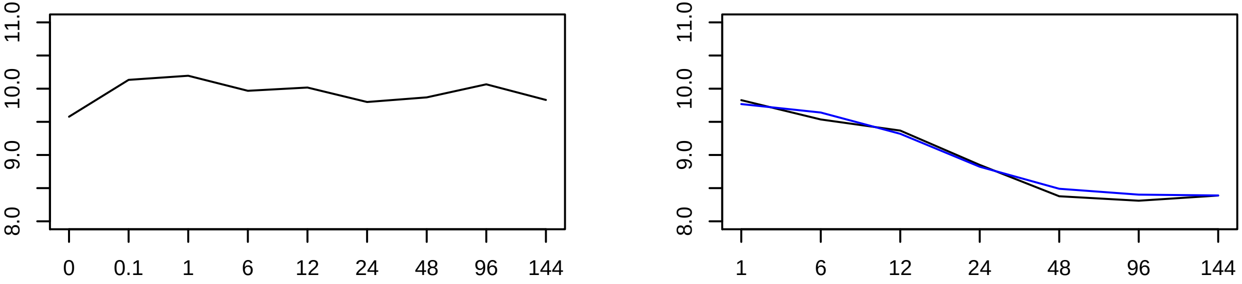

A_24_P747721 A_24_P747721 NA

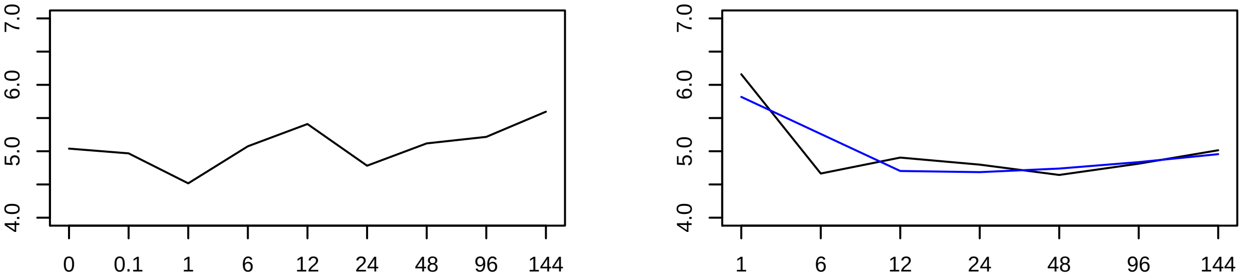

A_32_P221799 HIST1H2AM 6p22.1

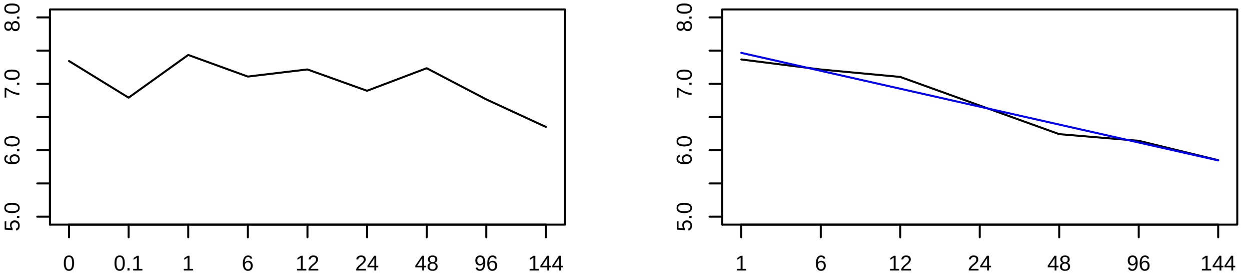

A_24_P192727 KAZALD1 10q24.31

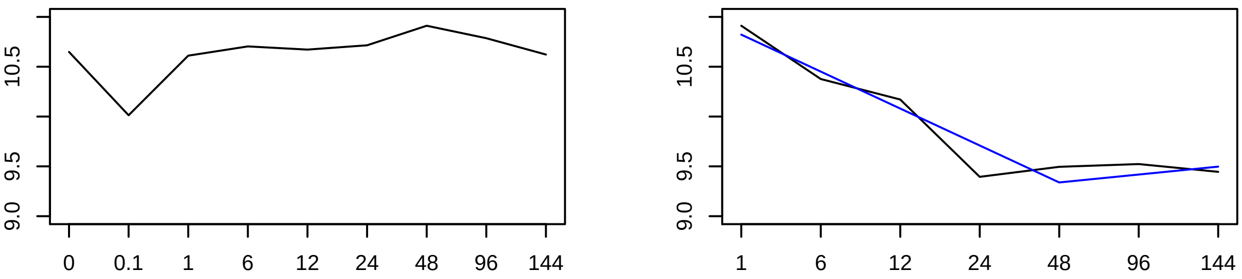

A_24_P170384 ENST00000313760 NA

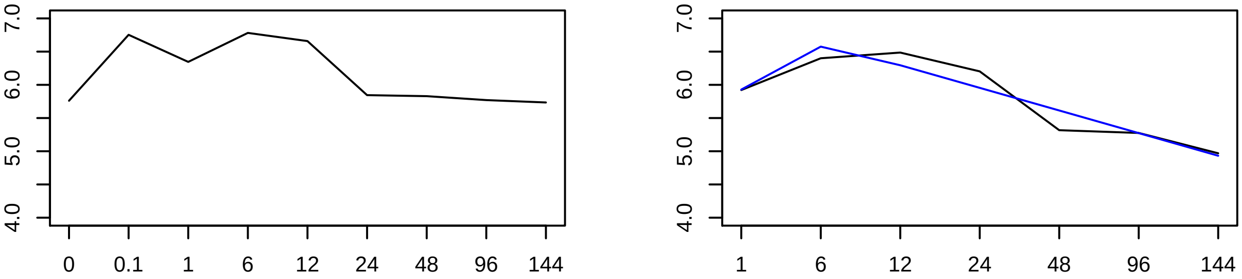

A_32_P148085 TOB1 17q21.33

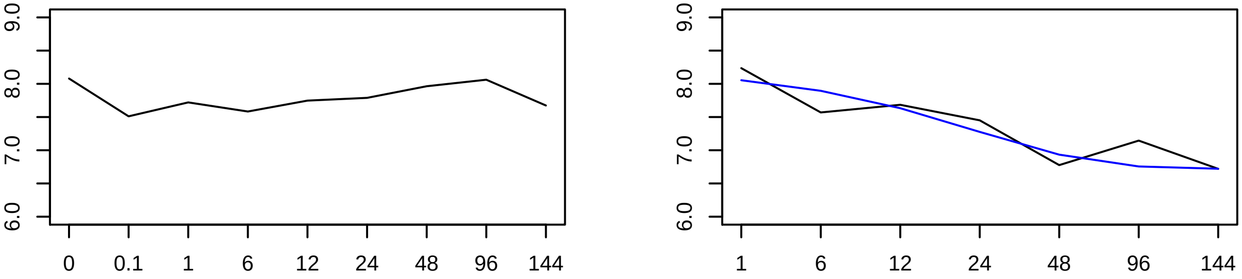

A_23_P66867 GEMIN4 17p13.3

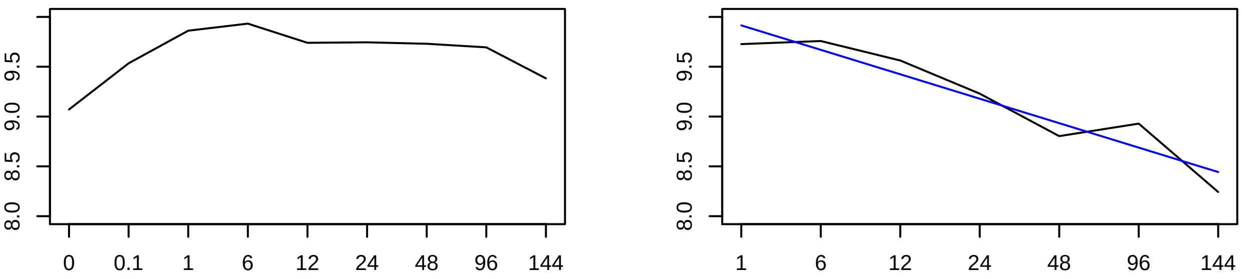

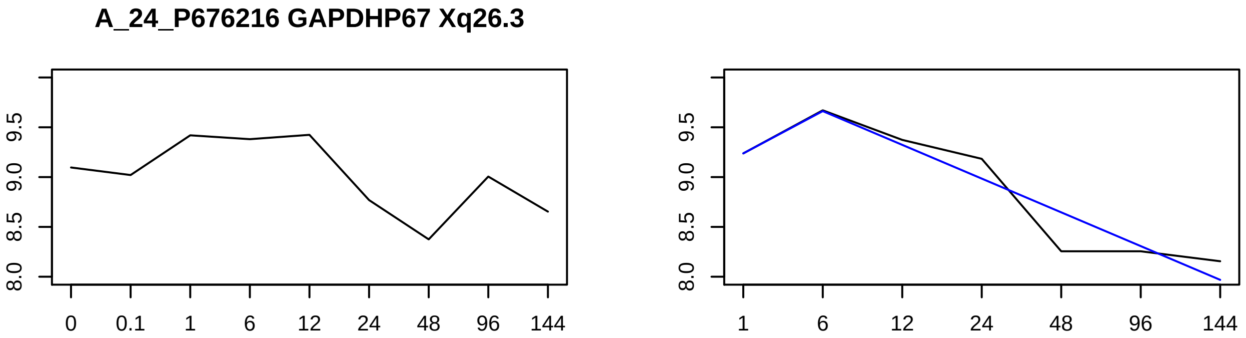
A_24_P676216 GAPDHP67 Xq26.3
8.0
8.5
9.0
9.5
0
0.1
1
6
12
24
48
96
144
1
6
12
24
48
96
144

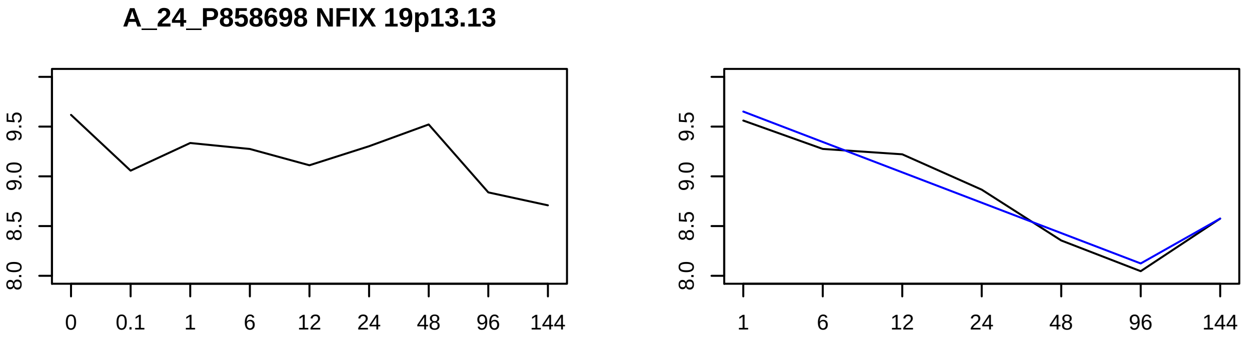
A_24_P858698 NFIX 19p13.13
8.0
8.5
9.0
9.5
0
0.1
1
6
12
24
48
96
144
1
6
12
24
48
96
144

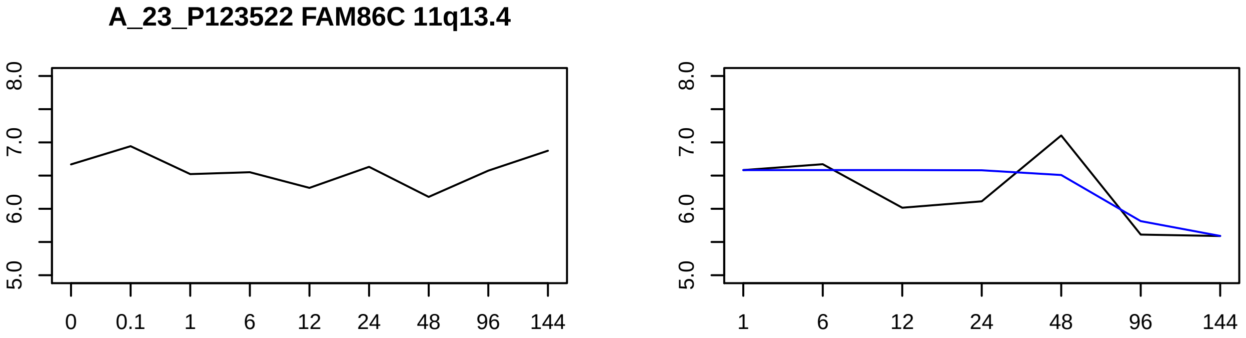
A_23_P123522 FAM86C 11q13.4
5.0
6.0
7.0
8.0
0
0.1
1
6
12
24
48
96
144
1
6
12
24
48
96
144

A_24_P169645 A_24_P169645 NA

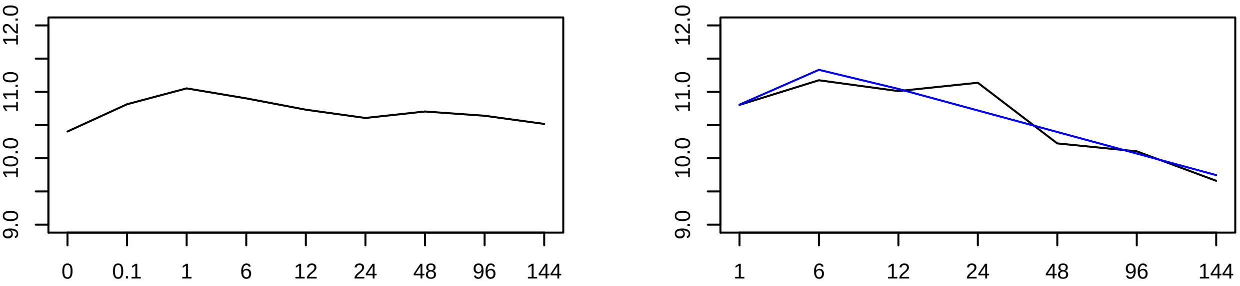

A_32_P44775 C9orf85 9q21.13

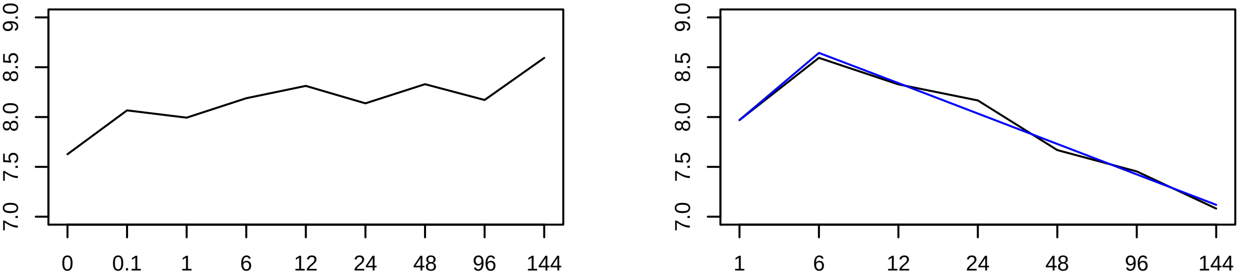

A_24_P823011 AMMECR1 NA

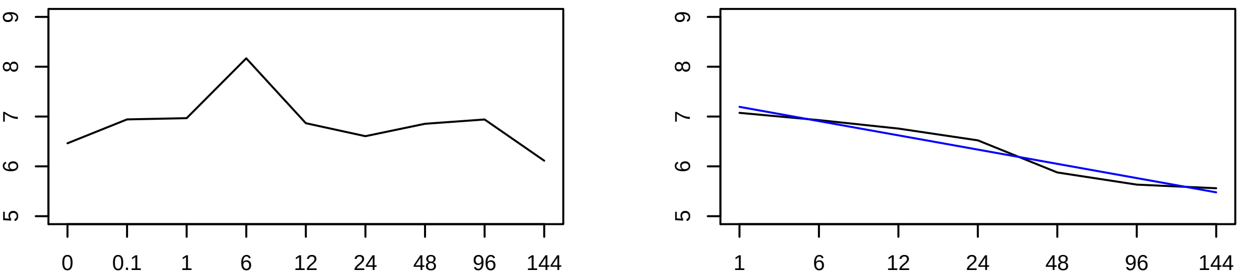

A_23_P416395 STC2 5q35.2

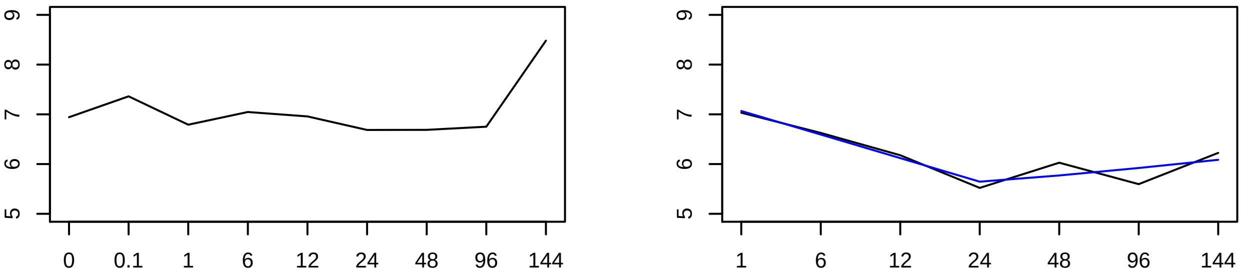

A_23_P103180 TYW3 1p31.1

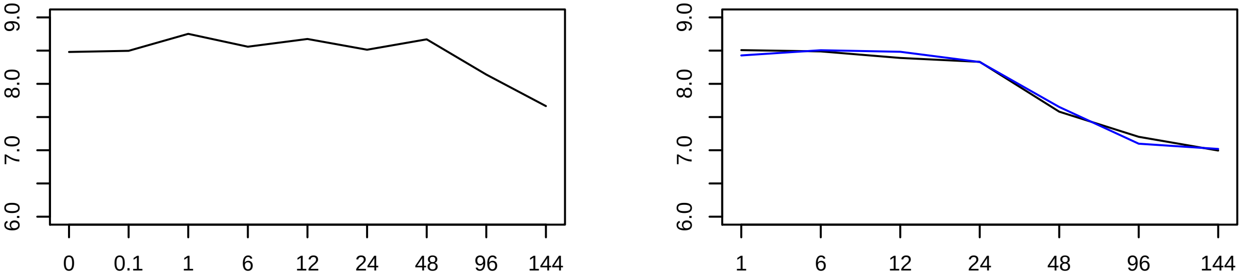

A_23_P121564 GUCY1B3 4q32.1

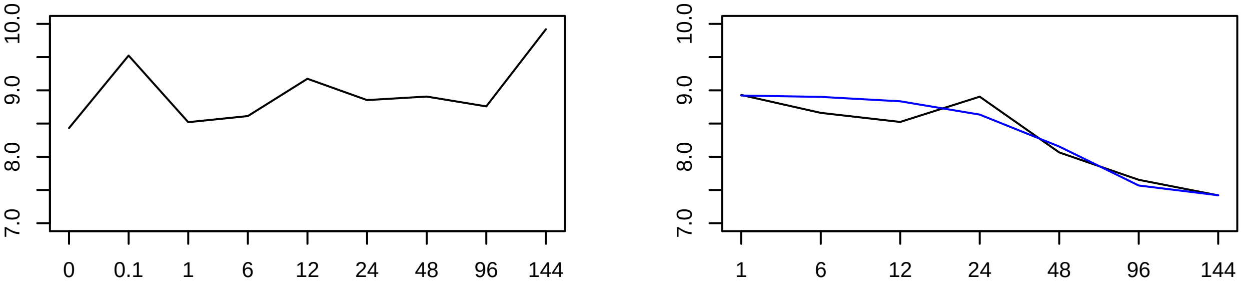

A_23_P80752 PLXND1 3q21.3

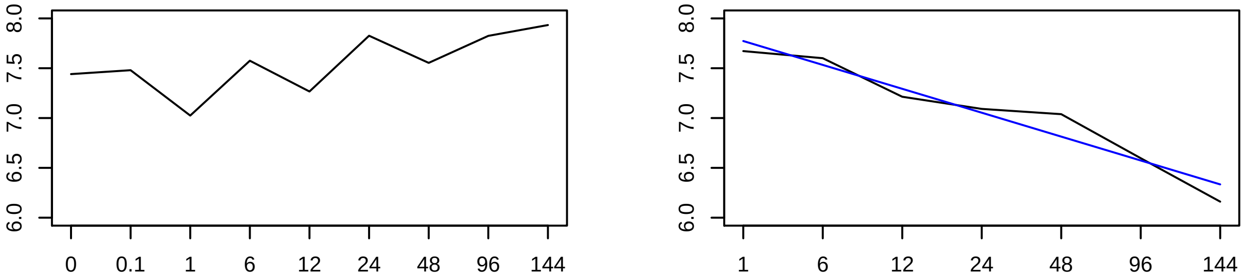

A_23_P211835 AGTR1 3q24

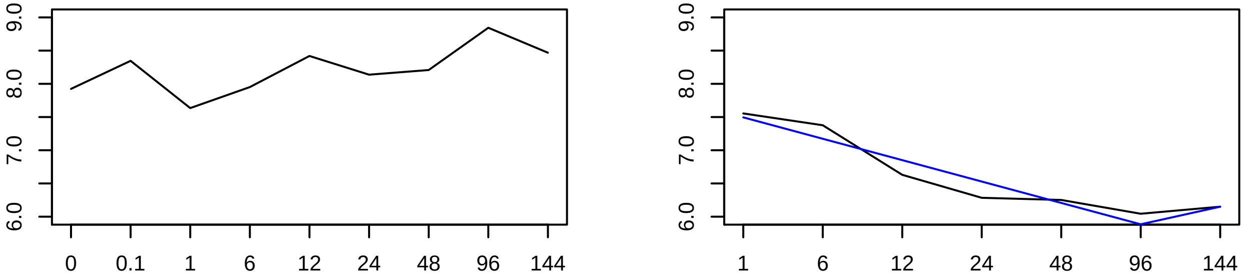

A_24_P273823 NPAT 11q22.3

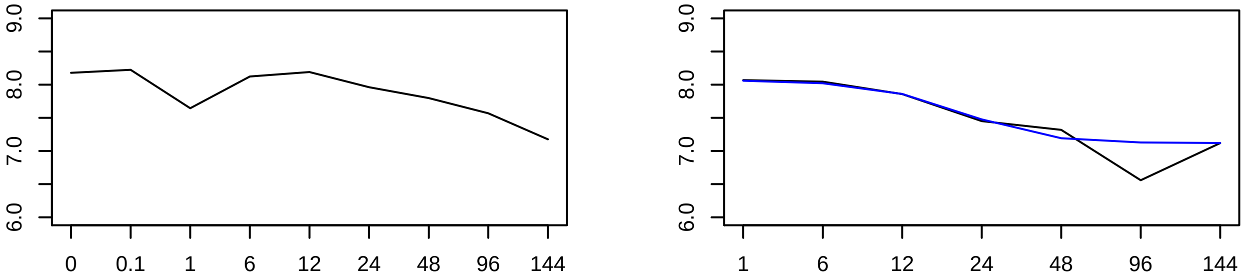

A_24_P919460 RPS2 16p13.3

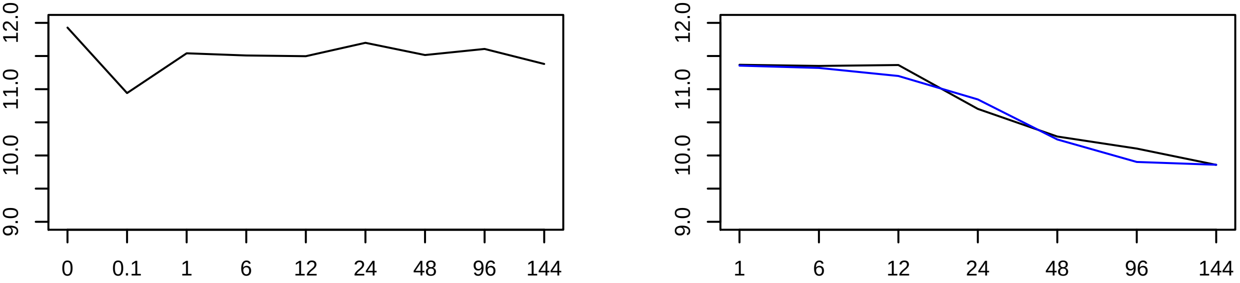

A_24_P607195 A_24_P607195 NA

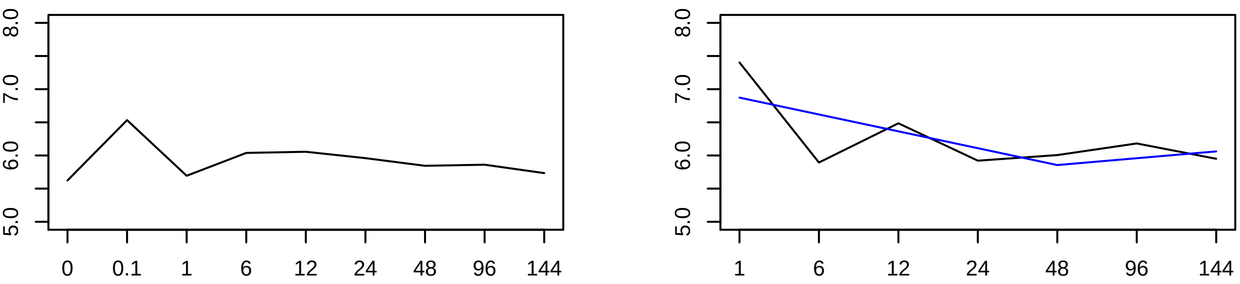

A_32_P210252 RPL22 1p36.31

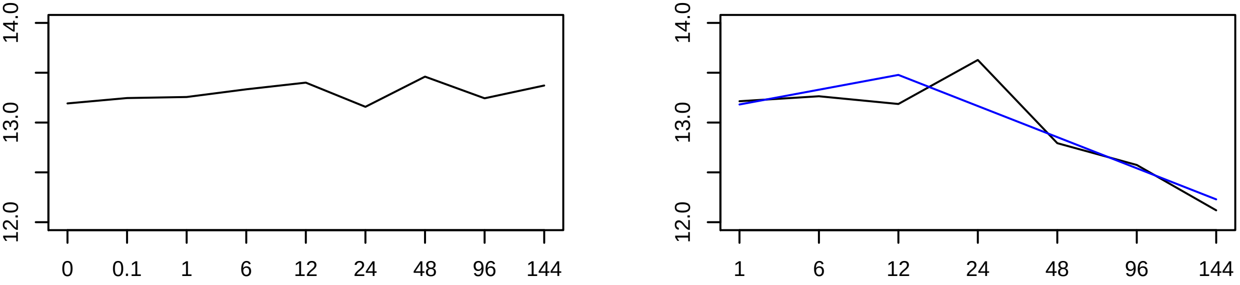

A_24_P913384 BQ323258 NA

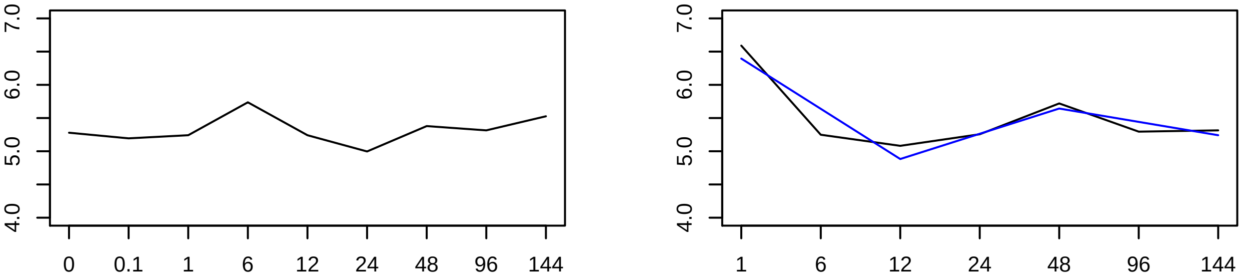

A_23_P218331 CYB561 17q23.3

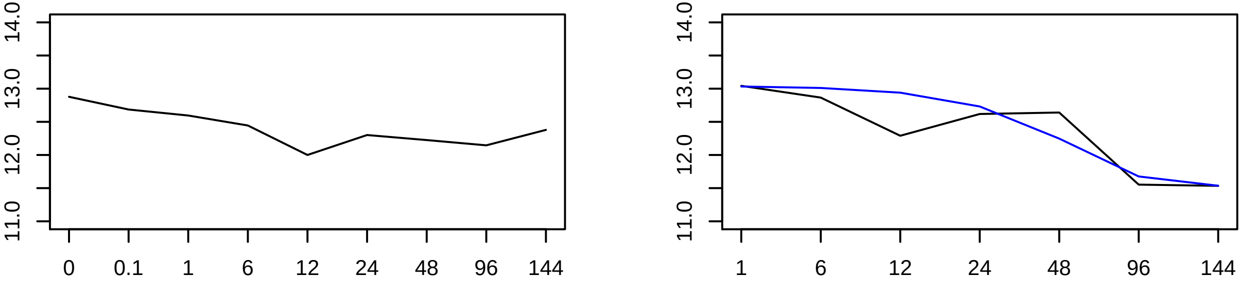

A_23_P7636 PTTG1 5q33.3

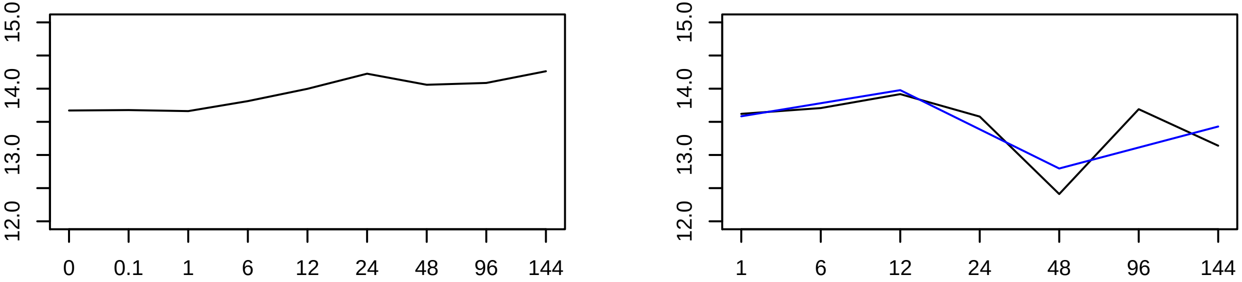

A_24_P264106 RPL23A 17q11.2

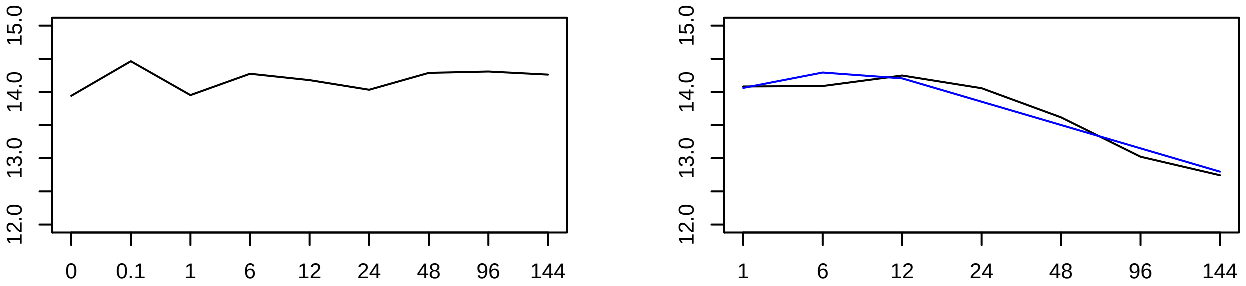

A_24_P30206 BCCIP 10q26.2

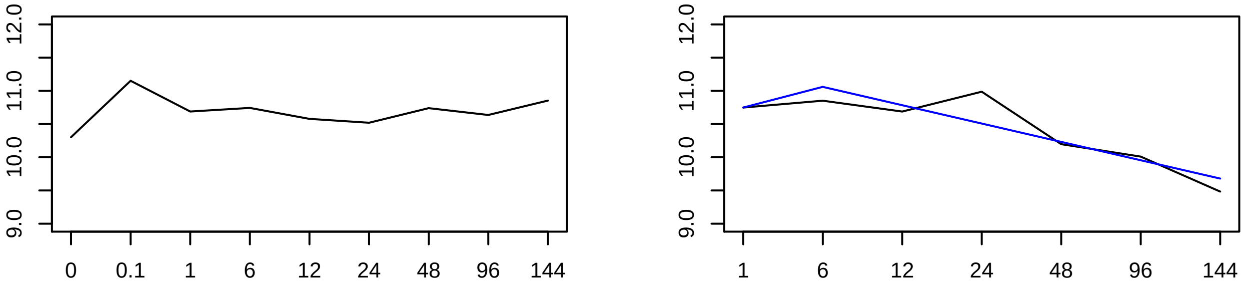

A_32_P162306 NPM1P24 1p36.11

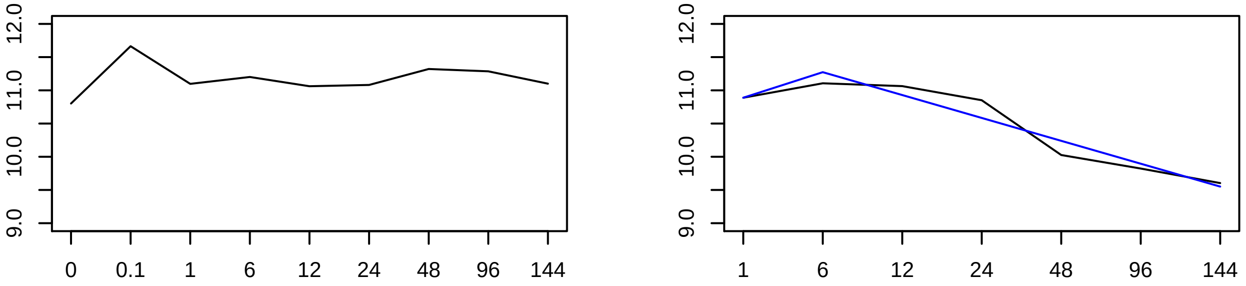

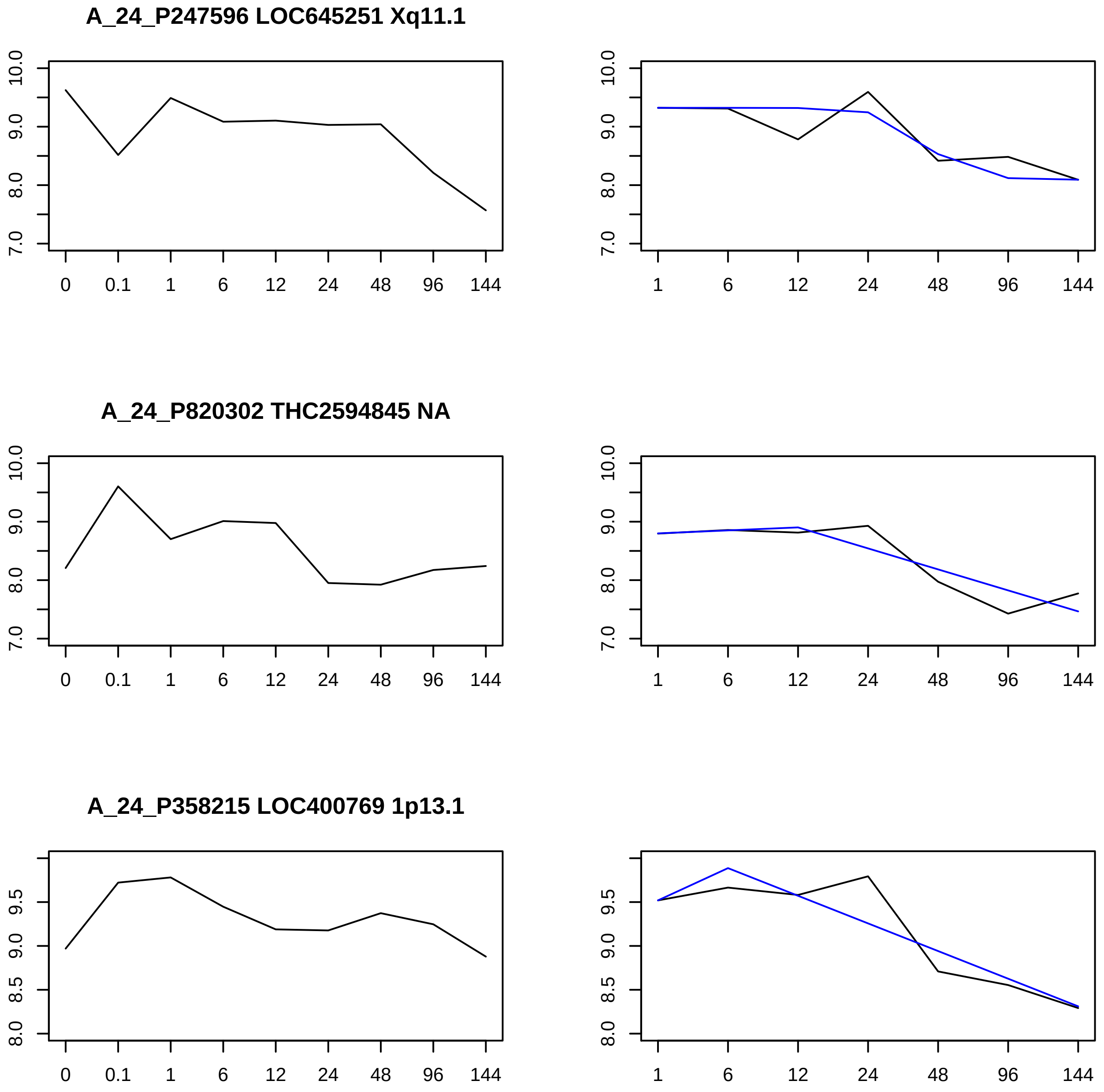
A_24_P247596 LOC645251 Xq11.1
7.0
8.0
9.0
10.0
0
0.1
1
6
12
24
48
96
144
1
6
12
24
48
96
144
A_24_P820302 THC2594845 NA
A_24_P358215 LOC400769 1p13.1
8.0
8.5
9.0
9.5

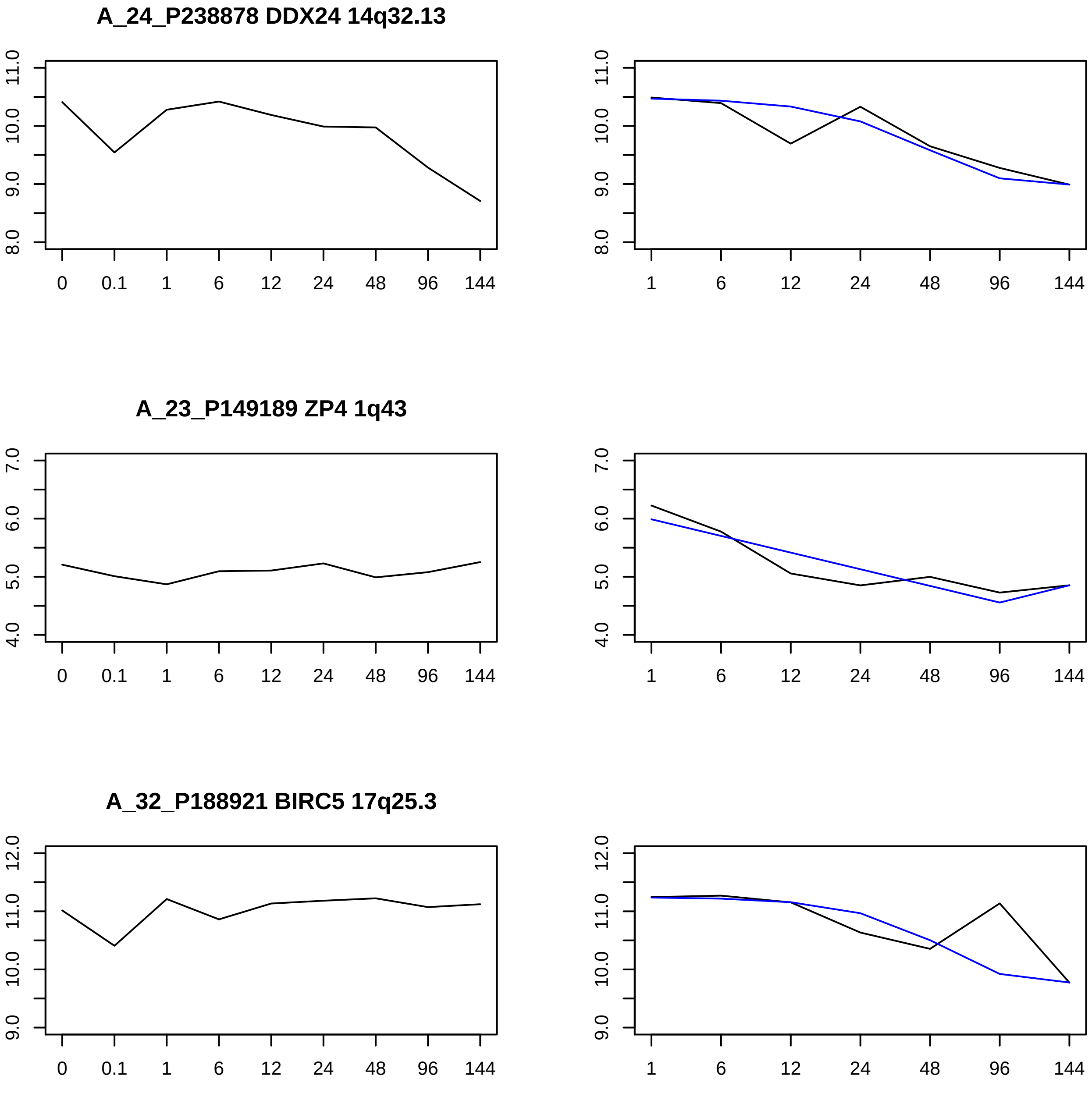
A_24_P238878 DDX24 14q32.13
8.0
9.0
10.0
11.0
0
0.1
1
6
12
24
48
96
144
1
6
12
24
48
96
144
A_23_P149189 ZP4 1q43
4.0
5.0
6.0
7.0
A_32_P188921 BIRC5 17q25.3
9.0
10.0
11.0
12.0

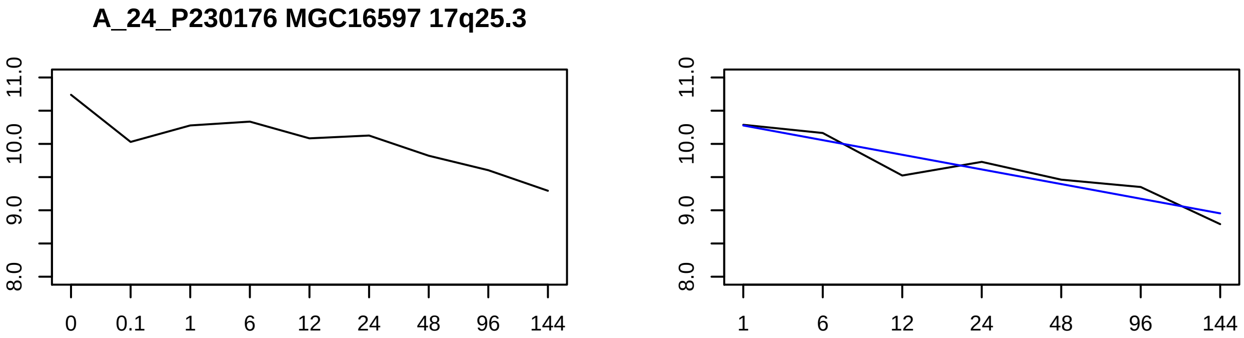
A_24_P230176 MGC16597 17q25.3
8.0 9.0 10.0 11.0
0 0.1 1 6 12 24 48 96 144
1 6 12 24 48 96 144

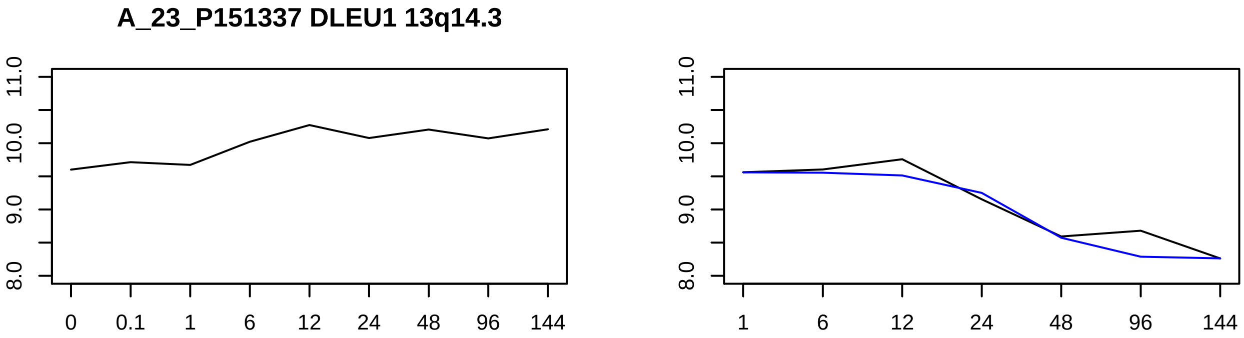
A_23_P151337 DLEU1 13q14.3
8.0 9.0 10.0 11.0
0 0.1 1 6 12 24 48 96 144
1 6 12 24 48 96 144

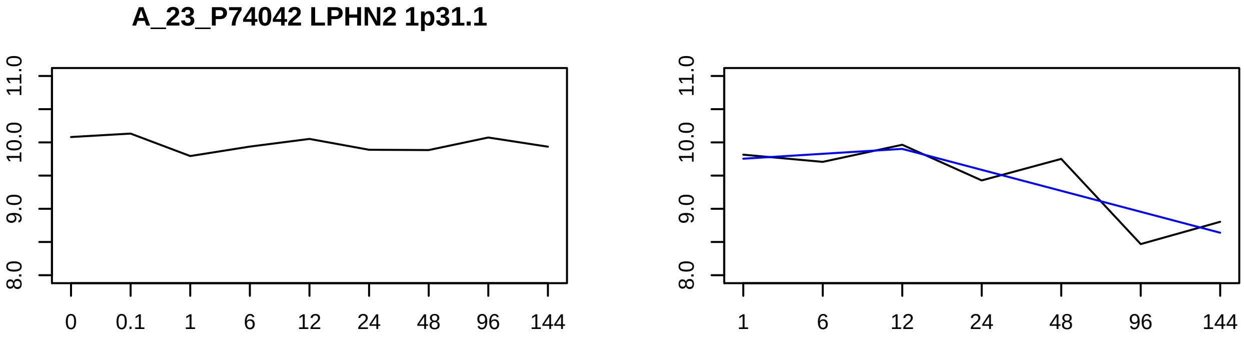
A_23_P74042 LPHN2 1p31.1
8.0 9.0 10.0 11.0
0 0.1 1 6 12 24 48 96 144
1 6 12 24 48 96 144

A_24_P137545 WWC2 4q35.1

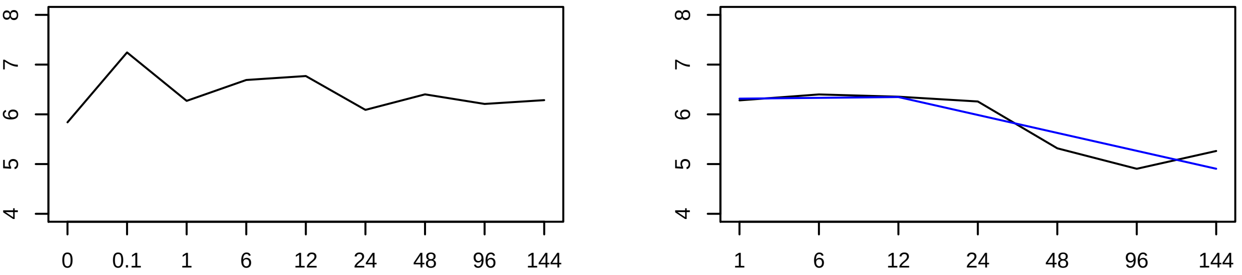

A_32_P84714 A_32_P84714 NA

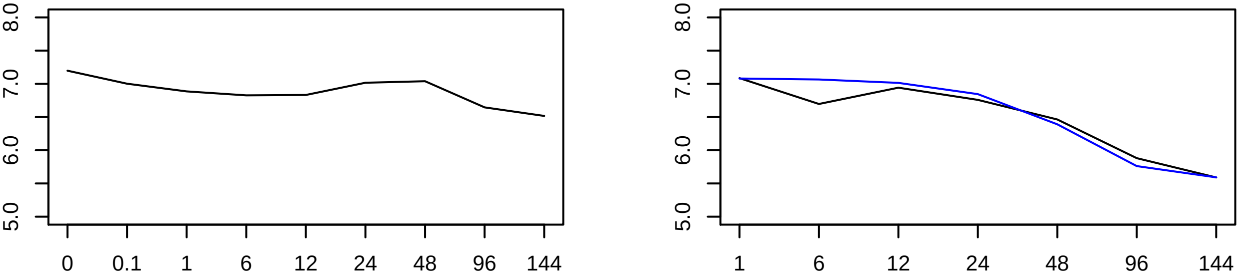

A_24_P479065 THC2504848 NA

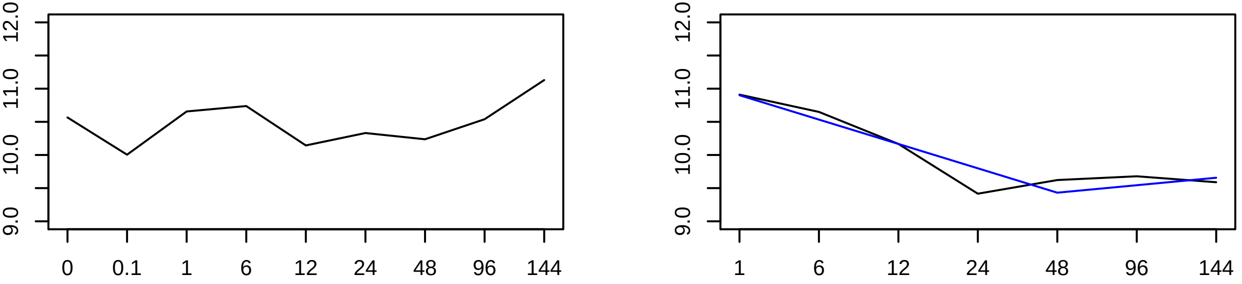

A_23_P37391 C14orf65 14q32.2

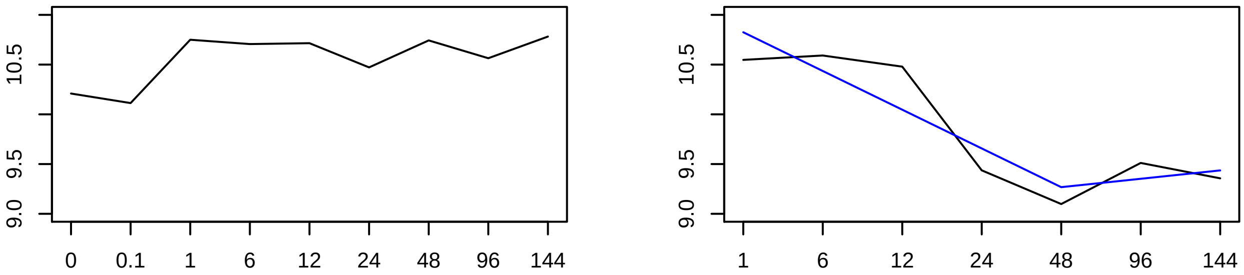

A_23_P255750 C17orf41 17q11.2

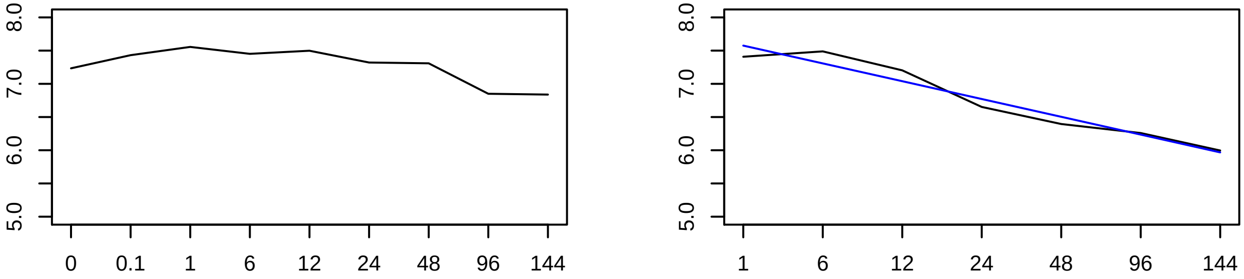

A_23_P115703 PCGF6 10q24.33

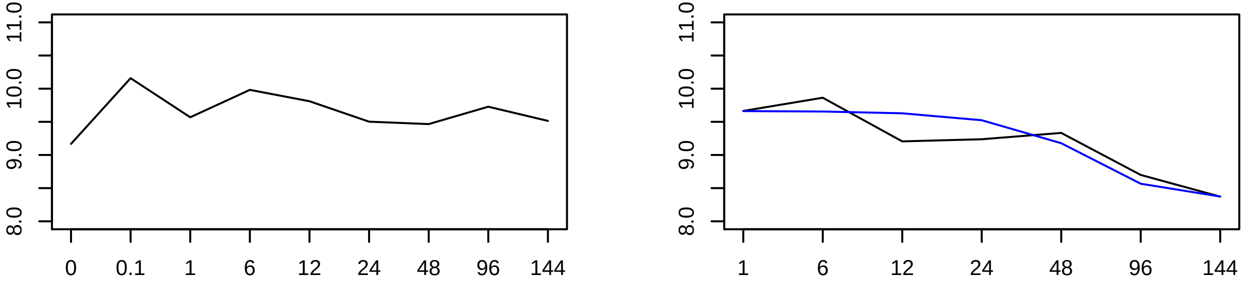

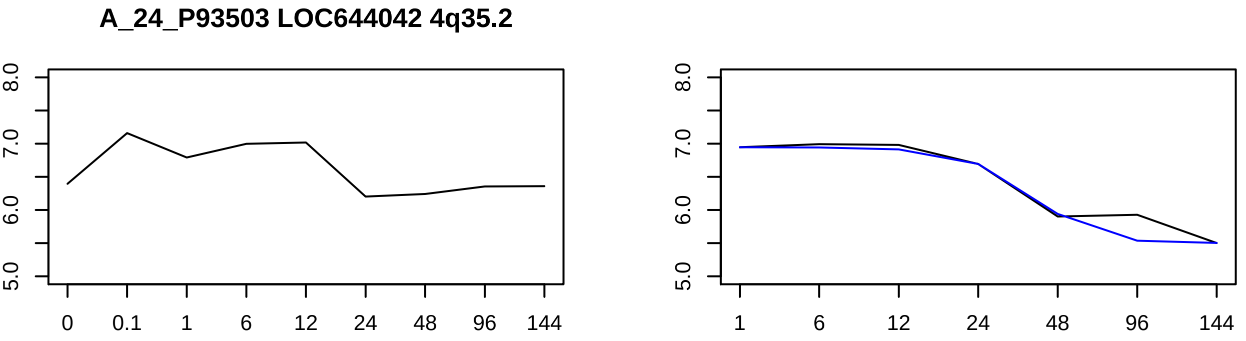
A_24_P93503 LOC644042 4q35.2
5.0 6.0 7.0 8.0
0 0.1 1 6 12 24 48 96 144
1 6 12 24 48 96 144

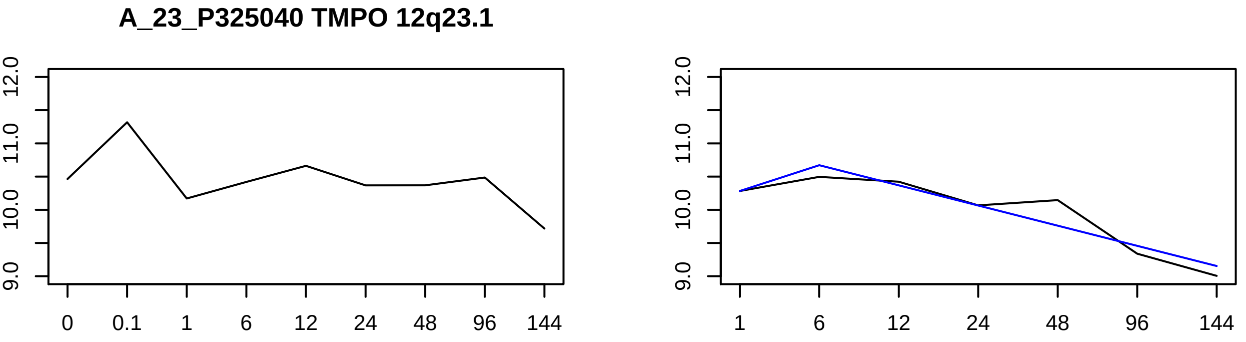
A_23_P325040 TMPO 12q23.1
9.0 10.0 11.0 12.0
0 0.1 1 6 12 24 48 96 144
1 6 12 24 48 96 144

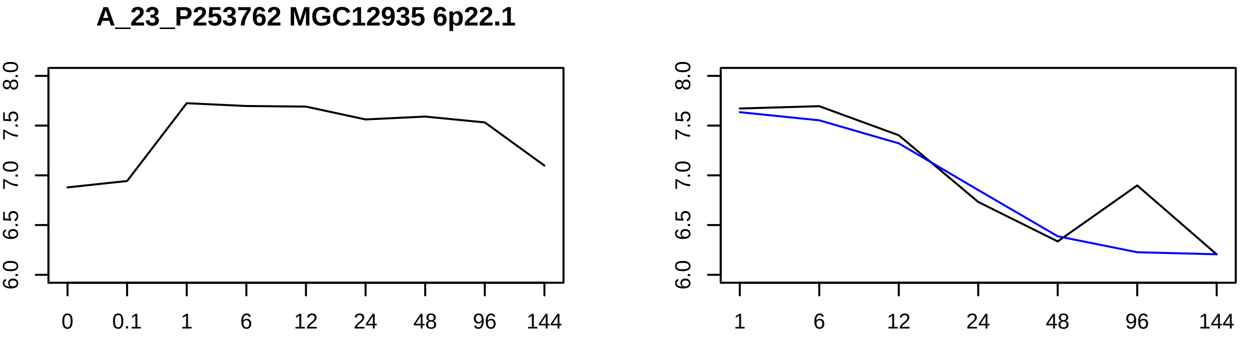
A_23_P253762 MGC12935 6p22.1
6.0 6.5 7.0 7.5 8.0
0 0.1 1 6 12 24 48 96 144
1 6 12 24 48 96 144

A_23_P82088 NRN1 6p25.1

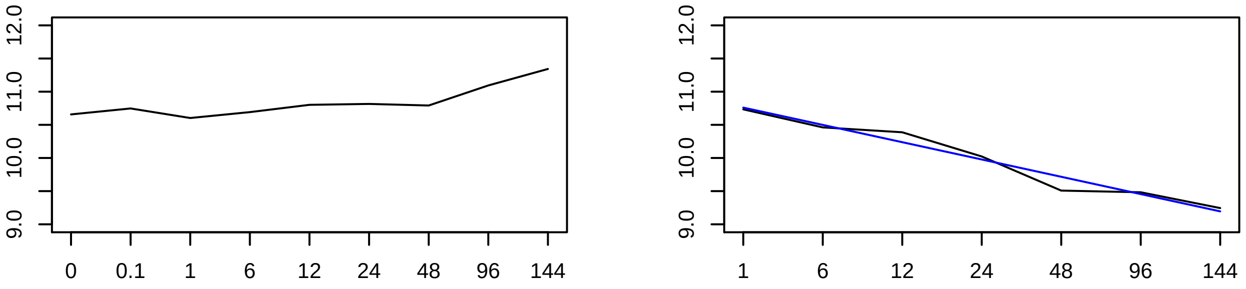

A_24_P654066 LOC730211 3q27.1

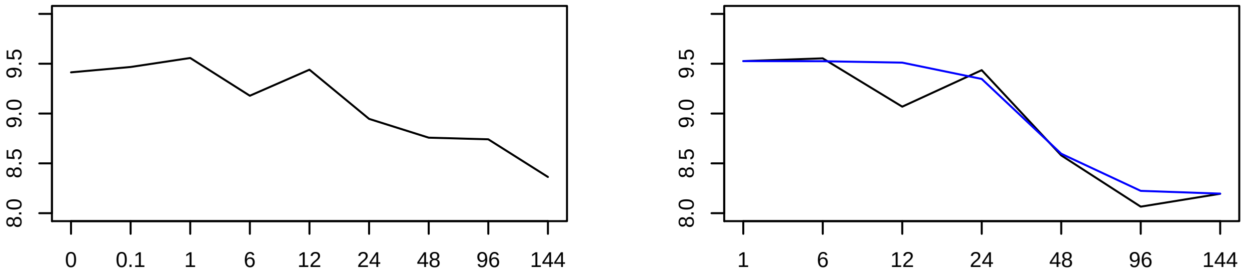

A_23_P54840 MT1A 16q13

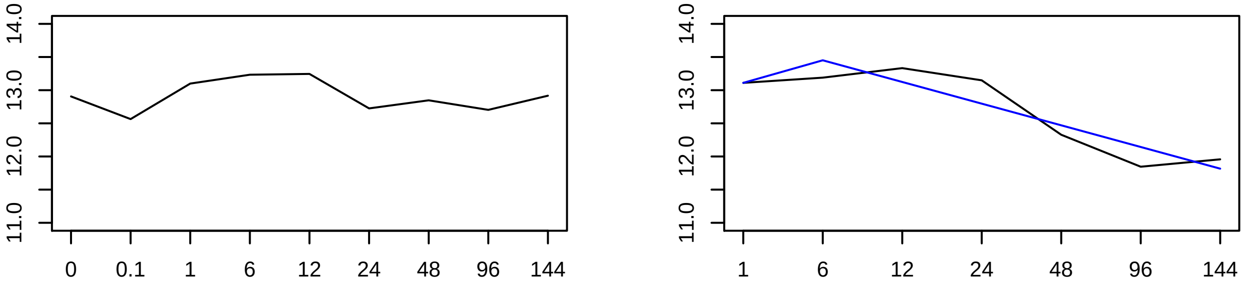

A_32_P108722 AP000337.1 NA

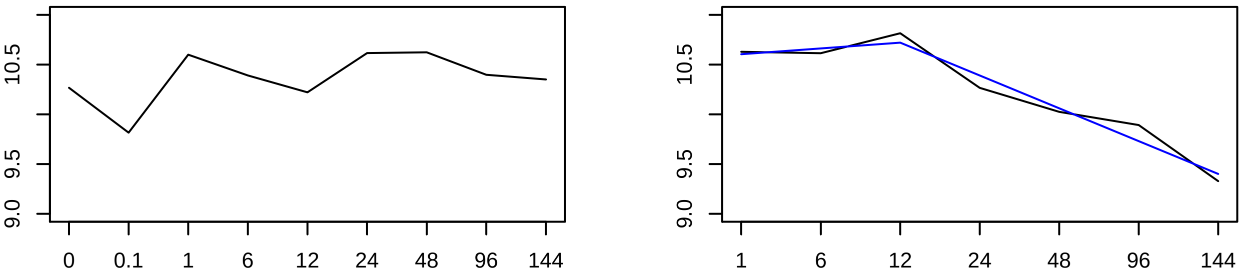

A_24_P117803 NP414444 NA

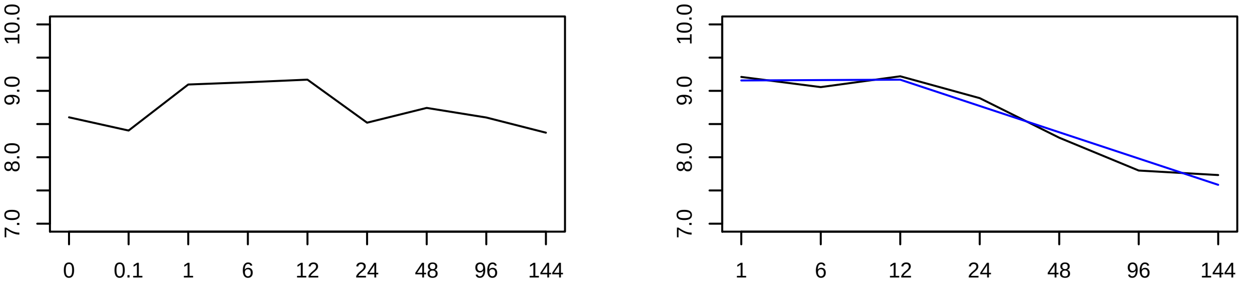

A_23_P75800 RAB3IL1 11q12.3

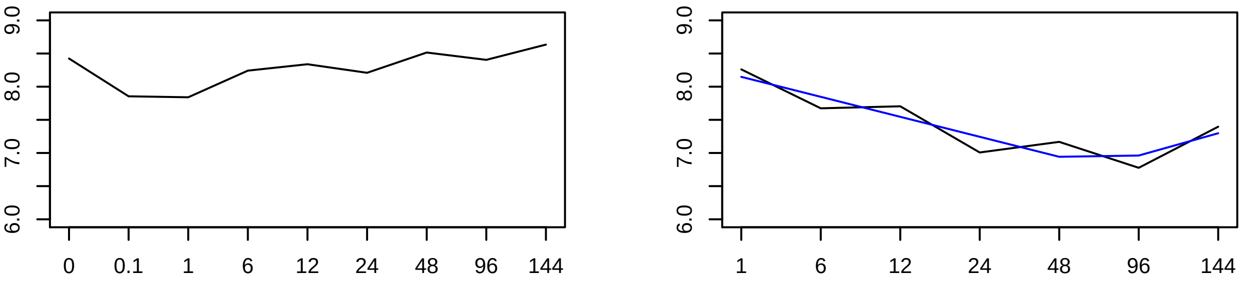

A_32_P190648 THC2651723 NA

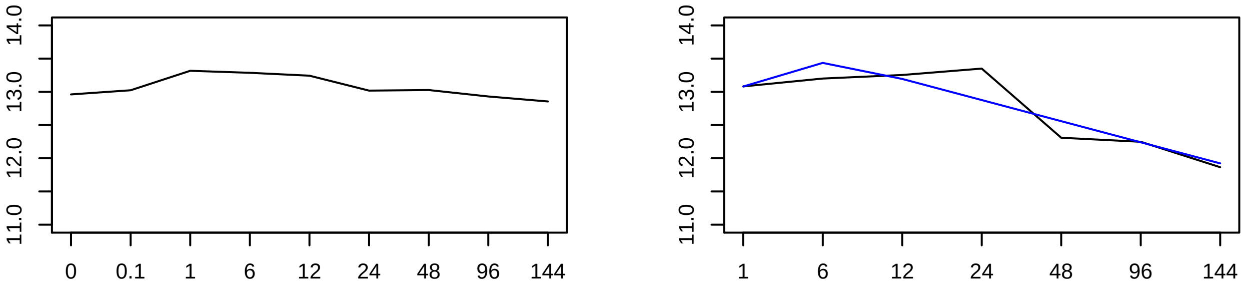

A_24_P202154 RPS2P45 16q22.1

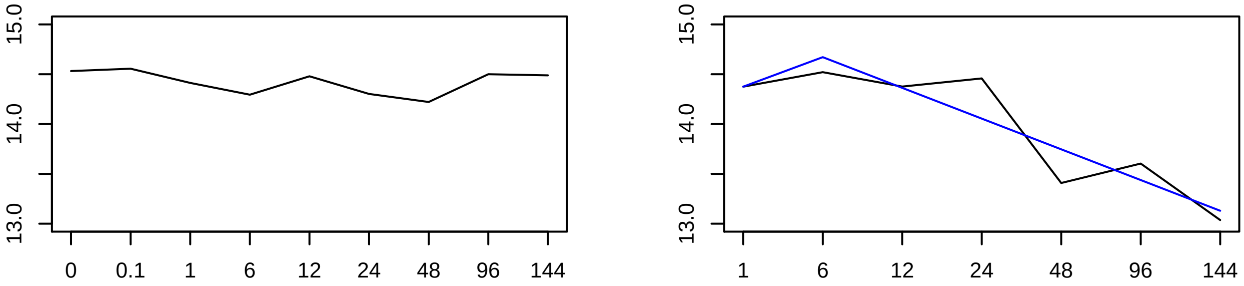

A_24_P110403 BF931515 NA

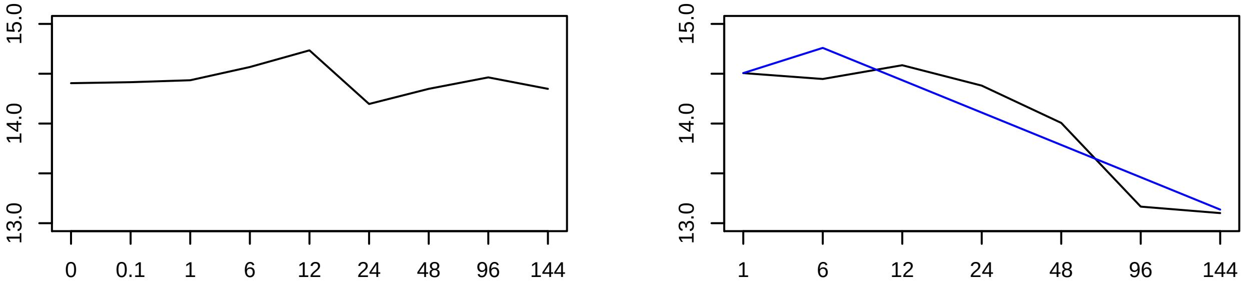

A_24_P304458 NAP1L1 12q21.2

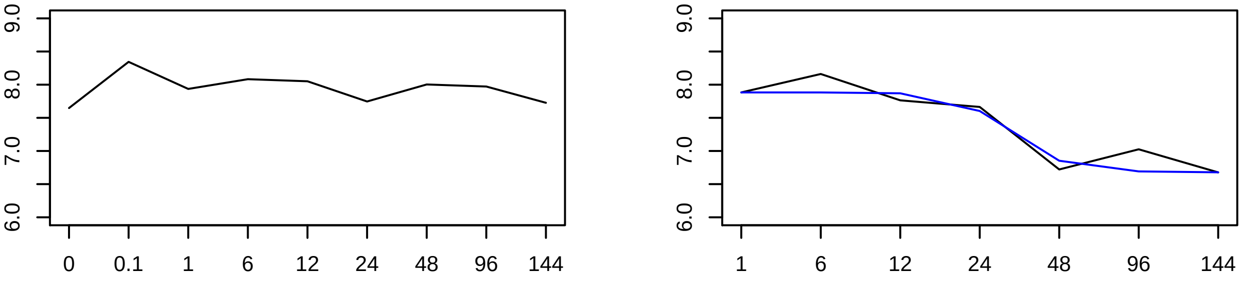

A_23_P216655 TRIM14 9q22.33

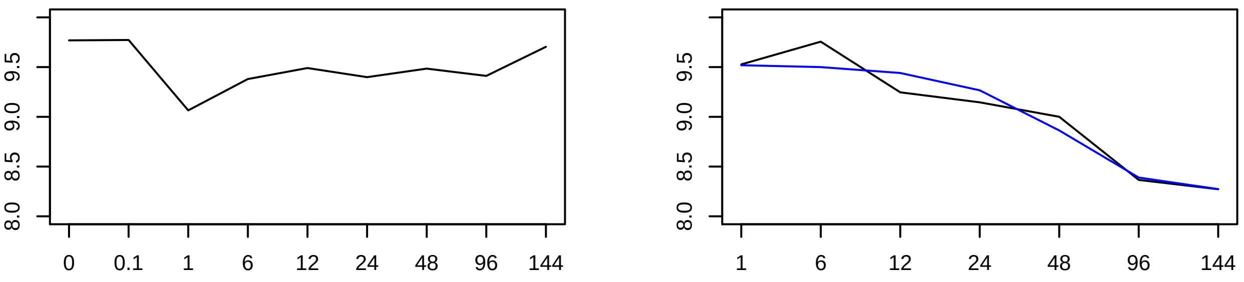

A_24_P383130 LOC341511 12p13.33

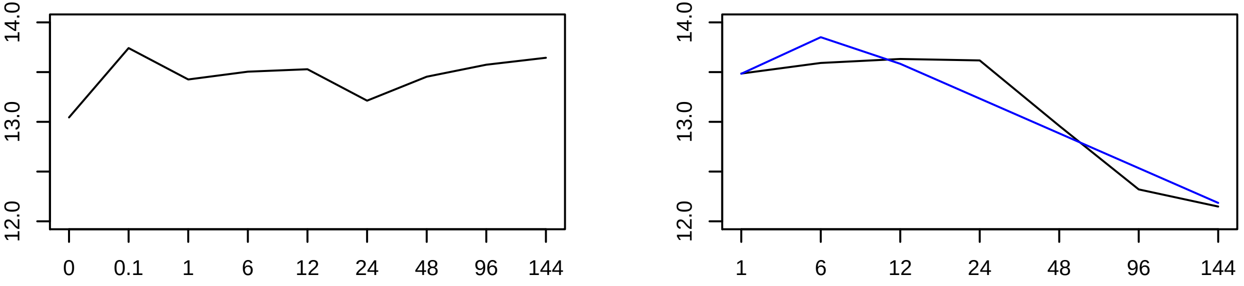

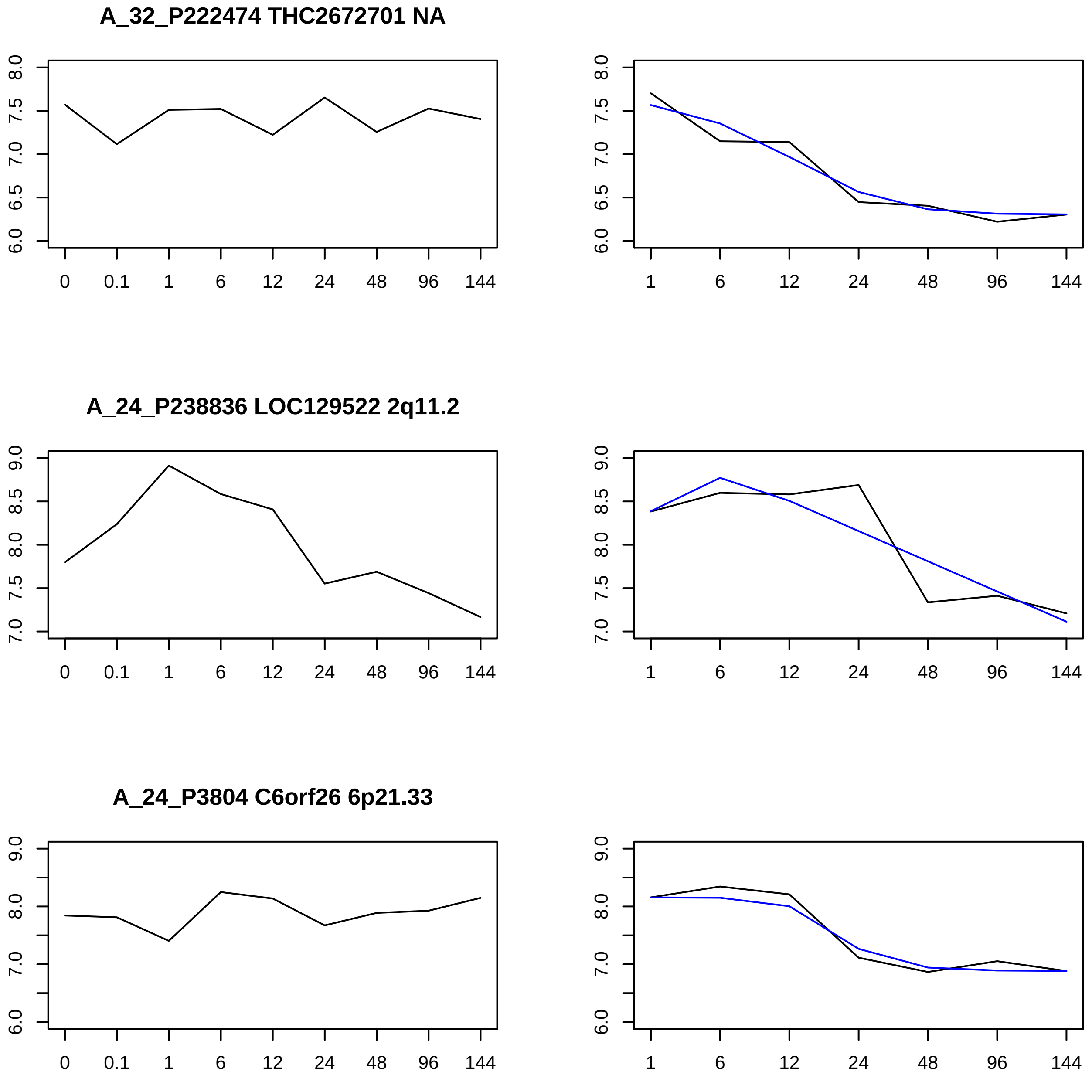
A_32_P222474 THC2672701 NA
A_24_P238836 LOC129522 2q11.2
A_24_P3804 C6orf26 6p21.33
0
0.1
1
6
12
24
48
96
144

A_24_P290481 VIL2 6q25.3

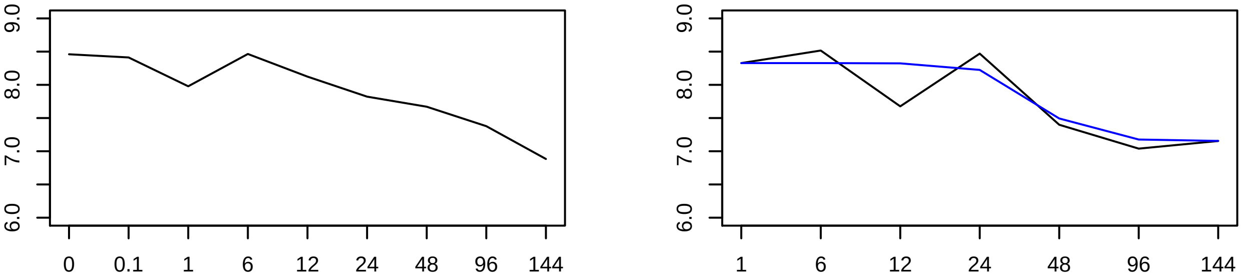

A_32_P66756 ENST00000379528 NA

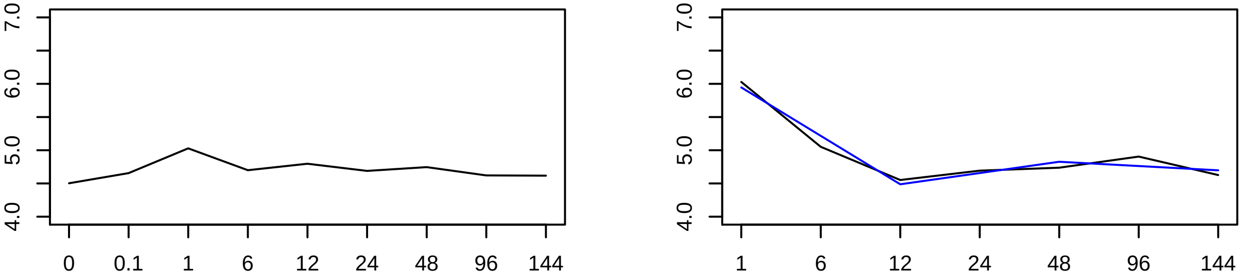

A_24_P393336 HMCN2 9q34.11

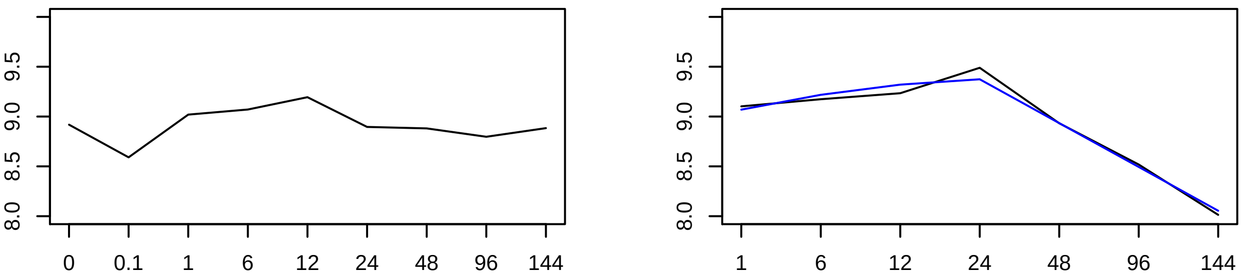

A_24_P457021 A_24_P457021 NA

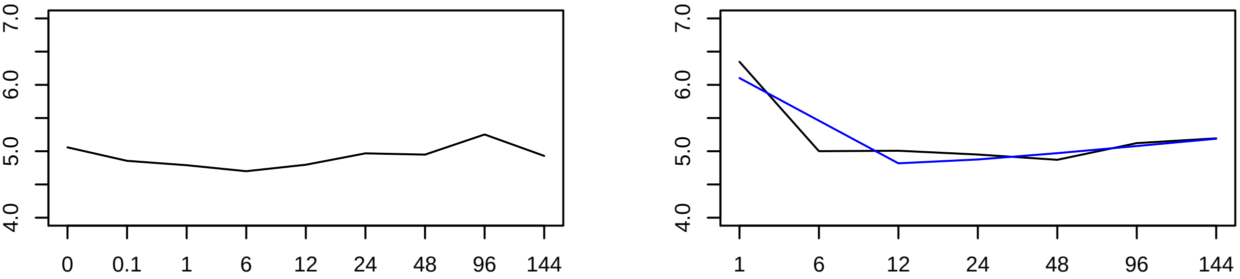

A_23_P111297 RPP40 6p25.1

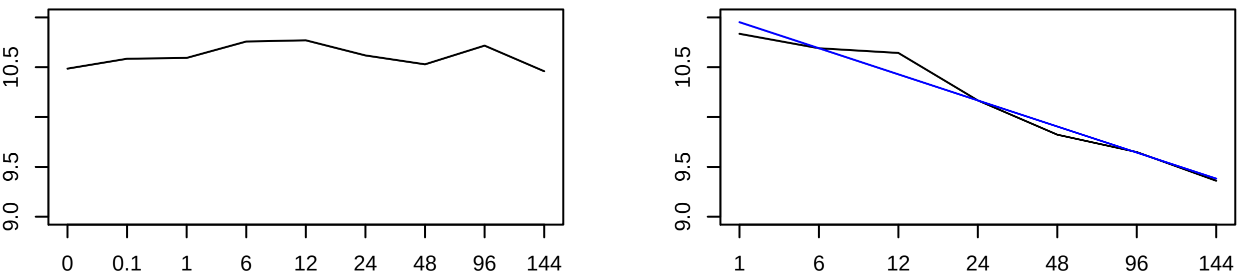

A_23_P30377 DDX46 5q31.1

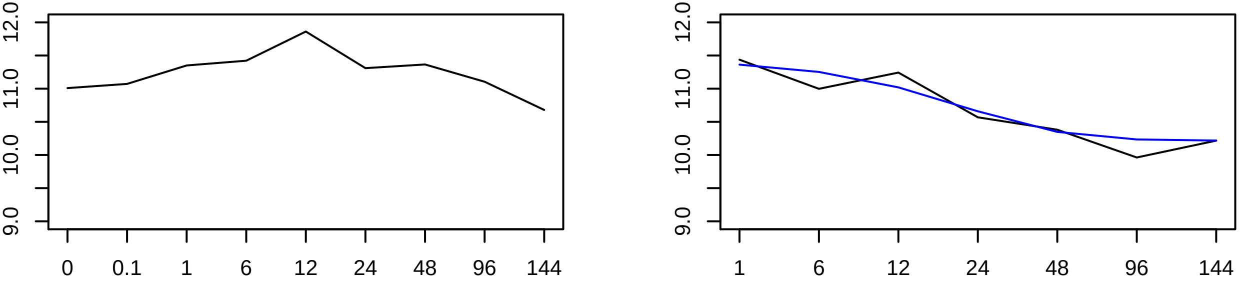

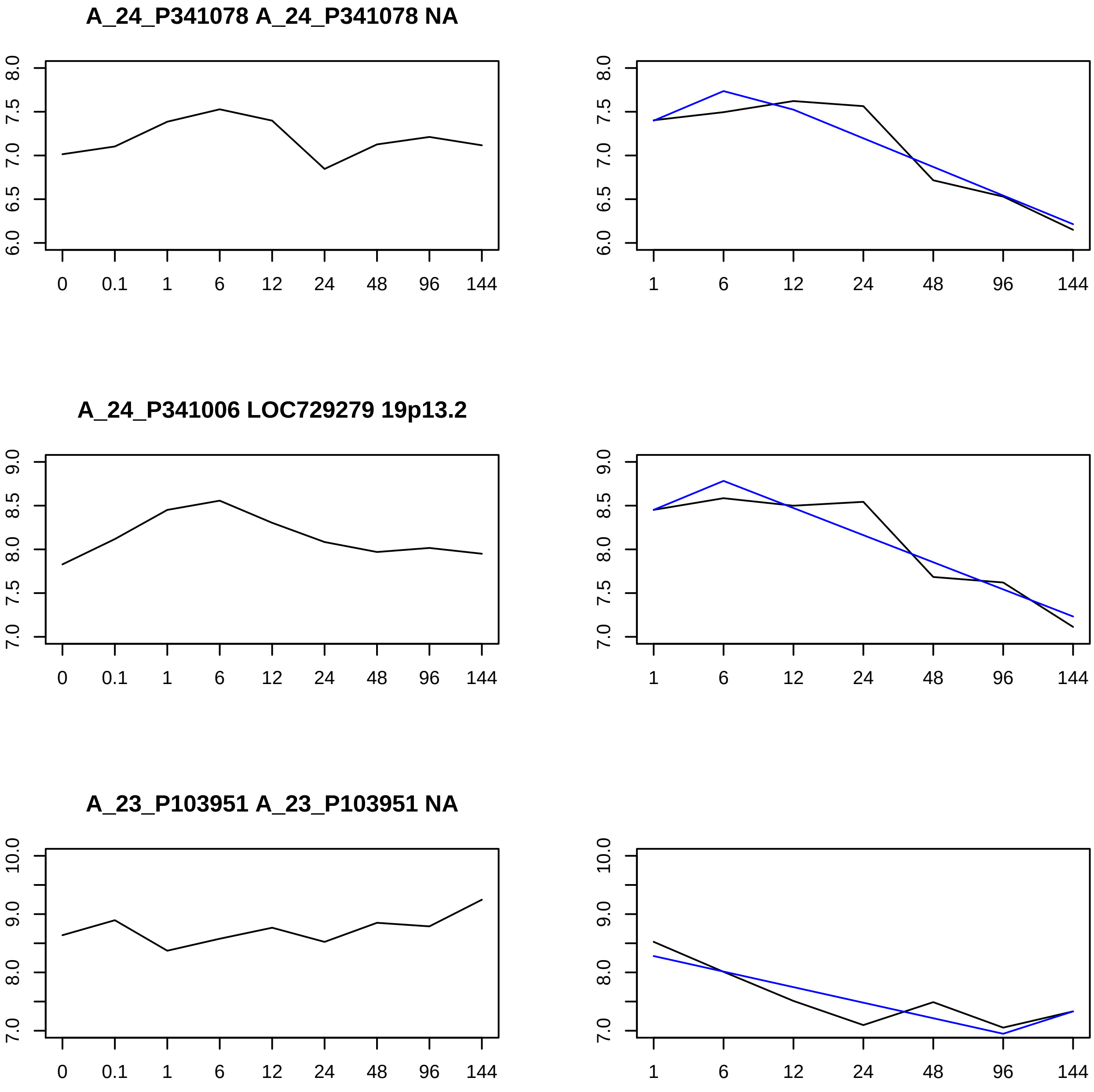
A_24_P341078 A_24_P341078 NA
6.0 6.5 7.0 7.5 8.0
0 0.1 1 6 12 24 48 96 144
1 6 12 24 48 96 144
A_24_P341006 LOC729279 19p13.2
7.0 7.5 8.0 8.5 9.0
0 0.1 1 6 12 24 48 96 144
1 6 12 24 48 96 144
A_23_P103951 A_23_P103951 NA
7.0 8.0 9.0 10.0
0 0.1 1 6 12 24 48 96 144
1 6 12 24 48 96 144

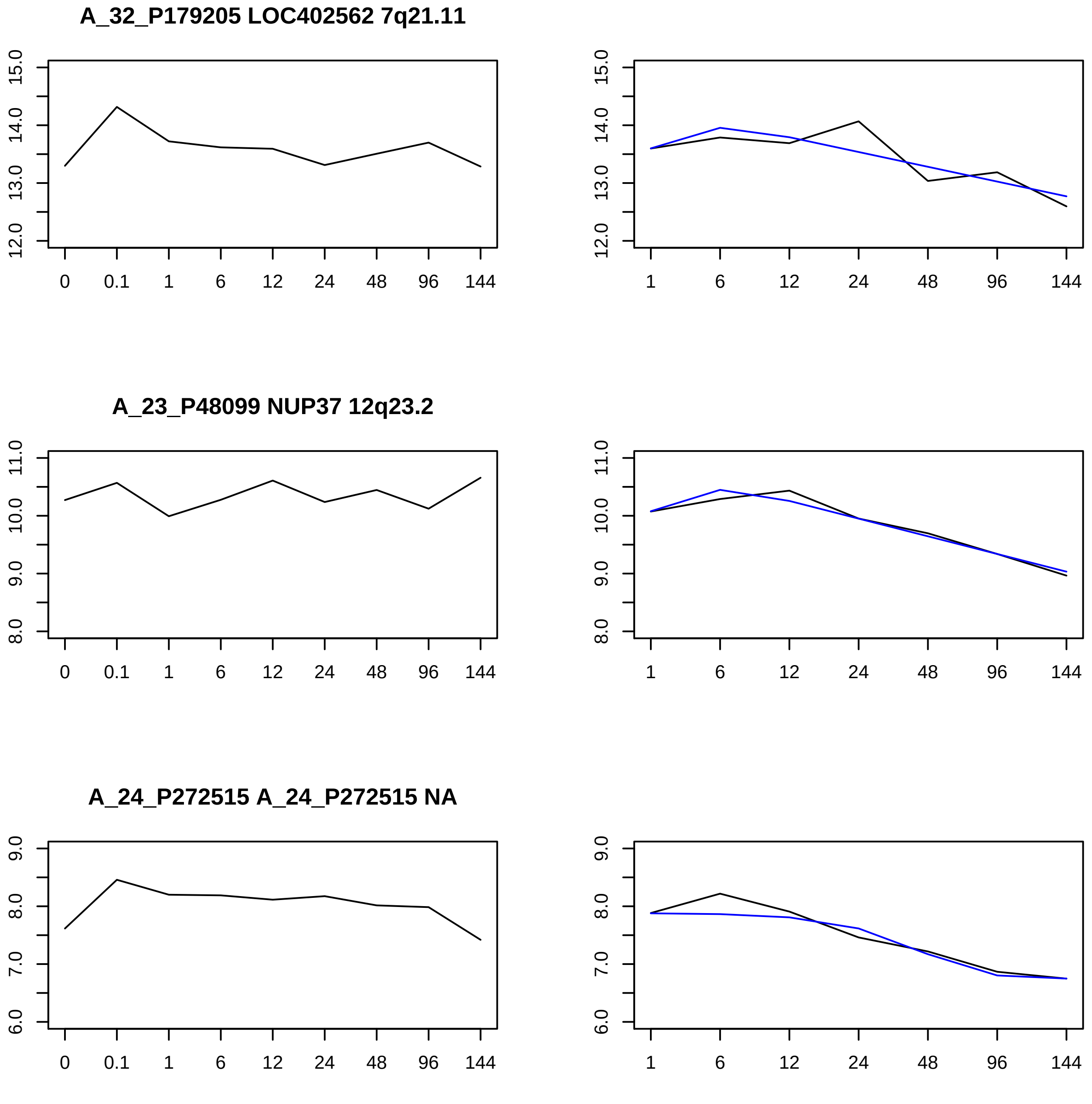

A_32_P179205 LOC402562 7q21.11
A_23_P48099 NUP37 12q23.2
A_24_P272515 A_24_P272515 NA
12.0
13.0
14.0
15.0
8.0
9.0
10.0
11.0
6.0
7.0
8.0
9.0
0
0.1
1
6
12
24
48
96
144

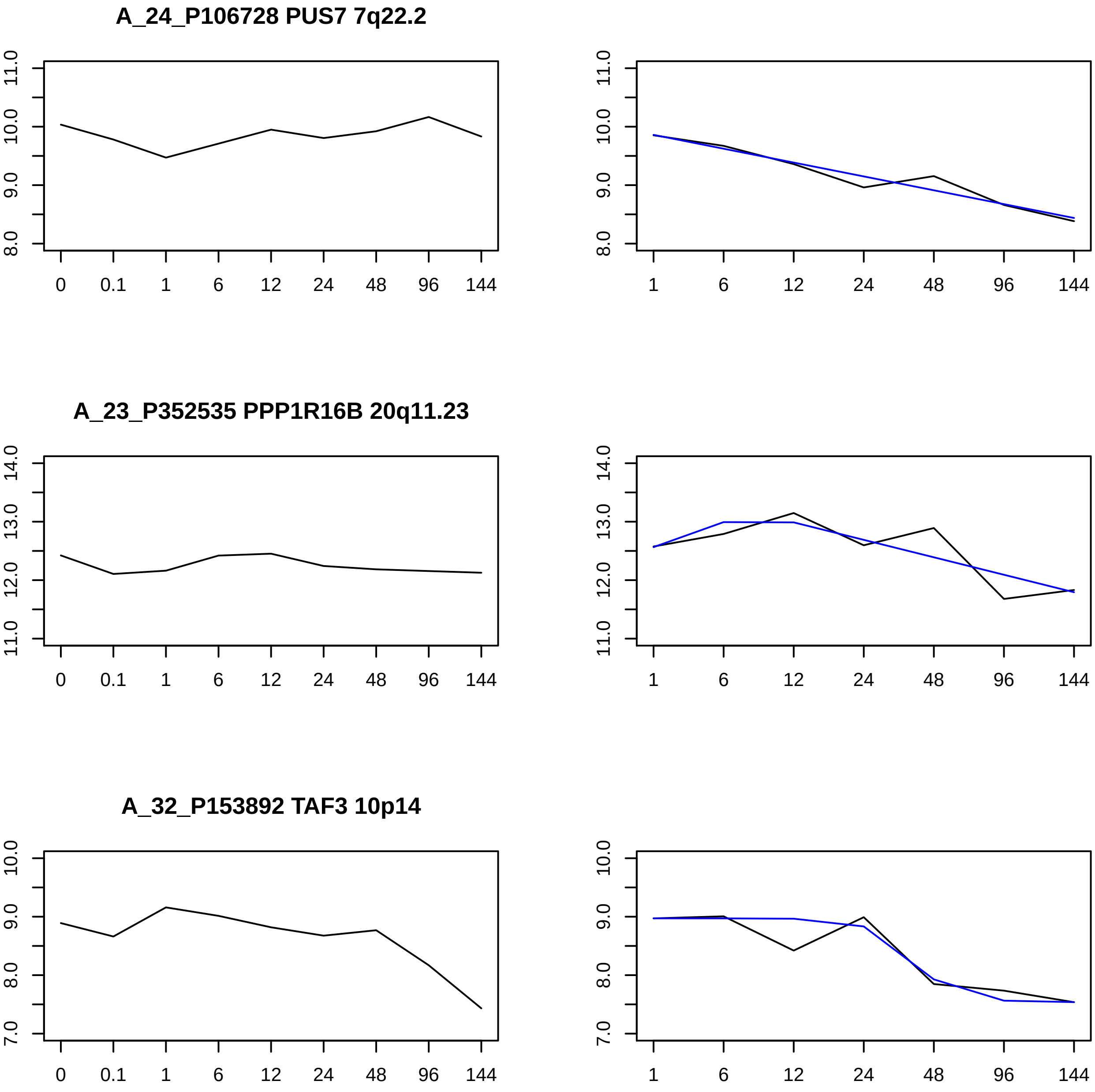
A_24_P106728 PUS7 7q22.2
8.0
9.0
10.0
11.0
0
0.1
1
6
12
24
48
96
144
1
6
12
24
48
96
144
A_23_P352535 PPP1R16B 20q11.23
11.0
12.0
13.0
14.0
A_32_P153892 TAF3 10p14
7.0
8.0
9.0
10.0

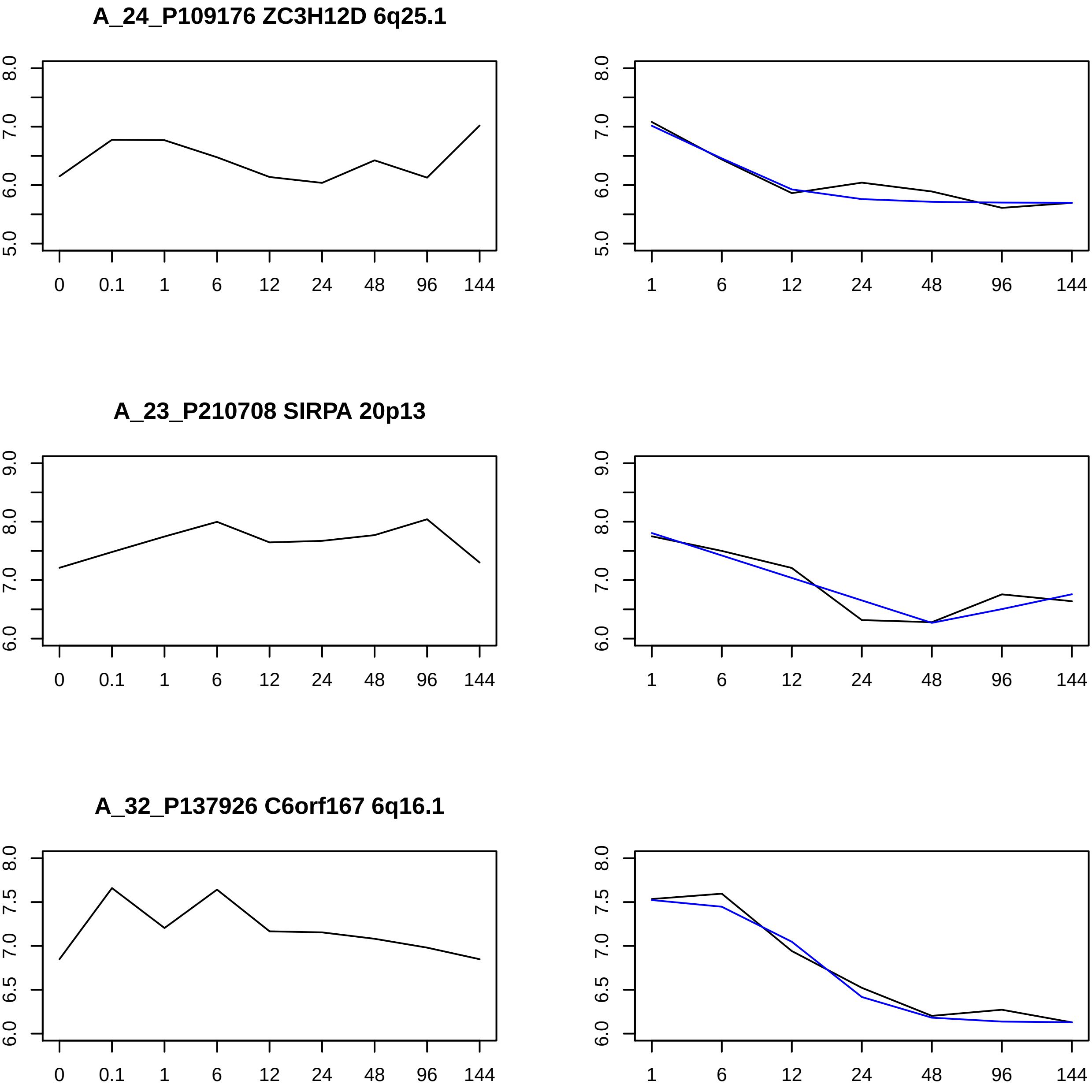
A_24_P109176 ZC3H12D 6q25.1
A_23_P210708 SIRPA 20p13
A_32_P137926 C6orf167 6q16.1
0
0.1
1
6
12
24
48
96
144

A_24_P632230 A_24_P632230 NA

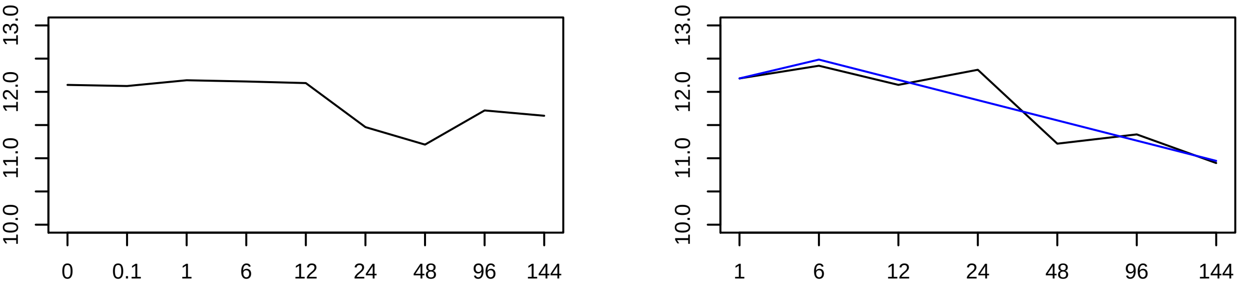

A_24_P73920 CR609948 NA

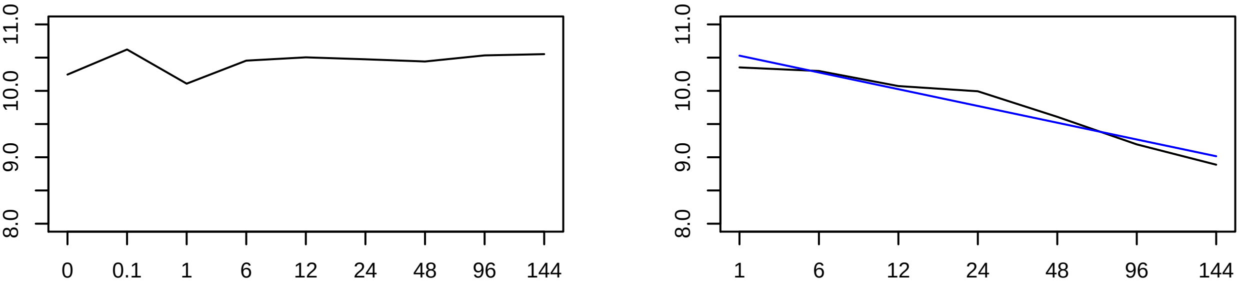

A_24_P401381 A_24_P401381 NA

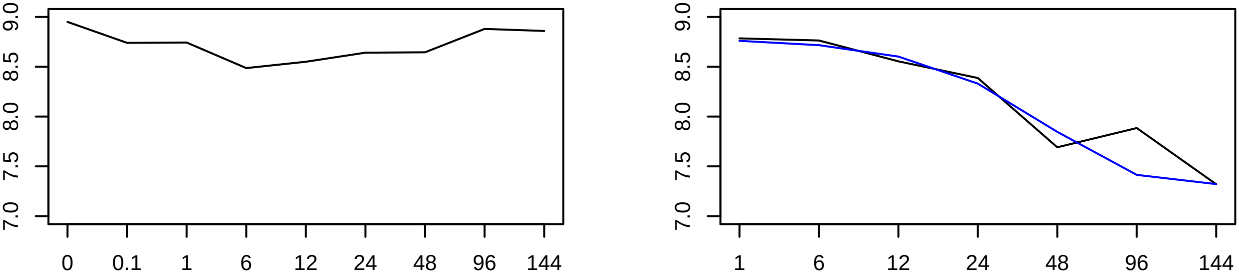

A_24_P250614 SF3A1 22q12.2

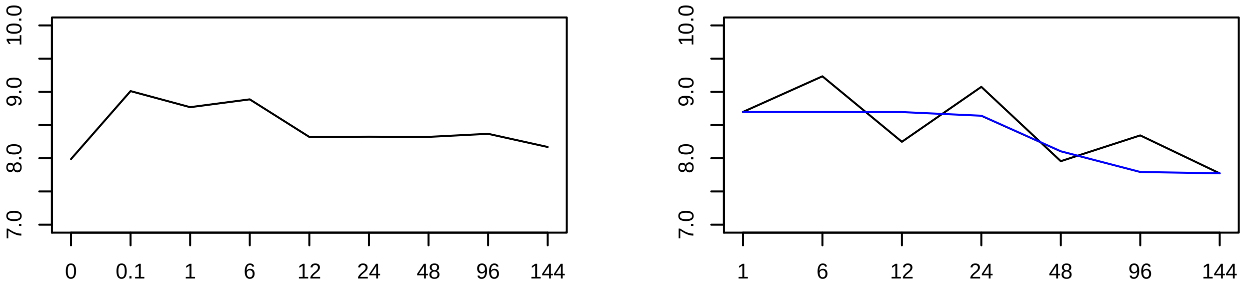

A_24_P932785 GATA4 8p23.1

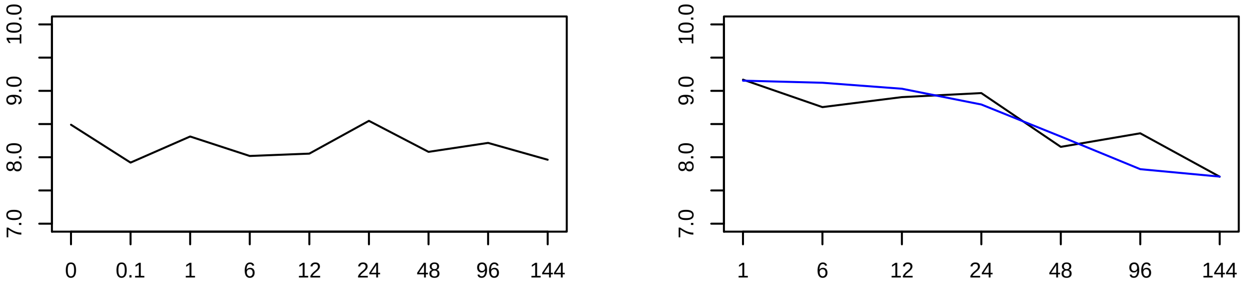

A_23_P28878 C20orf27 20p13

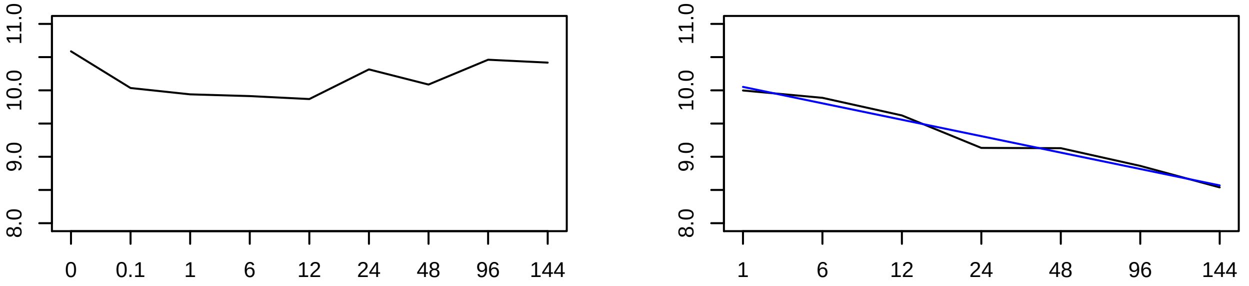

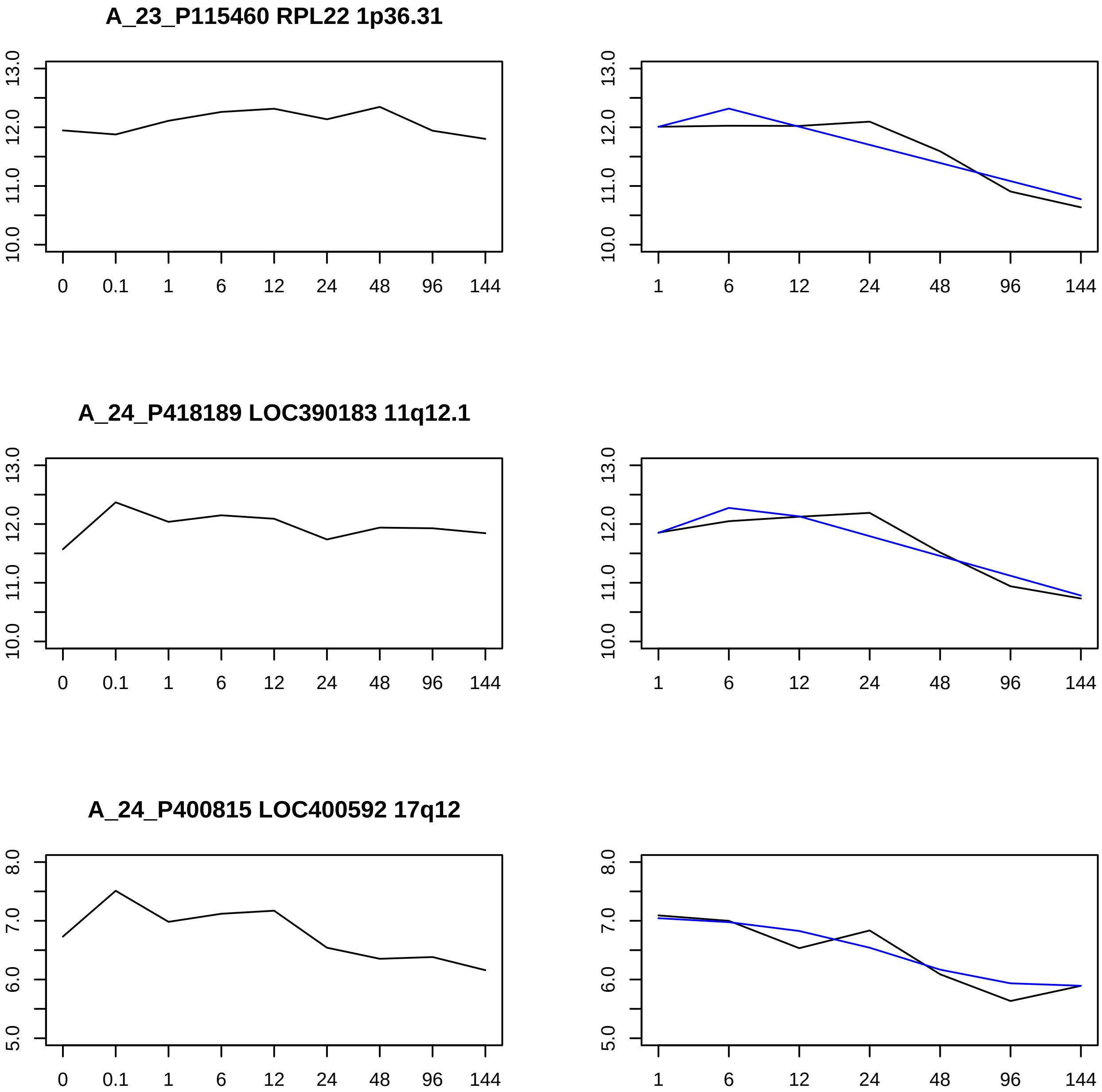
A_23_P115460 RPL22 1p36.31
A_24_P418189 LOC390183 11q12.1
A_24_P400815 LOC400592 17q12
10.0
11.0
12.0
13.0
5.0
6.0
7.0
8.0
0
0.1
1
6
12
24
48
96
144

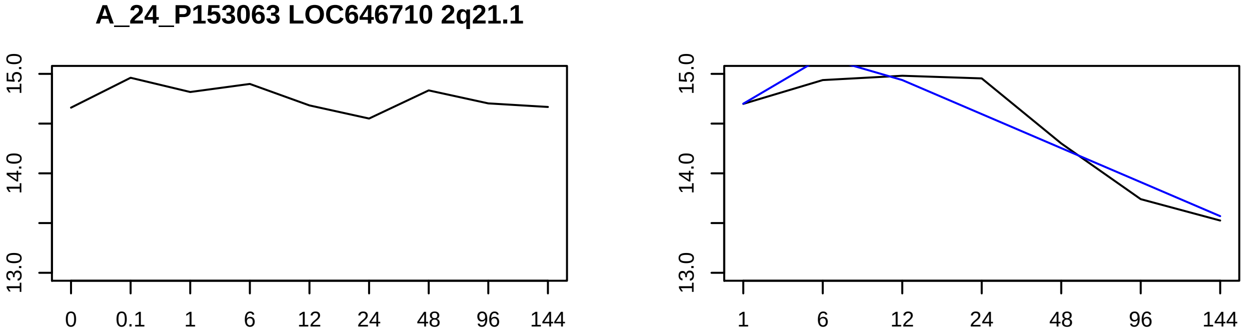
A_24_P153063 LOC646710 2q21.1
13.0
14.0
15.0
0
0.1
1
6
12
24
48
96
144

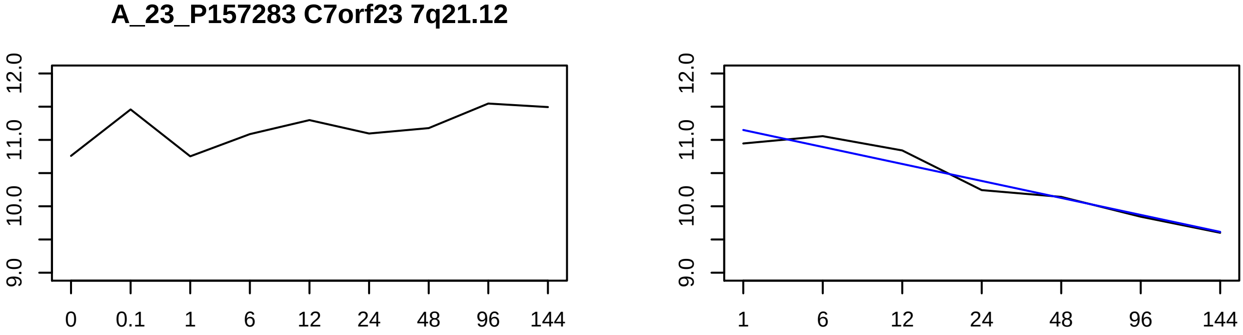
A_23_P157283 C7orf23 7q21.12
9.0
10.0
11.0
12.0
0
0.1
1
6
12
24
48
96
144

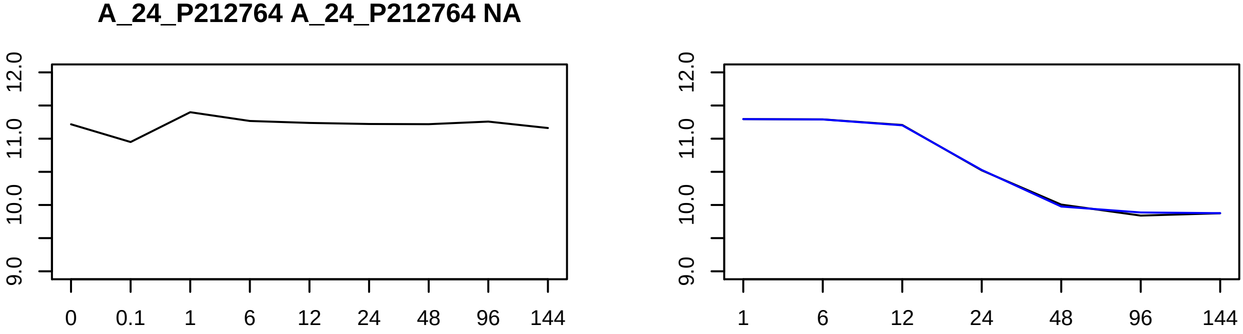
A_24_P212764 A_24_P212764 NA
9.0
10.0
11.0
12.0
0
0.1
1
6
12
24
48
96
144

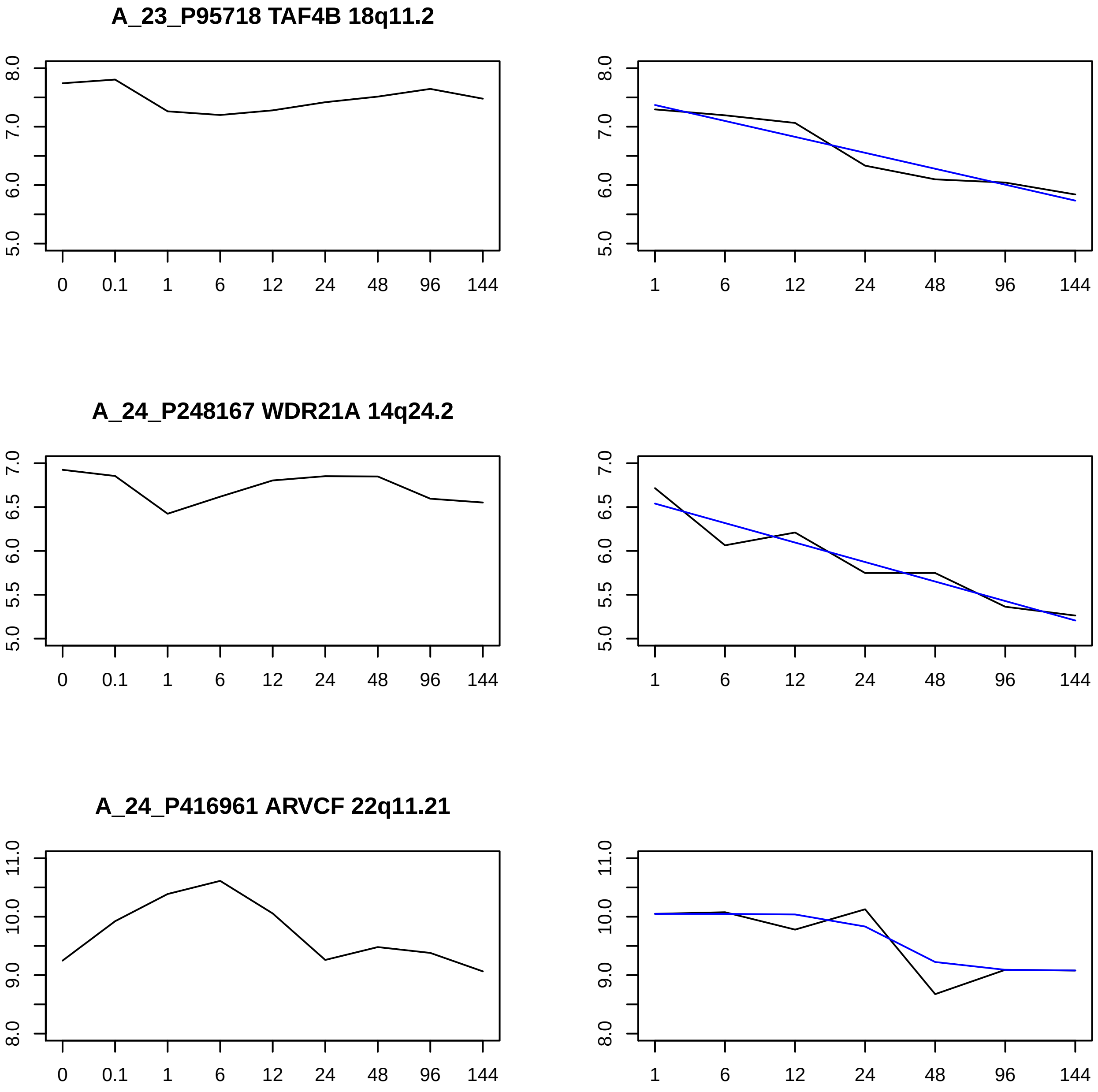
A_23_P95718 TAF4B 18q11.2
A_24_P248167 WDR21A 14q24.2
A_24_P416961 ARVCF 22q11.21
0
0.1
1
6
12
24
48
96
144
5.0
6.0
7.0
8.0
5.5
6.5
9.0
10.0
11.0

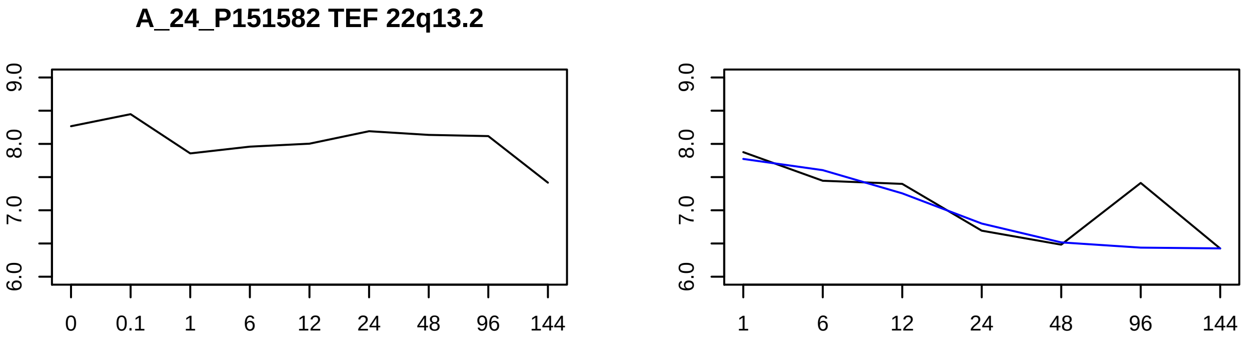
A_24_P151582 TEF 22q13.2
9.0
8.0
7.0
6.0
0
0.1
1
6
12
24
48
96
144
1
6
12
24
48
96
144

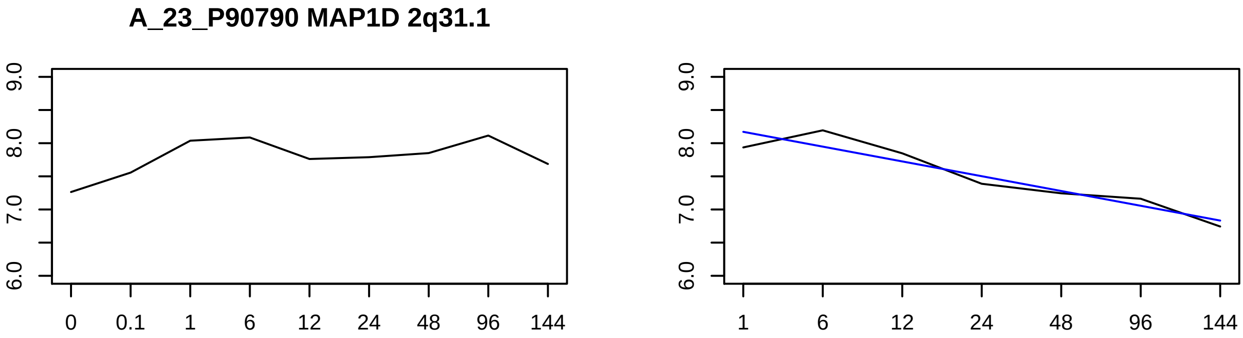
A_23_P90790 MAP1D 2q31.1
9.0
8.0
7.0
6.0
0
0.1
1
6
12
24
48
96
144
1
6
12
24
48
96
144

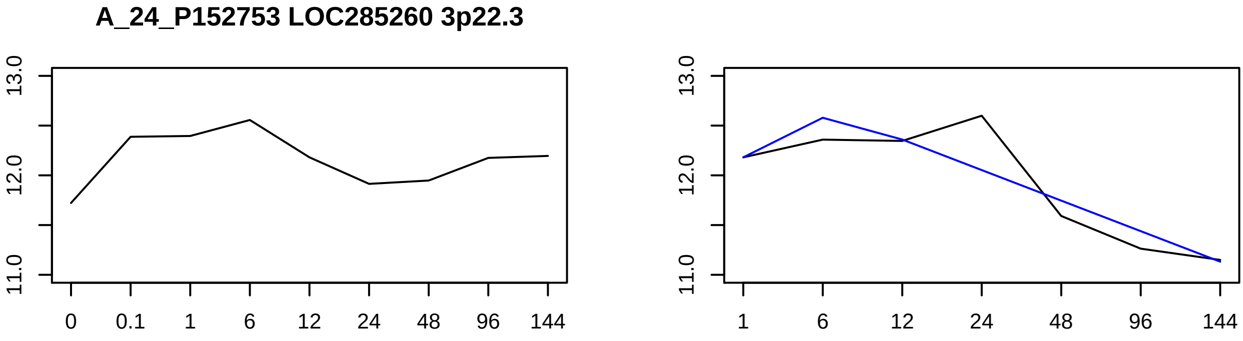
A_24_P152753 LOC285260 3p22.3
13.0
12.0
11.0
0
0.1
1
6
12
24
48
96
144
1
6
12
24
48
96
144

A_24_P359545 RBMXL1 1p22.2

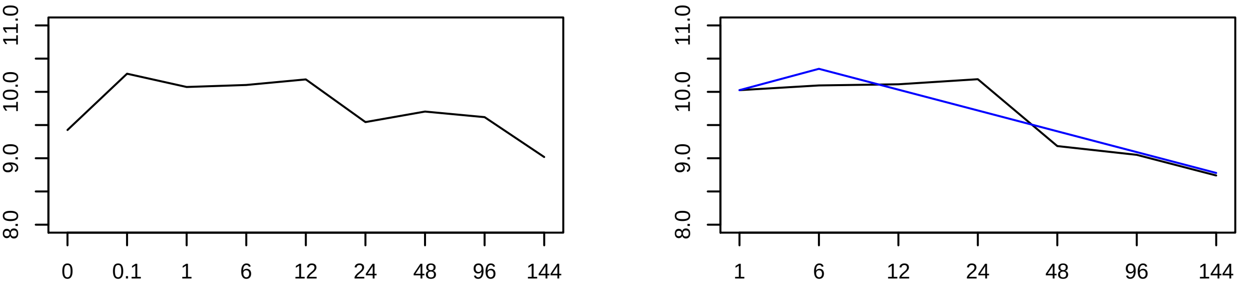

A_23_P93282 HIST1H3J 6p22.1

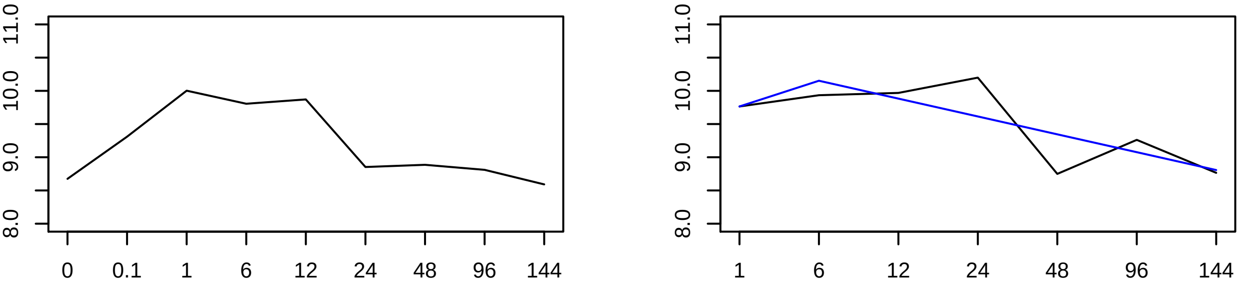

A_24_P136807 RFC1 4p14

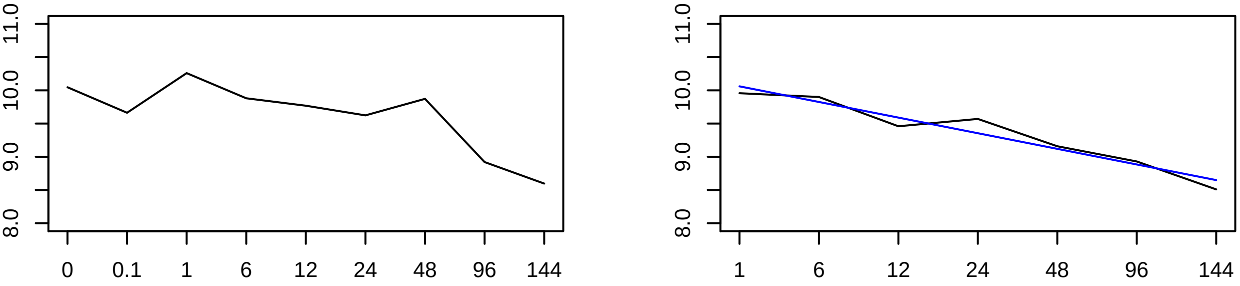

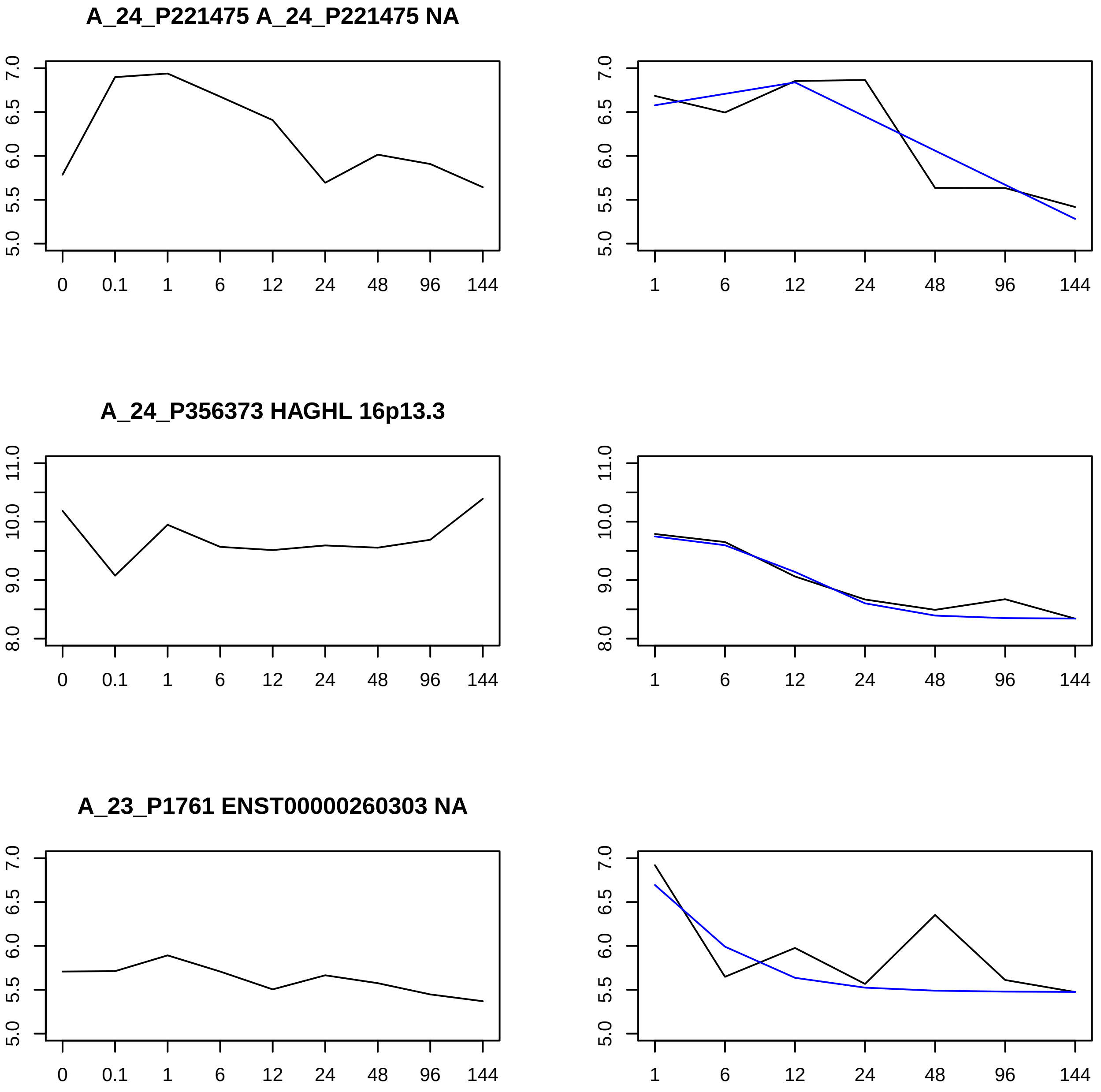
A_24_P221475 A_24_P221475 NA
A_24_P356373 HAGHL 16p13.3
A_23_P1761 ENST00000260303 NA

A_24_P54178 TMED5 1p22.1

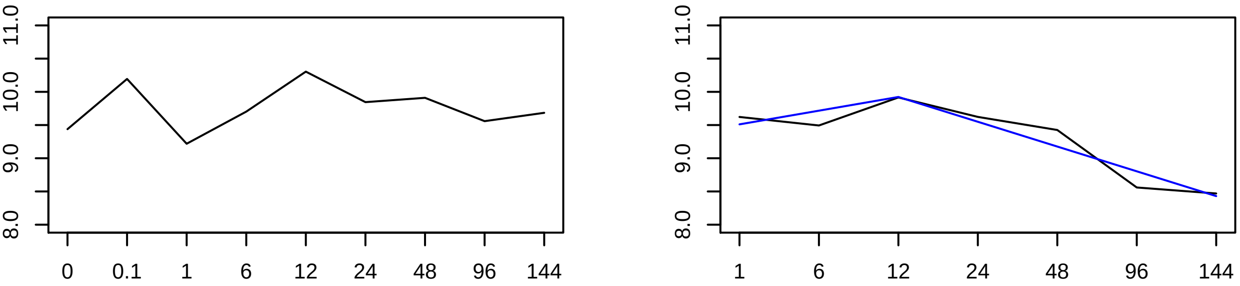

A_24_P375949 LOC645326 3p22.3

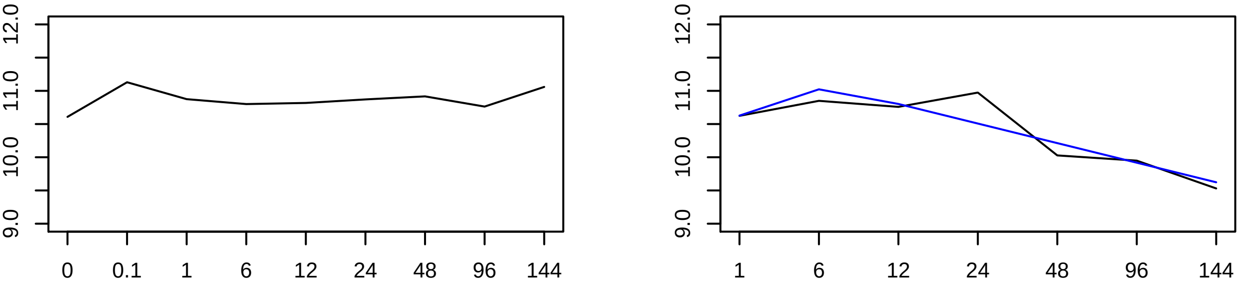

A_24_P846810 A_24_P846810 NA

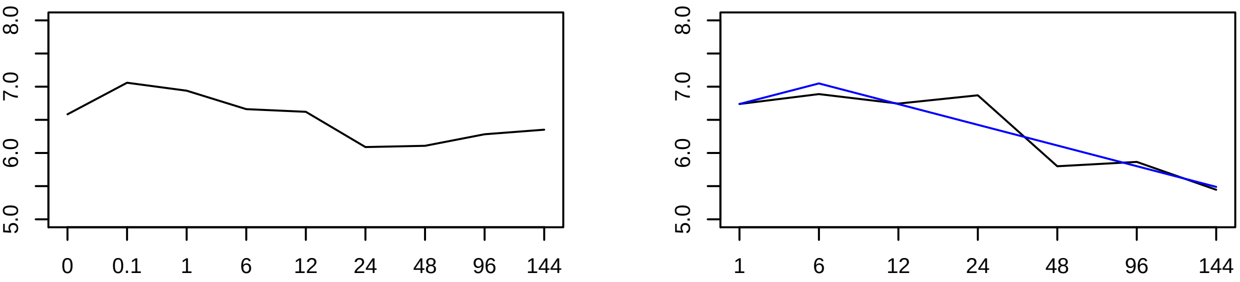

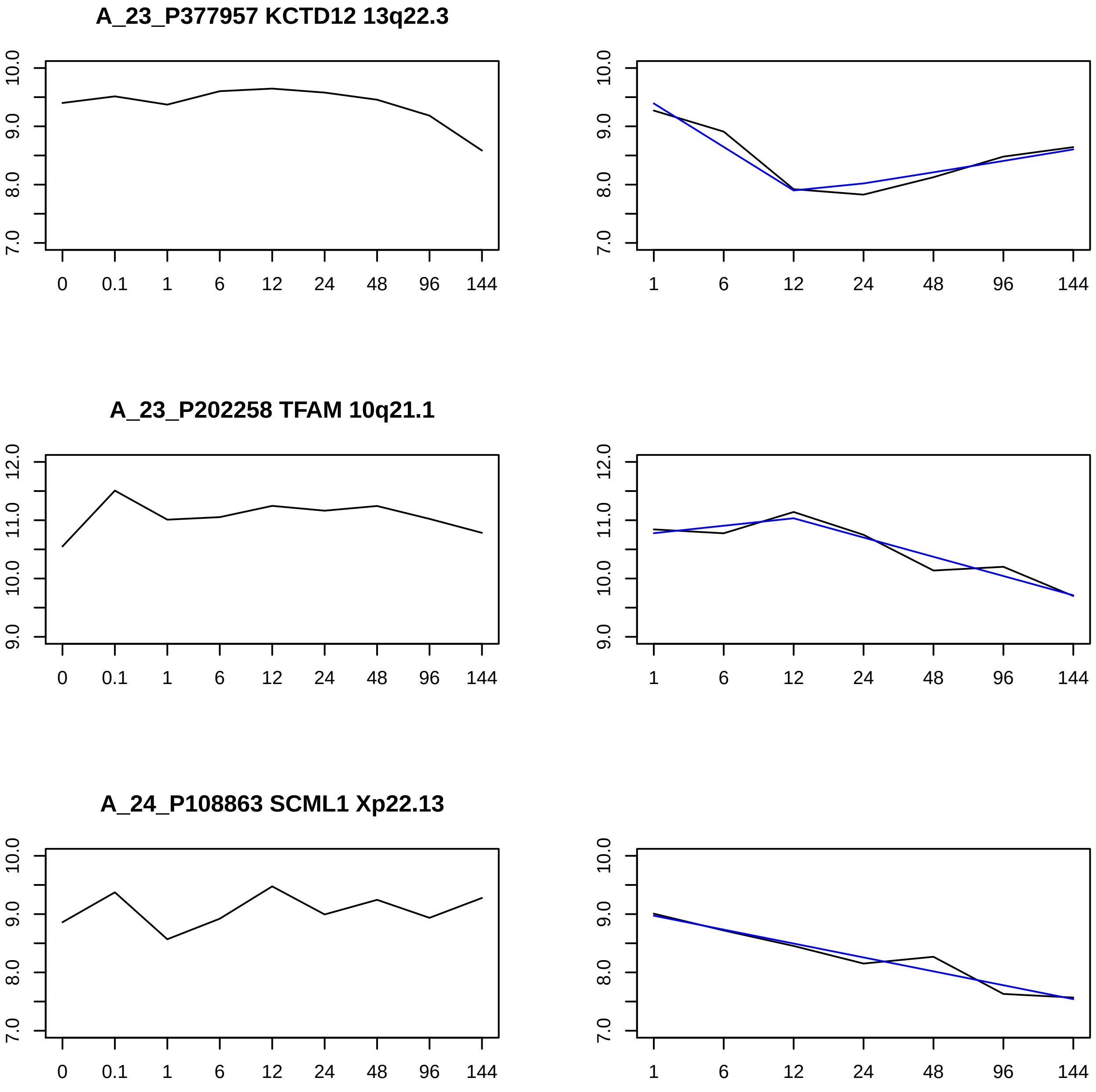
A_23_P377957 KCTD12 13q22.3
7.0
8.0
9.0
10.0
0
0.1
1
6
12
24
48
96
144
1
6
12
24
48
96
144
A_23_P202258 TFAM 10q21.1
9.0
10.0
11.0
12.0
A_24_P108863 SCML1 Xp22.13

A_23_P130537 ICAM4 19p13.2

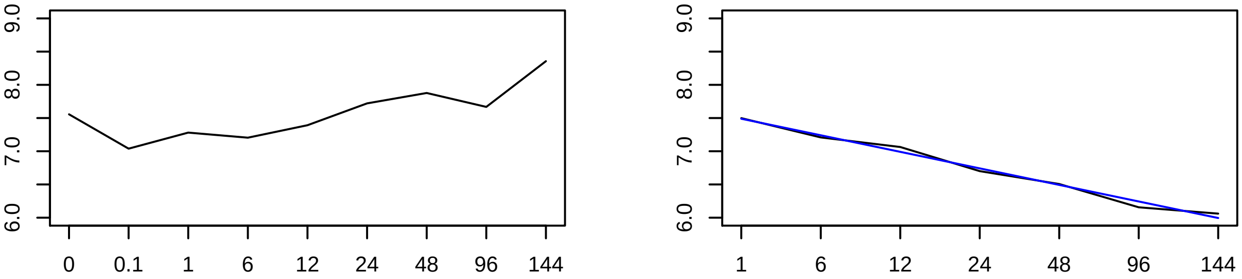

A_24_P491643 THC2506377 NA

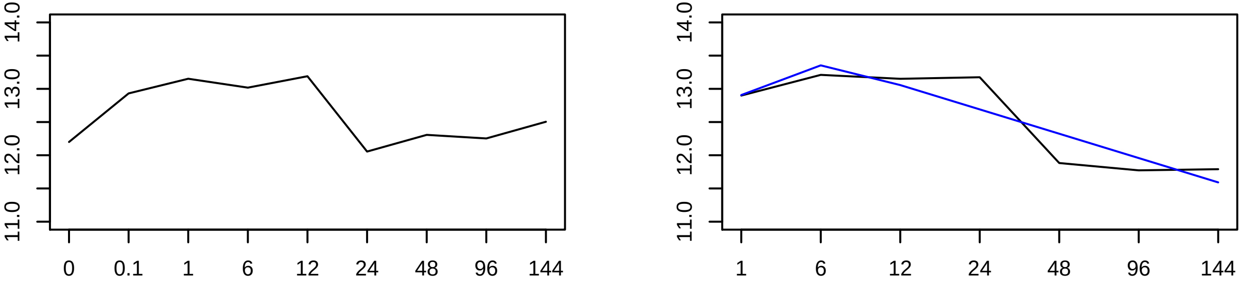

A_23_P149668 KIF14 1q32.1

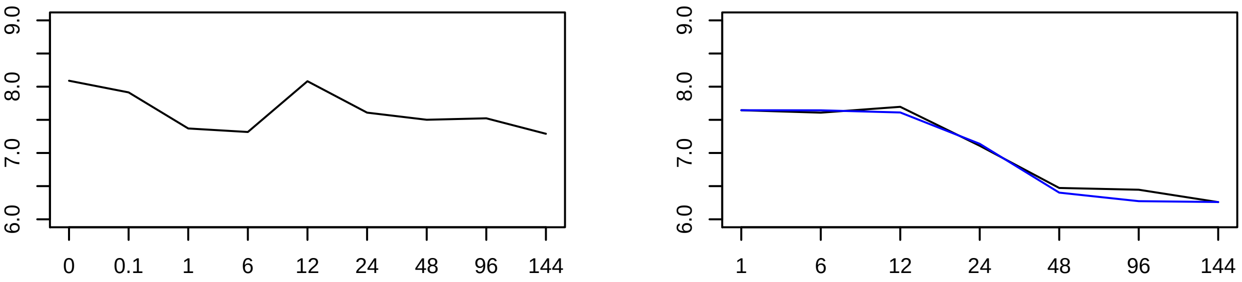

A_23_P215765 NRCAM 7q31.1

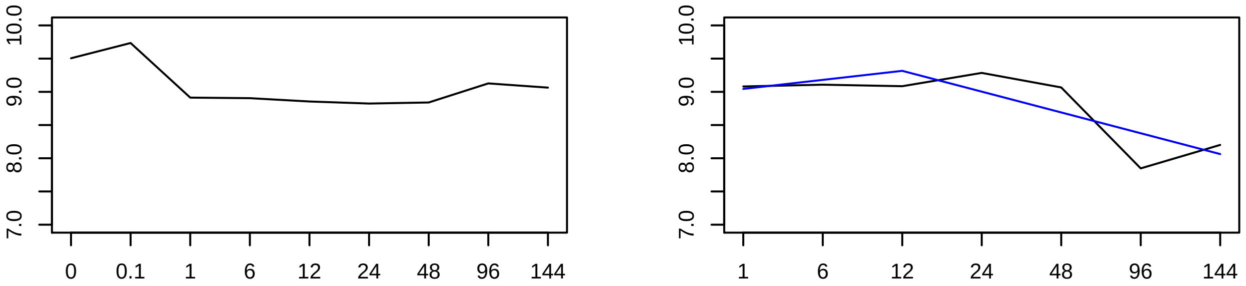

A_24_P358578 CTD−2503O16.2 5q13.3

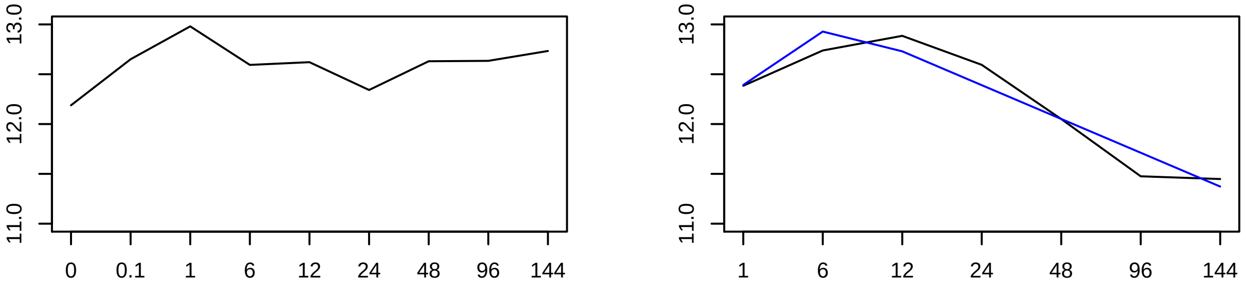

A_32_P49764 LOC728176 2p25.3

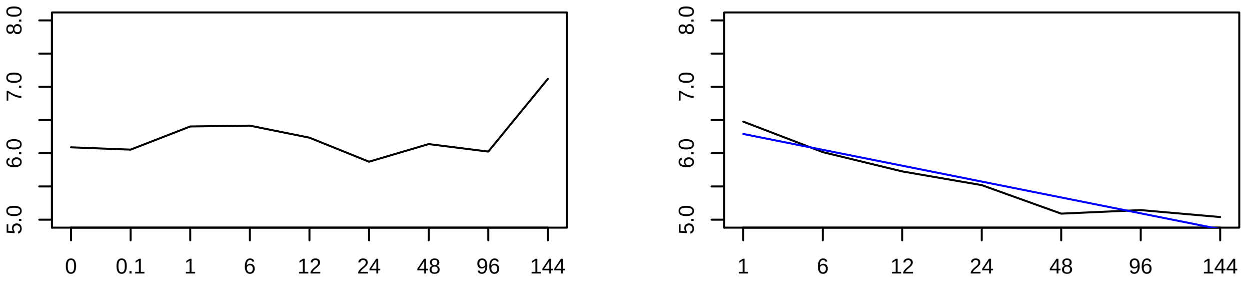

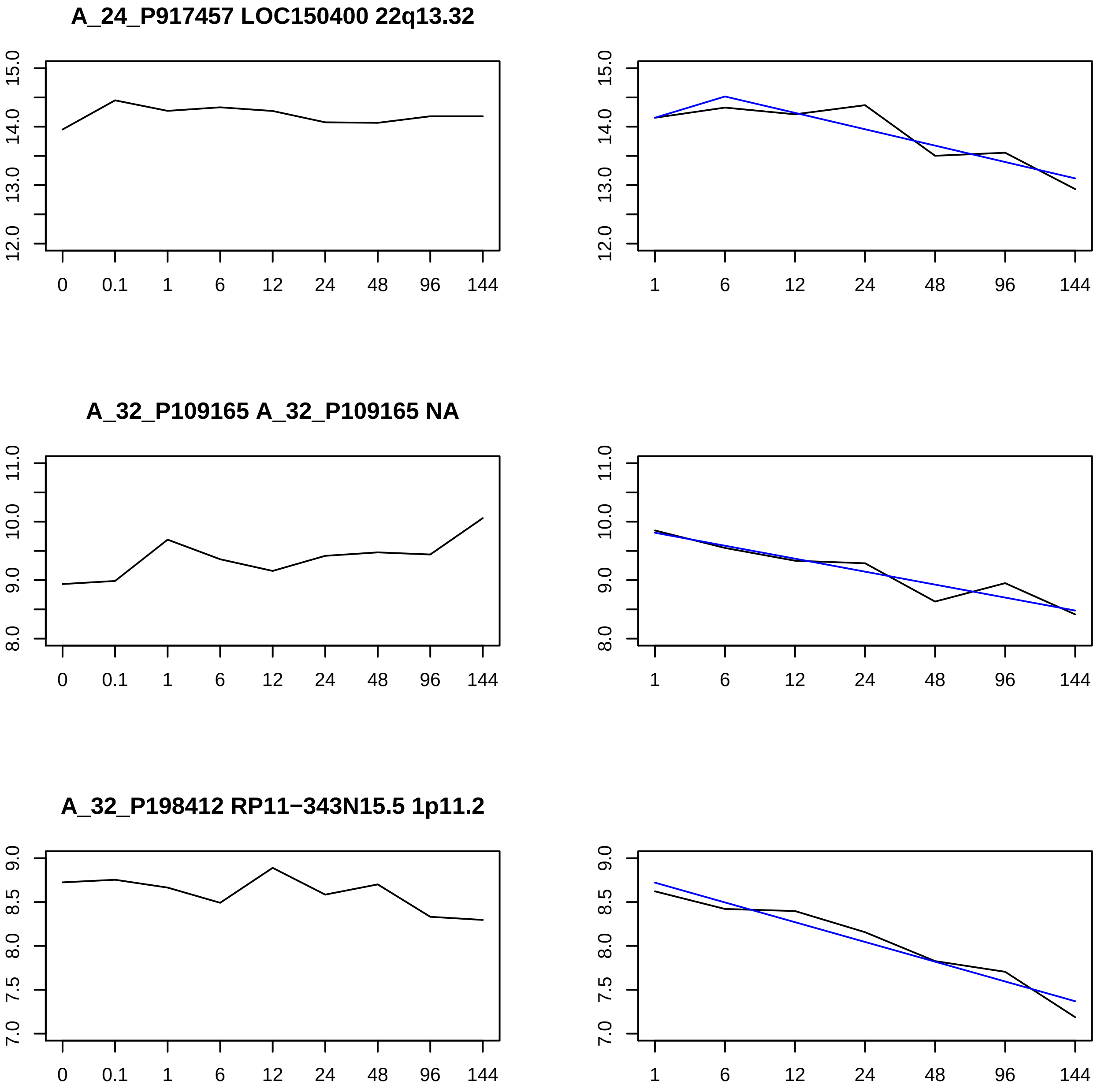
A_24_P917457 LOC150400 22q13.32
A_32_P109165 A_32_P109165 NA
A_32_P198412 RP11−343N15.5 1p11.2

A_24_P521662 A_24_P521662 NA

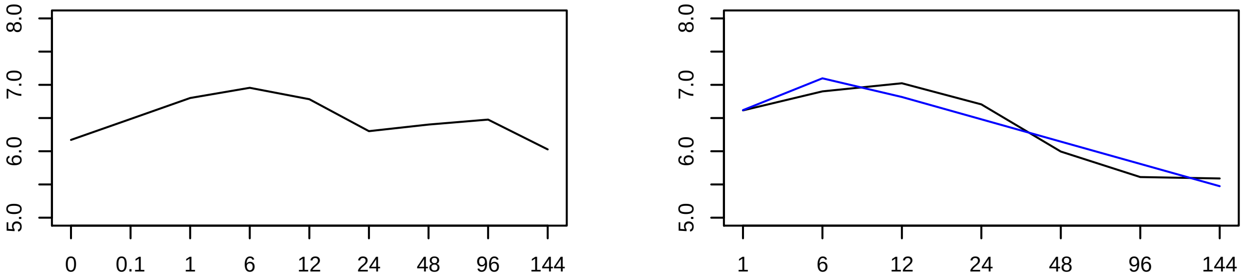

A_24_P375932 AC020983.5 NA

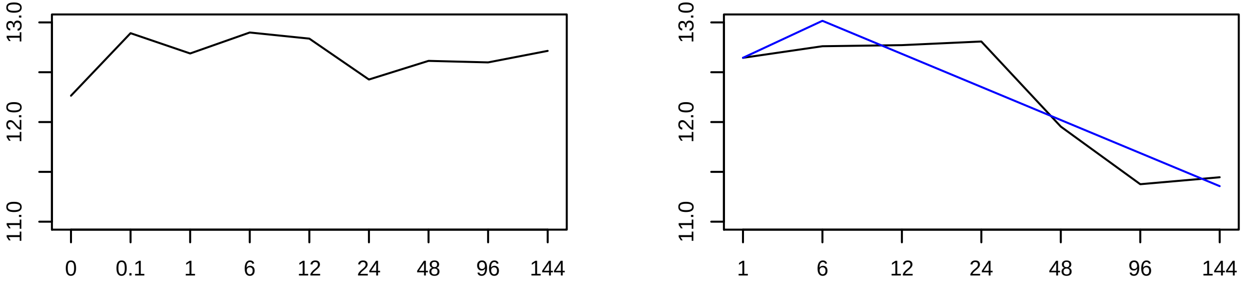

A_32_P165297 MBNL3 Xq26.2

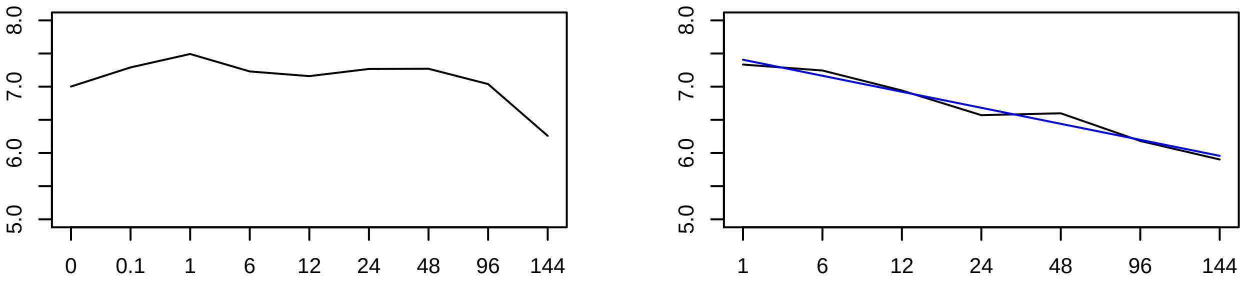

A_24_P941773 METTL7A 12q13.13

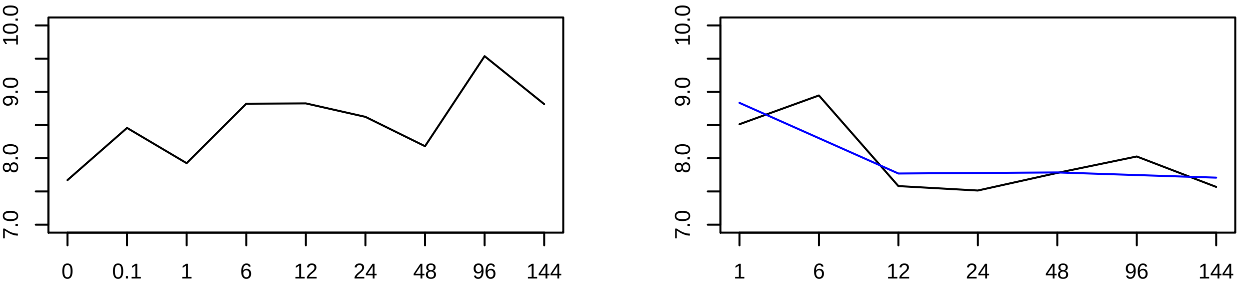

A_24_P926053 EEF1D NA

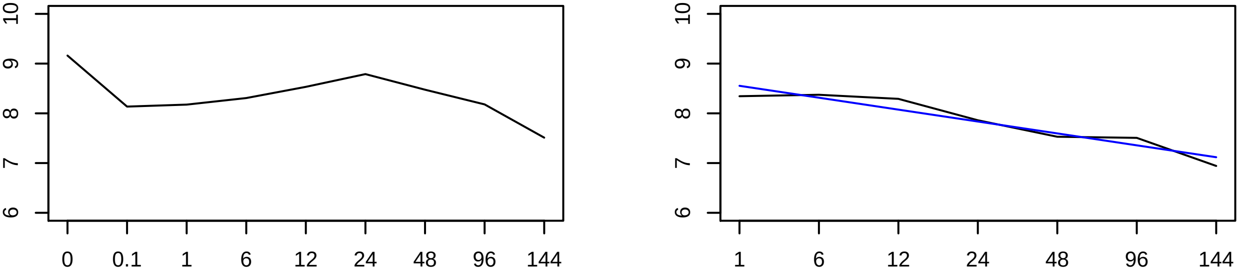

A_24_P212726 EEF1B2P2 5q13.1

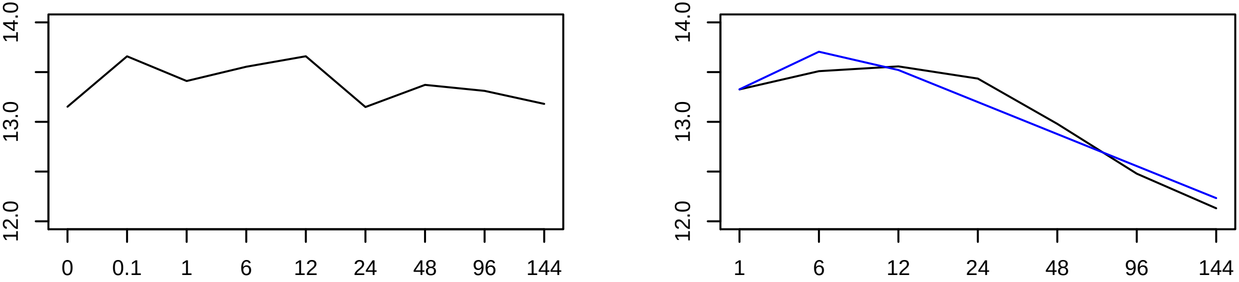

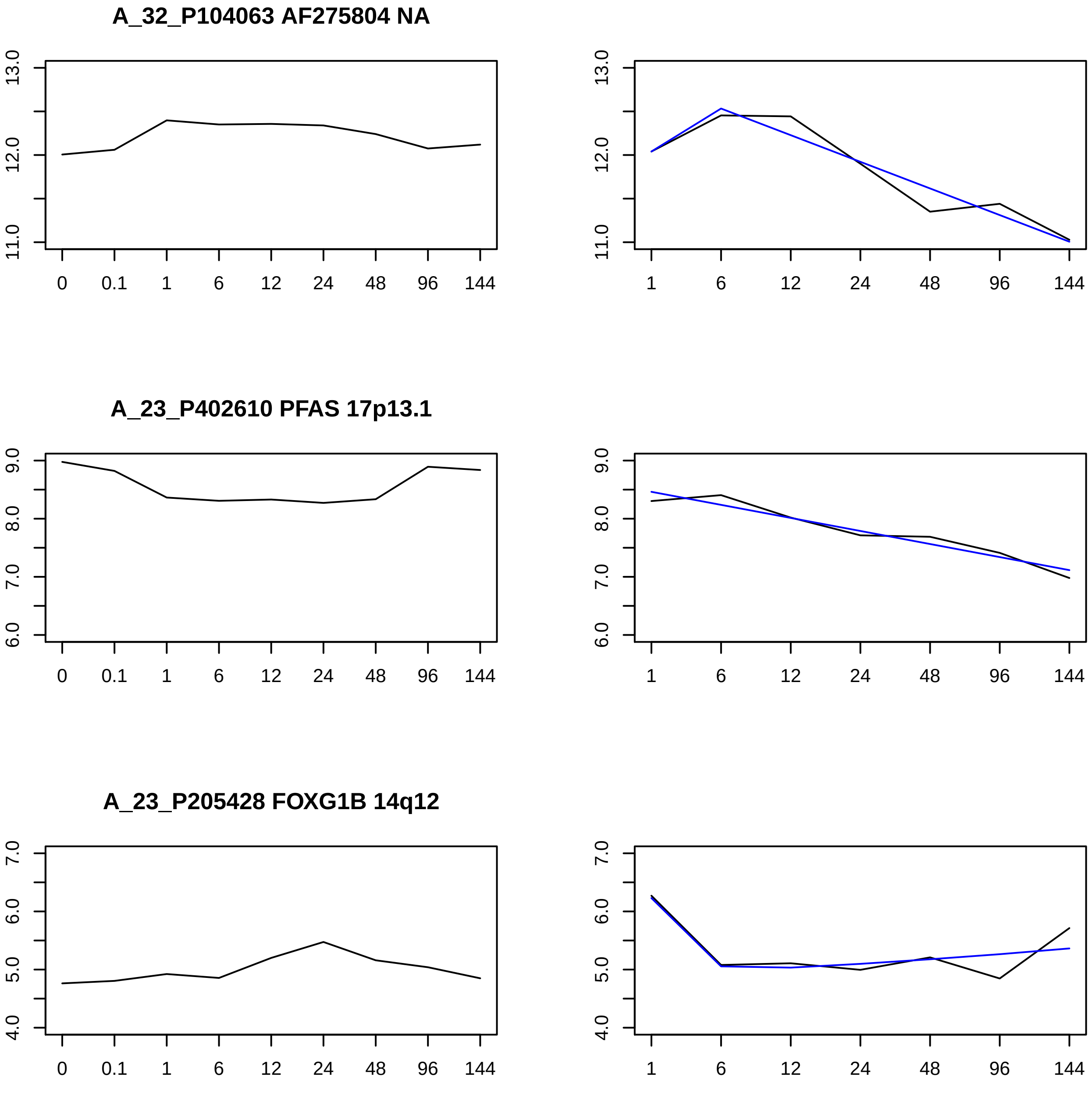
A_32_P104063 AF275804 NA
A_23_P402610 PFAS 17p13.1
A_23_P205428 FOXG1B 14q12

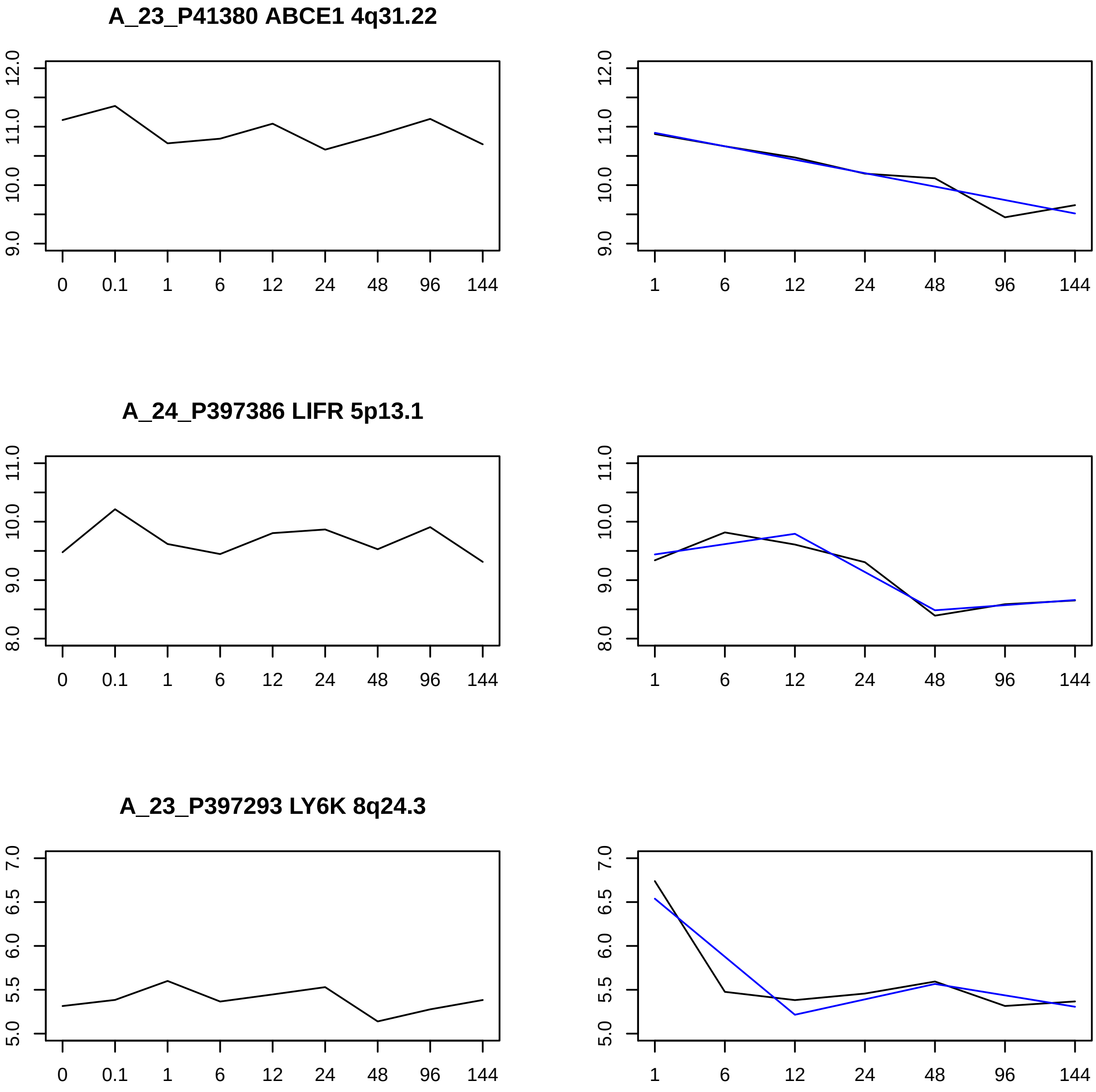
A_23_P41380 ABCE1 4q31.22
9.0
10.0
11.0
12.0
0
0.1
1
6
12
24
48
96
144
1
6
12
24
48
96
144
A_24_P397386 LIFR 5p13.1
8.0
9.0
10.0
11.0
A_23_P397293 LY6K 8q24.3
5.0
5.5
6.0
6.5
7.0

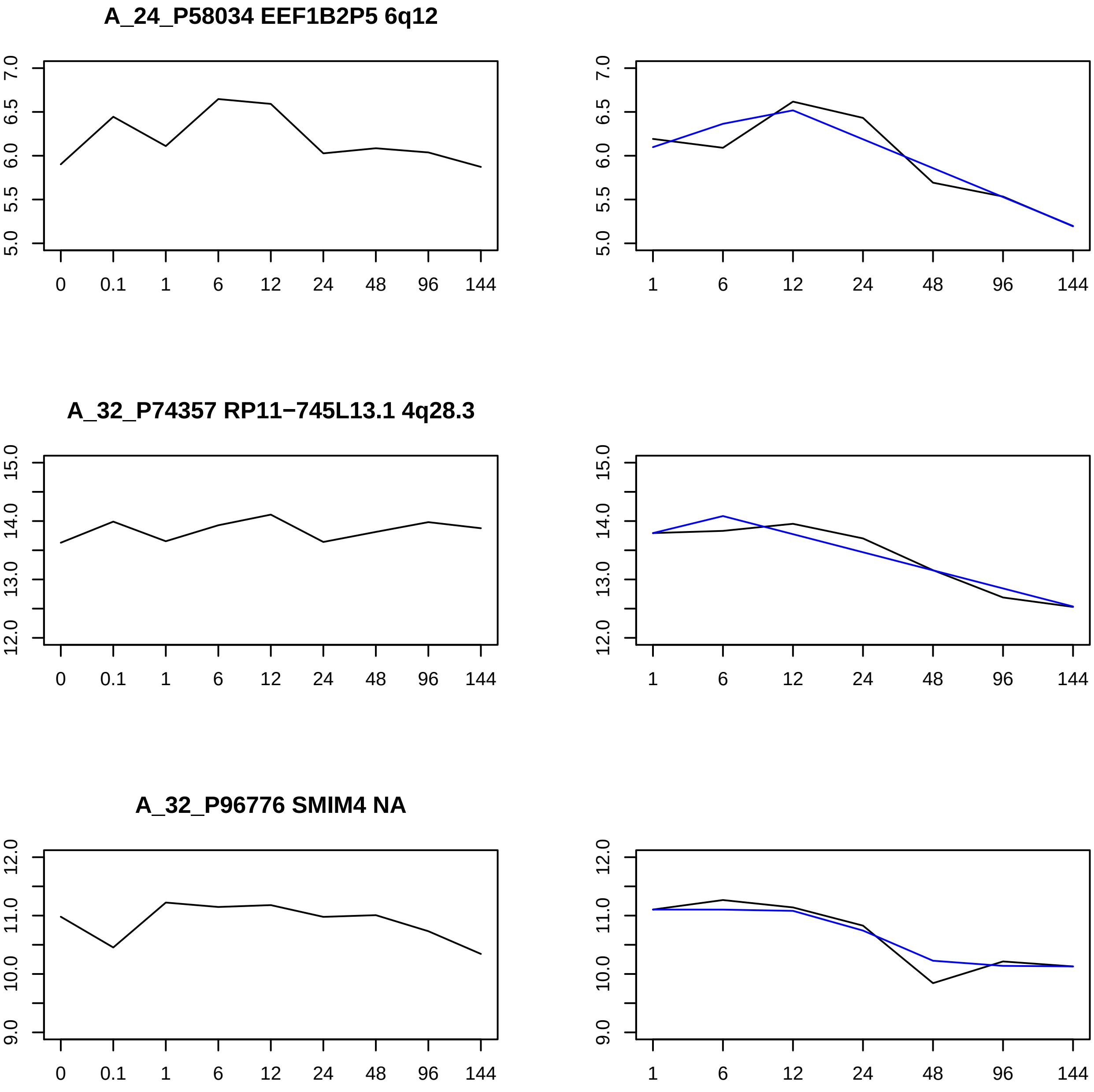

A_24_P58034 EEF1B2P5 6q12
5.0
5.5
6.0
6.5
7.0
0
0.1
1
6
12
24
48
96
144
A_32_P74357 RP11−745L13.1 4q28.3
12.0
13.0
14.0
15.0
A_32_P96776 SMIM4 NA
9.0
10.0
11.0
12.0

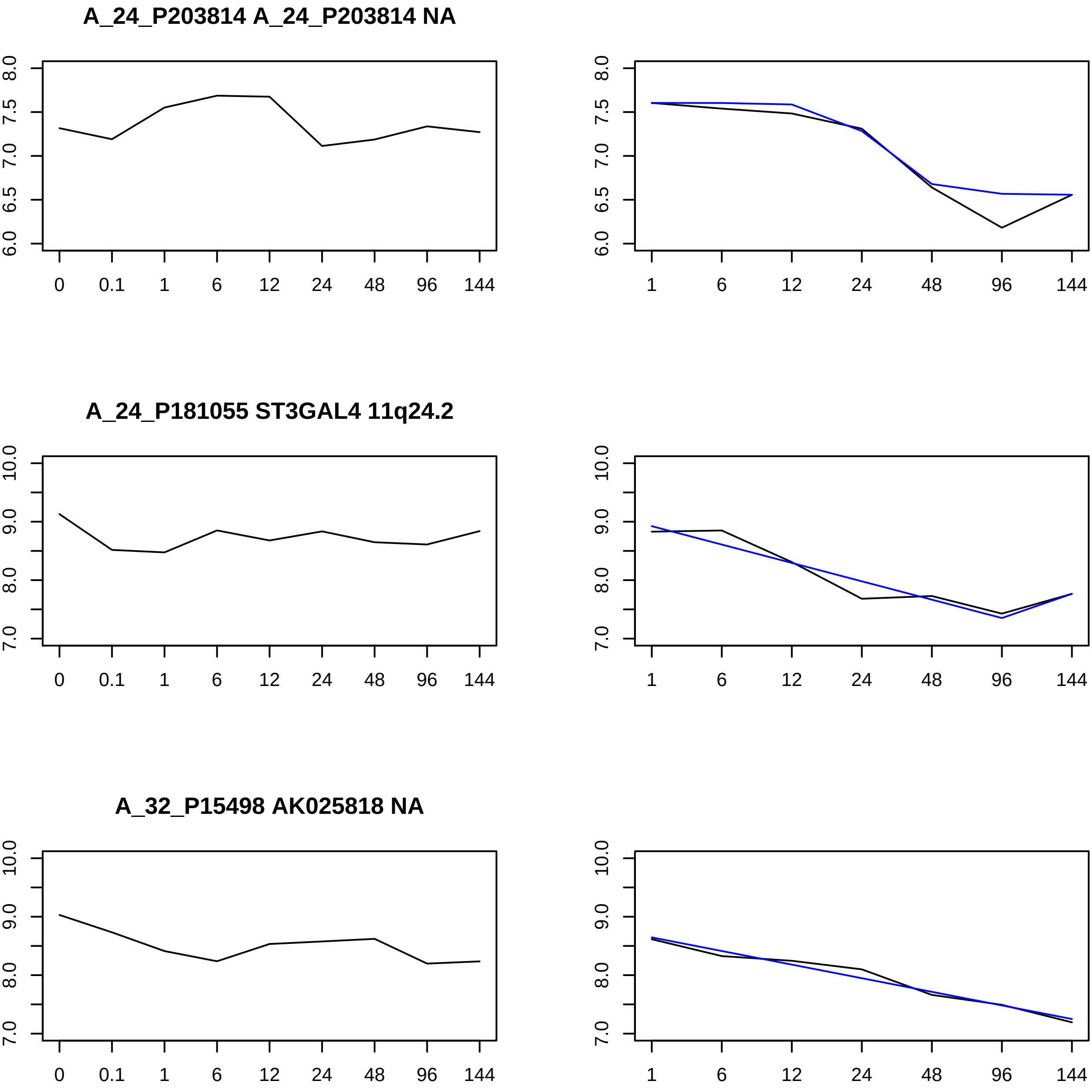
A_24_P203814 A_24_P203814 NA
6.0
6.5
7.0
7.5
8.0
0
0.1
1
6
12
24
48
96
144
1
6
12
24
48
96
144
A_24_P181055 ST3GAL4 11q24.2
7.0
8.0
9.0
10.0
A_32_P15498 AK025818 NA

A_24_P307443 LOC441641 12q14.1

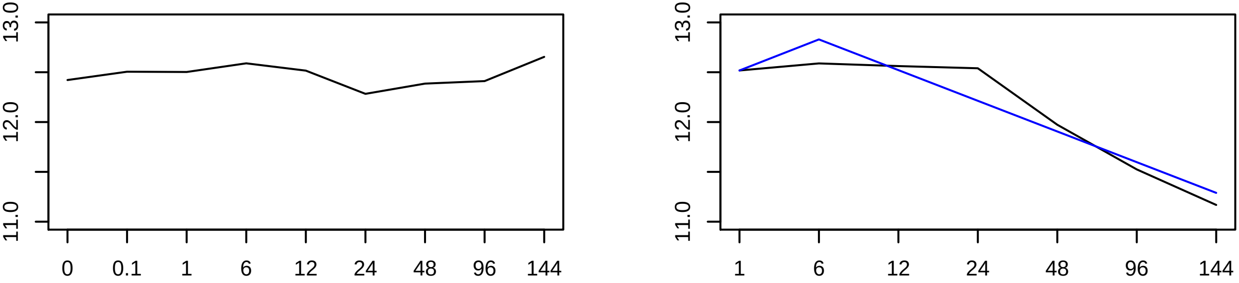

A_23_P200936 MTR 1q43

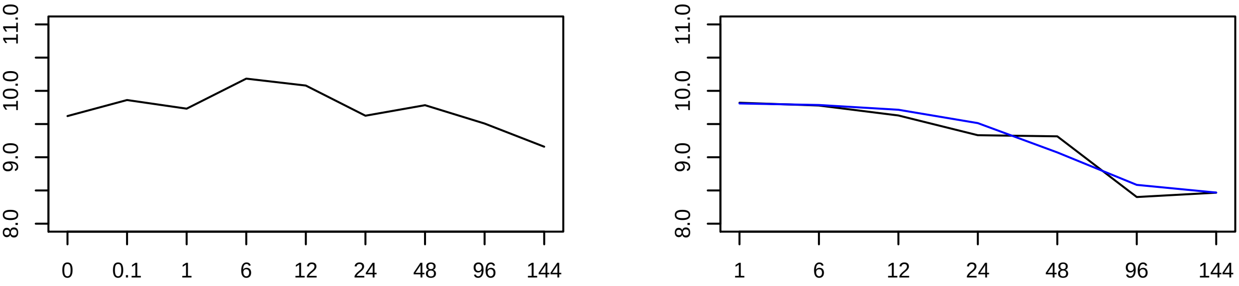

A_24_P158536 RHOBTB1 10q21.2

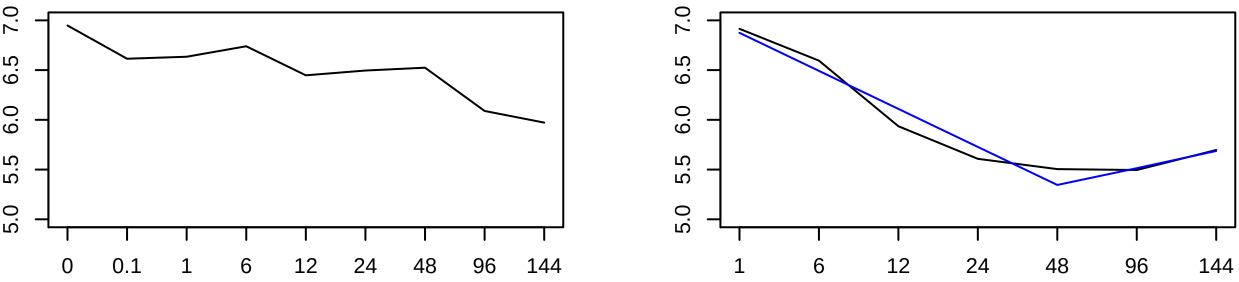

A_24_P58453 NOL8 9q22.31

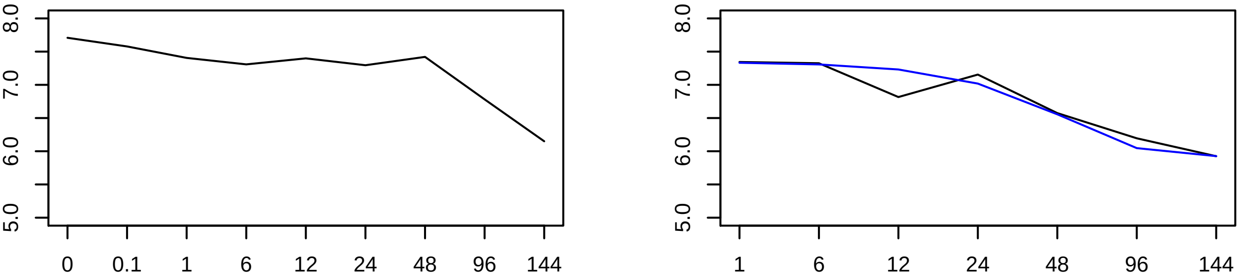

A_24_P239664 BCKDHB 6q14.1

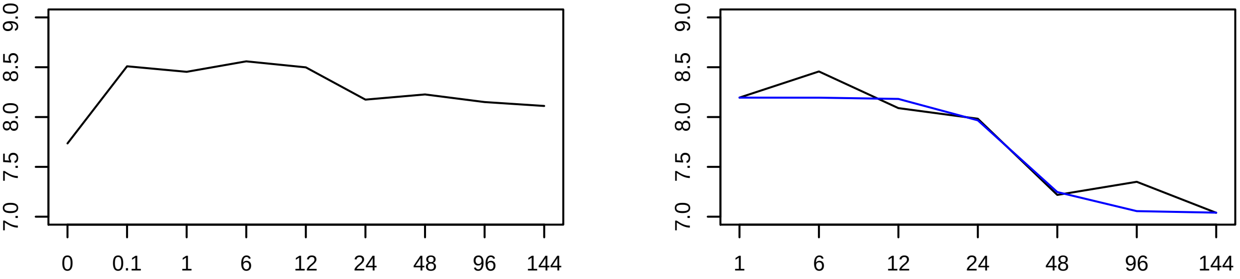

A_24_P84711 A_24_P84711 NA

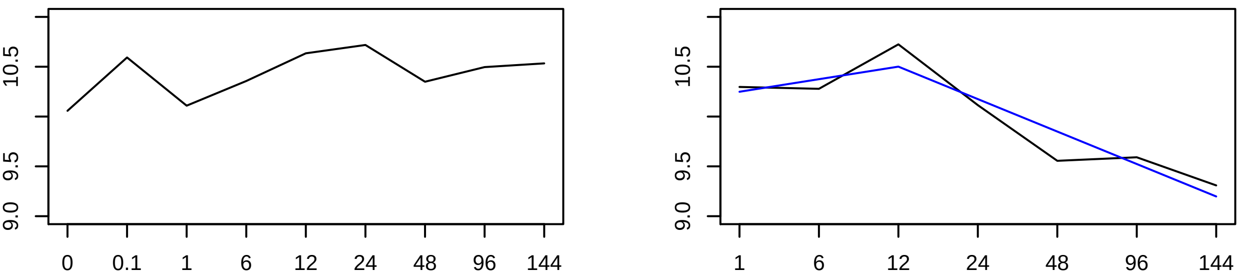

A_23_P213359 PCDH1 5q31.3

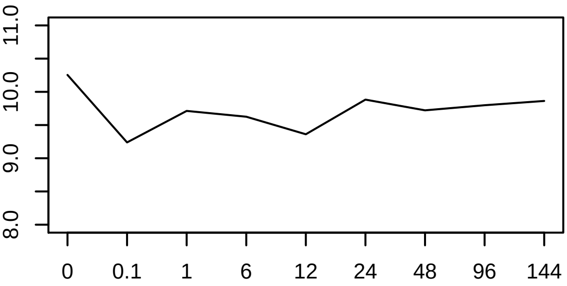

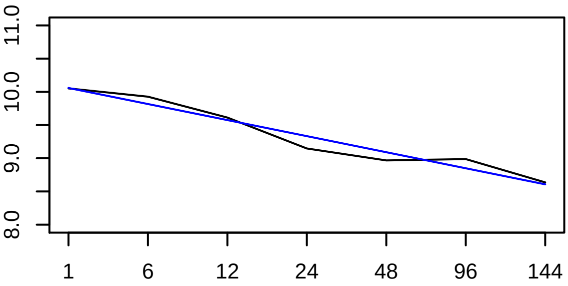

A_23_P41476 SHISA3 4p13

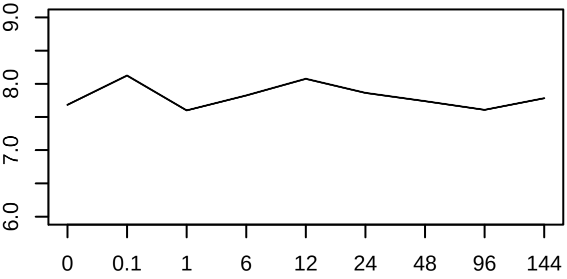

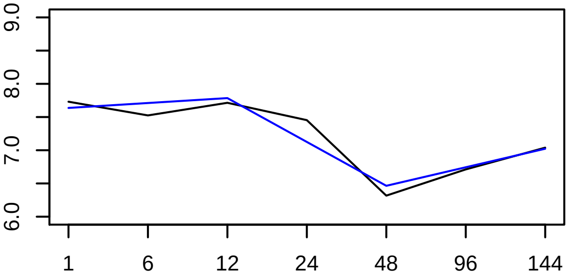

A_24_P938465 THC2727494 NA

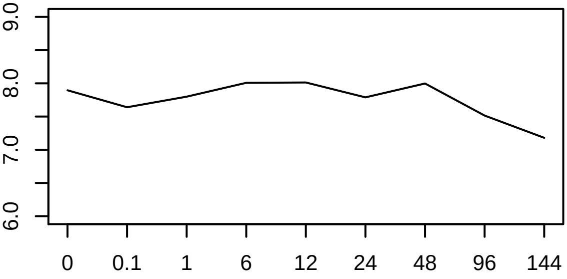

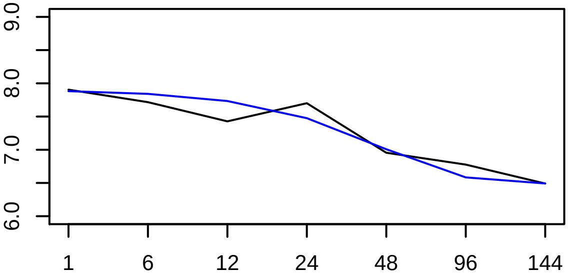

A_24_P631948 RPL6P29 Xq21.33

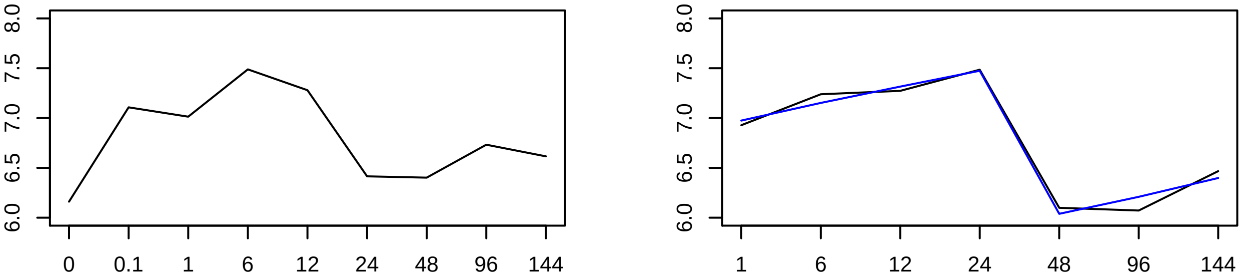

A_23_P337422 SERBP1 1p31.3

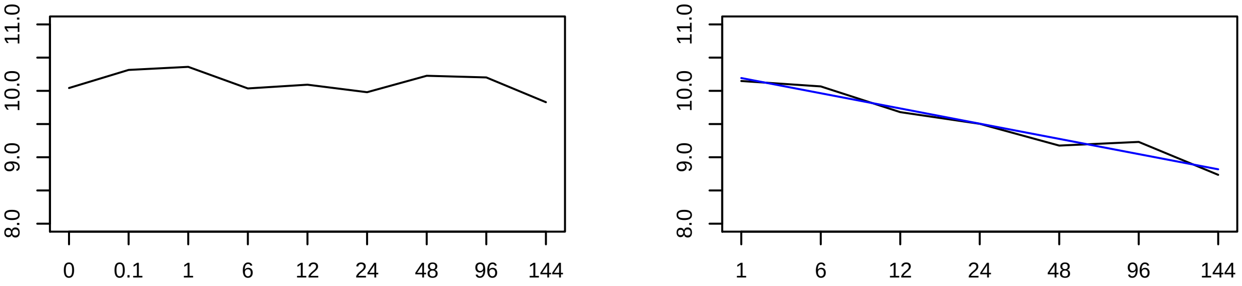

A_32_P164593 ZMAT4 8p11.21

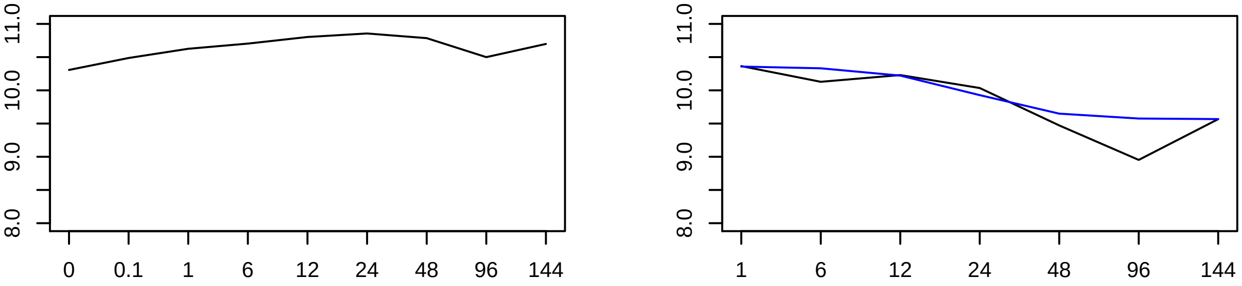

A_24_P196024 A_24_P196024 NA

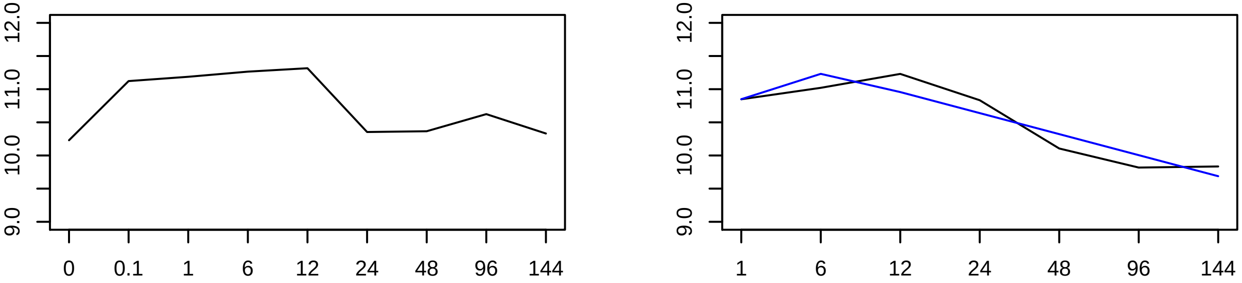

A_23_P82588 HSPC268 7q34

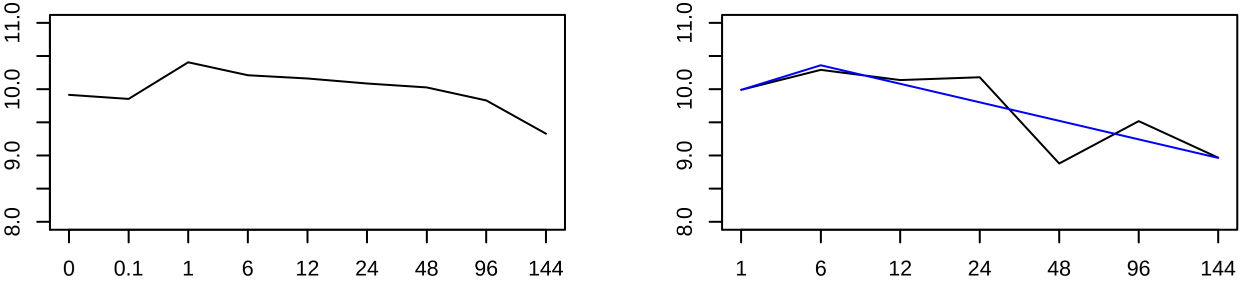

A_24_P238266 LOC402219 5q14.3

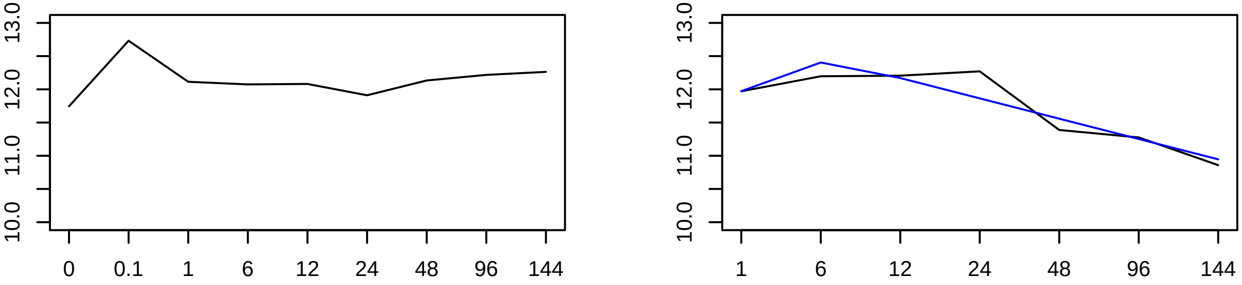

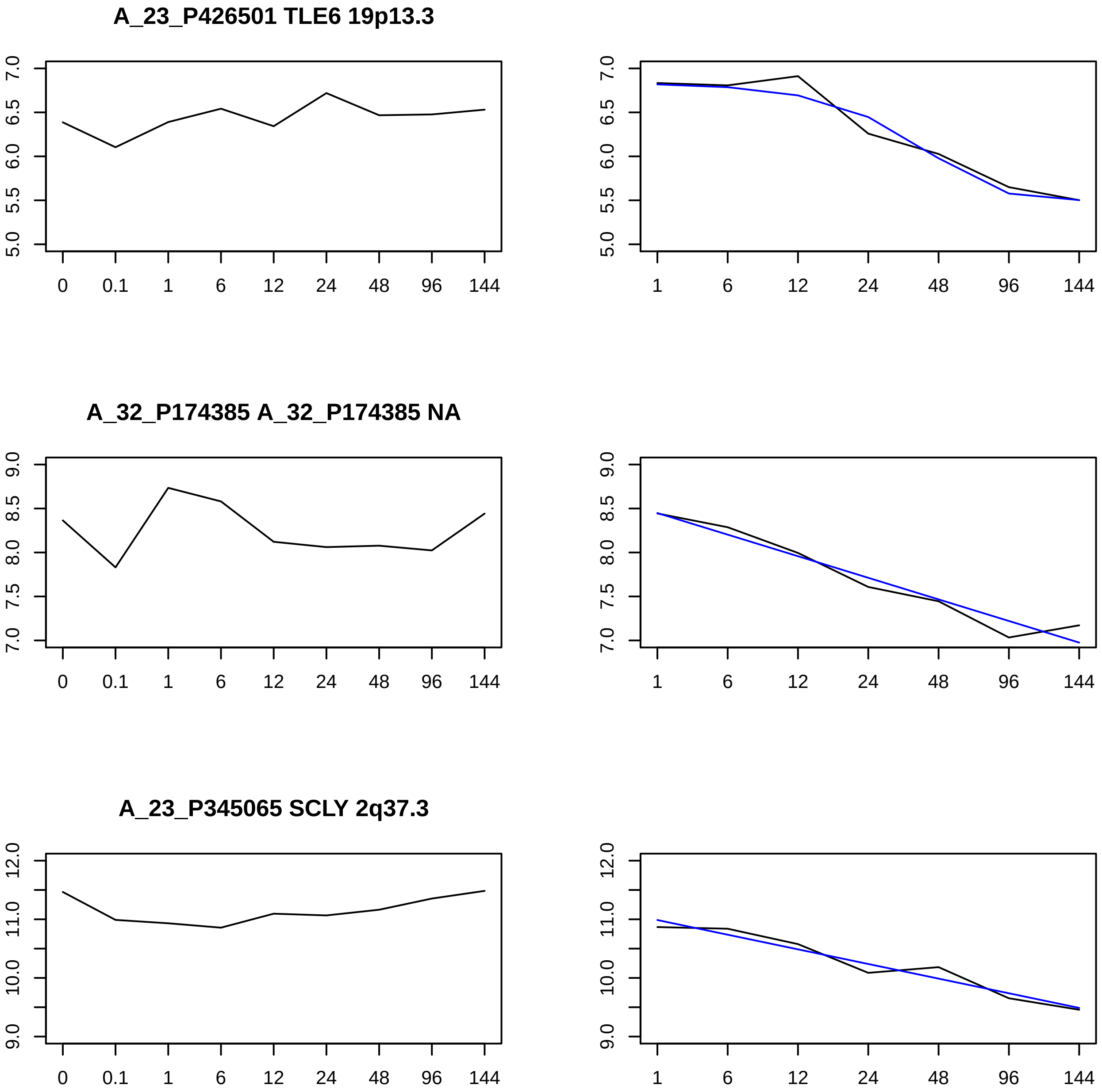

A_23_P426501 TLE6 19p13.3
A_32_P174385 A_32_P174385 NA
A_23_P345065 SCLY 2q37.3
0 0.1 1 6 12 24 48 96 144
1 6 12 24 48 96 144
5.0 5.5 6.0 6.5 7.0
7.0 7.5 8.0 8.5 9.0
9.0 10.0 11.0 12.0

A_23_P21882 A_23_P21882 NA

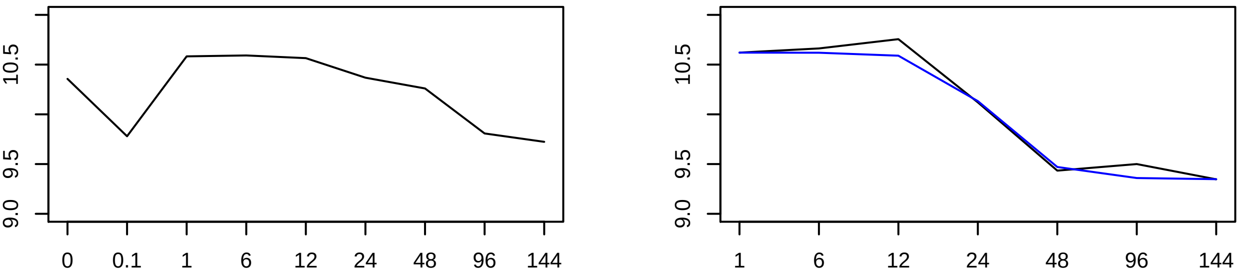

A_32_P218143 THC2627432 NA

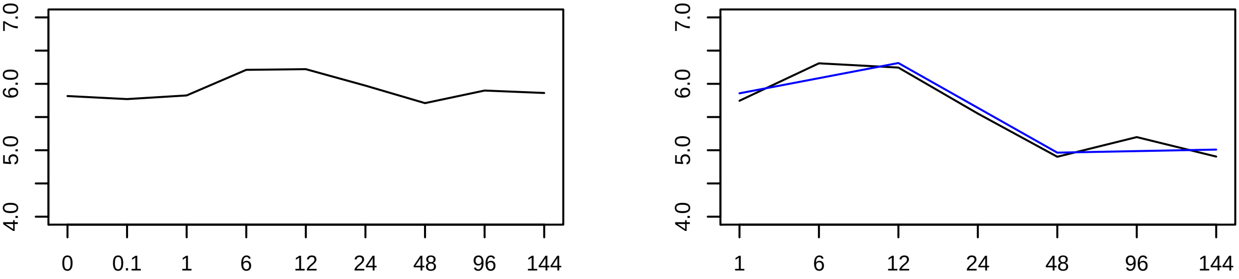

A_24_P375683 LOC731113 3q13.33

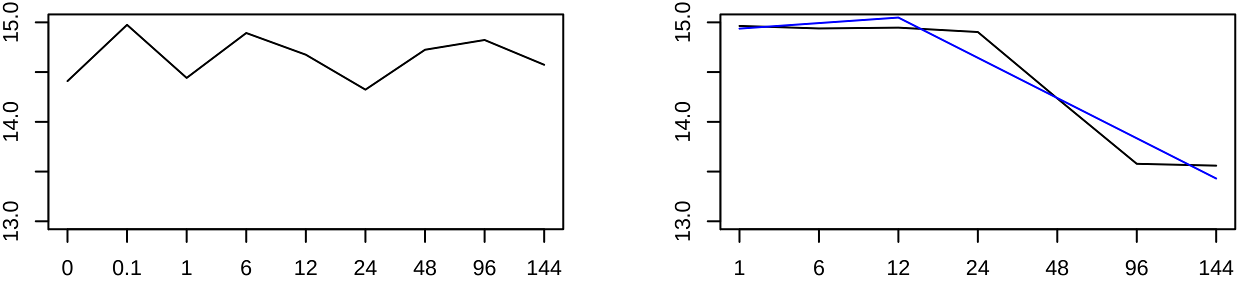

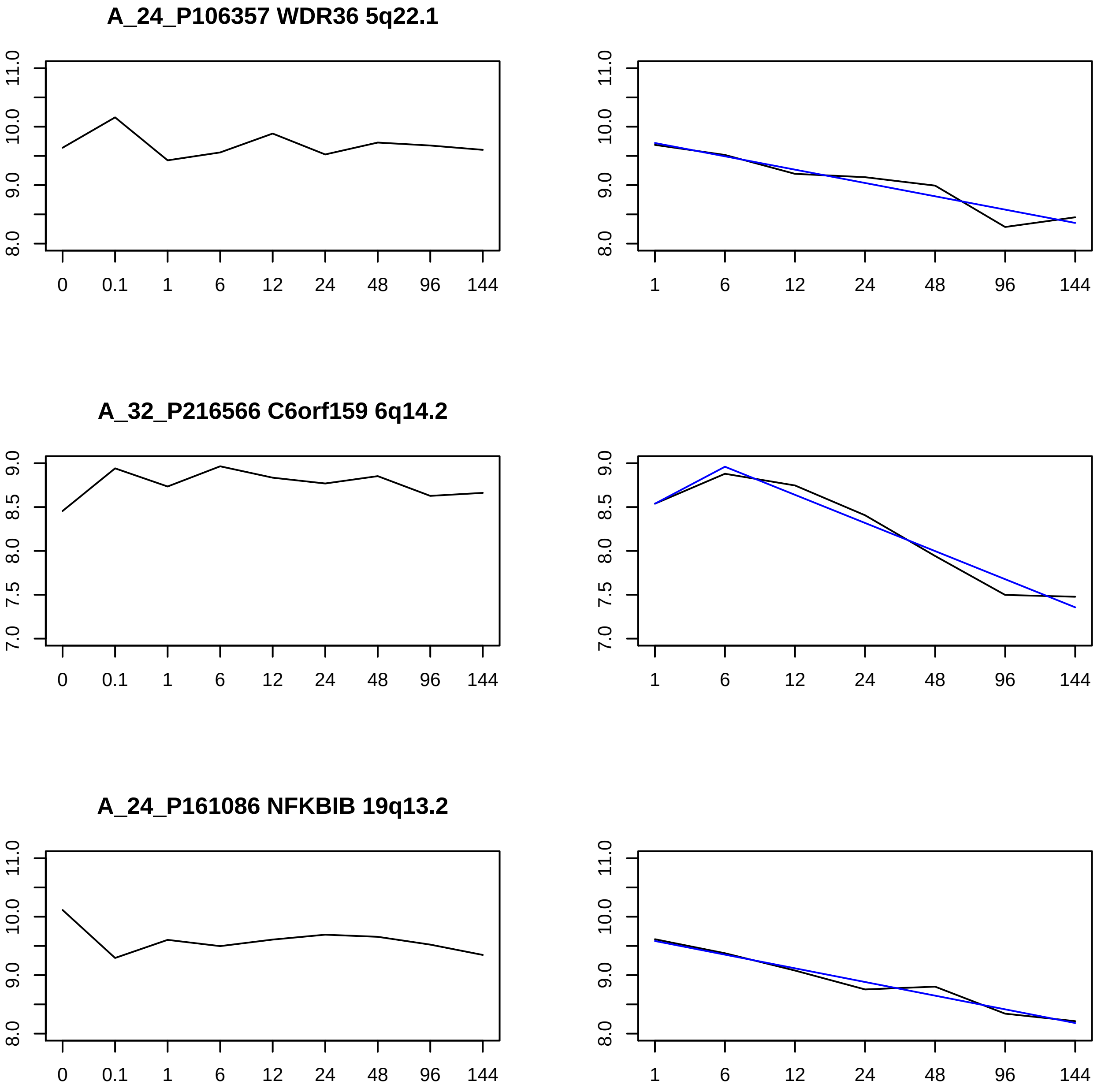
A_24_P106357 WDR36 5q22.1
A_32_P216566 C6orf159 6q14.2
A_24_P161086 NFKBIB 19q13.2
0 0.1 1 6 12 24 48 96 144
1 6 12 24 48 96 144

A_23_P100660 SERPINF1 17p13.3

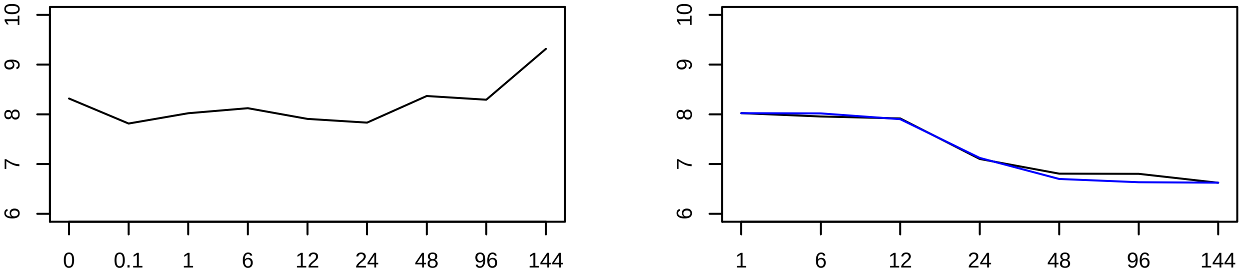

A_23_P156198 PHF15 5q31.1

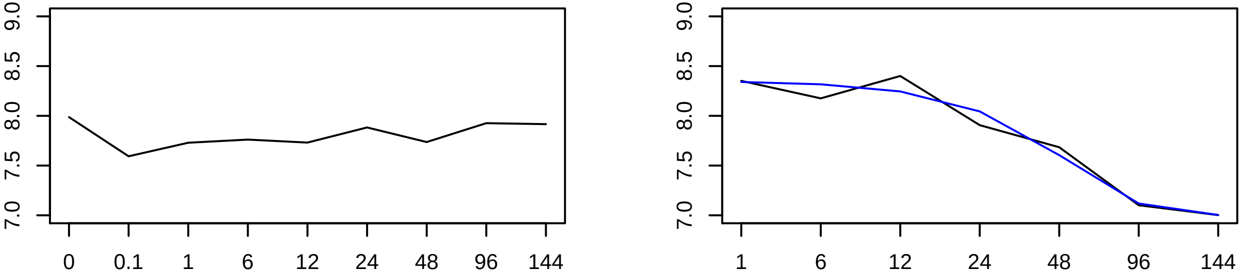

A_23_P145068 MTO1 6q13

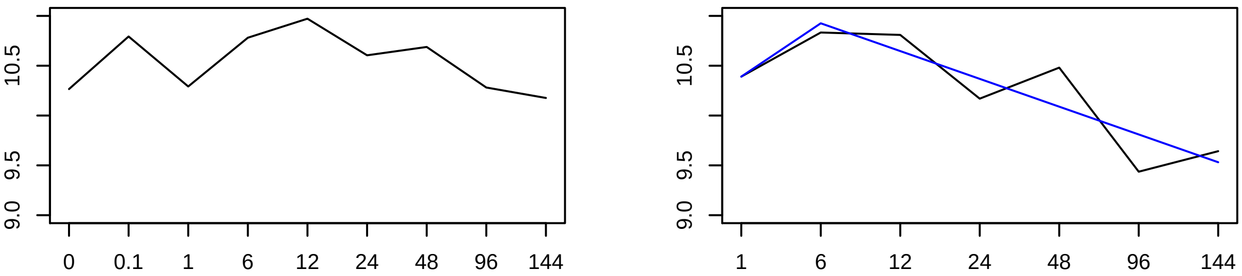

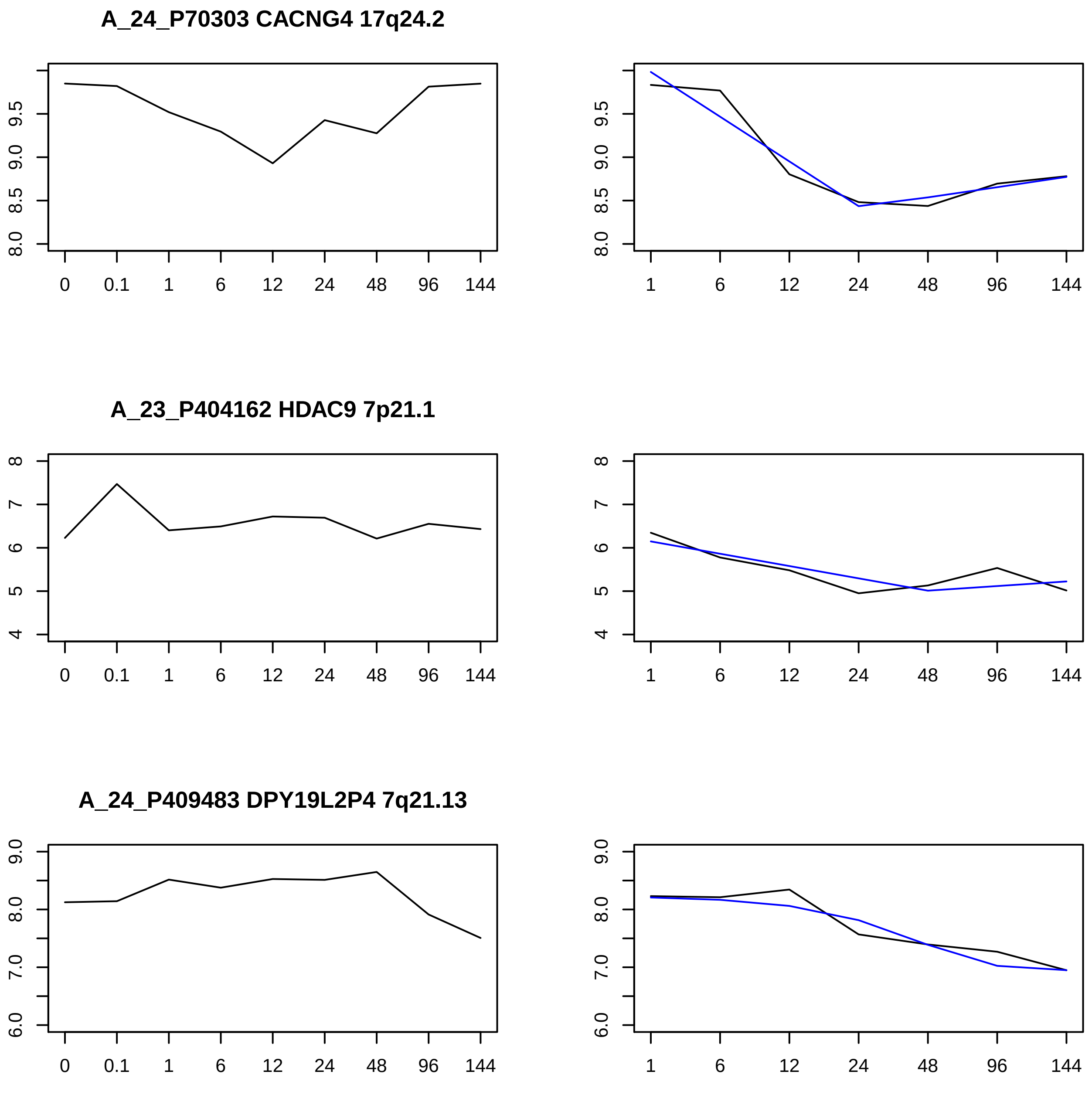
A_24_P70303 CACNG4 17q24.2
8.0
8.5
9.0
9.5
0
0.1
1
6
12
24
48
96
144
A_23_P404162 HDAC9 7p21.1
4
5
6
7
8
A_24_P409483 DPY19L2P4 7q21.13
6.0
7.0
8.0
9.0

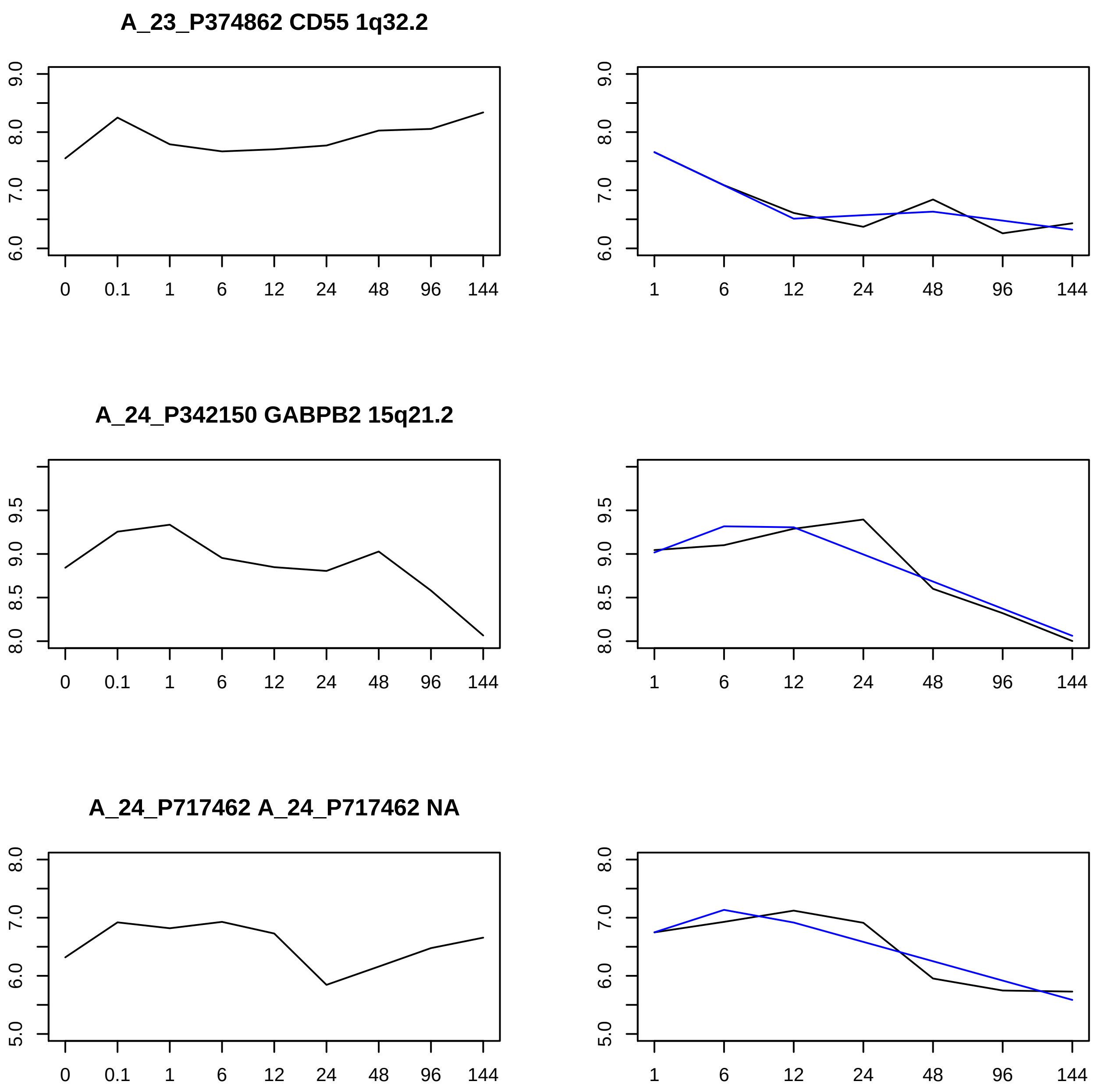
A_23_P374862 CD55 1q32.2
6.0 7.0 8.0 9.0
0 0.1 1 6 12 24 48 96 144
6.0 7.0 8.0 9.0
1 6 12 24 48 96 144
A_24_P342150 GABPB2 15q21.2
8.0 8.5 9.0 9.5
0 0.1 1 6 12 24 48 96 144
8.0 8.5 9.0 9.5
1 6 12 24 48 96 144
A_24_P717462 A_24_P717462 NA
5.0 6.0 7.0 8.0
0 0.1 1 6 12 24 48 96 144
5.0 6.0 7.0 8.0
1 6 12 24 48 96 144

A_32_P175580 RPS15A 16p12.3

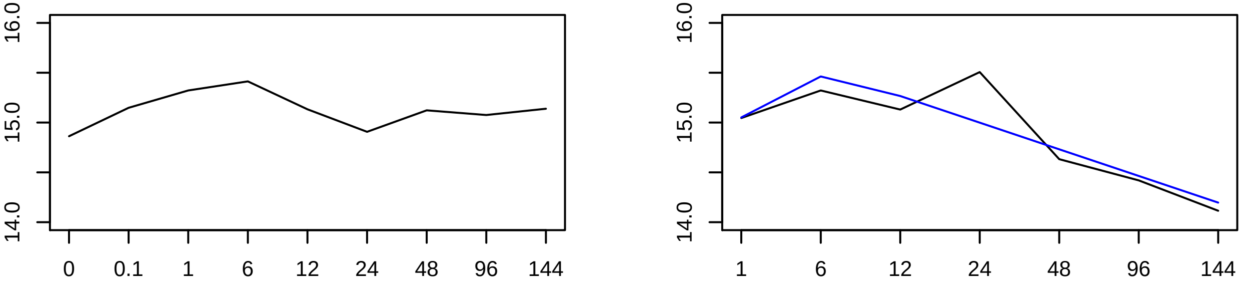

A_24_P865 C6orf204 6q22.31

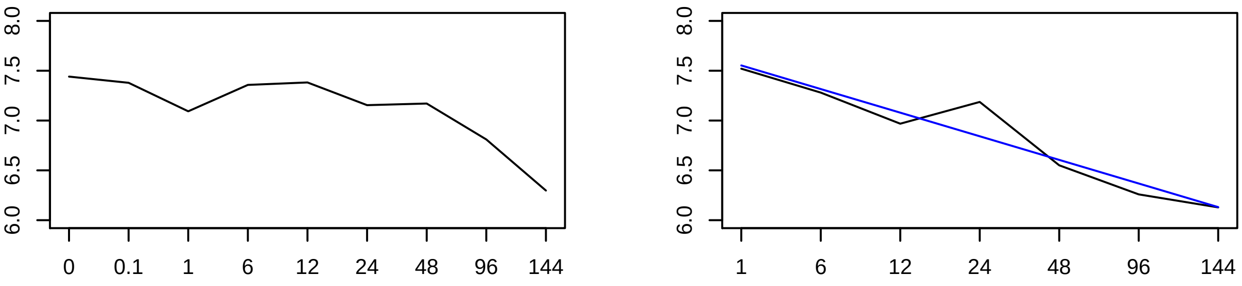

A_24_P169593 A_24_P169593 NA

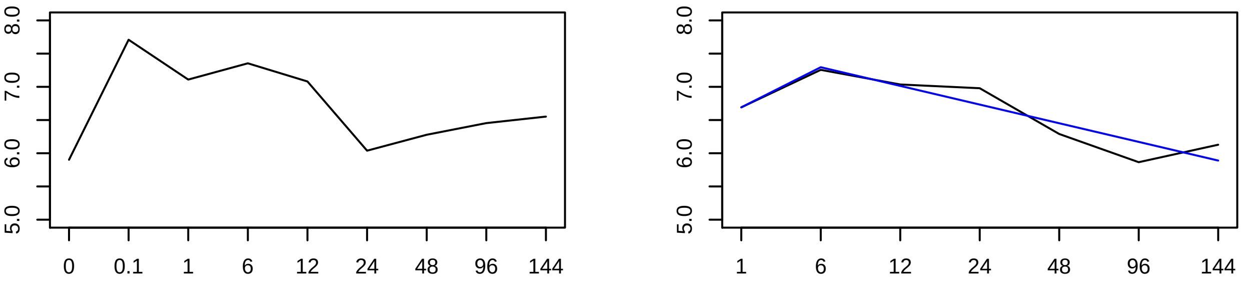

A_23_P218086 TPCN1 12q24.13

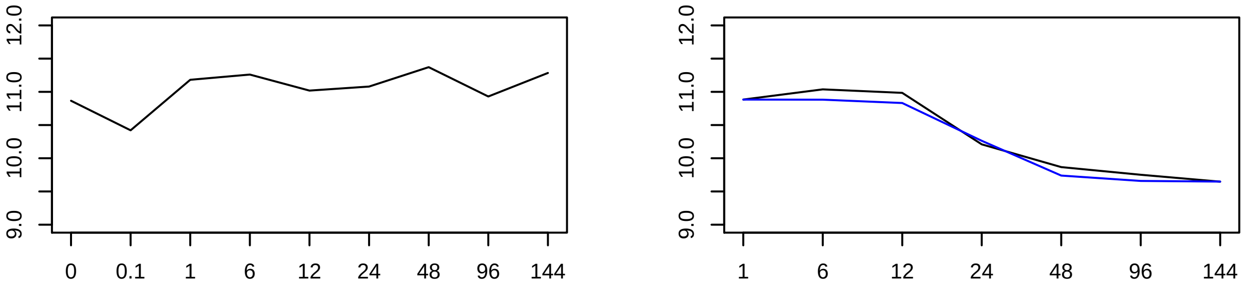

A_32_P2452 TMTC1 12p11.22

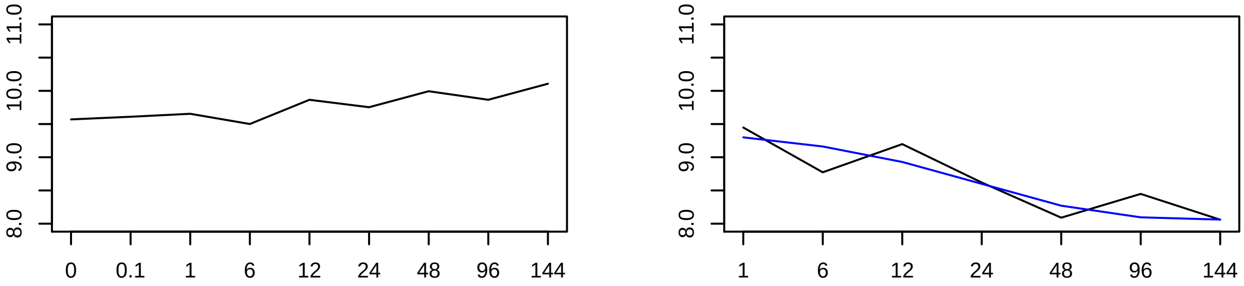

A_24_P281468 A_24_P281468 NA

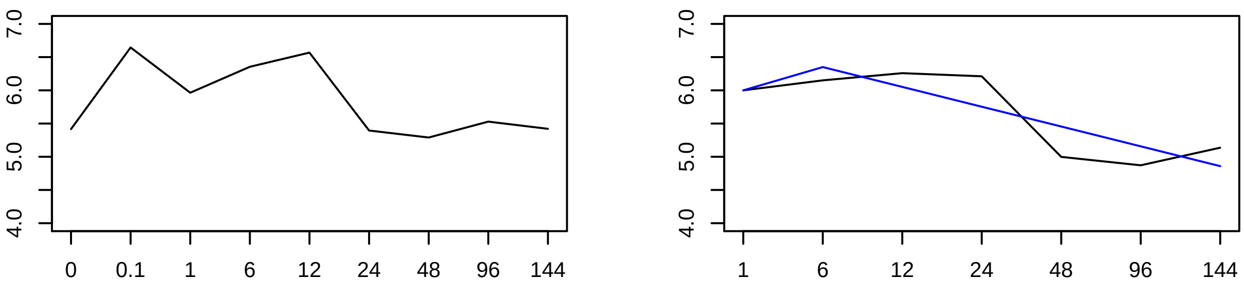

A_24_P271985 TNRC15 2q37.1

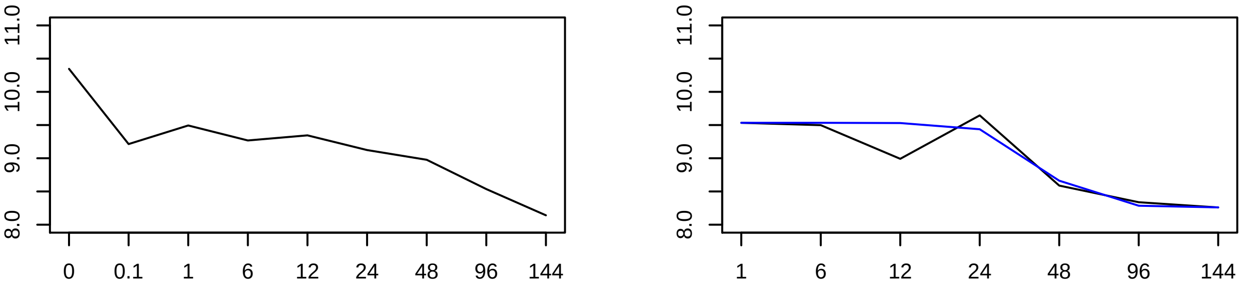

A_24_P625898 A_24_P625898 NA

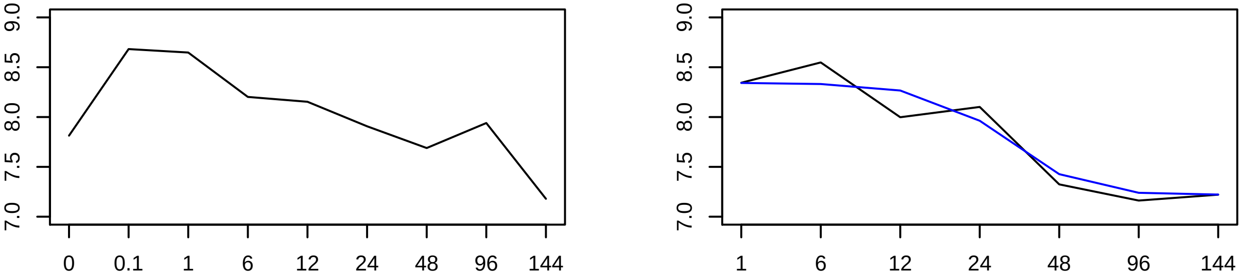

A_23_P351295 HS3ST5 6q22.1

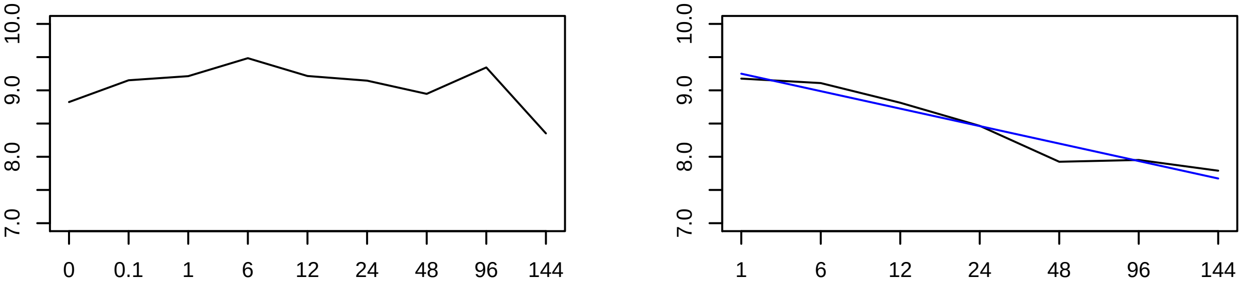

A_24_P549518 AHCYP3 2q12.1

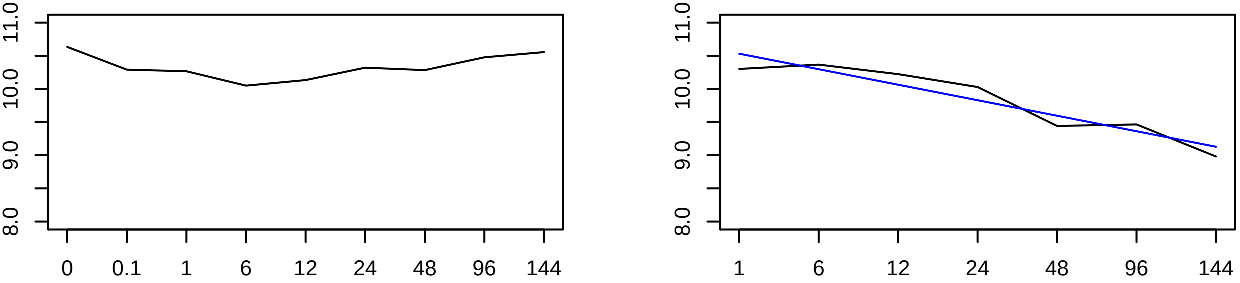

A_23_P64184 A_23_P64184 NA

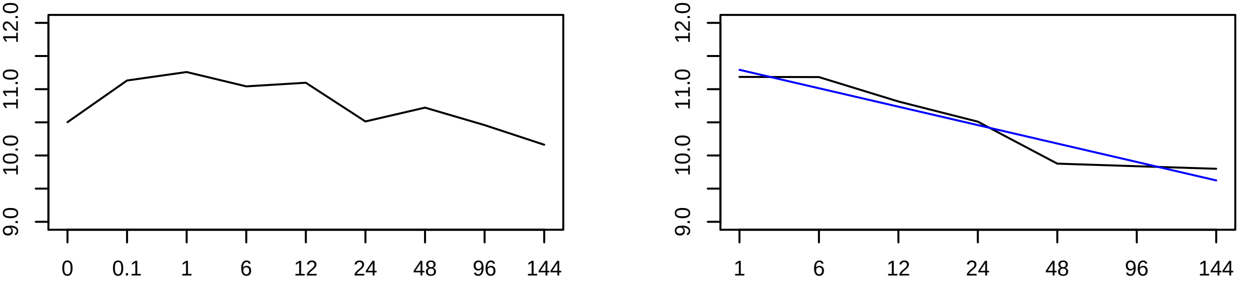

A_23_P405088 CLDN11 3q26.2

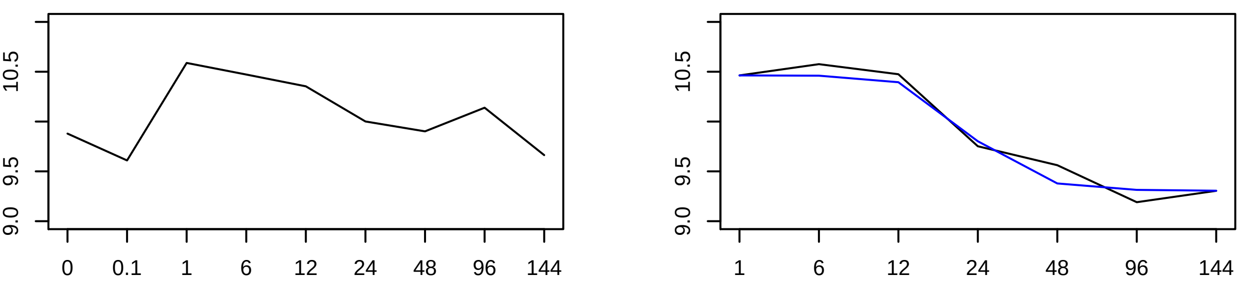

A_23_P166508 BC038245 NA

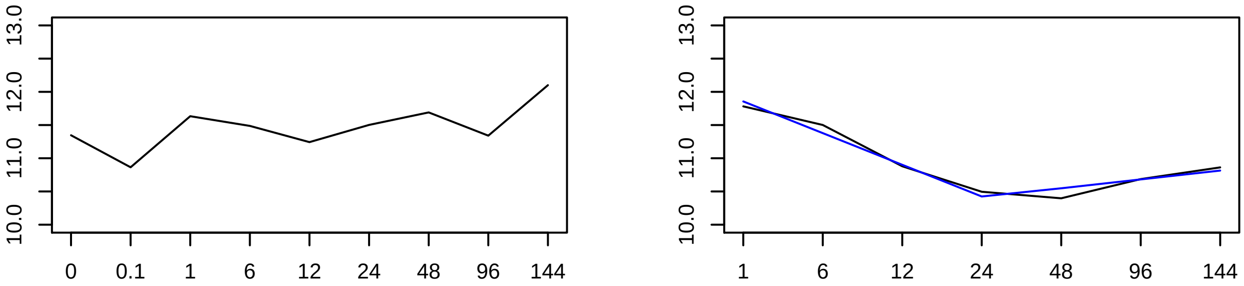

A_24_P683011 RP11−122A3.2 8q21.3

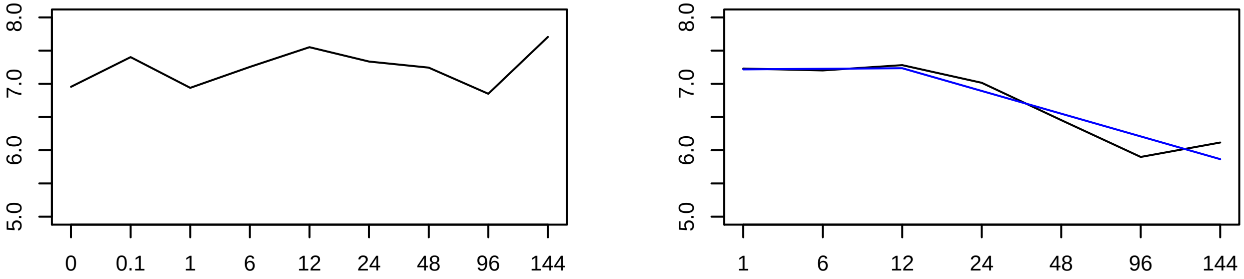

A_24_P106166 A_24_P106166 NA

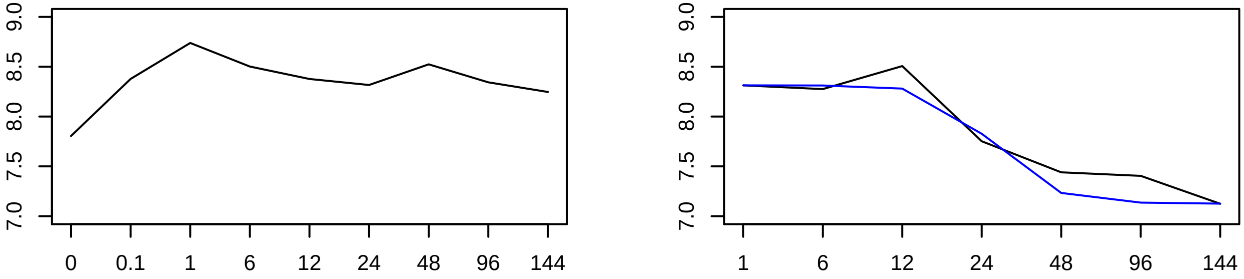

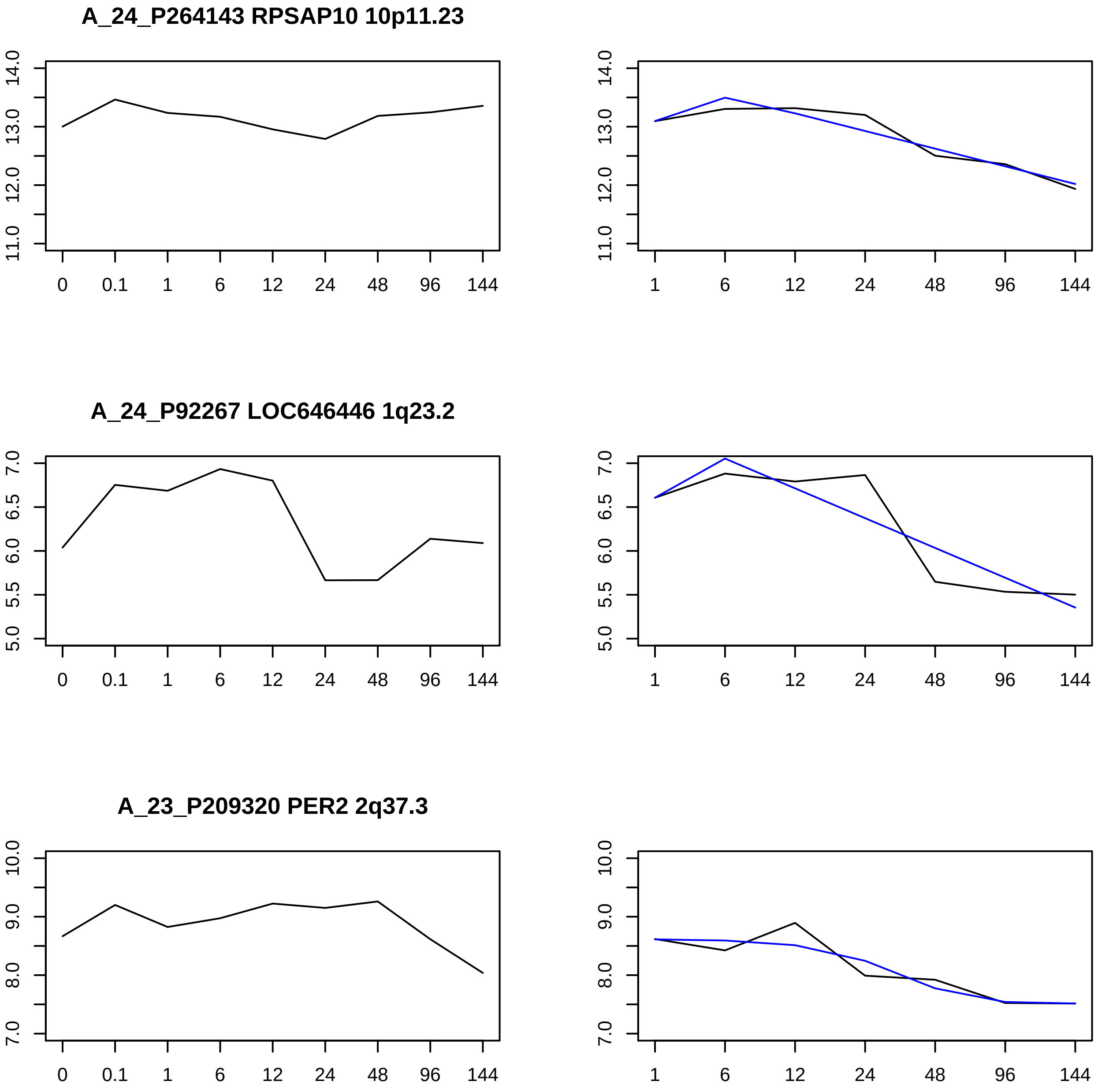
A_24_P264143 RPSAP10 10p11.23
11.0
12.0
13.0
14.0
0
0.1
1
6
12
24
48
96
144
A_24_P92267 LOC646446 1q23.2
5.0
5.5
6.0
6.5
7.0
A_23_P209320 PER2 2q37.3
7.0
8.0
9.0
10.0

A_24_P50028 RAB39 11q22.3

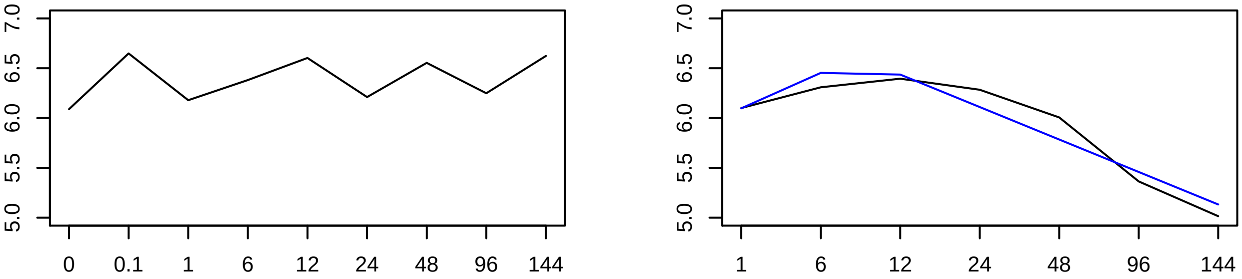

A_32_P923011 RP11−34P13.4 1p36.33

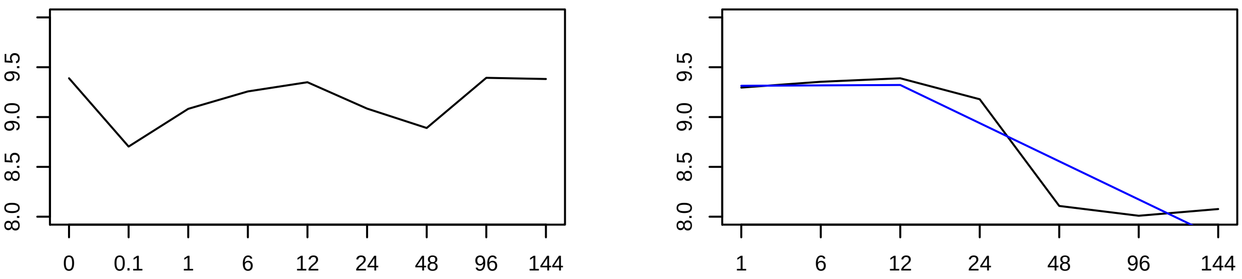

A_32_P14978 CEP152 15q21.1

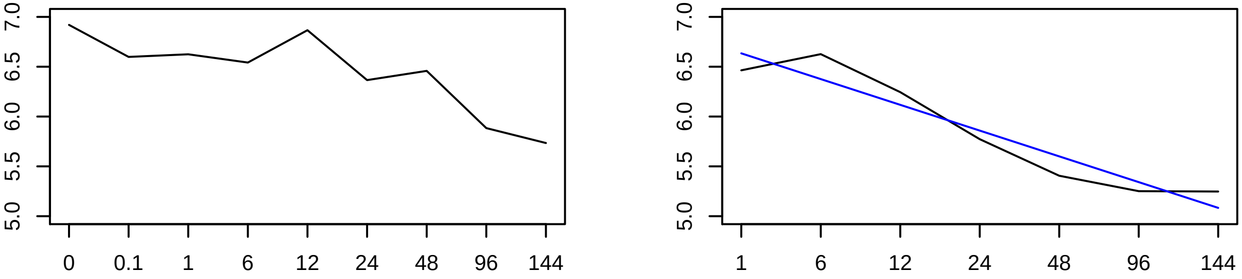

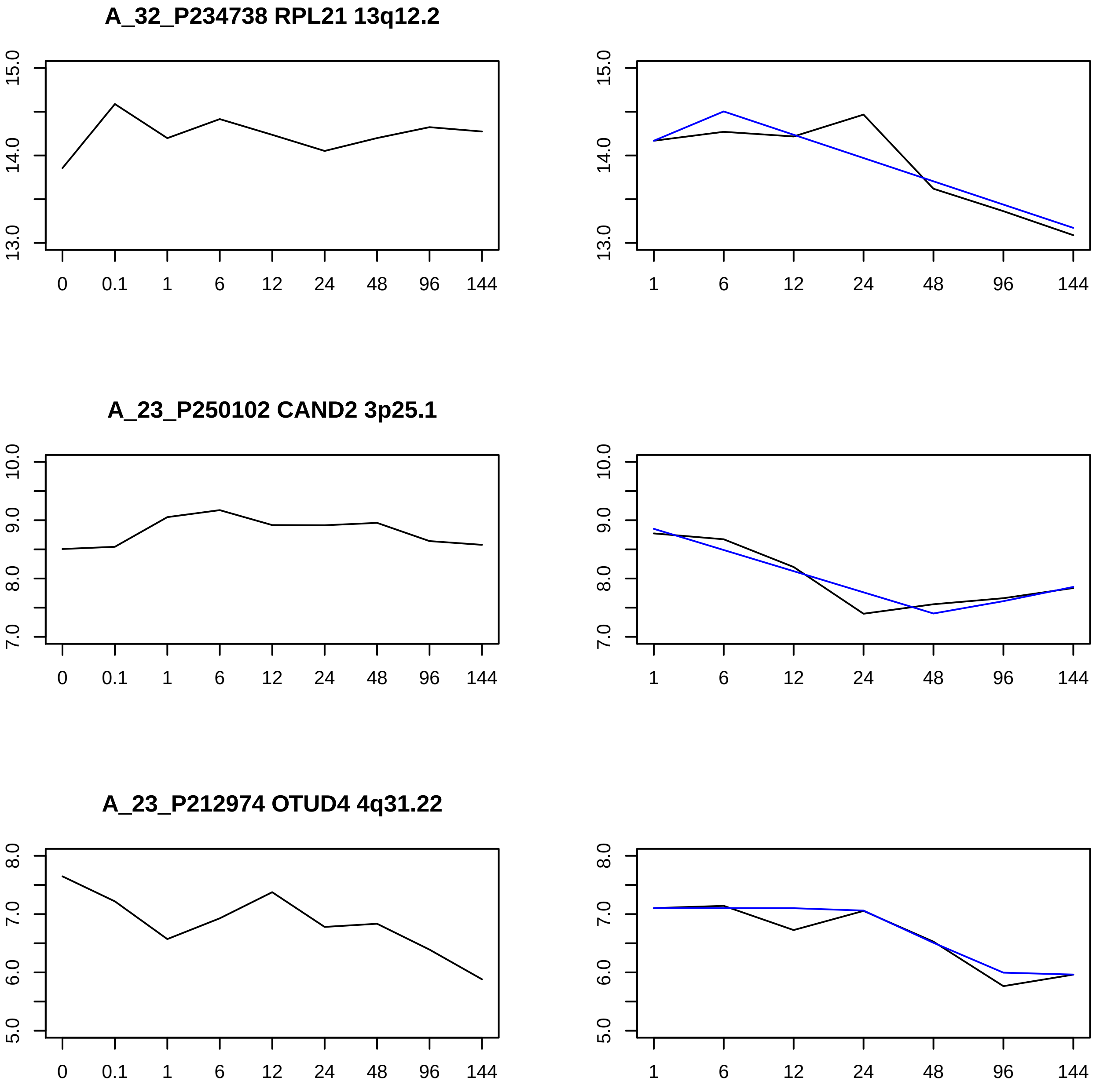
A_32_P234738 RPL21 13q12.2
13.0 14.0 15.0
0 0.1 1 6 12 24 48 96 144
1 6 12 24 48 96 144
A_23_P250102 CAND2 3p25.1
7.0 8.0 9.0 10.0
0 0.1 1 6 12 24 48 96 144
1 6 12 24 48 96 144
A_23_P212974 OTUD4 4q31.22
5.0 6.0 7.0 8.0
0 0.1 1 6 12 24 48 96 144
1 6 12 24 48 96 144

A_24_P779258 BCCIP NA

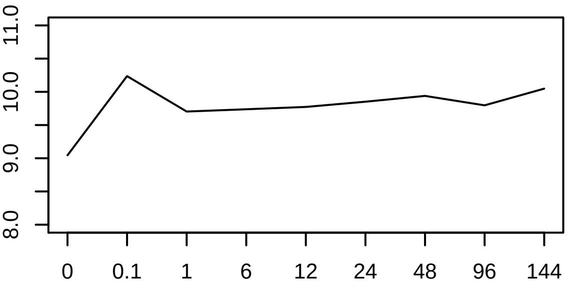

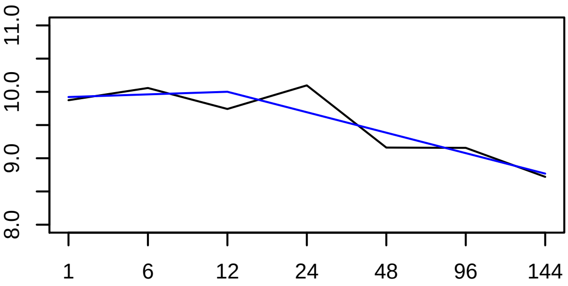

A_23_P145978 VIPR2 7q36.3

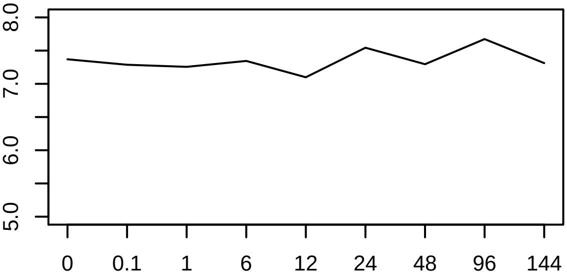

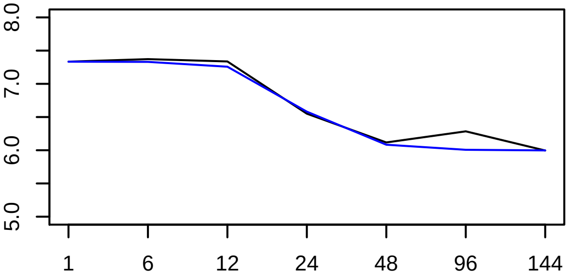

A_24_P84558 LOC652411 6p12.1

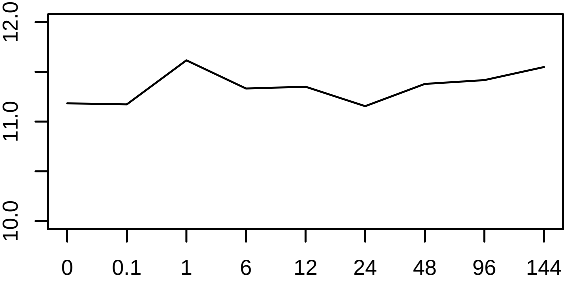

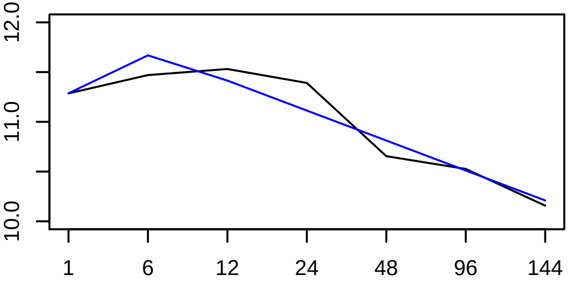

A_24_P166042 IMPDH2 3p21.31

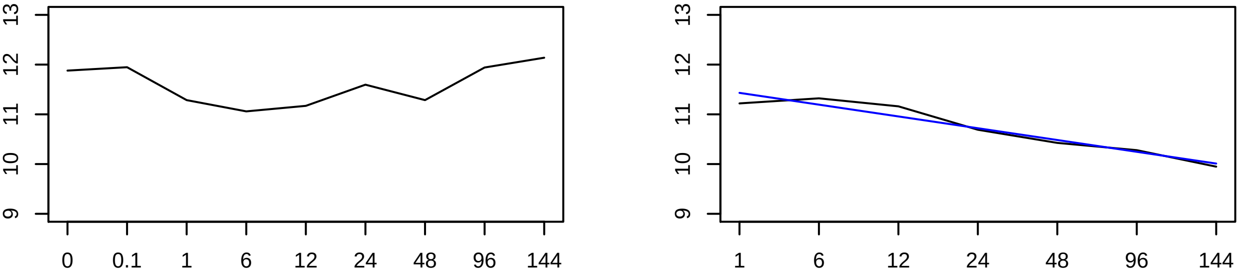

A_23_P77135 ATPBD4 15q14

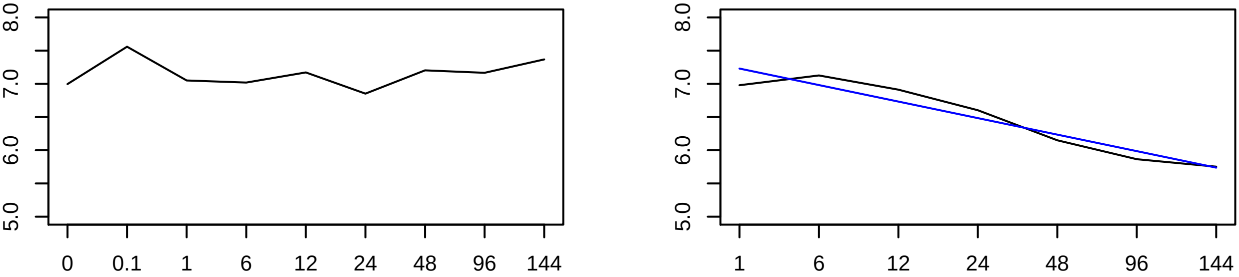

A_24_P289884 A_24_P289884 NA

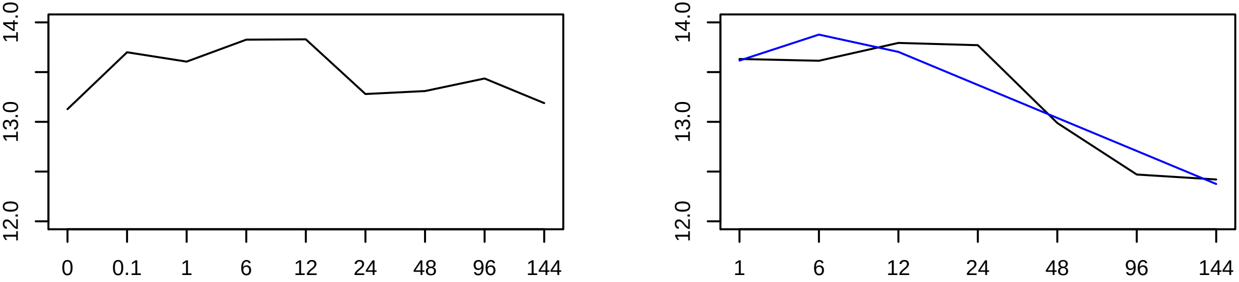

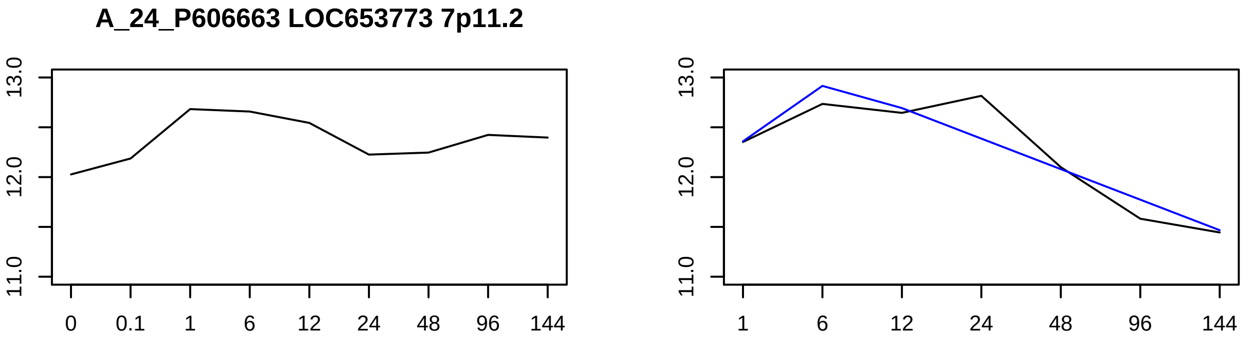
A_24_P606663 LOC653773 7p11.2
11.0
12.0
13.0
0
0.1
1
6
12
24
48
96
144
11.0
12.0
13.0
1
6
12
24
48
96
144

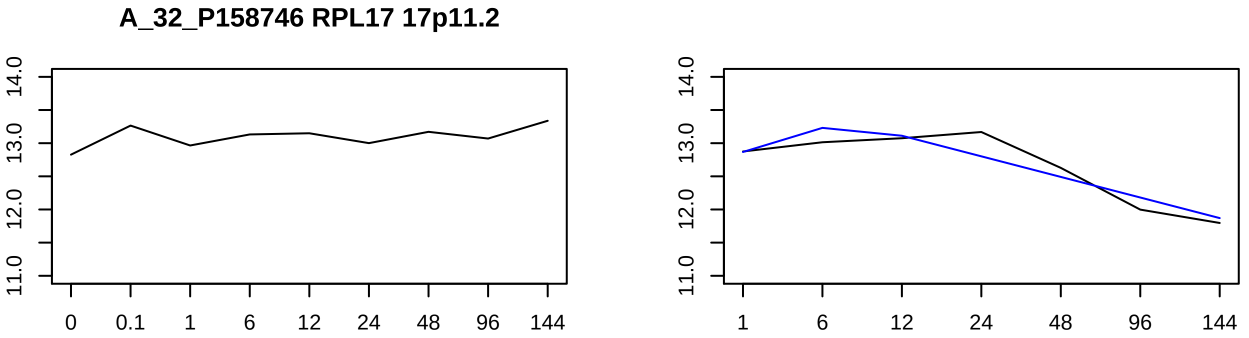
A_32_P158746 RPL17 17p11.2
11.0
12.0
13.0
14.0
0
0.1
1
6
12
24
48
96
144
11.0
12.0
13.0
14.0
1
6
12
24
48
96
144

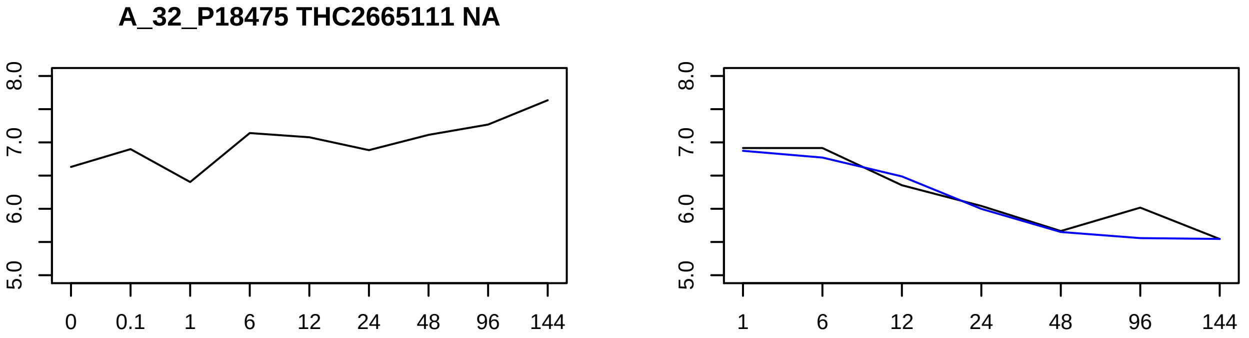
A_32_P18475 THC2665111 NA
5.0
6.0
7.0
8.0
0
0.1
1
6
12
24
48
96
144
5.0
6.0
7.0
8.0
1
6
12
24
48
96
144

A_24_P136211 CR619482 NA

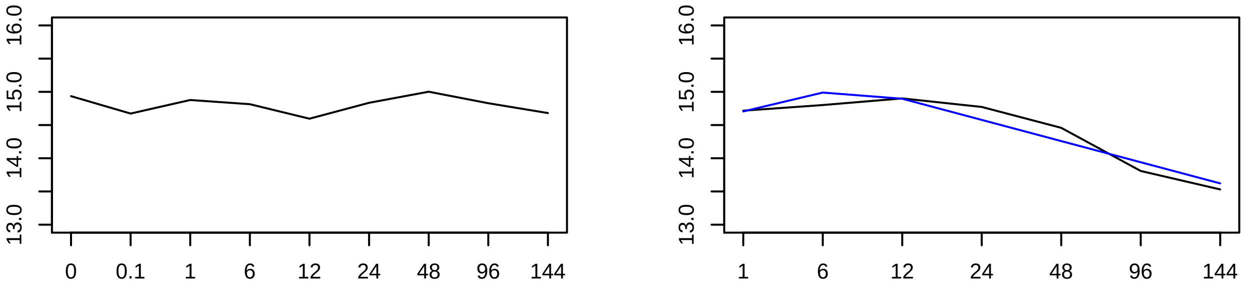

A_24_P33213 A_24_P33213 NA

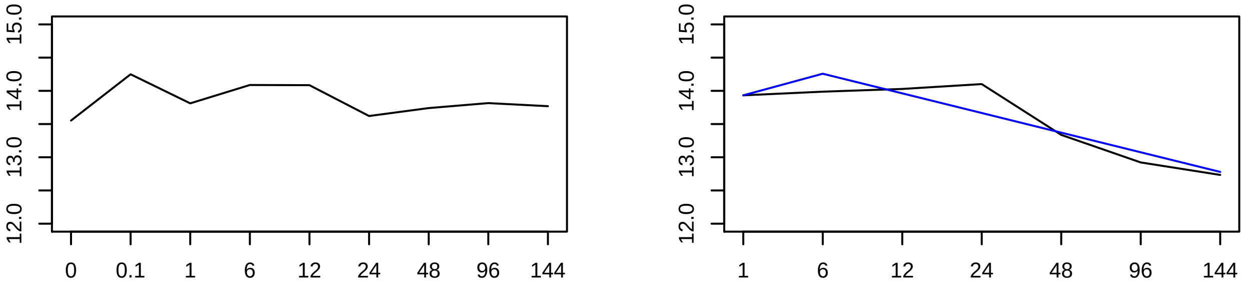

A_32_P65571 BX648855 NA

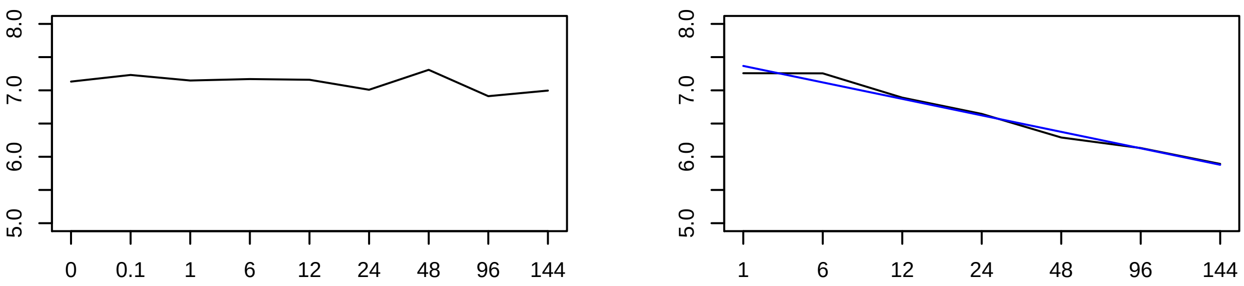

A_32_P69987 NRP1 10p11.22

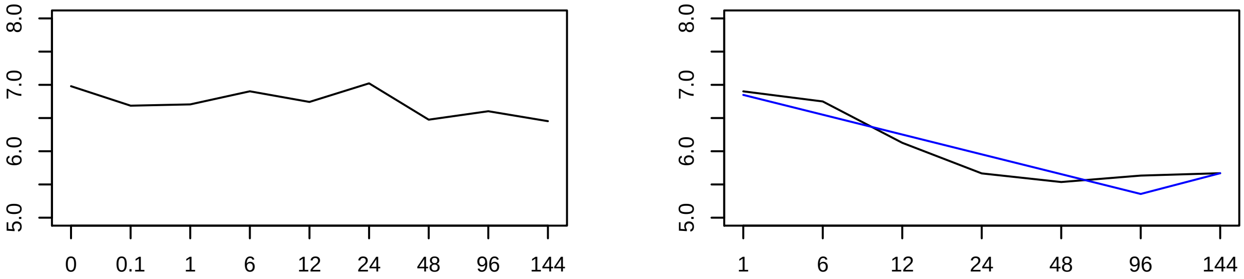

A_24_P140569 LRRTM2 5q31.2

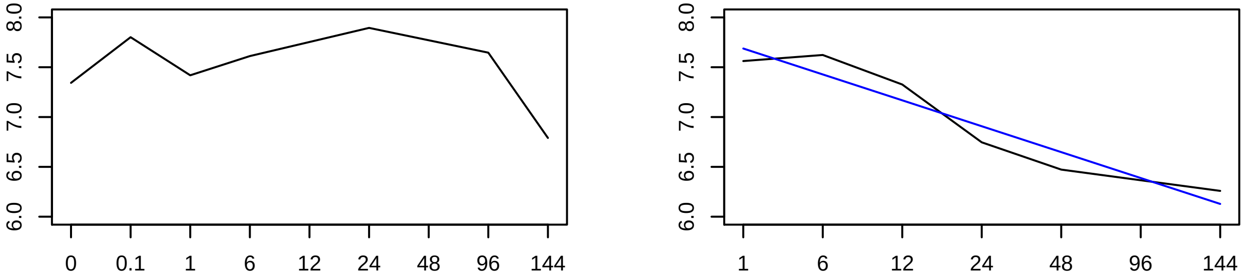

A_23_P259632 TMPIT 7q11.23

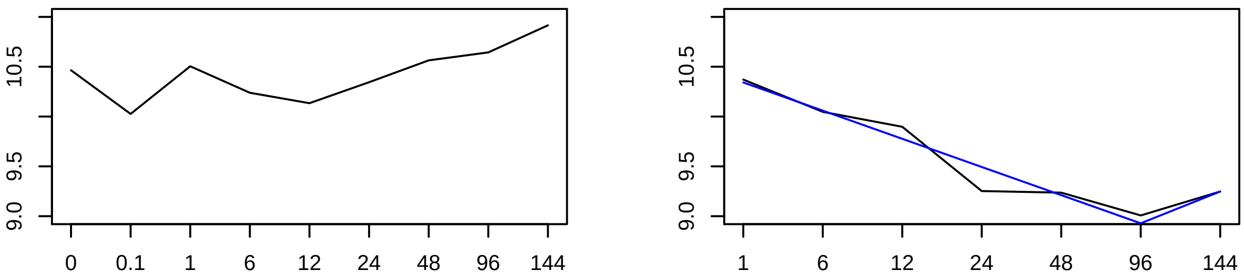

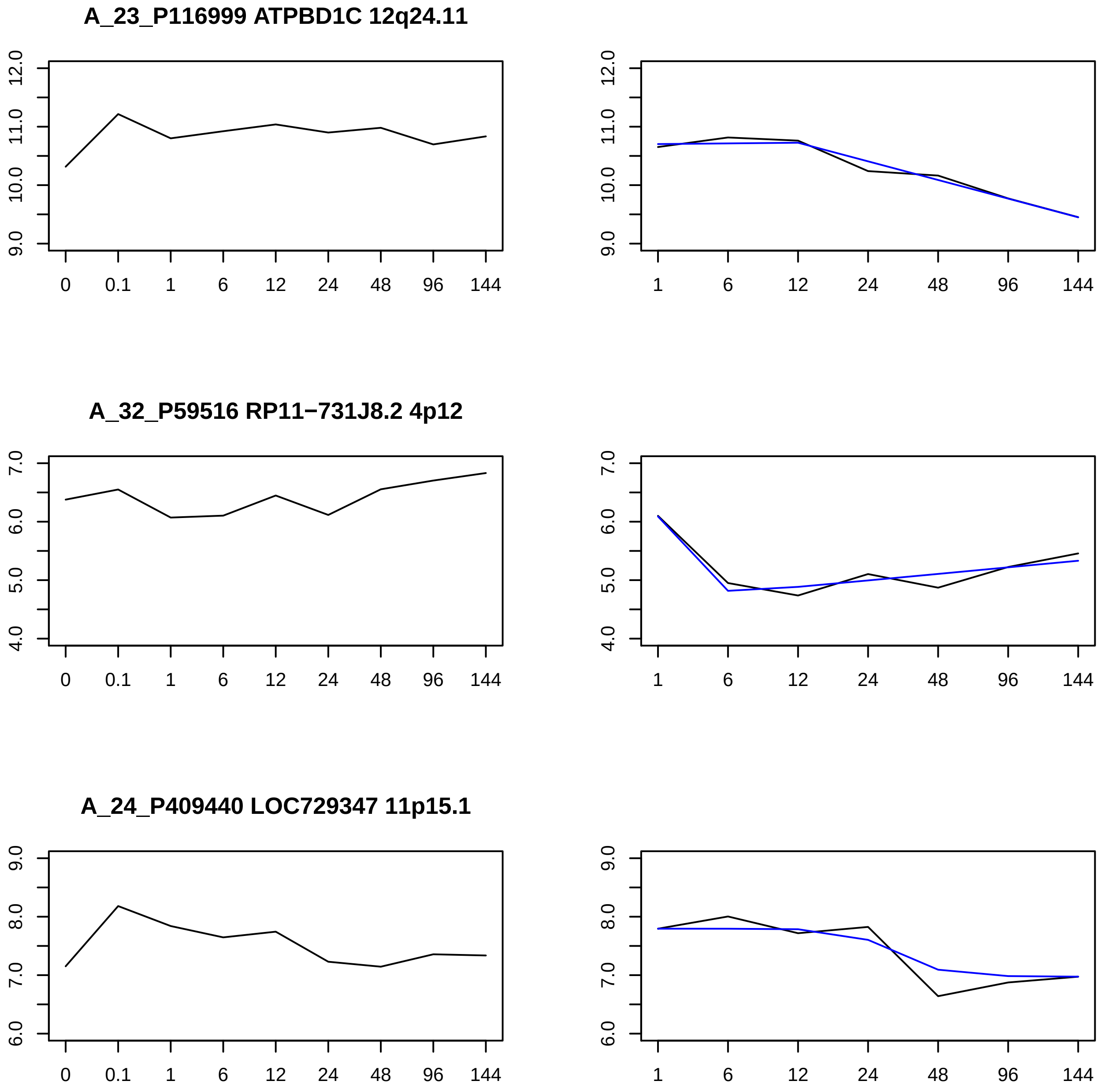
A_23_P116999 ATPBD1C 12q24.11
9.0
10.0
11.0
12.0
0
0.1
1
6
12
24
48
96
144
A_32_P59516 RP11−731J8.2 4p12
4.0
5.0
6.0
7.0
A_24_P409440 LOC729347 11p15.1
6.0
7.0
8.0
9.0

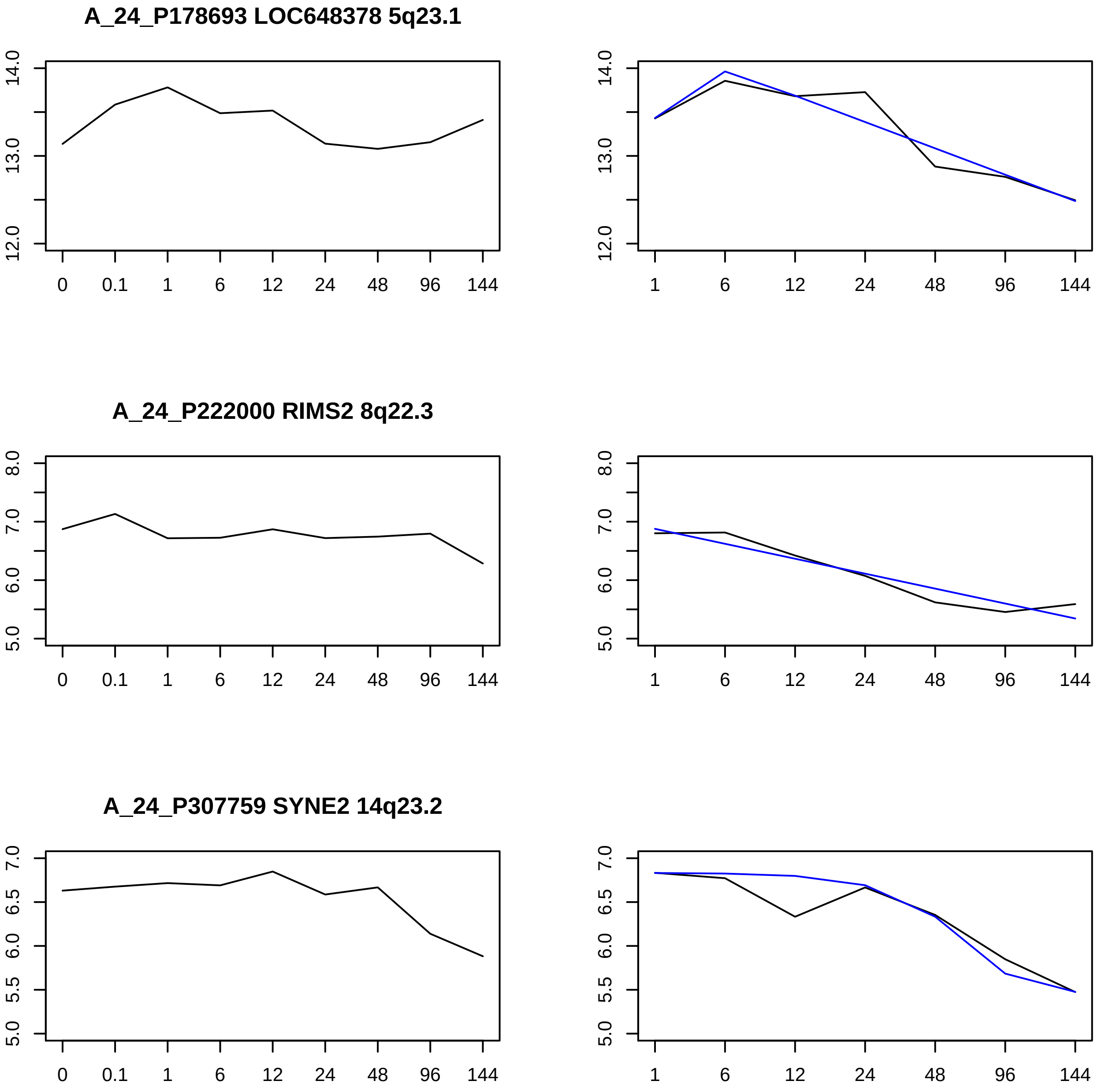

A_24_P178693 LOC648378 5q23.1
12.0
13.0
14.0
0
0.1
1
6
12
24
48
96
144
A_24_P222000 RIMS2 8q22.3
5.0
6.0
7.0
8.0
A_24_P307759 SYNE2 14q23.2
5.0
5.5
6.0
6.5
7.0

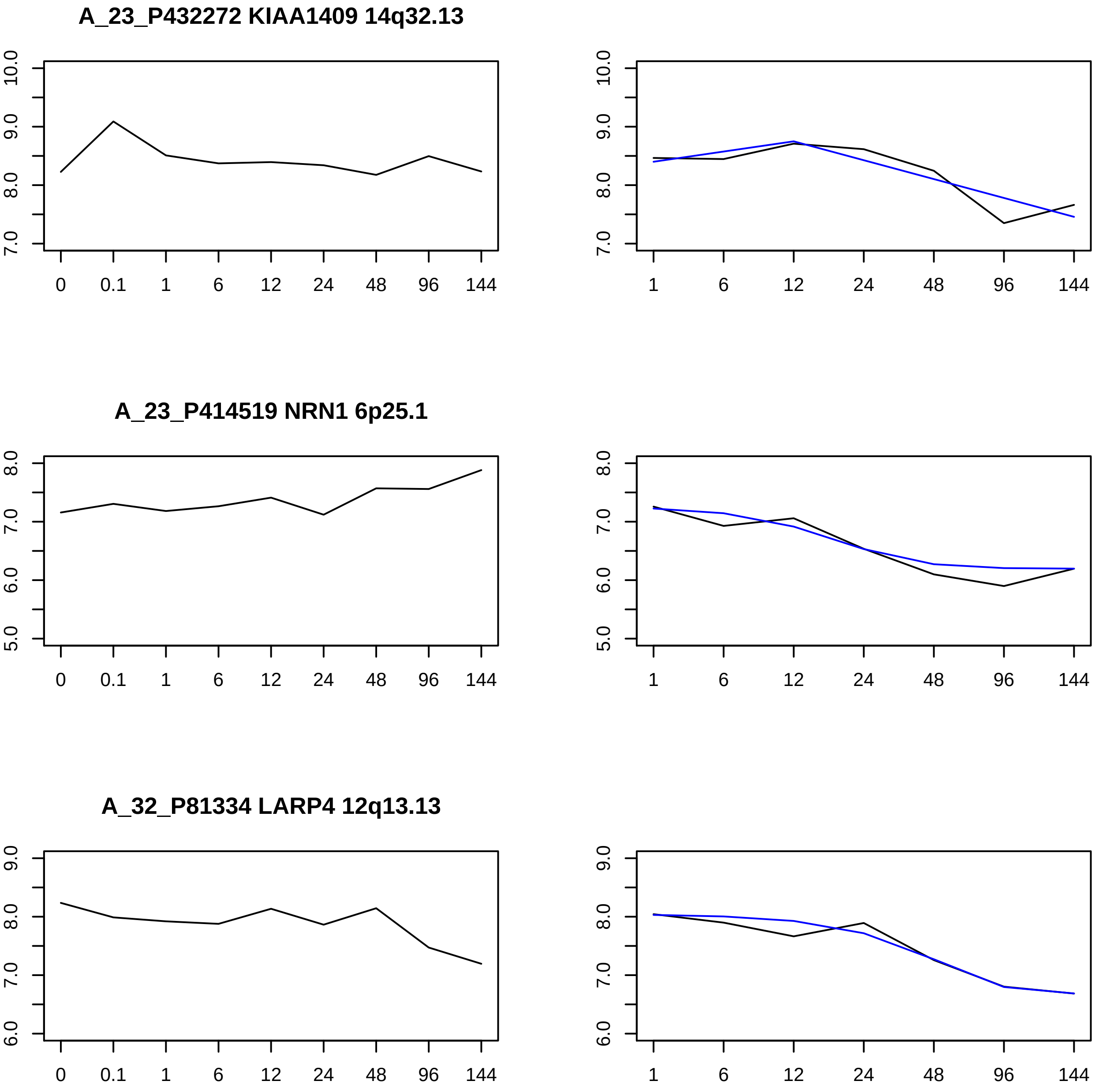
A_23_P432272 KIAA1409 14q32.13
10.0
9.0
8.0
7.0
0
0.1
1
6
12
24
48
96
144
1
6
12
24
48
96
144
A_23_P414519 NRN1 6p25.1
8.0
7.0
6.0
5.0
A_32_P81334 LARP4 12q13.13
9.0
8.0
7.0
6.0

A_23_P344853 WDR43 2p23.2

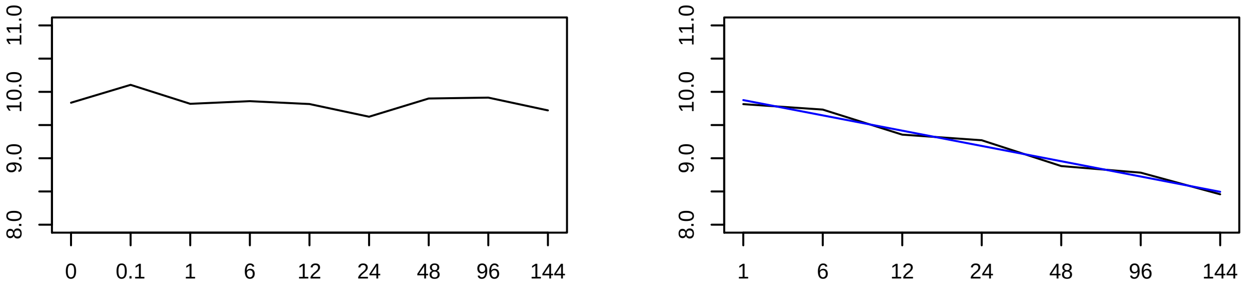

A_32_P199292 FAM60A 12p11.21

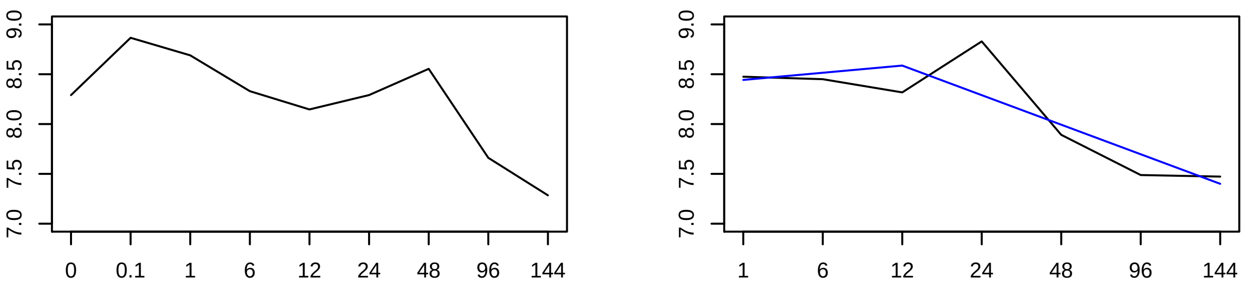

A_23_P315320 IL27 16p11.2

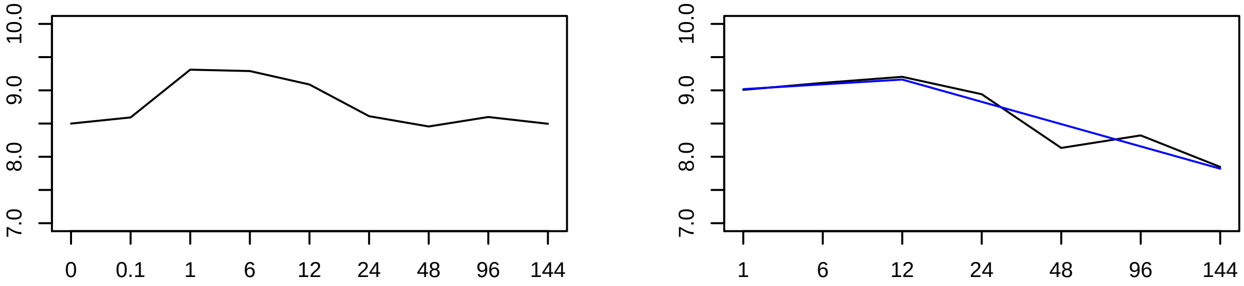

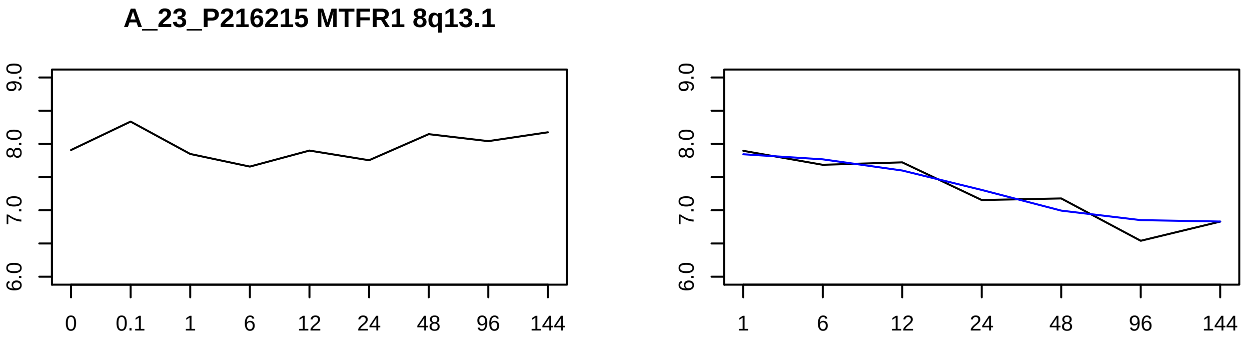
A_23_P216215 MTFR1 8q13.1
6.0
7.0
8.0
9.0
0
0.1
1
6
12
24
48
96
144
1
6
12
24
48
96
144

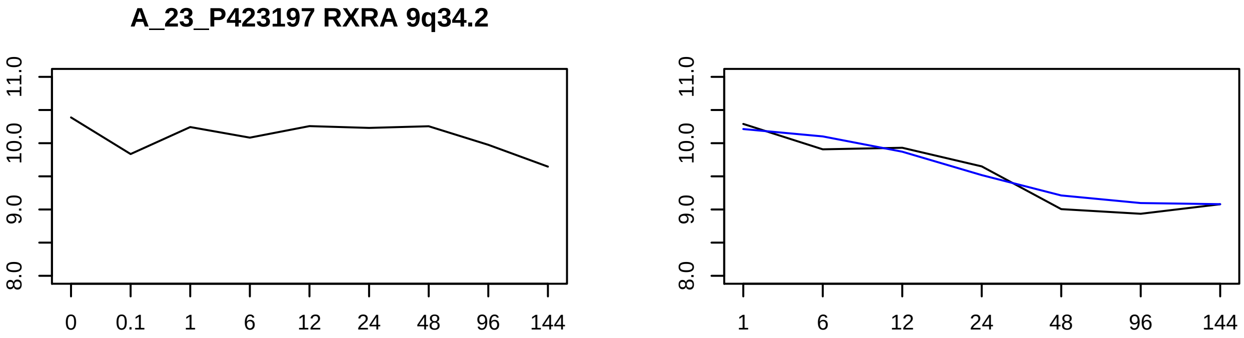
A_23_P423197 RXRA 9q34.2
8.0
9.0
10.0
11.0
0
0.1
1
6
12
24
48
96
144
1
6
12
24
48
96
144

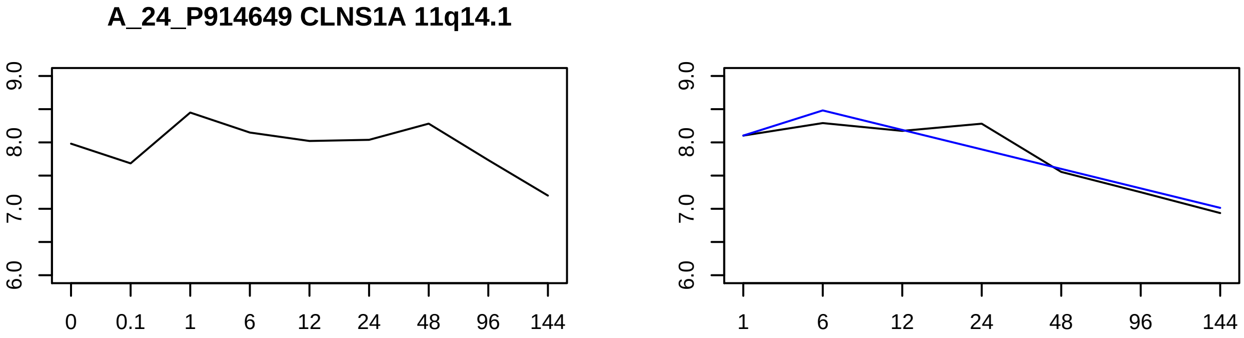
A_24_P914649 CLNS1A 11q14.1
6.0
7.0
8.0
9.0
0
0.1
1
6
12
24
48
96
144
1
6
12
24
48
96
144

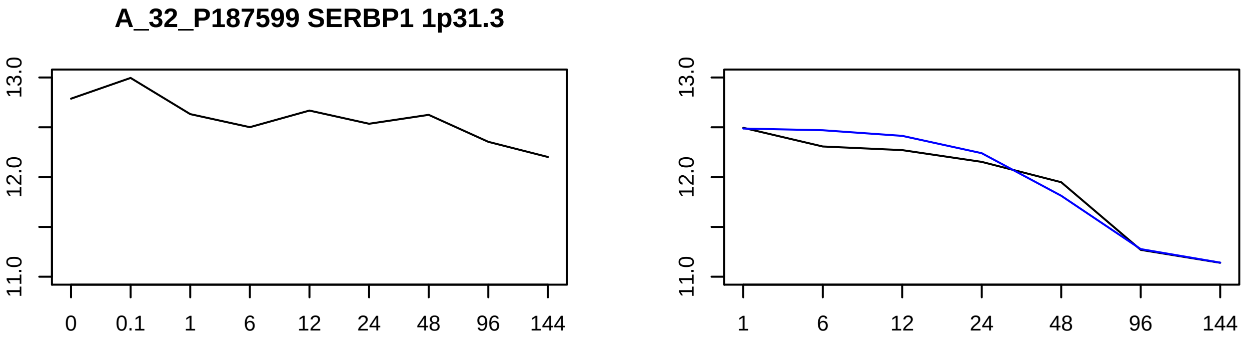
A_32_P187599 SERBP1 1p31.3
11.0
12.0
13.0
0
0.1
1
6
12
24
48
96
144
11.0
12.0
13.0
1
6
12
24
48
96
144

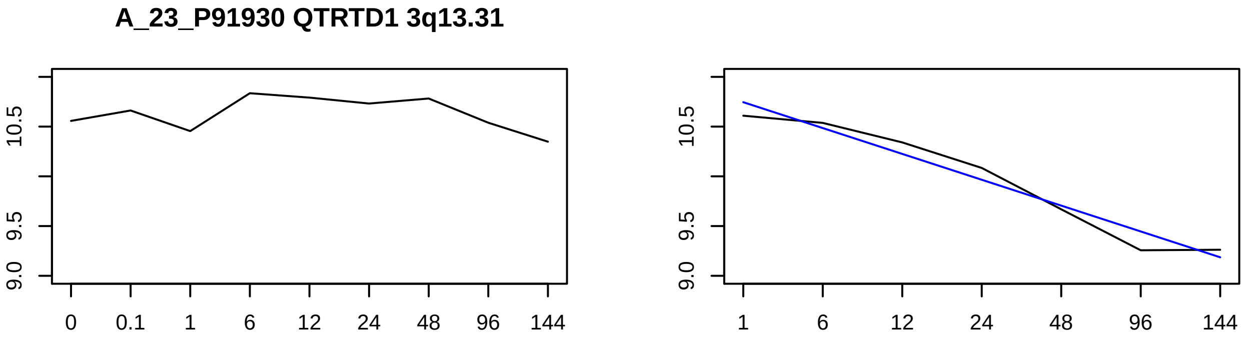
A_23_P91930 QTRTD1 3q13.31
9.0
9.5
10.5
0
0.1
1
6
12
24
48
96
144
9.0
9.5
10.5
1
6
12
24
48
96
144

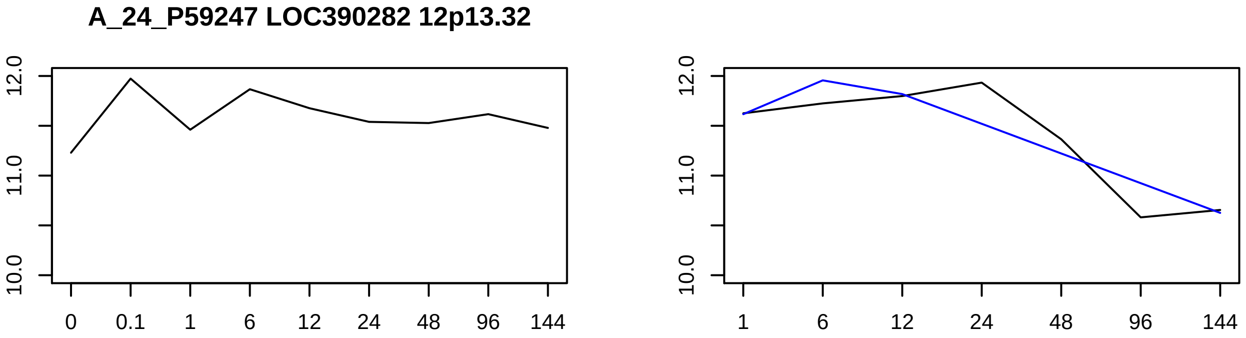
A_24_P59247 LOC390282 12p13.32
10.0
11.0
12.0
0
0.1
1
6
12
24
48
96
144
10.0
11.0
12.0
1
6
12
24
48
96
144

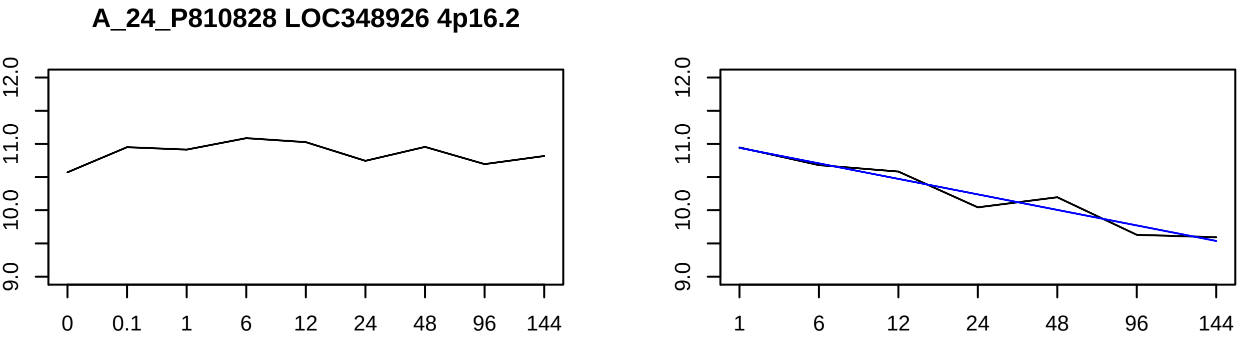
A_24_P810828 LOC348926 4p16.2
12.0
11.0
10.0
9.0
0
0.1
1
6
12
24
48
96
144
1
6
12
24
48
96
144

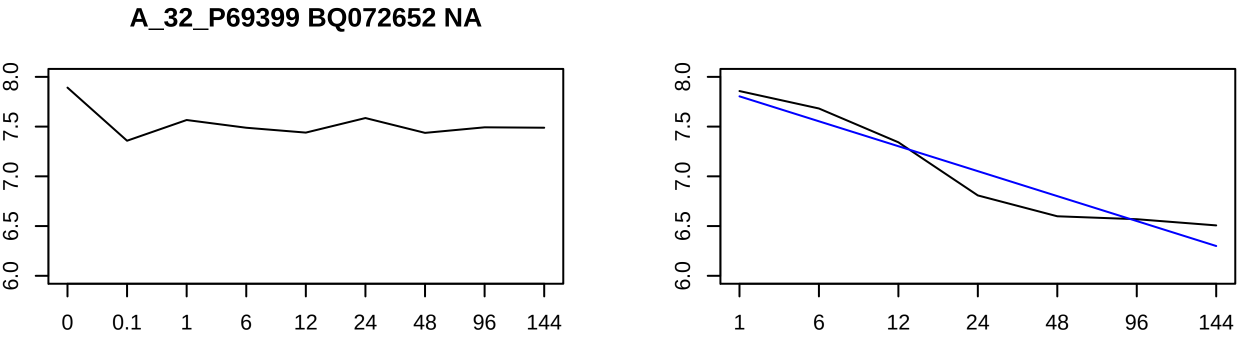
A_32_P69399 BQ072652 NA
8.0
7.5
7.0
6.5
6.0
0
0.1
1
6
12
24
48
96
144
1
6
12
24
48
96
144

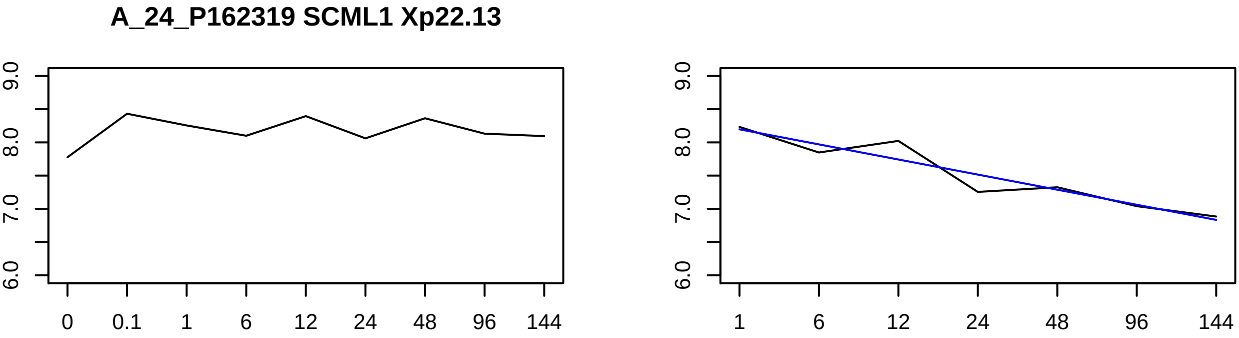
A_24_P162319 SCML1 Xp22.13
9.0
8.0
7.0
6.0
0
0.1
1
6
12
24
48
96
144
1
6
12
24
48
96
144

A_23_P92520 ANP32C 4q32.3

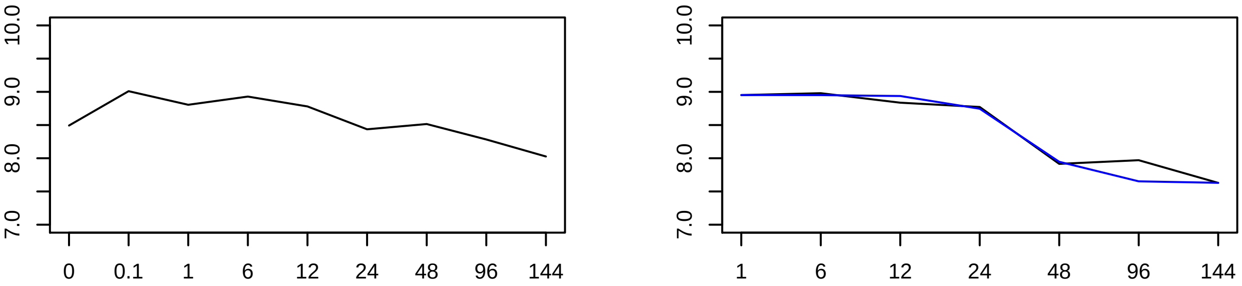

A_32_P59486 SDHD 11q23.1

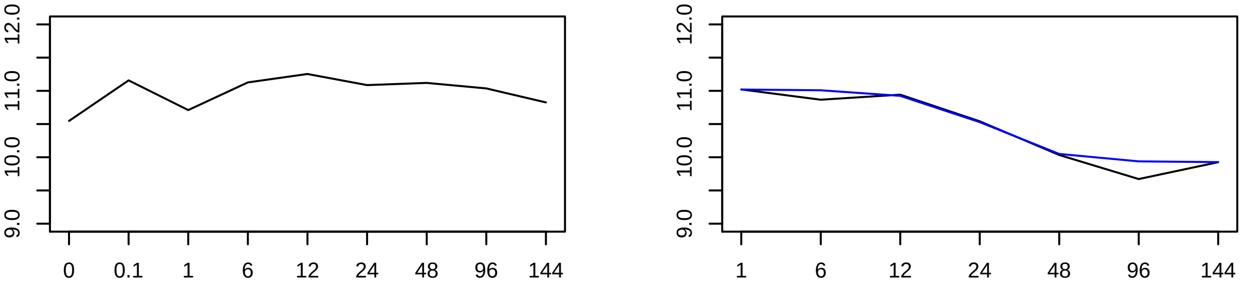

A_24_P50829 TRPM7 15q21.2

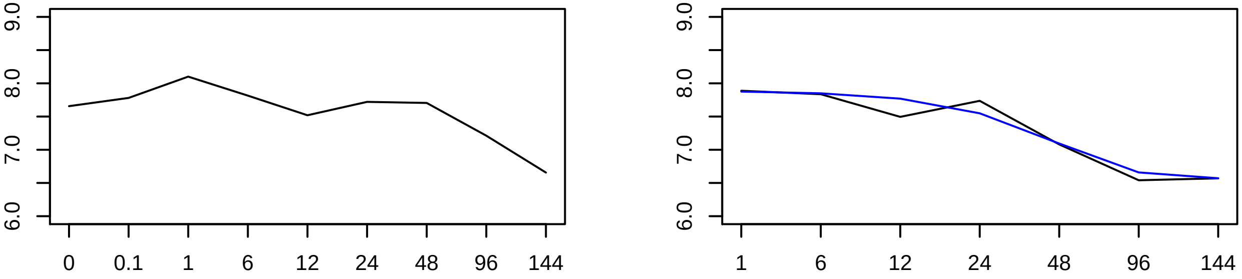

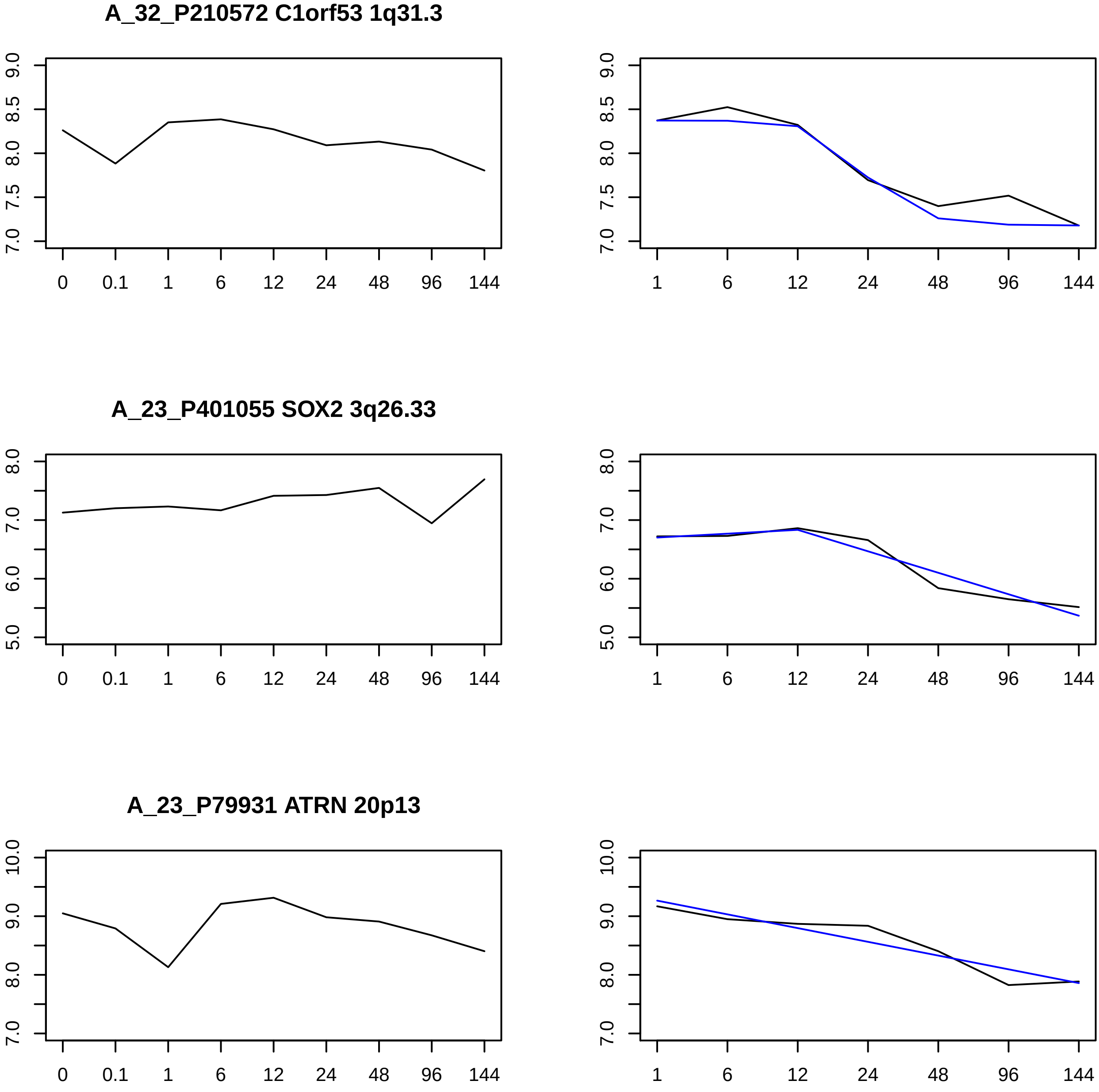
A_32_P210572 C1orf53 1q31.3
7.0
7.5
8.0
8.5
9.0
0
0.1
1
6
12
24
48
96
144
1
6
12
24
48
96
144
A_23_P401055 SOX2 3q26.33
5.0
6.0
7.0
8.0
A_23_P79931 ATRN 20p13
7.0
8.0
9.0
10.0

A_24_P122524 WDR3 1p12

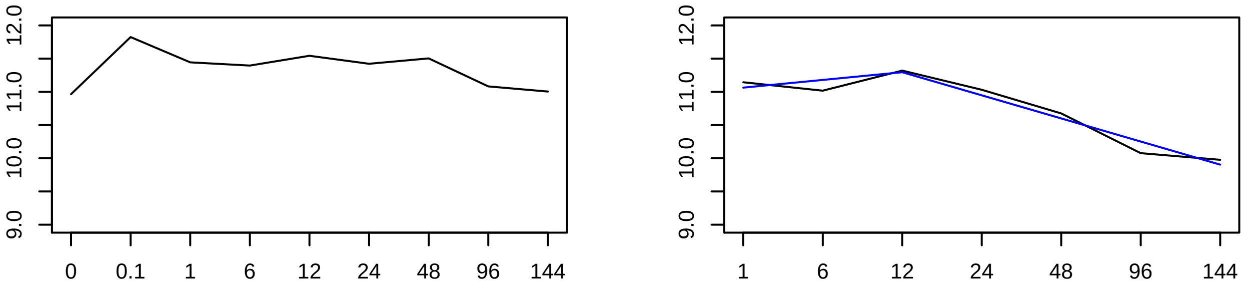

A_32_P115277 THC2638025 NA

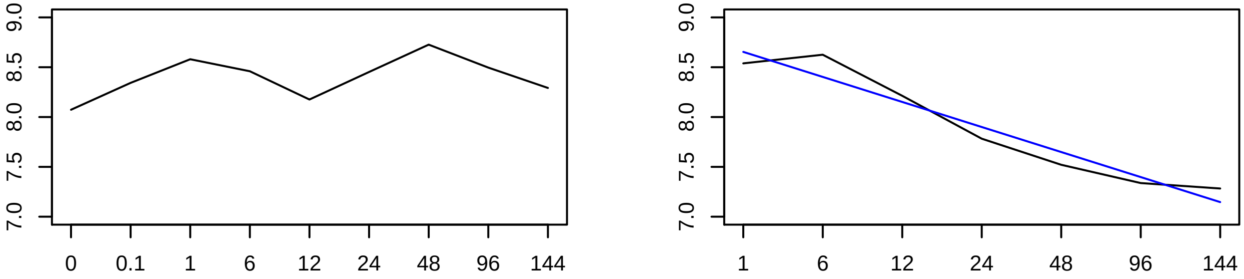

A_23_P138805 CHORDC1 11q14.3

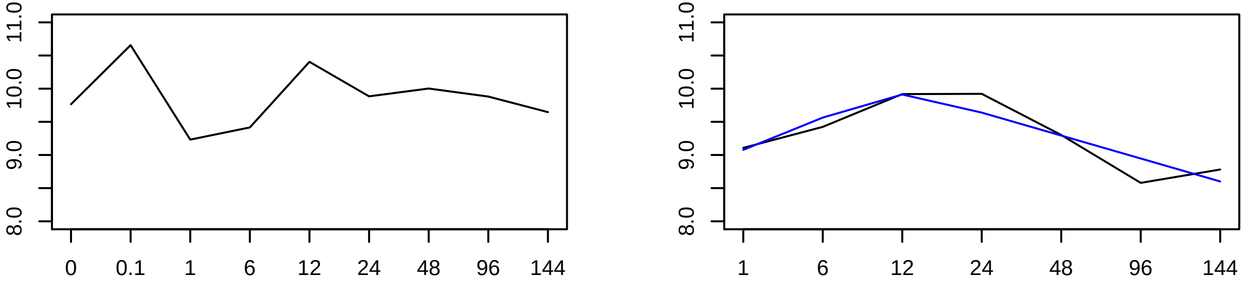

A_24_P127621 A_24_P127621 NA

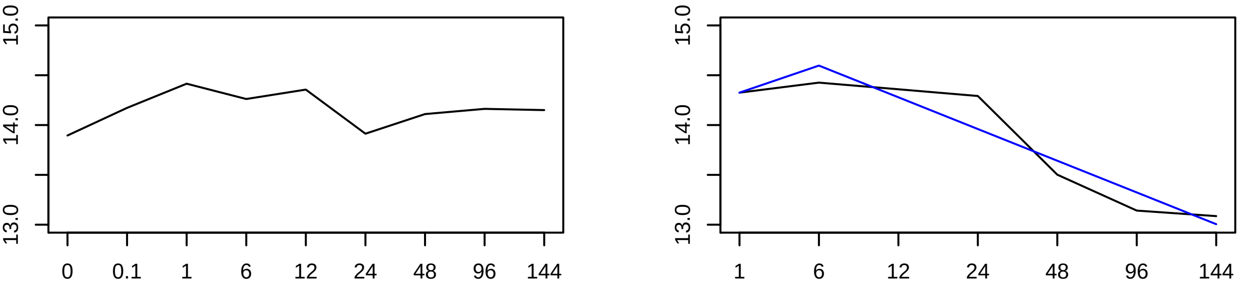

A_23_P77776 SFRS2 17q25.2

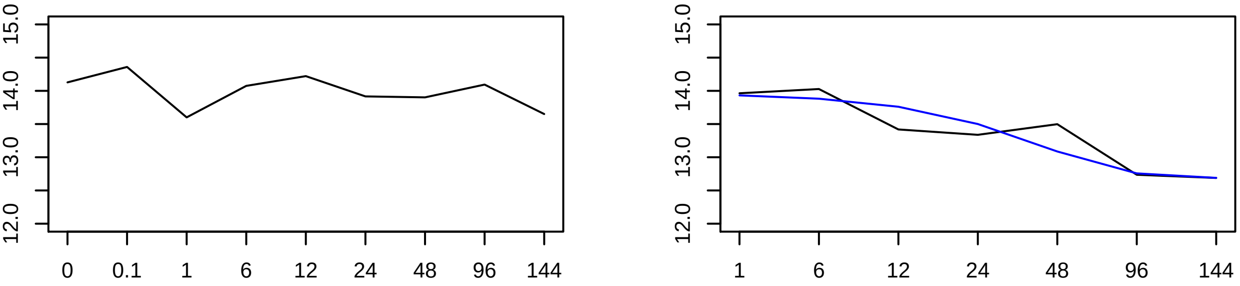

A_23_P90089 GCDH 19p13.13

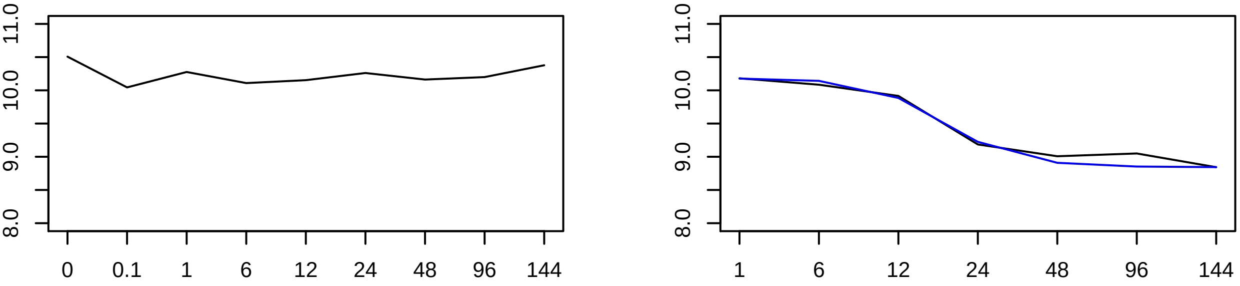

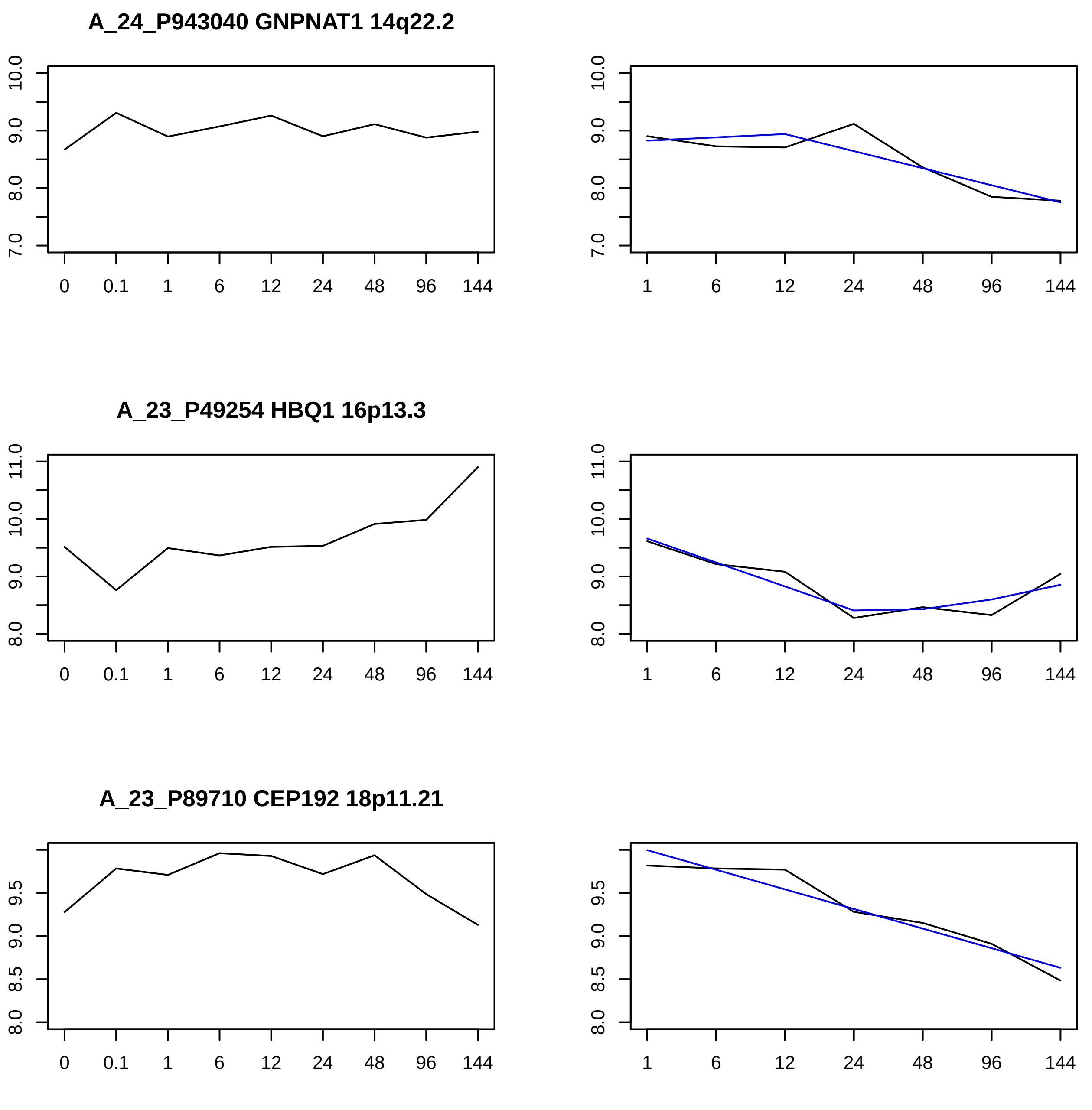
A_24_P943040 GNPNAT1 14q22.2
7.0
8.0
9.0
10.0
0
0.1
1
6
12
24
48
96
144
A_23_P49254 HBQ1 16p13.3
8.0
9.0
10.0
11.0
A_23_P89710 CEP192 18p11.21
8.0
8.5
9.0
9.5

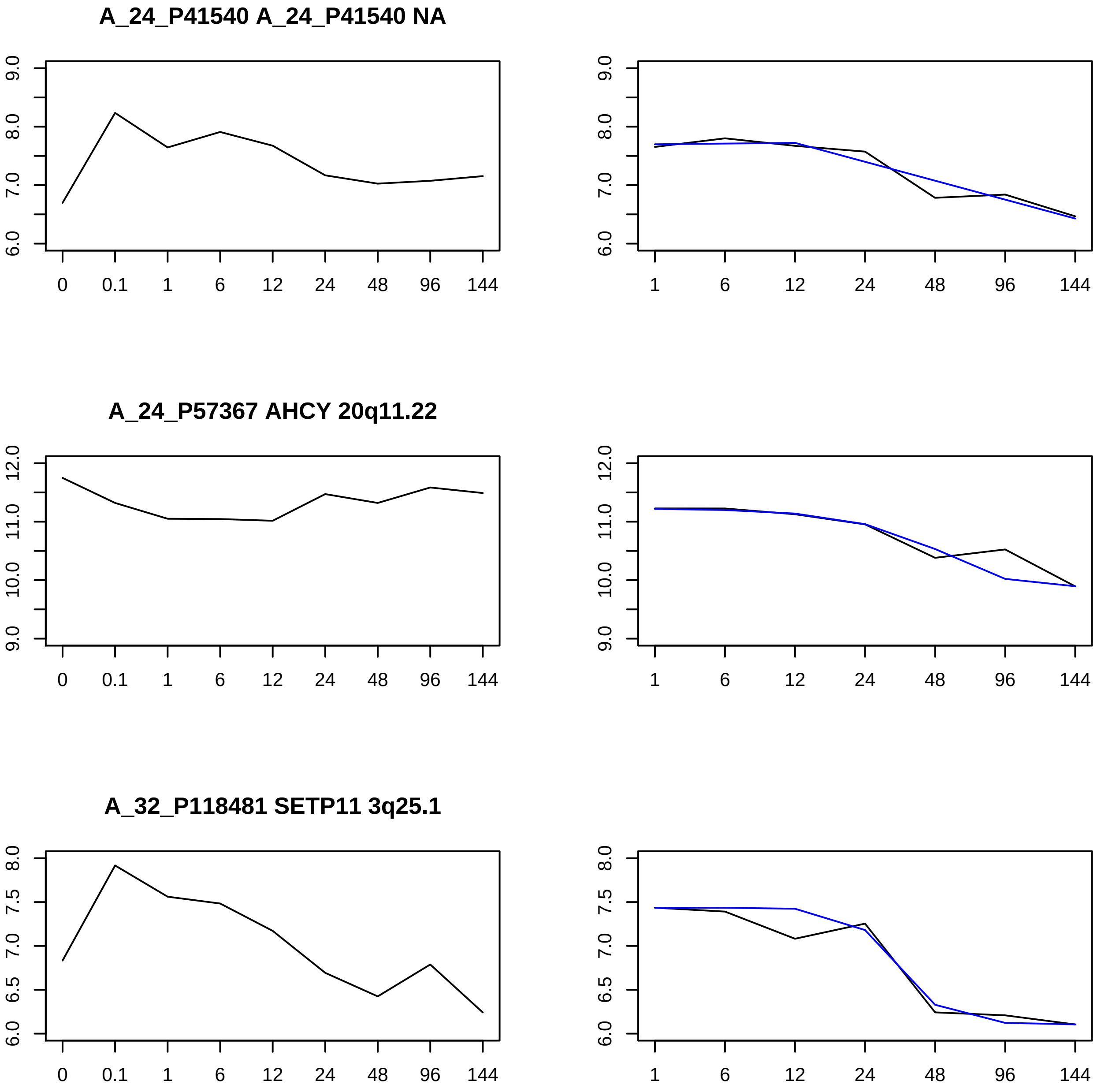
A_24_P41540 A_24_P41540 NA
6.0
7.0
8.0
9.0
0
0.1
1
6
12
24
48
96
144
1
6
12
24
48
96
144
A_24_P57367 AHCY 20q11.22
9.0
10.0
11.0
12.0
0
0.1
1
6
12
24
48
96
144
1
6
12
24
48
96
144
A_32_P118481 SETP11 3q25.1
6.0
6.5
7.0
7.5
8.0
0
0.1
1
6
12
24
48
96
144
1
6
12
24
48
96
144

A_23_P6514 A_23_P6514 NA

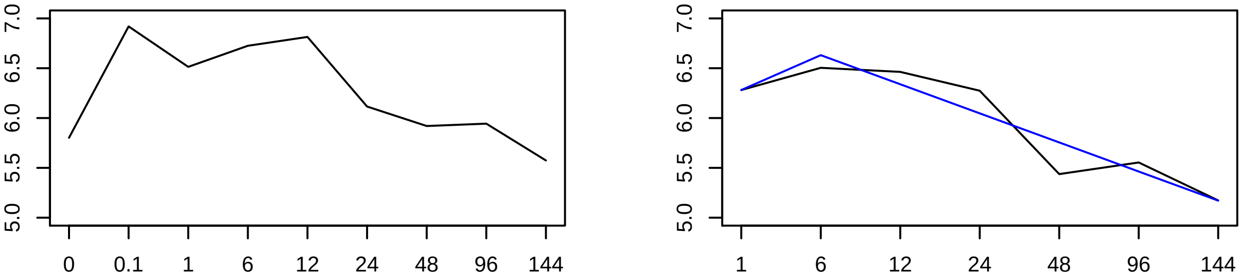

A_23_P40072 MTIF2 2p16.1

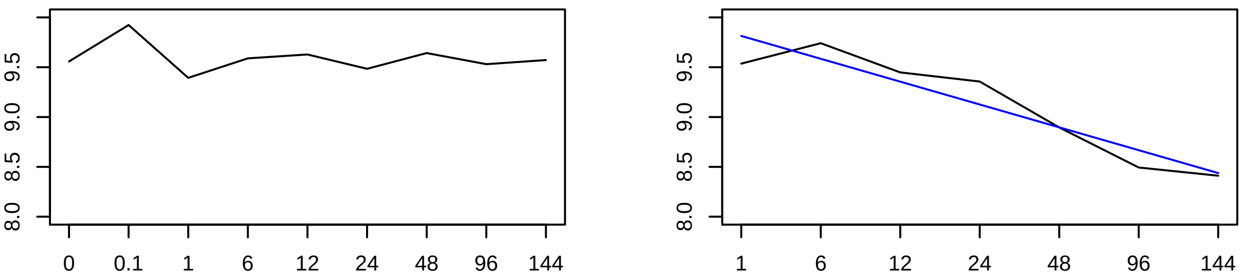

A_32_P32413 SETBP1 NA

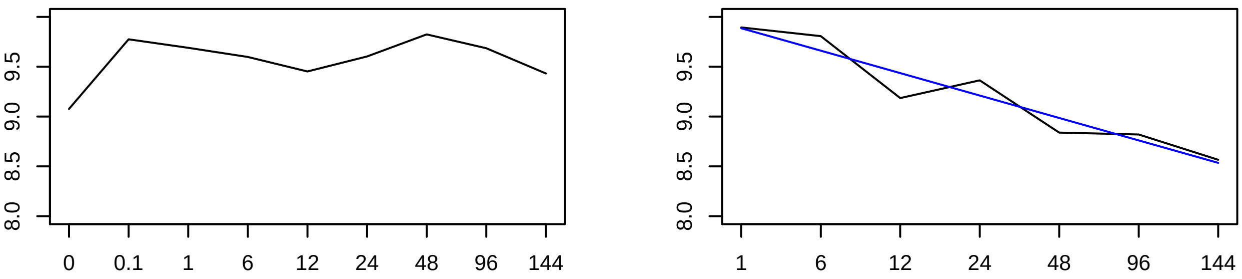

A_23_P167920 DLL1 6q27

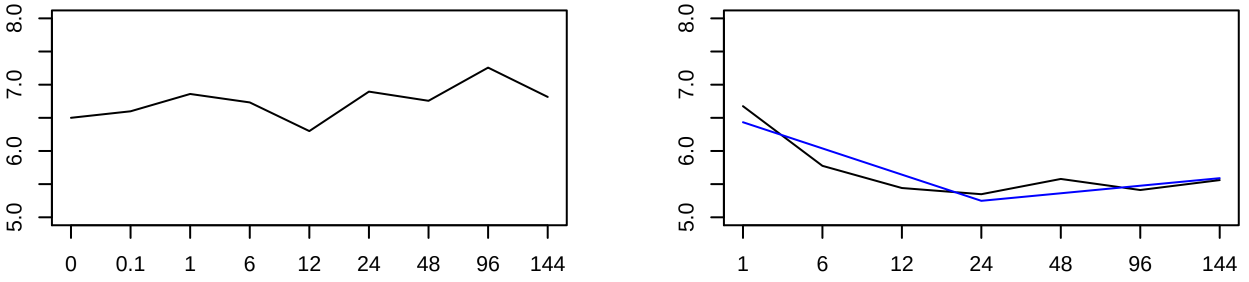

A_24_P7820 A_24_P7820 NA

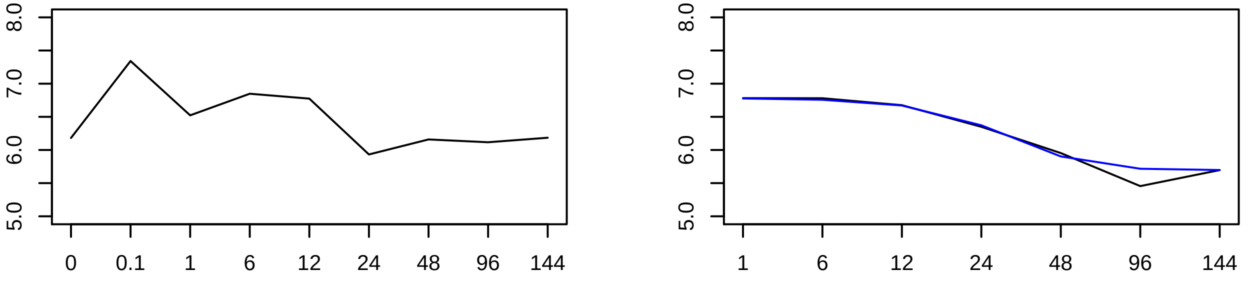

A_24_P531074 A_24_P531074 NA

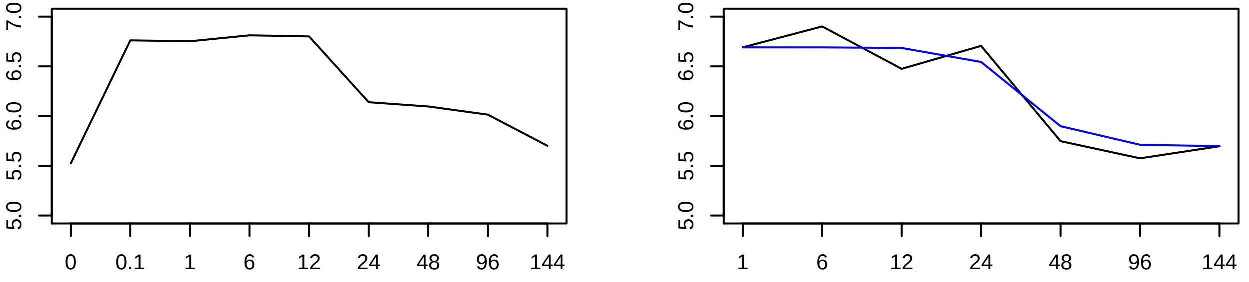

A_32_P76811 RP3−377H14.5 6p22.1

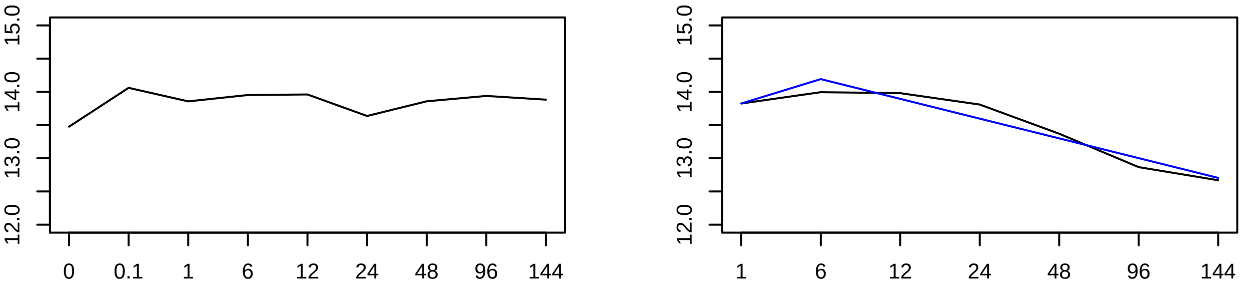

A_23_P204721 AQP2 12q13.13

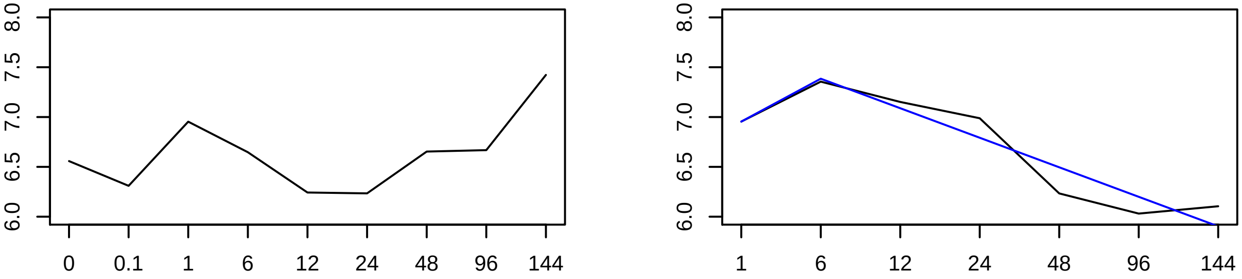

A_32_P117313 DKFZP779L1068 8q22.1

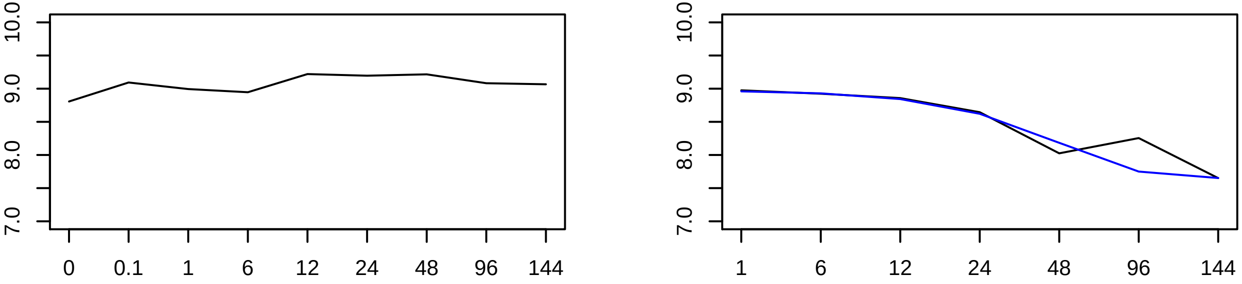

A_23_P356598 SLC6A15 12q21.31

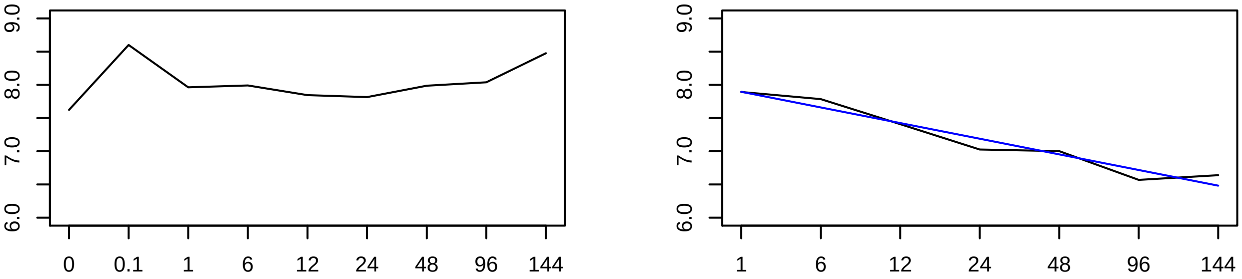

A_32_P58606 ONECUT2 18q21.31

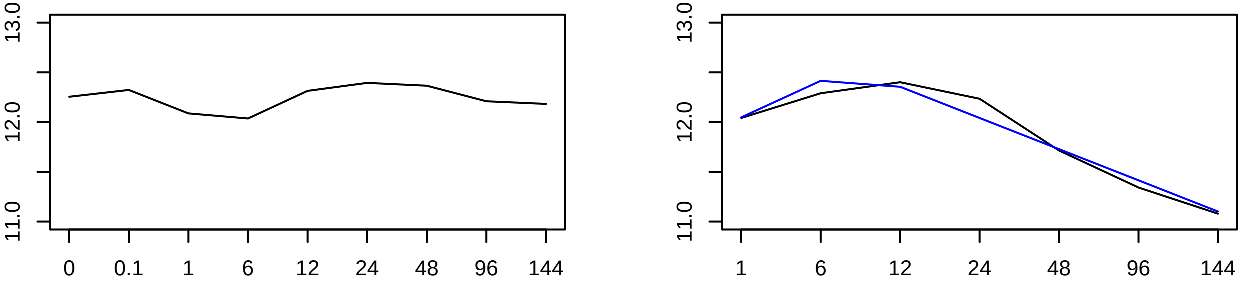

A_24_P565496 A_24_P565496 NA

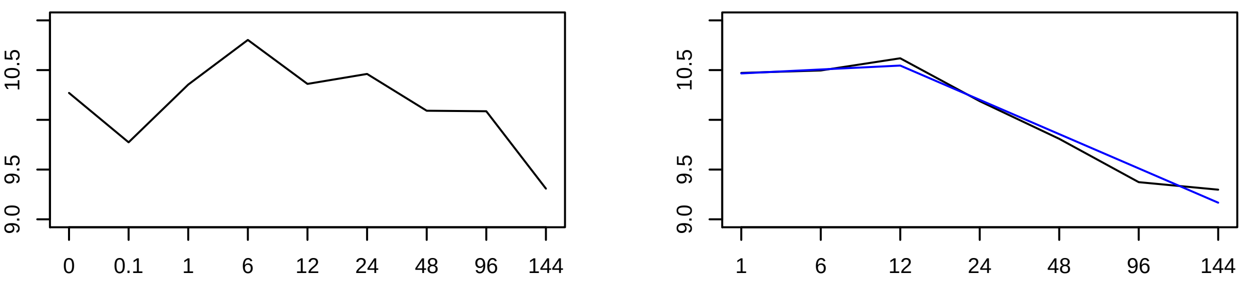

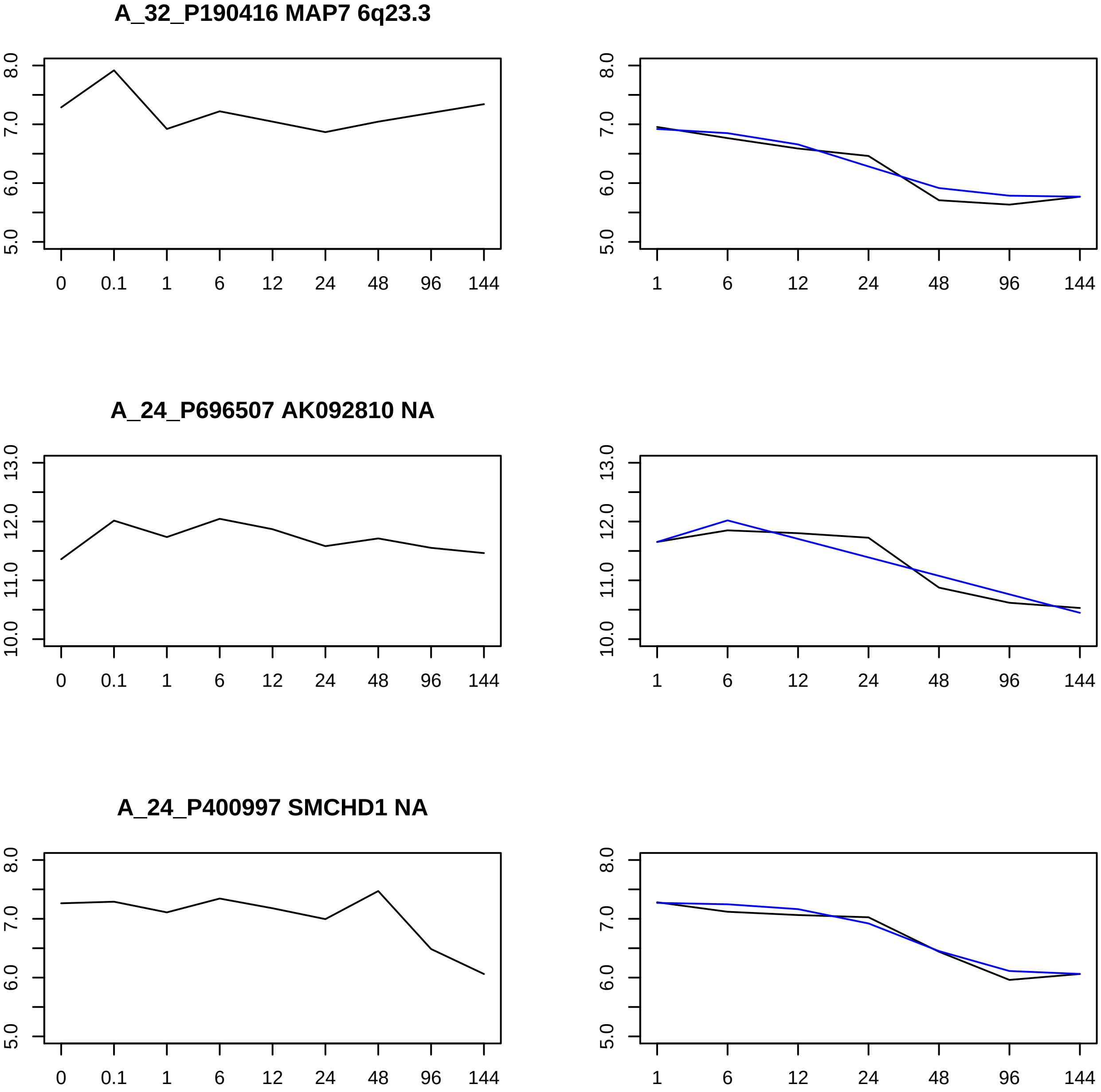
A_32_P190416 MAP7 6q23.3
5.0
6.0
7.0
8.0
0
0.1
1
6
12
24
48
96
144
1
6
12
24
48
96
144
A_24_P696507 AK092810 NA
10.0
11.0
12.0
13.0
0
0.1
1
6
12
24
48
96
144
1
6
12
24
48
96
144
A_24_P400997 SMCHD1 NA
5.0
6.0
7.0
8.0
0
0.1
1
6
12
24
48
96
144
1
6
12
24
48
96
144

A_24_P367139 A_24_P367139 NA

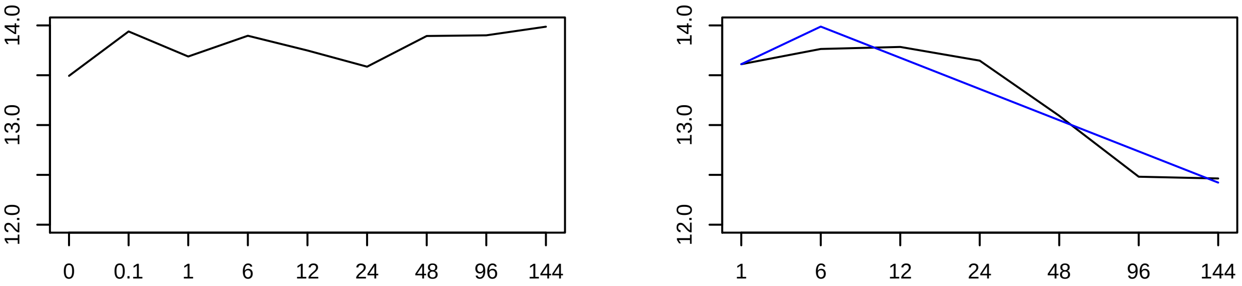

A_23_P339818 ARRDC4 15q26.3

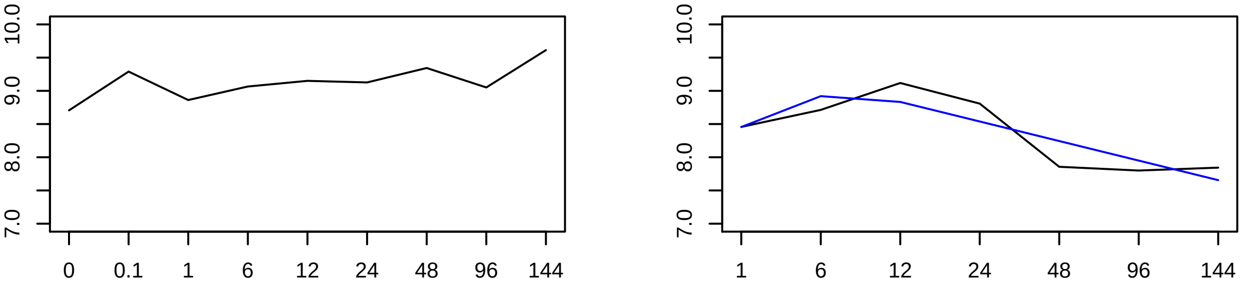

A_23_P121939 PTCD2 NA

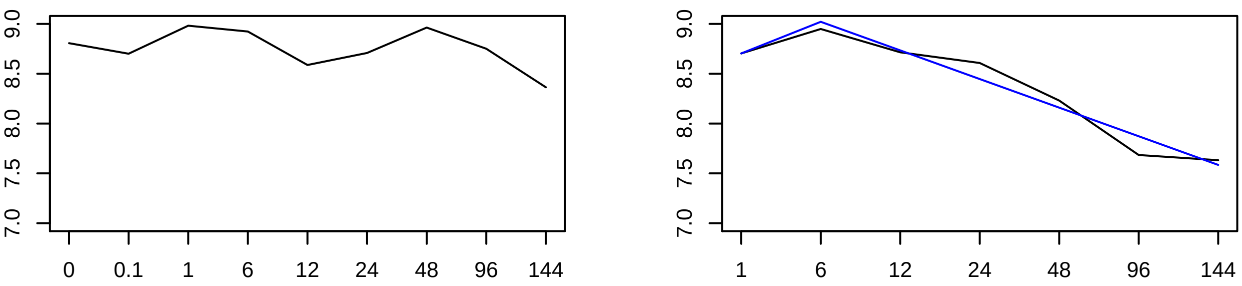

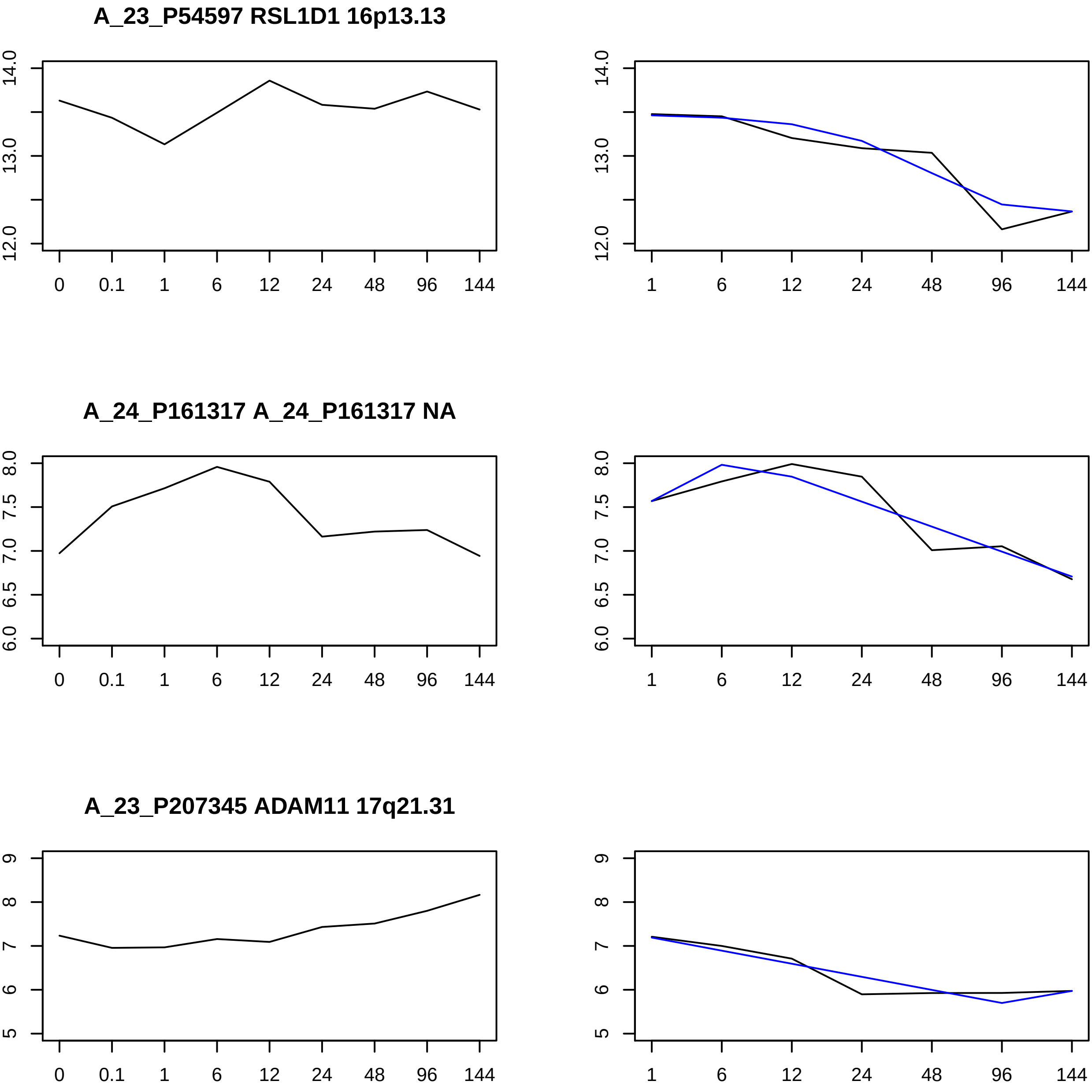

A_23_P54597 RSL1D1 16p13.13
12.0
13.0
14.0
0
0.1
1
6
12
24
48
96
144
A_24_P161317 A_24_P161317 NA
6.0
6.5
7.0
7.5
8.0
A_23_P207345 ADAM11 17q21.31
5
6
7
8
9

A_23_P254842 HDHD1A Xp22.31

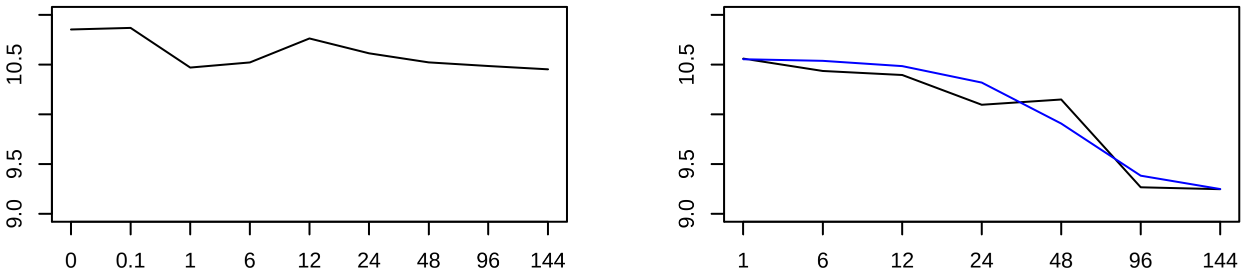

A_24_P121642 LOC441743 16p13.3

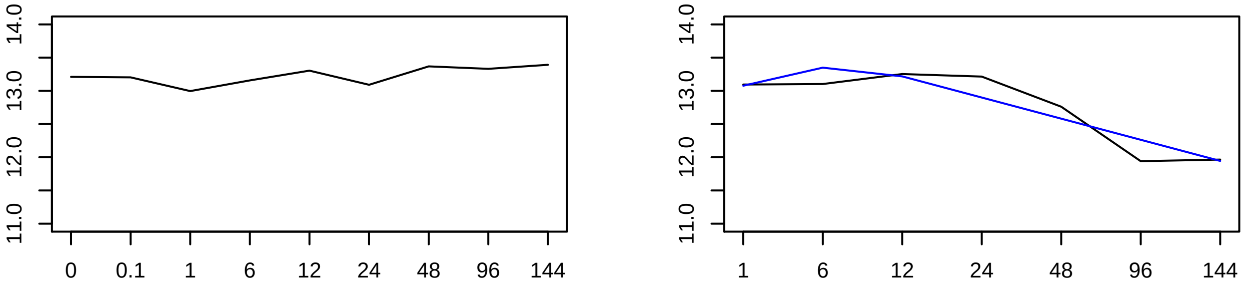

A_32_P203013 RPS10P7 1q32.1

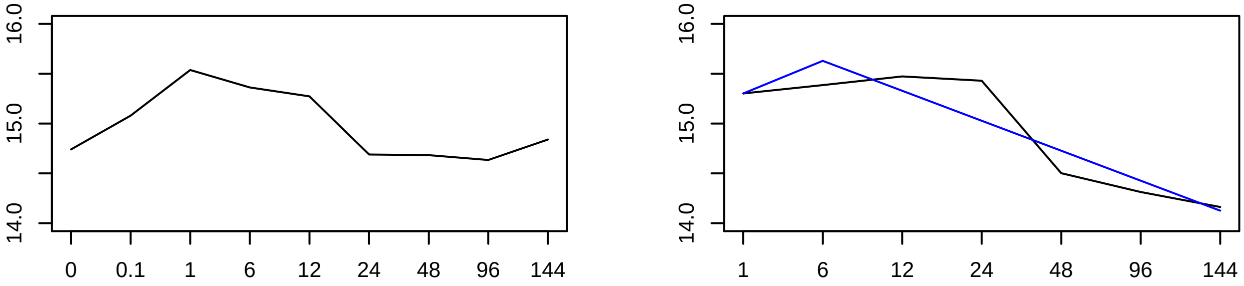

A_24_P261383 JOSD3 11q21

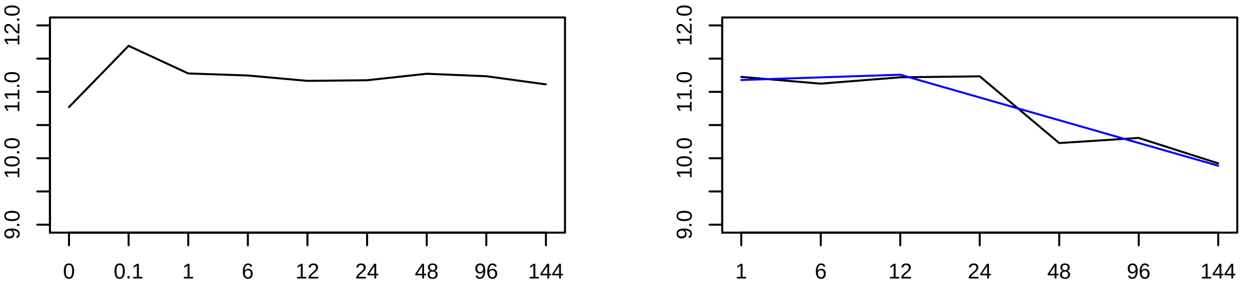

A_32_P124728 THC2645398 NA

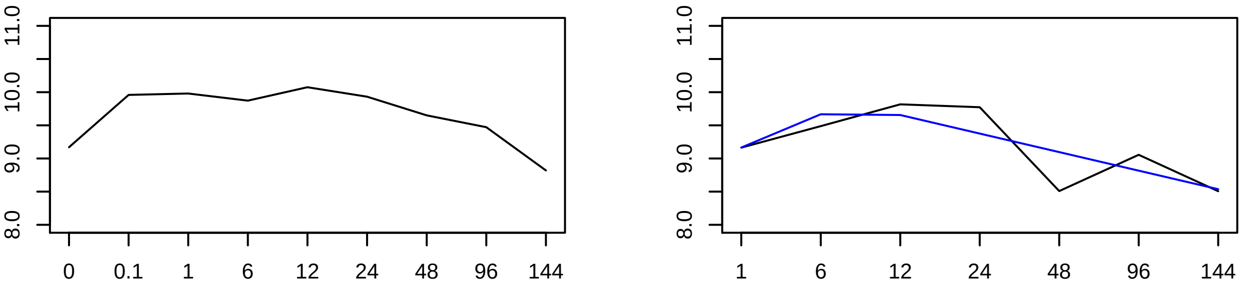

A_32_P15799 HMGN2 1p36.11

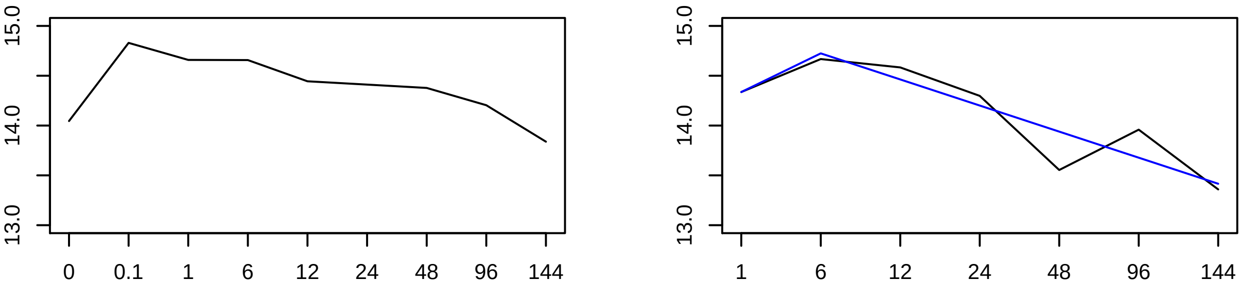

A_32_P88310 BM932296 NA

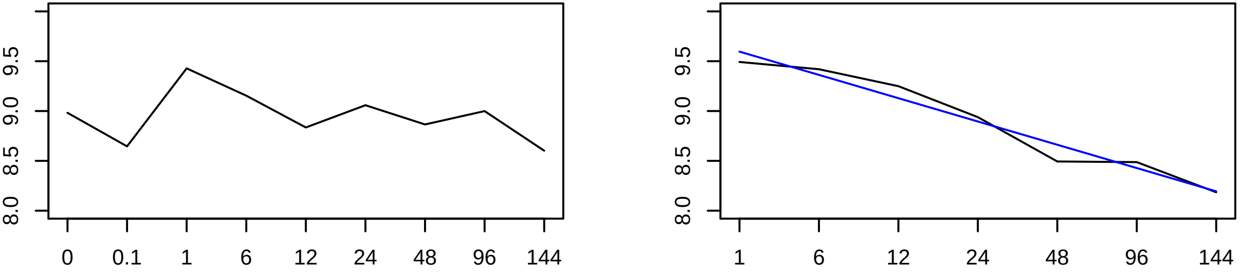

A_24_P530900 A_24_P530900 NA

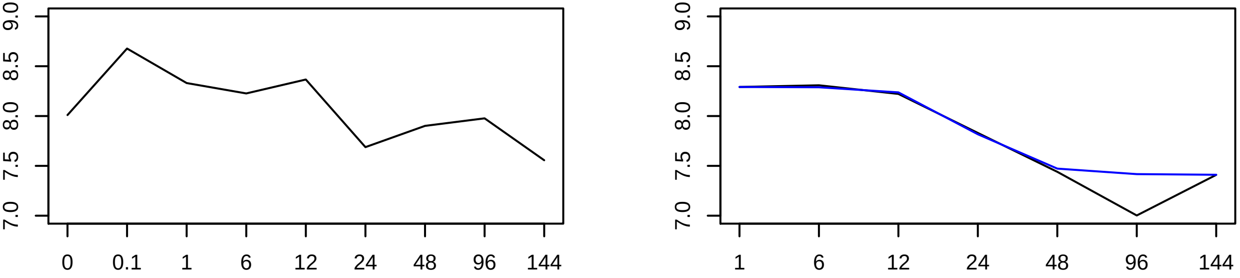

A_32_P101490 ANKRD18A 9p13.1

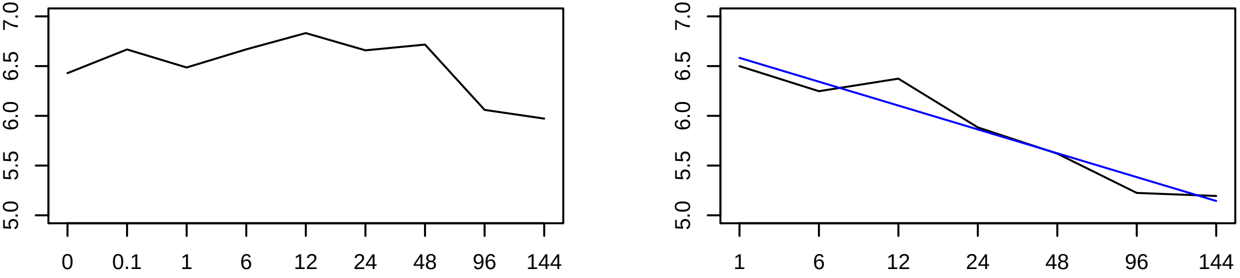

A_32_P137399 KTN1−AS1 14q22.3

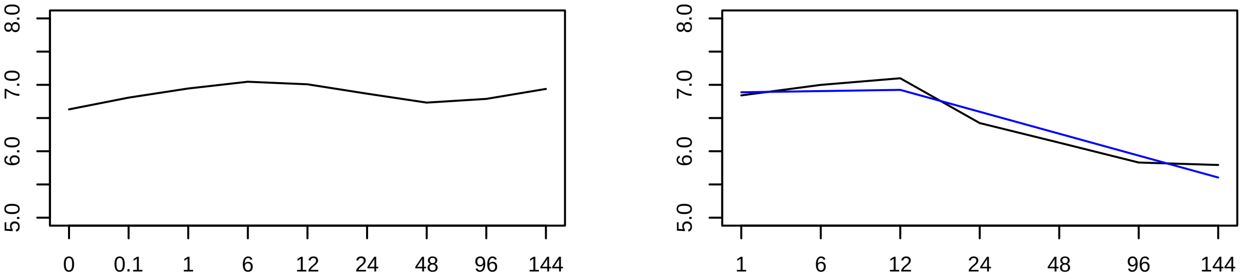

A_23_P155939 ZNF595 4p16.3

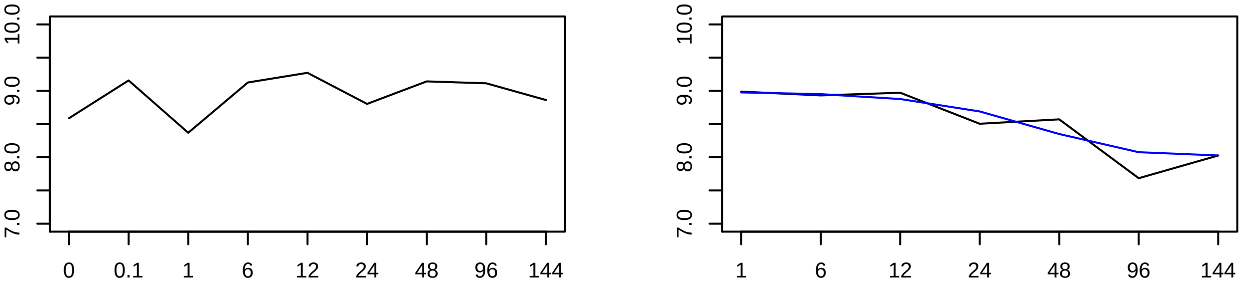

A_32_P27135 CR613436 NA

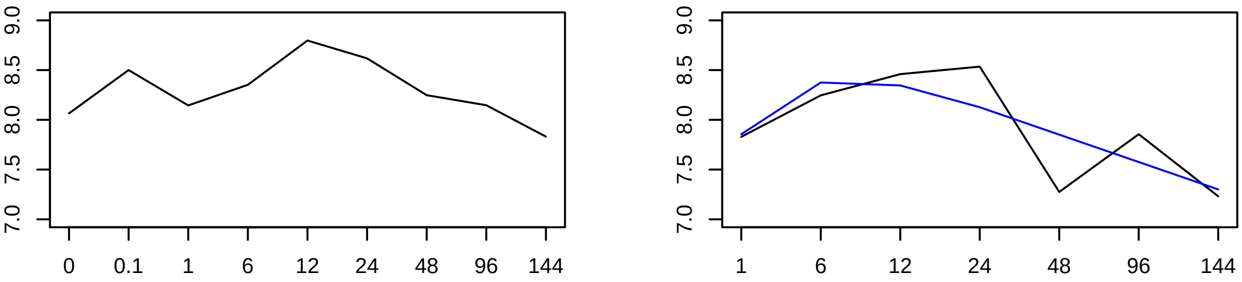

A_23_P336218 MGC27345 7q32.1

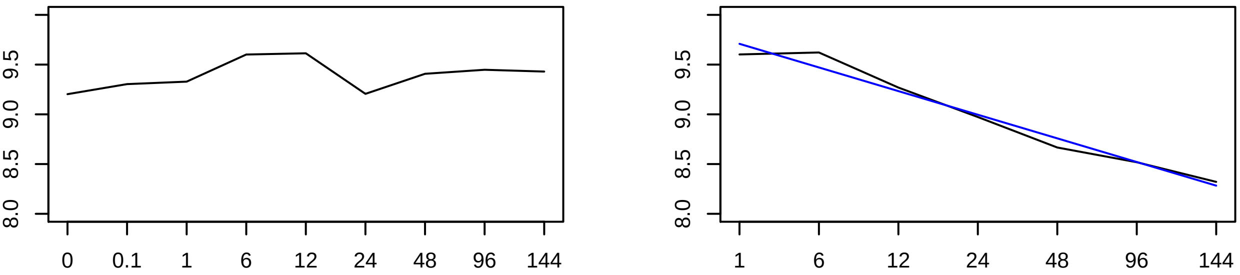

A_24_P248251 HLTF 3q24

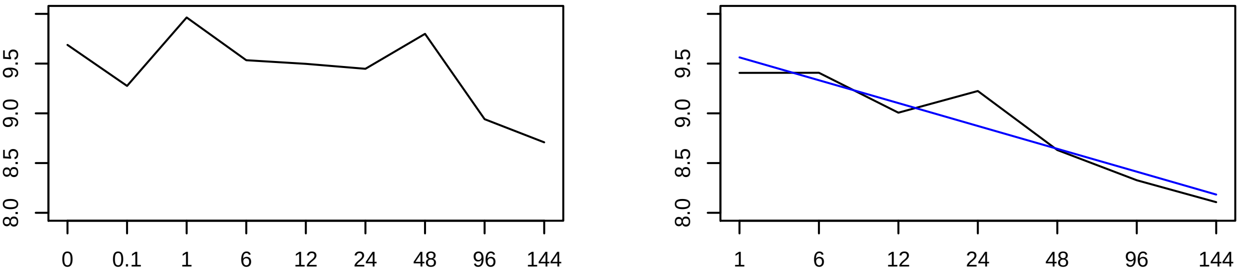

A_24_P306964 A_24_P306964 NA

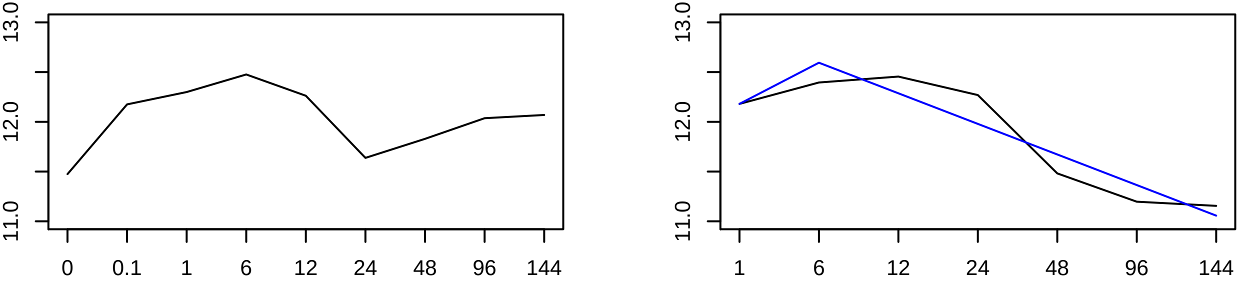

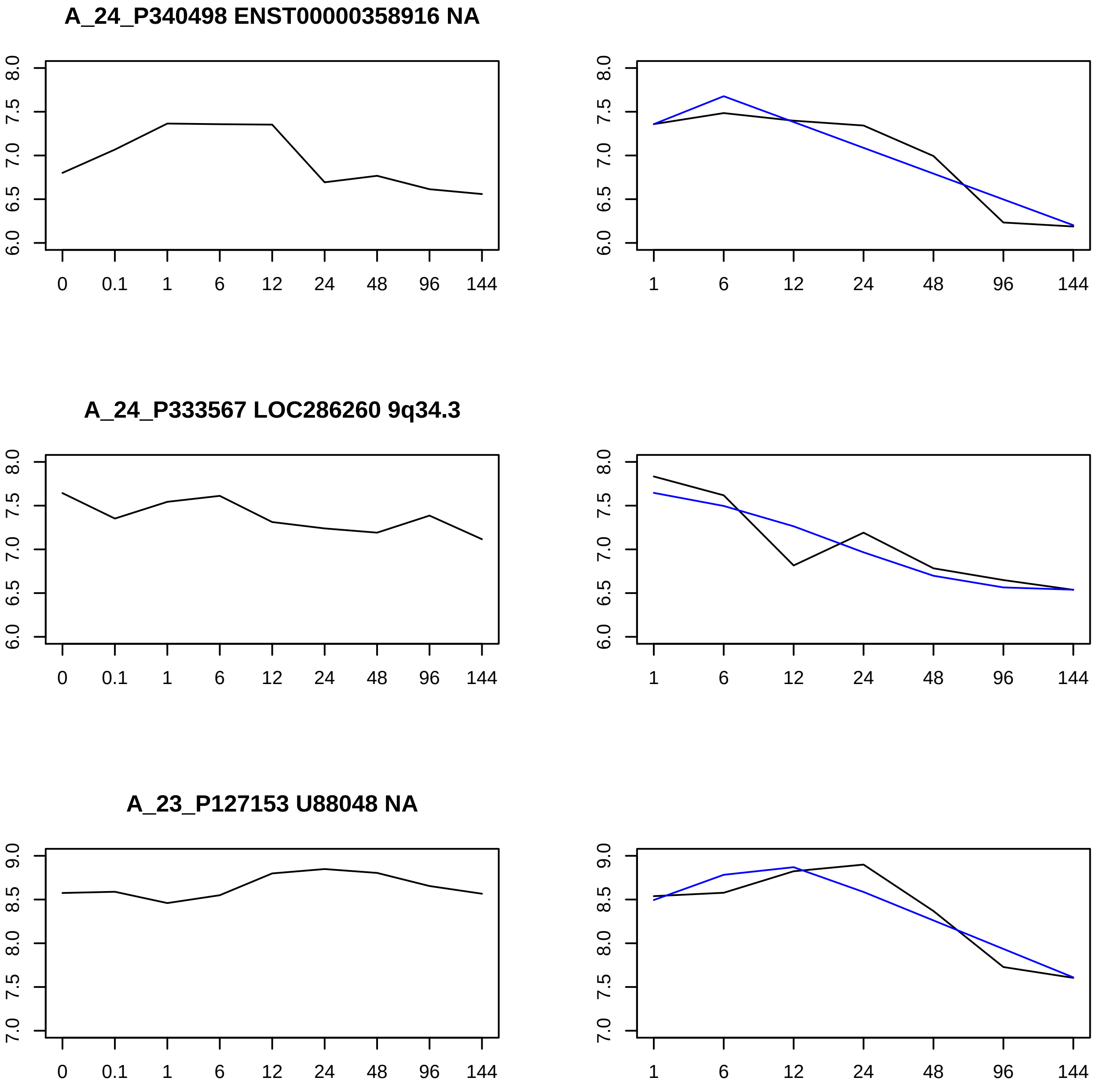
A_24_P340498 ENST00000358916 NA
6.0 6.5 7.0 7.5 8.0
0 0.1 1 6 12 24 48 96 144
1 6 12 24 48 96 144
A_24_P333567 LOC286260 9q34.3
6.0 6.5 7.0 7.5 8.0
0 0.1 1 6 12 24 48 96 144
1 6 12 24 48 96 144
A_23_P127153 U88048 NA
7.0 7.5 8.0 8.5 9.0
0 0.1 1 6 12 24 48 96 144
1 6 12 24 48 96 144

A_23_P134014 DEADC1 6q24.2

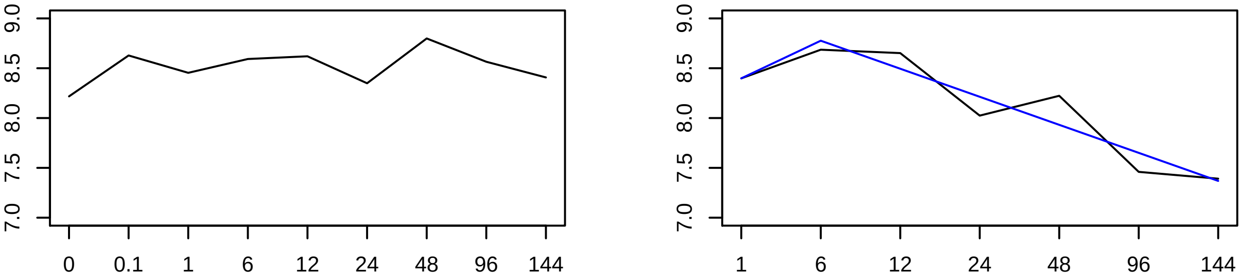

A_23_P52311 TAF5 10q24.33

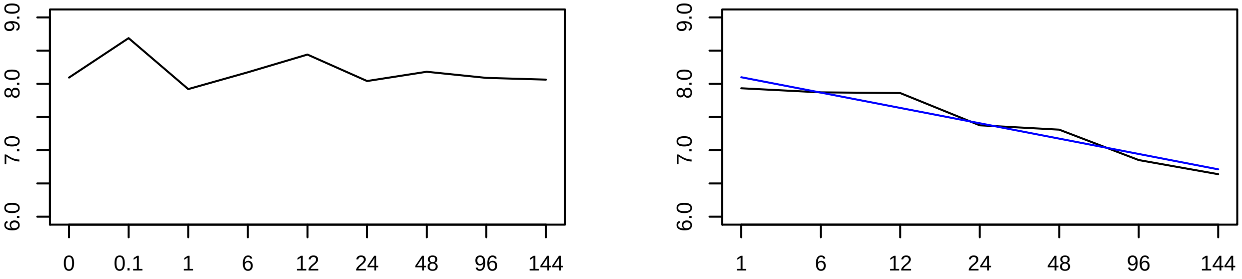

A_23_P76034 PVRL1 11q23.3

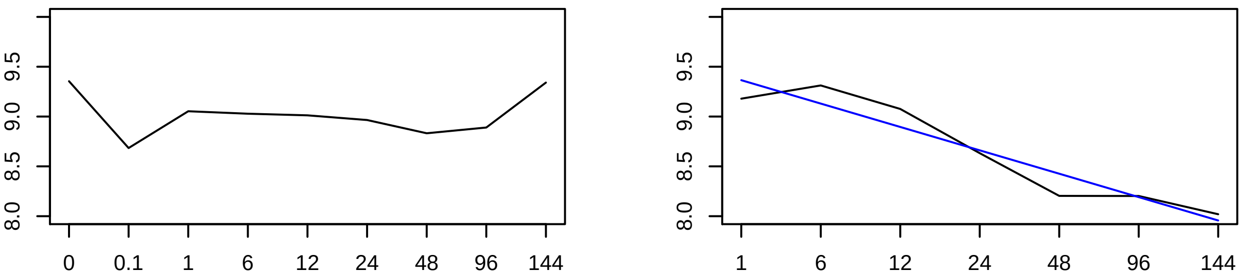

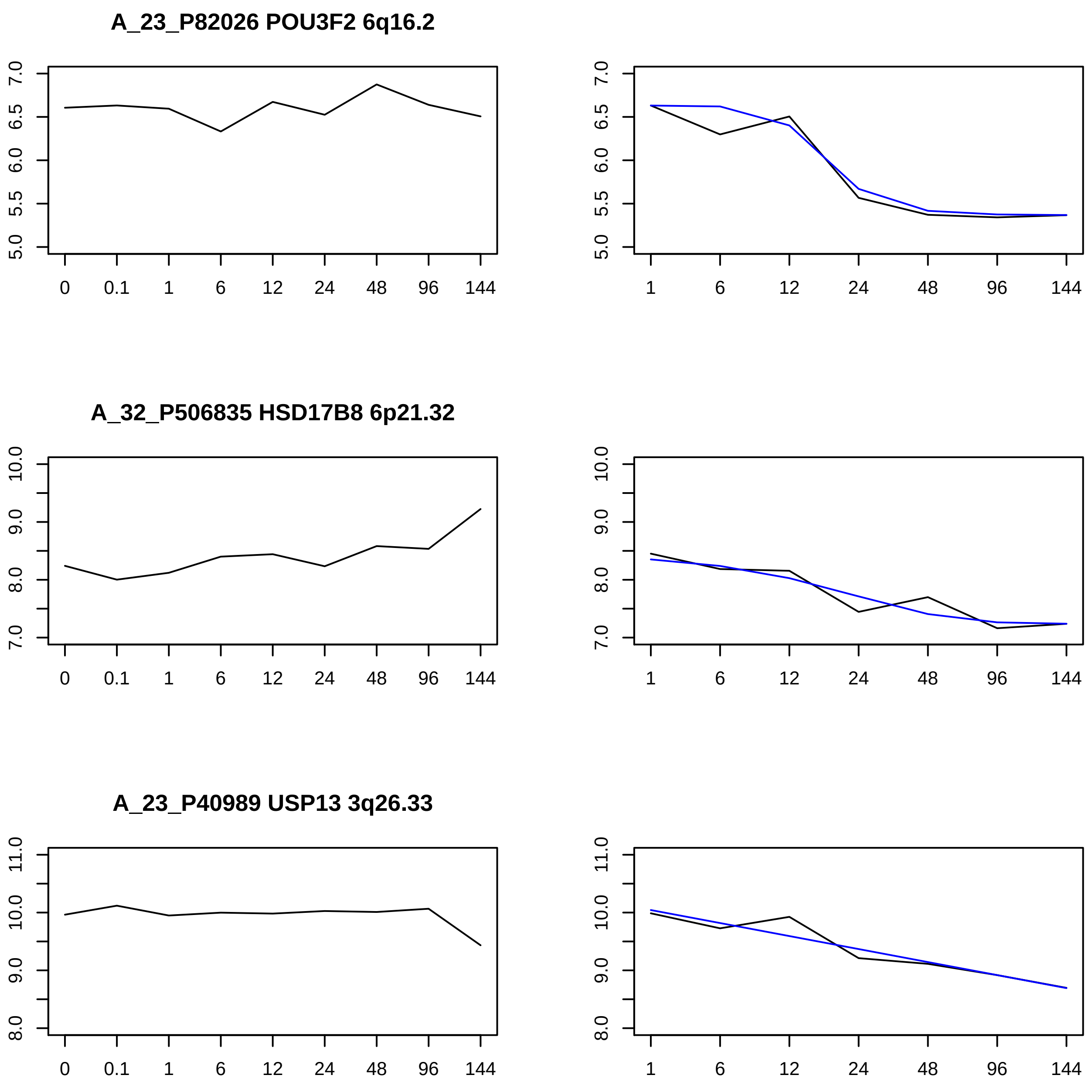
A_23_P82026 POU3F2 6q16.2
A_32_P506835 HSD17B8 6p21.32
A_23_P40989 USP13 3q26.33
5.0 5.5 6.0 6.5 7.0
7.0 8.0 9.0 10.0
8.0 9.0 10.0 11.0
0 0.1 1 6 12 24 48 96 144
1 6 12 24 48 96 144

A_23_P28485 GCA 2q24.2

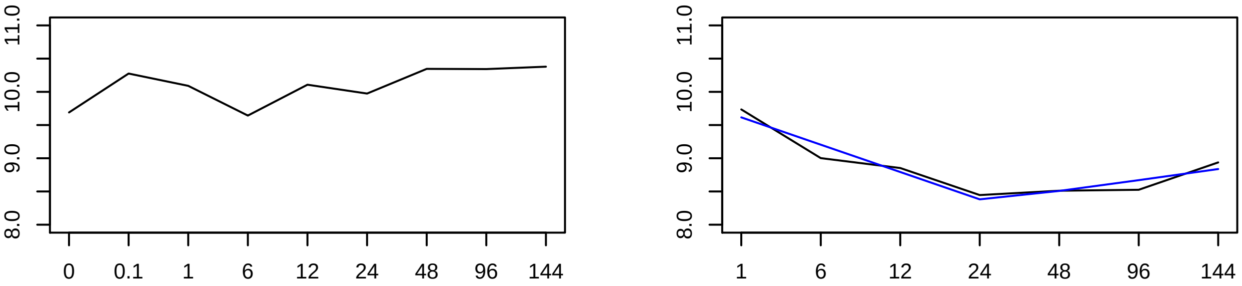

A_23_P92642 ANKHD1 5q31.3

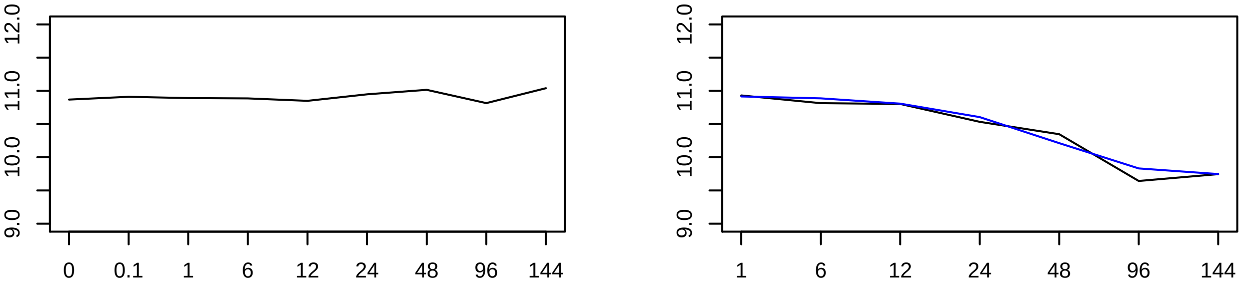

A_24_P136011 A_24_P136011 NA

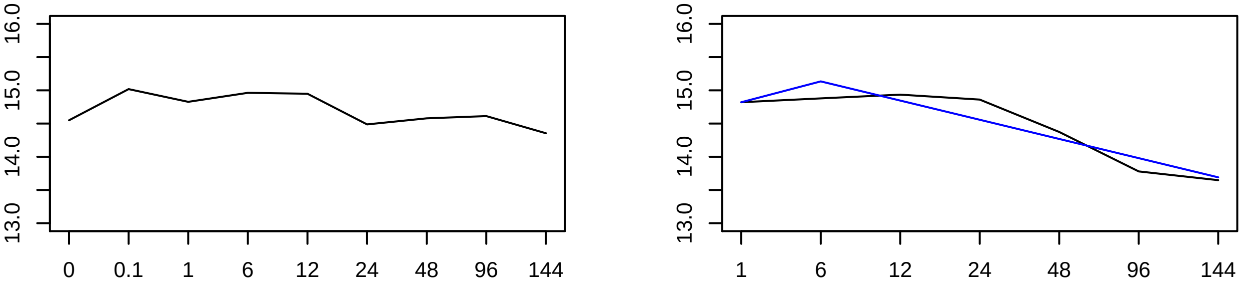

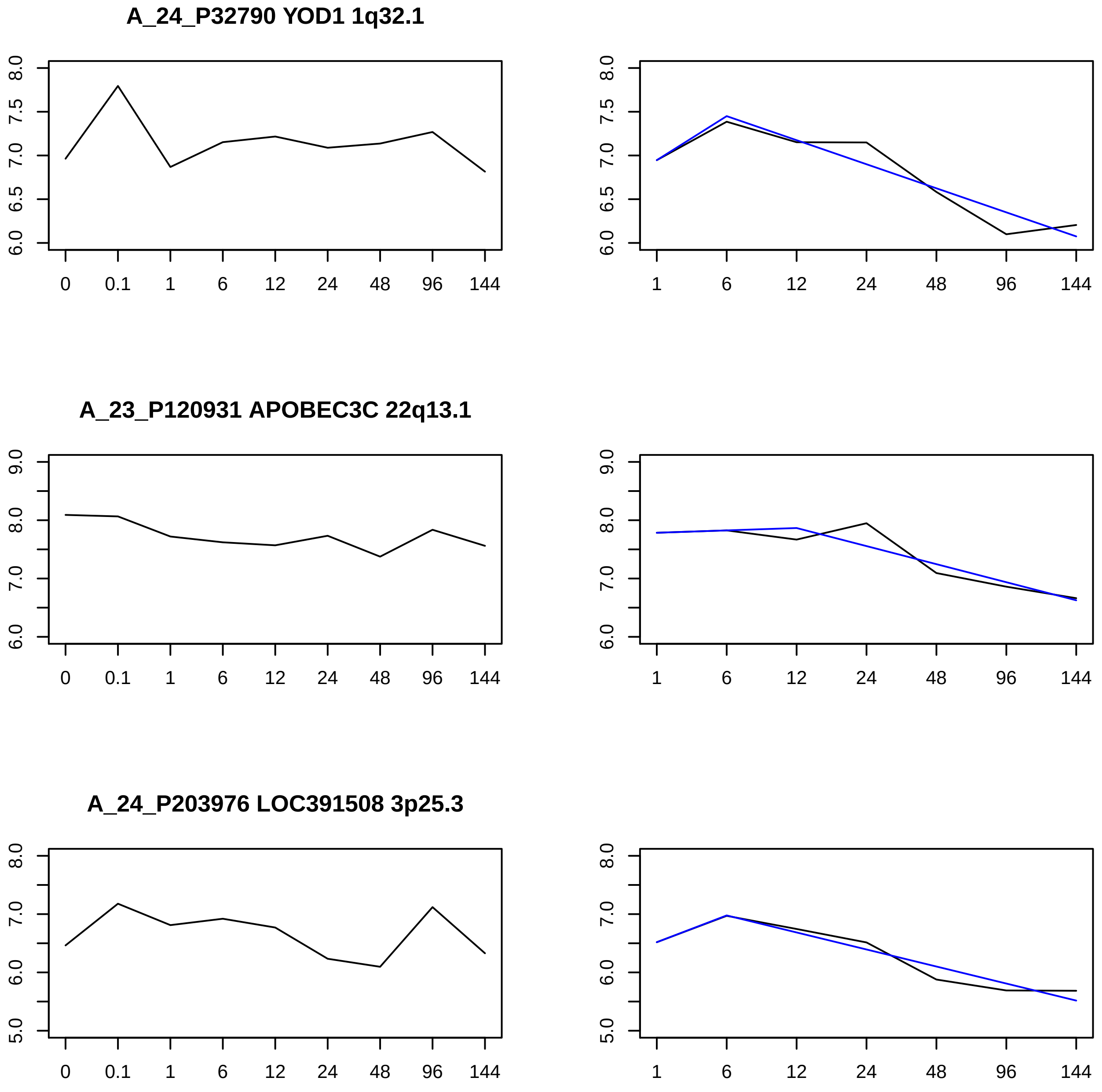
A_24_P32790 YOD1 1q32.1
6.0
6.5
7.0
7.5
8.0
0
0.1
1
6
12
24
48
96
144
1
6
12
24
48
96
144
A_23_P120931 APOBEC3C 22q13.1
6.0
7.0
8.0
9.0
A_24_P203976 LOC391508 3p25.3
5.0
6.0
7.0
8.0

A_23_P110557 UNC5A 5q35.2

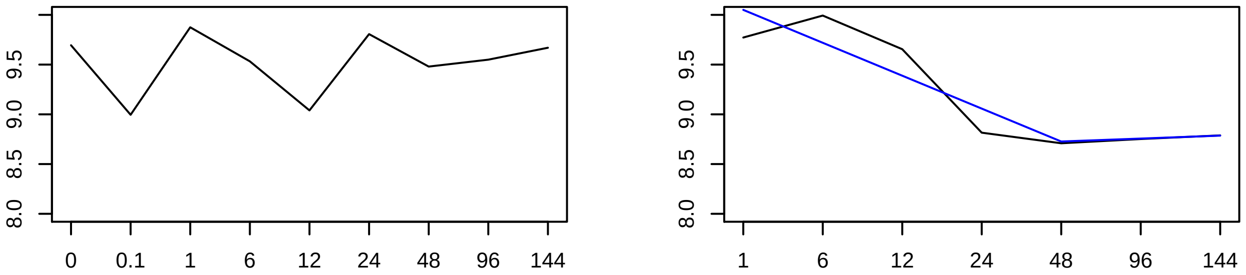

A_24_P306896 UBE2CP4 15q13.3

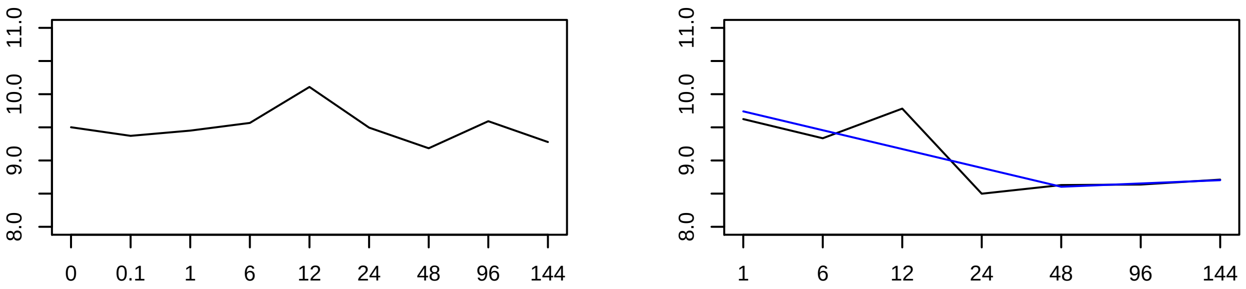

A_24_P324538 A_24_P324538 NA

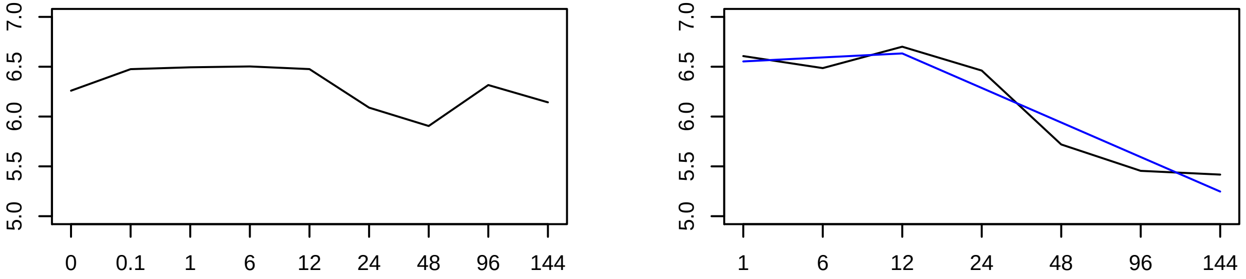

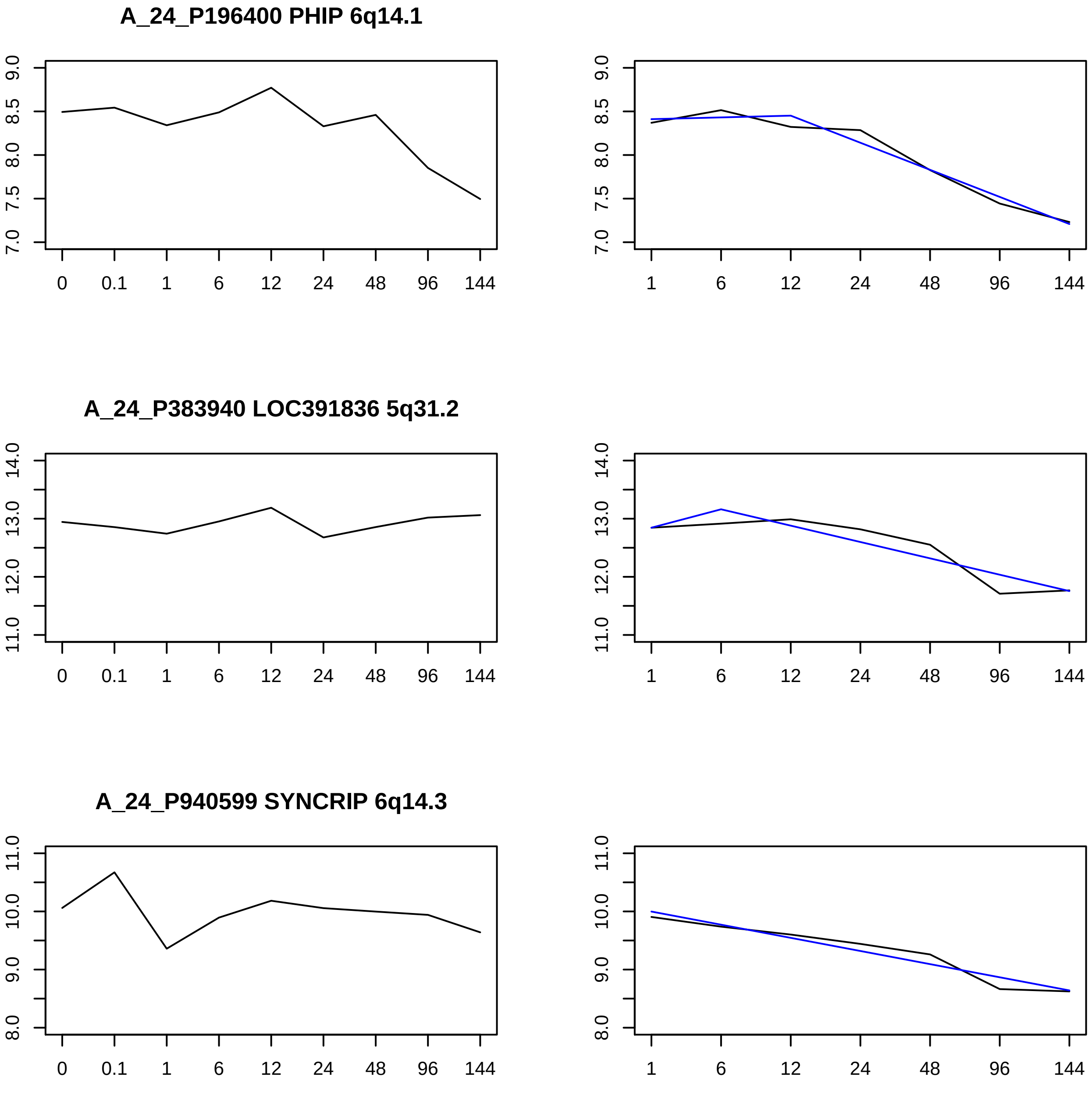

A_24_P196400 PHIP 6q14.1
7.0
7.5
8.0
8.5
9.0
0
0.1
1
6
12
24
48
96
144
A_24_P383940 LOC391836 5q31.2
11.0
12.0
13.0
14.0
A_24_P940599 SYNCRIP 6q14.3
8.0
9.0
10.0
11.0

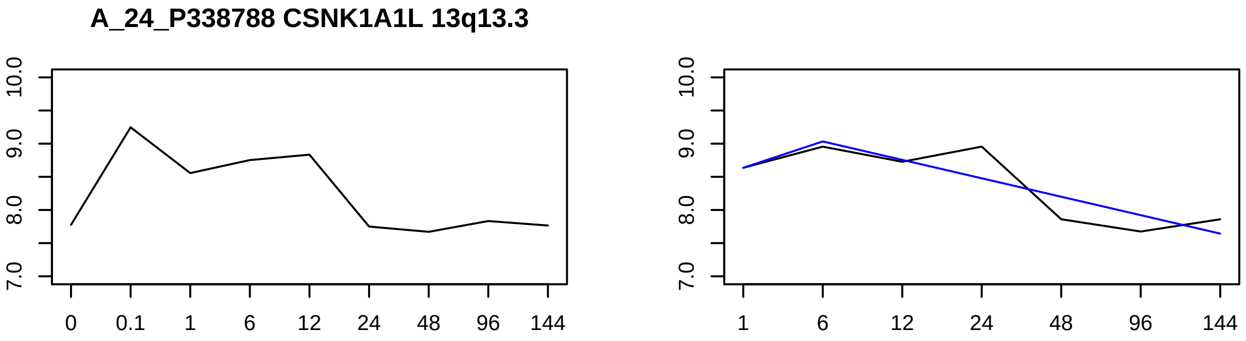
A_24_P338788 CSNK1A1L 13q13.3

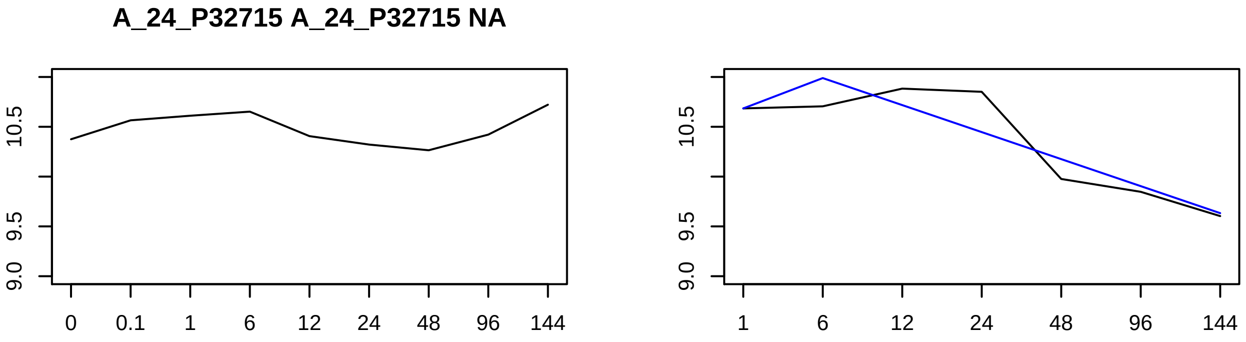
A_24_P32715 A_24_P32715 NA

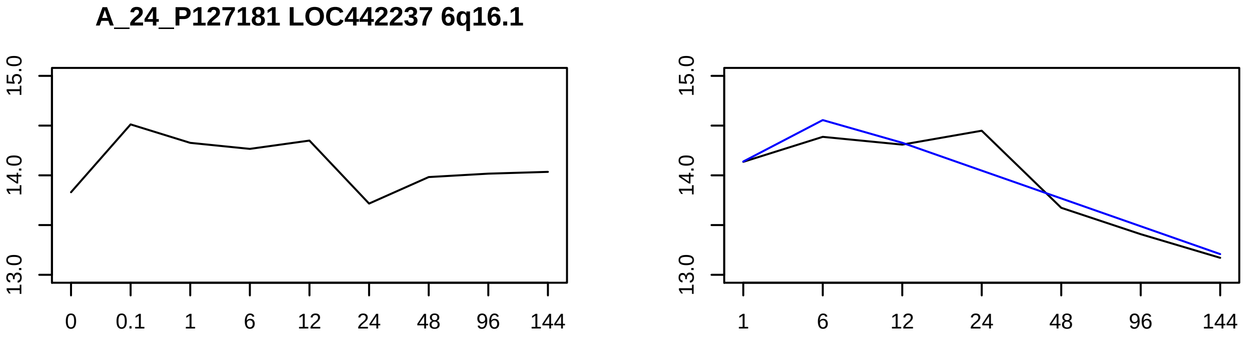
A_24_P127181 LOC442237 6q16.1

A_24_P335263 NUDT4 12q22

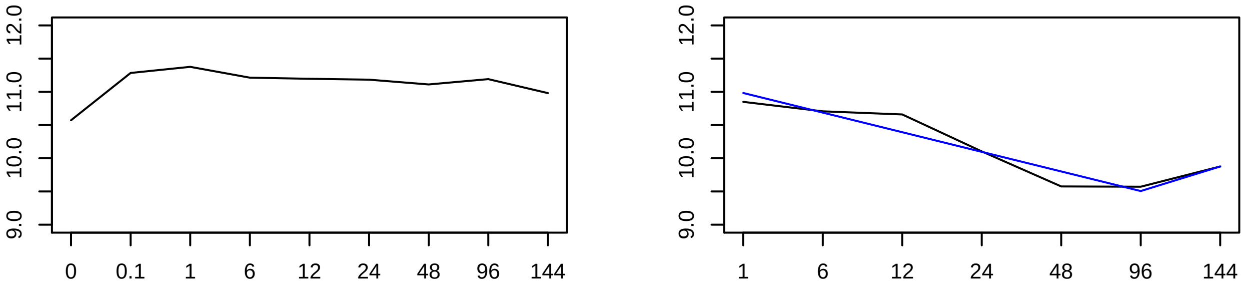

A_24_P109614 RP11−344H11.4 1p36.11

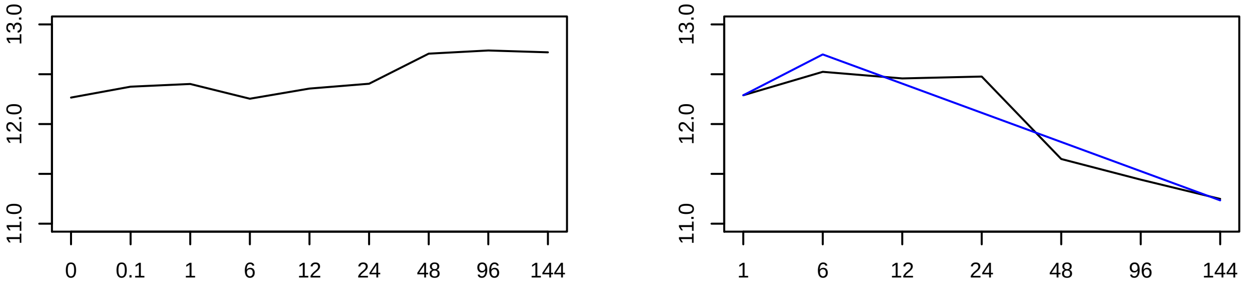

A_23_P48628 ZBTB25 14q23.3

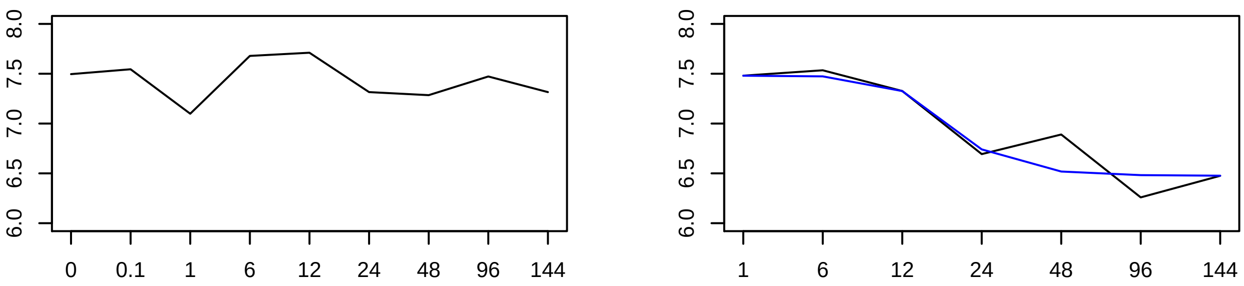

A_24_P501698 EIF3H NA

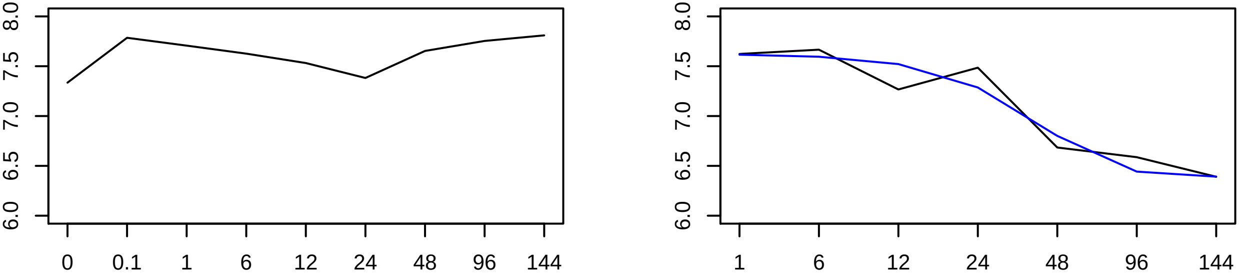

A_24_P67946 NUDT4 12q22

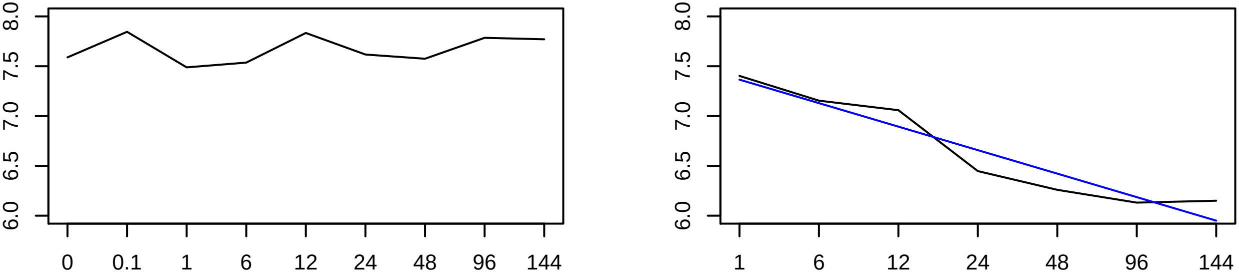

A_24_P48204 SECTM1 17q25.3

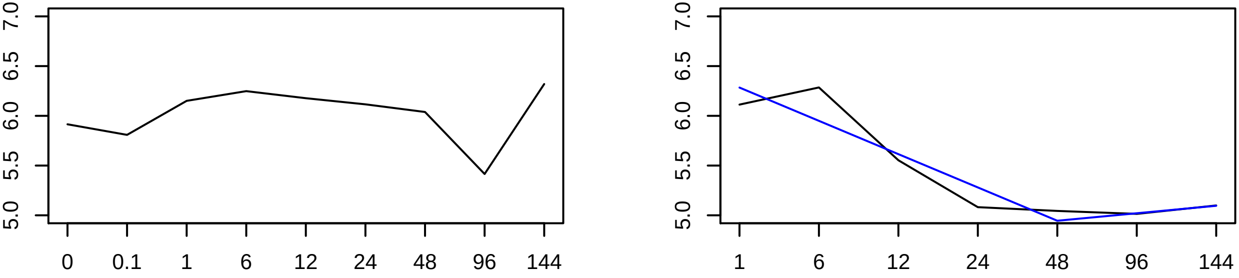

A_24_P110601 A_24_P110601 NA

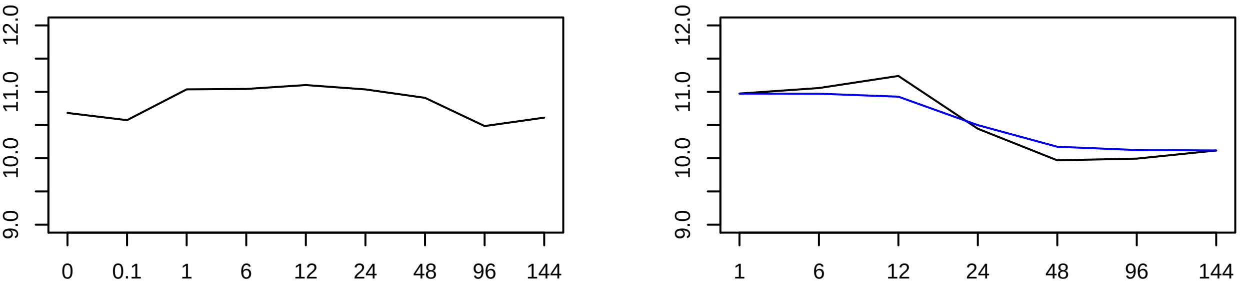

A_23_P157679 C8orf53 8q24.11

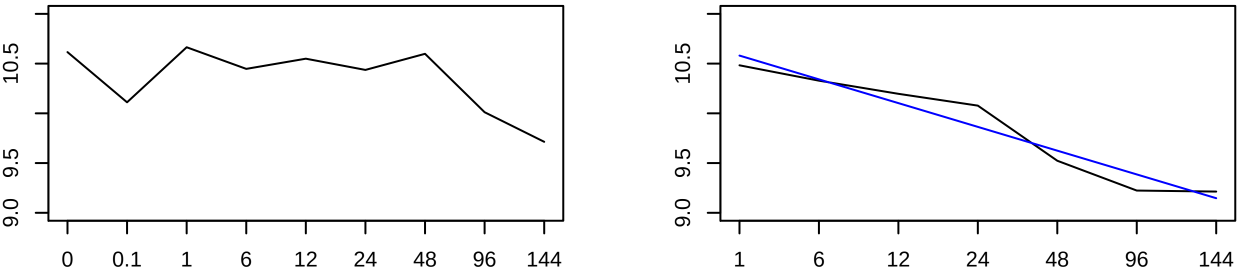

A_24_P187218 PCDH9 13q21.32

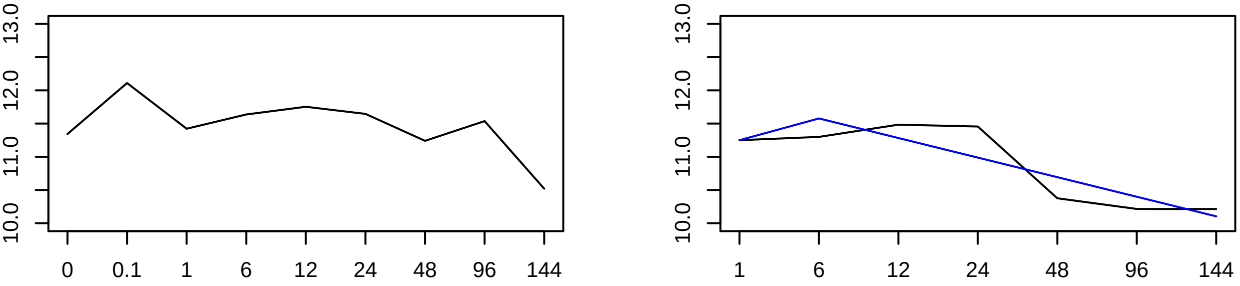

A_24_P217804 FGFR1OP 6q27

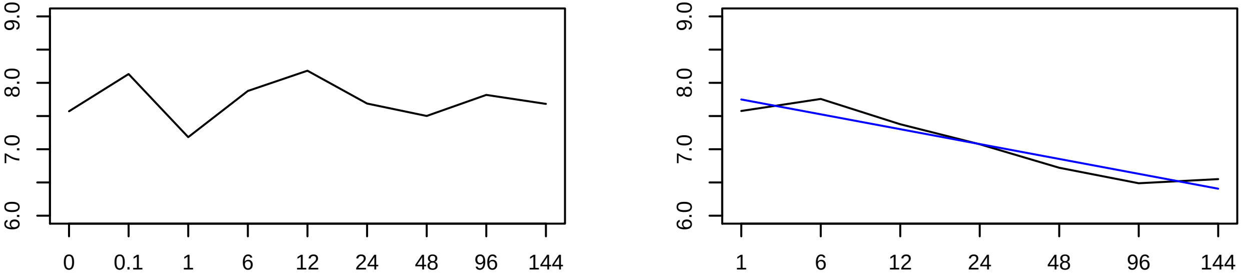

A_24_P349539 HMGB1P46 8q23.1

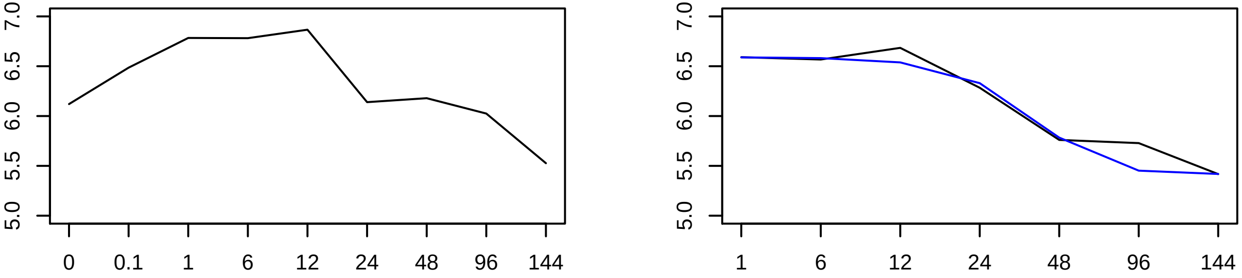

A_23_P86230 MYCBP 1p34.3

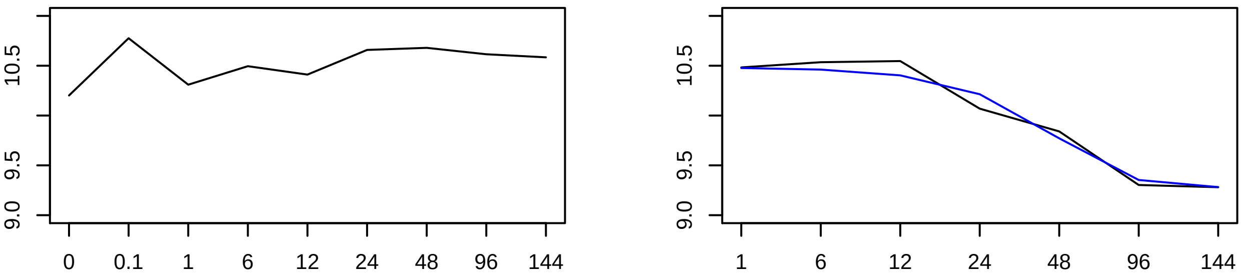

A_23_P79803 VSTM2L 20q11.23

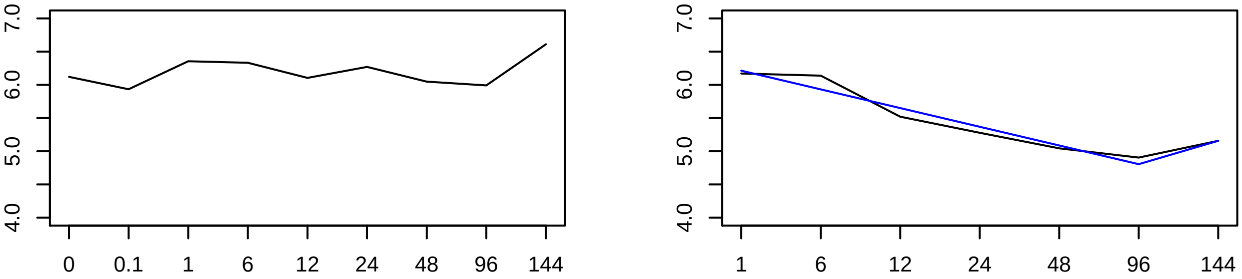

A_24_P91916 NXT2 Xq22.3

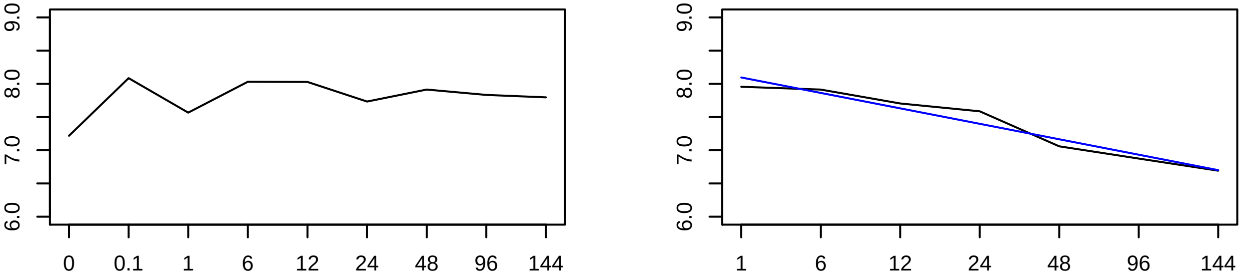

A_23_P169428 TRUB2 9q34.11

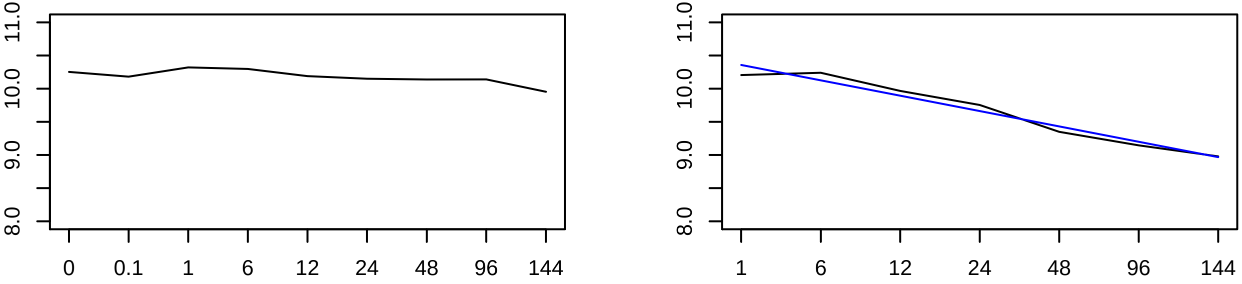

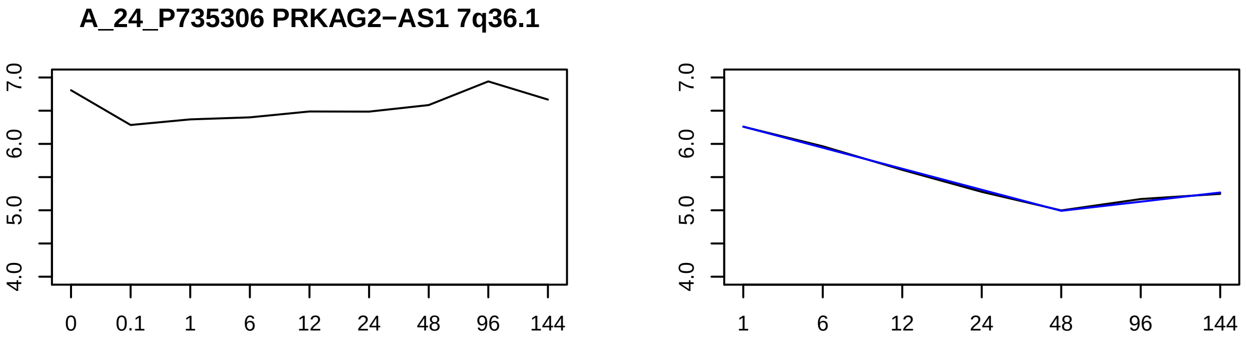
A_24_P735306 PRKAG2−AS1 7q36.1
4.0
5.0
6.0
7.0
0
0.1
1
6
12
24
48
96
144

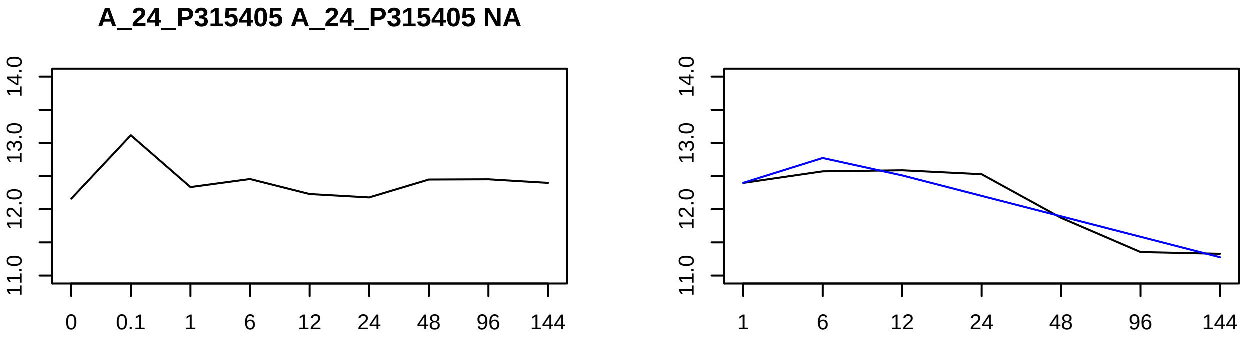
A_24_P315405 A_24_P315405 NA
11.0
12.0
13.0
14.0
0
0.1
1
6
12
24
48
96
144

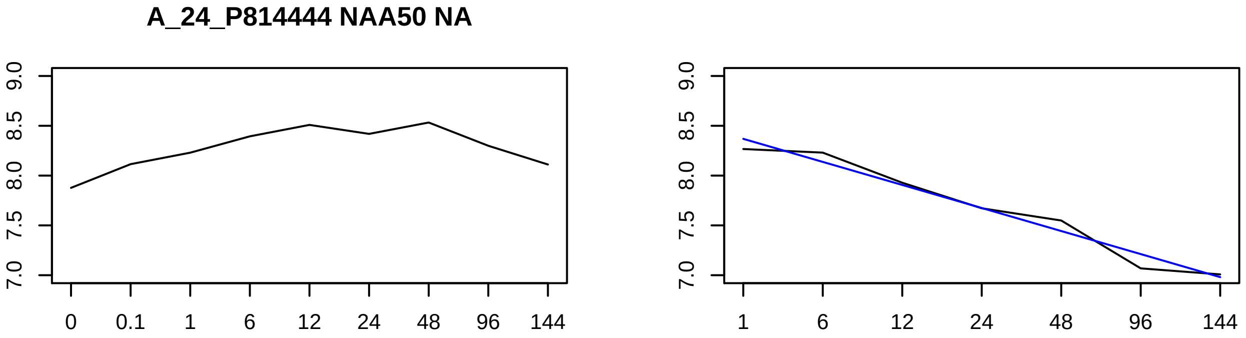
A_24_P814444 NAA50 NA
7.0
7.5
8.0
8.5
9.0
0
0.1
1
6
12
24
48
96
144

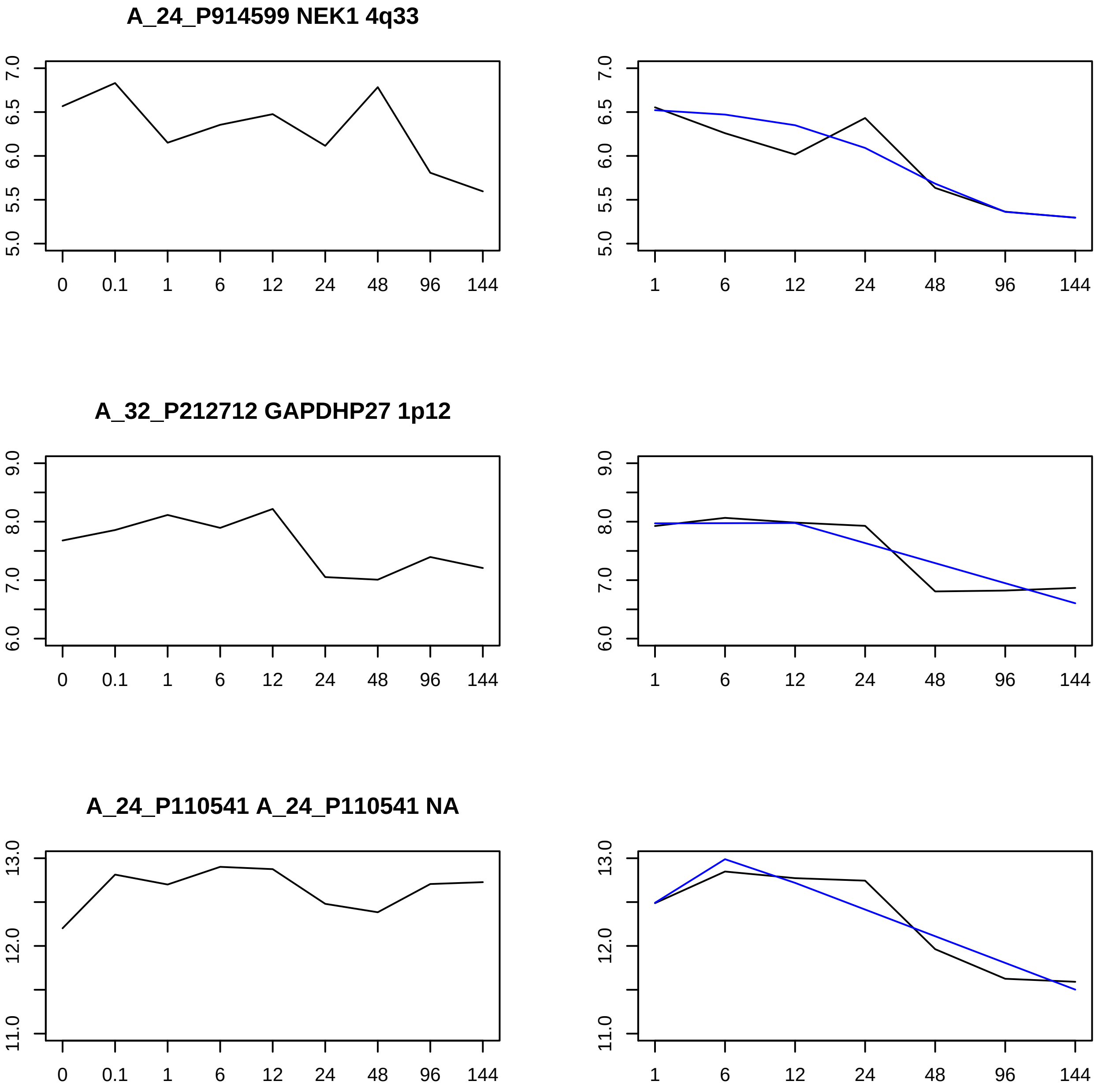

A_24_P914599 NEK1 4q33
5.0
5.5
6.0
6.5
7.0
0
0.1
1
6
12
24
48
96
144
A_32_P212712 GAPDHP27 1p12
6.0
7.0
8.0
9.0
A_24_P110541 A_24_P110541 NA
11.0
12.0
13.0

A_24_P575336 LOC391181 1q43

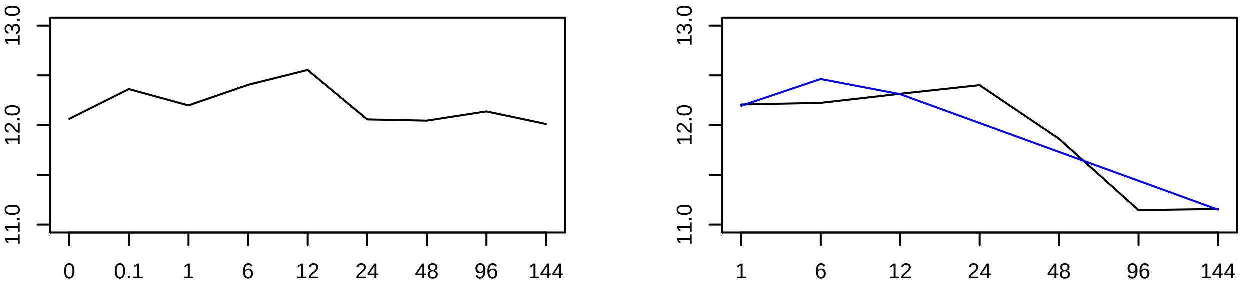

A_23_P859 ADSS 1q44

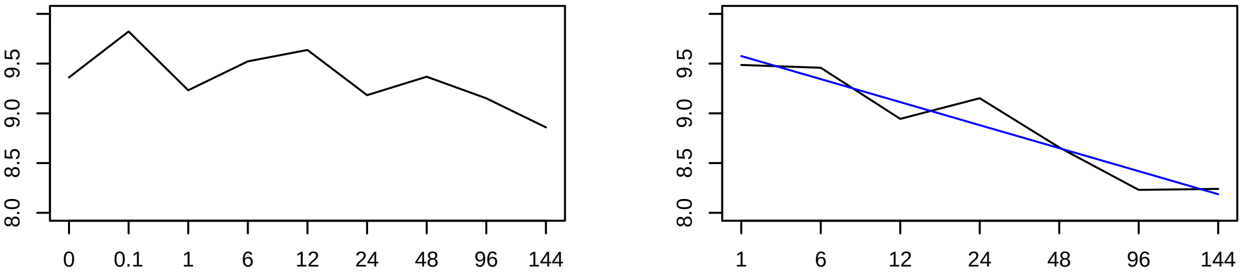

A_24_P106306 RPL26L1 5q35.2

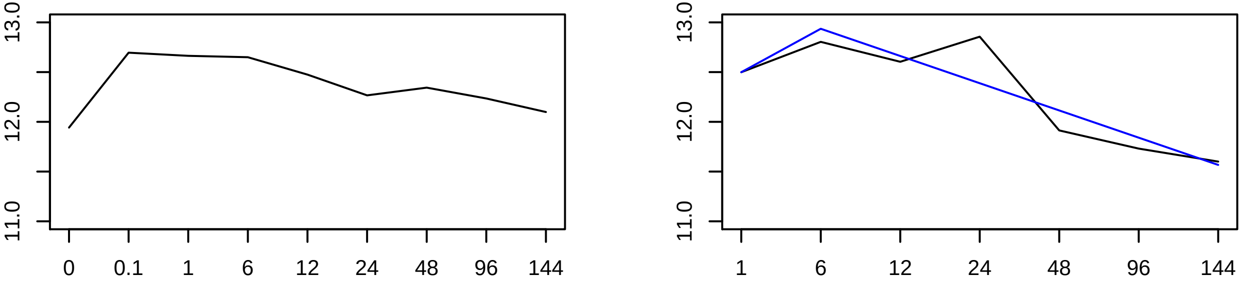

A_24_P931503 PHIP 6q14.1

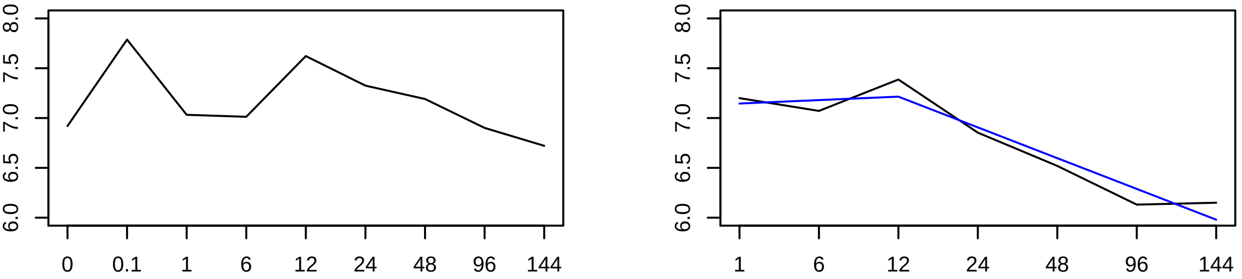

A_24_P247536 MGC40405 7q21.2

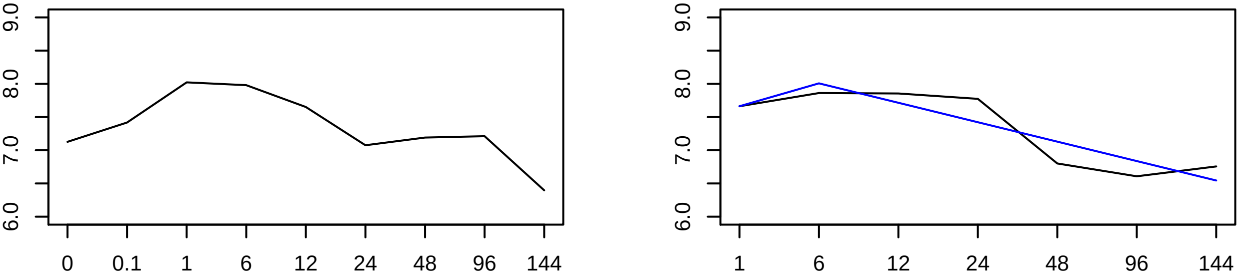

A_24_P372672 DLAT 11q23.1

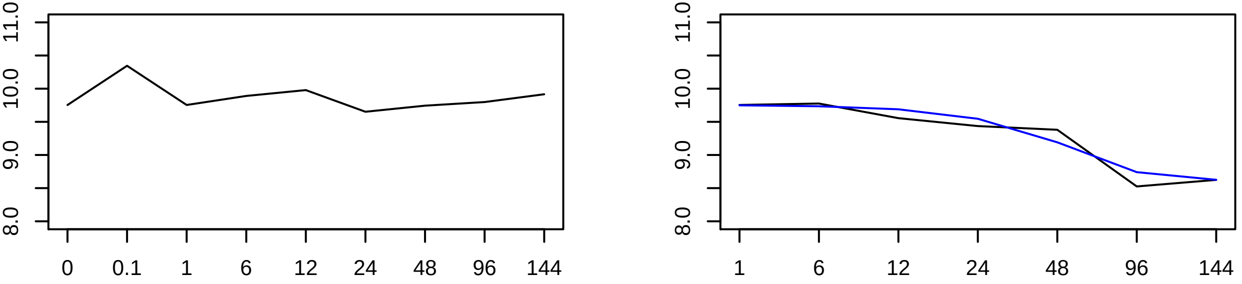

A_24_P84498 EFCAB10 7q22.3

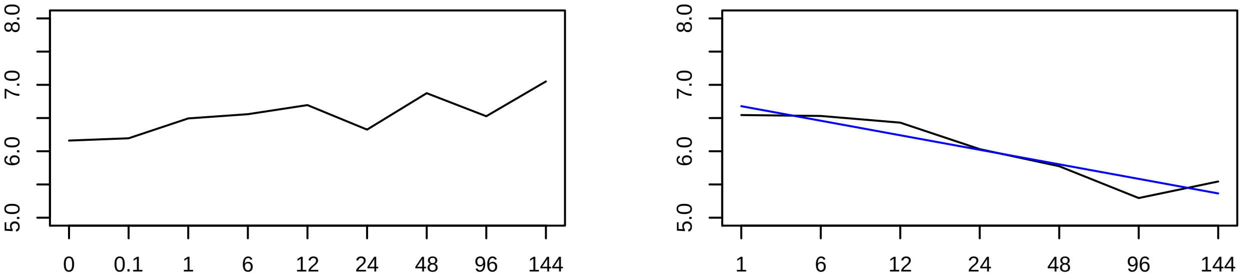

A_23_P137825 LOC128192 1p34.1

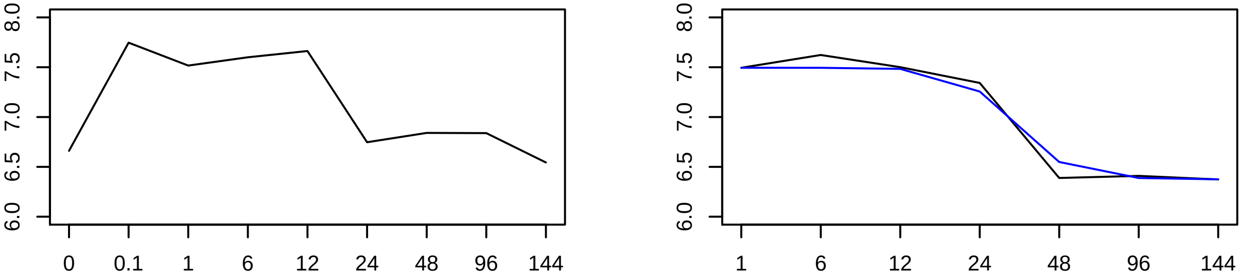

A_23_P309996 BCL2L11 2q13

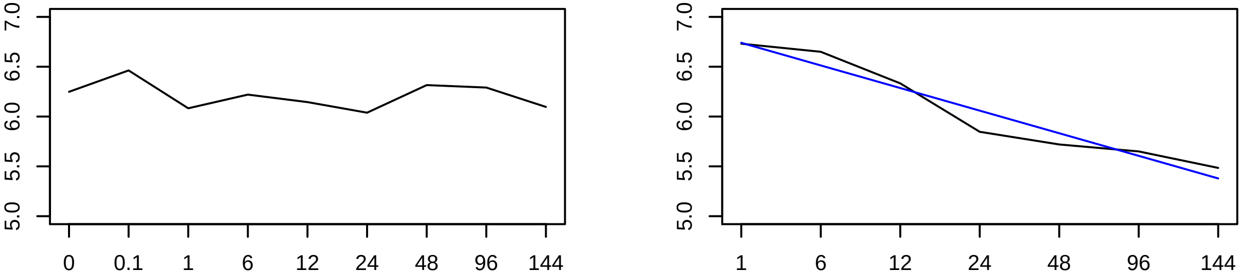

A_24_P75840 LOC129560 2q22.1

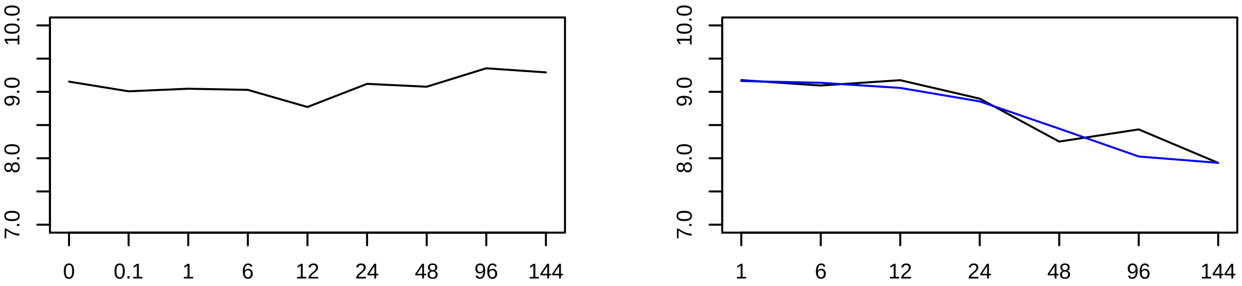

A_23_P376686 BTNL2 6p21.32

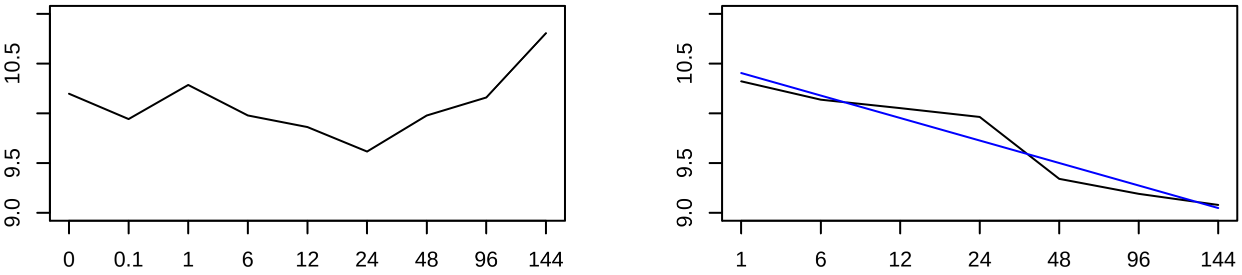

A_24_P306209 GAPVD1 9q33.3

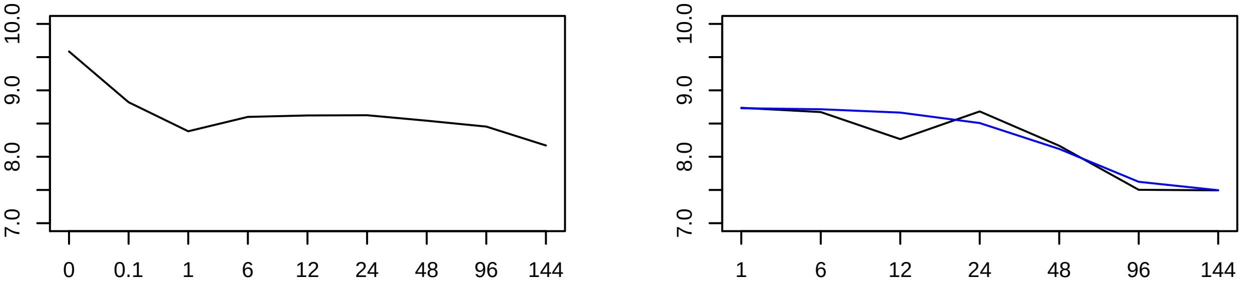

A_24_P270376 NUFIP1 13q14.12

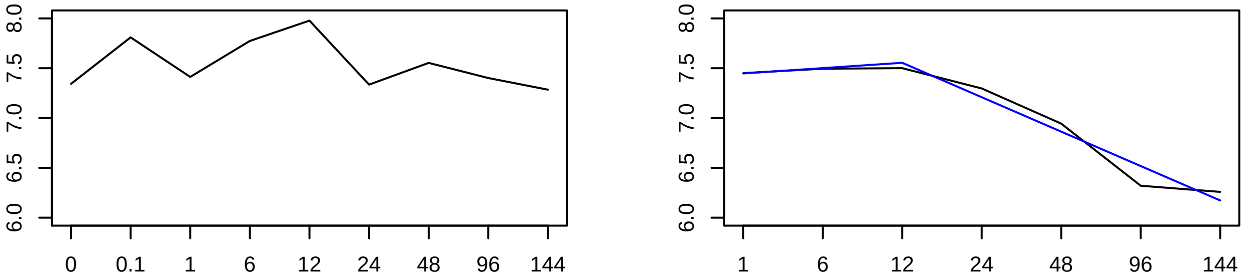

A_32_P196483 RPS3A 4q31.3

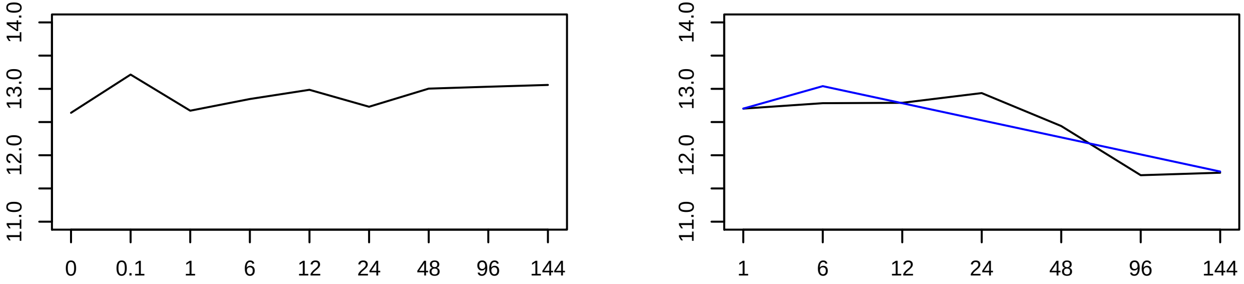

A_23_P354798 COQ10A 12q13.2

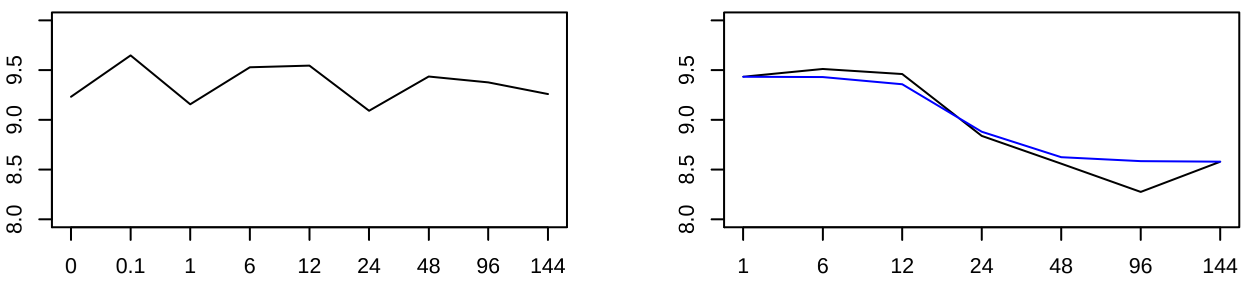

A_24_P925292 RSBN1L 7q11.23

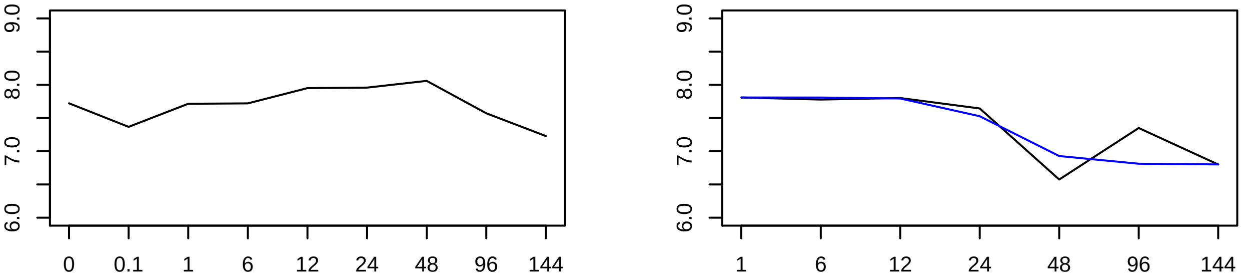

A_24_P913760 INTS6 13q14.3

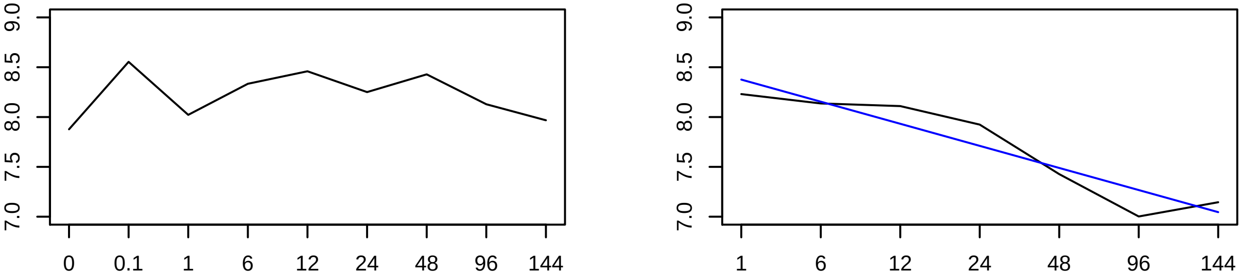

A_23_P125016 A_23_P125016 NA

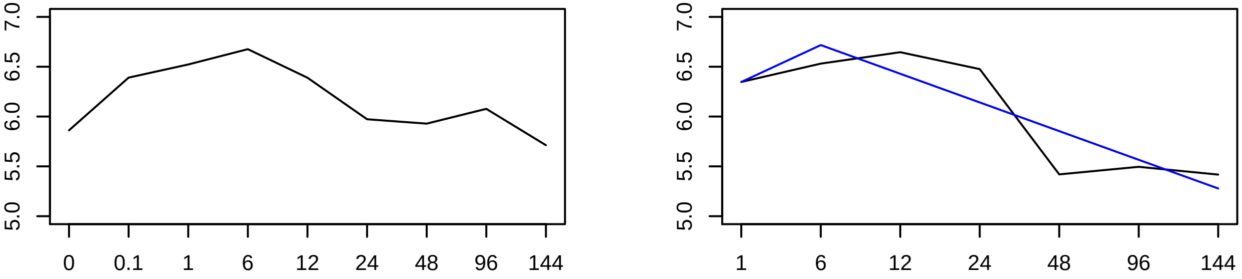

A_23_P169558 CSPP1 8q13.2

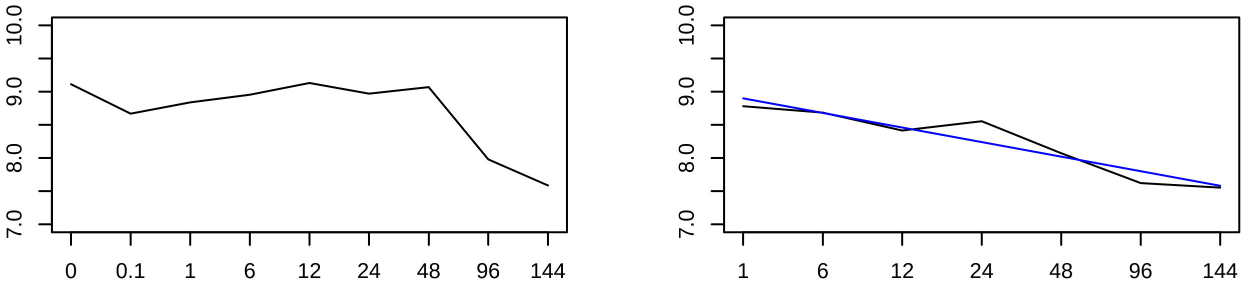

A_32_P56037 RGS5 NA

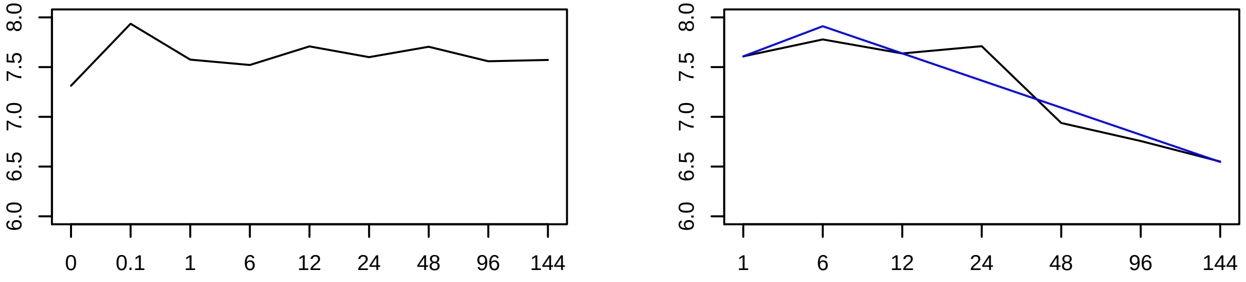

A_32_P129540 LOC732221 12q23.3

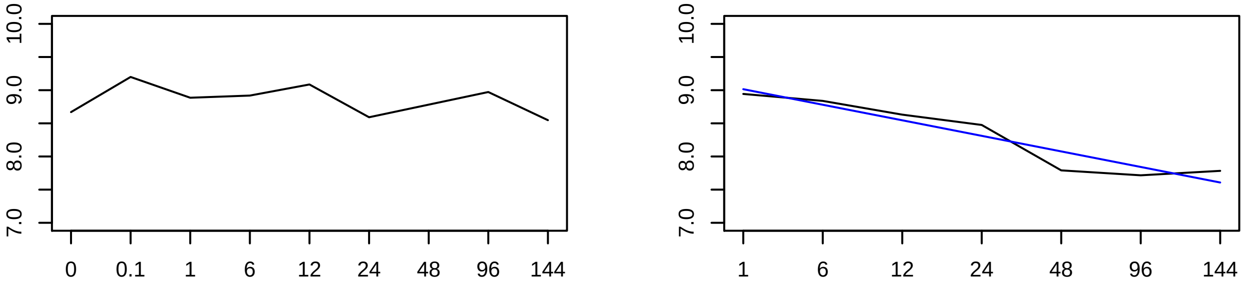

A_23_P153084 RALBP1 18p11.22

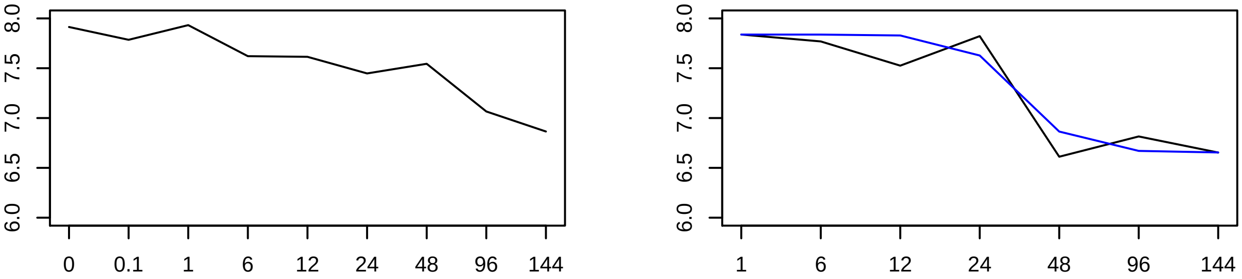

A_32_P139021 A_32_P139021 NA

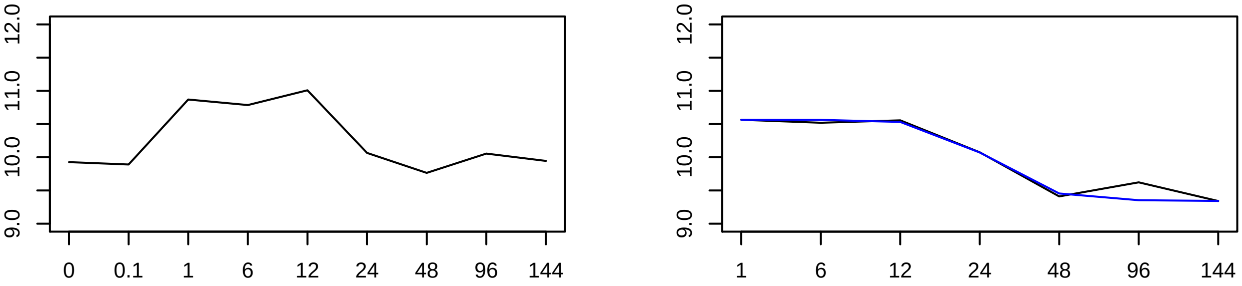

A_24_P24142 LOC643960 2q31.1

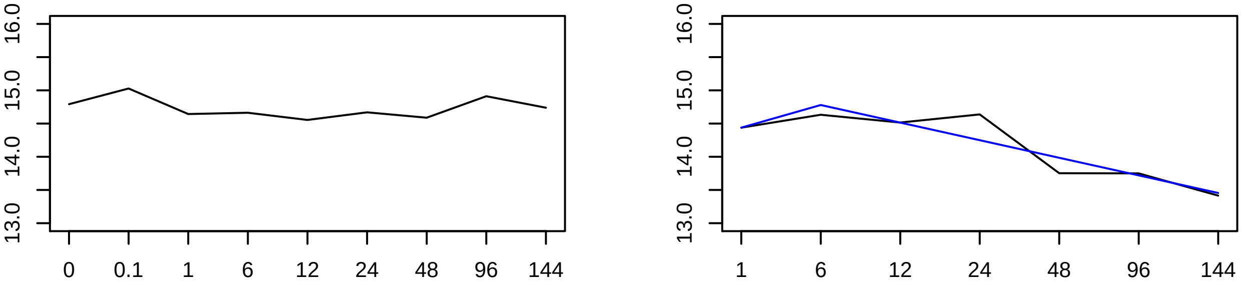

A_24_P272073 ENST00000335078 NA

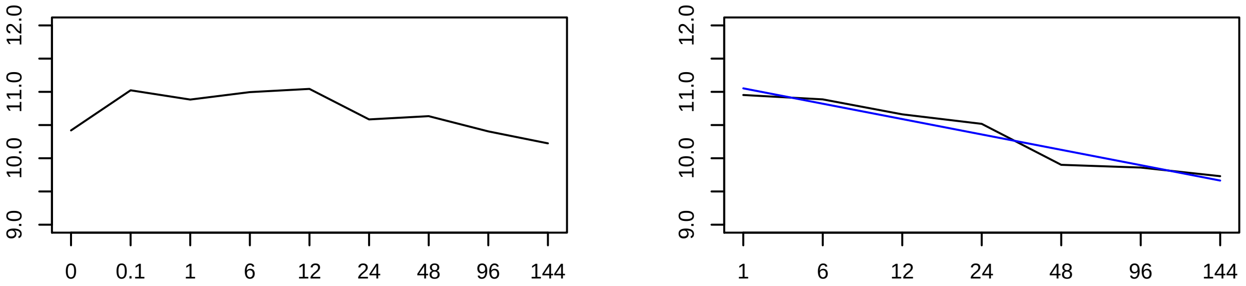

A_24_P247920 KIAA1652 22q11.21

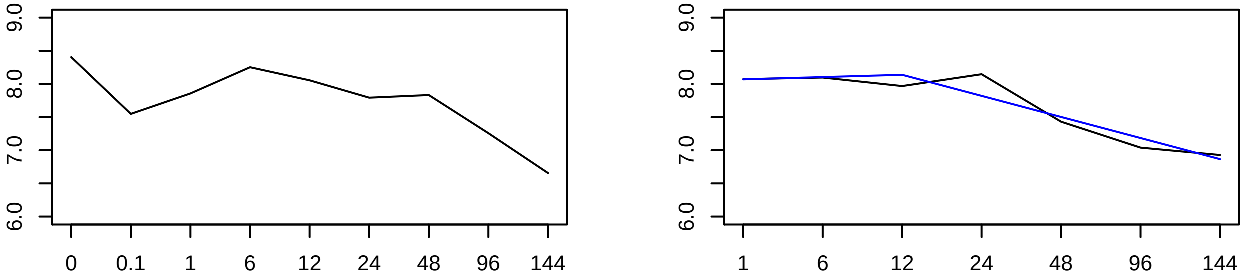

A_23_P78392 ST8SIA3 18q21.31

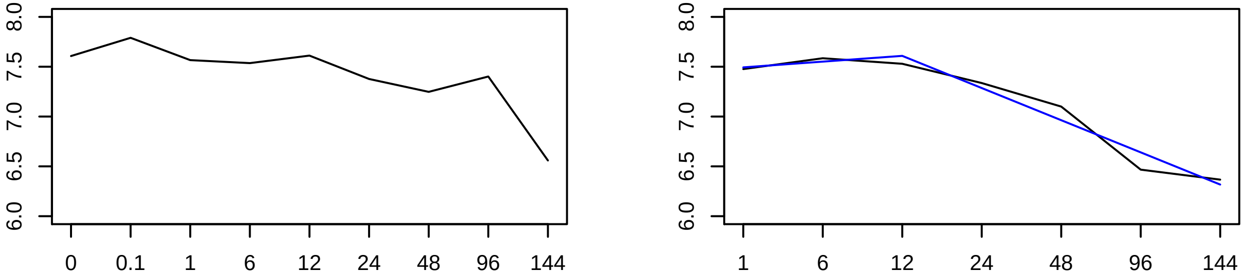

A_24_P375237 RPL12 9q33.3

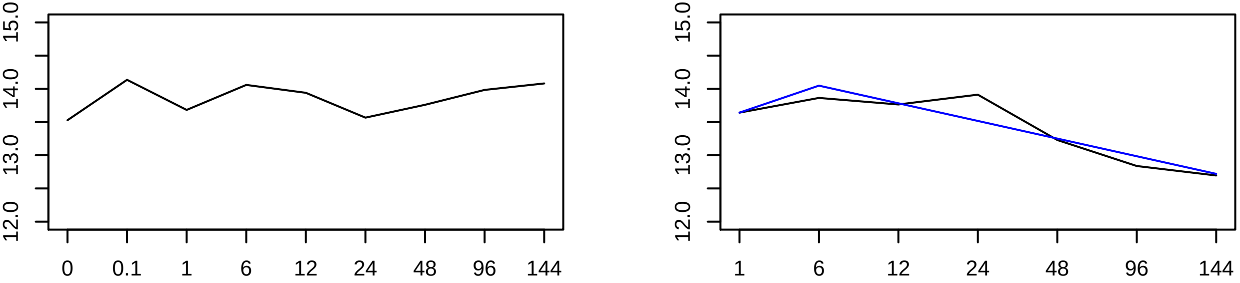

A_24_P317450 LOC202459 6q25.3

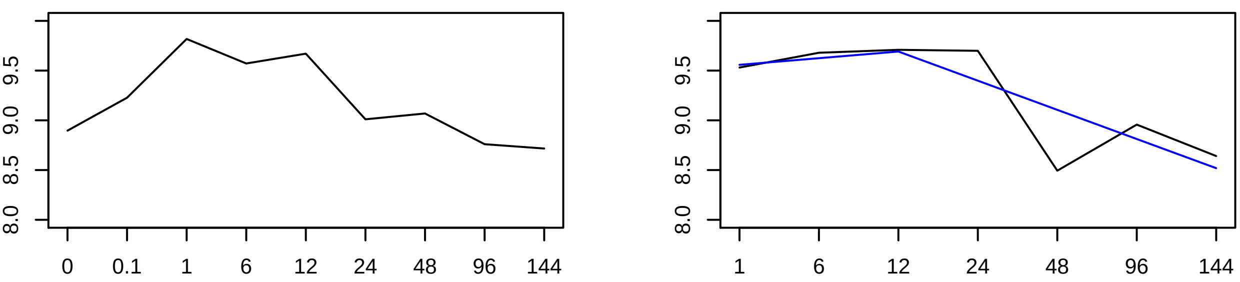

A_23_P62159 FAM120C Xp11.22

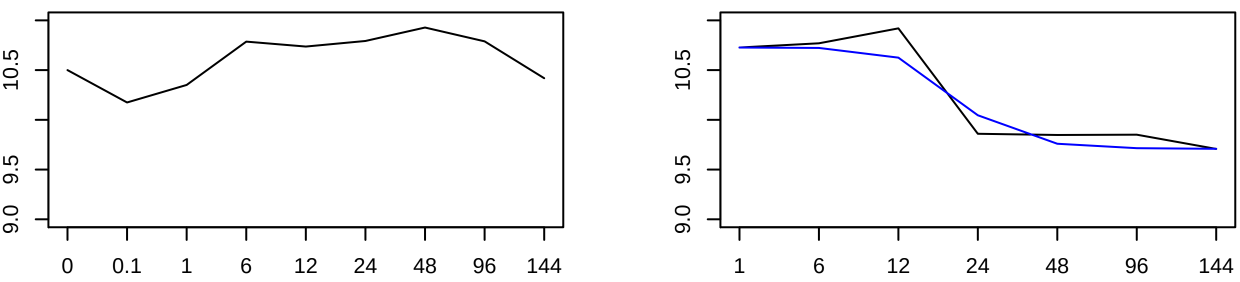

A_24_P921402 THC2484646 NA

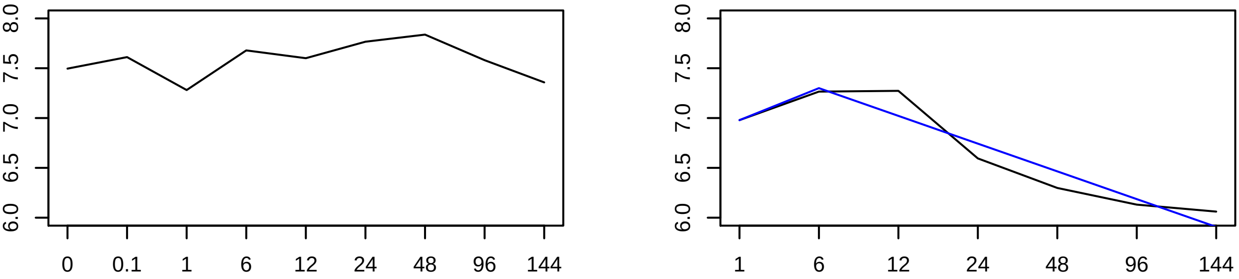

A_24_P144254 A_24_P144254 NA

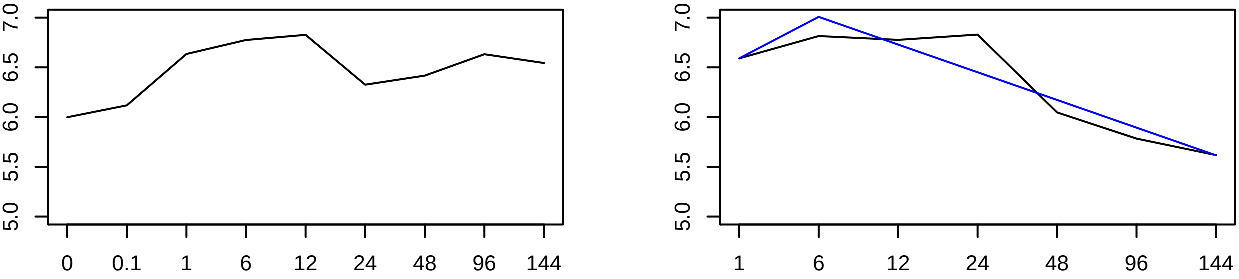

A_24_P341176 A_24_P341176 NA

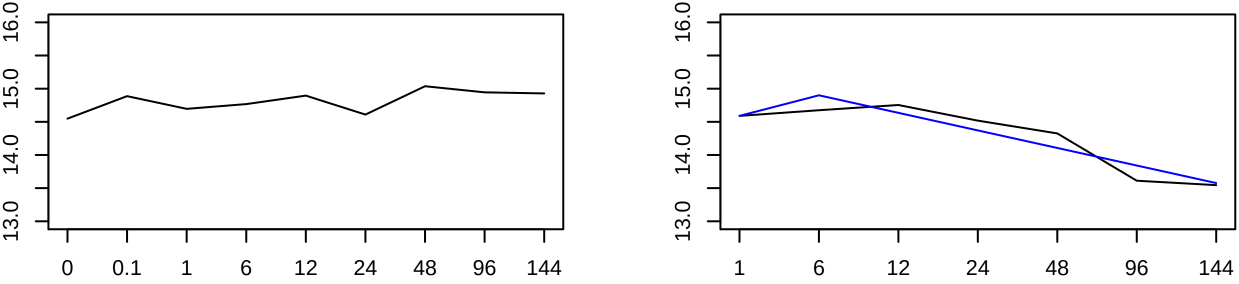

A_23_P45409 UPF3B Xq24

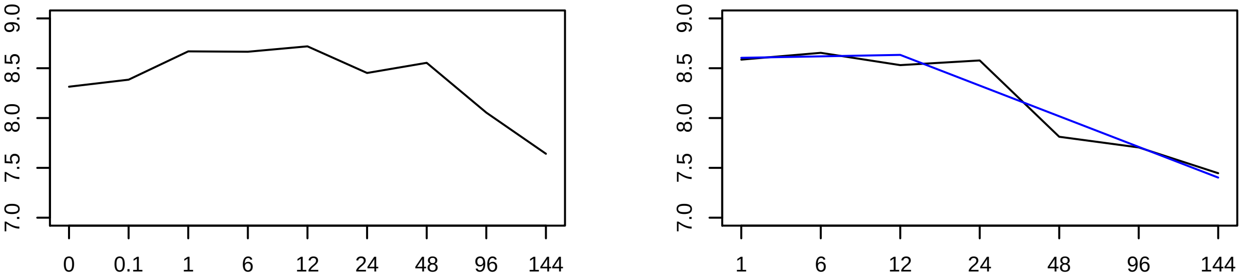

A_23_P87580 ANP32D 12q13.11

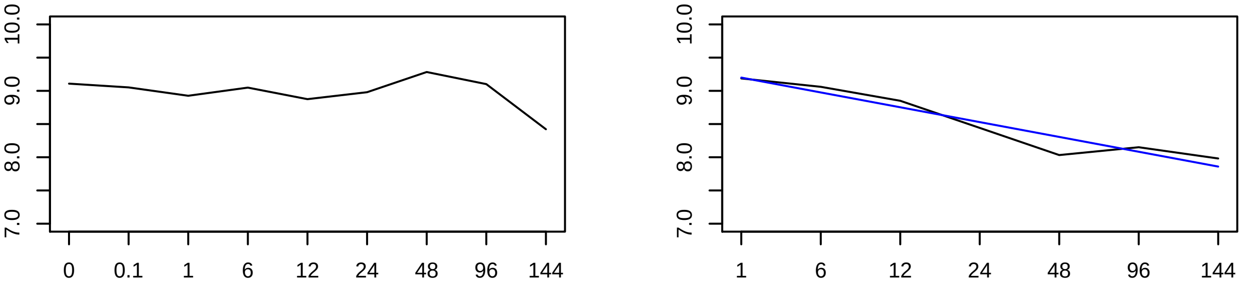

A_23_P412990 HRH3 20q13.33

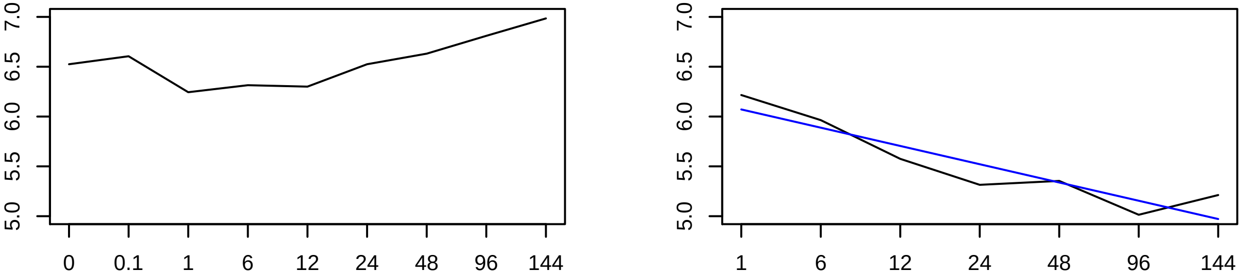

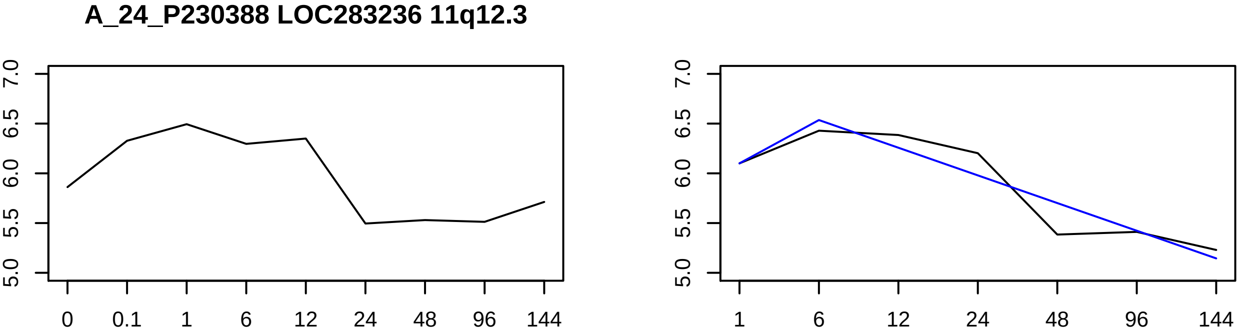
A_24_P230388 LOC283236 11q12.3
5.0
5.5
6.0
6.5
7.0
0
0.1
1
6
12
24
48
96
144
1
6
12
24
48
96
144

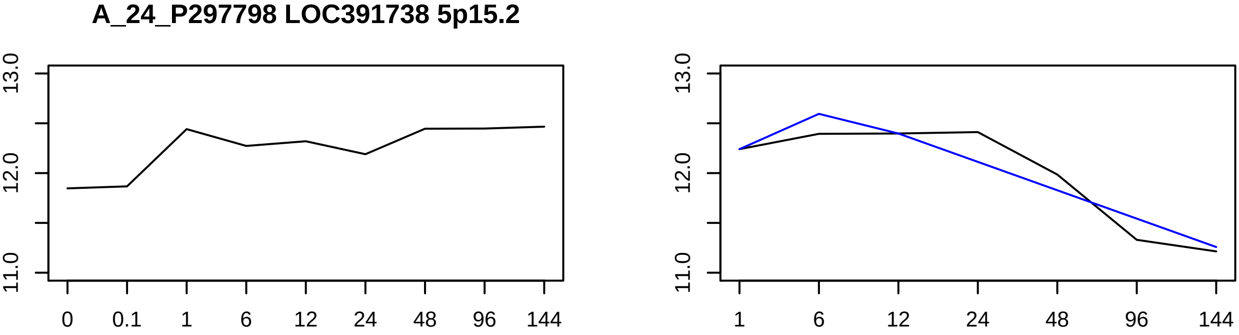
A_24_P297798 LOC391738 5p15.2
11.0
12.0
13.0
0
0.1
1
6
12
24
48
96
144
1
6
12
24
48
96
144

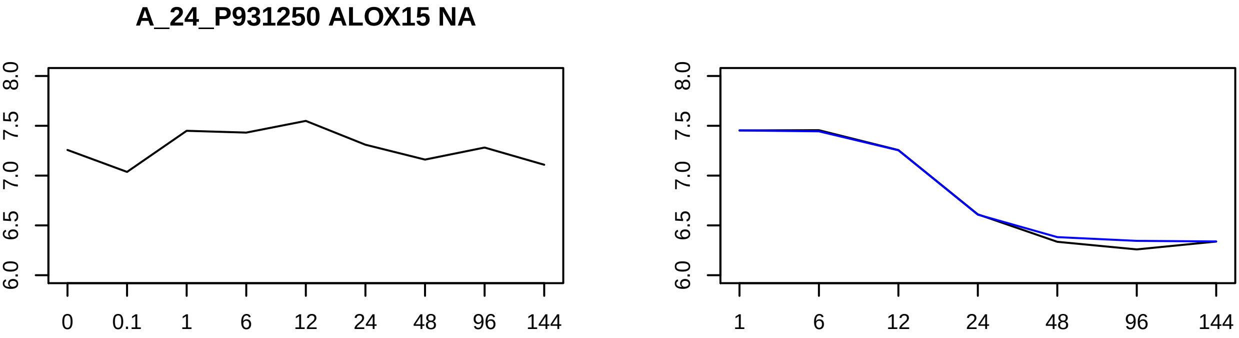
A_24_P931250 ALOX15 NA
6.0
6.5
7.0
7.5
8.0
0
0.1
1
6
12
24
48
96
144
1
6
12
24
48
96
144

A_24_P298928 AC000362.1 7q31.33

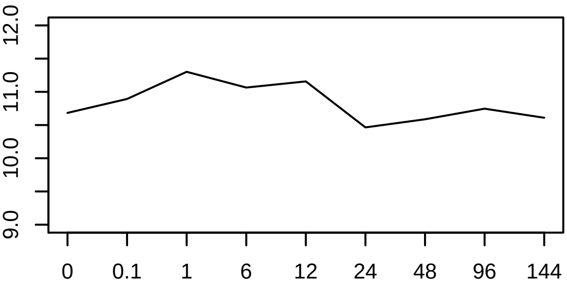

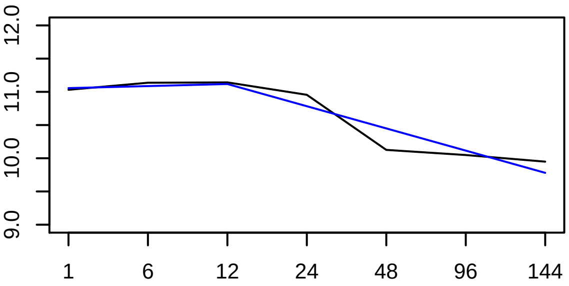

A_23_P33947 EFHC2 Xp11.3

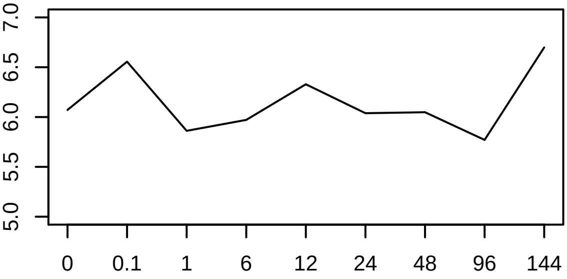

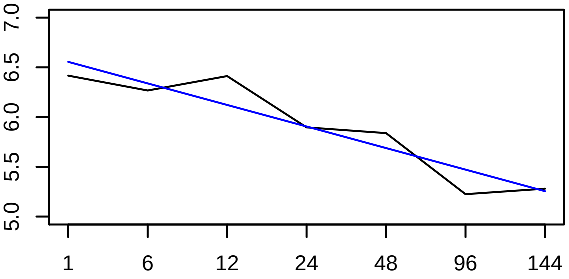

A_23_P216556 EPB41L4B 9q31.3

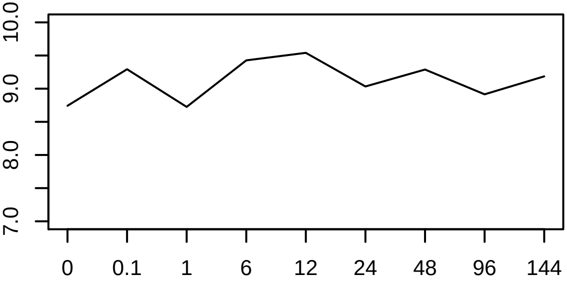

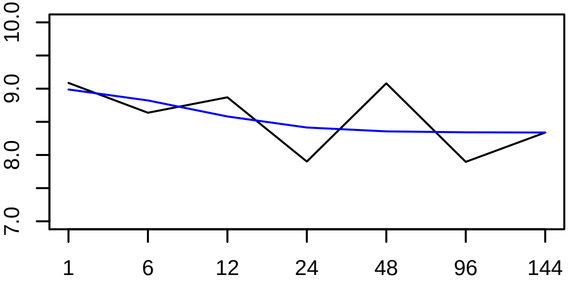

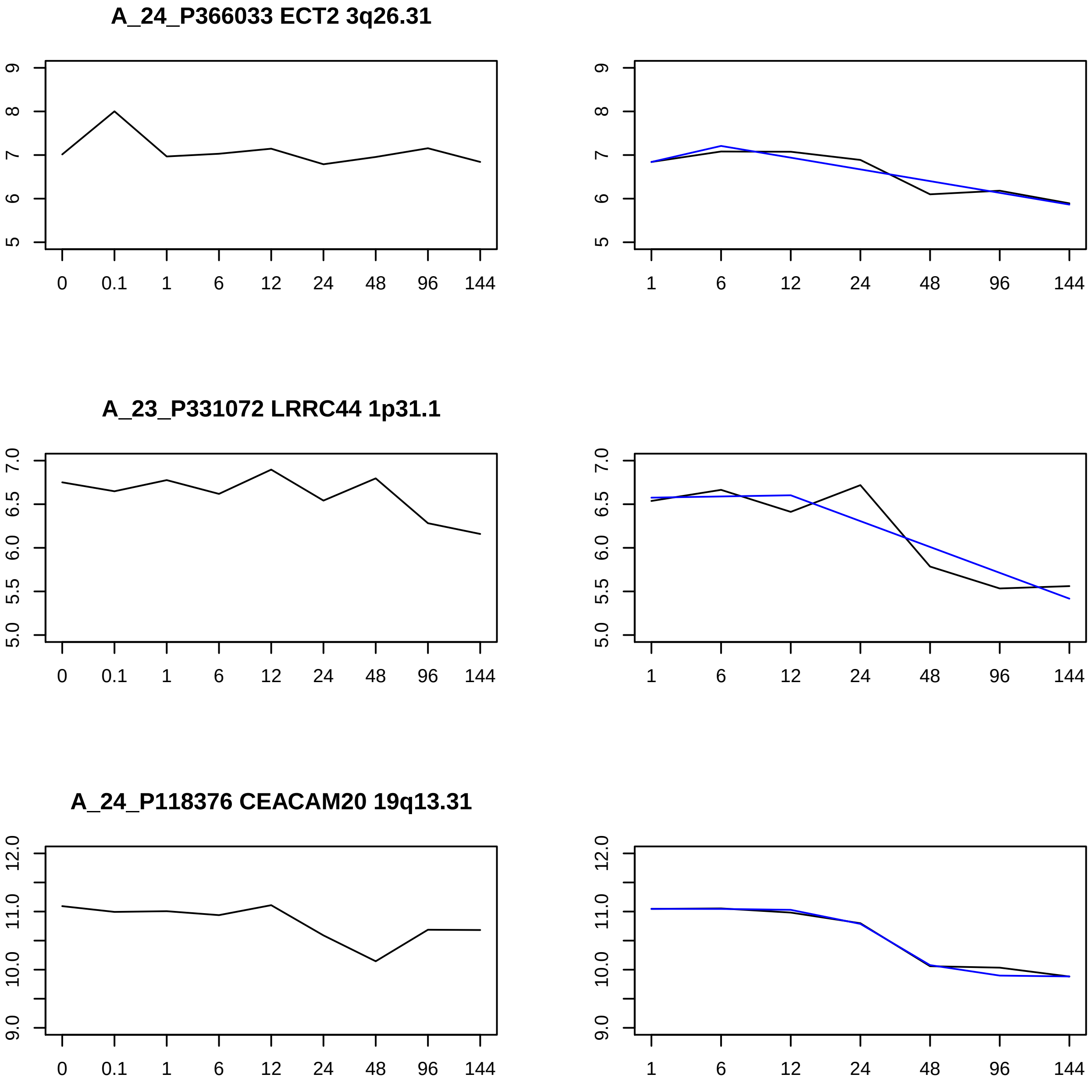

A_24_P366033 ECT2 3q26.31
A_23_P331072 LRRC44 1p31.1
A_24_P118376 CEACAM20 19q13.31
0 0.1 1 6 12 24 48 96 144
1 6 12 24 48 96 144
5 6 7 8 9
5.0 5.5 6.0 6.5 7.0
9.0 10.0 11.0 12.0

A_24_P325006 LOC494150 12q13.13

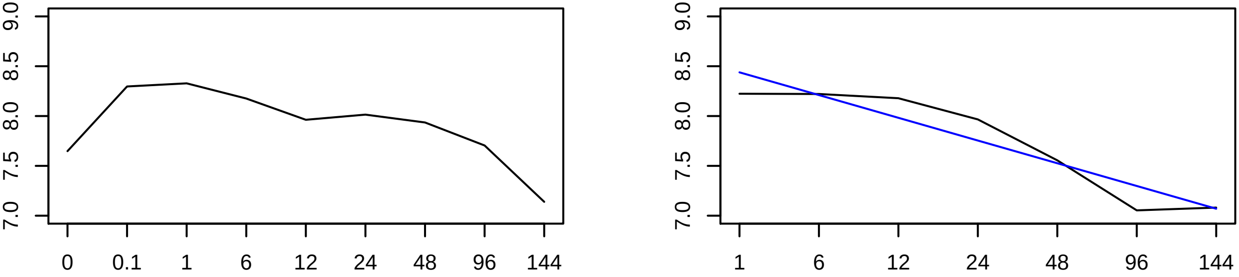

A_24_P418019 A_24_P418019 NA

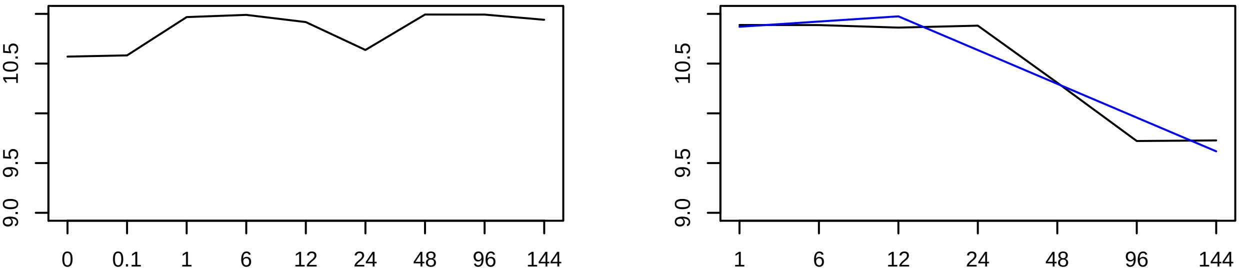

A_24_P277367 CXCL5 4q13.3

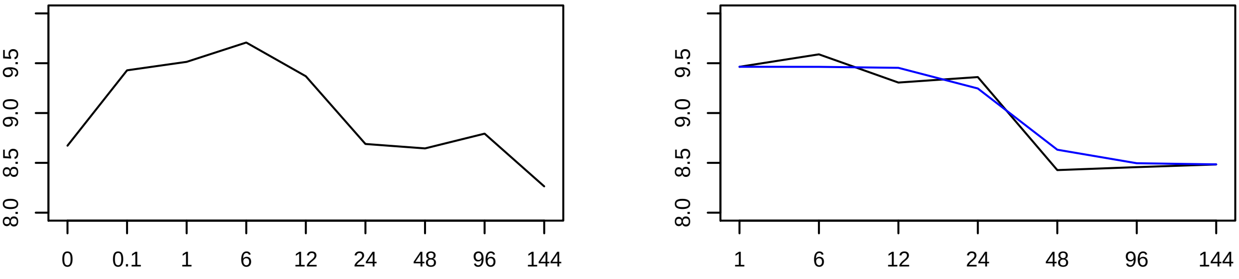

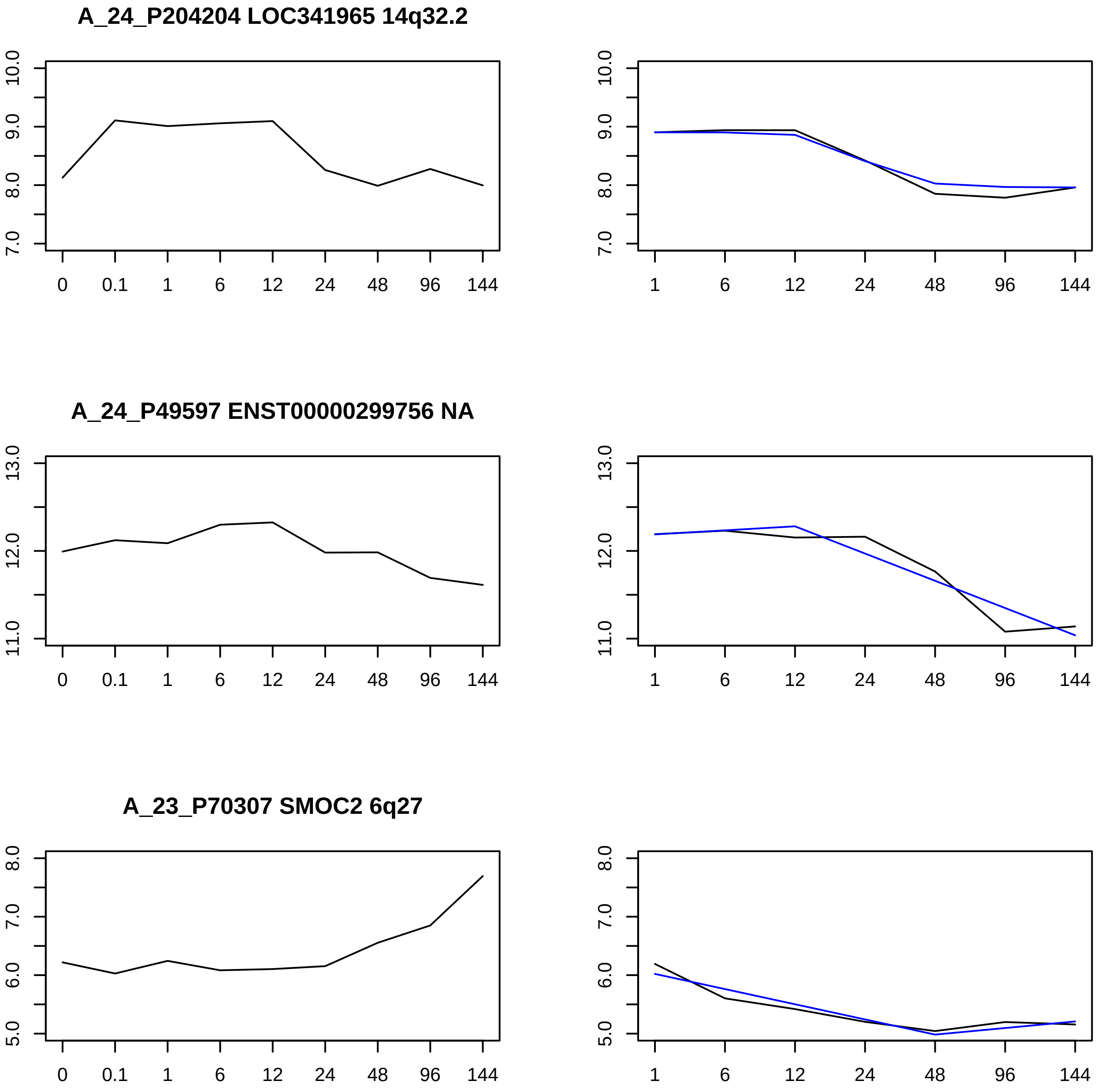
A_24_P204204 LOC341965 14q32.2
7.0
8.0
9.0
10.0
0
0.1
1
6
12
24
48
96
144
A_24_P49597 ENST00000299756 NA
11.0
12.0
13.0
A_23_P70307 SMOC2 6q27
5.0
6.0
7.0
8.0

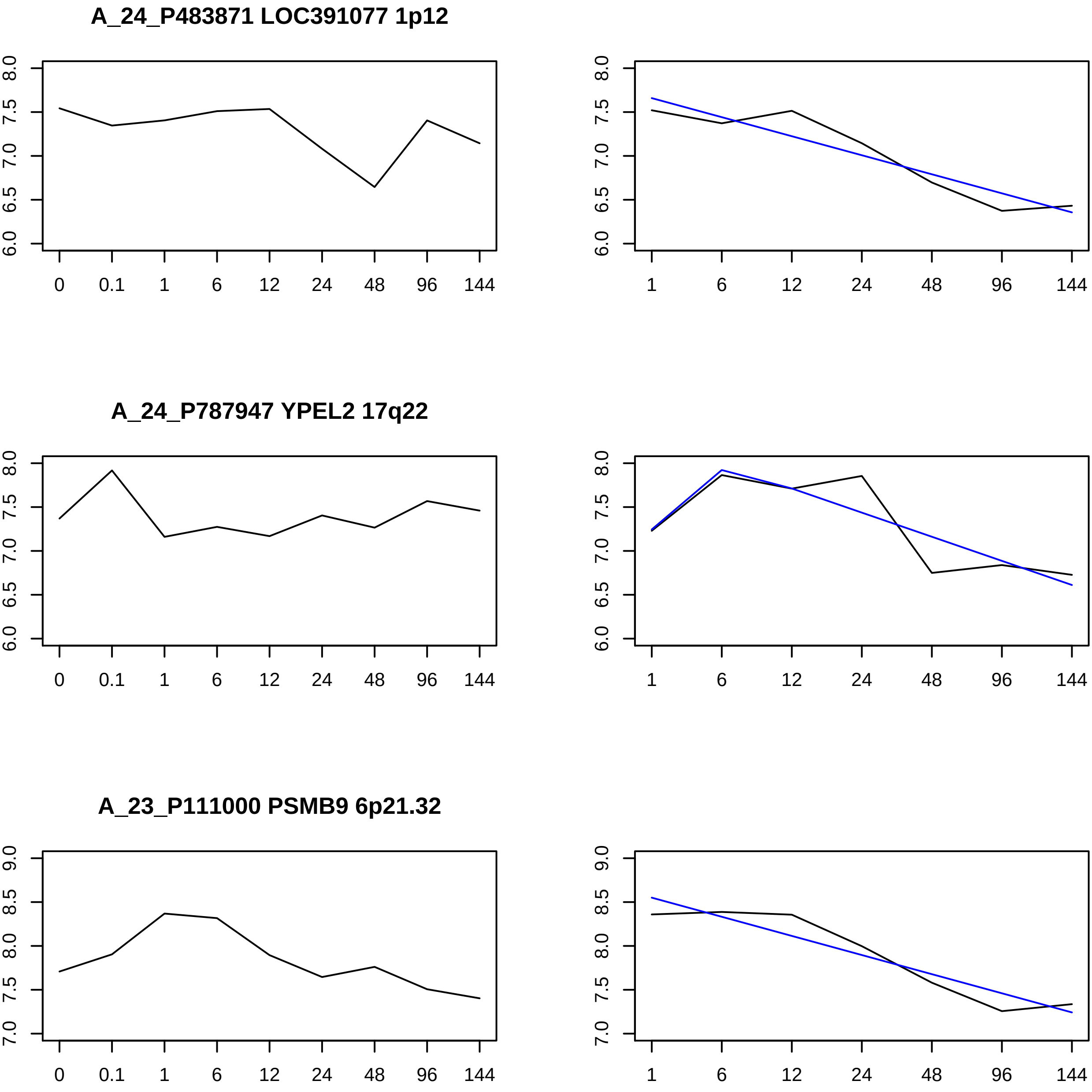
A_24_P483871 LOC391077 1p12
A_24_P787947 YPEL2 17q22
A_23_P111000 PSMB9 6p21.32
6.0 6.5 7.0 7.5 8.0
7.0 7.5 8.0 8.5 9.0
0 0.1 1 6 12 24 48 96 144
1 6 12 24 48 96 144

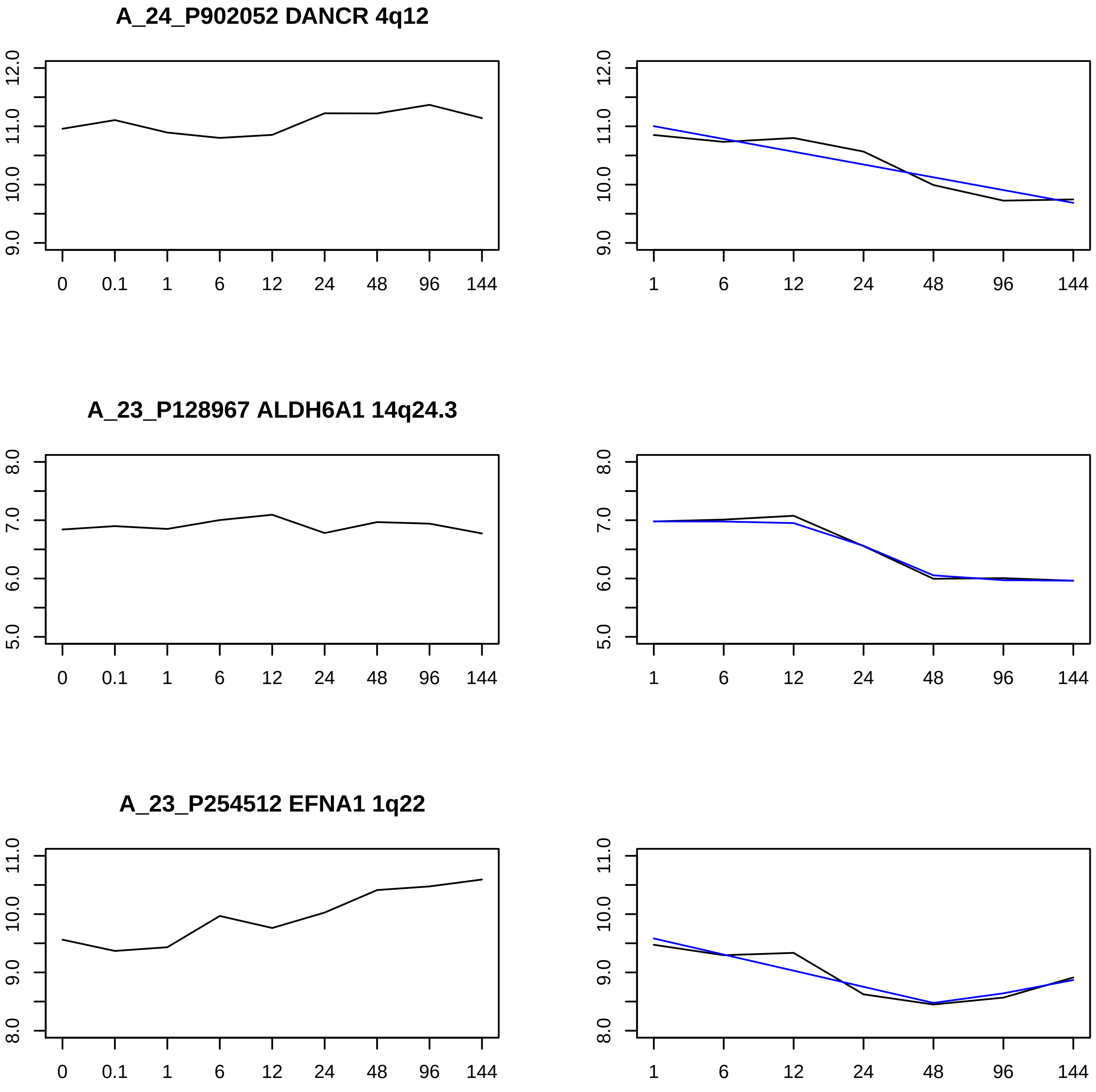
A_24_P902052 DANCR 4q12
9.0
10.0
11.0
12.0
0
0.1
1
6
12
24
48
96
144
1
6
12
24
48
96
144
A_23_P128967 ALDH6A1 14q24.3
5.0
6.0
7.0
8.0
A_23_P254512 EFNA1 1q22
8.0
9.0
10.0
11.0
