# Supplementary material for: SwitchFinder – a novel method and query facility for discovering dynamic gene expression patterns
Source: BMC Bioinformatics. 2016 Dec 15;17:532. doi: 10.1186/s12859-016-1391-0 (PMC5160026; doi:10.1186/s12859-016-1391-0)

**A_24_P119745 FN1 2q35**

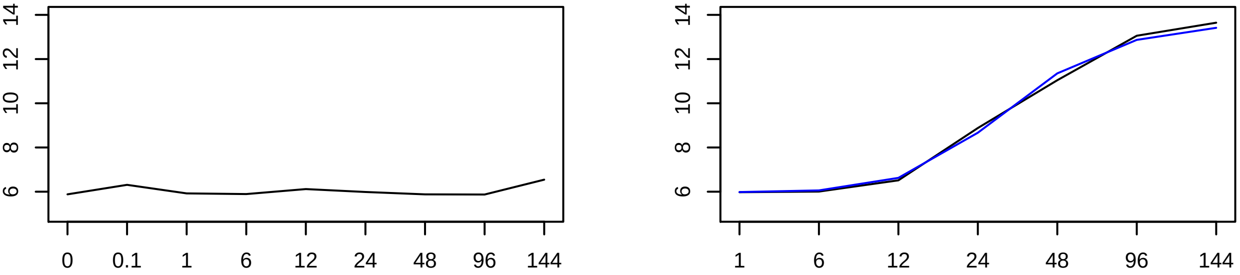


**A_23_P31064 MOXD1 6q23.2**

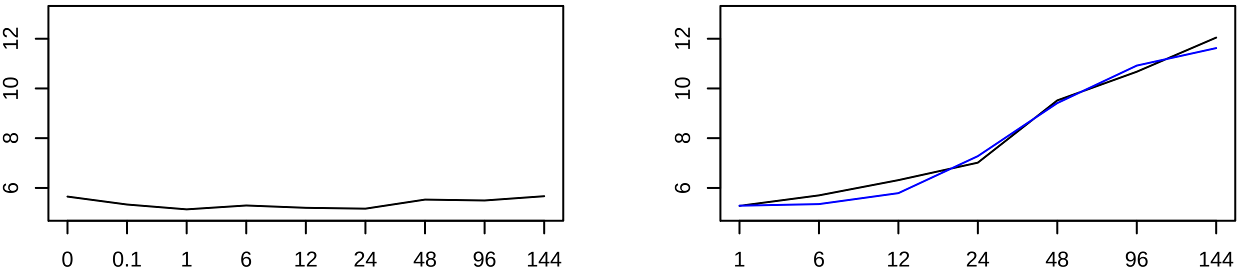


**A_23_P149121 DIRAS3 1p31.3**

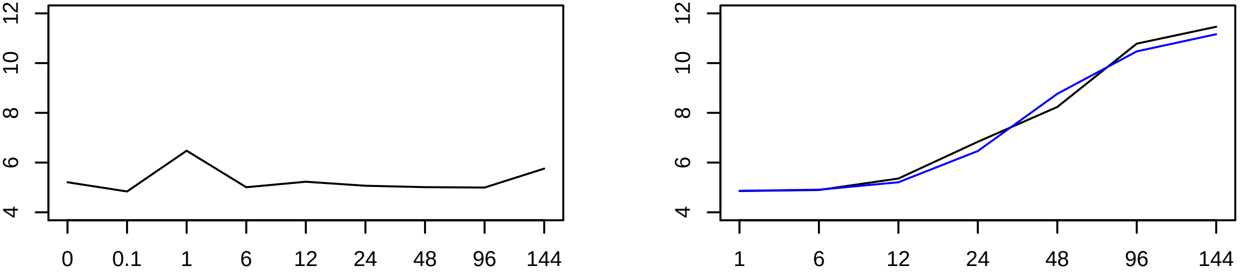

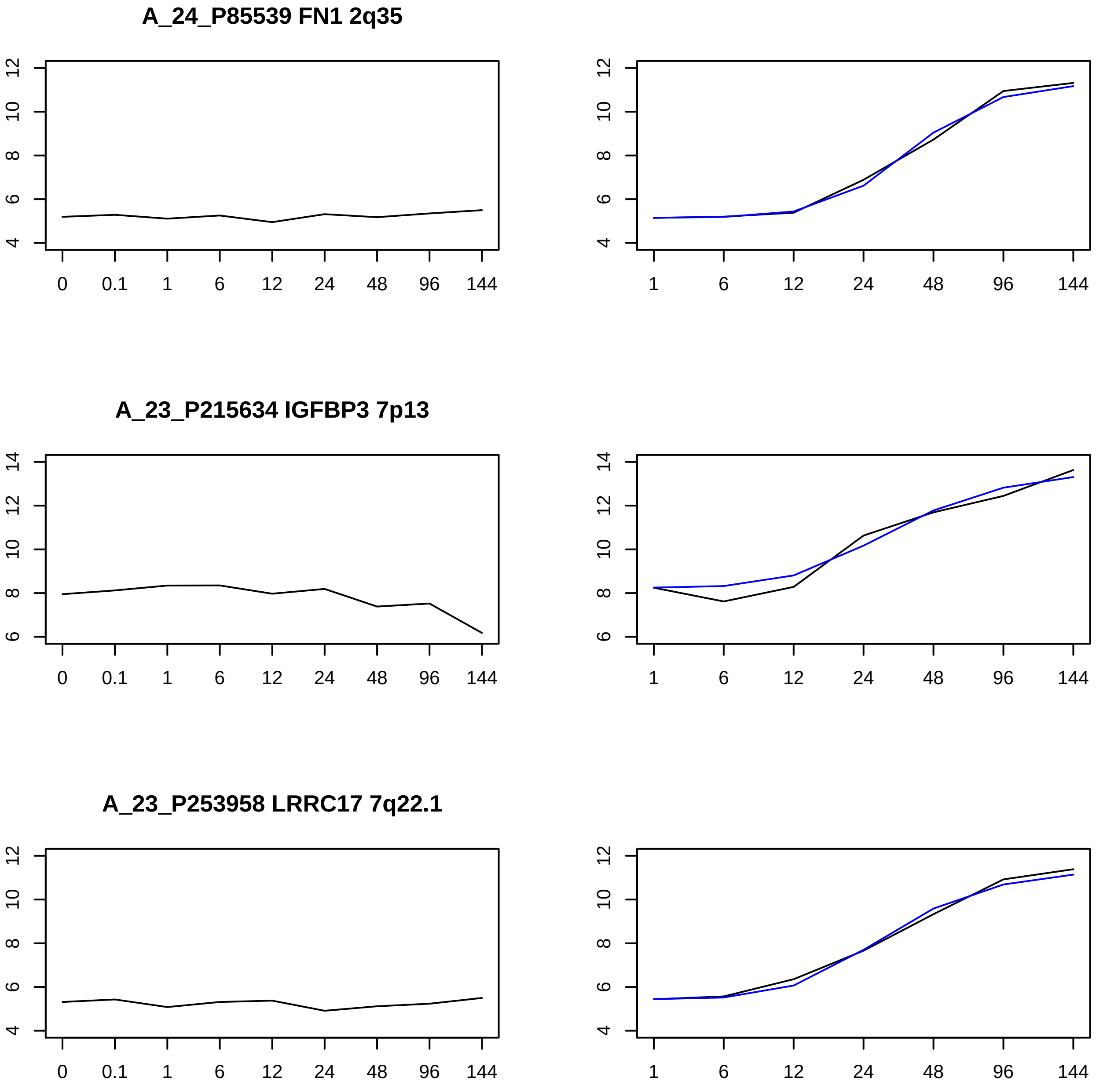
A_24_P85539 FN1 2q35
A_23_P215634 IGFBP3 7p13
A_23_P253958 LRRC17 7q22.1
0
0.1
1
6
12
24
48
96
144

**A_24_P109661 LOC121054 12q23.3**

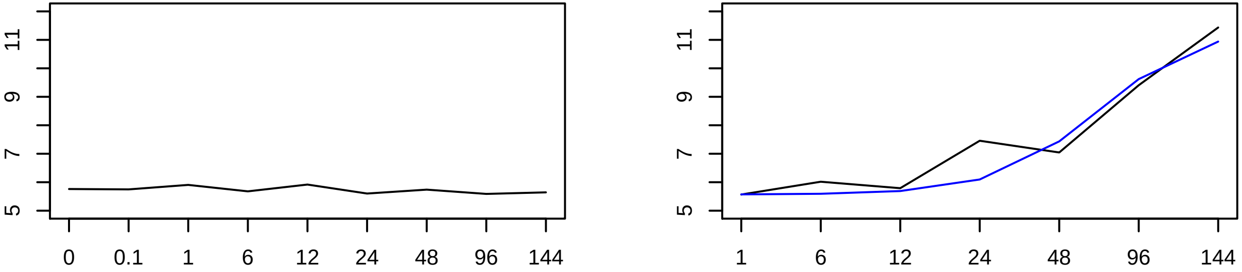


**A_23_P122924 INHBA 7p14.1**

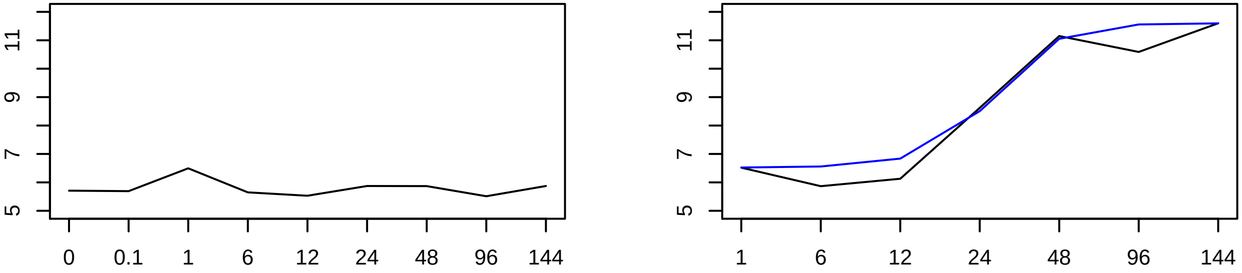


**A_23_P207520 COL1A1 17q21.33**

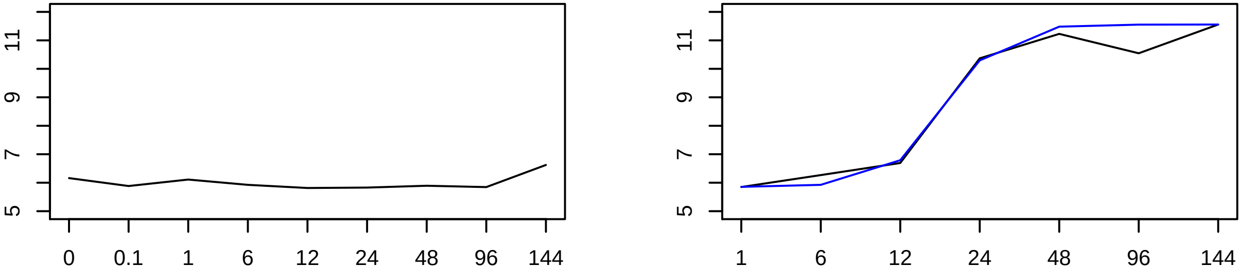

A_23_P1331 COL13A1 10q22.1

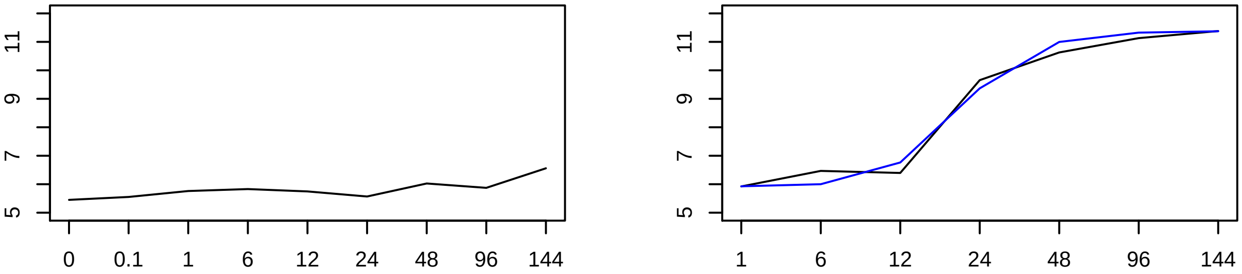


A_23_P337934 FBLIM1 1p36.21

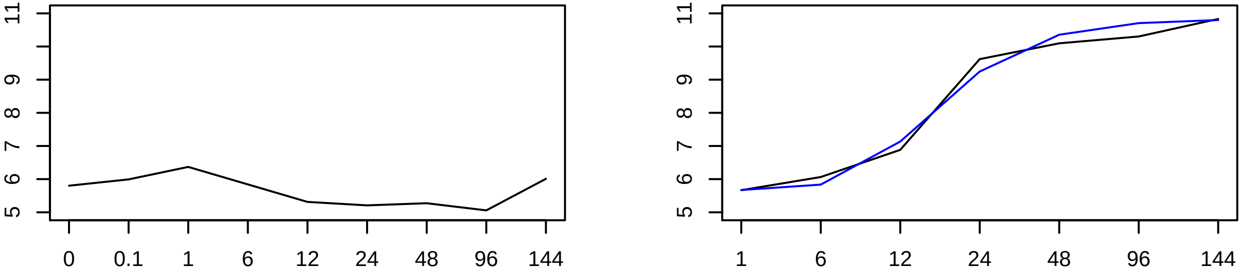


A_23_P49338 TNFRSF12A 16p13.3

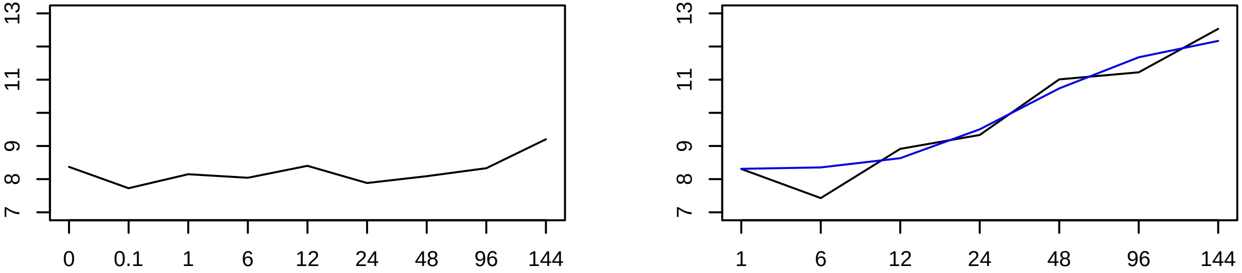

**A_32_P65473 KIAA1244 NA**

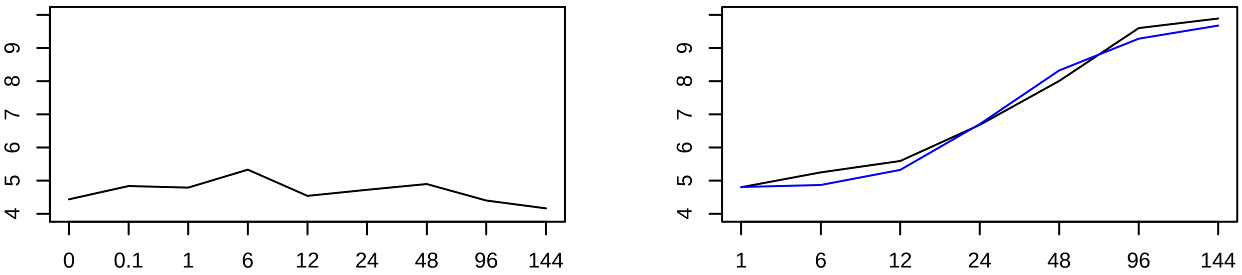


**A_23_P217866 IFI16 1q23.1**

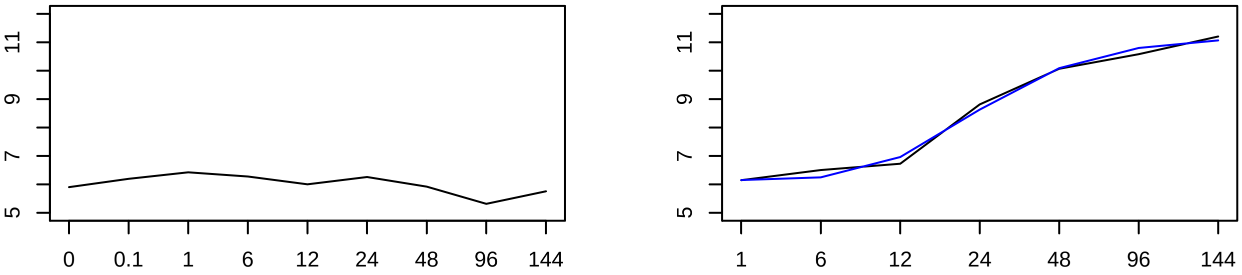


**A_23_P148249 THSD4 15q23**

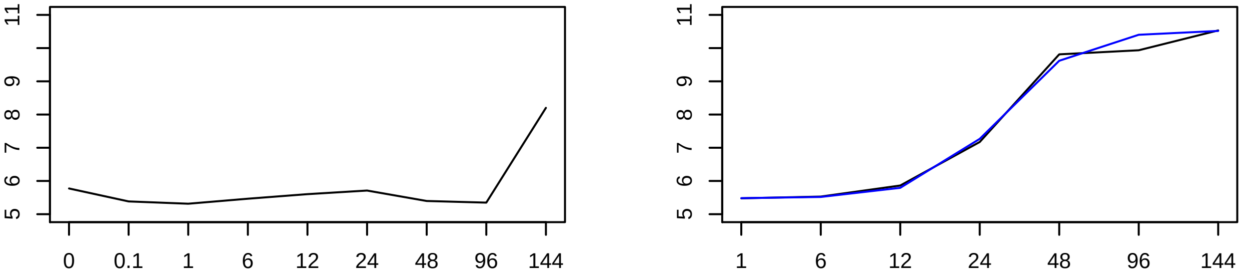

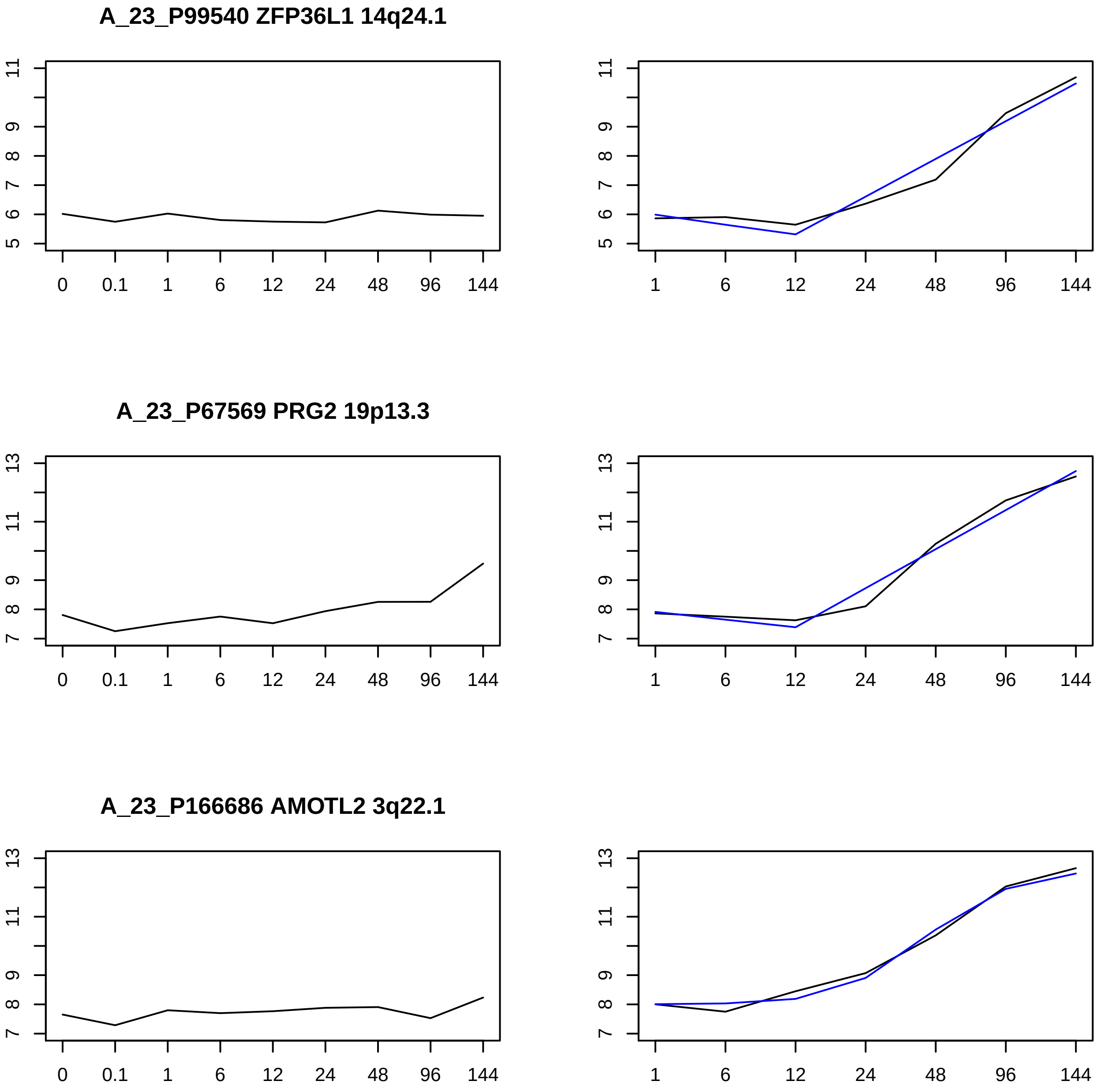
A_23_P99540 ZFP36L1 14q24.1
5
6
7
8
9
11
0
0.1
1
6
12
24
48
96
144
1
6
12
24
48
96
144
A_23_P67569 PRG2 19p13.3
7
8
9
11
13
A_23_P166686 AMOTL2 3q22.1
7
8
9
11
13

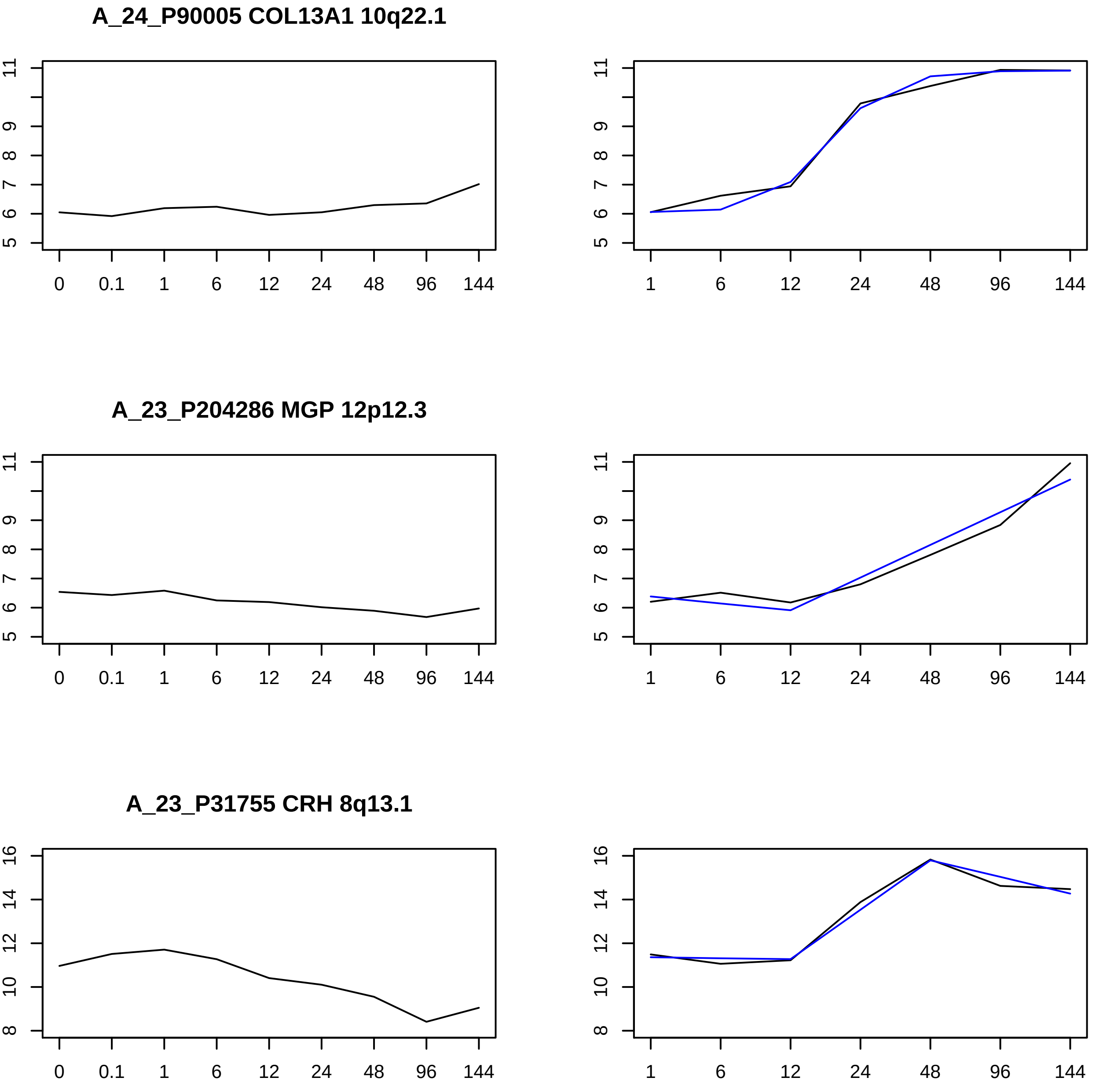
A_24_P90005 COL13A1 10q22.1
A_23_P204286 MGP 12p12.3
A_23_P31755 CRH 8q13.1
5 6 7 8 9 11
8 10 12 14 16
0 0.1 1 6 12 24 48 96 144
1 6 12 24 48 96 144

**A_23_P58082 CCDC80 3q13.2**

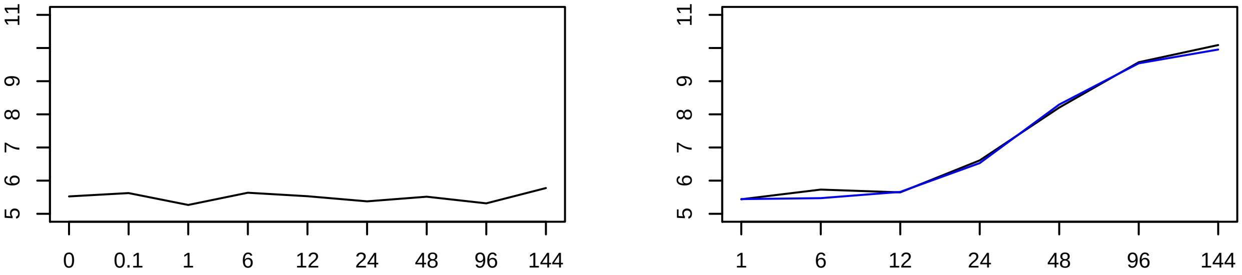


**A_32_P85360 THC2770932 NA**

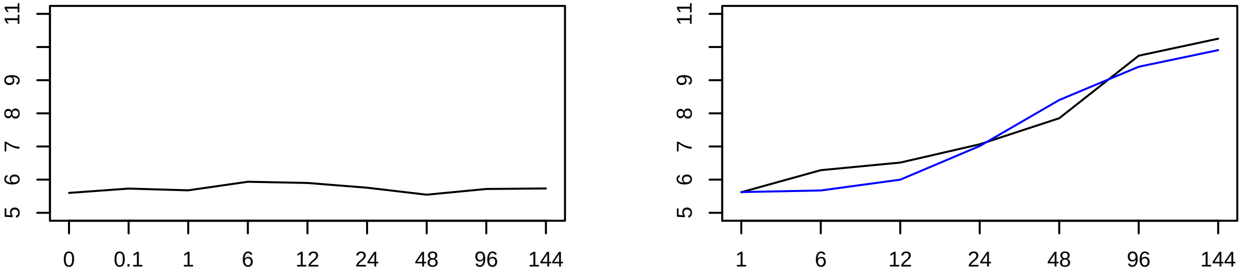


**A_23_P64792 KCNMB4 12q15**

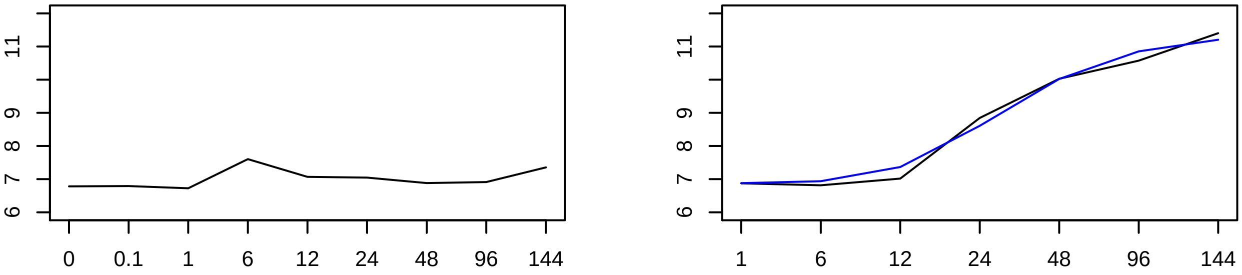

**A_23_P144476 SPRY1 4q28.1**

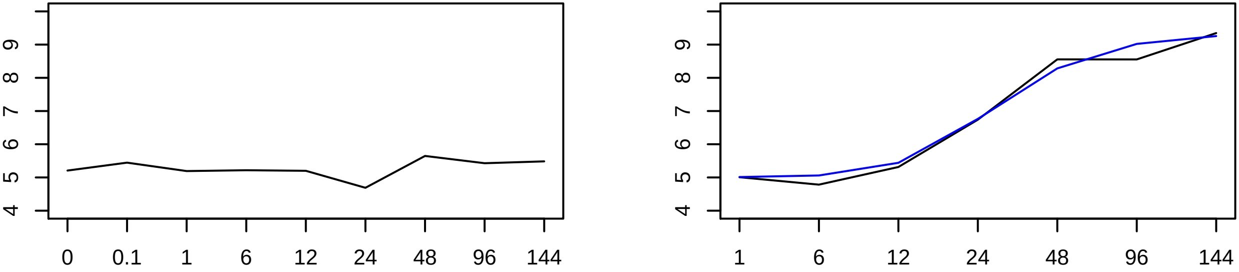


**A_23_P31739 STMN4 8p21.2**

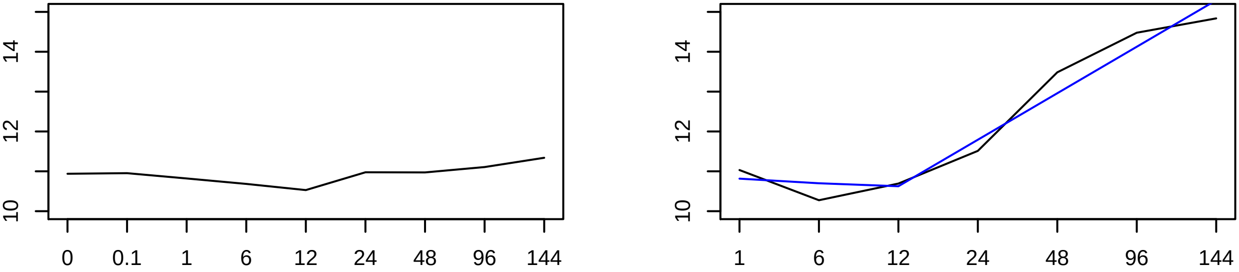


**A_23_P348257 NUAK1 12q23.3**

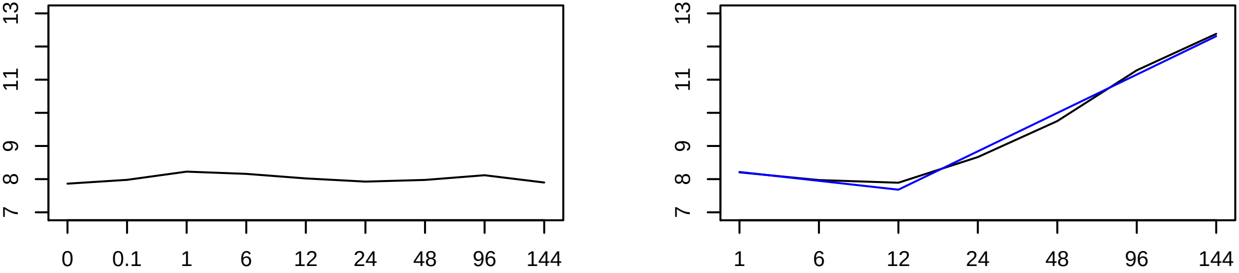

A_32_P941771

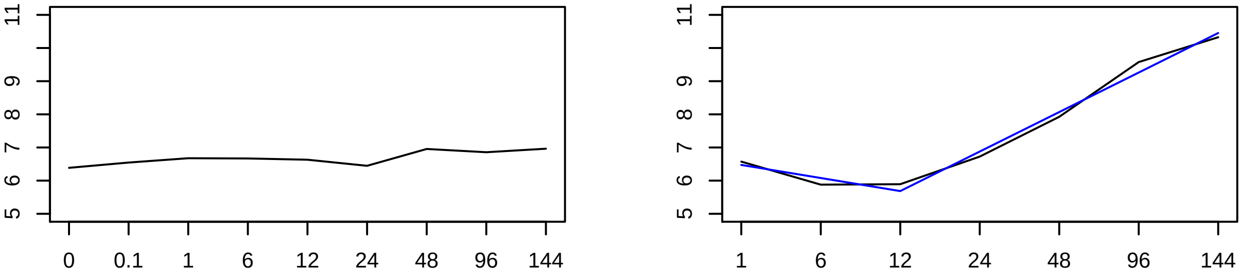


A_23_P39364 HOMER3 19p13.11

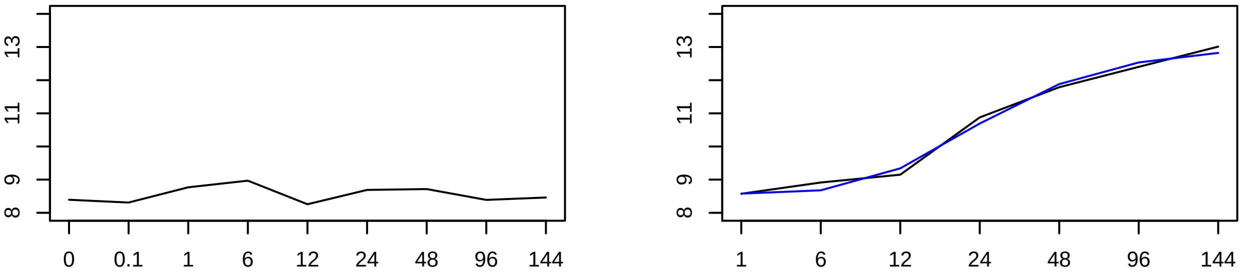


A_24_P358406 A_24_P358406 NA

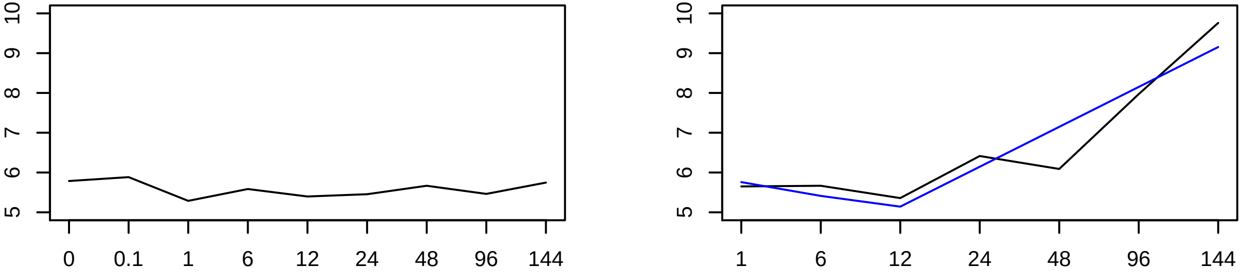

**A_23_P36531 TSPAN8 12q21.1**

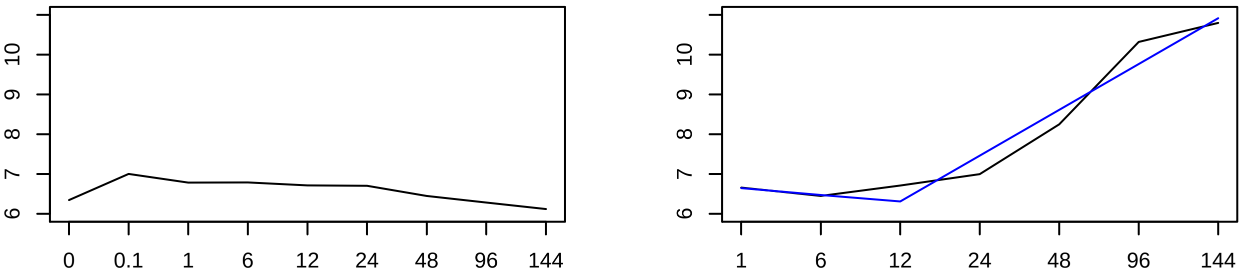


**A_32_P72351 AK026140 NA**

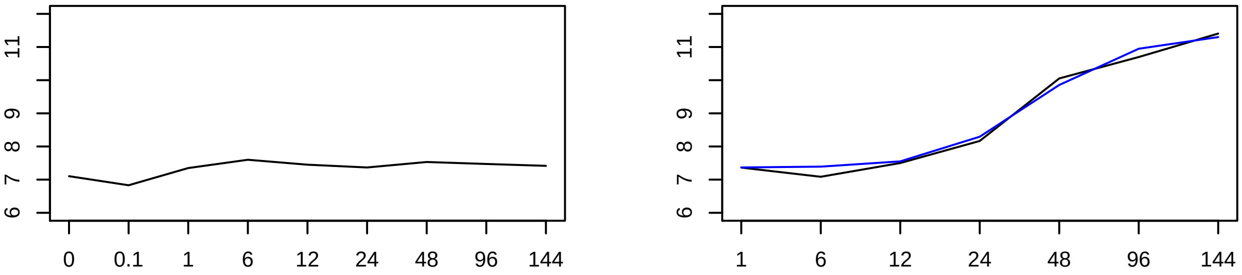


**A_23_P426021 KIAA0746 4p15.2**

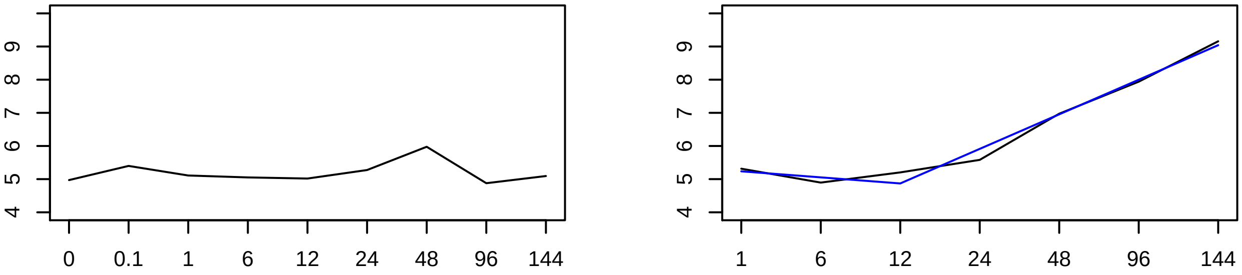

**A_23_P161563 RAB38 11q14.2**

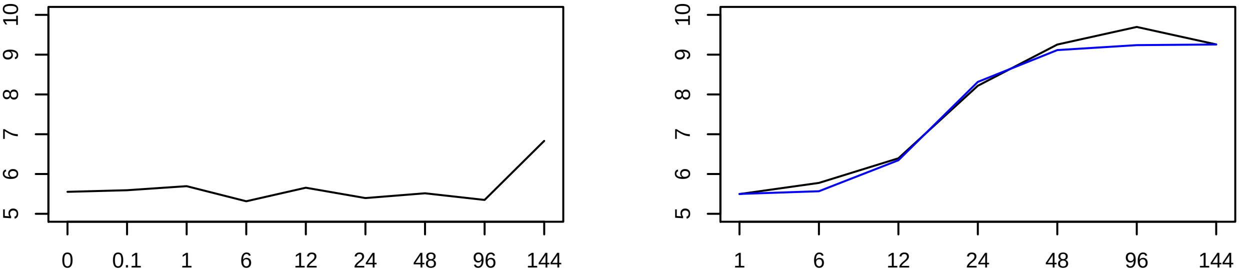


**A_23_P137984 S100A10 1q21.3**

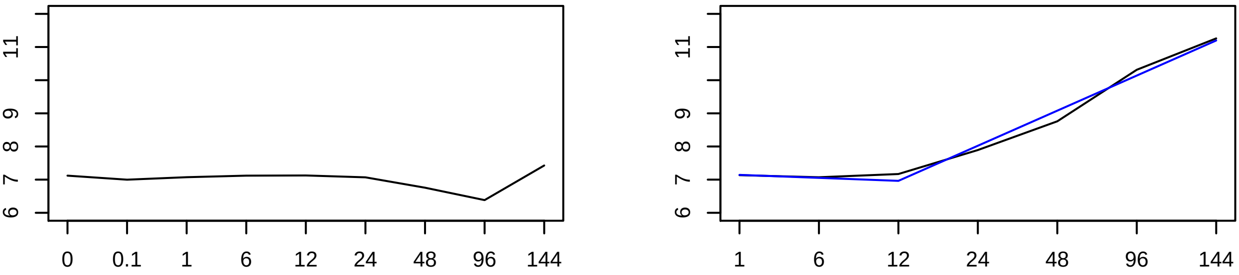


**A_23_P321501 DHRS2 14q11.2**

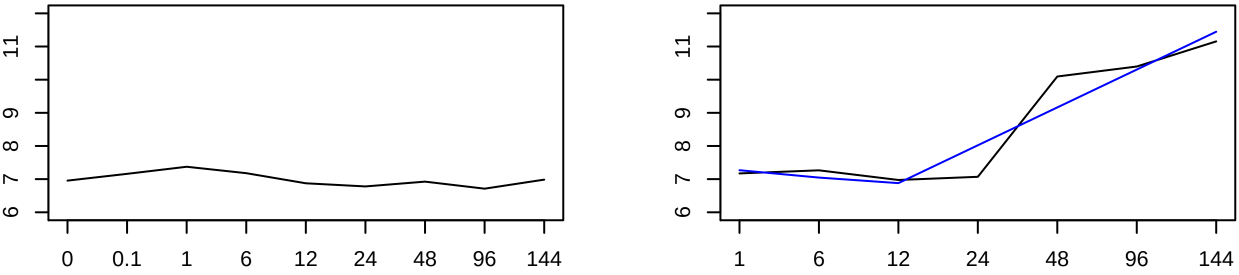

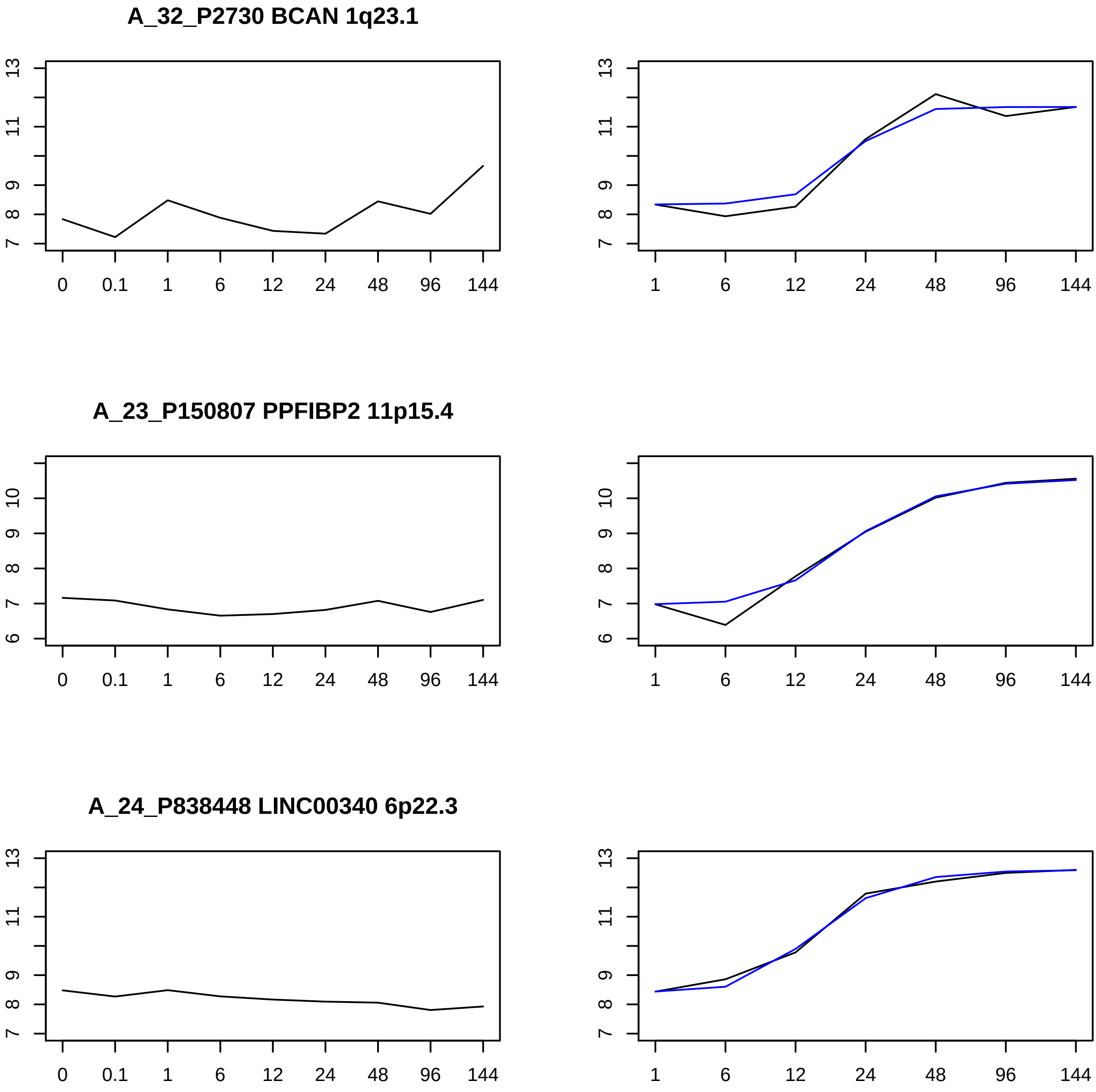
A_32_P2730 BCAN 1q23.1
A_23_P150807 PPFIBP2 11p15.4
A_24_P838448 LINC00340 6p22.3
0 0.1 1 6 12 24 48 96 144
1 6 12 24 48 96 144
7 8 9 11 13
6 7 8 9 10

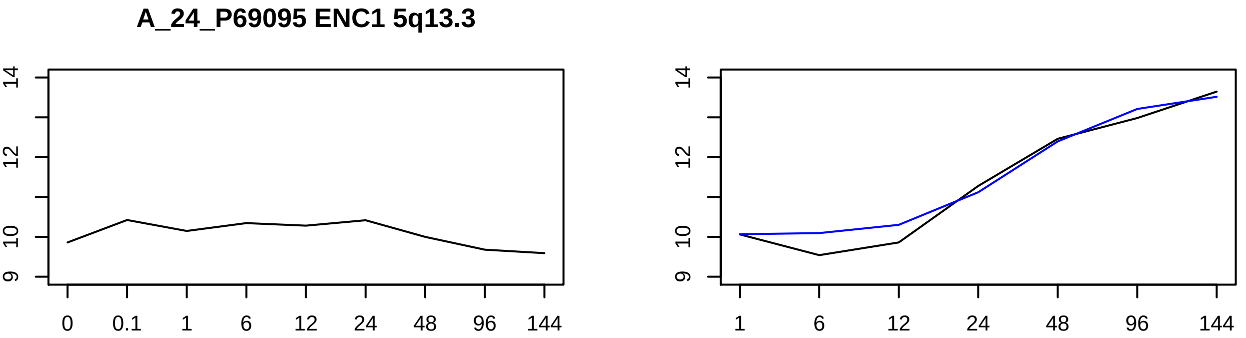
A_24_P69095 ENC1 5q13.3
9
10
12
14
0
0.1
1
6
12
24
48
96
144

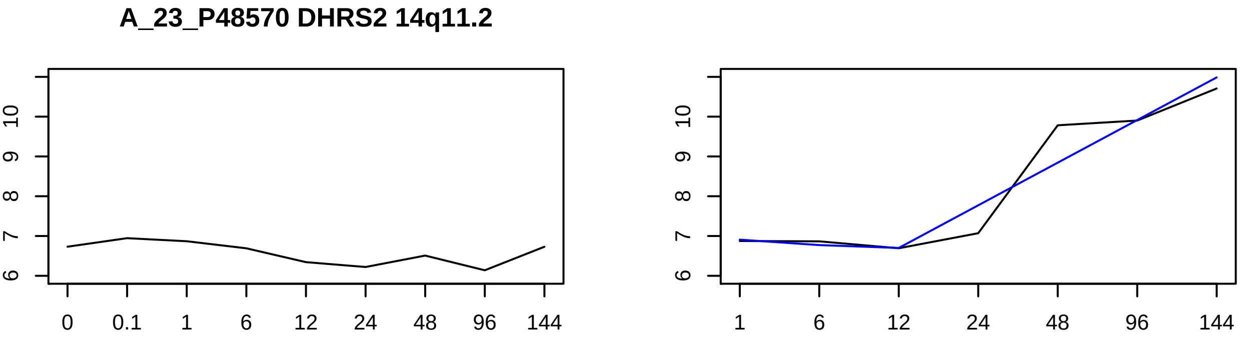
A_23_P48570 DHRS2 14q11.2
6
7
8
9
10
0
0.1
1
6
12
24
48
96
144

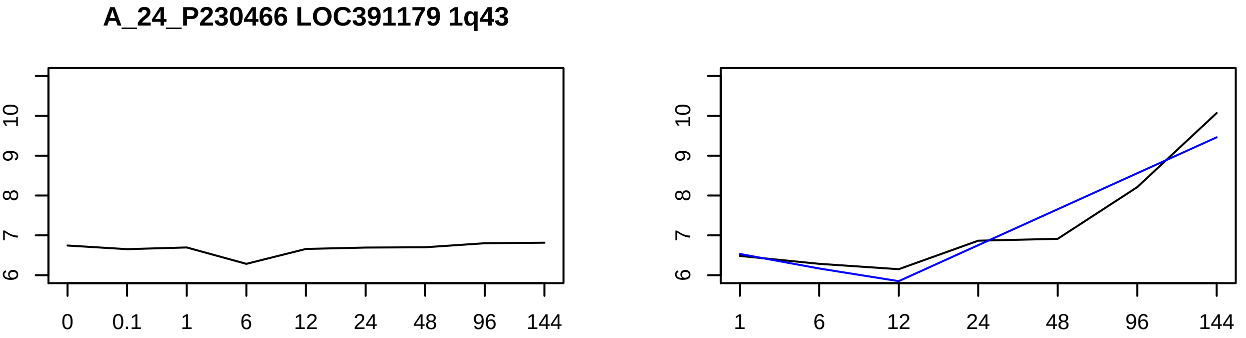
A_24_P230466 LOC391179 1q43
6
7
8
9
10
0
0.1
1
6
12
24
48
96
144

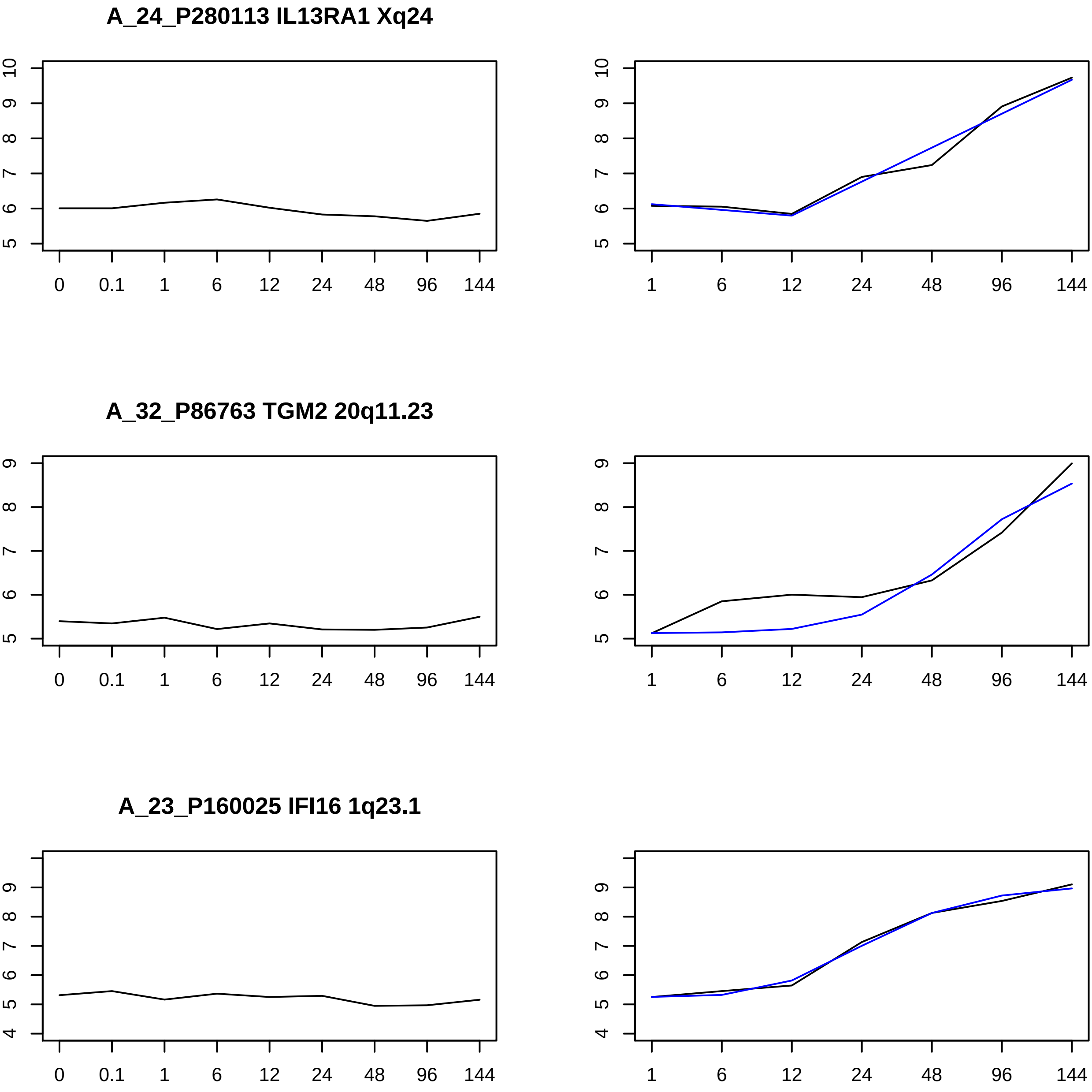
A_24_P280113 IL13RA1 Xq24
A_32_P86763 TGM2 20q11.23
A_23_P160025 IFI16 1q23.1
0 0.1 1 6 12 24 48 96 144
1 6 12 24 48 96 144

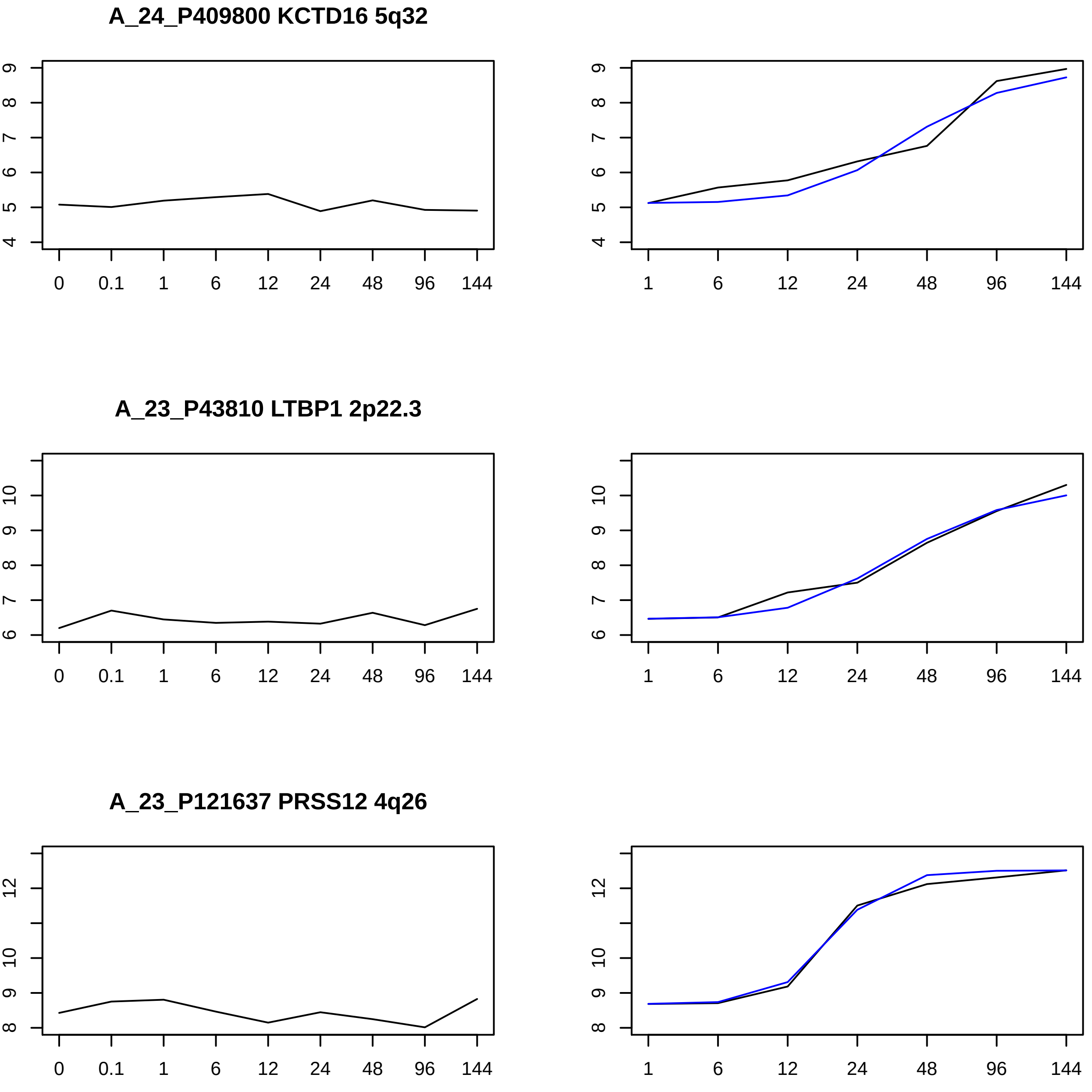
A_24_P409800 KCTD16 5q32
4 5 6 7 8 9
0 0.1 1 6 12 24 48 96 144
1 6 12 24 48 96 144
A_23_P43810 LTBP1 2p22.3
6 7 8 9 10
0 0.1 1 6 12 24 48 96 144
1 6 12 24 48 96 144
A_23_P121637 PRSS12 4q26
8 9 10 12
0 0.1 1 6 12 24 48 96 144
1 6 12 24 48 96 144

**A_23_P27035 TMEM98 17q11.2**

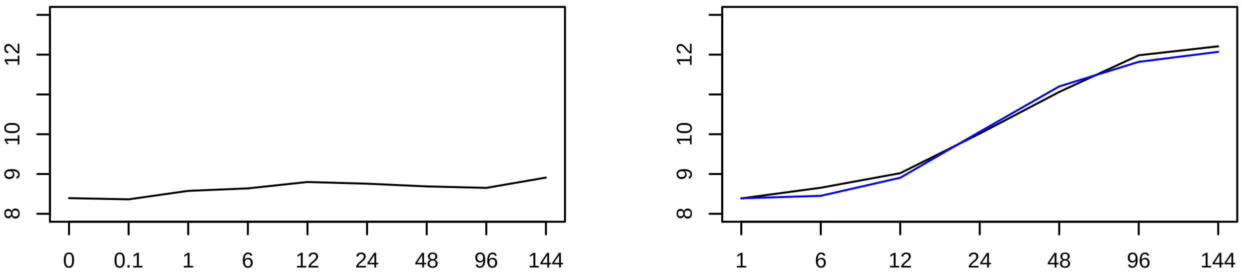


**A_23_P142533 COL3A1 2q32.2**

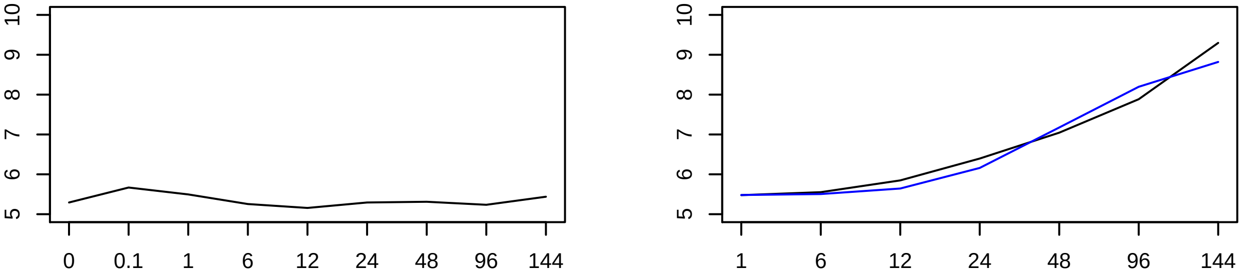


**A_23_P19650 VIP 6q25.2**

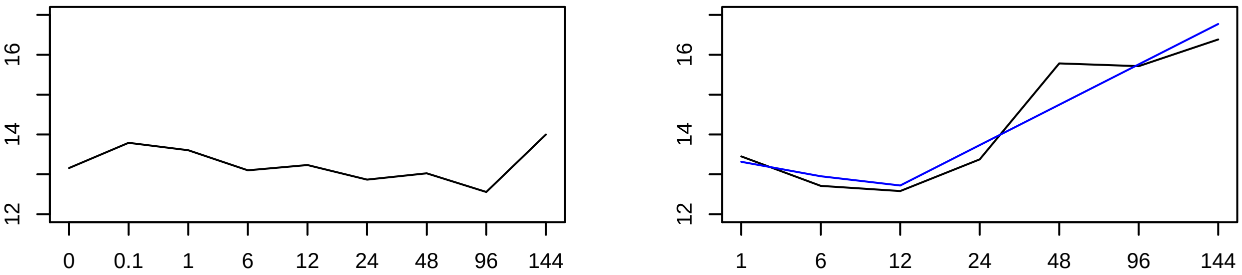

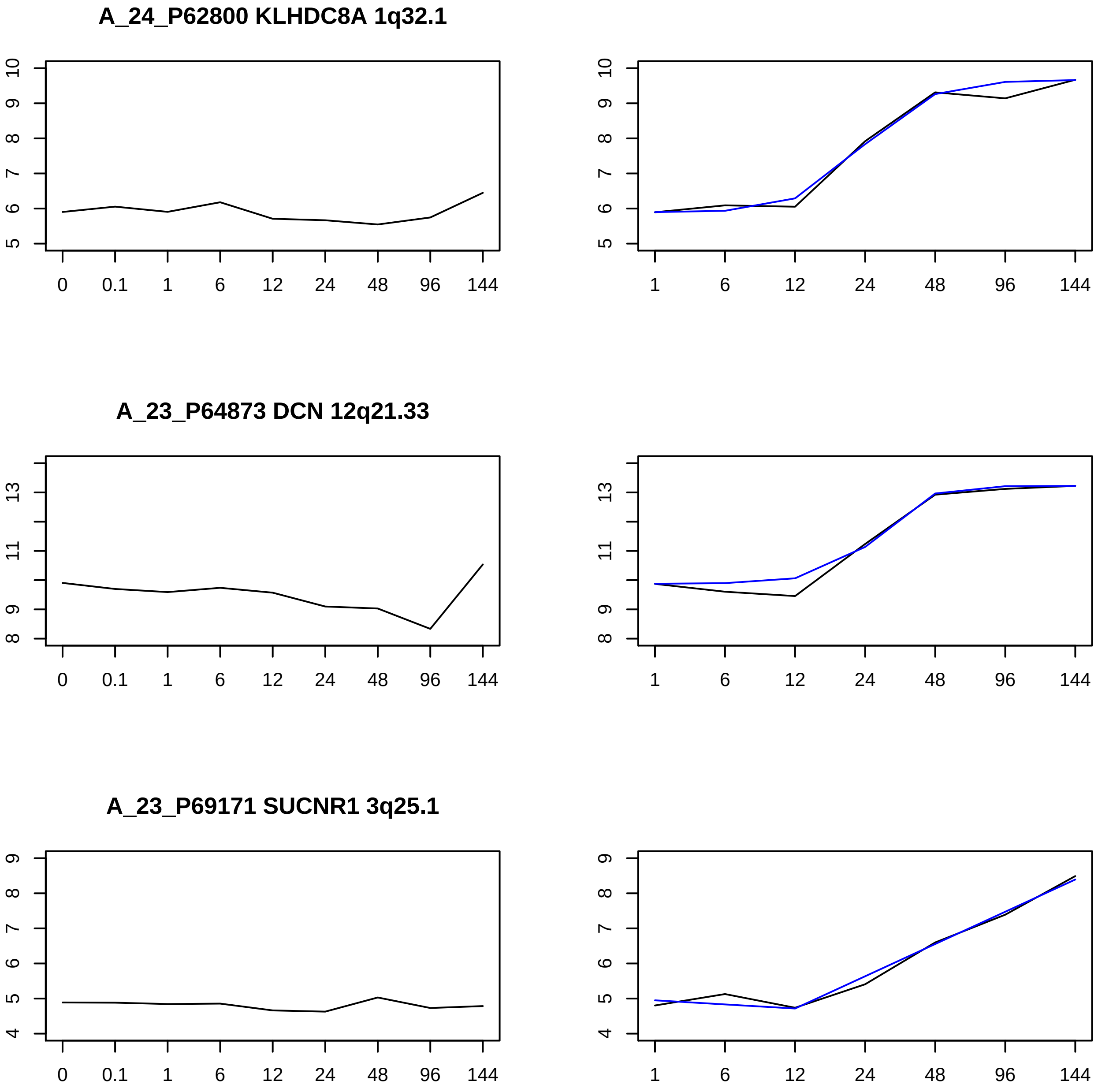
A_24_P62800 KLHDC8A 1q32.1
A_23_P64873 DCN 12q21.33
A_23_P69171 SUCNR1 3q25.1
0 0.1 1 6 12 24 48 96 144
1 6 12 24 48 96 144

**A_23_P213424 ENC1 5q13.3**

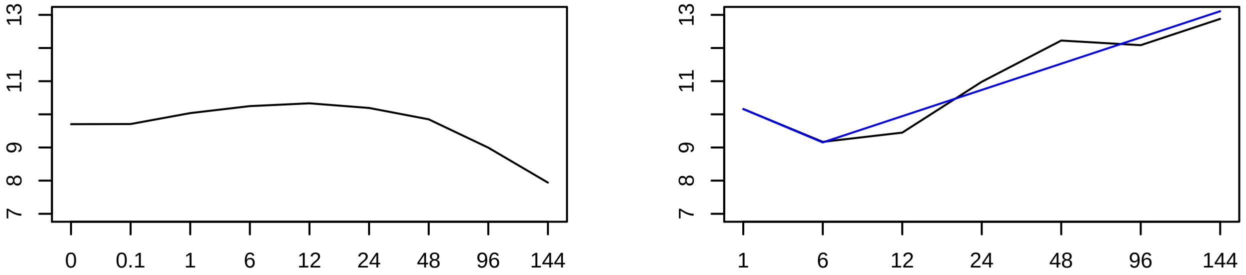


**A_23_P74799 SLC25A24 1p13.3**

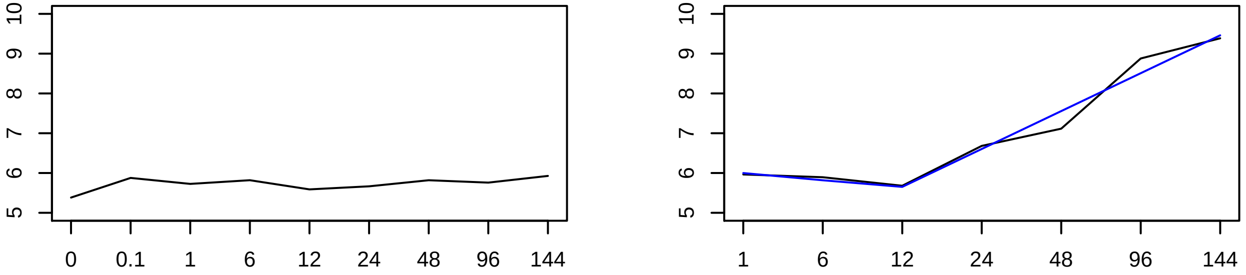


**A_23_P117694 CORO2B 15q23**

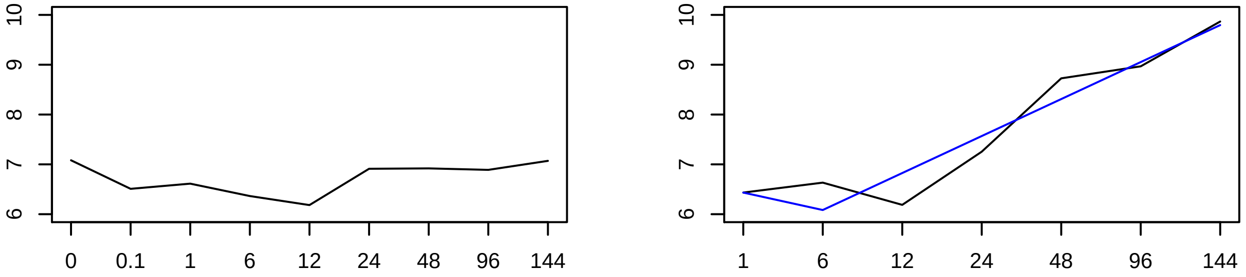

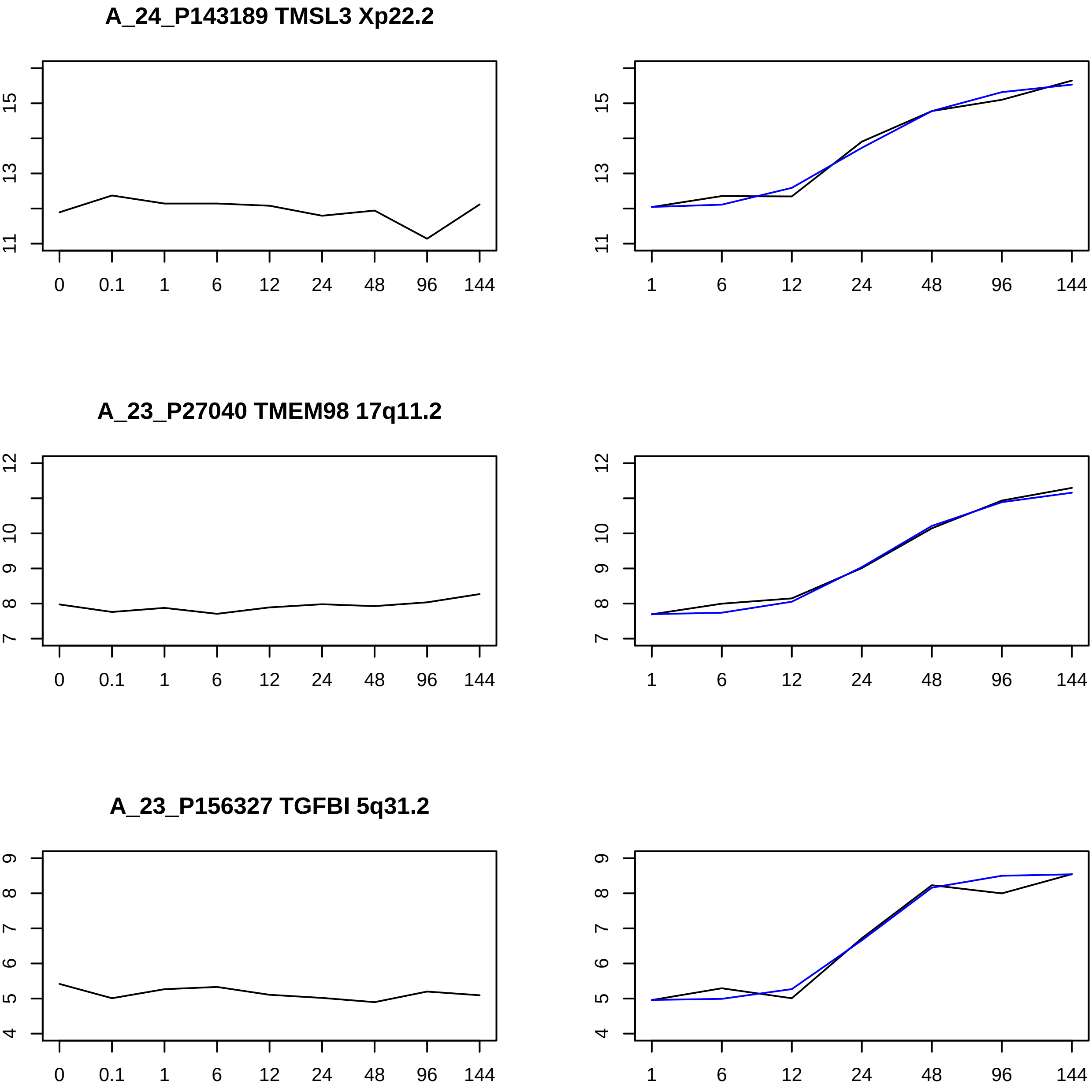
A_24_P143189 TMSL3 Xp22.2
11
13
15
0
0.1
1
6
12
24
48
96
144
A_23_P27040 TMEM98 17q11.2
7
8
9
10
12
A_23_P156327 TGFBI 5q31.2
4
5
6
7
8
9

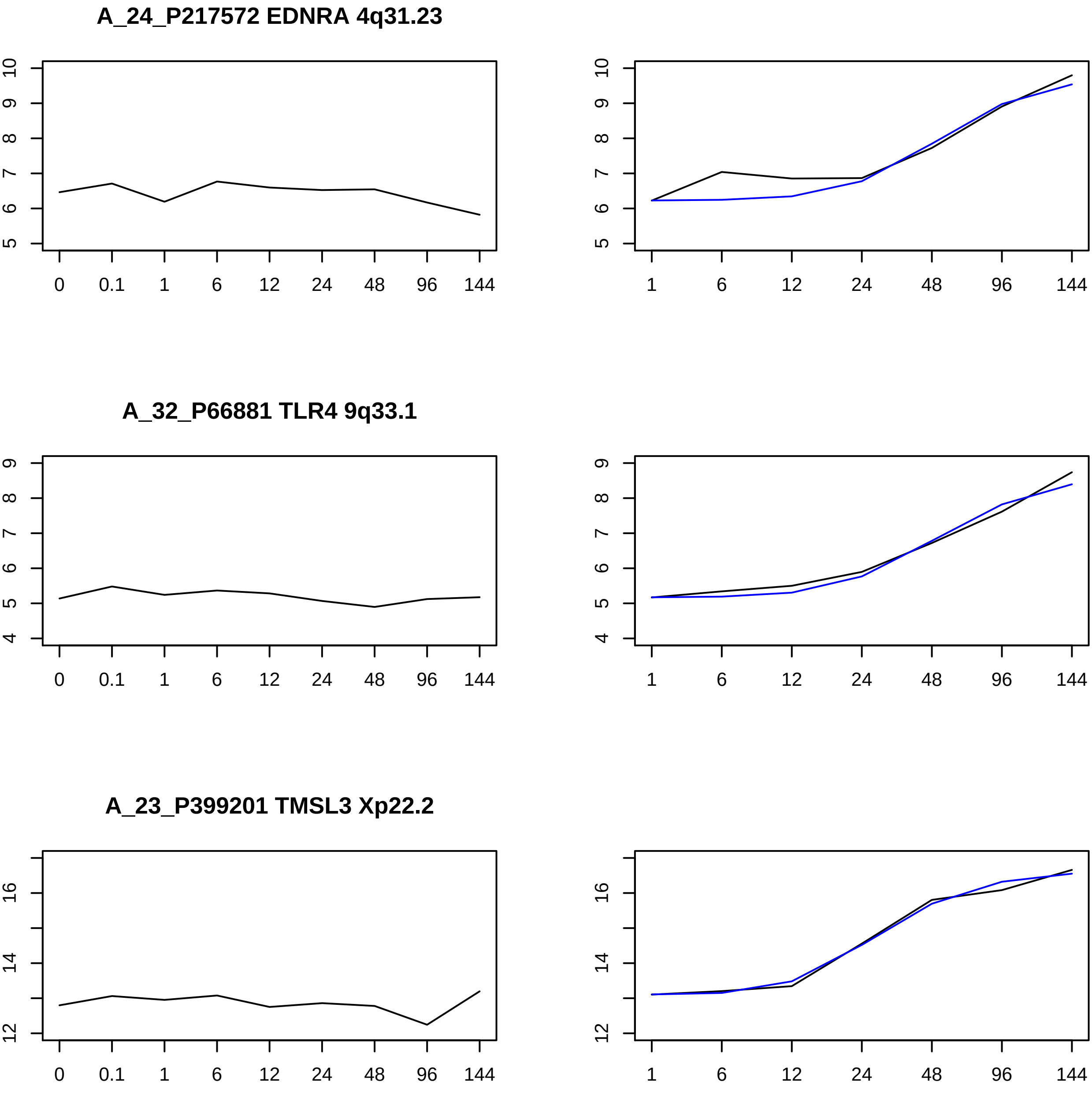
A_24_P217572 EDNRA 4q31.23
A_32_P66881 TLR4 9q33.1
A_23_P399201 TMSL3 Xp22.2
0
0.1
1
6
12
24
48
96
144
5
6
7
8
9
10
4
12
14
16

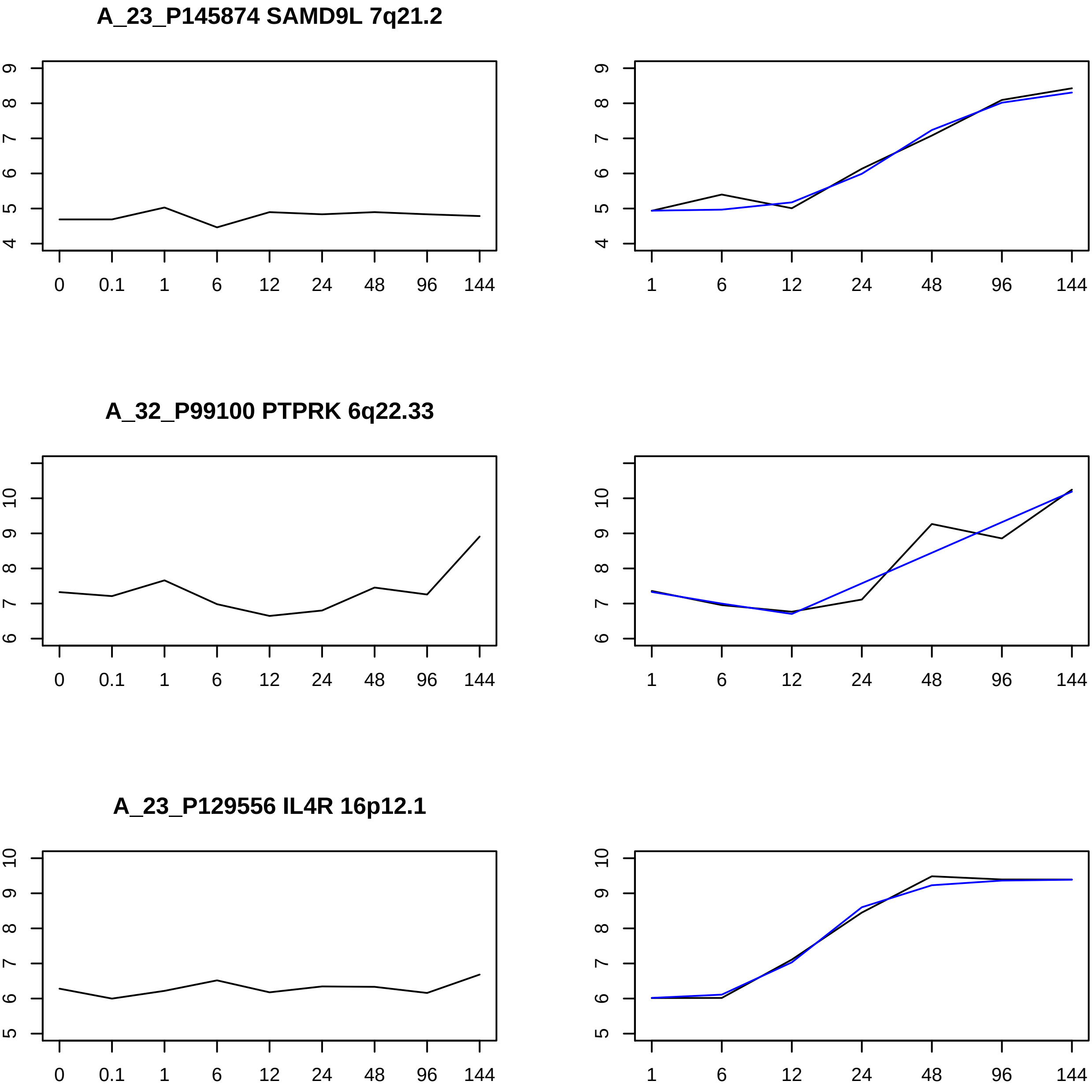
A_23_P145874 SAMD9L 7q21.2
A_32_P99100 PTPRK 6q22.33
A_23_P129556 IL4R 16p12.1
0 0.1 1 6 12 24 48 96 144
1 6 12 24 48 96 144
4 5 6 7 8 9
6 7 8 9 10
5 6 7 8 9 10

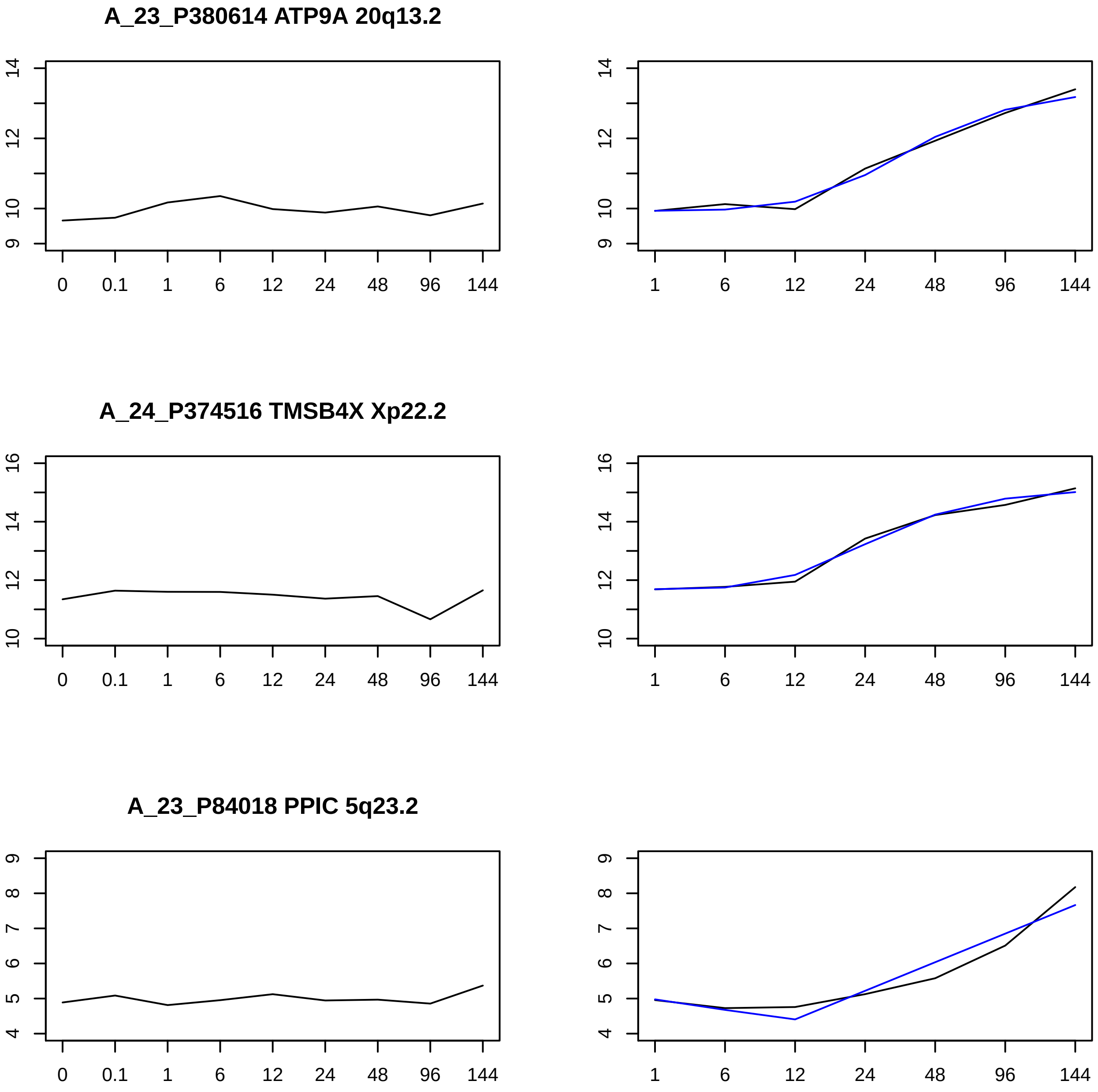
A_23_P380614 ATP9A 20q13.2
9
10
12
14
0
0.1
1
6
12
24
48
96
144
A_24_P374516 TMSB4X Xp22.2
10
12
14
16
A_23_P84018 PPIC 5q23.2
4
5
6
7
8
9

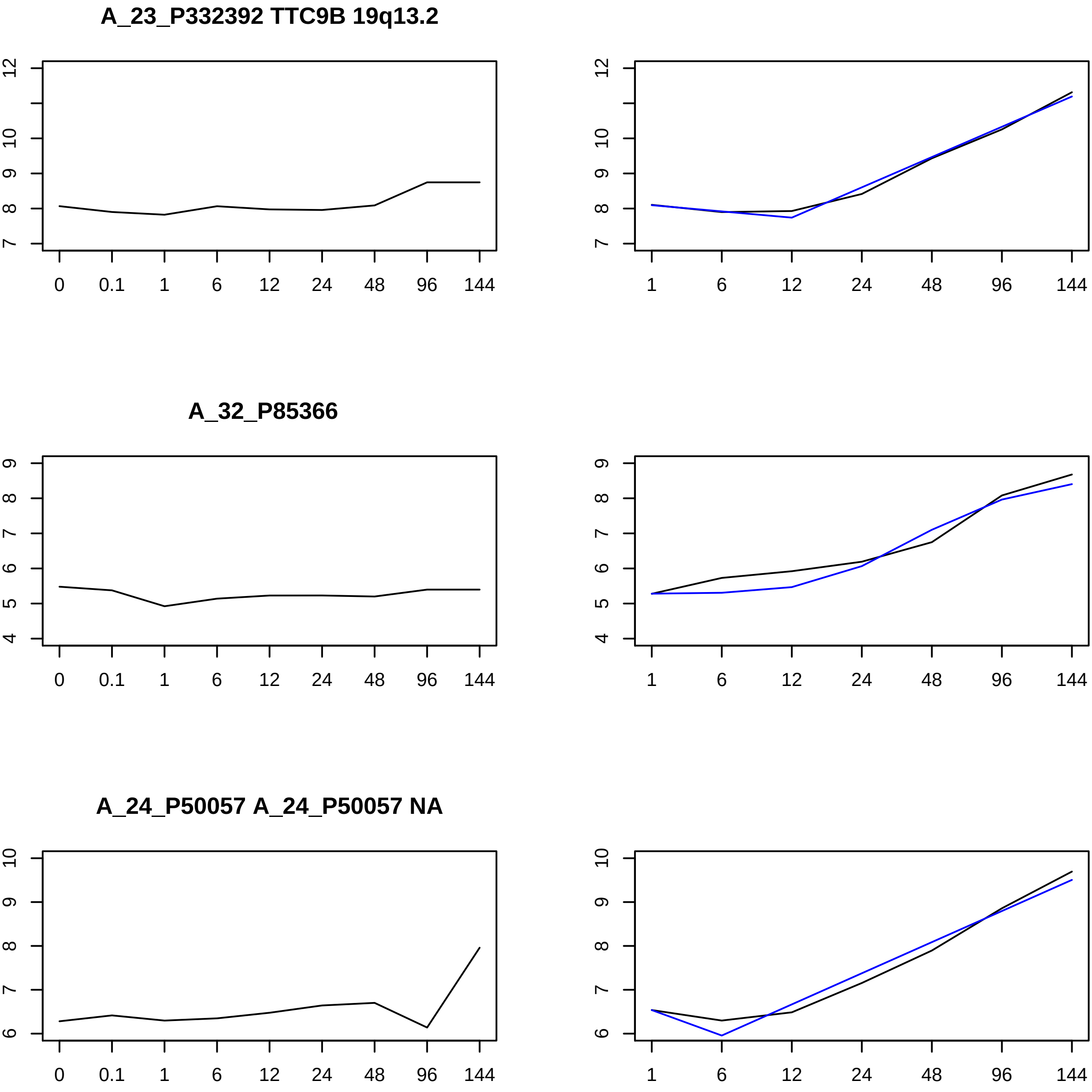
A_23_P332392 TTC9B 19q13.2
7 8 9 10 12
0 0.1 1 6 12 24 48 96 144
7 8 9 10 12
1 6 12 24 48 96 144
A_32_P85366
4 5 6 7 8 9
0 0.1 1 6 12 24 48 96 144
4 5 6 7 8 9
1 6 12 24 48 96 144
A_24_P50057 A_24_P50057 NA
6 7 8 9 10
0 0.1 1 6 12 24 48 96 144
6 7 8 9 10
1 6 12 24 48 96 144

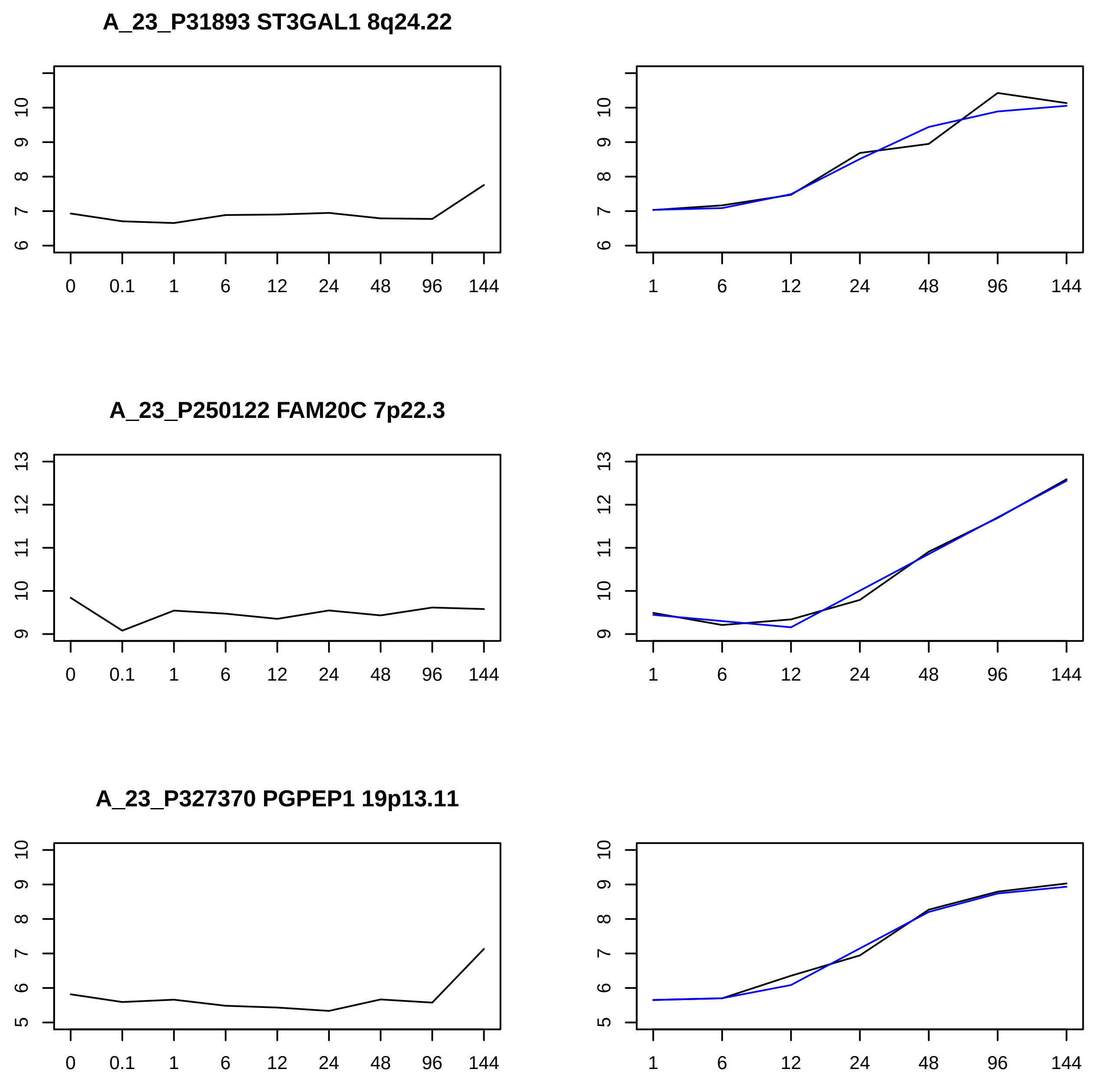
A_23_P31893 ST3GAL1 8q24.22
A_23_P250122 FAM20C 7p22.3
A_23_P327370 PGPEP1 19p13.11
0
0.1
1
6
12
24
48
96
144

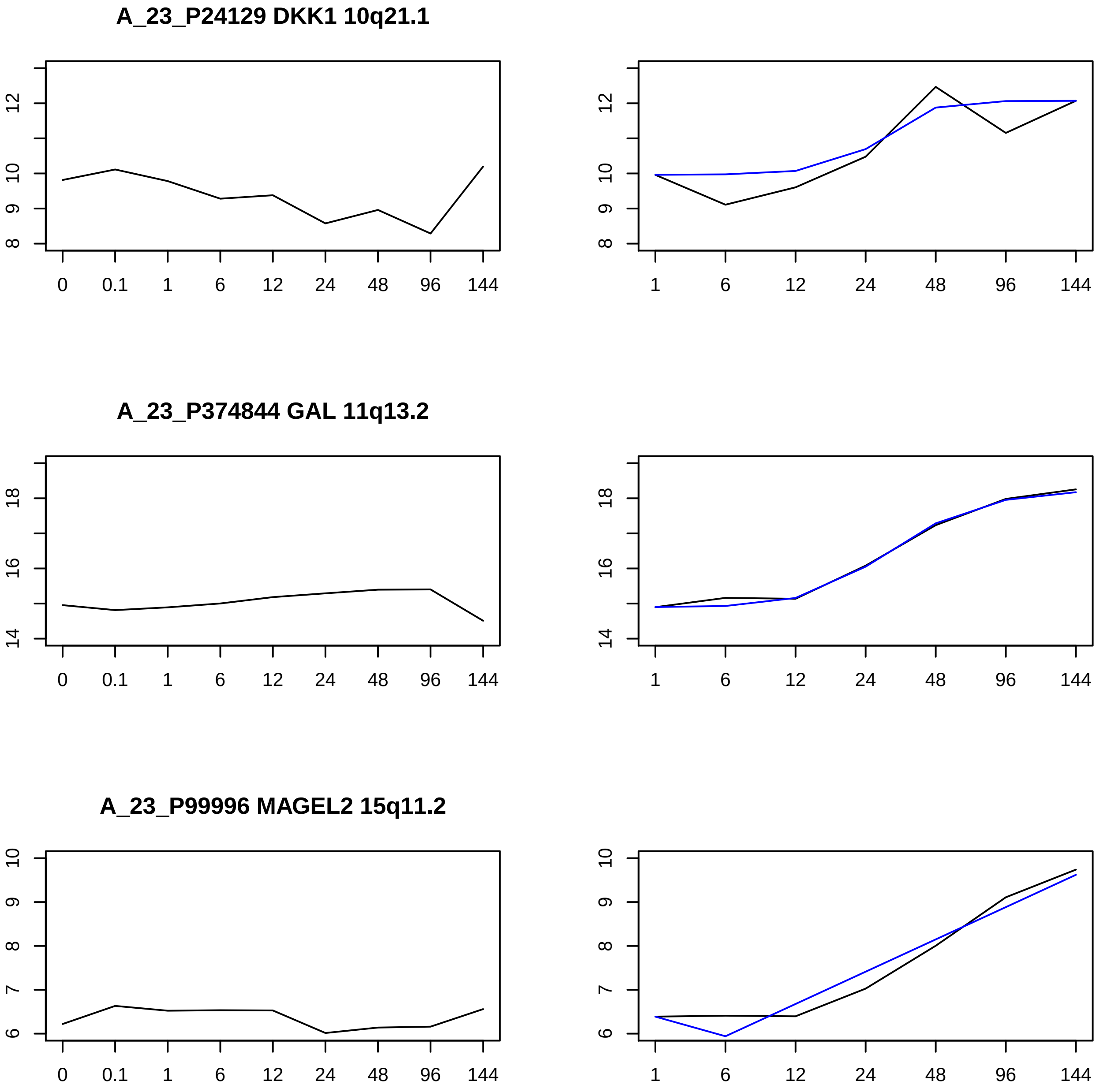

A_23_P24129 DKK1 10q21.1
8 9 10 12
0 0.1 1 6 12 24 48 96 144
8 9 10 12
1 6 12 24 48 96 144
A_23_P374844 GAL 11q13.2
14 16 18
0 0.1 1 6 12 24 48 96 144
14 16 18
1 6 12 24 48 96 144
A_23_P99996 MAGEL2 15q11.2
6 7 8 9 10
0 0.1 1 6 12 24 48 96 144
6 7 8 9 10
1 6 12 24 48 96 144

**A_23_P142631 FKBP1B 2p23.3**

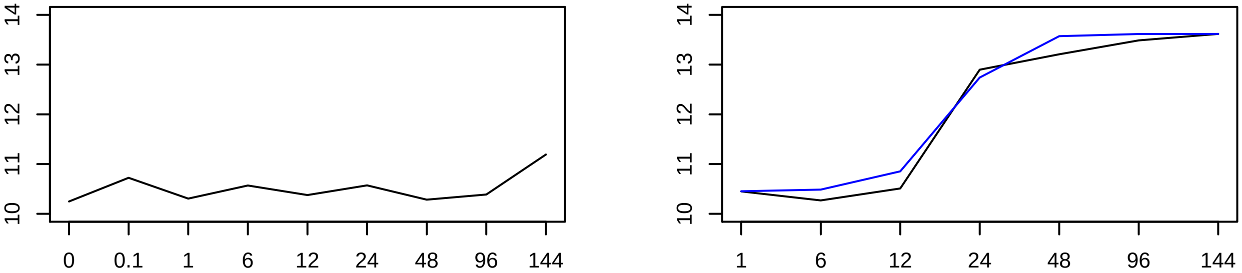


**A_23_P125809 ZCCHC12 Xq24**

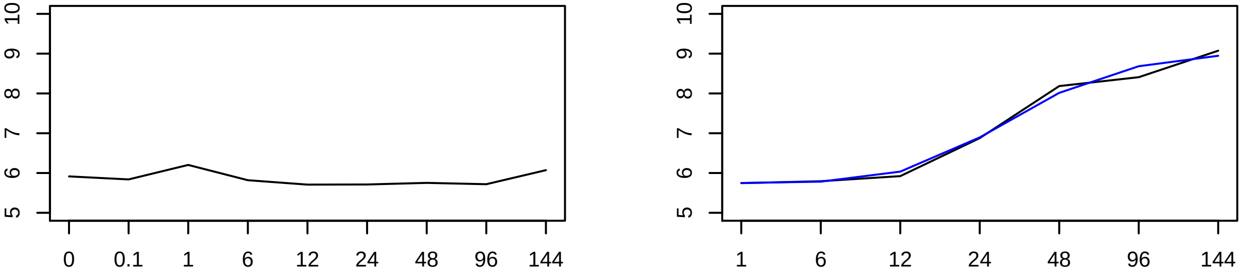


**A_24_P374513 ENST00000334647 NA**

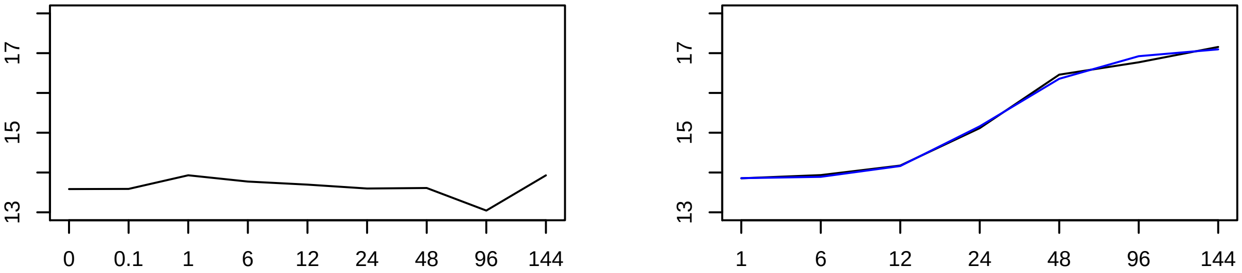

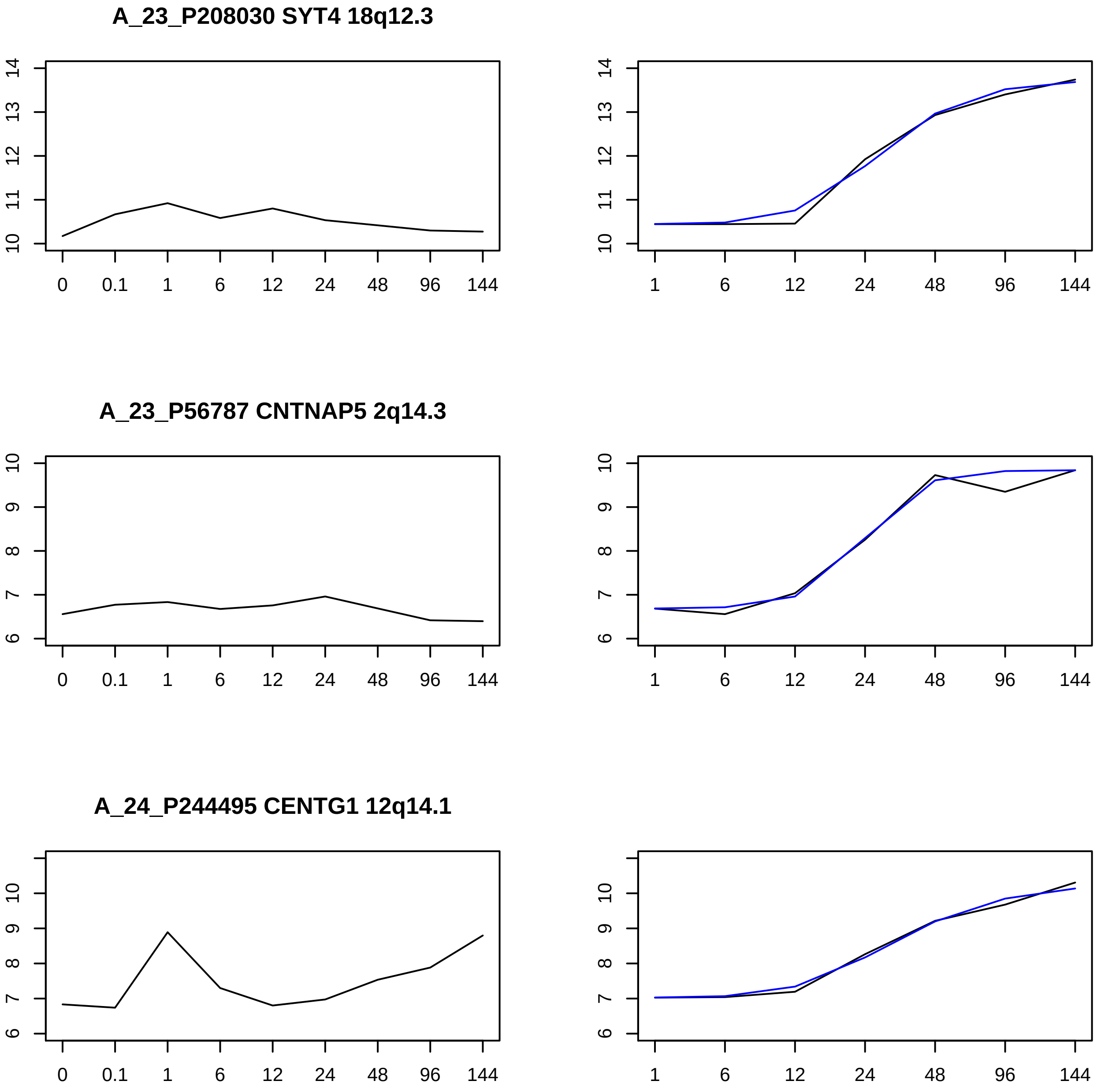
A_23_P208030 SYT4 18q12.3
10
11
12
13
14
0
0.1
1
6
12
24
48
96
144
1
6
12
24
48
96
144
A_23_P56787 CNTNAP5 2q14.3
6
7
8
9
10
A_24_P244495 CENTG1 12q14.1

**A_32_P71032 THC2638839 NA**

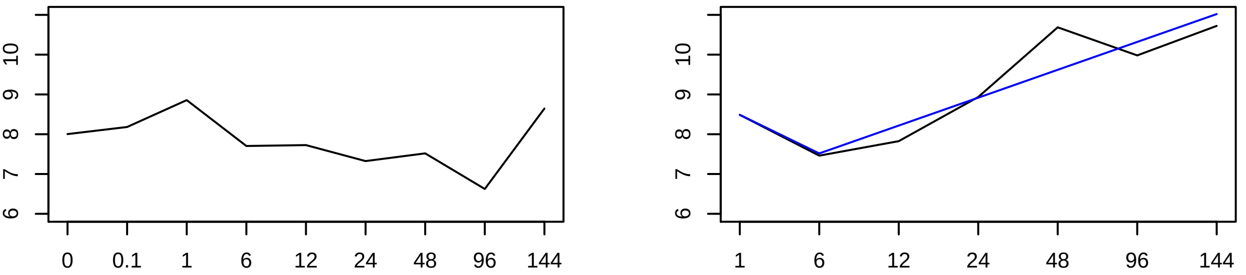


**A_23_P307544 PLXNA2 1q32.2**

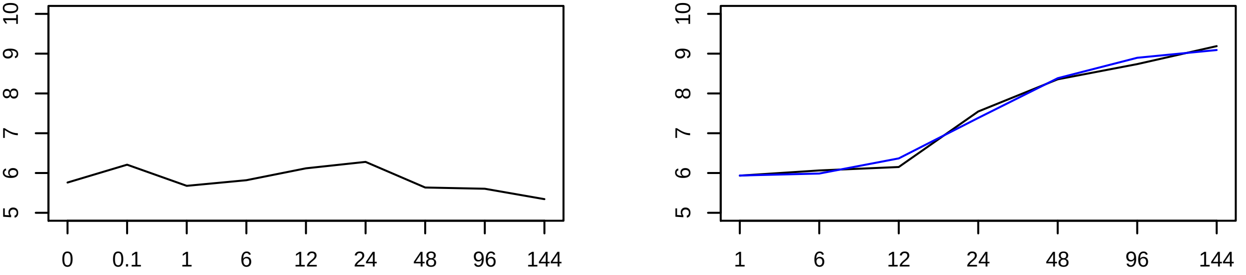


**A_23_P163682 RHBDF1 16p13.3**

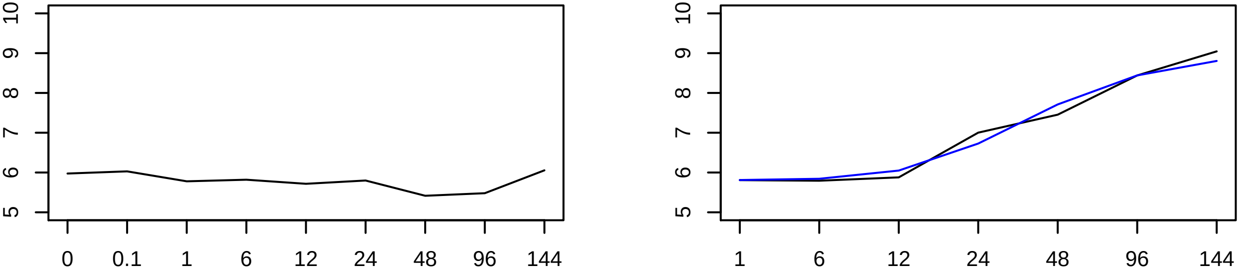

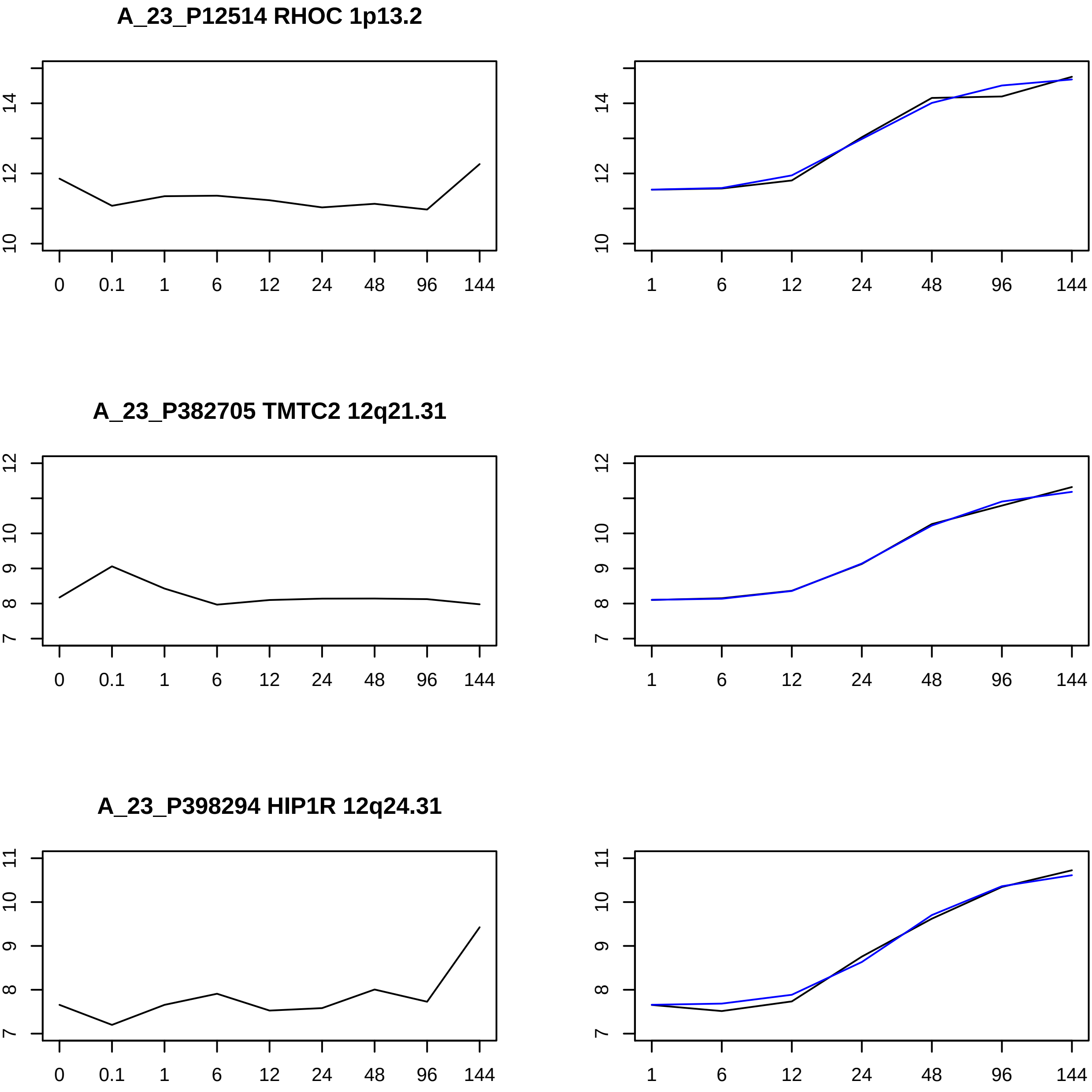
A_23_P12514 RHOC 1p13.2
10
12
14
0
0.1
1
6
12
24
48
96
144
1
6
12
24
48
96
144
A_23_P382705 TMTC2 12q21.31
7
8
9
10
12
A_23_P398294 HIP1R 12q24.31
7
8
9
10
11

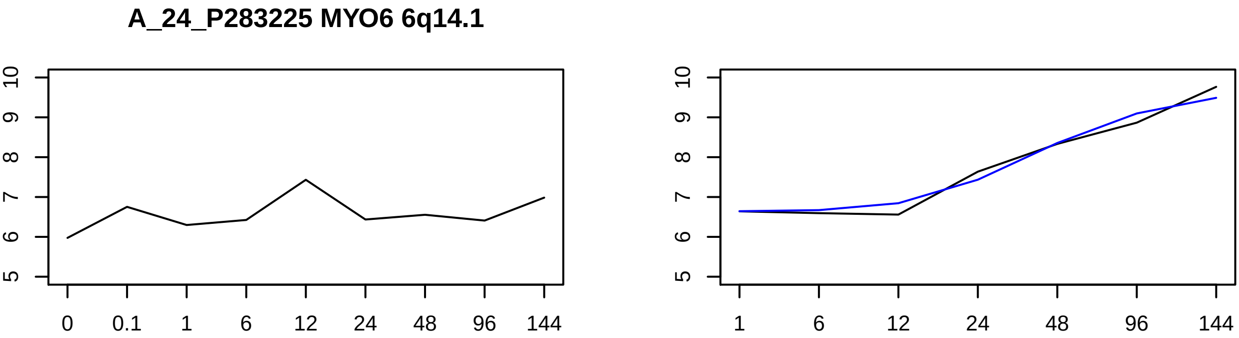
A_24_P283225 MYO6 6q14.1
5 6 7 8 9 10
0 0.1 1 6 12 24 48 96 144
1 6 12 24 48 96 144

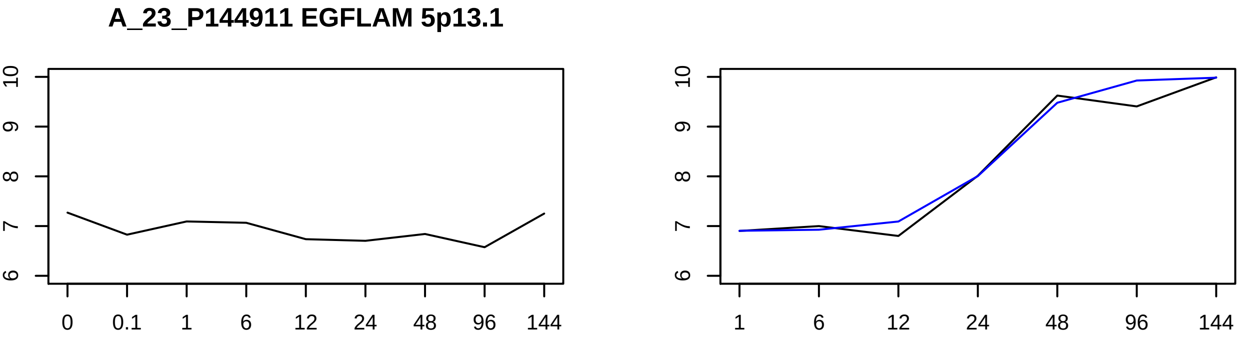
A_23_P144911 EGFLAM 5p13.1
6 7 8 9 10
0 0.1 1 6 12 24 48 96 144
1 6 12 24 48 96 144

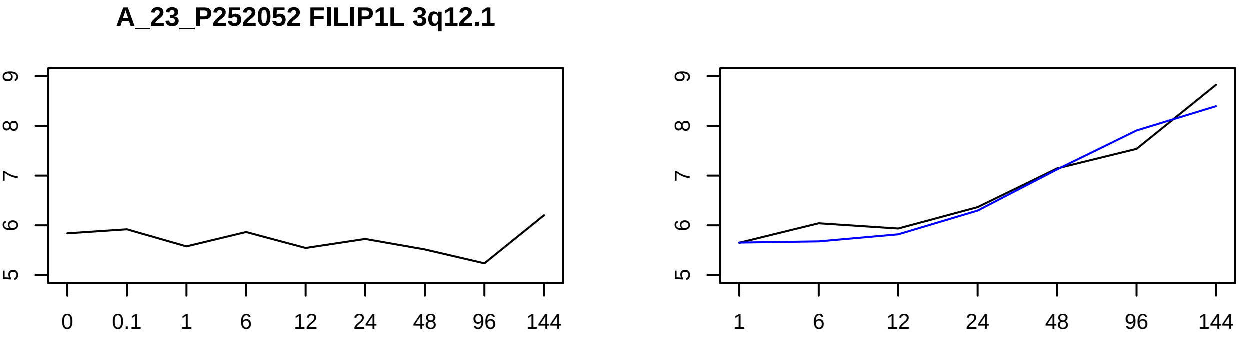
A_23_P252052 FILIP1L 3q12.1
5 6 7 8 9
0 0.1 1 6 12 24 48 96 144
1 6 12 24 48 96 144

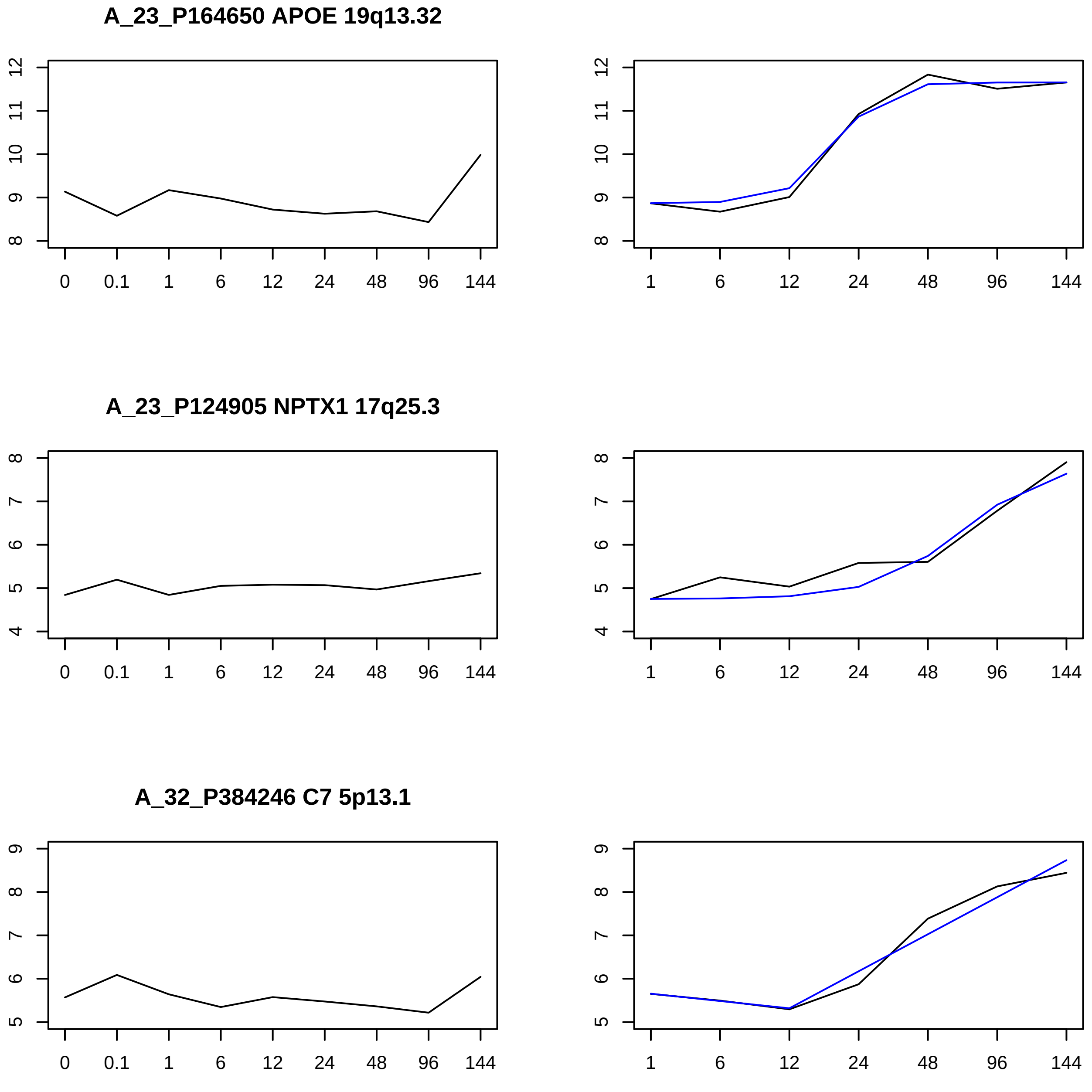
A_23_P164650 APOE 19q13.32
A_23_P124905 NPTX1 17q25.3
A_32_P384246 C7 5p13.1

**A_23_P255952 MYO6 6q14.1**

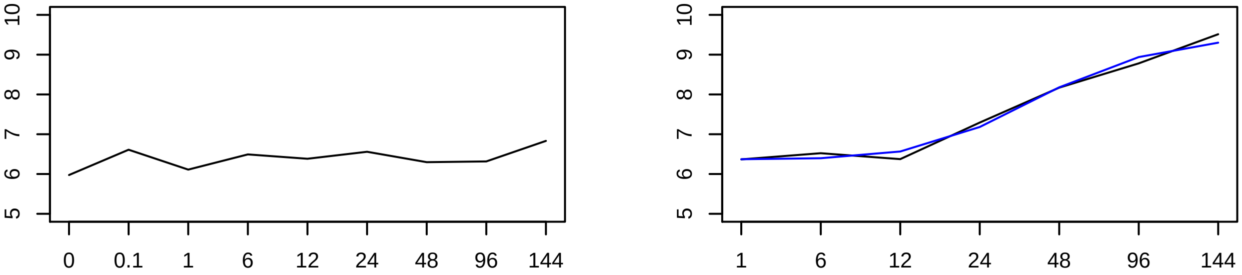


**A_23_P158470 C3orf54 3p21.31**

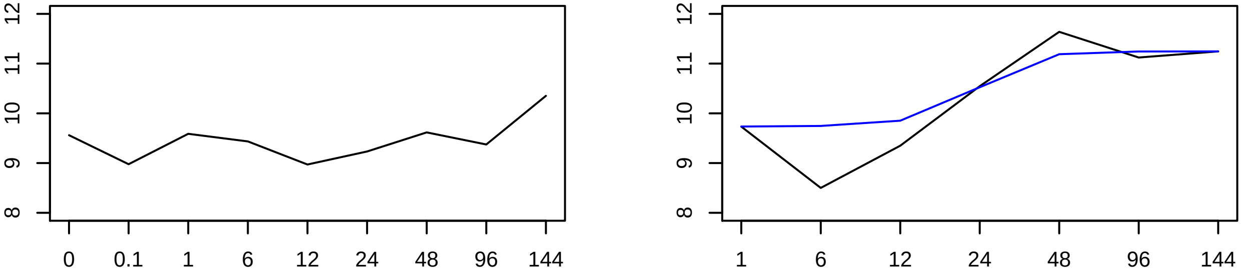


**A_24_P535256 INHBA NA**

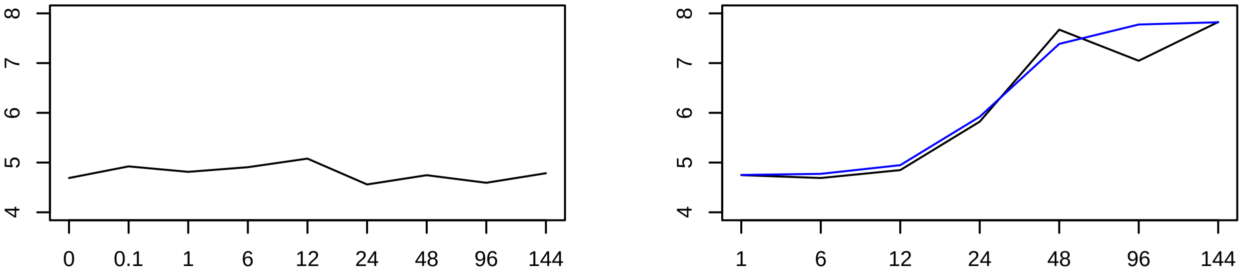

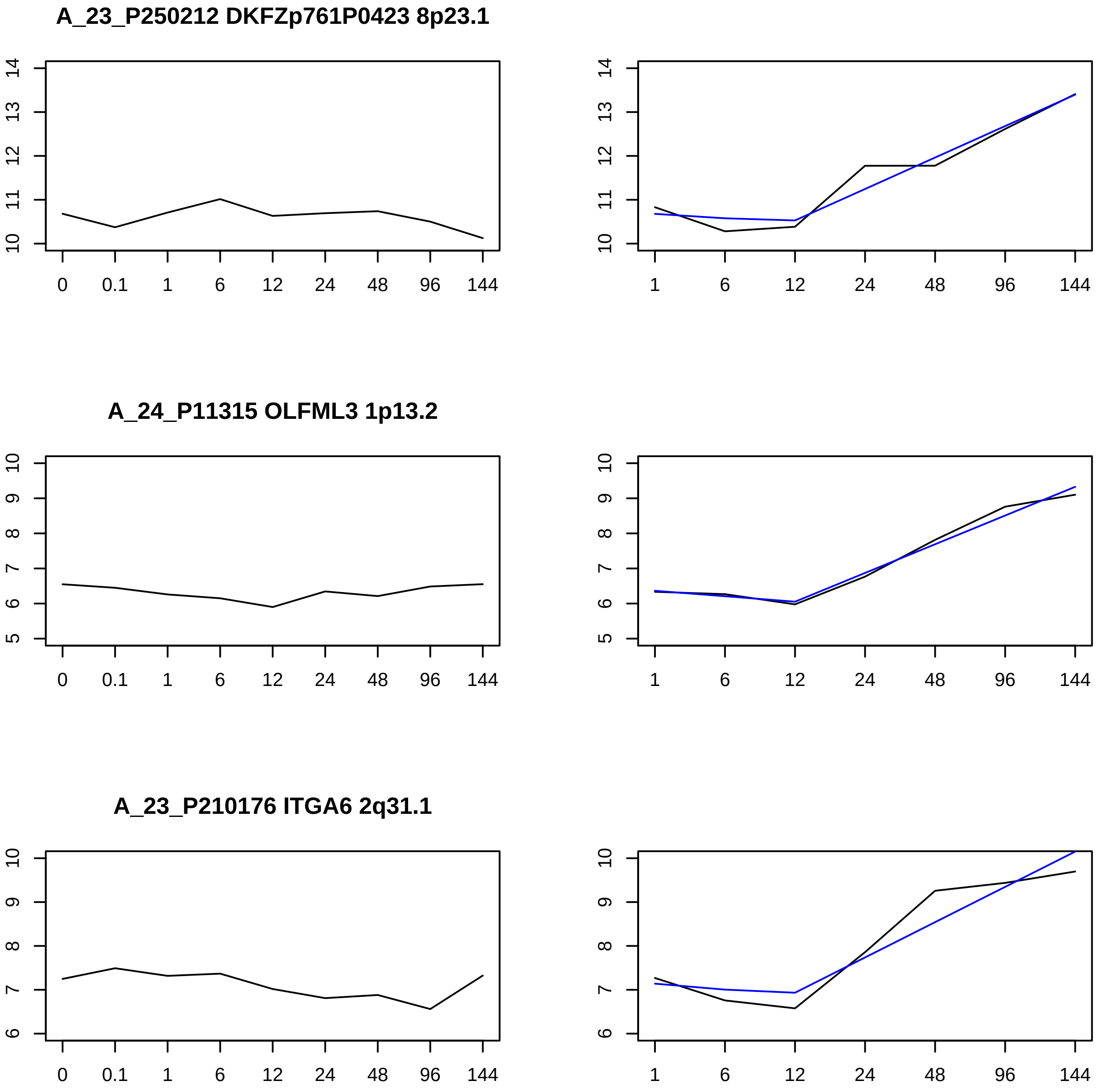
A_23_P250212 DKFZp761P0423 8p23.1
10
11
12
13
14
0
0.1
1
6
12
24
48
96
144
A_24_P11315 OLFML3 1p13.2
5
6
7
8
9
10
A_23_P210176 ITGA6 2q31.1

**A_23_P130149 ENO3 17p13.2**

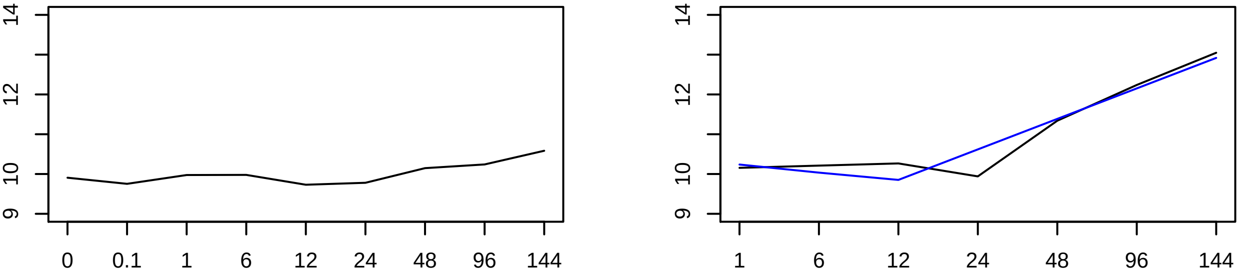


**A_23_P38630 SSTR2 17q25.1**

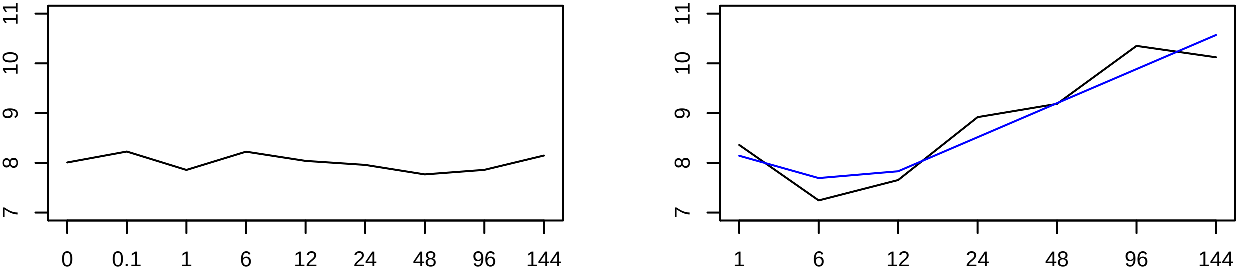


**A_24_P85888 SCN3A 2q24.3**

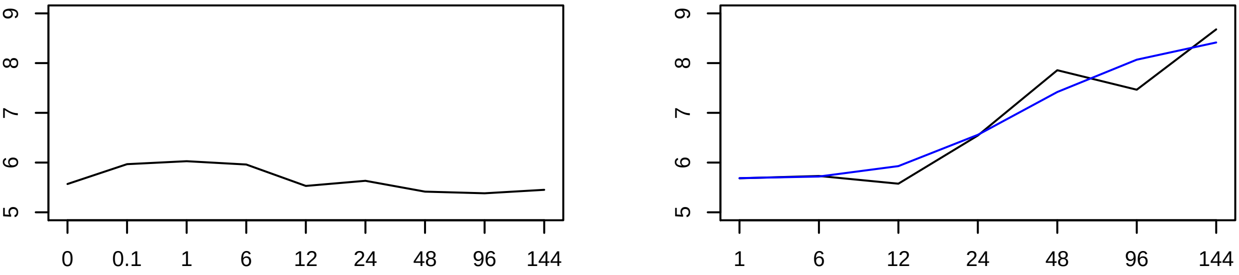

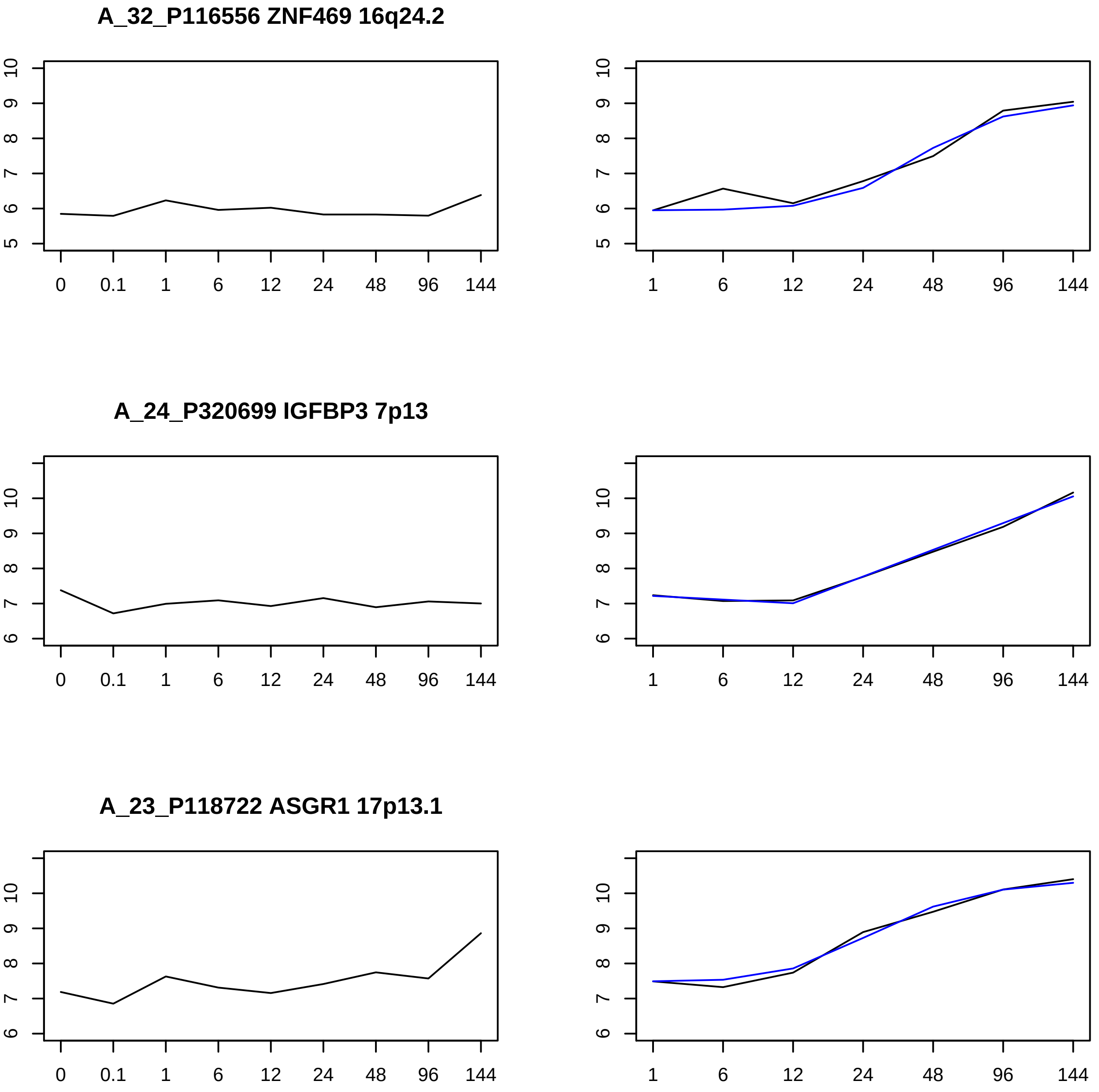
A_32_P116556 ZNF469 16q24.2
A_24_P320699 IGFBP3 7p13
A_23_P118722 ASGR1 17p13.1
0 0.1 1 6 12 24 48 96 144
1 6 12 24 48 96 144
5 6 7 8 9 10

**_P40437 KLRD1\KLRC3\KLRC4\KLRC4−KLRK1\KLRK**

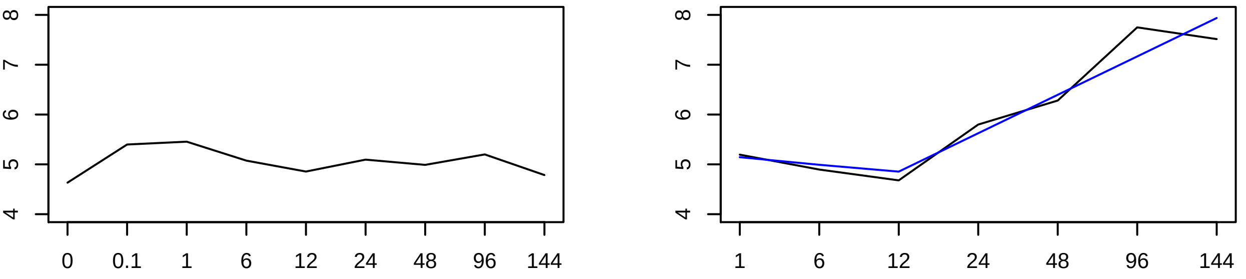


**A_24_P218265 TNFRSF10B 8p21.3**

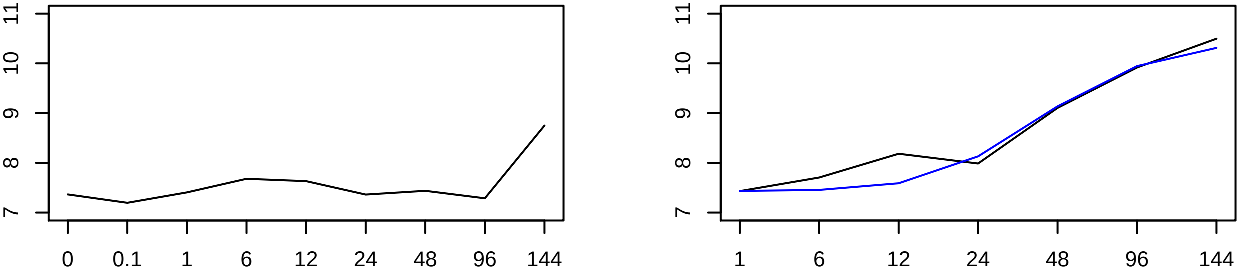


**A_23_P211504 KDELR3 22q13.1**

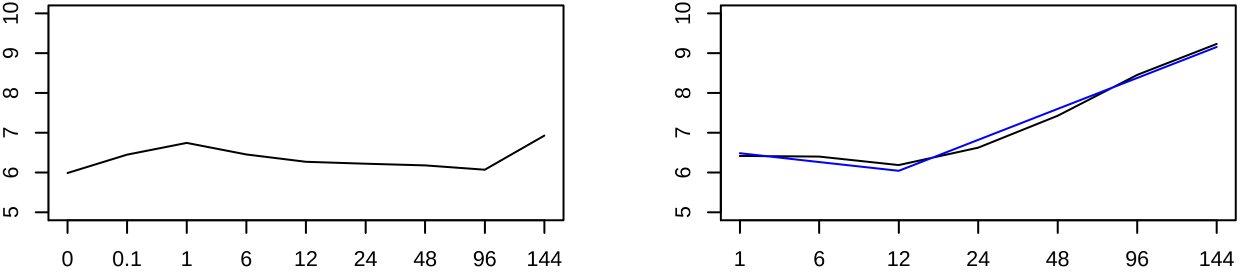

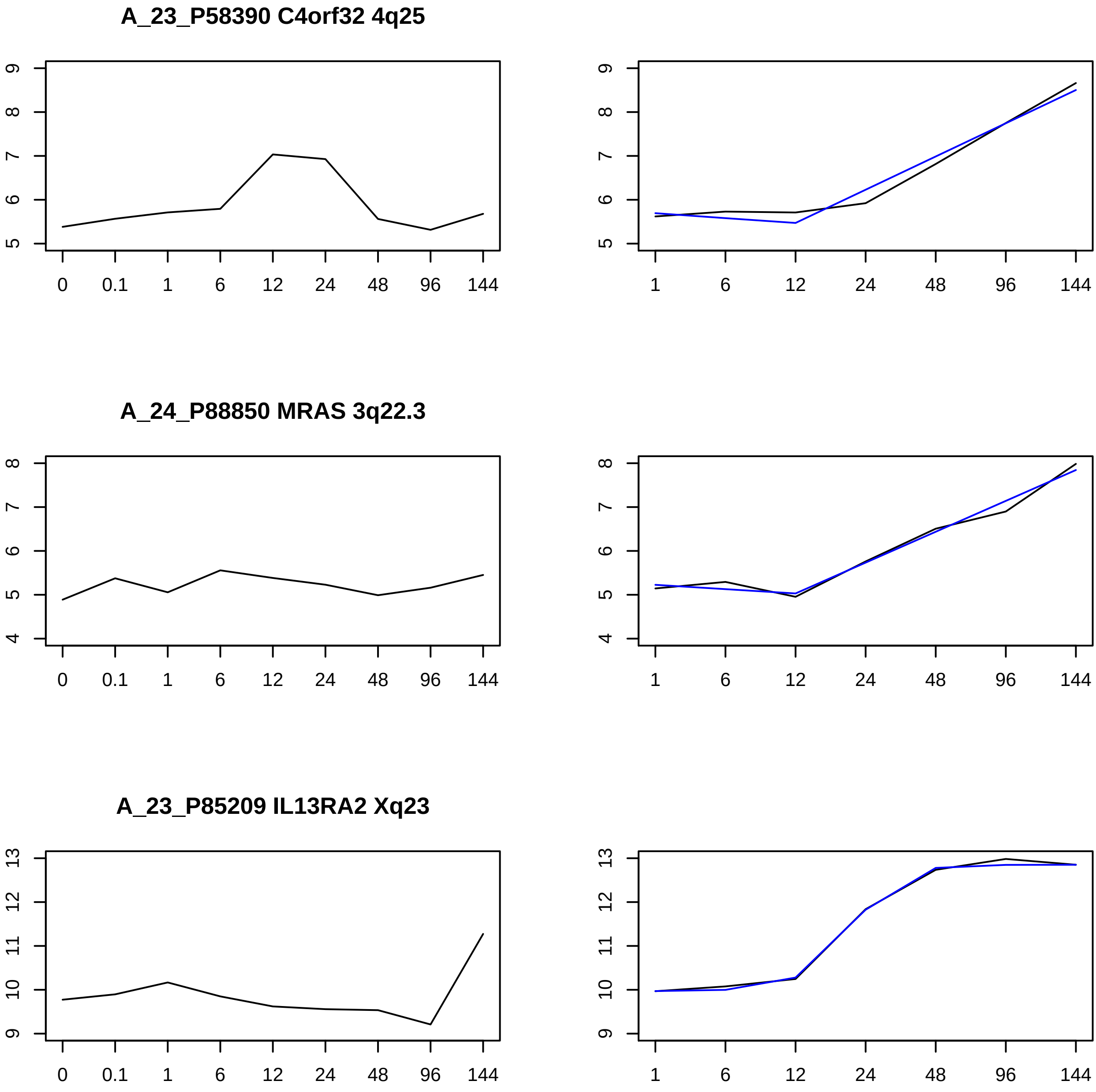

A_23_P58390 C4orf32 4q25
A_24_P88850 MRAS 3q22.3
A_23_P85209 IL13RA2 Xq23
0
0.1
1
6
12
24
48
96
144

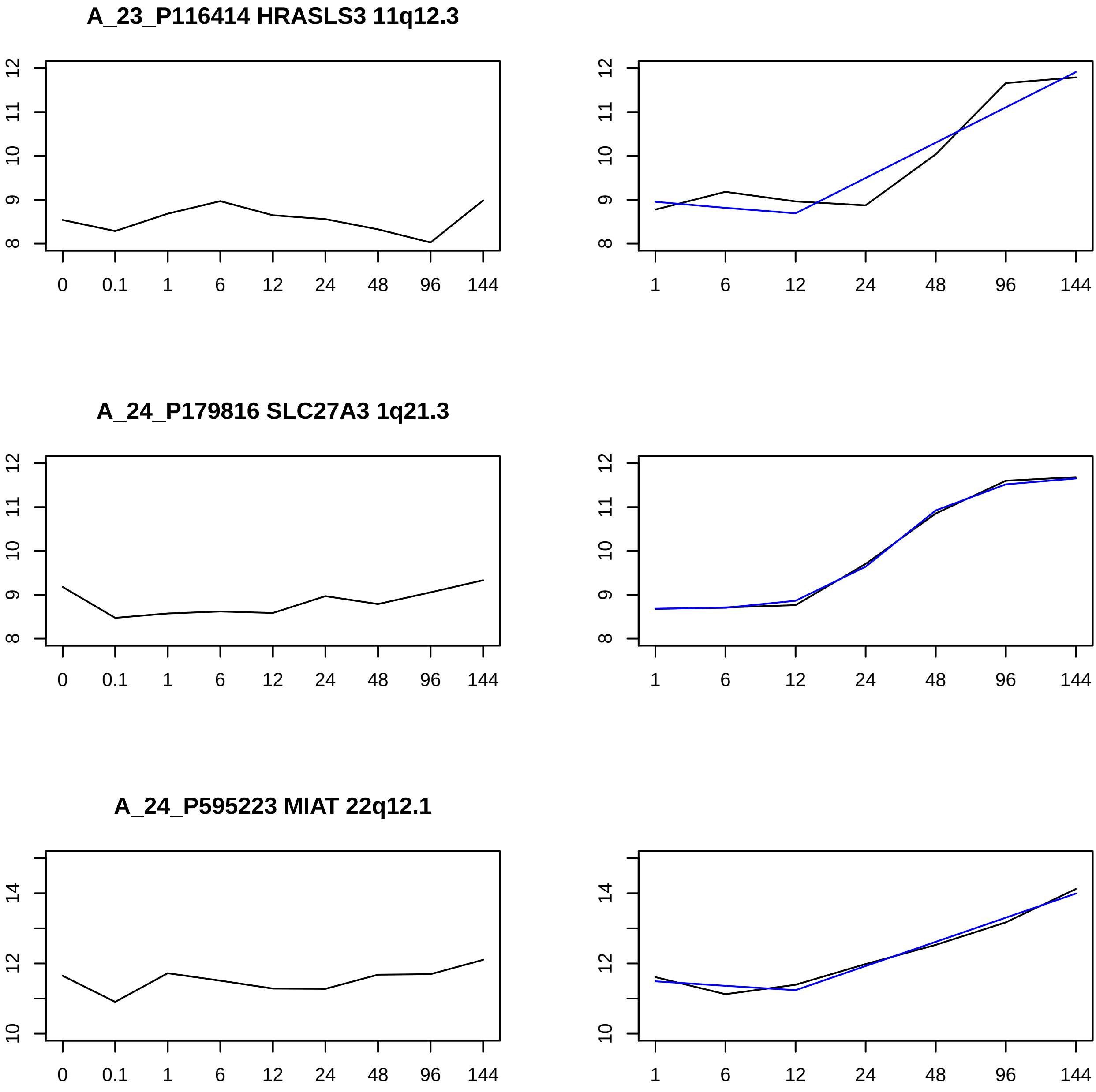

A_23_P116414 HRASLS3 11q12.3
A_24_P179816 SLC27A3 1q21.3
A_24_P595223 MIAT 22q12.1
8
9
10
11
12
14
0
0.1
1
6
12
24
48
96
144

**A_24_P79054 TGFB1 19q13.2**

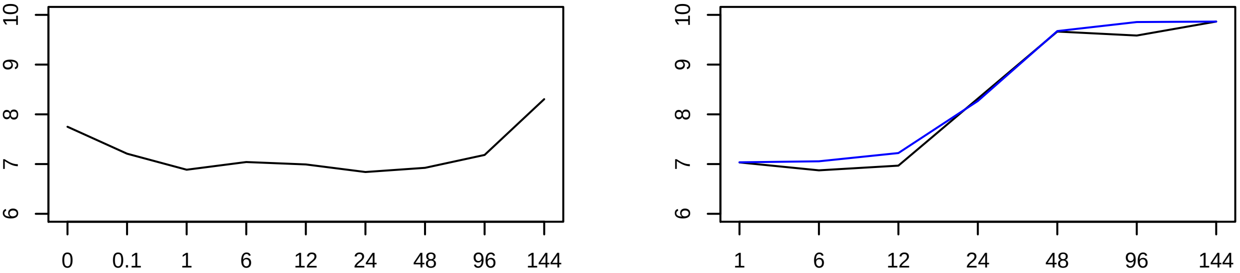


**A_23_P374082 ADAM19 5q33.3**

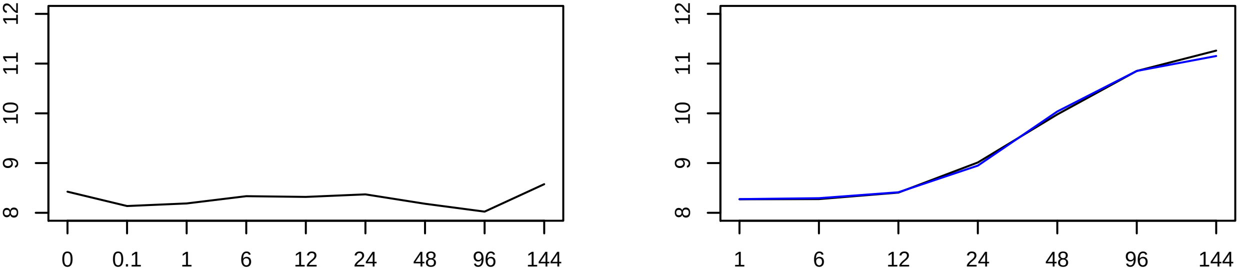


**A_32_P24832 OLFML3 1p13.2**

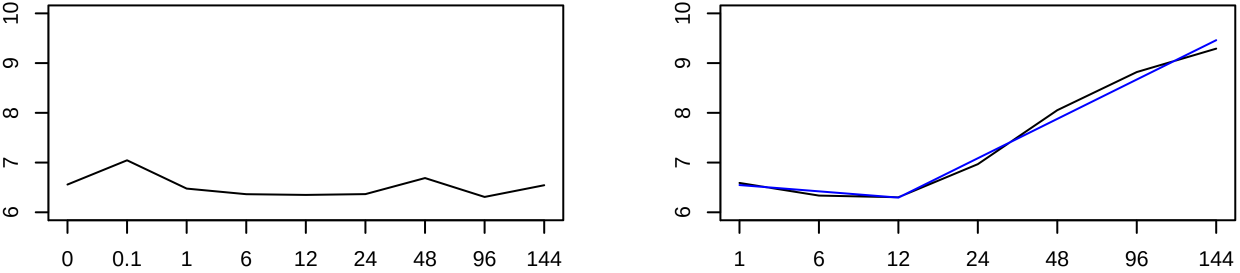

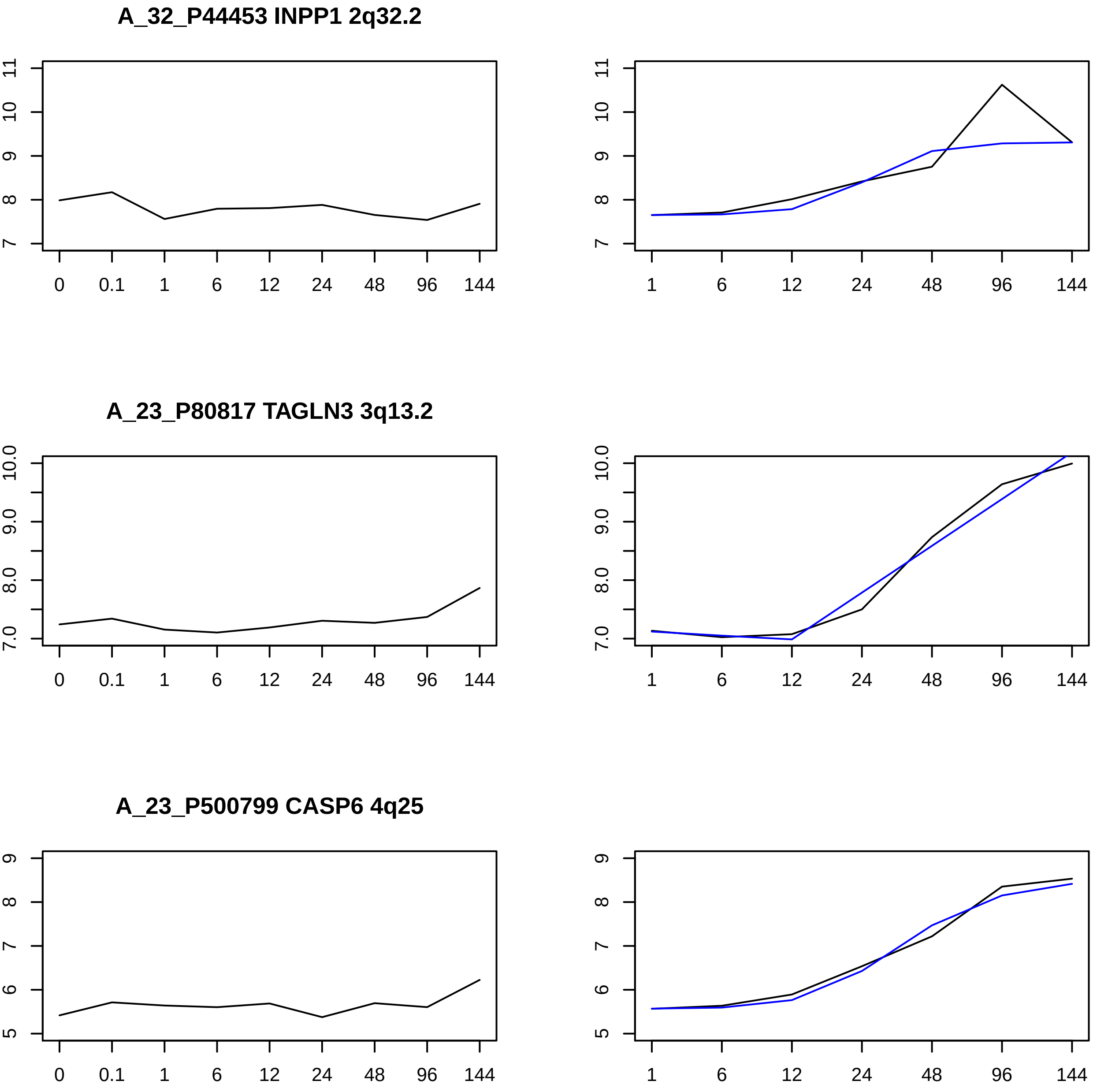
A_32_P44453 INPP1 2q32.2
A_23_P80817 TAGLN3 3q13.2
A_23_P500799 CASP6 4q25
0
0.1
1
6
12
24
48
96
144
7
8
9
10
11
7.0
8.0
9.0
10.0
5
6

**A_32_P6408 DKFZp547K054 7p22.1**

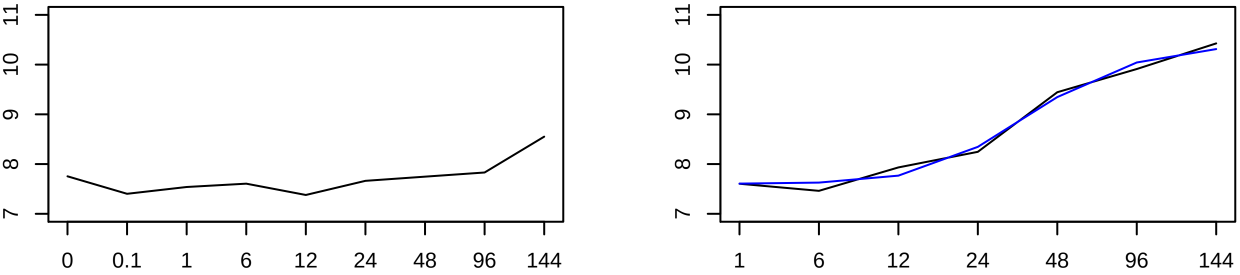


**A_23_P29124 SEPT5 22q11.21**

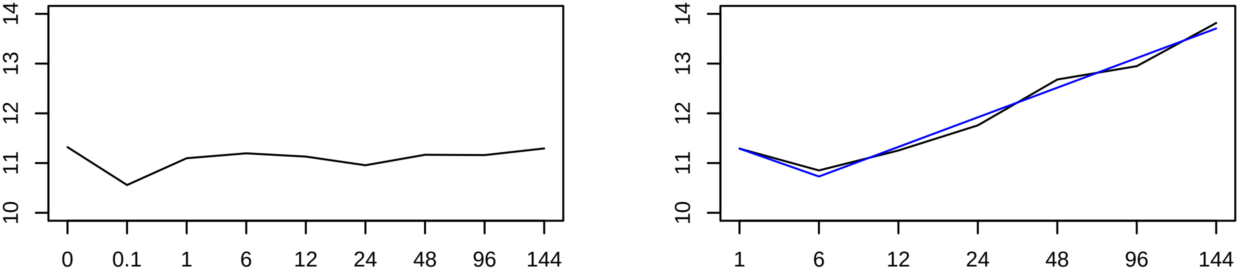


**A_23_P18372 B3GNT5 3q27.1**

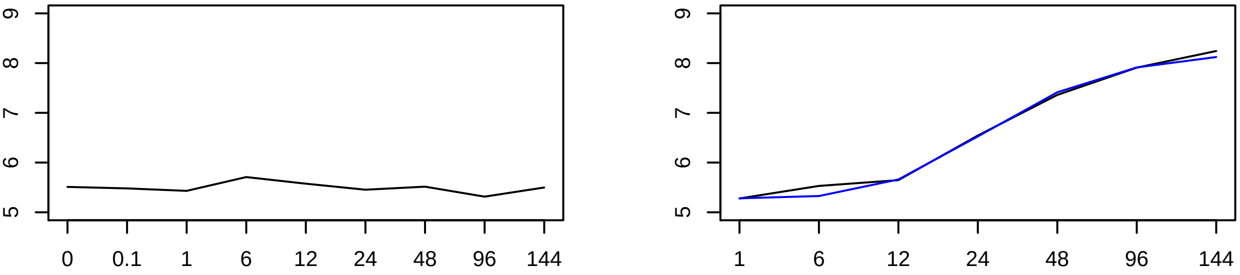

**A_23_P409623 PPFIBP2 11p15.4**

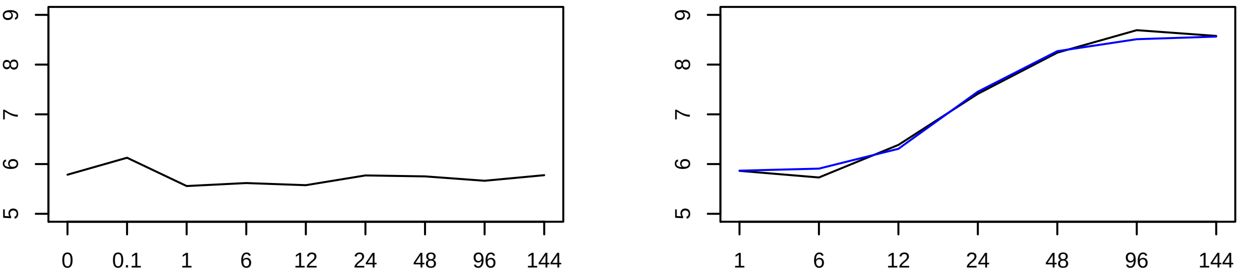


**A_23_P94552 TMEM2 9q21.13**

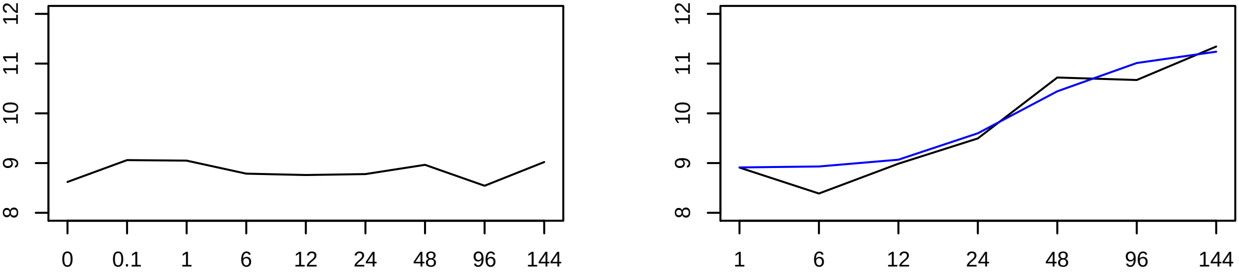


**A_24_P399220 HOXB3 17q21.32**

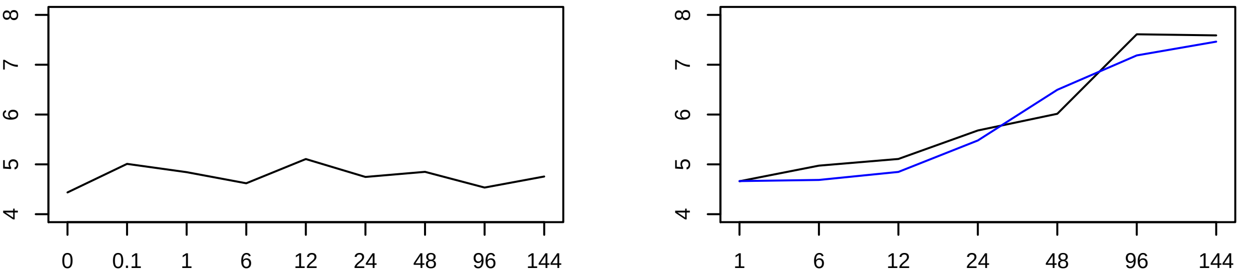

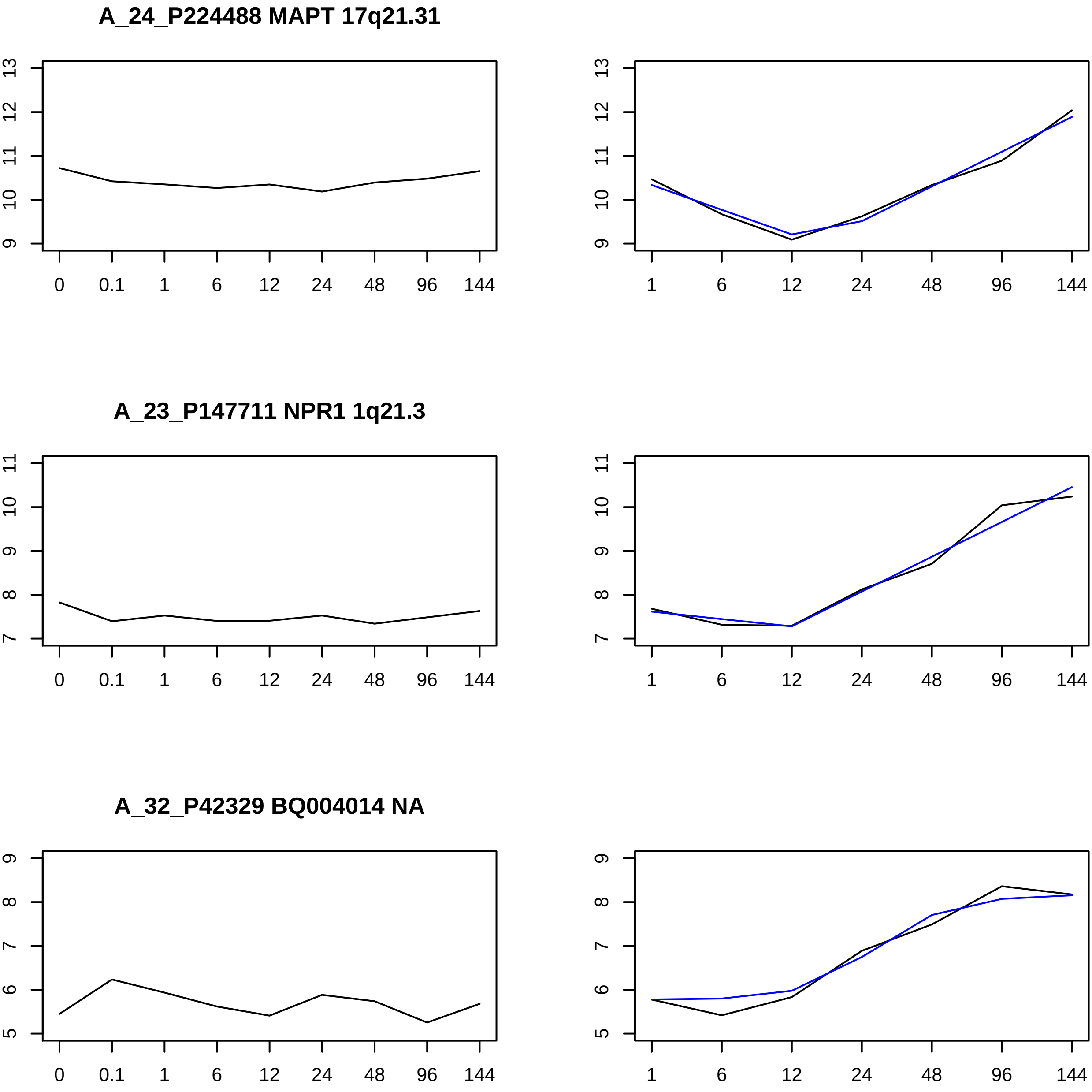
A_24_P224488 MAPT 17q21.31
A_23_P147711 NPR1 1q21.3
A_32_P42329 BQ004014 NA
0 0.1 1 6 12 24 48 96 144
1 6 12 24 48 96 144

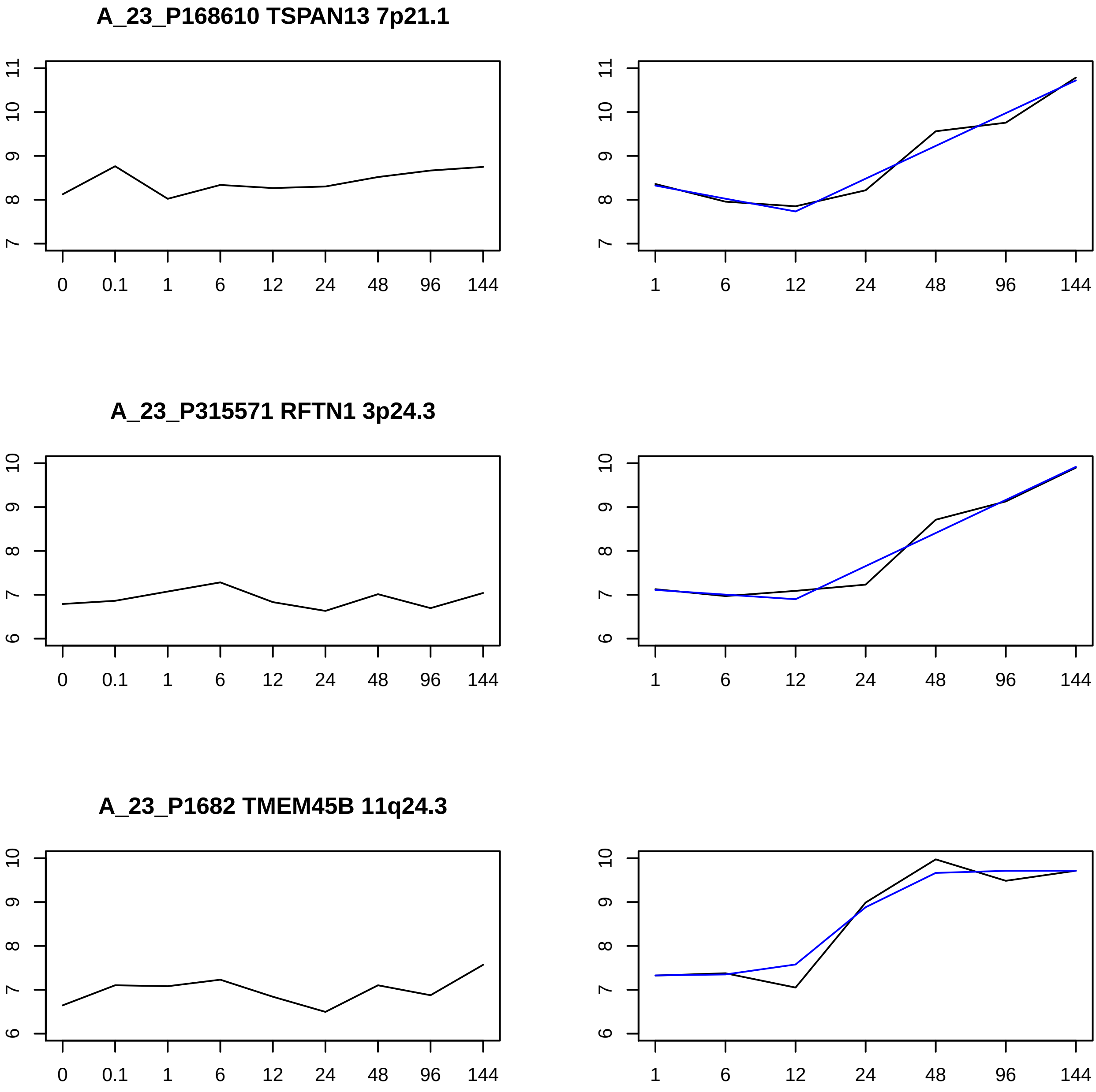
A_23_P168610 TSPAN13 7p21.1
7 8 9 10 11
0 0.1 1 6 12 24 48 96 144
7 8 9 10 11
1 6 12 24 48 96 144
A_23_P315571 RFTN1 3p24.3
6 7 8 9 10
0 0.1 1 6 12 24 48 96 144
6 7 8 9 10
1 6 12 24 48 96 144
A_23_P1682 TMEM45B 11q24.3
6 7 8 9 10
0 0.1 1 6 12 24 48 96 144
6 7 8 9 10
1 6 12 24 48 96 144

**A_23_P19291 TUBB2A 6p25.2**

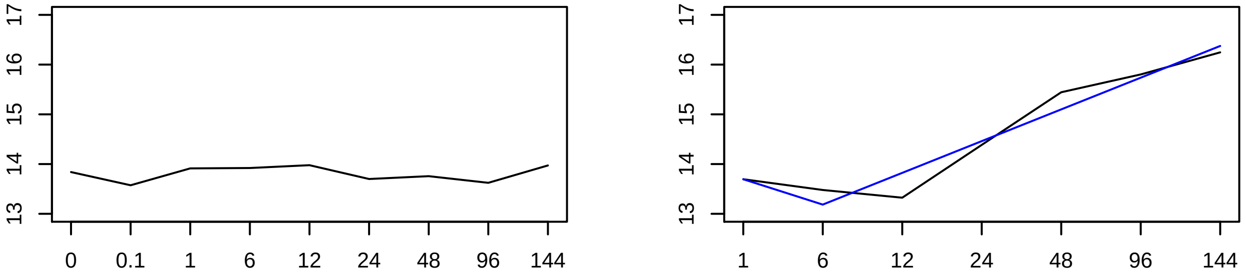


**A_23_P393880 KIAA1244 6q23.3**

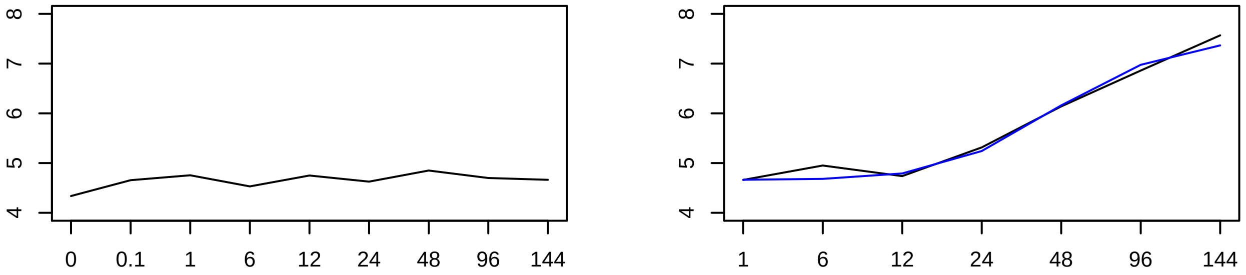


**A_23_P145863 S100A11 1q21.3**

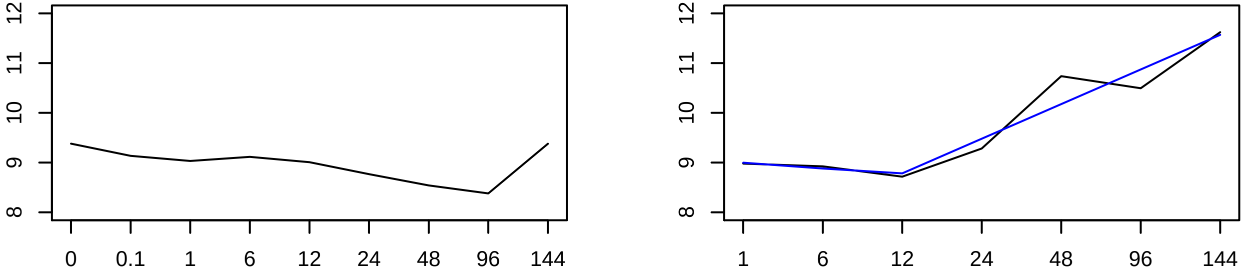

**A_24_P817236 ENST00000366569 NA**

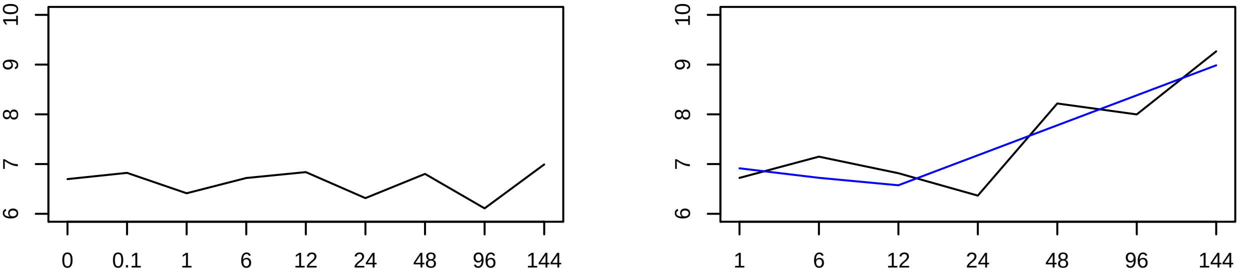


**A_32_P87491 U79293 NA**

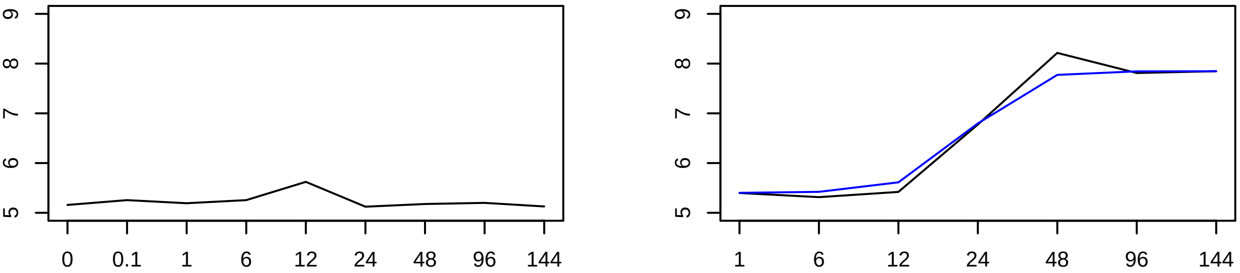


**A_23_P55251 ITGA3 17q21.33**

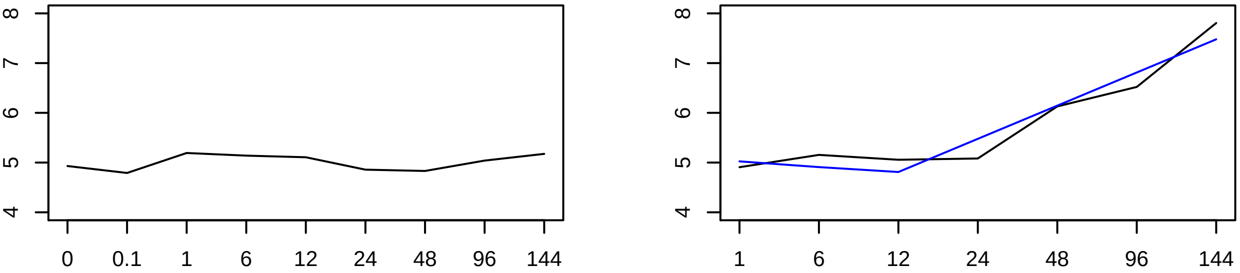

**A_23_P215024 LRRC1 6p12.1**

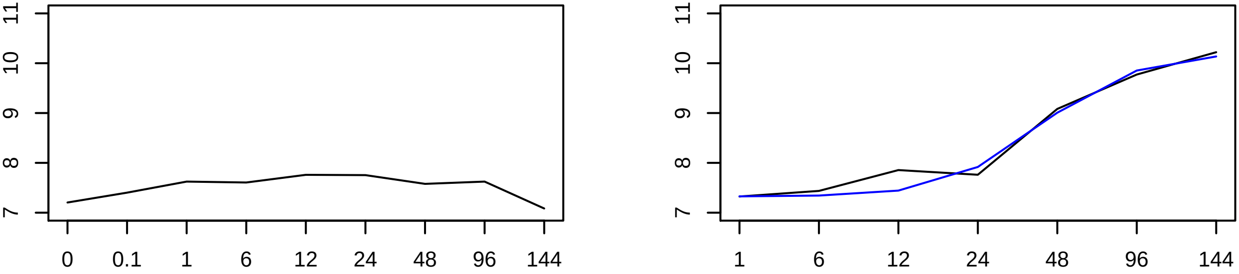


**A_32_P231097 RNF182 6p23**

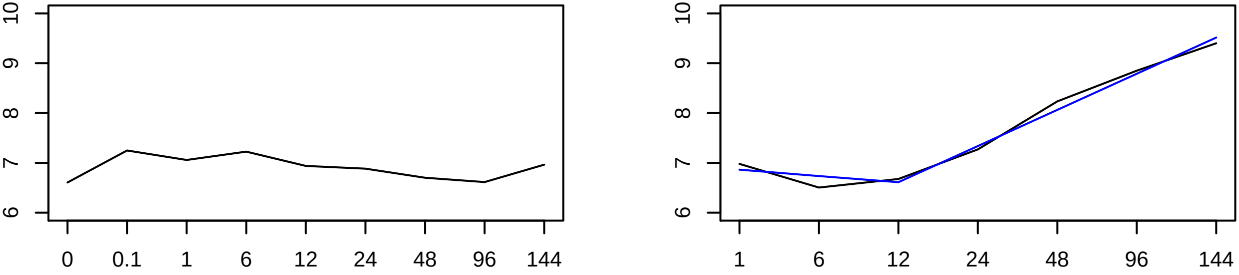


**A_23_P166459 LGALS1 22q13.1**

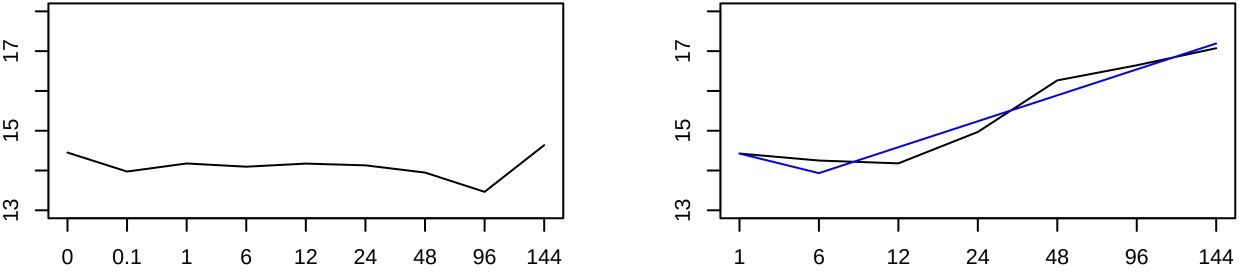

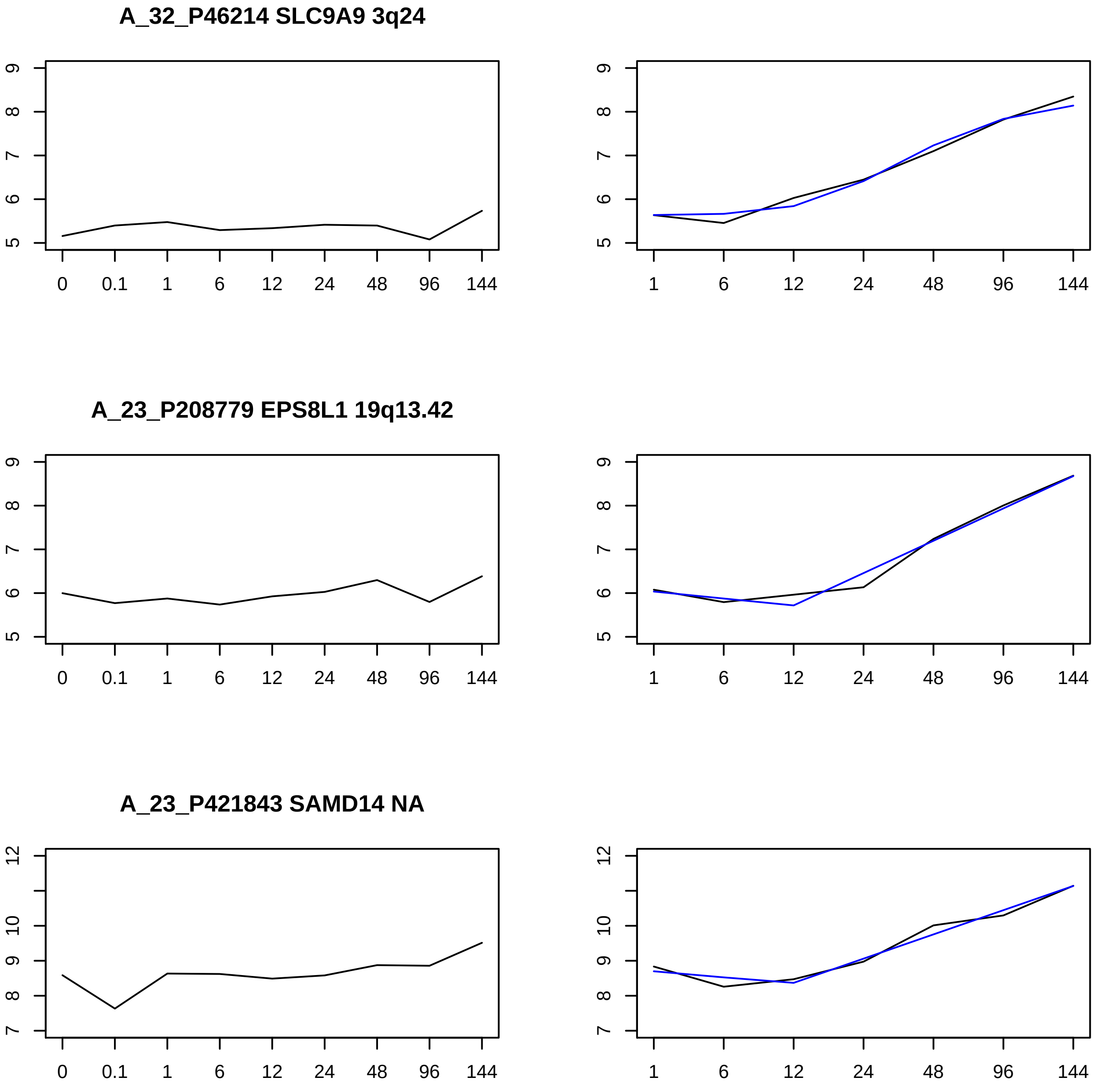
A_32_P46214 SLC9A9 3q24
A_23_P208779 EPS8L1 19q13.42
A_23_P421843 SAMD14 NA
0 0.1 1 6 12 24 48 96 144
1 6 12 24 48 96 144

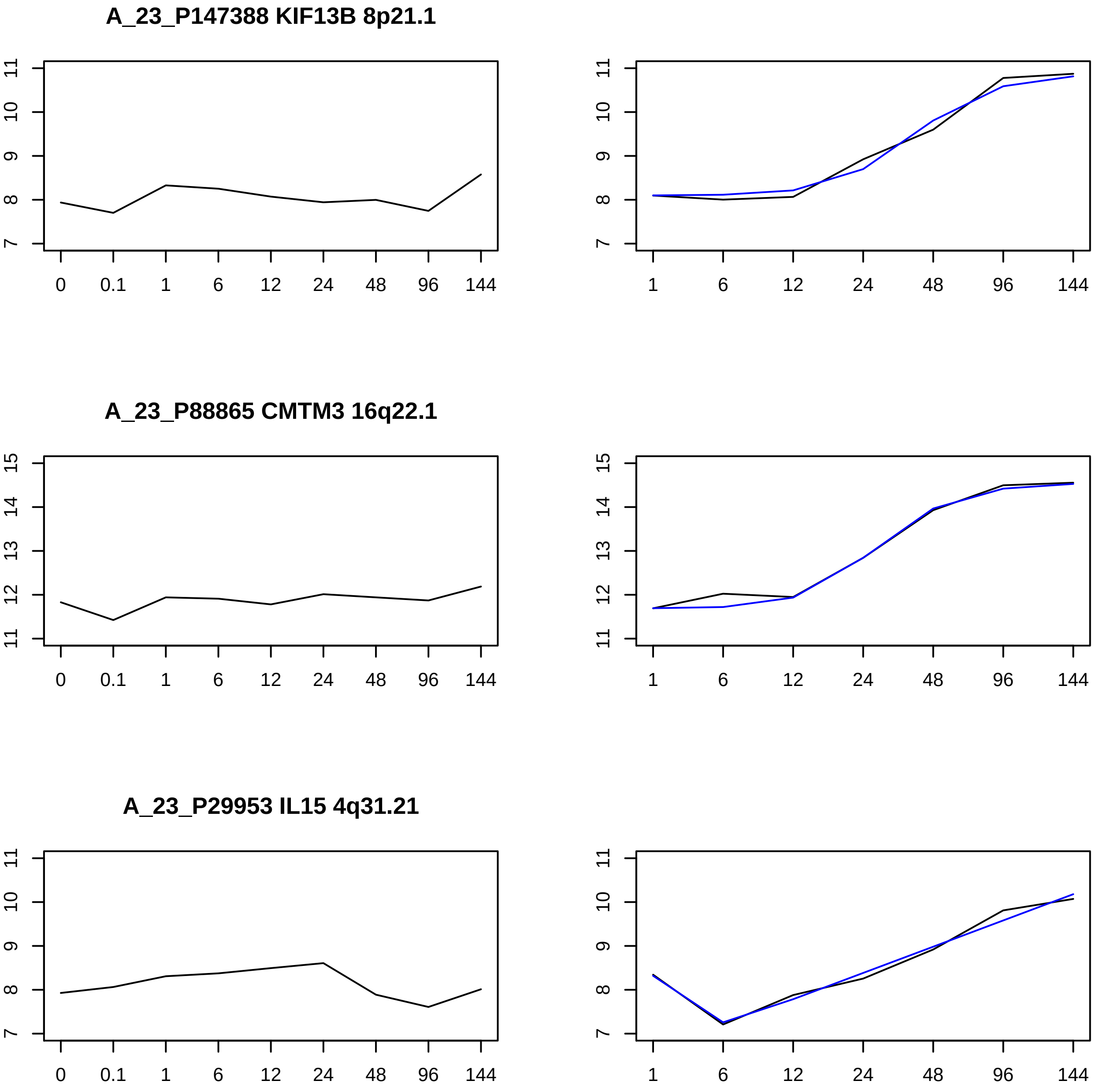
A_23_P147388 KIF13B 8p21.1
A_23_P88865 CMTM3 16q22.1
A_23_P29953 IL15 4q31.21
7 8 9 10 11
11 12 13 14 15
0 0.1 1 6 12 24 48 96 144
1 6 12 24 48 96 144

**A_23_P300220 YPEL4 11q12.1**

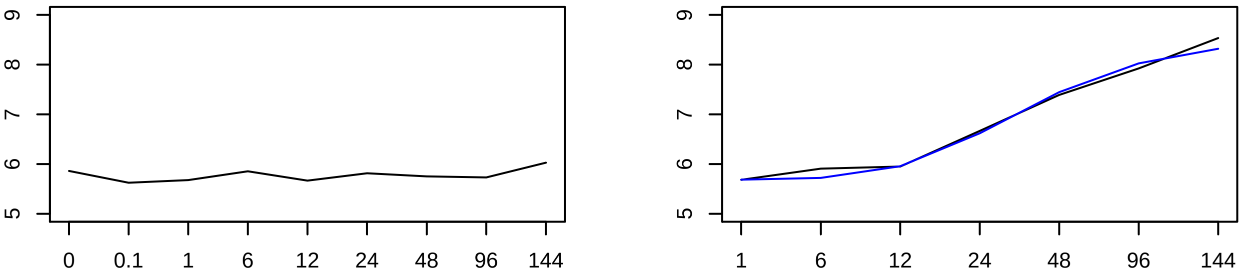


**A_23_P108673 TMEM166 2p12**

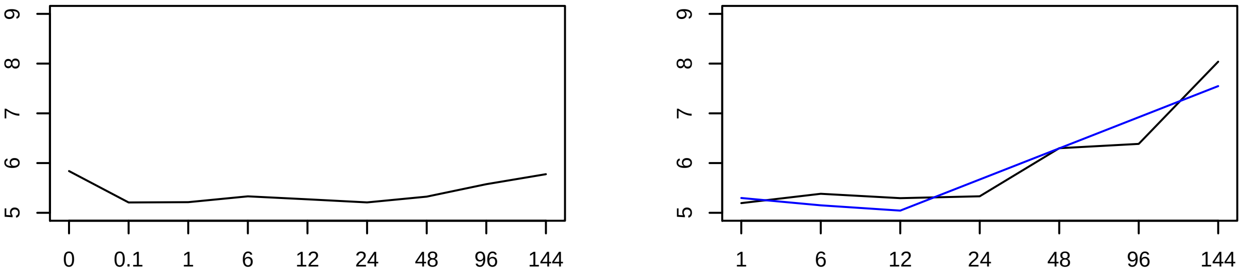


**A_23_P436369 FILIP1 6q14.1**

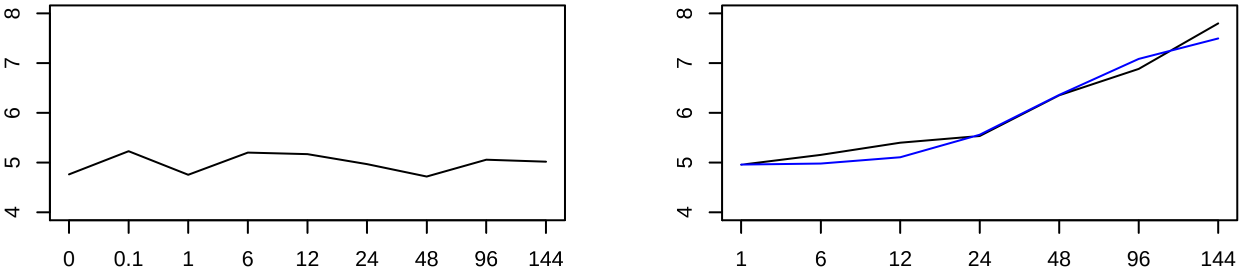

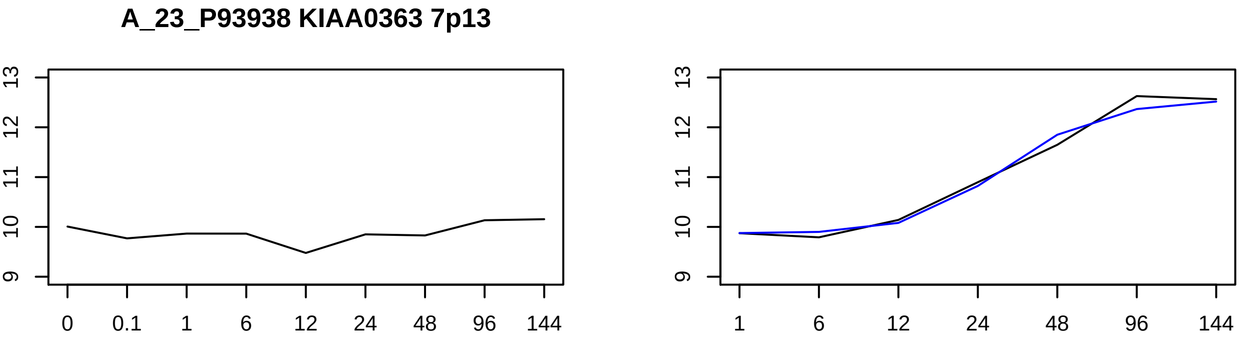
A_23_P93938 KIAA0363 7p13
9
10
11
12
13
0
0.1
1
6
12
24
48
96
144
9
10
11
12
13
1
6
12
24
48
96
144

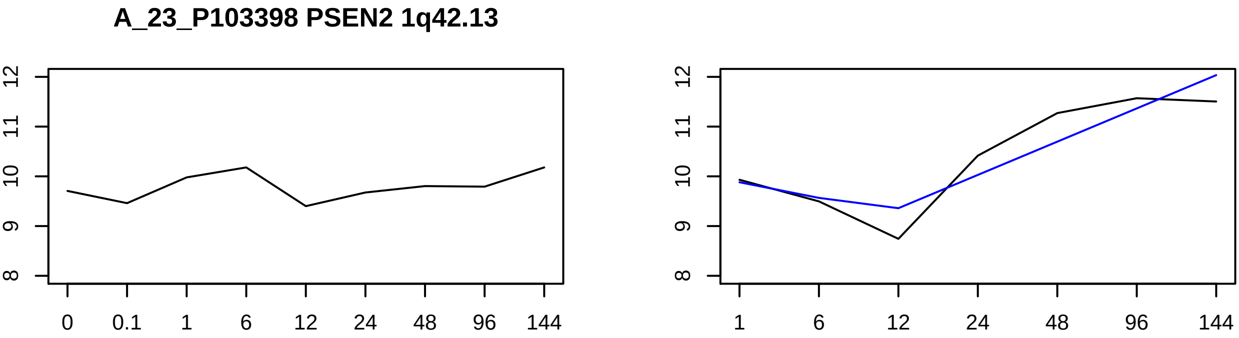
A_23_P103398 PSEN2 1q42.13
8
9
10
11
12
0
0.1
1
6
12
24
48
96
144
8
9
10
11
12
1
6
12
24
48
96
144

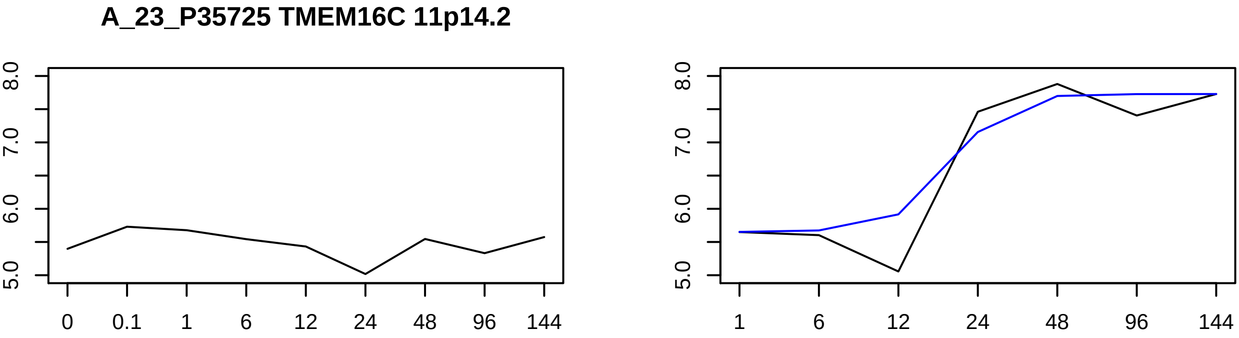
A_23_P35725 TMEM16C 11p14.2
5.0
6.0
7.0
8.0
0
0.1
1
6
12
24
48
96
144
5.0
6.0
7.0
8.0
1
6
12
24
48
96
144

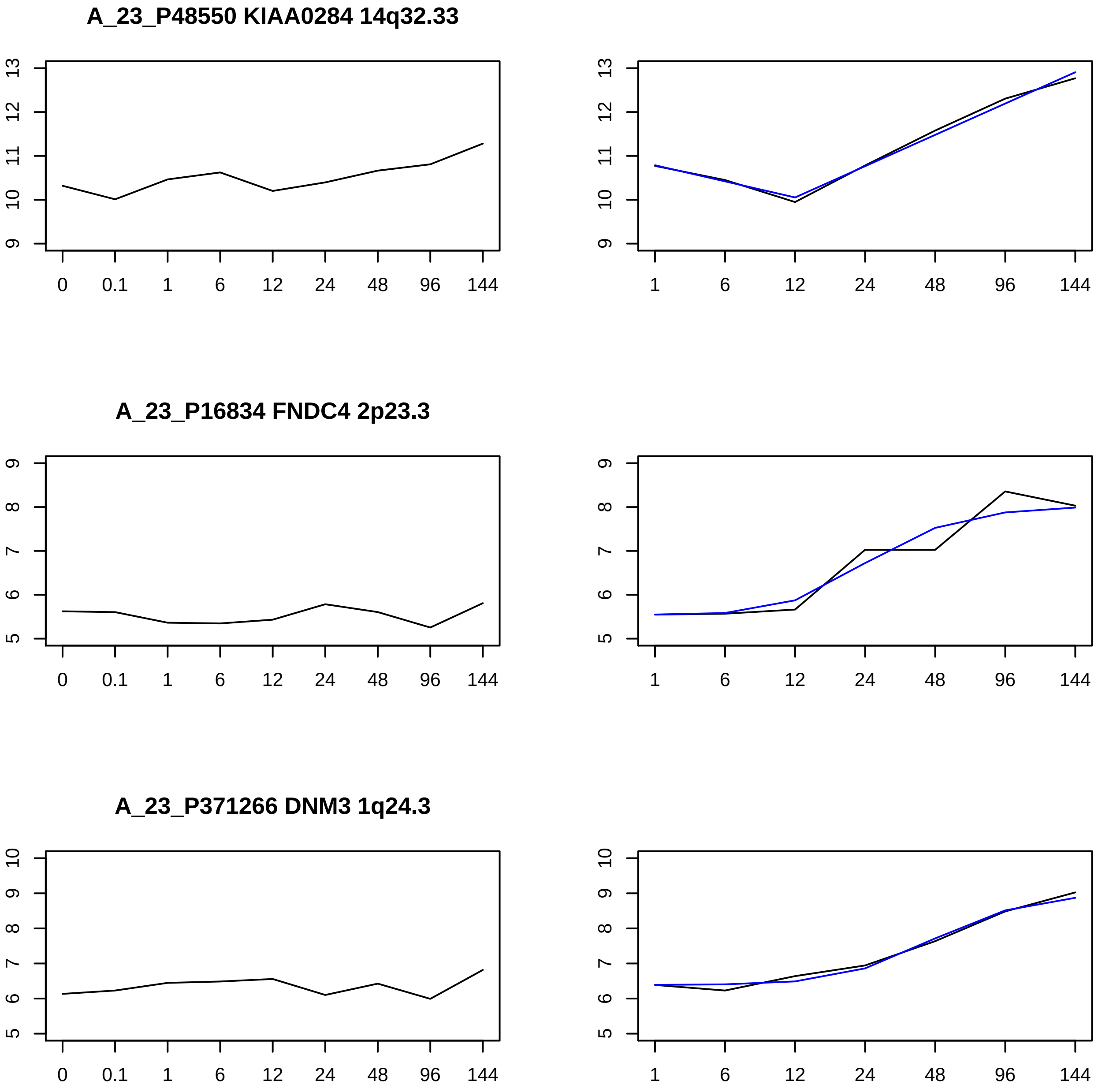
A_23_P48550 KIAA0284 14q32.33
9
10
11
12
13
0
0.1
1
6
12
24
48
96
144
1
6
12
24
48
96
144
A_23_P16834 FNDC4 2p23.3
5
6
7
8
9
0
0.1
1
6
12
24
48
96
144
1
6
12
24
48
96
144
A_23_P371266 DNM3 1q24.3
5
6
7
8
9
10
0
0.1
1
6
12
24
48
96
144
1
6
12
24
48
96
144

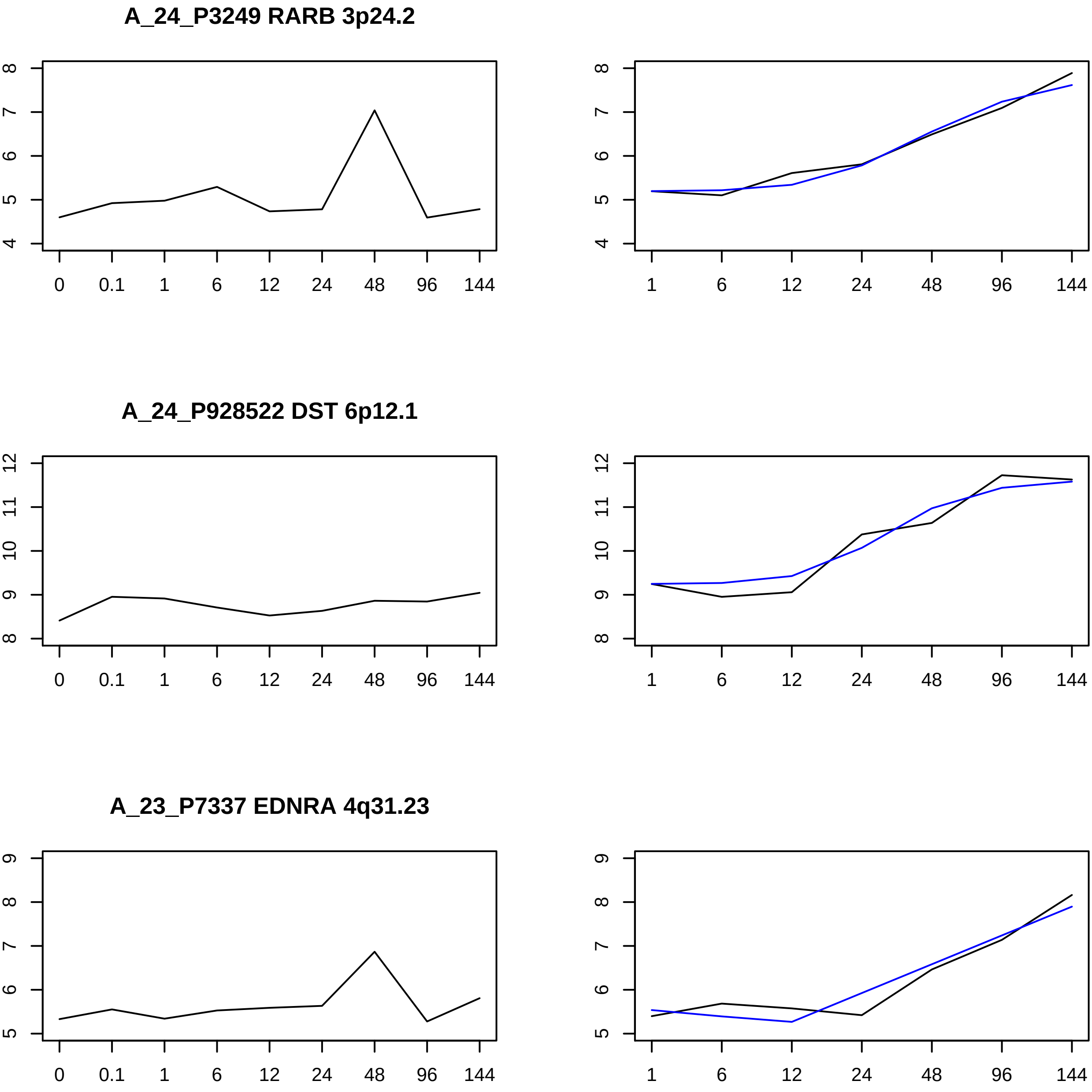
A_24_P3249 RARB 3p24.2
A_24_P928522 DST 6p12.1
A_23_P7337 EDNRA 4q31.23
0
0.1
1
6
12
24
48
96
144

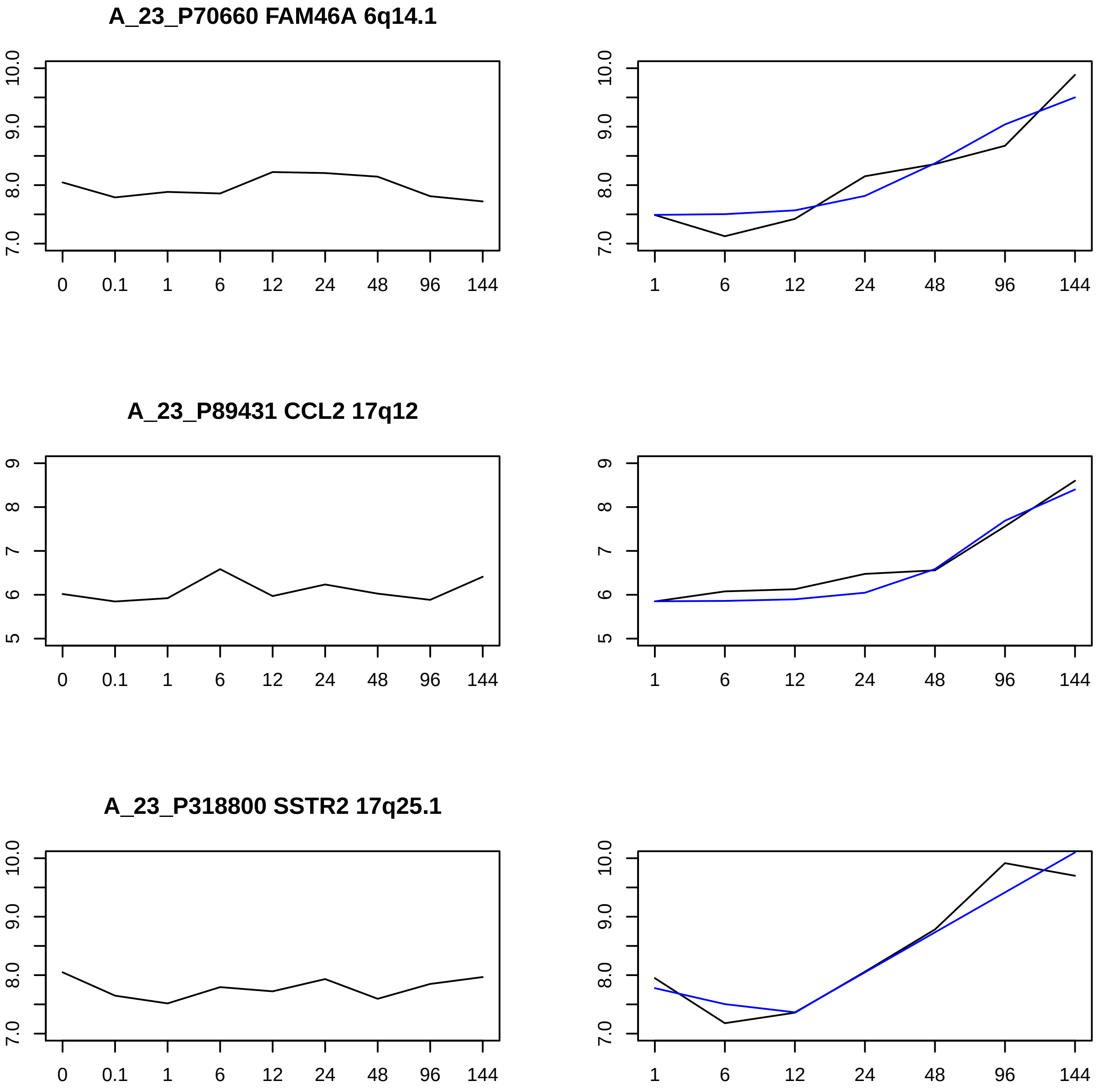
A_23_P70660 FAM46A 6q14.1
A_23_P89431 CCL2 17q12
A_23_P318800 SSTR2 17q25.1
7.0 8.0 9.0 10.0
5 6 7 8 9
0 0.1 1 6 12 24 48 96 144
1 6 12 24 48 96 144

**A_32_P9368 EFNA2 NA**

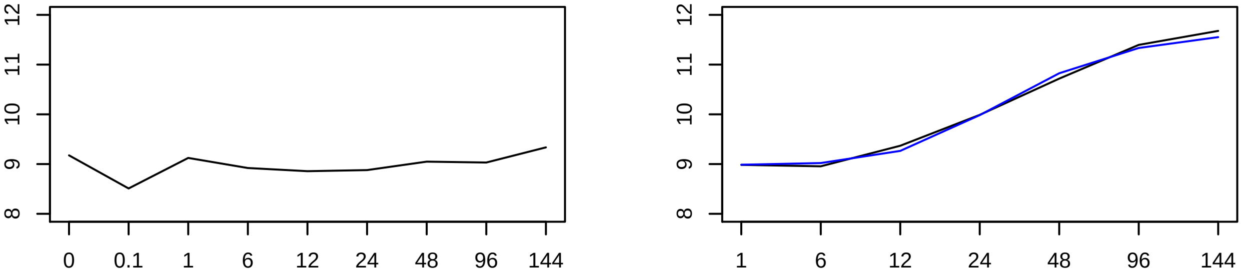


**A_23_P126593 S100A11 1q21.3**

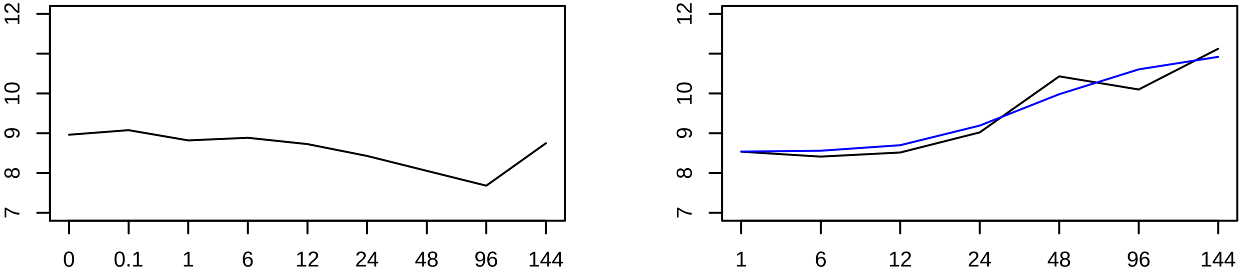


**A_32_P94798 ANXA2 15q22.2**

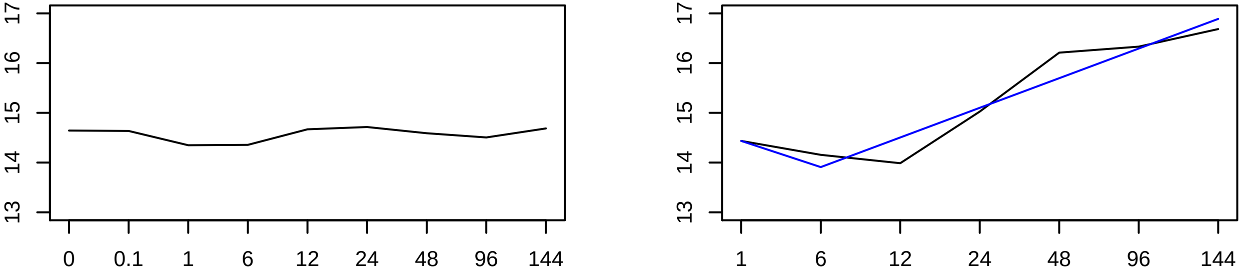

**A_23_P369328 C10orf35 10q21.3**

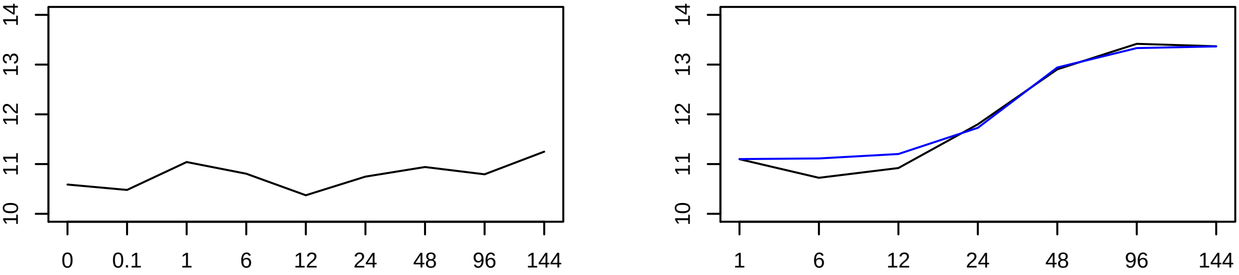


**A_24_P204244 ANXA2P1 4q31.3**

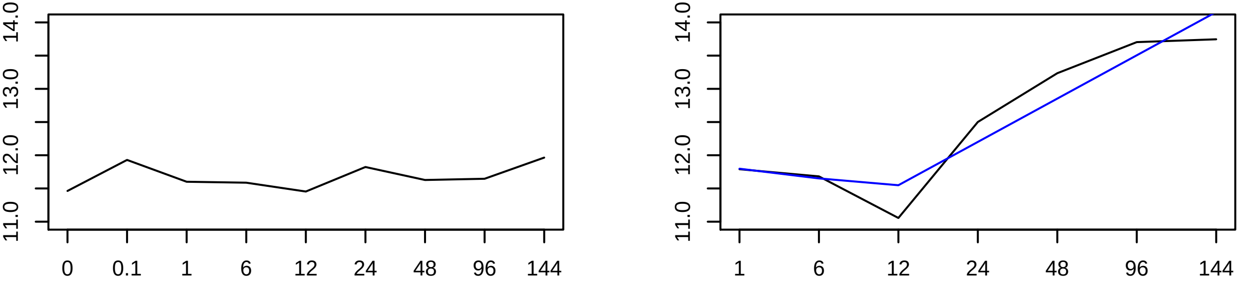


**A_23_P31896 ST3GAL1 8q24.22**

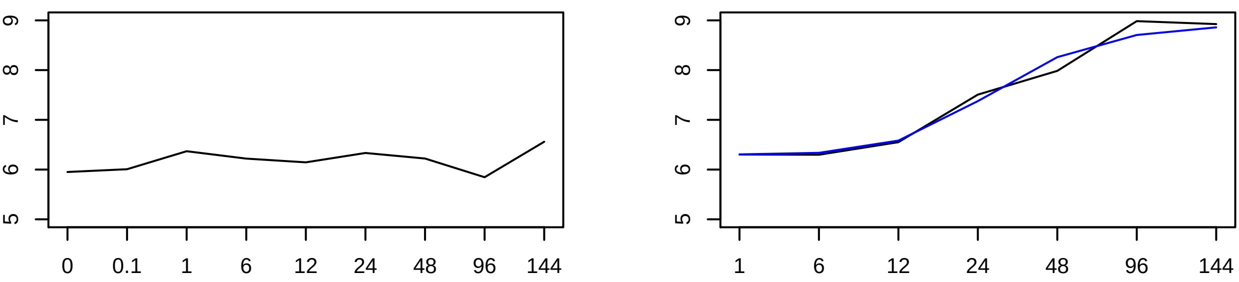

**A_24_P854492 MIAT 22q12.1**

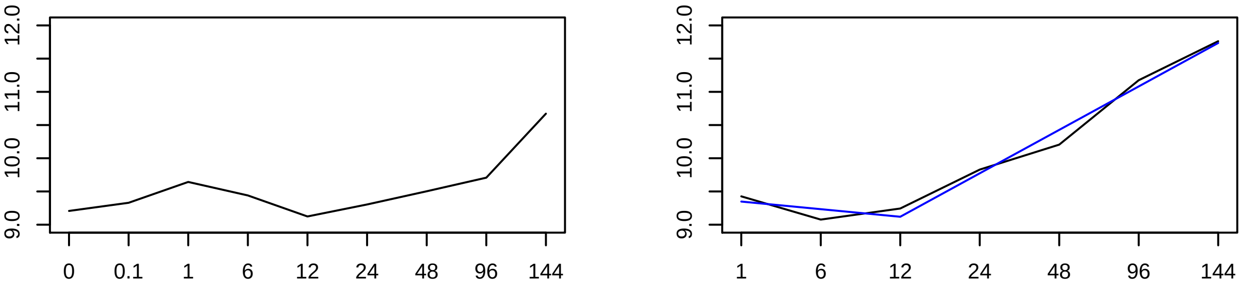


**A_23_P397120 LOC284422 19p13.3**

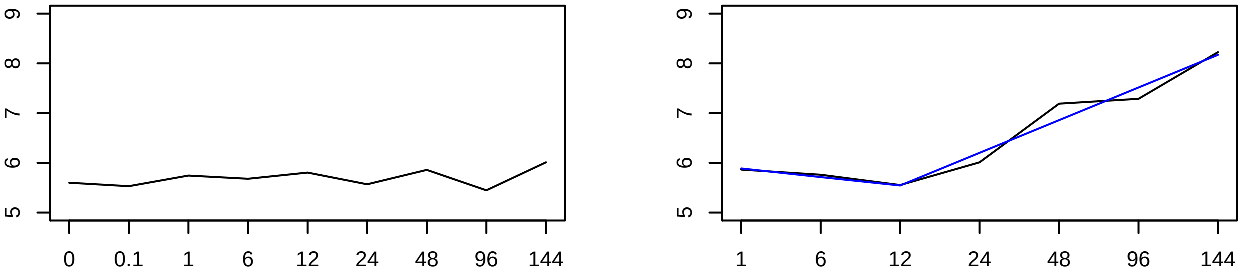


**A_23_P134744 RNF122 8p12**

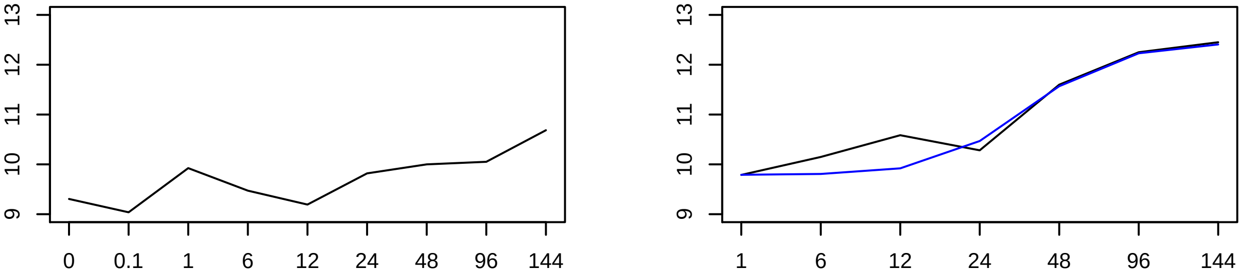

**A_23_P63447 THC2641682 NA**

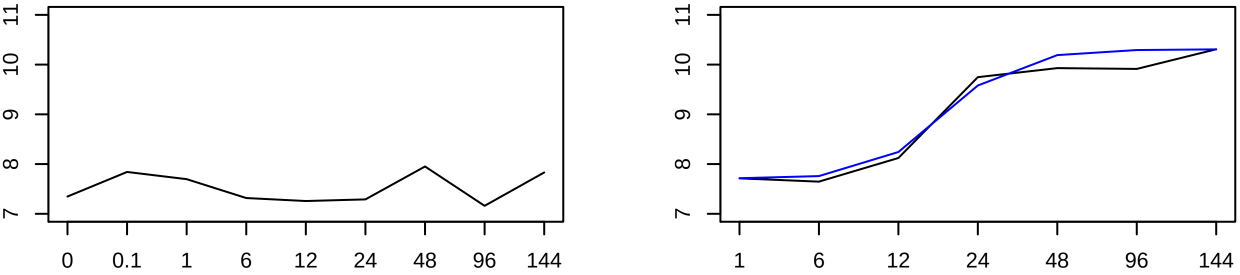


**A_23_P301021 ASTN1 1q25.2**

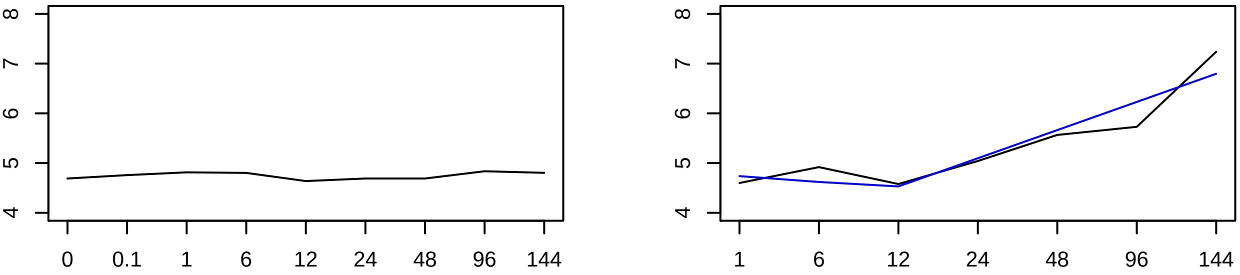


**A_24_P230869 PRSS12 4q26**

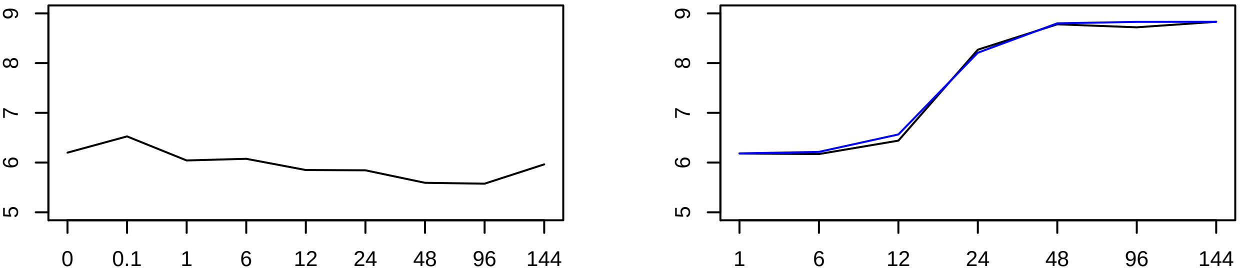

**A_23_P112726 SCN9A 2q24.3**

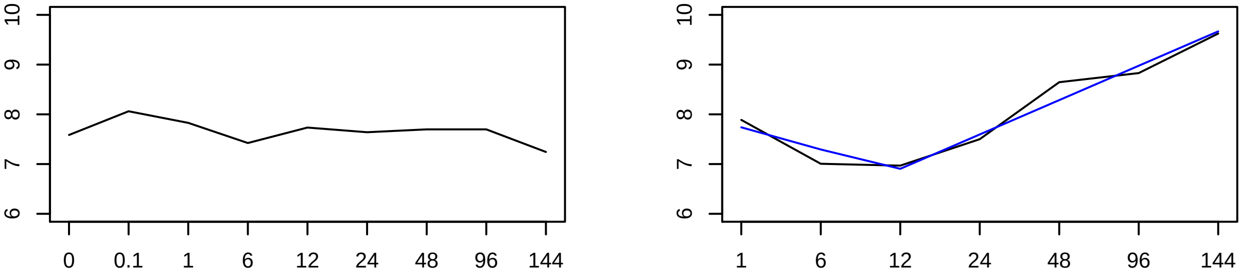


**A_23_P70818 SMO 7q32.1**

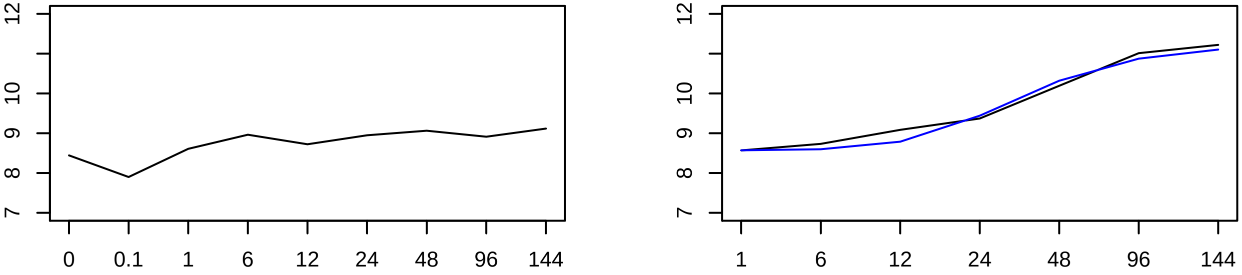


**A_32_P98423 C5orf13 5q22.1**

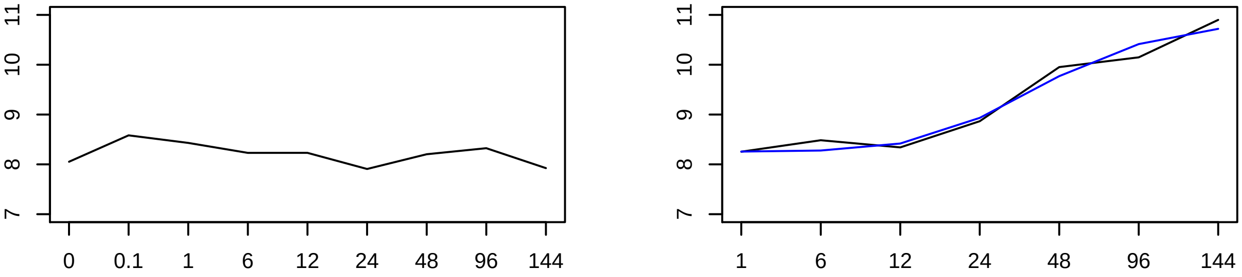

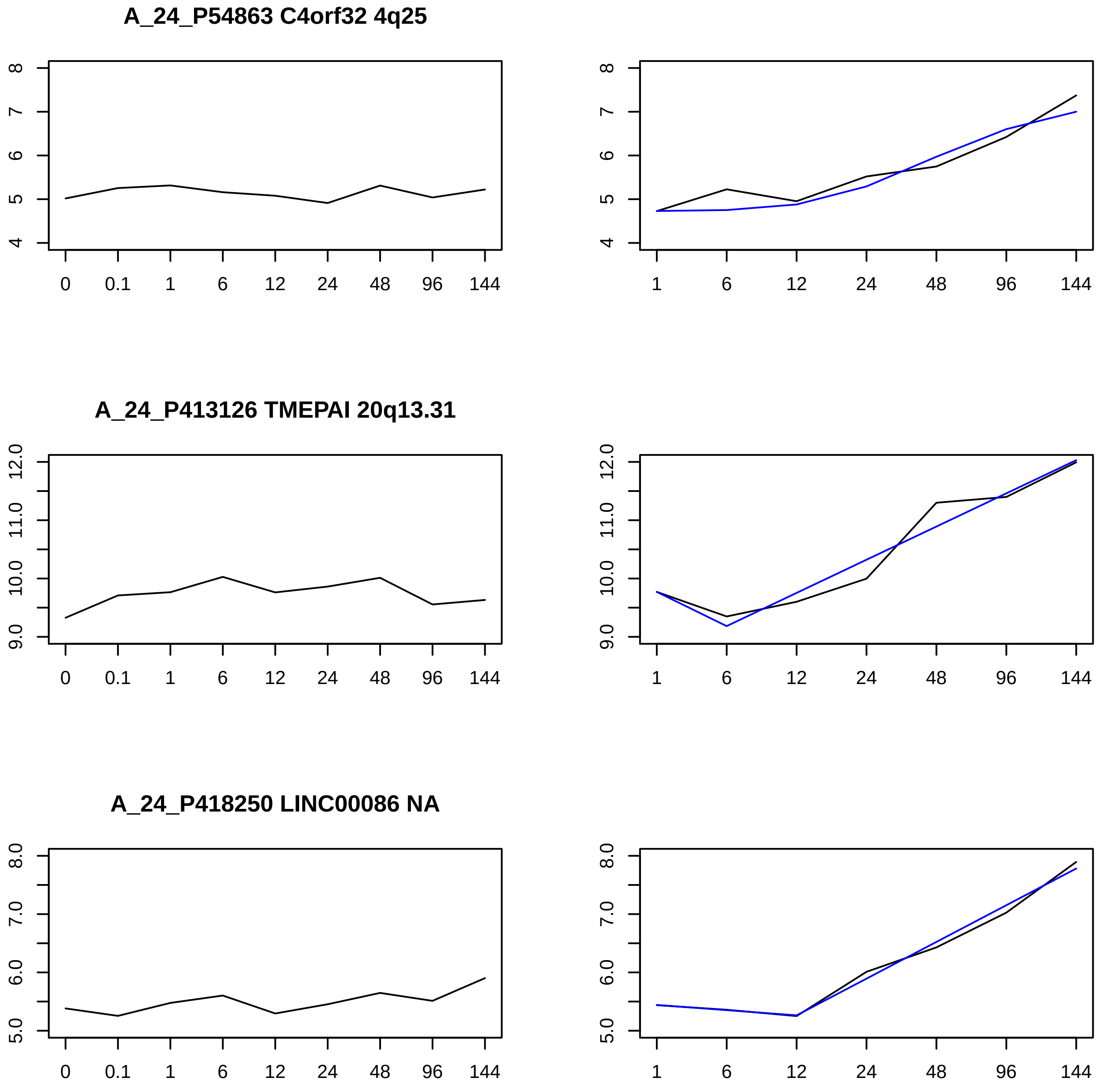
A_24_P54863 C4orf32 4q25
4
5
6
7
8
0
0.1
1
6
12
24
48
96
144
A_24_P413126 TMEPAI 20q13.31
9.0
10.0
11.0
12.0
A_24_P418250 LINC00086 NA
5.0
6.0
7.0
8.0

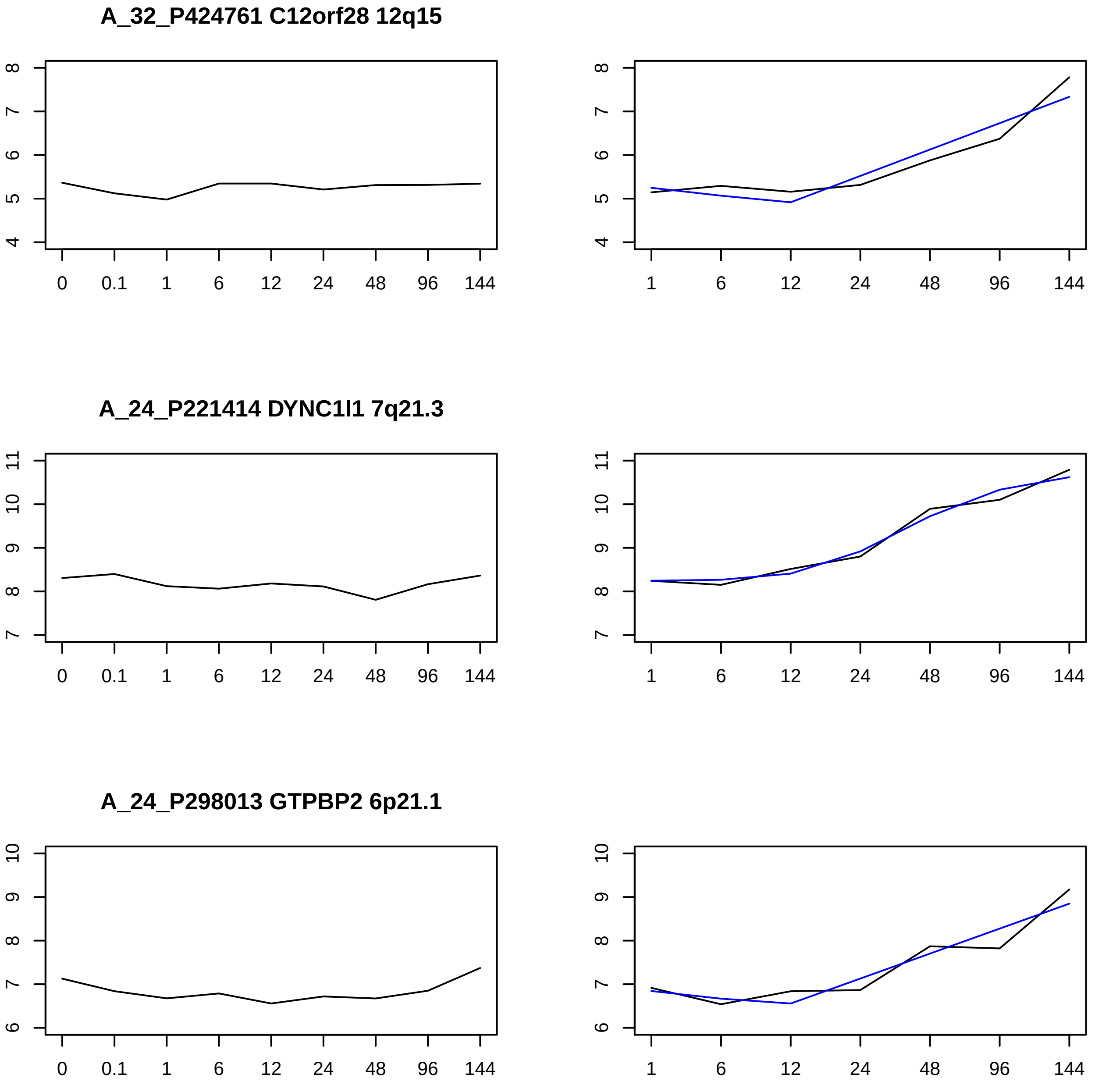
A_32_P424761 C12orf28 12q15
4 5 6 7 8
0 0.1 1 6 12 24 48 96 144
1 6 12 24 48 96 144
A_24_P221414 DYNC1I1 7q21.3
7 8 9 10 11
0 0.1 1 6 12 24 48 96 144
1 6 12 24 48 96 144
A_24_P298013 GTPBP2 6p21.1
6 7 8 9 10
0 0.1 1 6 12 24 48 96 144
1 6 12 24 48 96 144

**A_32_P104432 LINC00087 Xq26.3**

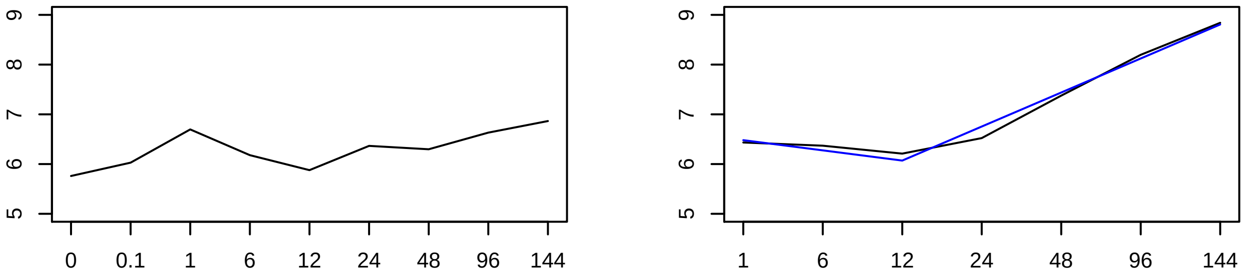


**A_23_P24414 EFEMP2 11q13.1**

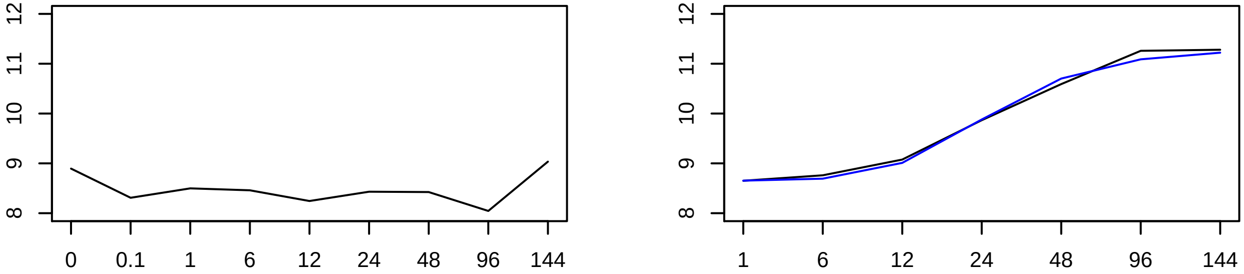


**A_23_P303455 GPR161 1q24.2**

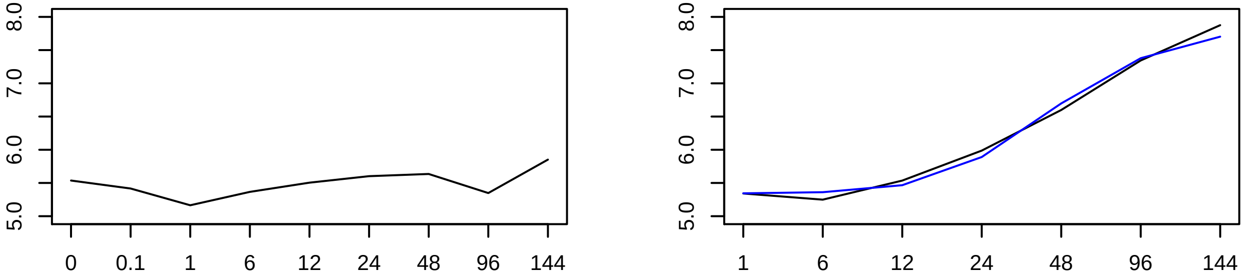

**A_23_P211957 TGFBR2 3p24.1**

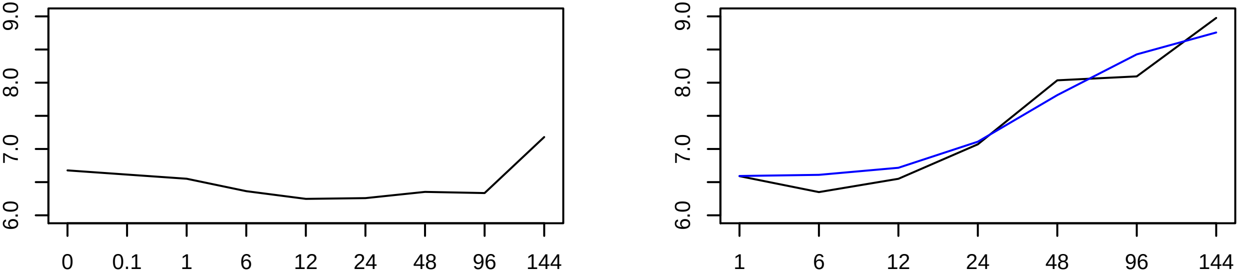


**A_23_P120125 COLEC11 2p25.3**

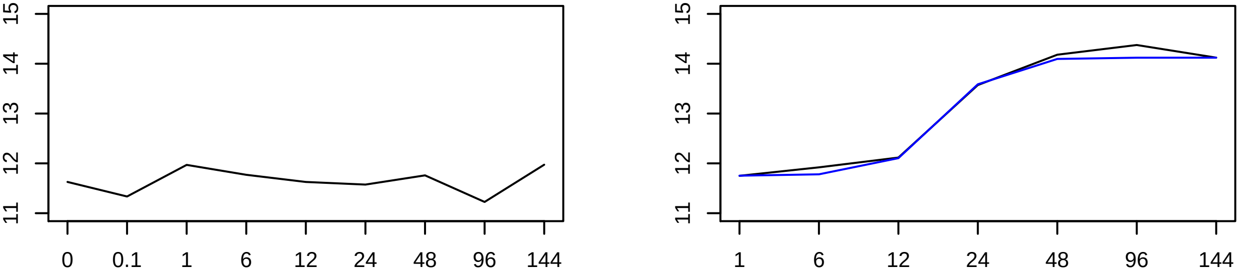


**A_23_P110571 MAST4 NA**

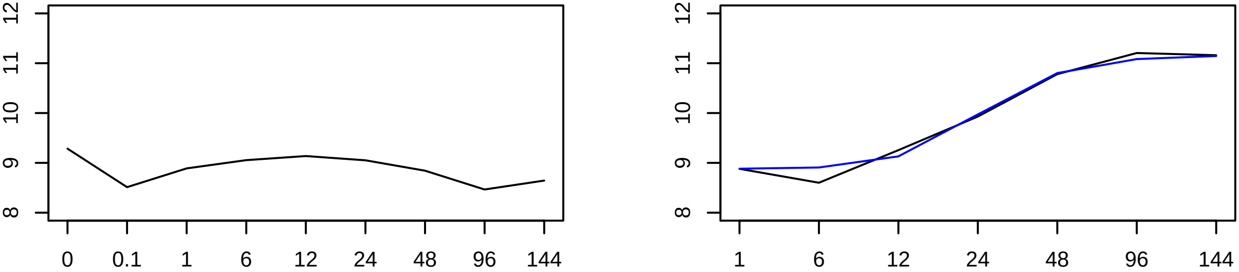

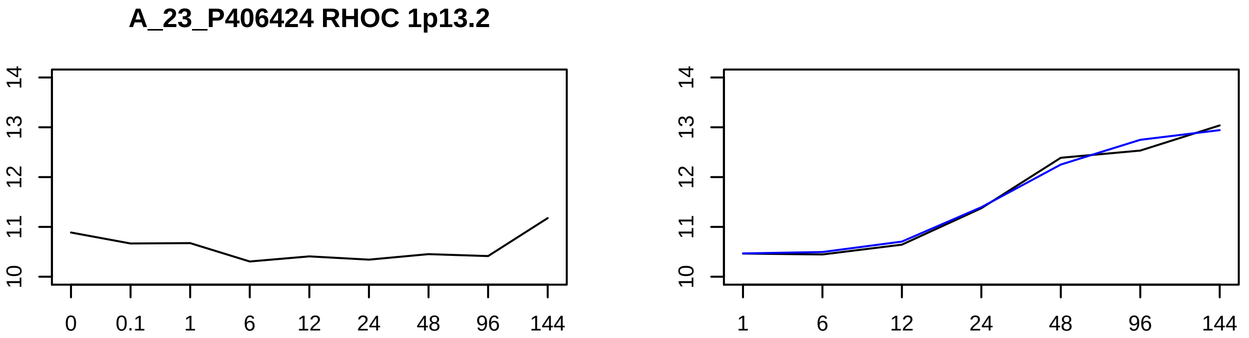
A_23_P406424 RHOC 1p13.2
10
11
12
13
14
0
0.1
1
6
12
24
48
96
144
1
6
12
24
48
96
144

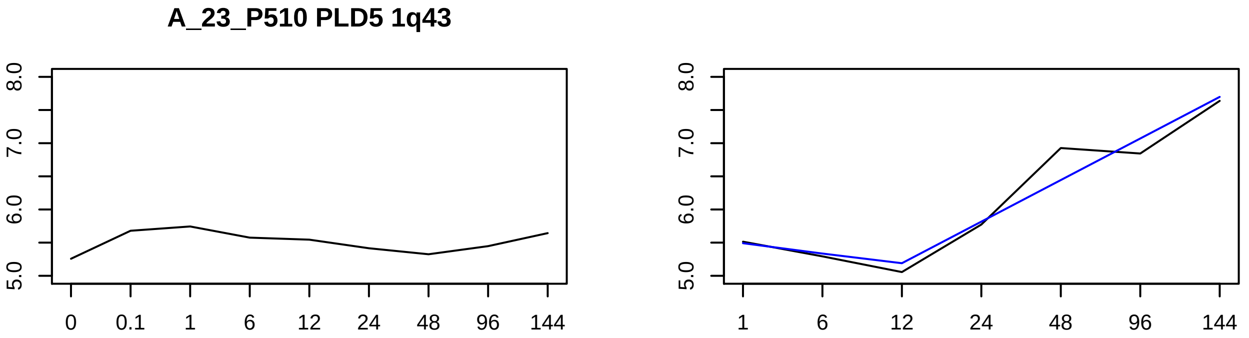
A_23_P510 PLD5 1q43
5.0
6.0
7.0
8.0
0
0.1
1
6
12
24
48
96
144
1
6
12
24
48
96
144

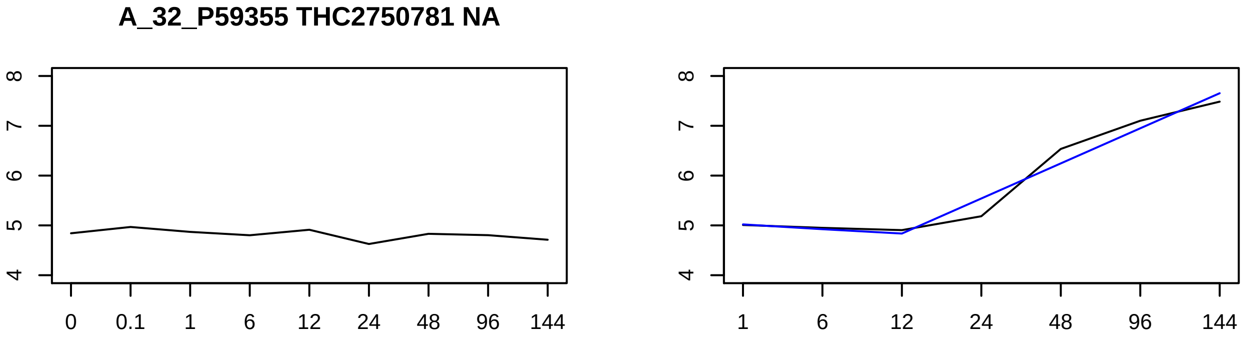
A_32_P59355 THC2750781 NA
4
5
6
7
8
0
0.1
1
6
12
24
48
96
144
1
6
12
24
48
96
144

**A_23_P84106 IL1RAPL1 Xp21.2**

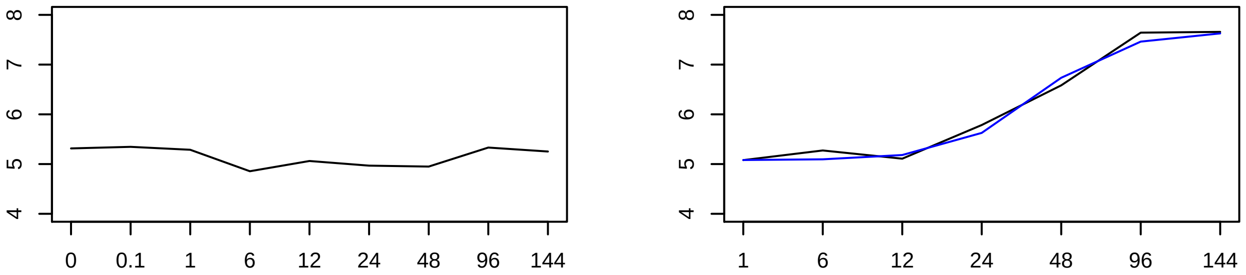


**A_23_P501822 JUP 17q21.2**

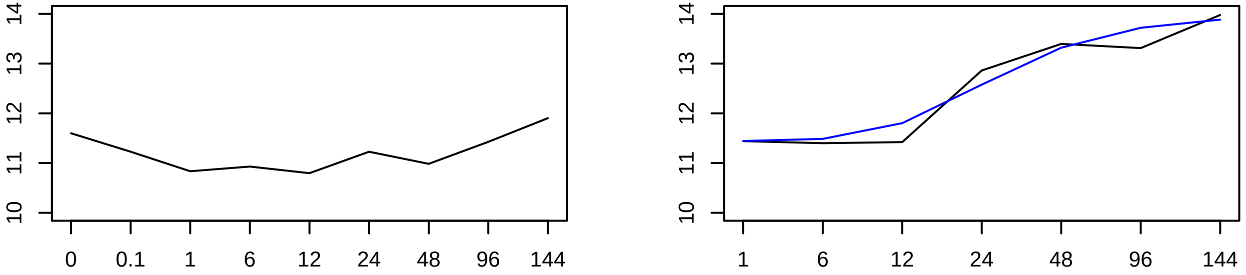


**A_23_P399255 RNF182 6p23**

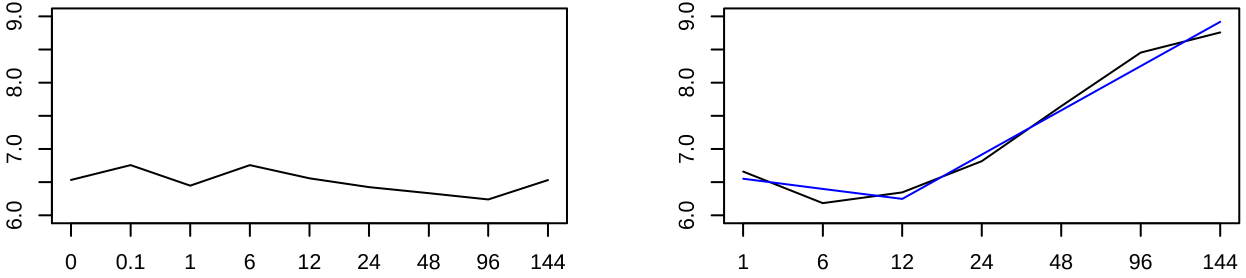

**A_23_P68106 TMSB10 2p11.2**

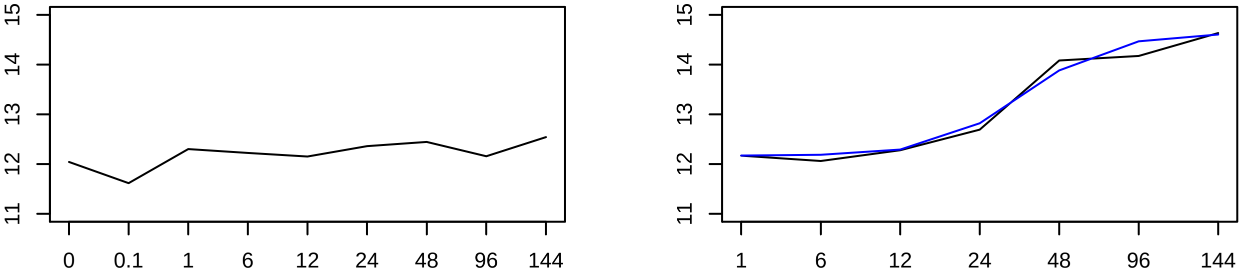


**A_23_P251795 GPC2 7q22.1**

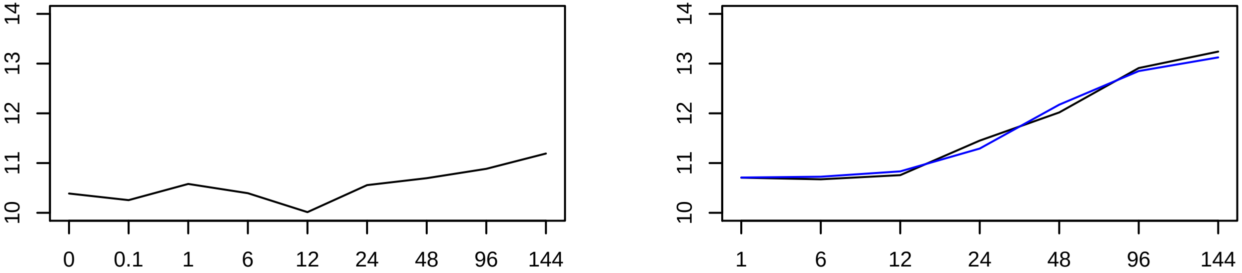


**A_23_P5392 TP53I3 2p23.3**

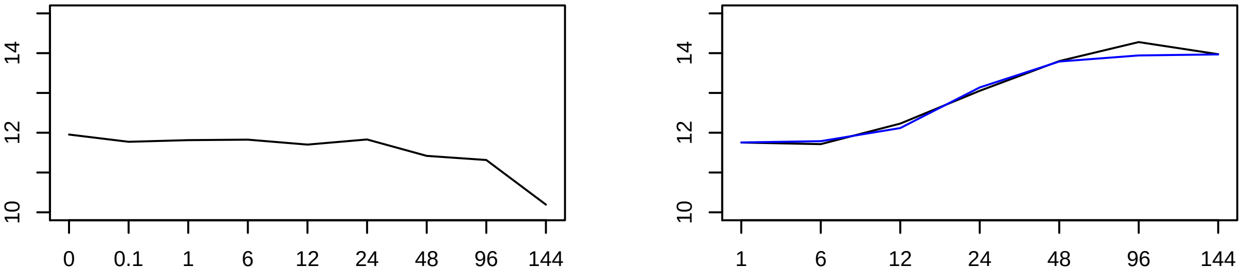

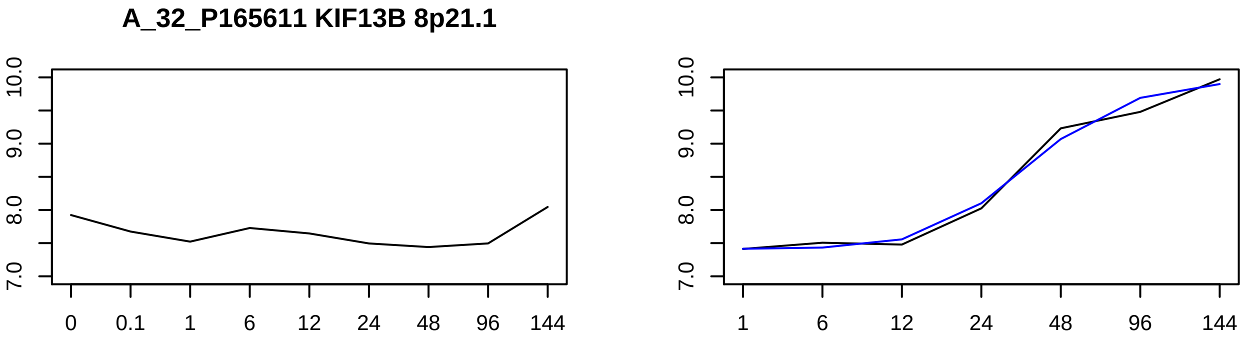
A_32_P165611 KIF13B 8p21.1
7.0
8.0
9.0
10.0
0
0.1
1
6
12
24
48
96
144
1
6
12
24
48
96
144

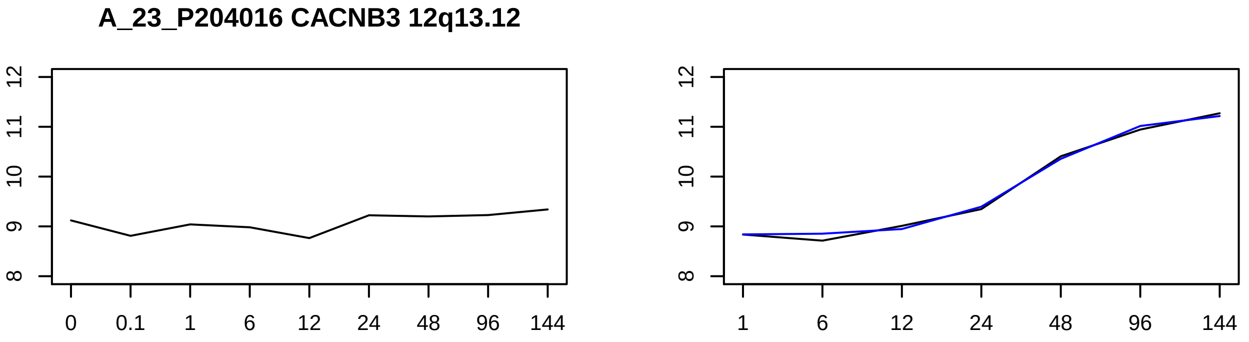
A_23_P204016 CACNB3 12q13.12
8
9
10
11
12
0
0.1
1
6
12
24
48
96
144
1
6
12
24
48
96
144

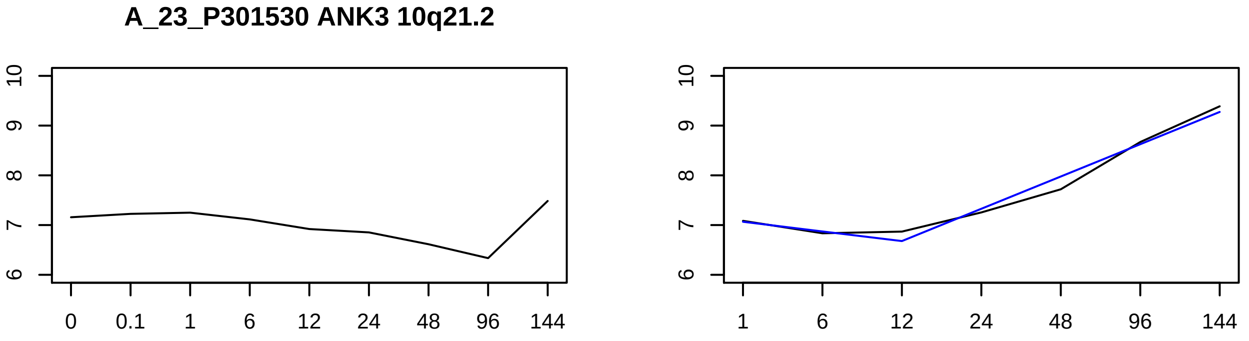
A_23_P301530 ANK3 10q21.2
6
7
8
9
10
0
0.1
1
6
12
24
48
96
144
1
6
12
24
48
96
144

**A_23_P155848 DKK2 4q25**

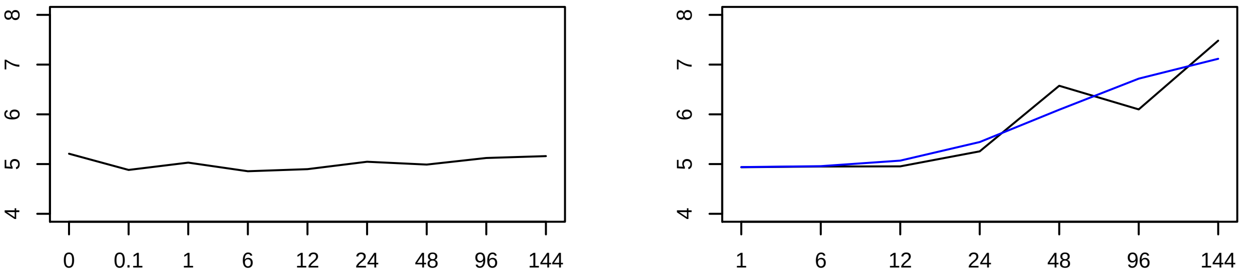


**A_23_P259621 LAT2 7q11.23**

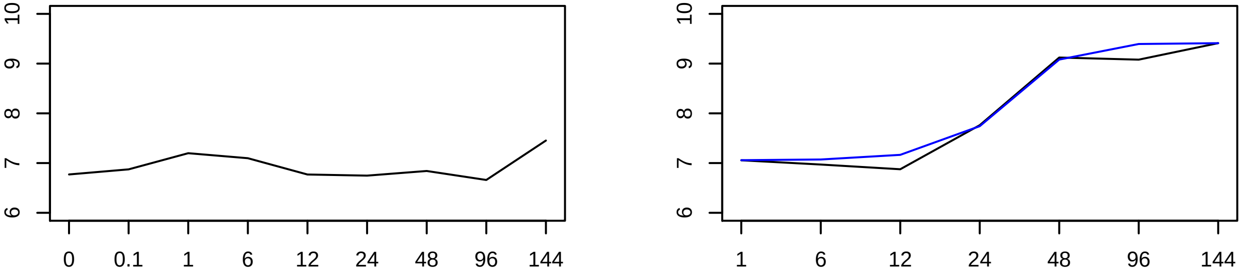


**A_24_P62521 PSEN2 1q42.13**

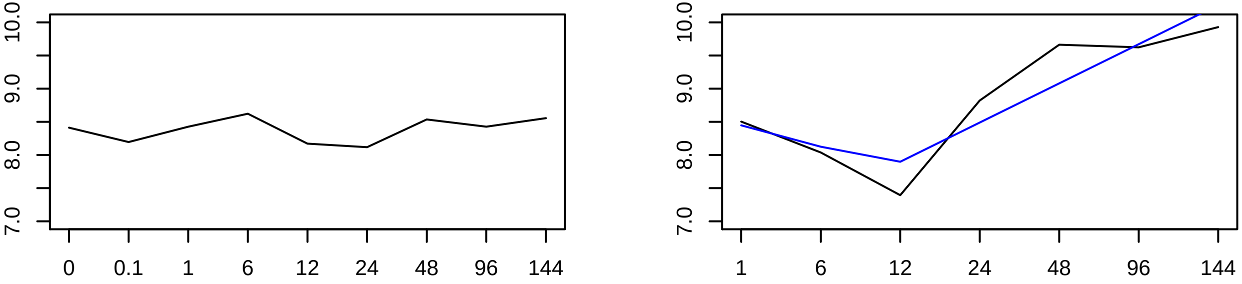

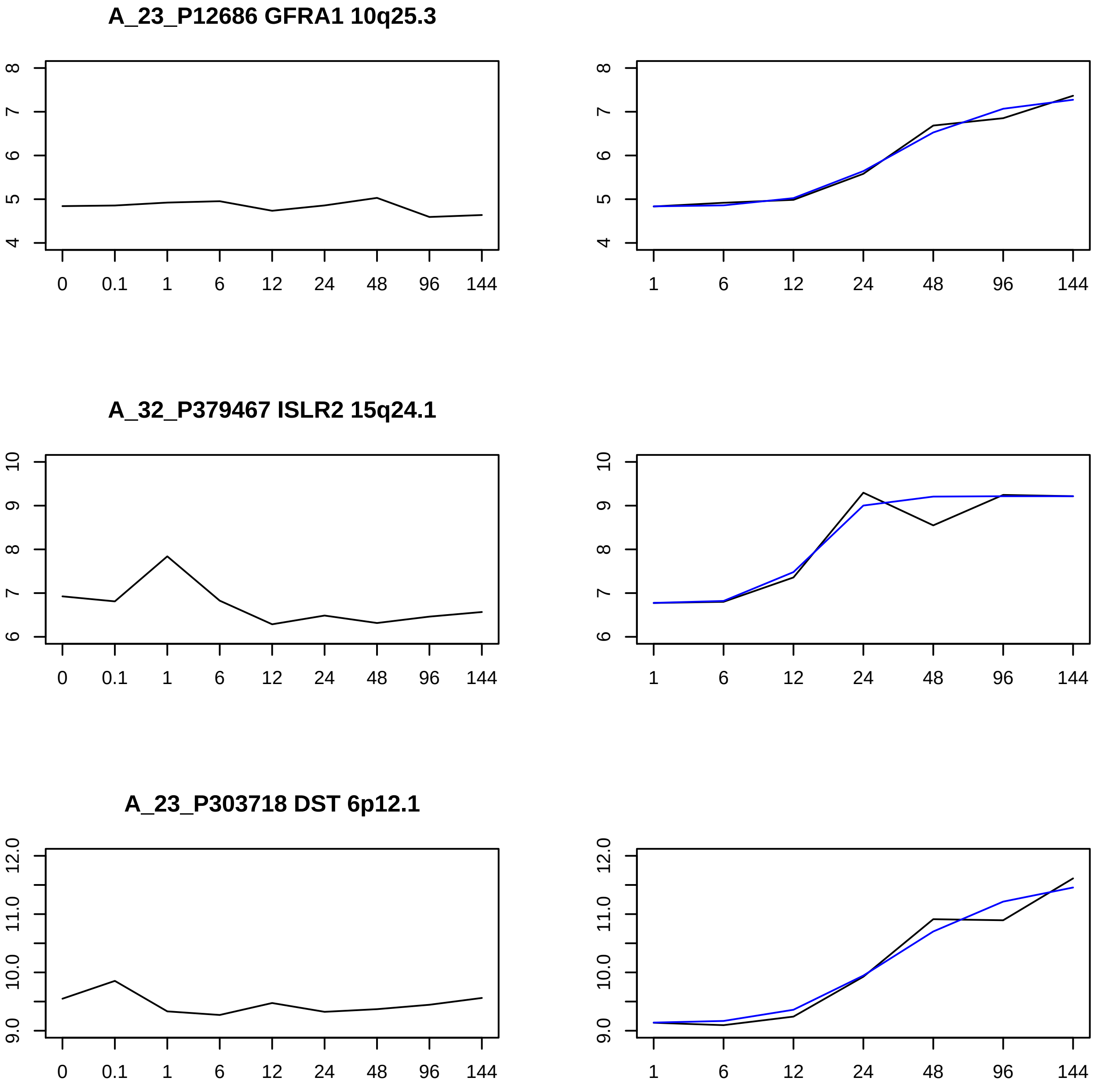

A_23_P12686 GFRA1 10q25.3
A_32_P379467 ISLR2 15q24.1
A_23_P303718 DST 6p12.1
0
0.1
1
6
12
24
48
96
144

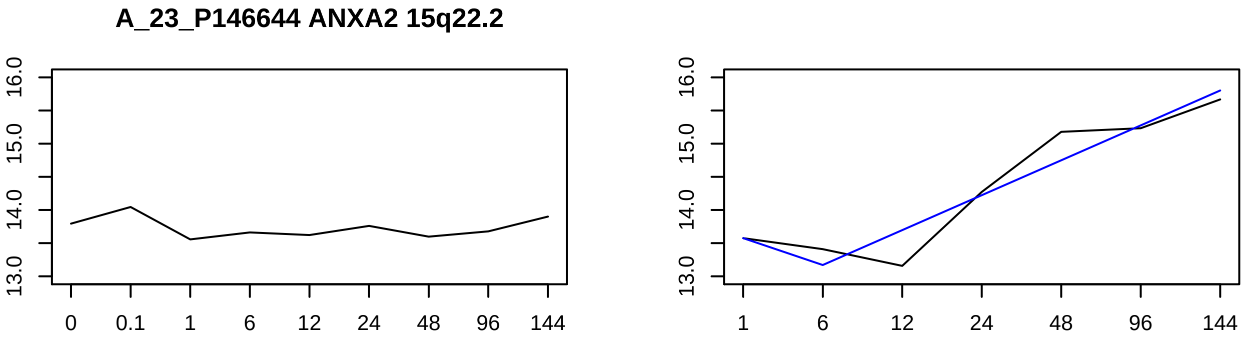
A_23_P146644 ANXA2 15q22.2
13.0
14.0
15.0
16.0
0
0.1
1
6
12
24
48
96
144

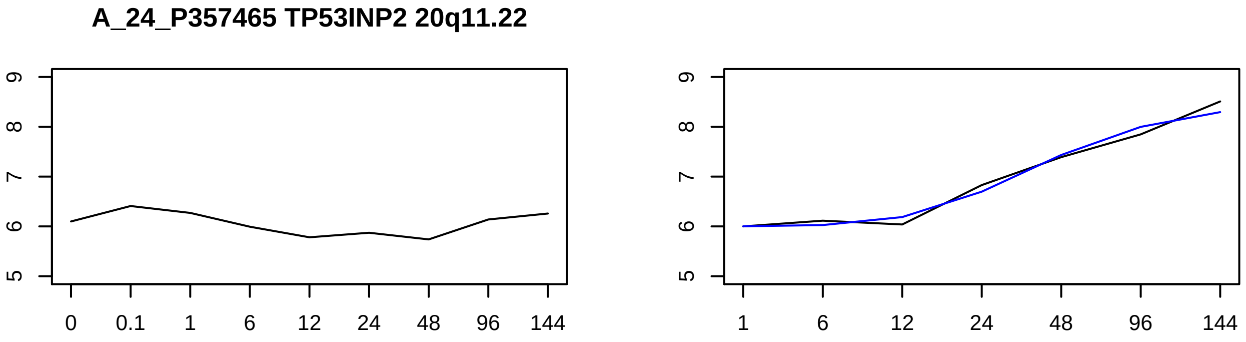
A_24_P357465 TP53INP2 20q11.22
5
6
7
8
9
0
0.1
1
6
12
24
48
96
144

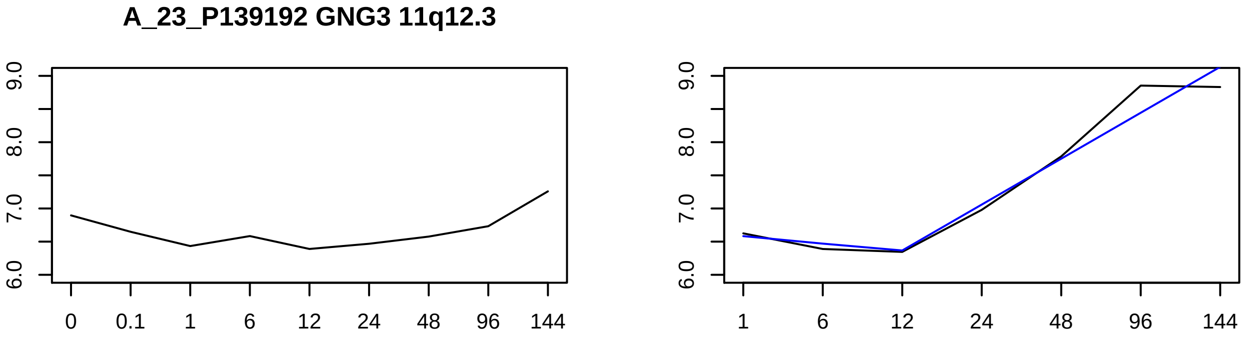
A_23_P139192 GNG3 11q12.3
6.0
7.0
8.0
9.0
0
0.1
1
6
12
24
48
96
144

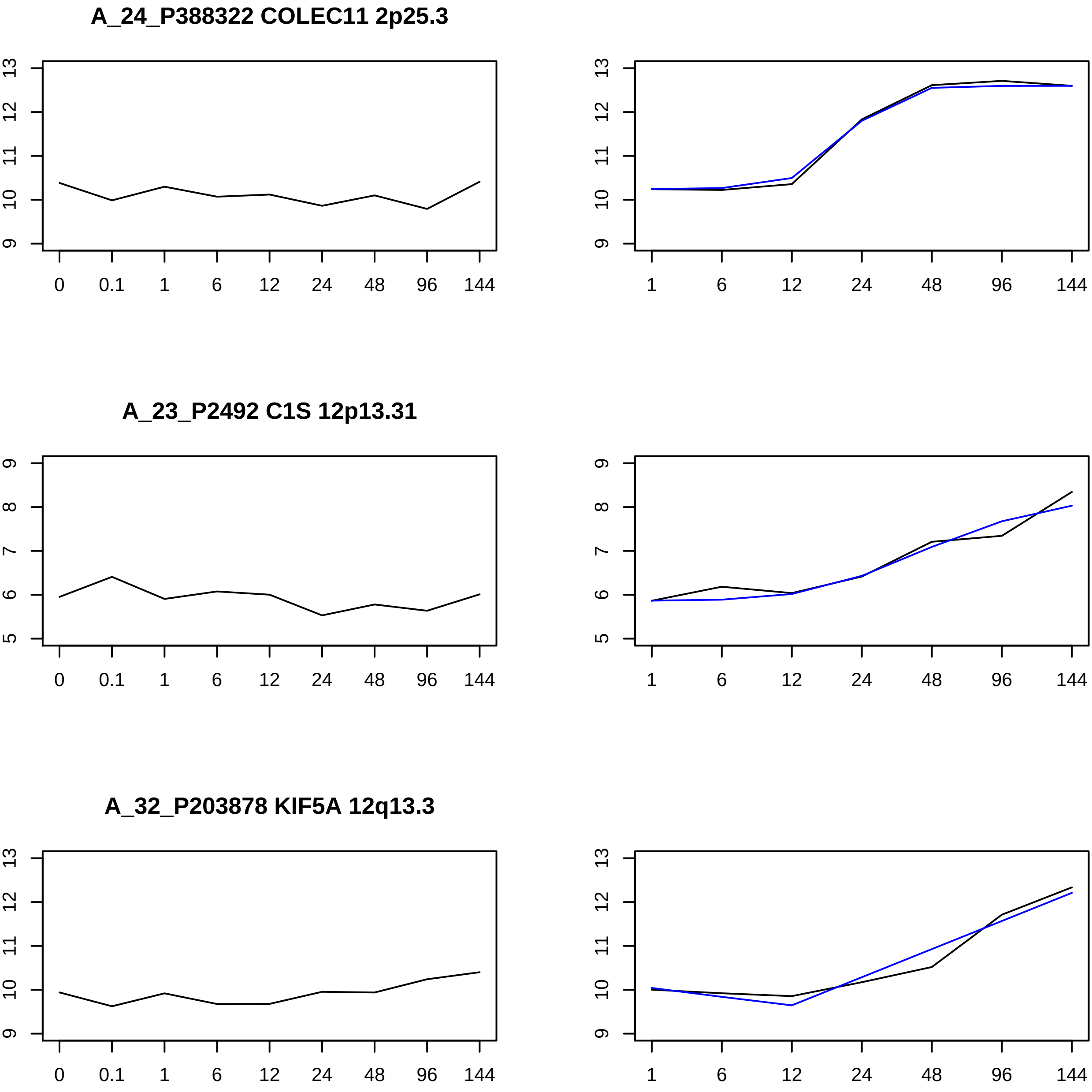
A_24_P388322 COLEC11 2p25.3
A_23_P2492 C1S 12p13.31
A_32_P203878 KIF5A 12q13.3
0 0.1 1 6 12 24 48 96 144
1 6 12 24 48 96 144
9 10 11 12 13
5 6 7 8 9

**A_23_P211233 COL6A2 21q22.3**

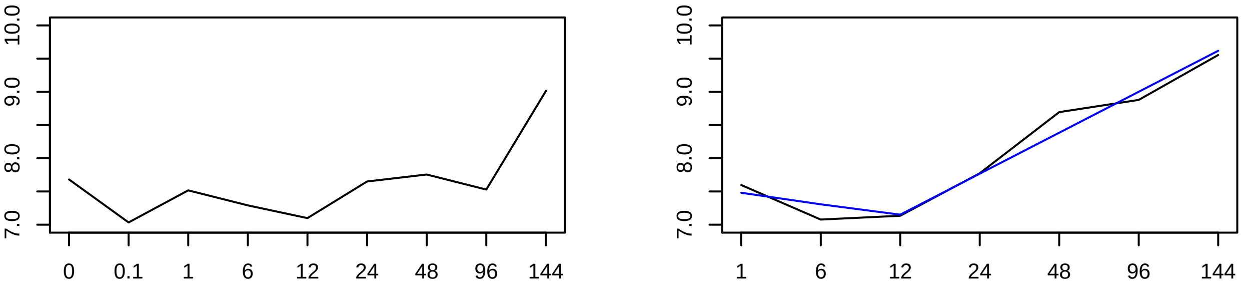


**A_23_P57836 A_23_P57836 NA**

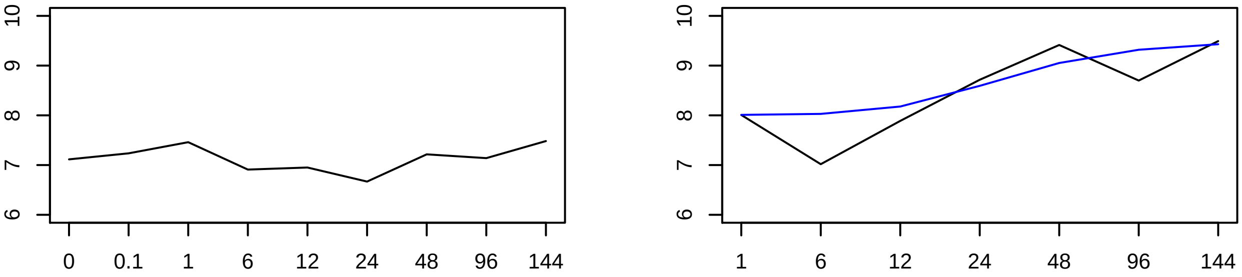


**A_23_P378364 PCDH7 4p15.1**

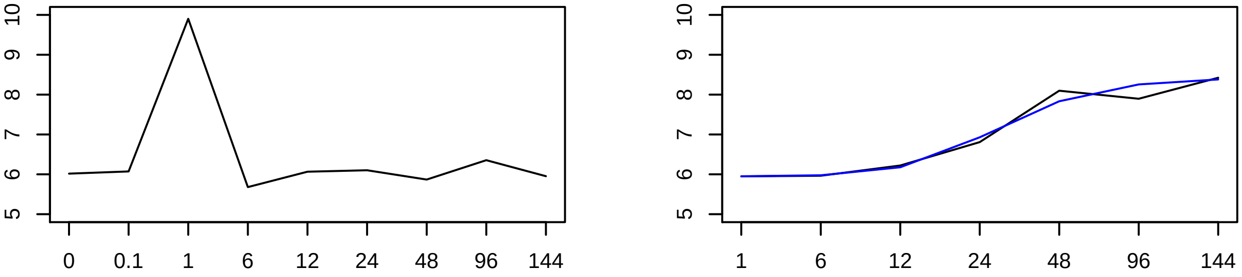

**A_23_P23346 MLLT11 1q21.2**

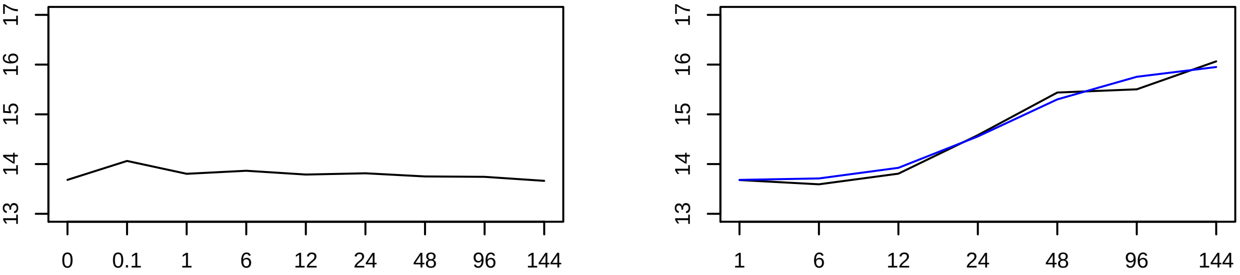


**A_23_P62115 TIMP1 Xp11.23**

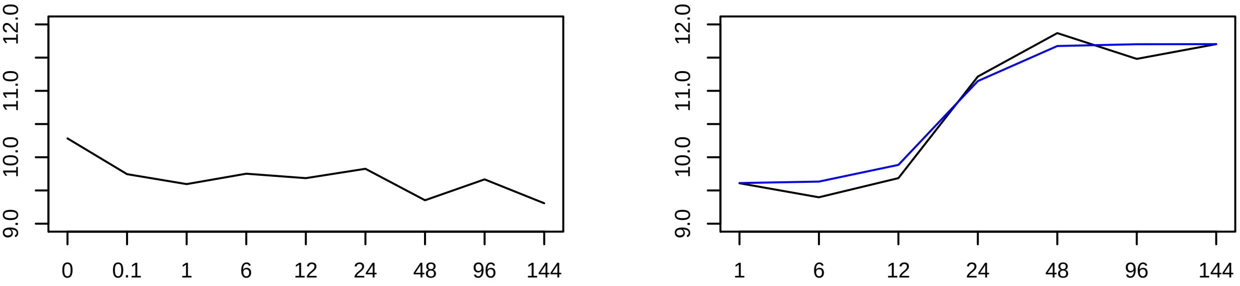


**A_24_P140608 HBEGF 5q31.3**

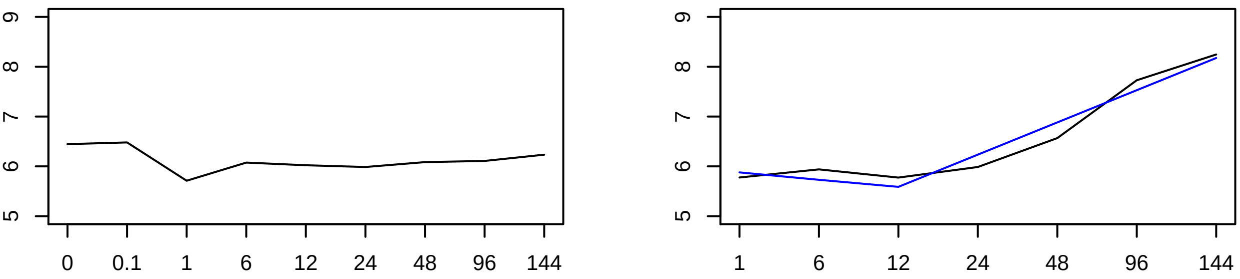

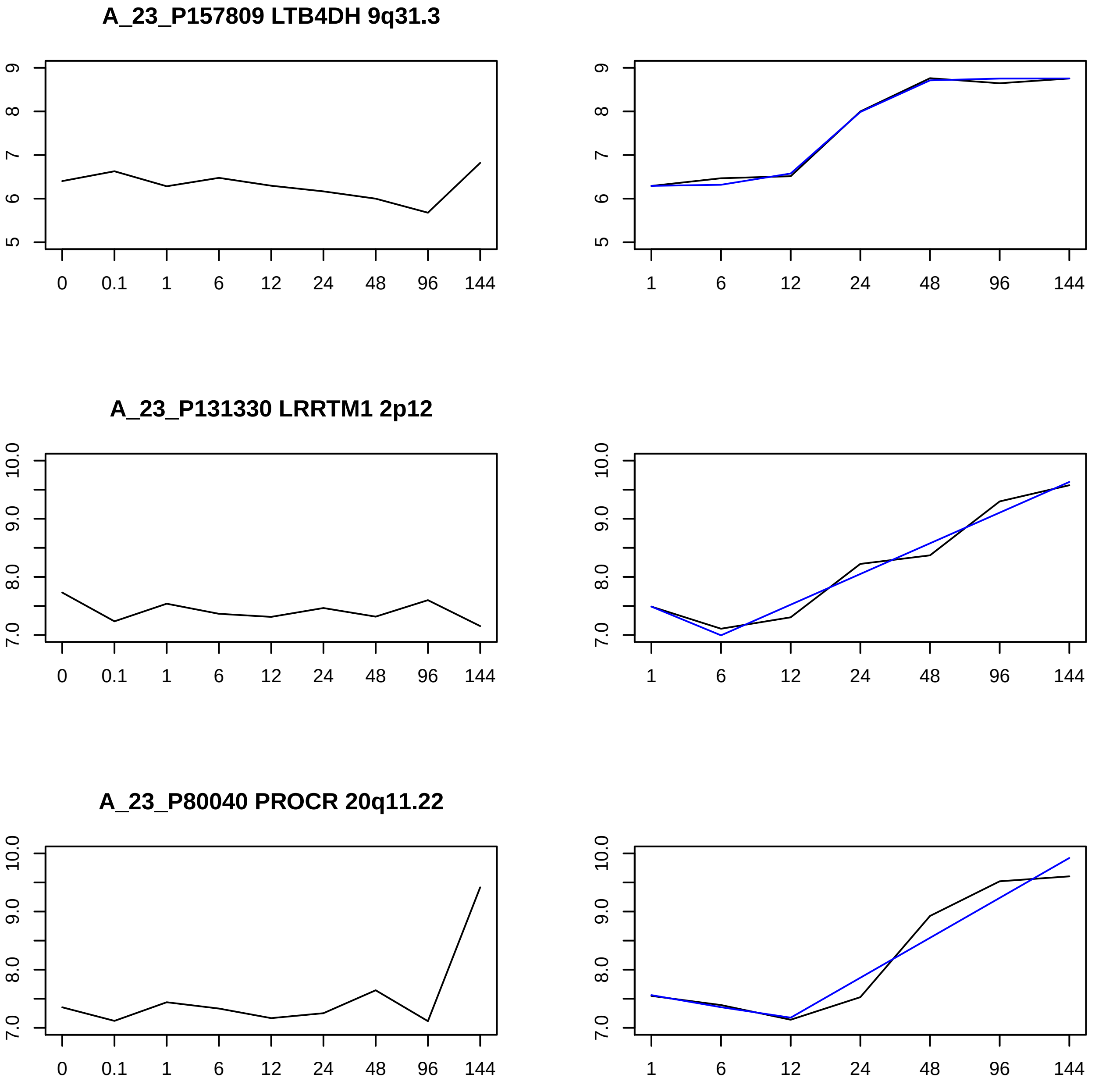

A_23_P157809 LTB4DH 9q31.3
A_23_P131330 LRRTM1 2p12
A_23_P80040 PROCR 20q11.22
0
0.1
1
6
12
24
48
96
144

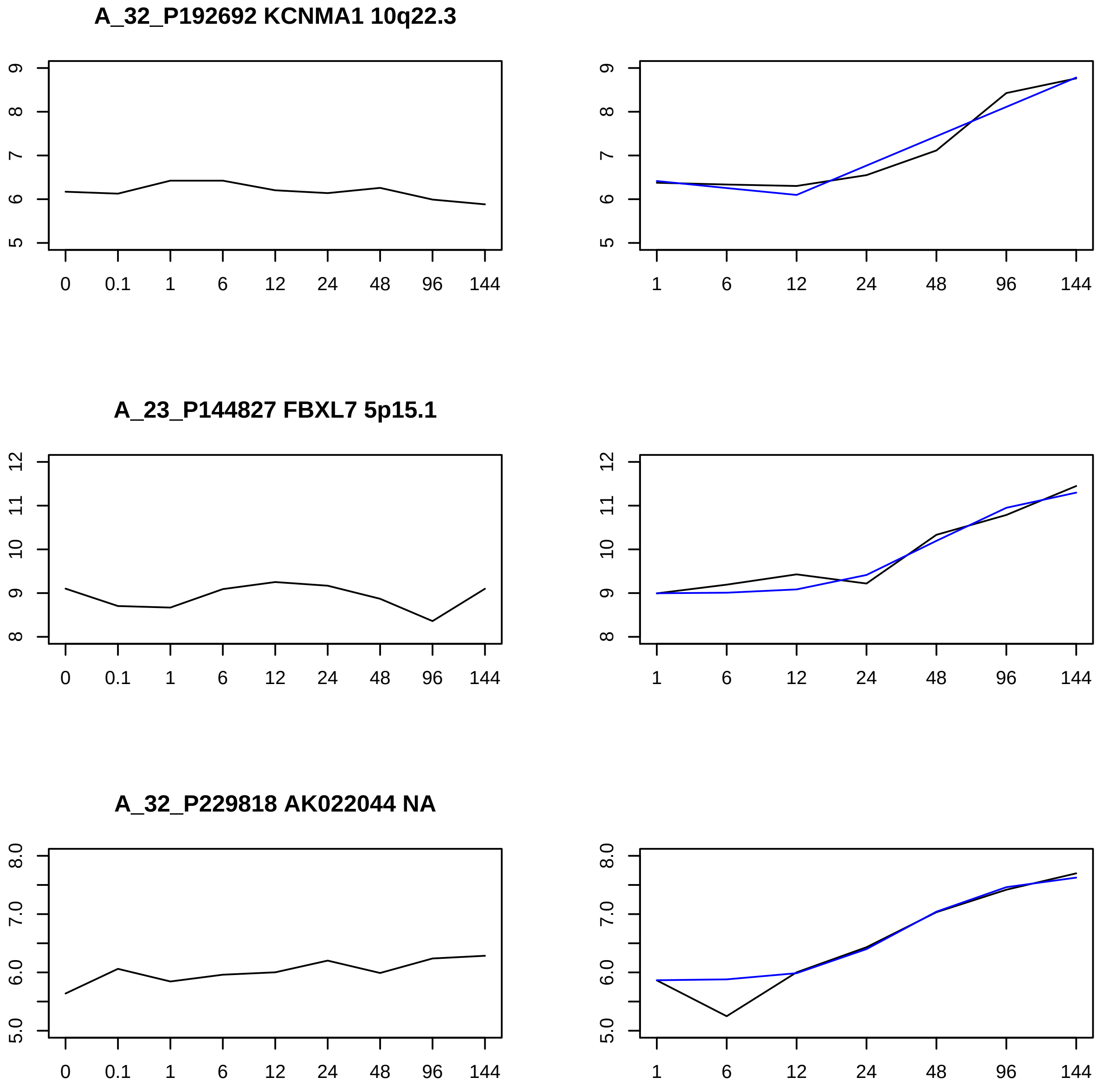
A_32_P192692 KCNMA1 10q22.3
5
6
7
8
9
0
0.1
1
6
12
24
48
96
144
A_23_P144827 FBXL7 5p15.1
8
9
10
11
12
A_32_P229818 AK022044 NA
5.0
6.0
7.0
8.0

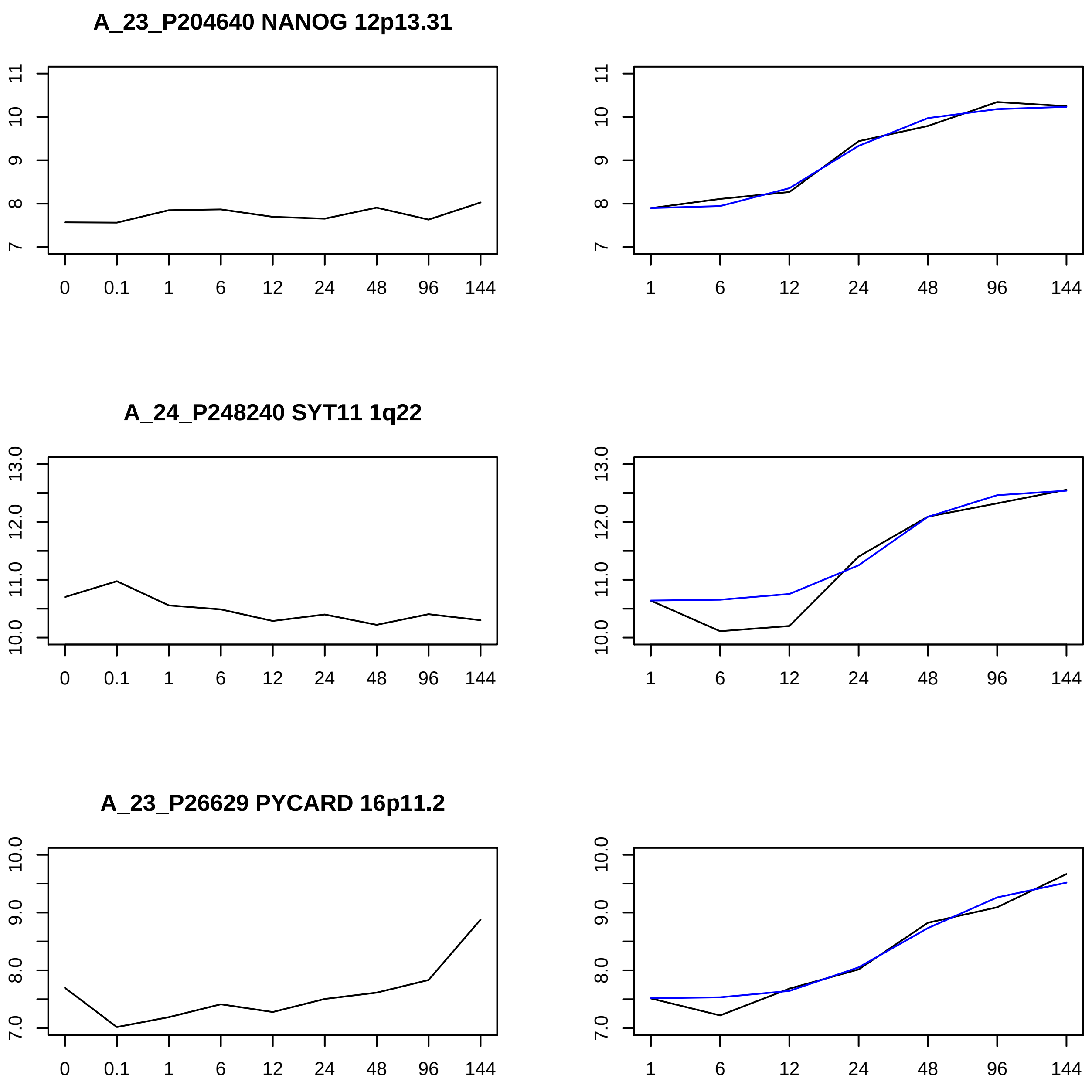
A_23_P204640 NANOG 12p13.31
7
8
9
10
11
0
0.1
1
6
12
24
48
96
144
A_24_P248240 SYT11 1q22
10.0
11.0
12.0
13.0
A_23_P26629 PYCARD 16p11.2
7.0
8.0
9.0
10.0

**A_23_P200260 PCNXL2 1q42.2**

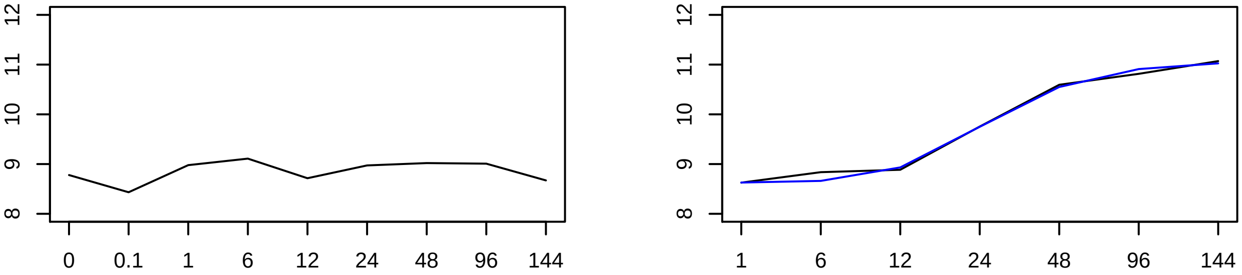


**A_24_P903680 FAM90A10 8p23.1**

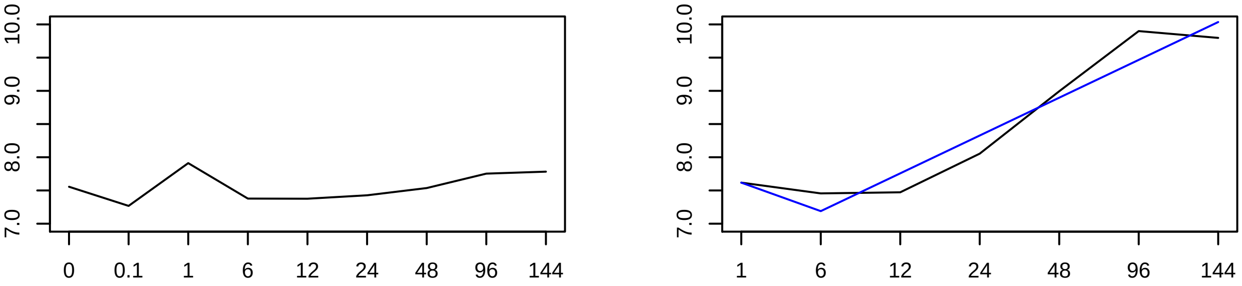


**A_23_P3312 ISLR 15q24.1**

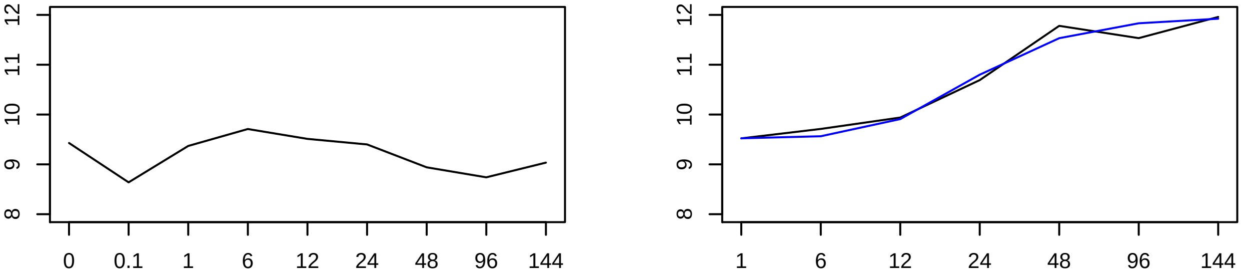

**A_23_P103672 NES 1q23.1**

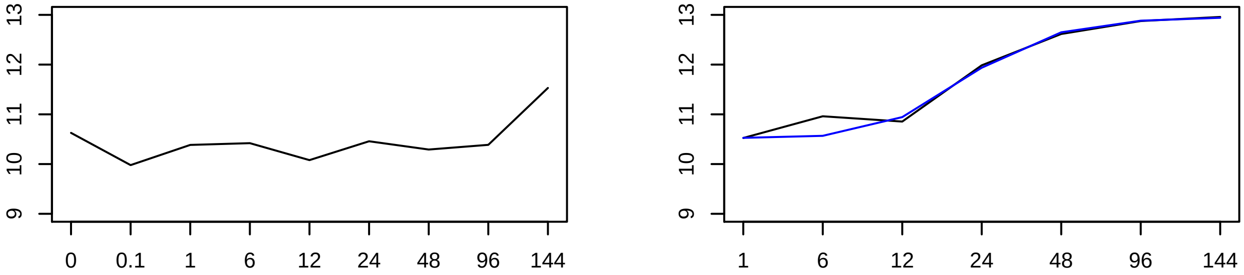


**A_24_P268676 BHLHB2 3p26.2**

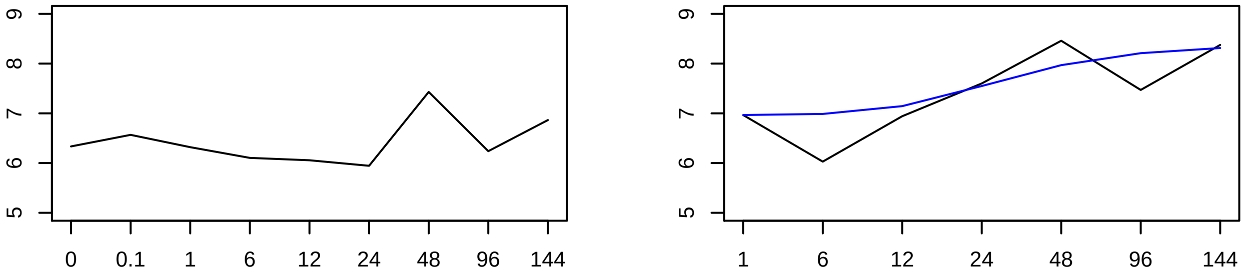


**A_24_P255471 SYT9 11p15.4**

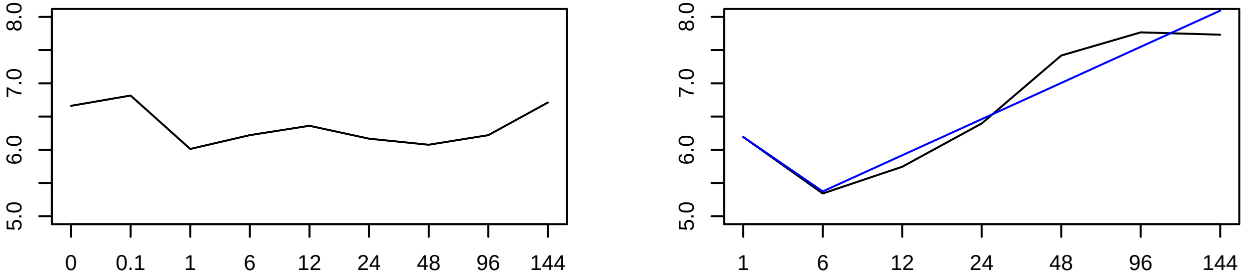

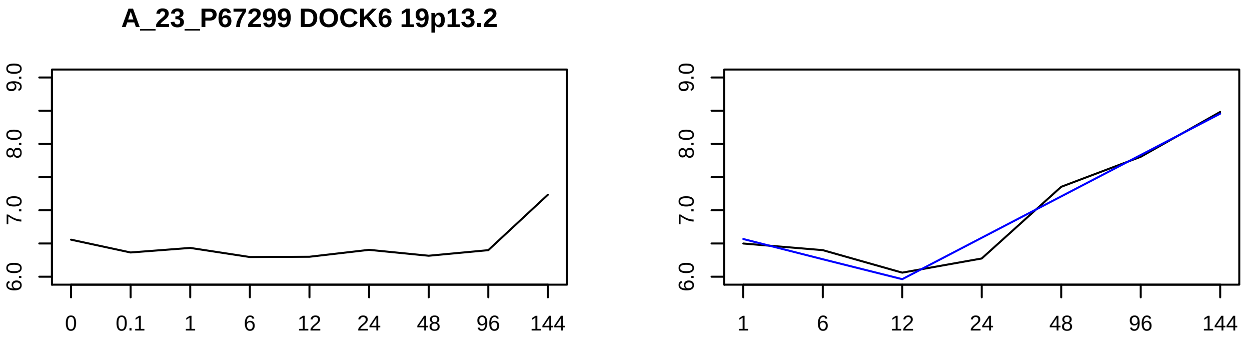
A_23_P67299 DOCK6 19p13.2
6.0
7.0
8.0
9.0
0
0.1
1
6
12
24
48
96
144
1
6
12
24
48
96
144

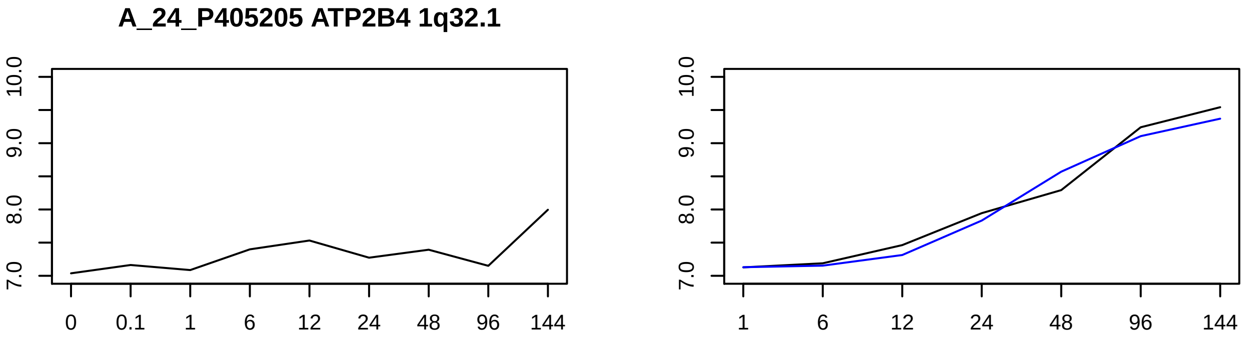
A_24_P405205 ATP2B4 1q32.1
7.0
8.0
9.0
10.0
0
0.1
1
6
12
24
48
96
144
1
6
12
24
48
96
144

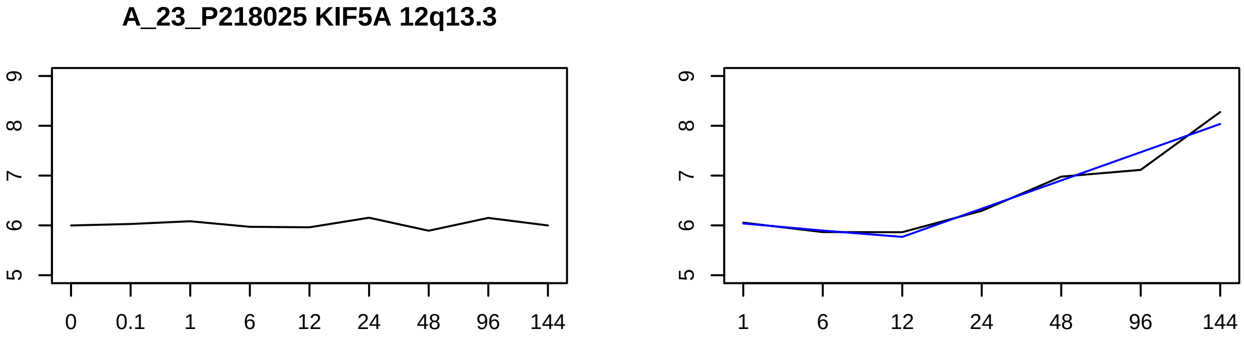
A_23_P218025 KIF5A 12q13.3
5
6
7
8
9
0
0.1
1
6
12
24
48
96
144
1
6
12
24
48
96
144

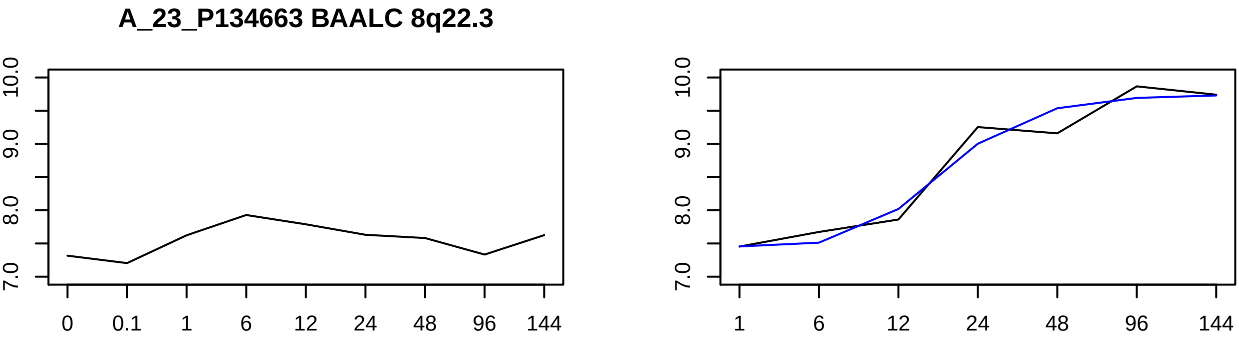
A_23_P134663 BAALC 8q22.3
7.0
8.0
9.0
10.0
0
0.1
1
6
12
24
48
96
144

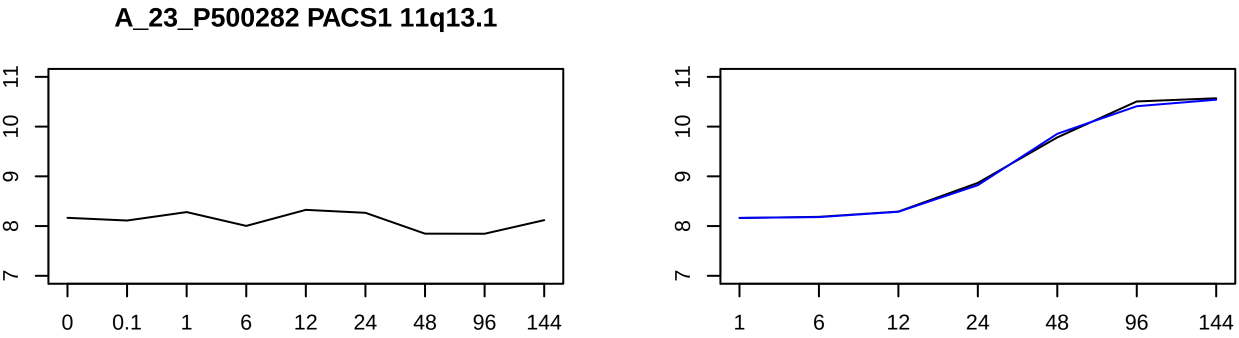
A_23_P500282 PACS1 11q13.1
7
8
9
10
11
0
0.1
1
6
12
24
48
96
144

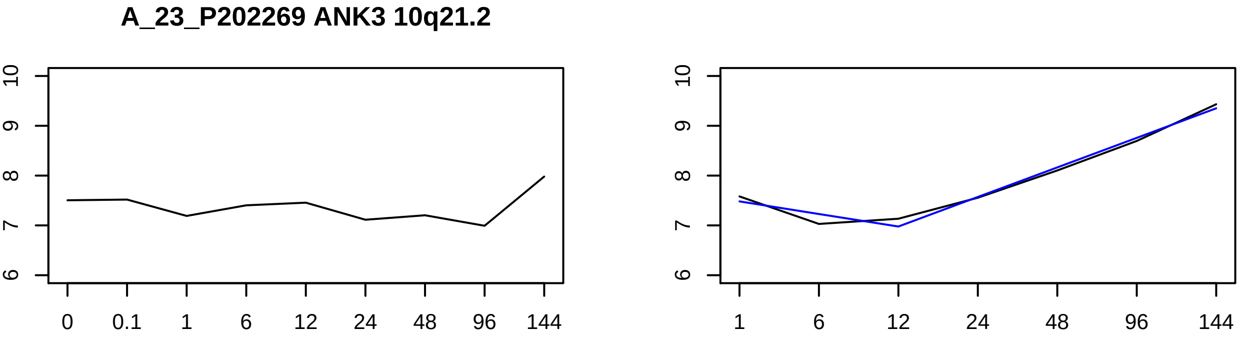
A_23_P202269 ANK3 10q21.2
6
7
8
9
10
0
0.1
1
6
12
24
48
96
144

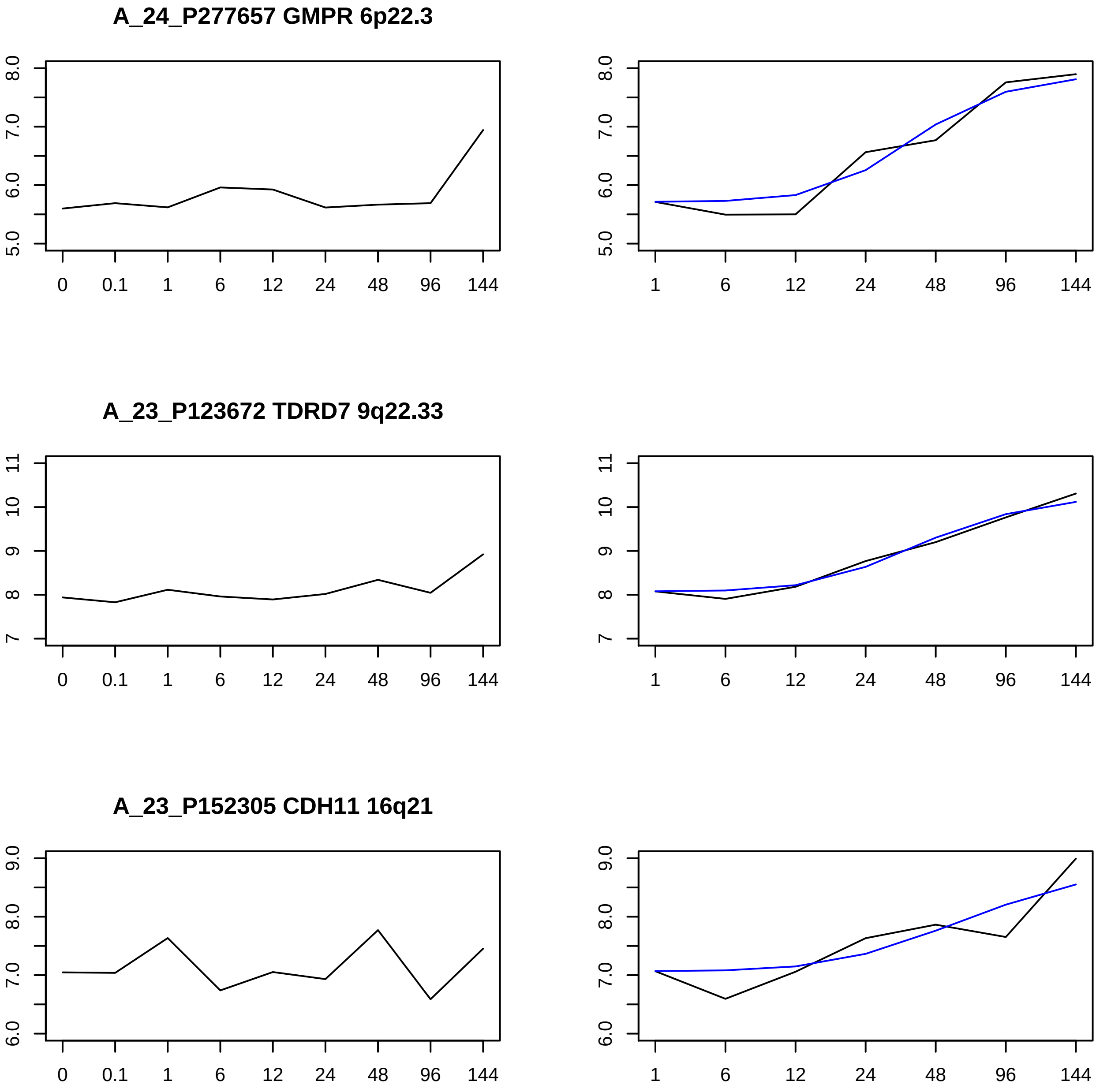
A_24_P277657 GMPR 6p22.3
5.0 6.0 7.0 8.0
0 0.1 1 6 12 24 48 96 144
1 6 12 24 48 96 144
A_23_P123672 TDRD7 9q22.33
7 8 9 10 11
0 0.1 1 6 12 24 48 96 144
1 6 12 24 48 96 144
A_23_P152305 CDH11 16q21
6.0 7.0 8.0 9.0
0 0.1 1 6 12 24 48 96 144
1 6 12 24 48 96 144

**A_23_P71316 RBPMS 8p12**

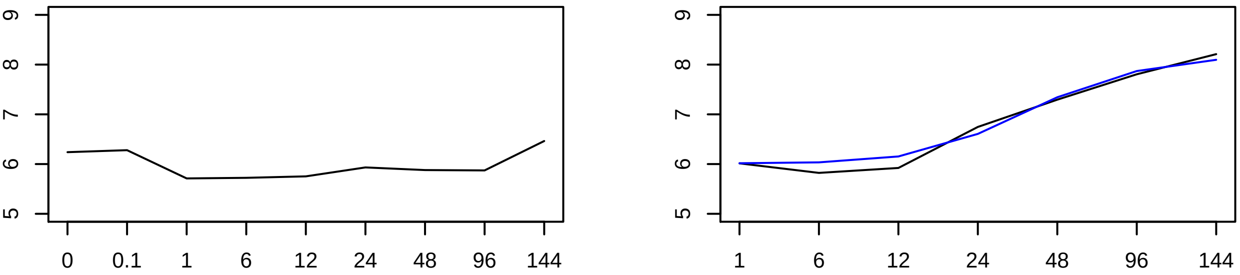


**A_23_P18649 FAT4 4q28.1**

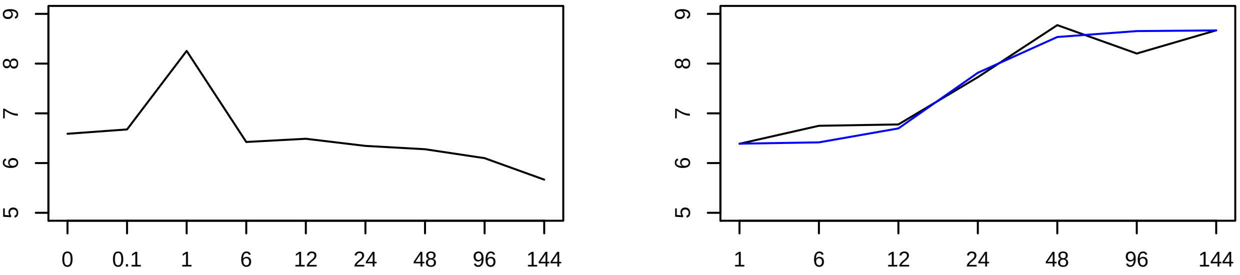


**A_23_P206018 TPM1 15q22.2**

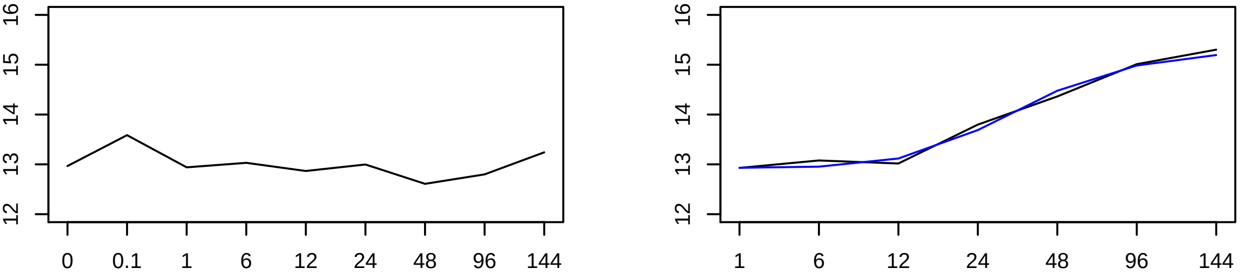

**A_23_P46618 PLXNA2 1q32.2**

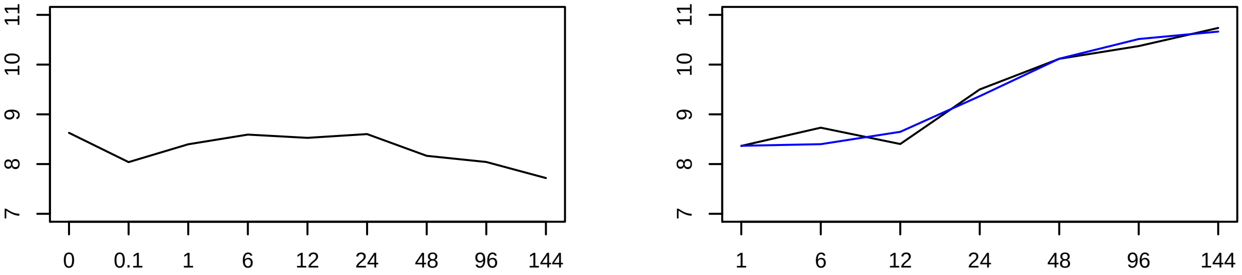


**A_23_P98645 DCHS1 11p15.4**

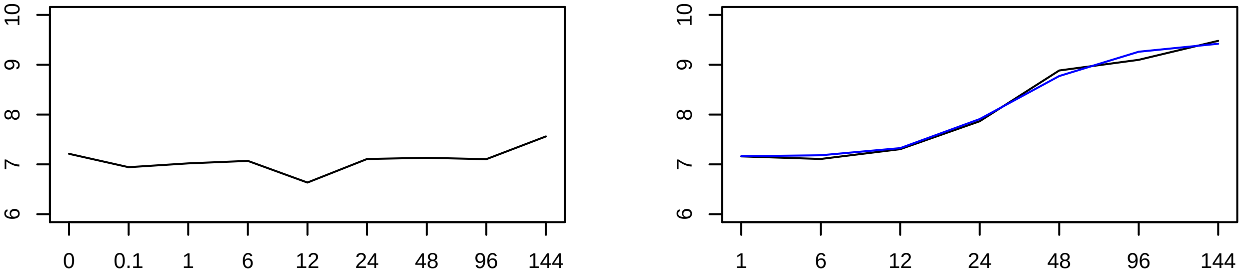


**A_23_P217319 FGF13 Xq26.3**

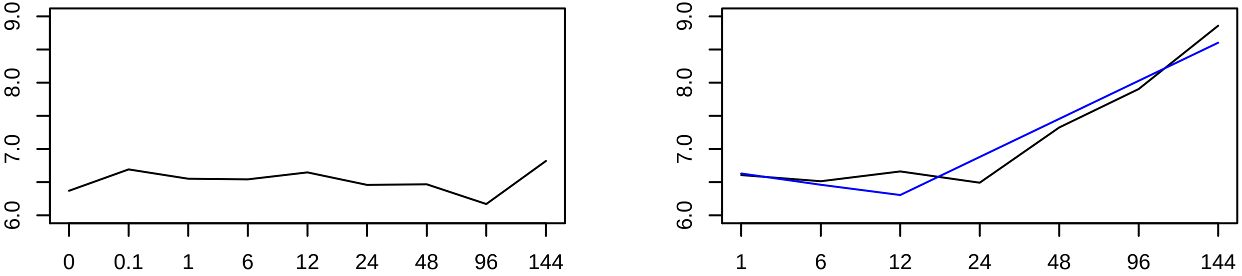

**A_23_P19176 MGC23985 5q33.1**

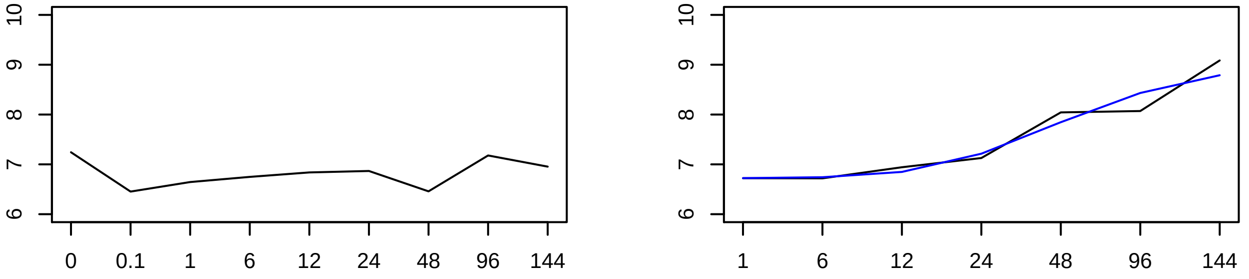


**A_23_P34045 EDA Xq13.1**

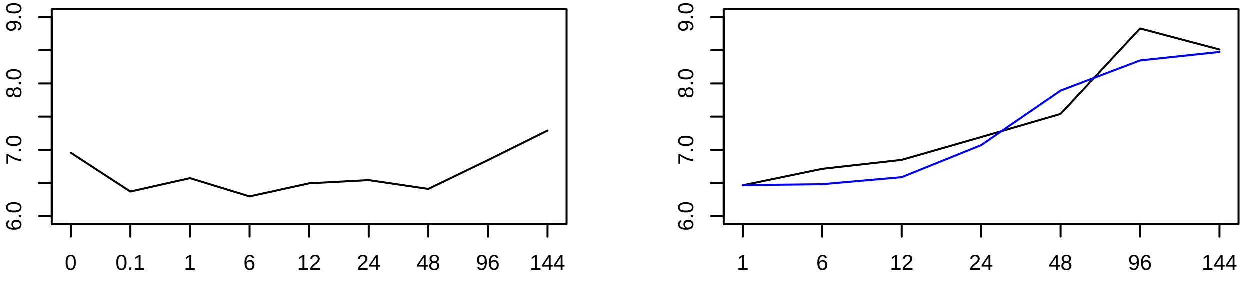


**A_23_P157117 CREB5 7p15.1**

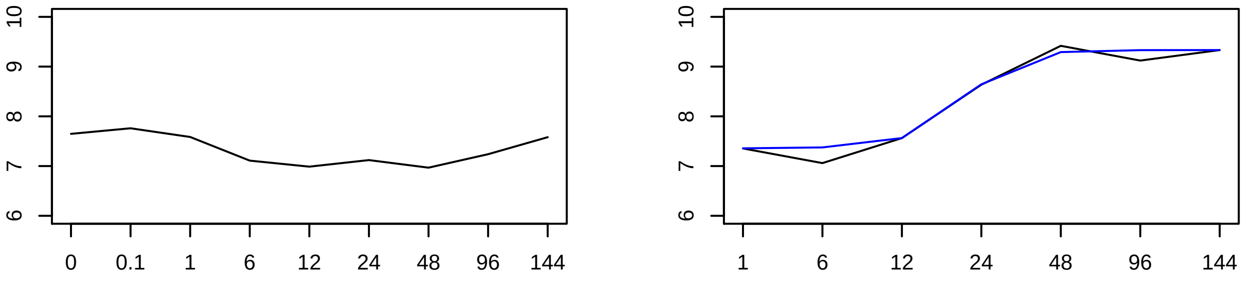

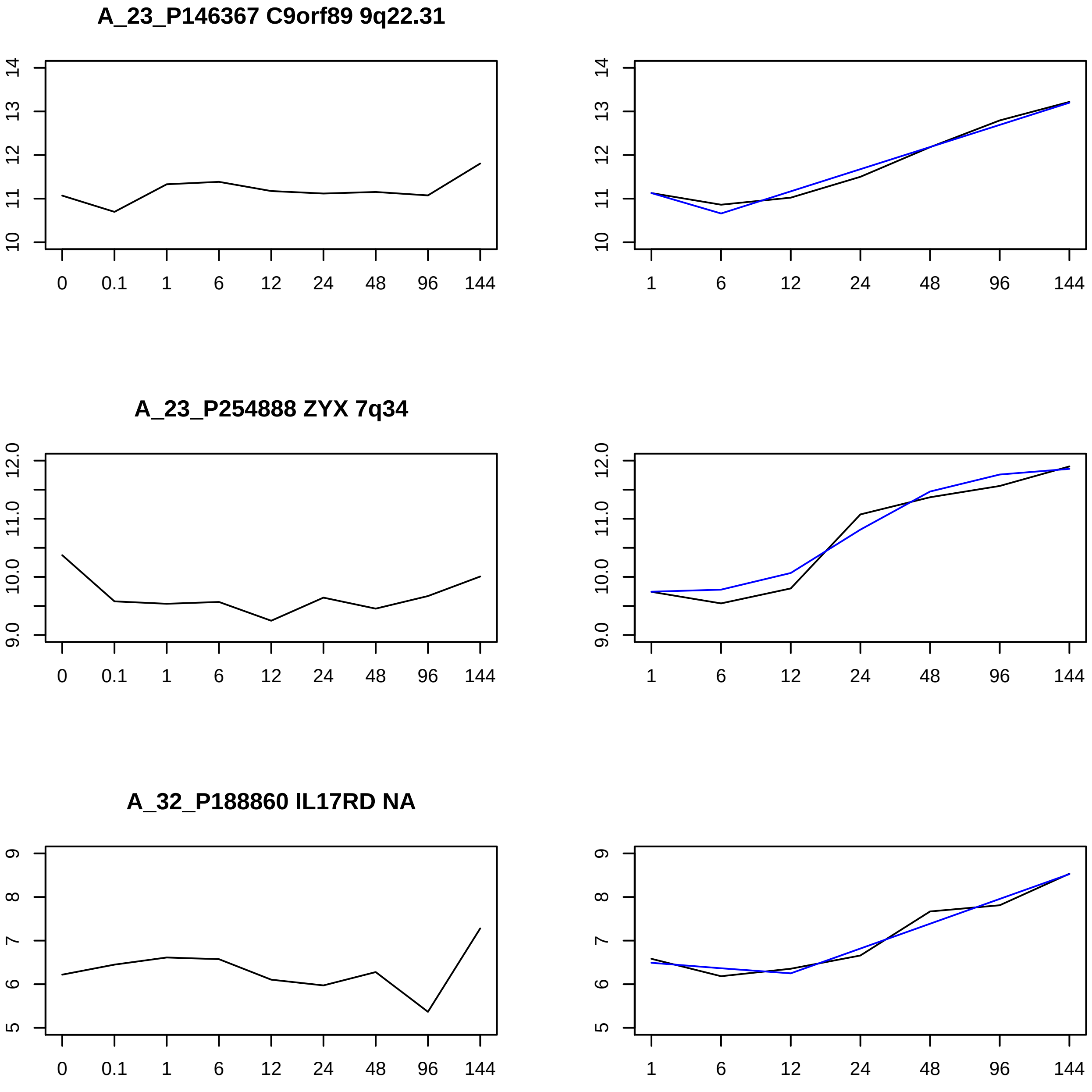
A_23_P146367 C9orf89 9q22.31
10
11
12
13
14
0
0.1
1
6
12
24
48
96
144
A_23_P254888 ZYX 7q34
9.0
10.0
11.0
12.0
A_32_P188860 IL17RD NA
5
6
7
8
9

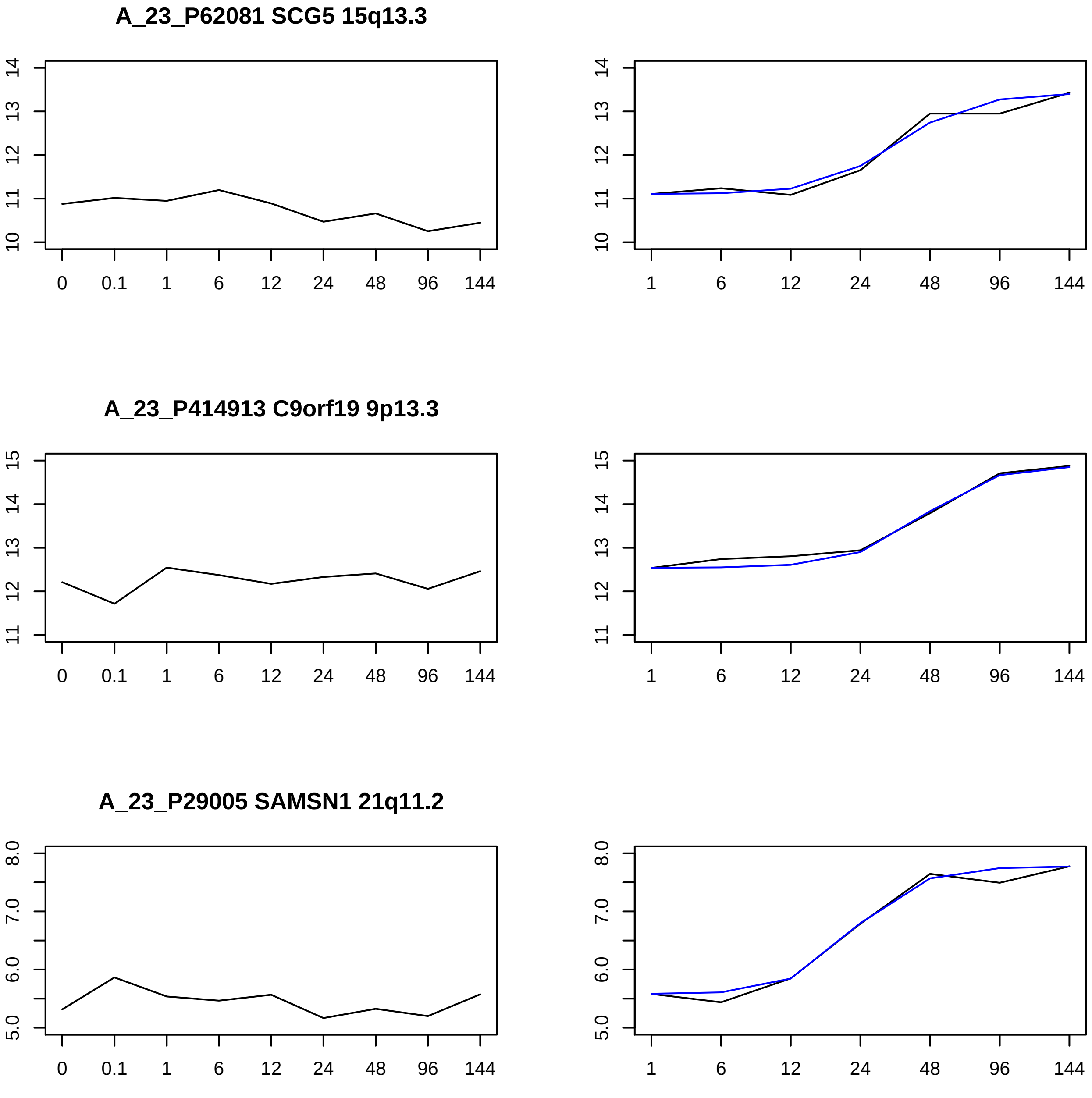

A_23_P62081 SCG5 15q13.3
10
11
12
13
14
0
0.1
1
6
12
24
48
96
144
A_23_P414913 C9orf19 9p13.3
11
12
13
14
15
A_23_P29005 SAMSN1 21q11.2
5.0
6.0
7.0
8.0

**A_23_P121702 OCIAD2 4p12**

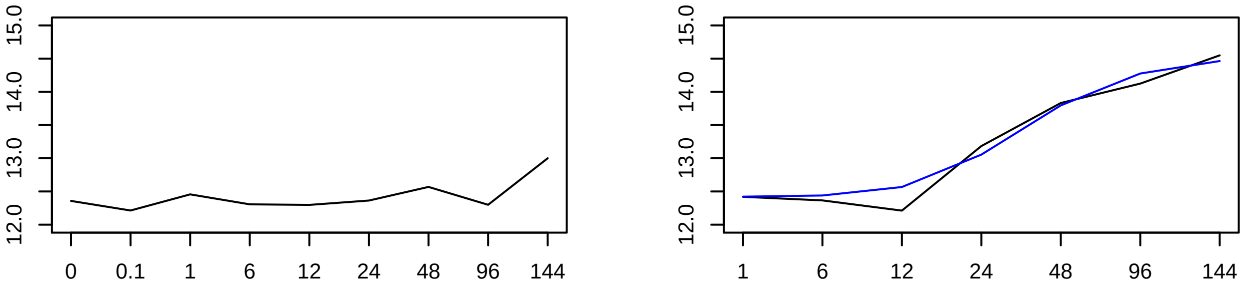


**A_23_P97780 LOC338620 10p11.22**

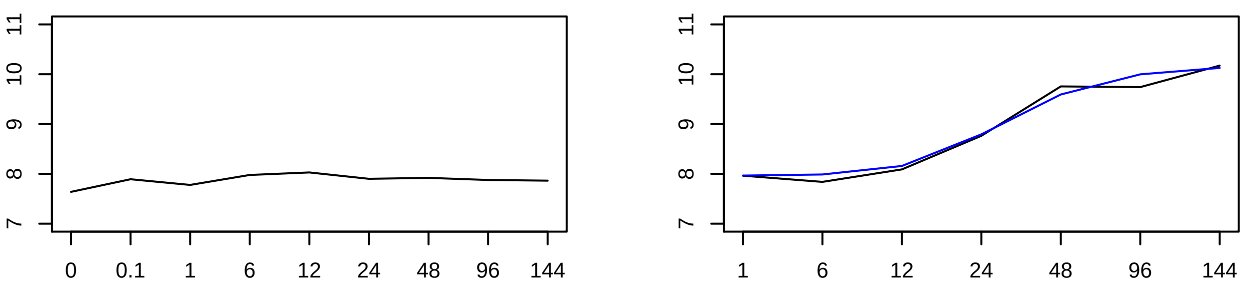


**A_23_P121716 ANXA3 4q21.21**

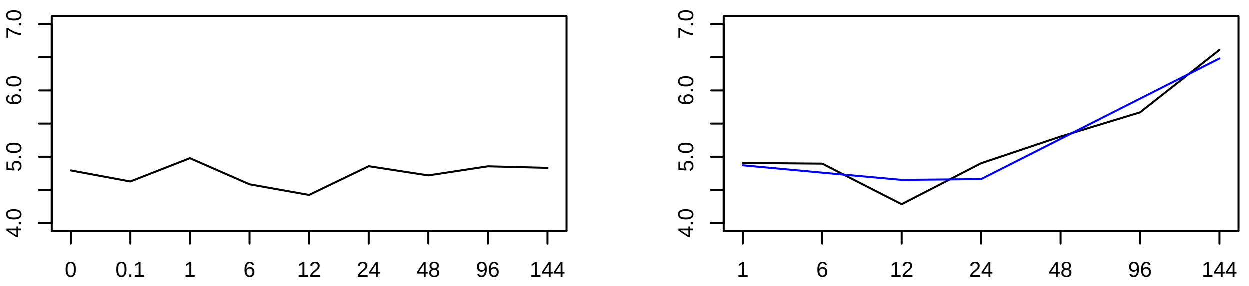

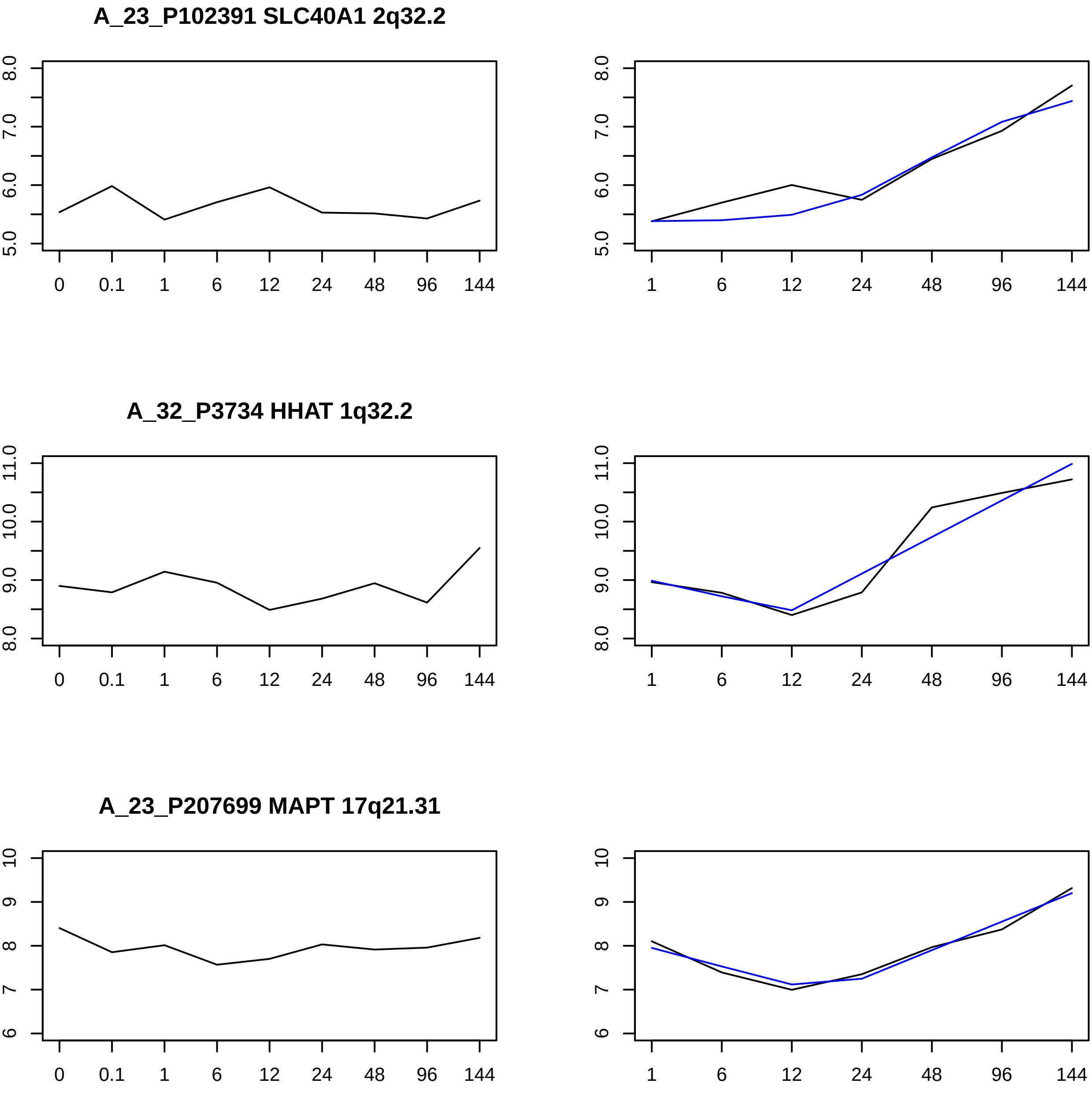
A_23_P102391 SLC40A1 2q32.2
5.0
6.0
7.0
8.0
0 0.1 1 6 12 24 48 96 144
1 6 12 24 48 96 144
A_32_P3734 HHAT 1q32.2
8.0
9.0
10.0
11.0
0 0.1 1 6 12 24 48 96 144
1 6 12 24 48 96 144
A_23_P207699 MAPT 17q21.31
6
7
8
9
10
0 0.1 1 6 12 24 48 96 144
1 6 12 24 48 96 144

**A_23_P35796 PPP2R5B 11q13.1**

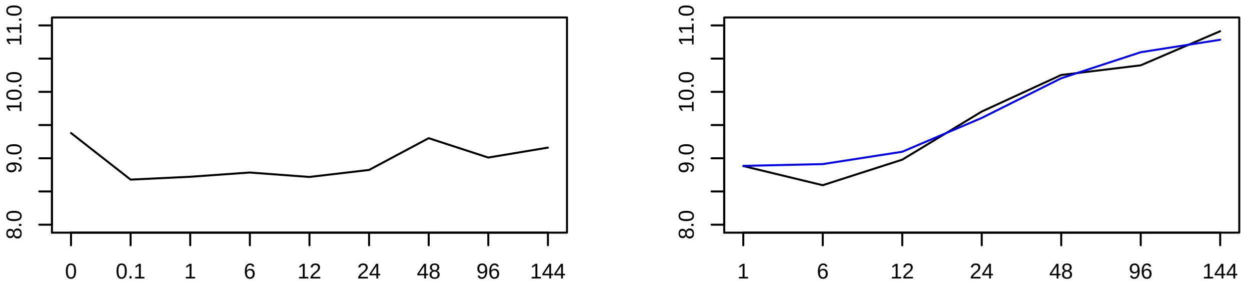


**A_23_P395404 FGF20 8p22**

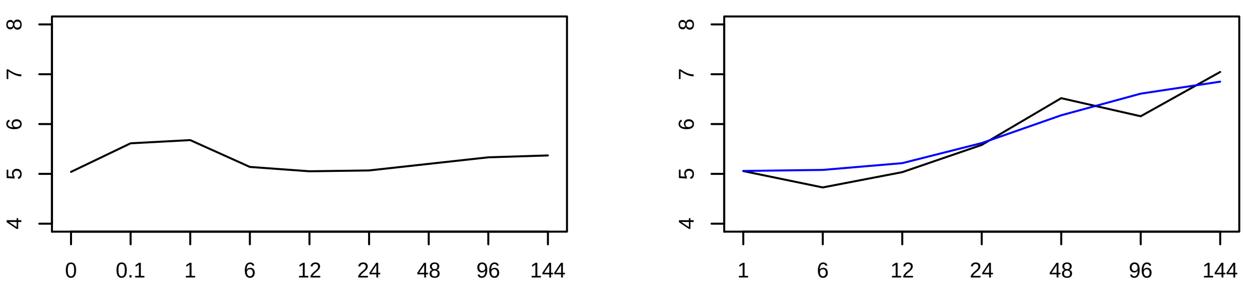


**A_23_P76071 B3GNT4 12q24.31**

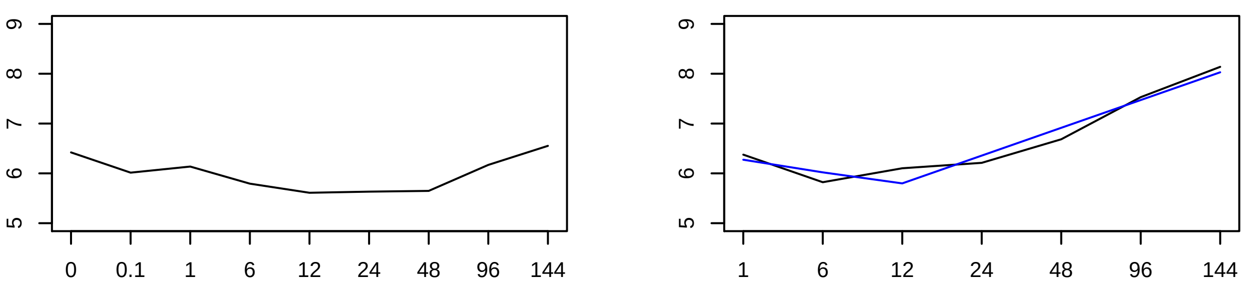

**A_24_P763655 THC2596442 NA**

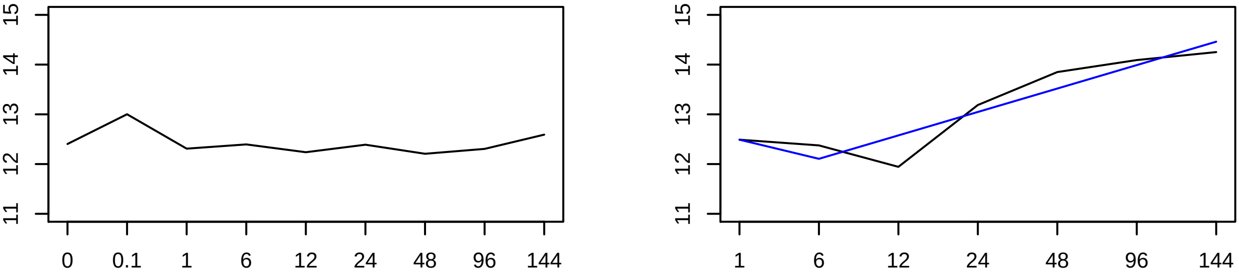


**A_24_P229728 LOC440181 14q23.1**

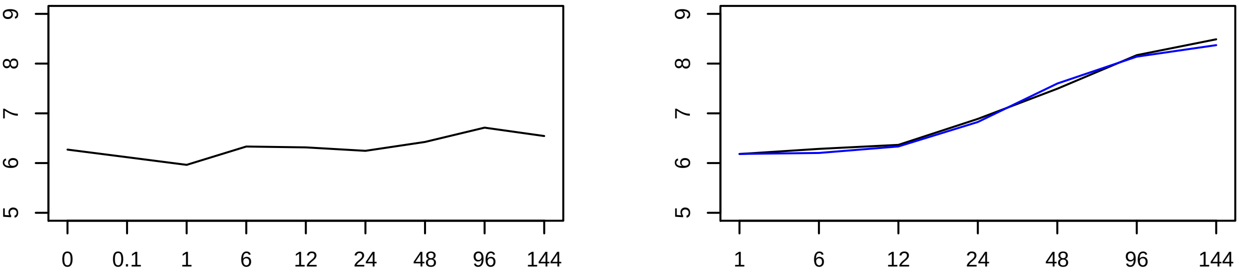


**A_32_P365574**

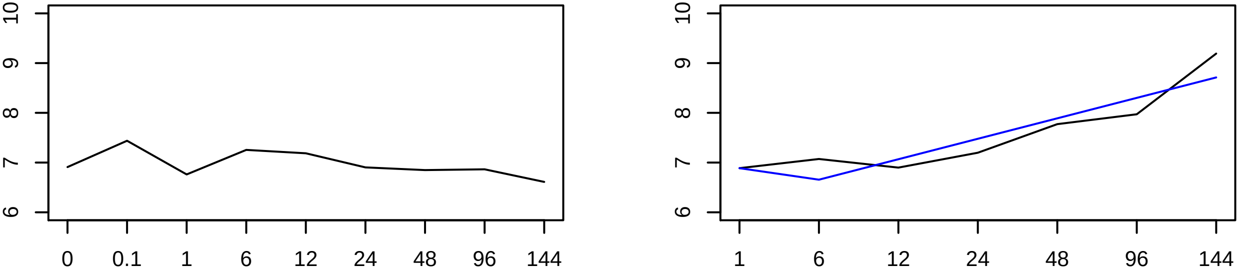

**A_23_P210015 PTPN18 2q21.1**

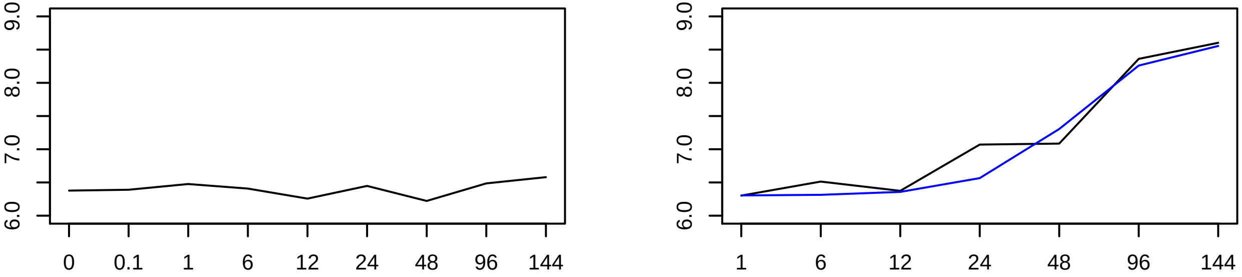


**A_23_P352870 PVRL2 19q13.32**

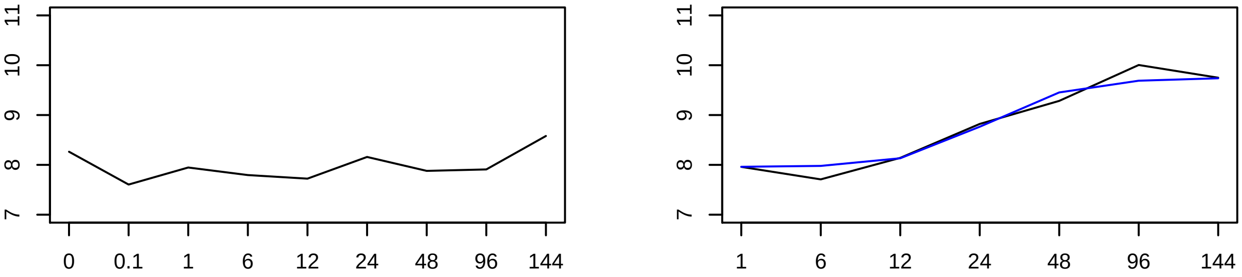


**A_23_P125705 NAP1L2 Xq13.2**

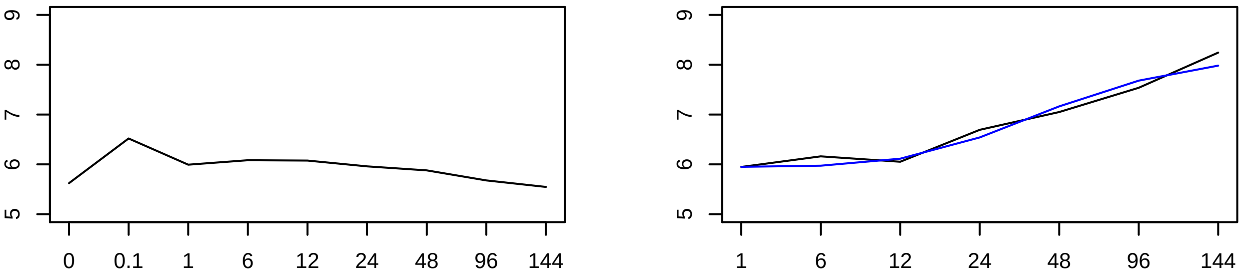

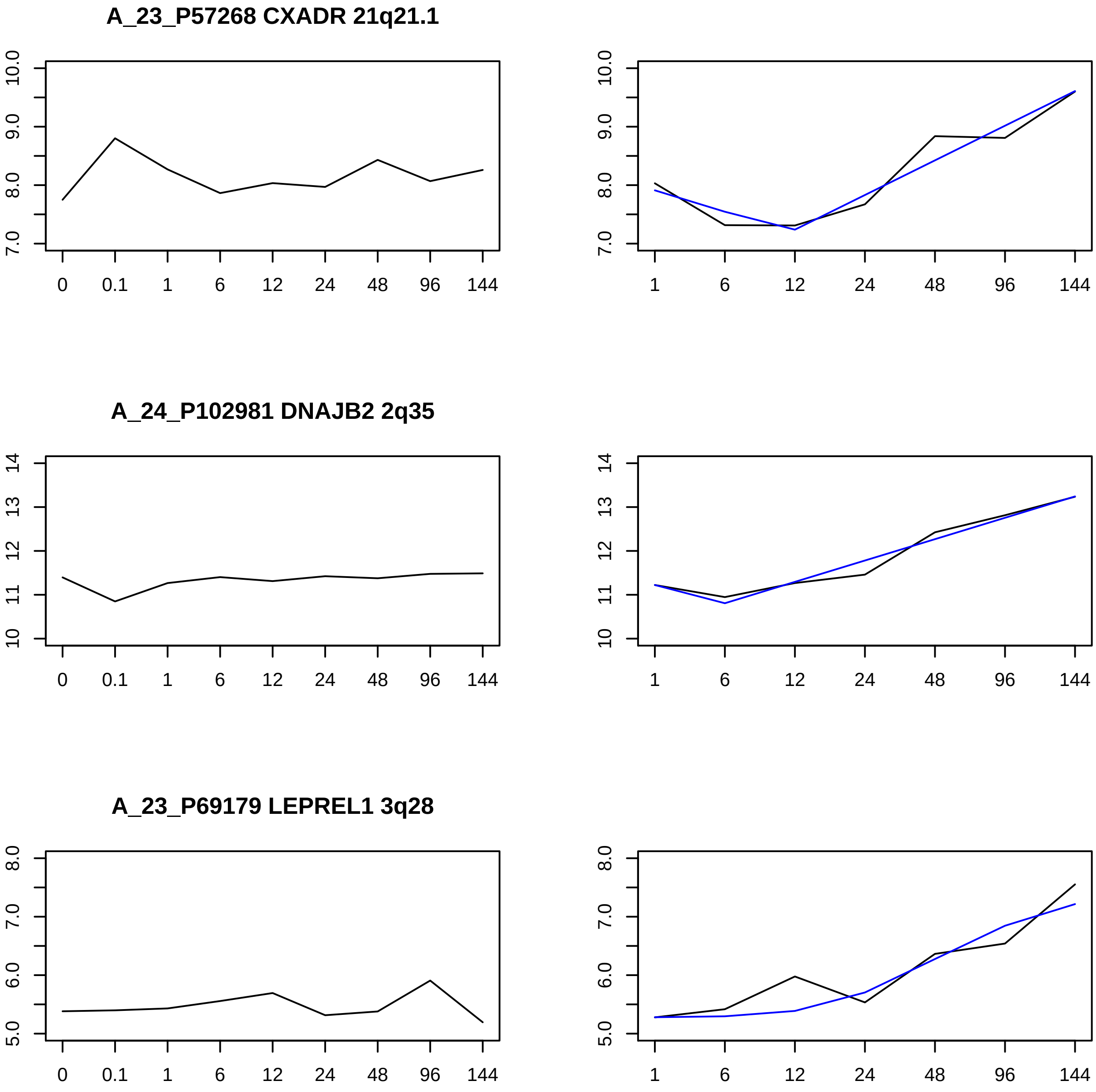
A_23_P57268 CXADR 21q21.1
7.0
8.0
9.0
10.0
0
0.1
1
6
12
24
48
96
144
A_24_P102981 DNAJB2 2q35
10
11
12
13
14
A_23_P69179 LEPREL1 3q28
5.0
6.0
7.0
8.0

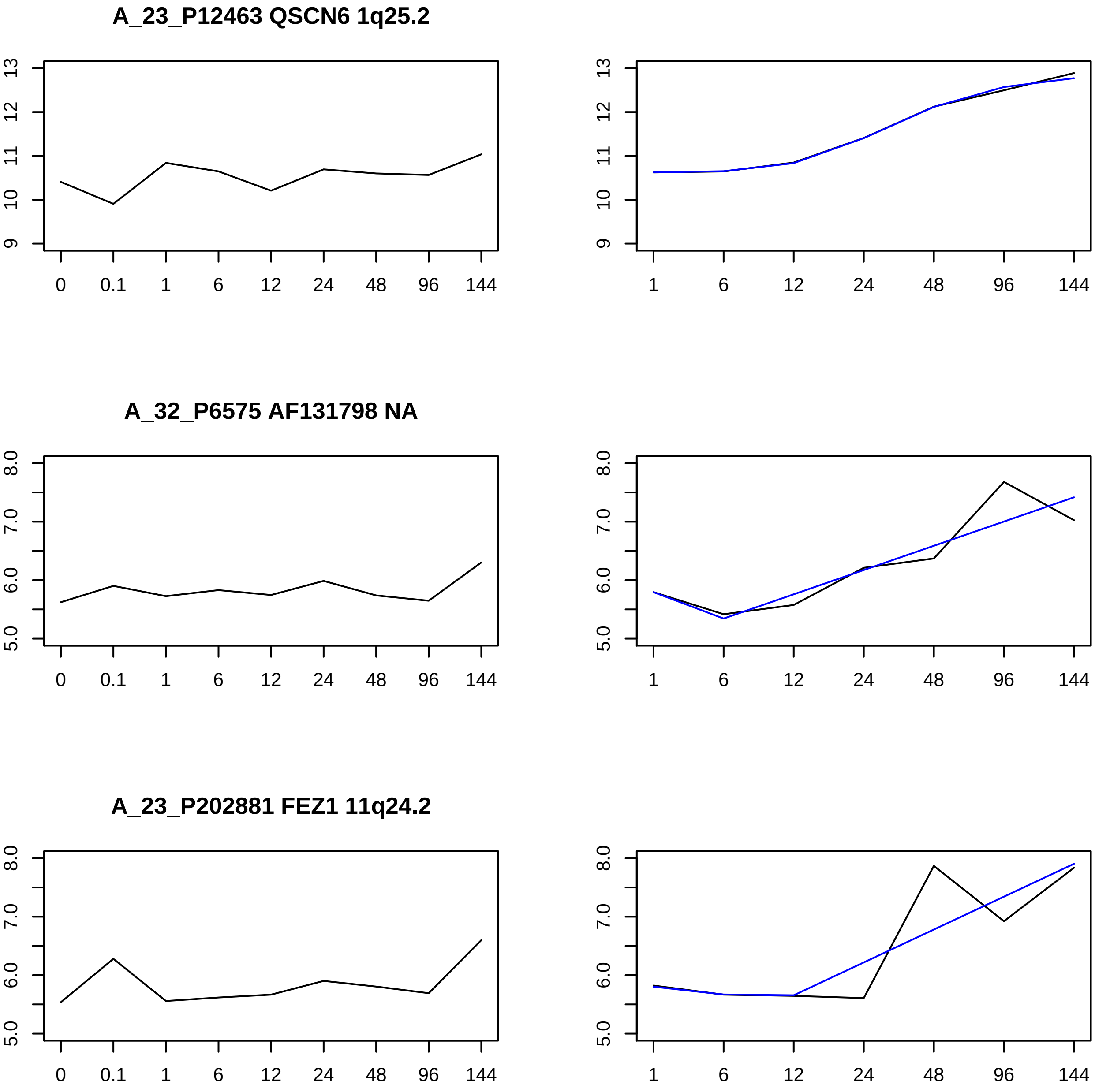
A_23_P12463 QSCN6 1q25.2
9
10
11
12
13
0
0.1
1
6
12
24
48
96
144
1
6
12
24
48
96
144
A_32_P6575 AF131798 NA
5.0
6.0
7.0
8.0
A_23_P202881 FEZ1 11q24.2

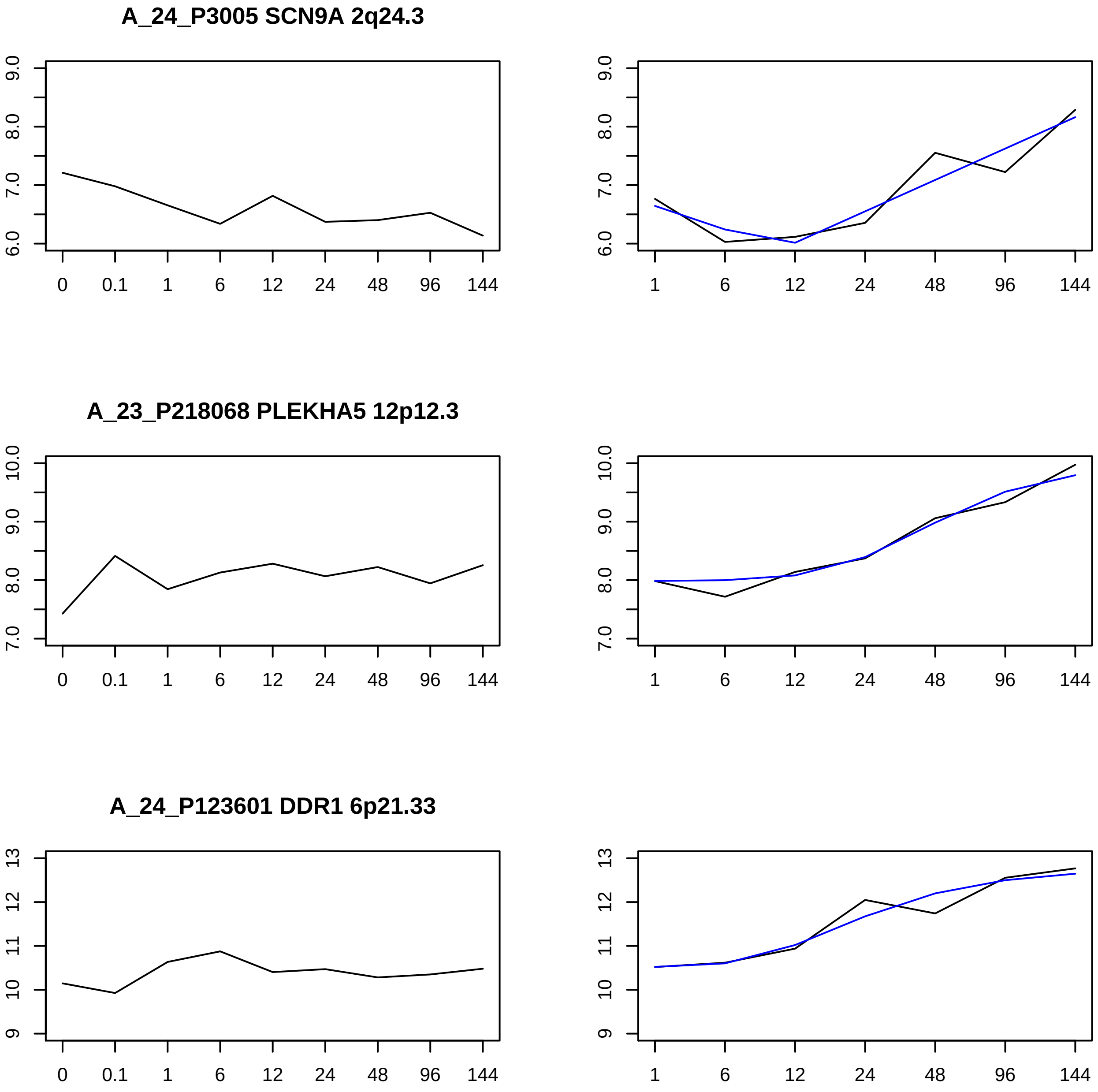

A_24_P3005 SCN9A 2q24.3
6.0
7.0
8.0
9.0
0
0.1
1
6
12
24
48
96
144
1
6
12
24
48
96
144
A_23_P218068 PLEKHA5 12p12.3
7.0
8.0
9.0
10.0
A_24_P123601 DDR1 6p21.33
9
10
11
12
13

A_23_P121480 CD200 3q13.2

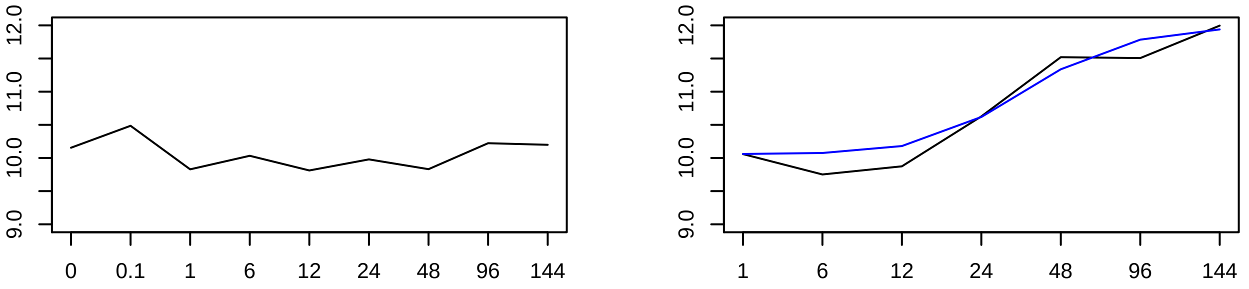


A_23_P18966 P4HA2 5q31.1

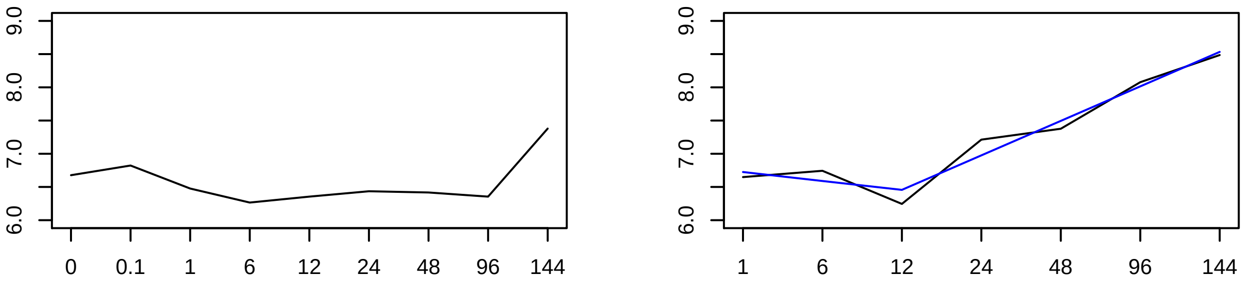


A_23_P340333 KIAA1754 10q25.1

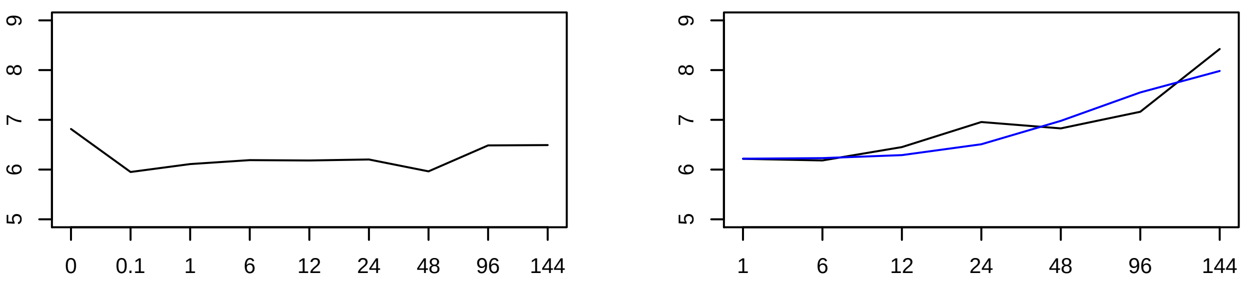

**A_32_P94444 PRSS2 7q34**

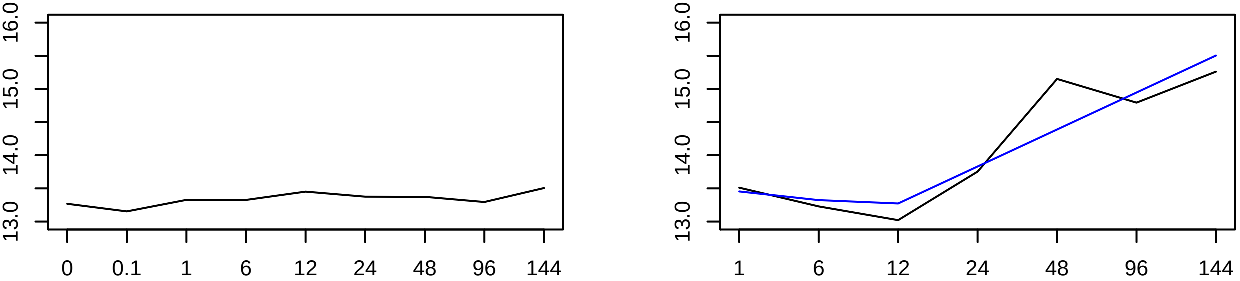


**A_23_P366254 SLC10A3 Xq28**

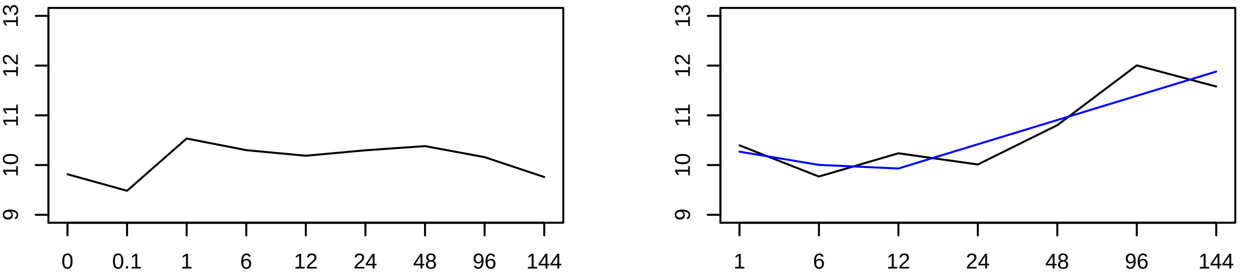


**A_32_P226205 ZFHX2 14q11.2**

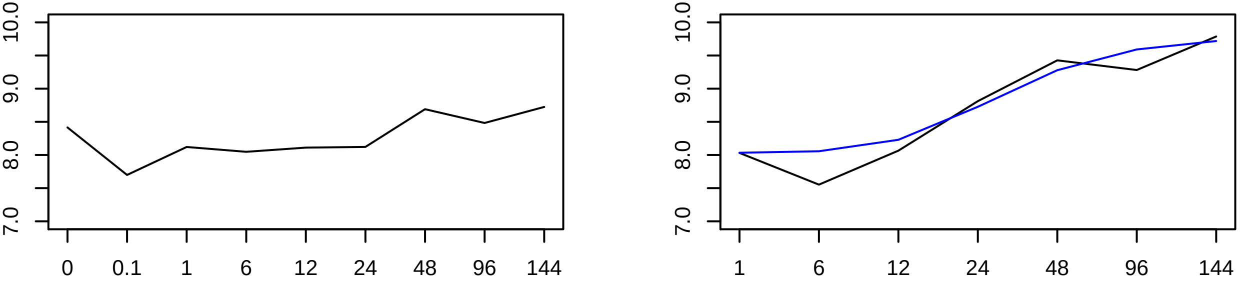

### A_24_P57977 SNIP 17q12

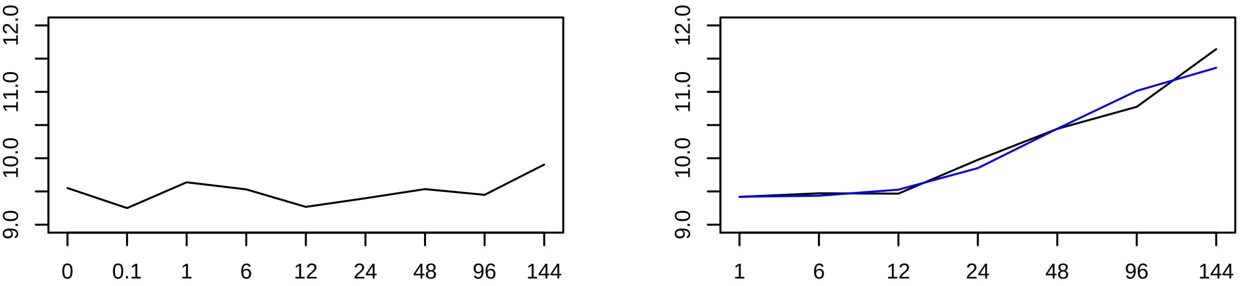


### A_32_P3113 AF070529 NA

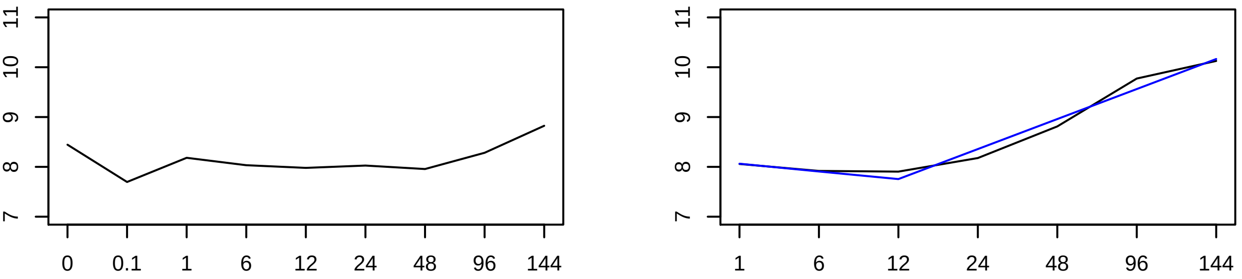


### A_23_P202138 SFXN3 10q24.31

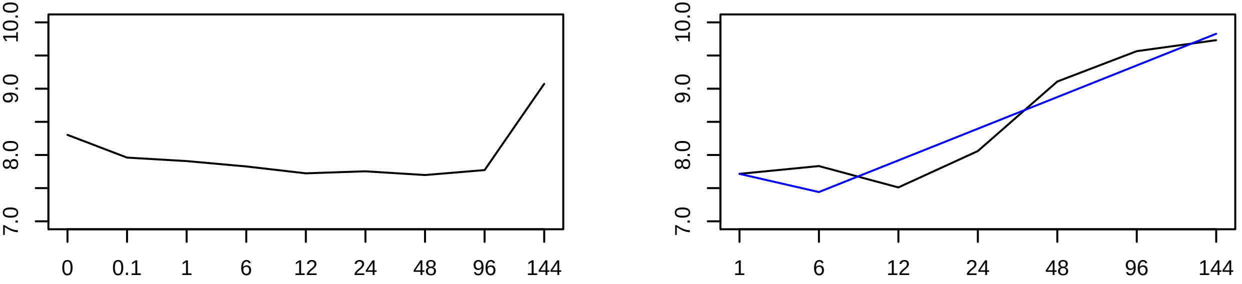

**A_23_P107401 TIMP2 17q25.3**

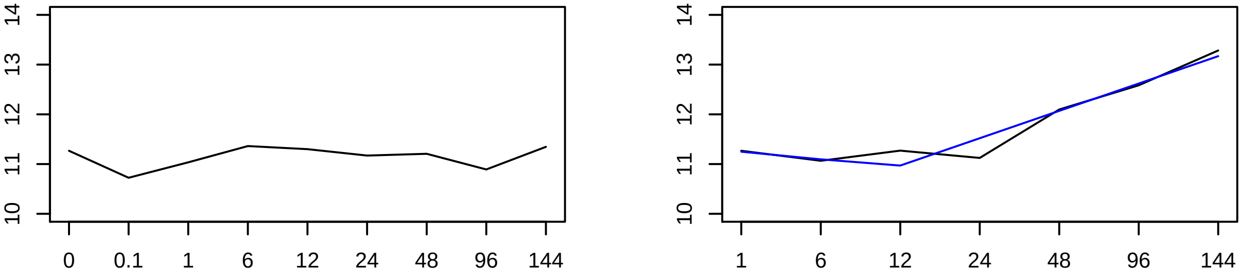


**A_23_P331049 DPYSL4 10q26.3**

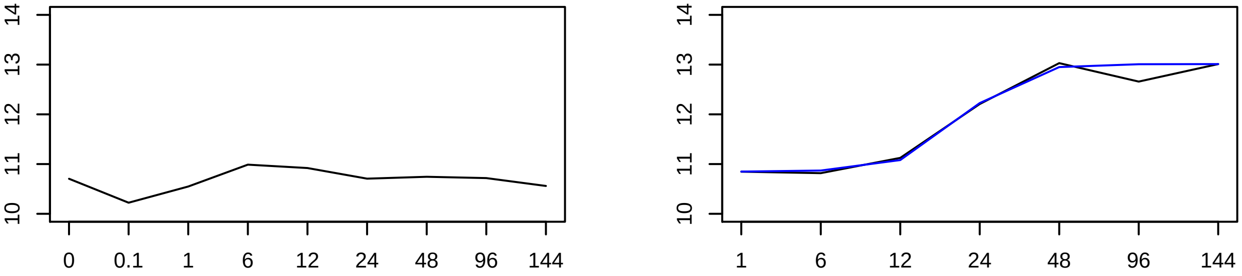


**A_32_P29632 CXADR 21q21.1**

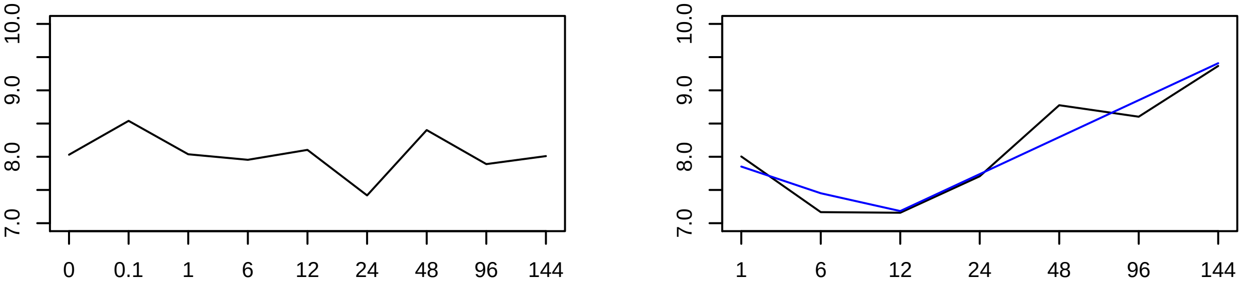

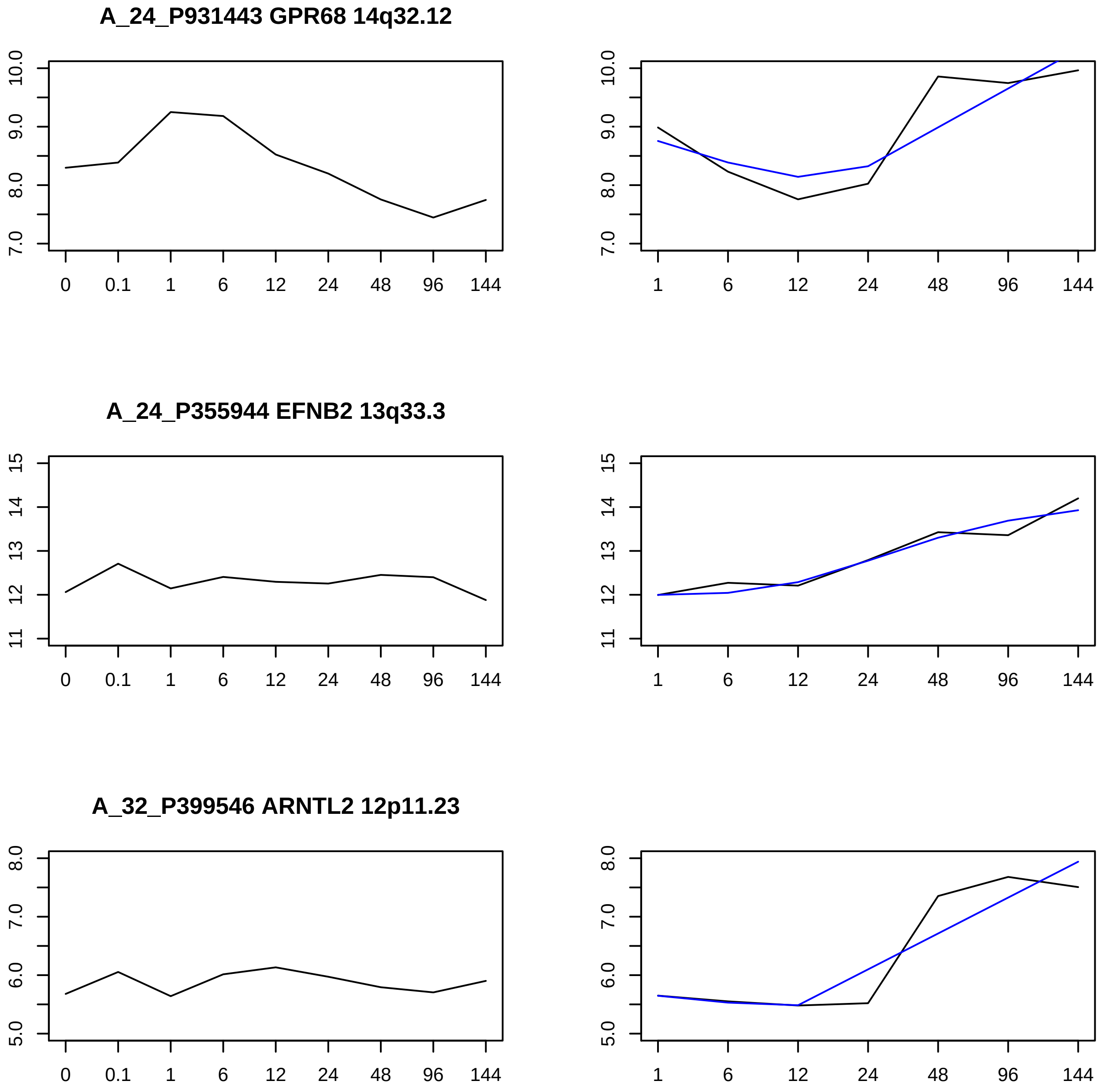
A_24_P931443 GPR68 14q32.12
7.0
8.0
9.0
10.0
0
0.1
1
6
12
24
48
96
144
A_24_P355944 EFNB2 13q33.3
11
12
13
14
15
A_32_P399546 ARNTL2 12p11.23
5.0
6.0
7.0
8.0

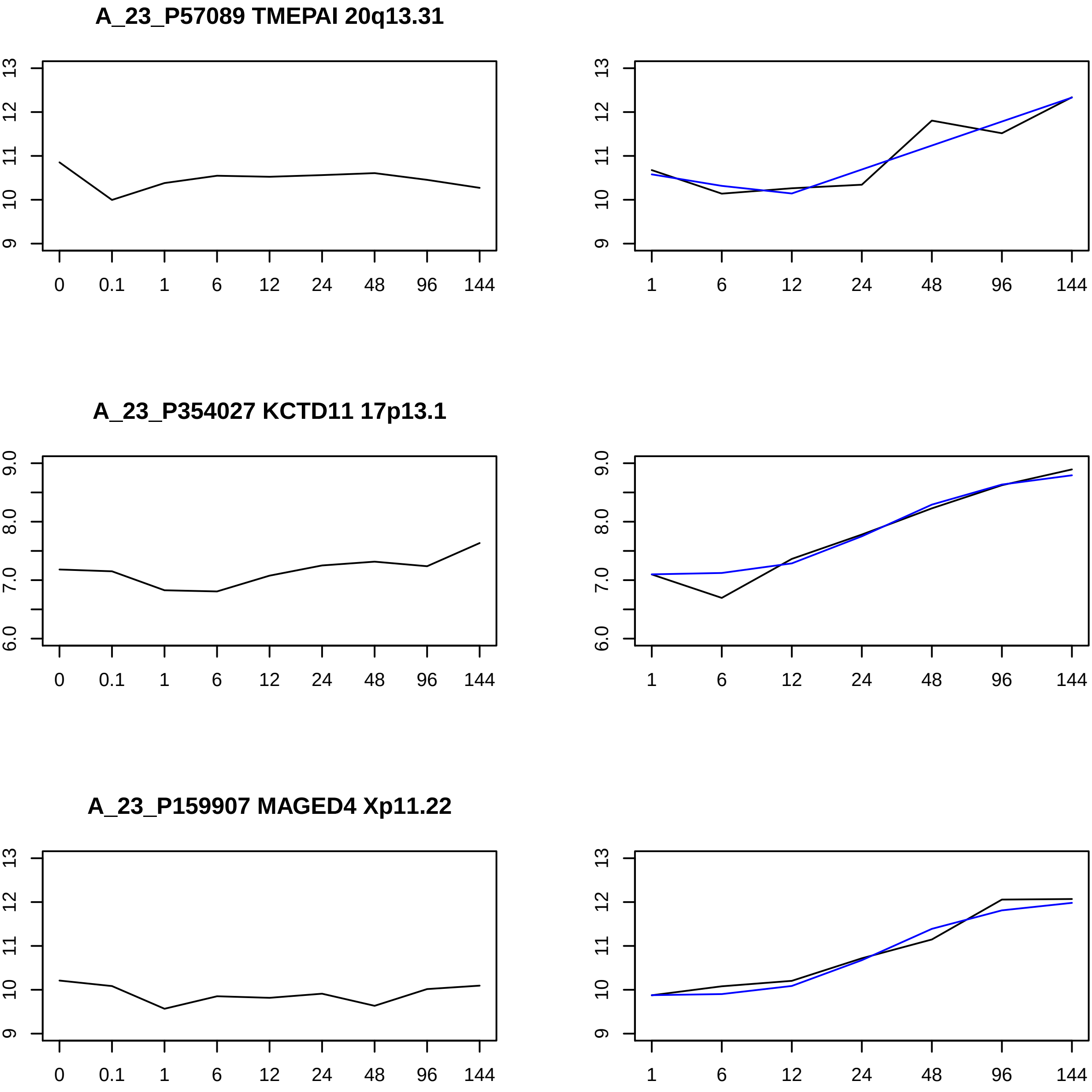
A_23_P57089 TMEPAI 20q13.31
A_23_P354027 KCTD11 17p13.1
A_23_P159907 MAGED4 Xp11.22
9
10
11
12
13
6.0
7.0
8.0
9.0
0
0.1
1
6
12
24
48
96
144

A_23_P161194 VIM 10p12.33

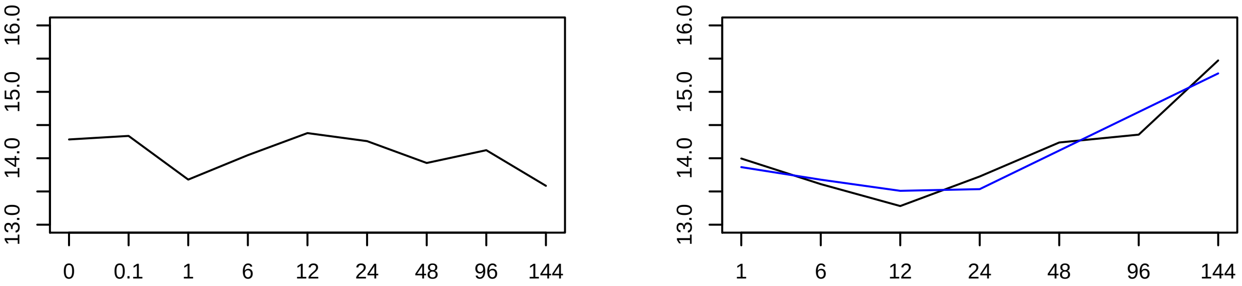


A_32_P83049 EFR3B NA

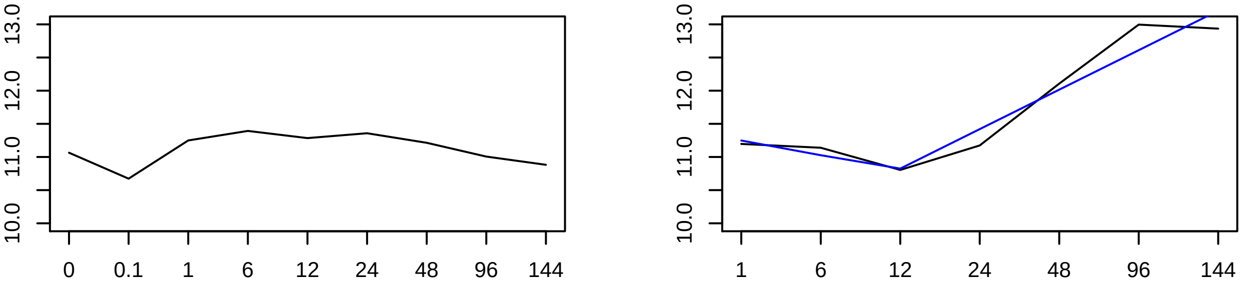


A_32_P69956 CR621698 NA

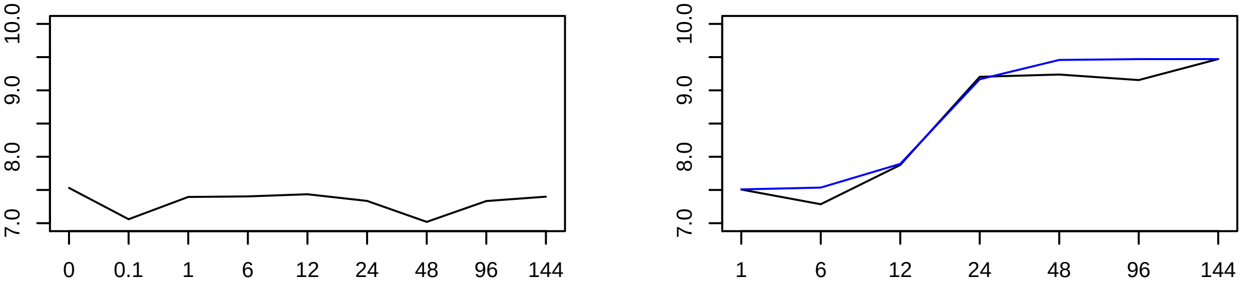

**A_23_P157299 AEBP1 7p13**

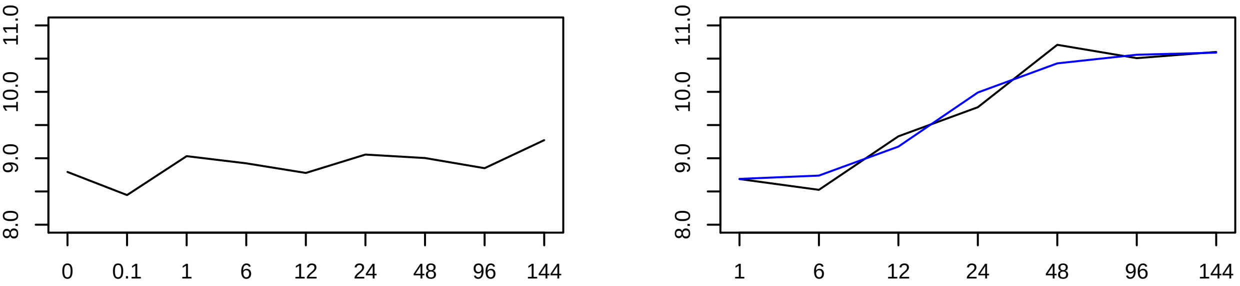


**A_23_P169278 AGTPBP1 9q21.33**

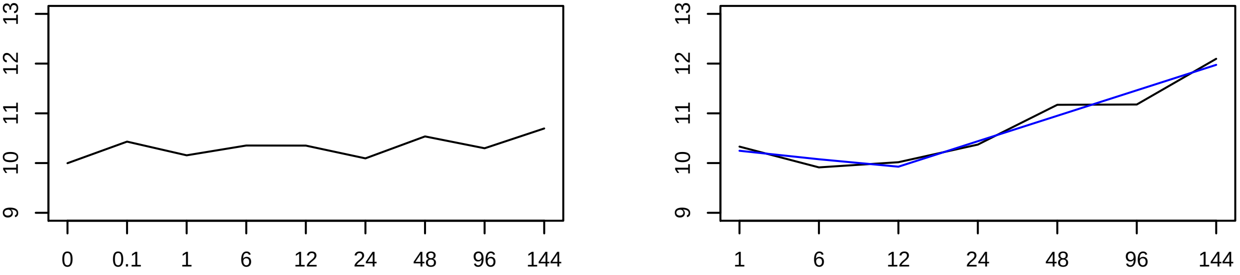


**A_23_P161190 VIM 10p12.33**

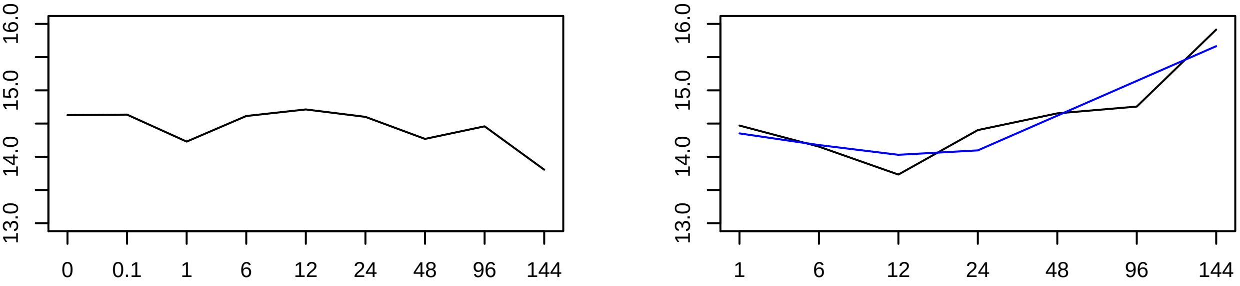

**A_32_P23659 BCAS4 20q13.13**

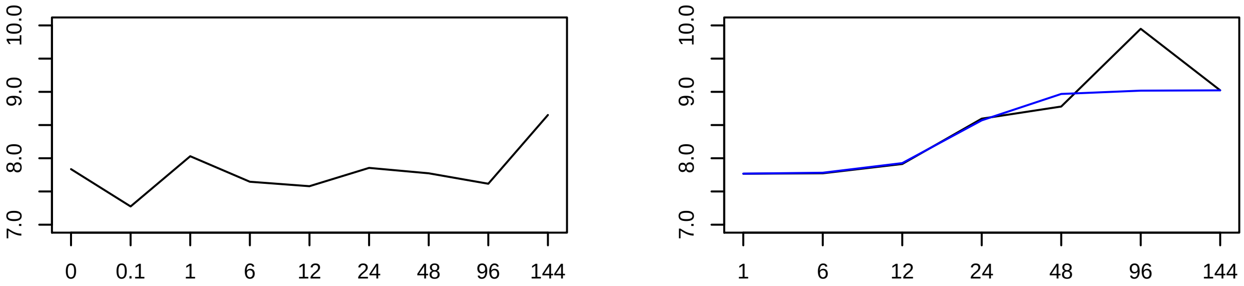


**A_23_P90470 A_23_P90470 NA**

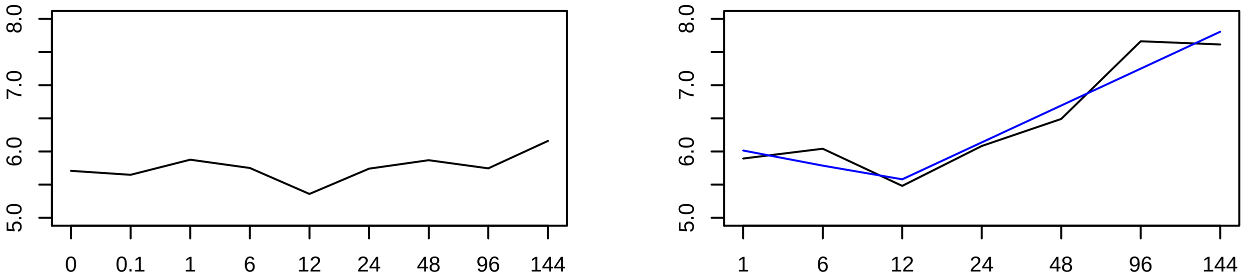


**A_23_P99063 LUM 12q21.33**

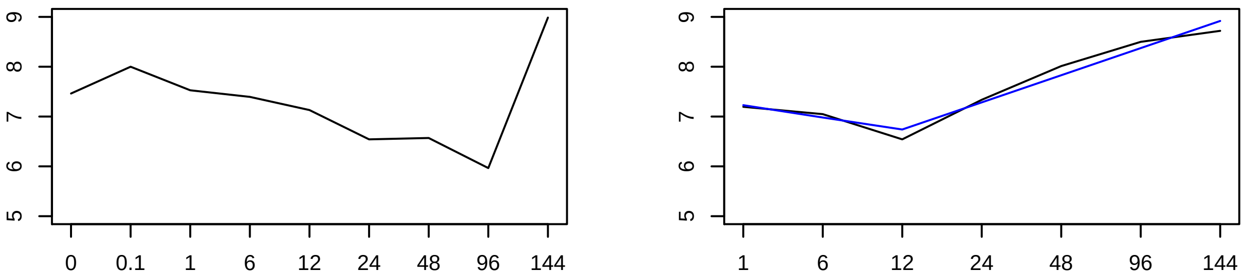

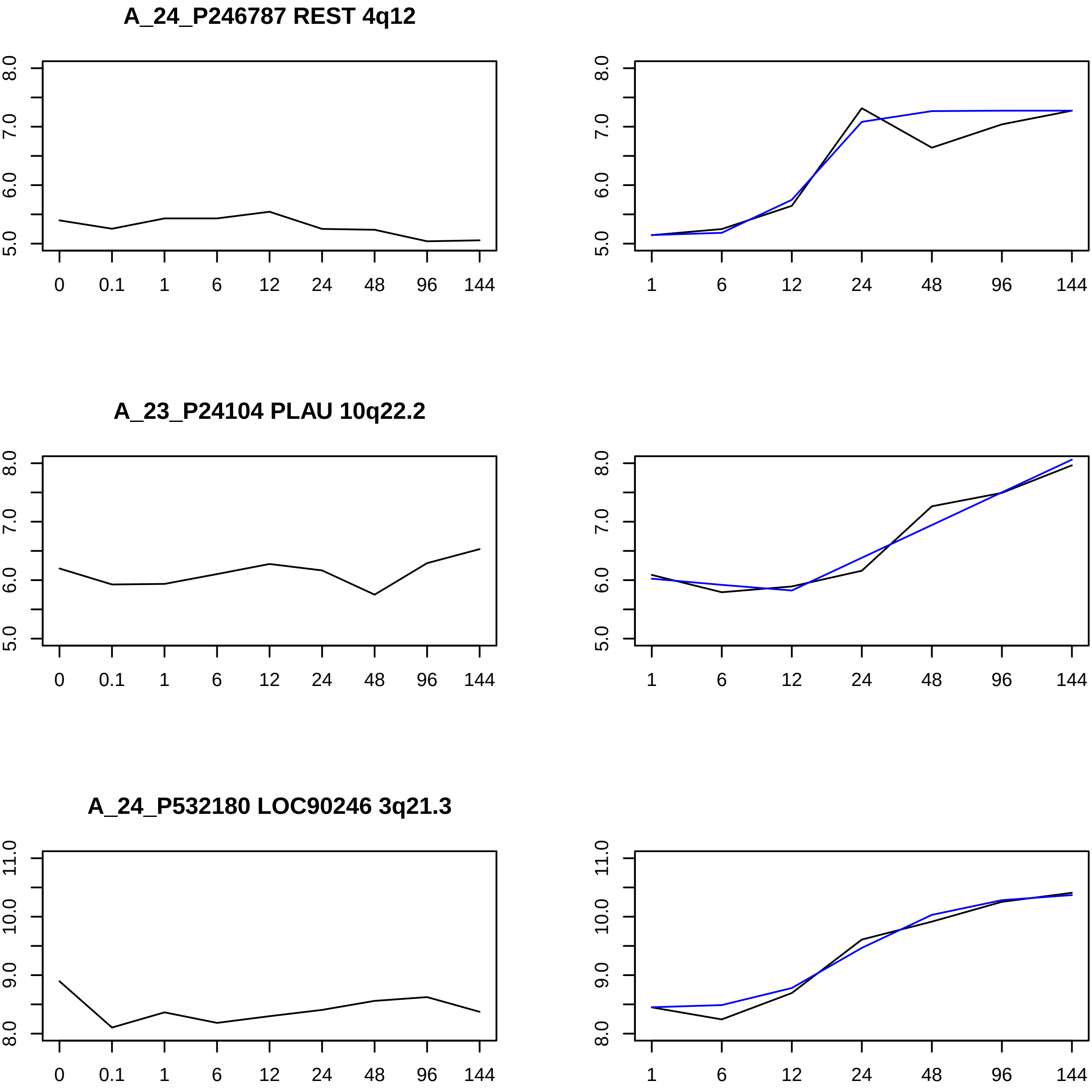
A_24_P246787 REST 4q12
5.0
6.0
7.0
8.0
0
0.1
1
6
12
24
48
96
144
1
6
12
24
48
96
144
A_23_P24104 PLAU 10q22.2
5.0
6.0
7.0
8.0
0
0.1
1
6
12
24
48
96
144
1
6
12
24
48
96
144
A_24_P532180 LOC90246 3q21.3
8.0
9.0
10.0
11.0
0
0.1
1
6
12
24
48
96
144
1
6
12
24
48
96
144

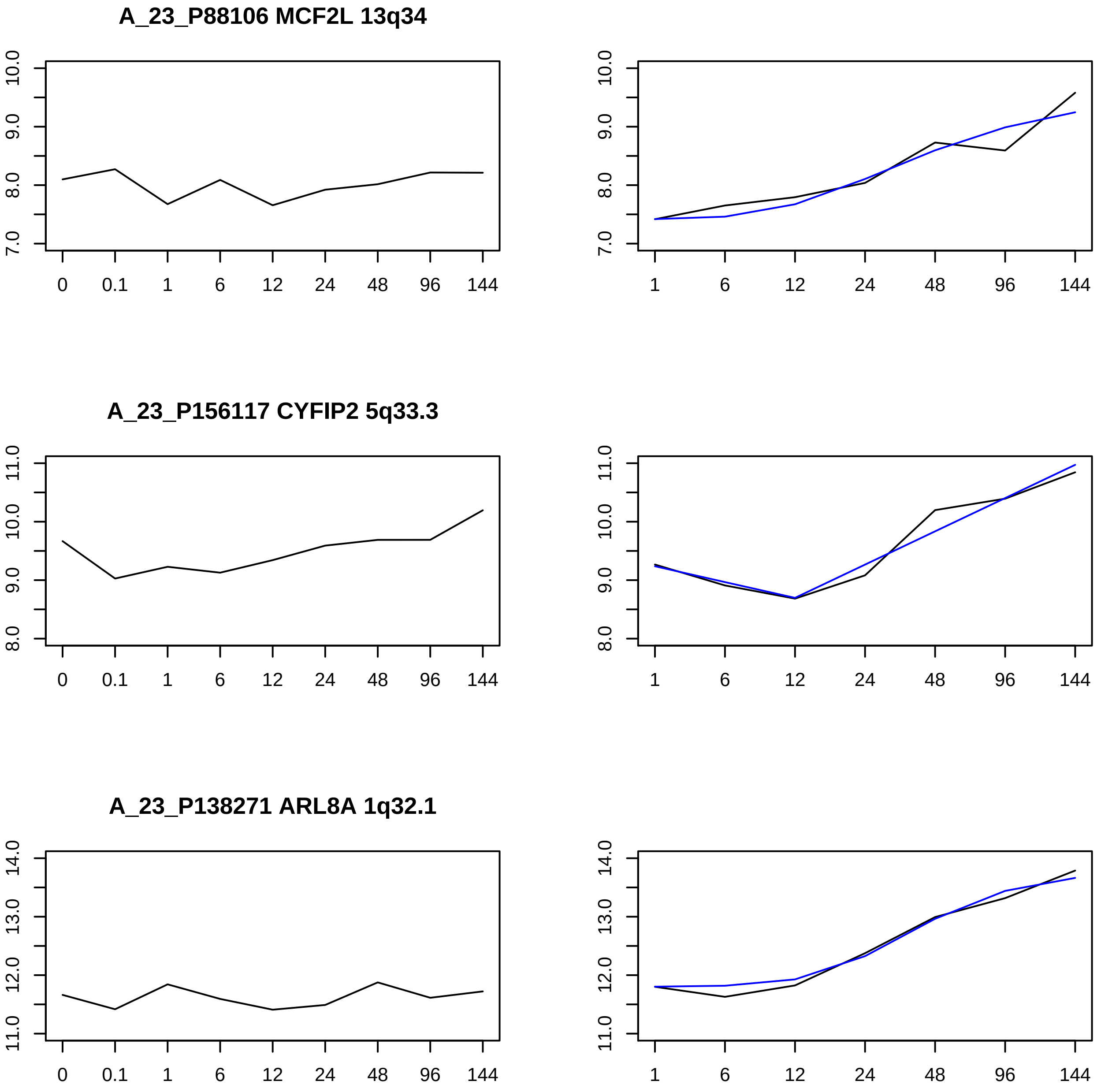
A_23_P88106 MCF2L 13q34
A_23_P156117 CYFIP2 5q33.3
A_23_P138271 ARL8A 1q32.1
7.0
8.0
9.0
10.0
11.0
12.0
13.0
14.0
0
0.1
1
6
12
24
48
96
144

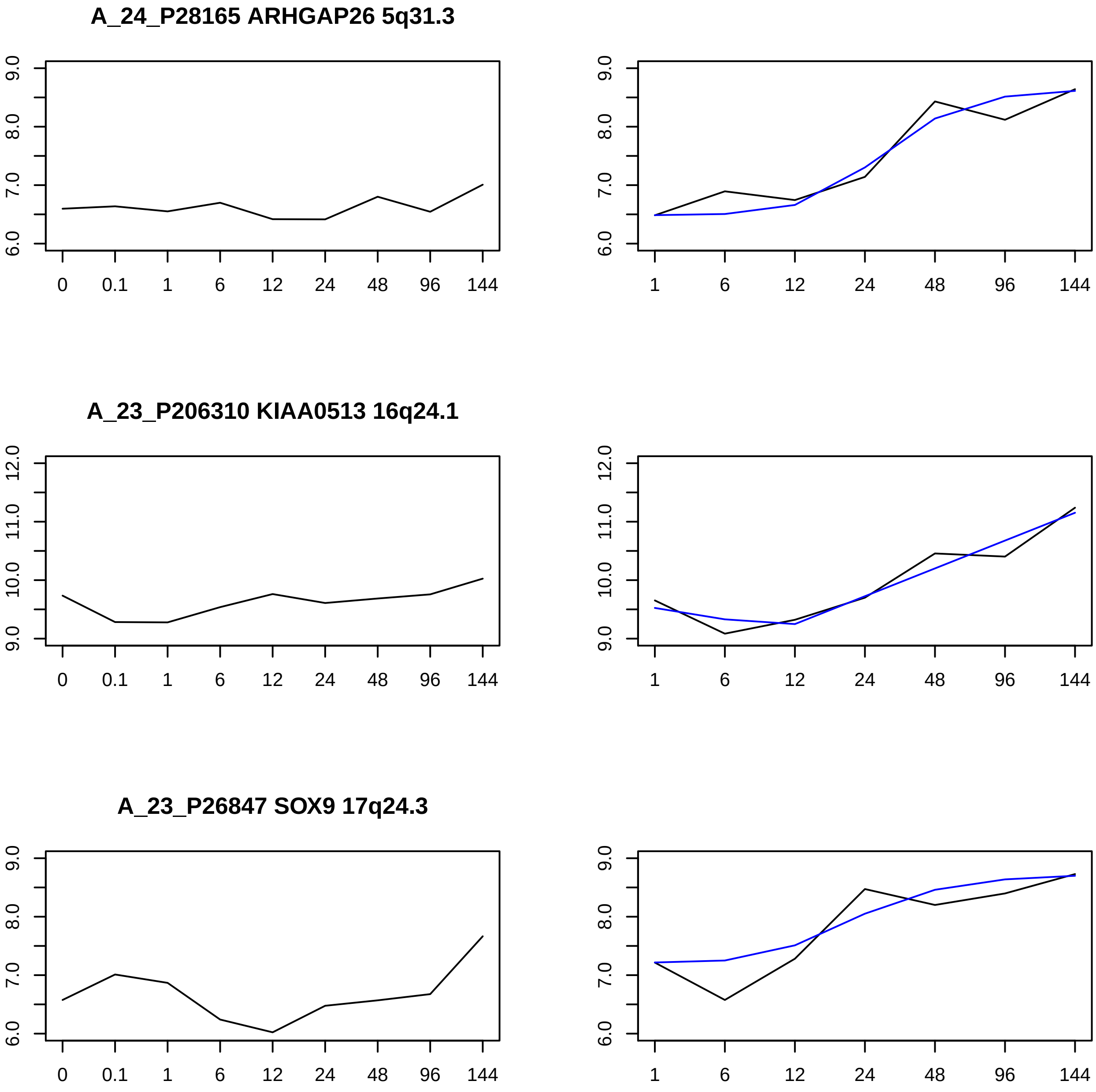
A_24_P28165 ARHGAP26 5q31.3
A_23_P206310 KIAA0513 16q24.1
A_23_P26847 SOX9 17q24.3
6.0 7.0 8.0 9.0
9.0 10.0 11.0 12.0
0 0.1 1 6 12 24 48 96 144
1 6 12 24 48 96 144

**A_23_P40548 YPEL1 22q11.21**

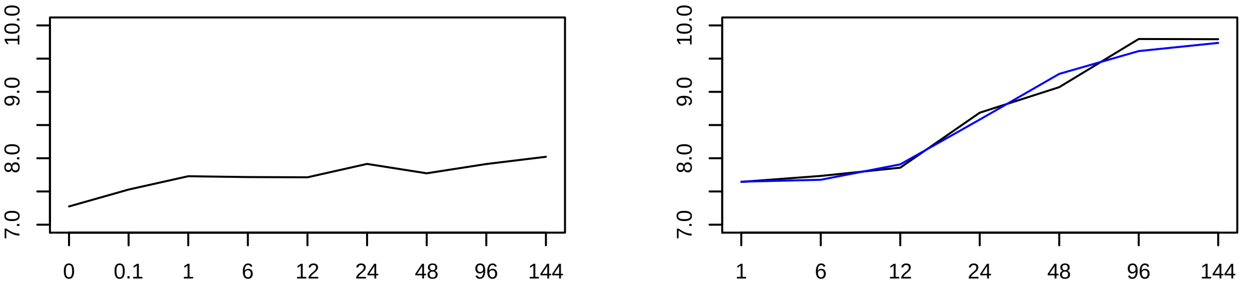


**A_23_P73097 RGS20 8q11.23**

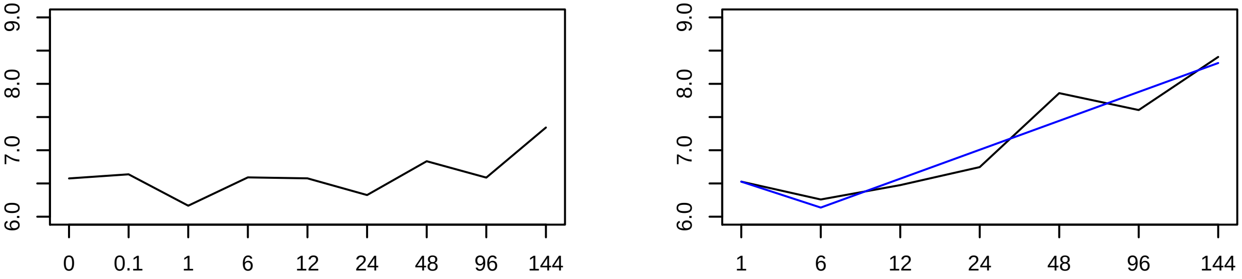


**A_23_P322 EFNA4 1q22**

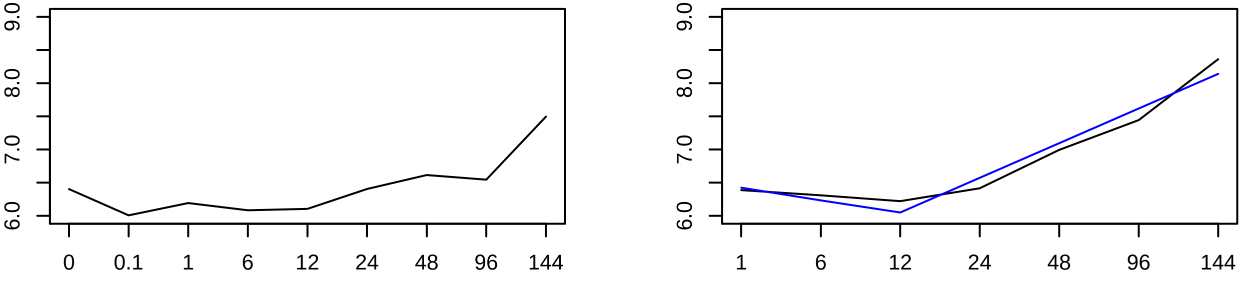

**A_23_P61919 DGKB 7p21.2**

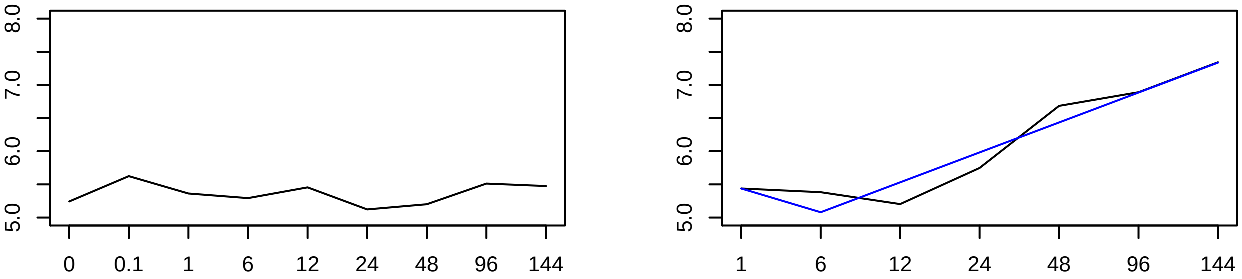


**A_23_P351275 UPP1 7p12.3**

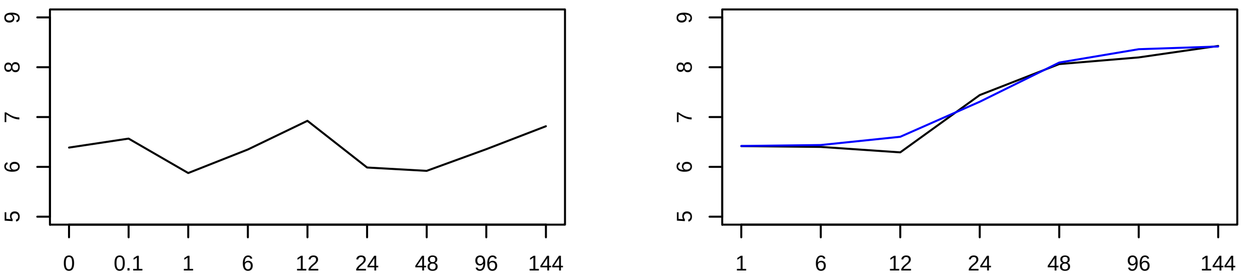


**A_32_P88987 AK022346 NA**

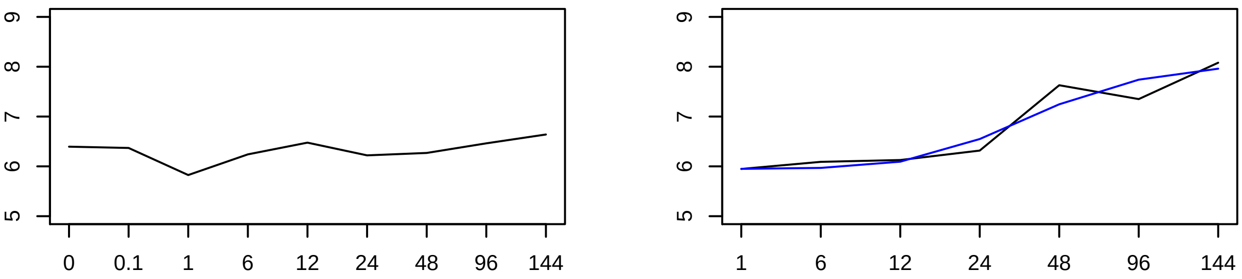

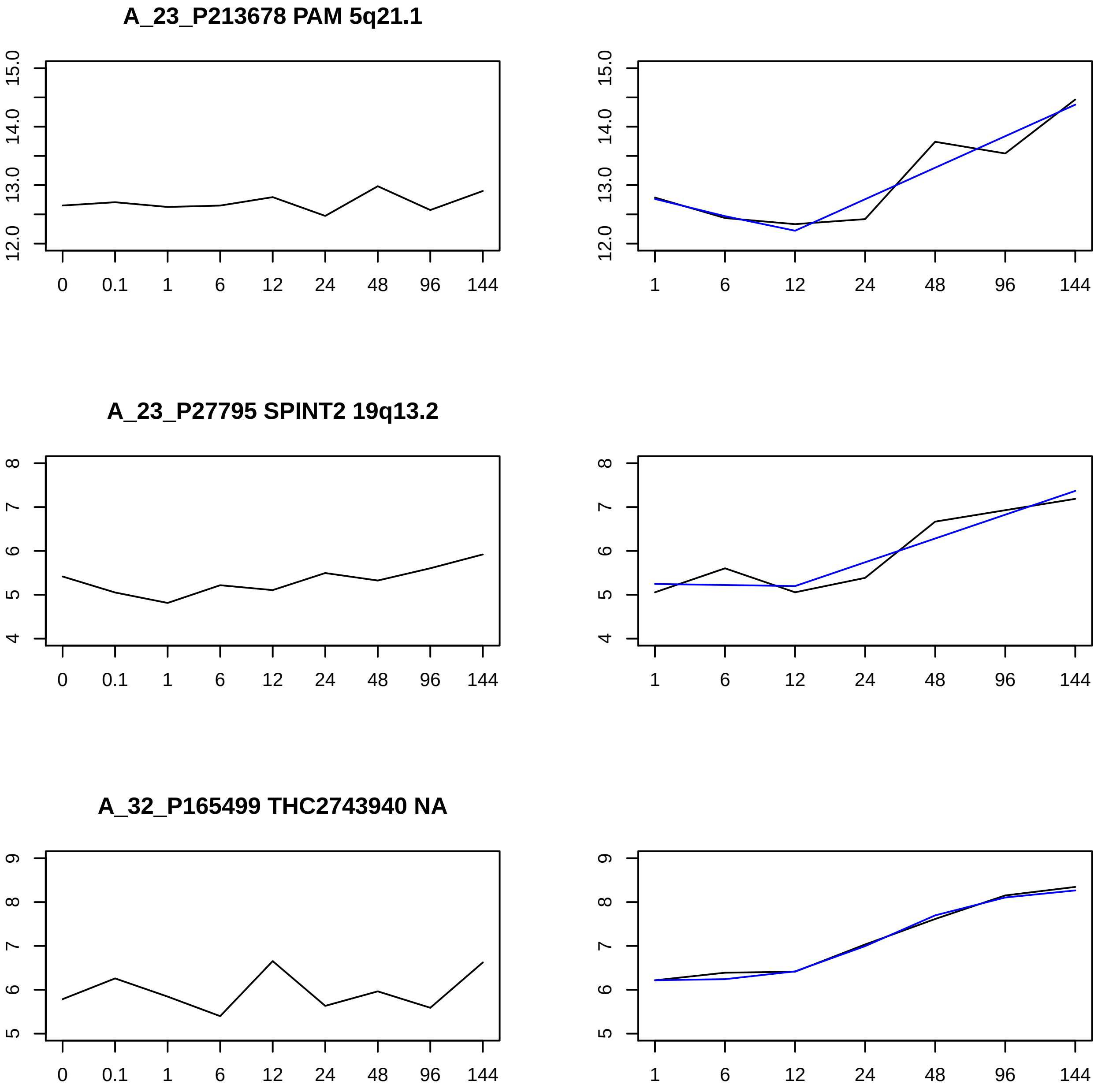

A_23_P213678 PAM 5q21.1
12.0
13.0
14.0
15.0
0 0.1 1 6 12 24 48 96 144
1 6 12 24 48 96 144
A_23_P27795 SPINT2 19q13.2
4
5
6
7
8
0 0.1 1 6 12 24 48 96 144
1 6 12 24 48 96 144
A_32_P165499 THC2743940 NA
5
6
7
8
9
0 0.1 1 6 12 24 48 96 144
1 6 12 24 48 96 144

**A_32_P201530 SNIP 17q12**

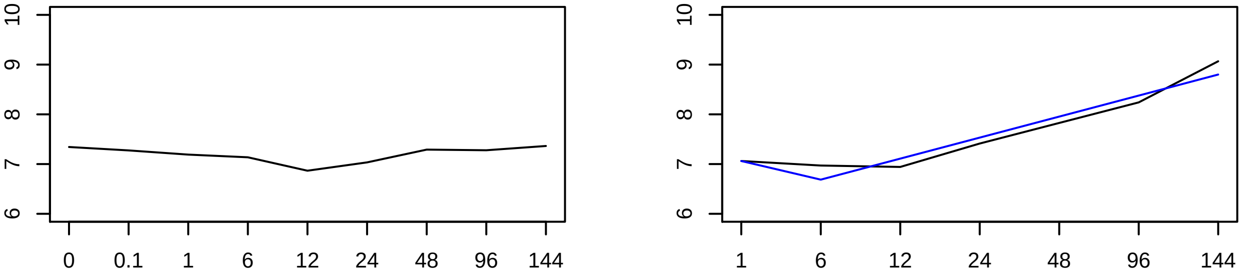


**A_23_P134914 LY6H 8q24.3**

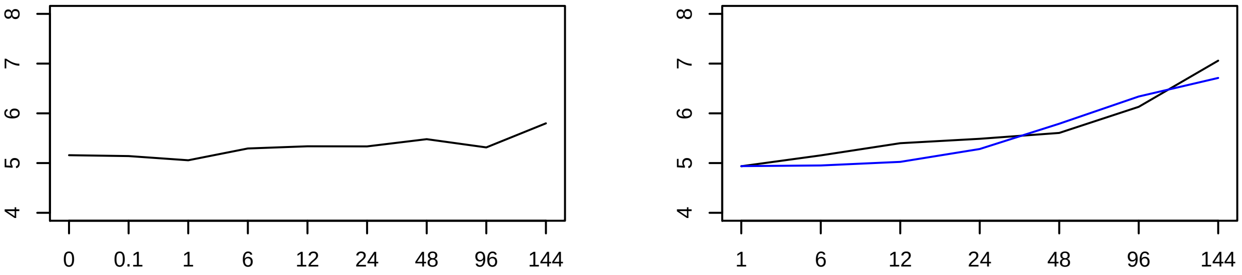


**A_23_P26223 ASL 7q11.21**

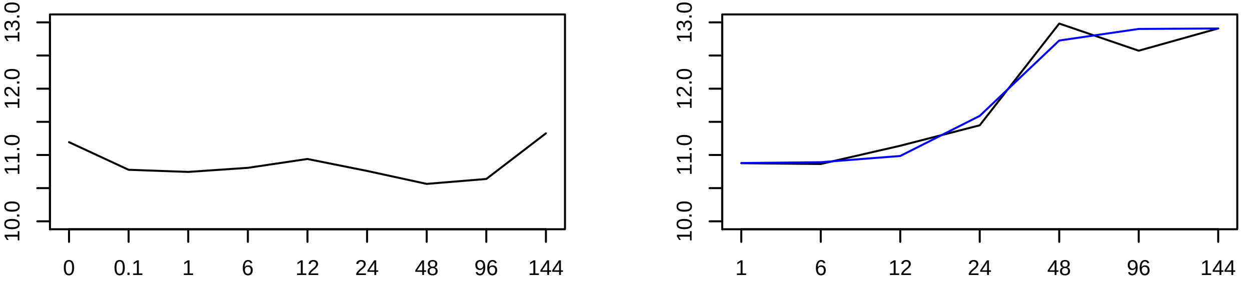

**A_23_P147805 UPP1 7p12.3**

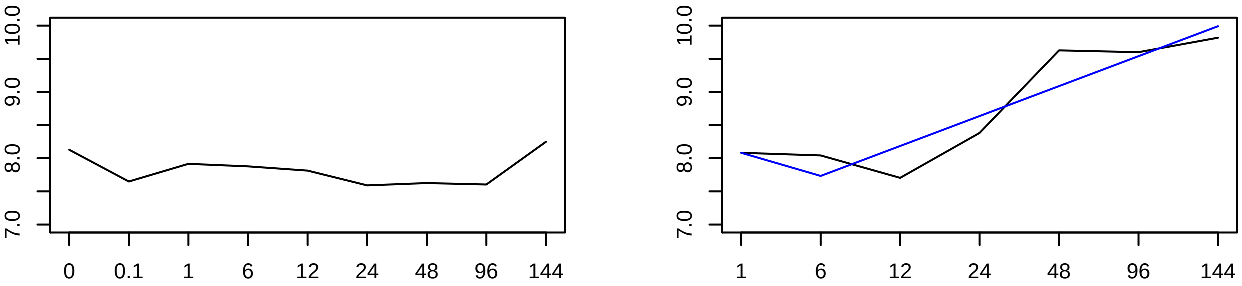


**A_32_P187919 AF086156 NA**

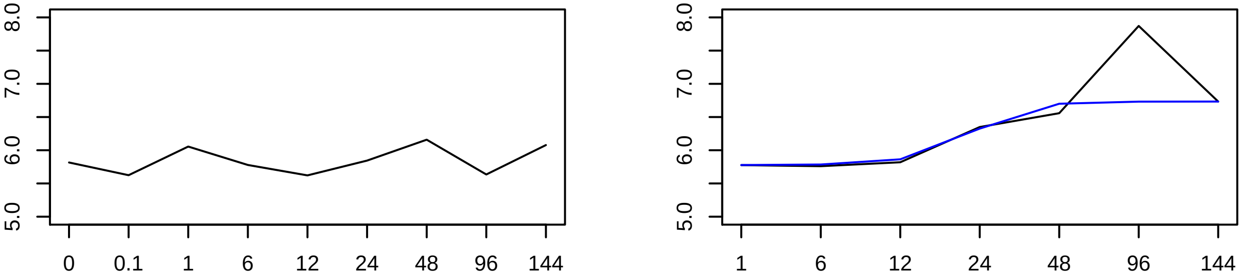


**A_23_P218928 C4orf18 4q32.1**

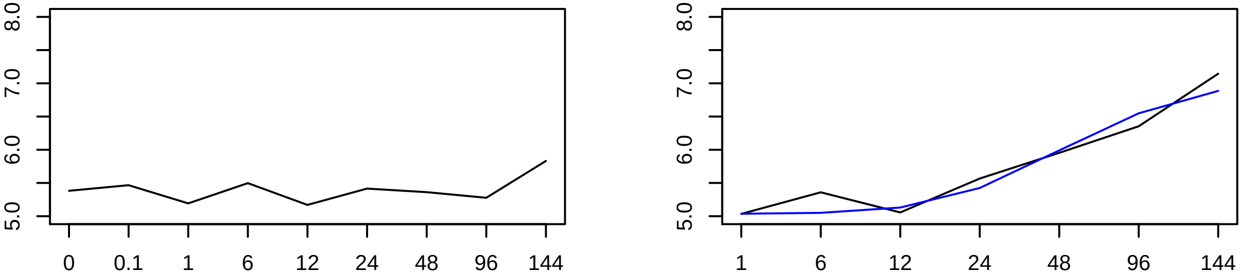

**A_23_P104193 ITGB1 10p11.22**

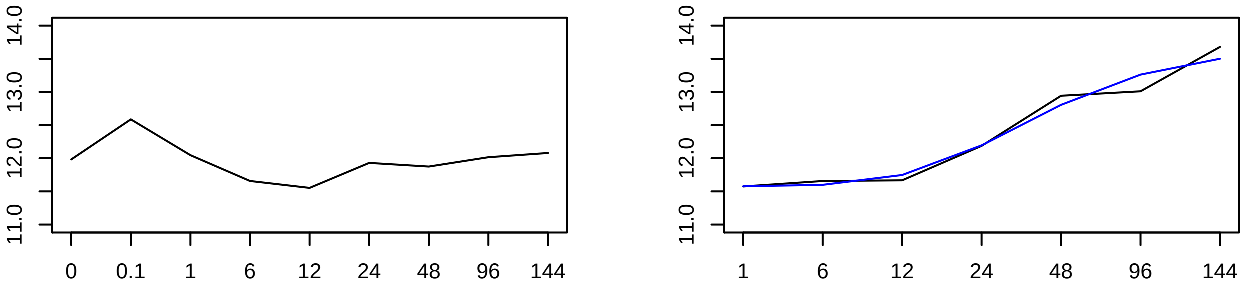


**A_24_P362540 DDEF2 2p25.1**

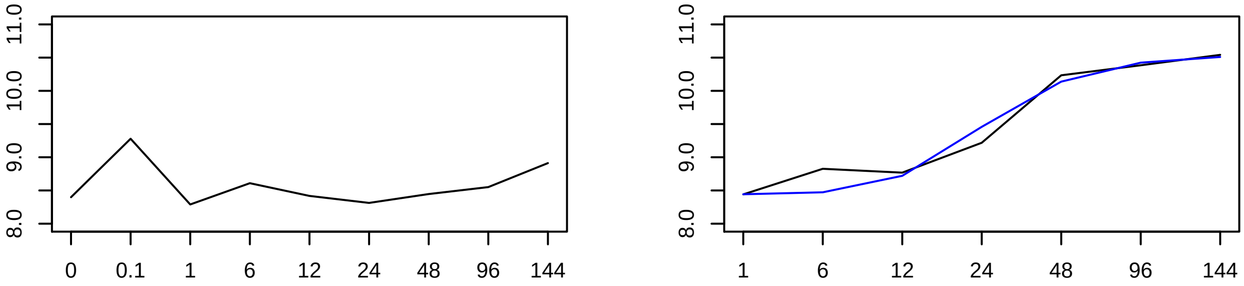


**A_23_P11543 FUCA1 1p36.11**

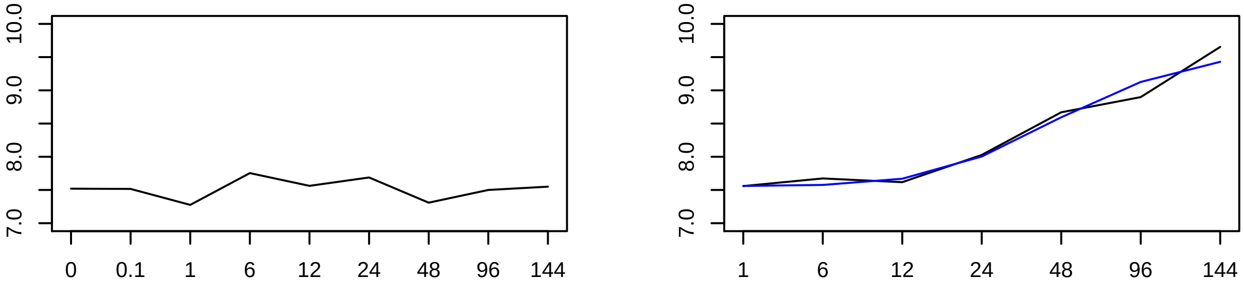

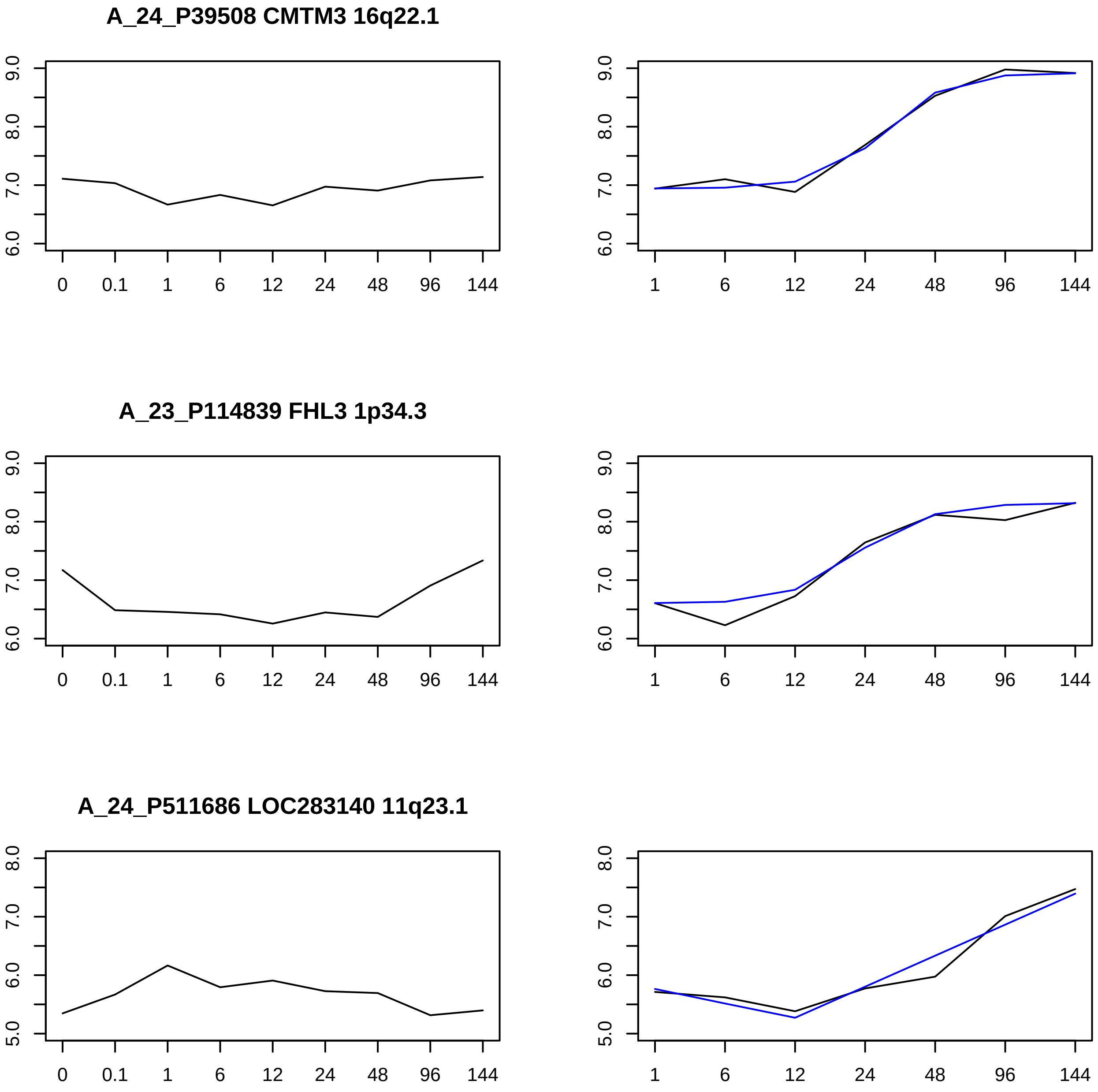
A_24_P39508 CMTM3 16q22.1
A_23_P114839 FHL3 1p34.3
A_24_P511686 LOC283140 11q23.1
6.0
7.0
8.0
9.0
5.0
0
0.1
1
6
12
24
48
96
144

**A_23_P396765 PGM2L1 11q13.4**

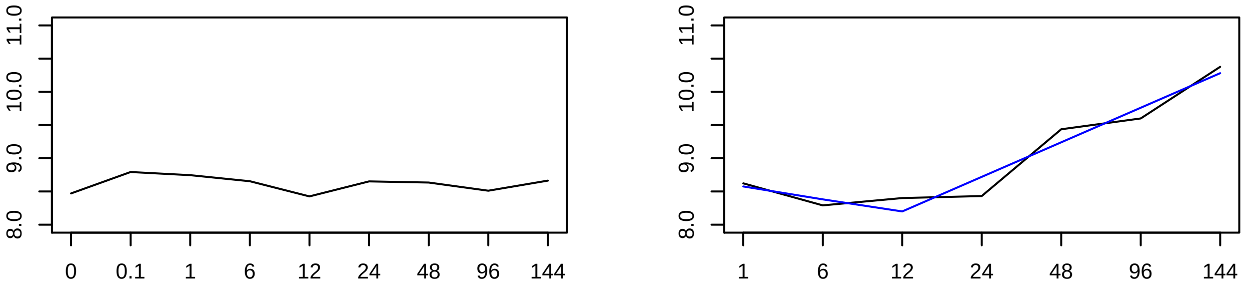


**A_32_P209582 THC2663167 NA**

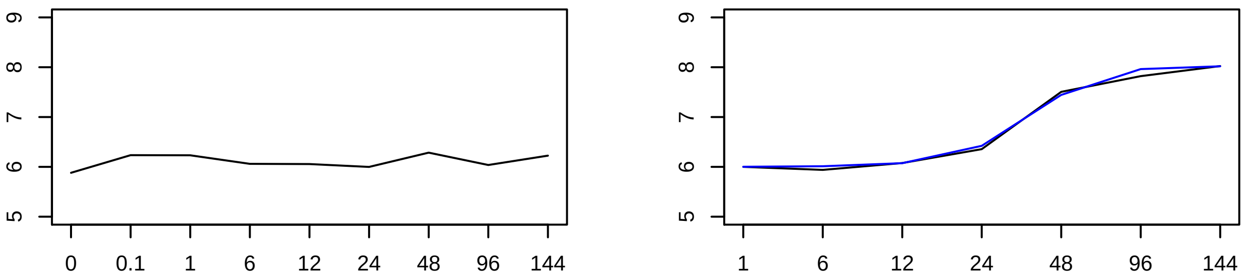


**A_23_P97932 MSRB2 10p12.2**

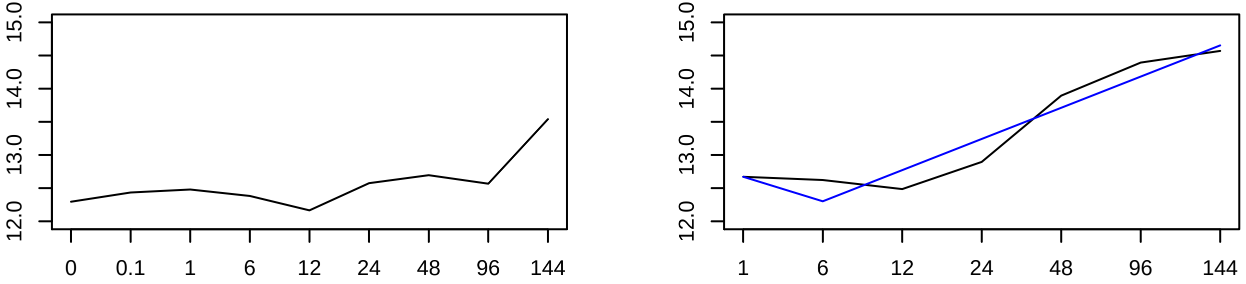

**A_24_P230486 A_24_P230486 NA**

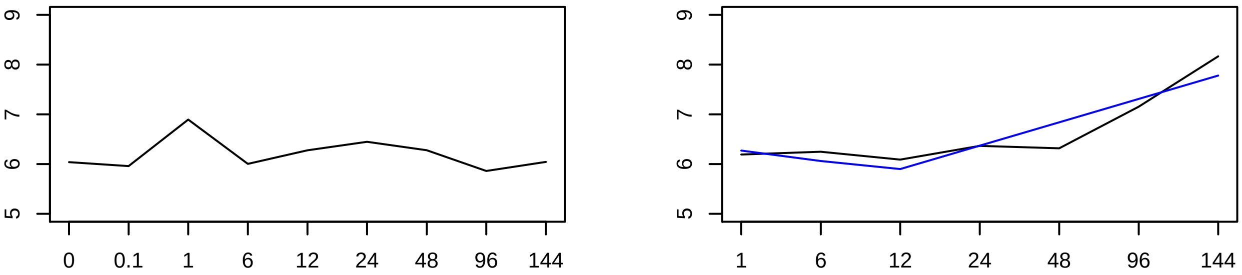


**A_24_P6825 ENST00000299903 NA**

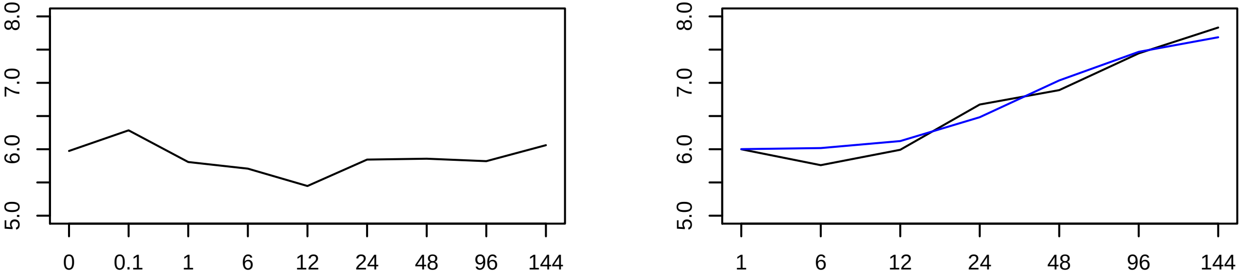


**A_23_P145485 ULBP2 6q25.1**

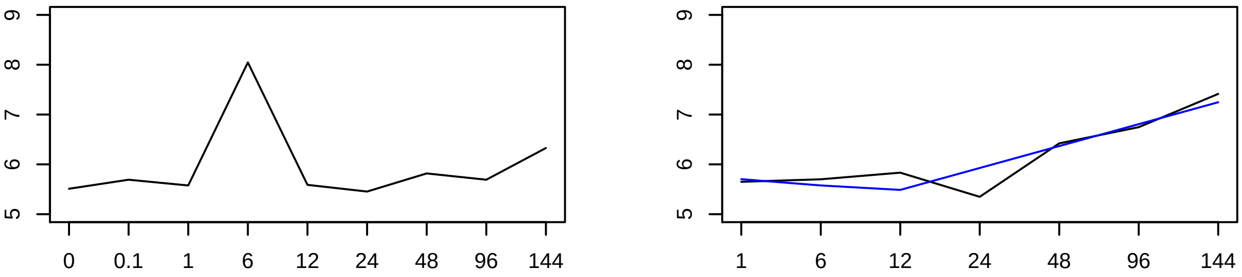

**A_23_P257335 KHDRBS3 8q24.23**

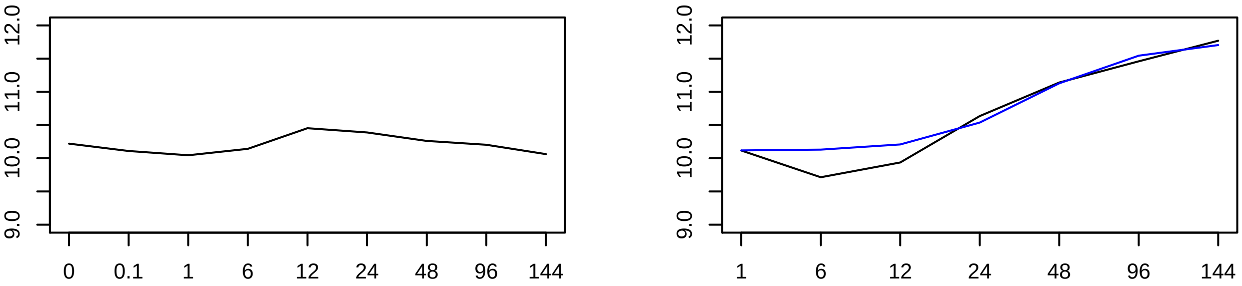


**A_23_P104199 ITGB1 10p11.22**

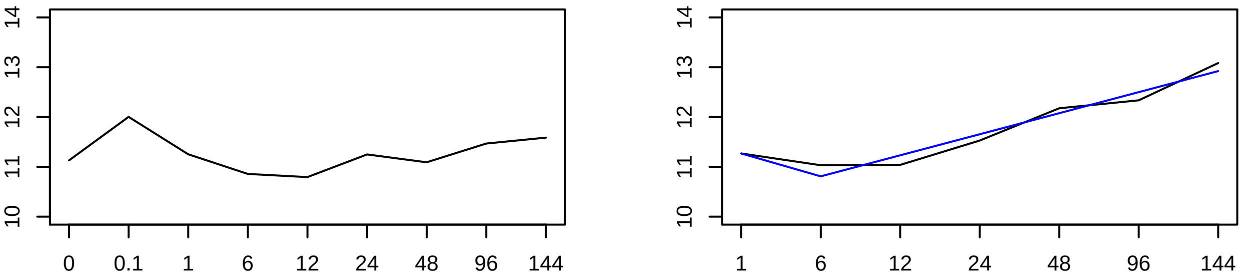


**A_23_P166807 PCBP4 3p21.1**

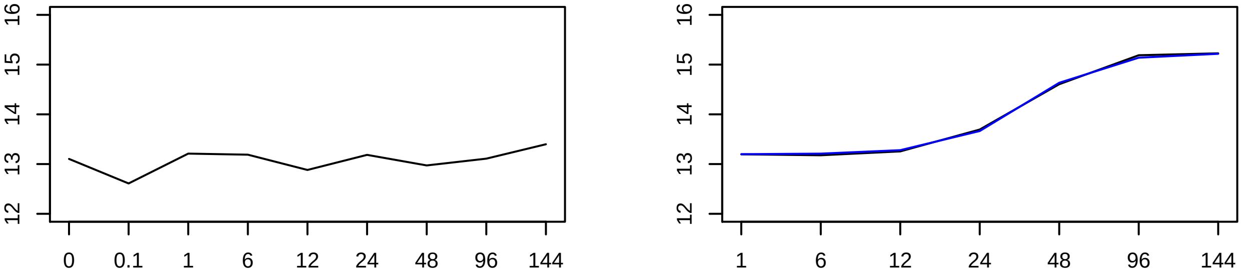

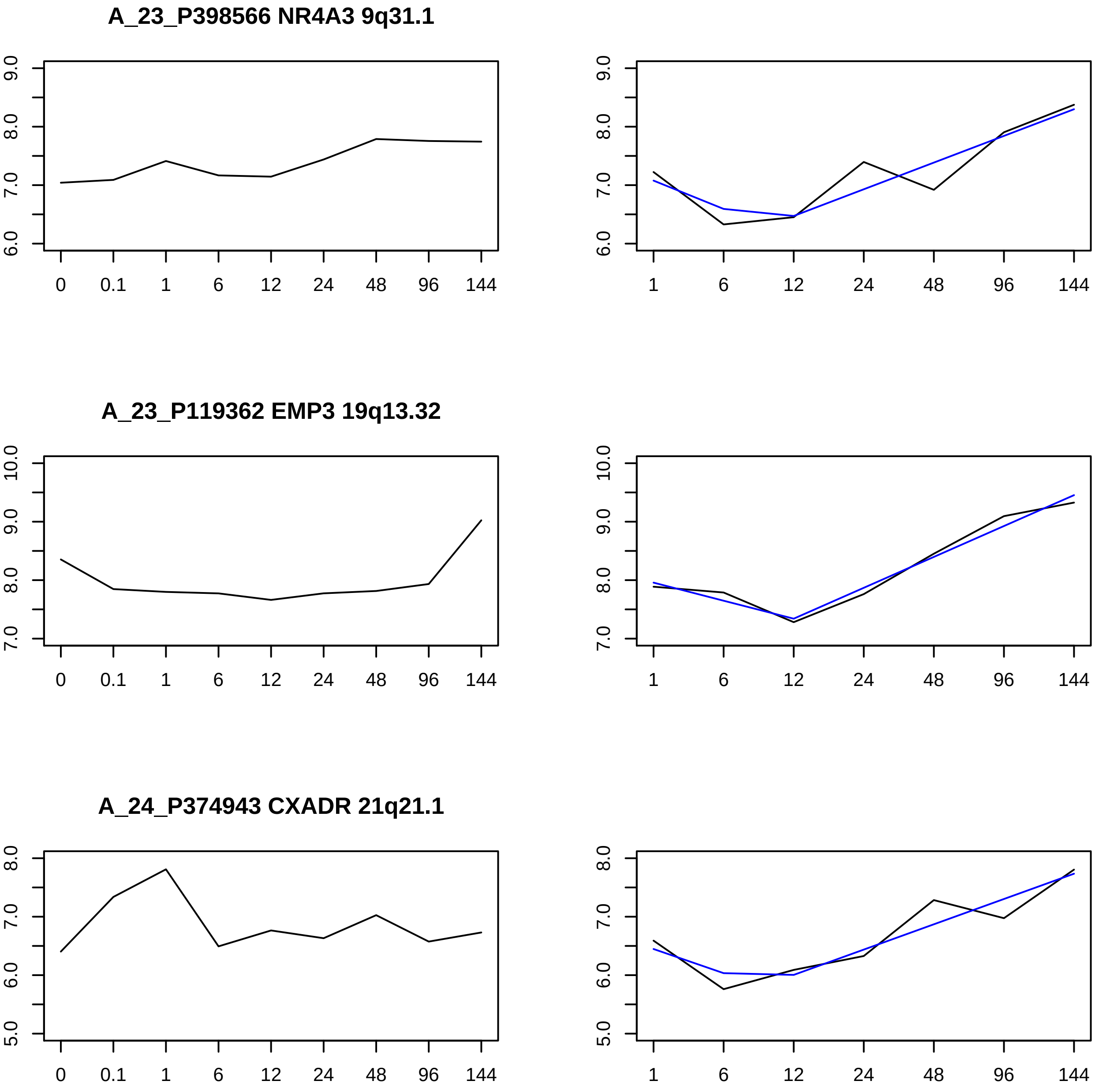
A_23_P398566 NR4A3 9q31.1
6.0
7.0
8.0
9.0
0
0.1
1
6
12
24
48
96
144
A_23_P119362 EMP3 19q13.32
7.0
8.0
9.0
10.0
A_24_P374943 CXADR 21q21.1
5.0
6.0
7.0
8.0

**A_23_P14508 TTC9 14q24.2**

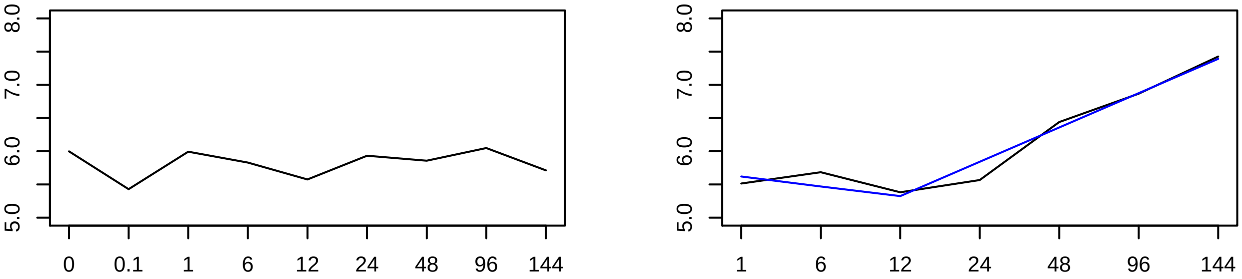


**A_32_P222695 FLJ41603 5q33.1**

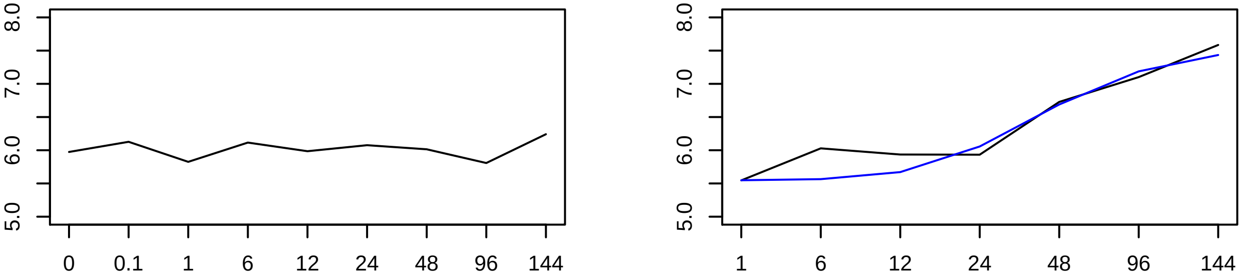


**A_23_P128319 ATP2B1 12q21.33**

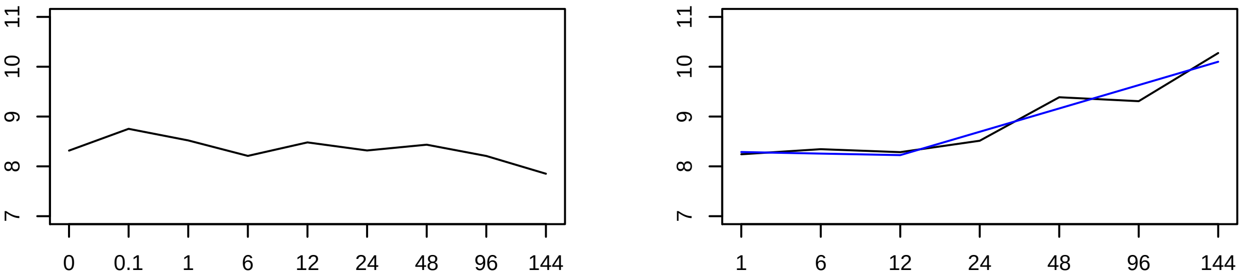

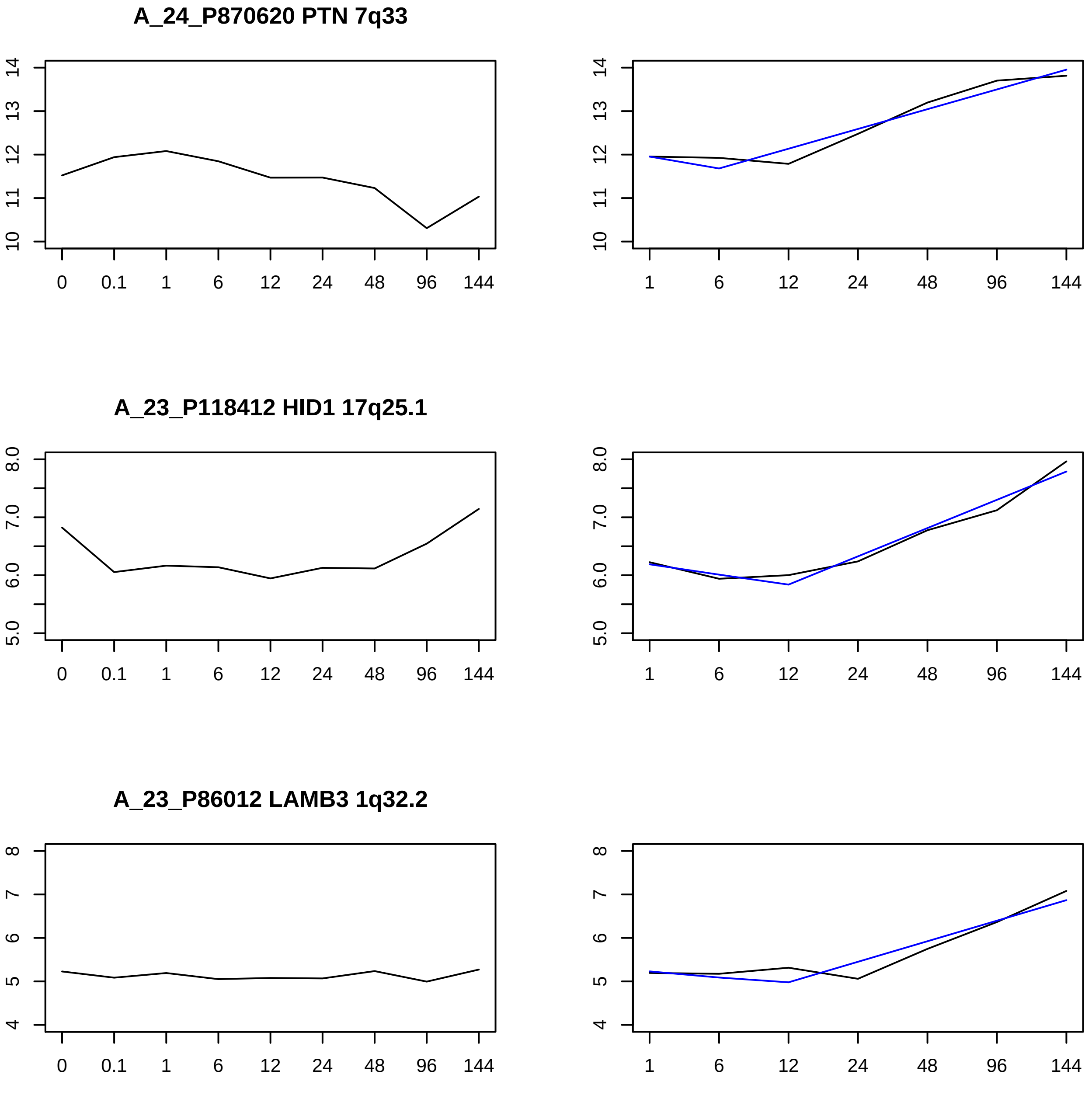

A_24_P870620 PTN 7q33
10
11
12
13
14
0
0.1
1
6
12
24
48
96
144
1
6
12
24
48
96
144
A_23_P118412 HID1 17q25.1
5.0
6.0
7.0
8.0
0
0.1
1
6
12
24
48
96
144
1
6
12
24
48
96
144
A_23_P86012 LAMB3 1q32.2
4
5
6
7
8
0
0.1
1
6
12
24
48
96
144
1
6
12
24
48
96
144

**A_24_P185186 SAMD14 17q21.33**

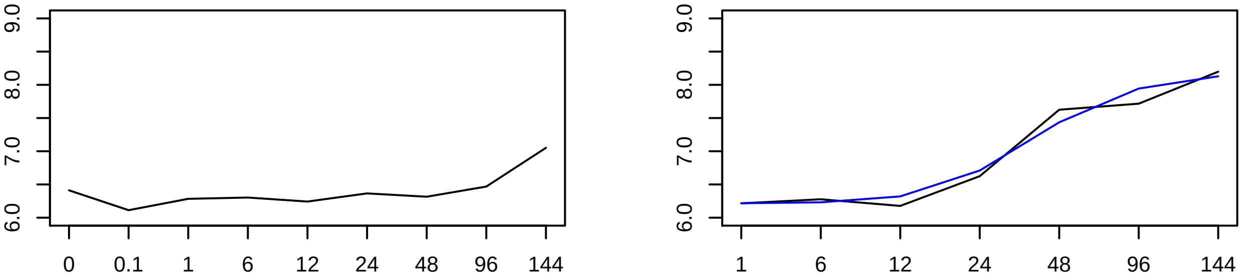


**A_32_P92188 CNTN5 NA**

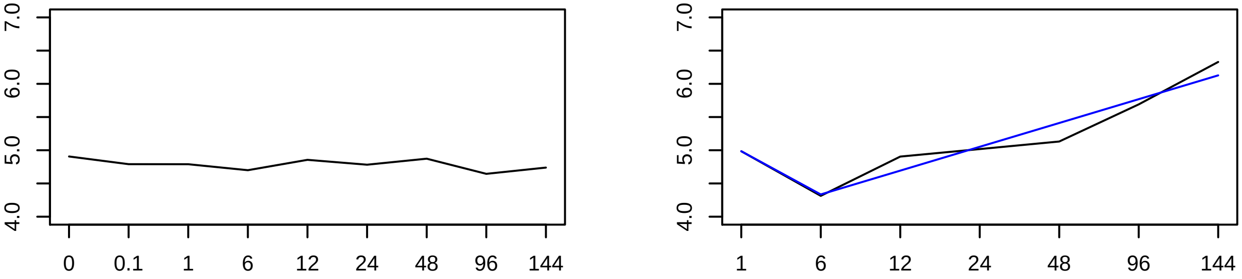


**A_23_P66311 DNASE1 16p13.3**

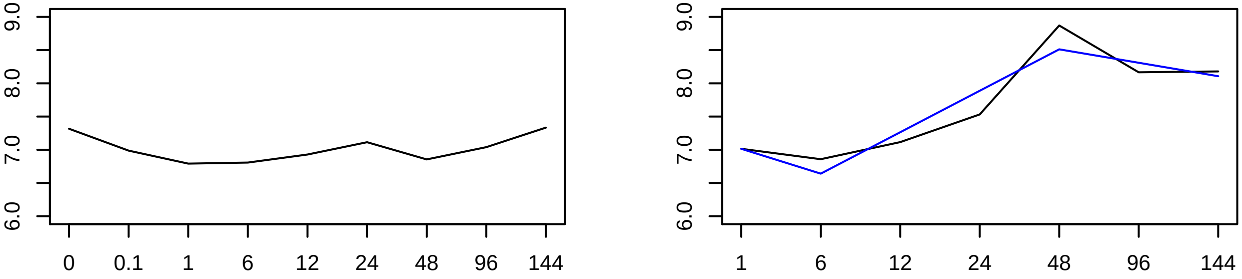

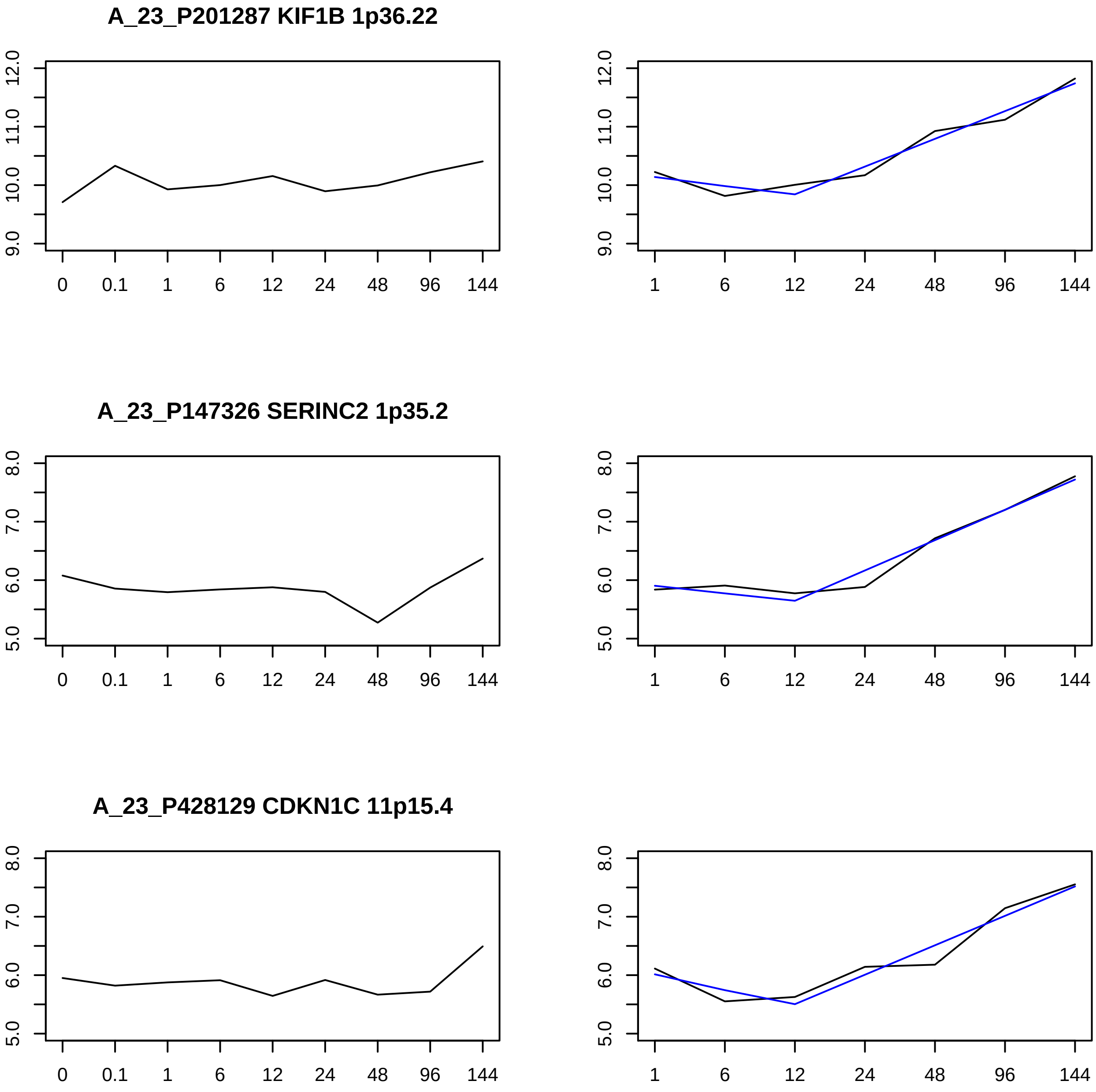
A_23_P201287 KIF1B 1p36.22
9.0
10.0
11.0
12.0
0
0.1
1
6
12
24
48
96
144
A_23_P147326 SERINC2 1p35.2
5.0
6.0
7.0
8.0
A_23_P428129 CDKN1C 11p15.4

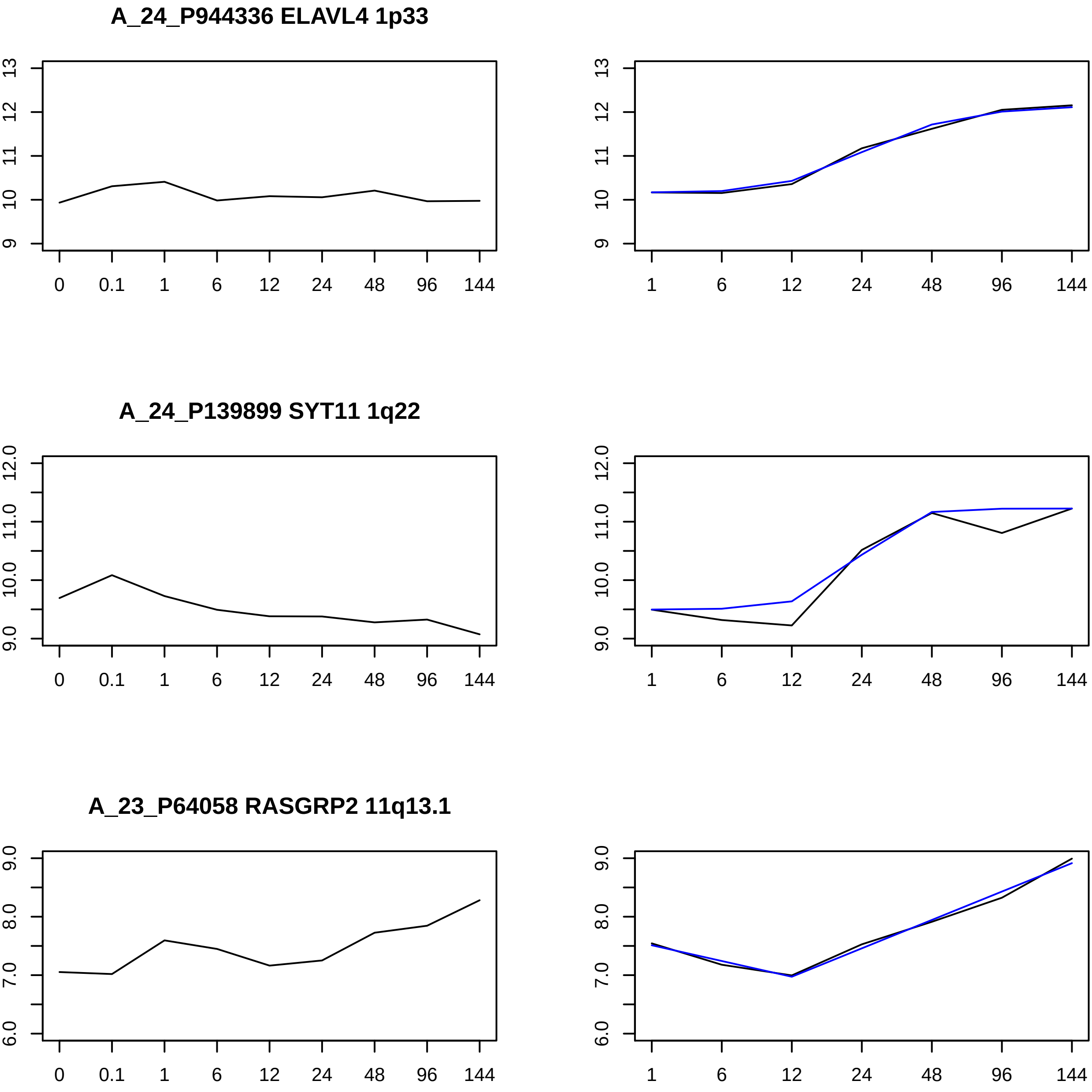
A_24_P944336 ELAVL4 1p33
9
10
11
12
13
0
0.1
1
6
12
24
48
96
144
1
6
12
24
48
96
144
A_24_P139899 SYT11 1q22
9.0
10.0
11.0
12.0
A_23_P64058 RASGRP2 11q13.1
6.0
7.0
8.0
9.0

A_32_P154473 KIF5C 2q23.1

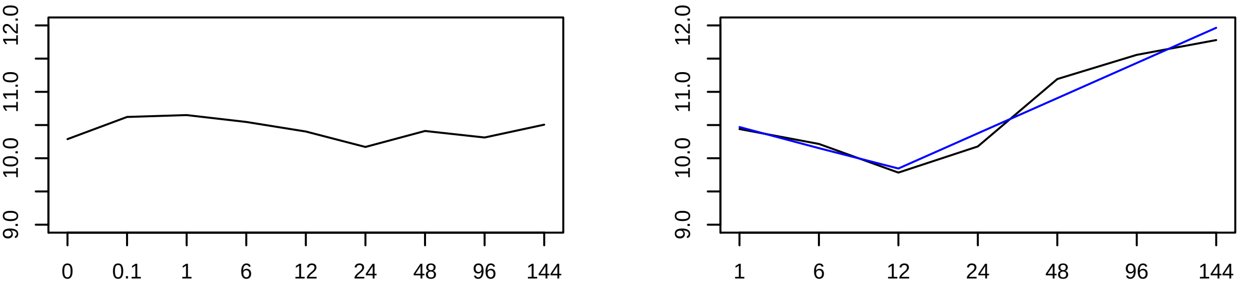


A_32_P234145 SHC4 15q21.1

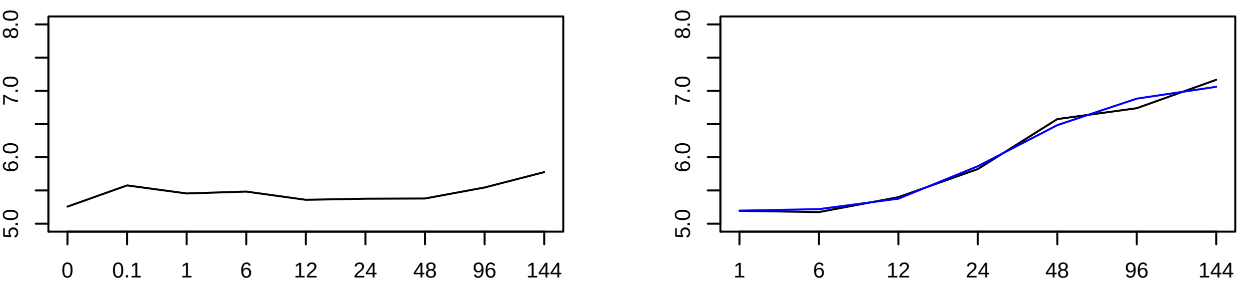


A_24_P598836 ITGB1 8q24.3

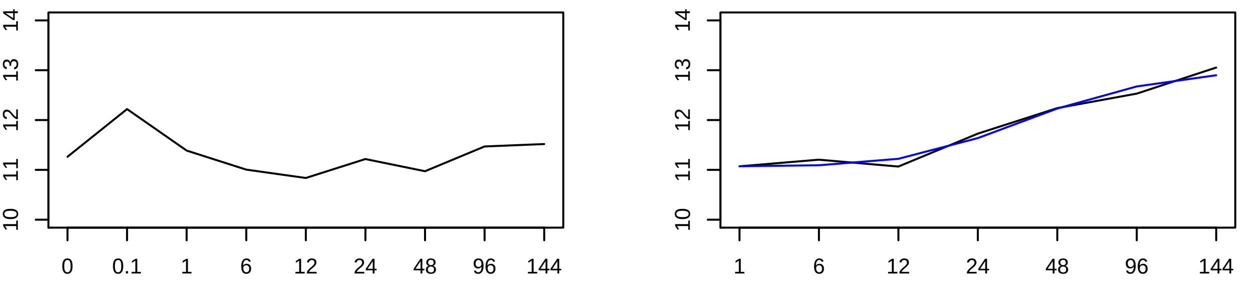

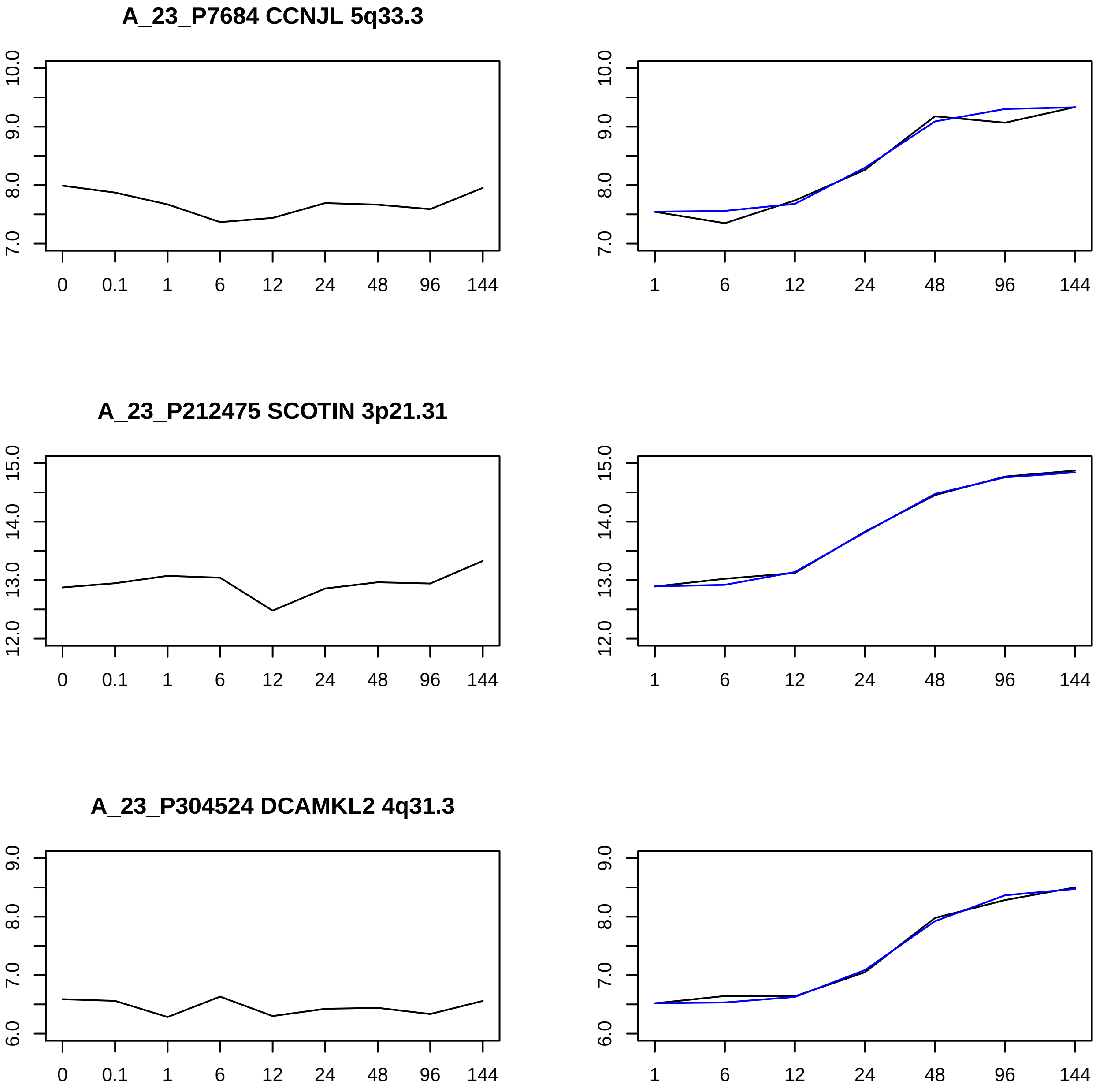
A_23_P7684 CCNJL 5q33.3
7.0
8.0
9.0
10.0
0
0.1
1
6
12
24
48
96
144
A_23_P212475 SCOTIN 3p21.31
12.0
13.0
14.0
15.0
A_23_P304524 DCAMKL2 4q31.3
6.0
7.0
8.0
9.0

**A_23_P39840 VAMP5 2p11.2**

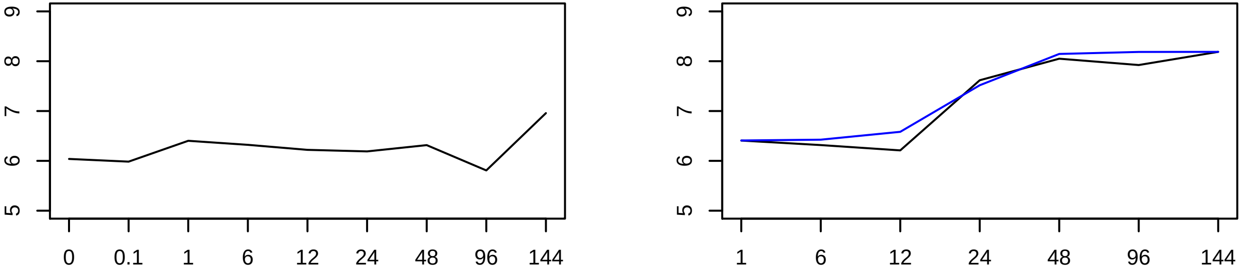


**A_23_P166929 SERPINI1 3q26.1**

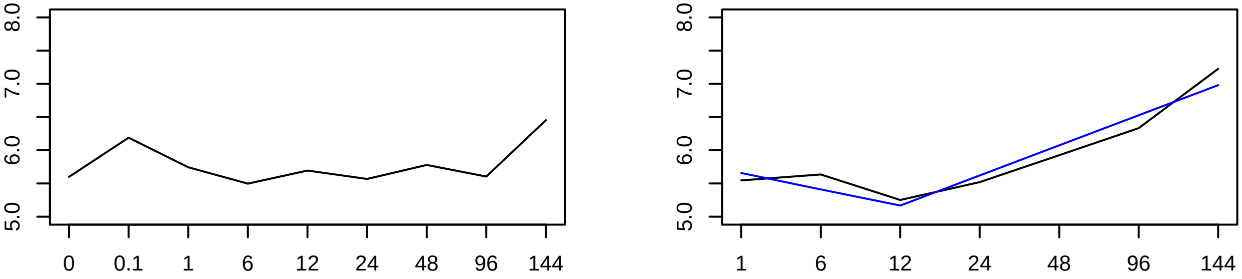


**A_23_P308722 C2orf39 2p23.3**

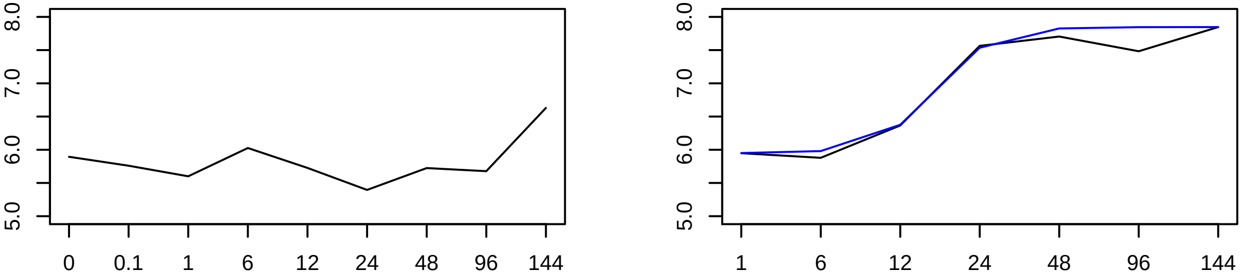

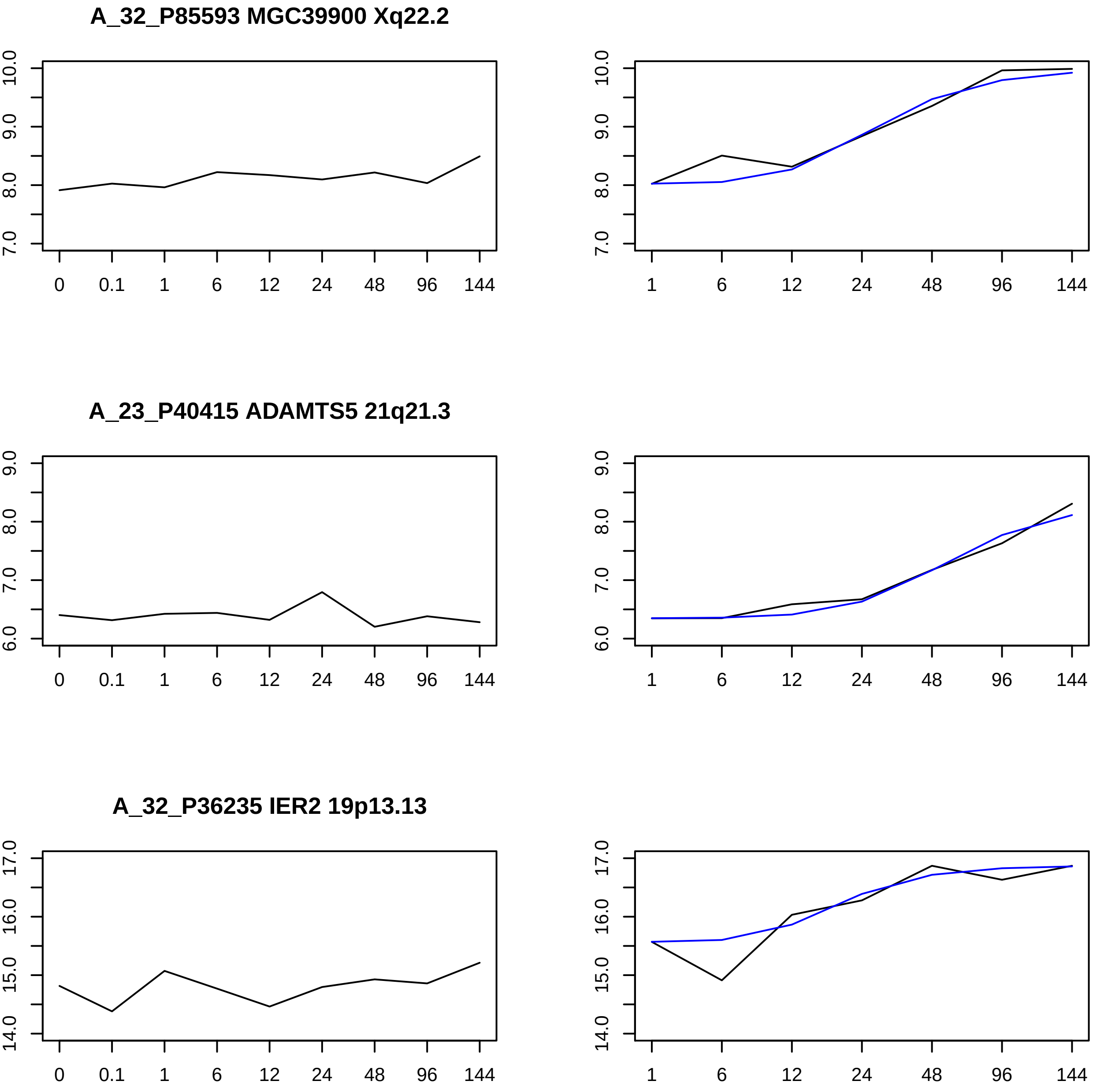
A_32_P85593 MGC39900 Xq22.2
A_23_P40415 ADAMTS5 21q21.3
A_32_P36235 IER2 19p13.13
7.0
8.0
9.0
10.0
6.0
14.0
15.0
16.0
17.0
0
0.1
1
6
12
24
48
96
144

**A_23_P58647 CTNNA1 5q31.2**

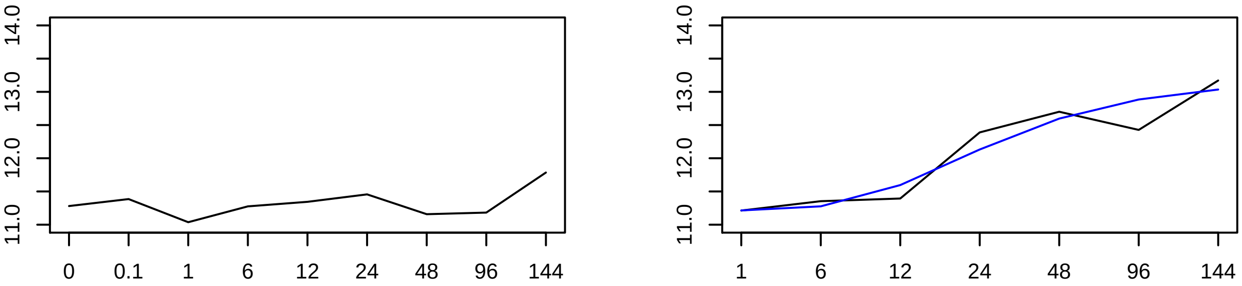


**A_24_P110914 TPT1−AS1 13q14.13**

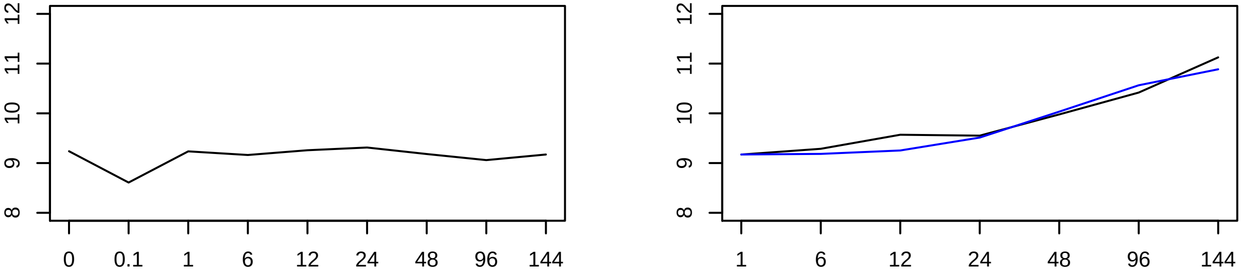


**A_24_P302584 SOX11 2p25.2**

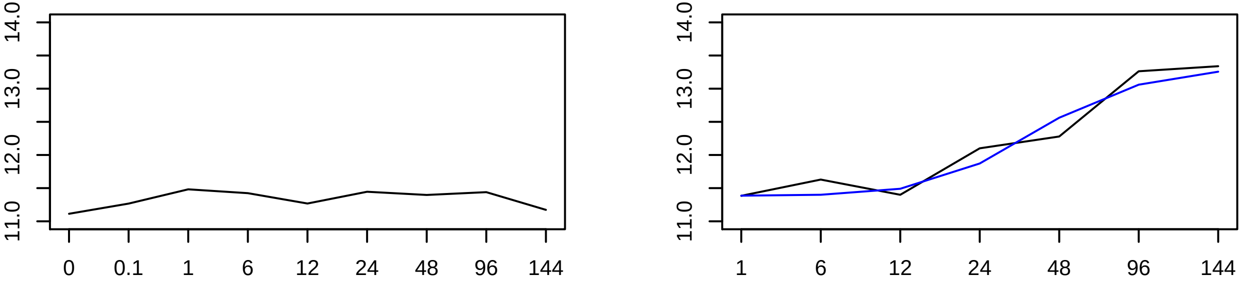

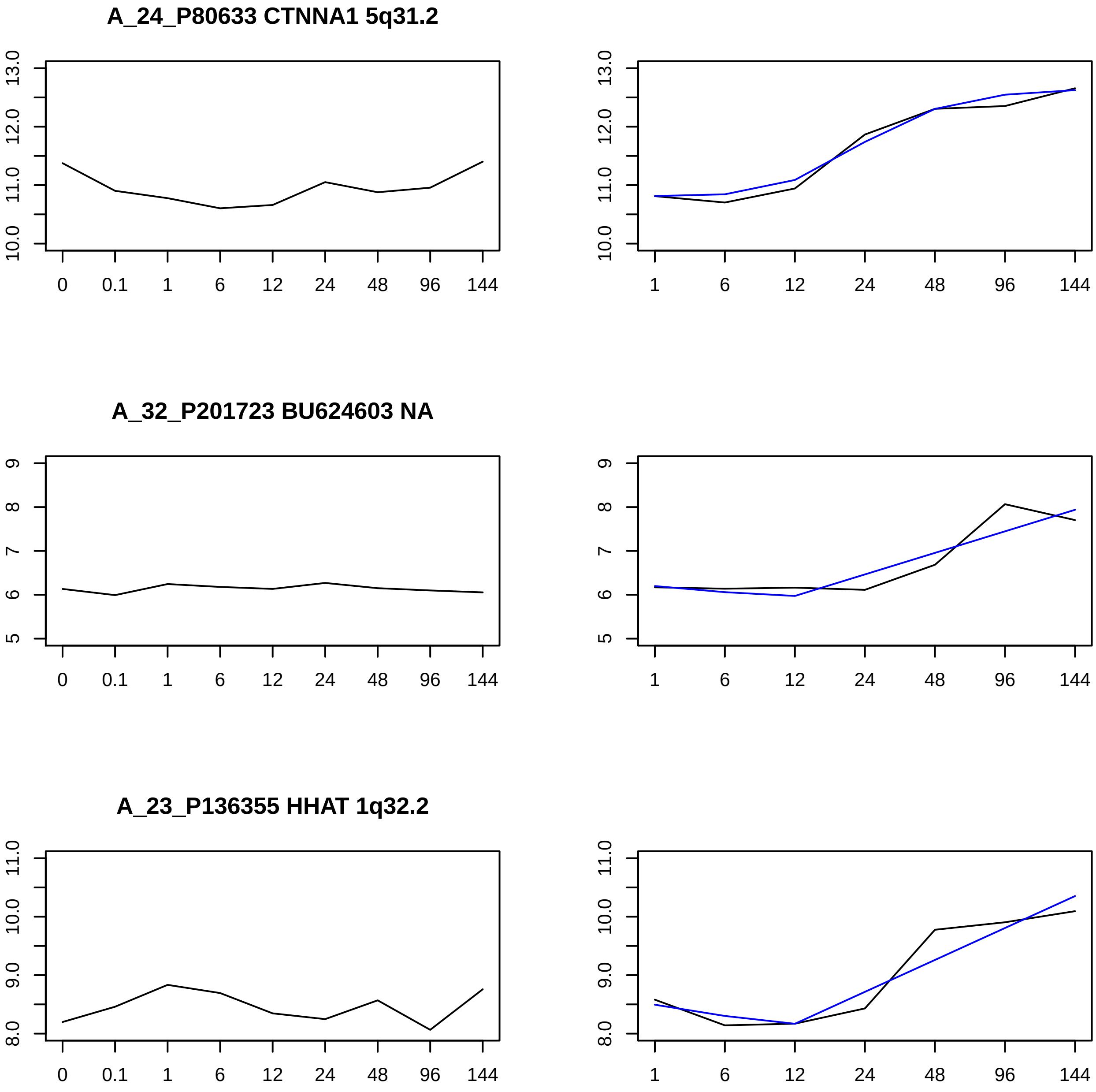
A_24_P80633 CTNNA1 5q31.2
10.0
11.0
12.0
13.0
0
0.1
1
6
12
24
48
96
144
1
6
12
24
48
96
144
A_32_P201723 BU624603 NA
5
6
7
8
9
A_23_P136355 HHAT 1q32.2
8.0
9.0
10.0
11.0

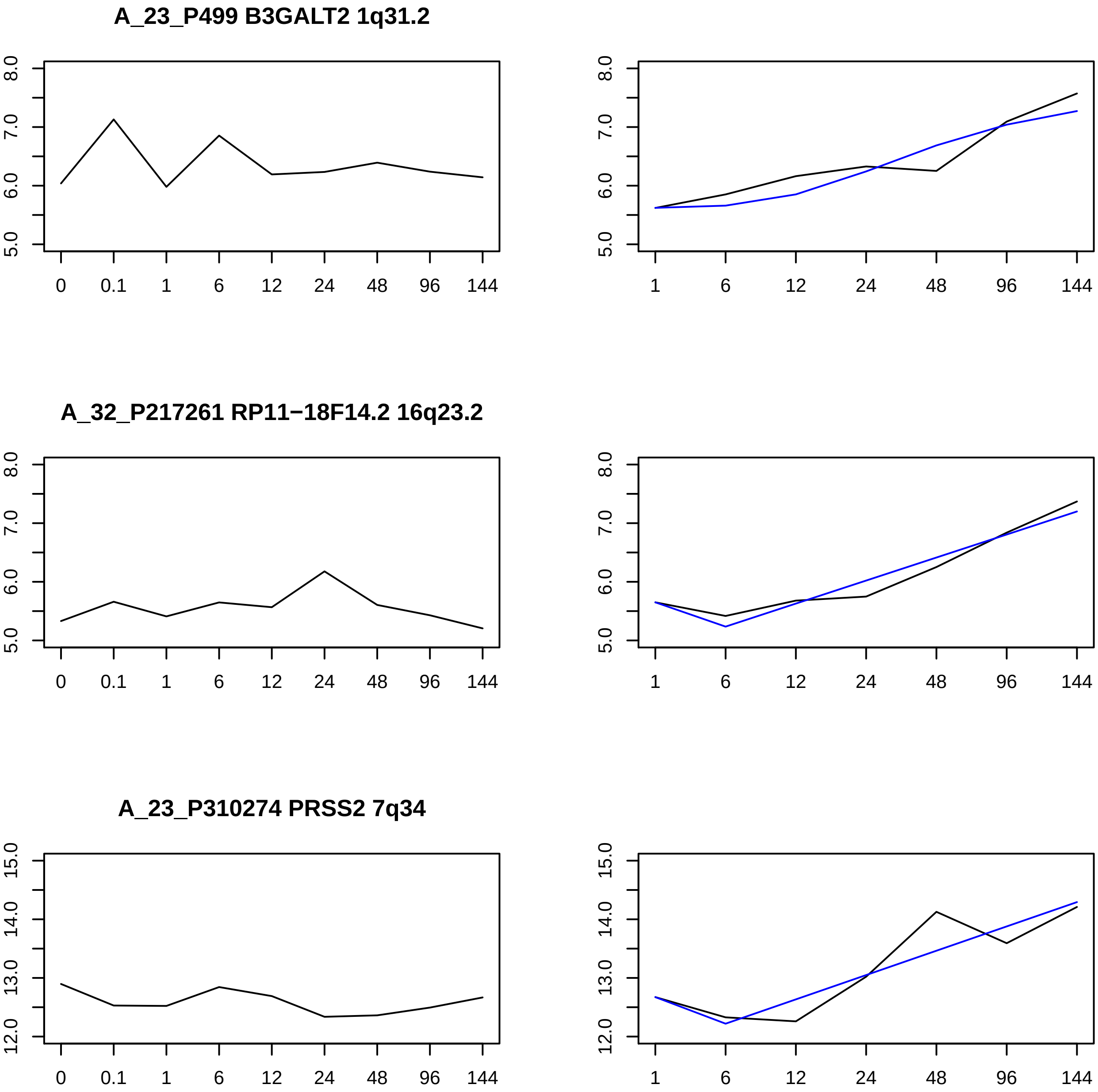
A_23_P499 B3GALT2 1q31.2
A_32_P217261 RP11−18F14.2 16q23.2
A_23_P310274 PRSS2 7q34
5.0
6.0
7.0
8.0
12.0
13.0
14.0
15.0
0
0.1
1
6
12
24
48
96
144

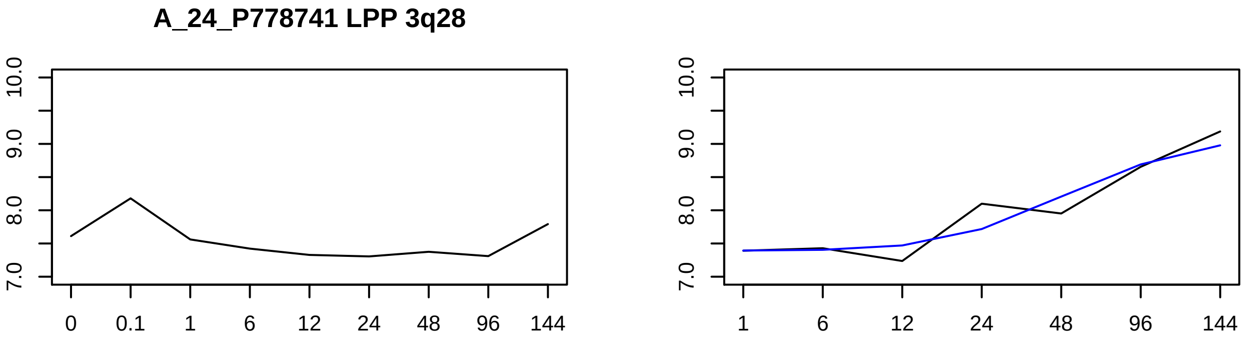
A_24_P778741 LPP 3q28
7.0
8.0
9.0
10.0
0
0.1
1
6
12
24
48
96
144
7.0
8.0
9.0
10.0
1
6
12
24
48
96
144

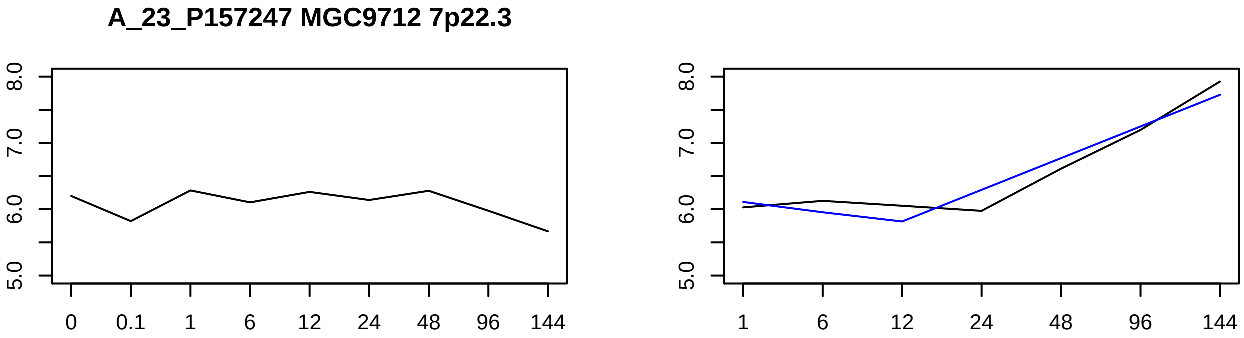
A_23_P157247 MGC9712 7p22.3
5.0
6.0
7.0
8.0
0
0.1
1
6
12
24
48
96
144
5.0
6.0
7.0
8.0
1
6
12
24
48
96
144

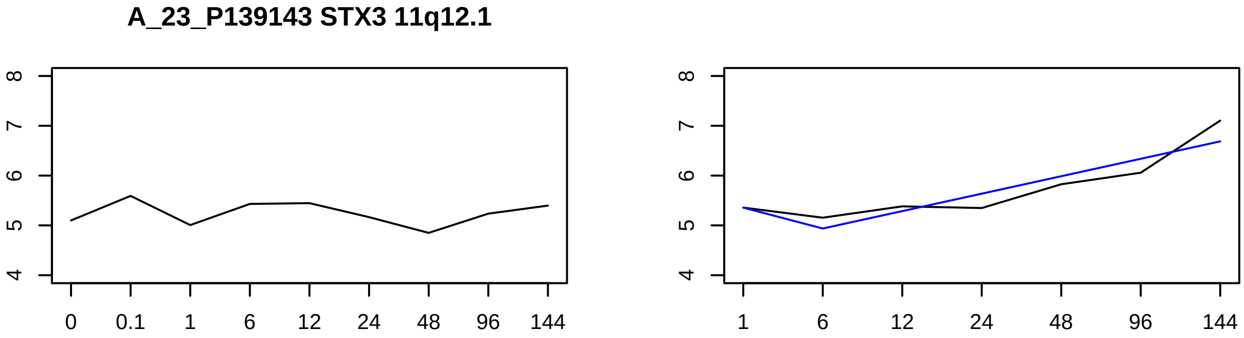
A_23_P139143 STX3 11q12.1
4
5
6
7
8
0
0.1
1
6
12
24
48
96
144
4
5
6
7
8
1
6
12
24
48
96
144

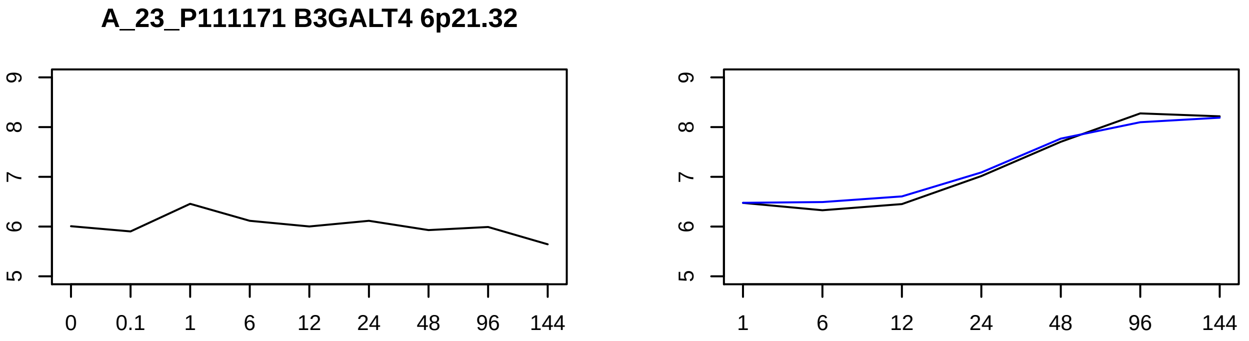
A_23_P111171 B3GALT4 6p21.32
5
6
7
8
9
0
0.1
1
6
12
24
48
96
144
1
6
12
24
48
96
144

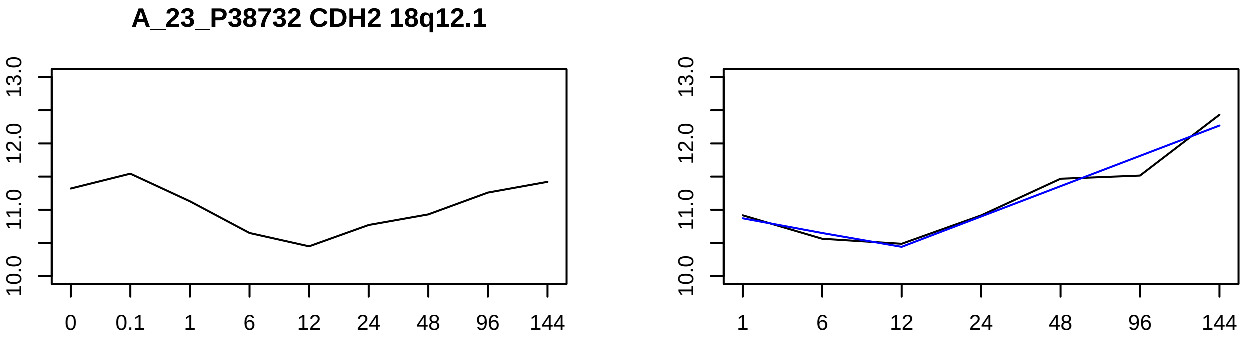
A_23_P38732 CDH2 18q12.1
10.0
11.0
12.0
13.0
0
0.1
1
6
12
24
48
96
144
1
6
12
24
48
96
144

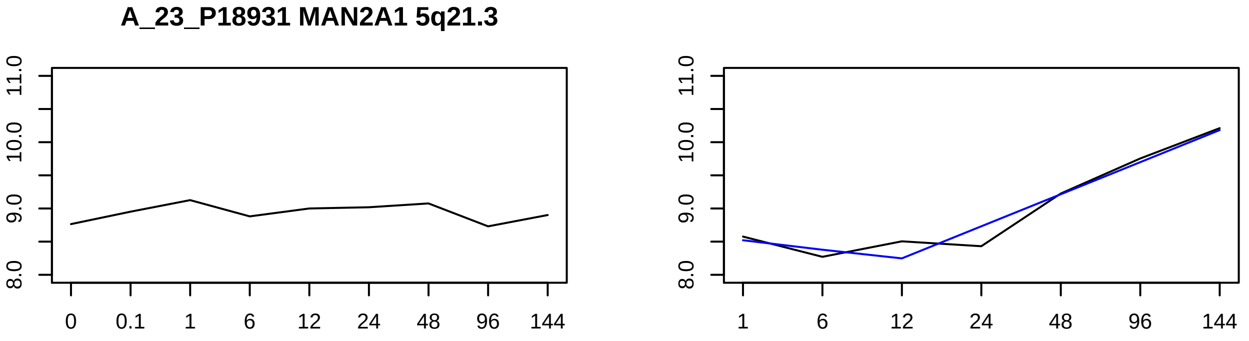
A_23_P18931 MAN2A1 5q21.3
8.0
9.0
10.0
11.0
0
0.1
1
6
12
24
48
96
144
1
6
12
24
48
96
144

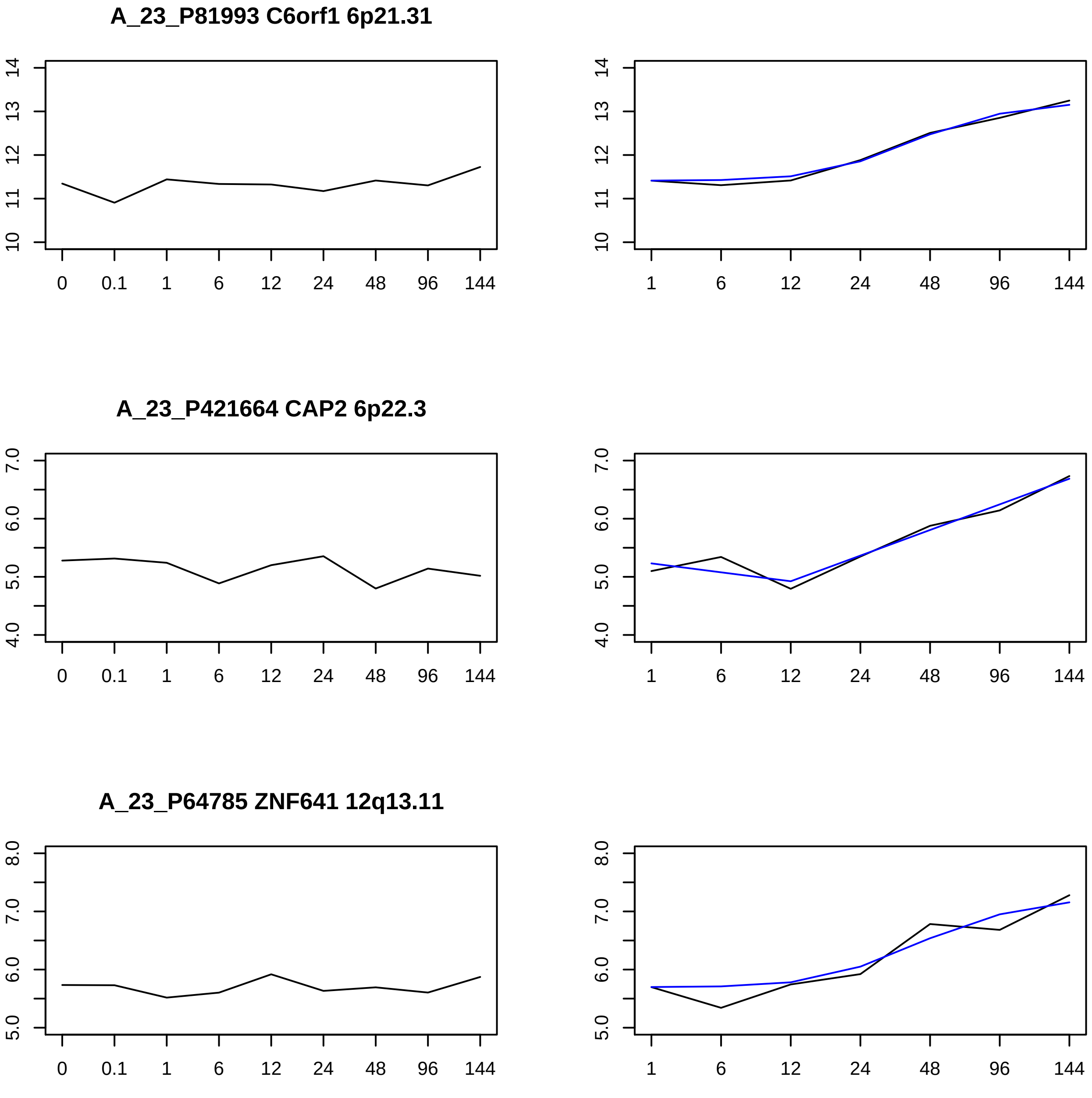
A_23_P81993 C6orf1 6p21.31
10
11
12
13
14
0
0.1
1
6
12
24
48
96
144
A_23_P421664 CAP2 6p22.3
4.0
5.0
6.0
7.0
A_23_P64785 ZNF641 12q13.11
5.0
6.0
7.0
8.0

**A_23_P219117 EPB49 8p21.3**

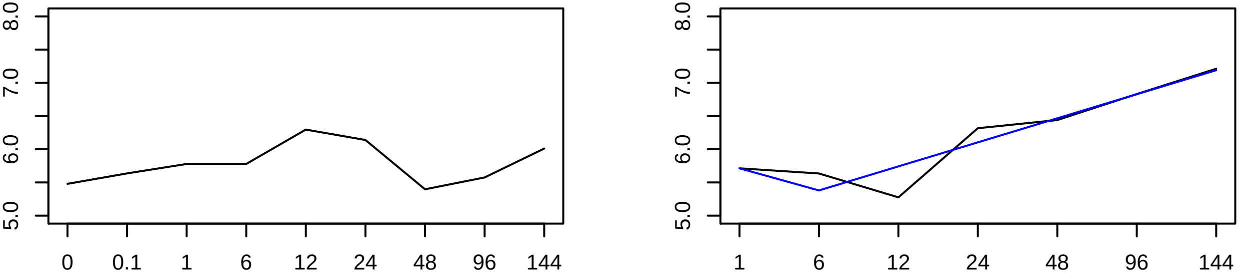


**A_24_P88696 SCG2 2q36.1**

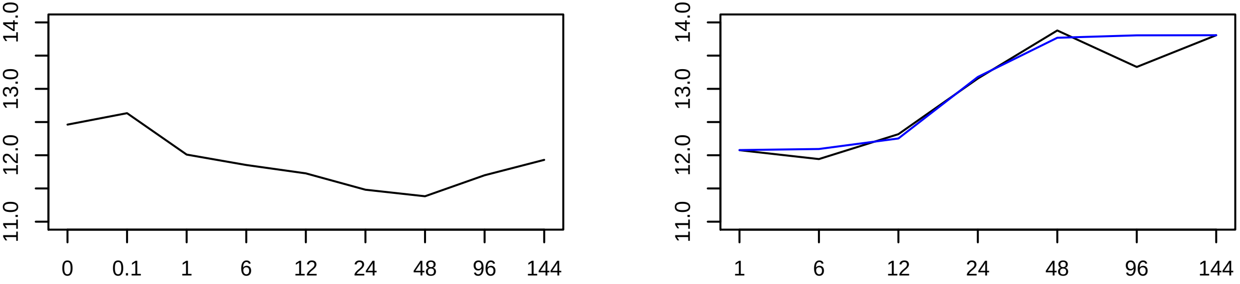


**A_23_P135257 PRSS3 9p13.3**

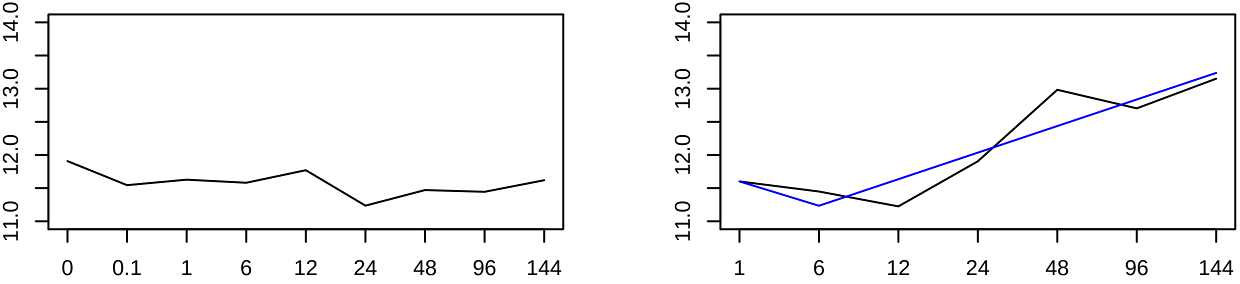

**A_24_P37355 AMER3 NA**

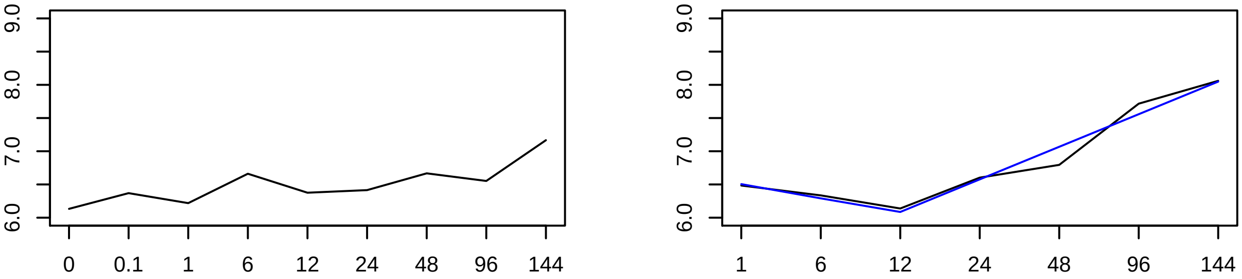


**A_32_P502567 AKAP6 14q13.1**

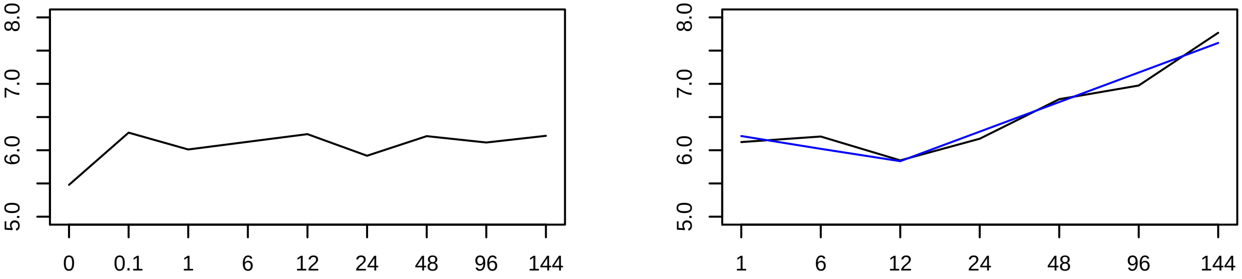


**A_23_P138495 PTPRE 10q26.2**

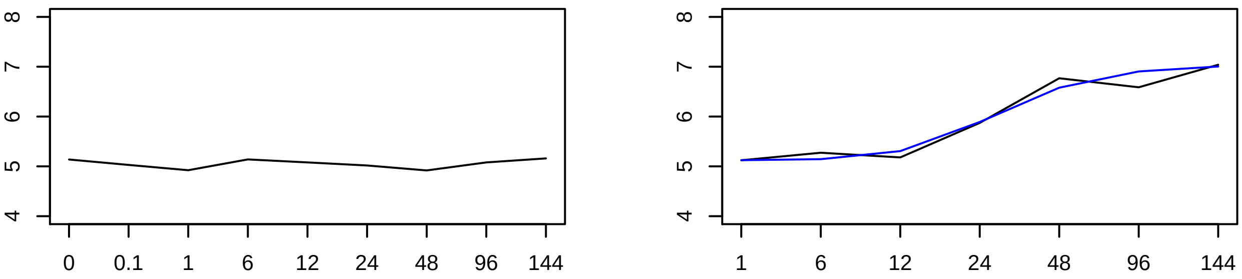

**A_23_P88099 MCF2L 13q34**

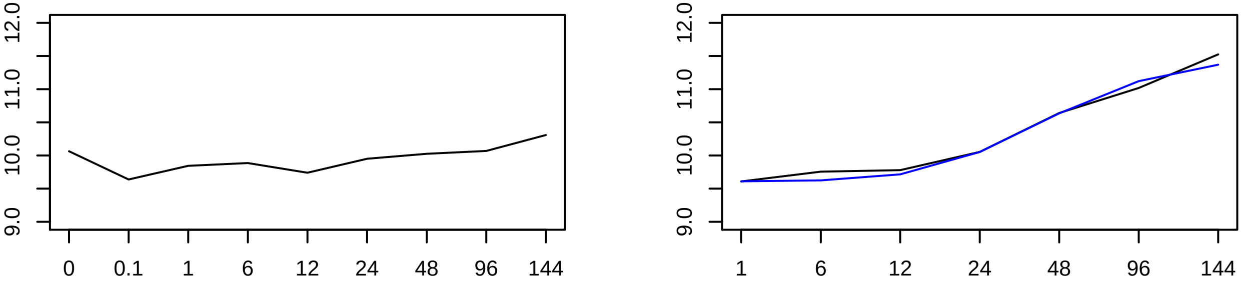


**A_23_P406227 MGC23284 16q24.3**

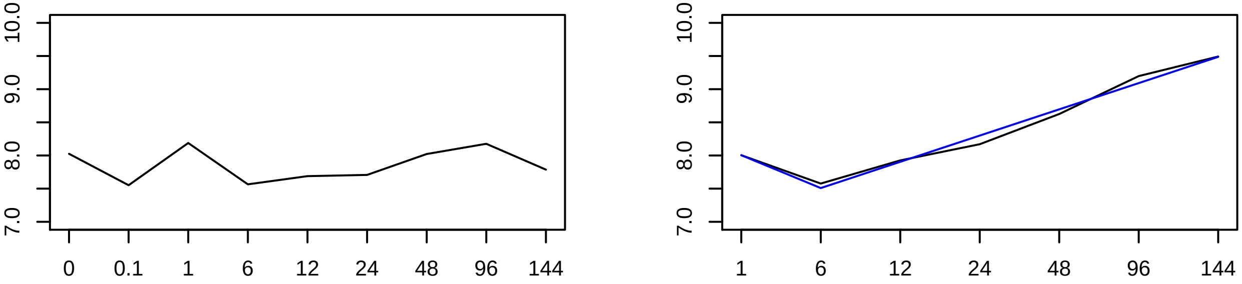


**A_23_P136623 ADCY5 3q21.1**

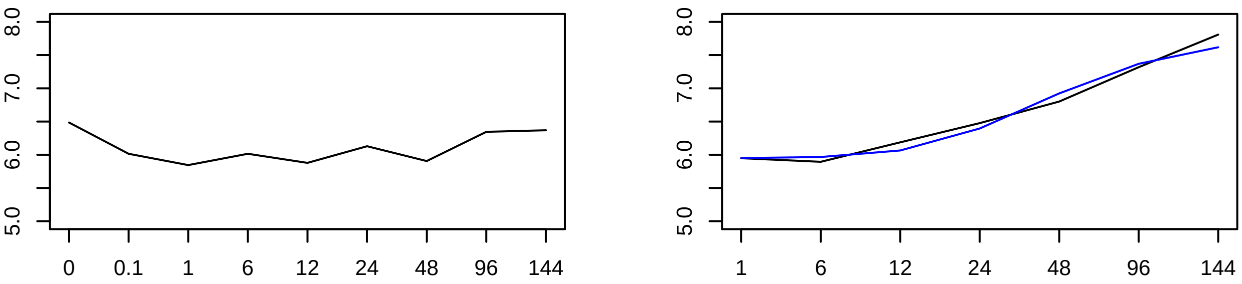

**A_24_P11900 MYH15 3q13.13**

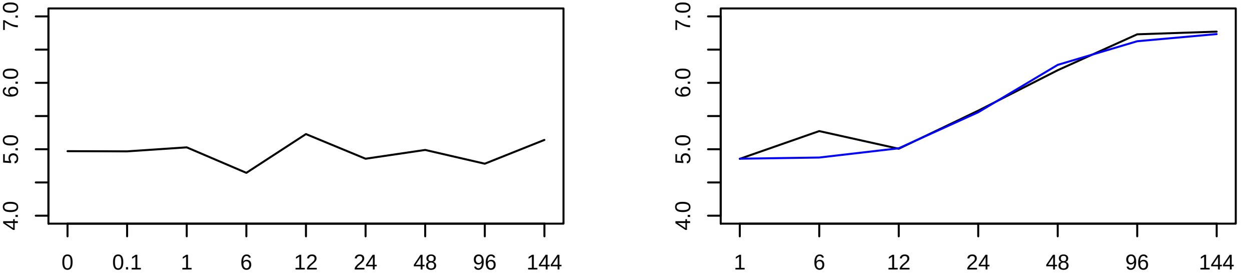


**A_24_P318656 ITGB3 17q21.32**

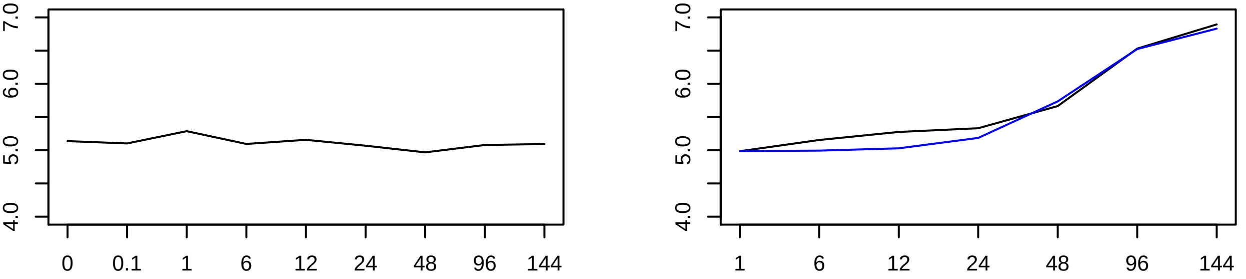


**A_23_P122959 KIAA0644 7p15.1**

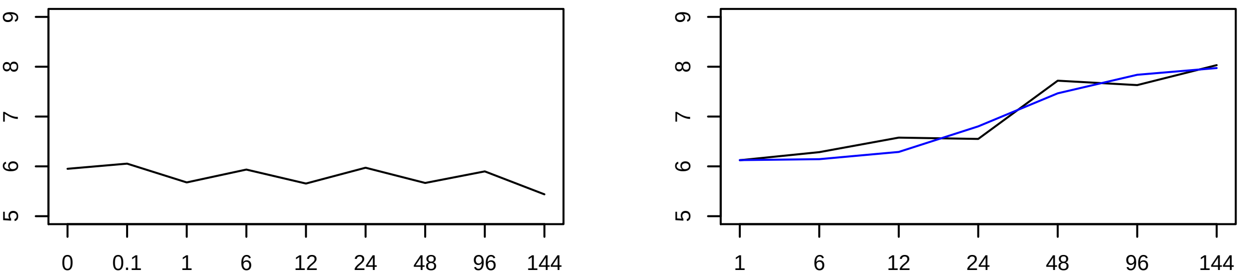

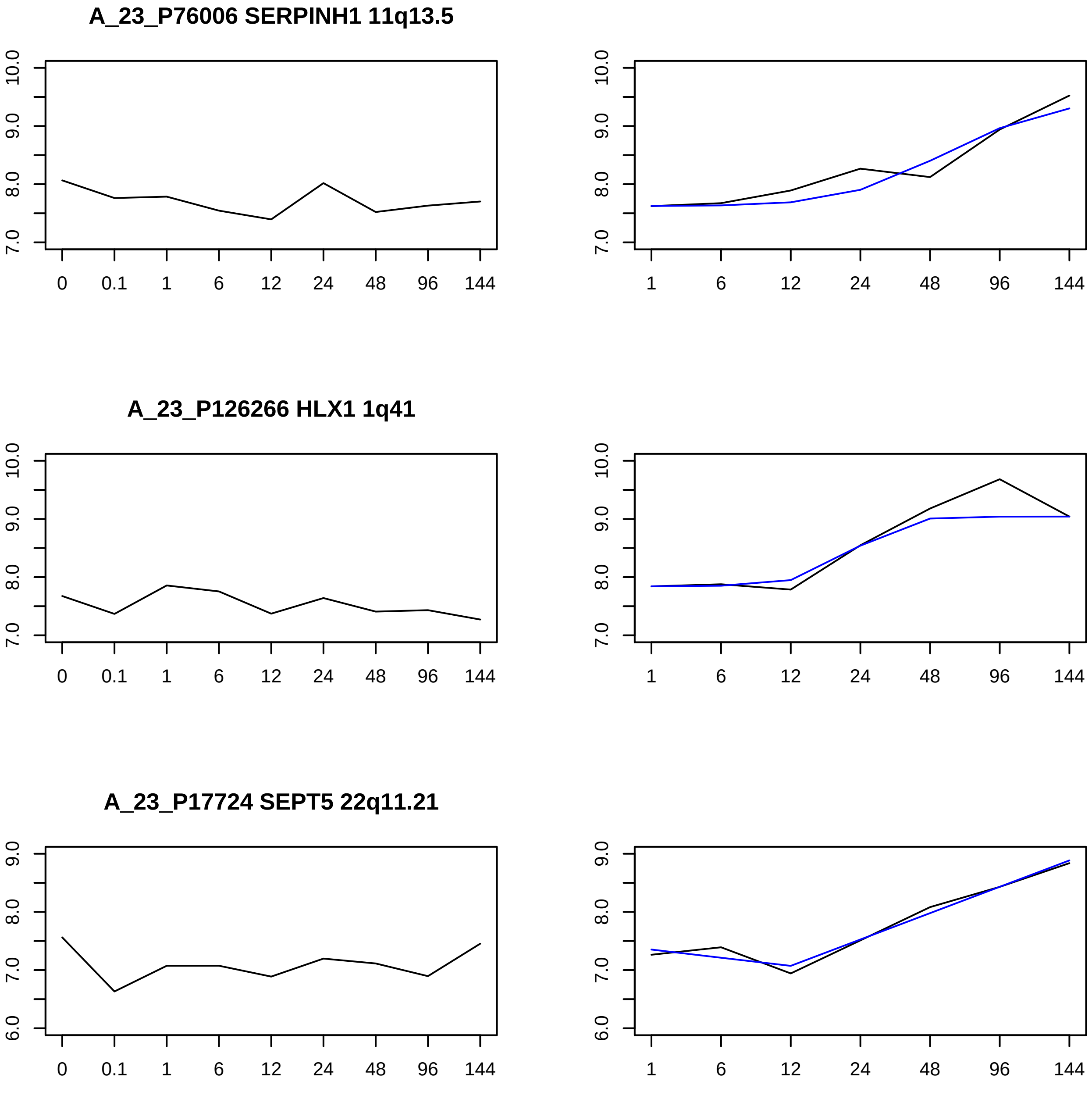
A_23_P76006 SERPINH1 11q13.5
A_23_P126266 HLX1 1q41
A_23_P17724 SEPT5 22q11.21
10.0
9.0
8.0
7.0
6.0
0
0.1
1
6
12
24
48
96
144

**A_24_P160263 MAGED2 Xp11.21**

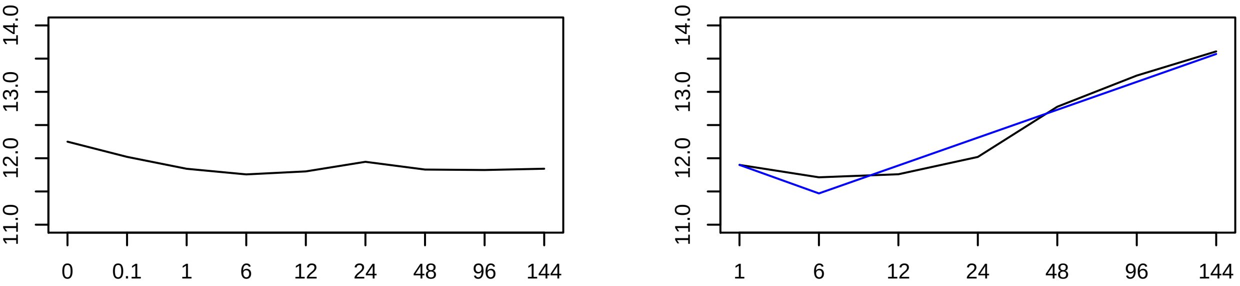


**A_32_P124269 THC2730628 NA**

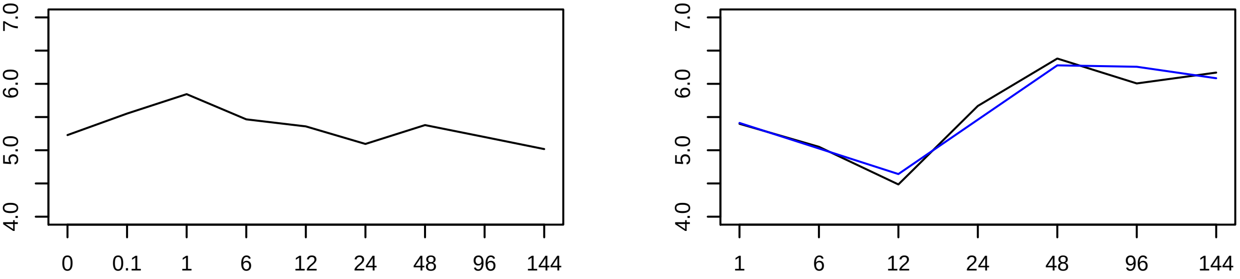


**A_23_P111766 A_23_P111766 NA**

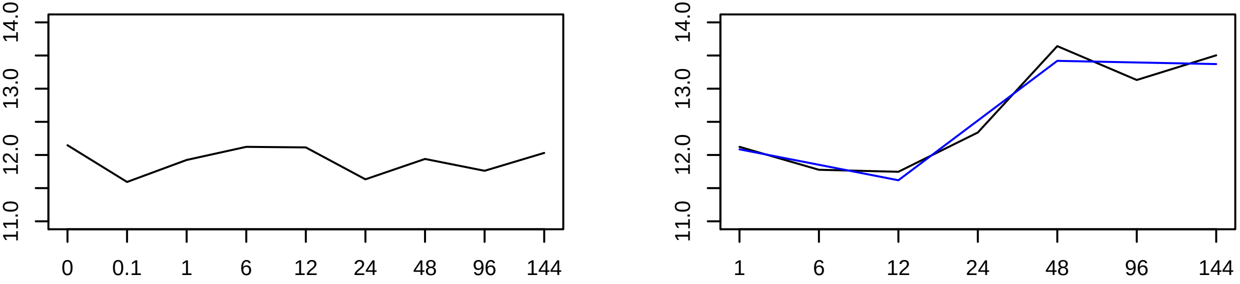

**A_32_P147622 MGC2780 13q34**

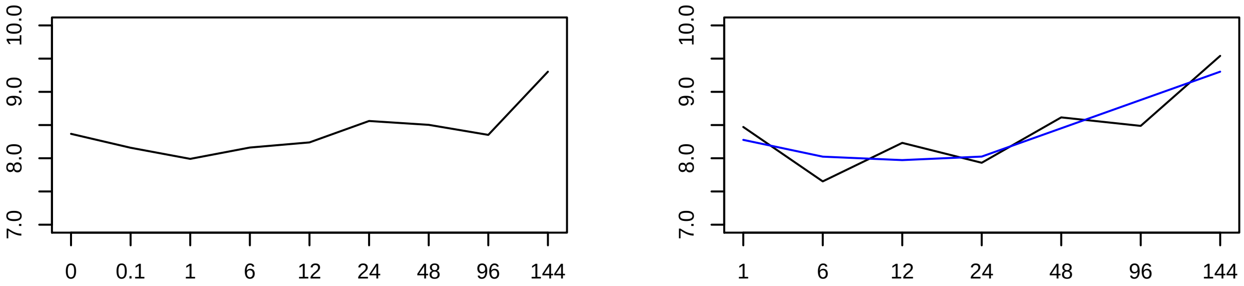


**A_24_P364363 TNFRSF1A 12p13.31**

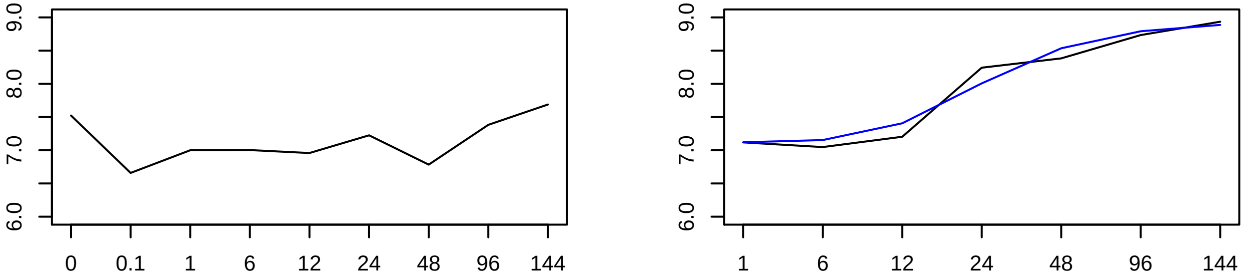


**A_24_P350832 AGTPBP1 9q21.33**

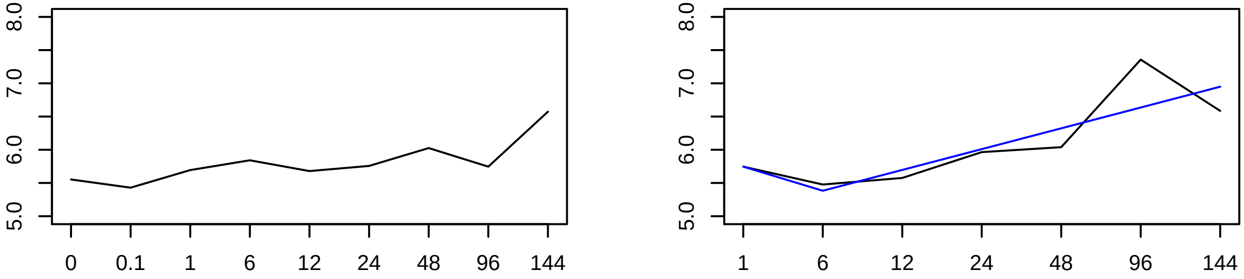

**A_24_P188447 ELAVL4 1p33**

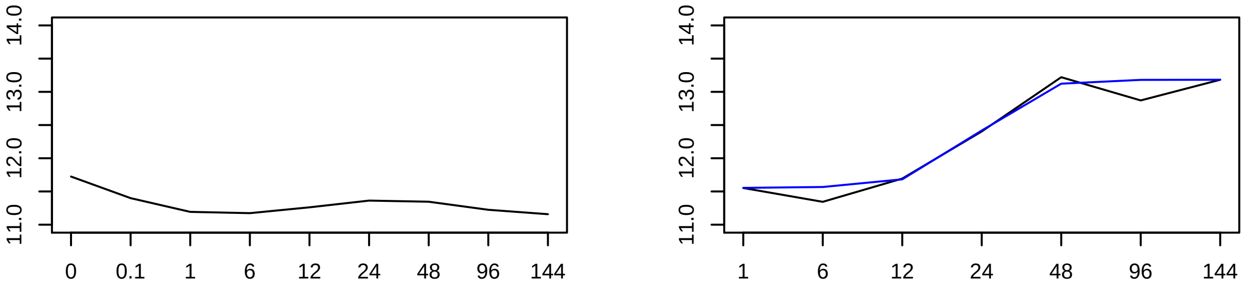


**A_32_P200947 RP11−553L6.5 3q13.31**

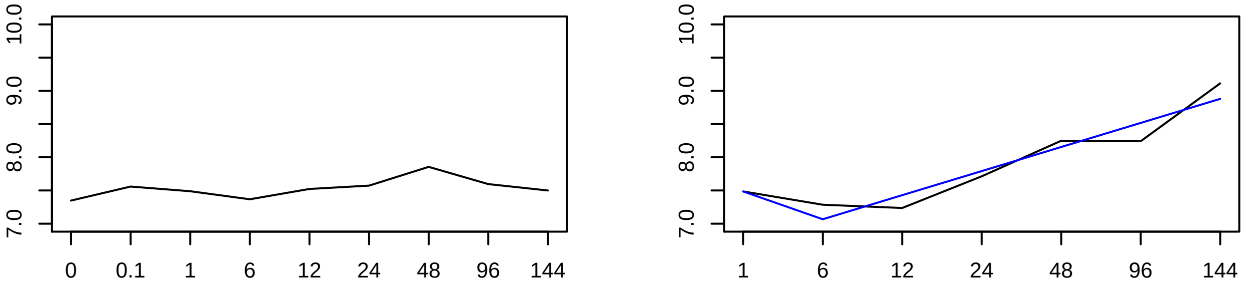


**A_23_P138567 AIFM2 10q22.1**

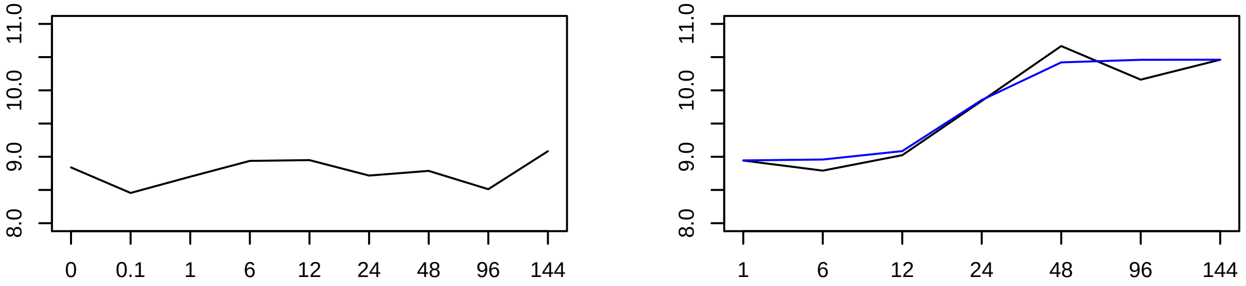

## A_23_P137196 IL13RA1 Xq24

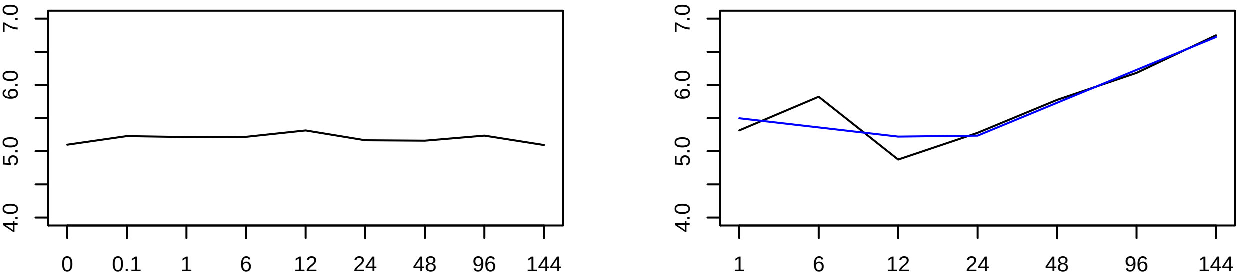


## A_23_P392384 C9orf58 9q34.13

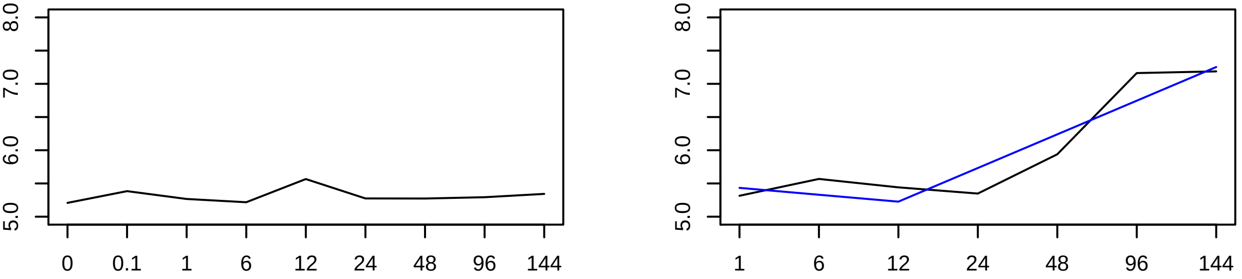


## A_24_P137434 DCBLD2 3q12.1

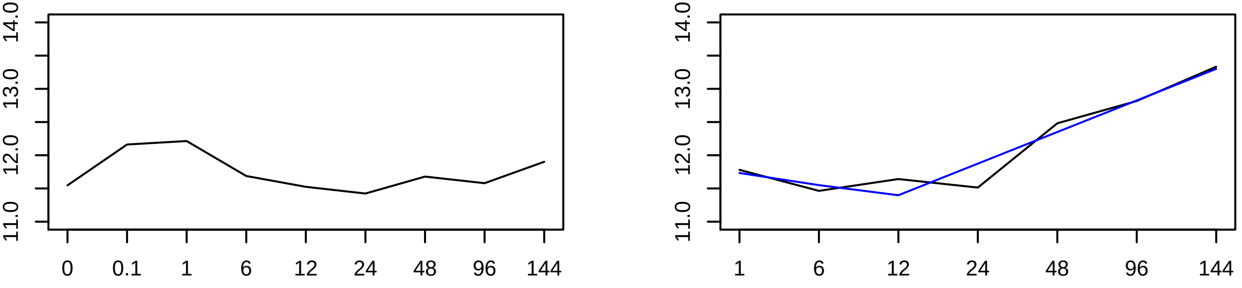

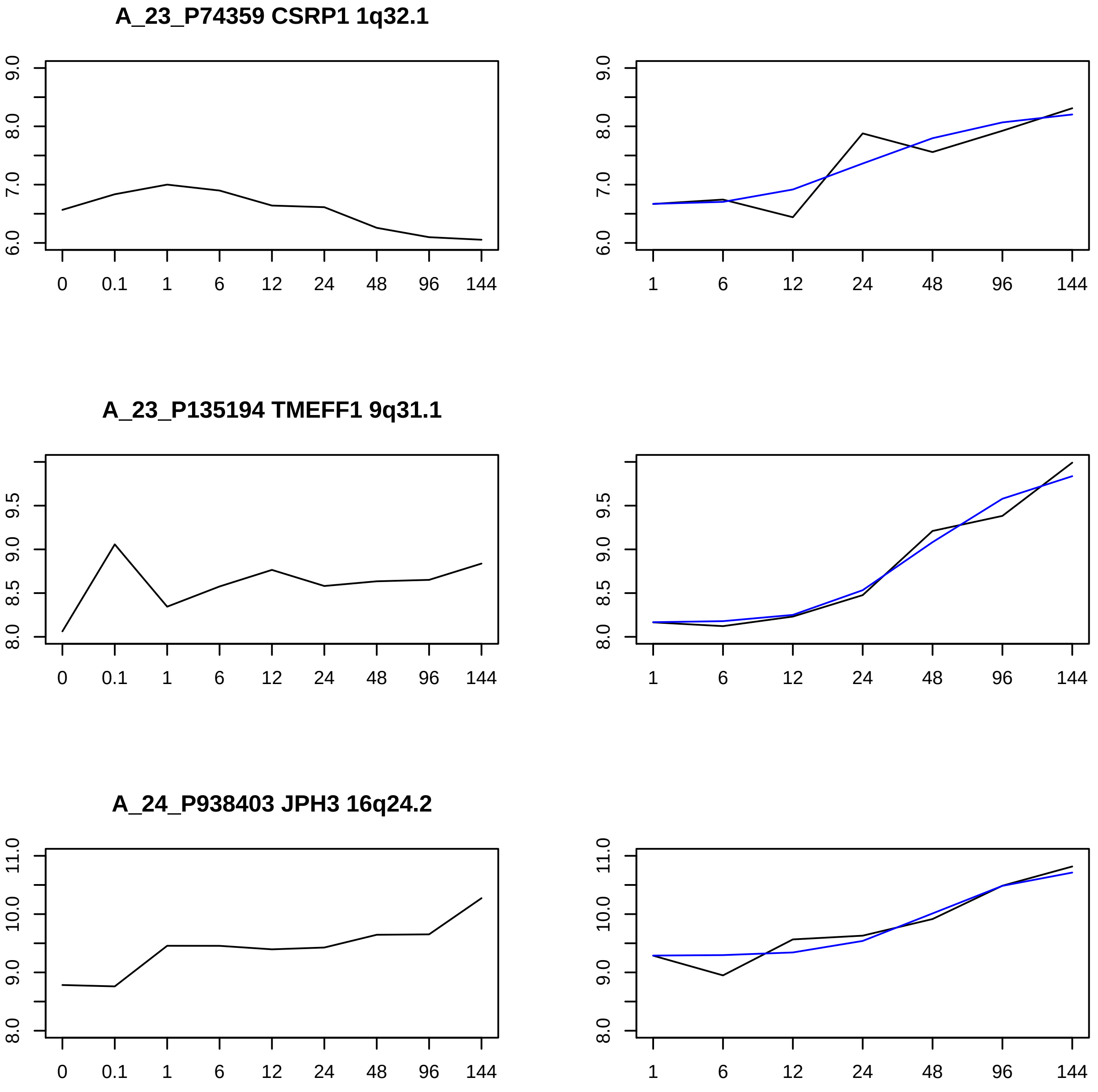

A_23_P74359 CSRP1 1q32.1
A_23_P135194 TMEFF1 9q31.1
A_24_P938403 JPH3 16q24.2
6.0 7.0 8.0 9.0
8.0 8.5 9.0 9.5
8.0 9.0 10.0 11.0
0 0.1 1 6 12 24 48 96 144
1 6 12 24 48 96 144

**A_23_P85250 CD24 Yq11.222**

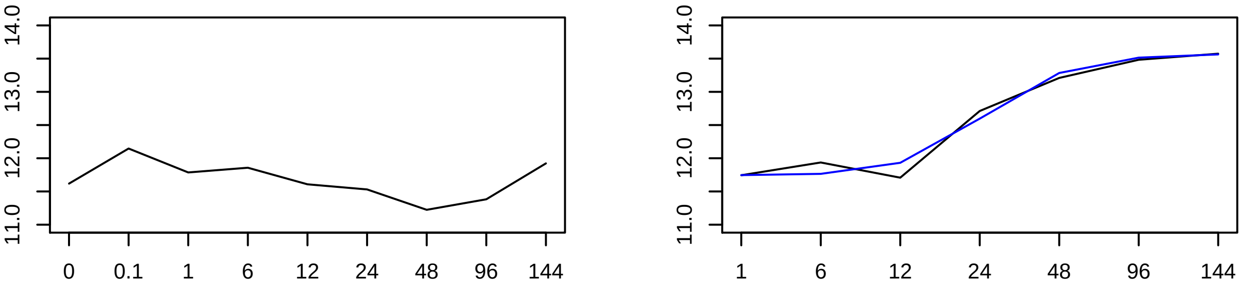


**A_32_P7146 AK123168 NA**

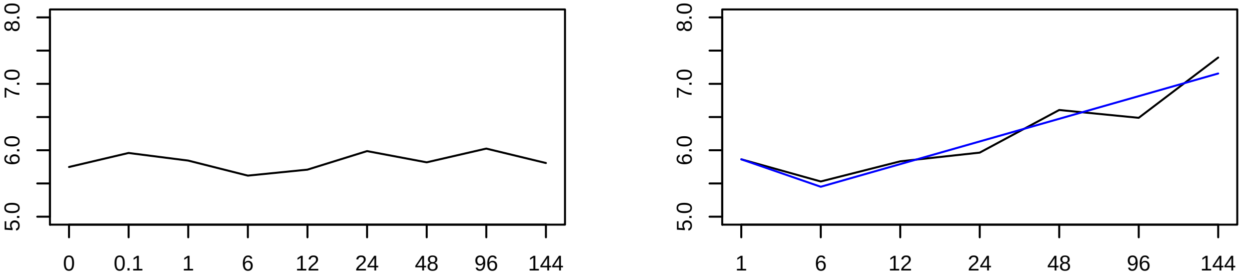


**A_23_P501276 TUBB2A 6p25.2**

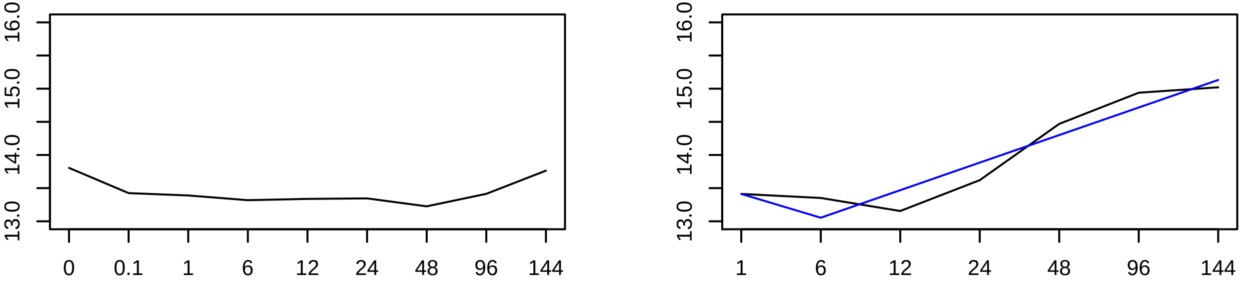

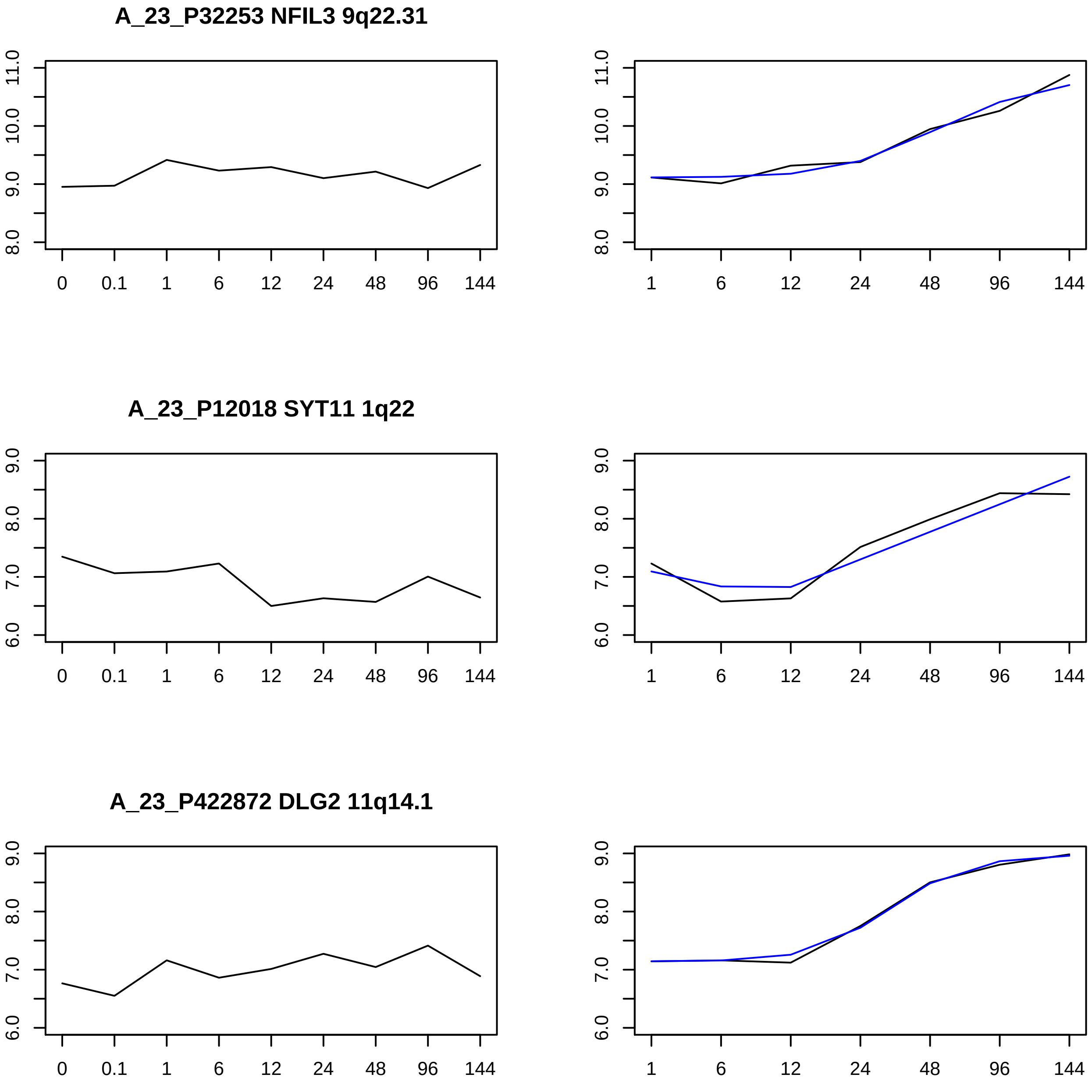
A_23_P32253 NFIL3 9q22.31
8.0
9.0
10.0
11.0
0
0.1
1
6
12
24
48
96
144
A_23_P12018 SYT11 1q22
6.0
7.0
8.0
9.0
A_23_P422872 DLG2 11q14.1

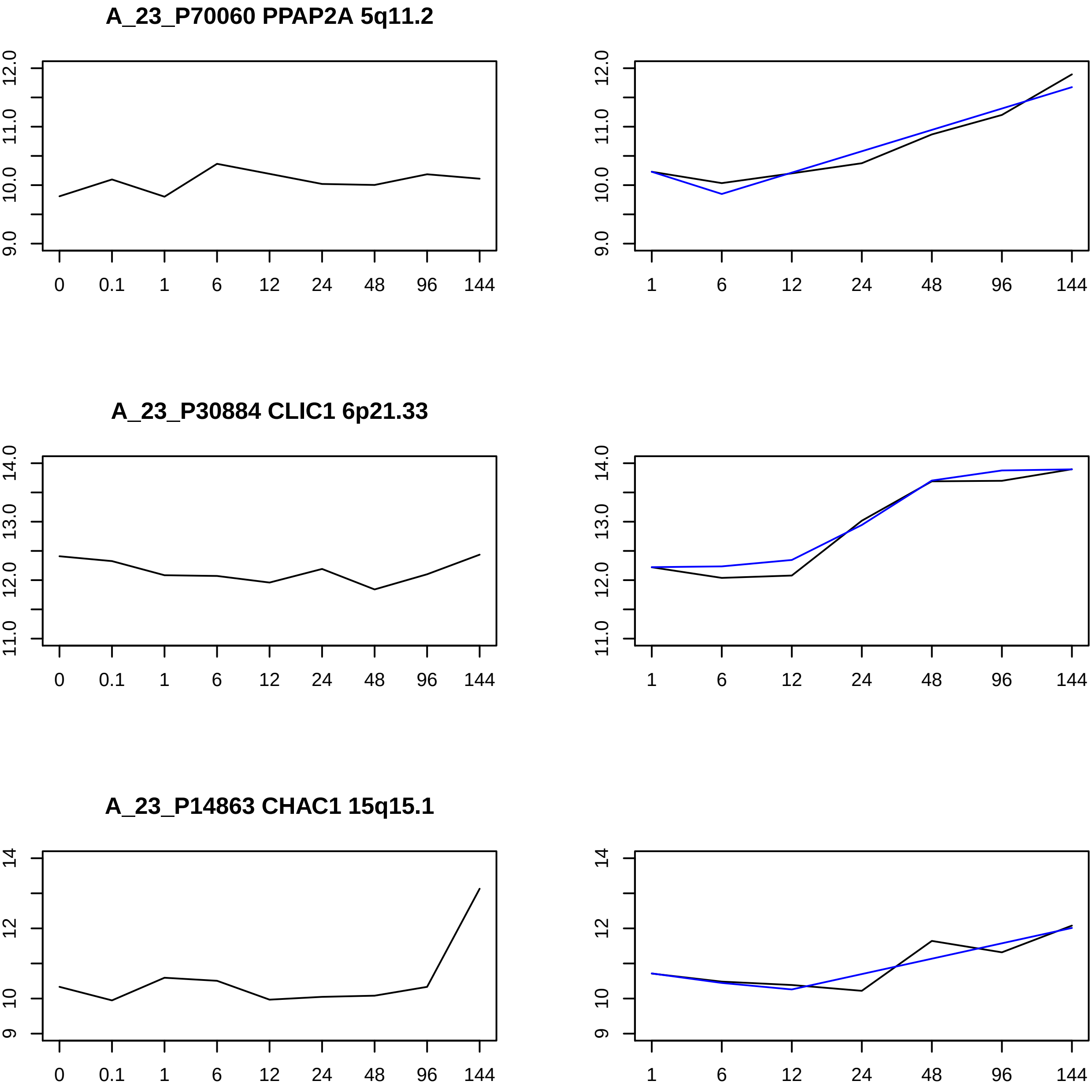
A_23_P70060 PPAP2A 5q11.2
9.0
10.0
11.0
12.0
0
0.1
1
6
12
24
48
96
144
A_23_P30884 CLIC1 6p21.33
11.0
12.0
13.0
14.0
A_23_P14863 CHAC1 15q15.1
9
10
12
14

A_24_P382489 SLC27A1 19p13.11

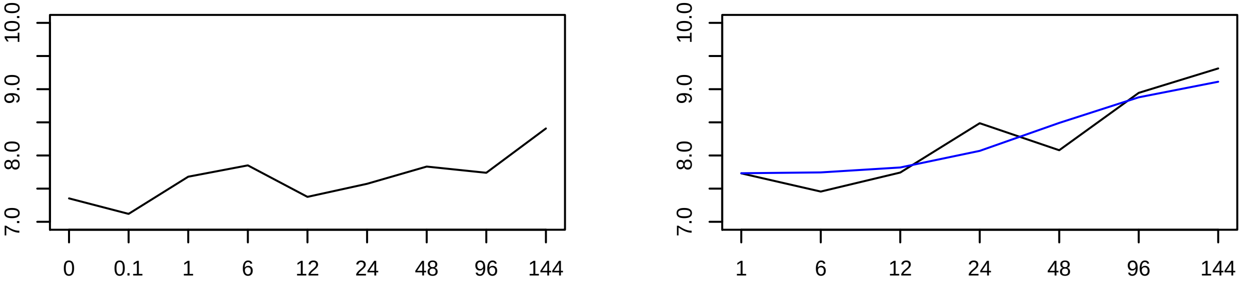


A_23_P4561 SERPINB8 18q22.1

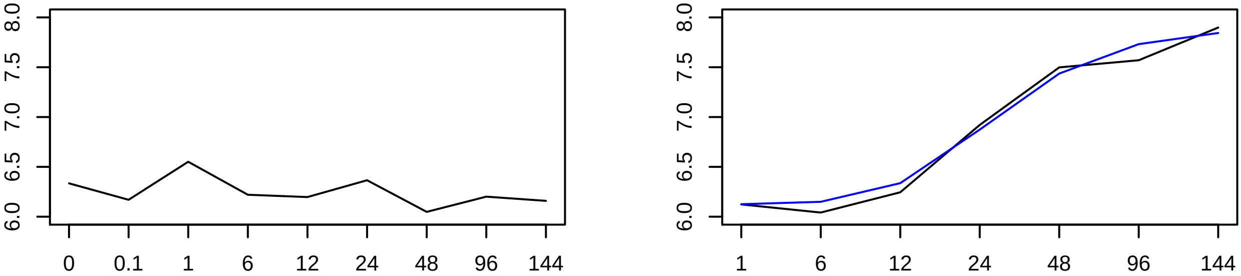


A_32_P221590 THC2643233 NA

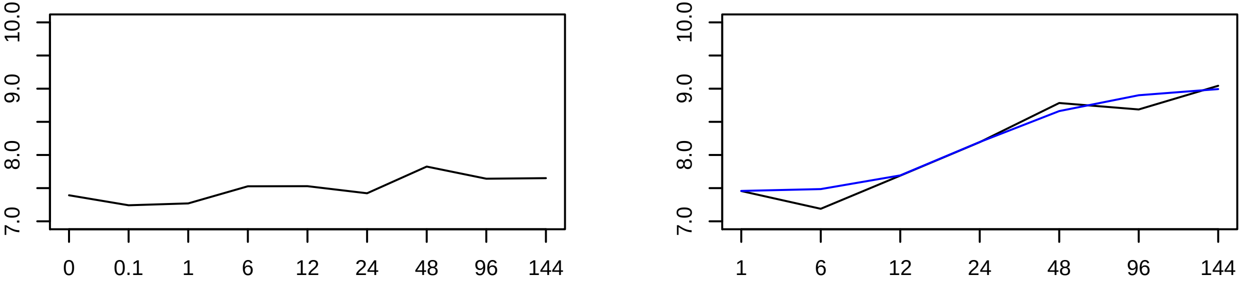

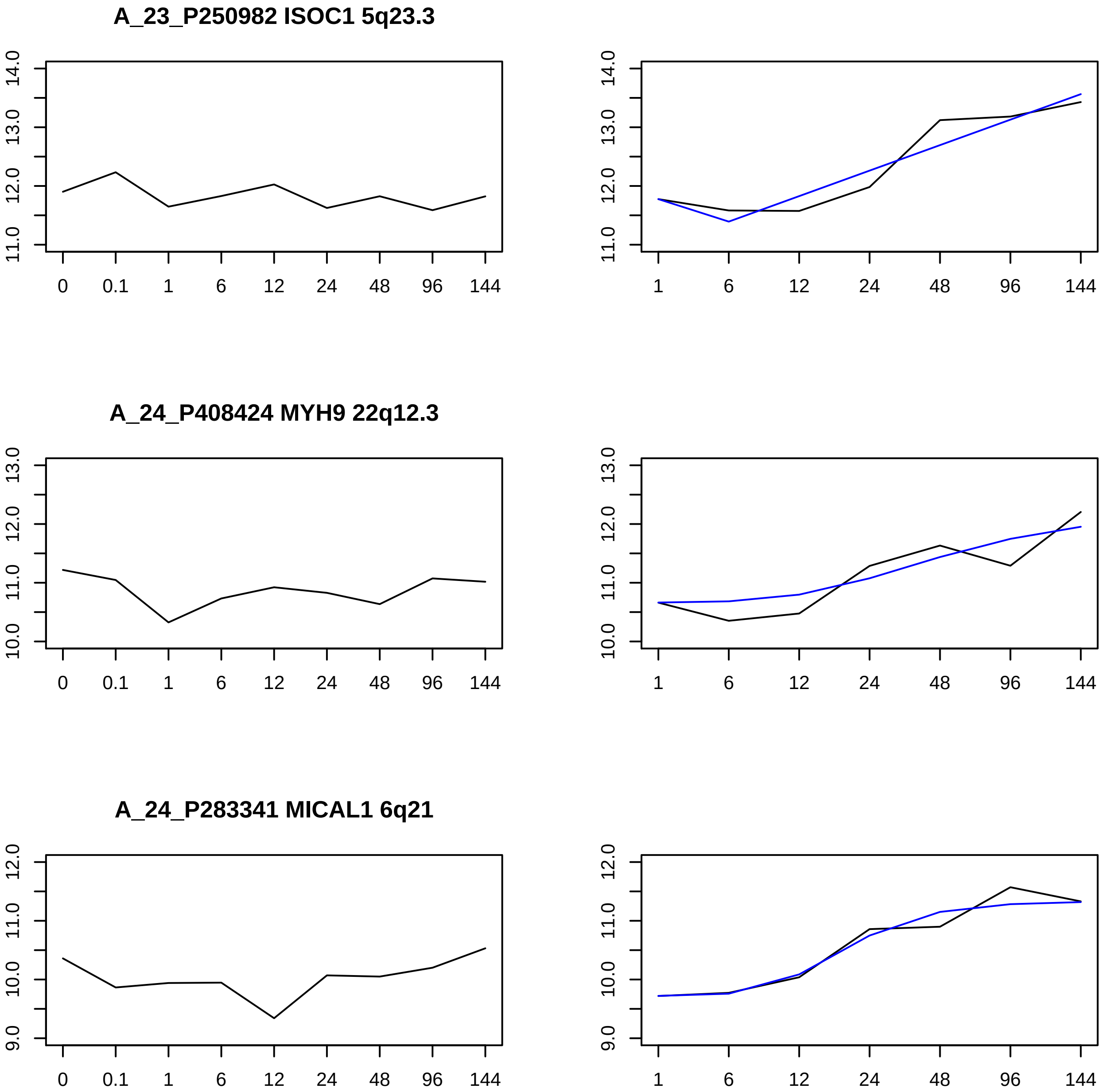

A_23_P250982 ISOC1 5q23.3
A_24_P408424 MYH9 22q12.3
A_24_P283341 MICAL1 6q21
0
0.1
1
6
12
24
48
96
144

**A_32_P49284 THC2568453 NA**

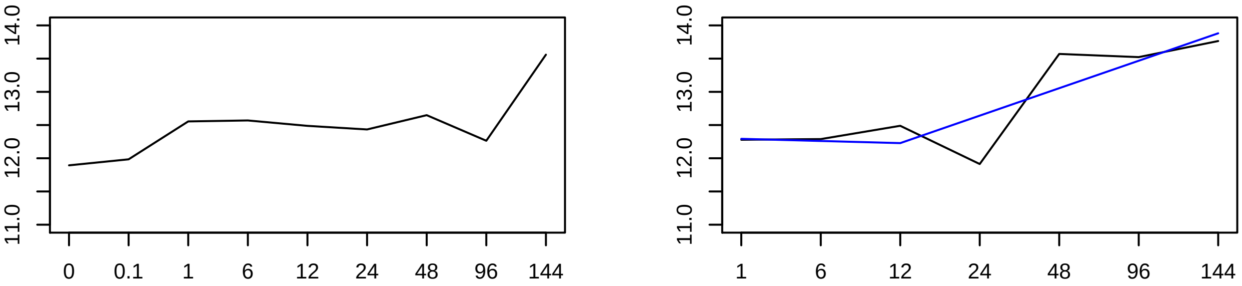


**A_23_P205273 PPP1R13B 14q32.33**

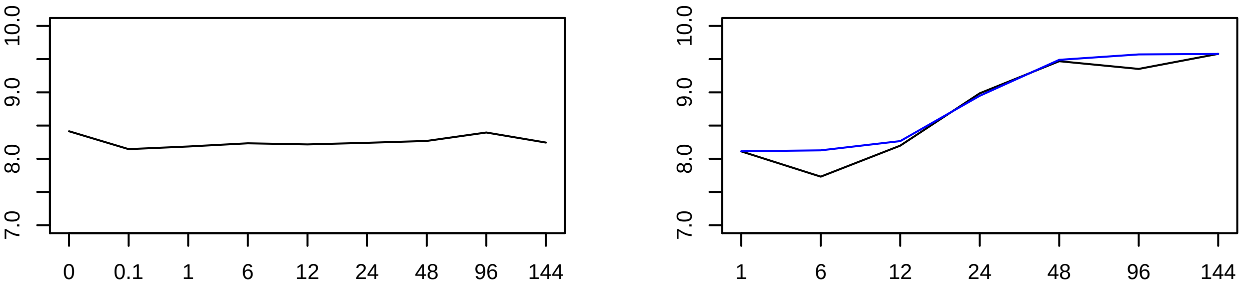


**A_23_P502274 MAPK11 22q13.33**

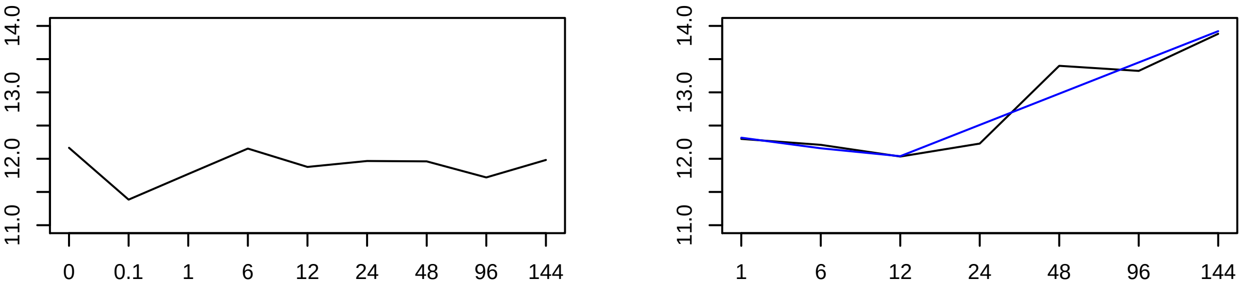

**A_23_P43283 GPR124 8p12**

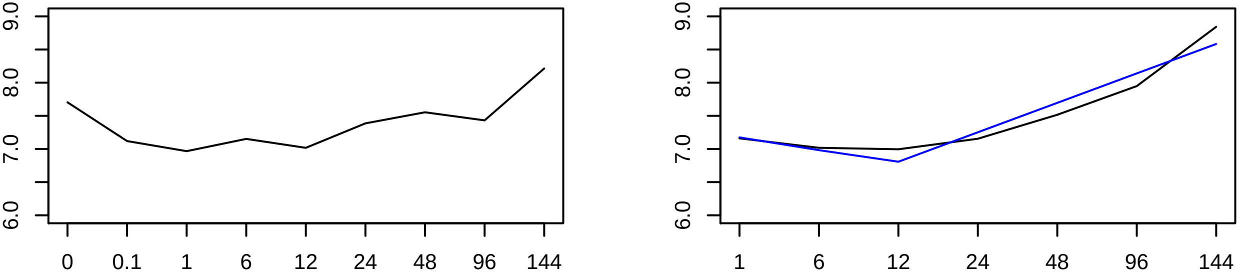


**A_24_P156113 EHD2 19q13.32**

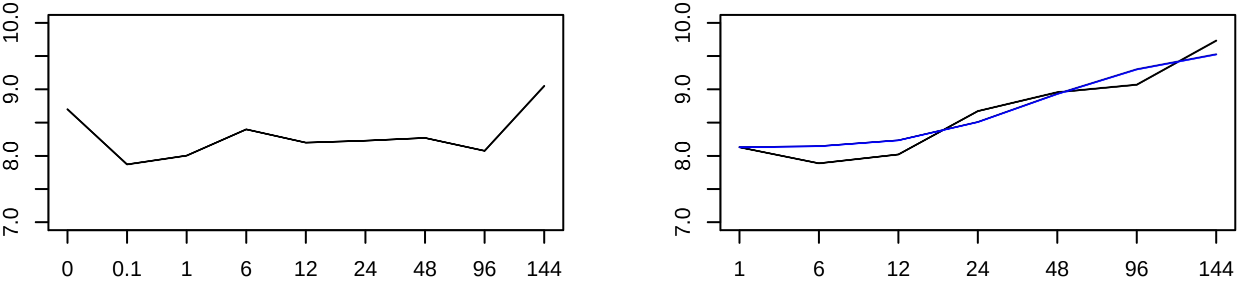


**A_23_P90172 PPP1R15A 19q13.33**

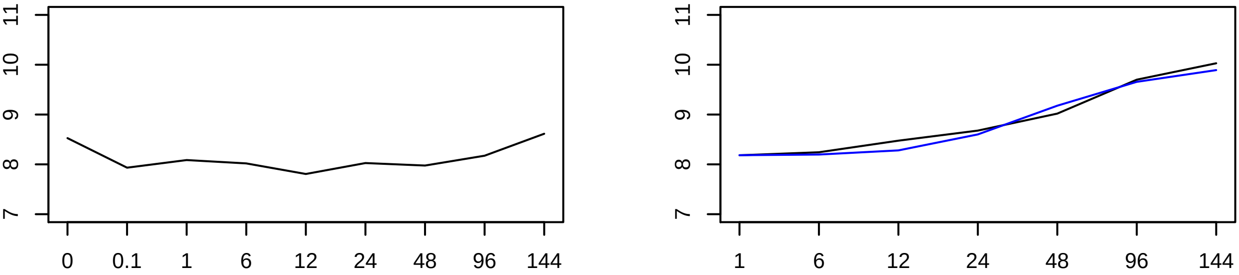

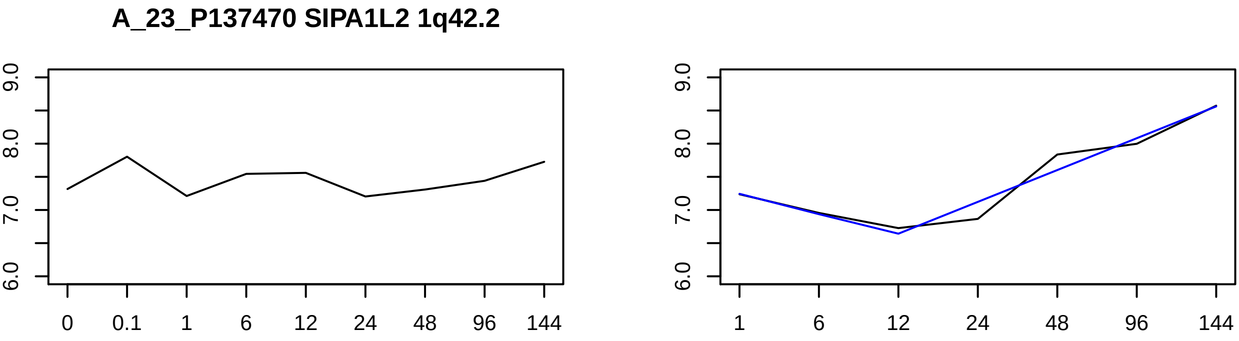
A_23_P137470 SIPA1L2 1q42.2
6.0
7.0
8.0
9.0
0
0.1
1
6
12
24
48
96
144
1
6
12
24
48
96
144

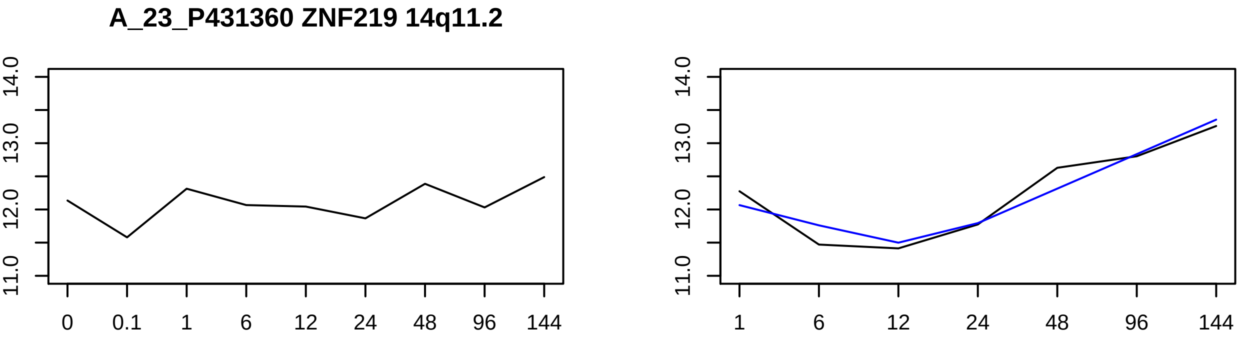
A_23_P431360 ZNF219 14q11.2
11.0
12.0
13.0
14.0
0
0.1
1
6
12
24
48
96
144
1
6
12
24
48
96
144

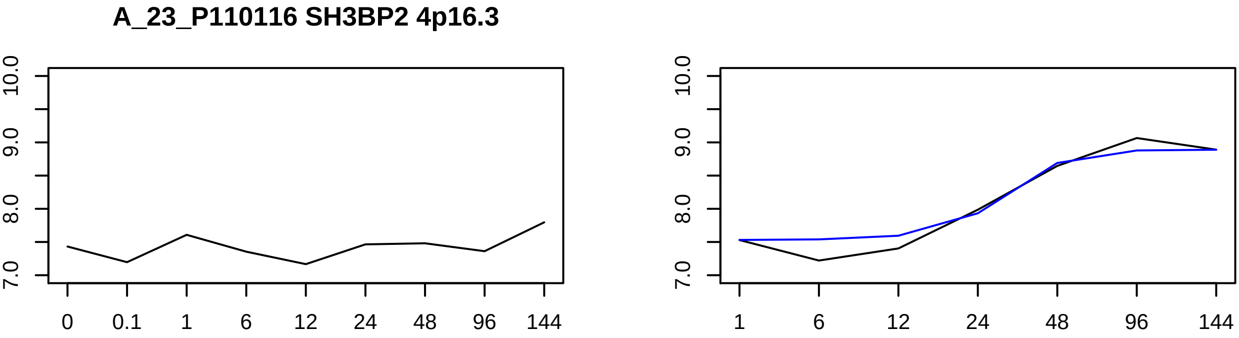
A_23_P110116 SH3BP2 4p16.3
7.0
8.0
9.0
10.0
0
0.1
1
6
12
24
48
96
144
1
6
12
24
48
96
144

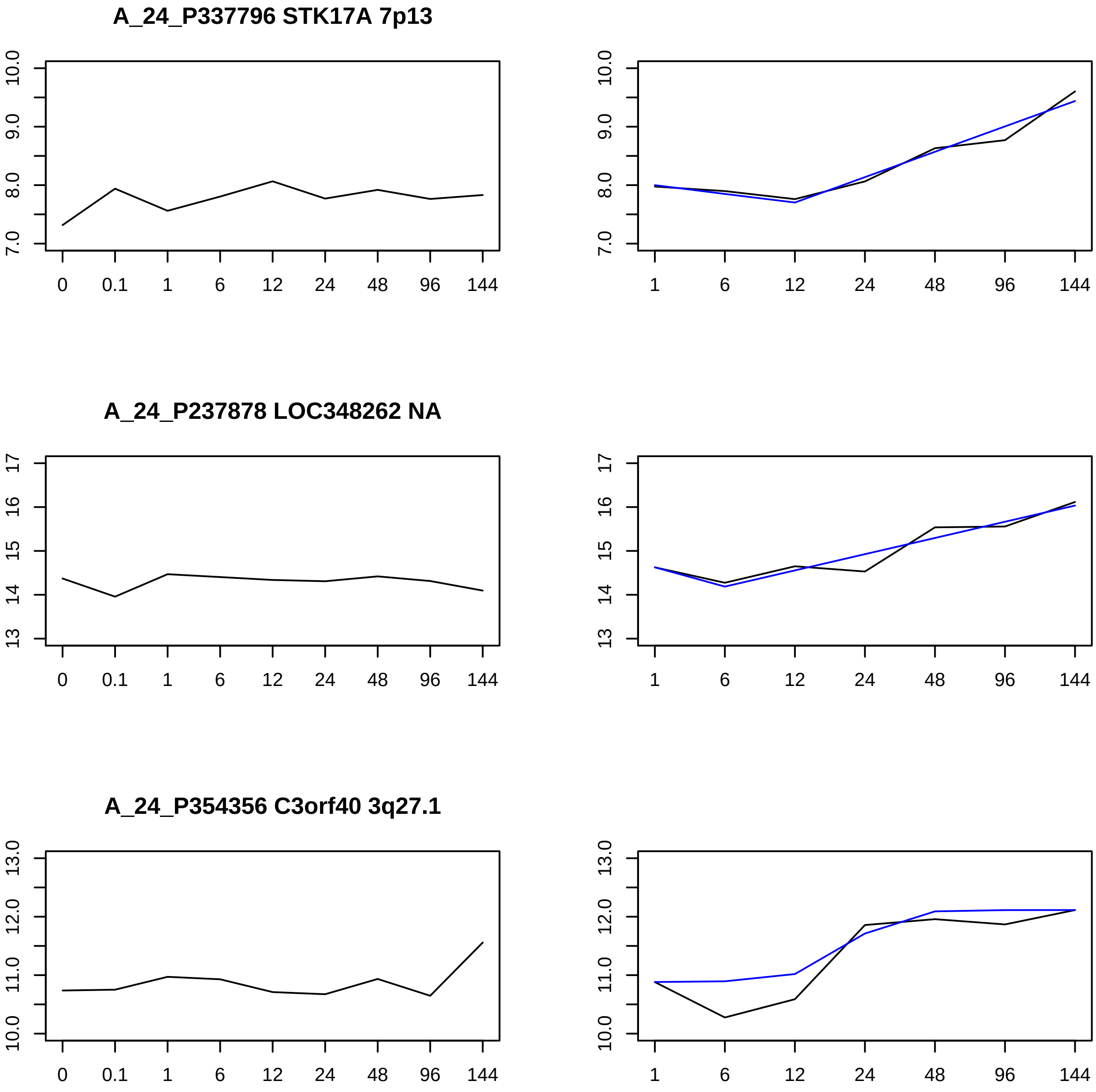
A_24_P337796 STK17A 7p13
7.0
8.0
9.0
10.0
0
0.1
1
6
12
24
48
96
144
A_24_P237878 LOC348262 NA
13
14
15
16
17
A_24_P354356 C3orf40 3q27.1
10.0
11.0
12.0
13.0

**A_32_P90080 SVH 7q22.1**

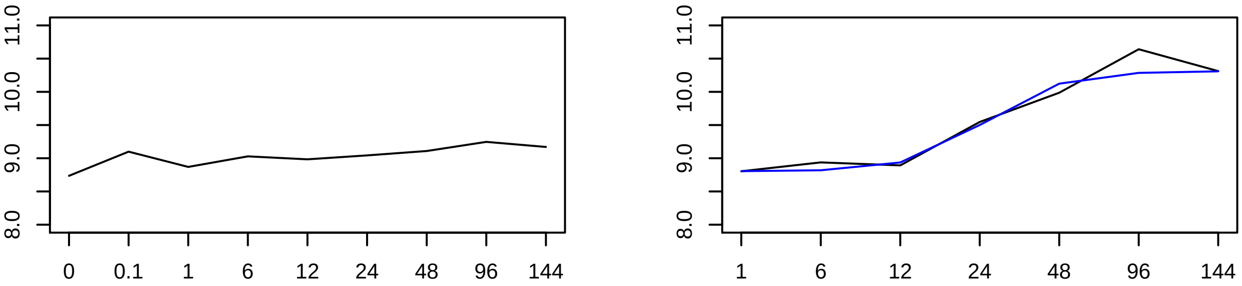


**A_23_P128215 SOCS2 12q22**

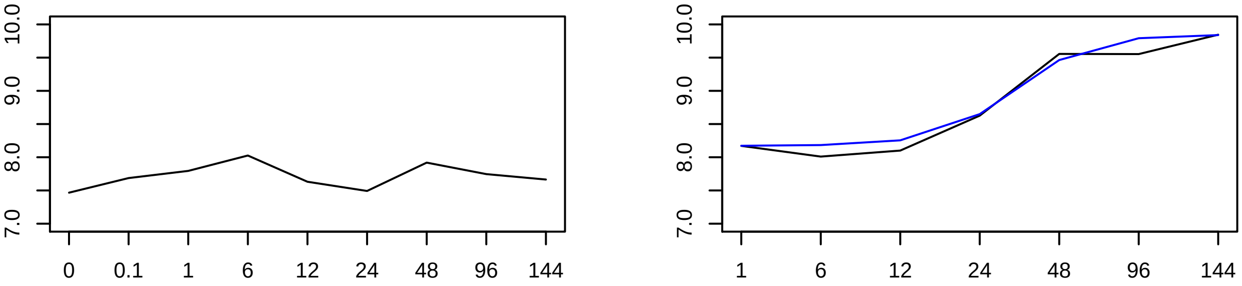


**A_23_P37088 RDH12 14q24.1**

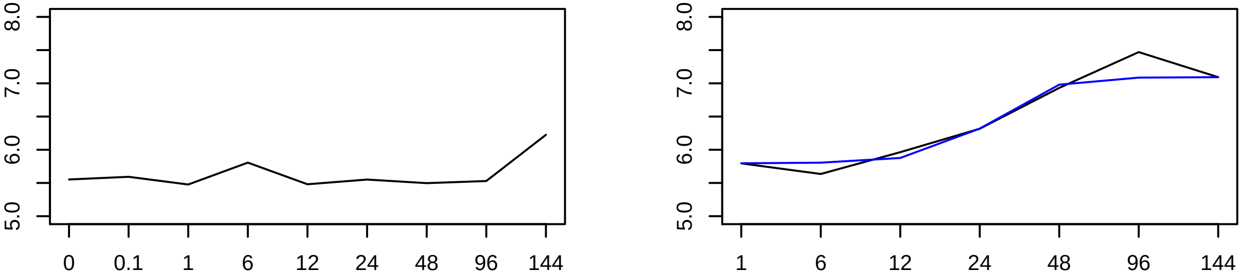

**A_32_P228206 THC2463424 NA**

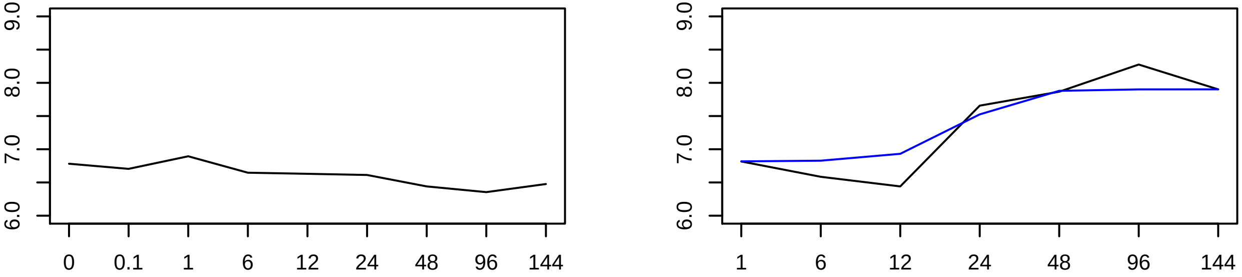


**A_23_P87461 B4GALNT1 12q14.1**

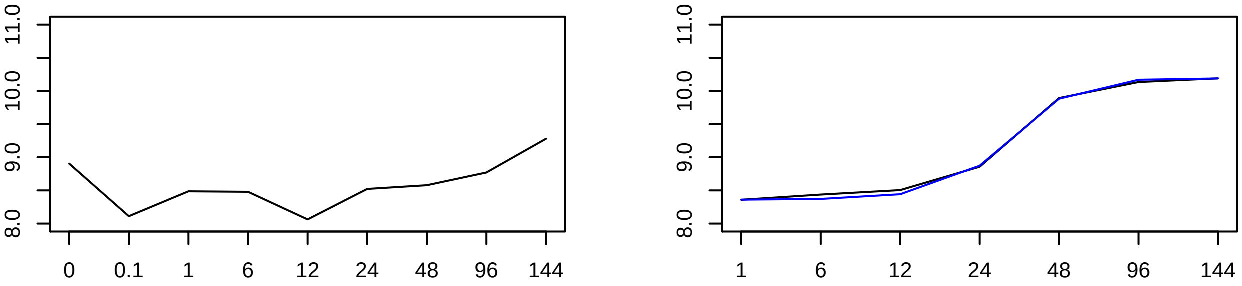


**A_23_P251075 CXorf6 Xq28**

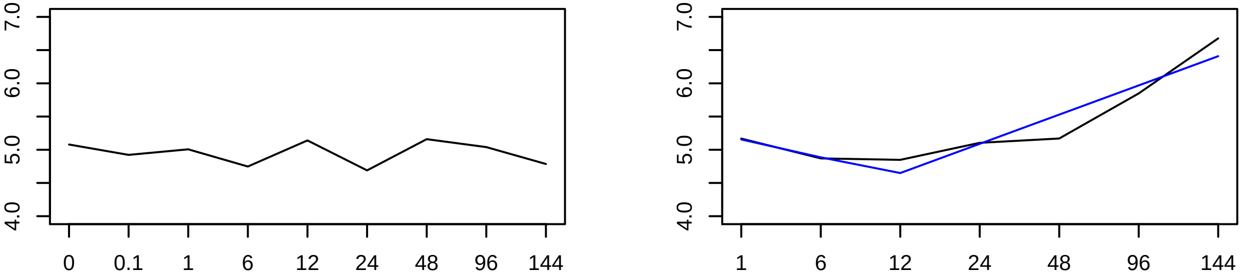

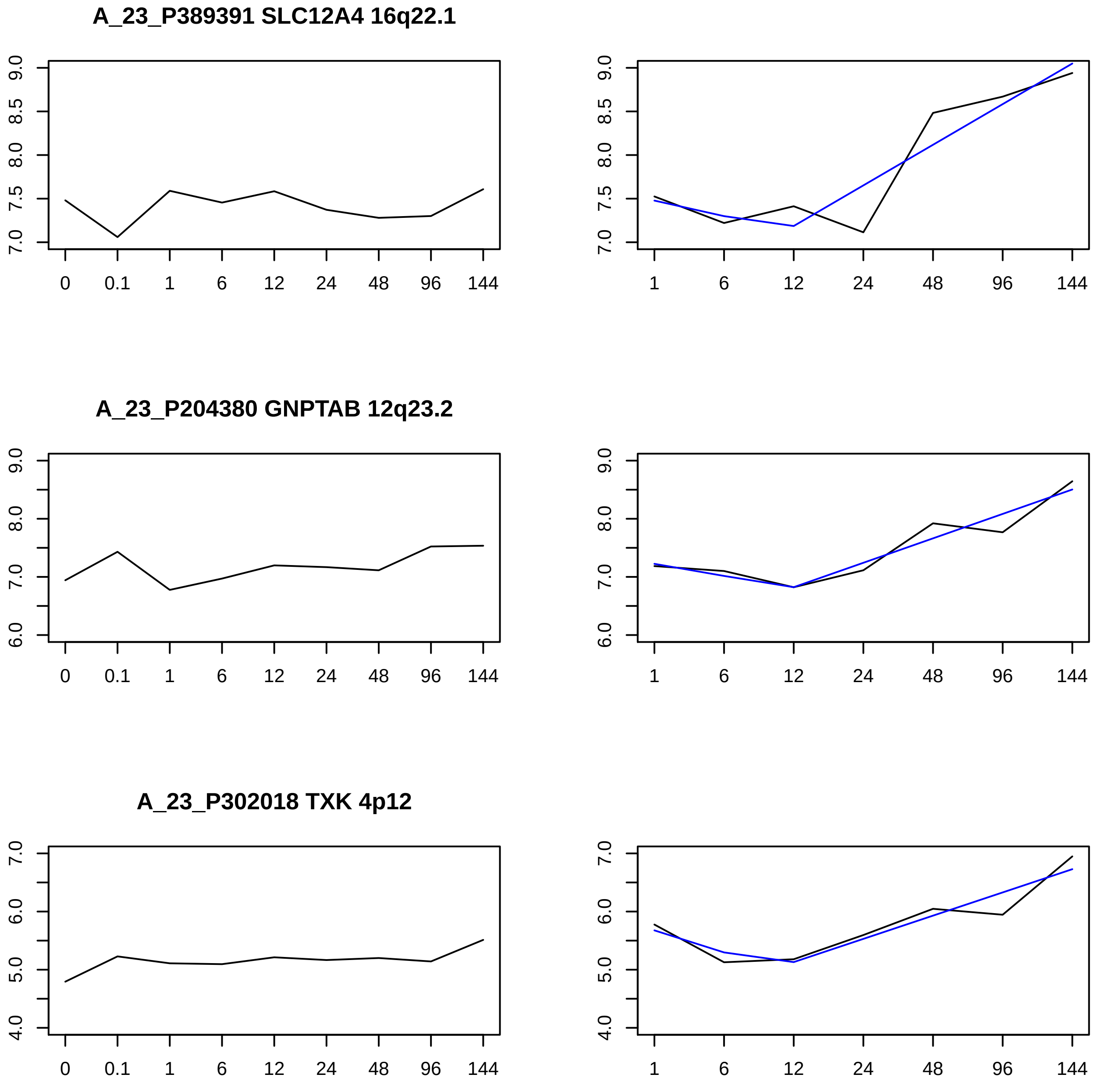
A_23_P389391 SLC12A4 16q22.1
7.0 7.5 8.0 8.5 9.0
0 0.1 1 6 12 24 48 96 144
7.0 7.5 8.0 8.5 9.0
1 6 12 24 48 96 144
A_23_P204380 GNPTAB 12q23.2
6.0 7.0 8.0 9.0
0 0.1 1 6 12 24 48 96 144
6.0 7.0 8.0 9.0
1 6 12 24 48 96 144
A_23_P302018 TXK 4p12
4.0 5.0 6.0 7.0
0 0.1 1 6 12 24 48 96 144
4.0 5.0 6.0 7.0
1 6 12 24 48 96 144

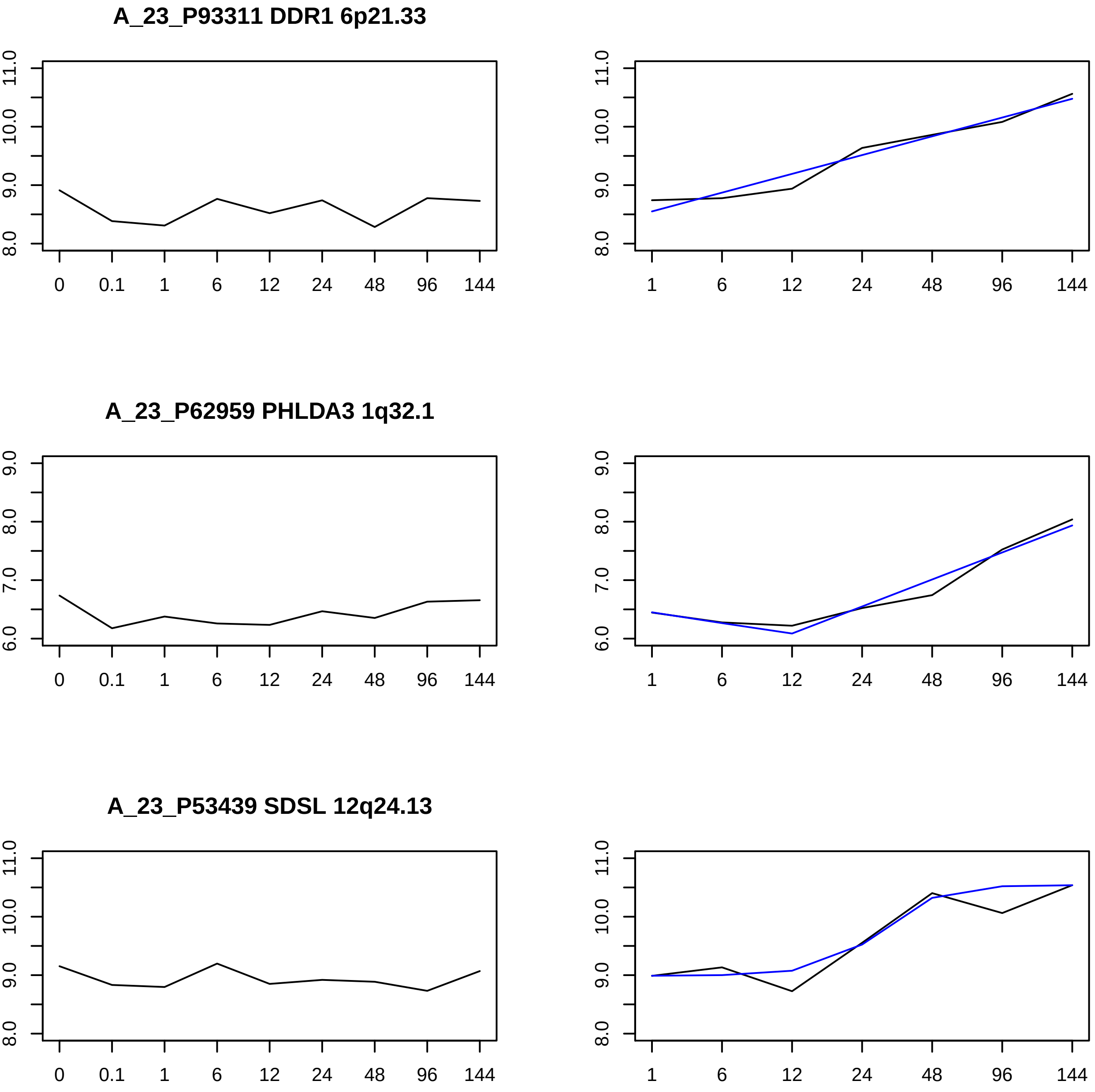
A_23_P93311 DDR1 6p21.33
8.0
9.0
10.0
11.0
0
0.1
1
6
12
24
48
96
144
A_23_P62959 PHLDA3 1q32.1
6.0
7.0
8.0
9.0
A_23_P53439 SDSL 12q24.13

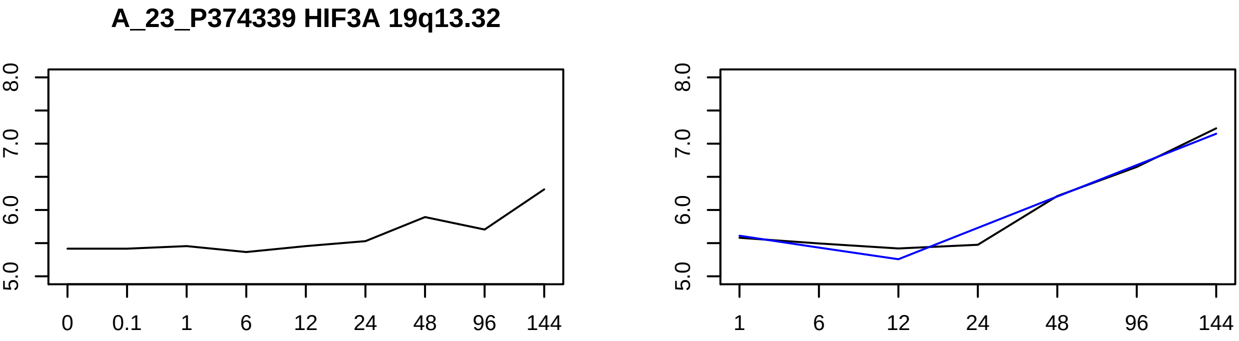
A_23_P374339 HIF3A 19q13.32
5.0
6.0
7.0
8.0
0
0.1
1
6
12
24
48
96
144
1
6
12
24
48
96
144

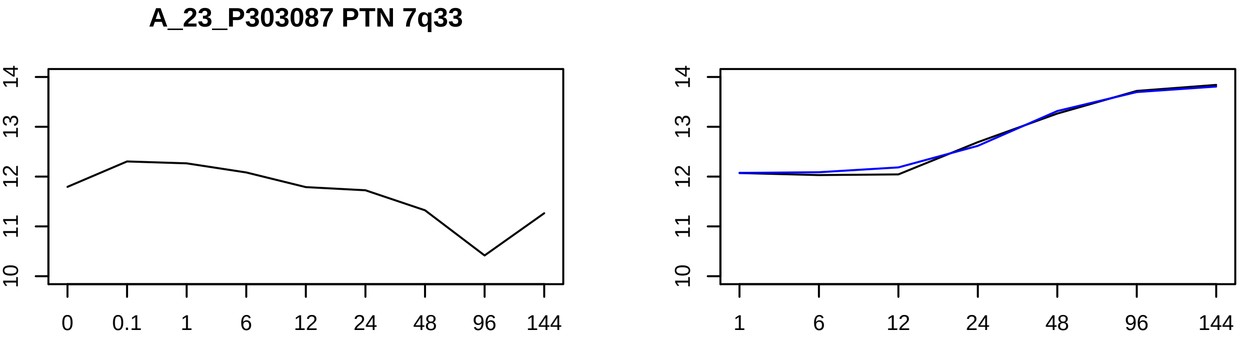
A_23_P303087 PTN 7q33
10
11
12
13
14
0
0.1
1
6
12
24
48
96
144
1
6
12
24
48
96
144

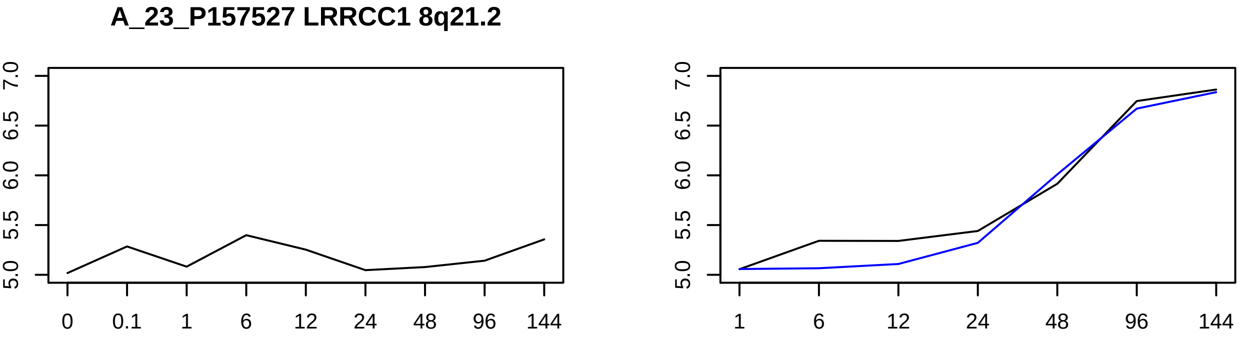
A_23_P157527 LRRCC1 8q21.2
5.0
5.5
6.0
6.5
7.0
0
0.1
1
6
12
24
48
96
144
1
6
12
24
48
96
144

**A_24_P184555 PXN 12q24.31**

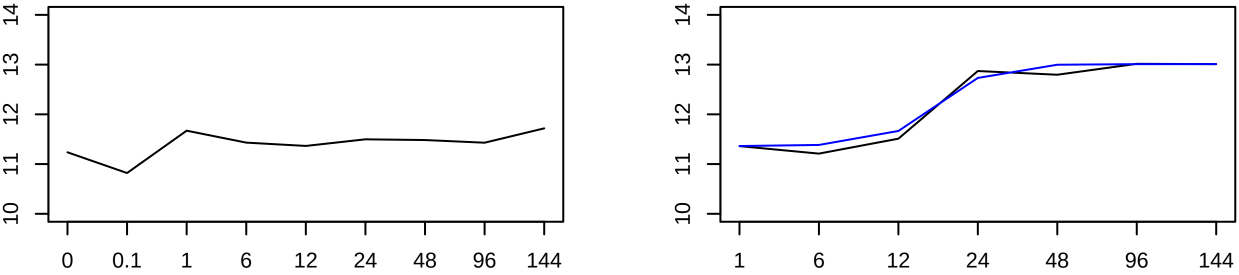


**A_24_P910169 A_24_P910169 NA**

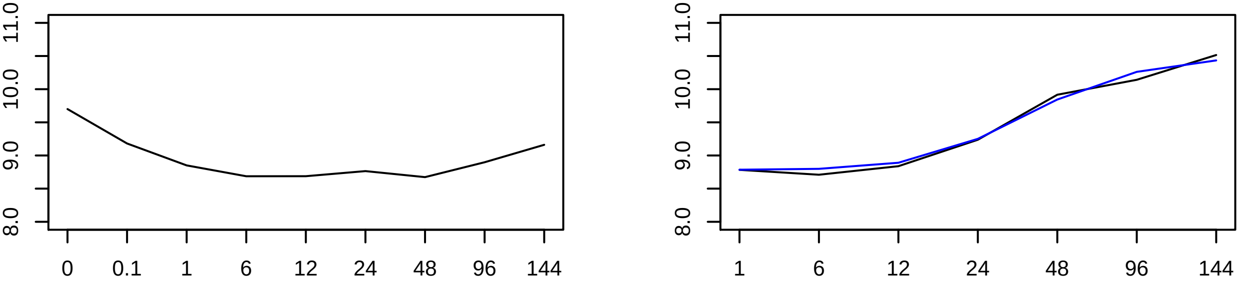


**A_24_P940694 SLC44A5 1p31.1**

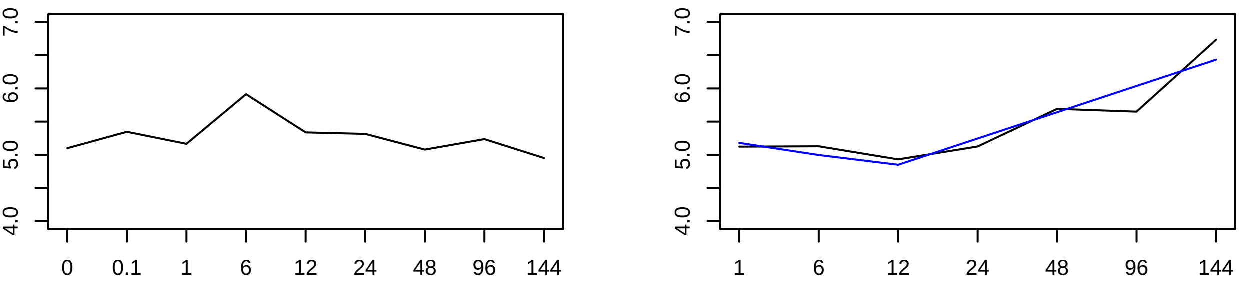

**A_24_P44462 TPM1 15q22.2**

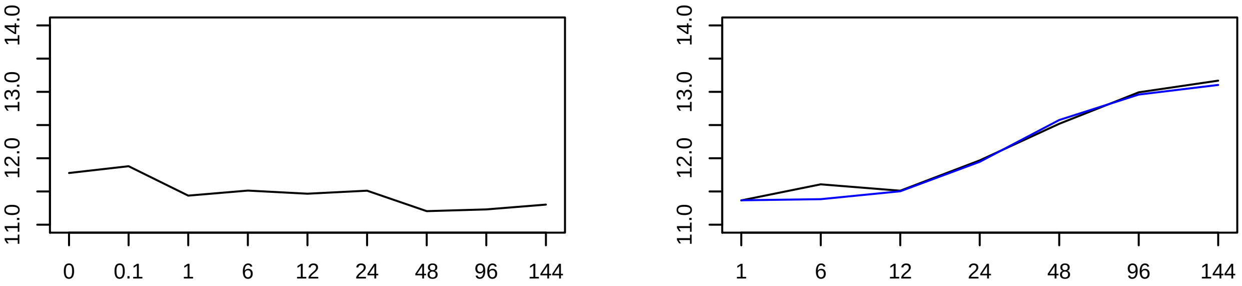


**A_23_P22352 FRMD4A 10p13**

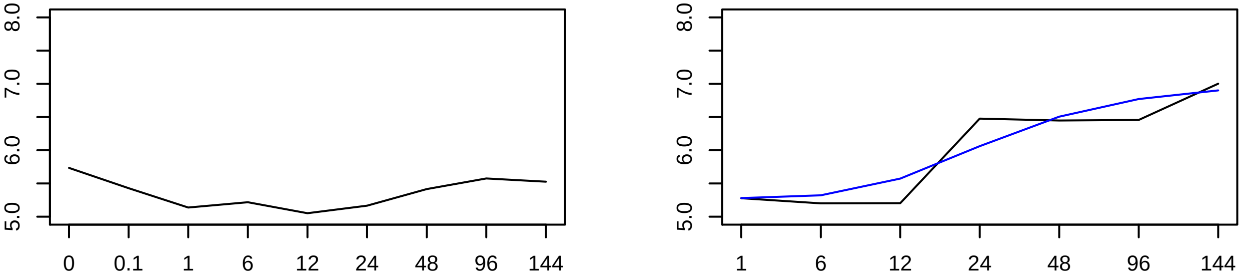


**A_23_P205828 TJP1 15q13.1**

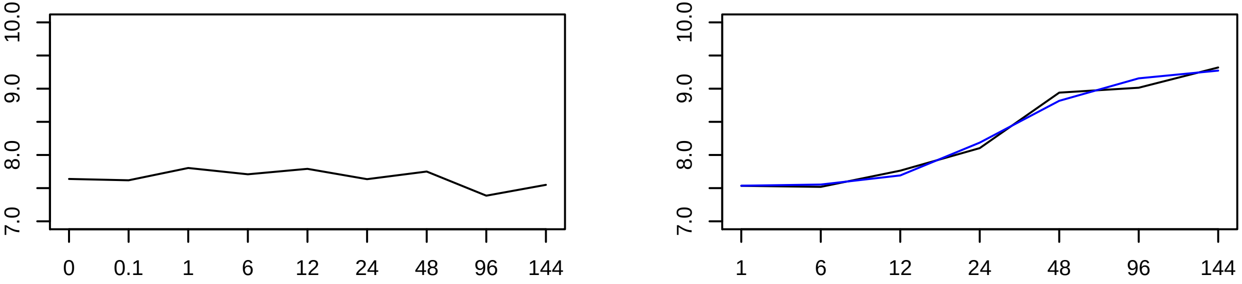

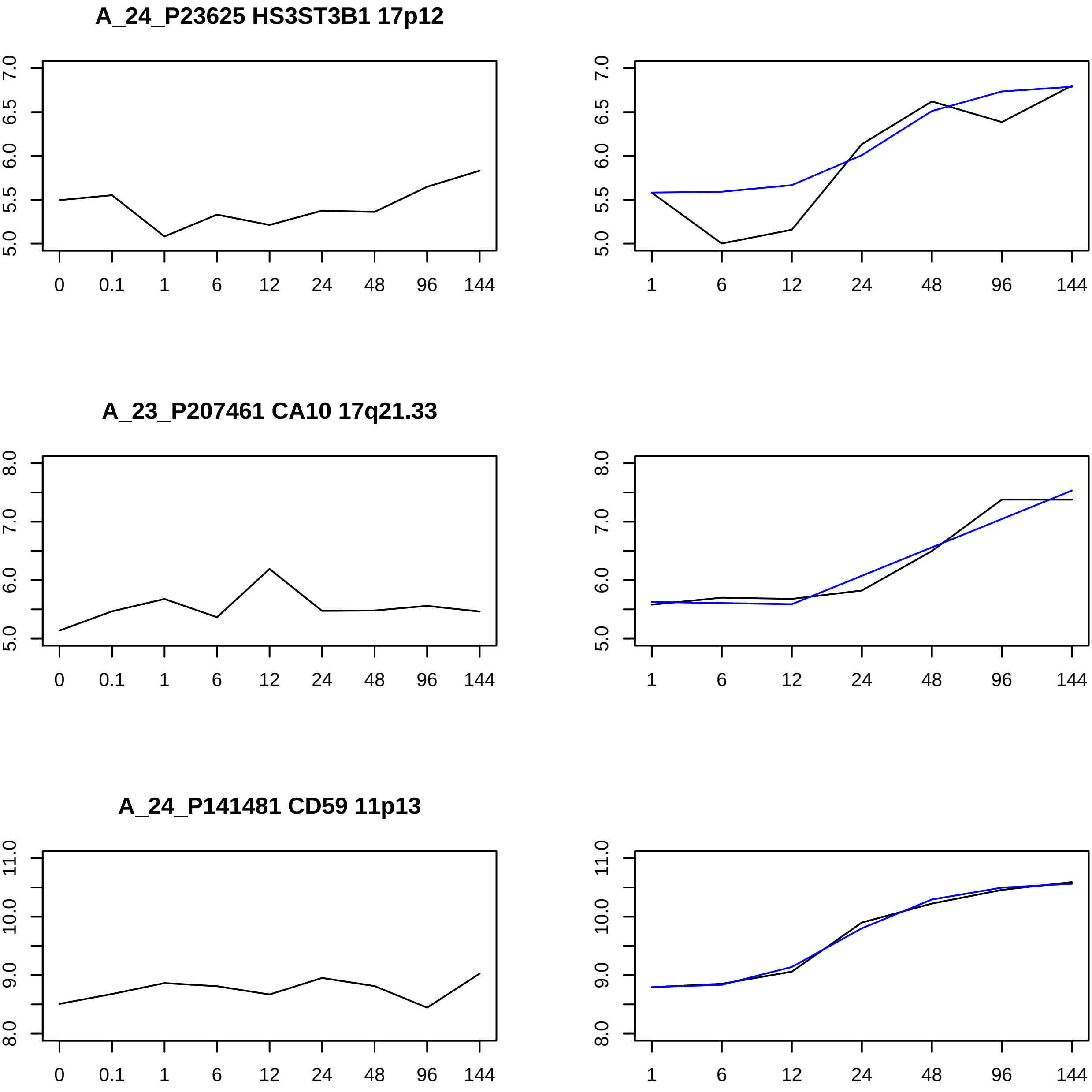
A_24_P23625 HS3ST3B1 17p12
5.0 5.5 6.0 6.5 7.0
0 0.1 1 6 12 24 48 96 144
1 6 12 24 48 96 144
A_23_P207461 CA10 17q21.33
5.0 6.0 7.0 8.0
0 0.1 1 6 12 24 48 96 144
1 6 12 24 48 96 144
A_24_P141481 CD59 11p13
8.0 9.0 10.0 11.0
0 0.1 1 6 12 24 48 96 144
1 6 12 24 48 96 144

**A_23_P59616 GTF2IRD2 7q11.23**

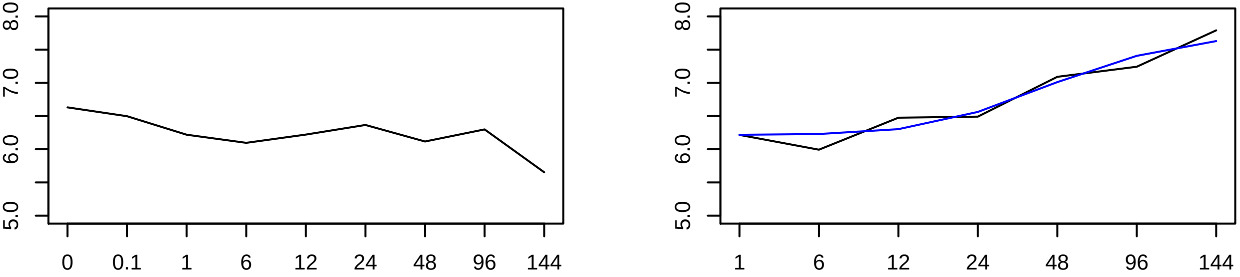


**A_23_P50974 HECW2 2q32.3**

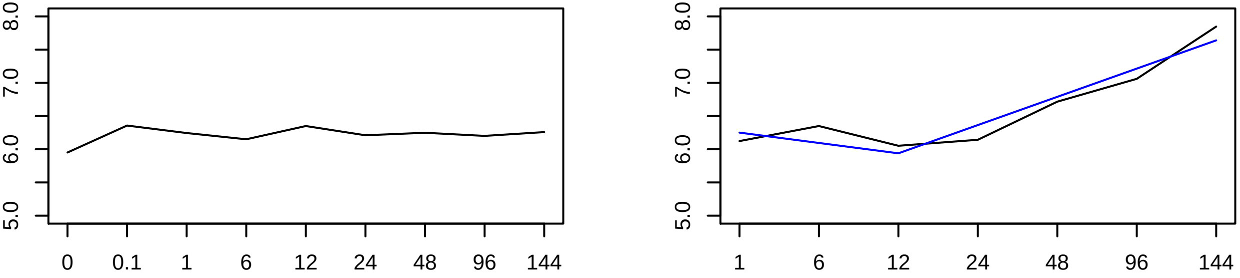


**A_23_P96985 PSEN2 1q42.13**

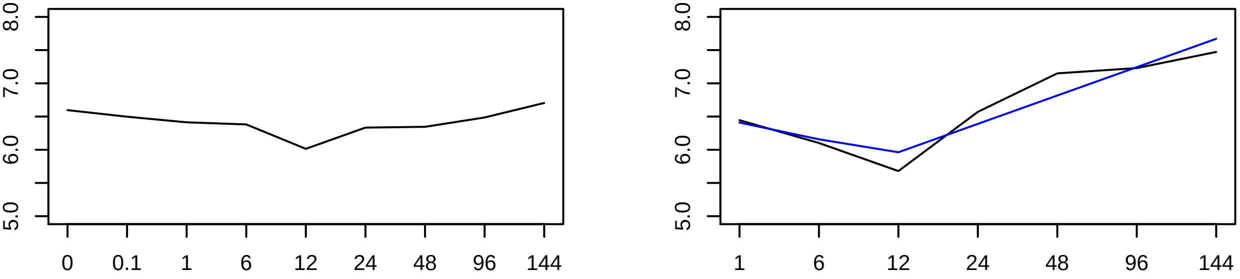

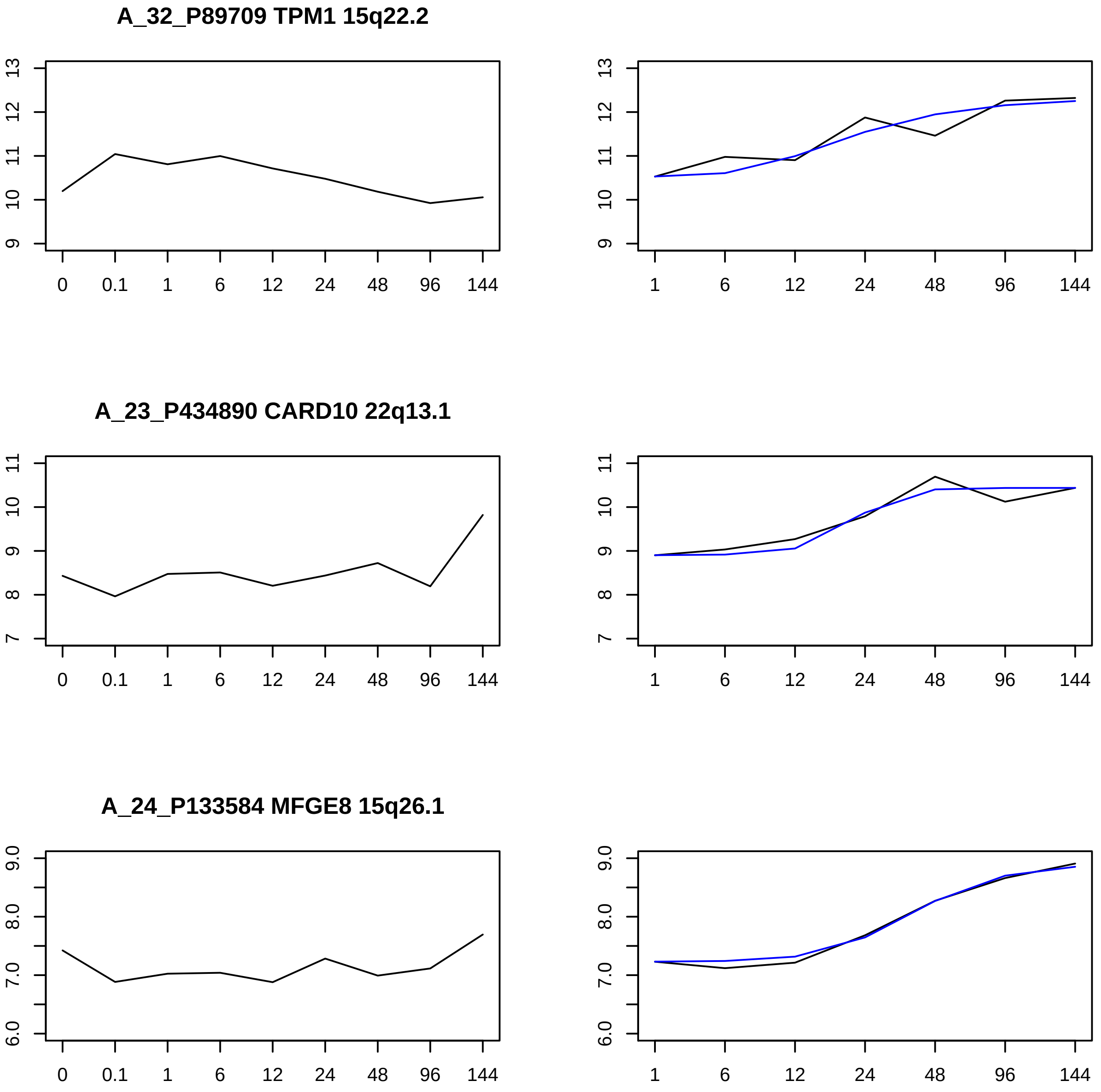
A_32_P89709 TPM1 15q22.2
9
10
11
12
13
0
0.1
1
6
12
24
48
96
144
1
6
12
24
48
96
144
A_23_P434890 CARD10 22q13.1
7
8
9
10
11
A_24_P133584 MFGE8 15q26.1
6.0
7.0
8.0
9.0

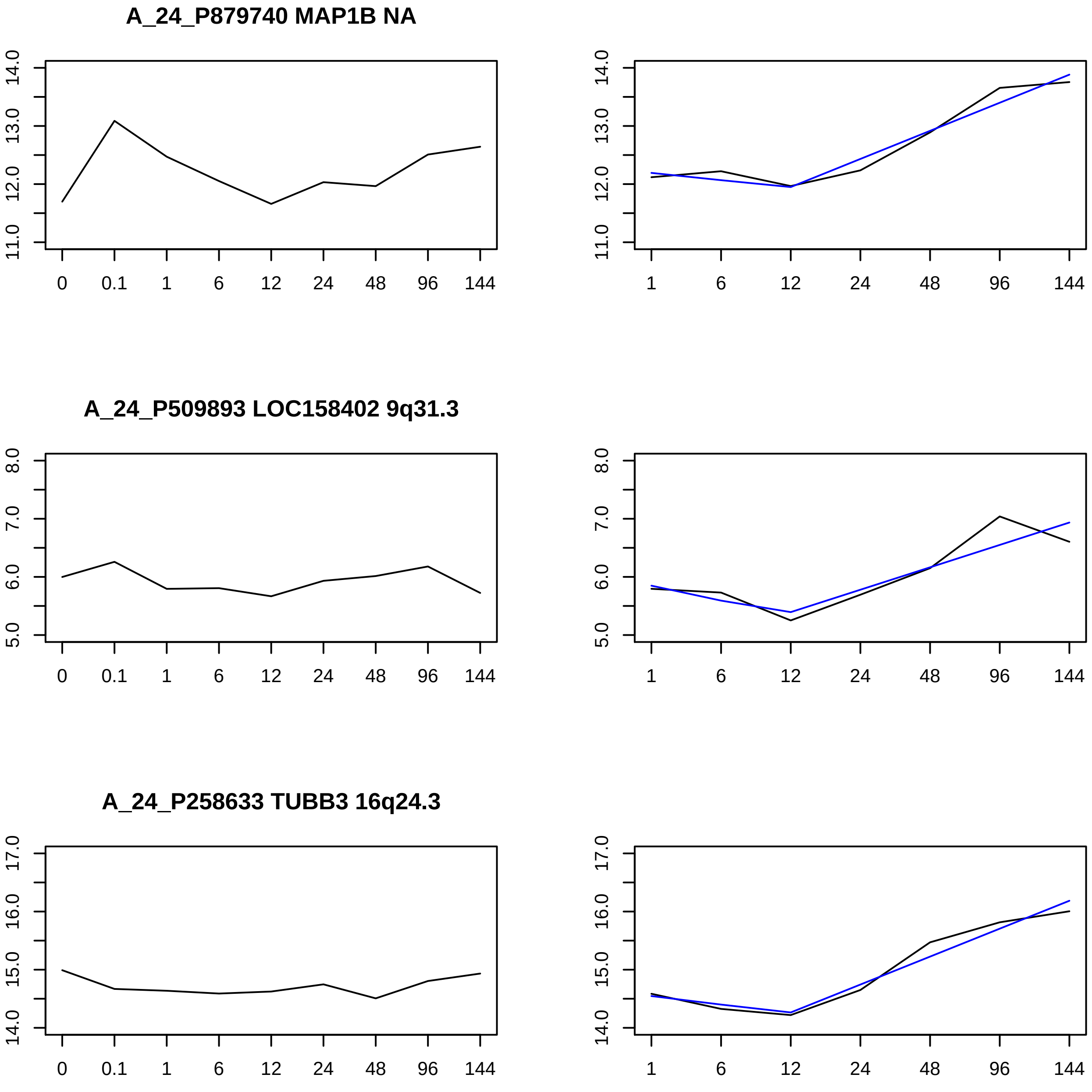
A_24_P879740 MAP1B NA
A_24_P509893 LOC158402 9q31.3
A_24_P258633 TUBB3 16q24.3
11.0 12.0 13.0 14.0
5.0 6.0 7.0 8.0
14.0 15.0 16.0 17.0
0 0.1 1 6 12 24 48 96 144
1 6 12 24 48 96 144

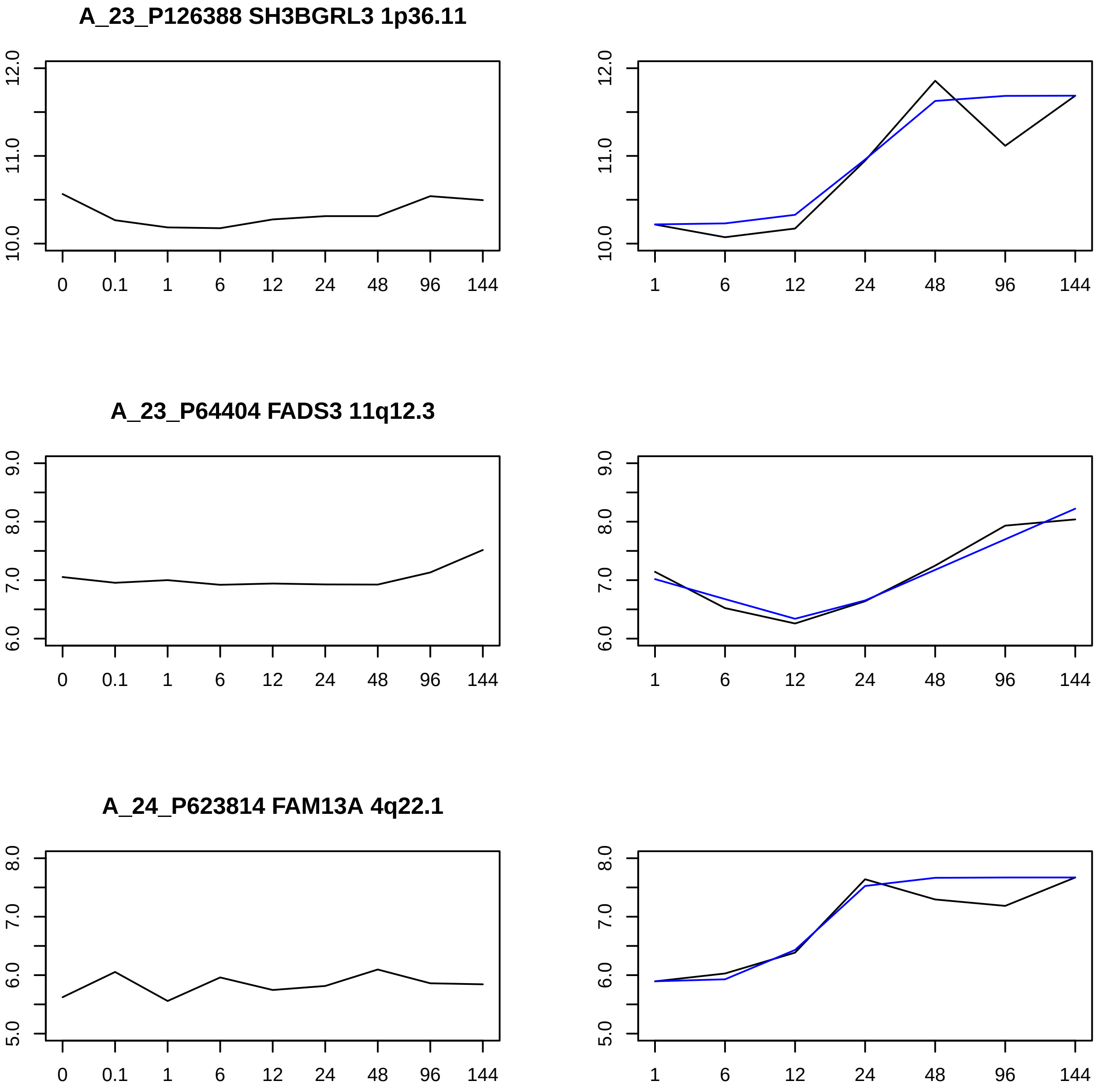
A_23_P126388 SH3BGRL3 1p36.11
10.0
11.0
12.0
0
0.1
1
6
12
24
48
96
144
1
6
12
24
48
96
144
A_23_P64404 FADS3 11q12.3
6.0
7.0
8.0
9.0
A_24_P623814 FAM13A 4q22.1
5.0
6.0
7.0
8.0

**A_23_P10025 NELL2 12q12**

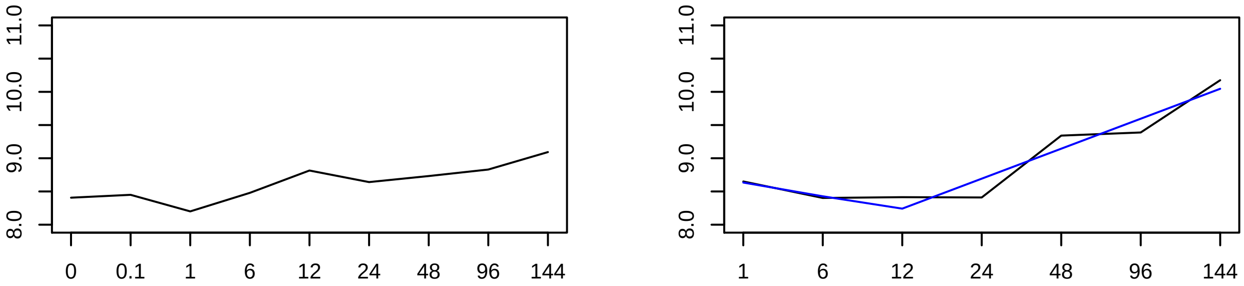


**A_32_P742644 ADAMTS10 19p13.2**

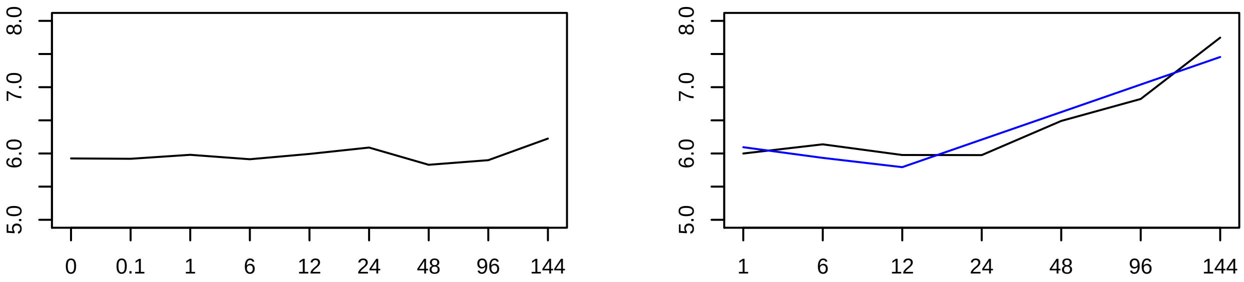


**A_23_P89187 LASP1 17q12**

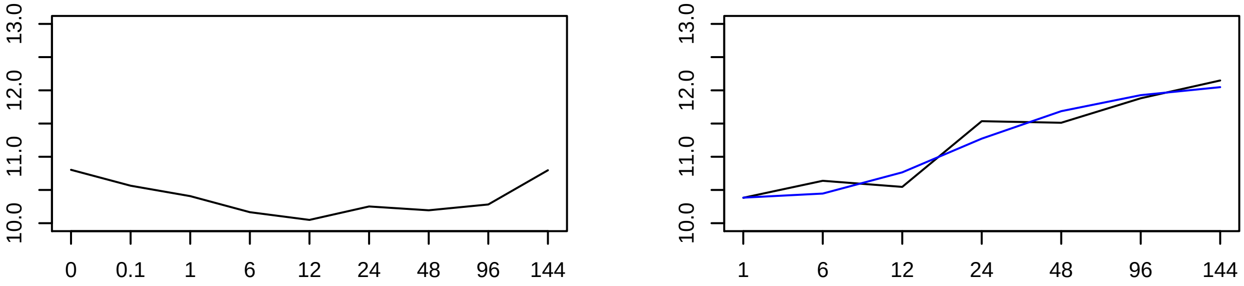

A_23_P58960 AGPAT4 6q26

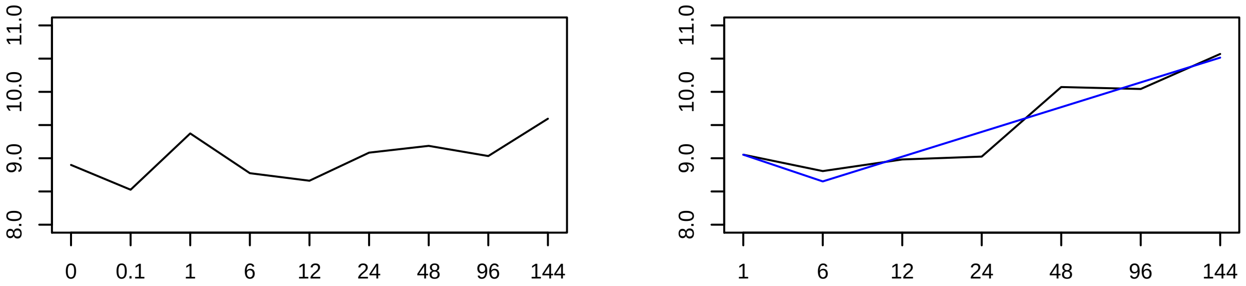


A_32_P12388 THC2582291 NA

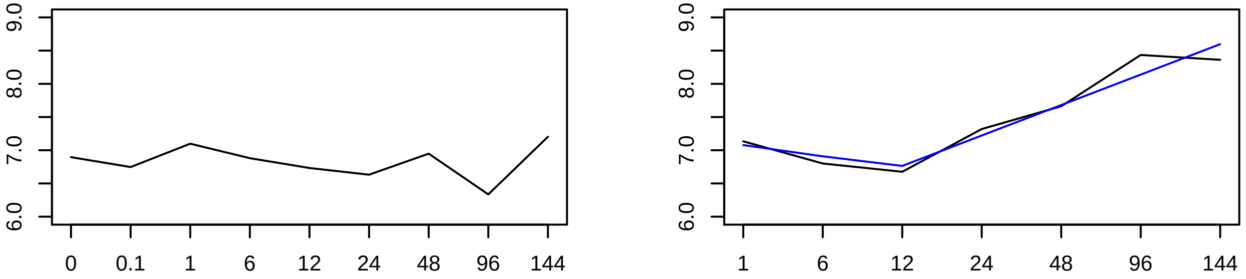


A_24_P71938 SMAD1 4q31.22

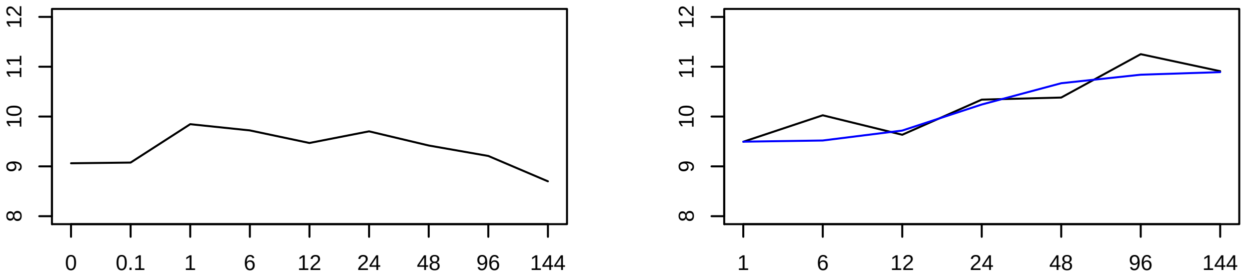

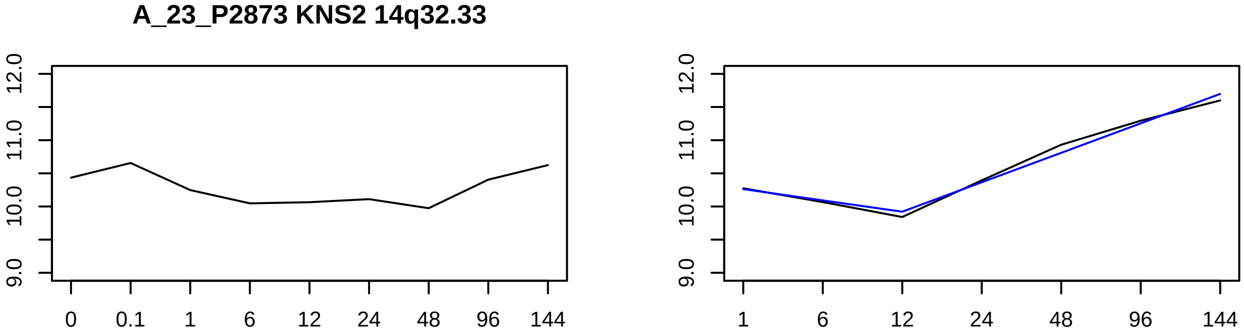
A_23_P2873 KNS2 14q32.33
9.0
10.0
11.0
12.0
0
0.1
1
6
12
24
48
96
144
1
6
12
24
48
96
144

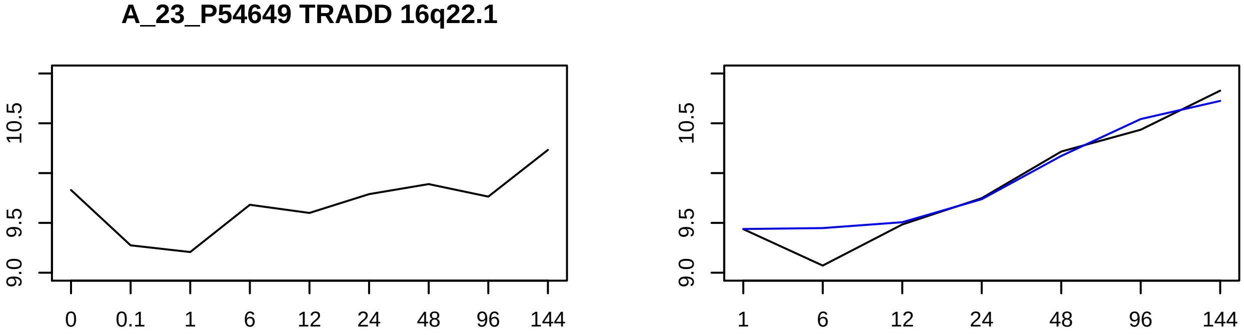
A_23_P54649 TRADD 16q22.1
9.0
9.5
10.5
0
0.1
1
6
12
24
48
96
144
1
6
12
24
48
96
144

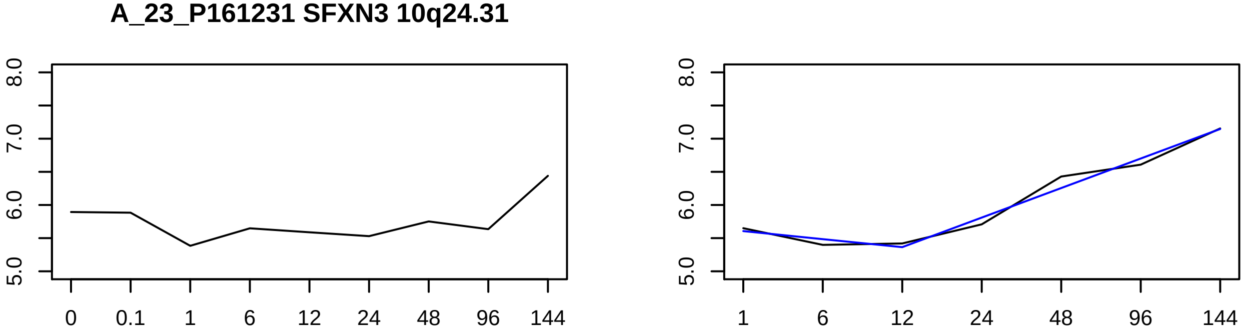
A_23_P161231 SFXN3 10q24.31
5.0
6.0
7.0
8.0
0
0.1
1
6
12
24
48
96
144
1
6
12
24
48
96
144

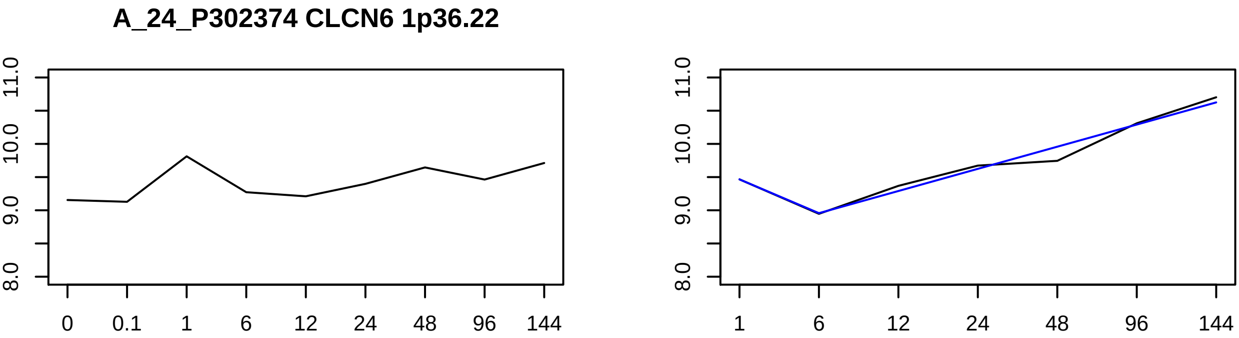
A_24_P302374 CLCN6 1p36.22
8.0
9.0
10.0
11.0
0
0.1
1
6
12
24
48
96
144

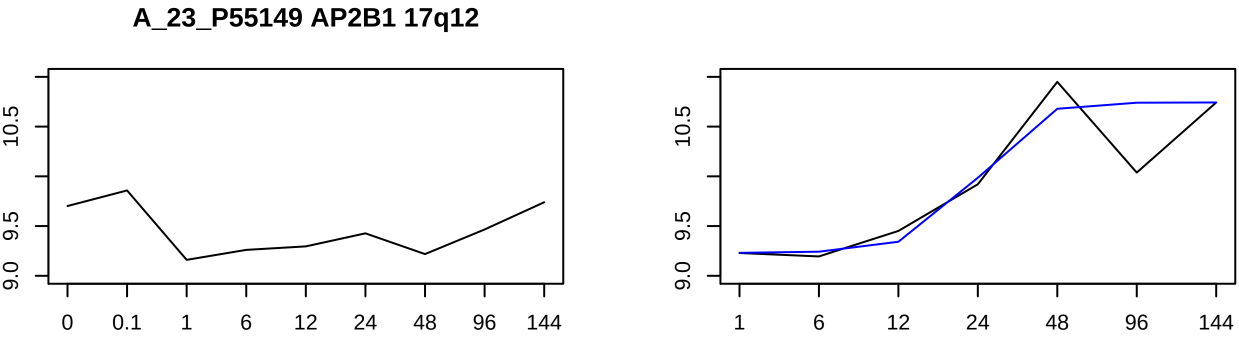
A_23_P55149 AP2B1 17q12
9.0
9.5
10.5
0
0.1
1
6
12
24
48
96
144

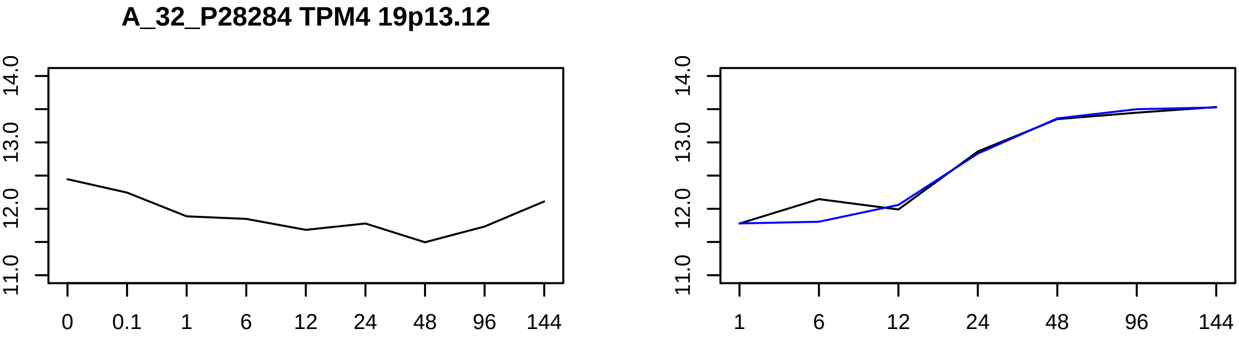
A_32_P28284 TPM4 19p13.12
11.0
12.0
13.0
14.0
0
0.1
1
6
12
24
48
96
144

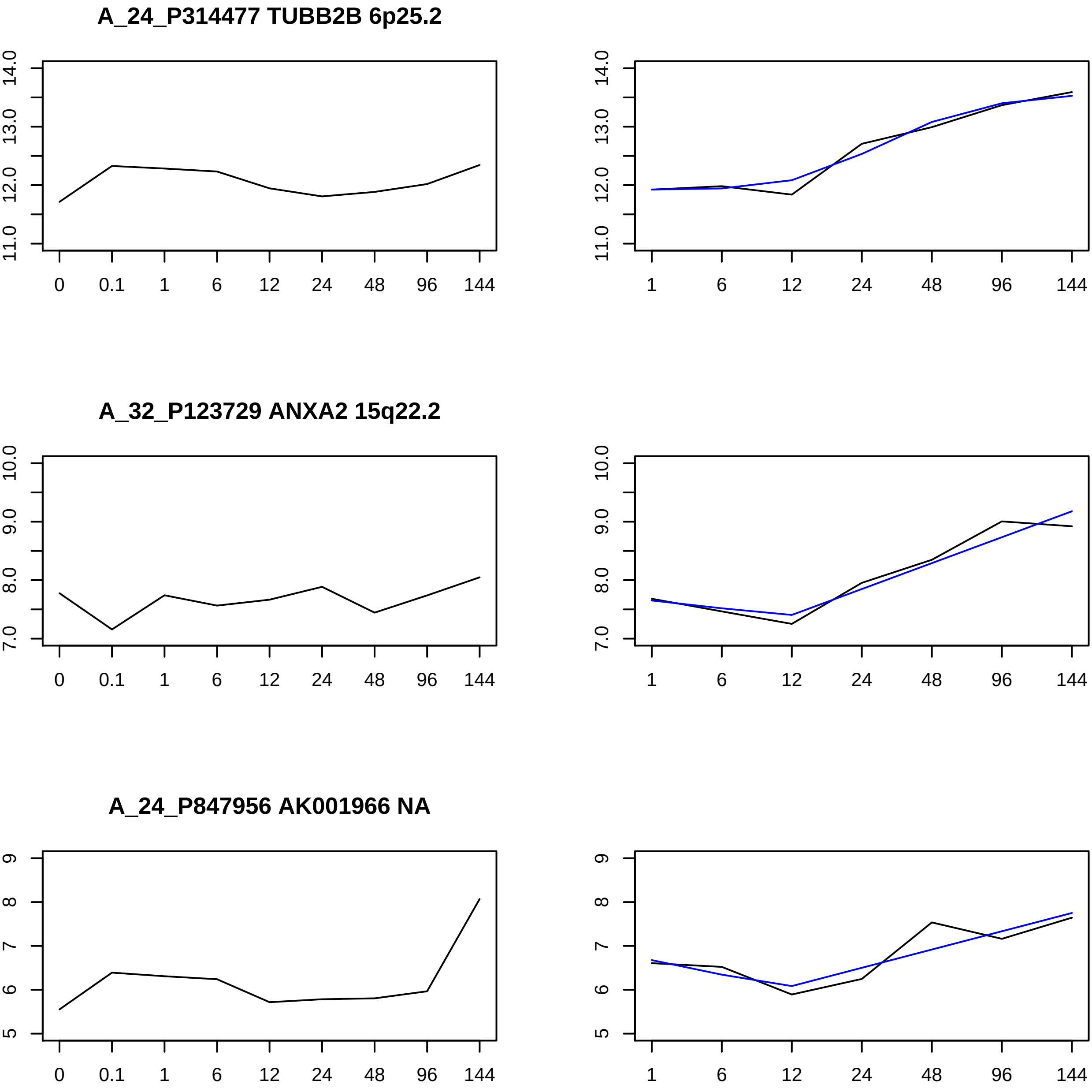
A_24_P314477 TUBB2B 6p25.2
11.0
12.0
13.0
14.0
0
0.1
1
6
12
24
48
96
144
A_32_P123729 ANXA2 15q22.2
7.0
8.0
9.0
10.0
A_24_P847956 AK001966 NA
5
6
7
8
9

**A_23_P409417 VPS37D 7q11.23**

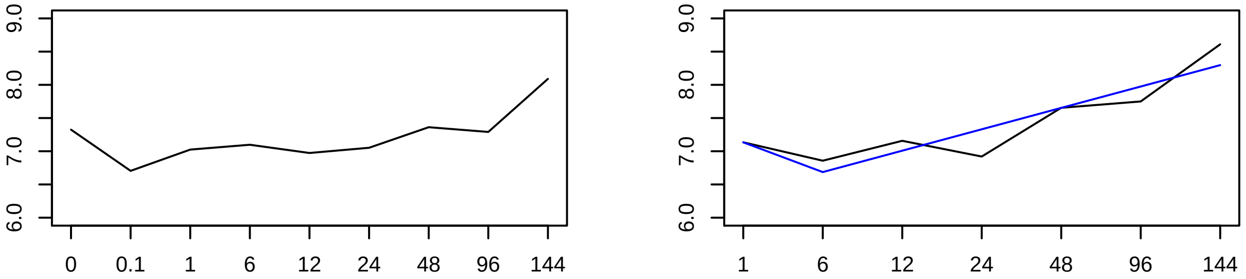


**A_23_P145916 AEBP1 7p13**

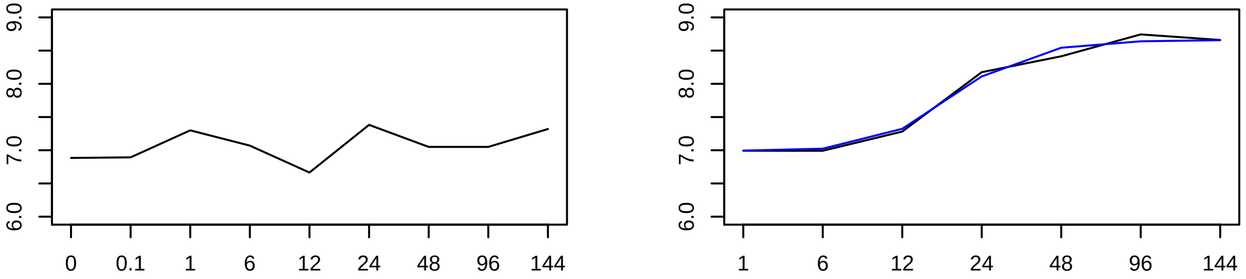


**A_24_P843020 LOC729111 19q13.2**

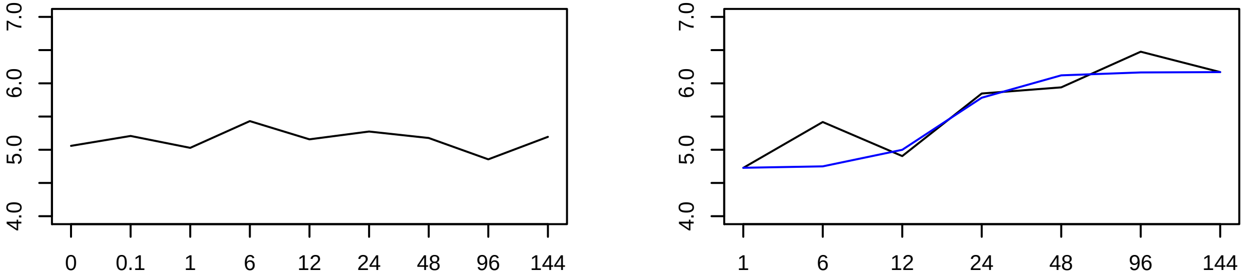

**A_32_P77369 SC4MOL 4q32.3**

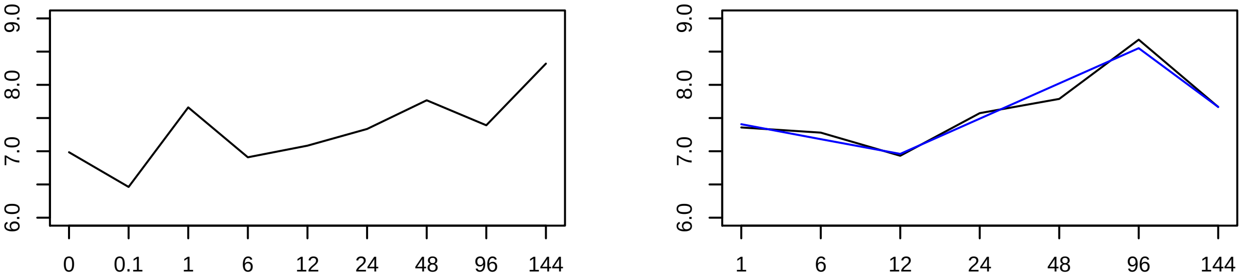


**A_32_P61708 A_32_P61708 NA**

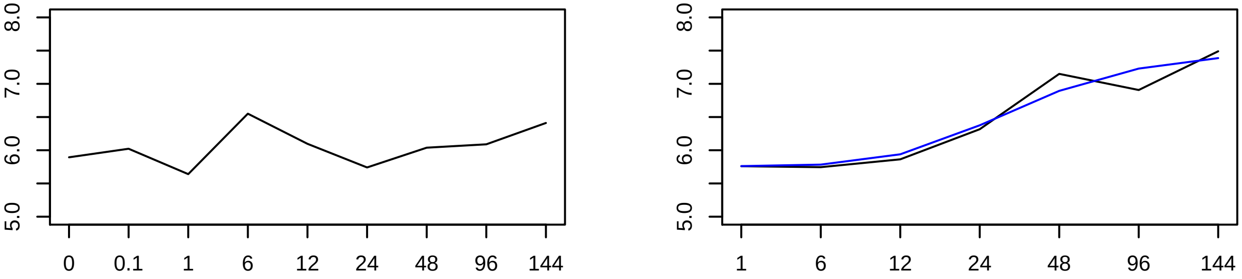


**A_23_P89902 RTN2 19q13.32**

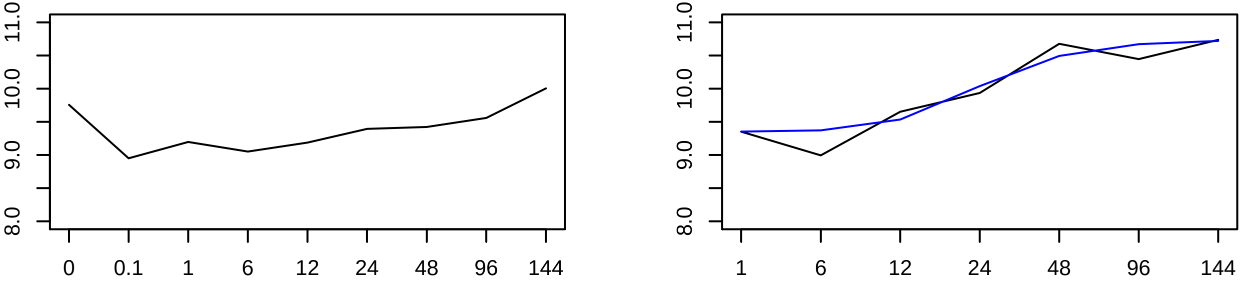

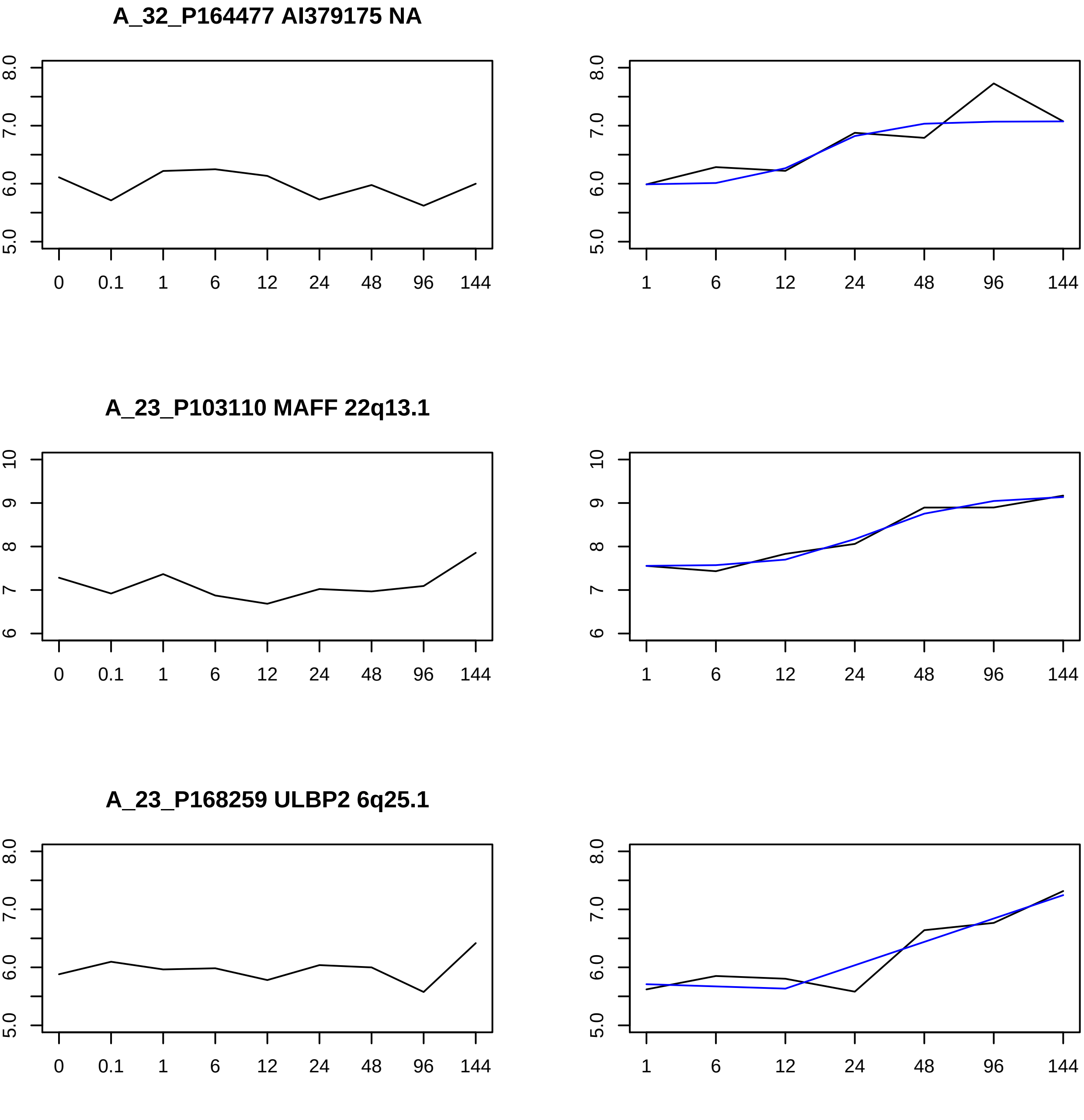

A_32_P164477 AI379175 NA
5.0
6.0
7.0
8.0
0
0.1
1
6
12
24
48
96
144
5.0
6.0
7.0
8.0
1
6
12
24
48
96
144
A_23_P103110 MAFF 22q13.1
6
7
8
9
10
0
0.1
1
6
12
24
48
96
144
6
7
8
9
10
1
6
12
24
48
96
144
A_23_P168259 ULBP2 6q25.1
5.0
6.0
7.0
8.0
0
0.1
1
6
12
24
48
96
144
5.0
6.0
7.0
8.0
1
6
12
24
48
96
144

**A_23_P348138 MKL1 22q13.1**

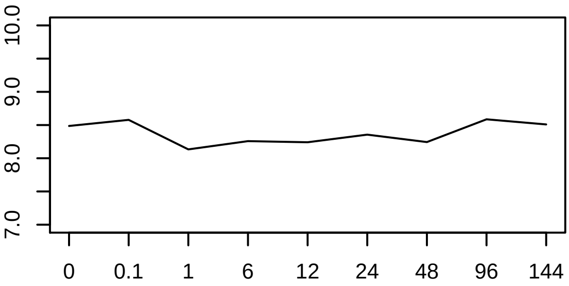


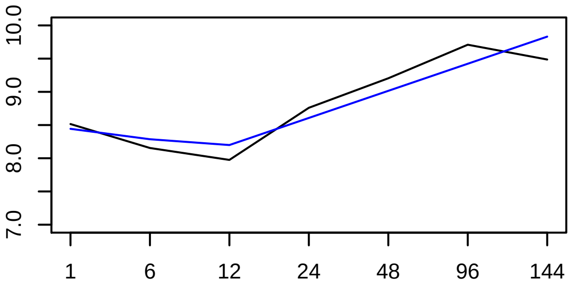


**A_23_P135568 TNIK NA**

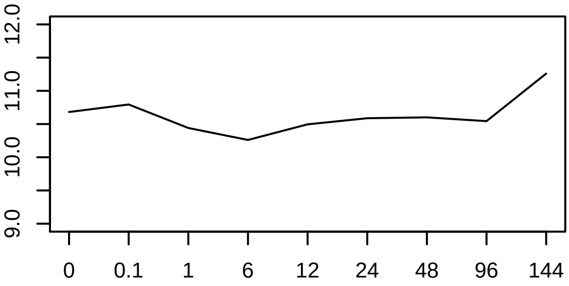


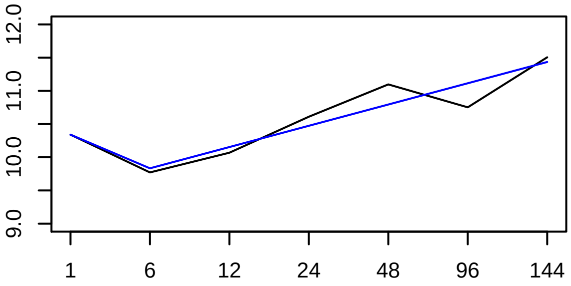


**A_24_P753760 THC2563307 NA**

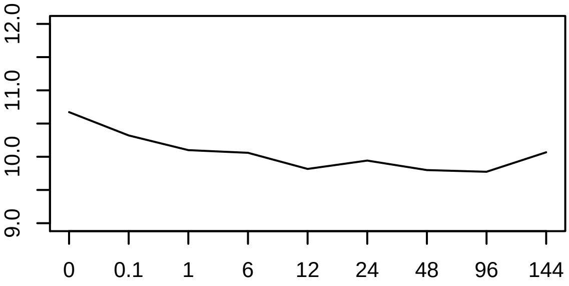


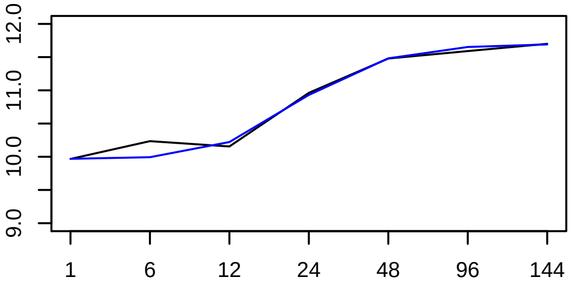

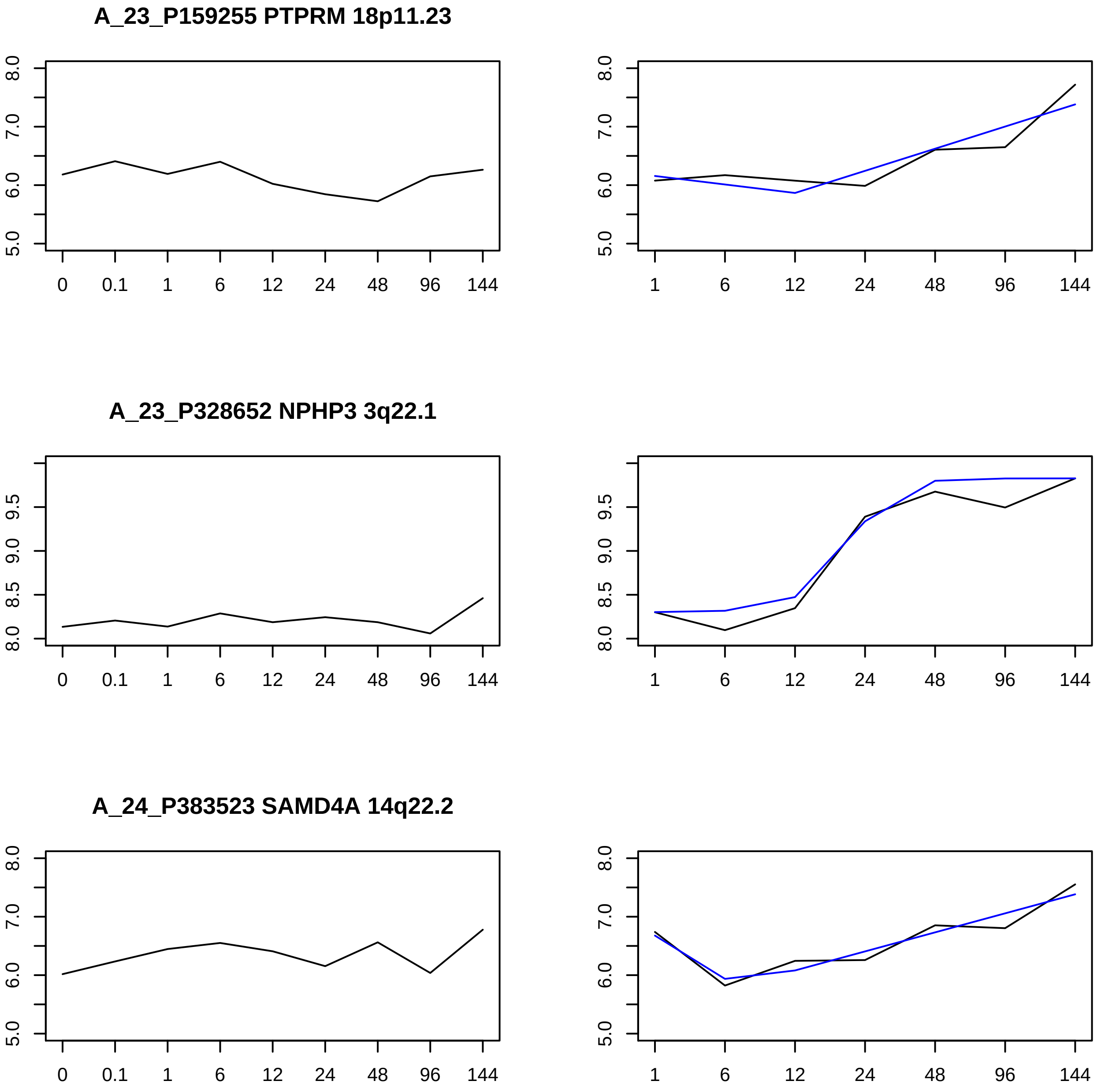
A_23_P159255 PTPRM 18p11.23
5.0
6.0
7.0
8.0
0
0.1
1
6
12
24
48
96
144
1
6
12
24
48
96
144
A_23_P328652 NPHP3 3q22.1
8.0
8.5
9.0
9.5
A_24_P383523 SAMD4A 14q22.2

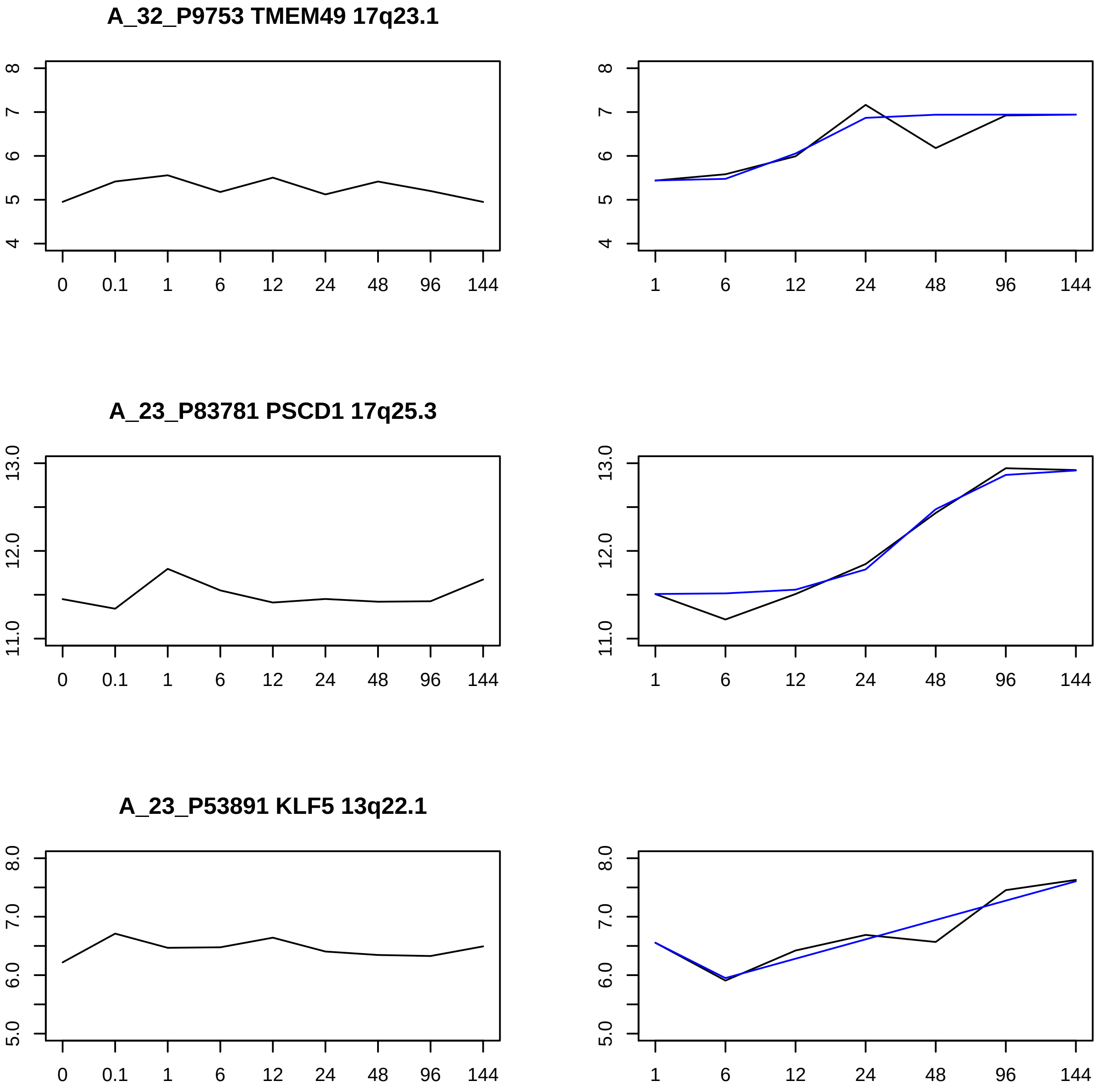
A_32_P9753 TMEM49 17q23.1
4
5
6
7
8
0
0.1
1
6
12
24
48
96
144
1
6
12
24
48
96
144
A_23_P83781 PSCD1 17q25.3
11.0
12.0
13.0
0
0.1
1
6
12
24
48
96
144
1
6
12
24
48
96
144
A_23_P53891 KLF5 13q22.1
5.0
6.0
7.0
8.0
0
0.1
1
6
12
24
48
96
144
1
6
12
24
48
96
144

**A_23_P49627 MRC2 17q23.2**

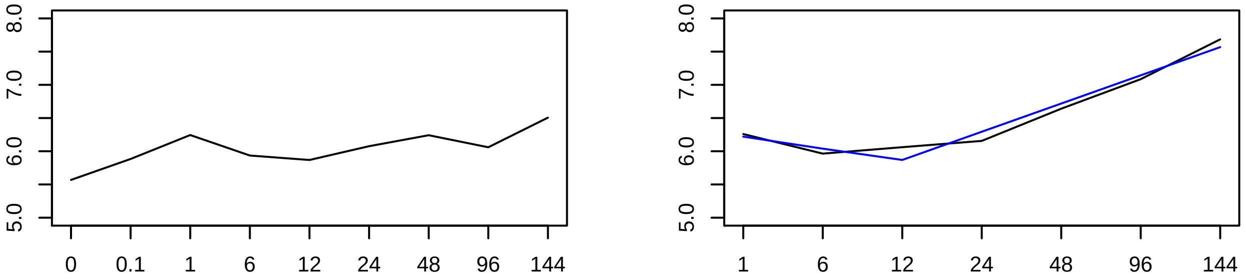


**A_23_P327519 STARD4 5q22.1**

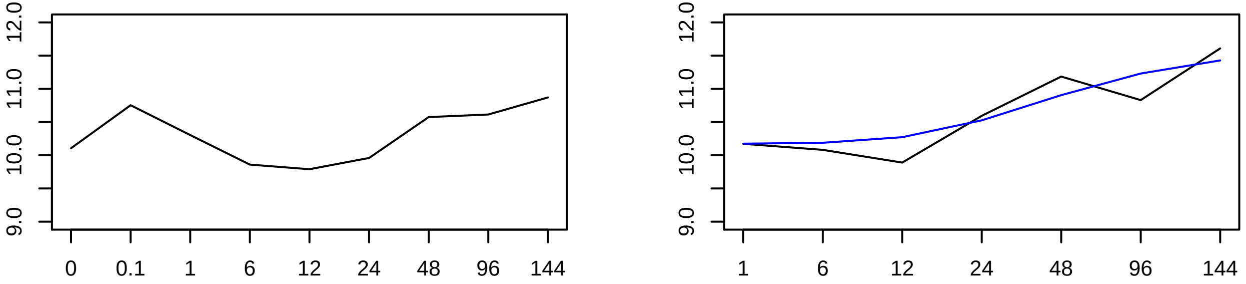


**A_23_P93431 AIG1 6q24.2**

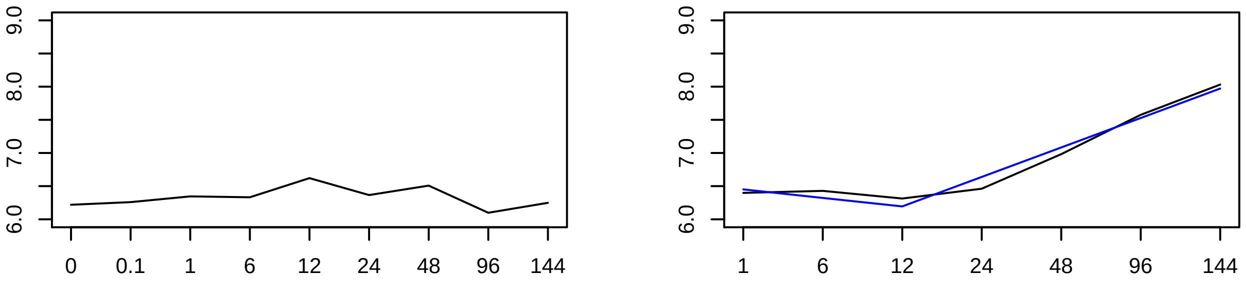

**A_24_P378019 IRF7 11p15.5**

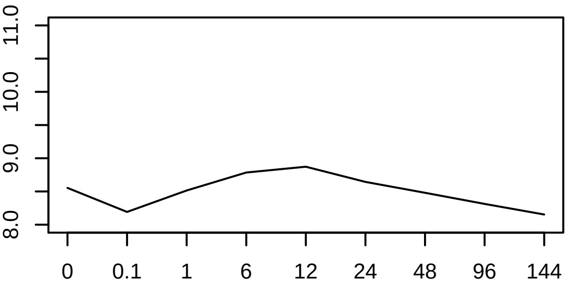


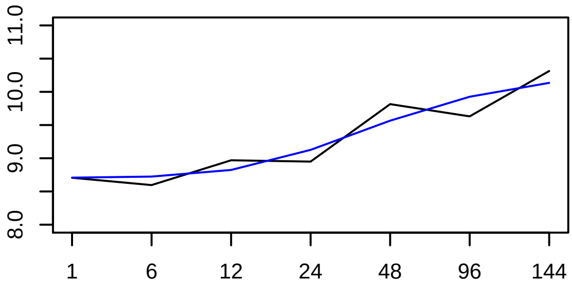


**A_23_P8981 STAR 8p11.23**

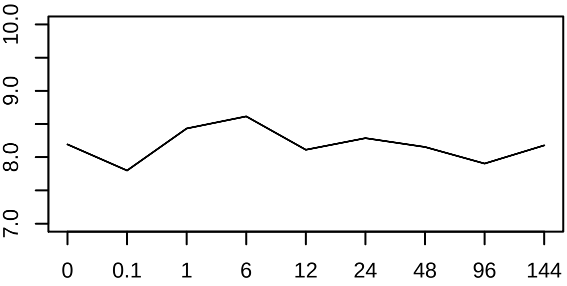


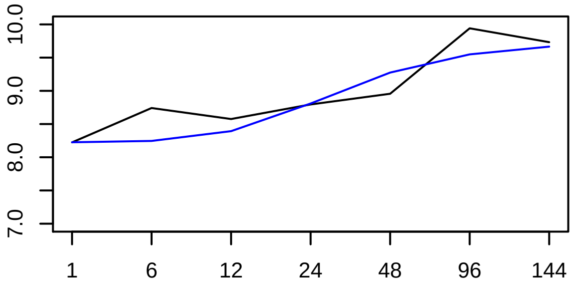


**A_23_P140907 TMEM8 16p13.3**

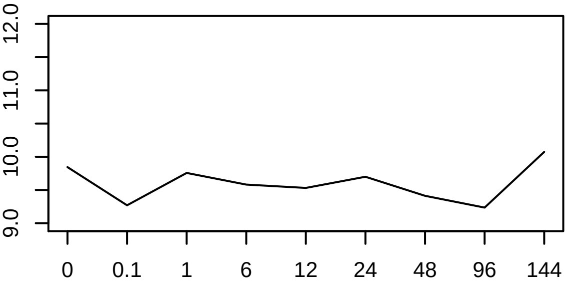


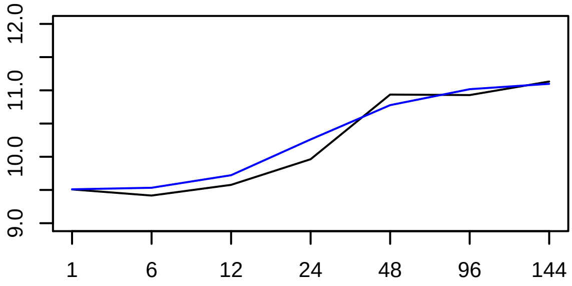

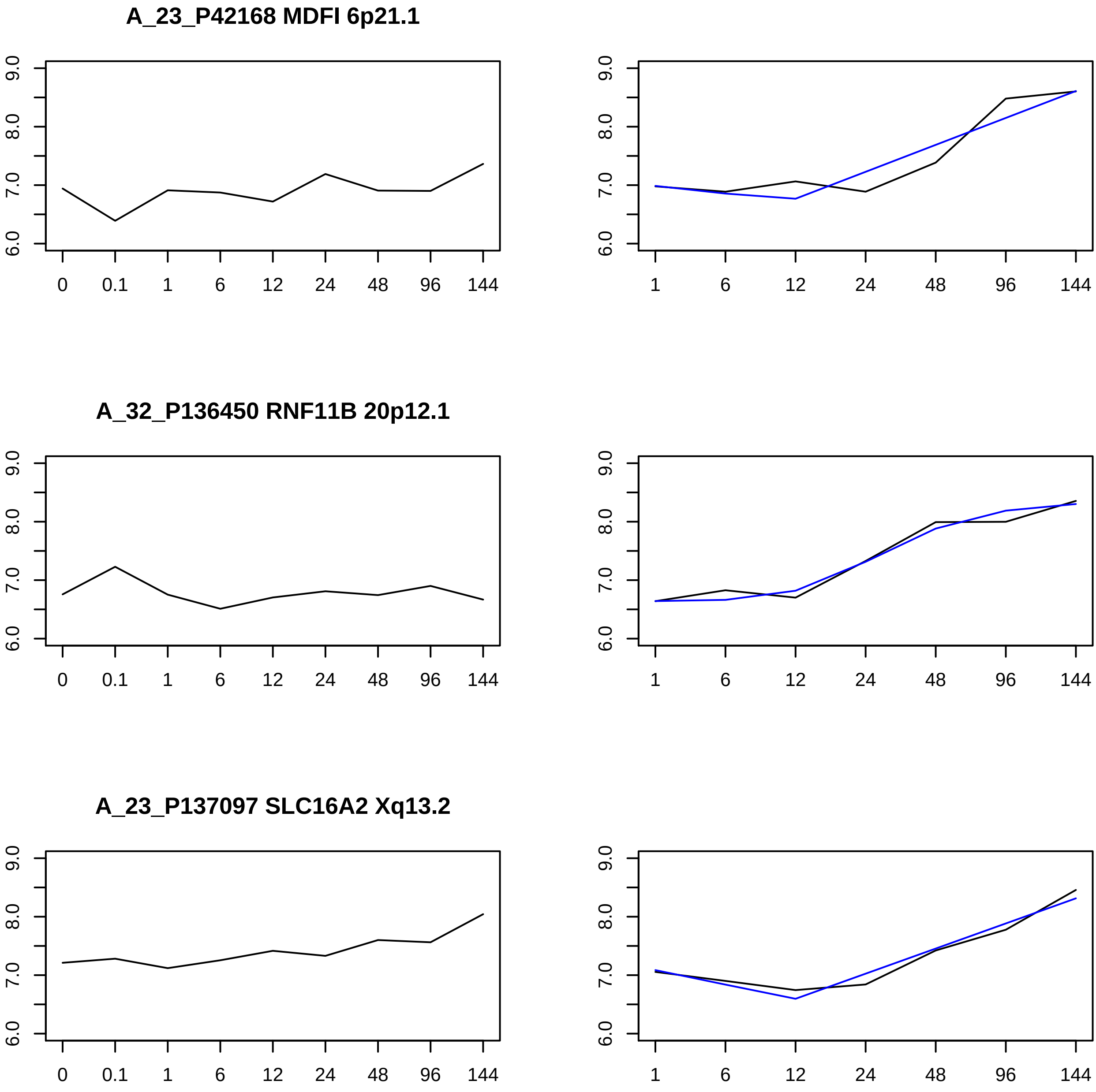
A_23_P42168 MDFI 6p21.1
A_32_P136450 RNF11B 20p12.1
A_23_P137097 SLC16A2 Xq13.2
6.0
7.0
8.0
9.0
0
0.1
1
6
12
24
48
96
144

**A_23_P39881 C2orf14 2q21.1**

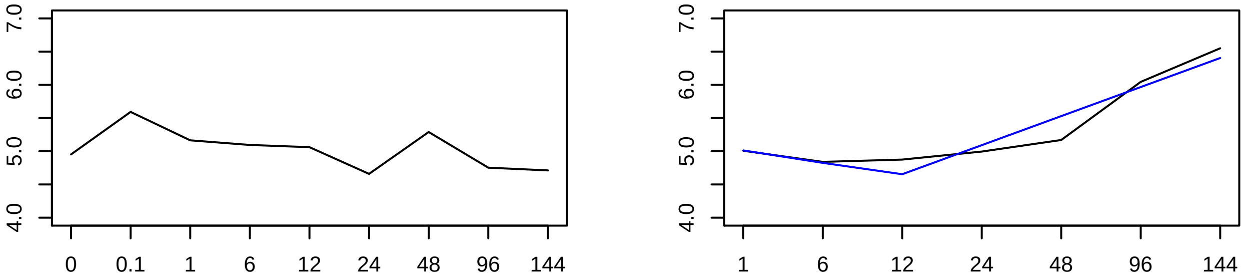


**A_32_P165477 SLC7A11 4q28.3**

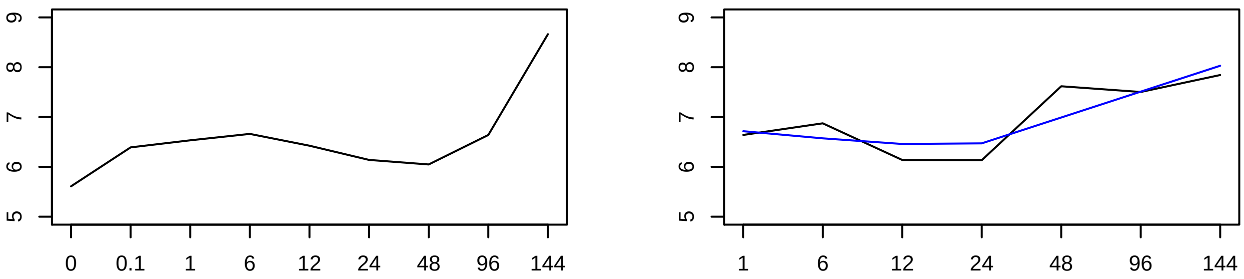


**A_23_P48951 MFGE8 15q26.1**

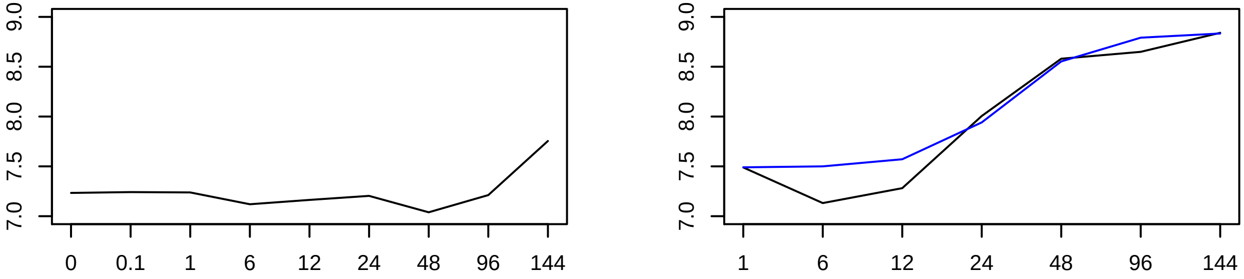

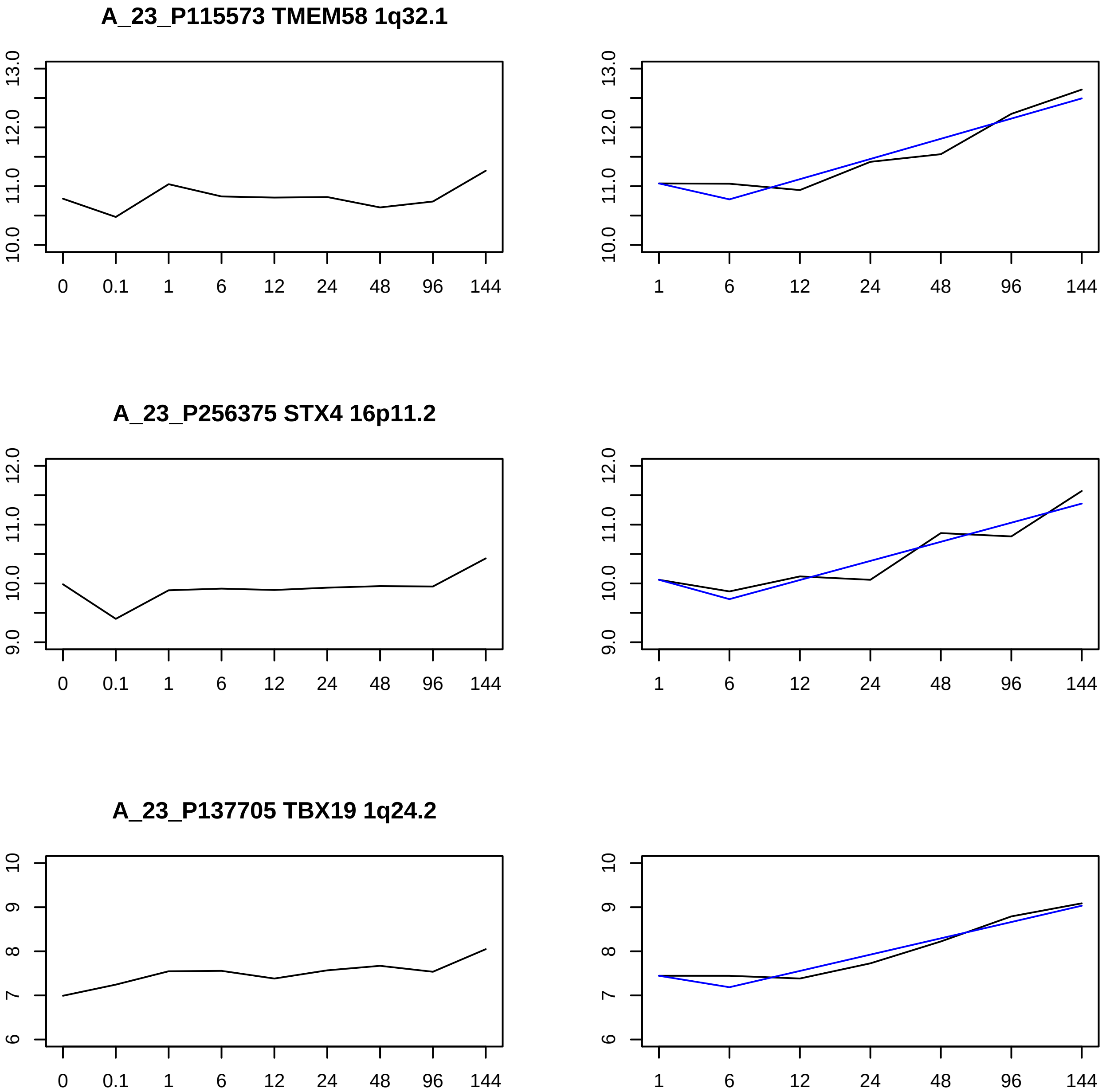
A_23_P115573 TMEM58 1q32.1
10.0
11.0
12.0
13.0
0
0.1
1
6
12
24
48
96
144
1
6
12
24
48
96
144
A_23_P256375 STX4 16p11.2
9.0
10.0
11.0
12.0
A_23_P137705 TBX19 1q24.2
6
7
8
9
10

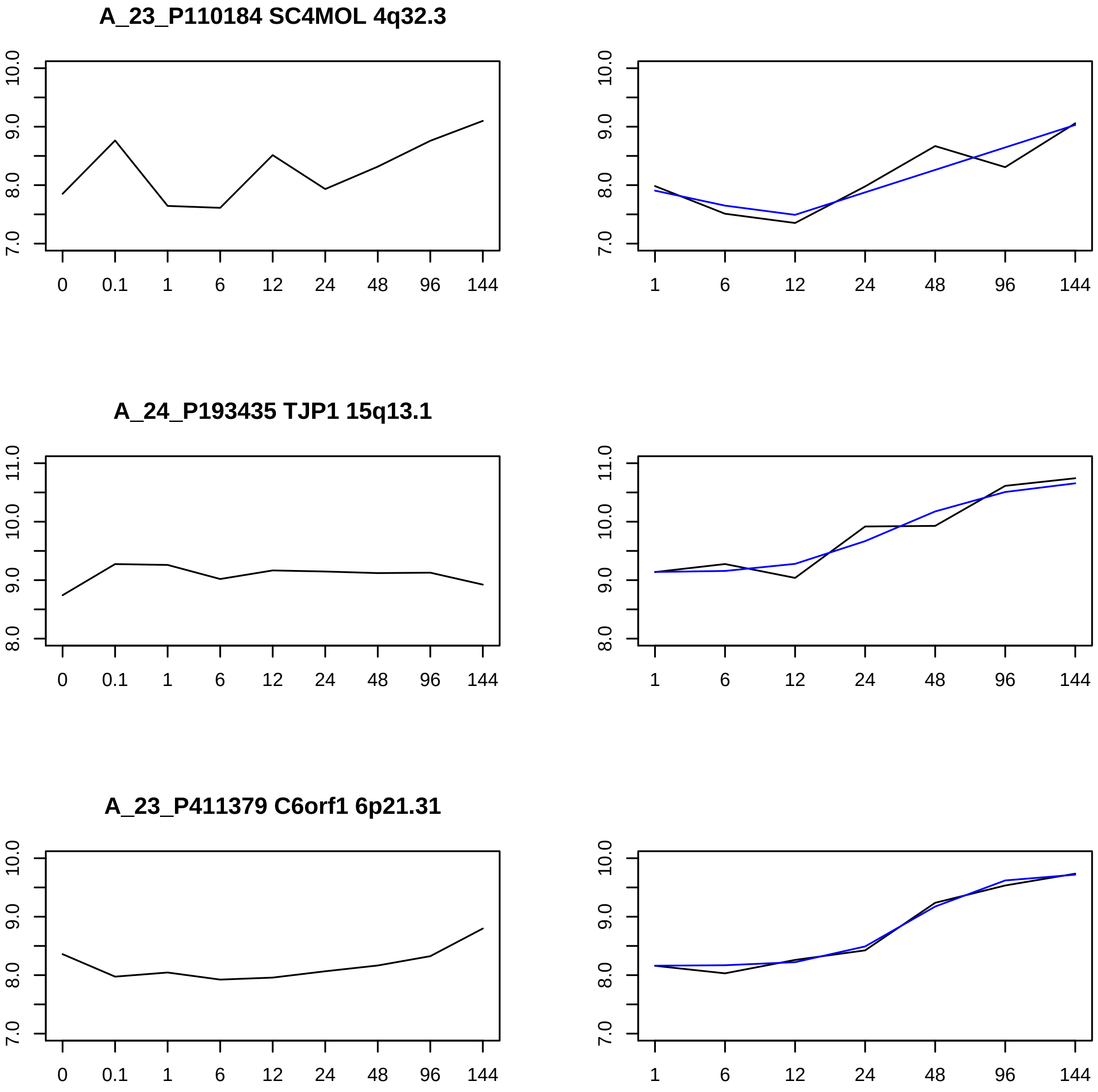
A_23_P110184 SC4MOL 4q32.3
A_24_P193435 TJP1 15q13.1
A_23_P411379 C6orf1 6p21.31
7.0 8.0 9.0 10.0
8.0 9.0 10.0 11.0
0 0.1 1 6 12 24 48 96 144
1 6 12 24 48 96 144

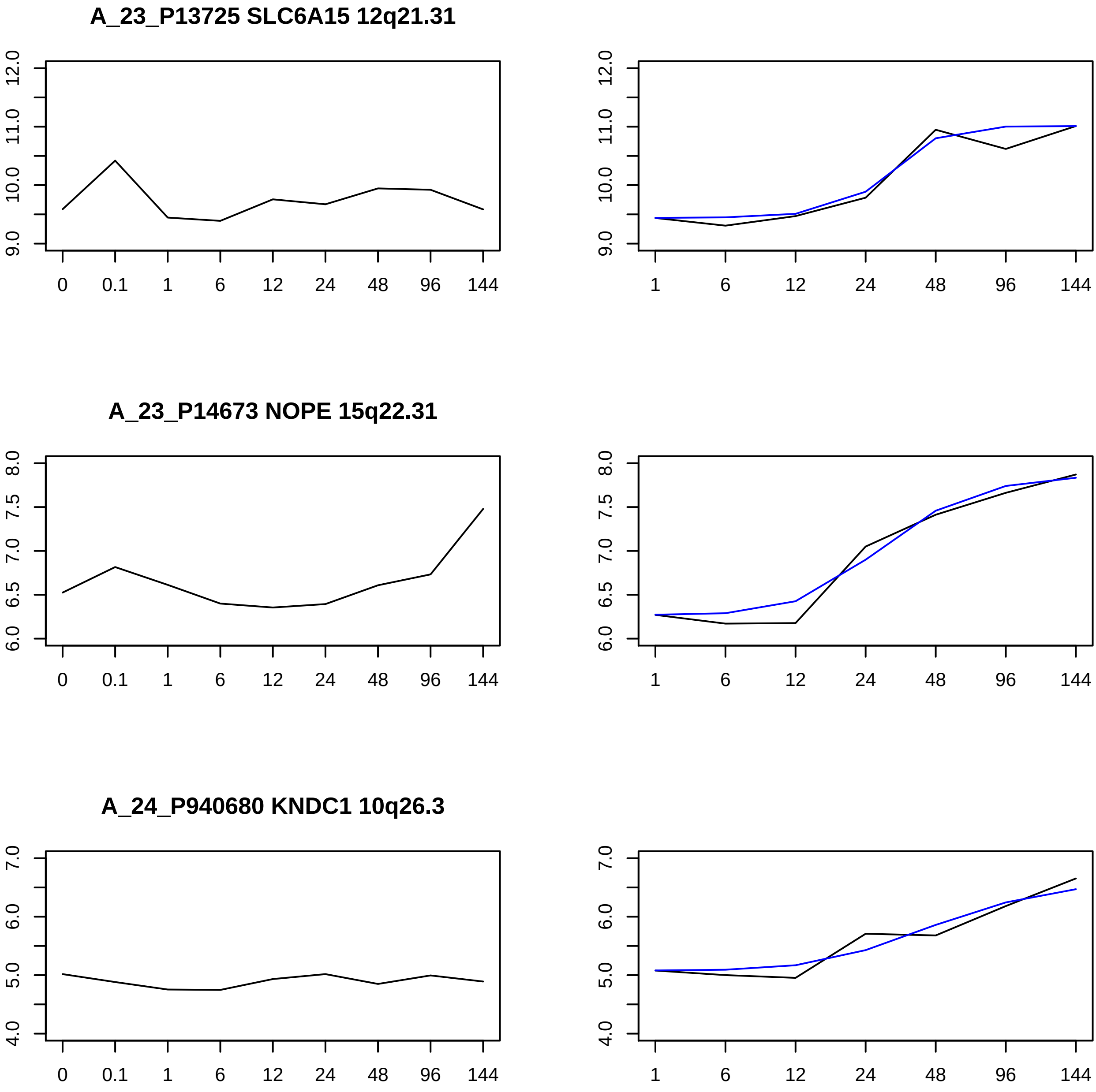
A_23_P13725 SLC6A15 12q21.31
9.0
10.0
11.0
12.0
0
0.1
1
6
12
24
48
96
144
1
6
12
24
48
96
144
A_23_P14673 NOPE 15q22.31
6.0
6.5
7.0
7.5
8.0
A_24_P940680 KNDC1 10q26.3
4.0
5.0
6.0
7.0

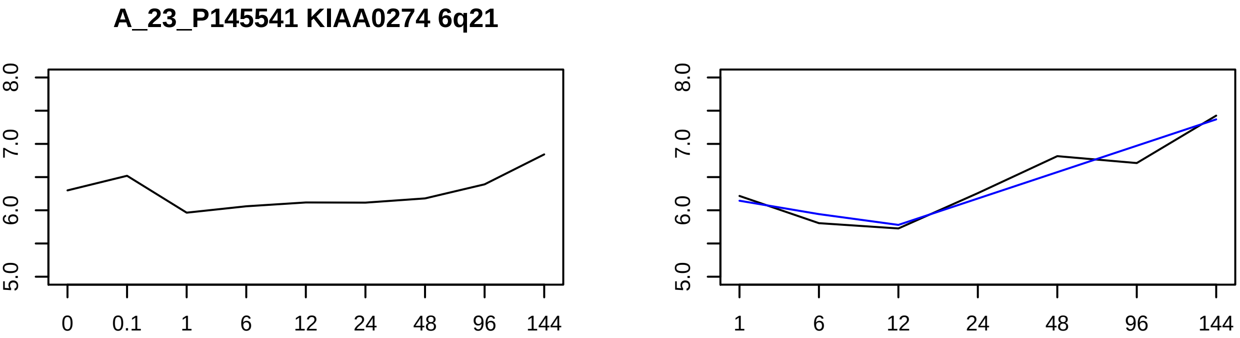
A_23_P145541 KIAA0274 6q21
5.0 6.0 7.0 8.0
0 0.1 1 6 12 24 48 96 144
1 6 12 24 48 96 144

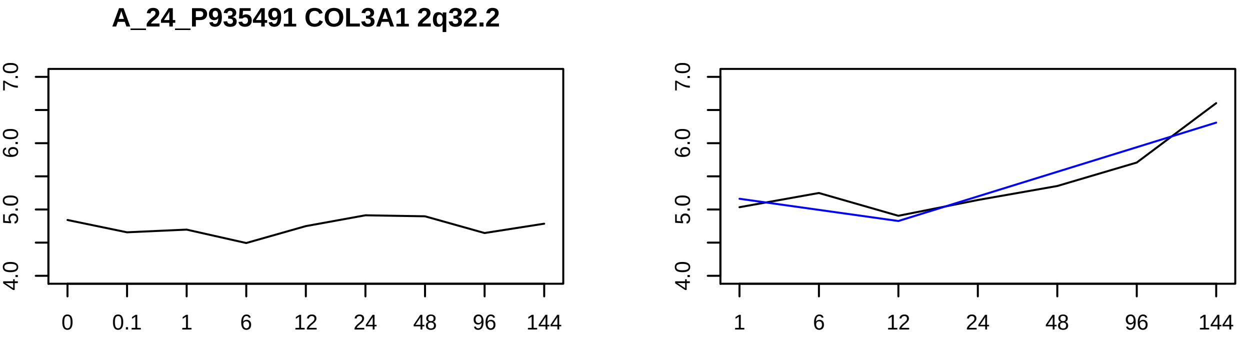
A_24_P935491 COL3A1 2q32.2
4.0 5.0 6.0 7.0
0 0.1 1 6 12 24 48 96 144
1 6 12 24 48 96 144

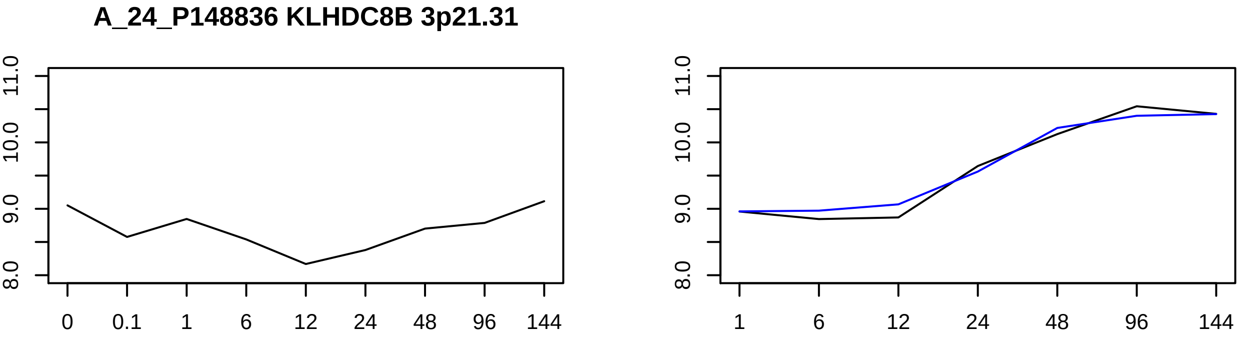
A_24_P148836 KLHDC8B 3p21.31
8.0 9.0 10.0 11.0
0 0.1 1 6 12 24 48 96 144
1 6 12 24 48 96 144

**A_23_P393620 TFPI2 7q21.3**

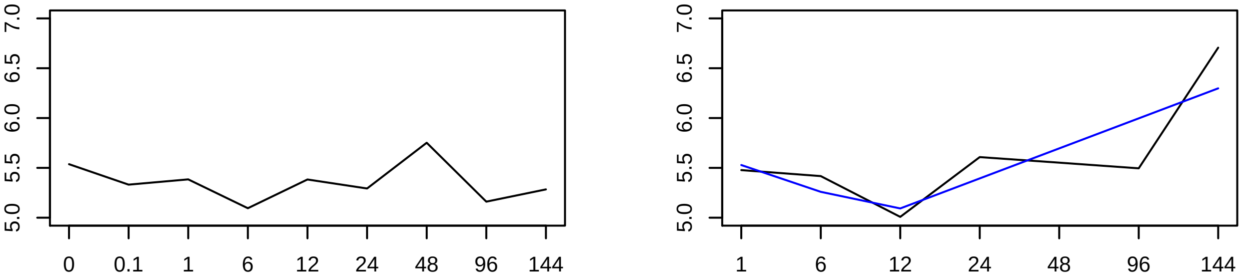


**A_32_P190181 THC2740317 NA**

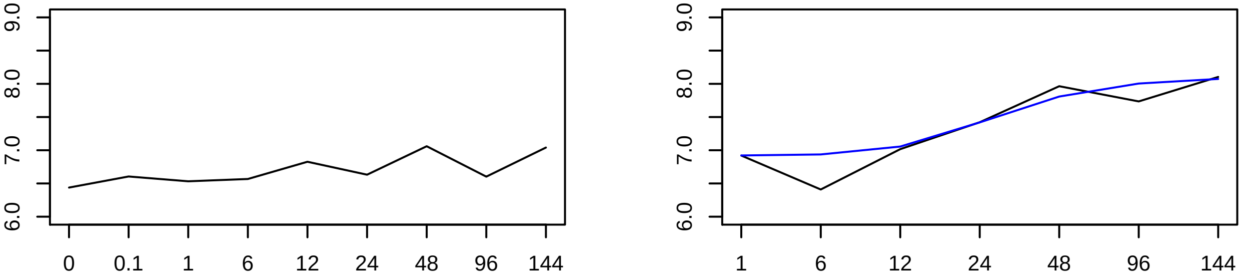


**A_23_P142075 ACP5 19p13.2**

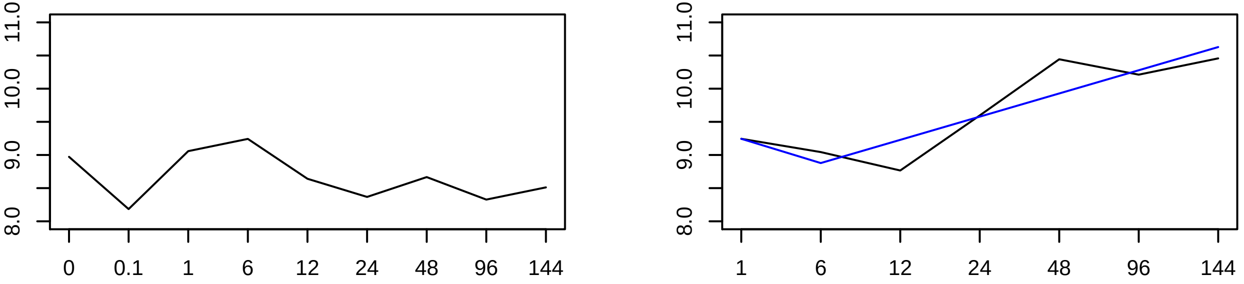

**A_23_P19369 LRRC16 6p22.2**

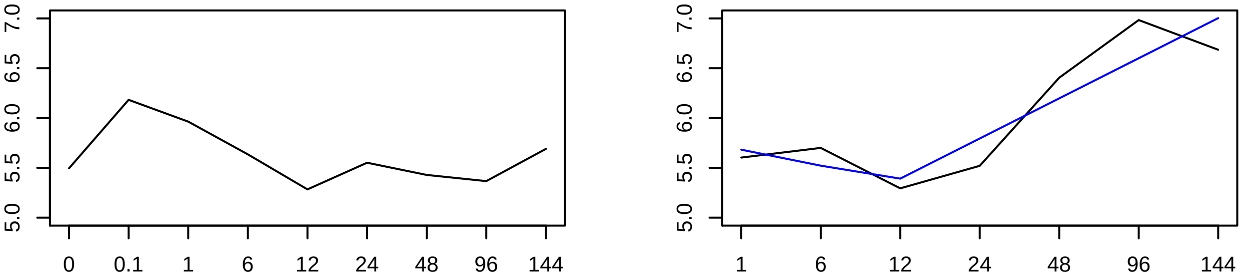


**A_23_P385322 STAMBPL1 10q23.31**

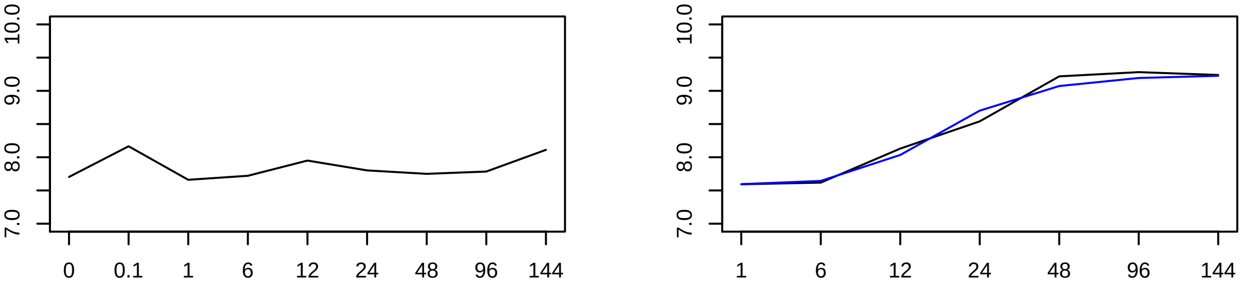


**A_23_P46045 RGS5 1q23.3**

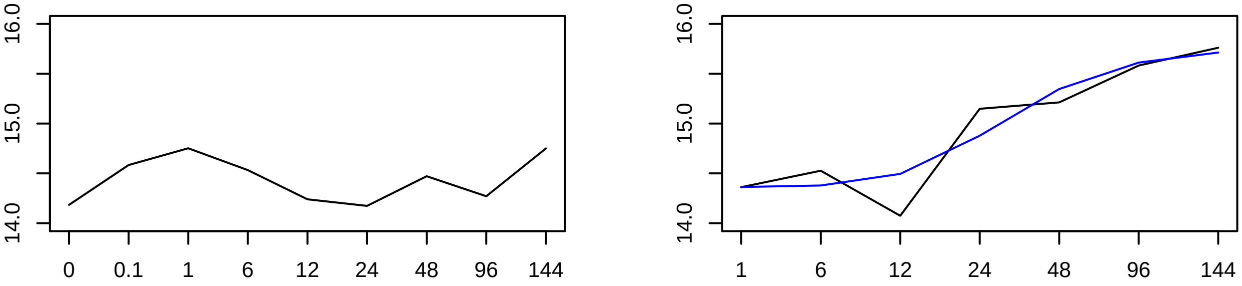

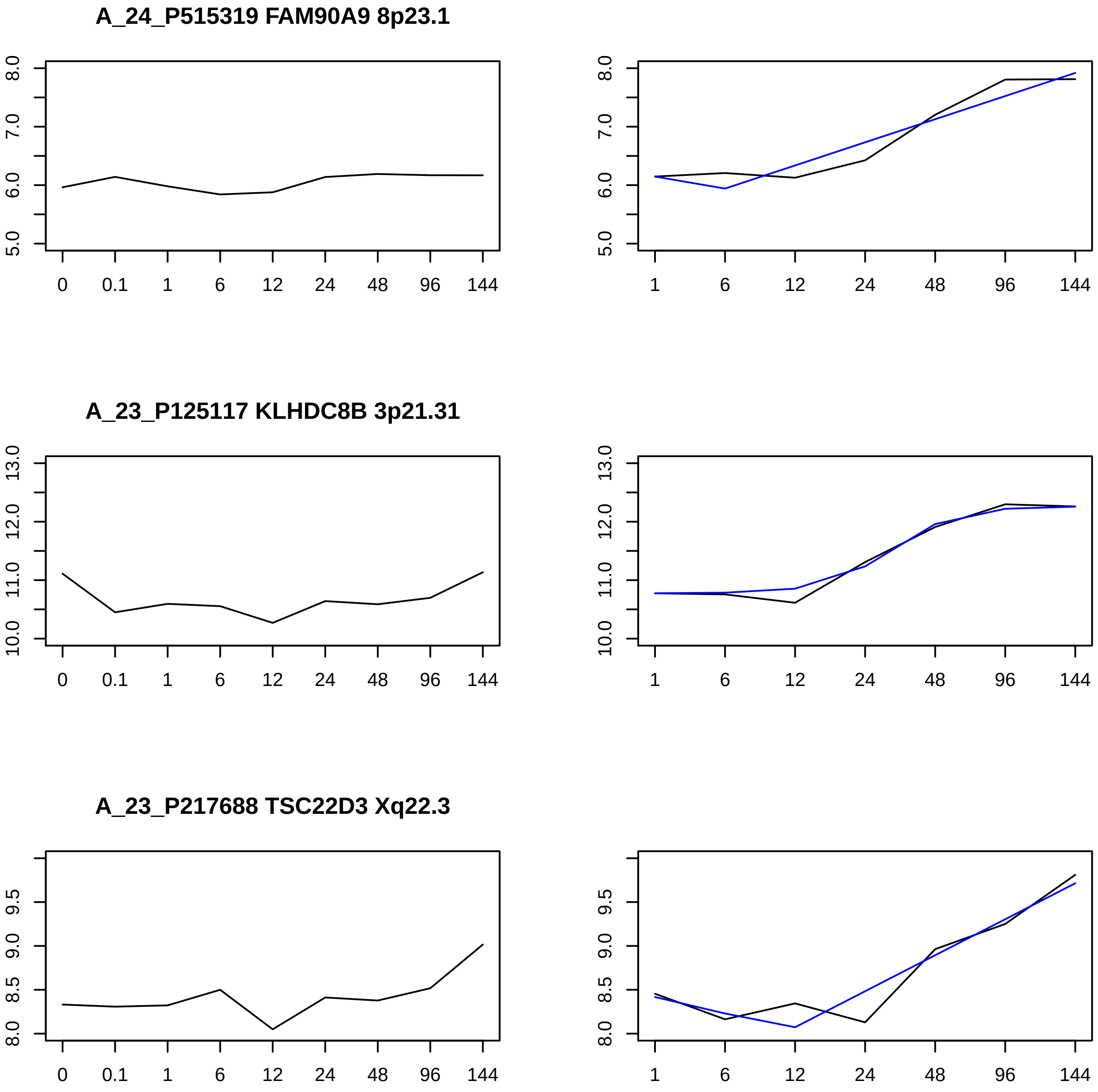
A_24_P515319 FAM90A9 8p23.1
5.0
6.0
7.0
8.0
0
0.1
1
6
12
24
48
96
144
A_23_P125117 KLHDC8B 3p21.31
10.0
11.0
12.0
13.0
A_23_P217688 TSC22D3 Xq22.3
8.0
8.5
9.0
9.5

**A_23_P61761 NRG3 NA**

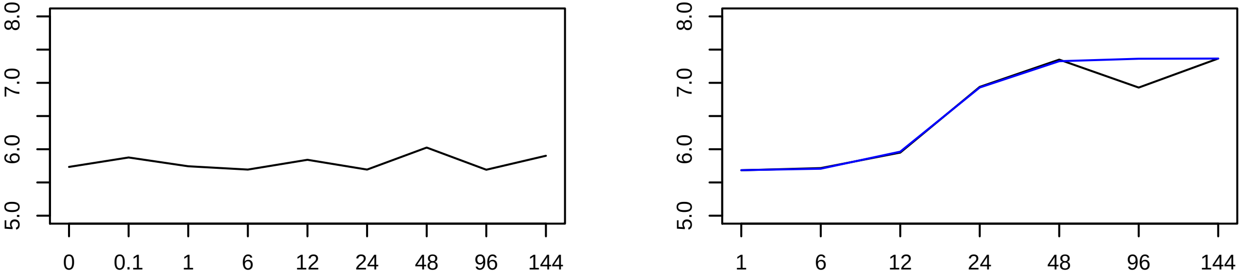


**A_23_P27724 SEPW1 19q13.32**

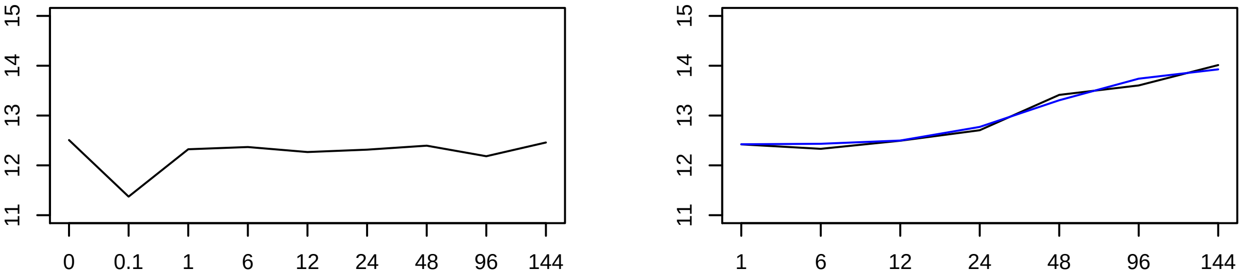


**A_23_P159382 SEC24D 4q26**

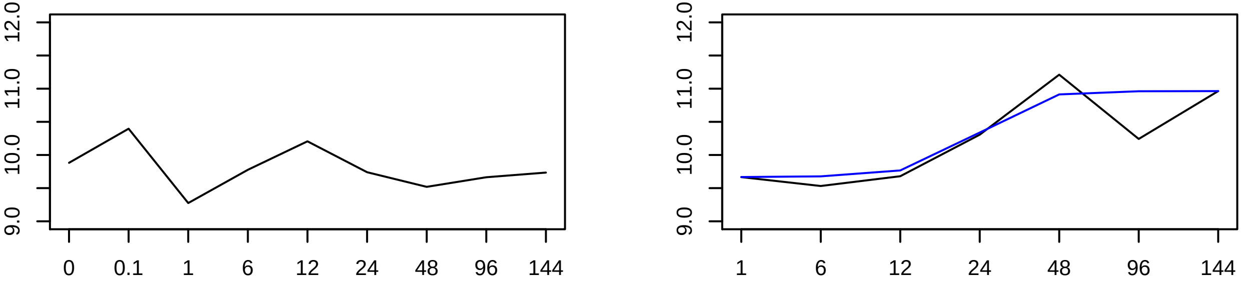

**A_23_P403335 EXPH5 11q22.3**

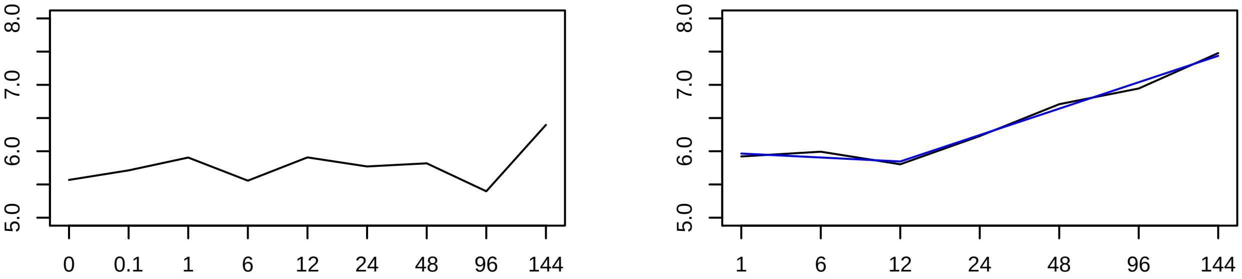


**A_32_P145502 BE710618 NA**

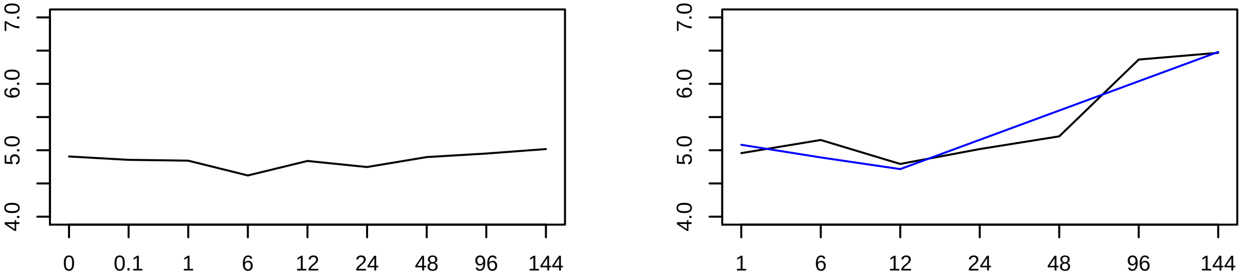


**A_23_P112241 DNAJB5 9p13.3**

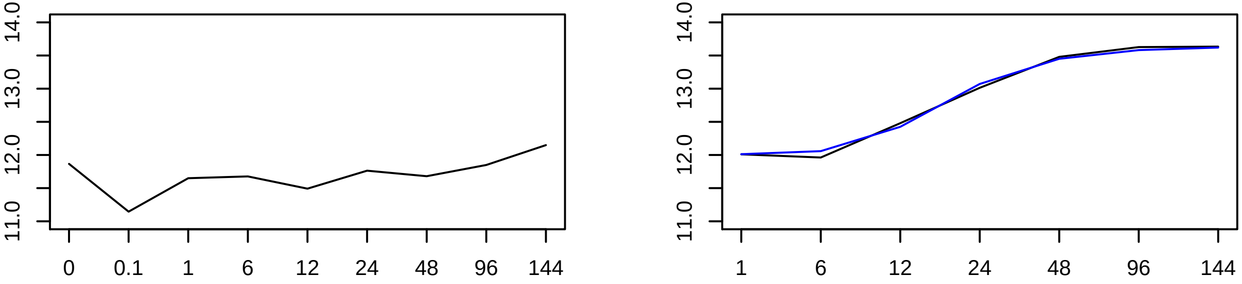

**A_24_P941572 FAM114A1 4p14**

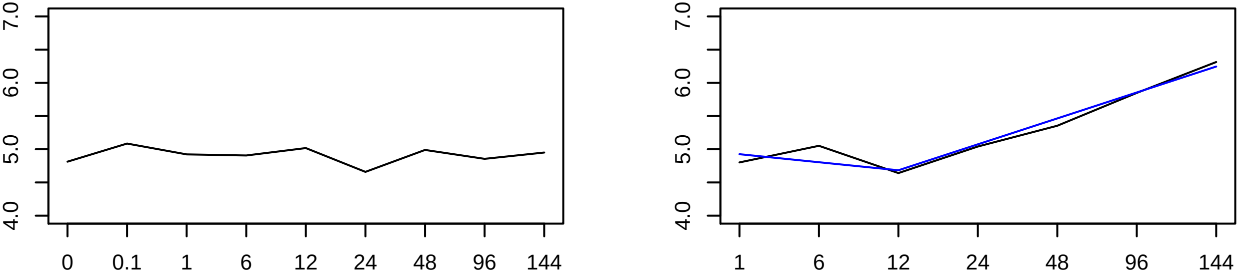


**A_32_P125128 CXADRP1**

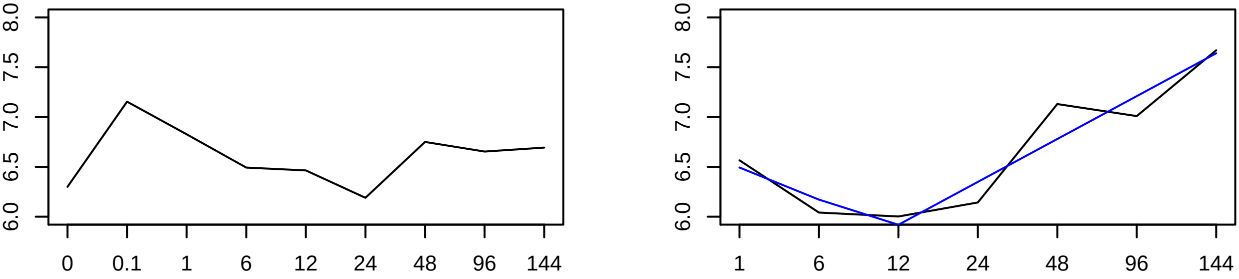


**A_32_P212483 CAPN6\DCX\PAK3 Xq23**

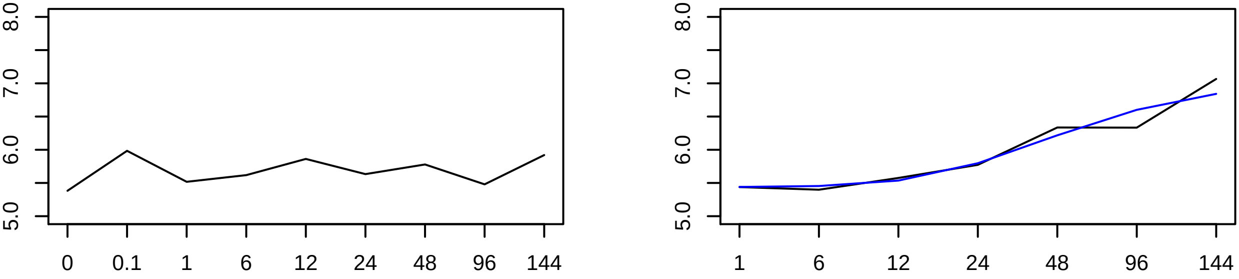

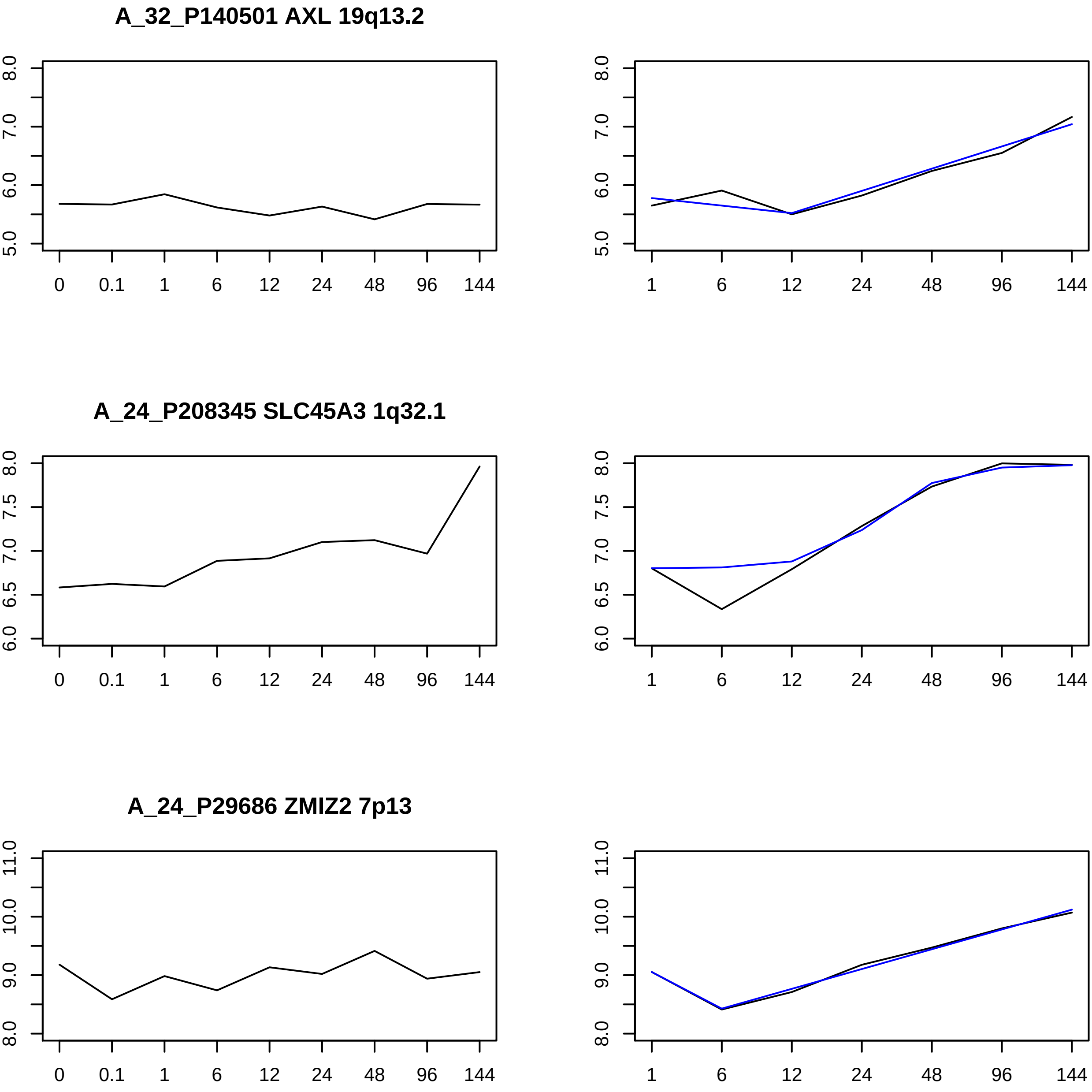
A_32_P140501 AXL 19q13.2
5.0 6.0 7.0 8.0
0 0.1 1 6 12 24 48 96 144
1 6 12 24 48 96 144
A_24_P208345 SLC45A3 1q32.1
6.0 6.5 7.0 7.5 8.0
0 0.1 1 6 12 24 48 96 144
1 6 12 24 48 96 144
A_24_P29686 ZMIZ2 7p13
8.0 9.0 10.0 11.0
0 0.1 1 6 12 24 48 96 144
1 6 12 24 48 96 144

**A_23_P104762 YAP1 11q22.2**

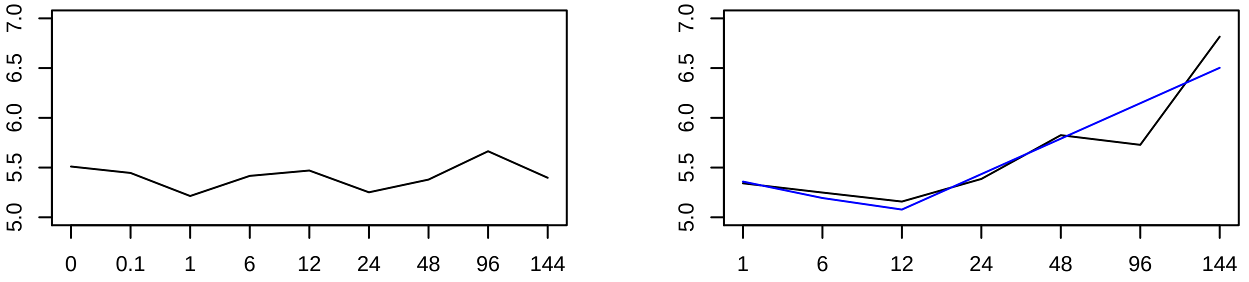


**A_23_P50338 TUBB4 19p13.3**

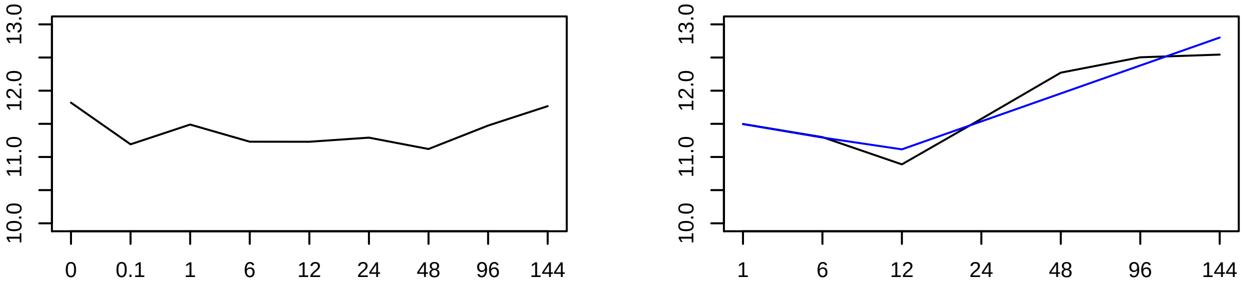


**A_23_P80377 PACSIN2 22q13.2**

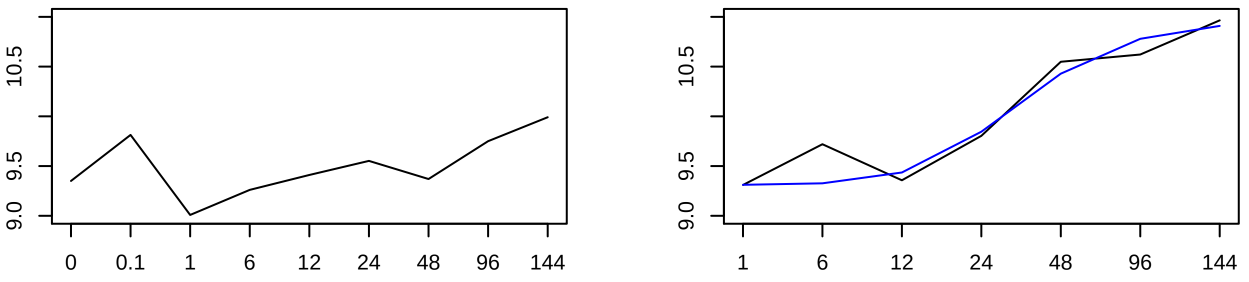

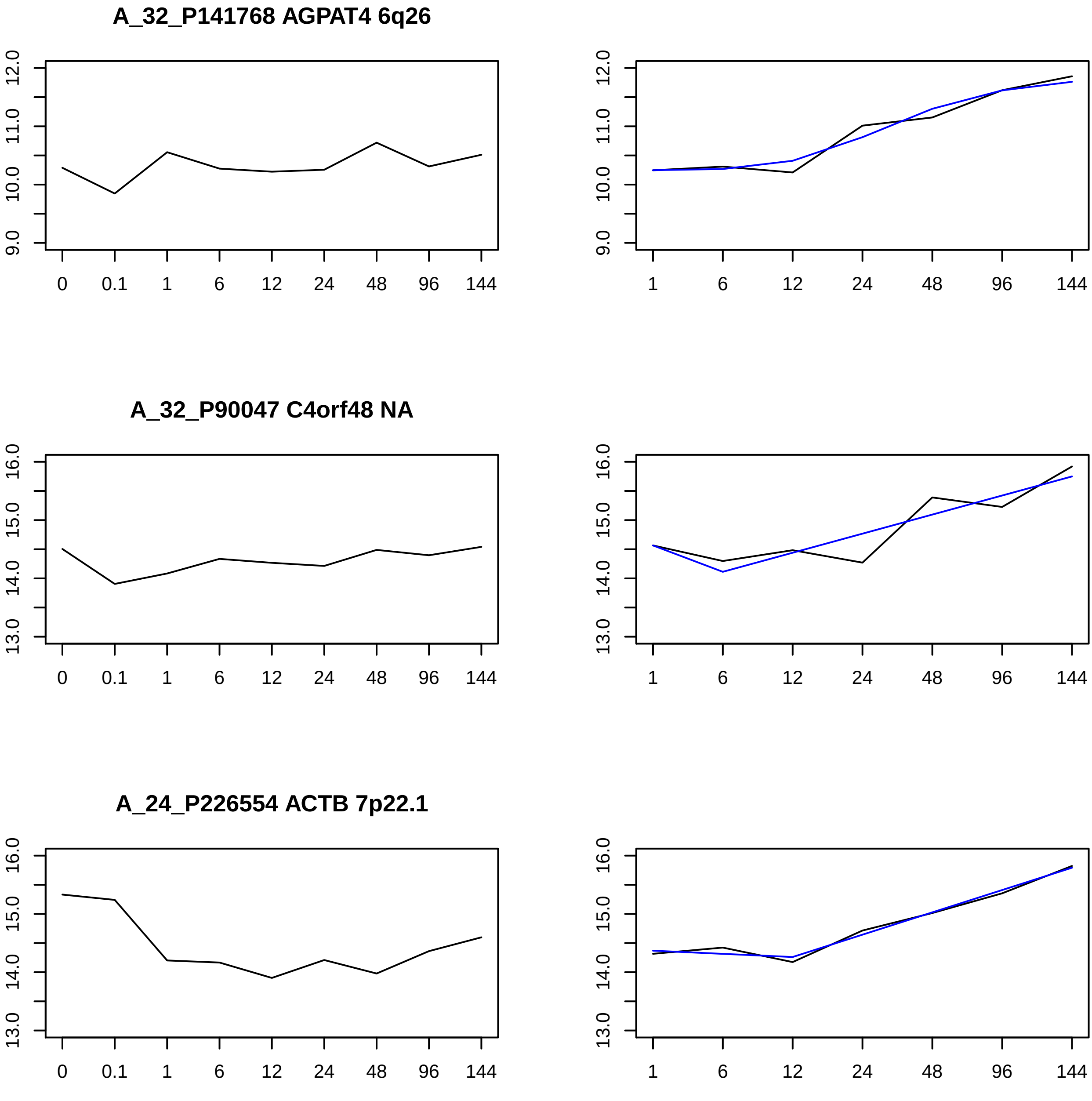

A_32_P141768 AGPAT4 6q26
9.0
10.0
11.0
12.0
0
0.1
1
6
12
24
48
96
144
A_32_P90047 C4orf48 NA
13.0
14.0
15.0
16.0
A_24_P226554 ACTB 7p22.1

**A_23_P348063 SYNGR1 22q13.1**

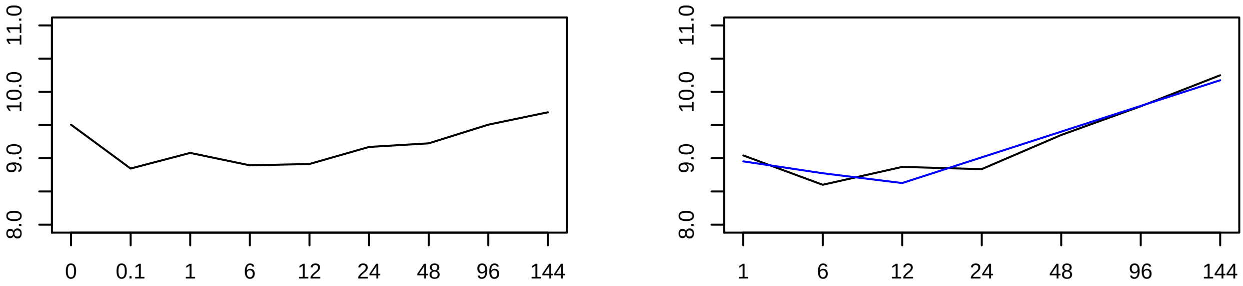


**A_23_P120227 LBH 2p23.1**

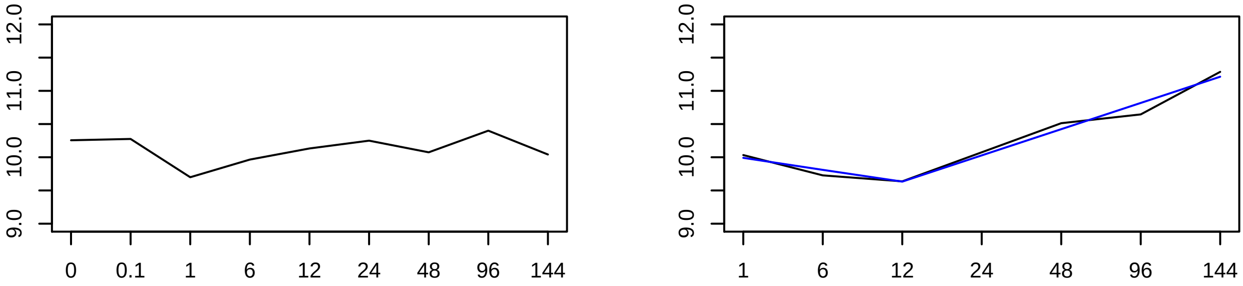


**A_24_P334726 DENND2A 7q34**

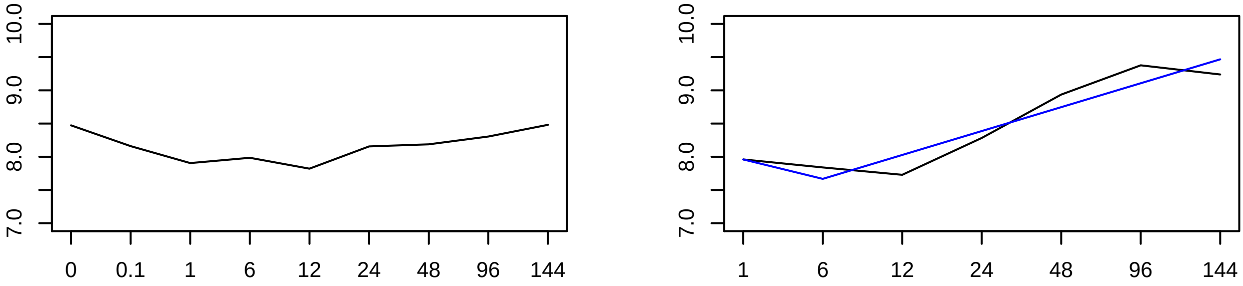

**A_32_P72553 A_32_P72553 NA**

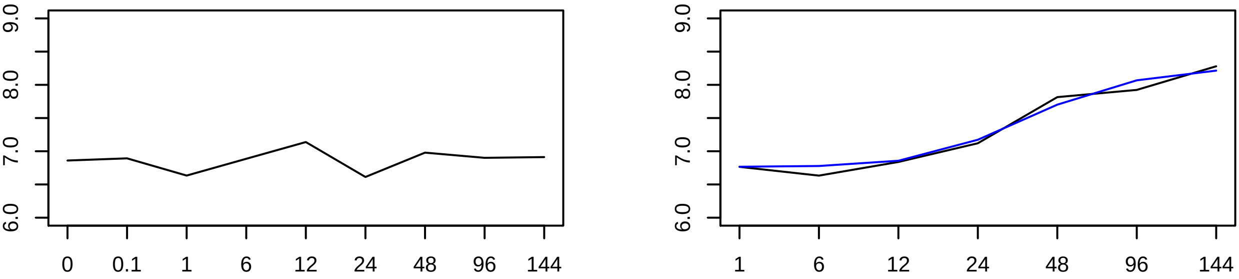


**A_24_P94222 FBLIM1 1p36.21**

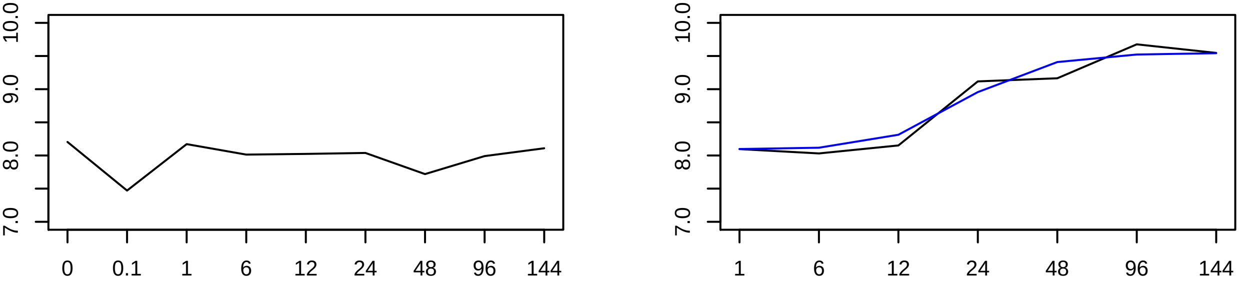


**A_32_P38145 PACSIN2 22q13.2**

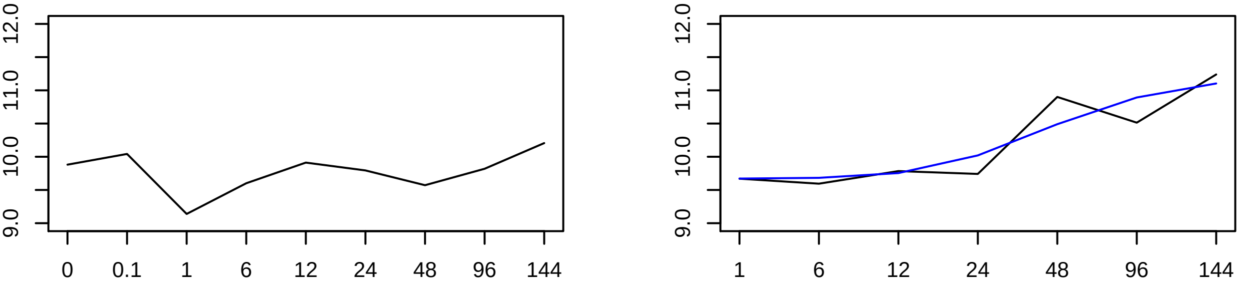

**A_32_P147001 THC2655314 NA**

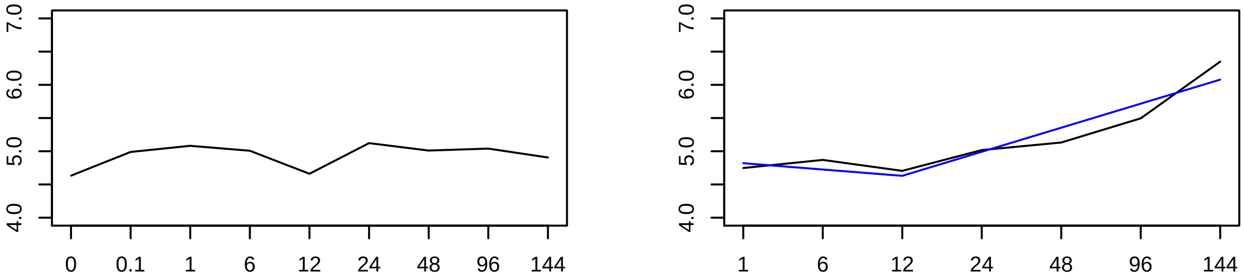


**A_32_P147514 RP11−412P11.1 4p15.2**

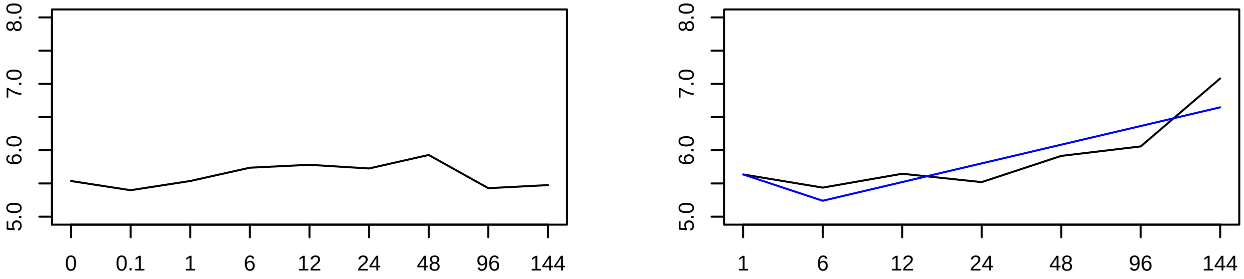


**A_32_P185766 BX103476 NA**

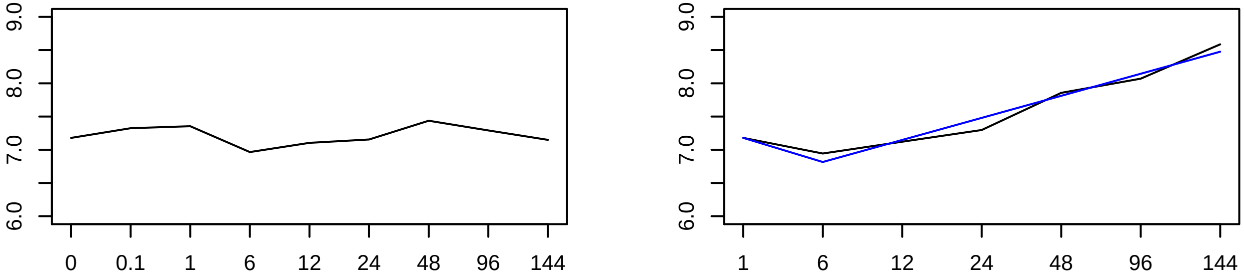

A_23_P37685 TMEM204 16p13.3

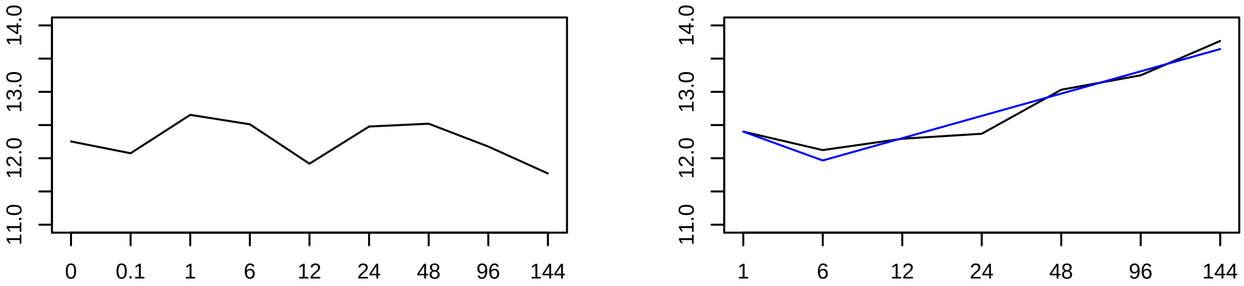


A_23_P410859 ZSWIM6 5q12.1

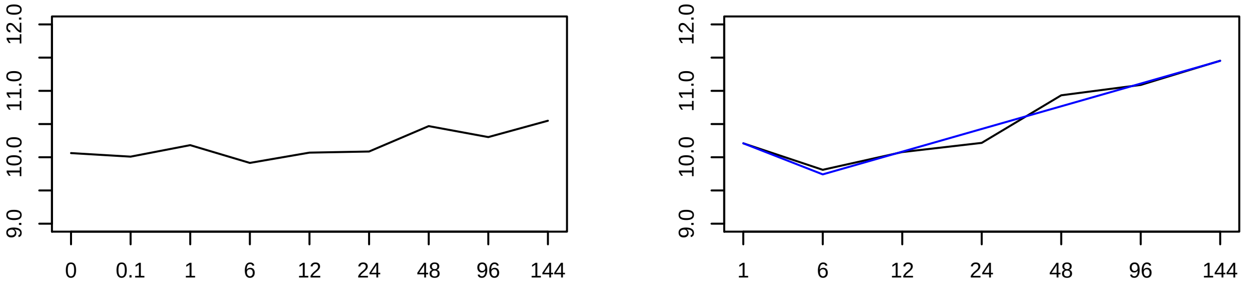


A_24_P340866 A_24_P340866 NA

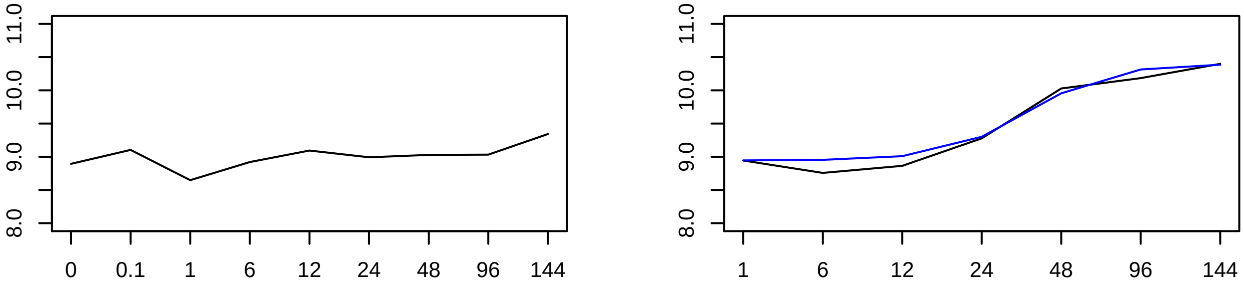

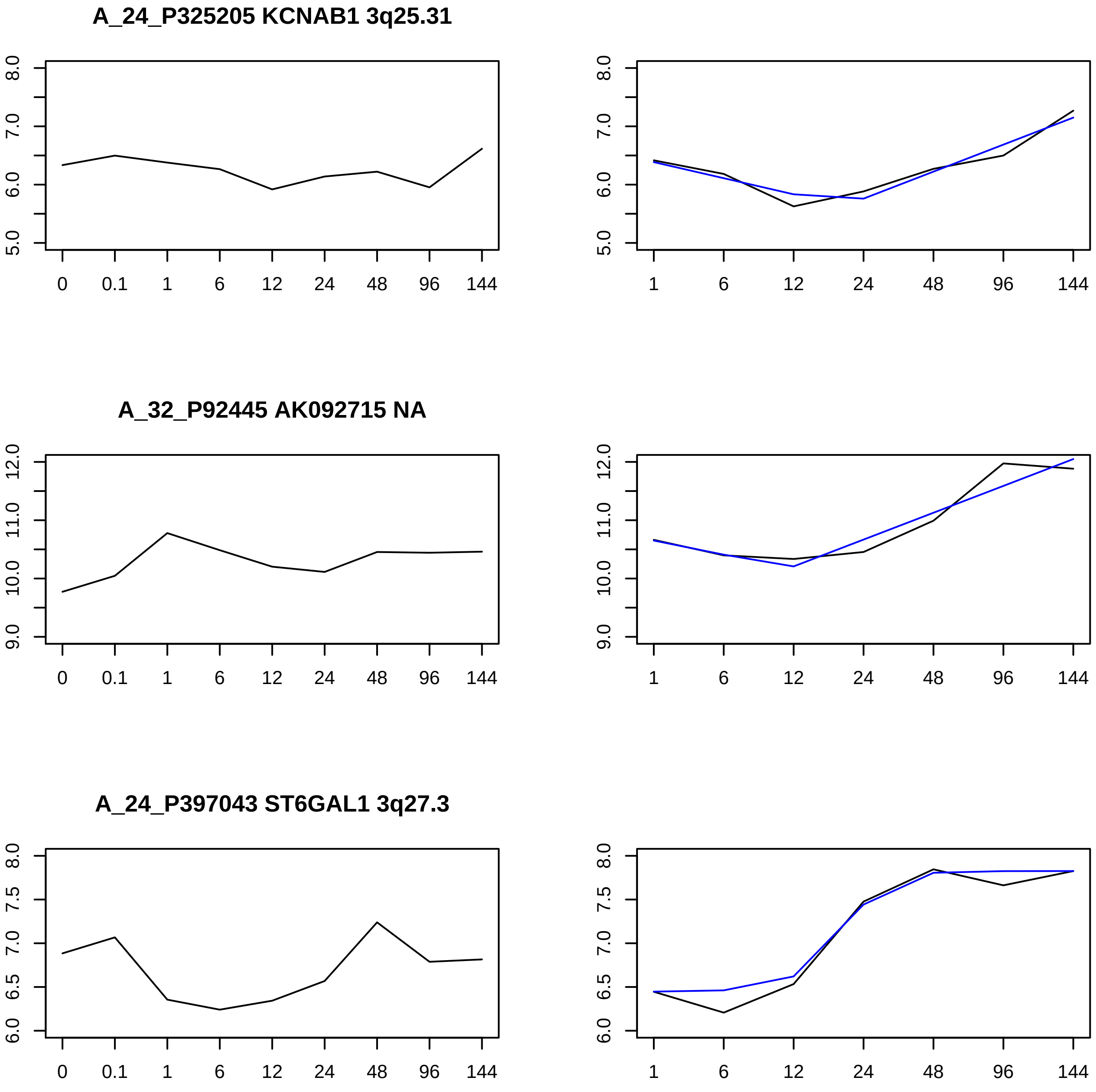
A_24_P325205 KCNAB1 3q25.31
A_32_P92445 AK092715 NA
A_24_P397043 ST6GAL1 3q27.3
5.0 6.0 7.0 8.0
9.0 10.0 11.0 12.0
6.0 6.5 7.0 7.5 8.0
0 0.1 1 6 12 24 48 96 144
1 6 12 24 48 96 144

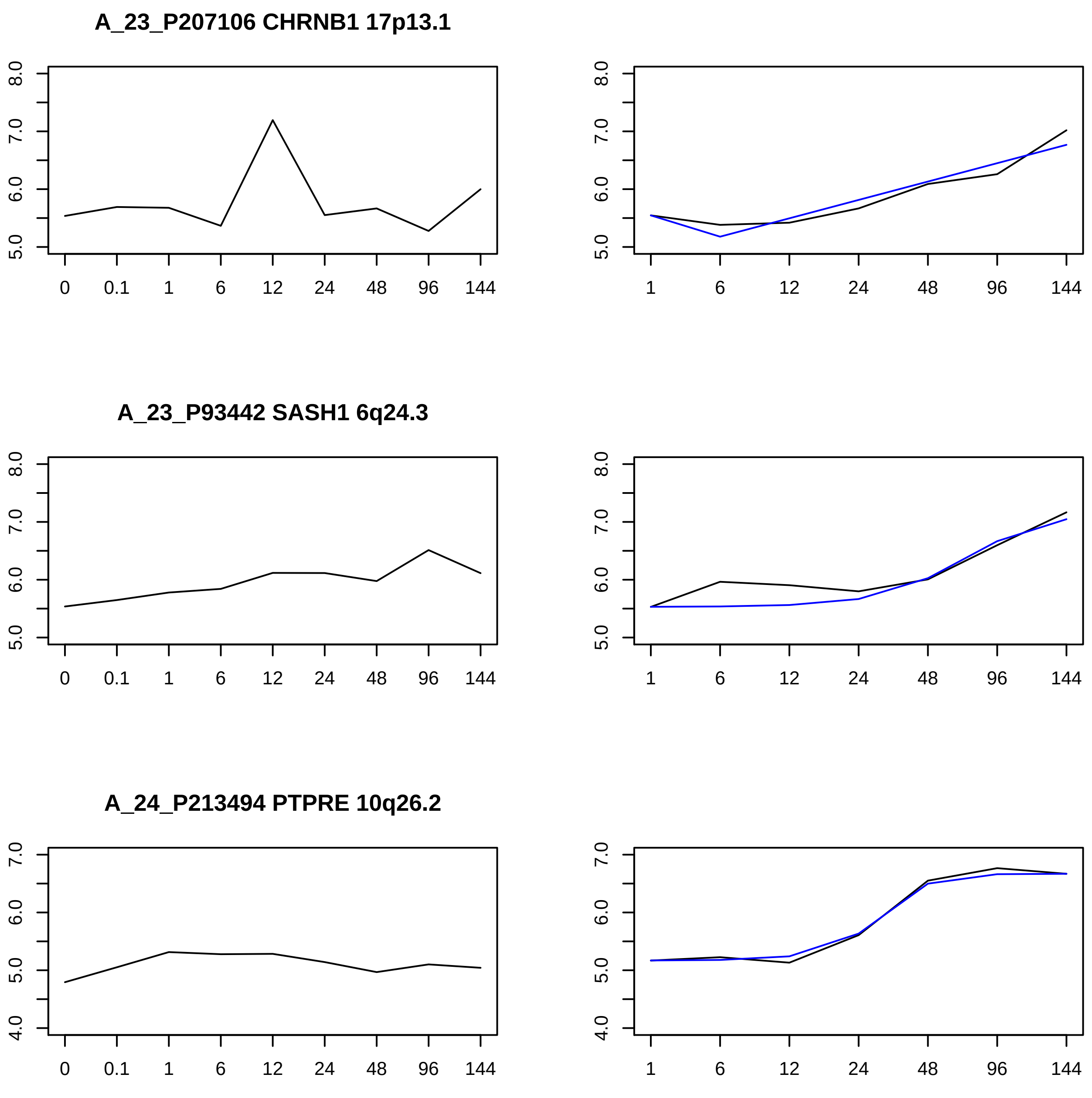
A_23_P207106 CHRNB1 17p13.1
A_23_P93442 SASH1 6q24.3
A_24_P213494 PTPRE 10q26.2
4.0
5.0
6.0
7.0
8.0
0
0.1
1
6
12
24
48
96
144

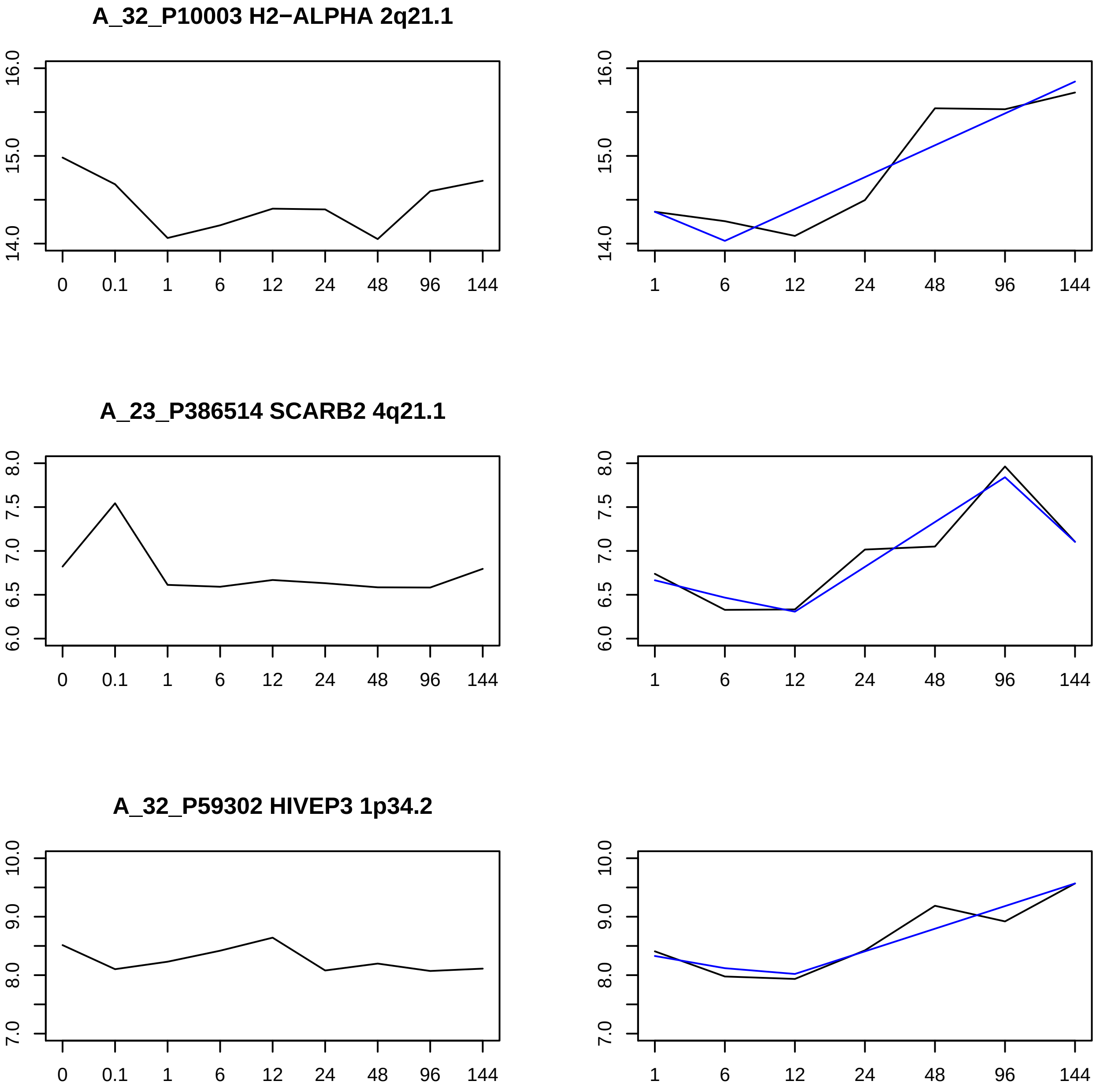
A_32_P10003 H2−ALPHA 2q21.1
14.0
15.0
16.0
0
0.1
1
6
12
24
48
96
144
A_23_P386514 SCARB2 4q21.1
6.0
6.5
7.0
7.5
8.0
A_32_P59302 HIVEP3 1p34.2
7.0
8.0
9.0
10.0

A_23_P111267 SH3BGRL2 6q14.1

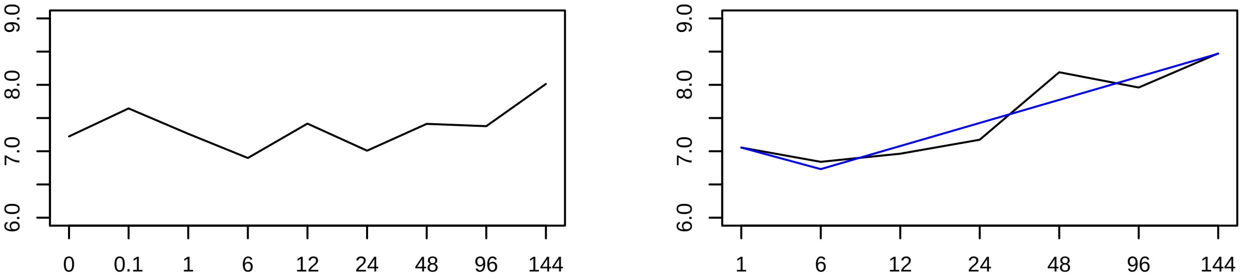


A_24_P58565 AK057935 NA

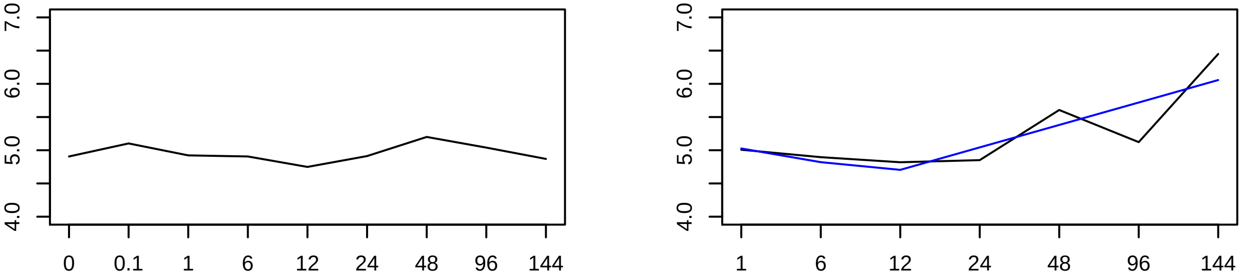


A_23_P259506 C5orf32 5q31.3

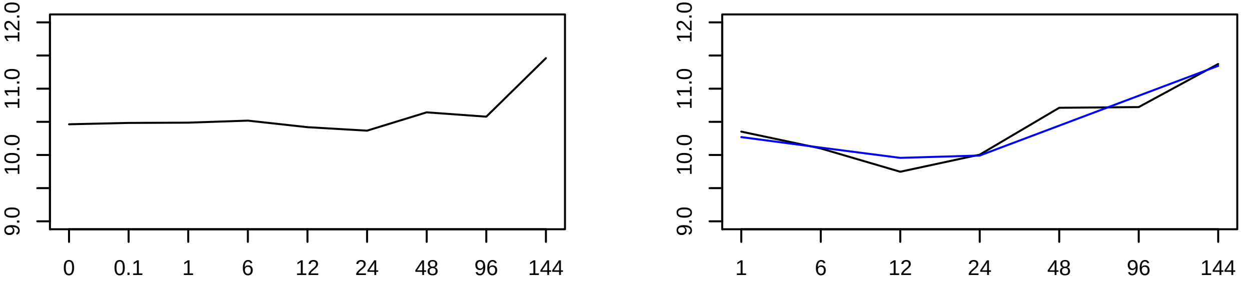

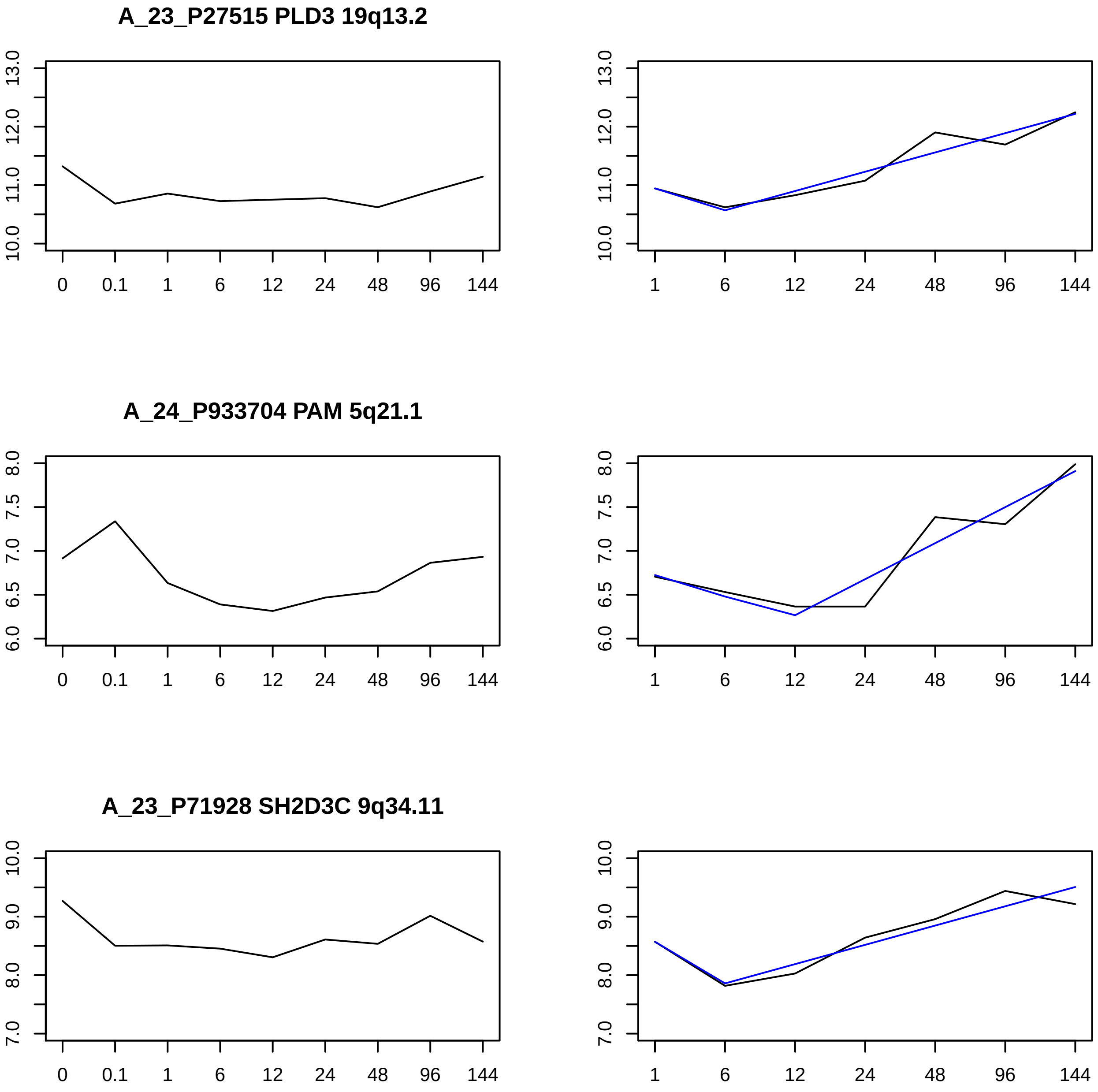
A_23_P27515 PLD3 19q13.2
10.0
11.0
12.0
13.0
0
0.1
1
6
12
24
48
96
144
A_24_P933704 PAM 5q21.1
6.0
6.5
7.0
7.5
8.0
A_23_P71928 SH2D3C 9q34.11
7.0
8.0
9.0
10.0

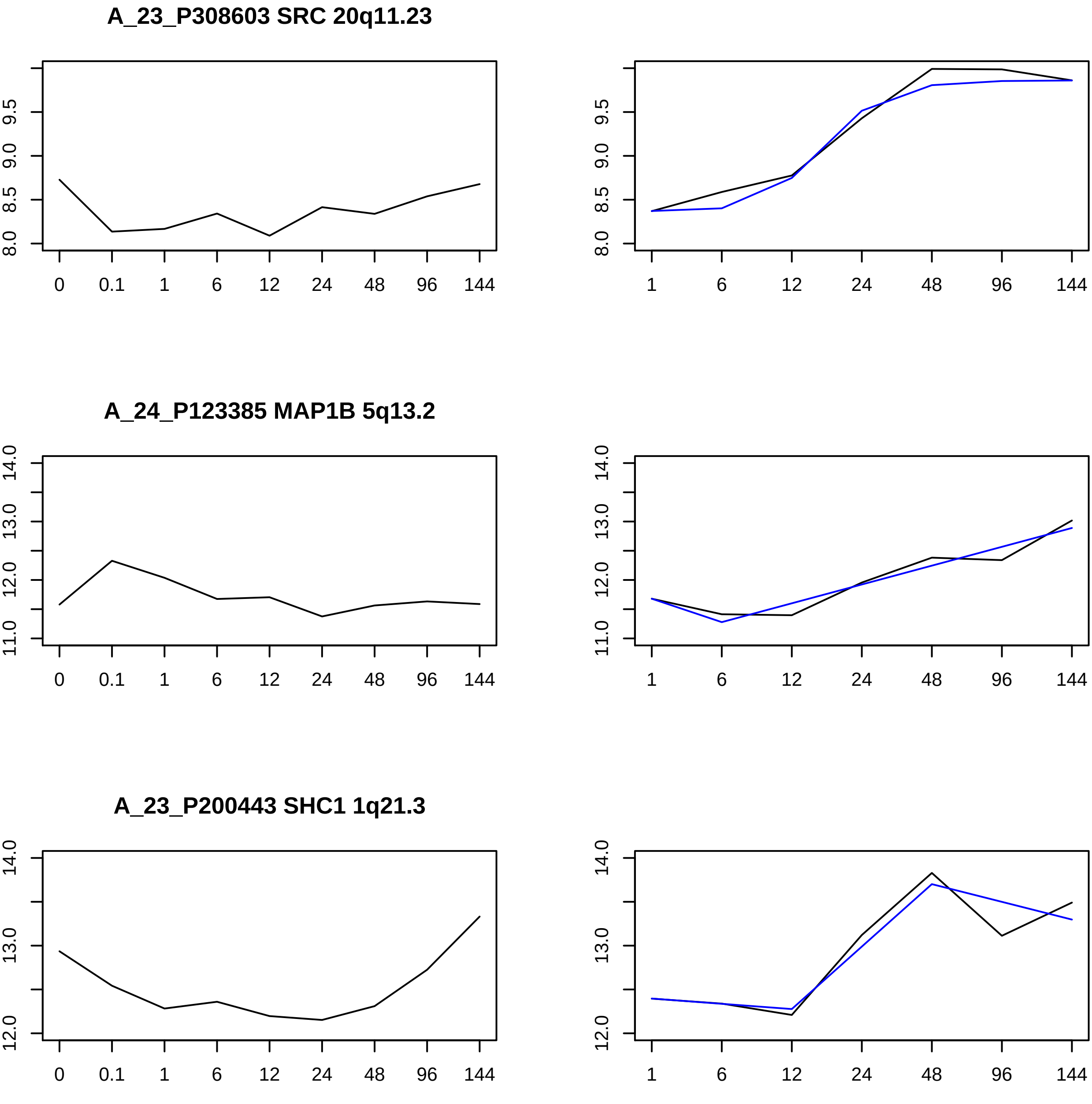
A_23_P308603 SRC 20q11.23
8.0
8.5
9.0
9.5
0 0.1 1 6 12 24 48 96 144
1 6 12 24 48 96 144
A_24_P123385 MAP1B 5q13.2
11.0
12.0
13.0
14.0
0 0.1 1 6 12 24 48 96 144
1 6 12 24 48 96 144
A_23_P200443 SHC1 1q21.3
12.0
13.0
14.0
0 0.1 1 6 12 24 48 96 144
1 6 12 24 48 96 144

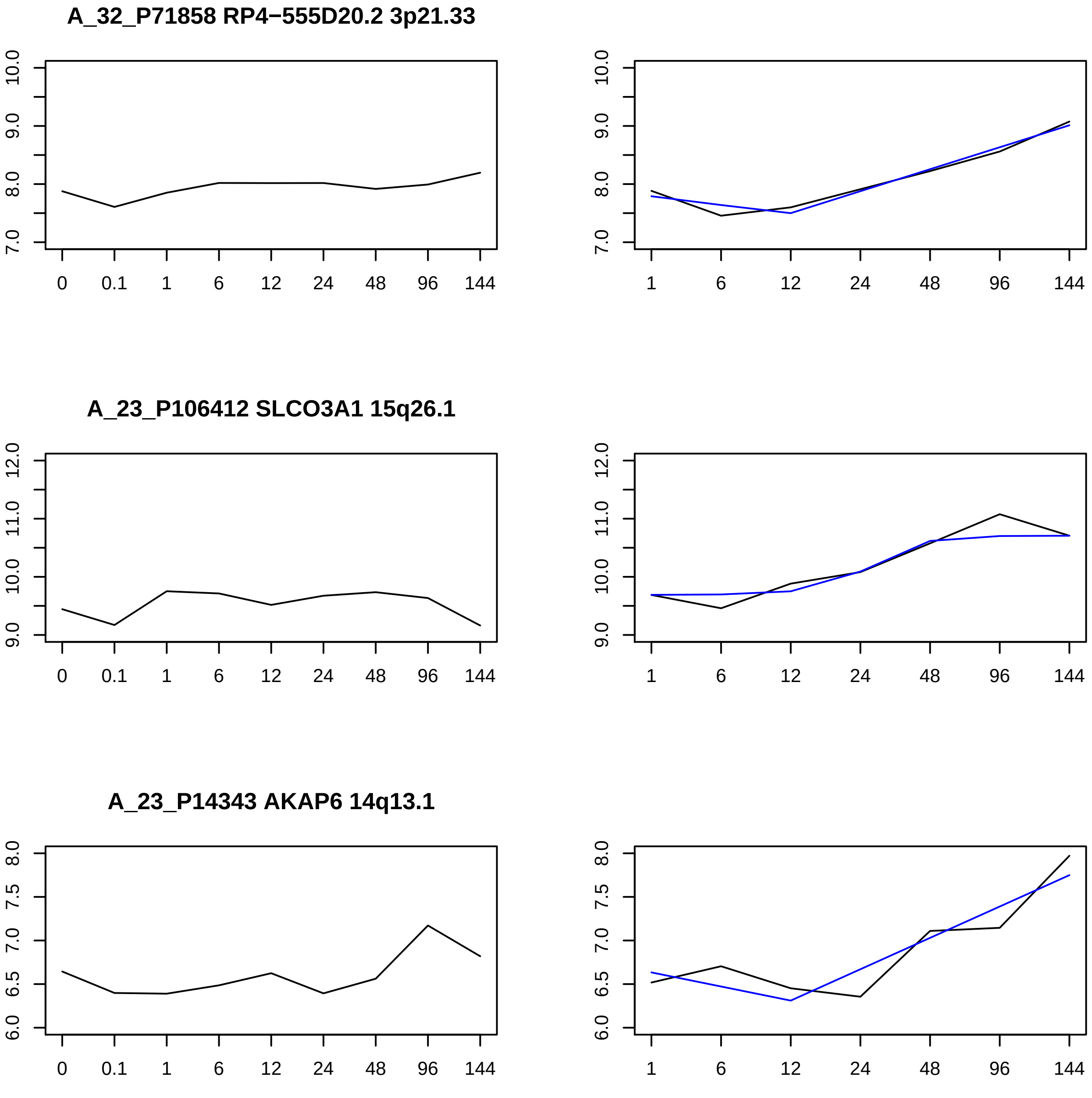

A_32_P71858 RP4−555D20.2 3p21.33
7.0
8.0
9.0
10.0
0
0.1
1
6
12
24
48
96
144
A_23_P106412 SLCO3A1 15q26.1
9.0
10.0
11.0
12.0
A_23_P14343 AKAP6 14q13.1
6.0
6.5
7.0
7.5
8.0

**A_23_P17955 FBXL2 3p22.3**

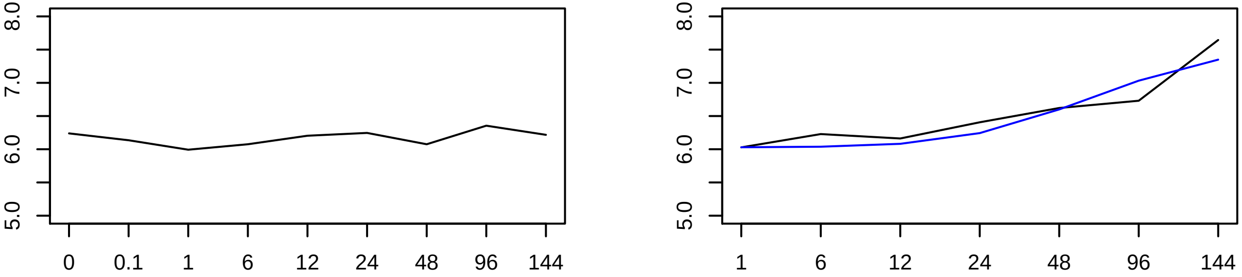


**A_24_P406870 ILK 11p15.4**

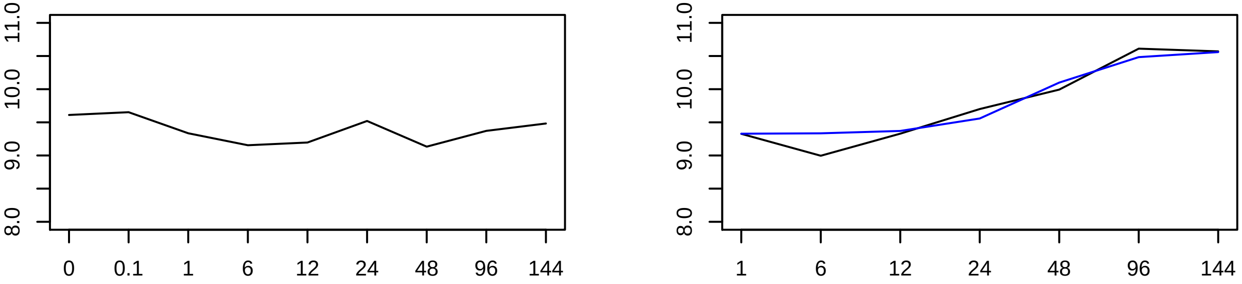


**A_23_P400847 IDS Xq28**

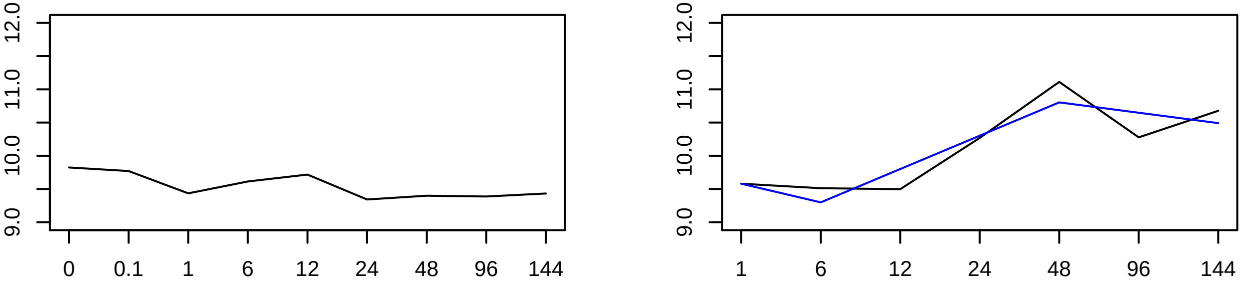

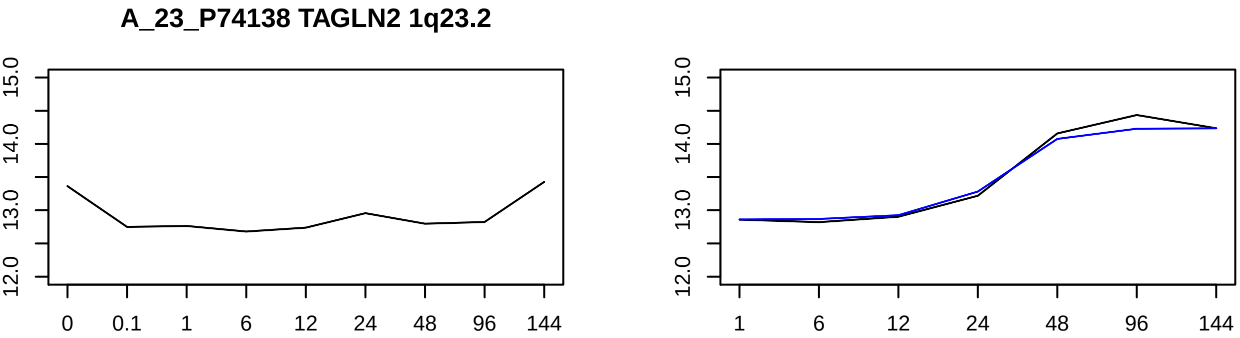
A_23_P74138 TAGLN2 1q23.2
12.0
13.0
14.0
15.0
0
0.1
1
6
12
24
48
96
144

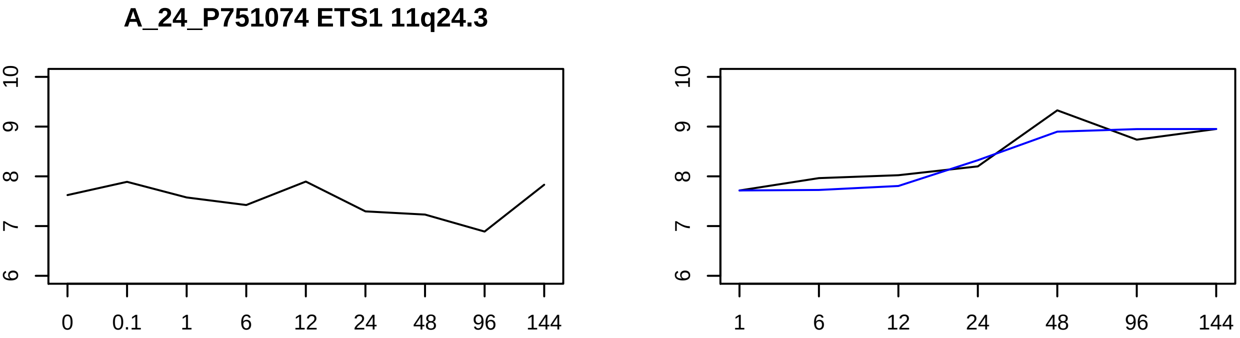
A_24_P751074 ETS1 11q24.3
6
7
8
9
10
0
0.1
1
6
12
24
48
96
144

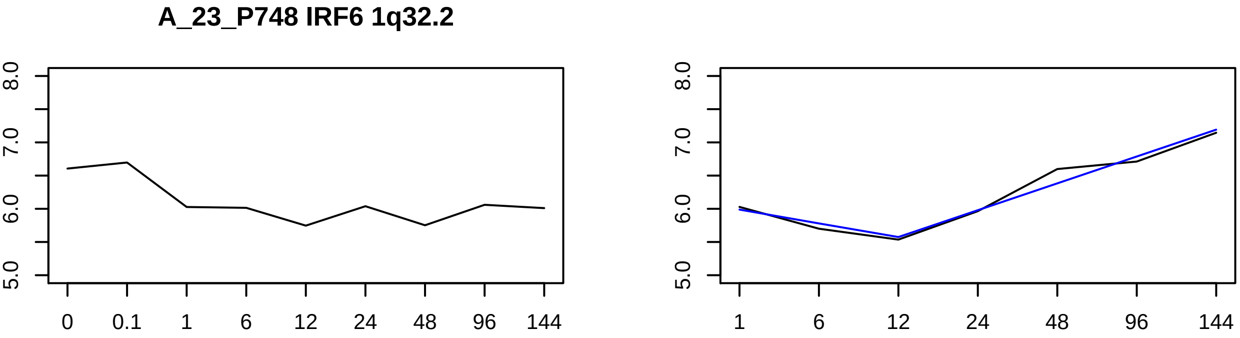
A_23_P748 IRF6 1q32.2
5.0
6.0
7.0
8.0
0
0.1
1
6
12
24
48
96
144

**A_23_P31143 TPD52L1 6q22.31**

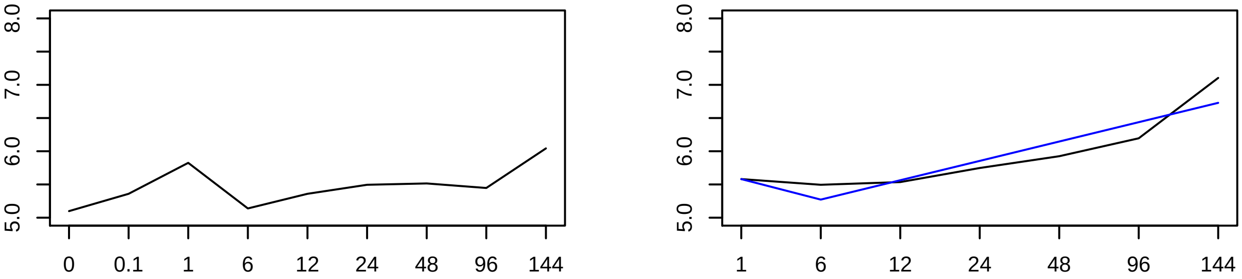


**A_23_P422071 B3GALT4 6p21.32**

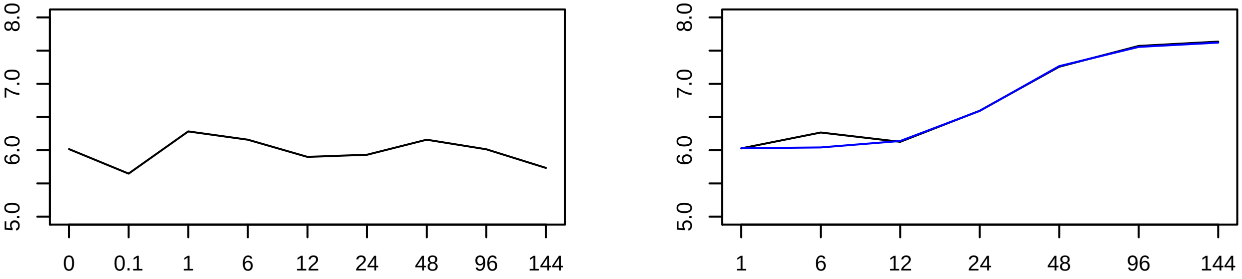


**A_23_P358714 KIAA1324 1p13.3**

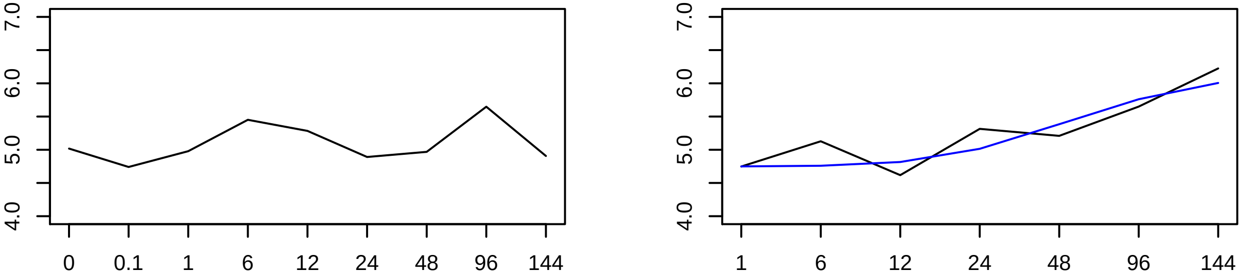

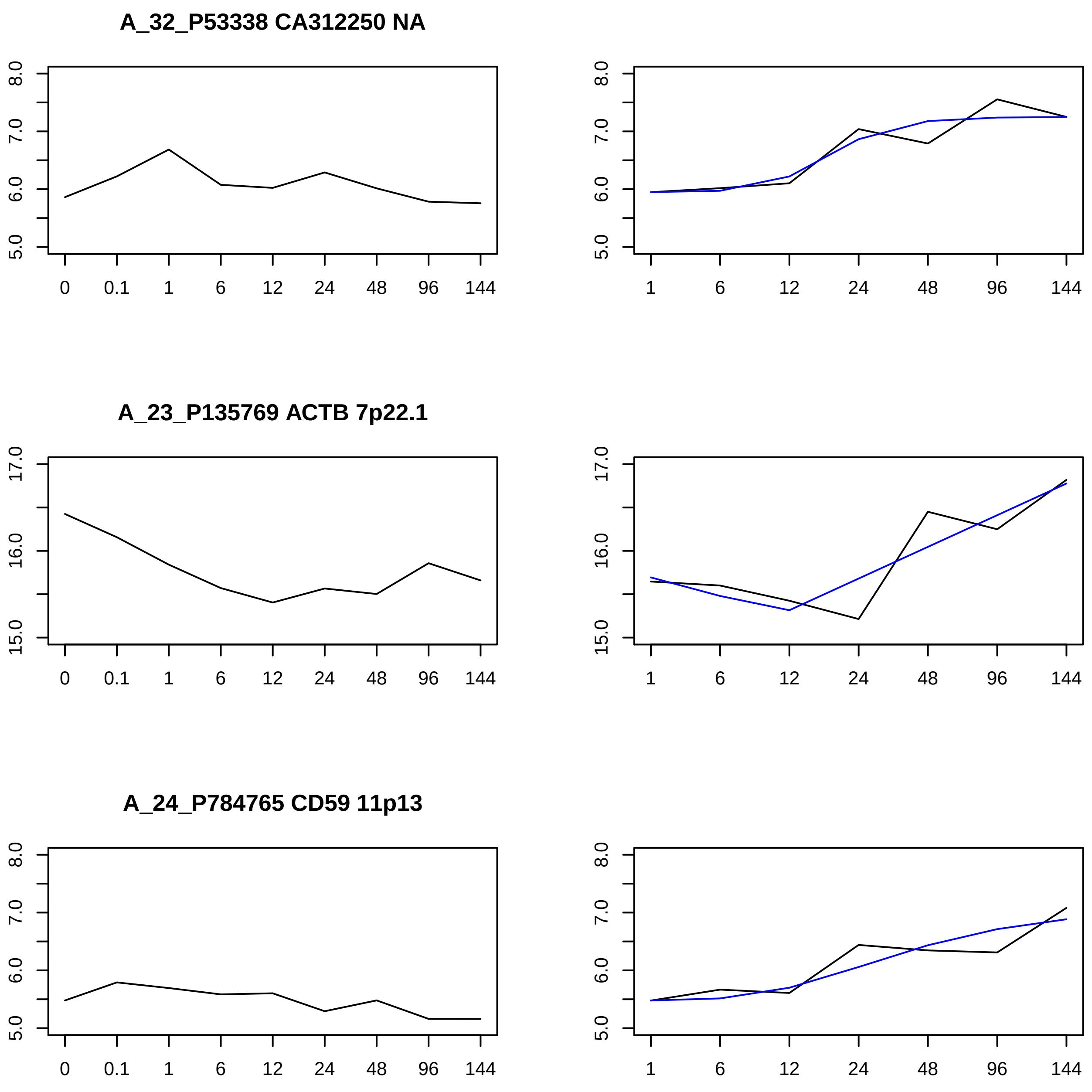
A_32_P53338 CA312250 NA
A_23_P135769 ACTB 7p22.1
A_24_P784765 CD59 11p13

**A_23_P202837 CCND1 11q13.2**

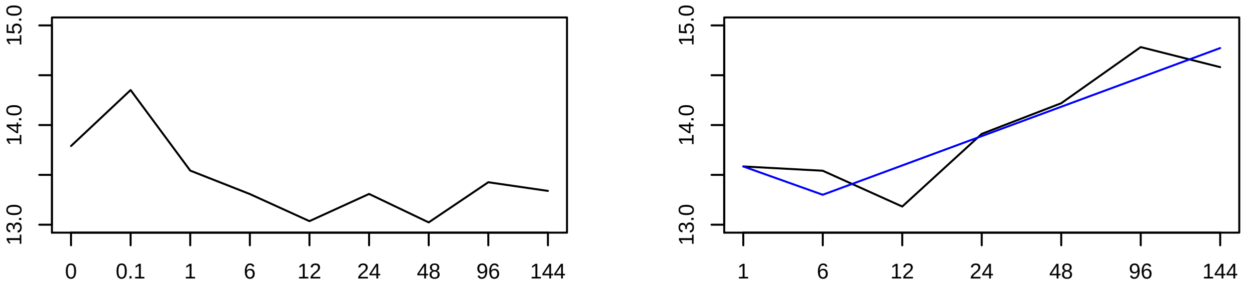


**A_32_P56415 THC2557134 NA**

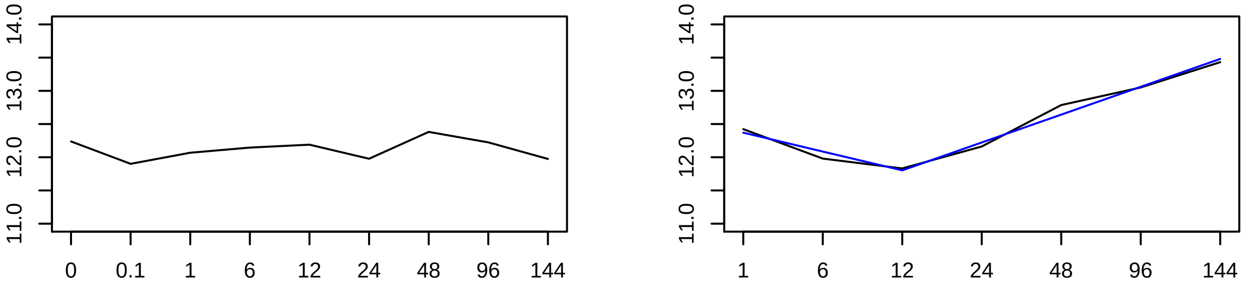


**A_24_P225448 CSRP1 1q32.1**

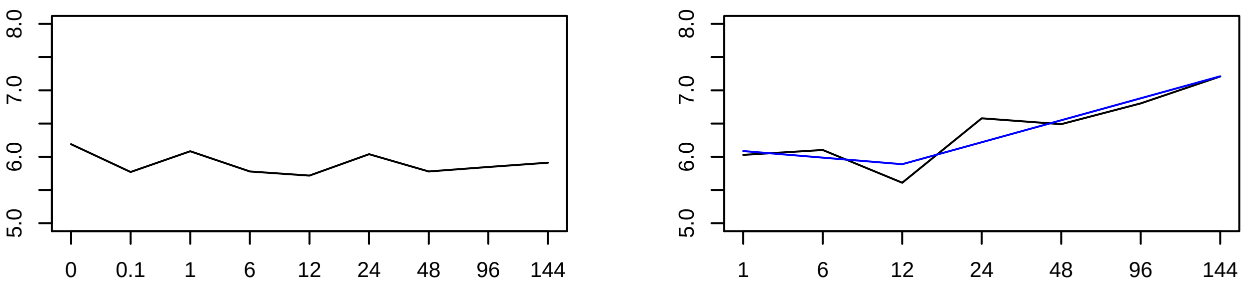

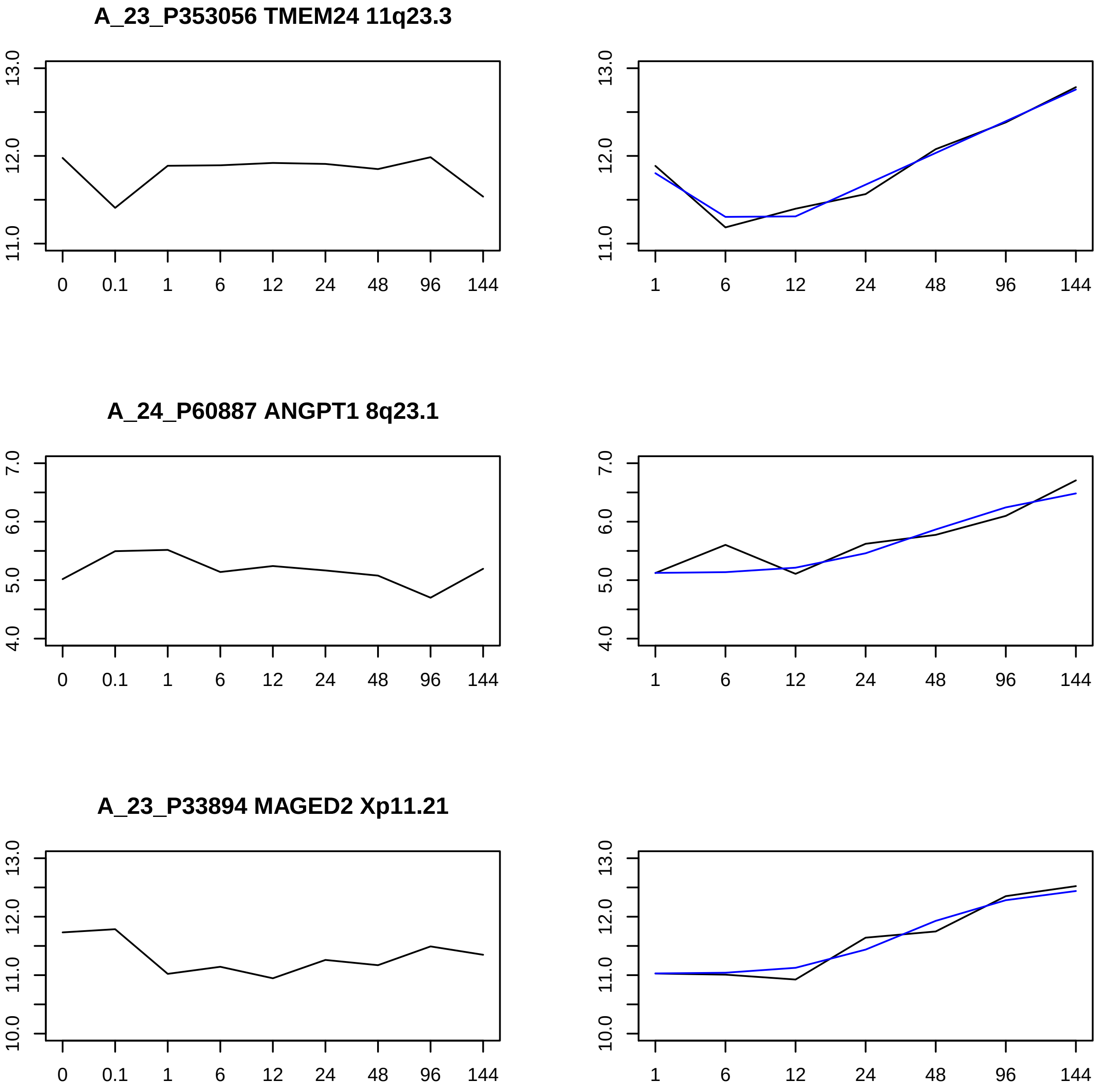
A_23_P353056 TMEM24 11q23.3
A_24_P60887 ANGPT1 8q23.1
A_23_P33894 MAGED2 Xp11.21
0 0.1 1 6 12 24 48 96 144
1 6 12 24 48 96 144
11.0 12.0 13.0
4.0 5.0 6.0 7.0
10.0 11.0 12.0 13.0

**A_24_P290751 DTX1 12q24.13**

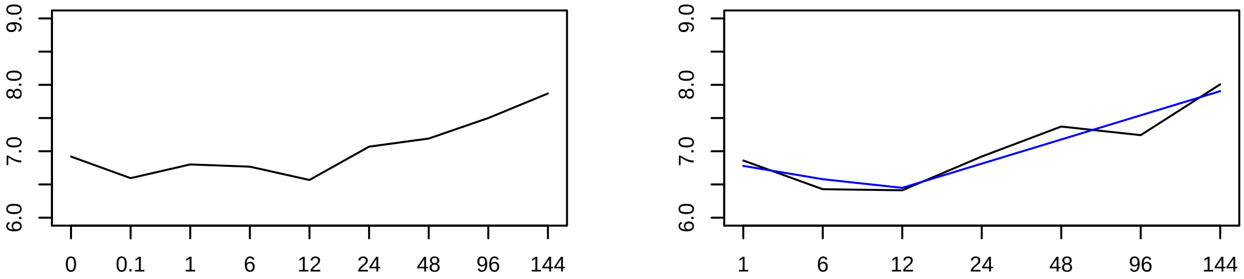


**A_32_P201579 BC020911 NA**

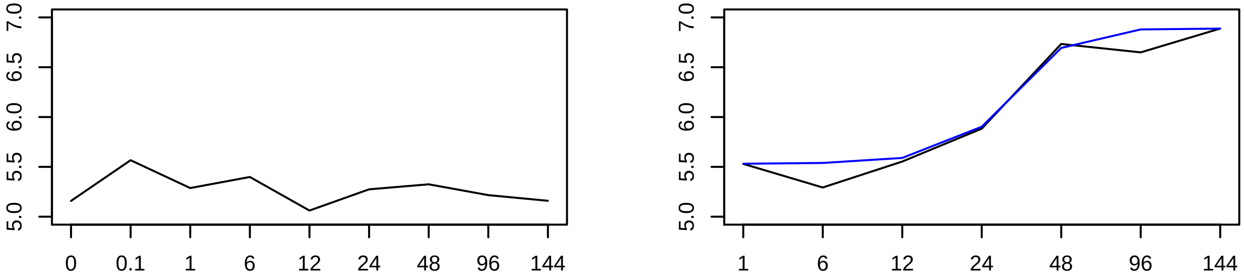


**A_32_P195065 SEMA4F 2p13.1**

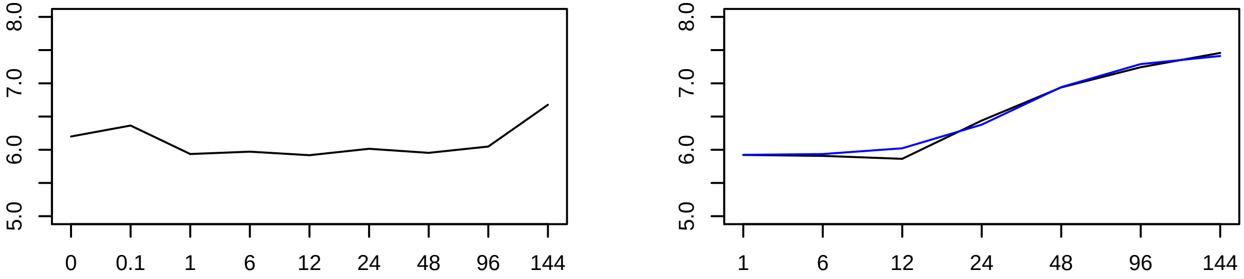

**A_32_P102627 A_32_P102627 NA**

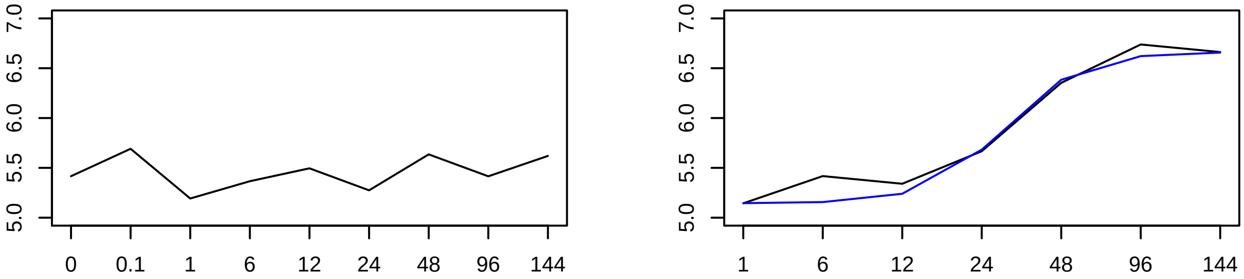


**A_23_P251825 IFRD1 7q31.1**

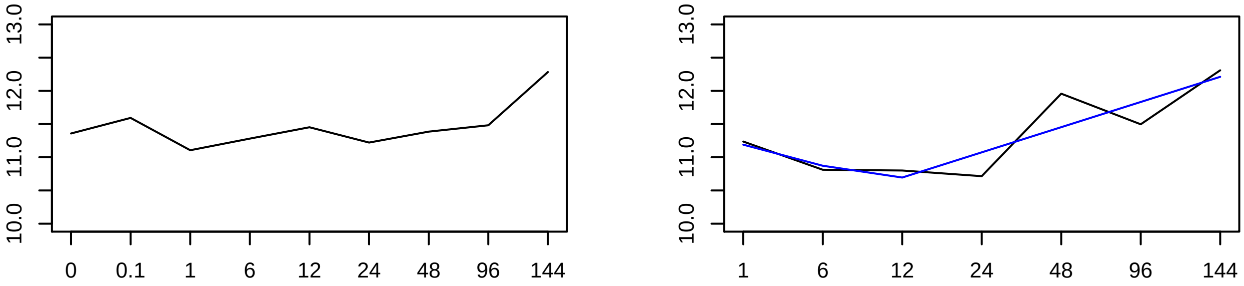


**A_23_P130995 FXYD5 19q13.12**

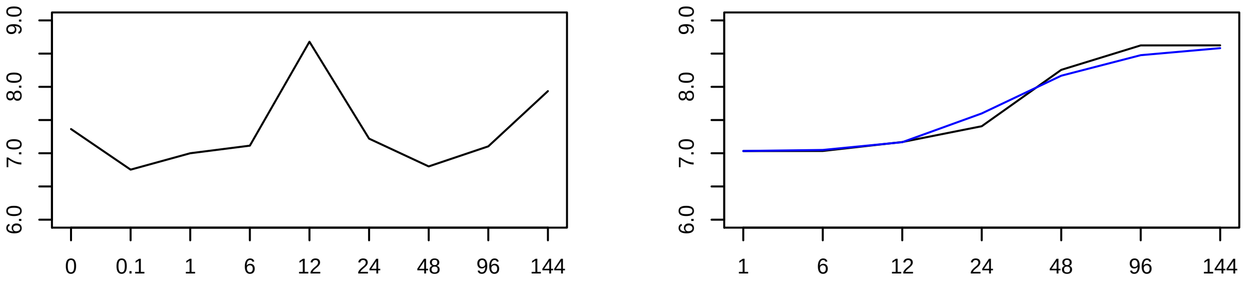

**A_23_P314760 PRKAG2 7q36.1**

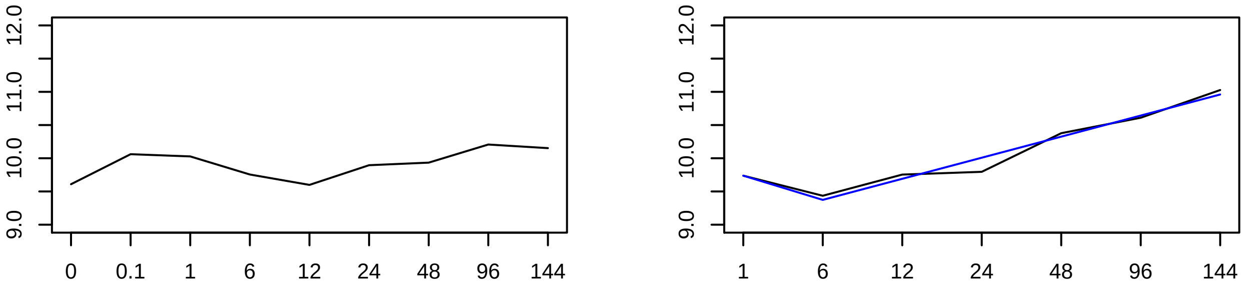


**A_24_P571824 A_24_P571824 NA**

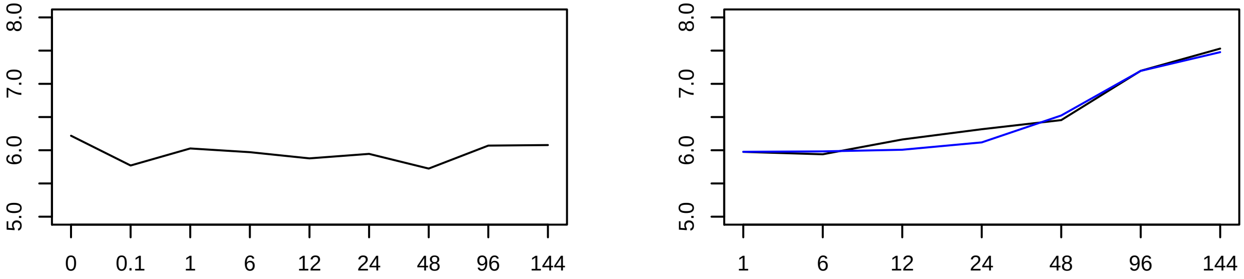


**A_23_P96568 FLNA Xq28**

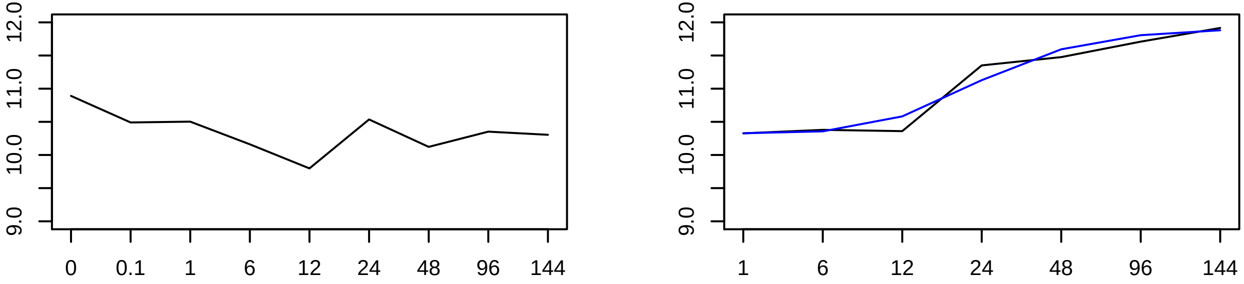

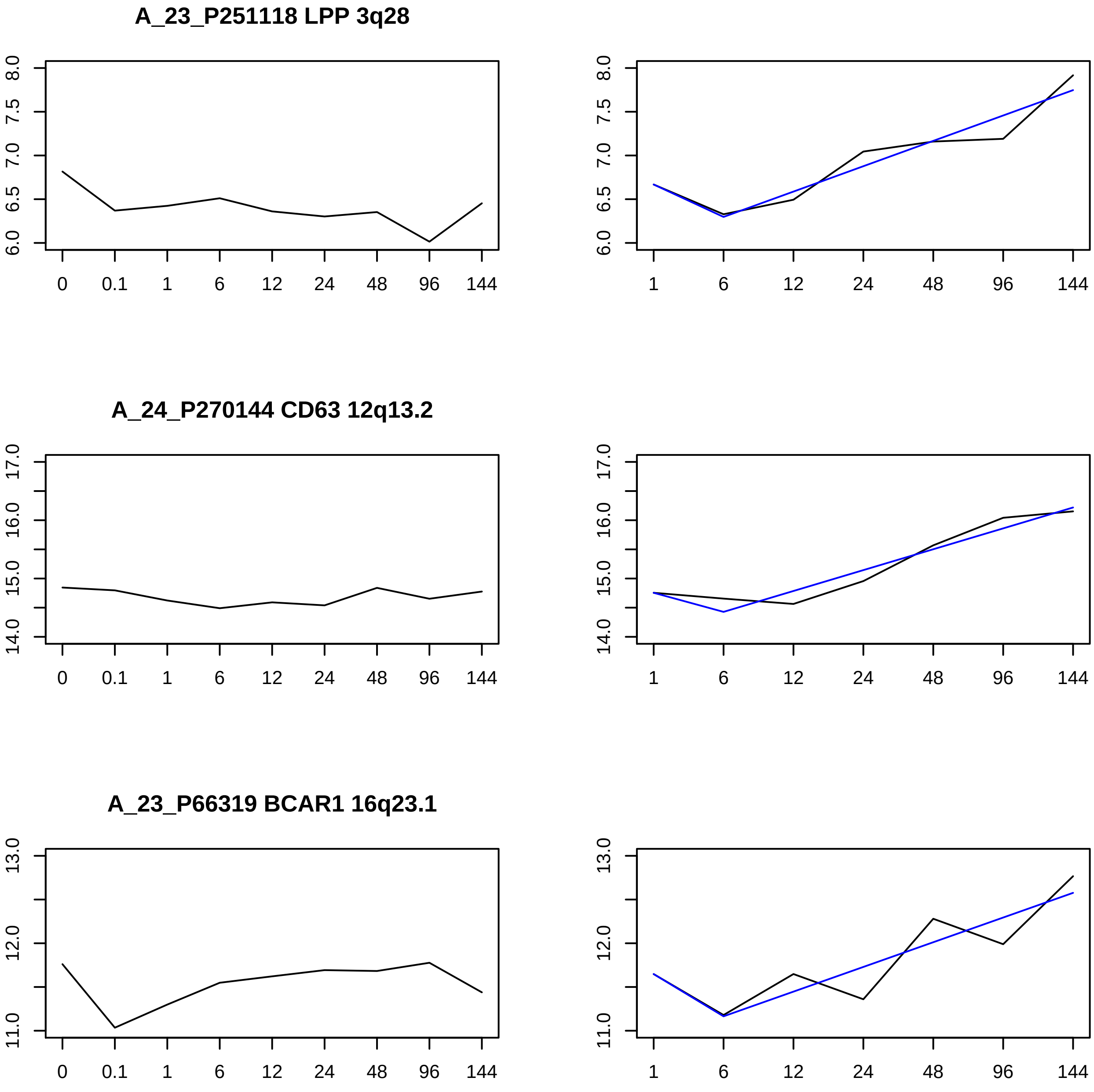

A_23_P251118 LPP 3q28
6.0
6.5
7.0
7.5
8.0
0
0.1
1
6
12
24
48
96
144
1
6
12
24
48
96
144
A_24_P270144 CD63 12q13.2
14.0
15.0
16.0
17.0
A_23_P66319 BCAR1 16q23.1
11.0
12.0
13.0

**A_23_P163402 CYP1A1 15q24.1**

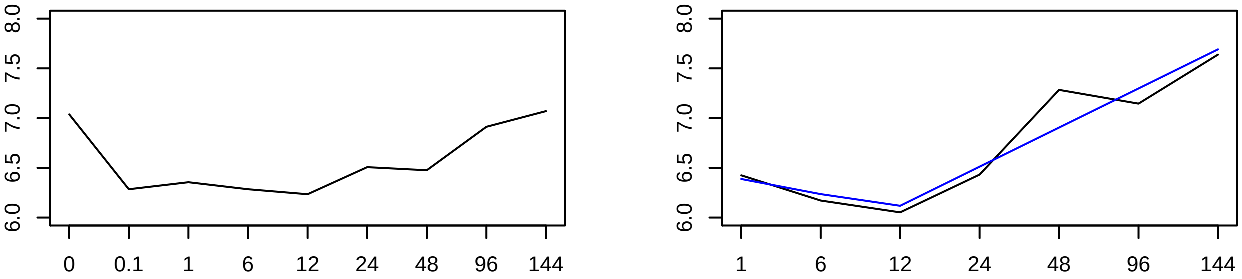


**A_24_P614261 LOC153811 5q35.3**

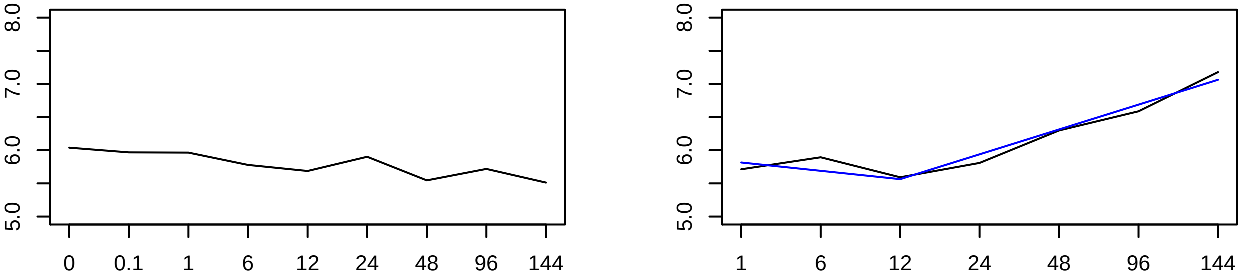


**A_24_P111106 FGF1 5q31.3**

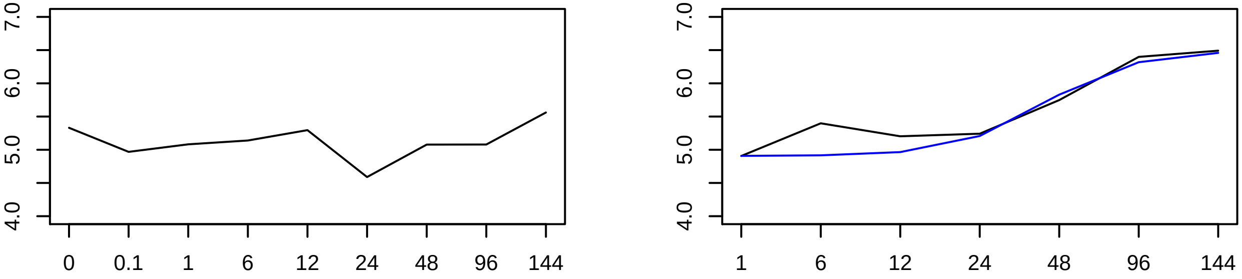

**A_23_P3532 LITAF 16p13.13**

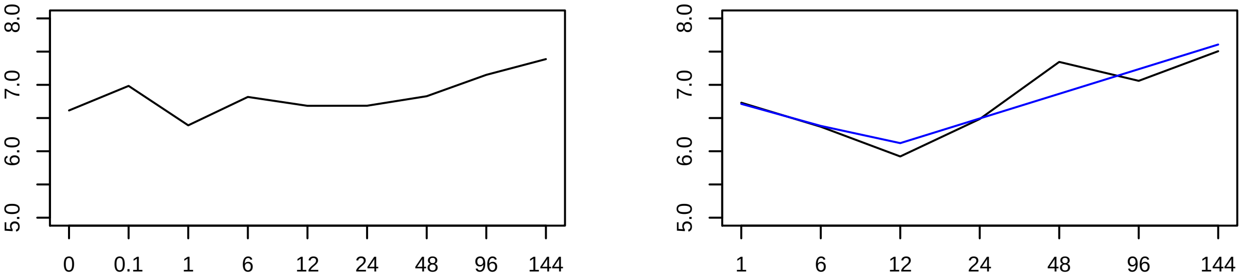


**A_24_P14731 PCSK1N Xp11.23**

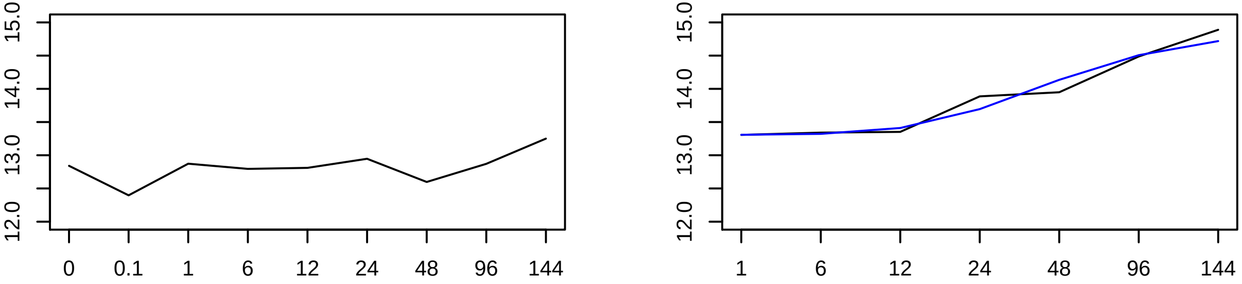


**A_32_P456318 SRSF12 NA**

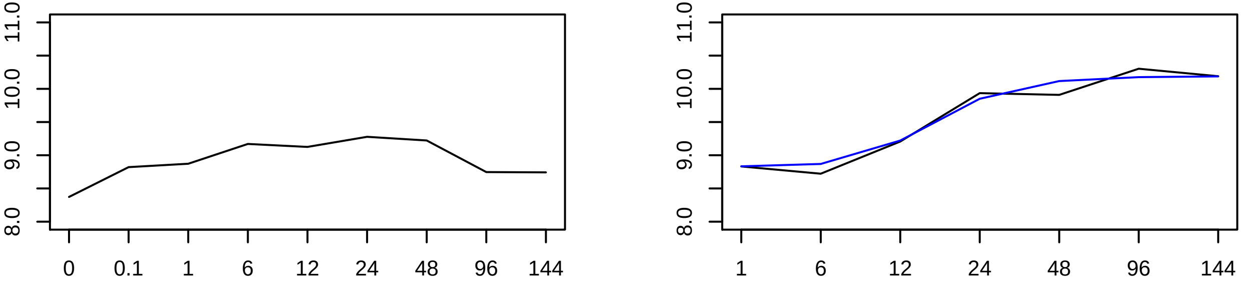

**A_32_P84242 FAM169A NA**

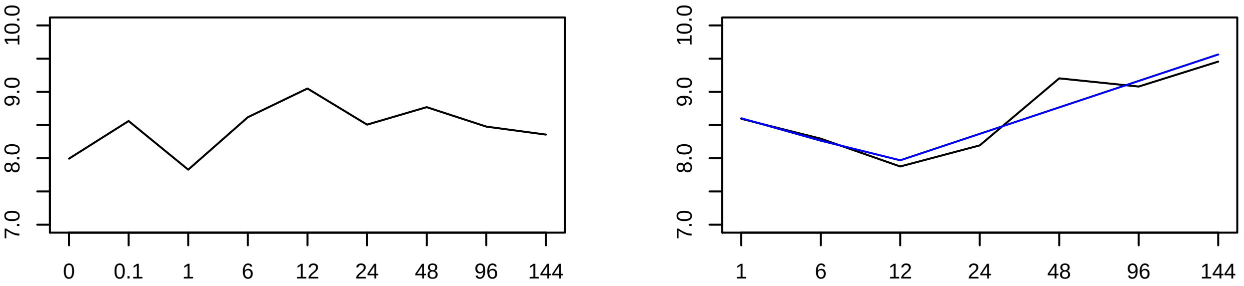


**A_32_P225916 THC2588453 NA**

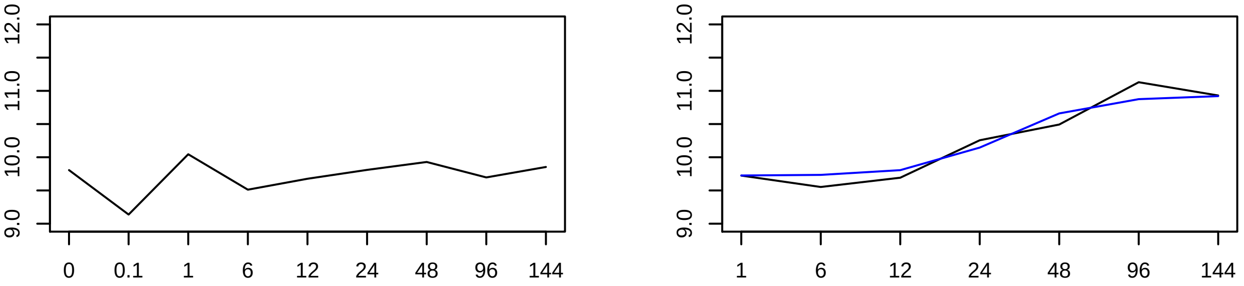


**A_32_P87582 THC2766373 NA**

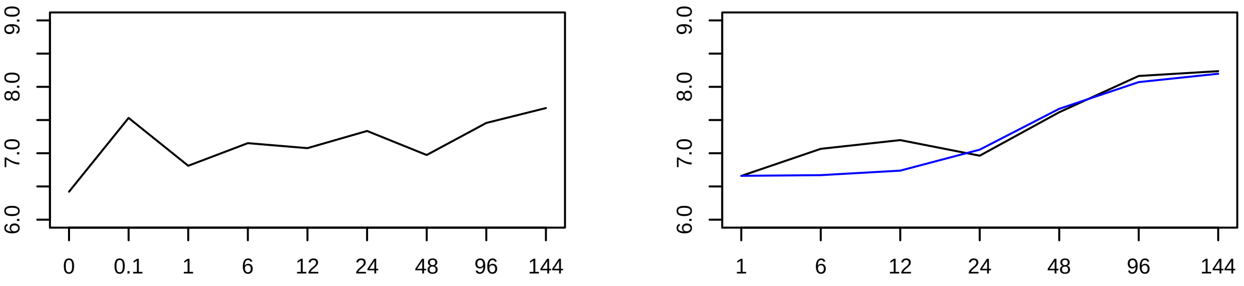

**A_24_P932319 CLASP2 NA**

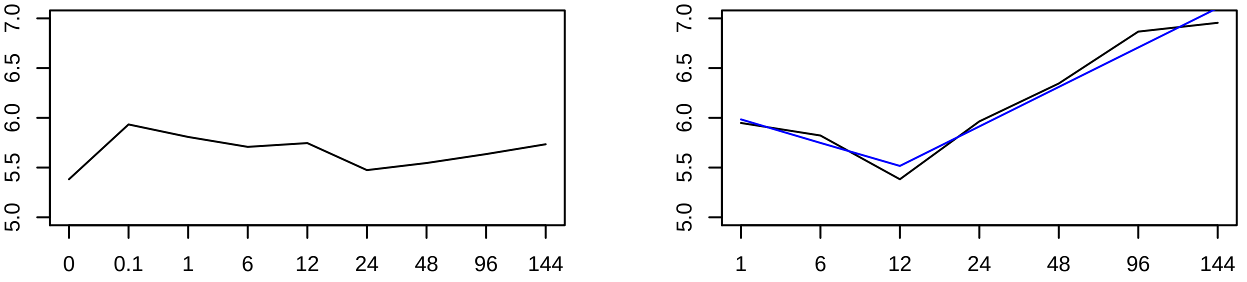


**A_24_P912985 A_24_P912985 NA**

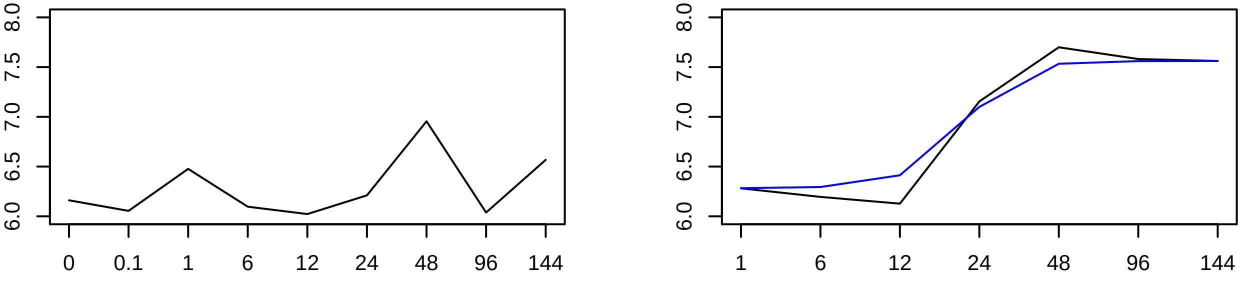


**A_23_P432444 MCF2L2 3q27.1**

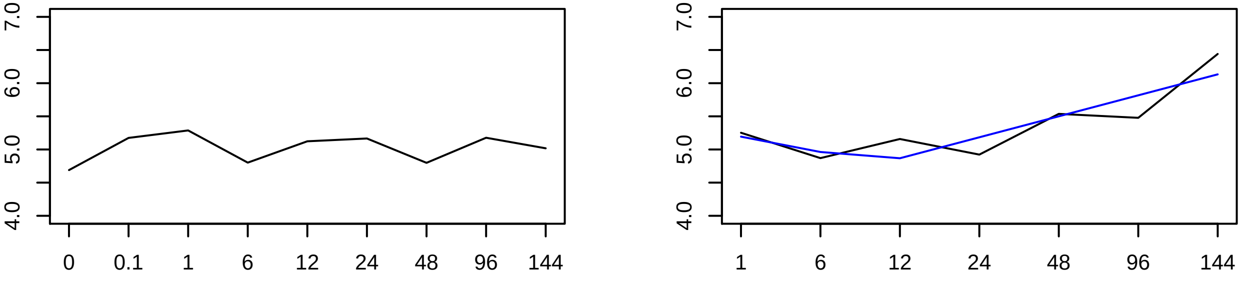

**A_23_P379026 GTPBP2 6p21.1**

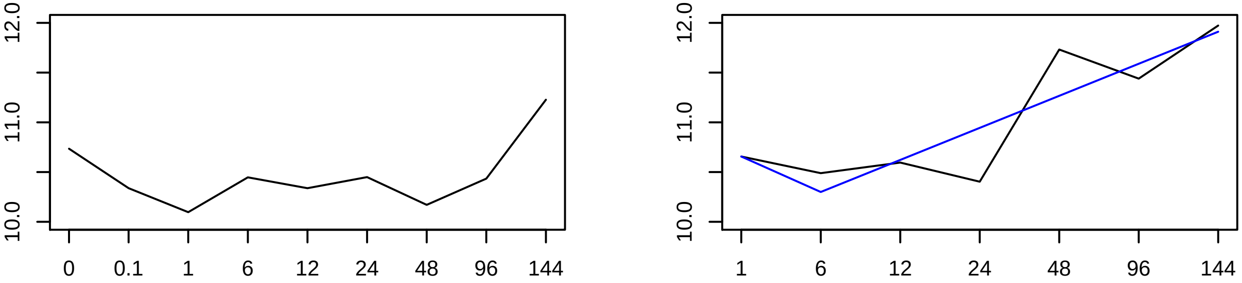


**A_23_P113161 C1orf21 1q25.3**

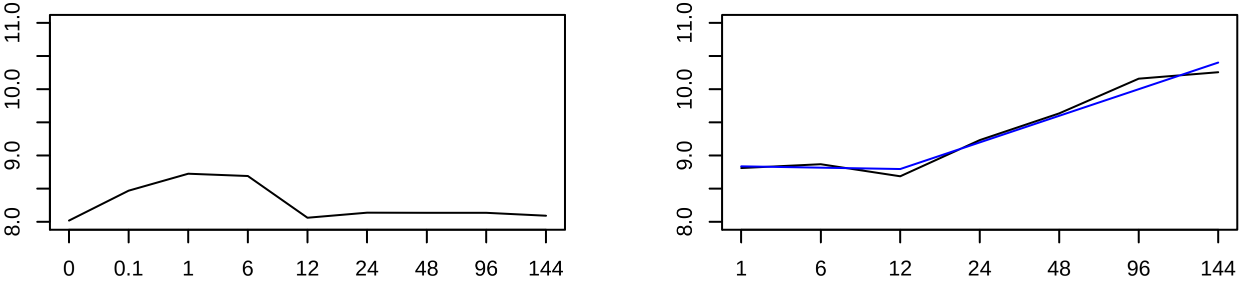


**A_23_P147918 S100A16 1q21.3**

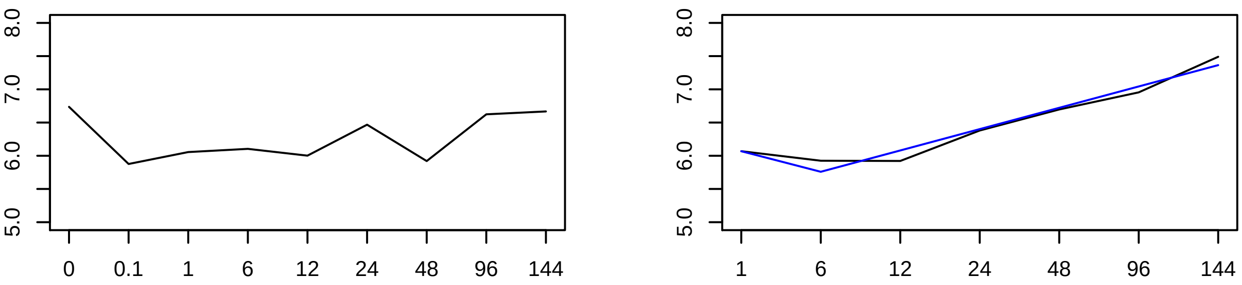

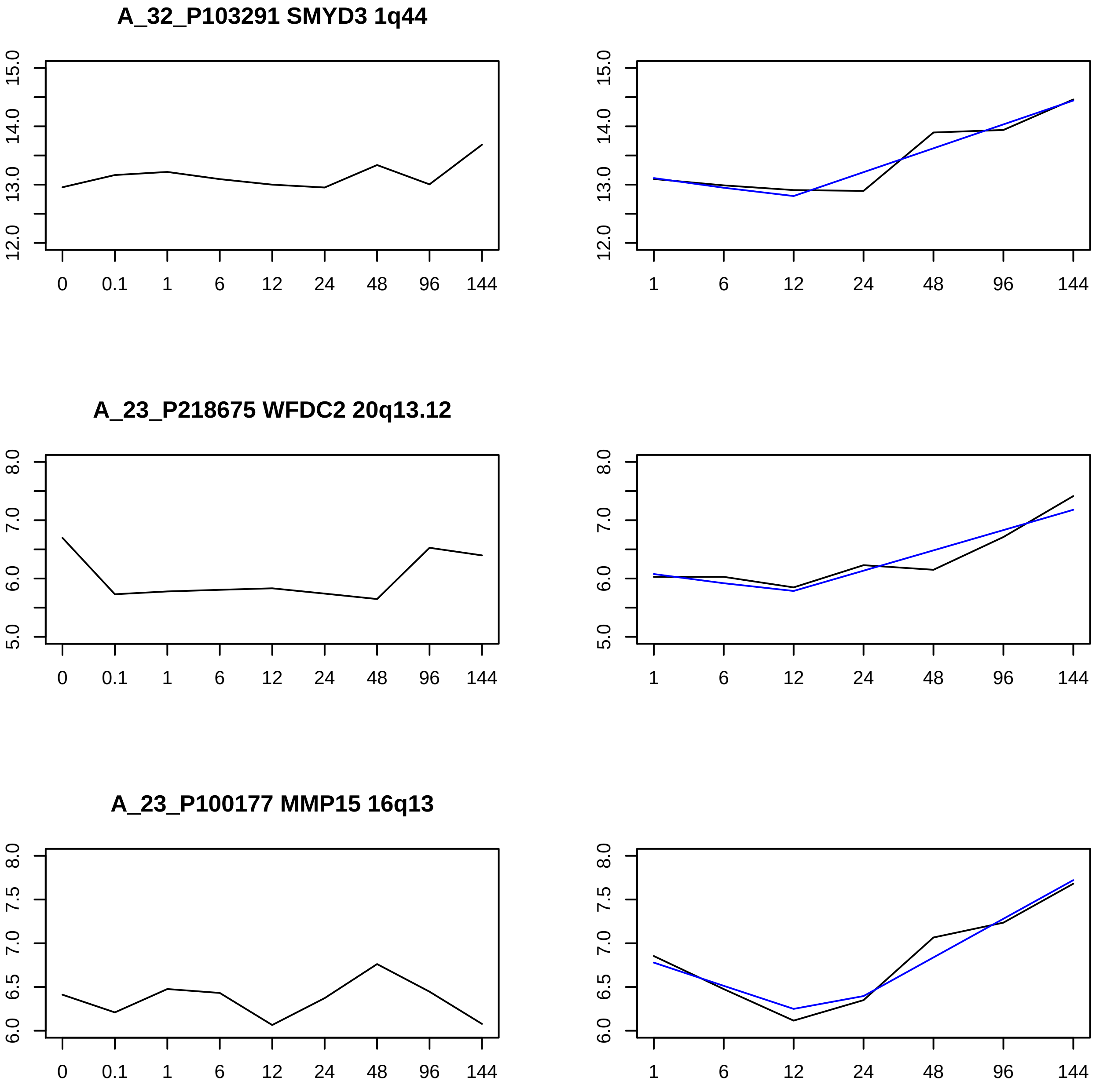
A_32_P103291 SMYD3 1q44
12.0 13.0 14.0 15.0
0 0.1 1 6 12 24 48 96 144
1 6 12 24 48 96 144
A_23_P218675 WFDC2 20q13.12
5.0 6.0 7.0 8.0
0 0.1 1 6 12 24 48 96 144
1 6 12 24 48 96 144
A_23_P100177 MMP15 16q13
6.0 6.5 7.0 7.5 8.0
0 0.1 1 6 12 24 48 96 144
1 6 12 24 48 96 144

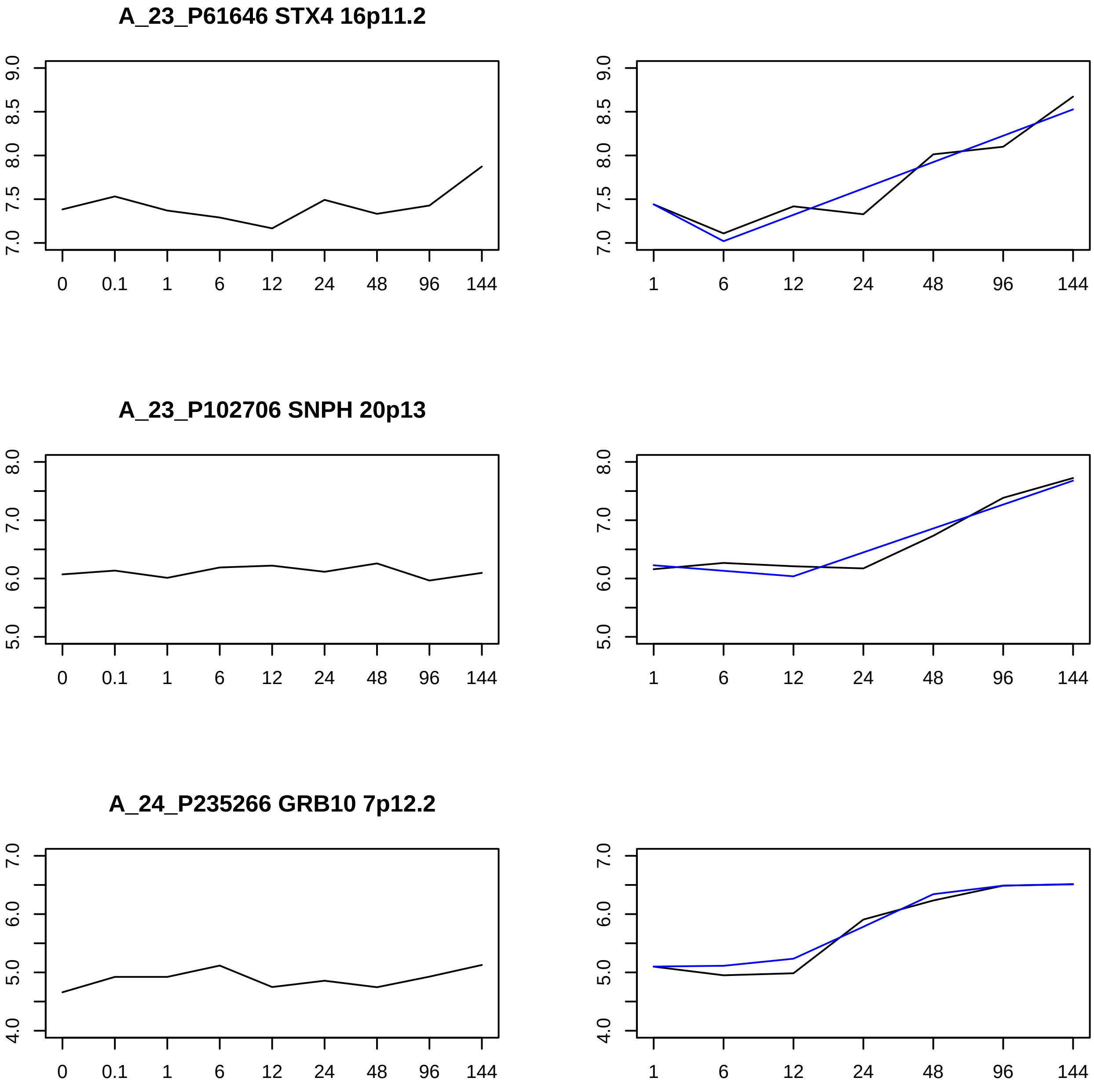
A_23_P61646 STX4 16p11.2
7.0 7.5 8.0 8.5 9.0
0 0.1 1 6 12 24 48 96 144
1 6 12 24 48 96 144
A_23_P102706 SNPH 20p13
5.0 6.0 7.0 8.0
0 0.1 1 6 12 24 48 96 144
1 6 12 24 48 96 144
A_24_P235266 GRB10 7p12.2
4.0 5.0 6.0 7.0
0 0.1 1 6 12 24 48 96 144
1 6 12 24 48 96 144

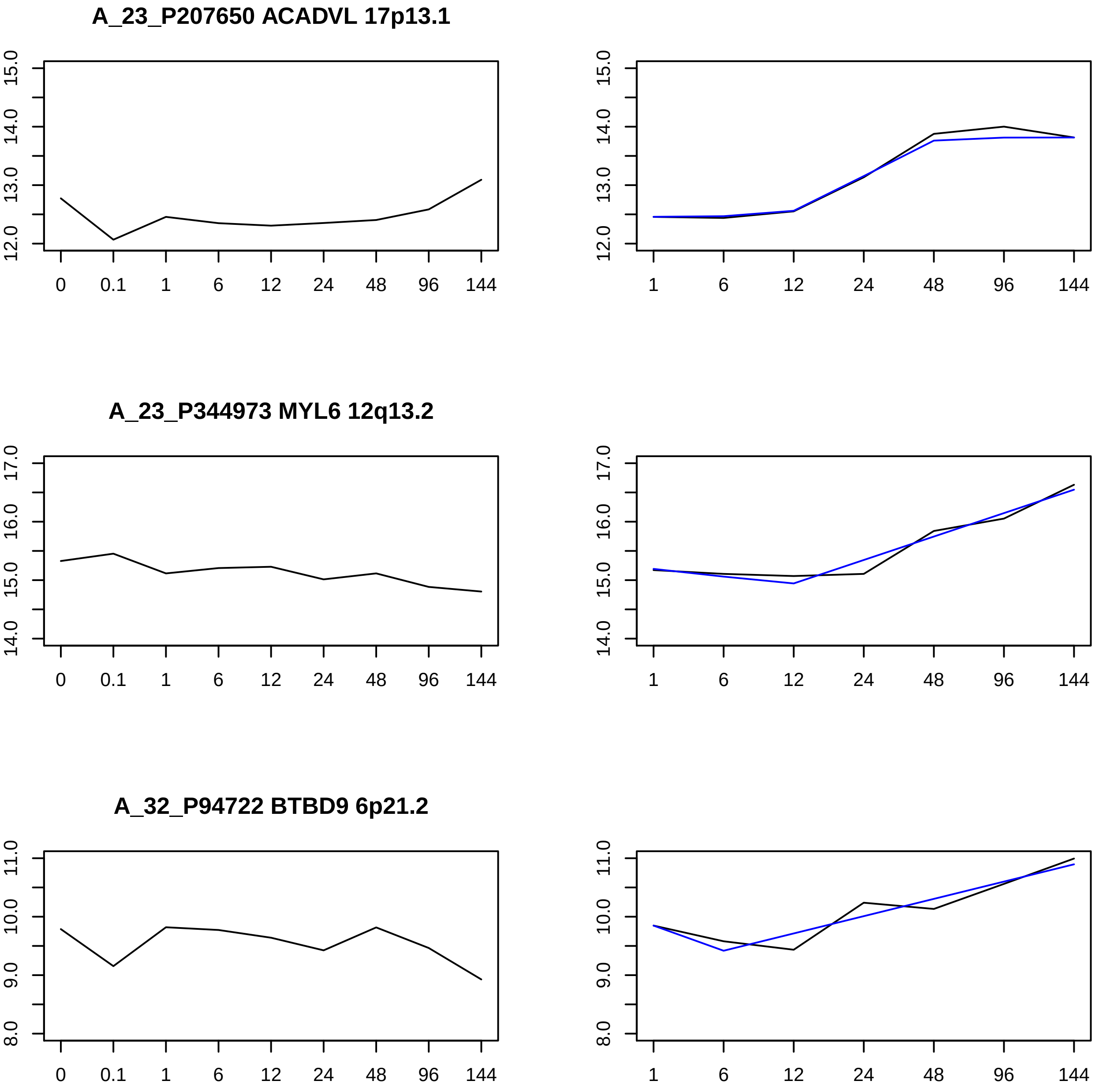
A_23_P207650 ACADVL 17p13.1
A_23_P344973 MYL6 12q13.2
A_32_P94722 BTBD9 6p21.2
12.0 13.0 14.0 15.0
14.0 15.0 16.0 17.0
8.0 9.0 10.0 11.0
0 0.1 1 6 12 24 48 96 144
1 6 12 24 48 96 144

**A_24_P405621 NISCH 3p21.1**

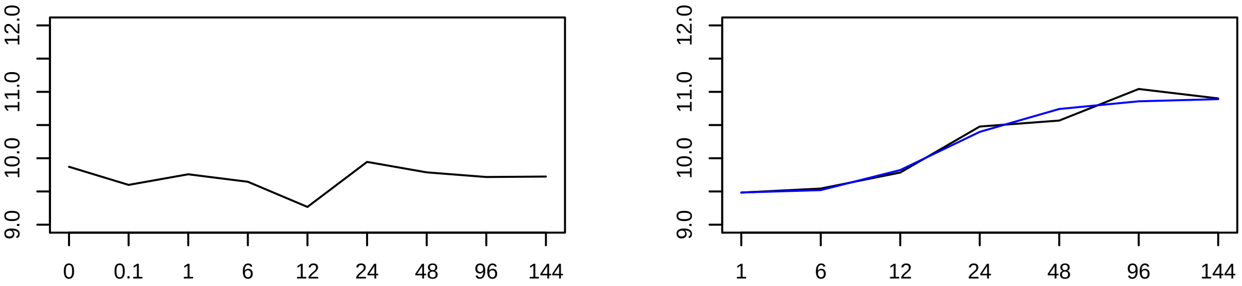


**A_32_P210744 A_32_P210744 NA**

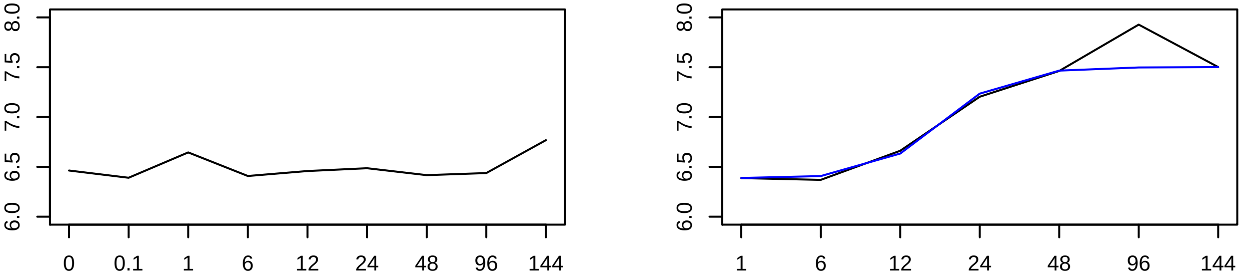


**A_24_P150791 JPH3 16q24.2**

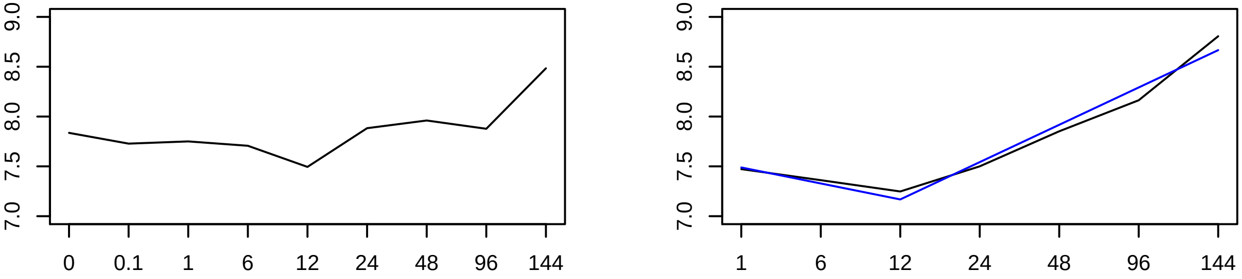

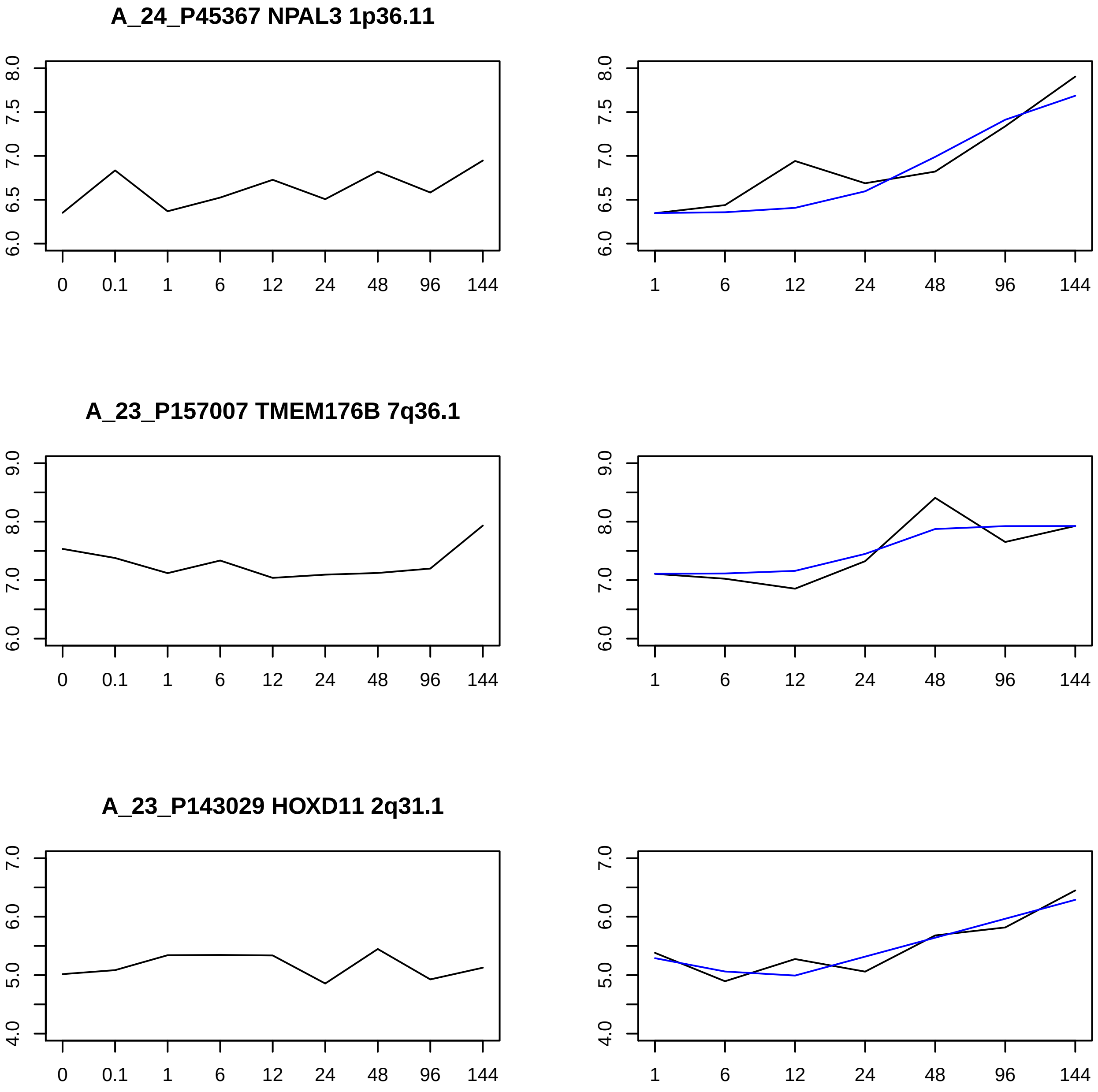
A_24_P45367 NPAL3 1p36.11
6.0 6.5 7.0 7.5 8.0
0 0.1 1 6 12 24 48 96 144
1 6 12 24 48 96 144
A_23_P157007 TMEM176B 7q36.1
6.0 7.0 8.0 9.0
0 0.1 1 6 12 24 48 96 144
1 6 12 24 48 96 144
A_23_P143029 HOXD11 2q31.1
4.0 5.0 6.0 7.0
0 0.1 1 6 12 24 48 96 144
1 6 12 24 48 96 144

**A_32_P133840 TMCC2 1q32.1**

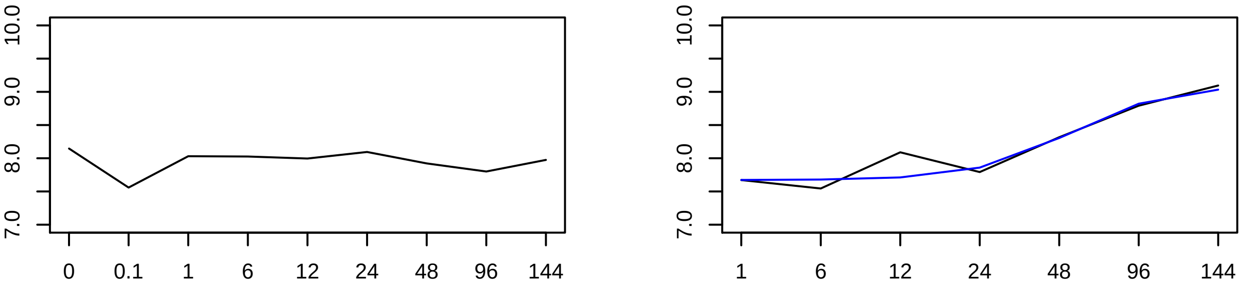


**A_23_P360777 NRG1 8p12**

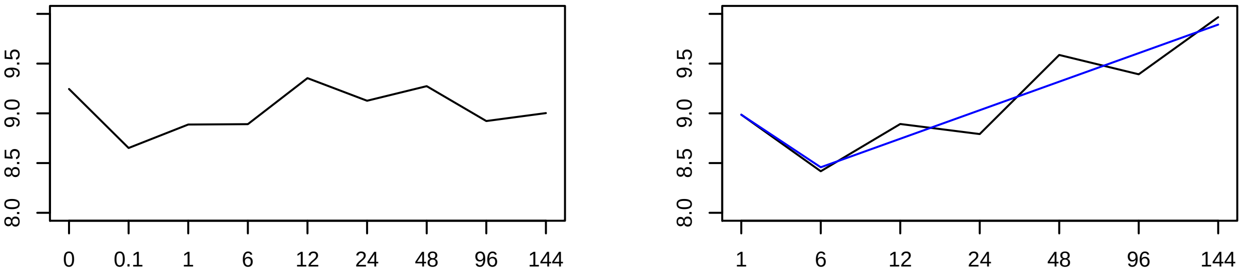


**A_23_P119353 RASIP1 19q13.33**

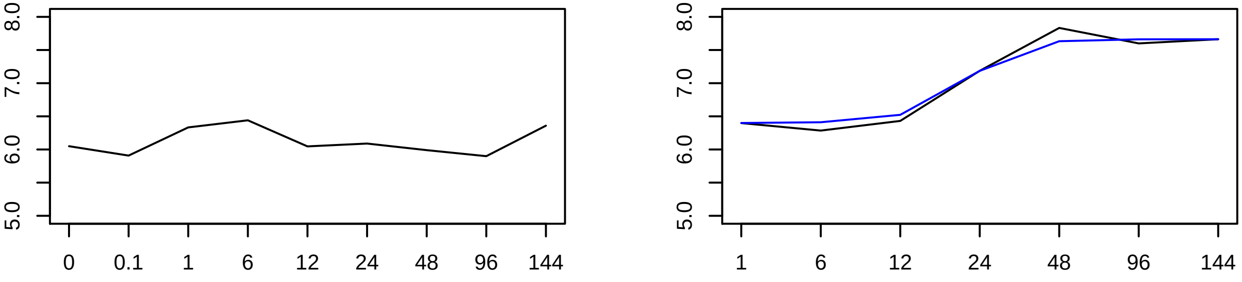

**A_23_P24444 DHCR7 11q13.4**

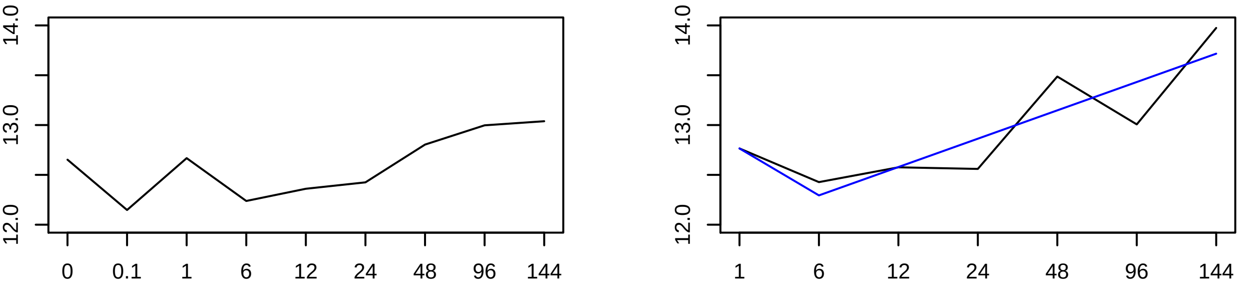


**A_32_P69849 NGFRAP1L1 Xq22.1**

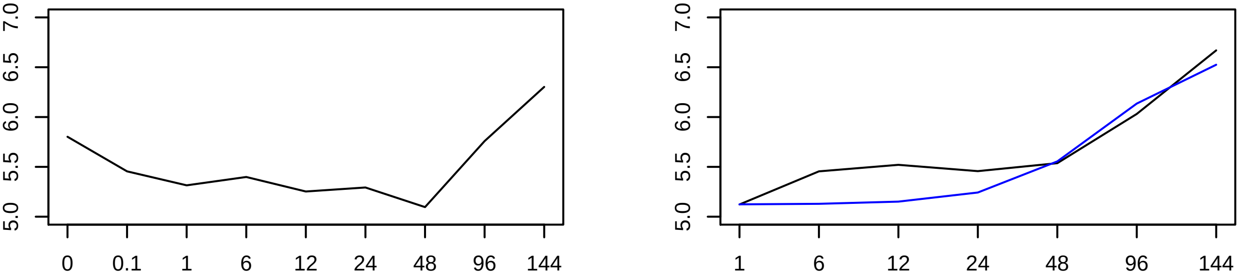


**A_24_P602871 RP5−875H10.1 6q24.3**

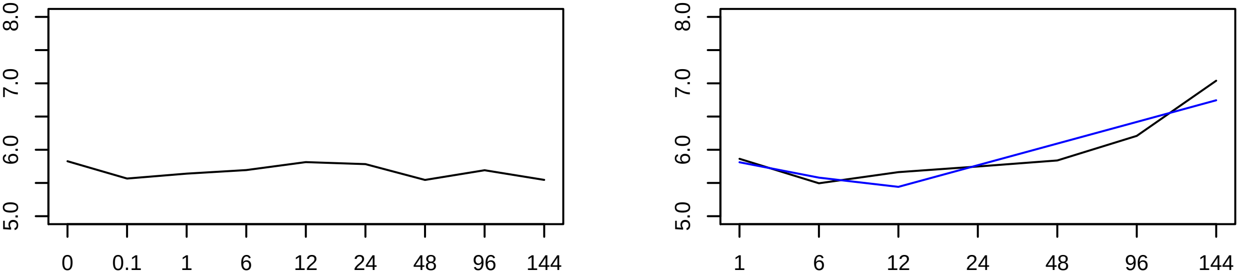

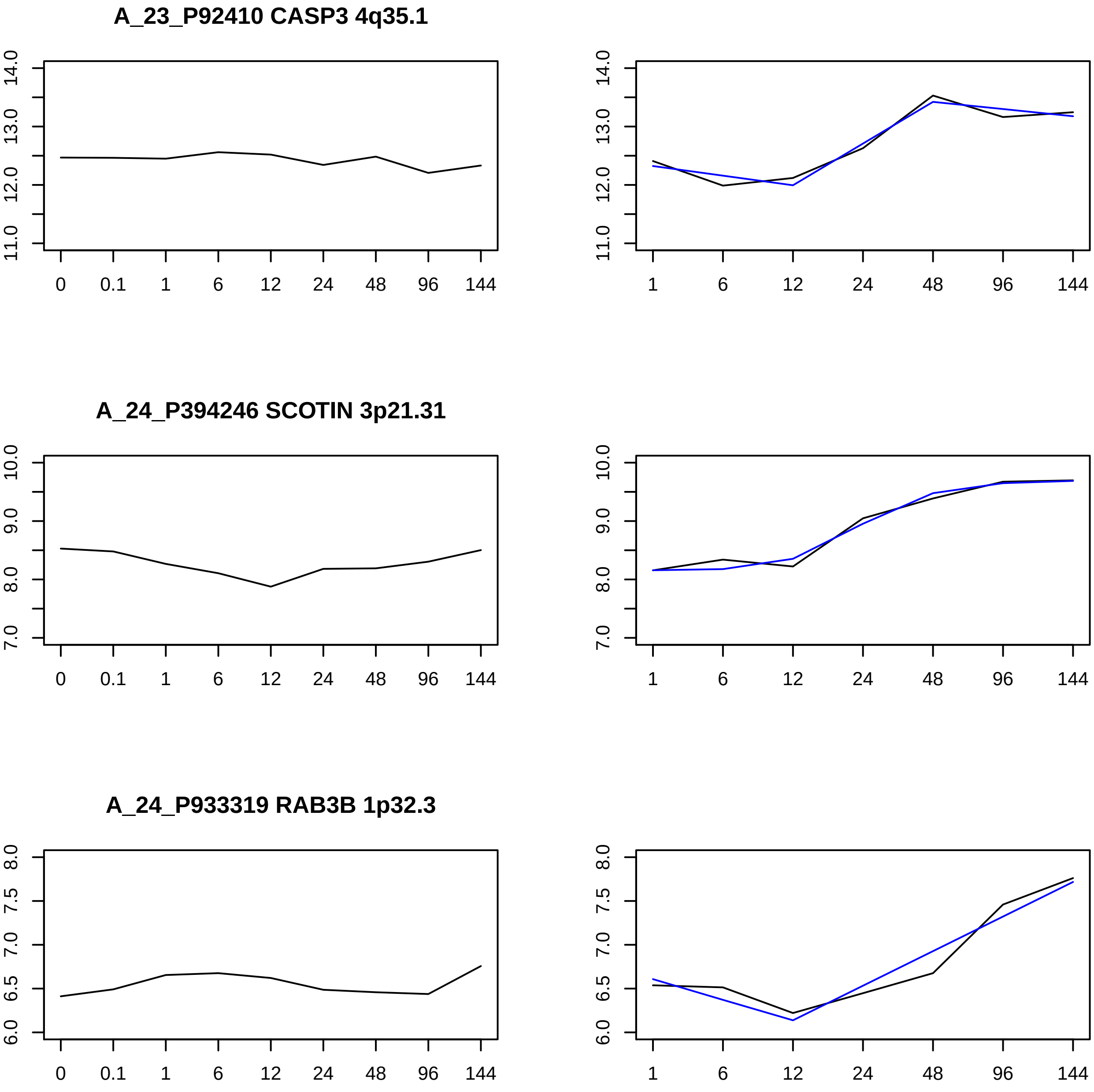

A_23_P92410 CASP3 4q35.1
11.0
12.0
13.0
14.0
0
0.1
1
6
12
24
48
96
144
A_24_P394246 SCOTIN 3p21.31
7.0
8.0
9.0
10.0
A_24_P933319 RAB3B 1p32.3
6.0
6.5
7.0
7.5
8.0

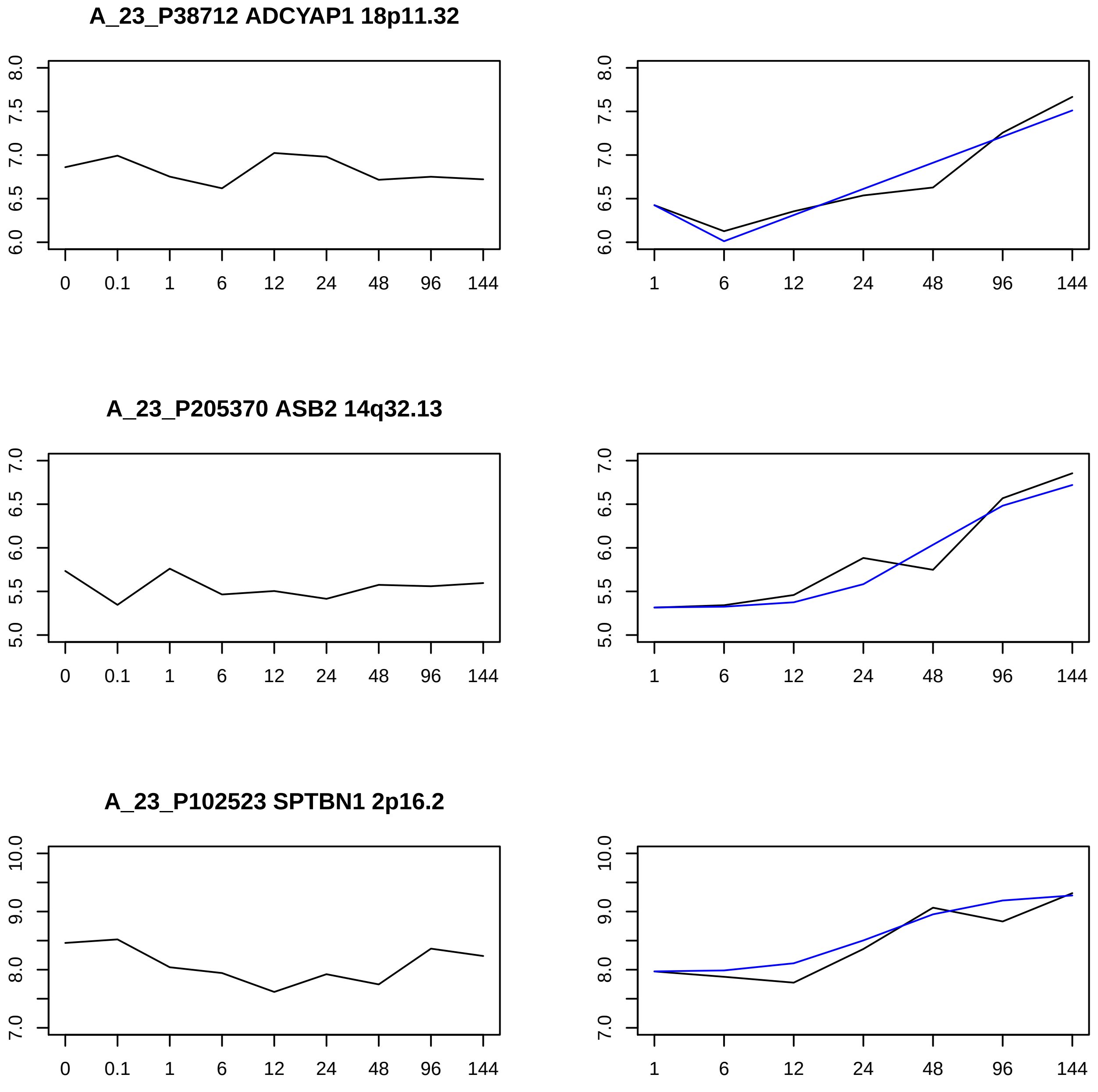
A_23_P38712 ADCYAP1 18p11.32
6.0
6.5
7.0
7.5
8.0
0 0.1 1 6 12 24 48 96 144
1 6 12 24 48 96 144
A_23_P205370 ASB2 14q32.13
5.0
5.5
6.0
6.5
7.0
0 0.1 1 6 12 24 48 96 144
1 6 12 24 48 96 144
A_23_P102523 SPTBN1 2p16.2
7.0
8.0
9.0
10.0
0 0.1 1 6 12 24 48 96 144
1 6 12 24 48 96 144

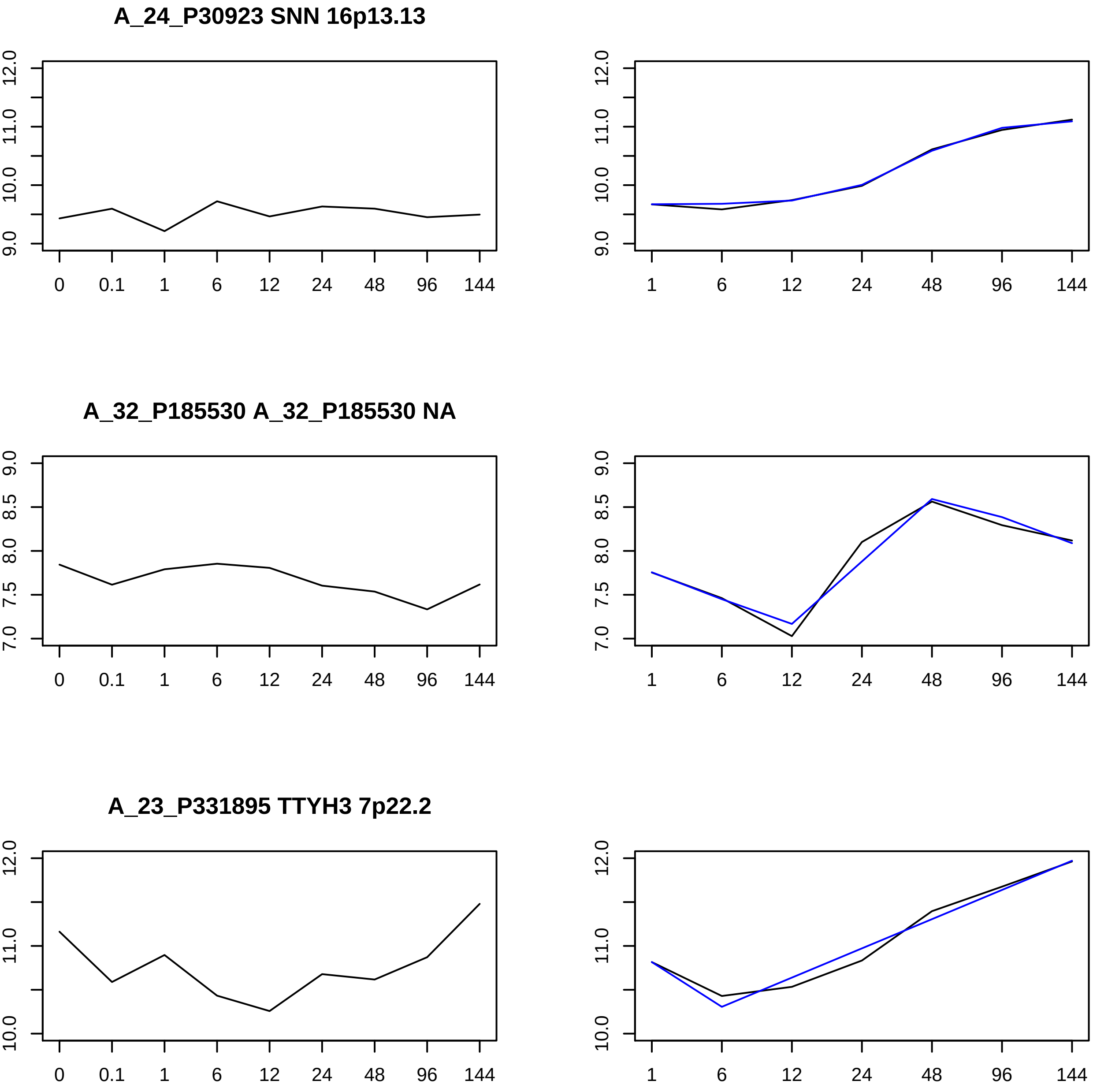
A_24_P30923 SNN 16p13.13
9.0
10.0
11.0
12.0
0
0.1
1
6
12
24
48
96
144
A_32_P185530 A_32_P185530 NA
7.0
7.5
8.0
8.5
9.0
A_23_P331895 TTYH3 7p22.2
10.0
11.0
12.0

**A_23_P39647 SLC4A3 2q35**

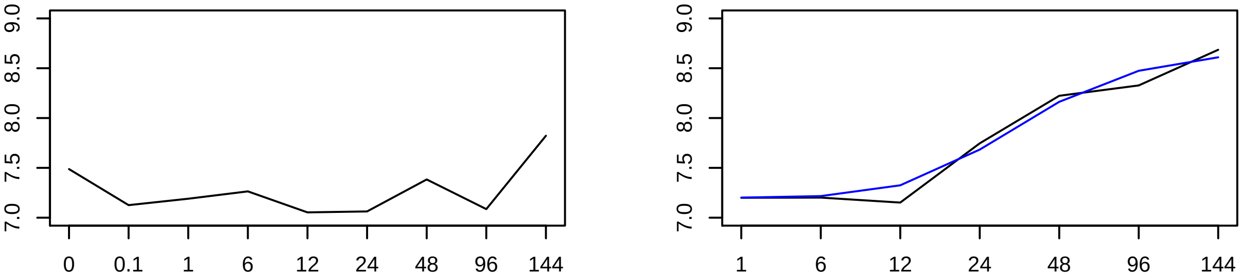


**A_23_P83579 ARNT2 15q25.1**

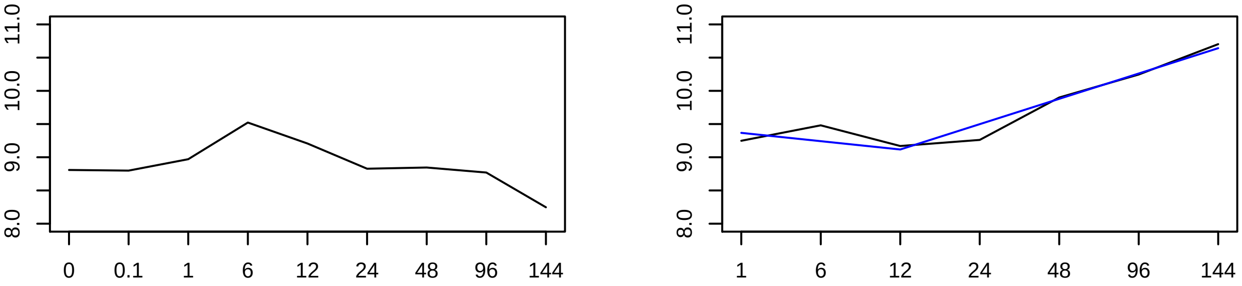


**A_23_P307968 CDH23 10q22.1**

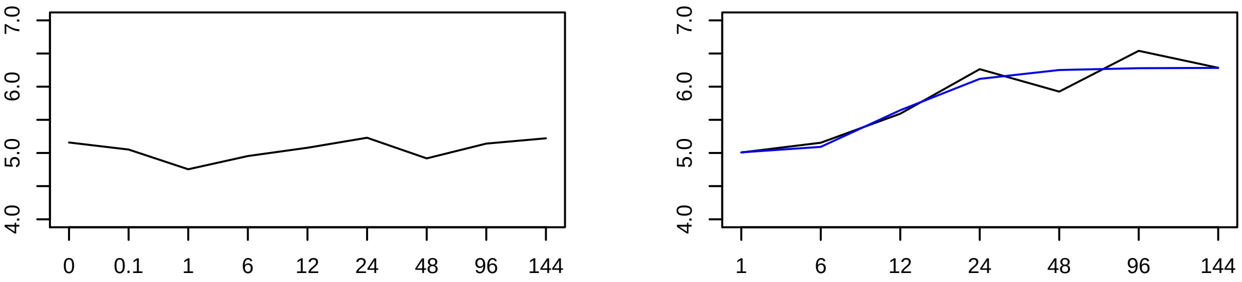

**A_24_P772103 PITPNC1 17q24.2**

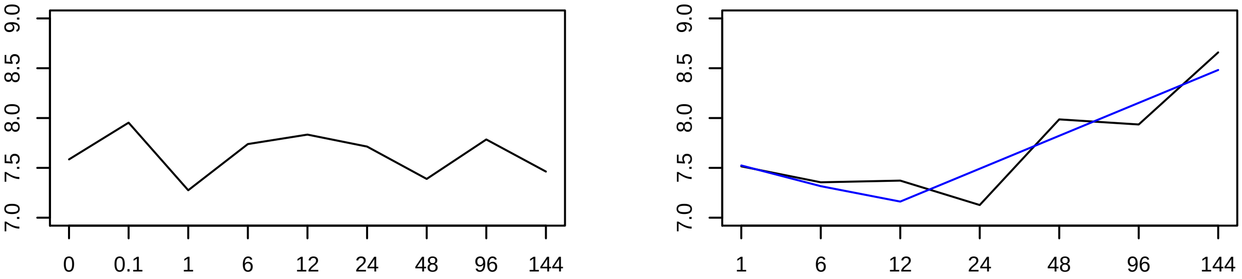


**A_32_P155035 AK096500 NA**

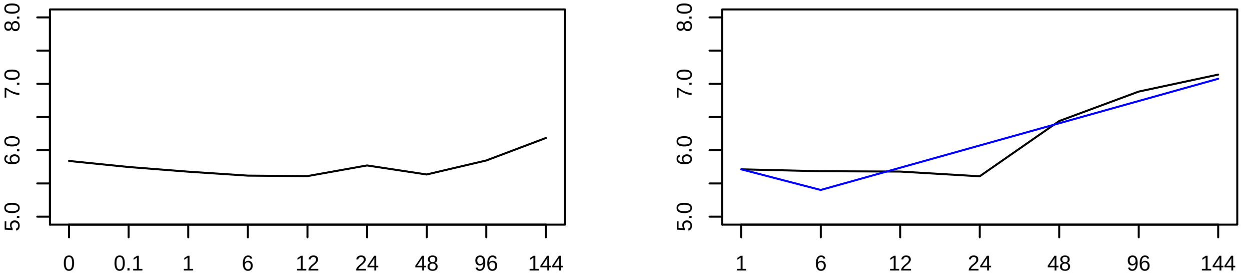


**A_23_P372874 S100A13 1q21.3**

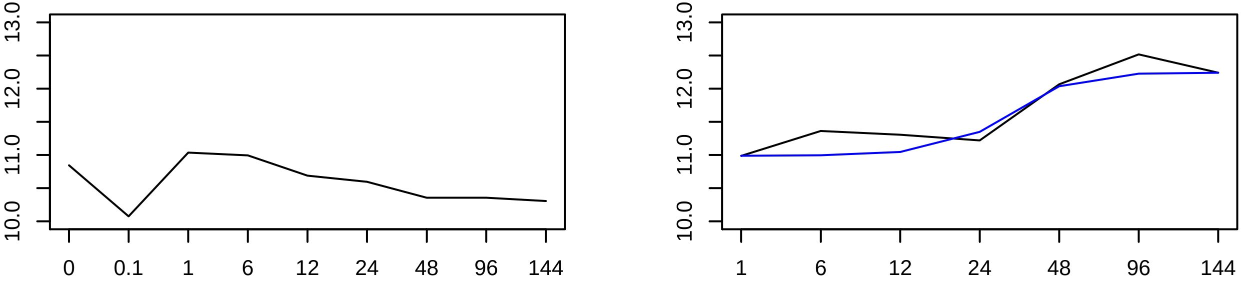

**A_24_P658427 NFIB 9p23**

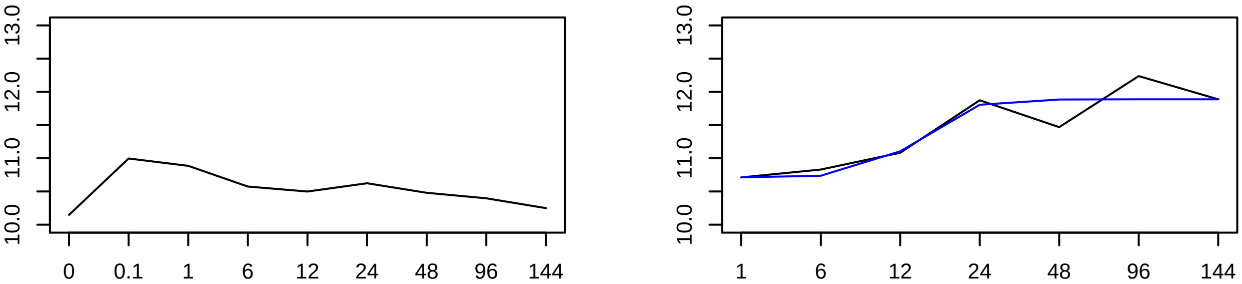


**A_23_P57497 MYH9 22q12.3**

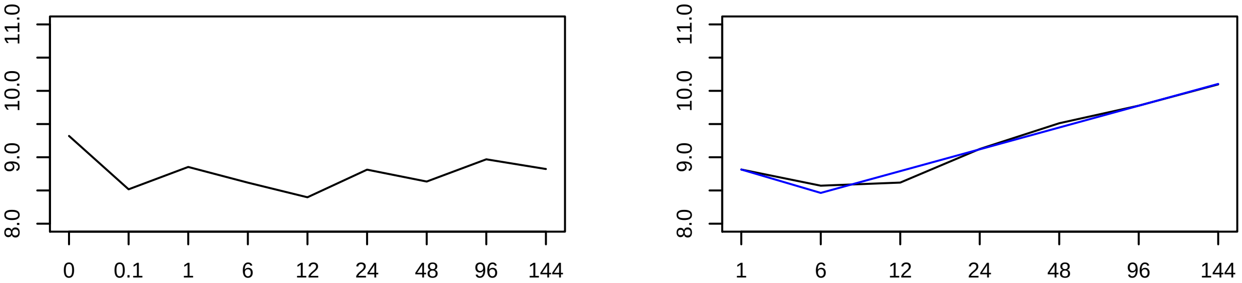


**A_32_P105465 AK093713 NA**

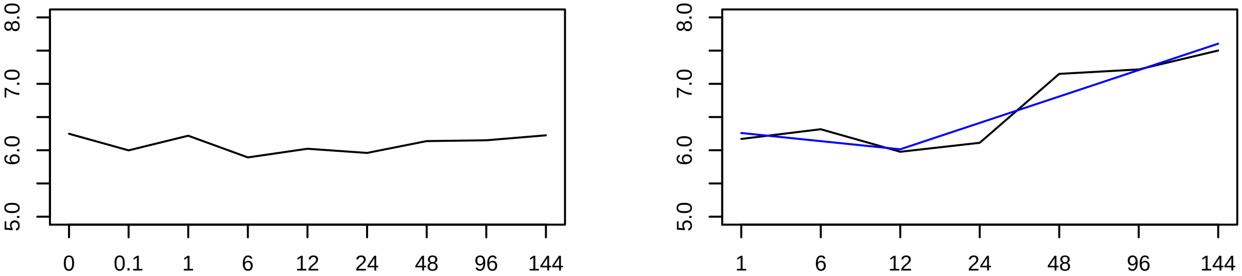

**A_24_P304629 VASH1 14q24.3**

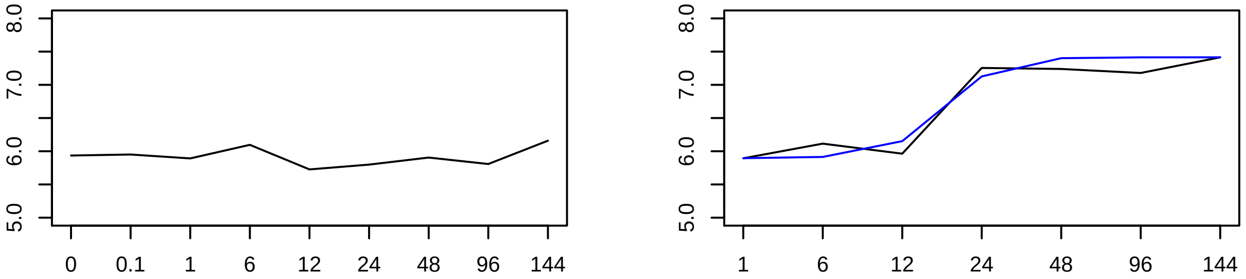


**A_23_P200928 NID1 1q42.3**

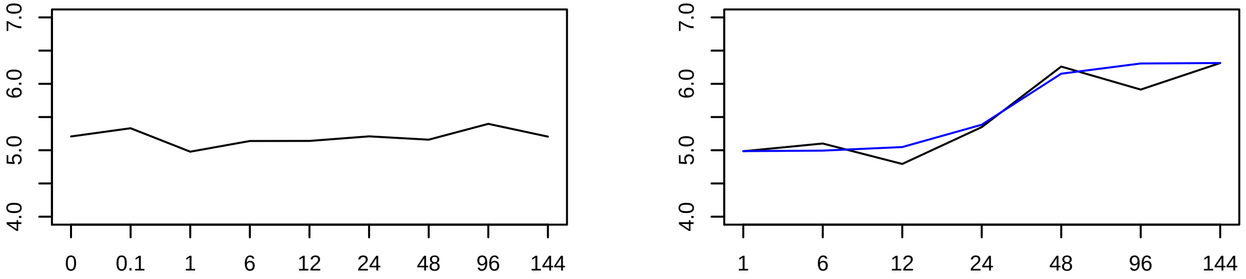


**A_23_P78802 PRKD2 19q13.32**

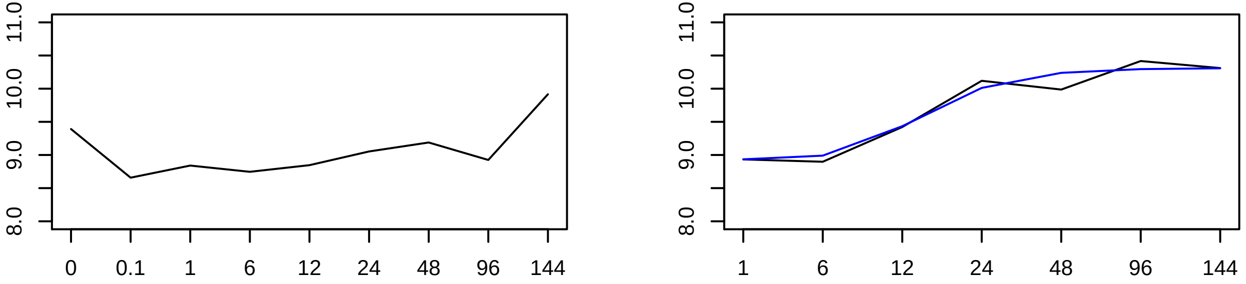

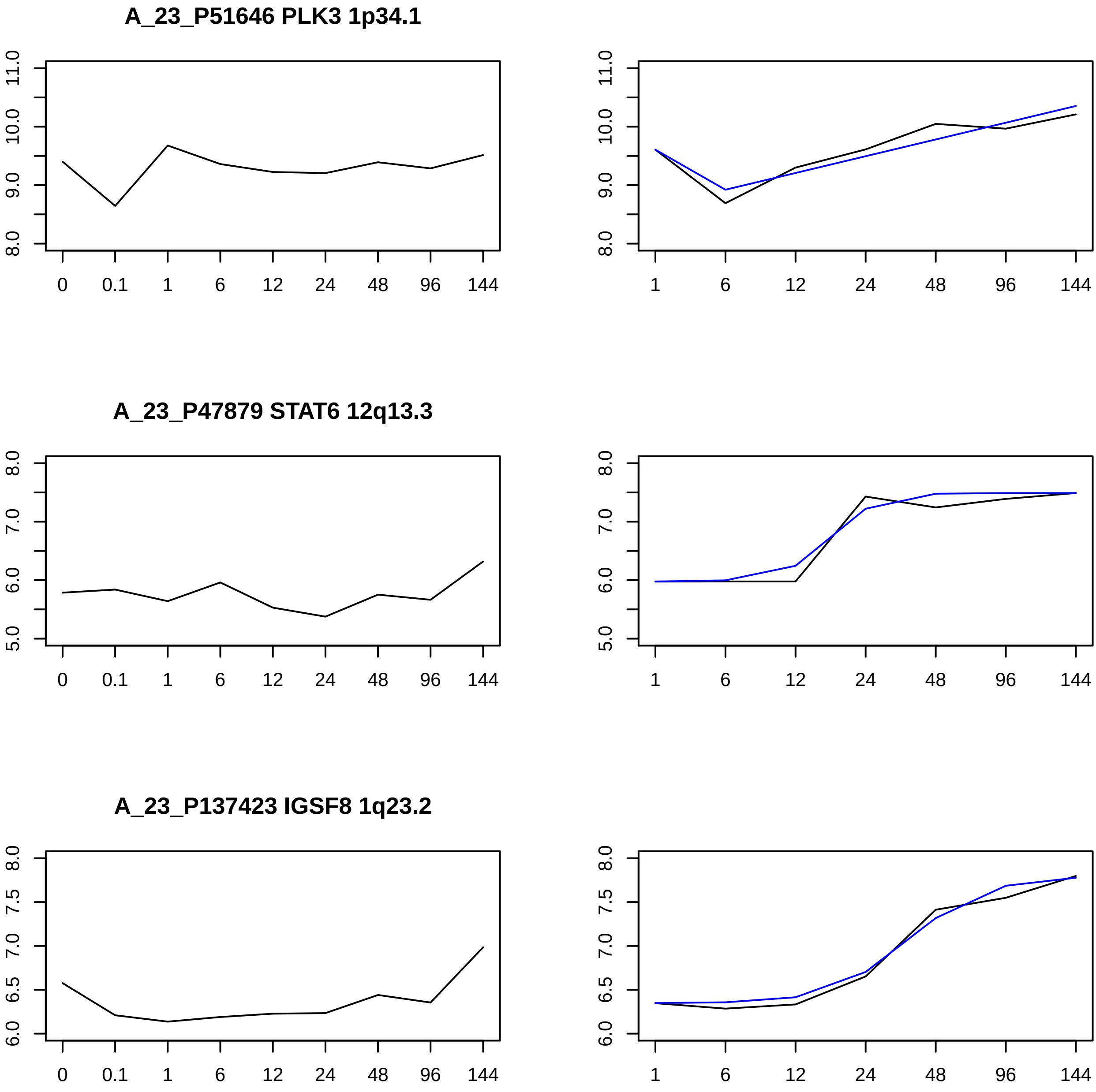
A_23_P51646 PLK3 1p34.1
8.0
9.0
10.0
11.0
0
0.1
1
6
12
24
48
96
144
1
6
12
24
48
96
144
A_23_P47879 STAT6 12q13.3
5.0
6.0
7.0
8.0
A_23_P137423 IGSF8 1q23.2
6.0
6.5
7.0
7.5
8.0

**A_23_P368484 FAM211A 17p11.2**

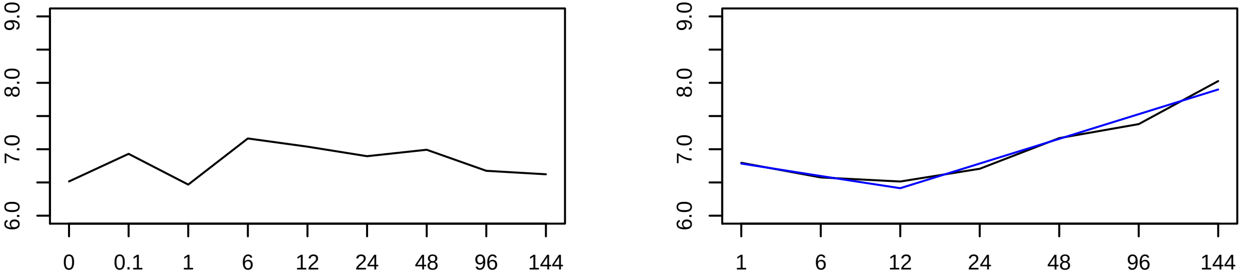


**A_23_P425917 C6orf188 6q22.1**

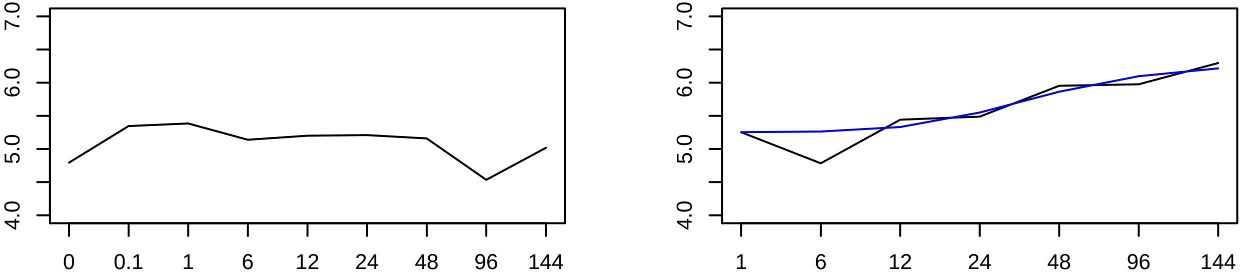


**A_24_P50543 TRIM69 15q21.1**

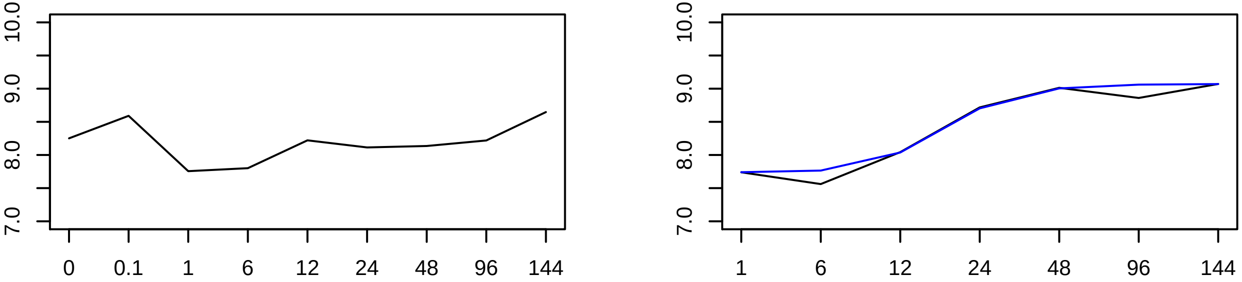

**A_23_P41227 ALCAM 3q13.11**

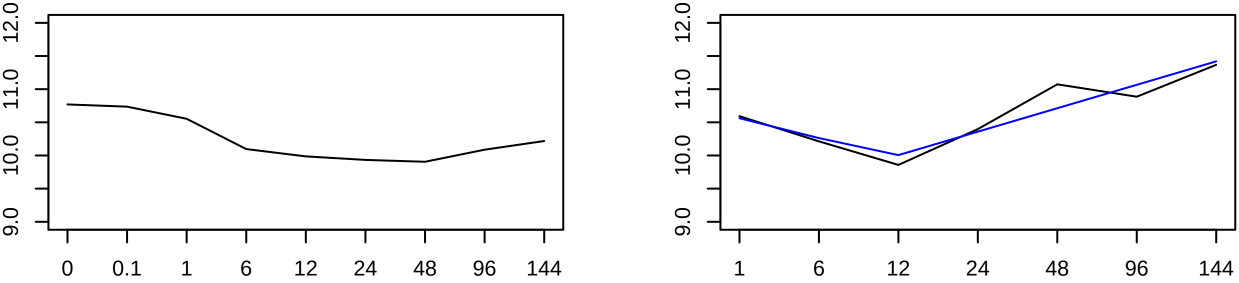


**A_23_P435904 LRCH3 NA**

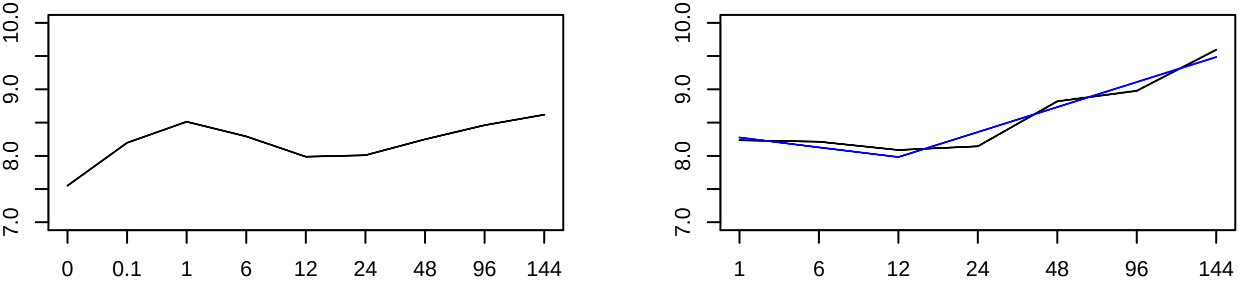


**A_23_P257583 DENND2A 7q34**

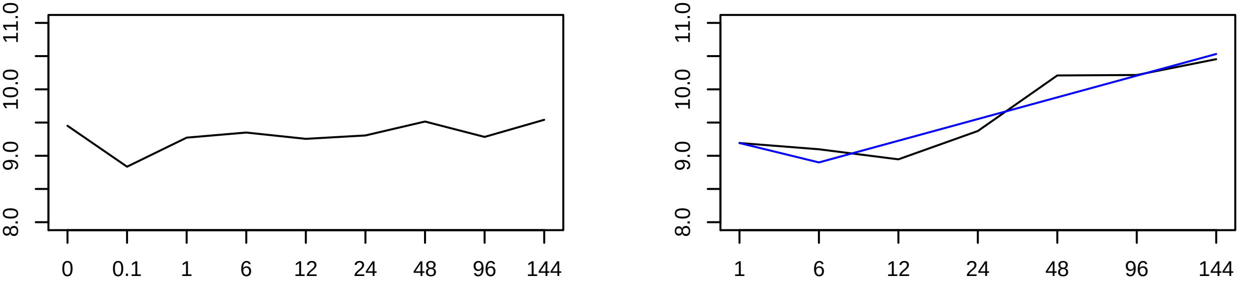

**A_23_P35684 INPP5F 10q26.11**

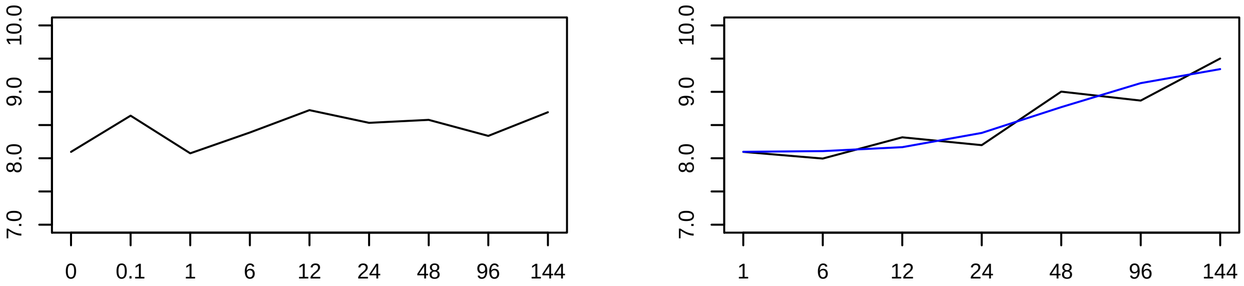


**A_32_P157465 AK091904 NA**

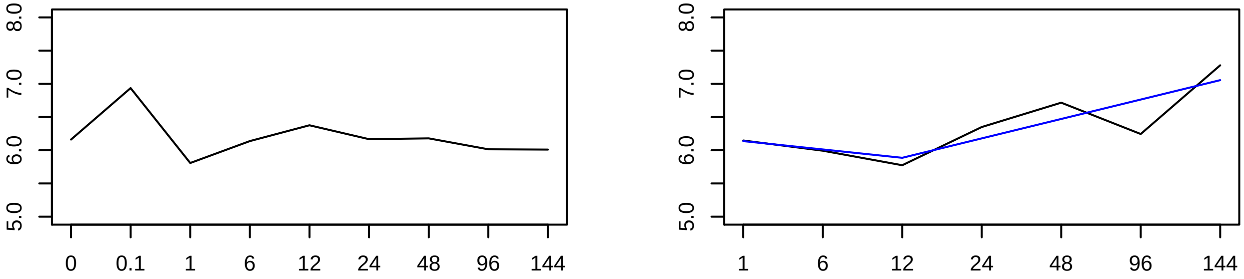


**A_23_P38446 TNFAIP1 17q11.2**

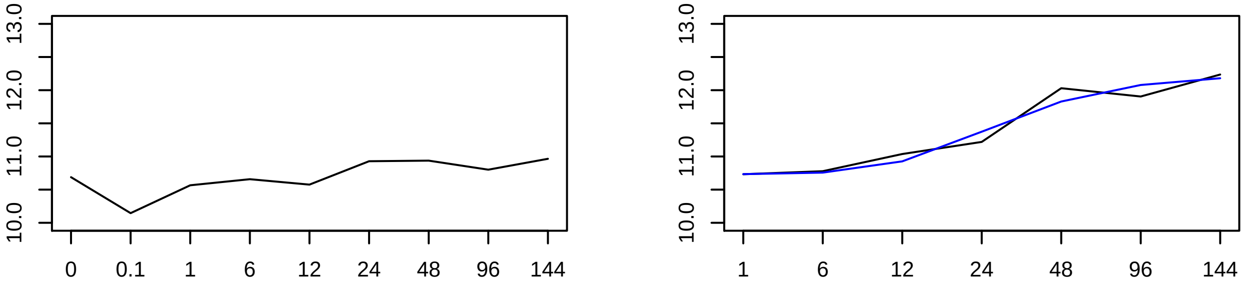

**A_23_P157371 FAM3C 7q31.31**

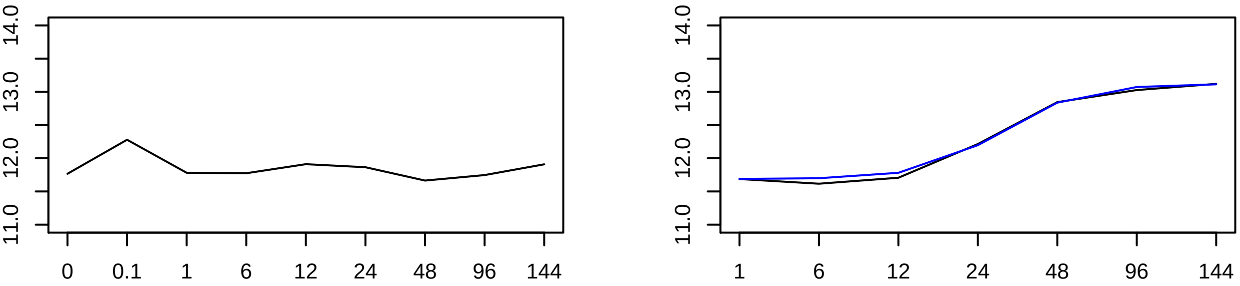


**A_23_P408376 HSPA12A 10q25.3**

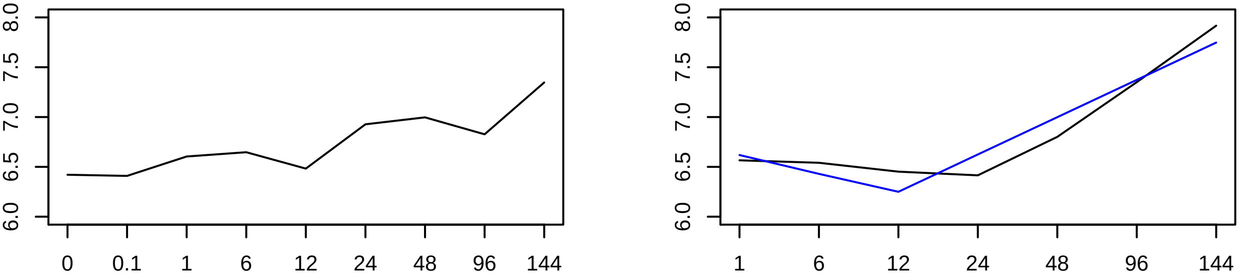


**A_24_P904845 AK021804 NA**

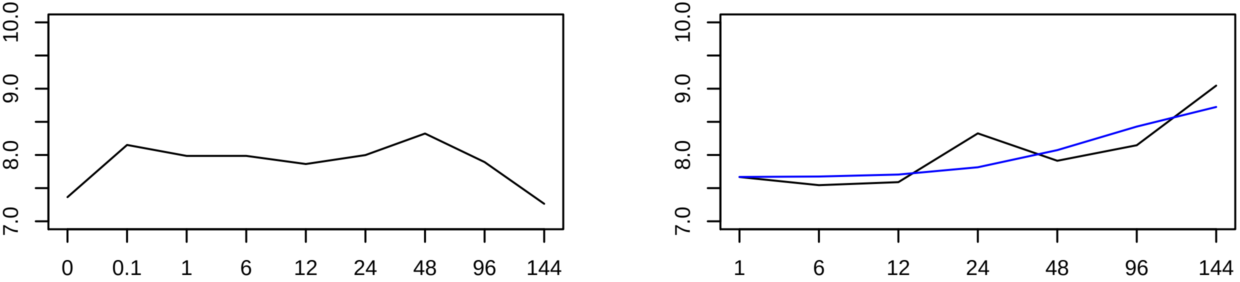

**A_23_P32913 SVH 7q22.1**

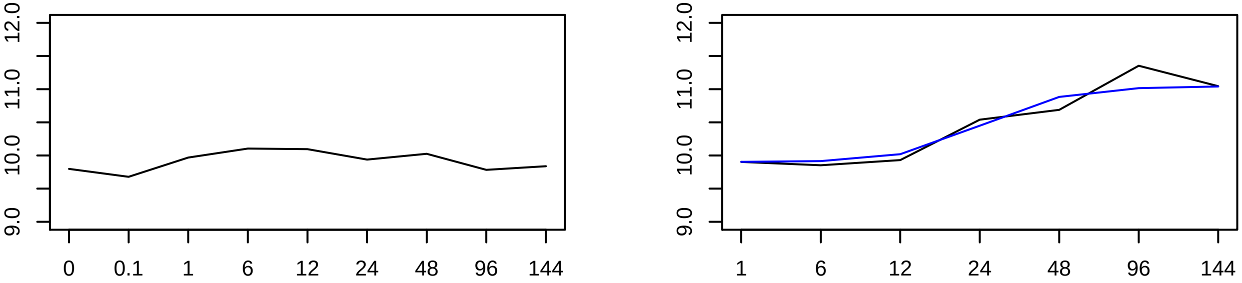


**A_32_P39049 AK055214 NA**

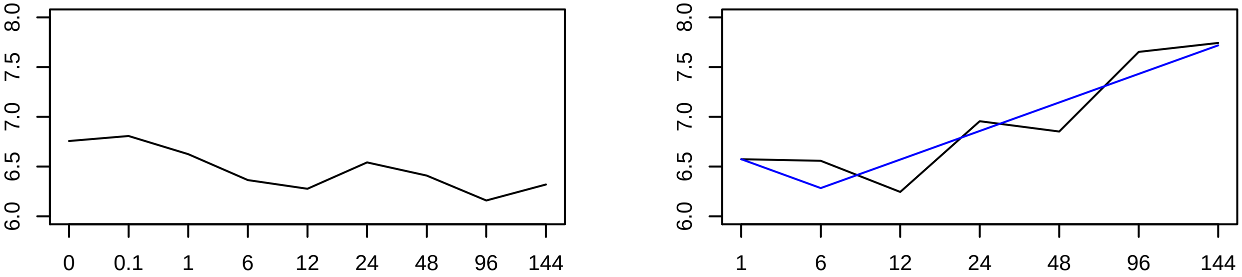


**A_32_P158376 A_32_P158376 NA**

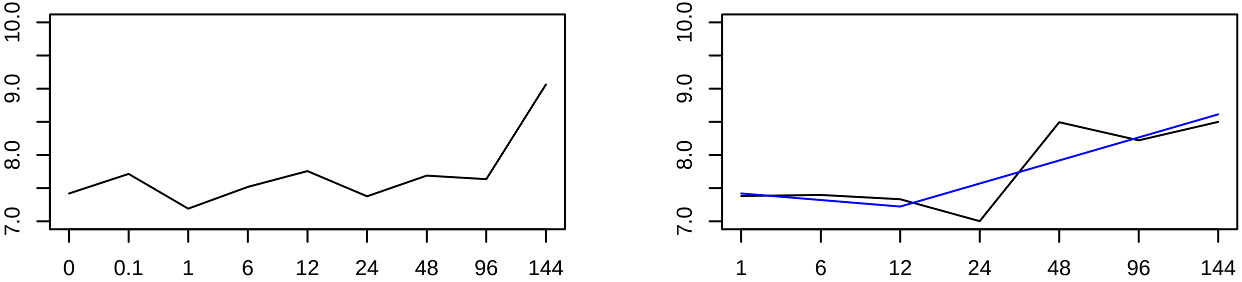

**A_24_P54879 SCARB2 4q21.1**

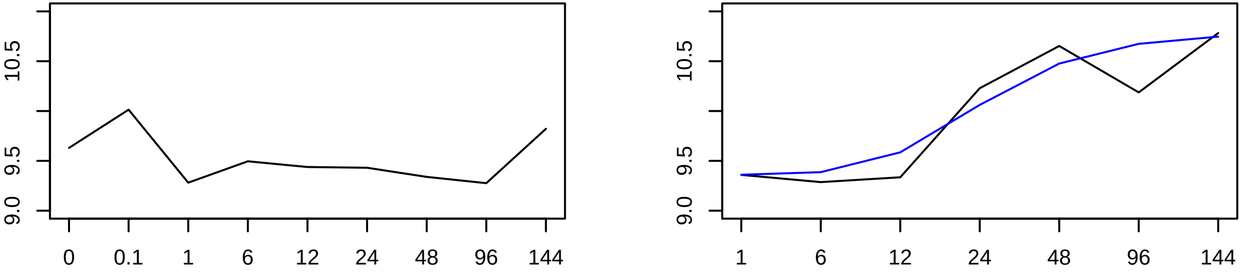


**A_23_P371824 TUFT1 1q21.3**

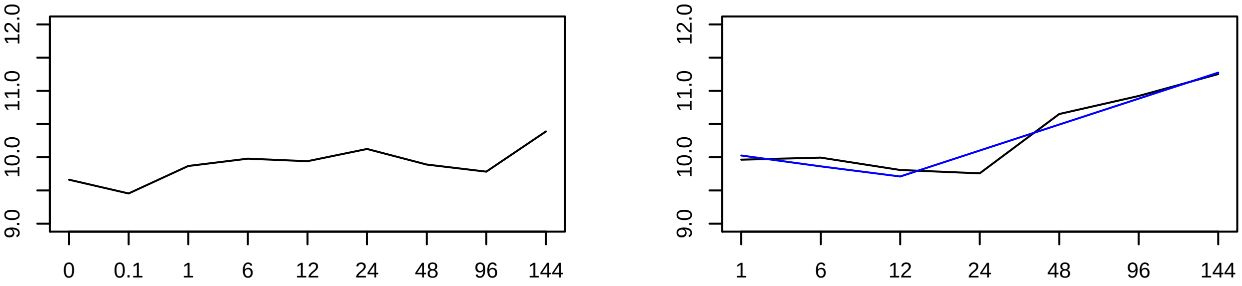


**A_23_P165608 SEMA4F 2p13.1**

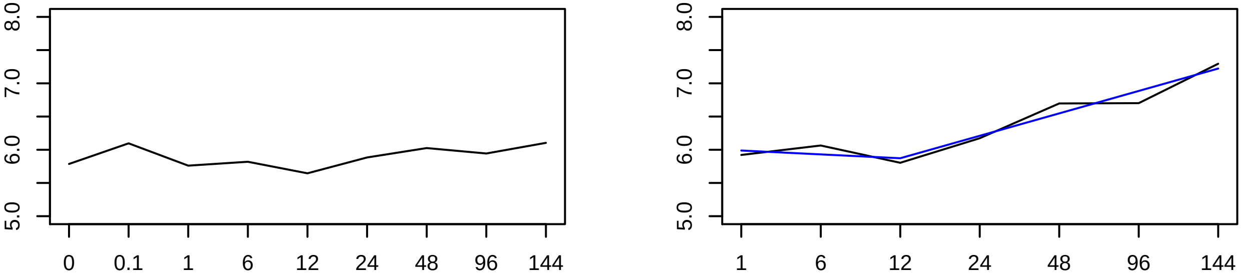

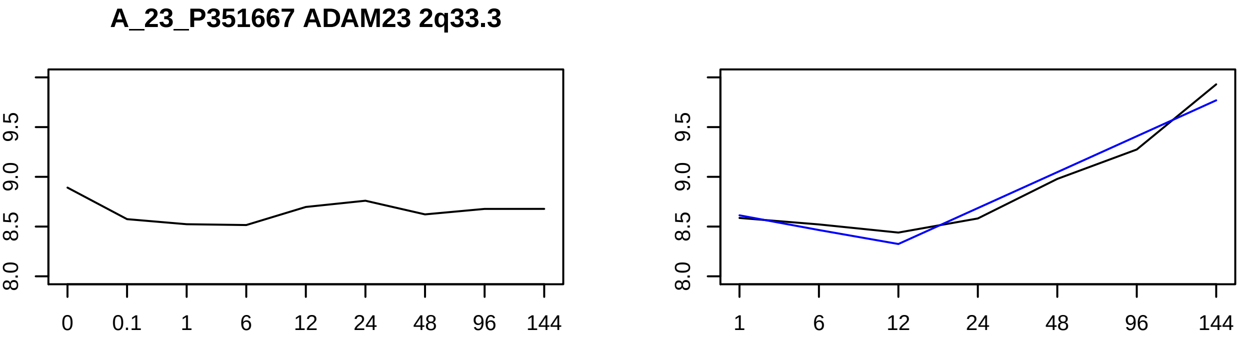
A_23_P351667 ADAM23 2q33.3
8.0
8.5
9.0
9.5
0 0.1 1 6 12 24 48 96 144
1 6 12 24 48 96 144

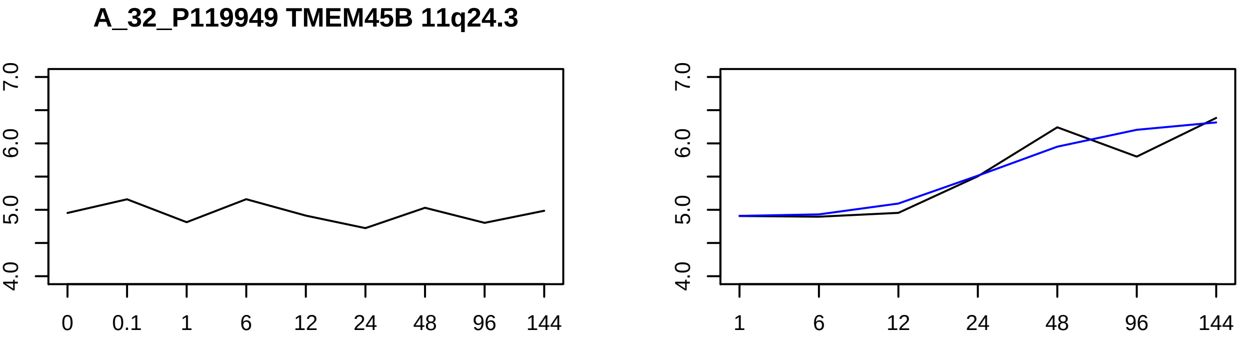
A_32_P119949 TMEM45B 11q24.3
4.0
5.0
6.0
7.0
0 0.1 1 6 12 24 48 96 144
1 6 12 24 48 96 144

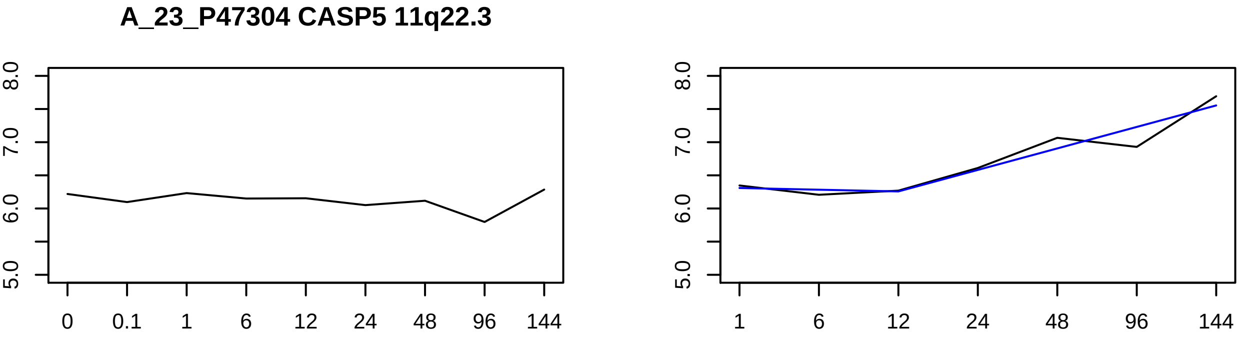
A_23_P47304 CASP5 11q22.3
5.0
6.0
7.0
8.0
0 0.1 1 6 12 24 48 96 144
1 6 12 24 48 96 144

**A_23_P216549 RUSC2 9p13.3**

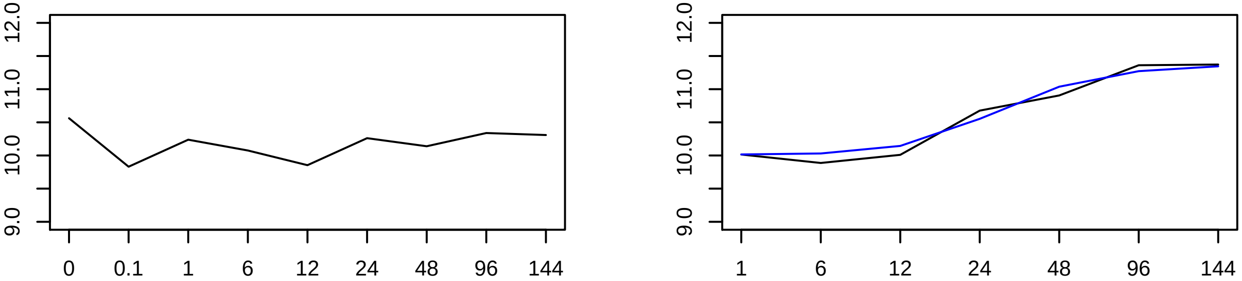


**A_23_P387000 XKR6 8p23.1**

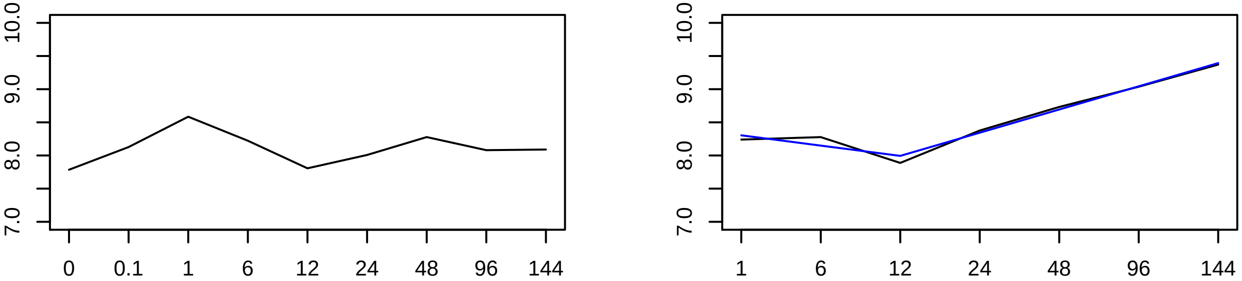


**A_23_P259094 ZNF435 6p22.1**

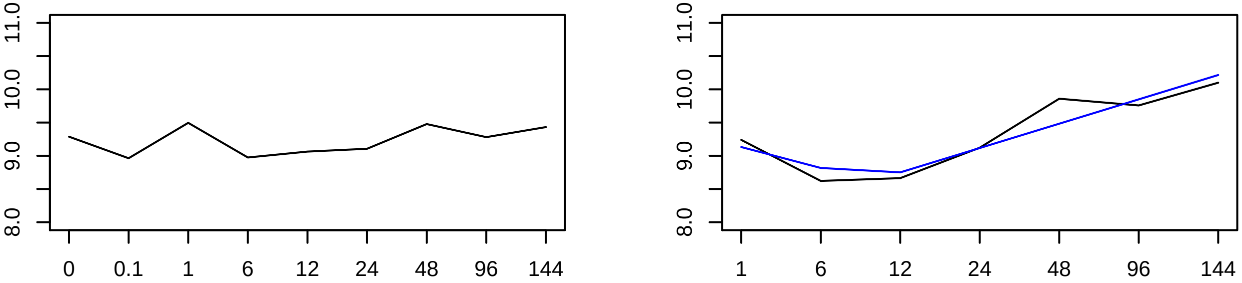

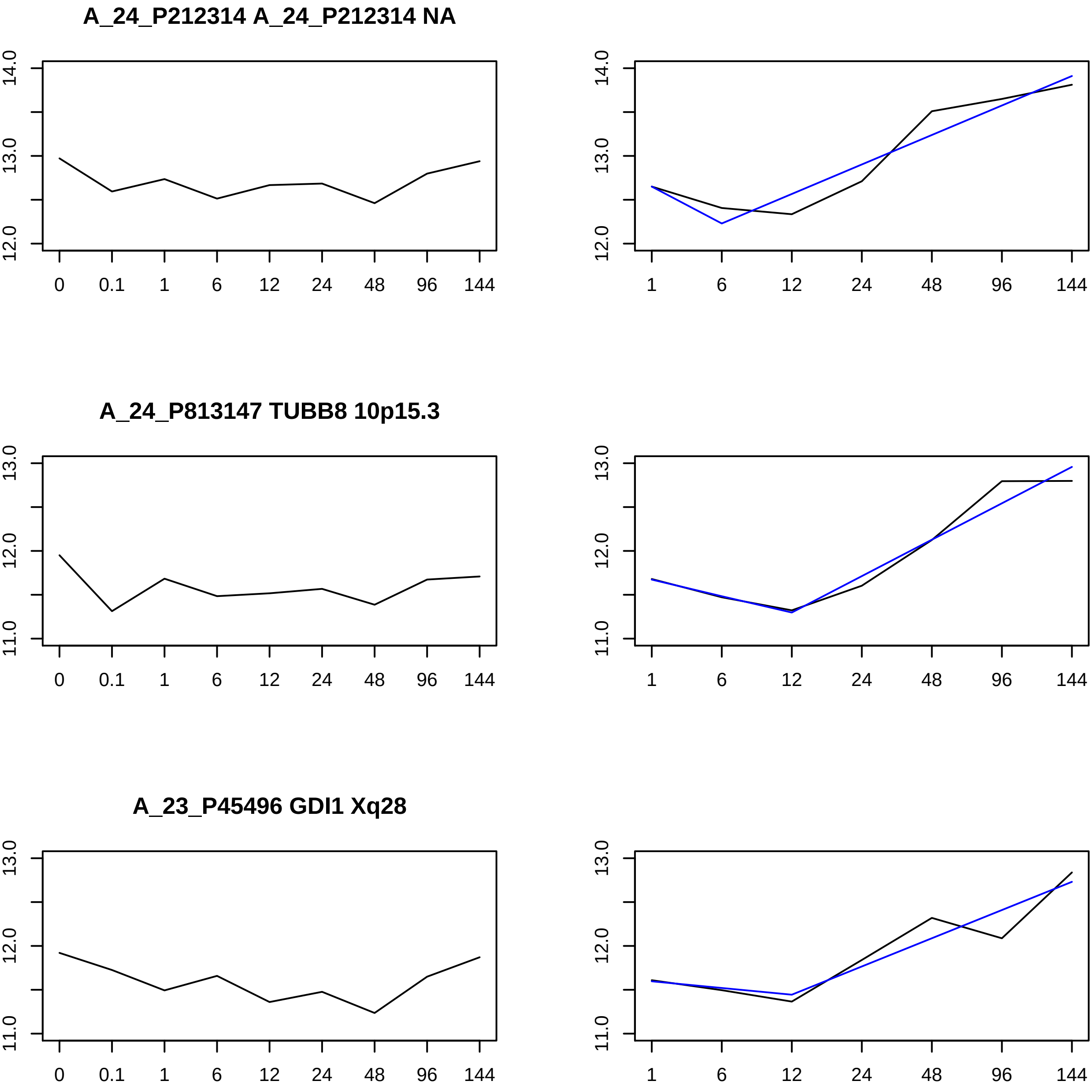
A_24_P212314 A_24_P212314 NA
12.0
13.0
14.0
0
0.1
1
6
12
24
48
96
144
A_24_P813147 TUBB8 10p15.3
11.0
12.0
13.0
A_23_P45496 GDI1 Xq28
1
6
12
24
48
96
144

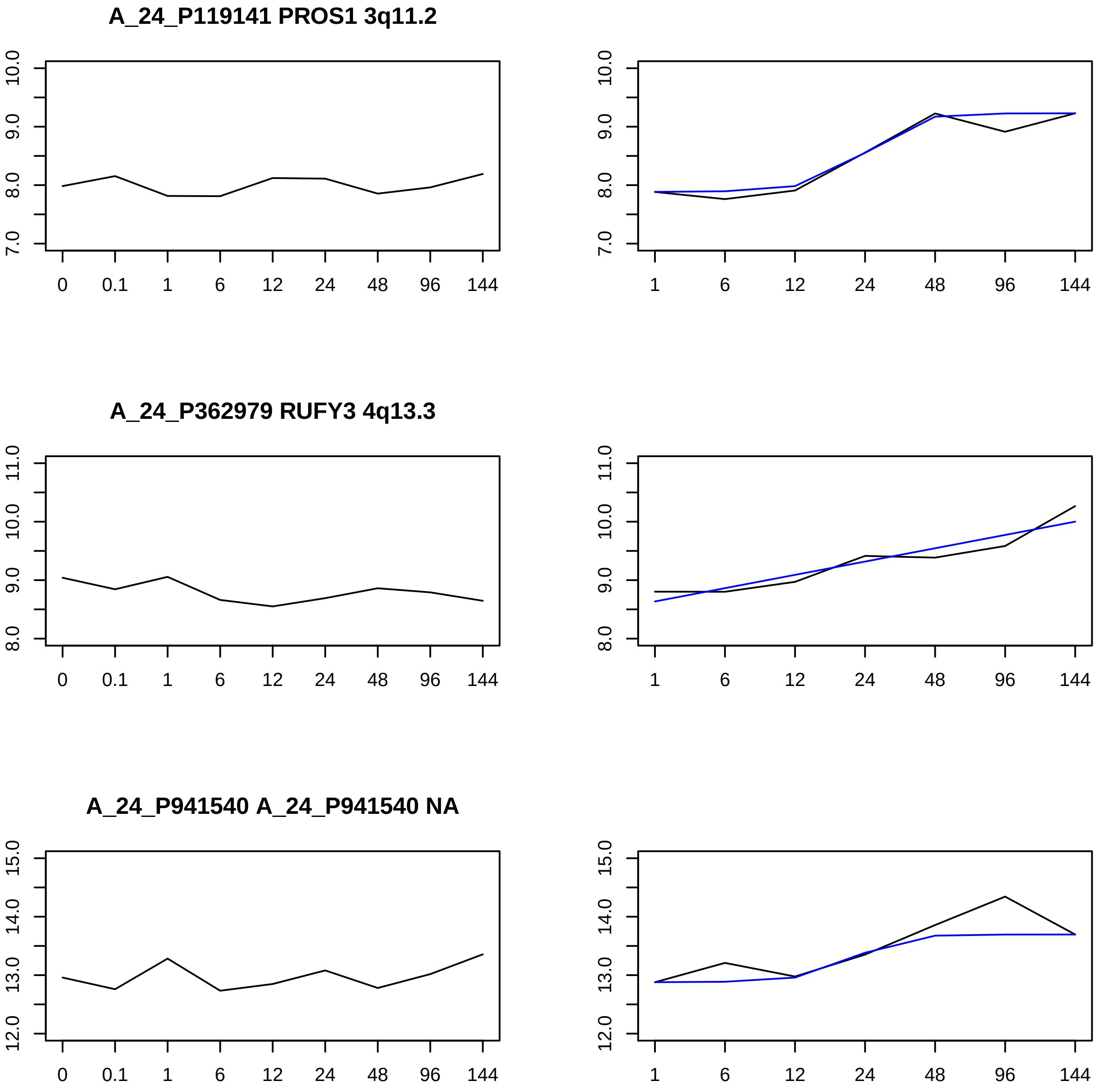
A_24_P119141 PROS1 3q11.2
A_24_P362979 RUFY3 4q13.3
A_24_P941540 A_24_P941540 NA
0
0.1
1
6
12
24
48
96
144

**A_23_P26037 FRMD5 15q15.3**

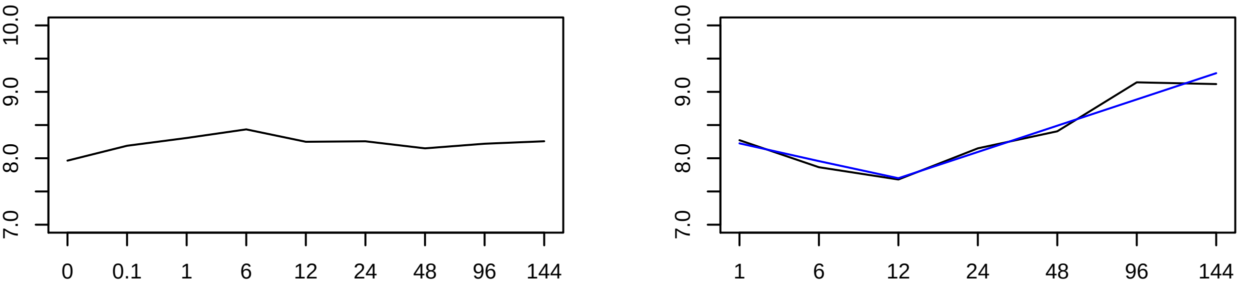


**A_23_P125717 NAP1L3 Xq21.32**

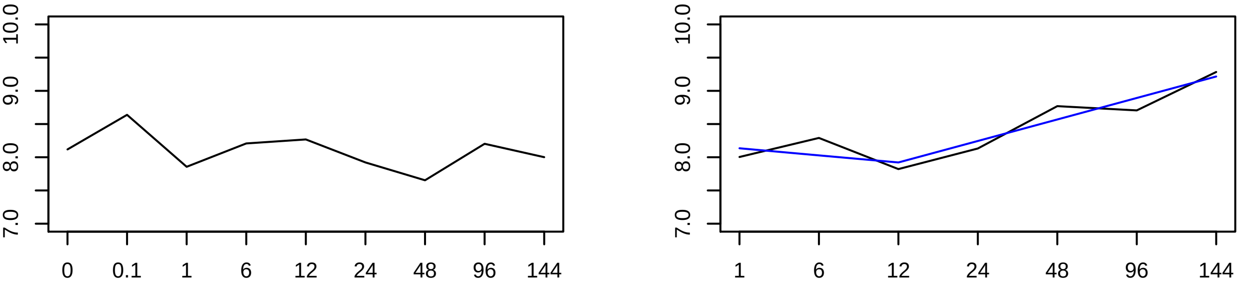


**A_23_P141974 TPM4 19p13.12**

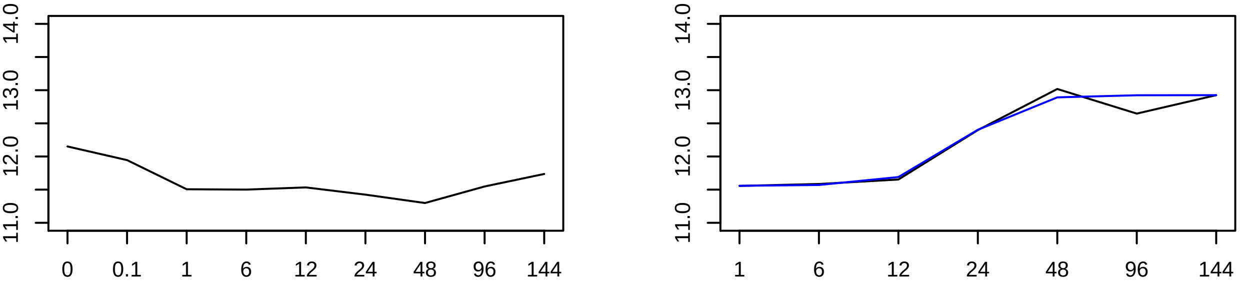

A_23_P51548 MGST3 1q24.1

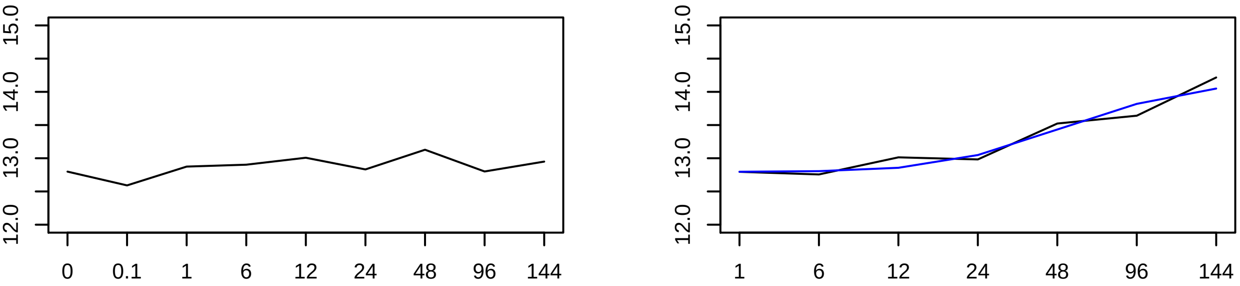


A_23_P319617 CHST7 Xp11.3

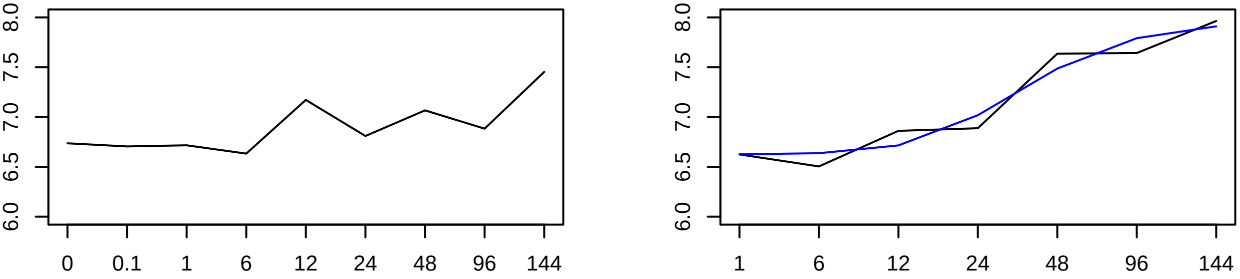


A_24_P367289 DDR1 6p21.33

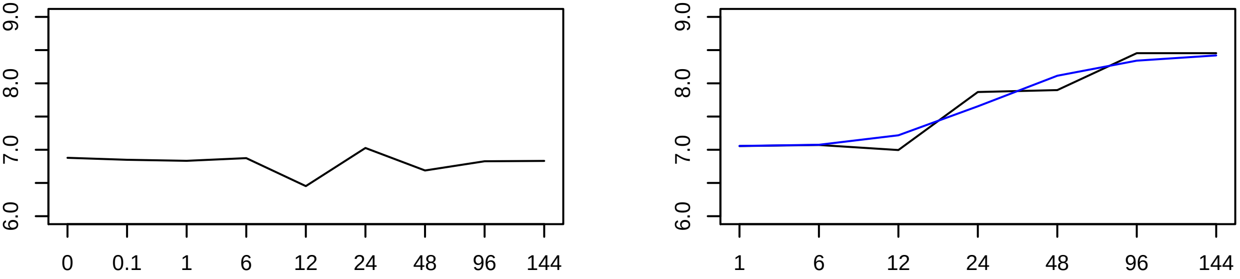

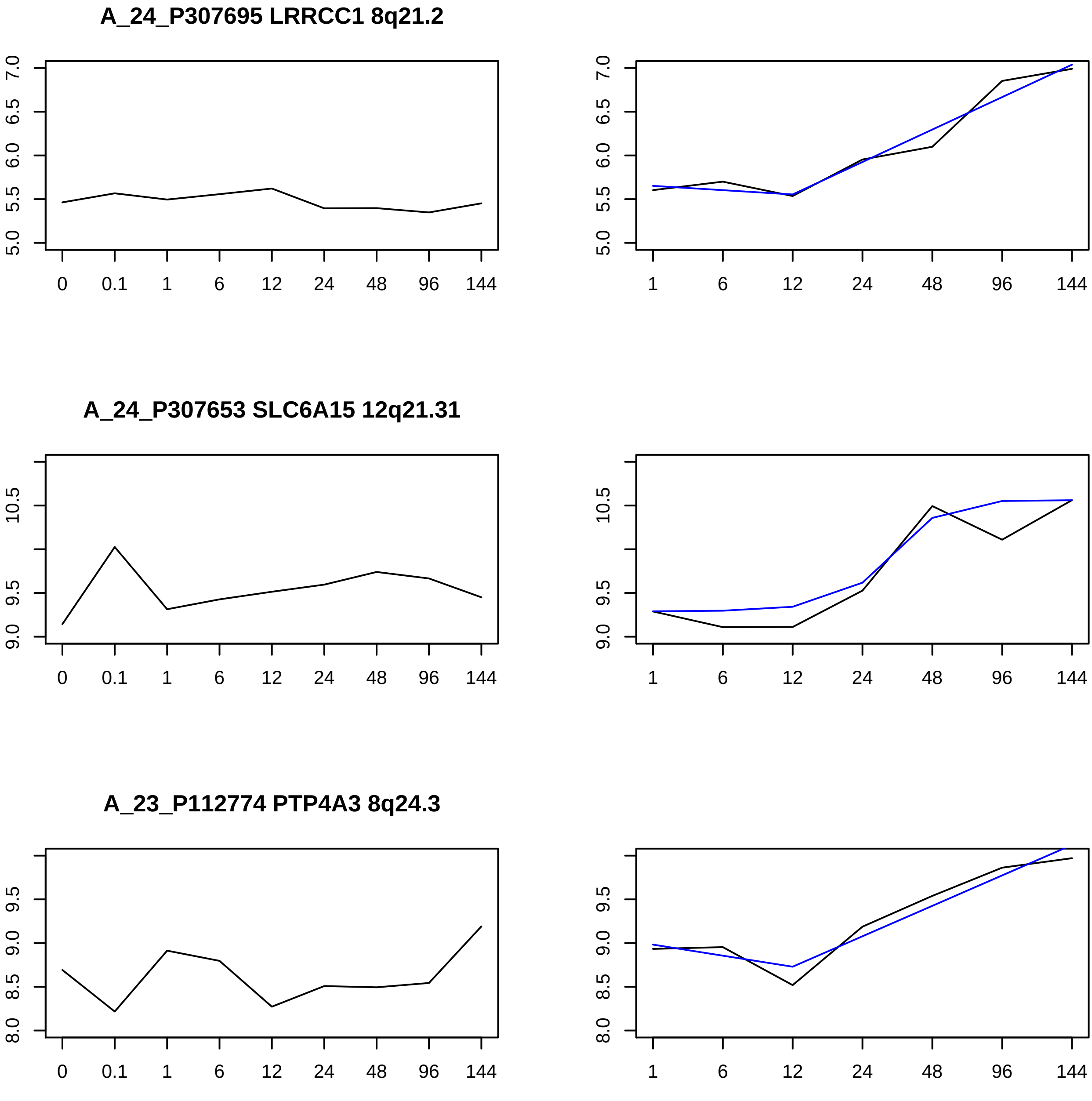

A_24_P307695 LRRCC1 8q21.2
5.0
5.5
6.0
6.5
7.0
0
0.1
1
6
12
24
48
96
144
A_24_P307653 SLC6A15 12q21.31
9.0
9.5
10.5
A_23_P112774 PTP4A3 8q24.3
8.0
8.5
9.0
9.5

**A_32_P11325 THC2505770 NA**

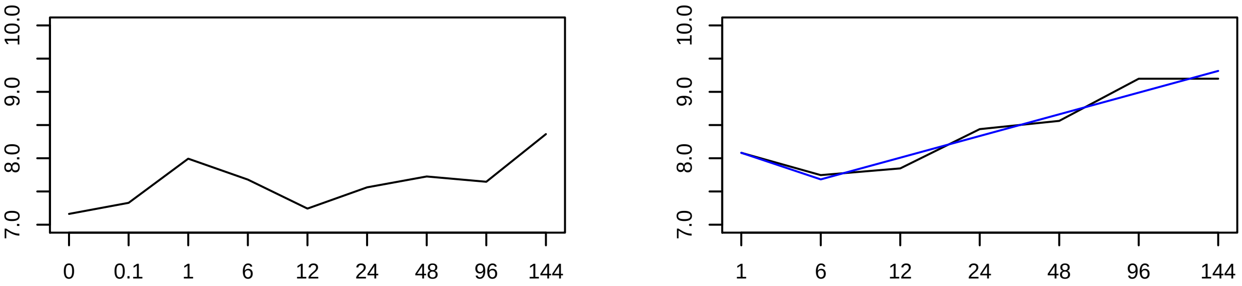


**A_24_P193295 RAB15 14q23.3**

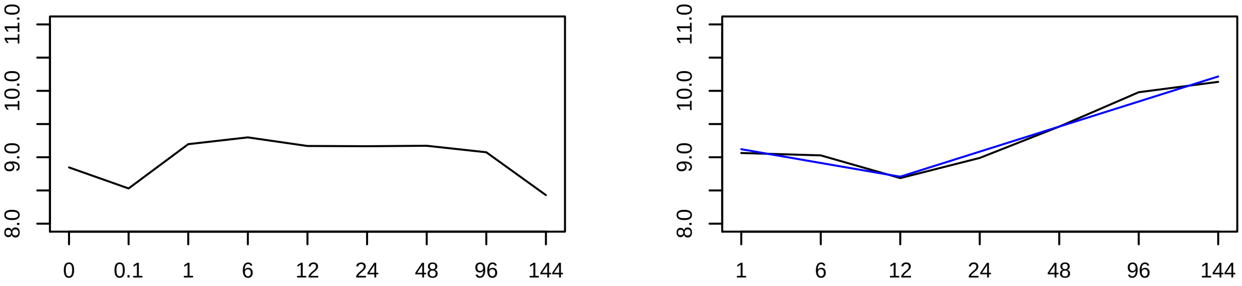


**A_32_P116989 ZCCHC18 NA**

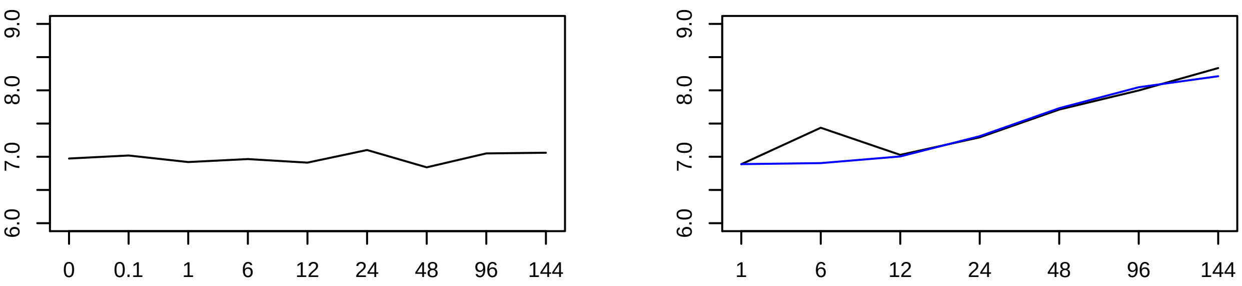

**A_32_P33304 ANK3 10q21.2**

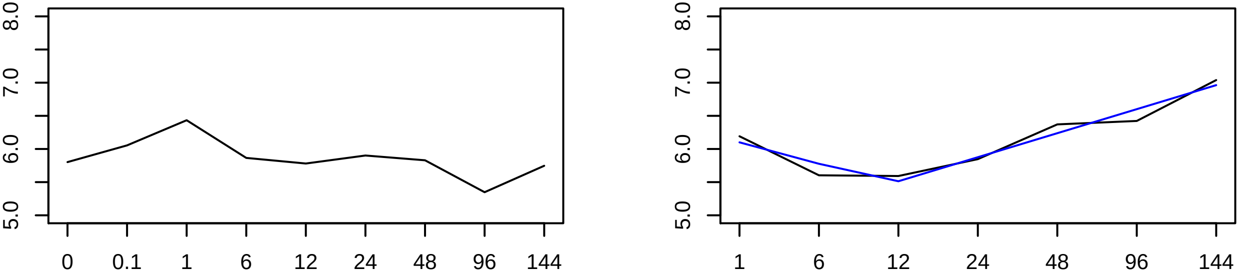


**A_23_P203505 TRIM5 11p15.4**

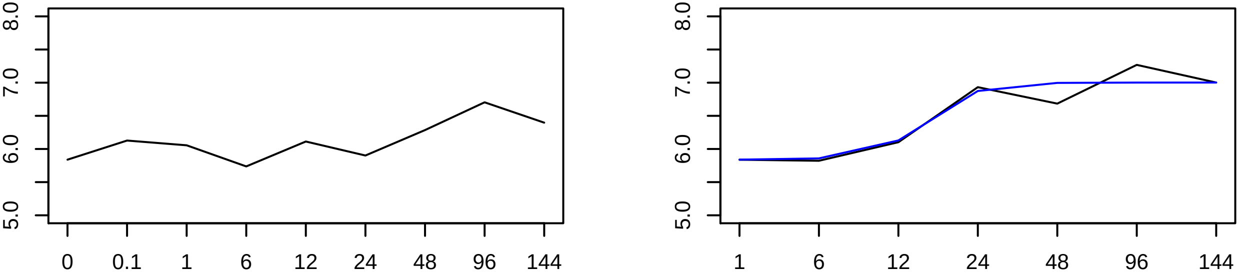


**A_23_P111745 ZMIZ2 7p13**

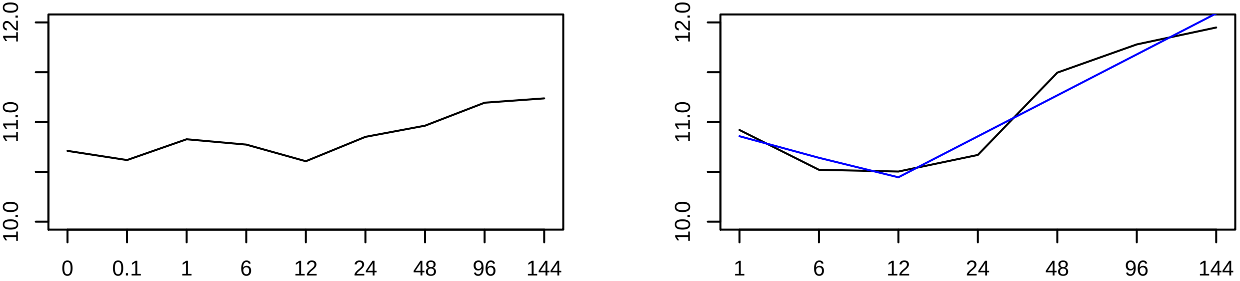

**A_23_P215625 ADAM22 7q21.12**

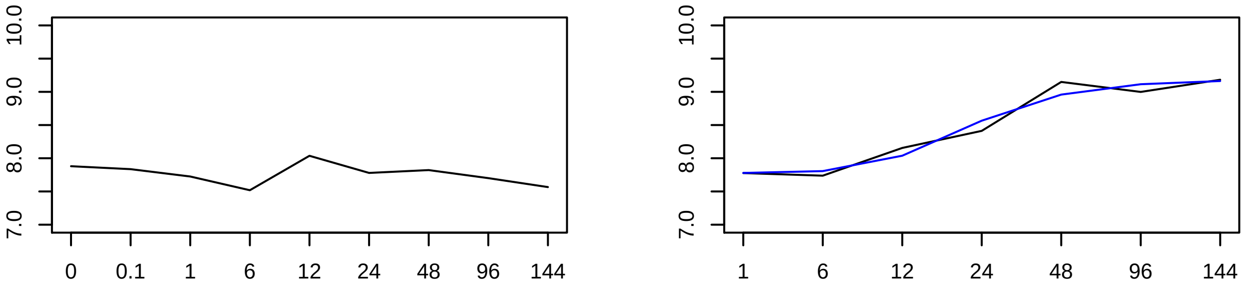


**A_23_P415033 ARL8A 1q32.1**

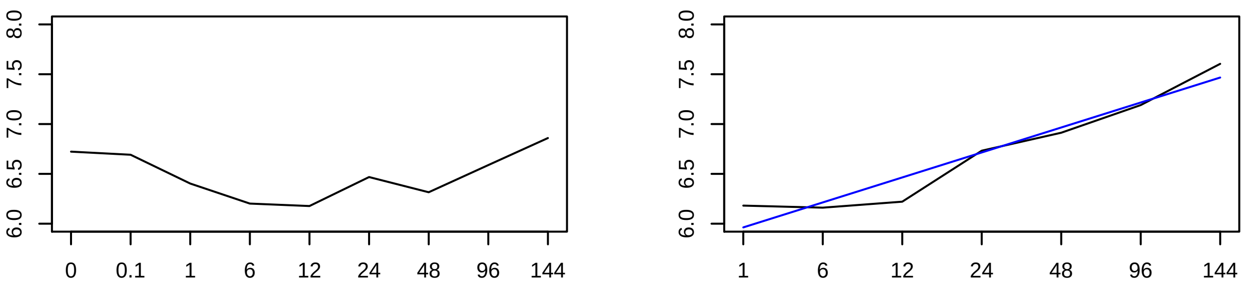


**A_32_P181222 KCNMA1 10q22.3**

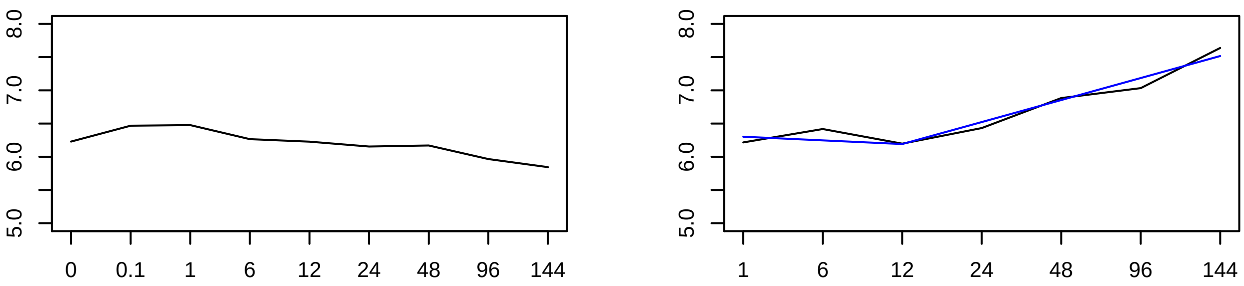

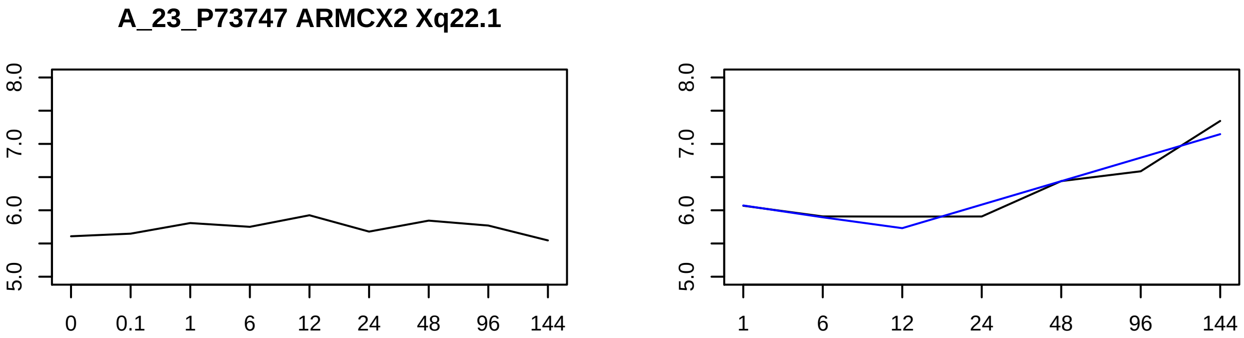
A_23_P73747 ARMCX2 Xq22.1
5.0 6.0 7.0 8.0
0 0.1 1 6 12 24 48 96 144
1 6 12 24 48 96 144

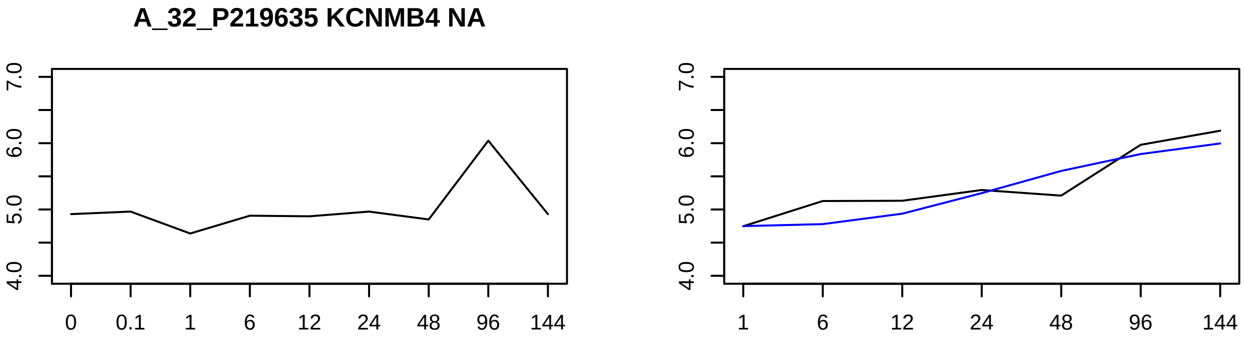
A_32_P219635 KCNMB4 NA
4.0 5.0 6.0 7.0
0 0.1 1 6 12 24 48 96 144
1 6 12 24 48 96 144

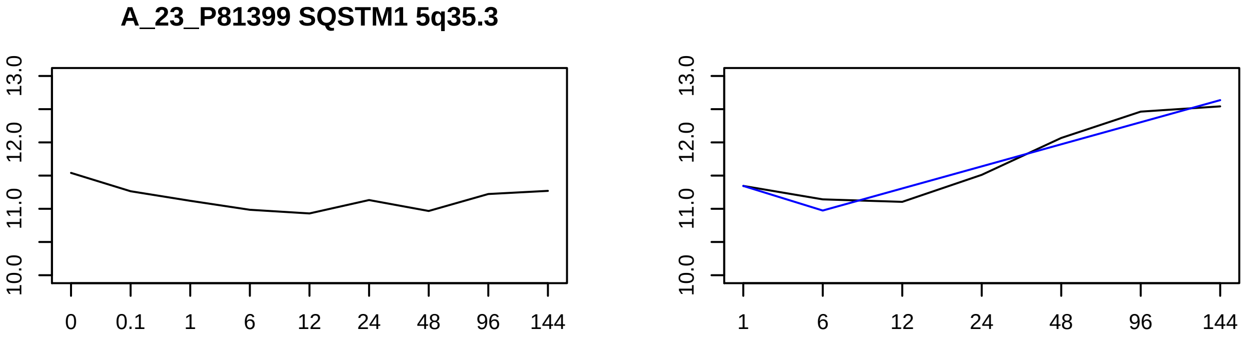
A_23_P81399 SQSTM1 5q35.3
10.0 11.0 12.0 13.0
0 0.1 1 6 12 24 48 96 144
1 6 12 24 48 96 144

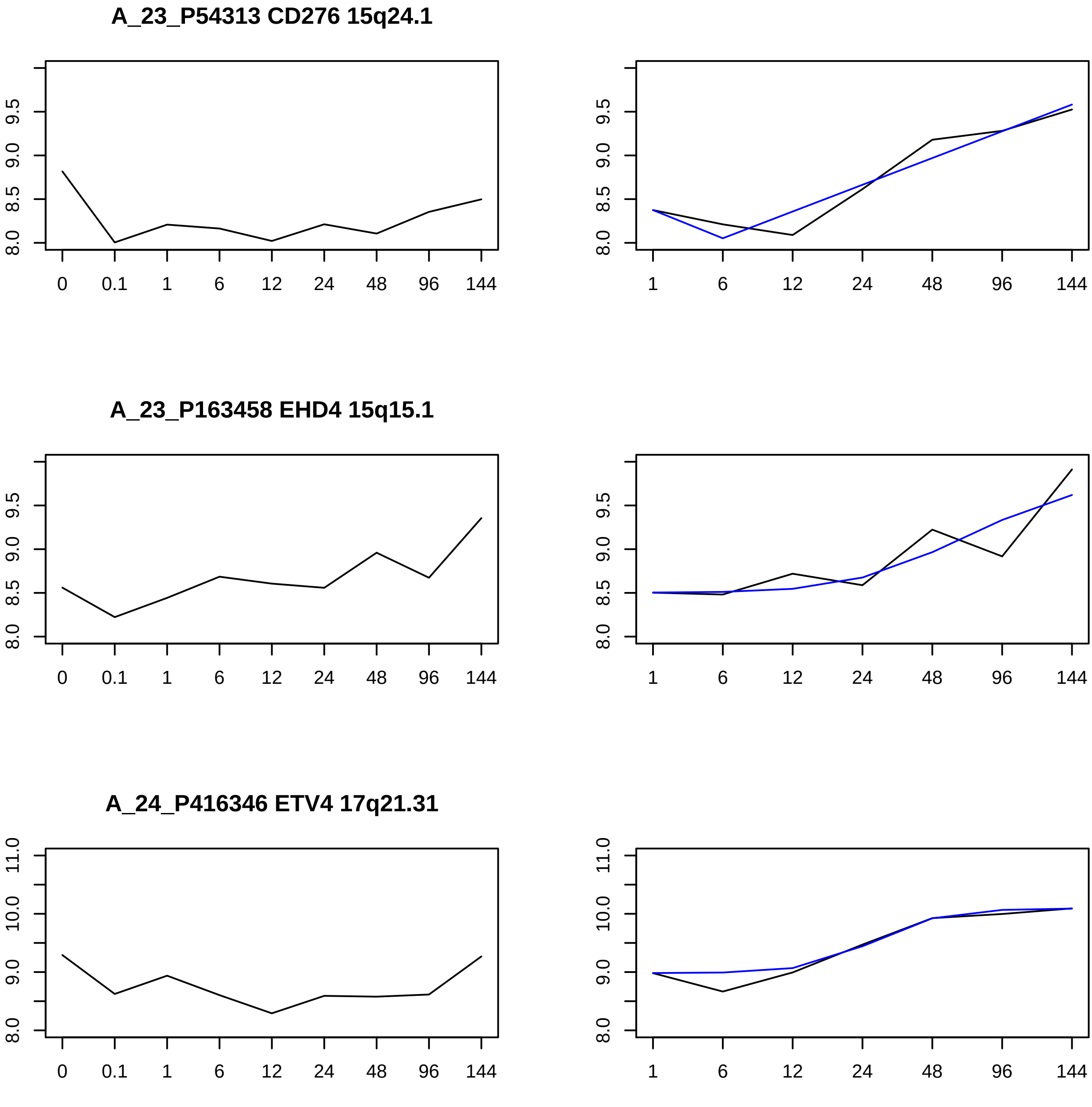
A_23_P54313 CD276 15q24.1
A_23_P163458 EHD4 15q15.1
A_24_P416346 ETV4 17q21.31
8.0
8.5
9.0
9.5
10.0
11.0
0
0.1
1
6
12
24
48
96
144

**A_23_P254271 TUBB6 18p11.21**

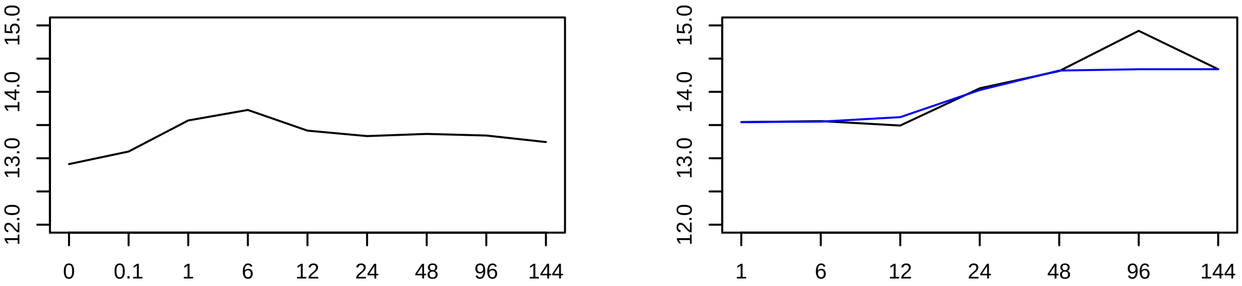


**A_23_P65230 TMTC4 13q32.3**

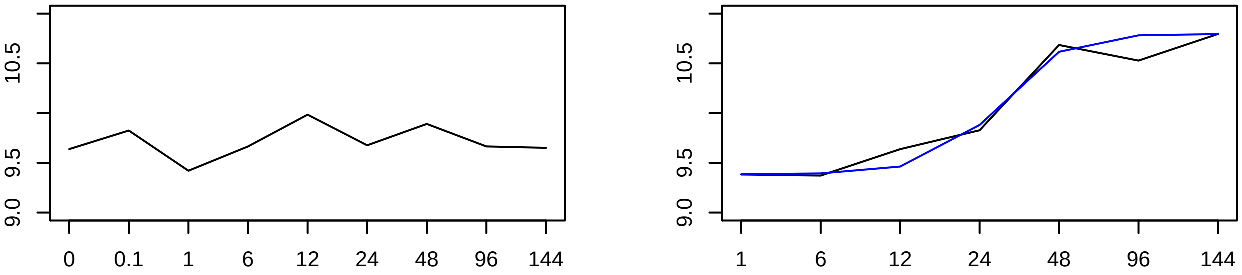


**A_24_P322908 USP27X NA**

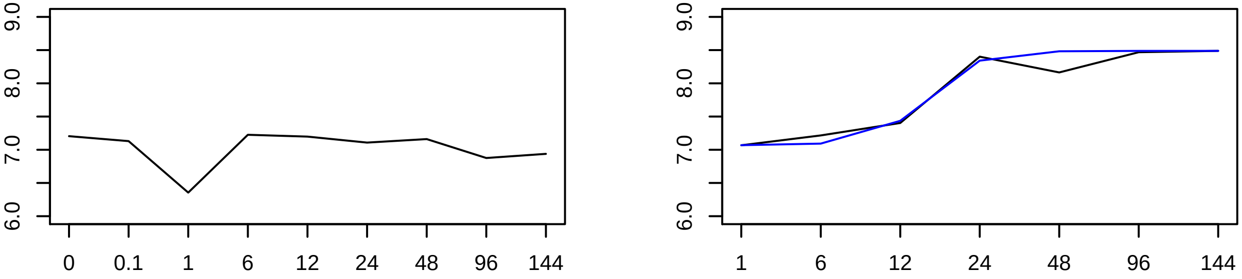

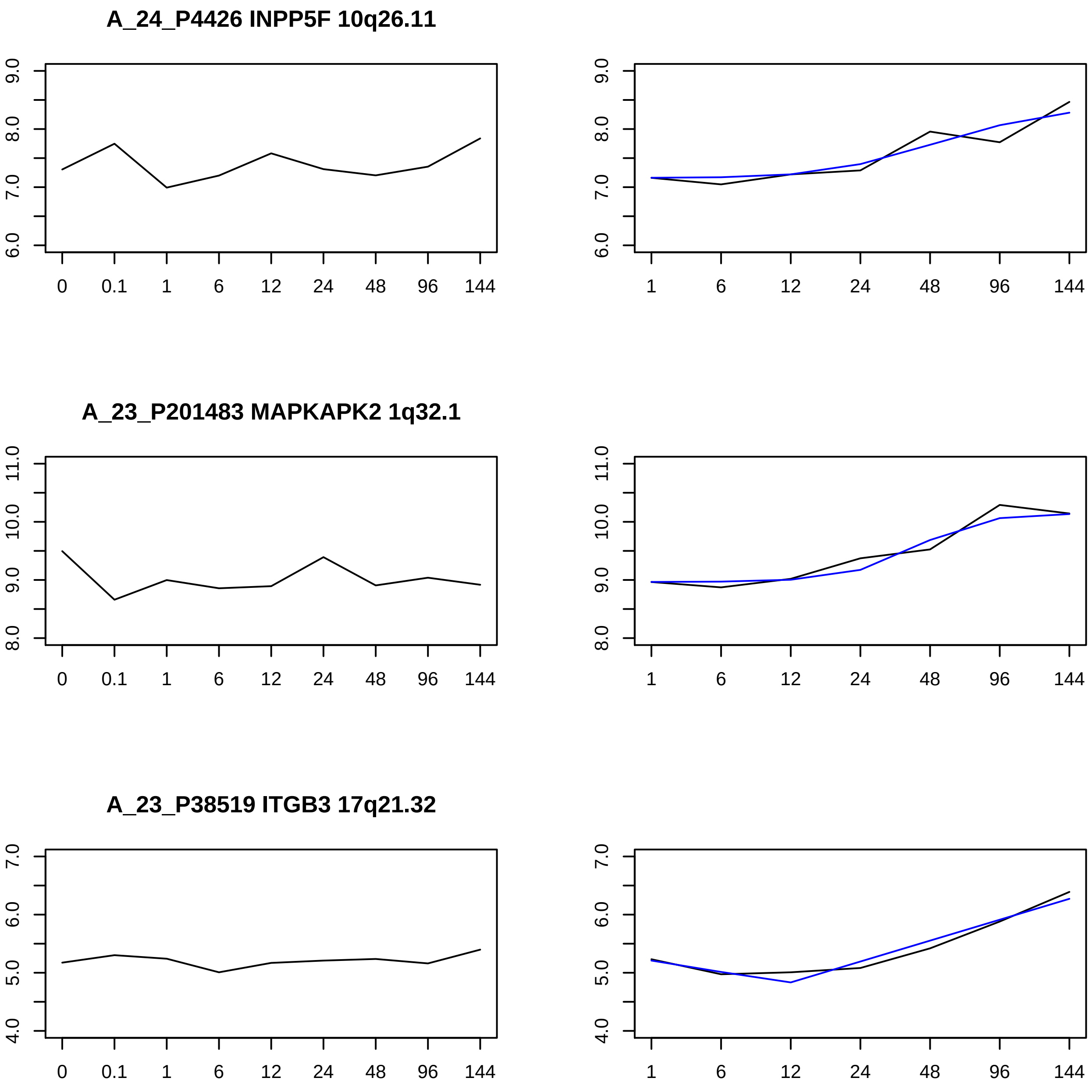
A_24_P4426 INPP5F 10q26.11
A_23_P201483 MAPKAPK2 1q32.1
A_23_P38519 ITGB3 17q21.32
0
0.1
1
6
12
24
48
96
144

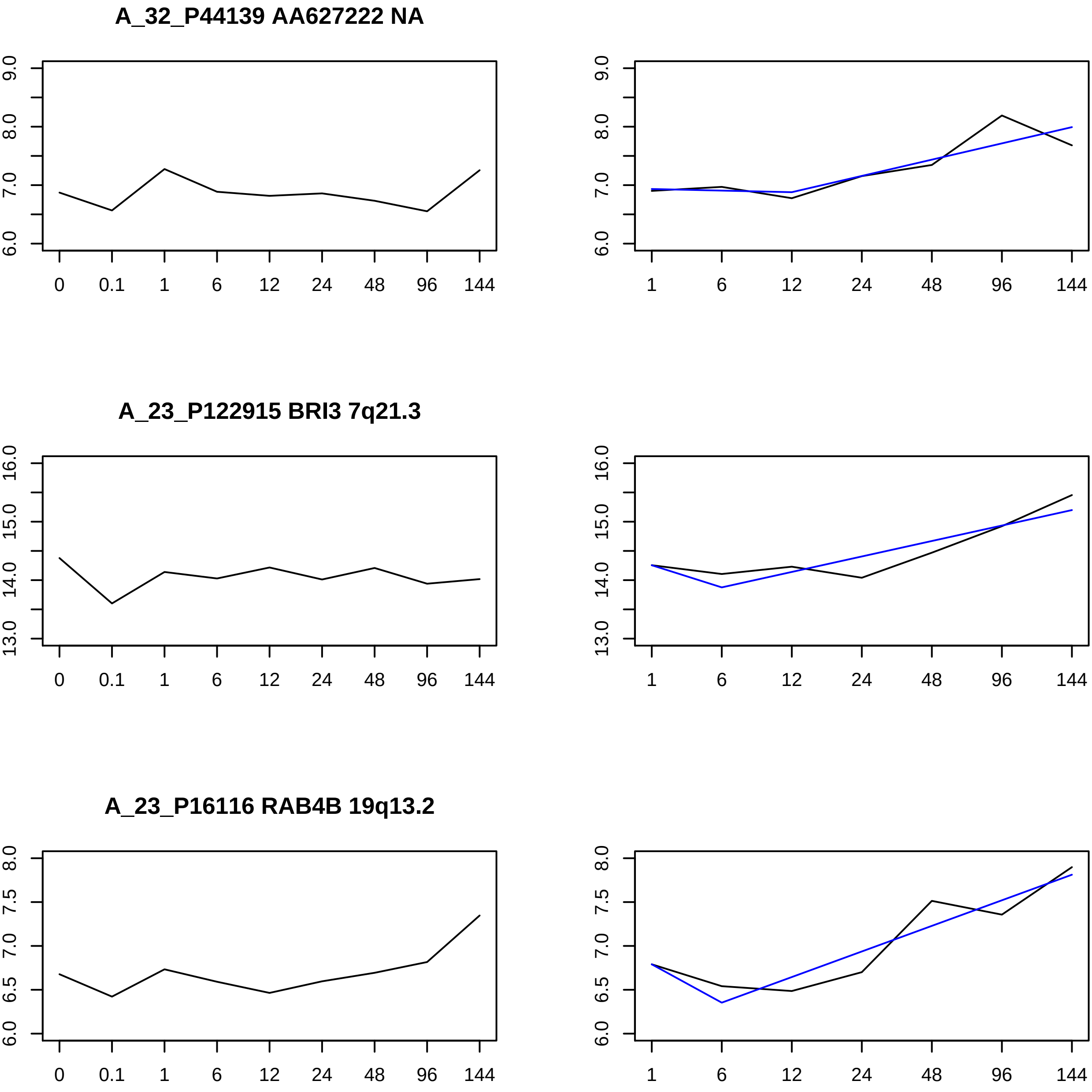

A_32_P44139 AA627222 NA
6.0
7.0
8.0
9.0
0
0.1
1
6
12
24
48
96
144
A_23_P122915 BRI3 7q21.3
13.0
14.0
15.0
16.0
A_23_P16116 RAB4B 19q13.2
6.0
6.5
7.0
7.5
8.0

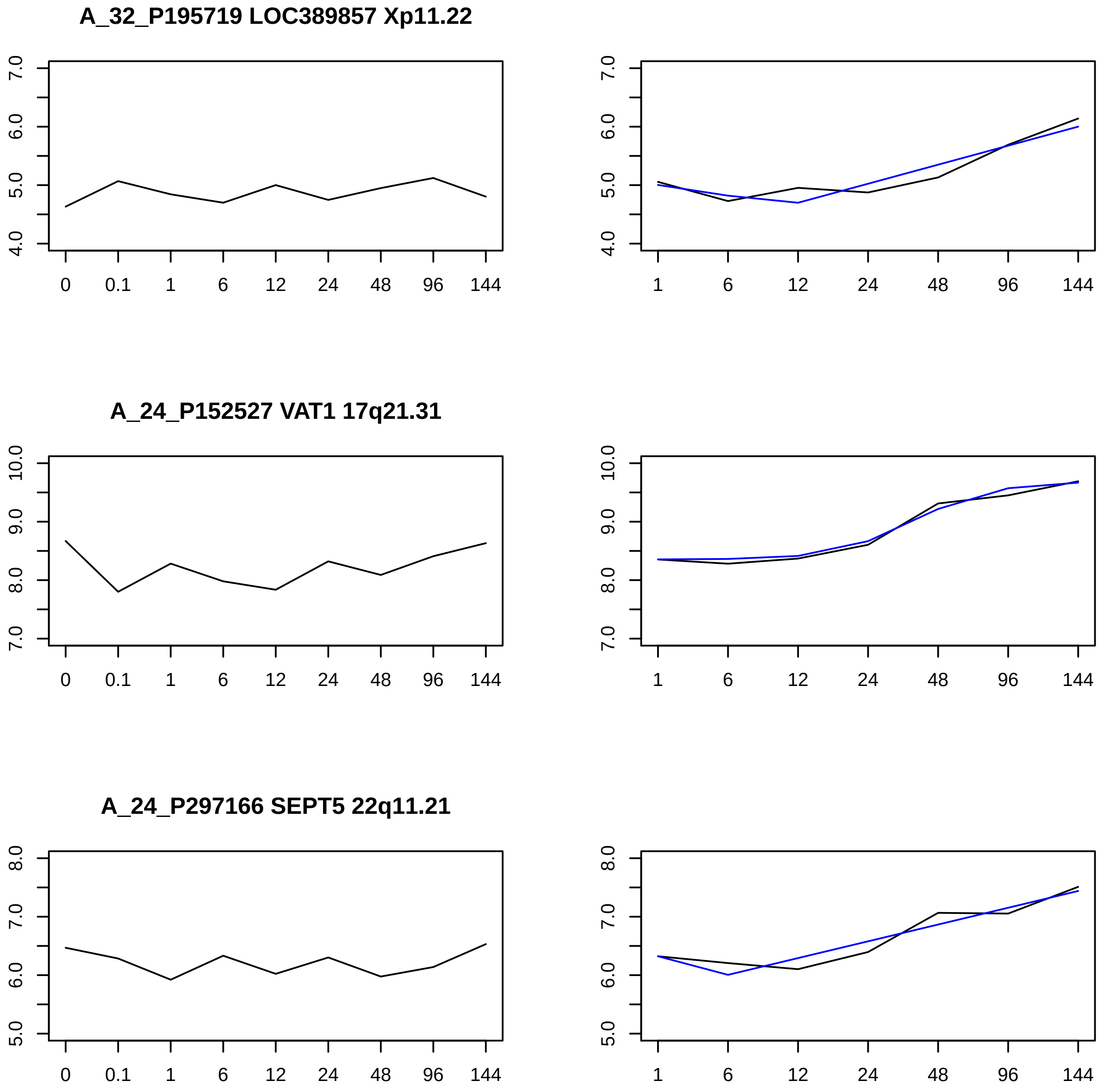

A_32_P195719 LOC389857 Xp11.22
4.0
5.0
6.0
7.0
0
0.1
1
6
12
24
48
96
144
A_24_P152527 VAT1 17q21.31
7.0
8.0
9.0
10.0
A_24_P297166 SEPT5 22q11.21
5.0
6.0
7.0
8.0

A_24_P71468 QPCT 2p22.2

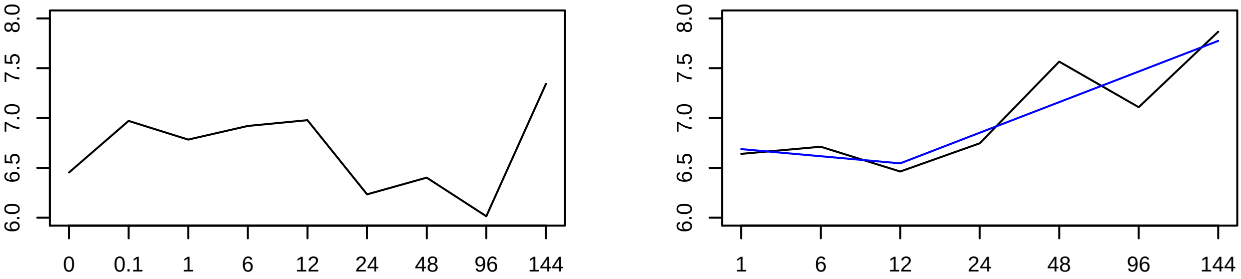


A_23_P154108 DYNC1I2 2q31.1

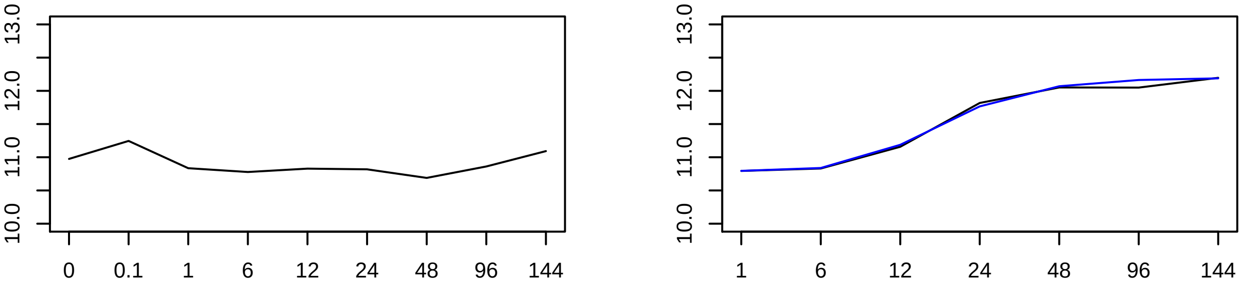


A_24_P373877 SYT5 19q13.42

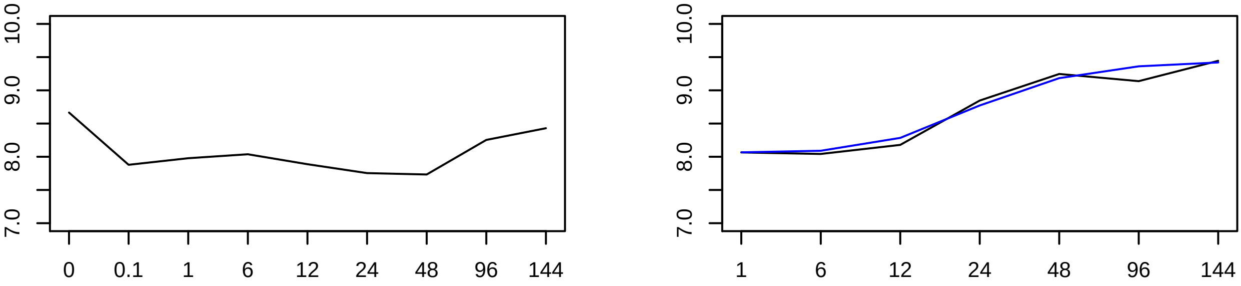

**A_24_P313756 TUBB6 18p11.21**

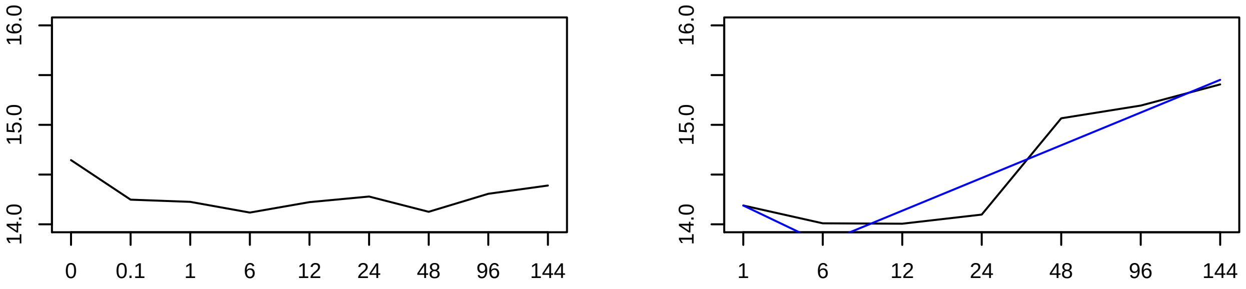


**A_32_P179131 THC2649467 NA**

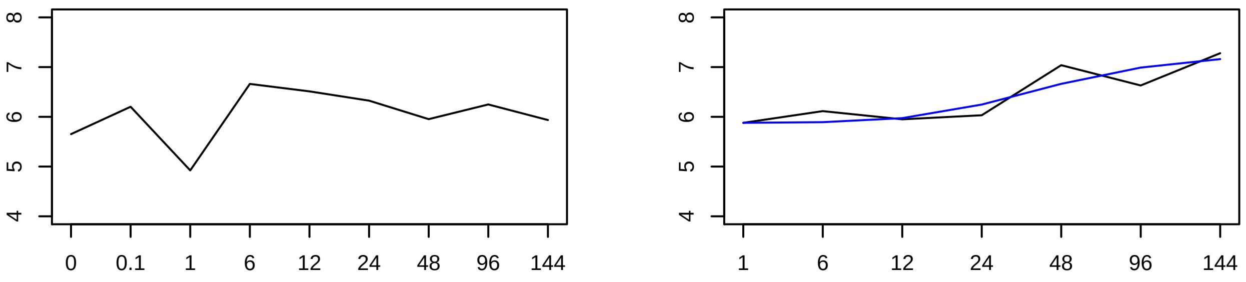


**A_23_P108835 YPEL5 2p23.1**

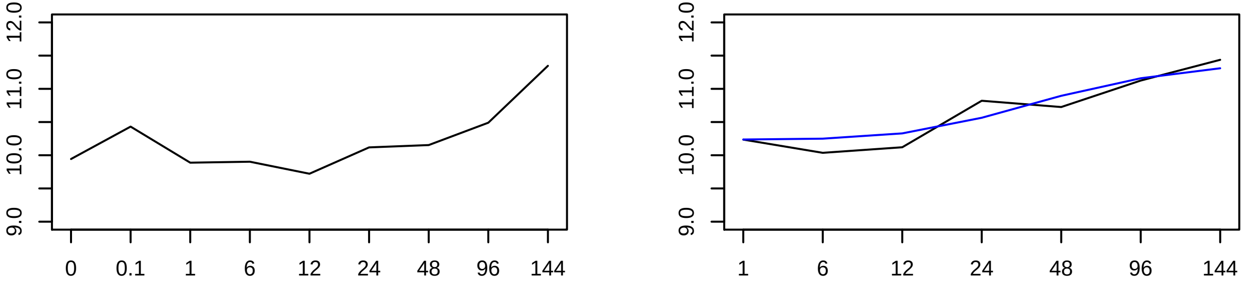

A_23_P371787 KIAA0247 14q24.1

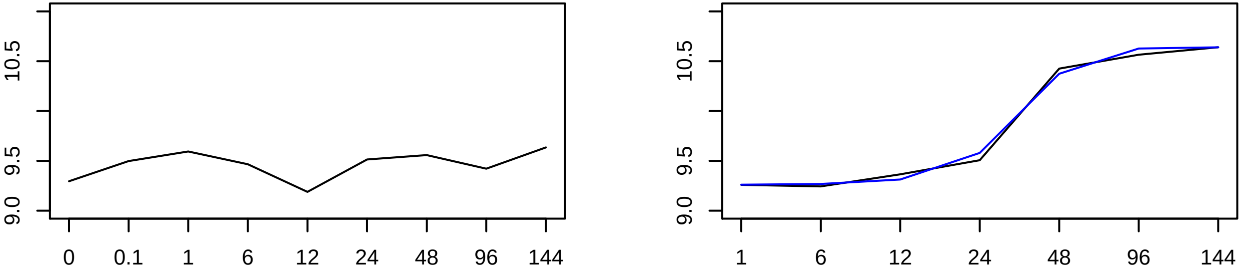


A_23_P125788 TCEAL7 Xq22.1

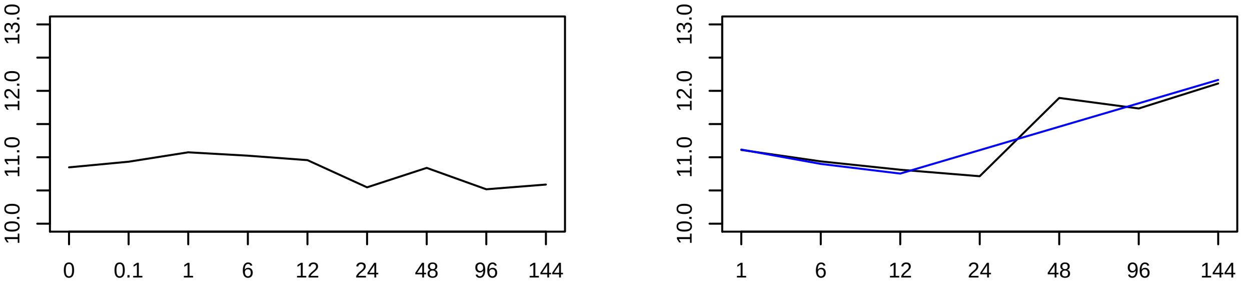


A_23_P90523 OCEL1 19p13.11

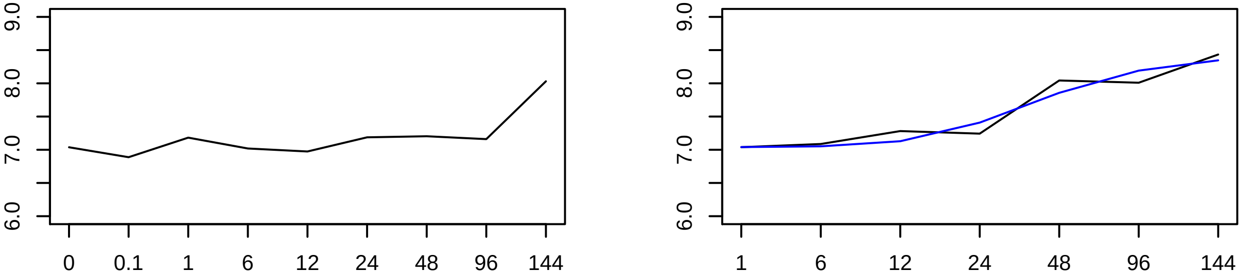

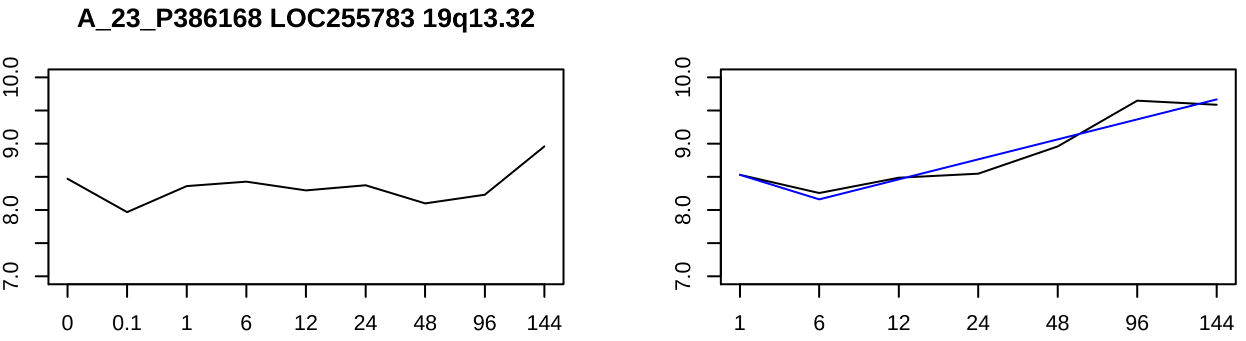
A_23_P386168 LOC255783 19q13.32
7.0
8.0
9.0
10.0
0
0.1
1
6
12
24
48
96
144
1
6
12
24
48
96
144

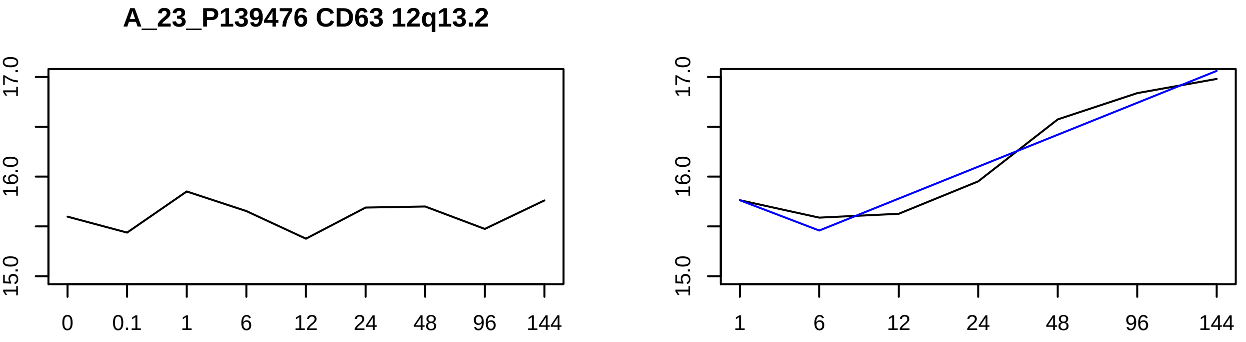
A_23_P139476 CD63 12q13.2
15.0
16.0
17.0
0
0.1
1
6
12
24
48
96
144
1
6
12
24
48
96
144

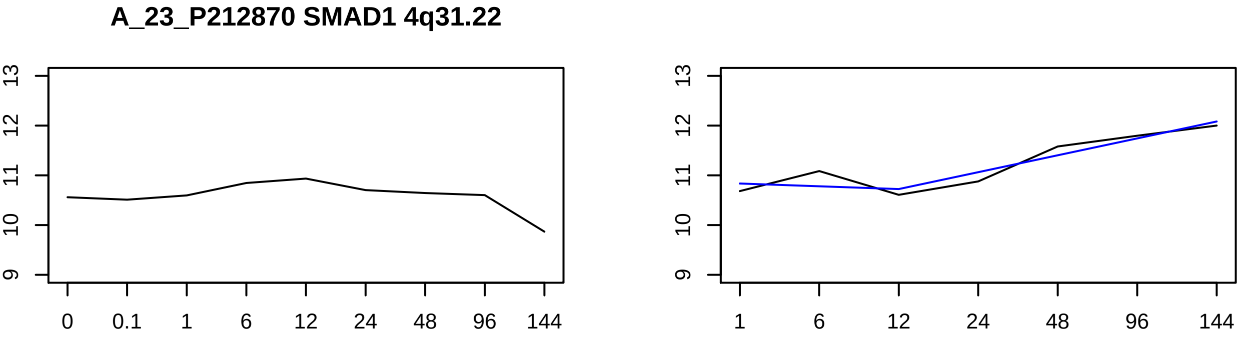
A_23_P212870 SMAD1 4q31.22
9
10
11
12
13
0
0.1
1
6
12
24
48
96
144
1
6
12
24
48
96
144

A_23_P139722 TNFRSF1A 12p13.31

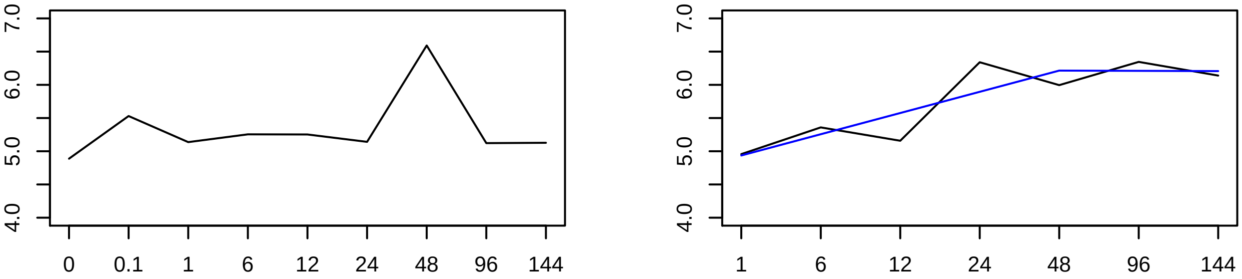


A_24_P327886 TCEA3 1p36.12

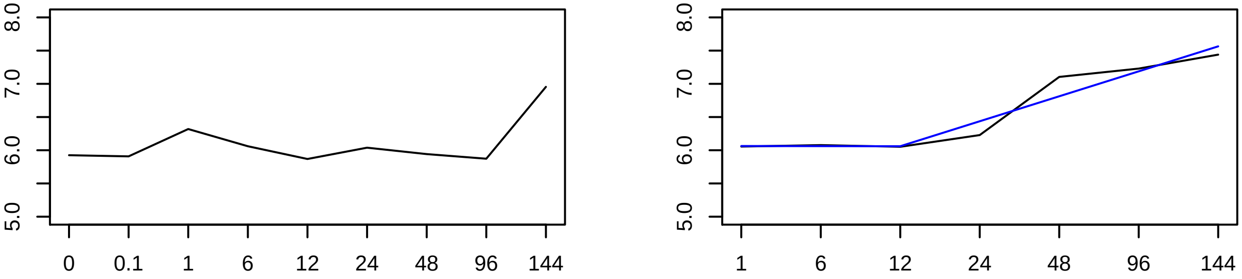


A_23_P60971 SESTD1 2q31.2

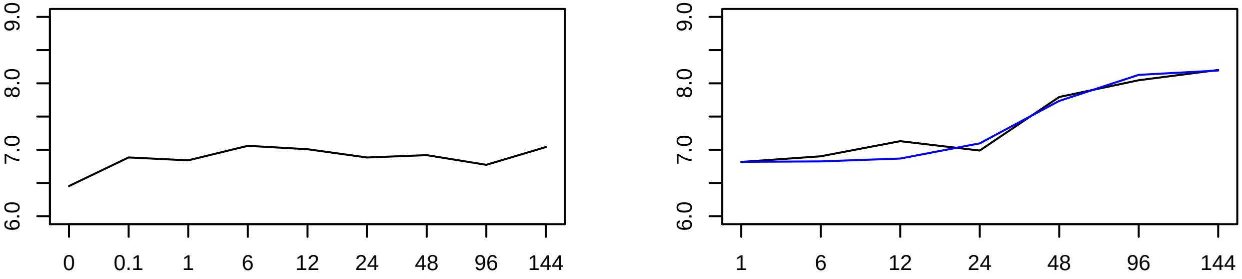

**A_23_P373724 PPFIBP1 12p11.22**

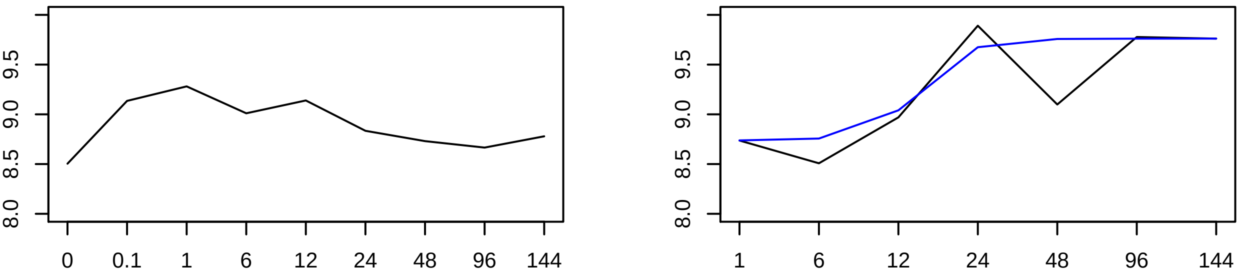


**A_23_P333228 MARCH4 2q35**

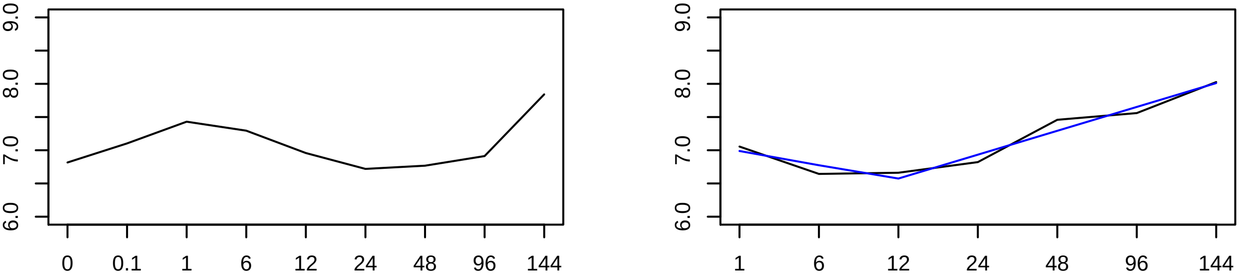


**A_24_P137897 IFRD1 7q31.1**

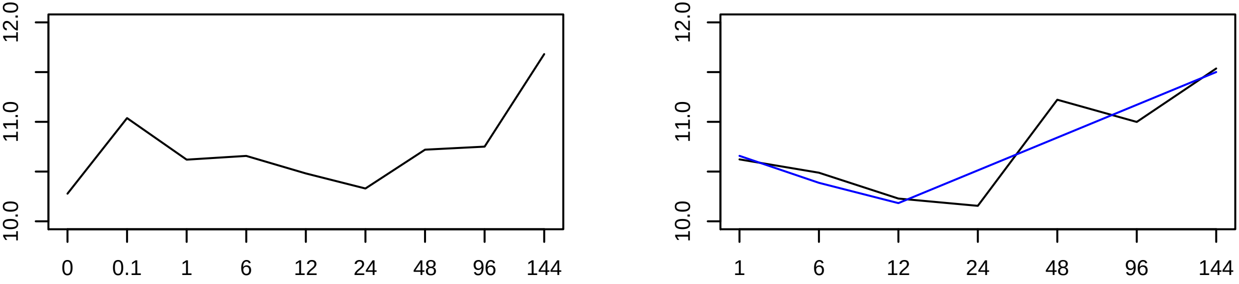

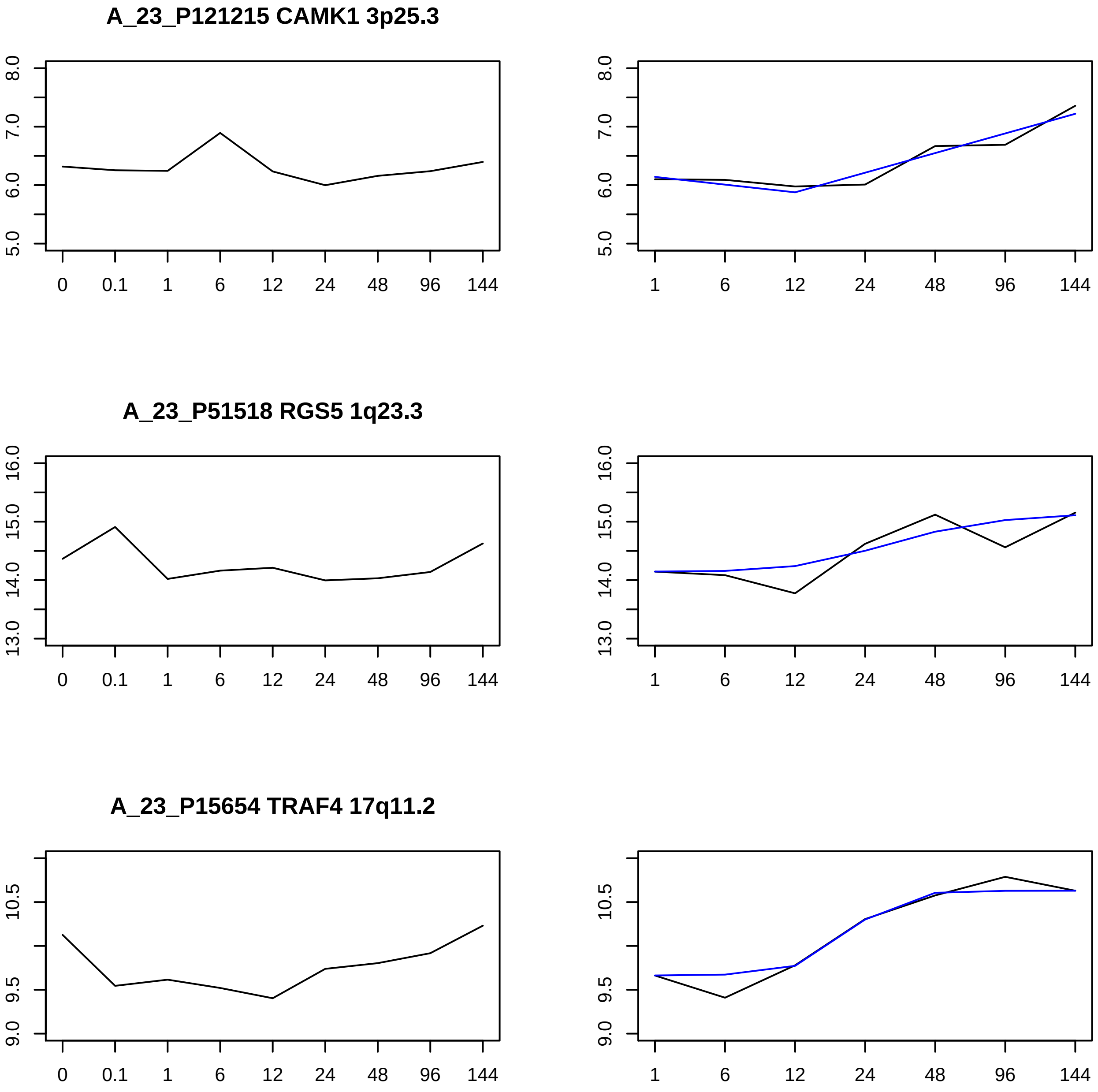
A_23_P121215 CAMK1 3p25.3
5.0
6.0
7.0
8.0
0
0.1
1
6
12
24
48
96
144
1
6
12
24
48
96
144
A_23_P51518 RGS5 1q23.3
13.0
14.0
15.0
16.0
A_23_P15654 TRAF4 17q11.2
9.0
9.5
10.5

A_23_P128161 TUBA1B 12q13.12

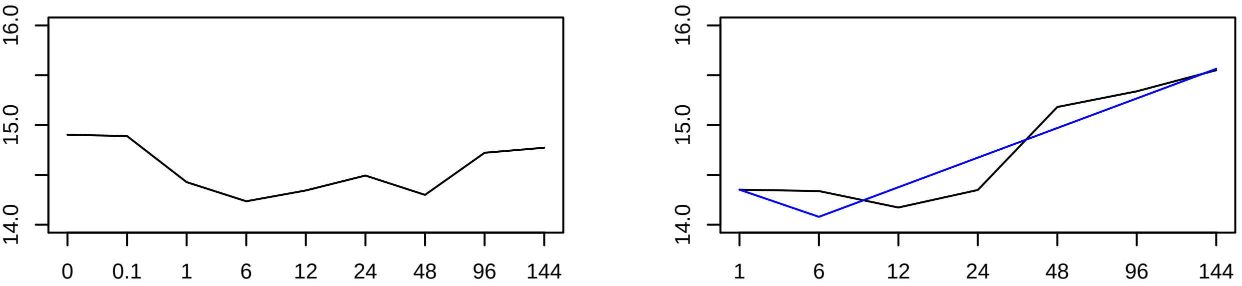


A_24_P63522 HMGCS1 5p12

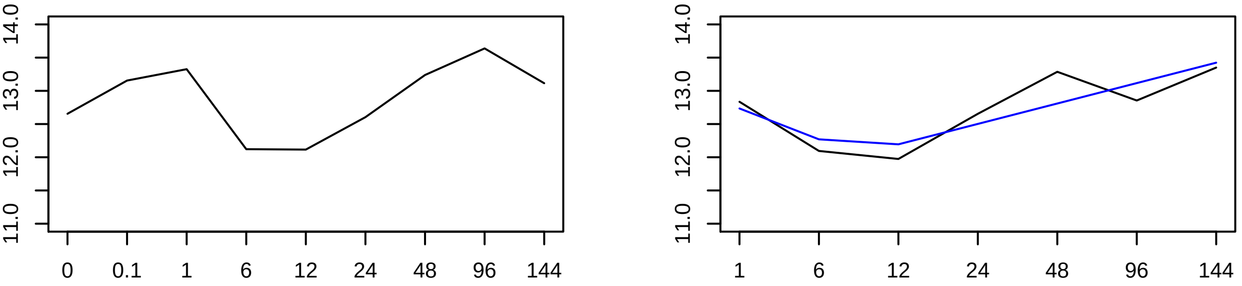


A_23_P360769 MAN2A1 5q21.3

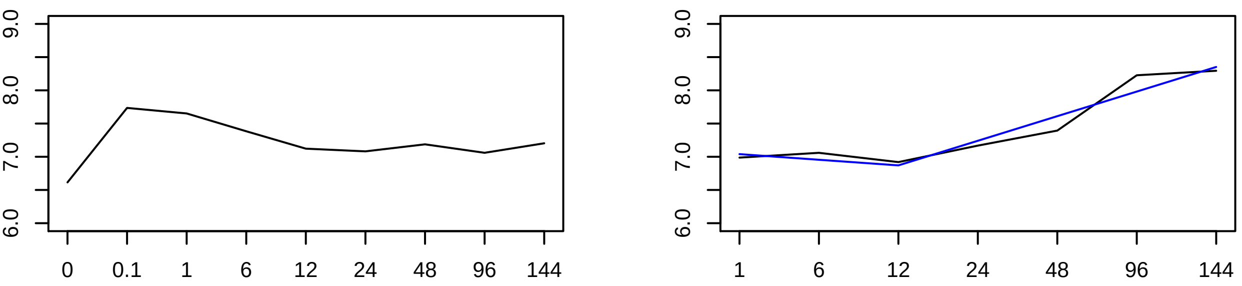

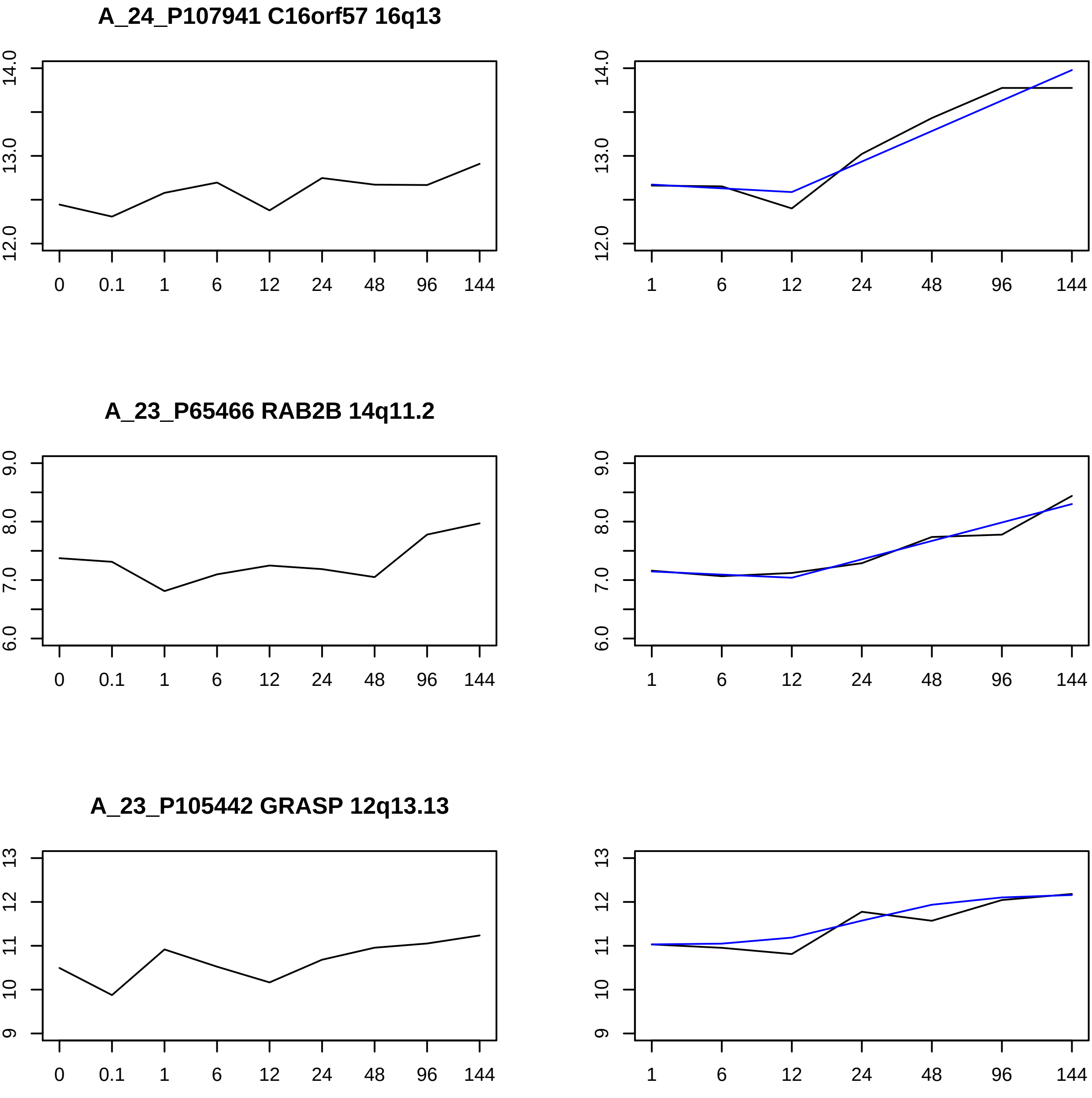
A_24_P107941 C16orf57 16q13
12.0
13.0
14.0
0
0.1
1
6
12
24
48
96
144
A_23_P65466 RAB2B 14q11.2
6.0
7.0
8.0
9.0
A_23_P105442 GRASP 12q13.13
9
10
11
12
13

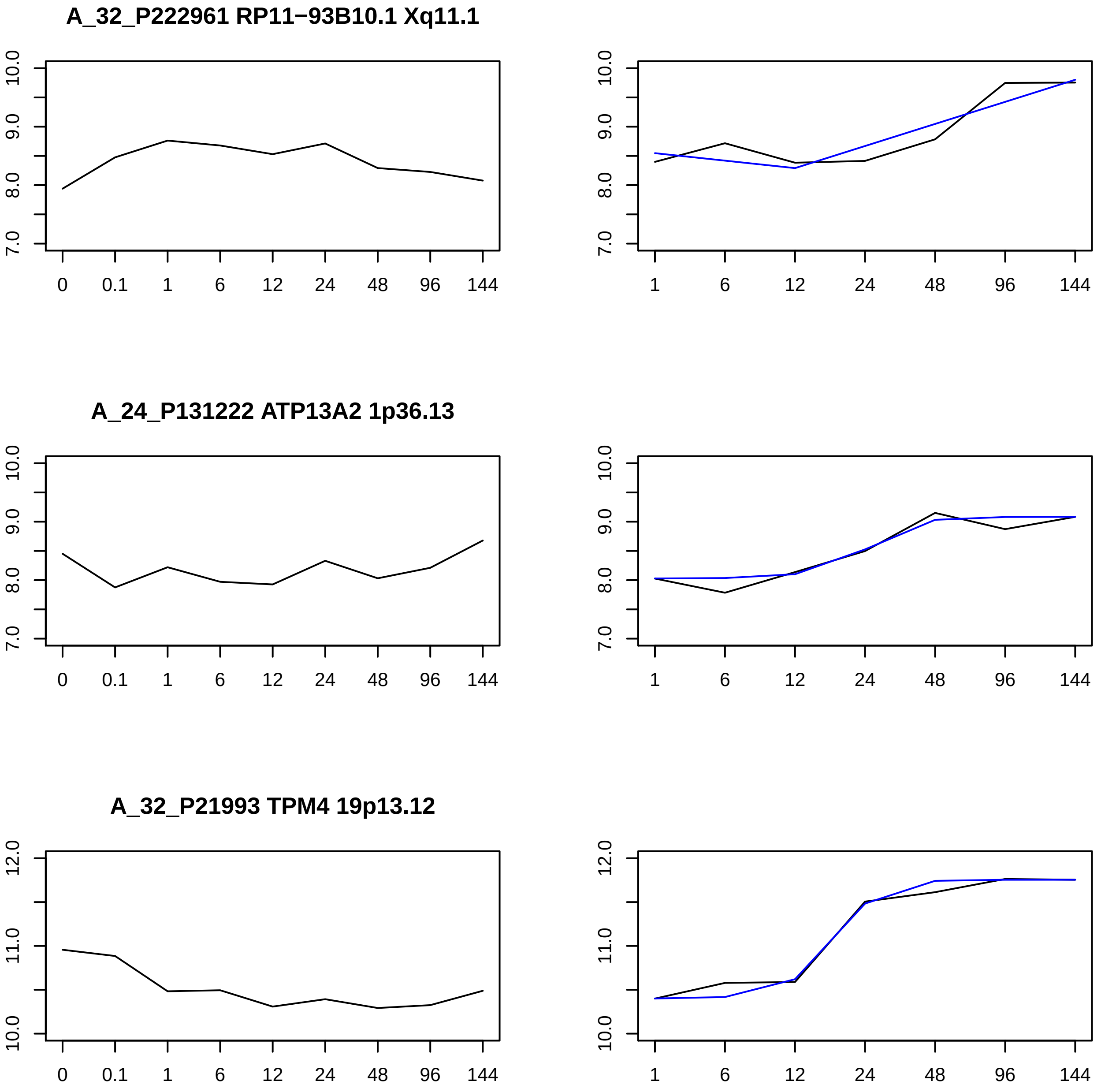
A_32_P222961 RP11−93B10.1 Xq11.1
7.0
8.0
9.0
10.0
0
0.1
1
6
12
24
48
96
144
A_24_P131222 ATP13A2 1p36.13
A_32_P21993 TPM4 19p13.12
10.0
11.0
12.0

**A_23_P398854 DOK7 4p16.2**

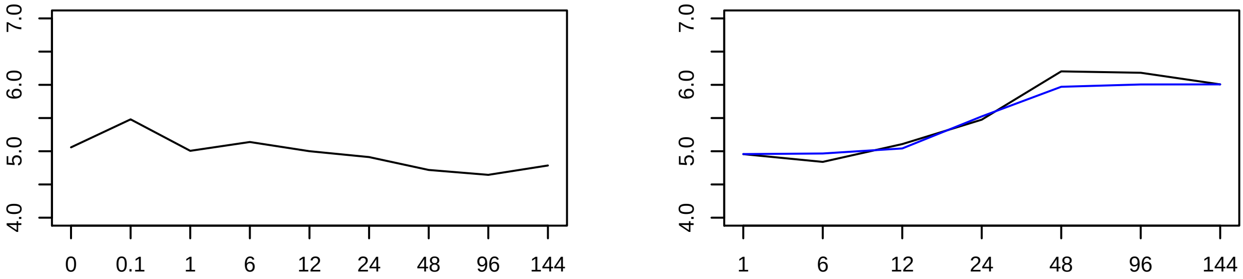


**A_24_P886515 THC2654231 NA**

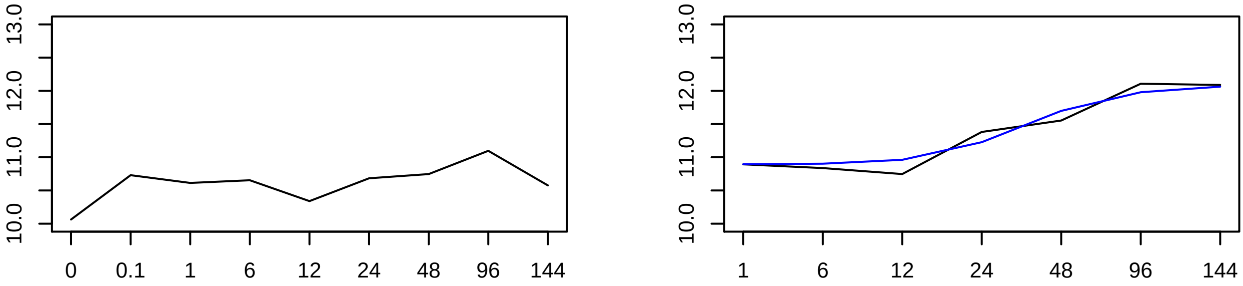


**A_24_P279489 VPS53 NA**

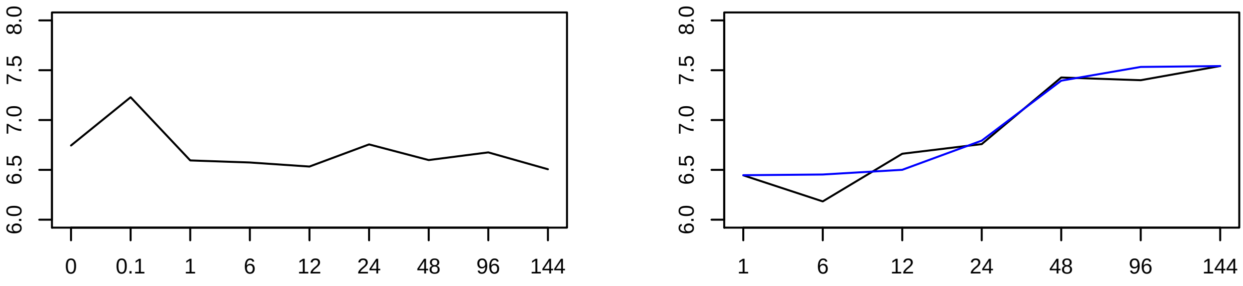

**A_24_P588897 SLCO3A1 15q26.1**

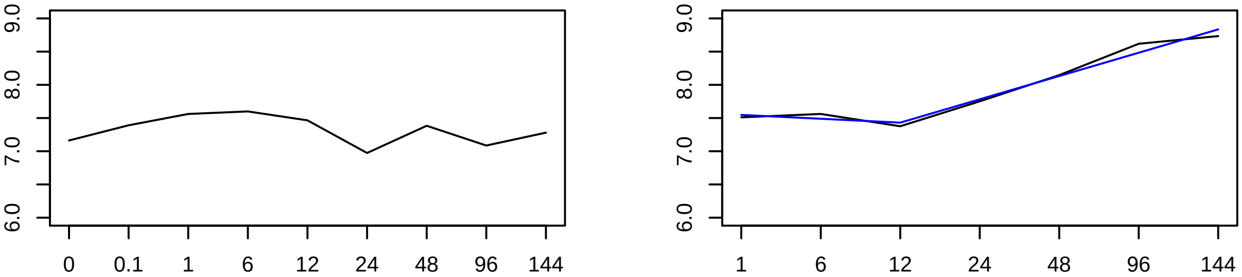


**A_32_P188193 THC2677780 NA**

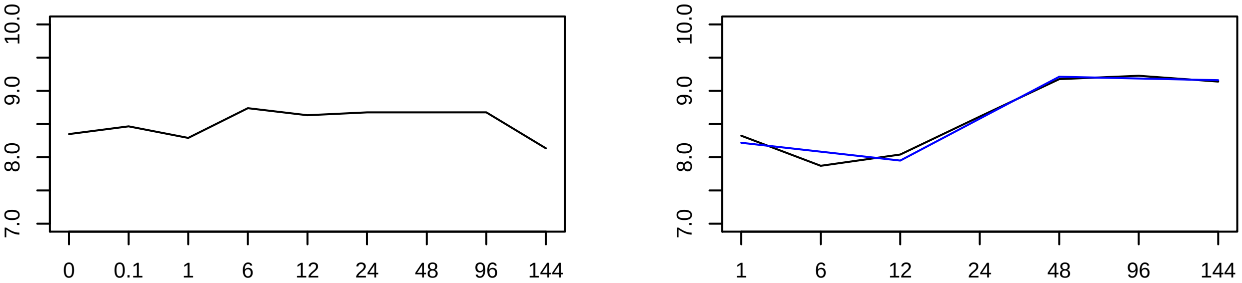


**A_23_P95470 CD151 11p15.5**

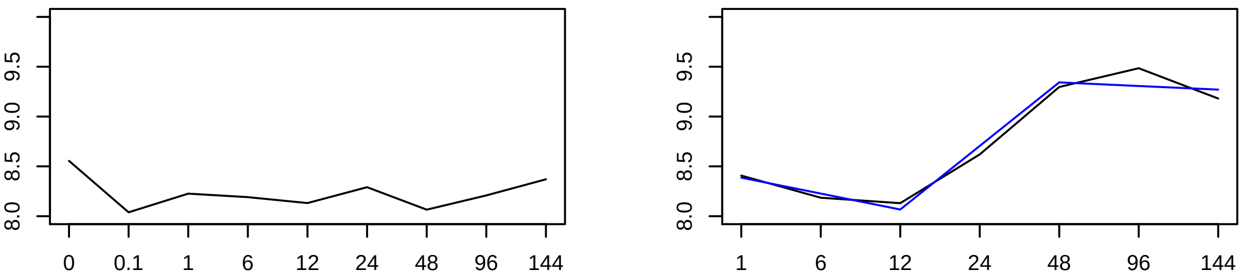

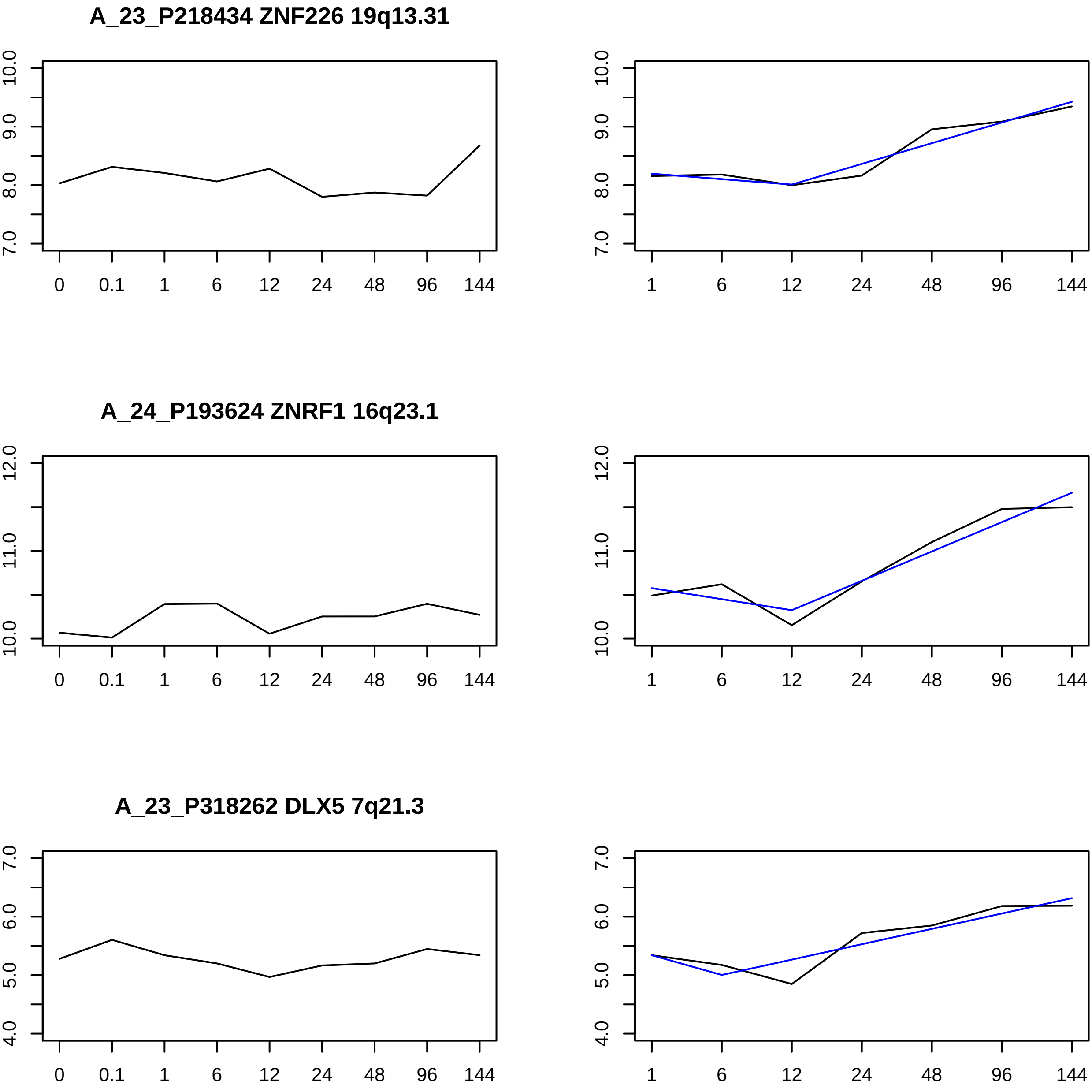
A_23_P218434 ZNF226 19q13.31
7.0
8.0
9.0
10.0
0
0.1
1
6
12
24
48
96
144
1
6
12
24
48
96
144
A_24_P193624 ZNRF1 16q23.1
10.0
11.0
12.0
A_23_P318262 DLX5 7q21.3
4.0
5.0
6.0
7.0

**A_32_P44204**

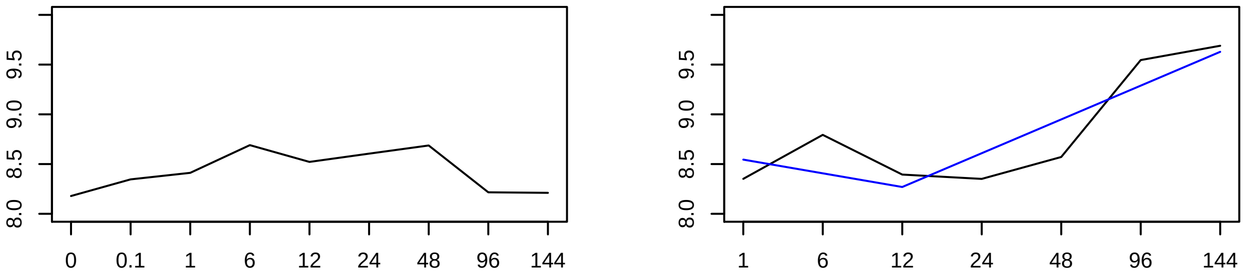


**A_23_P128154 TUBA1B 12q13.12**

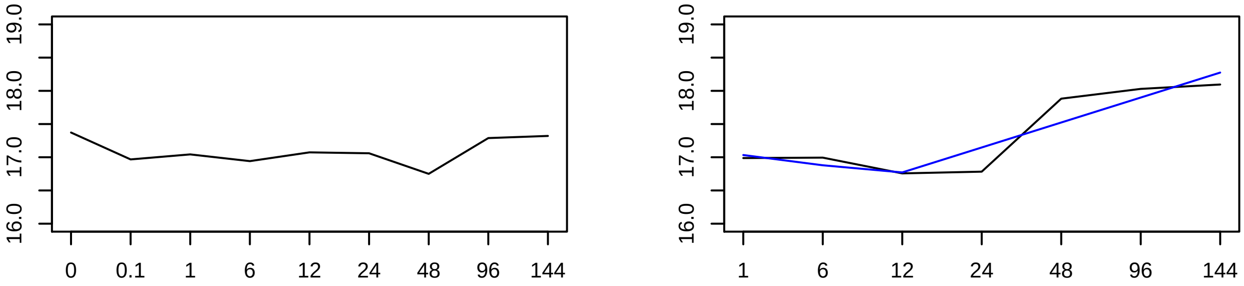


**A_24_P33477 CTNNA2 2p12**

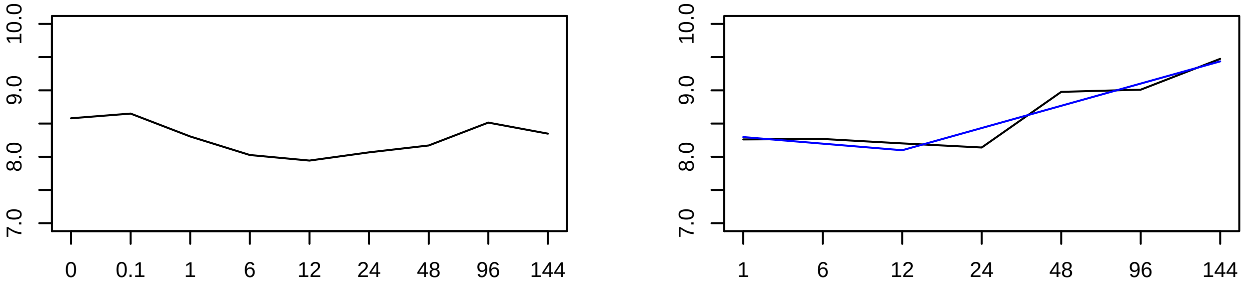

**A_23_P128919 LGALS3 14q22.3**

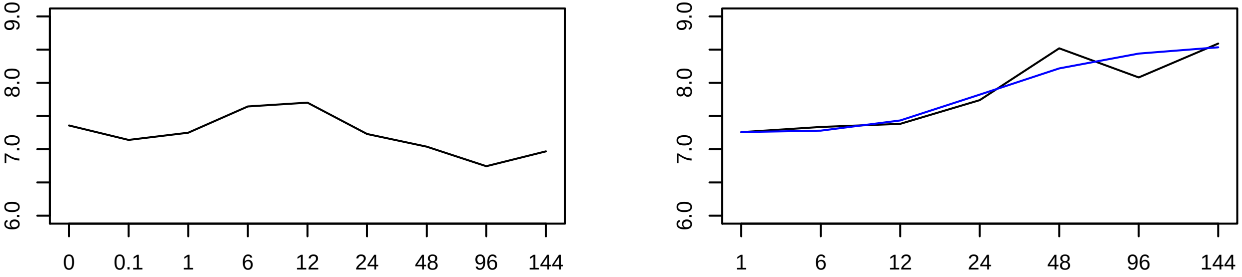


**A_24_P208909 TRIM2 4q31.3**

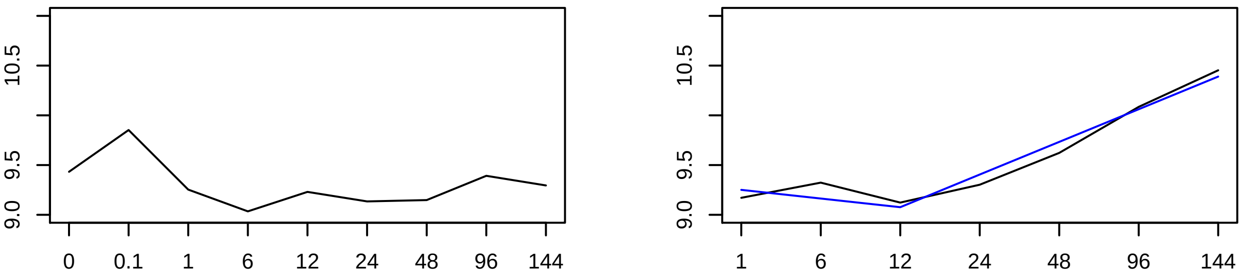


**A_32_P226801 THC2780391 NA**

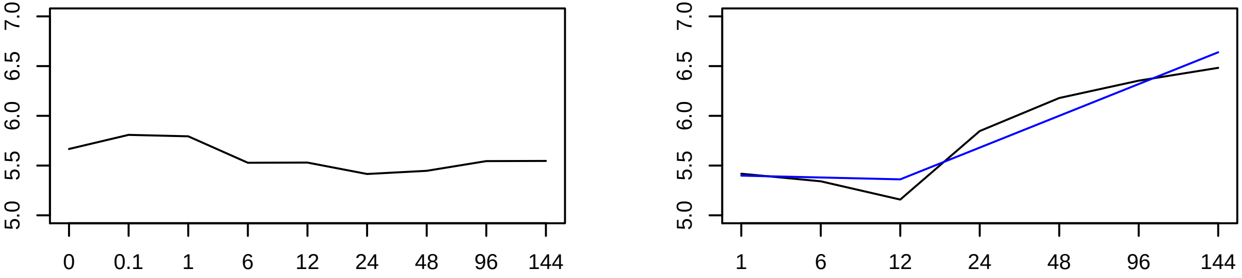

**A_23_P109072 SALL4 20q13.2**

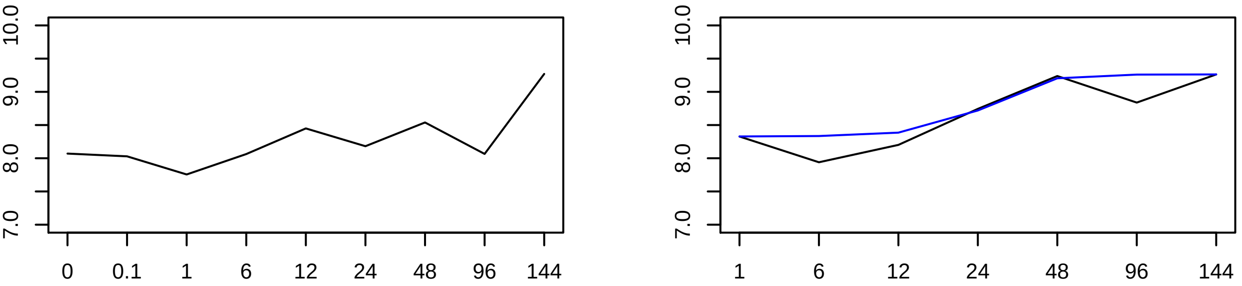


**A_23_P423309 PCDH12 5q31.3**

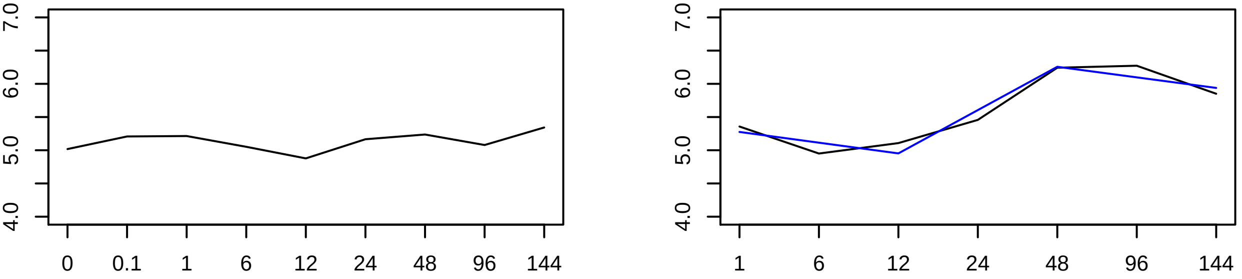


**A_23_P428842 TMEM44 3q29**

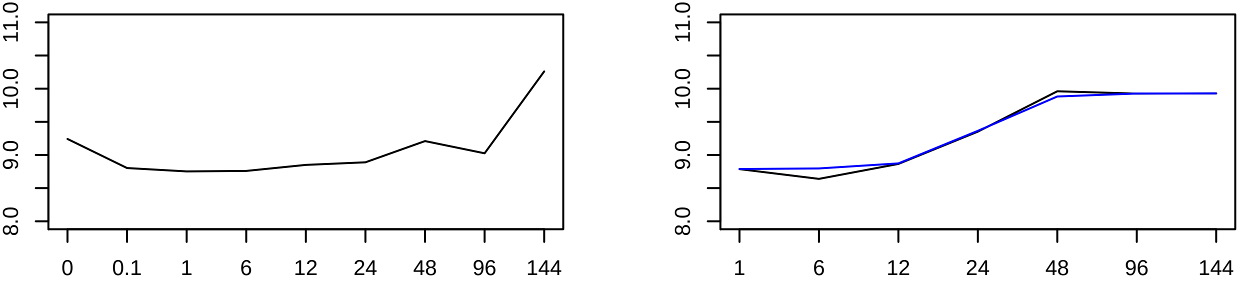

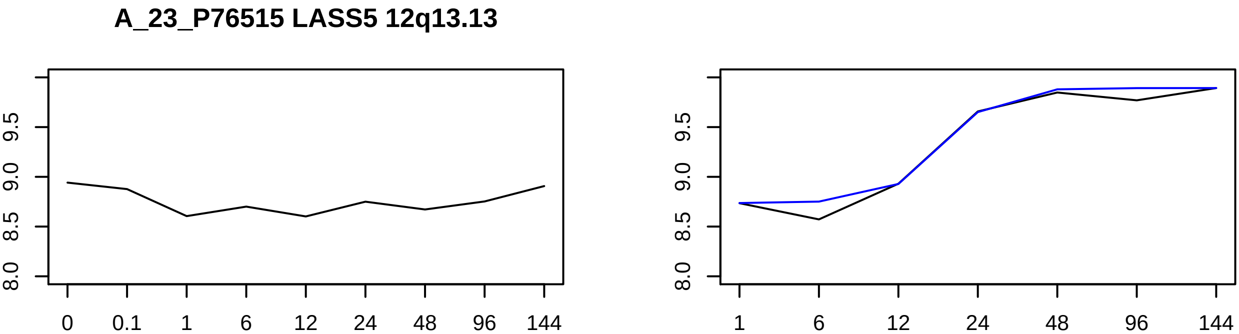
A_23_P76515 LASS5 12q13.13
8.0
8.5
9.0
9.5
0
0.1
1
6
12
24
48
96
144
1
6
12
24
48
96
144

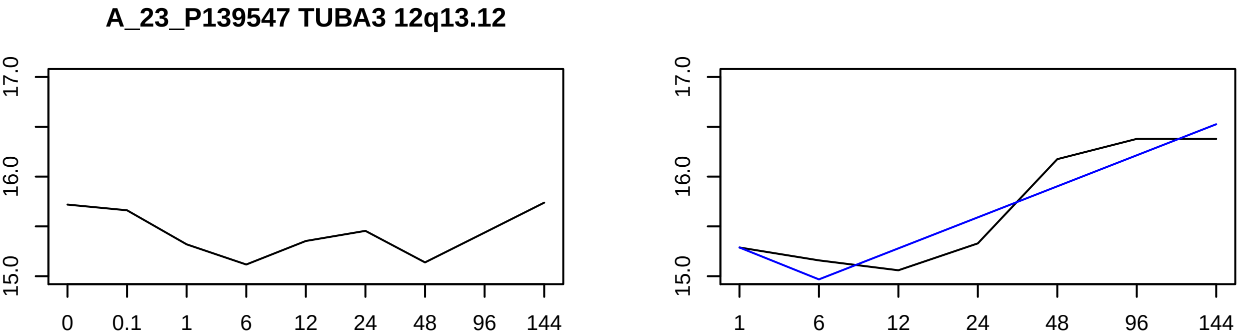
A_23_P139547 TUBA3 12q13.12
15.0
16.0
17.0
0
0.1
1
6
12
24
48
96
144
1
6
12
24
48
96
144

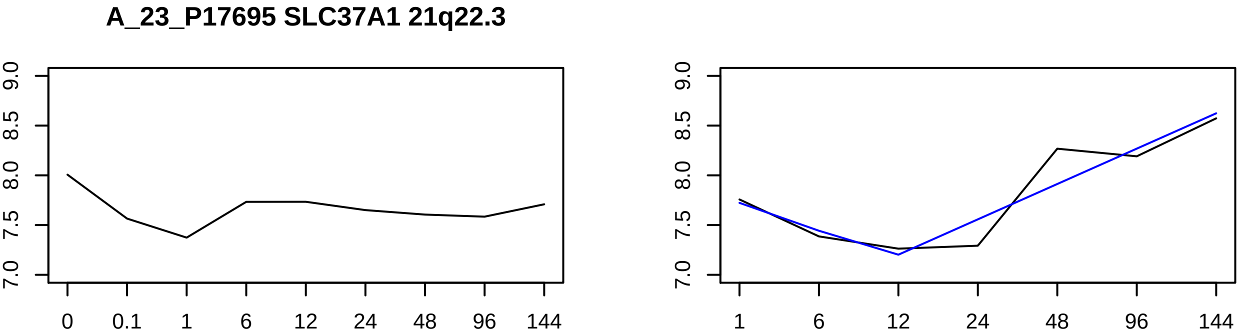
A_23_P17695 SLC37A1 21q22.3
7.0
7.5
8.0
8.5
9.0
0
0.1
1
6
12
24
48
96
144
1
6
12
24
48
96
144

**A_32_P75284 KATNAL1 13q12.3**

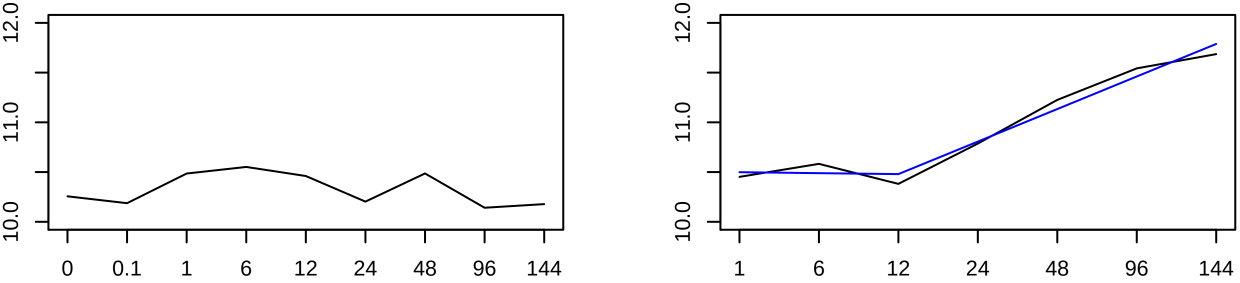


**A_32_P88479 KIAA0930 22q13.31**

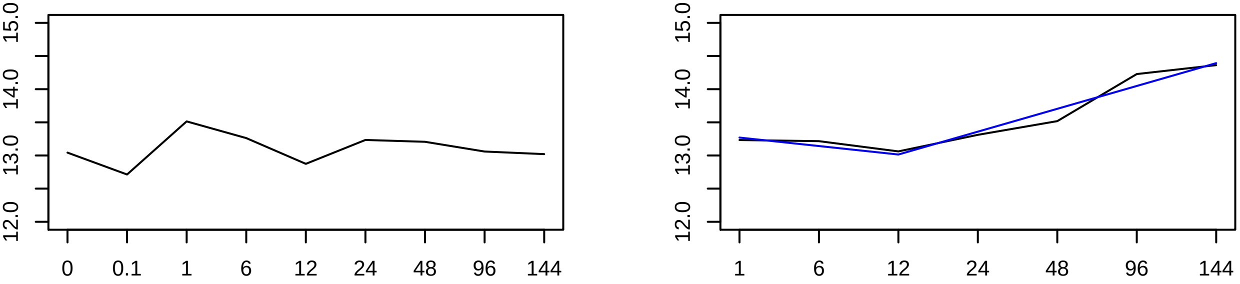


**A_23_P357104 ANXA6 5q33.1**

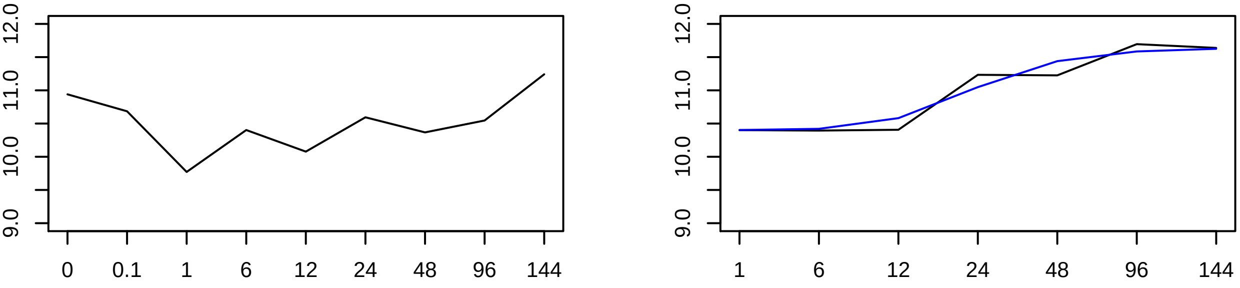

**A_23_P209098 GDF1 19p13.11**

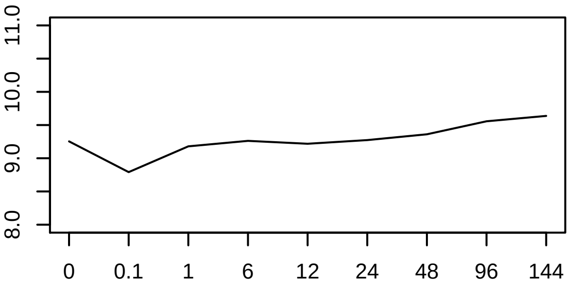


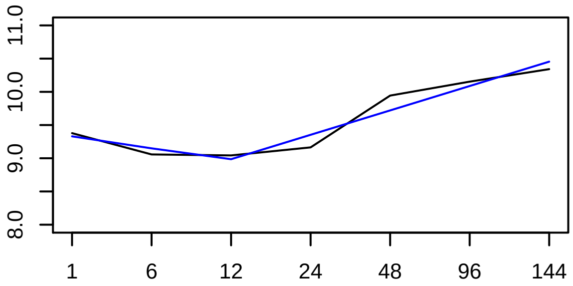


**A_23_P88351 SPG3A 14q22.1**

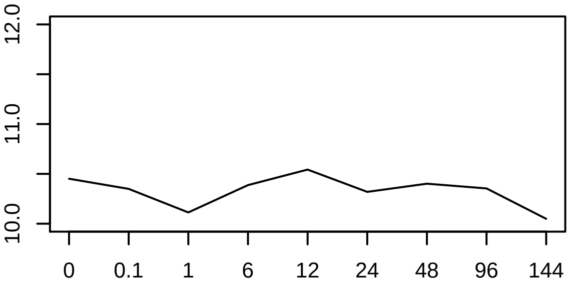


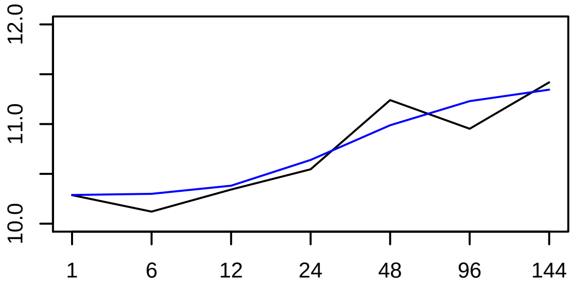


**A_23_P99496 MCF2L 13q34**

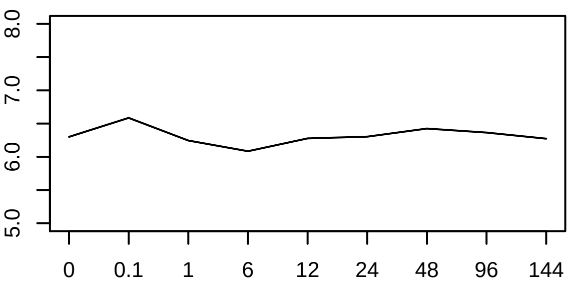


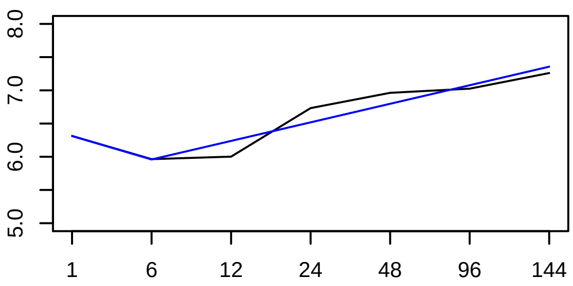

**A_23_P322704 C14orf24 14q13.2**

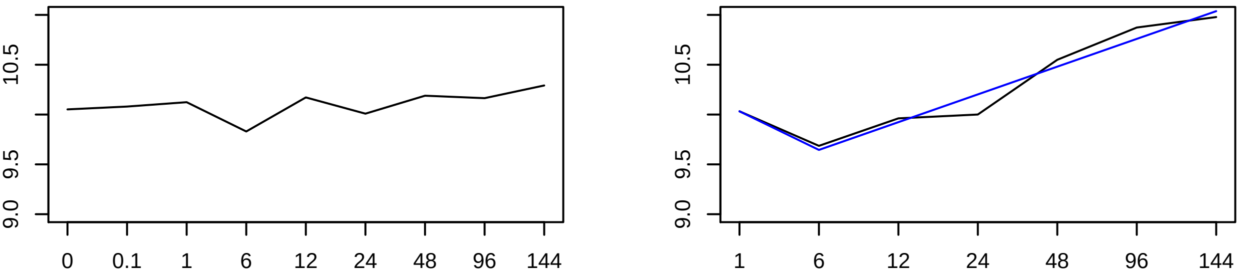


**A_23_P301855 LSAMP 3q13.31**

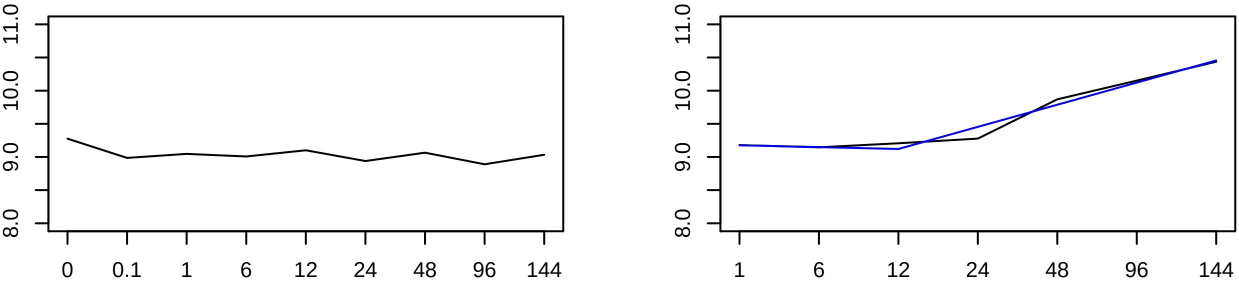


**A_32_P846696 LOC349196 8p23.1**

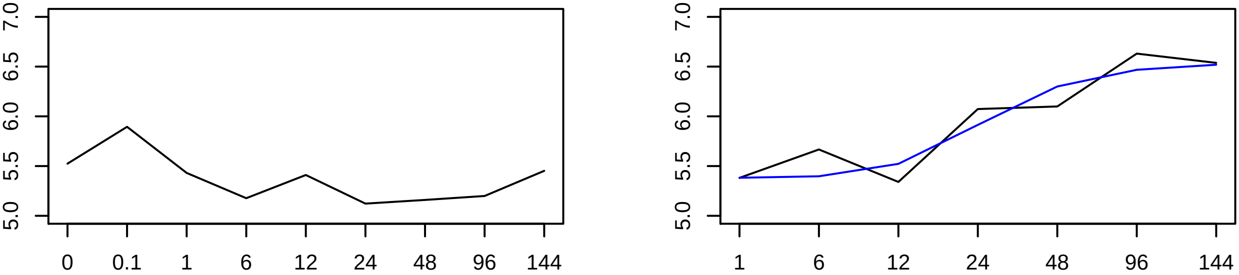

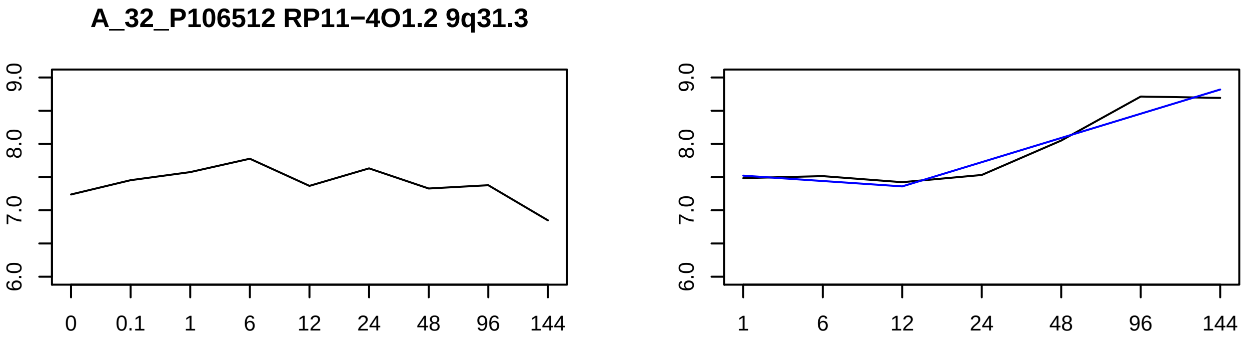
A_32_P106512 RP11−4O1.2 9q31.3
6.0 7.0 8.0 9.0
0 0.1 1 6 12 24 48 96 144
1 6 12 24 48 96 144

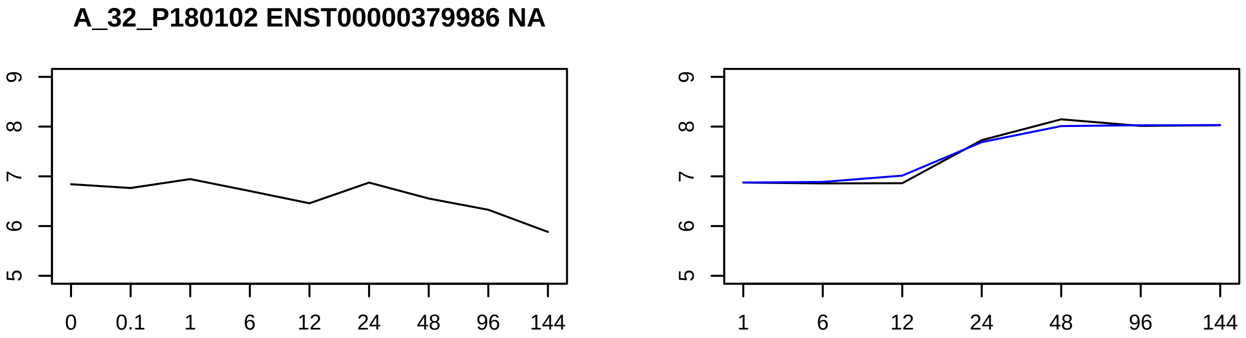
A_32_P180102 ENST00000379986 NA
5 6 7 8 9
0 0.1 1 6 12 24 48 96 144
1 6 12 24 48 96 144

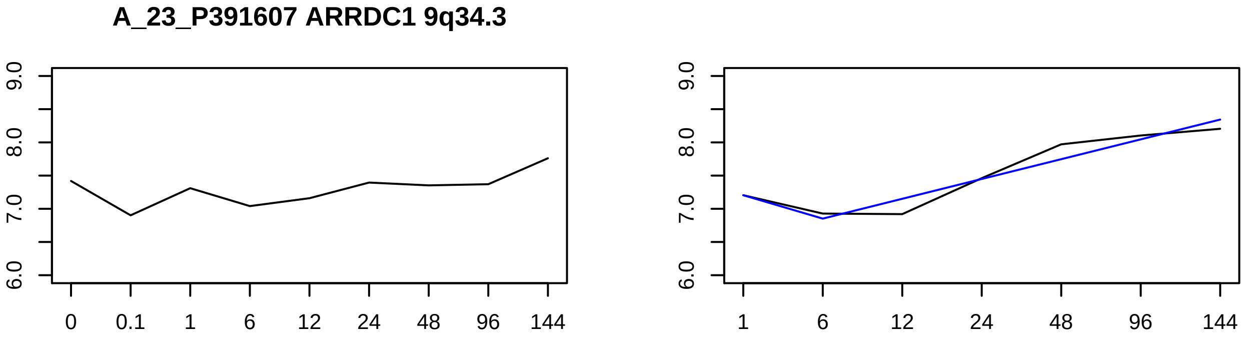
A_23_P391607 ARRDC1 9q34.3
6.0 7.0 8.0 9.0
0 0.1 1 6 12 24 48 96 144
1 6 12 24 48 96 144

**A_32_P42925 MRLC2 18p11.31**

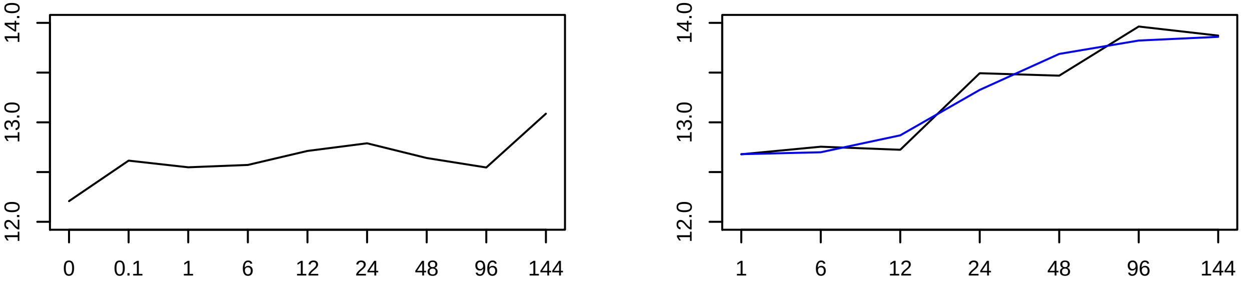


**A_32_P194372 AK129547 NA**

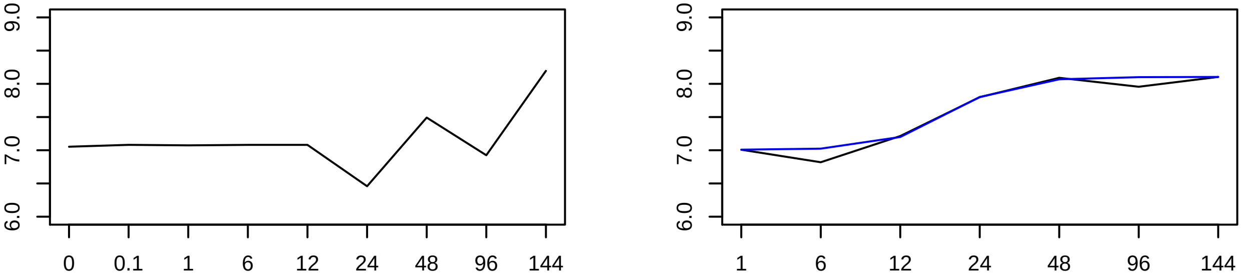


**A_24_P336276 SLCO3A1 15q26.1**

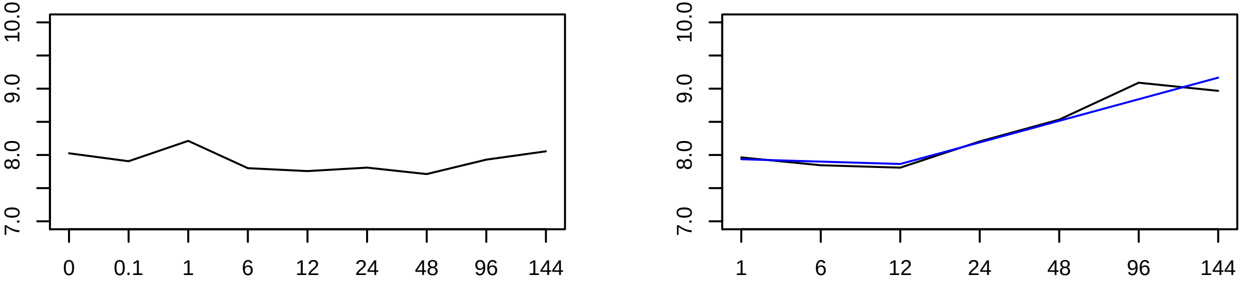

A_23_P154070 TUBA1 2q35

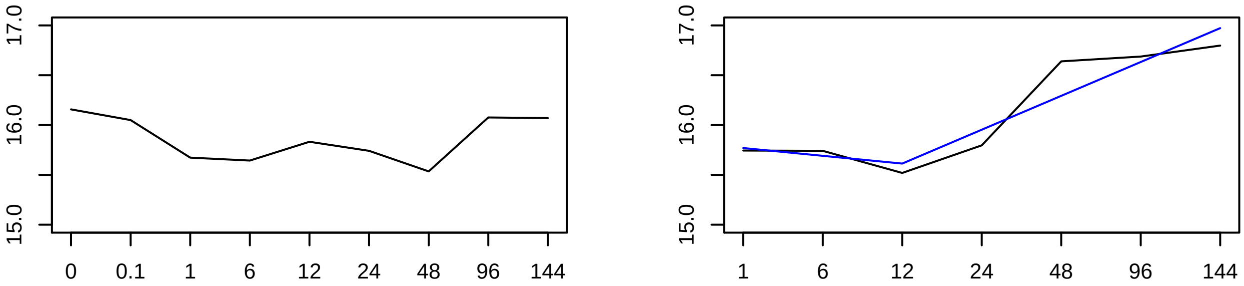


A_32_P55427 THC2701763 NA

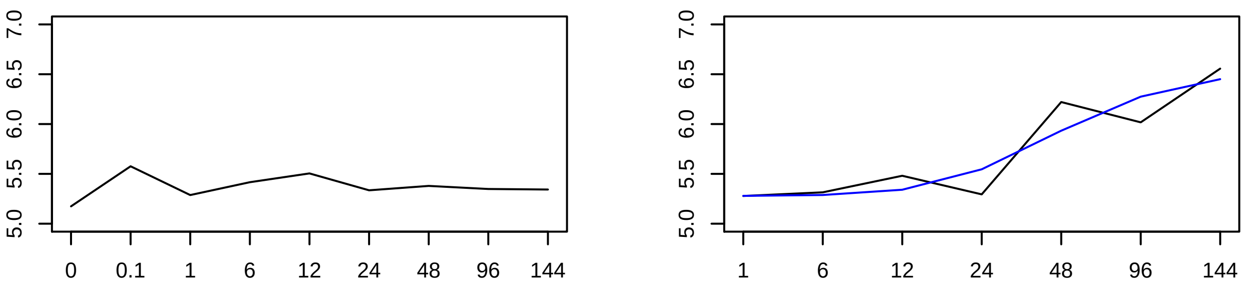


A_32_P117464 C3orf59 3q29

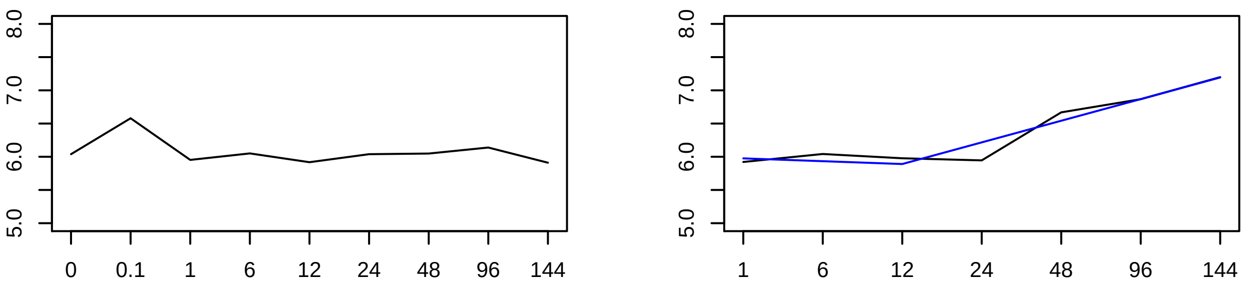

**A_24_P236008 SCYL2 12q23.1**

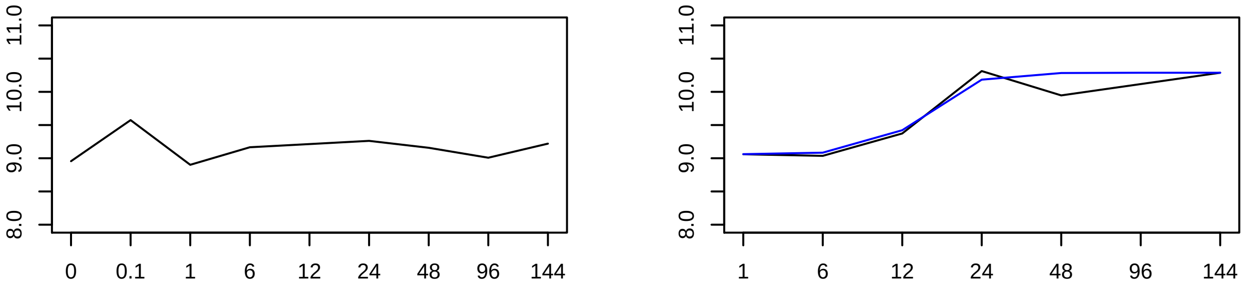


**A_24_P146188 ANKRD6 6q15**

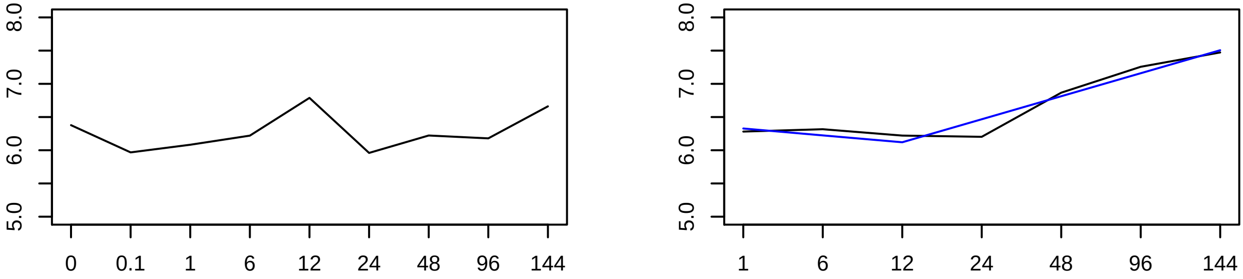


**A_23_P88580 ARID3B 15q24.1**

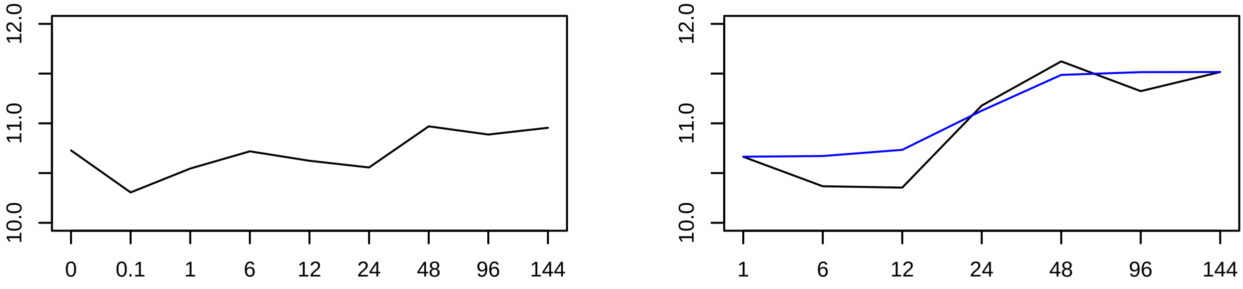

**A_23_P121011 AXUD1 3p22.2**

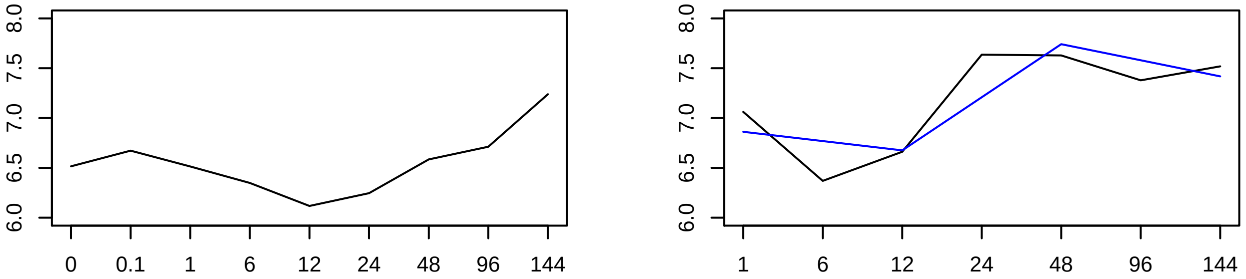


**A_24_P307808 KCTD16 5q32**

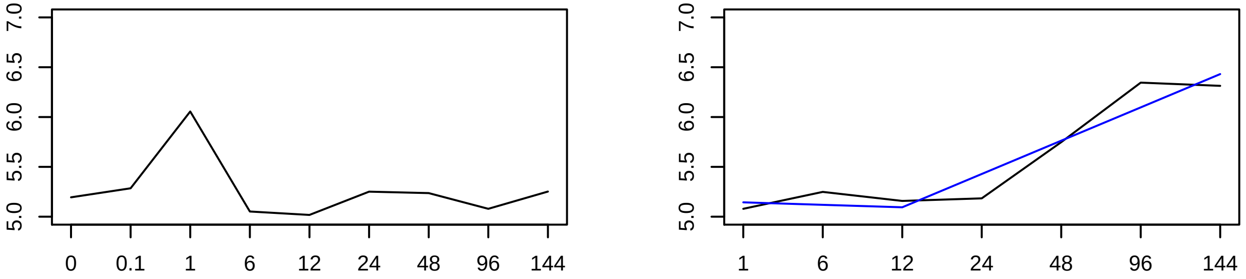


**A_24_P243741 ADAM22 7q21.12**

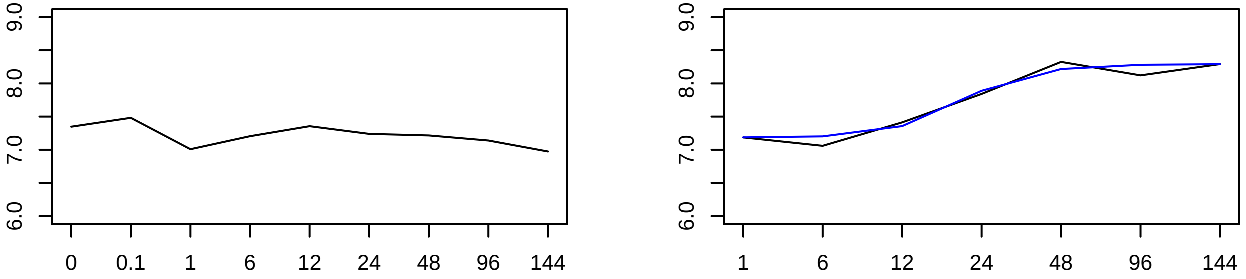

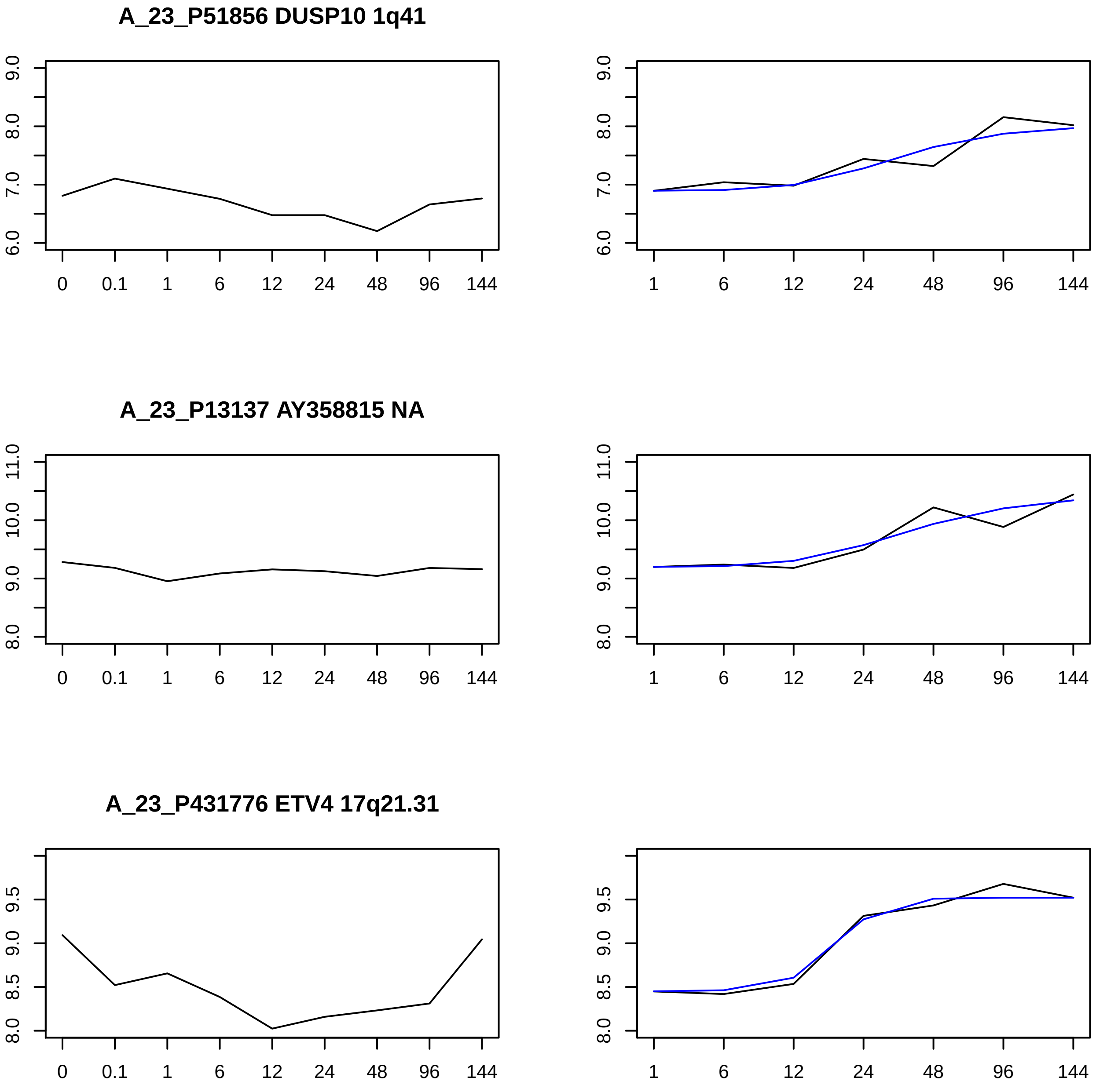
A_23_P51856 DUSP10 1q41
6.0
7.0
8.0
9.0
0
0.1
1
6
12
24
48
96
144
A_23_P13137 AY358815 NA
8.0
9.0
10.0
11.0
A_23_P431776 ETV4 17q21.31
8.0
8.5
9.0
9.5

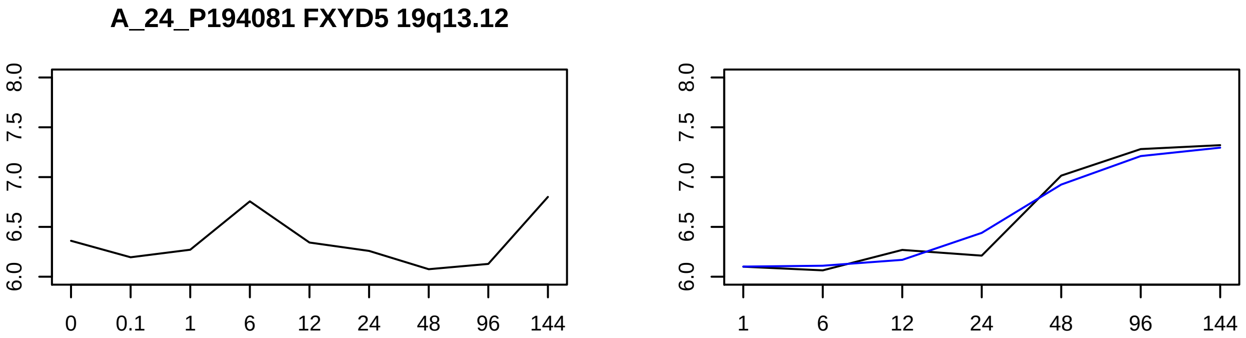
A_24_P194081 FXYD5 19q13.12
6.0
6.5
7.0
7.5
8.0
0
0.1
1
6
12
24
48
96
144
1
6
12
24
48
96
144

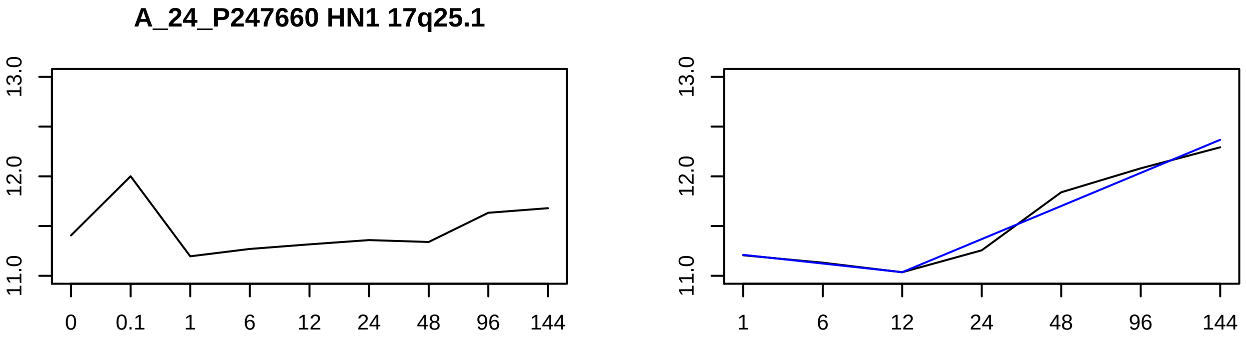
A_24_P247660 HN1 17q25.1
11.0
12.0
13.0
0
0.1
1
6
12
24
48
96
144
1
6
12
24
48
96
144

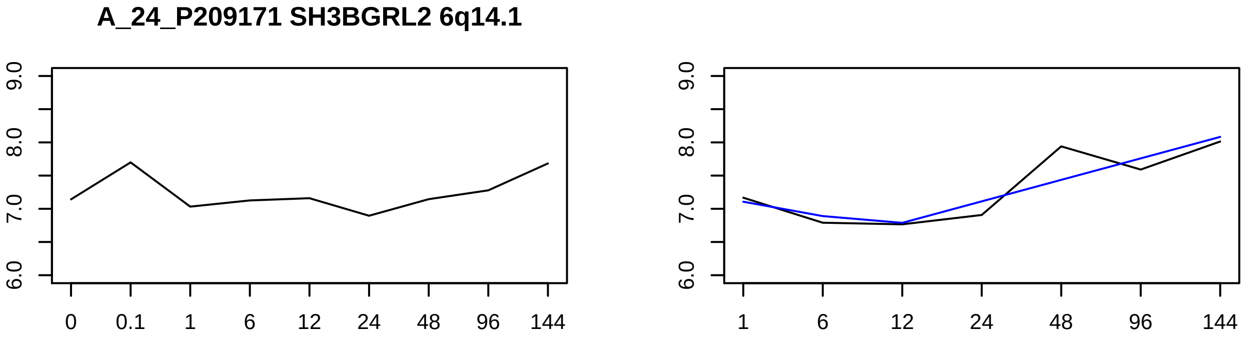
A_24_P209171 SH3BGRL2 6q14.1
6.0
7.0
8.0
9.0
0
0.1
1
6
12
24
48
96
144
1
6
12
24
48
96
144

**A_23_P503182 ABR 17p13.3**

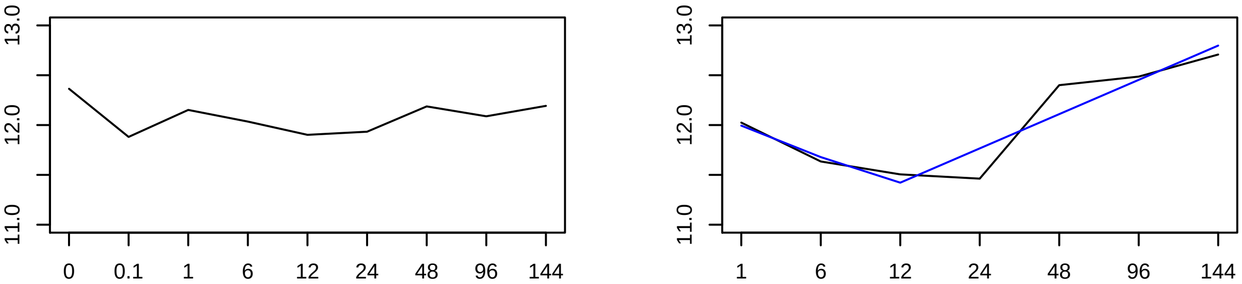


**A_23_P167093 IDUA 4p16.3**

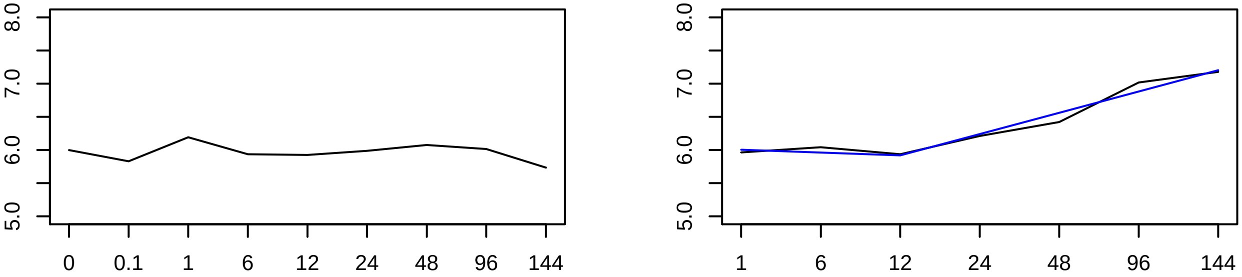


**A_23_P255436 ARSA 22q13.33**

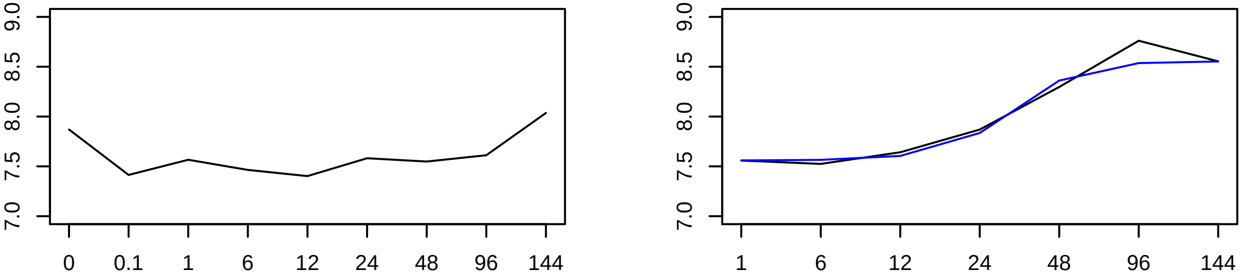

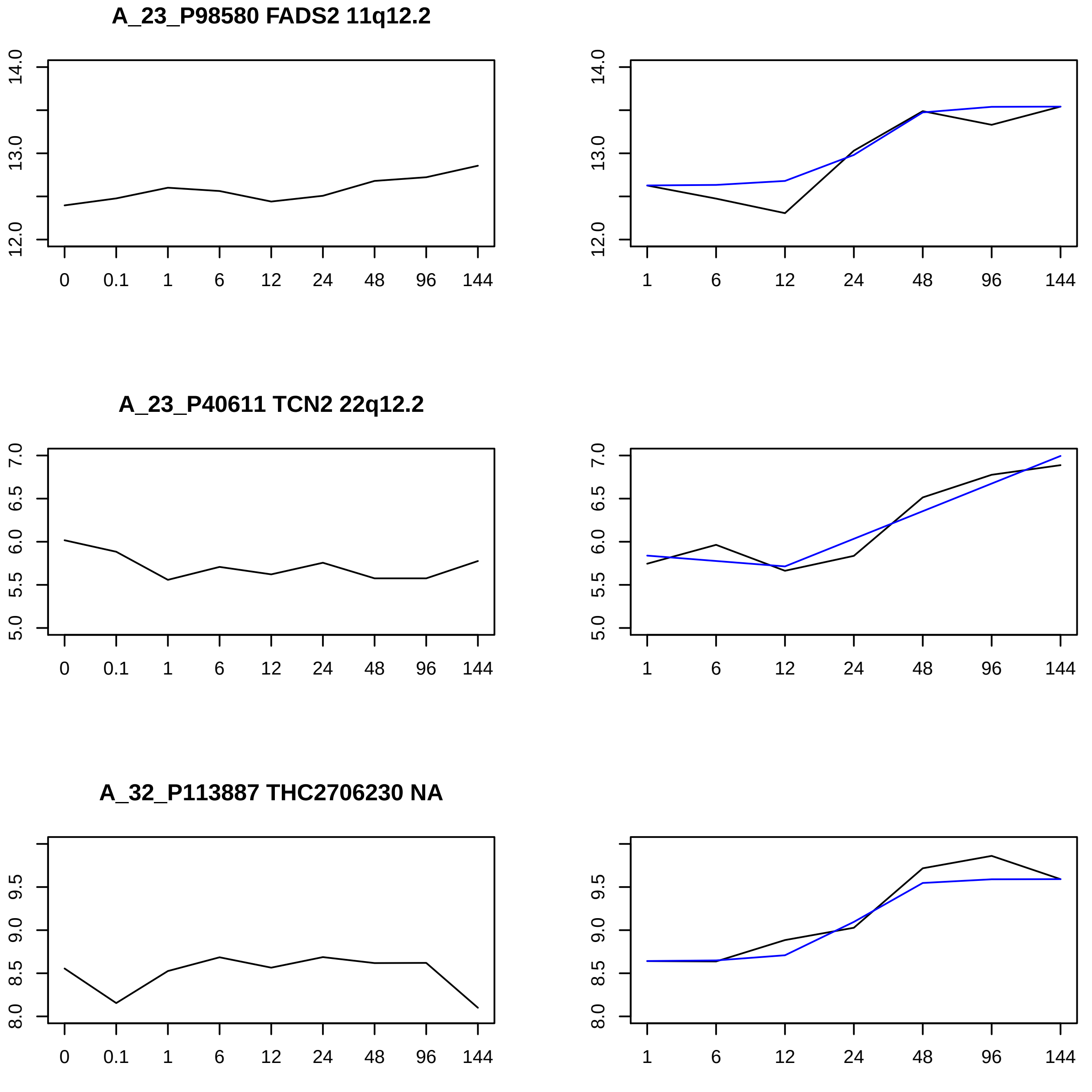
A_23_P98580 FADS2 11q12.2
12.0
13.0
14.0
0
0.1
1
6
12
24
48
96
144
A_23_P40611 TCN2 22q12.2
5.0
5.5
6.0
6.5
7.0
A_32_P113887 THC2706230 NA
8.0
8.5
9.0
9.5

**A_32_P128391 AL162073 NA**

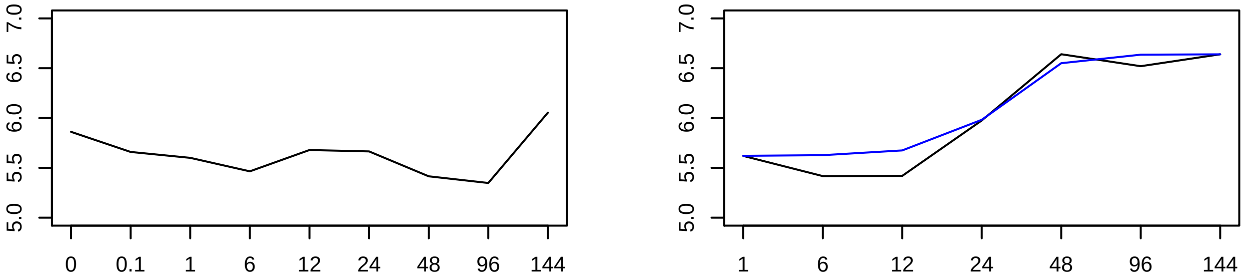


**A_24_P102362 ZNF553 16p11.2**

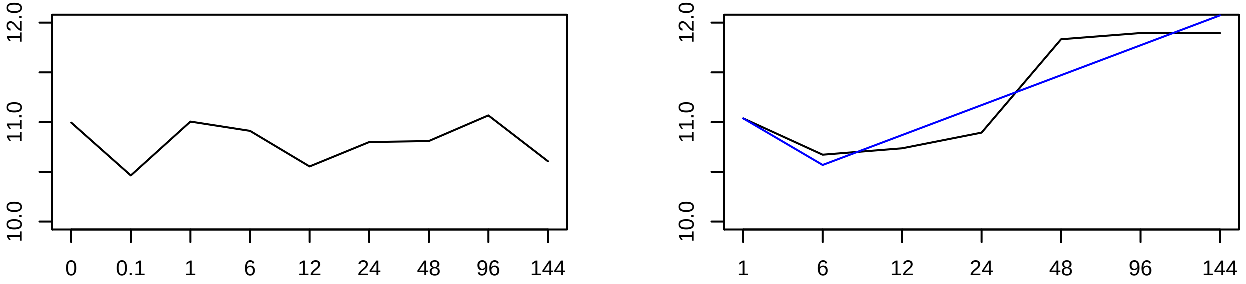


**A_23_P200267 PCNXL2 1q42.2**

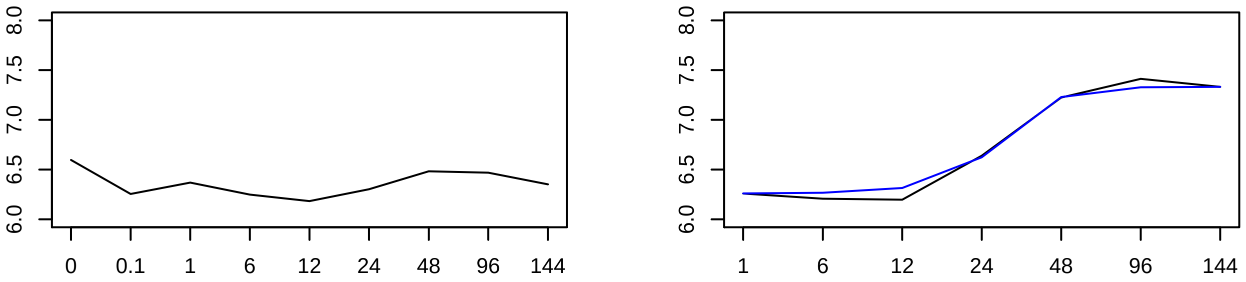

**A_32_P221631 THC2635591 NA**

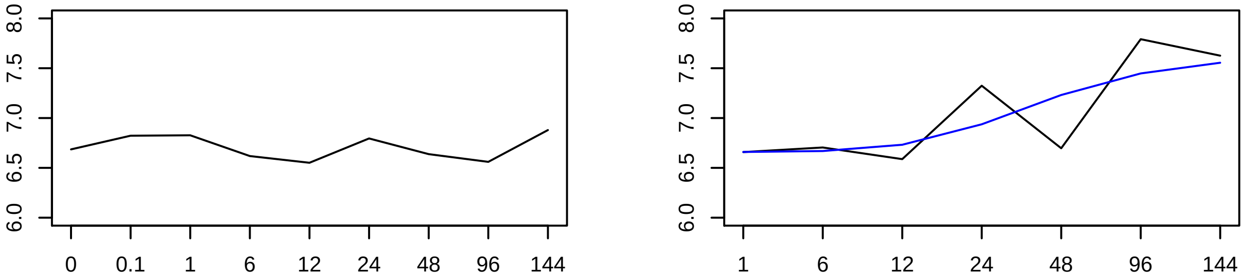


**A_23_P209519 DNAJB2 2q35**

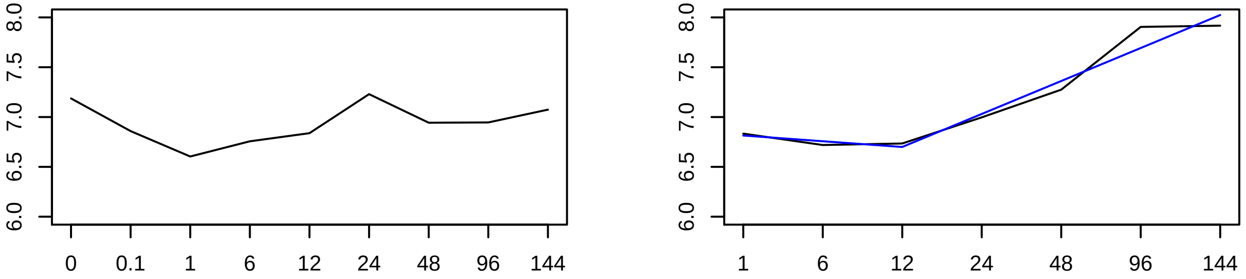


**A_23_P253052 CD99L2 Xq28**

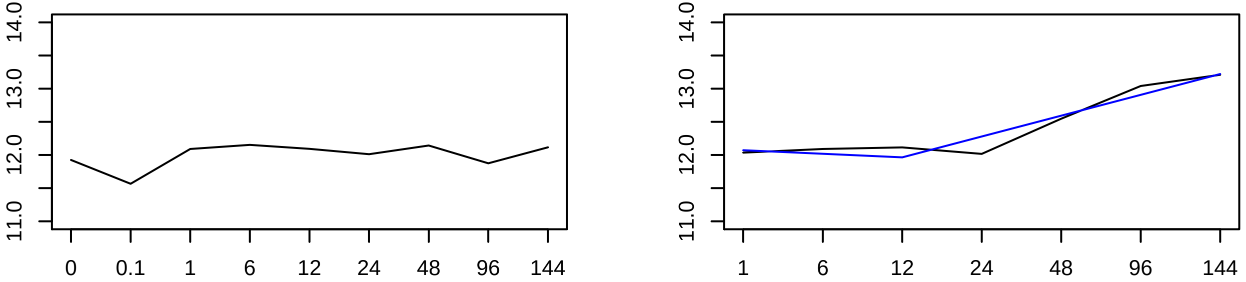

**A_23_P128597 TUBA3C 13q12.11**

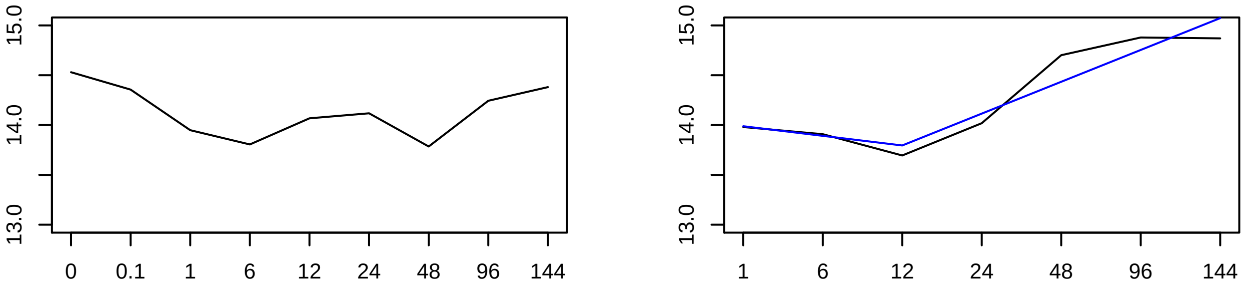


**A_32_P180825 FMN1 15q13.3**

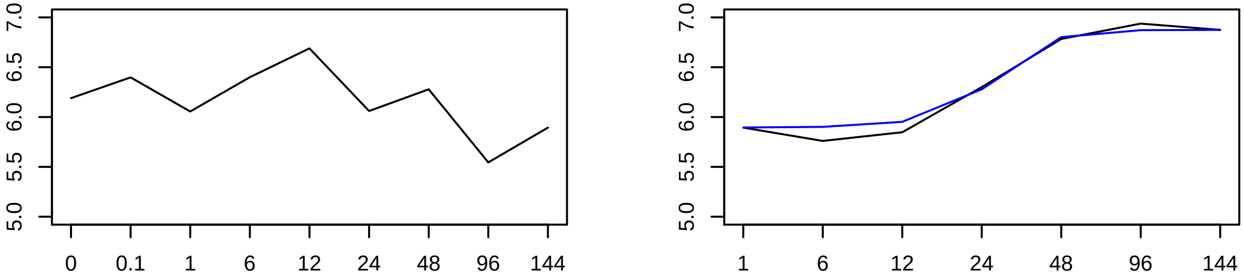


**A_32_P194848 TAGLN2 1q23.2**

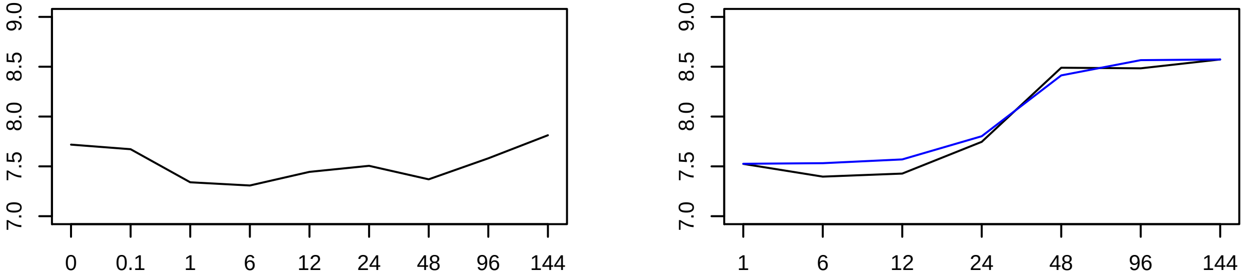

**A_32_P220463 FBLL1 NA**

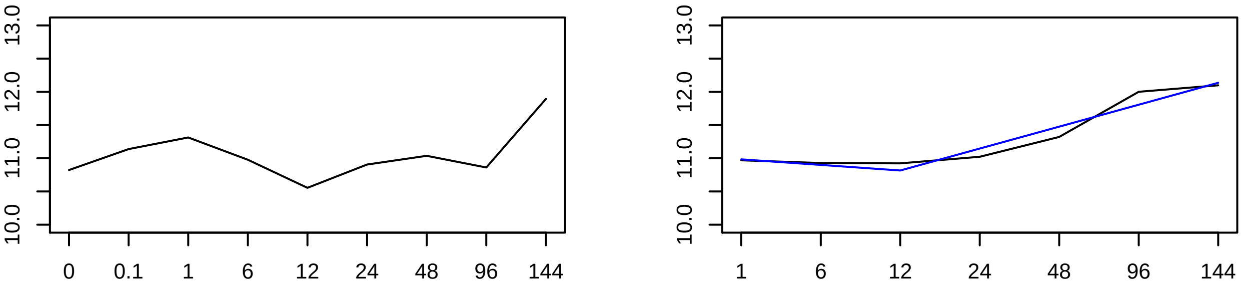


**A_23_P92765 CCDC112 5q22.3**

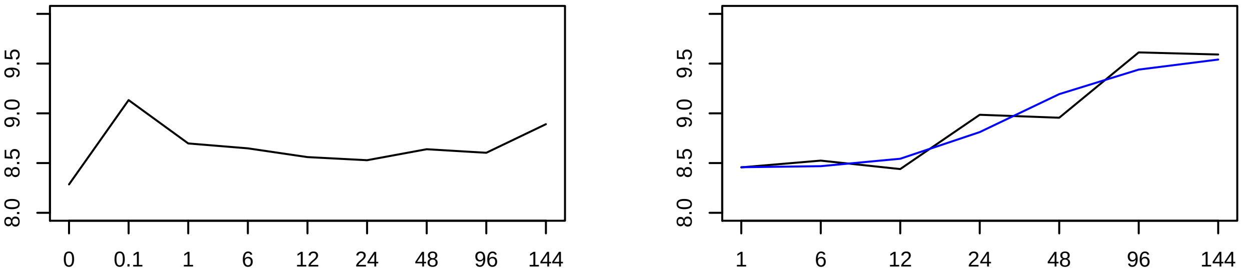


**A_24_P230675 SOCS2 12q22**

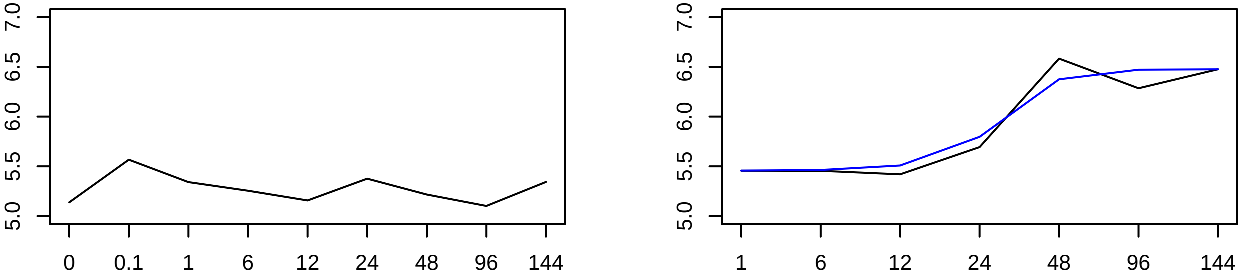

**A_24_P29260 MGAT4B 5q35.3**

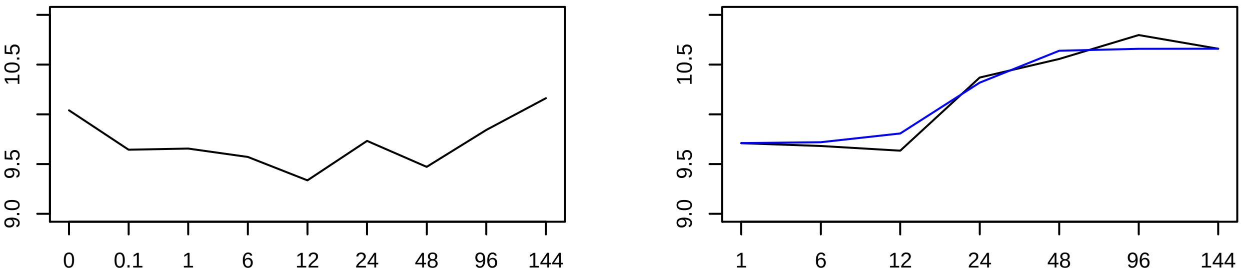


**A_23_P163711 FAM57B 16p11.2**

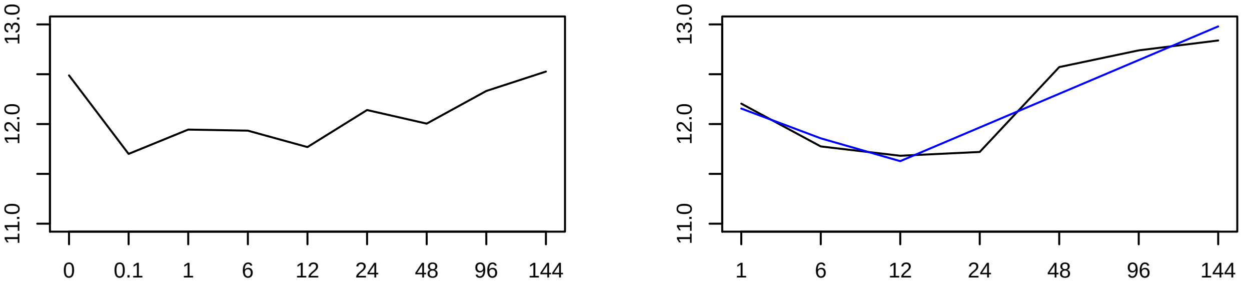


**A_24_P396753 TRIB2 2p25.1**

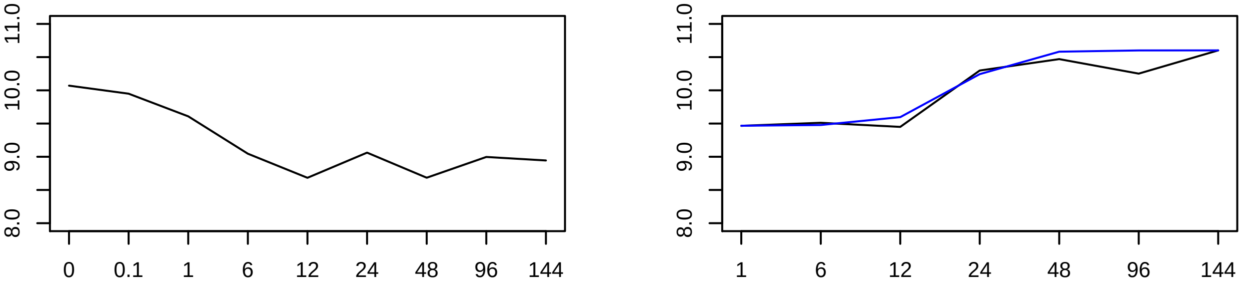

**A_23_P36364 THY1 11q23.3**

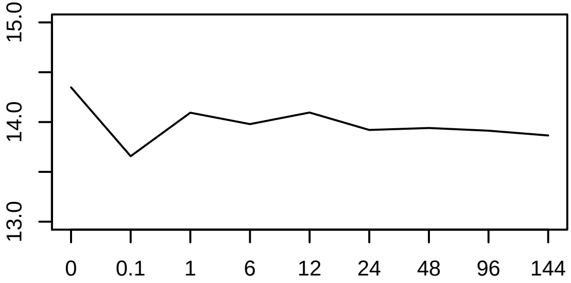


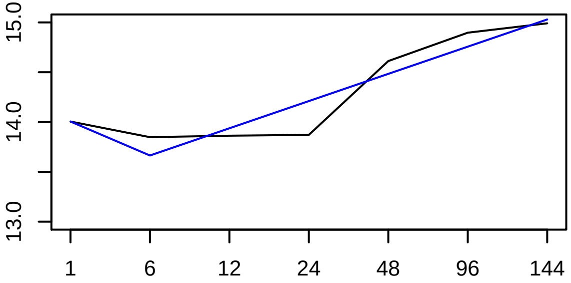

Supplement: Additional file 3 — Additional file A-H. These files contain the fitting results for the genes from the groups A-H, deduced by SwitchFinder, which represent eight dynamic patterns of the gene expression response to ATRA in neuroblastoma cell line. (ZIP 2457 kb) [file 12859_2016_1391_MOESM3_ESM.zip › AdditionalFile_B.pdf]
