# Supplementary material for: SwitchFinder – a novel method and query facility for discovering dynamic gene expression patterns
Source: BMC Bioinformatics. 2016 Dec 15;17:532. doi: 10.1186/s12859-016-1391-0 (PMC5160026; doi:10.1186/s12859-016-1391-0)
Supplement: Additional file 3 — Additional file A-H. These files contain the fitting results for the genes from the groups A-H, deduced by SwitchFinder, which represent eight dynamic patterns of the gene expression response to ATRA in neuroblastoma cell line. (ZIP 2457 kb) [file 12859_2016_1391_MOESM3_ESM.zip › AdditionalFile_C.pdf]

A_24_P240166 PHLDB2 3q13.2

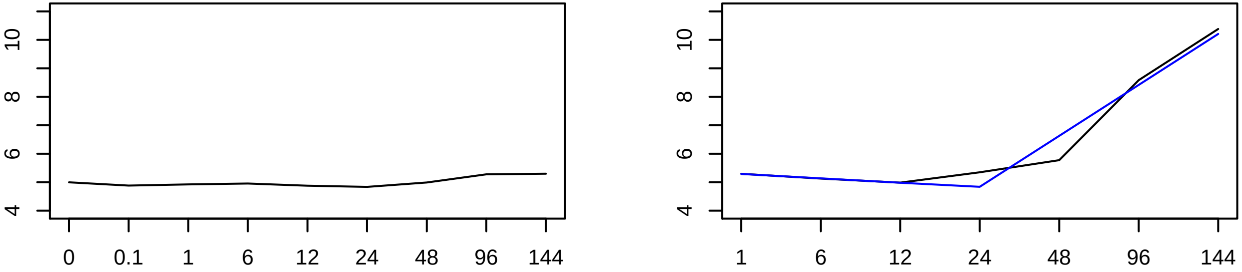

A_23_P150789 PRSS23 11q14.2

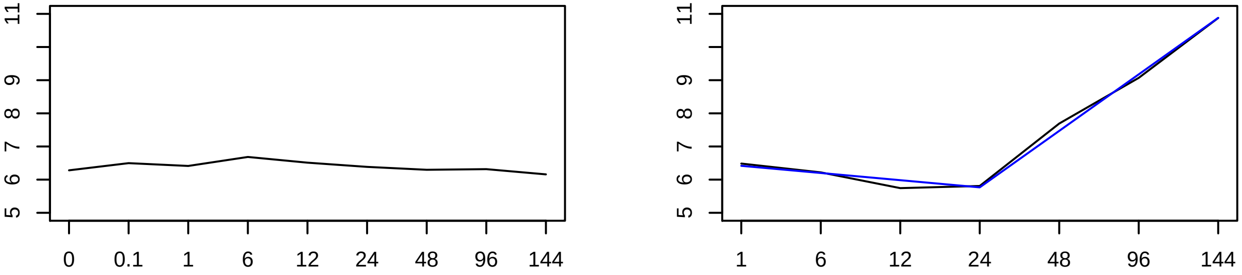

A_24_P281443 LOC649375 Xq25

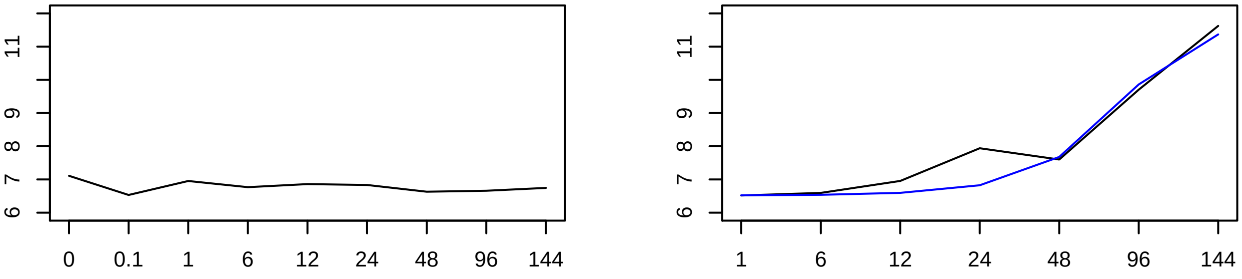

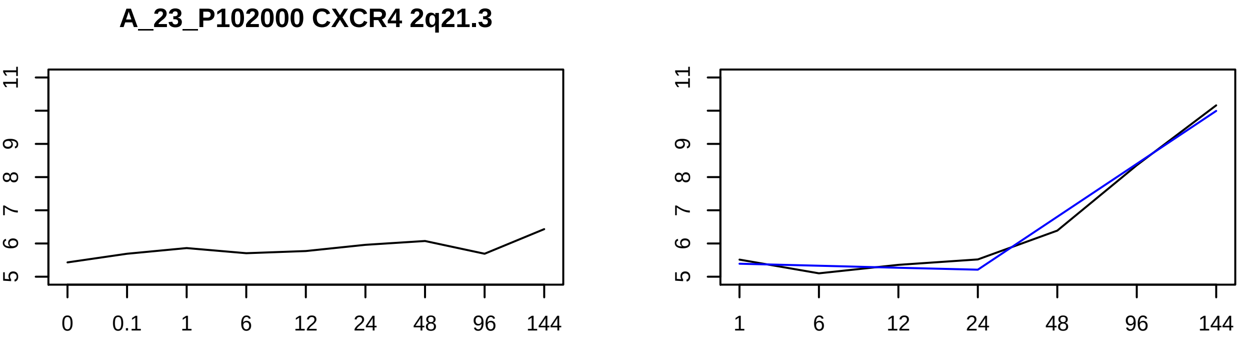
A_23_P102000 CXCR4 2q21.3
5
6
7
8
9
11
0
0.1
1
6
12
24
48
96
144
5
6
7
8
9
11
1
6
12
24
48
96
144

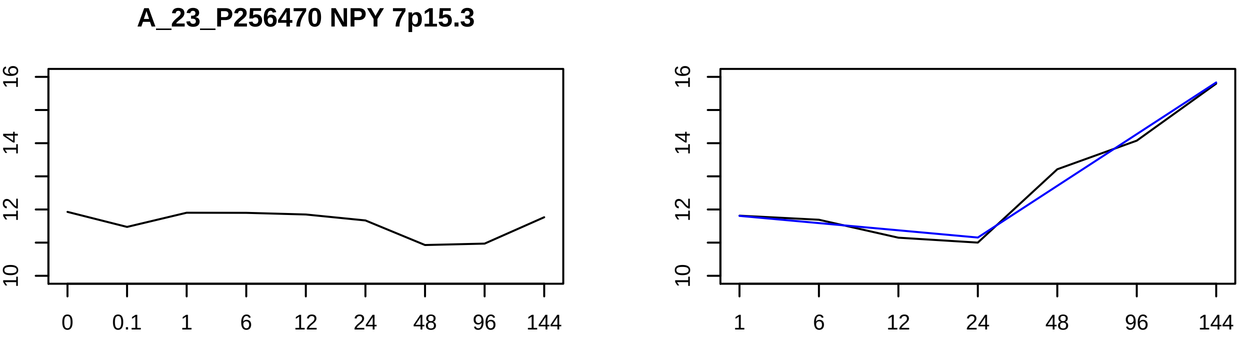
A_23_P256470 NPY 7p15.3
10
12
14
16
0
0.1
1
6
12
24
48
96
144
10
12
14
16
1
6
12
24
48
96
144

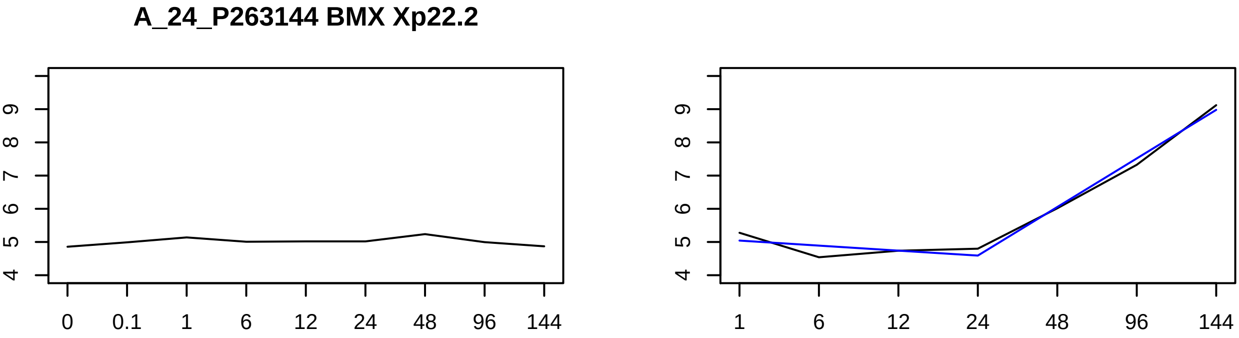
A_24_P263144 BMX Xp22.2
4
5
6
7
8
9
0
0.1
1
6
12
24
48
96
144
4
5
6
7
8
9
1
6
12
24
48
96
144

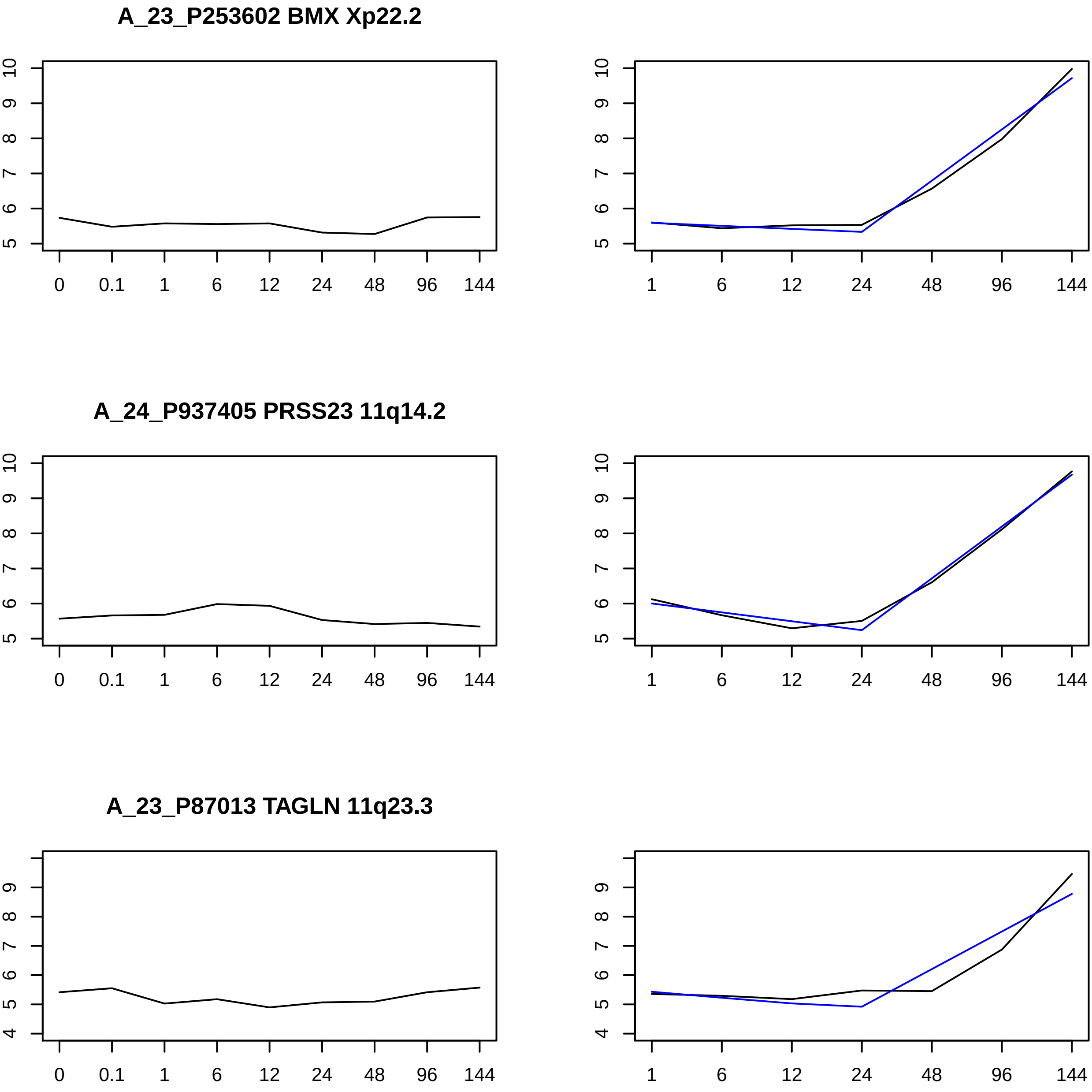
A_23_P253602 BMX Xp22.2
A_24_P937405 PRSS23 11q14.2
A_23_P87013 TAGLN 11q23.3
0 0.1 1 6 12 24 48 96 144
1 6 12 24 48 96 144
5 6 7 8 9 10
4 5 6 7 8 9

A_23_P401472 CHRM3 1q43

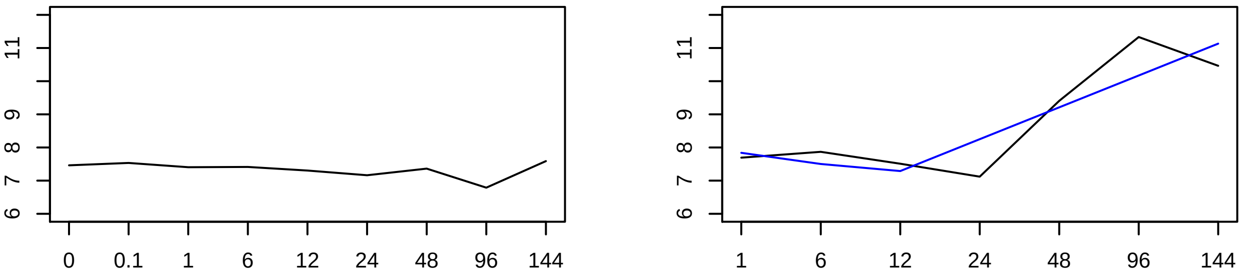

A_23_P167159 SCRG1 4q34.1

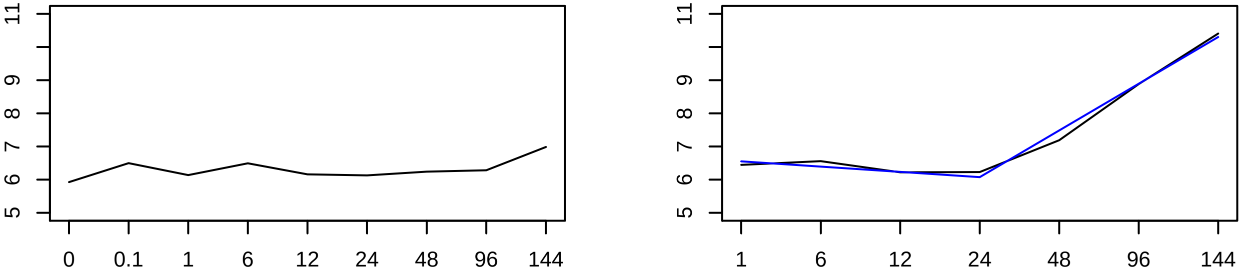

A_23_P253692 GPR64 Xp22.13

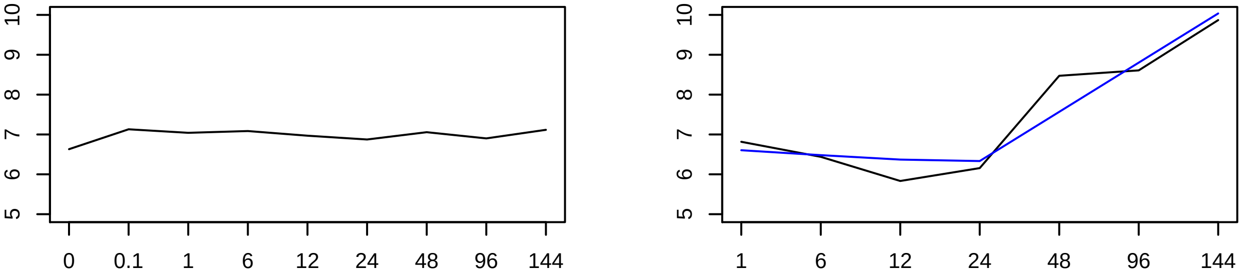

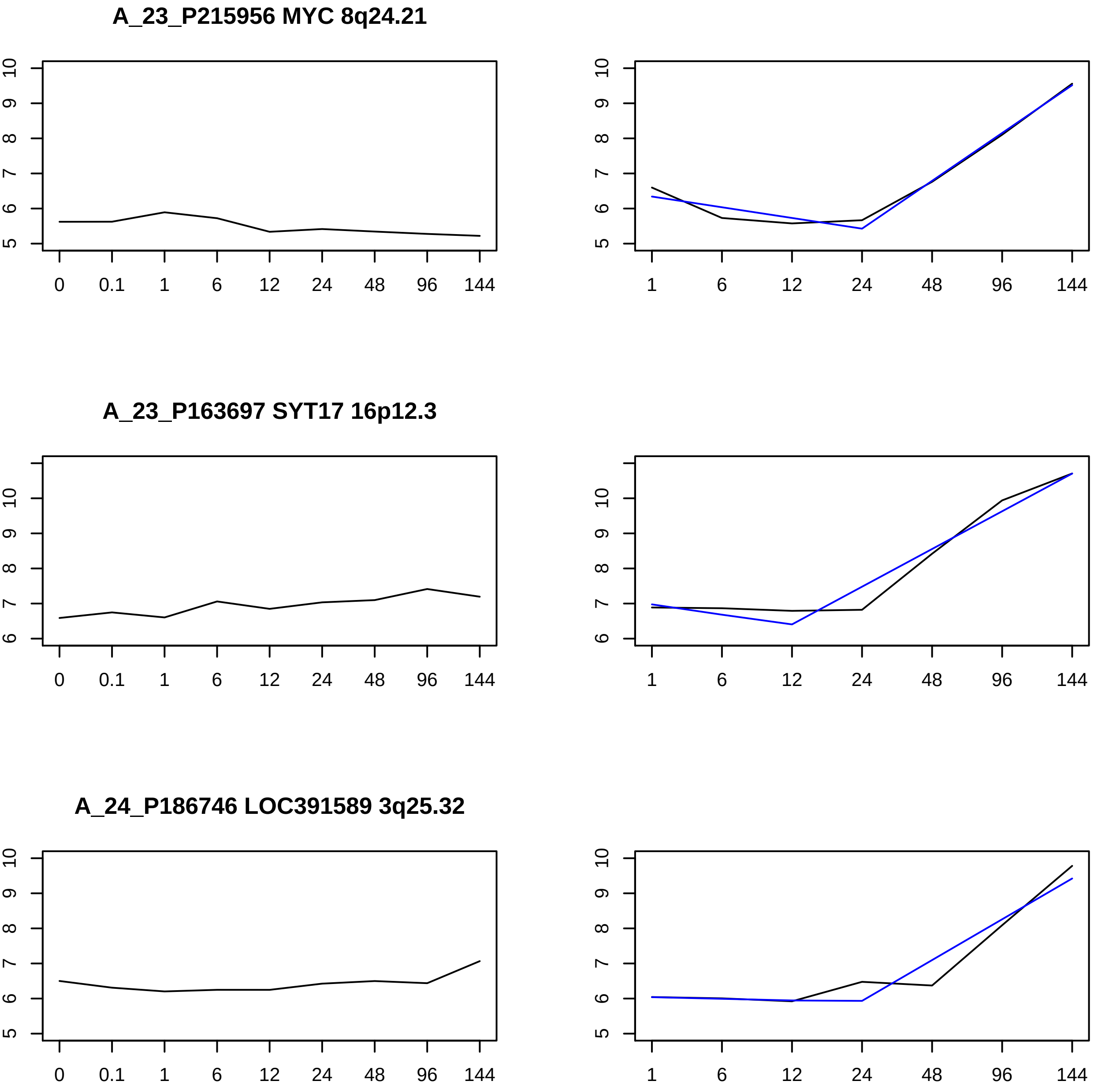

A_23_P215956 MYC 8q24.21
A_23_P163697 SYT17 16p12.3
A_24_P186746 LOC391589 3q25.32
0 0.1 1 6 12 24 48 96 144
1 6 12 24 48 96 144
5 6 7 8 9 10

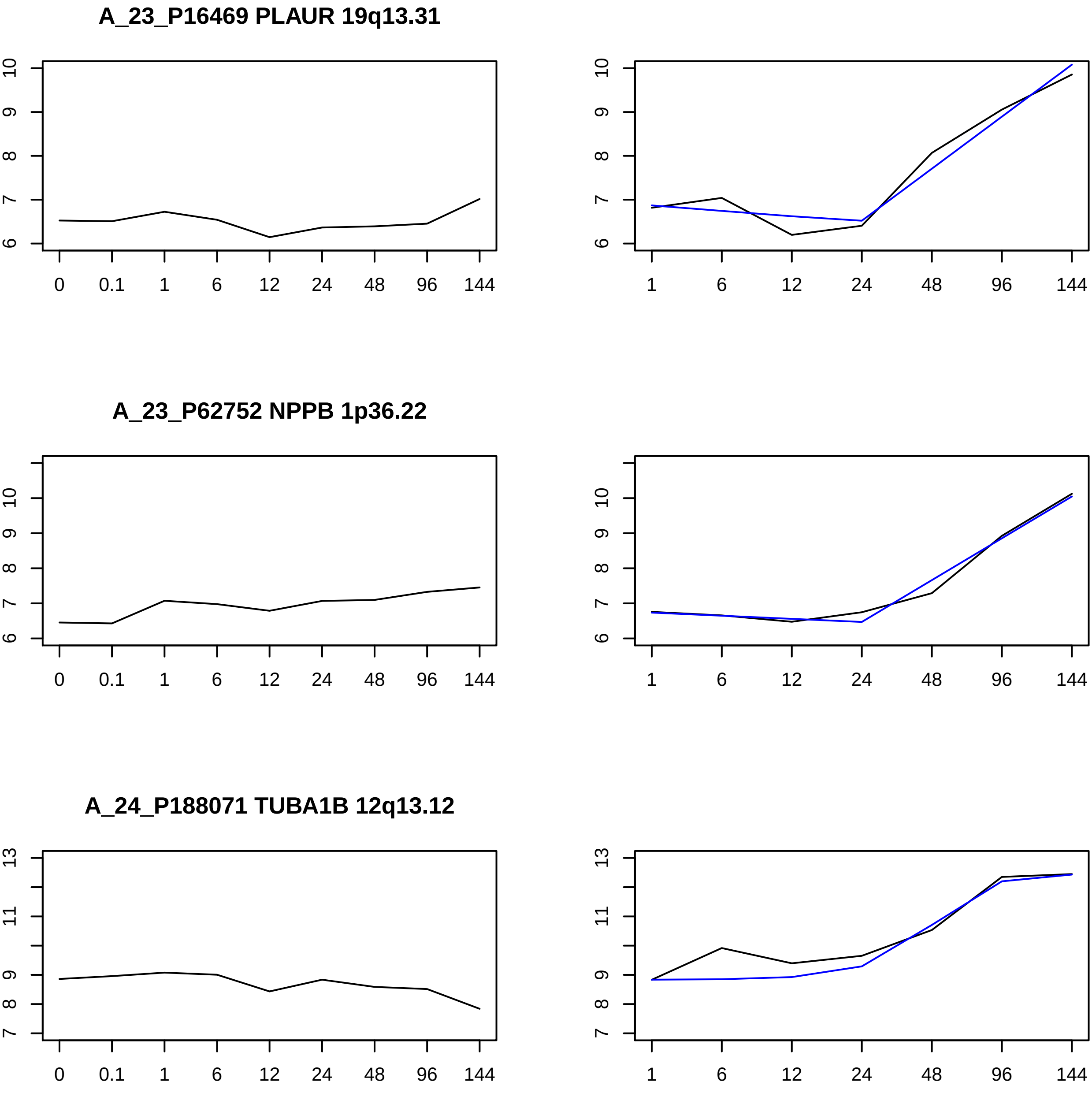
A_23_P16469 PLAUR 19q13.31
A_23_P62752 NPPB 1p36.22
A_24_P188071 TUBA1B 12q13.12
0
0.1
1
6
12
24
48
96
144

A_23_P55477 ADORA2B 17p12

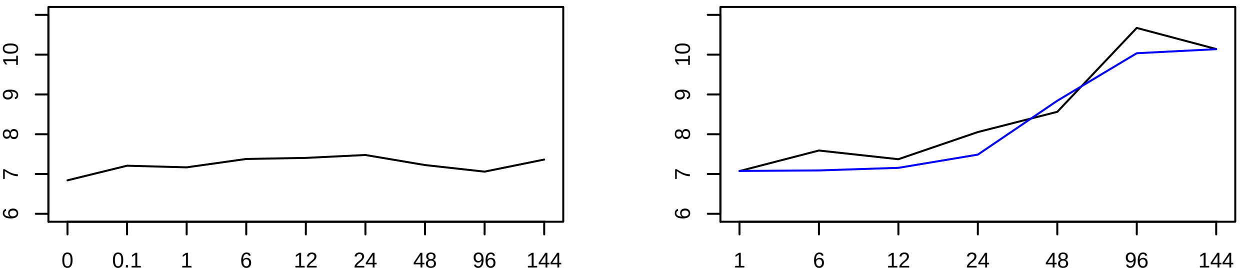

A_23_P373598 MAFK 7p22.3

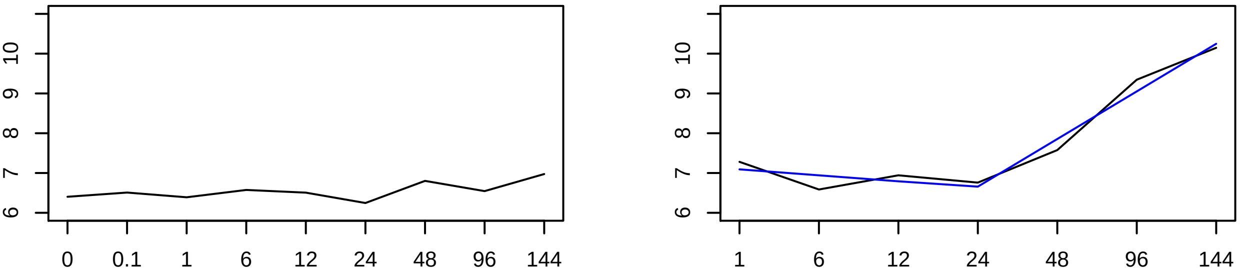

A_32_P134756 CDH6 5p13.3

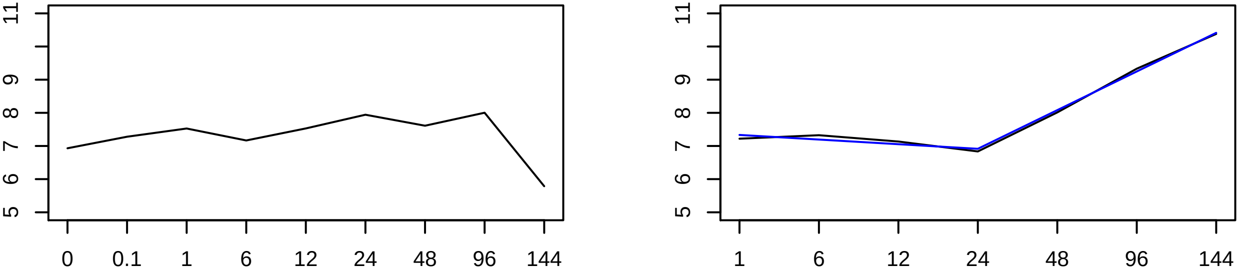

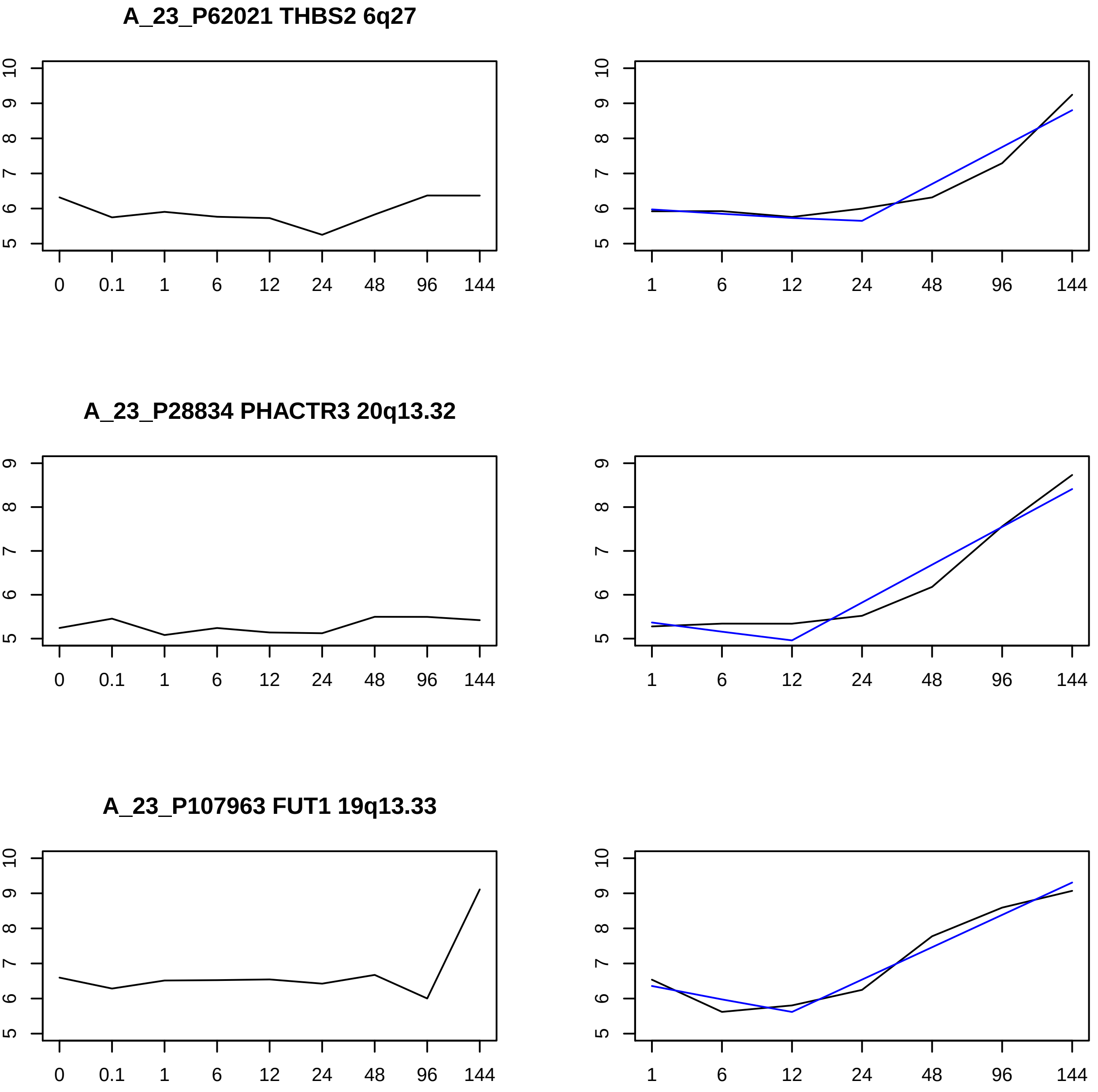
A_23_P62021 THBS2 6q27
5 6 7 8 9 10
0 0.1 1 6 12 24 48 96 144
1 6 12 24 48 96 144
A_23_P28834 PHACTR3 20q13.32
5 6 7 8 9
0 0.1 1 6 12 24 48 96 144
1 6 12 24 48 96 144
A_23_P107963 FUT1 19q13.33
5 6 7 8 9 10
0 0.1 1 6 12 24 48 96 144
1 6 12 24 48 96 144

A_32_P16323 POTEM 14q11.1

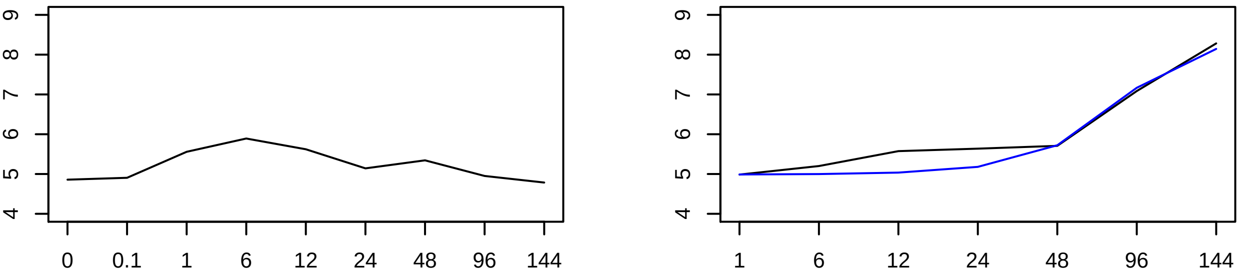

A_23_P92727 RAI14 5p13.2

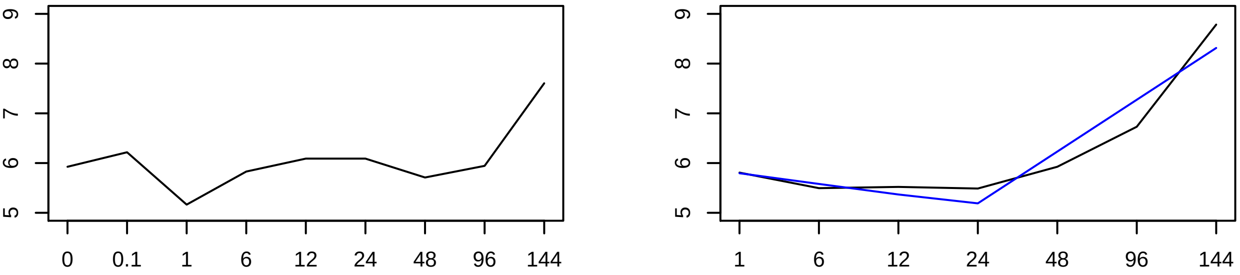

A_23_P144959 CSPG2 5q14.3

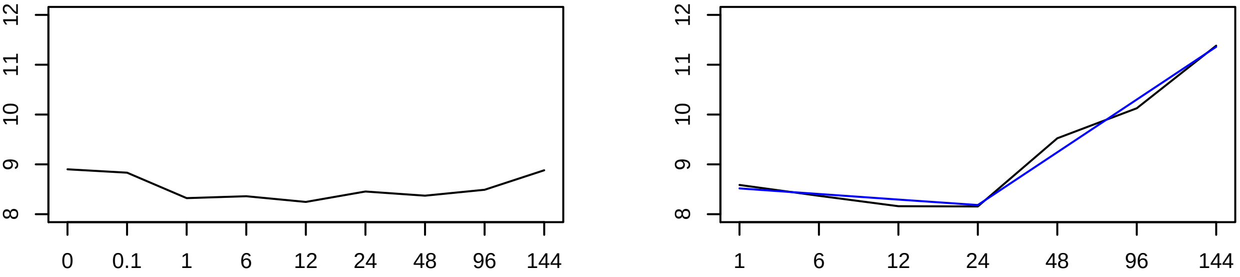

A_23_P407112 SPATA18 4q12

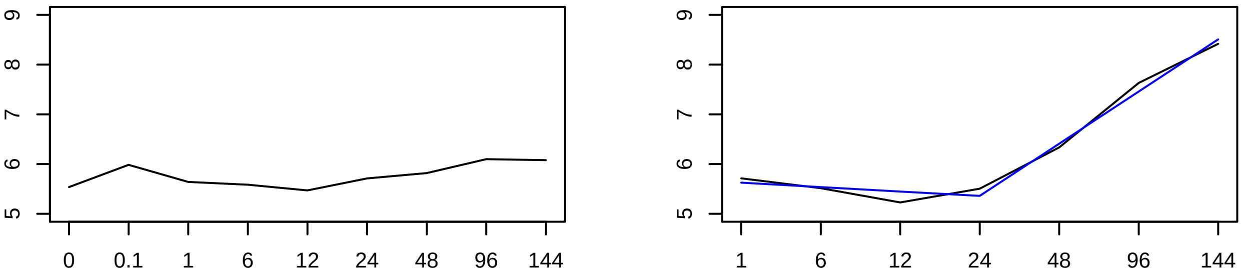

A_23_P209167 FSTL3 19p13.3

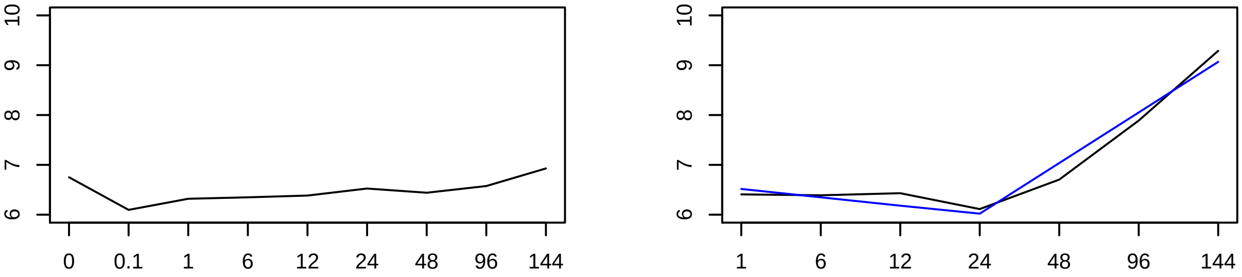

A_23_P145606 CHRM2 7q33

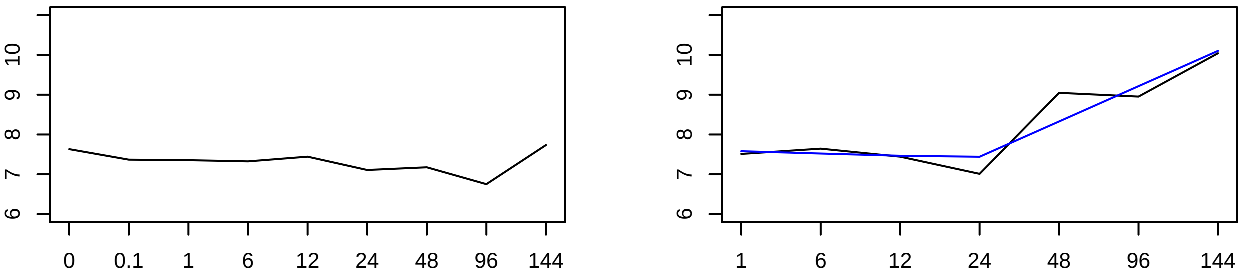

A_24_P164718 MARCH2 19p13.2

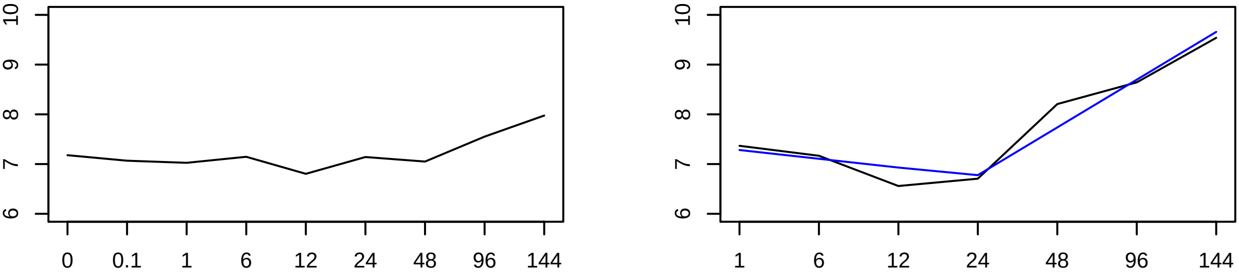

A_24_P475349 RAB6B 3q22.1

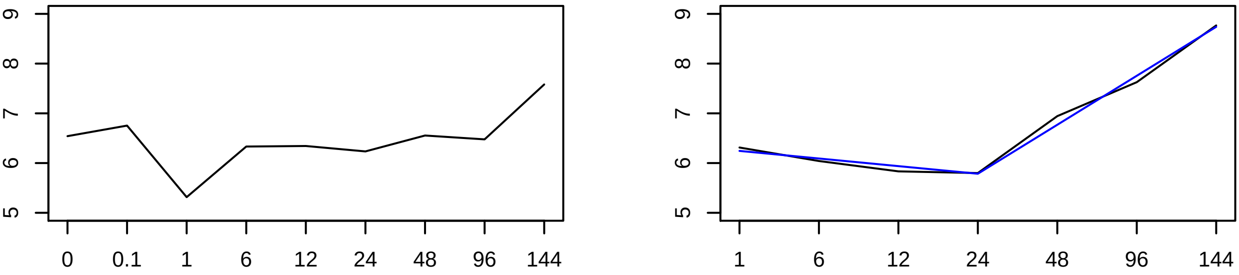

A_23_P320242 KIAA1324L 7q21.12

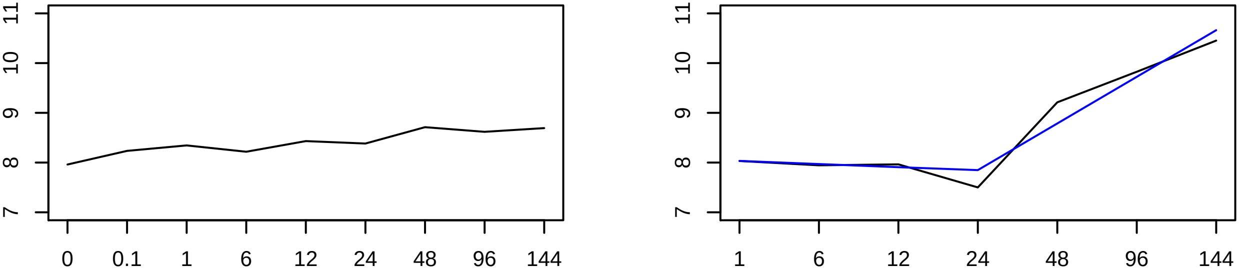

A_23_P122852 SMARCD3 7q36.1

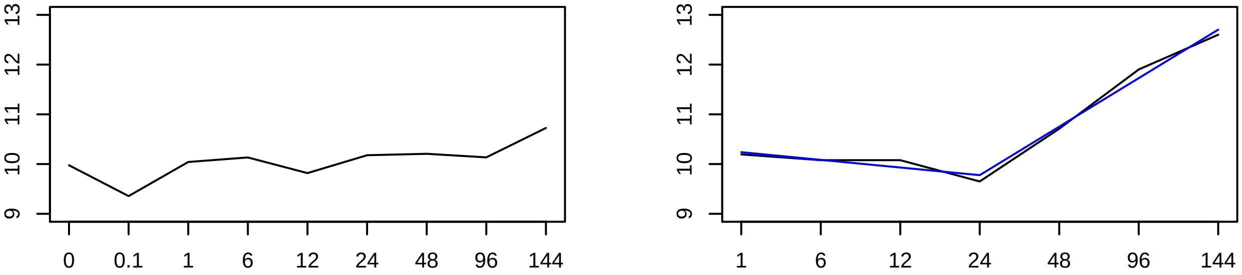

A_23_P112874 GPC5 13q31.3

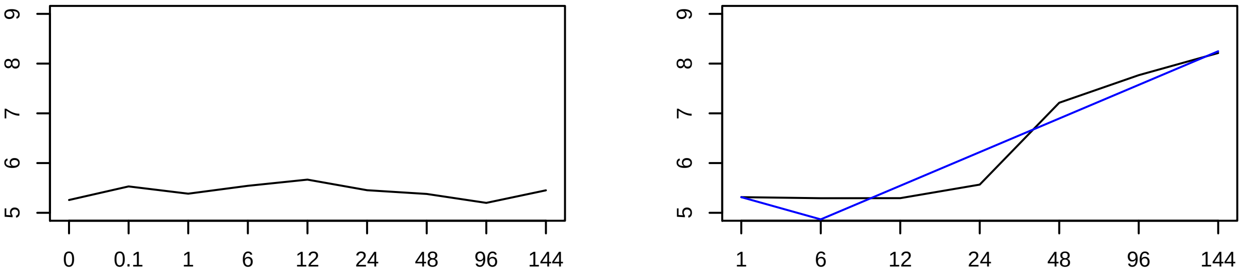

A_23_P134426 GPNMB 7p15.3

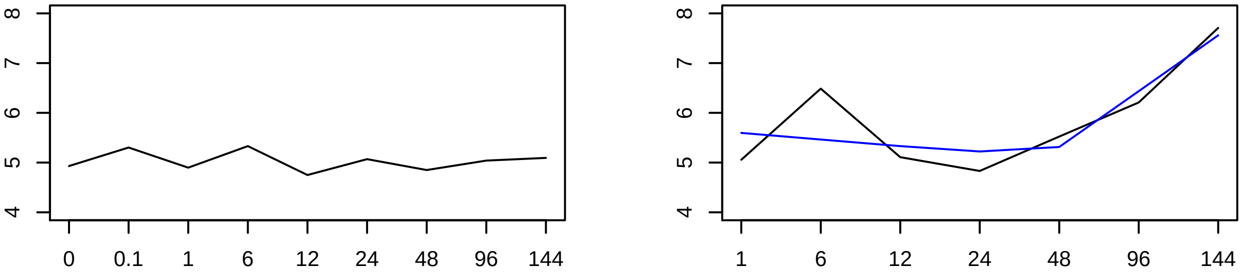

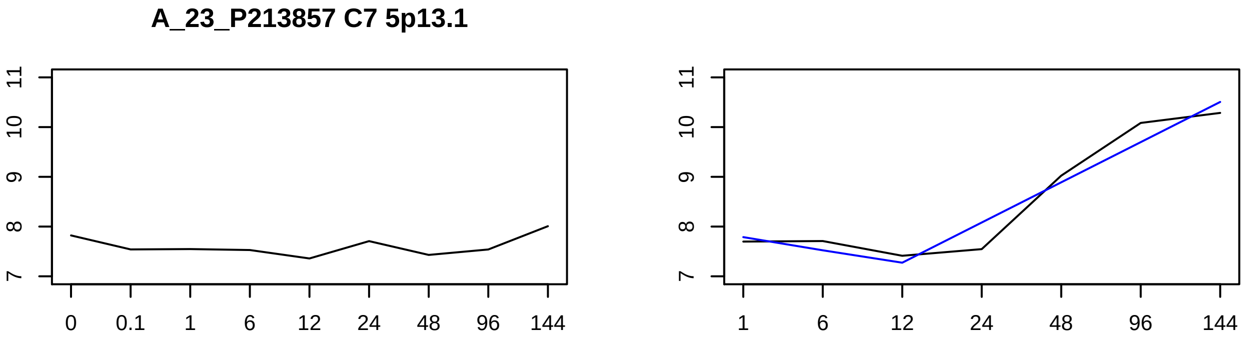
A_23_P213857 C7 5p13.1
7
8
9
10
11
0
0.1
1
6
12
24
48
96
144
7
8
9
10
11
1
6
12
24
48
96
144

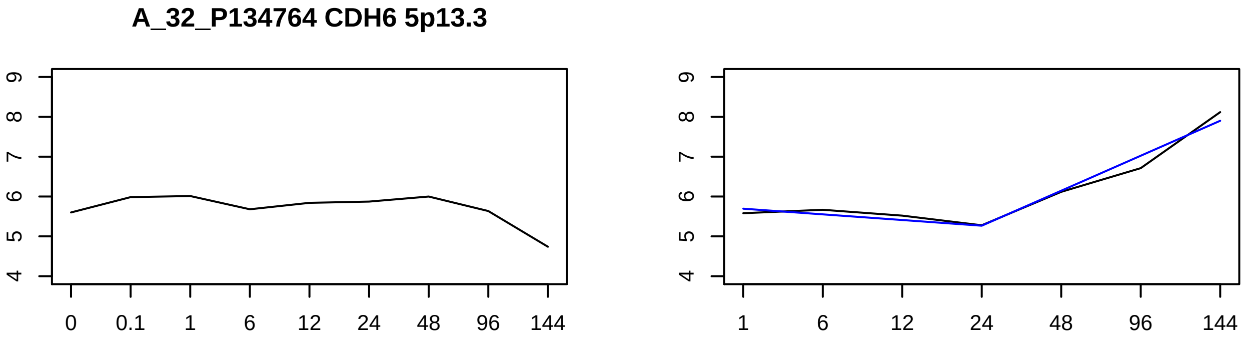
A_32_P134764 CDH6 5p13.3
4
5
6
7
8
9
0
0.1
1
6
12
24
48
96
144
4
5
6
7
8
9
1
6
12
24
48
96
144

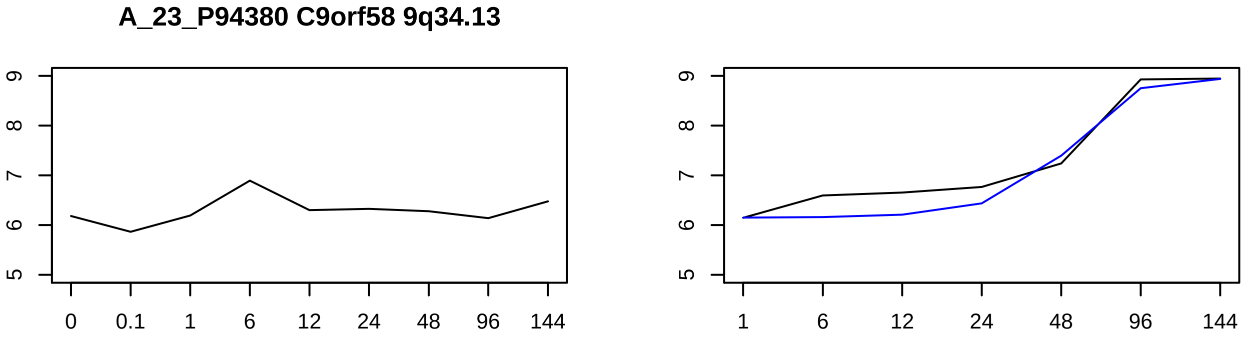
A_23_P94380 C9orf58 9q34.13
5
6
7
8
9
0
0.1
1
6
12
24
48
96
144
5
6
7
8
9
1
6
12
24
48
96
144

A_24_P334130 FN1 2q35

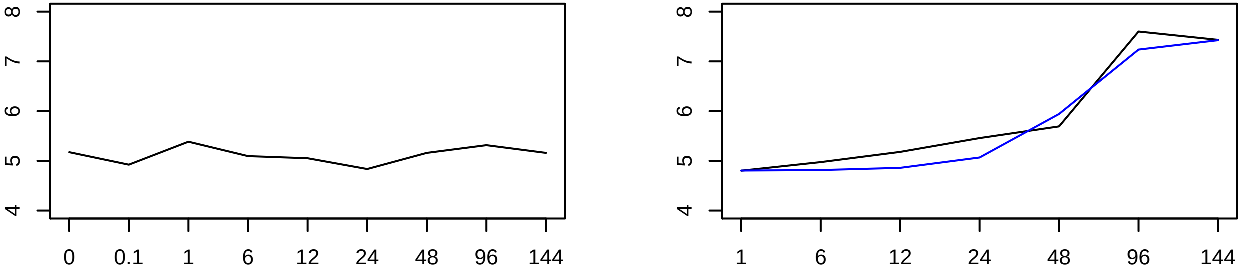

A_23_P109974 RAB6B 3q22.1

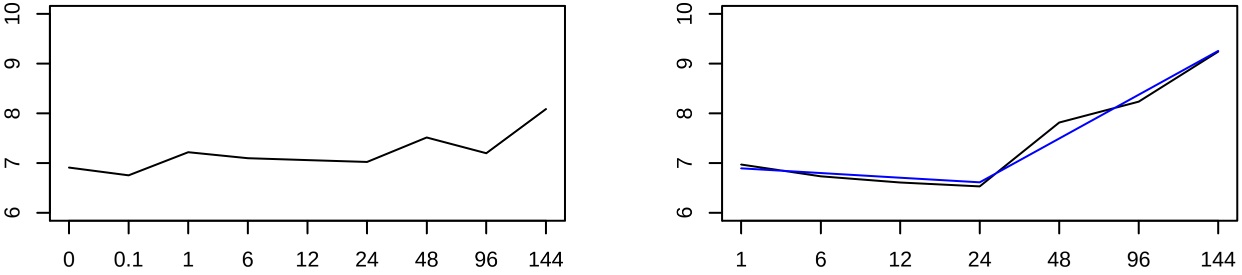

A_24_P62783 FABP3 1p35.2

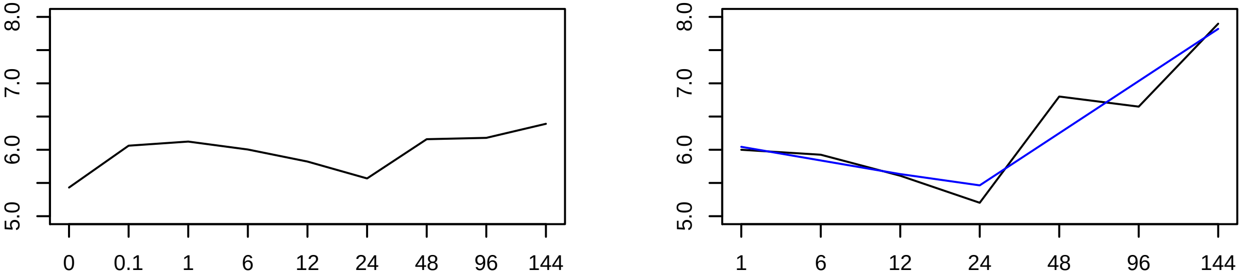

A_32_P205624 SHC2 19p13.3

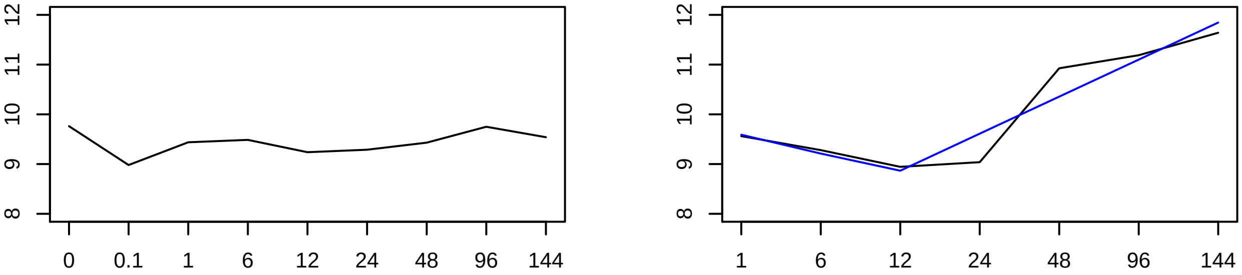

A_23_P47614 PHLDA2 11p15.4

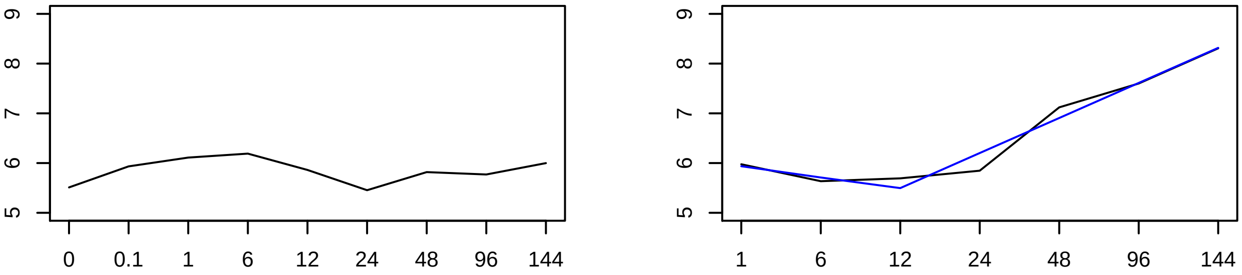

A_32_P231617 TM4SF1 3q25.1

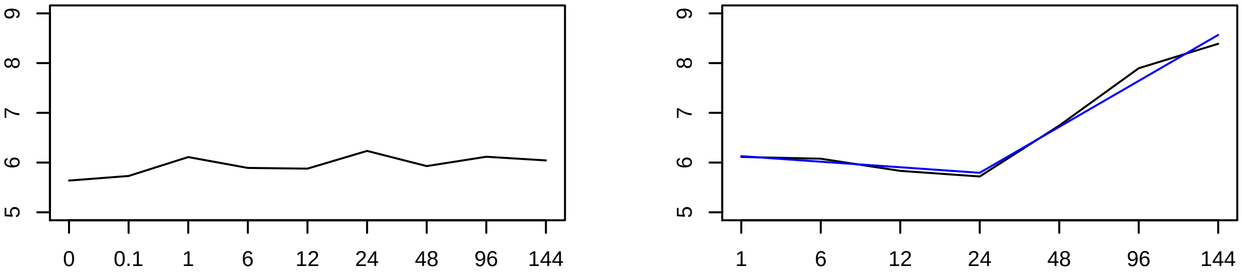

A_23_P8812 W60781 NA

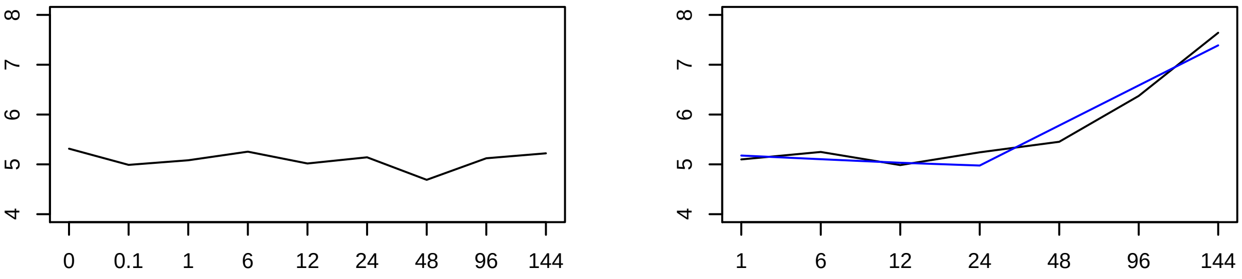

A_23_P83134 GAS1 9q21.33

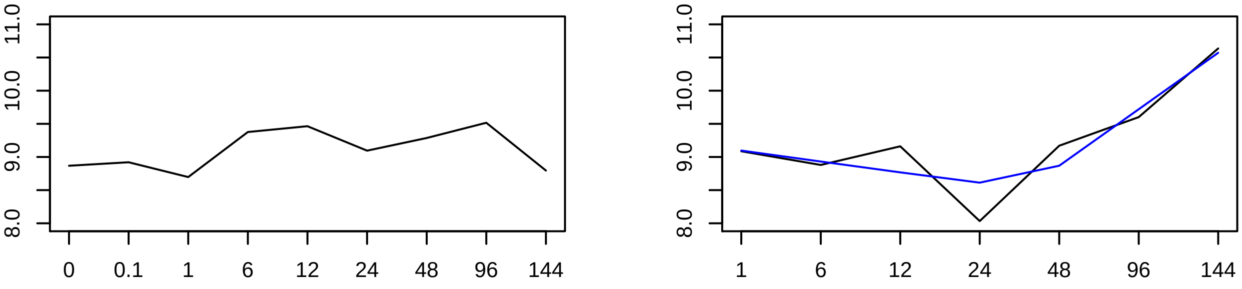

A_23_P65629 KCNK10 14q31.3

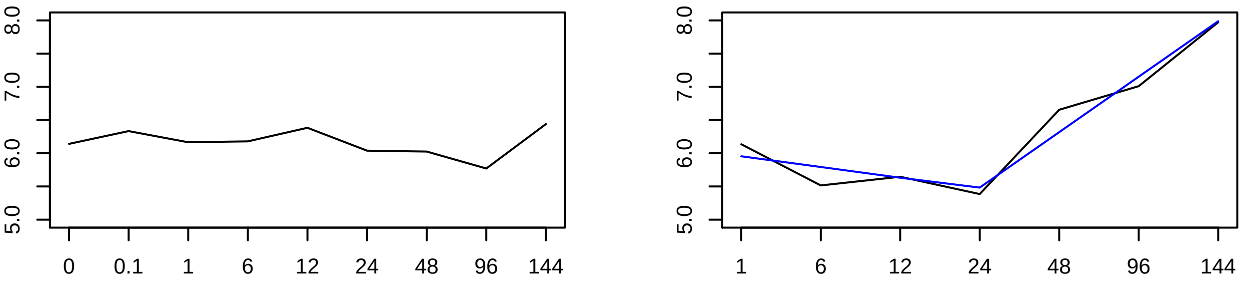

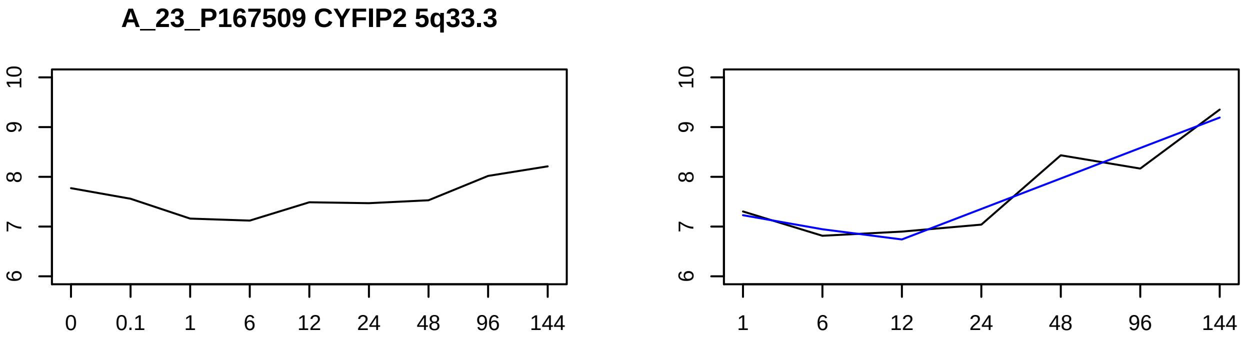
A_23_P167509 CYFIP2 5q33.3
6
7
8
9
10
0
0.1
1
6
12
24
48
96
144

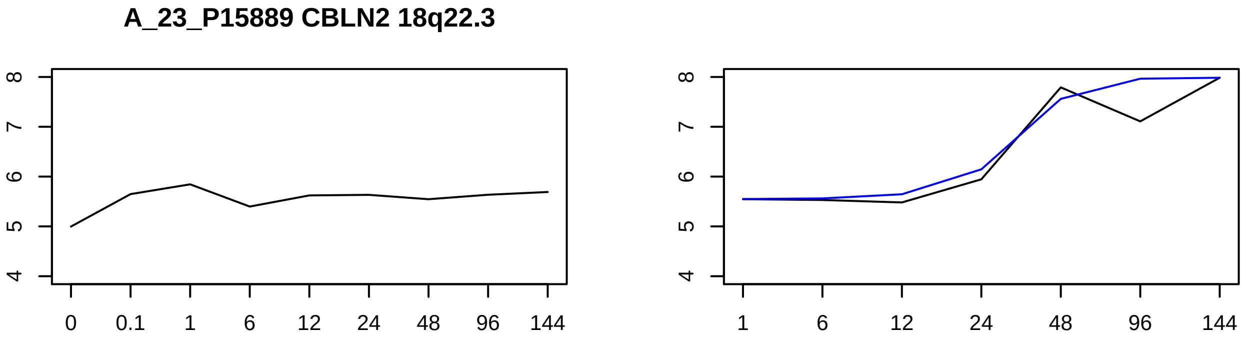
A_23_P15889 CBLN2 18q22.3
4
5
6
7
8
0
0.1
1
6
12
24
48
96
144

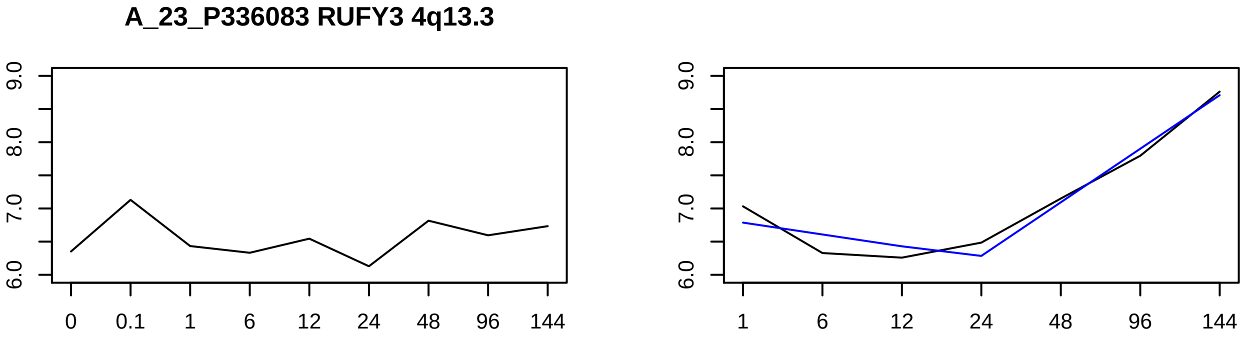
A_23_P336083 RUFY3 4q13.3
6.0
7.0
8.0
9.0
0
0.1
1
6
12
24
48
96
144

A_24_P703830 NANOS3 19p13.12

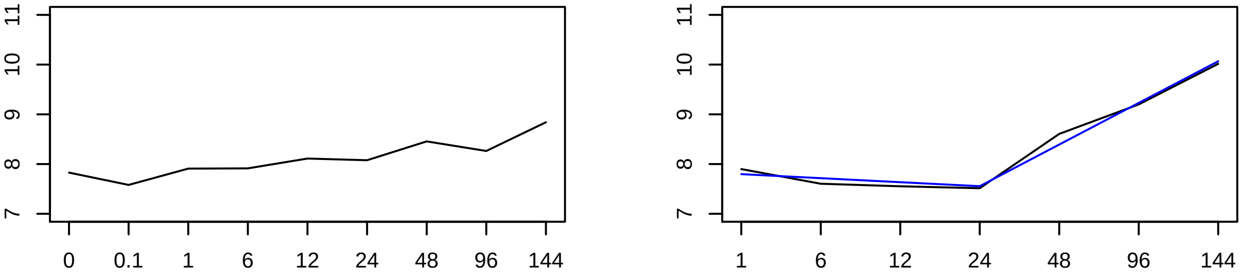

A_32_P82895 C2CD4C 19p13.3

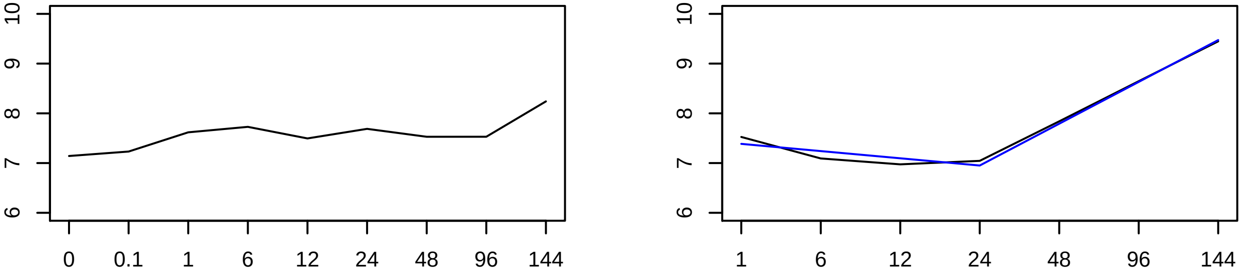

A_24_P11462 ADC 1p35.1

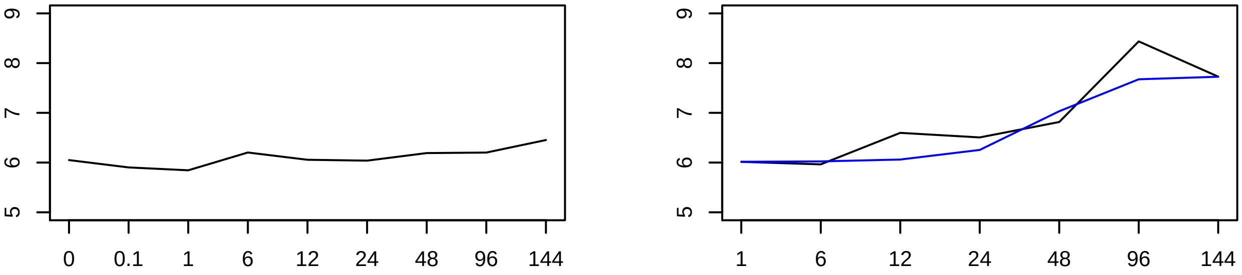

A_23_P33683 MARCH2 19p13.2

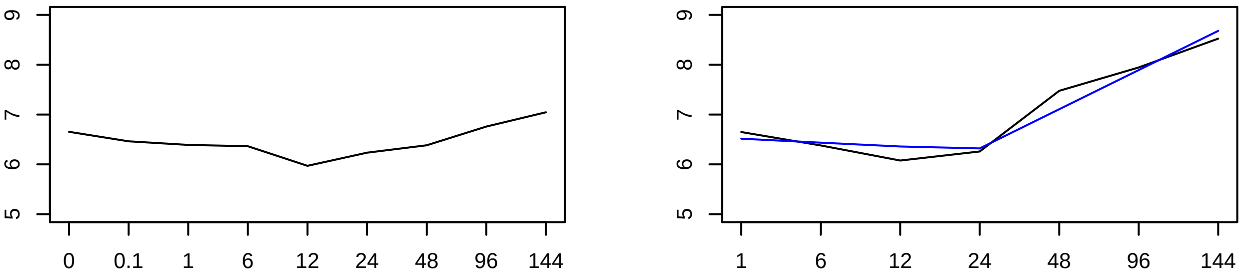

A_32_P43878 THC2676139 NA

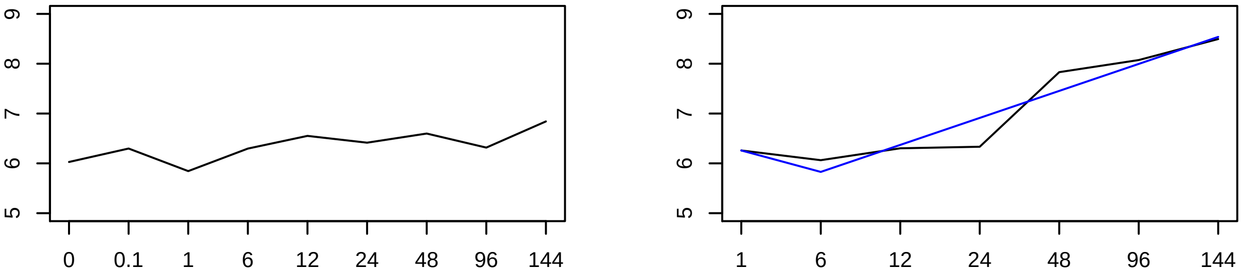

A_23_P141345 MPP3 17q21.31

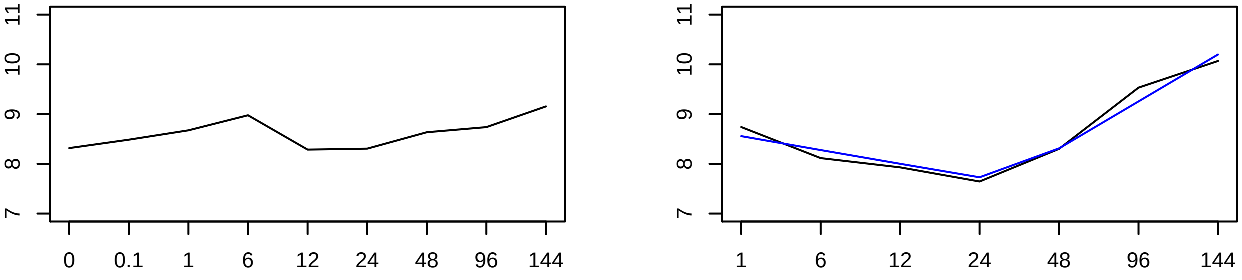

A_23_P201193 TSPAN2 1p13.2

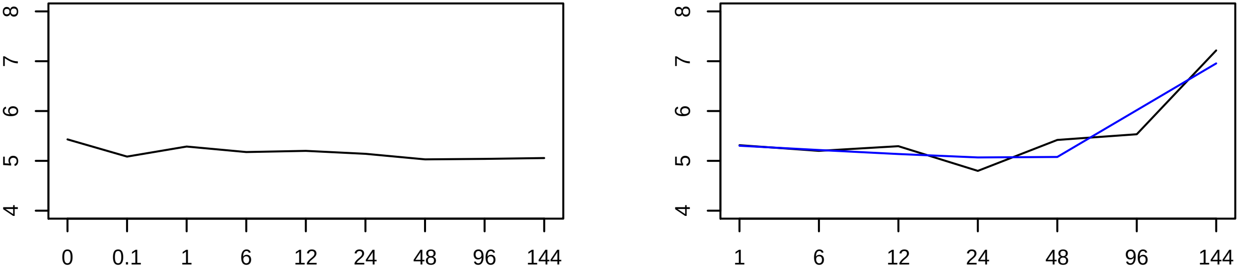

A_24_P303524 MICALL2 7p22.3

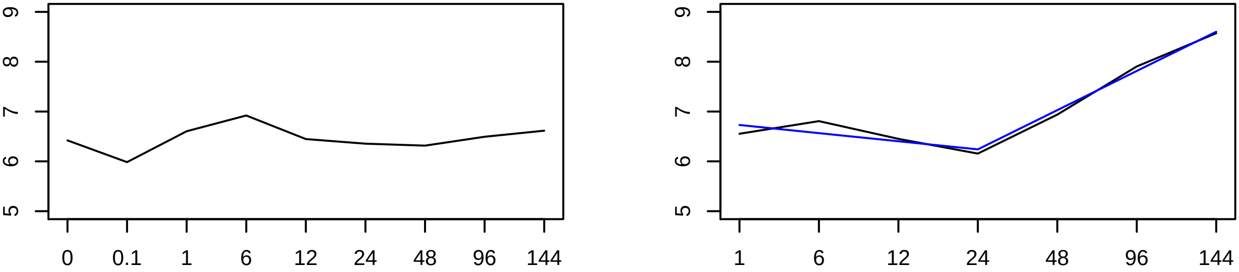

A_23_P43276 GPR124 8p12

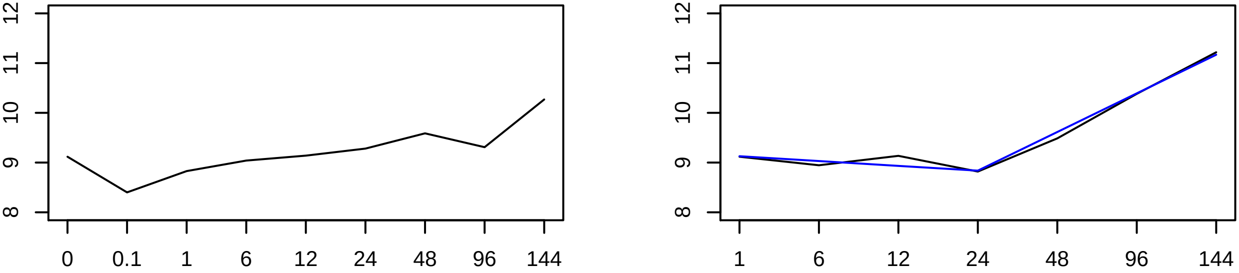

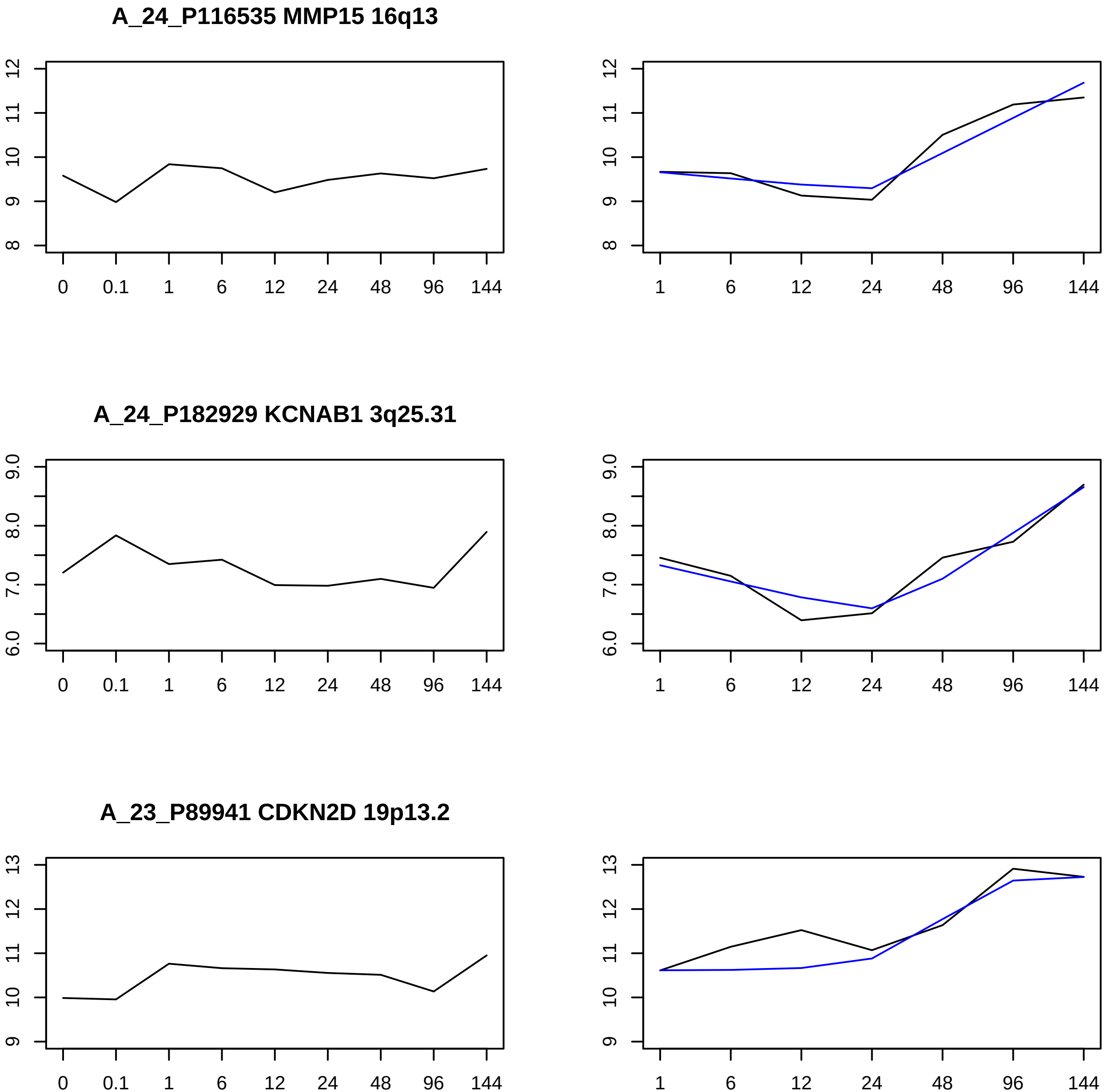

A_24_P116535 MMP15 16q13
8
9
10
11
12
0
0.1
1
6
12
24
48
96
144
A_24_P182929 KCNAB1 3q25.31
6.0
7.0
8.0
9.0
A_23_P89941 CDKN2D 19p13.2
9
10
11
12
13

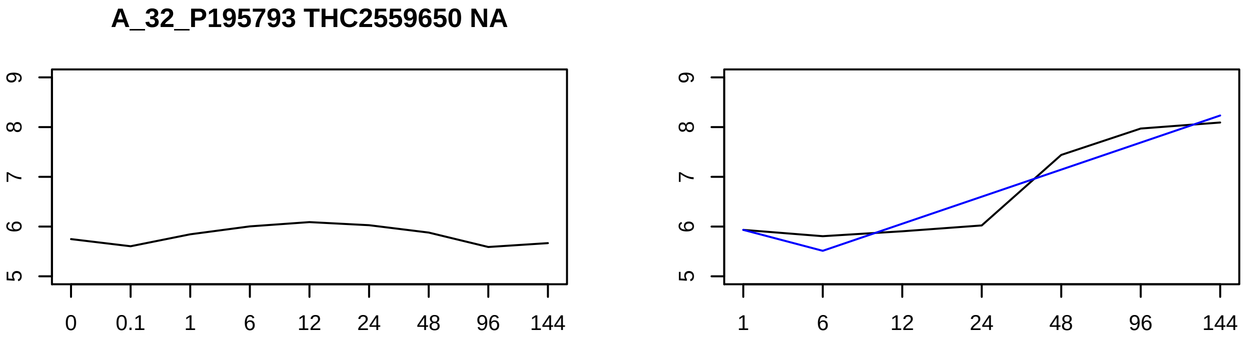
A_32_P195793 THC2559650 NA
5
6
7
8
9
0
0.1
1
6
12
24
48
96
144
1
6
12
24
48
96
144

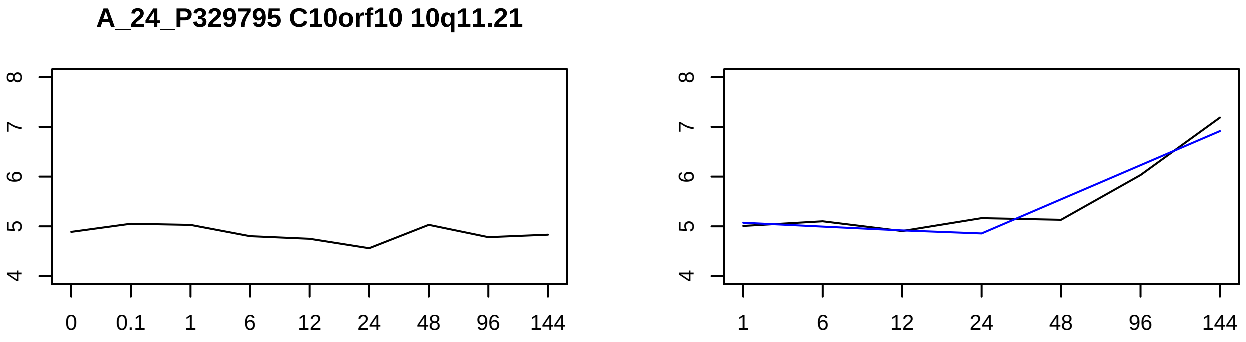
A_24_P329795 C10orf10 10q11.21
4
5
6
7
8
0
0.1
1
6
12
24
48
96
144
1
6
12
24
48
96
144

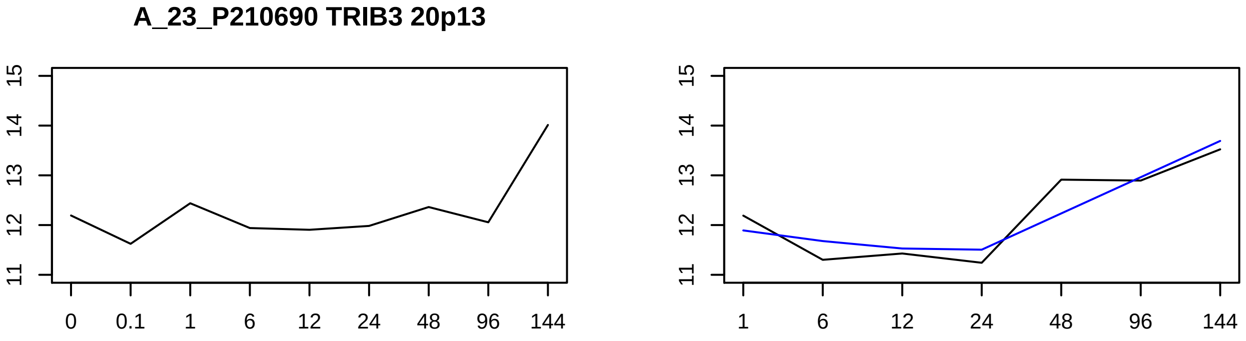
A_23_P210690 TRIB3 20p13
11
12
13
14
15
0
0.1
1
6
12
24
48
96
144
1
6
12
24
48
96
144

A_32_P122703 PGM2L1 11q13.4

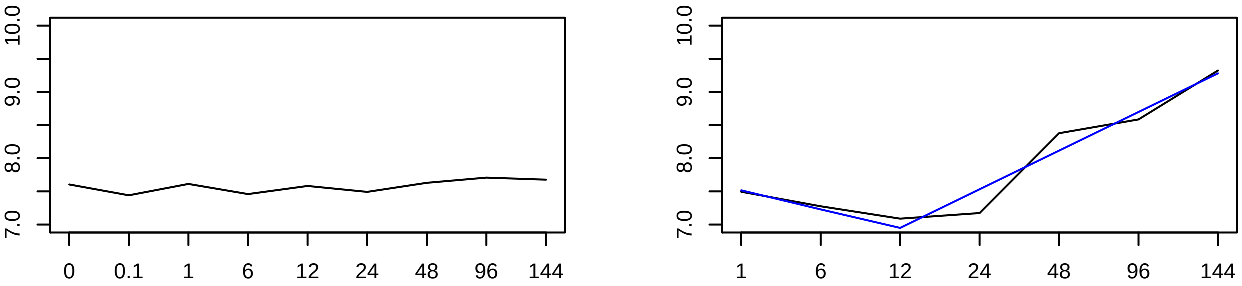

A_23_P12884 GRK5 10q26.11

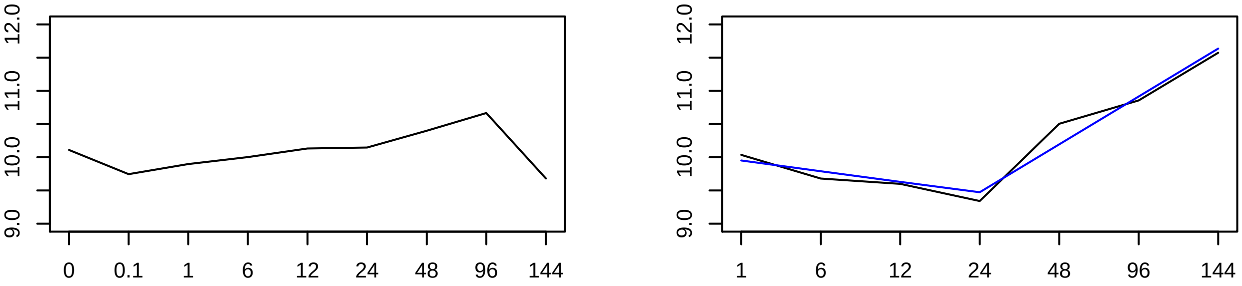

A_32_P153773 CACNA1B 9q34.3

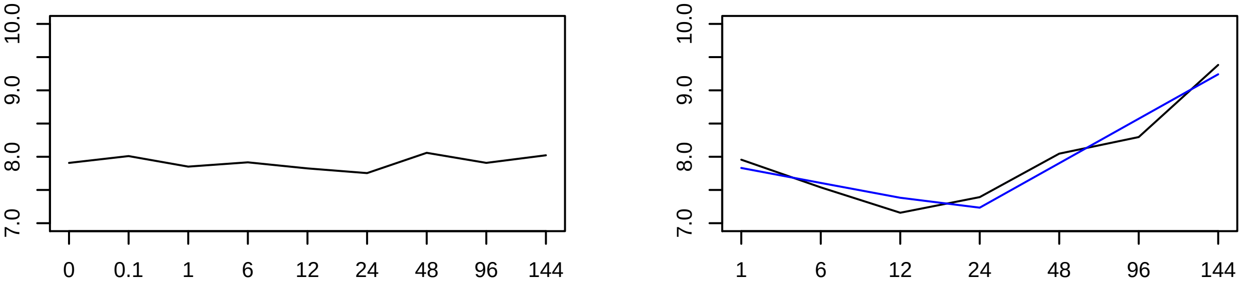

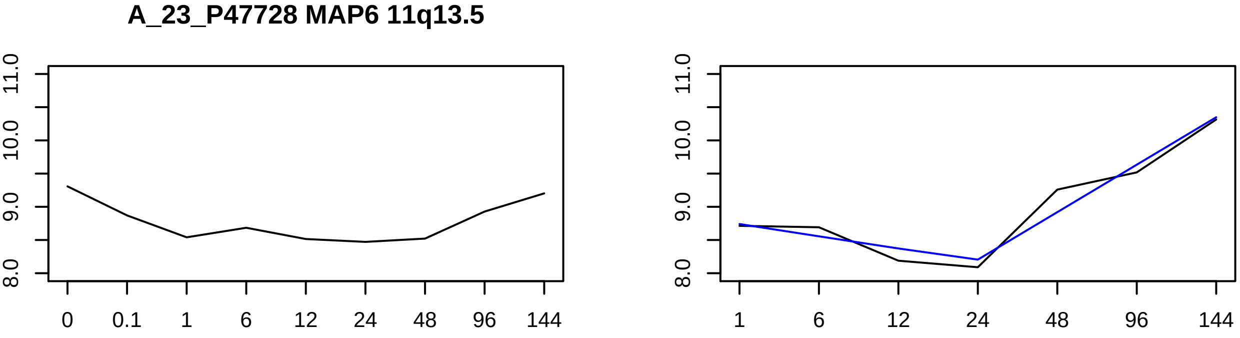
A_23_P47728 MAP6 11q13.5
8.0
9.0
10.0
11.0
0
0.1
1
6
12
24
48
96
144
8.0
9.0
10.0
11.0
1
6
12
24
48
96
144

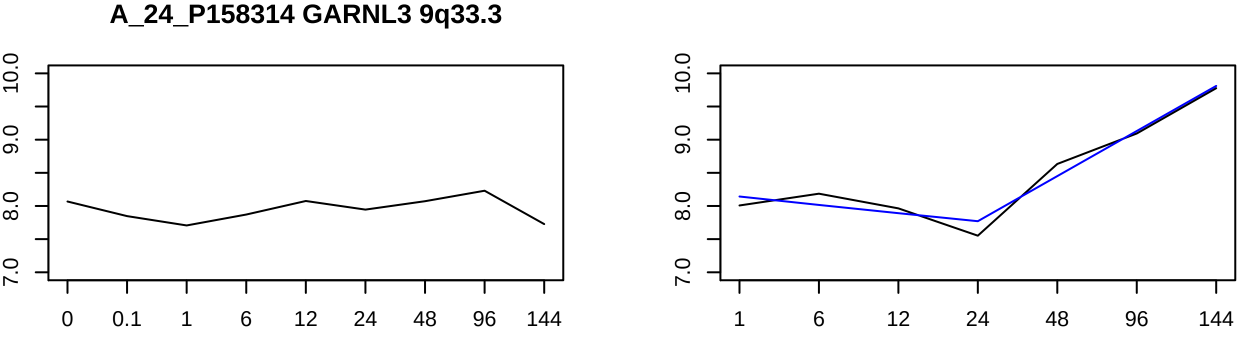
A_24_P158314 GARNL3 9q33.3
7.0
8.0
9.0
10.0
0
0.1
1
6
12
24
48
96
144
7.0
8.0
9.0
10.0
1
6
12
24
48
96
144

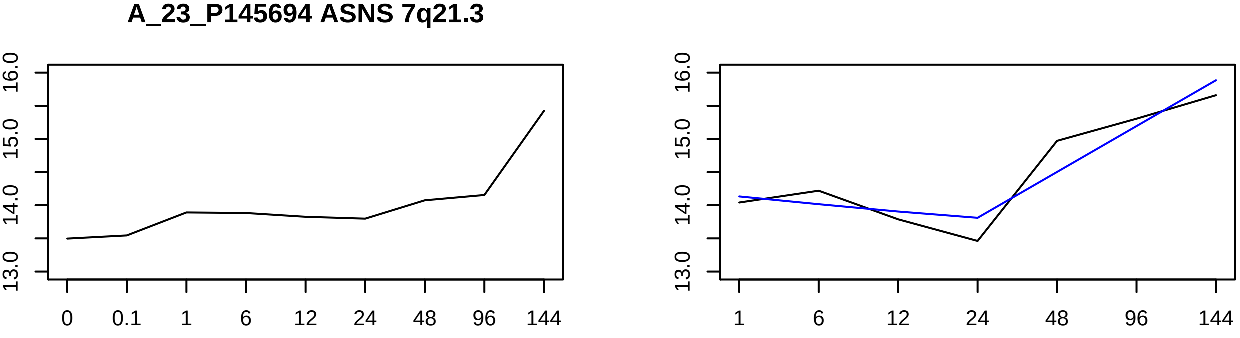
A_23_P145694 ASNS 7q21.3
13.0
14.0
15.0
16.0
0
0.1
1
6
12
24
48
96
144
13.0
14.0
15.0
16.0
1
6
12
24
48
96
144

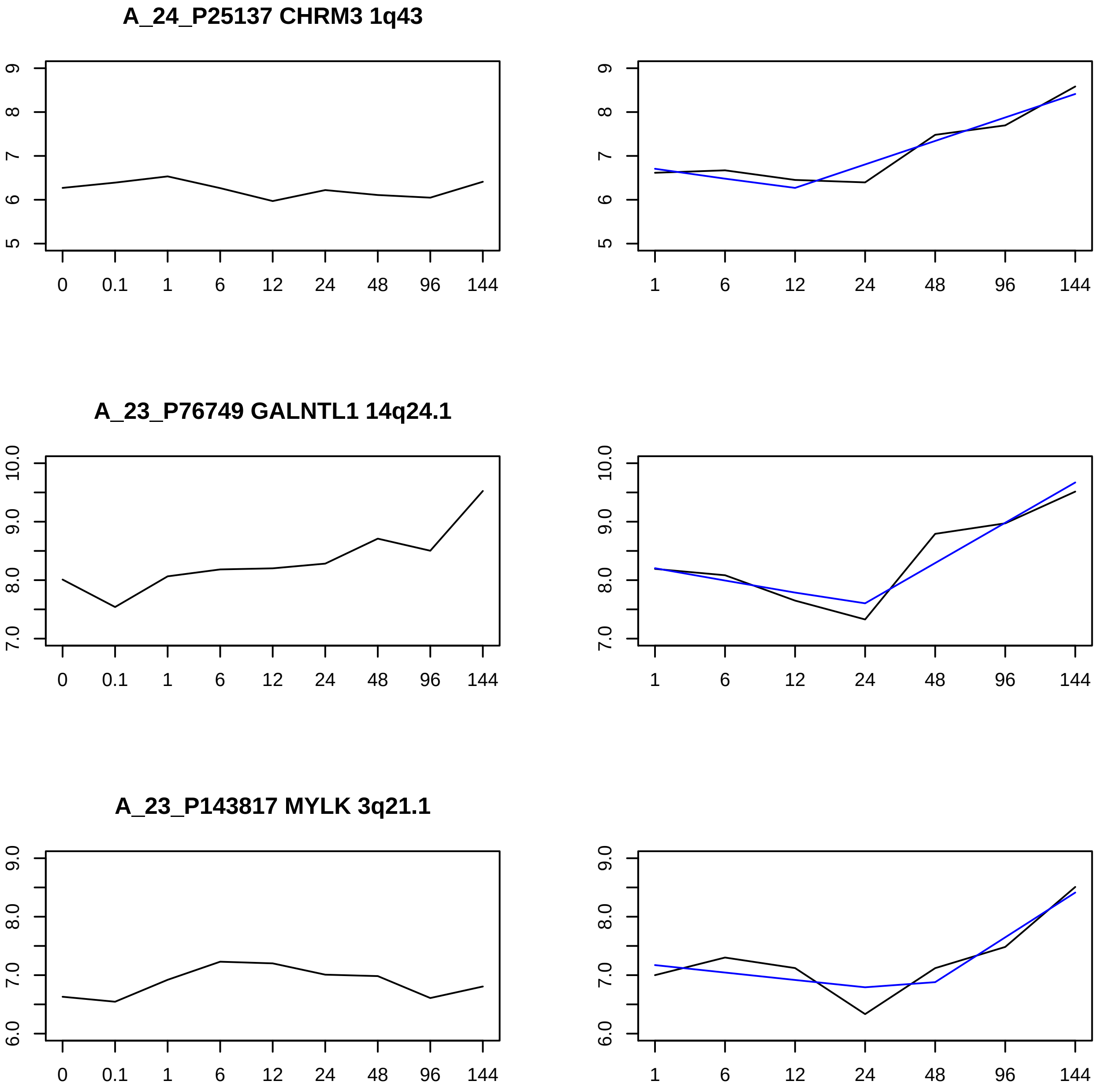
A_24_P25137 CHRM3 1q43
5
6
7
8
9
0
0.1
1
6
12
24
48
96
144
A_23_P76749 GALNTL1 14q24.1
7.0
8.0
9.0
10.0
A_23_P143817 MYLK 3q21.1
6.0
7.0
8.0
9.0

A_23_P347070 PAG1 8q21.13

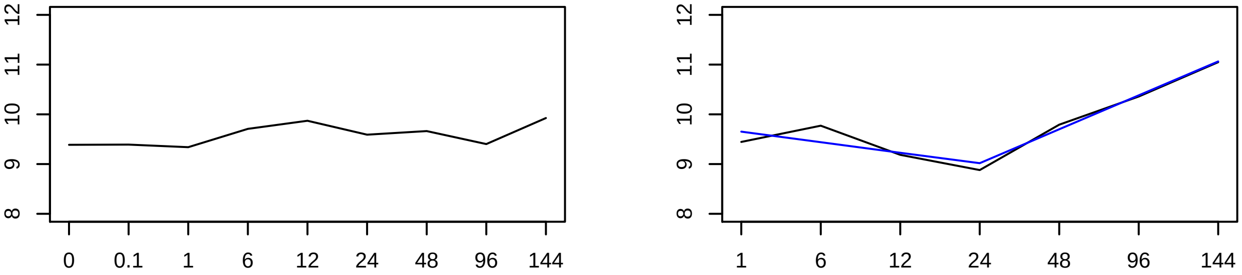

A_32_P88120 YPEL1 22q11.21

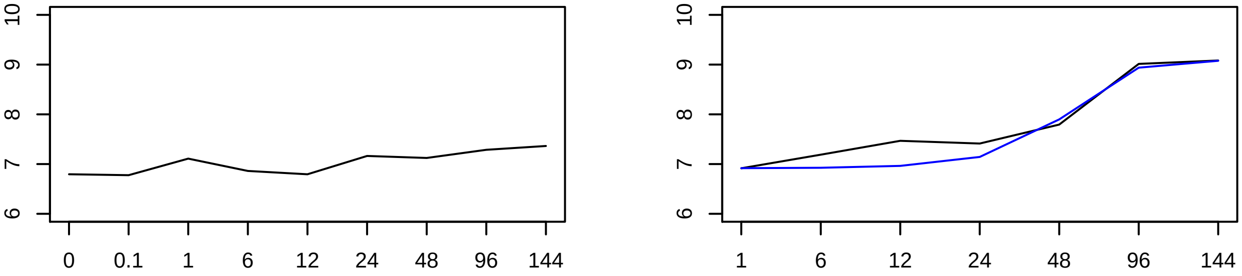

A_24_P918500 ITSN1 21q22.11

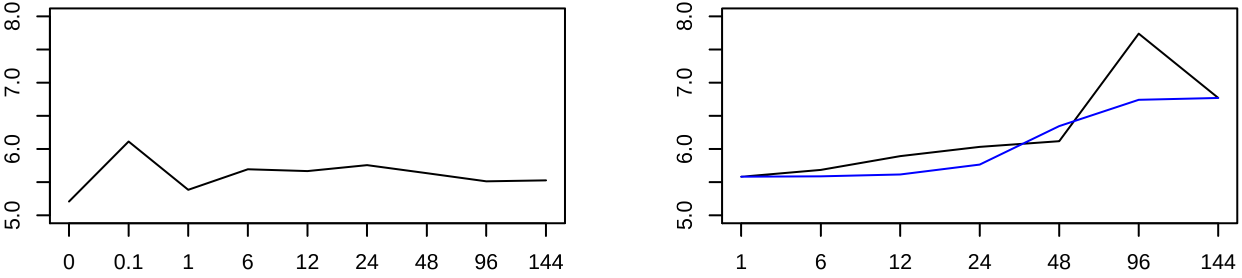

A_23_P141992 HSD11B1L 19p13.3

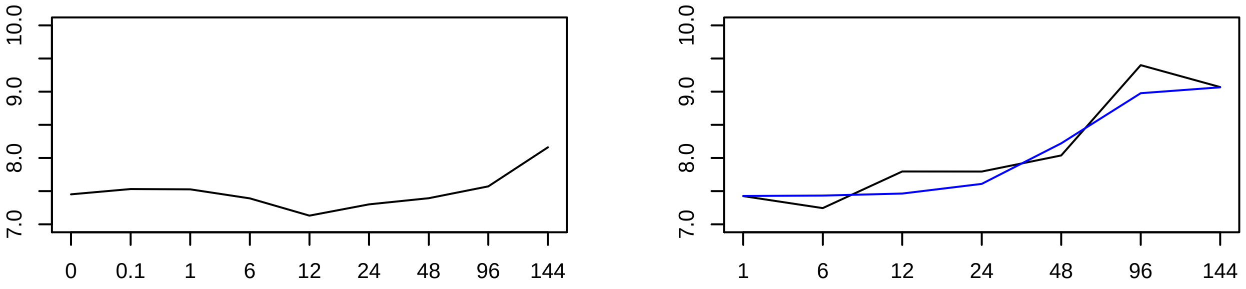

A_32_P122705 PGM2L1 11q13.4

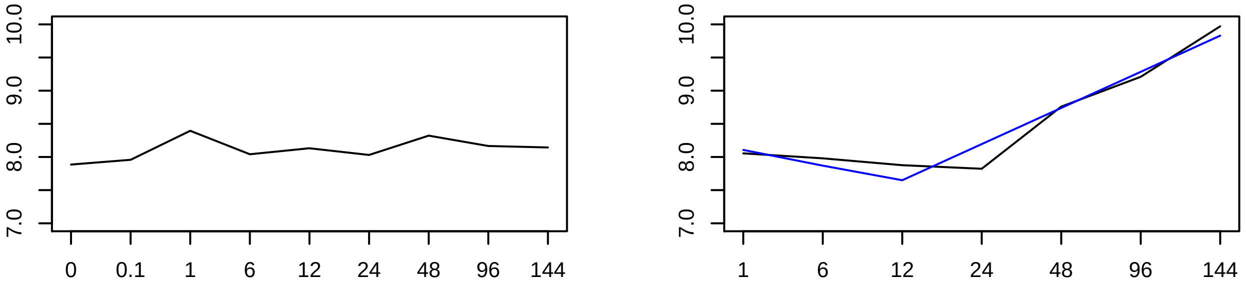

A_23_P50919 SERPINE2 2q36.1

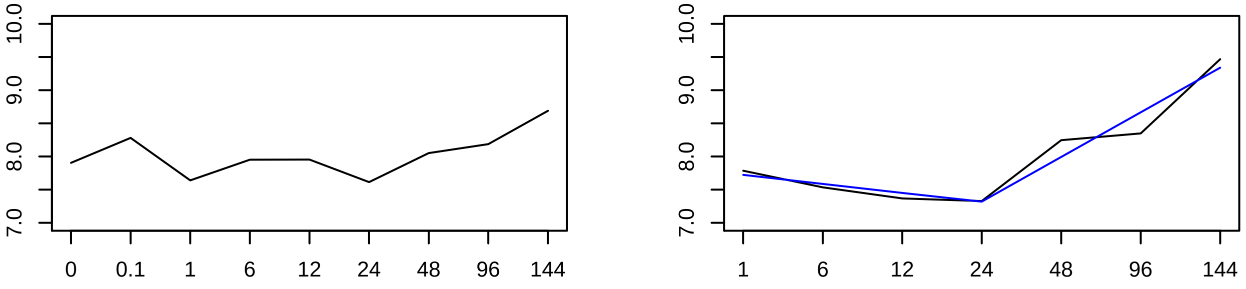

A_24_P375360 LOC651439 14q13.2

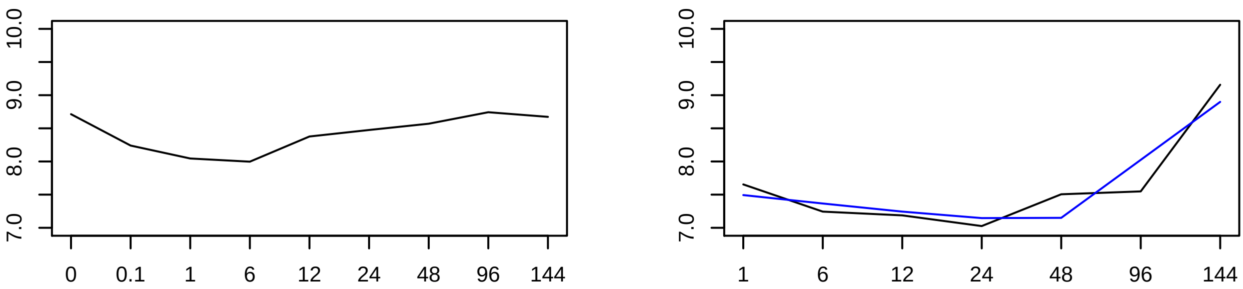

A_23_P33511 SCXA NA

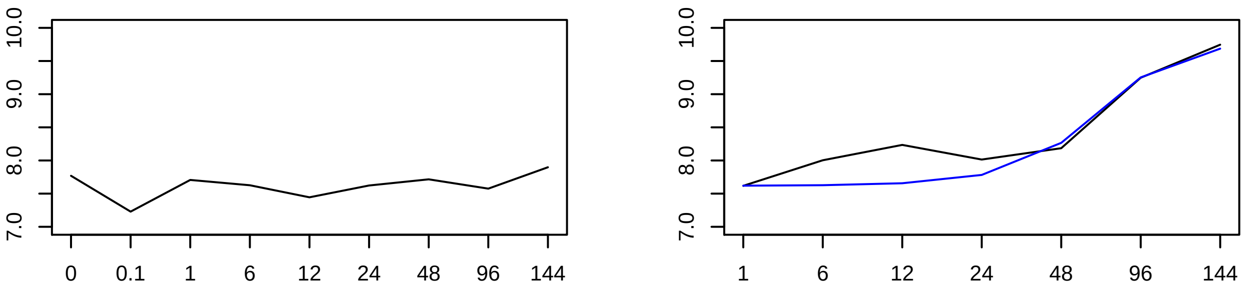

A_24_P320727 KIAA1324L 7q21.12

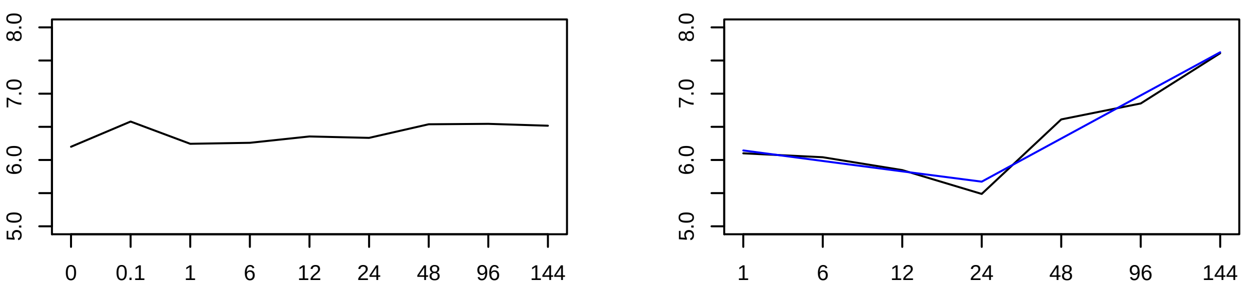

A_23_P386 ARHGEF10L 1p36.13

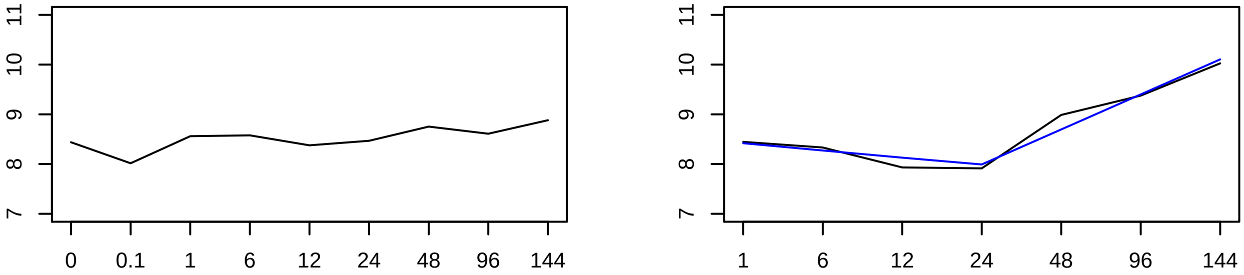

A_23_P309155 C19orf4 19p13.11

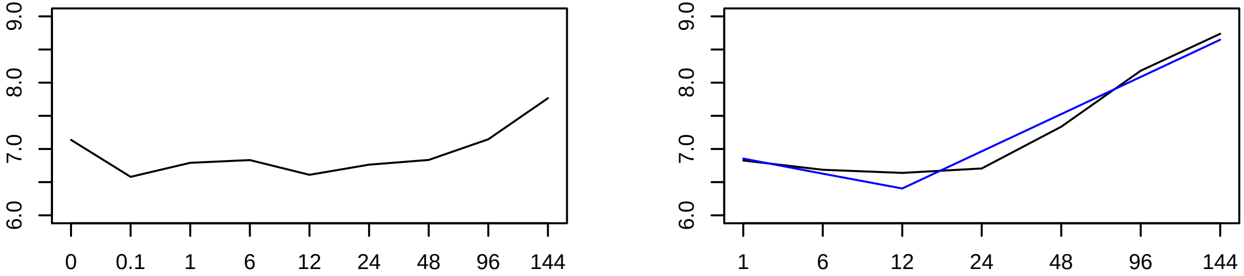

A_23_P335920 RPS6KA2 6q27

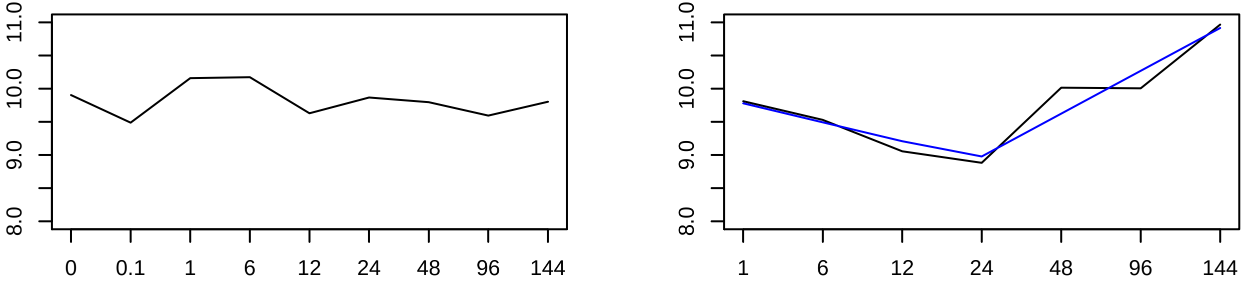

A_23_P200843 CHRM3 NA

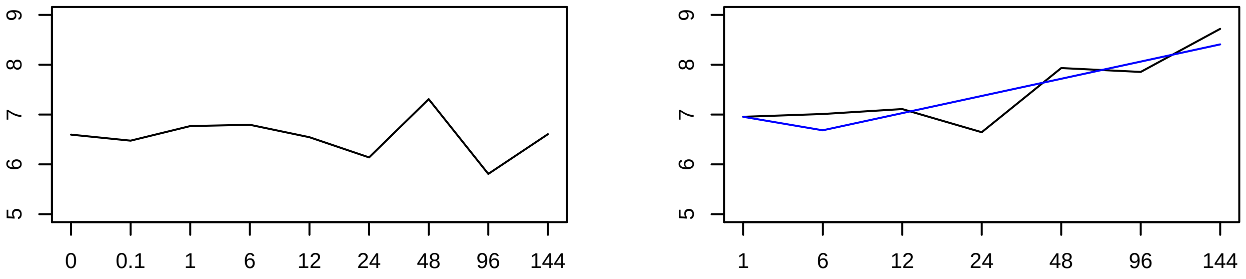

A_23_P163567 SMPD3 16q22.1

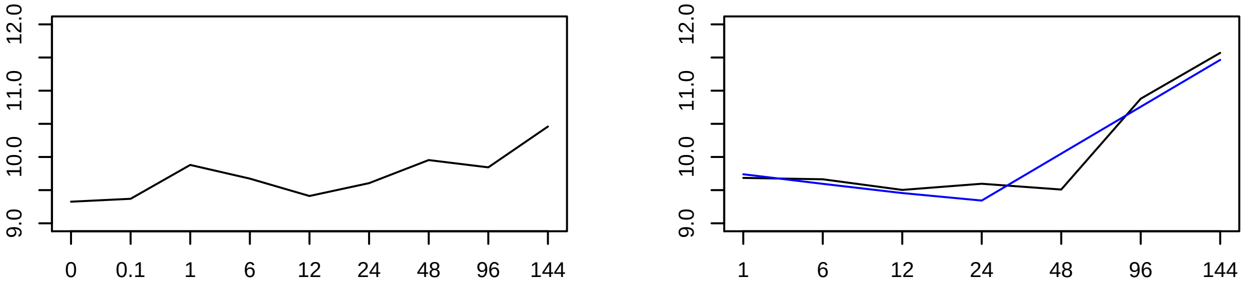

A_32_P111394 THC2643957 NA

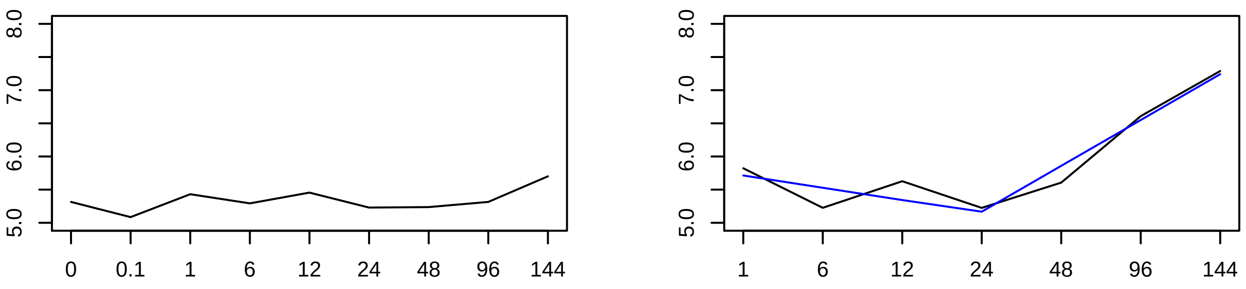

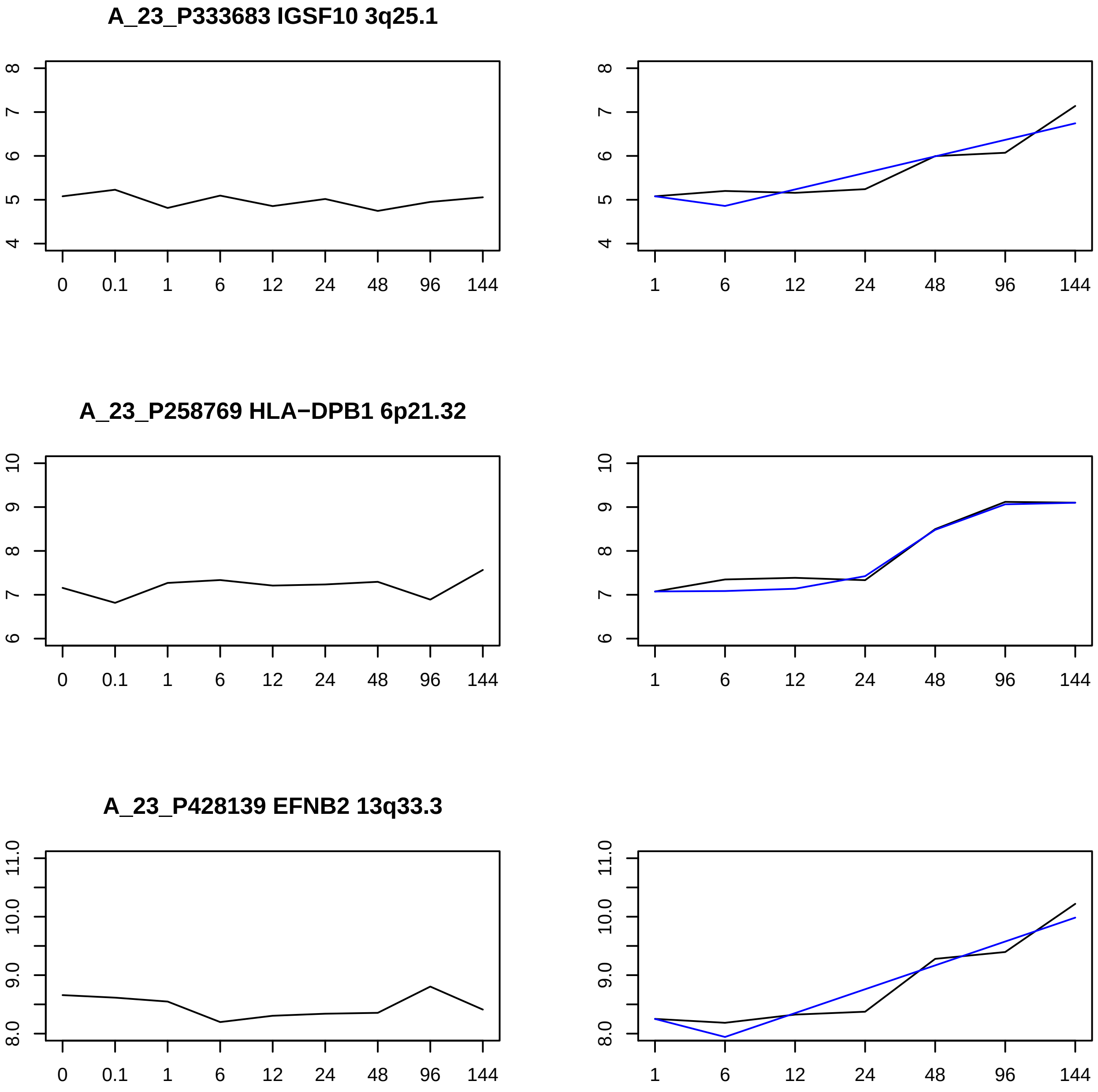

A_23_P333683 IGSF10 3q25.1
4
5
6
7
8
0
0.1
1
6
12
24
48
96
144
1
6
12
24
48
96
144
A_23_P258769 HLA−DPB1 6p21.32
6
7
8
9
10
A_23_P428139 EFNB2 13q33.3
8.0
9.0
10.0
11.0

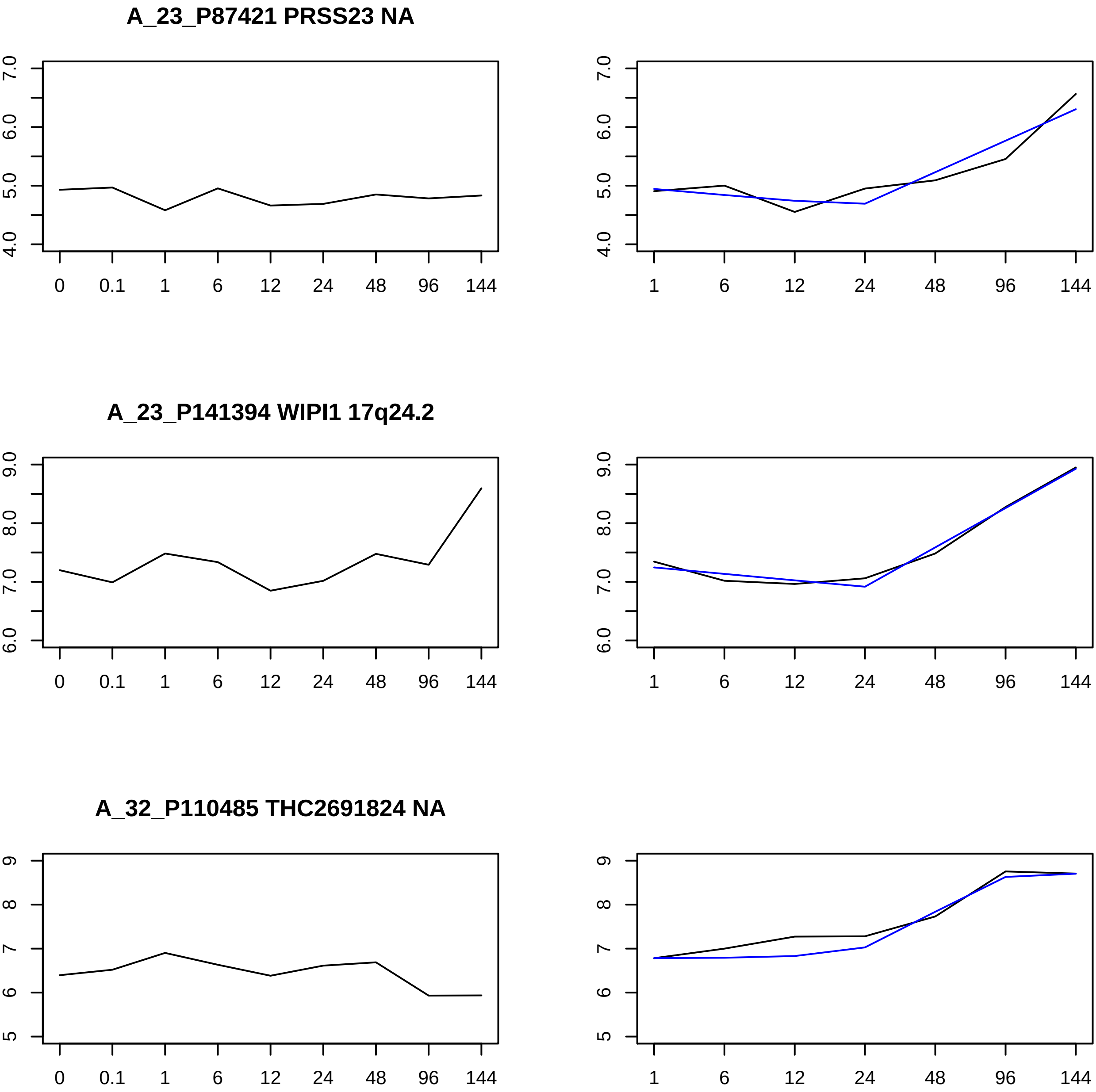
A_23_P87421 PRSS23 NA
A_23_P141394 WIPI1 17q24.2
A_32_P110485 THC2691824 NA
4.0 5.0 6.0 7.0
6.0 7.0 8.0 9.0
5 6 7 8 9
0 0.1 1 6 12 24 48 96 144
1 6 12 24 48 96 144

A_32_P42574 C1orf198 1q42.2

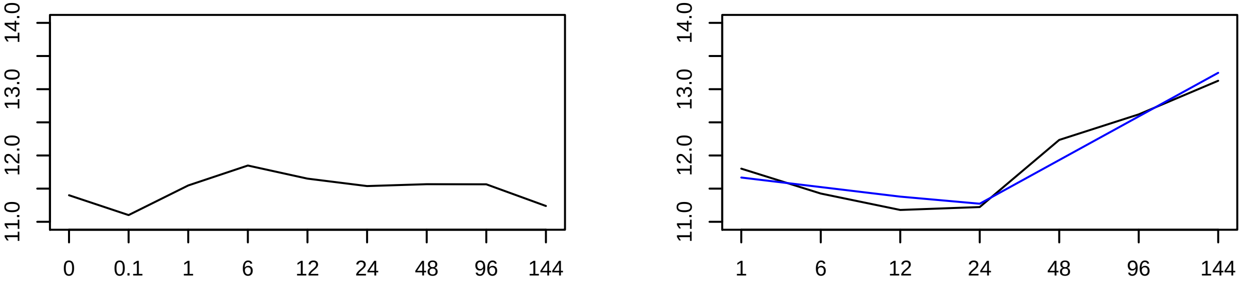

A_32_P113784 BM680083 NA

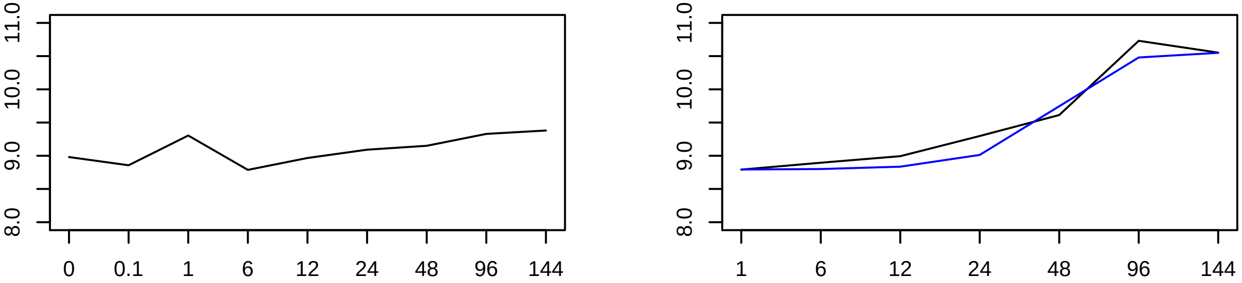

A_24_P274987 TMEFF1 9q31.1

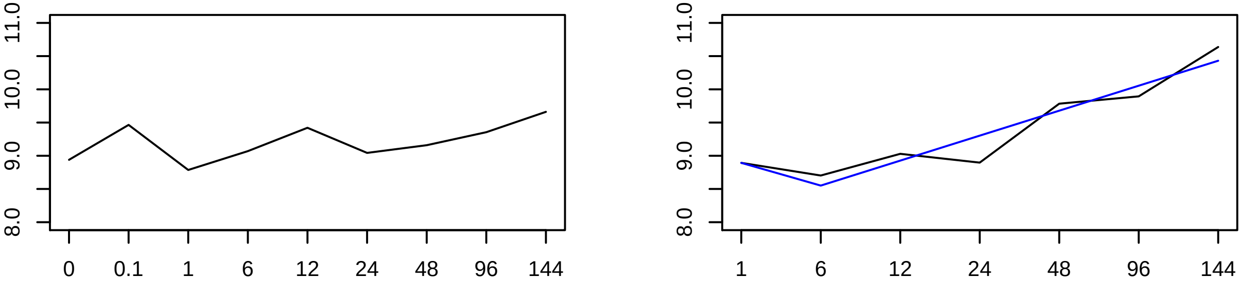

A_23_P204958 LATS2 13q12.11

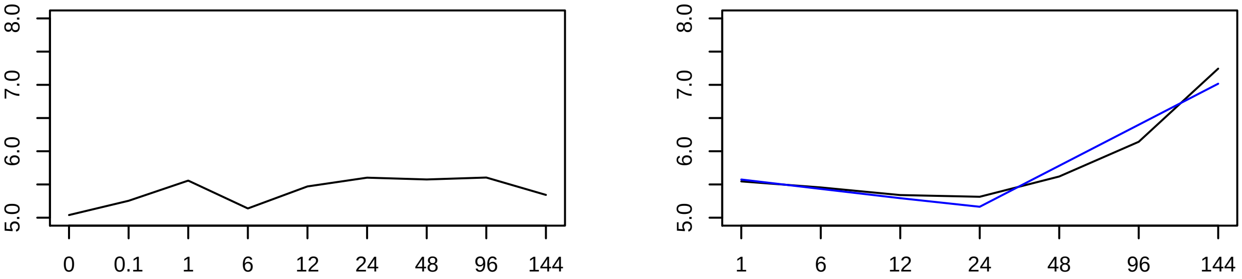

A_23_P118122 RGS11 16p13.3

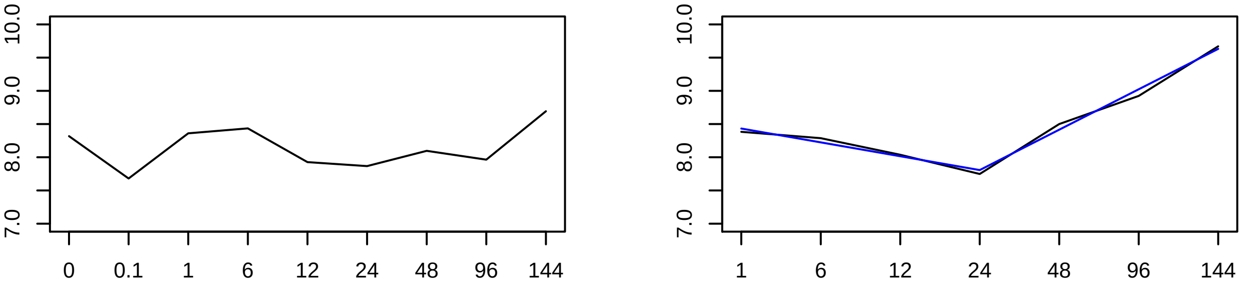

A_32_P115749 CD104030 NA

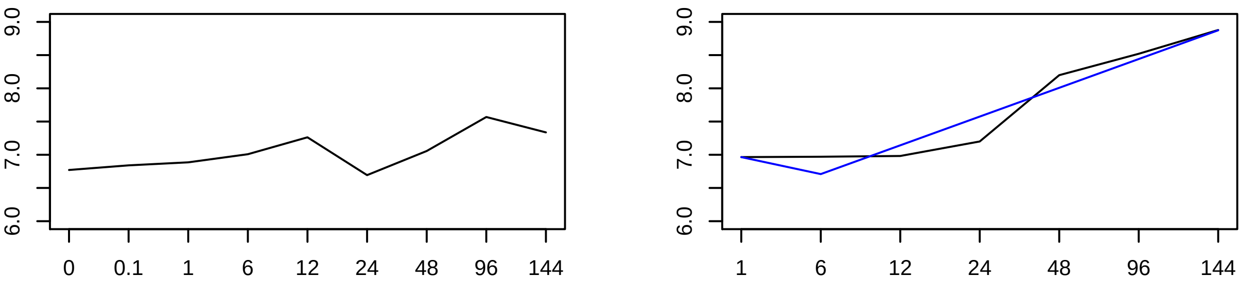

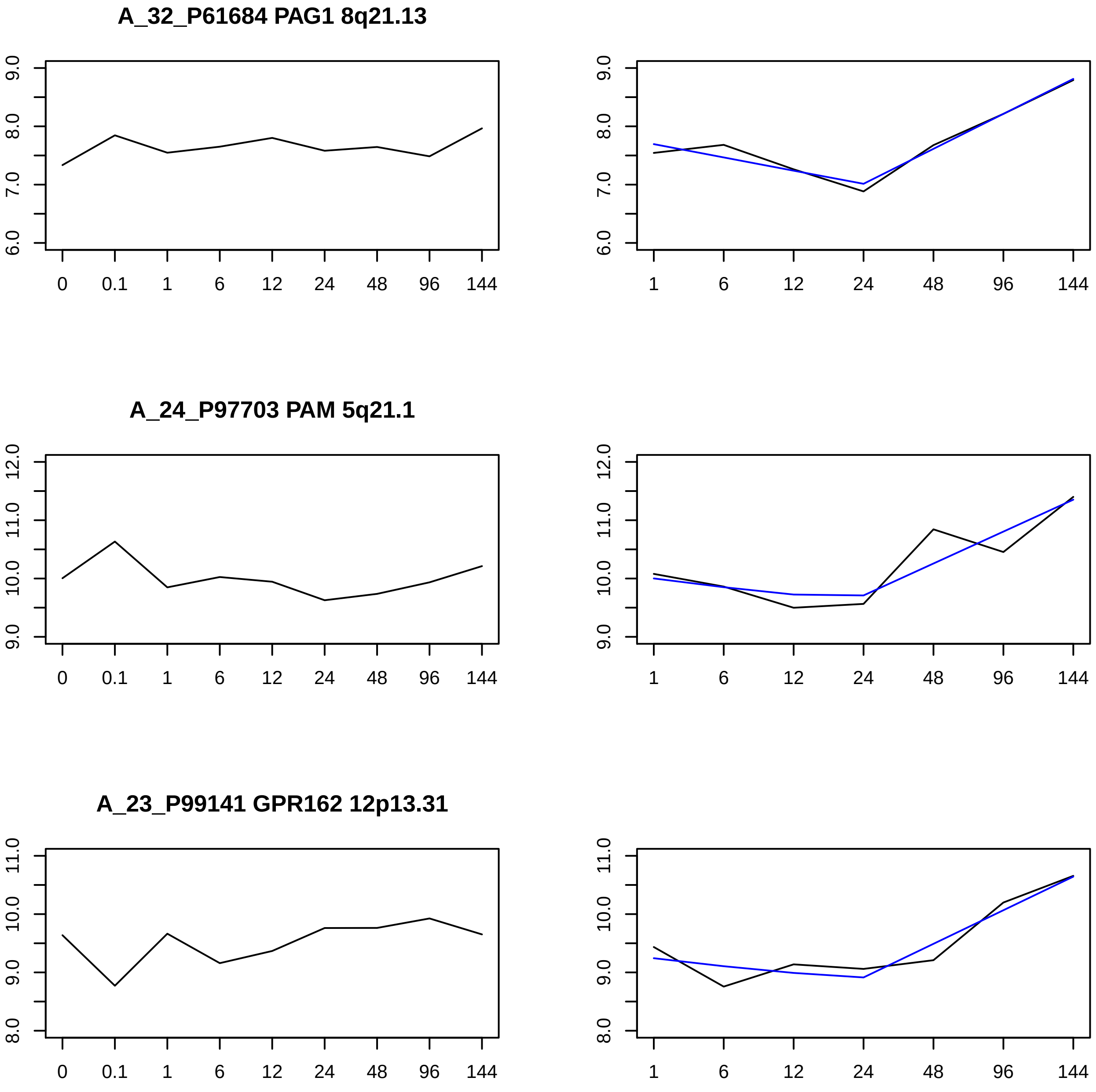
A_32_P61684 PAG1 8q21.13
6.0
7.0
8.0
9.0
0
0.1
1
6
12
24
48
96
144
1
6
12
24
48
96
144
A_24_P97703 PAM 5q21.1
9.0
10.0
11.0
12.0
A_23_P99141 GPR162 12p13.31
8.0
9.0
10.0
11.0

A_23_P212779 DKFZP564O0823 4q13.3

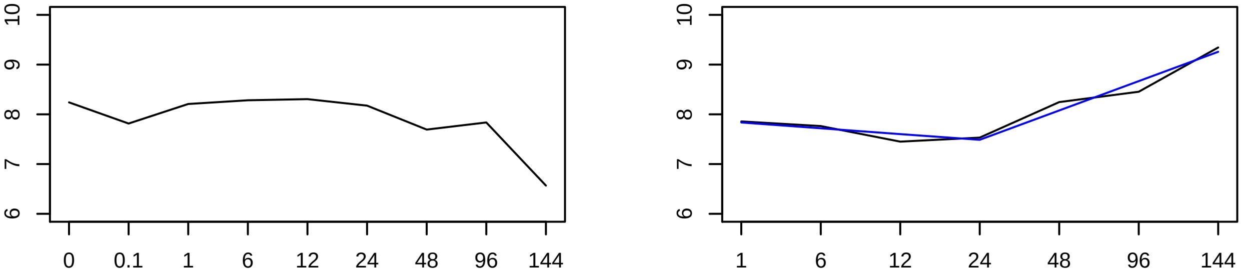

A_23_P10121 SFRP1 8p11.21

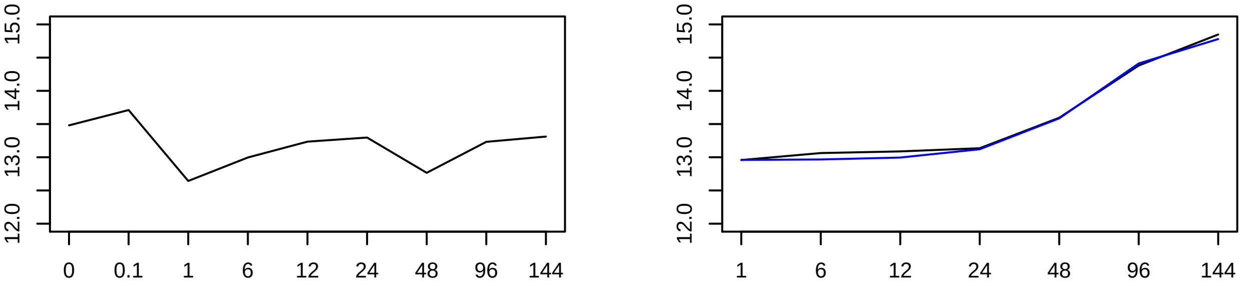

A_23_P153070 BCAS3 17q23.2

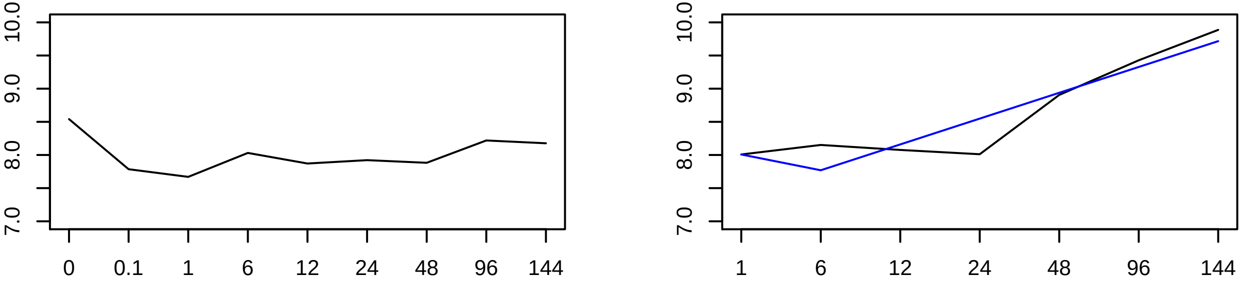

A_24_P191781 DKFZP564O0823 4q13.3

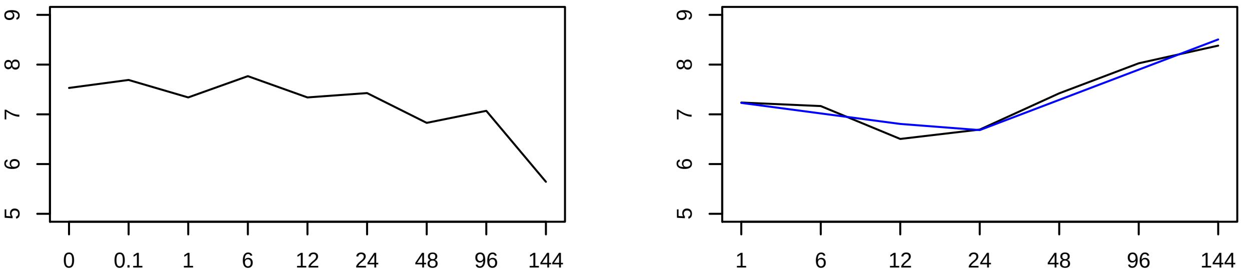

A_23_P32414 RP6−213H19.1 Xq26.2

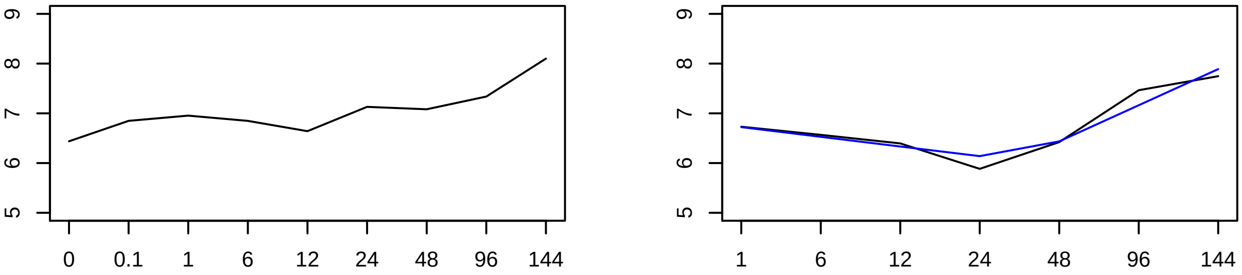

A_24_P917384 LOC158960 Xq28

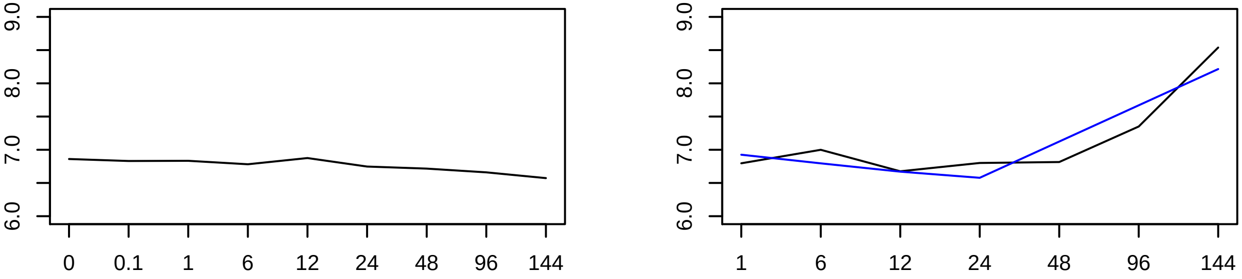

A_32_P196896 SEL1L3 NA

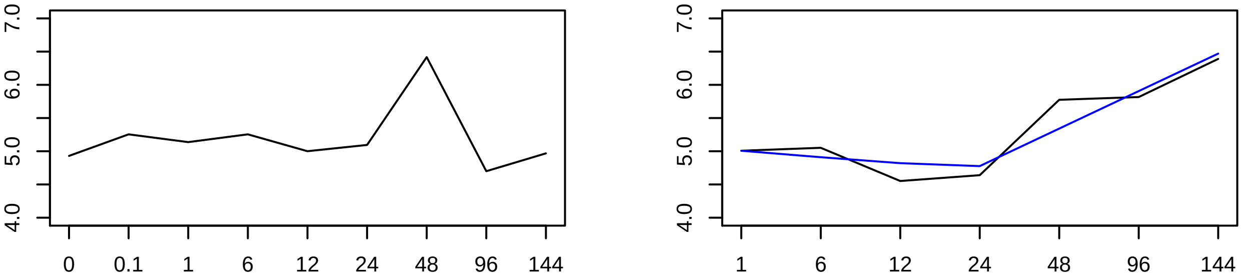

A_32_P219135 CREB5 7p15.1

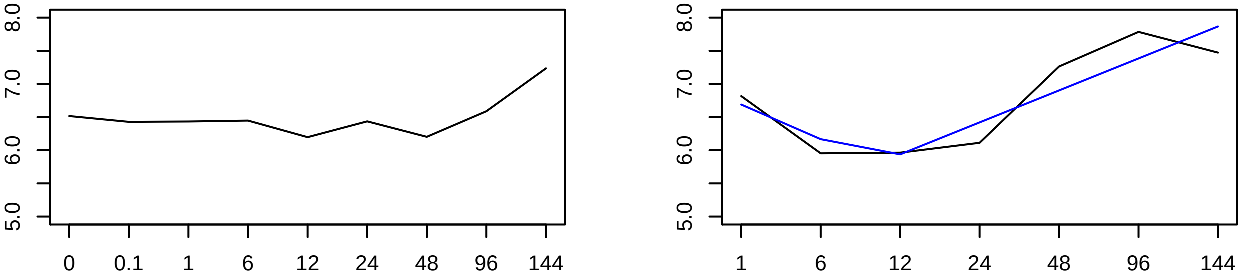

A_23_P110624 CTNND2 5p15.2

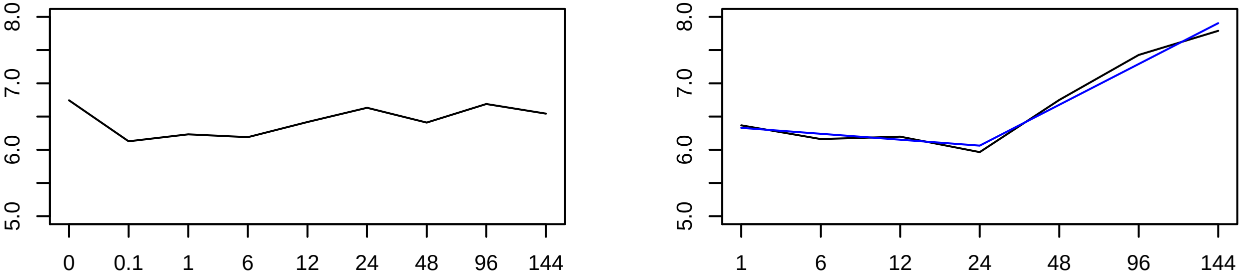

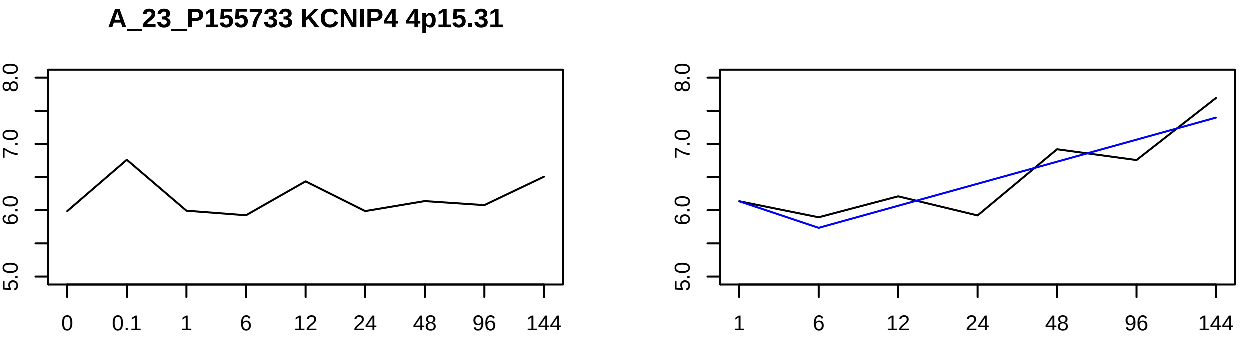
A_23_P155733 KCNIP4 4p15.31
5.0
6.0
7.0
8.0
0
0.1
1
6
12
24
48
96
144
1
6
12
24
48
96
144

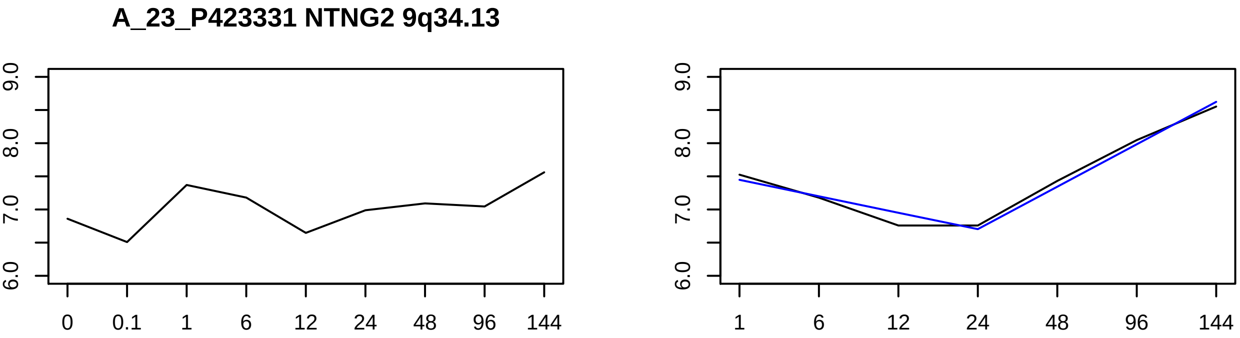
A_23_P423331 NTNG2 9q34.13
6.0
7.0
8.0
9.0
0
0.1
1
6
12
24
48
96
144
1
6
12
24
48
96
144

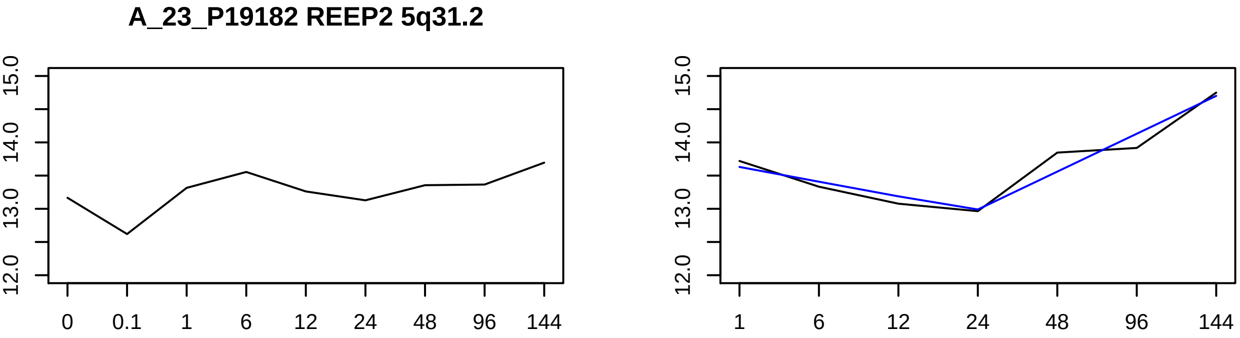
A_23_P19182 REEP2 5q31.2
12.0
13.0
14.0
15.0
0
0.1
1
6
12
24
48
96
144
1
6
12
24
48
96
144

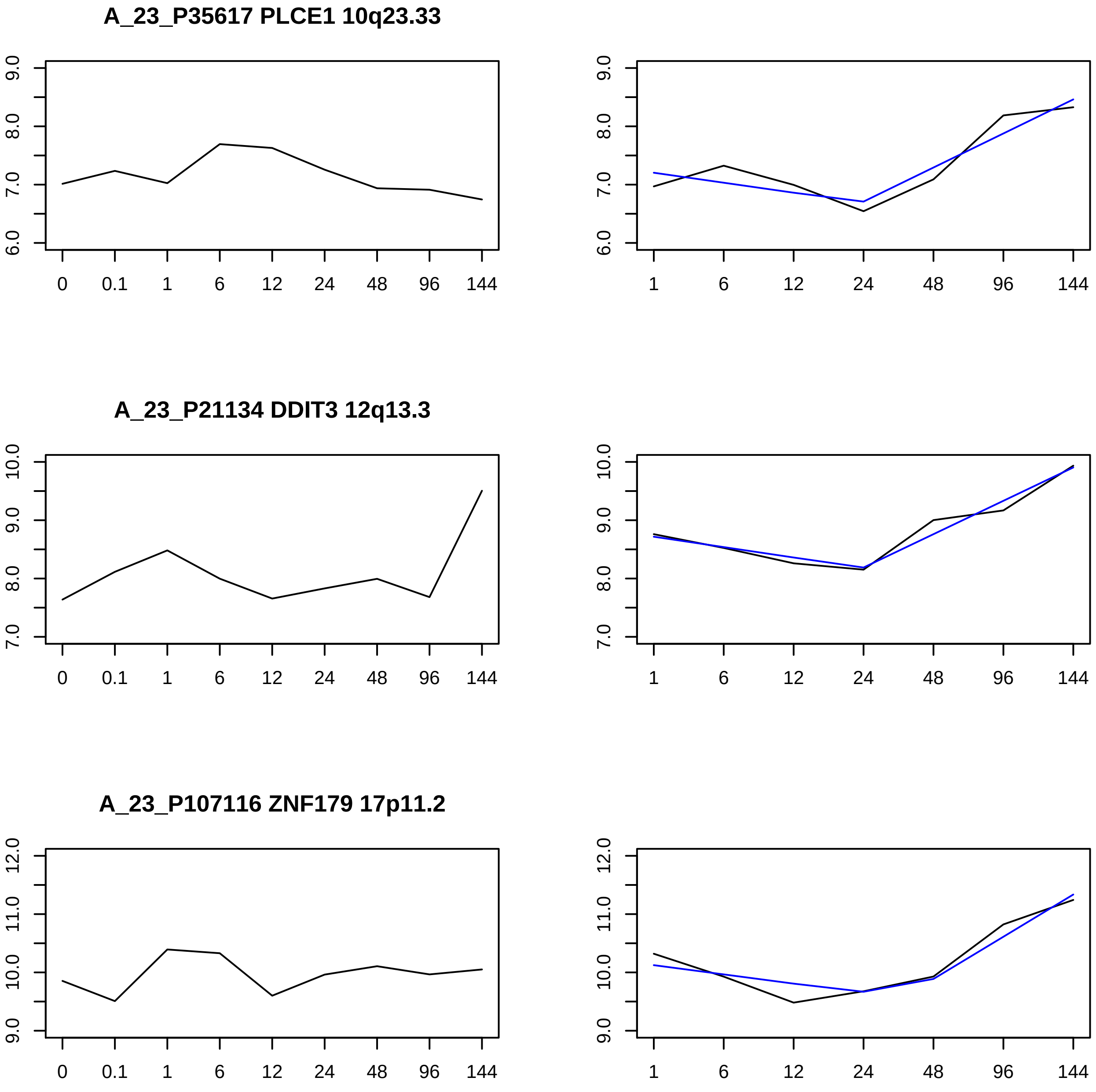

A_23_P35617 PLCE1 10q23.33
6.0
7.0
8.0
9.0
0
0.1
1
6
12
24
48
96
144
A_23_P21134 DDIT3 12q13.3
7.0
8.0
9.0
10.0
A_23_P107116 ZNF179 17p11.2
9.0
10.0
11.0
12.0

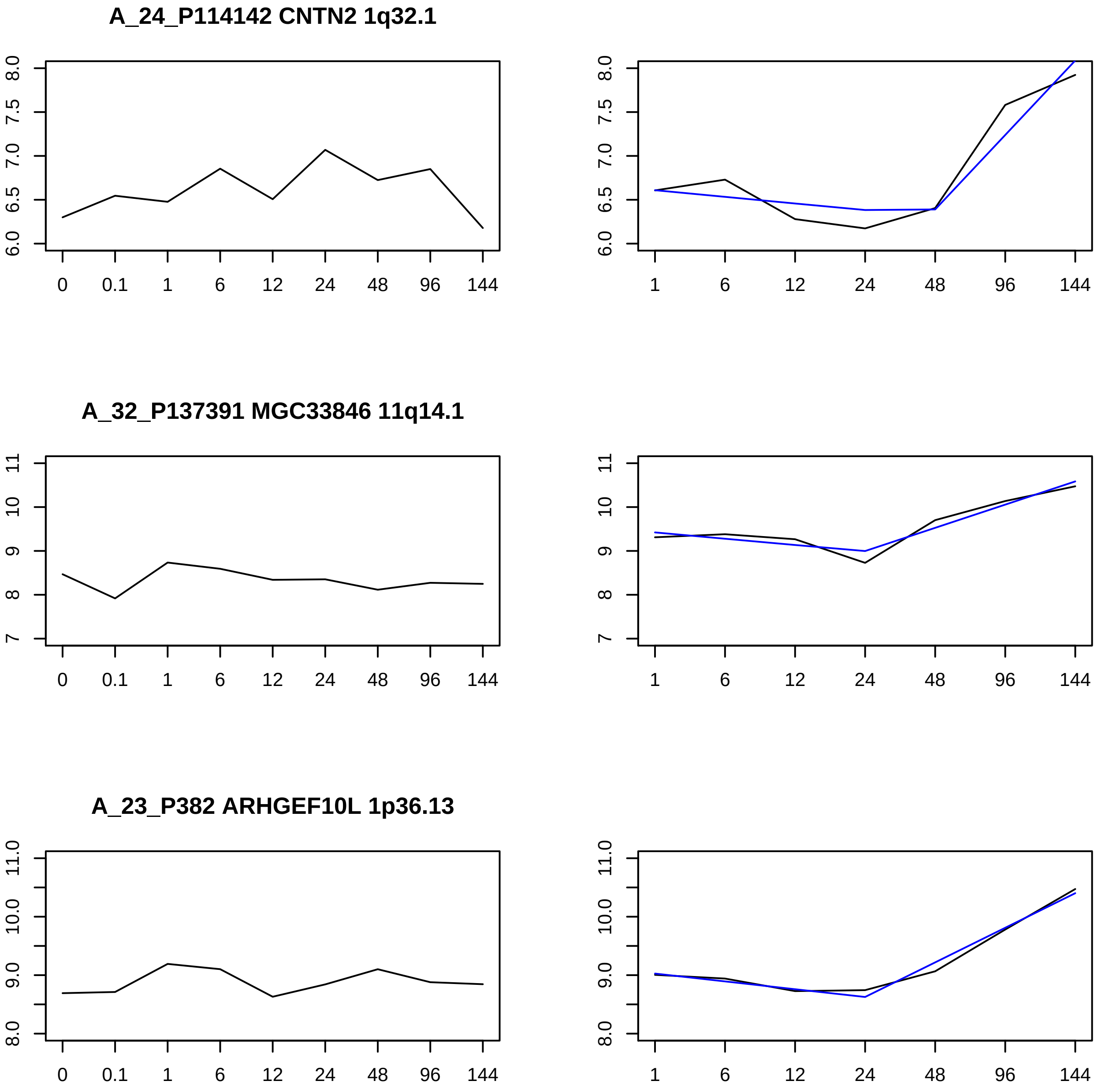
A_24_P114142 CNTN2 1q32.1
6.0 6.5 7.0 7.5 8.0
0 0.1 1 6 12 24 48 96 144
1 6 12 24 48 96 144
A_32_P137391 MGC33846 11q14.1
7 8 9 10 11
0 0.1 1 6 12 24 48 96 144
1 6 12 24 48 96 144
A_23_P382 ARHGEF10L 1p36.13
8.0 9.0 10.0 11.0
0 0.1 1 6 12 24 48 96 144
1 6 12 24 48 96 144

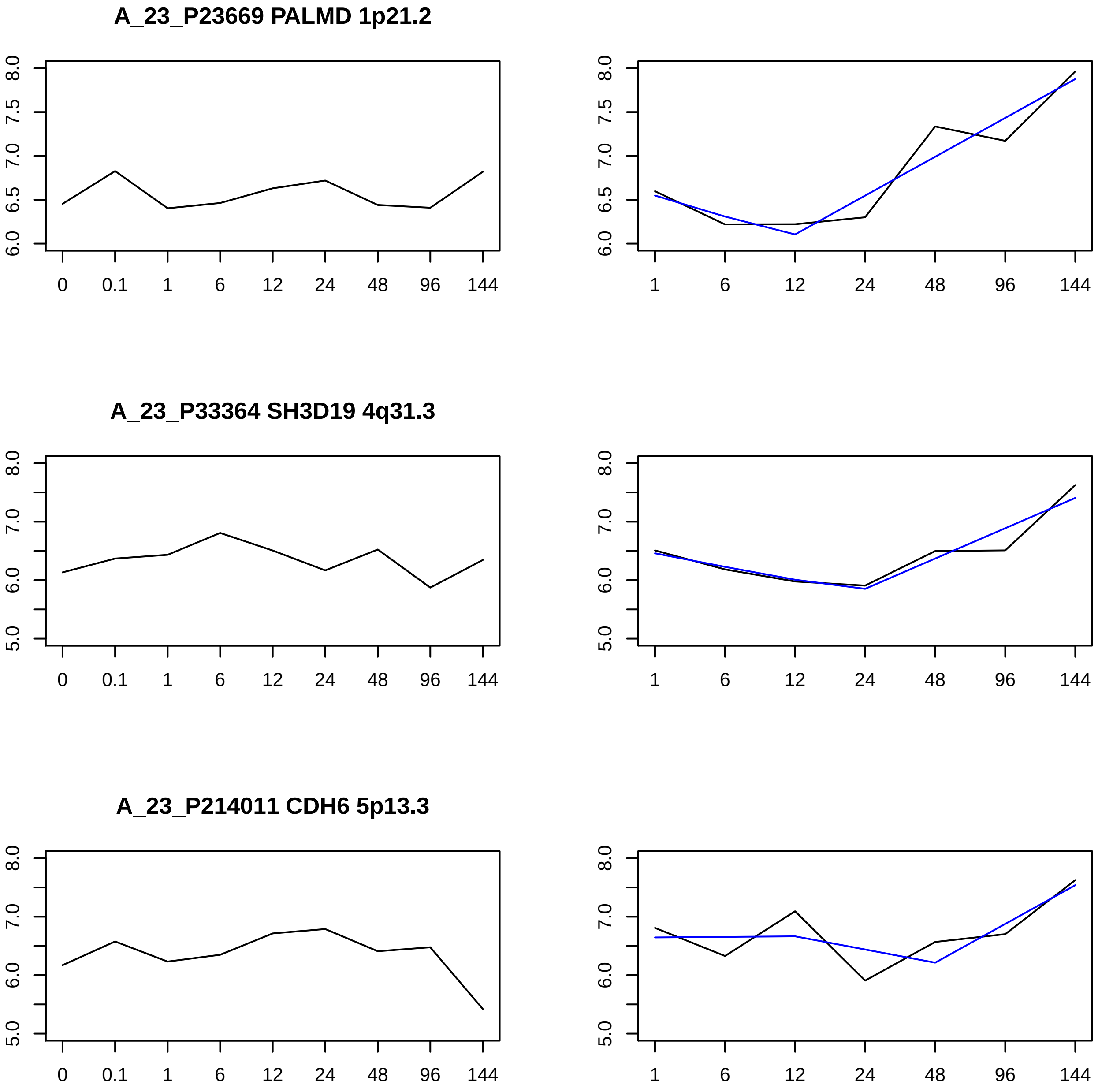
A_23_P23669 PALMD 1p21.2
6.0
6.5
7.0
7.5
8.0
0
0.1
1
6
12
24
48
96
144
1
6
12
24
48
96
144
A_23_P33364 SH3D19 4q31.3
5.0
6.0
7.0
8.0
A_23_P214011 CDH6 5p13.3

A_32_P98072 TCHH NA

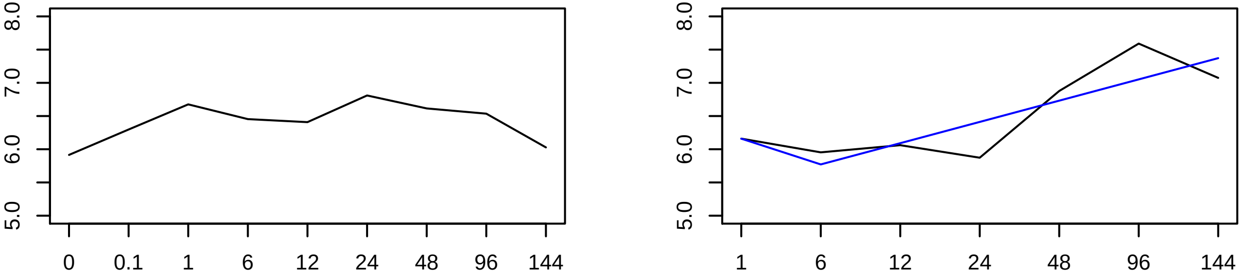

A_24_P273726 MSRB3 12q14.3

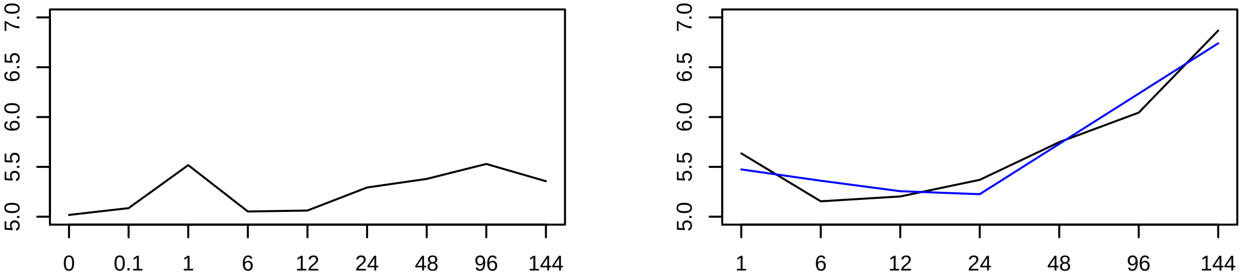

A_24_P246406 FAM69A 1p22.1

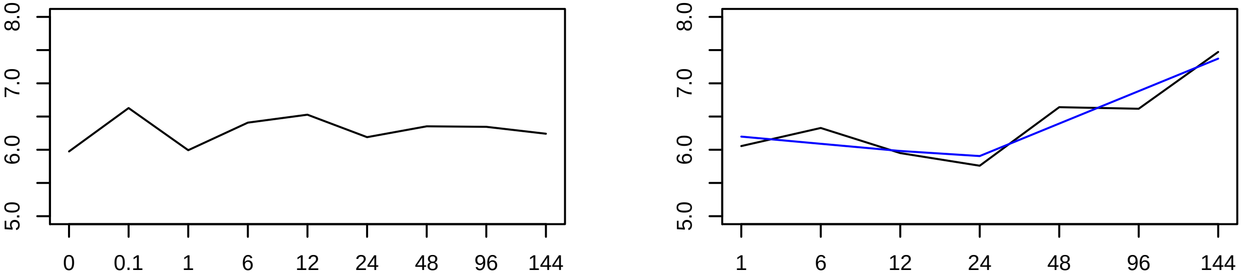

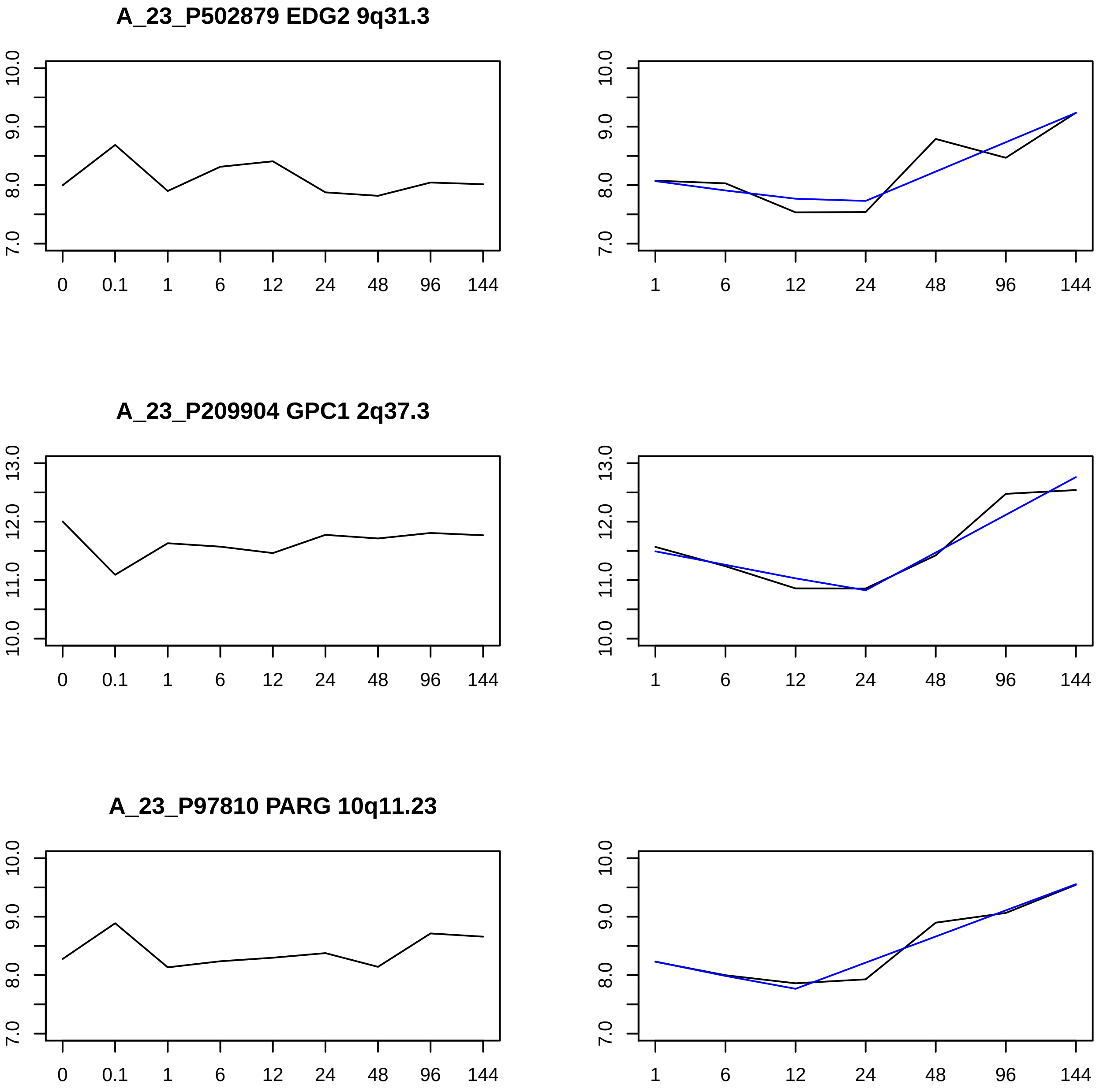
A_23_P502879 EDG2 9q31.3
7.0
8.0
9.0
10.0
0
0.1
1
6
12
24
48
96
144
A_23_P209904 GPC1 2q37.3
10.0
11.0
12.0
13.0
A_23_P97810 PARG 10q11.23

A_23_P216307 RUNX1T1 8q21.3

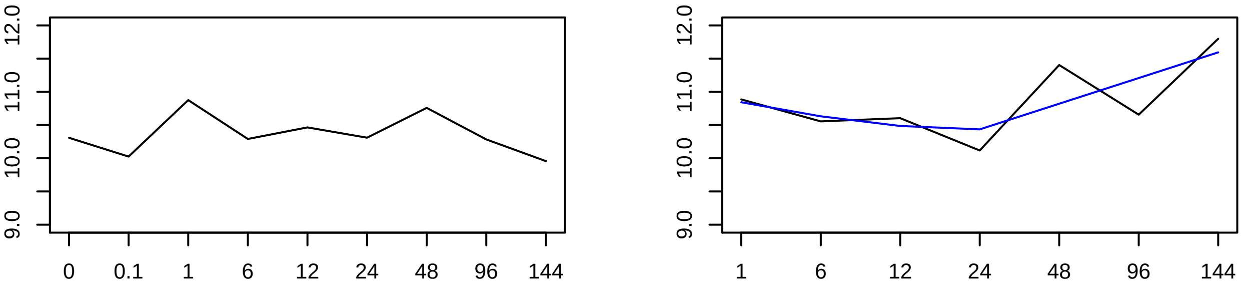

A_23_P168229 TXNDC5 6p24.3

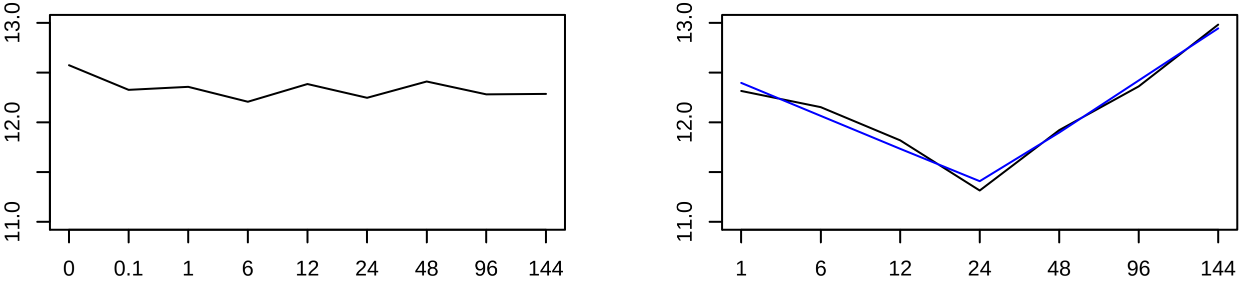

A_32_P192842 BM129308 NA

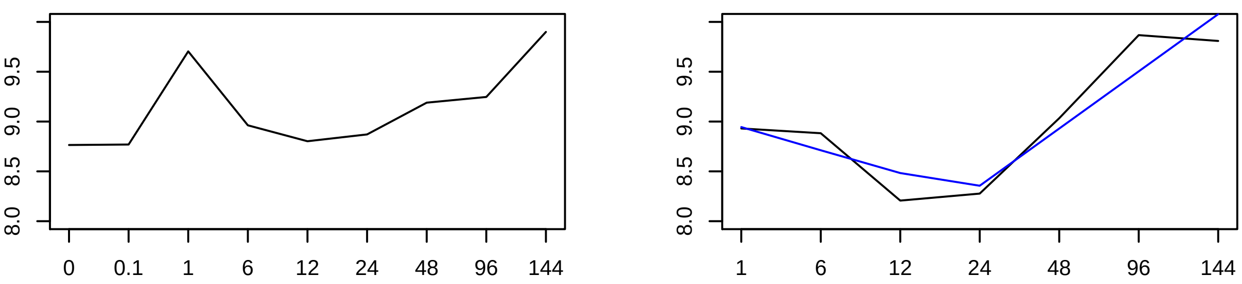

A_32_P77252 THC2608799 NA

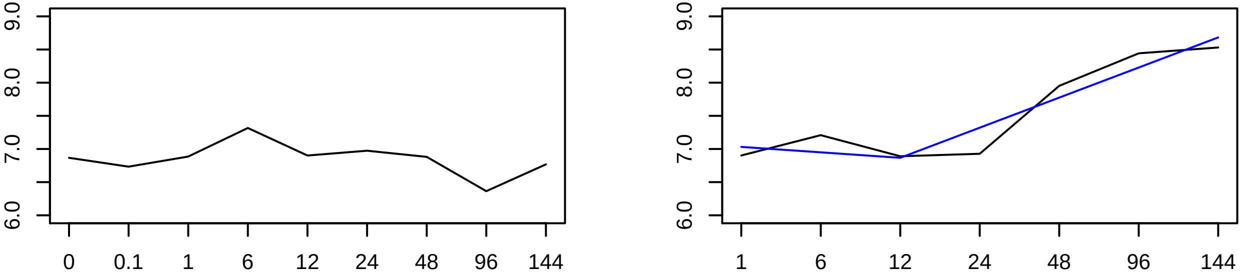

A_23_P166336 DKFZp434N035 22q11.21

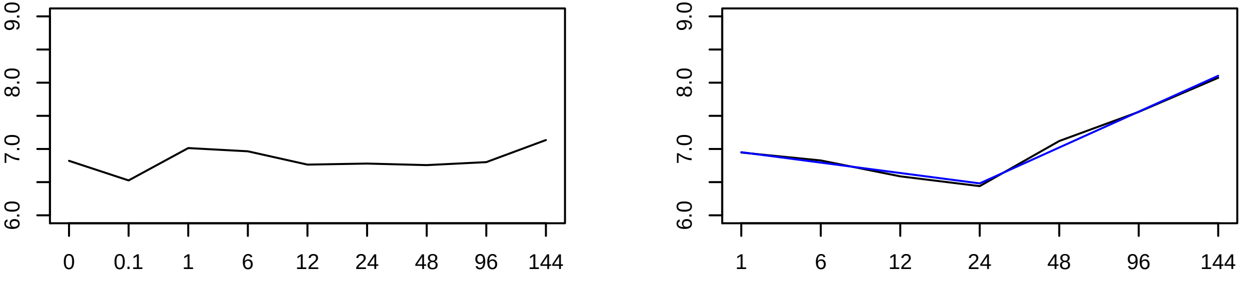

A_32_P172864 FAM102B 1p13.3

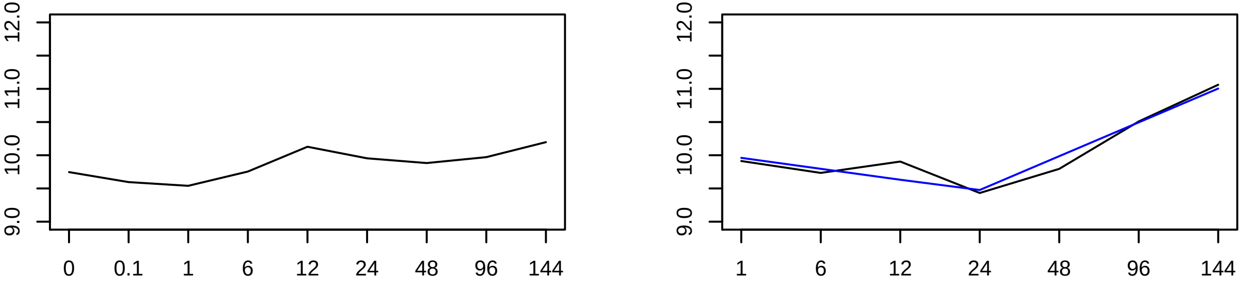

A_24_P402242 COL3A1 2q32.2

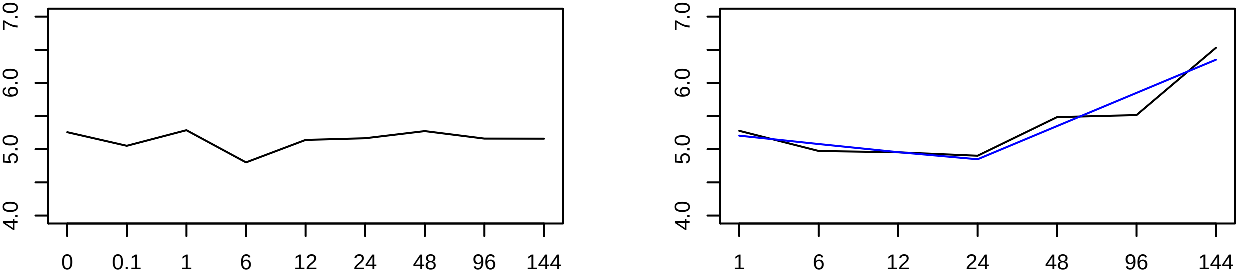

A_23_P67151 OLFM2 19p13.2

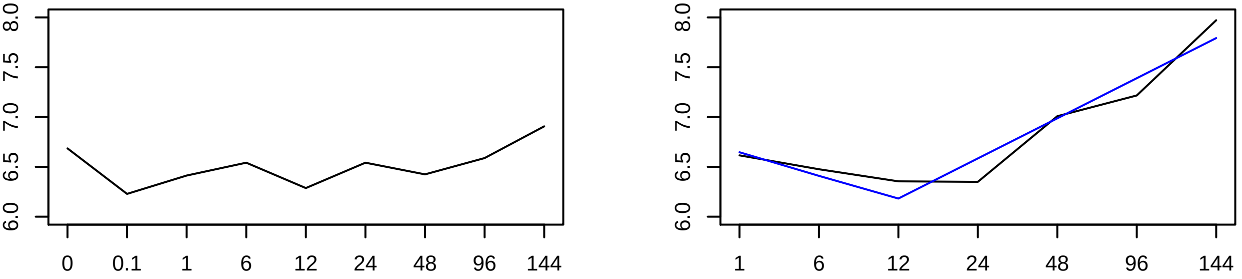

A_24_P18137 NEFL 8p21.2

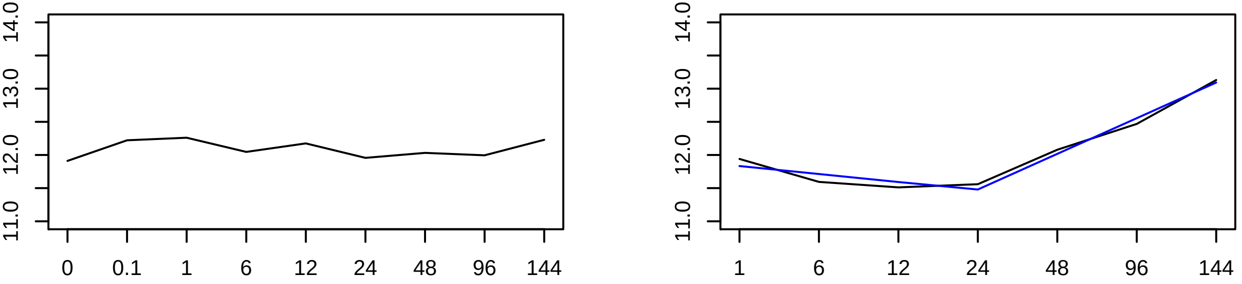

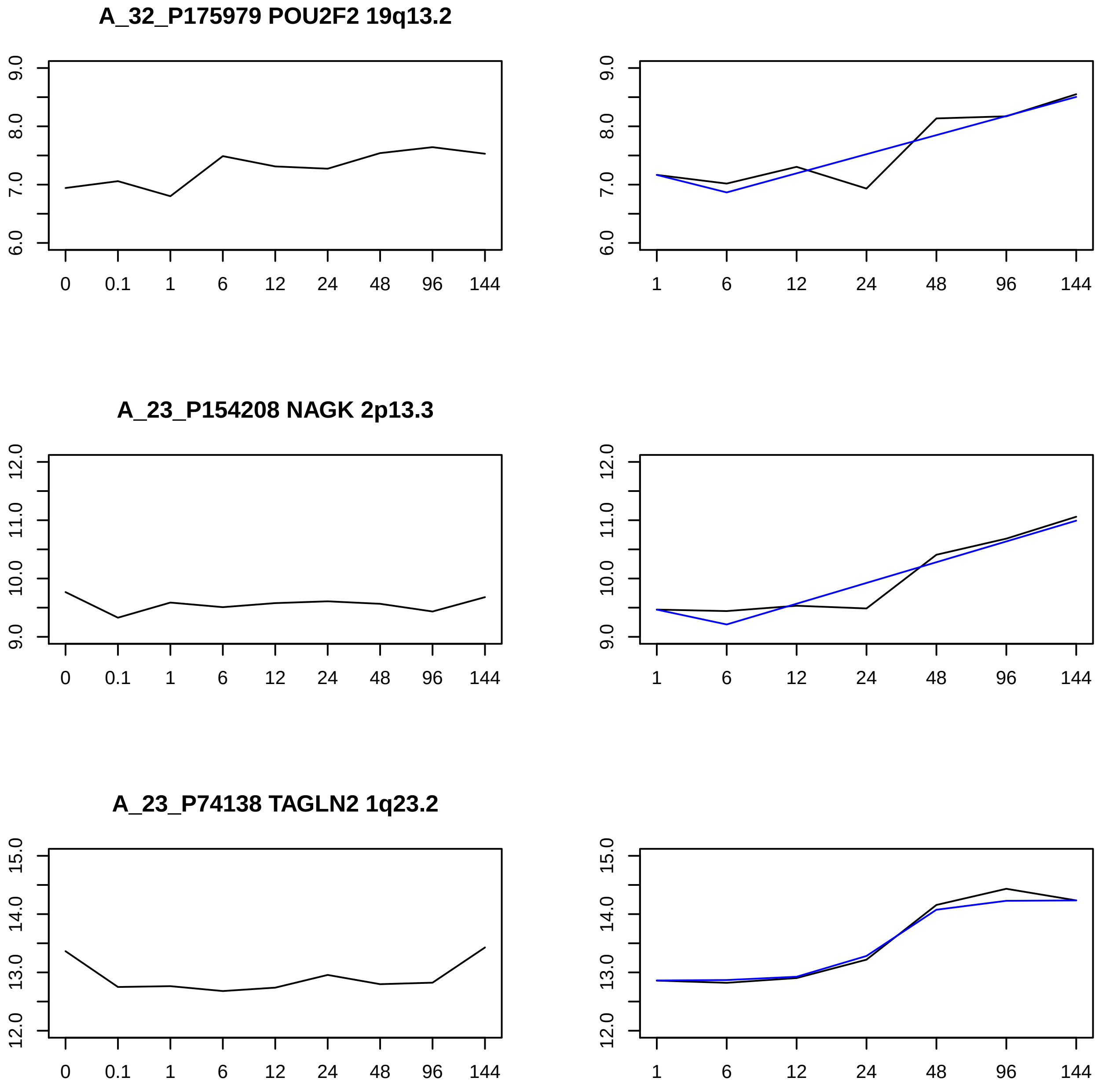
A_32_P175979 POU2F2 19q13.2
A_23_P154208 NAGK 2p13.3
A_23_P74138 TAGLN2 1q23.2
6.0 7.0 8.0 9.0
9.0 10.0 11.0 12.0
12.0 13.0 14.0 15.0
0 0.1 1 6 12 24 48 96 144
1 6 12 24 48 96 144

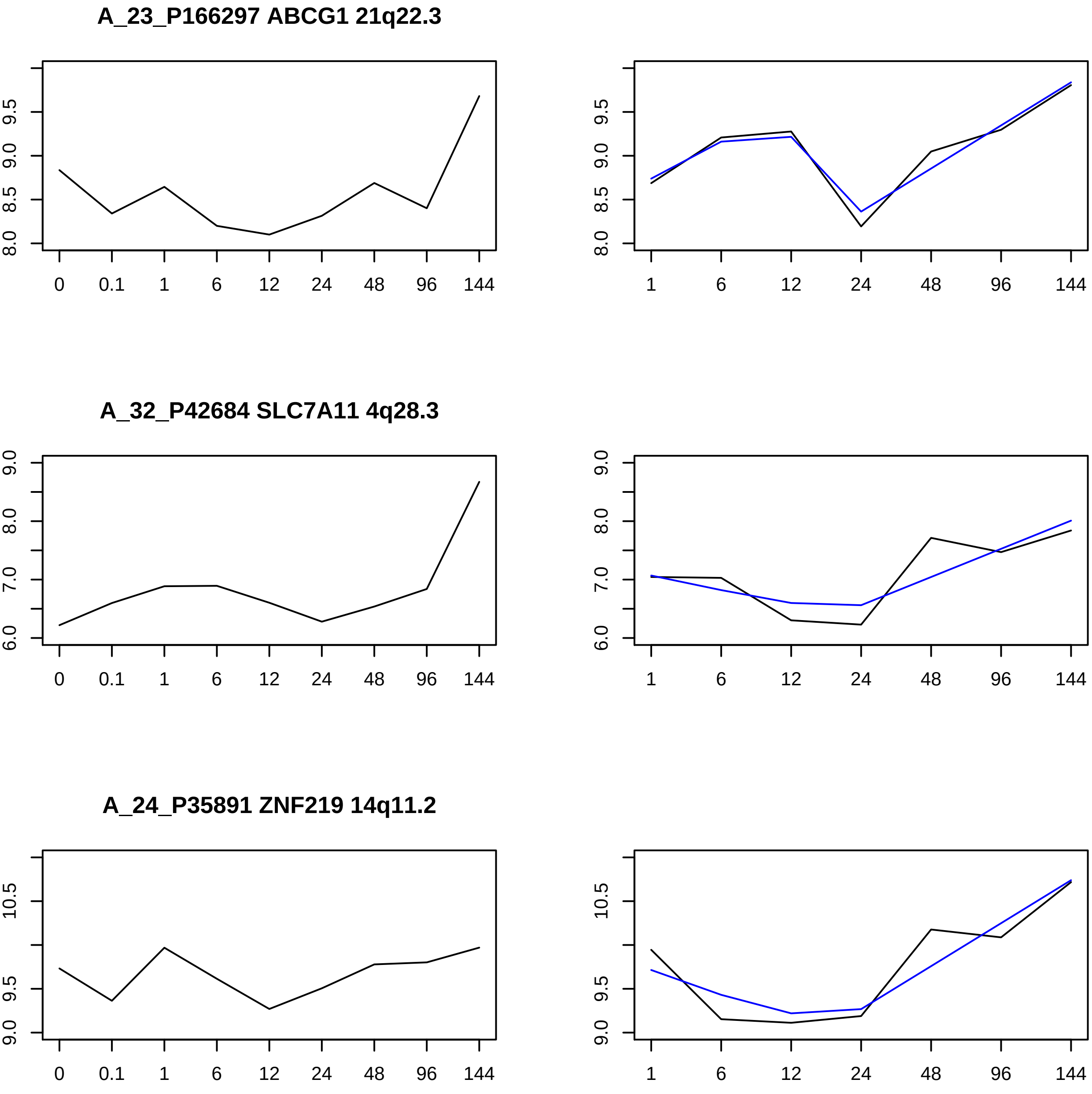
A_23_P166297 ABCG1 21q22.3
8.0
8.5
9.0
9.5
0
0.1
1
6
12
24
48
96
144
A_32_P42684 SLC7A11 4q28.3
6.0
7.0
8.0
9.0
A_24_P35891 ZNF219 14q11.2
9.0
9.5
10.5

A_23_P367610 SESTD1 2q31.2

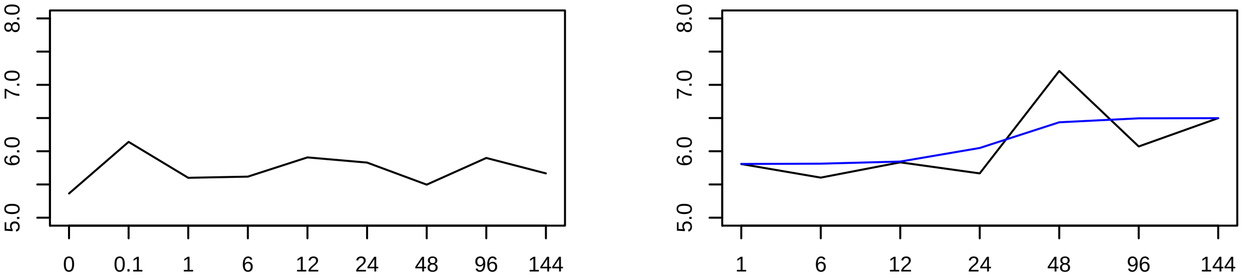

A_23_P62768 TMEM54 1p35.1

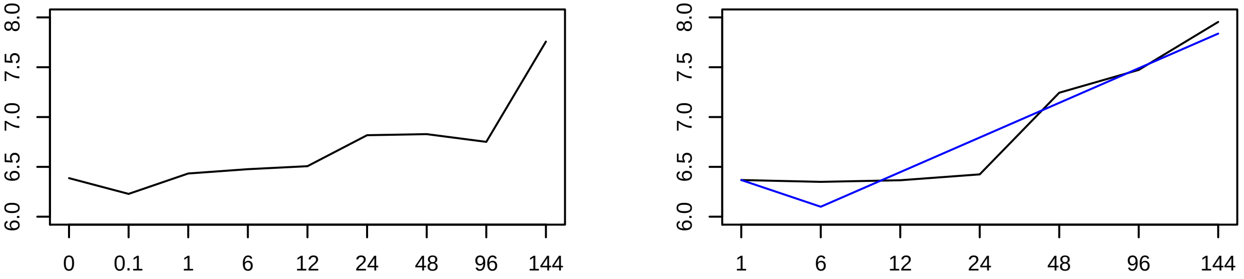

A_23_P335661 SAMD4A 14q22.2

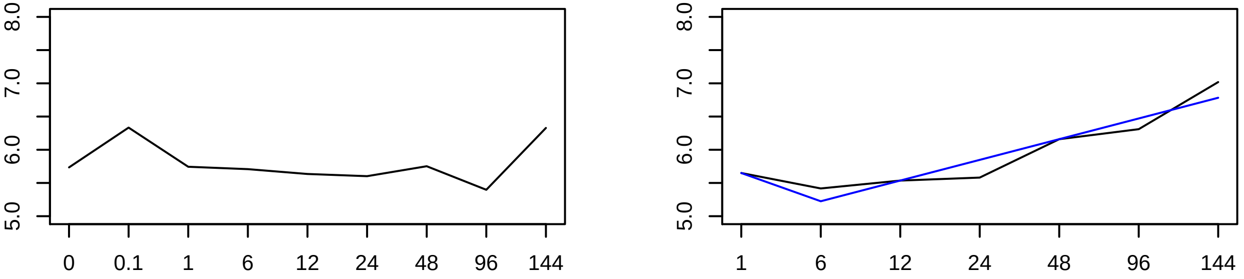

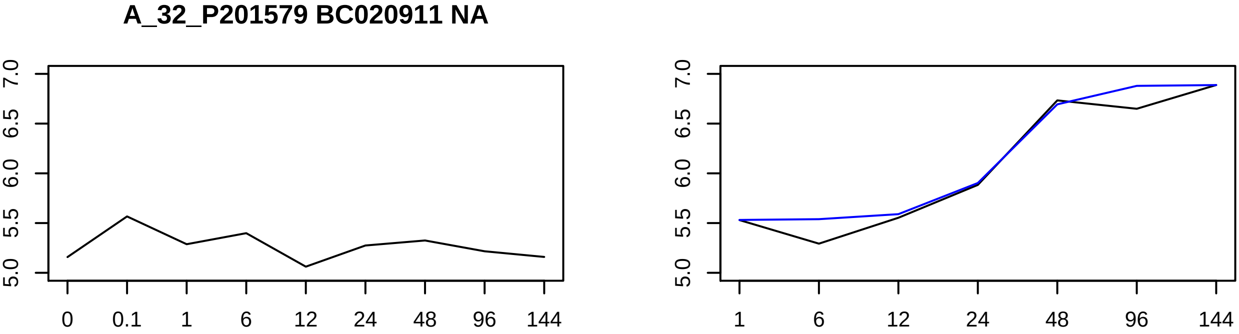
A_32_P201579 BC020911 NA
5.0
5.5
6.0
6.5
7.0
0
0.1
1
6
12
24
48
96
144
1
6
12
24
48
96
144

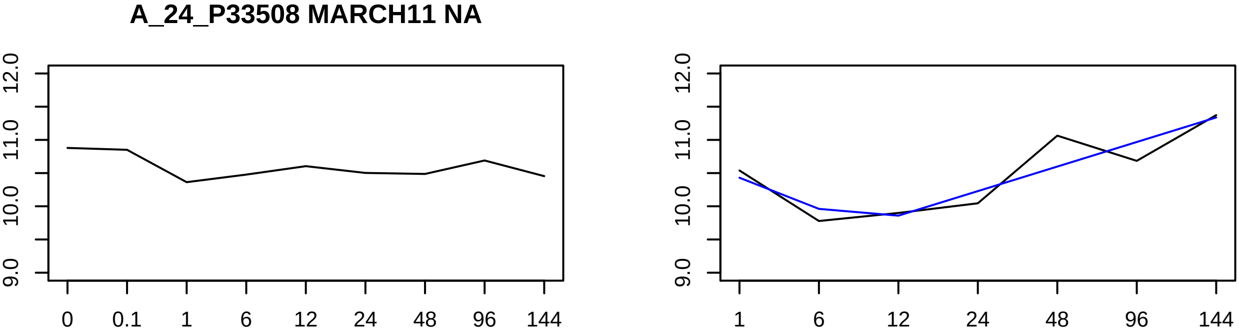
A_24_P33508 MARCH11 NA
9.0
10.0
11.0
12.0
0
0.1
1
6
12
24
48
96
144
1
6
12
24
48
96
144

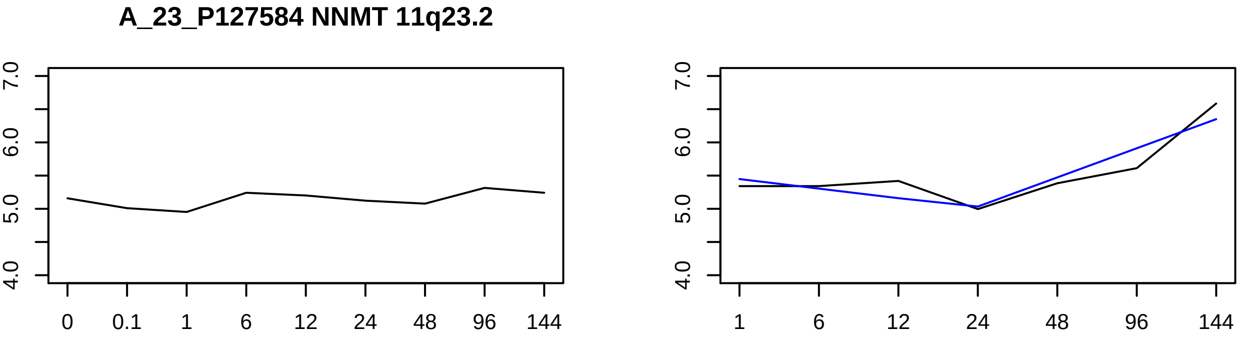
A_23_P127584 NNMT 11q23.2
4.0
5.0
6.0
7.0
0
0.1
1
6
12
24
48
96
144
1
6
12
24
48
96
144

A_32_P79351 THC2614991 NA

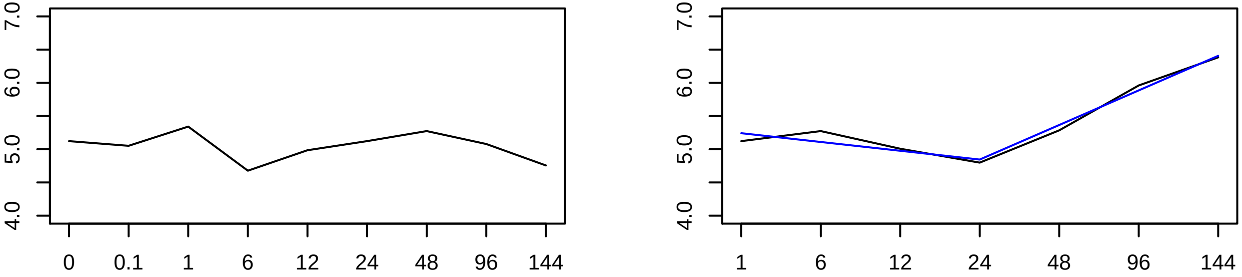

A_24_P586264 PPM1K NA

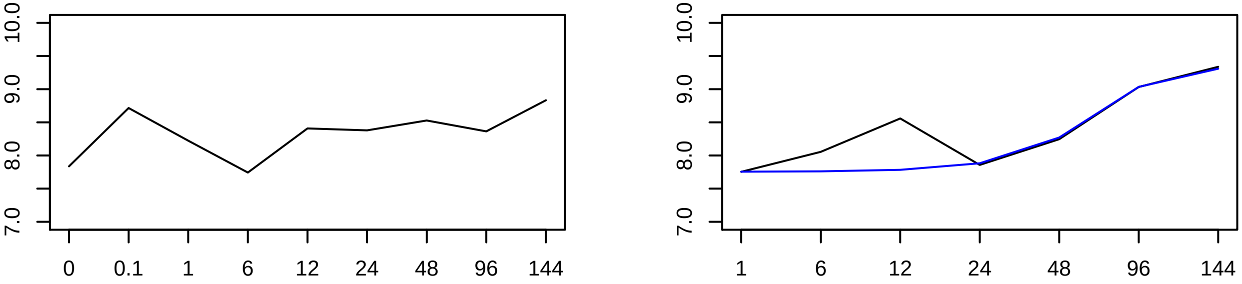

A_23_P112883 NRBP2 8q24.3

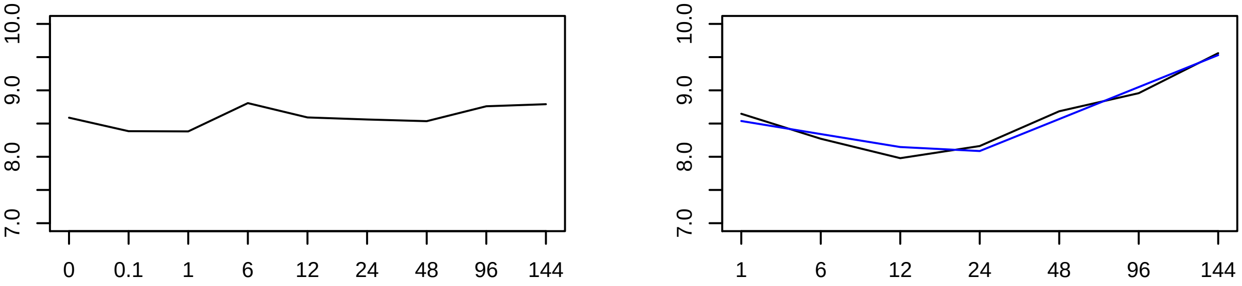

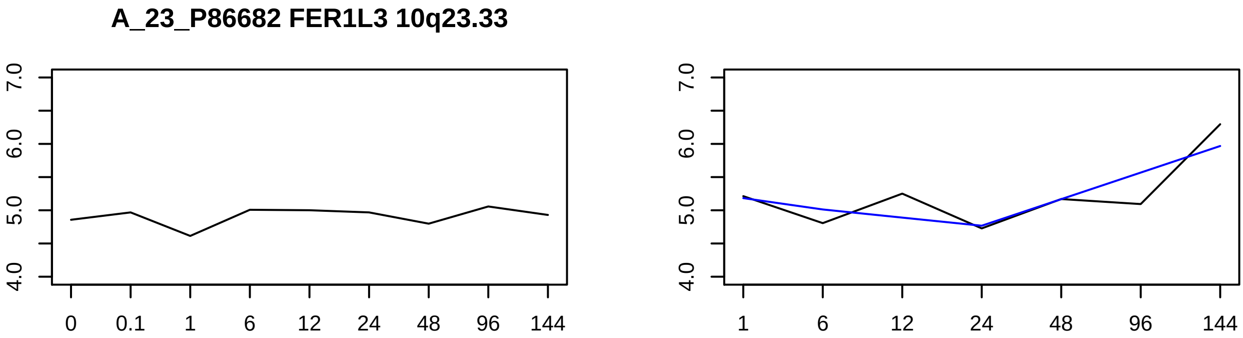
A_23_P86682 FER1L3 10q23.33
4.0
5.0
6.0
7.0
0
0.1
1
6
12
24
48
96
144
1
6
12
24
48
96
144

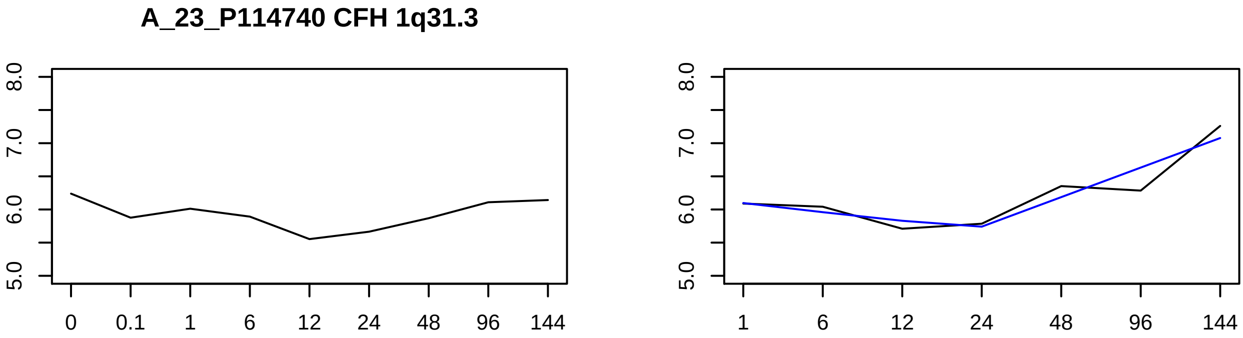
A_23_P114740 CFH 1q31.3
5.0
6.0
7.0
8.0
0
0.1
1
6
12
24
48
96
144
1
6
12
24
48
96
144

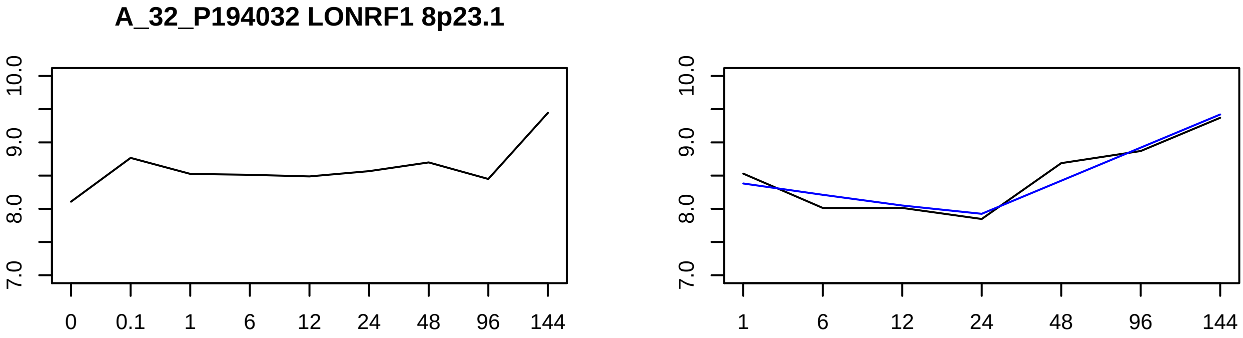
A_32_P194032 LONRF1 8p23.1
7.0
8.0
9.0
10.0
0
0.1
1
6
12
24
48
96
144
1
6
12
24
48
96
144

A_23_P394043 KIAA1543 19p13.2

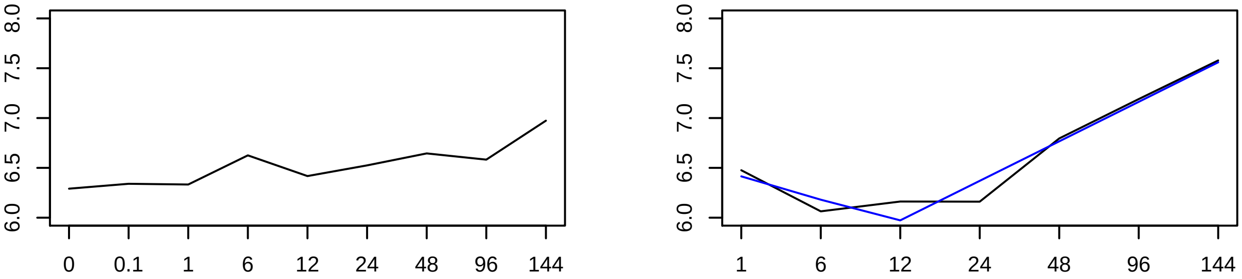

A_23_P397999 FZD5 2q33.3

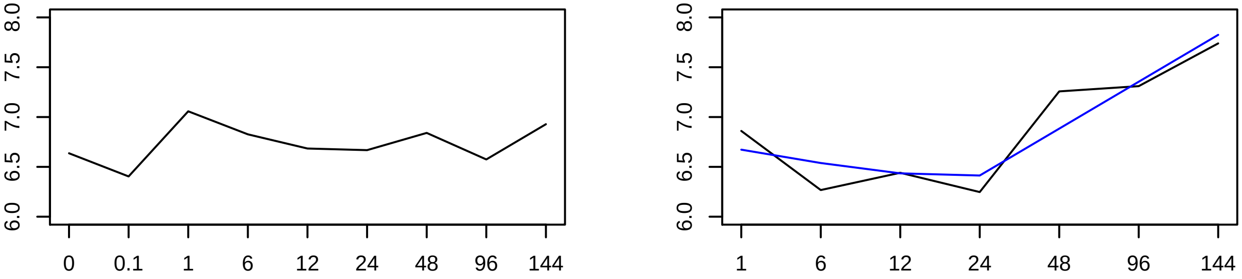

A_23_P31041 MYLIP 6p22.3

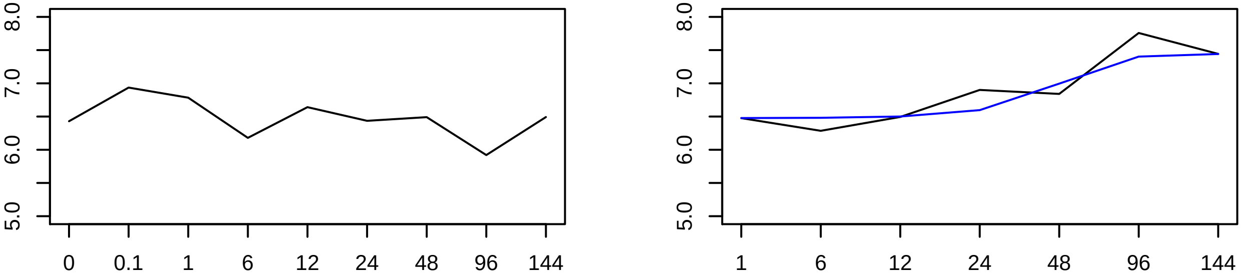

A_23_P6066 CPXM1 20p13

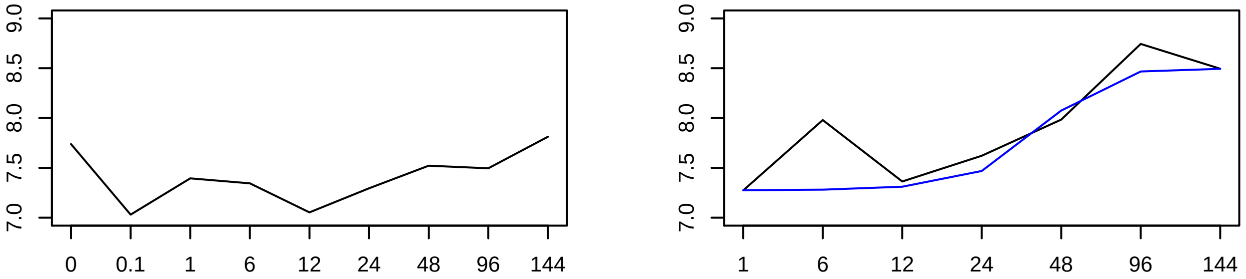

A_23_P8640 GPR30 7p22.3

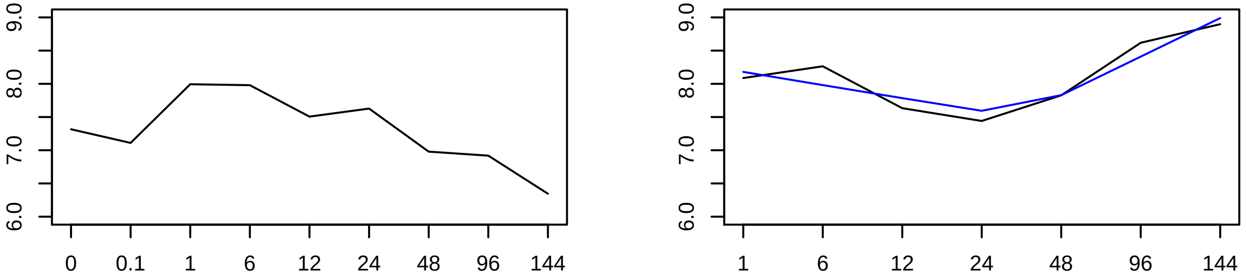

A_24_P366526 SYNGR2 17q25.3

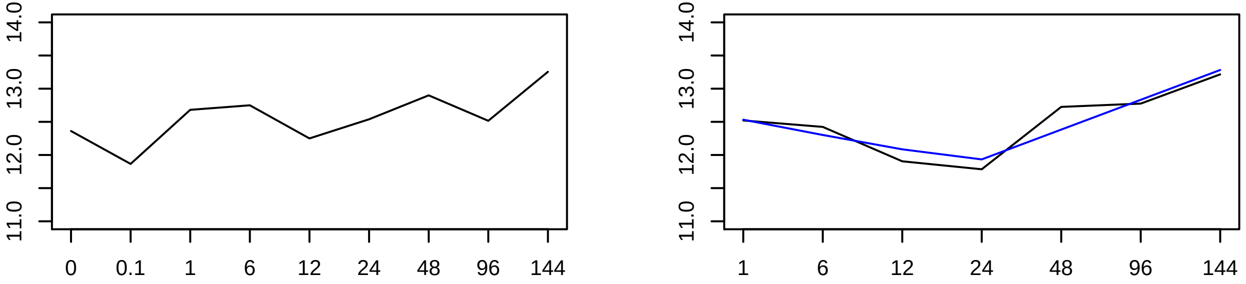

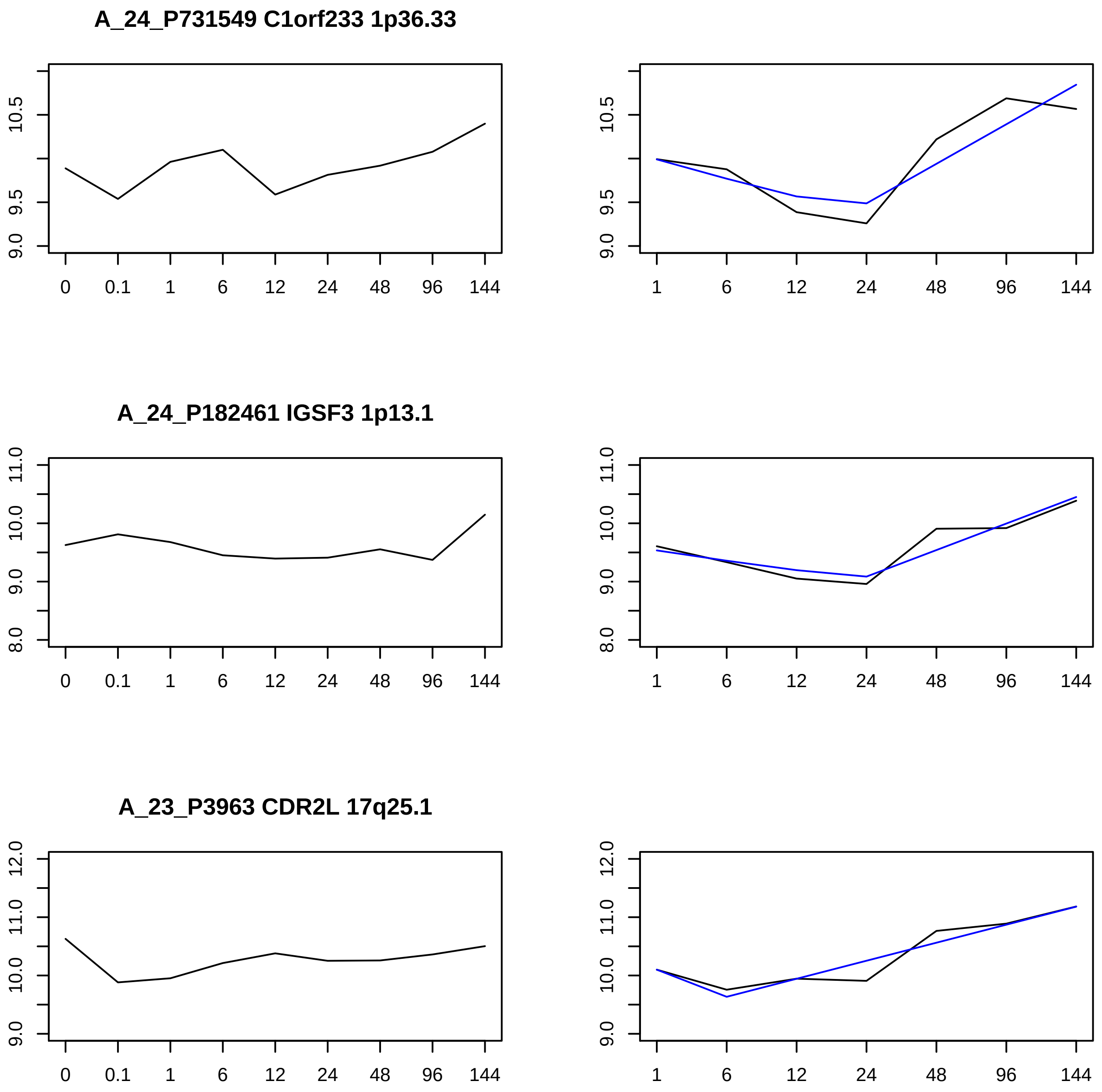

A_24_P731549 C1orf233 1p36.33
9.0
9.5
10.5
0
0.1
1
6
12
24
48
96
144
A_24_P182461 IGSF3 1p13.1
8.0
9.0
10.0
11.0
A_23_P3963 CDR2L 17q25.1
9.0
10.0
11.0
12.0

A_23_P91829 DCBLD2 3q12.1

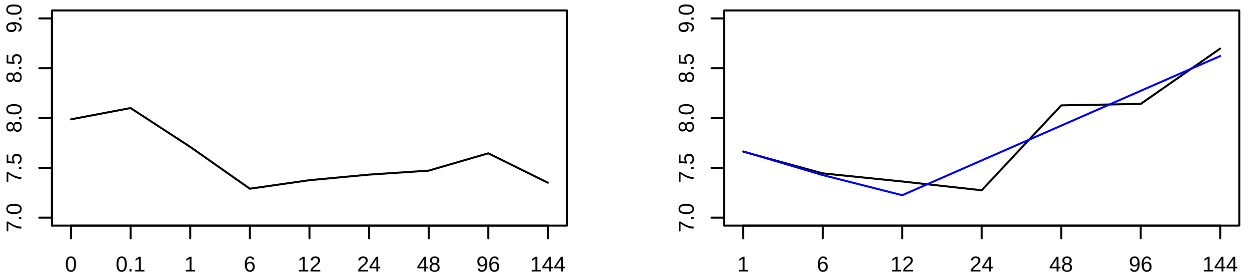

A_24_P304636 RPS6KA5 14q32.11

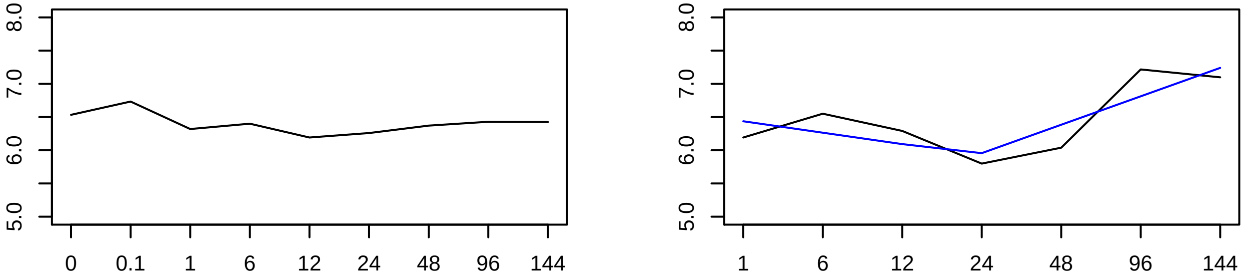

A_24_P100761 BCAS3 17q23.2

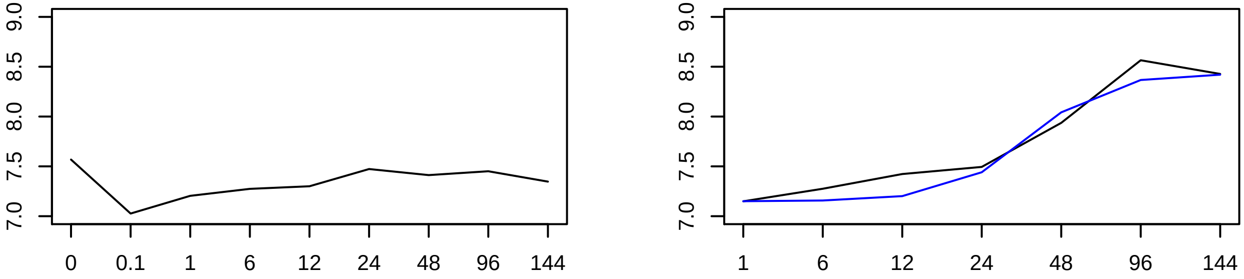

A_32_P188186 KIAA1244 6q23.3

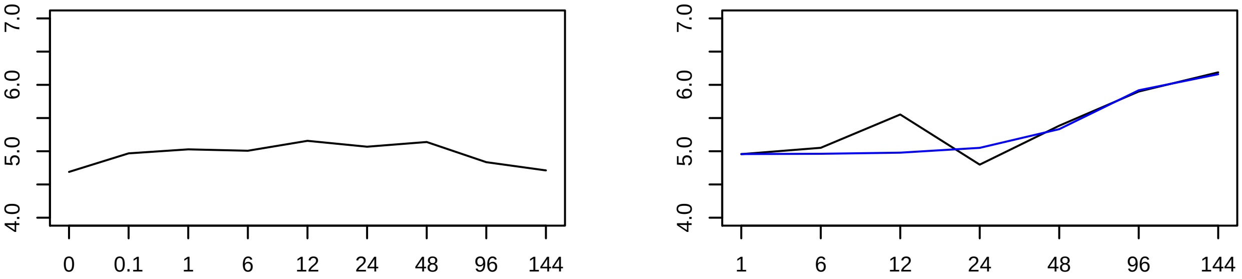

A_24_P893239 COX7B Xq21.1

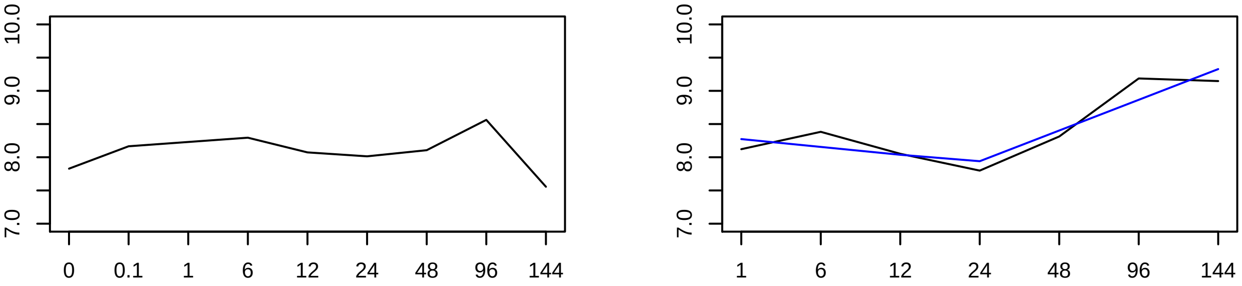

A_23_P301942 NPPC 2q37.1

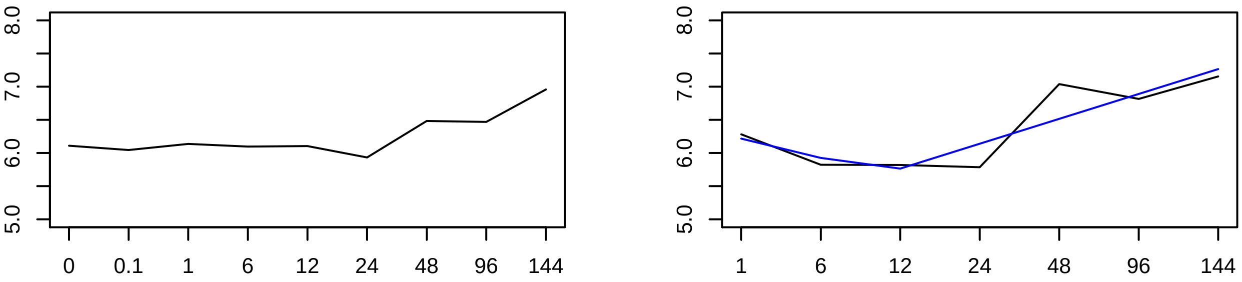

A_24_P236091 ENO2 12p13.31

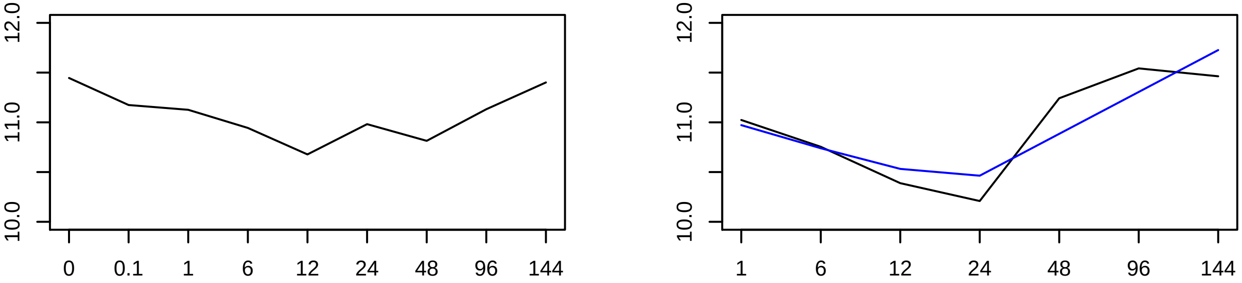

A_24_P49106 TCEAL7 Xq22.1

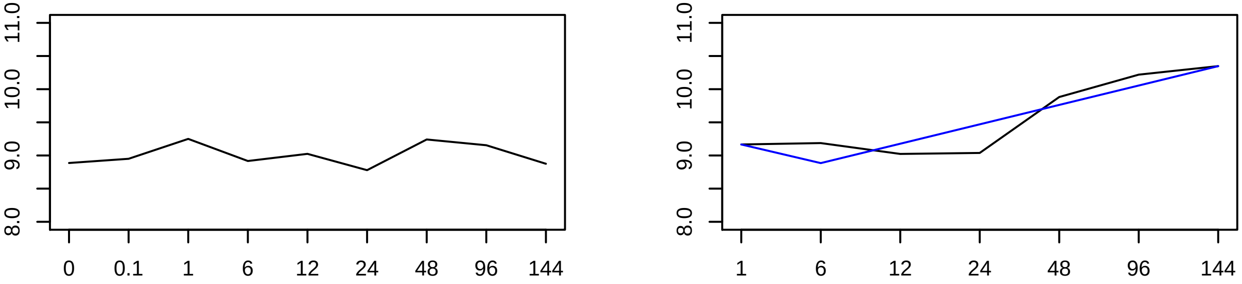

A_23_P387045 CCDC107 9p13.3

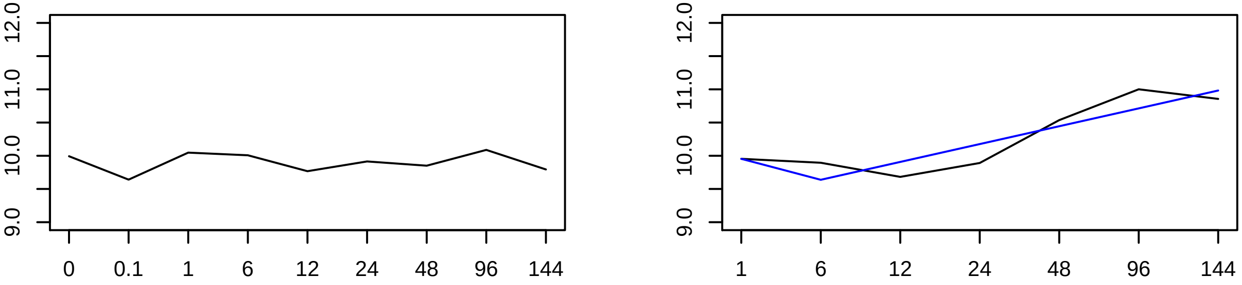

A_23_P77669 LOC55565 16q22.3

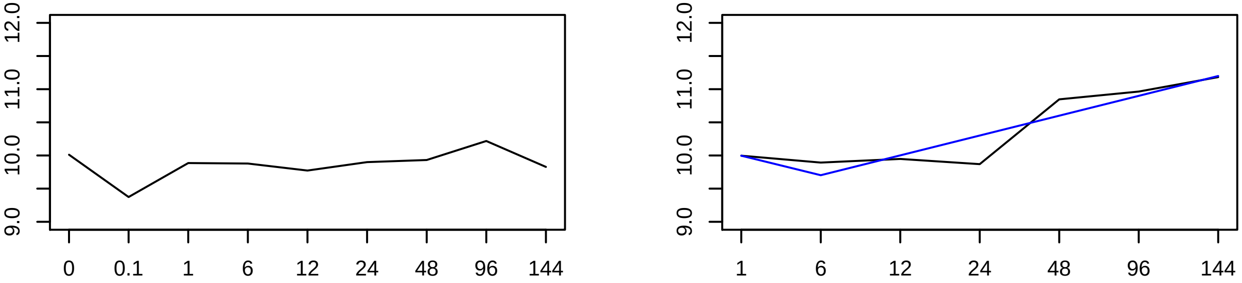

A_23_P503115 BCR 22q11.23

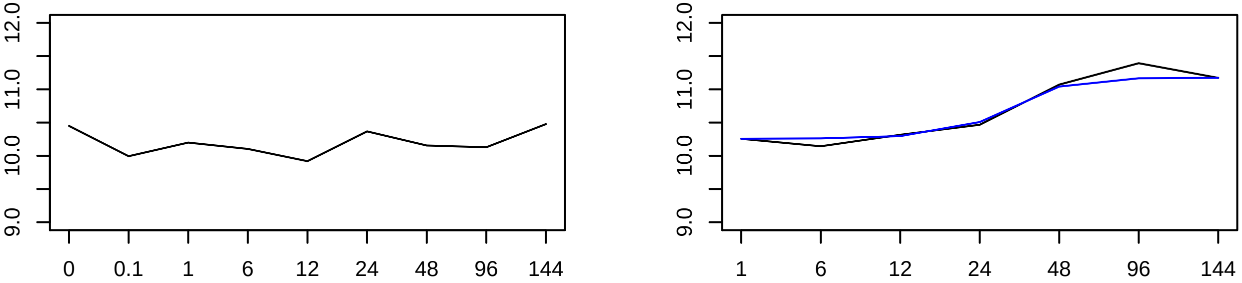

A_23_P34402 NCSTN 1q23.2

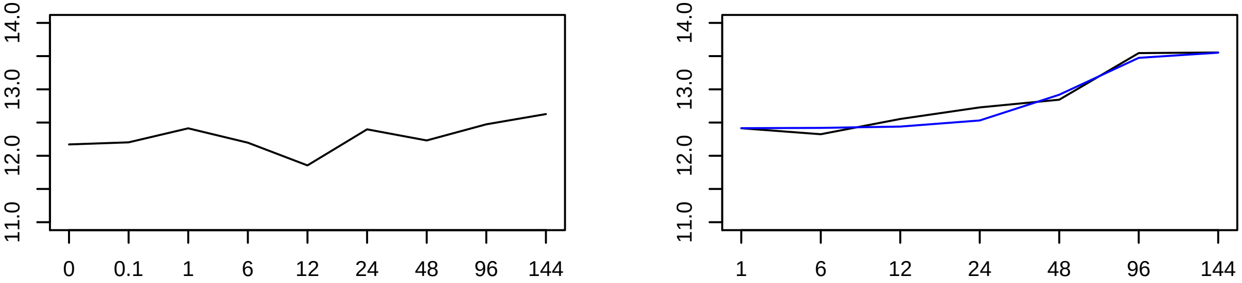

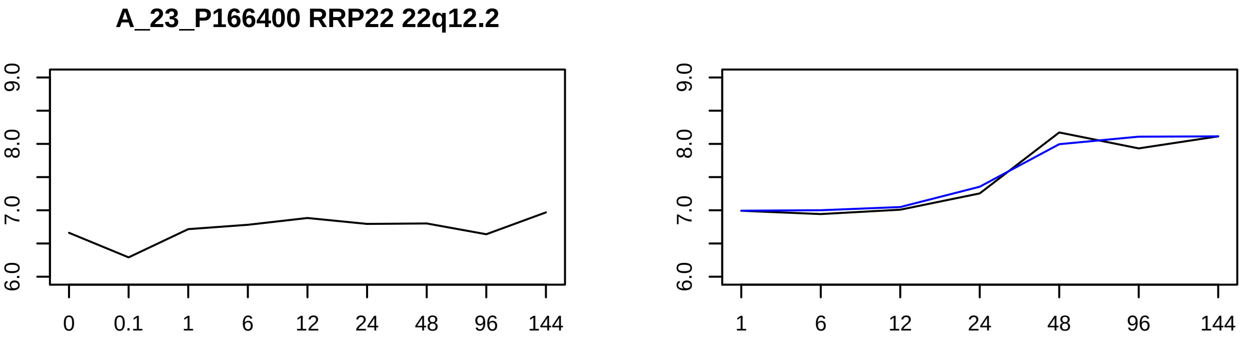
A_23_P166400 RRP22 22q12.2
6.0 7.0 8.0 9.0
0 0.1 1 6 12 24 48 96 144
6.0 7.0 8.0 9.0
1 6 12 24 48 96 144

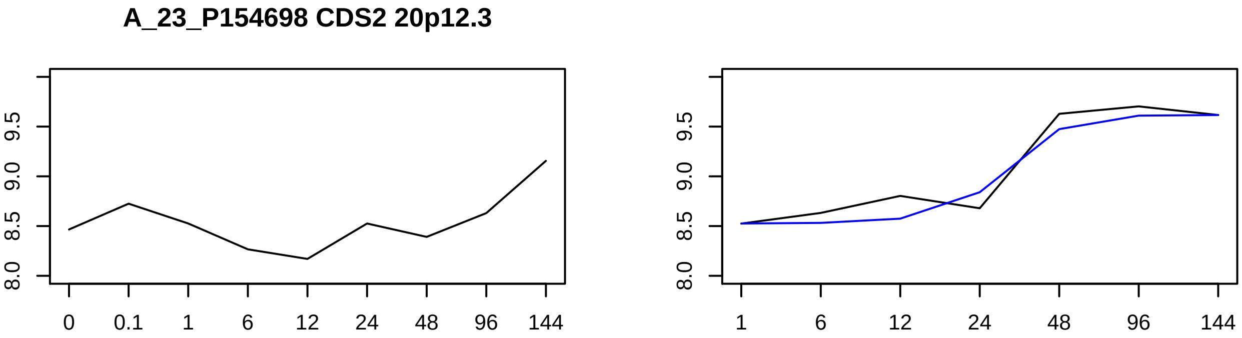
A_23_P154698 CDS2 20p12.3
8.0 8.5 9.0 9.5
0 0.1 1 6 12 24 48 96 144
8.0 8.5 9.0 9.5
1 6 12 24 48 96 144
