# Supplementary material for: SwitchFinder – a novel method and query facility for discovering dynamic gene expression patterns
Source: BMC Bioinformatics. 2016 Dec 15;17:532. doi: 10.1186/s12859-016-1391-0 (PMC5160026; doi:10.1186/s12859-016-1391-0)
Supplement: Additional file 3 — Additional file A-H. These files contain the fitting results for the genes from the groups A-H, deduced by SwitchFinder, which represent eight dynamic patterns of the gene expression response to ATRA in neuroblastoma cell line. (ZIP 2457 kb) [file 12859_2016_1391_MOESM3_ESM.zip › AdditionalFile_D.pdf]

A_23_P156970 MEST 7q32.2

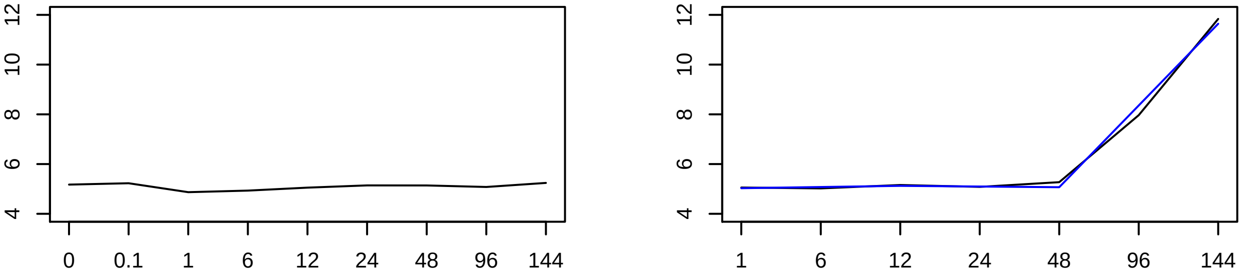

A_23_P302568 SLC30A3 2p23.3

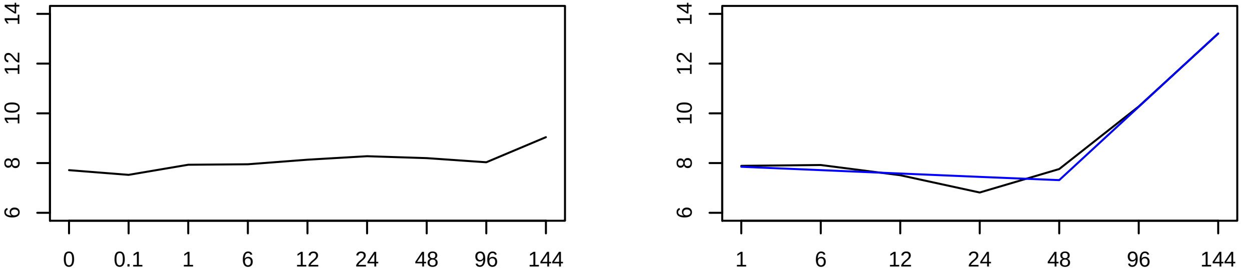

A_23_P121064 PTX3 3q25.32

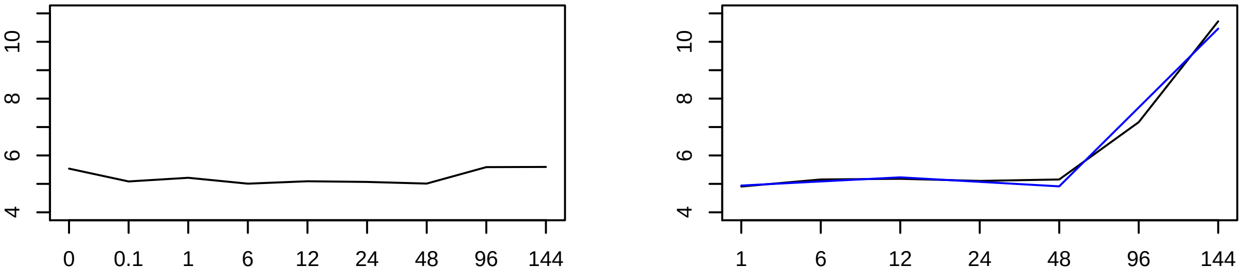

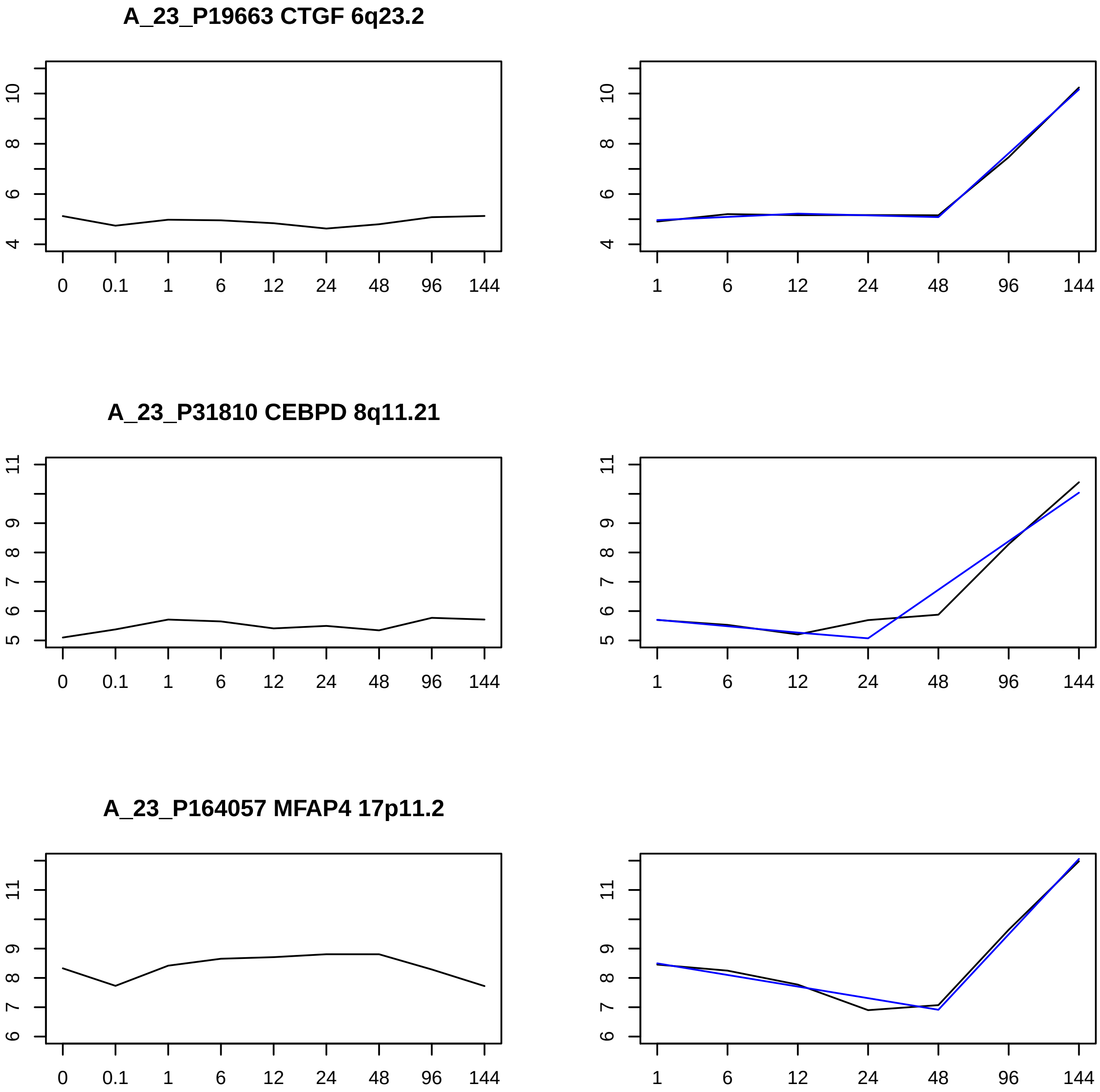
A_23_P19663 CTGF 6q23.2
A_23_P31810 CEBPD 8q11.21
A_23_P164057 MFAP4 17p11.2
0 0.1 1 6 12 24 48 96 144
1 6 12 24 48 96 144
4 6 8 10
5 6 7 8 9 11
6 7 8 9 11

A_23_P150053 ACTA2 10q23.31

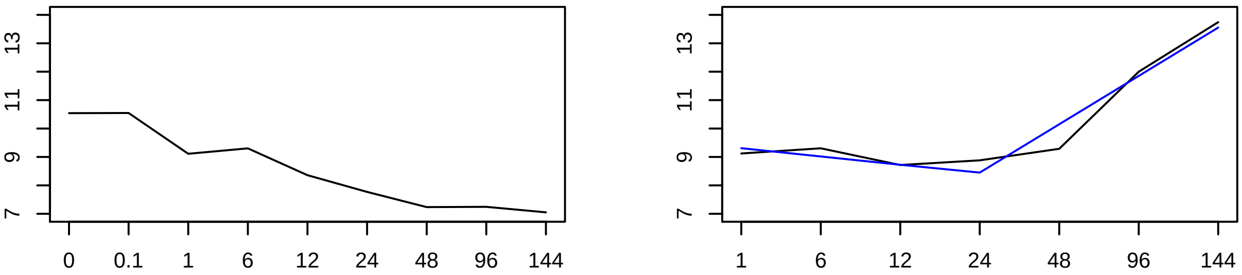

A_23_P39955 ACTG2 2p13.1

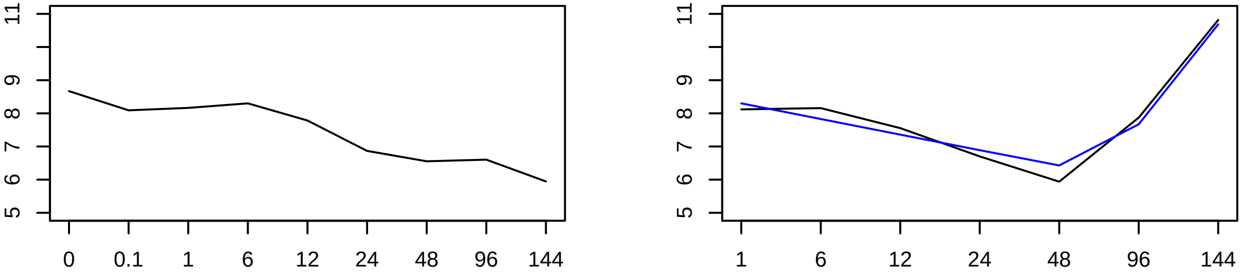

A_23_P383009 IGFBP5 2q35

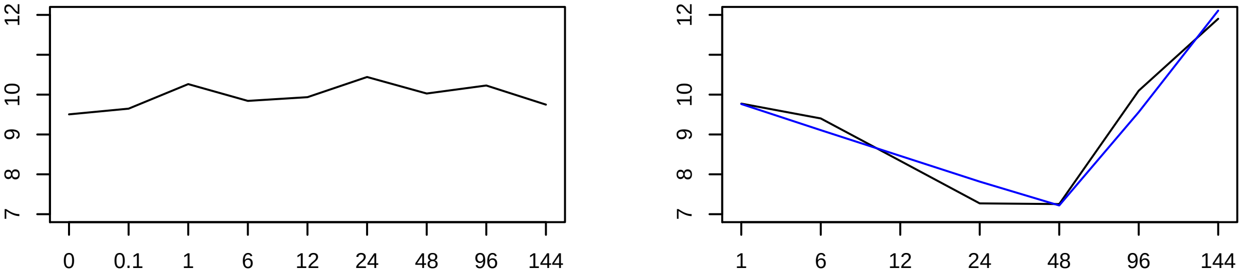

A_24_P24645 LOC132391 4q26

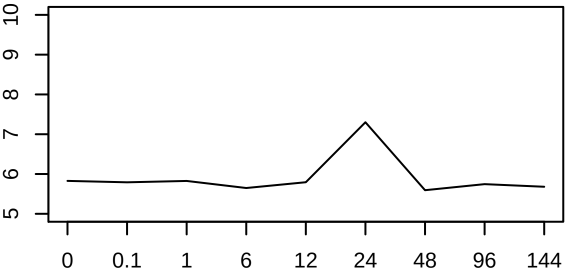

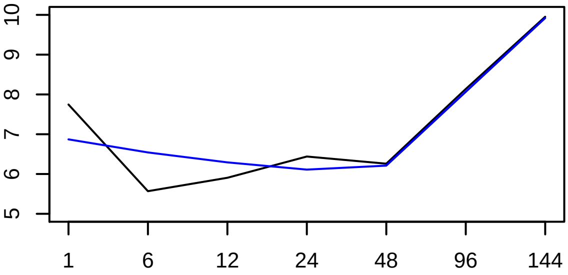

A_24_P169843 LOC343326 1q25.3

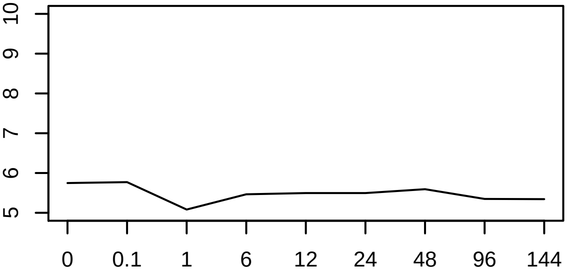

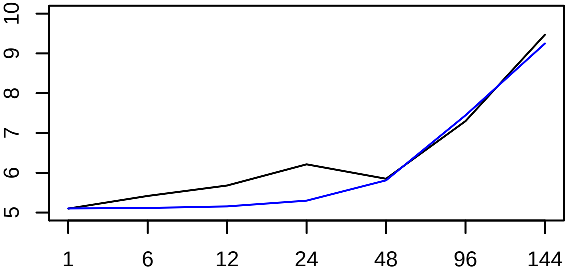

A_24_P289665 LOC389332 5q31.2

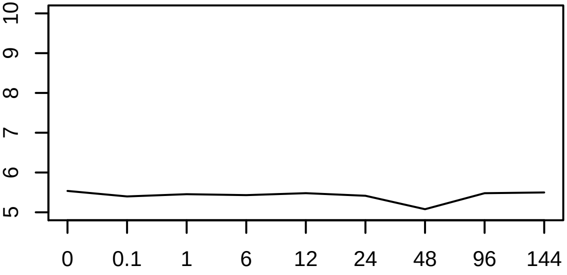

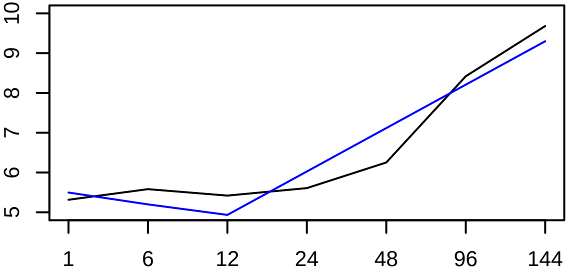

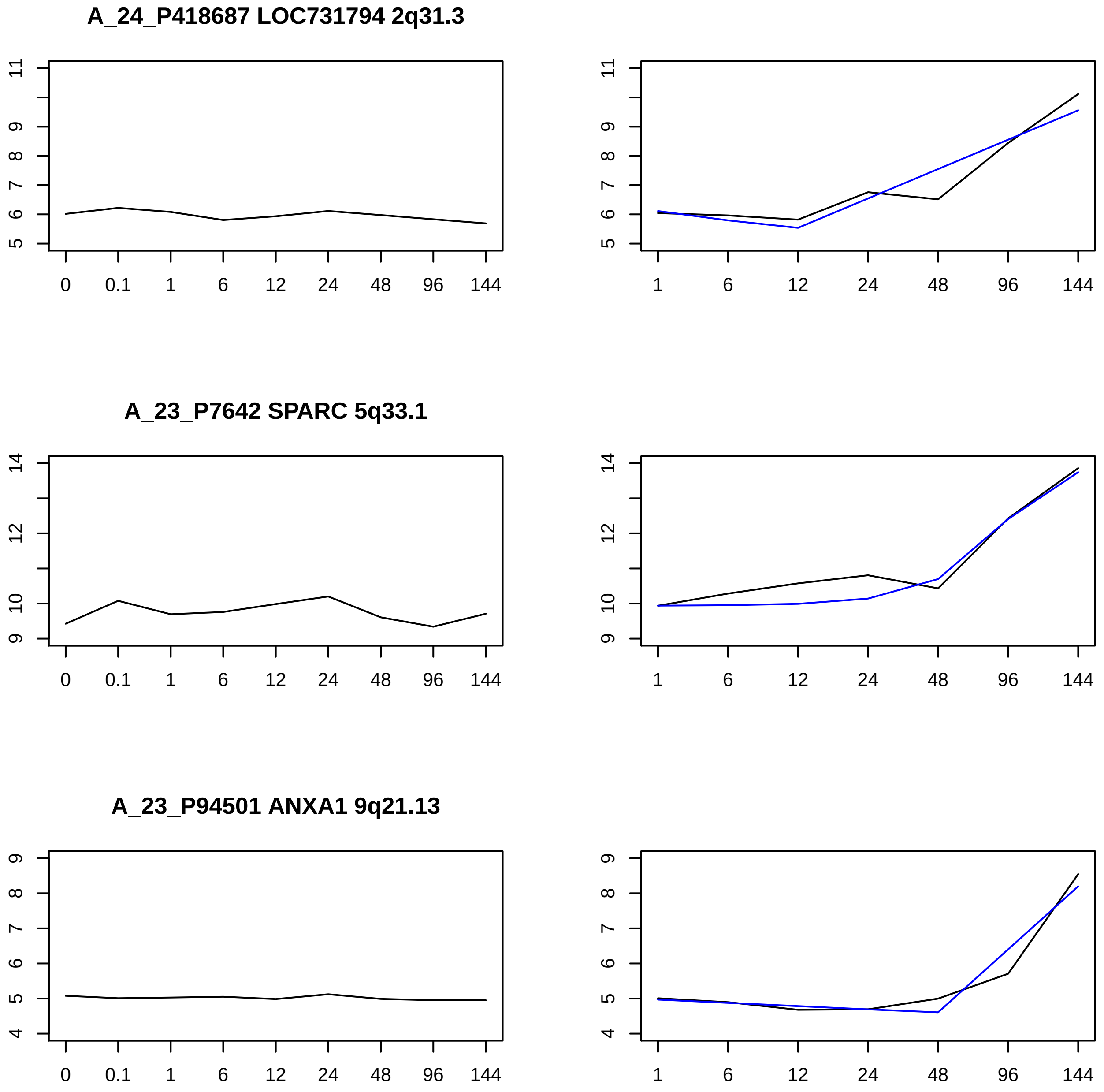
A_24_P418687 LOC731794 2q31.3
5 6 7 8 9 11
0 0.1 1 6 12 24 48 96 144
1 6 12 24 48 96 144
A_23_P7642 SPARC 5q33.1
9 10 12 14
0 0.1 1 6 12 24 48 96 144
1 6 12 24 48 96 144
A_23_P94501 ANXA1 9q21.13
4 5 6 7 8 9
0 0.1 1 6 12 24 48 96 144
1 6 12 24 48 96 144

A_32_P24140 GAS2 11p14.3

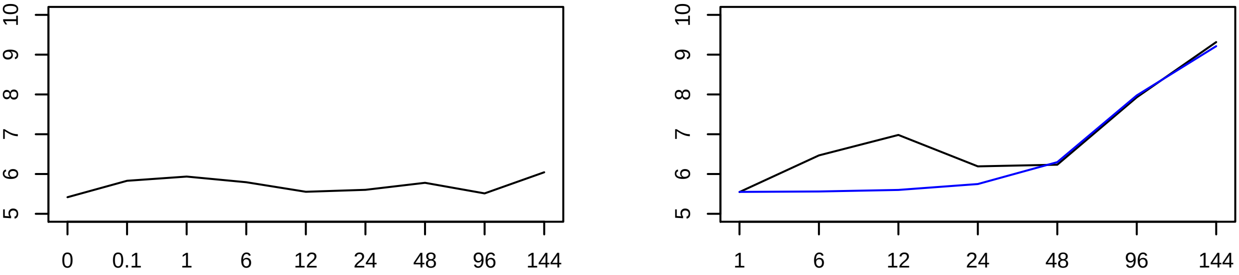

A_23_P202448 CXCL12 10q11.21

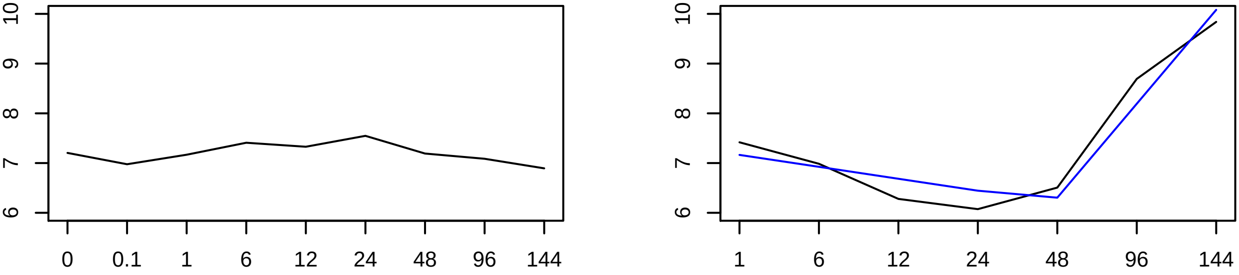

A_24_P785894 THC2651263 NA

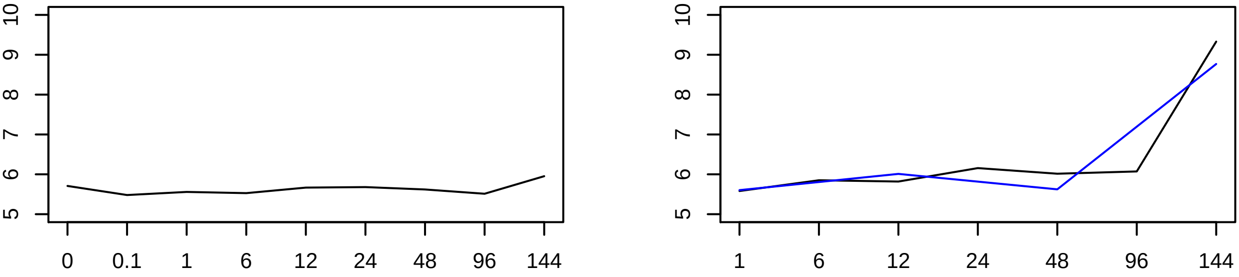

A_24_P372189 WISP1 8q24.22

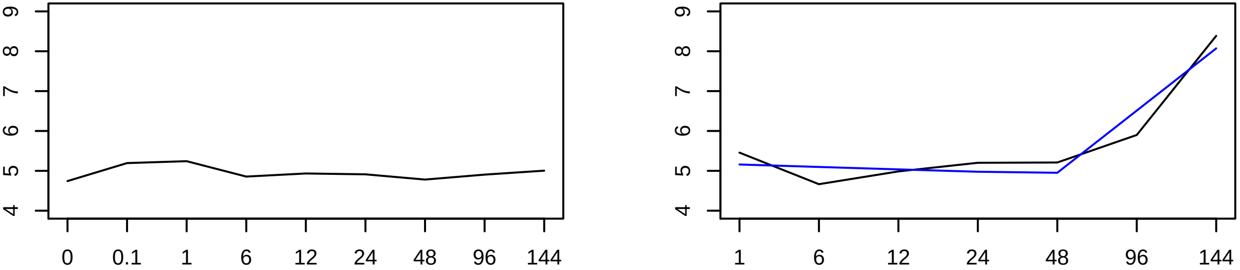

A_23_P125233 CNN1 19p13.2

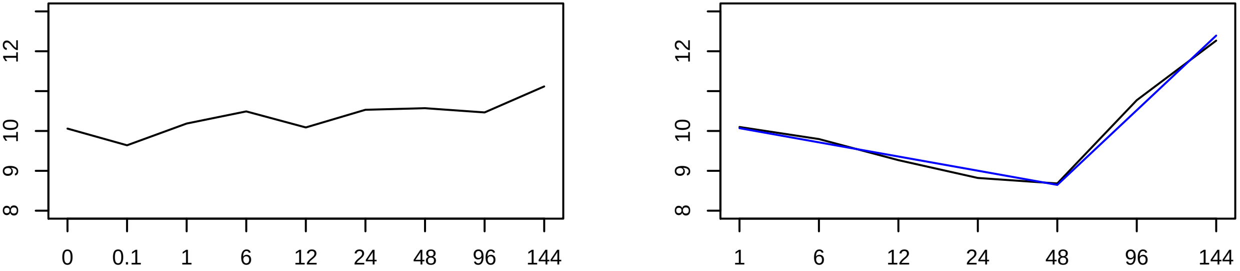

A_24_P409420 A_24_P409420 NA

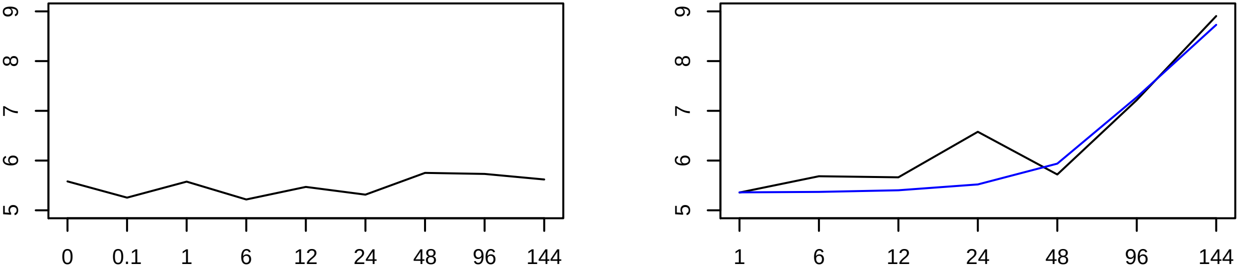

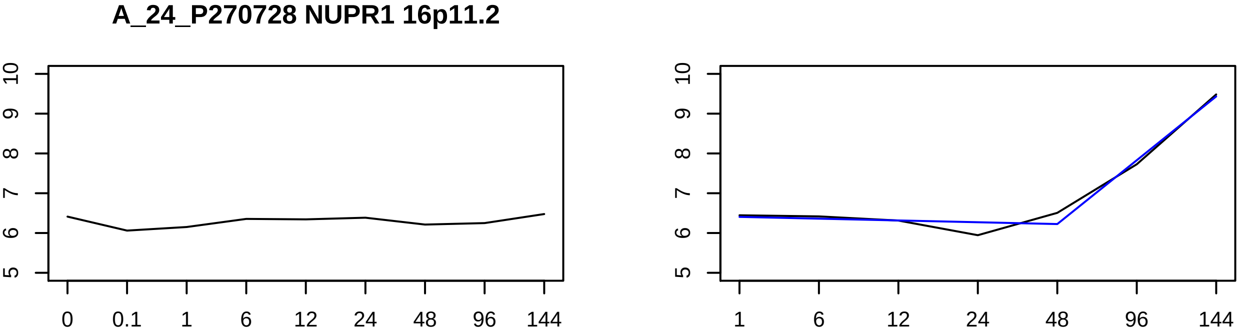
A_24_P270728 NUPR1 16p11.2
5
6
7
8
9
10
0
0.1
1
6
12
24
48
96
144
1
6
12
24
48
96
144

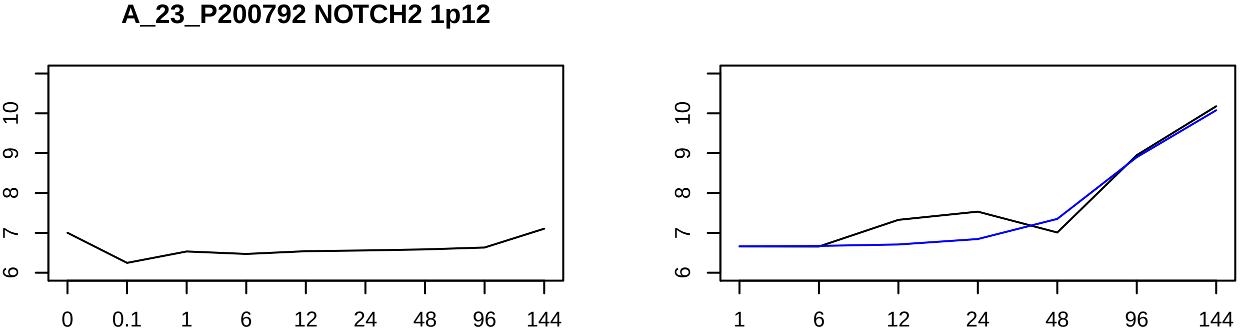
A_23_P200792 NOTCH2 1p12
6
7
8
9
10
0
0.1
1
6
12
24
48
96
144
1
6
12
24
48
96
144

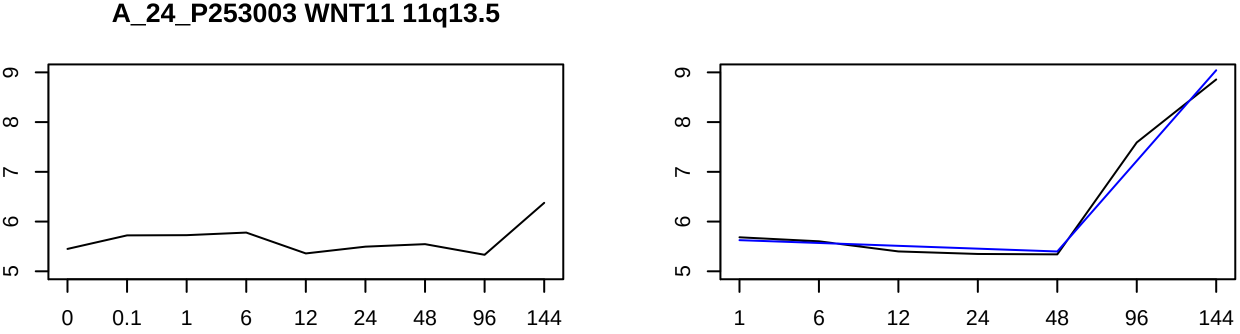
A_24_P253003 WNT11 11q13.5
5
6
7
8
9
0
0.1
1
6
12
24
48
96
144
1
6
12
24
48
96
144

A_32_P209365 A_32_P209365 NA

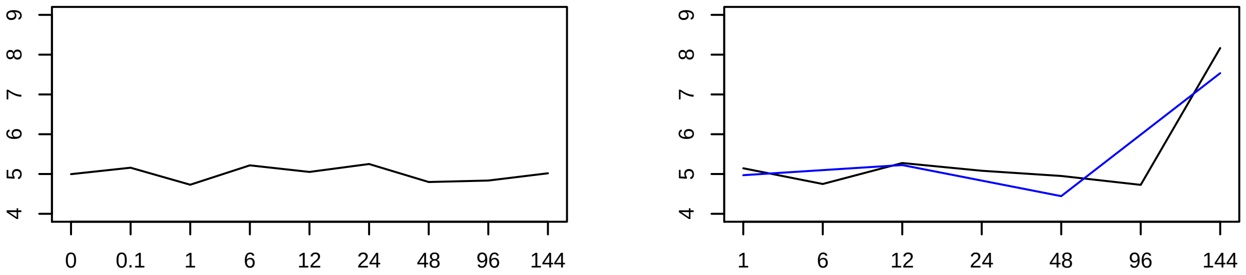

A_23_P382065 EMCN 4q23

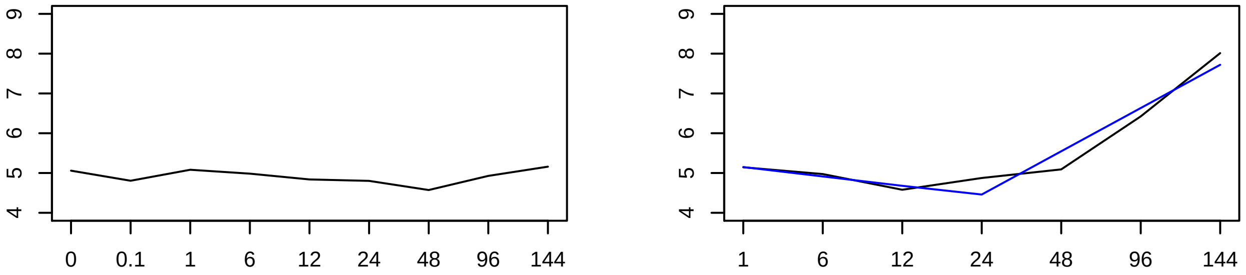

A_24_P358131 LOC651696 2p22.3

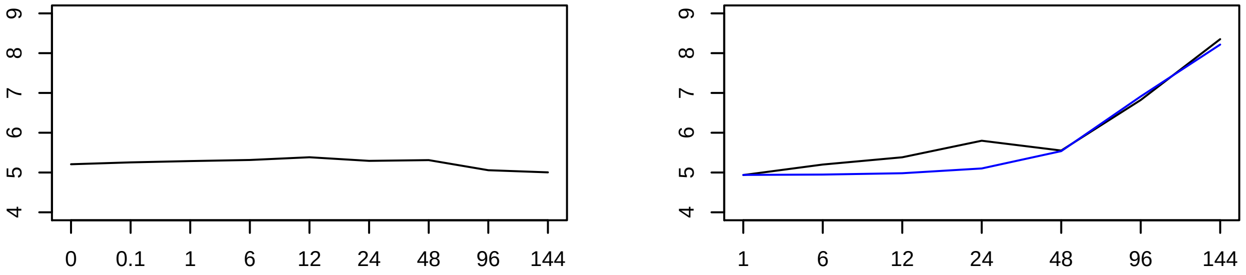

A_24_P6850 A_24_P6850 NA

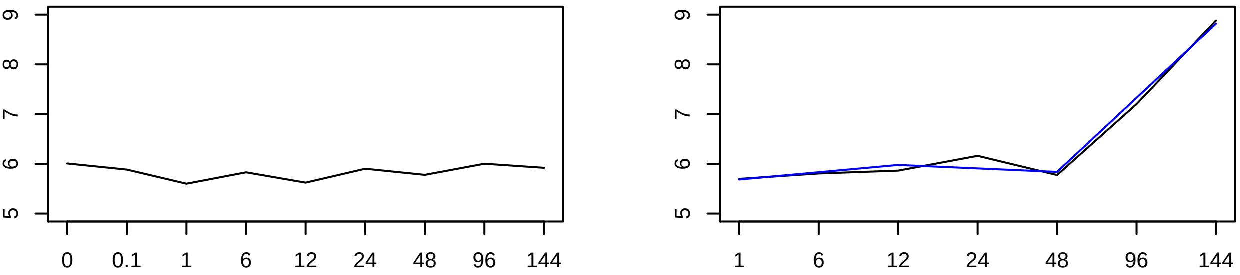

A_24_P401842 HHIP 4q31.22

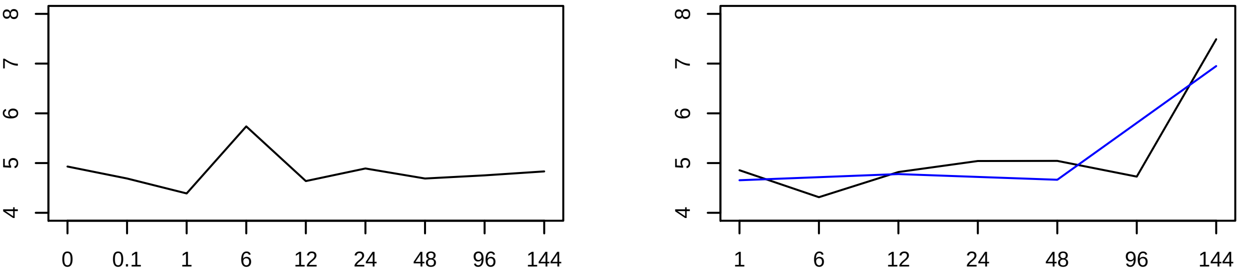

A_23_P59738 MYL7 7p13

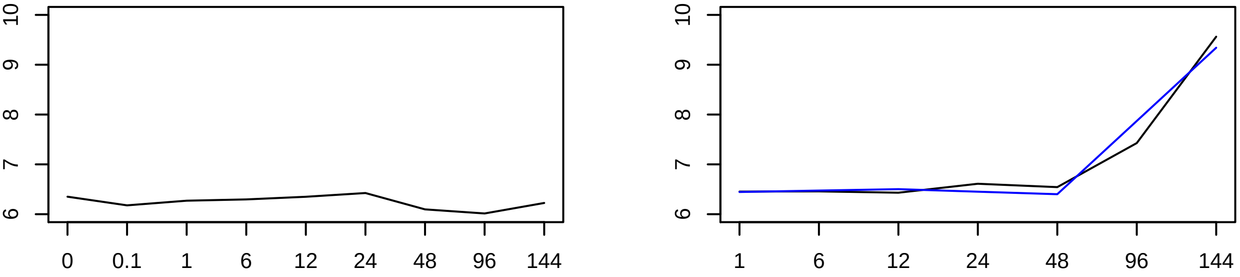

A_24_P92472 CFI 4q25

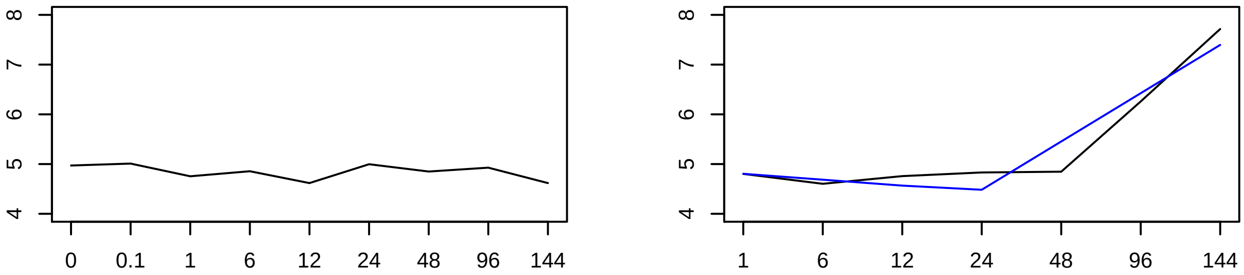

A_23_P154115 IGFBP5 2q35

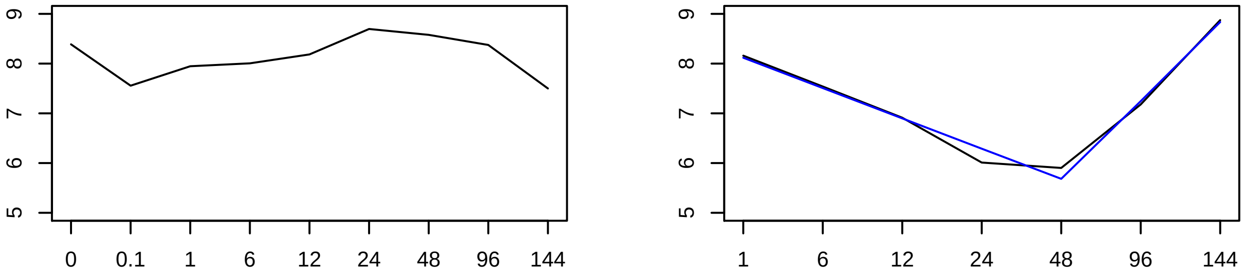

A_23_P50121 MC4R 18q21.32

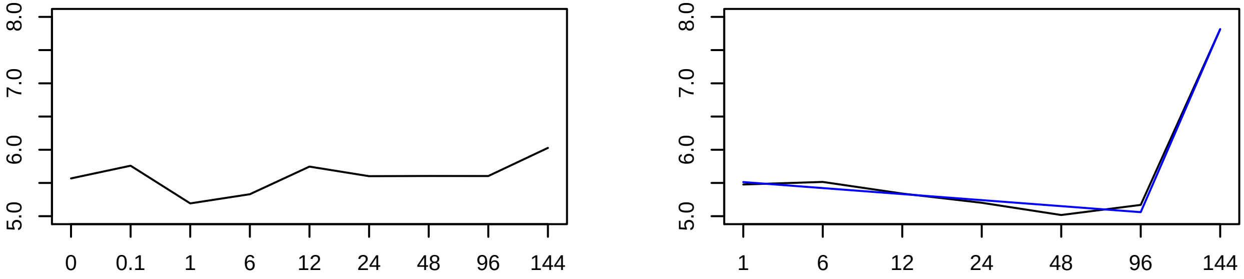

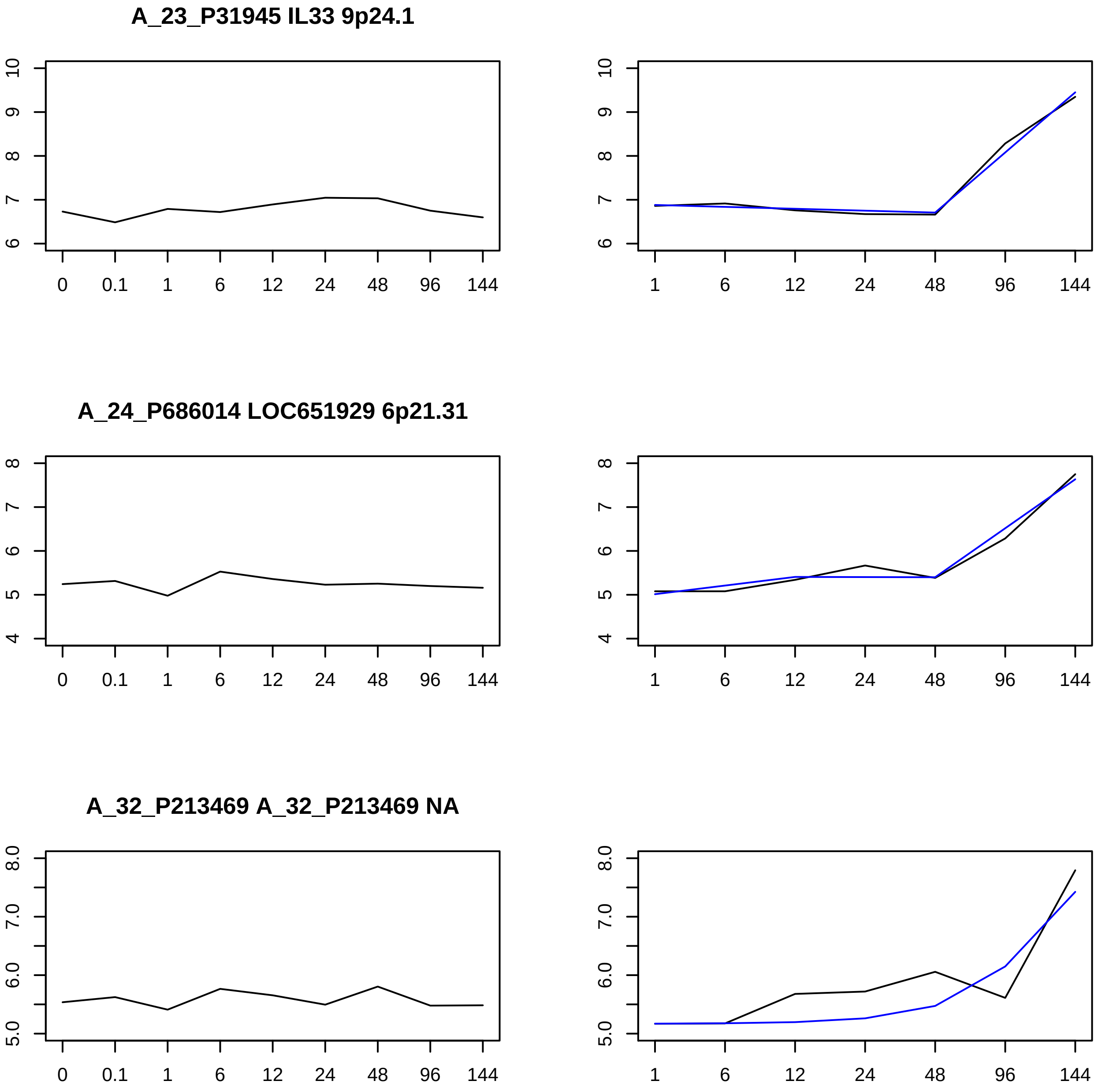
A_23_P31945 IL33 9p24.1
6
7
8
9
10
0
0.1
1
6
12
24
48
96
144
A_24_P686014 LOC651929 6p21.31
4
5
6
7
8
A_32_P213469 A_32_P213469 NA
5.0
6.0
7.0
8.0

A_24_P912074 INPP5D 2q37.1

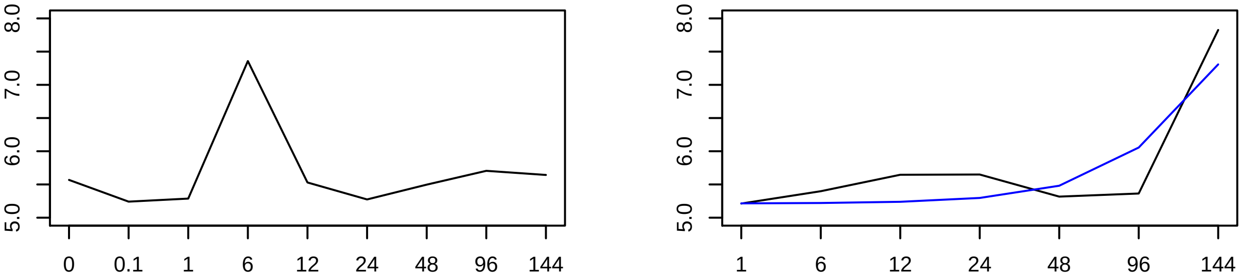

A_24_P401601 LOC390904 19p12

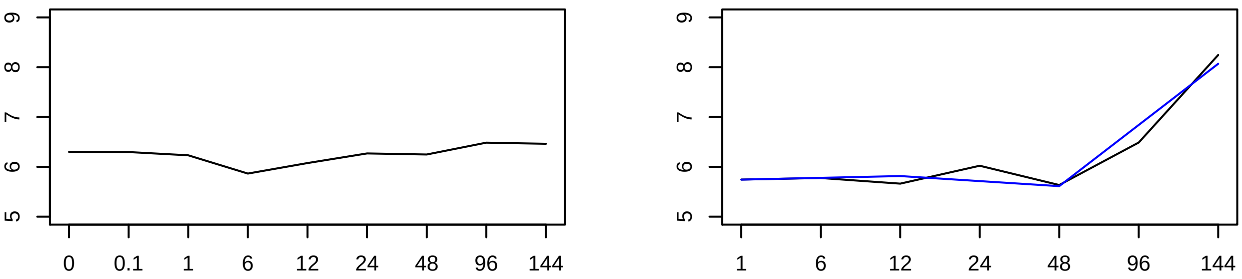

A_24_P57631 GPC3 Xq26.2

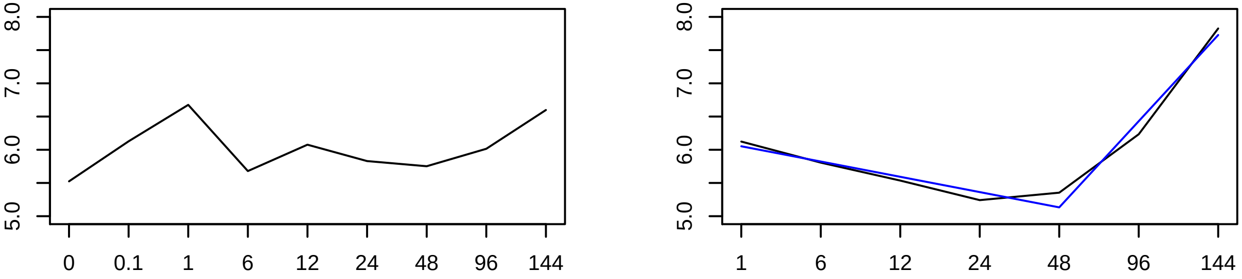

A_24_P393312 KIRREL 1q23.1

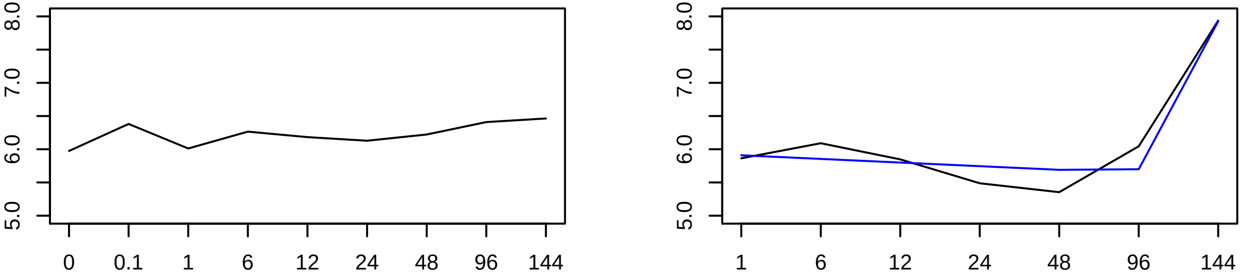

A_23_P7376 SGTB 5q12.3

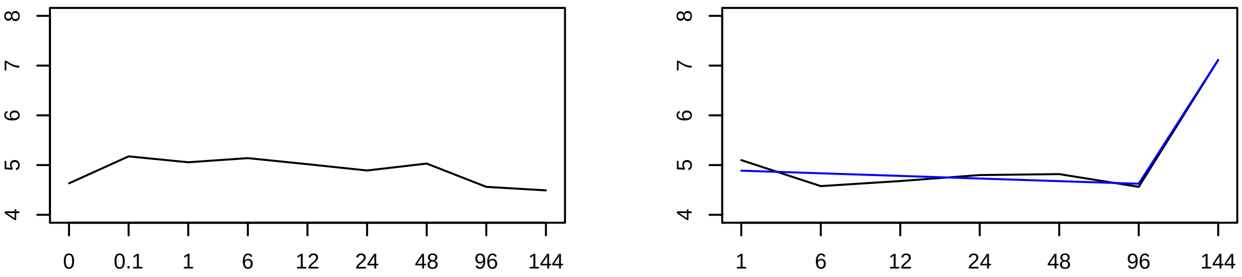

A_32_P101031 LYPD1 2q21.2

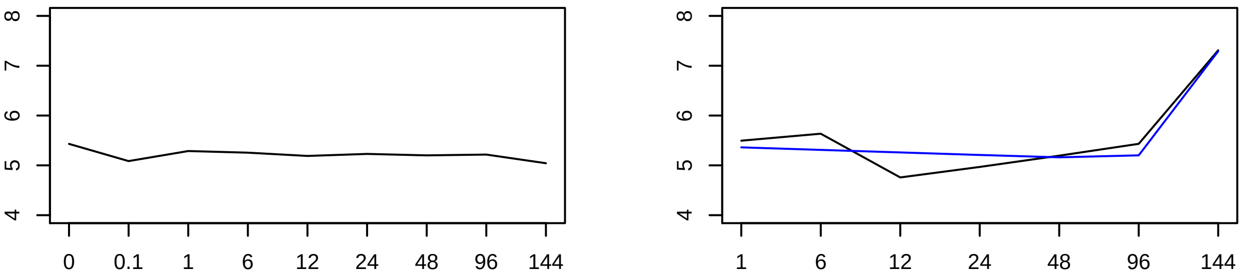

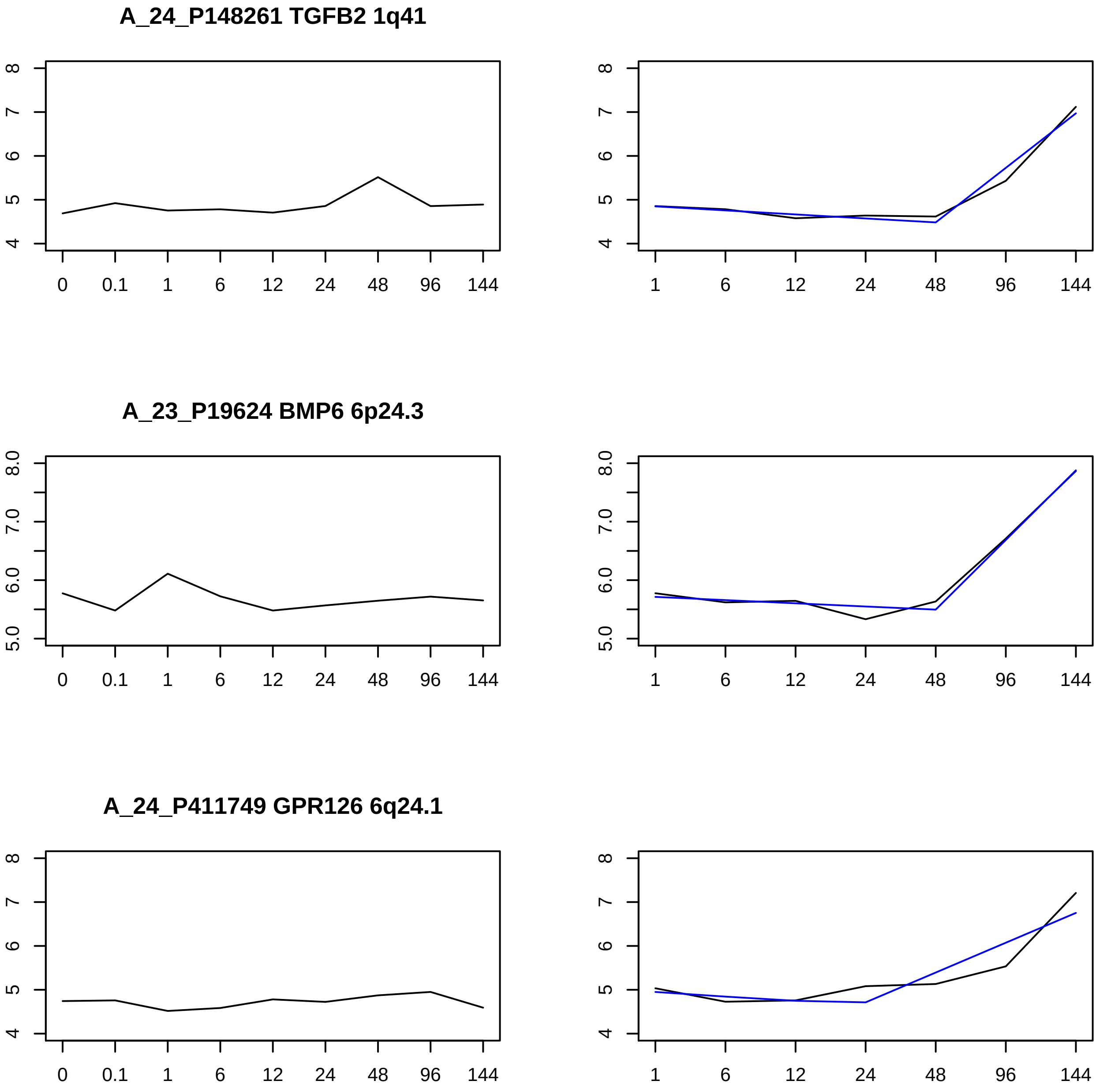
A_24_P148261 TGFB2 1q41
4
5
6
7
8
0
0.1
1
6
12
24
48
96
144
4
5
6
7
8
1
6
12
24
48
96
144
A_23_P19624 BMP6 6p24.3
5.0
6.0
7.0
8.0
0
0.1
1
6
12
24
48
96
144
5.0
6.0
7.0
8.0
1
6
12
24
48
96
144
A_24_P411749 GPR126 6q24.1
4
5
6
7
8
0
0.1
1
6
12
24
48
96
144
4
5
6
7
8
1
6
12
24
48
96
144

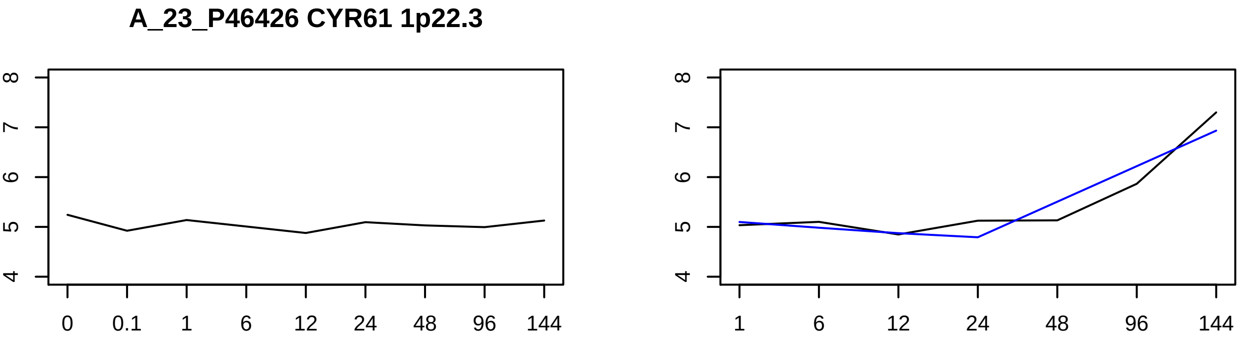
A_23_P46426 CYR61 1p22.3
4 5 6 7 8
0 0.1 1 6 12 24 48 96 144
1 6 12 24 48 96 144

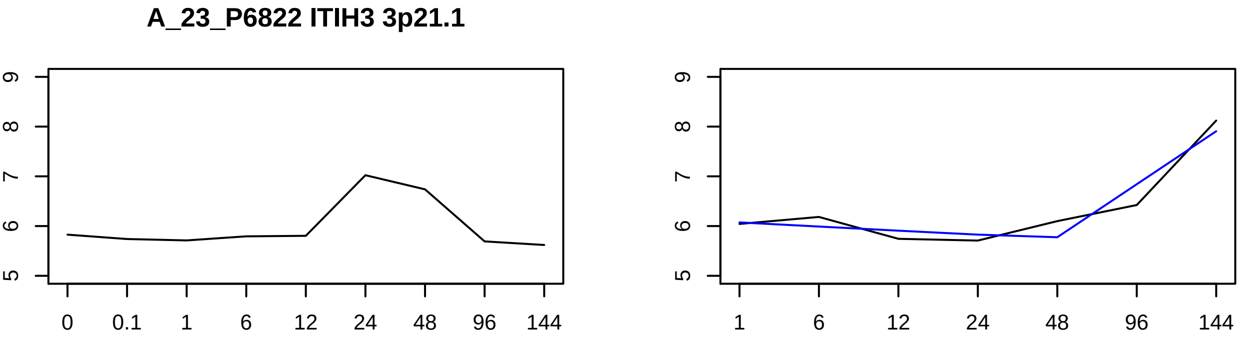
A_23_P6822 ITIH3 3p21.1
5 6 7 8 9
0 0.1 1 6 12 24 48 96 144
1 6 12 24 48 96 144

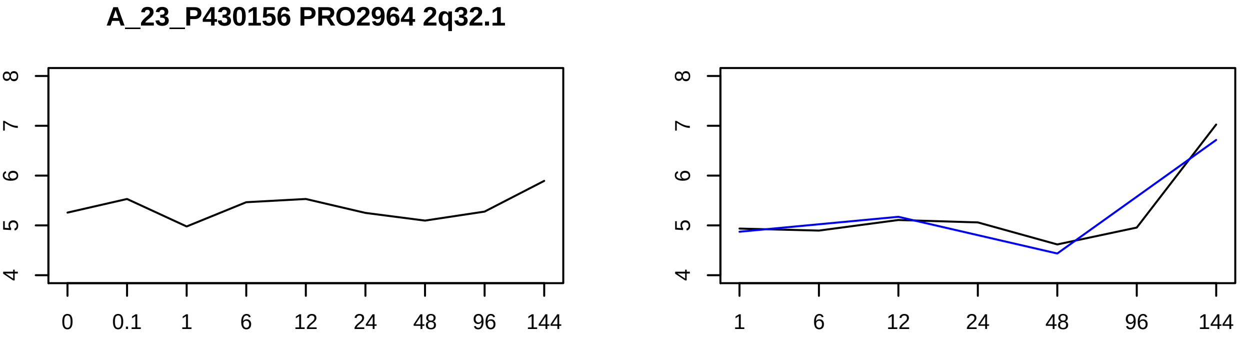
A_23_P430156 PRO2964 2q32.1
4 5 6 7 8
0 0.1 1 6 12 24 48 96 144
1 6 12 24 48 96 144

A_24_P912258 THC2500250 NA

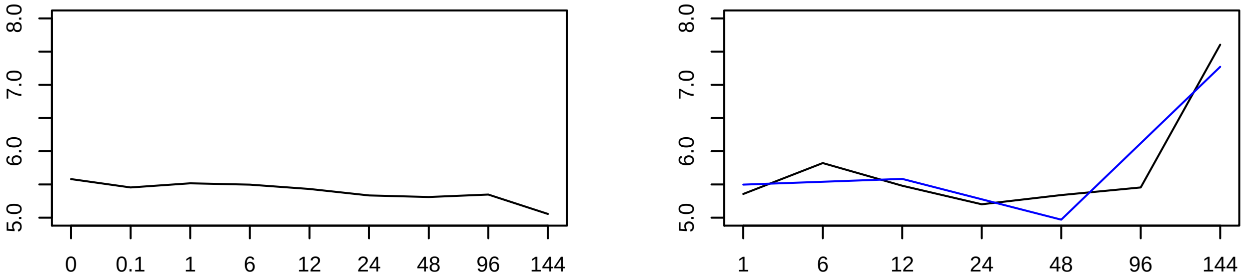

A_32_P82462 LOC554202 9p21.3

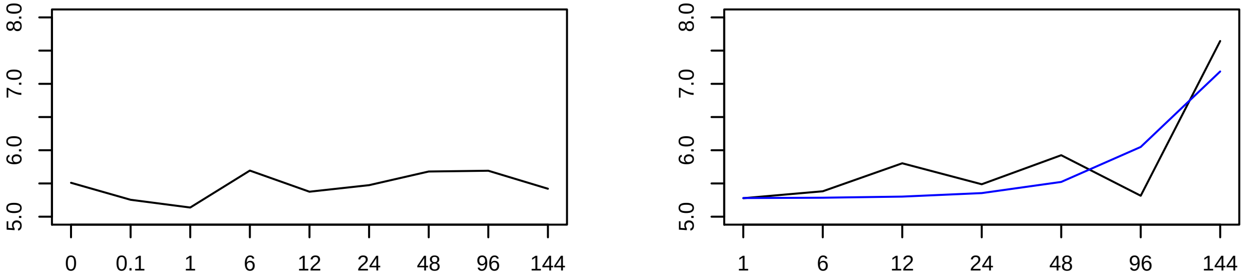

A_23_P127267 LGI1 10q23.33

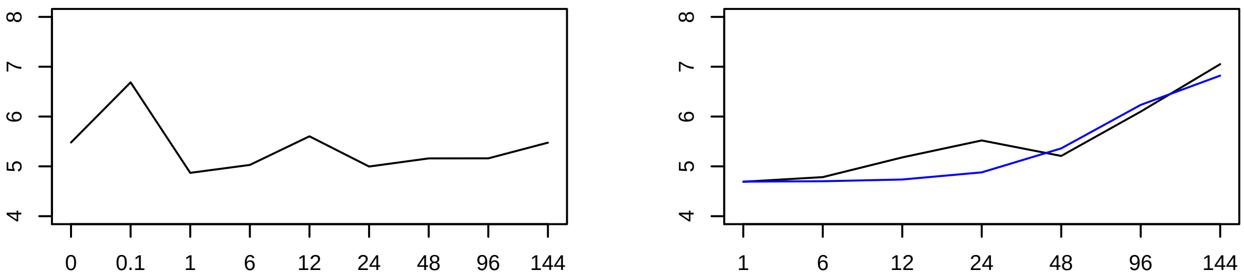

A_24_P471242 A_24_P471242 NA

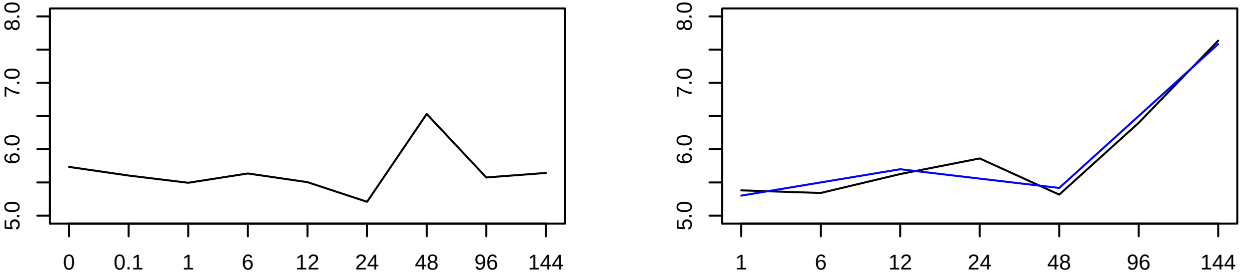

A_23_P13465 ELF5 11p13

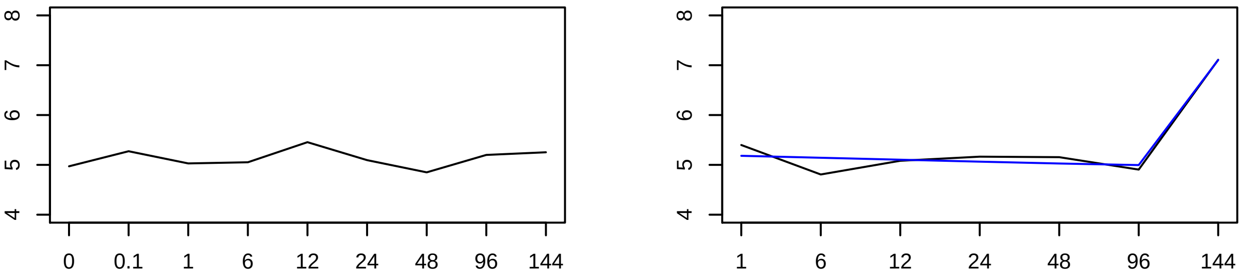

A_32_P213521 EMCN NA

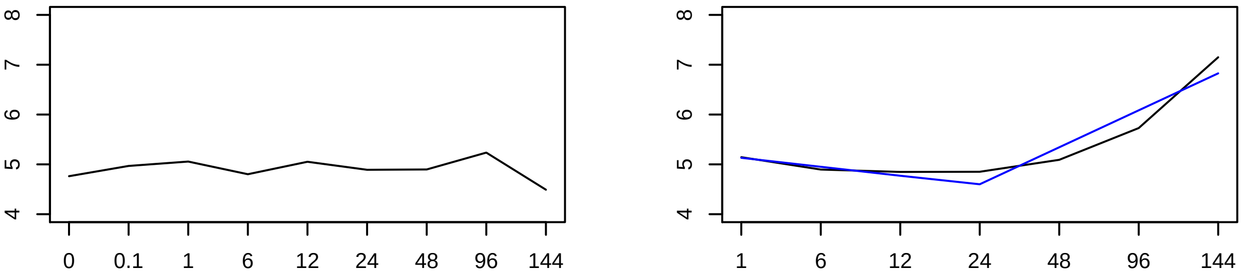

A_32_P302205 SI 3q26.1

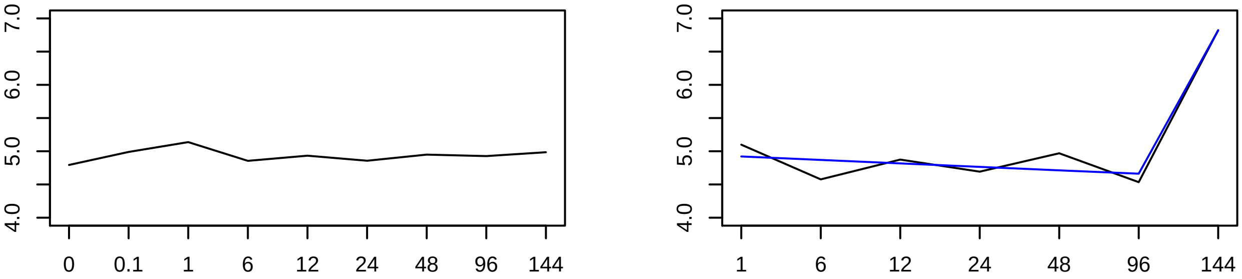

A_24_P70002 LATS2 13q12.11

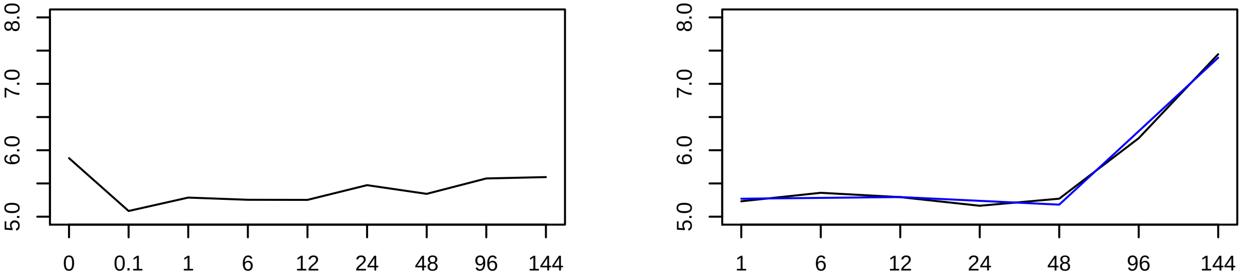

A_23_P140290 RTN1 14q23.1

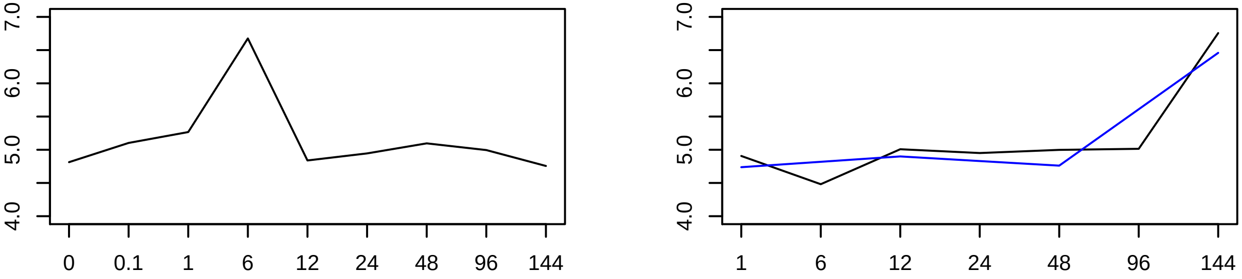

A_23_P97990 HTRA1 10q26.13

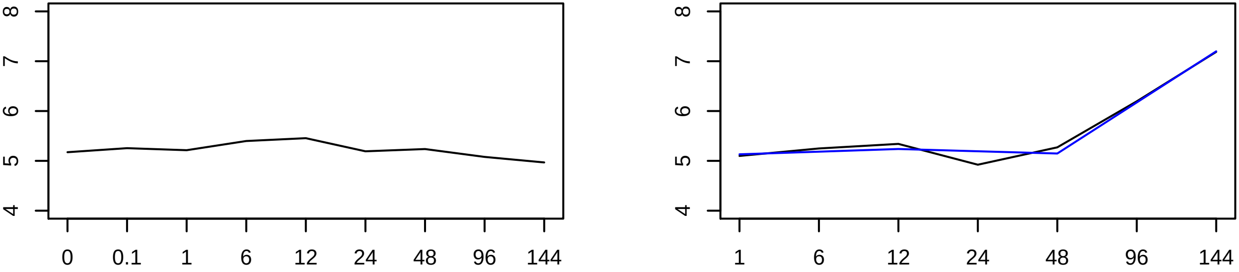

A_24_P247303 A_24_P247303 NA

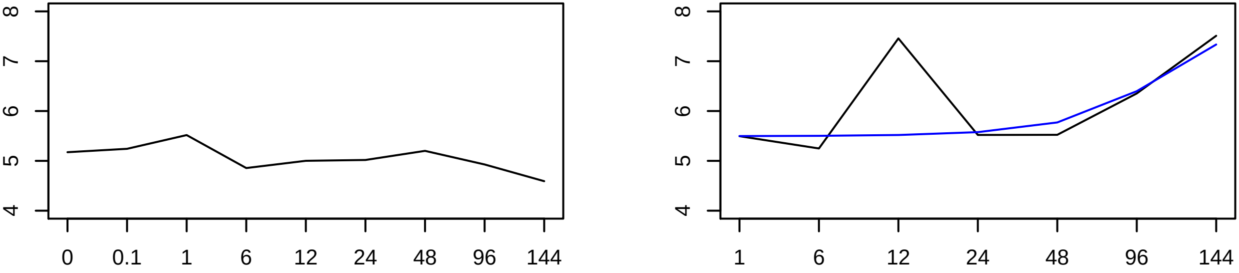

A_24_P384369 LOC646723 5p13.2

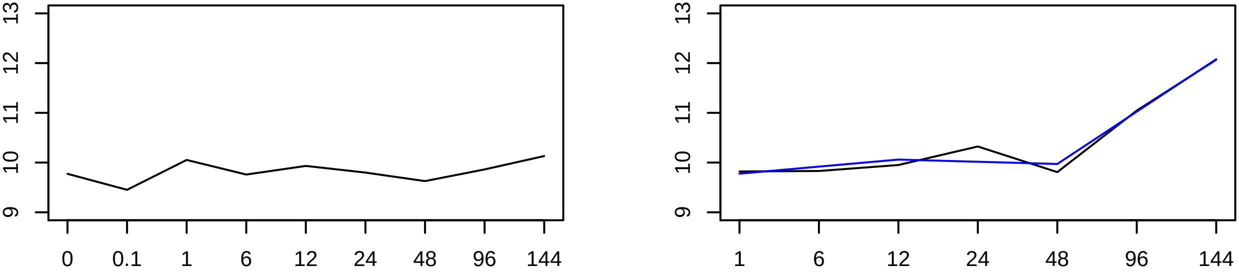

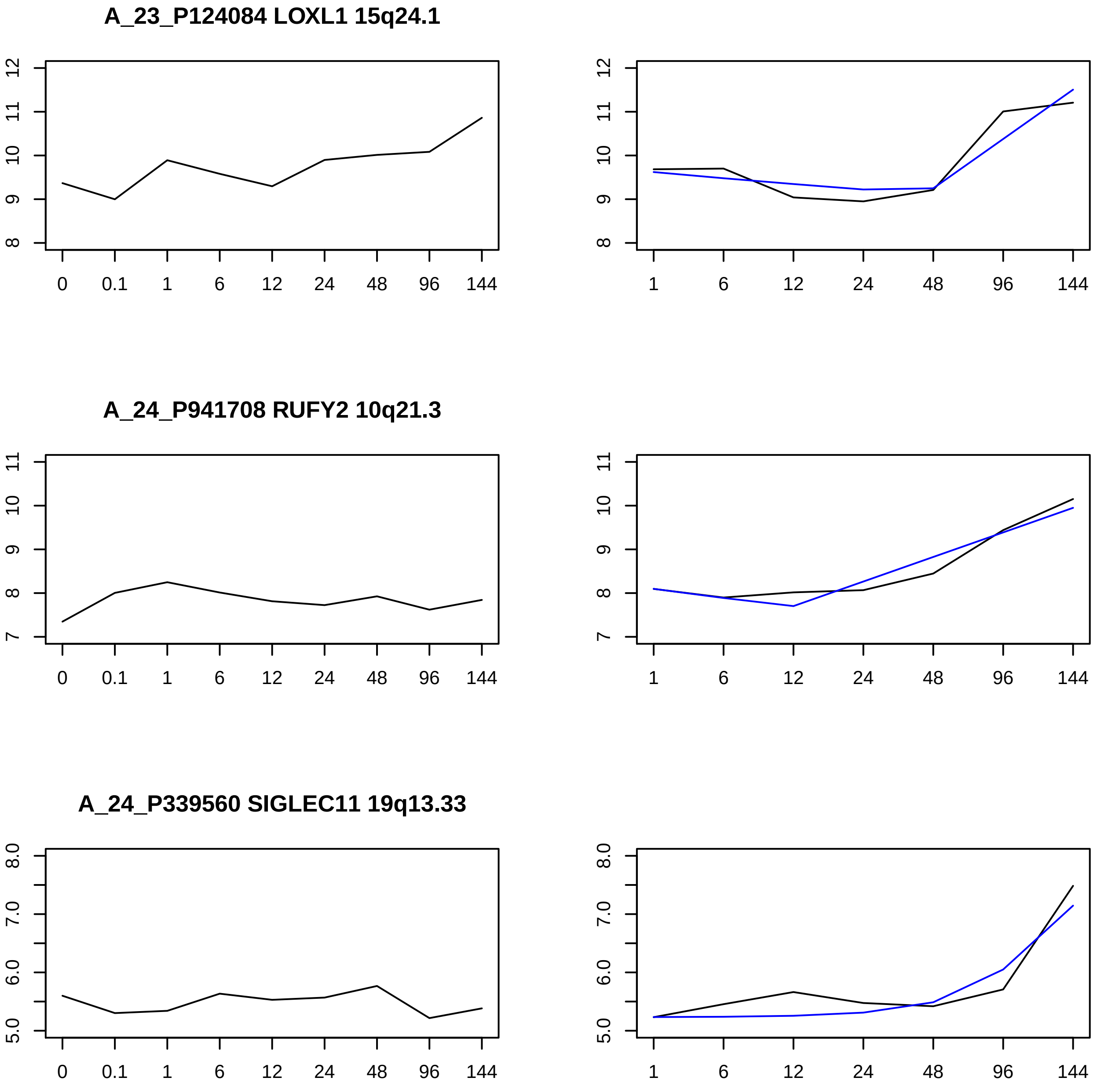

A_23_P124084 LOXL1 15q24.1
8
9
10
11
12
0
0.1
1
6
12
24
48
96
144
1
6
12
24
48
96
144
A_24_P941708 RUFY2 10q21.3
7
8
9
10
11
0
0.1
1
6
12
24
48
96
144
1
6
12
24
48
96
144
A_24_P339560 SIGLEC11 19q13.33
5.0
6.0
7.0
8.0
0
0.1
1
6
12
24
48
96
144
1
6
12
24
48
96
144

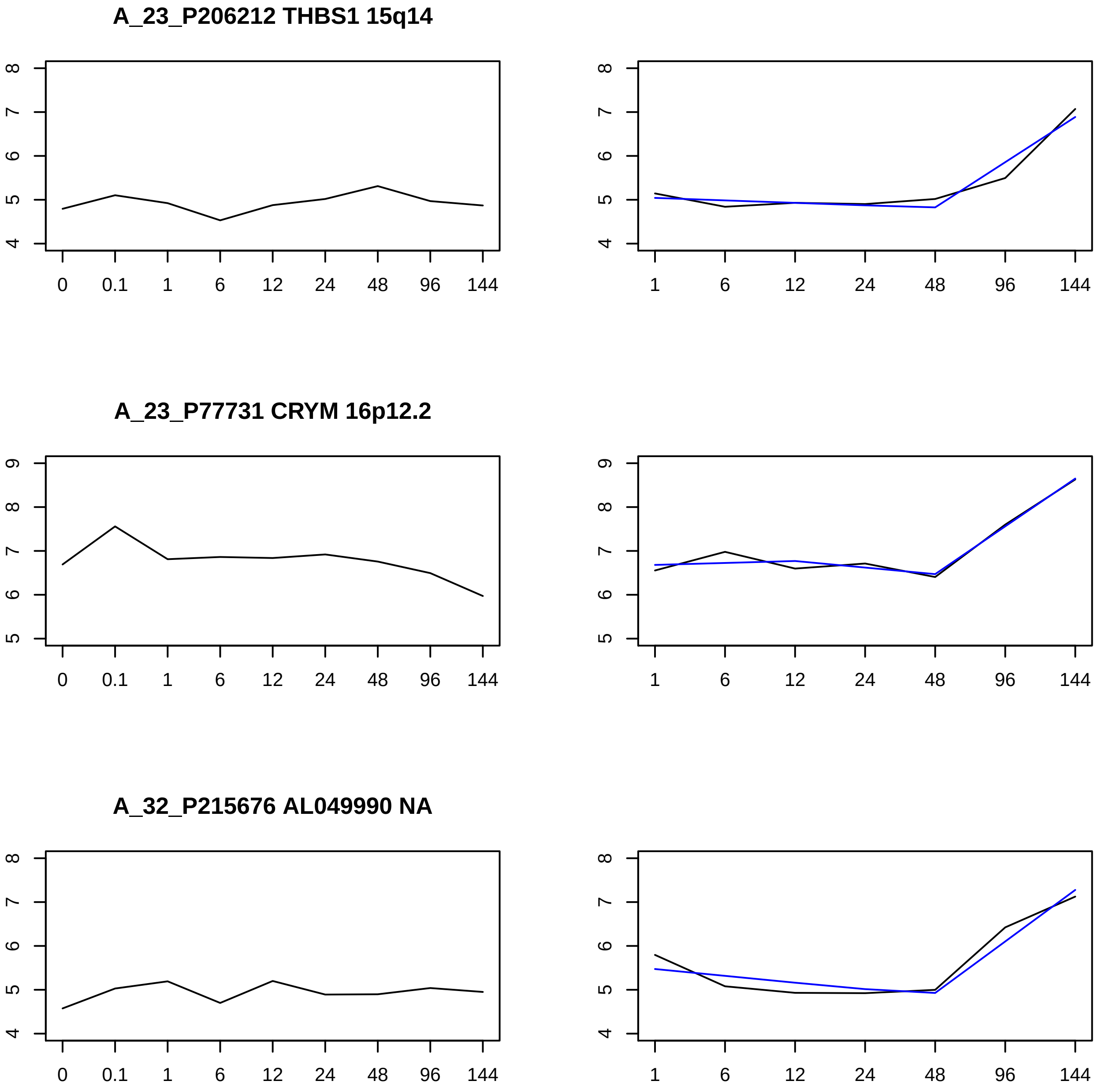
A_23_P206212 THBS1 15q14
A_23_P77731 CRYM 16p12.2
A_32_P215676 AL049990 NA

A_24_P238819 A_24_P238819 NA

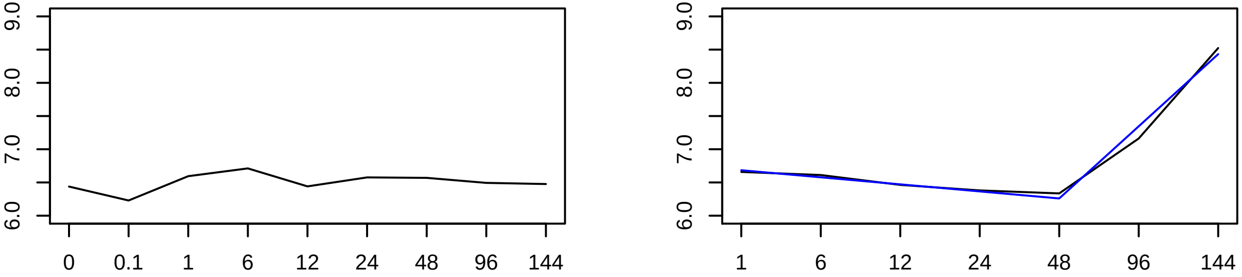

A_23_P216429 ASPN 9q22.31

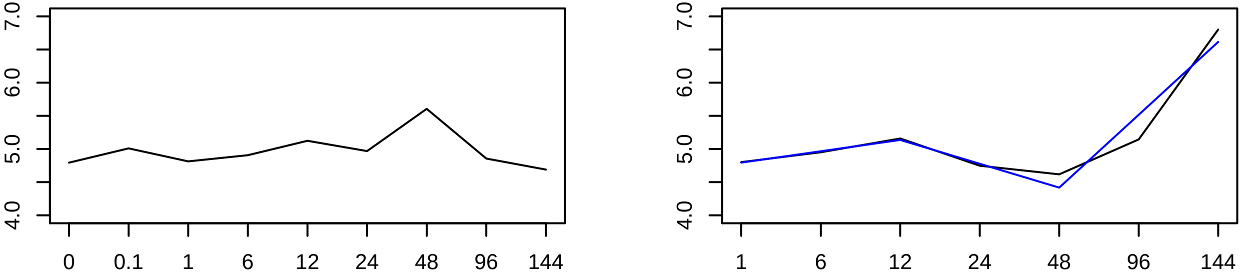

A_24_P272290 C6orf145 6p25.2

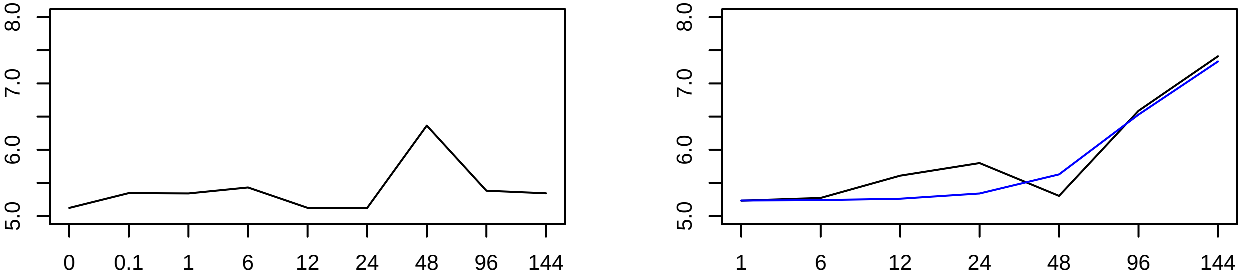

A_24_P355649 FLI1 11q24.3

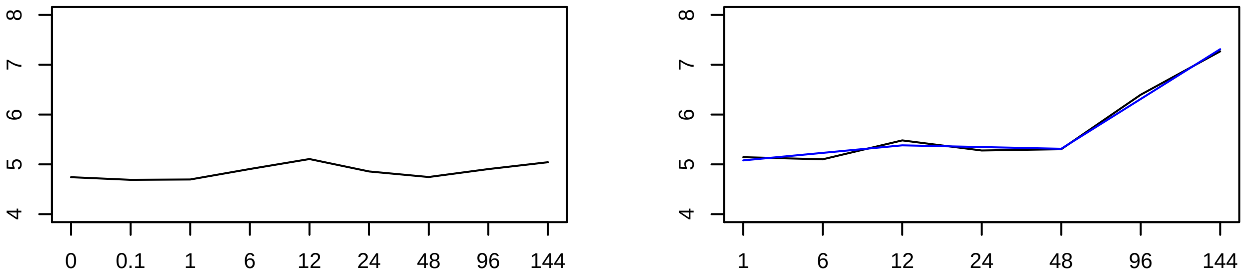

A_32_P215866 THC2661496 NA

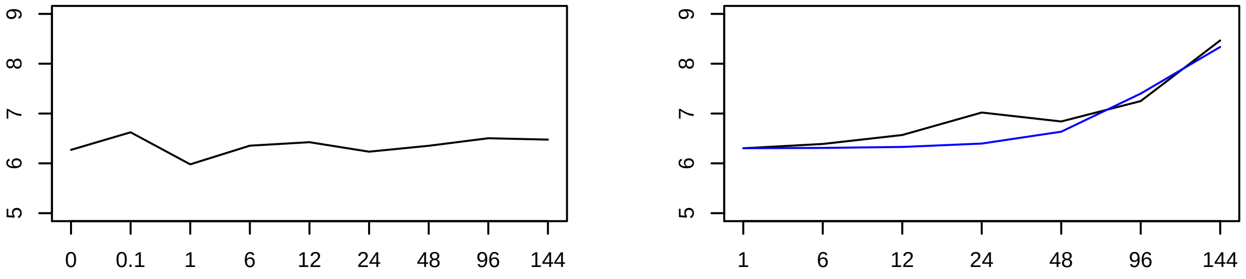

A_24_P76414 AF217970 NA

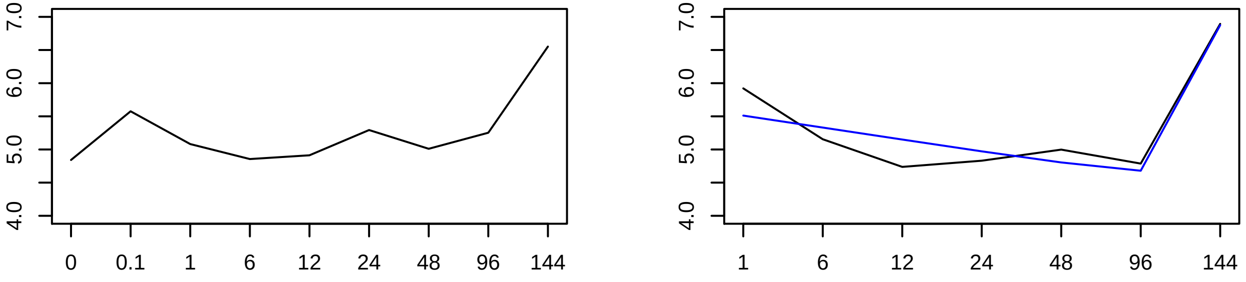

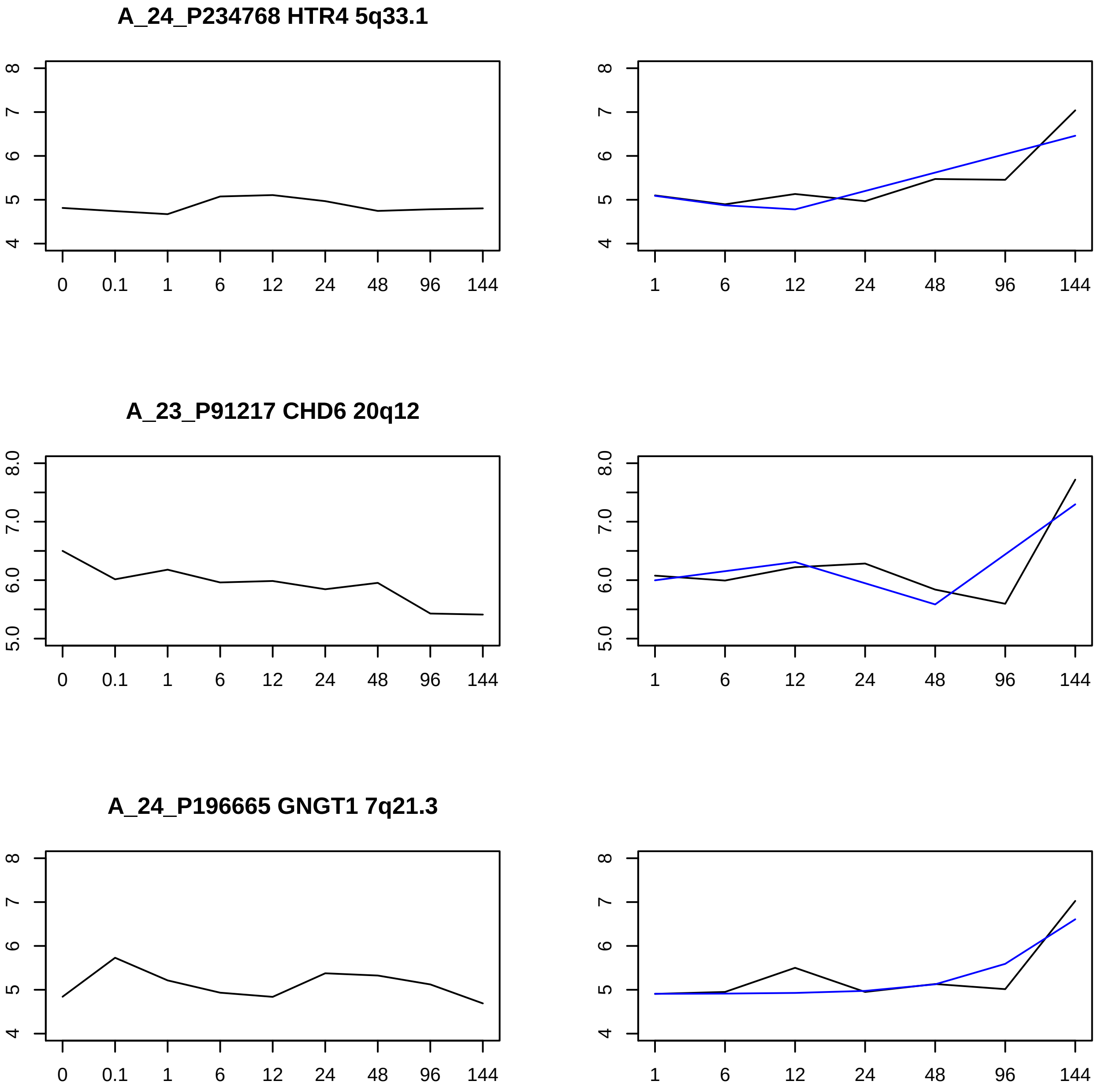
A_24_P234768 HTR4 5q33.1
A_23_P91217 CHD6 20q12
A_24_P196665 GNGT1 7q21.3
0 0.1 1 6 12 24 48 96 144
1 6 12 24 48 96 144
4 5 6 7 8
5.0 6.0 7.0 8.0

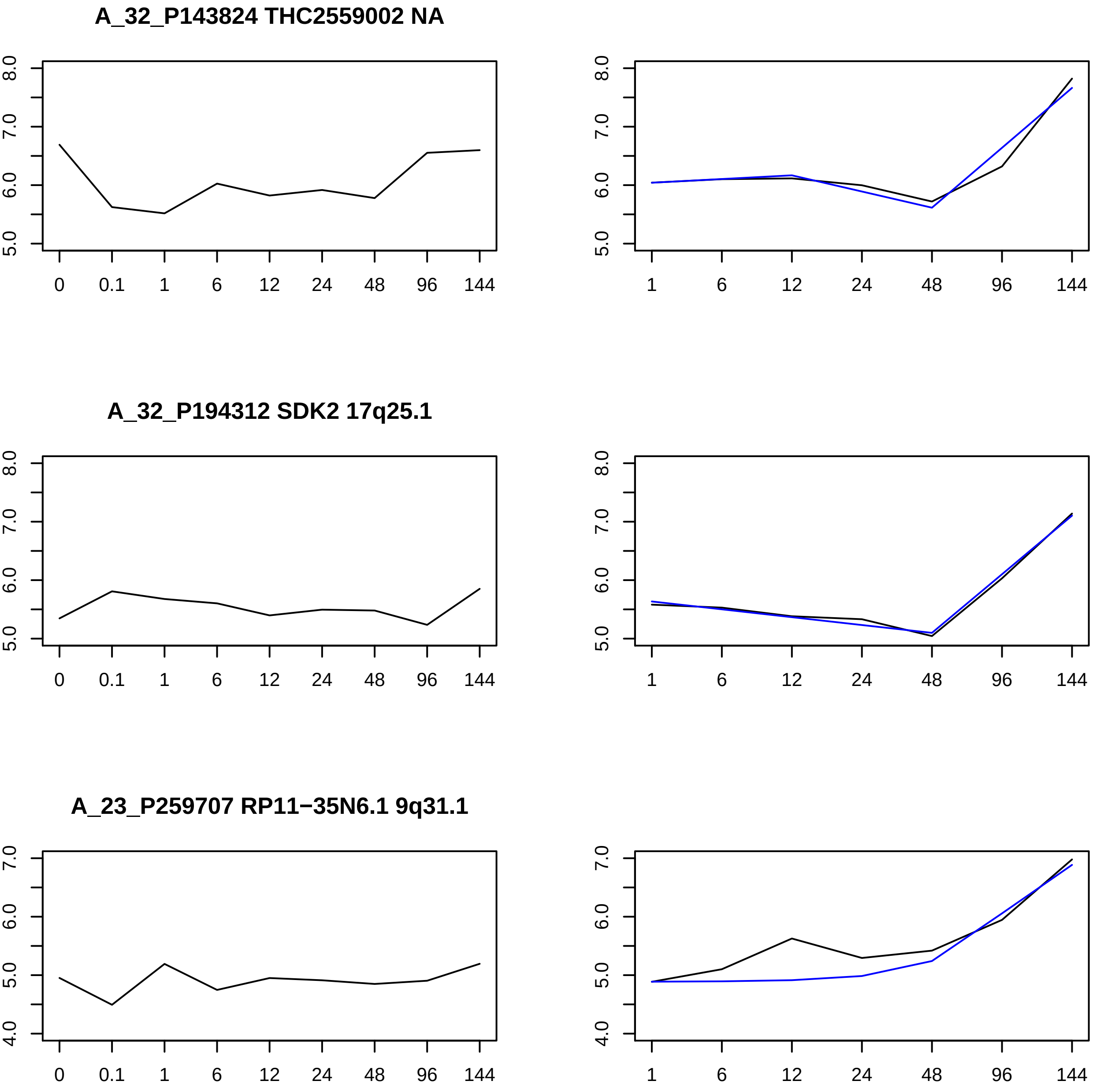
A_32_P143824 THC2559002 NA
5.0 6.0 7.0 8.0
0 0.1 1 6 12 24 48 96 144
5.0 6.0 7.0 8.0
1 6 12 24 48 96 144
A_32_P194312 SDK2 17q25.1
5.0 6.0 7.0 8.0
0 0.1 1 6 12 24 48 96 144
5.0 6.0 7.0 8.0
1 6 12 24 48 96 144
A_23_P259707 RP11−35N6.1 9q31.1
4.0 5.0 6.0 7.0
0 0.1 1 6 12 24 48 96 144
4.0 5.0 6.0 7.0
1 6 12 24 48 96 144

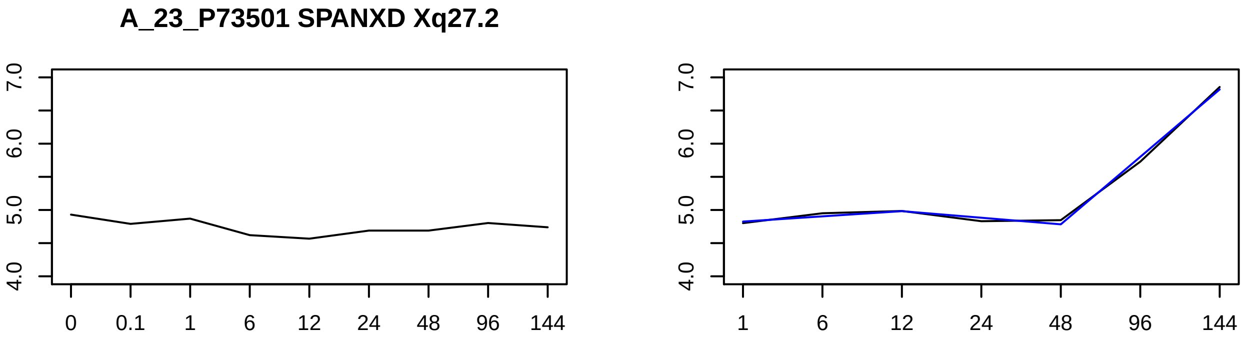
A_23_P73501 SPANXD Xq27.2
4.0 5.0 6.0 7.0
0 0.1 1 6 12 24 48 96 144
4.0 5.0 6.0 7.0
1 6 12 24 48 96 144

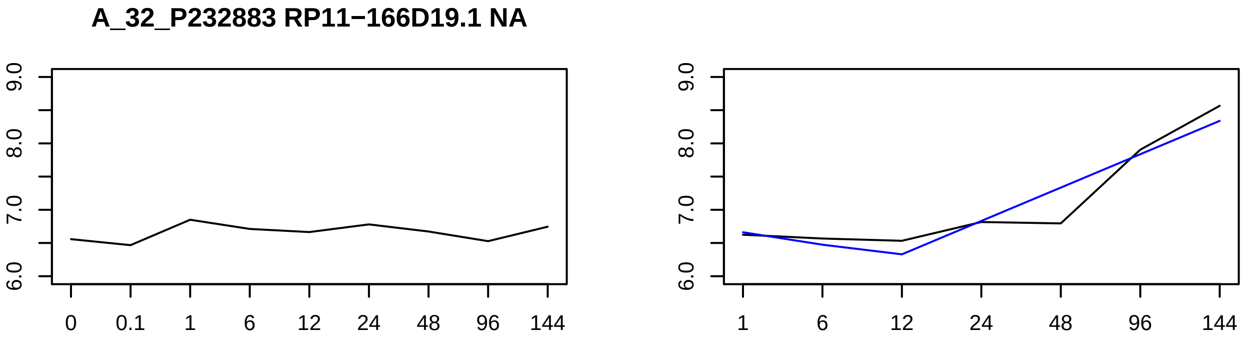
A_32_P232883 RP11−166D19.1 NA
6.0 7.0 8.0 9.0
0 0.1 1 6 12 24 48 96 144
6.0 7.0 8.0 9.0
1 6 12 24 48 96 144

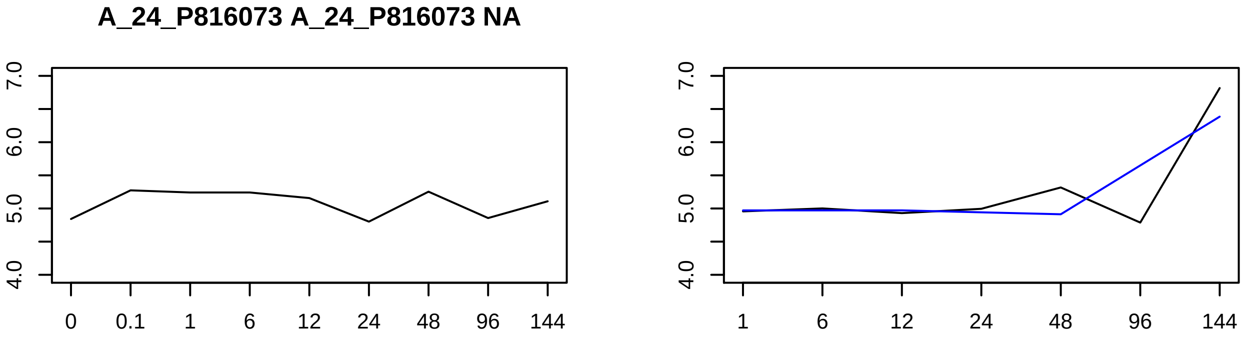
A_24_P816073 A_24_P816073 NA
4.0 5.0 6.0 7.0
0 0.1 1 6 12 24 48 96 144
4.0 5.0 6.0 7.0
1 6 12 24 48 96 144

A_23_P2543 CUTL2 12q24.12

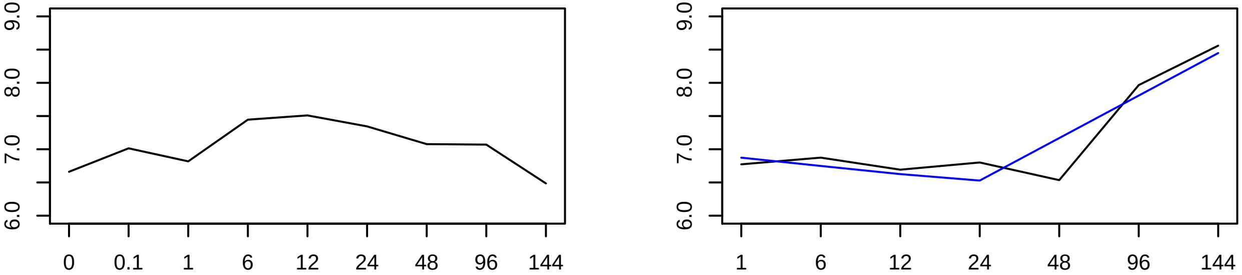

A_32_P29083 AK000992 NA

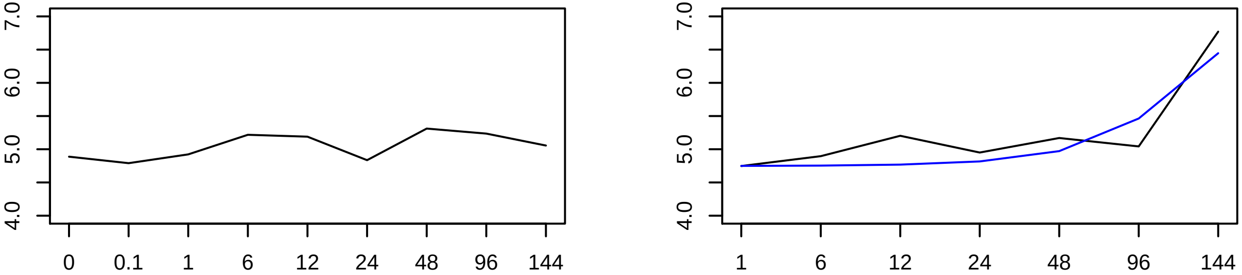

A_23_P96383 SRPX Xp11.4

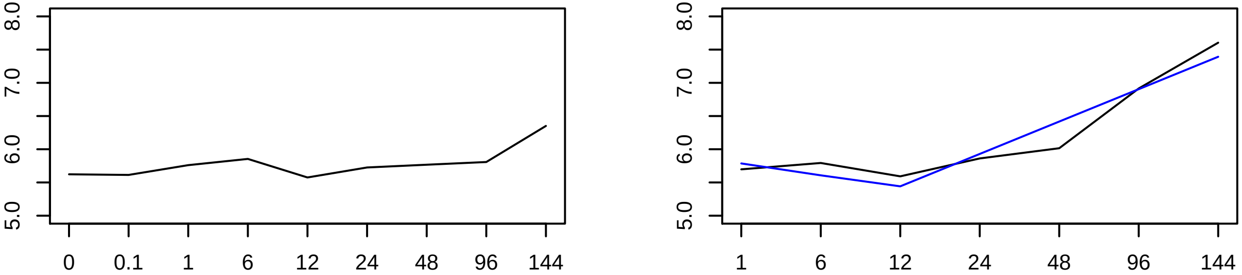

A_32_P128741 LOC723809 7q22.1

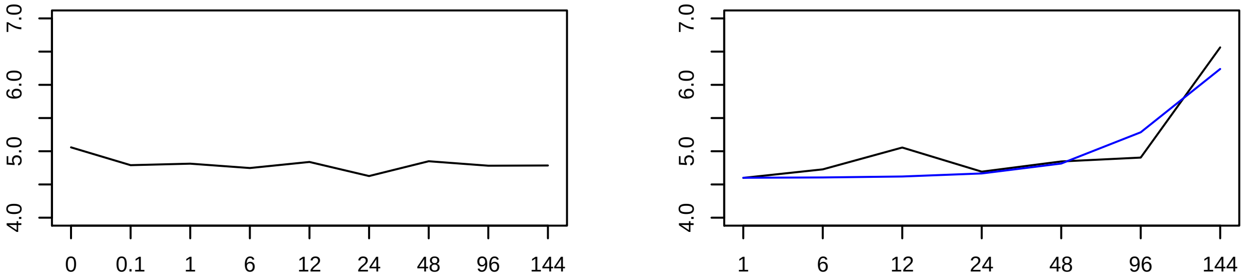

A_23_P88069 LHFP 13q13.3

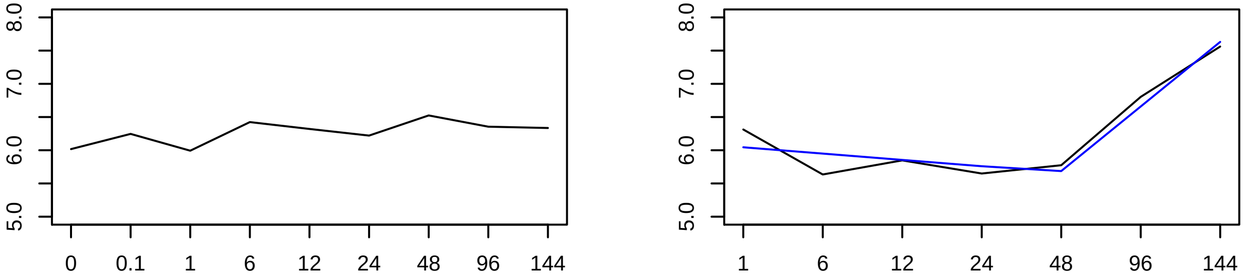

A_23_P399078 TIMP3 22q12.3

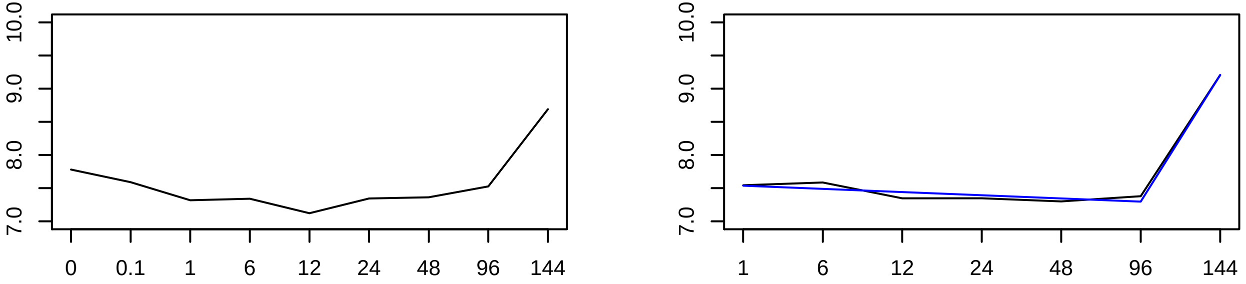

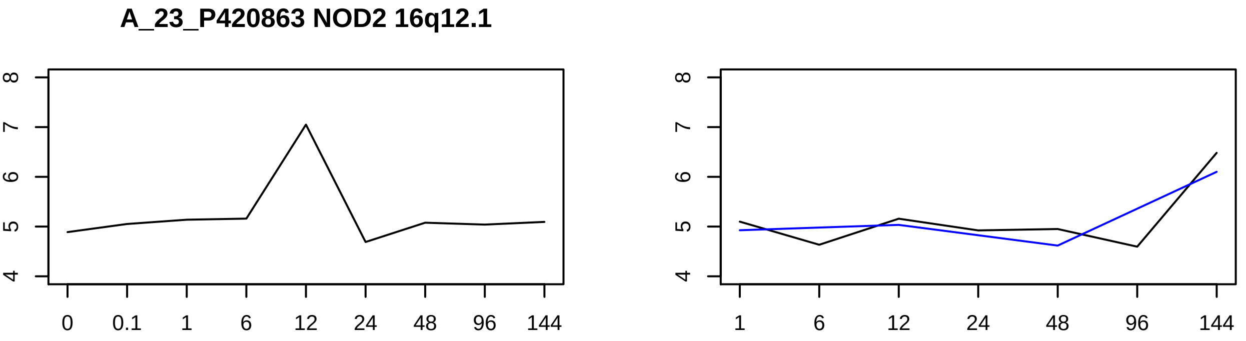
A_23_P420863 NOD2 16q12.1
4
5
6
7
8
0
0.1
1
6
12
24
48
96
144
1
6
12
24
48
96
144

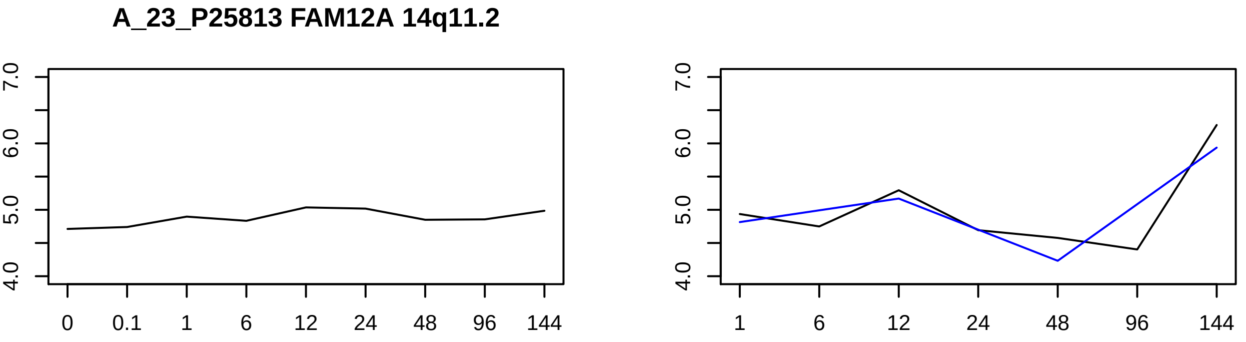
A_23_P25813 FAM12A 14q11.2
4.0
5.0
6.0
7.0
0
0.1
1
6
12
24
48
96
144
1
6
12
24
48
96
144

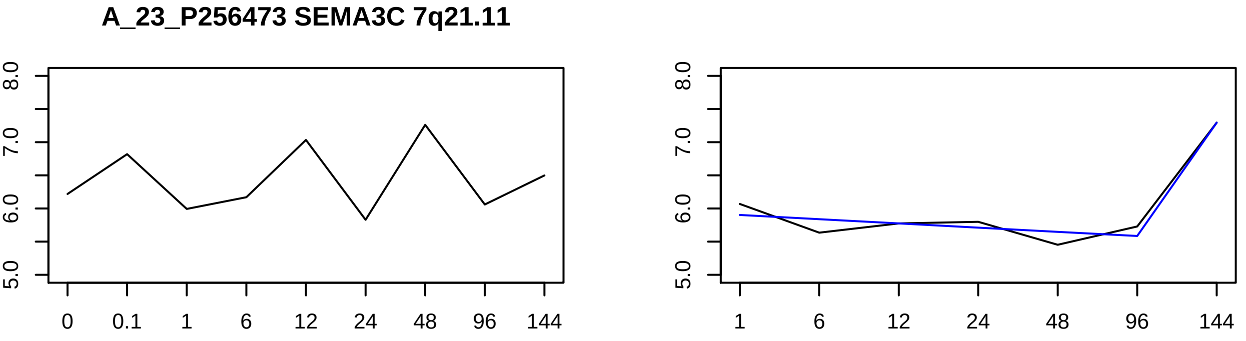
A_23_P256473 SEMA3C 7q21.11
5.0
6.0
7.0
8.0
0
0.1
1
6
12
24
48
96
144
1
6
12
24
48
96
144

A_23_P10127 SFRP1 8p11.21

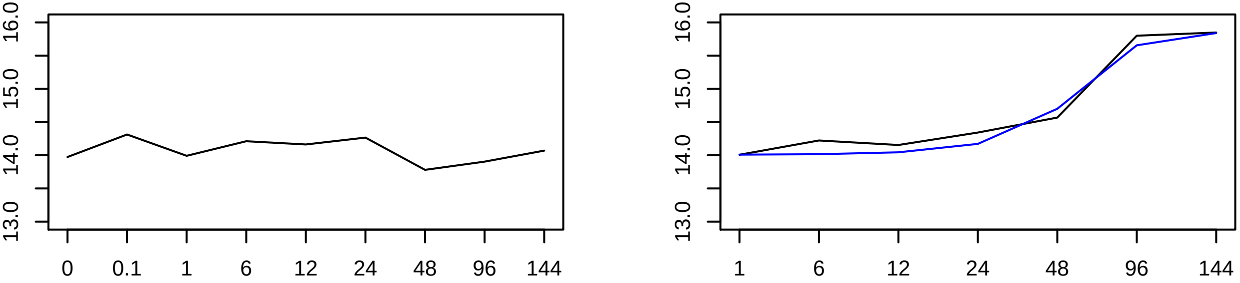

A_23_P216501 TPM2 9p13.3

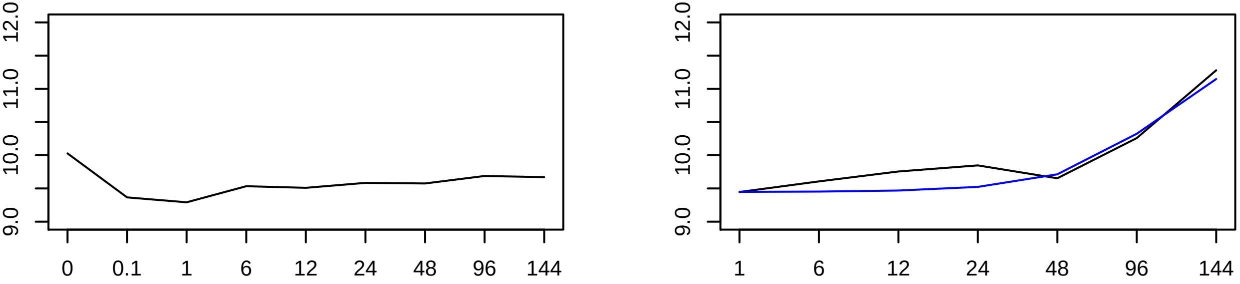

A_23_P19020 SNCAIP 5q23.2

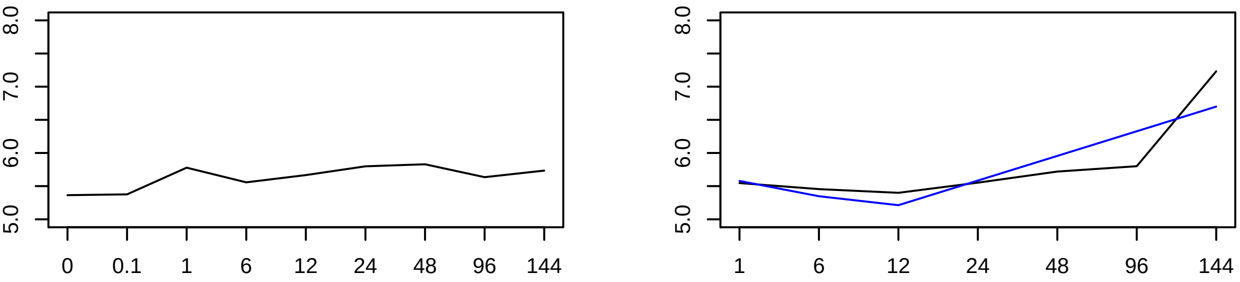

A_24_P508103 FLJ30428 2q21.1

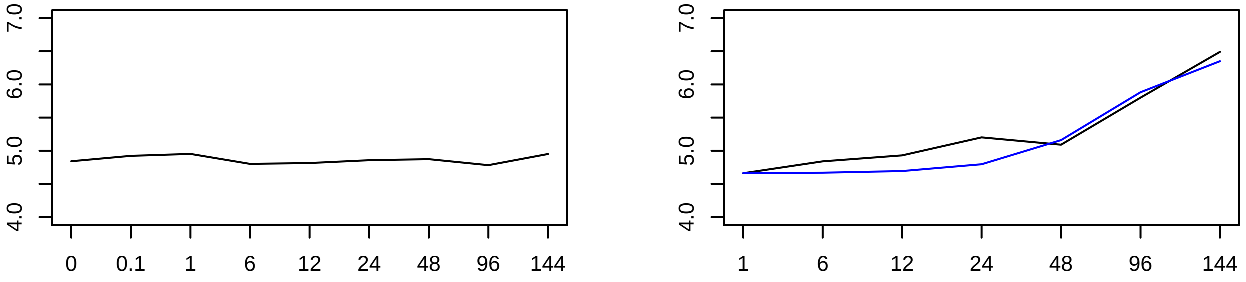

A_24_P489649 LOC131572 3p24.1

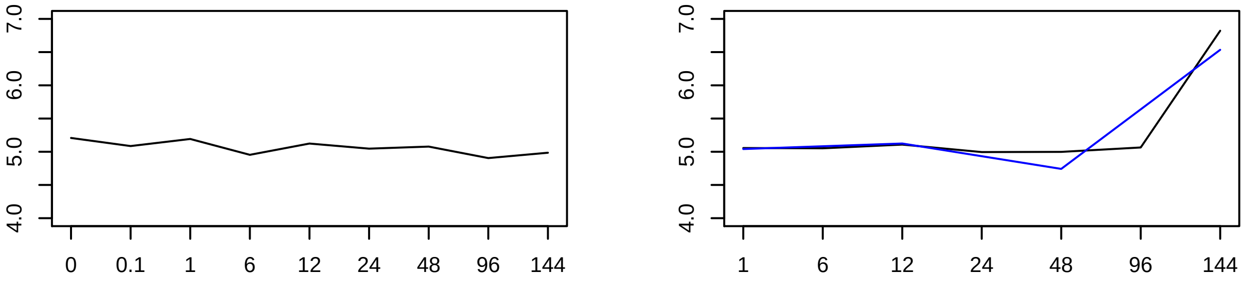

A_32_P39944 LOC399959 11q24.1

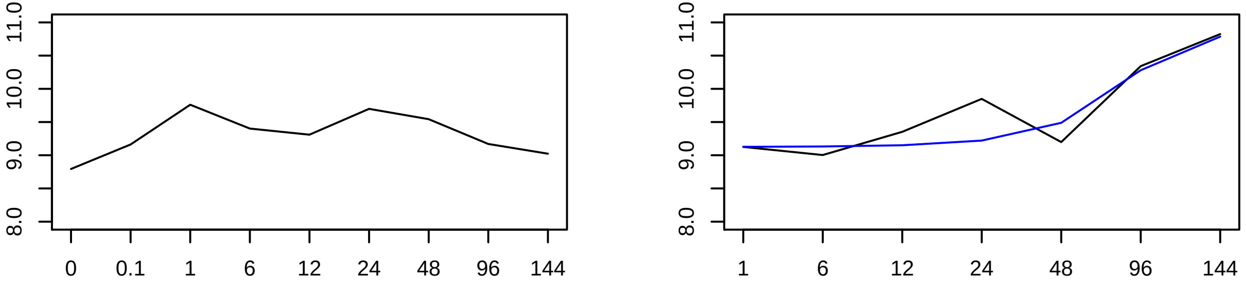

A_24_P360674 CDKN2B 9p21.3

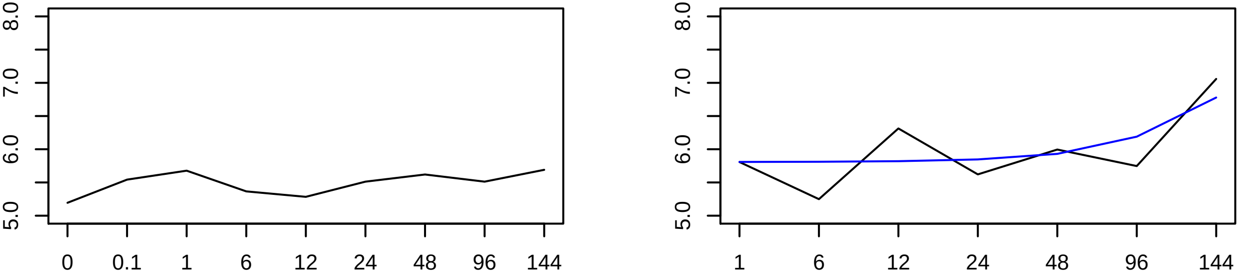

A_24_P252310 KIAA0773 7q34

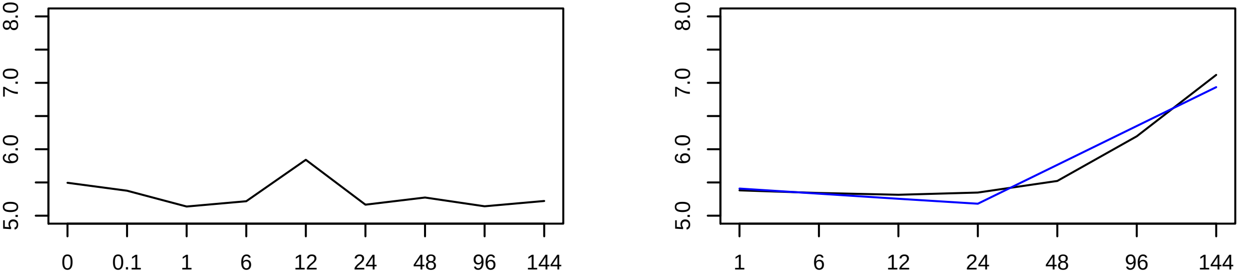

A_24_P929678 A_24_P929678 NA

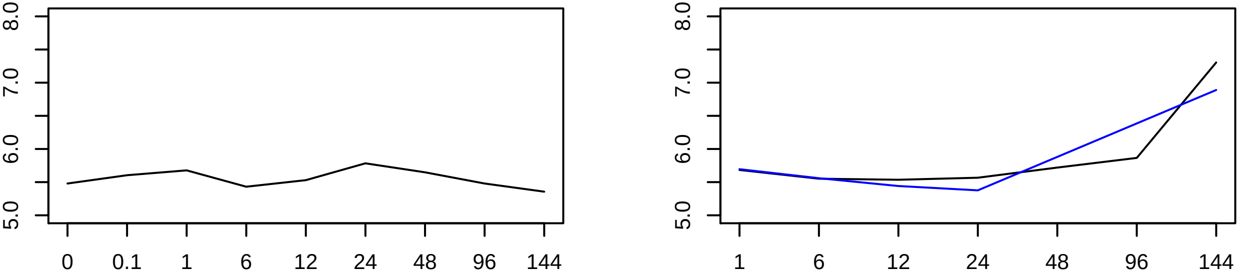

A_32_P127339 THC2550353 NA

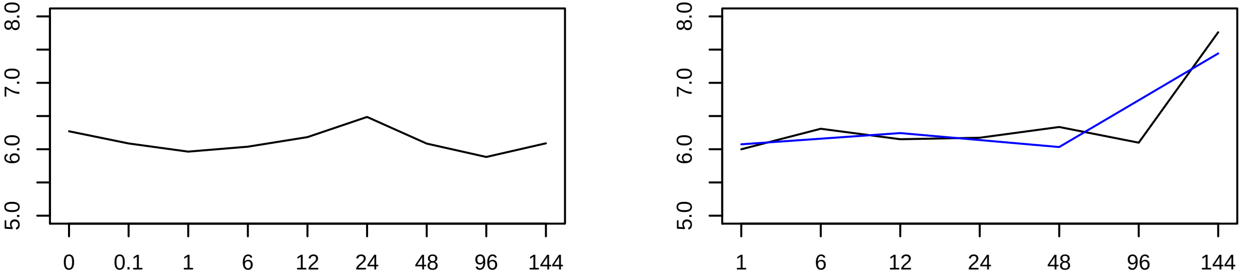

A_24_P165205 MORN1 1p36.33

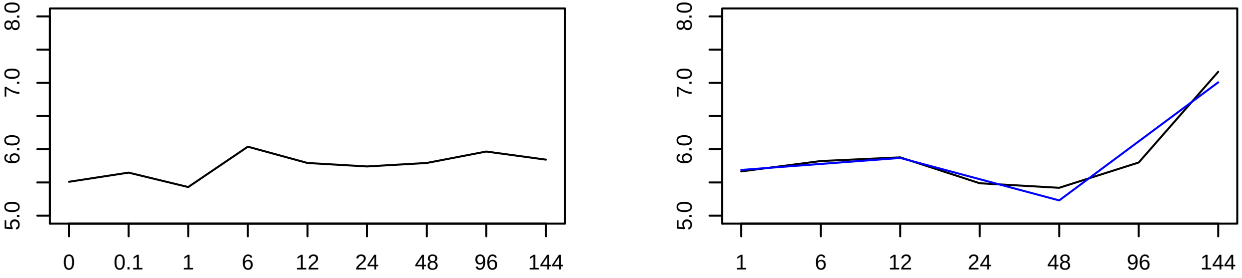

A_23_P19102 GALNT10 5q33.2

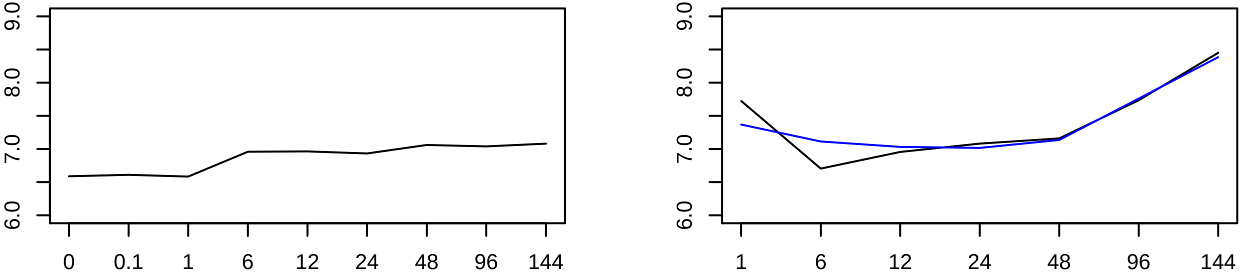

A_24_P132019 LNPEP 5q15

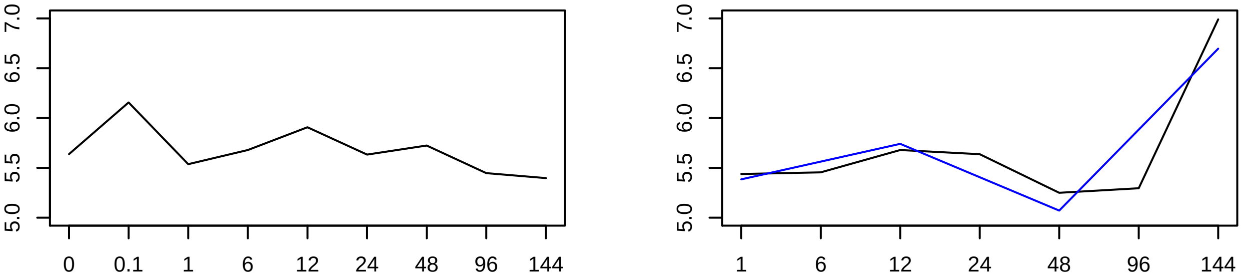

A_23_P23584 CTNNBIP1 1p36.22

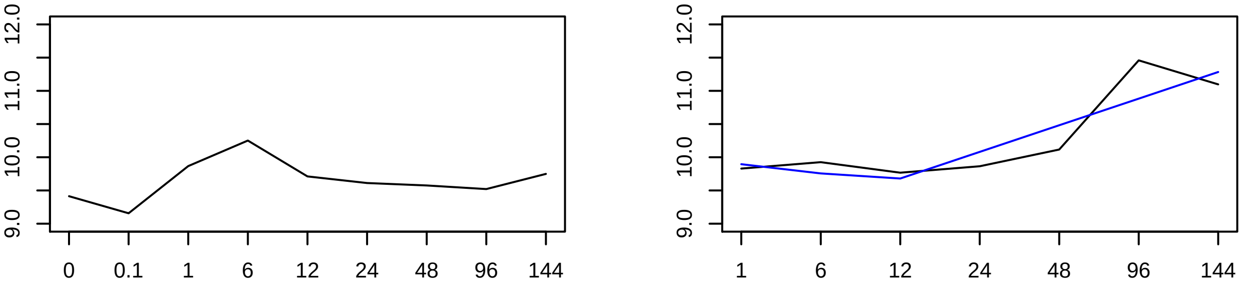

A_23_P153971 SGPP2 2q36.1

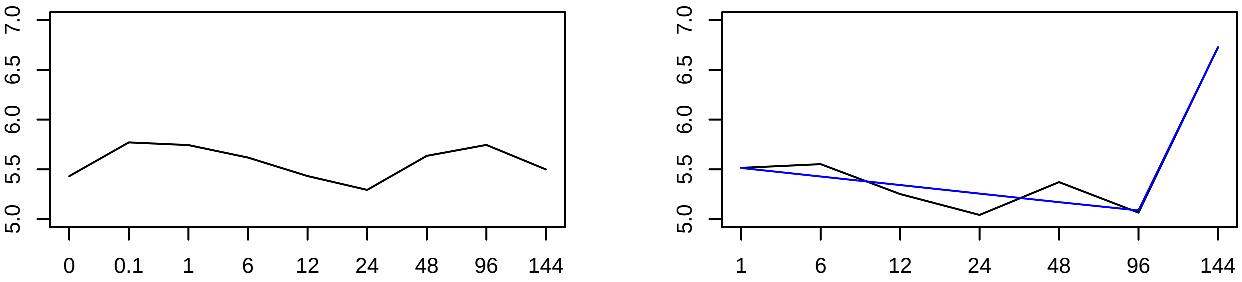

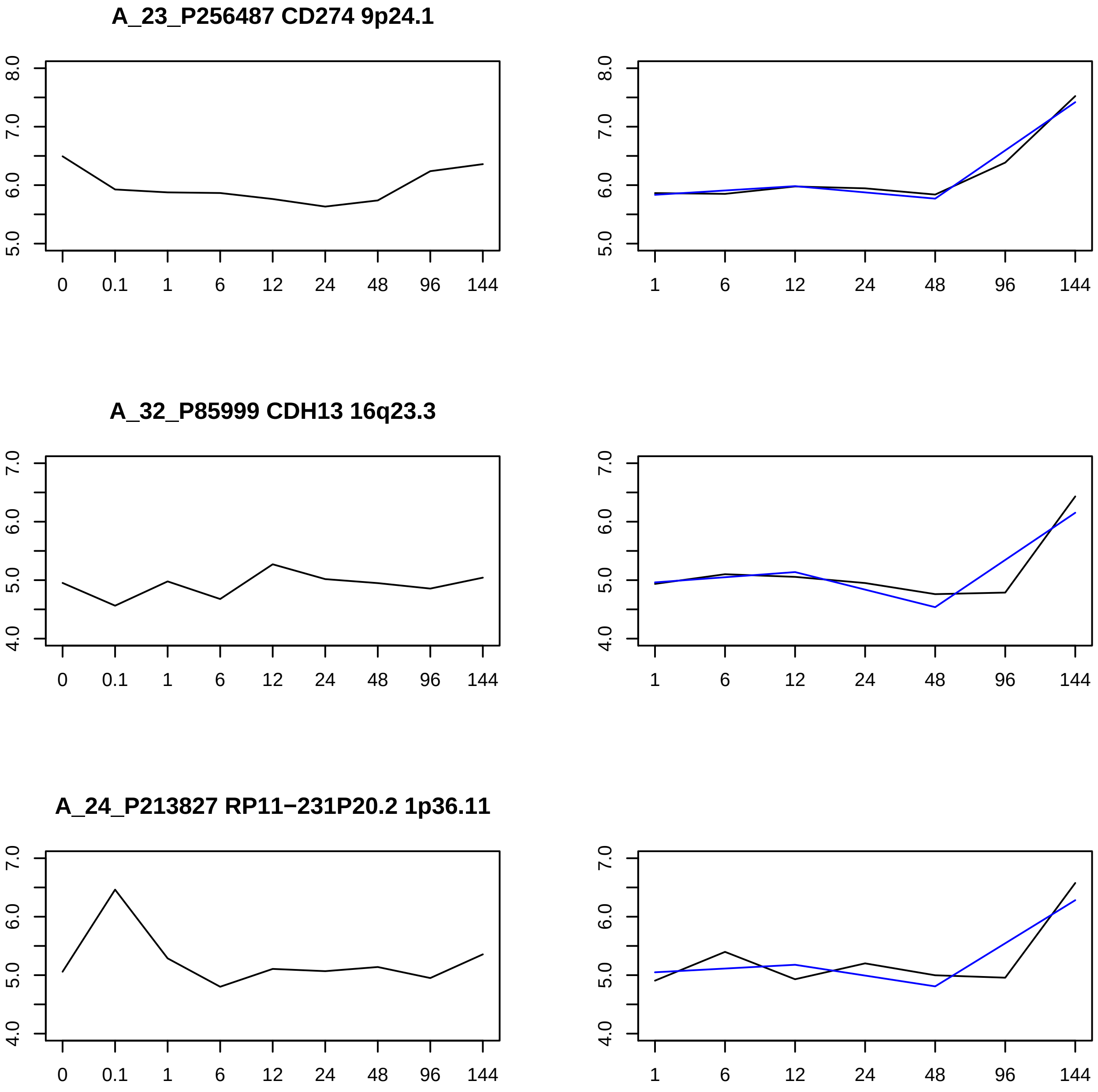
A_23_P256487 CD274 9p24.1
5.0
6.0
7.0
8.0
0
0.1
1
6
12
24
48
96
144
1
6
12
24
48
96
144
A_32_P85999 CDH13 16q23.3
4.0
5.0
6.0
7.0
A_24_P213827 RP11−231P20.2 1p36.11

A_23_P26583 NLRC5 16q13

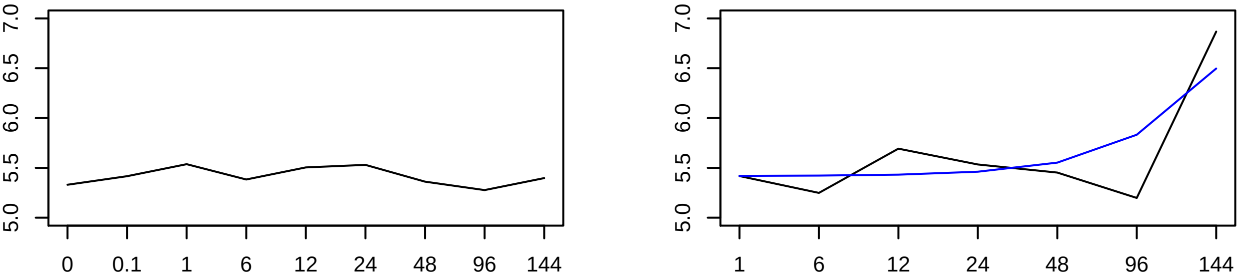

A_24_P758010 AJ318805 NA

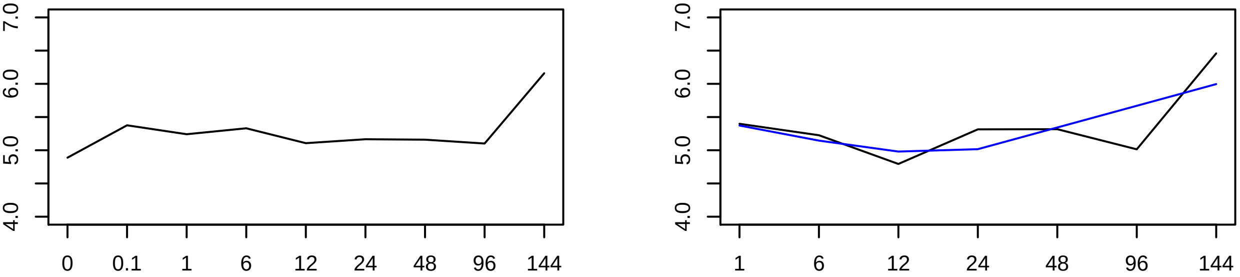

A_32_P161855 KIAA1199 15q25.1

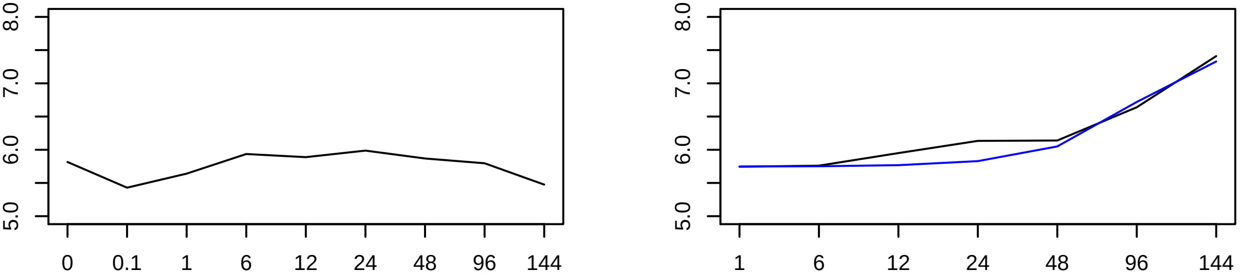

A_23_P76322 PIK3C2G 12p12.3

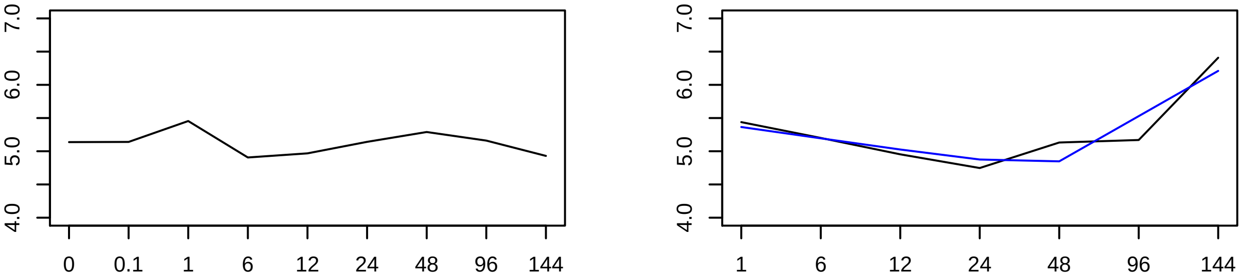

A_23_P252671 CLTB 5q35.2

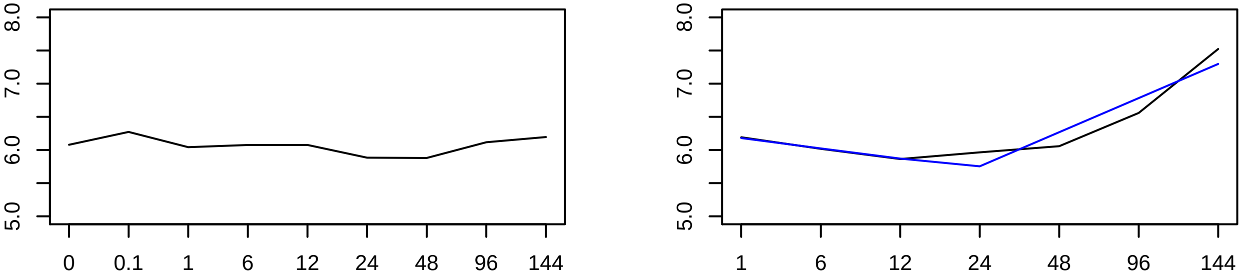

A_23_P117882 CRABP1 15q25.1

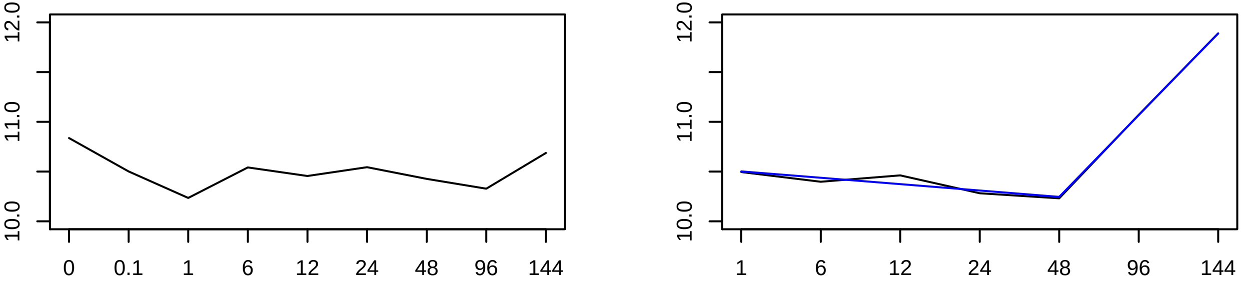

A_32_P146890 THC2695809 NA

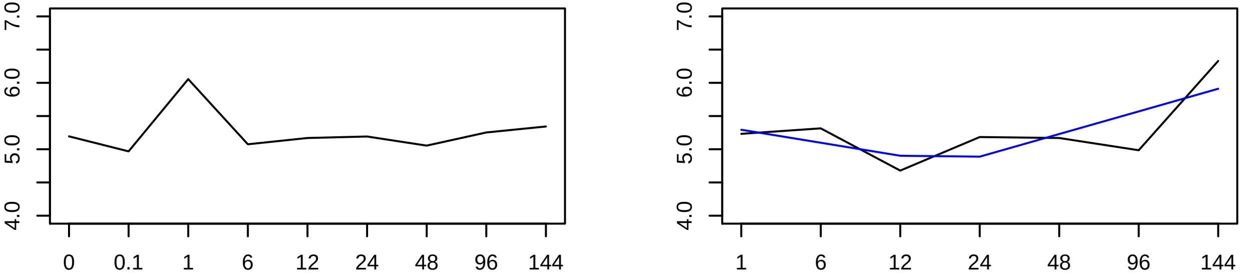

A_23_P406025 KIAA0367 9q21.13

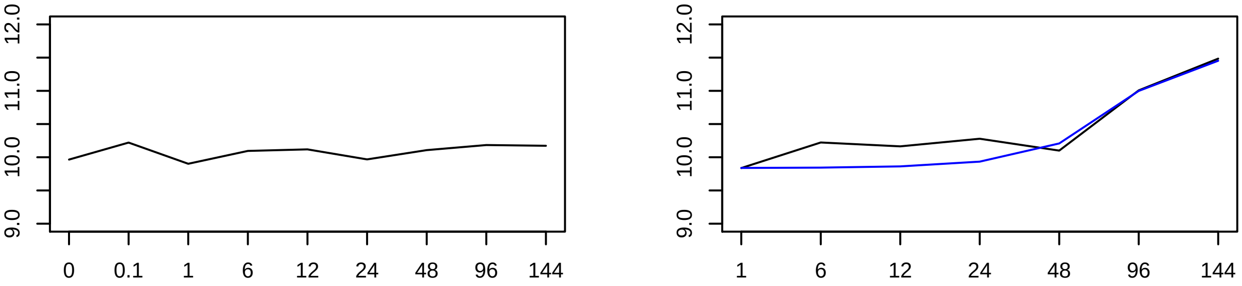

A_24_P935647 A_24_P935647 NA

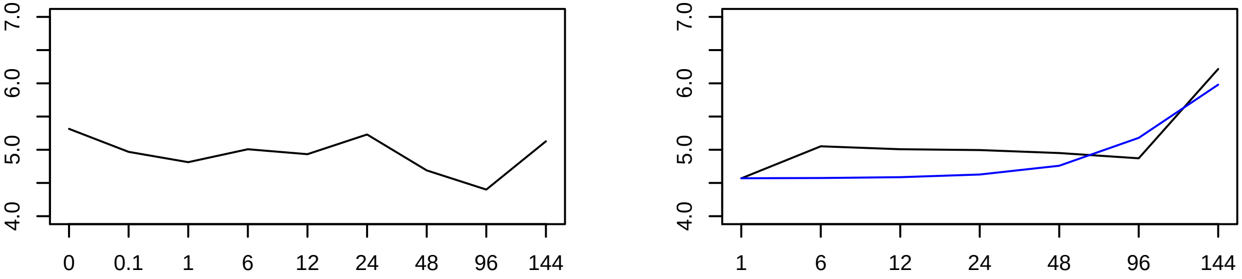

A_23_P397248 CLCA2 1p22.3

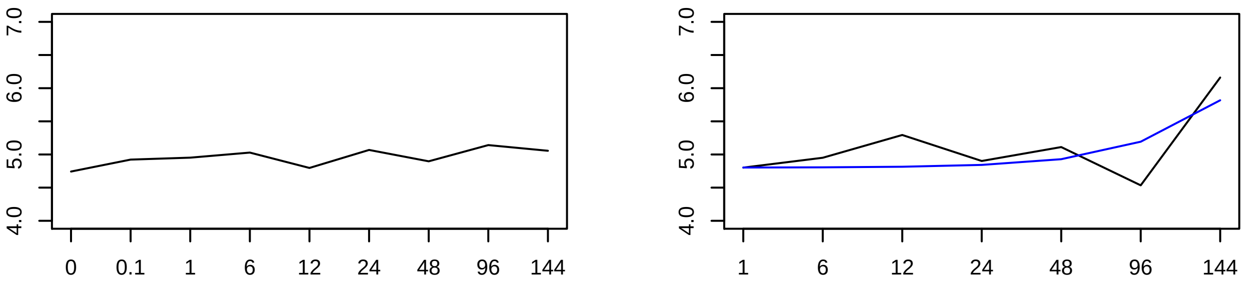

A_23_P110569 TRIM36 5q22.3

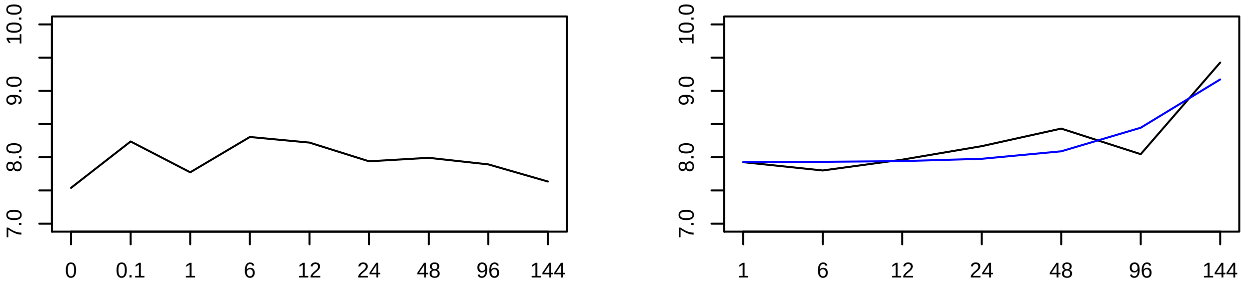

A_24_P398370 PCSK7 NA

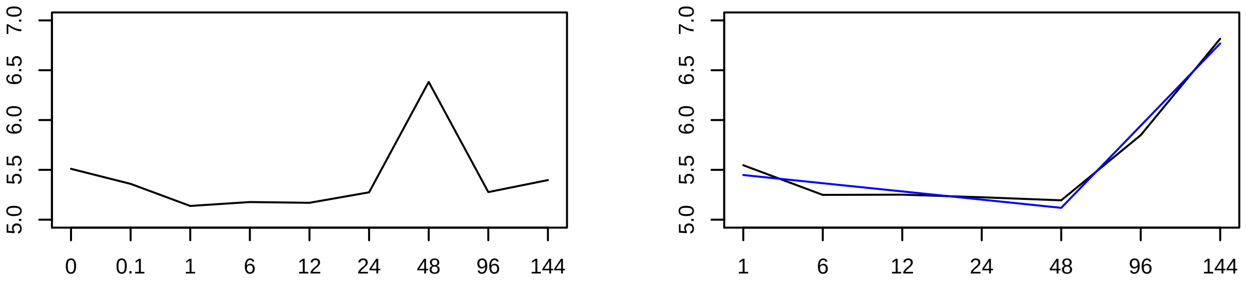

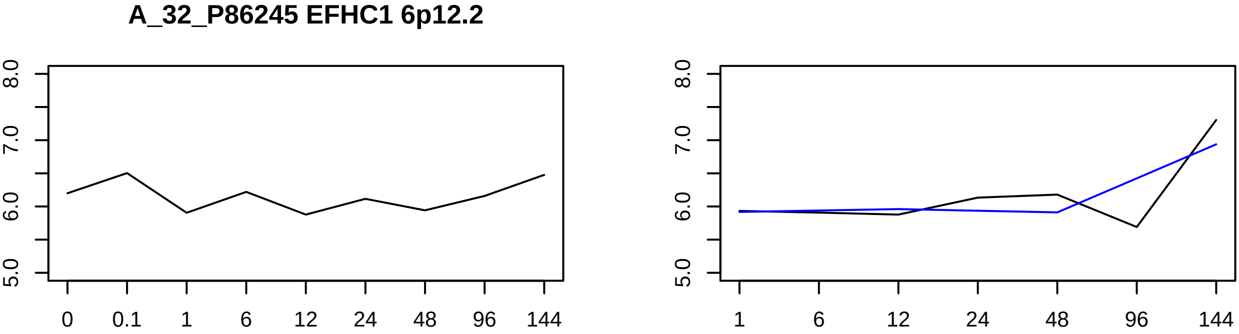
A_32_P86245 EFHC1 6p12.2
5.0
6.0
7.0
8.0
0
0.1
1
6
12
24
48
96
144
1
6
12
24
48
96
144

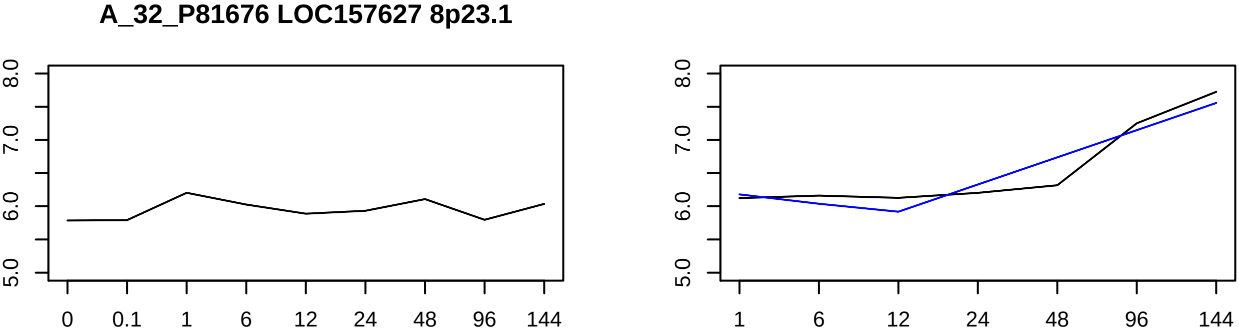
A_32_P81676 LOC157627 8p23.1
5.0
6.0
7.0
8.0
0
0.1
1
6
12
24
48
96
144
1
6
12
24
48
96
144

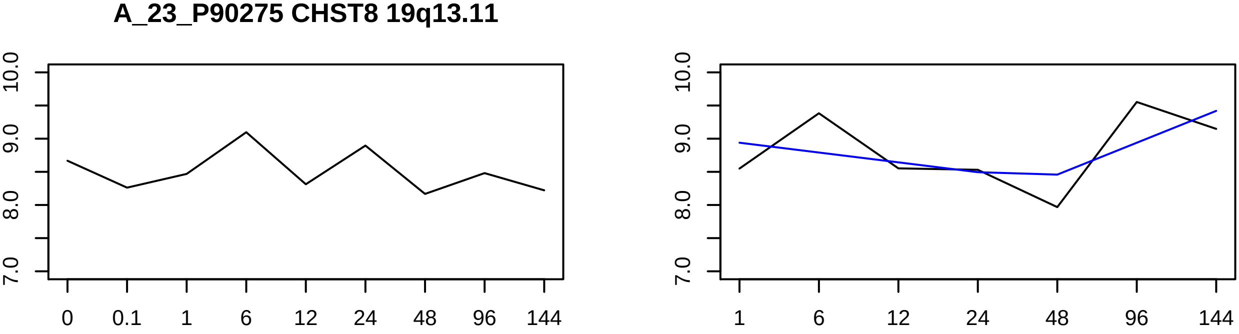
A_23_P90275 CHST8 19q13.11
7.0
8.0
9.0
10.0
0
0.1
1
6
12
24
48
96
144
1
6
12
24
48
96
144

A_24_P100613 LAMA1 18p11.31

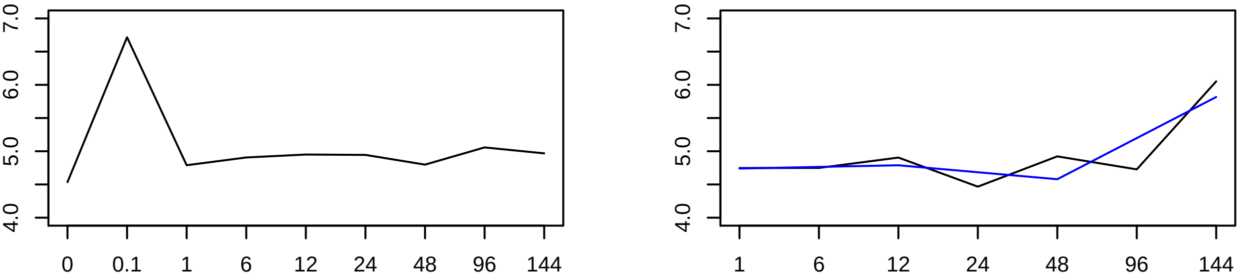

A_24_P293530 CYP4X1 1p33

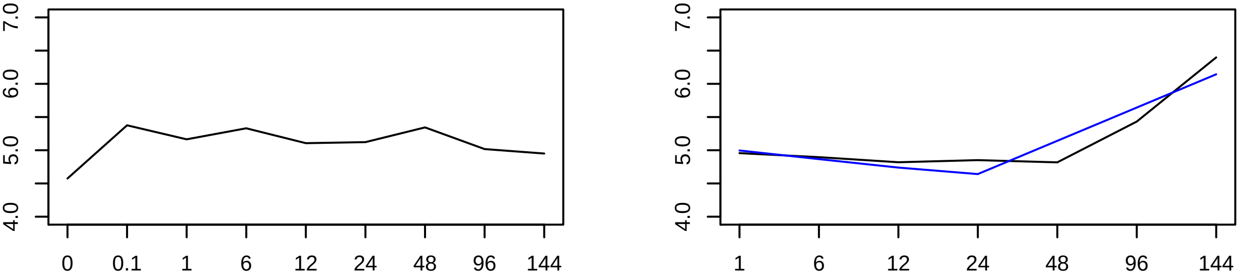

A_32_P140489 GDF6 8q22.1

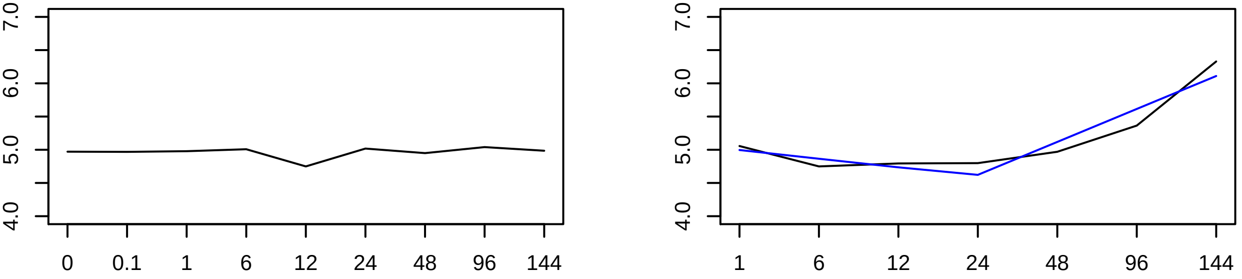

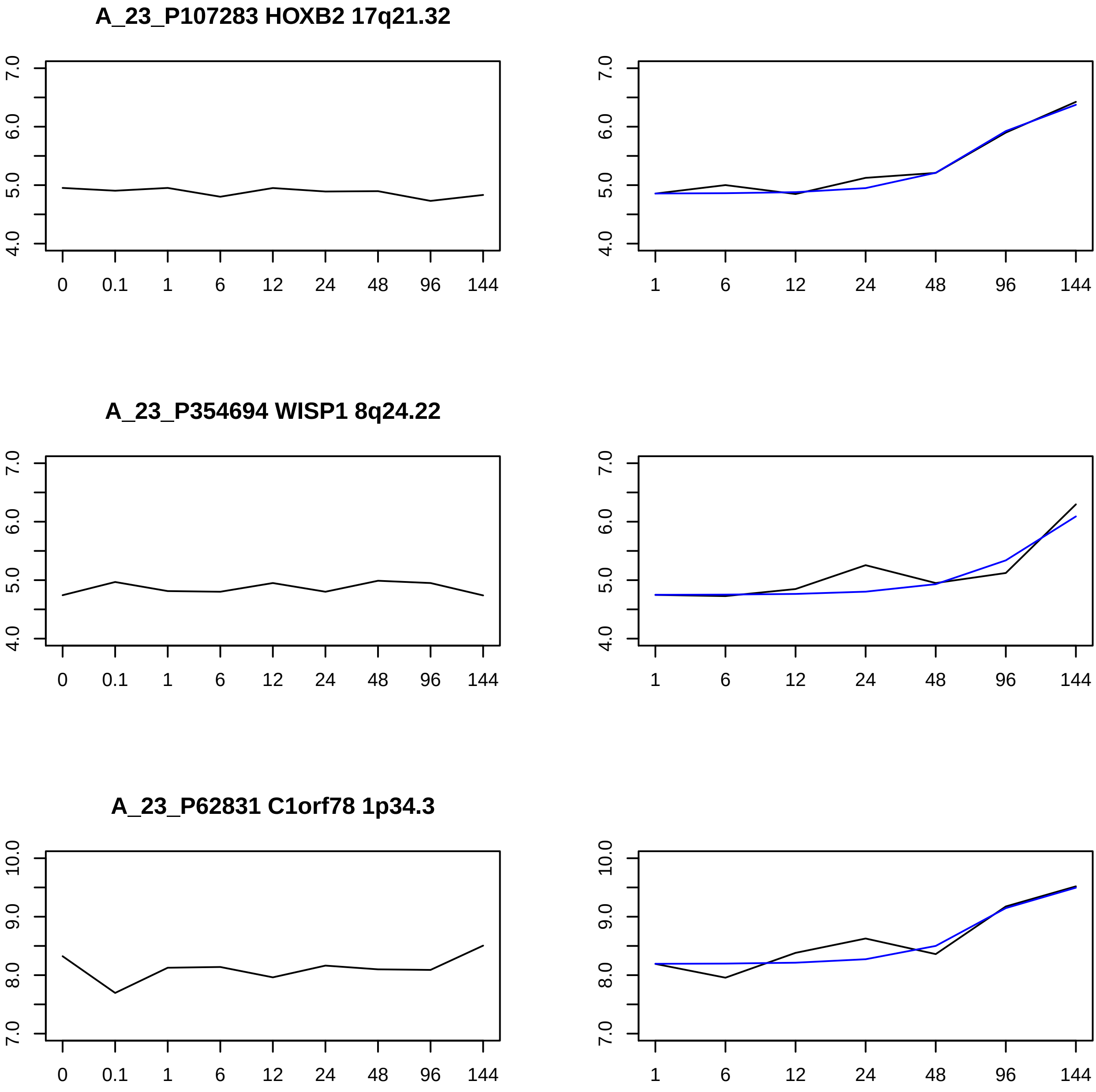
A_23_P107283 HOXB2 17q21.32
A_23_P354694 WISP1 8q24.22
A_23_P62831 C1orf78 1p34.3
4.0
5.0
6.0
7.0
7.0
8.0
9.0
10.0
0
0.1
1
6
12
24
48
96
144

A_32_P203046 AK098548 NA

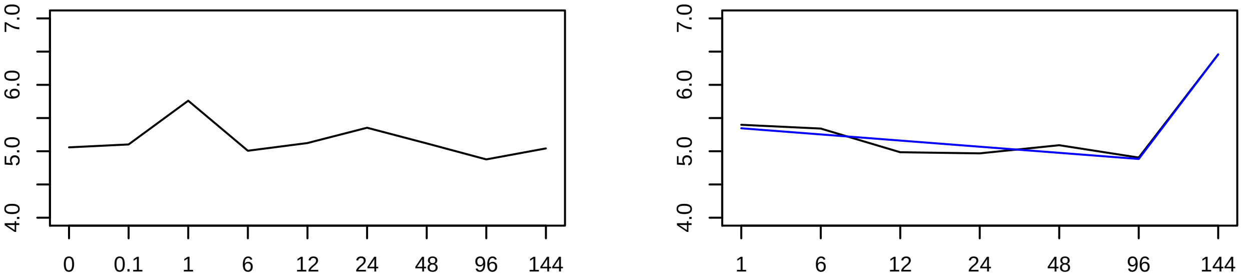

A_23_P3911 PLXDC1 17q12

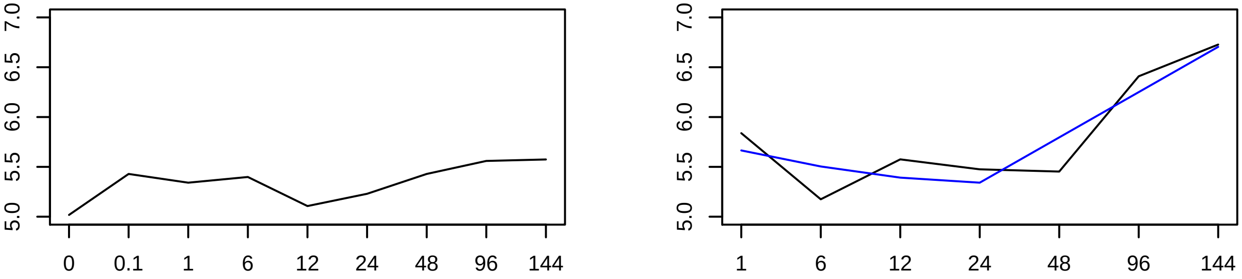

A_23_P51487 GBP3 1p22.2

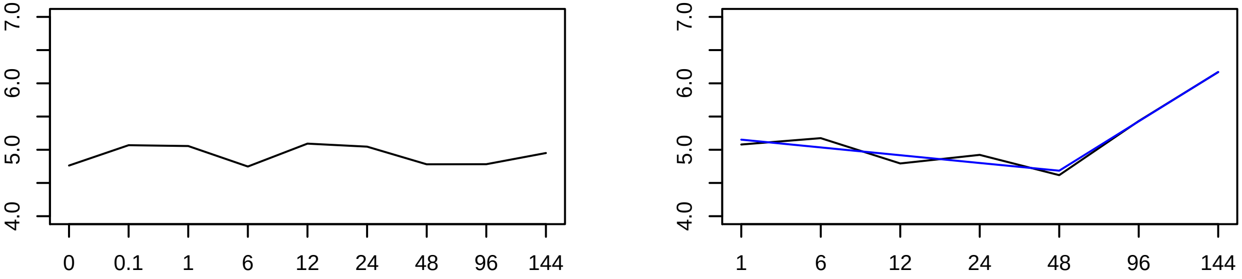

A_24_P717609 A_24_P717609 NA

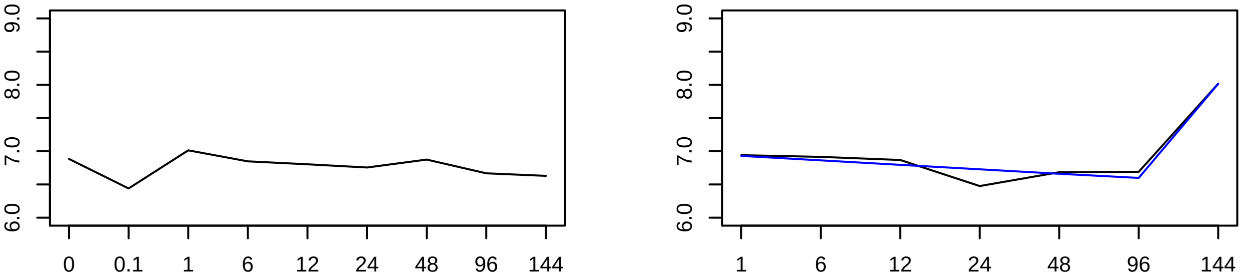

A_23_P20494 NDRG1 8q24.22

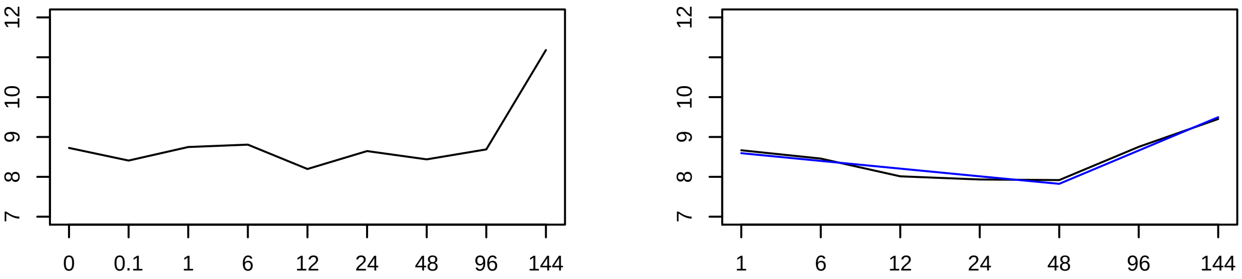

A_24_P185854 DMD Xp21.2

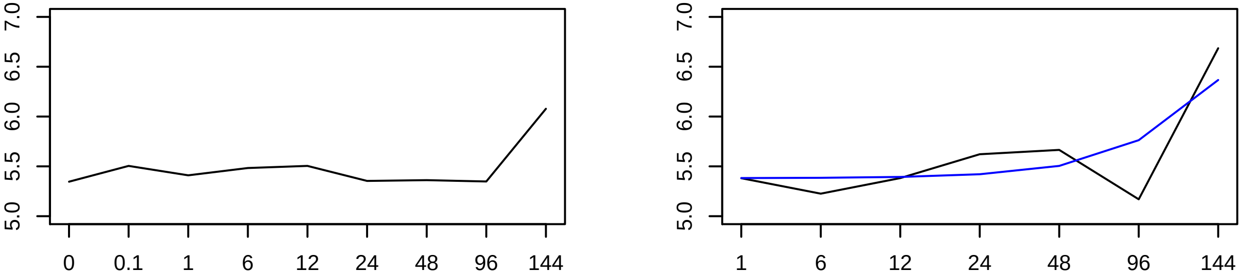

A_24_P222291 ABCC4 13q32.1

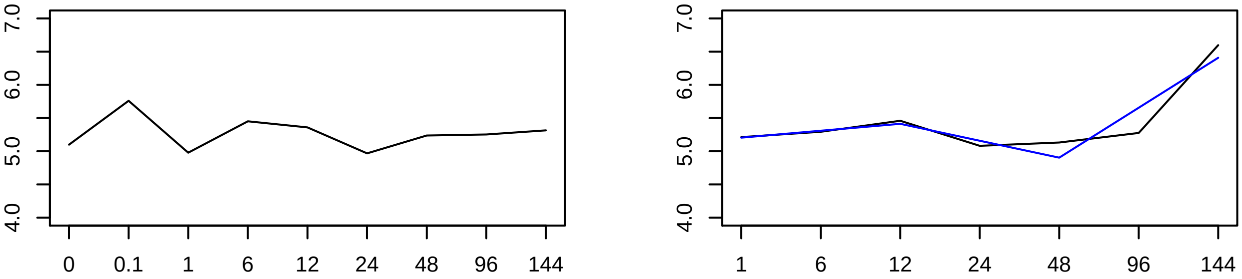

A_23_P385017 G6PC 17q21.31

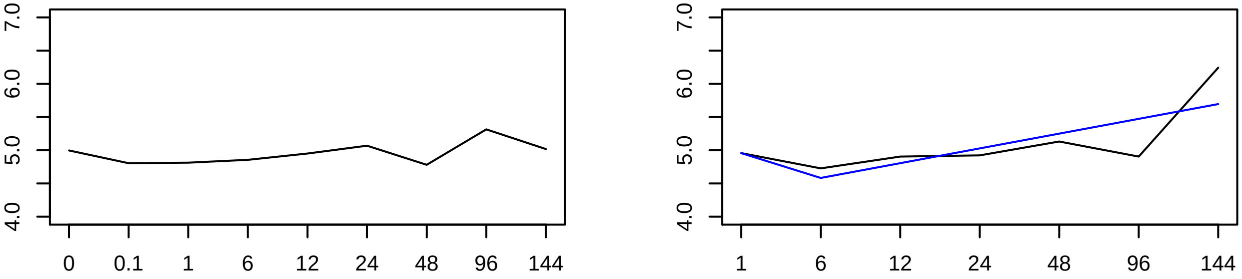

A_24_P545030 BC042091 NA

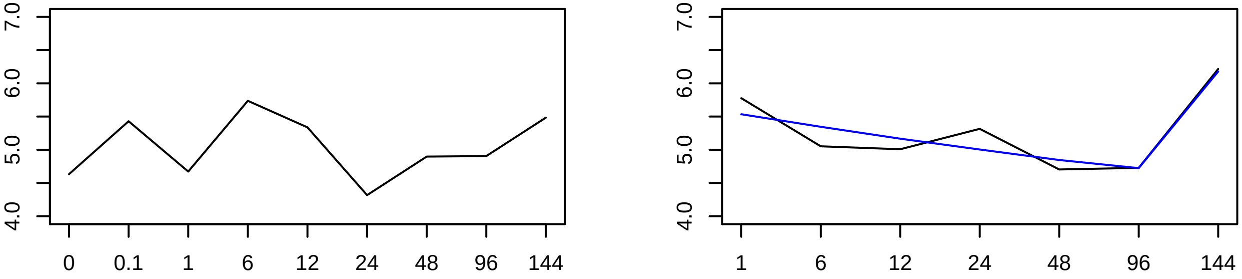

A_32_P132827 OPHN1 Xq12

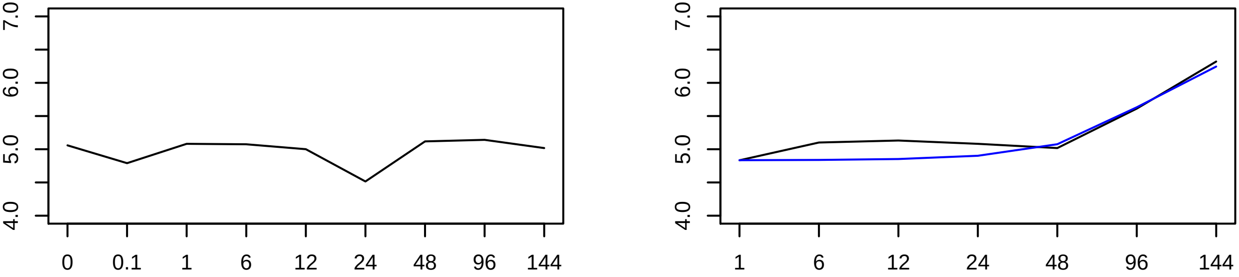

A_24_P876522 GPX8 NA

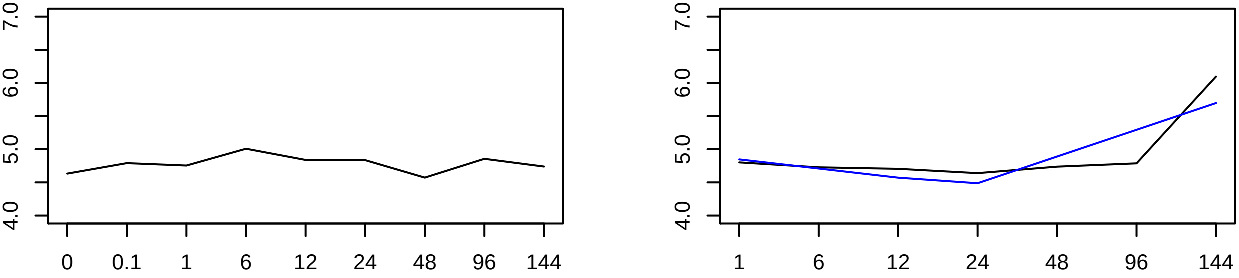

A_23_P85693 GBP2 1p22.2

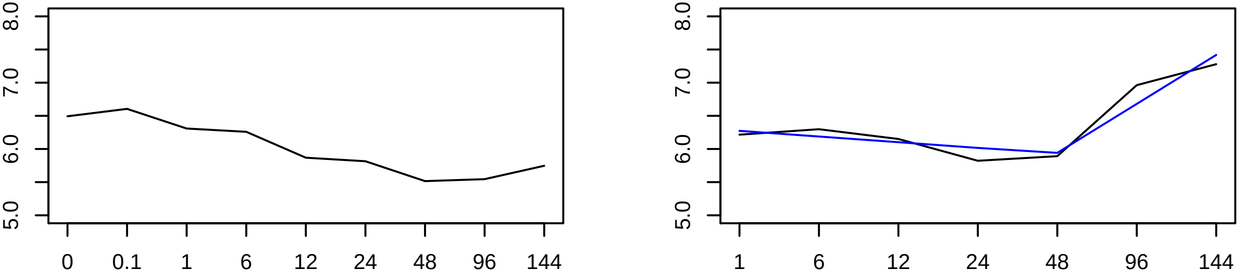

A_23_P114857 PLA2G2E 1p36.13

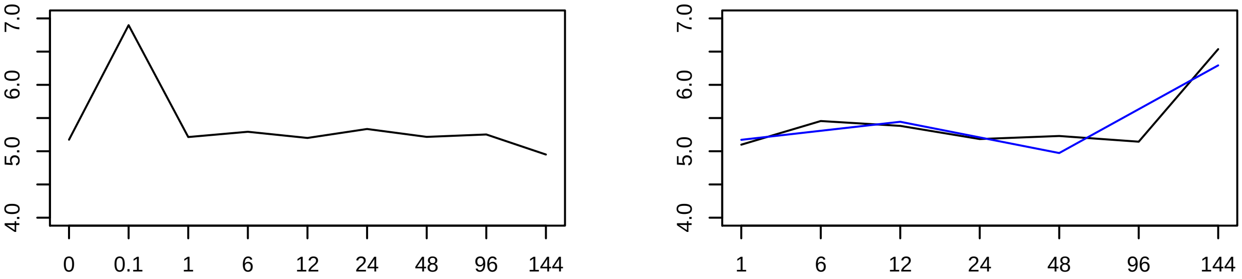

A_23_P128084 ITGA7 12q13.2

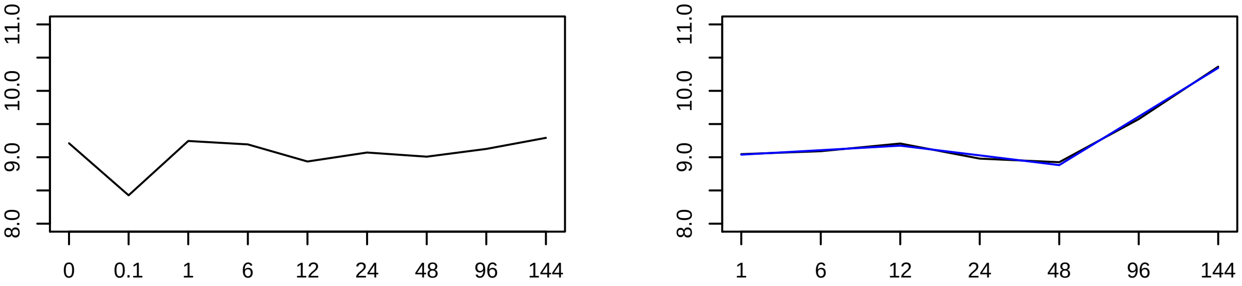

A_24_P412156 CXCL12 10q11.21

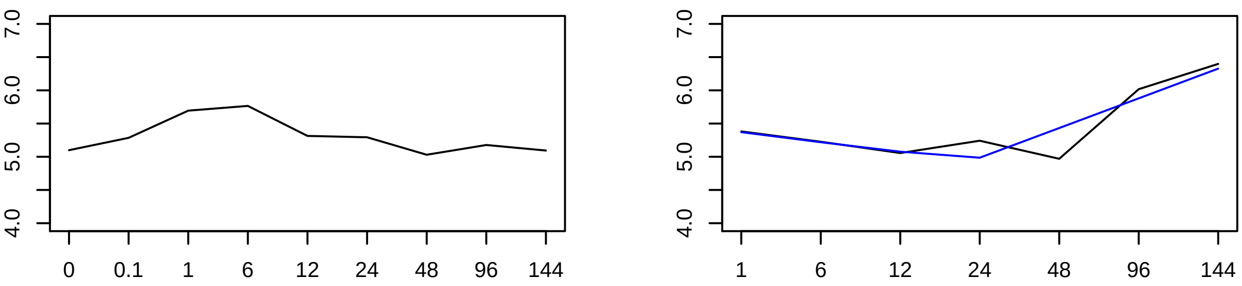

A_24_P929640 THC2504595 NA

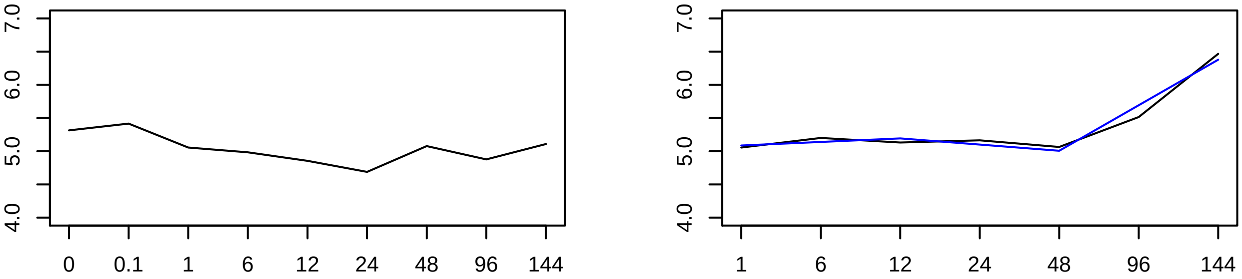

A_24_P56130 MYL6 12q13.2

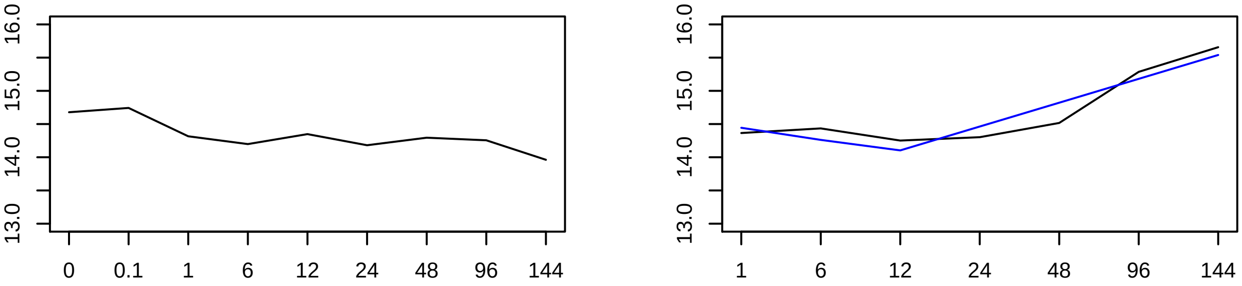

A_24_P68008 DRAXIN 1p36.22

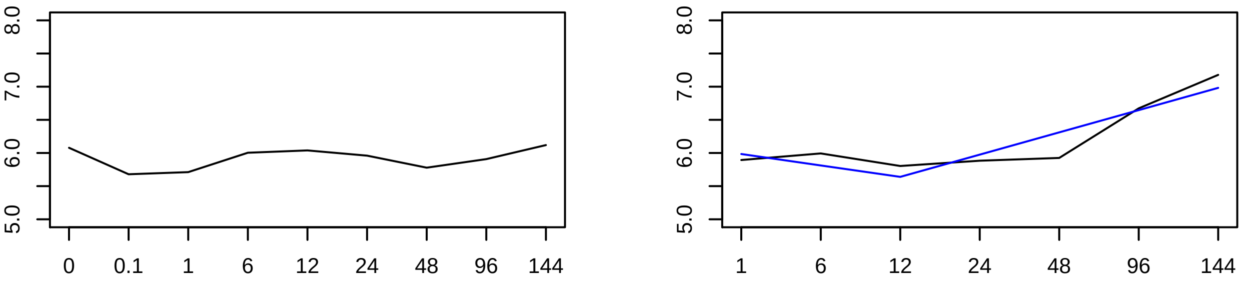

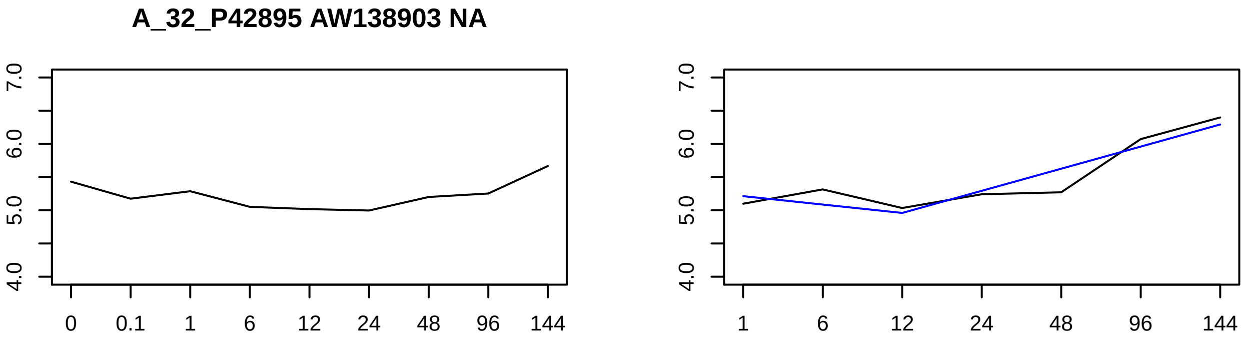
A_32_P42895 AW138903 NA
4.0
5.0
6.0
7.0
0
0.1
1
6
12
24
48
96
144
1
6
12
24
48
96
144

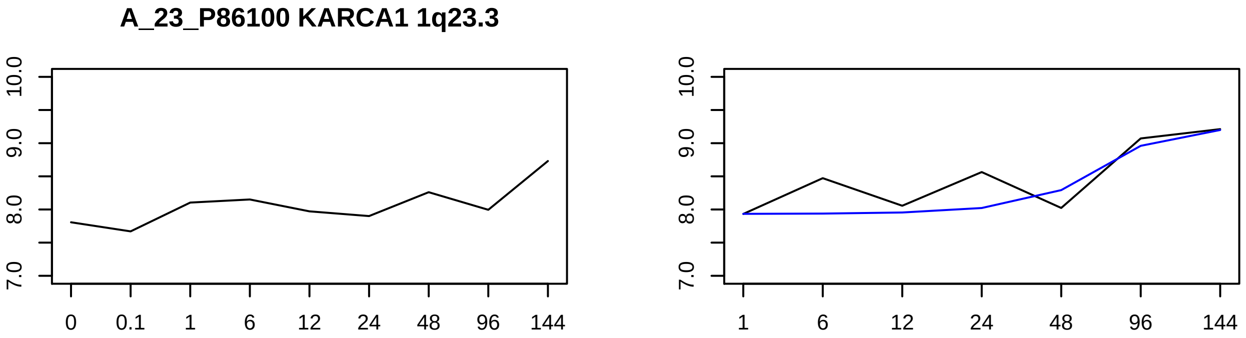
A_23_P86100 KARCA1 1q23.3
7.0
8.0
9.0
10.0
0
0.1
1
6
12
24
48
96
144
1
6
12
24
48
96
144
