# Supplementary material for: SwitchFinder – a novel method and query facility for discovering dynamic gene expression patterns
Source: BMC Bioinformatics. 2016 Dec 15;17:532. doi: 10.1186/s12859-016-1391-0 (PMC5160026; doi:10.1186/s12859-016-1391-0)

A_23_P210100 CYP26B1 2p13.3

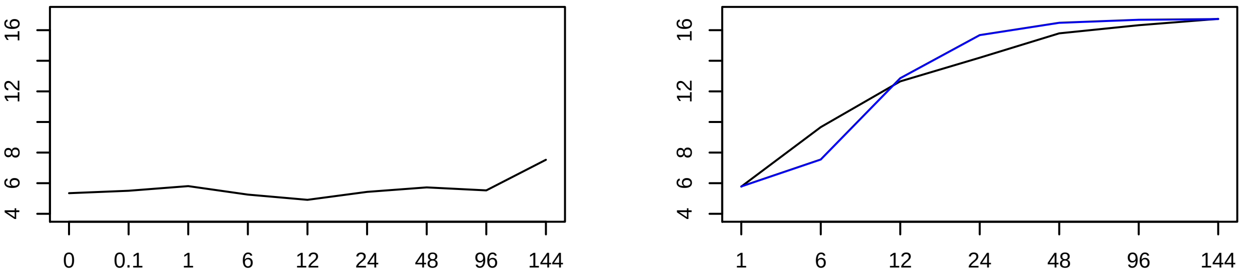

A_23_P138655 CYP26A1 10q23.33

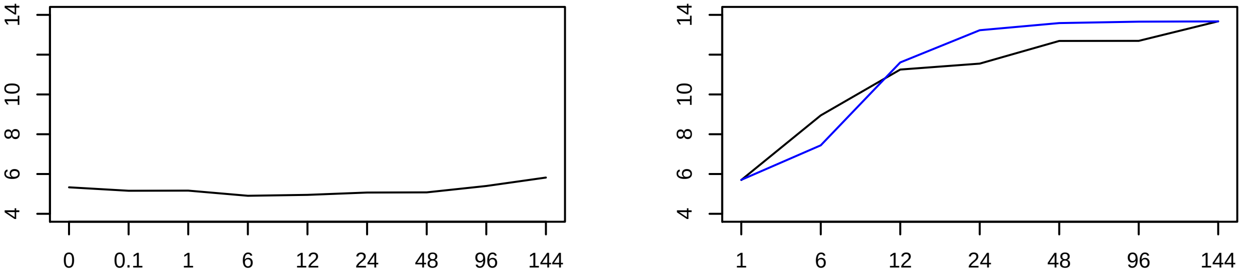

A_23_P73632 NR0B1 Xp21.2

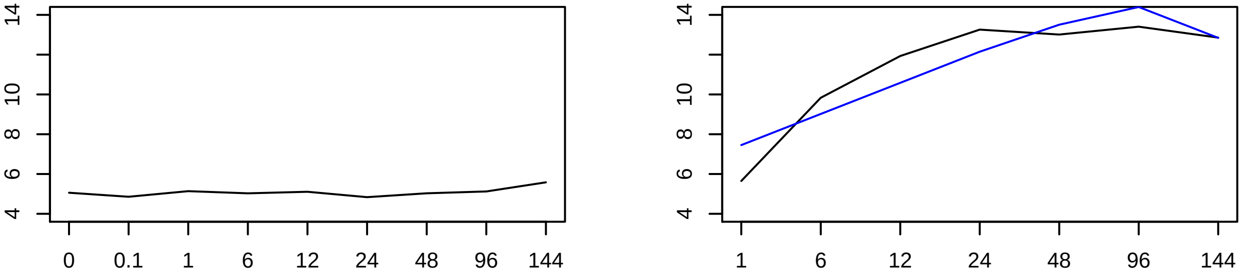

A_23_P115064 CRABP2 1q23.1

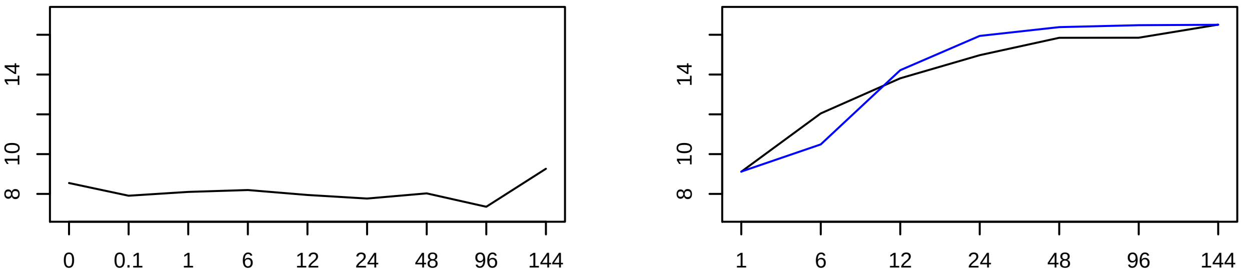

A_24_P198178 CALCA 11p15.2

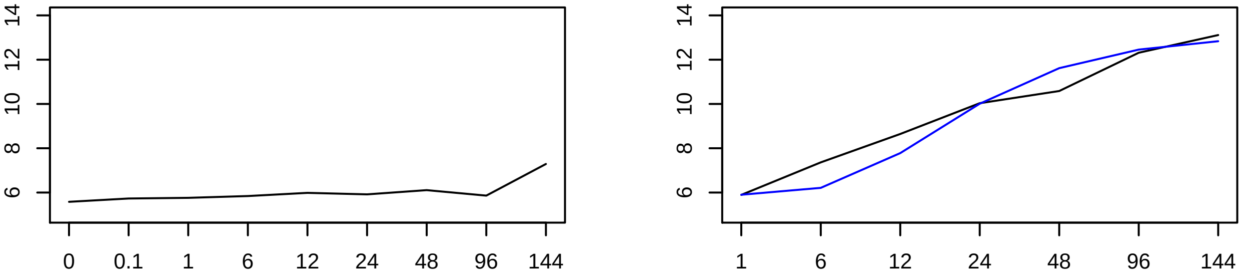

A_23_P30254 PLK2 5q11.2

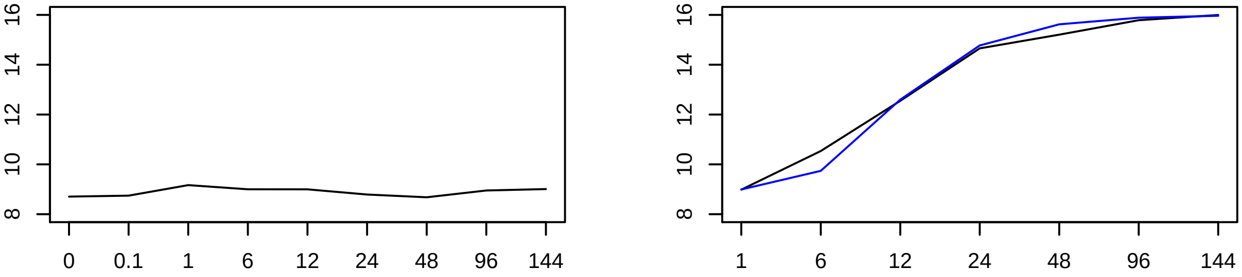

A_32_P229618 DLG2 11q14.1

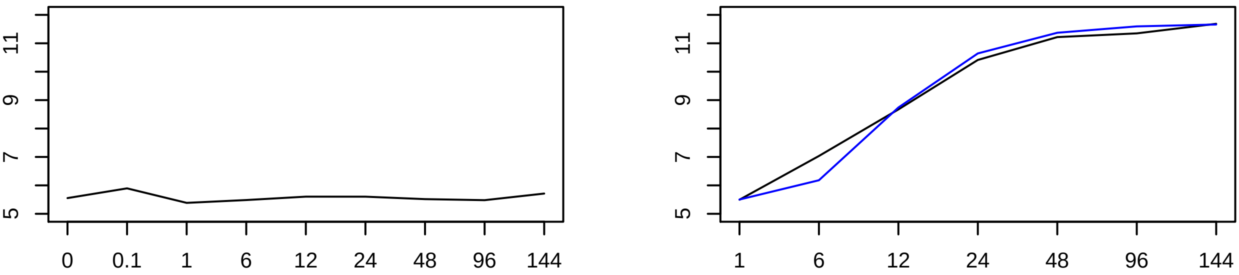

A_23_P16523 GDF15 19p13.11

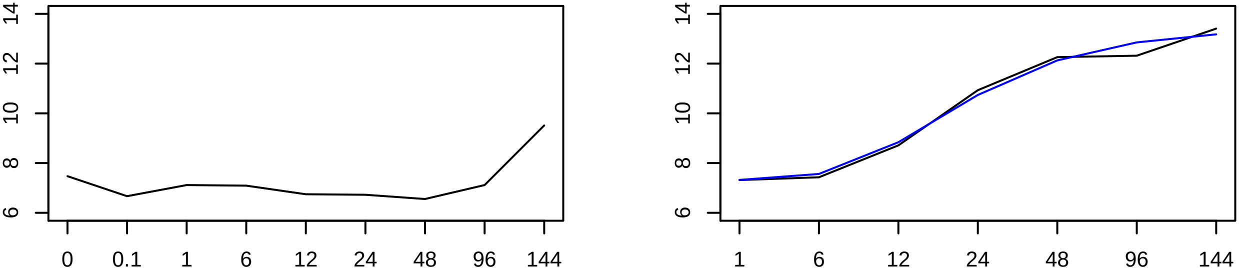

A_23_P16953 HTR2B 2q37.1

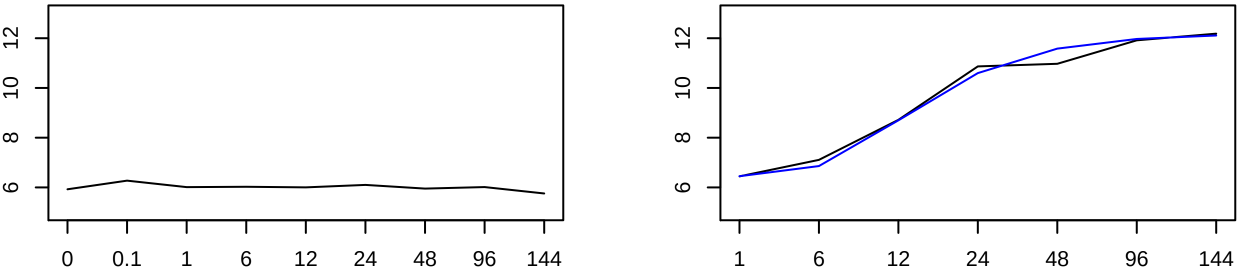

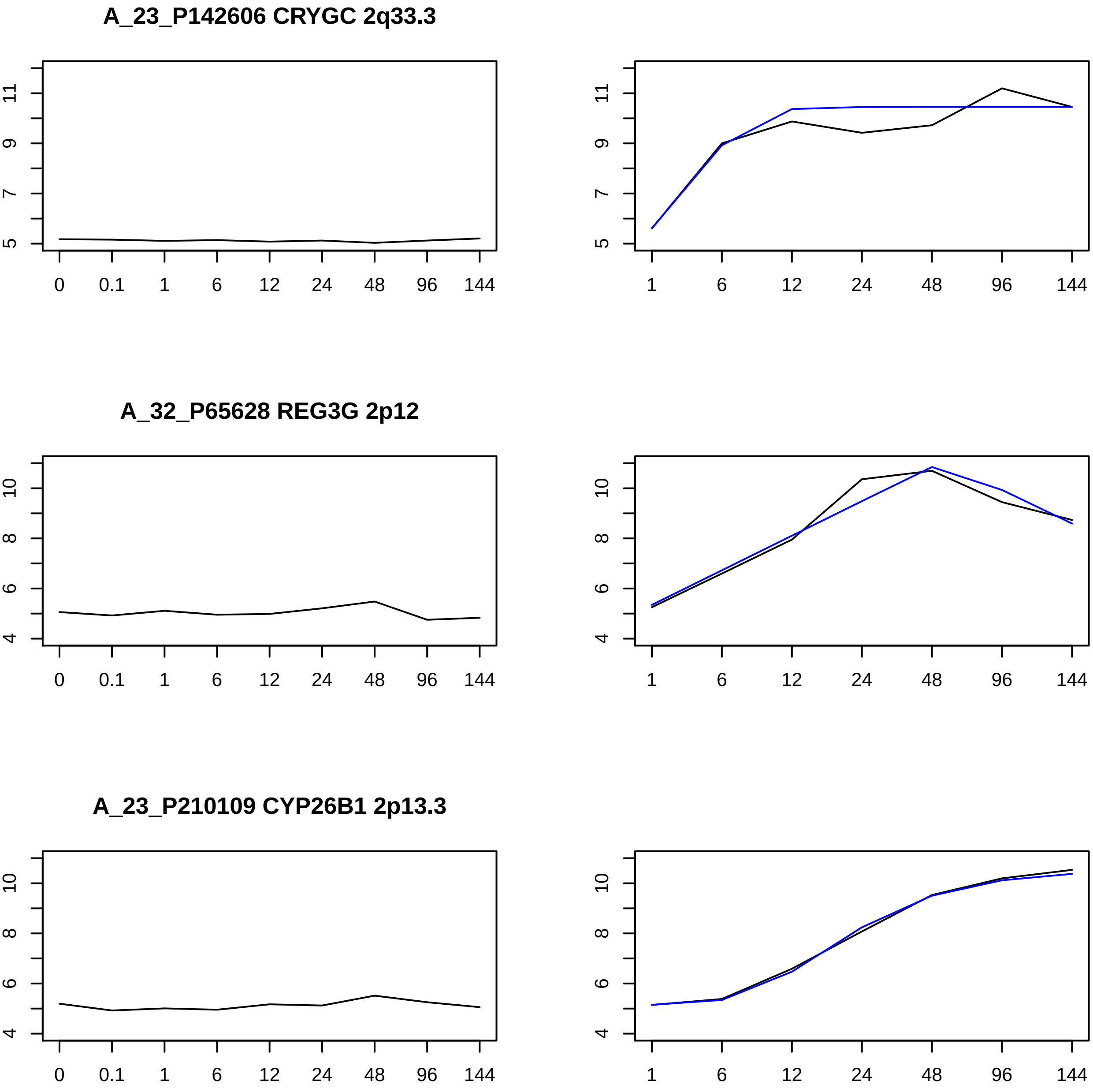
A_23_P142606 CRYGC 2q33.3
A_32_P65628 REG3G 2p12
A_23_P210109 CYP26B1 2p13.3
5 7 9 11
4 6 8 10
0 0.1 1 6 12 24 48 96 144
1 6 12 24 48 96 144

A_23_P256334 ITGA1 5q11.2

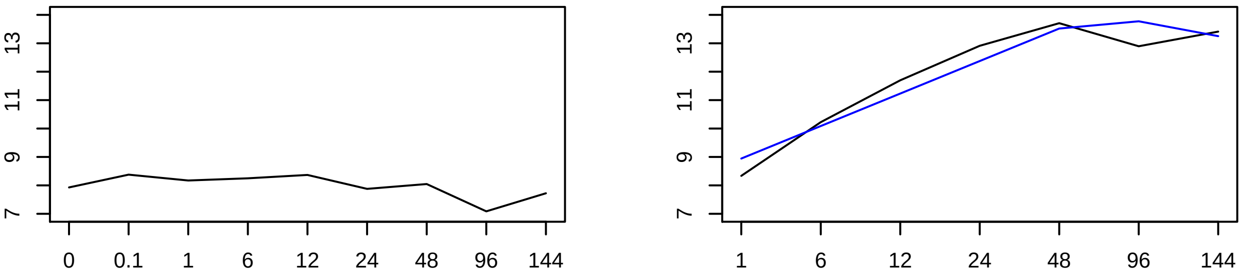

A_23_P33759 DHRS3 1p36.22

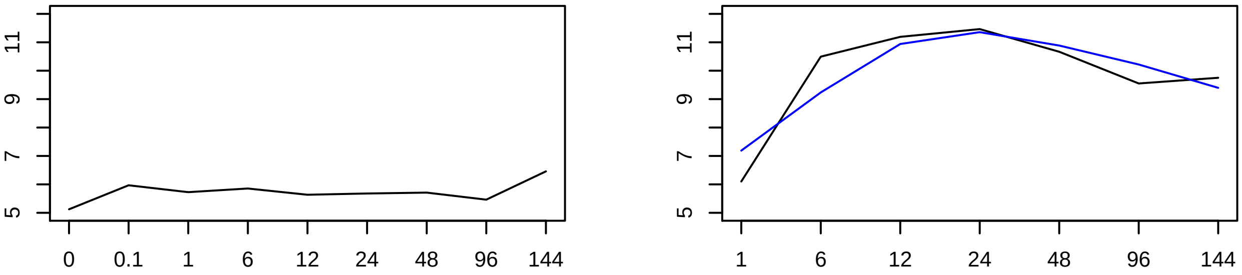

A_23_P101642 PTPRH 19q13.42

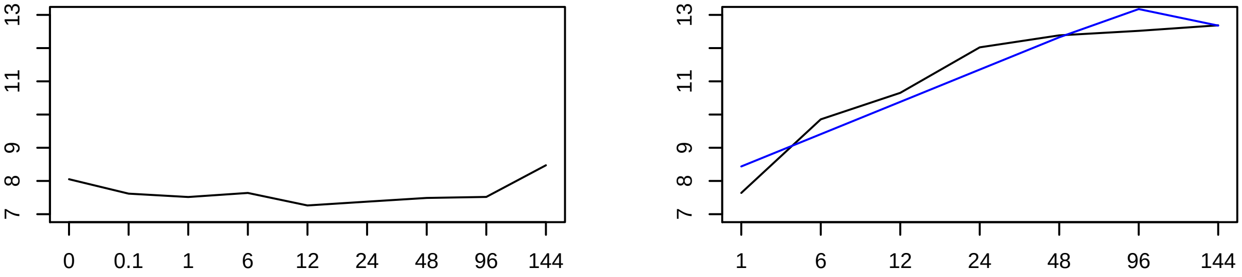

A_23_P414273 MST150 5q33.1

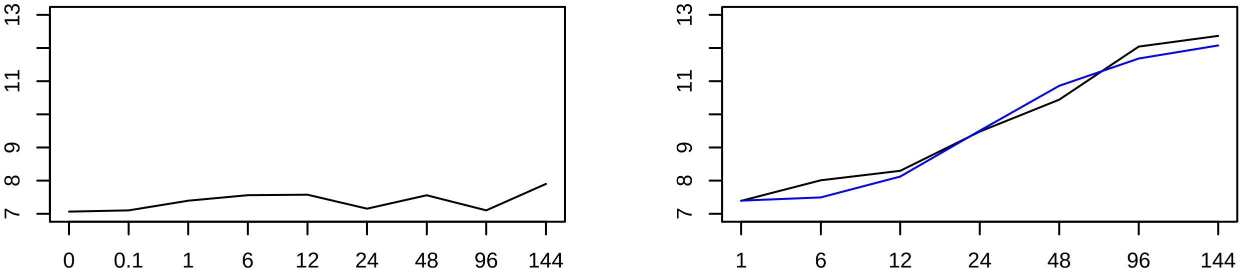

A_23_P420326 FNDC5 1p35.1

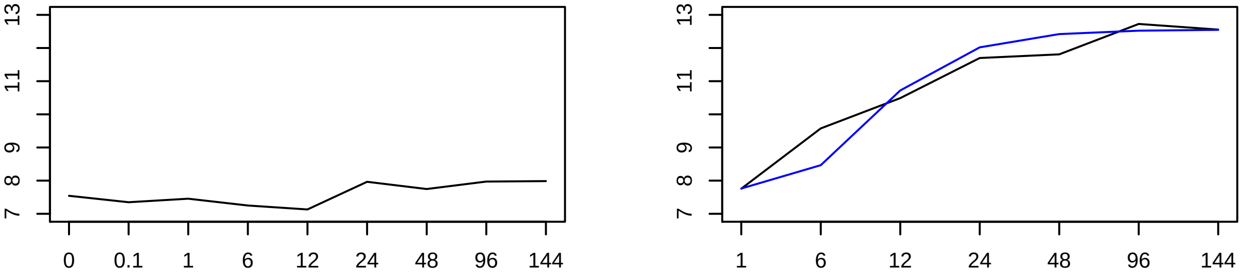

A_24_P810290 PPAPDC1A 10q26.12

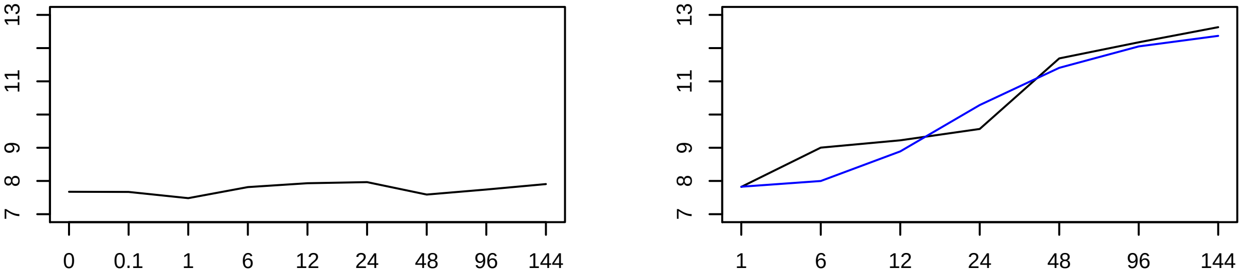

A_23_P369994 DCAMKL1 13q13.3

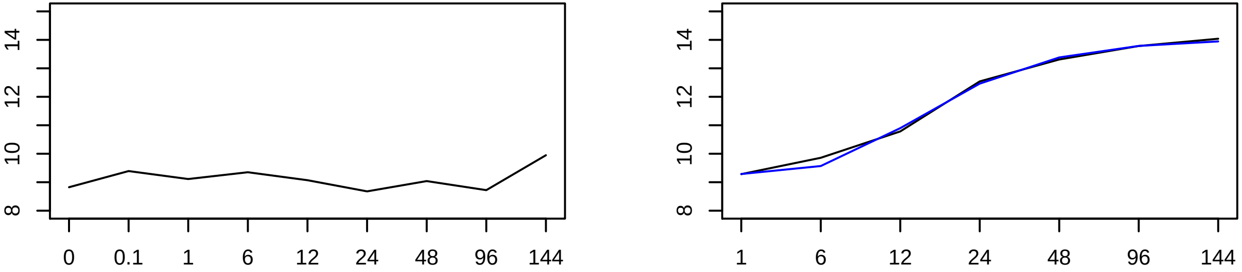

A_32_P116857 PDE11A 2q31.2

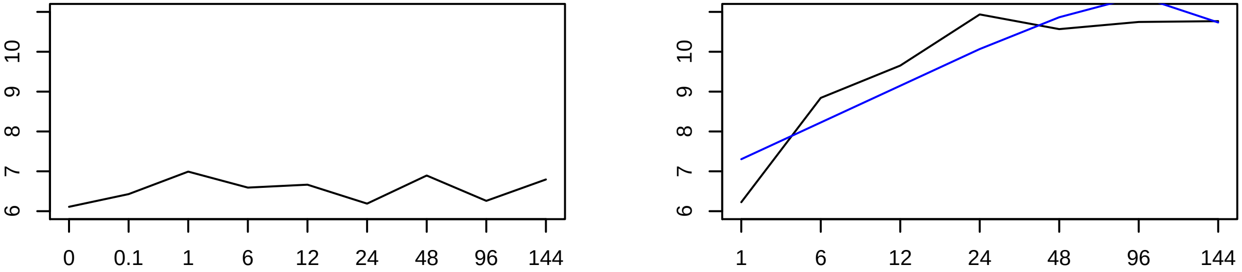

A_32_P538830 PPAPDC1A 10q26.12

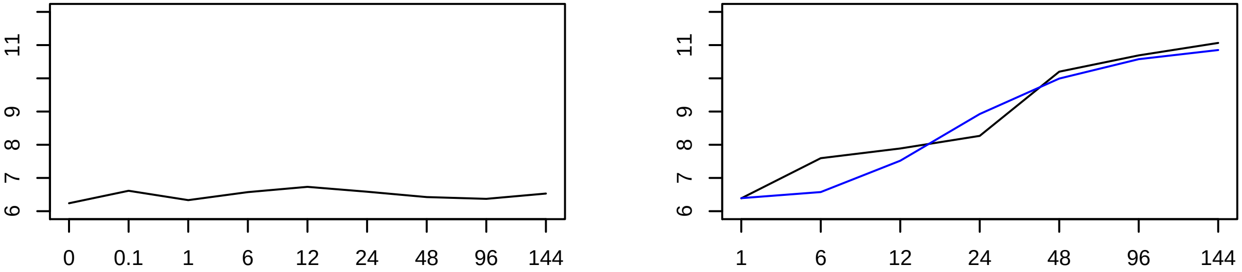

A_23_P139704 DUSP6 12q21.33

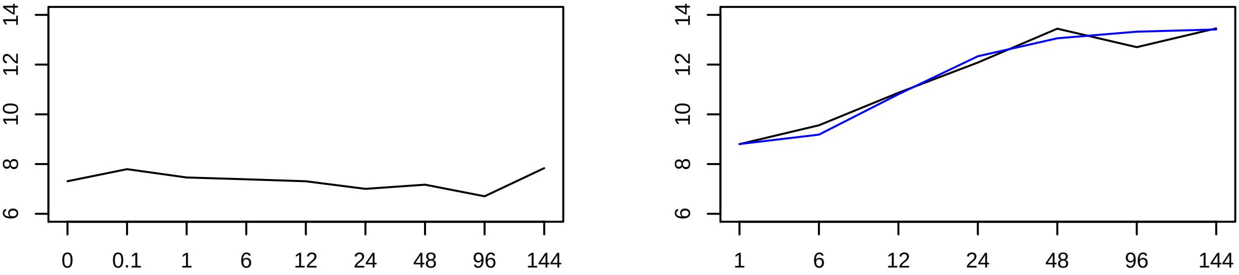

A_32_P205859 RAB43 3q21.3

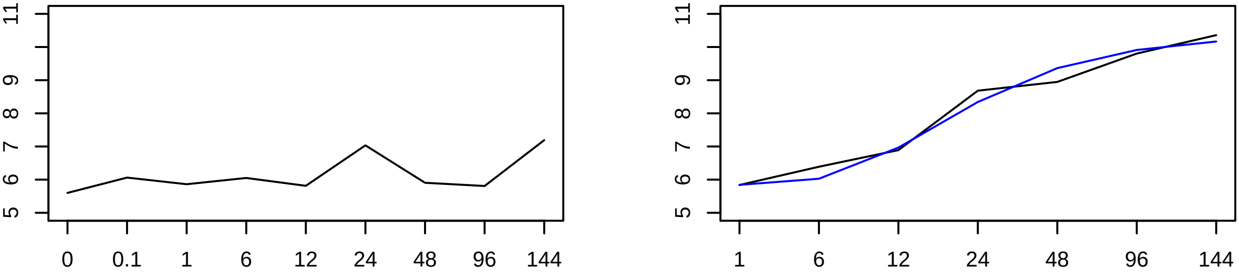

A_32_P108889 DCLK1 13q13.3

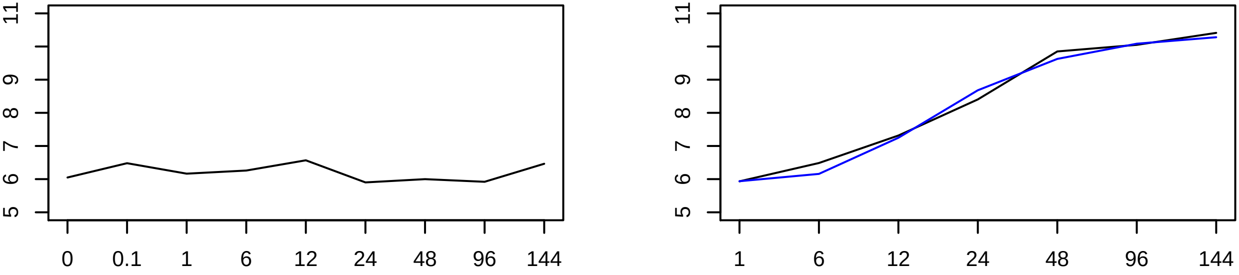

A_23_P7048 C4orf6 4p16.1

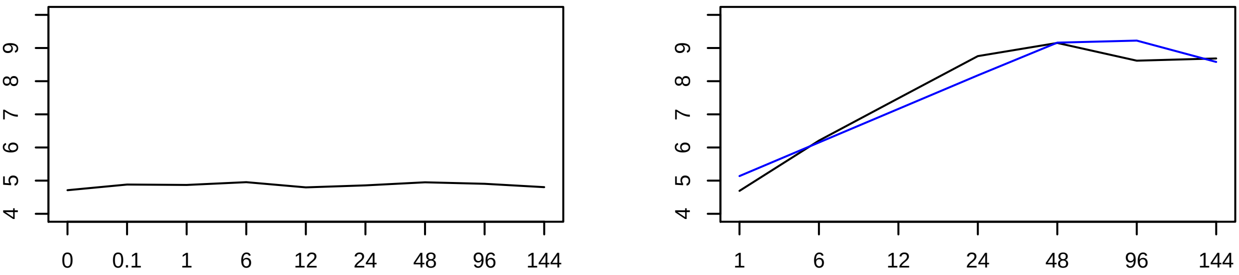

A_32_P16204 LOC375295 2q31.1

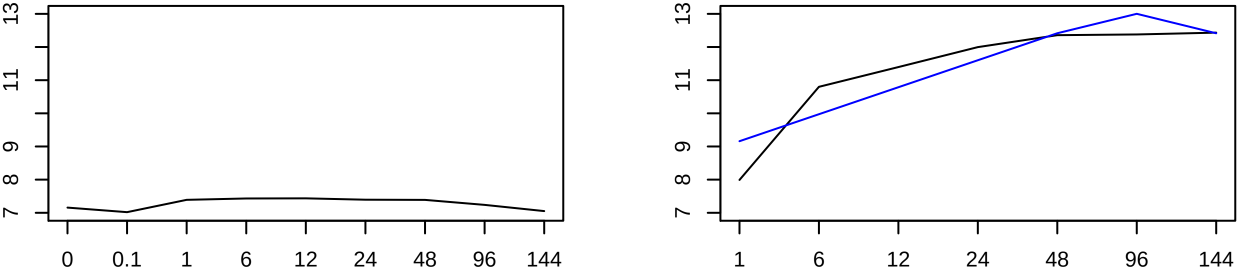

A_23_P159325 ANGPTL4 19p13.2

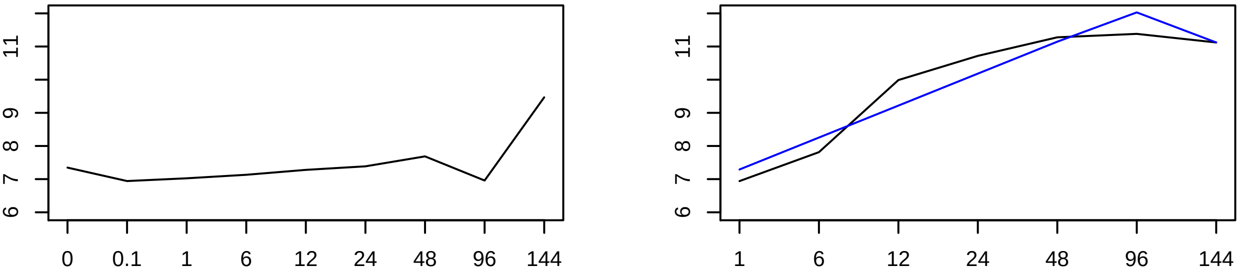

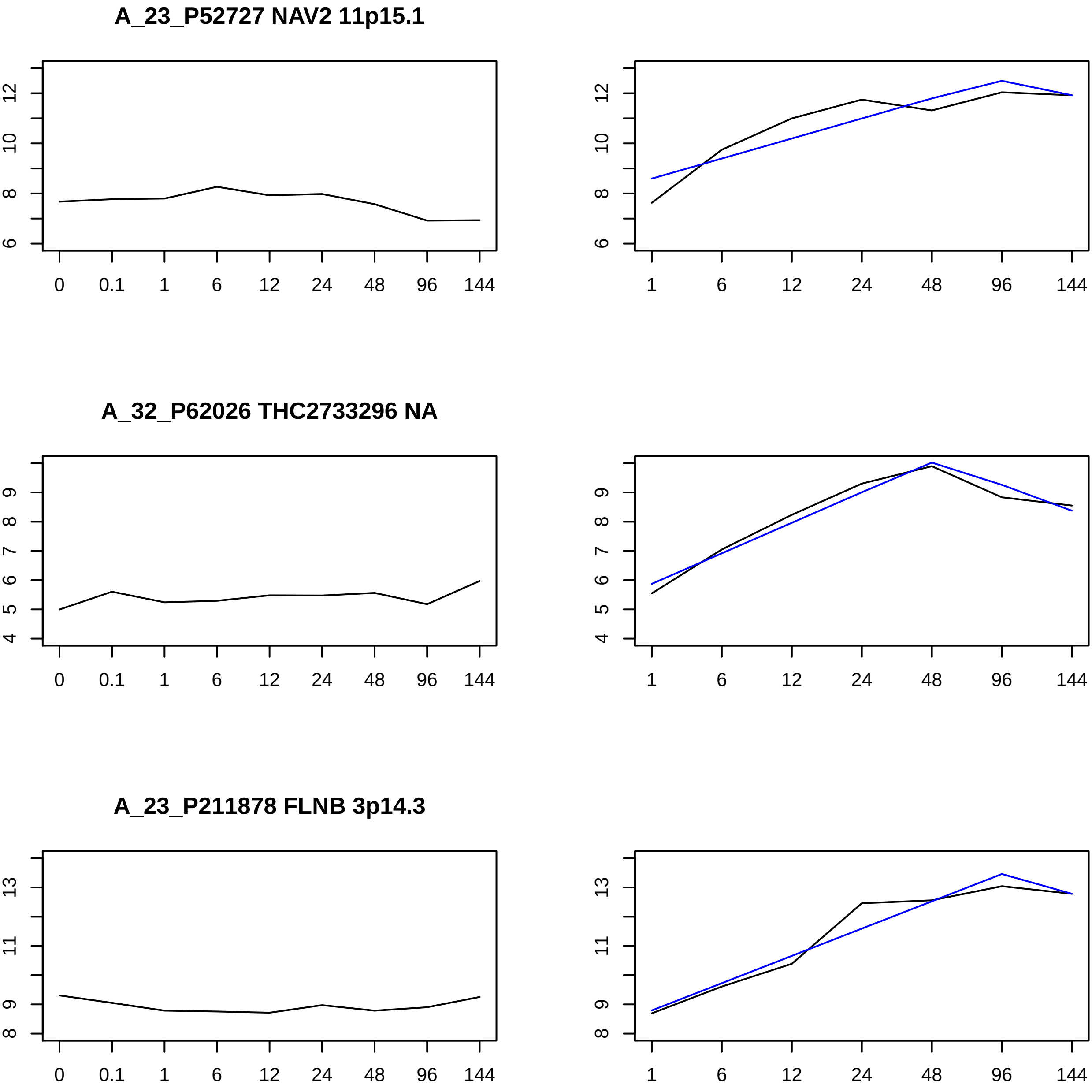
A_23_P52727 NAV2 11p15.1
A_32_P62026 THC2733296 NA
A_23_P211878 FLNB 3p14.3
0 0.1 1 6 12 24 48 96 144
1 6 12 24 48 96 144

A_23_P139912 IGFBP6 12q13.13

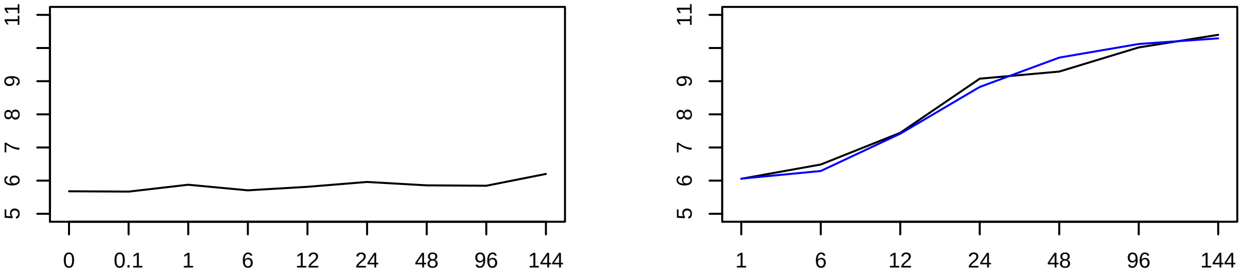

A_32_P139708 THC2664742 NA

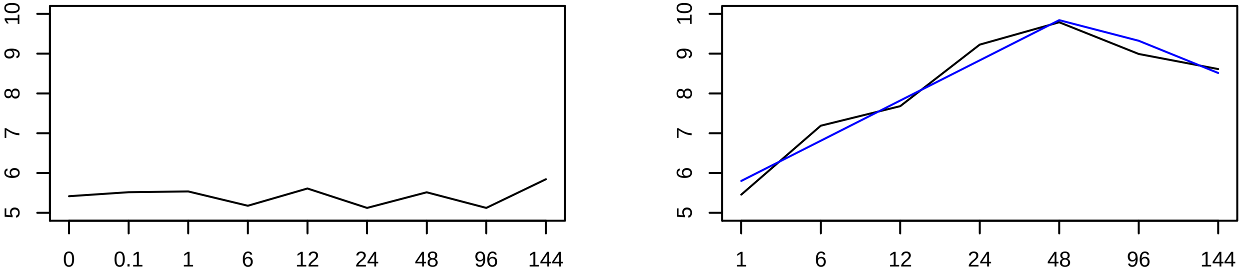

A_23_P302787 LOC375295 2q31.1

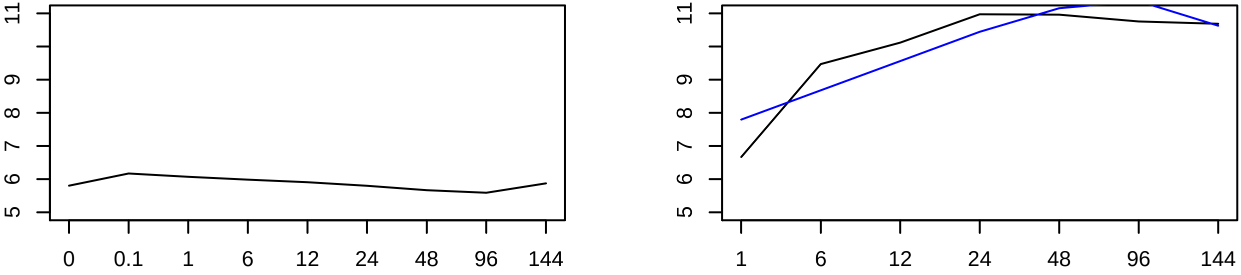

A_23_P205567 PRKCH 14q23.1

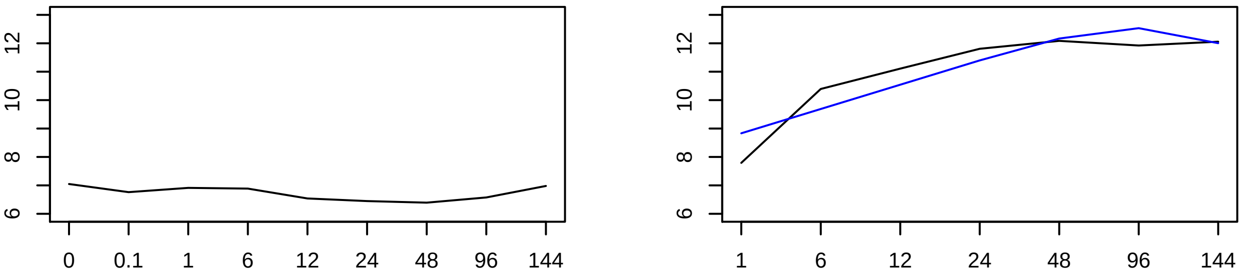

A_23_P170733 ANTXR2 4q21.21

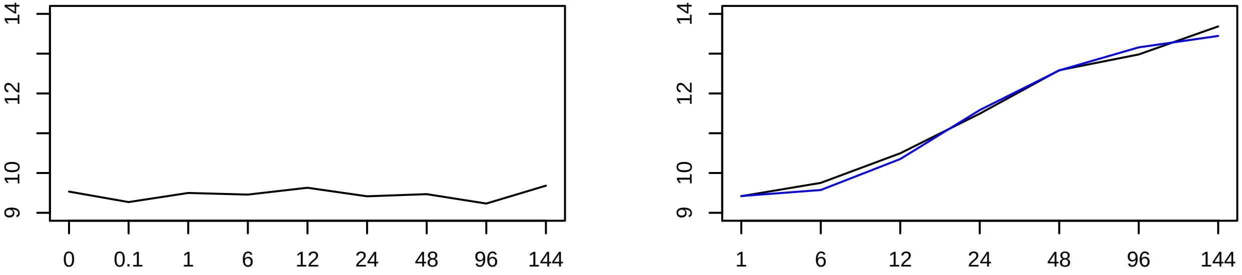

A_32_P228037 PDE11A 2q31.2

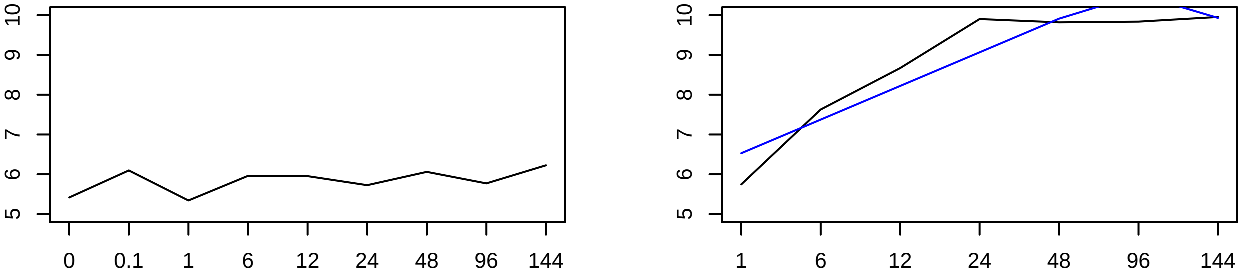

A_24_P397928 CTSB 8p23.1

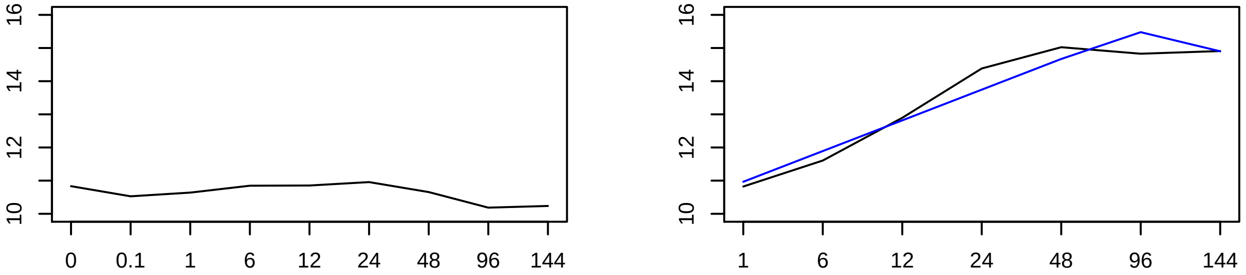

A_24_P861099 FLJ22536 6p22.3

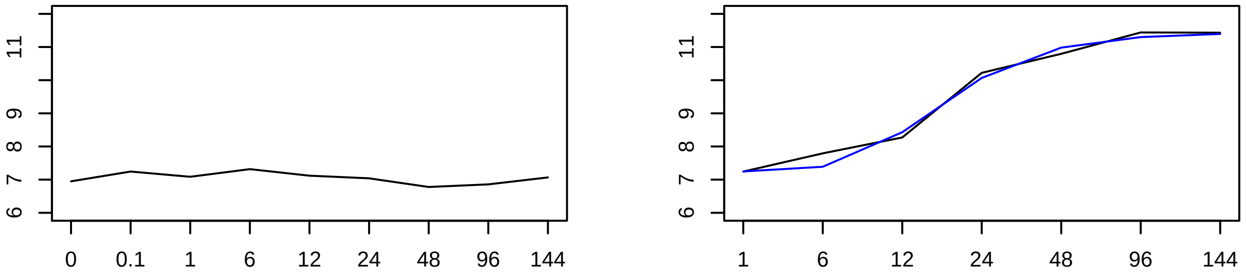

A_23_P257649 RBP1 3q23

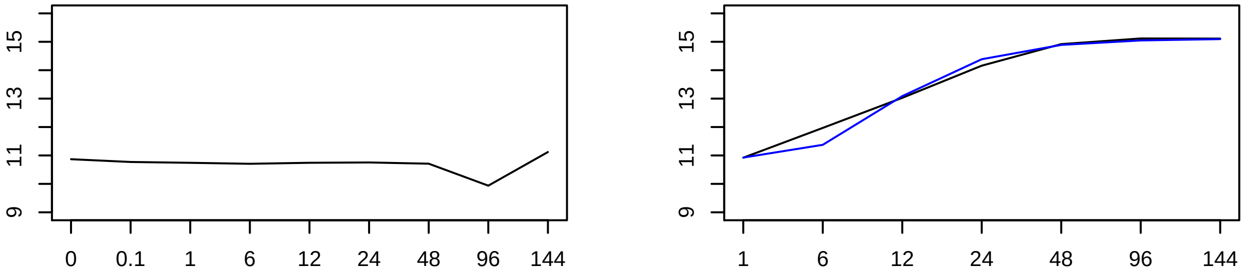

A_24_P346431 TNS3 7p12.3

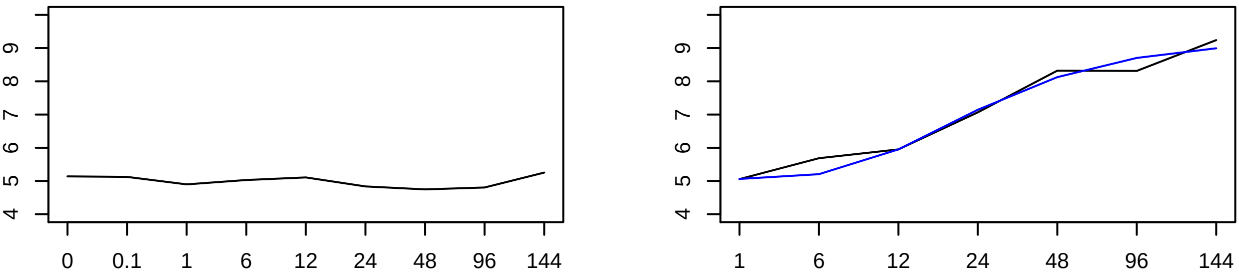

A_32_P340999 SYT2 1q32.1

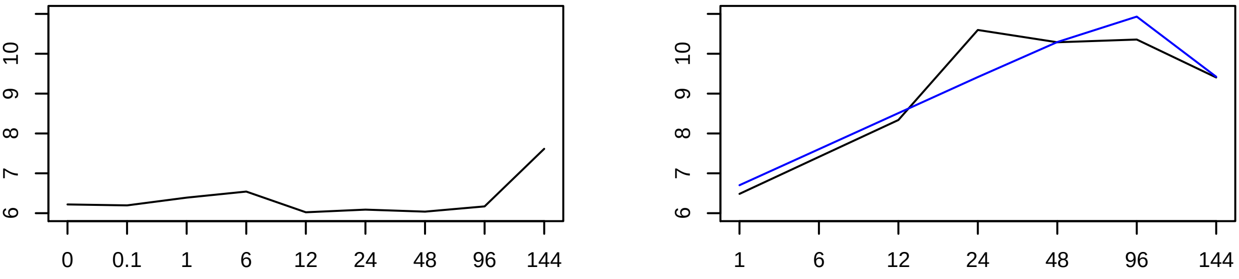

A_23_P43337 FREM1 9p22.3

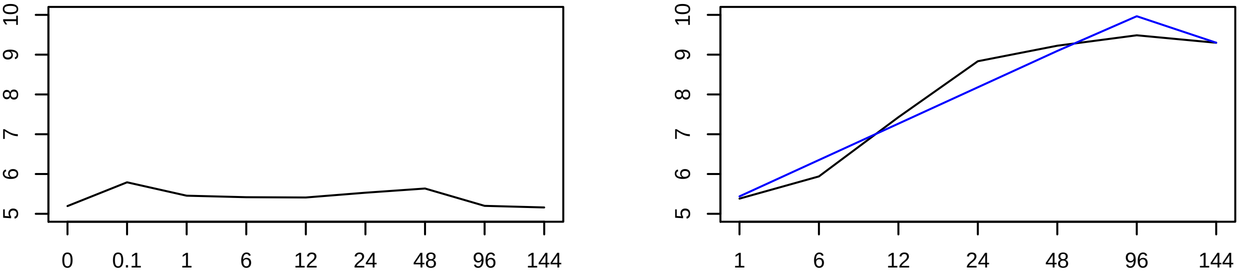

A_24_P592012 ZBTB46 20q13.33

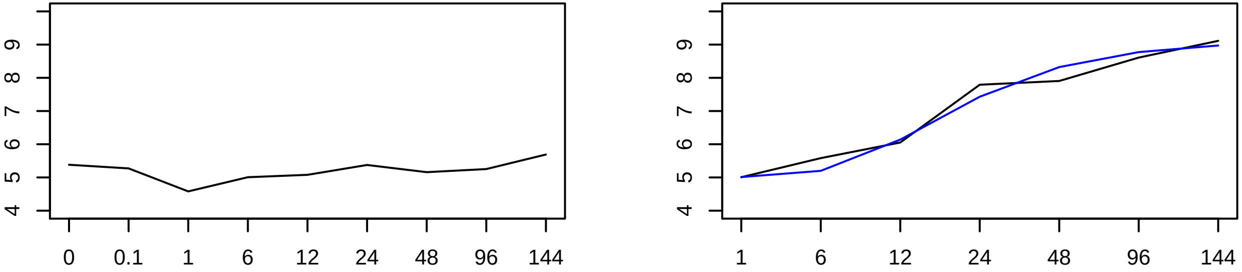

A_23_P82868 PLAT 8p11.21

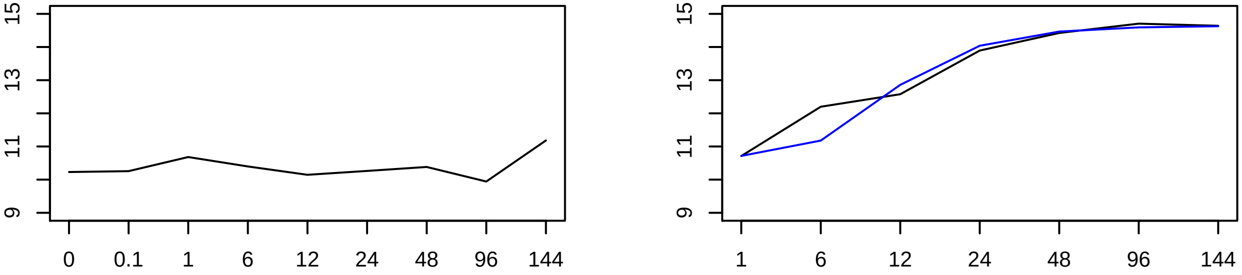

A_32_P186865 THC2663329 NA

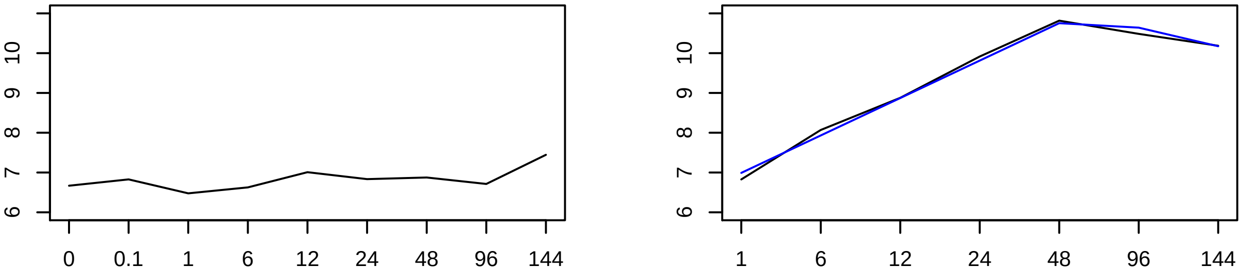

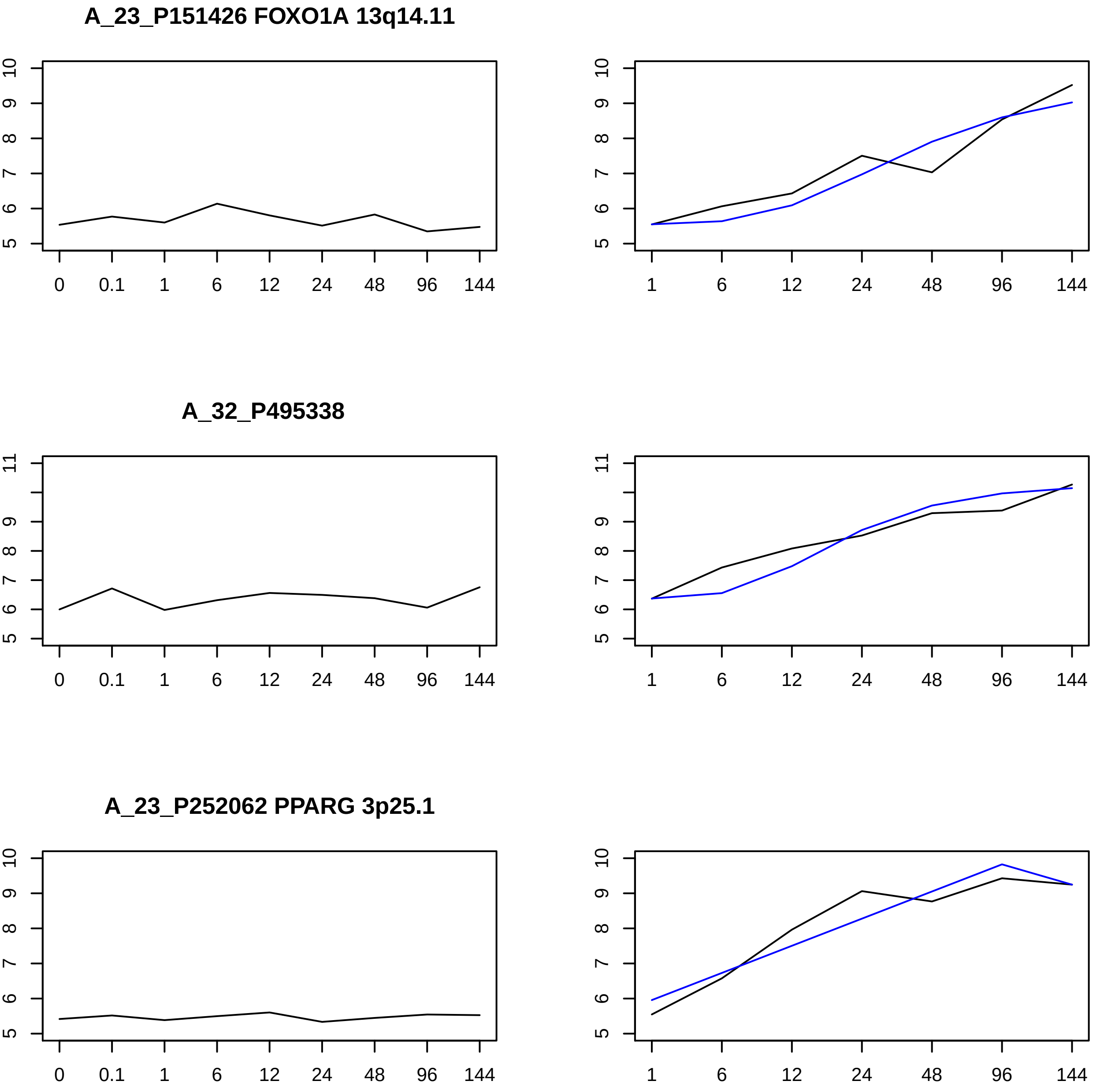
A_23_P151426 FOXO1A 13q14.11
5 6 7 8 9 10
0 0.1 1 6 12 24 48 96 144
5 6 7 8 9 10
1 6 12 24 48 96 144
A_32_P495338
5 6 7 8 9 11
0 0.1 1 6 12 24 48 96 144
5 6 7 8 9 11
1 6 12 24 48 96 144
A_23_P252062 PPARG 3p25.1
5 6 7 8 9 10
0 0.1 1 6 12 24 48 96 144
5 6 7 8 9 10
1 6 12 24 48 96 144

A_32_P91385 THC2622086 NA

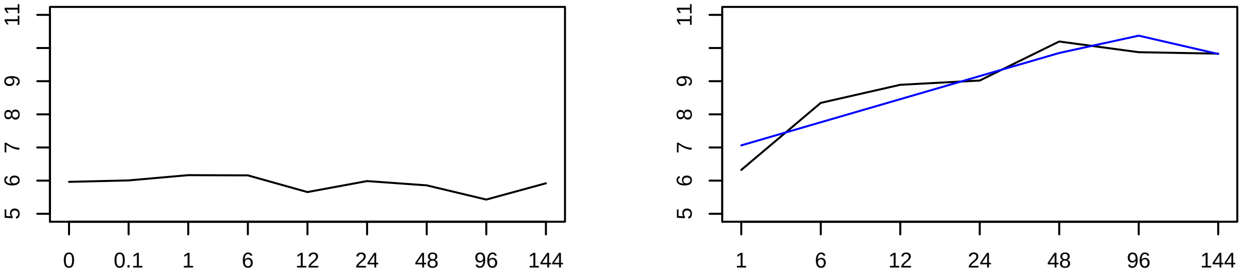

A_23_P170719 A_23_P170719 NA

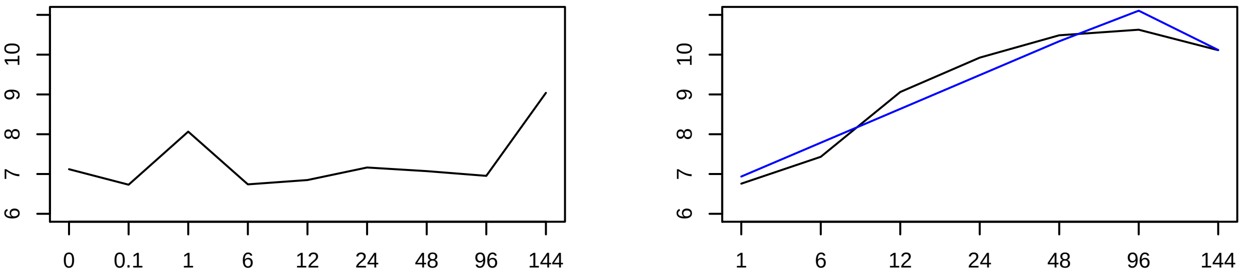

A_24_P303770 CTSB 8p23.1

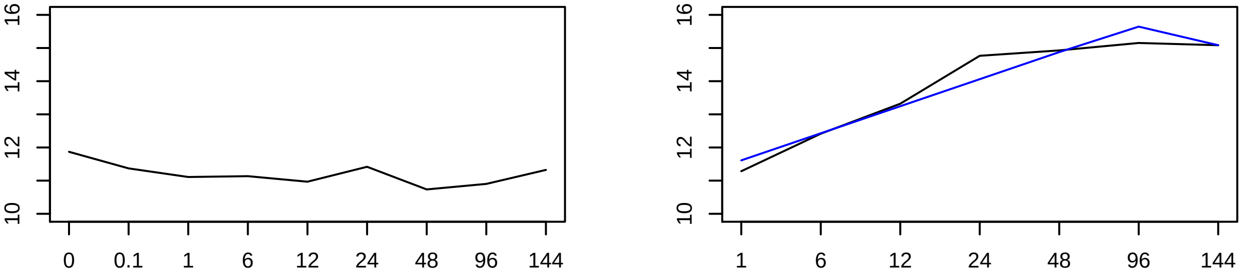

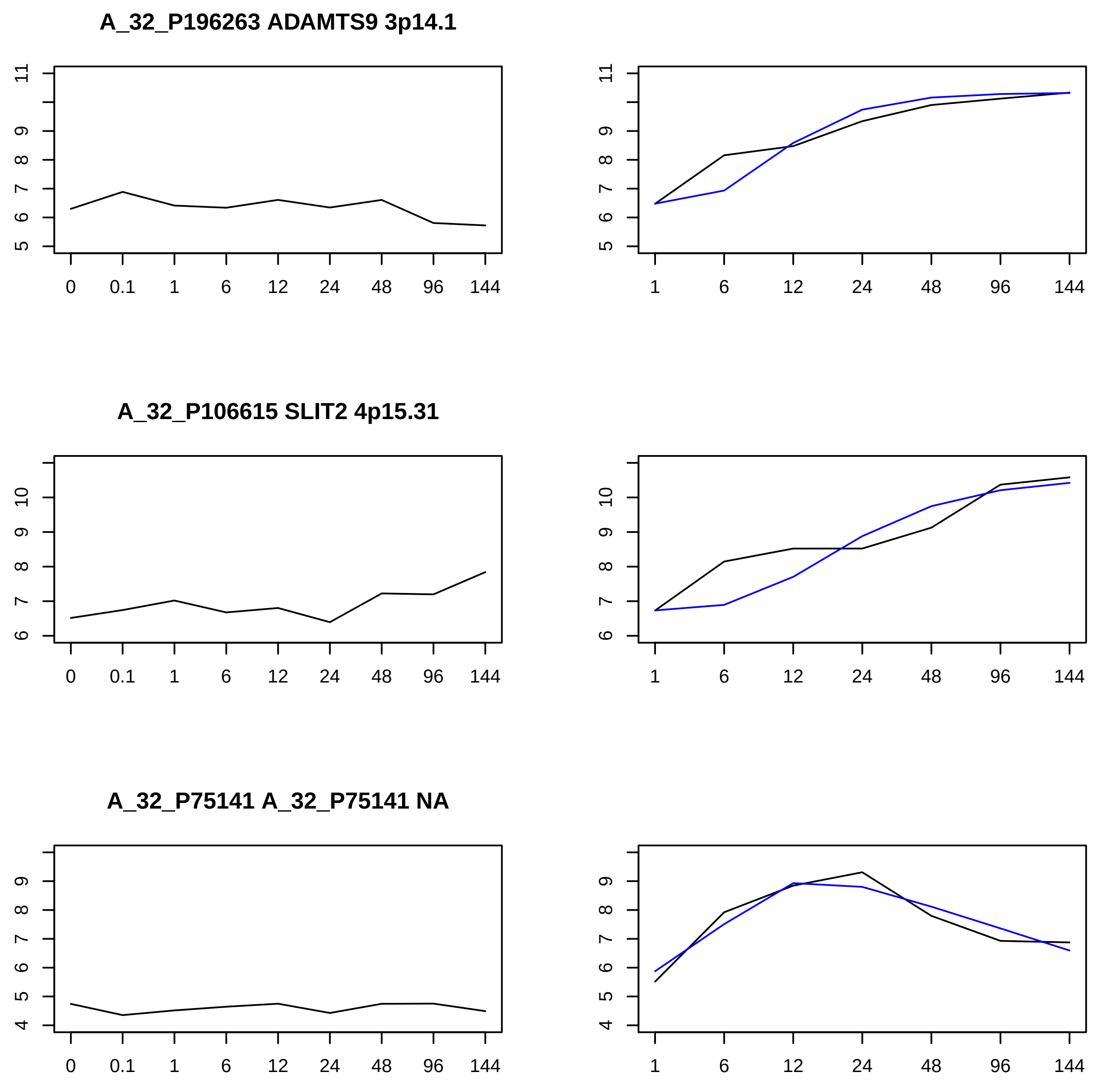
A_32_P196263 ADAMTS9 3p14.1
A_32_P106615 SLIT2 4p15.31
A_32_P75141 A_32_P75141 NA
0 0.1 1 6 12 24 48 96 144
1 6 12 24 48 96 144

A_23_P144348 SLIT2 4p15.31

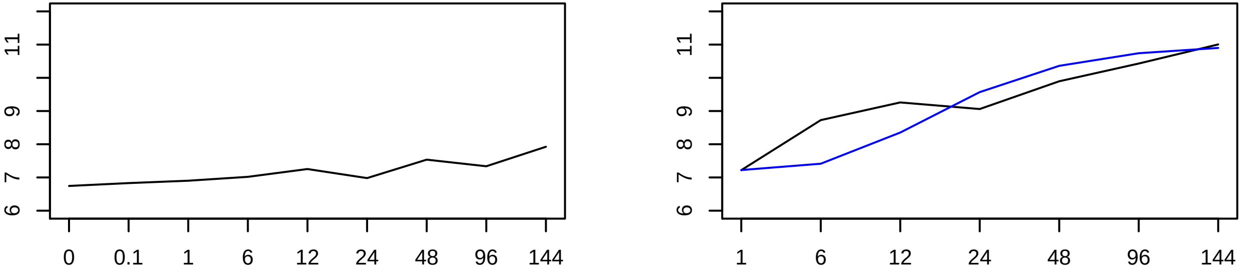

A_24_P365349 CACNG7 19q13.42

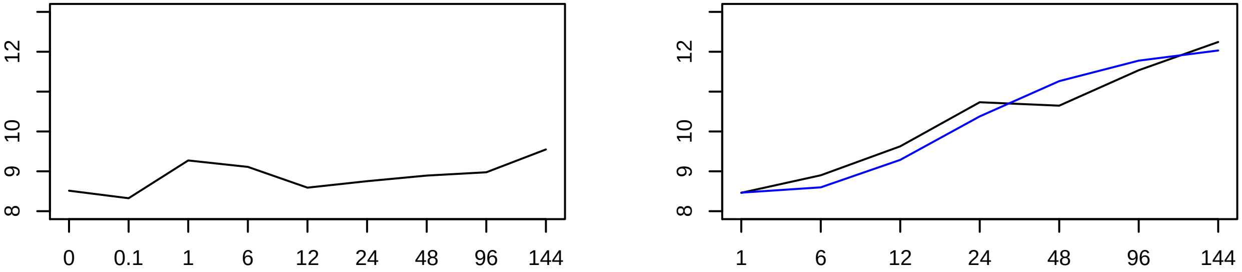

A_24_P530977 A_24_P530977 NA

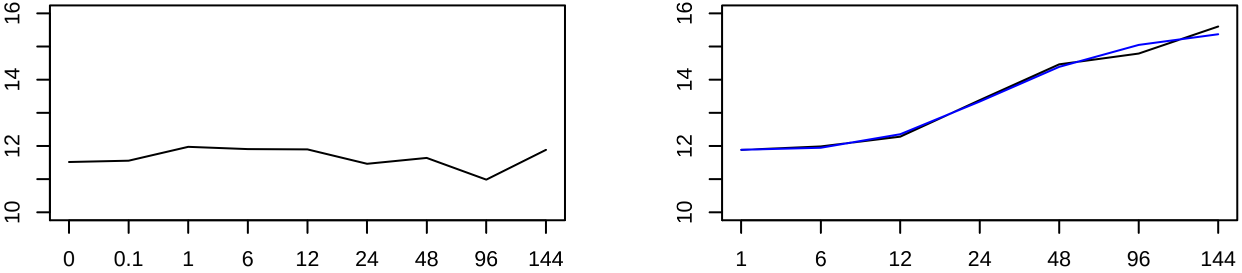

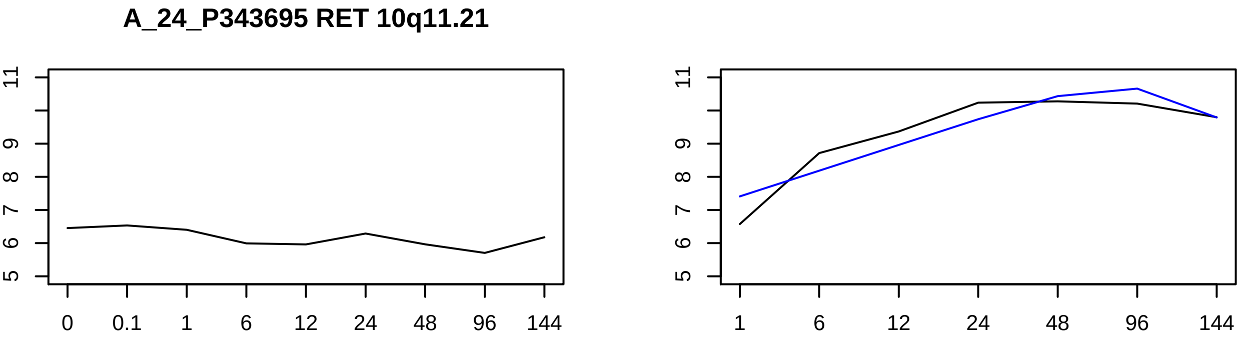
A_24_P343695 RET 10q11.21
5 6 7 8 9 11
0 0.1 1 6 12 24 48 96 144
5 6 7 8 9 11
1 6 12 24 48 96 144

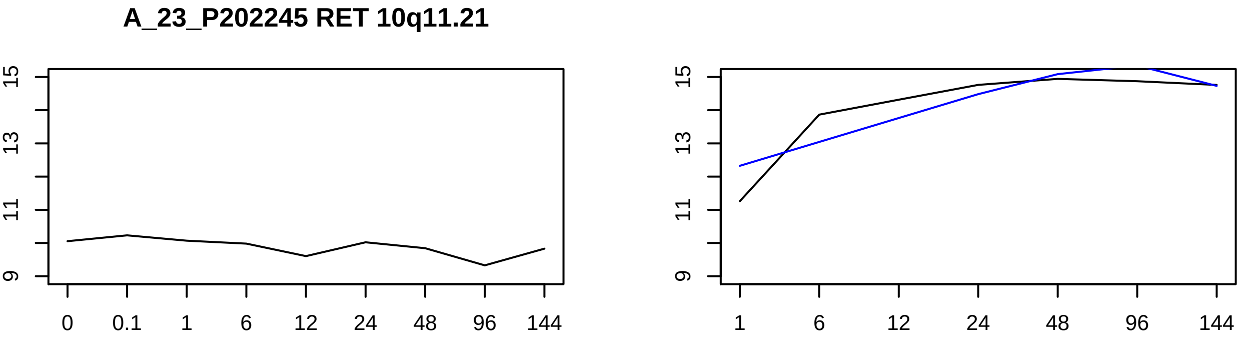
A_23_P202245 RET 10q11.21
9 11 13 15
0 0.1 1 6 12 24 48 96 144
9 11 13 15
1 6 12 24 48 96 144

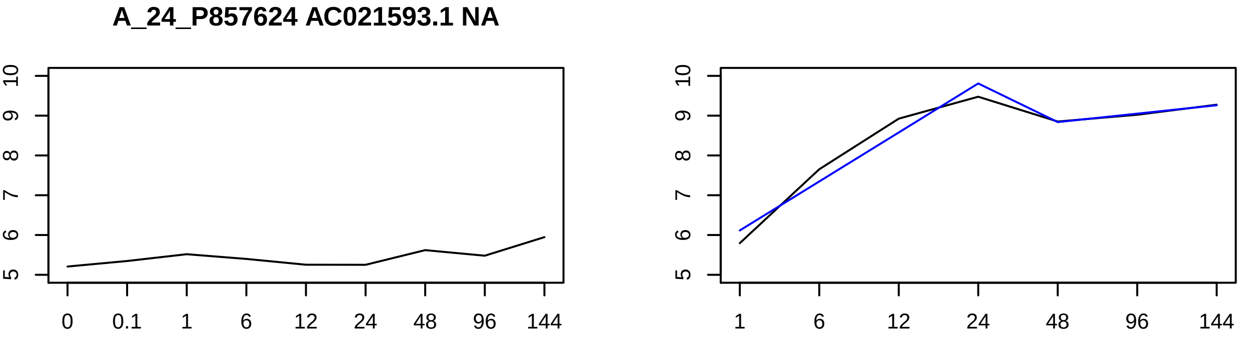
A_24_P857624 AC021593.1 NA
5 6 7 8 9 10
0 0.1 1 6 12 24 48 96 144
5 6 7 8 9 10
1 6 12 24 48 96 144

A_23_P215944 CTSB 8p23.1

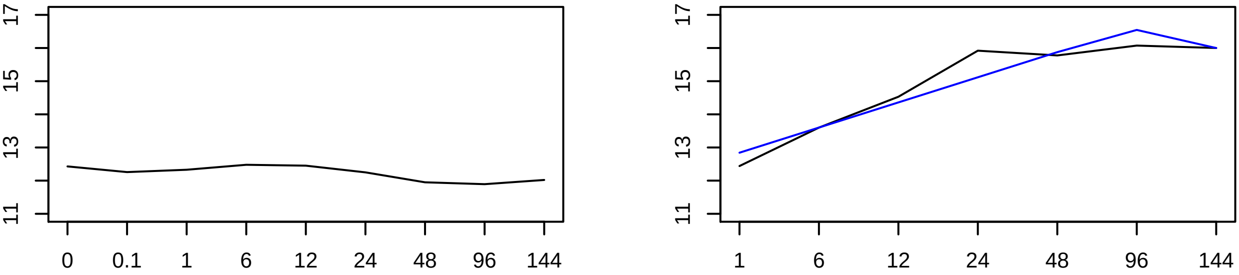

A_23_P116187 BCDO2 11q23.1

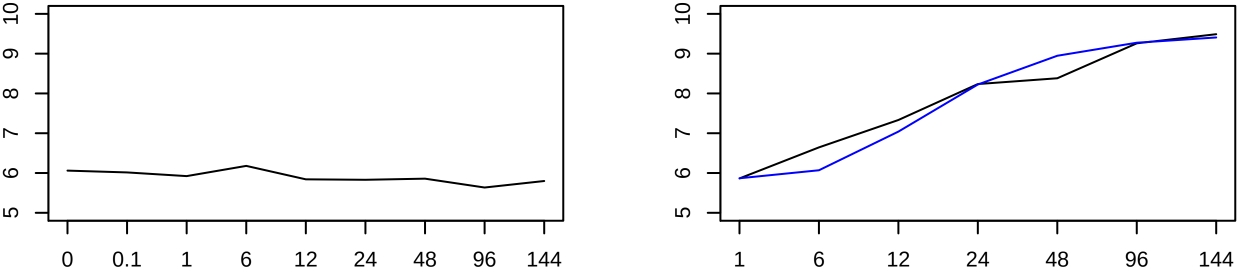

A_23_P216225 EGR3 8p21.3

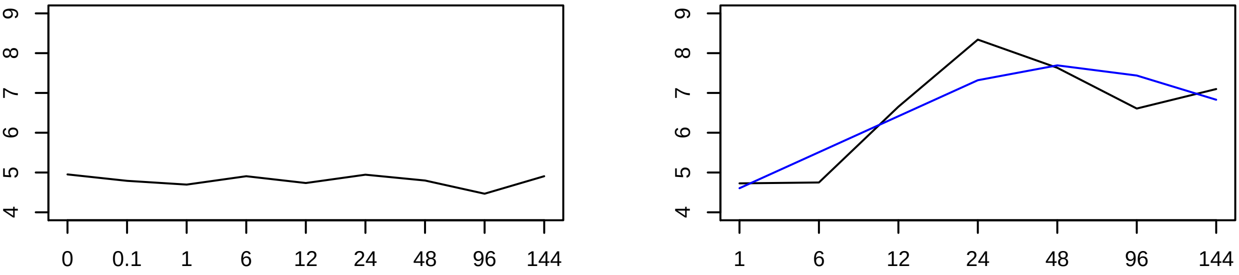

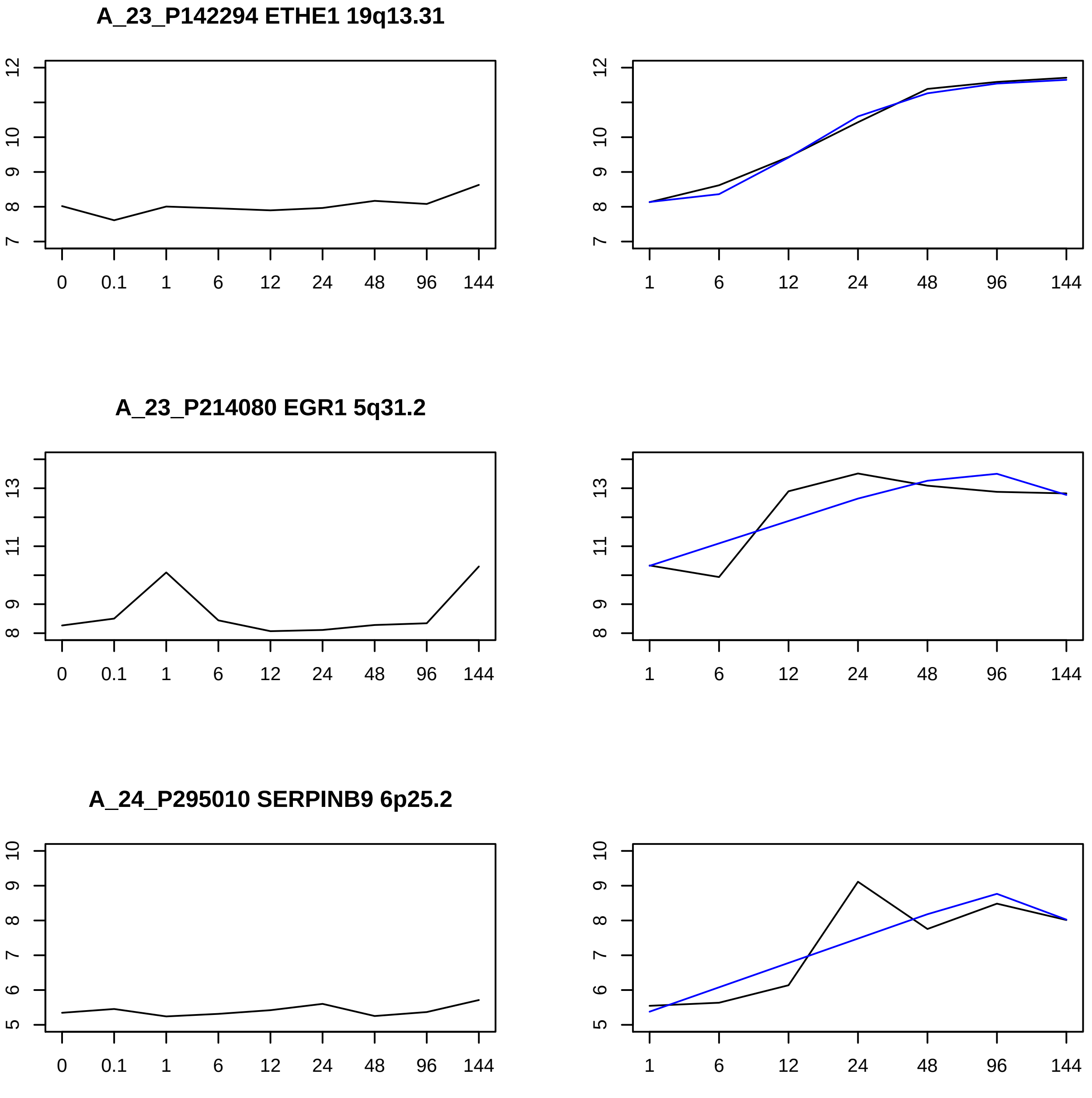
A_23_P142294 ETHE1 19q13.31
7 8 9 10 12
0 0.1 1 6 12 24 48 96 144
1 6 12 24 48 96 144
A_23_P214080 EGR1 5q31.2
8 9 11 13
0 0.1 1 6 12 24 48 96 144
1 6 12 24 48 96 144
A_24_P295010 SERPINB9 6p25.2
5 6 7 8 9 10
0 0.1 1 6 12 24 48 96 144
1 6 12 24 48 96 144

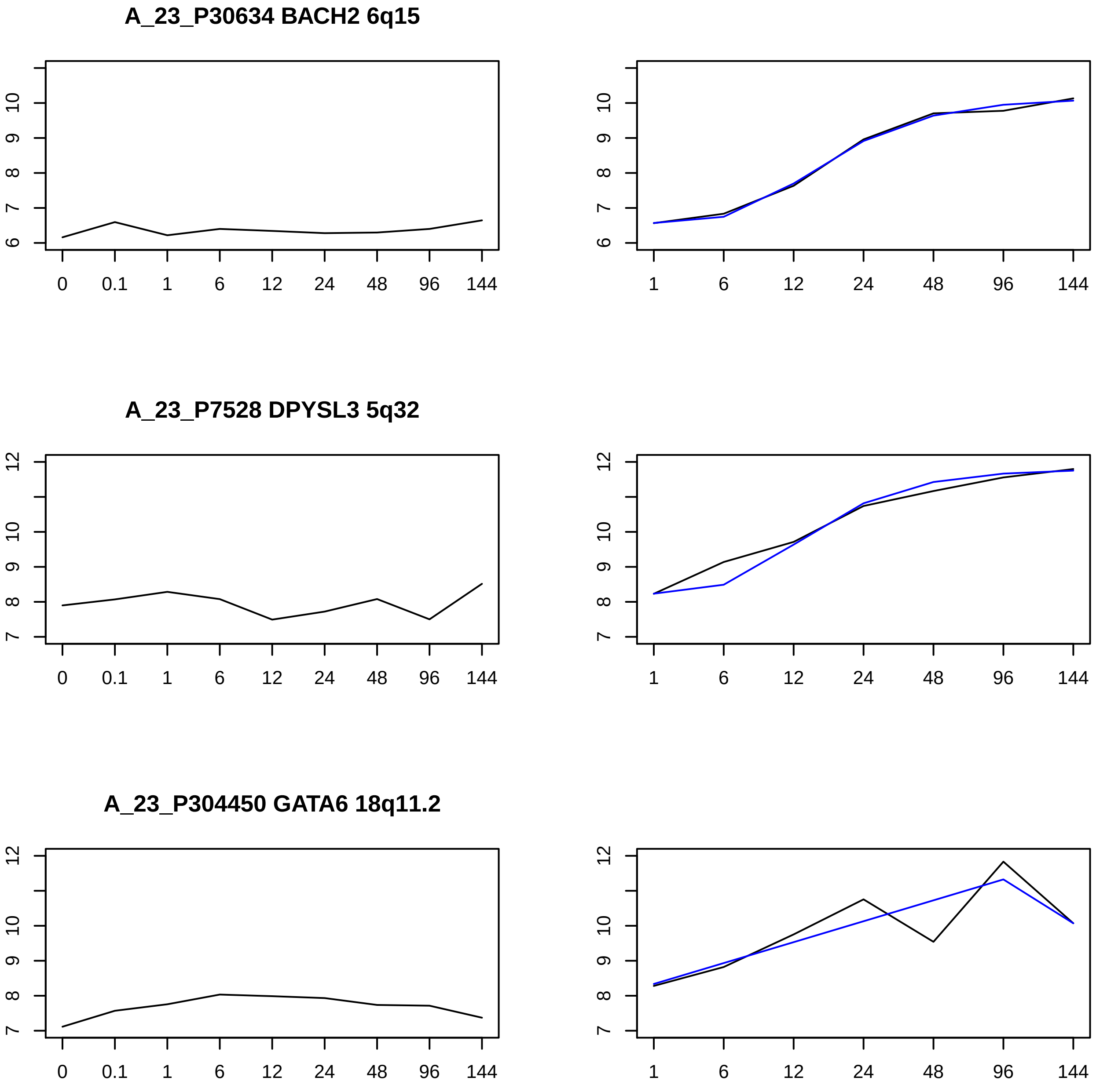
A_23_P30634 BACH2 6q15
6
7
8
9
10
0
0.1
1
6
12
24
48
96
144
1
6
12
24
48
96
144
A_23_P7528 DPYSL3 5q32
7
8
9
10
12
A_23_P304450 GATA6 18q11.2

A_32_P188953 LINC00478 21q21.1

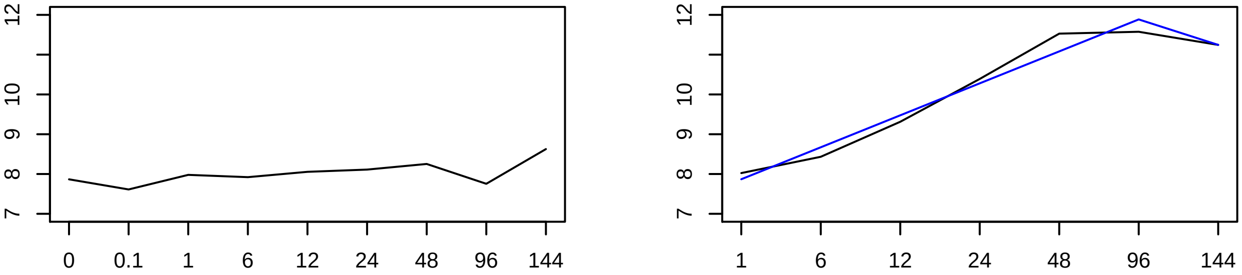

A_23_P7313 SPP1 4q22.1

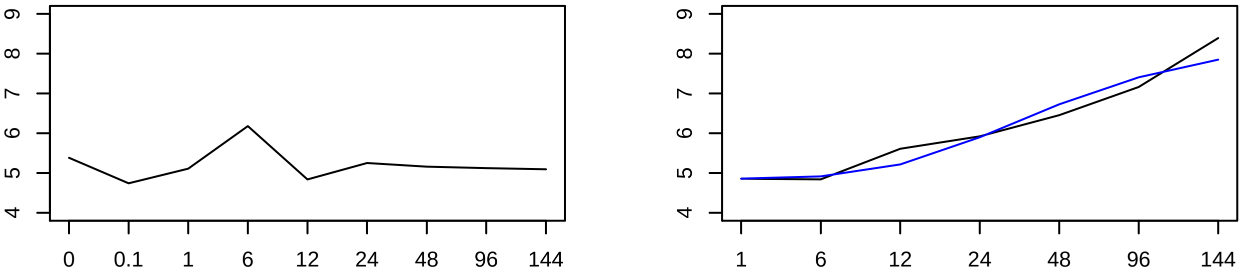

A_23_P32955 MERTK 2q13

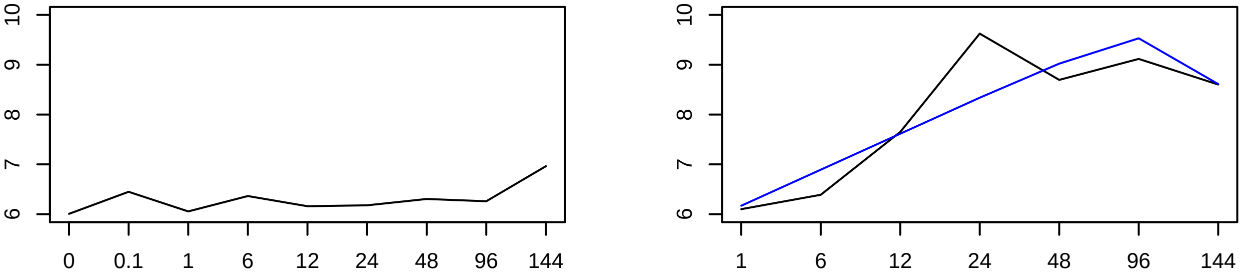

A_23_P381368 HOXD10 2q31.1

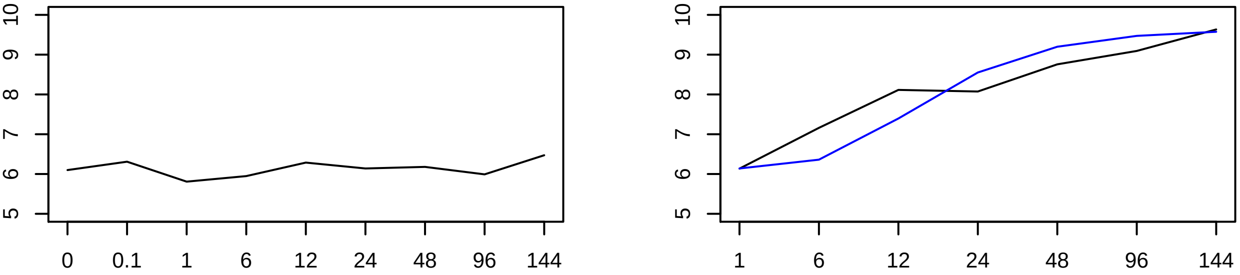

A_23_P101013 TMC6 17q25.3

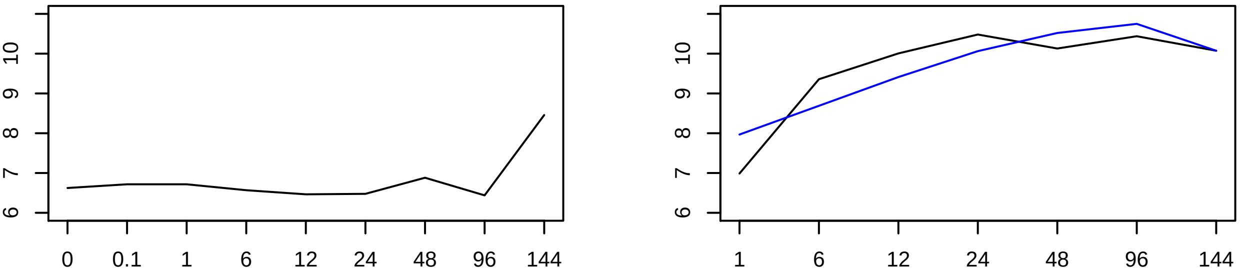

A_23_P26386 CGI−38 16q22.1

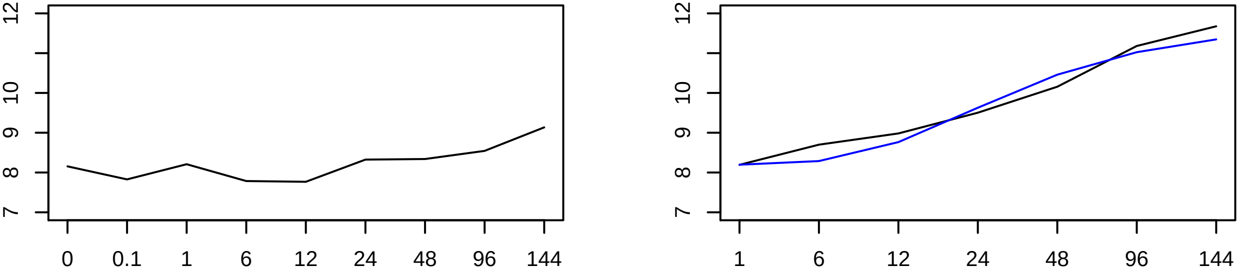

A_23_P371729 GJA5 1q21.1

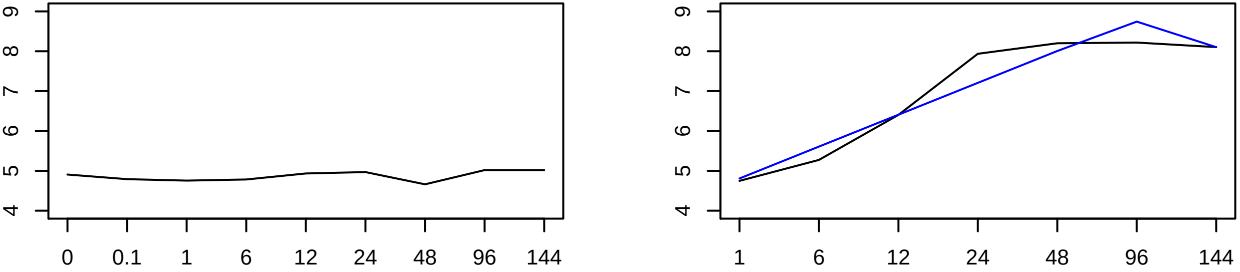

A_32_P93517 THC2725553 NA

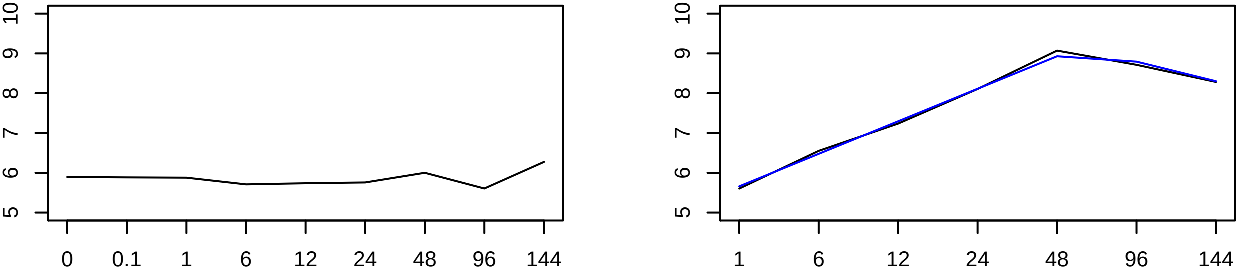

A_23_P217737 ATP7A NA

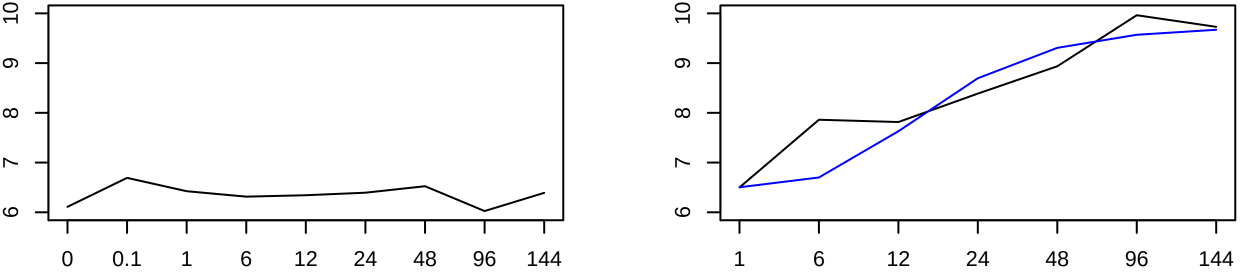

A_23_P389897 NGFR 17q21.33

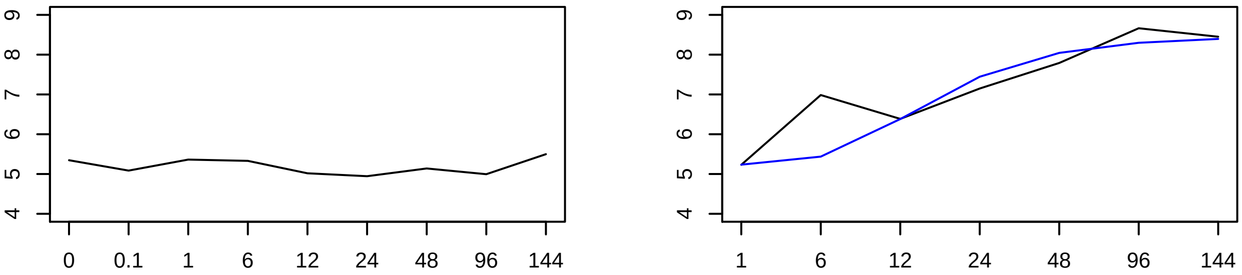

A_24_P928627 DCLK1 13q13.3

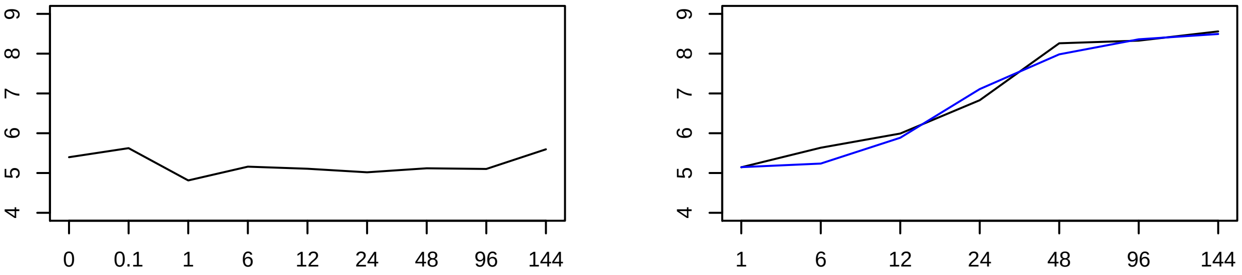

A_24_P408047 PLEKHA4 19q13.33

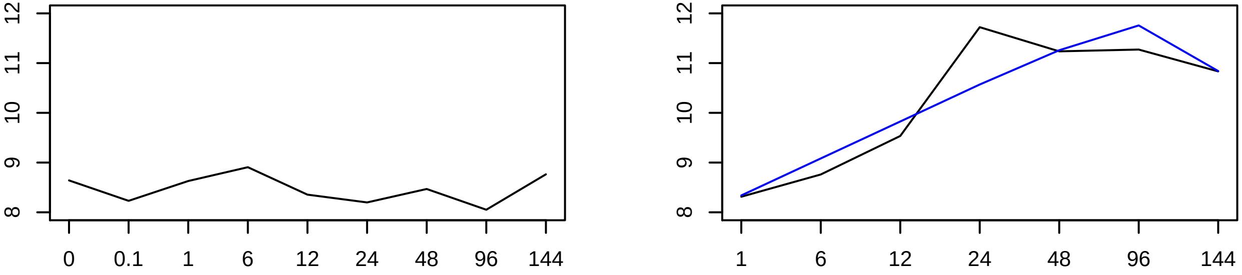

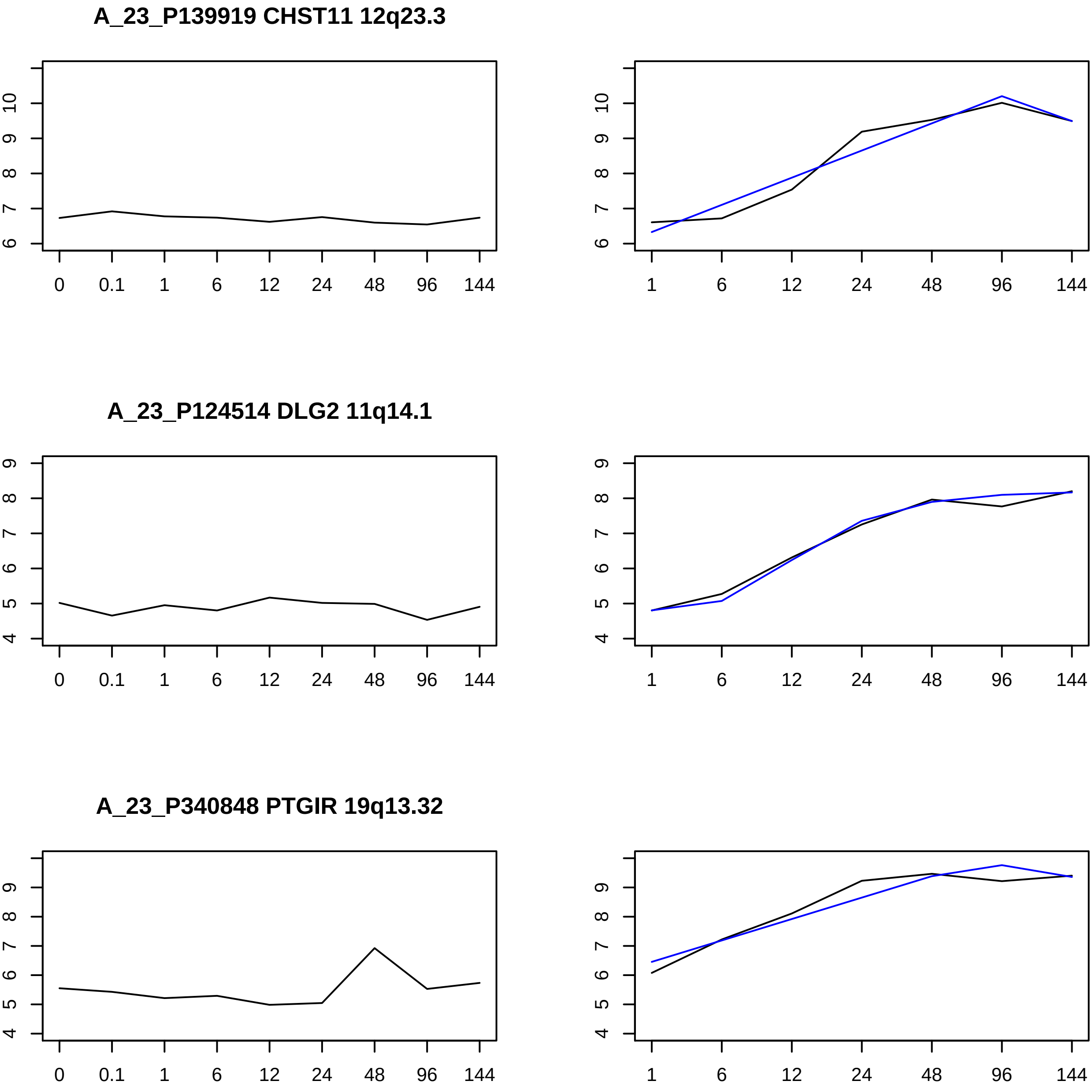
A_23_P139919 CHST11 12q23.3
6
7
8
9
10
0
0.1
1
6
12
24
48
96
144
1
6
12
24
48
96
144
A_23_P124514 DLG2 11q14.1
4
5
6
7
8
9
A_23_P340848 PTGIR 19q13.32
4
5
6
7
8
9

A_32_P207848 THC2669541 NA

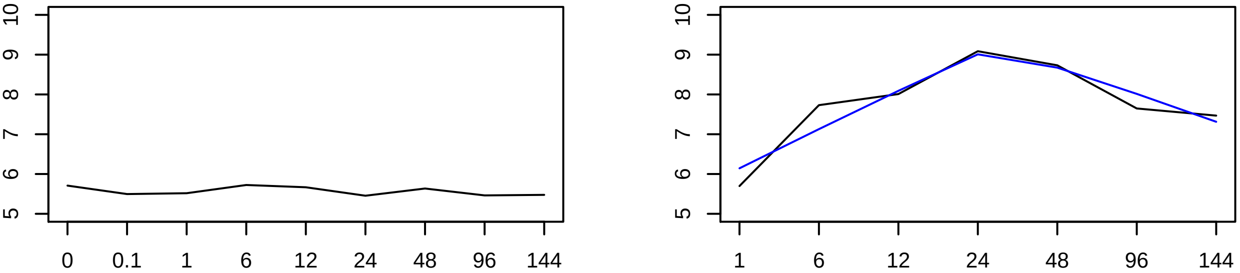

A_23_P140190 KIAA0125 14q32.33

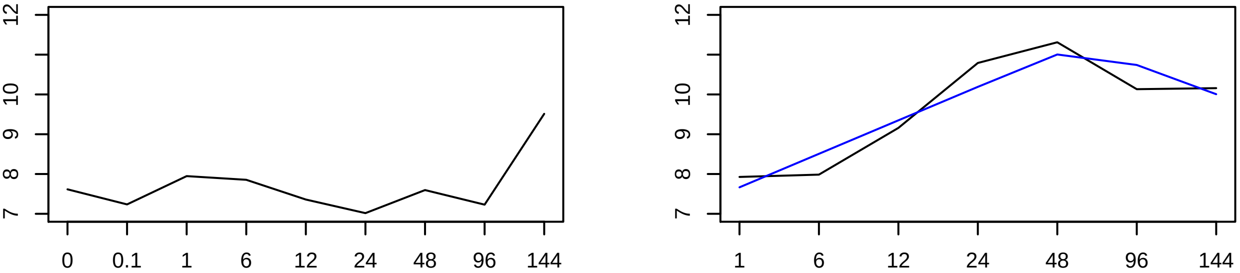

A_23_P96432 MAGEB3 Xp21.2

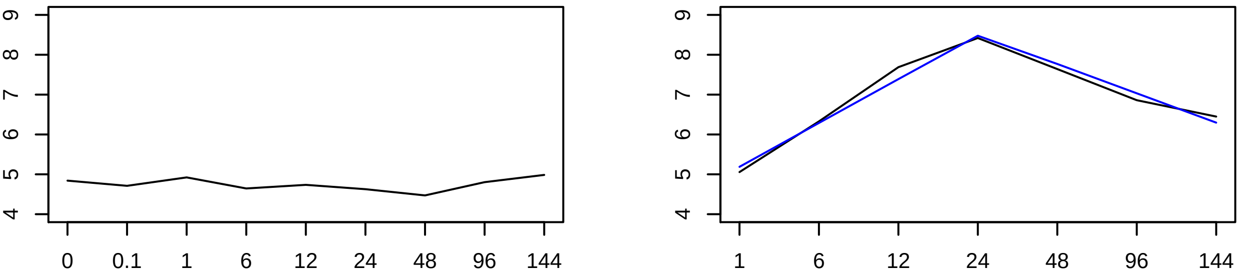

A_24_P277295 RAB43 3q21.3

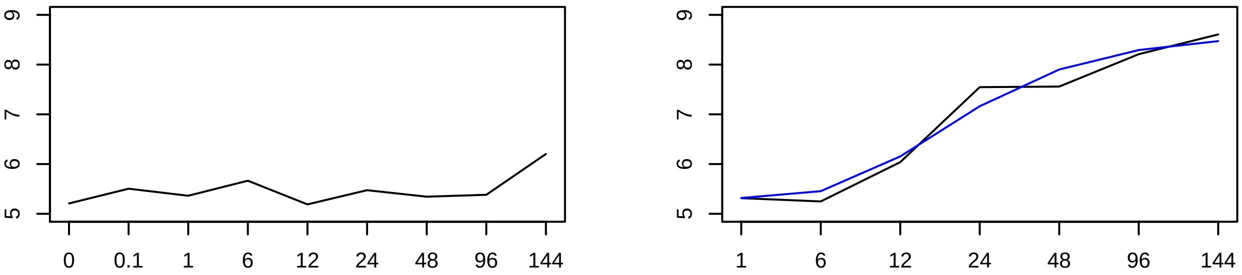

A_23_P54055 JUB 14q11.2

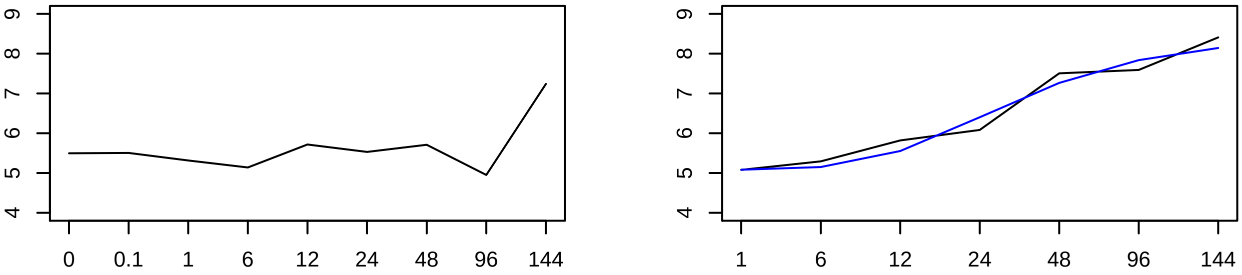

A_32_P36292 AI523154 NA

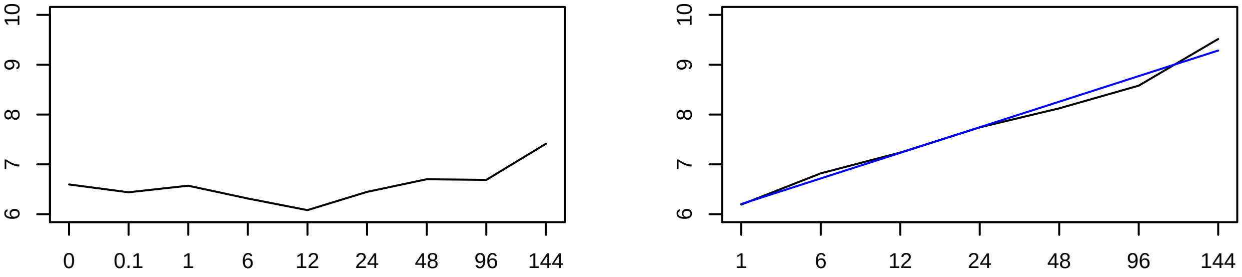

A_23_P431388 SPOCD1 1p35.2

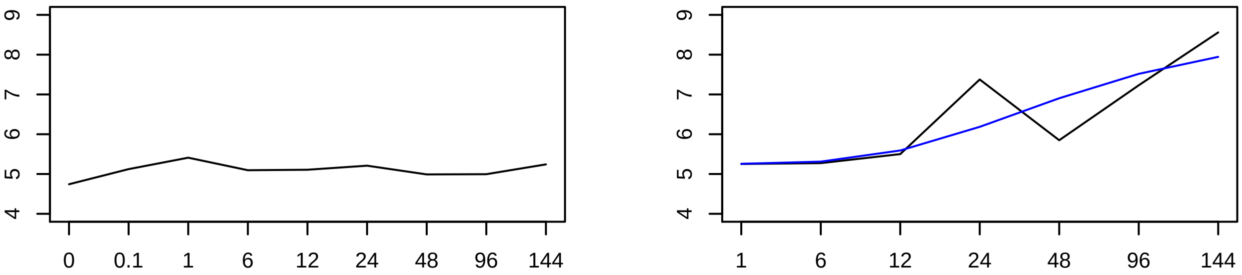

A_23_P326760 MYRIP 3p22.1

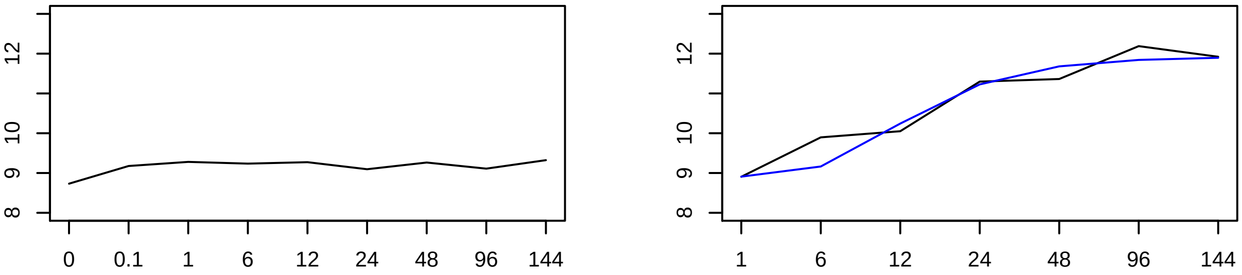

A_32_P56320 THC2722614 NA

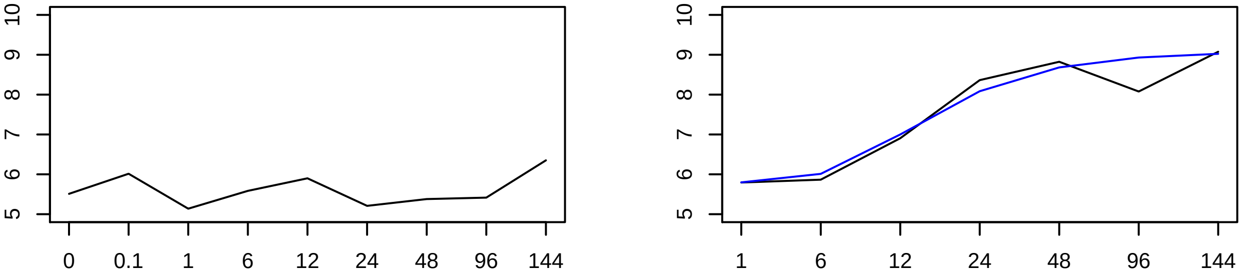

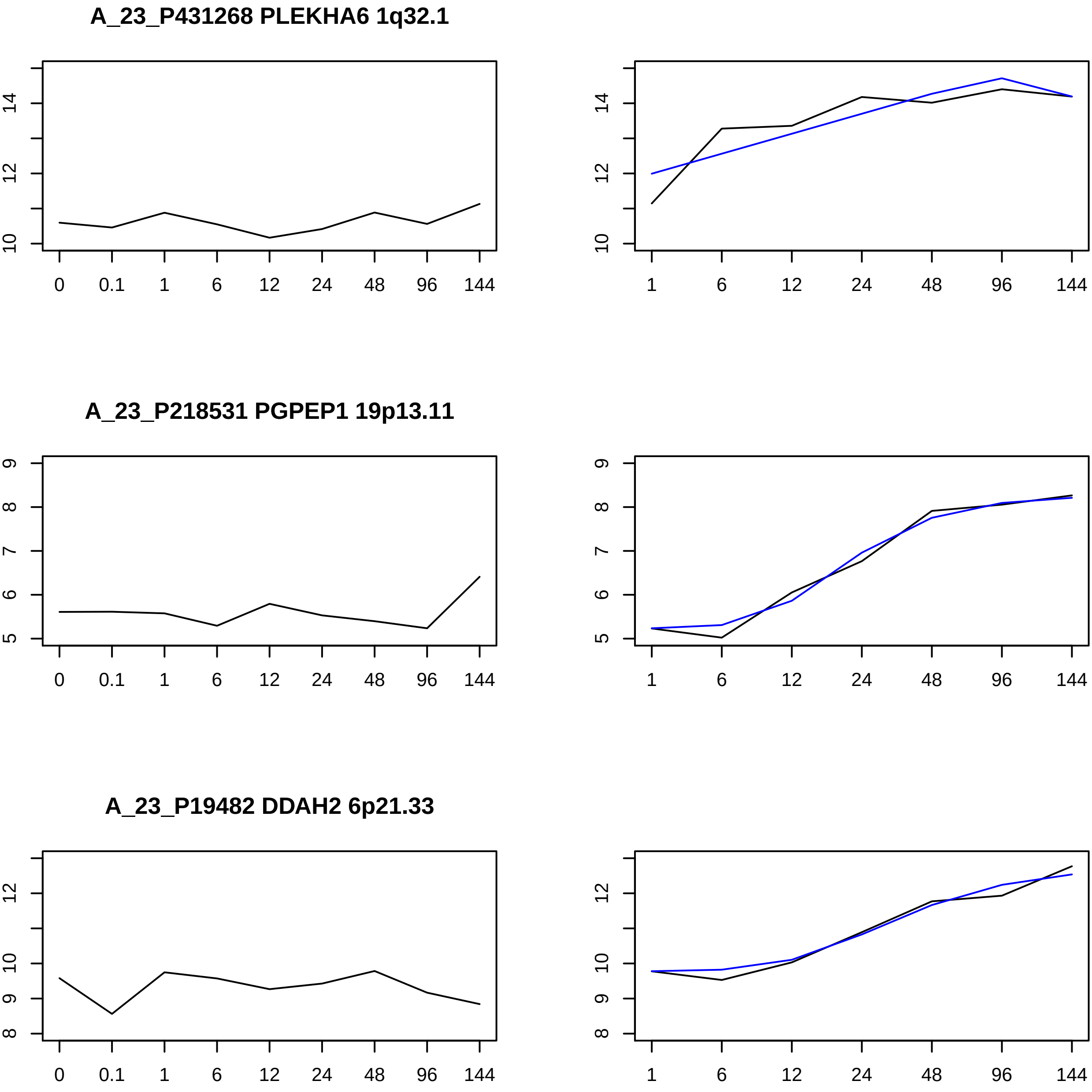
A_23_P431268 PLEKHA6 1q32.1
A_23_P218531 PGPEP1 19p13.11
A_23_P19482 DDAH2 6p21.33
0 0.1 1 6 12 24 48 96 144
1 6 12 24 48 96 144
10 12 14
5 6 7 8 9
8 9 10 12

A_23_P16673 CNN2 19p13.3

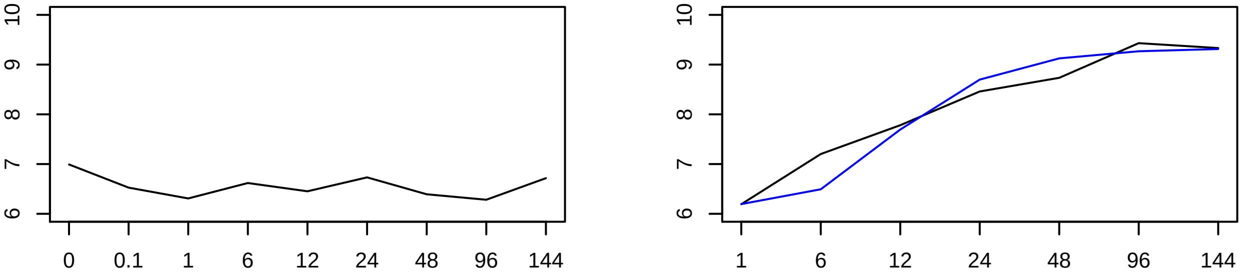

A_32_P208403 GNG2 14q22.1

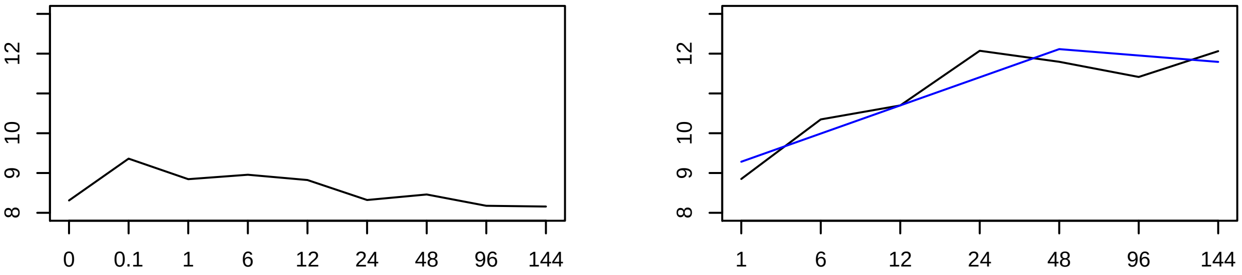

A_24_P196528 CRB1 1q31.3

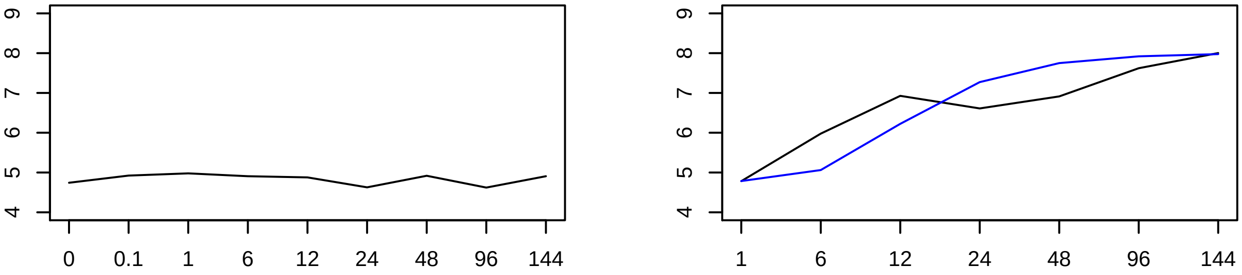

A_23_P148753 PLEKHA6 1q32.1

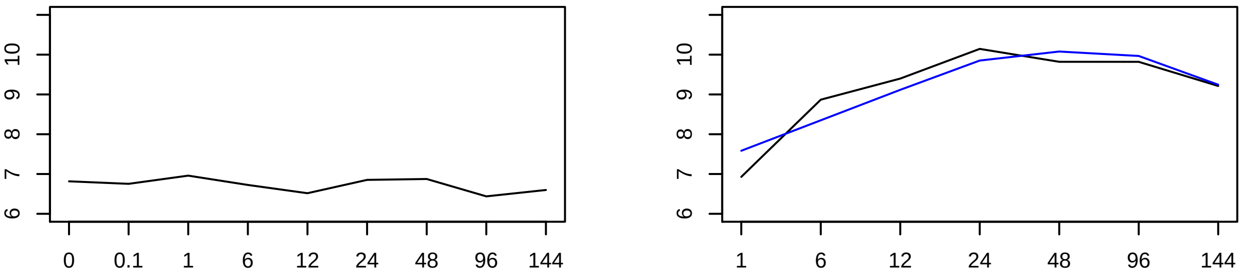

A_24_P149124 NREP 5q22.1

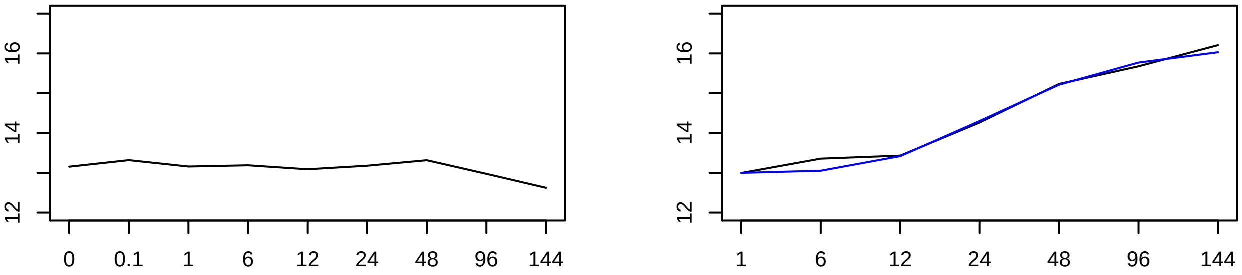

A_32_P81674 LOC157627 8p23.1

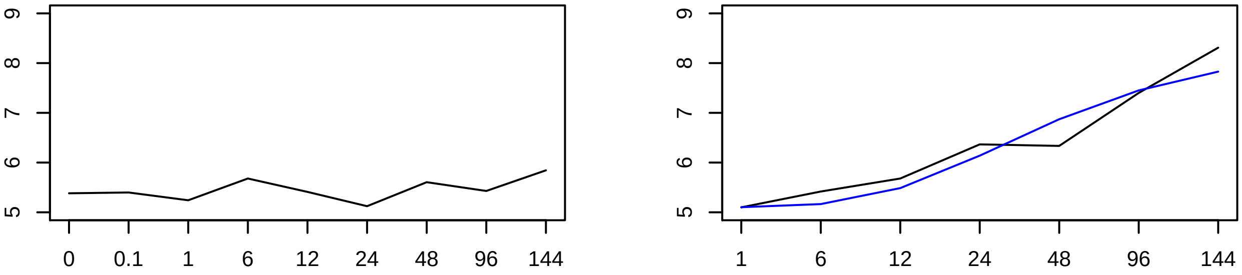

A_32_P104478 FGD6 12q22

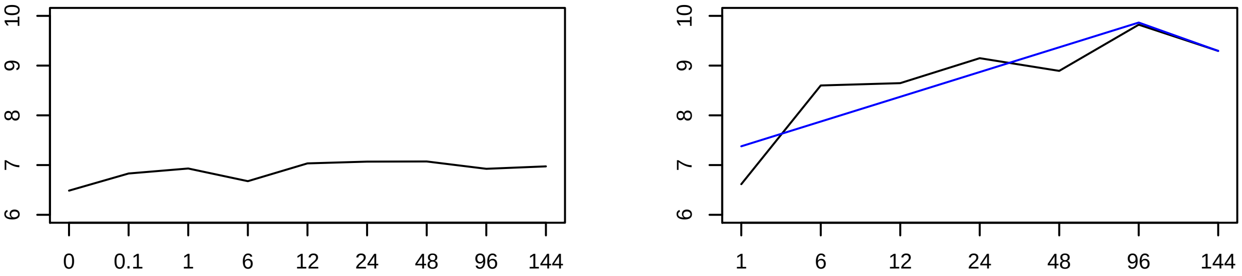

A_24_P341985 BCDO2 11q23.1

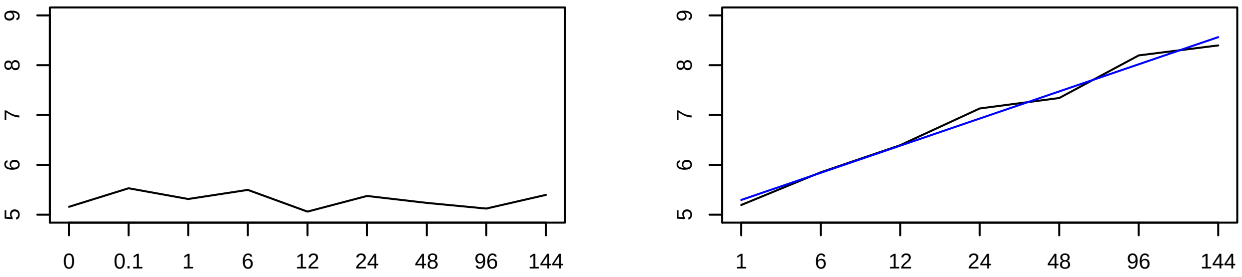

A_23_P151075 ARHGDIB 12p12.3

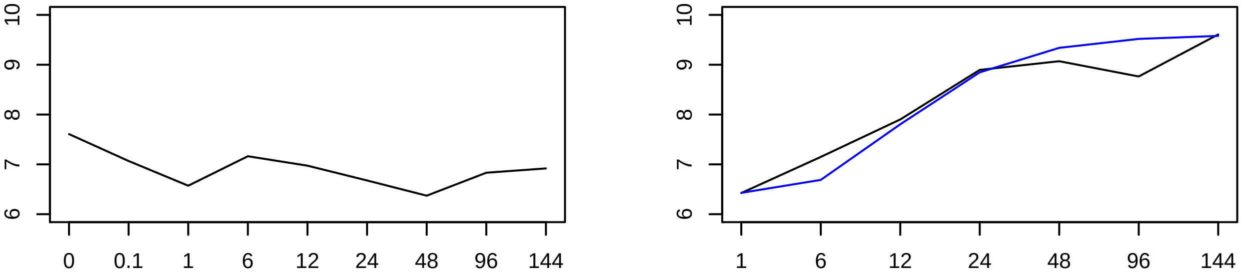

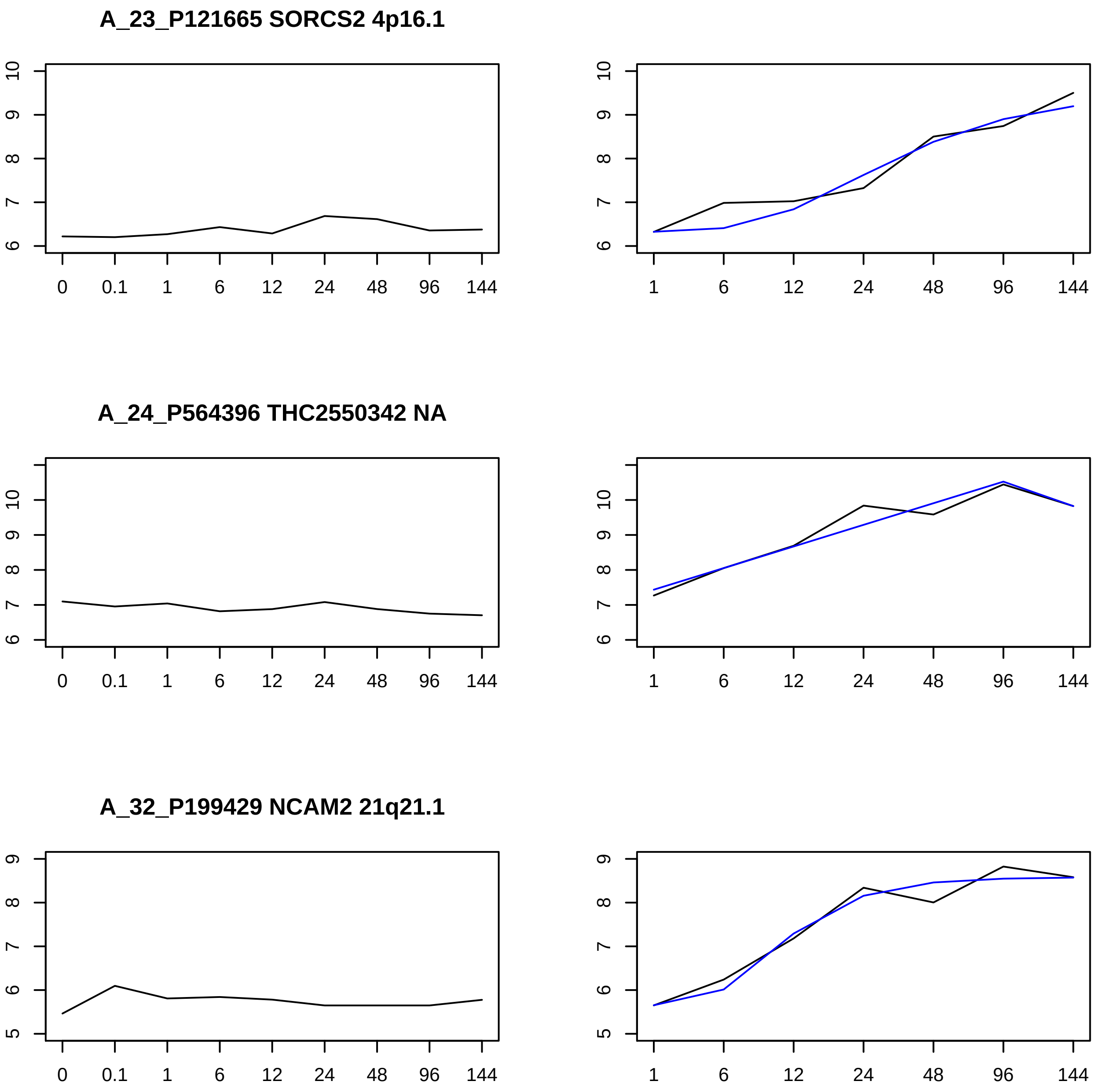
A_23_P121665 SORCS2 4p16.1
6 7 8 9 10
0 0.1 1 6 12 24 48 96 144
6 7 8 9 10
1 6 12 24 48 96 144
A_24_P564396 THC2550342 NA
6 7 8 9 10
0 0.1 1 6 12 24 48 96 144
6 7 8 9 10
1 6 12 24 48 96 144
A_32_P199429 NCAM2 21q21.1
5 6 7 8 9
0 0.1 1 6 12 24 48 96 144
5 6 7 8 9
1 6 12 24 48 96 144

A_23_P77000 VASH1 14q24.3

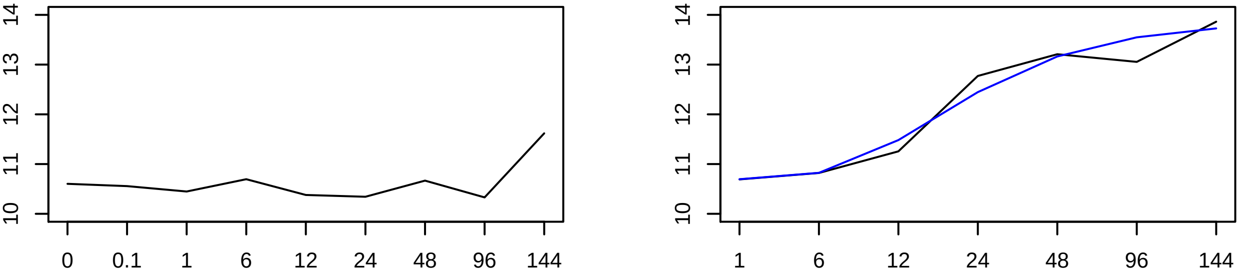

A_24_P149036 DPYSL3 5q32

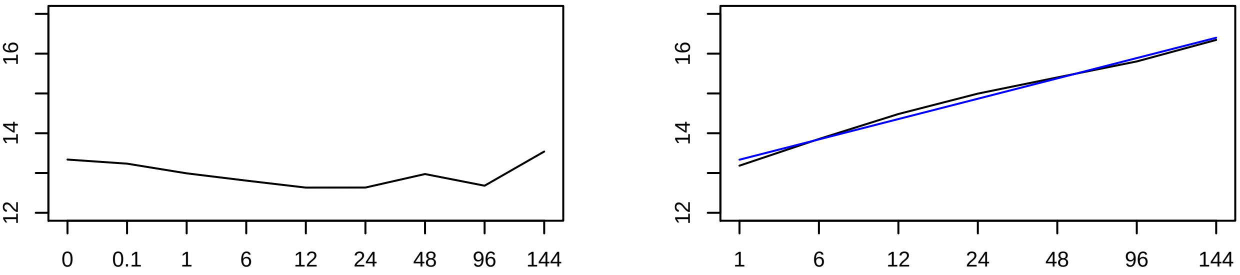

A_24_P355816 DRAM 12q23.2

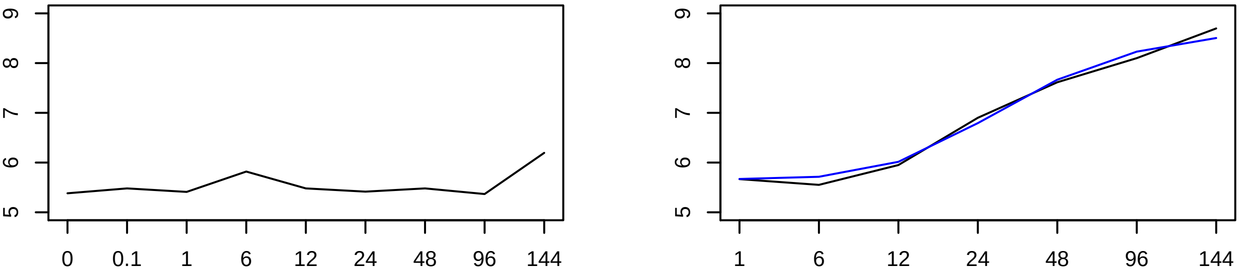

A_23_P210400 KCNQ2 20q13.33

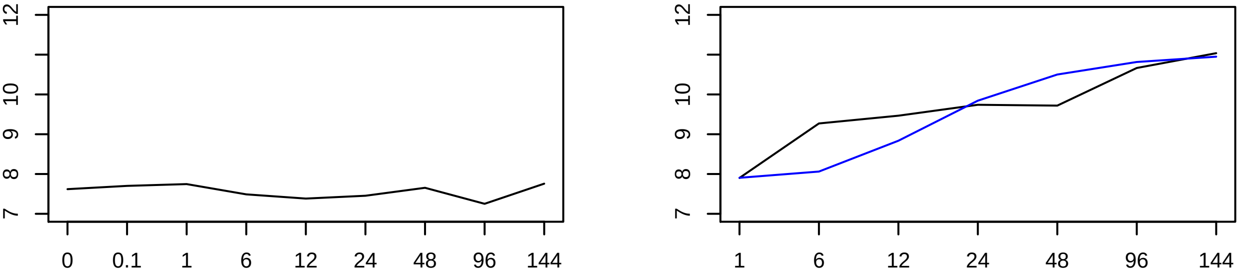

A_23_P370651 FAM13A 4q22.1

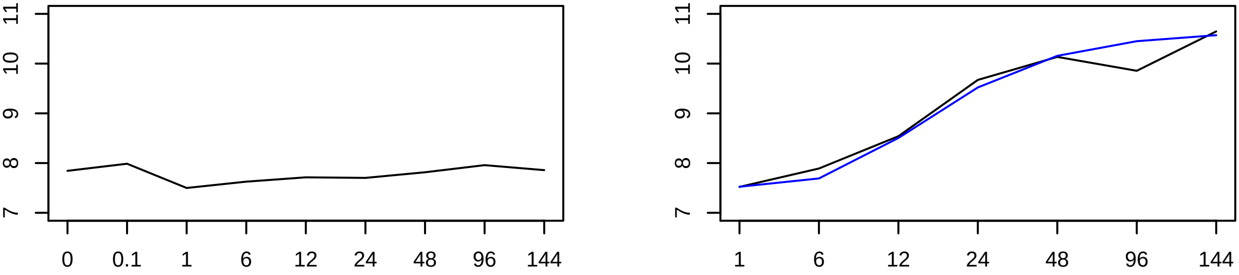

A_23_P14853 LTK 15q15.1

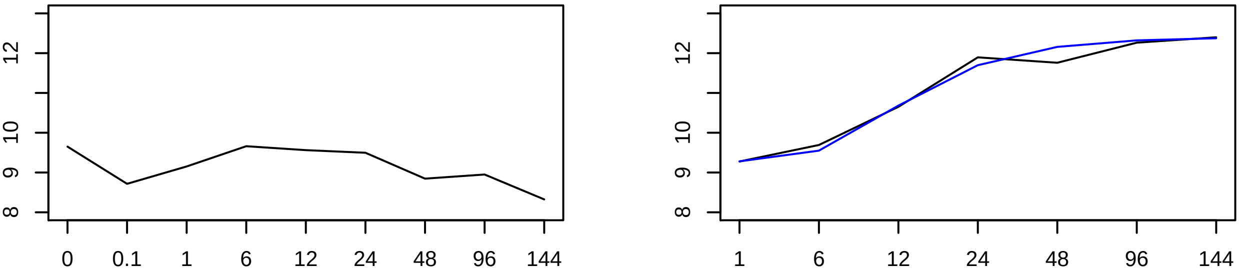

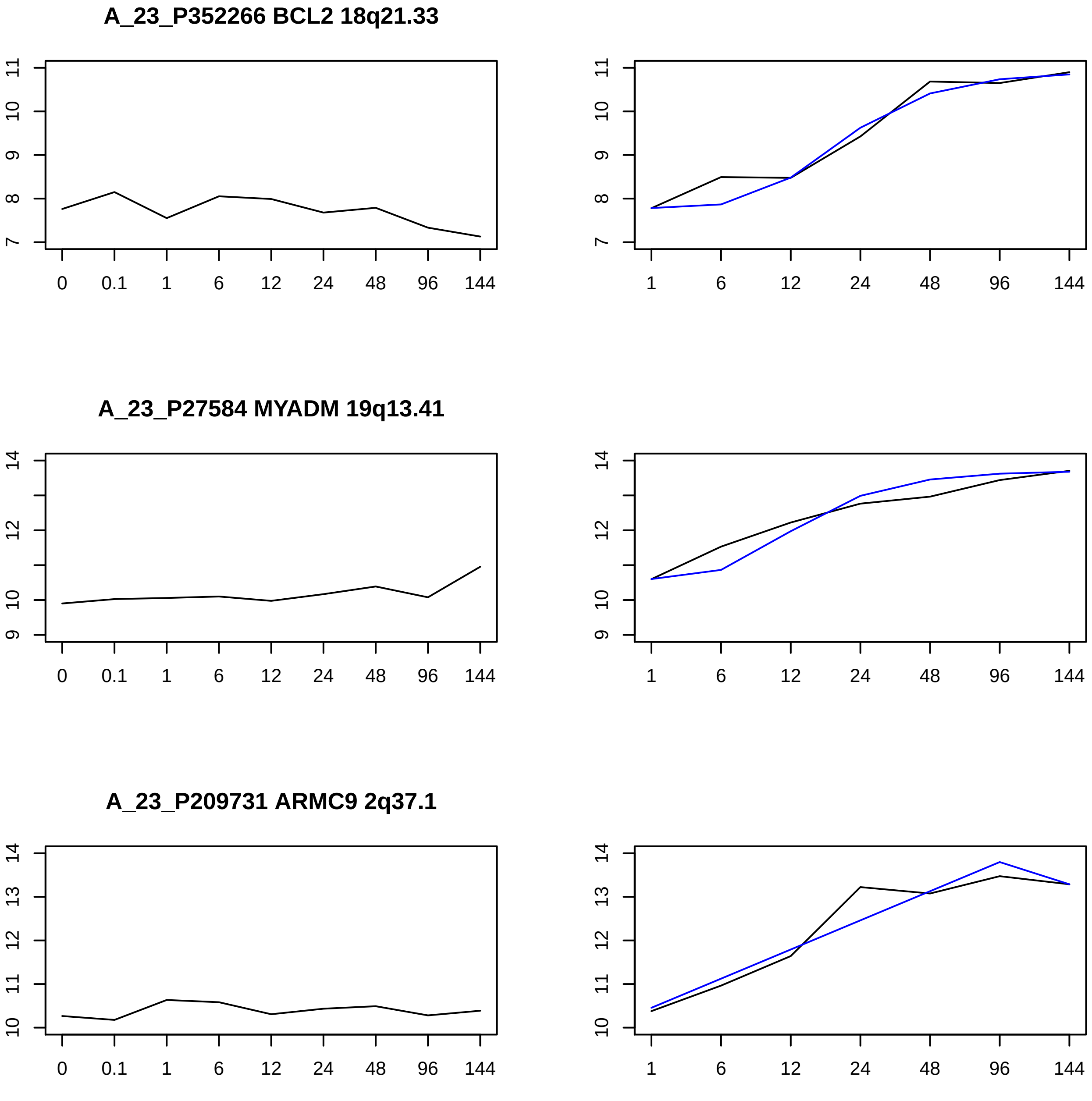
A_23_P352266 BCL2 18q21.33
A_23_P27584 MYADM 19q13.41
A_23_P209731 ARMC9 2q37.1
0 0.1 1 6 12 24 48 96 144
1 6 12 24 48 96 144
7 8 9 10 11
9 10 12 14
10 11 12 13 14

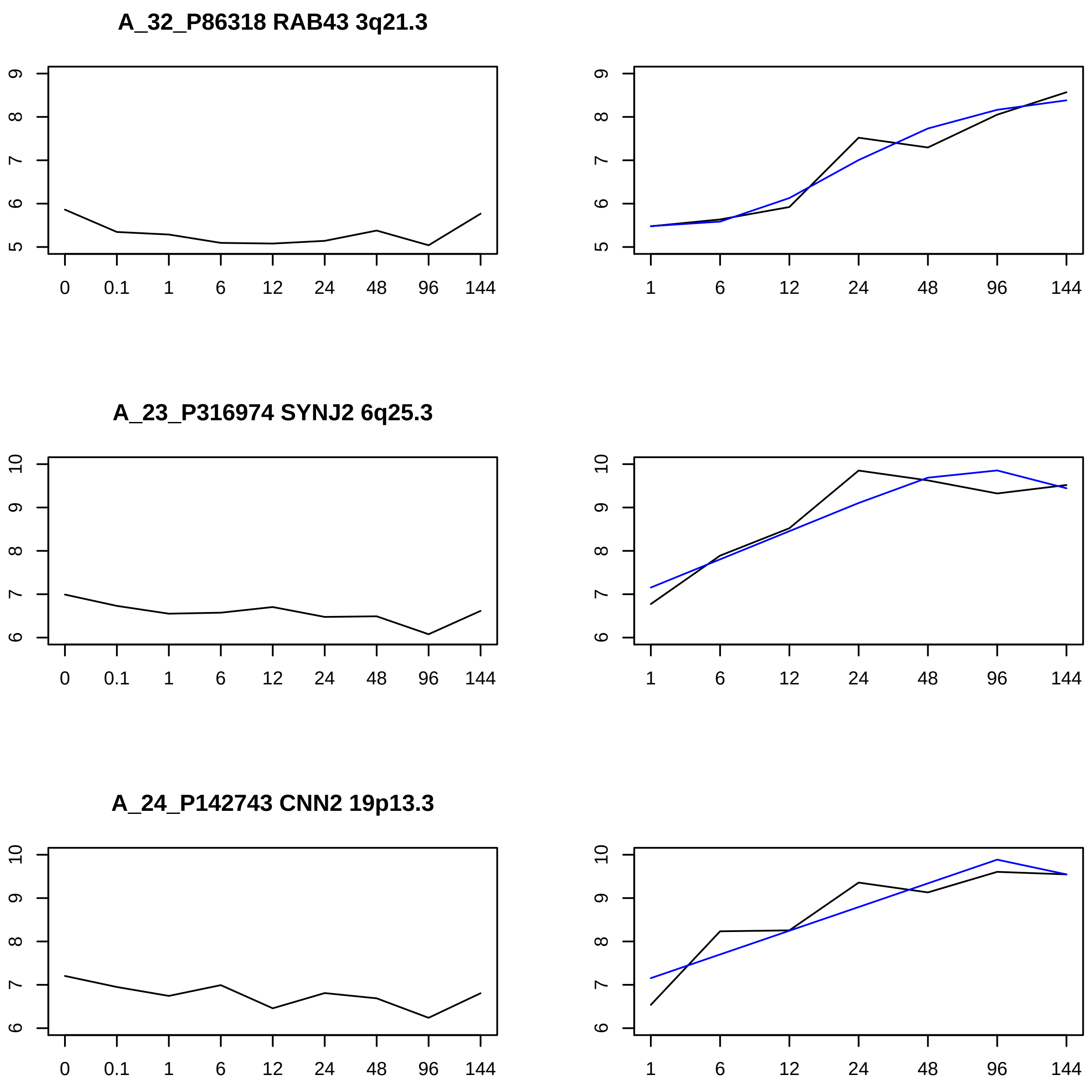
A_32_P86318 RAB43 3q21.3
5
6
7
8
9
0
0.1
1
6
12
24
48
96
144
1
6
12
24
48
96
144
A_23_P316974 SYNJ2 6q25.3
6
7
8
9
10
A_24_P142743 CNN2 19p13.3

A_32_P497742 GPR161 1q24.2

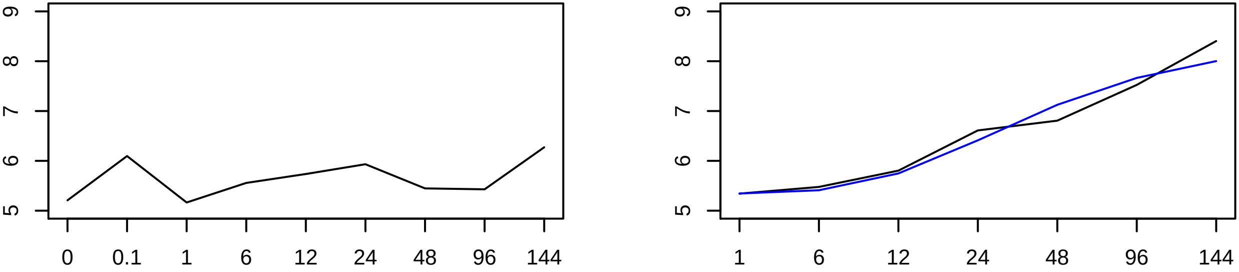

A_23_P35456 SH3PXD2A 10q24.33

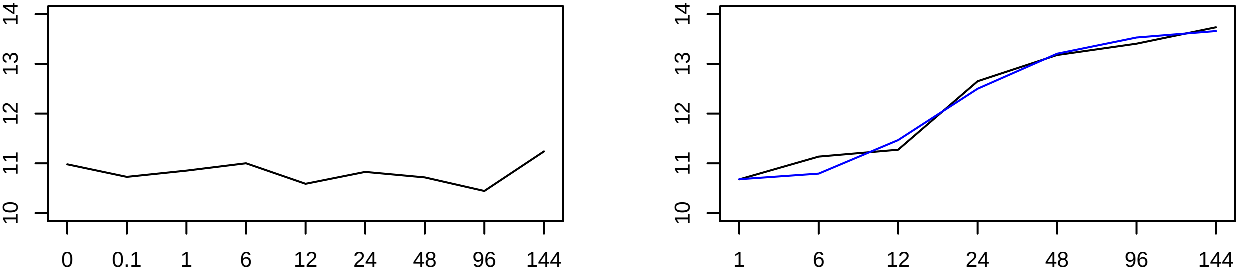

A_24_P129326 VGF 7q22.1

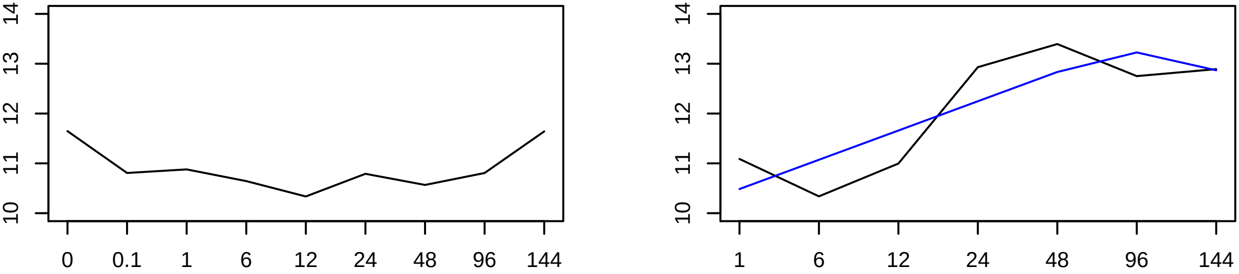

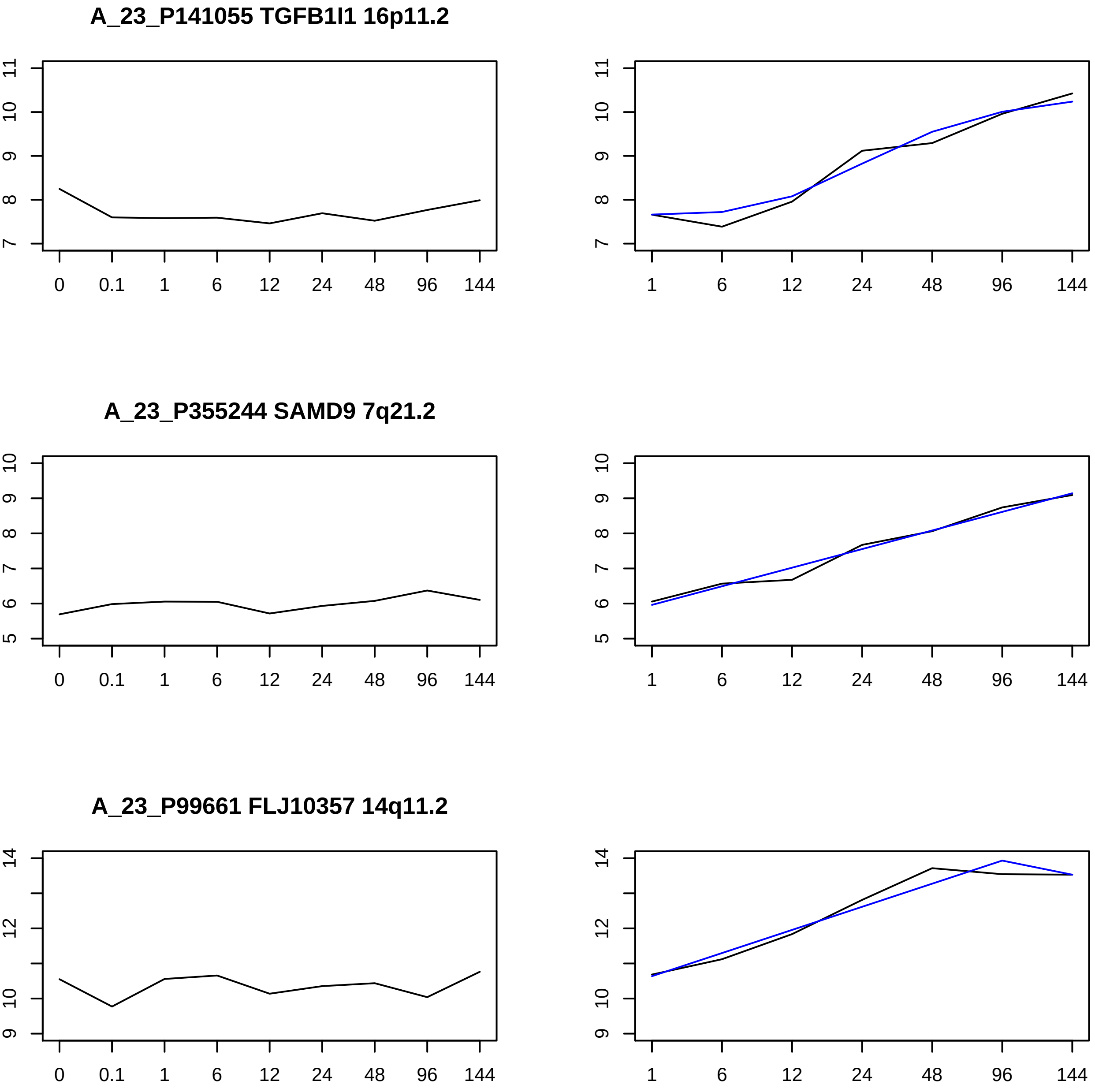
A_23_P141055 TGFB1I1 16p11.2
7 8 9 10 11
0 0.1 1 6 12 24 48 96 144
1 6 12 24 48 96 144
A_23_P355244 SAMD9 7q21.2
5 6 7 8 9 10
0 0.1 1 6 12 24 48 96 144
1 6 12 24 48 96 144
A_23_P99661 FLJ10357 14q11.2
9 10 12 14
0 0.1 1 6 12 24 48 96 144
1 6 12 24 48 96 144

A_24_P637651 THC2657355 NA

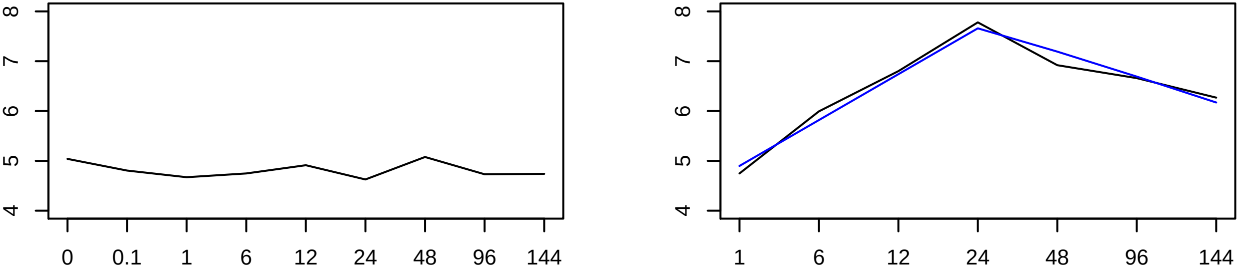

A_23_P42257 IER3 6p21.33

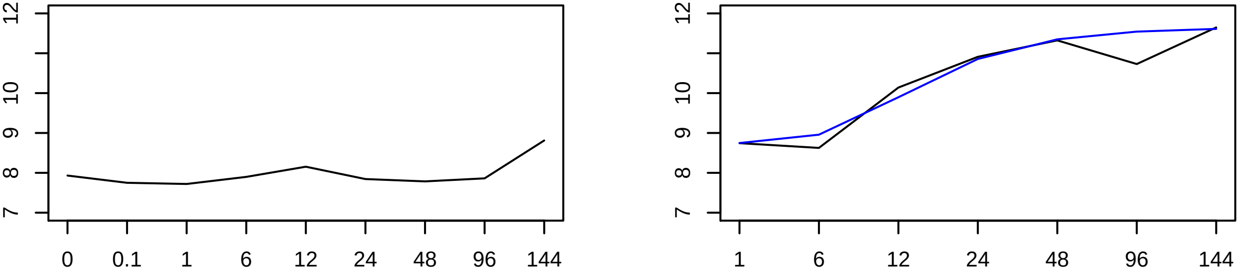

A_23_P500328 DCX Xq22.3

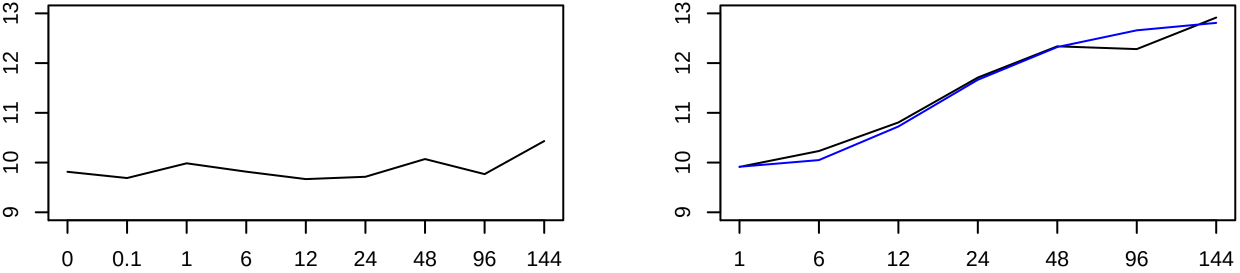

A_24_P328524 KALRN 3q21.2

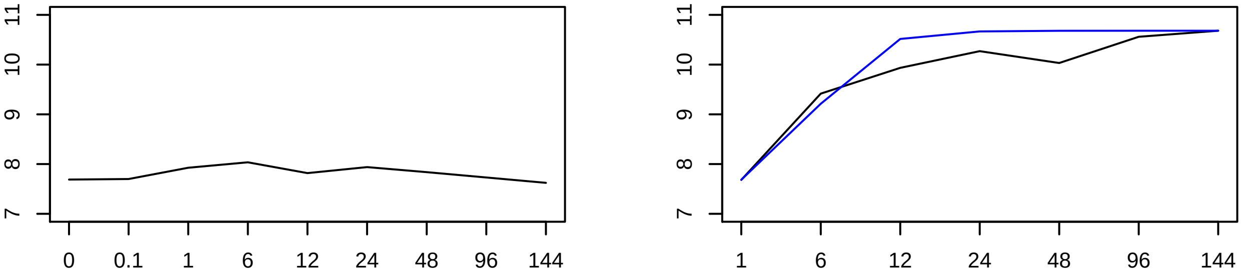

A_23_P99163 DRAM 12q23.2

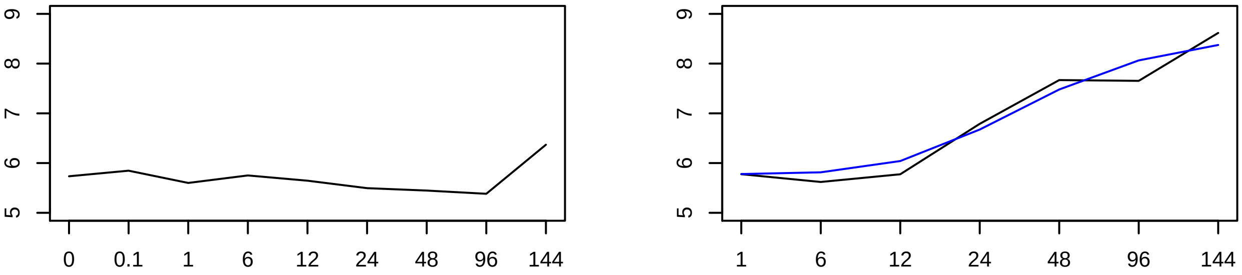

A_23_P46149 GPR137B 1q42.3

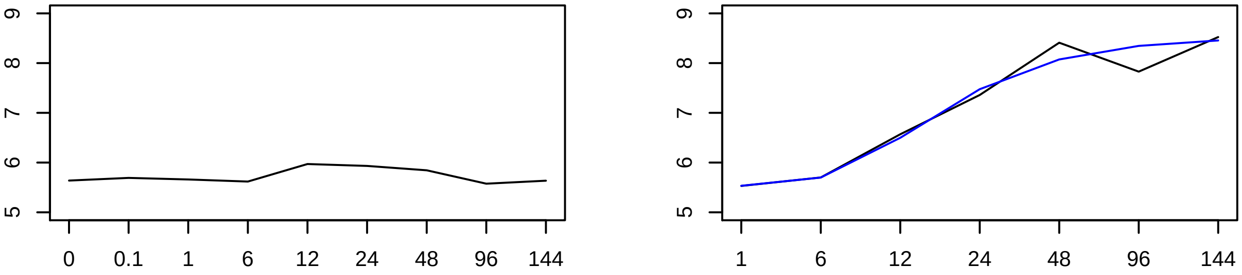

A_24_P945408 ARMC9 NA

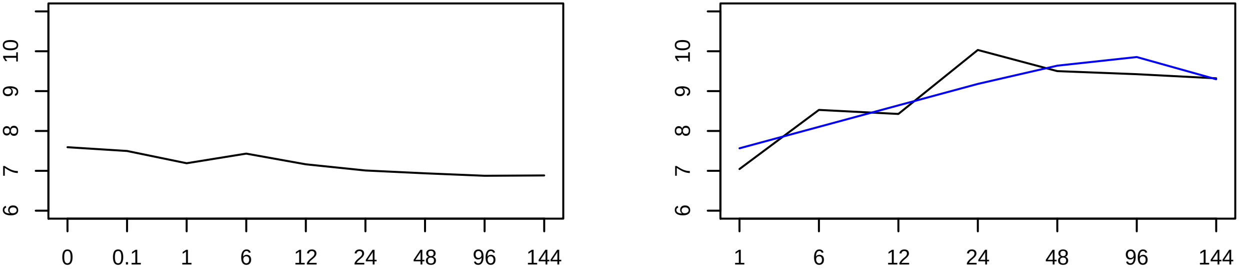

A_23_P10647 CYTL1 4p16.2

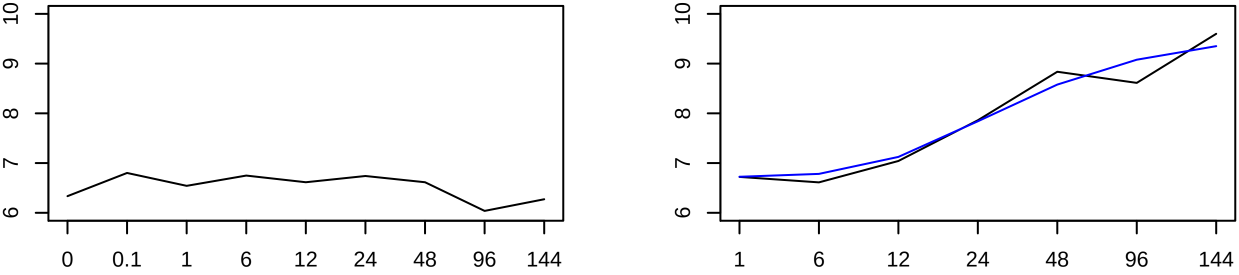

A_24_P284959 PCDH18 4q28.3

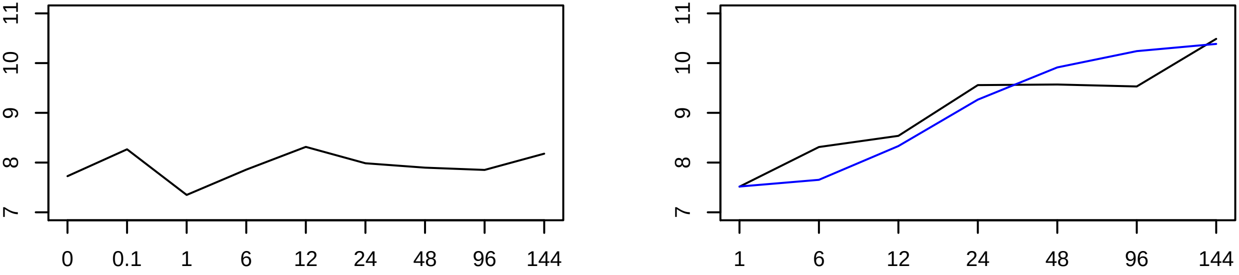

A_23_P83388 EPPK1 8q24.3

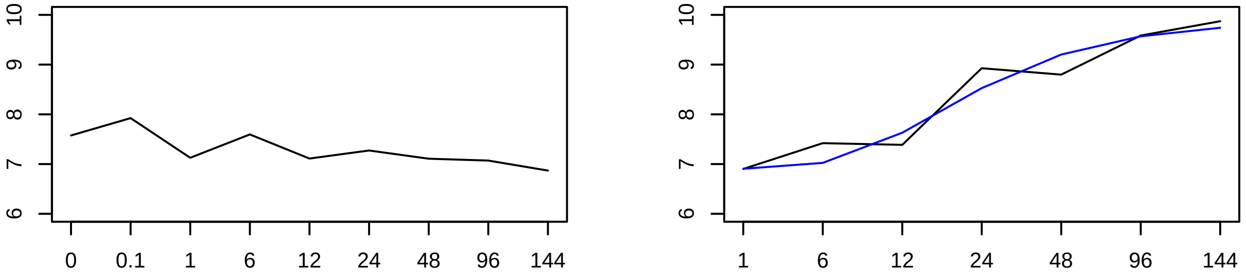

A_32_P113380 THC2677659 NA

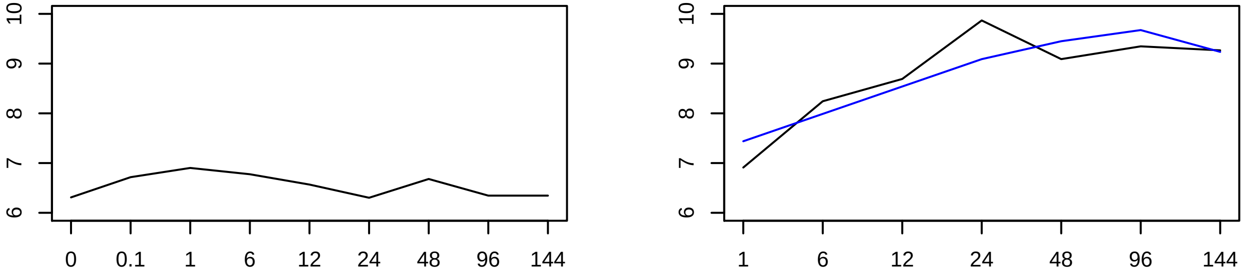

A_23_P32536 GNG2 14q22.1

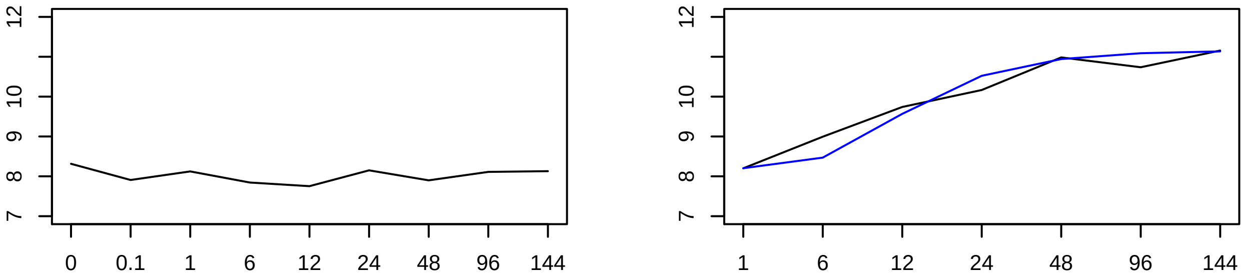

A_23_P148584 DOCK11 Xq24

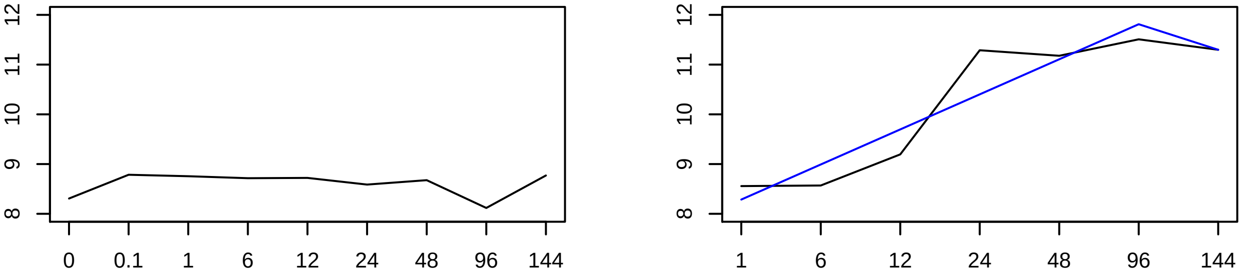

A_24_P201739 SH2B3 12q24.12

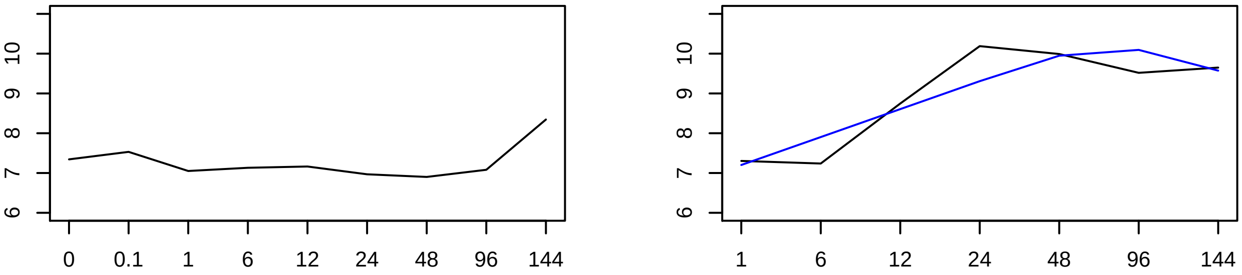

A_23_P216023 ANGPT1 8q23.1

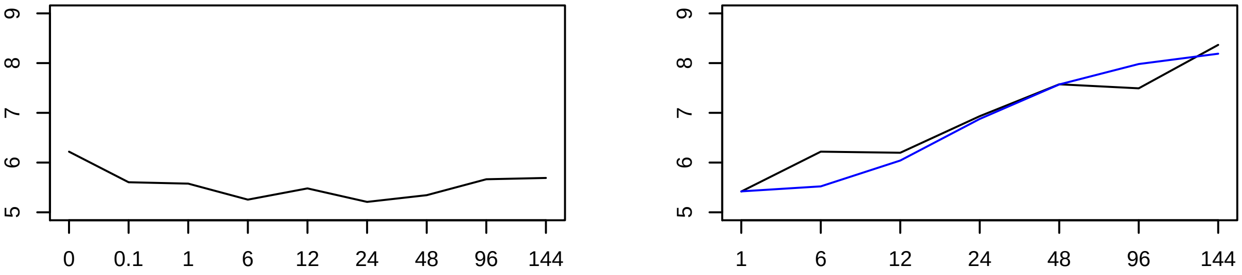

A_23_P144896 PDLIM7 5q35.3

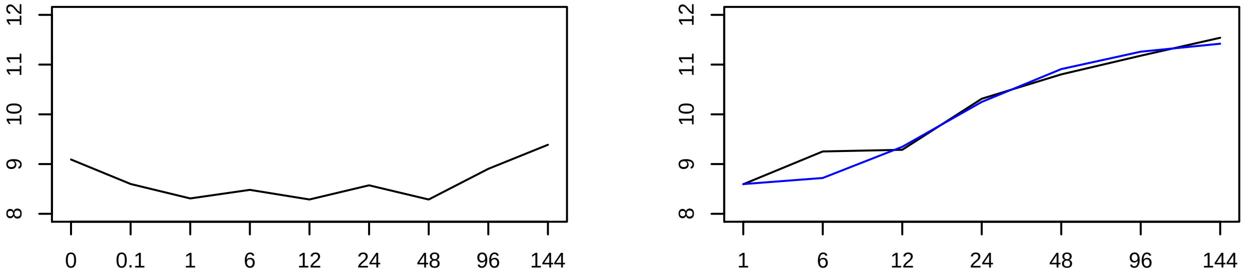

A_23_P209735 ARMC9 2q37.1

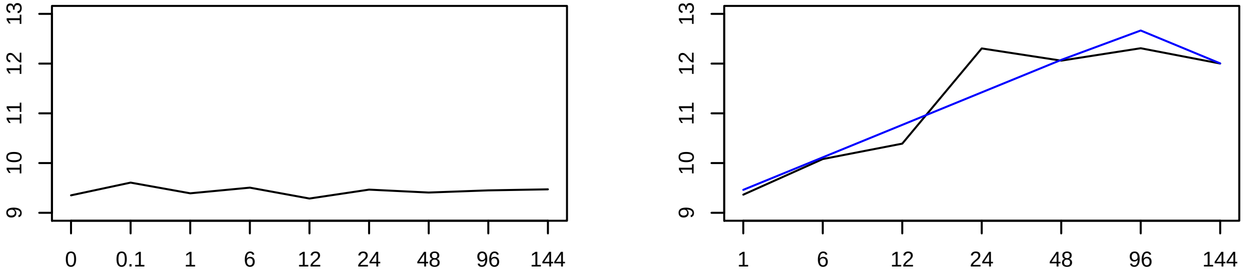

A_23_P353667 C19orf30 19p13.3

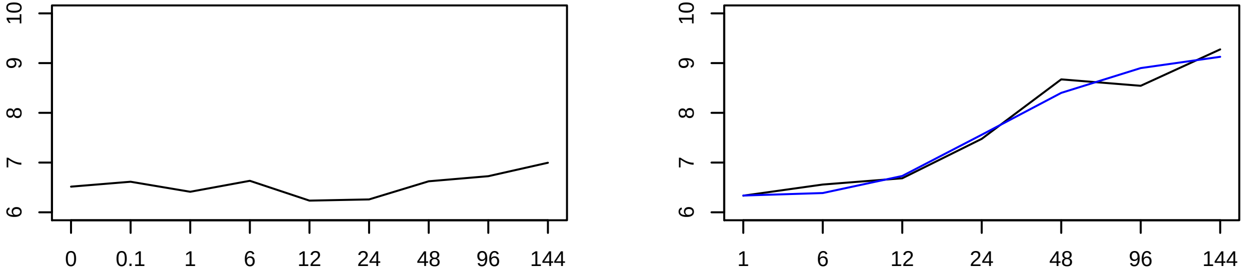

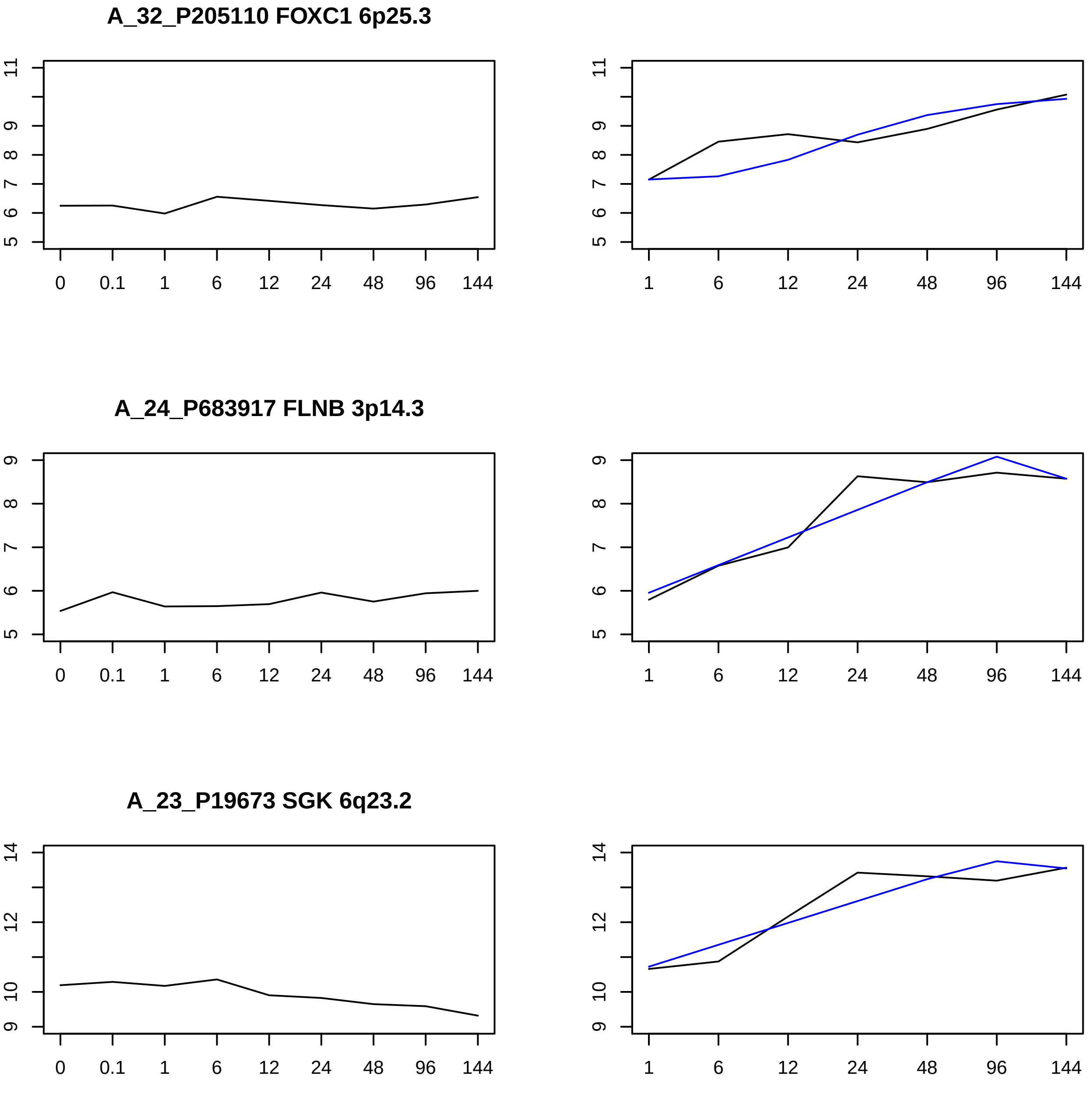
A_32_P205110 FOXC1 6p25.3
A_24_P683917 FLNB 3p14.3
A_23_P19673 SGK 6q23.2
0
0.1
1
6
12
24
48
96
144

A_32_P171253 THC2674306 NA

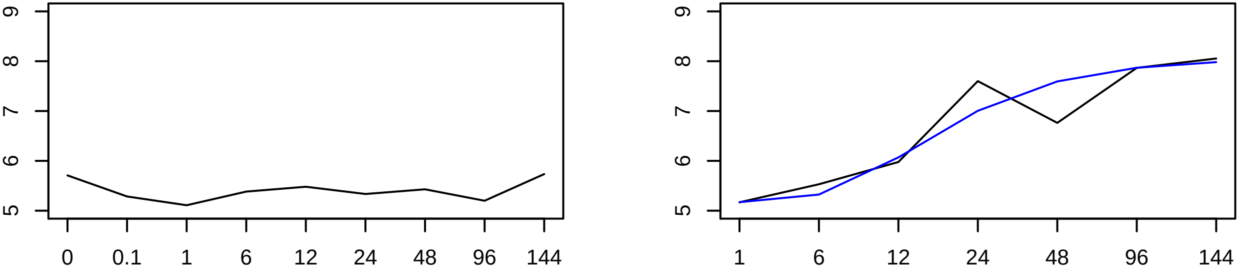

A_32_P83845 HEY1 8q21.13

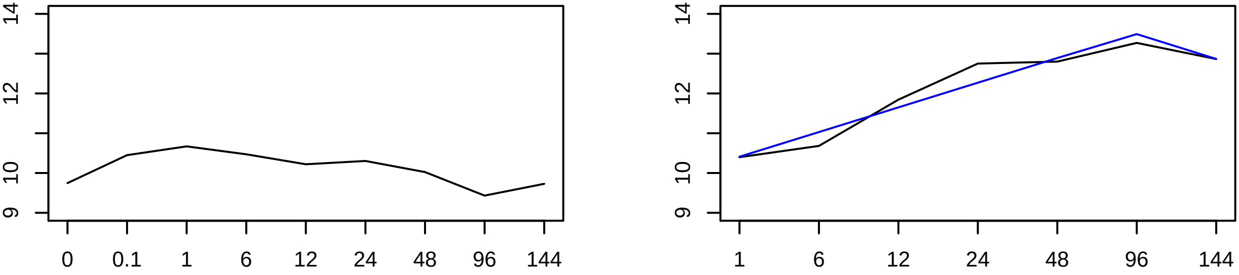

A_24_P22050 RAB20 13q34

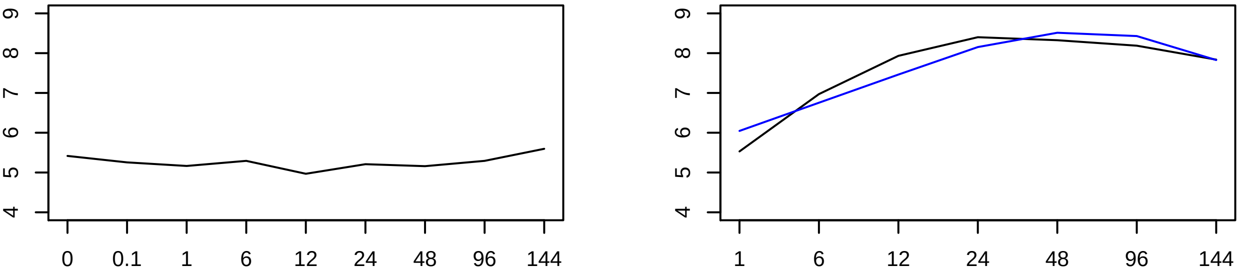

A_23_P145965 TPST1 7q11.21

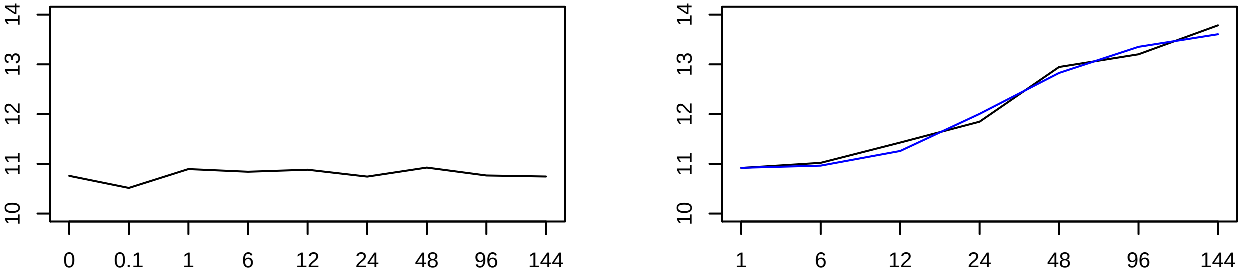

A_32_P109242 CSRNP3 NA

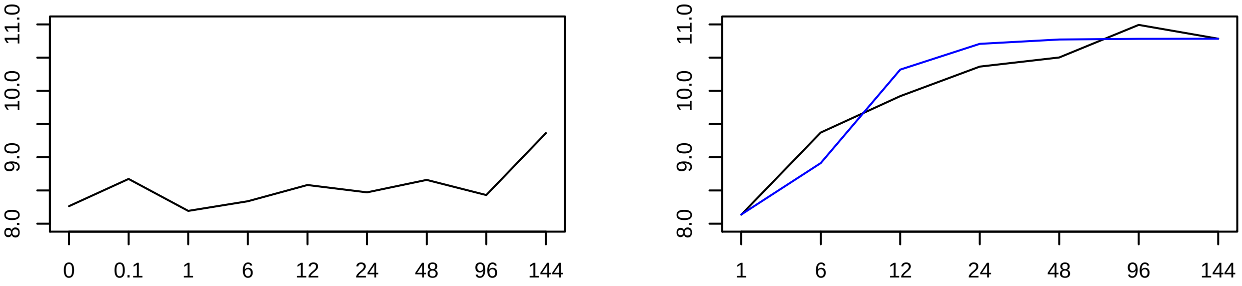

A_23_P6771 LMCD1 3p26.1

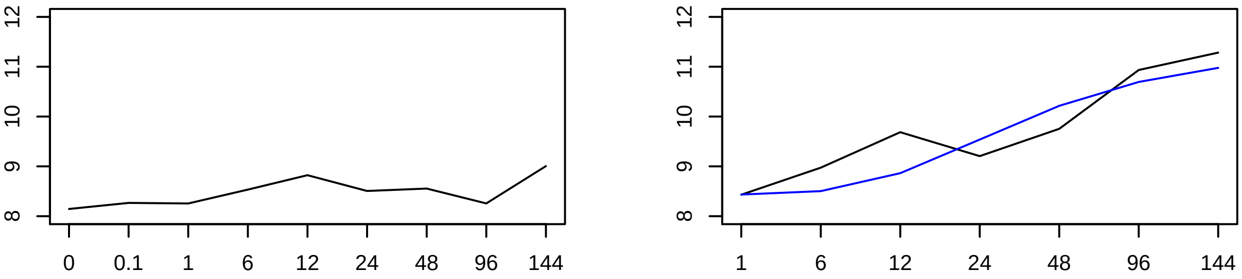

A_32_P99171 CHST11 NA

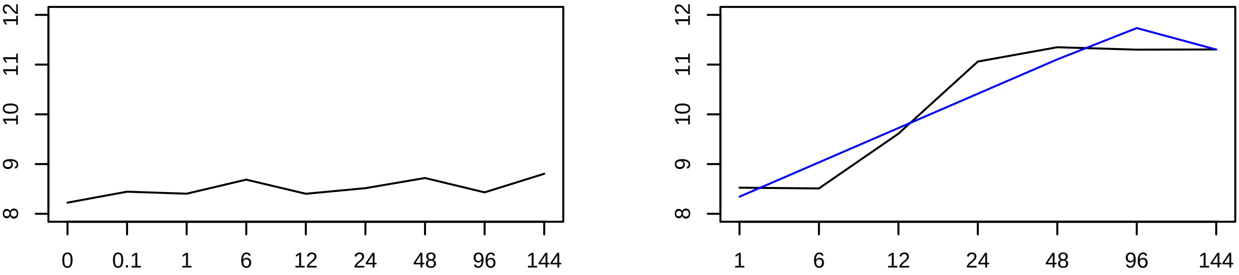

A_23_P130974 KIAA1683 19p13.11

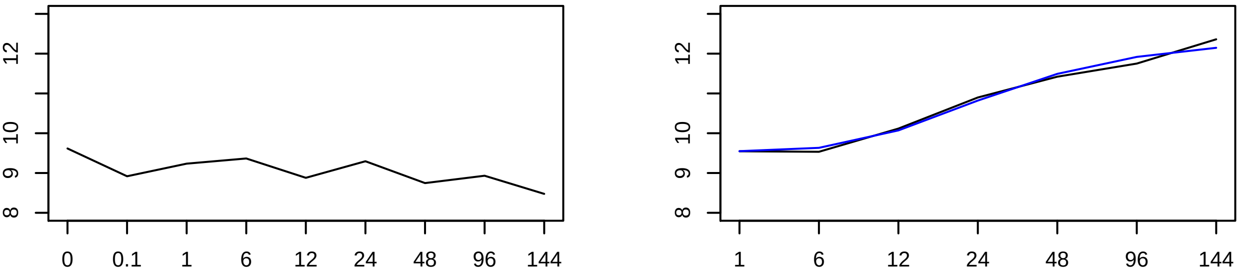

A_32_P19840 CK300181 NA

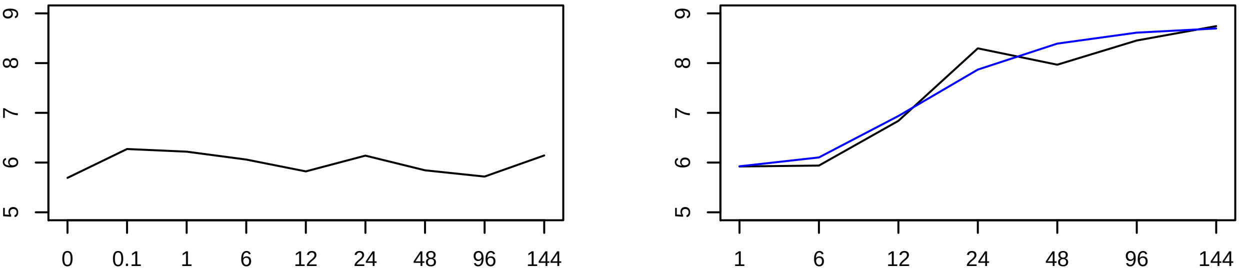

A_23_P66881 RGS9 17q24.1

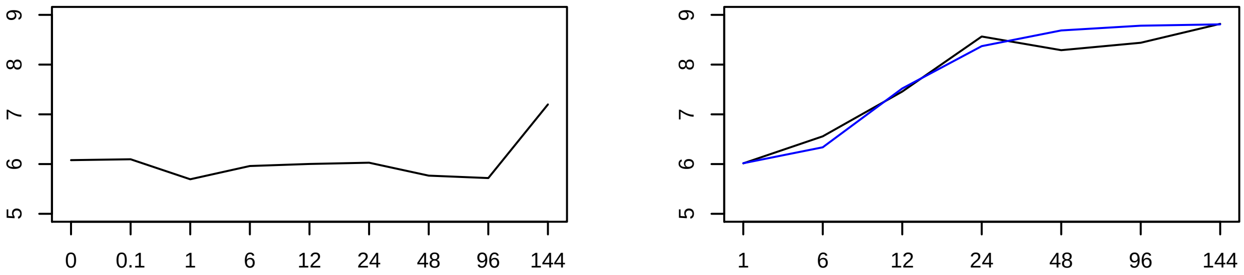

A_24_P627874 CR623787 NA

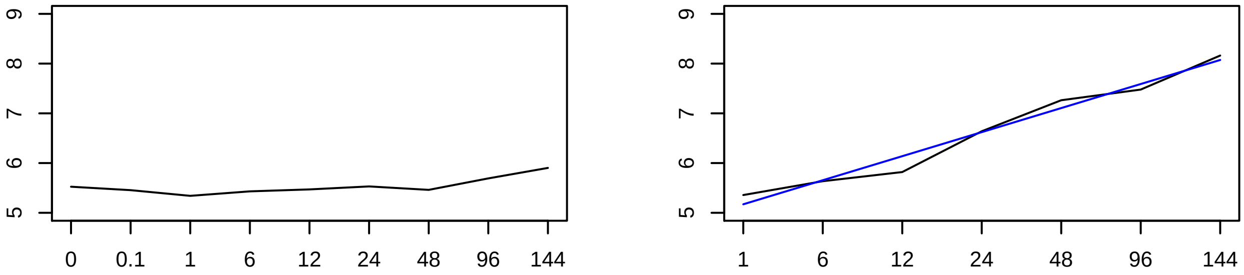

A_23_P337849 TNRC4 1q21.3

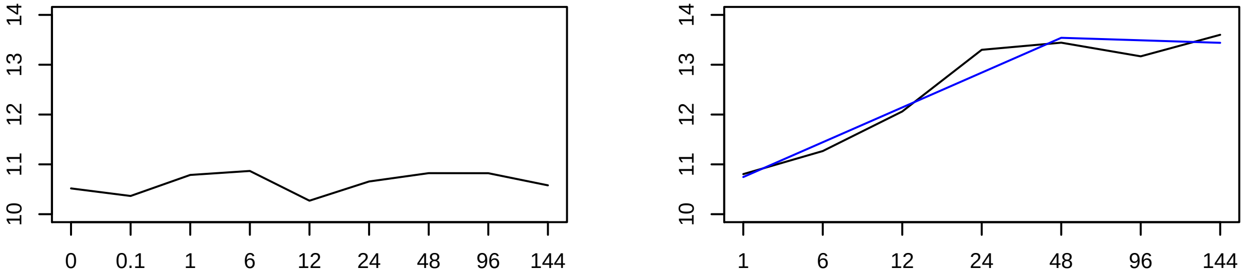

A_23_P255104 LHFPL2 5q14.1

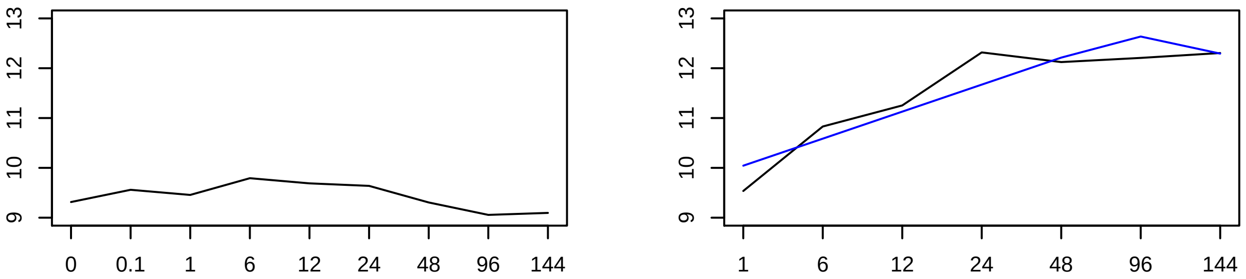

A_24_P294233 GLS 2q32.2

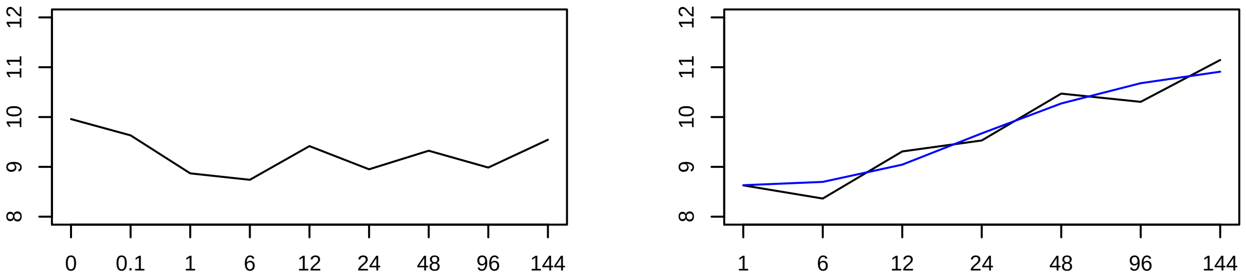

A_23_P66137 SOX8 16p13.3

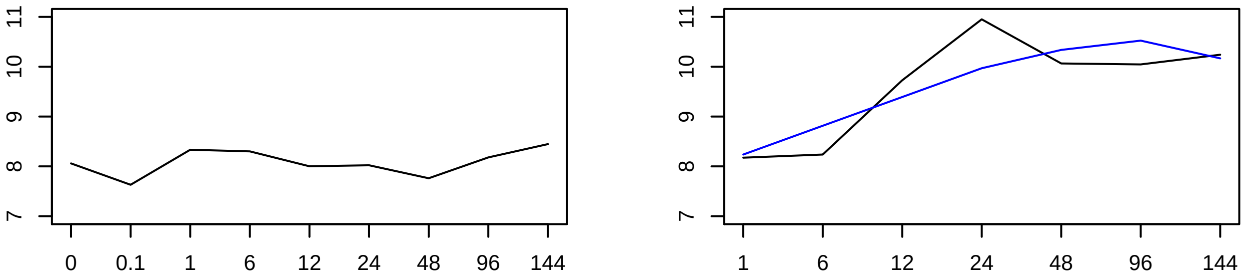

A_23_P57417 MMP11 22q11.23

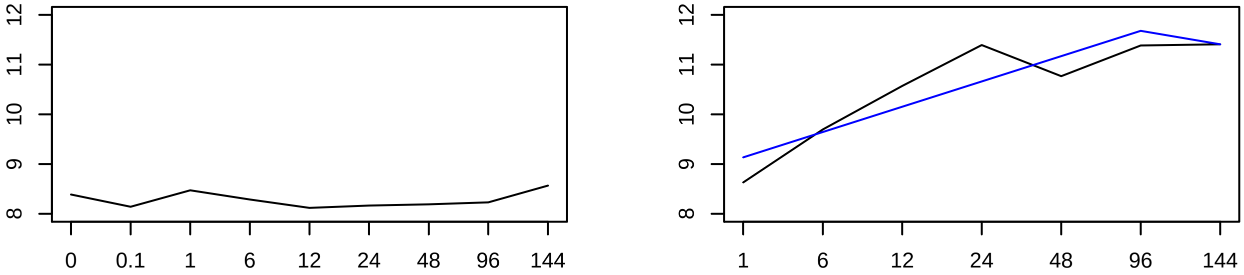

A_23_P35912 CASP4 11q22.3

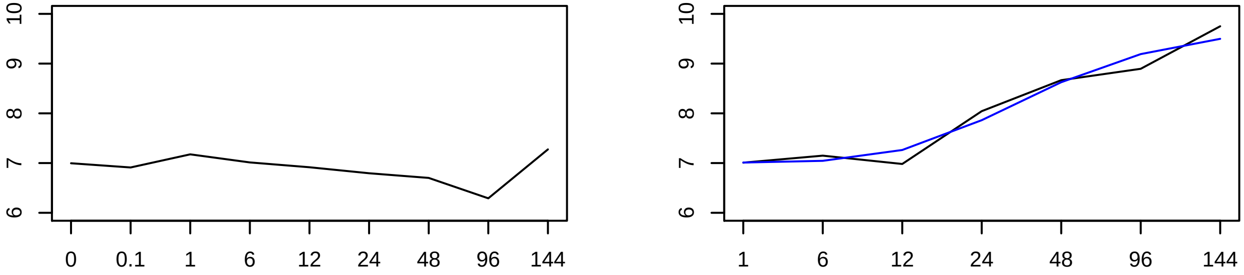

A_23_P390504 FOXC1 6p25.3

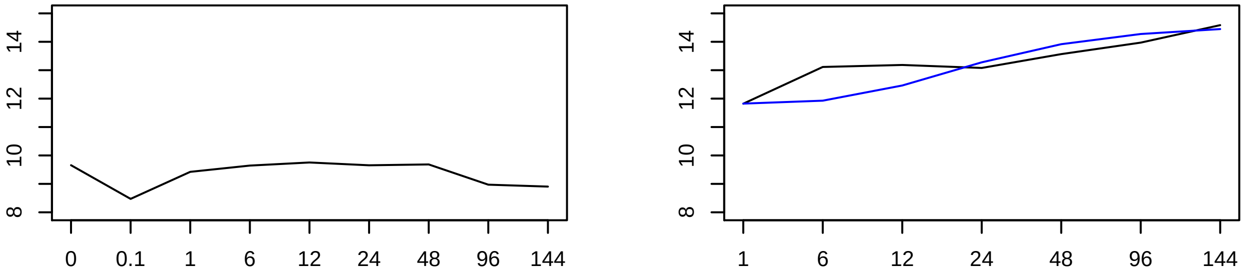

A_23_P62932 ATP1B1 1q24.2

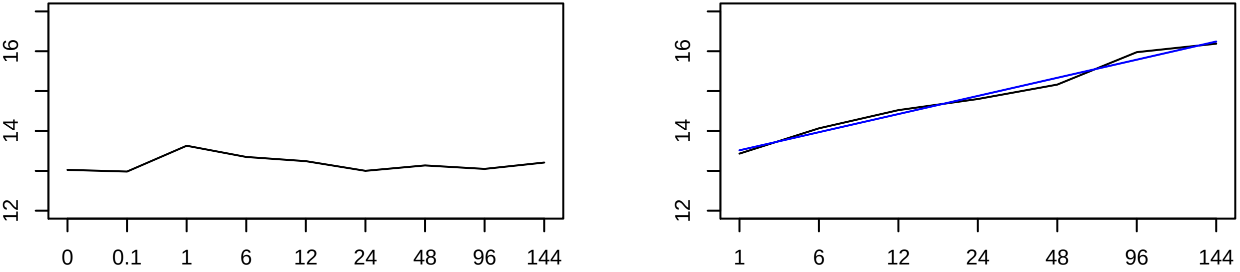

A_24_P307395 A_24_P307395 NA

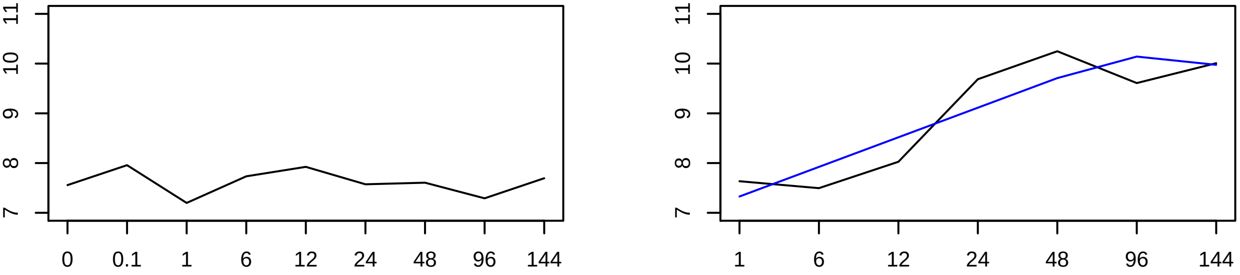

A_23_P128698 SPRY2 13q31.1

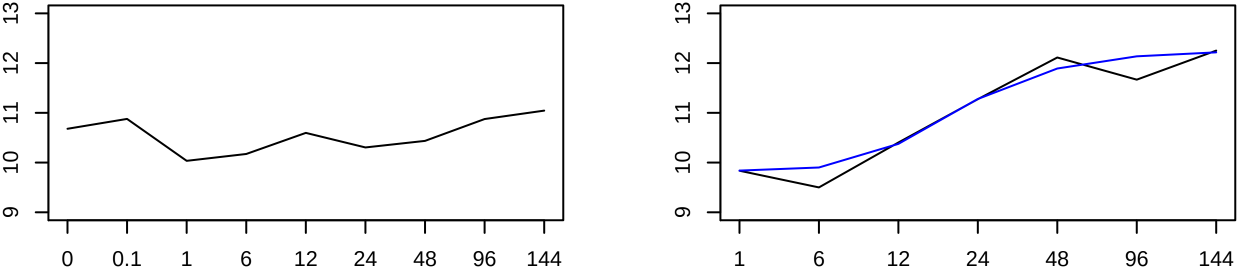

A_23_P76136 TSPAN11 NA

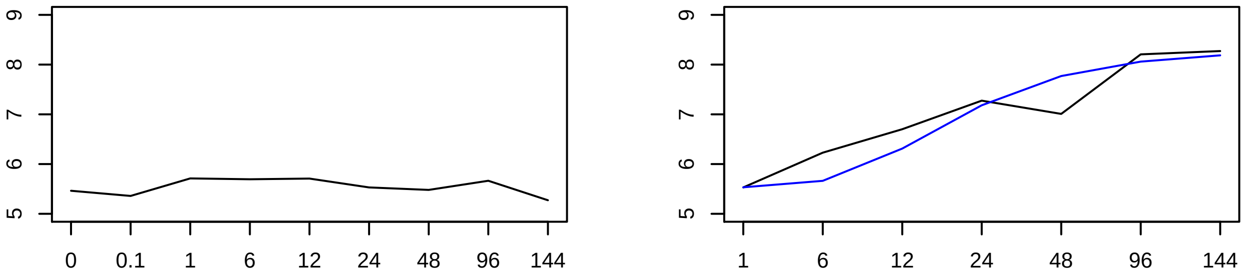

A_24_P80135 PTPN18 2q21.1

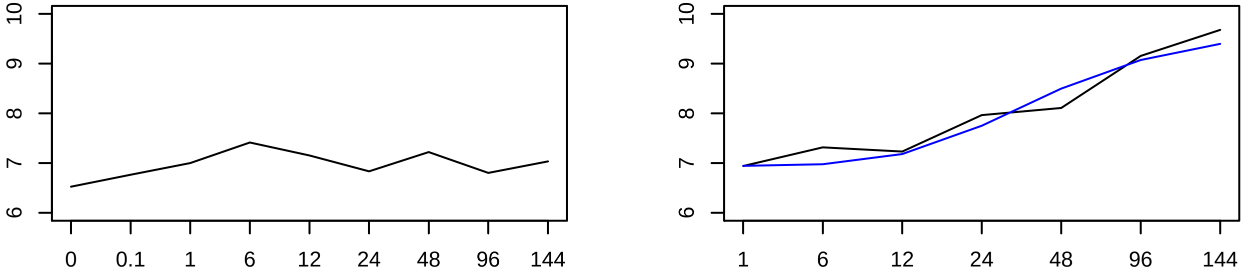

A_24_P24281 ENST00000344015 NA

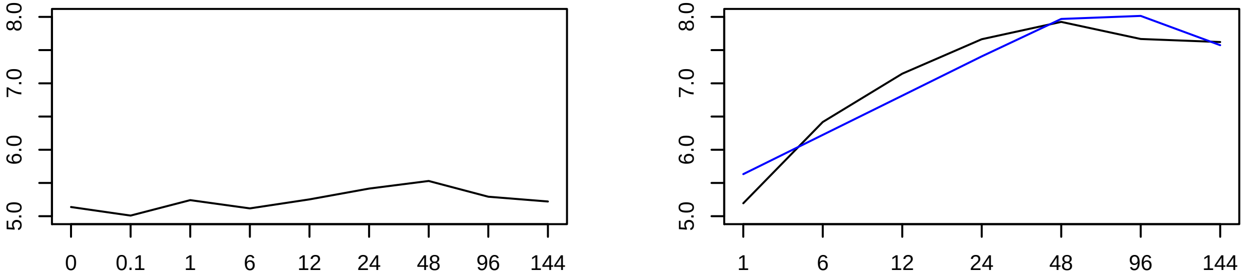

A_23_P162640 GABARAPL1 12p13.2

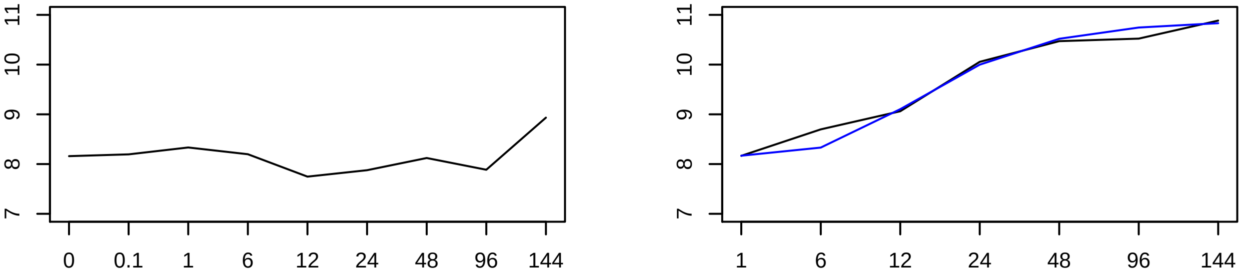

A_23_P19691 HEBP2 6q23.3

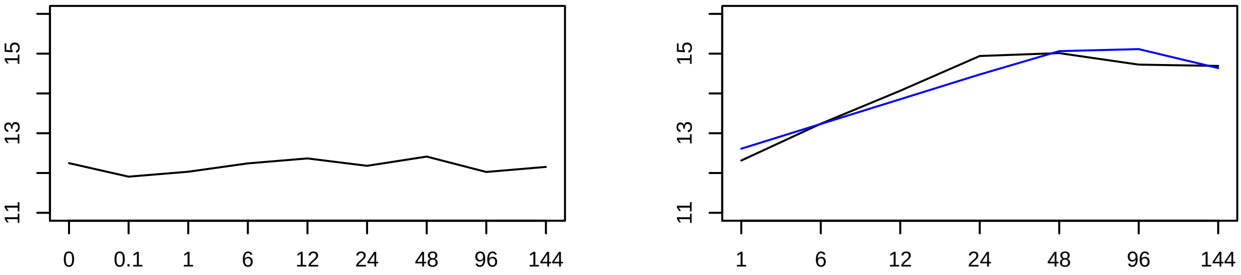

A_23_P215479 CYLN2 7q11.23

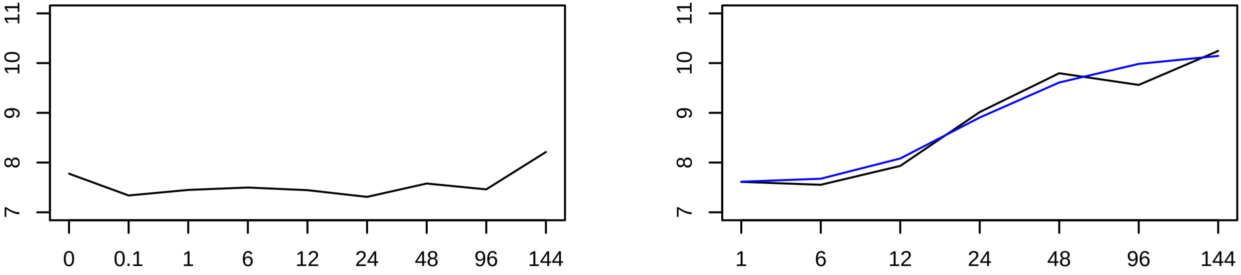

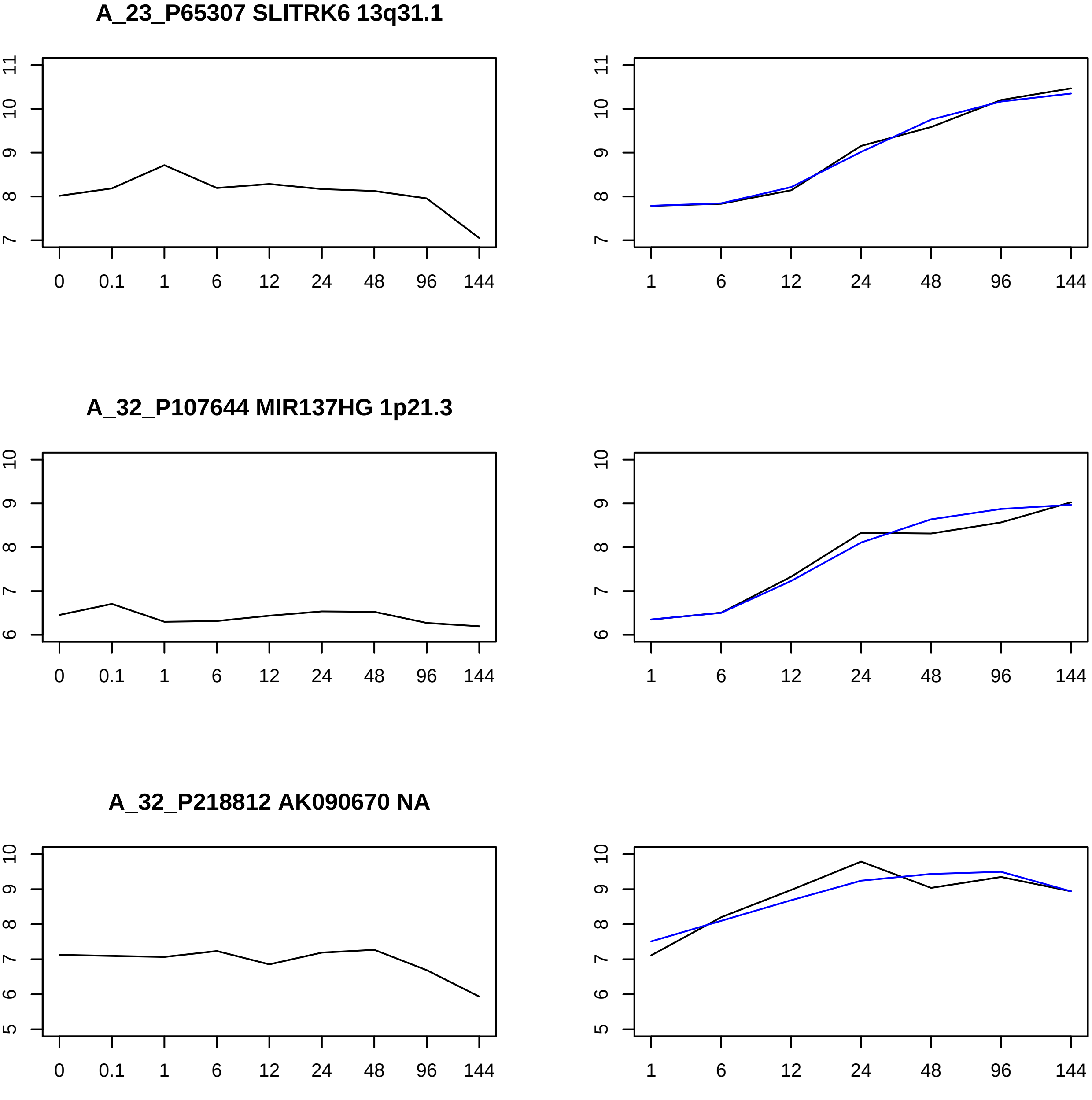
A_23_P65307 SLITRK6 13q31.1
7 8 9 10 11
0 0.1 1 6 12 24 48 96 144
7 8 9 10 11
1 6 12 24 48 96 144
A_32_P107644 MIR137HG 1p21.3
6 7 8 9 10
0 0.1 1 6 12 24 48 96 144
6 7 8 9 10
1 6 12 24 48 96 144
A_32_P218812 AK090670 NA
5 6 7 8 9 10
0 0.1 1 6 12 24 48 96 144
5 6 7 8 9 10
1 6 12 24 48 96 144

A_23_P50517 ZNF541 NA

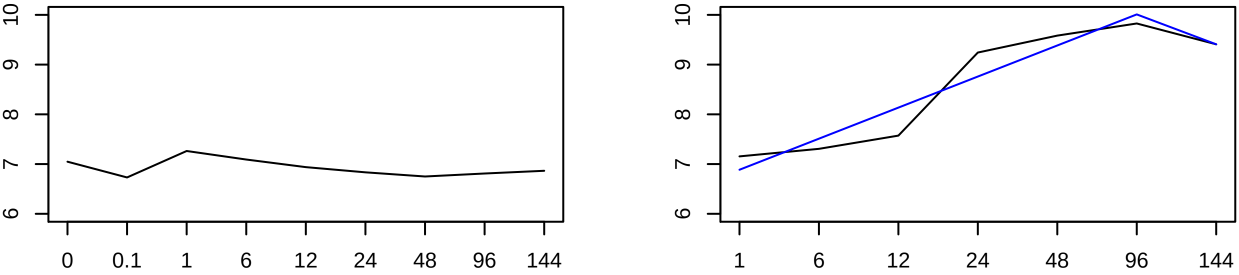

A_23_P129856 HIC1 NA

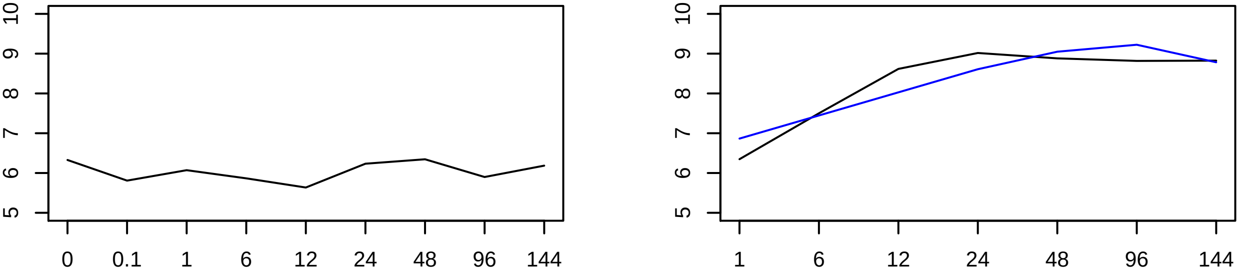

A_23_P51699 ARHGEF2 1q22

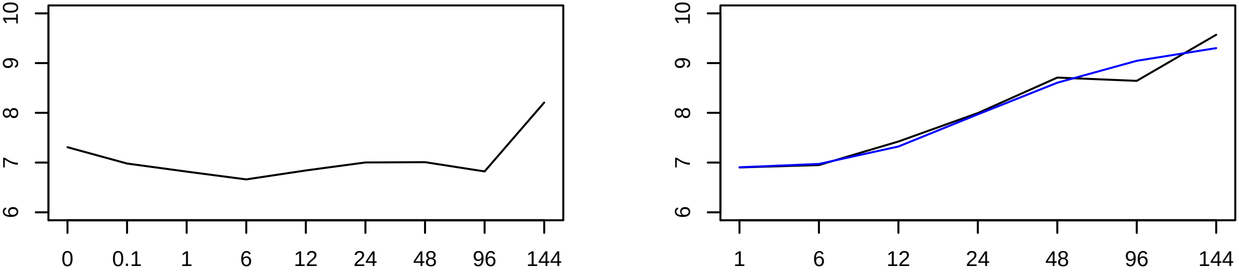

A_24_P354337 ARL6 3q11.2

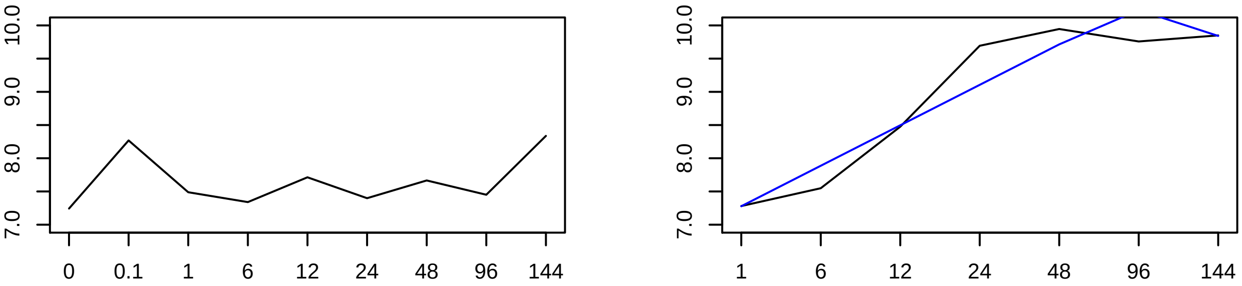

A_24_P90097 ADD3 10q25.2

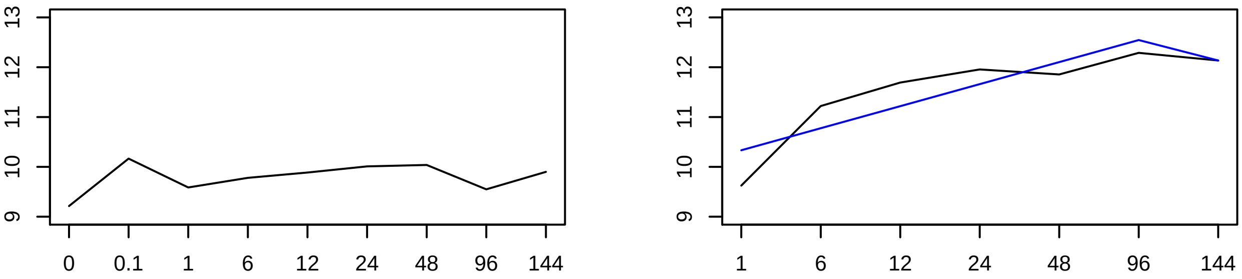

A_32_P827528 S1PR2 NA

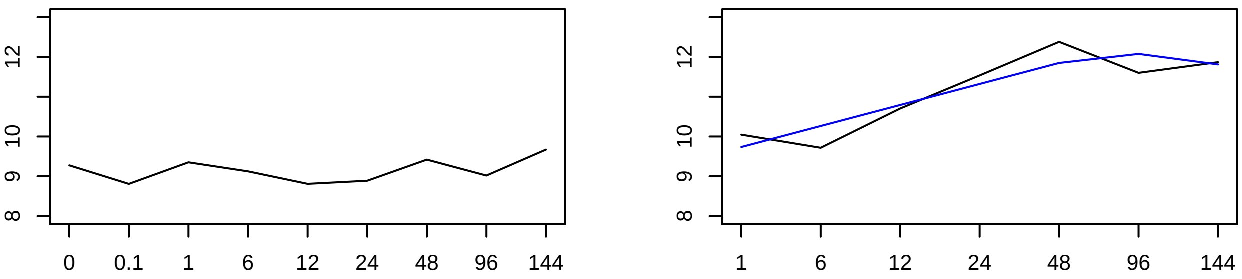

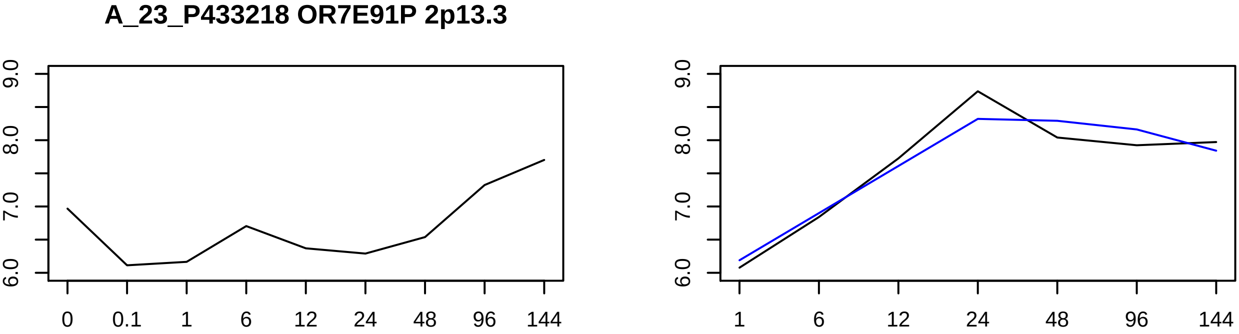
A_23_P433218 OR7E91P 2p13.3
6.0
7.0
8.0
9.0
0
0.1
1
6
12
24
48
96
144
1
6
12
24
48
96
144

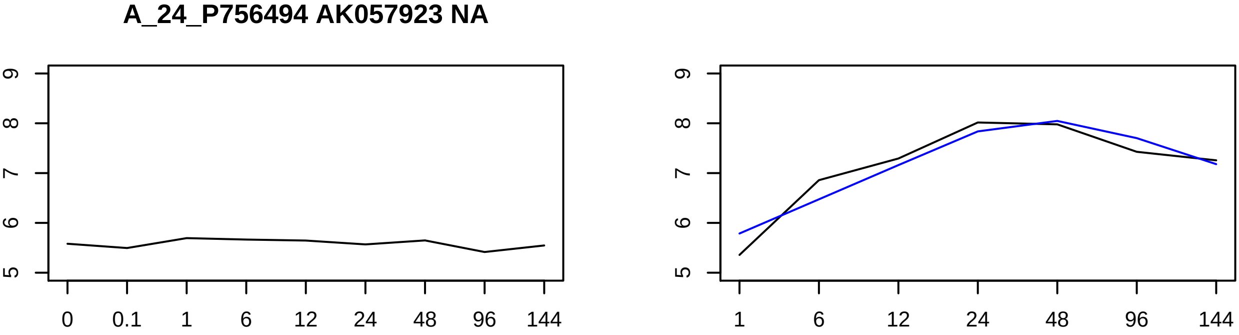
A_24_P756494 AK057923 NA
5
6
7
8
9
0
0.1
1
6
12
24
48
96
144
1
6
12
24
48
96
144

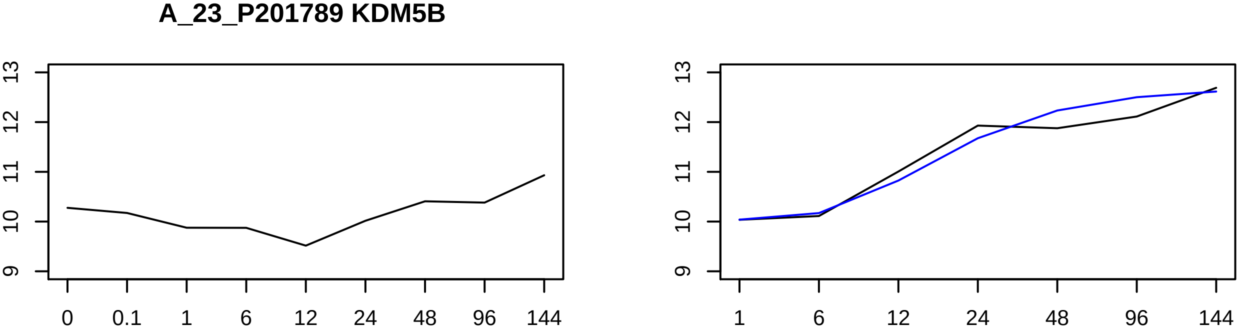
A_23_P201789 KDM5B
9
10
11
12
13
0
0.1
1
6
12
24
48
96
144
1
6
12
24
48
96
144

A_32_P16258 EXOC6B 2p13.3

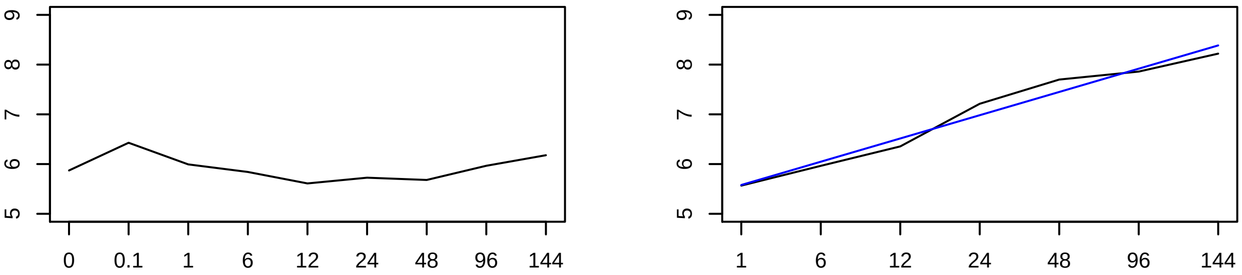

A_32_P148914 LOC389634 8p23.1

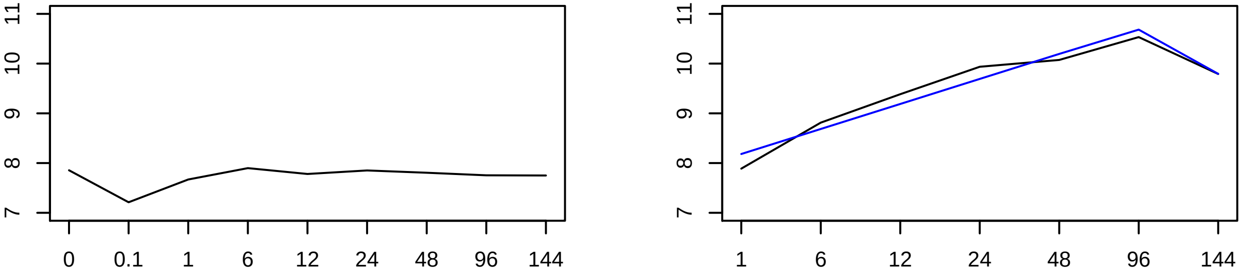

A_23_P100642 PNMT 17q12

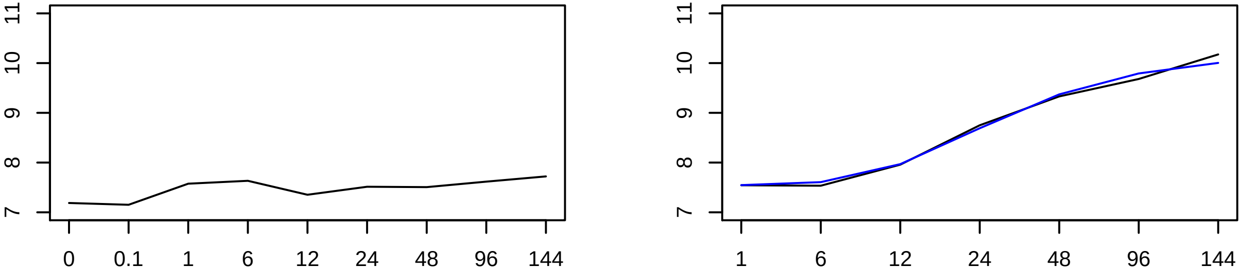

A_23_P379071 FBXL13 7q22.1

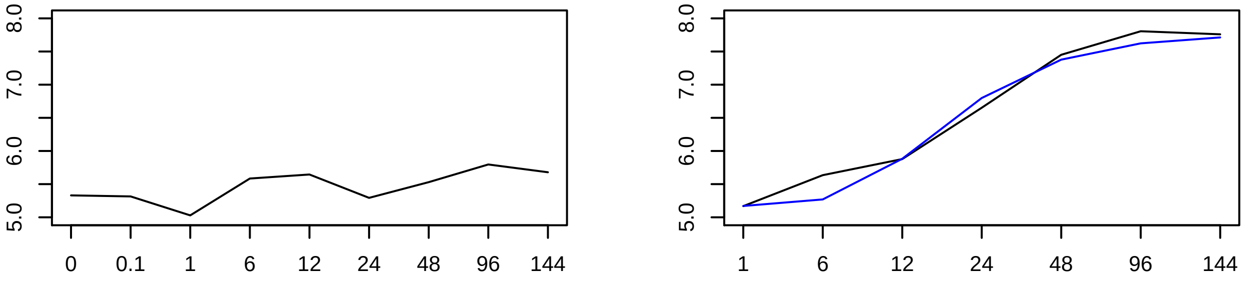

A_24_P788772 THC2582296 NA

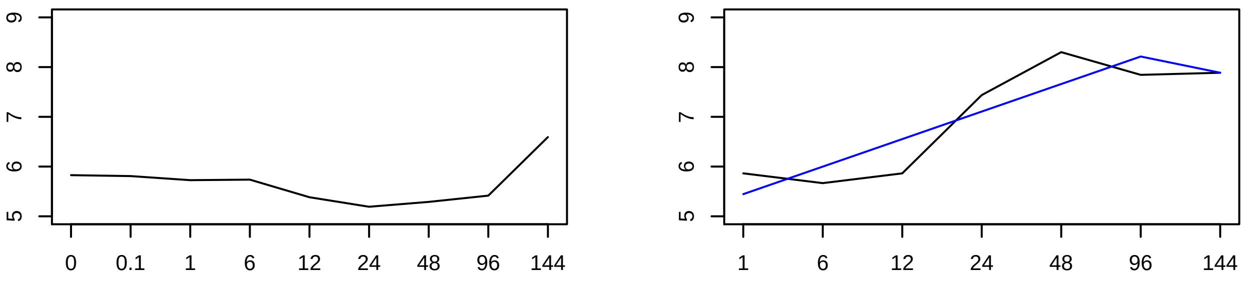

A_23_P351467 CMAH 6p22.2

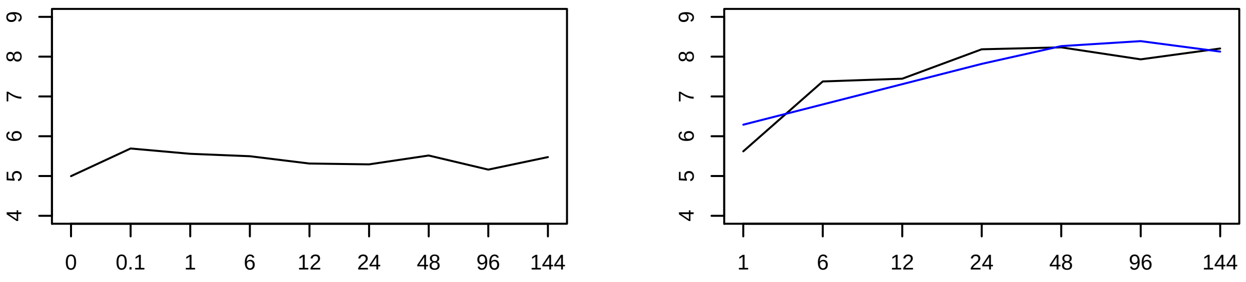

A_23_P217009 C9orf24 9p13.3

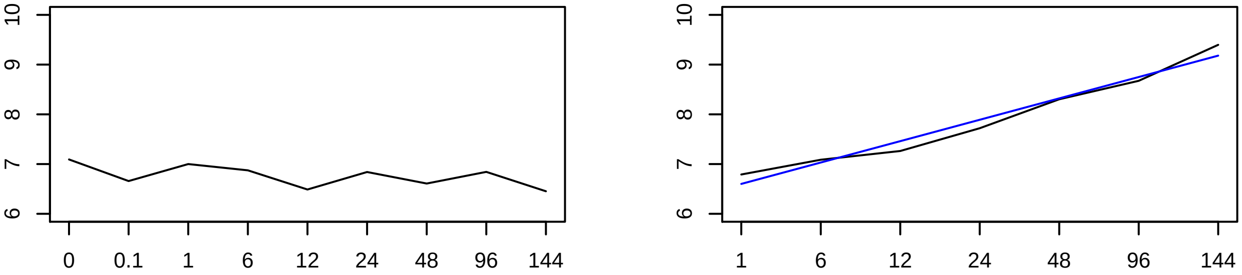

A_24_P183664 KIAA0644 7p15.1

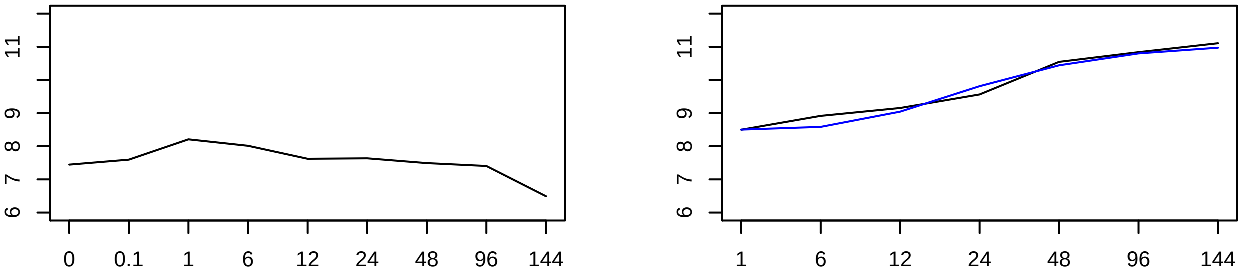

A_23_P300033 PDGFRA 4q12

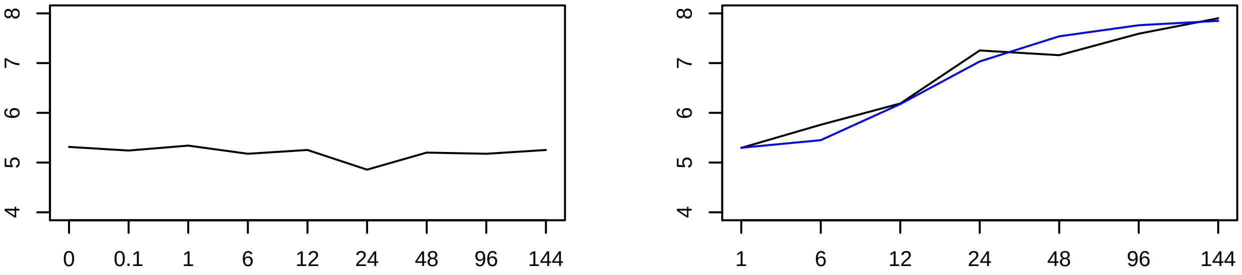

A_23_P65442 ISGF3G 14q12

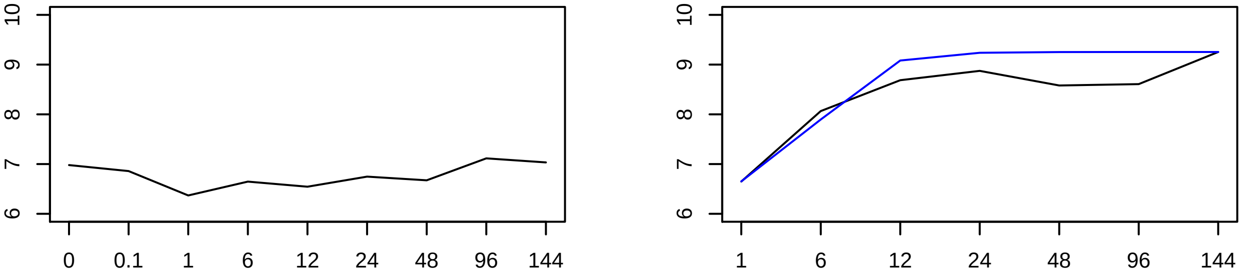

A_24_P355626 ABCG4 11q23.3

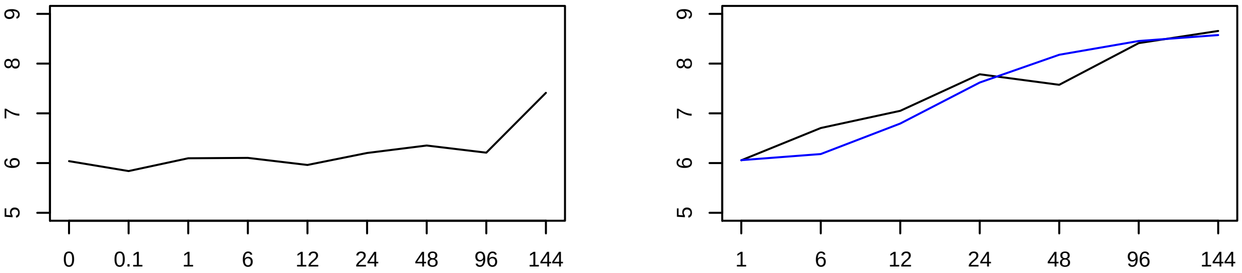

A_23_P75989 PAK1 11q14.1

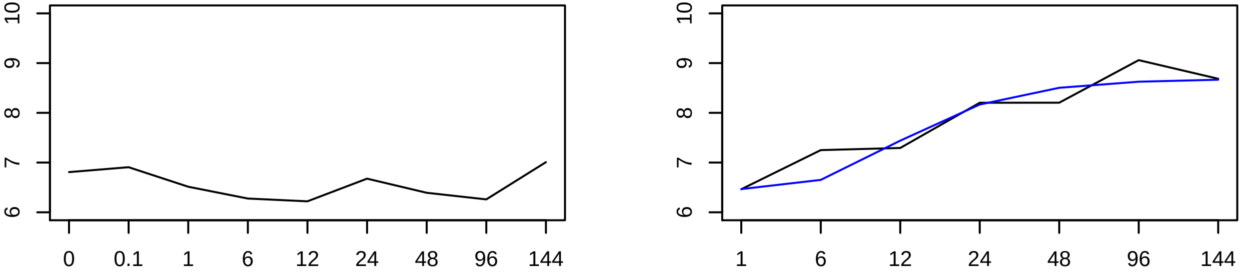

A_23_P106194 FOS 14q24.3

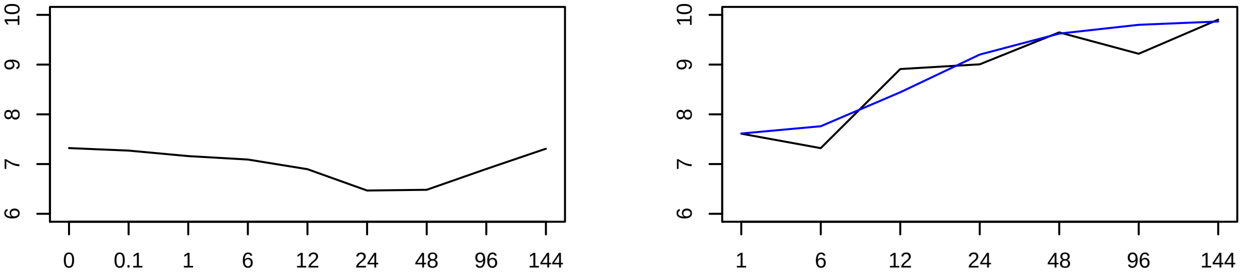

A_23_P35066 SNX7 1p21.3

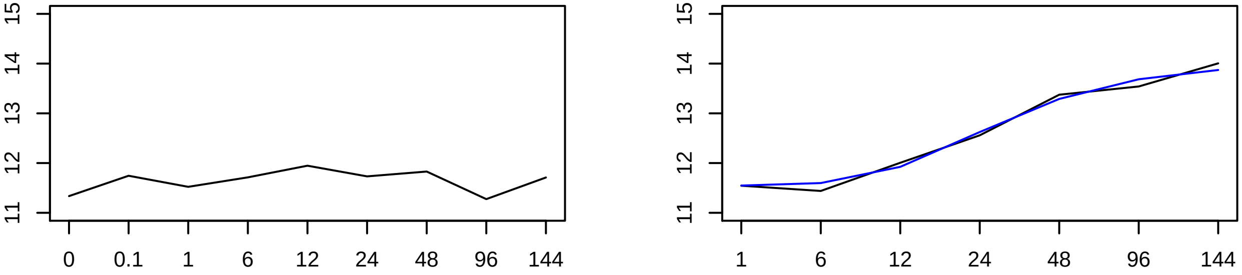

A_32_P70724 JARID1B 1q32.1

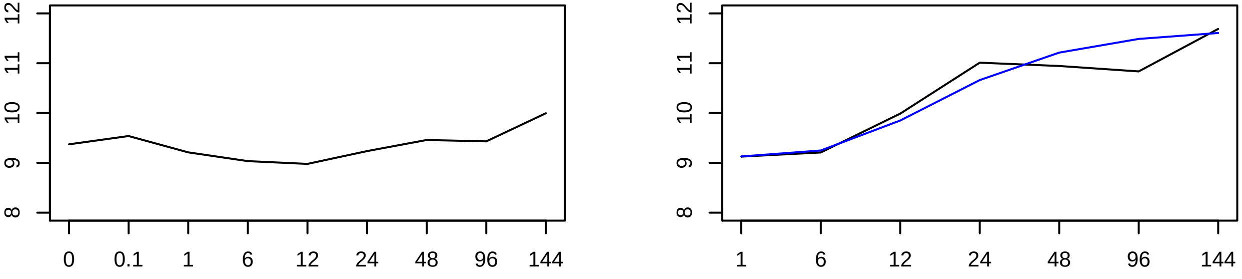

A_32_P132393 H40632 NA

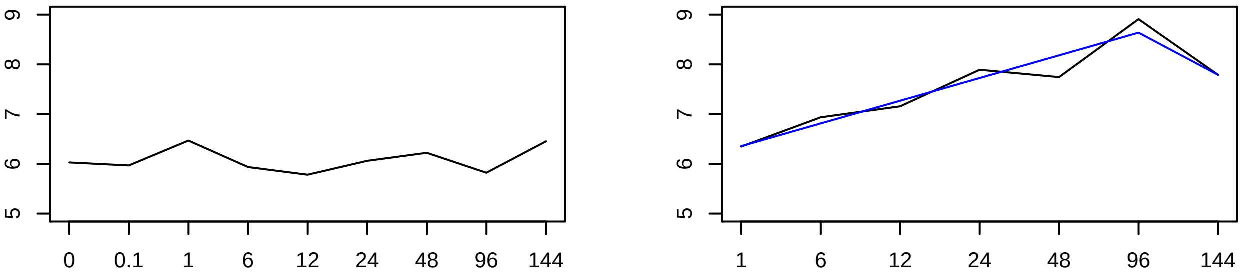

A_23_P5831 HPCAL1 2p25.1

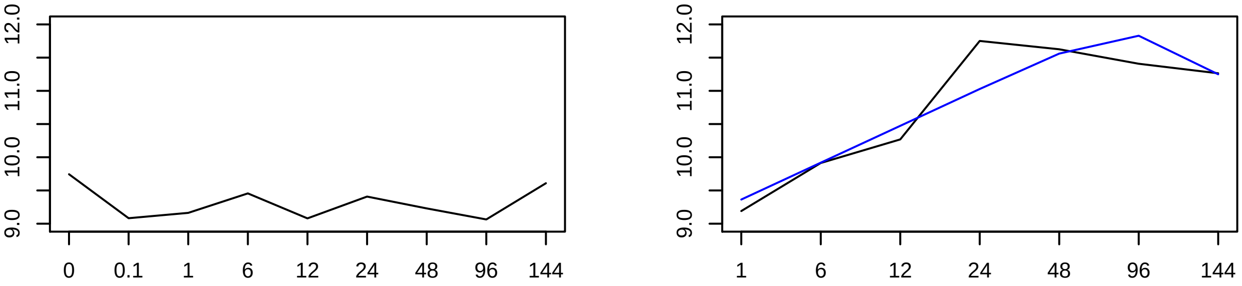

A_23_P93737 DYNC1I1 7q21.3

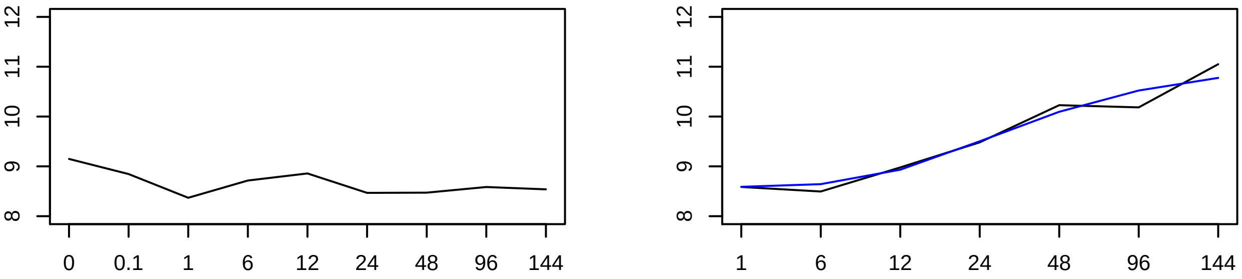

A_23_P8801 CYP3A5 7q22.1

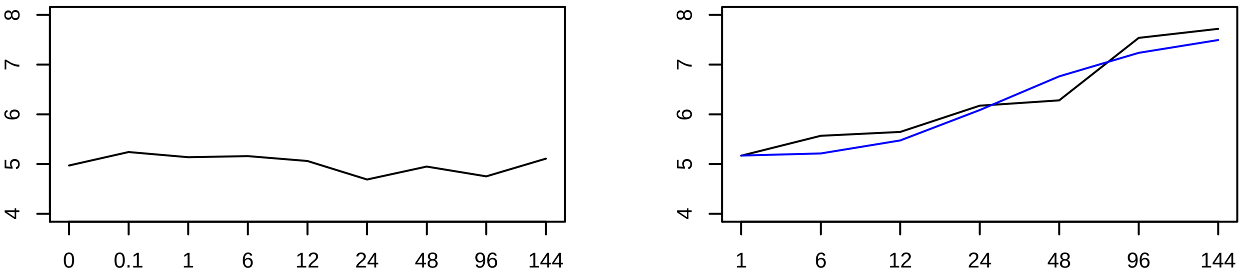

A_24_P47182 VCL 10q22.2

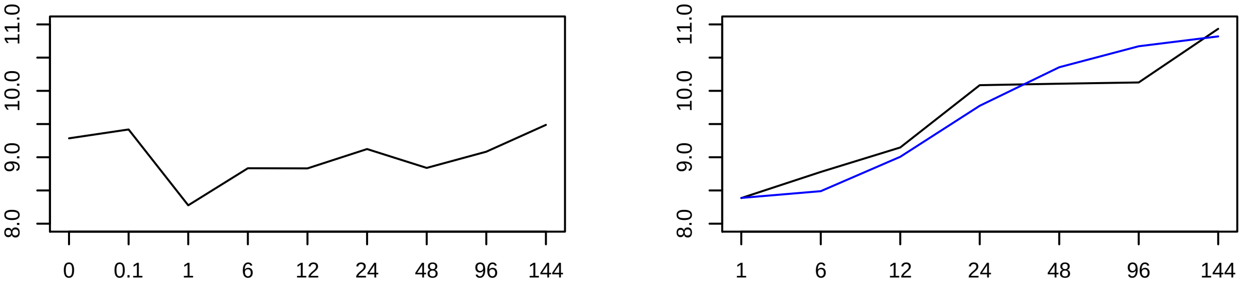

A_23_P170888 DPP6 7q36.2

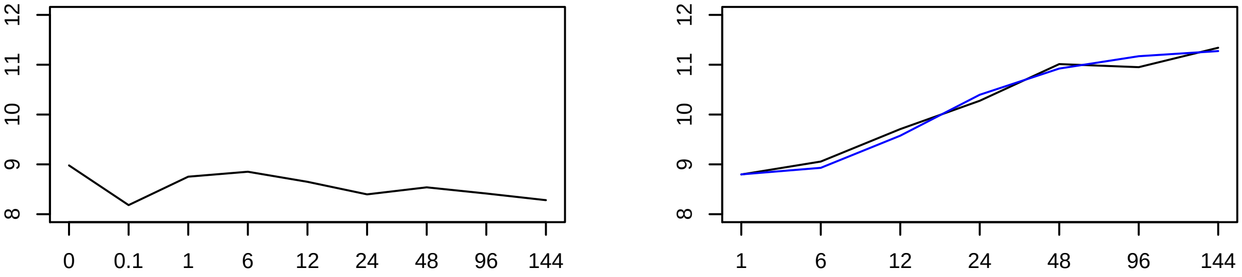

A_23_P26954 VAT1 17q21.31

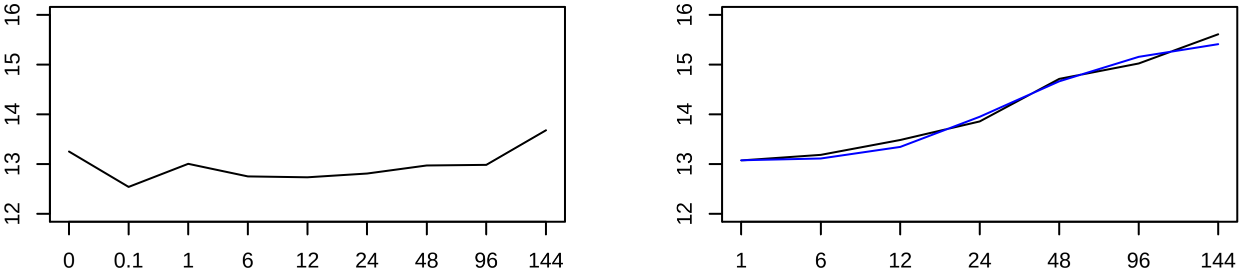

A_23_P18798 PCDHB9 5q31.3

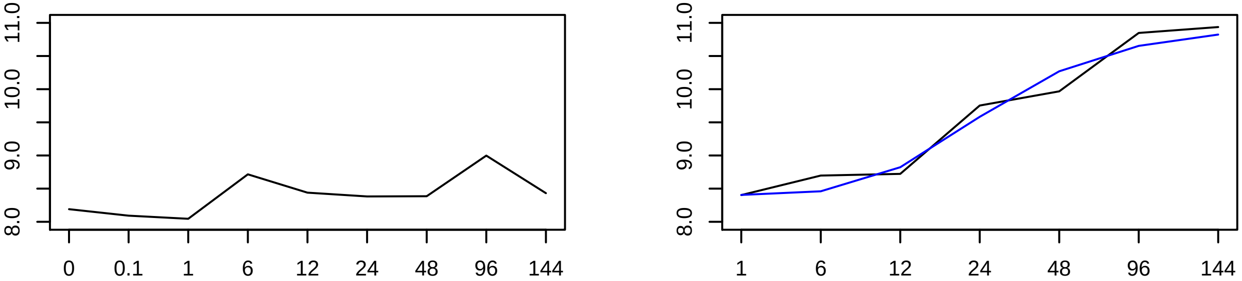

A_24_P154037 IRS2 13q34

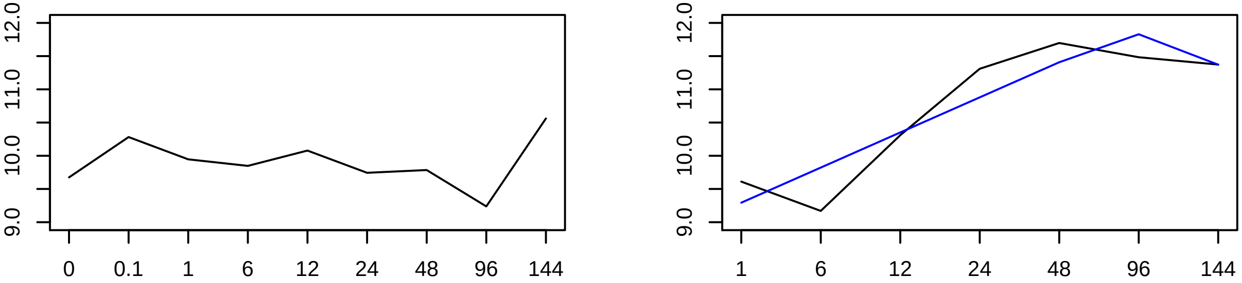

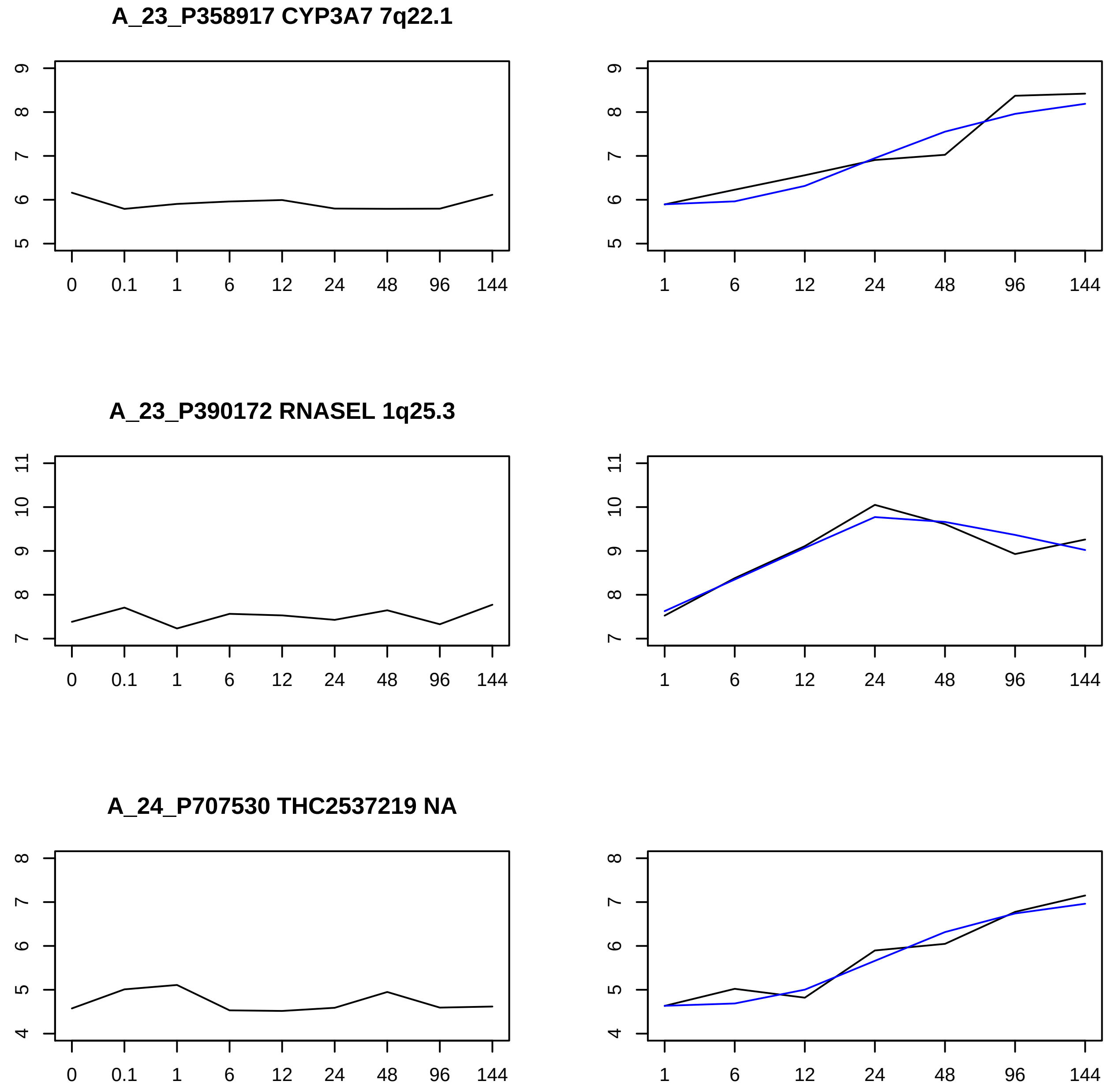
A_23_P358917 CYP3A7 7q22.1
A_23_P390172 RNASEL 1q25.3
A_24_P707530 THC2537219 NA
0 0.1 1 6 12 24 48 96 144
1 6 12 24 48 96 144

A_23_P344531 SYNPO 5q33.1

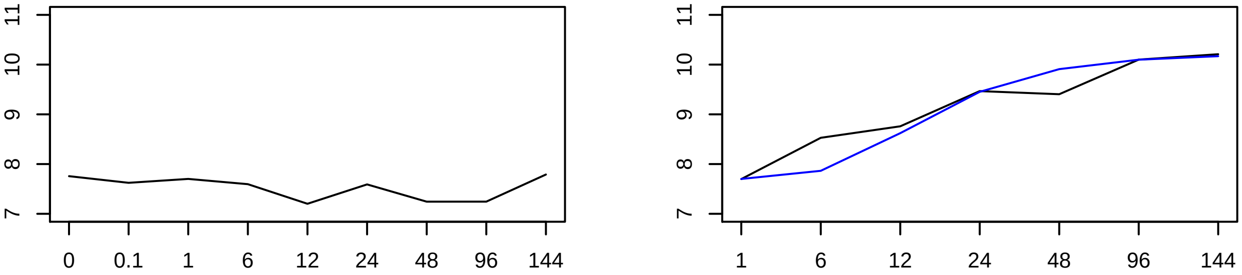

A_32_P184488 PHLDB3 19q13.31

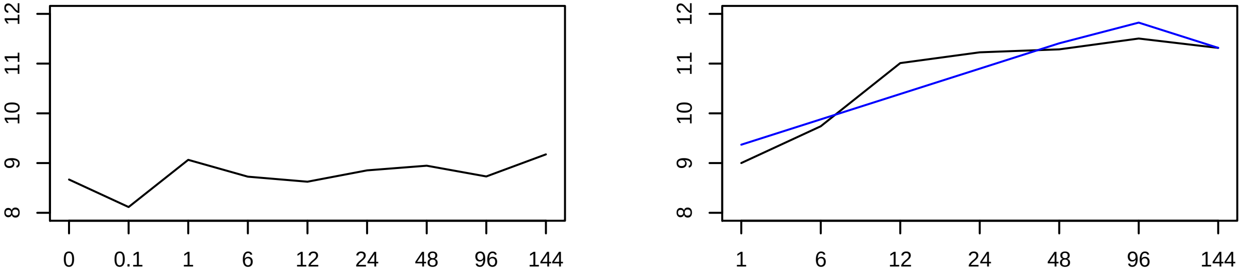

A_24_P382319 CEACAM1 19q13.2

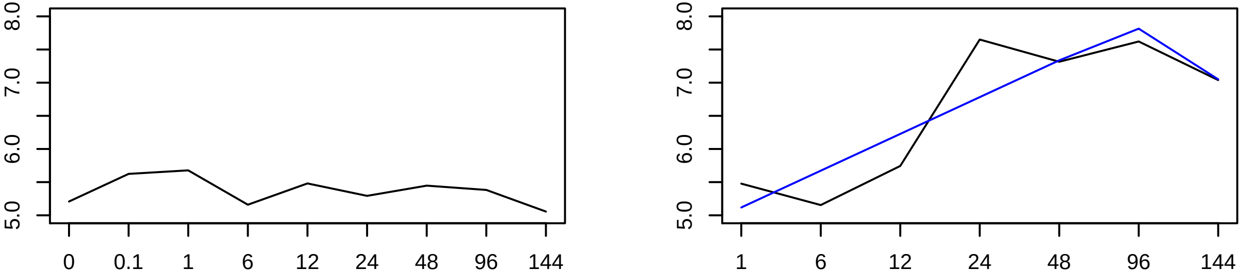

A_23_P70297 ANKRD6 6q15

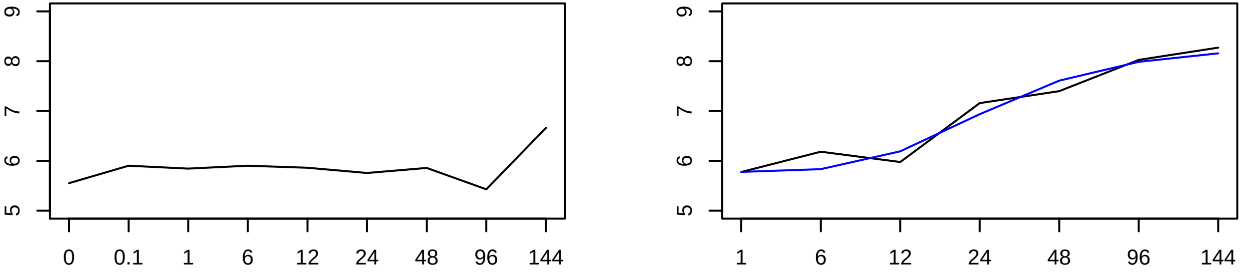

A_23_P211028 NCAM2 21q21.1

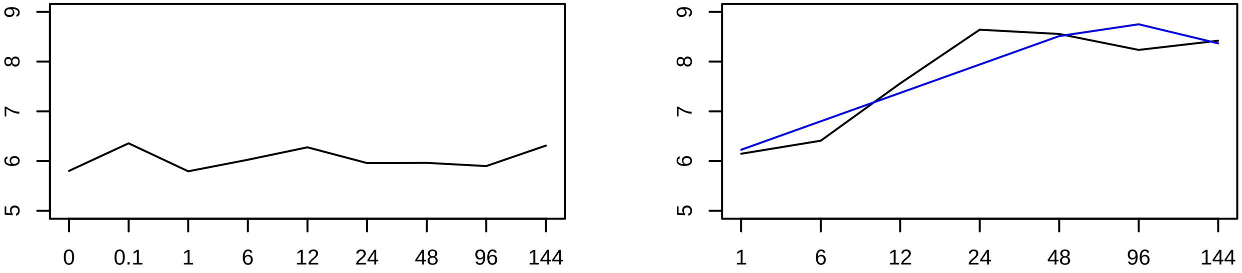

A_23_P160377 DNALI1 1p34.3

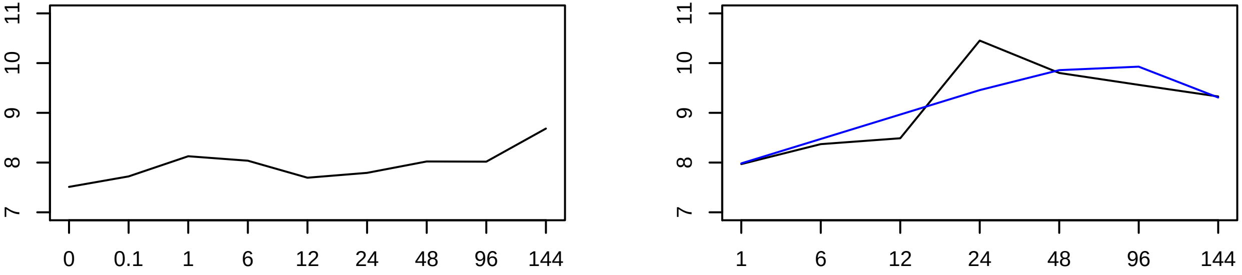

A_23_P67360 PLEKHA4 19q13.33

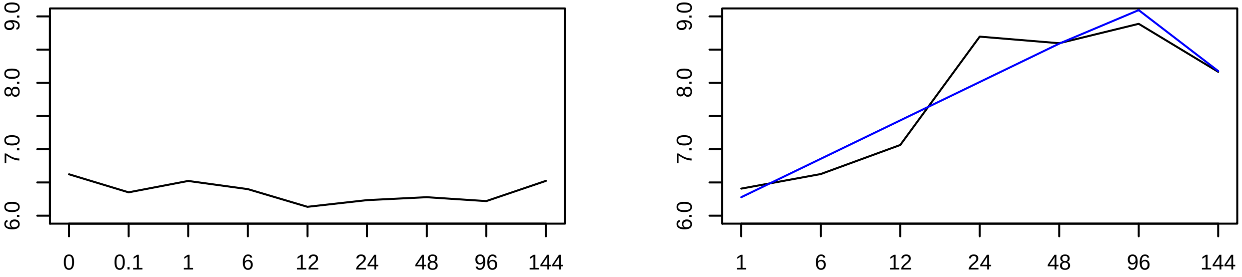

A_23_P23457 FBLIM1 1p36.21

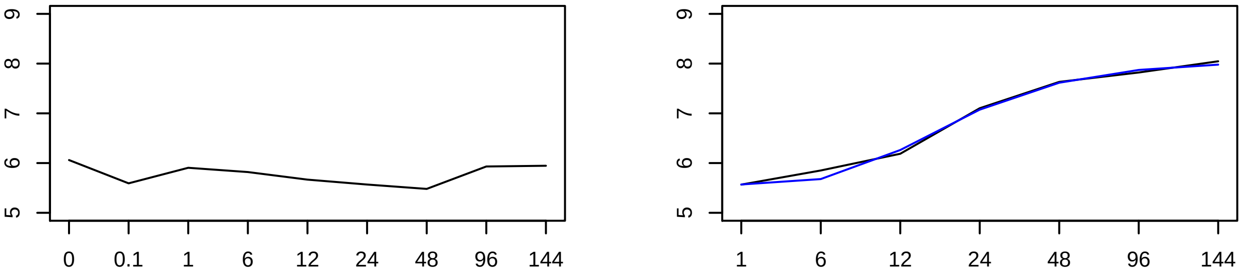

A_23_P142849 RND3 2q23.3

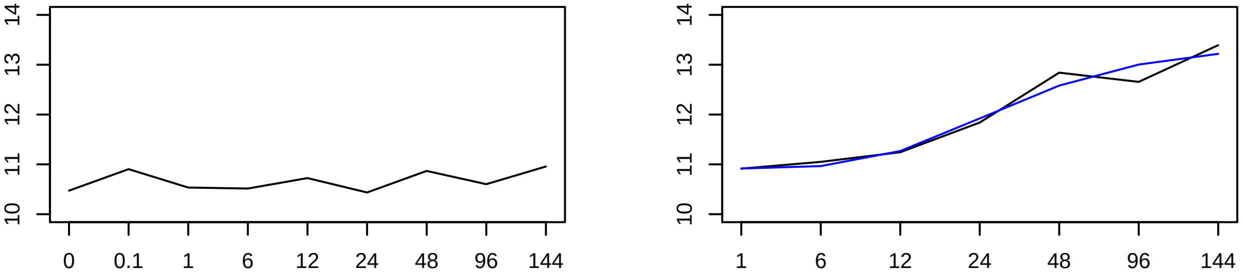

A_23_P394367 PDE11A 2q31.2

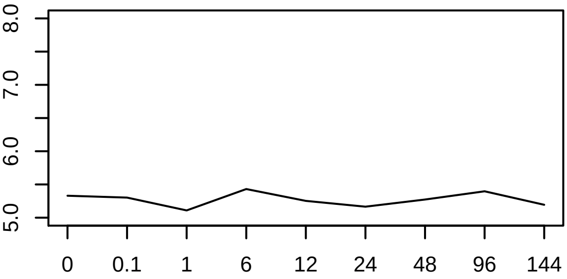

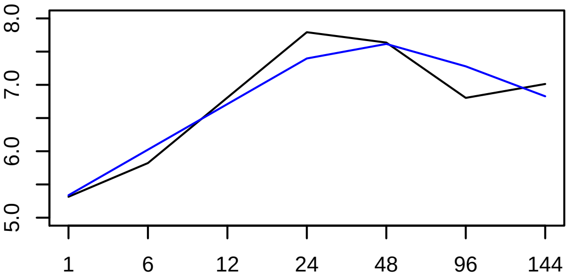

A_24_P79070 GNG8 19q13.32

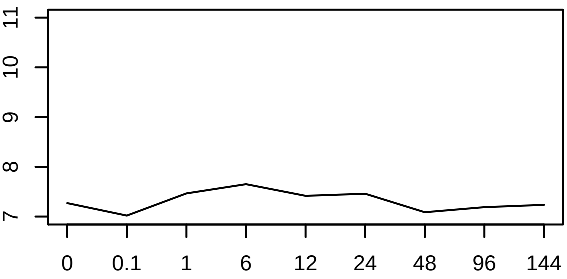

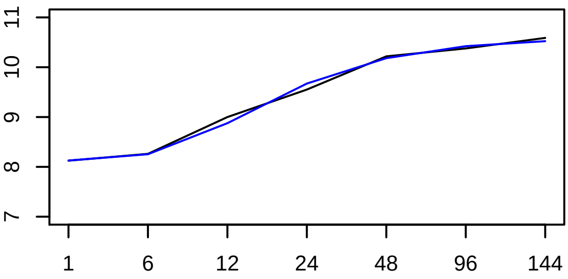

A_24_P930963 LMF1 NA

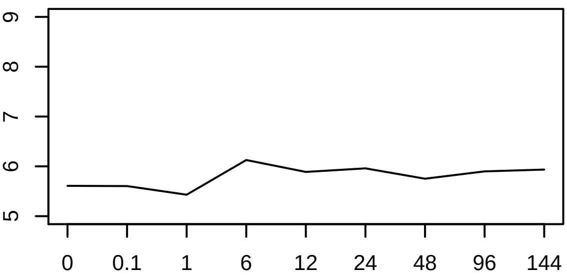

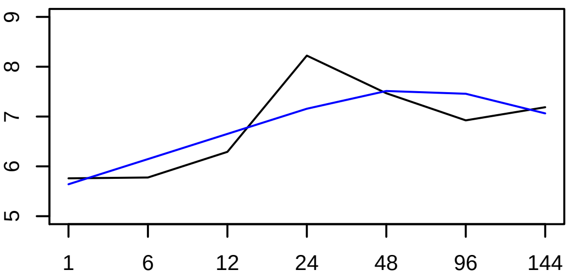

A_32_P207481 RP11−71H17.7 3q21.2

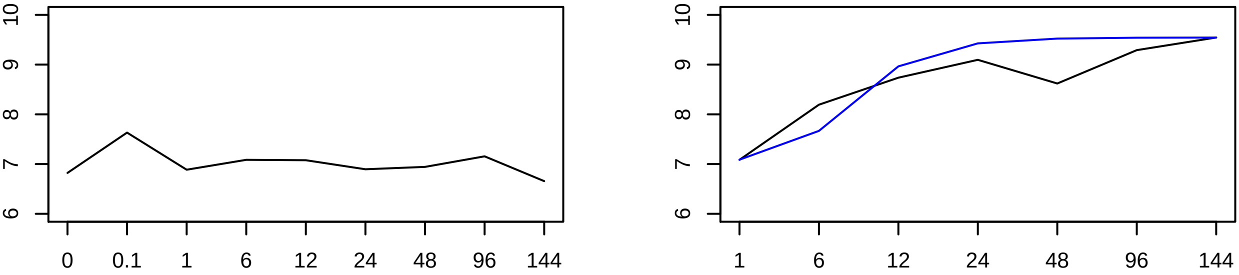

A_23_P150018 DUSP5 10q25.2

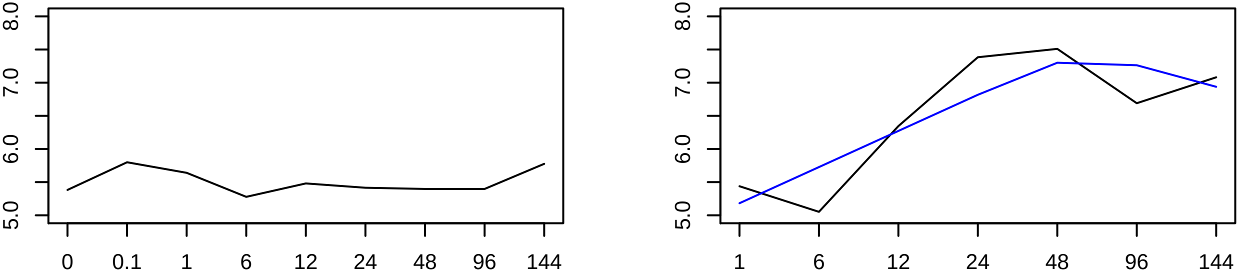

A_32_P176819 CMAH 6p22.2

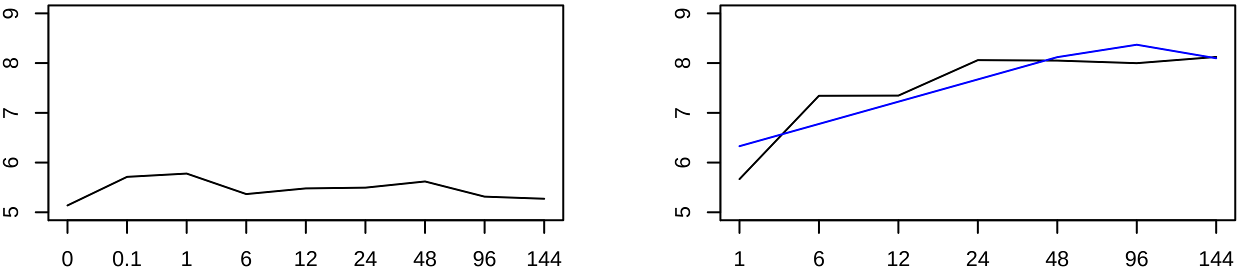

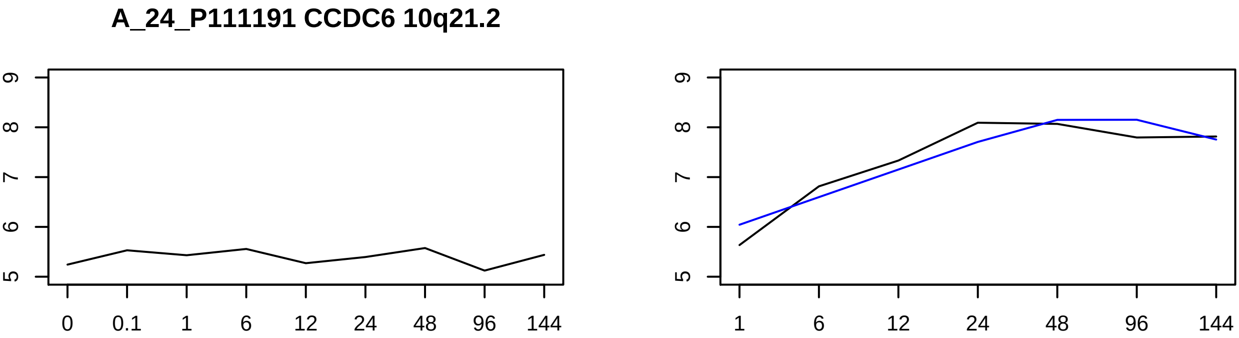
A_24_P111191 CCDC6 10q21.2
5
6
7
8
9
0
0.1
1
6
12
24
48
96
144
1
6
12
24
48
96
144

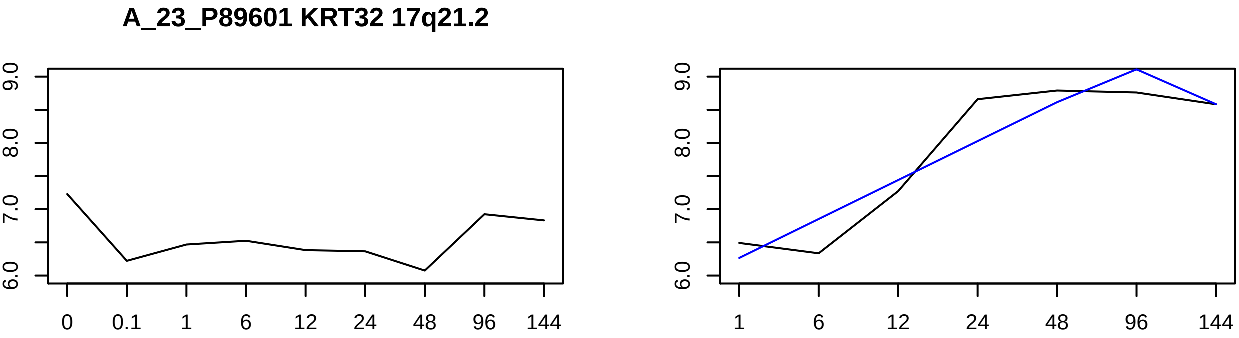
A_23_P89601 KRT32 17q21.2
6.0
7.0
8.0
9.0
0
0.1
1
6
12
24
48
96
144
1
6
12
24
48
96
144

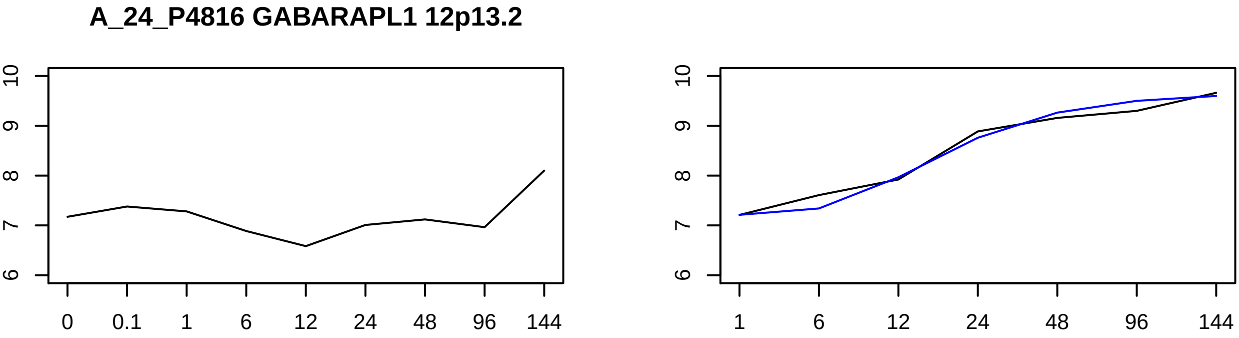
A_24_P4816 GABARAPL1 12p13.2
6
7
8
9
10
0
0.1
1
6
12
24
48
96
144
1
6
12
24
48
96
144

A_24_P106145 WDR1 4p16.1

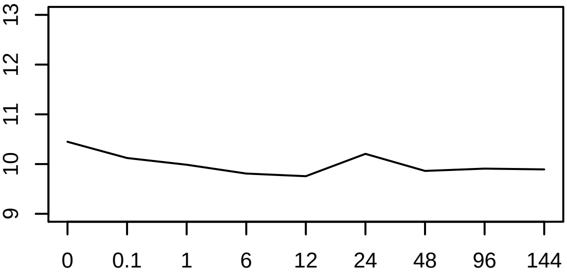

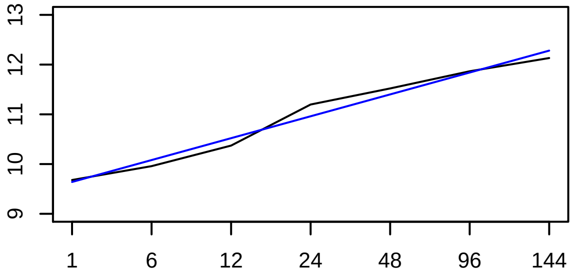

A_23_P157580 SDCBP 8q12.1

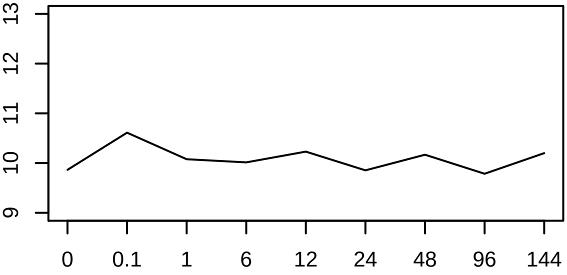

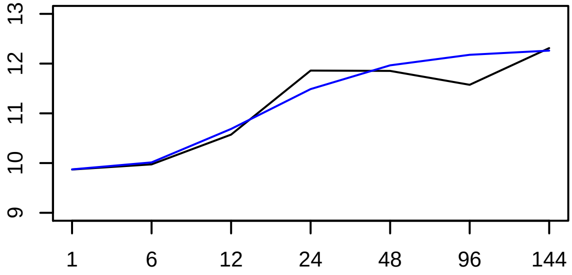

A_24_P333857 SGIP1 1p31.3

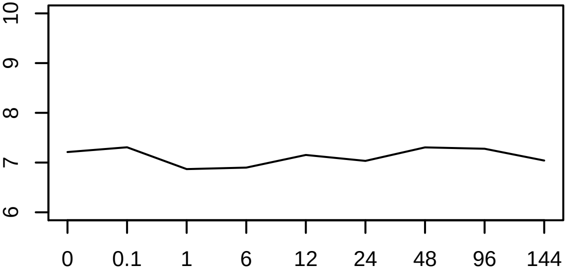

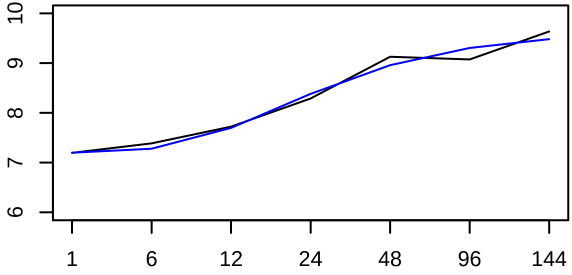

A_24_P229536 C21orf34 21q21.1

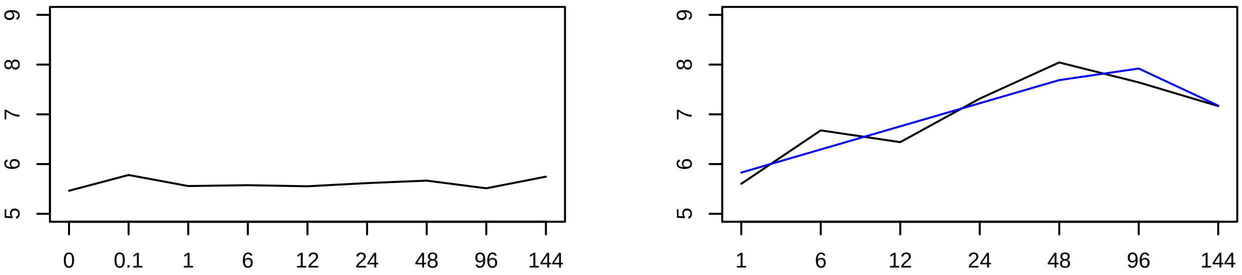

A_32_P190303 LONRF2 2q11.2

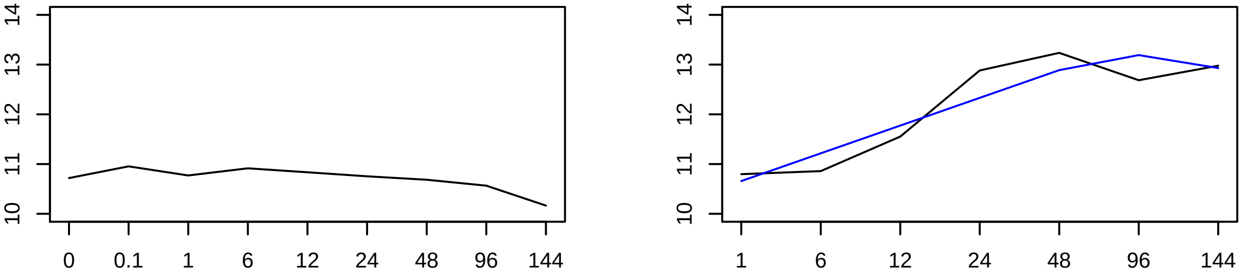

A_23_P82169 SOX4 6p22.3

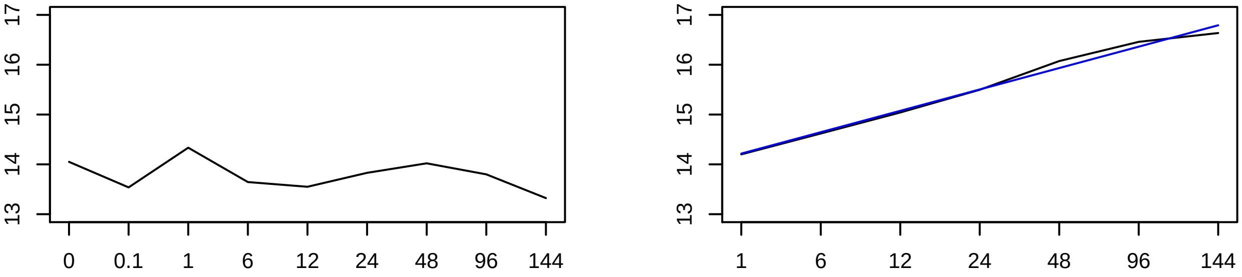

A_23_P404481 EDG1 1p21.2

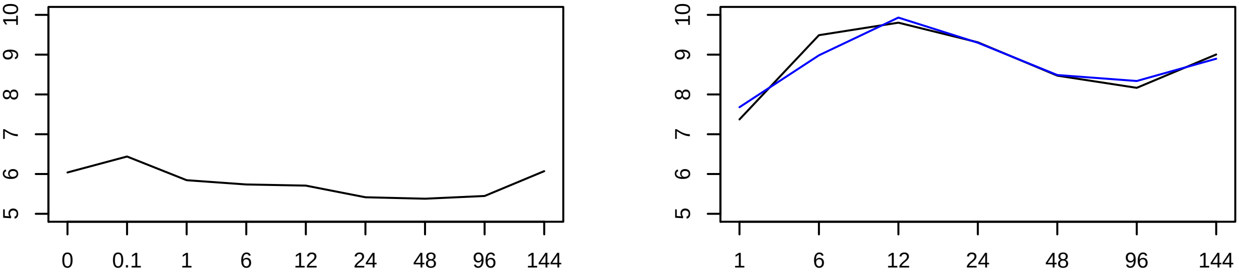

A_23_P167051 EVC 4p16.1

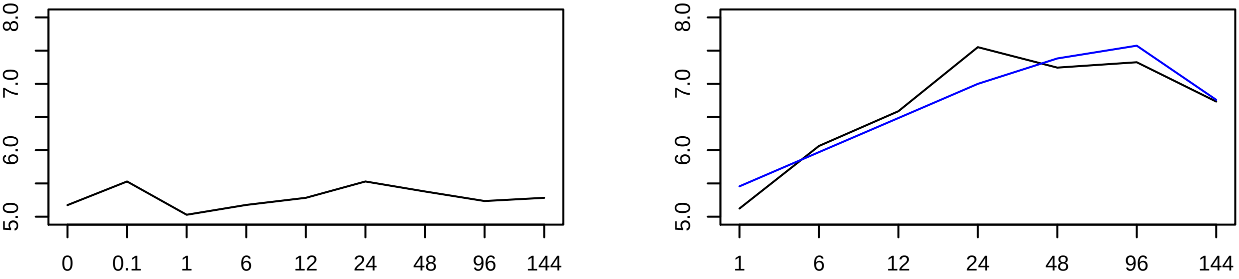

A_24_P229726 LOC440181 14q23.1

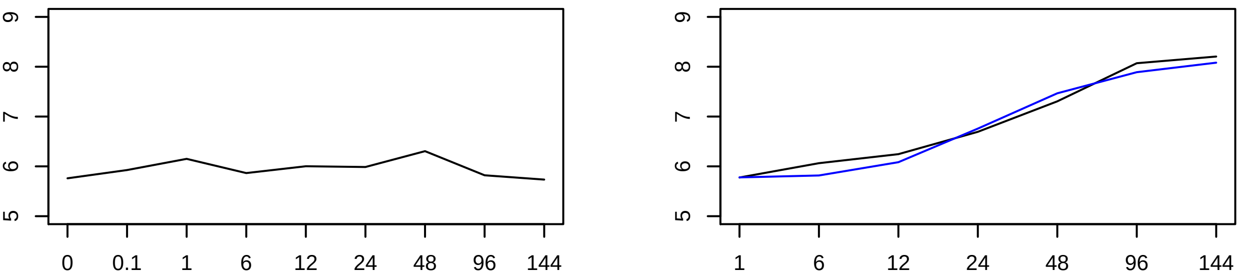

A_24_P273799 ZNF641 12q13.11

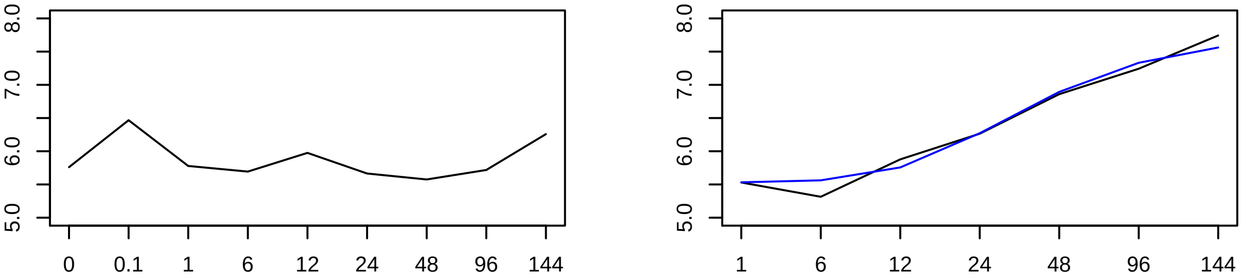

A_23_P1461 OPTN 10p13

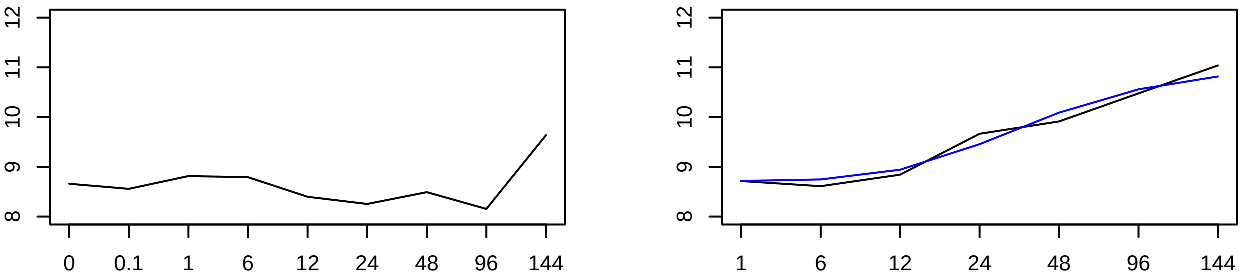

A_24_P377144 ANTXR2 4q21.21

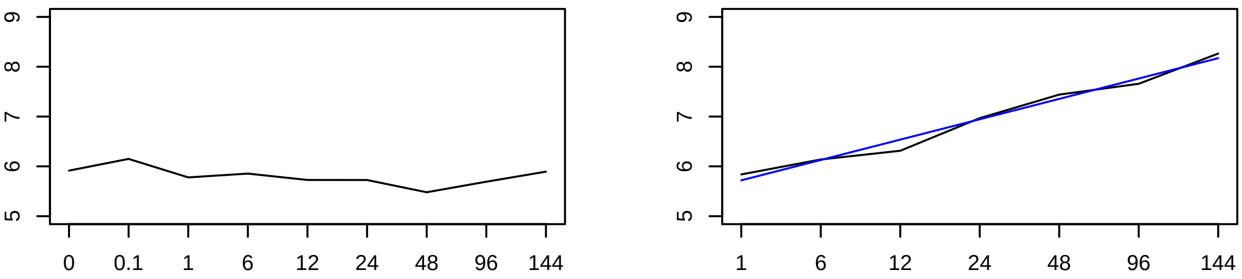

A_23_P250607 PLS3 Xq23

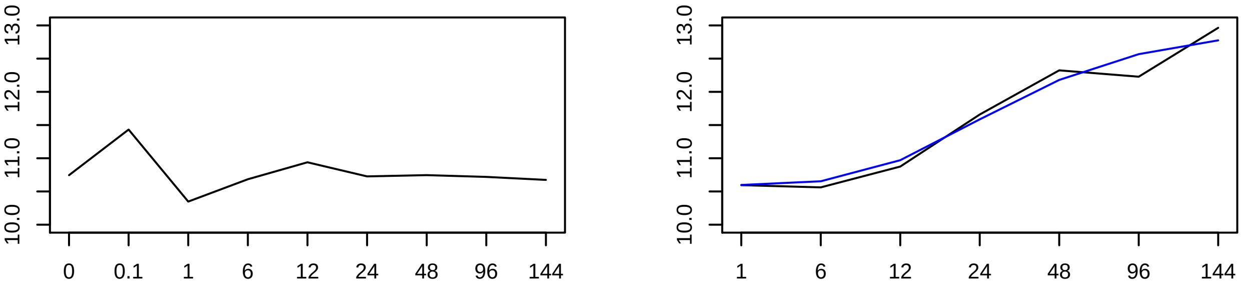

A_23_P34804 NTRK1 1q23.1

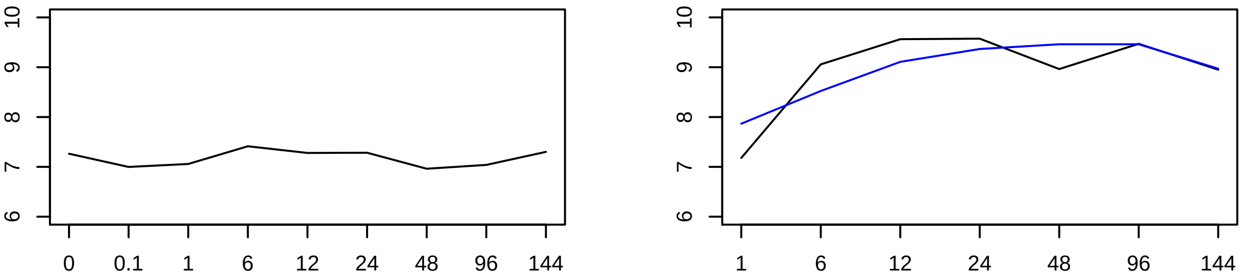

A_32_P442998 PCDH7 4p15.1

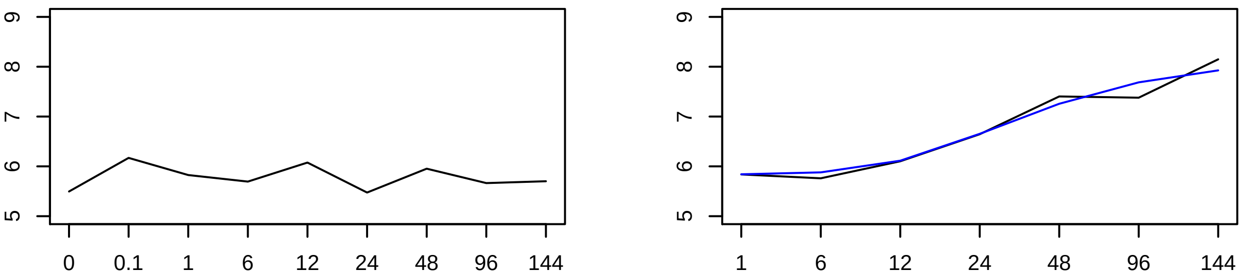

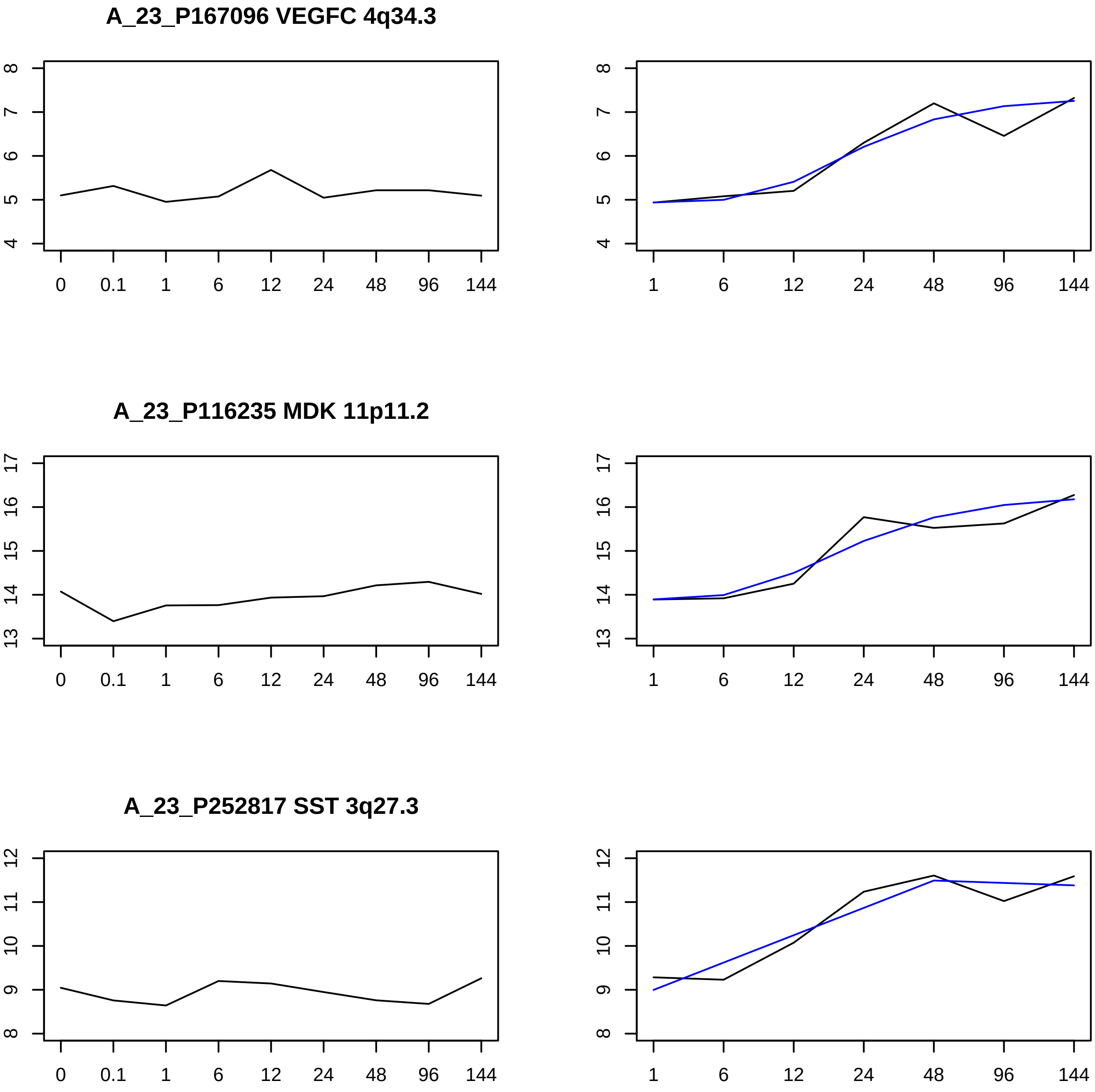
A_23_P167096 VEGFC 4q34.3
A_23_P116235 MDK 11p11.2
A_23_P252817 SST 3q27.3
0 0.1 1 6 12 24 48 96 144
1 6 12 24 48 96 144

A_32_P164376

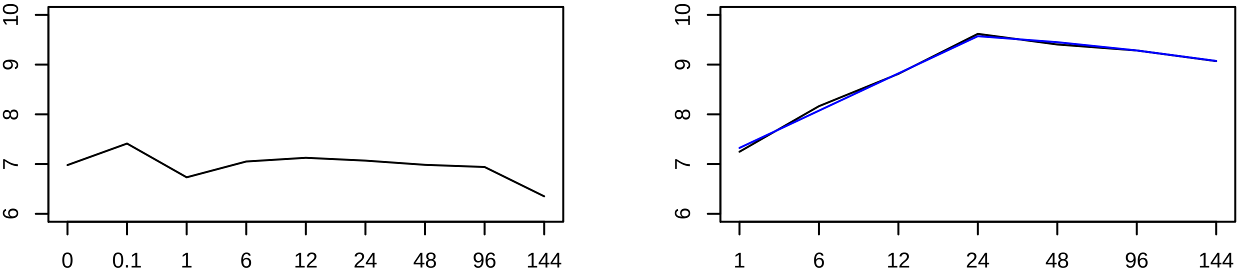

A_32_P88776 RP11−355I22.7 14q23.2

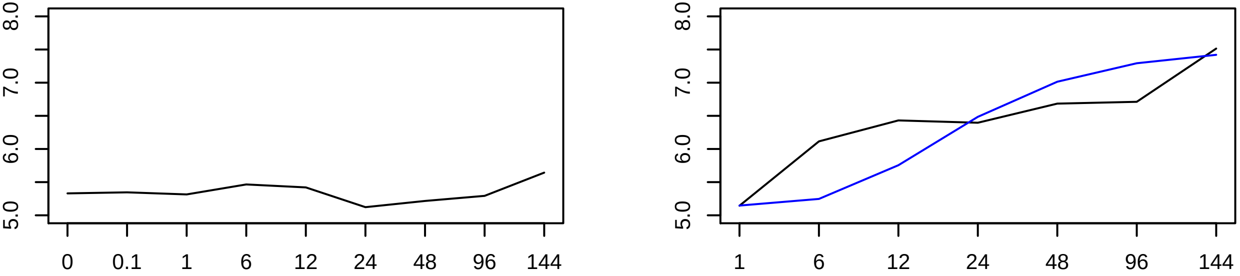

A_23_P203743 GAB2 11q14.1

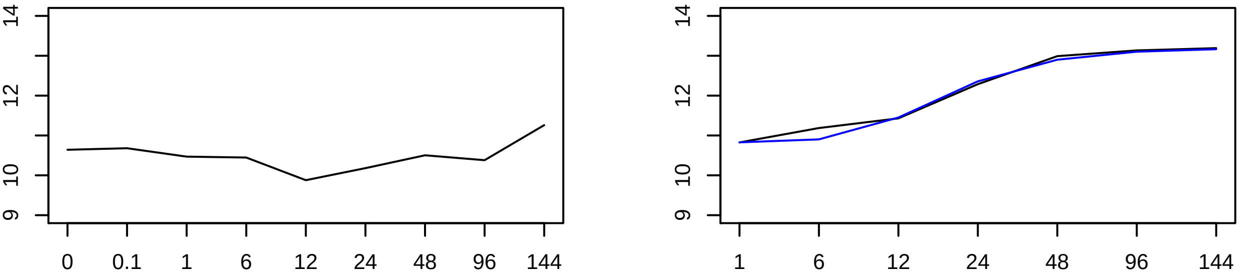

A_32_P171386 THC2696662 NA

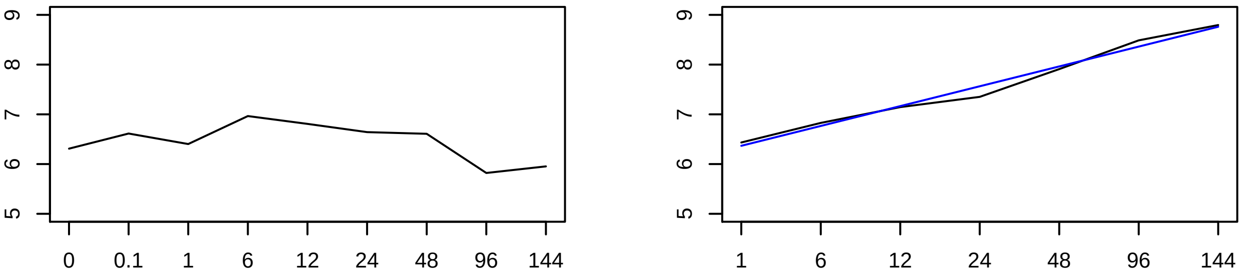

A_23_P391586 TPM1 15q22.2

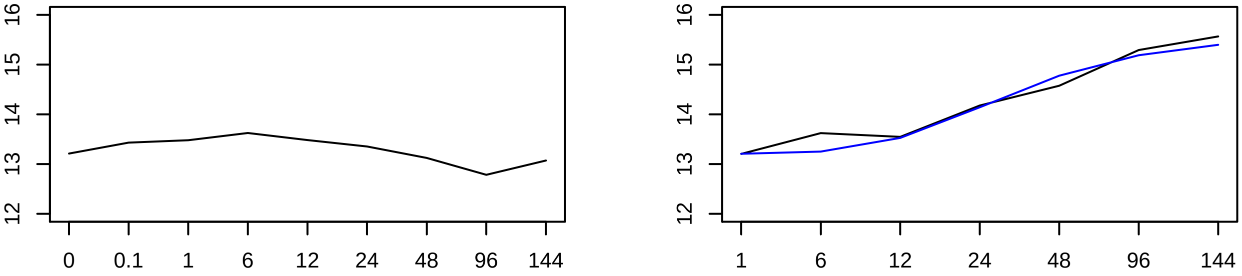

A_23_P30363 P4HA2 5q31.1

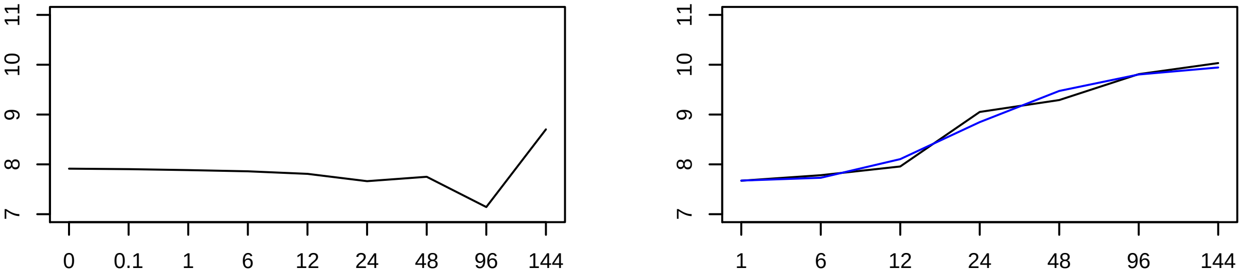

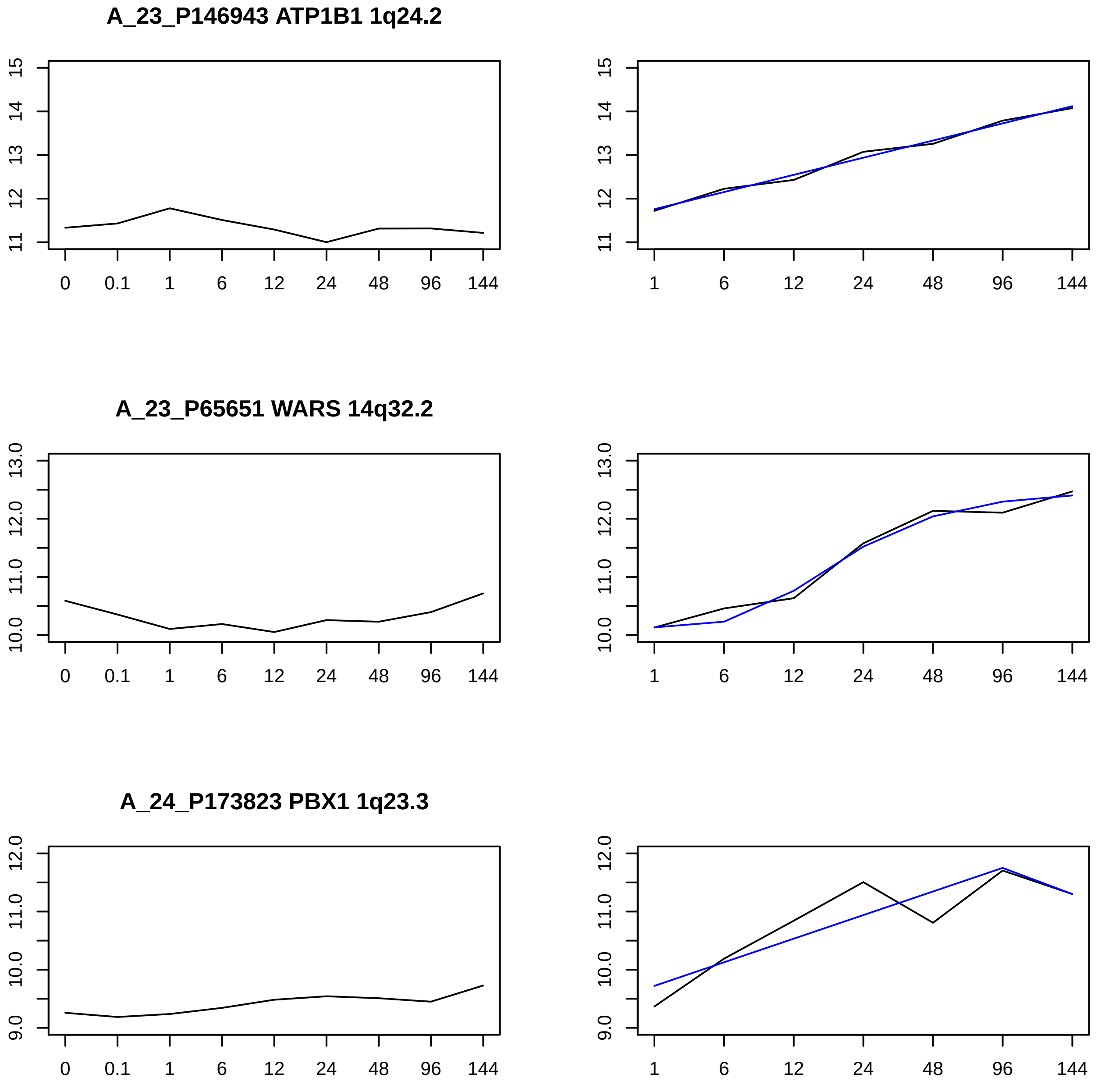
A_23_P146943 ATP1B1 1q24.2
11
12
13
14
15
0
0.1
1
6
12
24
48
96
144
A_23_P65651 WARS 14q32.2
10.0
11.0
12.0
13.0
A_24_P173823 PBX1 1q23.3
9.0
10.0
11.0
12.0

A_32_P60459 OTUD1 10p12.2

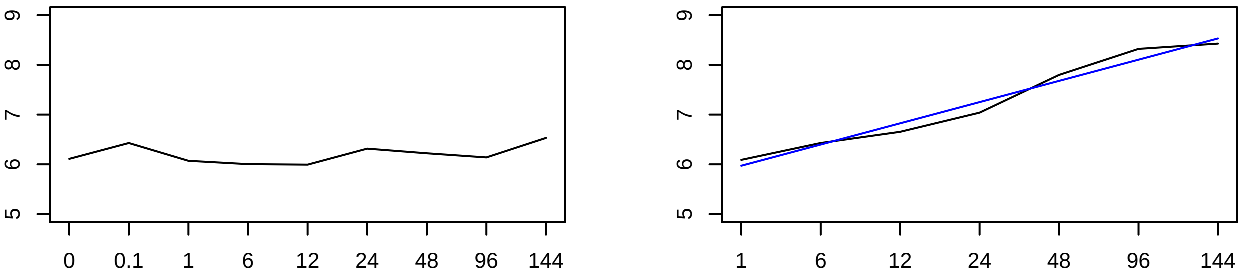

A_23_P21382 LAMB2 3p21.31

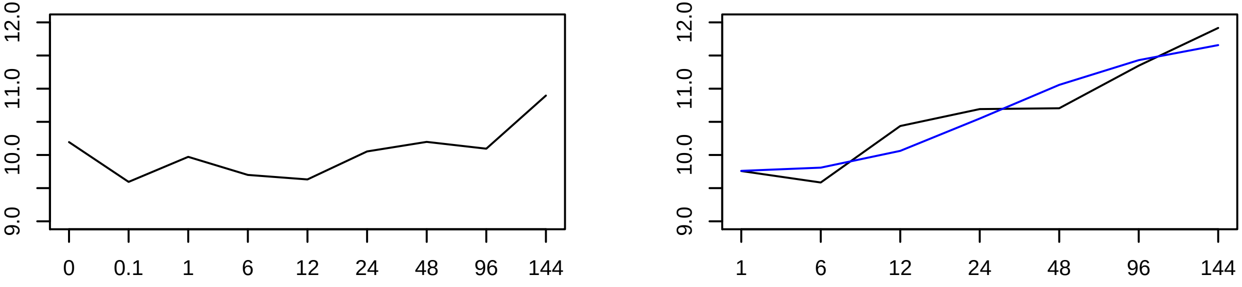

A_23_P126159 HPCA 1p35.1

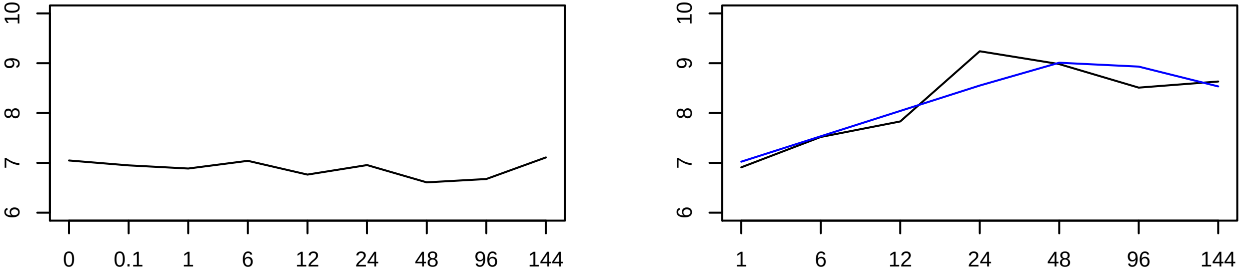

A_23_P39766 GLS 2q32.2

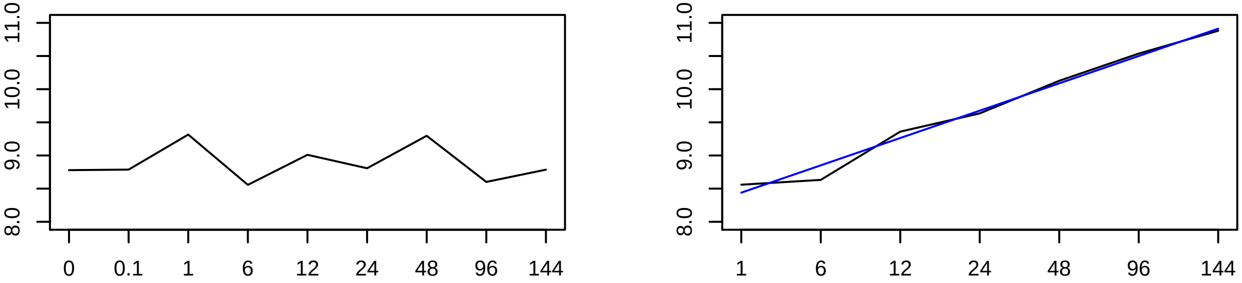

A_32_P164378 THC2703271 NA

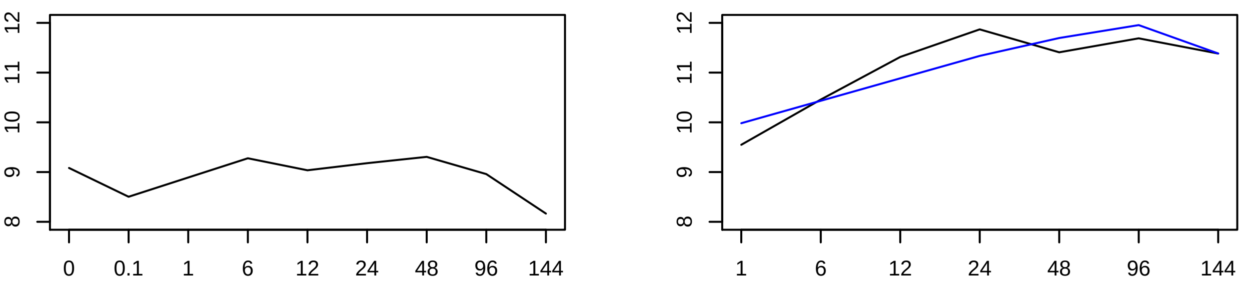

A_24_P79808 PBXIP1 1q21.3

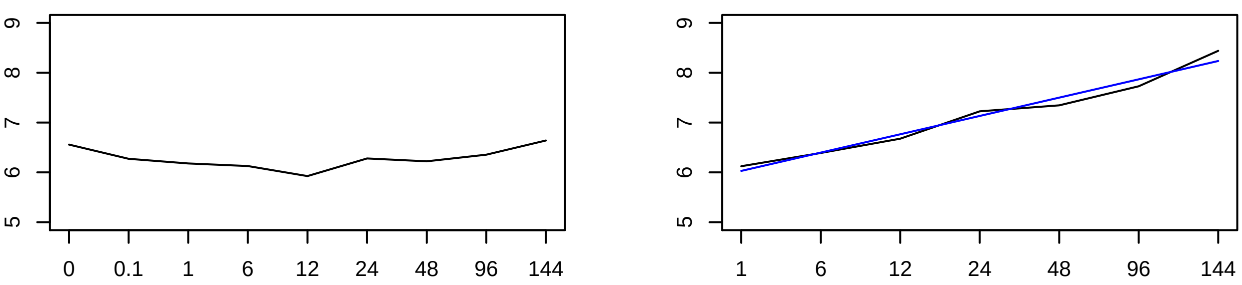

A_23_P344719 SYNJ2 6q25.3

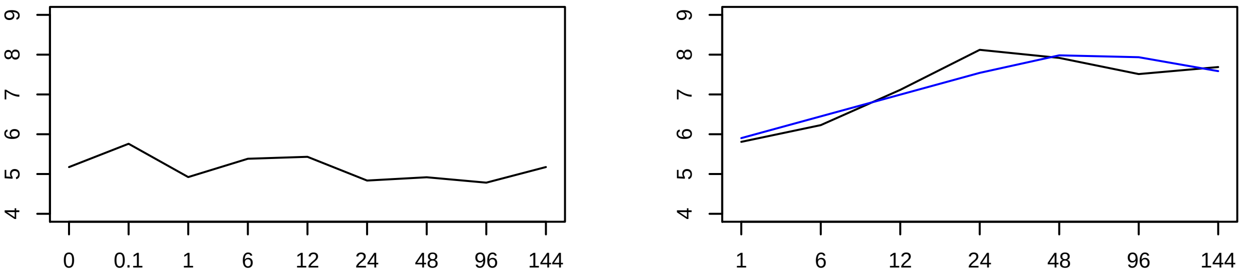

A_23_P65918 ITPKA 15q15.1

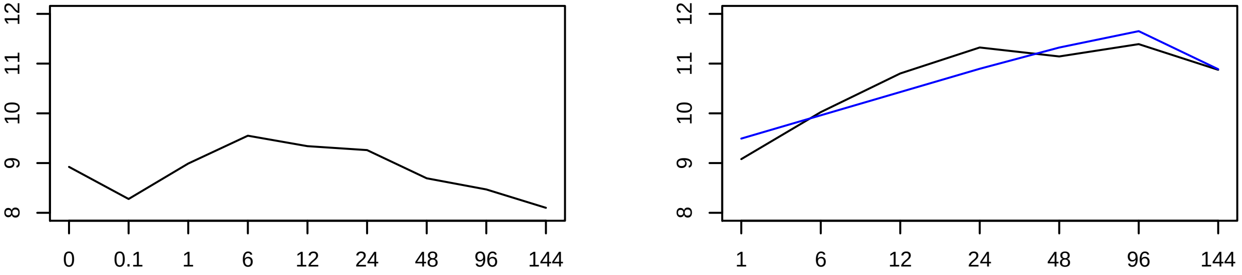

A_32_P5251 RARA 17q21.2

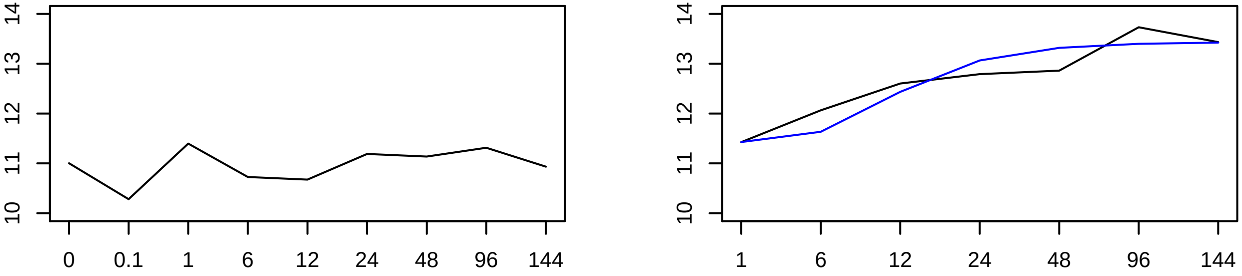

A_24_P252130 PPARD 6p21.31

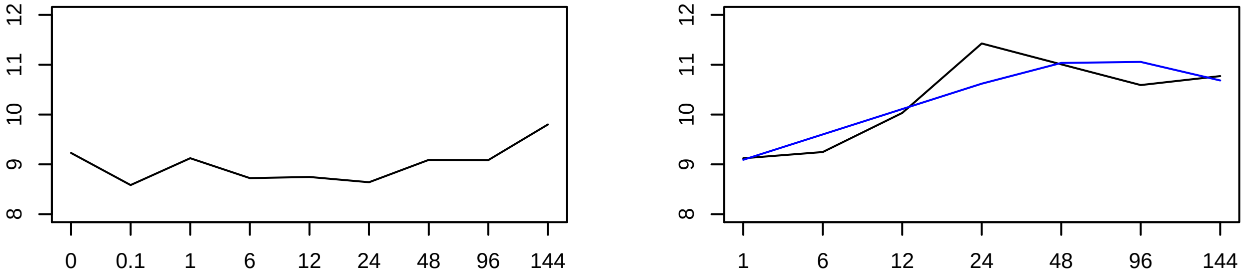

A_23_P215461 LIMK1 7q11.23

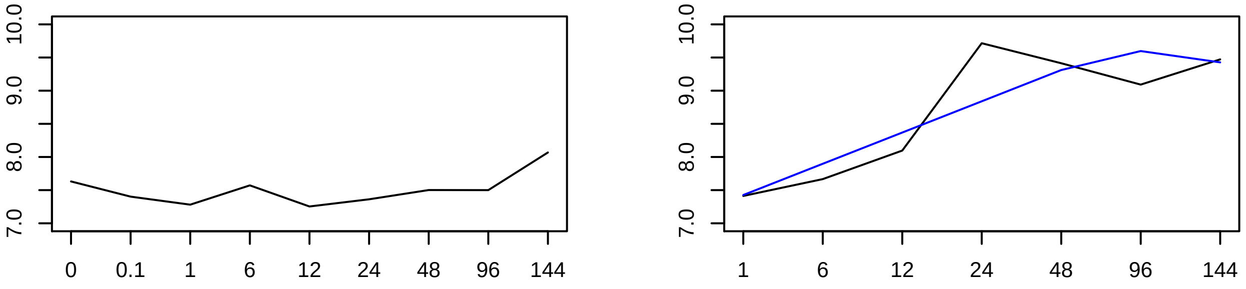

A_32_P25050 RDH10 8q21.11

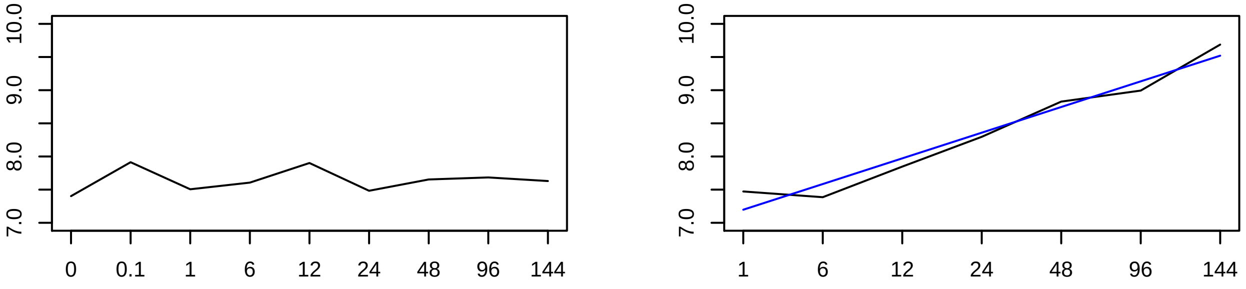

A_23_P370682 BATF2 11q13.1

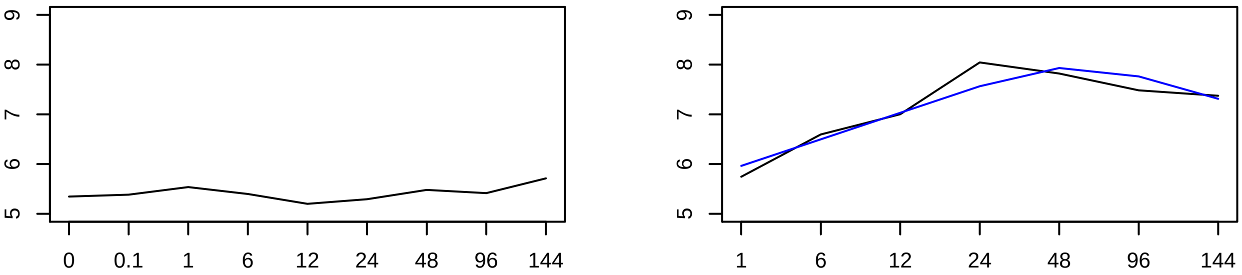

A_32_P157213 A_32_P157213 NA

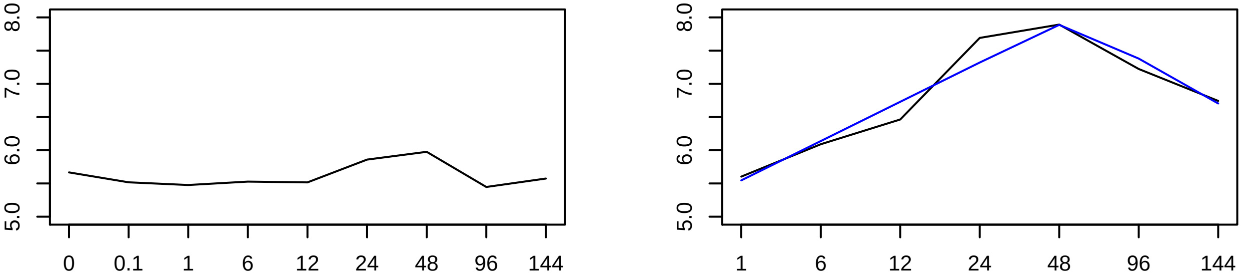

A_32_P181638 BVES 6q21

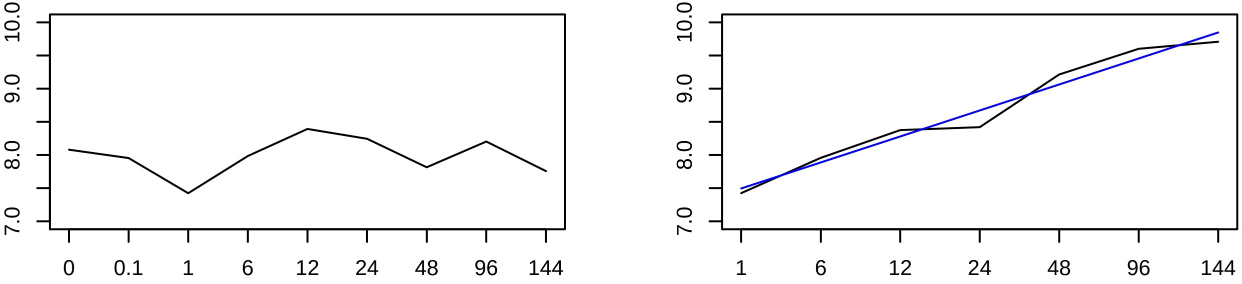

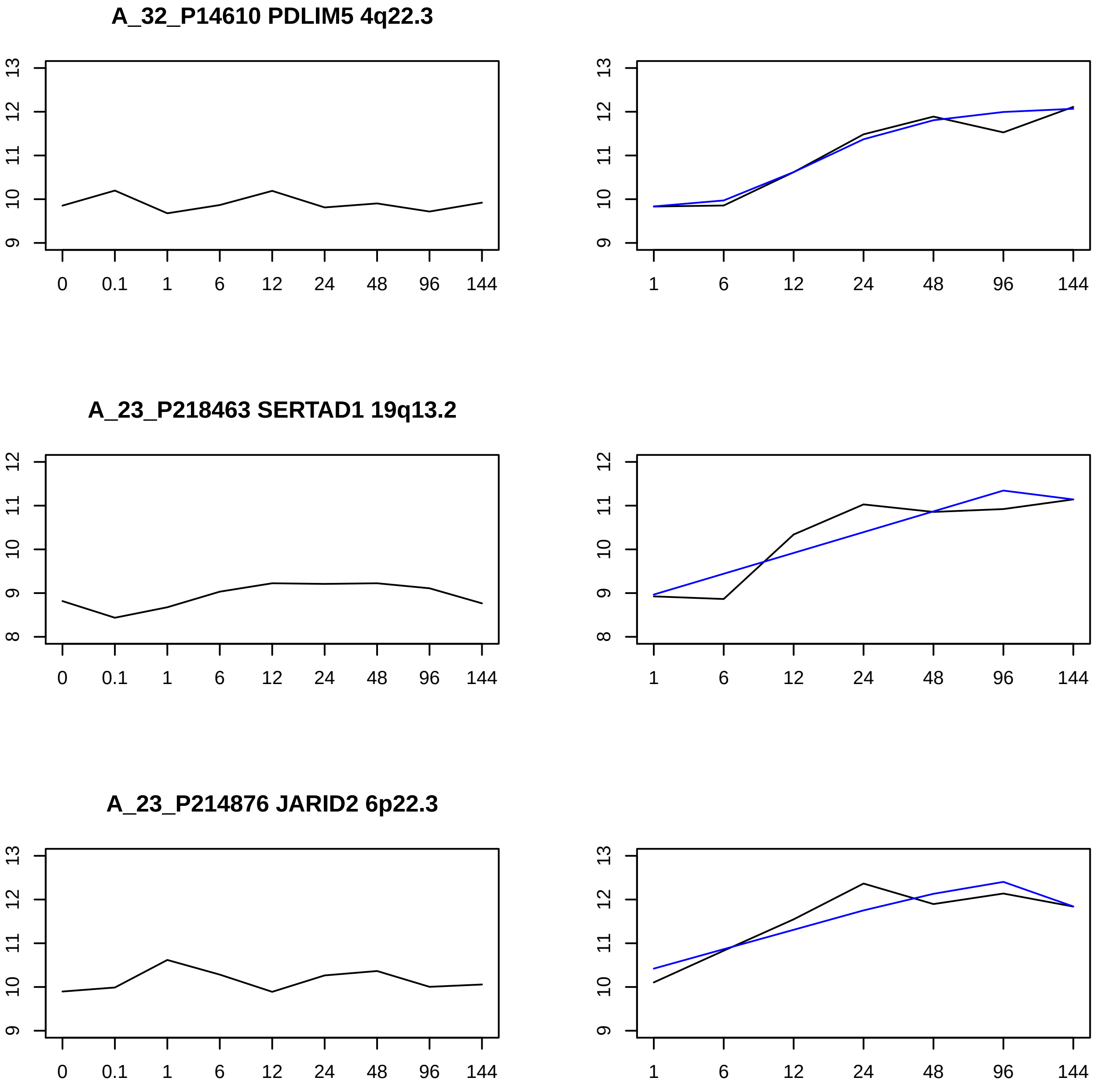
A_32_P14610 PDLIM5 4q22.3
9
10
11
12
13
0
0.1
1
6
12
24
48
96
144
1
6
12
24
48
96
144
A_23_P218463 SERTAD1 19q13.2
8
9
10
11
12
A_23_P214876 JARID2 6p22.3

A_24_P913431 TRIO 5p15.2

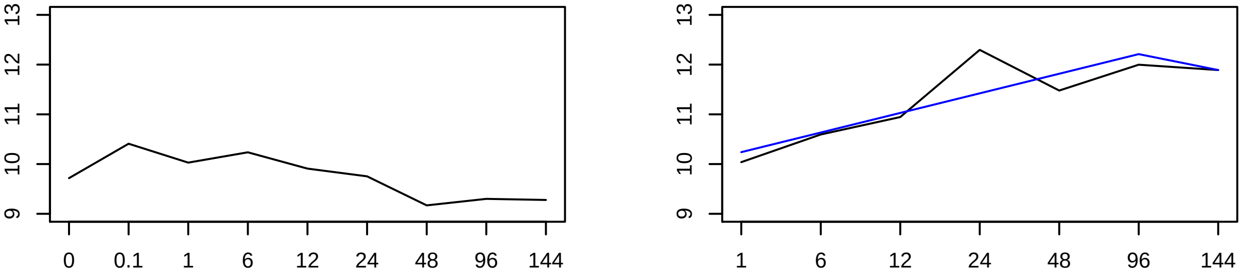

A_24_P319736 MEIS1 2p14

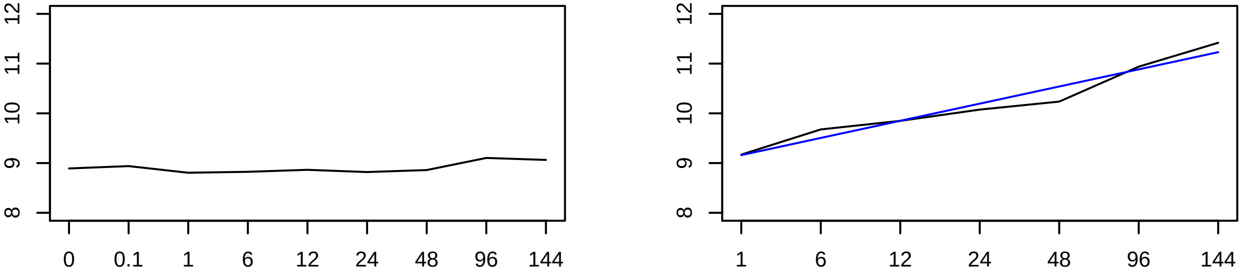

A_23_P210158 HOXD4 2q31.1

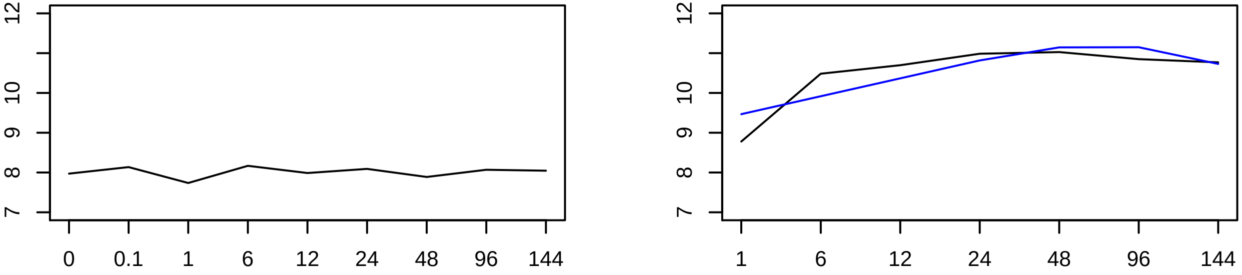

A_32_P121326 THC2701970 NA

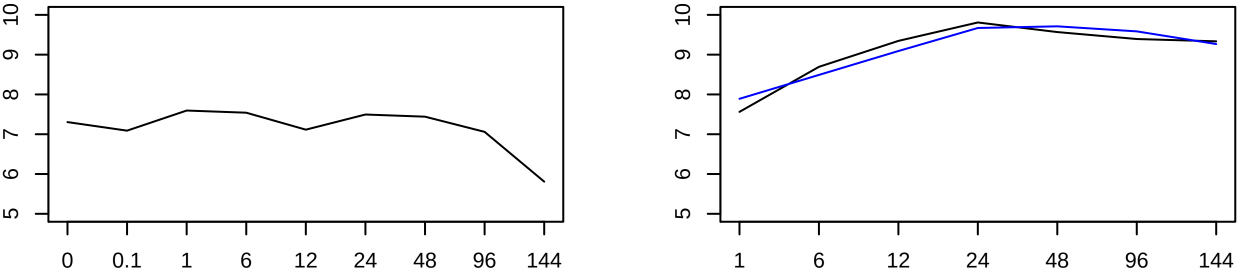

A_32_P175900 DPP6 7q36.2

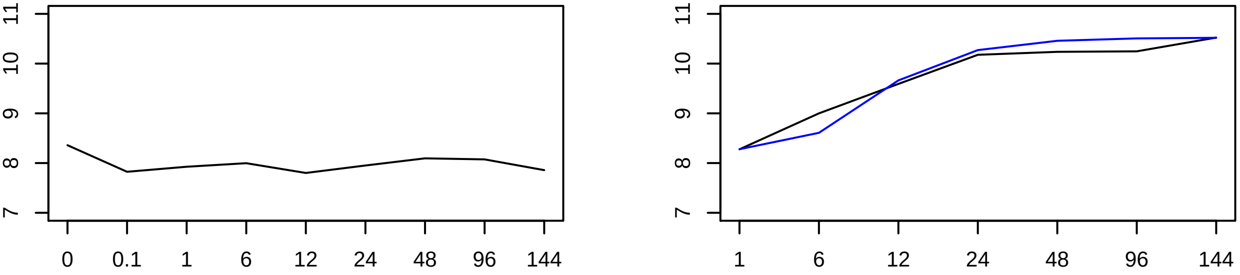

A_23_P107351 NLRP1 17p13.2

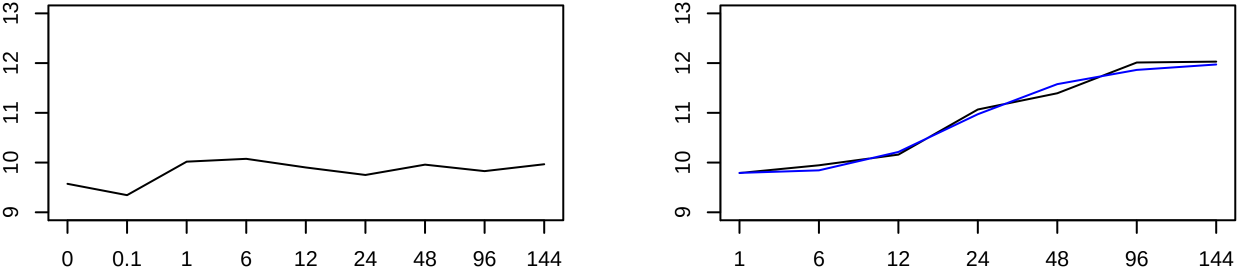

A_23_P408285 PRICKLE1 12q12

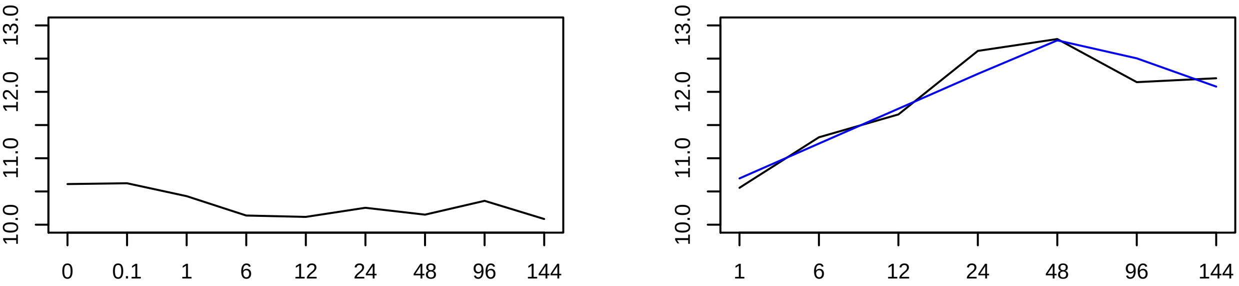

A_23_P351215 SKIL 3q26.2

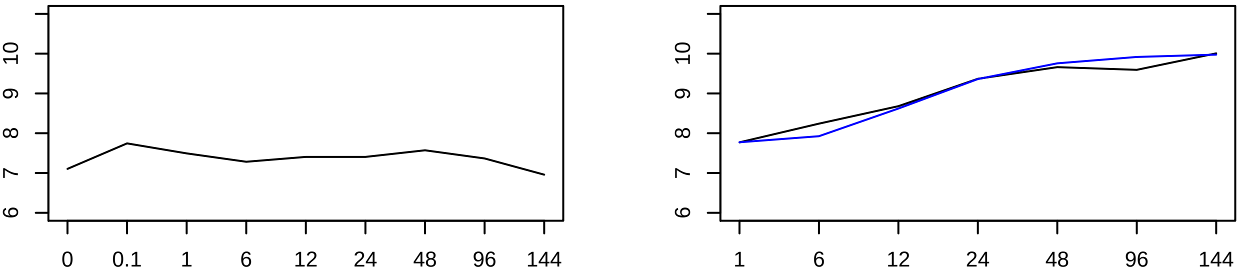

A_23_P343104 FLJ30901 22q13.2

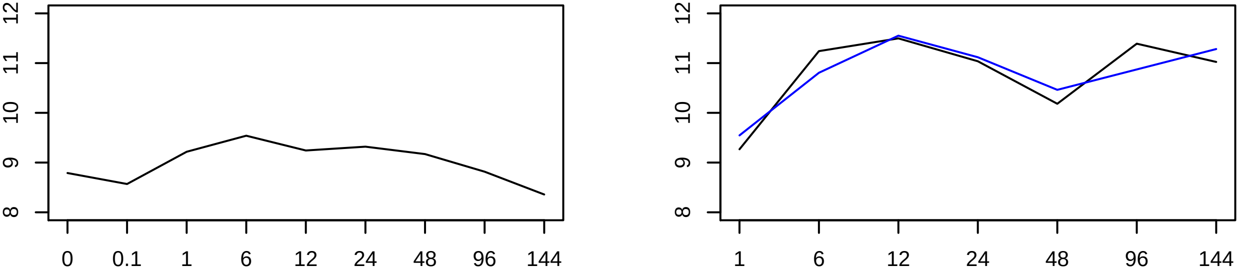

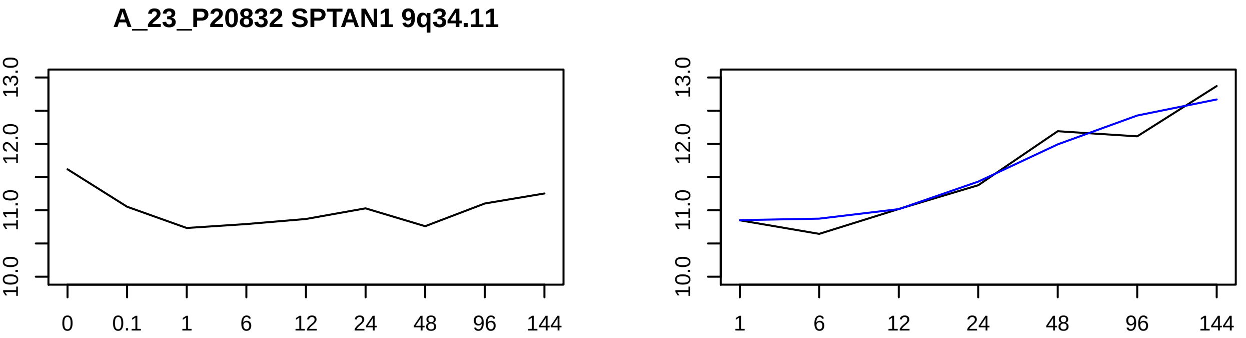
A_23_P20832 SPTAN1 9q34.11
10.0
11.0
12.0
13.0
0
0.1
1
6
12
24
48
96
144

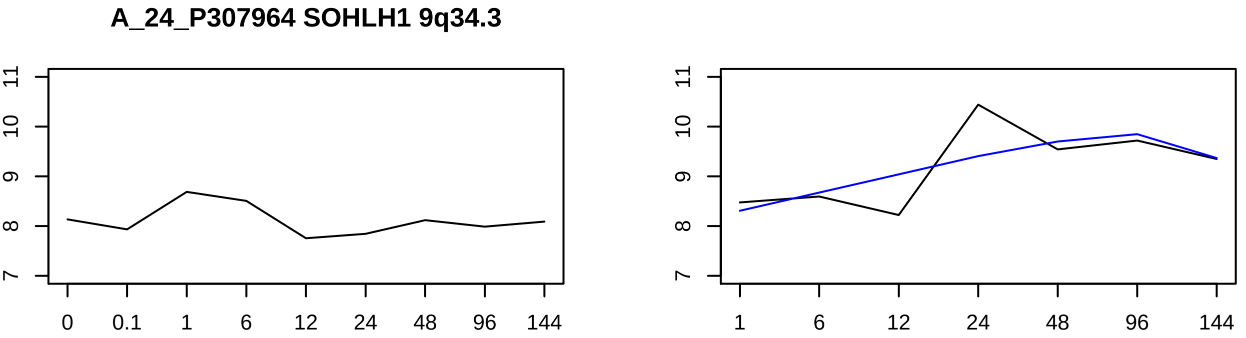
A_24_P307964 SOHLH1 9q34.3
7
8
9
10
11
0
0.1
1
6
12
24
48
96
144

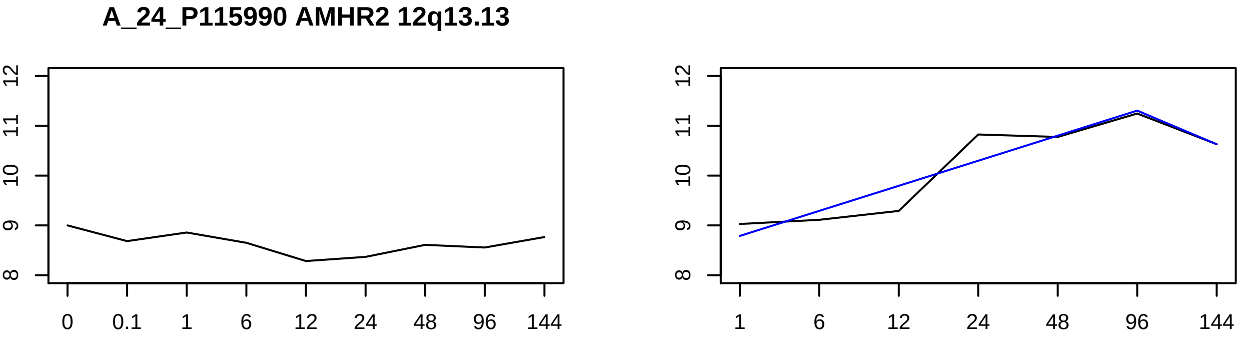
A_24_P115990 AMHR2 12q13.13
8
9
10
11
12
0
0.1
1
6
12
24
48
96
144

A_23_P141180 TOM1L2 17p11.2

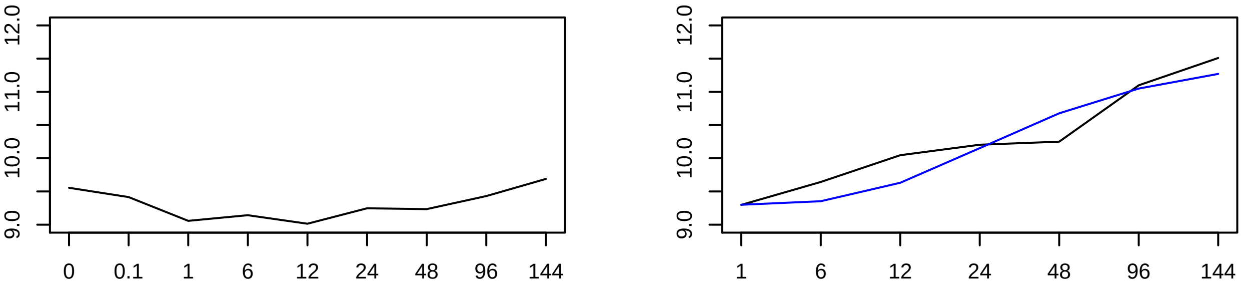

A_23_P138541 AKR1C3 10p15.1

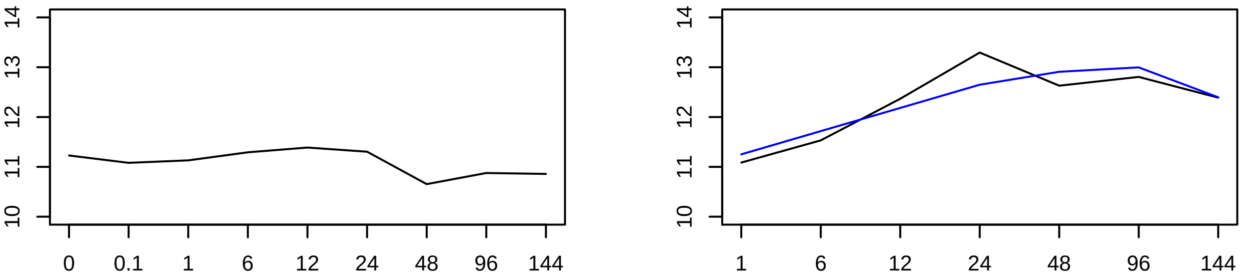

A_24_P298360 LTBP3 11q13.1

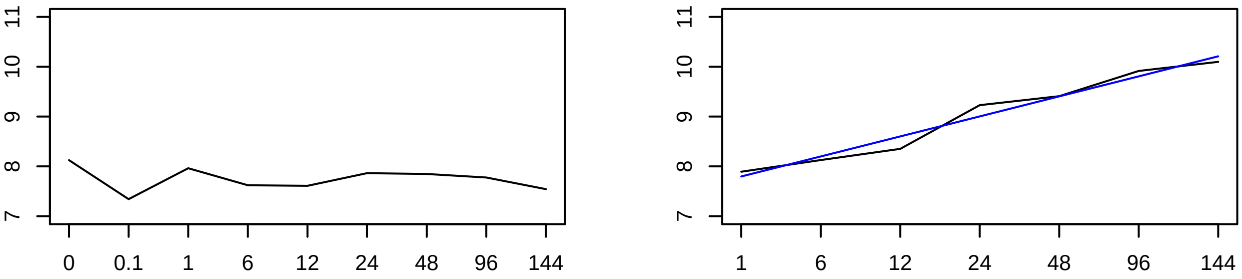

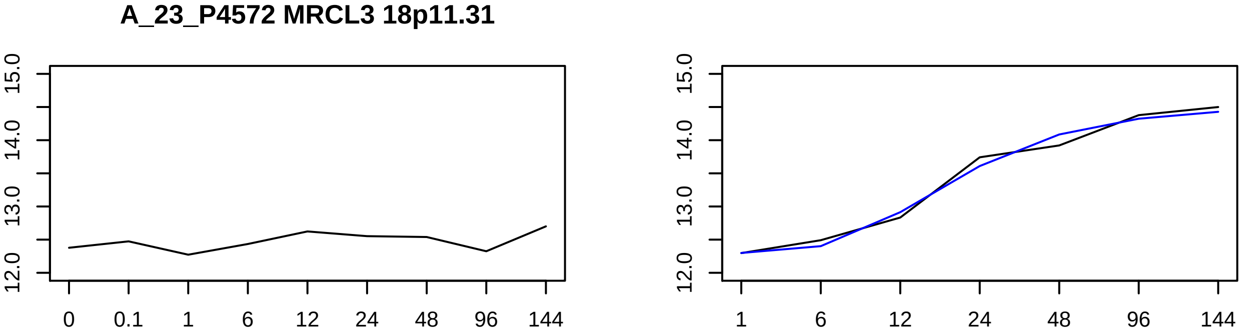
A_23_P4572 MRCL3 18p11.31
12.0
13.0
14.0
15.0
0
0.1
1
6
12
24
48
96
144
1
6
12
24
48
96
144

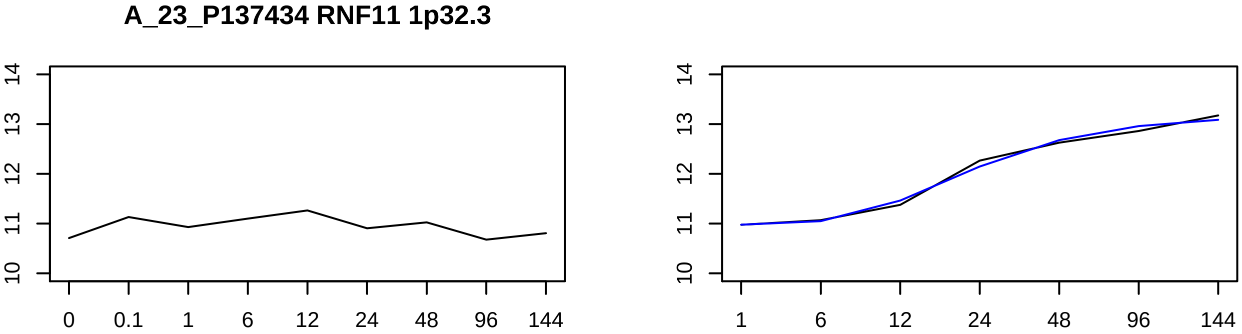
A_23_P137434 RNF11 1p32.3
10
11
12
13
14
0
0.1
1
6
12
24
48
96
144
1
6
12
24
48
96
144

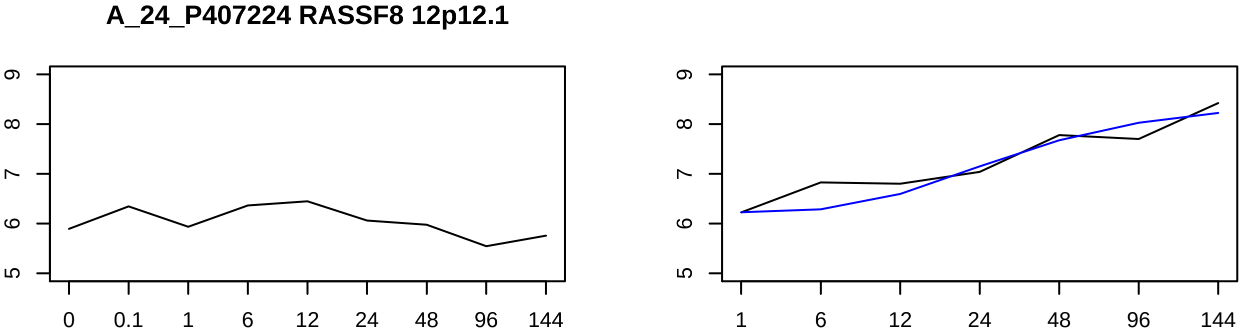
A_24_P407224 RASSF8 12p12.1
5
6
7
8
9
0
0.1
1
6
12
24
48
96
144
1
6
12
24
48
96
144

A_23_P112634 C4orf34 4p14

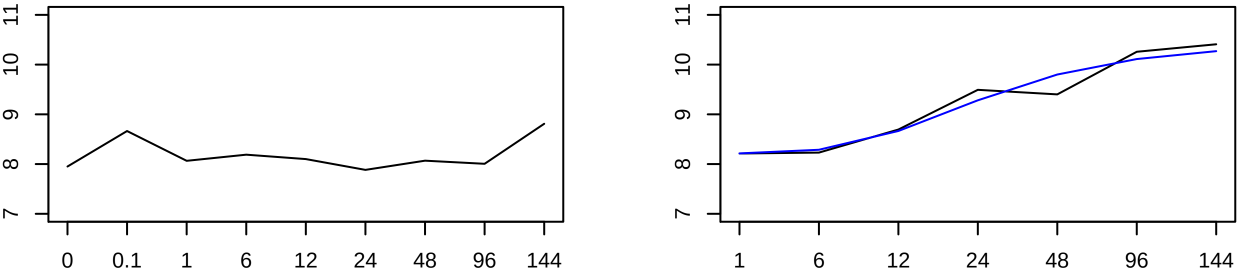

A_23_P59261 TPBG 6q14.1

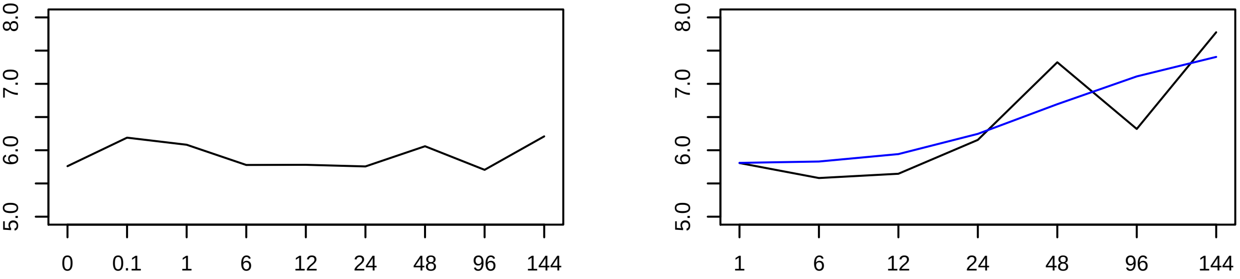

A_23_P54144 BMP4 14q22.2

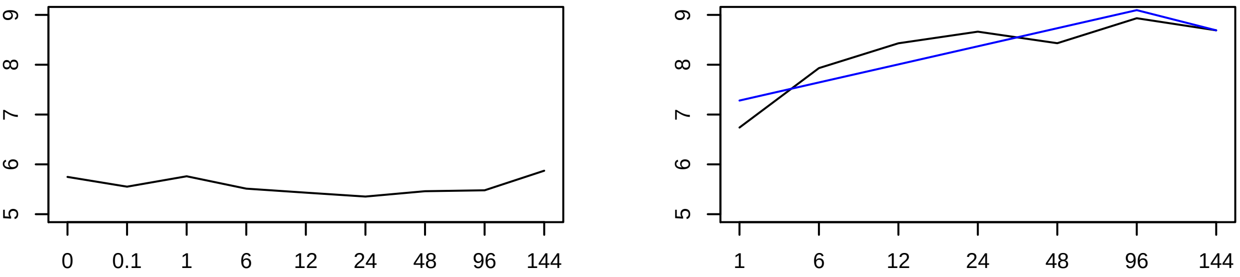

A_24_P944640 EPB41L5 2q14.2

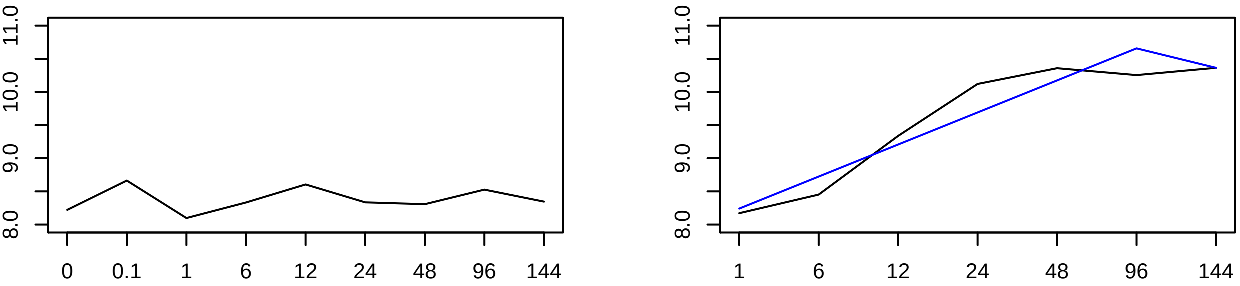

A_24_P835500 ITPRIPL2 NA

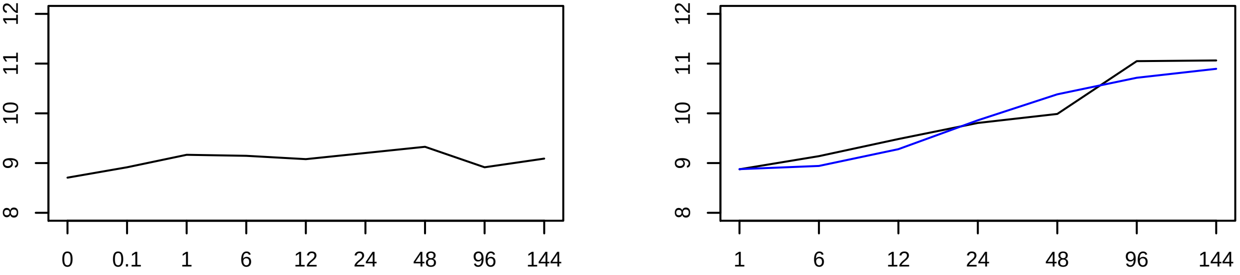

A_23_P157545 NKX3−1 8p21.2

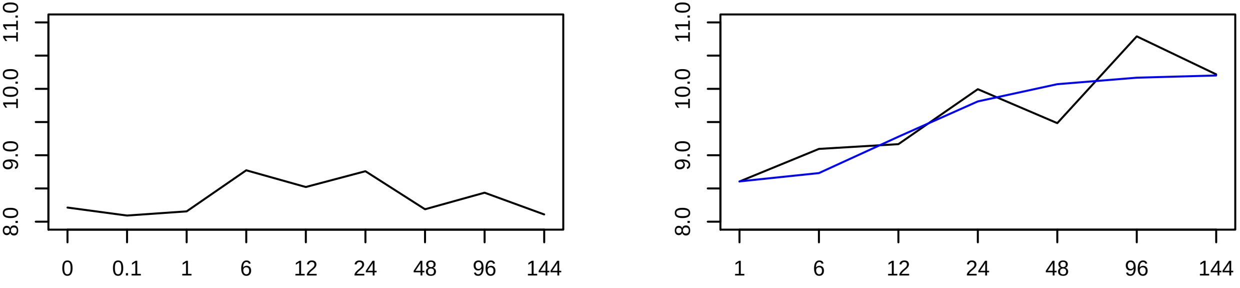

A_23_P156852 PECI 6p25.2

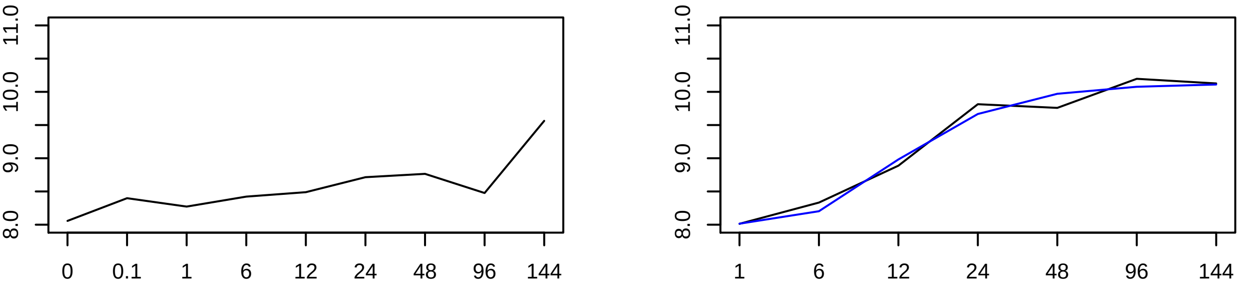

A_23_P100022 SV2B 15q26.1

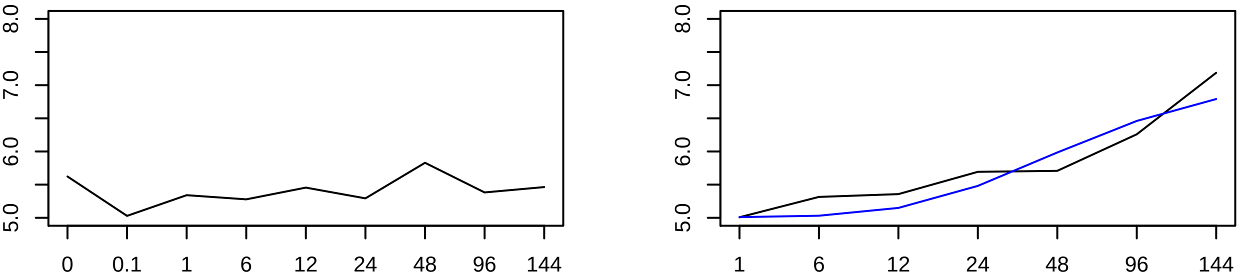

A_23_P420442 SEMA6D 15q21.1

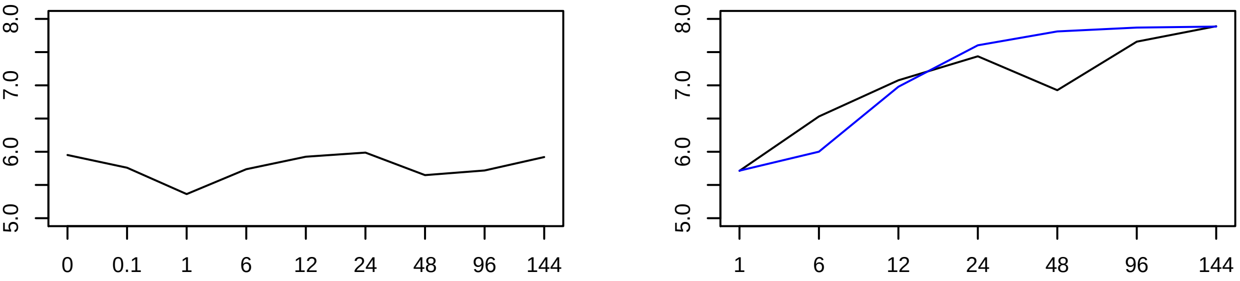

A_23_P32577 DACH1 13q21.33

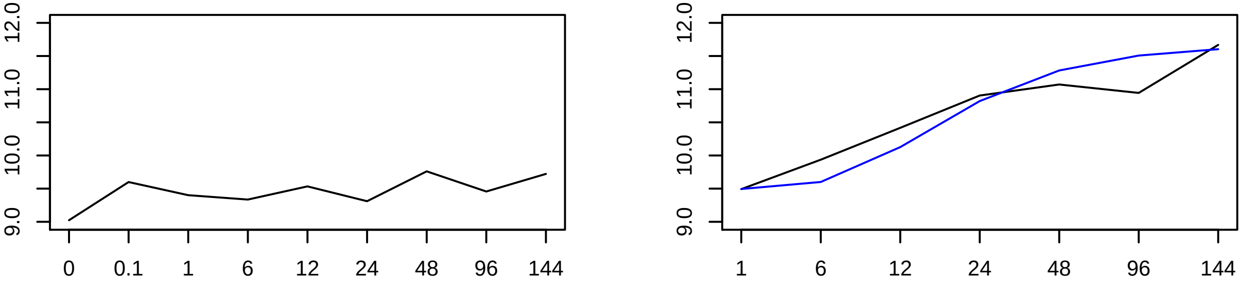

A_23_P111701 GNG11 7q21.3

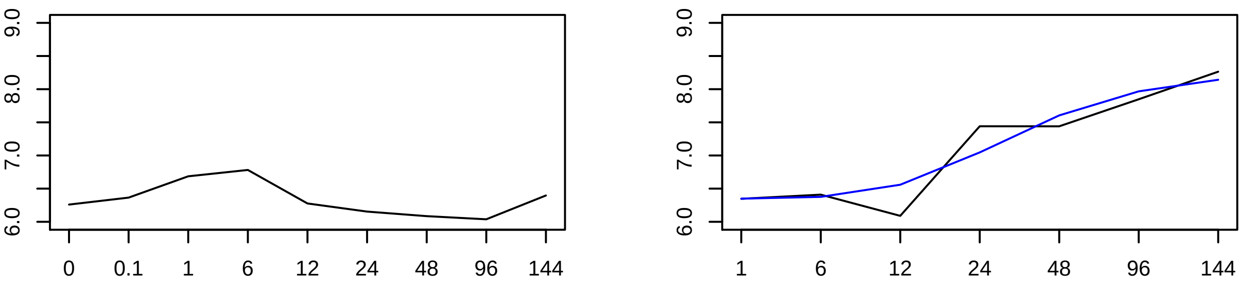

A_32_P162250 ARHGAP18 6q22.33

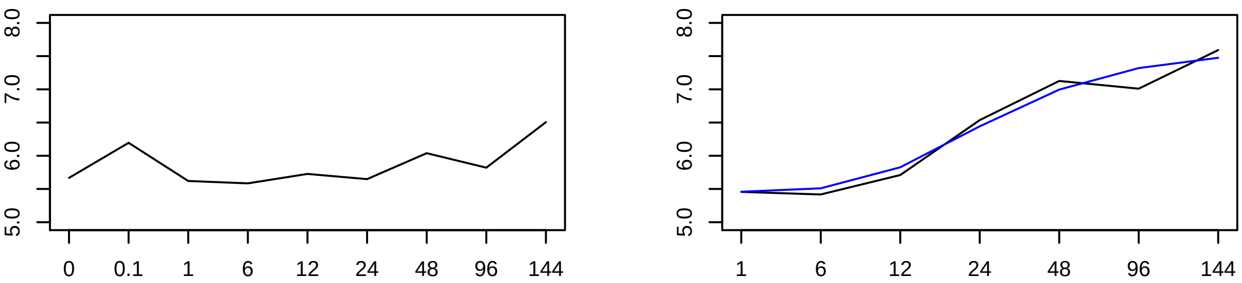

A_23_P202435 ADD3 10q25.2

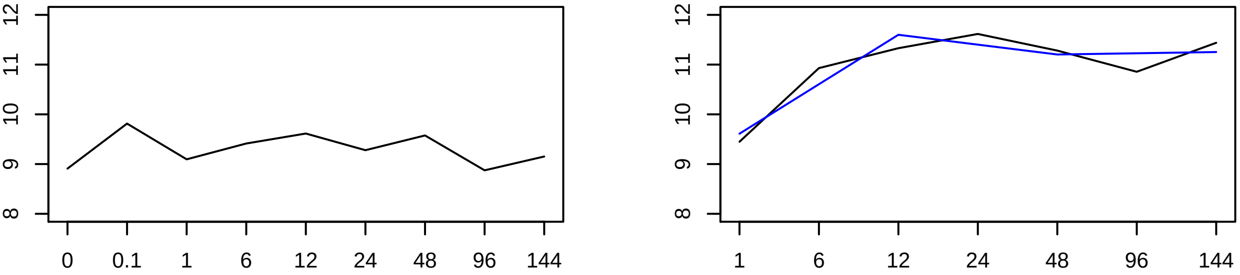

A_23_P212688 EVI1 3q26.2

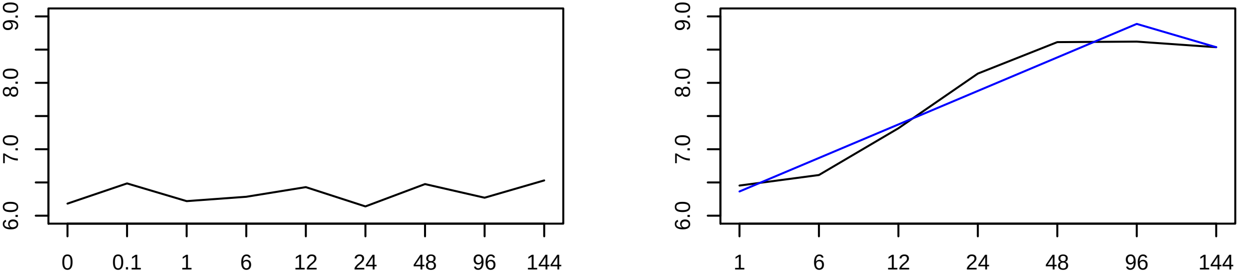

A_23_P202327 ADAM12 10q26.2

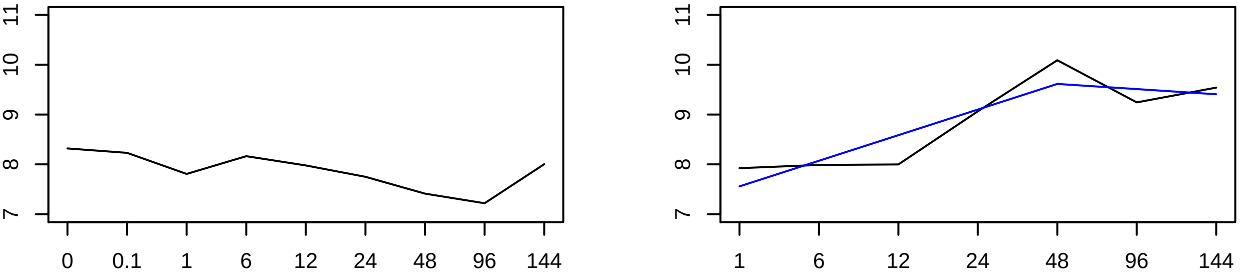

A_23_P127367 POLD4 11q13.1

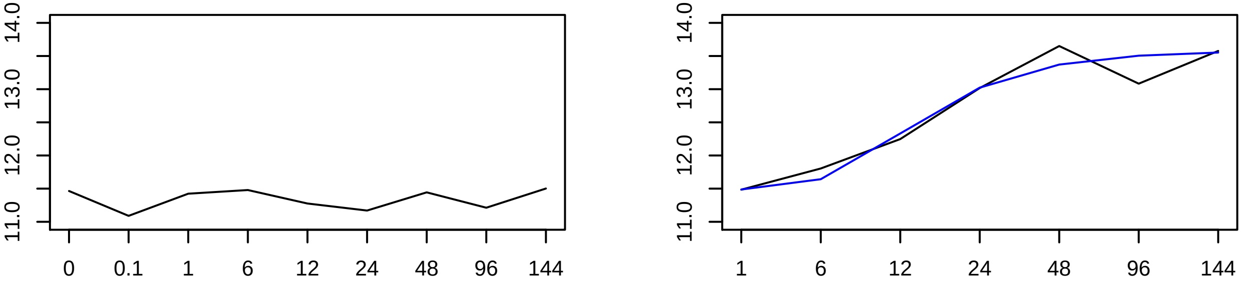

A_24_P20630 LEF1 4q25

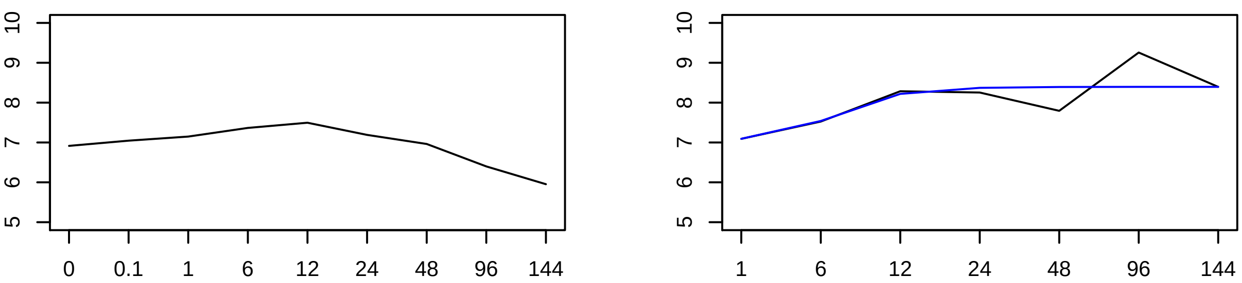

A_23_P41390 SH3TC1 4p16.1

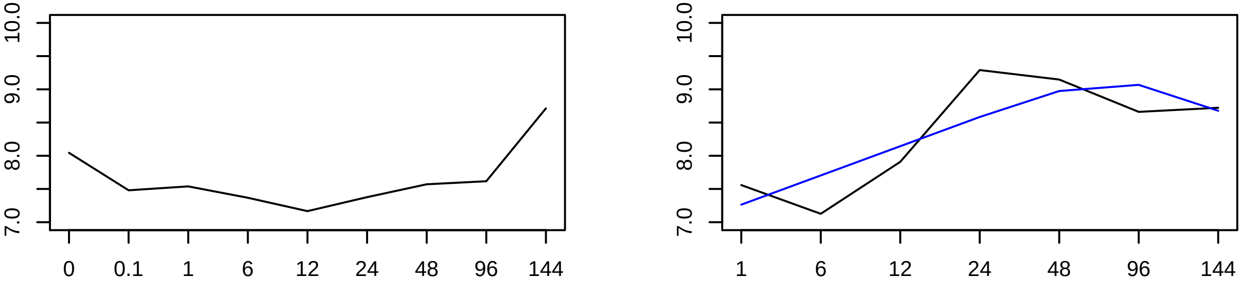

A_23_P151297 TENC1 12q13.13

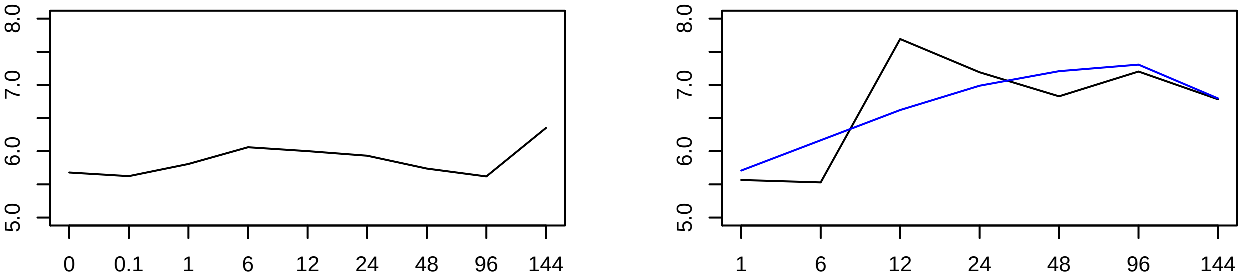

A_23_P2181 CYB5R2 11p15.4

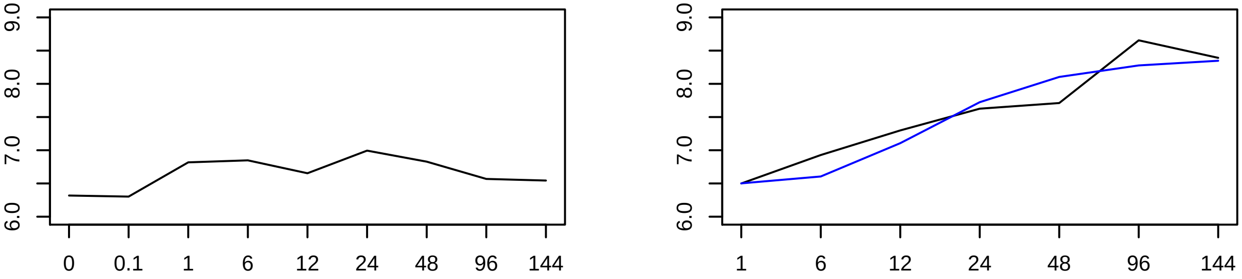

A_32_P232495 DPP6 7q36.2

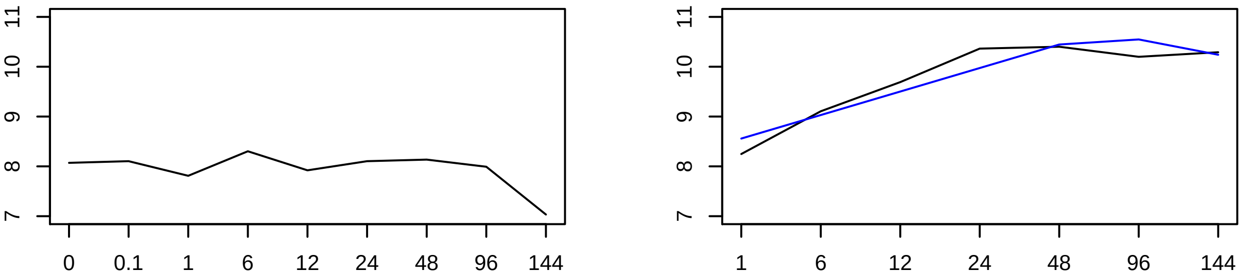

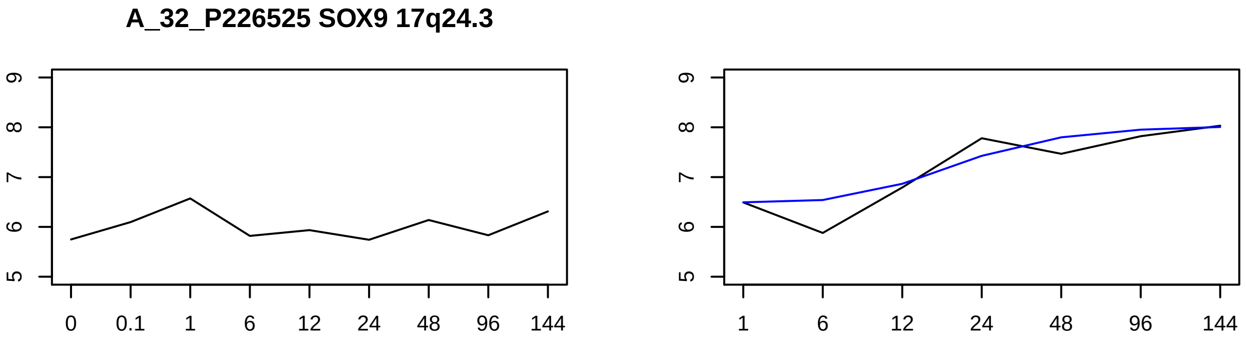
A_32_P226525 SOX9 17q24.3
5
6
7
8
9
0
0.1
1
6
12
24
48
96
144
1
6
12
24
48
96
144

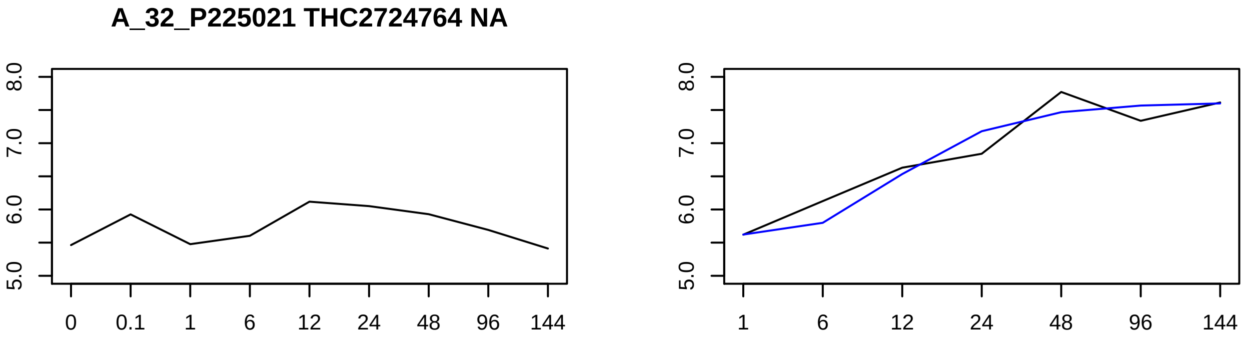
A_32_P225021 THC2724764 NA
5.0
6.0
7.0
8.0
0
0.1
1
6
12
24
48
96
144
1
6
12
24
48
96
144

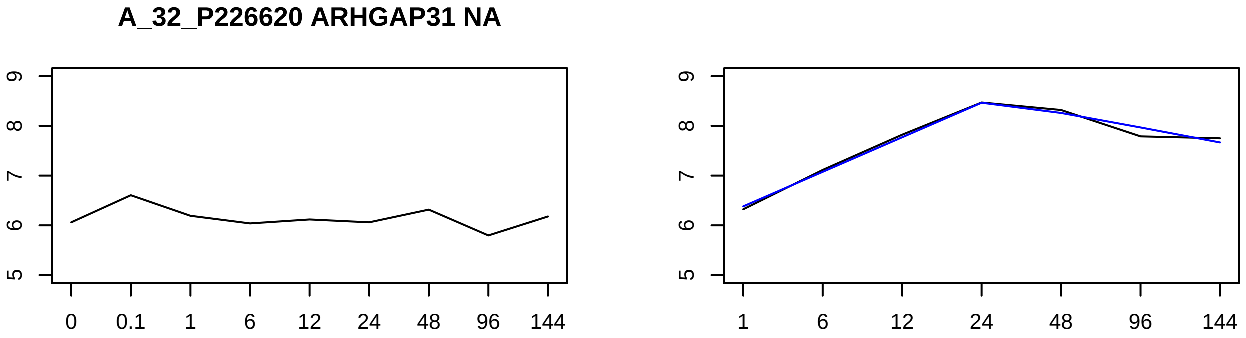
A_32_P226620 ARHGAP31 NA
5
6
7
8
9
0
0.1
1
6
12
24
48
96
144
1
6
12
24
48
96
144

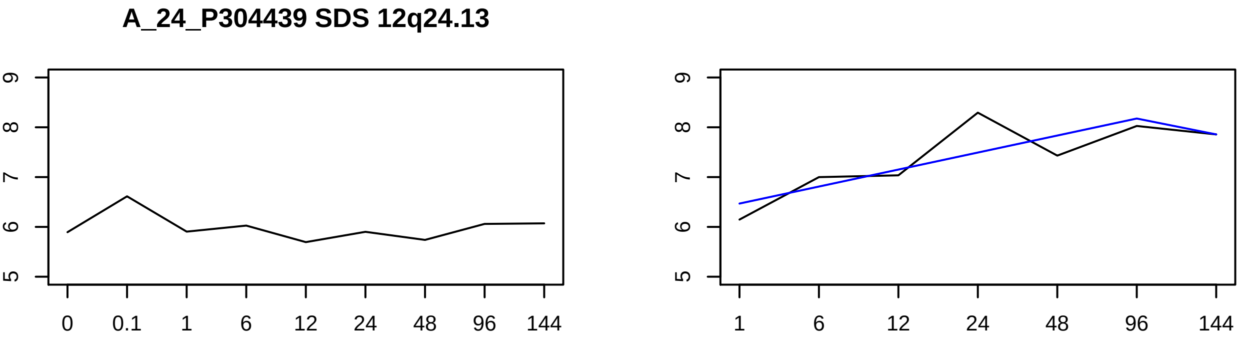
A_24_P304439 SDS 12q24.13
5
6
7
8
9
0 0.1 1 6 12 24 48 96 144
1 6 12 24 48 96 144

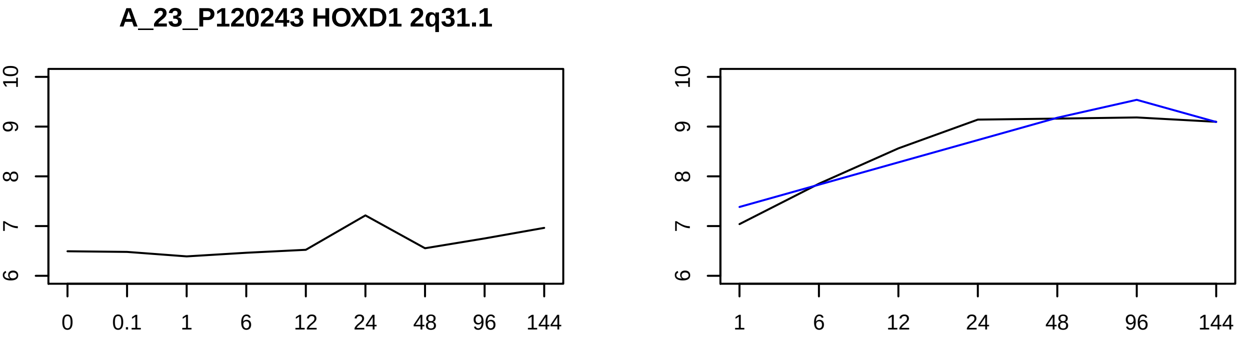
A_23_P120243 HOXD1 2q31.1
6
7
8
9
10
0 0.1 1 6 12 24 48 96 144
1 6 12 24 48 96 144

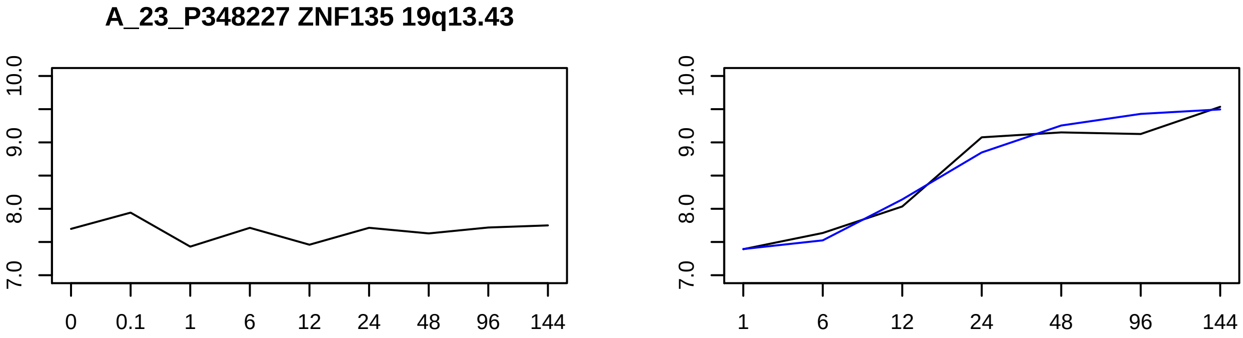
A_23_P348227 ZNF135 19q13.43
7.0
8.0
9.0
10.0
0 0.1 1 6 12 24 48 96 144
1 6 12 24 48 96 144

A_24_P911676 SOX4 6p22.3

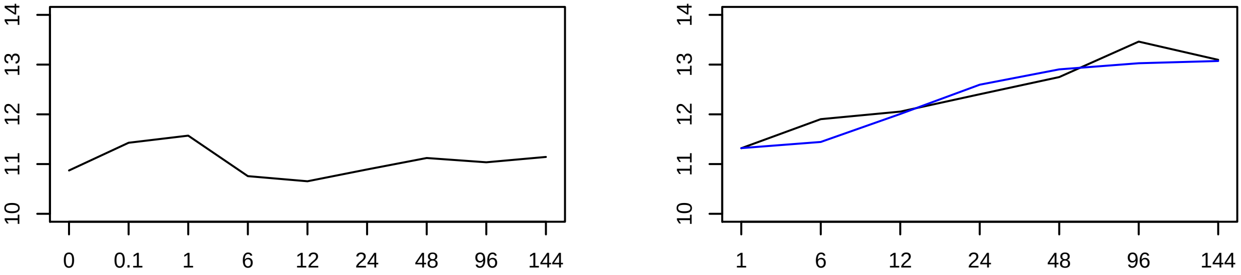

A_24_P261032 RDH10 8q21.11

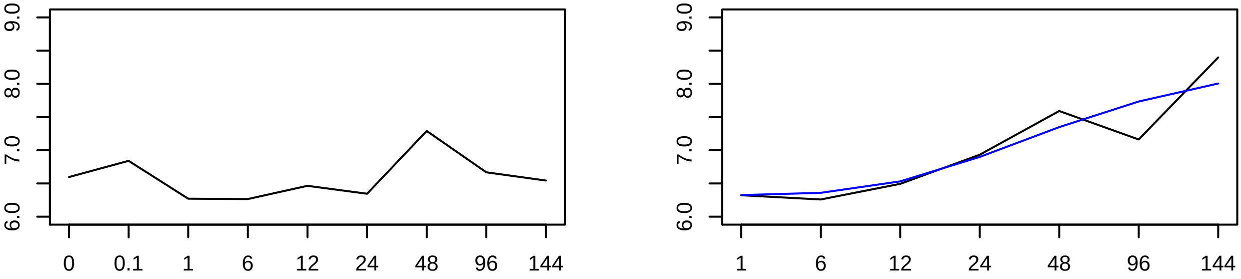

A_23_P214168 COL12A1 6q13

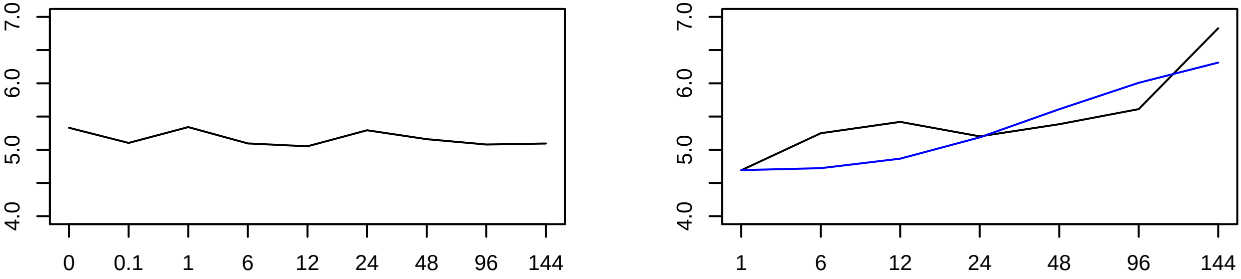

A_32_P191895 BC045716 NA

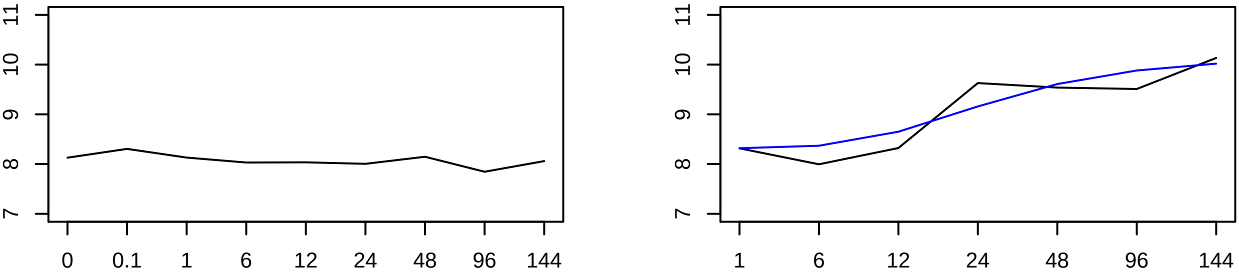

A_23_P136405 PDCD1 2q37.3

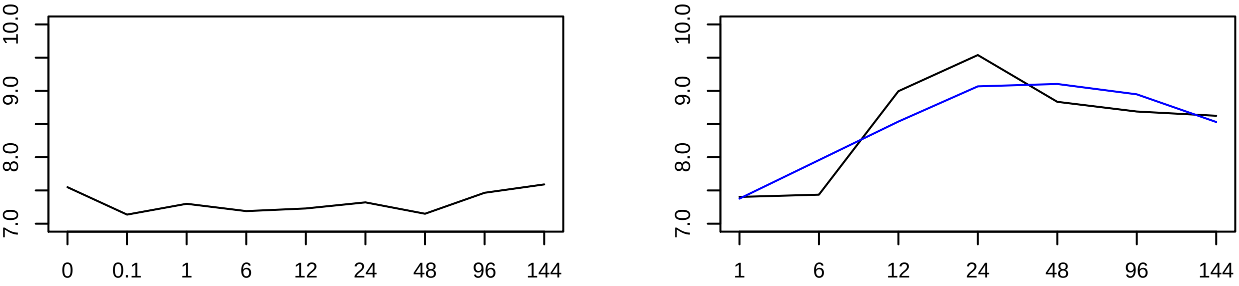

A_23_P502915 WDR1 4p16.1

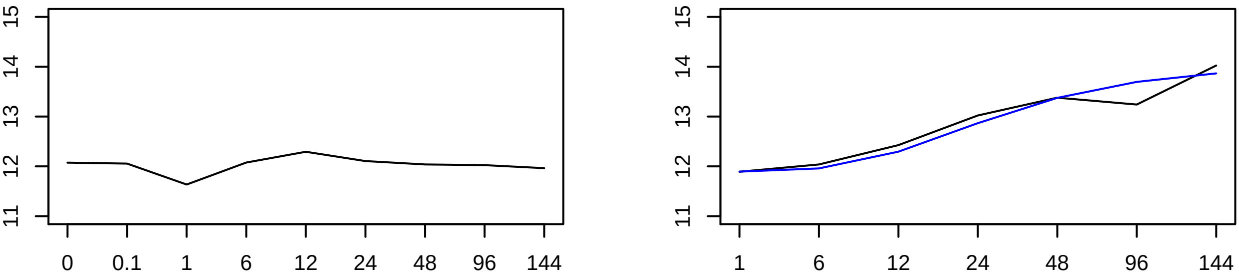

A_24_P317827 C9orf127 9p13.3

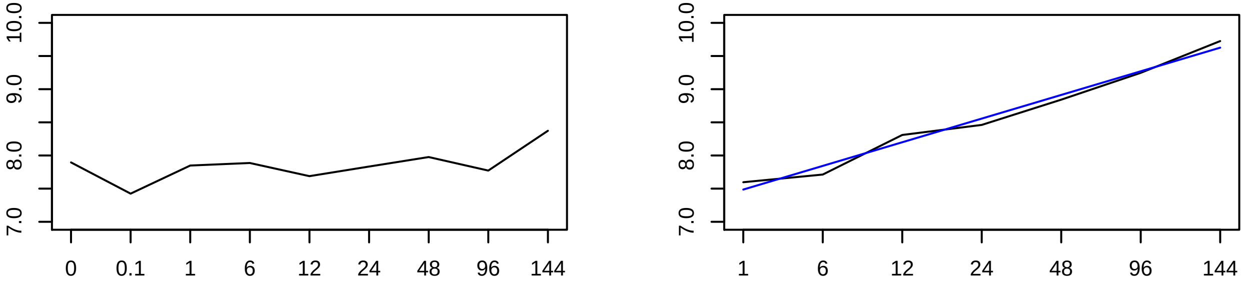

A_24_P668351 THC2618883 NA

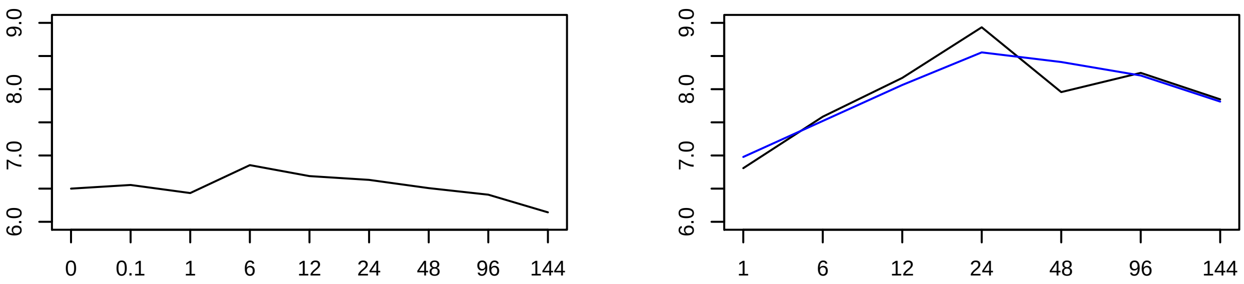

A_24_P76158 DOCK11 Xq24

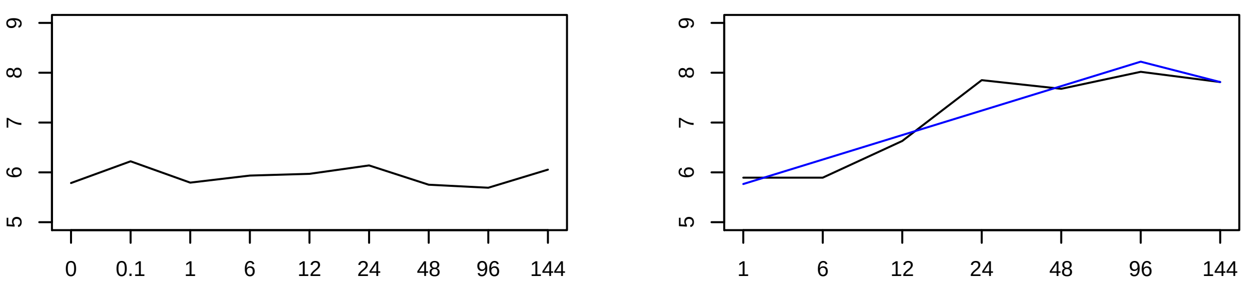

A_24_P29595 STXBP5 6q24.3

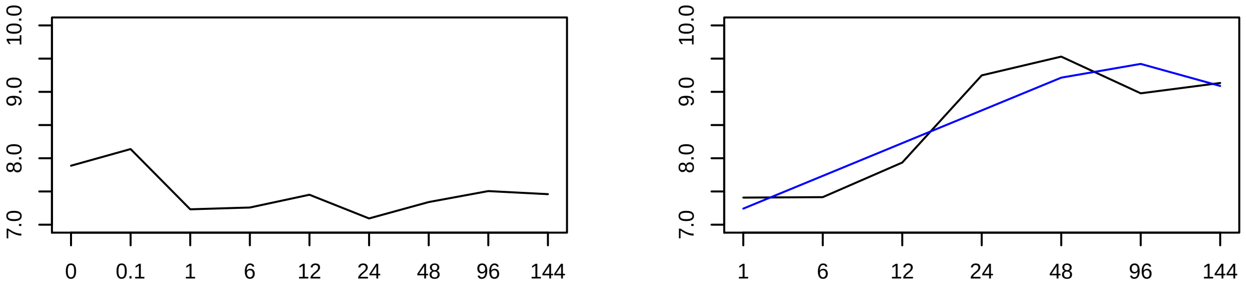

A_23_P62881 SGIP1 1p31.3

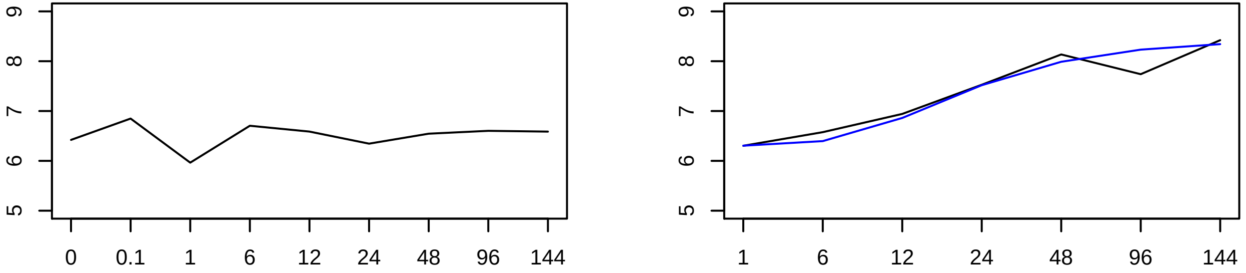

A_23_P416711 ST6GALNAC3 1p31.1

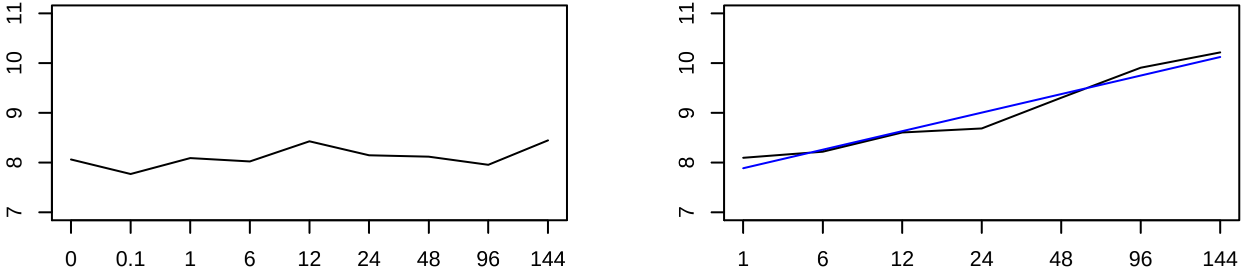

A_23_P117782 LARP6 15q23

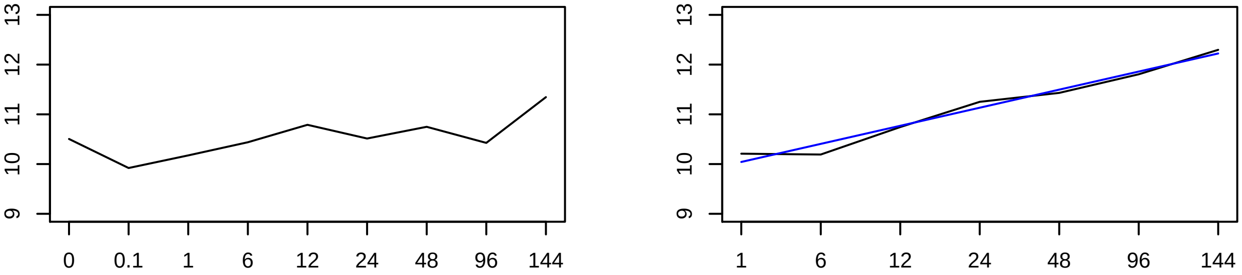

A_23_P82449 DFNA5 7p15.3

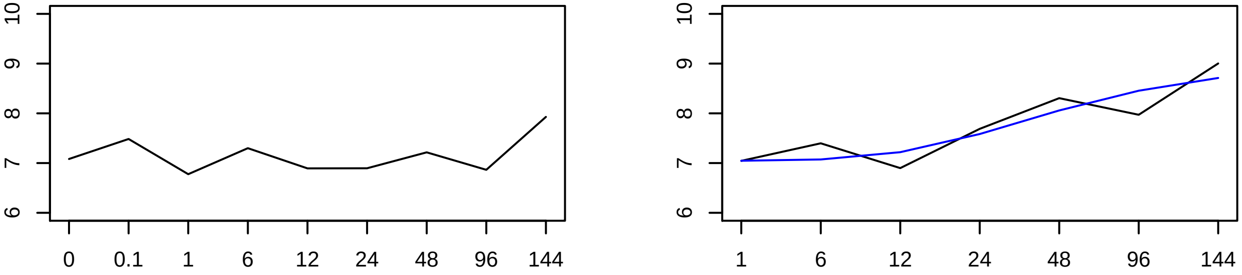

A_23_P52207 BAMBI 10p11.23

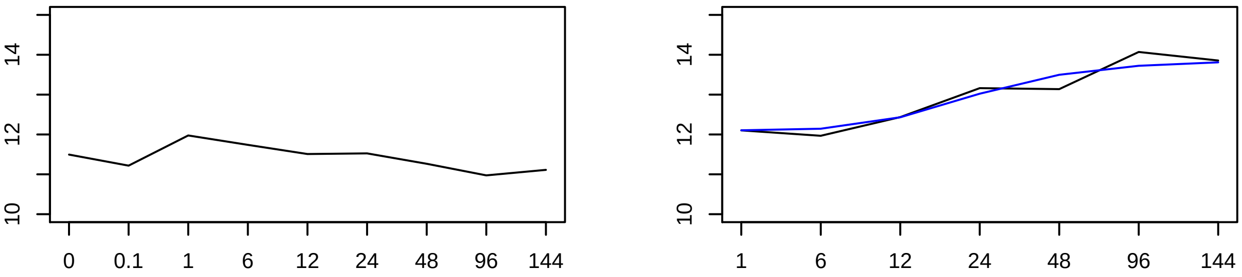

A_23_P151307 RAPGEF3 12q13.11

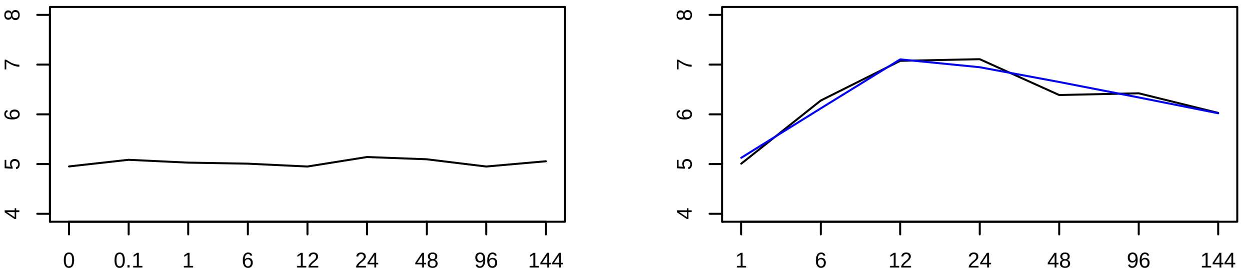

A_23_P84344 SIGIRR 11p15.5

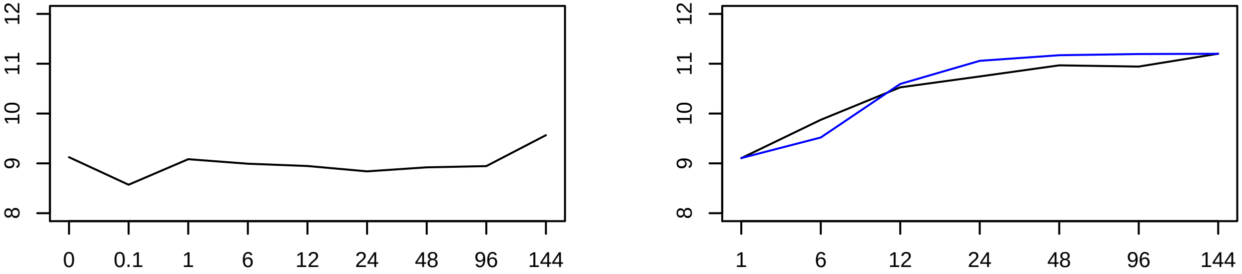

A_23_P60296 OSTF1 9q21.13

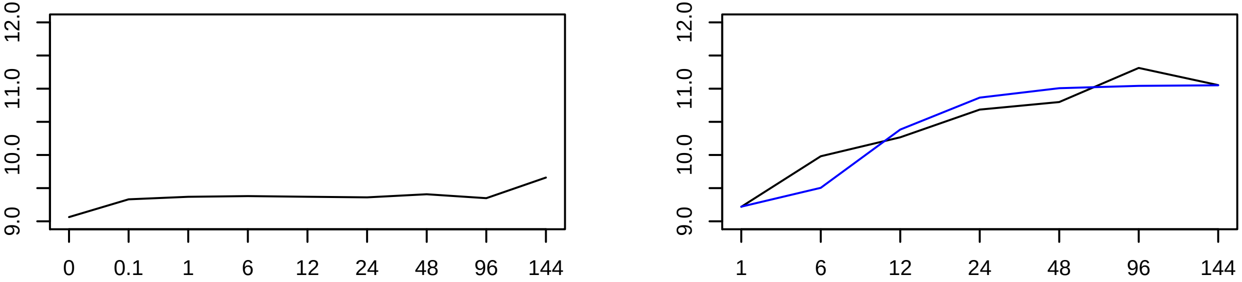

A_23_P82651 NPTX2 7q22.1

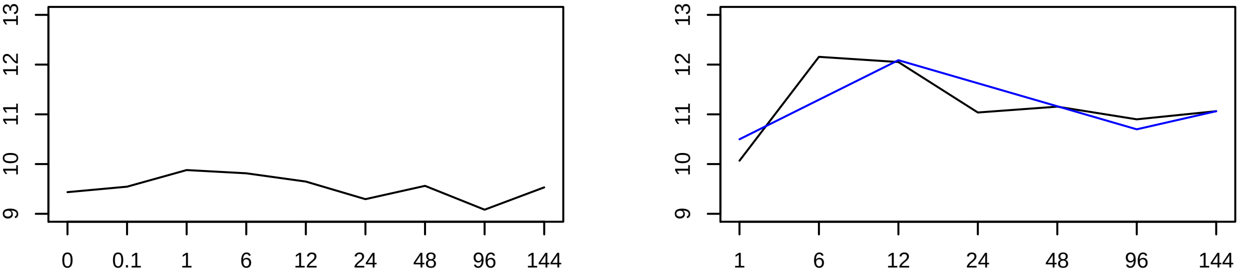

A_23_P47924 PTPRR 12q15

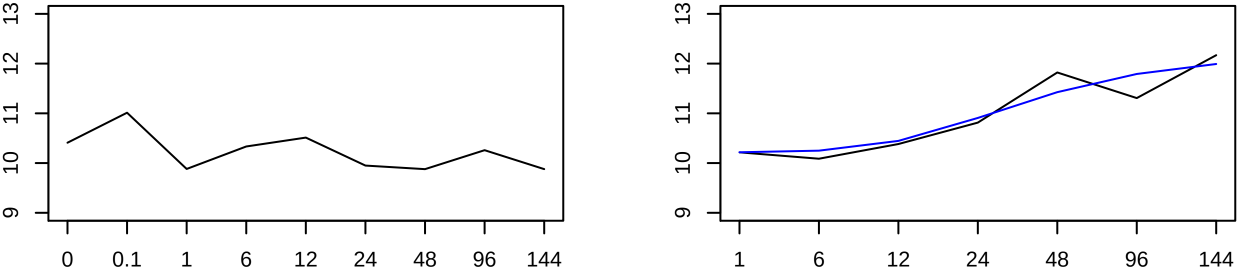

A_32_P95397 ITGB1 10p11.22

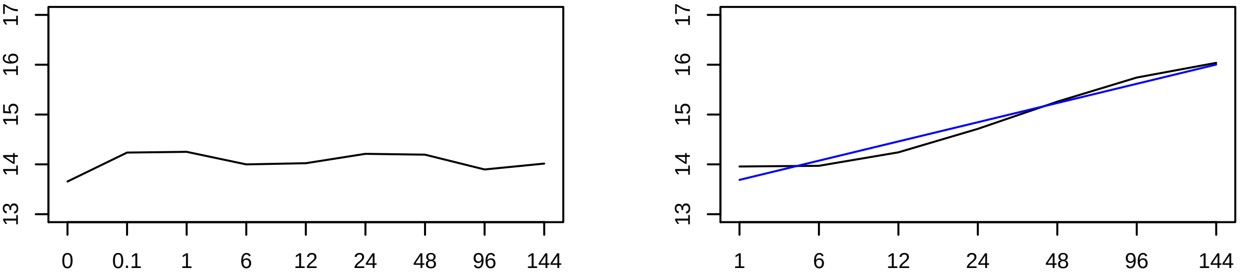

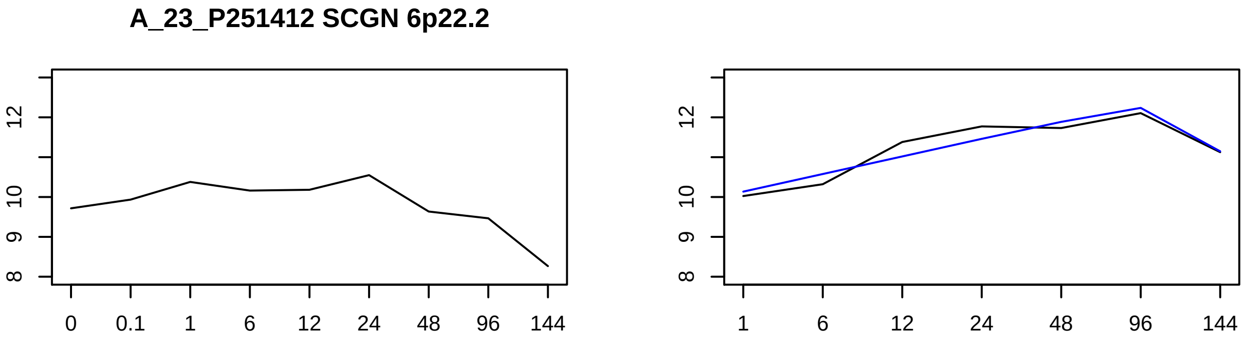
A_23_P251412 SCGN 6p22.2
8
9
10
12
0
0.1
1
6
12
24
48
96
144
8
9
10
12
1
6
12
24
48
96
144

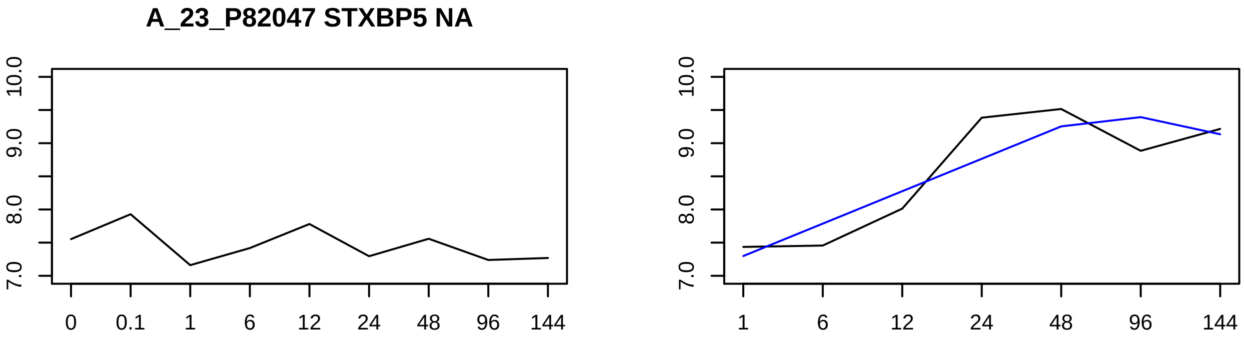
A_23_P82047 STXBP5 NA
7.0
8.0
9.0
10.0
0
0.1
1
6
12
24
48
96
144
7.0
8.0
9.0
10.0
1
6
12
24
48
96
144

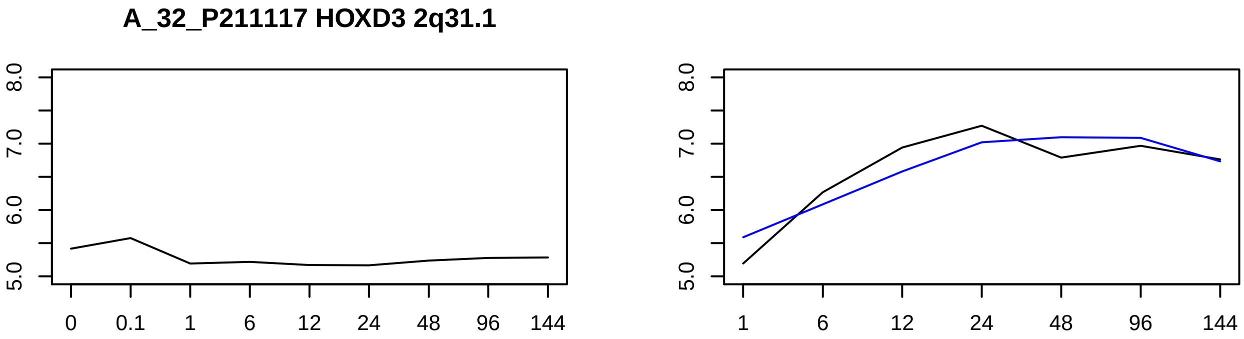
A_32_P211117 HOXD3 2q31.1
5.0
6.0
7.0
8.0
0
0.1
1
6
12
24
48
96
144
5.0
6.0
7.0
8.0
1
6
12
24
48
96
144

A_23_P22487 PCSK1N Xp11.23

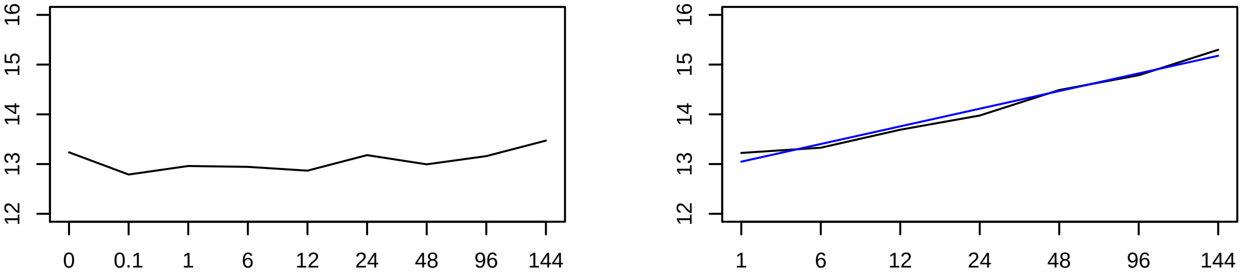

A_24_P915692 PHLDA1 12q21.2

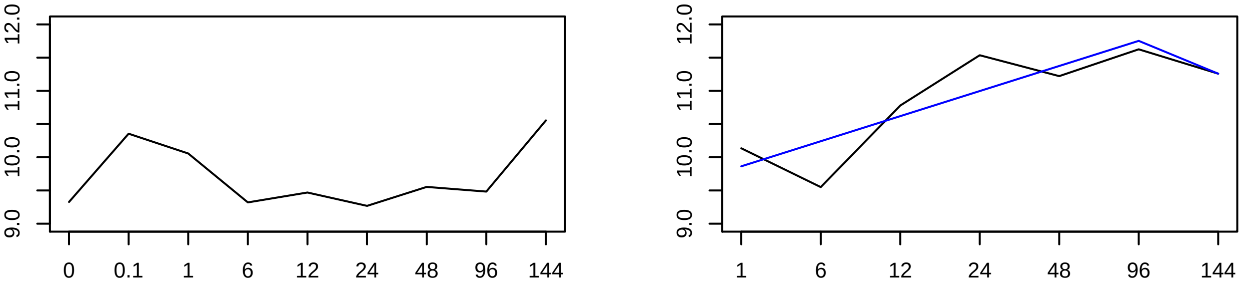

A_23_P75523 CD59 11p13

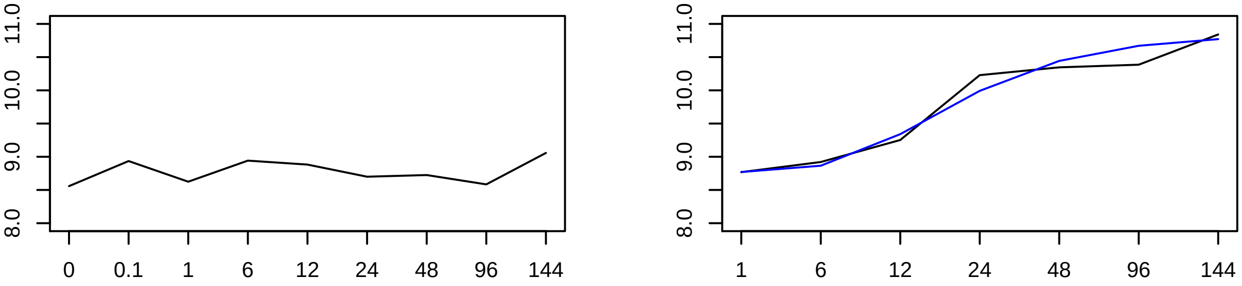

A_23_P11081 AKAP4 Xp11.22

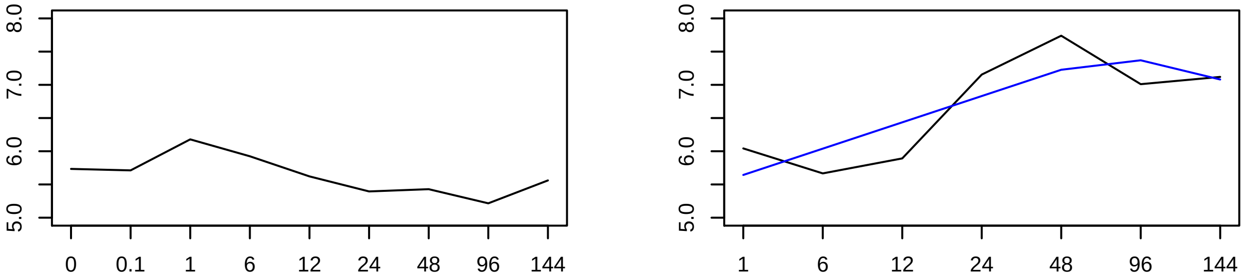

A_24_P923251 TGM2 20q11.23

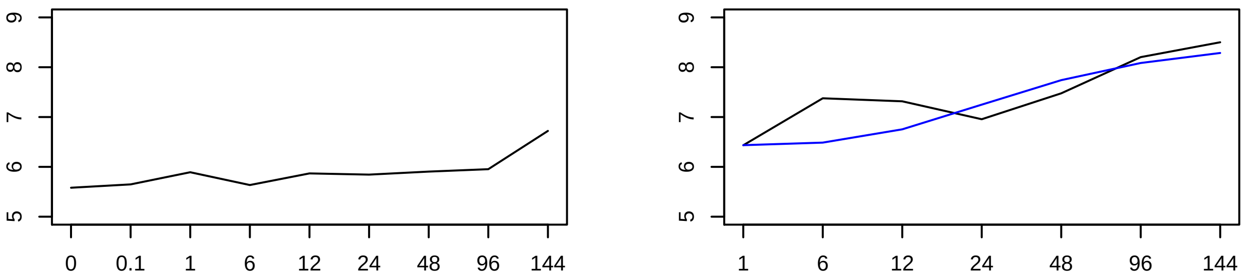

A_24_P282343 CDKL5 Xp22.13

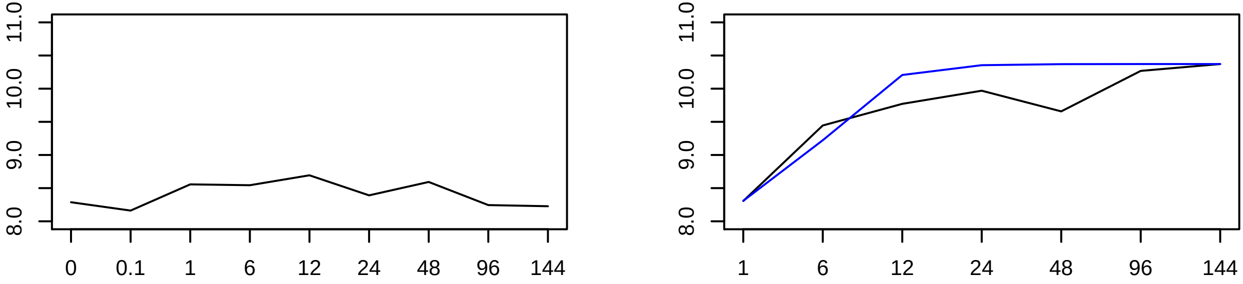

A_24_P40626 GREM2 1q43

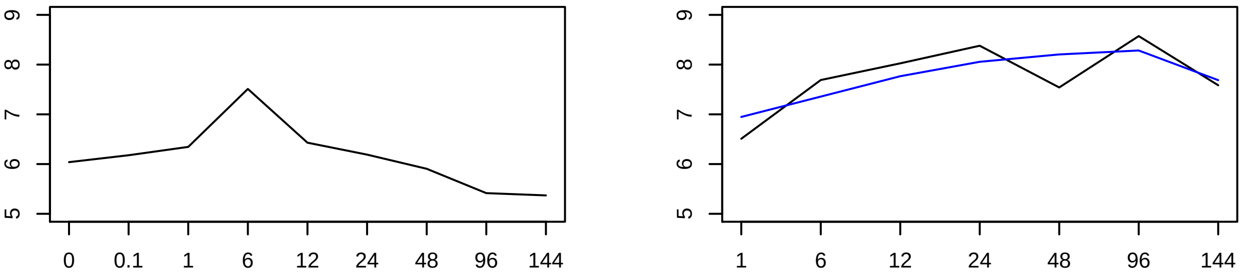

A_32_P50943 SGIP1 1p31.3

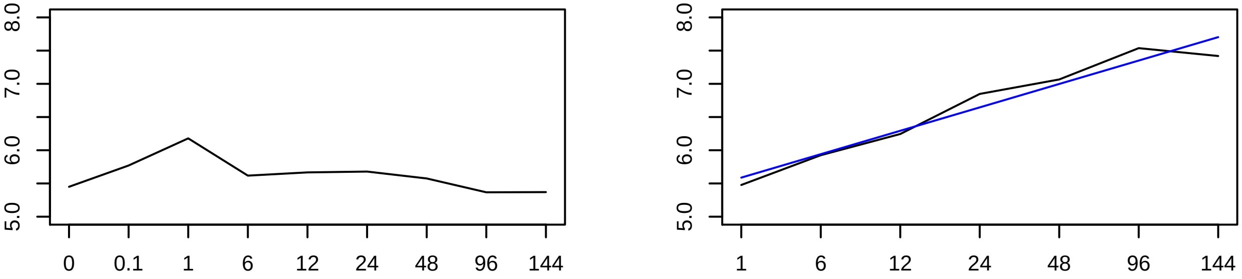

A_23_P92490 BDH2 4q24

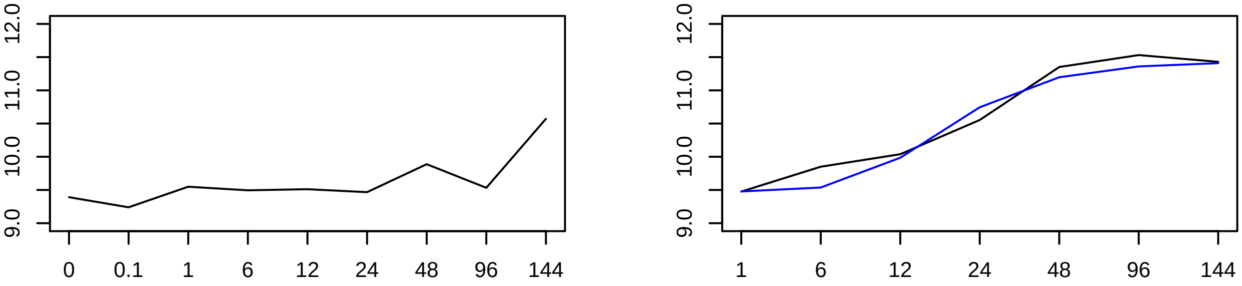

A_23_P203120 IGSF4 11q23.2

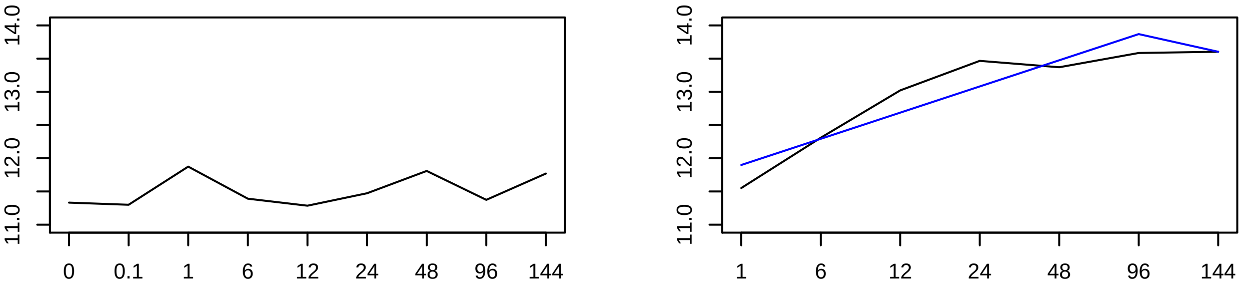

A_23_P143964 SH3BP5 3p24.3

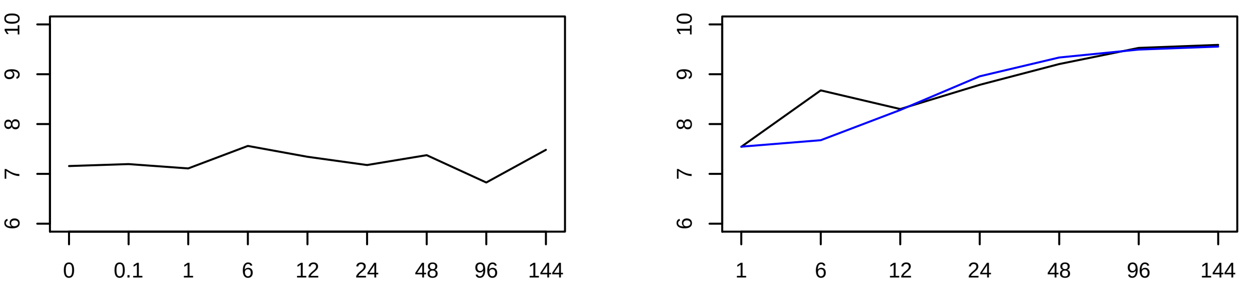

A_23_P354074 LYST 1q42.3

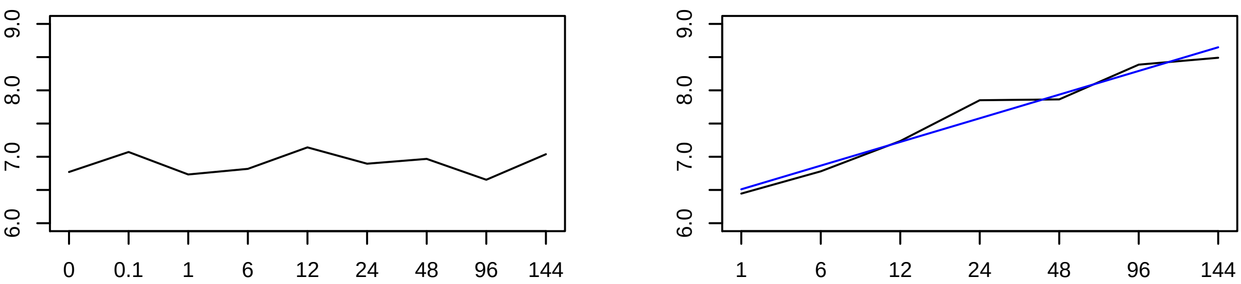

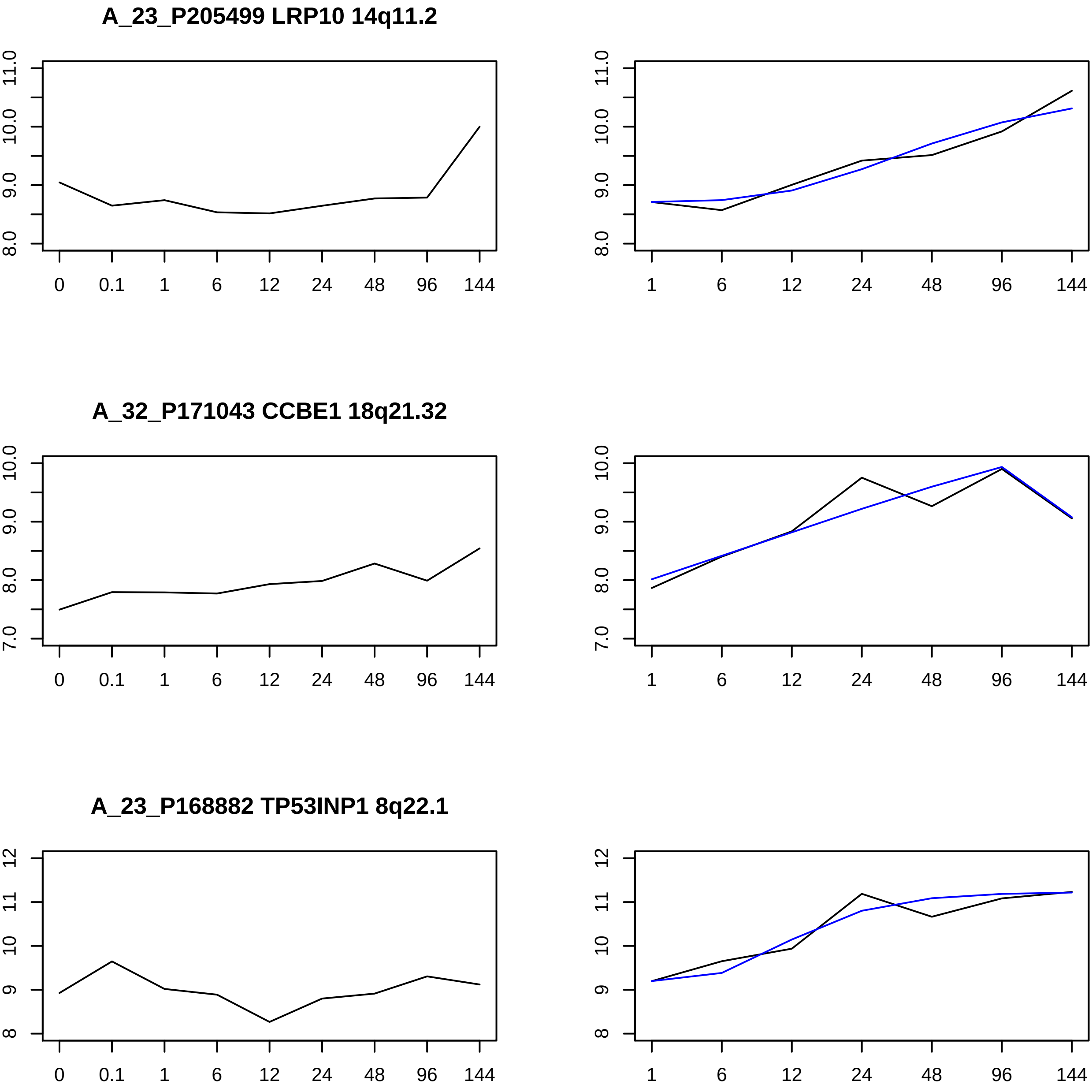

A_23_P205499 LRP10 14q11.2
8.0
9.0
10.0
11.0
0
0.1
1
6
12
24
48
96
144
1
6
12
24
48
96
144
A_32_P171043 CCBE1 18q21.32
7.0
8.0
9.0
10.0
A_23_P168882 TP53INP1 8q22.1
8
9
10
11
12

A_23_P253029 BOK 2q37.3

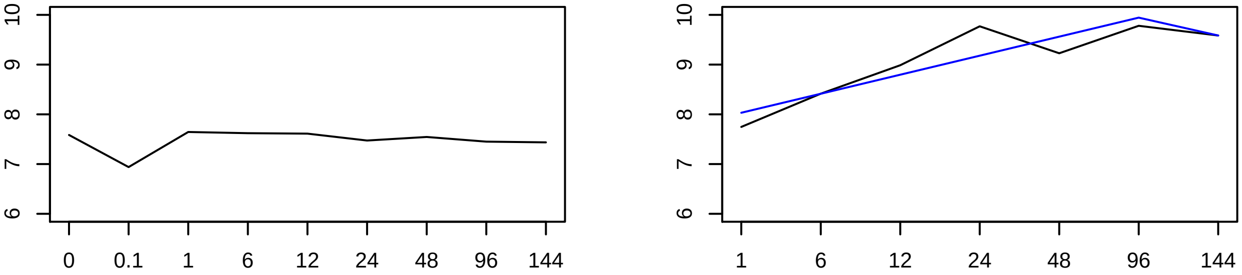

A_23_P313560 NCOA3 20q13.12

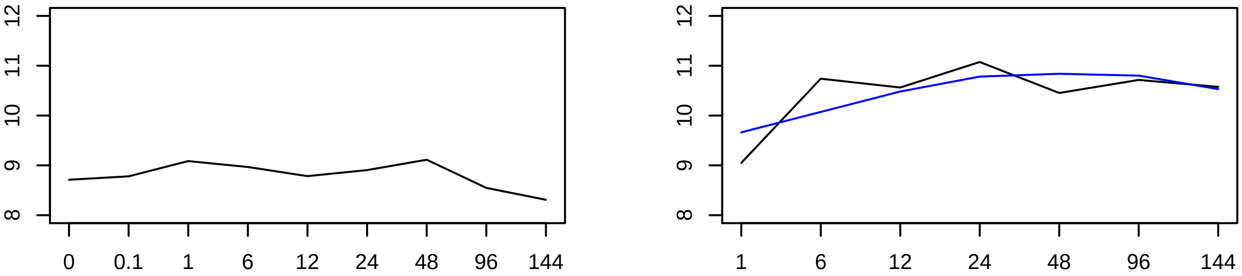

A_24_P357169 EPPK1 8q24.3

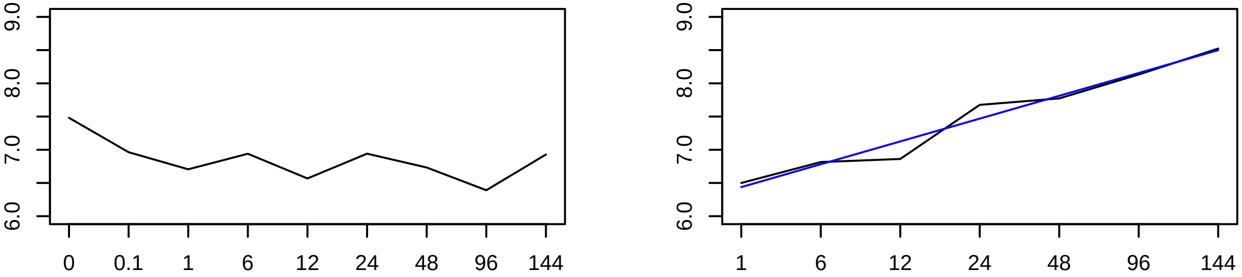

A_23_P251937 CPEB4 5q35.2

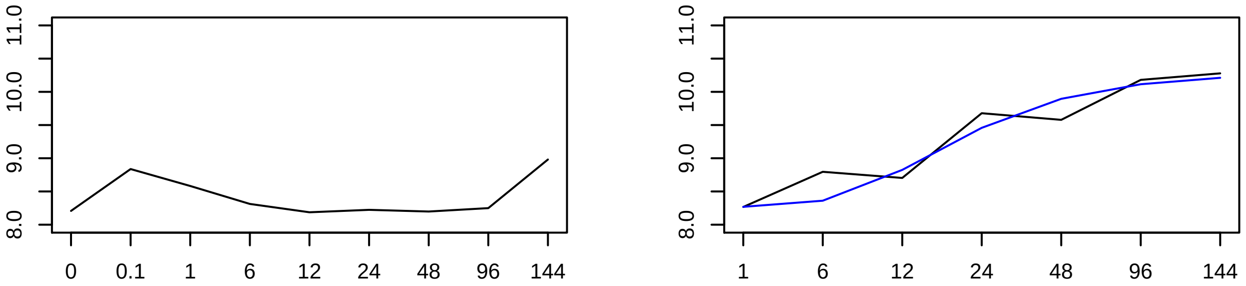

A_23_P56197 CRLF1 19p13.11

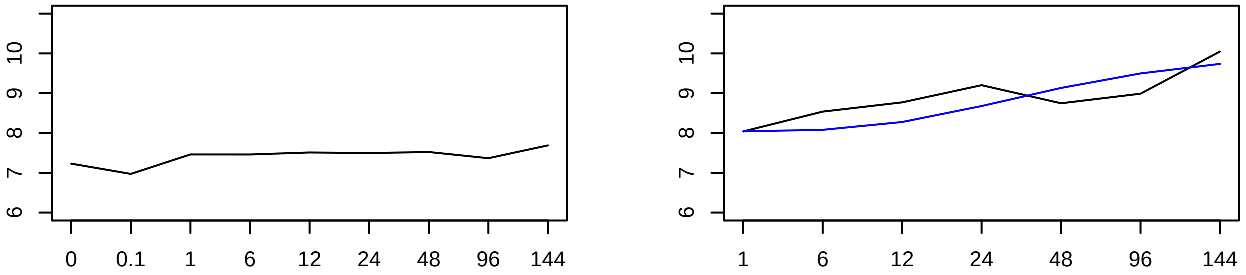

A_32_P75094 AIFM2 10q22.1

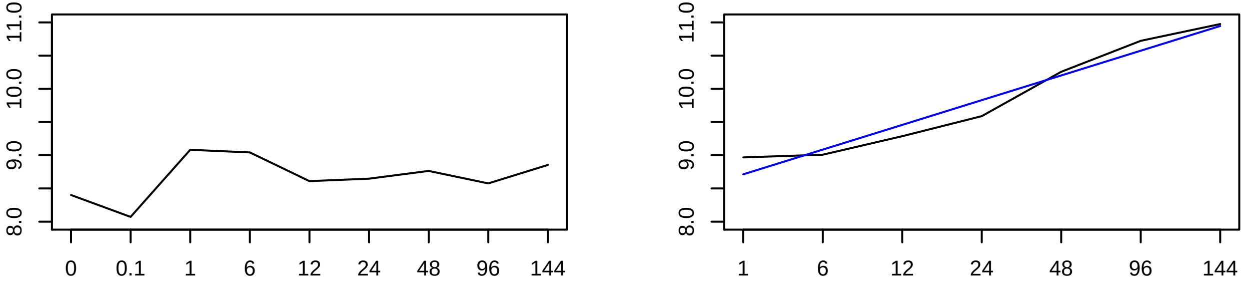

A_23_P417942 FNBP1L 1p22.1

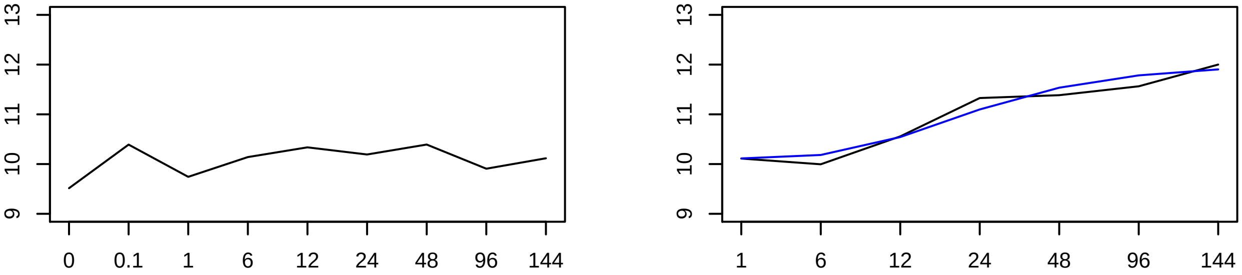

A_32_P123143 HCN1 5p12

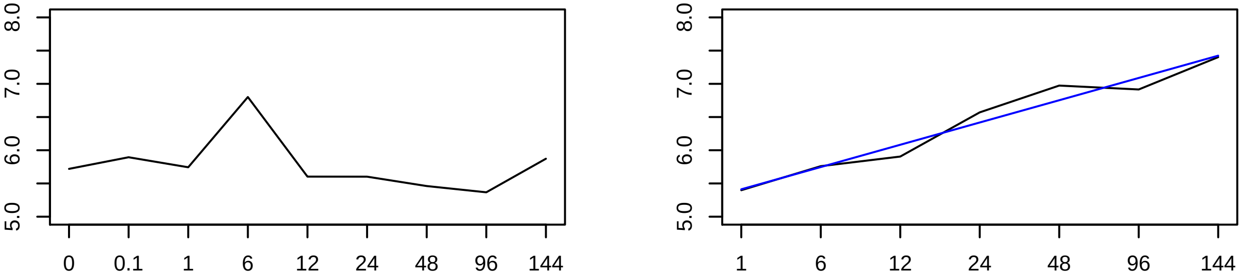

A_23_P360215 POLD4 11q13.1

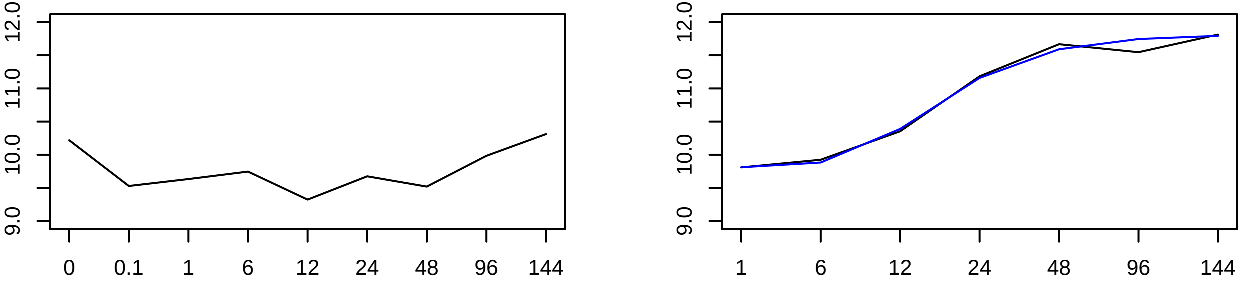

A_23_P133236 PCDHB14 5q31.3

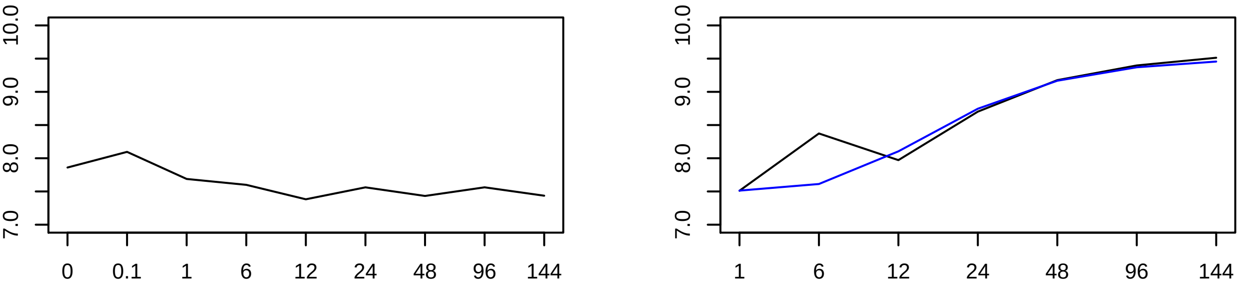

A_23_P360964 DACT3 19q13.32

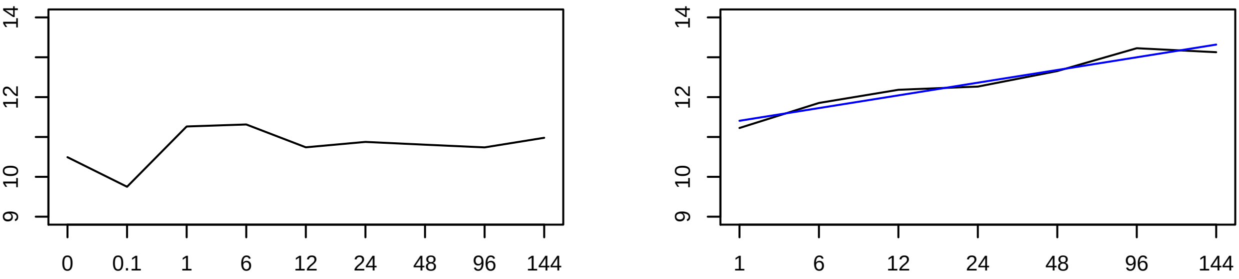

A_24_P944788 HGF 7q21.11

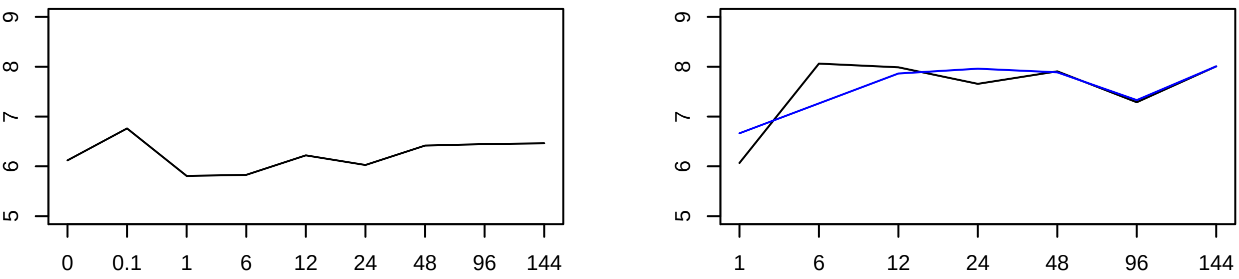

A_24_P943597 PHLDA1 12q21.2

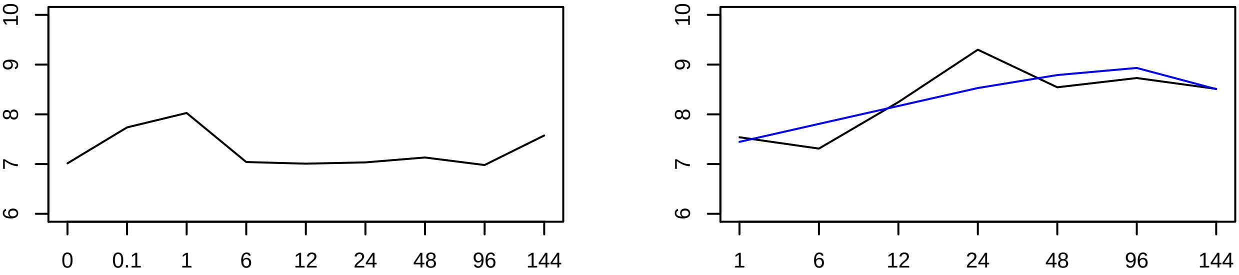

A_23_P259201 THNSL2 2p11.2

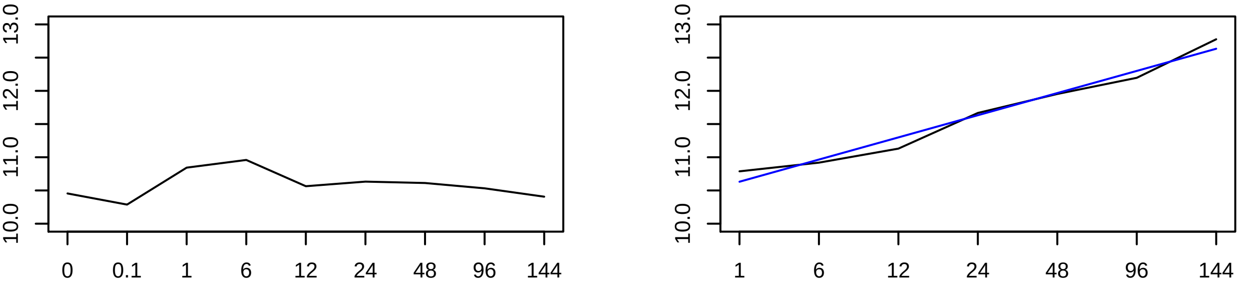

A_24_P56388 HIF1A 14q23.2

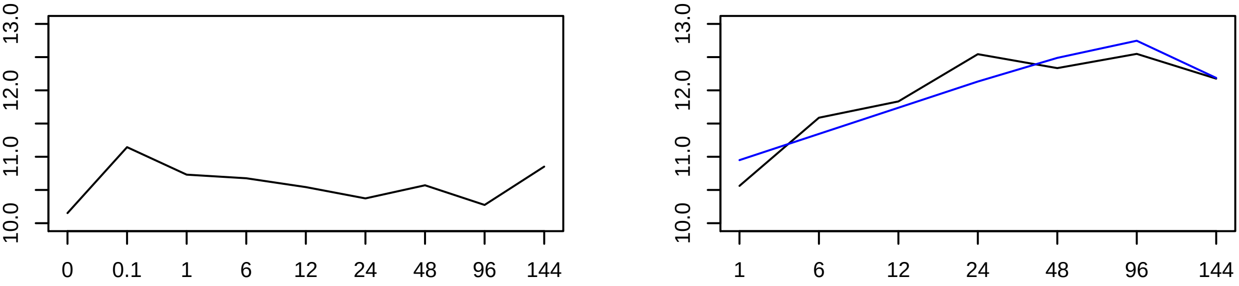

A_23_P314584 MAPKAPK3 3p21.31

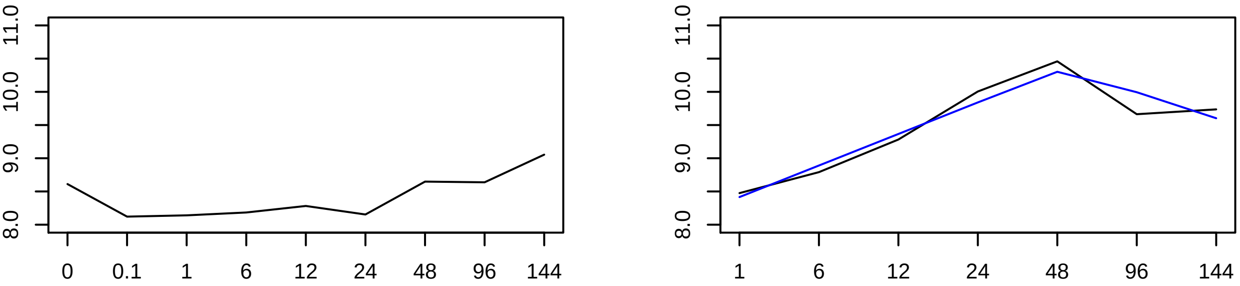

A_24_P194688 EFHA2 8p22

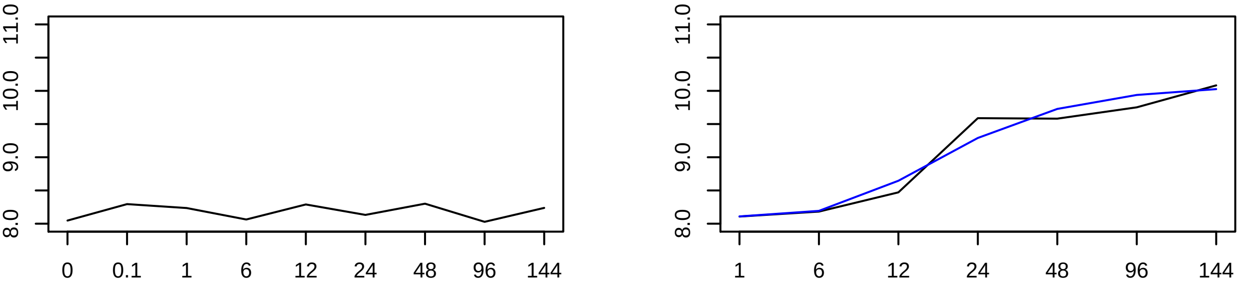

A_32_P63086 SKIL NA

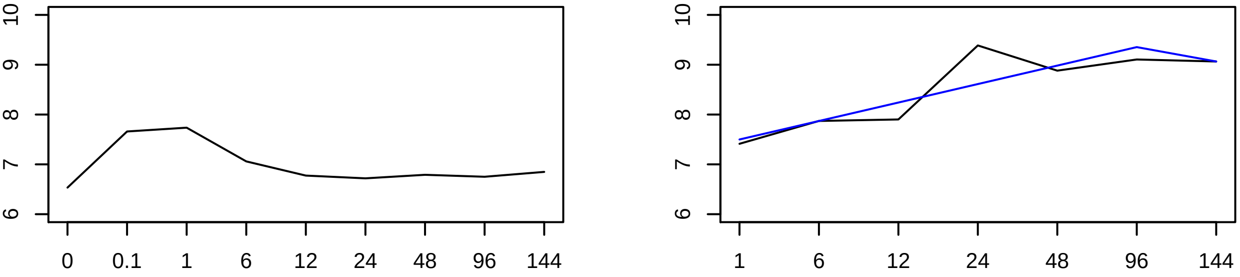

A_23_P53390 PTPRB 12q15

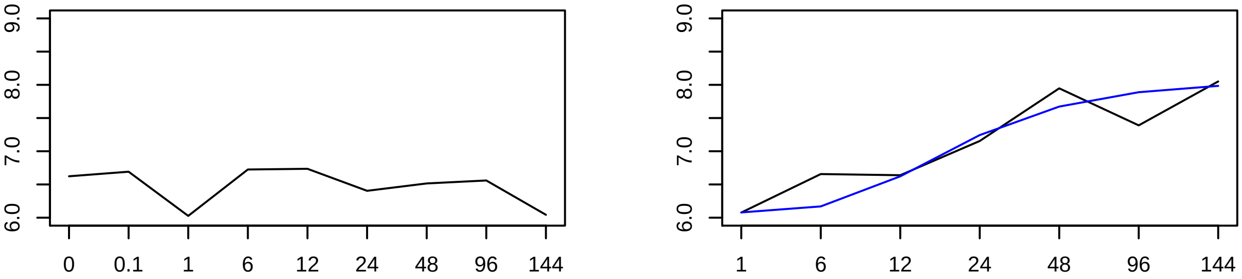

A_24_P227230 IGSF4 11q23.2

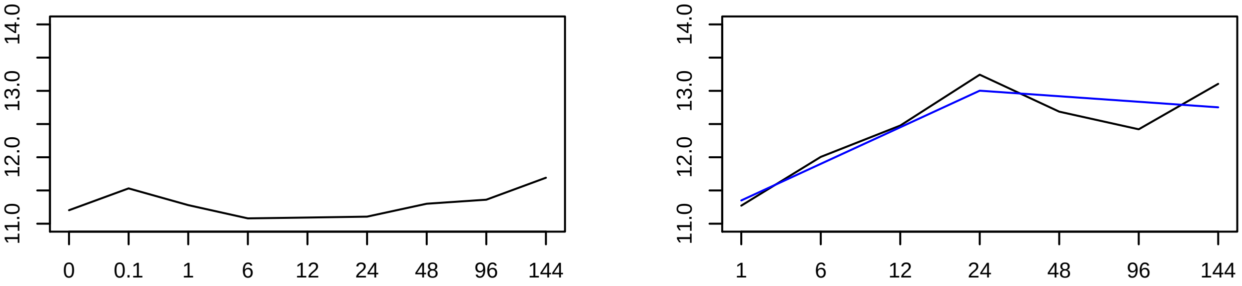

A_32_P198325 C22orf36 22q11.23

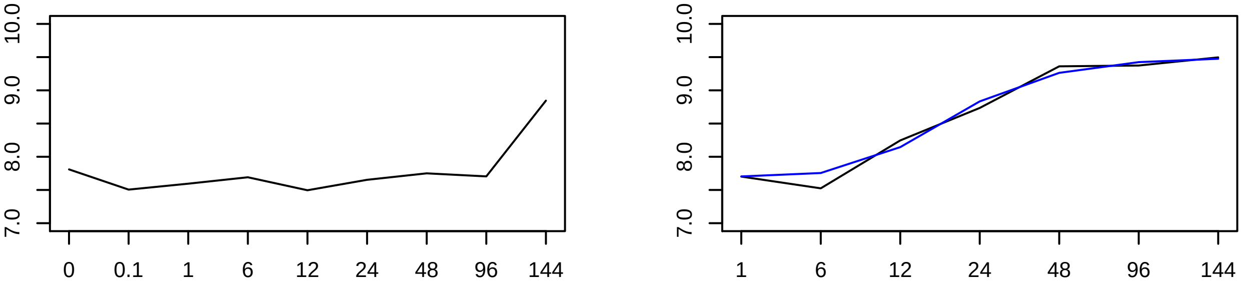

A_23_P201790 PPP1R12B 1q32.1

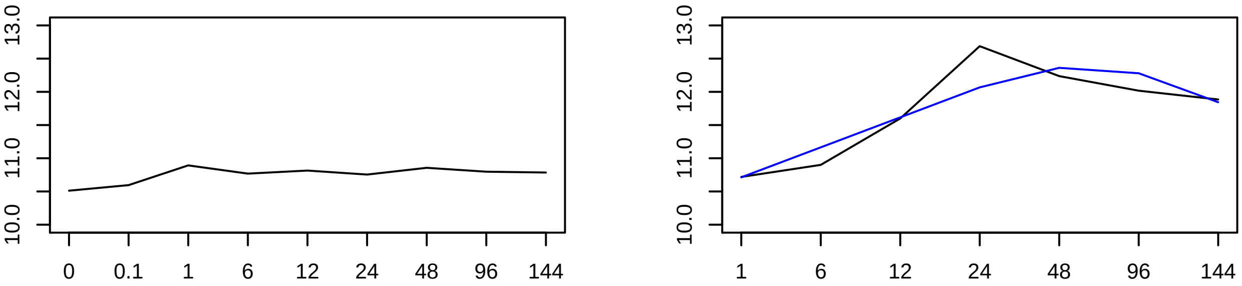

A_23_P1038 JARID1B 1q32.1

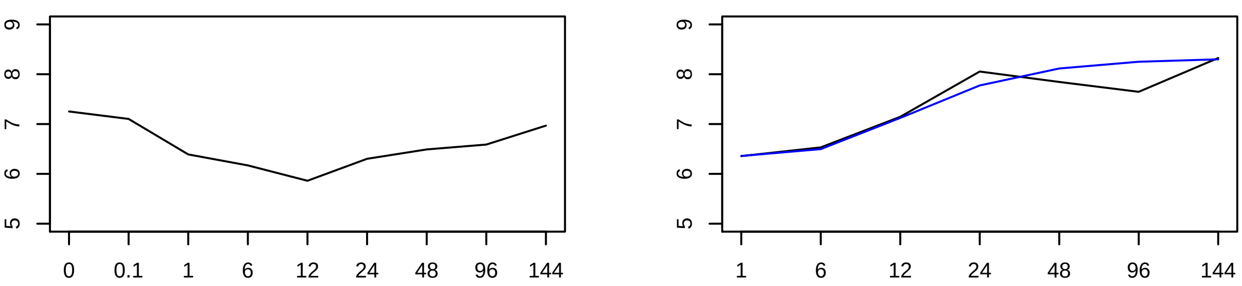

A_23_P90696 TRIB2 2p25.1

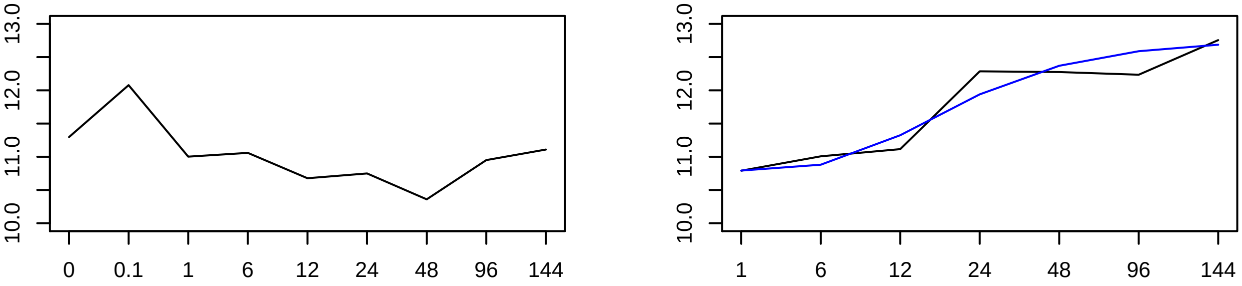

A_23_P317324 EVI1 3q26.2

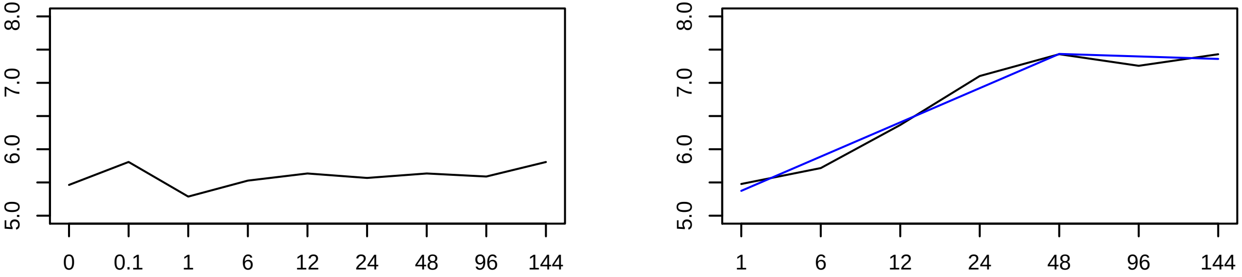

A_32_P405759 COL22A1 8q24.23

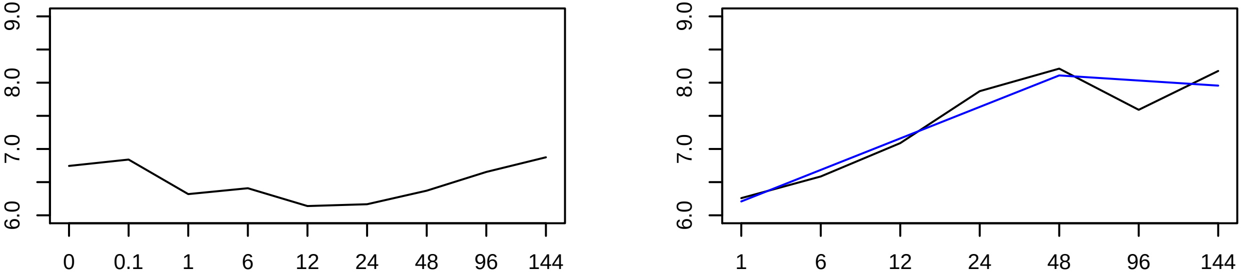

A_32_P164203 RNF217 6q22.31

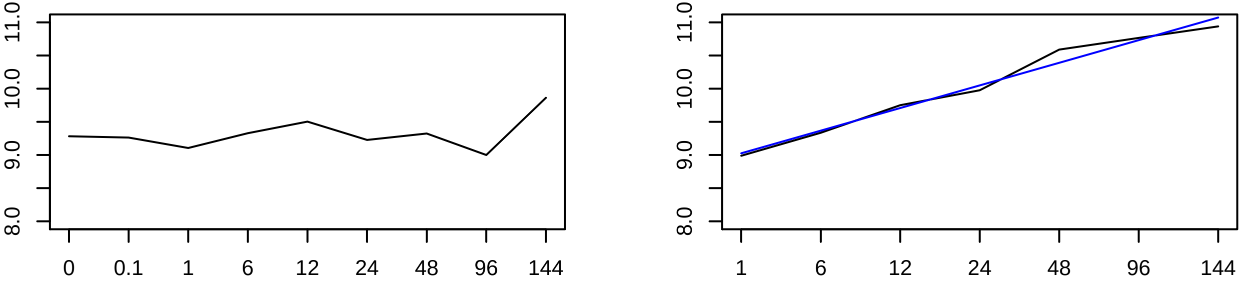

A_23_P50786 CLIPR−59 19q13.12

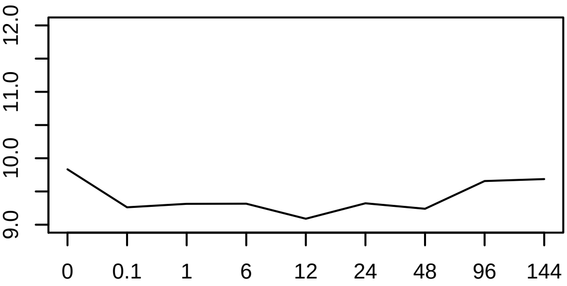

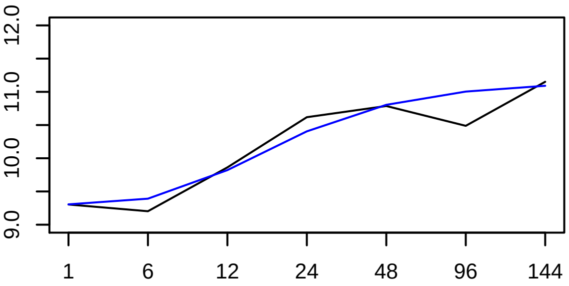

A_24_P544269 AI732190 NA

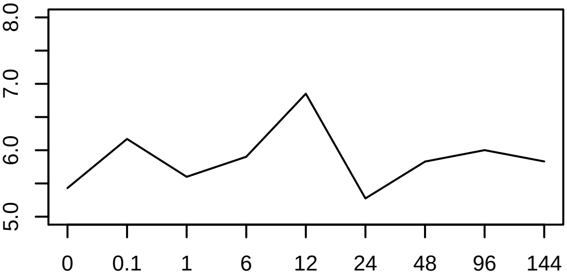

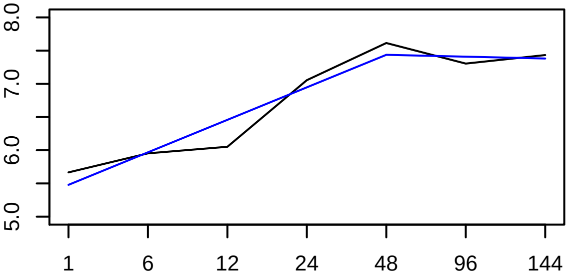

A_23_P80473 CHST13 3q21.3

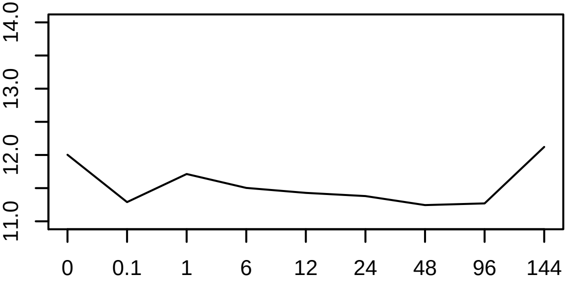

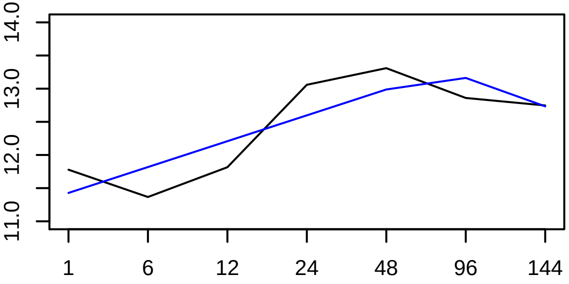

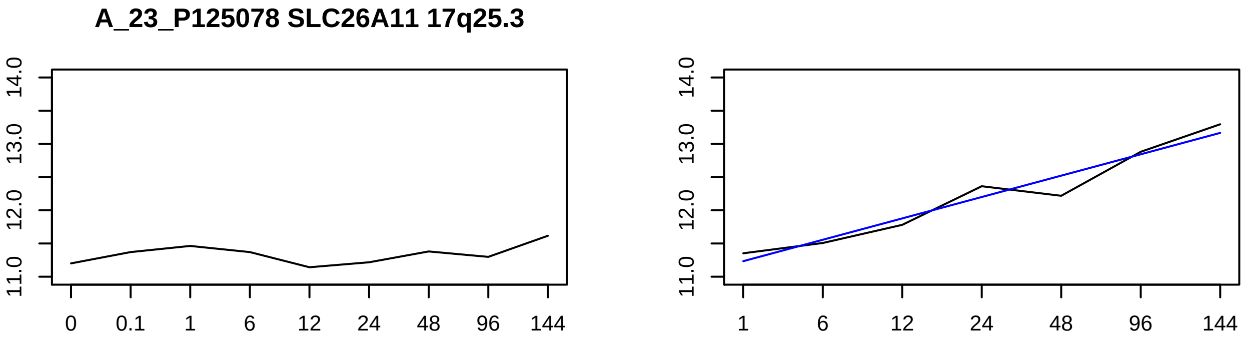
A_23_P125078 SLC26A11 17q25.3
11.0
12.0
13.0
14.0
0
0.1
1
6
12
24
48
96
144

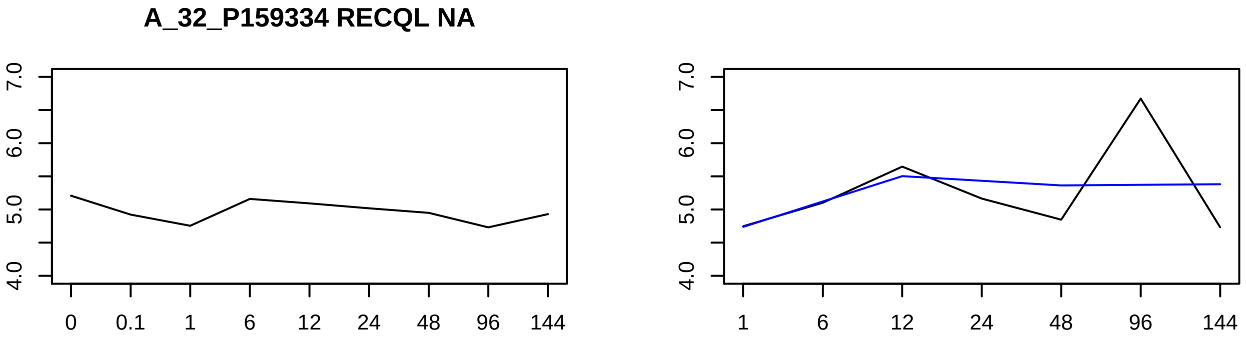
A_32_P159334 RECQL NA
4.0
5.0
6.0
7.0
0
0.1
1
6
12
24
48
96
144

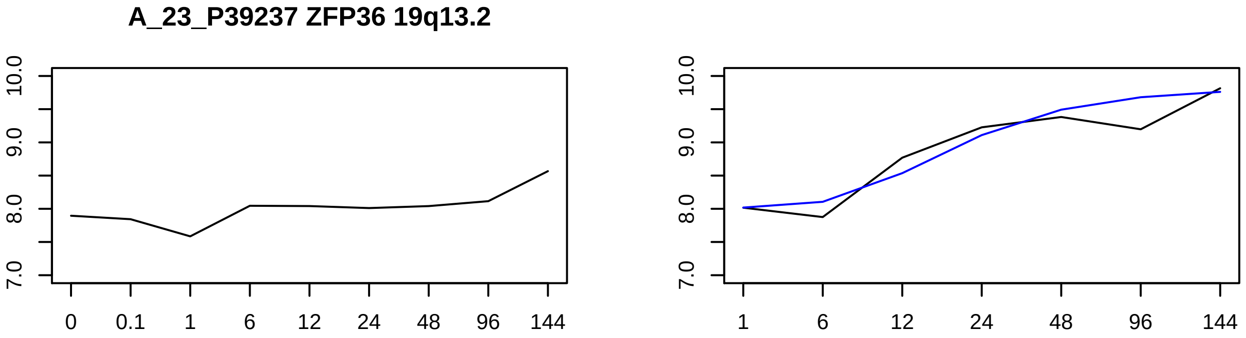
A_23_P39237 ZFP36 19q13.2
7.0
8.0
9.0
10.0
0
0.1
1
6
12
24
48
96
144

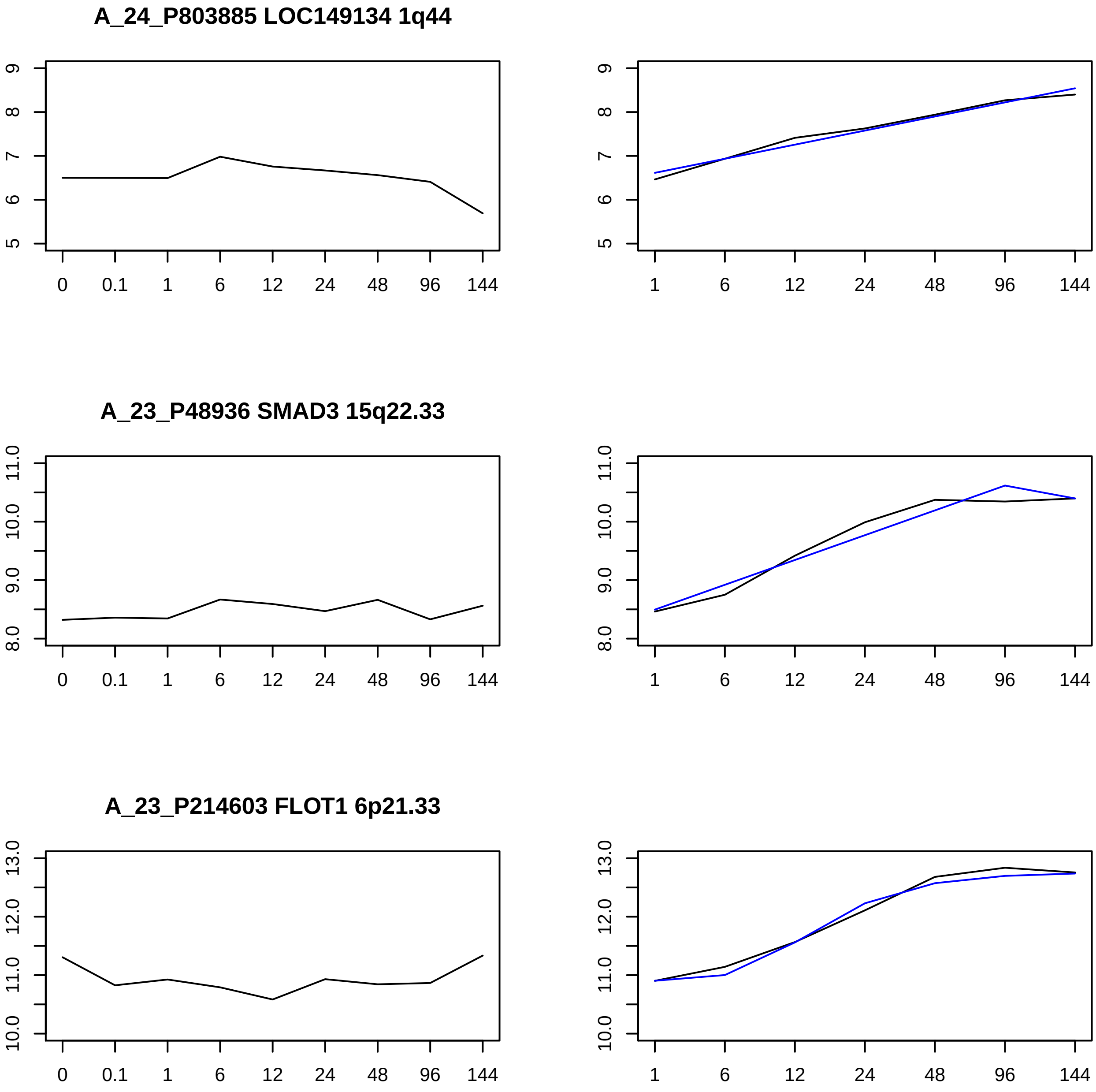
A_24_P803885 LOC149134 1q44
A_23_P48936 SMAD3 15q22.33
A_23_P214603 FLOT1 6p21.33
0 0.1 1 6 12 24 48 96 144
1 6 12 24 48 96 144
5 6 7 8 9
8.0 9.0 10.0 11.0
10.0 11.0 12.0 13.0

A_24_P794447 LOC399959 11q24.1

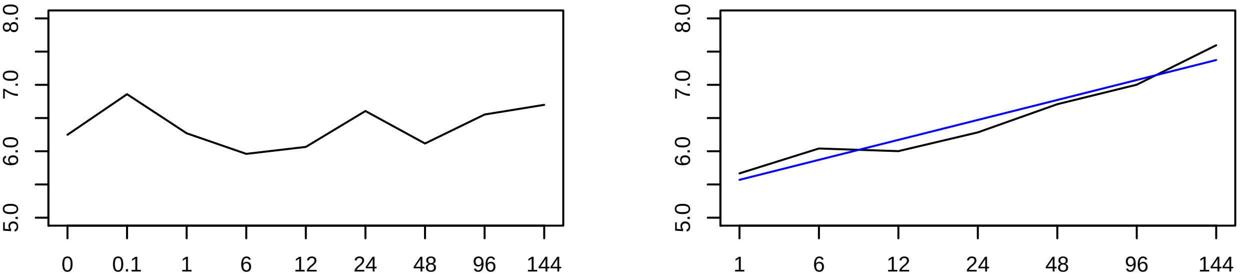

A_23_P125423 C1R 12p13.31

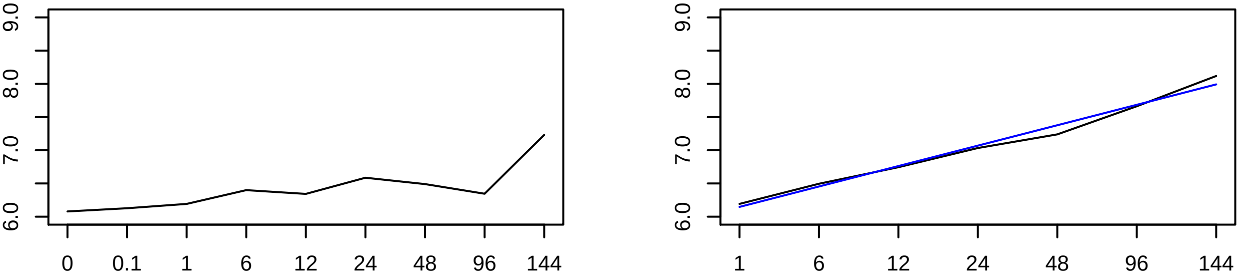

A_24_P920319 ZBED5 11p15.3

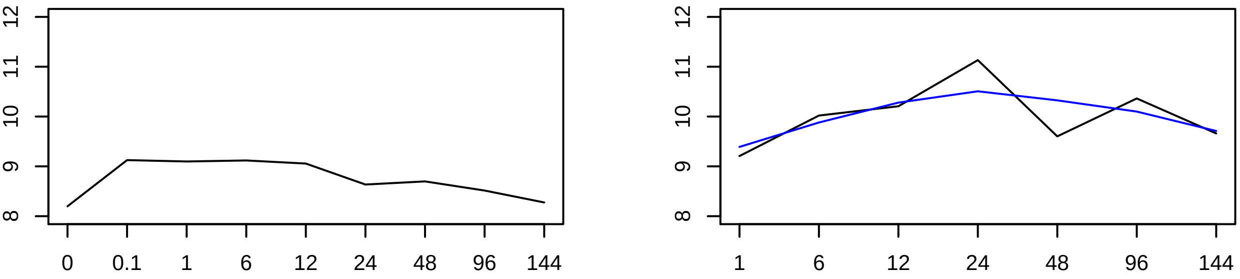

A_24_P398147 NEBL 10p12.31

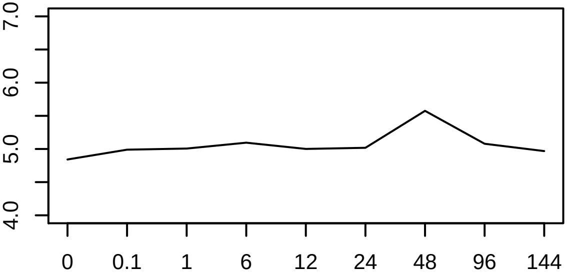

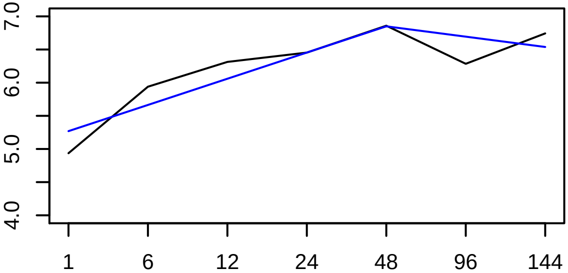

A_23_P13946 SH2B3 12q24.12

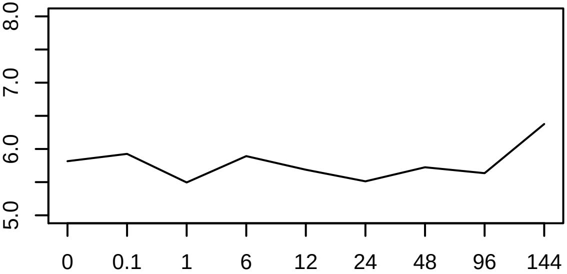

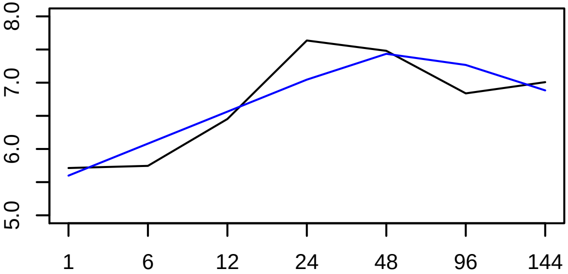

A_24_P287043 IFITM2 11p15.5

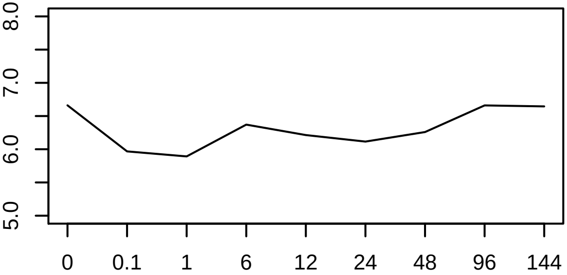

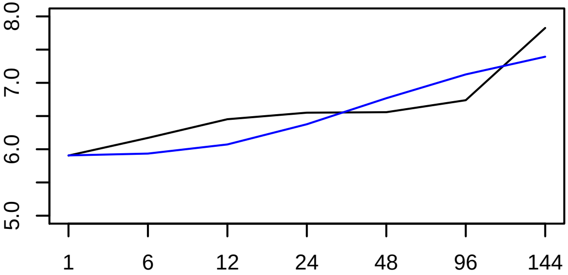

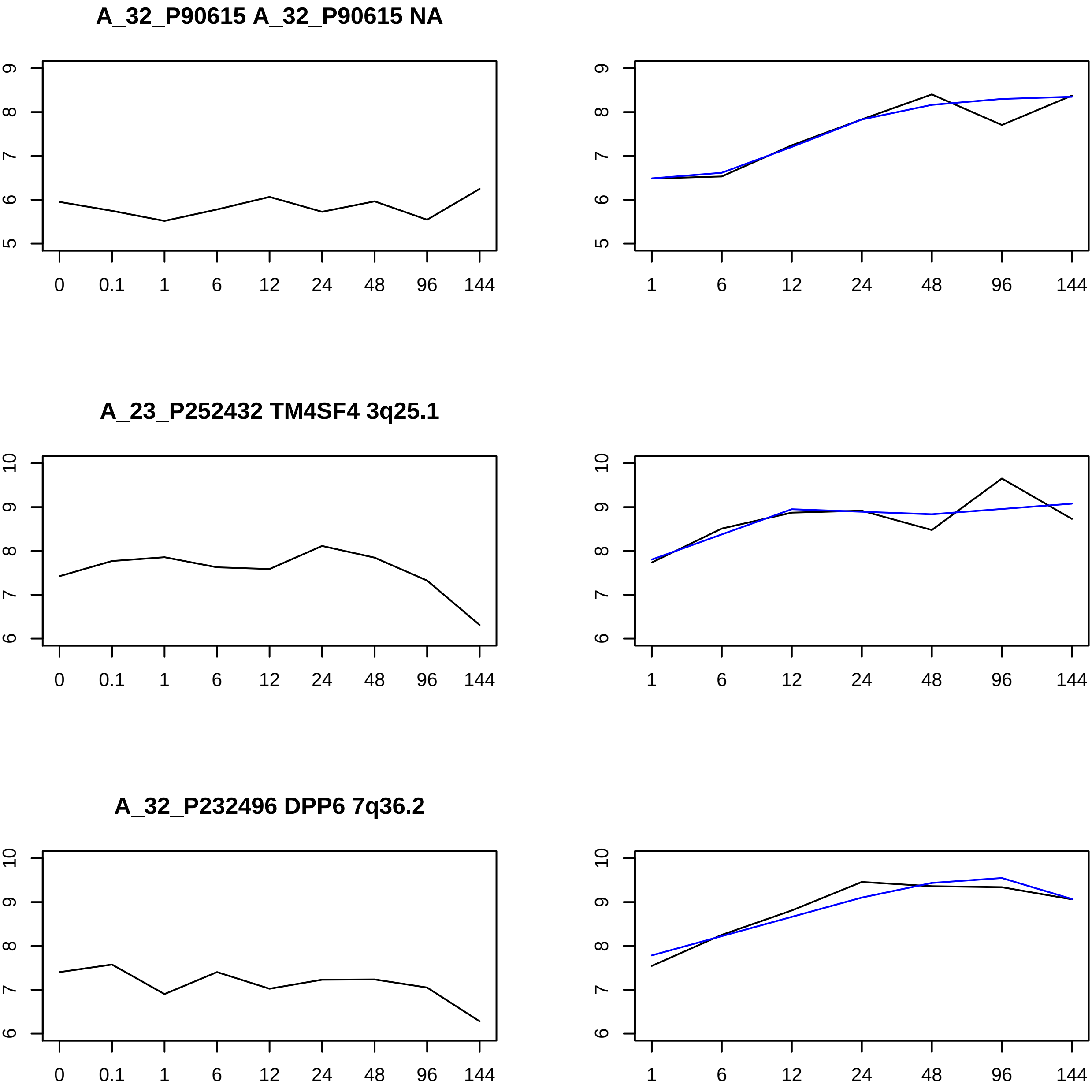

A_32_P90615 A_32_P90615 NA
A_23_P252432 TM4SF4 3q25.1
A_32_P232496 DPP6 7q36.2
0
0.1
1
6
12
24
48
96
144

A_23_P91076 TMEM87B NA

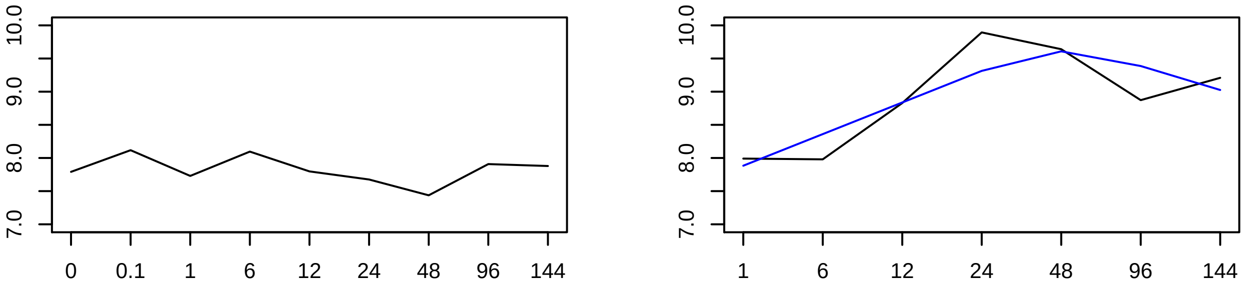

A_23_P83028 RECK 9p13.3

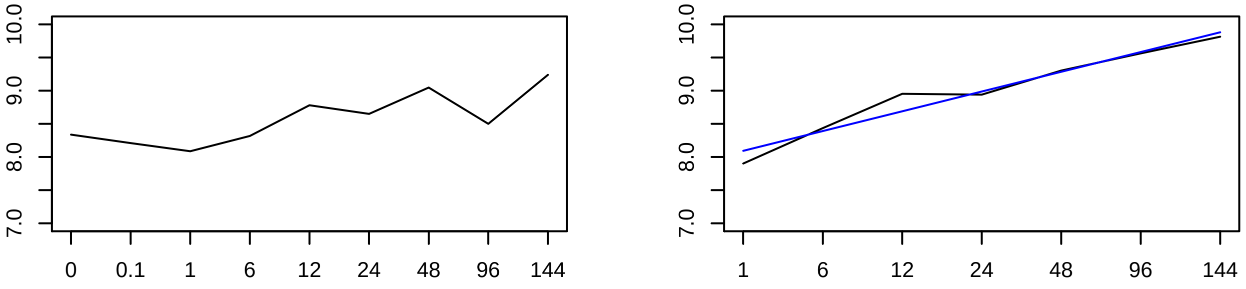

A_23_P24987 TSPAN31 12q14.1

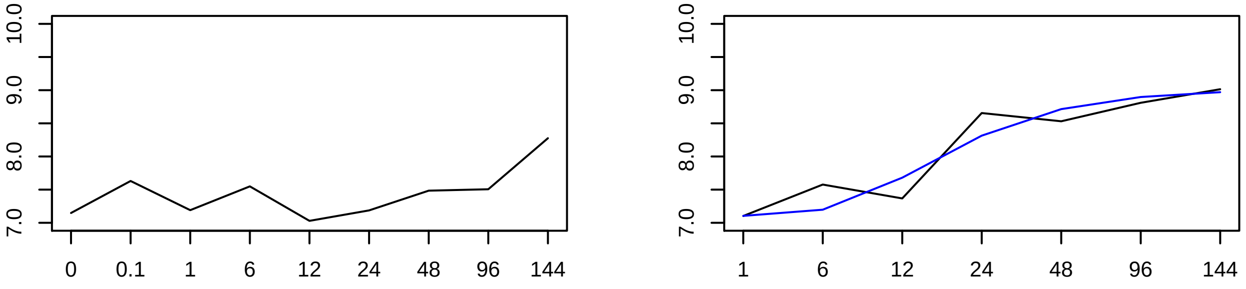

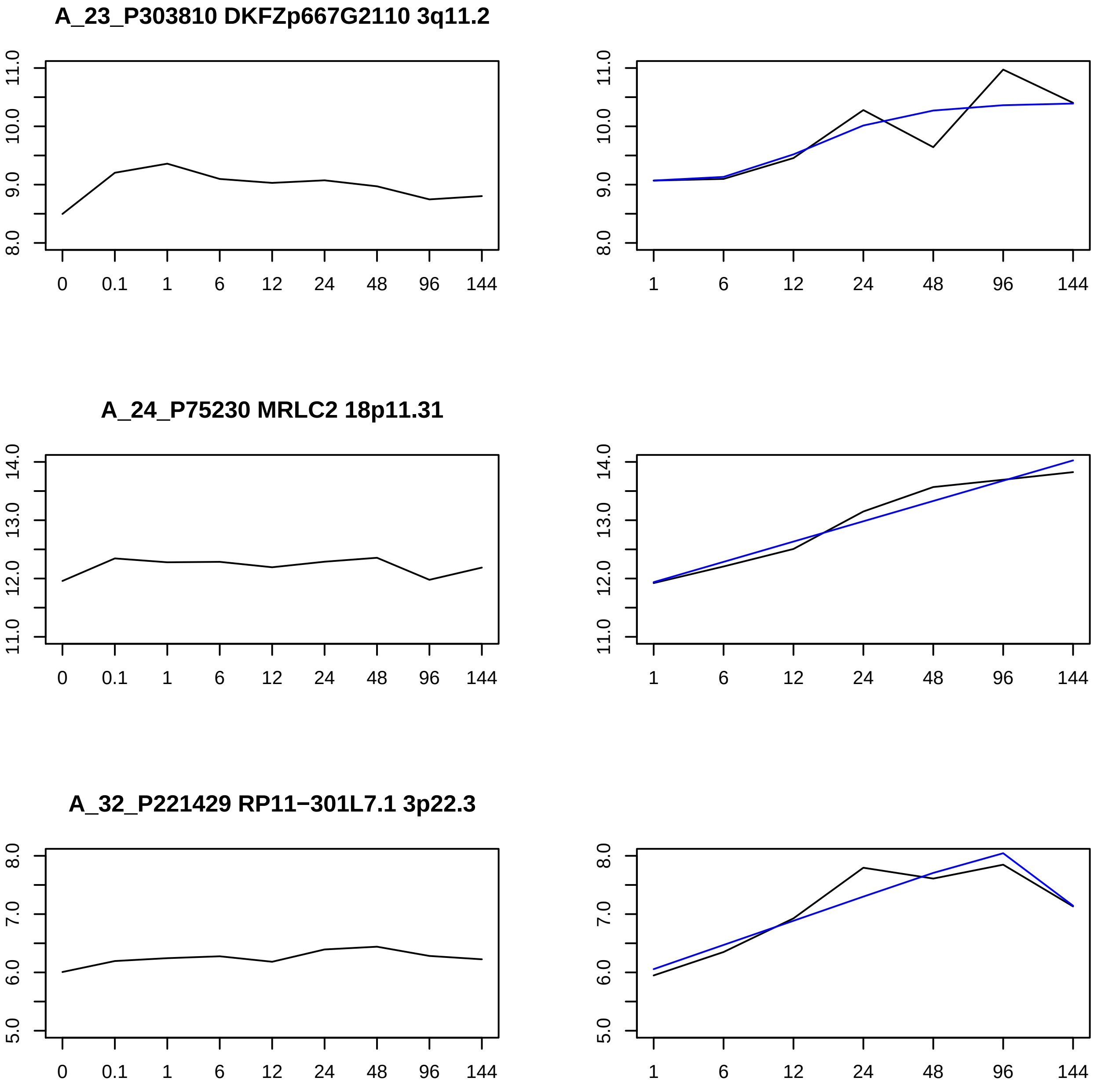
A_23_P303810 DKFZp667G2110 3q11.2
8.0
9.0
10.0
11.0
0
0.1
1
6
12
24
48
96
144
A_24_P75230 MRLC2 18p11.31
11.0
12.0
13.0
14.0
A_32_P221429 RP11−301L7.1 3p22.3
5.0
6.0
7.0
8.0

A_24_P223124 FNDC3B 3q26.31

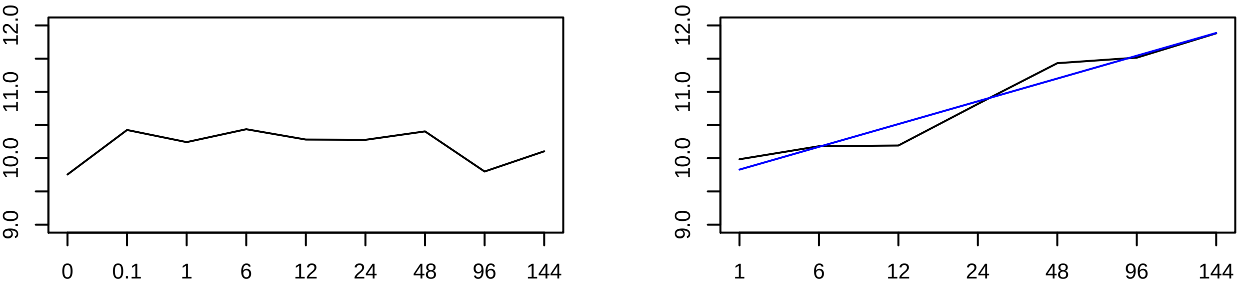

A_24_P360206 PCDHA9 5q31.3

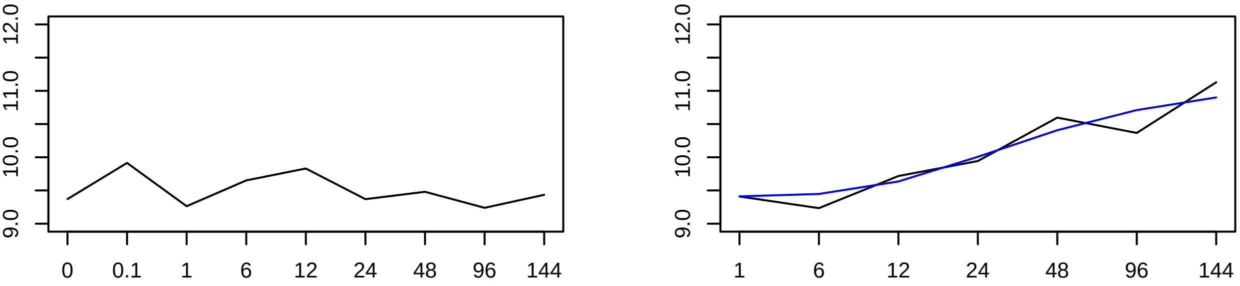

A_32_P219704 A_32_P219704 NA

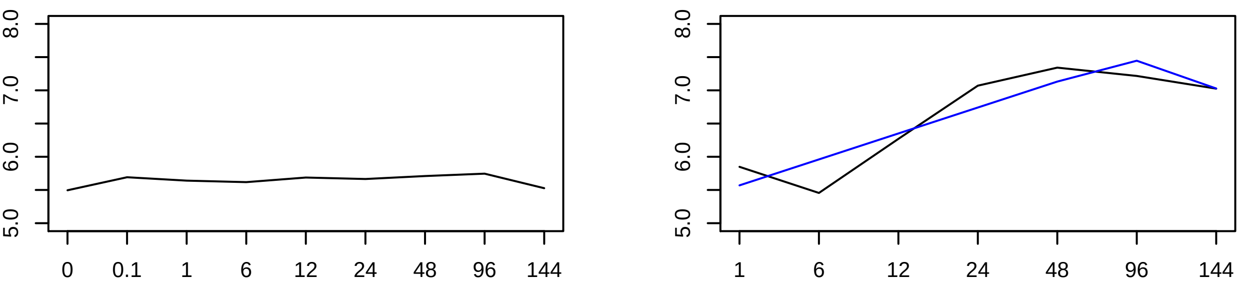

A_32_P156746 BE825944 NA

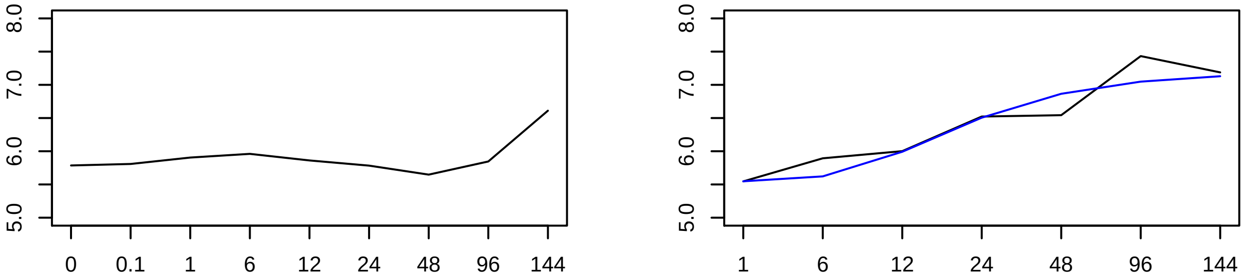

A_32_P224302 ZNF135 19q13.43

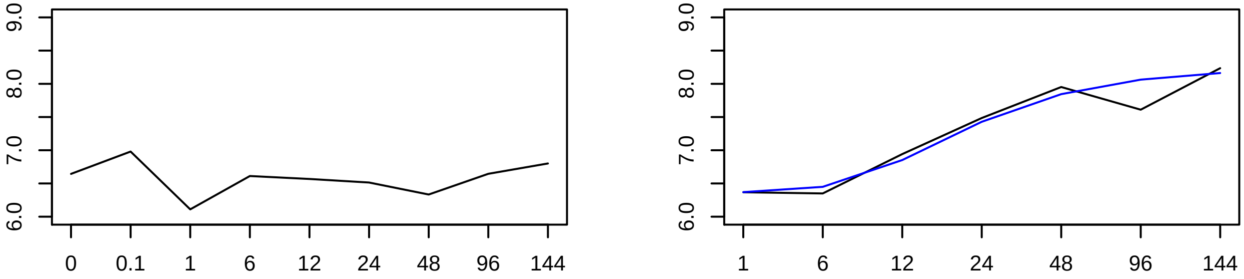

A_23_P37244 SNAPC1 14q23.2

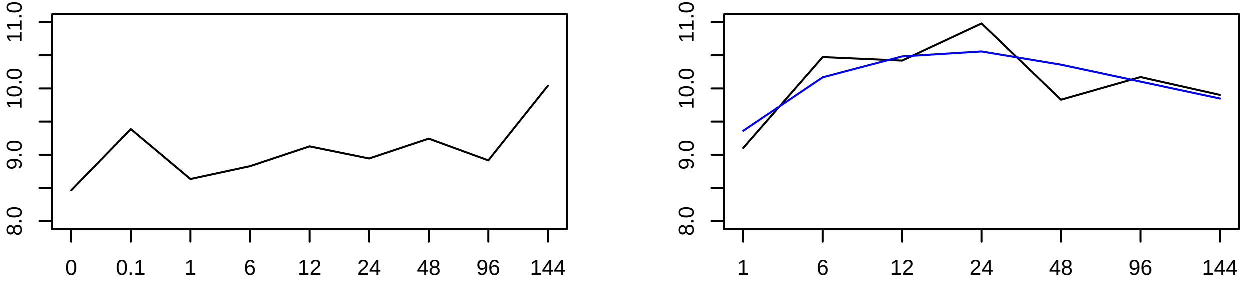

A_23_P58031 MAP3K13 3q27.2

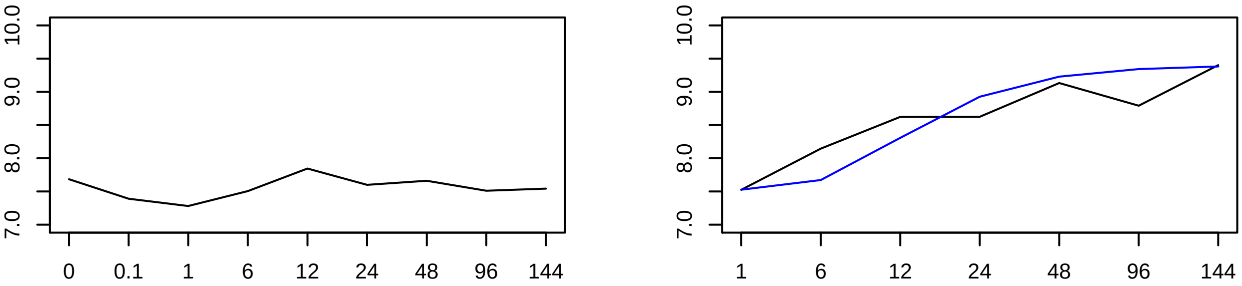

A_32_P15288 BX114329 NA

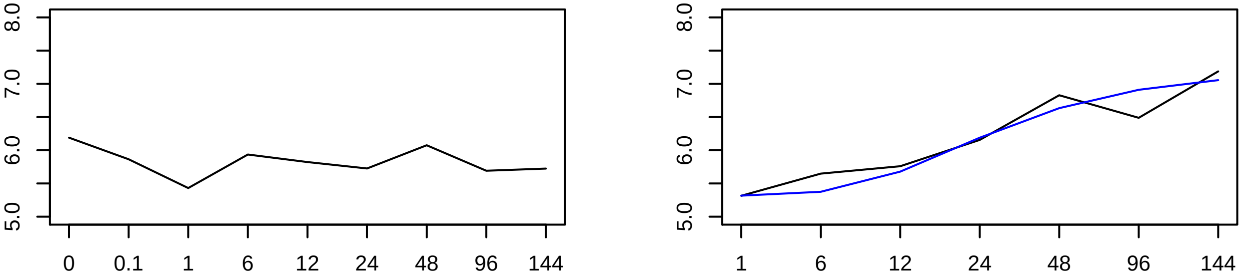

A_23_P208991 PALM 19p13.3

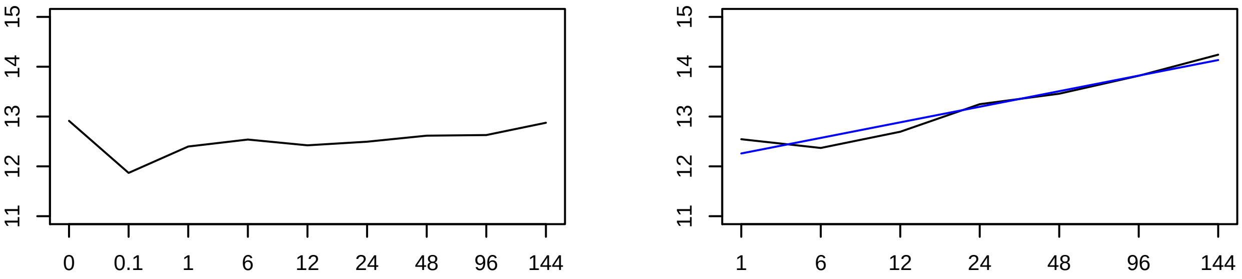

A_23_P77031 PTPN21 14q31.3

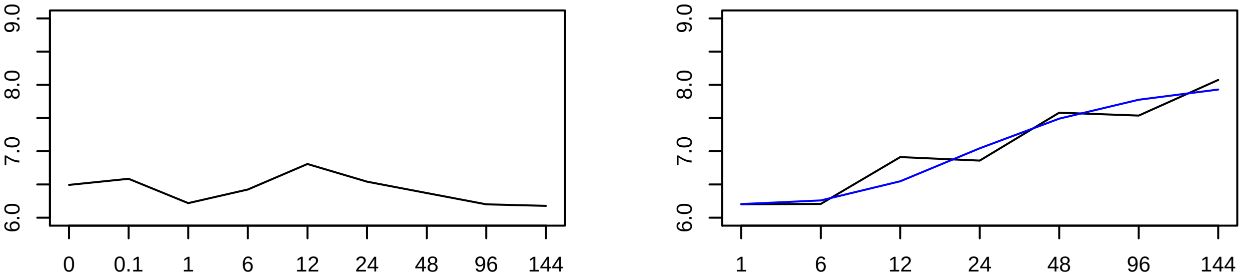

A_23_P80048 FER1L4 20q11.22

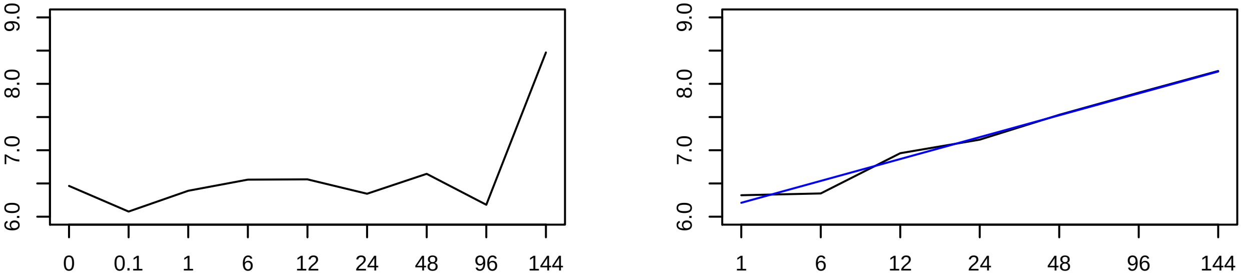

A_23_P140146 FAM14A 14q32.13

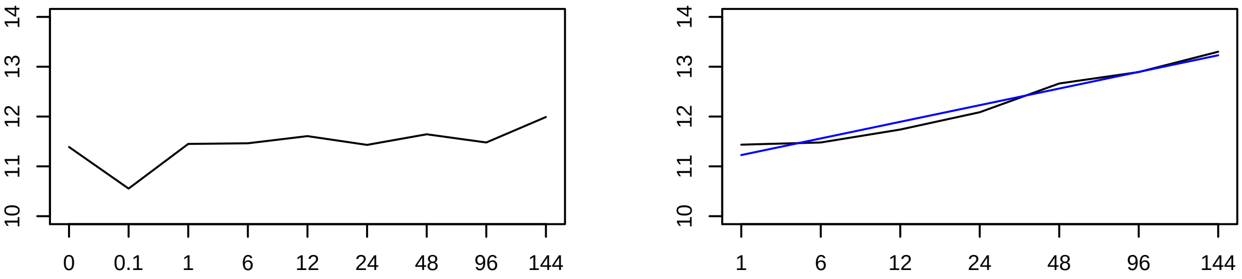

A_23_P133359 ZFP2 5q35.3

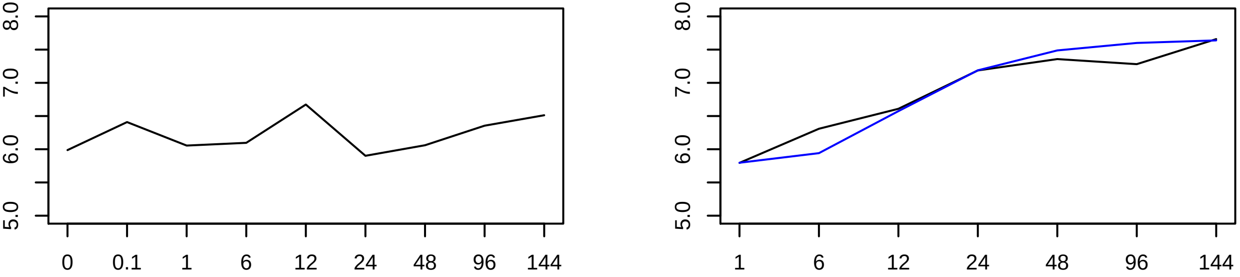

A_24_P111342 CASP9 1p36.21

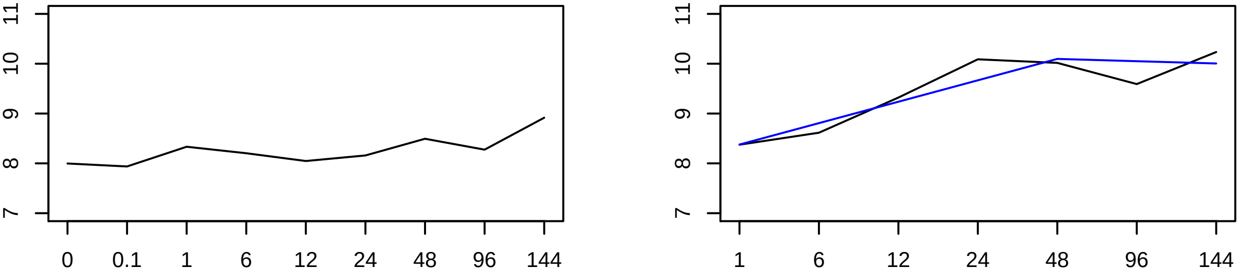

A_24_P171075 CREM 10p11.21

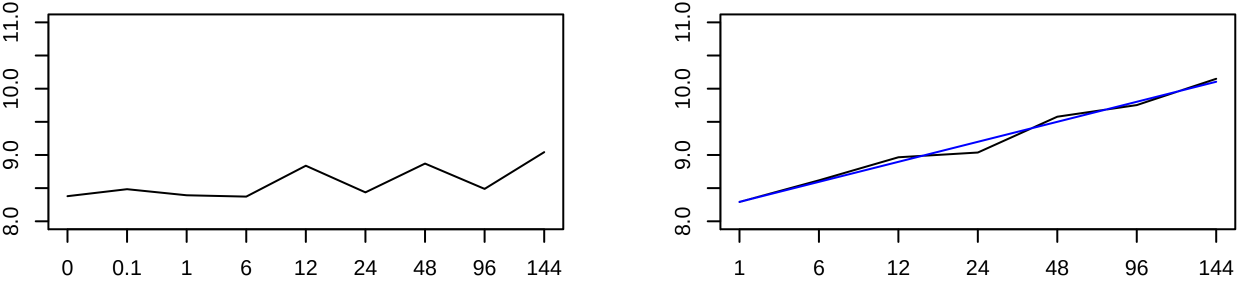

A_24_P182494 DUSP10 1q41

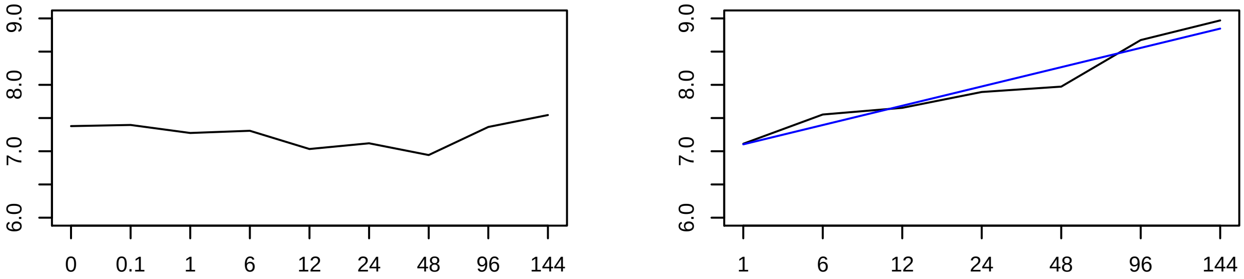

A_23_P128956 ZFYVE1 14q24.2

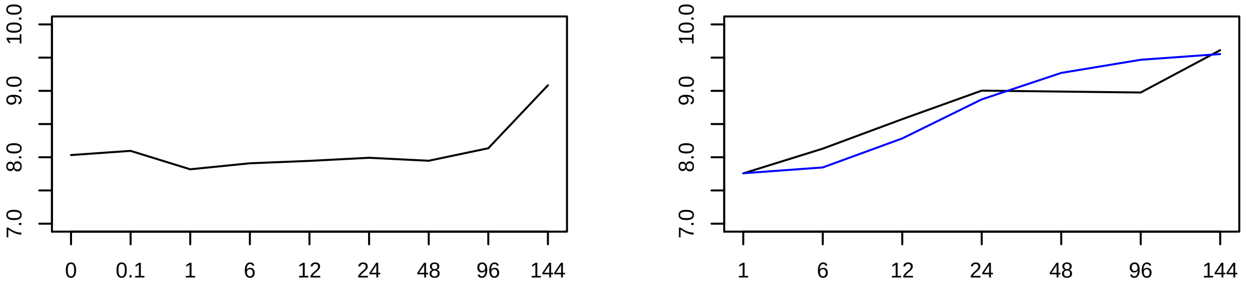

A_24_P333421 LOC643641 7q36.1

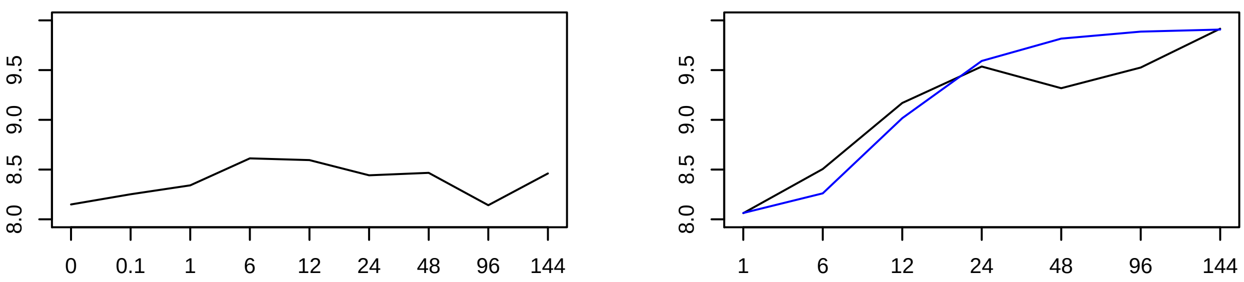

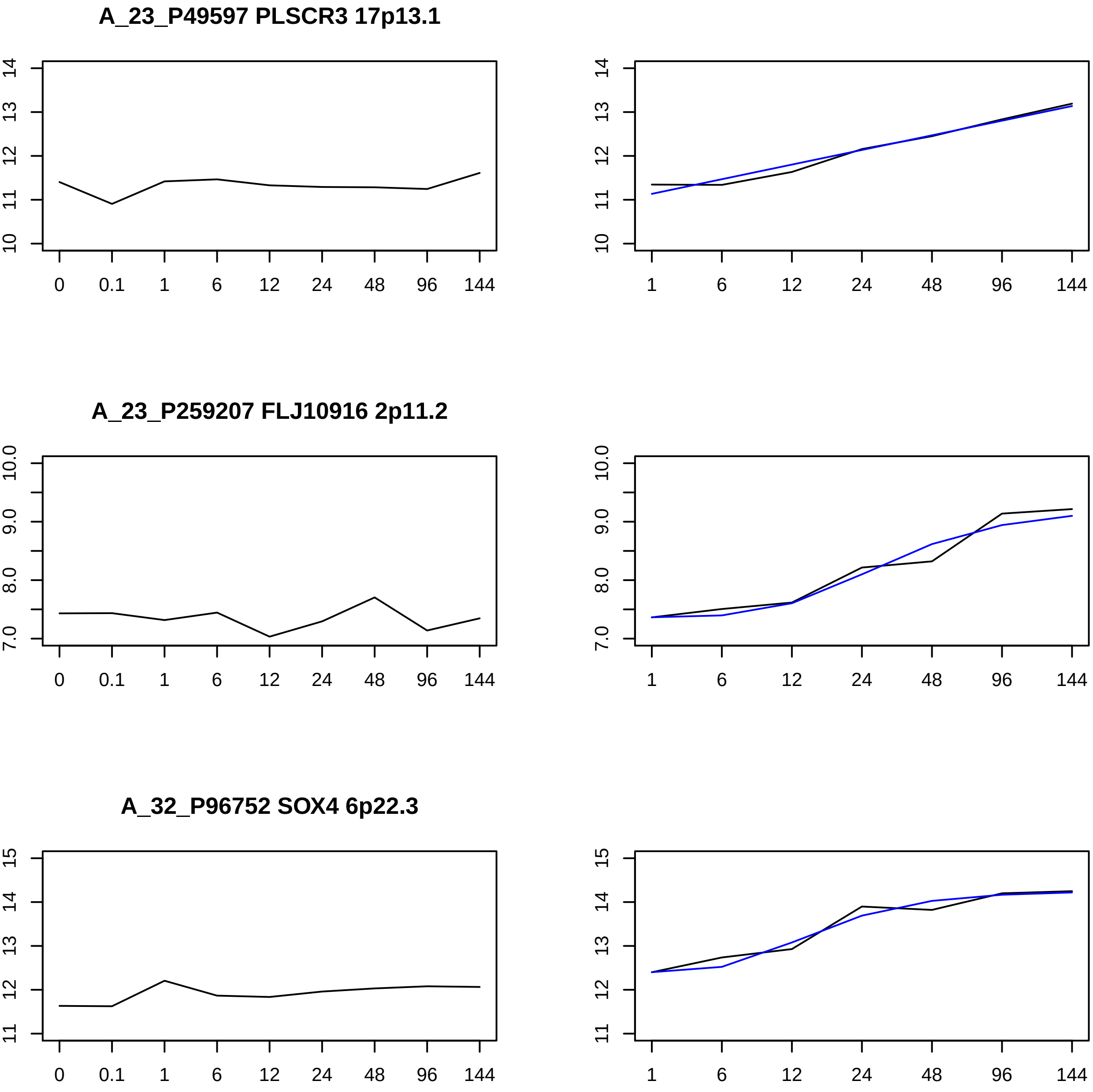
A_23_P49597 PLSCR3 17p13.1
10
11
12
13
14
0
0.1
1
6
12
24
48
96
144
A_23_P259207 FLJ10916 2p11.2
7.0
8.0
9.0
10.0
A_32_P96752 SOX4 6p22.3
15

A_23_P251002 A_23_P251002 NA

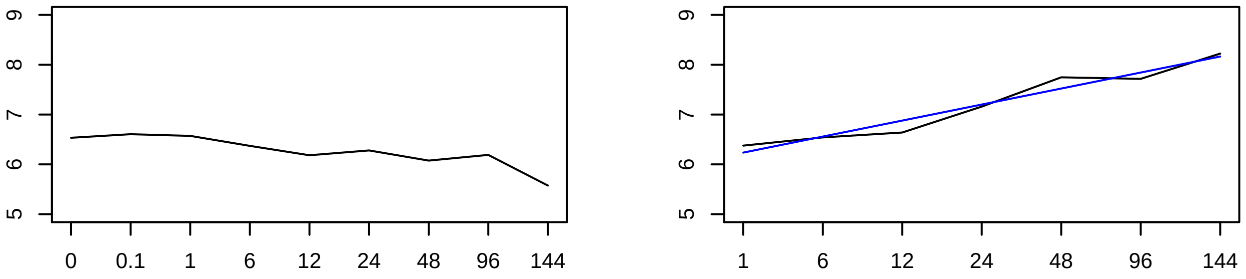

A_23_P351204 KCNQ2 20q13.33

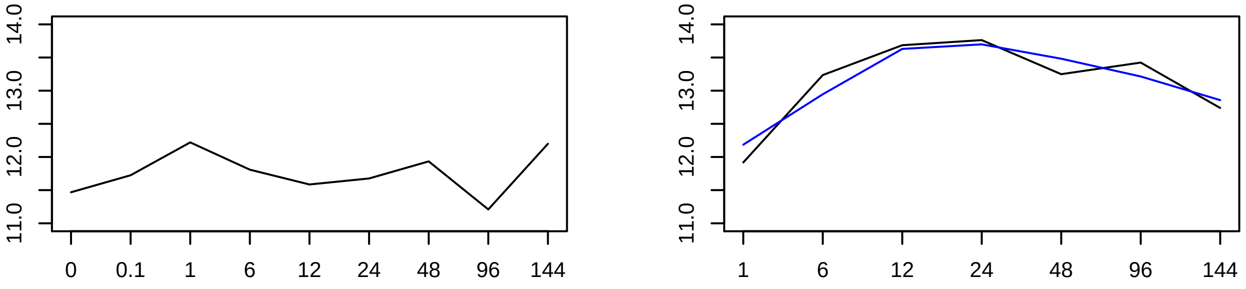

A_24_P108311 NEDD4L 18q21.31

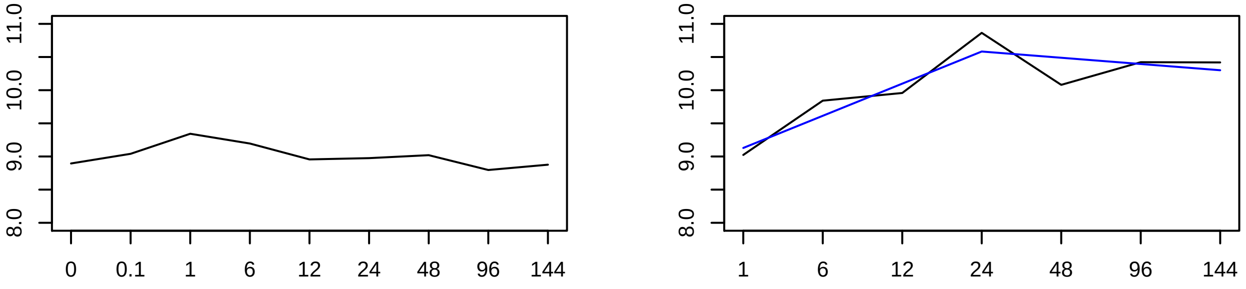

A_24_P323715 ENST00000383061 NA

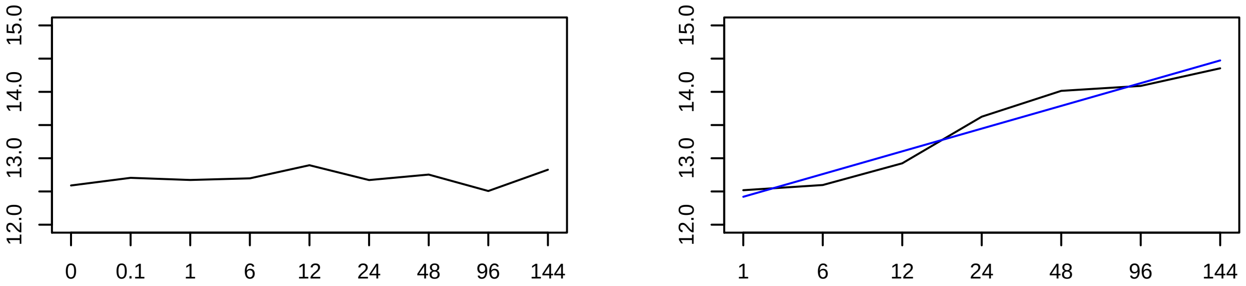

A_24_P772488 PLXNA4A 7q32.3

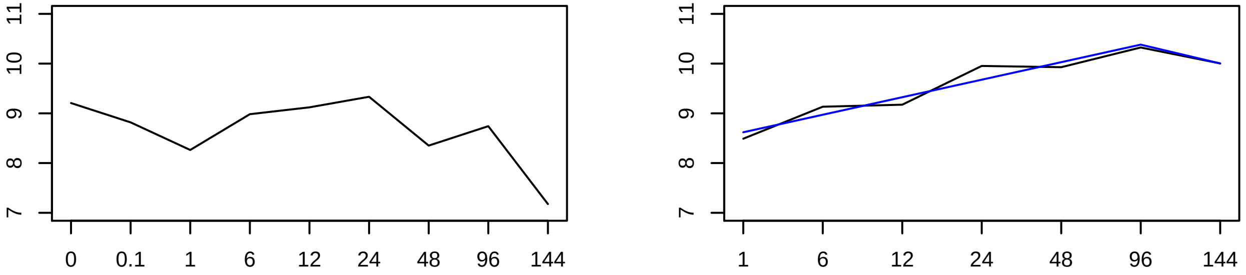

A_23_P73220 FGD6 12q22

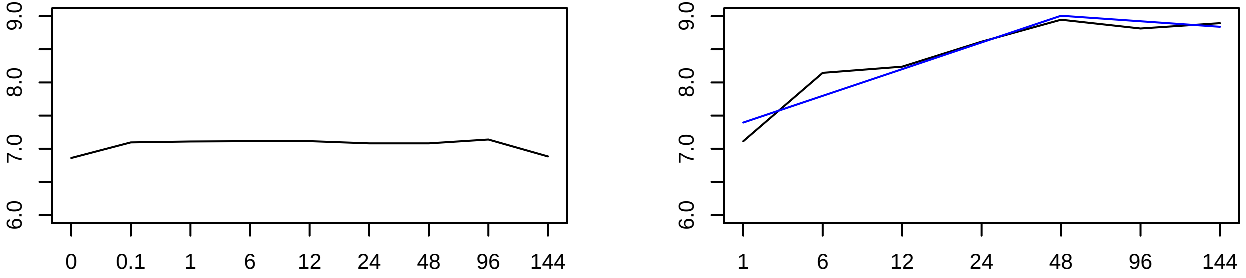

A_23_P203115 TMEM25 11q23.3

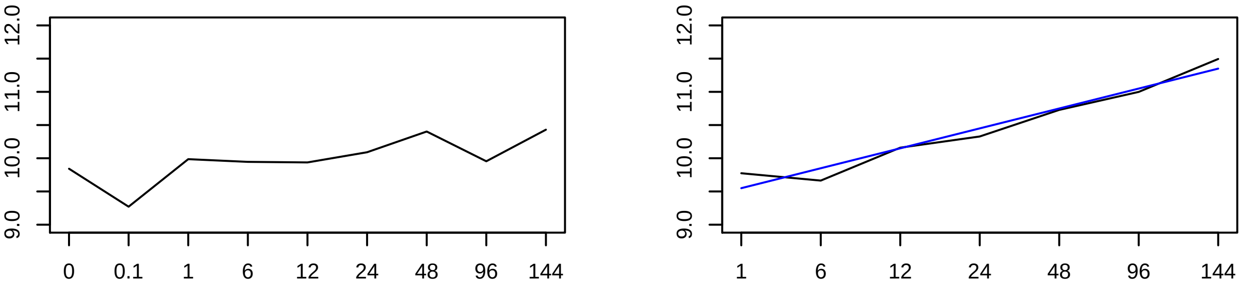

A_23_P204847 LCP1 13q14.12

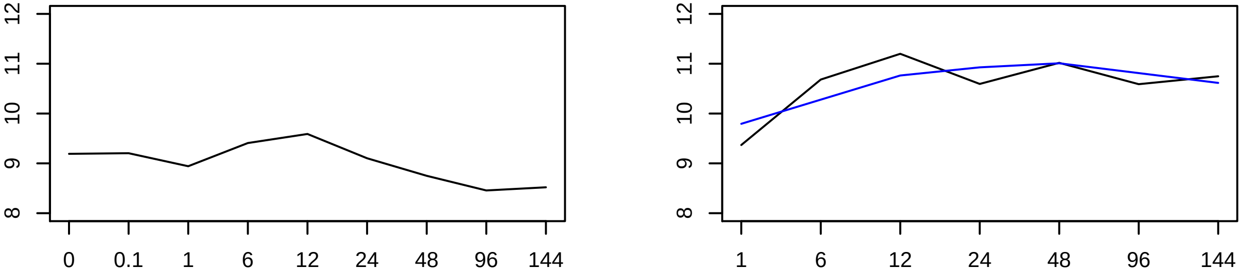

A_23_P382045 TULP4 6q25.3

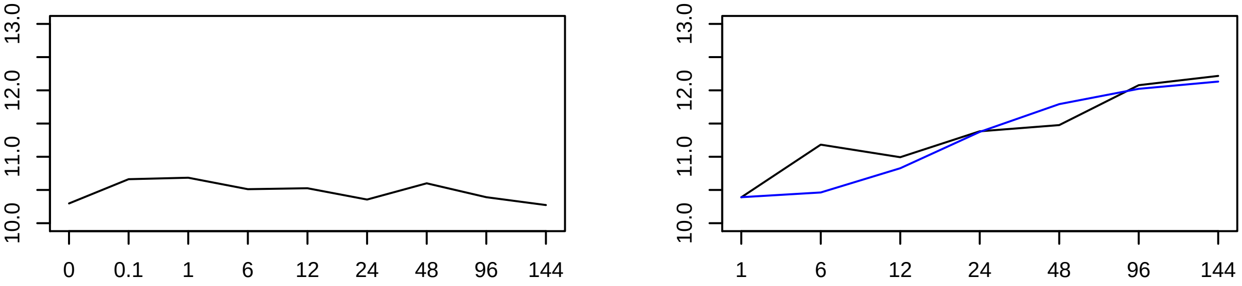

A_23_P257003 PCSK5 9q21.13

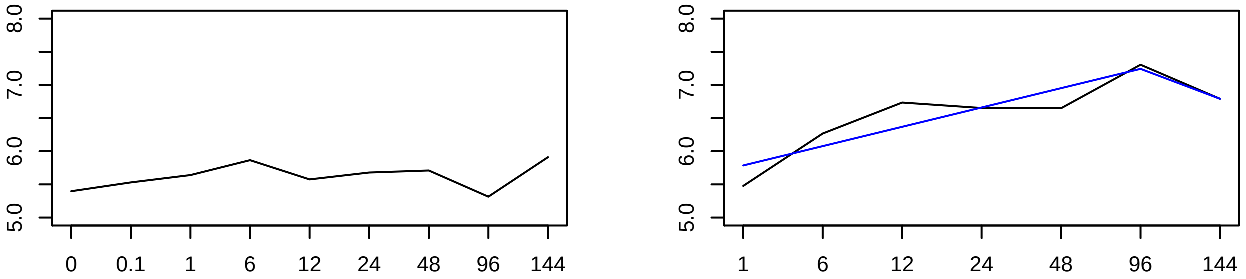

A_23_P336342 SLC4A8 12q13.13

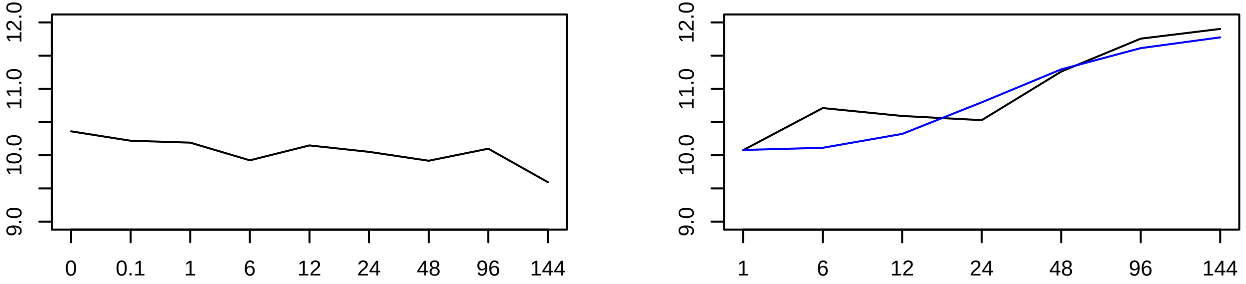

A_23_P127964 PRCP 11q14.1

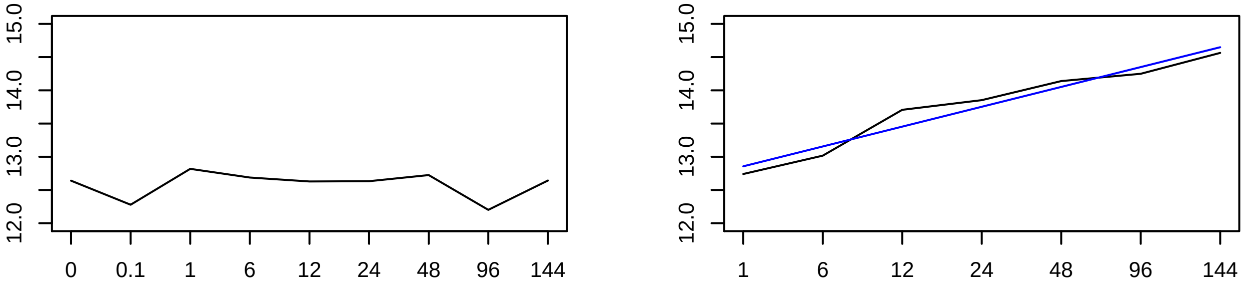

A_24_P484699 AK021467 NA

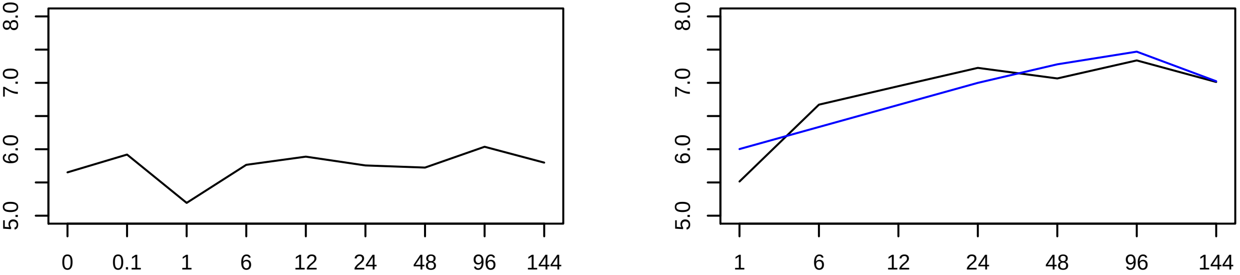

A_32_P159023 BX114329 NA

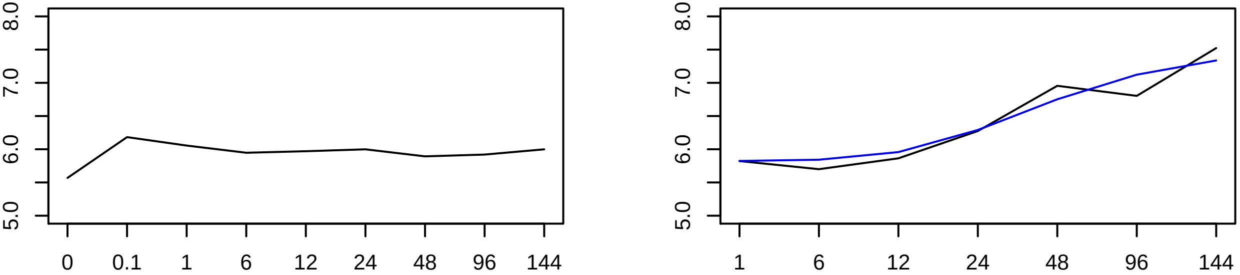

A_24_P13682 TLE3 15q23

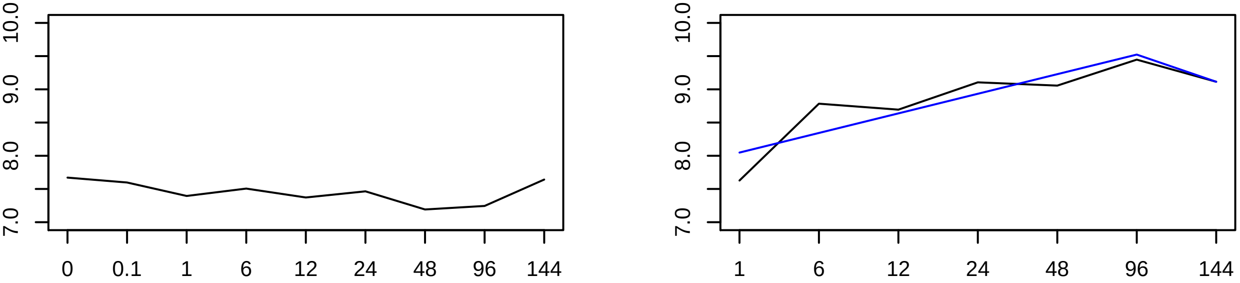

A_32_P160972 C6orf115 6q24.1

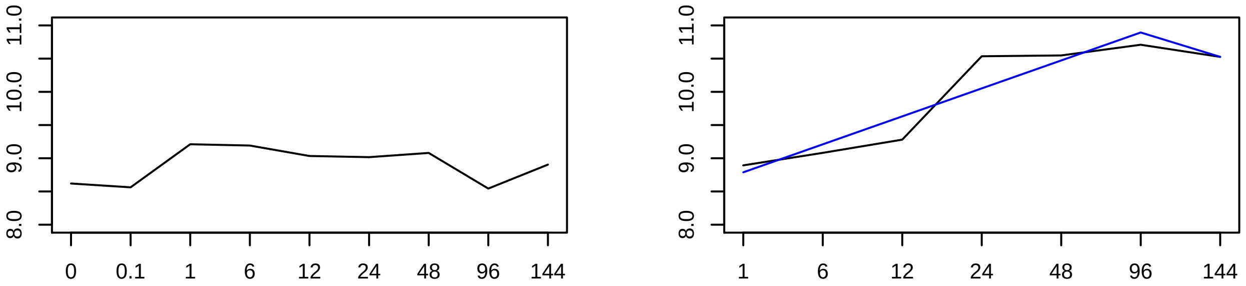

A_23_P213000 WDR1 4p16.1

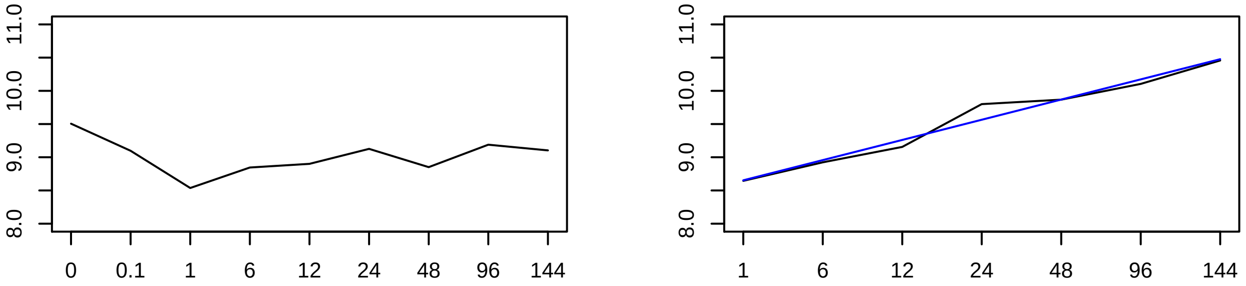

A_24_P174641 PDLIM5 4q22.3

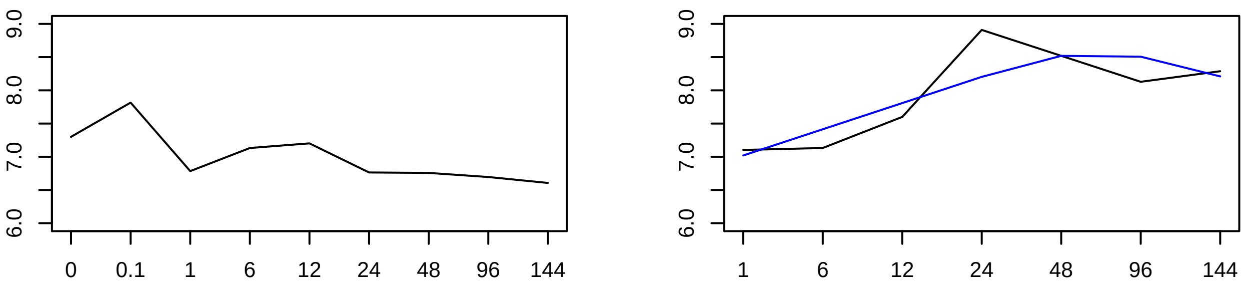

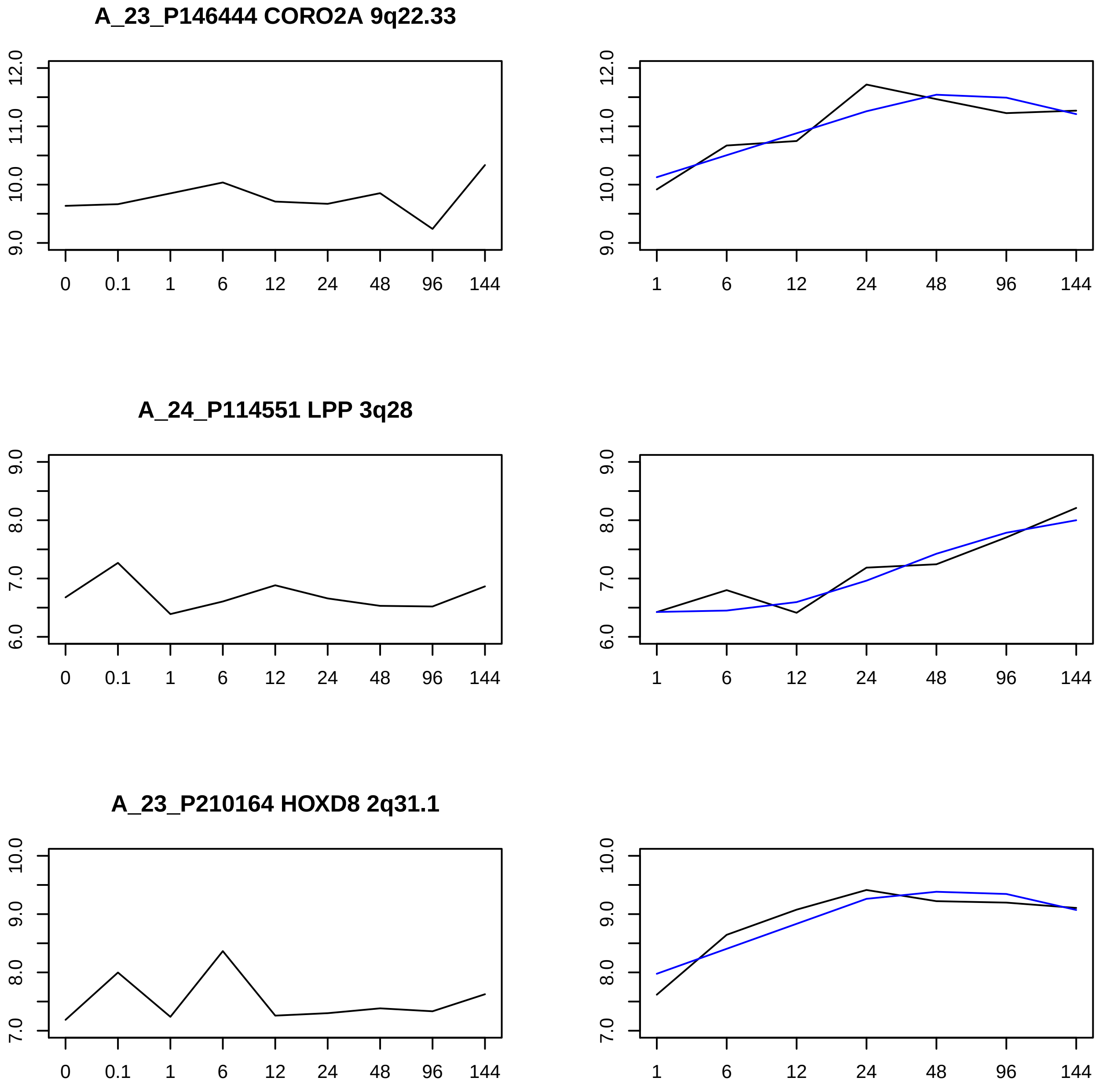
A_23_P146444 CORO2A 9q22.33
9.0
10.0
11.0
12.0
0
0.1
1
6
12
24
48
96
144
A_24_P114551 LPP 3q28
6.0
7.0
8.0
9.0
A_23_P210164 HOXD8 2q31.1
7.0
8.0
9.0
10.0

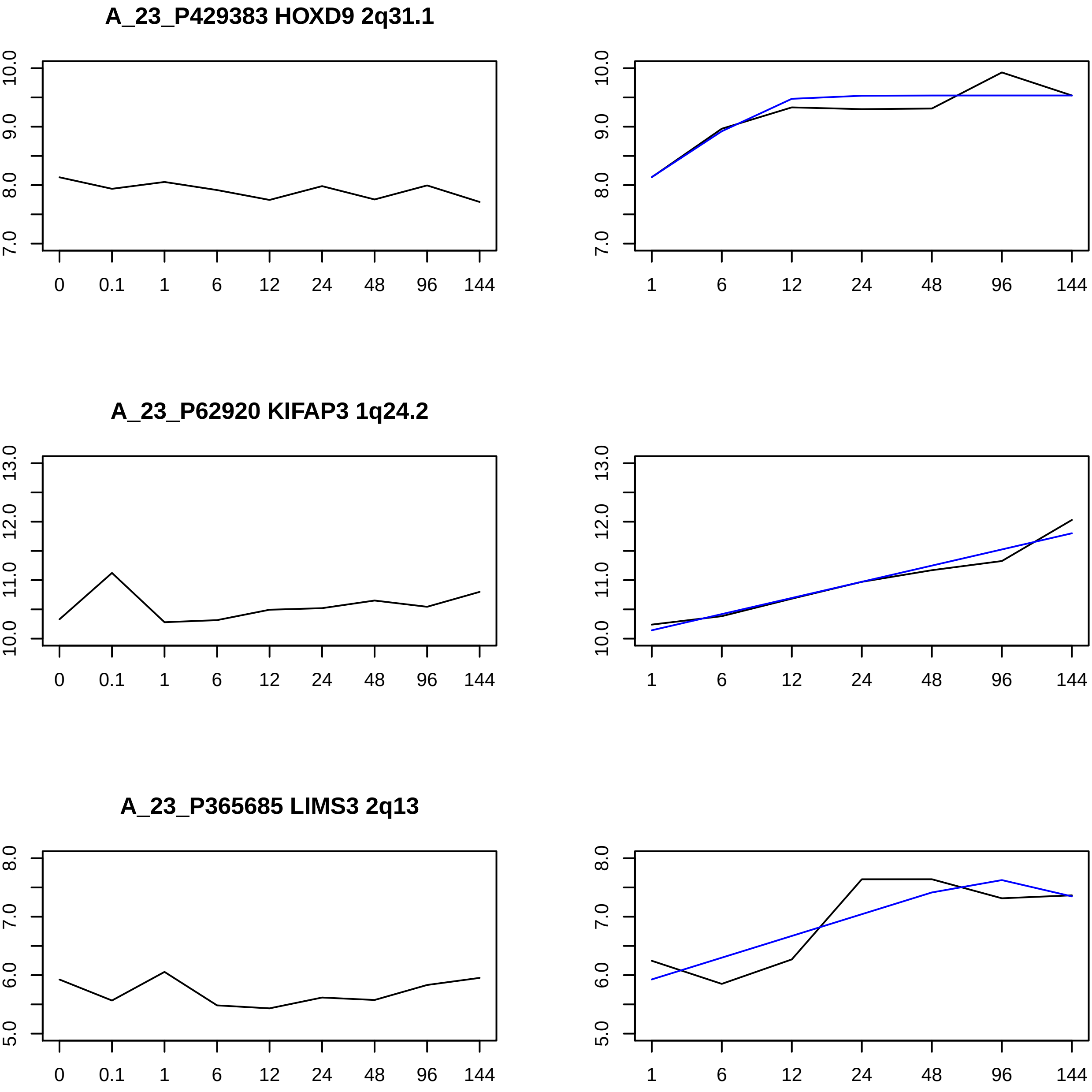

A_23_P429383 HOXD9 2q31.1
7.0
8.0
9.0
10.0
0
0.1
1
6
12
24
48
96
144
7.0
8.0
9.0
10.0
1
6
12
24
48
96
144
A_23_P62920 KIFAP3 1q24.2
10.0
11.0
12.0
13.0
0
0.1
1
6
12
24
48
96
144
10.0
11.0
12.0
13.0
1
6
12
24
48
96
144
A_23_P365685 LIMS3 2q13
5.0
6.0
7.0
8.0
0
0.1
1
6
12
24
48
96
144
5.0
6.0
7.0
8.0
1
6
12
24
48
96
144

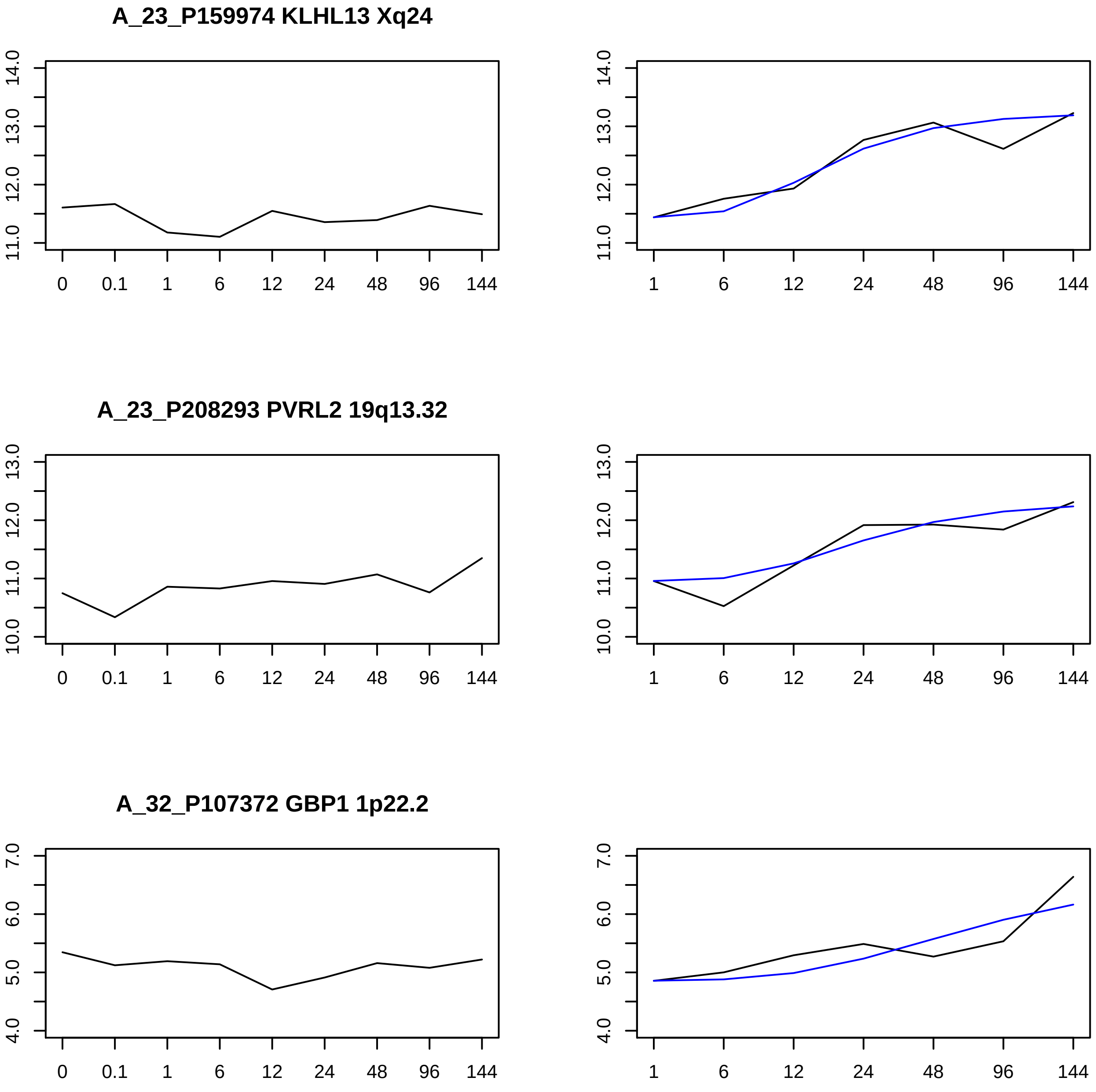
A_23_P159974 KLHL13 Xq24
A_23_P208293 PVRL2 19q13.32
A_32_P107372 GBP1 1p22.2
0 0.1 1 6 12 24 48 96 144
1 6 12 24 48 96 144
11.0 12.0 13.0 14.0
10.0 11.0 12.0 13.0
4.0 5.0 6.0 7.0

A_24_P388528 ST6GAL1 3q27.3

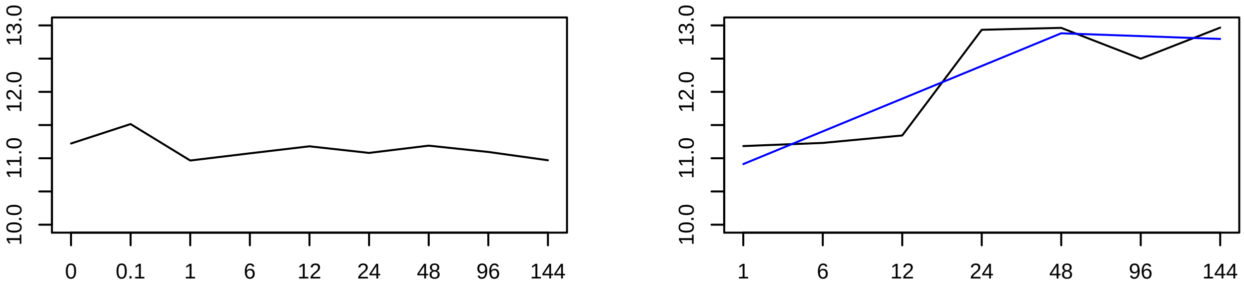

A_23_P81131 CORIN 4p12

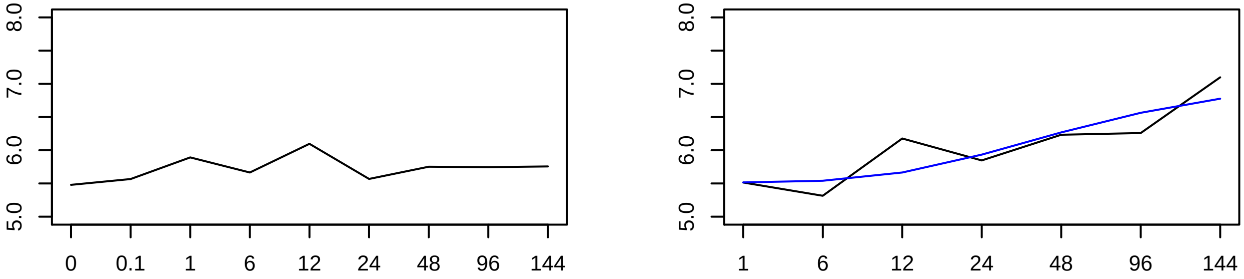

A_32_P52153 LOC730999 10q22.1

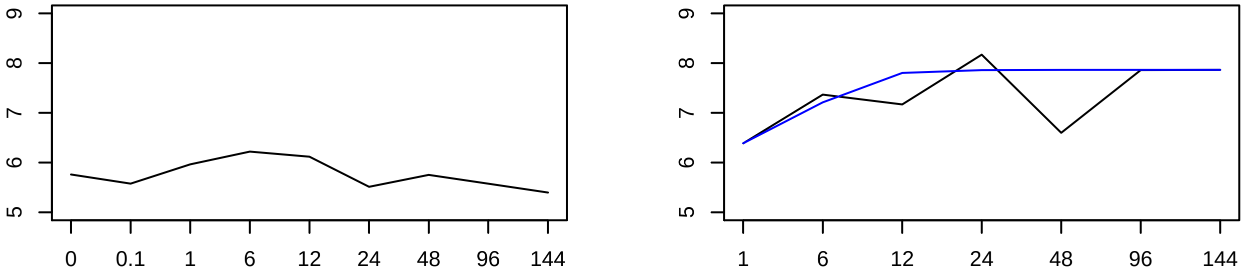

A_23_P79251 EHD3 2p23.1

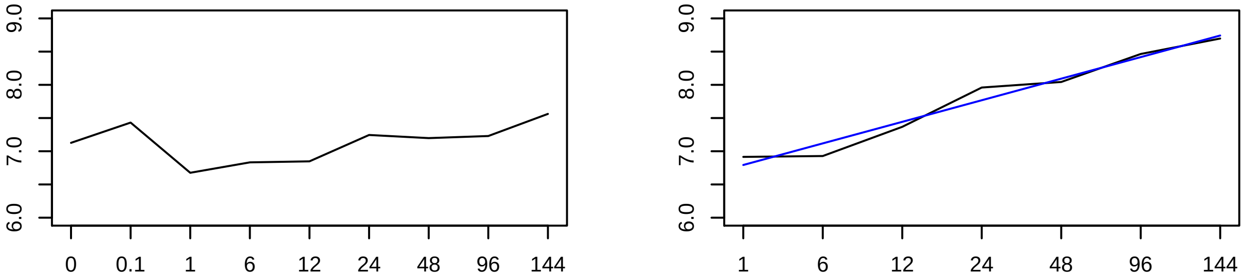

A_23_P303260 STX7 6q23.2

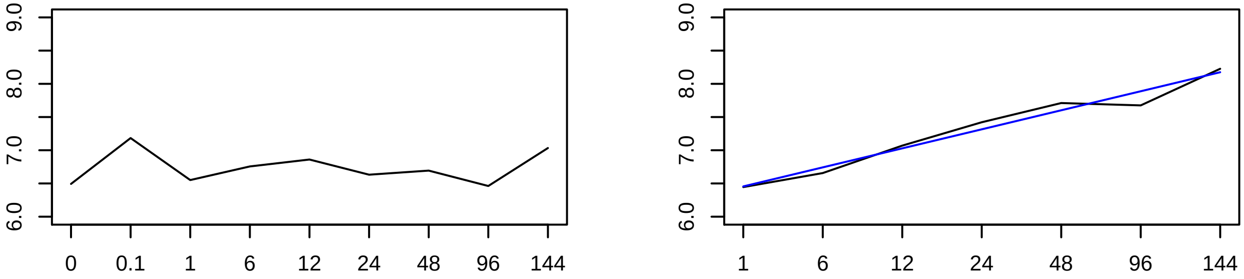

A_24_P350223 SHC4 15q21.1

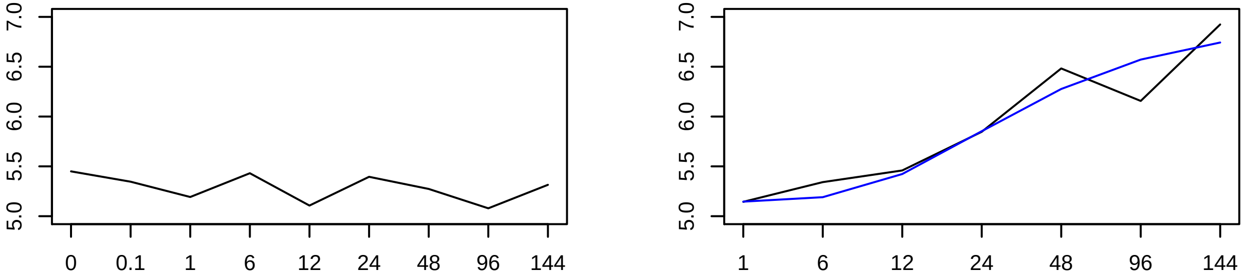

A_23_P323563 PLEKHG2 19q13.2

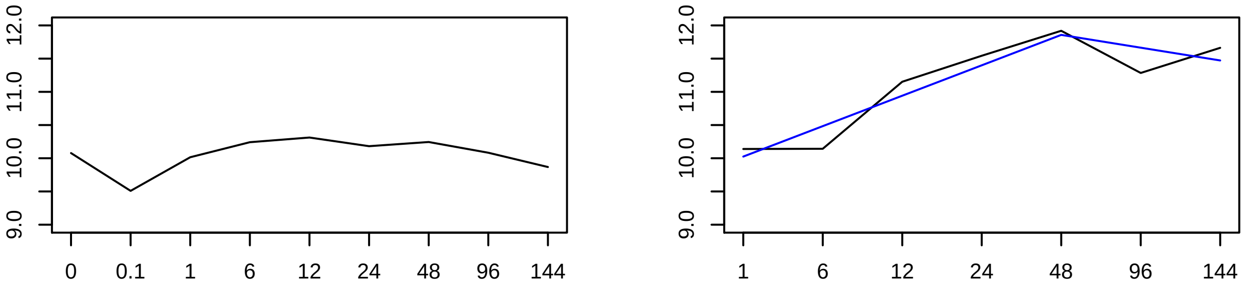

A_32_P126846 AI435603 NA

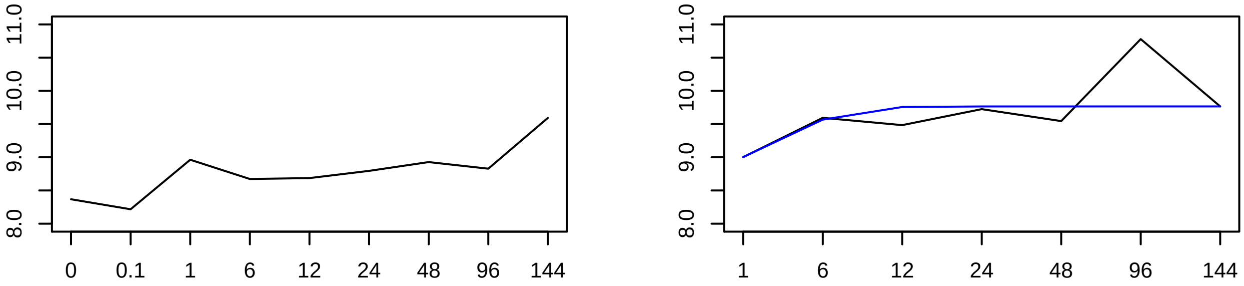

A_23_P325093 GGTL3 20q11.22

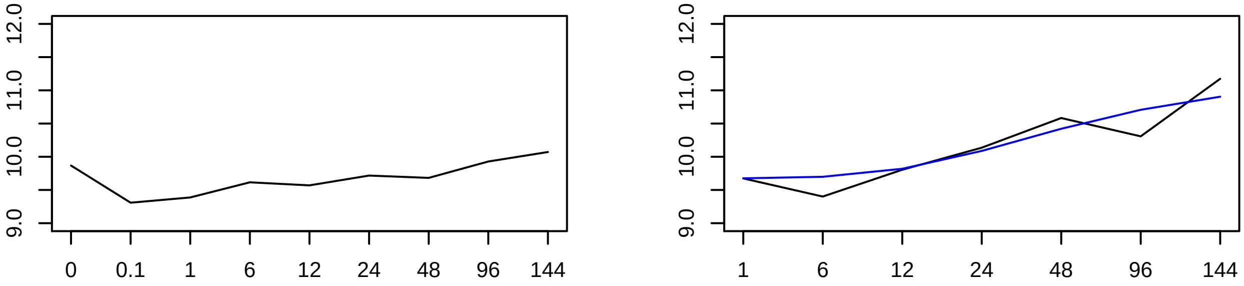

A_23_P76151 NAB2 12q13.3

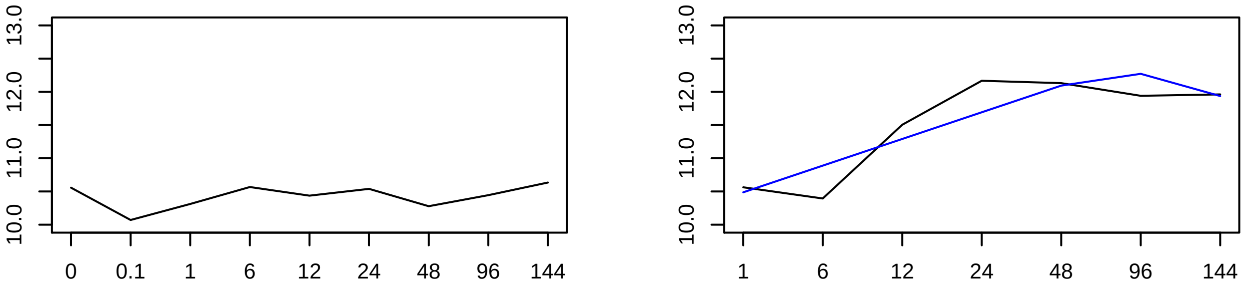

A_24_P794833 FLOT1 NA

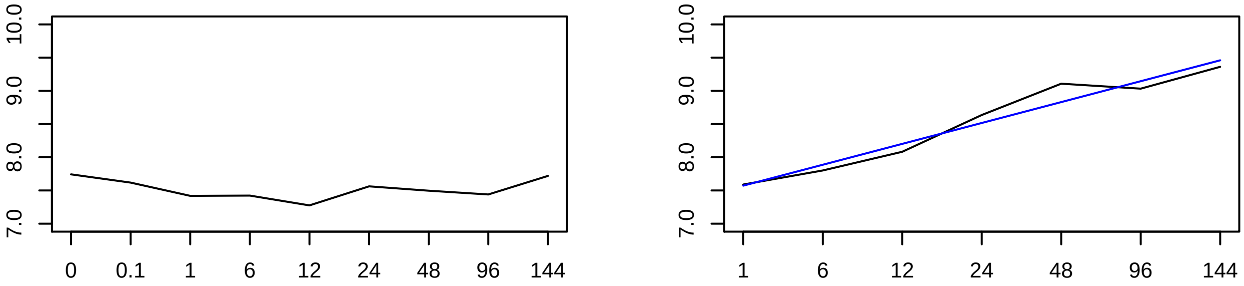

A_32_P213948 A_32_P213948 NA

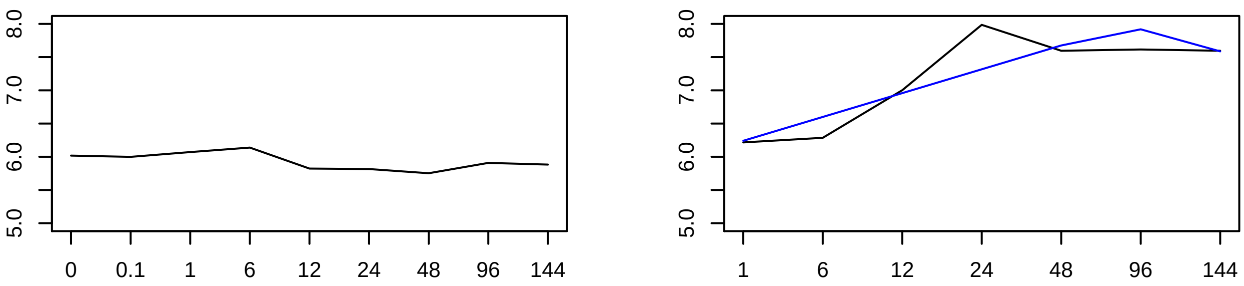

A_24_P59220 ENST00000375923 NA

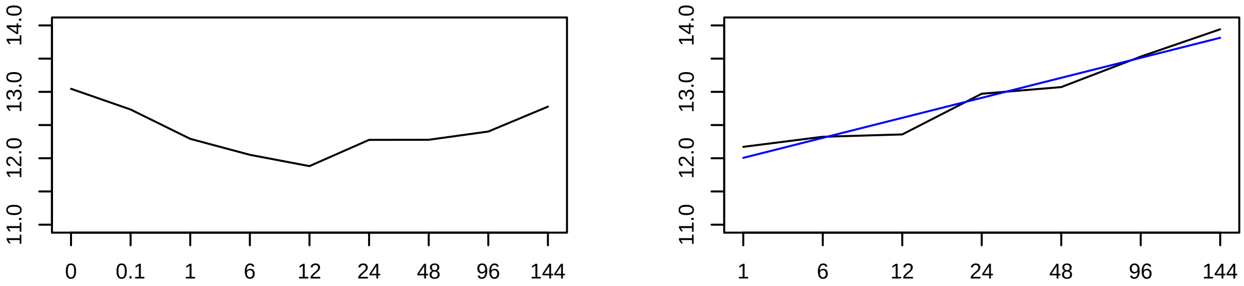

A_24_P312578 AKR1C1 10p15.1

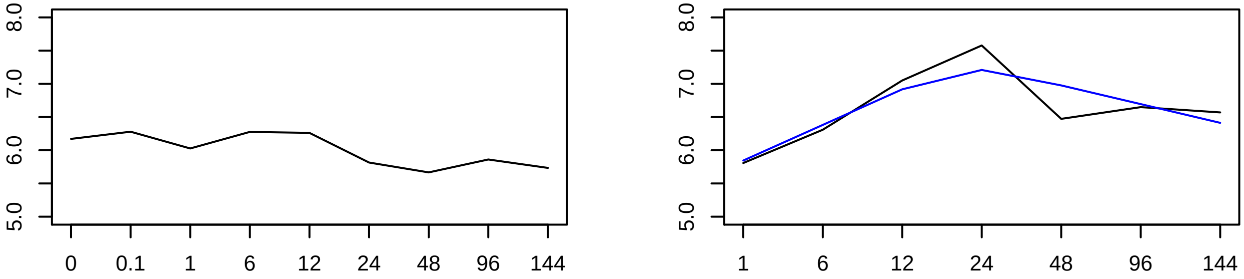

A_23_P382128 C8orf31 8q24.3

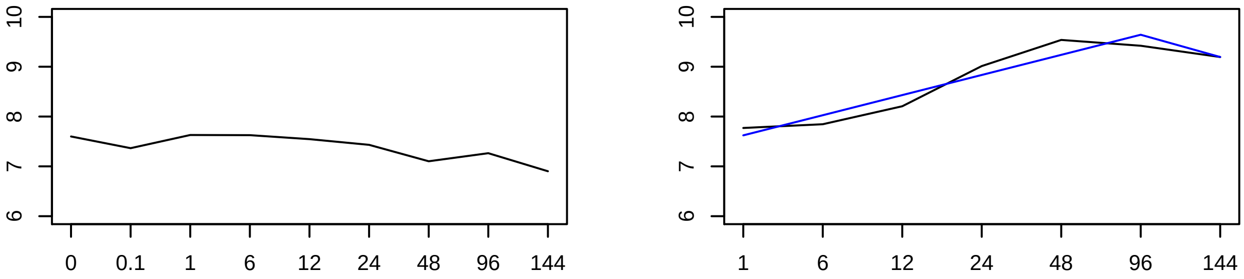

A_23_P82351 PTHB1 7p14.3

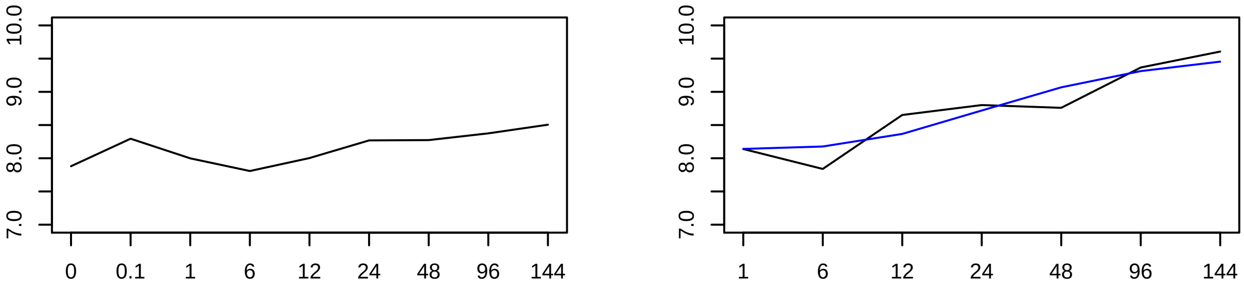

A_23_P47867 PPFIBP1 12p11.22

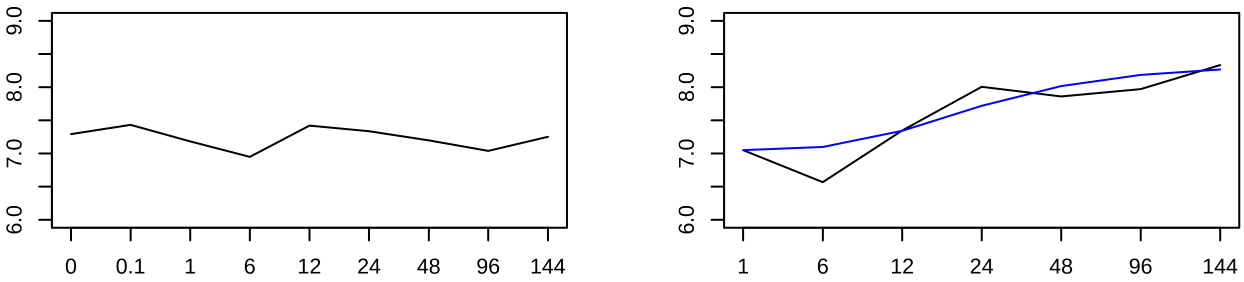

A_32_P823874 RP11−429B14.4 15q26.1

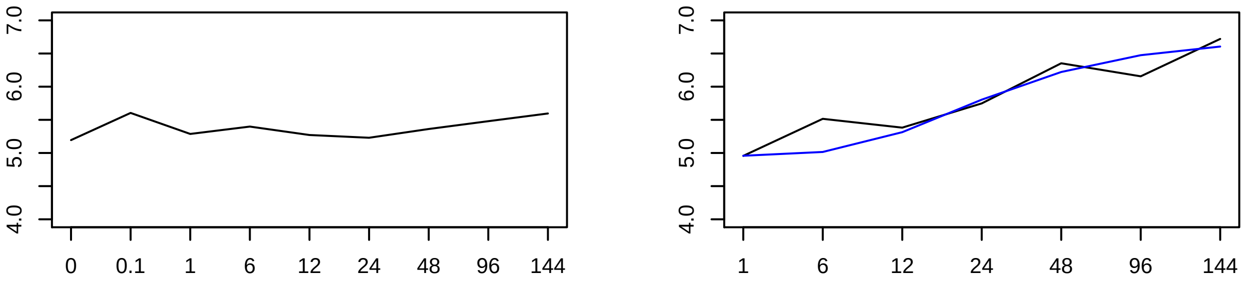

A_32_P144220 ENST00000383061 NA

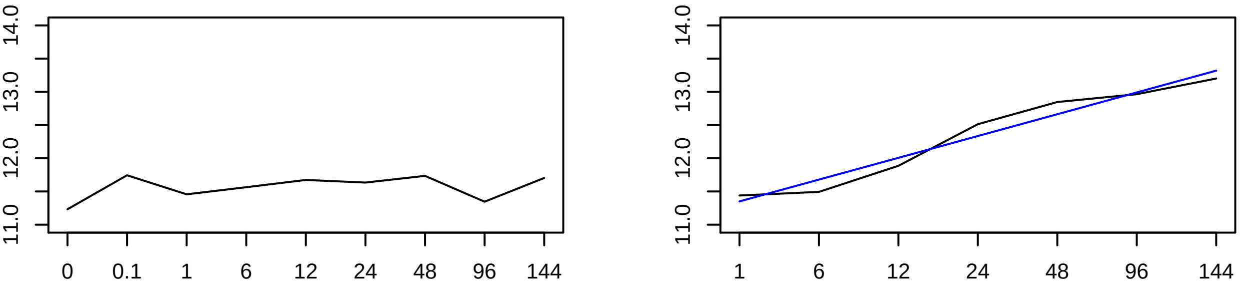

A_24_P265506 NTRK1 1q23.1

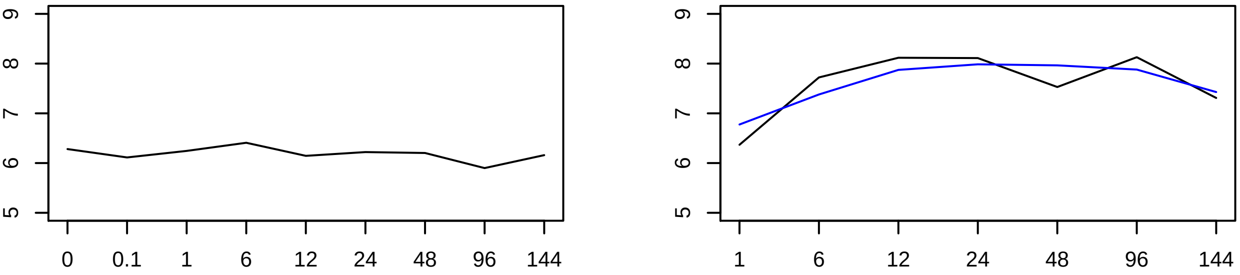

A_23_P65779 STRA6 15q24.1

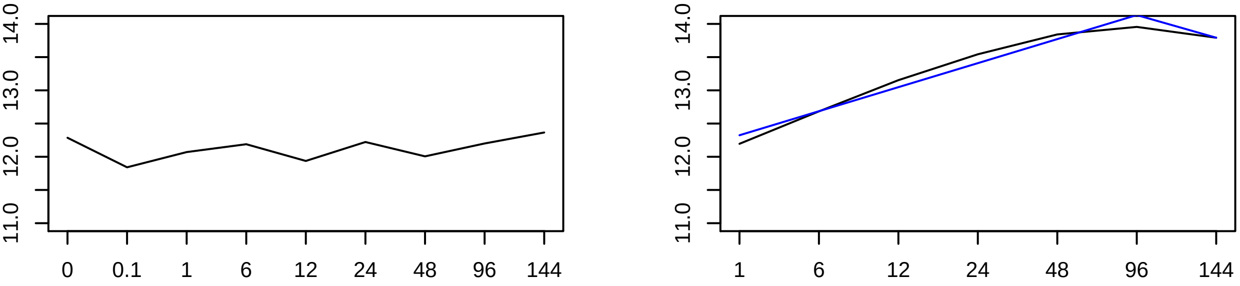

A_23_P132595 VGLL4 3p25.2

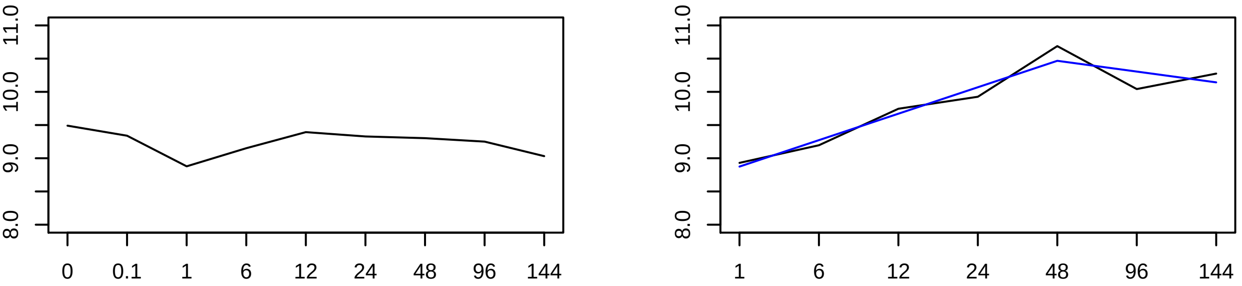

A_23_P171359 WDR44 Xq24

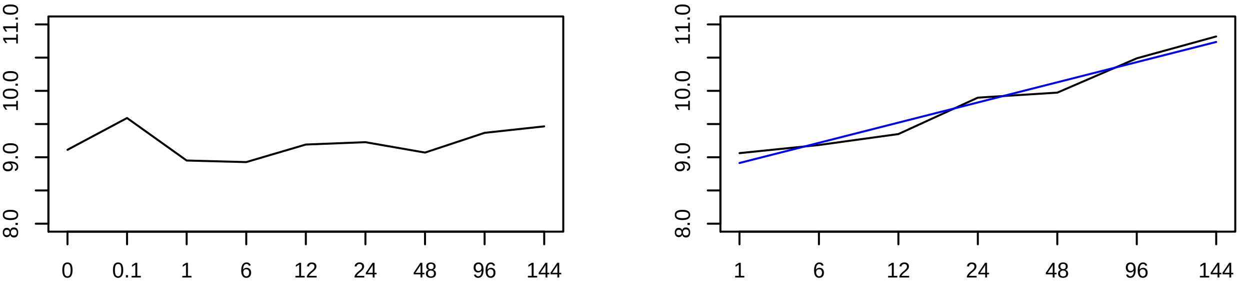

A_32_P227921 CNKSR3 6q25.2

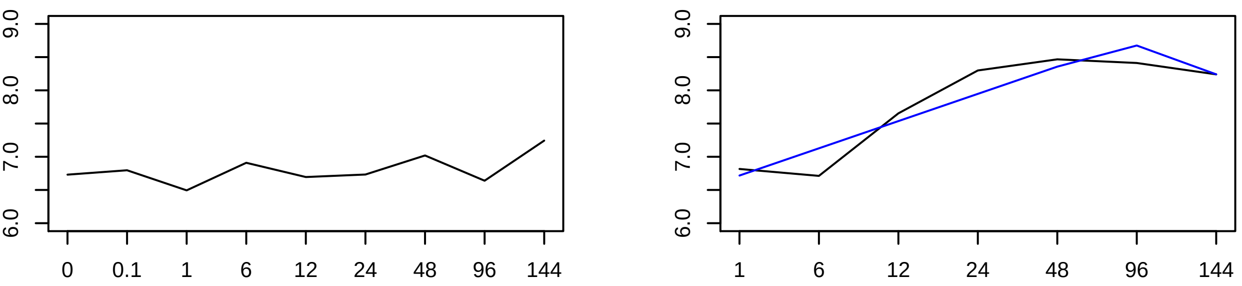

A_24_P759477 ITGB8 7p15.3

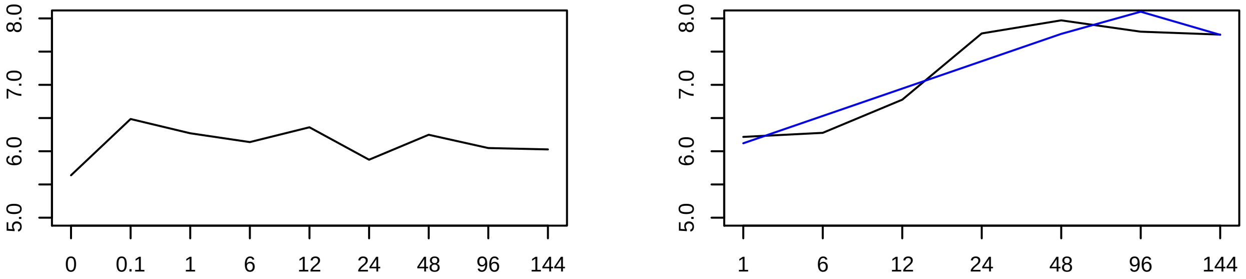

A_32_P85732 C9orf135 9q21.11

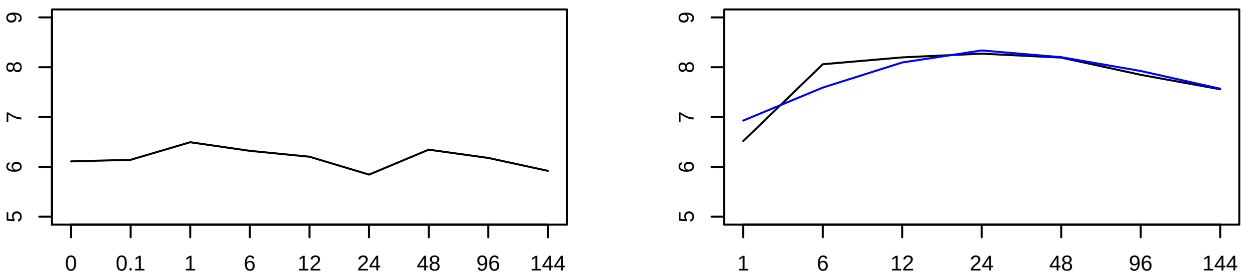

A_23_P73809 LRCH2 Xq23

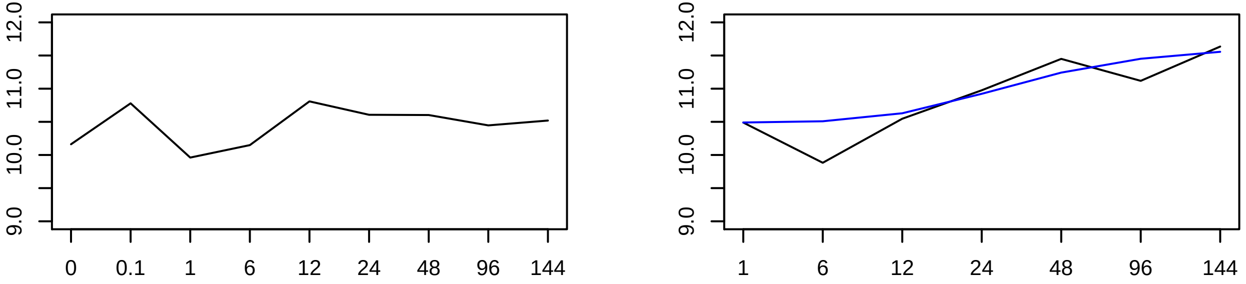

A_32_P19716 ZNF697 1p12

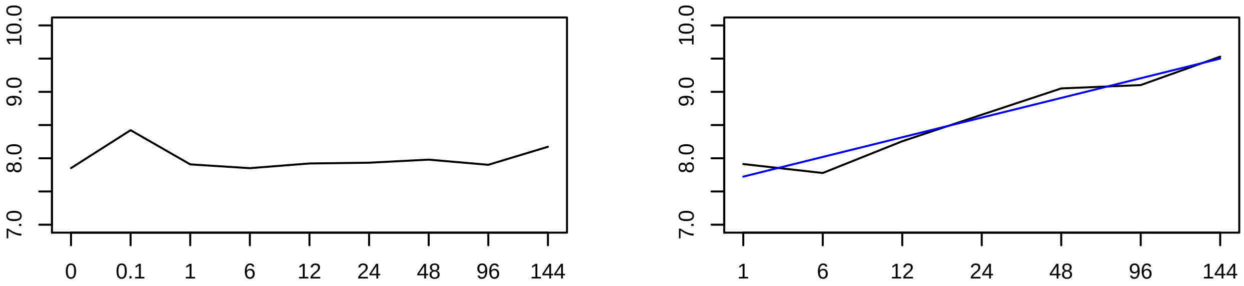

A_23_P97795 ACBD5 10p12.1

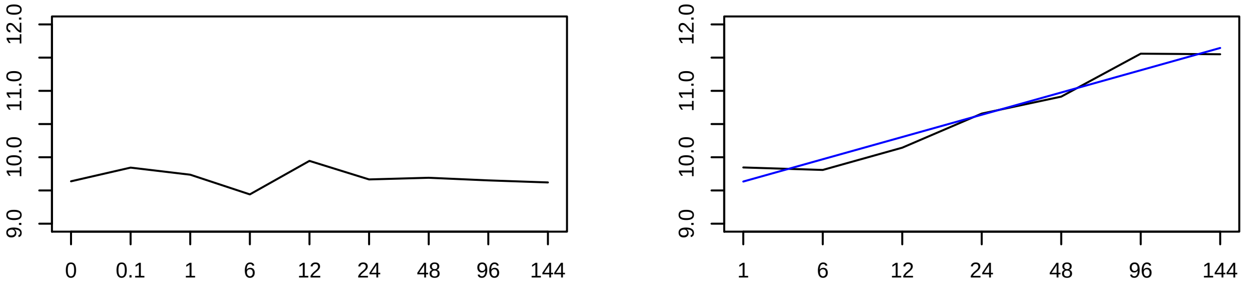

A_23_P717 C1orf75 1q32.3

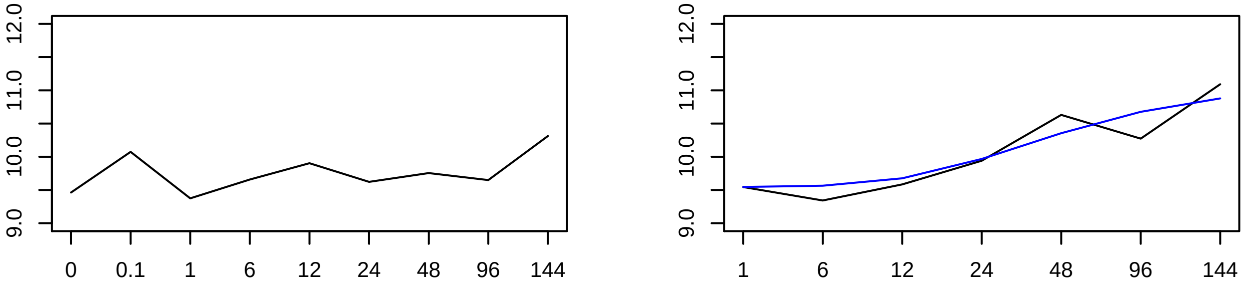

A_23_P501831 FAXDC2 5q33.2

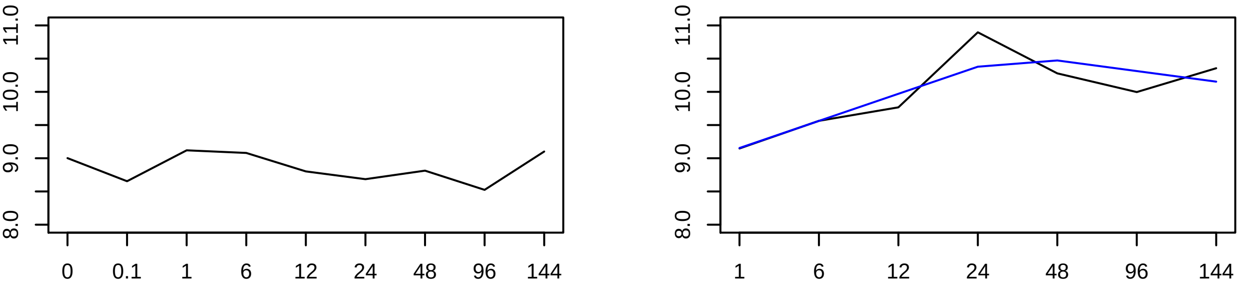

A_32_P4647 BE703462 NA

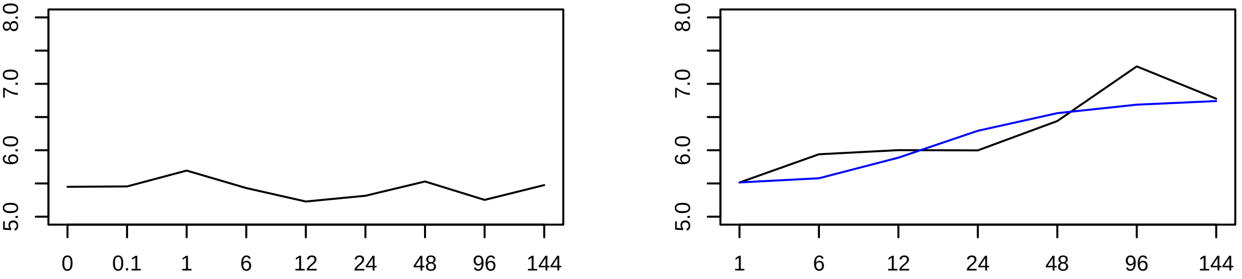

A_24_P269062 SPRY4 5q31.3

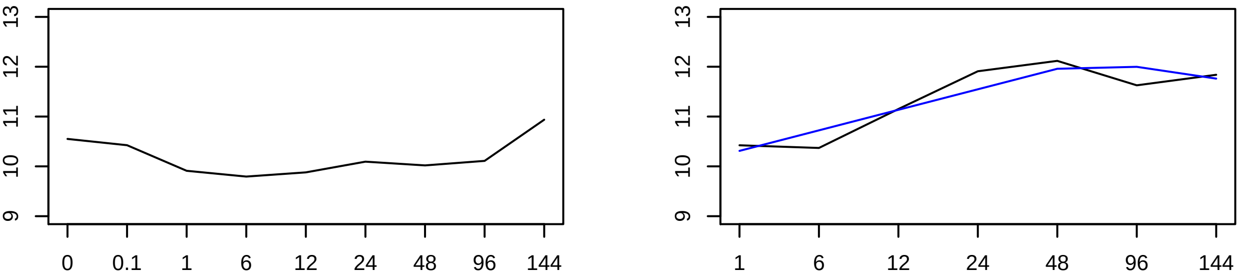

A_24_P374634 STAU2 8q21.11

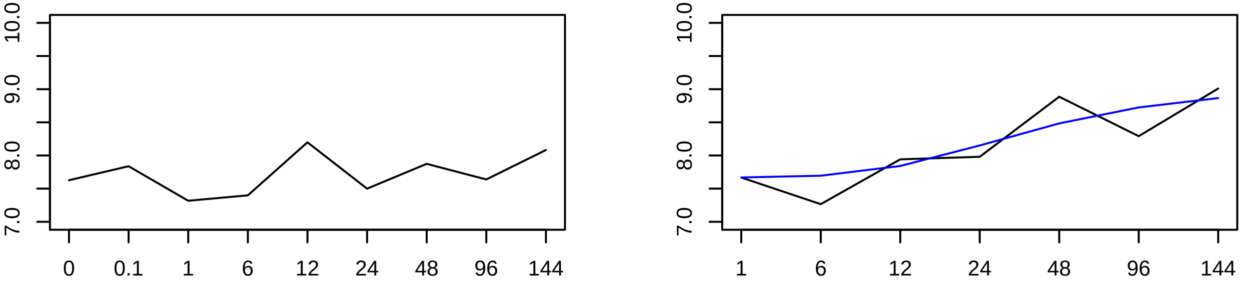

A_24_P349039 CDGAP 3q13.33

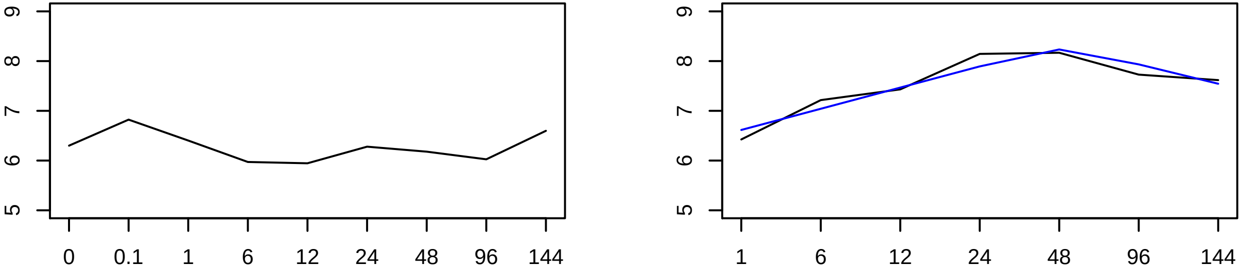

A_23_P401774 ELMOD1 11q22.3

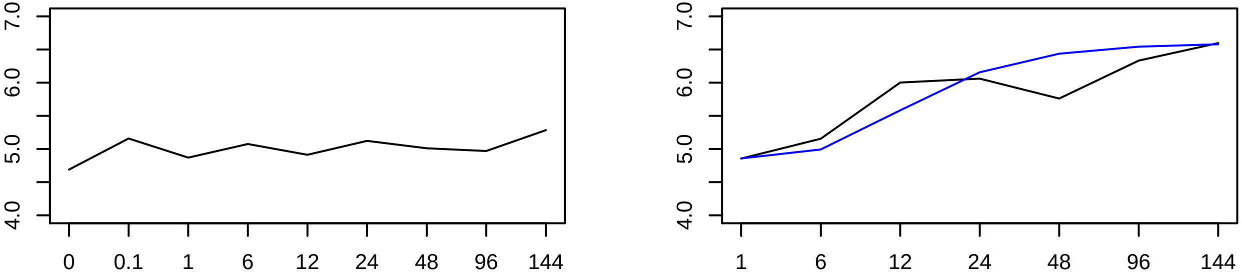

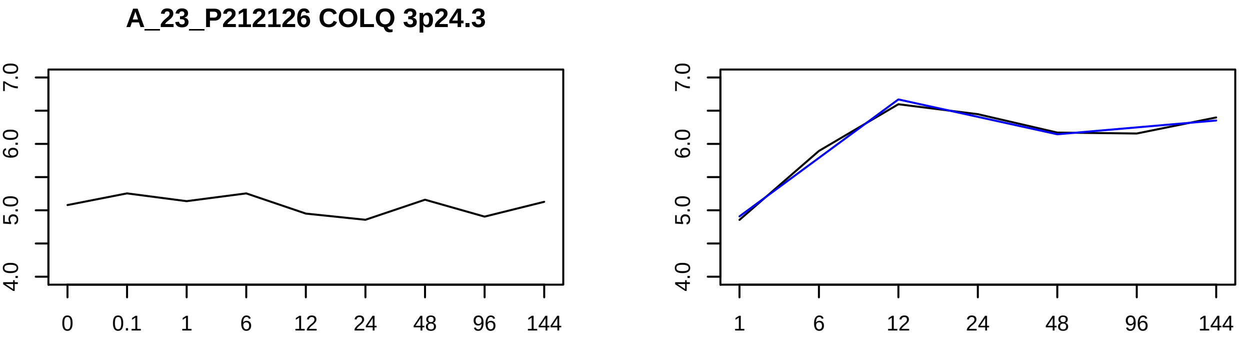
A_23_P212126 COLQ 3p24.3
4.0
5.0
6.0
7.0
0
0.1
1
6
12
24
48
96
144
1
6
12
24
48
96
144

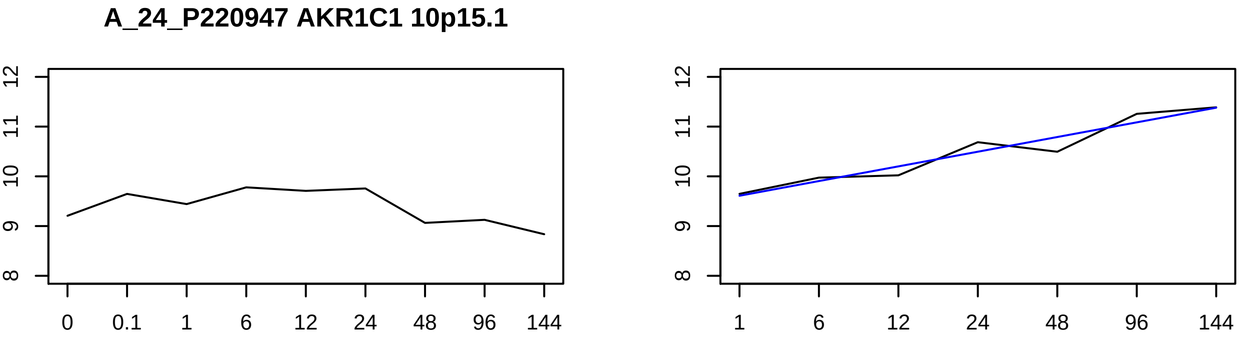
A_24_P220947 AKR1C1 10p15.1
8
9
10
11
12
0
0.1
1
6
12
24
48
96
144
1
6
12
24
48
96
144

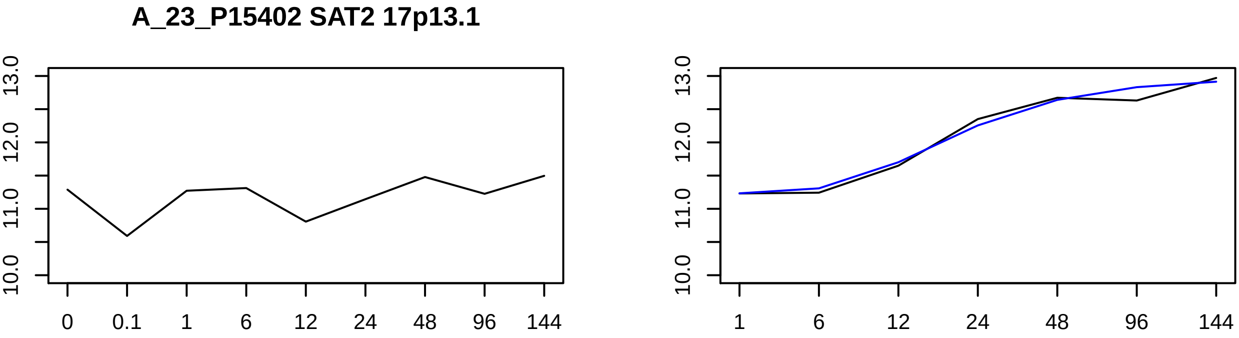
A_23_P15402 SAT2 17p13.1
10.0
11.0
12.0
13.0
0
0.1
1
6
12
24
48
96
144
1
6
12
24
48
96
144

A_23_P65823 GABARAPL3 15q26.1

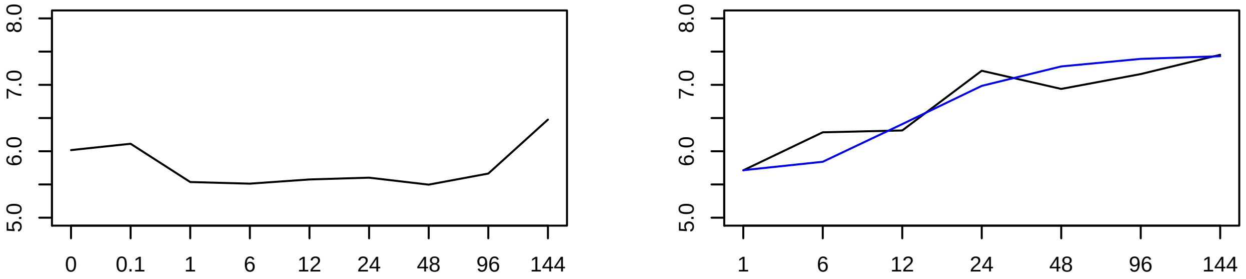

A_23_P41789 SLC27A6 5q23.3

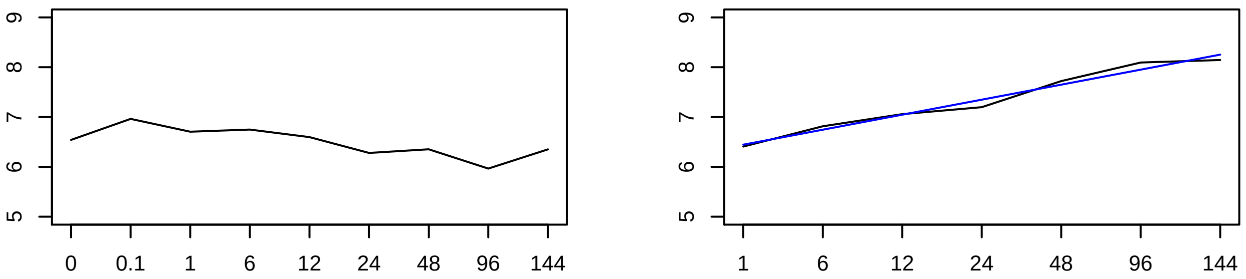

A_24_P251962 PCDHB2 5q31.3

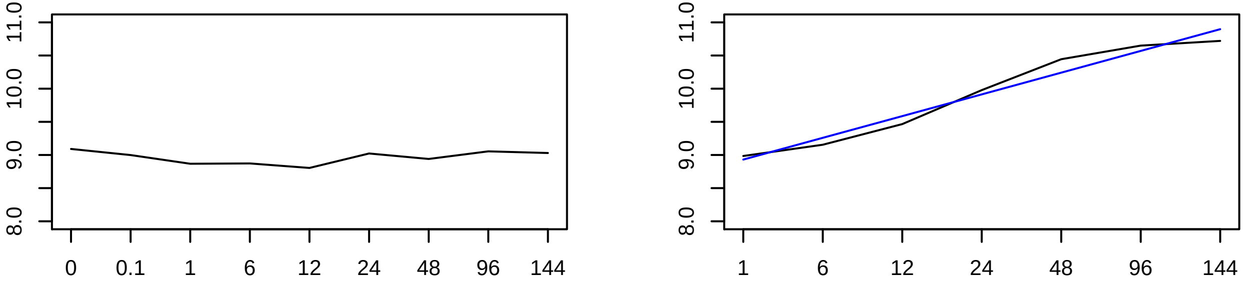

A_24_P49657 CR620043 NA

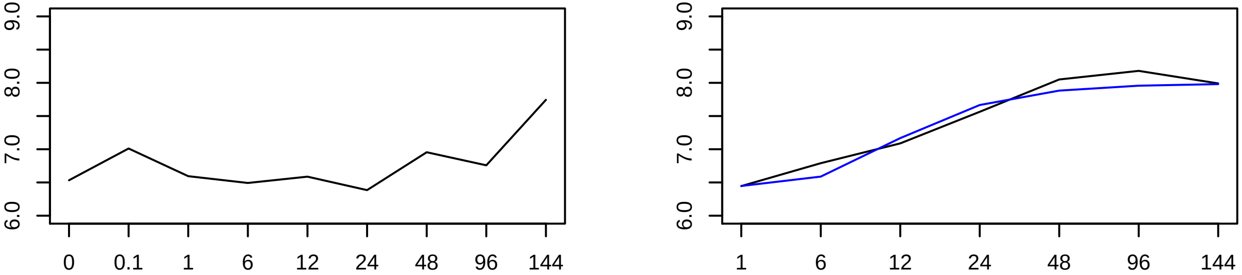

A_23_P14105 RCBTB2 13q14.2

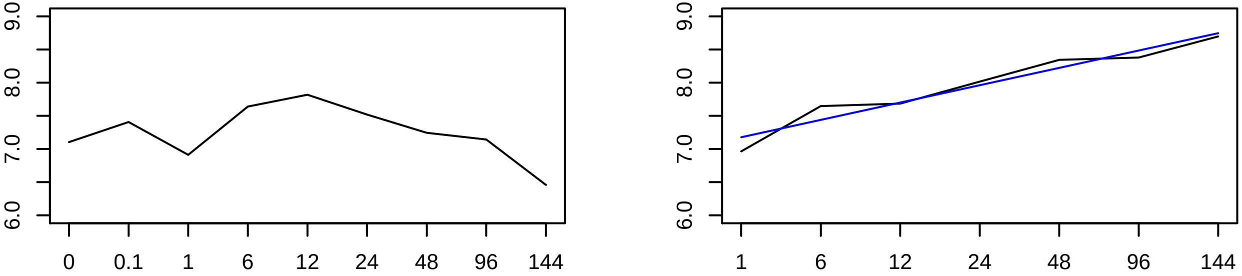

A_23_P213375 PCDHB2 5q31.3

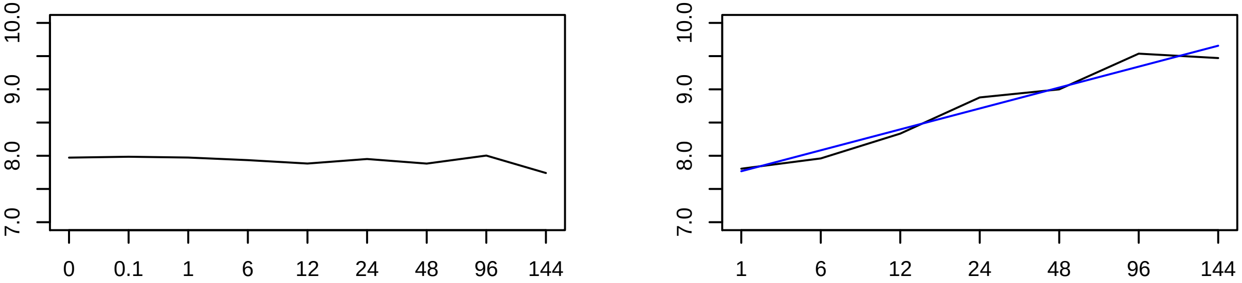

A_23_P427083 CDRT4 17p12

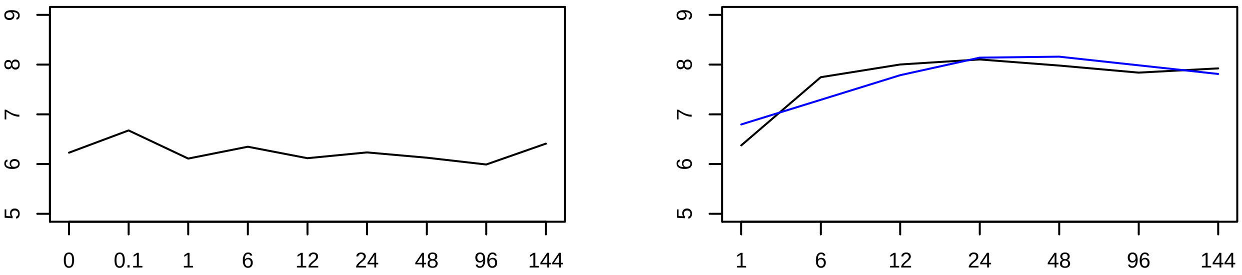

A_23_P120002 SP110 2q37.1

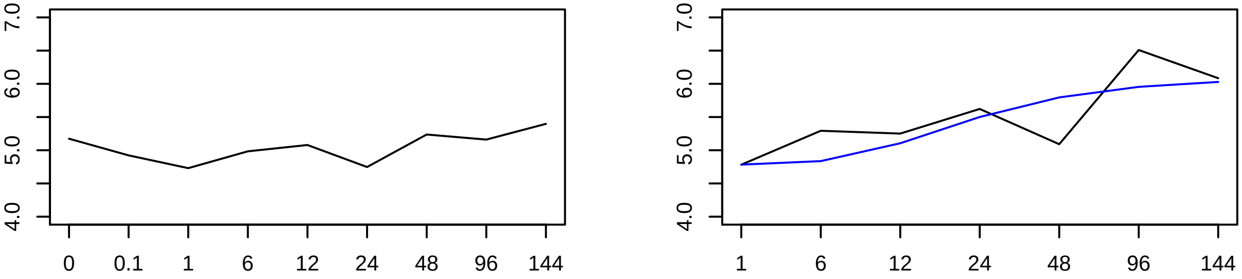

A_23_P117506 DHRS7 14q23.1

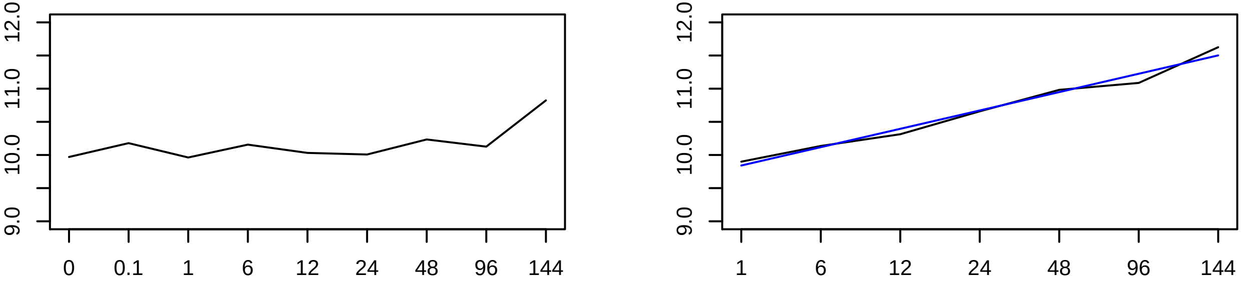

A_24_P238525 ENST00000324709 NA

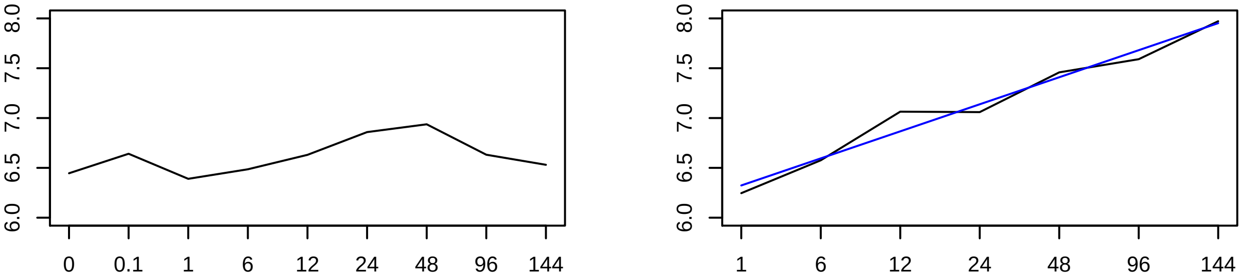

A_23_P406341 KIAA1914 10q25.3

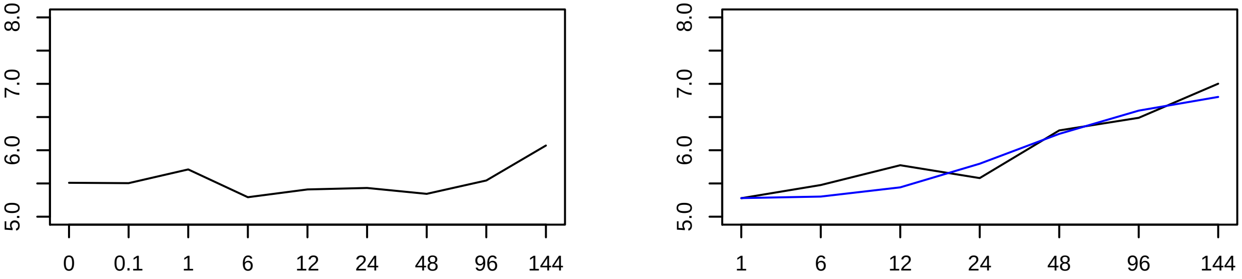

A_23_P421306 SYT12 11q13.1

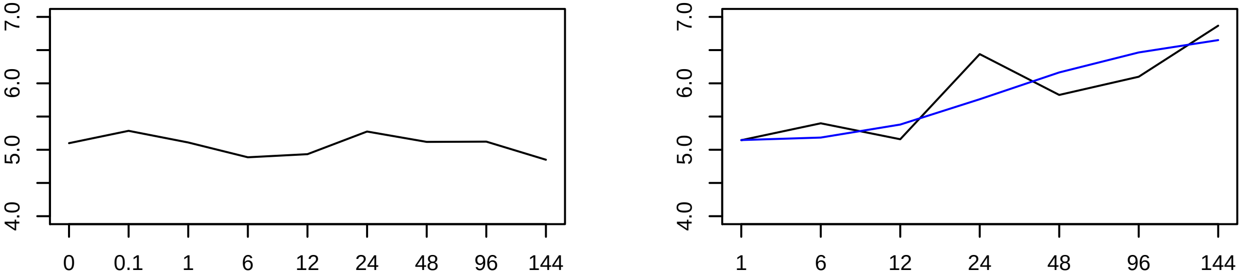

A_24_P247175 LOC649003 1q42.11

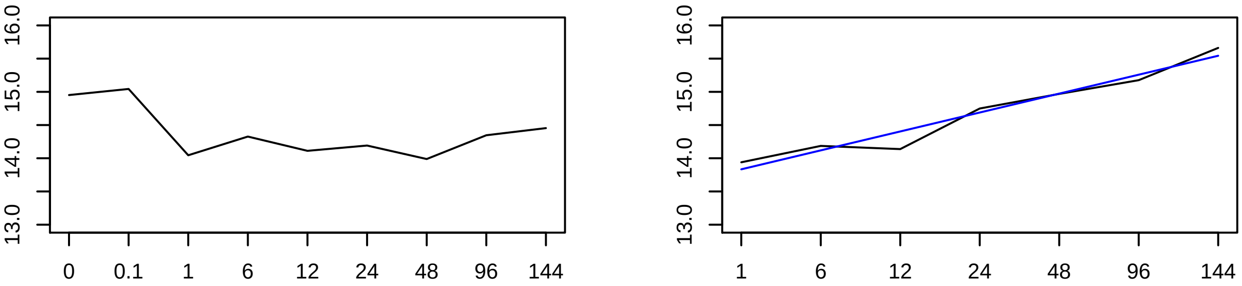

A_24_P880043 PCGF5 10q23.32

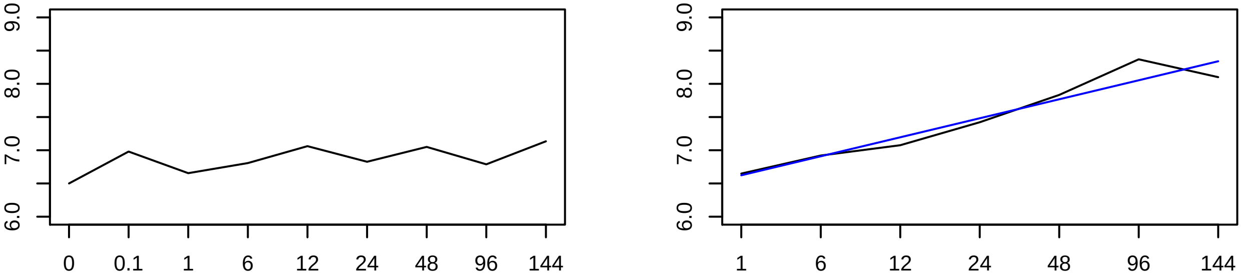

A_23_P205686 PSEN1 14q24.2

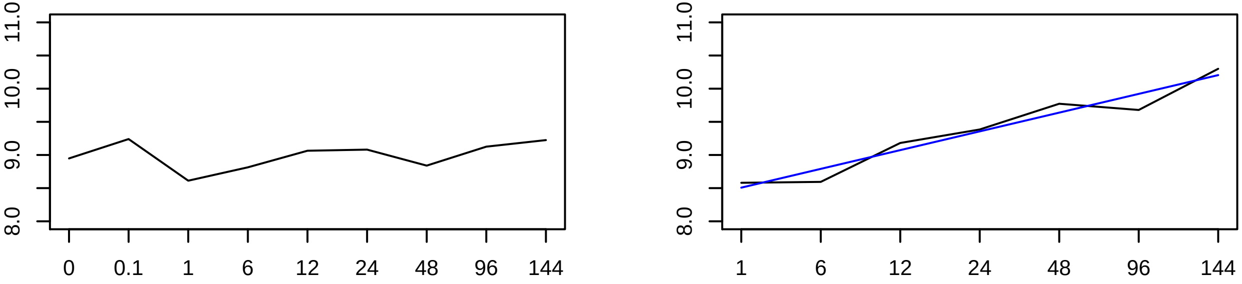

A_24_P83118 DUSP18 22q12.2

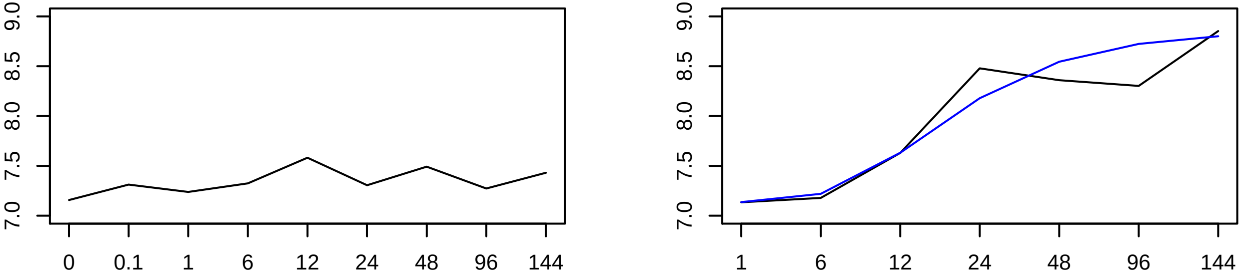

A_24_P281975 GNPTAB 12q23.2

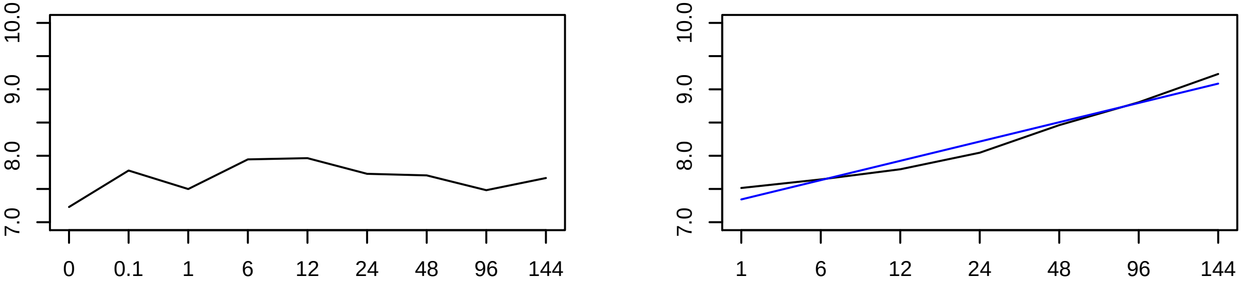

A_23_P53724 CLSTN3 12p13.31

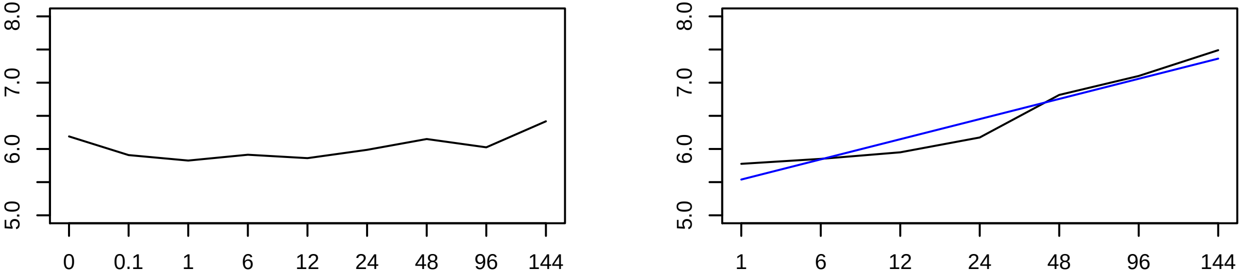

A_23_P257971 AKR1C1 10p15.1

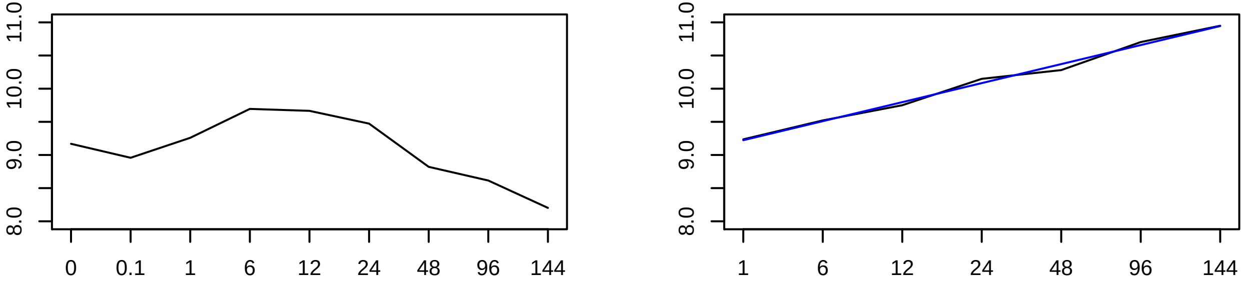

A_23_P134085 CNKSR3 6q25.2

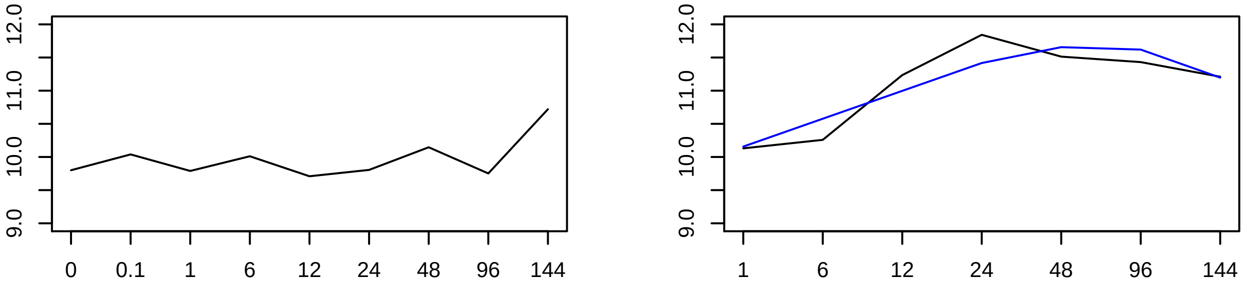

A_23_P345820 WDFY3 4q21.23

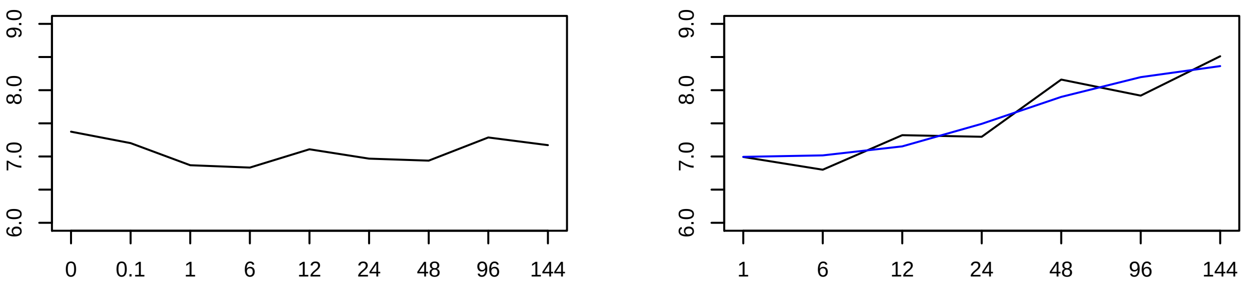

A_24_P355006 ADAM22 NA

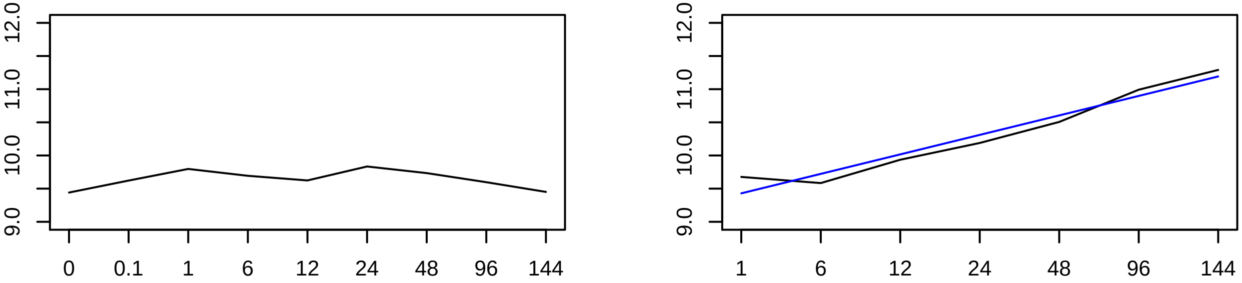

A_24_P173754 C1orf21 1q25.3

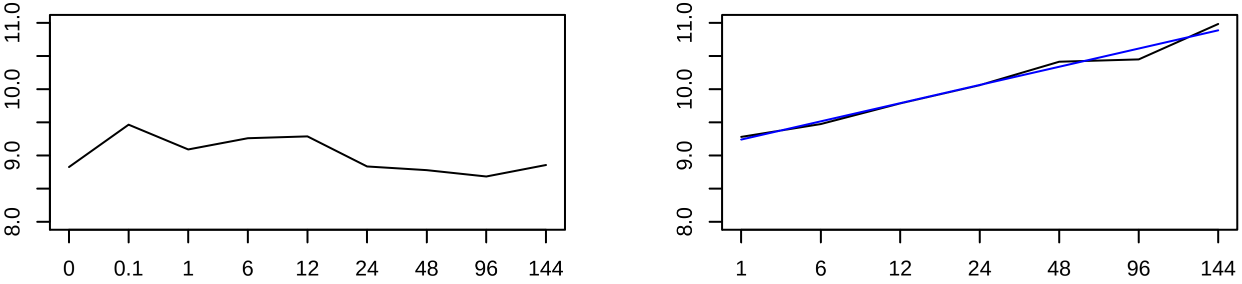

A_23_P49391 TP53TG3 16p11.2

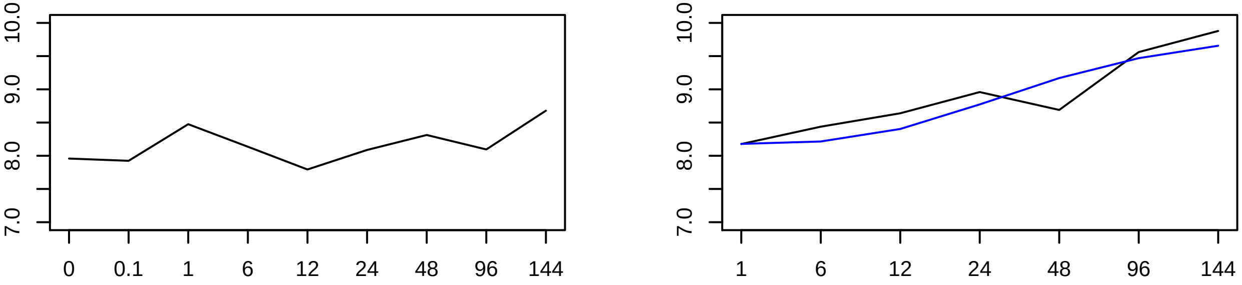

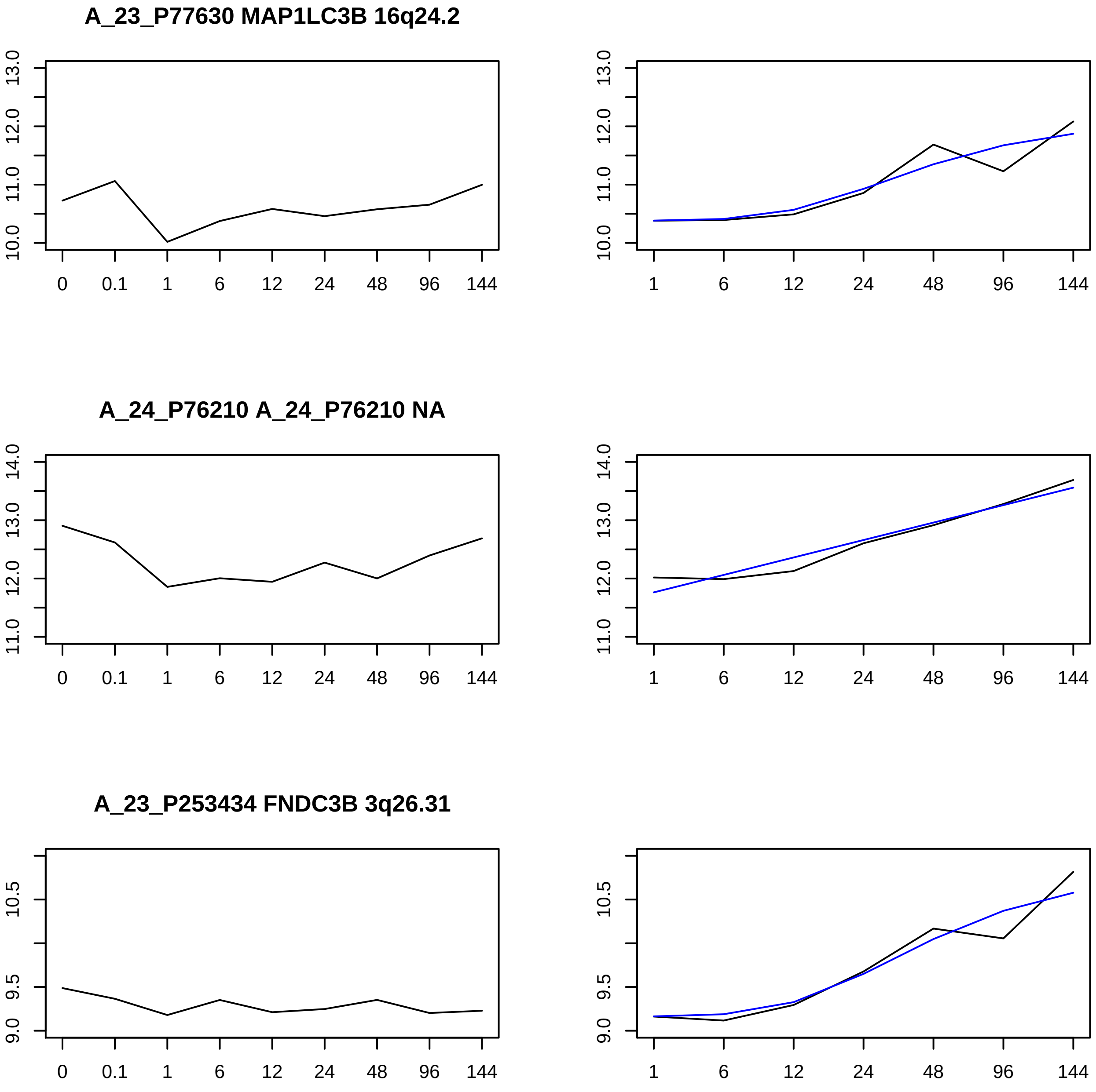
A_23_P77630 MAP1LC3B 16q24.2
10.0
11.0
12.0
13.0
0
0.1
1
6
12
24
48
96
144
A_24_P76210 A_24_P76210 NA
11.0
12.0
13.0
14.0
A_23_P253434 FNDC3B 3q26.31
9.0
9.5
10.5

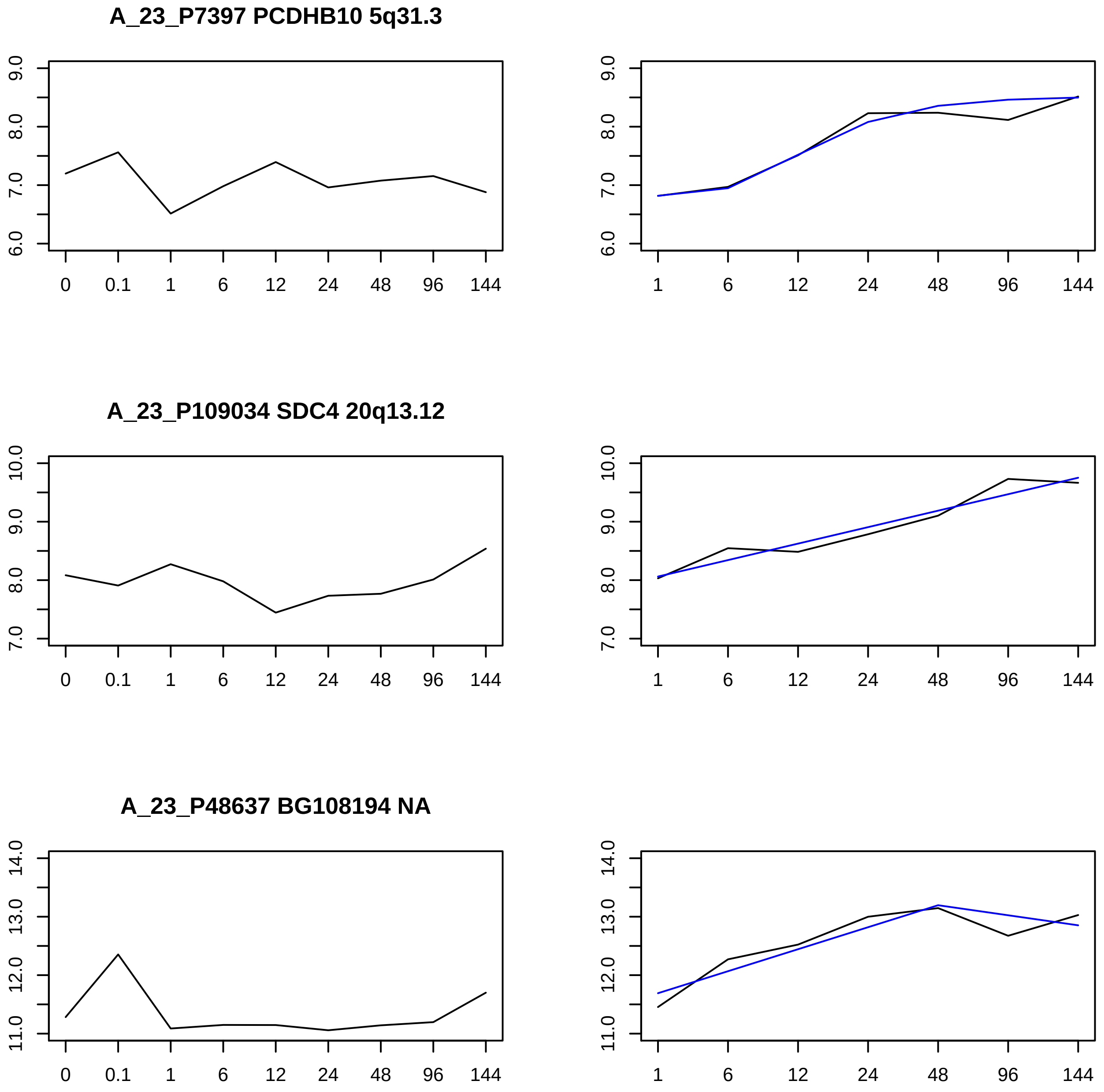

A_23_P7397 PCDHB10 5q31.3
A_23_P109034 SDC4 20q13.12
A_23_P48637 BG108194 NA
0
0.1
1
6
12
24
48
96
144

A_23_P167401 PCDHB11 5q31.3

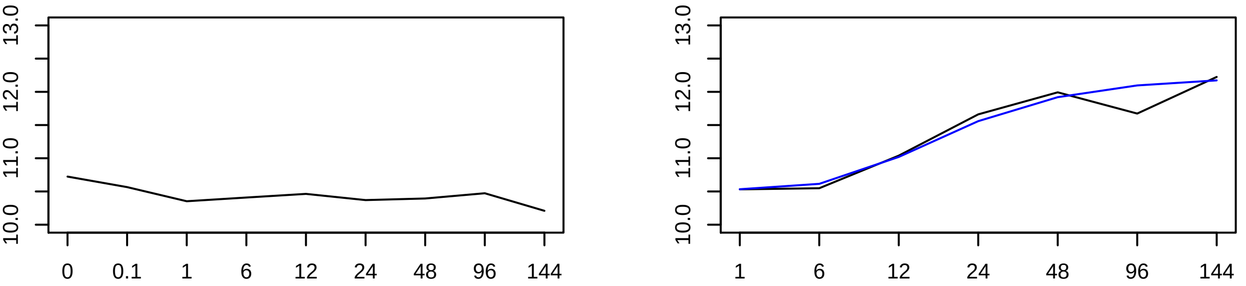

A_23_P212089 NFKBIZ 3q12.3

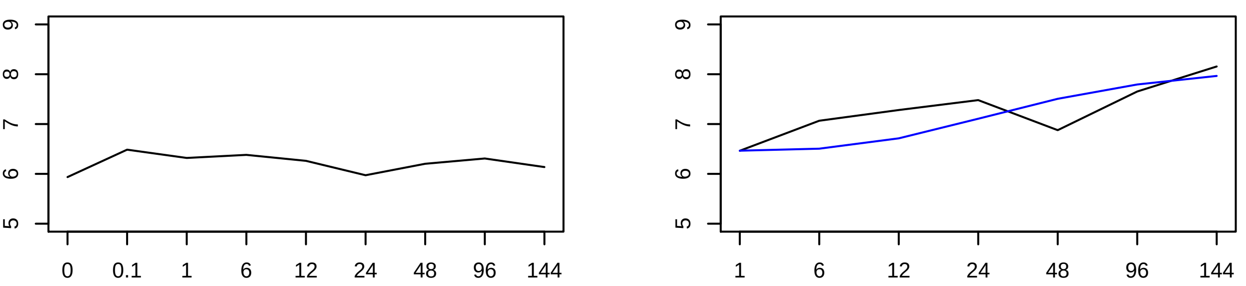

A_24_P417959 C14orf43 14q24.3

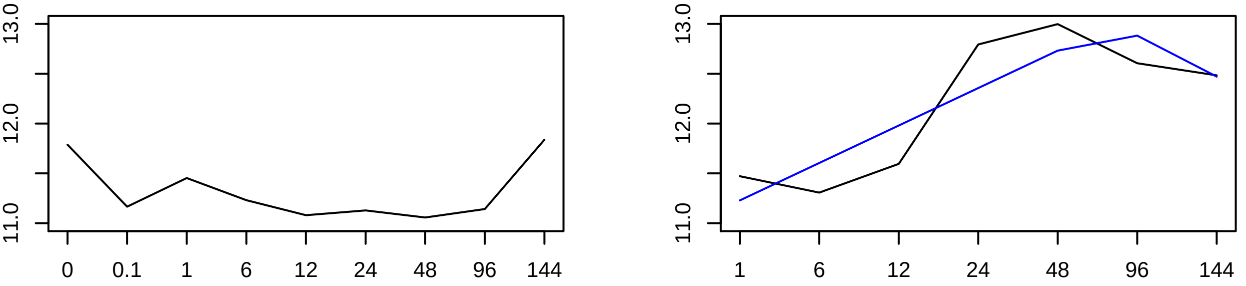

A_23_P67618 ZNF792 19q13.11

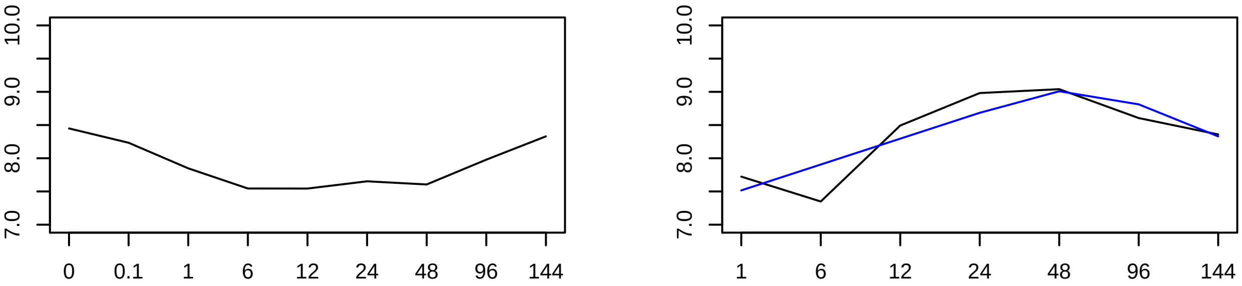

A_23_P257201 RNF146 6q22.33

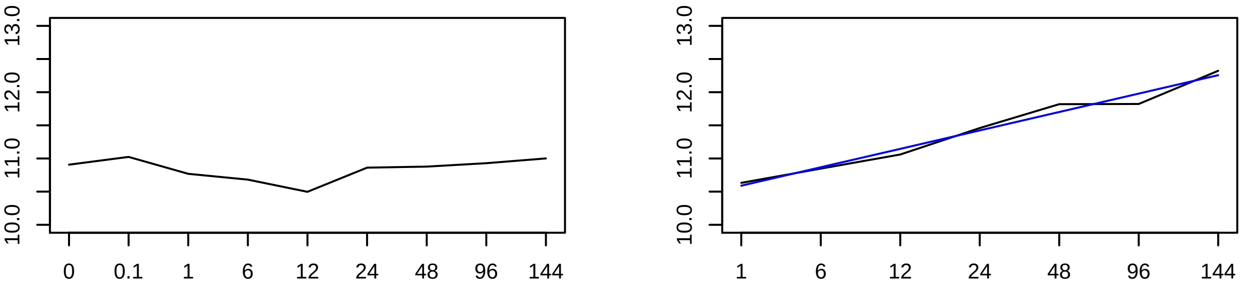

A_23_P332399 GULP1 2q32.2

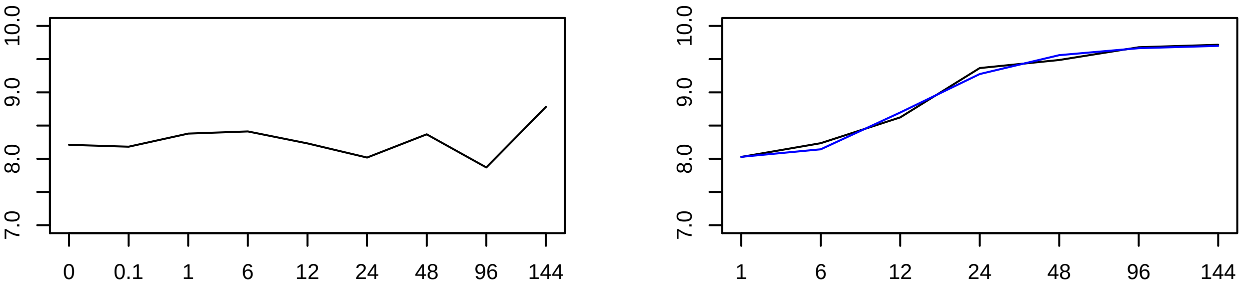

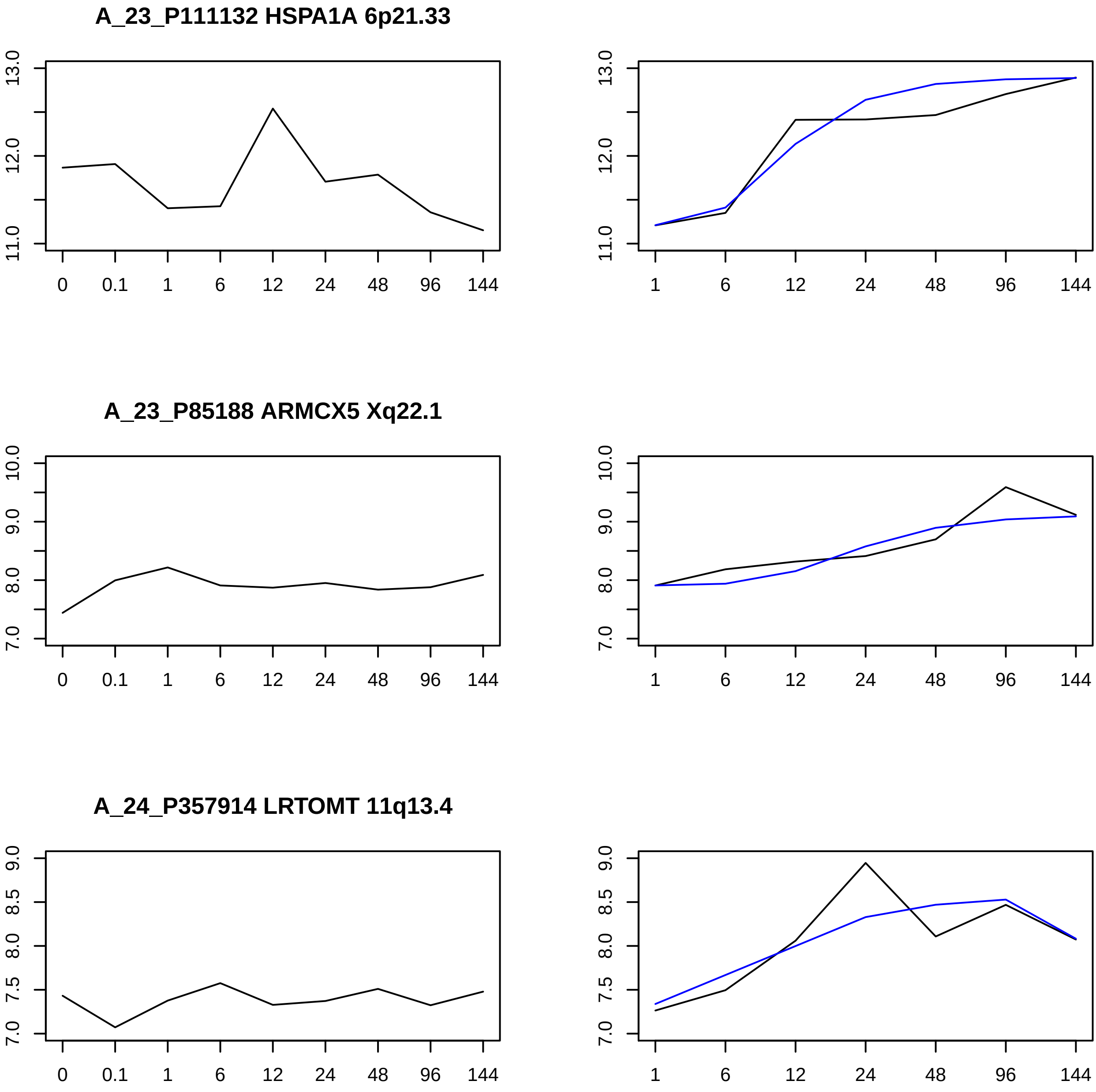

A_23_P111132 HSPA1A 6p21.33
A_23_P85188 ARMCX5 Xq22.1
A_24_P357914 LRTOMT 11q13.4
0
0.1
1
6
12
24
48
96
144

A_23_P65262 N4BP2L2 13q13.1

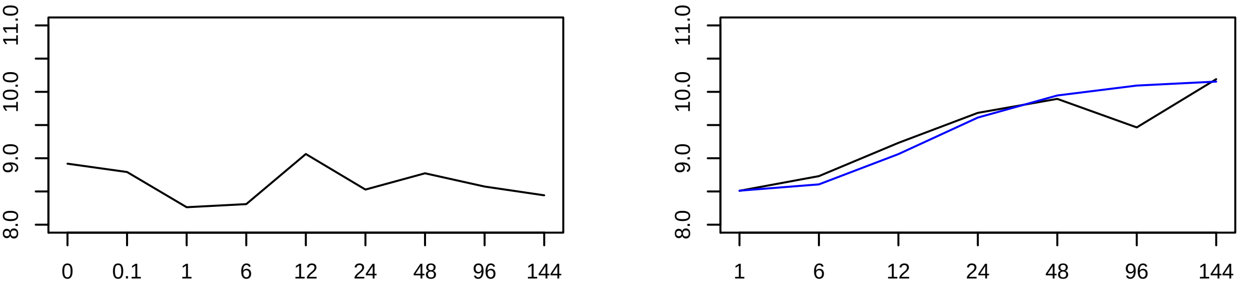

A_23_P5376 IDH1 2q33.3

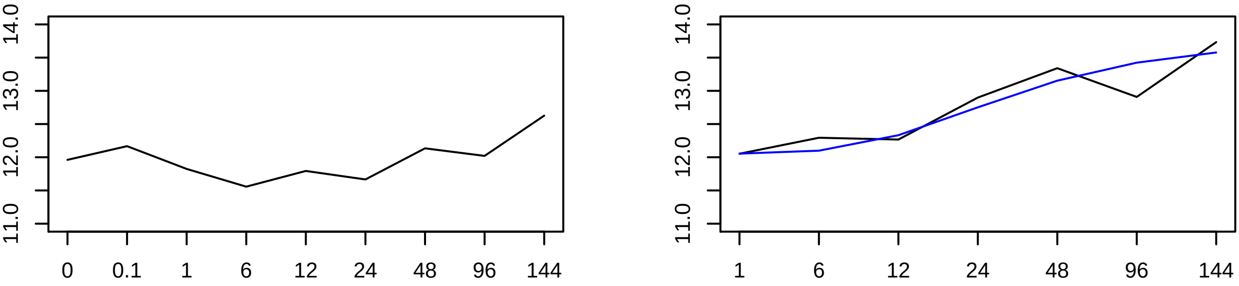

A_24_P108005 MAP1LC3B 16q24.2

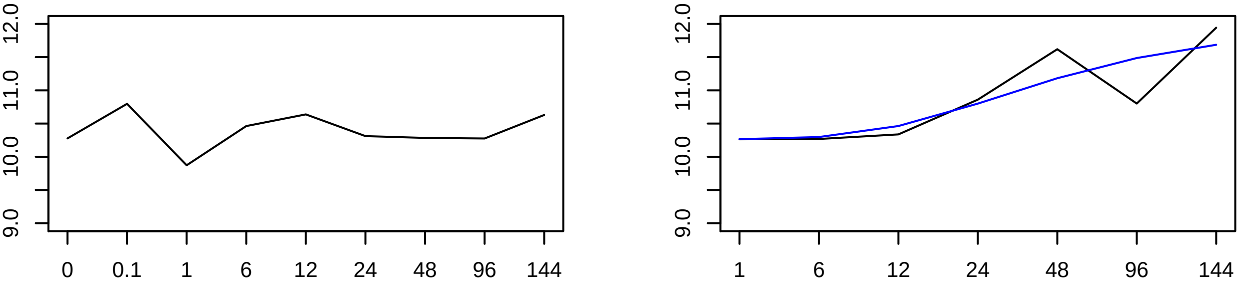

A_23_P212339 FYCO1 3p21.31

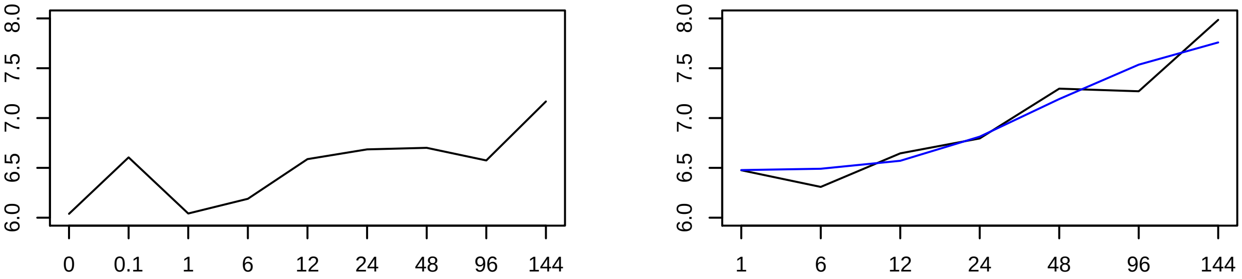

A_23_P171237 ACRC Xq13.1

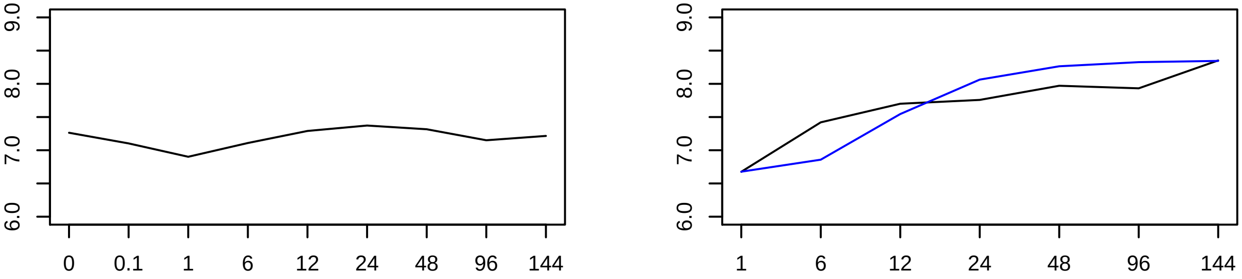

A_23_P16733 RALB 2q14.2

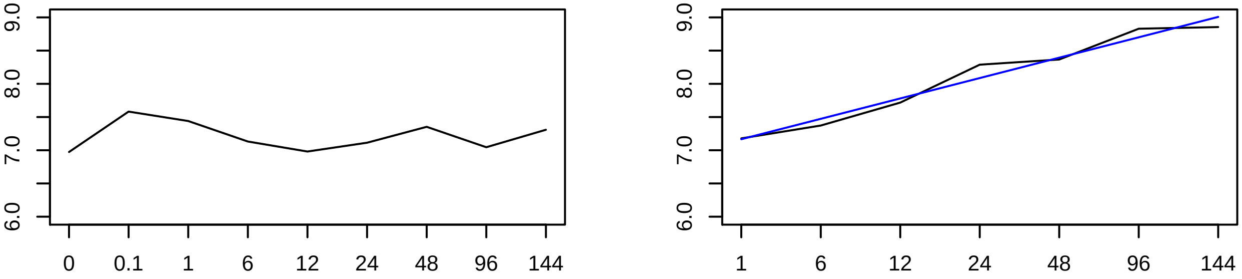

A_24_P305541 TRIB3 20p13

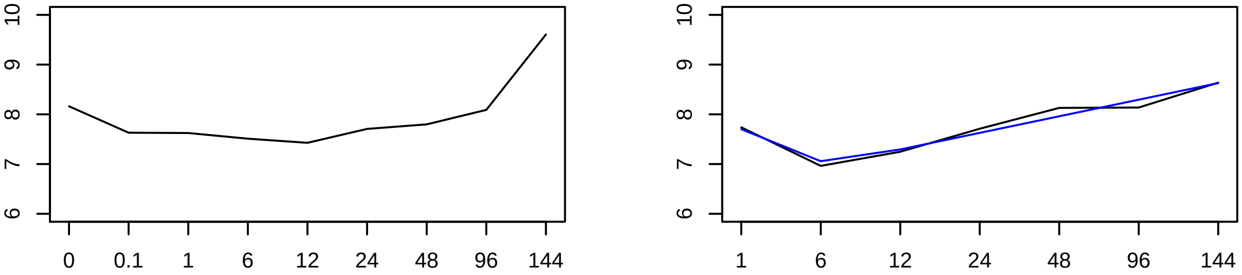

A_24_P148750 SH3BP5 3p24.3

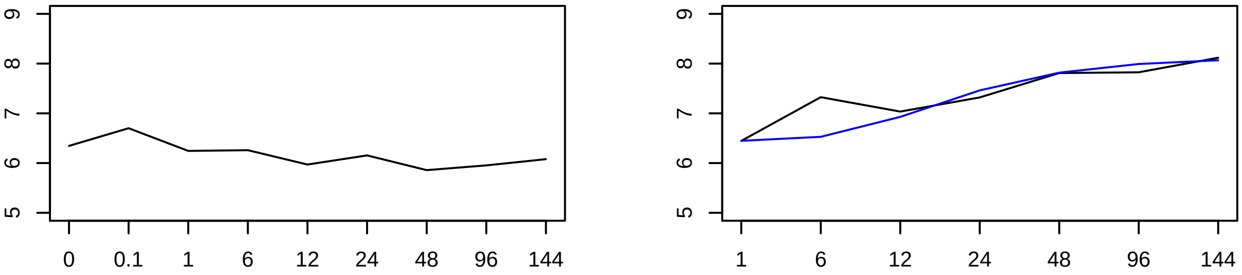

A_32_P162862 AY831680 NA

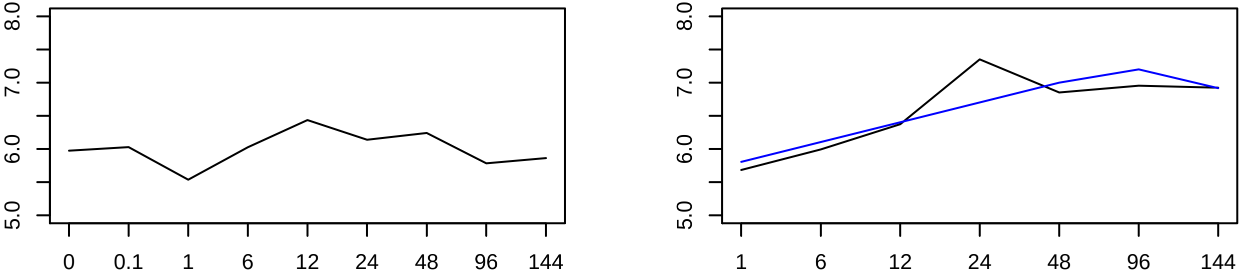

A_23_P5281 LYL1 19p13.13

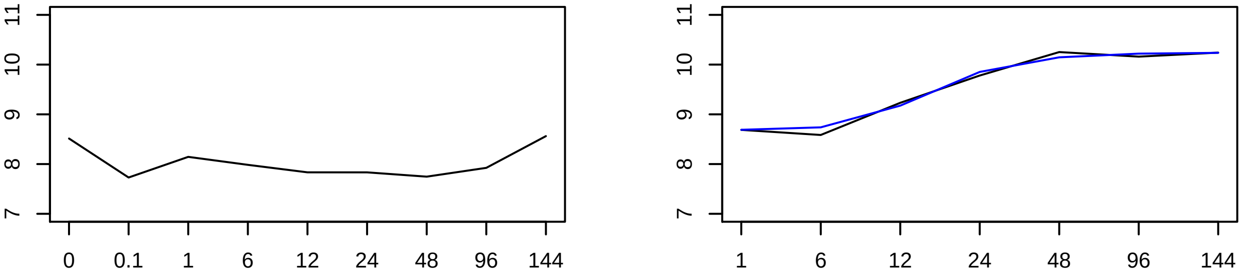

A_23_P101905 APC2 19p13.3

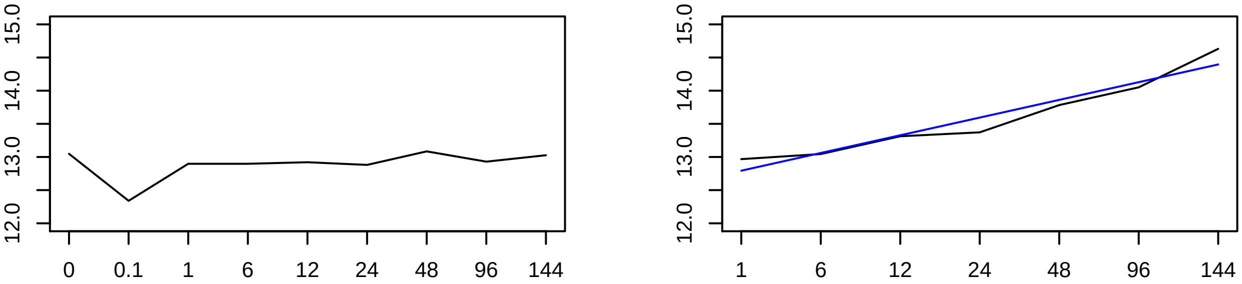

A_23_P418083 C6orf152 6q14.1

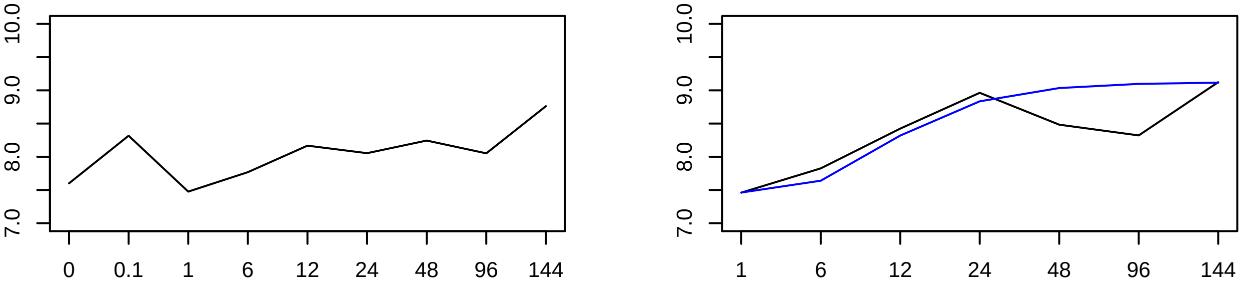

A_23_P369574 CLASP2 3p22.3

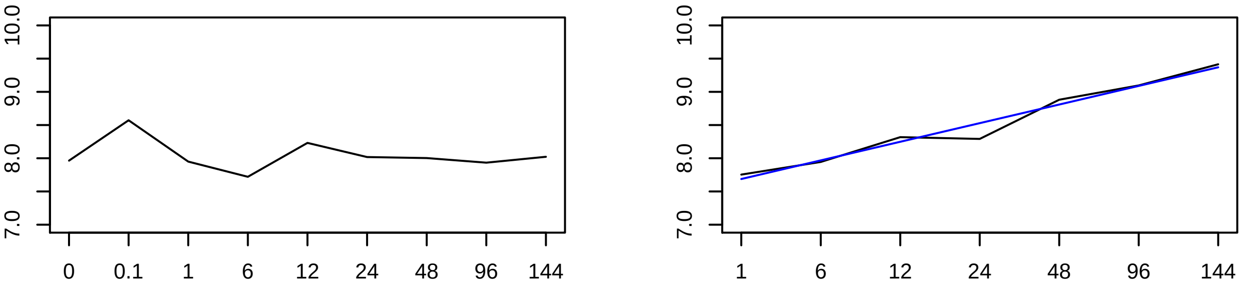

A_24_P933458 NAV2 11p15.1

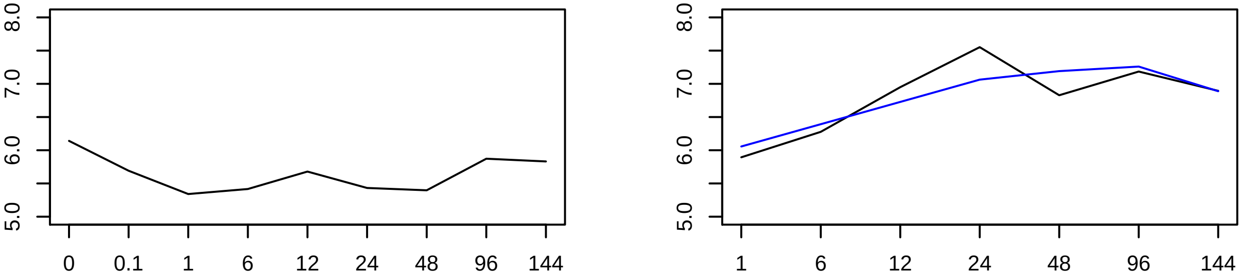

A_23_P502913 WDR1 4p16.1

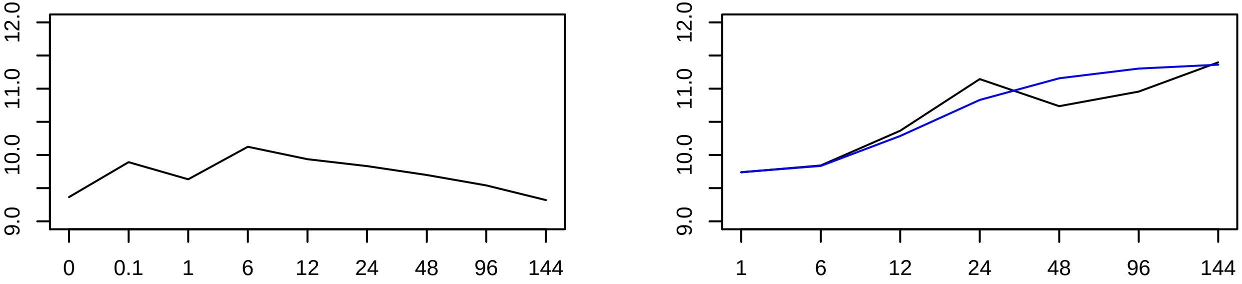

A_32_P147969 THC2526509 NA

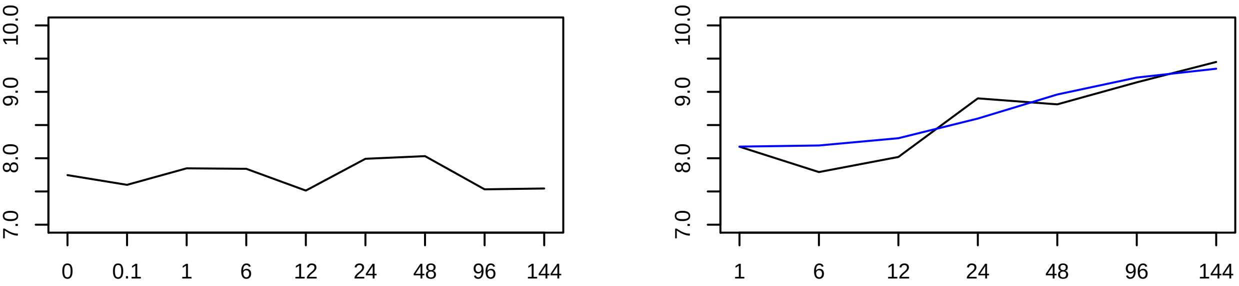

A_24_P41882 PDLIM7 5q35.3

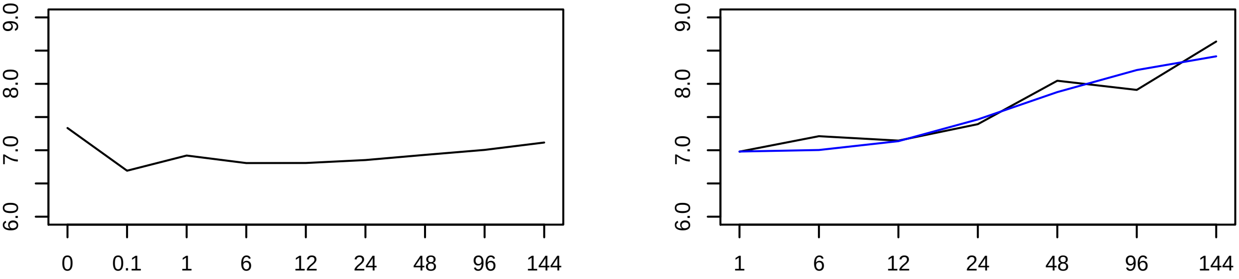

A_23_P39465 BST2 19p13.11

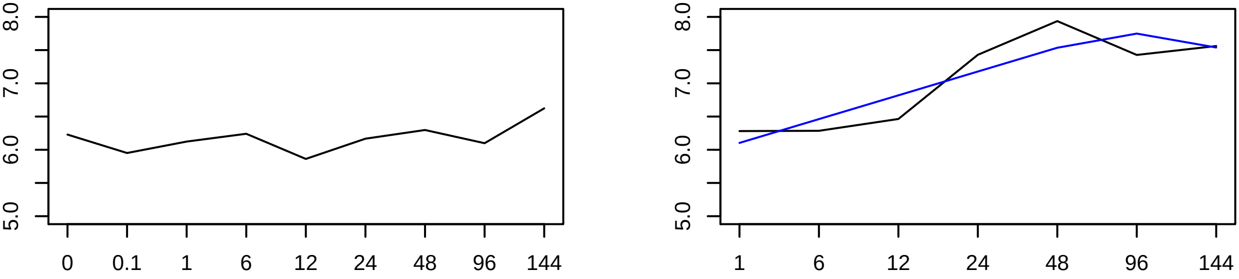

A_23_P159211 AK123096 NA

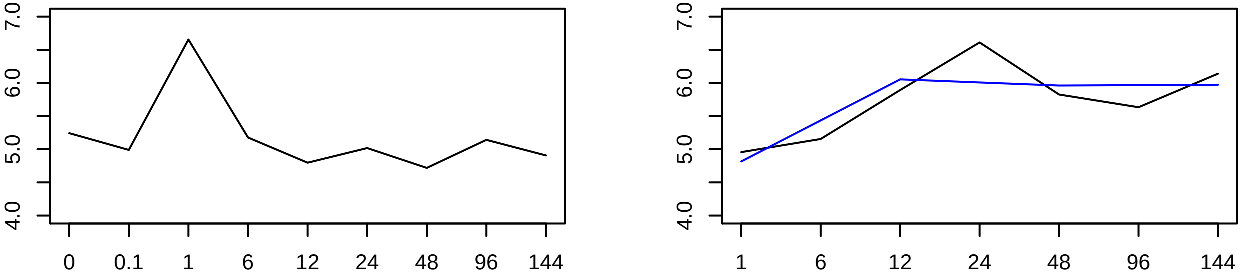

A_23_P122464 ZNF193 6p22.1

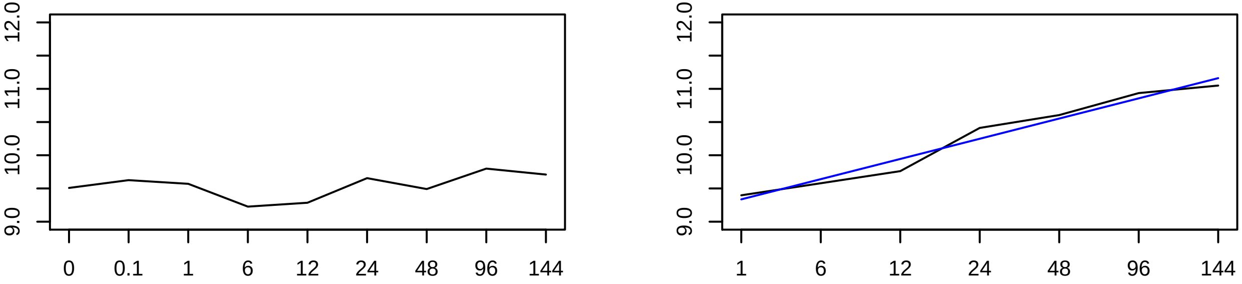

A_24_P179407 FGD6 12q22

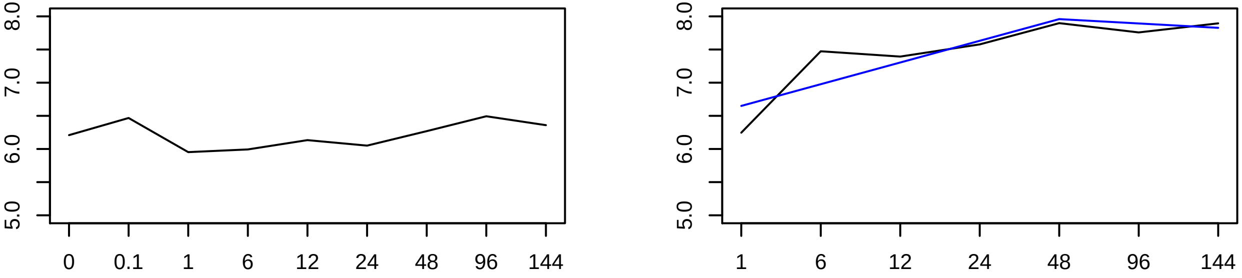

A_32_P151152 THC2593596 NA

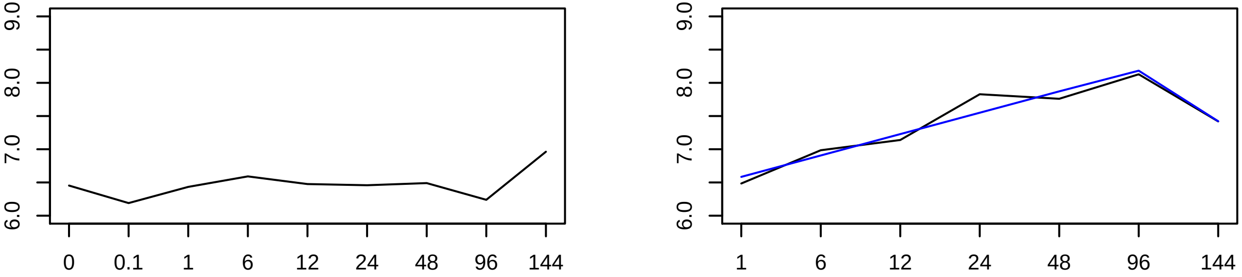

A_23_P32029 SLC35D2 9q22.32

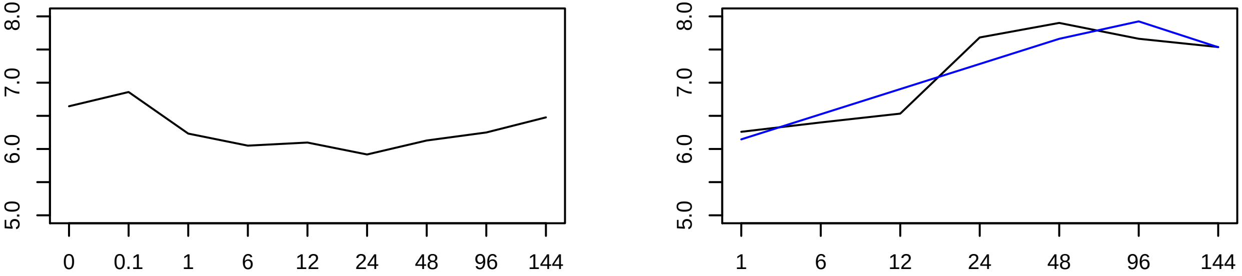

A_23_P65481 TEP1 14q11.2

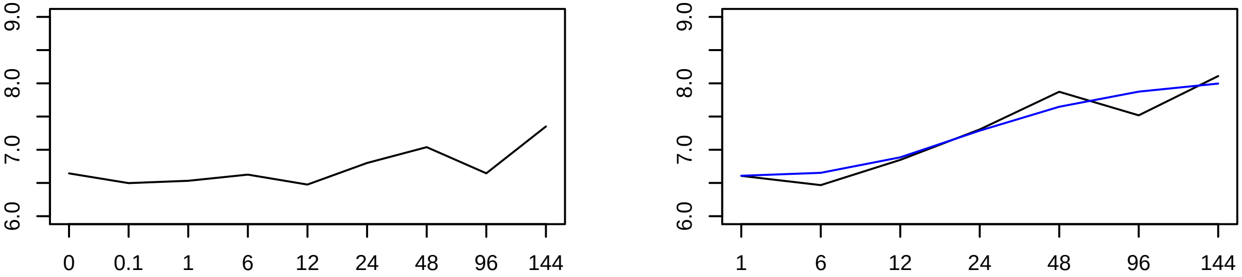

A_23_P399169 HIC1 17p13.3

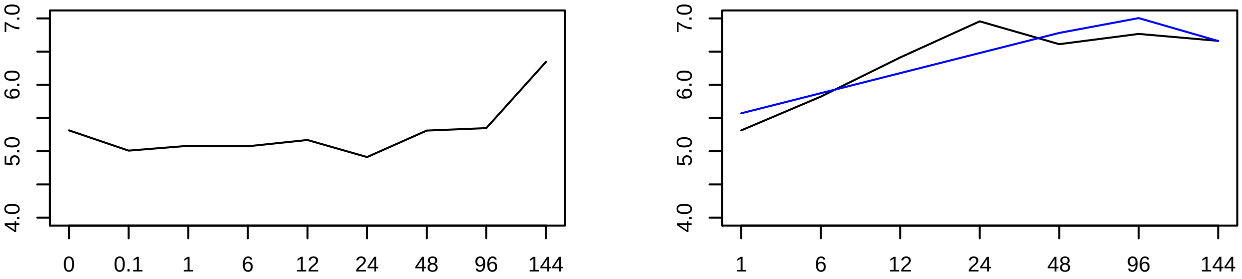

A_32_P47754 SLC2A14 12p13.31

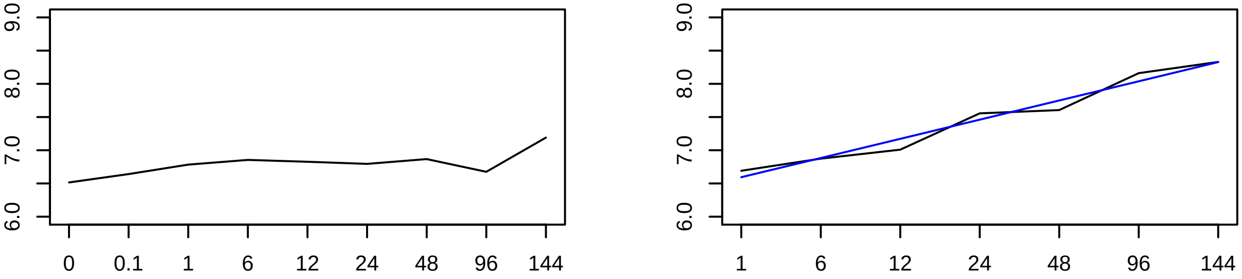

A_23_P425880 TRIO 5p15.2

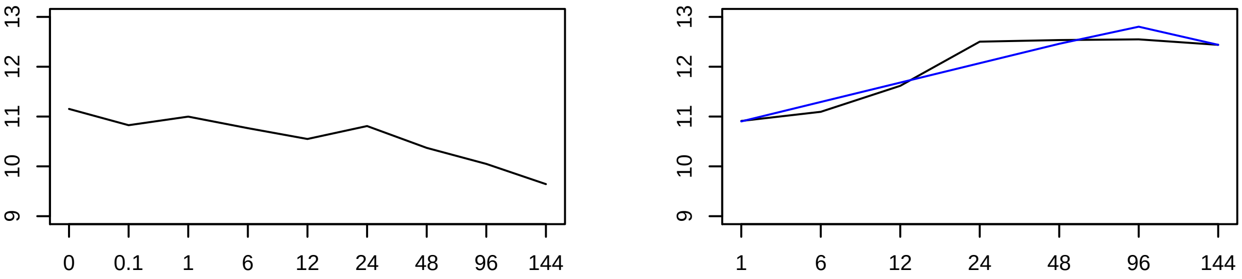

A_23_P44295 CLASP2 3p22.3

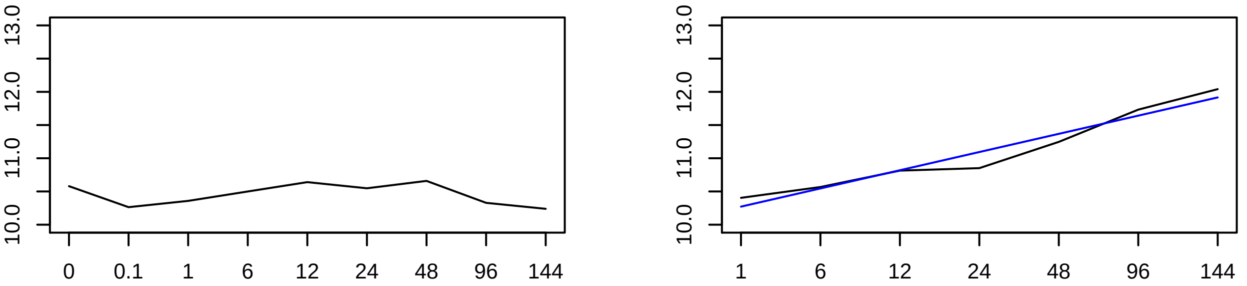

A_24_P389916 LRRC32 11q13.5

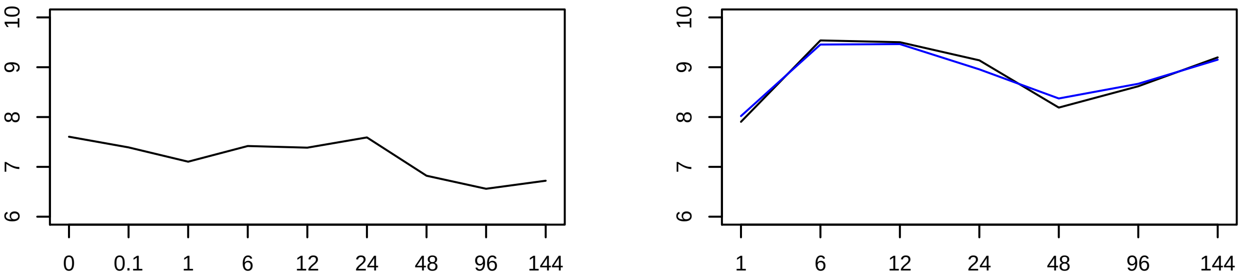

A_23_P201319 DISP1 1q41

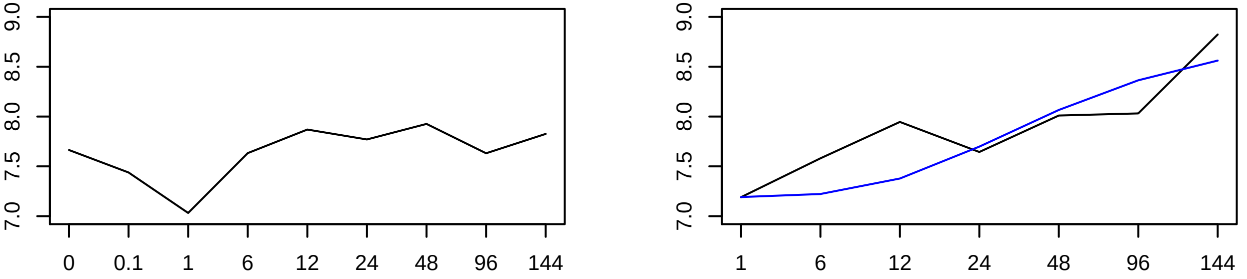

A_23_P20196 ARPC1B 7q22.1

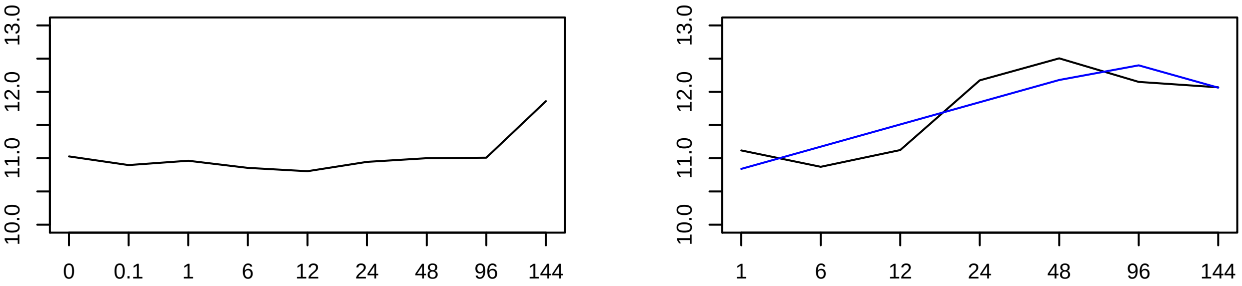

A_23_P52634 PANX1 11q21

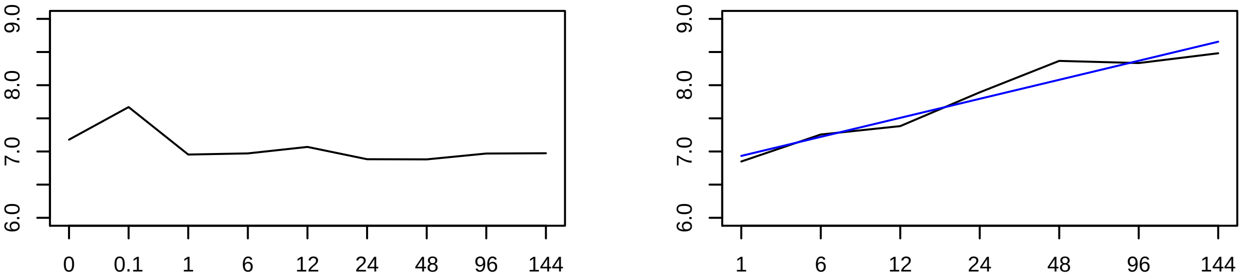

A_32_P45009 IDH1 2q33.3

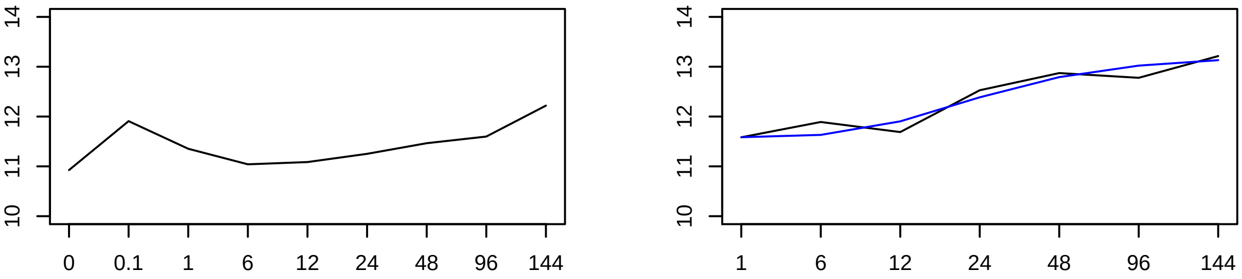

A_23_P87049 SORL1 11q24.1

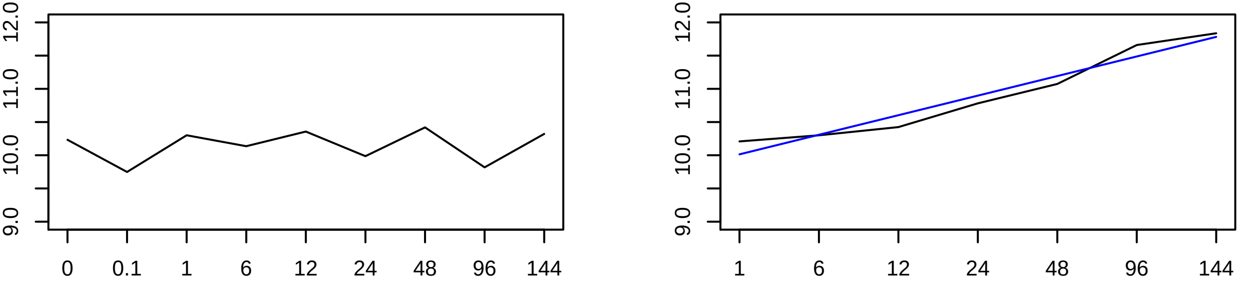

A_23_P210900 ACSS2 20q11.22

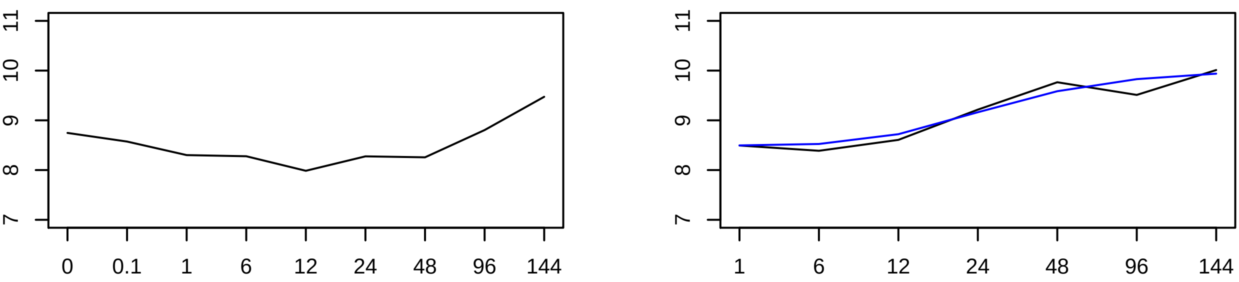

A_23_P151710 PTGER2 14q22.1

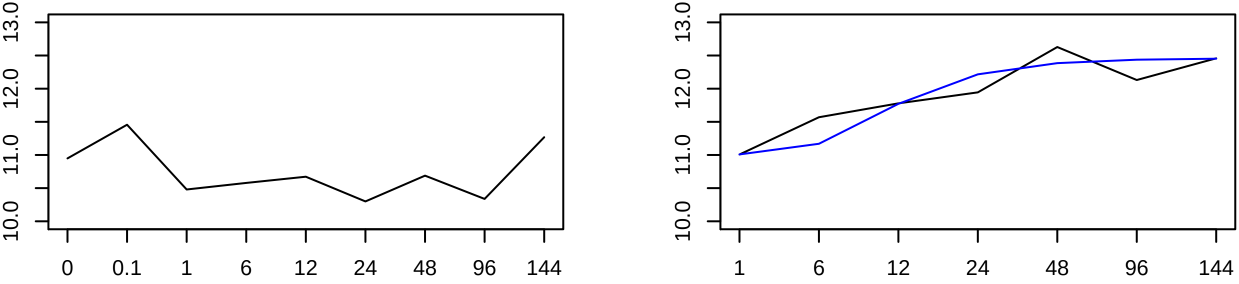

A_24_P37253 LYPD6 2q23.2

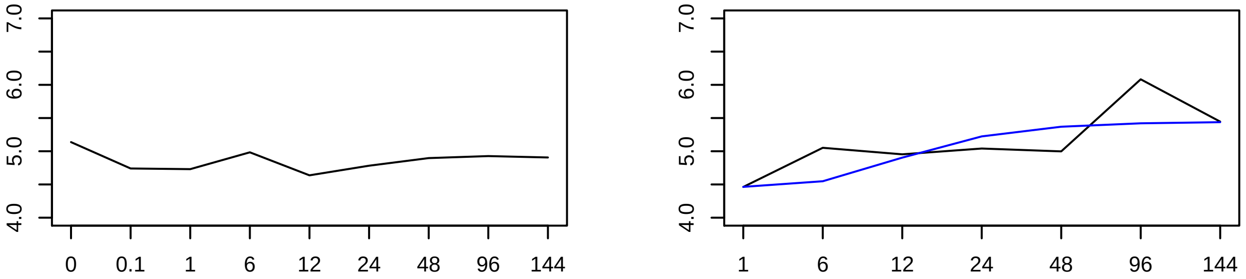

A_24_P152968 AKR1C1 10p15.1

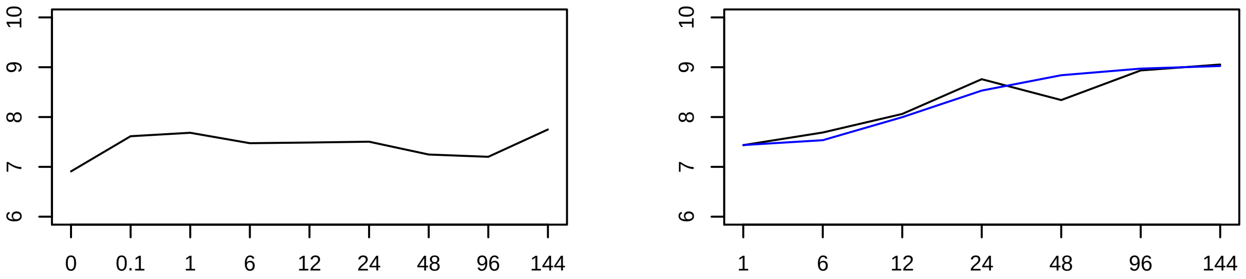

A_24_P890536 LOC728649 9q12

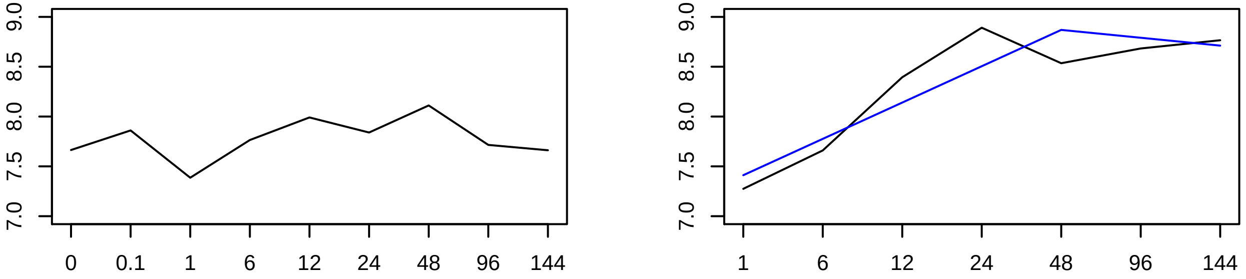

A_23_P116602 USP35 11q14.1

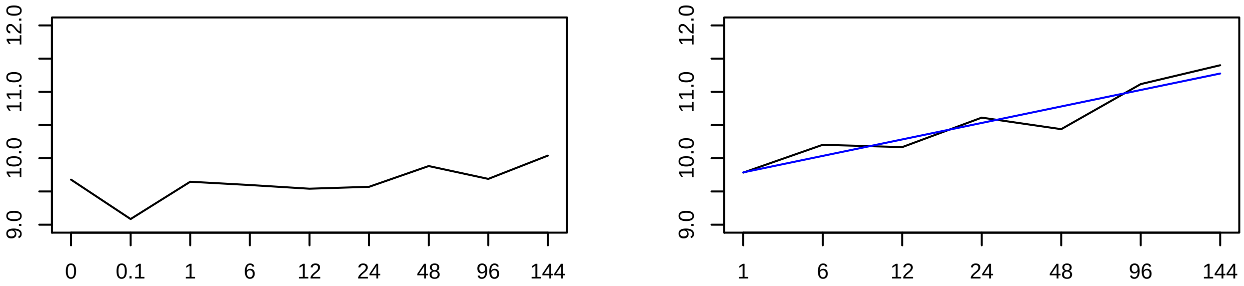

A_23_P32036 C9orf95 9q21.13

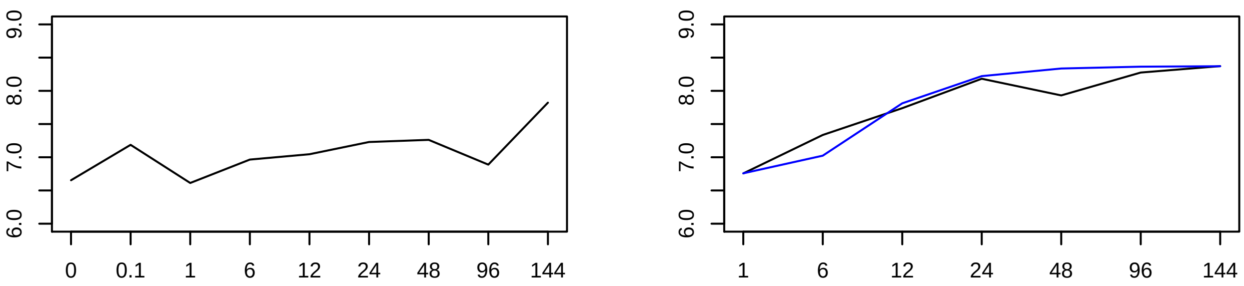

A_32_P18034 THC2708710 NA

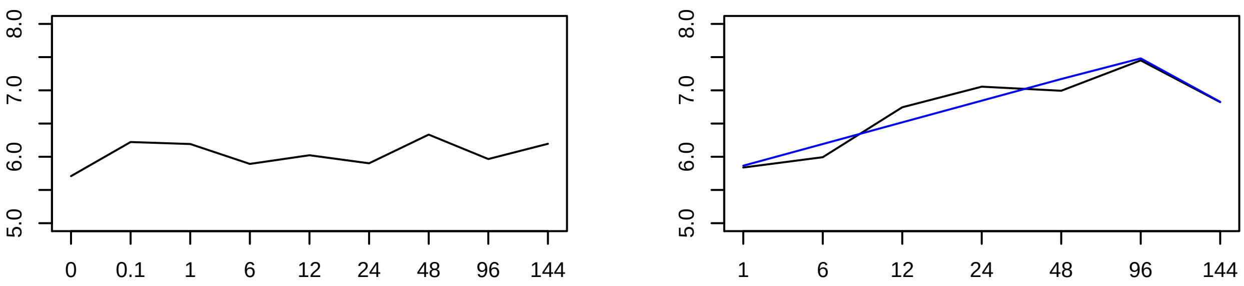

A_24_P460763 AK022443 NA

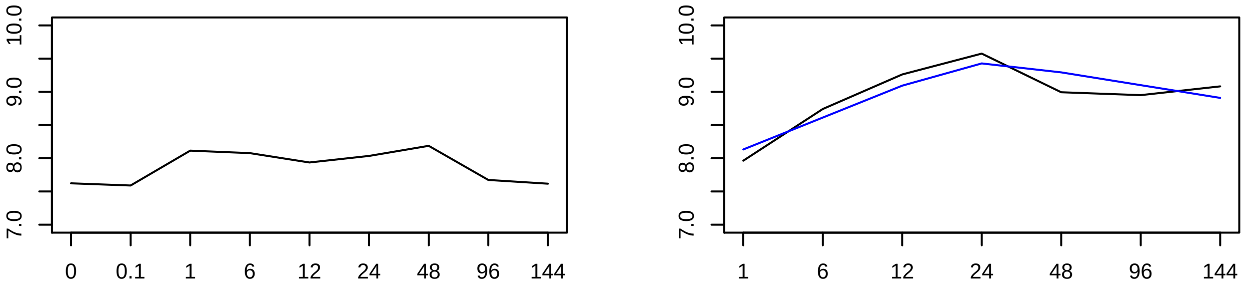

A_23_P114057 SEMA4C 2q11.2

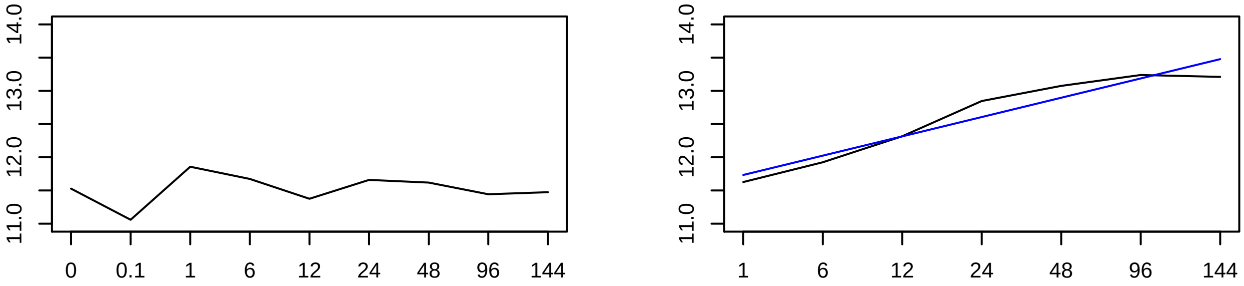

A_23_P316601 RIT1 1q22

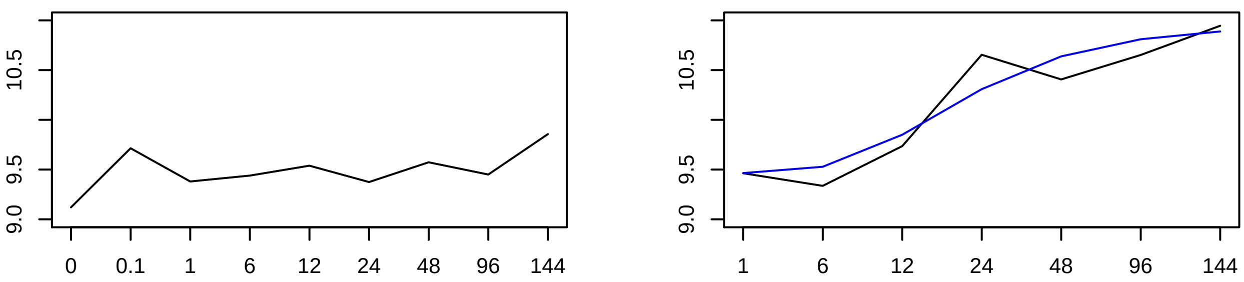

A_23_P55682 ZNF447 19q13.43

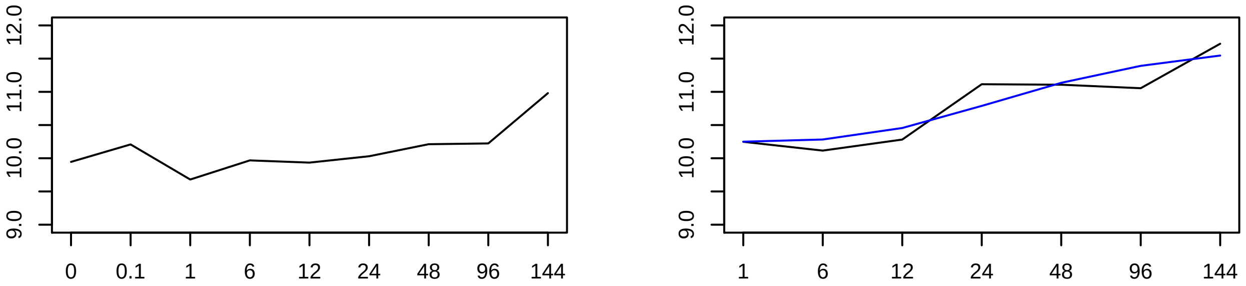

A_32_P144018 BC004962 NA

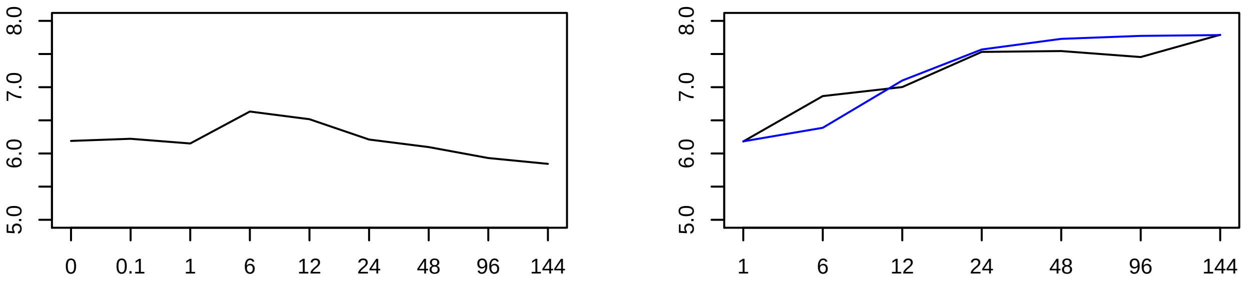

A_23_P147423 ADAMTS9 3p14.1

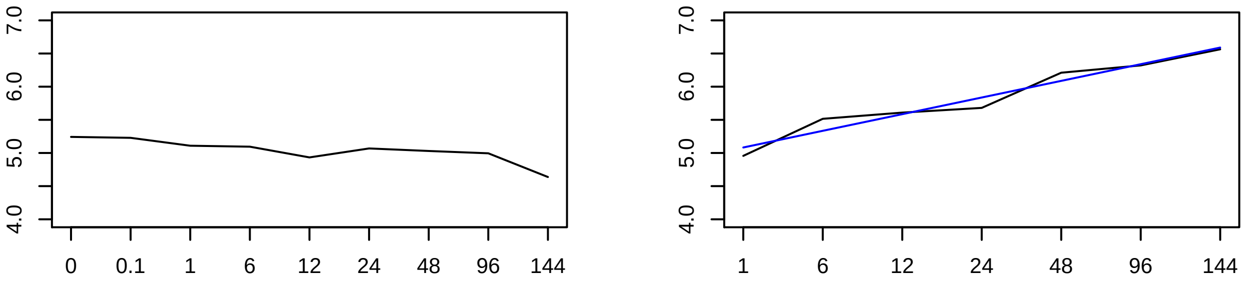

A_23_P361049 MYO1B 2q32.3

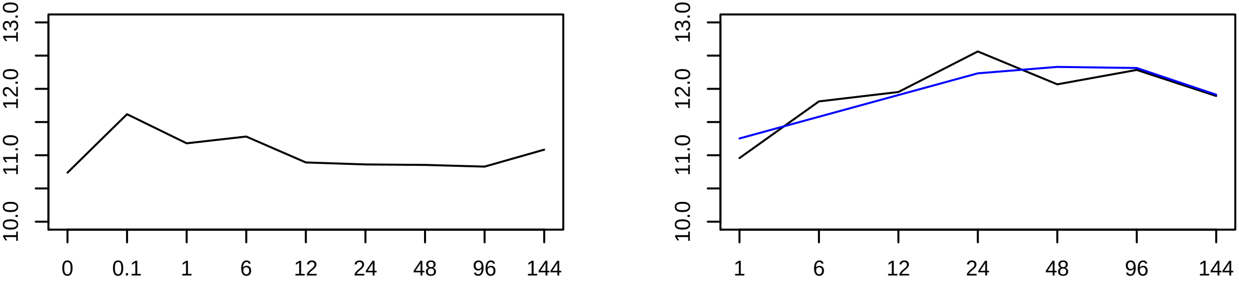

A_23_P108734 ALLC 2p25.3

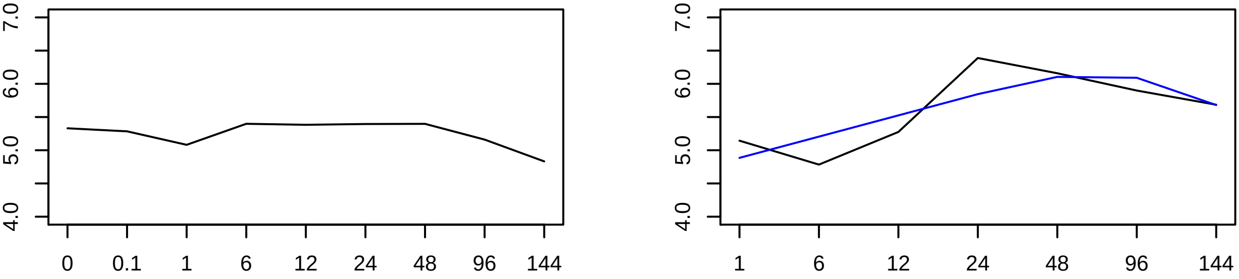

A_23_P153155 GALR1 18q23

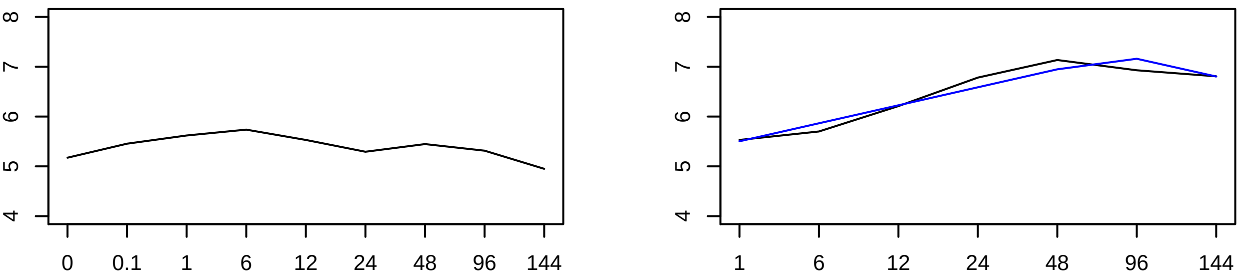

A_24_P798709 NPAL2 8q22.2

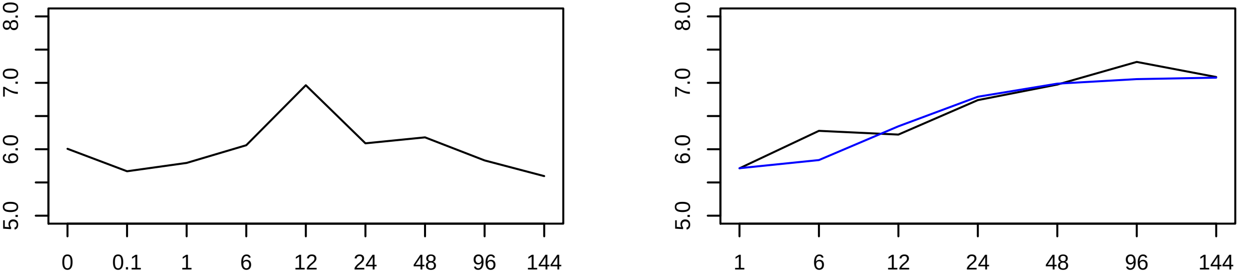

A_23_P205713 STXBP6 14q12

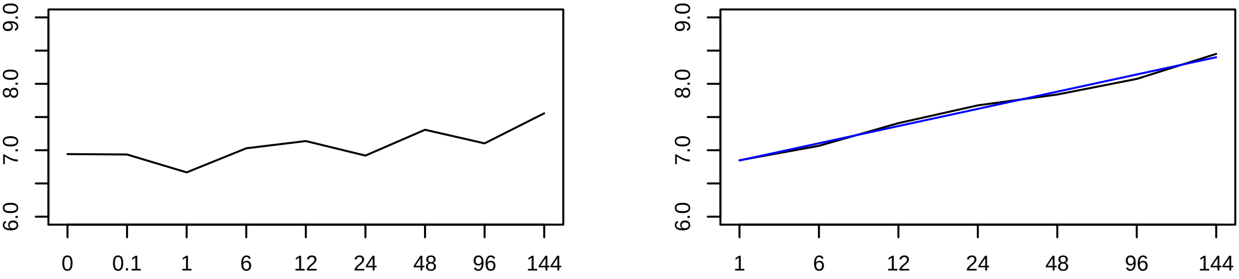

A_32_P161913 TMEM240 1p36.33

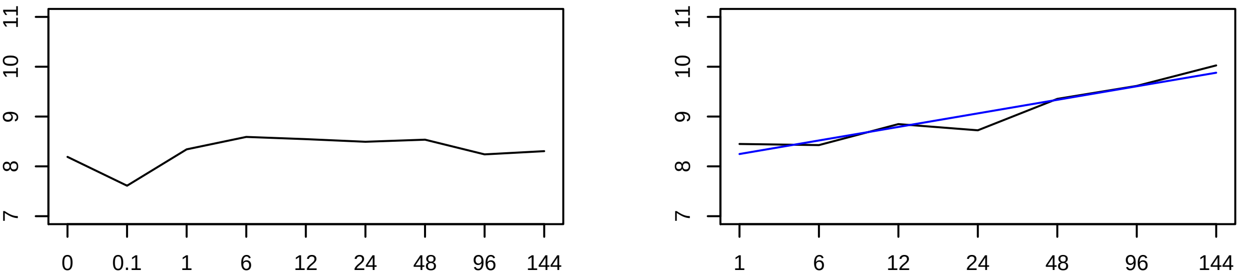

A_23_P37205 NDRG2 14q11.2

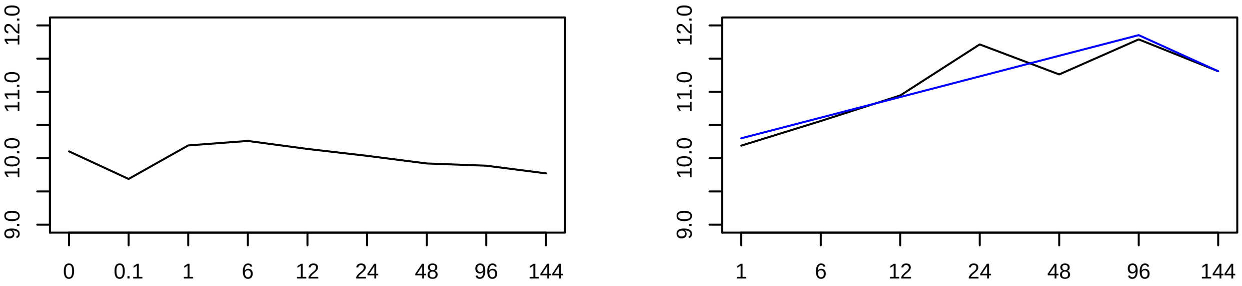

A_23_P97309 CASP9 1p36.21

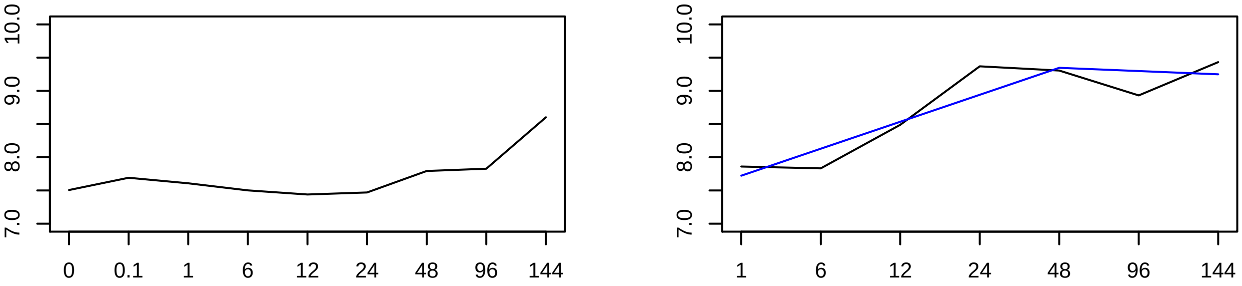

A_23_P94255 TRPA1 8q13.3

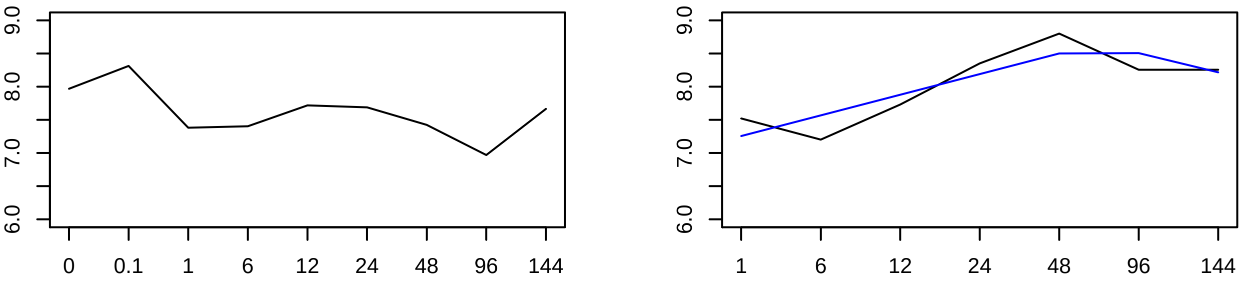

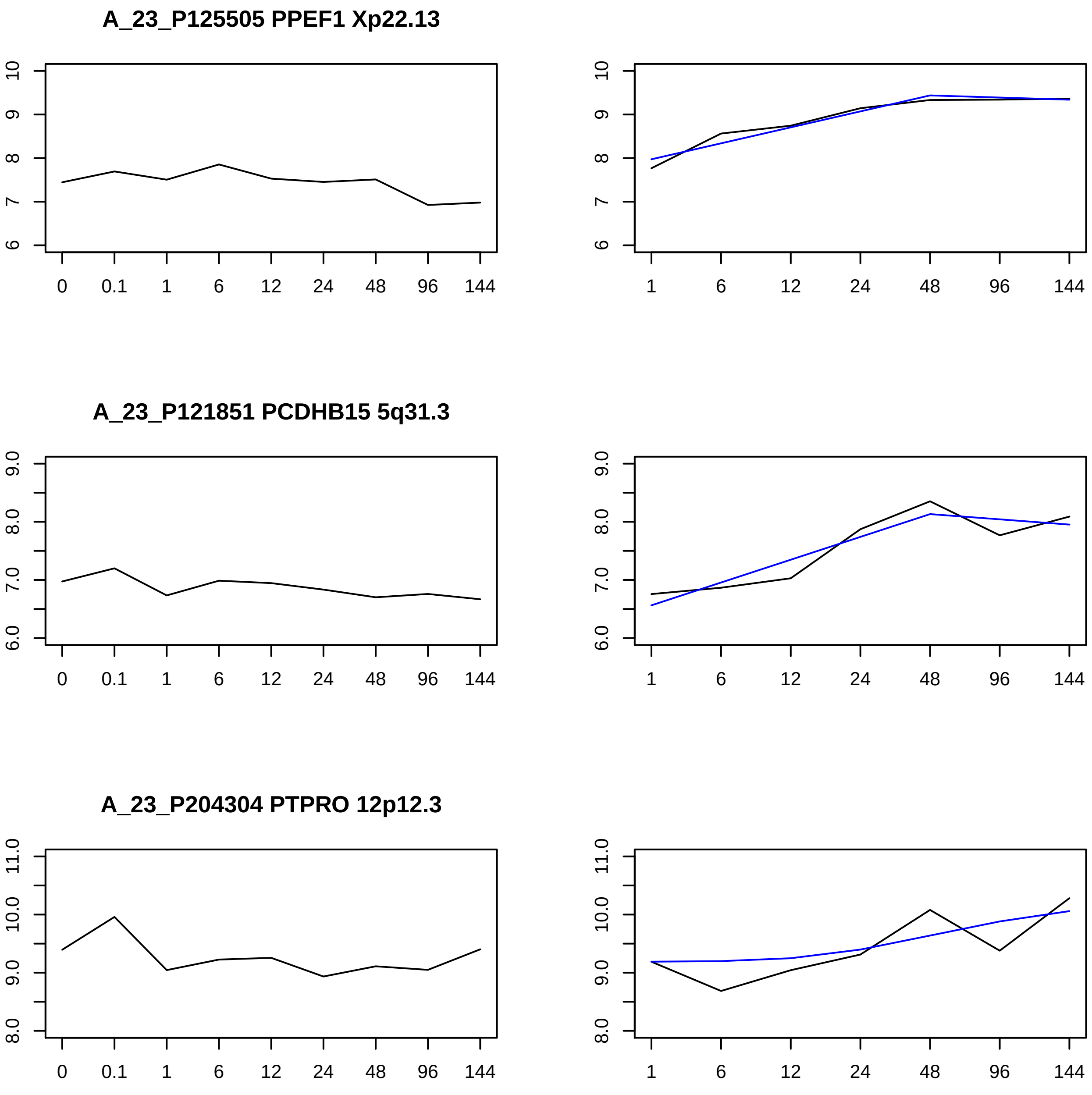
A_23_P125505 PPEF1 Xp22.13
6
7
8
9
10
0
0.1
1
6
12
24
48
96
144
A_23_P121851 PCDHB15 5q31.3
6.0
7.0
8.0
9.0
A_23_P204304 PTPRO 12p12.3
8.0
9.0
10.0
11.0

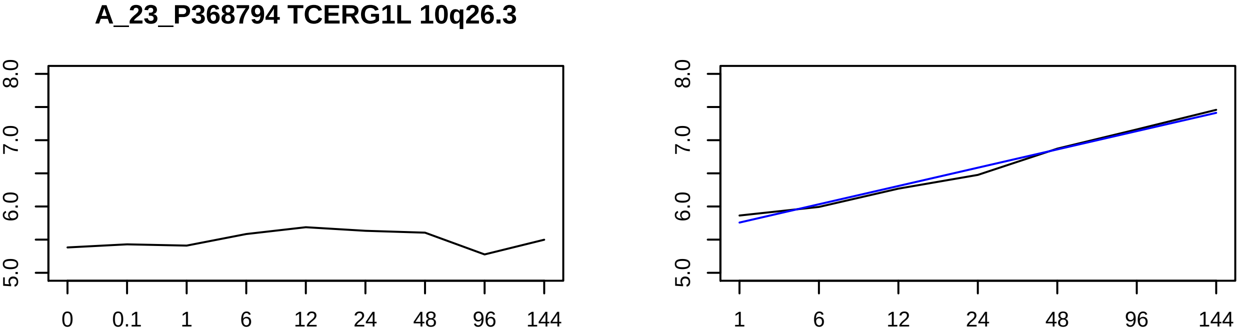

A_23_P368794 TCERG1L 10q26.3
5.0
6.0
7.0
8.0
0
0.1
1
6
12
24
48
96
144

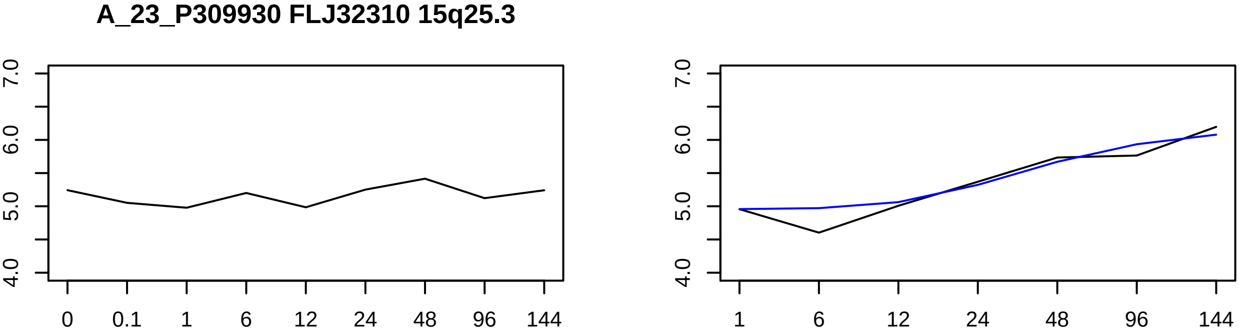

A_23_P309930 FLJ32310 15q25.3
4.0
5.0
6.0
7.0
0
0.1
1
6
12
24
48
96
144

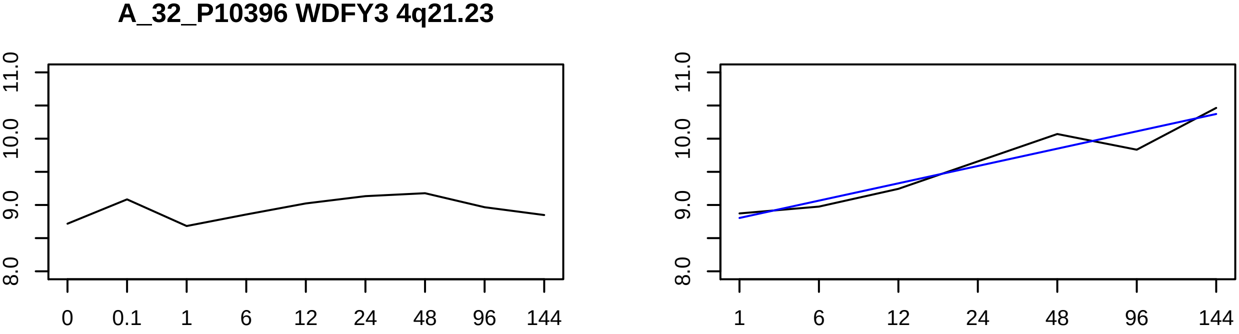

A_32_P10396 WDFY3 4q21.23
8.0
9.0
10.0
11.0
0
0.1
1
6
12
24
48
96
144

A_23_P143127 EML4 2p21

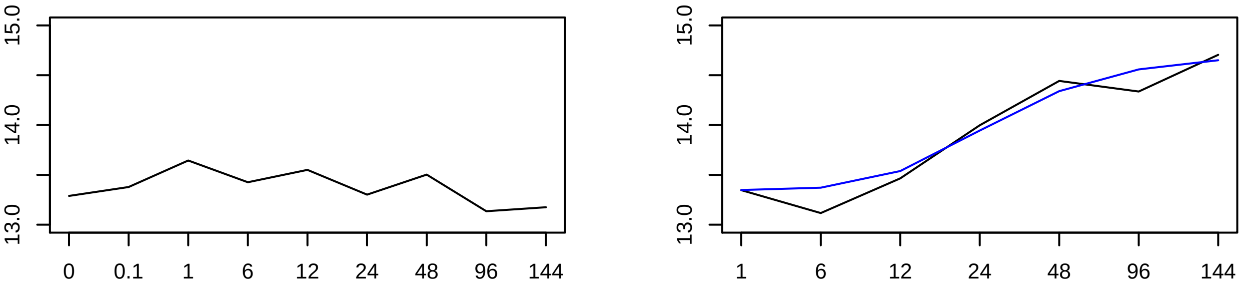

A_32_P194115 UNC84B 22q13.1

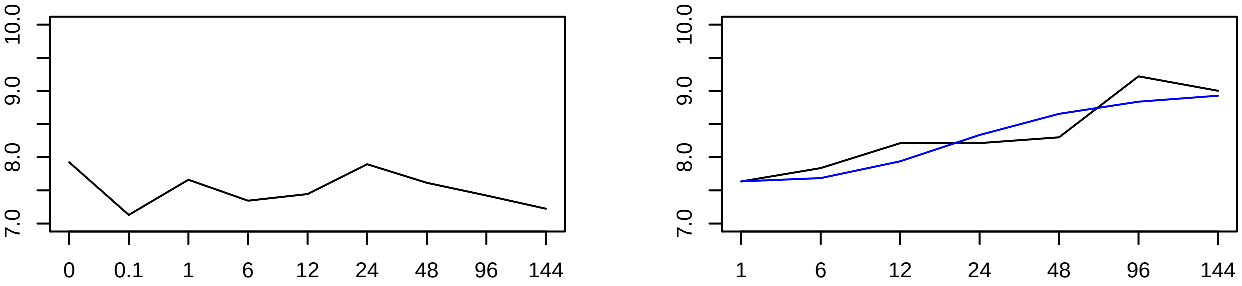

A_24_P932760 KLC1 NA

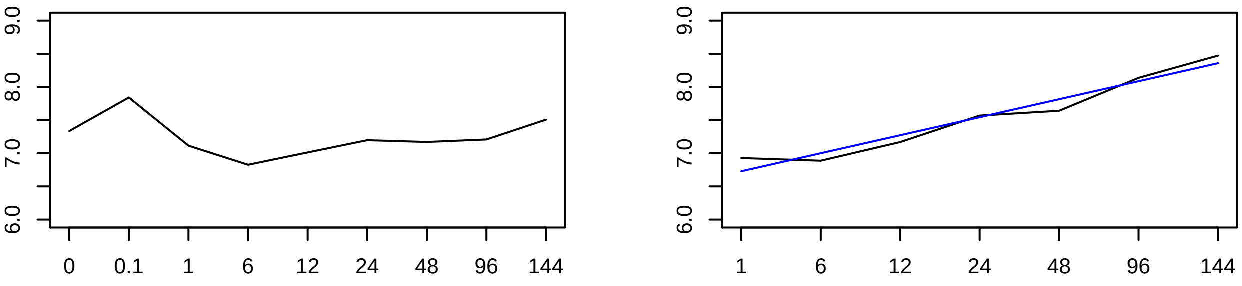

A_23_P344673 HOXD−AS1 2q31.1

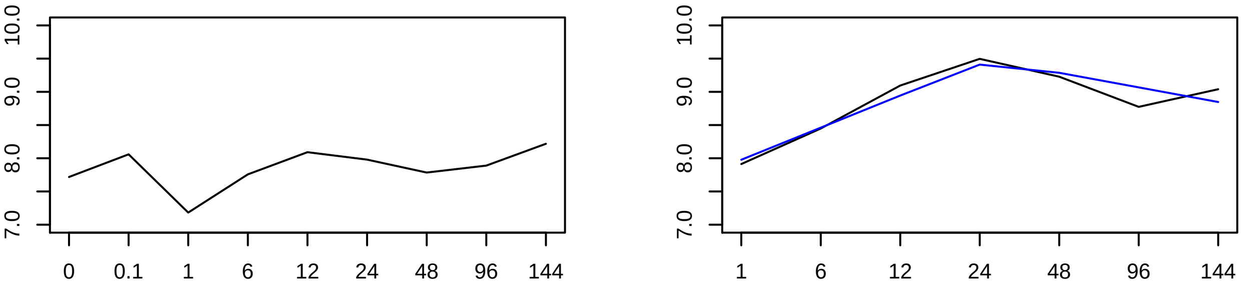

A_23_P30283 TMEM157 5q21.1

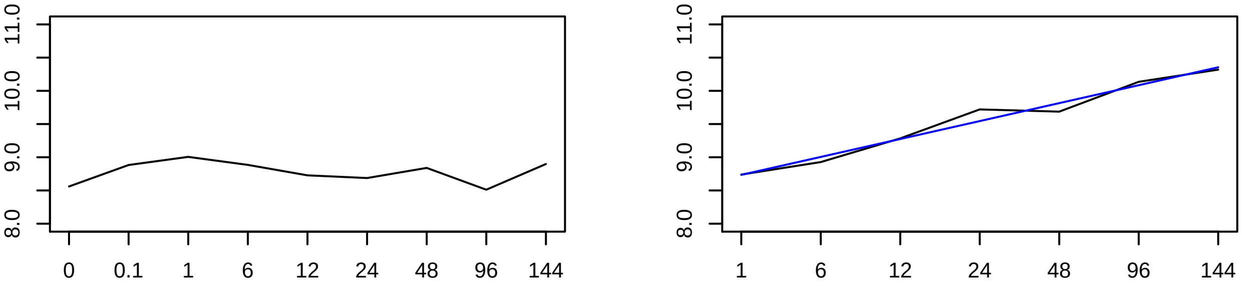

A_23_P119344 TEAD2 19q13.33

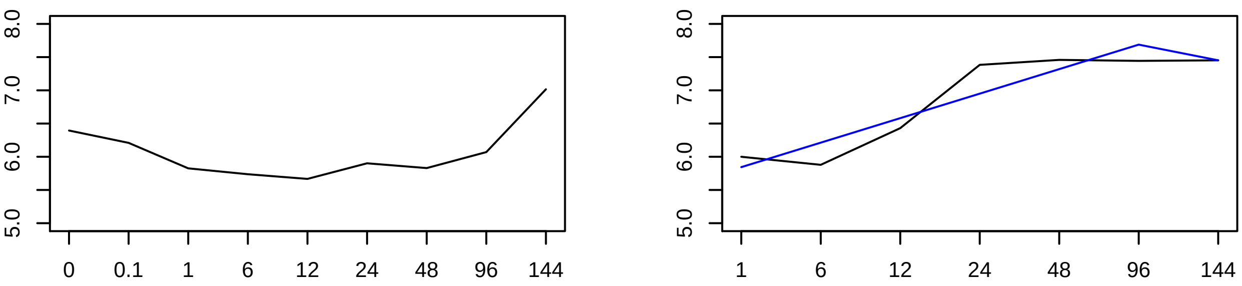

A_32_P155776 FKSG30 2q21.1

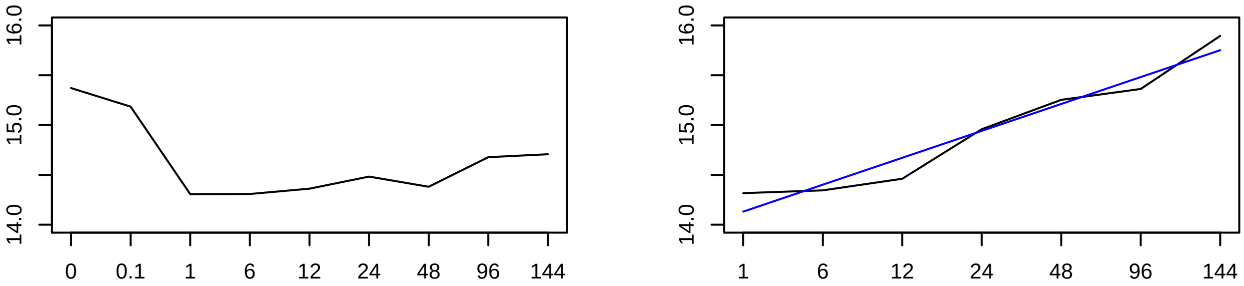

A_23_P34233 QPRT 16p11.2

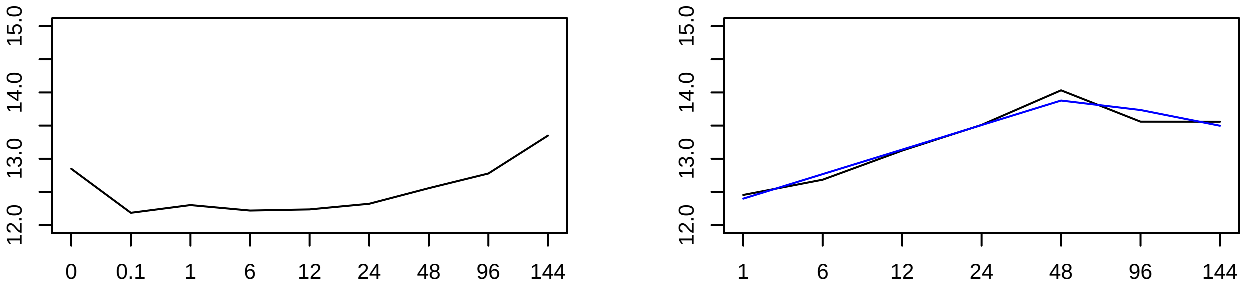

A_23_P14798 NEO1 15q24.1

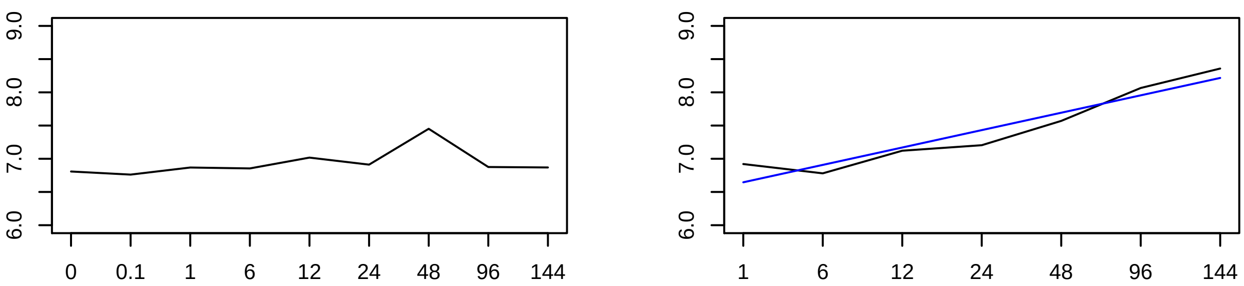

A_32_P61693 CACNG8 NA

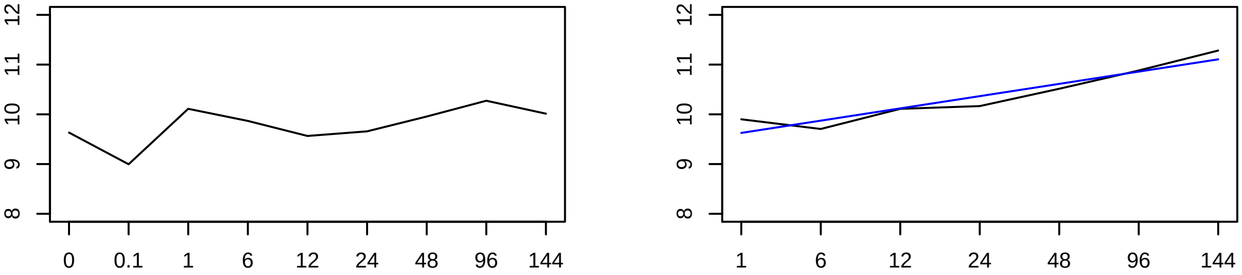

A_32_P20582 VPS13D 1p36.22

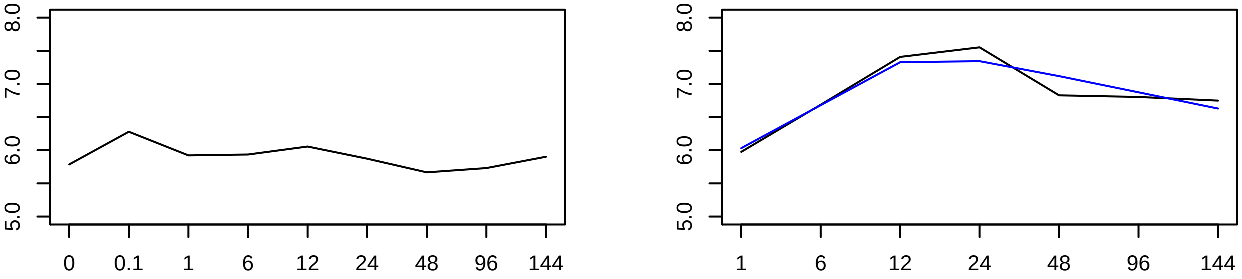

A_23_P124962 FAM89B 11q13.1

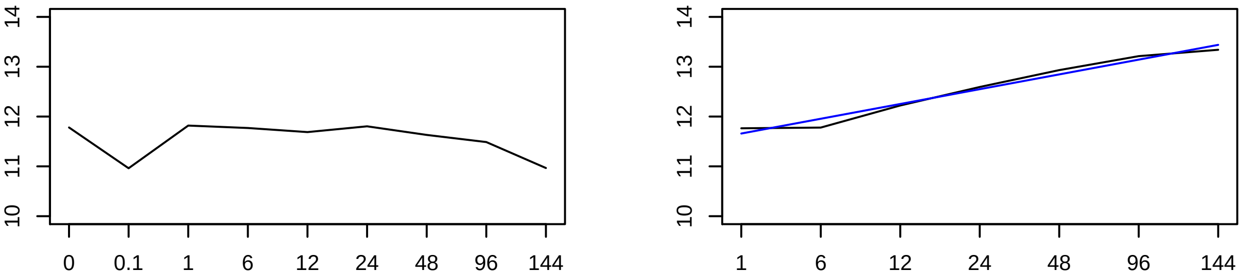

A_23_P122906 AUTS2 7q11.22

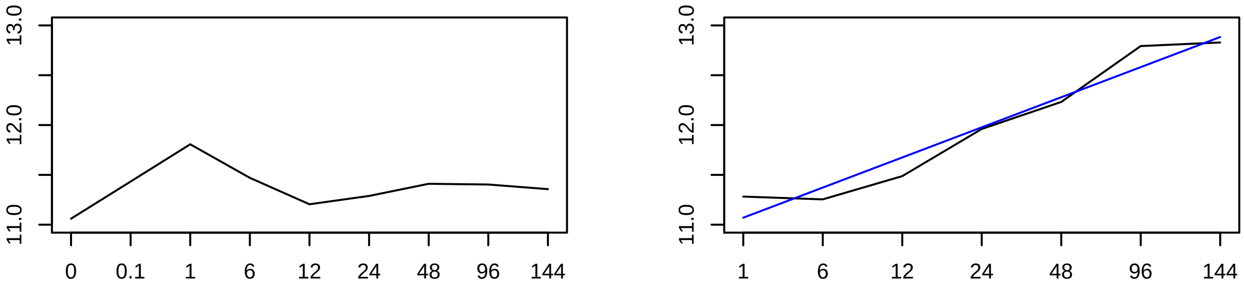

A_32_P103678 THC2729899 NA

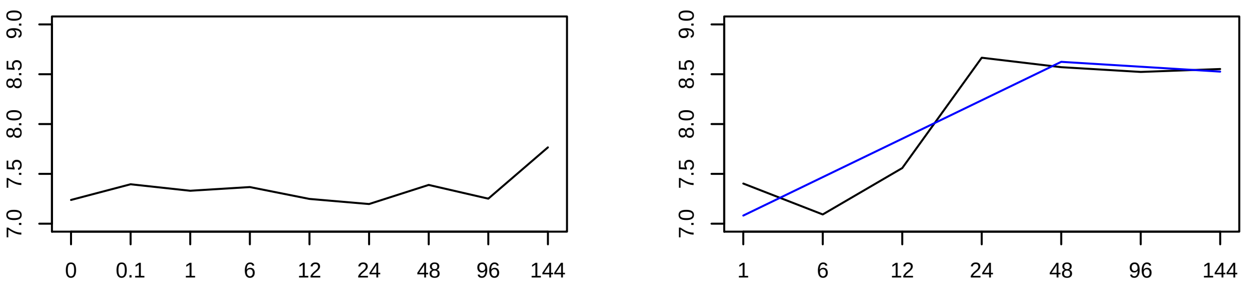

A_23_P102731 SMOX 20p13

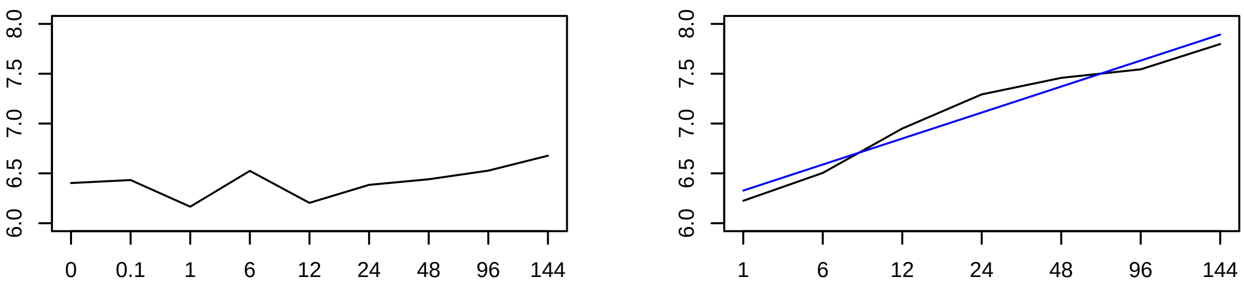

A_32_P179646 A_32_P179646 NA

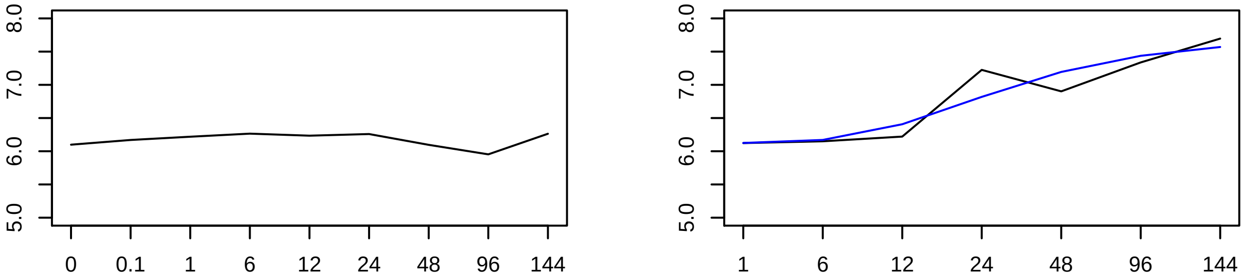

A_32_P197561 EBF1 5q33.3

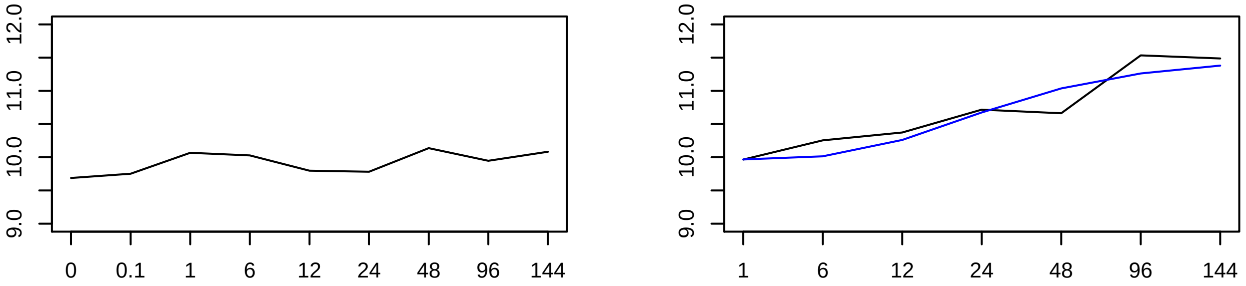

A_32_P122089 A_32_P122089 NA

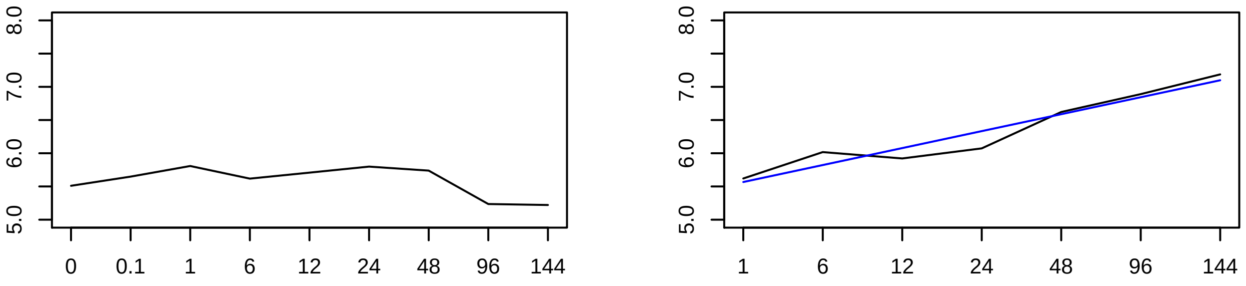

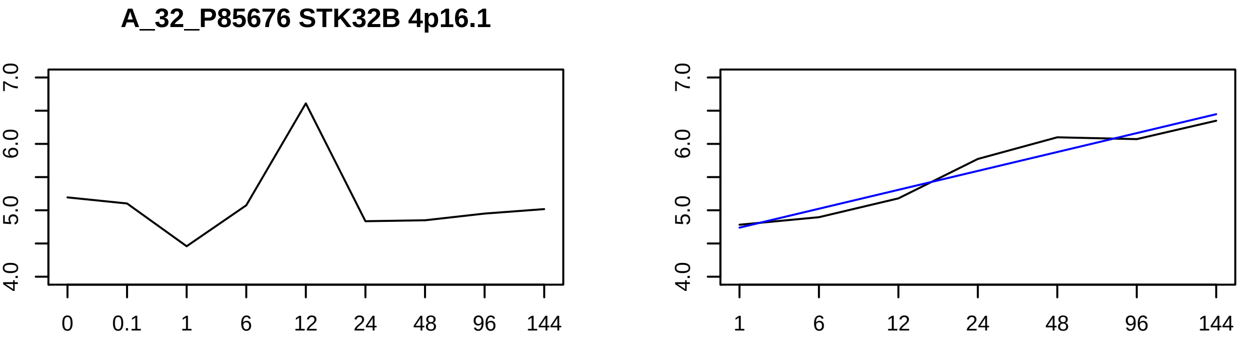
A_32_P85676 STK32B 4p16.1
4.0
5.0
6.0
7.0
0
0.1
1
6
12
24
48
96
144
1
6
12
24
48
96
144

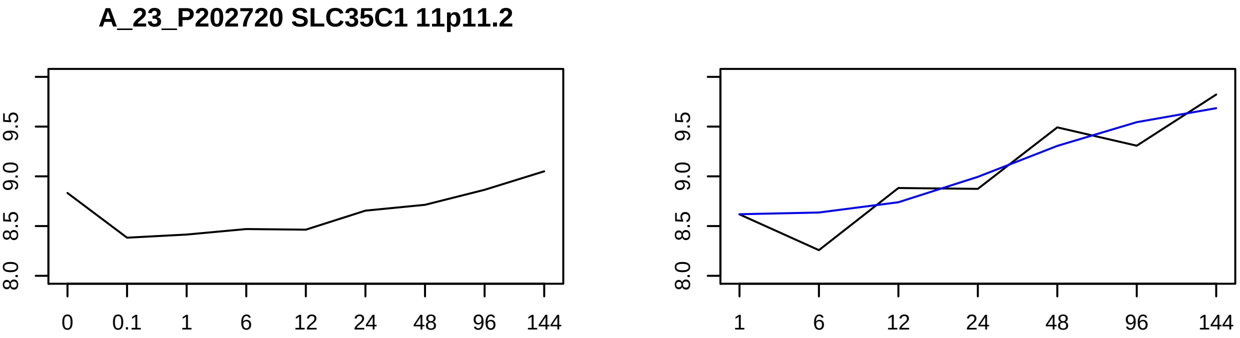
A_23_P202720 SLC35C1 11p11.2
8.0
8.5
9.0
9.5
0
0.1
1
6
12
24
48
96
144
1
6
12
24
48
96
144

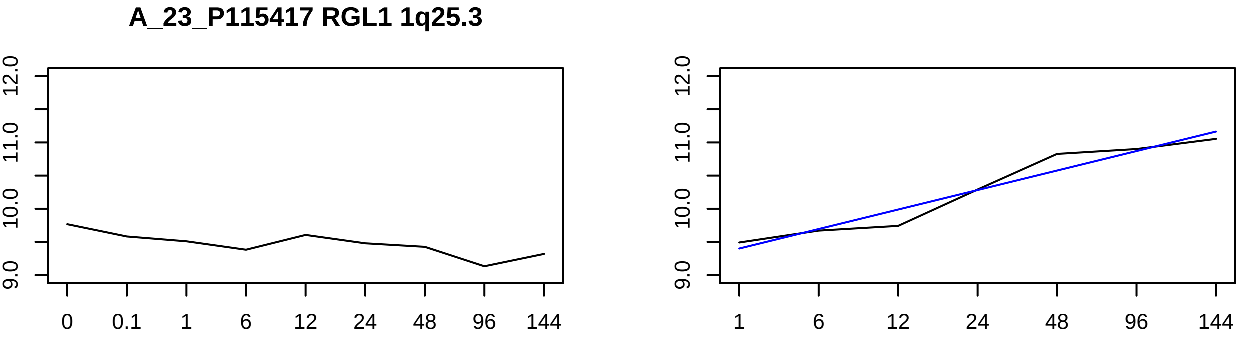
A_23_P115417 RGL1 1q25.3
9.0
10.0
11.0
12.0
0
0.1
1
6
12
24
48
96
144
1
6
12
24
48
96
144

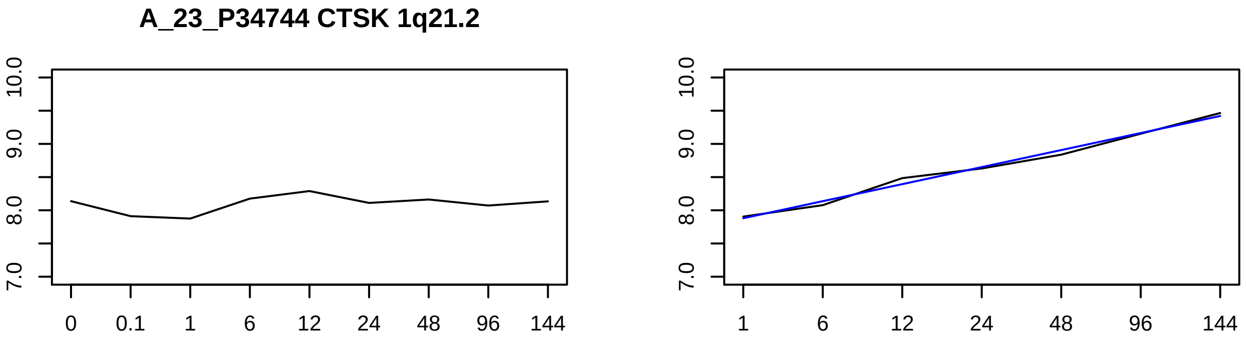
A_23_P34744 CTSK 1q21.2
7.0
8.0
9.0
10.0
0
0.1
1
6
12
24
48
96
144

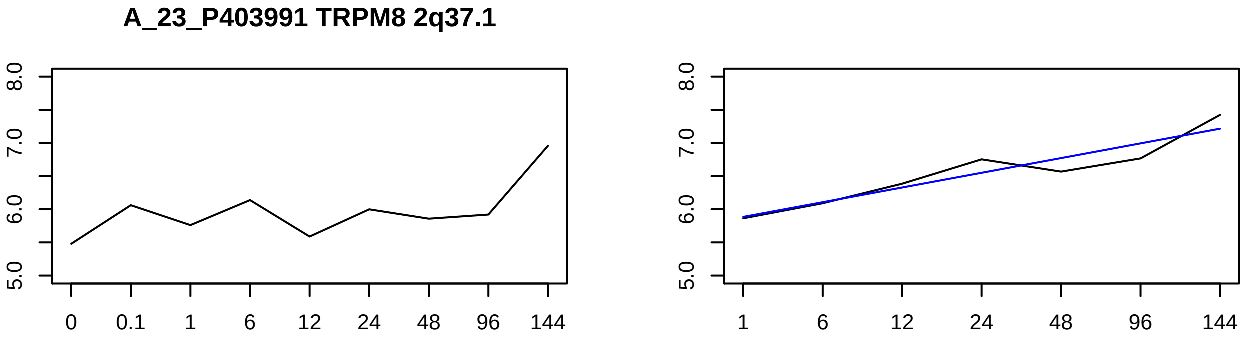
A_23_P403991 TRPM8 2q37.1
5.0
6.0
7.0
8.0
0
0.1
1
6
12
24
48
96
144

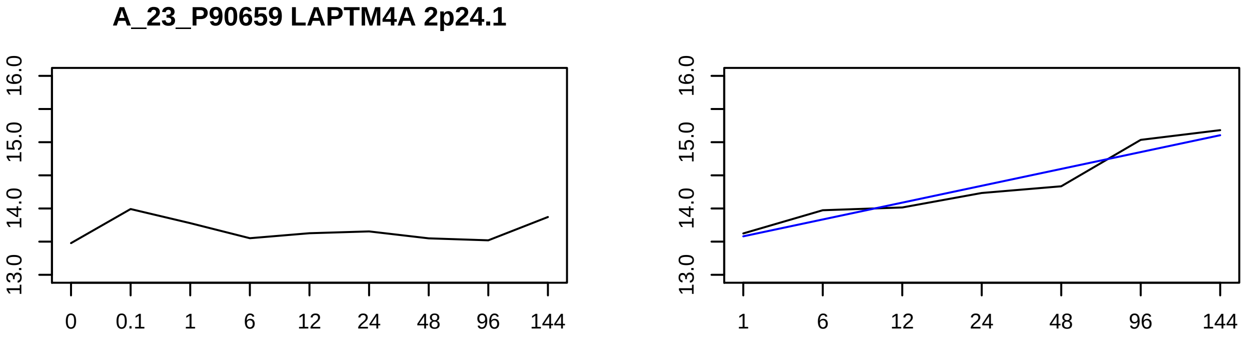
A_23_P90659 LAPTM4A 2p24.1
13.0
14.0
15.0
16.0
0
0.1
1
6
12
24
48
96
144

A_23_P4628 ZNF606 19q13.43

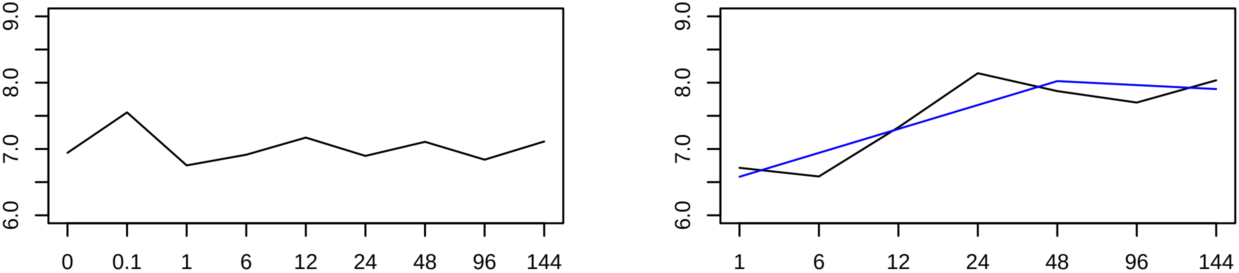

A_23_P24004 IFIT2 10q23.31

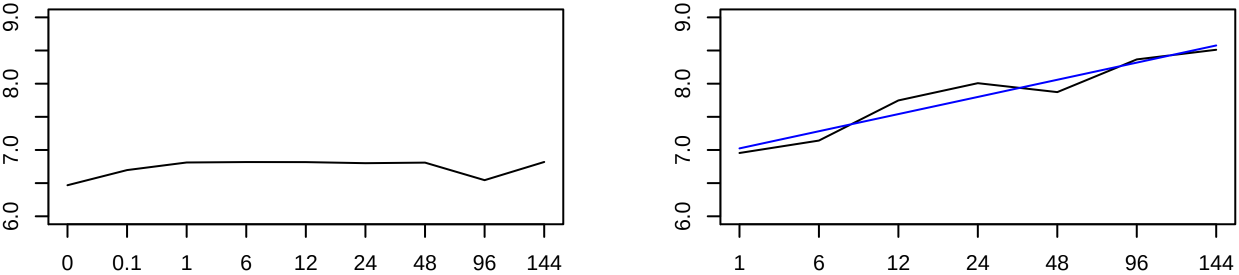

A_23_P406131 TMEM159 16p12.2

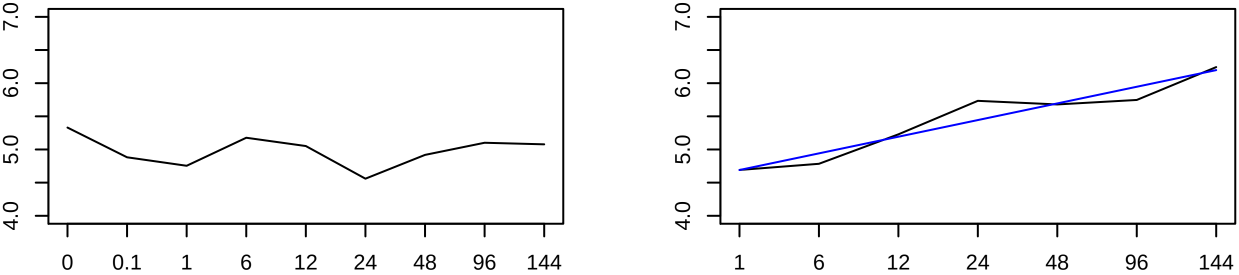

A_24_P295050 KLHL7 7p15.3

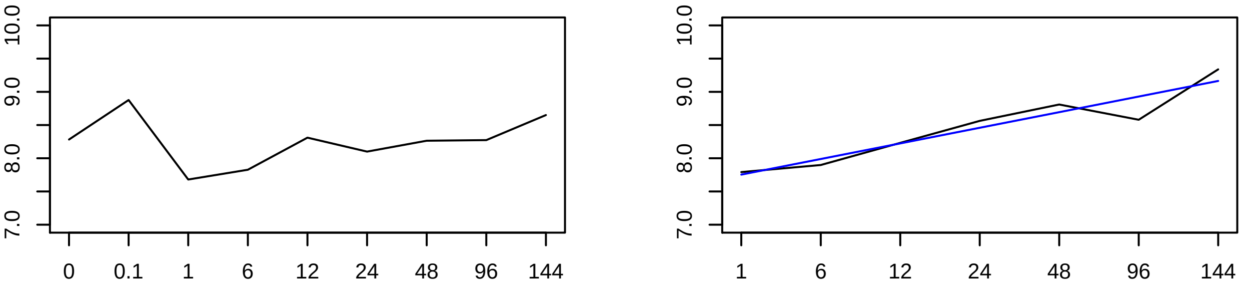

A_32_P139260 A_32_P139260 NA

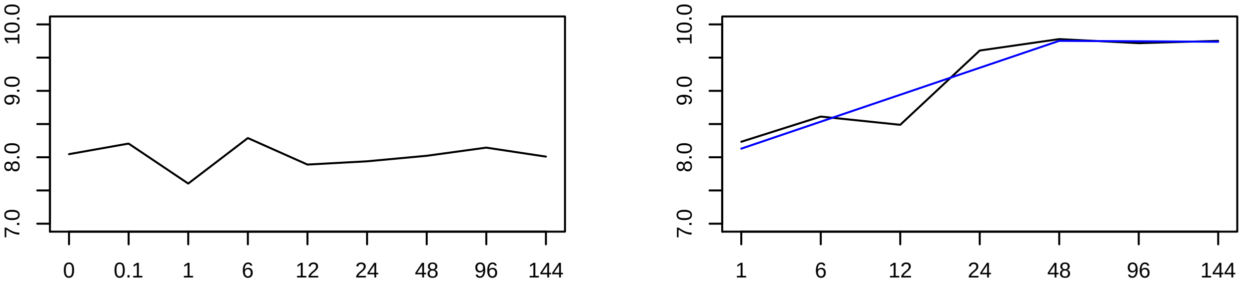

A_23_P313568 NCOA3 20q13.12

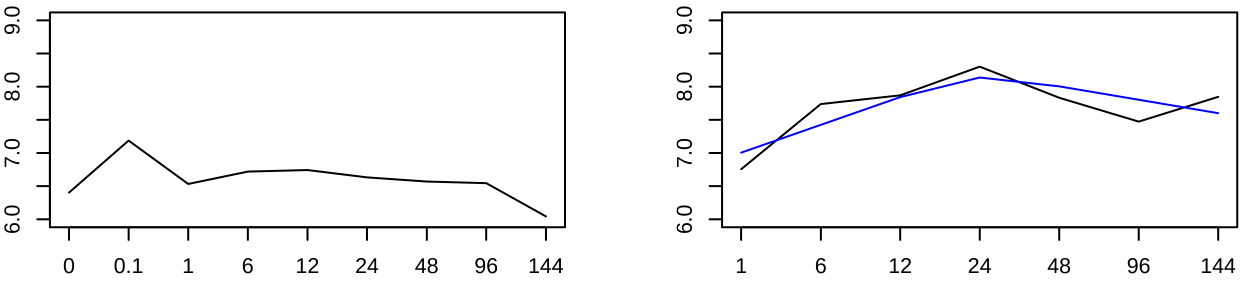

A_23_P502783 BVES 6q21

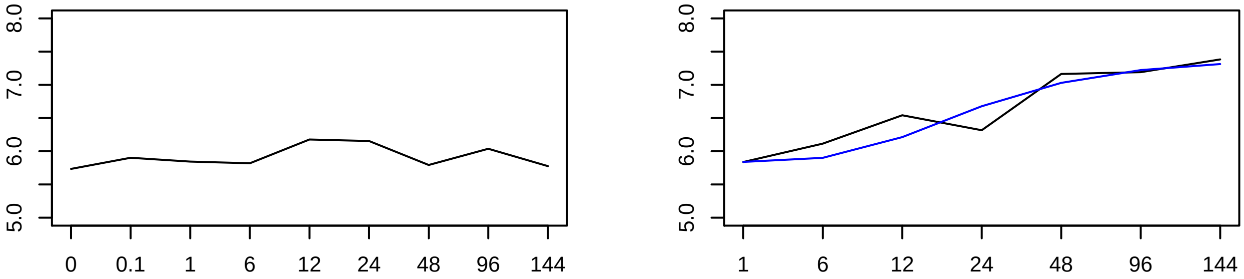

A_32_P418453 FLOT1 6p21.33

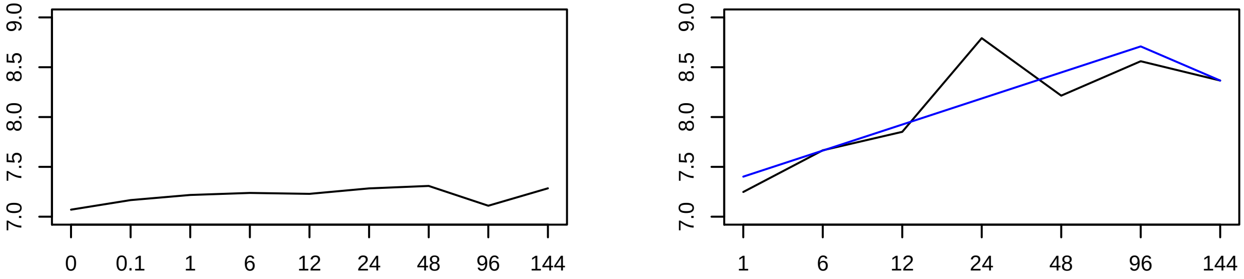

A_23_P361984 ST8SIA4 5q21.1

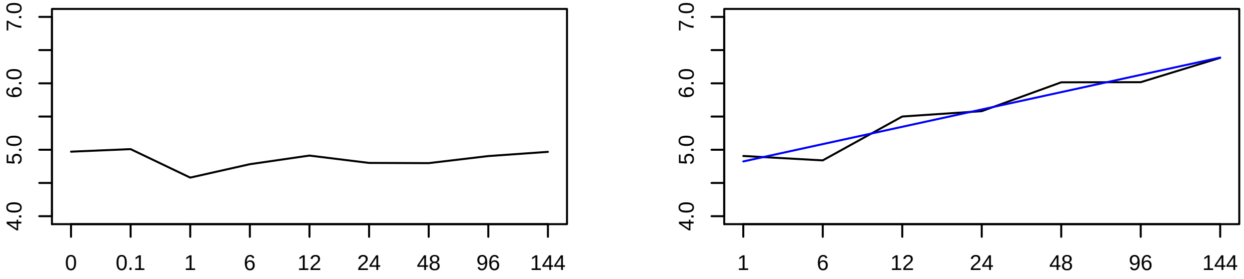

A_23_P904 C1orf165 1p33

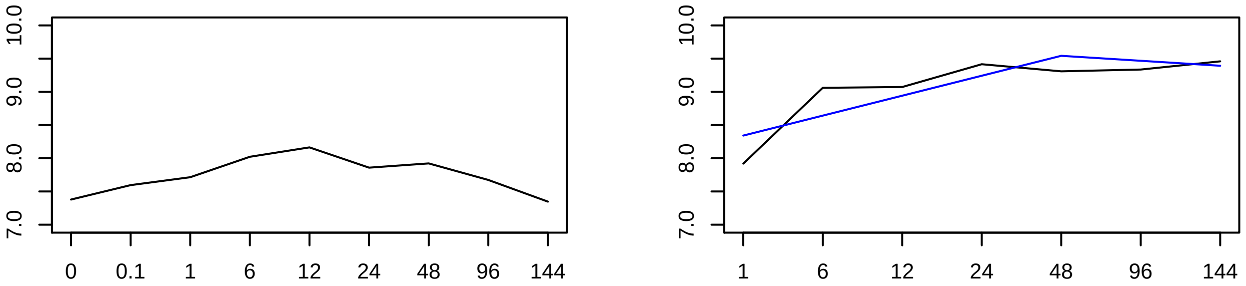

A_23_P130347 KCTD1 18q11.2

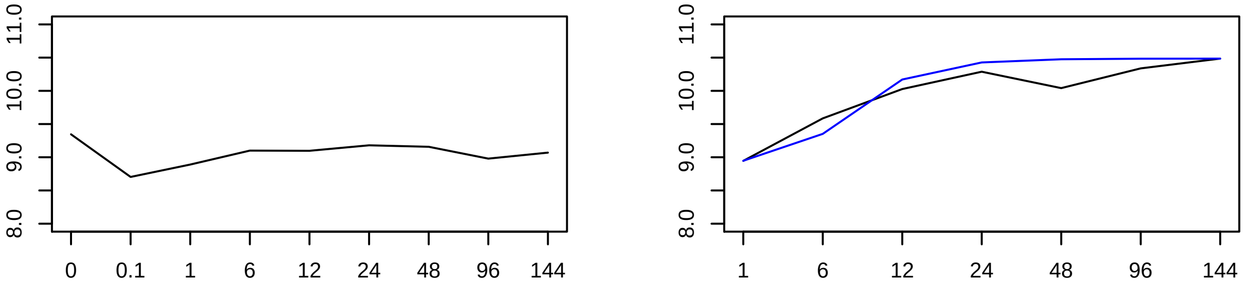

A_23_P354314 GPR161 1q24.2

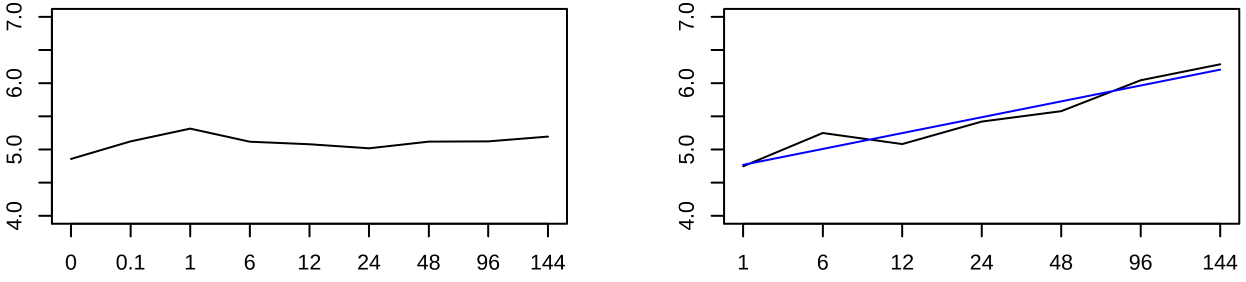

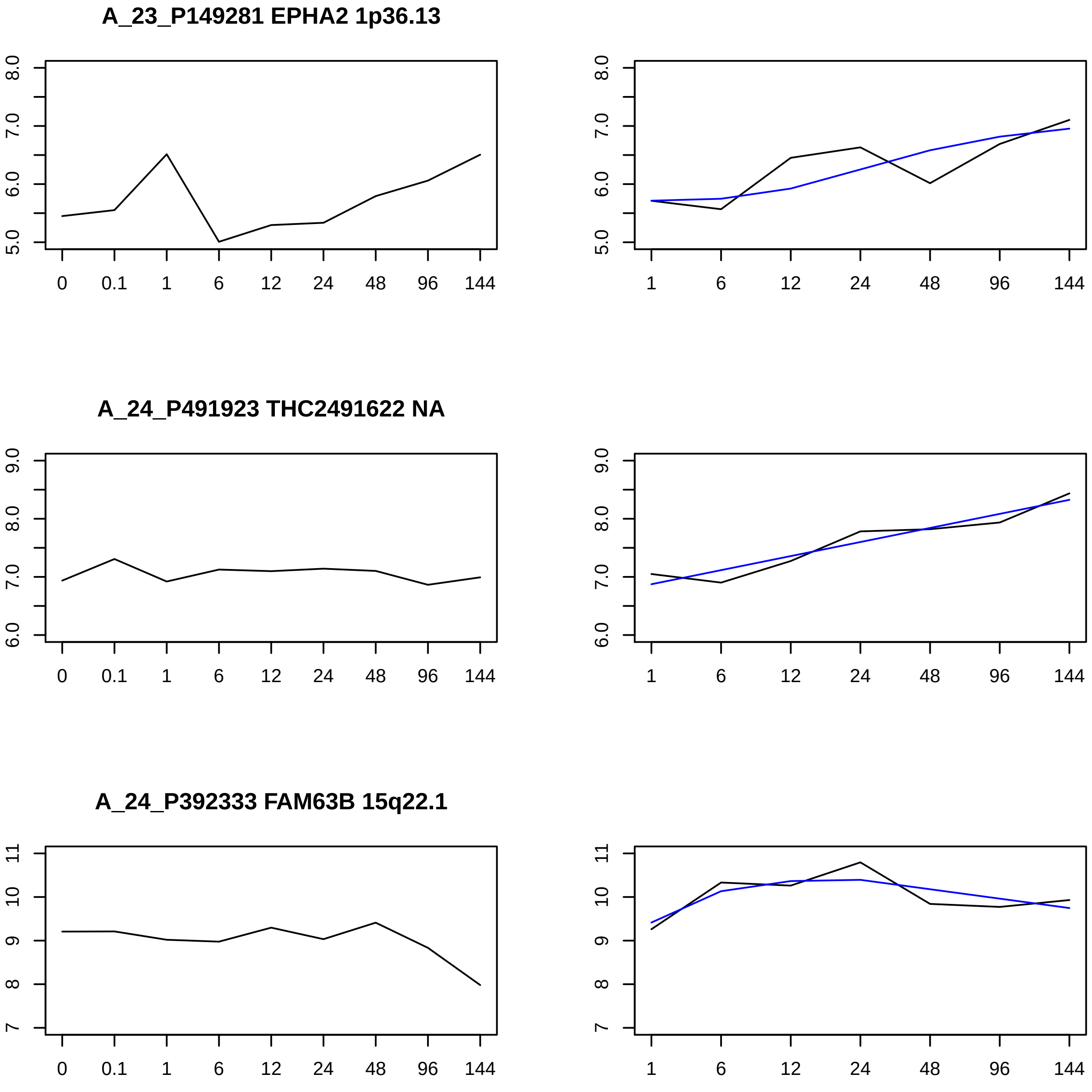
A_23_P149281 EPHA2 1p36.13
A_24_P491923 THC2491622 NA
A_24_P392333 FAM63B 15q22.1
0 0.1 1 6 12 24 48 96 144
1 6 12 24 48 96 144
5.0 6.0 7.0 8.0
6.0 7.0 8.0 9.0
7 8 9 10 11

A_23_P120325 KIF3C 2p23.3

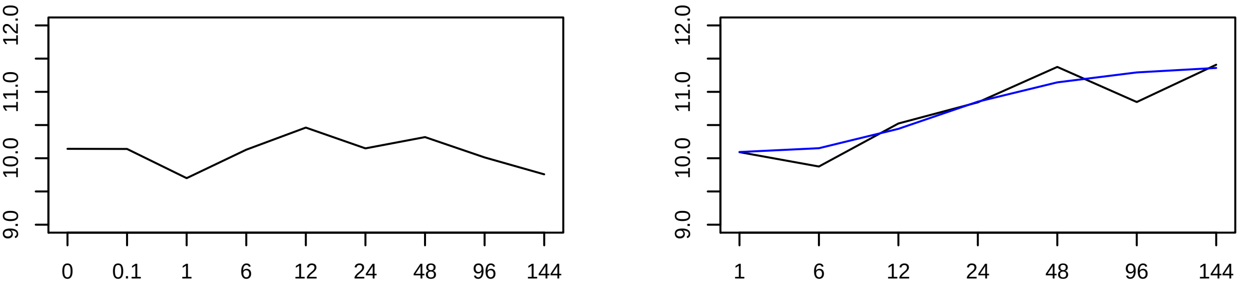

A_23_P20363 C8orf70 8q21.12

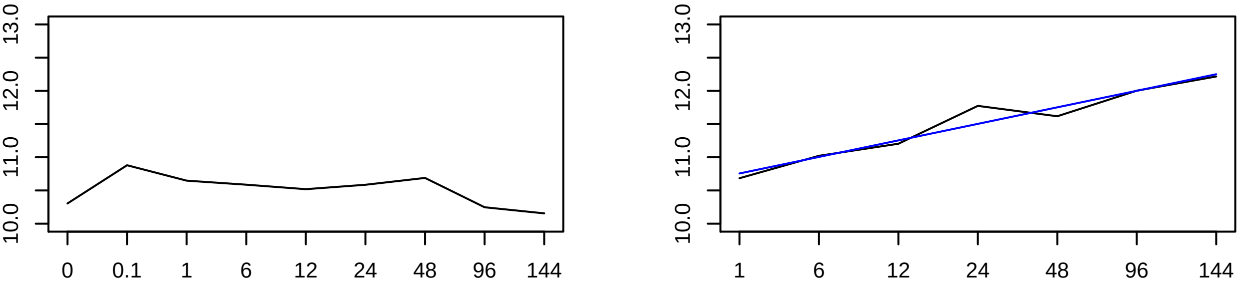

A_24_P264943 COMP 19p13.11

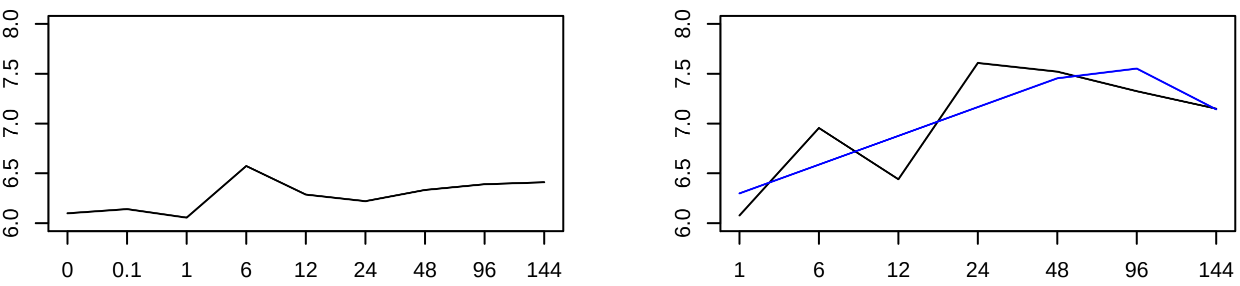

A_32_P125589 THC2649341 NA

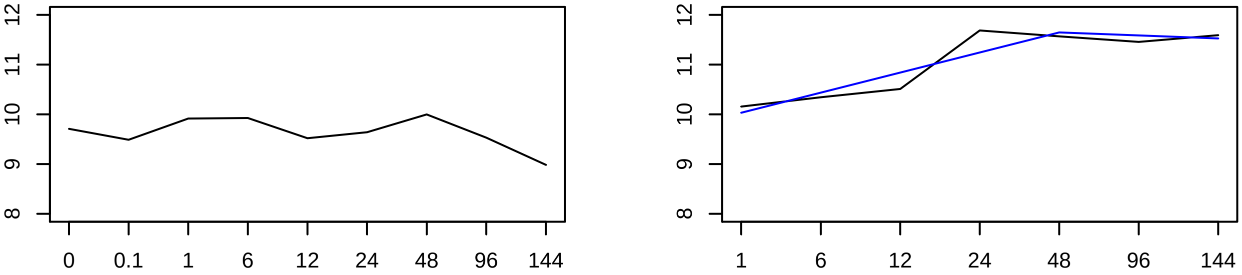

A_24_P131522 ANTXR1 2p14

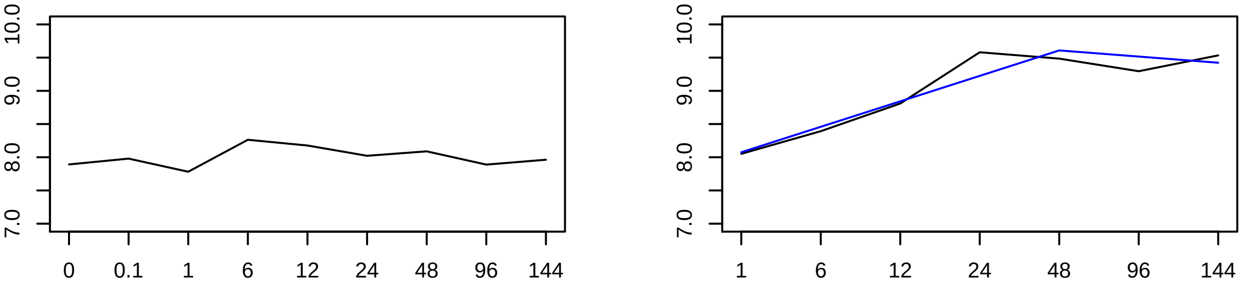

A_23_P354805 KLF12 13q22.1

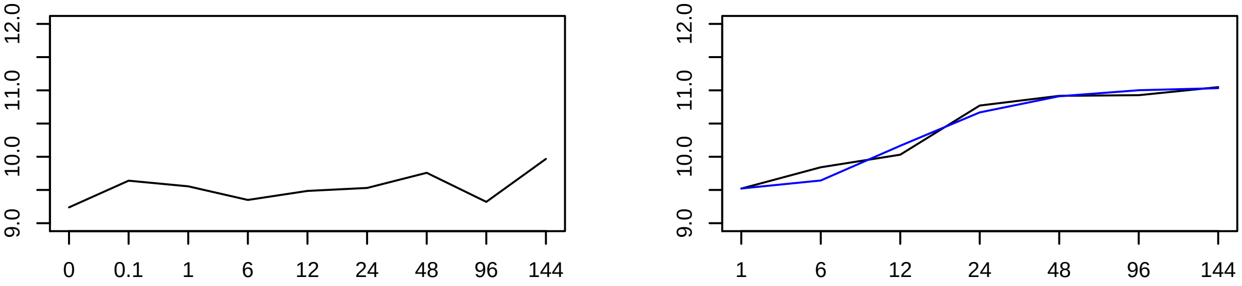

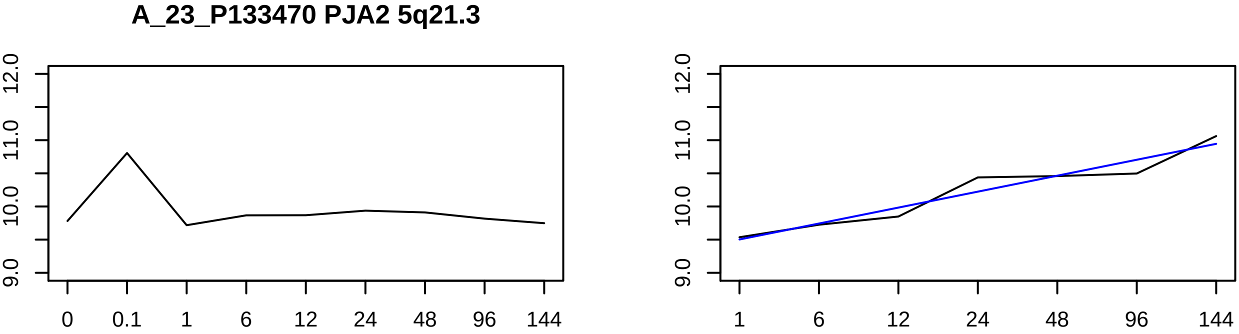
A_23_P133470 PJA2 5q21.3
9.0
10.0
11.0
12.0
0
0.1
1
6
12
24
48
96
144
9.0
10.0
11.0
12.0
1
6
12
24
48
96
144

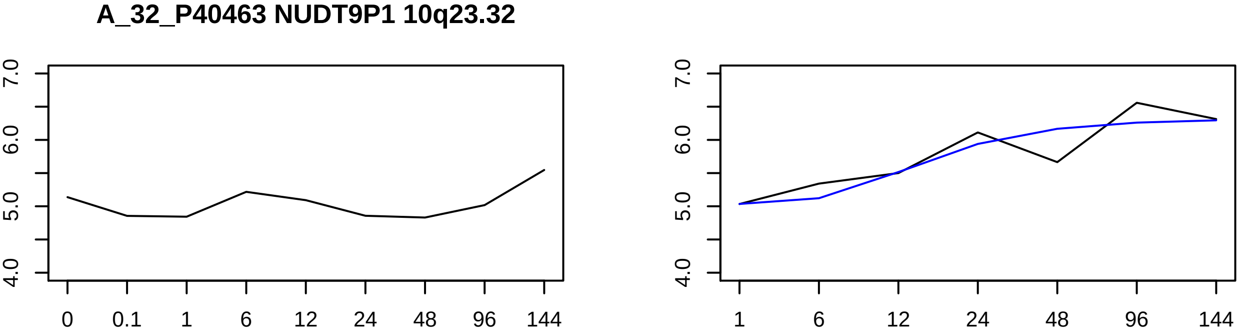
A_32_P40463 NUDT9P1 10q23.32
4.0
5.0
6.0
7.0
0
0.1
1
6
12
24
48
96
144
4.0
5.0
6.0
7.0
1
6
12
24
48
96
144

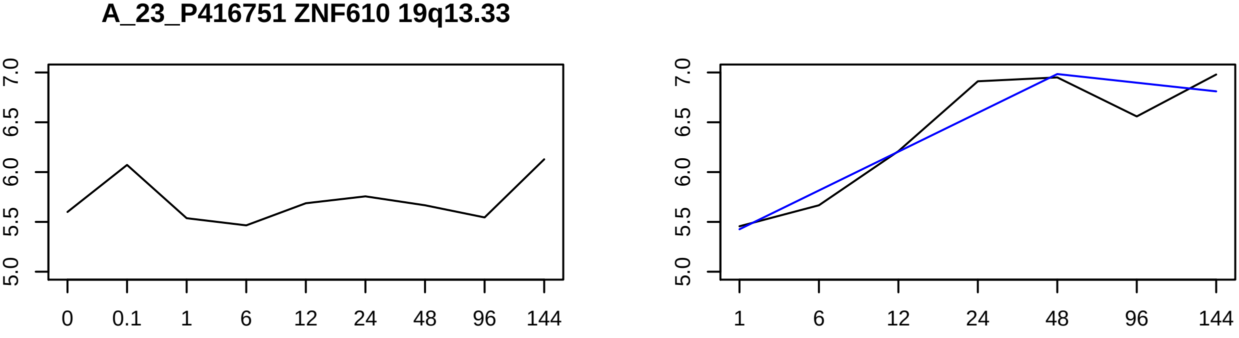
A_23_P416751 ZNF610 19q13.33
5.0
5.5
6.0
6.5
7.0
0
0.1
1
6
12
24
48
96
144
5.0
5.5
6.0
6.5
7.0
1
6
12
24
48
96
144

A_23_P58763 PELO 5q11.2

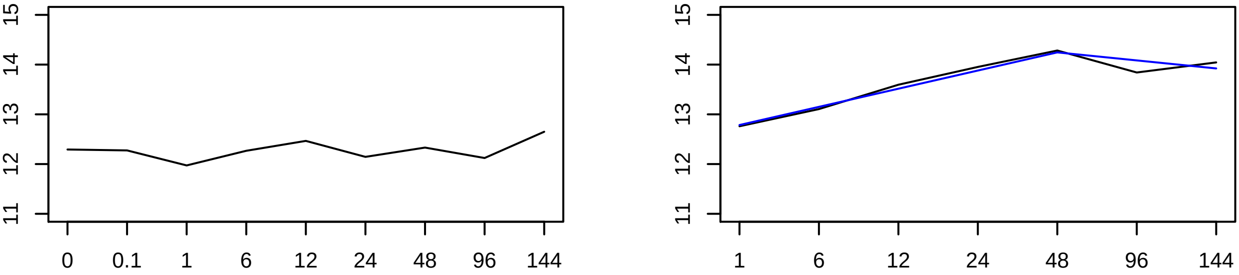

A_23_P35906 CASP4 11q22.3

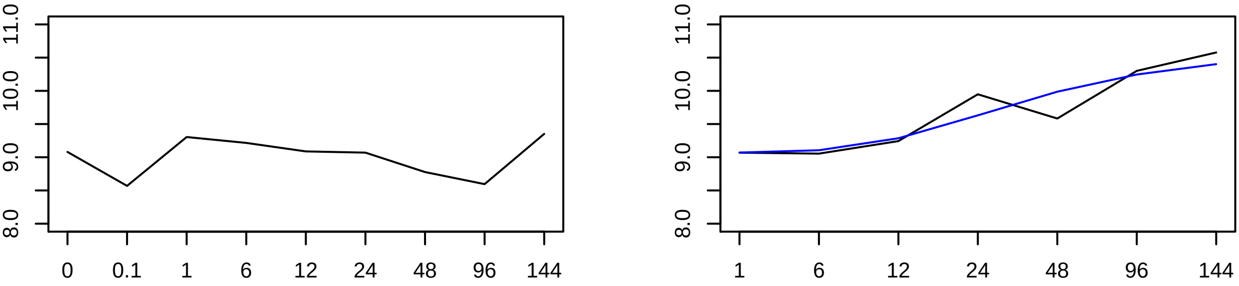

A_23_P17345 MAFB 20q12

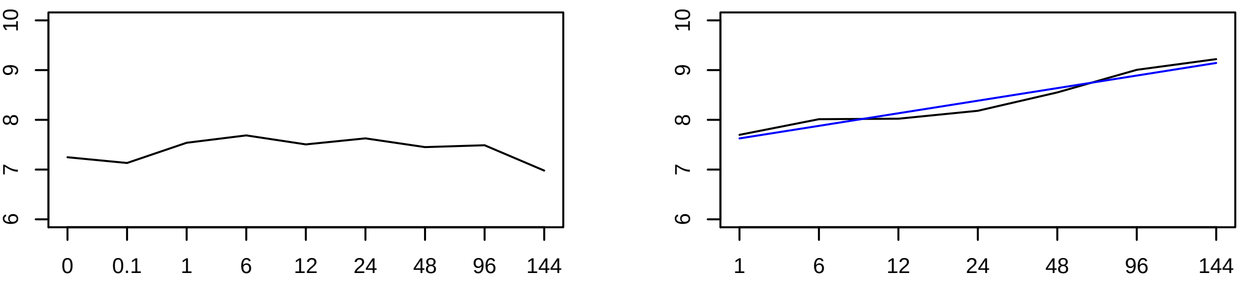

A_24_P345846 ANTXR2 4q21.21

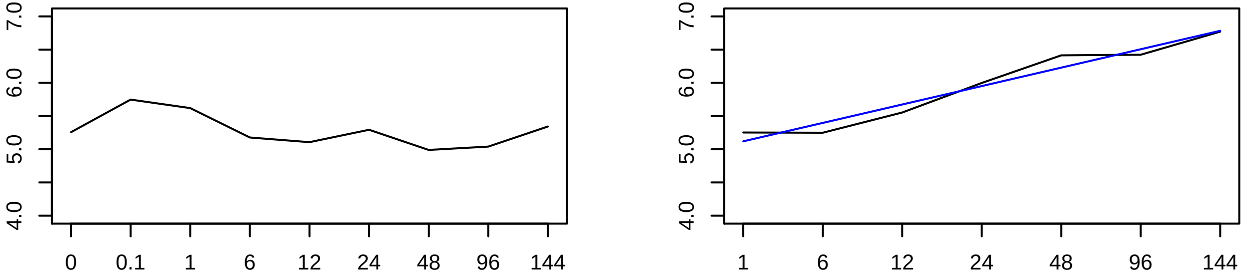

A_23_P70938 STARD3NL 7p14.1

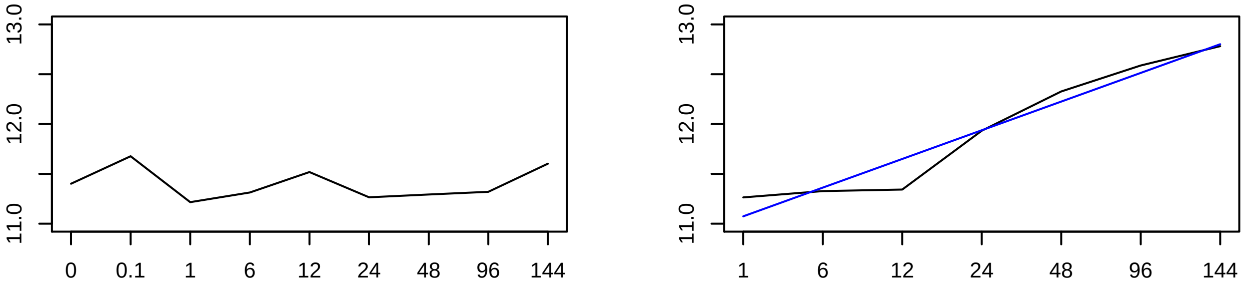

A_23_P369210 CLCN5 Xp11.22

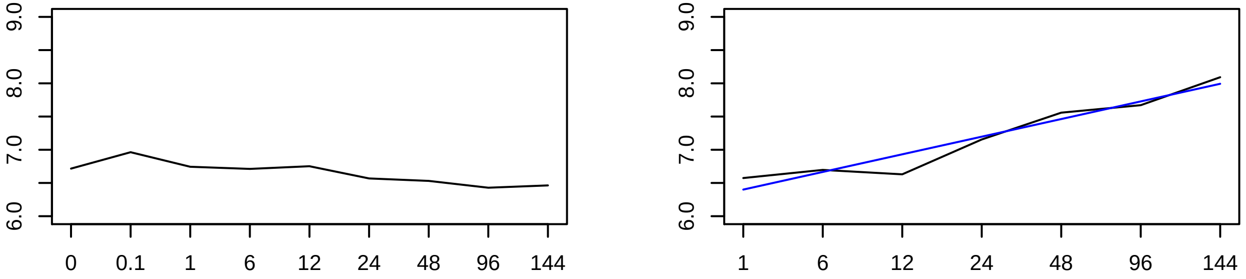

A_24_P133171 AK026078 NA

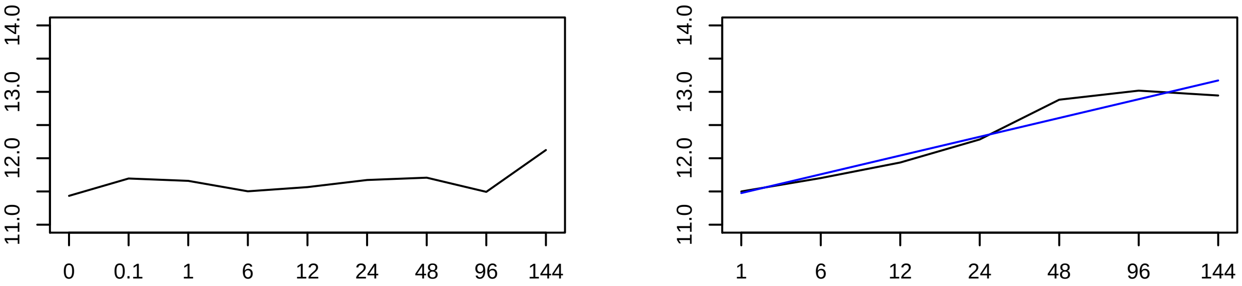

A_23_P52552 BAG3 10q26.11

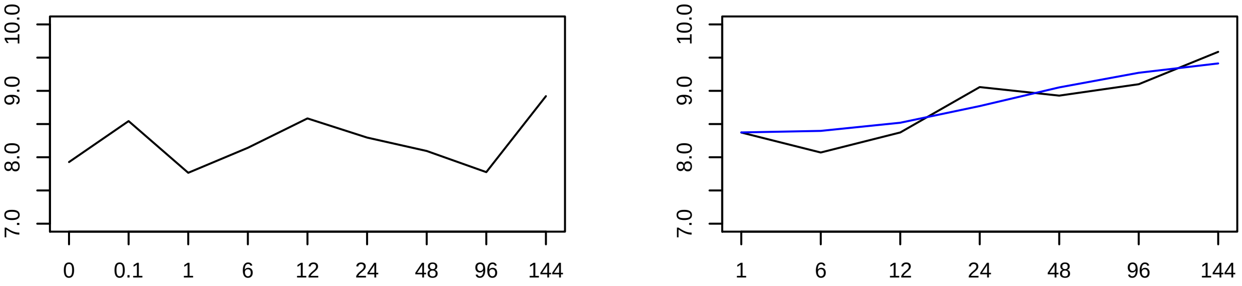

A_23_P310094 SYNPO2 4q26

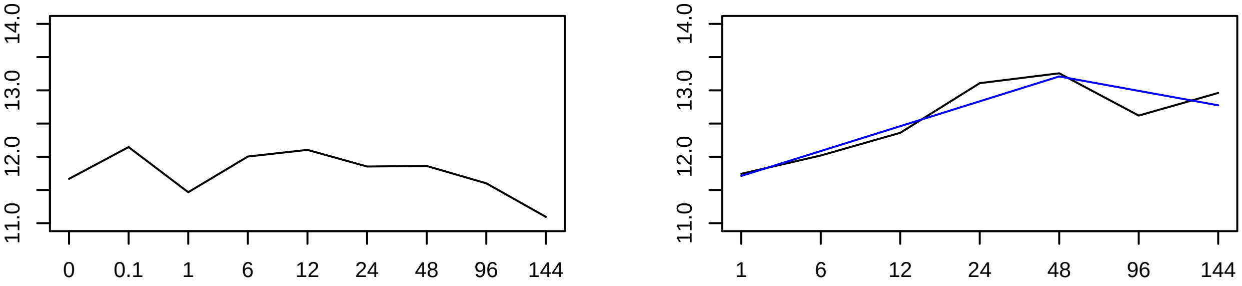

A_24_P296689 AK124426 NA

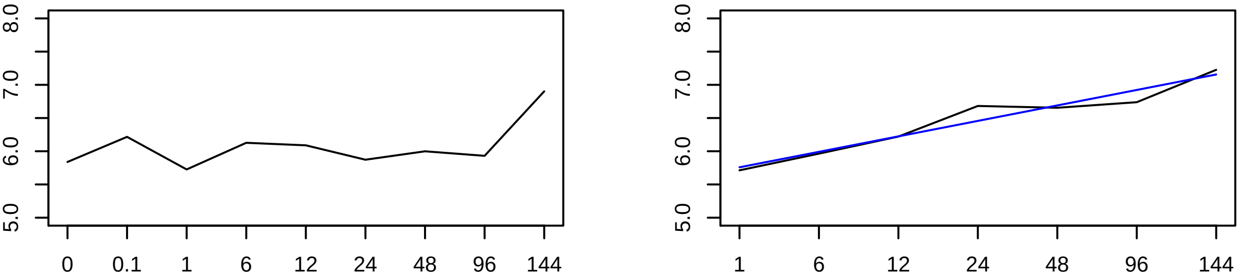

A_32_P211026 THC2645562 NA

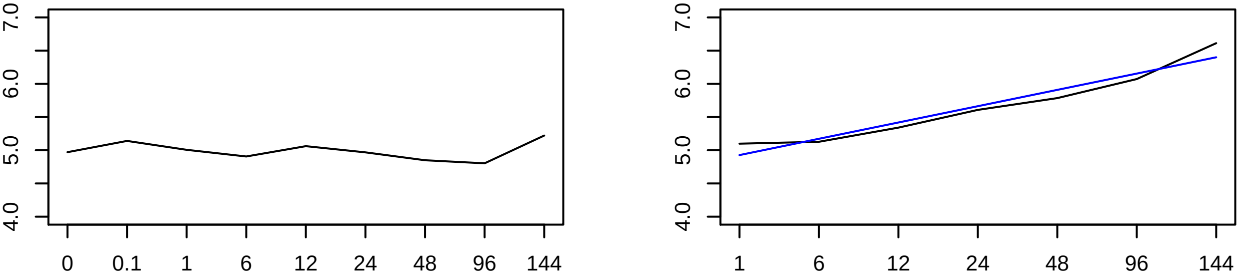

A_23_P94128 NEIL2 8p23.1

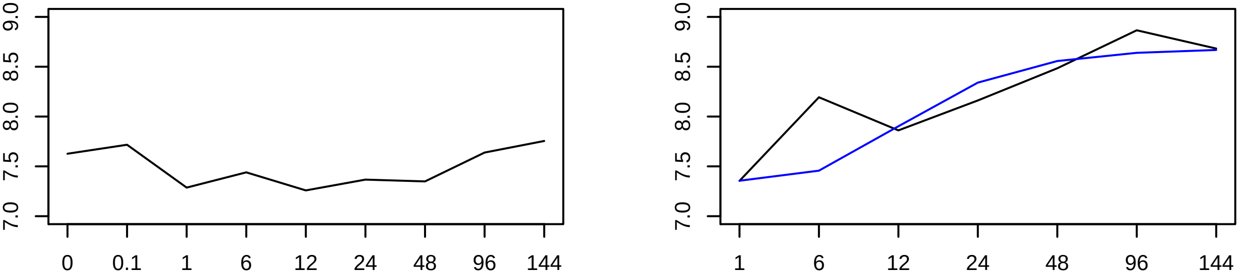

A_23_P31584 RABL5 7q22.1

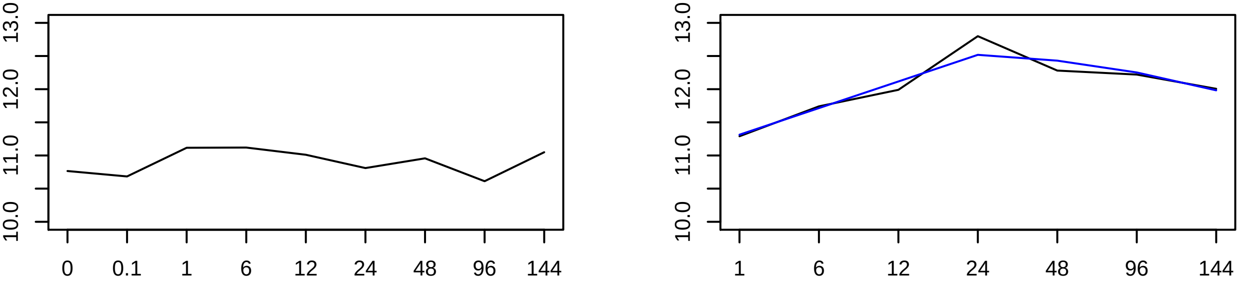

A_23_P314526 FLOT1 6p21.33

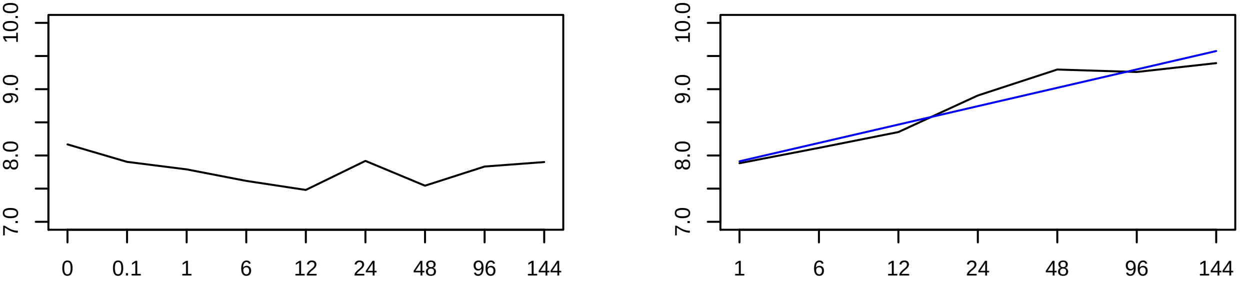

A_23_P392126 LOC201229 17q11.2

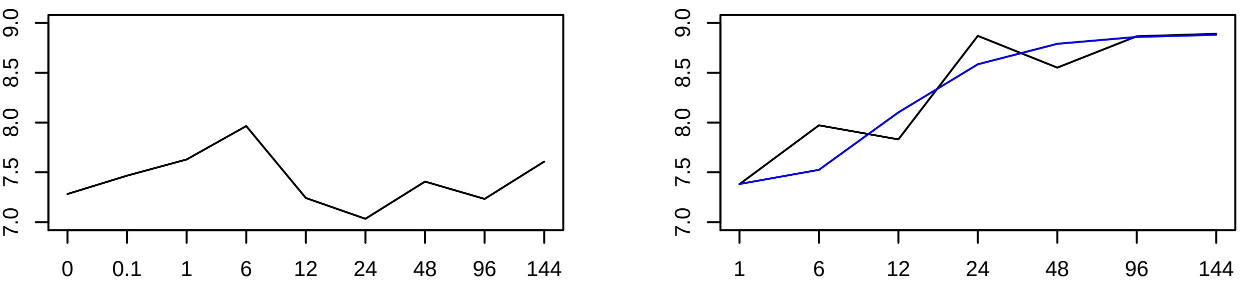

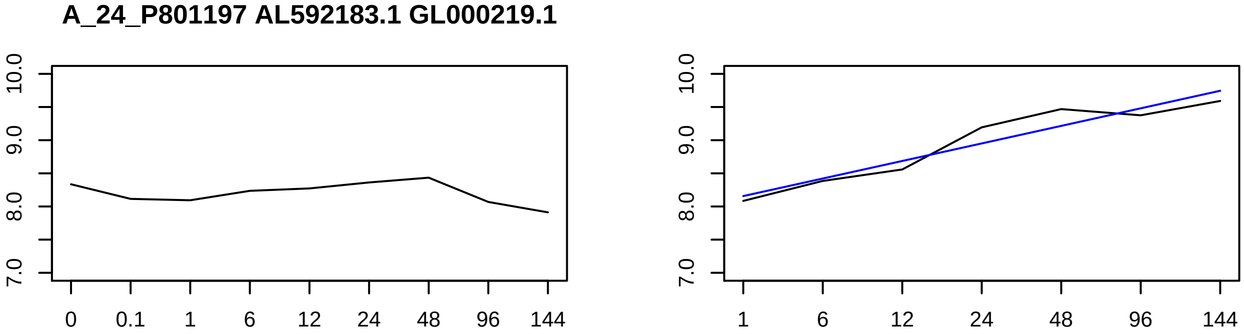
A_24_P801197 AL592183.1 GL000219.1
7.0
8.0
9.0
10.0
0
0.1
1
6
12
24
48
96
144
1
6
12
24
48
96
144

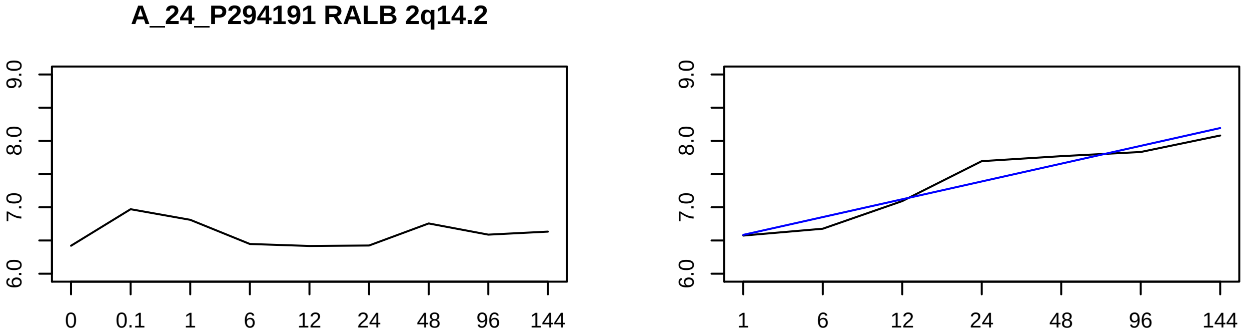
A_24_P294191 RALB 2q14.2
6.0
7.0
8.0
9.0
0
0.1
1
6
12
24
48
96
144
1
6
12
24
48
96
144

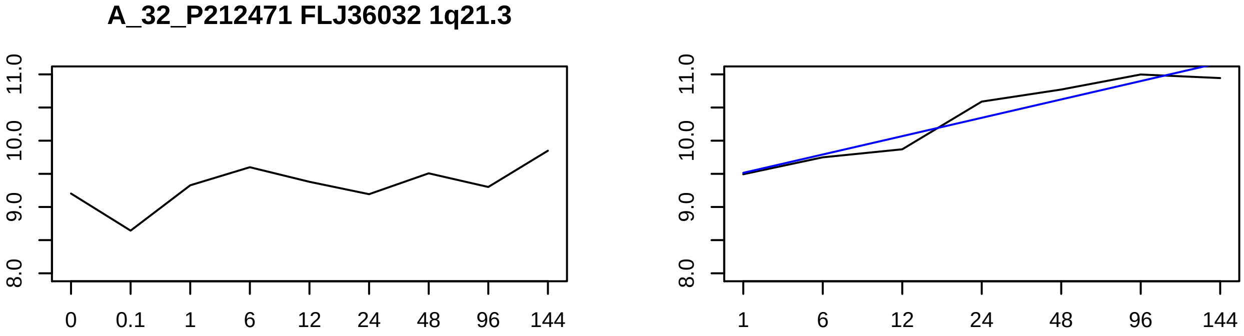
A_32_P212471 FLJ36032 1q21.3
8.0
9.0
10.0
11.0
0
0.1
1
6
12
24
48
96
144
1
6
12
24
48
96
144

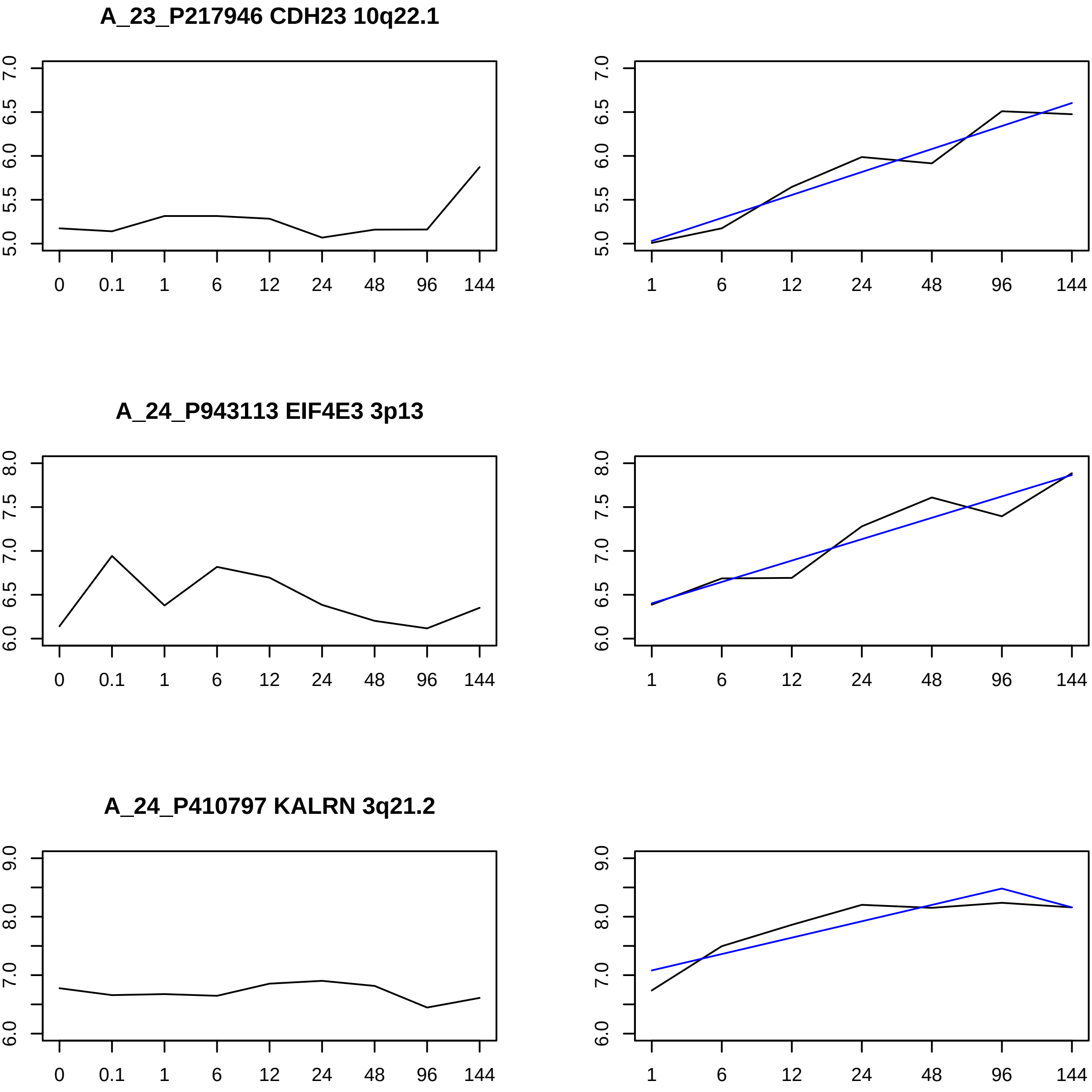
A_23_P217946 CDH23 10q22.1
5.0
5.5
6.0
6.5
7.0
0
0.1
1
6
12
24
48
96
144
A_24_P943113 EIF4E3 3p13
6.0
6.5
7.0
7.5
8.0
A_24_P410797 KALRN 3q21.2
6.0
7.0
8.0
9.0

A_23_P351232 ZNF775 7q36.1

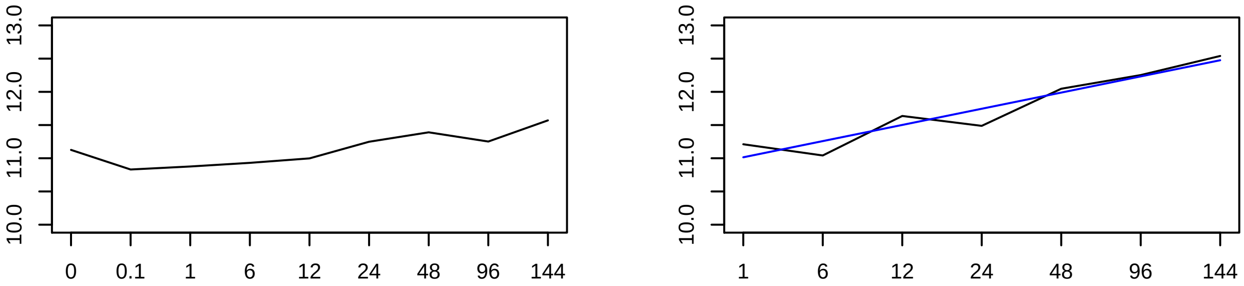

A_32_P118657 AK022044 NA

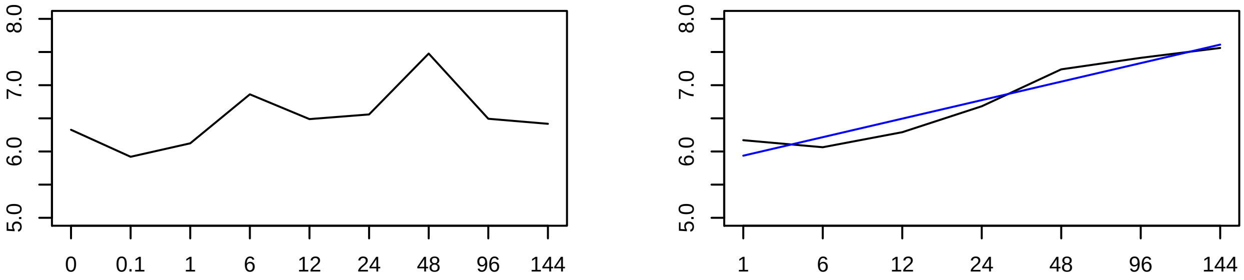

A_24_P463989 THC2633081 NA

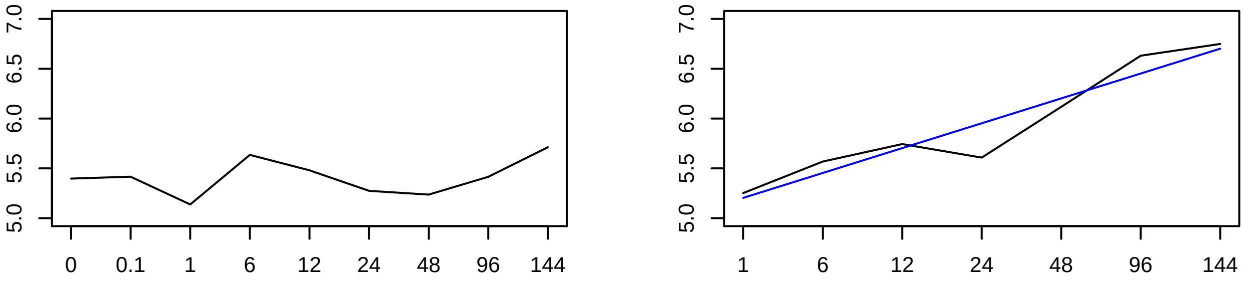

A_32_P187817 THC2648398 NA

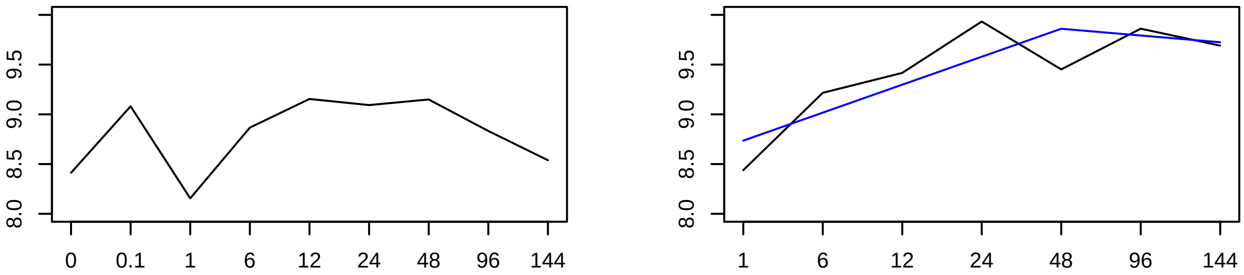

A_23_P46936 EGR2 10q21.2

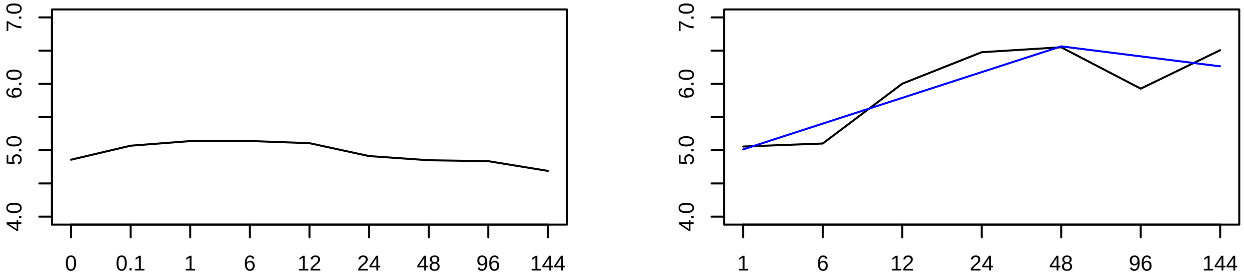

A_24_P133253 KITLG 12q21.32

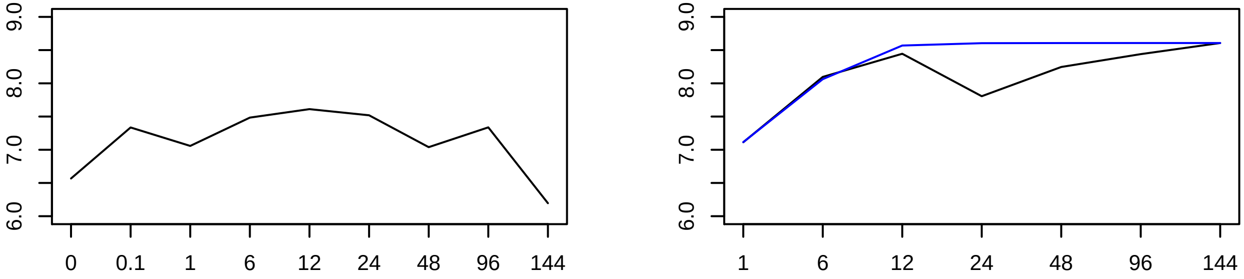

A_24_P123616 HSPA1A 6p21.33

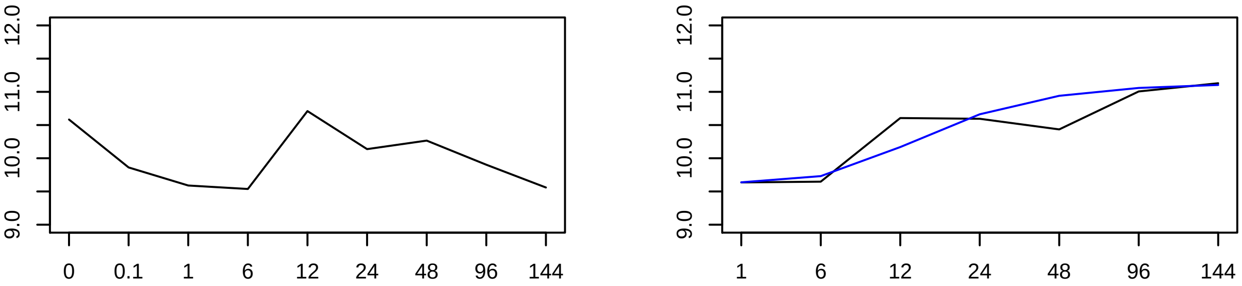

A_23_P331479 KIAA1949 6p21.33

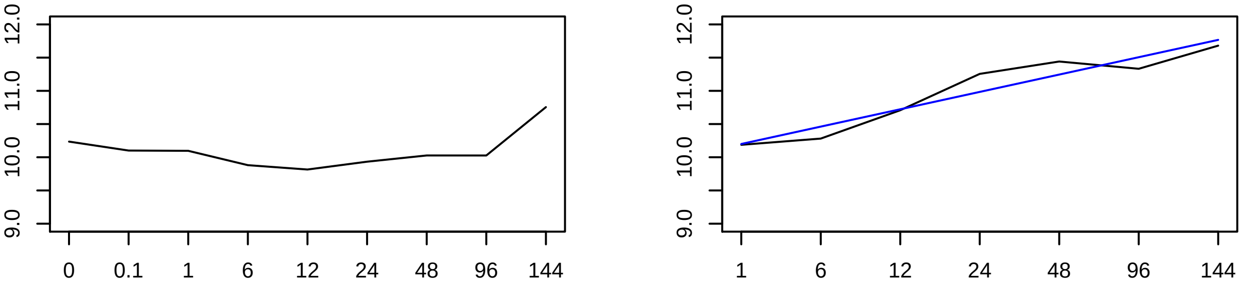

A_32_P21640 MIR137HG\MIR137\MIR2682 1p21.3

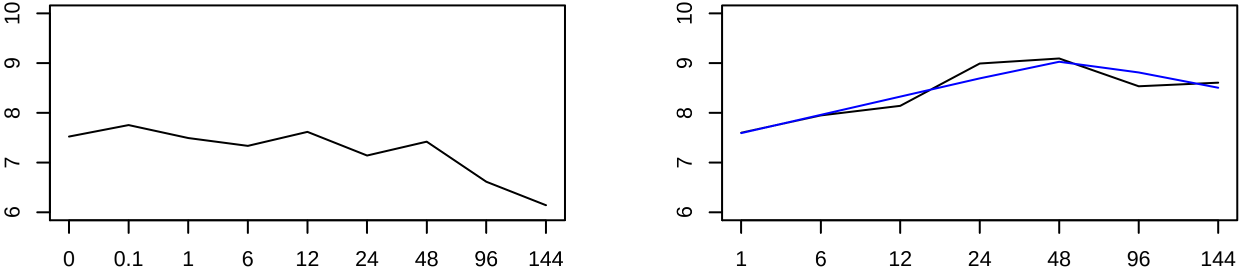

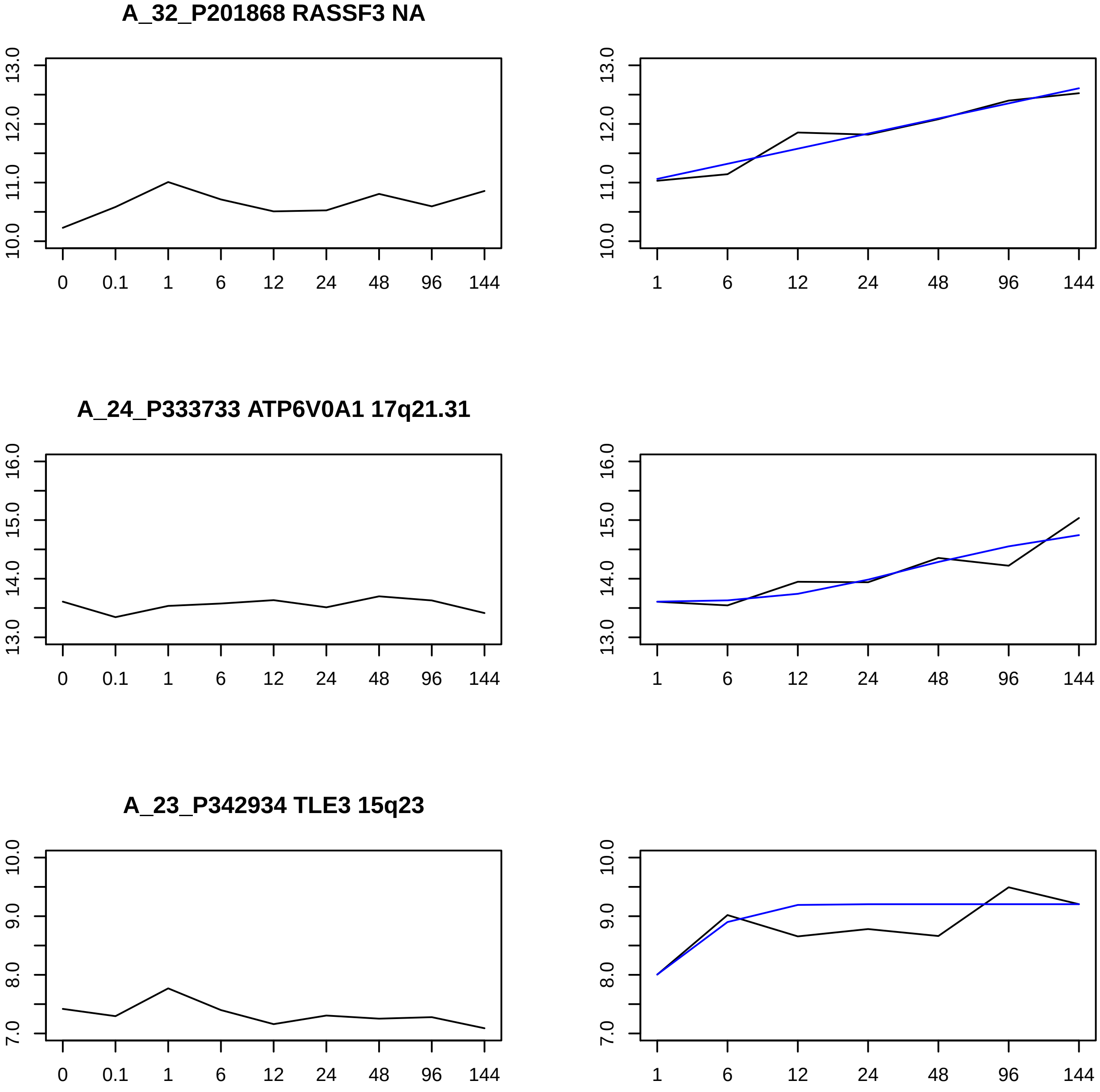
A_32_P201868 RASSF3 NA
10.0
11.0
12.0
13.0
0
0.1
1
6
12
24
48
96
144
A_24_P333733 ATP6V0A1 17q21.31
13.0
14.0
15.0
16.0
A_23_P342934 TLE3 15q23
7.0
8.0
9.0
10.0

A_24_P396980 PFN2 3q25.1

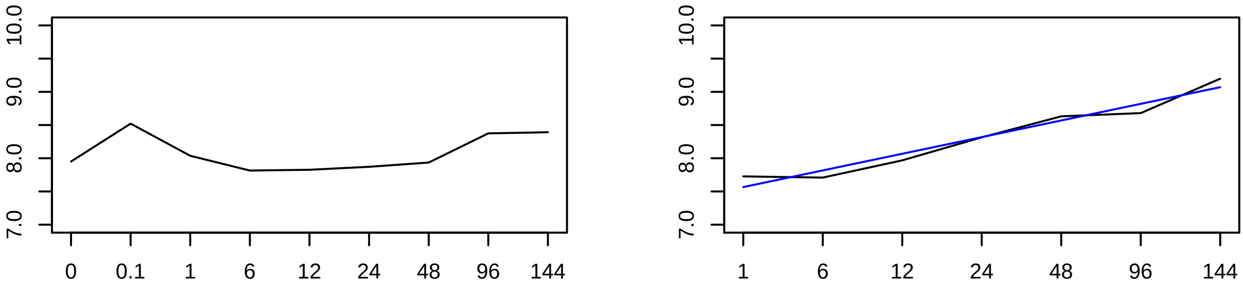

A_32_P154726 HOXC−AS2 12q13.13

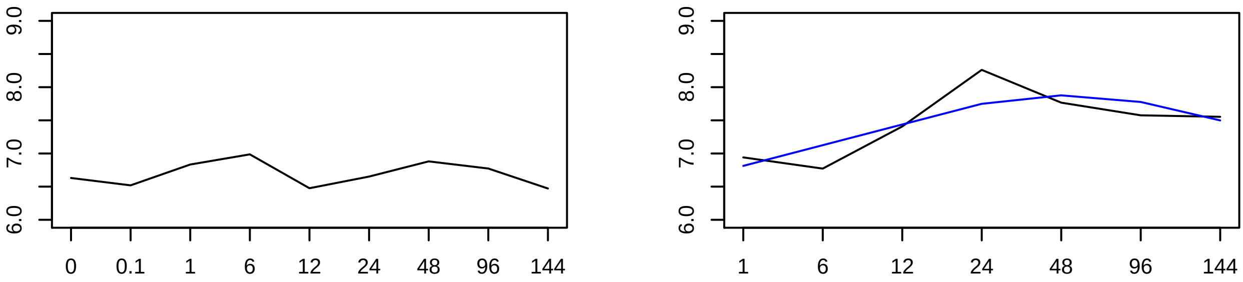

A_23_P82523 ABCB1 7q21.12

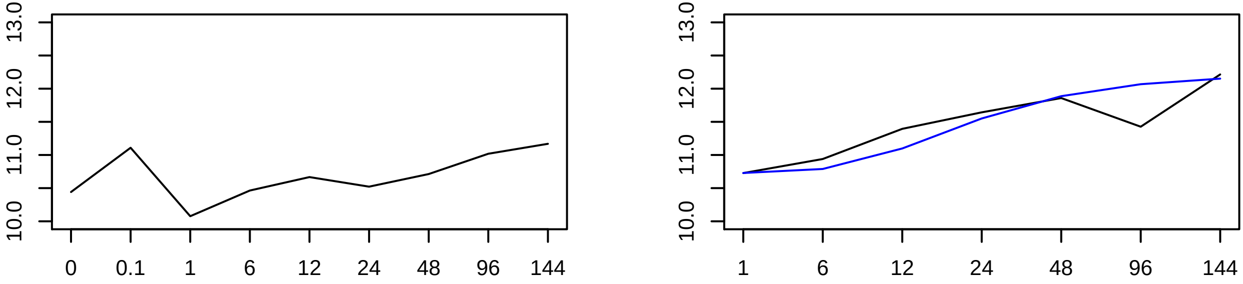

A_32_P838639

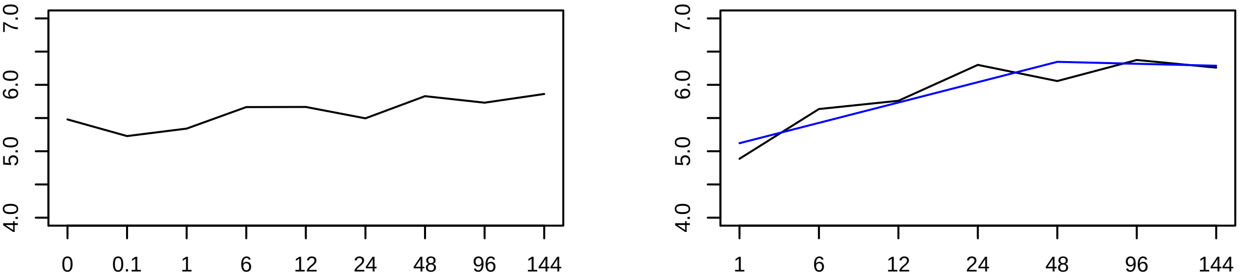

A_24_P42603 TRIO 5p15.2

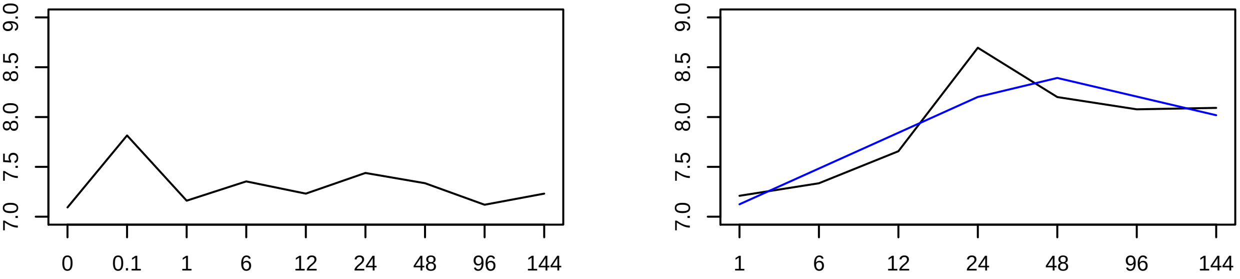

A_23_P53257 AVIL 12q14.1

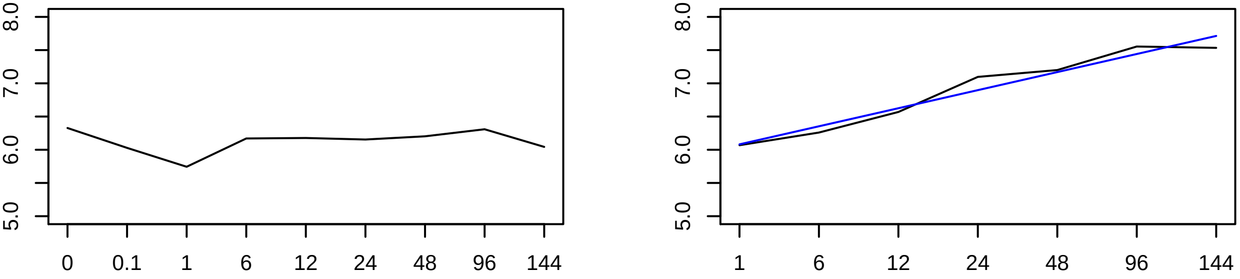

A_23_P209712 SP100 2q37.1

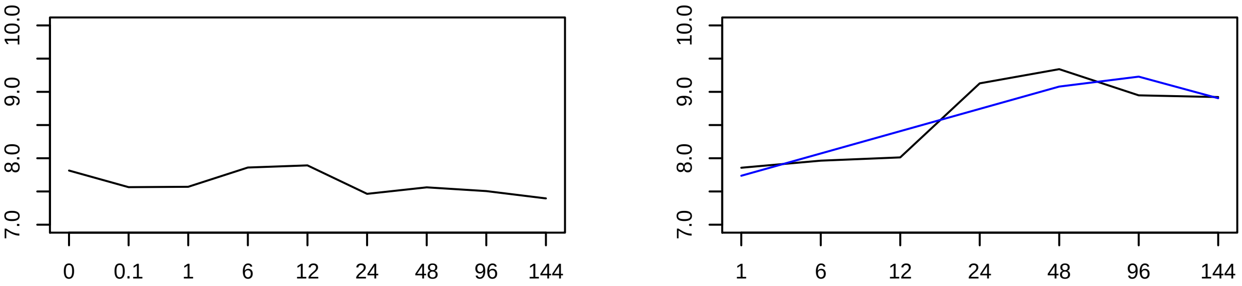

A_24_P380679 LSMEM1 7q31.1

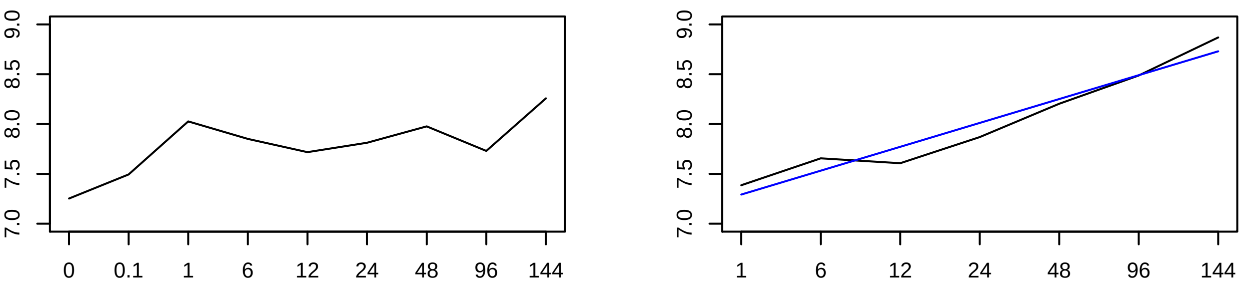

A_23_P21673 KIAA1797 9p21.3

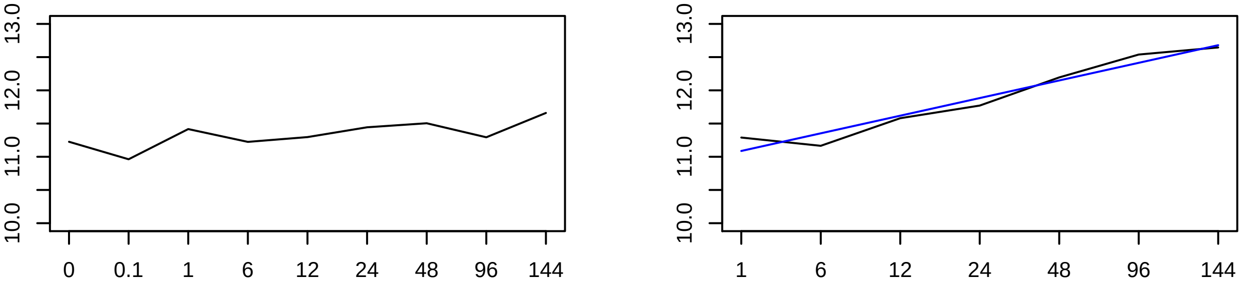

A_23_P164706 ZNF177 19p13.2

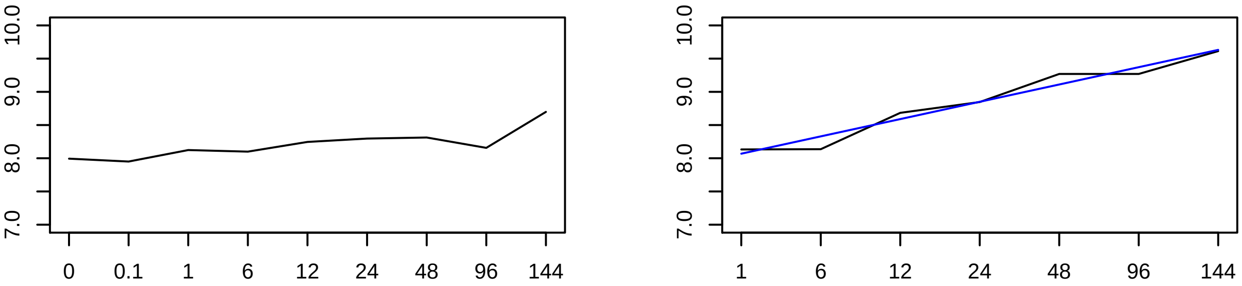

A_23_P324490 KIAA0355 19q13.11

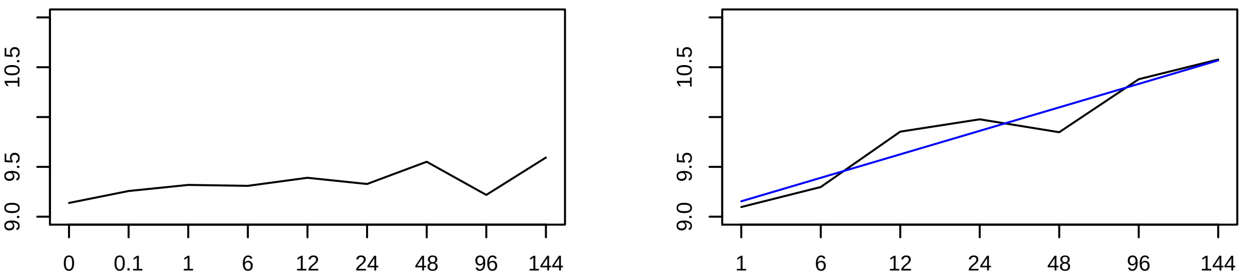

A_32_P119744 LINC00883 3q13.12

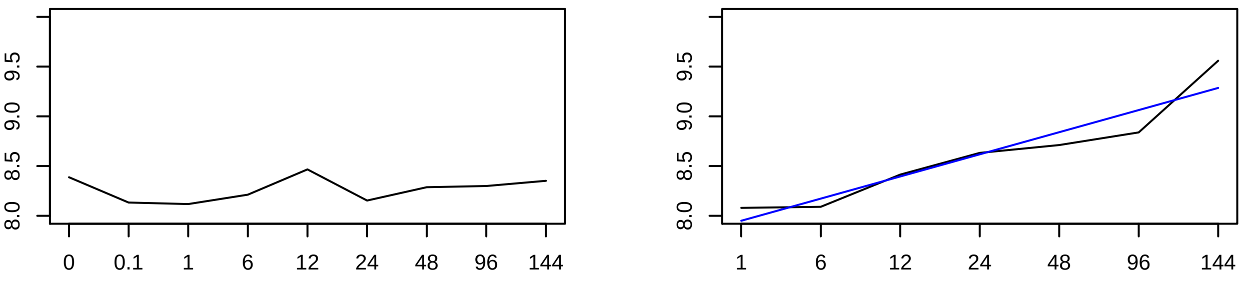

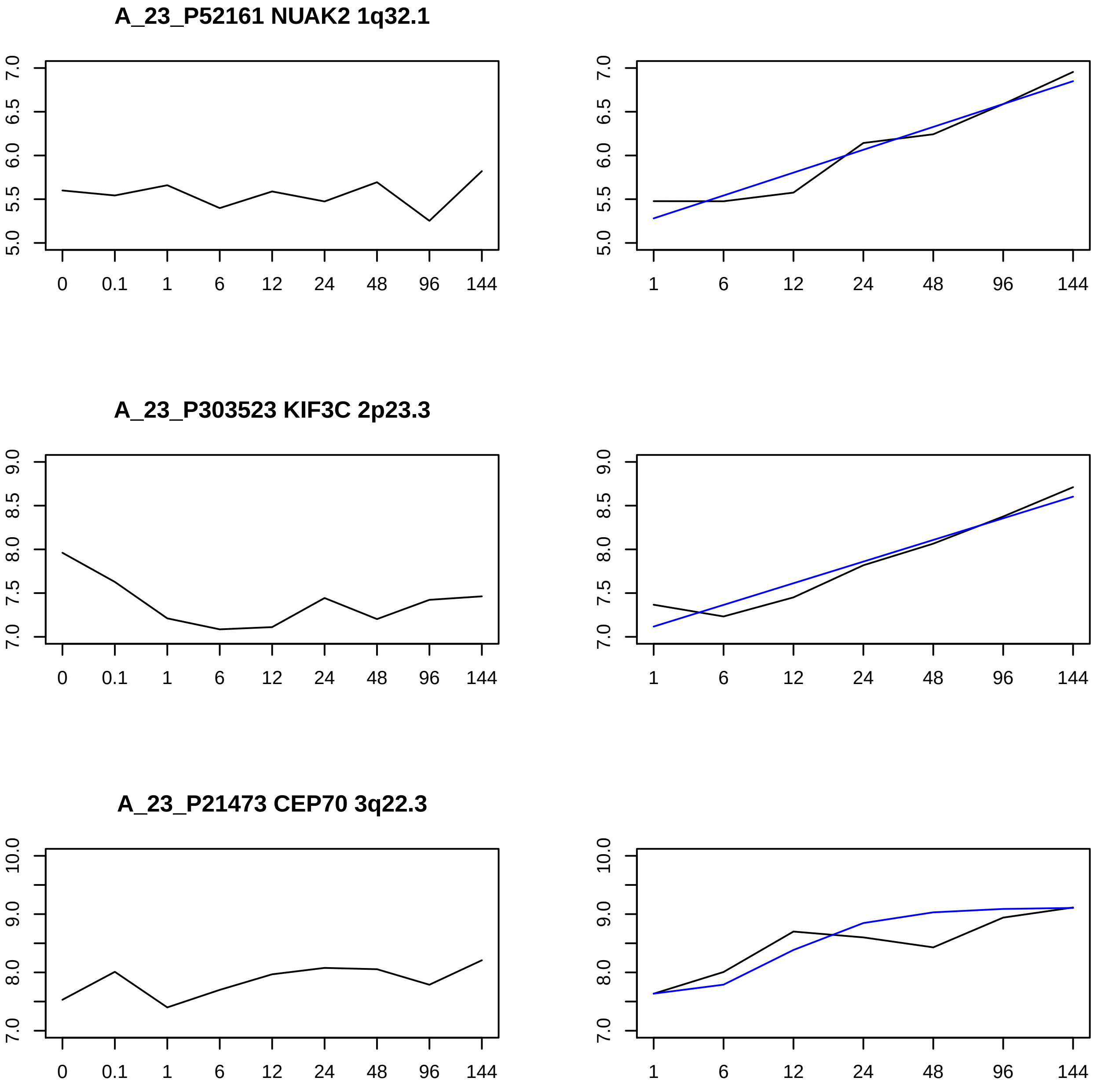

A_23_P52161 NUAK2 1q32.1
5.0
5.5
6.0
6.5
7.0
0
0.1
1
6
12
24
48
96
144
A_23_P303523 KIF3C 2p23.3
7.0
7.5
8.0
8.5
9.0
A_23_P21473 CEP70 3q22.3
7.0
8.0
9.0
10.0

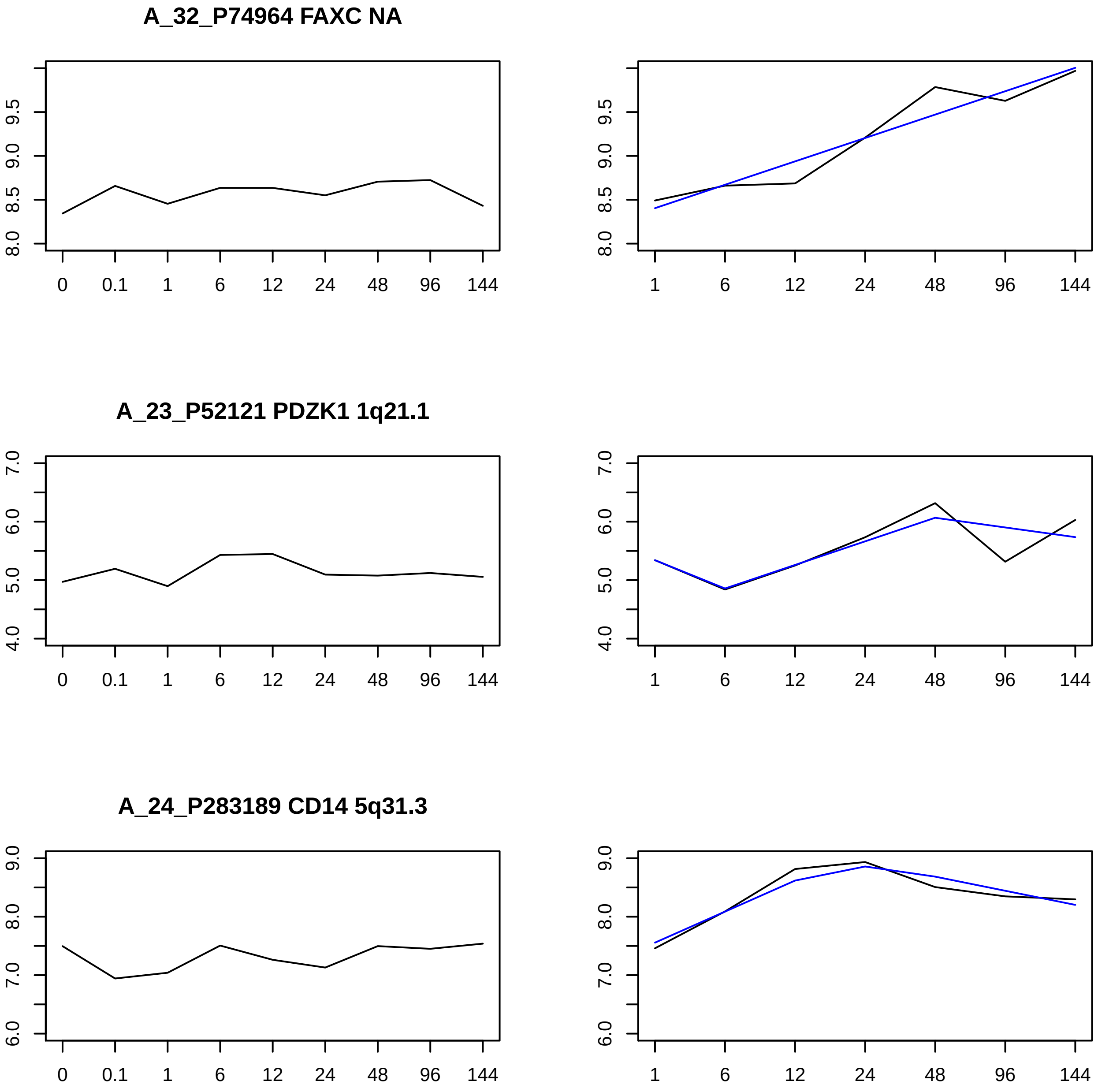
A_32_P74964 FAXC NA
A_23_P52121 PDZK1 1q21.1
A_24_P283189 CD14 5q31.3
0 0.1 1 6 12 24 48 96 144
1 6 12 24 48 96 144
8.0 8.5 9.0 9.5
4.0 5.0 6.0 7.0
6.0 7.0 8.0 9.0

A_23_P70355 SERPINB6 6p25.2

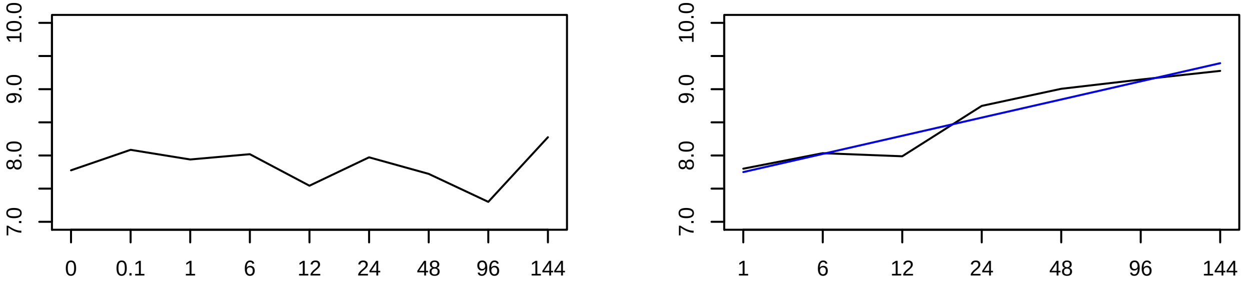

A_23_P51986 MTMR11 1q21.2

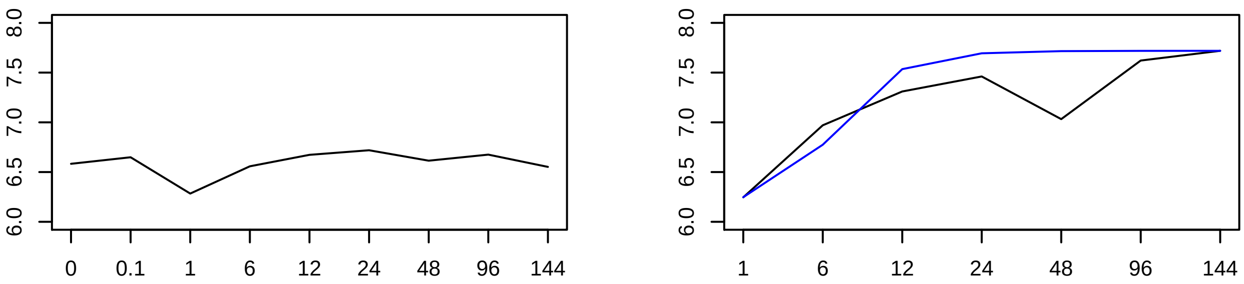

A_23_P155027 MORC2 22q12.2

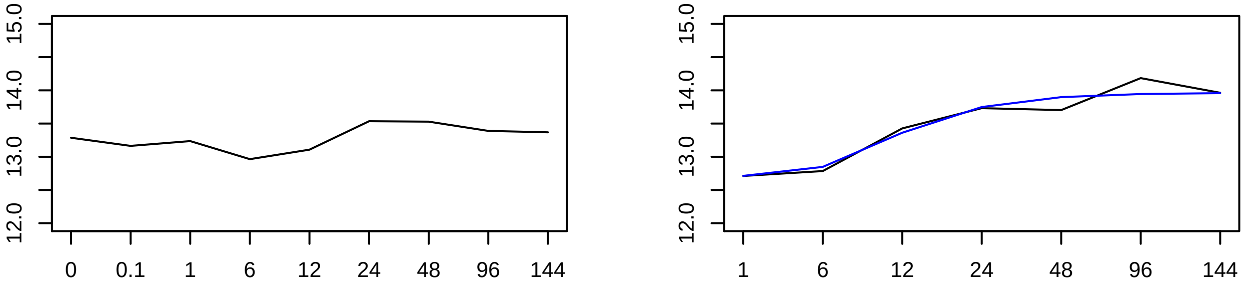

A_23_P40866 ZBTB20−AS1 3q13.31

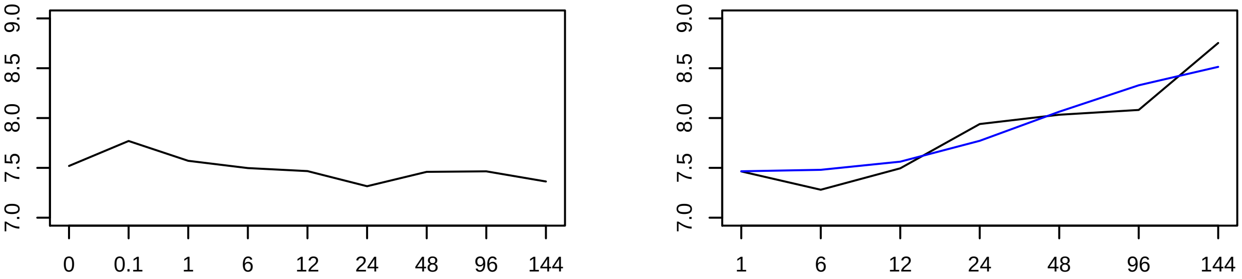

A_32_P106646 FAM36A 1q44

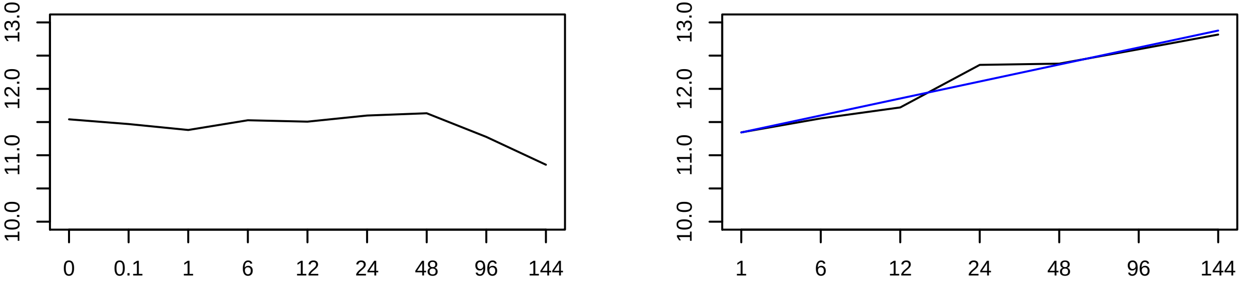

A_32_P146659 ATP6V0E2−AS1 7q36.1

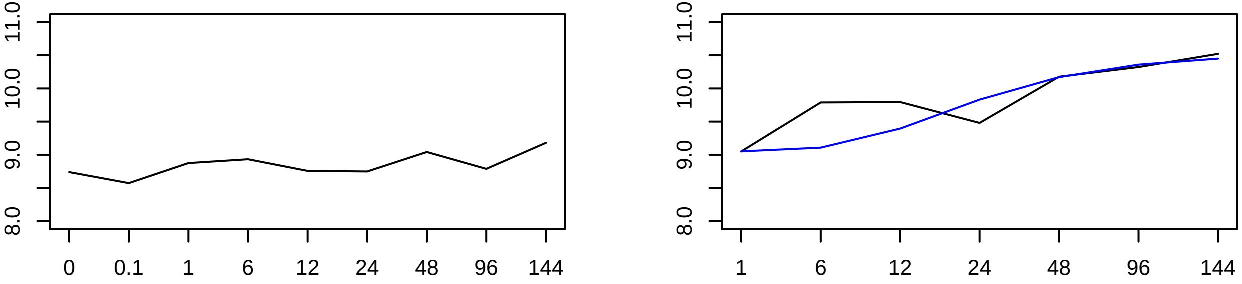

A_23_P105264 ETV6 12p13.2

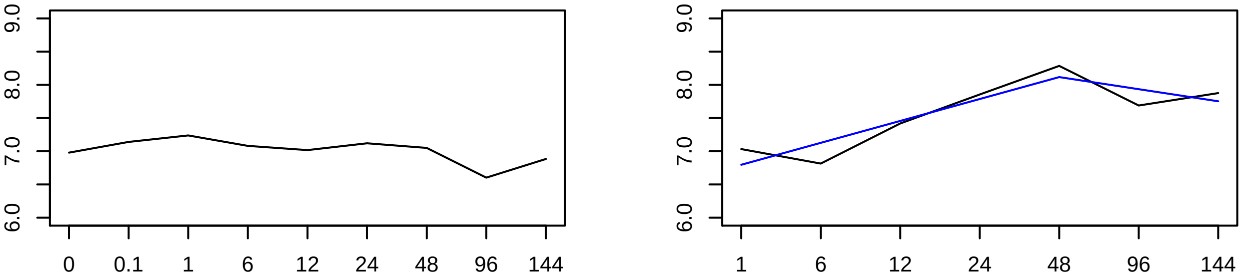

A_23_P139965 C13orf21 13q14.11

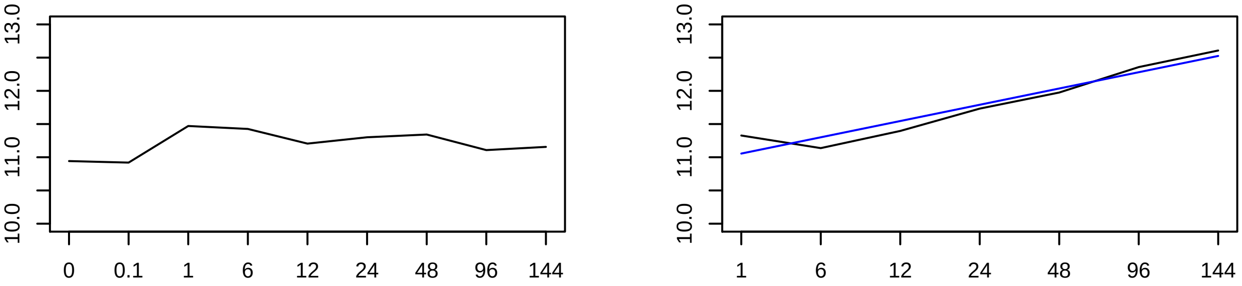

A_23_P368187 NAB2 12q13.3

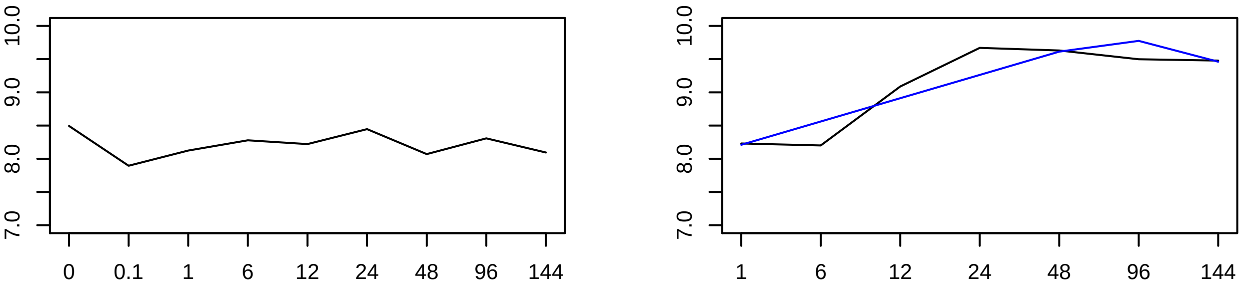

A_23_P81262 PCDHB4 5q31.3

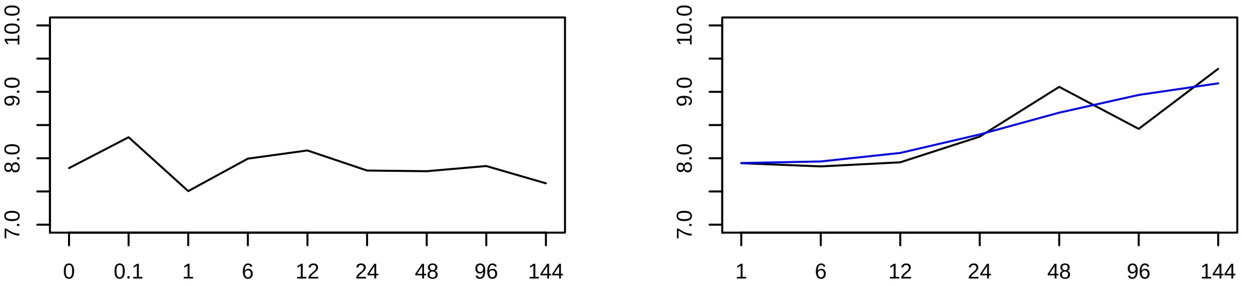

A_23_P41487 TBC1D9 4q31.21

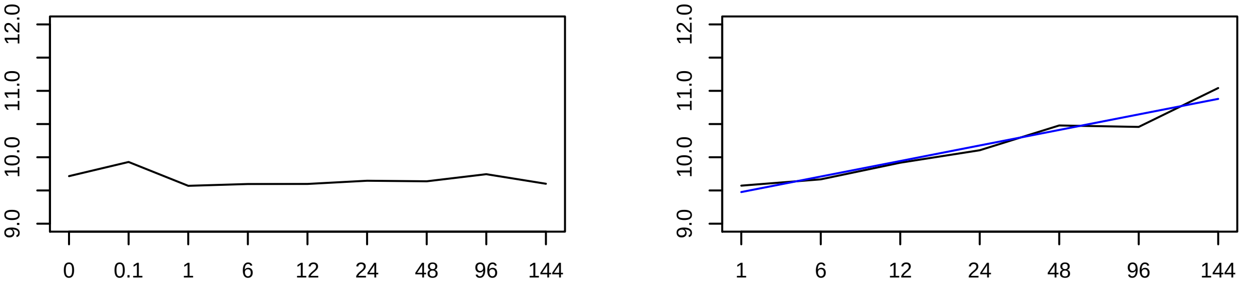

A_24_P24263 PDLIM5 4q22.3

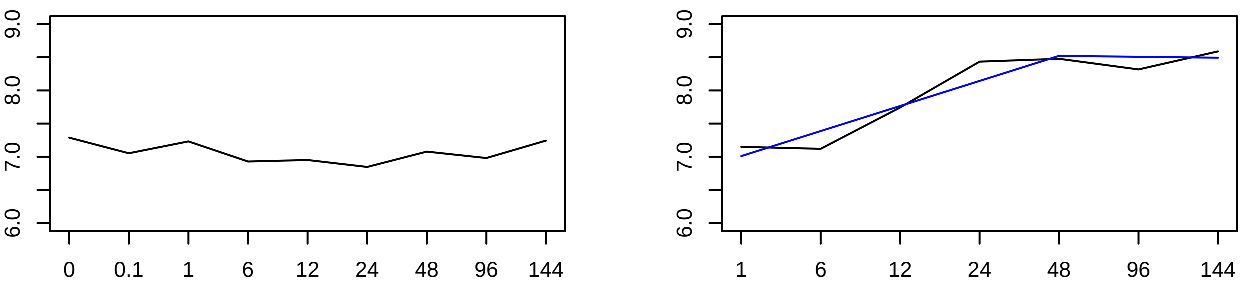

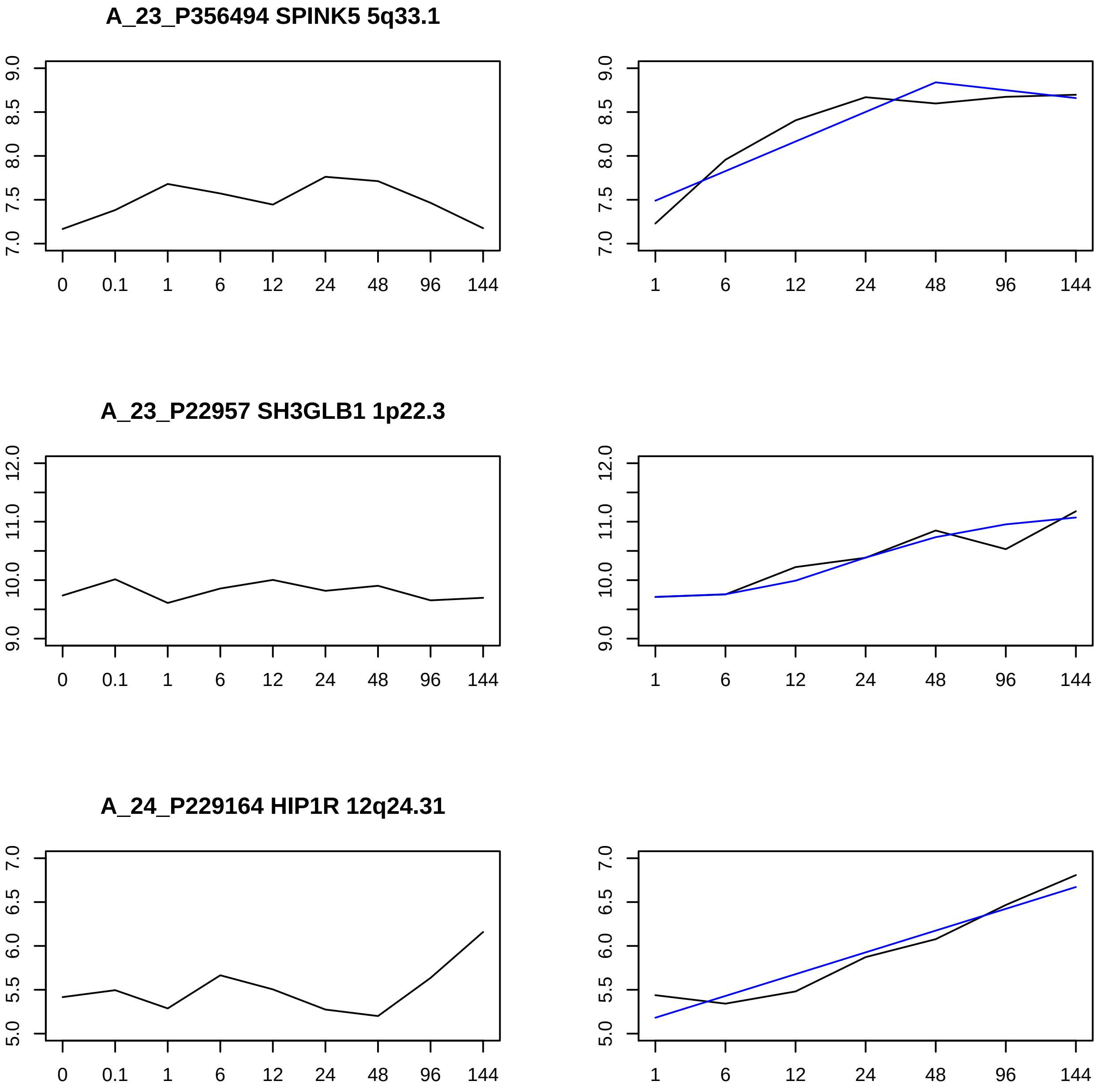
A_23_P356494 SPINK5 5q33.1
7.0 7.5 8.0 8.5 9.0
0 0.1 1 6 12 24 48 96 144
1 6 12 24 48 96 144
A_23_P22957 SH3GLB1 1p22.3
9.0 10.0 11.0 12.0
0 0.1 1 6 12 24 48 96 144
1 6 12 24 48 96 144
A_24_P229164 HIP1R 12q24.31
5.0 5.5 6.0 6.5 7.0
0 0.1 1 6 12 24 48 96 144
1 6 12 24 48 96 144

A_23_P45025 MAPK10 4q21.3

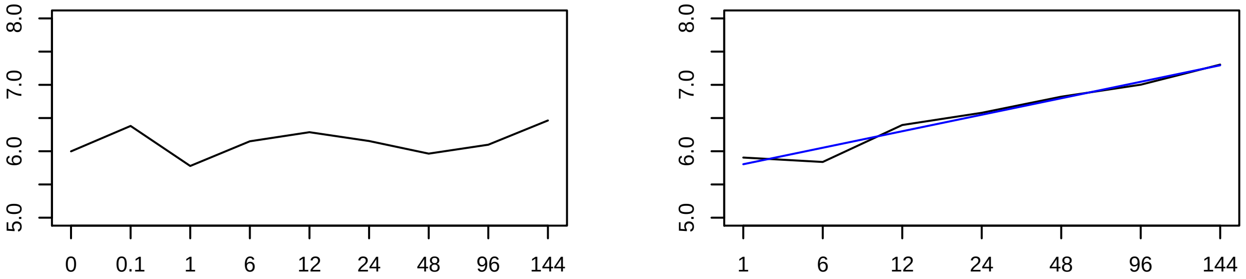

A_24_P12435 NCOA7 6q22.32

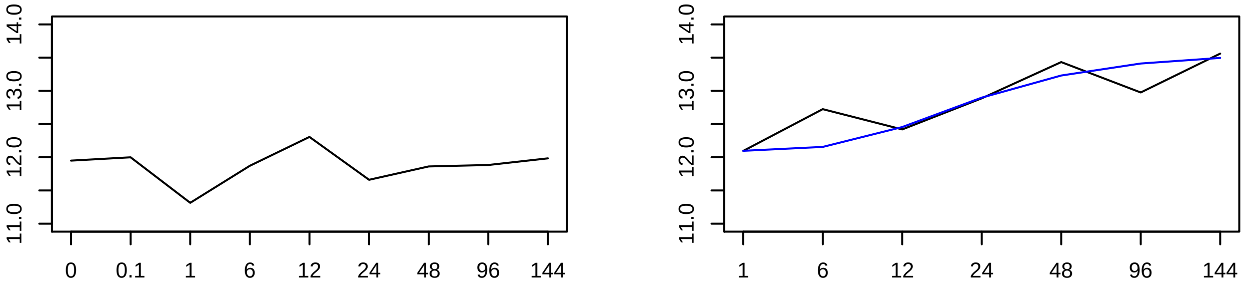

A_23_P204801 SLC41A2 12q23.3

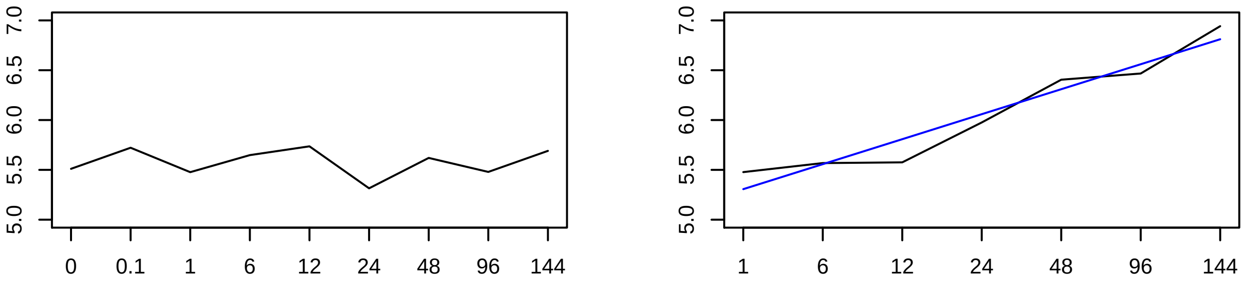

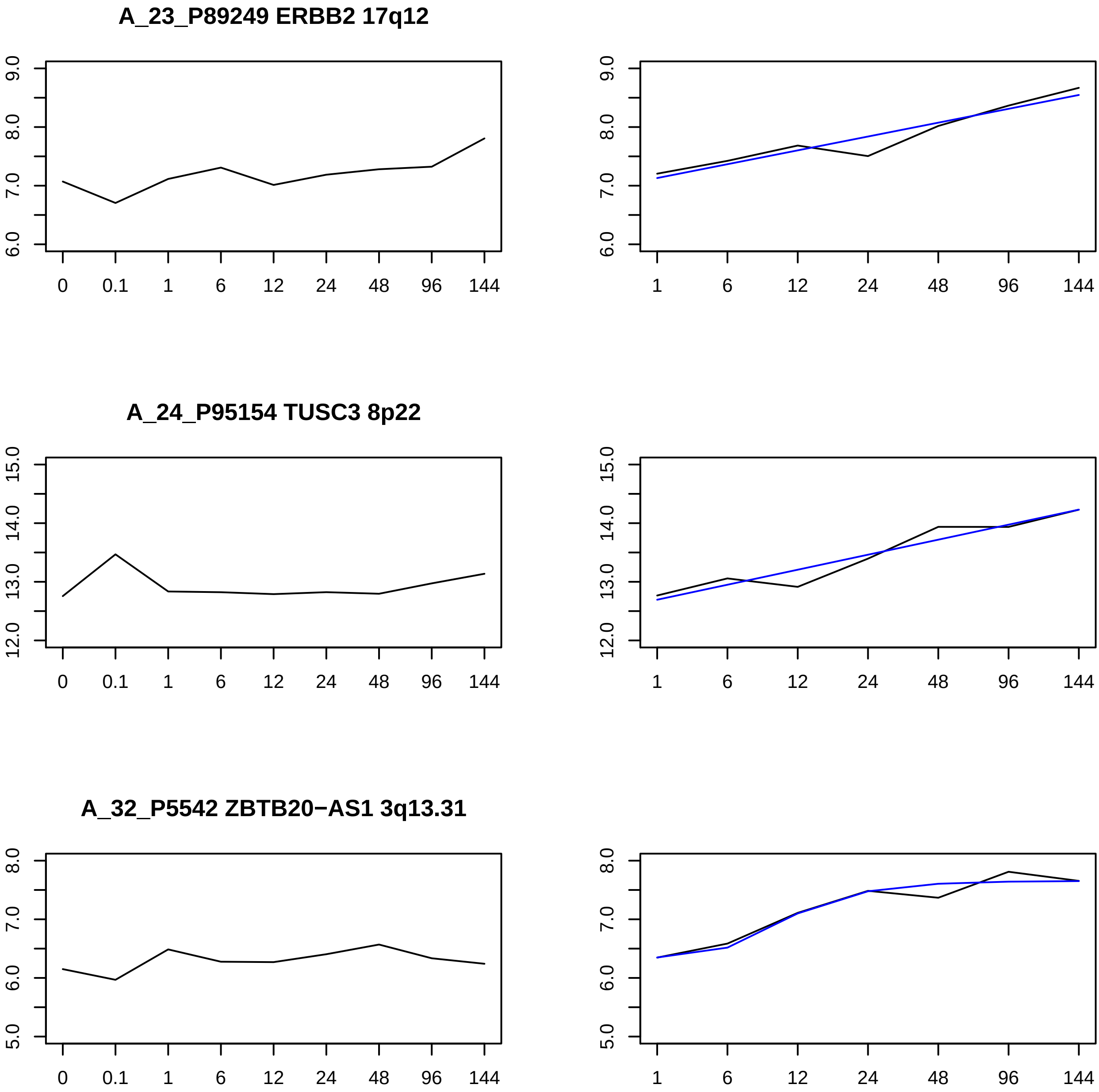
A_23_P89249 ERBB2 17q12
A_24_P95154 TUSC3 8p22
A_32_P5542 ZBTB20−AS1 3q13.31
0
0.1
1
6
12
24
48
96
144
6.0
7.0
8.0
9.0
12.0
13.0
14.0
15.0
5.0

A_23_P421401 PDGFRB 5q33.1

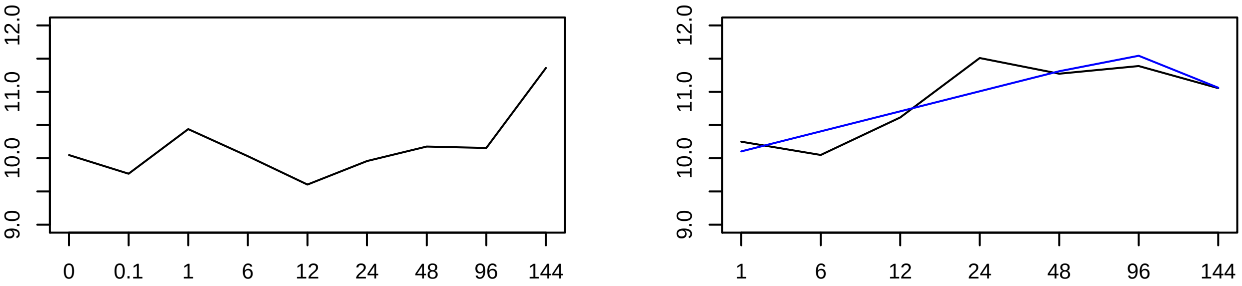

A_23_P204550 SCYL2 12q23.1

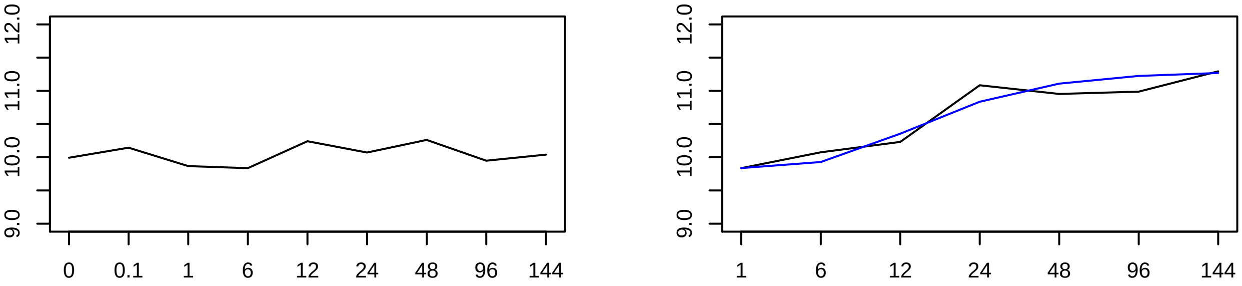

A_23_P25615 SOHLH2 13q13.3

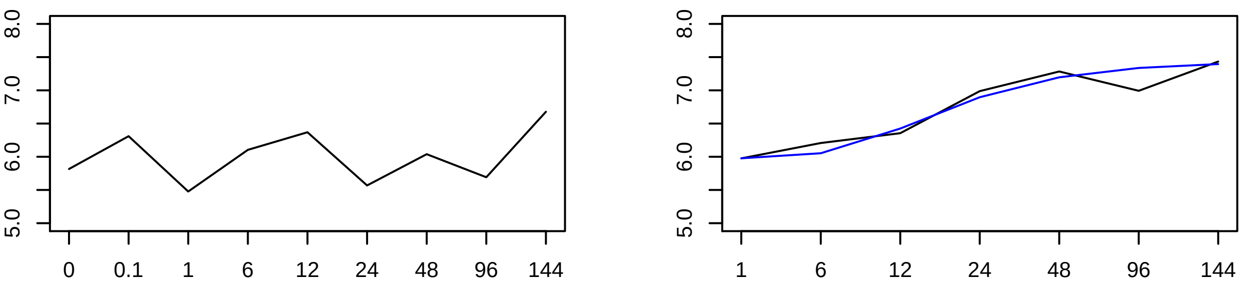

A_24_P227121 THC2685096 NA

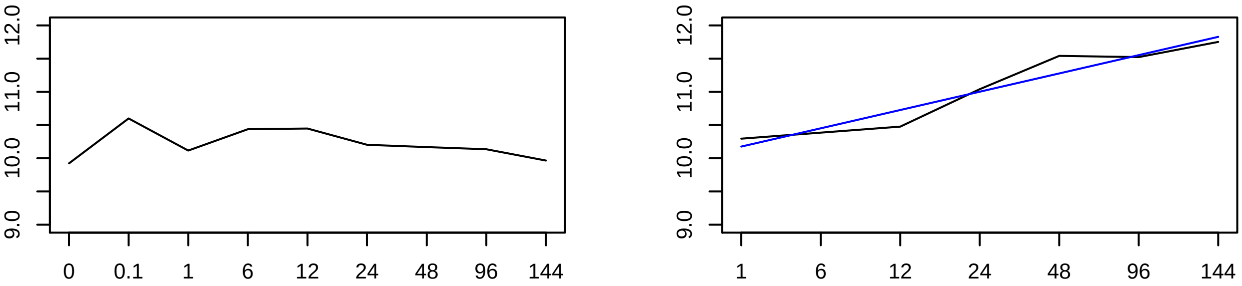

A_23_P150931 LMBR1L 12q13.12

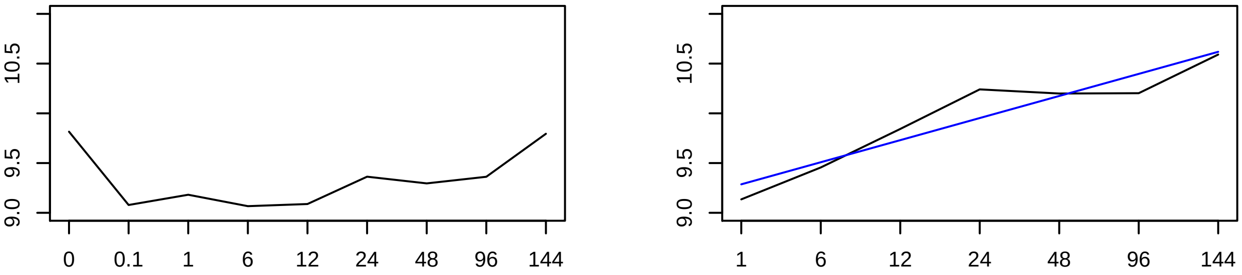

A_23_P213385 BASP1 5p15.1

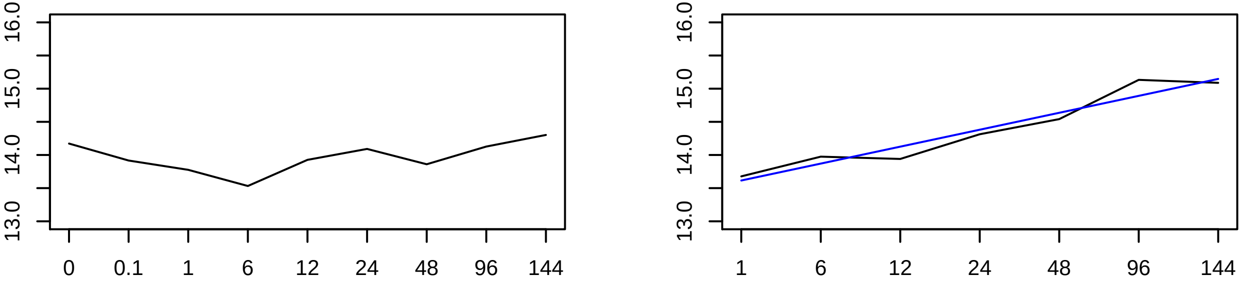

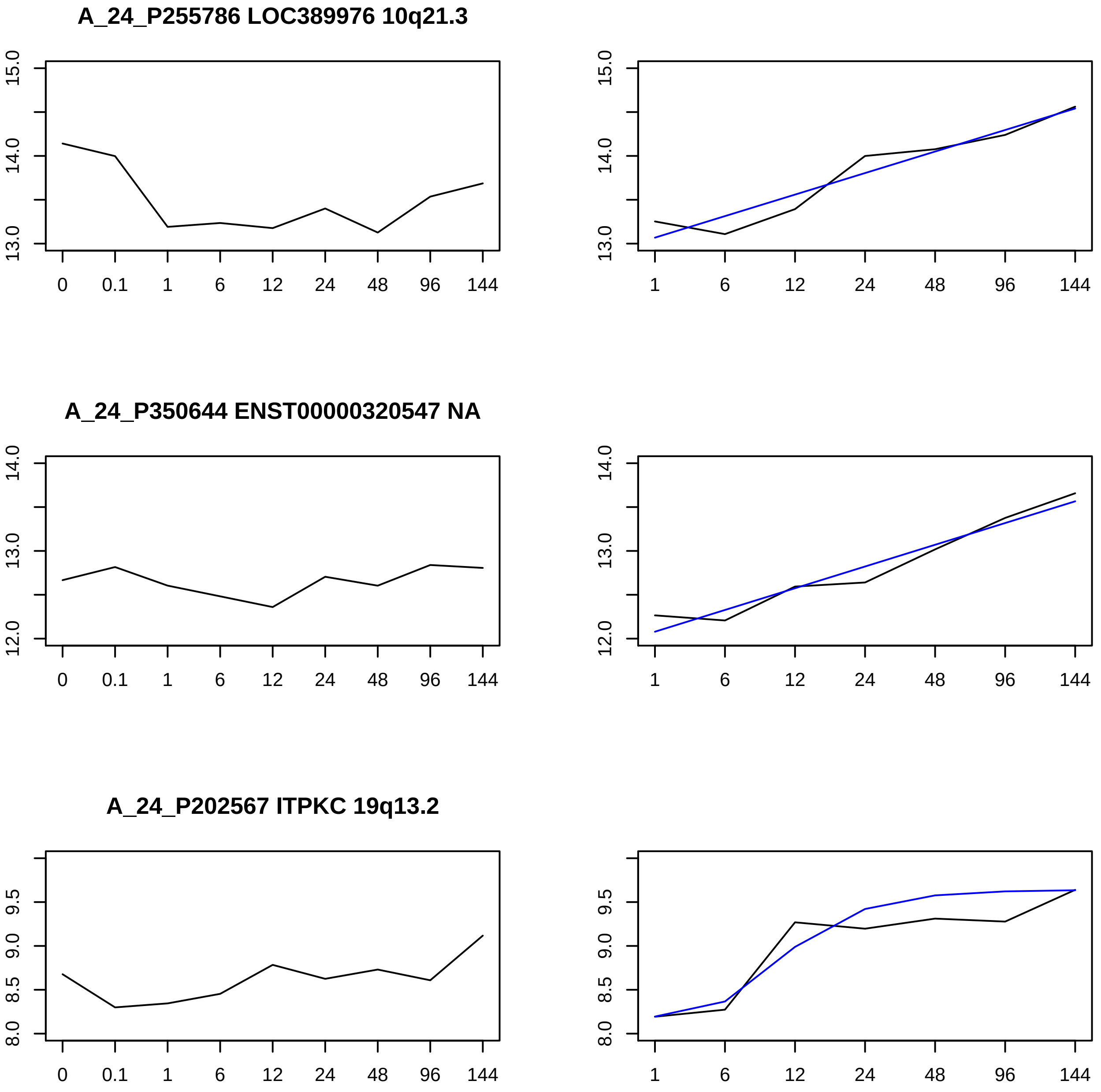

A_24_P255786 LOC389976 10q21.3
13.0
14.0
15.0
0
0.1
1
6
12
24
48
96
144
1
6
12
24
48
96
144
A_24_P350644 ENST00000320547 NA
12.0
13.0
14.0
A_24_P202567 ITPKC 19q13.2
8.0
8.5
9.0
9.5

A_24_P161393 A_24_P161393 NA

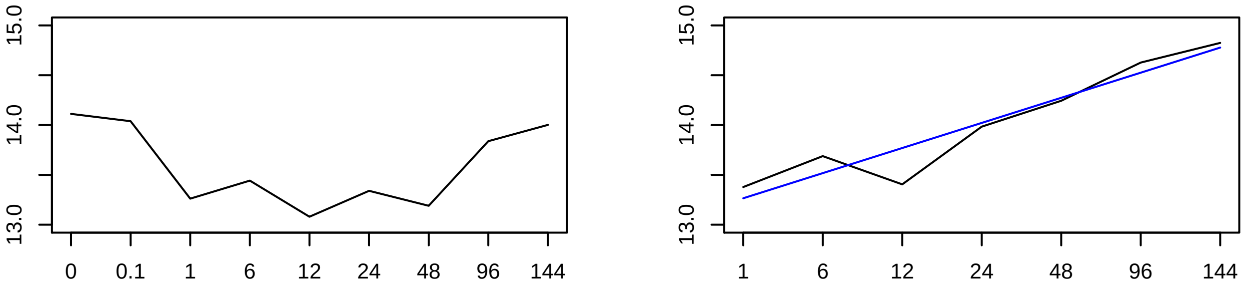

A_23_P141484 C17orf63 17q11.2

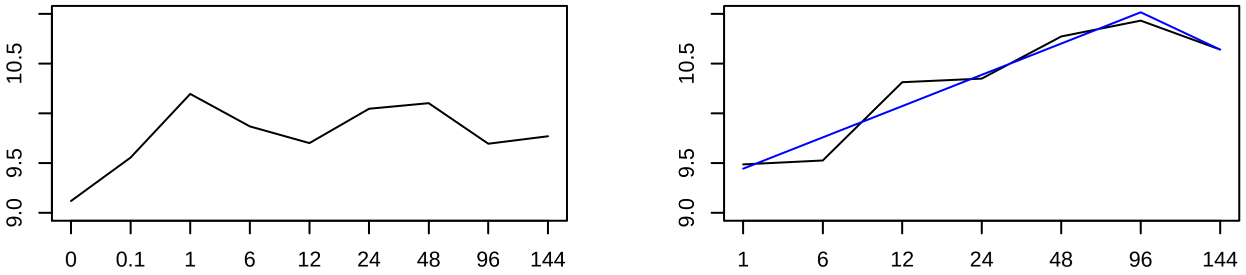

A_23_P86330 IER5 1q25.3

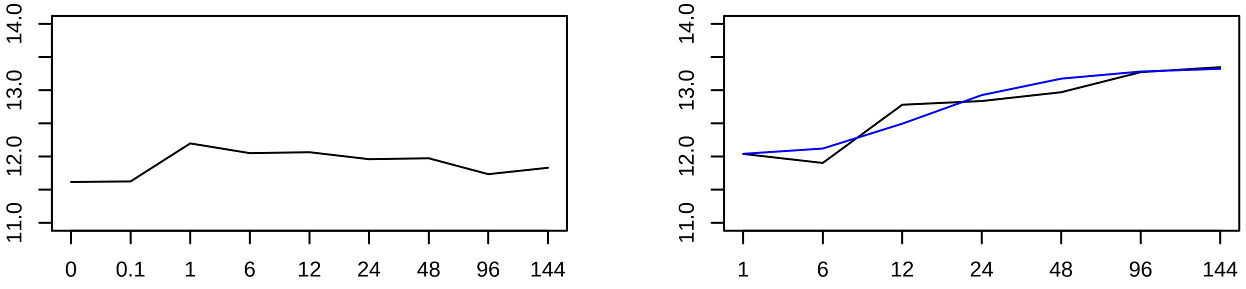

A_24_P682285 HSPA1A 6p21.33

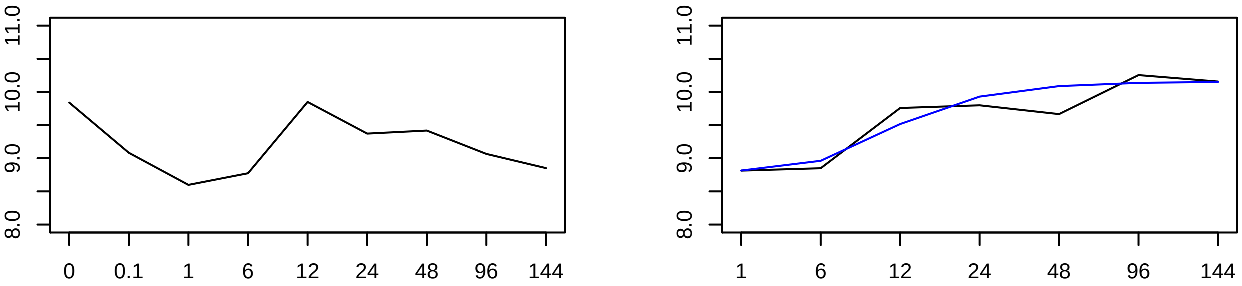

A_23_P335452 C10orf56 10q22.3

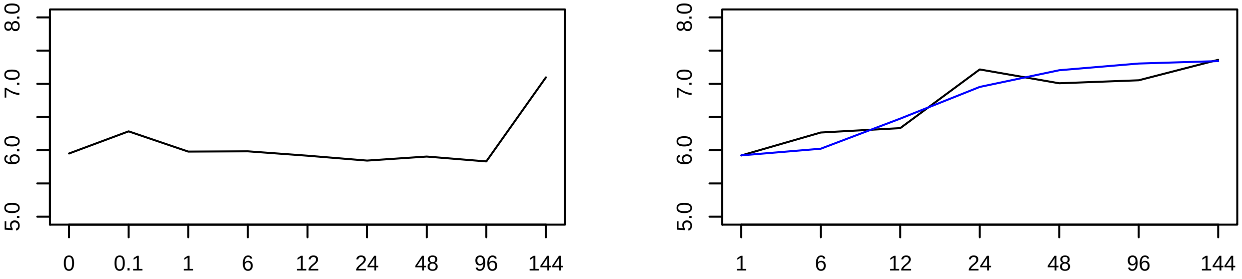

A_23_P326319 C16orf45 16p13.11

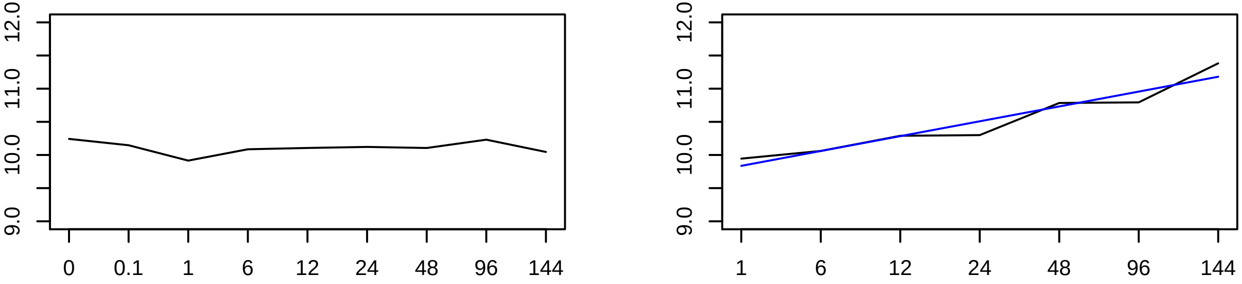

A_32_P153833 AK098597 NA

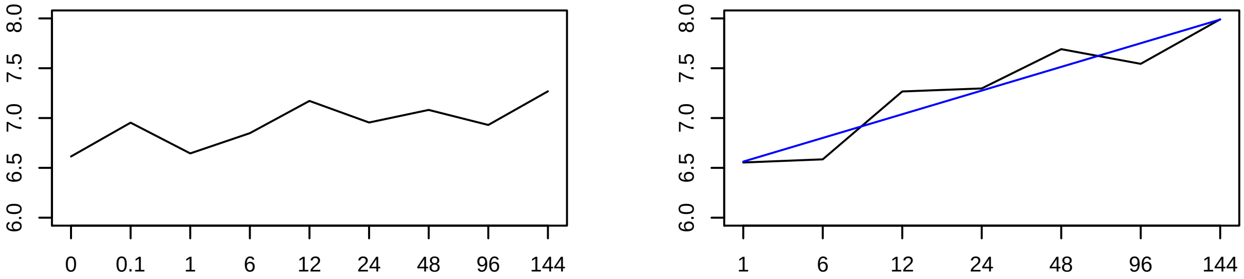

A_24_P14595 POFUT2 21q22.3

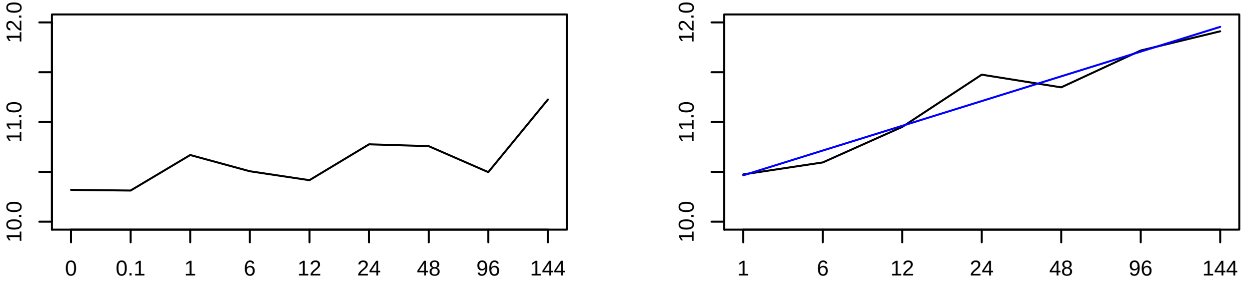

A_23_P203891 NCOR2 12q24.31

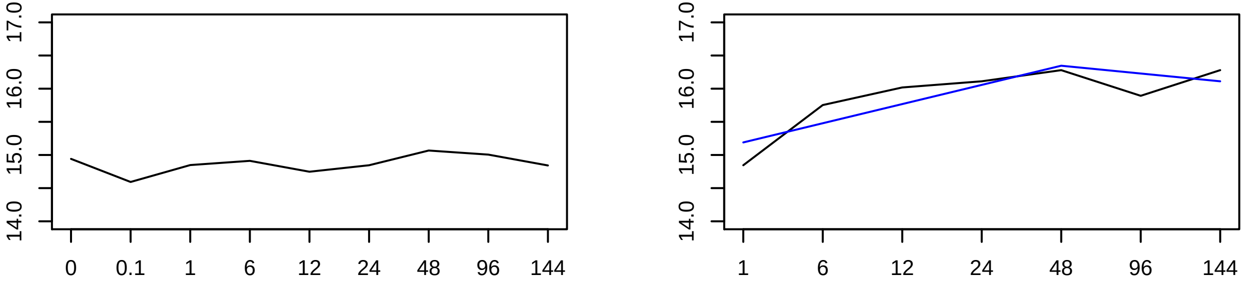

A_23_P207414 AARSD1 17q21.31

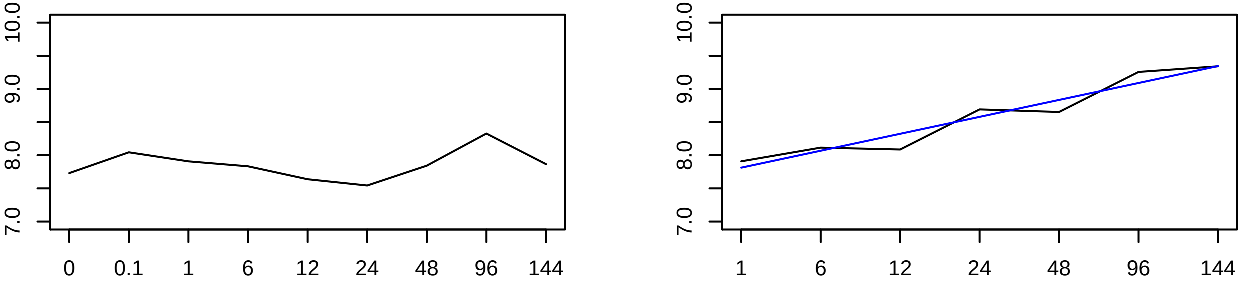

A_24_P380284 PCDHB9 5q31.3

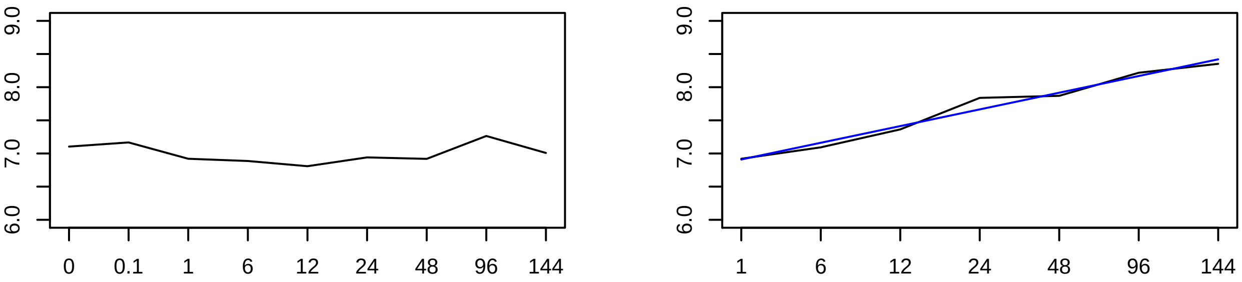

A_24_P374834 OTUD1 10p12.2

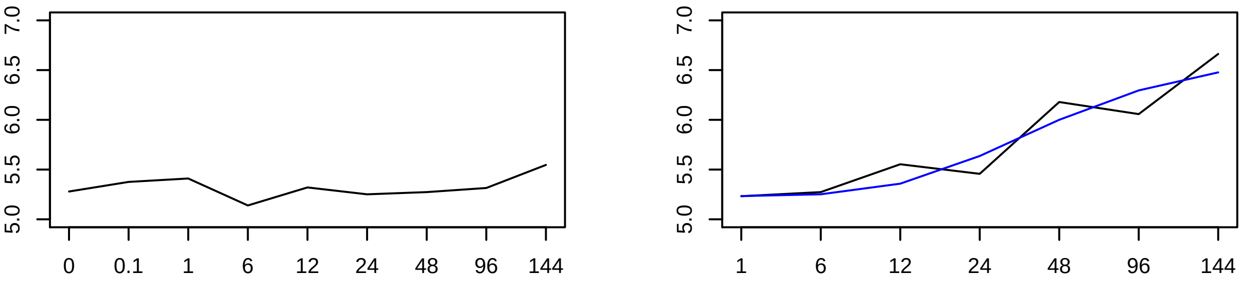

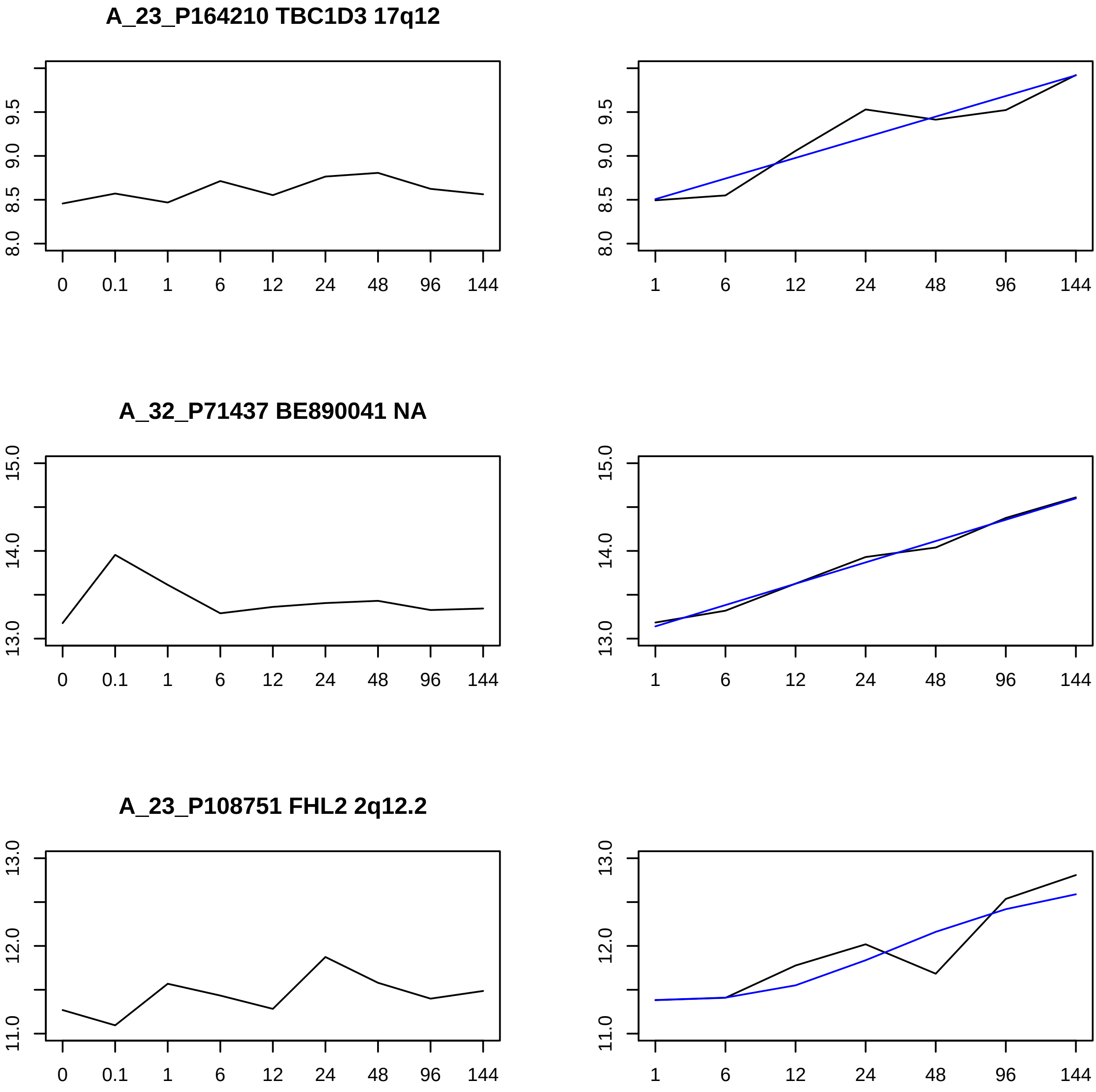
A_23_P164210 TBC1D3 17q12
8.0
8.5
9.0
9.5
0
0.1
1
6
12
24
48
96
144
1
6
12
24
48
96
144
A_32_P71437 BE890041 NA
13.0
14.0
15.0
A_23_P108751 FHL2 2q12.2
11.0
12.0
13.0

A_24_P838743 SERPINB6 6p25.2

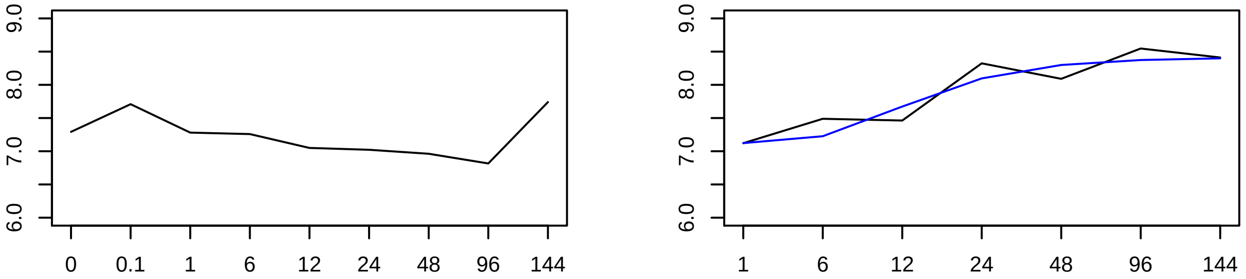

A_23_P135857 EIF2AK3 2p11.2

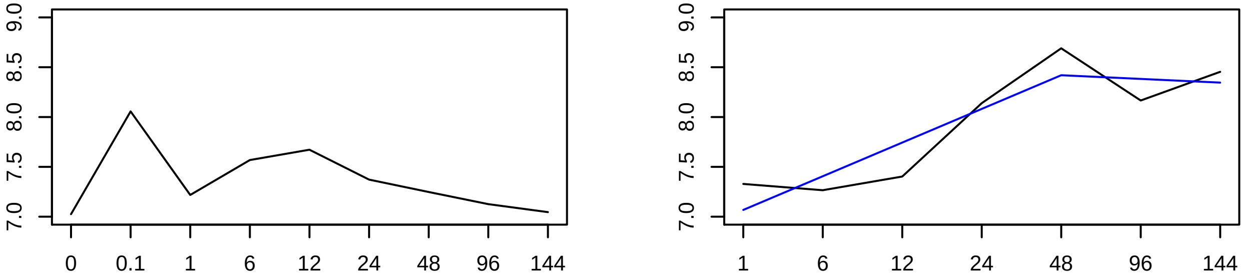

A_32_P355396 KIAA0329 14q32.31

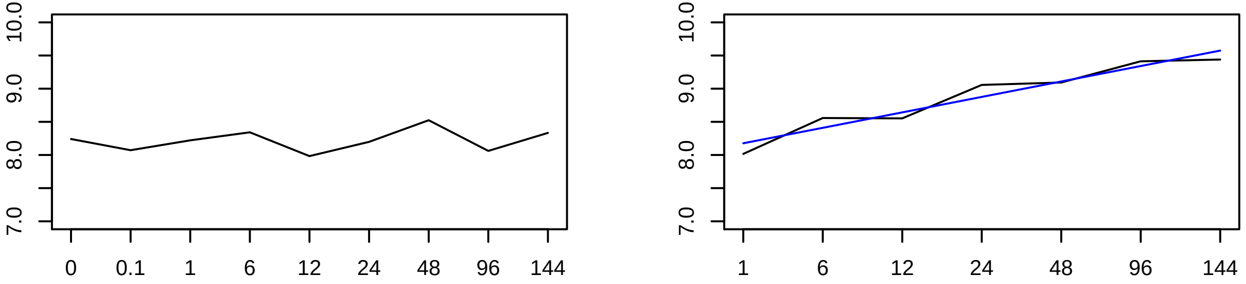

A_24_P6552 DPP6 7q36.2

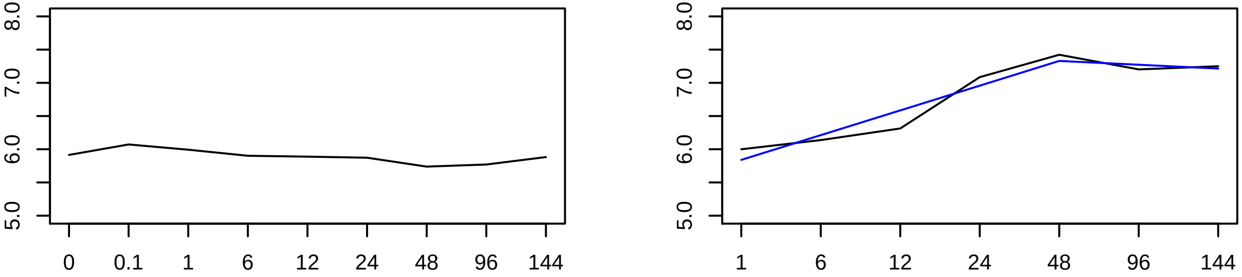

A_23_P259272 WSB2 12q24.23

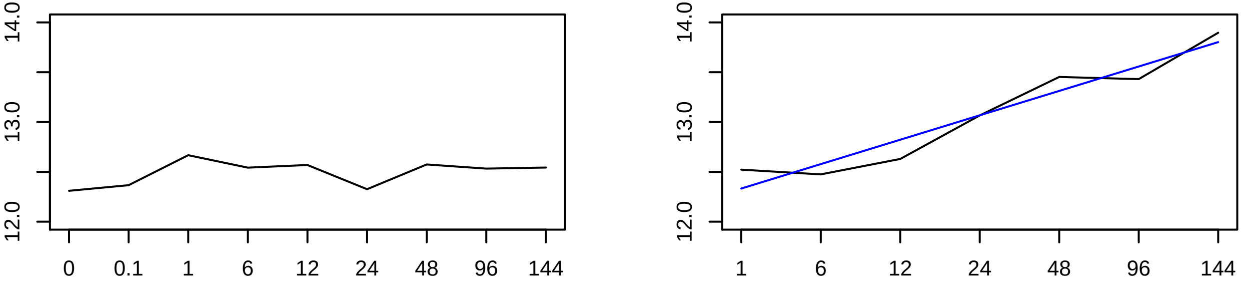

A_24_P264416 A_24_P264416 NA

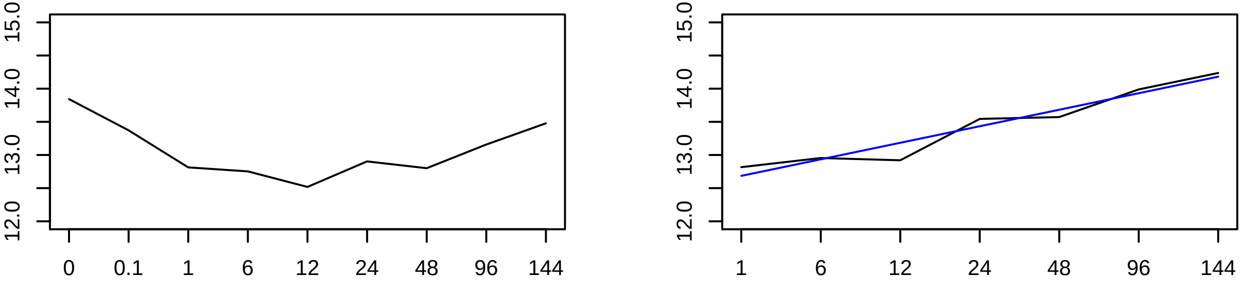

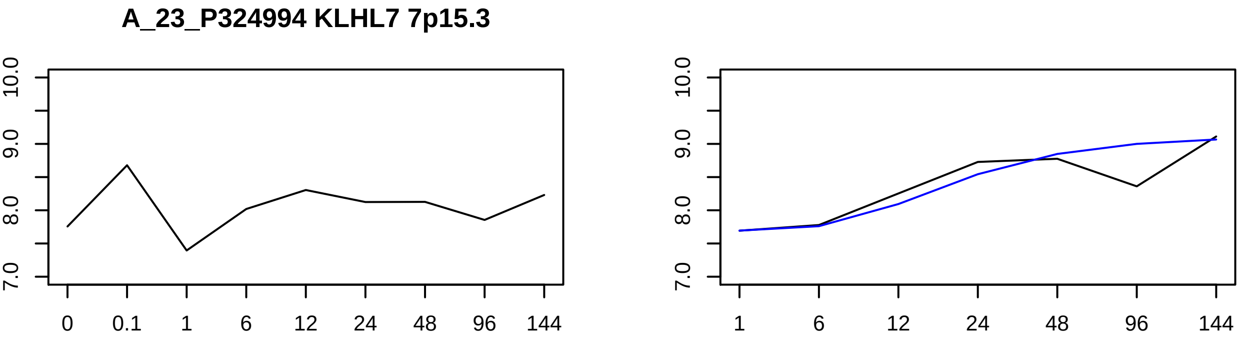
A_23_P324994 KLHL7 7p15.3
7.0
8.0
9.0
10.0
0
0.1
1
6
12
24
48
96
144

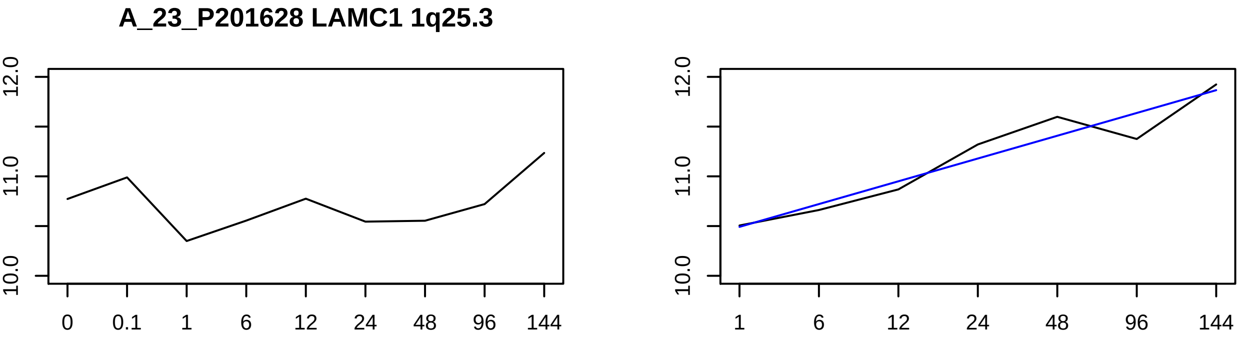
A_23_P201628 LAMC1 1q25.3
10.0
11.0
12.0
0
0.1
1
6
12
24
48
96
144

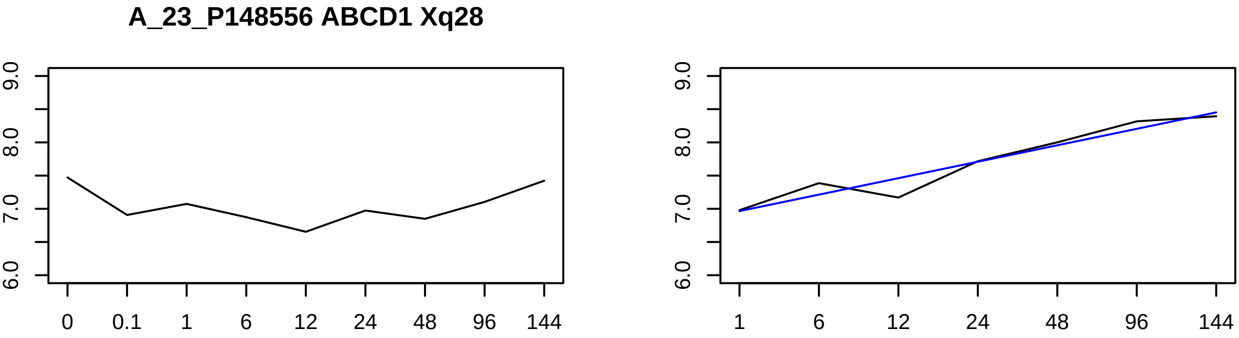
A_23_P148556 ABCD1 Xq28
6.0
7.0
8.0
9.0
0
0.1
1
6
12
24
48
96
144

A_23_P133656 LAMA4 6q21

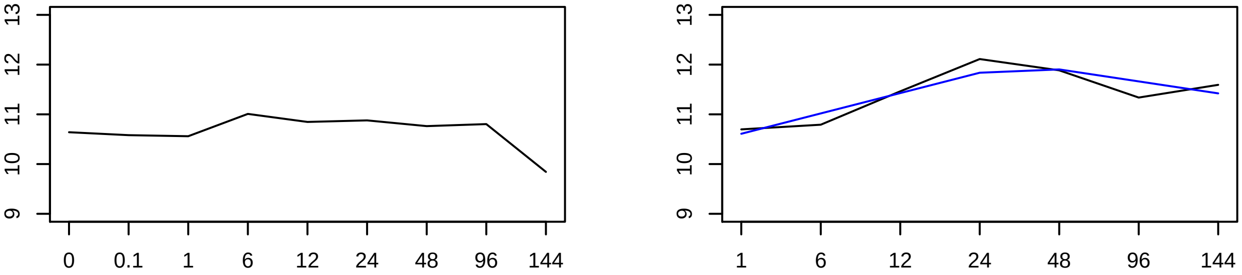

A_23_P201979 CREM 10p11.21

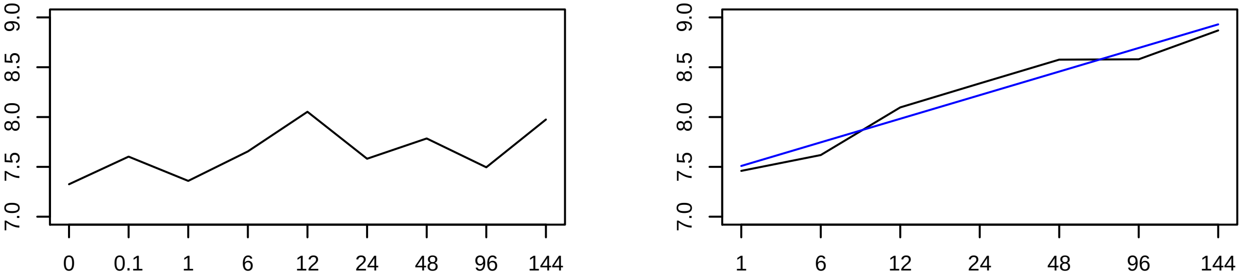

A_23_P2322 TSPAN19 12q21.31

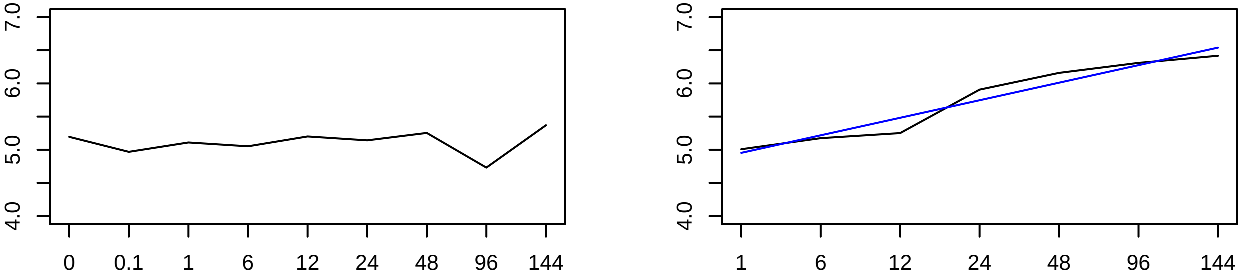

A_23_P130352 KCTD1 18q11.2

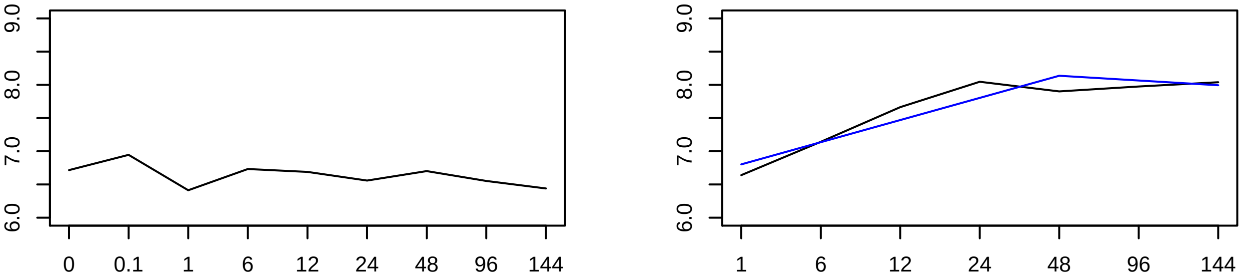

A_24_P64362 C10orf26 10q24.32

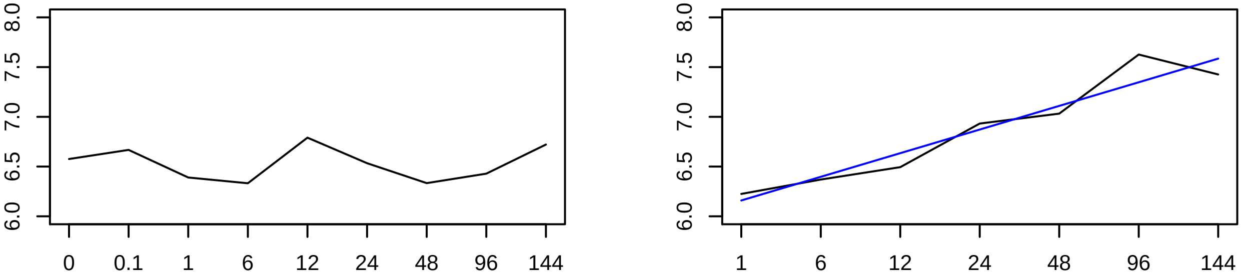

A_24_P639441 CD59 11p13

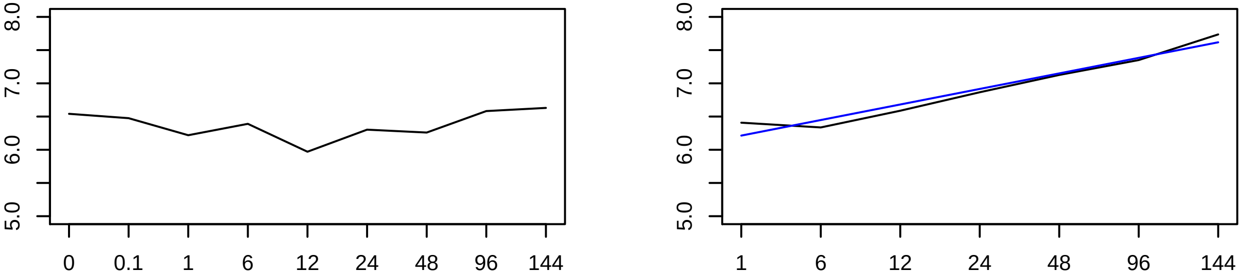

A_23_P138000 SEMA6C 1q21.2

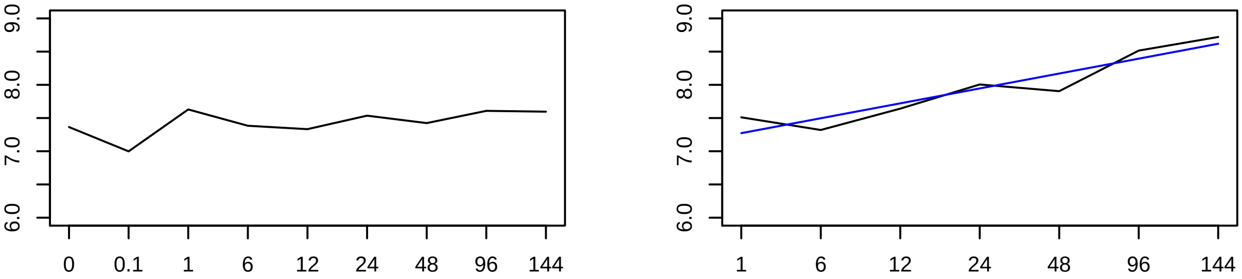

A_23_P47155 PANX1 11q21

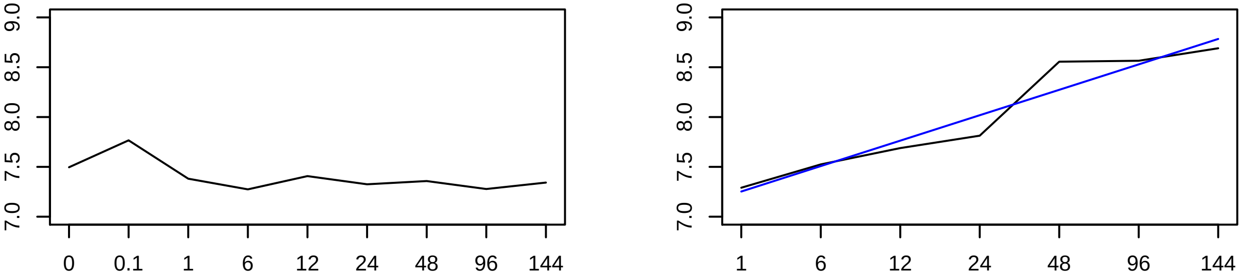

A_32_P98914 AW389821 NA

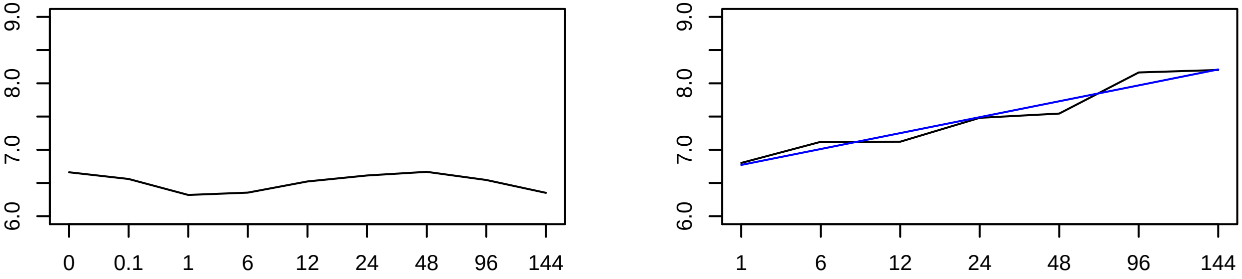

A_24_P146138 PCDHA9 5q31.3

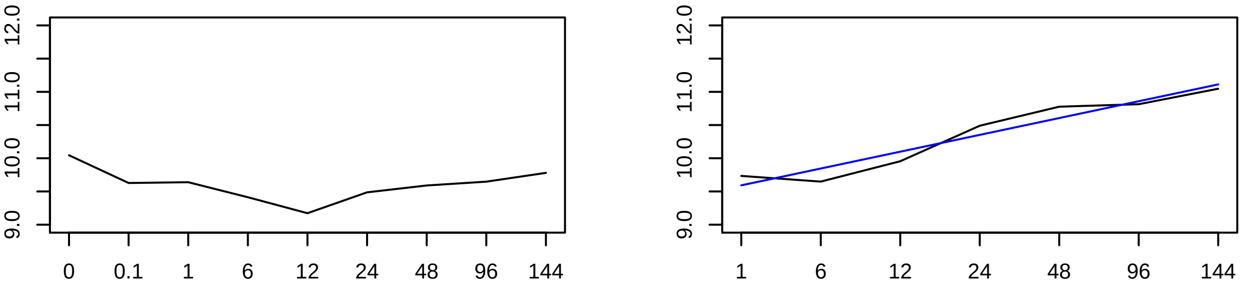

A_32_P15829 ST6GAL1 3q27.3

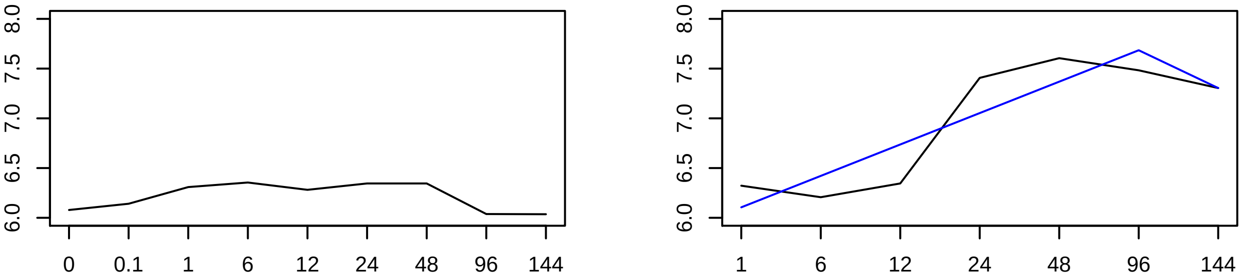

A_23_P217228 TRO Xp11.21

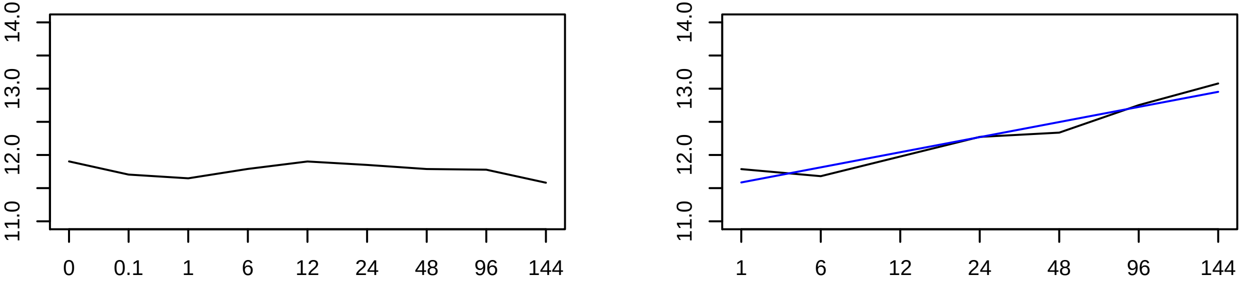

A_24_P551302 BC038432 NA

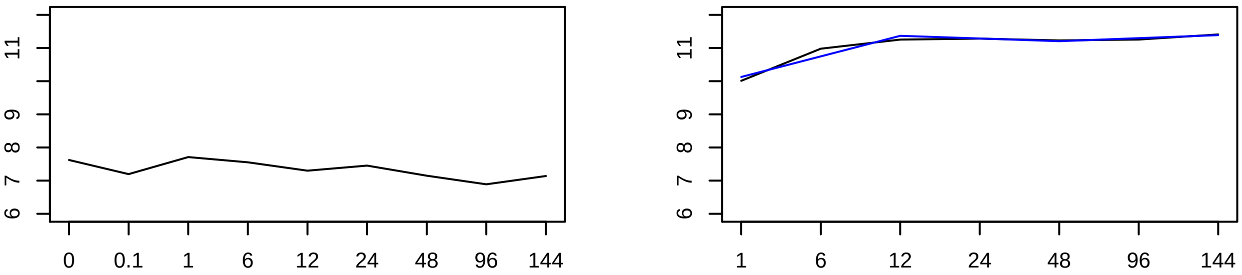

A_24_P350437 THAP2 12q21.1

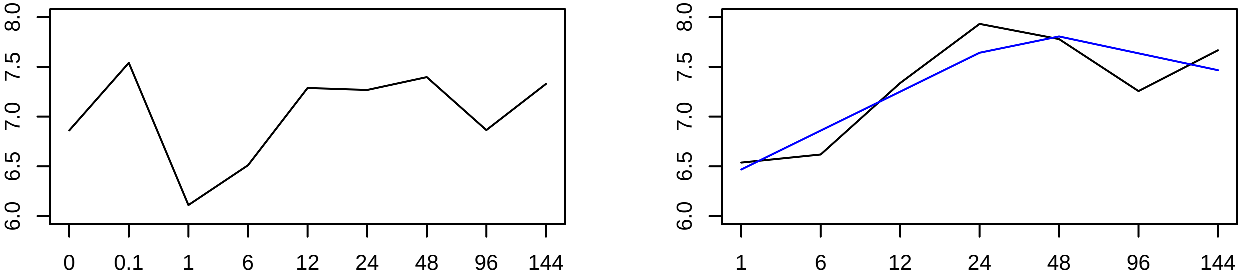

A_23_P28015 ZNF558 19p13.2

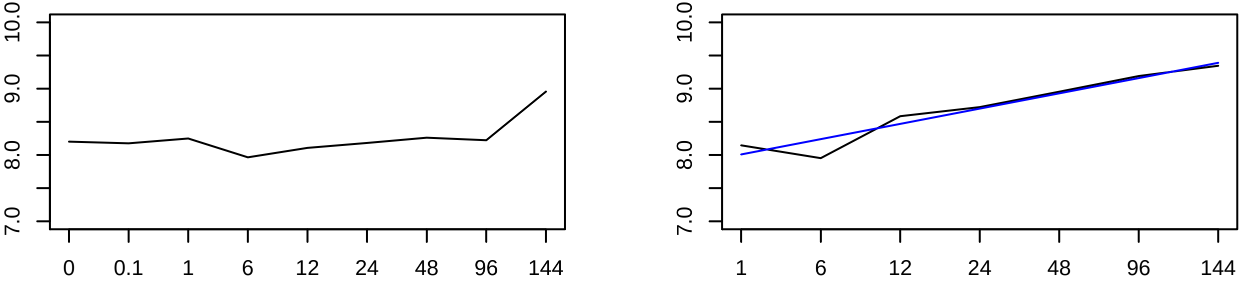

A_23_P334045 PCDHA9 5q31.3

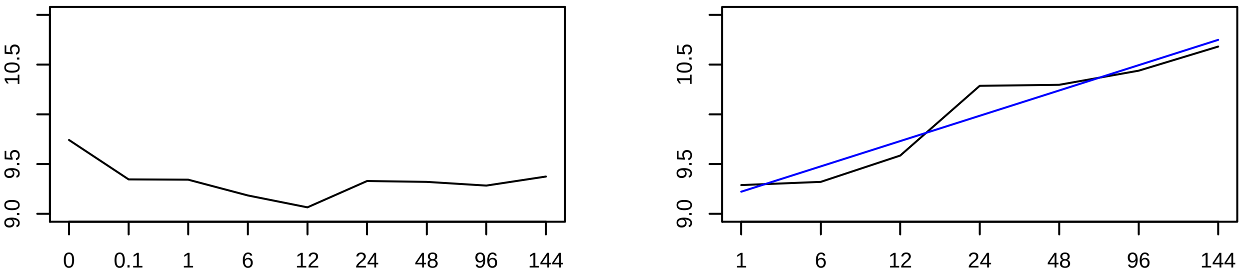

A_23_P13438 BTBD10 11p15.2

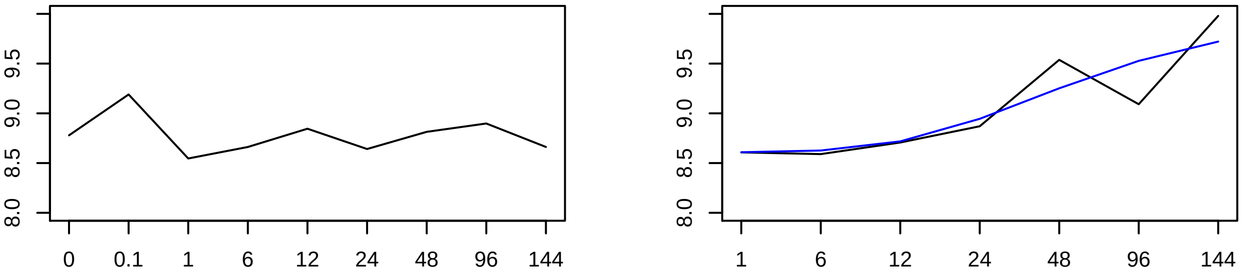

A_23_P31323 ACTB 7p22.1

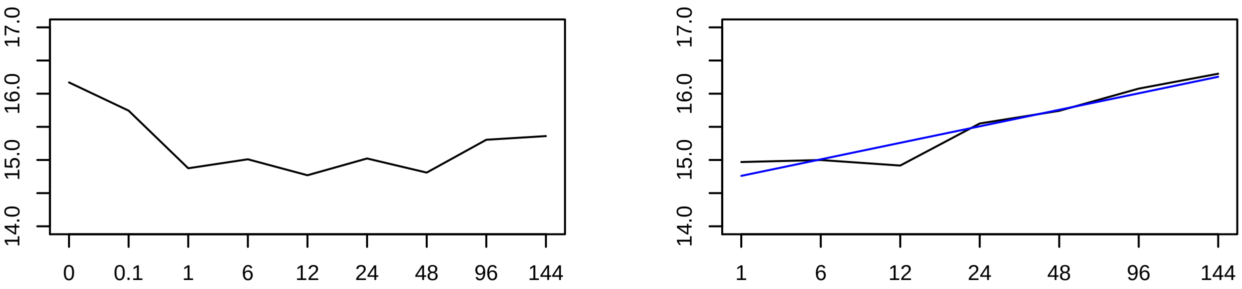

A_23_P169039 SNAI2 8q11.21

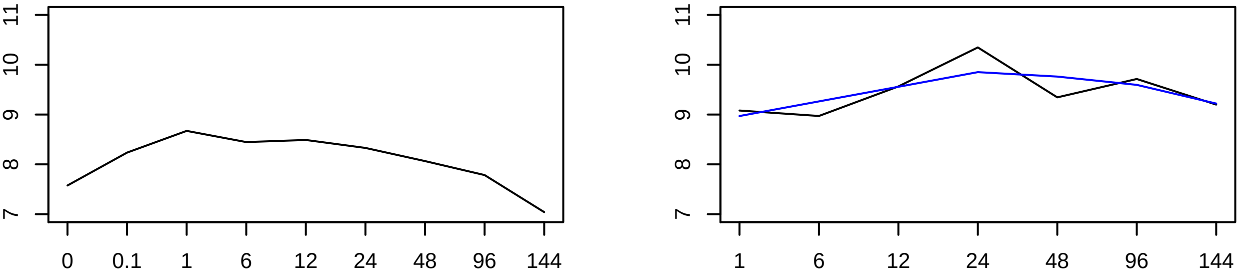

A_23_P149992 PDLIM1 10q23.33

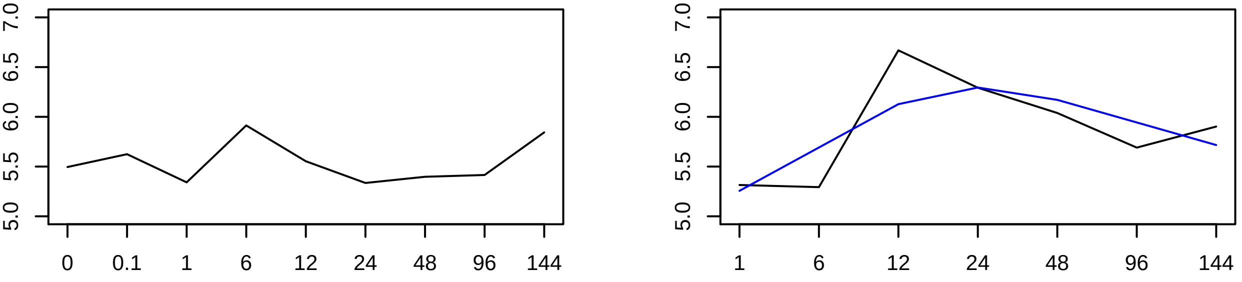

A_23_P93383 RGL2 6p21.32

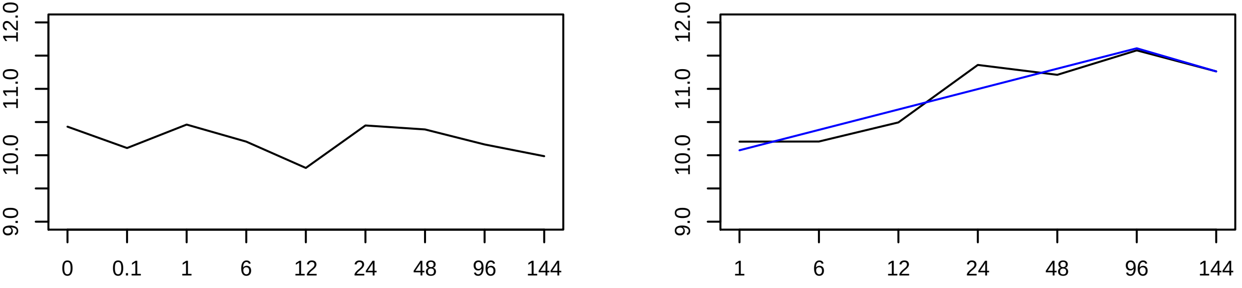

A_23_P203488 SMPD1 11p15.4

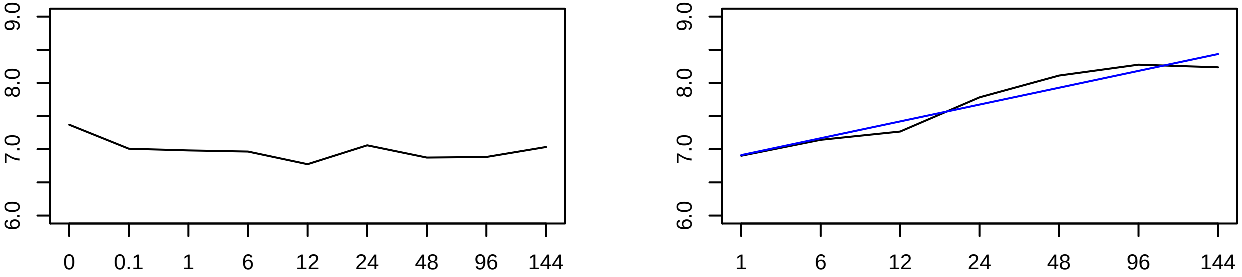

A_23_P218225 QPRT 16p11.2

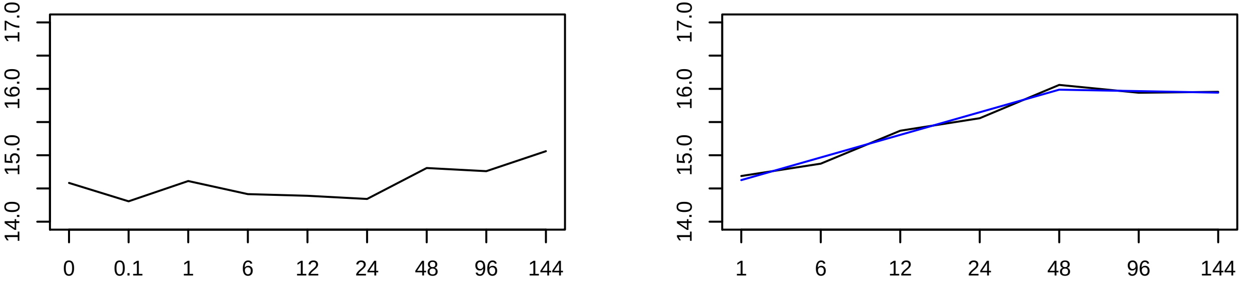

A_32_P172002 A_32_P172002 NA

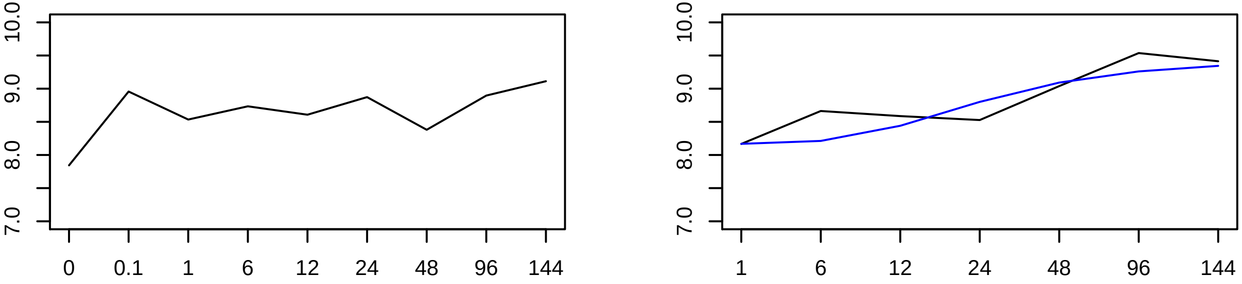

A_24_P892402 AK057652 NA

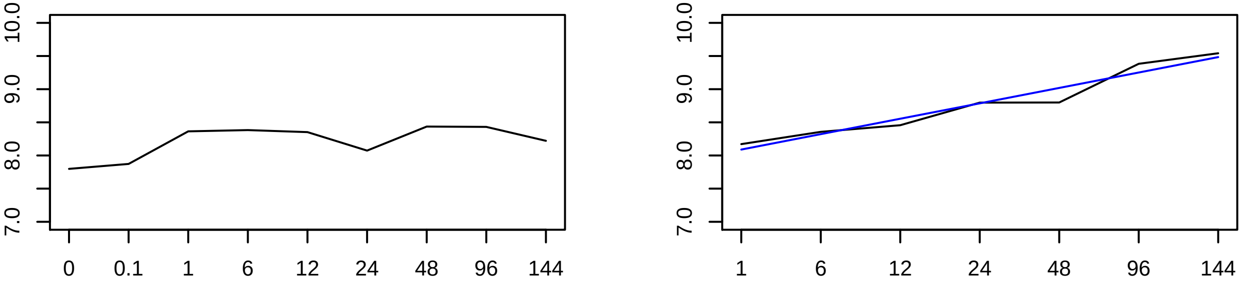

A_23_P394216 KIAA0329 14q32.31

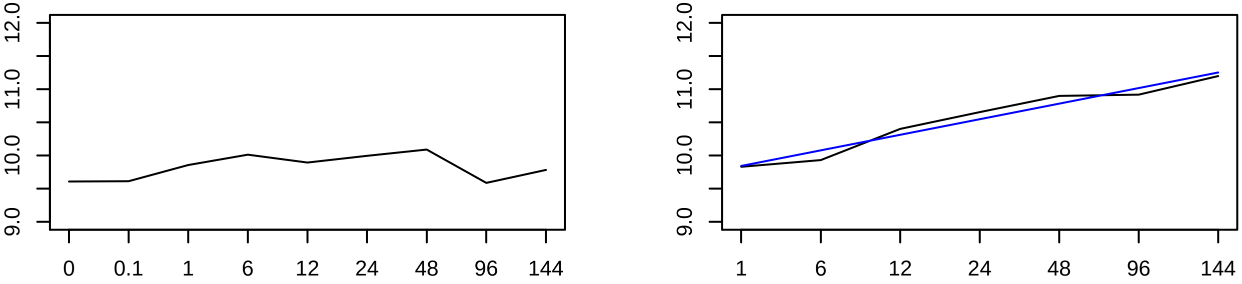

A_23_P94319 KBTBD11 8p23.3

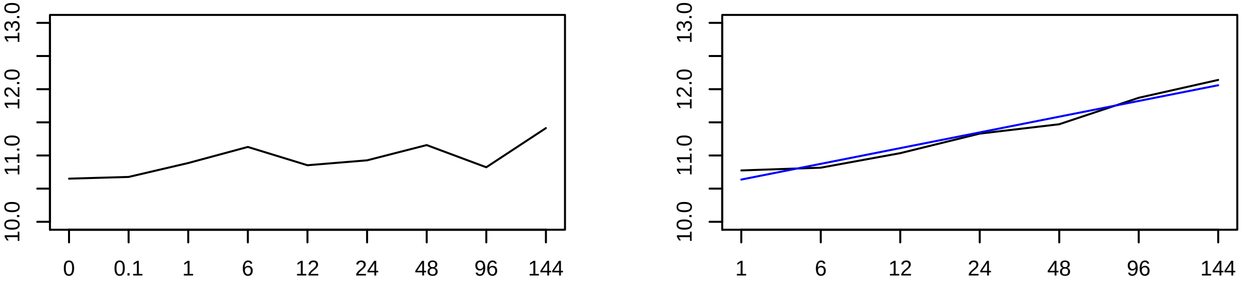

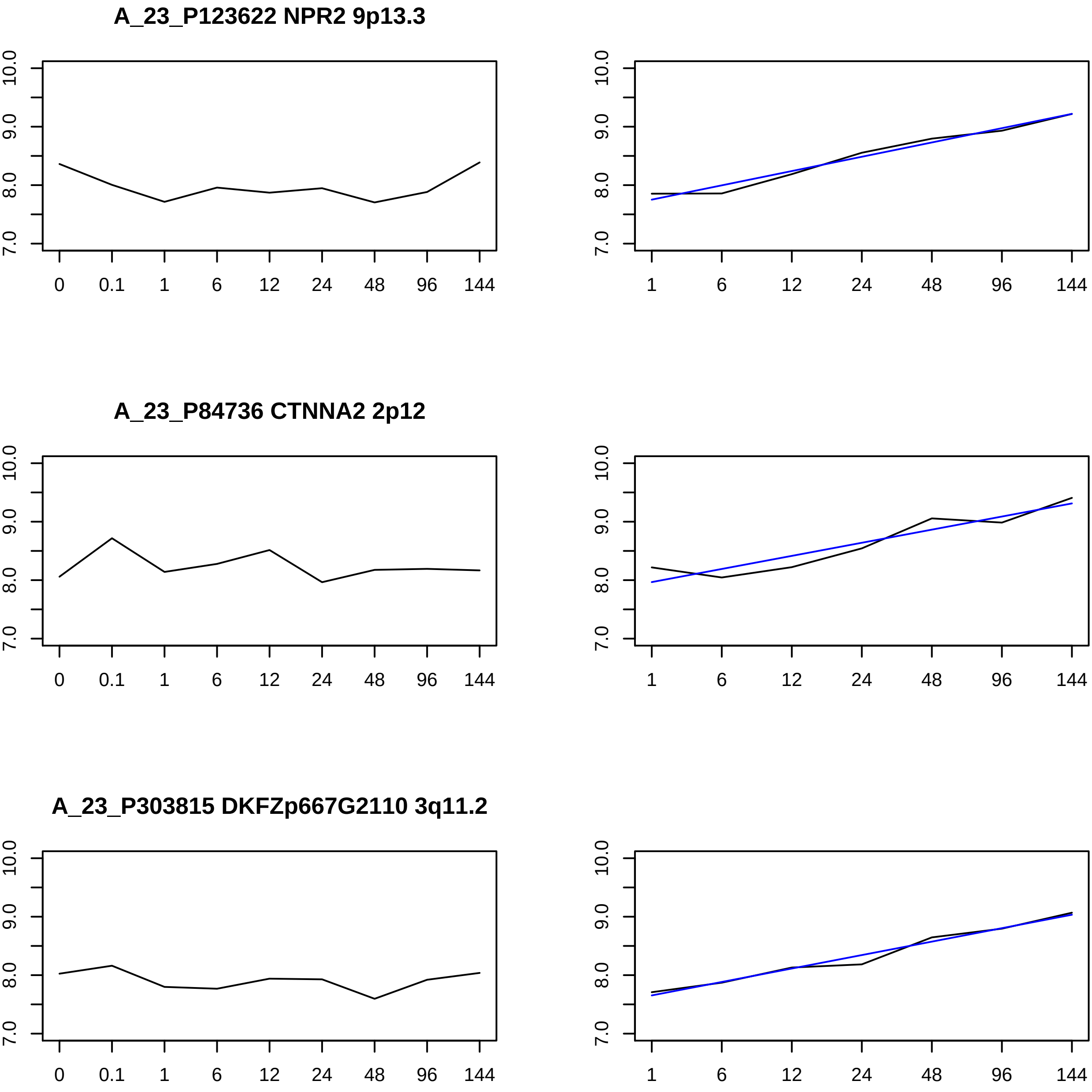
A_23_P123622 NPR2 9p13.3
A_23_P84736 CTNNA2 2p12
A_23_P303815 DKFZp667G2110 3q11.2
7.0 8.0 9.0 10.0
0 0.1 1 6 12 24 48 96 144
1 6 12 24 48 96 144

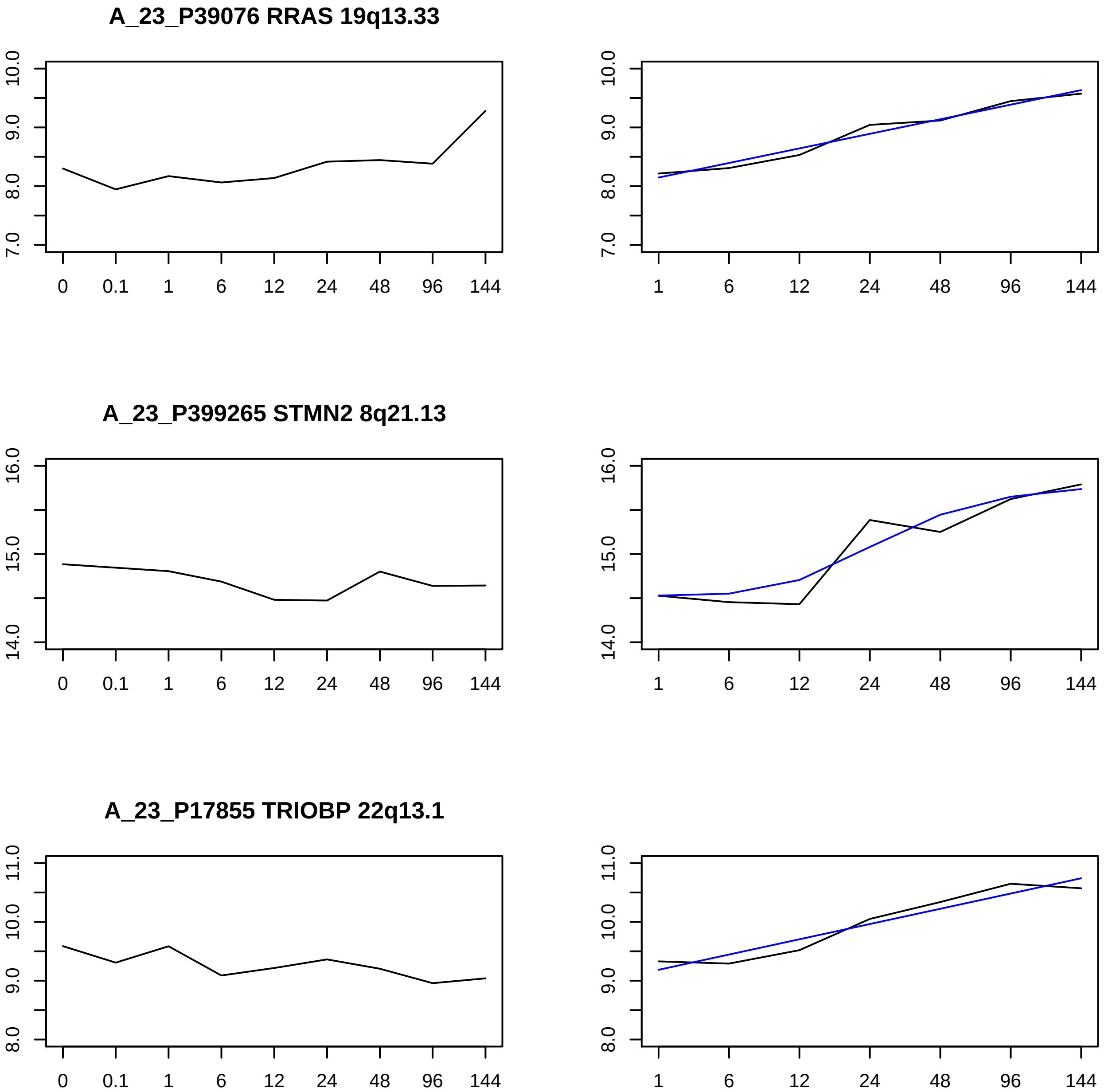
A_23_P39076 RRAS 19q13.33
7.0
8.0
9.0
10.0
0
0.1
1
6
12
24
48
96
144
1
6
12
24
48
96
144
A_23_P399265 STMN2 8q21.13
14.0
15.0
16.0
A_23_P17855 TRIOBP 22q13.1
8.0
9.0
10.0
11.0

A_23_P23748 WDR47 1p13.3

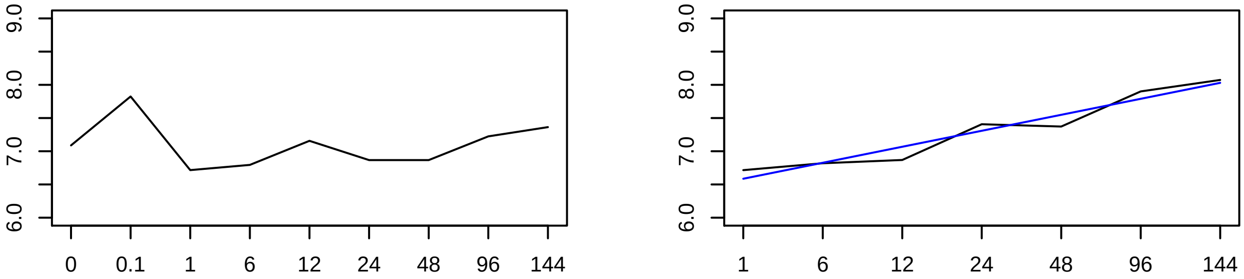

A_23_P12733 H2AFY2 10q22.1

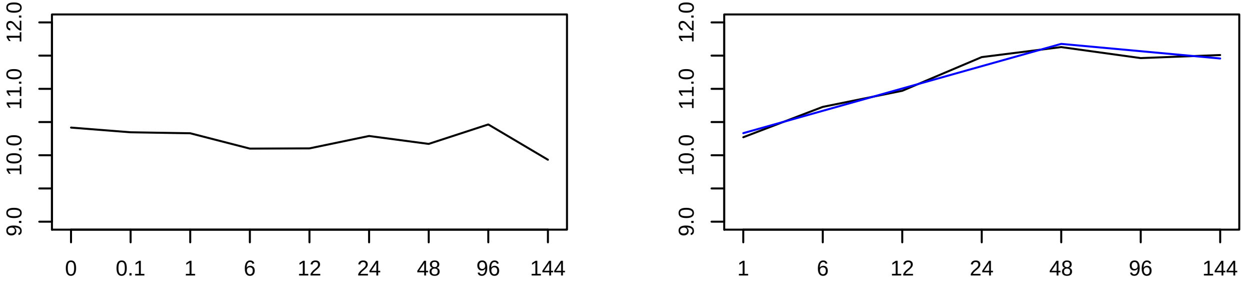

A_23_P120970 TTLL1 22q13.2

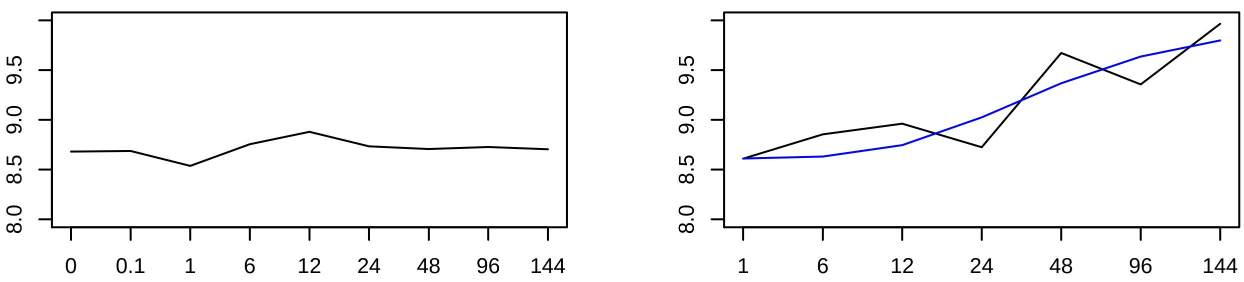

A_23_P18713 ABCG2 4q22.1

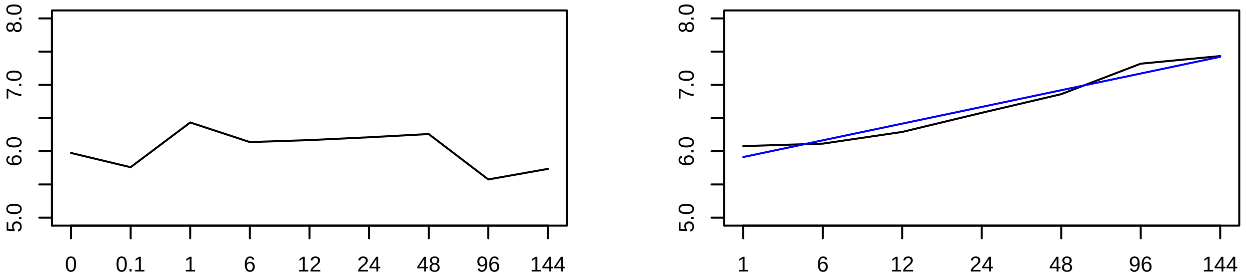

A_32_P201390 C2orf14 2q21.1

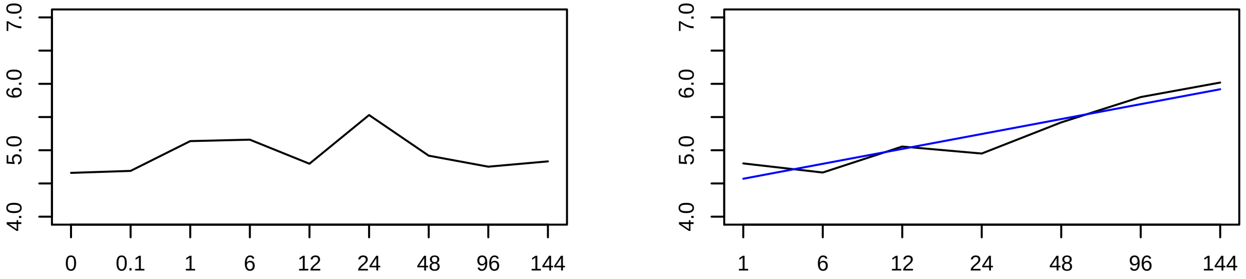

A_32_P30926 DYNC1I2 2q31.1

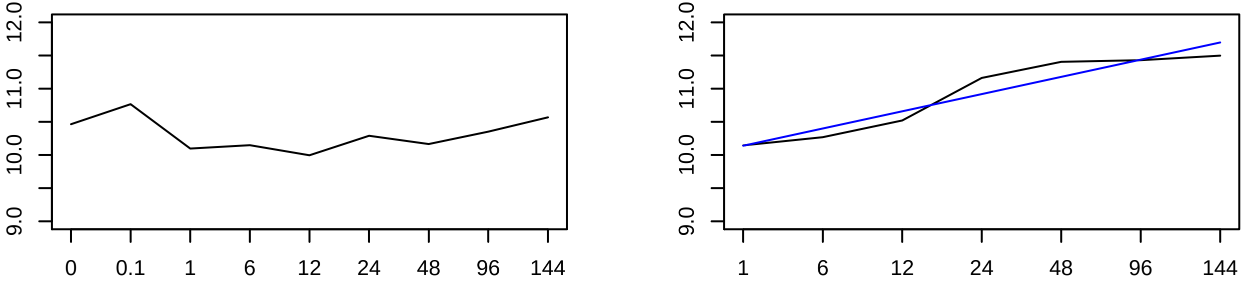

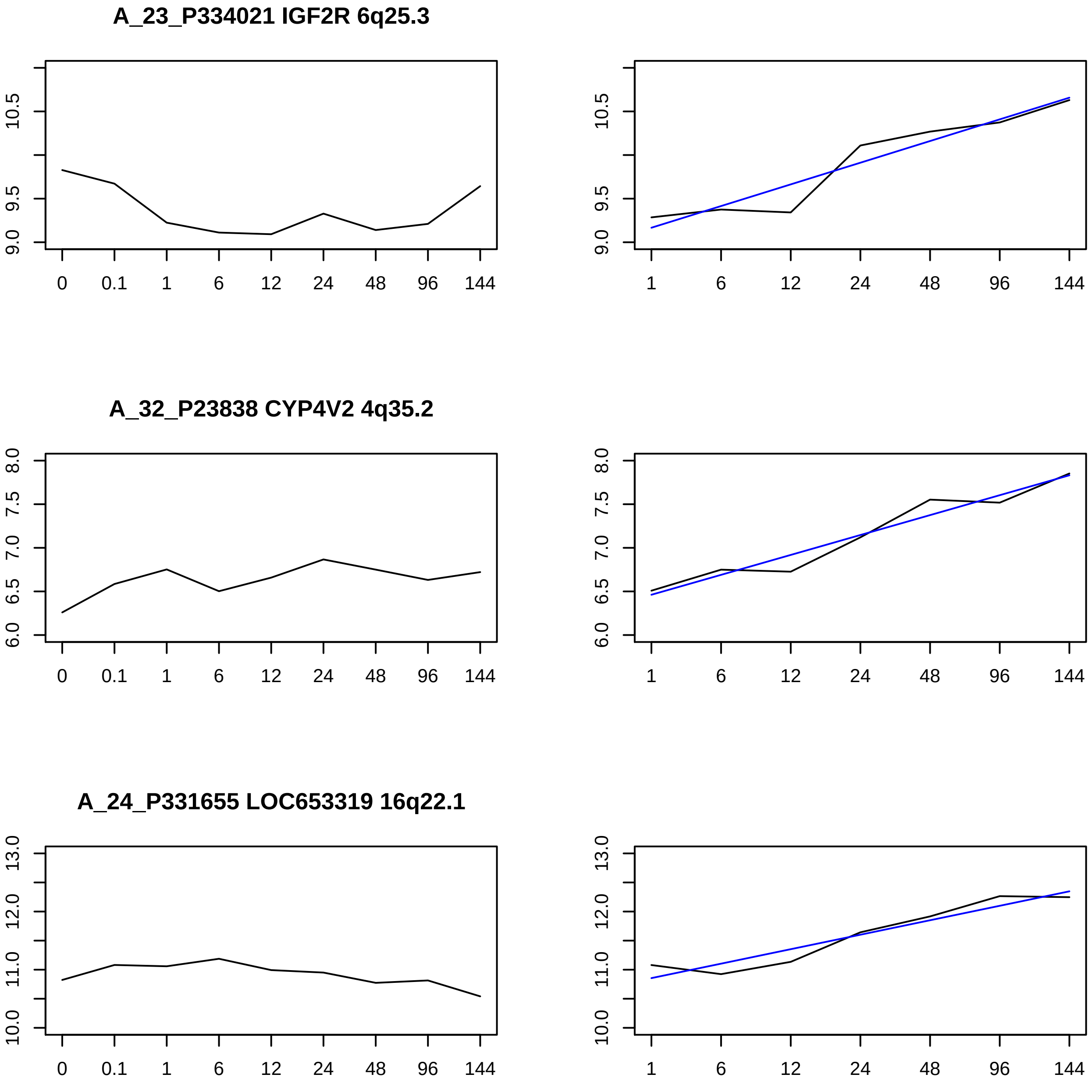
A_23_P334021 IGF2R 6q25.3
9.0
9.5
10.5
0
0.1
1
6
12
24
48
96
144
1
6
12
24
48
96
144
A_32_P23838 CYP4V2 4q35.2
6.0
6.5
7.0
7.5
8.0
A_24_P331655 LOC653319 16q22.1
10.0
11.0
12.0
13.0

A_23_P93780 HGF 7q21.11

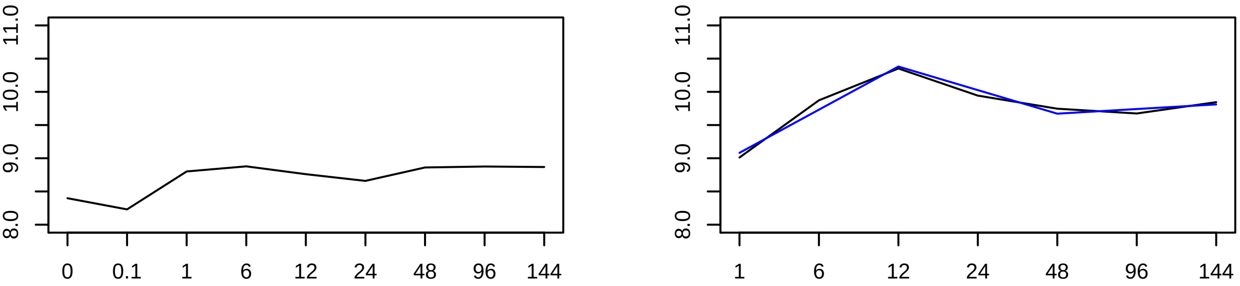

A_23_P58464 PCDHB6 5q31.3

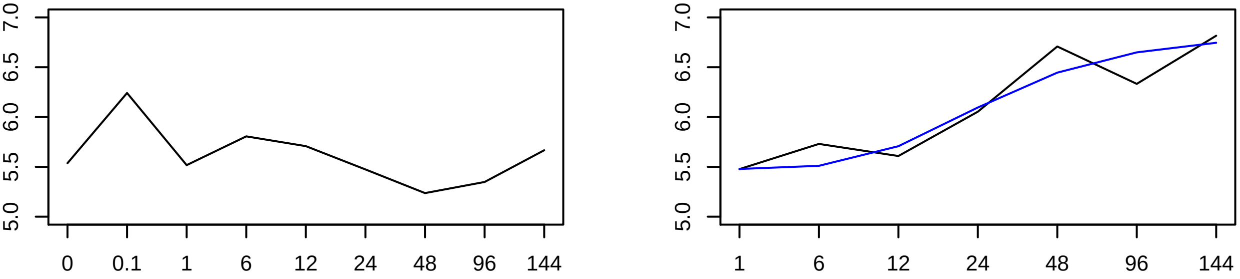

A_32_P220472 ZFAND6 15q25.1

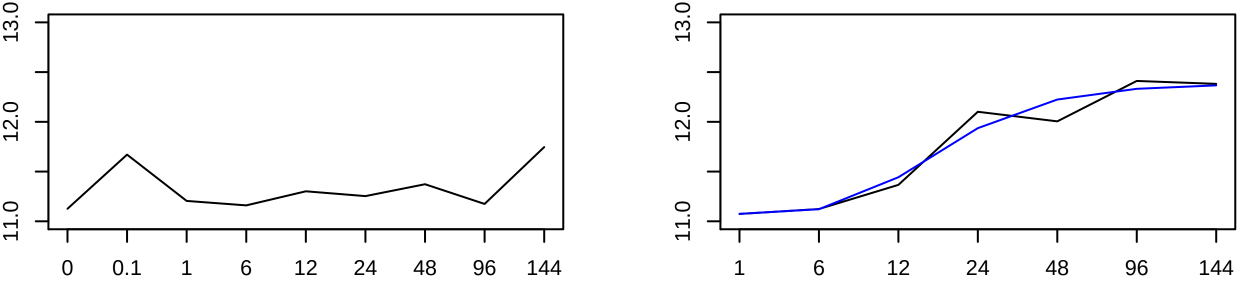

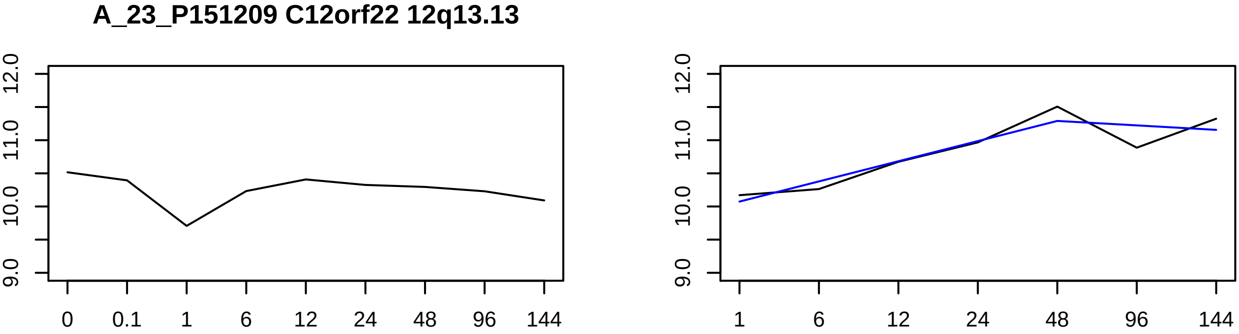
A_23_P151209 C12orf22 12q13.13
9.0
10.0
11.0
12.0
0
0.1
1
6
12
24
48
96
144

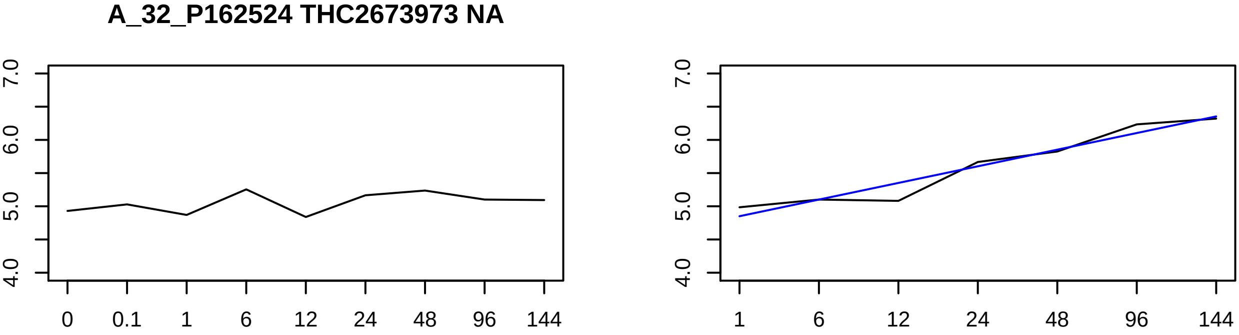
A_32_P162524 THC2673973 NA
4.0
5.0
6.0
7.0
0
0.1
1
6
12
24
48
96
144

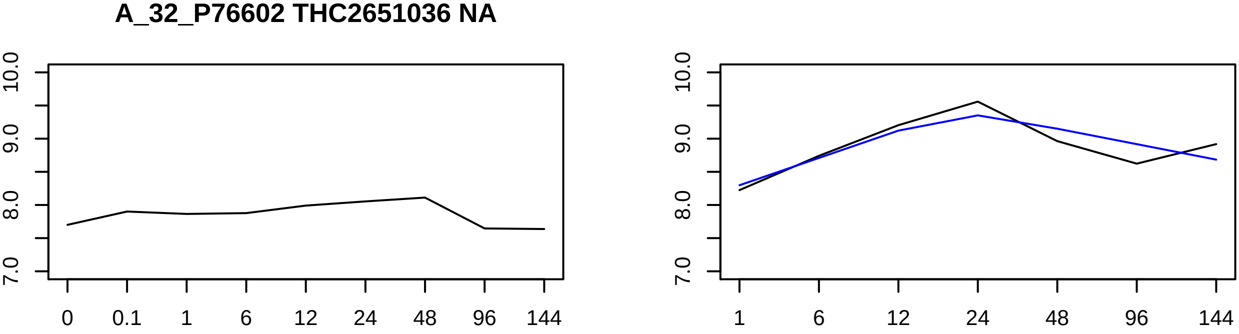
A_32_P76602 THC2651036 NA
7.0
8.0
9.0
10.0
0
0.1
1
6
12
24
48
96
144

A_23_P213562 F2R 5q13.3

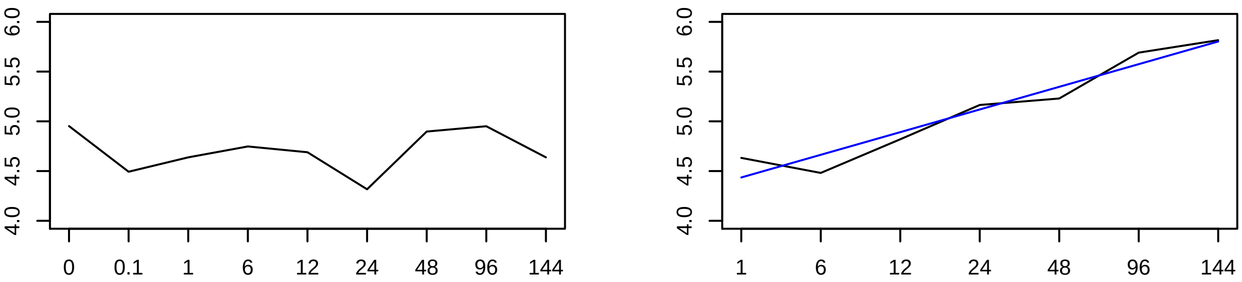

A_23_P59397 RSHL2 6q25.3

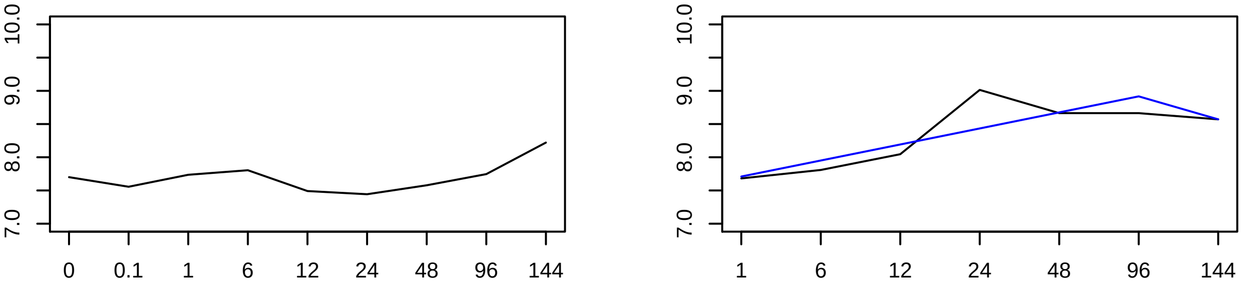

A_24_P98613 TSPAN14 10q23.1

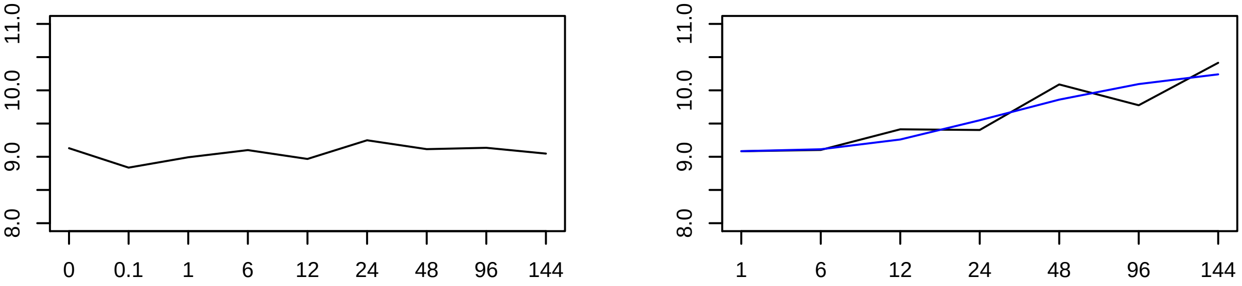

A_23_P129174 LRRC49 15q23

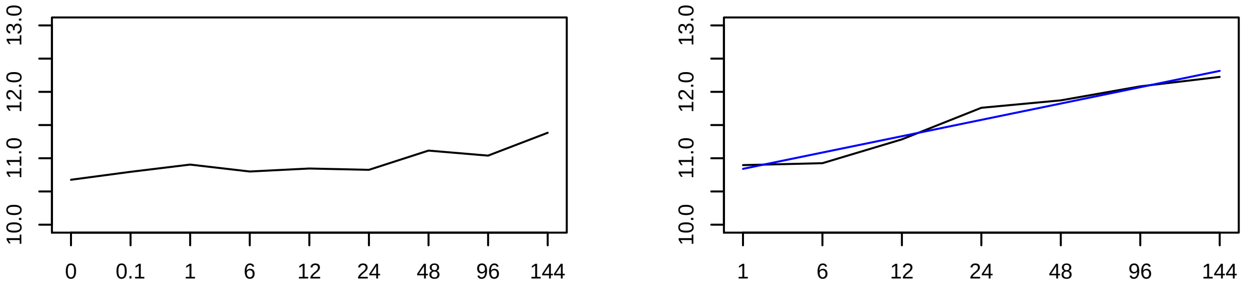

A_32_P124580 SAMD8 NA

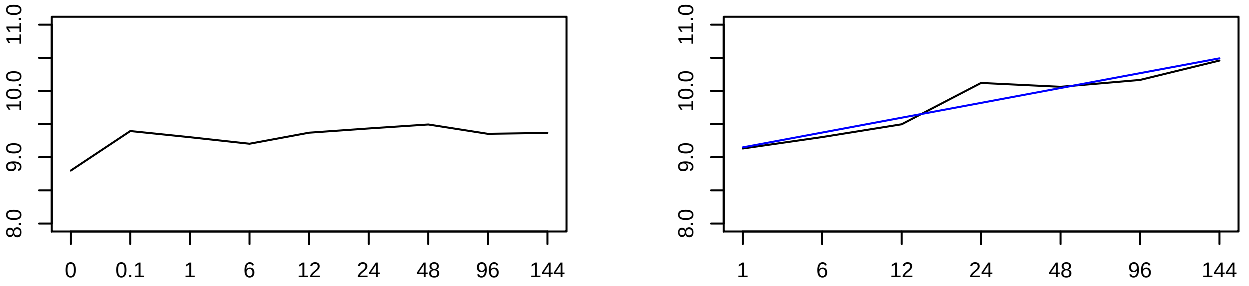

A_32_P67533 L3MBTL3 6q23.1

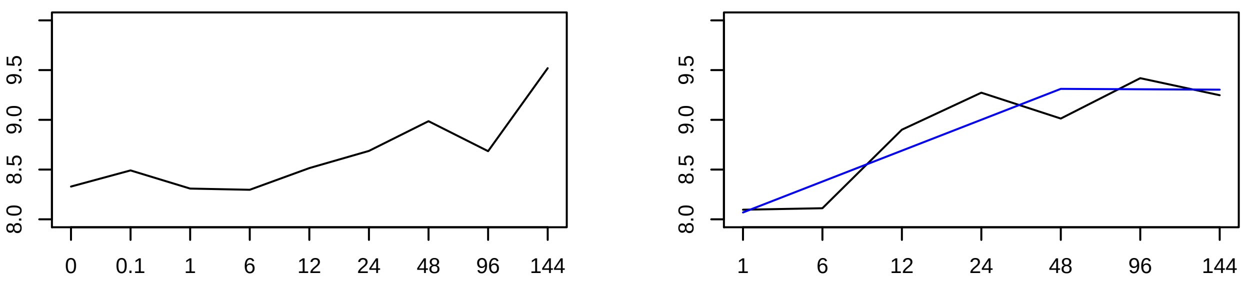

A_32_P146286 ZP4 1q43

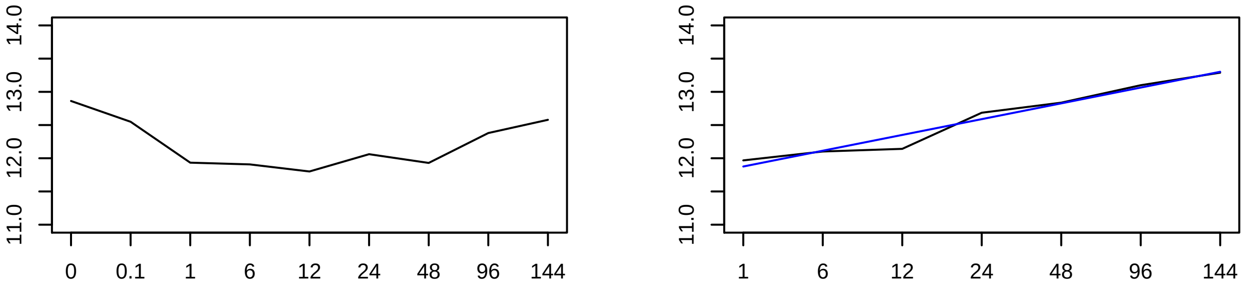

A_23_P17998 HES1 3q29

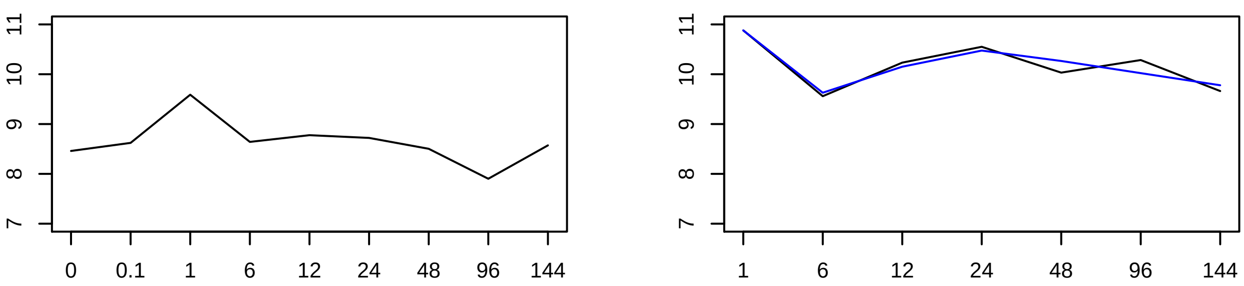

A_23_P217367 ATG4A Xq22.3

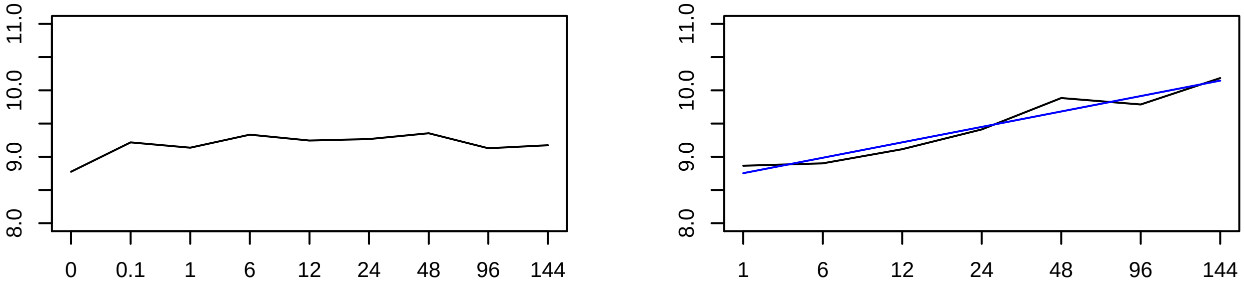

A_24_P410017 ENST00000375923 NA

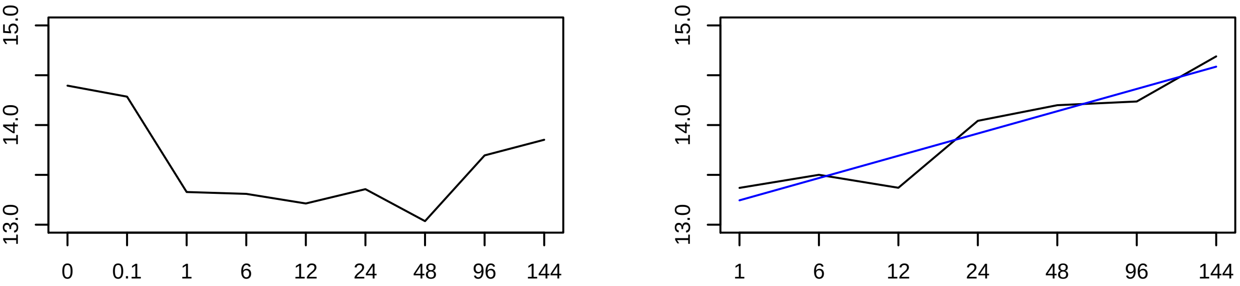

A_23_P113966 EXOC1 4q12

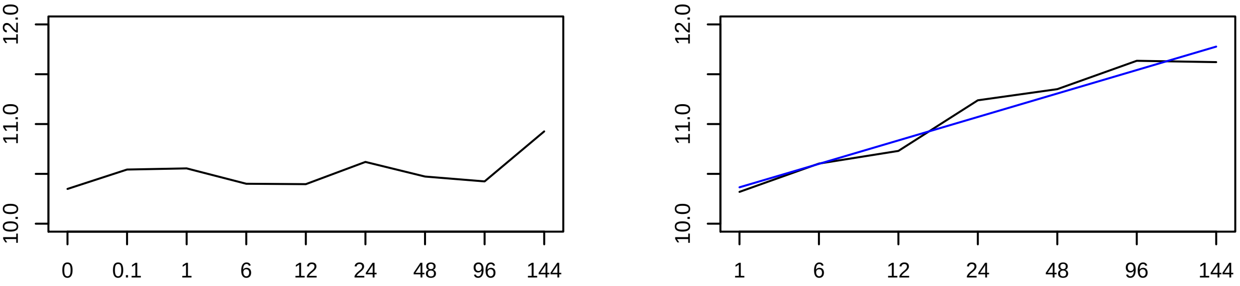

A_32_P133926 A_32_P133926 NA

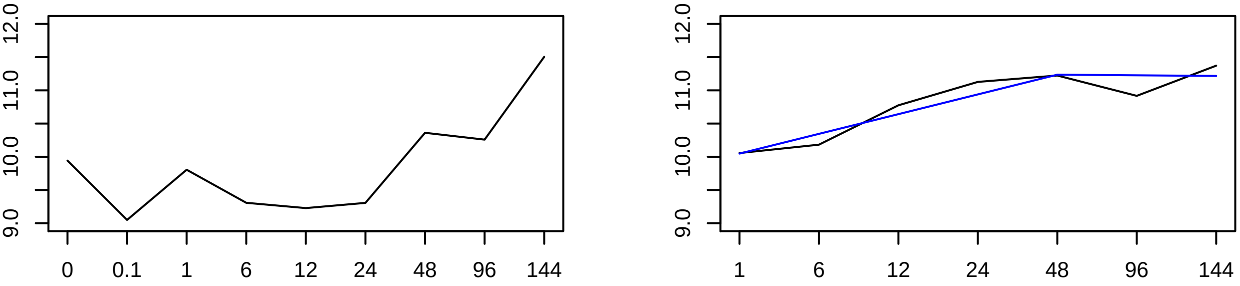

A_23_P345674 ZNF71 19q13.43

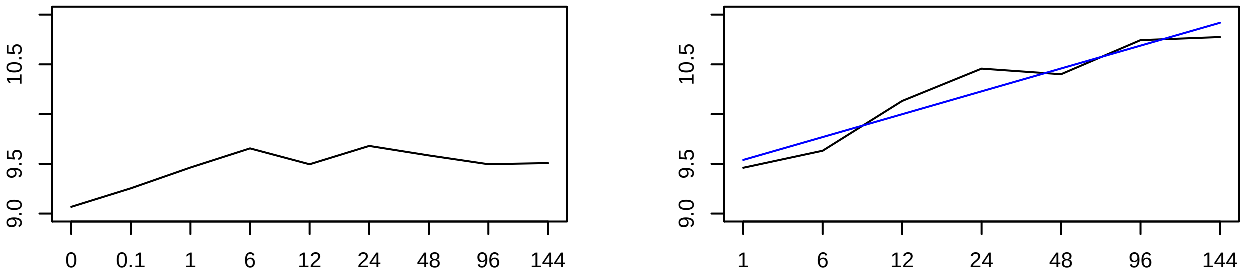

A_23_P170761 PDLIM5 4q22.3

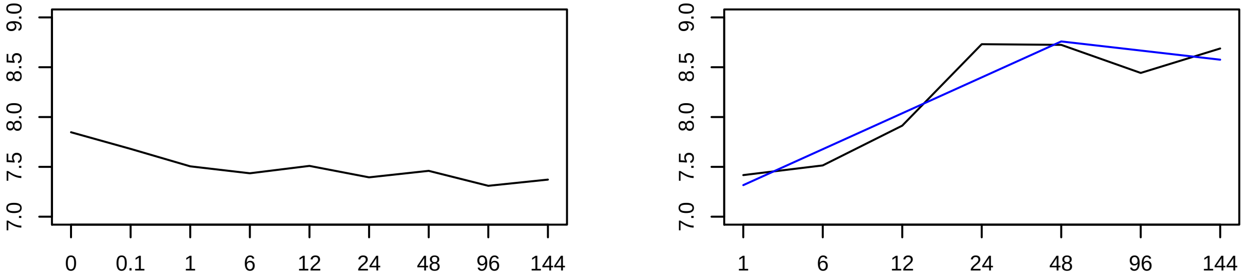

A_23_P142255 SHD 19p13.3

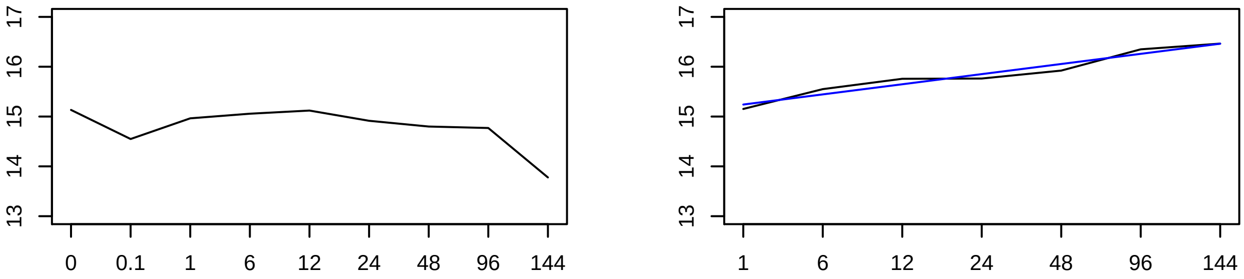

A_23_P95060 EPHB3 3q27.1

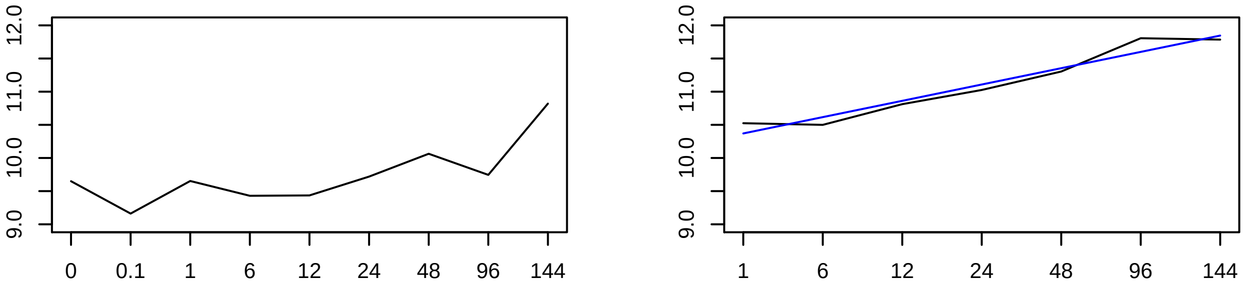

A_24_P930337 THC2503773 NA

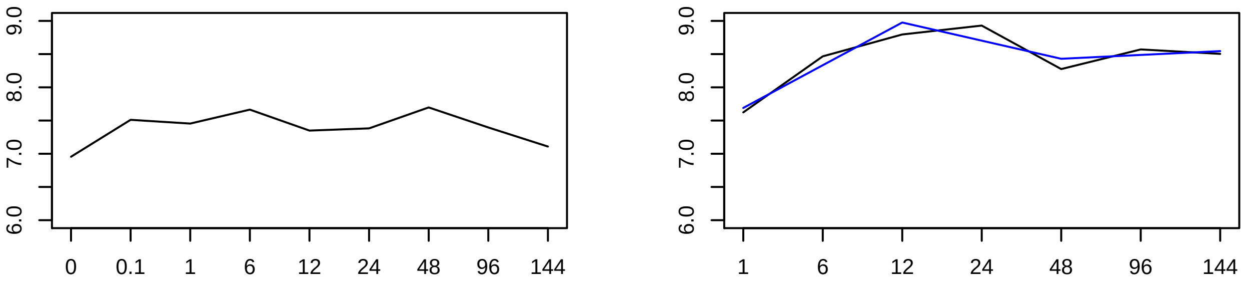

A_23_P163787 MMP2 16q12.2

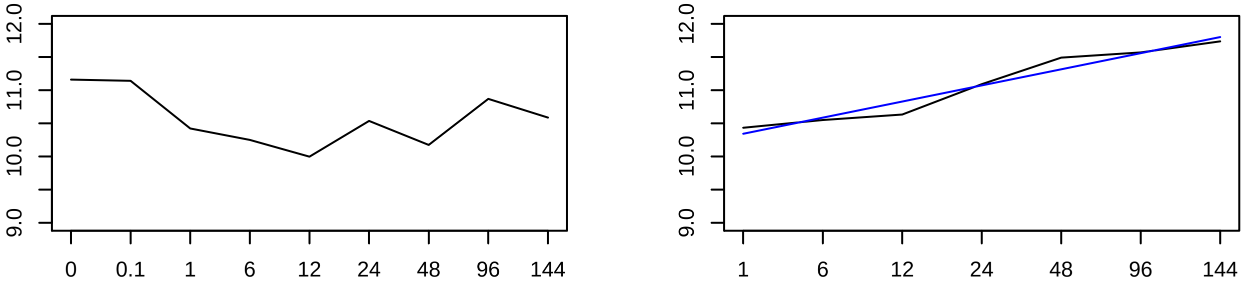

A_23_P374944 ZNF507 19q13.11

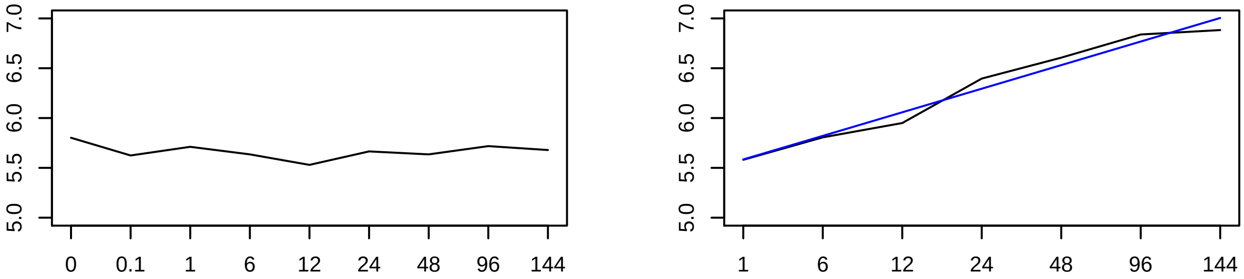

A_23_P97181 GREM2 1q43

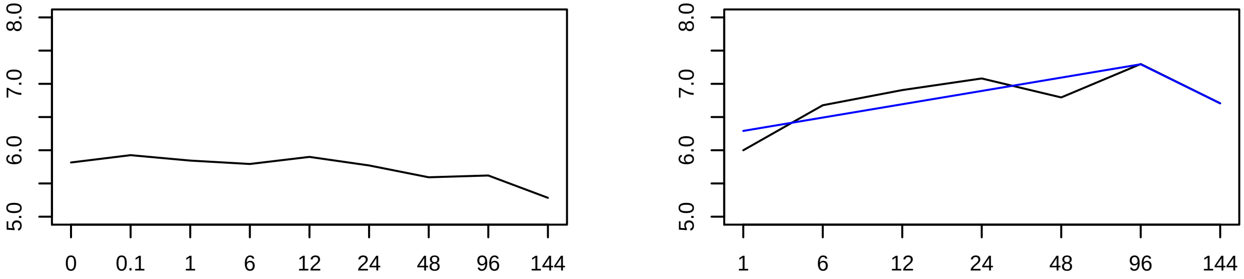

A_23_P417404 FAM129B 9q34.11

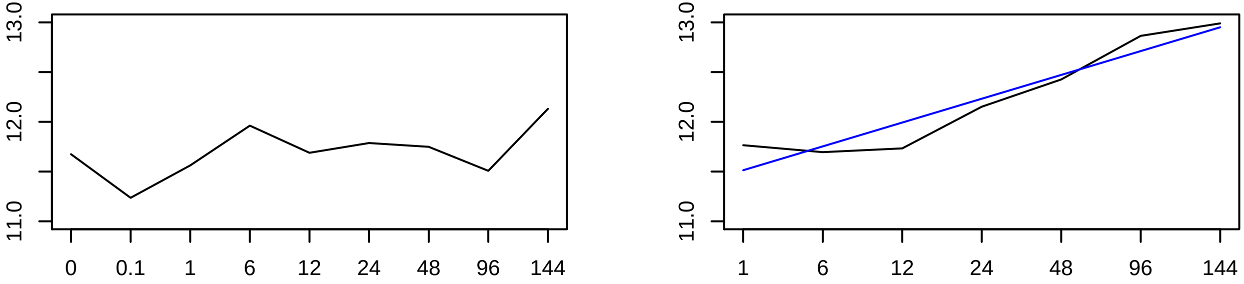

A_23_P318420 CTTNBP2NL 1p13.2

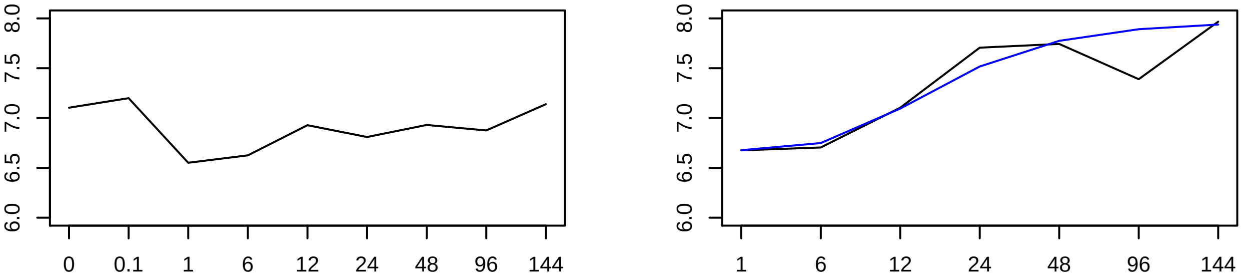

A_24_P938293 HES1 3q29

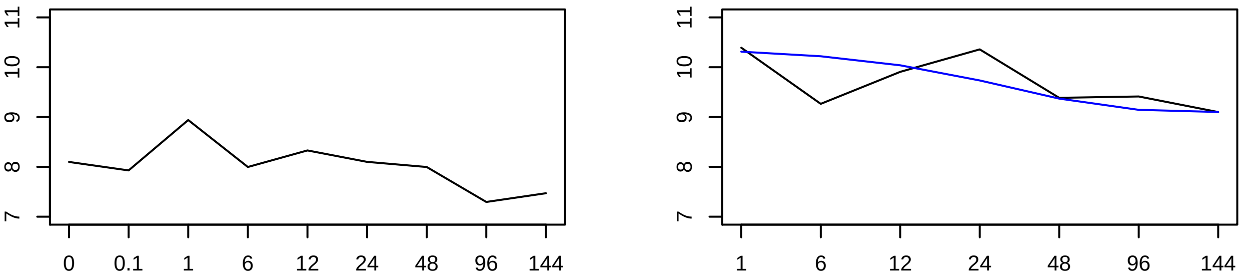

A_32_P2354 QPRT 16p11.2

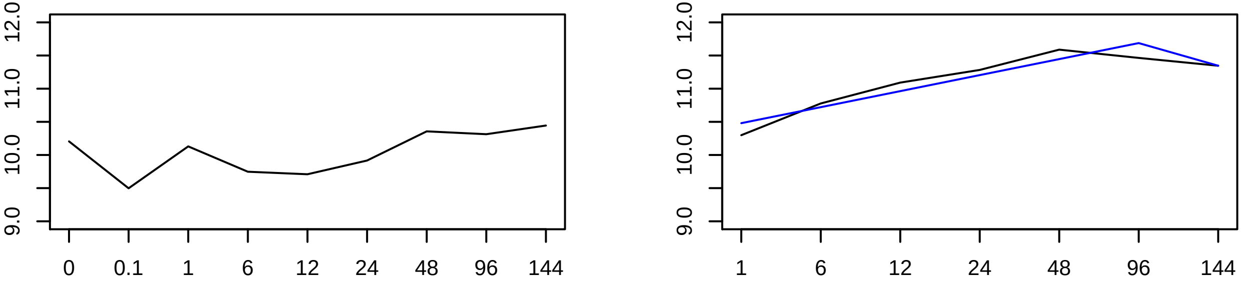

A_23_P59988 AMAC1L2 8p23.1

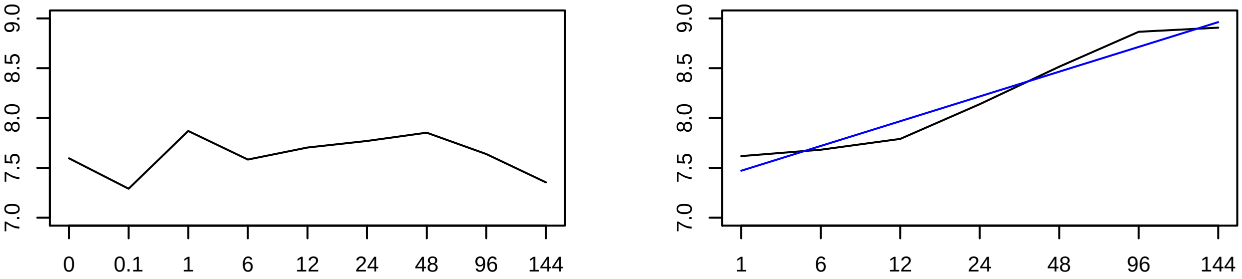

A_23_P148121 EHBP1L1 11q13.1

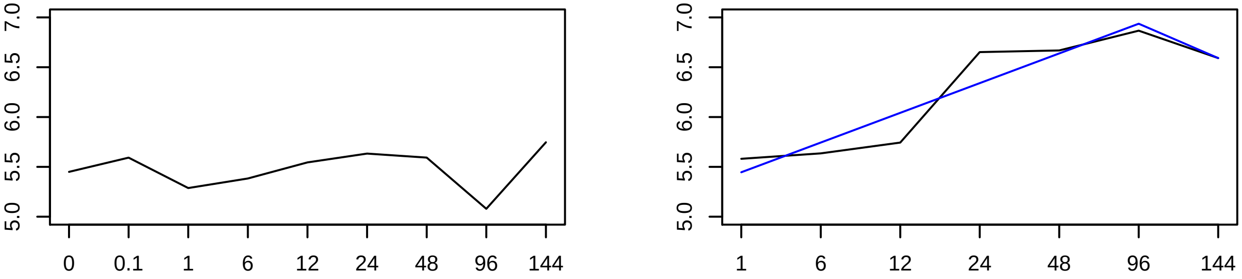

A_23_P51202 ZNF436 1p36.12

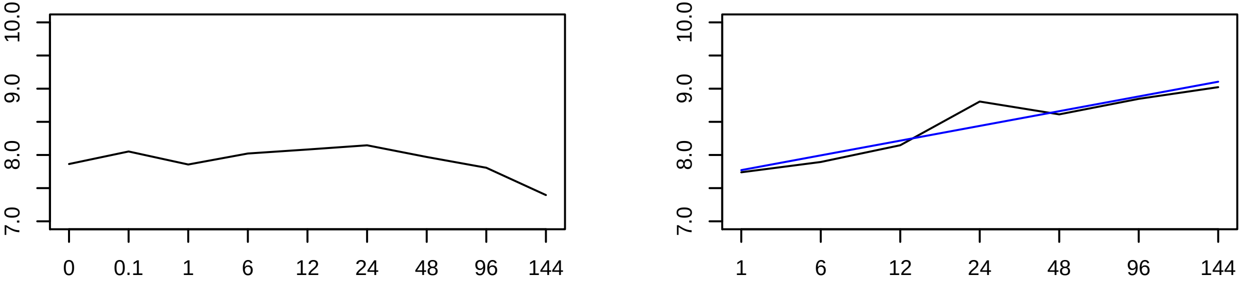

A_23_P56213 GRAMD1A 19q13.11

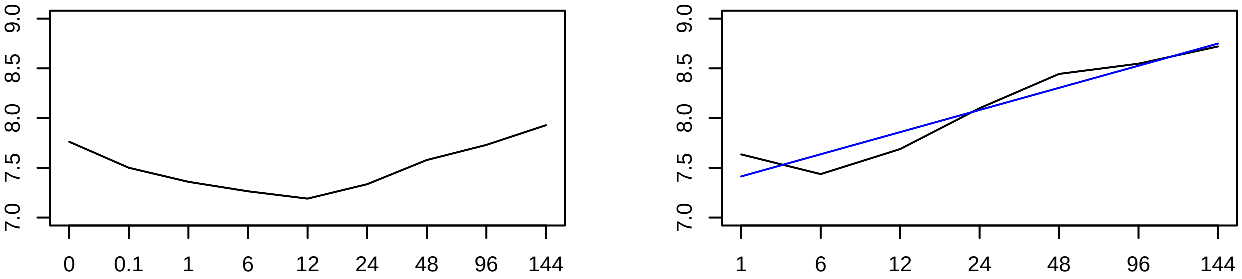

A_24_P703642 ANAPC1 2p11.2

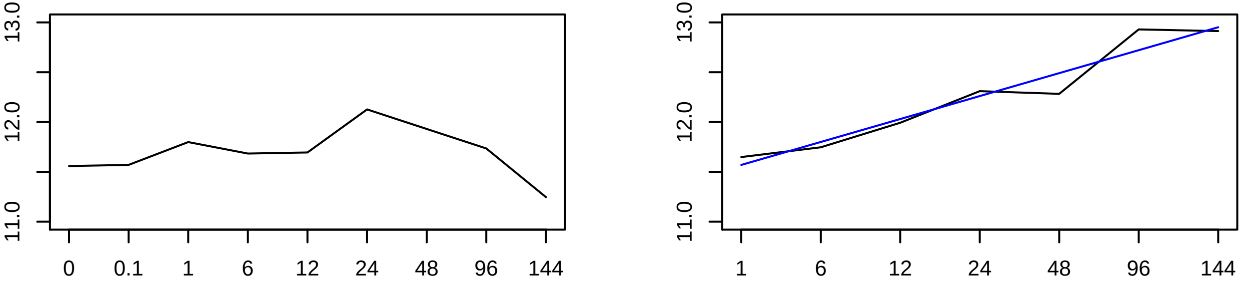

A_24_P333019 RNF24 20p13

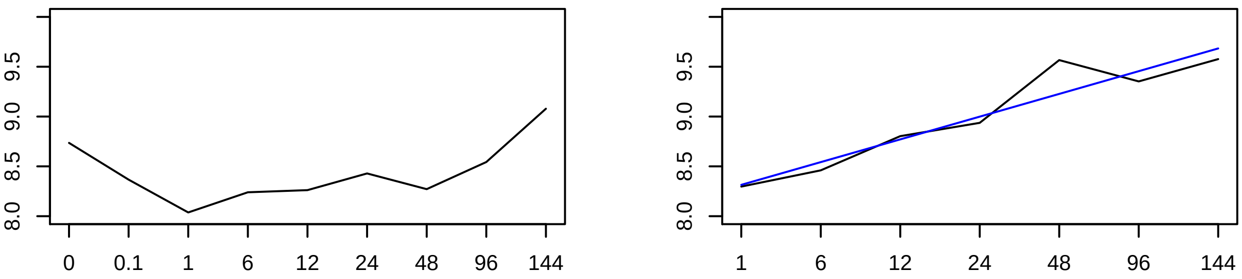

A_23_P322845 PPAPDC1B 8p12

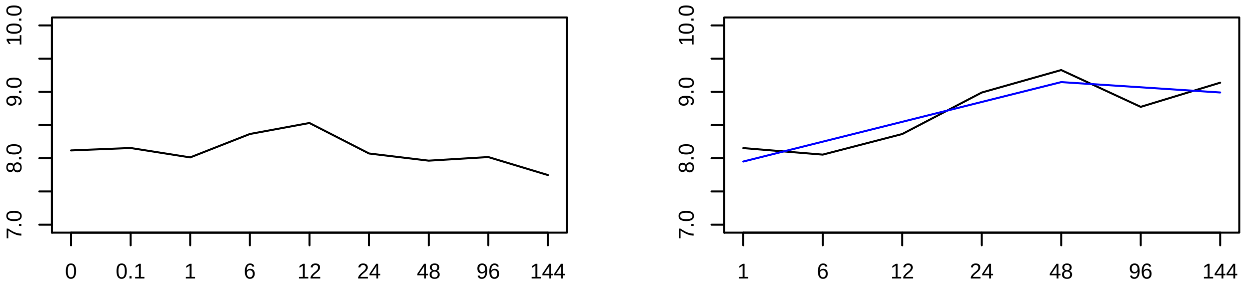

A_24_P935682 AY358248 NA

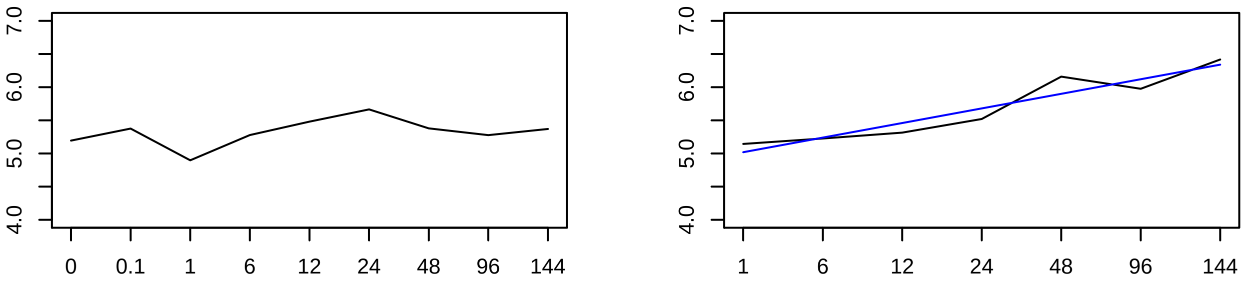

A_32_P120043 C6orf188 6q22.1

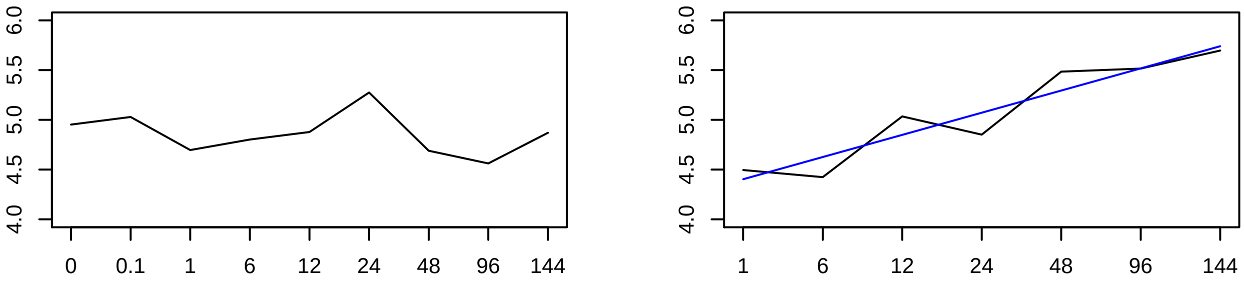

A_23_P98483 ZBED5 11p15.3

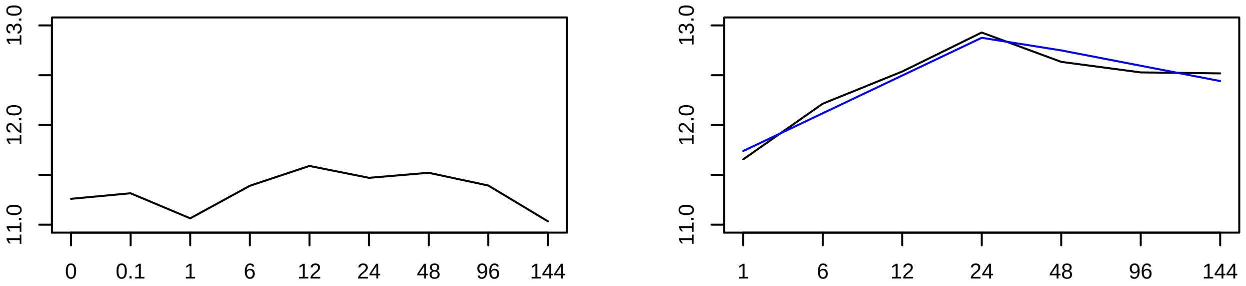

A_23_P217028 USP20 9q34.11

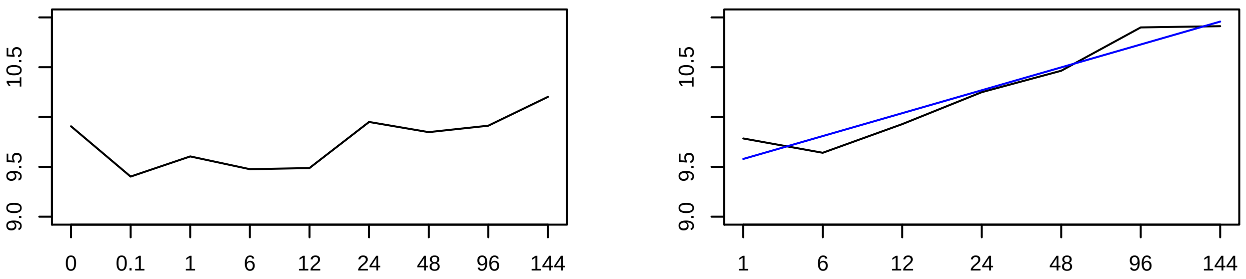

A_23_P159012 ANKRA2 5q13.2

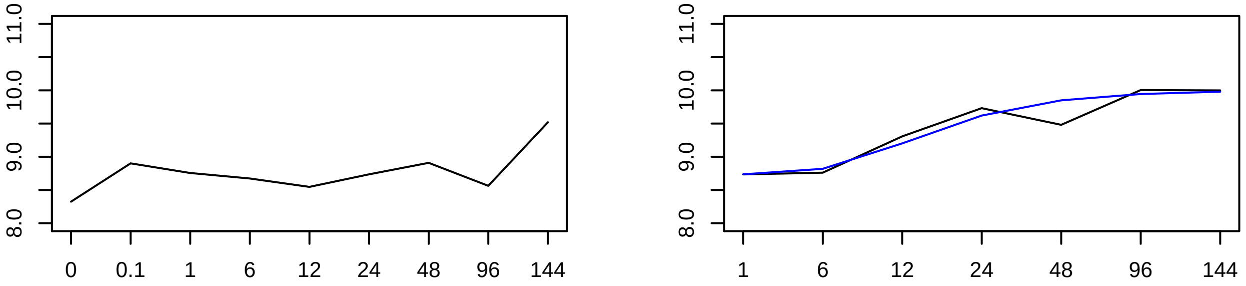

A_23_P33791 SSBP2 5q14.1

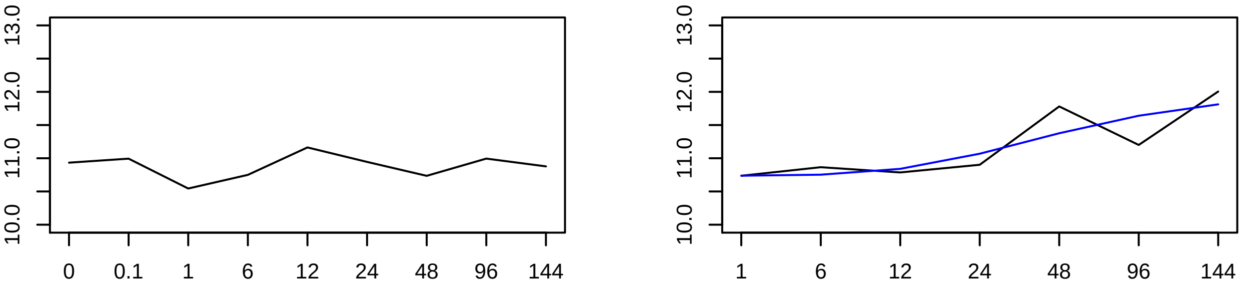

A_23_P144627 PCDHB13 5q31.3

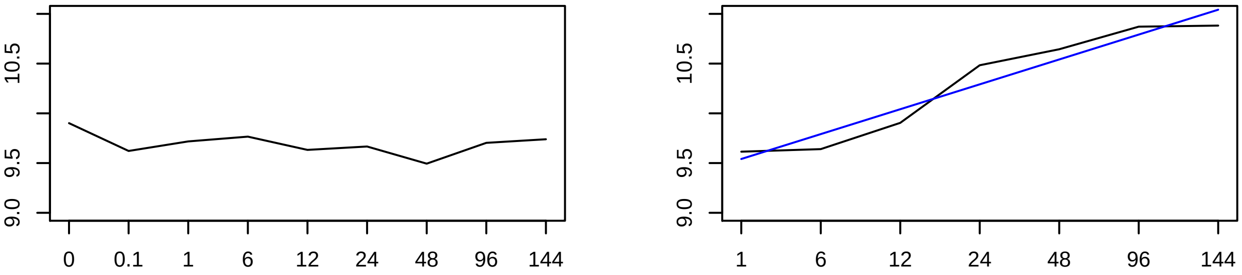

A_23_P30163 FLJ13197 4p14

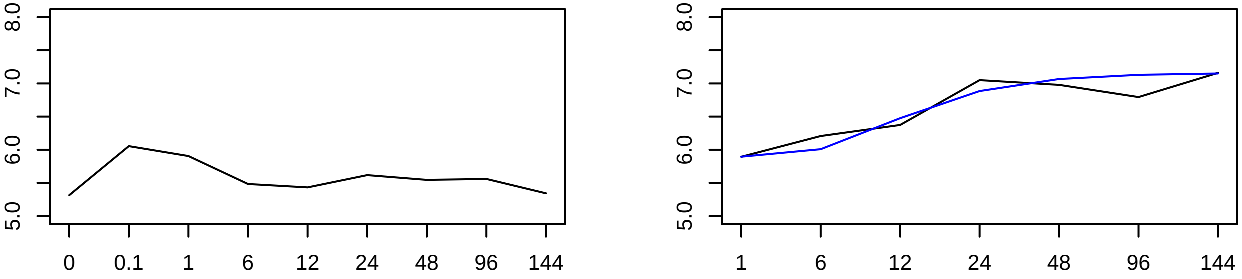

A_24_P49800 A_24_P49800 NA

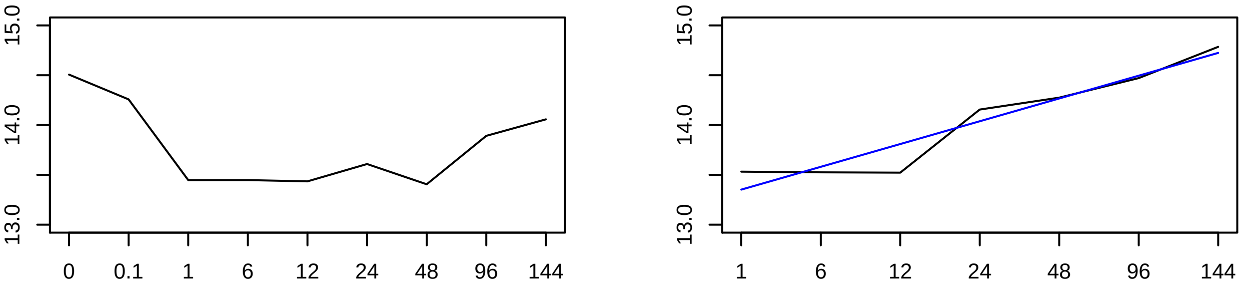

A_23_P83159 KLHL9 9p21.3

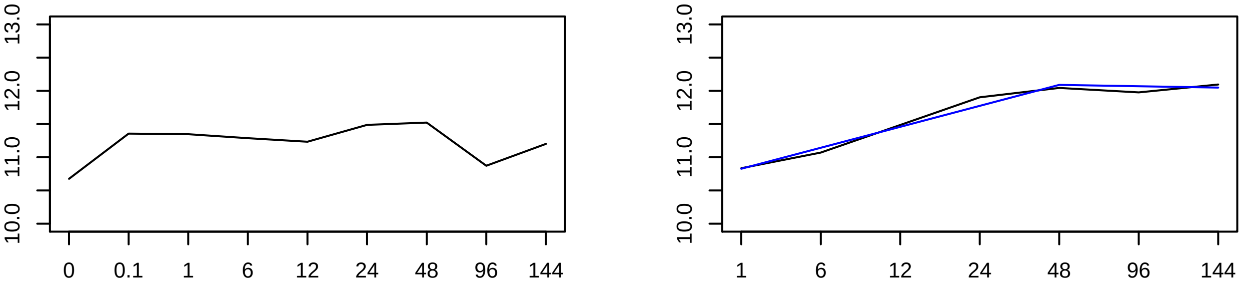

A_32_P122595 THC2702250 NA

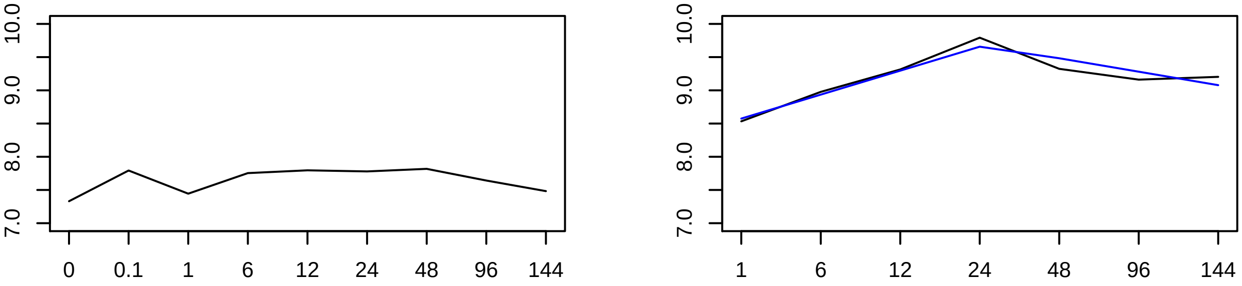

A_24_P291826 SYTL3 6q25.3

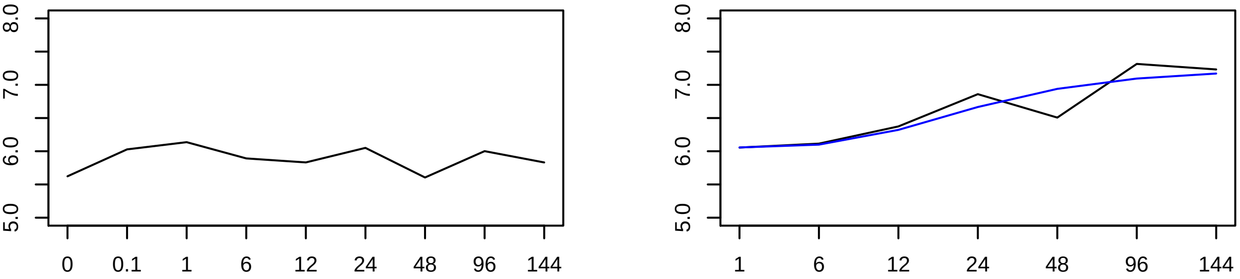

A_23_P78099 VTN 17q11.2

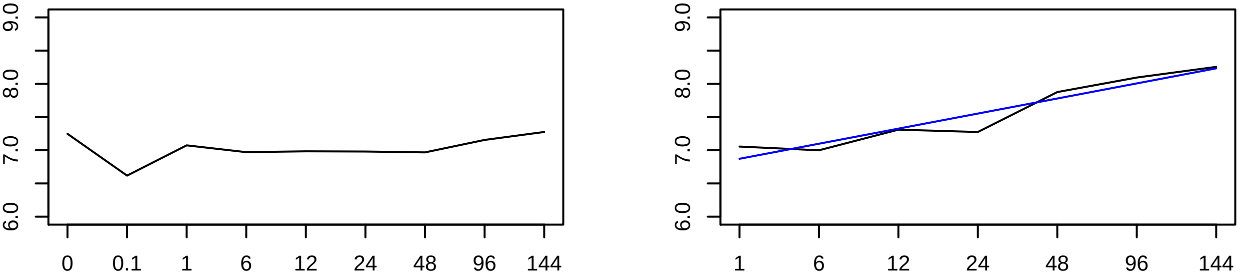

A_23_P164451 TBX2 17q23.2

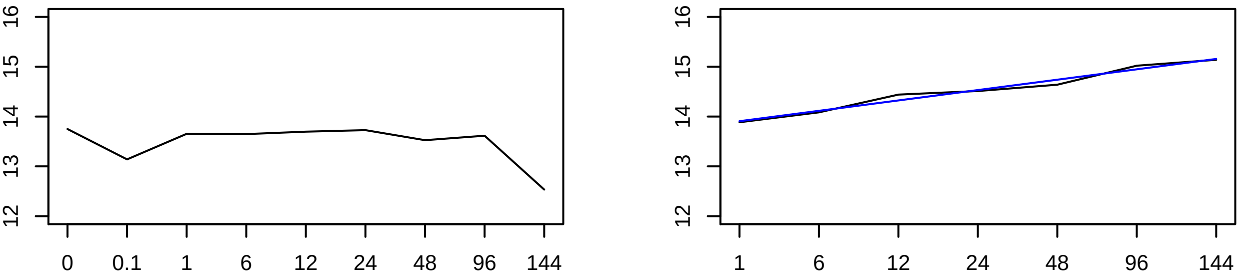

A_23_P202280 VCL 10q22.2

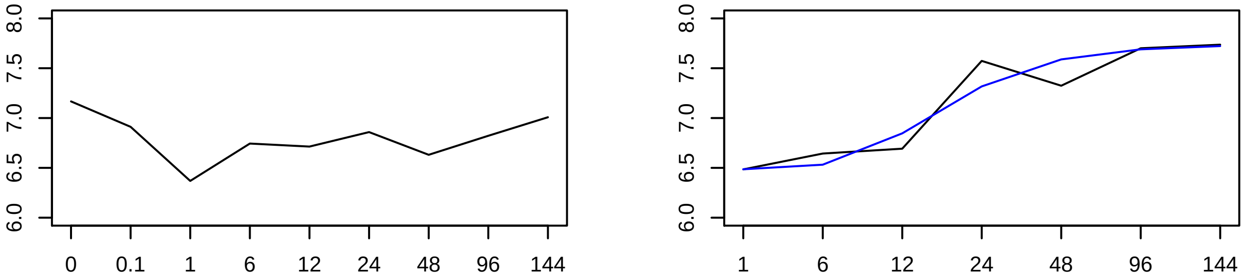

A_23_P123086 KIAA1908 7p22.3

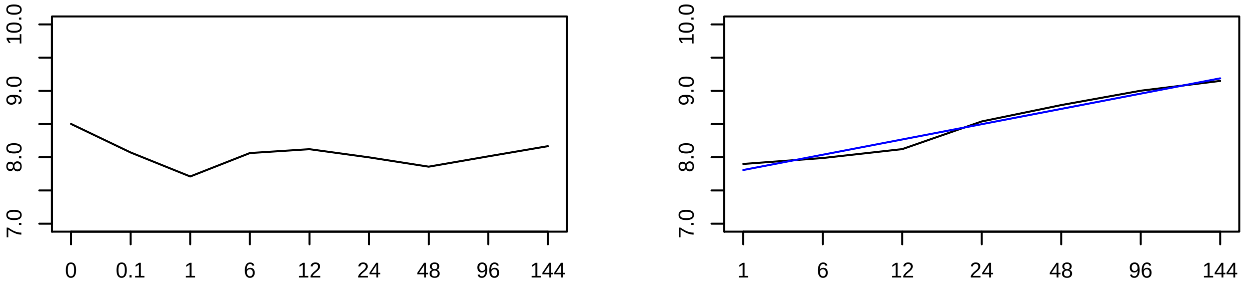

A_23_P37727 CX3CL1 16q13

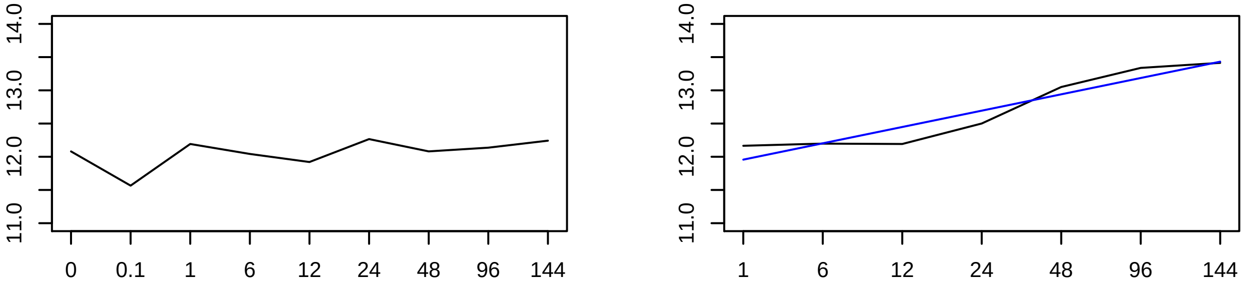

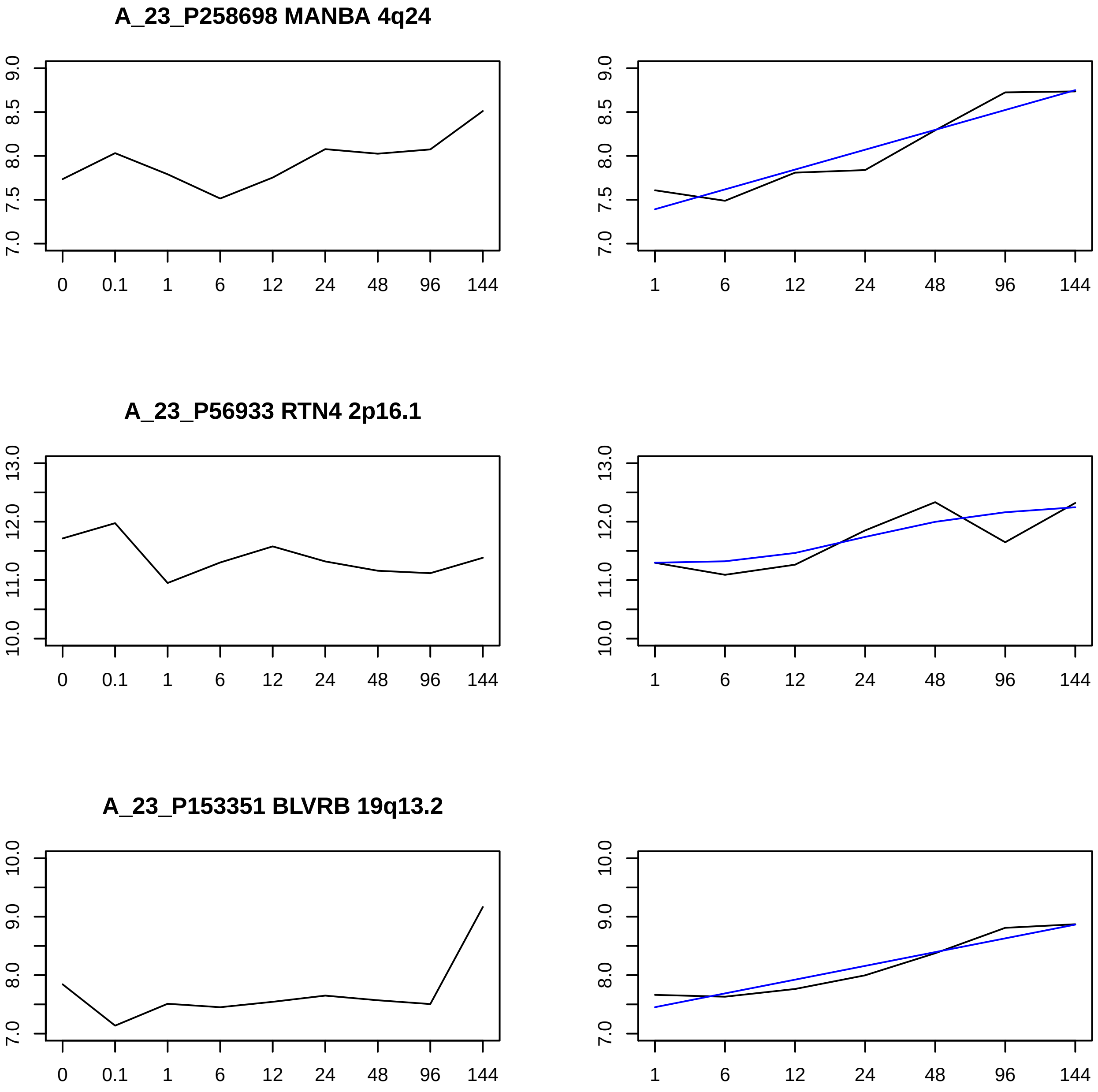
A_23_P258698 MANBA 4q24
9.0
8.5
8.0
7.5
7.0
0
0.1
1
6
12
24
48
96
144
A_23_P56933 RTN4 2p16.1
13.0
12.0
11.0
10.0
A_23_P153351 BLVRB 19q13.2
10.0
9.0
8.0
7.0

A_24_P786025 CR936711 NA

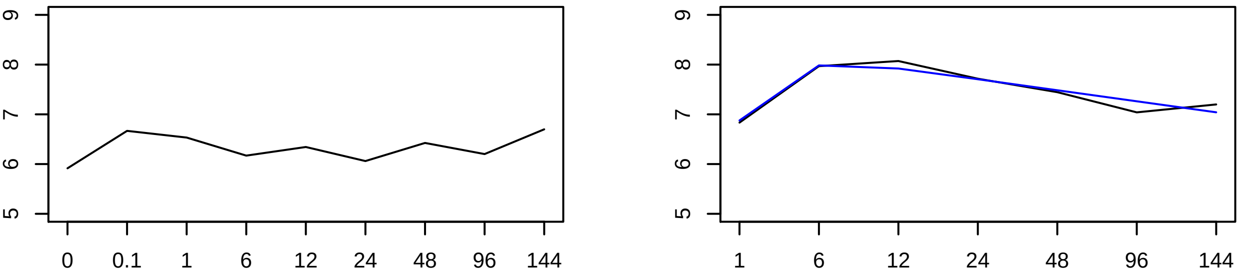

A_32_P21354 THC2688038 NA

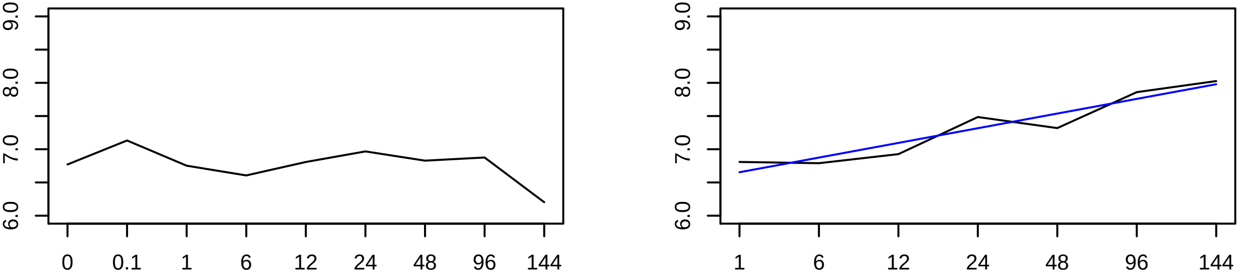

A_32_P180101 C14orf144 14q32.33

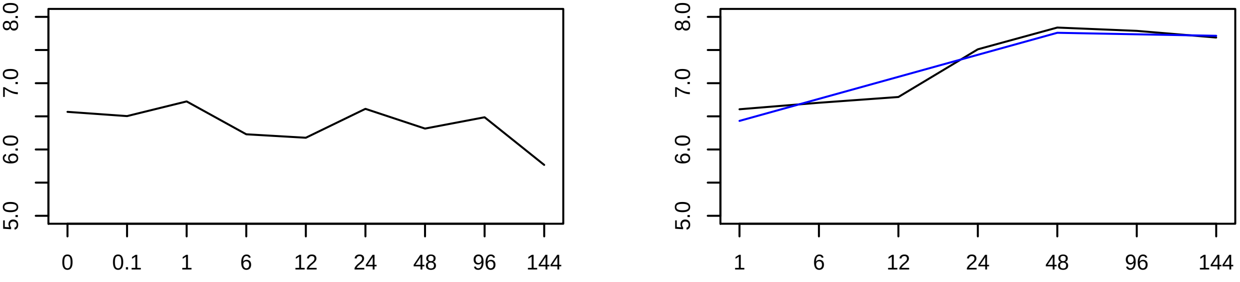

A_32_P94685 BC042589 NA

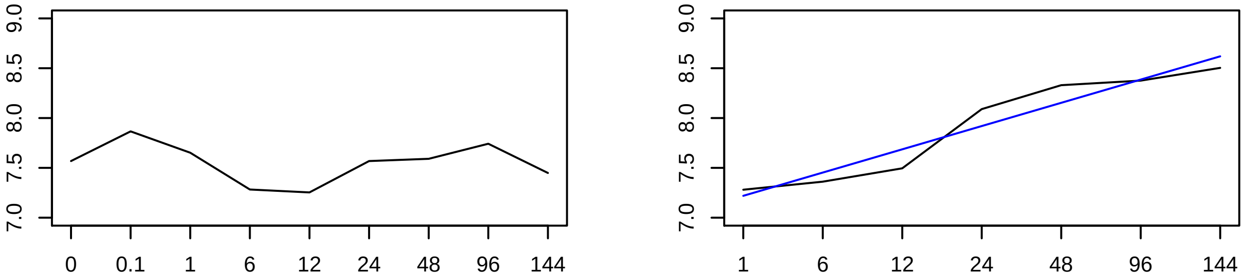

A_23_P52697 CD248 11q13.1

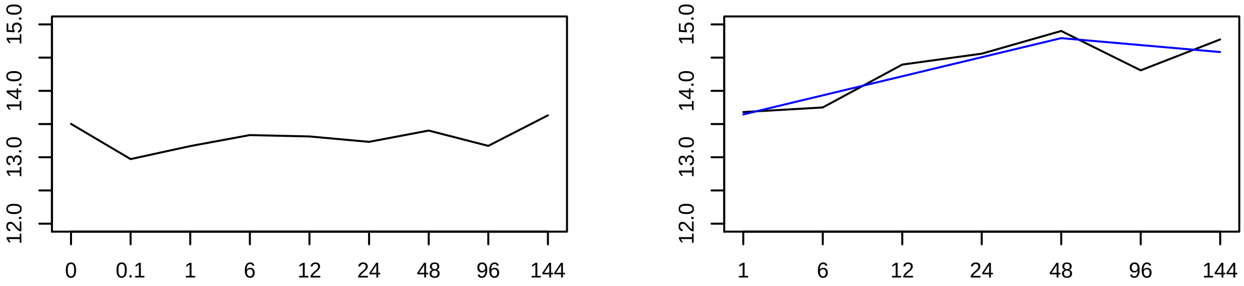

A_23_P426636 AHNAK 11q12.3

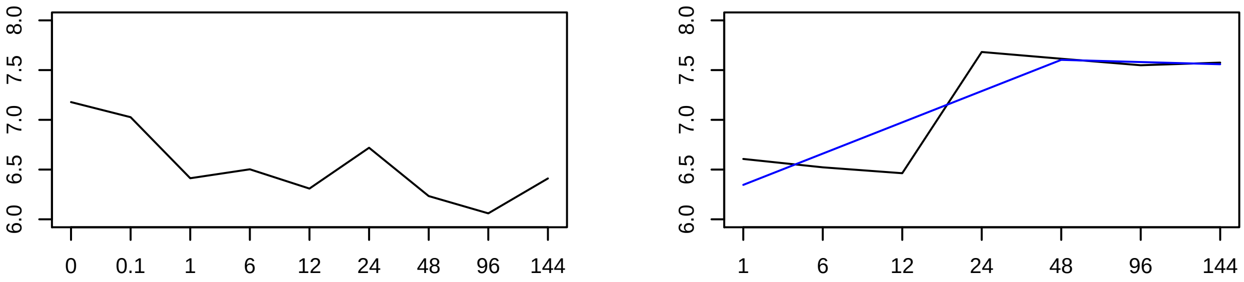

A_24_P857404 LOC92312 1q22

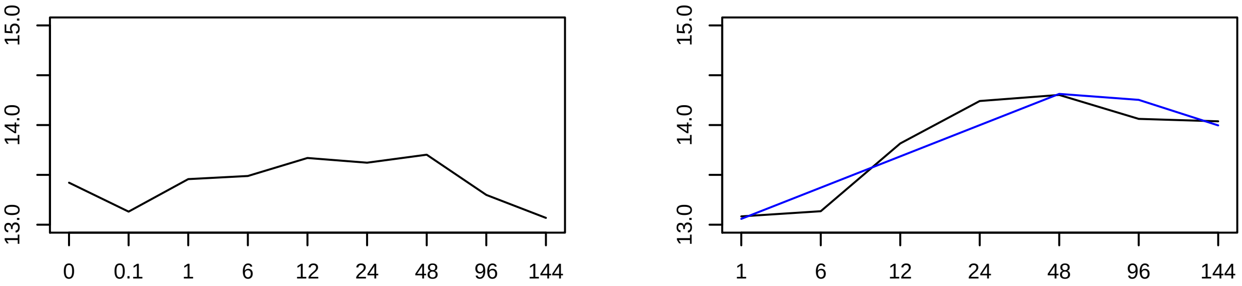

A_32_P136871 THC2712372 NA

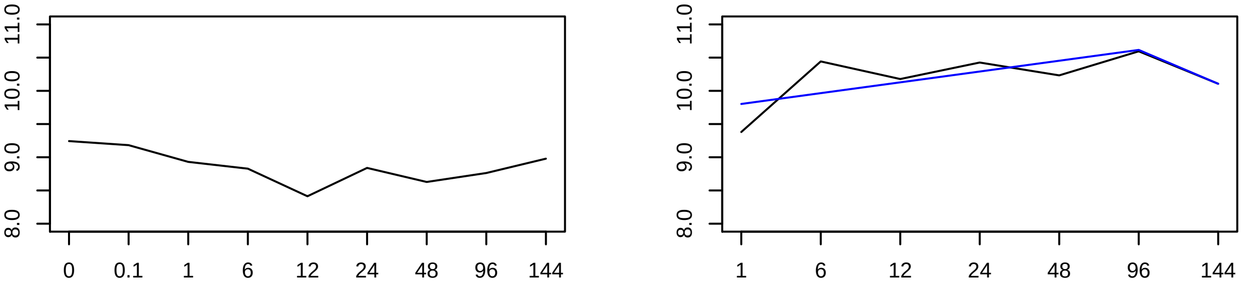

A_23_P258048 AK056630 NA

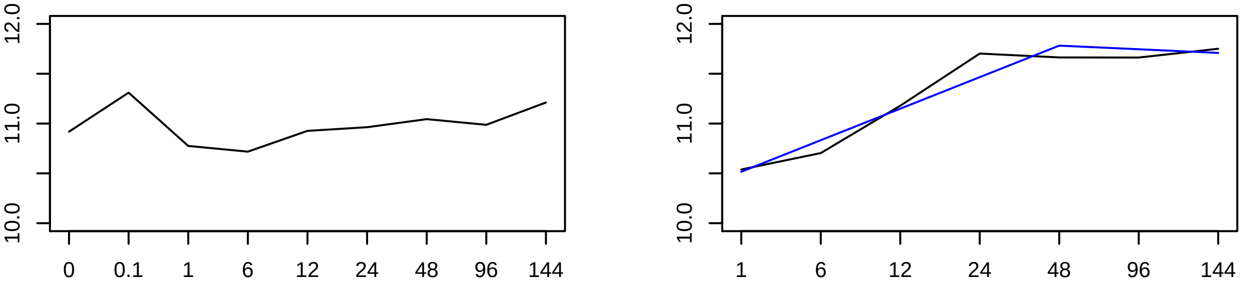

A_24_P347418 LRCH1 NA

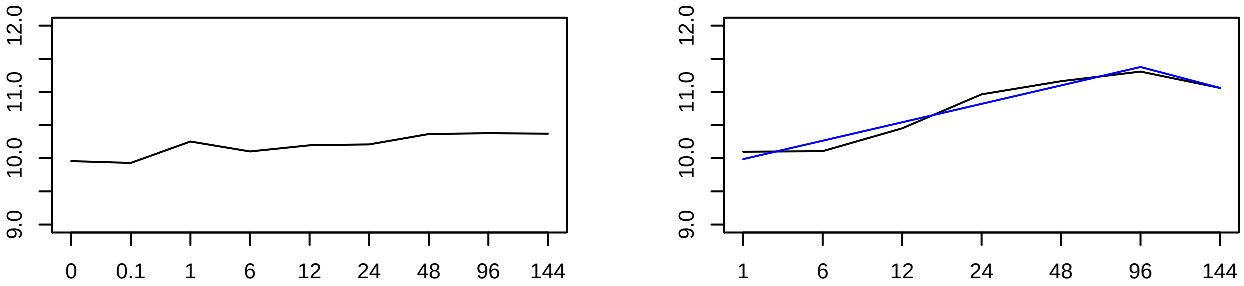

A_23_P167017 POPDC2 3q13.33

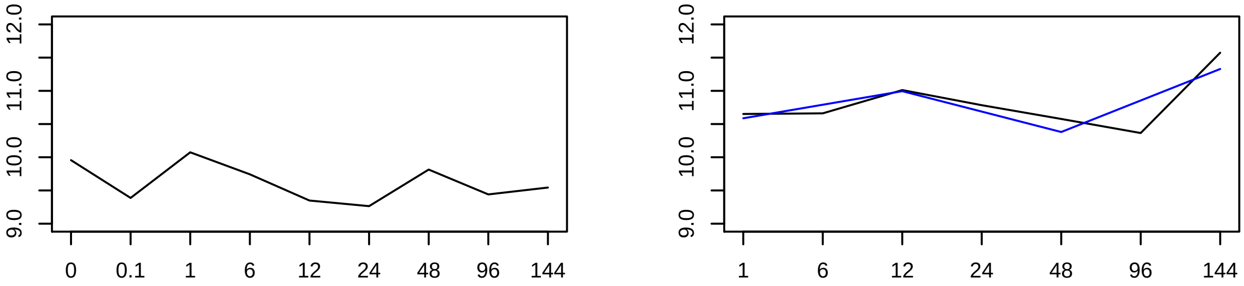

A_24_P181295 C14orf37 14q23.1

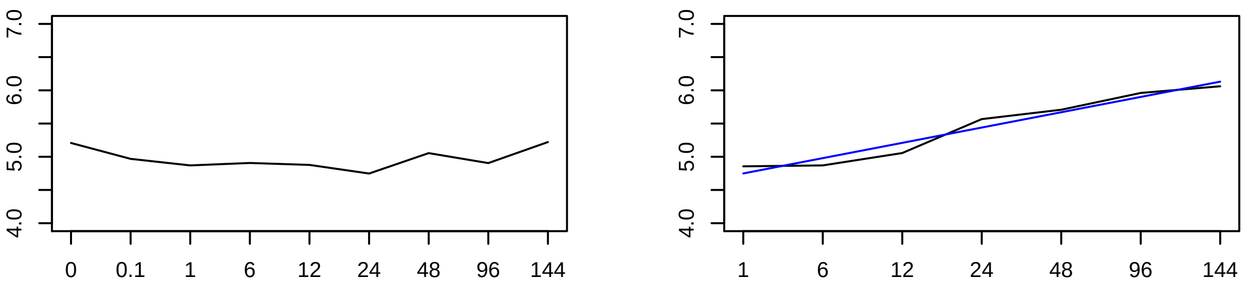

A_24_P769977 THC2701748 NA

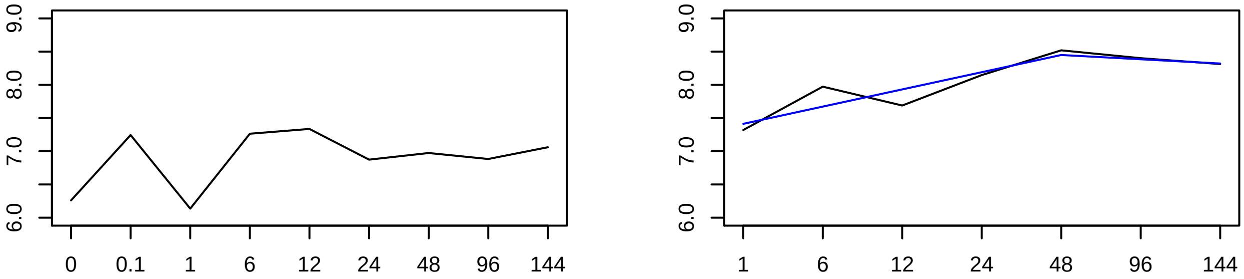

A_32_P100707 THC2505910 NA

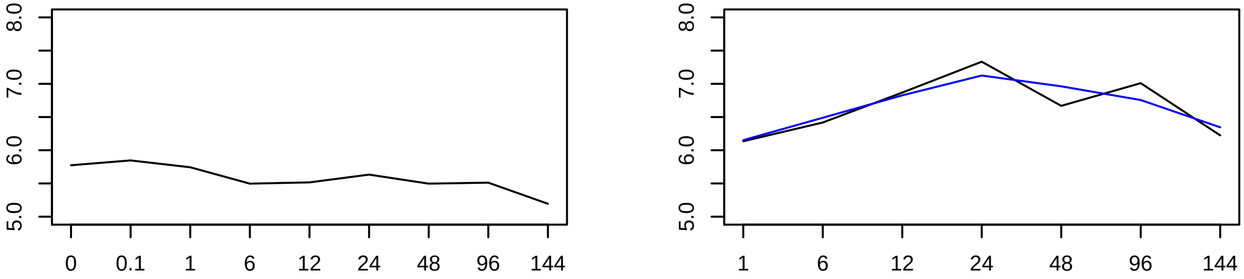

A_23_P118392 RASD1 17p11.2

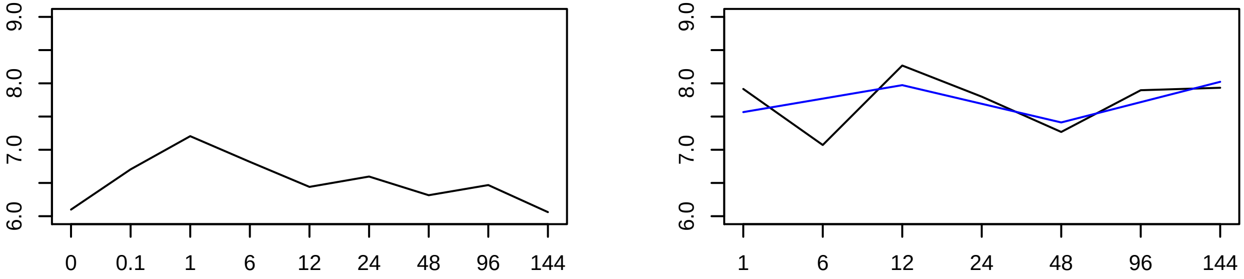

A_24_P152743 TMC6 17q25.3

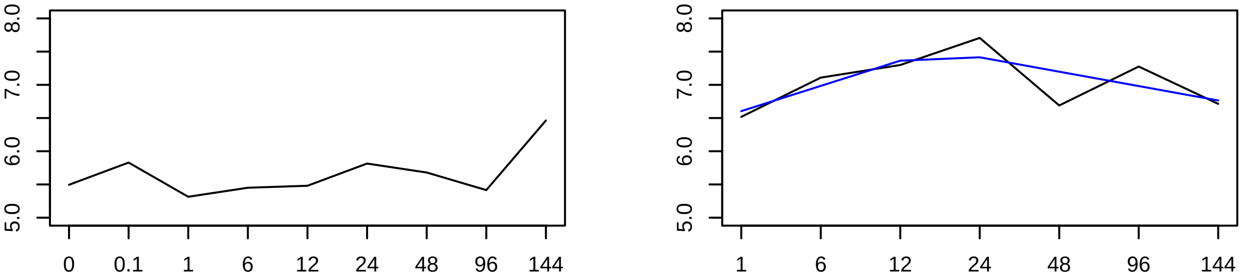

A_32_P6442 A_32_P6442 NA

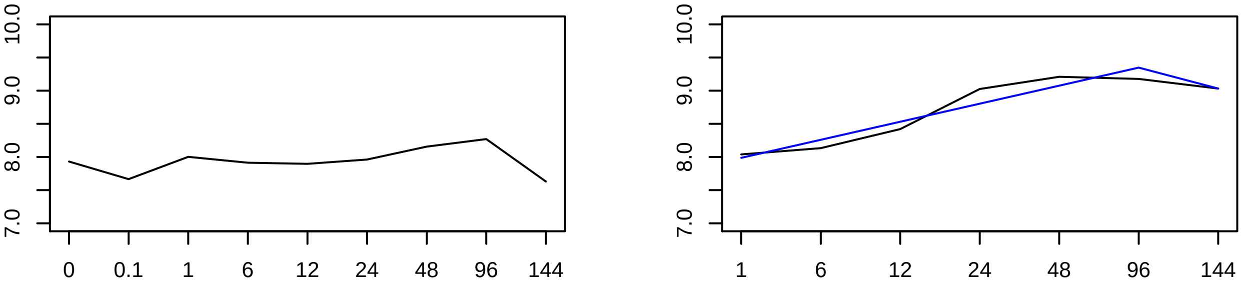

A_24_P158946 FGD4 12p11.21

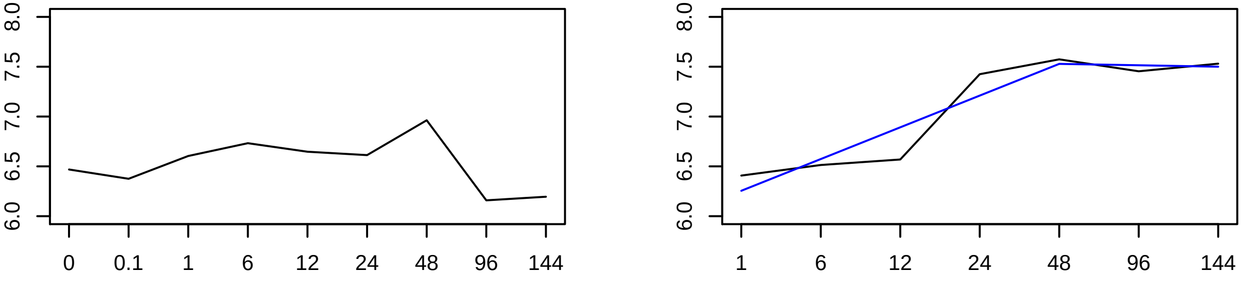

A_23_P6935 CD47 3q13.12

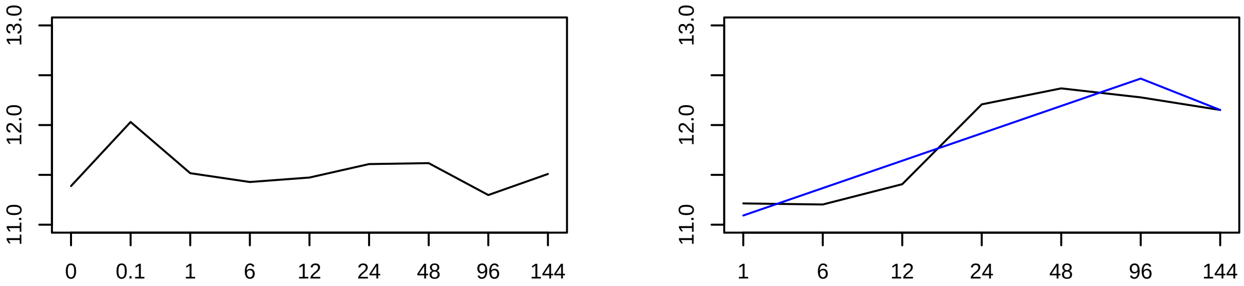

A_23_P13772 TBX3 12q24.21

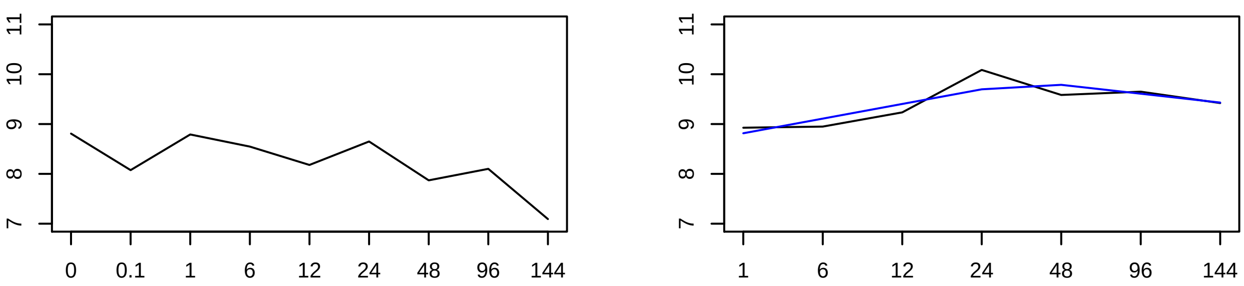

A_24_P174313 FLJ10081 2q11.2

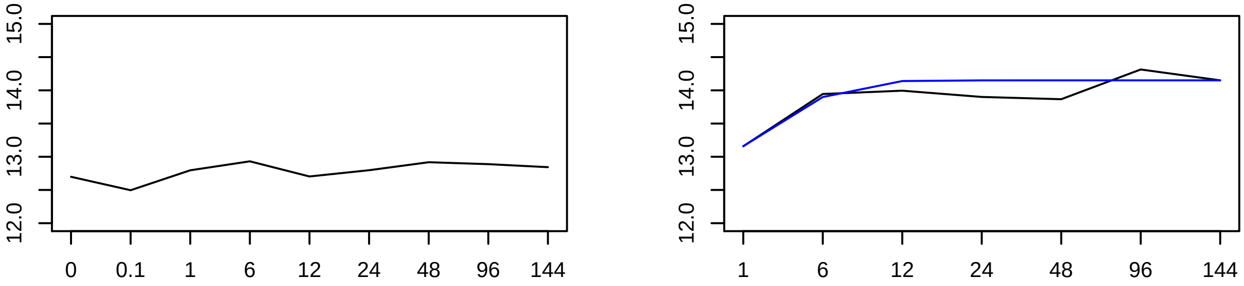

A_32_P219148 LOC497257 8p23.1

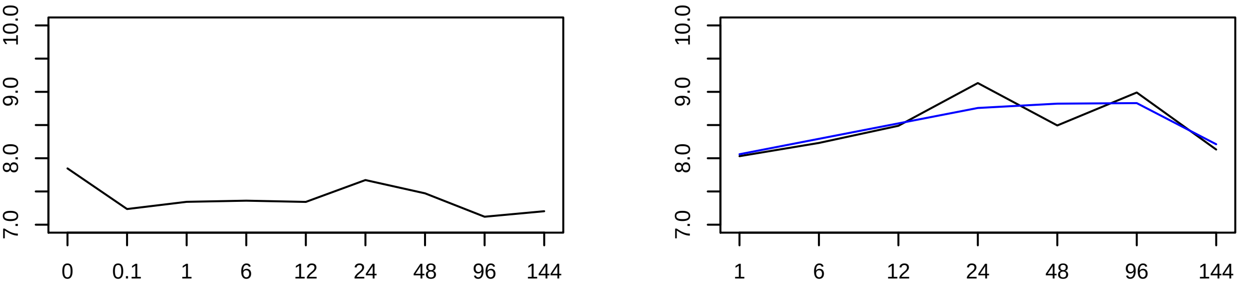

A_24_P937915 PRKCH NA

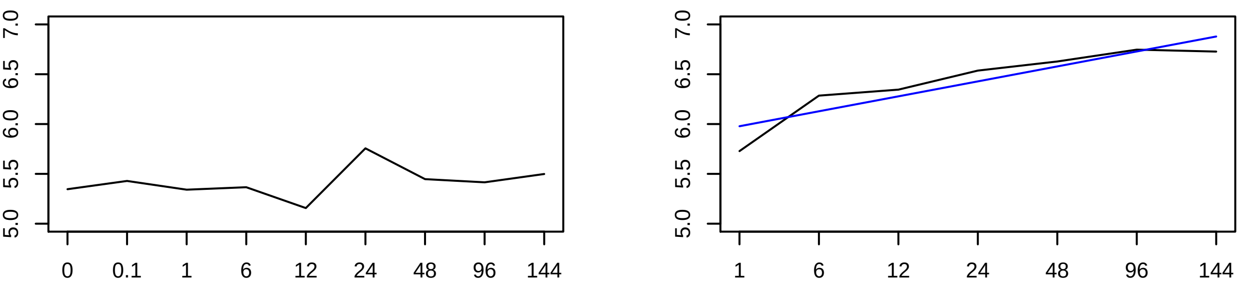

A_23_P320705 CRYGD 2q33.3

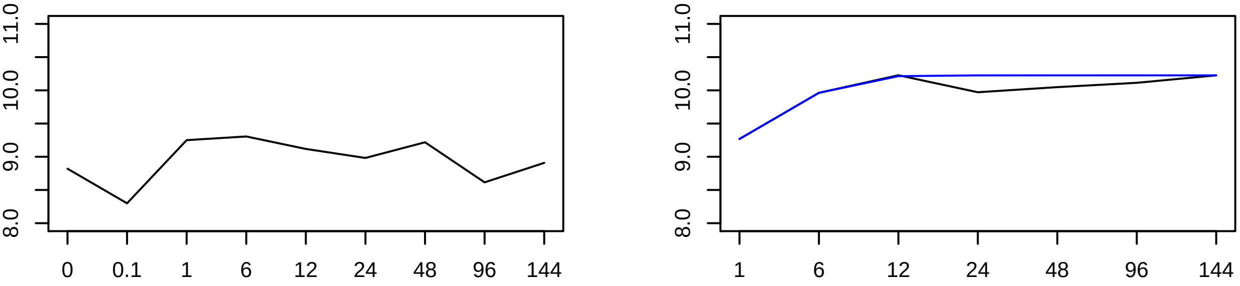

A_32_P775170

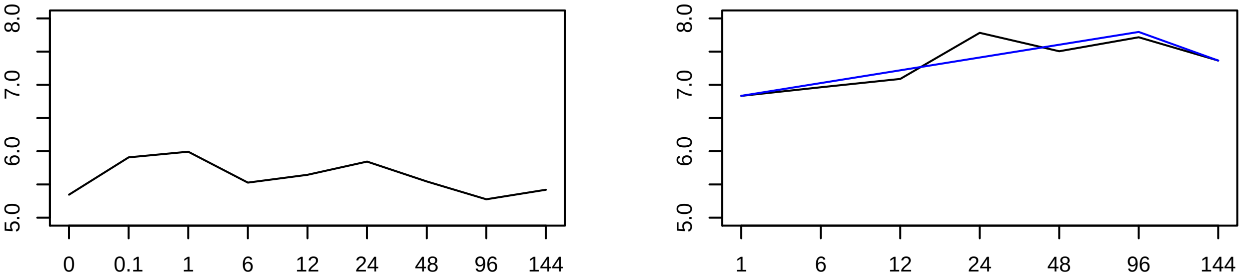

A_23_P24903 P2RY2 11q13.4

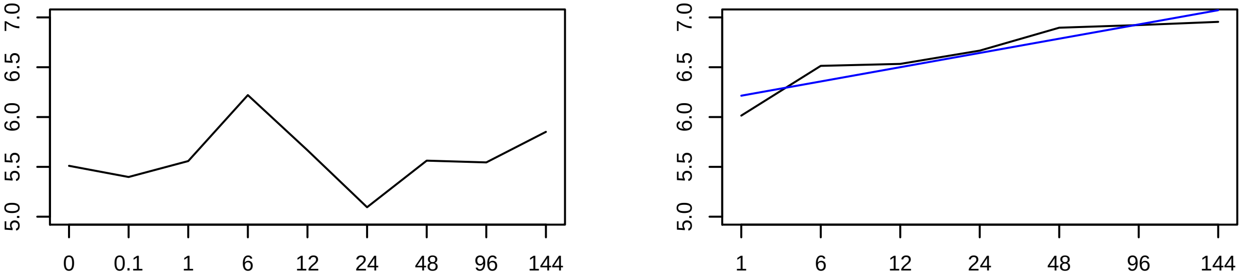

A_23_P252175 MYO1B 2q32.3

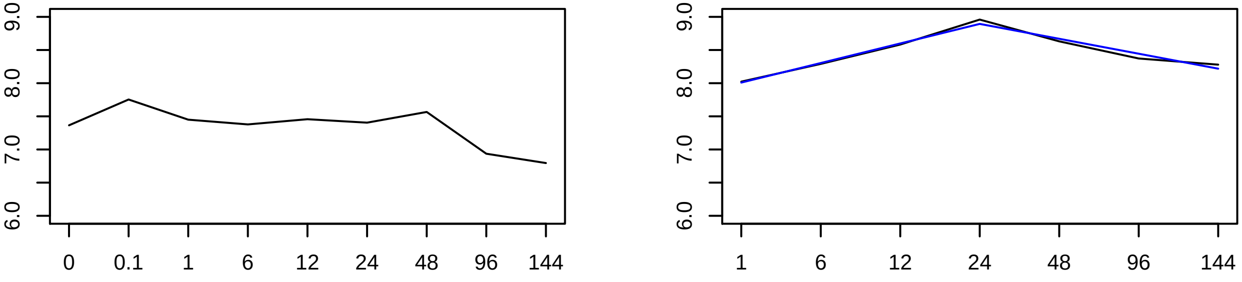

A_23_P389987 TLX2 2p13.1

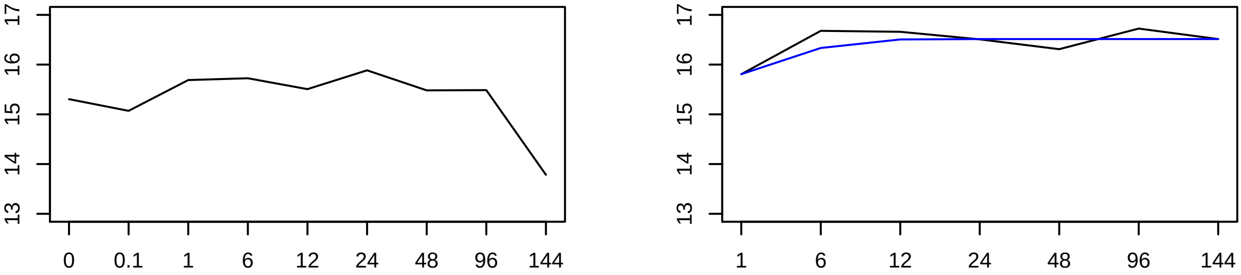

A_23_P165796 HOXD13 2q31.1

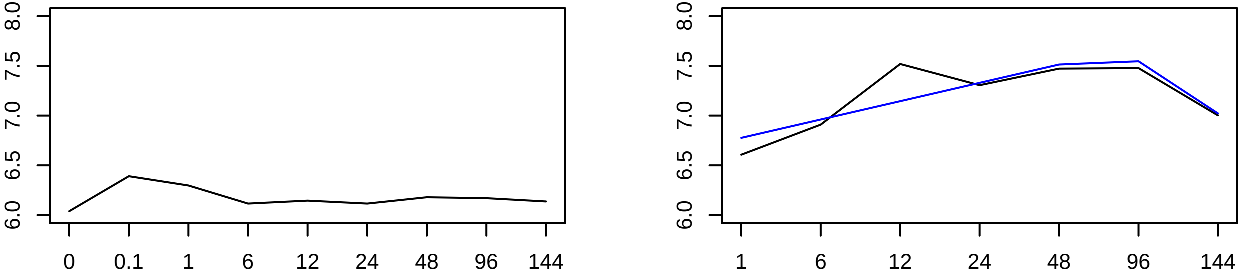

A_24_P190873 C1orf76 1q25.2

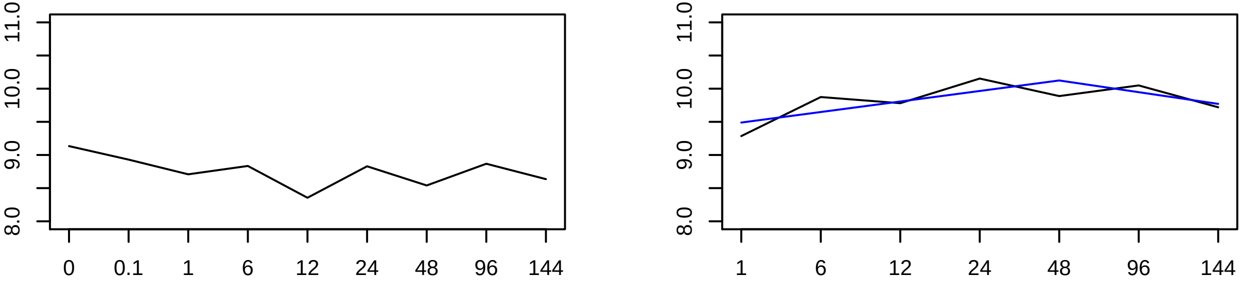

A_23_P33356 ADAMTS9 NA

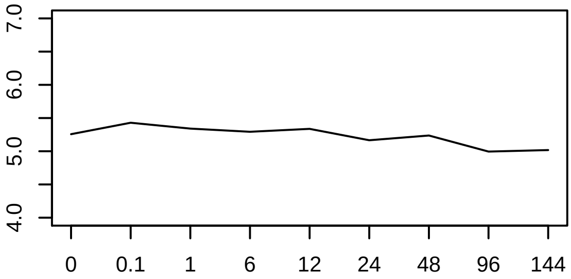

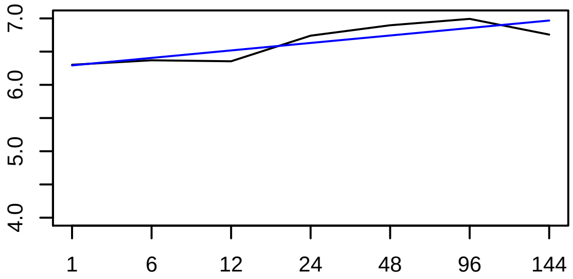

Supplement: Additional file 3 — Additional file A-H. These files contain the fitting results for the genes from the groups A-H, deduced by SwitchFinder, which represent eight dynamic patterns of the gene expression response to ATRA in neuroblastoma cell line. (ZIP 2457 kb) [file 12859_2016_1391_MOESM3_ESM.zip › AdditionalFile_A.pdf]
