# Supplementary material for: SwitchFinder – a novel method and query facility for discovering dynamic gene expression patterns
Source: BMC Bioinformatics. 2016 Dec 15;17:532. doi: 10.1186/s12859-016-1391-0 (PMC5160026; doi:10.1186/s12859-016-1391-0)
Supplement: Additional file 4 — Tables S1-S8. Demonstrating genes from the groups A-H and their functional annotations. (PDF 63 kb) [file 12859_2016_1391_MOESM4_ESM.pdf]

Supplementary Table 1

A INDUCED_IMMEDIATELY	
BACH2, BATF2, CREM, CSRNP3, DACH1, EBF1, EGR1/2/3, FOS, FOXC1, GATA6, HES1, HEY1, HIC1, HIF1A, HOXD1/3/8/9/10/13, KDM5B(JARID1B), KLF12, LEF1, MAFB, NCOA3/7, NKX3-1, NR0B1, PBX1, PPARG/D, RARA, SMAD3, SOX4/8/9, TBX2/3, TEAD2, TLE3, TLX2, TULP4, ZFP2, ZNF71/135/436/606/641	GO:0003700 sequence-specific DNA binding transcription factor activity; GO:0006355 regulation of transcription, DNA-templated; GO:0030154 cell differentiation
AKR1C1/3, BCDO2, CRABP2, CYP26A1/B1, DHRS3, RARA, RBP1, RDH10, SDC4, SP100, STRA6, PPARD/G	GO:0001523 retinoid metabolic process; GO:0042573 retinoic acid metabolic process; GO:0001972 retinoic acid binding; GO:0032526 response to retinoic acid
BMP4, EGR1, GREM2, LEF1	GO:0030509 BMP signaling pathway
DACT3, LEF1, PSEN1, SOX4	GO:0016055 Wnt signaling pathway
FOXC1, HES1, HEY1, HIF1A, MDK, NCOR2, PSEN1, TLE3	GO:0007219 Notch signaling pathway; GO:0005112 Notch binding
ERBB2, IRS2, KITLG, PDGFRA/B, SPRY2/4	GO:0007173 epidermal growth factor receptor signaling
PDGFRA/B, PLAT	GO:0048008 platelet-derived growth factor receptor signaling pathway
NGFR, NTRK1, PCSK5, PLEKHG2, RALB, RIT1	GO:0048011 neurotrophin TRK receptor signaling pathway; GO:0038180 nerve growth factor signaling pathway
DISP1	GO:0007224 smoothened signaling pathway; GO:0008158 hedgehog receptor activity; GO:0009880 embryonic pattern specification
APC2, EML4, KIFAP3, LYST, NEIL2, SPTAN1	GO:0015630 microtubule cytoskeleton
AHNAK, ARPC1B, AVIL, CORO2A, CTTNBP2NL, FAM129B, FGD4/6, FHL2, FLNB, KALRN, LCP1, MYRIP, PDLIM5/7, PPP1R12B, SYNPO/2, TRIOBP, VCL	GO:0015629 actin cytoskeleton
ARHGDIB, CLASP2, CNN2, LIMK1, NUAK2, PAK1, PALM, PFN2, PLK2, RND3, SDCBP, SOX9	GO:0007010 cytoskeleton organization
CEACAM1, GAB2, ITGA1, ITGB8;<br>ADD3, LIMK1, MYADM, MRCL3(MYL12A), TRIO	GO:0007229 integrin-mediated signaling pathway; GO:0005911 cell-cell junction; GO:0040011 locomotion; GO:0016477 cell migration
ANTXR1, ATP1B1, BVES, CALCA, CDH23, CEACAM1, CLSTN3, COL12A1, COMP, FBLIM1, KITLG, NCAM2, NEO1, PCDHB2/4/6/9-11/13/14, PPFIBP1, PSEN1, PVRL2, RET, RND3, SPP1, TGFB1I1, TPBG, TRO,VTN	GO:0007155 cell adhesion; GO:0007411 axon guidance
HIF1A, HTR2B, KITLG, LEF1, RET, SOX8	GO:0001755 neural crest cell migration
EGR2/EGR3, ERBB2, SOX8	GO:0007422 peripheral nervous system development
JARID1B, JARID2	GO:0016568 chromatin modification; GO:0048863 stem cell differentiation
SLIT2, SLITRK6, FLOT1	GO:0035385 Roundabout signaling pathway; GO:0050772 positive regulation of axonogenesis
EPHA2, EPHB3; SEMA6C, SEMA6D	GO:0048013 ephrin receptor signaling pathway; GO:0030215 semaphorin receptor binding; GO:0007411 axon guidance
DCX, DPYSL3, ERBB2, KCNQ2, PSEN1, PTPRO, RRAS, SPTAN1, ST8SIA4; STMN2, TEAD2	GO:0007411 axon guidance; GO:0030426 growth cone; GO:0048666 neuron development
LAMB2, LAMC1	GO:0005605 basal lamina; GO:0031175 neuron projection development
DLG2, GLS, GNG2/8, HCN1, KCNQ2, PANX, RRAS, SDCBP, SST, SYNJ2, SYT2; STX7, STXBP5/6	GO:0007268 synaptic transmission; GO:0019905 syntaxin binding; GO:0045202 synapse
HTR2B, FOS,KALRN, NAB2, NAV2, DCX, RGS9, RTN4, VCL	GO:0007399 nervous system development; neurite branching; GO:0030334 regulation of cell migration
CDKL5	GO:0001764 neuron migration; GO:0050773 regulation of dendrite development; GO:0051726 regulation of cell cycle
BCL2, BOK, CASP4/9, CTSB, NLRP1, SKIL; ANGPT1, CPEB4, CRLF1, F2R, HIF1A, MDK, NTRK1, PSEN1	GO:0006915 apoptotic process; GO:0043524 negative regulation of neuron apoptotic process
ADAM12, ADAMTS9, MMP2/11	GO:0008237 metallopeptidase activity
F2R, GALR1, GPR161, HTR2B, IGF2R, P2RY2, PTGER2, PTGIR	GO:0004930 G-protein coupled receptor activity; GO:0004966 galanin receptor activity; GO:0007218 neuropeptide signaling pathway; GO:0007189 adenylate cyclase-activating G-protein coupled receptor signaling pathway
CRLF1	GO:0005127 ciliary neurotrophic factor receptor binding

Supplementary Table 2

B INDUCED_12	
AEBP1, ARNT2/L2, CREB5, ETS1, ETV4, HIF3A, HOXB3, IRF7, KLF5, MAFF, MKL1, MSRB2, MYH9/15, NANOG, NFIB, NFIL3, RARB, REST, SALL4, SMAD1, SOX9/11, STAT6, TBX19, TSC22D3, ZFP36L1, ZNF219/226/641	GO:0003700 sequence-specific DNA binding transcription factor activity; GO:0009790 embryo development
APOE, GPC2, RDH12	GO:0001523 retinoid metabolic process
AMER3, DKK1/2, MDFI, NPHP3, SFRP1, SMO	GO:0017147 Wnt-protein binding; GO:0007224 smoothened signaling pathway; GO:0048663 neuron fate commitment; GO:0001755 neural crest cell migration
TUBA3C, TUBB2A/2B/3/4/6/8; DST, MAP1B, MAPT; KIF1B/13B/5A/5C	GO:0007017 microtubule-based process; GO:0008017 microtubule binding; GO:0000226 microtubule cytoskeleton organization; GO:0005871 kinesin complex;
AMOTL2, DCHS1, TJP1/2, YAP1	GO:0035329 hippo signalling; GO:0030054 cell junction; GO:0005516 calmodulin binding; GO:0005923 tight junction; cell migration GO:0022008 neurogenesis
RAB15/2B/38/3B/4B, RASGRP2, RHOC, SH2D3C; DIRAS3, ABR, ARHGAP26, ARL8A, DOCK6, GDI1, KNDC1, MCF2L, MRAS	GO:0007264 small GTPase mediated signal transduction
ADCYAP1, CAP2, EDNRA	GO:0007190 activation of adenylate cyclase activity; GO:0007163 establishment or maintenance of cell polarity; GO:0007411 axon guidance
EDNRA, GPR68/124/161, SSTR2, SUCNR1	GO:0086100 endothelin receptor signaling pathway; GO:0004930 G-protein coupled receptor activity;
ACTB, ALCAM, ANK3, COL1A1/13A1/3A1/6A2, CORO2B, LSAMP, SPTBN1, SRC, TPM1/4	GO:0007010 cytoskeleton organization; GO:0030424 axon GO:0007411 axon guidance; GO:0010718 positive regulation of epithelial to mesenchymal transition; GO:0048706 embryonic skeletal system development; GO:0007399 nervous system development
CTNNA1, CD59, FN1, FBLIM1, PTPRK, PXN, BCAN	GO:0007155 cell adhesion; GO:0048870 cell motility; GO:0030198 extracellular matrix organization
PLAU, PRSS3/12; SCG2/5	GO:0004252 serine-type endopeptidase activity; GO:0014909 smooth muscle cell migration; GO:0043542 endothelial cell migration
SOCS2	growth hormone receptor signaling
INHBA, DLX5, DPYSL4, ENC1, FEZ1, FGF13, GAL, IGSF8, JUP, LY6H, NES, NRG1, PTN, TAGLN2/3, TIMP1/2; GFRA1	GO:0007399 nervous system development; GO:0035860 glial cell-derived neurotrophic factor receptor signaling
PLK3	GO:0007093 mitotic cell cycle checkpoint
SEMA4F; EFNA4; PLXNA2	GO:0030215 semaphorin receptor binding; GO:0048013 ephrin receptor signaling pathway; GO:0021915 neural tube development; neurogenesis
B3GALT2/4, B3GNT4/5	GO:0008378 galactosyltransferase activity; GO:0006486 protein glycosylation
ANXA2/3/6, CCDC80, CXADR, DCN, HOMER3, EGFLAM, LGALS1/3, LTBP1, MFGE8, MGP, MRC2, NID1, TGFB1, TGFBI; ITGA3/6/B1/B3	GO:0005544 calcium-dependent phospholipid binding; GO:0030054 cell junction; GO:0007160 cell-matrix adhesion; GO:0031012 extracellular matrix; GO:0005178 integrin binding; GO:0005518 collagen binding; GO:0001649 osteoblast differentiation
CHAC1, DTX1	GO:0005112 Notch binding; GO:0008593 regulation of Notch signaling pathway
NRG1; NFIB	GO:0048011 neurotrophin TRK receptor signaling pathway; GO:0045595 regulation of cell differentiation; GO:0009790 embryo development; GO:0030154 cell differentiation
ADAM19/22/23, ADAMTS5/10, MMP15, THSD4	GO:0004222 metalloendopeptidase activity; GO:0031012 extracellular matrix
APOE, CASP3/5/CASP6, CYFIP2, LITAF, NISCH, SCOTIN (SHISA5), TRADD; IFI16, PHLDA3, PYCARD	GO:1901214 regulation of neuron death; GO:0042981 regulation of apoptotic process; GO:0072332 intrinsic apoptotic signaling pathway by p53 class mediator
SYNGR1, SYT4/5/9/11	GO:0008021 synaptic vesicle; GO:0043005 neuron projection; GO:0030425 dendrite; GO:0030426 growth cone
FBXL2/7	GO:0004842 ubiquitin-protein transferase activity
ADCY5, ADCYAP1, AKAP6; CRH, GAL, VIP	GO:0008179 adenylate cyclase binding; GO:0005184 neuropeptide hormone activity;
TTYH3	GO:1902476 chloride transmembrane transport
NANOG, SMAD1, SOX9, SOX11	GO:0019827 stem cell maintenance; GO:0009880 embryonic pattern specification; GO:0014032 neural crest cell development
DDR1, SSTR2	GO:0008285 negative regulation of cell proliferation

Supplementary Table 3

C INDUCED_24	
DDIT3, MAFK, MYC, SMARCD3, RUNX1T1	GO:0003700 sequence-specific DNA binding transcription factor activity; GO:0007399 nervous system development
RAI14	Retinoic Acid-Induced protein 14
PHLDA2, PHLDB2	GO:0030334 regulation of cell migration; GO:0045995 regulation of embryonic development
PRSS23	GO:0008233 peptidase activity
ADORA2B, CHRM2/3, GPR64/124/126, NPY	GO:0004930 G-protein coupled receptor activity; GO:0007188 adenylate cyclase-modulating G-protein coupled receptor signaling pathway; GO:0016907 G-protein coupled acetylcholine receptor activity; GO:0007218 neuropeptide signaling pathway;
MYLIP, SCRG1	GO:0008092 cytoskeletal protein binding; GO:0007399 nervous system development
MARCH2	GO:0004842 ubiquitin-protein transferase activity;
PLAUR	GO:0030377 urokinase plasminogen activator receptor activity; GO:0005925 focal adhesion
GPC1/5	GO:0001523 retinoid metabolic process; GO:0014037 Schwann cell differentiation;
PARG, SPATA18	GO:0006974 cellular response to DNA damage stimulus
NCSTN	GO:0007219 Notch signaling pathway; GO:0007220 Notch receptor processing
EFNB2	GO:0048013 ephrin receptor signaling pathway; GO:0007411 axon guidance
CDKN2D; LATS2	GO:0000082 G1/S transition of mitotic cell cycle; GO:0035329 hippo signaling
NEFL, MAP6, MICALL2, PLCE1, SH3D19, TCHH, WIPI1	GO:0005883 neurofilament; GO:0007010 cytoskeleton organization
NRBP2	GO:0030182 neuron differentiation; GO:0030154 cell differentiation
FZD5, GRK5, SOSTDC1	GO:0016055 Wnt signaling pathway
DDIT3	GO:0090090 negative regulation of canonical Wnt signaling pathway
BCAS3	GO:0035035 histone acetyltransferase binding; GO:0003682 chromatin binding; GO:0042393 histone binding
ENO2	GO:0043025 neuronal cell body
SYNGR2, SYT17	GO:0008021 synaptic vesicle;
NANOS3	GO:0051726 regulation of cell cycle; GO:2001234 negative regulation of apoptotic signaling pathway; GO:0007275 multicellular organismal development
CXCR4	GO:0061351 neural precursor cell proliferation; GO:0001764 neuron migration; GO:0070098 chemokine-mediated signaling pathway; GO:0030334 regulation of cell migration; GO:0043217 myelin maintenance
BMX, CDH6, CTNND2, DCBLD2, THBS2	GO:0007155 cell adhesion
ASNS, CYR61, GRK5, TXNDC5	GO:0043066 negative regulation of apoptotic process
PHACTR3, TAGLN2	GO:0003779 actin binding
NPPC	GO:0003418 growth plate cartilage chondrocyte differentiation
FSTL3	GO:0001503 ossification; GO:0002244 hematopoietic progenitor cell differentiation

Supplementary Table 4

D INDUCED_LATE	
CEBPD, CUTL2, ELF5, FLI1, HOXB2	GO:0001071 nucleic acid binding transcription factor activity; transcription factor activity
BMP6	GO:0030509 BMP signaling pathway; GO:0030154 cell differentiation; GO:0045666 positive regulation of neuron differentiation
BMP6, MEST; CRABP1	GO:0032526 response to retinoic acid; GO:0001972 retinoic acid binding
HHIP	GO:0097108 hedgehog family protein binding; GO:0045879 negative regulation of smoothened signaling pathway; GO:0007405 neuroblast proliferation
	GO:0048706 embryonic skeletal system development
LGI1, MYL6/7, OPHN1, EFHC1, SNCAIP	GO:0016459 myosin complex; GO:0007519 skeletal muscle tissue development; GO:0007411 axon guidance; GO:0043025 neuronal cell body; GO:0007399 nervous system development
SEMA3C	GO:0030215 semaphorin receptor binding; GO:0021915 neural tube development; GO:0001755 neural crest cell migration
MFAP4	GO:0030198 extracellular matrix organization
ACTA2, ACTG2	GO:0015629 actin cytoskeleton
CTGF	GO:0032330 regulation of chondrocyte differentiation
ITGA7	GO:0005178 integrin binding; GO:0030198 extracellular matrix organization; GO:0016477 cell migration; GO:0030154 cell differentiation
NOTCH2	GO:0007219 Notch signaling pathway; GO:0030154 cell differentiation;
CTNNBIP1, SFRP1, WISP1, WNT11	GO:0016055 Wnt signaling pathway; GO:0030877 beta-catenin destruction complex
NDRG1 (N-Myc Downstream Regulated 1)	GO:0015630 microtubule cytoskeleton; GO:0032287 peripheral nervous system myelin maintenance; GO:0030330 DNA damage response, signal transduction by p53 class mediator
LATS2 (Large Tumor Suppressor Kinase 2)	GO:0035329 hippo signalling; GO:0046620 regulation of organ growth
THBS1	GO:0007155 cell adhesion; GO:0001968 fibronectin binding; GO:0030198 extracellular matrix organization; GO:0007050 cell cycle arrest;
CYR61	cell adhesion, proliferation, differentiation, angiogenesis, apoptosis, GO:0031012 extracellular matrix
IL33, CD274, GBP2/3	GO:0006955 immune response
CXCL12	GO:0070098 chemokine-mediated signaling pathway; GO:0001764 neuron migration
ASPN	GO:0030512 negative regulation of transforming growth factor beta receptor signalling pathway
ANXA1, BMP6, PTX3, TGFB2	GO:0006954 inflammatory response; GO:0030154 cell differentiation
GAS2	GO:0006915 apoptotic process; GO:0007050 cell cycle arrest; GO:0008360 regulation of cell shape
SPARC	GO:0001503 ossification; GO:0071363 cellular response to growth factor stimulus; GO:0022604 regulation of cell morphogenesis
CRYM	GO:0070324 thyroid hormone binding; GO:0050877 neurological system process
MC4R	GO:0004977 melanocortin receptor activity; GO:0004980 melanocyte-stimulating hormone receptor activity
ITIH3	GO:0004866 endopeptidase inhibitor activity; GO:0030212 hyaluronan metabolic process
HTRA1	GO:0001558 regulation of cell growth
SDK2	GO:0007155 cell adhesion

Text

Supplementary Table 5

E REPRESSED	
E2F2, EBF3, FOXN4, GATA3/4, HAND1, INSM1, KLF9/15, LZTS1, MSX2, MXD4, NFIX, ONECUT2, PA2G4, PCGF6, POU3F2, PROX1, PTTG1, RFXAP, RXRA, SCML1, SHOX2, SIX3, SNAI1, SOX2/7, TAF3/4B/5, TEF, TFAM, TLE1/2, TRERF1, VDR, ZBTB25, ZFPM2, ZNF287	GO:0001071 nucleic acid binding transcription factor activity; GO:0000122 negative regulation of transcription from RNA polymerase II promoter; GO:0030154 cell differentiation; GO:0001701 in utero embryonic development; GO:0048935 peripheral nervous system neuron development; GO:0030182 neuron differentiation
CCNC, DLK1, DLL1, HDAC9, LFNG, TLE1/2	GO:0007219 Notch signaling pathway;
KREMEN2, SIX3, SOX2, TLE1	GO:0030178 negative regulation of Wnt signaling pathway; GO:0043124 negative regulation of I-kappaB kinase/NF-kappaB signalling;
FGFR2, HDAC9, MXD4, NFIX, PER2, RFC1	GO:0042127 regulation of cell proliferation;
ASPM, CAMK4, CAMKK1, CAMKV, PCNT, RYR2	GO:0005516 calmodulin binding;
NRP1/2	GO:1901166 neural crest cell migration involved in autonomic nervous system development;
EFNA1, NRP1/2, NPPA, PLXND1, GFRA3, MGLL, NDRG1, PSD3, PVRL1, SEMA3A/6A, SPTB	GO:0046875 ephrin receptor binding; GO:0071526 semaphorin-plexin signaling pathway;
BCL2L11, ECT2, FGF5, FGFR2, ITSN1	neurotrophin TRK receptor signaling
RXRA	GO:0048384 retinoic acid receptor signaling pathway;
RALBP1, ECT2; DUSP5P(RHOU)	GO:0043089 positive regulation of Cdc42 GTPase activity
MSX2, RYR2	GO:0030509 BMP signaling pathway
ACE, ARHGAP22, BCCIP, CDON, CENPF, FABP5, FGFR2, HDAC9, HRH3, INSM1/2, KAZALD1, LYN, MICALCL, NELL1, NLGN1, PEG10, PER2, PSD3, RPS3A, RSL1D1, SYNCRIP, TPD52, UNC45B, USP13	GO:0030154 cell differentiation; GO:0061101 neuroendocrine cell differentiation; GO:2000179 positive regulation of neural precursor cell proliferation;
BCL2L11, BCL11B, BFAR, BIRC5, CLN3, ECT2, HSPD1, ING5, ITSN1, MSX2, NRP1, PRKDC, SET, STEAP3, UNC5A; CROP, FGFR2, FKSG2, PEG10, PIM1, PSMB9, TOX3	GO:0006915 apoptotic process; GO:0043066 negative regulation of apoptotic process; GO:0043524 negative regulation of neuron apoptotic process
SIX3	GO:0014016 neuroblast differentiation; GO:0097402 neuroblast migration
ASPM, DLL1, PROX1, SOX2	GO:0097150 neuronal stem cell maintenance
FAM60A, PTPRU	GO:0030336 negative regulation of cell migration;
ALK, DSCAML1, FGF5, GFRA3, MTR, NELL1, NLGN1, NRN1, PCDH1, SHOX2, SLITRK1	nervous system development
WWC2	GO:0035331 negative regulation of hippo signalling
AKT3, ARVCF, CDH4/7/18/22, CDON, CLDN11, DSCAML1, ICAM4, IGFBP7, KIF14, NLGN1, NRCAM, NRP1/2, PCDH1/9/17, PKD1L1, PTPRT, PTPRU, PVRL1, SIRPA, SNAI1, SORBS2	GO:0007155 cell adhesion; GO:0007158 neuron cell-cell adhesion; GO:0016477 cell migration
ASPM, GATA3, GFRA3, NRCAM, NRP1/2, PCNT, SEMA3A/6A	GO:0001764 neuron migration;
ADM, BIRC5, CXCL5, FGF5, FGFR1OP, FGFR2, LIFR, LYN, NAP1L1, PHIP, POU3F2, PROX1, RPS15A, TBC1D8, TPD52	GO:0008284 positive regulation of cell proliferation;
RAB37/39	GO:0007264 small GTPase mediated signal transduction
AGTR1, DBH, FGFR2, RPA3, SIX3	GO:0042127 regulation of cell proliferation
SMOC2, SPOCK3	GO:0005539 glycosaminoglycan binding; GO:0005509 calcium ion binding; metalloendopeptidase inhibitor;
AGTR1,IGFBP4/7, IGFBPL1, KAZALD1; FBP1, NPPA, RERG, ZC3H12D	GO:0001558 regulation of cell growth; GO:0030308 negative regulation of cell growth
CHGA	neuroendocrine activity
AGTR1, CACNA2D2, CACNG4, NUDT4, PKD1L1, RYR2, TRPM7, TPCN1	GO:0005245 voltage-gated calcium channel activity; GO:0019722 calcium-mediated signaling
CABC1(ADCK3)	suppresses p53-induced apoptosis
NXPH1	promote adhesion between dendrites and axons
ABCA12, ABCB10, ABCC9, CACNG4, CAMK4, CAMKK1, CYB561, CYGB, DBH, EDG7, GABRB1, GRIA4, GRID1, GRIK3, HTR3A, KCNC1/G1/J12, PDE7B, SLC1A3, SLITRK1/5	GO:0007268 synaptic transmission; GO:0035249 synaptic transmission, glutamatergic; GO:0007267 cell-cell signalling; GO:0022857 transmembrane transporter activity; GO:0005249 voltage-gated potassium channel activity
COLEC12, CXCL5, DEFB1, HAMP, IKBKE, PVRL1, SECTM1	GO:0006935 chemotaxis; GO:0005125 cytokine activity
HIST1H4B/4D/4L	GO:0035575 histone demethylase activity; GO:0000723 telomere maintenance

Supplementary Table 6

F REPRESSED_CYCLIC	
E2F1, E2F2, E2F7, E2F8, MYBL2, LMO1, PSMC3IP, SOX7, TCF7/19, TFDP1/3, UHRF1	GO:0001071 nucleic acid binding transcription factor activity; GO:0005667 transcription factor complex; GO:0051726 regulation of cell cycle;
MYCNOS	MYCN opposite strand; neuroblastoma derived
ATAD2, AURKA/B, BIRC5, BUB1B, CABLES1, CASC5, CDC2/6/25A/C, CDCA2/4/5/7/8, CCNA2/E2, CDT1, CENPF/M/O/P/Q, CEP152, CHAF1A/B, CHTF18, CKS1B, DBF4, DHFR, DSN1, ESCO2, ESPL1, EXO1, FEN1, GINS1/2, GMNN, H2AFX, ITGB3BP, JAG2, KIF2C, KNTC1, MAD2L1, MASTL, MCM2/3/4/10, MLF1IP(CENPU), MND1, NASP, NCAPD2/3/G/G2/H, NUF2, NUP210, PCNA, PKMYT1, PLK1/4, POLA2/D1/D3/E/E2, PRIM1, ORC1L/6L, RBL1, RFC2/3/5, RPA1/3, RRM1/2, SAC3D1, SKP2, SMC2, TIPIN, TYMS, VRK1, XRCC2, ZWINT	GO:0007049 cell cycle; GO:0000082 G1/S transition of mitotic cell cycle; GO:0000086 G2/M transition of mitotic cell cycle; GO:0007093 mitotic cell cycle checkpoint; GO:0006260 DNA replication; GO:0007059 chromosome segregation; GO:0051297 centrosome organization; GO:0051225 spindle assembly; spindle pole; GO:0015630 microtubule cytoskeleton; histone binding;
BARD1, BLM, BRCA1/2, DDX11, DKC1, DTL, FANCA/D2/E/G, MRE11A, NEIL3, NTHL1, RAD9A /51/54B, TICRR, TOPBP1, TERT, TREX1, UNG, USP1, XRCC2	GO:0006259 DNA metabolic process; G2 DNA damage checkpoint; GO:0006281 DNA repair; GO:0000723 telomere maintenance; GO:0043240 Fanconi anaemia nuclear complex
HELLS	GO:0008283 cell proliferation; GO:0006342 chromatin silencing; GO:0006306 DNA methylation; GO:0043066 negative regulation of apoptotic process
EXOSC2/5, GEMIN4, NOLC1, SUV39H1	GO:0006364 rRNA processing;
CNTNAP2	GO:0007155 cell adhesion; GO:0008038 neuron recognition; GO:0050877 neurological system process
KCNH4, KCNH6; PCSK1	GO:0005249 voltage-gated potassium channel activity; GO:0007268 synaptic transmission
ABCB10, CPNE7, FABP5, SCARB1	GO:0005215 transporter activity
PRPS2	nucleotide biosynthetic process
IRX5/6	GO:0050877 neurological system process; GO:0048856 anatomical structure development
ENDOG, ICMT, JAG2, POLE, RNASEH2B, RPA1, XRCC2	GO:0001701 in utero embryonic development; GO:0043065 positive regulation of apoptotic process
CIT	GO:0000910 cytokinesis; GO:0048699 generation of neurons; GO:0043025 neuronal cell body;
GART, ICMT, METTL1, PRMT3, SUV39H1, TYMS, WDR4	GO:0008168 methyltransferase activity
PPIF	GO:0042981 regulation of apoptotic process
DGAT2	GO:0042572 retinol metabolic process; GO:0001523 retinoid metabolic process
SDC1	GO:0060070 canonical Wnt signaling pathway
MYOHD1	GO:0016459 myosin complex; GO:0005741 mitochondrial outer membrane
ABCB10, ATAD3A, HADH, MTP18, PPIF, SFXN2, SLC25A10, TYMS	GO:0005743 mitochondrial inner membrane;
MGME1	GO:0000002 mitochondrial genome maintenance; GO:0043504 mitochondrial DNA repair
CAD, TK1, TYMS	GO:0046134 pyrimidine nucleoside biosynthetic process; GO:0006206 pyrimidine nucleobase metabolic process

Text

Supplementary Table 7

G SPIKED	
KLF10, ZNF37A	GO:0001071 nucleic acid binding transcription factor activity
CMKLR1, P2RY6, RGS16	GO:0004930 G-protein coupled receptor activity; GO:0007186 G-protein coupled receptor signaling pathway
PIK3R1	GO:0048015 phosphatidylinositol-mediated signalling; GO:0008286 insulin receptor signaling pathway; GO:0048011 neurotrophin TRK receptor signaling pathway
IGF2	GO:0008083 growth factor activity; GO:0008286 insulin receptor signaling pathway
GHR	GO:0004903 growth hormone receptor activity; GO:0060396 growth hormone receptor signaling pathway
RAC2	GO:0007264 small GTPase mediated signal transduction;
BTG2	GO:2000178 negative regulation of neural precursor cell proliferation; GO:0030182 neuron differentiation
CDKN1A	GO:0008285 negative regulation of cell proliferation
S100B	GO:0007409 axonogenesis; GO:0005509 calcium ion binding; GO:0050877 neurological system process
TSPYL2	GO:0045786 negative regulation of cell cycle; GO:0016568 chromatin modification
WNT6	GO:0016055 Wnt signaling pathway
CD52, CD177	GO:0005887 integral component of plasma membrane; GO:0002376 immune system process
IGHA2, IGHM	GO:0003823 antigen binding; GO:0006955 immune response
TEX101	GO:0031225 anchored component of membrane
IL2RB, IL10RB	GO:0004911 interleukin-2 receptor activity; GO:0019221 cytokine-mediated signaling pathway
AQP6	GO:0055085 transmembrane transport;
TTC7A, TTC30A, TTC30B	GO:0042073 intraciliary transport
DDIT4L	GO:0009968 negative regulation of signal transduction
ANGPT2	GO:0001525 angiogenesis
HSD17B1	GO:0006694 steroid biosynthetic process
BTBD9	GO:0042428 serotonin metabolic process
PIP5K2A	GO:0016740 transferase activity; GO:0006650 glycerophospholipid metabolic process
NID2	GO:0030198 extracellular matrix organization;
TPCN2	GO:0005245 voltage-gated calcium channel activity; GO:0005216 ion channel activity
PSCD4	GO:0032012 regulation of ARF protein signal transduction

Supplementary Table 8

H CLEFT	
ASCL1, ATF5, CEBPA, CITED4, FOSL2, HDAC5, HES6, JDP2	GO:0003700 sequence-specific DNA binding transcription factor activity; GO:0008134 transcription factor binding
GPR56/155	GO:0004930 G-protein coupled receptor activity; GO:0007218 neuropeptide signaling pathway; GO:0021801 cerebral cortex radial glia guided migration; GO:0035025 positive regulation of Rho protein signal transduction; GO:2001223 negative regulation of neuron migration
NANOS1	GO:0017148 negative regulation of translation; GO:0010631 epithelial cell migration
NOS1AP	GO:0050999 regulation of nitric-oxide synthase activity; nNOS signaling at neuronal synapses; neurotransmission; neurotoxicity; GO:0042981 regulation of apoptotic process
MICAL2	GO:0030042 actin filament depolymerization;
ASCL1	GO:0048663 neuron fate commitment; GO:0050769 positive regulation of neurogenesis; GO:0030182 neuron differentiation; GO:0048485 sympathetic nervous system development; neural crest differentiation; GO:0008593 regulation of Notch signaling pathway
SEMA3D	GO:0007399 nervous system development
PARD6A	GO:0017048 Rho GTPase binding; GO:0045216 cell-cell junction organization; cell polarization; GO:0001837 epithelial-to-mesenchymal transition (EMT)
CD9, COL5A1/18A1, LAYN, PODXL	GO:0001726 ruffle; GO:0007155 cell adhesion; GO:0005925 focal adhesion; GO:0048870 cell motility; GO:0016477 cell migration
TG	GO:0006590 thyroid hormone generation; GO:0031641 regulation of myelination
FBN1	GO:0001501 skeletal system development; GO:0035582 sequestering of BMP in extracellular matrix; GO:0035583 sequestering of TGFbeta in extracellular matrix
CXCR7(ACKR3)	GO:0007186 G-protein coupled receptor activity; GO:0006935 chemotaxis
NRG1	GO:0048663 neuron fate commitment; GO:0014032 neural crest cell development; GO:0007399 nervous system development; GO:0009790 embryo development; GO:0007416 synapse assembly; GO:0031594 neuromuscular junction
AGRIN	GO:0045213 neurotransmitter receptor metabolic process; GO:0050808 synapse organization;
IGFBP5	GO:0031994 insulin-like growth factor I binding
BBC3, GADD45B/G, HRK, PPP2R2B, TNFRSF19	GO:0006915 apoptotic process; GO:0043065 positive regulation of apoptotic process; GO:0097193 intrinsic apoptotic signaling pathway; GO:0043525 positive regulation of neuron apoptotic process
IL17D	GO:0005125 cytokine activity; GO:0006954 inflammatory response
LGR5, NEFL, ISG15/20	G-protein coupled receptor signaling
DTNBP1, KIF1A, NEFL, SNAP25	GO:0043005 neuron projection
DKK3	GO:0016055 Wnt signaling pathway;
NEFL	GO:0005883 neurofilament; GO:0050772 positive regulation of axonogenesis
ADRA2A, DTNBP1, MICAL2, NEFL	GO:0007010 cytoskeleton organization
MATN2	GO:0007411 axon guidance; GO:0031175 neuron projection development; GO:0008347 glial cell migration
GNAZ	GO:0007193 adenylate cyclase-inhibiting G-protein coupled receptor signaling pathway; GO:0003924 GTPase activity
ARG2	GO:0006809 nitric oxide biosynthetic process; GO:0006525 arginine metabolic process; GO:0005759 mitochondrial matrix
